# LncSTPred: a predictive model of lncRNA subcellular localization and decipherment of the biological determinants influencing localization

**DOI:** 10.3389/fmolb.2024.1452142

**Published:** 2024-09-05

**Authors:** Si-Le Hu, Ying-Li Chen, Lu-Qiang Zhang, Hui Bai, Jia-Hong Yang, Qian-Zhong Li

**Affiliations:** ^1^ School of Physical Science and Technology, Inner Mongolia University, Hohhot, China; ^2^ The State Key Laboratory of Reproductive Regulation and Breeding of Grassland Livestock, Inner Mongolia University, Hohhot, China

**Keywords:** lncRNA, subcellular localization, oversampling method, algorithm improvements, model biological interpretation

## Abstract

**Introduction:**

Long non-coding RNAs (lncRNAs) play crucial roles in genetic markers, genome rearrangement, chromatin modifications, and other biological processes. Increasing evidence suggests that lncRNA functions are closely related to their subcellular localization. However, the distribution of lncRNAs in different subcellular localizations is imbalanced. The number of lncRNAs located in the nucleus is more than ten times that in the exosome.

**Methods:**

In this study, we propose a new oversampling method to construct a predictive dataset and develop a predictive model called LncSTPred. This model improves the Adaboost algorithm for subcellular localization prediction using 3-mer, 3-RF sequence, and minimum free energy structure features.

**Results and Discussion:**

By using our improved Adaboost algorithm, better prediction accuracy for lncRNA subcellular localization was obtained. In addition, we evaluated feature importance by using the F-score and analyzed the influence of highly relevant features on lncRNAs. Our study shows that the ANA features may be a key factor for predicting lncRNA subcellular localization, which correlates with the composition of stems and loops in the secondary structure of lncRNAs.

## 1 Introduction

Messenger RNAs (mRNAs) encode proteins that underlie various organismal phenomena, although they only represent about 2% of the total RNA. The remaining 98% consists of non-coding RNAs (ncRNAs), whose functions are still poorly understood (Birney et al., 2007; Wang and Li, 2013). Studies suggest that ncRNAs, especially long non-coding RNAs (lncRNAs) exceeding 200 base pairs in length, play vital roles in regulating biological activities (Atianand et al., 2017; Batista and Chang, 2013). The research on lncRNAs has expanded beyond their traditional biological functions to disease studies, particularly in cancer development and diagnosis (Esguerra and Eliasson, 2014; Flynn and Chang, 2014; Kameswaran and Kaestner, 2014; Li et al., 2014; Wang and Chang, 2011; Yan et al., 2015). Some lncRNAs are considered biomarkers for carcinogenesis and show differential expression between cancer and normal tissues (Harries, 2012; Kitagawa et al., 2012). For instance, overexpression of H19 can accelerate bladder and prostate cancer metastasis (Luo et al., 2013; Zhu et al., 2014a; Zhu et al., 2014b), while AFAP1-AS1 is highly expressed in esophageal adenocarcinoma (Wu et al., 2013), and *Gas5* is underexpressed in breast cancer (Mourtada-Maarabouni et al., 2009; Wu et al., 2013). Even the Oncotype Dx genes has been used as a strong evidence for the risk classification in NCCN clinical guidelines (Sparano et al., 2018).

Based on the relative abundance of lncRNAs (Derrien et al., 2012), 17% of lncRNAs are enriched in the nucleus and 4% in the cytoplasm, each with distinct functions. In the nucleus, lncRNAs can act as molecular scaffolds (Clemson et al., 2009), assist in alternative splicing (Gonzalez et al., 2015), regulate chromatin remodeling (Jiang et al., 2015; Kugel and Goodrich, 2012; Martens et al., 2004; Melé and Rinn, 2016; Saxena and Carninci, 2011), and modify DNA/RNA methylation (Yang et al., 2015). In the cytoplasm, they can regulate translation, promote or inhibit mRNA degradation (Gong and Maquat, 2011; Wilusz, 2016), and affect gene expression by binding to miRNAs (Lauressergues et al., 2015; Paraskevopoulou et al., 2013; Wang et al., 2010; K; Wang et al., 2015; Winter et al., 2009). Therefore, studying the subcellular localization and functions of lncRNAs is crucial (Bridges et al., 2021; Miao et al., 2019). Although experimental methods like lncRNA-FISH have been widely used, their utilization is hindered by their time-consuming nature, high cost, and low efficiency (L. Wang et al., 2015; Xiao et al., 2015). Consequently, many researchers have directed their efforts toward developing more reliable and efficient predictive models to address these shortcomings.

In recent decades, several predictive models for lncRNA subcellular localization have been proposed. For instance, lncLocation incorporates sequential, physicochemical, and structural features in its predictive model construction. It leverages the SVM algorithm along with binomial distribution and iterative feature selection techniques to create predictive models (Feng et al., 2020). In iLoc-lncRNA 2.0, the predictive model is built using 8-mer features and the mRMR method, which results in 1407 features and subsequently submitted to the SVM algorithms for model construction (Zhang et al., 2022). DeepLncLoc transforms sequence information into a matrix using word2vec, then uses a CNN to construct the DeepLncLoc model (Zeng et al., 2022).

**FIGURE 1:**
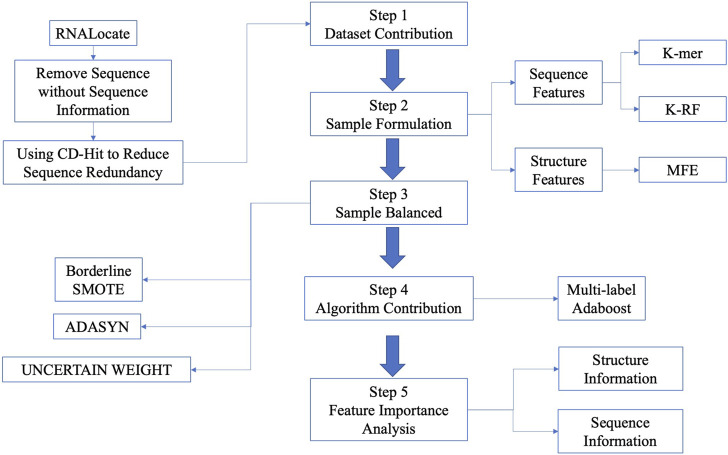
The flowchart of predictive model construction.

In this paper, we presented the LncSTPred, an Adaboost-based model for predicting and interpreting lncRNA subcellular localization based on primary RNA sequences in the RNALocate database. Our model supports five localization types including nucleus, cytoplasm, cytosol, ribosome, and exosome, accommodating both sequence and structure features. To solve the imbalance of categories, we employed Borderline-SMOTE, ADASYN, and UNCERTAIN WEIGHT on the training set. We enhanced Adaboost using maximum likelihood estimation and selected key features through the F-score. Subsequently, we conducted bioinformatics analyses of sequence and structure distributions of these features. An overview of the research process is depicted in Figure 1.

## 2 Materials and methods

### 2.1 Collection and preprocessing of dataset

The data used in this study related to subcellular localization of LncRNA in mammals, including *Homo sapiens* and *Mus musculus*, and were extracted from the RNALocate database (https://www.rnalocate.org/download) (Cui et al., 2022). Researchers can download all raw lncRNAs in the “Download and API” page. Subsequently, we retained lncRNAs related to *H. sapiens* and *M. musculus*, and excluded lncRNAs without sequence annotation information and those with multiple subcellular localizations. Redundant sequences were then removed using the CD-HIT program (Huang et al., 2010; Li and Godzik, 2006) with a threshold set at 80%. Subsequently, categories with sample sizes below 10 were excluded, resulting in 1342 lncRNAs across five distinct categories. These categories included 673 lncRNAs located in the nucleus, 407 in the cytoplasm, 152 associated with ribosomal localization, 94 within the cytosol, and 16 originating from exosomes, as depicted in Table 1.

**TABLE 1:** Number of lncRNA in each subcellular localization.

Subcellular localization	Mammals	*Homo sapiens*	Li’s Dataset Zeng et al. (2022)	Lin’s Dataset Su et al. (2018)
Nucleus	673	342	325	156
Cytoplasm	407	71	328	426
Cytosol	94	69	88	
Ribosome	152	132	88	43
Exosome	16	16	28	30

### 2.2 Nucleotide composition features

The nucleotide compositional features from lncRNA are significant features used to characterize the biological function of RNA and their species. Sequences are represented by Equation 1. The traditional method of sequence feature extraction is K-mer. Additionally, we aimed to extract sufficient information from the sequences. Therefore, we used reading frame (RF) features to further characterize the sequences, following the methodology outlined by Rainey et al. (Rainey and Repka, 2013). For convenience of description, we defined the 3-RF by Equation 2.

$$\mathrm{Sequence}=\left\{N_{1}{,N}_{2},N_{3},N_{4},N_{5},\ldots ,N_{M‐4}{,N}_{M‐3}{,N}_{M‐2},N_{M‐1},N_{M}\right\}$$ (1)

$$3-{RF}_{1}^{y}=\left\{\left[N_{1}\,{,N}_{2},N_{3}\right],\left[N_{4},N_{5},N_{6}\right]\ldots ,\left[N_{M-5},N_{M-4}\,{,N}_{M-3}\right],\left[N_{M-2},N_{M-1},N_{M}\right]\right\}$$

$$\;3-{RF}_{2}^{y}=\left\{N_{1},\left[N_{2},N_{3},N_{4}\right],\left[N_{5},N_{6},N_{7}\right],\ldots ,\left[N_{M-4}{,N}_{M-3}{,N}_{M-2}\right],N_{M-1},N_{M}\right\}$$ (2)

$$\;3-{\mathrm{RF}}_{3}^{y}=\left\{N_{1},N_{2},\left[N_{3},N_{4},N_{5}\right],\left[N_{6},N_{7},N_{8}\right],\ldots \left[{,N}_{M‐3}\,{,N}_{M‐2},N_{M‐1}\right],N_{M}\right\}$$

Where the sequence described a lncRNA with a length of M base pairs. *N*_*i*_ denoted the type of nucleotide at position *i*. {*N*_1_ *, N*_2_ *, N*_3_ *, … , N*_K_} was called k-mer, and there were $4^{k}$ combinations in total. $3-{RF}_{x}^{y}$ represented the combination of three reading frames in different starting points. *x* = {1,2,3} represented the first, second and third position respectively, *y* = {1,2, 3, 64} were the 64 combinations of 3-mer respectively.

### 2.3 Minimum free energy

The formation of base pairs can reduce the energy of RNA molecules and make the structure more stable. Therefore, based on the core idea of the minimum free energy (MFE) and the Zuker algorithm, we defined the global minimum value of the overall energy as Equation 3 (Zuker and Stiegler, 1981; Zuker and Sankoff, 1984).

$$\begin{array}{l} E_{ij}=\left[E_{\left(i+1,j-1\right)}+\alpha_{ij},\min\left(E_{i+k}+\beta_{k}\right),\min\left(E_{i+k,j-l}+\gamma_{k+l}\right),\right.\\ \;\left.\min\;\left(E_{i+k,j}+E_{i,j-l}+\varepsilon_{k+j+i-l}\right),\delta_{j-l}\right] \end{array}$$ (3)

Where, $\alpha_{ij}$ represents the stacking energy when *i* and *j* are paired. $\beta_{k},\gamma_{k},\varepsilon_{k},\text{and }\delta_{k}$ describe the energy of the bulge loop, interior loop, multi-branched loop, and hairpin loop, respectively. In the actual calculation, Zuker’s algorithm uses four free energy functions and five dynamic programming matrices. The minimum free energy of the RNA sequence is similar to the backtracking process of the Nussinov base pair maximization algorithm.

### 2.4 Feature importance

The F-score is a simple and effective feature selection method which measures the discriminative power of features across categories (Xie et al., 2010). The F-score of the *i*th feature in a multi-classification problem can be defined as Equation 4:

$$F\left(i\right)=\frac{\sum_{j=1}^{l}{\left({\bar{x}}_{i}^{\left(j\right)}-{\bar{x}}_{i}\right)}^{2}}{\sum_{j}^{l}\frac{1}{n_{j}-1}\sum_{k=1}^{n_{j}}{\left(x_{k,i}^{\left(j\right)}-{\bar{x}}_{i}\right)}^{2}}$$ (4)

Where *l* denotes the total number of categories. $n_{j}$ represents the number of samples in the *j*th class. The ${\bar{x}}_{i}$ and ${\bar{x}}_{i}^{\left(j\right)}$ are the average values of the *i*th feature across the entire dataset and the *j*th data subset, respectively. $x_{k,i}^{\left(j\right)}$ is the *k*th observation of the *i*th feature in the *j*th class

### 2.5 Sample balance process

Machine learning algorithms generally assume that positive and negative datasets are balanced. However, when the ratio of positive to negative sets exceeds 1:3, the results will be affected. To address this imbalance, two main approaches can be employed. One is adjusting sample weights in the algorithm, such as using the class-weight parameter in the XGBoost algorithm to balance different categories. The other is oversampling the minority categories to equalize sample numbers across categories. In this study, the ratio of nuclear to ribosome samples exceeds 1:4, and the ratio of nuclear to exosome samples is more than 1:40, making it challenging to find a suitable class-weight to characterize this complex distribution. Therefore, we constructed a predictive dataset using two oversampling methods, Borderline-SMOTE (Han et al., 2005) and ADASYN (He et al., 2008), for sample balancing. By focusing on borderline instances, Borderline-SMOTE generally produces better classification results compared to SMOTE, particularly when the minority class is at high risk of misclassification. It also reduces the risk of overfitting by concentrating on the most informative samples. ADASYN focuses on the more difficult minority class samples. This targeted approach ensures that the classifier is better trained on challenging examples, improving its robustness and generalizability. Finally, we filtered with the UNCERTAIN WEIGHT (Kendall et al., 2018) method.

### 2.6 Predictive model algorithm

The Boosting algorithm constructs high-accuracy classifiers by combining several base classifiers, each with moderate accuracy. Adaboost exemplifies this strategy and is known for its high accuracy and ability to model complex split interfaces through nonlinear combination. In the Adaboost algorithm, each base classifier generates a predicted classification result and a self-correction factor to estimate the reliability of the classification (Schapire and Singer, 1999).

In the original binary classification problem solved in the Adaboost algorithm, the coefficients $\alpha_{t}$ corrected for the basic classifier during iteration are shown in Equation 5:

$$\alpha_{t}=\frac{1}{2}\log\frac{\left(1-\varepsilon_{t}\right)}{\varepsilon_{t}}$$ (5)

$\varepsilon_{t}$ is the classification error rate of the base classifier on the training dataset. This coefficient ensures that, in each round, the classifier’s accuracy is at least greater than random probability, which is more than 1/2 in a binary classification problem. To extend this to a multi-classification problem, after ensuring the training data is balanced, the accuracy of the base classifier must be at least 1/k, where k is the number of categories. In this paper, k = 5. To ensure that each round prioritizes minimizing the classification error of the base classifier with the highest weight in the final classifier, we refined it as Equation 6:

$$\alpha_{t}=\frac{1}{2}\log\frac{\left(1-\varepsilon_{t}\right)}{\varepsilon_{t}}+\log\left(k-1\right)$$ (6)

In multi-classification Adaboost, we update the sample weights and decrease the weight of the previously classified base classifier. The weight of correctly classified samples is shown as Equation 7:

$$\omega_{t+1,i}^{\prime }=\omega_{t,i}\times \exp\left({-\alpha }_{t}\right)$$ (7)

The weight of wrongly classified samples is shown as Equation 8:

$$\omega_{t+1,i}^{\prime }=\omega_{t,i}\times \exp\left(\alpha_{t}\right)$$ (8)

To ensure the weights sum to 1, the weights $\omega_{t+1,i}$ of round t+1 in the training set after the round t are shown as Equation 9:

$$\omega_{t+1,i}=\frac{\omega_{t+1,i}^{\prime }}{\sum_{i}^{N}\omega_{t+1,i}^{\prime }}$$ (9)

### 2.7 Performance evaluation

We use the Specificity (*Sp*), Sensitivity (*Sn*), Accuracy (*ACC*), and Matthews Correlation Coefficient (*MCC*) to measure the performance of the predictive model. Evaluation indicators can be written as Equation 10:

$$S_{n}=\frac{TP}{TP+FN}\;;\;S_{p}=\frac{TN}{TN+FP}ACC=\frac{TP+FN}{TP+TN+FP+FN}$$

$$\;MCC=\frac{TP\times TN-FP\times FN}{\sqrt{\left(TP+FP\right)\left(TP+FN\right)\left(TN+FP\right)\left(TN+FN\right)}}$$ (10)

In the context of classification issue, *TP* represents the number of correctly recognized positives, *FN* represents the number of positives recognized as negatives, *FP* represents the number of negatives recognized as positives, and *TN* represents the number of correctly recognized negatives. Additionally, the ROC (Receiver Operating Characteristic) curve is established to evaluate the model’s robustness. The AUC value, ranging from 0 to 1, represents the area under the ROC curve. A larger AUC value indicates better model performance.

## 3 Results

### 3.1 Description of predictive dataset

**FIGURE 2:**
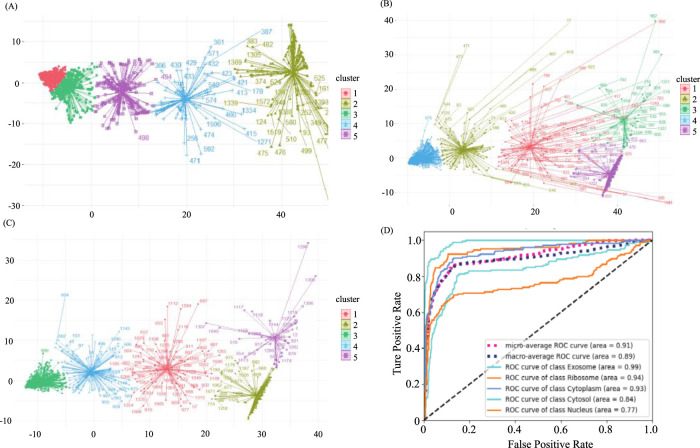
Demonstration of sample distribution at various stages of the balancing process and predictive model results **(A)**. The distribution of the original dataset through K-means clustering. *X*-axis and *Y*-axis were projection of the data onto the first dimension of a reduced-dimensional space after using PCA (Principal Component Analysis) **(B)** The distribution of the balanced dataset through K-means clustering. **(C)** The distribution of the predictive dataset through K-means Clustering. **(D)** ROC curve of all categories and averaged cases.

In our original dataset, the ratio of ribosome samples, cytosol samples, and exosome samples to nucleus samples exceeded 1:3, the distribution of original dataset is shown in Figure 2A. To address this imbalance, we oversampled cytoplasm, ribosomes, and exosomes. The balanced dataset was analyzed using the K-means clustering method with all features, including k-mer, k-RF, and MFE. Figure 2B illustrates the K-means clustering results after data balancing. Clusters three and five corresponded to nuclear and cytoplasmic localization, while clusters 1, 2, and four represented ribosome, cytosol, and exosome localizations, respectively. After preprocessing, which involved removing outliers and de-linearizing the dataset, the revised K-means clustering results were shown in Figure 2C. In this updated dataset, clusters 1 to five denoted ribosome, cytoplasm, exosome, cytosol, and nuclear localizations, respectively, the processing of the dataset is shown in Table 2.

**TABLE 2:** LncRNA subcellular localization dataset.

Name	Origin Dataset	Balanced Dataset	Predictive Dataset
Nucleus	673	673	673
Cytoplasm	407	407	407
Cytosol	94	407	394
Ribosome	152	407	363
Exosome	16	320	302

### 3.2 Predictive modelling process

The predicted results, both before and after sample balancing, were shown in Table 3. From Table 4, we found that exosomes, cytosols, and ribosomes were well-identified in the new predictive dataset. Notably, despite the limited data in the sample, these categories exhibited improved predictive results compared to those that were not oversampled. This result suggested that oversampling may enhance the model’s ability to capture more biological characteristics, thus improving prediction accuracy. This aligns with the concept of biological diversity. Furthermore, the ROC curve was shown in Figure 2D. From Figure 2D, we found that the AUC values for exosome, ribosome, cytoplasm, cytosol, and nucleus are 0.99, 0.94, 0.93, 0.84, and 0.77, respectively. These precise predictive outcomes indicate that the model has a commendable generalization ability across diverse subcellular localization categories.

**TABLE 3:** The predictive results of each subcellular localization.

Sample Balance	Subcellular localization	*S*_*n*_(%)	*S*_*p*_(%)	*MCC*	*ACC* (%)
Before	Nucleus	98.36	89.28	0.885	86.58
	Cytoplasm	95.33	94.97	0.891	
	Cytosol	47.38	98.51	0.604	
	Ribosome	32.97	98.87	0.456	
	Exosome	6.25	99.92	0.173	
After	Nucleus	58.54	90.22	0.606	94.14
	Cytoplasm	75.81	93.25	0.684	
	Cytosol	98.39	99.59	0.965	
	Ribosome	99.57	98.34	0.885	
	Exosome	99.19	99.79	0.989	

**TABLE 4:** Predictive results using different feature combinations in 10-fold cross validation.

Feature	*S*_*n*_(%)	*S*_*p*_(%)	*ACC* (%)	*MCC*
3-mer	54.31	70.23	68.35	0.467
3-RF	62.53	81.23	80.35	0.673
3-mer+3-RF	84.74	95.05	92.81	0.771
3-mer+3-RF + MFE	86.38	96.67	94.14	0.829

### 3.3 Predictive modelling process

The predicted results, both before and after sample balancing, were shown in Table 3. In Table 3, we found that exosomes, cytosols, and ribosomes were well-identified in the new predictive dataset. Notably, despite the limited data in the sample, these categories exhibited improved predictive results compared to those not oversampled. This result suggested that oversampling may enhance the model’s ability to capture more biological characteristics, thus improving prediction accuracy. This aligns with the concept of biological diversity. Furthermore, ROC curve analysis is conducted, and the results were shown in Figure 2D. In Figure 2D, we found that the AUC values for exosome, ribosome, cytoplasm, cytosol, and nucleus are 0.91, 0.89, 0.99, 0.93, and 0.77, respectively. These precise predictive outcomes indicate that the model has a commendable generalization ability across diverse subcellular localization categories.

To identify which feature categories most influence the predictive model’s results, we conducted separate experiments on 3-mer, 3-RF, MFE, and various combinations of these features. Table 4 shown the predictive results of different feature combinations in the LncSTPred model. We observed that the model’s performance using sequential features closely resembles that of other theoretical models. However, a substantial improvement in efficacy was achieved by adding the MFE feature.

### 3.4 Comparison with other researchers’ methods

Over the past few decades, numerous lncRNA predictive models have emerged. In this study, we conducted a comparative analysis between LncSTPred and existing theoretical algorithms, as well as other scholars’ predictive models to assess LncSTPred’s performance. Our comparison included Support Vector Machine (SVM), Random Forest, and XGBoost, with results presented in Table 5. *MCC* is a comprehensive performance metric that effectively reflects the classification ability of the model, especially when dealing with unbalanced datasets. Despite both XGBoost and LncSTPred employing the boosting integration strategy, LncSTPred displayed higher accuracy and generalization ability, particularly when using class-weight parameters for predicting lncRNA subcellular localization. This improvement is attributed to optimized error recognition and adjustments in error recognition point weights within LncSTPred.

**TABLE 5:** Comparison with existing theoretical algorithm.

Method	Subcellular Localization	*S*_*p*_(%)	*S*_*n*_(%)	*MCC*	*ACC* (%)
SVM	Exosome	66.92	65.53	0.262	73.37
	Cytosol	73.74	64.17	0.306	
	Cytoplasm	92.15	48.32	0.445	
	Ribosome	88.47	59.23	0.451	
	Nucleus	94.11	25.85	0.274	
Random Forest	Exosome	85.91	75.95	0.550	84.77
	Cytosol	85.91	72.85	0.427	
	Cytoplasm	95.85	76.70	0.604	
	Ribosome	91.69	68.06	0.557	
	Nucleus	96.85	48.32	0.553	
Xgboost	Exosome	96.97	94.44	0.906	90.22
	Cytosol	92.31	93.54	0.810	
	Cytoplasm	92.78	89.47	0.804	
	Ribosome	91.26	78.13	0.680	
	Nucleus	93.88	51.35	0.519	
LncSTPred	Exosome	99.79	99.19	0.989	94.14
	Cytosol	99.58	98.39	0.965	
	Cytoplasm	93.25	75.81	0.684	
	Ribosome	98.34	99.57	0.885	
	Nucleus	90.22	58.54	0.606	

We compared LncSTPred with machine learning-based models, specifically iLoc-lncRNA 2.0 and LightGBM-LNCLOC, using datasets from these models for performance validation (Table 6). Both iLoc-lncRNA 2.0 and LncSTPred demonstrated high precision in ribosome and exosome categories, with superior sensitivity in predicted cytoplasmic and nuclear localizations. In contrast, lncLocation, which did not use oversampling, showed comparable accuracy in nuclear and cytoplasmic categories but lower performance in ribosomal and exosomal predictions. This indicated that oversampling enhanced lncRNA subcellular localization prediction, particularly improved sensitivity in the cytoplasmic category compared to lncLocator. The analysis of classification datasets highlighted LncSTPred’s robust adaptability across various datasets, affirming its reliability as a classification model.

**TABLE 6:** Comparison with previous state-of-the-art methods.

Method	Subcellular Localization	*S*_*p*_(%)	*S*_*n*_(%)	*MCC*	*ACC* (%)
iLoc-lncRNA 2.0	Nucleus	95.59	91.03	86.59	91.60
	Cytoplasm	98.96	94.37	94.59	
	Ribosome	99.01	83.72	85.71	
	Exosome	99.36	66.67	83.33	
lncLocation	Nucleus	—	74.19	95.83	87.78
	Cytoplasm	—	100	85.00	
	Ribosome	—	55.56	100	
	Exosome	—	33.33	100	
LncSTPred in Lin’s dataset	Nucleus	78.74	90.06	60.48	90.76
	Cytoplasm	95.20	75.12	67.06	
	Ribosome	99.84	97.67	97.53	
	Exosome	99.84	96.67	92.90	
LightGBM-LNCLOC	Nucleus	—	90.00	42.90	70.60
	Cytoplasm	—	40.00	66.67	
	Cytosol	—	50.00	100	
	Ribosome	—	40.00	40.00	
	Exosome	—	42.90	100	
LncSTPred in Li’s dataset	Nucleus	81.25	81.29	61.39	87.68
	Cytoplasm	87.82	60.49	50.92	
	Cytosol	93.58	53.62	45.31	
	Ribosome	94.51	47.69	43.03	
	Exosome	99.83	6.25	75.43	

### 3.5 Analysis of feature importance

**FIGURE 3:**
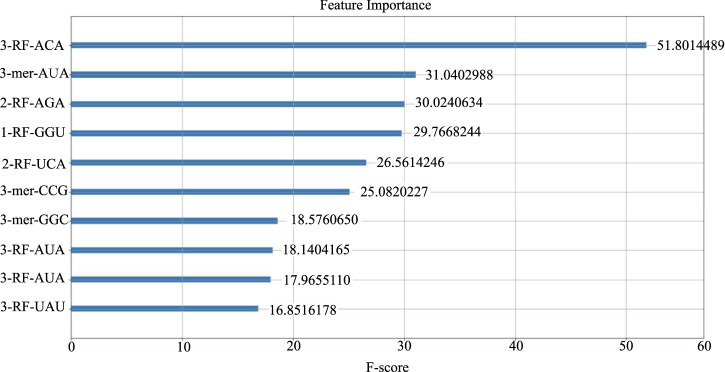
Top 10 features of the LncSTPred model.

We computed the feature importance in LncSTPred using the F-score described in Section 2.4, and the results were depicted in Figure 3. The top 10 combinations were “ACA” and “AUA” in the third RF, “AUA”, “AGA”, and “UCA” in the second RF, and “GGU” in the first RF. Additionally, “AUA”, “UAU”, “CGG”, and “GGC” were prominent in the 3-mer. The triplex nucleotide “ANA” had a significant impact on predictive modeling. To explore the potential effects of these 10 triplexes on the subcellular localization of lncRNAs, we analyzed their distribution in lncRNA sequences and secondary structure substructures.

**FIGURE 4:**
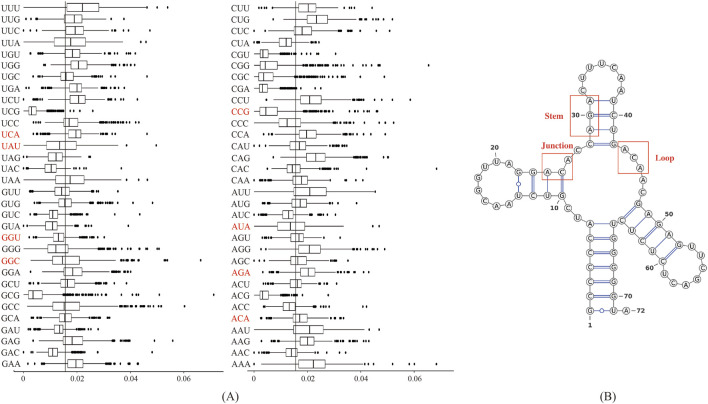
Biological analysis of the 10 most important features: **(A)** Frequency distribution of triplex nucleotides in lncRNA sequences. The *X*-axis represents 64 types of triplexes nucleotides, and the *Y*-axis represents the frequency of triplexes. **(B)** Three types of substructures in the secondary structure of lncRNAs.

Figure 4A displayed the frequency of triplex nucleotides in the original dataset. Interestingly, ACA and AGA frequencies exceeded the random probability, whereas AUA and UAU frequencies were below the random probability in the lncRNA sequences.

We used the RNAfold software to predict the secondary structure of lncRNA (Gruber et al., 2008). The output was in dot-bracket format, where '...' denoted loop structures, ')))' or '(((' represented stem structures, and '..(', '..)',').. ', or '(..' indicated junction structures. Figure 4B displayed three distinct substructures observed in the secondary structure. A frequency analysis of these substructure types was conducted, and the results were presented in Table 7. Analysis of Table 7 revealed specific enrichments: ACA in the junction, and AGA and TAT in the stem structures. While the frequency of ATA in the predictive dataset was lower than that of random combinations, ATA, ACA, and AGA predominantly appeared at critical positions within the junction and stem substructures of the lncRNA secondary structure. These findings suggested the significant contributory role of ANA in the construction of RNA secondary structures.

**TABLE 7:** Frequency distribution of triplex nucleotide in secondary structure components.

	AUA (%)	ACA (%)	AGA (%)	UAU (%)	Random combination (%)
Junction	1.632	3.081	2.998	1.382	1.563
Loop	1.414	2.691	1.938	1.415	1.563
Stem	1.613	2.232	3.347	2.101	1.563

## 4 Discussion

In recent years, the recognition of lncRNA subcellular localization has garnered increasing attention, as researchers have realized its potential for discovering the function of LncRNA. In this paper, we propose an improved algorithm for predicting lncRNA subcellular localizations, called LncSTPred.

During the establishment of the predictive model, we recognized the significant impact of the predictive dataset on the results. To address this, we utilized three oversampling methods—Borderline-SMOTE, ADASYN, and UNCERTAIN WEIGHT—to oversample the sample sets. This ensured that the predictive model for each category of samples could be sufficiently trained to achieve the best predictive results. Constructing LncSTPred using the improved Adaboost algorithm, we achieved 94.14% accuracy in the 5-categorical dataset and 90.76% accuracy in Lin’s 4-categorical dataset. This demonstrates that our improvements to the Adaboost algorithm, combined with data balancing, can provide better results than using the class-weight parameter in other algorithms.

Several studies have explored the impact of specific nucleotide combinations on RNA secondary structure. For example, the predictions of Smith et al. accurately identify mascRNA and a conserved hairpin upstream of Evolutionarily Conserved Structures (ECS). They observed that “ANA” triplex nucleotides predominantly appear at the stem-loop junction in ECS (Smith et al., 2013). Novikova et al., through biochemical probing, delineated a complex, two-dimensional structure comprising distinct sub-domains, including helical segments, terminal loops, internal loops, and linker regions. This study underscores that purine-rich sequences are highly conserved and often situated in single-stranded regions such as terminal and internal loops (Novikova et al., 2012). These findings corroborate the involvement of “ANA” triplex nucleotide composition in lncRNA secondary structure. Additionally, we performed a quantitative analysis of feature importance to identify the most significant features. By analyzing the frequency of triplex nucleotides and the stem-loop structures of lncRNA, we aimed to understand the relationship between significant features and lncRNA subcellular localization. Our analysis revealed a bias in the frequency of ANA nucleotide combinations within triplex nucleotides and the substructural frequency of stem-loop structures. These findings suggest that ANA nucleotide combinations play key roles in the composition of lncRNA secondary structures. In previous studies, Constanty et al. found that conserved U-rich and A-rich motifs were associated with specific processing and localization functions of lncRNAs like NEAT1 and MALAT1 (Constanty and Shkumatava, 2021). Furthermore, Cai et al. provided evidence that specific triplexes, including ACA, ATA, and AGA, significantly influence localization patterns by analyzing various sequences (Cai et al., 2023). Lyu et al. emphasized the relevance of trinucleotide propensity and position-specific features in recognizing lncRNA subcellular localization, demonstrating that specific triplexes like UAU could play a role in these predictions (Lyu et al., 2023).

Although LncSTPred has achieved better results in predicting lncRNA subcellular localization, we still face some challenges. On the one hand, the training process of AdaBoost is more complex, leading to significantly higher computing time compared to other prediction models. On the other hand, LncSTPred currently only accurately predicts lncRNAs only localized to a single subcellular localization, whereas many lncRNAs are localized in multiple subcellular localizations. Therefore, we will focus on lncRNA subcellular localization prediction in the future, further enhancing the accuracy of predicting subcellular localization of lncRNAs and the prediction of multi-localized lncRNAs using deep learning algorithms.

## Data Availability

The datasets presented in this study can be found in online repositories. The names of the repository/repositories and accession number(s) can be found in the article/Supplementary Material.
