# Supplementary material for: LncSTPred: a predictive model of lncRNA subcellular localization and decipherment of the biological determinants influencing localization
Source: Front Mol Biosci. 2024 Sep 5;11:1452142. doi: 10.3389/fmolb.2024.1452142 (PMC11411566; doi:10.3389/fmolb.2024.1452142)
Supplement: Supplementary file 1 [file DataSheet1.PDF]

Supporting information S1

In this paper, the original dataset created through the RNAlocate database(Zhang et al., 2016) contains homo sapiens and mus musculus. The sequence numbers of Homo sapiens and mus musculus are 625 and 717 respectively. In the origin dataset, there are 673 belong to nucleus, 407 to cytoplasm, 94 to cytosol, 152 to ribosome and 16 to exosome. Both the accession numbers and sequences are given. None of the lncRNA included here has ≥80% sequence identity to any other in a same subset.

Zhang, T., Tan, P., Wang, L., Jin, N., Li, Y., Zhang, L., Yang, H., Hu, Z., Zhang, L., & Hu, C. (2016). RNALocate: a resource for RNA subcellular localizations. *Nucleic acids research*, *45*(D1), D135-D138.

Homo sapiens

Exosome

ID|184|gene_id|100124412|transcript_id|NR_026819 Homo sapiens lncRNA
CACACAACGGGGTTTCGGGGCTGTGGACCCTGTGCCAGGAAAGGAAGGGCGCAGCTCCT
GCAATGCGGAGCAGCCAGGGCAGTGGGCACCAGGCTTTAGCCTCCCTTTCTCACCCTACAGAG
GGCAGGCCCTTCAGCTCCATTCTCCTCCAAGGCTGCAGAGGGGGCAGGAATTGGGGGTGACAG
GAGAGCTGTAAGGTCTCCAGTGGGTCATTCTGGGCCCAGAGATGGGTGCTGAAGCTCCCACGC
CTGCCTGTGAAAATGGAGTCCTCTCTCACCTGGGAGAGCCAGGTGCTGCCCCGAGAAGGATGC
ATTTATGGCTTCATGAAGTCTTTCCTGACCCCCGATGCTGCTGACTATAGAGACAAAGACTCACT
ATGTTGCTCAGGCTGGTCTTGAACTCCTGGCCTCAAGCGATCCTCCCACCTCAGCCTCCCAAAG
TGTTGGGATTATAGACATGAGCCACTGCACCTGGCCGACCTTGGGCAAGTTCTTAAACCCTTCA
AAGCCTCATTTTTCTCCAATCACAAAAGGGAAAGATGGTAATATTTTCCCCACCAAATTCTTGTC
GGATGCCCTCACAGAATTGAGATTATGTACGTAAAACACAAGGTGCCTAACCCGGCACAGAGC
AGGAGGGCTAAGCGTGACATCCAGCACGTGGTCAGTGGAATCCAGTATTCCTACCCACCTCTCT
AGTCTCCCCTCCACCCCTCTCCCTTTCAGAGGCACCAAGCTGCTTGTGGTCTTGTCTGTTCCCAC
TCCCTGCCTGACTGAACATTTTCTCCACCTCCTGATCATCAGCAGCAGAAACTGGCTGCTCTTCC
TGCTGGGTAGACAGCCAGACTGTATTTCCCAGCTGCCCCTGCAGTGAGATGTGGCCATCGGAG
CCAGCATTGGCCAATGGACTCTGCATGGGAGTGACGCATGCTGCCTCCAGGCTTGTCCCTAAAA
CCTCCCACGTGTCCTCCGCCTGCTCTTCCCACCTCCAAGGAGCACGGCAATTGTGGAAGACCCA
GATTAGTGATGGCAGAACCATAGATGGGAGGAACCTGGGTCCCTGACTTAAAGTATCATGGATT
TGGATGTTCCCTTAGTGAGAAATAAACTTCCATTGTGTTTAAGCCTTTA
ID|196|gene_id|100129196|transcript_id|NR_034182 Homo sapiens lncRNA
GATGACGCTGTAGCTCTCCACGTAATCGACGTGTTCGTCCTGCGGCTCGCTGGCGATCTGC
CGCAGCGTGGCCTTGTCCACGCTGCCCACTCCGATGGCGAACAGCTCGACGCCGCTGGCCCGG
GCCCGCGCAGACACGTCCTGCACGCTGTCCTGGGGCCTCCCGTCTGTCACCACGATGACCACC
TGCGACACGCGGGGCGGTGTGACACCGGGCTCCCGGAAGCCCACGGGACTGTCCAGGAGAGG
AGGCTGAGGCCCGGAGAGGCGGGAGGACTGCCGGGTCGCACAGCCAGGCGGCACCCGGGGG

AAGAGGACCCGGCTGGCCGAAGAGGAGACCCGTTTCCTCTGACTGAGAGACGTGGCTGGGGG
GCTTTCCATCTGCTTCGCCTCCCTCGGGCAGGCAGCTCTGAGATGCCCGGAGGGATGTGATGCC
CCATCGCCAAATTGGGGTCTCTAAGGTCAGGGCCATGCCTTCCCCTCAGACTGAGGCGGCTGA
GGGCAGGGTCCTCAGGCTGATGGCTGTGGGAGTCGTGCCACGCCCCTCGTGCCCCCGGGAGG
CCCGGCCGGGGCTGGGTGGGGGGATCCGGTGGTTCCACATGGTGCAGTTCTGCGCTTTGGCCA
CCAGATGGCAGCCGAAGCTAAGGGATGGGGCTGCCGTCTCCCGTCTTGCTGTCCTTGTCCCGGT
AAGACCGGGGACCCTGGACCAGGGAACCGACGGAGGGCCACTCCCATGTGGATGCCCAAGGG
ACCAACCCAAGAAAGATGCCGCCCTTATCCTCCTCCACTCCCCGCTATGAAATGGCCACCTCAC
GGGGCATCATGGGAATCAAAGTGTCTGCAGGCAAAGCTCATAACTCCGAGCGGGAGCCTCCAA
ACAGCAGCATCCTAGCAGGGTGGCGTGCACGTACCTTGCTGATGTCAGGGGACCTGGAACGAC
CACCCTCTGCATCGCCGAAGGCTTTGGTGATAGCGAACTGGATGGCCAGGCCGGTCATGGTGC
CTGTGGACAGCGGCTGGATACGGCGCACAGCCTGCAGCAGTGCGGCCTTGGAGACATGAGCC
CGCAGCGAGAACTCCTGCTTCACGGTGCTGGCATAGTTGACCATGCCCACCCGGGTGGCATTG
GGCCCCACGTCCAGCGACTCGATGACCTGGGACAGGAATACCTTCACTTTCTCAAATTCAACAG
GCCGAACGCTGCGAGAGCTGTCGACAACAAACACCAGGGTCACACAAGGGCCATCCACGGTG
ACCAGCGGTGAGAAACGCAATGCTGTCCTCGTGCCATGCTGGAGGTGAACGAGGAGAACGTG
AGCTGGGGTCTCCTCACCAACTGCTCCCCCCACCCCAACCCCCCTGACGGCCAGGGCTGTGTC
CAGGGCTCAGGGCACAAGGCTCCGTTAGCTGGGCAGCAGCTGGCCCATGAAACAGCCCTTTCA
GCTCCACCTCTGAATGGCAGTTCTATGGGCTCCTCGGAAGAACACAGGCGCCTTCTCTGTGTCA
CTTCTCCGCTCATTAGCAGAGCTGCAGAAATACACTACAGGACTCAAAATTGAGAACAGTGTGT
CCGGTATGAGCCAGAAGGAAAAAAAAATGCACGAAGGGGCCTTCTGCAAGGTTGCTCACAAGT
CAAACAGGCAGCTGTGAAGGGCTCTGCTGTTCCCTCCTCCTGTTTTCACATCCCTCGGGCTTCCA
GAAACTTAAAGCCGTTAGTGTTTCATGCAAGAGAGAGCATCTGTGATCCACGGAGAGGCCCCT
GGGCAGTGTGGTTCAGTGGGAAGCACACAGGACTCCCTCCCCAGTGCTCCCAGGATCAGCTCT
TTCCCTAAAGAGCAAACCTGGTTATCTCGCAATGACCTAGCTAACACAAATGCAACATCAGCCG
GGGCAGCCTGCCCCCTTCACTCTTTGCCCACCATCAGCTGTTTGGCAGTATCCATATACGCCTTT
ACCAGTATTTATTTAATCTTTTTGTTTAATTCACTCTAAACCAACTCACTTCTTAAAAATTTCTCTG
CCTTATAAGAAATAATAGCCATGAAATCACAAAAAAAAAAAAAAA
ID|4406|gene_id|148898|transcript_id|NR_033690 Homo sapiens lncRNA
CTCGGGAGTGCGGTCGGCAGTAGAGCCTGGCGGTGTCTTGGCTTCGGCGCTTTAACCCTG
TAGGGGCGTGCGACTGCGTCCTTCTCAAGGGGGCGCCCCTCGAGCGGCGGGCTGAGTGGCGT
CCAGCGGCGGGGAGGCGAAAACCCTGGGGGACTCAGAAGCGGGTGGCGGTGTAGGGCGTGT
AAGGAGGAGATAGCCAGGCCCCTGCGGGGGGGGCGGGCCCTCCTTAGCGTCCCAGAGGGCTT
GTCTGCTACAGGGGCTCCCAGCGGCCTCCCGCACCGAAGGGACCCTGGGCGTCTGAGGCCCTG
ATCCGGGCGGGCCTTCAGGATGTTAGCAGTCCCTGTCCGGTTGAAGGTAGGAAGCCGAAAACC
CGAGTGGGGGACGAACAGACTTACCTCCTGTCCCGCAAAAGATCCACTAGATCGTCGTCTCCA
AAACCTGAGAGACCGCGAACGGGTTCCAGAGCCGCAGCGTTCTCTCAGACCTGGCGTTCAGGA
AGATTCTAGAGAGCACGGGCAGGTCCCGGAGGTCTCAGACCCGCAGGTAGATCTCGAATTCGT
AGACTTGCAGGCGAAGCCCAGATATCGTAGGCTGATCCTAAAGACTCAGATTCCCGAGGCCTC
AGACTCGCAGGCAGCTCAGAAACCACAGGCTCATAGGCAGATCCCTGAGACCACAGAGGCTG
GCCGAGAAACCACCAGCAACTAAGCTGCATTCAGCTGGAGTTCCTCGGAACGGGAGGACTCGC
AGCTCTCCCTTTCCCCAGCCAGGATGAGGGAATTAGACAATGGAAGTCGAGCACTGGGGAAAG
CAGGCATTCTGCGTGGGGAAGAGCTCCCAGCTCGCTGTAGCCTTGGAGAGGGAAGTGATGGAA
AAAGAATATAGCAGCTTATTAGGGACTCCCACCCTCTTCAAGTTATAAATATCCCCAGAACCTTG

GCTACGTGCCCGGAAGCCTGTTGTCTCTAACTGTACAGGAGACTTCTCTGCCTGGGCTGGGAAA
GGGGCCTTTCTTTTGTTTTGCAGATAGATCCACCCACTGAGGAACTTGAAAATTTTTTTCAAACAT
AACTCCAGGCCTTGCTTTTATTAGTGAGTGTCCTGTTCCCAGTGCCCGGTTATTTATGATTAAATT
ACTGAAGTGTCTAAAAAGAACAGAAGGACCCACCCCAGCCATAATGCAGACTTTCCCATTCCTT
CTCTTTCCACCCGCAACTGTGGGGCTAAGAATTCTTAAGATGTCACAGAATTATACCATCCTACC
TCATCTAAAGTCGATCCGAGGCTGGTCCGATGGTAGTGGGTTACCAGAACTTAATAACATTAAT
AACTCTAAAGTTAGTATACAACCCCCCACTGCTAAATTTGGCTATAAAAATAAAATTGATCTGAG
CACCATATAAAATGGAATCCTGGAGGGTCCCCTAGAGGCAGTATCCCCAGTGGAAAGAGCGTG
GGCCTCAAGGTCAGACAAGCCTGGTTTGGAATCCAGGCTGTGTATCCTTTGGAAAGTCATTGAA
TCCAGGCCCATTTCCTTTTCTCTAAAATGAGGGTCATCATACCTAAGGATGTTGTGAGGCGTGAG
TGAGATGATTAAAGCGTTCCAGAGAGTGCGCTCTTGGATGATAGCCGTTACTTTTTATTAACCCT
GGCTTTGGTGGTAAGGGAAACTTCCAGAAATGCTTGTGGGTATATTGGATAGCTCAGAAAATCT
GGGAAATCAAAGTGGCTCAATAGCTGTGAGGAACTGTGGGTGACTTTATCCTCACATCGTGTTT
CTTCCAGAAATTCAGACGCAACAGAGGTTAGGCCAGTTTGGTGGGTTCCAGTGAGCGGTGTGTT
GAGCCTAGTAGAGCATAGGTACTTGGGGTTGCCAAAACCCCAGCAAGTTGAGGCTTCACGCTC
AGGGAAACTTCTGGGTCAGAGGTTAATAACCACGCTCCTTGGAGCAAGGAGGAGGGGAGCCTT
TCTGACTAGCTGAAGTAATTGCAGGTTTCTAACTGGGCCTAAGGTGAGCTGAGGGCTTGAGCCC
CTCAGCCCAGCGGGGGTCCCTTTTCATCCCTTCTCTGACAGATTGCTTTGTAAACTTTCTTAGGC
CTTCCCCCCACCCCTTTGCCCCAGTGCTTTAAGCCCTTCTTTGTCTTCTTGCTGTTTCTTTTATTCC
TCAGGCCTGCGGGGCGGGGGCGGGGTGGCGCCCAGGACGACTCCCCGGGCTCAGCTTGGCTG
CCTGCCTCCTTCTGTAAGTGCTTTTTTTTTCTTCACCTGGGACCCTCTAGAGGTTGGAAAGAGAA
GAGAGGCTGGGAGCGGATGGAAAGCATGACTGCATCTGGAGCCCCTGGGGGGAGTGGGGAAG
AGGGAGTGGAAGGACAGTGGCTGAGGGGCTTCCTGTTGCAGCCTTCAGCTGGACTCAGGGTTG
GGGTCACTGGAGGAGGAGGTGGCTTCCTGCCCCATTGTTCTGGACTGCAGTGTTTCTTGGGAAT
ACTTACTGTATGTGGATTTTCAGTAACCAACTTGGAAAGCTGTATTTGTTCCCATGATTCAGCAA
ACAATGAGTACCTACTGTGTGTAGAAGCAGCAGGACAGCCTGTCATCACTGGGAAAGAGGCCA
GAAGAGCCGTTTGGCCAGGGTCTCCAATTTAGGCTTTCAACATTATCTCTAAAGAAGGTTATACA
TTATGTCGGCTCCACTGGCTGATGGGATCAAAGACCCTCCTTGTATATCCTTAAACCTGTACTCT
ATTACCAGGCCACTGTGCCTGTGGAGCACTGTAAGTTCCTACCTTTTAATAAATTTTATAATTATC
TTGTGAGCAGCACAATTGTCATAGTTTTTTGTATCAGAAACATTAATTTGTTAAACATTTTAAAAA
AAAAAAAAA

ID|4407|gene_id|148898|transcript_id|NR_033691 Homo sapiens lncRNA

CTGCTACAGGGGCTCCCAGCGGCCTCCCGCACCGAAGGGACCCTGGGCGTCTGAGGCCC
TGATCCGGGCGGGCCTTCAGGATGTTAGCAGTCCCTGTCCGGTTGAAGGTAGGAAGCCGAAAA
CCCGAGTGGGGGACGAACAGACTTACCTCCTGTCCCGCAAAAGATCCACTAGATCGTCGTCTCC
AAAACCTGAGAGACCGCGAACGGGTTCCAGAGCCGCAGCGTTCTCTCAGACCTGGCGTTCAGG
AAGATTCTAGAGAGCACGGGCAGGTCCCGGAGGTCTCAGACCCGCAGGCCTGCGGGGCGGGG
GCGGGGTGGCGCCCAGGACGACTCCCCGGGCTCAGCTTGGCTGCCTGCCTCCTTCTCAAACAA
TGAGTACCTACTGTGTGTAGAAGCAGCAGGACAGCCTGTCATCACTGGGAAAGAGGCCAGAAG
AGCCGTTTGGCCAGGGTCTCCAATTTAGGCTTTCAACATTATCTCTAAAGAAGGTTATACATTAT
GTCGGCTCCACTGGCTGATGGGATCAAAGACCCTCCTTGTATATCCTTAAACCTGTACTCTATTA
CCAGGCCACTGTGCCTGTGGAGCACTGTAAGTTCCTACCTTTTAATAAATTTTATAATTATCTTGT
GAGCAGCACAATTGTCATAGTTTTTTGTATCAGAAACATTAATTTGTTAAACATTTTAAAAAAAAA
AAAAA

ID|9222|gene_id|252948|transcript_id|NR_001552 Homo sapiens lncRNA
GAGTGTGGATTATGCCCTGGCCATGTCCTCATGTTTCCCAACGGCTGGGAGTCAGAAATTG
AGTGACCTGGGAGCACCTTTGTTGTGCCATCTATAGCAAAAAGACACTTGGGTGCAAGTGGGAA
TCTTGAGTCACTTTGAGGAGCATTGCACAAAGCCCTATGTCTTCAGCCAAGTGCACCCTTTCCTT
TT
ID|9223|gene_id|252952|transcript_id|NR_001549 Homo sapiens lncRNA
GACAGTCCCATTGAGCAACACTTCAATGAATGCACCCCTCCAAATTGAGAAGGCCATGAA
GATGAAATGAAATAGTCTAGATTCCCAGATGAAGTTGCAAGACATCTTCTACCCCTCAACAGAC
TGTATCTTCACCTCTATCTGACCTTATTGCTGCTCACATTATTTGTCTCAAGATAAAATCCCAAAA
CTCTGGAGGGGTGCTGCCTCACAAGATGCATCACCTGCTCAGTTGGGAACCAAATTTGAGGTAA
ATTCAAGGTGCCCTG
ID|16671|gene_id|503693|transcript_id|NR_024061 Homo sapiens lncRNA
GGCTACGCAAGACCTTCAGTTCCGGATTAGGAGGCCCCGCCCCCCGGCCCGAGGGAGGG
GCGGAGAGACCCGCTCCTGCGACTTAGGGCGATGCCACCTTAAAGGGCTTGACCTCCTCGAAG
CCAGAACTGCGGAAGAGGATGGAGAAAGAAACGCTAGATAGACCCAGGATTCGAACCCACAA
CAGCTTGGATGCAAAGCTCATTTTGAATTCTGAAGAGCTGGGAGTTTAGCAGTGGACGACCAAA
CAAAAAAATACCAGAAGACGGTGAAAGCAGCAGCTGGAAATGAGAGAAAAATTAAGAAATAG
AAAATGCTGTTTCATGTATTAGGGAGTGCACATCTTTAAAGAGAAGTGAAGAACCTTTTTGGCTA
GGCGCTTTCTGGGGACCTTGGCAGTTATCAACAAGTCACTCTGAAACTAACAGAAGGTGAGGA
GGGAGTAATCCAGAAAGGAAACTGCCATTTTTGCTAGGGCAAGAAAGTAGACCTAGCAATGCA
ATGCCATCAAGGAGTTAGCTGGCTCTAGCGTTCTCCGAACTTTGCAACTCATTTTATATTACATTG
TCTGCGTCAAGAAATTTCAAGTAAATGCCTTGAGATTTTATATGTATAAAATGTAGTCTCTGGCC
AGGCGGGGTGGCTCACGCCTGTAATCCCAGCACTTTGGGAGGCTGAGGCGAGTAGATCACGAT
GTCAGGAGATCAAGACCATCCTGGCTAACATGGTGAAACCCCGTCTCTACTAAAAAATACAAAA
AATTAGCCGGGCATGATGGTGGGCGCCTGTAGTCCCAGCTACTCGGGAGGCTGAGACAGGAGA
ATGGTGTGAACCCGGGAGGCAGAGCTTGCAGTGAGCCGAGATCGCGCCACTGCACTCCAGCCT
GGGCGACAGAGCGAGACTCCGTCTCAAAAAAAAAAAAAAAAAAAAAAAGTCTCAGAAGAAGTG
TCAAGTTGACTAAATGATTTTTAAGTCCCTTCCACTCTACTATATCACAATAATTTCCCCTATTTTA
CCTTATTTAATTTTCACAACAACCCTGCAGCCTGTAAGTAGGTGACTCCTCCATGGTCAGCAGGT
GGTCAGTGGTCGAACTATTTAGGACTATCTGATGCTCTTTCCTCTGCTCACCATTGCAATACAAT
GAGAAAAATGCAAATGAATCTAAGTACAGTGTTCTCTGGGAATATGAAGATAGAACTAAAAACT
GCCTGAGAGATCAGGGCAGGCTTCTAGAAAGTTGTGTCTAACTAGTCTTGAGGTTTACATAAAC
CACACCAGGCTGATGGGACTGGGAAGGGCCCACTAAGCAGTGAGACCATTCCCTTTTGGAGAG
TCCTTGCATCCCCTCCCAGATTTCCTCTTTGAGGGGAAGGTGAGAGAGGAGGTAAAAGGGGTG
AGGAATGGAGAATAACTCATTCTGGTTCTTGTTTCCCCTTTTCCACATAAAAGTATATTTGTCTTG
TGTTCCATACACCAGTCCATACTGATGTGATGGTGTTTTTTATGCTTCCTTTTGAATAAACATTTCA
TTCTTAA
ID|18016|gene_id|54067|transcript_id|NR_024622 Homo sapiens lncRNA
GAGATGAGAAGAAAGAGTAGGCTCTGTTGTCCCTCAGACGGCGCTGCTGCAGTGCGTCTT
CGCACTTACGCGGAGCGGTAATGTGAGGAAGCCTCCCGCCAAACAGATGAGGGAGGAGGAGG
AGGGGAGAGGCCAGAGAGAGAAGCGGCTTTCGGGAAGGAAAGTGTGATCTAGTAGGTGGAAT
AGTCCTCTCCAAACTTTGACCTGAGGTGTGACTATTGAATGACCCTCTATTTGGATTTGGATTAC
ACAAAGTTTAACGTTTTTTGGAAAAATCCTACCTAGGTAATGTGTAGTTCTCAATGCATCATGTCA
GGCAGCTTATGATGCCAATTTGTCCCATGGCCTTAAATTCCACAACCTCATCATCTACCACCTTT

GGTGCTTTCAGAATCATGACTCTCAATGTTGAAGAATGGGCTACTGCATGGAAGGTGCTGATCC
TTTTAGAGGCAGCCGTGGAAGAGGAAAAGAGATCAGAAGAGAAAAGGATATTGGTTTGCGGAA
CATGTGGGACAAGAAGTTCCCAGAAGAATTTGTGAGCAACTTCCCGACAGTAAGGCCCTGGCT
AGACCTCAGGATGGTCCCTGCCTCCTGGACATTAGGAAGCCCAAAGGCCAGAACAAAAACACA
TGCCTAGTGGGGGAAGGCTCACTAAGAGGGCACCAAGTGGGGCAAATACCCCTGGTAACCCAT
TTATGGAGGCTGCCACAGAAATGCTAGTTGGAAATTTTCCTCCTTCAGTCTATCATGAATTTCTTT
TTTCTCTTTTGAGATGAAGTCGCCCGGGCTGCAGTTCAGTGGTGCAATCTCAGCTCACTGCAAGC
TCTGCCTCCCAGGTTCCAACGATTGTCTTGTCTCAGCCTCCTGAGTAGCTGAGATTATAAGCACG
CACCATCATACCCGACTAATTTTTGTATTTATAGTAGAAATGGGGTTTTGCCATGTTGGCCAGGC
TGGTCTTGAACTCCTGACCTTAGGGTGATCCACCCATCTTGGCCTCCAAAAGTGCTGGGATTACA
GGCATGAGCCACTGCACTCGGCTGTACCGTGATTATTAGTTCAAGGTTCTCTTTCACTGGGGTAA
AGACCTTAAGAGAGCAGTTTTTGGGCTTTATATTCTCAATTTCCCGCACAGTGCCTAGCACATAG
CAAGGACTCAACAAATTTCATTGAATGAATGAAAAAACATAGTTTACCAAAAAAAAAAAAAAA

ID|18017|gene_id|54067|transcript_id|NR_024623 Homo sapiens lncRNA

GAGATGAGAAGAAAGAGTAGGCTCTGTTGTCCCTCAGACGGCGCTGCTGCAGTGCGTCTT
CGCACTTACGCGGAGCGGTAATGTGAGGAAGCCTCCCGCCAAACAGATGAGGGAGGAGGAGG
AGGGGAGAGGCCAGAGAGAGAAGCGGCTTTCGGGAAGGAAAGTGTGATCTAGTAGGTGGAAT
AGTCCTCTCCAAACTTTGACCTGAGAATGACCCTCTATTTGGATTTGGATTACACAAAGTTTAAC
GTTTTTTGGAAAAATCCTACCTAGGTAATGTGTAGTTCTCAATGCATCATGTCAGGCAGCTTATG
ATGCCAATTTGTCCCATGGCCTTAAATTCCACAACCTCATCATCTACCACCTTTGGTGCTTTCAGA
ATCATGACTCTCAATGTTGAAGAATGGGCTACTGCATGGAAGGTGCTGATCCTTTTAGAGGCAG
CCGTGGAAGAGGAAAAGAGATCAGAAGAGAAAAGGATATTGGTTTGCGGAACATGTGGGACA
AGAAGTTCCCAGAAGAATTTGTGAGCAACTTCCCGACAGTAAGGCCCTGGCTAGACCTCAGGA
TGGTCCCTGCCTCCTGGACATTAGGAAGCCCAAAGGCCAGAACAAAAACACATGCCTAGTGGG
GGAAGGCTCACTAAGAGGGCACCAAGTGGGGCAAATACCCCTGGTAACCCATTTATGGAGGCT
GCCACAGAAATGCTAGTTGGAAATTTTCCTCCTTCAGTCTATCATGAATTTCTTTTTTCTCTTTTGA
GATGAAGTCGCCCGGGCTGCAGTTCAGTGGTGCAATCTCAGCTCACTGCAAGCTCTGCCTCCCA
GGTTCCAACGATTGTCTTGTCTCAGCCTCCTGAGTAGCTGAGATTATAAGCACGCACCATCATAC
CCGACTAATTTTTGTATTTATAGTAGAAATGGGGTTTTGCCATGTTGGCCAGGCTGGTCTTGAAC
TCCTGACCTTAGGGTGATCCACCCATCTTGGCCTCCAAAAGTGCTGGGATTACAGGCATGAGCC
ACTGCACTCGGCTGTACCGTGATTATTAGTTCAAGGTTCTCTTTCACTGGGGTAAAGACCTTAAG
AGAGCAGTTTTTGGGCTTTATATTCTCAATTTCCCGCACAGTGCCTAGCACATAGCAAGGACTCA
ACAAATTTCATTGAATGAATGAAAAAACATAGTTTACCAAAAAAAAAAAAAAA

ID|21927|gene_id|654412|transcript_id|NR_026821 Homo sapiens lncRNA

CACACAACGGGGTTTCGGGGCTGTGGACCCTGTGCCAGGAAAGGAAGGGCGCAGCTCCT
GCAATGCGGAGCAGCCAGGGCAGTGGGCACCAGGCTTTAGCCTCCCTTTCTCACCCTACAGAG
GGCAGGCCCTTCAGCTCCATTCTCCTCCAAGGCTGCAGAGGGGGCAGGAATTGGGGGTGACAG
GAGAGCTGTAAGGTCTCCAGTGGGTCATTCTGGGCCCAGAGATGGGTGCTGAAGCTCCCACGC
CTGCCTGTGAAAATGGAGTCCTCTCTCACCTGGGAGAGCCAGGTGCTGCCCCGAGAAGGATGC
ATTTATGGCTTCATGAAGTCTTTCCTGACCCCCGATGCTGCTGACTATAGAGACAAAGTCTCACT
ATGTTGCTCAGGCTGGTCTTGAACTCCTGGCCTCAAGCGATCCTCCCACCTTAGCCTCCCAAAG
AGTTGGGATTATAGACATGAGCCACTGCACCTGGCCGACCTTGGGCAAGTTCTTAAACCCTTCA
AAGCCTCATTTTTCTCCAATCATAAAAGGGAAAGATGGTAATATTTTCCCCTCCAAATTCTTGTCG
GATGCCCTCACAGAATTGAGATTATGTACGTAAAACACCAGGTGCCTAACCCGGCACAGAGCA

GGAGGGCTAAGCGTGACATCCAGCACGTGGTCAGTGGAATCCAGTATTCCTACCCACCTCTCTA
GTCTCCCCTCCACCCCTCTCCCTTTCAGAGGCACCAAGCTGCTTGTGGTCTTGTCTATTCCCACT
CCCTGCCTGACTGAACATTTTCTCCACCTCCTGATCATCAGCAGCAGAAACTGGCTGCTCTTCCT
CCTGGGTAGACAGCCAGACTGTATTTCCCAGCTGCCCCTGCAGTGAGATGTGGCCATCGGAGC
CAGCATTGGCCAATGGACTCTGCATGGGAGTGACGCATGCTGCCTCCAGGCTTGTCCCTAAAAC
CTCCCACGTGTCCTCCGCCTGCTCTTCCCACTTCCAAGGAGCACGGCAATTGTGGAAGACCCAG
ATTAGTGATGGCAGAACCATAGATGGGAGGAACCTGGGTCCCTGACTTAAAGTATCATGGATTT
GGATGTTCCCTTAGTGAGAAATAAACTTCCATTGTGTTTAAGCCTTTA

ID|22817|gene_id|677784|transcript_id|NR_026823 Homo sapiens lncRNA

CACACAACGGGGTTTCGGGGCTGTGGACCCTGTGCCAGGAAAGGAAGGGCGCAGCTCCT
GCAATGCGGAGCAGCCAGGGCAGTGGGCACCAGGCTTTAGCCTCCCTTTCTCACCCTACAGAG
GGCAGGCCCTTCAGCTCCATTCTCCTCCAAGGCTGCAGAGGGGGCAGGAATTGGGGGTGACAG
GAGAGCTGTAAGGTCTCCAGTGGGTCATTCTGGGCCCAGAGATGGGTGCTGAAGCTCCCACAC
CTGCCTGTGAAAATGGATTCCTCTCTCACCTGGGAGAGCCAGGTGCTGCCCCGAGAAGGATGC
ATTTATGGCTTCATGAAGTCTTTCCTGACCCCCGATGCTGCTGACTATAGACAAAGTCTCACTAT
GTTGCTCAGGCTGGTCTTGAACTCCTGGCCTCAAGCGATCCTCCCACCTTAGCCTCCCAAAGTGT
TGGGATTATAGACATGAGCCACTGCACCTGGCCAACCTTGGGCAAGTTCTTAAACCCTTCAAAG
CCTCATTTTTCTCCAATCATAAAAGGGAAAGATGGTAATATTTTCCCCGCCAAATTCTTGTCGGAT
GCCCTCACAGAATTGAGATTATGTACGTAAAACACCAGGTGCCTAACCCGGCACAGAGCAGGA
GGGCTAAGCGTGACATCCAGCATGTGGTCAGTGGAATCCAGTATTCCTACCCACCTCTCTAGTC
TCCCCTCCACCCCTCTCCCTTTCAGAGGCACCAAGCTGCTTGTGGTCTTGTCTATTCCCACTCCCT
GCCCGACTGAACATTTTCTCTACCTCCTGATCATCAGCAGCAGAAACTGGCTGCTCTTCCTCCTG
GGTAGACAGCCAGACTGTATTTCCCAGCTGCCCCTGCAGTGAGATGTGGCCATCGGAGCCAGC
ATTGGCCAATGGACTCTGCATGGGAGTGACGCATGCTGCCTCCAGGCTTGTCTCTAAAACCTCC
CACGTGTCCTCCACCTGCTCTTCCCACCTCCAAGGAGCACGGCAATTGTGGAAGACCCAGATTA
GTGATGGCAGAACCATAGATGGGAGGAACCTGGGTCCCTGACTTAAAGTATCATGGATTTGGAT
GTTCCCTTAGTGAGAAATAAACTTCCATTGTGTTTAAGCCTT

ID|24160|gene_id|728409|transcript_id|NR_024102 Homo sapiens lncRNA

TGGACAGCAGCAGAGGGAGGAGGAGGCAGGACAGACCAGAAGAGAGACAATGAGGTTC
TGAACCTAGATGTTGGCCAAGGGAGCAGAGGAGGGCAGGTCAGGAGGCCCTCGCCCTGCAAT
CACCCTTCCTGGGAGCCTTCACTTCACCTGTGATCTCAAGACCTCTCCCTACTGCCTGACTCGCG
CCGAGCTTATGGAACATCTTCCCTTGAGGGTCGCAGTCCATTCGATGAGCCCTTGTCACAGGTC
CTGCTTCTGTGGGGAGCTCAAGCGCGGACACCCTTGGAACACTCCCCAAGTCTCTTCCTTCCCA
TCCAGCACCACTTCCCTAAGTCACAGCTGCACCACCTCTCATCTGGACTGCTCACAGCAGGTGG
AGTCAGGATCAAAATAGCCAAAGAAAAAGTAAGAATCCGTATACAGGAAACAAATATGTGATG
ATGACCGGAGCAGCTCAGAATCAGAACTTGAGCAAGACCTGGGCTGCTTTGATTTTCTGAAGGT
CTCAAAATATTAGGGGCAAAAAAGAAAATAAAAAAAGAAAAAAAAGAAGGAATACCAAAAGC
AAGTTACATAAAGTATGAGATATATTACAAAAAAAAAAAAAAA

ID|25577|gene_id|79469|transcript_id|NR_002771 Homo sapiens lncRNA

ACCTGATCTCATCAATCTAGCGGAAGAGACAGGATAACCTATCCAAGAGTATAGCGCCAC
TATGACTCCGCCGGAAAAATTACTTTAAAAATCGCCAAAAATTACTTGGAGCAAAGGGCAGTAG
GCGGAGCTTCGCCAAGGCTGGCGCAGTCGGTTTTGACCTGTAGCAGAGAACCAATTCTGGAGA
ACAGCCTCACTTCTTTGATTGAATACTCACATAATGCTTTGGAACAGGACATGAGATTAAGGTTT
AATAATGATAGAATGAAGACCATAATAAAAGAGACCTCTACTTACCTCAGCAATTCTTACCTTTC

TTACCTATTTGATGAAGATGTCTTTTGAAAGGTGTACTGCAAGGAACAAAATGTTTGTAAATTCT
GCTTTTACCAAGGTTTCTTTTTTAGTTGATGCCAAAGAGTTCCAATATTGAACATCTTAAGTCTGT
TACTTGGAGTATGGATTGAGTTTGGAGCTTACTCAGAGGACTACAGGAGAGTATCCAGGAGGTG
GATAATTACTGTACCTTCCTCATGGAAAAAGTTTTATTTAAAGTGTTATTTCTCGTTGAATACTATC
AAAAAGGAAAAAAAAATGACCTAAACTTTTGAGACAGATTTGGCTCTAGTAAGTATTTAGATAT
ATCGCTTGCATATCTGGGAGAAGAAATAAGAGACTATCATCAATACATTCCCATCTACTAAAAA
ATTTTATTTTACACATGTCAAGGGATTACTTATAACTTCCATTGTCTTTTTTTAAGCCTATATGCA
ATGAAAAATATATTGGCAAAATAAATATTTAAACCTTTTATGTTAAAATTACTGTGAAAGATGAC
AAGTTAGCTGCTGTTTTTGTCTACATTATACTGAAATTAAATGTTTATAATTTATATTTTGGGTTTA
TTTATTTATAAATCATGGAATTTATGCAAAAAACATGAGTAGTACAGATTCTCCTCTAATTCTGTA
GGACTTTGAATAATGTGATATTTTTCTTATAGTTGGACCCTTGTGTTTTGAAGAAATGCCAACTGC
TTGAAGAATTTCCTTGTTATTTGTATTATTTGCTATAGGGTTAGATGTTGAGAAATTCTTCTGACAA
AAAATTTTAAGCCAGTTTTACACTAAATATTCCTTAGTCTGATTAATTTGTTATTGGATGTATTCTG
TATCTTTCTTTTGTAATTTGTAACTTTTATCCACTTAGCACGAATGATTCTATTGAAGAAAATCTTT
AGGAAGTGGTAGAAACTTTAAATCGCCCCATAGTTTGCCTGTTTCCACATTTTATTATCTTATAAT
CTTTGGGAGTGCTTACACTTATGGAGCTAACATTTTCAGAGATACAGGTTCTCATAGTACTACTA
AAACTTTTTTCCTCTTTGGACTGAATACCTATGATTATAACTATACAGTAGTTTAAGTTTCCTTGTG
ATTAGTCAAAAATACCATTTTAGTATGAAGCAATGAAGTCTATTATTTGTTGTCCCATAATTGAGA
AACTTAGATACACCTTTTATTAAGAGTTTGTAAATTCTAGCTTAGTCTACACAGATTTTTATATCAA
TTTGTTTATATTTTTATTAATGTCATTTCTGGAATTGTGAAAATGTTAATGTTCGACAAGCAACATT
AAAAATAGATTTGAAACATT

ID|30050|gene_id|BC038538|transcript_id|BC038538 Homo sapiens lncRNA

CAAGGACAGCAGTAGGAGCTGAATGCAGTGACTGTATCATAACTGCTGGTTATGAGGATT
GGATTTCTTTTTTGCAGATGTCACGTGCCGGGGTGAACTGTTATTAACTAAAAGGAGTCAGGGCT
TAGCTGCAAGAAAGGAGGAATTGCTCTTAAAGTGCTTGGAAGTCACCGATCTAACCAACGGAC
AAAAAATTTCCTGAGGACAGACCCTCCCCTCTGGAGGCCTCCTTCTATCATTGGCTCCTGACTCC
CCGAAGCAAGGTCAAGTGGGAGTTTAATAGCTGTTCCTGGGACTGTGCAGAGGGCAGCCCTTT
GAACTCAGTGTCTCAATCCCACAGGTGGGAGGTTCCCACCACTGCGTAATTTCGAGAGGGAGG
GCCGAGACTGCCTGGGGGGAAGTCCCATGGCTACCTGGTTCGAGGCAGCTCTTTGATATCTCCT
GGAGCACTCCTGGTCCTAGAACCAGACAGCTCTCAACTGAACTGACACAAGTAACAAACTCAG
CATCCCCTCTCCTGGGCATTCCTACAAGCCATGCGAGCCCCGTGTTGGTGGAGACACTGCCTTC
TCCACCGCAGACACCAACAAGTTAGAATGGAGAAACTGTCTATGGCCCTACCACTCTGAACAC
GCCGGATGTCATCTCATCTCGGAAGCTAAGCAGGGTCGGGCCTGCTTAGGACTTGGGTGGGAG
AATGGAGAAATTGATGGACTTTTGACGATGCAGGTTTTAACATCAGATCTGAGCCCTAATAGCT
GTGTGTCTCAGCTGTCTGGGCCACACCATTAACAGGGAAAAGTTTCCTCCACTTAAAGAAAGAA
AAAGACAAAGAGGAGAAGGAAGGCAATACGGAGCTCTCCTGGAAGGTACATTAAATCATTAAA
TGCCAAAAAAAAAAAAAAAAAAAAAAAAAA

ID|30141|gene_id|BX647987|transcript_id|BX647987 Homo sapiens lncRNA

GGCTGCTAAGTTGTGCGTTCCAGGGTGCACTCGCCGGCGAGGCTGCACGGCGCCGGTTTC
ATTGCTAAAATCCGACGGACTTGTTGCAGAACTCGTTTTTTTTGTCGGGAAAGGGCTTCCCCGTG
TAACTTTACAGCAGTTATCGTAGATAAGACTTGCAAATTACAGATGTGGACGGTGAGGGAACGG
GAATTTCACTGAAAGCGCCTGAGAACCAGTAAGAGACCTGCAACAGCTTTGTGCTCCACTGGCG
AATGCTTCCTCCCCTAATACACATTATGTGTGCTTGGAGACACAAGGAGTAAGATACAGCCCCT

CAAGGGACTCGTCATAGGGAGAGGACACAGCTTTGGGAATAAAGGGGCCACAAAGGAGGAGA
GAGGAGTTTGCTGAAACTTCCCAAGCTCCCATTCCAGCAGTTCCTGAAGGATTCATTTCTCTCTG
AGGATCTCATGAGGTGGCTTAGCAGCTAGAATCTCTTCCTTAACAGTGGGAAGAACAACTCCTG
GAAATTTAGGTCGGCTTTGACTGGATTTAAAAGAAAGCTGTATAATTCCACTTCTGTATATACAA
CCAAAGGAAATGAGATGTGCATACCCATGTTCAATGAAGCATTATTCACAATAGCCAAAATGTG
GAACCAATCCTTAAACAAAAGGAAAACAGAAAATCTGCTCAAGTCCCTGCAGGCACCTGAGAA
AATCACAACCCACACTTGCGAACCCCTAGGAGACCTAAAGCTGTGGTTTTACAAATAAACAGAT
AAGTAACATTCCTACTCTTTCCTATAGGTACTCAATCTTCTTAACAACTCCCATCCCACACAGGTT
TGCATCCCCAGCAGATACATATGGCTATGTGGTCACCCATTCGATCAGCCTATAAAAGACTTGTC
TTATTTGATATTGTGAAACCCATGGTTATCAGTGTATATGAAAATGTTTGGTTCAGGGGTCAATGT
ACAATATGACAAGCAAAGTCCTCTAATACACAGTTATATAAAATCACTCACCTGAGAGCTAAGT
GGACAATTGGCCTTATATTGGCTGGAGTTGGAGCCCCAGAGGGAAAATTTGCCTATTATTAGGC
AACTCTTCAAAACTTTACTTATTCGTTTCTAAACACTGTCTCATAAAATGGGGGATGCTTTAGATG
AATTAAGATTAGCACTAGATTACTTATTCGTTGAAAATTAGCACTAGATTTCTTATTGGTTGAACA
AGGAGGAGTGTGCACCATAATCAATATAACCTGCTGCAGTTATGTAAACACCTCAGGTCACATA
GAAGAAGATATACAGAAAATACATGAACAAGCTGTGTGGTTGCGTAAATATAACCAAGGCACC
AATCCCAACTGTATCTGGTCAGCCATTAGAAGCACCCTCCTGAGTCTCACTTGGTTGTTGCCCTT
CCTAGGAACTTTGATAGTTGTCTTATTATTAATCTTTGGCCCTTGCTTGTTTAAATTCTTGGTAAAG
TTTGTGTTTTTTAGATTACAACAATTCCACATAAAGATGATACTGACACAAAGCTTCCAATCCATC
CCGTCTTCTGAACCGGGAAATAAAGACACCCTGCCTTTAGGTGTCTTTAGATCTAGATCAGGCAT
CTAGAGATTTTTACTCCTCCAATACTTGGCAGGGTCTATGCCCGTAGACTCATCAGGATACCATG
TAAATGAGGACCTCCCTTGGTACCCCAGAGGCCAATTGTAGACCCTTATGAGAAAATCCTGTCC
CCAAAATGAACATCCACTGTCAGCAGGAAGAAGTTACAGAAGACCAGCCTTTGCCCTTACGATT
AAGGGTCCTCTTGTAAGTAGAGAGGGGAATGTTAGGAAAGCAGGAGCCTAGGTGATCCAGAAT
GACACCATTTTAAAATAAACTCCACCTGAAAACTAGCAAGGCATCTGCACACATCTGTTTAGCA
AGTATATCCTTCTGTAACTGGAATAAAAAGCGTTATTTTAGAATATGCCTATAGTTTGTAATAATT
AGCCGAGGGAAAAAAAGTTCACATTGGTGTAGGAAAAGTGCCCTAATGGTCATGGGTAATAAT
GGGACCGTGGTTCTGGTCAGTGATTTGATGGAAGGGGTGTAGATAATATATTGTTGTAAATAATA
AAAATAAAAAATTGTTGTAAGTAATAAATAAAATAATATTGTTGTAAGTAATAAAGTAGGAGAAA
TATTTGGTCTTTTGCTTTTGGGTTAGTGCAACAGTAGTACCATTACTTGGAGGAGAAGTTAGAGA
AGGACAAGAATAGGCTTGAATAAGATGAGTGAGAAGAGAGAAAAATTCCGTAAGTGGAGATG
GCCAGGGAACACCAGGGCAGCAGAATCTAACTCTGAATTTAATGCTGTTGTCTGTTCATATTGC
ACTGCGCTGTAATCTCCCTATCTTTCCTAAACCATTGGCAAAGTCTGCTATCCACAGGGGCCTTG
GGTCGGTGGTGCTTTGGGAAATACCAGAAGAGGGAAAGTACGGCAAATGTGCAGTGTCACAGA
TGATAGGAGGAAAGCTGGCCAGTGGGATGATTAAAACAATTTCGGTGGGAGTGGGAATTCAGA
CCATGTAAATGGTCTGAATTAAGAAAATTTCAAGAAACAAAAAATGTTTCTAATGAGCCAAAGT
GAAAGCTCTTCATGATTTCTTTATAGAGGCTCTCTAGTGCTTGTCCTAATTTCCTGAAATCTCATG
GGTATCTTACTCTCCTGGTGCCCGCCTCAAACATTCACTGTAGCAATTATATGAGCAGCCTGGAG
AATCCCAATCCCAGTGGCCTTGCTCCCTGGATCTACTTTCTCTTAAAAATCATTCCCCTAAGTTTC
CGTCACTTGGTTGAAGTTTAGTAGATATCTCCACATCTCCAGCTAGAACAGCACCGGAATTTCAG
GACTAGGGCAATAGAGTCTGATATTAAATTCTATTTCTATCTATCTATCTATCTATCTATCATCTAT
CTATCTATCTATCTATCTATCTATCTATCTATCCACAGAACAATTTGTGGGATAGTAGCTTTGGGG
TCCCAGATCTAGGATGGGCCTGGTCCTCCATGCTGGTTCTCCTGGGTGATATTTCTGTTAGTTTG
AACAGTAATCACACTTTTATTACTGTTTCATCTTTTTTTTTTTCACTCTTAGACTGATTTTATCATGT

GTATAAAACATACACACACCTACATCTCAAGGATAAACATTAATCCTTGAAAAATATGTGGTCTA
AAACTATATTGAGGAGAGAAAGATAGGACACTTTAATTGCCACATGATTAACACGAGGTCACCA
CACAGCTTTCACAAATCTACAAGTCATTCTATTTAGGCTGAGTCAAGTTCTCCCTTGGGCCAGCC
AGAGCACAAATAATGGAGCAATGTGACTCTTCAATGACAGATCTCTGATTTCCAGAAAAGAGAC
TTTGAAATACCCAATTCTAGTAAATATGCAATTAAATTTCCAGCTAAATGACCTTTGCTCCTGTAG
TCTTCATCTTGGGGAAGGGAAAGAAGTCTTATTCTGTTTAATAGTTTGATTTTTATAATAGGGTTT
GTCTCTGTTATAAATCTTGTTTGATATTTAAAATGTTAGCTAAGAGAAAACTTCTGTTTGTTTTTAT
TTAGTGGGAAGAAACTAACCCTCATTGAGTGCCTAGTTAACATCAGGCACTTTGTACACATAATC
AATTGAATCTTGACCACAACCCTGTGAGGTGAAAAATGAGAAGATTAAATAAGCCATGGGAAG
ATTTTTTAAAATGACATGAAACTCCAAGGGTAAAAAGTAGGCTGGGAAATCTTAGGATGGTGAA
CCATCATGTTTCCTGAATGTGAATCCTGTACCTGGTTTGACTGTAGTCACCCCTTCTCCAACACTC
TCCACGATGCCGGCTGCCTCATGGCCTAAAATCACAGGAAGTGGGGTCACCATGGTACCACTA
ACCACGTGGTCATCTGTGCCACAGATTCCTACAGCCACCATCTACAGAGTGAAGAGAAGATGTT
TATAAAAGATTGTGAGTCTCAGGATGTTCAACATGATCAATGCCAAATAAATATCAGAATACTTG
GAAGTAAAGTACTAATAGTAGGAGTTCCTGATTATCAGGTGCCTATGATGTGACAGGCACATTG
TGGCCTGTGCTCGACCAGGAGACTGCTTTTCCAGGCTGAGATCAAAGACGGTGATGTGCCTACC
CGCAGATCCTAGATGGCCATGTTTGCAACAGAGGTAATGATGAGCAGTGCGAAGATGTGACTC
CAAAGCCTGGAGAAGGACAGACCAGCATCCCTGCAAGAGATCCAACTGTCTTCTTTTCCGCAG
AGGTCCATAATTTACATGGGCCTGACAAAGAATACTGTAACTTGCAGAAATTATCTTCAGTTCAA
ATCCTTCATTTTGGGGGTTAAATAACTTGCCCAAGATGTCGAGGGAGCGGAAATACTTGCTGGT
CTTGCAGCGGCTGTGGAGCCTGGAATCCTGCCAAGAAGAGCATGTGTGAGAGATCAAAATGTT
TGCTTACATTCCTCCTGTGAAGGGAGTTGGGAAATAACCTAAGAAGCCCAATCCAAAAACAAAG
ATGTACCATCCAGTCTTCTCTGTGAGAACCAAACTACAAACCATCTGAATGTTCCAAAAGTGTTT
TGATGATGTATTGATGTATTTTAAATTATCCTGAAAATGTTCTTGGCAGATGCTTAAACATGTAAT
TACTGAGATGATGTAGAAATACCAAGTGTTTATTAAAACAATACAGAGTAATACAAATGGTATGT
TTGTCGCAATATAAAAGTATCTAAAAATTAAAGAGTTCTTTAATGGTGAAAAAAAAAAAAAAAA
AAA
ID|30602|gene_id|X15624|transcript_id|X15624 Homo sapiens lncRNA
ATGGGCGGAGGGAAGCTCATCAGTGGGGCCACGAGCTGAGTGCGTCCTGTCACTCCACTC
CCATGTCCCTTGGGAAGGTCTGAGACTAGGGCCAGAGGCGGCCCTAACAGGGCTCTCCCTGAG
CTTCAGGGAGGTGAGTTCCCAGAGAACGGGGCTCCGCGCGAGGTCAGACTGGGCAGGAGATG
CCGTGGACCCCGCCCTTCGGGGAGGGGCCCGGCGGATGCCTCCTTTGCCGGAGCTTGGAACAG
ACTCACGGCCAGCGAAGTGAGTTCAATGGCTGAGGTGAGGTACCCCGCAGGGGACCTCATAAC
CCAATTCAGACCACTCTCCTCCGCCCATT

Cytosol

ID|198|gene_id|100130581|transcript_id|NR_027412 Homo sapiens lncRNA
GGAGGAGGGTAAAGGAATCCACGTCCCAAGCAGAGAAGCAGCTTTCCCTACACAGCACA
GGACACGGTCCGCGCACAGAAGCCGCAGGAGACGCAGGCACAGGGGCTGGGGAGAATCCTT
GCTGGGCCCTCGCCGTCCATGCGCCGCCTCCCTCTGCCCGGTGTCTGGTGTCAGCCTCCTGCCT
GGCAGAGGAACTCCAGCCCCTGCTCCCCGAAGCTCCTCCAGGCCTTCTGCTTCCCTGACTCGGA
ATGGGCCCGCTGTCCAGTGGGTACAAGGCTGGTCTCCCCGCCCACTGGTGACGACGTAAAGAT
CCAGCTCAGCCTGGTTTCCCCTGGTGATGATATCTTGCGACTCGTCTTTCTCCAGGAAAACAGG
GACCGCTCTCCGCCTACAGGTCTCTTCCACAGACCTATCTTGTTTTCACCCCCTGTCTCTTTTGGT

TCAGTCTTTTCGCTCATACAAACAAGGAAGTCGCCCCTAGCCCAGCCGCGGCTCCTTAGCTGGA
GGCGGGCCCGGGGGTGGAGTCAACCGCGGTGGCCACGCCTCCTGGGAAAGGGCAGGGCATG
CAAATTCGAAATGAAAGCCCGGAAACCCCGGAACTAGAACTGGTATCTCTTCAACTACCTGTGA
AAACTGATGTGATGAAAAGGGGAATTTGAAGGAGCCATTCCAGAAGACAGGGCGAAAACTGAA
GTGCAATCAGGGCCAAGAAAAACAGAAATAGCAGGACCTGGAGTTGCATAGGTTGAATAGTTG
AATAGGCTGCTCTCCTCAGCTGGCAGGAAACCTGGCAGCCTCCTGGTGCCCAAGGACTGAAAA
CCTTAGAAGCACCTGGAGTTGGCAGCCTTGGCATGGTCAGGTTGGCACCTCTGGAGGTGCCCA
GGCTTCCCTGGCAGCATTGTGAGCAGTGGATGGTGTTGAAGGGCAGCCAGAGGAGGAATGGAA
CACATGCTCCTTGCTAACCACACGGACAAGGCCACGTTCACAGGTACACAAAGGCAACGCAGT
TGCTCAGGTGCTTCGGTATCACAGCCAAGACCCCTTCGGGGGAAGCTAGTCGGATACTGGGAC
CCACATTCCAGACTACTGAGCCGCGGTCGCGCCCTCGGCTCCGTTTCTGCTCCCTCCACCCCAC
GAGGACGGGGGTGGAAGGCCACCTTCGATGGGTGCATCCTCCACGATGACCTGCTAACAAAGG
TGCATGGATTTCAGAGTCTGATTGGCCTACAACAGCATTTGGCTTGTGGAGACAGTGGTTCCCTG
ATGAAAAACTGCCATGATGGGAATAGAAACAATCAGAGGAAACTTCCGTGAGTTCCCAGGACA
CATCCACCCACCTCCTCCACGTGTAACCACCACCTCTACCTTCCCCTCTGGTGCTGTGGATGAGC
CATCCGTGCTCCTGGCAAAGGCCCACCTGCCACTTGGGCACAGGAACCCATCCATCCCTCCTTA
CCTCTGGAAAACTCCTCCAAACACTTGTCTATATTTGTCCCTGTCATTCTCTCTCTCTCCCCCCTCT
CTCTATTTTTTATTTTATTTTATTTTATTTATTTATTTATTTGAGACGGAGTCTCACTCTGTCACCCA
GGCTGGAGTGCAGTGGCATAATCTTGGCTCACTGGAACCCCTACCTCTTGTGTTAAAGCAATTCT
CCTGCCTCAACCACCTGAGTAGCTGGGATTACAGGCGCCCACCACCTCGCTGGGCTAATTTTTG
AATTTGTAATTGAGATGGGGTTTCACCATGGTAGCCAGGCTGGTCTCAAACTCCTGACCTCAAG
CGATCTGCCCACCTCCGTCTCTCAAAGTGCTGGGATTAGAGGTGTGAGCCACCACGCCCGGCCT
ATTTATTTTTTTGAGACAGAGTCTCCCTCTGTTGTCCAAGCTAGAGTGCAGTGATACCATCTCGG
CTCACTGCCACCTCTGCCTCCCAGGTTCAAGTGACTCTCCTGCCTCAGCCTCCCTAGTAGCTGGG
ATTACAGCCATGCACCACCACACCTGGCTAATTTTTGTATTTTTAGTAGAGATGGGGTTTCACCA
CGTTGGCCAGGGTGGTCTTGAGCTCCTGACCTCAGGTGATCTGCCCACCTCAGCCTCTCAAAGT
GCTGGGATTACGGGCATGAGCTGCTGTGCCCAGCCTCAATTATTTTAATCTTTGAATAACTTCGG
ACTTGCAGAAAAGTTAAAAGACTTACATGGAGAACTTCTCTACCCTCTTCCCCATCCCCGCAAG
GTACACAGTTGGTAAAGCGAGAAGTCTGGGGTTCAGTGACACACTTCTTAACTCCCAAGTTCGT
GCTCTTTCTTTTCTCTCTCTCTCTCTCTCTGTTGTCTCTCCCTCCCTCCTTCACTCCCTCTCTCTCCC
CTTGATGGCCACATTTACTTTATAATTTTCTCTCTCACTCTTTCTCTGTCTCACTCTCTCTTACACAA
CACACACACTCATAAGAAGACACCTATATACATTTTTTTCCTGAACCATTGGTAAGTAATTTGCA
CACAGGATGTCCCTTCACCCCCCAGTCCACCAATACTTCGGTGTGTTTCCTAAGAACAAAGGCC
TTCTGGAAGTTTCACATTAATTCCATACTGGATCTACAGTCCGAGTTCAGATTTCACCAATTGTCC
CAATAAAGTCCTTTAGGTTTTTCTGGAAAAAAAAAAAAAAAAAAAAAAAAAAAAAAAAAA
ID|199|gene_id|100130581|transcript_id|NR_027413 Homo sapiens lncRNA
GGAGGAGGGTAAAGGAATCCACGTCCCAAGCAGAGAAGCAGCTTTCCCTACACAGCACA
GGACACGGTCCGCGCACAGAAGCCGCAGGAGACGCAGGCACAGGGGCTGGGGAGAATCCTT
GCTGGGCCCTCGCCGTCCATGCGCCGCCTCCCTCTGCCCGGTGTCTGGTGTCAGCCTCCTGCCT
GGCAGAGGAACTCCAGCCCCTGCTCCCCGAAGCTCCTCCAGGCCTTCTGCTTCCCTGACTCGGA
ATGGGCCCGCTGTCCAGTGGGTACAAGGCTGGTCTCCCCGCCCACTGGTGACGACGTAAAGAT
CCAGCTCAGCCTGGTTTCCCCTGGTGATGATATCTTGCGACTCGTCTTTCTCCAGGAAAACAGG
GACCGCTCTCCGCCTACAGGTCTCTTCCACAGACCTATCTTGTTTTCACCCCCTGTCTCTTTTGGT
TCAGTCTTTTCGCTCATACAAACAAGGAAGTCGCCCCTAGCCCAGCCGCGGCTCCTTAGCTGGA

GGCGGGCCCGGGGGTGGAGTCAACCGCGGTGGCCACGCCTCCTGGGAAAGGGCAGGGCATG
CAAATTCGAAATGAAAGCCCGGAAACCCCGGAACTAGAACTGGTATCTCTTCAACTACCTGTGA
AAACTGATGTGATGAAAAGGGGAATTTGAAGGAGCCATTCCAGAAGACAGGGCGAAAACTGAA
GTGCAATCAGGGCCAAGAAAAACAGAAATAGCAGGACCTGGAGTTGCATAGGTTGAATAGTTG
AATAGGCTGCTCTCCTCAGCTGGCAGGAAACCTGGCAGCCTCCTGGTGCCCAAGGACTGAAAA
CCTTAGAAGCACCTGGAGTTGGCAGCCTTGGCATGGTCAGGTTGGCACCTCTGGAGGTGCCCA
GGCTTCCCTGGCAGCATTGTGAGCAGTGGATGGTGTTGAAGGGCAGCCAGAGGAGGAATGGAA
CACATGCTCCTTGCTAACCACACGGACAAGGCCACGTTCACAGGTACACAAAGGCAACGCAGT
TGCTCAGGTGCTTCGGTATCACAGCCAAGACCCCTTCGGGGGAAGCTAGTCGGATACTGGGAC
CCACATTCCAGACTACTGAGCCGCGGTCGCGCCCTCGGCTCCGTTTCTGCTCCCTCCACCCCAC
GAGGACGGGGGTGGAAGGCCACCTTCGATGGGTGCATCCTCCACGATGACCTGCTAACAAAGG
TGCATGGATTTCAGAGTCTGATTGGCCTACAACAGCATTTGGCTTGTGGAGACAGTGGTTCCCTG
ATGAAAAACTGCCATGATGTAAGGAAGAGCCTGTCAGAGCGAGGCTGGGGTGCTGCGTGTTGG
GGAGGTGGAGGTGTGGCTTCCCGGGAGAAGCTCCACCCGCTGGCTGAGTCTGGCACATAAACC
AGTCTGTGAGGGGATGGATGTGGGTGTAATGGGGGCAATTACAGTAGGAAGGAGCCCACGTG
GAGCCTGCATTCTCTGGGACAGGGCATTACTGCATTCTCTGGGACAGGCTAAGGCCCAGATCCT
ACCTTCCCAGGTGGCTGGATGGGTCATAGATGTATGAACCGGTCCCCTCATTTTCTGATTGCCCT
GTGCTTAACGTTTCTGTACCTTTACTGAGGCTCTTTCCTCCAACTCCAGTGCCCAGACCCCCCTTC
TCCTGAACATGAATGCCTGTCCATGGAAATTCGAGTCTCTCTCTCTCACCCAGGCTGGAGTGCA
GTGATGCAATCTCAACTCACTGCAACCTCTGCCTCCCAGGTTCAAGTGATTCTTGTGCCTCAGCC
TCTGGAGTATCTAGGATCACAGGTGCGTGCCACCATGTCTGGCTAATGTTTTGTATTTATAGTAG
AGATGGGTTTCGACATATTGGCCAGGCTGGTCTTGATCTCCTGGCCTCAAAGTGATCTACCCAC
CTGGGCCTCCCAAATTGCTGGGATTACAGTTGTGAGCCACCACACCCAGCCTGTCCCTGAAATT
CTAATGAAATGTGCGATAAAGTTGTTTTGTTTTTCTTTTTGTTTTCCCTTCTTGGCAAAGCCTGGTG
TTTCTATTTTAGTGGATTTGCCTGGCACTGAGGACTGCTATGGTGGTCTTCAGAGGCTCCTGGTA
TTGACTGCTTGTGAAACCGCTTTTGCAAAATTATGACTGAGACAGTGAAAGAGATCTAACTTAAC
CGACCCAATCTTGCTTCTAACCTCCAAATTGTCCTTATTCATTCCTGAGCATAGCCTGAACTAACT
TTGGGAGAAGCTTAGTTTATATTTTATTTTATAGTTTAAAACAAAGATGTTAACAGCCCTTTCCCA
AGGCAGACTTCCTTCTTGCCTGGGGACTAGGTTGCCTTTGGAGGACTAACATTAGCCACGAGAT
TAGAAATTATGGGCTGGGCCTCGTGGCTCACCCCTGTAATCCCAGCACTTTGGGAGGCCACGGC
AGGTAGATCACCTGAGGTCAGGAGTTCAAGACCAGCCTGGCCAACGTGGTGAAACCCCATCTC
TACTAAAGAATACAAAAATTAGCCGGTTATGGTGGCACATGCCTATACTGCCAGCTACTTGGGA
AGCTGAGGTGGGAGGATCGCTTGAACCTGGGAGGCGGCGTGGAGGTTGCAGTGAGCCAGGAT
CTTGCCACTGCACTCCAGCTTGGGCGACAGAGTGAGACTCTGTCTCAAAAAAAAAAAAGTTTAGA
AATTATGCTTTAGGAGTCATGCAGCTGGAGGCTACAAGATTCTGACCCTCCCTAAACTGCTCCTA
AGATCAGTGCTTGAGATATTTTGCAGACCCTGCACTTGATGGATCAGCTGGCACCACCCAGACT
GATTAACTGGCTCATGTGATCTTGTGGTCCCCACCCAGGAACTTAATCAGCACAAGGAGACAGC
TTCAACTCCCTATGATTTCATCCCTGACCAATCAGCACTCCTGGGCTCACTGGCTTCCCCCTACC
CACCAAGTTGTCCTTAAAAAGTCTGCTCCCCAAATGCTCGGGTAGACTGATTTGGGTAATAATAA
AACTCCGGTCTCCCACACAGCCA
ID|349|gene_id|100505641|transcript_id|NR_046251 Homo sapiens lncRNA
AATTAGCCAGCCCATCCGTCGCTCACGAGGTCGCGCCTCGCACCCGCCTTCCTCCTTTTCT
TTTACCCTCCCCTTCAGAAAAAACGGCGGTTGGGCTGCGACGGCCGCCAAAGGCGGACTAGAA
GCGGAGGGGTGAAAATCCCGGCAGAGAAGGAAGAAGGGACTGCAGGCGGGAGGAGGAGGA

GGATAAGGAGGAAGGGAGCCCGCCCAGCCGGAGCCATCTCCGTCGAGAACAAAATGGCGGCG
CTGGCGGAGGGCCAACTATAAGGCGGGGCCGCGGCCATTCCCCCTCCACCCCCACCCTTGCGC
CGGCCGGGCCGGTCAGGGGGAACCCGCTCGCTTCGCCCGGCCGCGGGCGGGGAGGGGAGGG
GAGCGGCCCGGCCCACTATGCAAAGCGCCGGGCGCCGCCGCCGCCACCCCGTGGCAAAGAAT
ATGCTTTTTGTCTAATGATGTTAGATAAAAAGCAAATTTGGAGCAGTTTTCTTATTCGAGTTCAAA
ATGGTTCATAAAGCAGCGAAGACAACTCAAAACATCAGTAACACATTTGGCCCAGGAACTGCTA
ACAAACATACAGTGCAGTGGTGGCTTAAGAAGTTTTGCAAGGAAGAGAGCCTTGAAGATGAGG
AACGTGATATGGGCCATGGGAAGTTGACAACGACCAATTGAGAGCAATCATTGAACTACATGA
AAAATTACAGAAGAACTCAGCGTTGACTATTCTACGGTCATTAGGGTCGTTCAGCATTCGAAGC
AAATTGGAAAGGTGAAAAAGCTTGATAAGTGGGTGCCTCATGAGCTGAGCGGAAATCAAAACT
ATCGTTTTGAAGTGTAGTCTTCTTTTATGCTACGCAACAACAAACCATTTCTCAATCGGATTGTGA
CATGTAATGAAAAGTGAATTTTATACAACAACCAGCTCAGTGGTTGGACCAAGAGGCAGCTCCA
AAGCAGTTCCCAAAGCCAAACTTGCACCAAAAAAAAAGGTCATGGTCACTGTTCGGTGGTCTGCT
GCCCATCTGATCCACTACAGTTTTCTGAATCCTGGTGAAACCATTACATCTGAGAAGTATGCTCA
GCAAATTGGTAAGATGCACCGAAAACTGCAATACCTGCTGCCTGCATTGGTCAACAGAAAGGG
CCCAATTCTTCTCCACGACAACACCTGACTGCGTGTCGCACAACCAGTGCTTCAAAAGTTGAAG
GAATTGGGCTACAAAGTTTTGCCTCATCCACCATATTCACCTGACCTTTCGCCAACTGACTACCA
CTTCTGCTAGCATCTTGACAACTTTTTGCAGGGAAAACACTTCAGCATGGTGCAGAAAATGCTTT
CCAAGAGTTCGTCAAATCCTGAAGCACGGATTTTTATGCTACAGGAATAAACAAACATTTCTCAT
TGGCAAAAATGTGTTGATTGTAATGGTTCCTGTTTTGATTAATAGAATATGTTTTAGCGTAGTTAC
AATGATTTAAAATTCGCAGTCTGGAAATTTAATTTTTGCATCAACCTAATATTTCTATGGTAAATC
CTTGCAAACATGGAAACAATGCATTTGGCCCAGTGCTTTGTGGTTGTGTACTCTTTTTCTTTGTTT
TTTTAATAGATGGCATTGGCCGGGCATGGTGGCTCATGCCTGTAATCCCAGCATTTTGGGAAGG
TGAGGTGGGTGGATCACCTGAGGTCAGGAGTTCAAGACCAGCCTGACTAACATGGTAAAACCC
CATCTCTACTAAAAATACAAAAAAATTAGCTAGGCGGGGTGGCGGGCATCTAATTCCAGCTACT
TCATGAGGCTGCGACAGGAGAATCATTTGAACTCGGGAGGCAGAGGTTGCAGTGAGCCGAGAT
CACACCATTGCACTCCAGCCTGGGCGATGAGCGAAACTGTCTCAAAAAAAAAAAAAAGTTGGGG
TCTCGCTGTGTTGACCAGGCTGGTCTCGAACTCCTGGCCTCAAGTGATCCTCCCATCTTGGCCTC
CCAAAGTGTTAGGATTACAGGCGTGAGCCACCACACCCTGCCTGGTTGTGTACTCTTTTAAATAC
TAAGTTTTTAATGTTAAATGCTGCTTTTAGATACACTGTAAAAATACACCTATCAATGAGTTTTTTT
ATTAAAAACATTGCAATTGTACTAGACTTTAAATACTAAGCAATAATTCAGGCTTCAATGTTGGTT
TATAGTTTTCTCATTTCTTTCATTTAATACCTCTGTAAAATGAAGCAGTTACTTCCATTTTCCTGAG
GTGAGATAAGTGCCCTGCACAATGTTATAGGCCCAGTAAGTGAGACTGGAGCTCTGATCTAATC
CCTTTGACATTAAAGTTTGTGCAGTAAACCAGCTATGTCAGTTTGCCACATTTGTAATAGTGTTCA
ATAAATTTTCACTTAAAGGTTTAATATTAAAGTAGTAGCAAGAATTCATAACCTTAATTGGCTTTA
ACAGCTTTTCTTTCCCTGAAAACAGATGCATATGTACACATGCACACAGATTGCTTACAATATCA
GCACGTAAAAGGCCTTTGCACATTGAAGTCGGCACTGCTTTGGTGCCTTTTTTGTTTTTTGGCTCG
GTGTTTTGACTGCAAGTCTTTTTGGATAGAATTTTATAGTTAGAAAGTAGCTAACACTTGGGTTTT
ATAGGCACAAAAAACAAGTCTTATACTAGCTGTACTTTATTTTTTGAGTTCTTATTAATGAGGAAC
ATCCACTTTTGCATTGACAGTGATTTCAAGATTGCTTTATCAGCCTTTAAAGGATTCTTGACTAGT
CGTGCACATCAGAACTGCCAGGTCCCCAGTGGTTCTGAAGCAGTAAGCTTTGGGTGGGCTCTGG
CATCAGCACTTTCACTAAGCTTCACAGATAATTCTGATGCATACTCCAGGCCTGAACCACTGATC
AATTTGAAACATGCATAACAAAGCAAAAAAAGTTTTGTTTCACCTTTTGAAATACAGTTAACTCTT
TTACCATGCCAGAGATCATTCAGAGAGACAGGTCGTTGCTCCGGAGTGATACAGATCTGGCAGT

ACCCAGCCCTTGTGTGTGTGCGTTAGCTCAGCACCTGCCCACACTGCGAGCCCCCGTAGGATGT
GCCTTGTCCTTCCCTGTTTCAGCACTTAACACACTACCTGGTACAGAGTATGTAGTGGGCATCTG
TTGAATGAATGCTTTTCCCAGTAGCAGTGTATTCATACAATATTAATATAATTGTCCCCTGGCTTA
CAGATAAAAATGAAAGCATCAAGTGCCCAGTGAGTGAGACCCAGGTGTTCTTCCTCCACCCCTA
GTGGTCCCCTGGGCAGGTCTTTTTTTTTTTGTAACACTCACCAGTCTGTTCTGTAGTCAATCATTG
ATTGACTTGTCTGTGAACTTGGAGGAACTGTTTCATAGTTTCATTAGCACAGAGTAAACATGTTT
GCCATGCAAGGTTATTTTGCATCTGCATTTAAGTGATAATGTTGAATCAATGAAAAGTGTTGATT
AAGCAGTAGTTGTAGATATGCTAAGTTTTTCAAATTACTAATATCAAGTGGAGATTGTTTTTACTT
TTAAGGGTATTGCTTTTGTGATAGCATAAATAATGGTTTTCCTTTTTTGTAATGTAAATTAATTGCT
GGCAACTTTTGTATTCCCATAGACTGGGGAAGCTTAATTGCCTTTACAAGTACTTATGTACAACTT
TGTATCAAATTTTCTGTAATAGTTTATGCTTTAGTACTATATATGTACTAATAATTTTATCTGACTTC
TGTTTATATCATTTGTACAATTACATGGTTGTAAAATAAACTTTTAAACCTCACGTAA
ID|350|gene_id|100505641|transcript_id|NR_046252 Homo sapiens lncRNA
GCACCGGAACGCCGCAGCCGGCGCTCGGTCCATCCGGCCCGTGGGCTGTGGGGCCCCGG
GCGGCGGCGGCTACGTAGCTGCCGCCGGCCTTGGCCGCGGCCGGGCGGGCGGATATGTGGCT
TAAATGGCGCAGGCGTCGGGGCCTAGAGGCTGAAGGGCTGGTCGTCCTAAAAACTCGTGGACG
TCGCGCACGGTTACCCGGACGCAGGCCCGCCCCTCTGGGGAACCGCGAGCTCCTCGCAGCTG
GGTGGGGACCGTCCTCGCAGTGGCGGCCTCCACCCTGCCTTCTCGCTTAGCCAAGGCTTCTGGA
AGGCTCGCAAAGTGTCTGTTGTTGGAAGAAGCCGGGCCCTGACTGGCGCGACGGATTCTCCCA
GCCGCTCAGCAGAGCGTCTGTAGGTCGGAAGTGGGGGTTGTATTGGAATATGCTTTTTGTCTAA
TGATGTTAGATAAAAAGCAAATTTGGAGCAGTTTTCTTATTCGAGTTCAAAATGGTTCATAAAGC
AGCGAAGACAACTCAAAACATCAGTAACACATTTGGCCCAGGAACTGCTAACAAACATACAGT
GCAGTGGTGGCTTAAGAAGTTTTGCAAGGAAGAGAGCCTTGAAGATGAGGAACGTGATATGGG
CCATGGGAAGTTGACAACGACCAATTGAGAGCAATCATTGAACTACATGAAAAATTACAGAAG
AACTCAGCGTTGACTATTCTACGGTCATTAGGGTCGTTCAGCATTCGAAGCAAATTGGAAAGGT
GAAAAAGCTTGATAAGTGGGTGCCTCATGAGCTGAGCGGAAATCAAAACTATCGTTTTGAAGTG
TAGTCTTCTTTTATGCTACGCAACAACAAACCATTTCTCAATCGGATTGTGACATGTAATGAAAA
GTGAATTTTATACAACAACCAGCTCAGTGGTTGGACCAAGAGGCAGCTCCAAAGCAGTTCCCAA
AGCCAAACTTGCACCAAAAAAAAAGGTCATGGTCACTGTTCGGTGGTCTGCTGCCCATCTGATCC
ACTACAGTTTTCTGAATCCTGGTGAAACCATTACATCTGAGAAGTATGCTCAGCAAATTGGTAAG
ATGCACCGAAAACTGCAATACCTGCTGCCTGCATTGGTCAACAGAAAGGGCCCAATTCTTCTCC
ACGACAACACCTGACTGCGTGTCGCACAACCAGTGCTTCAAAAGTTGAAGGAATTGGGCTACA
AAGTTTTGCCTCATCCACCATATTCACCTGACCTTTCGCCAACTGACTACCACTTCTGCTAGCATC
TTGACAACTTTTTGCAGGGAAAACACTTCAGCATGGTGCAGAAAATGCTTTCCAAGAGTTCGTC
AAATCCTGAAGCACGGATTTTTATGCTACAGGAATAAACAAACATTTCTCATTGGCAAAAATGTG
TTGATTGTAATGGTTCCTGTTTTGATTAATAGAATATGTTTTAGCGTAGTTACAATGATTTAAAATT
CGCAGTCTGGAAATTTAATTTTTGCATCAACCTAATATTTCTATGGTAAATCCTTGCAAACATGGA
AACAATGCATTTGGCCCAGTGCTTTGTGGTTGTGTACTCTTTTTCTTTGTTTTTTTAATAGATGGCA
TTGGCCGGGCATGGTGGCTCATGCCTGTAATCCCAGCATTTTGGGAAGGTGAGGTGGGTGGATC
ACCTGAGGTCAGGAGTTCAAGACCAGCCTGACTAACATGGTAAAACCCCATCTCTACTAAAAAT
ACAAAAAAATTAGCTAGGCGGGGTGGCGGGCATCTAATTCCAGCTACTTCATGAGGCTGCGAC
AGGAGAATCATTTGAACTCGGGAGGCAGAGGTTGCAGTGAGCCGAGATCACACCATTGCACTC
CAGCCTGGGCGATGAGCGAAACTGTCTCAAAAAAAAAAAAAAGTTGGGGTCTCGCTGTGTTGAC
CAGGCTGGTCTCGAACTCCTGGCCTCAAGTGATCCTCCCATCTTGGCCTCCCAAAGTGTTAGGA

TTACAGGCGTGAGCCACCACACCCTGCCTGGTTGTGTACTCTTTTAAATACTAAGTTTTTAATGTT
AAATGCTGCTTTTAGATACACTGTAAAAATACACCTATCAATGAGTTTTTTTATTAAAAACATTGC
AATTGTACTAGACTTTAAATACTAAGCAATAATTCAGGCTTCAATGTTGGTTTATAGTTTTCTCATT
TCTTTCATTTAATACCTCTGTAAAATGAAGCAGTTACTTCCATTTTCCTGAGGTGAGATAAGTGCC
CTGCACAATGTTATAGGCCCAGTAAGTGAGACTGGAGCTCTGATCTAATCCCTTTGACATTAAA
GTTTGTGCAGTAAACCAGCTATGTCAGTTTGCCACATTTGTAATAGTGTTCAATAAATTTTCACTT
AAAGGTTTAATATTAAAGTAGTAGCAAGAATTCATAACCTTAATTGGCTTTAACAGCTTTTCTTTC
CCTGAAAACAGATGCATATGTACACATGCACACAGATTGCTTACAATATCAGCACGTAAAAGGC
CTTTGCACATTGAAGTCGGCACTGCTTTGGTGCCTTTTTTGTTTTTTGGCTCGGTGTTTTGACTGCA
AGTCTTTTTGGATAGAATTTTATAGTTAGAAAGTAGCTAACACTTGGGTTTTATAGGCACAAAAA
ACAAGTCTTATACTAGCTGTACTTTATTTTTTGAGTTCTTATTAATGAGGAACATCCACTTTTGCAT
TGACAGTGATTTCAAGATTGCTTTATCAGCCTTTAAAGGATTCTTGACTAGTCGTGCACATCAGA
ACTGCCAGGTCCCCAGTGGTTCTGAAGCAGTAAGCTTTGGGTGGGCTCTGGCATCAGCACTTTC
ACTAAGCTTCACAGATAATTCTGATGCATACTCCAGGCCTGAACCACTGATCAATTTGAAACATG
CATAACAAAGCAAAAAAAGTTTTGTTTCACCTTTTGAAATACAGTTAACTCTTTTACCATGCCAG
AGATCATTCAGAGAGACAGGTCGTTGCTCCGGAGTGATACAGATCTGGCAGTACCCAGCCCTTG
TGTGTGTGCGTTAGCTCAGCACCTGCCCACACTGCGAGCCCCCGTAGGATGTGCCTTGTCCTTC
CCTGTTTCAGCACTTAACACACTACCTGGTACAGAGTATGTAGTGGGCATCTGTTGAATGAATGC
TTTTCCCAGTAGCAGTGTATTCATACAATATTAATATAATTGTCCCCTGGCTTACAGATAAAAATG
AAAGCATCAAGTGCCCAGTGAGTGAGACCCAGGTGTTCTTCCTCCACCCCTAGTGGTCCCCTGG
GCAGGTCTTTTTTTTTTTGTAACACTCACCAGTCTGTTCTGTAGTCAATCATTGATTGACTTGTCTG
TGAACTTGGAGGAACTGTTTCATAGTTTCATTAGCACAGAGTAAACATGTTTGCCATGCAAGGTT
ATTTTGCATCTGCATTTAAGTGATAATGTTGAATCAATGAAAAGTGTTGATTAAGCAGTAGTTGTA
GATATGCTAAGTTTTTCAAATTACTAATATCAAGTGGAGATTGTTTTTACTTTTAAGGGTATTGCTT
TTGTGATAGCATAAATAATGGTTTTCCTTTTTTGTAATGTAAATTAATTGCTGGCAACTTTTGTATT
CCCATAGACTGGGGAAGCTTAATTGCCTTTACAAGTACTTATGTACAACTTTGTATCAAATTTTCT
GTAATAGTTTATGCTTTAGTACTATATATGTACTAATAATTTTATCTGACTTCTGTTTATATCATTTG
TACAATTACATGGTTGTAAAATAAACTTTTAAACCTCACGTAA
ID|351|gene_id|100505641|transcript_id|NR_046253 Homo sapiens lncRNA
GCACCGGAACGCCGCAGCCGGCGCTCGGTCCATCCGGCCCGTGGGCTGTGGGGCCCCGG
GCGGCGGCGGCTACGTAGCTGCCGCCGGCCTTGGCCGCGGCCGGGCGGGCGGATATGTGGCT
TAAATGGCGCAGGCGTCGGGGCCTAGAGGCTGAAGGGCTGGTCGTCCTAAAAACTCGTGGACG
TCGCGCACGGTTACCCGGACGCAGGCCCGCCCCTCTGGGGAACCGCGAGCTCCTCGCAGCTG
GGTGGGGACCGTCCTCGCAGTGGCGGCCTCCACCCTGCCTTCTCGCTTAGCCAAGGCTTCTGGA
AGGCTCGCAAAGTGTCTGTTGTTGGAAGAAGCCGGGCCCTGACTGGCGCGACGGATTCTCCCA
GCCGCTCAGCAGAGCGTCTGTAGAATATGCTTTTTGTCTAATGATGTTAGATAAAAAGCAAATTT
GGAGCAGTTTTCTTATTCGAGTTCAAAATGGTTCATAAAGCAGCGAAGACAACTCAAAACATCA
GTAACACATTTGGCCCAGGAACTGCTAACAAACATACAGTGCAGTGGTGGCTTAAGAAGTTTTG
CAAGGAAGAGAGCCTTGAAGATGAGGAACGTGATATGGGCCATGGGAAGTTGACAACGACCA
ATTGAGAGCAATCATTGAACTACATGAAAAATTACAGAAGAACTCAGCGTTGACTATTCTACGG
TCATTAGGGTCGTTCAGCATTCGAAGCAAATTGGAAAGGTGAAAAAGCTTGATAAGTGGGTGCC
TCATGAGCTGAGCGGAAATCAAAACTATCGTTTTGAAGTGTAGTCTTCTTTTATGCTACGCAACA
ACAAACCATTTCTCAATCGGATTGTGACATGTAATGAAAAGTGAATTTTATACAACAACCAGCTC
AGTGGTTGGACCAAGAGGCAGCTCCAAAGCAGTTCCCAAAGCCAAACTTGCACCAAAAAAAAG

GTCATGGTCACTGTTCGGTGGTCTGCTGCCCATCTGATCCACTACAGTTTTCTGAATCCTGGTGA
AACCATTACATCTGAGAAGTATGCTCAGCAAATTGGTAAGATGCACCGAAAACTGCAATACCTG
CTGCCTGCATTGGTCAACAGAAAGGGCCCAATTCTTCTCCACGACAACACCTGACTGCGTGTCG
CACAACCAGTGCTTCAAAAGTTGAAGGAATTGGGCTACAAAGTTTTGCCTCATCCACCATATTCA
CCTGACCTTTCGCCAACTGACTACCACTTCTGCTAGCATCTTGACAACTTTTTGCAGGGAAAACA
CTTCAGCATGGTGCAGAAAATGCTTTCCAAGAGTTCGTCAAATCCTGAAGCACGGATTTTTATGC
TACAGGAATAAACAAACATTTCTCATTGGCAAAAATGTGTTGATTGTAATGGTTCCTGTTTTGATT
AATAGAATATGTTTTAGCGTAGTTACAATGATTTAAAATTCGCAGTCTGGAAATTTAATTTTTGCA
TCAACCTAATATTTCTATGGTAAATCCTTGCAAACATGGAAACAATGCATTTGGCCCAGTGCTTT
GTGGTTGTGTACTCTTTTTCTTTGTTTTTTTAATAGATGGCATTGGCCGGGCATGGTGGCTCATGC
CTGTAATCCCAGCATTTTGGGAAGGTGAGGTGGGTGGATCACCTGAGGTCAGGAGTTCAAGAC
CAGCCTGACTAACATGGTAAAACCCCATCTCTACTAAAAATACAAAAAAATTAGCTAGGCGGGG
TGGCGGGCATCTAATTCCAGCTACTTCATGAGGCTGCGACAGGAGAATCATTTGAACTCGGGAG
GCAGAGGTTGCAGTGAGCCGAGATCACACCATTGCACTCCAGCCTGGGCGATGAGCGAAACTG
TCTCAAAAAAAAAAAAAAGTTGGGGTCTCGCTGTGTTGACCAGGCTGGTCTCGAACTCCTGGCCT
CAAGTGATCCTCCCATCTTGGCCTCCCAAAGTGTTAGGATTACAGGCGTGAGCCACCACACCCT
GCCTGGTTGTGTACTCTTTTAAATACTAAGTTTTTAATGTTAAATGCTGCTTTTAGATACACTGTAA
AAATACACCTATCAATGAGTTTTTTTATTAAAAACATTGCAATTGTACTAGACTTTAAATACTAAG
CAATAATTCAGGCTTCAATGTTGGTTTATAGTTTTCTCATTTCTTTCATTTAATACCTCTGTAAAAT
GAAGCAGTTACTTCCATTTTCCTGAGGTGAGATAAGTGCCCTGCACAATGTTATAGGCCCAGTA
AGTGAGACTGGAGCTCTGATCTAATCCCTTTGACATTAAAGTTTGTGCAGTAAACCAGCTATGTC
AGTTTGCCACATTTGTAATAGTGTTCAATAAATTTTCACTTAAAGGTTTAATATTAAAGTAGTAGC
AAGAATTCATAACCTTAATTGGCTTTAACAGCTTTTCTTTCCCTGAAAACAGATGCATATGTACAC
ATGCACACAGATTGCTTACAATATCAGCACGTAAAAGGCCTTTGCACATTGAAGTCGGCACTGC
TTTGGTGCCTTTTTTGTTTTTTGGCTCGGTGTTTTGACTGCAAGTCTTTTTGGATAGAATTTTATAGT
TAGAAAGTAGCTAACACTTGGGTTTTATAGGCACAAAAAACAAGTCTTATACTAGCTGTACTTTA
TTTTTTGAGTTCTTATTAATGAGGAACATCCACTTTTGCATTGACAGTGATTTCAAGATTGCTTTAT
CAGCCTTTAAAGGATTCTTGACTAGTCGTGCACATCAGAACTGCCAGGTCCCCAGTGGTTCTGA
AGCAGTAAGCTTTGGGTGGGCTCTGGCATCAGCACTTTCACTAAGCTTCACAGATAATTCTGATG
CATACTCCAGGCCTGAACCACTGATCAATTTGAAACATGCATAACAAAGCAAAAAAAGTTTTGT
TTCACCTTTTGAAATACAGTTAACTCTTTTACCATGCCAGAGATCATTCAGAGAGACAGGTCGTT
GCTCCGGAGTGATACAGATCTGGCAGTACCCAGCCCTTGTGTGTGTGCGTTAGCTCAGCACCTG
CCCACACTGCGAGCCCCCGTAGGATGTGCCTTGTCCTTCCCTGTTTCAGCACTTAACACACTACC
TGGTACAGAGTATGTAGTGGGCATCTGTTGAATGAATGCTTTTCCCAGTAGCAGTGTATTCATAC
AATATTAATATAATTGTCCCCTGGCTTACAGATAAAAATGAAAGCATCAAGTGCCCAGTGAGTG
AGACCCAGGTGTTCTTCCTCCACCCCTAGTGGTCCCCTGGGCAGGTCTTTTTTTTTTTGTAACACT
CACCAGTCTGTTCTGTAGTCAATCATTGATTGACTTGTCTGTGAACTTGGAGGAACTGTTTCATA
GTTTCATTAGCACAGAGTAAACATGTTTGCCATGCAAGGTTATTTTGCATCTGCATTTAAGTGATA
ATGTTGAATCAATGAAAAGTGTTGATTAAGCAGTAGTTGTAGATATGCTAAGTTTTTCAAATTACT
AATATCAAGTGGAGATTGTTTTTACTTTTAAGGGTATTGCTTTTGTGATAGCATAAATAATGGTTT
TCCTTTTTTGTAATGTAAATTAATTGCTGGCAACTTTTGTATTCCCATAGACTGGGGAAGCTTAAT
TGCCTTTACAAGTACTTATGTACAACTTTGTATCAAATTTTCTGTAATAGTTTATGCTTTAGTACTA
TATATGTACTAATAATTTTATCTGACTTCTGTTTATATCATTTGTACAATTACATGGTTGTAAAATA
AACTTTTAAACCTCACGTAA

ID|352|gene_id|100505641|transcript_id|NR_046254 Homo sapiens lncRNA
GCACCGGAACGCCGCAGCCGGCGCTCGGTCCATCCGGCCCGTGGGCTGTGGGGCCCCGG
GCGGCGGCGGCTACGTAGCTGCCGCCGGCCTTGGCCGCGGCCGGGCGGCGGATATGTGGCT
TAAATGGCGCAGGCGTCGGGGCCTAGAGGCTGAAGGGCTGGTCGTCCTAAAAACTCGTGGACG
TCGCGCACGGTTACCCGGACGCAGGCCCGCCCCTCTGGGGAACCGCGAGCTCCTCGCAGCTG
GGTGGGGACCGTCCTCGCAGTGGCGGCCTCCACCCTGCCTTCTCGCTTAGCCAAGGCTTCTGGA
AGGCTCGCAAAGTGTCTGTTGTTGGAAGAAGCCGGGCCCTGACTGGCGCGACGGATTCTCCCA
GCCGCTCAGCAGAGCGTCTAATATGCTTTTTGTCTAATGATGTTAGATAAAAAGCAAATTTGGAG
CAGTTTTCTTATTCGAGTTCAAAATGGTTCATAAAGCAGCGAAGACAACTCAAAACATCAGTAAC
ACATTTGGCCCAGGAACTGCTAACAAACATACAGTGCAGTGGTGGCTTAAGAAGTTTTGCAAGG
AAGAGAGCCTTGAAGATGAGGAACGTGATATGGGCCATGGGAAGTTGACAACGACCAATTGAG
AGCAATCATTGAACTACATGAAAAATTACAGAAGAACTCAGCGTTGACTATTCTACGGTCATTA
GGGTCGTTCAGCATTCGAAGCAAATTGGAAAGGTGAAAAAGCTTGATAAGTGGGTGCCTCATG
AGCTGAGCGGAAATCAAAACTATCGTTTTGAAGTGTAGTCTTCTTTTATGCTACGCAACAACAAA
CCATTTCTCAATCGGATTGTGACATGTAATGAAAAGTGAATTTTATACAACAACCAGCTCAGTGG
TTGGACCAAGAGGCAGCTCCAAAGCAGTTCCCAAAGCCAAACTTGCACCAAAAAAAAGGTCAT
GGTCACTGTTCGGTGGTCTGCTGCCCATCTGATCCACTACAGTTTTCTGAATCCTGGTGAAACCA
TTACATCTGAGAAGTATGCTCAGCAAATTGGTAAGATGCACCGAAAACTGCAATACCTGCTGCC
TGCATTGGTCAACAGAAAGGGCCCAATTCTTCTCCACGACAACACCTGACTGCGTGTCGCACAA
CCAGTGCTTCAAAAGTTGAAGGAATTGGGCTACAAAGTTTTGCCTCATCCACCATATTCACCTGA
CCTTTCGCCAACTGACTACCACTTCTGCTAGCATCTTGACAACTTTTTGCAGGGAAAACACTTCA
GCATGGTGCAGAAAATGCTTTCCAAGAGTTCGTCAAATCCTGAAGCACGGATTTTTATGCTACA
GGAATAAACAAACATTTCTCATTGGCAAAAATGTGTTGATTGTAATGGTTCCTGTTTTGATTAATA
GAATATGTTTTAGCGTAGTTACAATGATTTAAAATTCGCAGTCTGGAAATTTAATTTTTGCATCAA
CCTAATATTTCTATGGTAAATCCTTGCAAACATGGAAACAATGCATTTGGCCCAGTGCTTTGTGG
TTGTGTACTCTTTTTCTTTGTTTTTTTAATAGATGGCATTGGCCGGGCATGGTGGCTCATGCCTGT
AATCCCAGCATTTTGGGAAGGTGAGGTGGGTGGATCACCTGAGGTCAGGAGTTCAAGACCAGC
CTGACTAACATGGTAAAACCCCATCTCTACTAAAAATACAAAAAAATTAGCTAGGCGGGGTGGC
GGGCATCTAATTCCAGCTACTTCATGAGGCTGCGACAGGAGAATCATTTGAACTCGGGAGGCA
GAGGTTGCAGTGAGCCGAGATCACACCATTGCACTCCAGCCTGGGCGATGAGCGAAACTGTCT
CAAAAAAAAAAAAAAGTTGGGGTCTCGCTGTGTTGACCAGGCTGGTCTCGAACTCCTGGCCTCAA
GTGATCCTCCCATCTTGGCCTCCCAAAGTGTTAGGATTACAGGCGTGAGCCACCACACCCTGCC
TGGTTGTGTACTCTTTTAAATACTAAGTTTTTAATGTTAAATGCTGCTTTTAGATACACTGTAAAAA
TACACCTATCAATGAGTTTTTTTATTAAAAACATTGCAATTGTACTAGACTTTAAATACTAAGCAA
TAATTCAGGCTTCAATGTTGGTTTATAGTTTTCTCATTTCTTTCATTTAATACCTCTGTAAAATGAA
GCAGTTACTTCCATTTTCCTGAGGTGAGATAAGTGCCCTGCACAATGTTATAGGCCCAGTAAGTG
AGACTGGAGCTCTGATCTAATCCCTTTGACATTAAAGTTTGTGCAGTAAACCAGCTATGTCAGTT
TGCCACATTTGTAATAGTGTTCAATAAATTTTCACTTAAAGGTTTAATATTAAAGTAGTAGCAAGA
ATTCATAACCTTAATTGGCTTTAACAGCTTTTCTTTCCCTGAAAACAGATGCATATGTACACATGC
ACACAGATTGCTTACAATATCAGCACGTAAAAGGCCTTTGCACATTGAAGTCGGCACTGCTTTG
GTGCCTTTTTTGTTTTTTGGCTCGGTGTTTTGACTGCAAGTCTTTTTGGATAGAATTTTATAGTTAG
AAAGTAGCTAACACTTGGGTTTTATAGGCACAAAAAACAAGTCTTATACTAGCTGTACTTTATTTT
TTGAGTTCTTATTAATGAGGAACATCCACTTTTGCATTGACAGTGATTTCAAGATTGCTTTATCAG
CCTTTAAAGGATTCTTGACTAGTCGTGCACATCAGAACTGCCAGGTCCCCAGTGGTTCTGAAGC

AGTAAGCTTTGGGTGGGCTCTGGCATCAGCACTTTCACTAAGCTTCACAGATAATTCTGATGCAT
ACTCCAGGCCTGAACCACTGATCAATTTGAAACATGCATAACAAAGCAAAAAAAGTTTTGTTTC
ACCTTTTGAAATACAGTTAACTCTTTTACCATGCCAGAGATCATTCAGAGAGACAGGTCGTTGCT
CCGGAGTGATACAGATCTGGCAGTACCCAGCCCTTGTGTGTGTGCGTTAGCTCAGCACCTGCCC
ACACTGCGAGCCCCCGTAGGATGTGCCTTGTCCTTCCCTGTTTCAGCACTTAACACACTACCTGG
TACAGAGTATGTAGTGGGCATCTGTTGAATGAATGCTTTTCCCAGTAGCAGTGTATTCATACAAT
ATTAATATAATTGTCCCCTGGCTTACAGATAAAAATGAAAGCATCAAGTGCCCAGTGAGTGAGA
CCCAGGTGTTCTTCCTCCACCCCTAGTGGTCCCCTGGGCAGGTCTTTTTTTTTTTGTAACACTCAC
CAGTCTGTTCTGTAGTCAATCATTGATTGACTTGTCTGTGAACTTGGAGGAACTGTTTCATAGTTT
CATTAGCACAGAGTAAACATGTTTGCCATGCAAGGTTATTTTGCATCTGCATTTAAGTGATAATG
TTGAATCAATGAAAAGTGTTGATTAAGCAGTAGTTGTAGATATGCTAAGTTTTTCAAATTACTAAT
ATCAAGTGGAGATTGTTTTTACTTTTAAGGGTATTGCTTTTGTGATAGCATAAATAATGGTTTTCCT
TTTTTGTAATGTAAATTAATTGCTGGCAACTTTTGTATTCCCATAGACTGGGGAAGCTTAATTGCC
TTTACAAGTACTTATGTACAACTTTGTATCAAATTTTCTGTAATAGTTTATGCTTTAGTACTATATA
TGTACTAATAATTTTATCTGACTTCTGTTTATATCATTTGTACAATTACATGGTTGTAAAATAAACT
TTTAAACCTCACGTAA

ID|353|gene_id|100505641|transcript_id|NR_046255 Homo sapiens lncRNA

GCCAAAGCGGCCTCGAAGCGGGTCCCGTGTCTGCGGCGGGCGGCCCCCGGCGCGGAGG
GCAGGGCGCGGGGAGCCCGGGCTCCCGGTGTCGGCGGATGGCGGGAGCCCGGGGGCCGCGG
GCTCGCGGGGCAGCGGGCGAGAGACGAGACGCCGAGAATATGCTTTTTGTCTAATGATGTTAG
ATAAAAAGCAAATTTGGAGCAGTTTTCTTATTCGAGTTCAAAATGGTTCATAAAGCAGCGAAGA
CAACTCAAAACATCAGTAACACATTTGGCCCAGGAACTGCTAACAAACATACAGTGCAGTGGTG
GCTTAAGAAGTTTTGCAAGGAAGAGAGCCTTGAAGATGAGGAACGTGATATGGGCCATGGGAA
GTTGACAACGACCAATTGAGAGCAATCATTGAACTACATGAAAAATTACAGAAGAACTCAGCGT
TGACTATTCTACGGTCATTAGGGTCGTTCAGCATTCGAAGCAAATTGGAAAGGTGAAAAAGCTT
GATAAGTGGGTGCCTCATGAGCTGAGCGGAAATCAAAACTATCGTTTTGAAGTGTAGTCTTCTTT
TATGCTACGCAACAACAAACCATTTCTCAATCGGATTGTGACATGTAATGAAAAGTGAATTTTAT
ACAACAACCAGCTCAGTGGTTGGACCAAGAGGCAGCTCCAAAGCAGTTCCCAAAGCCAAACTT
GCACCAAAAAAAAAGGTCATGGTCACTGTTCGGTGGTCTGCTGCCCATCTGATCCACTACAGTTTT
CTGAATCCTGGTGAAACCATTACATCTGAGAAGTATGCTCAGCAAATTGGTAAGATGCACCGAA
AACTGCAATACCTGCTGCCTGCATTGGTCAACAGAAAGGGCCCAATTCTTCTCCACGACAACAC
CTGACTGCGTGTCGCACAACCAGTGCTTCAAAAGTTGAAGGAATTGGGCTACAAAGTTTTGCCT
CATCCACCATATTCACCTGACCTTTCGCCAACTGACTACCACTTCTGCTAGCATCTTGACAACTTT
TTGCAGGGAAAACACTTCAGCATGGTGCAGAAAATGCTTTCCAAGAGTTCGTCAAATCCTGAAG
CACGGATTTTTATGCTACAGGAATAAACAAACATTTCTCATTGGCAAAAATGTGTTGATTGTAAT
GGTTCCTGTTTTGATTAATAGAATATGTTTTAGCGTAGTTACAATGATTTAAAATTCGCAGTCTGG
AAATTTAATTTTTGCATCAACCTAATATTTCTATGGTAAATCCTTGCAAACATGGAAACAATGCAT
TTGGCCCAGTGCTTTGTGGTTGTGTACTCTTTTTCTTTGTTTTTTTAATAGATGGCATTGGCCGGGC
ATGGTGGCTCATGCCTGTAATCCCAGCATTTTGGGAAGGTGAGGTGGGTGGATCACCTGAGGTC
AGGAGTTCAAGACCAGCCTGACTAACATGGTAAAACCCCATCTCTACTAAAAATACAAAAAAAT
TAGCTAGGCGGGGTGGCGGGCATCTAATTCCAGCTACTTCATGAGGCTGCGACAGGAGAATCA
TTTGAACTCGGGAGGCAGAGGTTGCAGTGAGCCGAGATCACACCATTGCACTCCAGCCTGGGC
GATGAGCGAAACTGTCTCAAAAAAAAAAAAAAGTTGGGGTCTCGCTGTGTTGACCAGGCTGGTCT
CGAACTCCTGGCCTCAAGTGATCCTCCCATCTTGGCCTCCCAAAGTGTTAGGATTACAGGCGTG

AGCCACCACACCCTGCCTGGTTGTGTACTCTTTTAAATACTAAGTTTTTAATGTTAAATGCTGCTT
TTAGATACACTGTAAAAATACACCTATCAATGAGTTTTTTTATTAAAAACATTGCAATTGTACTAG
ACTTTAAATACTAAGCAATAATTCAGGCTTCAATGTTGGTTTATAGTTTTCTCATTTCTTTCATTTA
ATACCTCTGTAAAATGAAGCAGTTACTTCCATTTTCCTGAGGTGAGATAAGTGCCCTGCACAATG
TTATAGGCCCAGTAAGTGAGACTGGAGCTCTGATCTAATCCCTTTGACATTAAAGTTTGTGCAGT
AAACCAGCTATGTCAGTTTGCCACATTTGTAATAGTGTTCAATAAATTTTCACTTAAAGGTTTAAT
ATTAAAGTAGTAGCAAGAATTCATAACCTTAATTGGCTTTAACAGCTTTTCTTTCCCTGAAAACAG
ATGCATATGTACACATGCACACAGATTGCTTACAATATCAGCACGTAAAAGGCCTTTGCACATTG
AAGTCGGCACTGCTTTGGTGCCTTTTTTGTTTTTTGGCTCGGTGTTTTGACTGCAAGTCTTTTTGGA
TAGAATTTTATAGTTAGAAAGTAGCTAACACTTGGGTTTTATAGGCACAAAAAACAAGTCTTATA
CTAGCTGTACTTTATTTTTTGAGTTCTTATTAATGAGGAACATCCACTTTTGCATTGACAGTGATTT
CAAGATTGCTTTATCAGCCTTTAAAGGATTCTTGACTAGTCGTGCACATCAGAACTGCCAGGTCC
CCAGTGGTTCTGAAGCAGTAAGCTTTGGGTGGGCTCTGGCATCAGCACTTTCACTAAGCTTCAC
AGATAATTCTGATGCATACTCCAGGCCTGAACCACTGATCAATTTGAAACATGCATAACAAAGC
AAAAAAAGTTTTGTTTCACCTTTTGAAATACAGTTAACTCTTTTACCATGCCAGAGATCATTCAGA
GAGACAGGTCGTTGCTCCGGAGTGATACAGATCTGGCAGTACCCAGCCCTTGTGTGTGTGCGTT
AGCTCAGCACCTGCCCACACTGCGAGCCCCCGTAGGATGTGCCTTGTCCTTCCCTGTTTCAGCA
CTTAACACACTACCTGGTACAGAGTATGTAGTGGGCATCTGTTGAATGAATGCTTTTCCCAGTAG
CAGTGTATTCATACAATATTAATATAATTGTCCCCTGGCTTACAGATAAAAATGAAAGCATCAAG
TGCCCAGTGAGTGAGACCCAGGTGTTCTTCCTCCACCCCTAGTGGTCCCCTGGGCAGGTCTTTTT
TTTTTGTAACACTCACCAGTCTGTTCTGTAGTCAATCATTGATTGACTTGTCTGTGAACTTGGAG
GAACTGTTTCATAGTTTCATTAGCACAGAGTAAACATGTTTGCCATGCAAGGTTATTTTGCATCTG
CATTTAAGTGATAATGTTGAATCAATGAAAAGTGTTGATTAAGCAGTAGTTGTAGATATGCTAAG
TTTTTCAAATTACTAATATCAAGTGGAGATTGTTTTTACTTTTAAGGGTATTGCTTTTGTGATAGCA
TAAATAATGGTTTTCCTTTTTTGTAATGTAAATTAATTGCTGGCAACTTTTGTATTCCCATAGACTG
GGGAAGCTTAATTGCCTTTACAAGTACTTATGTACAACTTTGTATCAAATTTTCTGTAATAGTTTA
TGCTTTAGTACTATATATGTACTAATAATTTTATCTGACTTCTGTTTATATCATTTGTACAATTACAT
GGTTGTAAAATAAACTTTTAAACCTCACGTAA
ID|356|gene_id|100505817|transcript_id|NR_038340 Homo sapiens lncRNA
GCTGACTCTCTTTTCGGACTCAGCCCGCCTGCACCCAGGTGAAATAAACAGCCATGTTGCT
CACACAAAGCCTGTTTGGTGGTCTCTTCACAGGGACACGGATGAAATTTGGTGCCGTGACTCGG
ATCGGGGAACCTCCCTTAGGAGATCAATCCCCTGTACTCCTTTTCTTTGCCCTGTGAGAAAGATC
CACCTATGACCTCAGGTCCTCAGACCGACCAGCCCAAGGAACATCTCACCAATTTTAAATCAGA
CCTTGAAGATTTGTTGTTCAAGGAGAAACTGAAGAGCAAGAAGGAAAGTGAGAGCCAGCAATA
CCAGCAGAGCCAGATCTGAGCTGGGAGAAGGGGAGAAAGTTTGTGAAGAGGAGATCGGTGAC
CTGGGCTCCTTATGTGCCTGAAAGAGTTTGAGTTTCCTGTTAACTCCAAATCAACAGTATTTTCAA
CAAGAAATGTGCAATTGAAATCAAGTGCTGTTTAAGTGCAGCTAGGATTTCCACAGGAAGACAC
TTGCAGTGAACAGAGTTATGGAGCAGCAAAAACACAGATCTATTTGGAAAAAGAGAAAACATA
TGCGTTGTATTTTGCTTCAATTATAAAATACCATCCTCTCAAAGGTGGTTCTAAATTACAAAGGAC
TTTGATTTCTAGGTAGATTCTGGGTAGAGACTTCCTTTCATATTGAGGCATTAATGACACCTTTTA
ACCTGGGAAGCAATATGACTGGAGTTGTACTTTGAGAAGATTAATCAGGTTTGGTTGCAGAATG
AAAGAGAAGATGAAGTCAAGAGATTGGTTTAGAGGCTCTAGCAGAAGCTTAGTCATATTTCAAA
ATGATCAAATATCAAGAAAAATTCTGAGCTGCATAACTTGTATAAAGTAATTTTCAGTGATTTTTT
TCATGGTTATGATAAAAGAACTGGATTAGCAGAAACTTTTACCCTGAATCAAGATTTAATTTTTCT

TTGAGCTCATCTTAAGGATATCGGAACATAGGGAGCAAACGATGGTGTGGCTGCCTCAGTGCTT
GATTTTTAACGGTTTTGAAGAGAATAGTTACATTTCTTCTCCTAGTAAGAACTAATAAATACATTA
ACAGAAATGAATTCCCTATCCCTTTGTACACTGGTCTATTTCTTCAAAACATTAAATACTATTGAT
AAGATATCACCTTTTAAAAAA

ID|375|gene_id|100507246|transcript_id|NR_038108 Homo sapiens lncRNA

GCGTTCTTTTCGAGGTCGGCCGCGTGGCTGGAAGACATGGCCACTCCAGTCGGTGTTGAG
CACGGCGAGCAGTCTCAGGCCTTTAGTGATGATGATTAAAGGTGGTGGCTGTGGCCTTGAAAAC
AGTCATGTGAAAACTCATCACCTTAAGGTGTTAAGTGTAAGGATCTTCATGATGAAATTTCTGTA
AATGGTGCAGTCAGCCTCAGTTTCCAAAGCCGGAAAAGGATCCTCTAGTAGCCACGGTGTGGC
AGCTGCTCTGAACCAGGACCTGGACCCGGACCCAAAGTGCCATGTCTTTAATGTGAGTTAGCTC
CCAGCGATGCCAGATGGGATCAGCACAGCCCTGCCTCTGCTGCTAATTGTTCCTCTAAAGTAAT
CGCCATGCGTTCTTTGGGCTTCATCTTTAAAGGAATGAAGCAACTGAGATTATTCTGGAAAACCT
TTTGGCAGTTAGTGAAATTAGAGTACAACTAAGAACATTTTCAGACCTCCACTGTGGATGACCTG
GGTATAATCTCACAAATCGATGGGACTGCAAGGATTGTAAACTGAAATGAACATGATTATACTC
TGTTGGAAGAGCCTAAGAGGAAACTGATGCCATGAGTTTCAGAGAGTAATGCTTAACCCCAGTT
ACACAGGATGCCGTCTTGTGTTTCCTCTTGTTTAGTTACCCACTACAGTGATTTTGTGATCTGCTA
ATGGGTTGCCACCCACAACCATTGCTTTAGCACTTTTACTTCAAATCAATGAAGGATTGATAAAA
GTTCTCCTGGTGTCTCCGCAGAGTGCCTTCCAGGAACAGATCTTTGCATAGAATATCAGTGGTTT
CCTTTTTTGTTTCAAATAGTGGTCAGAAAATACCCAGTGTTGACTCACCAAGGCAATCAGCTTCC
TTTTTCCCTTTTTTTGTTTTTTTTTTAACATTTTATATTTTTGCTTTATTTTATTTTATTTTATTTTATTTT
ATTTTATTTTATTTTATTTTTTGAGACGGAGTTCCACTCTGTCGCCAGGCTGGAGTGAAGTGGTAC
AATCTTGGCTCACTGCAACCTCCACCTCCCGGGTTCAAGCAATTCTCCTGGCTCAGCCTCCTGAG
TGCTGGGACTACAGGCGCGTACCTTCTTTAGTAGAGACTGGGTTTCACCATGTTGGCCAGGATG
GTCTCTATCTCCTGACCTTGTGATCTGCCTGCCTCAGCTTCCCAAAGTGCTGAGATGACAGGTGT
GAGCCATCAGACCCAGCATTTTTTTTTTTAATTTAAATTTAAATTTTTTTTCATTTTTTTGAGAGGTT
TTTTTTGTTTTGTTTTGTTGTTGTTGTTGTTGTTGTTTTTGAGACAGTCTTGCTCTGTCACCCAGGCT
GGGAGTGCAGTGGCATGATCTCTGCAACCTCTACCTCCCAGGTTCAAGCAATTCTTGTGCCTCA
GCCTCCCAAGTAACTGGGACTACAGGTGCACGCTACCACACCTGGCTGATTTTTTTTGTTTTAGT
AGAGACAGGGTTTCAACCATGTTGCCCAGGTTGGTCTCAAACTCCTGAGCTCAGGCAATCCACC
CGCCTTGGCCTCCCAAAGTGCTAGGATTACAGGTGTGAGCCACCACACCCAGCTATTTTTTCTTT
CGTTTTTTAATTTTAAAGTTGGGGGGGGGTCTCAATTTGTTACCCTGGCTGGTCTCGAACTCCCG
GACTTAAGCGATCCTCTGGCTCCAAGCCCACTACCAGTCTCAGGTTTCTTTACTAAAAGATCACT
ACCTTTTTTTCTCTTATCTGCTGCCATGTGAGATGTGGCTTTCACCTTCCGCCATGATTGTGAGGC
CTTCCCAGCCATGTAGAACTGTAAGTCCAATAAACCTCTTTTGTAAATTAAAAAAAAAAAATCAC
TATTTAAGATACTAGGATGGATTGTGACTGTTGAGGAGTACTTACATATCCTACATTTGACTACAT
TATTTCCAAACCAAGTATTCCATCCAAAGGAACATACTGCTATCATAGAGACCAAGGAGGGACT
GTTTAAGGTTGCCAAGGTGAAGCGAGCTGAGAGGCTTTGTCCTCGTGCCAGTAACTCTGAAATC
TCTCTTAATTCCTGCTGTCCAGGCAGCAGAATGCCATGGTTTCCCCAAGTAGGTAGCTGCTTTAG
CAGTTAAAGCCCAAATGTCTGTTCTGTTGATCAGAGGTCTCTGAATTTCTGAAGTGGTGTTTCGTT
TCTGGTGACTGAGTTAATCCTTTACAATCCCTCTTGTAAAGTGTGCTAATAGAAAGAATCCACCT
TTCAAAGCTGCAGAACCAGACCGTGCCCTAAATTGACCAACGTAACTGATGTGCCTCAGGAAGT
CTCTTGCCAGCTGTCCCTGTGAAGACCCCCCTCCTCCCCCCCAGCTGCTGCCTTGCACACTGAA
GCATCTCAGACTGTGCAAAGCCGTGTAGTCATCAAGACAGTAAATCCCAGGGCTTGGTTAAGTG
CTGTGTGATAACTTGTTTGGATGAGACTTAACTTAAAACCACTTACAATAAACTTGGGAAACTAC

CGTCA
ID|376|gene_id|100507246|transcript_id|NR_038109 Homo sapiens lncRNA
GCGTTCTTTTCGAGGTCGGCCGCGTGGCTGGAAGACATGGCCACTCCAGTCGGTGTTGAG
CACGGCGAGCAGTCTCAGGCCTTTAGTGATGATGATTAAAGGTGGTGGCTGTGGCCTTGAAAAC
AGTCATGTGAAAACTCATCACCTTAAGGTGTTAAGTGTAAGGATCTTCATGATGAAATTTCTGTA
AATGGTGCAGTCAGCCTCAGTTTCCAAAGCCGGAAAAGGATCCTCTAGTAGCCACGGTGTGGC
AGCTGCTCTGAACCAGGACCTGGACCCGGACCCAAAGTGCCATGTCTTTAATGTTAGCTCCCAG
CGATGCCAGATGGGATCAGCACAGCCCTGCCTCTGCTGCTAATTGTTCCTCTAAAGTAATCGCC
ATGCGTTCTTTGGGCTTCATCTTTAAAGGAATGAAGCAACTGAGATTATTCTGGAAAACCTTTTG
GCAGTTAGTGAAATTAGAGTACAACTAAGAACATTTTCAGACCTCCACTGTGGATGACCTGGGT
ATAATCTCACAAATCGATGGGACTGCAAGGATTGTAAACTGAAATGAACATGATTATACTCTGTT
GGAAGAGCCTAAGAGGAAACTGATGCCATGAGTTTCAGAGAGTAATGCTTAACCCCAGTTACA
CAGGATGCCGTCTTGTGTTTCCTCTTGTTTAGTTACCCACTACAGTGATTTTGTGATCTGCTAATG
GGTTGCCACCCACAACCATTGCTTTAGCACTTTTACTTCAAATCAATGAAGGATTGATAAAAGTT
CTCCTGGTGTCTCCGCAGAGTGCCTTCCAGGAACAGATCTTTGCATAGAATATCAGTGGTTTCCT
TTTTTGTTTCAAATAGTGGTCAGAAAATACCCAGTGTTGACTCACCAAGGCAATCAGCTTCCTTTT
TCCCTTTTTTTGTTTTTTTTTAACATTTTATATTTTTGCTTTATTTTATTTTATTTTATTTTATTTTATTT
TATTTTATTTTATTTTTTGAGACGGAGTTCCACTCTGTCGCCAGGCTGGAGTGAAGTGGTACAATC
TTGGCTCACTGCAACCTCCACCTCCCGGGTTCAAGCAATTCTCCTGGCTCAGCCTCCTGAGTGCT
GGGACTACAGGCGCGTACCTTCTTTAGTAGAGACTGGGTTTCACCATGTTGGCCAGGATGGTCT
CTATCTCCTGACCTTGTGATCTGCCTGCCTCAGCTTCCCAAAGTGCTGAGATGACAGGTGTGAG
CCATCAGACCCAGCATTTTTTTTTTTAATTTAAATTTAAATTTTTTTTCATTTTTTTGAGAGGTTTTTT
TTGTTTTGTTTTGTTGTTGTTGTTGTTGTTGTTTTTGAGACAGTCTTGCTCTGTCACCCAGGCTGGG
AGTGCAGTGGCATGATCTCTGCAACCTCTACCTCCCAGGTTCAAGCAATTCTTGTGCCTCAGCCT
CCCAAGTAACTGGGACTACAGGTGCACGCTACCACACCTGGCTGATTTTTTTTGTTTTAGTAGAG
ACAGGGTTTCAACCATGTTGCCCAGGTTGGTCTCAAACTCCTGAGCTCAGGCAATCCACCCGCC
TTGGCCTCCCAAAGTGCTAGGATTACAGGTGTGAGCCACCACACCCAGCTATTTTTTCTTTCGTT
TTTTAATTTTAAAGTTGGGGGGGGGTCTCAATTTGTTACCCTGGCTGGTCTCGAACTCCCGGACT
TAAGCGATCCTCTGGCTCCAAGCCCACTACCAGTCTCAGGTTTCTTTACTAAAAGATCACTACCT
TTTTTTCTCTTATCTGCTGCCATGTGAGATGTGGCTTTCACCTTCCGCCATGATTGTGAGGCCTTC
CCAGCCATGTAGAACTGTAAGTCCAATAAACCTCTTTTGTAAATTAAAAAAAAAAAAATCACTATT
TAAGATACTAGGATGGATTGTGACTGTTGAGGAGTACTTACATATCCTACATTTGACTACATTATT
TCCAAACCAAGTATTCCATCCAAAGGAACATACTGCTATCATAGAGACCAAGGAGGGACTGTTT
AAGGTTGCCAAGGTGAAGCGAGCTGAGAGGCTTTGTCCTCGTGCCAGTAACTCTGAAATCTCTC
TTAATTCCTGCTGTCCAGGCAGCAGAATGCCATGGTTTCCCCAAGTAGGTAGCTGCTTTAGCAGT
TAAAGCCCAAATGTCTGTTCTGTTGATCAGAGGTCTCTGAATTTCTGAAGTGGTGTTTCGTTTCTG
GTGACTGAGTTAATCCTTTACAATCCCTCTTGTAAAGTGTGCTAATAGAAAGAATCCACCTTTCA
AAGCTGCAGAACCAGACCGTGCCCTAAATTGACCAACGTAACTGATGTGCCTCAGGAAGTCTCT
TGCCAGCTGTCCCTGTGAAGACCCCCCTCCTCCCCCCCAGCTGCTGCCTTGCACACTGAAGCAT
CTCAGACTGTGCAAAGCCGTGTAGTCATCAAGACAGTAAATCCCAGGGCTTGGTTAAGTGCTGT
GTGATAACTTGTTTGGATGAGACTTAACTTAAAACCACTTACAATAAACTTGGGAAACTACCGTC
A
ID|377|gene_id|100507246|transcript_id|NR_038110 Homo sapiens lncRNA
GCGTTCTTTTCGAGGTCGGCCGCGTGGCTGGAAGACATGGCCACTCCAGTCGGTGTTGAG

CACGGCGAGCAGTCTCAGGCCTTTAGTGATGATGGTGCAGTCAGCCTCAGTTTCCAAAGCCGG
AAAAGGATCCTCTAGTAGCCACGGTGTGGCAGCTGCTCTGAACCAGGACCTGGACCCGGACCC
AAAGTGCCATGTCTTTAATGTGAGTTAGCTCCCAGCGATGCCAGATGGGATCAGCACAGCCCTG
CCTCTGCTGCTAATTGTTCCTCTAAAGTAATCGCCATGCGTTCTTTGGGCTTCATCTTTAAAGGAA
TGAAGCAACTGAGATTATTCTGGAAAACCTTTTGGCAGTTAGTGAAATTAGAGTACAACTAAGA
ACATTTTCAGACCTCCACTGTGGATGACCTGGGTATAATCTCACAAATCGATGGGACTGCAAGG
ATTGTAAACTGAAATGAACATGATTATACTCTGTTGGAAGAGCCTAAGAGGAAACTGATGCCAT
GAGTTTCAGAGAGTAATGCTTAACCCCAGTTACACAGGATGCCGTCTTGTGTTTCCTCTTGTTTA
GTTACCCACTACAGTGATTTTGTGATCTGCTAATGGGTTGCCACCCACAACCATTGCTTTAGCAC
TTTTACTTCAAATCAATGAAGGATTGATAAAAGTTCTCCTGGTGTCTCCGCAGAGTGCCTTCCAG
GAACAGATCTTTGCATAGAATATCAGTGGTTTCCTTTTTTGTTTCAAATAGTGGTCAGAAAATACC
CAGTGTTGACTCACCAAGGCAATCAGCTTCCTTTTTCCCTTTTTTTGTTTTTTTTTTAACATTTTATA
TTTTTGCTTTATTTTATTTTATTTTATTTTATTTTATTTTATTTTATTTTATTTTTTGAGACGGAGTTCC
ACTCTGTCGCCAGGCTGGAGTGAAGTGGTACAATCTTGGCTCACTGCAACCTCCACCTCCCGGG
TTCAAGCAATTCTCCTGGCTCAGCCTCCTGAGTGCTGGGACTACAGGCGCGTACCTTCTTTAGTA
GAGACTGGGTTTCACCATGTTGGCCAGGATGGTCTCTATCTCCTGACCTTGTGATCTGCCTGCCT
CAGCTTCCCAAAGTGCTGAGATGACAGGTGTGAGCCATCAGACCCAGCATTTTTTTTTTTAATTT
AAATTTAAATTTTTTTTCATTTTTTTGAGAGGTTTTTTTTGTTTTGTTTTGTTGTTGTTGTTGTTGT
TTTTGAGACAGTCTTGCTCTGTCACCCAGGCTGGGAGTGCAGTGGCATGATCTCTGCAACCTCTA
CCTCCCAGGTTCAAGCAATTCTTGTGCCTCAGCCTCCCAAGTAACTGGGACTACAGGTGCACGC
TACCACACCTGGCTGATTTTTTTTGTTTTAGTAGAGACAGGGTTTCAACCATGTTGCCCAGGTTG
GTCTCAAACTCCTGAGCTCAGGCAATCCACCCGCCTTGGCCTCCCAAAGTGCTAGGATTACAGG
TGTGAGCCACCACACCCAGCTATTTTTTCTTTCGTTTTTTAATTTTAAAGTTGGGGGGGGGTCTCA
ATTTGTTACCCTGGCTGGTCTCGAACTCCCGGACTTAAGCGATCCTCTGGCTCCAAGCCCACTAC
CAGTCTCAGGTTTCTTTACTAAAAGATCACTACCTTTTTTTCTCTTATCTGCTGCCATGTGAGATGT
GGCTTTCACCTTCCGCCATGATTGTGAGGCCTTCCCAGCCATGTAGAACTGTAAGTCCAATAAA
CCTCTTTTGTAAATTAAAAAAAAAAAAATCACTATTTAAGATACTAGGATGGATTGTGACTGTTGA
GGAGTACTTACATATCCTACATTTGACTACATTATTTCCAAACCAAGTATTCCATCCAAAGGAAC
ATACTGCTATCATAGAGACCAAGGAGGGACTGTTTAAGGTTGCCAAGGTGAAGCGAGCTGAGA
GGCTTTGTCCTCGTGCCAGTAACTCTGAAATCTCTCTTAATTCCTGCTGTCCAGGCAGCAGAATG
CCATGGTTTCCCCAAGTAGGTAGCTGCTTTAGCAGTTAAAGCCCAAATGTCTGTTCTGTTGATCA
GAGGTCTCTGAATTTCTGAAGTGGTGTTTCGTTTCTGGTGACTGAGTTAATCCTTTACAATCCCTC
TTGTAAAGTGTGCTAATAGAAAGAATCCACCTTTCAAAGCTGCAGAACCAGACCGTGCCCTAAA
TTGACCAACGTAACTGATGTGCCTCAGGAAGTCTCTTGCCAGCTGTCCCTGTGAAGACCCCCCT
CCTCCCCCCCAGCTGCTGCCTTGCACACTGAAGCATCTCAGACTGTGCAAAGCCGTGTAGTCAT
CAAGACAGTAAATCCCAGGGCTTGGTTAAGTGCTGTGTGATAACTTGTTTGGATGAGACTTAACT
TAAAACCACTTACAATAAACTTGGGAAACTACCGTCA

ID|378|gene_id|100507246|transcript_id|NR_038111 Homo sapiens lncRNA

GCGTTCTTTTCGAGGTCGGCCGCGTGGCTGGAAGACATGGCCACTCCAGTCGGTGTTGAG
CACGGCGAGCAGTCTCAGGCCTTTAGTGATGATGGTGCAGTCAGCCTCAGTTTCCAAAGCCGG
AAAAGGATCCTCTAGTAGCCACGGTGTGGCAGCTGCTCTGAACCAGGACCTGGACCCGGACCC
AAAGTGCCATGTCTTTAATGTTAGCTCCCAGCGATGCCAGATGGGATCAGCACAGCCCTGCCTC
TGCTGCTAATTGTTCCTCTAAAGTAATCGCCATGCGTTCTTTGGGCTTCATCTTTAAAGGAATGAA
GCAACTGAGATTATTCTGGAAAACCTTTTGGCAGTTAGTGAAATTAGAGTACAACTAAGAACATT

TTCAGACCTCCACTGTGGATGACCTGGGTATAATCTCACAAATCGATGGGACTGCAAGGATTGT
AAACTGAAATGAACATGATTATACTCTGTTGGAAGAGCCTAAGAGGAAACTGATGCCATGAGTT
TCAGAGAGTAATGCTTAACCCCAGTTACACAGGATGCCGTCTTGTGTTTCCTCTTGTTTAGTTACC
CACTACAGTGATTTTGTGATCTGCTAATGGGTTGCCACCCACAACCATTGCTTTAGCACTTTTACT
TCAAATCAATGAAGGATTGATAAAAGTTCTCCTGGTGTCTCCGCAGAGTGCCTTCCAGGAACAG
ATCTTTGCATAGAATATCAGTGGTTTCCTTTTTTGTTTCAAATAGTGGTCAGAAAATACCCAGTGT
TGACTCACCAAGGCAATCAGCTTCCTTTTTCCCTTTTTTTGTTTTTTTTTTAACATTTTATATTTTTGC
TTTATTTTATTTTATTTTATTTTATTTTATTTTATTTTATTTTATTTTTTGAGACGGAGTTCCACTCTGT
CGCCAGGCTGGAGTGAAGTGGTACAATCTTGGCTCACTGCAACCTCCACCTCCCGGGTTCAAGC
AATTCTCCTGGCTCAGCCTCCTGAGTGCTGGGACTACAGGCGCGTACCTTCTTTAGTAGAGACT
GGGTTTCACCATGTTGGCCAGGATGGTCTCTATCTCCTGACCTTGTGATCTGCCTGCCTCAGCTT
CCCAAAGTGCTGAGATGACAGGTGTGAGCCATCAGACCCAGCATTTTTTTTTTTAATTTAAATTT
AAATTTTTTTTCATTTTTTTGAGAGGTTTTTTTTGTTTTGTTTTGTTGTTGTTGTTGTTGTTTTTGA
GACAGTCTTGCTCTGTCACCCAGGCTGGGAGTGCAGTGGCATGATCTCTGCAACCTCTACCTCC
CAGGTTCAAGCAATTCTTGTGCCTCAGCCTCCCAAGTAACTGGGACTACAGGTGCACGCTACCA
CACCTGGCTGATTTTTTTTGTTTTAGTAGAGACAGGGTTTCAACCATGTTGCCCAGGTTGGTCTCA
AACTCCTGAGCTCAGGCAATCCACCCGCCTTGGCCTCCCAAAGTGCTAGGATTACAGGTGTGAG
CCACCACACCCAGCTATTTTTTCTTTCGTTTTTTAATTTTAAAGTTGGGGGGGGGTCTCAATTTGTT
ACCCTGGCTGGTCTCGAACTCCCGGACTTAAGCGATCCTCTGGCTCCAAGCCCACTACCAGTCT
CAGGTTTCTTTACTAAAAGATCACTACCTTTTTTTCTCTTATCTGCTGCCATGTGAGATGTGGCTTT
CACCTTCCGCCATGATTGTGAGGCCTTCCCAGCCATGTAGAACTGTAAGTCCAATAAACCTCTTT
TGTAAATTAAAAAAAAAAAAATCACTATTTAAGATACTAGGATGGATTGTGACTGTTGAGGAGTAC
TTACATATCCTACATTTGACTACATTATTTCCAAACCAAGTATTCCATCCAAAGGAACATACTGCT
ATCATAGAGACCAAGGAGGGACTGTTTAAGGTTGCCAAGGTGAAGCGAGCTGAGAGGCTTTGT
CCTCGTGCCAGTAACTCTGAAATCTCTCTTAATTCCTGCTGTCCAGGCAGCAGAATGCCATGGTT
TCCCCAAGTAGGTAGCTGCTTTAGCAGTTAAAGCCCAAATGTCTGTTCTGTTGATCAGAGGTCTC
TGAATTTCTGAAGTGGTGTTTCGTTTCTGGTGACTGAGTTAATCCTTTACAATCCCTCTTGTAAAG
TGTGCTAATAGAAAGAATCCACCTTTCAAAGCTGCAGAACCAGACCGTGCCCTAAATTGACCAA
CGTAACTGATGTGCCTCAGGAAGTCTCTTGCCAGCTGTCCCTGTGAAGACCCCCCTCCTCCCCC
CCAGCTGCTGCCTTGCACACTGAAGCATCTCAGACTGTGCAAAGCCGTGTAGTCATCAAGACAG
TAAATCCCAGGGCTTGGTTAAGTGCTGTGTGATAACTTGTTTGGATGAGACTTAACTTAAAACCA
CTTACAATAAACTTGGGAAACTACCGTCA
ID|487|gene_id|100874051|transcript_id|NR_047509 Homo sapiens lncRNA
CAAGGACAGCAGTAGGAGCTTACTGCAGTGACTGTATCATAACTGCTGGTTATGAGGATTG
GATTTCTTTTTTGCAGATGTCACGTGCCGGGGTGAACTGTTATTAACTAAAAGGAGTCAGGGCTT
AGCTGCAAGAAAGGAGGAATTGCTCTTAAAGTGCTTGGAAGTCACCGATCTAACCAACGGACA
AAAAATTTCCTGAGGACAGACCCTCCCCTCTGGAGGCATCCTTCTATCATTGGCTCCTGACTCCC
CGAAGCAAGGTCAAGTGGGAGTTTAATAGCTGTTCCTGGGACTGTGCAGAGGGCAGCCCTTTG
AACTCAGTGTCTCAATCCCACAGGTGGGAGGTTCCCACCACTGCGTAATTTCGAGAGGGAGGG
CCGAGACTGCCTGGGGGGAAGTCCCATGGCTACCTGGTTCGAGGCAGCTCTTTGATATCTCCTG
GAGCACTCCTGGTCCTAGAACCAGACAGCTCTCAACTGAACTGACACAAGTAACAAACTCAGC
ATCCCCTCTCCTGGGCATTCCTACAAGCCATGCGAGCCCCGTGTTGGTGGAGACACTGCCTTCT
CCACCGCAGACACCAACAAGTTAGAATGGAGAAACTGTCTATGGCCCTACCACTCTGAACACG
CCGGATGTCATCTCATCTCGGAAGCTAAGCAGGGTCGGGCCTGCTTAGGACTTGGGTGGGAGA

ATGGAGAAATTGATGGACTTTTGACGATGCAGGTTTTAACATCAGATCTGAGCCCTAATAGCTGT
GTGTCTCAGCTGTCTGGGCCACACCATTAACAGGGAAAAGTTTCCTCCACTTAAAGAAAGAAAA
AGACAAAGAGGAGAAGGAAGGCAATACGGAGCTCTCCTGGAAGGTACATTAAATCATTAAATG
CCA
ID|694|gene_id|101926933|transcript_id|NR_110002 Homo sapiens lncRNA
ATCTGAAGAACGCCGTTGAGAGACTACACCGCCCATGAAACCCTGCAGTATAACCTTGAA
GTACCATCACGGAGAGAAACAGTGCCATCTCAGGGCCAGTGGCGCAATGGATAACGCGTCTGA
CTACGGATCAGAAGATTCCAGGAGCCAATGACCAATAGGCCAGAGCACTGGAGACTTTCTGGA
AGAAAGAGGTACTGAAGCATCCAATATGGAAATAGTCAAGATATAGAAAATGCAAGCTCTTGA
AGGATTCAAGATATAGAAAGGATCTGAGATATGGAAAGAACTCAGGATATAGAAGGATCTGAA
ATTTTGGAGACTAAGATATGAGAATATTTAAGACACAGGTAGGAAGGACACCTTCAAGGAATTT
GCTGGCTACCCTTATTTTTATAATATGCTTGCTAACATCAGCATGGGAGCATCTTGAGTTTTTAGC
CTACTACACAAGCAGATATTGAACCAACATTAACAGGAATTAGTGTAGTCTGTTTTCCAATAAGA
GAGTGTTCAAGGATCTTCATGTATTCCAGTTTCCTCAGAAACACATGCT
ID|695|gene_id|101927167|transcript_id|NR_125398 Homo sapiens lncRNA
AGGAAATGAACTTTTTAATAATCCGAGGAGGGAGGAAAGCCCTCGGTCCCCTCGGCTCCG
GGGGCGCCCGGCTGGGCCCAGCTCCGCGGGCGCGCCCCCGTCACCCCCTCCCAGGCCAGTTC
AGGGCAGCGCCAAGGGGCCGCGGGCTCAGGCTGAGGCCTCCTCCTGCCTAGGCGTCTGGCGT
CCGTTTGTCTGTCCGAGGCCTCAGCGCAGGTAAAGCGCGGGGCTGGGGGGCGCAGAGCTCCAT
TGGGCTGGGAACCAGCGCGTCTCGTGCCCTTGGGGGACCCGCCTGCCCGAGCCGCCTGCTAAG
CCTCCCGCTCCCCTCCACGCTGGAGCGCGCGGAGCCCGGTCTGCTCGGAACTCCACTCTGCGG
GAGCGGAGGCCGGCGCCAGCCCGGGAAGCCGCGCAGGGGGCGGGAGGCCGAGCGCGAGGC
CCTTGGCGCCGGGCCTCCGCGGCCAGGCCGGGCAGCTTACGATTCTTCATCAACTCGTAAAATG
AGATCGCCGCGGAGCTCGAGCCTATTTGGCGTCTCCGGAGACTAGCAAGACCCTCTGAAAGAC
ACCGCGTCCCCAGGGATAATCCCCCGTGTCCGGCAAAGCTGCCCCGCTCGAGCGCGAACCCTG
GGACCCAGCGCGGGAGGCAGACCAGCAGCTGGCCGCTGGGCTGTGAACGCCAGGACCGAGC
GGAAGCTTCCCGCCCGGCCGCGATCGGTGCCGCGGCTCTCAGGGAAGTGGCTACGCGCGTCC
CTCGGGAAAGCAGAGTAATTTGCCTGTTTCTGGCAAATCTGAAGATTGGAATGCCACACGGCCC
AGCCATTCCTTTTCCAGGCATATGAGAAGCCCTCACACATGAGCAAGAAAGAGATGTGGCCAA
AGACATTCATGGAGCCCTGTTTGTTAAAACAGAGAAAAAGAAATTAAGAAATACCCAGCCTGG
GCAACATAGGGAGACCCTGTCTCTACTAAAAATAAAAATTTATACTTGGGAGGCTAAGGCAGGA
GGATCACTTGAGCTCAGAAGATCGAAGCTTGCAATCAGCTGTGATCGTGTCACTGCATGCCAGC
CTGGGTGACAGAGAGTGAGACCCTGTATCAATAAAGAAAGAAAGAAAGAAAAGAAACAGAAA
TAAACTTAGGACACAGCAGACGGCCCATCCTGAGGGTACCACATAGCAGATGCTGGGACCTTTT
TTCAGGTGGACGCCACATGACCTTTCCTTTGCAGGCATCCATGCTGATGTTCACAAGGCACACA
GCATTTCAGCTGAAGAAAGAACTGGCAGGATTTTCATCACGAAAGGGCACTGTCTATCGTTTAT
GAAAATGAAGCATATGAAGTAAAAGTTTGGAAACAGATGGAATTAAATCTTCTGACAGATTTTA
AAATAAGATCTAAAAAAGAGCAAGACATGCTCTTGGATGGGGAAACTTAATATCAGAAAAATGT
CAATTTTCCCATTTTTTCCATATAAATTTAATGTAATTCAAATTCCATTACCAACAGGGTGATTTTT
TAAAAAACTAGATAAAATGATCTGAAAGATTATGCAGCAGAATAAATGCCCAAGAATTACCAGG
GAAGACTGGAAAAAAGACTGCTGTGGGGGTTTTGCCTTCCTGGAAATAAGACCATACTGCAAA
GTCACTGTAATCAGATCAATATGGCATTGGCAGAGGACTAGACAATGGAAAGGAATGGAGAAT
ACAAAAATAAGTTTCAGGATATATGAGAATGAGATATATGCCAAAGGTAGTATTTTGATTCGGTG
GGAGAAGGGCAGACTGTGAAGTAAGTGGTACCAGCCACAACTGAATATCCATCTGGGATAAAA

TAAAATTGCACTCGTCTTAGAGATCCAAATCAACTTCAGATGGATTAAAACTTTGAATGTAAAAA
ACATAAATGACTAACAGTCCTGCAAAATATCTTGGAGACAACCTGTGCCATCTGGAGAGTGGGA
AGAGCACATGCAAAGGCCAAGGGGTGGAGCAGCCCAGCATGTTCTGGAAAAGGTAGGGCTCC
CCAAGGCTGGGATGATGGTGGAGACCTGGGTGTGTGGGAGCACAGGGGTGGGGGCCCGTGGG
CCAGGAATGCACAGAGAGGGGCTGGTGCTCTGCCGCAGGCCCAAGCCCCCAAAGCCCGGTCA
TTCCCAGCACCATCTTCACGGGTTTCTGCCCAGGTCTTTCTGCTGCATCTCTTCCTCCCCCGATTC
CTTAATCATTTTTTTTAAAATCAGTTCATGTCTTTGTAAACCAAATTATTTCTAAAAGGCAAATTTA
TATTACTGCCGAAATCAAGGGTCAGTGAGCTAGTTGTGTATGTTCTGAATCACATGGAAATAAAT
GCATCGAACATTAA

ID|813|gene_id|102577426|transcript_id|NR_108031 Homo sapiens lncRNA

GTGCCACGGACGCACGGGCAAGATGGCTGCGGCCGTGGGAGGAGCTTGGGTCCAGCAGC
CACAGAACTCGGCCGAGGGTTCAGCGGACTGCGGCTGCGCGGCGCCGGCGACAGCGTCAACT
GCTTTTGTGTCAAAAGGAAACCAACAGCCGGCTCCATAGCTCAGGGGGGGAGACGATGGCACT
ACCGCGCCCCTTCCCGCCATGCGTTTGTTTGCGGCGTTCCCCGACGCGCGCGCTGAGGCTCTGG
TCTGCGGCTCTGCGCTTGGCGGCTGCGCGCCCTCGGCCTTCGGGTCCCCGCCGCCTCCGTGCTG
CAAGGTCTGATTTCTTCCTGTGGAGTTAACACGGAAAAGCGCAGACGGCCACATTCATGACCCG
GGAGTAACGGCTCTACCTGTCACATTCGCTTTCAGCCTAAAACAAAATTTTAGTTTATTTTAGAC
AAAATATATAACTAAGGTATGCTGGC

ID|2596|gene_id|114915|transcript_id|NR_015370 Homo sapiens lncRNA

TGTCGGGTCCCAAAATACGTGGTCTAAAGTCCTTTGGGCTCCTGCCGCTGCCAGCACTGAT
CATATGCTCTTGACCCTGGTCGGGTGGGGGGATGTCTGGATACCTCCTCTGTGGAAAGAACTGG
CCACACATCCGCAGAGGTGACTCGGGCTGAGAACCCAGGCTCCTCCTCCCTTTGGTGACACGC
CCCTCGGTCCCTCACTGGCACTTCTCCCTCCGGCCACACGGCGGCGTCTCGCCATAGCGCAGC
GGCCGATGGTACAGCCCGCTCCCCCCTCGCGCTCTCGGACAGTGGGTCCTTCCACTTGTAGAAA
AGCATTGTGGGACGGAAGCCTGTCCTTTCTTCCTTTTGGTGCGAGCTTGCTGTGGTTTTTGCTCTG
GGTCCTCTGGGATGGCGCCTGGCTGTGGCCGCGTGGTCTCTCACGCAGGGGCGCCGGGCGGG
GGAACGCGGCCACCCTGAGTCTGGTGAGTCGACTGCGGCGGCCTGTGTCCGAAGTGTCCGGGG
CCGTGAACAAGGGCAGCGGCCTGGCCTCAGGCCTGCGTTCCCACGTTTGGAAACGGGGAGCTT
CGTCGATTTGTGTTTACATCATCGACTATGCCAGGGAGTTCTCCAGATAAGCCTGGTTTTATTTTC
GTCAGTGAAAAGGCCTTACCGTATAACTGACTTTATGCTTGCCCTGCCCCCGTATAAAATAACTT
AAAAGCAGCGTGCCTGGTTACAGCTGTTTCCACGTGCGGTGCTCGTCGGGAGTGATCACCTACC
CTACAGGTGGAAGATGGATGCCTGAAGTGTAGACTGCTGCTAGCTGAATACCATCTGGGAGCA
TAAAGGTGACCTGAAGGATGTCCTTGGTGAGGATTTTGAAAATTTGATCTTCACAAGAGTTGCCT
GGATCATTTGAAATTTCTGGGAGTCTGAGGAGTACTGACATAATTACCTGCTGGAGTCTGTAAAT
ACACATTTAAGACAGTGAGGATGTGAATAAATATATTAATGCACTTTGGCATTTGTGTTTTAAGT
GATTAACTGCCAGAAACAGCTATTTCTAAAAAGTTATAAGGGAGGAGGGGTTCTTTTTTGATCAG
TATTCACTGCTGTCACCATAATTAATAGCCTTAAAATAGCTTGTGTTTGGCCCAAAGAGAAATGT
ACTTTCTTCCAGTGACTCAAAAATCGTGGCTAGAACTAGACT

ID|10595|gene_id|283104|transcript_id|NR_036485 Homo sapiens lncRNA

CAGGTTCCAGCCCCGACCCGGGCGCGCGGGGCCGACTAGGGTCGGGTCCAGTGTGCGGT
GGTCGCTCCGCTCCGGGCCGCTCCGCTCTGGGCGTCAGGGCGCGGGGAGCTGCCCCGGGGTT
CTGTCCACCGGGGAGGAAAGCCACGAGCACTGAGCGCCTCCTGAGAGCCAGCCCTGACGTGA
ACTCATTTTATCTGCCACGACCCAGAAGGAGTCTACTGCTAAGATTTCAGCATGTCCTGTGGCTG
AGTTAATCAGAGTTATGACAGGAAGGTACCGGGCACACCATCGCAATGCTCCATCAATGCTAGT

ATGTTGTGTTCTTTCCTTCATATCAAGTCAACTCAAGCTTGCTCTACTTACCTGGTGTACACAGTC
TAAGAACTGTAAGAAGACTGGAGCAAAACCACTCCCCTGACAGTTGAGGGTCAAGCTGCTCCT
CTGACTGAATTTGTGACCAAAAGAGAGCCACTCTTTTTCAACCAACATCTGGAAGCCTTCAAGT
GTCCTATAAAAGGGATCACTGAGTAACTGAACCAGGGATGTCACCTAGGGCATAAGCAGGATG
GATTGTCATTAATTTTAGTTCTGAAAAAGGCCTATTACTAAGATAAAAGCACTTCCTTCTGATGAT
AGCTAATTCACAAATTTACCTGGACAGCAAATTTGTTCACTAACCATTCCAGGATGGCCAATAAA
ATTAATTTTGTAAACTTGCCAGTAAAAACTAAAGCTCAAATTCATTTTGGCTACAAGTTTACTTTC
AGGGAATTGAGACTTTAATCCTTAACTGGCCAGTTTGCTGACAGCAACCTTTTCCTAAGTTAATC
AGAGGCCAGAGGATCAGTTAAAATCAATTCTACTTTTTGGCAGATGCAGATTAATGTCTTAATGT
AGTCTGAACTCACACTTTTCACTCTGGAGTGAGTTAAGGCGGGGCTTATAGAGAGCCAGGGGAT
GGACTGACAAAACCCAGGAAGGATGGTCCACGCCTCACACTGCTGGTCAAGGGCTCCATCCGC
AAGCCTGCATGGTACATCTGTGGATGGGAGAGCTTGATGCCCGGAGCCAGAGCTCCAGGCTGC
GTAGGGGCTAAGAGCTCATGGTACGGAGTTTTCTGATGGATACCAGGTGCTGTTTAACACAGGA
CTTTCTCTGTCCATAAACACATTAGTCATTATACAAAAGAGTCATCTGAAGAATTTAAAAATGAC
CAAATGGAAATATAAATATAAAATATCAAAAAATATGTCTAATAGTTTACTTGTGCAGTGATGTC
CCAGCCTTCCTCCAAACAGACCAAAACTGAGGAACCATTCTCAAGTACTTCTGATACAACCACA
GCCAGCTGCATTATCCTGTGAAGCTAAGAGACAGGAAGGAGAACACAATTAGACCAAGCGCTT
TCTGAGTGCAGAAGTCAGAGAGAGTTGCTTTCAACCCTGGATAATCAAACGACTTAGGGATAAA
GCGCTTGCACAAAAGAACCACATAATGTTTTAAATACCTAAAGTTCTTTCAAATCAAGATTAAAC
AATGACACTTTTGACTGGATGCAATGACTCATGCCCATAATCCCAGCACTTTGGGAGGCCGAGC
CGGGCAGGTCATTTGAGGTTCGGAGTTCGAGACCAGCCTGGGCAACATAAGCAAGACCCTGTC
TCGAAAAATAAAAATAAAAAAGATACTCCTATTTGTCTATACTGTGTACTAAATGAGAAAAAAAC
TTTCAATATATTTGTGCTAAGTTTTTAAAGCCACATCAATTGGCTCAAAGTCCTTAATAAACAATT
AAGGTATGAGAATAGTATATAGATTTTAAAACCAGATGTATAACATTGTAATTTTAAAAAATATTT
TAATGATCATCAGACATAATTTGCAATTTGAATCCAACTCTGAACAAGAACAAAAATGAAAAAA
CCCATCTCTTAAACAGAGCTAGGCTACTTTAAACACAGGAATAAGGCAAATGCCCTACTCAGTA
ATGAGAAGCTCCTCATGTAACTGTTTCTACAACATAGCTATCACCAGGCCTGAATTCTTTTGGGC
TCAGAATTTGTTCTAAGAAATGGCTATTATTAAAAAGTCAAAAAATAACAGATGCTGGCGAGCC
TGTGGAGAAAAGGGAATGTATATACATTTCTGGTGGGAATGTAAATTAGTTCAGCCATTGTAGA
AAGTAGTTTGGTGATTTCTCAAATAACTTAAAACAGAATTACCATTTGACCTAGCAATCCCATTA
CTGAGTATATACCCAAAGGAATATAAATTGTTCTACCATAAAGTCACATGCACACACATGTTCAG
TGCTGTACTATTCACAATAGCAAAGACATGGAGTCAACCTATATGCCCATCAACGGTAGACTGG
ATAAAGAAAATATGGTACATATACACCATGGAATACTACACAGCCATAAAAAGAATGAGCTTAT
GTCCTTTGCAGTAACACAGCTGAAGCAAACTAACACAGGAATGGAAAACCAAATACTGCATATT
CTCACTTATAAGTGGGAGCTAAACATTGAGTACACATGGACACAAAGAAAGGAATAGACGCCT
GGGGCGTATTTGAGGTTGGAGGTGAGAGGAGGGTGAGGATAGAAAAACTACCTATCAGGTACT
ATGCTTATTACCTGGGTGATGAAATAATCTGTACACCAAACTCCCAAGATATGCAATGTACCTAT
ACAACAAACCTACAT

ID|10646|gene_id|284029|transcript_id|NR_026951 Homo sapiens lncRNA

ATGGGTTGGGCATTAGGAACGGCCTGGCCCCACAAATCACACAACTGGGGTGTAGAACAA
AATGGTTCCACAGATGACACCAGGCTGGTCCAGGGCCGATGCAGGCCAGAACCCCGCTGGGA
GGACTCCCCAGAACAGGCACTGAGCTGAGGAAAGGCAGGACACGTGGTCTGCTACCGACCCC
ACCAGATCTTGCTGGGACTCTGGGCAGCTCCGGAGTAAGAGCAGCATGCTCTGCAACGAAGAG
CTAGGTCCAAGGACCGCAGGCGGAGGCAGGAAGTGTCAAGATCCCAGGTTACGGAATGGCAC

CAAGTCGGTAACCAAAGGAAGCAACAACCCGGGTGTTCAAGGCAGTCGTGCAAGGCGGCGCG
GGGGCGAGGCCGAGGCCACTCCCTCCCACTTCCTTGACGCTATTTCCTGGGAGGAGCCGGAGC
TCGCGGCCCGGAGCCAAGAGATTCCCAGCCCAGTGCTCCAAAATACTCGGCCTGGGGGAGGCT
GGTCCGTGAGCGTGCGCCCGGACCGTTCCGCCATGCATAGTGGACCCAGCACTACAGCAAGCC
TGGCACGACCTCTAAGGCGGTTCGCGGCAACGTCCGCACGTCGGCTCGTTGGTCTAGGGGTAT
GATTCTCGCTTCGGGTGCGAGAGGTCCCGGGTTCAAATCCCGGACGAGCCCTCCTTTACCTTTT
ACTGAGACAAGAGTGTCTTCAAGGAATAGGTTACCTGACCAGCGCTGCAGGAGGCTTGACTGT
AAGTCATCAATGTACAGTAAGCTCTTTTGTGGCACCGCGGTAGCAGAAAGCATCTCAATCCCCA
GACCCGCCTGGCATGTCAGGAAGAGACGAGGCACGCCCAGACTTGGGCAGGTCCCTCCCTTTC
TGCTTCCTGACTTCCTTCCTGGTGTCTCAACTCGGCCACCACAGCCTGGTTTCGCTTTGATTGAC
ACGCGTCAATCTATAGTTGTGGATGACAGTGTTCGGGAGAGCCCAGGAACTGTCAAAACTTGGT
TCTCTGCAGTCCTGAAGGCACTCTGTCTCTTTCGGTCACTCCATCTGACGATTGGTGCCTGAAAT
ACACCTACGGTTTCTGGTCAGCGTTTATCAGTGGTTGGAAGAACAGAACCCCTAGGAACCCTGC
TGTCAGCACGCAGAGTGTAAAGTCTCAGATCAAGGATTCGGAGAAAGGTCTCTACAGGTCCCTC
CTCGTTAGTATAGTGGTTGGTTGGGATGAGTCGGGTTGGACCCGTGCGGATCCAGGAAAACAG
CGATCGAGGTAAAAGTAATGGCTGCCGTCGTCTGCCCAGCGCGCAATTGTGAGTGACCTTCACC
GCTAGCGCGCGCCACCCACCCCACATGAACCTGTAGGGTTATGCCCAGTCTACAGACGAGGAA
ACCAAGATCCAGGGAAGCTGAGTCACCGGCAGAGCTGGGATTACGTCTAAGATTTGTCCAGGG
GGCGAGAACTGCAGCGACACCATGATTTATCATCTCCAGAAAACAAAAAAAAAGGCCTAGACA
CATAAAAATGGCTTCTCTGTAGAGTGAGCTCAACGAGCTCAATGTGATTCCAGTTAAGGAATTG
GGCCTTAAGCCAAATGCGCCTAAACGCAACTTGCCTCTCTATTATTTATCCAGAGGATGAATTTA
ATTCCTACGTACATGCGCCCACATCTCGTCAGCTGGTGCCTCAGAGAAATGCGCTGACAAATCT
TAAAGGTGGGGCTTCATGACAAAAGTCCGCCCGTTATTGCTGCTCAACAGACCCATCAAGTCAC
GCTGTTGGACAGGCCTCGTGGCGCAACGGTAGCGCGTCTGACTCCAGATCAGAAGGTTGCGTG
TTCAAATCACGTCGGGGTCAAACAAAACATTGTTTTCCCTAATCAAATAGACCTACGGTGACATT
TTTCAGGATAGGAAAAAAAAAAAAGCCTCCTCAGTAGAAGGATAGGAGGGGAATGAGACAAATA
TGAACGTTATTCAGAAGTTGGGTGCAGACTTTCAACATTATGTGATATATCCCTGTGGCAAAACT
GCGCATGTATCCCTAAGGTCTATAAAAGTAAAAATAAATTTAAAAATAAAAAAATTCCCTGTTGT
AAAAAAAAAAAAAAAAAAAAAA
ID|14396|gene_id|400960|transcript_id|NR_033872 Homo sapiens lncRNA
CGGGGGGGAGGGGCGGCGAGGCGAGGCCTGCGGAGCGGAGGAAGACAAACATAAAGA
TGAAGTGCTTAATTCAAGGTCACACAATGTCGGGGATGGACCTCCAGATTTCCTTACTGACCTG
CATTATCTTGTGGATACCTGAGTGAATTCCACACCACCCATTTTATAGATGAAACCACTGAGACA
TGGAGCAATTGACTGAGTAGTCACTGCCCCACCCCTGCCTGGAGCCTCACACATGACTCGCCTC
CTCTCAGTGACCACAGATGGAGTCTTGCTGTGTCACCAGGATGGAGTGCAGTGGCGCAATCTCG
GCTCACTGCAACCTCTGCTTCCCGGGTTCAAGTGATTCTCCTGCCTCAGCAACCCGAGTAGCTG
GCACTACAGGAAAACAAGCTCAGGGCTCCCACTGATTGTACATTATGGTGAGACGTGGTCTTGT
AACACAGTGGTTAAGAATGTGACTTTGGAGCCAGCCTGCTAGGGGCTGAATTCTGACTCAGCCA
TTTACTAACTGTGTGACCTTAGGGAGGAGGACCTGGTTGATAGAAGAATTTATCTCGTAGTCTTA
ACATCCACTGTAATTCCAAACTTTGAGGACGATAGAGATCAGTGGGCTTTGACTGCCCAGCATG
CAGTCTCCATTCTTAGGTCACAGAATTTTCCACACTCCTTGACAGAGGTGCTTAACCCTTATCCAT
CAGCATTCTCAGACAGCACCTCCGGTTAGGGACAGTATCTGACCGTCCGACATCCCTGCAGCCC
CCATGTGTCACACAGACTCAGCCTCAGGTAGATGGTTTCCGTAGATGGTGGCTTTCTTTGGACCC
TTGACATCAACCAGTTACTATTTGACTCTTCTGCAGAACCCCATGTTGACCCCTGCTTTGAGGTG

GCAGAGTTAACACTACTCTTCGTTATAAGCTCTCGTCTGGGGCAACTCAGAAGAACCCAGAGAC
AGACTCCCAGACTCGGCCAGAGGCTGATGCCAACTCCTTCCAGCTCTCACGGTAACCATGAGG
CCCCTGCGTGTTTCTGTTGTGGAGGTCCCAGAGGGAACTGTATGGACAGACACAGCCCGAAGG
GCCATCTGCCTCAAGCCACAGCAGCACAGAATTCTAAAGATTGTGGGGAAGGATGGGGCCAAG
GGGACCAGATGTGCAGAGGTAATGAATACCAGGGAATCAGGAAACCACTGGATCAGTTCTTTG
AGCCACAGAACAACACTCATCCCAGCAGCTGGGAAGCTATCTGCAAAAGTCCATCTGCAGAAA
GGGCTTCCTGGTCTTGCGTGTGGCTGGGCCATTTCTCTGGATGCAGGTTTTTCATTATACAGTCTT
CTCAAAGAACCACCTGCTCTCAACACCTTCTCGGTGACTCCATTTCCCCTCCTCTGGGCTCCTGG
AATGCCACAATGGCCCCACCTTAGCCAGGCAGCTGTGATGAGAGGTGTTGACCCAACTGCCTCT
CTGCTGGCTCACTGAATCAGGTGGAAGGGGCTGGGGACAGCACAGTCTTGCATTGTGGTTGGG
GACCTGGGCCATCCAGAGGCAGGACTGTCCAAAGGCAGGGTACTGCTGGGATTCCGCCAGGC
ACTGGGTGATGCATGGCATAGGGAGCTGAAGAGGGACAGGCTCTTGGCCAGGAAAAACAGCC
CCAGAGCCACAGGTGTGCCATCGAGTCTTCCTCCTCTCCCATCCTTAATCACTCCTGTTTGGTGA
CAGCACACAGCTAGGCCTGGAGCCCAGCAAAGAGCCCCTGGGTCCCAGAGGAAGTTGTTCTGG
GGGCTGGAGACAATCACAAATGCCAGGTTTGTGAGCCCCAGACTCTGGTTCATAGCAAGAGGC
AGACTTGTGAACAAACCACCAGATCACACCGTAAAACACGCTCAACGAGGAGTGGGTGCCCTT
TCTAGGGACAGGCAGAAGGCTCCATGGAGTGACCTTTGAATGGGGACATAAAAGTGGATCCCT
TCAACAGCCCCTGCACAGCCCCAAGTCAGGCCTCCAGCCCTTGGACTCACAGATGTCCTCTGTA
GACGACTGGATGTCCTCCCGTGCTACCCTTTACCCCTGCAGCGAGGGGCACAGAGCCAGAGCC
CTCCAGGTCCTCACCACTGGCATCACCAACATAGTGGACATTTTCCCTCTCAGAATGAAAAGGG
AGTGAAATTTCCCGTGATCTTTCCACAAATGACCAGCTTGGGGAGTGGGAGAGGAAGTGCTGC
AGTGCCTGGGGAAGGGGCCCTGGAACAGTTCCTGGAAATGGAGAGGGGTGGATAGGGTGGAG
ACAATGCTCATGGAGTGCAGTATGGGGCCTACCCAGAGTTGCTTGGCCACCCCTCTGCCTCTGA
ATCAGGGGTGATCAATGAAGGAAAATTACTGCAAAAAAAAAAAA
ID|14420|gene_id|401242|transcript_id|NR_033379 Homo sapiens lncRNA
TTTTCTCCCGCCTTTTCTCCTAGATGTGCCTTGTTTGCAAAATCATGTCTATCATGTCCACGA
CTCGCCTCAATTTTTTTTCTTTTGTTTTTGTGGCACTTTCATTCTCTCTCAATTCTTCAAATTCACAT
GGAAACCAAGGAAAACAAGCTCCGTTTTGCTTTCAATATTGATGCATTTCTGCTCTAAGGCTTTG
ACACCACCCTGGGTGGCTGTGTCAAGAGATGAAAGACAAAATGAAAGAGGCCGCGGAAGCAC
AGACTGGCAGAATTCCAAAACCCGAAAAACAAGCAAGGCACCAAATTAACAAGCCCAATCACA
TTGGTTACATTTCCCAGTATTATCGAAGATCAAGCAAGAAAGAAAAAGACAGAAAACAAATGCG
AAACCACAGCTTCCTTGGCTCTTTTTGCCCATATGCCGAAGAGCCGAGCATTGCCGCCAAGCAT
TGGCACCGGCCATGCGGAGACTAGCGAAAGCTTTGCAATTCCTCTACTTCTCAGTGATGACTCG
GGCTTTCTCCTTACATTCTTGATGGGCATGACCGGTCCTGTCAGCTGGGTGGCCAATTTCTGTTC
TTCAGCAGAACTGTCTCCCTTCTTGGGGGCCGAGGGCTTTCTGGGGAAGAGGATGAGTTTGGAG
CAATACTCATTCAGACGCTGCACATTGGCCTGCAGGGAATCGGTGGACTTGTTCTGCCTCCTCG
GATCCACAGAGATGCCAGTGGTCCGGGTCACCTTCTTGTGAATGCCCGCCACCCTGAGCTCCAG
GCTGAAGCCTCTGCCGGCGCGCACCTTGATGTGATAACGCACAGCAGGGCACAGCACAATGGG
CCAGATGGGTCCCGACTCTCCGGGCGCGGGGCGATGCAGCGCCCTTTGGCTTGCCGGGCCTTG
ATTCTGCGGATCTTCTGGTTGAACCACGTGGCCACACGCTGCTGCCAGTCCTTGTGGAAGTGGG
GCTTCAACATCATGCCATTCCTGCTGGGCGCCATGGCTGCCTACGGCCCTCCTACGAAGGAAAC
ACTTTATAGATTTCTATAGCAGTGTTACATAGTAAGGTTTTATGGCGAGTTTTGTAGCATGGATCA
TACTACCTTAGGACTGATGGTAAAGTTTTAGTCTAAGGAAAGCTCTGAAAATTTGAAAATGTGTA
ACCACCCAATGAGTTCAGCTTGCCCACTGCCTAGACAGAACCATTTATCAAGACGGGCATGCAA

AGCCGGCTGTGTGGGAGACCCGTTTTATTATTATTCAAATCAGTCTCCCCAAGTGGATTGCAGTT
TTTAAGGATAATTTGGTGGGTAGGGGACCAGTGAGTTGGGAAAGTTGATTAGTCGGGTCGGAG
ATGAAATCAGAGGGAGTCGAAGCTGTCCTCTTGTGTTGGCTTAATTTCTGGATGGGGGCCACAA
GACCAGATGAGCCAGTTTATCGTTATGGGTGGTGCTAGCTGATCCATAGAGTACAGGGGCTGCA
AAATATCTCAAGAACTGATCTTCGGTTTTACAATAGTGATGTTATCCTGAGGAGCAATTGGGGGA
GGTTTAGAATCTTGCAGCCTCCAGCTGCATGACTCCTAAACCATAATTTATAATCTTGTGGCTAA
TTTGTTAGTCCTGCGAAAGCAGTCTAGTCCCCAGGCAGGAAGCGGGTTTGTTTTGGAAAGGGCT
GCTATTGTCTTTGTTTCAAAGCTAAACTATAAACTAAGTTCCTCCCAAAGTTAGGTCAGCCTACG
ACCAGGAATGAACAAGGACAGCTTGGAGGTTAGAAGCAAGATGGAATTGGTTAGGTTAGATCT
CTCACTGTCTCAGTTGTAATTTTGCGATGGTGTTTTCAAATGTTGATTTAAAGAAGACCATAAATT
TGTGTTGCTTTCTCAGATTTTGAGAATAAGGACAAAAAGTGTATGTTTTAATGGGAGAATCTTAA
GAATTTCACTACTGAAGTGGATTTCTCTGTCCATTAACAGTGCTAAATGTTTTGTATTTCCCAGTT
GGAAAAACAAAATCTGCAAGCTTTGAATGATACTTGTTATTAGGTTTTGGTAACAGCTGTTGTAC
TATTCCCATTTCAATGCTCAATAGCCACACGTGGCATGTCATACCTACCAGGTGGACAGCACAG
ATATAGAACATTTTCATCATTGTAAAAAGCCCTATTTGACAACACTAACTTAAAGTATTAATCTTT
TAGAAGAGAAAGCTTCAGTCCAACCCAAATTACACAATTTTACTTTTGAAAATATTGTTGAAACA
ACGTTCAATTTTTTAAAATTTTGGAAAACTGAATGAAATCCATTTGTAAATGCGCAAATGGTACT
GGAGGTGGAAAACTGAAGCCTTGCTATAGTTTTAGTTTTTTGGAGGGTTATGATCAACACAGAG
AAGAAAAGATTCATCTACCCATAGAGCTTCTTGAGAAAAATTTTCTAACAATAATTTTGAAAATTT
GTAGTGAATTTGTTCTTGCCATTATTTATGGGTGCTTTCATGTGCGAGGCACATCAAAGTTCTGG
GGAATGACTTAGGCACAACCAAATGGCTATCTTGGGAAGACCAAAGTCCTTTGCTATATATGAG
AGTTTTGAGATGGCGACTACCTTTCTGTATGGTAGATTCTTGTTTTTGAATATGCCCTCTGGGTTT
TCAAAACAAATTGTTTTTACAATACCTATAGTAGTTATCTAGGGCATATTCTTTAGCTTCAAAAAT
TCTATCTTTAGAATGGGCTTCAATATTCATAACTGAGTTATTTTCCTCTAATAAATCATTAACATTT
TCCCATGT
ID|21584|gene_id|644714|transcript_id|NR_033947 Homo sapiens lncRNA
AAATATCTGAAATTTTGATGCCGCTTTGCTCACAATGTCATCCTTTATCCTTTAAACCTGGAG
TTGTCTGAGGTGGTTTGCAAACTGCAAAGCGCCCTGTTCCCAATGATGCACCTGAGAAGTCTGT
AAATGGGGTAAATTTAGGCAAACACACCTGGAAAAGTGGCATTTTGGAGTGGGGAATGCTGTG
GCCGGAGGTTAGCAACCGGATGATGCAAAAGCGTATTTAAGGCAGCAGTTCTGGCCTTTCCCC
GCCACGGAGCAGAAGCCCCCATAGTGATGTGAGAAGCGGCGCTGTCCCAGTTTACTAGGATGG
GATGGGCTTTGGATCTAACCAGCAGCGAAGAAAACCTGCGGTGCGGCTGCGCTCGGTAGTAGG
GGTCCACAAAGCGTCAGAATCTGCAGCAGCCCCGGCCTTTGCGAGCTCCAGCTCCGAAGTCAA
CCCGGCCCCCAAATTCCACTGGGAAACCTTAAGACACCGGTGTTTTTACCGTAAGAAGACCTCA
GAAGCCCATGGAAACAAGGCGATGACGGCGCTTGAGGGTGACCAGACCTCCTCAATCATTCCA
TCAGCCACCCAGGACTCAACAGCAGAACCTCAGAGGACTCAGCTGAAAGAGGTTTTCACTCGA
GCACTAACCACAGAGAAATGGGAAGTGCCATCCTGCTGTCATACGCAGCCGATGGCTGGGTAG
GACTAAATGCCCTTCTGTGGTGAGATGAACCAAGACGGCAGATGGGGGAATAACATCAGTGAG
AGAGAGATTACGAATGGATCCTTGTATTGCAGATCAAACTGGGTTGTCTCTTCTCAAACCACCCA
GGCAGTTTCTGAATGTCCCAAGATAGGGGTAGGAGAAAAGAAAAAAAAGACAAAACAGGAGA
CGGTGGGTAAGAAAACCTAAAAGGCACAATGAGGCAAAGGCCTAAGATGGAGGCTGGCTTTG
GGATCTGAAATCCGGCCACAATCCTCCTTGTTTTACCTCAAGTCCAAGAATTGTCAAAAAGAAA
ACCCAGAAGGGCTATTCATTCTAAATTTTAAAATCTTTTCTTCCTGGCCTACCAAGTCCTTGTGTT
TTCTTTCTAGGCGCCCATAAATACAACCCCATTACAGTTCTATTTGGCTAATAATTTACAGAACAT

TTAAAATACCGGGTAGGTGAACATCTCTTCATGGCTTTTGTGTAAGGGCCTGAAGCGAAGAGAA
AAACAACTGATTGGAGATTCCTATCTGGCGCCTTCTCCAGGGAAACTGACAACCAGAGAAATCT
GTTGCTAGGATTTCTAGGACATCTGGAAATTCCCTTTTTGTTCTTTCTCAACAATATAAAATGGTA
TATTCGCAAACCTATCTGAATCACCTCAATCTAGGCCACTTTGAAAATTTCATTTAAAAATGTAAT
CACAGATTTTTAAACTAATTACCCAATTTGCAAACAAATCCCAGCTACTTCTGGTCTTGAACATG
GAGCAAAGGTGTGTCAGTAAGGCCAACATTCTTAGGTTGACAGAGACAGGTTAACACATTGGCT
GGCTCAGCCTGTGAAAAAGAACCAATGATGATGCACACCCTGGCTGAAGTGACATCTGGCCAA
AGGATTCCAACAGAATGAATTCCATCAACATGGAAAAGATAATCCATGGCACCAATTCTGTGCT
GCTTACAGGCAATGGTTACCAAATGAGTCAAAACATGGCCTAGAAGTGGAGCCGGGCCCAATG
AAAAGCCTGAGTTCACACGTGCTAGGGGCTCCACTGACCGGCCTCTGCAGCCCAGCACCCAGT
CCTCTTGCTCTGGGTGCAAGGTGAGGAGGCAGAGTGCACACACCGGCTCCTGTGTCCCCGGCC
TGGCTGGCAATGGTAAAATGGGCTGCCTTTCTTTTGCAGCTTCTTCTAATCGAAATACCAAAGGG
TACTGACCTCTGGCCTCAGGGATGTGACTTGCAGTGCAAATTTTACCTCTTCAGTGCAGAGAGTG
GTCTTGAAATCTTGCCAGTGTGTGGCCCTCCCTCAGCTCCTGCCACCCTGGGGAGCCAGGATGC
TGAAATGCATAAGGCAAAAGCACTGAATCGGATCTATTCCCCACTGACCTTTAGTTACGATCACT
CTGCCCTGACCAGCTGGAGTTGGGCCTGTGTGAAGCCAGTGATTTCCAACACACTGAAGAAGG
TATCTGAGTCCAGTGTGAAAGCCAAATTCCTCCTGAATCTGTGATTTGGTGAAGCTCTGTGAGGT
ATTTTTTGTGTGTGGAAGACTGAAAGTGAAGGGAAACAACTAAAAAAAATTAAACAGACAAAAGC
AAAAAAAAAAAAAAAAA
ID|21930|gene_id|654434|transcript_id|NR_027058 Homo sapiens lncRNA
AAGTTGCTGACGGAGCTACTTCCGCCCAGGGGAGTGGGAAGTAAGTGGAGACACGTGCTT
TGGCCTGTTGGAGGGGAAACCCGCTCTCGCCTCCTGGTGGTCGCCGACTCGCAGTCCGCAGGA
TGACTCAGGGCAGCCCTGACCACAGTTCCGCCGCCATCGGCCCTAGCCTGCGGAATTGGGCTC
GCCCCGGGACGATAACAGAGCTCTGCCGGGGGCTGGAGGCACTGACCGGGTGACCAGAGACC
CAGAGACCAGACCCCCTCCACGGCGCCCGGGATTTCGGGGACGGCTTCTCCCATCGCAAGTTT
CAACAGAAAATGAGAAATATCCCCCGACGATTGGCTGAAAACATGCAGCAACCACTATTTTCTT
CCTGCCCCTCGTTGATGAGAGCATTCGAAGTGACCTCAGCAGGGCATCCAGGTCAGTTTCTGGA
AGACTTGTGTGTGTGATGAATGAATATCTGGTTTTGTCTCTGCTGGGCCTGTGTGCCTGGAAAGG
AATTGTTTTGGCCTAGGATCATCCAGGTTTGTTTGGTTTAGTTCTTGACCACATATTTTTTGAATGG
TGACTGCTTAAGACCCTGTTGTGTATGGCTATAAATAGATACACGCCAAGGTGACCACATACTCT
GTGGTTCCTGTGCCCGCCGGCCATGTGTCTGAGTGTGTAGCCTCTCATCATCTAAAGGAACTTTG
GCTGCAGAGGGGAGGCCTGGTCCCATGGGAAGTTTTGGGAGCGCAGCAGCAGGTGGGTTCAA
ACGACCCAGCAAGTGCCTCCTTAGCACTTAGAGGCGGAGGCCACCACGTTGTCCACGTGGGGT
TTCTGATCCCAGCTCCCCACCAGCCTGCTGGACCTCGGGCAGGTCCCTCCCTGTTTGTACCTCCA
TTACTTCTTCAGTAAGATGGGGACACTGAAGATGACCATGCCTCCCACCAGATTGGTGCTTTTTT
TGTTTTAATAAGGGACAGAGTCTCACTATGTTGCCTATAGGCTGGTCTCAAGCTCTTGGCCTCAG
TTTTCCTGCCTCAGCCTTCAAATATGCTGGGGTGACAGGCATGAGCCACTGCACTGGCCCAGAT
TGGTACATTTGAGGGTTAAATGAGCAAATCCTATAAAGCACTCAGGAGAGGGCTGTGGGGTTTG
GGCTGGGGCCTGGGAGCACTTTAGACGTGGTAGCTATGTTGTTGTCACCTTTCTCCTCTTTCAAA
GCATTTTTTGTTTGTTTGTTTTTGTTTTTGAGACAGAGTTTCGCTCTTGTGGCCCAGGCTGGAGTG
CGGTGGTGTGACCTCGGCTCACTGCATCCTCCGCCTCCCGGGTTCAAGTGATGGGATTACAGGT
GCCTGCCACAACGCCCAGCTAATTGTTTGTATTTTTAGTAGAGACGAGGTTTCACCATGTTGGCC
AGGCTGGTCTCGAACTCCTGACCTCAGGTGATCCACCTGCCTTGGCCTCCCAAAGTGCTGGGAT
TACAGGTATGAGCTACCGCGCCCGGCACAAAGCATTTTCAGCTGTGAAATTCCCTGGGGTGCTG

CAGTAGAGGAAGGTTTGGGGTCTTGACGGGTGCTATTTGCCACGGAAAGATGCCTTCCTGCTCC
CAGGGCAGGAGAGCCGAGGTAAGACTTACTGTAGGCTGTCGTTTTTTTTGTTTGTTTTTTGTCTTT
GCGATGGAGTCTCACTCTGTCGCCAGGCTGGAGTGCAGTGGCATGATCTTGGCTCACTGCAGAA
CCTCCACCTCCCAGGTTCAAGCGATTCTCCTGCCTCAGCCTCCCAAGTAACTGGGATTACAGGC
ACATGCCCCCACAACCAGCTAATTTTTTATTTTTAGTAGAGACAGGGTTTCACATGTTGGCCAGG
CTGGTCTTGAACTCCTGACCTCAGGTGATCCGCCCGCCTCGGCCTCCCAAAGTGCTGGGATTAC
AGACATGAGCCACTGCGCCCAGCCAGGCTGTTGTTTTTTTACCTCCTTGTTTGCACAATTTGGGC
CACTCACAAGAGTGTATACCCTGTGATAAACAGTTACCTACATTCTCCTCTGCATGCTTGTCTTTA
GAGGAAGGAAATGTATTAATTGCCCAAAGTAATATATTGTGTTAAGATGTGATATATACTGGGG
AAAAAAAAAGTGTATATTGACATTTCTGGAATAAACCACTTTGATTCCCAAAAAAAAAAAAAAA
ID|28631|gene_id|92070|transcript_id|NR_033339 Homo sapiens lncRNA

CCGCCCACGTCAGCGCCTGGCGCGGGCCCGAACAAACCACGGCATGGAGACGGAATGAT
GCAGACTCCTATCCGCGAGATACCTGCTGTAATTTCGCTGTGGTTCGTGAGGACGCGTCCTGGG
CTGCCCTGAGAAGCCTACGTCTCCCCTTCGAGGCCCGGGAAGACCTCCAACCCCGCTGACAAT
GCTGGGCCCTCAGCCAGACCTGCCCTGCGTGCCACGTTCTGTTCTAAGATCGGGCTGCCGAGCT
GTGGCCTGGAGGAAACCTGGCAGGTGTCACCAGAGCTTTGGGGAGGGTGAGGCCCTGTCGGG
GAAGCCCAGTGGGAGTCATGAAGGAGGGAACACGTGTGGAGCCCTTGTAGGGGGAGGGGCAG
CCCTGCAGAGATCTAAGAAAAAAATTCCAGAAAAATGAGCAGCAACCTCCAAGGCCAGGCATCG
GTGCAGGGGACAAGGGGTCGTAGCTGGAGGGGCCTAGGTGAGGCTGCCGGGAAGGGACGAT
GTGGTTGGTGGAGTGCACAGGCAGGGACCTCACTGGACTTTCCTGTCTGCTCAGCATGGACAG
GCAGCCCAGGAGAAGGCAGCACGTGGCCGGGTGCAGGGACGTACCACCCCCACTTCCCCAGG
GGAGCTGGGGTCAGACGAGTCCCAGGCACTCCATCCTCTGCAGCAAGTCAGGTTGTGATTTACT
AGGGGGTGGTGAATATAATGGAGAGACTTCTGGGGAGGAATTCCTGGCTCCCGCGTGGACTTG
CAGAGCTCAACAGGCAGCCACGTGGCTGAGTGTCCAGCAAACATCACATAAGGCTTTGGGTCC
TGCAGGTGGGGCTGCCATGAGCAGCAAGCTCAGTCCAGAAGAACAGTTCCTCTCCAGGATCCA
CTTCCTGCGCACTTTTATGTGCAGTGTAGCTGGAGCAGAGCTCCCCGGAATTCCACAGGCAACT
GAGAACGGAGAGGGATGCAGGCCAGCCAGGGATCCAGCGTCTTCCCCATCGTCACTCTCCATG
GCCTCCGTCTACACACAGTGTTCGTCTGCACAGCTTGTCAGCGCGTTATCATGACTTCTATTTGG
CACCGGCCCGTCTGTCCACTGTCCTGGCTTGTTCCAAGCGCTGCCTTCTCCAACTGGGGTCCTG
GCTGCAGAGCTGTCTGCTCCCCACGTTGGGCAACTCCAGCCAAGATTCCTACACCCAAATGTGA
CCGTGTTGCTCACGAAGAAGGCTCAGCTTTGCGTGTGCCCAGCCGTGTGCACAGCTCGTCCCAA
CTCCTGCGGGTGGCACCTGCCTCTCCCACCTCCAGTCTTTCCCCTGTGATGAGCAGATGACCAC
CGCCCTCCAGGGTCAGCGTTTGGCTGTTTGTGTGCCTGCCCACCCGCCTCCCTGTGCCTGGGCC
TGCCCTGTGCCTGCACACAGGAGTGGCTCATGGCGTTTGCCCTACACGGATGGGCTGTCCTGGG
AGCTACTGGACAGTCACCTTGGTGGGAATGCCAGAGGCATGGGCATTAGGTCCCCCCGGCCAG
CCTCCGTTGCCACATGGGCTATTTTTGTCCATCGCGTGGGACAACCTAGTATTGGGGGAAAACT
CAGTCCACTCTAAAGAAGCATCGGCGTTTTGGATGGACGAATGCTGCTTCATGCGACTCCATGT
CAATGGACTATTTTATTCAACTCGGGTATTTATGAGTGTCTCCTCTGTGCCAGCCACGTGTGAAA
GAAGAACTCCAAGTTACTGGACACCAGGACAAACCAGGTAGATCACTGCATCCGGTTCCTCCTC
TTTCTGCCGACTCTCCCGGGCAACCGTTCTGATTCCTGGCATGGAGTTGGGGGCGAGCTCATCA
GTACCCAGAGCGCCTGGTGTGCAGCCAAAGGCCCCCATTTTATTAGTCGGTGTGTTCAGTATCA
CGCTCAAGACAAGGGCCAACCTATTACTTCACACACCTCAAATACAAAAAGTGCCTGGGAACG
TCGATGAGGTATTAAAGGAAACCACTGGTCTTGTCAGTCATGTACACACAGAAAAAAAAACCAG
TCATTTTTATTTTTATTTTTTGAGACAGAGTTTCGCTCTTGTTGCCCAGGCTGGAGTGCAATGGTG

CAATTTCAGCTCACTGCAATCTCTGCCACCCGGATTCAAGCAATTCTCCTGTCTCAGCCTCCCGA
GTAGCTGGAATTGCAGACACCCATCACCACGCCCAGCTAATTTTATGTATTTTTAGTAGAGATGG
GGTTTCACCATGTTGGTCAGGCTGGTCTTGAACTCCTGACCTCAGGTGATCCACCCACGTCAGG
CTCCCAAAGTGCTGAGATTACAGGCATGAACCACTGTGCCCAGCCGAAAACAACCAAGAATTTT
TTAACAATAAAATGACCAGCAAGTAAACTTGAAACAAAAATGGACAAATATCTAAAATATTTTTA
AATGTATGTATTTTAATTGGCAAATAATACTTGTATATATTGTTTACAACTTGTTTTAAAGTATGAA
TACATTGTGGAATGGCTAAATCAAGTTAATTAACATGCATTAACTCACATACTTTTTGTTTTTTGG
TGTGGTGAGGACACTTAACATCTACTCTCTTAGCAATTTTCCAGTCACCATGTCCTACTATAGATC
CCTAGAACATATTCCTCCAATGTAACTGAAATGTATCCCTTGACCAACATCACTGCCCGGCCCCA
GCCCCAGGTAAACACCATTCTACCCTCTGCTTCTGTGAGTTCAGCTTTTCTAGATTTCACCTGTGA
GTGAGATTGTGCAGCATTTGTCTTTCTGTGCCTGGGTTTTTTCACATAATGCCCTTCTGGTTCATC
TATATTGTCATATATGGTAGCATTGTCTTCCTTTGTAAGGCTGACTGGTATACCATTGCGTATATA
CACCACATTGTTTACCTAAAATATTTTTTAATACAATCGCTGAAAACTGCCAAAAAAAAAAAAAA
AA

ID|30347|gene_id|ENSG00000228549|transcript_id|ENST00000438002 Homo sapiens lncRNA

AAACTCGATTCACCATCGCCAGCCACGGGAGGACTGGGAGGACCTCCAGAGGAGGTTAG
GTTGACTTCATGGTAACTTTAGATCCTGAAACCTCCCAGGATATTTCTTGTCTTCCCTTTGATCGC
TCTTCCGCCTACCCAACAGGACAGGACTCGACGCCTTTCTTTCCTGGCAGCAAGAGGTCCGTTG
CGGACAAGACCAAAGTGAGCAGCTGGTTTCCCCTACGTGTCCTTCCGGGCCTGGGCGTCTCTGG
AGCTCAGGCTGACCCGAGACCTGACTCCCGGCCAGTGGGACCAGCAGGAGCCTGGAAGAGCG
CGCCCACCGGGGTGGAGGTTGGTCGCCGGGGGTCGAGAACCGCAGTCAAACCCTCTTCTTCCC
CGGGCACCGCGCACCTGCCCCCGGGGATGCCGAAGGAAGTGGCCCATAAAGCTTCTCTGCAAC
CGAAAGAGGCCTGAAGCTCCAGGAGGGCCGAGAGGAGCCTCGTTGAGCGAACCCAGCCCTCT
GCCTGGCTGGCCCTGGTCAACAGGCTCGGAAGAGGCTGATTTGGAGGACAGAACGGAAGAAA
AGACCTAAAGGTTTCGAATCTCATGATGTAGAGATGTTAAAAGCCTCCAATCCTAAGGTCTGACT
GTGCGGGGGAGCGAGGGGGTCTCAAGCTGGATGGACCCCTGAGCCTTCATCTGGAGAGTCCTC
TGCACAAGCTCAGACAGCAGGACAACGCGCATCAGTGGTTCTCAAGAGGGGGCAACTTCGCCC
TTACACGCCTCTCACCTCCACGCTGGGACACTAGGTCACGAATGGGGGAAGCGGGGAGGGAG
AATGCTAACCCCCTGGCATGTATCTAGTCAGCAGAGGCGACGGCTGCTGCTAAACACCTTACAA
TCCACGGGAGGGCCCCTCCCCTACCCCGAAGTAGCCATTCCGCAGAGGTGGAGAGACTCTCGT
GTAGCTCAATGCCCACGCACTTAGCCGATGGGAAATTACGAATTGATGACCAGTTGGCTCTTGG
ATCTGAGGAAAAAACTCCAGAGTCAGAGGGAACTCTCGAAGTTTTGCCCGGAGCAAACGGAAG
GGTGGCTTTGCCATCGCCTAAGATGGGAAAATGGCAGGTGTCACAGGTTGCAGGGGAAGGTCG
GAGACCAGCTGAGGGCCCCGGAGCCTTCCTGGAAAGAGTTTCCCATCCAGCCCGTCTCGGTTTC
CGCATCCGTCTGATTCCTTATGATGTTGAGGGTGCTGGCGTCTGGGTCCTTTATGATGCAGAGGG
TGCCCCCGTCTCACCCTGGGCGCCTCCGCGCTCCCGCCTCCTCCTGGCAACCTGGTGCGCGGCT
CCGGACCTGGCGACCCACGACCGGCTGGTCACTTGCTGCCACCTCGCAAAGGCGCATCTCTAG
TTCAGTGGTGAGCTGCGGCCGGGTCGCTGCAACTCGCTCCAGGCCTCCGGACTCGTGGCCTCG
GTGTCCCTCGCGGAGCCCTCGGTGTGTCGCTTGCAGGCTCTTTTTTTGAAGAAAGCAGGGAGGG
AATGGCCTTGTGAGAGACTCCAGGAGCAAAGAGCGACCCTCACAAGGCCCAAGTCCTCCCAGA
GCTCAGGGAAGCTGTCGCTTCTGACAGAAGAAGGGAGACAAAGCTCCCTCCTGTGTGTCCCTG
GTGGTCTAGTGGCTAGGATTCGGCGCTTTCACCGCCGCGGCCCGGGTTCGATTCCCGGCCAGG
GAATTGTTTTACACTGGCCGCCCTCCCGCAGGAATCTTCCTTCACTACGCTGTCAGCCGGCCTGC

TCCAAGGGCCAGAAGCAGAACAGTCTCCGCAGCGGGGTTAAAGCCGGGCGAAGGAGGGCAA
GTGCTGGTGGACCACCTCTCACGACACACCGTTCCTGTTTATCTCAGTGTCCGTCATCCGCGGG
AGCAGCTTTAGAGAGCGACTGAGCGTCTCGCTCCGGTGTACACAGCCCGGCAAAGATGCCAGC
CCCCGTGGAGCTGCACCCAATAAGCCCACCTTCTTTCCCGTCGCCACCCCGGAGACGCCCATC
GGGCTGAGCTGCGAATAACTAAGAGAGAGGCCAAGCCAAGTCGTGGCGTTTGTGGCAGCCCC
GGACACGGGCACCAGCCAGTCAGCGGAGCCTCCTCACCTCCGTTGCCAGCGAAGGCGCTCGTT
AGGCCTTGGGAAGAGGCGACCGGAGGCGATGCCCGCGAATTTGTTAGGGGGGTAAGCGGCGG
GTGAGGTCCTCGAGGGCGGTCCCGTTTGCTGATTGAGCGGTAGACGGAGGCGATGTTCGCTGA
CCCAACAAGGACAGCAGGTGGAGTAGGCACAGATGGAAAACTGCTGCCGGTGCCCTAAGCAG
AAGGCAGGTGGAAAAATCAGCACTAGGACGTCGAAGCGATGGTACCACAGTCAAATCCCACG
ACGTCTACACTCTACCAAGCACTTGCGCACGCTCCCCCTTTTCCATTCAGTACTCCCAAGAGGG
GTTCGGAAGAACCCCGAGTCCATTGTAAGCTCAGGGGAGAGCGGGAGCCAGGGAGGTGAAGT
GCGCAGACTCGGCAGCGGCGGCGGGCAGAACCGCGGGGGGGTGAGAGGGCGCGGTGGCTGC
GGGGCGGGAGCCGCTGCTGAGAGGCGGCCTGGGTTGTCTTGTGGGGTGACTGTCGGTGGAATC
TTTGGTGGAGAGTGGTTTGGAAGAATGGCGAGGGGCGGCAGTGGGGAGGGTGGTGACCCTGA
GCGACCGGCCAGGGCGAGGAGGCTGTGCTGTCCCTGCAGGCCATGTGCTCATTTCCACTTACCT
GGCAGGGGAGAGACCGTGGTCACGAAGGGGGTTCTCCCAGAGTGAAGCTTCTTCATCTCACTC
TAGAGTTGCTGATCCCTGTGATTTCCTCCATGTGGGAAACGGTGTTTGTGCTAGAAGAGGCTGC
GCTCTTTACCTGACATAACGGGGTTCAAGACTGACAACGCCTCACGCCCACCCGAAAACGTTTA
CATGGCTTCCTTGTCTCTTTTTTTTTCTGTCCTAAAGTCGCCTCATCTTCACATCCCCTCTTTTTTTT
CTTCCACACTCGAGAGTGTCTCTCTCTCTCAGTAAAAGCTCCACCAAATATTTGAAATATCTCAA
CCAGAAAGACTGCAATAAATACATTATTTCATTCGTGG

ID|30348|gene_id|ENSG00000228549|transcript_id|ENST00000451828 Homo sapiens lncRNA

ACCTGGTGCGCGGCTCCGGACCTGGCGACCCACGACCGGCTGGTCACTTGCTGCCACCTC
GCAAAGGCGCATCTCTAGTTCAGTGGTGAGCTGCGGCCGGGTCGCTGCAACTCGCTCCAGGCC
TCCGGACTCGTGGCCTCGGTGTCCCTCGCGGAGCCCTCGGTGTGTCGCTTGCAGGCTCTTTTTTT
GAAGAAAGCAGGGAGGGAATGGCCTTGTGAGAGACTCCAGGAGCAAAGAGCGACCCTCACAA
GGCCCAAGTCCTCCCAGAGCTCAGGGAAGCTGTCGCTTCTGACAGAAGAAGGGAGACAAAGCT
CCCTCCTGTGTGTCCCTGGTGGTCTAGTGGCTAGGATTCGGCGCTTTCACCGCCGCGGCCCGGG
TTCGATTCCCGGCCAGGGAATTGTTTTACACTGGCCGCCCTCCCGCAGGAATCTTCCTTCACTAC
GCTGTCAGCCGGCCTGCTCCAAGGGCCAGAAGCAGAACAGTCTCCGCAGCGGGGTTAAAGCC
GGGCGAAGGAGGGCAAGTGCTGGTGGACCACCTCTCACGACACACCGTTCCTGTTTATCTCAGT
GTCCGTCATCCGCGGGAGCAGCTTTAGAGAGCGACTGAGCGTCTCGCTCCGGTGTACACAGCC
CGGCAAAGATGCCAGCCCCCGTGGAGCTGCACCCAATAAGCCCACCTTCTTTCCCGTCGCCAC
CCCGGAGACGCCCATCGGGCTGAGCTGCGAATAACTAAGAGAGAGGCCAAGCCAAGTCGTGG
CGTTTGTGGCAGCCCCGGACACGGGCACCAGCCAGTCAGCGGAGCCTCCTCACCTCCGTTGCC
AGCGAAGGCGCTCGTTAGGCCTTGGGAAGAGGCGACCGGAGGCGATGCCCGCGAATTTGTTA
GGGGGGTAAGCGGCGGGTGAGGTCCTCGAGGGCGGTCCCGTTTGCTGATTGAGCGGTAGACG
GAGGCGATGTTCGCTGACCCAACAAGGACAGCAGGTGGAGTAGGCACAGATGGAAAACTGCT
GCCGGTGCCCTAAGCAGAAGGCAGGTGGAAAAATCAGCACTAGGACGTCGAAGCGATGGTAC
CACAGTCAAATCCCACGACGTCTACACTCTACCAAGCACTTGCGCACGCTCCCCCTTTTCCATTC
AGTACTCCCAAGAGGGGTTCGGAAGAACCCCGAGTCCATTGTAAGCTCAGGGGAGAGCGGGA
GCCAGGGAGGTGAAGTGCGCAGACTCGGCAGCGGCGGCGGGCAGAACCGCGGGGGGGTGAG

AGGGCGCGGTGGCTGCGGGGCGGGAGCCGCTGCTGAGAGGCGGCCTGGGTTGTCTTGTGGGG
TGACTGTCGGTGGAATCTTTGGTGGAGAGTGGTTTGGAAGAATGGCGAGGGGCGGCAGTGGGG
AGGGTGGTGACCCTGAGCGACCGGCCAGGGCGAGGAGGCTGTGCTGTCCCTGCAGGCCATGT
GCTCATTTCCACTTACCTGGCAGGGGAGAGACCGTGGTCACGAAGGGGGTTCTCCCAGAGTGA
AGCTTCTTCATCTCACTCTAGAGTTGCTGATCCCTGTGATTTCCTCCATGTGGGAAACGGTGTTTG
TGCTAGAAGAGGCTGCGCTCTTTACCTGACATAACGGGGTTCAAGACTGACAACGCCTCACGCC
CACCCGAAAACGTTTACATGGCTTCCTTGTCTCTTTTTTTTTCTGTCCTAAAGTCGCCTCATCTTCA
CATCCCCTCTTTTTTTTCTTCCACACTCGAGAGTGTCTCTCTCTCTCAGTAAAAGCTCCACCAAAT
ATTTGAAATATCTCAA

ID|30349|gene_id|ENSG00000228549|transcript_id|ENST00000453554 Homo sapiens lncRNA

GACCCACGACCGGCTGGTCACTTGCTGCCACCTCGCAAAGGCGCATCTCTAGTTCAGTGG
TGAGCTGCGGCCGGGTCGCTGCAACTCGCTCCAGGCCTCCGGACTCGTGGCCTCGGTGTCCCT
CGCGGAGCCCTCGGTGTGTCGCTTGCAGGCTCTTTTTTTGAAGAAAGCAGGGAGGGAATGGCCT
TGTGAGAGACTCCAGGAGCAAAGAGCGACCCTCACAAGGCCCAAGTCCTCCCAGAGCTCAGG
GAAGCTGTCGCTTCTGACAGAAGAAGGGAGACAAAGCTCCCTCCTGTGTGTCCCTGGTGGTCTA
GTGGCTAGGATTCGGCGCTTTCACCGCCGCGGCCCGGGTTCGATTCCCGGCCAGGGAATTGTTT
TACACTGGCCGCCCTCCCGCAGGAATCTTCCTTCACTACGCTGTCAGCCGGCCTGCTCCAAGGG
CCAGAAGCAGAACAGTCTCCGCAGCGGGGTTAAAGCCGGGCGAAGGAGGGCAAGTGCTGGTG
GACCACCTCTCACGACACACCGTTCCTGTTTATCTCAGTGTCCGTCATCCGCGGGAGCAGCTTTA
GAGAGCGACTGAGCGTCTCGCTCCGGTGTACACAGCCCGGCAAAGATGCCAGCCCCCGTGGA
GCTGCACCCAATAAGCCCACCTTCTTTCCCGTCGCCACCCCGGAGACGCCCATCGGGCTGAGCT
GCGAATAACTAAGAGAGAGGCCAAGCCAAGTCGTGGCGTTTGTGGCAGCCCCGGACACGGGC
ACCAGCCAGTCAGCGGAGCCTCCTCACCTCCGTTGCCAGCGAAGGCGCTCGTTAGGCCTTGGG
AAGAGGCGACCGGAGGCGATGCCCGCGAATTTGTTAGGGGGGTAAGCGGCGGGTGAGGTCCT
CGAGGGCGGTCCCGTTTGCTGATTGAGCGGTAGACGGAGGCGATGTTCGCTGACCCAACAAGG
ACAGCAGGTGGAGTAGGCACAGATGGAAAACTGCTGCCGGTGCCCTAAGCAGAAGGCAGGTG
GAAAAATCAGCACTAGGACGTCGAAGCGATGGTACCACAGTCAAATCCCACGACGTCTACACT
CTACCAAGCACTTGCGCACGCTCCCCCTTTTCCATTCAGTACTCCCAAGAGGGGTTCGGAAGAA
CCCCGAGTCCATTGTAAGCTCAGGGGAGAGCGGGAGCCAGGGAGGTGAAGTGCGCAGACTCG
GCAGCGGCGGCGGGCAGAACCGCGGGGGGGTGAGAGGGCGCGGTGGCTGCGGGGCGGGAG
CCGCTGCTGAGAGGCGGCCTGGGTTGTCTTGTGGGGTGACTGTCGGTGGAATCTTTGGTGGAGA
GTGGTTTGGAAGAATGGCGAGGGGCGGCAGTGGGGAGGGTGGTGACCCTGAGCGACCGGCCA
GGGCGAGGAGGCTGTGCTGTCCCTGCAGGCCATGTGCTCATTTCCACTTACCTGGCAGGGGAG
AGACCGTGGTCACGAAGGGGGTTCTCCCAGAGTGAAGCTTCTTCATCTCACTCTAGAGTTGCTG
ATCCCTGTGATTTCCTCCATGTGGGAAACGGTGTTTGTGCTAGAAGAGGCTGCGCTCTTTACCTG
ACATAACGGGGTTCAAGACTGACAACGCCTCACGCCCACCCGAAAACGTTTACATGGCTTCCTT
GTCTCTTTTTTTTTCTGTCCTAAAGTCGCCTCATCTTCACATCCCCTCTTTTTTTTCTTCCACACTCG
AGAGTGTCTCTCTCTCTCAGTAAAAGCTCCACCAAATATTTGAAATATCTCAACCA

ID|30350|gene_id|ENSG00000228549|transcript_id|ENST00000457856 Homo sapiens lncRNA

CACCAGCCAGTCAGCGGAGCCTCCTCACCTCCGTTGCCAGCGAAGGCGCTCGTTAGGCCT
TGGGAAGAGGCGACCGGAGGCGATGCCCGCGAATTTGTTAGGGGGGTAAGCGGCGGGTGAGG
TCCTCGAGGGCGGTCCCGTTTGCTGATTGAGCGGTAGACGGAGGCGATGTTCGCTGACCCAAC

AAGGACAGCAGGTGGAGTAGGCACAGATGGAAAACTGCTGCCGGTGCCCTAAGCAGAAGGCA
GGTGGAAAAATCAGCACTAGGACGTCGAAGCGATGGTACCACAGTCAAATCCCACGACGTCTA
CACTCTACCAAGCACTTGCGCACGCTCCCCCTTTTCCATTCAGTACTCCCAAGAGGGGTTCGGA
AGAACCCCGAGTCCATTGTAAGCTCAGGGGAGAGCGGGAGCCAGGGAGGTGAAGTGCGCAGA
CTCGGCAGCGGCGGCGGGCAGAACCGCGGGGGGGTGAGAGGGCGCGGTGGCTGCGGGGCG
GGAGCCGCTGCTGAGAGGCGGCCTGGGTTGTCTTGTGGGGTGACTGTCGGTGGAATCTTTGGT
GGAGAGTGGTTTGGAAGAATGGCGAGGGGCGGCAGTGGGGAGGGTGGTGACCCTGAGCGACC
GGCCAGGGCGAGGAGGCTGTGCTGTCCCTGCAGGCCATGTGCTCATTTCCACTTACCTGGCAG
GGGAGAGACCGTGGTCACGAAGGGGGTTCTCCCAGAGTGAAGCTTCTTCATCTCACTCTAGAGT
TGCTGATCCCTGTGATTTCCTCCATGTGGGAAACGGTGTTTGTGCTAGAAGAGGCTGCGCTCTTT
ACCTGACATAACGGGGTTCAAGACTGACAACGCCTCACGCCCACCCGAAAACGTTTACATGGC
TTCCTTGTCTCTTTTTTTTTCTGTCCTAAAGTCGCCTCATCTTCACATCCCCTCTTTTTTTTCTTCCAC
ACTCGAGAGTGTCTCTCTCTCTCAGTAAAAGCTCCACCAAATATTTGAAATATCTCAACCAGAAA
GACTGCAATAAATACATTATTTCATTCGTGGAAGGTACAGACCA

ID|30351|gene_id|ENSG00000228549|transcript_id|ENST00000619677 Homo sapiens lncRNA

AGAATGCTAACCCCCTGGCATGTATCTAGTCAGCAGAGGCGACGGCTGCTGCTAAACACC
TTACAATCCACGGGAGGGCCCCTCCCCTACCCCGAAGTAGCCATTCCGCAGAGGTGGAGAGAC
TCTCGTGTAGCTCAATGCCCACGCACTTAGCCGATGGGAAATTACGAATTGATGACCAGTTGGC
TCTTGGATCTGAGGAAAAAACTCCAGAGTCAGAGGGAACTCTCGAAGTTTTGCCCGGAGCAAA
CGGAAGGGTGGCTTTGCCATCGCCTAAGATGGGAAAATGGCAGGTGTCACAGGTTGCAGGGGA
AGGTCGGAGACCAGCTGAGGGCCCCGGAGCCTTCCTGGAAAGAGTTTCCCATCCAGCCCGTCT
CGGTTTCCGCATCCGTCTGATTCCTTATGATGTTGAGGGTGCTGGCGTCTGGGTCCTTTATGATG
CAGAGGGTGCCCCCGTCTCACCCTGGGCGCCTCCGCGCTCCCGCCTCCTCCTGGCAACCTGGT
GCGCGGCTCCGGACCTGGCGACCCACGACCGGCTGGTCACTTGCTGCCACCTCGCAAAGGCGC
ATCTCTAGTTCAGTGGTGAGCTGCGGCCGGGTCGCTGCAACTCGCTCCAGGCCTCCGGACTCGT
GGCCTCGGTGTCCCTCGCGGAGCCCTCGGTGTGTCGCTTGCAGGCTCTTTTTTTGAAGAAAGCA
GGGAGGGAATGGCCTTGTGAGAGACTCCAGGAGCAAAGAGCGACCCTCACAAGGCCCAAGTC
CTCCCAGAGCTCAGGGAAGCTGTCGCTTCTGACAGAAGAAGGGAGACAAAGCTCCCTCCTGTG
TGTCCCTGGTG

ID|30354|gene_id|ENSG00000233421|transcript_id|ENST00000415386 Homo sapiens lncRNA

GACGCACGACCGACTGGTCACTTGCTGCCACCTCGCAAAGGCGCATCTCTAGTCCAGTGG
TGAGCTGCGGCCGGGTCGCTGCAACTCGCTCCAGGCCTTCGGACTCGTGGCCTCGGTGTCCCTC
GCGGAGCCCTCGGTGTGTCGCTTGCAGGCTCTTTTTTTGAAGAAAGCAGGGAGGGAATGGCCTT
GTGAGAGACTCCAGGAGCAAAGAGCGACCCTCACAAGGCCCAAGTCCTCCCAGAGCTCAGGG
AAGCTGTCGCTTCTGACAGAAGAAGGGAGAGAAAGCTCCCTCCTGTGTGTCCCTGGTGGTCTAG
TGGCTAGGATTCGGCGCTTTCACCGCCGCGGCCCGGGTTCGATTCCCGGTCAGGGAATTGTTTT
ACACTGGCCGCCCTCCCGCAGGAATCTTCCTTCACTACGCTGTCAGCCGGCCTGCTCCAAGGGC
CAGAAGCAGAACAGTCTCCGCAGCGGGGTTAAAGCCGGGCGAAGGAGGGCAAGTGCTTGTGG
ACCACCTCTCACGACACACCGTTCCTATTTGTCTCCGTGTCCGTCATCCGCGGGAGCAGCTTTAG
AGAGCGACTGAGCTTCTCGCTCAGGTGTACACAGCCCGGCAGAGATGCCAGCCCCCGTGGAGC
TGCACCCAATAAGCCCACCTTCTTTCCCGTCGCCACCCCGGAGACGCCCATCGGGCTGAGCTGC
GAATAACTAAGAGAGAGGCCAAGCCAAGTCGTGGCGTTTGTGGCAGCCCCGGACACGGGCAC

CAGCCAGTCAGCGGAGCCTCCTCACCTCCGTTGCCAGCGAAGGCGCTCGTTAGGCCTTGGGAA
GAGGCGACCGGAGGCGATGCCCGCGAATTTGTTAGGGGGGTAAGCGGCGGGTGAGGTCCTCG
AGGGCGGTCCCGTTTGCTGATTGAGCGGTAGAGGGAGGCGATGTTCGCTGACCCAACAAGGAC
AGCAGGTGGAGTAGGCACAGACGGAAAACTGCTGCCGGTGCCCTAAGCAGAAGGCAGGTGGA
AAAATCAGCACTAGGACGTCGAAGCGATTGTACCACAGTCAAATCCCACGACGTCTACACTCTA
CCAAGCACTTGCGCACGCTCCCCCTTTTCCATTCAGTACTCCCAAGAGGGGTTCGGAAGAACCC
CGAGTCCACTGTAAGCTCAGGGGAGAGCGGGAGCCAGGGAGGTGAAGTGCGCAGACTCGGCA
GAGGCGGCGGGCAGAACCGCGCGGGGGTGAGAGGGCGCGGTGGCTGCGGGGCGGGAGCCG
CTGCTGAGAGGCGGCCTGGGTTGTCTTGTGGGGTGACTGTCGGTGGAATCTTTGGTGGAGAGTG
GTTTGGAAGAATGGCGAGGGGCGGCAGTGGGGAGGGTGGTGACCCTGAGCGACCGGCCAGG
GCGAGGAGGCTGTGCTGTCCCTGCAAGCCATGTGCTCATTTCCACTTTACCTGGCAGGGGAGAG
ACCGTGGTCACGAAGGGGGTTCTCCCAGAGTGAAGCTTCTTCATCTCACTCTAGAGTTGCTGAT
CCCTGTGATTTCCTCCATGTGGGAAATGGTGTTTGTGGTAGAAGAGGCTGCGCTCTTTACCTGAC
ATAACGGGGTTCAAGACTGACAACGCCTCACGCCCATCCAAAAACGTTTACATGGCTTCCTTGT
CTCTTTTTTTTTTTCTGTCCTAAAGTCGCCTTATCTTCACATCCCCTCTTTTTTTTCTTCCACACTCG
AGAGTGTCTCTCTCATTAAAAGCTCCACCAAATATTTGAAATATCTCAACCAGAAA
ID|30355|gene_id|ENSG00000233421|transcript_id|ENST00000614408 Homo sapiens lncRNA

AGAATGCTAACCCCCTGGCATGTATCTAGTCAGCGGAGGCGACGGCTGCTGCTAAACACC
TTACAATCCACGGGAGGGCCCCTCCCCTACCCCGAAGTAGCCATTCCGCAGAGGTGGAGAGAC
TCGCGTGTAGCTCAATGCCCACGCACTTAGCCGATGGGAAATTACGAATTGATGACCAGTTGGC
TCTTGGATCTGAGGAAAAAACTCCAGAGTCAGAGGGAACTCTCGAAGTTTTGCCCGGAGCAAA
CGGAAGGGTGGCGTTGCCATCGCCTAAGATGGGAAAATGGCAGGTGTCACAGGTTGCAGGGG
AAGGTCGGAGACCAGCTGAGGGCCCCGGAGCCTTCCTGGAAAGAGTTTCCCATCCAGCCCGTC
TCGGTTTCCGCATCCGTCTGATTCCTTATGACGTTGAGGGTGCTGGCGTCTGGGTCCTTTATGAT
GCAGAGGGTGCCCCCGTCTCACCCCGGGCGCCTCCGCGCTCCCGCCTCCTCCTGGCAACCTGG
TGCGCGGCTCCGGACCTGGCGACGCACGACCGACTGGTCACTTGCTGCCACCTCGCAAAGGCG
CATCTCTAGTCCAGTGGTGAGCTGCGGCCGGGTCGCTGCAACTCGCTCCAGGCCTTCGGACTCG
TGGCCTCGGTGTCCCTCGCGGAGCCCTCGGTGTGTCGCTTGCAGGCTCTTTTTTTGAAGAAAGC
AGGGAGGGAATGGCCTTGTGAGAGACTCCAGGAGCAAAGAGCGACCCTCACAAGGCCCAAGT
CCTCCCAGAGCTCAGGGAAGCTGTCGCTTCTGACAGAAGAAGGGAGAGAAAGCTCCCTCCTGT
GTGTCCCTGGTG
ID|30372|gene_id|ENSG00000251629|transcript_id|ENST00000512688 Homo sapiens lncRNA

TGCCGCCATGTAAACGTTCTTTGCTCCTGCTTCGTCTTCTGCCGTGATTGTGAGGCCTCCCC
AGCCATGTGGAACTGTGAGTCCAGTAAACCTCTTTTTCTTTATAAATTACCCAGTTTCAGATACGT
CTTTATTAGCGGCGTGAGAATGGACTAATACACTAACTTTCTCAGTGAGCATCTTATTCCAGTTAT
AAAACTACCTCTGGTTCTGAAGATACAAAGATAGGATAATGCTAACTATTTCTTTGCATAAGGCA
AGTGAAAAAATACAGATGACCTGATATCTTCTATCTGCCTACTCTCTCTACCTTTTGAAAGCTCTC
TTTCTGTTGCTTTAATAGCATTGAATTGATACGCCATTATTGTGTCTATTCCTTTCACAATACCCTT
AAATAAAGTCTTACTTTTAACTTTTTCCAGTGCCGTTTTTGTTTTGCTGAACCTTAGAAAAATTAAT
AGTTTACAGTAAATTAGGTGGAGATGCTAGAACATCTGAGGCAAAATATTGAGAATGGGTTTTA
AAGGCTACATAAAAATATGCTGGCCGGGCGCGGTGGCTCACGCTTGTAATCCCAGCACTTTGG
GAGGCCGAGGCGGGCGGATCACGAGGTCAGGAGATCGAGACCACAGTGAAACCCAGTCTCTA

CTAAAAATACAAAAAATTAGCCTGGCGTGGTGGTGGGCGCCTGTAGTCCCAGCTACTCGGAGA
GGCTGAGGCAGGAGAATGGCGTGAACCCGGGAGGCGGAGCTTGCAGTGAGCCAGGATTGCAC
CACTGCACTCCAGCCTGGGTGACAGAGCGAGACTCCGTCTTAAAAAAAAAAAAGAAAAAAAAAA
TGCTAAAATGGATTATGTAAGAACAGATAATCCACGATTTAACTATGTCCCCGCAGAGAACTTA
GGAGAGATTCCCTTCACTAAAGTGGTGAGATATATGTCATGCCACTACCATCAAGATTTCAGATA
AAACAGGAGAGGTAAATCTTCCCCAAAACCCAGTCTACTGCCATCAAAGGTGCAGAAAATATAT
ATTGTCTATGAAATACAATTGATGTTCATCACATATAATCCTAATTTATTTTACCTTGCCTTTTGTG
TATTCCAGTAGACTATTTAAATATTTCATAATGACAGAAGCTATGAATAAAAACAAAATTAATTTT
ATTTTTCATATTTATATTCCATTTATTTCTAAATAGGATATTGCAACTTAATATTATTTTTTAAAGAT
GCAAACACAAACATCATTAAATTGTTTAAAAAATTCAGAAAAAAATTAAAATACATGGAATGAA
AAAGGCACCTATATGAGGGATAAAGATCCTACTTAAAATTAGTTGTGAGTATACTAGTGGACAA
CAAAAAAGAGATTGCAATTGGATATGTTGCTCCTGTTATCCAAAGTAGGAAGCCCATTCTTTCTG
TGGATCAGTGTCTTAGTTTTCTATCACTGCTGTAGCAAATAGTCACAAATTTCATTGTTTGGAACA
ACACAAACATATTTTCTTAAAATTCTGTAGGCCAAAAGTCTTGGTAGGCTCAACTGCTTCCCCTTT
TCCAAATCTCACAGGACTACAATCAAAGCATCGGTTAGCCTGGGCTCTTATTTCAAAGCTTTGAG
AGAGAATCTTCTTCCAAAATCATTCGTGTTTCTGGCAAAATTCCTGTGCTATCCTGGCAGGCTATT
GGCTGGGAGACCTAGCTGCTTTAGACCACCTGCATTCCTGATCGCTTCATCCTTTCATTCTTCAA
ATAAGCAATGTATGTTAAGTTCTTAAGCTTTAAGTCTCTCTAACATCTCTTTTGCCAGATTTCTTCT
TCTCCAGCTGAAGATAGCTCTCAGCTTTTAAAGGTTCCTGTGACTAGATTGGACCCCCTCATAAA
TTTCAAATTAATCTTTTAATTTTAAGATCTTCAACCTTAATTACATCTGCAAAATTCCTCTGTTCAA
GAAATATTATGTATTCAAAGGTTCCAGGAATTAGGGAGTAGATATCTTTAAGGGGAAATTCTGCC
TTCCATGGGGAGAAACTTGGAATGTCAAGCTTTCCTAGTACCAAAATATTGCCAAACACACAAG
CACAGCCTTTTCGAATATGACTTAAGTTGGGTCATAATAATAAATGTAATAATATTAAGCAATAA
TGTCCTTAATACCTCTGAAATTATTTAGGCATACTTCATTTCCTTTGGCAGTTGTAAATTCCCAAG
CAATCTTGTTTCATAATAATAAAATGTTTTACCCAGACAAGGGTAGTGTAGTTGCTTCTAGAAGT
AAATTGTCTCAAGATGTGTTGAAATTGTACTTTCTTTAGACAATGGTTCTGATATAATATTTTCTGA
CTTTTGATAGAGGCTAAAATTAGGTAGTTTTTTCTAAAAGTATTTAATTAGGGCTTTGGATACTGG
TAGTTTTATATTGTGAATTGGGAGATTTGTTCACTTTGCAATTAGATAAACAGAAGATGAGTTTGA
GCACCAATTGCATTTTTCTCTTCCTCTGTCAATAGAGTTTGTTAGGTAAAATCCCTCAAATATCAG
GTGAACTCAAAAGAATGTCTTTTGTATTTAAACTGGTTGCATAGATTCATGTATATCAAGTGCTTG
AAAATTAGCTAATATTTTCTTTAATATGATGAAGATGCCTCATTTTCATTCTTTCTAAATGTACTTC
AGTTGCTGAGAGAATTTTAGTATGCTGAGTGATAGTTCTGGCCTTTTGGCAAATCAGGCATATCC
TGAATGTAATGATAATCCCCACCCAGCAGGTATTTCTAACTGAATACAAACAAAATTAACTGTGT
GAAAATGTTCATATAATTATGGTGTGACAGGATGTGAGATAAGACACTTTATTTTCCTTATTGTCA
ATAGGCAGATTGCAGACTAATTTAGTGATTTAAAAGACATAAACAACTAATTTTTTAGGCATAAA
AAACCTAAATCAATGTTTGAGGCAACTAAATAACTAAATACTGTGAGGTAAACATTTTAAAATAA
AATATTTTTATATTCTTATTTTCTTTTATGTACTATTAACAGGCTTGGAAATATTGTTTCCTCTTCTT
AAATATACACAATAATACATATTTTGGTAGTTTATTCATCTTAAAAATAATGAAAGAATCCAGTAT
TTATTTTGAAGACATTGCATATGCATATACAGTTATATAAATATGTATTCTTTTTCAATATTTTCCG
ATACTCCAAGTCTCATGACTCATAGAATAAAATTGAAAATAAAAGCTGTTGACTAATATGATTTA
TTTCTGTTTGCAAATTTGTAAATAGGAAAGCCTCTCAAATGCTTATCAGCTCCAAGAATACATAA
ATACAGAATATCGAGCTCCTATTGCTGCATATTCCAACCCCCCACAAGCTGCCATCTGTTACTCA
TAGGTACAGCTTTCTAACATCAGGTCTCTATGAACTCGATGCTTATGTTTTACAAAATAAATGGT
GGAAAGAAACCAAAAATGGTTAGAGTCTCATCTCTACCACTGTCAAATGGGCTTCATTTTTCCCT

TTTCTCACAGCAAATAACAAAATATCCTCCAGAGAGTTGTGCTAAATGCTGCCTCTGGTCATATG
TTGCTTGGTGTGTAGGGAGCAGCCGCAATGGAGCAACAACATTATTTGCAGATGTTATTTTGTGT
TCTGAAGTGTTATTTTCTTATTAATTTATGCTAAGTCAAATTTGCACTGAAATACTAAAGTACTAA
ATACTGAATTTAGTATTTTGAAGAATATTGAGGAGTATAAGAAGGACATGTATCACAATTGCTTT
TATTTATTAATAAAATAATAGTGTTTATCTTAGTTCTTATATATAACTTTTTTAAAGTAACATGGAA
GCATGAGTAAAGTTAAAACTTTGAGTGACCATTTTCAGAAATAAAACTGTATTTAGGTACCTGGA
AACCAAAATTTAGACTTAAAGTGAAAGATAGGATTTGGATTATCTAGTTTTGATGTGAAGAAAGC
AAGCTGATCCAGGCTGCCATTTACTTGAACTACTCTTTGTAGGCCTCAGCCTCACGTTCAAAAGT
GTTCCACACAGCTACTGGGTACAGTTGTCTCTATTTAACCATACATGAAGGAGGGTCATAAAAG
CATGGGATTTAGTAAATCGGTATGCTGAGTCATATGTTGCAATAAACTGCATATAATAAATTAGT
TGAAATATGCAATGCACAGGTGGGGATGTATATAATATACATAAAACTATGTAATATATACCTAC
ATACATACATACGATATTACAGCAAAATTTGGAGCTATATTCCAATTGTTTTCCCCTTATACATTT
GAGGAACTCTTTGGTCAGCATATGGCTCTCTCATAAGGTCTCCCTGCCTTTTTTTAATGTCCAACC
CTCCTTTGCATTCCCACCTGGCCCAGAACGTGTGCTCATCCTGTACTTAAGTAATTATTGCATTCA
TTCAATCTTTATCCCAGAGCTTTTTTTTTAATTGTGCAGAGTTTTCACACTACCTACAATTTCTAGA
ATGTTCTGTCTACAAAGCATGAAGGATTCGTGTCTATTTATCTTGATCTAGCAGTTTCTTTTTCTAT
GGCACTGAATAAACTTATAAACATGCAGGATTTAAAAAATATGATTCAATTTTTCTAAAGATGGT
GAAAGTGATATTTTCTTTAGAATTTTTTCCTGATGGATATTATGTATAAATCTCTCTGATTTCACAT
GGCAAAAATAAGGTTAAGTTAAAAATTACCTGAGAGAATCCTTGAATTTATGACTTCTGGGCTTA
AGTTTTATTGTAACATCTCCACGTTTCAATATTTAACTATCTCTGGGCCACATAAAGCATGAGGA
CAGGTACTTTTGATATATTTGCTGTGTTGTGGGAGAAAAACAAATCTCACCATTTTCTTATAATTG
CCCCAAAATTTTAGTTTAAGAAATACTTTTAATAAAAGTAGTCTCCTTCCCAGATTAGTAAATGTA
TTCTAACTAAAGGGTTTTGTGTGTGTGTGTATGTGTATATGTATACACCTATATATATTCATGTATA
TATATACATCTATATATATACACACACACCTTTAGTTAGTCTATGTGTGTGTGTGTATATATATATA
TATATATATATAAAAGTTATAGTCAATTGAACTCAACGAATCAAATGATTGAATGTGCCCTATCT
GCCAGGTATTCGATAATATAATTGAGAGTGAATACAAGAAAAAATAAACTATACTTACTATCTCA
GGAAGTCCATTTTAATTGGGAAGTAAAAATGTAGCCACATAAACATGTTACATATTGATAGGATA
CATTTTGGTGGTGTTTGCCAGGTGCTGCAGTTGTGGAATTAGCAGCCATTAATTTTATCCTAGGG
GTTGAAGATACTGTTAGTGCTAGTTTTTGAAGAACAACTGGGGAATTTCCAGCTGAATTTGATAA
GGACAATACAGGGAATAGAAATACTATTGTCAGAGAGCTTTACAATGCAAAGTTTCTGTGTTCC
AGTAATGTGTCTGTACAAGAACAGAAAAACTATCGTGGATTCAAATTTGAAACTGTTTGTAAGCC
TGAACAAATAATTTTAAGCAGAGGCATTCACAGTGTGATTTATTTTATAAAGAATATTTGGGATCT
CAAATTGCAGAATGGATAGTTTATATTAGAAAAGTATTTTAACAGTTCAACGTAGGAATAATTGC
AAATTGAACTAGGGTAGAAATTGTGGGGAGAAGATAGATATGTATGCACTTATATGTACAGATG
TGCATACCTCCTTGTTTGGATGATGGGACCCAACACATGGGCACTTGCATAGACGAAAAGCAAA
TTCTATGTTACTTTCAACCCCAAAGGAGAGAAGACTGCCTGGCAGGGTACACAAGGTGGCCCAC
CCAGAGACAGAGTAACCTCAGGATGGAGCTGTAAGAGATAGGTAATGTATAGCAAGTGGGGAG
AGATGAGTGAGGTTTGGTATCTGGCTCTGGGGAAATTCAGGGAAGTGTTTGTGACTGAGCATGT
GAGAGCCTGATAAGGGAGCTGGTTGAGGTGGGCCTCTTCCAGCCAATTGGAGGTCTGAGTATA
ACAAAAAAGCAGAGTAAGAAGGAATTCCTCTGGCTGGACTGCCTTGAACTGCAATATAGGAATT
CTCCAGCCTGCAGACTTGAATTGAAACGTTGGCTCTTTTGGGGTAGAGCTTTCTAGTCTTTAGAT
TAGAGCTTTTTGCATCAGCTCTTCTGGGTCTCCAGCTTGCTGACTGCCCATCTTGGGACTTCTCTG
TCTGCATCTCTCTTATGTGTATACATAAGATTTGTTGTTAAATTTTACACACACACACACACACAA
TTGGAATCTGTTGGTTCTGTTTTTCTGAAGAATCCTATTACAGGTCCACATGGGGAAGTACTAGA

TTTCGGCAGAAGGAGAAGTAAGGAAAAAGTATAGATTCCTCAGGGATCTAGAACTAGAAATAC
CATTTGAACCAGCCATCCCATTACTGAGTATATACCCAAAGGACTATAAATCATGCTGCTATAAA
GACACATGCACACGTATGTTTATTGCAGCACTATTCACAATAGCAGACTTGGAACCAAGTCAAA
TGTCCAACAATGATAGACTGGATTAAGAAAATGTGGCACATATACACCATGGAATACTAGGCAG
CCTTAAAAAAATGATGAGTTCATGTCCTTTGTAGGCACATGGATGAAATTGGAAATCATCATTCTC
AGTAAACTATCGCAAGAACAAAAAACCAAACACCGCATATTCTCACTCATAGGTGGGAATTGAA
CAATGAGAACACATGGACACAGGAAGGGGAACATCACACTCTGGGGACTGTTGTGGGGTGGG
GGGAGGGGGGAGGGATAGCTTTAGGAGATATACCTAATGCTAAATGACGAGTTAATGGGTGCA
GCACACCAGCATGGCACATGTATACATATGTAAATACCCTGCACATTGTGCACATGTACCCGAA
AACTTAAAGTATAAAAATAATAAAATAAAATAAAATAAAATAAAATAAATAATATTTAGGAAGA
ACCATTACTTAATAAAAGATAAACCATTTATTCAAATATTTTCTTTTATTTCTGAATCCATCACTGT
GGGAAATCTGGAATTCTACATTTCACAAGACAATGAACTTTTGTTTGCCCTGGAGAAATATGTAA
CTTTTCACAGGCTTTTTCTATTTTAACATATATAGAGATAGTGTTTGGAAATTTAAGAACAAGAGT
GGACCTAAGTATGAAAAATACGTACAACTTTTTCTTTAGTTACTCTTGATACTTTTCATTGTTCAG
GTTTTATGTATAATAAAACCTTACTTTATTTCCTTTCTTTTTAGTTTTCTTCCTTTGTTTTGCTATTGC
TATAGCACAGGATAAAGAACTTACAGAATATACATTGGTTATGCAATTTAATTAATTTTTAGGAG
TTATGATTATTATAATCATCAAAGCCTTGGGCTCAAAATAAAAATAATGATTTTCATTTTTAAATA
TACTTTTATTTTAAGGAATCAGTGGCTATGATTAATTTAATAGCACTCCTTCAATTCTAATAATGTT
TTGTTACAAAGCTTACATGTCATTTCTTTAAGCCAGCTCTTTTTATGTTATTAATGTTAGAACATTT
TAAGTCAATGATTTCATTTGCTAATATTGGTTTATTTCTAACAGCATCCATCATTGTTTAACAGTCT
GTTGGAGTGTTGCAAATTTATGAATTTTTATTATGAGGGCTCCTTTTGGCAACAGATAATTTTTTG
TTAACTATCATCAAAAAAGATAGAGGCTTTCTTCTTTCAAGAACAATTTGTGTTTTGATAAAAACA
ATGTGTTATAATAGATTACATTTATTTTATATAATGTTATAACTATTAGGGATGAGGCTTTTATAGA
GATTATTATAATAACTTTGCTAGTCATTGATTTTTATTCAGTAATATATGTTGTCTTGTGTGTTTGTG
TGTGTGTATTCATTCCCATGGATACTTGTCCTATGGTTGTAAAAACATTAATGTCAAAATGCATGA
GTTGAATTTAAGACATGGAGAGACTGCAATTGTAGTAGCAATTAATTTTGTTCACTATGTATTCA
AATATGGTCACCTCACTGAATTCTGAAAATATAATAGAATTATCCTTCCTGATTCTGATGTAGGTG
ACTGCAGTCATAGGATTAGTTCTGAAAAATTATAAGTGCATAAAACATCTCACTTATAGGAAAGA
CATTTACTTAATAAGAAGAGCTTCAAGAACATTCTTTACTTACGCCACAATATCTGGCAGAATTT
GTGATATAGTTAGTAAACAAGCCCAGATACCTAAATGACTACAGTGAGCACAGGAGCCACCTC
CCATATACAAACTTACAACTGACATGTTCCTTGAGCAAGTAATAAACTTTTATTGTATCAAGACA
TTGTTATTTTATTTGGAGAATATTATAGAATAATCTCTGTTATCCTGATTTACATAACACAATTCAC
CTTTTTCATATGCATGTAAGAATACAGATCATTAACATGGTTATTTTCTATGAAAATTATAGTTCA
AGTTTTAATCTGATACTTGAAATAACAGTATGCCTTTGCCCAACTAAATATAACTTGCTTTTTTATT
GTCAGTGAGGATAATGATTTTTATCACTATATTTCTCAAAATACAAAGCAAGAAAGAATTCACCG
AGTCTGTAATTAGTTCAGGAAATATGTTGAAGAAACATAAGGGGGCCAGGCGCAATGGCTCAC
ACCTATAATCCCAGCACTTTGGGAAACTGTGACGGGTGGATCAATAGAGCTCAGGAGTTTGAGA
CTAGCCTGGGAAACATGGCAAAACCCCTGCTCTACAAAAAAATACAATAATAAAAGTTATAATA
ATCTGGGTGTGGTGGTCTGCACCTGTAAGTCCCAGCTATGTGGAAGGCTGAAGTGGGAGAATC
CAGGCCCAGGAGGTTTAGGATGCAGTGAGCCGTATTCCTGACACTGCACTCCAGCCTAGGTGTC
AGAGTGAGGCCCTGTCTCAAAACATAAAAAATAAAAAAATAAGGATTACATGAAATAGGATACT
GTTATTATTAAATGTAACACAAAATAAATCCCGATGAGATTATAATATGCTGATATTGATAATAA
CAATTATAATACCAGCAAAATTATATTTATTAAATATTTAAAATGTGCCTGTCACTATACTAAGTG
ATTTGCATGTTGTATCCCATTTATTCTCATAGCAATCTTGTCATATAAGTAATATTATCTCAATTTTT

TCAGAAAATAAAGCCAAACTAAGGTTTAGTGAGGTGTTTTGGGCTGAATTGTGTCACCCCAAAA
TTCAGAACCTCAGAATATAATGGTATTTAGAGATAAGGTCTTTCAAGAAATGATTATGTCAAAAT
GAGTCTGTTAGGGTAGGGTCTTAACCCATATGACTGGTGTCCTTATAAAAACAGGAGGAGACAC
CAGGGATGACACACACAGAGGAAAAGCCACGTGTGGACACAGTAAGAAGGCAGCCATCTGAA
AACCAACAAGAAAGATCTTATGGGAAACCAGCCCTGCCTGCACCTTGATCTTGGATTTCTAATCT
CCATAAATATGAGAAAAATATTTCTATCAAGTCACAGATCTGTGGCATTTTGTTATGGTAGTCAT
ATGGCAGTTATATTATTATCTTTAACTATAATAAGATAGAGGTAAAGATAACTTACGTGAAGTAA
CAAAAATAGTAAGTGACAGAGACAGCATTTACATCAGGAGTGTTTAACATTAGTTTACACTTGTA
TCCCAAGCACACATTGCAGTTTGGATATTTCTTTGTGGTTGCAACCTTTTTTGCTTAGCCTTTTTGT
TGCTTCTGTAACAGTTATTGTTCAACGATAAAATTGCCATAAGCCTATTTCACCATATTAGAATTT
AGATCACAAATTCATTCCCAAACATCTGTTGAGAACACATTGGATGTCTGAATTAAAATGACTTA
AGAAACACAGTTTTATGGTATGTGATCATCTGACTTAGTCTTCCAAACGGTTAAAAATTCCTTTAA
AAAATGGGAACTGTGCAGCTCATAAATCAACACAACATAGGTGAGGAATTTCCAAAGGTGATTA
AATTTTTATGCACATTTATATAATTTAGCACATTAGTATTCCTATGTCTGGGTAAAACTCTCCTGTG
GGCTTTAAACTGCTTAGTTGGTATCATCAAATTAACGATGACCAAATTAGTACATATTAGCAGTT
AGATTCTTATATTTAAGGTCAATGTCTTATTTCCTGTATAATGTTAACAAACTTACAATAATTTGTA
AGTCTTTGCTTGCATAAAATGCCACTCATAGTAAAGAATATTCTATAGCTTAAATGATAATGAGA
AATATTTTGGATTTATATTCCAATTTTATATTACCAAAGGCTAGATGGTATAGATTCCAGTTATGT
CATCTATACATGGTGCATCATTATTAACTTAAAGTAGAGCTAAAGCAAAACAACAGTAACAGAG
GCACTATTTATAACAGTGAAAACATGGCCAGGTATGGTGGTTTATGCCTGTAATCCCAGCACTTT
GGGAGGCTGAGGCAGGCAGATCATGAGGTCAAGAAGTCGAGACCATCCTGGCCAACATGGTG
AAACGCCGTCTCTACTAAAATTACAAAACTTAGCTGGGCATGGAGGCGTGTGCTTGTAGTCCCA
GCTACTCAGGAGGCTGAGGCAGAAGAATTGCTTGAACCCAGGAGGTGGAGGTTGCATTGAGCT
GAGATCGCACCACTATACTCTAGCCCGGCAATAGAGCAAGATTCTGTCTAAAAAAAAAGAAAA
AAGAAAAAAAAAAAAACAGTGAAAACATAACCTTCCCTTCATTACTGAAGATCTATCAAAACCAA
TTGTATATAATGCAAGCATCTATAGCAAAAGTTTACTTTTGTTTAGACATATCTAAACCAGTCTAA
TAAAAGTAGCAATGTTTTGTGATGGAGACAGATCACAAATTTAGATATTAGAAATTGTTGAGACT
ATTGGATAATTAATTAATTTAGGGACCCTAGCTATCTTATACATAAATTTTAATATGTATTATGATT
TATCAGTCATTTAAAAATGTCTACTTTTTCAACTCAAGATAATTTGTGAGCAGCATCAGAAAGGA
GGAAACTTCTTTGTCCTCTTCCTTTATAATAATTCCTTCCATTTGGTTTTCTACCTGTATTTACAAA
ACATTTATAAATCATCCATTGCTTCTTAATGGCTTACAGAAGCAATGATGAAACTGTGACAGGAT
AAAATTAACAAGATTTTCTCATAATATAAAACTAAGCTCCATTGTGTGCTAGATGGTTTTTGTCTC
AACGTTGTGATTTAACATAAATTTTAAACACAGAACACTATTTCAAGAATTCTTCTCTCATTCTGT
AAACAAATTTGAAGAGGAATGTATTAGTCCGTTCTCATGCTGCTAATAAAGACATACCTGAGACT
GAGTAATTTATTAAAAAAAAAAGATGTAATGGACTTATAGTTCCCCATGGCTGGGAAGGCCTTAC
AATTATGGCAGAAGGTGAAGGAGGAGCAGAGGCATGTCTTACATGGCTGCAGGCAAGAGAGT
GTTGGCGGGGGAAACTGCCCTTTATAAAACCATCAGATCTCATGAGACTTACTCATTTTTATGAG
AACAGCATGGGAAAACCTGCCCCCATGATTCAATTACATCCCACCAGGTCCCTTCTATGATACA
AGGGGATTATGGCAGCTACAATCAAGATGACATTTGGGTGATGTCACAGCCAAACCATACCATT
GTCCTTACATTTCAAAACACAATCATGCCTTCTCAACAGTCCCCGAAAGTCTCAACTCATTCCAG
CATTAACTCAAAATTCCCAGTCCAAAATCTCATCTGAGACAAGGCAAGTCCCTTCTGCCTGTAAA
ATCAAAAGCAAGTTAGTTACTTCCTAGATACAATGGGGGTACAAGCACTGGGTAAATATACACA
TTTCAAATTGGAGAAATCAGTCAAAACCAAAGGGGCTATAGGCCCAATGCAAGTCTGAAATCCA
ATAGGGCAGTCACTAAACCTTAAACTTCCAAAACGATCCCCTTTGACTCCATGTCTCACATTCAG

GTCATGCTGATGCAAGAGGTAGTTTCCTATAGTCTTGAGCAGCTCTGCCTTTGTGGCTTTGCATG
GTACAGGACCCCTCCTGGTTGCCTTCACGGGCTGGCGTTGTGTCTGCAGCACTTCCTGGCACAT
GGTGCAAGCTGCTGTTGGATCTACCATTCTGGGGTCTGGAATATGGTGGCCTTCTTCTTACTGCT
CCACCAGGCAGTGCCCCAGCGGGGATCCAACCTCACATTTCCCTTCCACACTGCCCTAGCAGA
GGTTCTCCATGAGGGCTCTGCCCCTGTAGCAGACTTCTGCCTGGGCATCCAGGTGTTCCCATAC
ATCTTCTGAAATCTAGGCAGAGGTTCTCAAACCTCAATTCTTGACTTCTGTCCATCTACAGGCTC
AACACCACGTGGAAGCTGCCAAGGCATGGGGCTTGCACCCTCTGAAACAATGGCCTGAGCTGT
ATGTTGCCCACTTTTTGTCATGGCTGGAGCATGTAGGTCACAGGACACCAAGTCCCAAGACTGC
ATACATCAGGGAACCCCTGAACCTGGCCCAGGAAACTATTTTTCCCTCTTAGACCTCTGGACCT
GTGATGGAAGGGGCTGCCTCAAATGTCTCTGACATGCTCTGGAGACATTTTCTCCATGGTCTTGG
TGATTAACATTTGGCTCCTCATTCATTATGCAAATTTCTGCAGCCAGCTTGAATTTCTCCTCAAAA
AATGTGGTTTTCTTTTCTCTCATATCATCAGGCCATAGAGTTTTCAAAGTTTTATGCTCTGCTTCTT
GTTGAATGCTTTGCCAATTAGAAATTTCTTCCAGCAGATACTAAATCATCTCTCTCAAGTTCAAAG
TTCCACAGATCTCTAGGGCAGGGGCAAAATGCCACCAGTCTCTTTGCATAGCAAAAGTGACCTT
TACTCCAGTTTCCAACAAGTTCCTCATCTCCATCTGAGACCACTTCAGCCTGGACTTTATTGTCCA
TATCACTATCAGCCTTTTGGTCAAAGCCATTAAACGAGTCTCTAGGAAGTTCCAAACTTTCCCAC
ATTTTTCTACCTTCTTTGGAGCCCTCCAAATGGTTCCAGCCTCCACCTGTTACCCAGTTCCAAAGT
CACTTCCATATTCTTGAGTATCTTTACAGCAGTGCTGCATTCTCTGTGCAACCAATTGACTATATT
AGTCTATTCTCATGCTTCTAATAAAGACATACCTGAGACTGGGTGATTTATTGGAAAAAAAAAAA
GGTTTAATGGACTCACAGTTCCACATAGCTAGAGAGGCCTCACAATCATGGTGGAAGGTGAAG
GAAGAGCAAAGACATGTCTTACGTAGCAGAAGGCAAGAGAGCGTGTGCAGGGAAACTGCCCTT
TATAAAACCCATCAGTTCTCATGAGATTTATTCACTATCATGAGAAAAGCATGGAAAAATCCCAC
CCATGATTCAATTACCTCCCATCAGATCTCTCCCATGACACATGGGGATTATGGATAGCTACAAT
TCAAGATGAGATTTGGGTAGGGTCACAGTCAAACCTTATCAAGGAATTGAATACAAAGAGATAA
TATACTAGTTATCATACTTTTGTGTGTTTACATAGGCAATATGAATATTTTCTGTTTATCTTTTGCAT
TTAGGTGCTTATCTTTGATTTTTTTTACCAGTTTCTTAAAATTACATAAGTAATCCACAAAAATCAT
CTCCAGTTTATAAAAAAGATTCAAATTGGTTGACATAGAAATGTGTGTTTTCAAAGGCCAGTTCA
CCTTCTTTTTTTCGTCTGTAACACTCTCTTATCCCTAAATAAATTATATTGTAAGTTGTTGGTATAC
CTACATGGATCTCAAATGTGGACAATTTTTACCCCAGAATAATATTTGGCAATGTATAGAGACAT
TTTTGATTGCCTTATCTAAATAGAGGGAGTGAAGAAAACCTCTAGTGTGTATTAGCCAGAGATAA
TGCTCAATCATTCAGTCCTCAGGACAGCCCCATCACAAAGAATTATTAGTCCATATGCTGGTATA
TATCATGCCAGTCTTTCTCTGGATCAACTGCATTTACACATATGCACATGGATGTTTTGTTGACTG
TAGTATGTCAAAATTTAAAGACATATCAGGCACACAGTAGATAAAACTCTTCAAGCTATATCCAT
ATGAATGAAGGTCAATCATTCATATTGATAGAATTAATAAATTTGTTGACTAATAAAGCTCTTTAA
ATACATGTTATTTTAGATAAAAATTAAGAGCTTCTCAATTTTGTAAAATAGTCATCTTAGTGTTTTA
GTTTTTGAATTATTAAAAAAAACACTGTTGAATTTTTTTTTAATAATAAAGCTCAGCTTTGCTACTA
CTTCTGGACTAGTCTATTTGCCAATTCTGAGATTTCTCAAATTTATGAGTCACATTAGATAATGTC
TGCTCTTTCAATTGTTCTAATTTGTTTTATTATCTGATCCAAATGCCTGAATCTCAATGGATTTATC
ATTAACTCTTTCTAACTCAGAAACTCTTGTGAGAGAGATGACAGACATATCACAAGATTATTGTG
CTGCACTGAACAAGGGCATGATACATGGTAAATAAATGAATGAAAAGAGAAGGAGAAGAGAG
CTATTTTGGGGTCTCATTTATACTCCACTTATATCATCTTTGATTTCTAATAATAAACTGATGAAAC
ATATTTTAAGTAATTTATAAATGTAAAATTCGTTTTTTATTTTACAATTTTAGCTACAGCACAGTAA
CTGTTATAATAGTTAAAACTTTTTTTCACATTAGAACCTTACTTTTTTAACAGTGAAAAGAATTATA
ATTATGTCTTTACATTTCTATCAGTATTTCATTGCAATATGTCCTGAATTTGACAGATGAACAATTA

TCTCTTTTTTTATTAATGGACTTTGCAGGAAACAAATTAAGGCCATTTGAGTAATTACAATAACTT
GATACATTTCTAATTTGCTATAGAATGTAACTCATGACTTGTATCTGAAAAAAAAGGAAGGAAGA
TAGAGAGACAAAAGATAGGAAGGGAGGGAAGAAGGAAACAAGGAAGGGAGGAAGGAAGGA
AGGGAGGGAGGGAGGAAAGAACGAAGGAAGGAAAGAGAAGAAGGAAGGAGGGAGGGAGGG
AAATAATCTAATGAGAAATGGGAAACAATAGTATCATTAATTTTTTTAAATTATACTTTATCTGGT
ACATGTGTAGAACGTGCGGGCTTGTTACATAGGTAAACACGTACCATGGTGGTTTGCTGCACCC
ATCAACCCGTCATCTACATTAGGTATTTCTCCTAATGCTATCACTCCCCCAGTCCCCCACCCCCC
AACAGGCCCCGGTGTCTGATGCCTGCCTCCCCGTGTCCATGTGTTCTCATTGTTCAGCTCCCATT
TATGAGTGAGAACATGCGGTGTTTGGTTTTCTGTTCTTGTGTTAGTTTGCTGAAAATGACTGGTAA
TGTTTAATAAGATGGGAGAGATGGATGTTACCTATTAAGATGCATATTGTTTTCCTCAAGGAATA
TTACCTTTTGAGCATTTTGCACTGTGGTCCAAAAATGGCTCAAATTCATCTGTTTCTTGCTGAATC
CCAGCTTTGCATGTGTGTCACTAAATCCACAAGGCAGCGATTCAGTGCTAATGAACTCTTCTGGT
GGTTACTAACTGCTCTATAAGCATTGTAGGTAGACCATAAATAAAATTTTACCAGTCAGTTATGA
AAGGAGAAAAATGAGAACAAAATAGGGAGTTTTCAATGAAGCTAATGTTAAAAGGAGTGATCTT
TATCTGCTCATATACATTTGCAGTTTTACAATATTAAAATAGCTGTGTATCATTGATATTTTATTAT
TTTGCTGTCCTTACTTTTCAAAACAATATATTGTAAACCTGTACTAGTTCTTACAAGTCTCTTGAAA
AATTACGGTTTGTGATACTGTATTTAATAGTCTATCAGGCCATGGTTTGCATTAATAAAATATGCC
GGGGACCCAGGGACACTACACTGCACCTCCTTTTTGTCTCAATTACATACCATGGTTAAGTCTTA
TTGCATTTTTATAAGTTTATTTAACAATTTAAGTTCGAAAGATATTACACAATTTCATAGTGCTCAA
TTTTAATACATTTCAAATGAAAGCAAAAAGGAAAAAATGAATCCCATTAGAATTTTATTCTGTTCA
TATATGCATACTCACAATATAATTAATATTCTGAGCATTATTCATTAACTAGGTGGCCAGTATATT
ATGGATTCAGATTTCATTTAAACTTTGAGGAGAAAAGAAAGTAACTTTATAAATAATAGCATTAA
ATAATGTTCATAGGAAATATAACTTTATTGAAGTATTCATAAAATATAAATTGGACCTCTCTAAGA
CGTTTATTTCCTGTATAACTCTTACTACACTAATTTCTTACTTTCCTGTTGATTTTTTAAGCTATAGT
TAAAATACATAGGGAAGGGCTTCAAGATGGCTGACTAAAGGCACCTAGCACTCGCCTCCTCTG
GAAAAAAGGACCAAAACAGTGAGTAGATAATAATAAGTCAAATAGTAGATAATAACAAGTCAA
ATAGAGAGTATAACACAGAACAGTAAAATTCAGCAGGGAAGTGTCAGGGAACCTCTTAGGCAG
AGAAGTAAAAGGAAGTGAAGCATCCTCCCAGGCTGGGACCAGCTGTGAACCAGGAGAAACCTT
CCAGTGCAGAAAATATGTAAGTGACAGATCCCAGTAGTTCACATTCCCACTGCAGACTACTGTG
GTACTAGCCACAGAGATAGCCTCAGCCATCATGGTCTCTGGGACCAGGTTAGGGAACTGCCAC
ACTCCCTAGGGAGAGTCCACACAATAACATGGTTCGAGAGAATGAATTTGTACTGGGCCCCGC
ACACTTCCCTGAGACACAAACGACTGCAGCCTGGTGCCATTTAGAGAGCCCAGCCCCAACCAG
ACTACATCCTGCCCTGGGACCCAAAGGGCCCCTGTGTCTCCACATCCCTGGATCCTCATTGACA
TCTCCGTATGTCTACCCAGAAAACTGTAGCATCACTGCACTGGGTGGACCAGCGGTGCAGATGA
ATCTCCAGCAGTCTAGCCCATGCAGTTTCGTATAGCCAAGGGAATTTGTGGTGAAACTCAGTAA
GAAGACTGTCCCTGGGACAAAGGGAGCTGAAGCACATGCCTCCCAGAGCCTGAGGGCAGTGT
GCACAGGAATACTGCCCCTGACAGCAACCCTGCCCCCTCCCAGCAGTAGGGCTGCCACATATC
TGTACAAGCTTTCAGGGGGCCTAGGGACCTGAAGCTCAGGTACTTTCCGTGGACCTAAGAGCA
GACCTGTCCCACCCACCATTGCTCCCACTGAATCTCACCATCTGGGGGCCTGGAAATCAATACC
TCTTGCCAGCCACAGCTGGTGCTTGCCTGCATCATCAGTGAGCCTGAGGACAGGCCCCCTCTGA
CCAGTGCTGTCCCTACCAGTGCCCACAAGTGTCGTCTAGGAGCCAGGAGATTGACTTACCCCAC
GTACCACAGACTGTACCAACATATACCATTGGTGGCCTAAGGCCCAGGCCTTTTCACCACTGCG
GATGTGAGGCCTAGATATCAGCCTTCCCTGCCTATCACTGCTGATGCACAAAGGTGCACCATCA
GGAGACCTGGAAACAGATCCAGCCTGCCCGCTGTCAGCACCTGAGAACACTGGTAAGGGACA

AGAGAATTTACATGTCCTGCCTACCACCACTTGCATCCTGATAAACTATCATGGGGATGCAGAA
AAAGACCACTCCTTTTGATACCTTTCTTGCCAGTGCCTGTGTGCATTATCTGGGAGCTTGGGGTT
GATCTGACTCCCCCGACTTCCATTAGTTCCCATGCATGCCTTCTGGAGGCCAGTGGATTGCTCCA
CCCAGTCTGCTCATGCCTACACACACGAGCCACGGGGCTGGGGATTGACAAAAACATCTTCCA
CAGTCTGTGTGCACGGTAAGGGGCCTGAGGACAGGCCCAGCTCACCTGGTGCCACCCCTGCCA
GTACCGGCATGTGTCTTCTATGGTCCTAGAGGTCGACCTGTCCACCACCACTGTCATCTGTGCAC
CCCTAACGAGAGCCTGGGGATGGACCCGCCAAGCTTGCTGCCACCACTGCTGCTGGCATGTTTC
CCAACAAGCACTACCTGGAAGCCTGAGGATTGGCACACCACTGTTATTTTGATCACAGATGCTG
TATAAGCTGCCTAGAGCTTGTCCAGTCTACTGGTGCCTAGAGGATCTCTCCAGCCTACTGGTGC
CAATGCAAACGCTTAAGAAAGCCACCTGGAGGCCCTCAAAATGGTCTATCTCTAGCTGCTACCA
CCAGTACCCATGTATACTGCATACACAAGGGCTAACATGCTCAGCCCACTGCTGCCACCACTGG
GGCCTGAGGACTGGCCCAGCTTGTGTTGCCATCCTTAGCAATGTTCCACCACAGTCTCCACTAA
CAACTGCAGCTGAAGCCACTGAATAACTCACAGGAACCACTGATAGTGATTACAGTTAAAGAA
ATCATACAGAGACTGCACTACTGCATGCACTGAAAATCAAAGCCAAAGCATCGTATTCAATCAA
CGTAATAGATACATCTGTAGAAAGTCTTTCCCTATGAAAGCCATTCCATAAATTGGAACAAGCAA
CTGTTACATCGATGCAGATTTCAATGTAAGGACACAAGAAATAAGAAAAAGCAAGGAGACATG
ACACCTCCAAAGGAAAAAAAGAGTATCTATCTATTCAGCAACAGATTTCAACAAAAAGGAAGTC
TATGAAATCCTTAAATAATAATTCAGAATAATAACATTAAAATAACTCAGTGAGATACAAGAGCA
CACAGATAAAAAATACAAAGAAATCAGAAAAAATTCATAATCTGAATAGGAAATTAAACAAATA
GATGTATATCATATAAAAGCAAACAGATATTCTGAACTTGAACAATTACATAAATAAAAATACAA
AAAGAGCTTCAATAAGACTAAATCAAGCAGAAGAAAAATTGTTGAACTTGAAAACAGGTATTTT
GAAATAATCTAGTCAGATCAAAAAAAAAAAAGAATAAAAAATAATGAAGAAAGCCTACAGGAC
ATATGAGACTCCACAGAGAGAACAAGTGTTTGAGTTTGGGCATTCCAGAGAAAGAAGAAACAA
AGTCATAGAAACCTACTTAATGAAATAATAGCCAAAAACTTCCCAAGTCTTGCAAGAAATAAAA
GCATCCAAATGCAGAAATTTCAAATAATTCCCAAATAGAATCAACCCAAAAATGTCTTCTCCAAT
ACACATTATAGTCAAACCATCAAAAGTCAAAACAGAATTTTAAAAACAGGAACAGAAAAGCACT
AAGTCACATTTAAGTGAACCCCCATCAGACTTACAGGGTTTTTCTAACAGAAACCTTACAAGCCA
GCAAAGAATGGGATGATACACTCAAAGTCCTGAAAGGAAAAAAAAATCAGCCAAGAAGCAAA
GAACACTATATTAGAAAGCTATTCTTTAAAAATGAAGAAAAACTCCTTTCCAGACAAGCACAAA
CTGAGAGAATTTGTCACAAGACTGATCCTACAAGAAGGGAGTCTTGGTCCTTCGTAAGTGAATT
CTACATCTAGATGTGAGGGCCAGTATTTGCCATCATGAAATCACATGAAGTATAATACTCACTTA
CAGAGCACACACACACAGATACAGAAATTTAAGAAAAAAATGGGCTTAAATATTACTATTACAGAA
AACCATCAAATTGCAATAAAAAACAAAGTGAAAGAAAGGAACAAAAGATACACAAAATAATCA
GAGAATAATTAACAAATTGACAGGAATAACTTTTTACATATCAATAATAAGCTGGAATGTAAATG
GATTCAGTTACCCCCTAAAAAGATATAGATTGTCTGAATGGATTAAGAAAAACATGACCCAACC
ATATGCTGTCCAGAAGAAACTCATCTCATCTATGAAGACAAATAAAATGAAAGTGAAAGGACGG
AAAAAGATATTCCATGCAAATGAAAACCAAAATTTAGCAGGAGTAGCTATATTTATACCAATAA
AAATGACTTTAGGTCAGAAAGAGTTAAAAGAGATGAAAAAGGTCATTATATAATAATAGAGGTA
TCAATTCAGCAAGACTATAACAATTGTAAATGTGTAGGCACCCAACACTACAACATCCAGATAG
ATACAACAAATATTATTAAATCTAAAGGCAGAGATAGACTCCAATACAATAATAGTTGAGGACT
TCAACACTCCACTCACATAATGTGTCAAATGCTGTGGGTGGAGTGTTGGAGTTTGAACTGAATTT
TAGACTAAGTGAACCTAACATTTACAGAATATTTTATCCAACAGTCACAGAATACACATTCTTCT
CAGGAGCATATGCAGCATTCTAGCAGGGTAACTCATATGTTAGGCTACAAAACAAGTCTCAACA
AATTTTAAAAAATCAAATTTTTTTCAAATATTTTCTCAGACTACGGTGCAACAAAACTAGATATCA

ATAGTAAGAGGAACTTTGAAAGCAATACAAATACATAGAAATTAACCAACAGGATCCTGAATGA
CCACTGGGTCAATGATGAAATTAAAAAGAAAATAATTCCAGAAACAAATAGAGAAAAAACACA
ACATATATACCTCTGGGATCCAACAAAACTAGTGCTAAGAGGGAAGTTTATAATAATGAATGCC
TACATAAAAAAATACAAATATGTCAAATAGACAATCTAACAATGCACCTCAAGGAATTAAGAAAG
CAAGGAATTAGAAAACCAAATCCGGCCGGGCGCGGTGGCTCACGCCTGTAATCCCAGCACTTT
GGGAGGCCGAGGCGGGCGGATCACGAGGTCAGGAGATCCAGACCATCCTGGCTAAAACTGTG
AAACCCTGTCTCTACTAAAAATACAAAAAATTAGCTGGGCGTGGTGGCGGGCGTCTGTAGTCCC
AGCTACTCGGGAGGCTGAGGCAGGAGAATGGCGTGAACCCGGGAGGCGGAGCTTGCAGTGAG
CTGAGATTGCGCCACTGCACTCCAGCCTGGGCGACAGAGCGAGACTCCGTCTCAAAAAAAAAA
AAAAGAAAACCAAATCCAACATTAGTAGAATGAAAGTAATAACACATATCAGAGCAAAACTATA
CAAAATGGAGACTAAACACCAACAAAAATACACTGCAACCTCTGTCTCCTTGGTTCAAGCAATT
CTCCTGCCTCAGCCTCTTGAGTAGCTGGGATTACAGGCGCCACCACCACGCATGGCTAATTTTT
ATATTTTTAGTAGGGATGGGTTTTCACCATGTTGGCCAGGCTGTTCTCAAAACTCCTGACCTCAG
GTGATCCGCCTGCCTCGGCCTCCCAAAGTGCTGGGATTACAGGCATGAGCCACGGTGCCCAGC
CCAAAAATGAATAGTGTTTCTGTACATCAATAATAAACTAACTGAAAAATAAAGAAGACAGATC
TGTTTACAGTAGCTACAGAAAATAAAATATCTAGAAGTACATTTAACCGAGTTAGTGAAATTTTT
ATGCAAGGATAATGATGAAACATGAATGAAAGAAATTAAAGAGGACACAAACTGAAAGATATC
TAATACTCATGGGTTGAAAGAATTAGCATTGTTAAAATGACTGTAATACCGAAAGCAATCTACA
GATTCAATGCAATCCCTATCAAAATACCAATAATTATTTTTTTTCACAGAAACAGGAAAGCAGTC
CCAAAATTTGTATGAAACCACAAAAGACCCTAAATAGCCAAAACAATCCTGAGTTAGAAGAATA
AAGCTGGAGGCACCACAACACCTGACTTCAAACTATTCTACAAAGCTGTAATAACCAAATCAGC
ATAATATTAGTATAAAAACAGACATATAGACCATATAGACCAATGAAACAGAATAGAGAATCCA
GAAATAAATTTATATATTTACAGCCAATTGATTCTCAGAAAAGGCACCAAGAACATGCACTGAG
GAAAGGATGCCCTCTTTAATAAATCGTACTGGGAATACGTGATGTCCATATGCAGAACCATGGA
ACTATTCACCTATCTCTCATCATATACAAAAATCAACTCAAAAGAGATTAAAGACTTACATGTAA
CATCTGAAACTATAAAACTACTAGAAGTAAACACAGGGAGAAACTTTCAGGGCATAGATCTAGG
CAAAGATTTTATGGCTAAGATTTCAAAATCATAGGCAAAAAAAAAAAAATAGACAAATGTAACTTT
ATAAAACTAAAAGTCTTCTGCGAAGAAAAGGAAATCAACAGAGTGAAGTGACAACCTGTAGAA
TGGGAGAAAATATTTGCAAACTATATGACAAGAAATGAATAACCAAAATAATTAACTCAACTAA
ACAGCAATATCATGACAATAAATAATATAATAATAATAATTCCATTAAATAGTGGCTAAAAGGTC
TGAGTAGACATGCAAATGGCCAACAGGTATATGAAAAAAACAGTGAACATCACTAACCATCAG
AAAATGCAATTCAGGACCACAAAAAGTTATCATCTTACCCCAGCTAGAACGGCTATTATCAAAA
ACAAAACAAAAAAACAAAACAAAACAAAACAAAAAAAGAATCCCAGAAAGGATGCAGAGAAA
AGGGAACTCTTATACATTGTTATTGGGAATATAAATTAATATAAATTATTACAGCCTTTATGGAAA
ACAGTATGGATGTTTCTCAAATAACTGAAAATAGAATGACTACACGATCTGGCAATACCACGAC
TGGGTATTTATTCAAAGGAAAGGAAATCAGCACATAAAAGGGGTACATGCACCCTCATGTTTAT
TACAGCACTGTAGCTGAAGTGTAGAATAAACATGTGTCCATCAATGAATGAATGCATAAAGAAA
ATGTTATATGTATACATATGTGTCTGTATGCCTATACCCACAAATATATACAATGTATTTTATATAT
ACAATGTATTTTTTAATCCAGCAATAAAATATGGTAAAATACTATCATTGGAAGCAATATGTATG
GAACTAGAGGTTATTATGCTAACTGAAATAAACCAGGCACAGAAAGACAAATGTTGCATGTTCT
CACTAATAGGTGAAAGCCGAAACATTTGATCTCATGGGAACAGAGTGTAAAATGTTGGTTACCA
GAGGCTGGGGAGGGTGAGAATGAAGAGTTGTTTAATTGGTACAGACATGCAGAAGAAATAAGT
GCTGGTGTTCAATAGTACAGTAAGATAACTCTAGTTAACATATTATATTGTATATTTTAAAATAGC
TGAAAGAGAATATCTGAAATATTCCCAGTGAATTTTCTTTTCTTTTCTTTTCTTTCTTTCTTTTTTTTT

TTTTTAATGGAGTCTCGCTCTGTCTCCCAGGCTGGAGTGCAGTGGCGCCATCTGGGCTCACTGC
AAGCTCCACCTCCTGGGTTCAAGCCGTTCTCCCTCCTCAGCCTCCTGAGTAGCTGGGACTATAG
GCGCCCGCCACCACGCCAGGCTGATTTTGTTTTTGTATTAGTAGAGACAGGGTTTCACCGTGTTA
GCCAGGATGGTGGGCTGCACCTAATCAGTCTGCATCAAACCTTCCCTAATTAAAAAATCTTGGC
CGGGCGCGGTGGCTTGTGCCTGTAATCCCAGCACTTTGAGAGGCTGAGGCAGGCAGATTATTTG
AGGTCAGGAGTTCGAGACCAGCCTGGCCAACATGATGAAACCCCTTCTCTACTAAAATTACAAA
AATTAACCGGACGCAGTGGCTCCTGCCTGTGGCCTCAGCTACTCGGGAGGCTGAGGCTGGAGA
ATCACTTGAACCTGGGAGGCAGAGATTGCAATGAGCCGAGATCGCGCCACTGCACTCCAGTCT
TGGCAACAGAGAGAGACTCTATCTAAAAAAAACCCAAAAAACAAAAAACAAACACAAGAAGTT
TTGGCTGCACCAAGATGCTAGCTGTGTGTGTTATTATTTTGACTACCATGTAGTTTAGTGAAGGT
GTTCAGTAAACAGATGACCAAGTTTAATTTTATAATTTTCAGGAGTATTATCCTGATTCTAGTATA
GAATTATCCTAATTCTAGTGTTAGAATTAGATTTTAGCAATATCTATTACTGTTCTTTGTTTGCCAT
TTTCCTATTGTACTATTGCTACATTTAGTGATATAAGGCCATCTGGATTCAAATTTCTAATTATTTT
CAACATGTATATTATTTTCAGTGTTGCTTTTGGTAATACATTTGAAATGATAATATTGAGCTACAT
GAAAAATAAATATATGATTATTCTATCTTTTATCAATTGCATGGATTAAATGTAGAGAAAGCTCGT
GAGTAATCCCATTATAAAAGATGAGAGAAATAAGAAATACATGCTTAGTGTATATAGAAGATAA
TGGGATATGGTGTTTATTTTAAAATATTTACGTACCTGTATTATTCACTACATATGATTACATTGAT
ACCTCAAACAAAATGTGTGAATATAATTGAATAAAATTATATCTAGAGGGAATTCATTTTCTAAA
GTGTGCATAGCAAAGGGTGCTATGTCAAAACATGTAAAATCATCACAGTGTACTTCAGAAACTT
CCAAGTATTGAATATACAAAATAAAAAACATGAATTTGTGCTGTTTTTCAGCCAAAGGCTCTTAT
CAACAAAGGTTTTCAGTTATTGTGGGAGAACCCTTTCTTGTTACCGTAGATCATCTTACTACATGA
TGGTATTTTATCTTTCTCTCGCTCACTTGACCTTATAGGGAAATCACCAGTAAGAATATACTTTTT
GTGACAATGTAAATATTATCCAGTCAAGATAGTAAATATTCATTCCTTAGCTATGATTGCCAGAA
TTTTCTTAAACTAAGATCAATGTGTGGAATGTCATCAGTATATAAAATCTTACTAATAACTTGGAA
AGACCATGATTTCTTTGTTTCTATATGTGATTTGTGGACGTTTACCTTCTCATGTGCAATCTTGGTA
AATCATAAAGTATGTTTAAATGTCAGTTTTCTCATCCATAAGCTGGGGATACCATTGTCTCCCTTG
CAGTATTGCTGTGGGTATGAAGACTGAAATTGAAGTGGCTGACATTTATAAGGCACATATGATA
GCTATTACATTTTTTATATTTCCATTTCCATTTGGGGAAATGTGGTTGATAGGGAGAATTCTTCTTT
TCTTTCTTCTTCTTCTTTTTTTTTTTTTTTTGGAAGGAATCTCGCTCTGTCATCCAGGCTGGAGTGCA
GTGGTGTGATCTTGGTTCGCTGCAACCTCTGCCTCCTGGGTTCAAGCAATTCTCCTGCCCCAGCC
TCAAGAGTAGCTTGGACTACAGGTGTGTGCCACCACACCCAGCTAATTTTTGAATTTTTAGTGGT
GACAGGGTTTTACCATATTGGTCAGGCTGGTCTCGAACTCCTGACCTCATATCTGCCCACCTTGG
CCTCCCAAAGTGCTGGGATTACAGGTGTGAGCCACTGCGCCTGGCAGACAGGGAGAATTCTTC
TGTTAAGACTTCCCTGAAATTCAAGGTAGGCTAATTGTCCCTTATGTGCTCACTTACCACTGGTG
GAAATATCCCTCTTTAAGACCTAATATAATATACTTTATGTCTTTCTACTGGTAATCAGTAAGTGC
TTGGAGGATAGCAAATGCATTTTATTAAAACTTTTACTCCCAGTACATAGCAGATTGCTGAAGTC
ACAATTGTTAAATAAATGTTTGTTGCCCTGCACTAAAATGTGTAAGATACTTGATGAGAAAATTT
AGAAAATATTTTAAAAGACTTTTTATGTGGCTATAGAATATTTAACTTAAGATCCATGTGTTTGCT
TTATGTGTCAGAGTTTGTTATGTACTATGCTTTTGTATCTTCATGGCTTTTTTTTTAAATCTAGTTTT
TAAACATAAATAATTTTCTGATCTTTTTTAAGTGAAATATTTAAAATACATTAAAAATGTTTTGACA
TTATGATTATTTGTTTACACATGGTTAAATTATATCTATCTGAATTATGTGAATTATTCTTTGACAA
GTGATGATTCTTCATCTCAAAAGCCTAATGAAGATAGTATATTAATTGGTTCTCTGGTCTTAAATG
ATAATCTTTCAATAGTAAATTGCAAAGAAGCTTGCTGATATTTCTCTTGCATCGGTTAAATAATAA
TTTCCAGTAATCAAGCCATGGCTAATGATGATGGAAGTAACACATTTATGCAACAAAAATTTGCA

CATATTTCTTCCAGAAGCCAATAAGCAGTTACATGAAGCAACATAAAAGCAATTTTTTCCATCTT
TCAAGCAACCAACAAAATTAAATTTCATTGTTATTATTTAATAAAAATAGTAATATTTATGACTTT
ATATTACAGTTGGTATACTAATCTTTTAGTTAATAAATTTACAAGCTTAGTGGGAAAAACAAAAC
AAAAGCATAAACGAAATCTGTATGCAGTATGGAACTTATTAGGTACATATGACCCGATTCTAATT
TAAAACCTGTAATGCAATTGAAATAATACCCAACATAAAGCTTATGACAAAACAAATGCCTACA
ATTTACTTTAGAAAGTAAAGCGCATGCAGTTATTGAAAAAAATAGACTGTTTACACAGAATGTTA
ACTGTTCCTTTTGCATTGTTGTATCAAAATAGGTGATAAGCCCCAGTGTGTGTTGTTCCCCTCTAT
GCGTTTATGTGTTCTTATCATTTAGCTCCCACTTACATGTGAGAACATGCCGTATTTGGTTTTCTGT
CCCGTGTTAGTTTGCTAAGGATAATGGTCTCCAGCTCCATCCATGTCCCTGCAAAGGACATGAA
CAACACACACTGGGGCCTAACTGAGGGTGGAAGGTGTGATGAGGGAGAGGATCAGGAAAAAT
AACTAATGAATACTAGGCTTGATACCTGAGTGATGTAACAATCTGCTCAACAAACCTTCATGACA
CTGGTTTACCTAGGCAACAAACCTGCACATCCTGCAAATATACCCCTGAACTTAAAAGCTAAAA
AACAGGCCCGGGTGCAGTGGCTCACGCCTGTAATCCCAGCACTTTGGGAGGCCAAGGCGGGTG
GACCACAAGGTCAGGAGATCGAGGCCATCCTGGCTAACACGGTGAAACCCCATCTCTACTAAA
AATGCAAAAAATCAGCCAGGCGTGGTGGTAGGCACCTGTAGTCCCAGCTACTCAGGAGGCTGA
GGCAGGAGAATGGCGTGAACCTGGGAGGTGGAGCTTGCAGTGAGCCGAGATCACACCACTGC
ACTCCAGCCTGGGCGACAGAGCGAGACTCCTCAAAAAAAAAAAAAAAGCTAAAAAACAGAAAT
AATGACAAGGAAGAAAACACGTACATATTCAATAATAATAATAATAGCCAACAGGATTACTATG
AAAAAAGATAGGTGATAACTCTTCAGCTAACATTAATCAGGCTCTGTGTGCATGACTTGACTTGT
TATTTCTTATATATGTACAAACTCATTTAATACTCATAACCCTACATGTACATAGTTTTGATGACCT
ATTTTACAGATGGGAAAACTGAGACCCAGAGAAGTTAAACAACTCCCCCAAGGTTAGAATCTGA
TAGTTGCTAGAATCTGGCGAATGCAGATGTGTTTCAAATTATATTCGTTATTATAATTATATTTCTT
ATTTGTTAAATAAAAACACATGGGGGTCCAAGATGGCGGACTAGAAGCCACTAGTATGCCACG
GTGCTCATGGAGAGAAATAAAAGGGGTATGTAAATACTACATTTTCTTTTTTTTTTTTTTTTTTTT
ATTATACTCTAAGTTTTAGGGTACATGTGCACATTGTGCAGGTTAGTTACATATGTATACATGTGC
CATGCTGGTGTGCTGCACCCACTAATGTGTCATCTAGCATTAGGTATATCTCCCAGTGCTATCCC
TCCCCCCTCCCCCGACCCCACCACAGTCCCCAGAGTGTGATATTCCCCTTCCTGTGTCCATGTGA
TCTCATTGTTCAATTCCCACCTATGAGTGAGAATATGCGGTGTTTGGTTTTTTGTTCTTGCGATAG
TTTACTGAGAATGATGGTTTCCAATTTCATCCATGTCCCTACAAAGGATATGAACTCATCATTTTT
TATGGCTGCATAGTATTCCATGGTGTATATGTGCCACATTTTCTTAATCCAGTCTATCATTGTTGG
ACATTTGGGTTGGTTCCAAGTCTTTGCTATTGTGAATAGTGCCGCAATAAACATACGTGTGCATG
TGTCTTTATAGCAGCATGATTTATAGTCCTTTGGGTATATACCCAGTAATGGGATGGCTGGGTCA
AATGGTATTTCTAGTTCTAGATCCCTGAGGAATCGCCACACTGACTTCCACAATGGTTGAACTAG
TTTACAGTCCCACCAACAGTGTAAAAGTGTTCCTATTTCTCCACATCCTCTCCAGCACCTGTTGTT
TCCTGACTTTTTAATGATTGCCATTCTAACTGGTGTGAAATGATATCTCATAGTGGTTTTGATTTGC
ATTTCTCTGATGGCCAGTGATGATGAGCATTTCTTCATGTGTTTTTTGGCTGCATAAATGTCTTCTT
TTGAGAAGTGTCTGTTCATGTCCTTCGCCCACTTTTTGATGGGGTTGTTTGTTTTTTTCCTGTAAAT
TTGTTTGAGTTCATTGTAGATTCTGGATATTAGCCCTTTGTCAGATGAGTAGGTTGCGAAAATTTT
CTCCCATGTTGTAGGTTGCCTGTTCACTCTGATGGTAGTTTCTTTTGCTGTGCAGAAGCTCTTTAG
TTTAATTAGATCCCATTTGTCAATTTTGGCTTTTGTTGCCATTGCTTTTGGTGTTTTGGACATGAAG
TCCTTGCCCACGCCTATGTCCTGAATGGTAATGCCTAGGTTTTCTTCTAGGGTTTTTATGGTTTTA
GGTCTAATGTTTAAATCTTTAATCCATCTTGAATTGATTTTTGTATAAGGTGTAAGGAAGGGATCC
AGTTTCAGCTTTCTACATATGGCTAGCCAGTTTTCCCAGCACCATTTATTAAATAGGGAATCCTTT
CCCCATTGCTTGTTTTTCTCAGGTTTGTCAAAGATCAGATAGTTGTAGATATGCGGCATTATTTCT

GAGGGCTCTGTTCTGTTCCATTGATCTATATCTCTGTTTTGGTACCAGTACCATGCTGTTTTGGTT
ACTGTAGCCTTGTAGTATAGTTTGAAGTCAGGTAGTGTGATGCCTCCAGCTTTGTTCTTTTGGCTT
AGGATTGACTTGGCAATGCGGGCTCTTTTTTGGTTCCATATGAACTTTAAAGTAGTTTTTTCCAAT
TCTGTGAAGAAAGTCATTGGTAGCTTGATGGGGATGGCATTGAATCTGTAAATTACCTTGGGCA
GTATGGCCATTTTCACGATATTGATTCTTCCTACCCATGAGCATGGAATGTTCTTCCATTTGTTTG
TGTCCTCTTTTATTTCCTTGAGCAGTGGTTTGTAGTTCTCCTTGAAGAGGTCCTTCACATCCGTTGT
AAGAAATGGATACATTCCTCGACACATACACTCTCCCAAGACTAAACCAGGAAGAAGTTGAATC
TCTGAATAGACCAATAACAGGCTCTGAAATTGTGGCAATAATCAATAGTTTACCAACCAAAAAG
AGTCCAGGACCAGATGGATTCACAGCCGAATTCTACCAGAGGTACAAGGAGGAACTGGTACCA
TTCCTTCTGAAACTATTCCAATCAATAGAAAAAGAGGGAATCCTCCCTAACTCATTTTATGAGGC
CAGCATCATTCTGATACCAAAGCCGGGCAGAGACACAACCAAAAAAGAGAATTTTAAACCAAT
ATCCTTGATGAACATTGAGACAAAAATCCTCAATAAAATACTGGCAAACCGAATCCAGCAGCAC
ATCAAAAAGCTTATCCACCATGATCAAGTGGGCTTCATCCCTGGGATGCAAGGCTGGTTCAATA
TACGCAAATCAATAAATGTAATCCAGCATATAAACAGAGCCAAAGACAAAAACCACATGATTAT
CTCAATAGATGCAGAAAAAGCCTTTGACAAAATTCAACAACCCTTCATGCTAAAAATTCTCAATA
AATTAGGTATTGATGGGACGTATTTCAAAATAATAAGAGCTATCTATGACAAACCCACAGCCAA
TATCATACTGAATGGGCAAAAACTGGAAGCATTCCCTTTGAAAACTGGCACAAGACAGGGATG
CCCTCTCTCACCGCTCCTATTCAACATAGTGTTGGAAGTTCTGGCCAGGGCAATCAGGCAGGAG
AAGGAAATAAAGGGTATTCAATTAGGAAAAGAGGAAGTCAAATTGTCCCTGTTTGCAGACGAC
ATGATTGTTTATCTAGAAAACCCCATCGTCTCAGCCCAAAATCTCCTTAAGCTGATAAGCAACTT
CAGCAAAGTCTCAGGATACAAAATCAATGTACAAAAATCACAAGCATTCTTATACACCAACAAC
AGACAAACAGAGAGCCAAATCATGGGTGAACTCCCATTCACTACATTTTCAACTAAAGCATATA
GGTGCATGGACTGGGAATAATCAAGAAAACAACTCGAGCCACAGATAAAGCAGAGGAGCAAG
GCAAAATGACTGTCCACCCTGGAGTGACATGGAGCTAGGGGAGGCTCTCCTGCCCAGGGAAAC
AGAGAGTGAATGAGAGACCGCGGGGACTTATGCTTCTTCCACTGATCTTTGCAACCCGGGGGTC
AGGAGATCCCCTCACTTGACTTACTCCACCAGGACCTTCAGTCTGACATGCACAGCTACCTAGA
GTCTTAGCAGAGACACCTCTATGGTACATTTGGAGTCACCACACATCCTAGCAGTAGCAGCTGC
CGCTCTGGCAGAGGGGGTGGTTAAAGTCCTATACATGCCCACAGGAAAGAGGCTGAATCCAGG
GGGCTGAGCAGTGATGGTCTGCAGGTCTGGCTTCCAGGGCAGCTCACAGGATAAGACCCACTG
TCCTGGGACTTCAGCCAGACTCCAGTAGCAGCCCTGAGCTCCCTGAGACAGAGCTCCCAGAGG
GAAAGATGGGCTGCTATCTTTGCTGTTTCACAGCCTTGGCTGTTGTAGTCTTCAGCGCAGCTGCC
CTACGAAAAAGGAGTCAGCCTGCTTCTTTATGCGAGTCCCTGATGAAATTTCTCCTCACTGGGCA
GGACCAGGGTCTCCAGTCACCCTTTGCTGGTGTTTTCAGCAGCCCCATCCCCCCGTGGGTTGGA
GCTCCCAGAGGGAGGGGCAGGTCATCATCTTTGCTGTTTTGCAGCCTTAGCTGTTGTTTGCCTTC
AGCATTTAGGGAGTACGAGGTGACTAGGGACTGGAGCAGACCCCCAGCACAGCACTGCTGCCC
TTTGAAAATGCAGTCAGACTGCAAAAAAACAACAACAACAACATAAAAAAACAACTGTGGGTC
ACCTATCTTATTTCTCCTCACTGGGCAGGACCACTCGCCTGGGTCTCAAACCACCCCCTGCTGAG
GCTATCAAGCCAGTAGCAGCTCTGCACTTATCTAGGACAGAACTCCAAGTGGGAGGGACAAGC
TGCCATCCTTGCTGTCTAGCAGCCCTTGCCTTTGCCATCTCCAAGCTTGGGAGAGTCTGGGGGG
ACAAGGGGCTGTTCTGGGCCCTCAGCACAGGGCAGCCGCCTCACAGAAAAGTTAATGGACTGT
CCTCCACACAGGTCCTGGTCCTCACTTCACCTCACTGAACAGGGTCACCTGACCTGGACTGTCC
TCCACACAGGTCCTGGTCCTCACTTCACCTCACTGAACAGGGTCACCTGACCTGGGACTGGAGC
ACAAATACCCTGTCTCTGTCTGATTACTTCAGTGAGTGTCAGCTCAGAATTTCTGAGGAGAAAAT
TCCAAAATCAAGCCACAAACCCTCTGGCAGTACAGCCCTAACAGCCCTTGAGCTGAGGAAAGA

ACAAAGGGCCTAGTCTCTAAGCTGGCTCTGCCAGCACAAAGCAGGCACCATATGGAGAGGAGT
CCAGCCCCTCTACCCTGGGAACCTGCACCCTCACTCTTAAGACAGGACCCCTGGTGCAAAACCA
CAGAACAGCCACCTCTACCAGTGGCTGAACATGCCCAGCAGAAGTGGCTCTGAGATTTTCTATG
GTAAGGCTCCTAGAGCCAACTGACTATCCCTCTGCCACTGCCATAGCAGTGGTCCTTCCCCTGC
TGCCCTCAGCCTGGGGAAGAAACAAAAAGCCTGAAGGCTGCACTCAAGCTTACAGCATGCCAC
AGATACCATATGAAAAGGAGCTCAGTCTCTCTTCCCTGTGAGCCCTGGAACCCCTGCCACCCAA
CAAGCAGAGGCTCTAGTTCACACCAGCAGTGCAACTGCCCCAGTCCCCCGACTCAACACCGCC
AGTAACAGCAACTCCATGTTGGAGGTGGAGTCACTCGAGGGGCCACTGAAAACCCCTCTGCCA
CTGCCTCTGCAGTGGTACTGCCCCTACTATTCTCTTGAAAGAGCAAAGATCCTAAATGCTGTATC
TTAGCCCCAGCAAGCTGCAGTCAACACAAAGAGAGGAGGCCAGTCTGTCTCCCATGGGCTGCA
GCCCTGCTCCCCCTTGCTCATCAATAGGCAGGGCCTCCCTGGCTTGGACAGACAACAGAGAATC
CCATCCCAGGCTGATTGCACTGAGTGATTGAT

ID|30372|gene_id|ENSG00000251629|transcript_id|ENST00000512688 Homo sapiens lncRNA

TGCCGCCATGTAAACGTTCTTTGCTCCTGCTTCGTCTTCTGCCGTGATTGTGAGGCCTCCCC
AGCCATGTGGAACTGTGAGTCCAGTAAACCTCTTTTTCTTTATAAATTACCCAGTTTCAGATACGT
CTTTATTAGCGGCGTGAGAATGGACTAATACACTAACTTTCTCAGTGAGCATCTTATTCCAGTTAT
AAAACTACCTCTGGTTCTGAAGATACAAAGATAGGATAATGCTAACTATTTCTTTGCATAAGGCA
AGTGAAAAAATACAGATGACCTGATATCTTCTATCTGCCTACTCTCTCTACCTTTTGAAAGCTCTC
TTTCTGTTGCTTTAATAGCATTGAATTGATACGCCATTATTGTGTCTATTCCTTTCACAATACCCTT
AAATAAAGTCTTACTTTTAACTTTTTCCAGTGCCGTTTTTGTTTTGCTGAACCTTAGAAAAATTAAT
AGTTTACAGTAAATTAGGTGGAGATGCTAGAACATCTGAGGCAAAATATTGAGAATGGGTTTTA
AAGGCTACATAAAAATATGCTGGCCGGGCGCGGTGGCTCACGCTTGTAATCCCAGCACTTTGG
GAGGCCGAGGCGGGCGGATCACGAGGTCAGGAGATCGAGACCACAGTGAAACCCAGTCTCTA
CTAAAAATACAAAAAATTAGCCTGGCGTGGTGGTGGGCGCCTGTAGTCCCAGCTACTCGGAGA
GGCTGAGGCAGGAGAATGGCGTGAACCCGGGAGGCGGAGCTTGCAGTGAGCCAGGATTGCAC
CACTGCACTCCAGCCTGGGTGACAGAGCGAGACTCCGTCTTAAAAAAAAAAAGAAAAAAAAAA
TGCTAAAATGGATTATGTAAGAACAGATAATCCACGATTTAACTATGTCCCCGCAGAGAACTTA
GGAGAGATTCCCTTCACTAAAGTGGTGAGATATATGTCATGCCACTACCATCAAGATTTCAGATA
AAACAGGAGAGGTAAATCTTCCCCAAAACCCAGTCTACTGCCATCAAAGGTGCAGAAAATATAT
ATTGTCTATGAAATACAATTGATGTTCATCACATATAATCCTAATTTATTTTACCTTGCCTTTTGTG
TATTCCAGTAGACTATTTAAATATTTCATAATGACAGAAGCTATGAATAAAAACAAAATTAATTTT
ATTTTTCATATTTATATTCCATTTATTTCTAAATAGGATATTGCAACTTAATATTATTTTTTAAAGAT
GCAAACACAAACATCATTAAATTGTTTAAAAAATTCAGAAAAAAATTAAAATACATGGAATGAA
AAAGGCACCTATATGAGGGATAAAGATCCTACTTAAAATTAGTTGTGAGTATACTAGTGGACAA
CAAAAAAGAGATTGCAATTGGATATGTTGCTCCTGTTATCCAAAGTAGGAAGCCCATTCTTTCTG
TGGATCAGTGTCTTAGTTTTCTATCACTGCTGTAGCAAATAGTCACAAATTTCATTGTTTGGAACA
ACACAAACATATTTTCTTAAAATTCTGTAGGCCAAAAGTCTTGGTAGGCTCAACTGCTTCCCCTTT
TCCAAATCTCACAGGACTACAATCAAAGCATCGGTTAGCCTGGGCTCTTATTTCAAAGCTTTGAG
AGAGAATCTTCTTCCAAAATCATTCGTGTTTCTGGCAAAATTCCTGTGCTATCCTGGCAGGCTATT
GGCTGGGAGACCTAGCTGCTTTAGACCACCTGCATTCCTGATCGCTTCATCCTTTCATTCTTCAA
ATAAGCAATGTATGTTAAGTTCTTAAGCTTTAAGTCTCTCTAACATCTCTTTTGCCAGATTTCTTCT
TCTCCAGCTGAAGATAGCTCTCAGCTTTTAAAGGTTCCTGTGACTAGATTGGACCCCCTCATAAA
TTTCAAATTAATCTTTTAATTTTAAGATCTTCAACCTTAATTACATCTGCAAAATTCCTCTGTTCAA

GAAATATTATGTATTCAAAGGTTCCAGGAATTAGGGAGTAGATATCTTTAAGGGGAAATTCTGCC
TTCCATGGGGAGAAACTTGGAATGTCAAGCTTTCCTAGTACCAAAATATTGCCAAACACACAAG
CACAGCCTTTTCGAATATGACTTAAGTTGGGTCATAATAATAAATGTAATAATATTAAGCAATAA
TGTCCTTAATACCTCTGAAATTATTTAGGCATACTTCATTTCCTTTGGCAGTTGTAAATTCCCAAG
CAATCTTGTTTCATAATAATAAAATGTTTTACCCAGACAAGGGTAGTGTAGTTGCTTCTAGAAGT
AAATTGTCTCAAGATGTGTTGAAATTGTACTTTCTTTAGACAATGGTTCTGATATAATATTTTCTGA
CTTTTGATAGAGGCTAAAATTAGGTAGTTTTTTCTAAAAGTATTTAATTAGGGCTTTGGATACTGG
TAGTTTTATATTGTGAATTGGGAGATTTGTTCACTTTGCAATTAGATAAACAGAAGATGAGTTTGA
GCACCAATTGCATTTTTCTCTTCCTCTGTCAATAGAGTTTGTTAGGTAAAATCCCTCAAATATCAG
GTGAACTCAAAAGAATGTCTTTTGTATTTAAACTGGTTGCATAGATTCATGTATATCAAGTGCTTG
AAAATTAGCTAATATTTTCTTTAATATGATGAAGATGCCTCATTTTCATTCTTTCTAAATGTACTTC
AGTTGCTGAGAGAATTTTAGTATGCTGAGTGATAGTTCTGGCCTTTTGGCAAATCAGGCATATCC
TGAATGTAATGATAATCCCCACCCAGCAGGTATTTCTAACTGAATACAAACAAAATTAACTGTGT
GAAAATGTTCATATAATTATGGTGTGACAGGATGTGAGATAAGACACTTTATTTTCCTTATTGTCA
ATAGGCAGATTGCAGACTAATTTAGTGATTTAAAAGACATAAACAACTAATTTTTTAGGCATAAA
AAACCTAAATCAATGTTTGAGGCAACTAAATAACTAAATACTGTGAGGTAAACATTTTAAAATAA
AATATTTTTATATTCTTATTTTCTTTTATGTACTATTAACAGGCTTGGAAATATTGTTTCCTCTTCTT
AAATATACACAATAATACATATTTTGGTAGTTTATTCATCTTAAAAATAATGAAAGAATCCAGTAT
TTATTTTGAAGACATTGCATATGCATATACAGTTATATAAATATGTATTCTTTTTCAATATTTTCCG
ATACTCCAAGTCTCATGACTCATAGAATAAAATTGAAAATAAAAGCTGTTGACTAATATGATTTA
TTTCTGTTTGCAAATTTGTAAATAGGAAAGCCTCTCAAATGCTTATCAGCTCCAAGAATACATAA
ATACAGAATATCGAGCTCCTATTGCTGCATATTCCAACCCCCCACAAGCTGCCATCTGTTACTCA
TAGGTACAGCTTTCTAACATCAGGTCTCTATGAACTCGATGCTTATGTTTTACAAAATAAATGGT
GGAAAGAAACCAAAAATGGTTAGAGTCTCATCTCTACCACTGTCAAATGGGCTTCATTTTTCCCT
TTTCTCACAGCAAATAACAAAATATCCTCCAGAGAGTTGTGCTAAATGCTGCCTCTGGTCATATG
TTGCTTGGTGTGTAGGGAGCAGCCGCAATGGAGCAACAACATTATTTGCAGATGTTATTTTGTGT
TCTGAAGTGTTATTTTCTTATTAATTTATGCTAAGTCAAATTTGCACTGAAATACTAAAGTACTAA
ATACTGAATTTAGTATTTTGAAGAATATTGAGGAGTATAAGAAGGACATGTATCACAATTGCTTT
TATTTATTAATAAAATAATAGTGTTTATCTTAGTTCTTATATATAACTTTTTTAAAGTAACATGGAA
GCATGAGTAAAGTTAAAACTTTGAGTGACCATTTTCAGAAATAAAACTGTATTTAGGTACCTGGA
AACCAAAATTTAGACTTAAAGTGAAAGATAGGATTTGGATTATCTAGTTTTGATGTGAAGAAAGC
AAGCTGATCCAGGCTGCCATTTACTTGAACTACTCTTTGTAGGCCTCAGCCTCACGTTCAAAAGT
GTTCCACACAGCTACTGGGTACAGTTGTCTCTATTTAACCATACATGAAGGAGGGTCATAAAAG
CATGGGATTTAGTAAATCGGTATGCTGAGTCATATGTTGCAATAAACTGCATATAATAAATTAGT
TGAAATATGCAATGCACAGGTGGGGATGTATATAATATACATAAAACTATGTAATATATACCTAC
ATACATACATACGATATTACAGCAAAATTTGGAGCTATATTCCAATTGTTTTCCCCTTATACATTT
GAGGAACTCTTTGGTCAGCATATGGCTCTCTCATAAGGTCTCCCTGCCTTTTTTTAATGTCCAACC
CTCCTTTGCATTCCCACCTGGCCCAGAACGTGTGCTCATCCTGTACTTAAGTAATTATTGCATTCA
TTCAATCTTTATCCCAGAGCTTTTTTTTTAATTGTGCAGAGTTTTCACACTACCTACAATTTCTAGA
ATGTTCTGTCTACAAAGCATGAAGGATTCGTGTCTATTTATCTTGATCTAGCAGTTTCTTTTTCTAT
GGCACTGAATAAACTTATAAACATGCAGGATTTAAAAAATATGATTCAATTTTTCTAAAGATGGT
GAAAGTGATATTTTCTTTAGAATTTTTTCCTGATGGATATTATGTATAAATCTCTCTGATTTCACAT
GGCAAAAATAAGGTTAAGTTAAAAATTACCTGAGAGAATCCTTGAATTTATGACTTCTGGGCTTA
AGTTTTATTGTAACATCTCCACGTTTCAATATTTAACTATCTCTGGGCCACATAAAGCATGAGGA

CAGGTACTTTTGATATATTTGCTGTGTTGTGGGAGAAAAACAAATCTCACCATTTTCTTATAATTG
CCCCAAAATTTTAGTTTAAGAAATACTTTTAATAAAAGTAGTCTCCTTCCCAGATTAGTAAATGTA
TTCTAACTAAAGGGTTTTGTGTGTGTGTGTATGTGTATATGTATACACCTATATATATTCATGTATA
TATATACATCTATATATATACACACACACCTTTAGTTAGTCTATGTGTGTGTGTGTATATATATATA
TATATATATATAAAAGTTATAGTCAATTGAACTCAACGAATCAAATGATTGAATGTGCCCTATCT
GCCAGGTATTCGATAATATAATTGAGAGTGAATACAAGAAAAAATAAACTATACTTACTATCTCA
GGAAGTCCATTTTAATTGGGAAGTAAAAATGTAGCCACATAAACATGTTACATATTGATAGGATA
CATTTTGGTGGTGTTTGCCAGGTGCTGCAGTTGTGGAATTAGCAGCCATTAATTTTATCCTAGGG
GTTGAAGATACTGTTAGTGCTAGTTTTTGAAGAACAACTGGGGAATTTCCAGCTGAATTTGATAA
GGACAATACAGGGAATAGAAATACTATTGTCAGAGAGCTTTACAATGCAAAGTTTCTGTGTTCC
AGTAATGTGTCTGTACAAGAACAGAAAAACTATCGTGGATTCAAATTTGAAACTGTTTGTAAGCC
TGAACAAATAATTTTAAGCAGAGGCATTCACAGTGTGATTTATTTTATAAAGAATATTTGGGATCT
CAAATTGCAGAATGGATAGTTTATATTAGAAAAGTATTTTAACAGTTCAACGTAGGAATAATTGC
AAATTGAACTAGGGTAGAAATTGTGGGGAGAAGATAGATATGTATGCACTTATATGTACAGATG
TGCATACCTCCTTGTTTGGATGATGGGACCCAACACATGGGCACTTGCATAGACGAAAAGCAAA
TTCTATGTTACTTTCAACCCCAAAGGAGAGAAGACTGCCTGGCAGGGTACACAAGGTGGCCCAC
CCAGAGACAGAGTAACCTCAGGATGGAGCTGTAAGAGATAGGTAATGTATAGCAAGTGGGGAG
AGATGAGTGAGGTTTGGTATCTGGCTCTGGGGAAATTCAGGGAAGTGTTTGTGACTGAGCATGT
GAGAGCCTGATAAGGGAGCTGGTTGAGGTGGGCCTCTTCCAGCCAATTGGAGGTCTGAGTATA
ACAAAAAAGCAGAGTAAGAAGGAATTCCTCTGGCTGGACTGCCTTGAACTGCAATATAGGAATT
CTCCAGCCTGCAGACTTGAATTGAAACGTTGGCTCTTTTGGGGTAGAGCTTTCTAGTCTTTAGAT
TAGAGCTTTTTGCATCAGCTCTTCTGGGTCTCCAGCTTGCTGACTGCCCATCTTGGGACTTCTCTG
TCTGCATCTCTCTTATGTGTATACATAAGATTTGTTGTTAAATTTTACACACACACACACACACAA
TTGGAATCTGTTGGTTCTGTTTTTCTGAAGAATCCTATTACAGGTCCACATGGGGAAGTACTAGA
TTTCGGCAGAAGGAGAAGTAAGGAAAAAGTATAGATTCCTCAGGGATCTAGAACTAGAAATAC
CATTTGAACCAGCCATCCCATTACTGAGTATATACCCAAAGGACTATAAATCATGCTGCTATAAA
GACACATGCACACGTATGTTTATTGCAGCACTATTCACAATAGCAGACTTGGAACCAAGTCAAA
TGTCCAACAATGATAGACTGGATTAAGAAAATGTGGCACATATACACCATGGAATACTAGGCAG
CCTTAAAAAATGATGAGTTCATGTCCTTTGTAGGCACATGGATGAAATTGGAAATCATCATTCTC
AGTAAACTATCGCAAGAACAAAAAACCAAACACCGCATATTCTCACTCATAGGTGGGAATTGAA
CAATGAGAACACATGGACACAGGAAGGGGAACATCACACTCTGGGGACTGTTGTGGGGTGGG
GGGAGGGGGGAGGGATAGCTTTAGGAGATATACCTAATGCTAAATGACGAGTTAATGGGTGCA
GCACACCAGCATGGCACATGTATACATATGTAAATACCCTGCACATTGTGCACATGTACCCGAA
AACTTAAAGTATAAAAATAATAAAATAAAATAAAATAAAATAAAATAAATAATATTTAGGAAGA
ACCATTACTTAATAAAAGATAAACCATTTATTCAAATATTTTCTTTTATTTCTGAATCCATCACTGT
GGGAAATCTGGAATTCTACATTTCACAAGACAATGAACTTTTGTTTGCCCTGGAGAAATATGTAA
CTTTTCACAGGCTTTTTCTATTTTAACATATATAGAGATAGTGTTTGGAAATTTAAGAACAAGAGT
GGACCTAAGTATGAAAAATACGTACAACTTTTTCTTTAGTTACTCTTGATACTTTTCATTGTTCAG
GTTTTATGTATAATAAAACCTTACTTTATTTCCTTTCTTTTTAGTTTTCTTCCTTTGTTTTGCTATTGC
TATAGCACAGGATAAAGAACTTACAGAATATACATTGGTTATGCAATTTAATTAATTTTTAGGAG
TTATGATTATTATAATCATCAAAGCCTTGGGCTCAAAATAAAAATAATGATTTTCATTTTTAAATA
TACTTTTATTTTAAGGAATCAGTGGCTATGATTAATTTAATAGCACTCCTTCAATTCTAATAATGTT
TTGTTACAAAGCTTACATGTCATTTCTTTAAGCCAGCTCTTTTTATGTTATTAATGTTAGAACATTT
TAAGTCAATGATTTCATTTGCTAATATTGGTTTATTTCTAACAGCATCCATCATTGTTTAACAGTCT

GTTGGAGTGTTGCAAATTTATGAATTTTTATTATGAGGGCTCCTTTTGGCAACAGATAATTTTTTG
TTAACTATCATCAAAAAAGATAGAGGCTTTCTTCTTTCAAGAACAATTTGTGTTTTGATAAAAACA
ATGTGTTATAATAGATTACATTTATTTTATATAATGTTATAACTATTAGGGATGAGGCTTTTATAGA
GATTATTATAATAACTTTGCTAGTCATTGATTTTTATTCAGTAATATATGTTGTCTTGTGTGTTTGTG
TGTGTGTATTCATTCCCATGGATACTTGTCCTATGGTTGTAAAAACATTAATGTCAAAATGCATGA
GTTGAATTTAAGACATGGAGAGACTGCAATTGTAGTAGCAATTAATTTTGTTCACTATGTATTCA
AATATGGTCACCTCACTGAATTCTGAAAATATAATAGAATTATCCTTCCTGATTCTGATGTAGGTG
ACTGCAGTCATAGGATTAGTTCTGAAAAATTATAAGTGCATAAAACATCTCACTTATAGGAAAGA
CATTTACTTAATAAGAAGAGCTTCAAGAACATTCTTTACTTACGCCACAATATCTGGCAGAATTT
GTGATATAGTTAGTAAACAAGCCCAGATACCTAAATGACTACAGTGAGCACAGGAGCCACCTC
CCATATACAAACTTACAACTGACATGTTCCTTGAGCAAGTAATAAACTTTTATTGTATCAAGACA
TTGTTATTTTATTTGGAGAATATTATAGAATAATCTCTGTTATCCTGATTTACATAACACAATTCAC
CTTTTTCATATGCATGTAAGAATACAGATCATTAACATGGTTATTTTCTATGAAAATTATAGTTCA
AGTTTTAATCTGATACTTGAAATAACAGTATGCCTTTGCCCAACTAAATATAACTTGCTTTTTTATT
GTCAGTGAGGATAATGATTTTTATCACTATATTTCTCAAAATACAAAGCAAGAAAGAATTCACCG
AGTCTGTAATTAGTTCAGGAAATATGTTGAAGAAACATAAGGGGGCCAGGCGCAATGGCTCAC
ACCTATAATCCCAGCACTTTGGGAAACTGTGACGGGTGGATCAATAGAGCTCAGGAGTTTGAGA
CTAGCCTGGGAAACATGGCAAAACCCCTGCTCTACAAAAAAATACAATAATAAAAGTTATAATA
ATCTGGGTGTGGTGGTCTGCACCTGTAAGTCCCAGCTATGTGGAAGGCTGAAGTGGGAGAATC
CAGGCCCAGGAGGTTTAGGATGCAGTGAGCCGTATTCCTGACACTGCACTCCAGCCTAGGTGTC
AGAGTGAGGCCCTGTCTCAAAACATAAAAAATAAAAAAATAAGGATTACATGAAATAGGATACT
GTTATTATTAAATGTAACACAAAATAAATCCCGATGAGATTATAATATGCTGATATTGATAATAA
CAATTATAATACCAGCAAAATTATATTTATTAAATATTTAAAATGTGCCTGTCACTATACTAAGTG
ATTTGCATGTTGTATCCCATTTATTCTCATAGCAATCTTGTCATATAAGTAATATTATCTCAATTTTT
TCAGAAAATAAAGCCAAACTAAGGTTTAGTGAGGTGTTTTGGGCTGAATTGTGTCACCCCAAAA
TTCAGAACCTCAGAATATAATGGTATTTAGAGATAAGGTCTTTCAAGAAATGATTATGTCAAAAT
GAGTCTGTTAGGGTAGGGTCTTAACCCATATGACTGGTGTCCTTATAAAAACAGGAGGAGACAC
CAGGGATGACACACACAGAGGAAAAGCCACGTGTGGACACAGTAAGAAGGCAGCCATCTGAA
AACCAACAAGAAAGATCTTATGGGAAACCAGCCCTGCCTGCACCTTGATCTTGGATTTCTAATCT
CCATAAATATGAGAAAAATATTTCTATCAAGTCACAGATCTGTGGCATTTTGTTATGGTAGTCAT
ATGGCAGTTATATTATTATCTTTAACTATAATAAGATAGAGGTAAAGATAACTTACGTGAAGTAA
CAAAAATAGTAAGTGACAGAGACAGCATTTACATCAGGAGTGTTTAACATTAGTTTACACTTGTA
TCCCAAGCACACATTGCAGTTTGGATATTTCTTTGTGGTTGCAACCTTTTTTGCTTAGCCTTTTTGT
TGCTTCTGTAACAGTTATTGTTCAACGATAAAATTGCCATAAGCCTATTTCACCATATTAGAATTT
AGATCACAAATTCATTCCCAAACATCTGTTGAGAACACATTGGATGTCTGAATTAAAATGACTTA
AGAAACACAGTTTTATGGTATGTGATCATCTGACTTAGTCTTCCAAACGGTTAAAAATTCCTTTAA
AAAATGGGAACTGTGCAGCTCATAAATCAACACAACATAGGTGAGGAATTTCCAAAGGTGATTA
AATTTTTATGCACATTTATATAATTTAGCACATTAGTATTCCTATGTCTGGGTAAAACTCTCCTGTG
GGCTTTAAACTGCTTAGTTGGTATCATCAAATTAACGATGACCAAATTAGTACATATTAGCAGTT
AGATTCTTATATTTAAGGTCAATGTCTTATTTCCTGTATAATGTTAACAAACTTACAATAATTTGTA
AGTCTTTGCTTGCATAAAATGCCACTCATAGTAAAGAATATTCTATAGCTTAAATGATAATGAGA
AATATTTTGGATTTATATTCCAATTTTATATTACCAAAGGCTAGATGGTATAGATTCCAGTTATGT
CATCTATACATGGTGCATCATTATTAACTTAAAGTAGAGCTAAAGCAAAACAACAGTAACAGAG
GCACTATTTATAACAGTGAAAACATGGCCAGGTATGGTGGTTTATGCCTGTAATCCCAGCACTTT

GGGAGGCTGAGGCAGGCAGATCATGAGGTCAAGAAGTCGAGACCATCCTGGCCAACATGGTG
AAACGCCGTCTCTACTAAAATTACAAAACTTAGCTGGGCATGGAGGCGTGTGCTTGTAGTCCCA
GCTACTCAGGAGGCTGAGGCAGAAGAATTGCTTGAACCCAGGAGGTGGAGGTTGCATTGAGCT
GAGATCGCACCACTATACTCTAGCCCGGCAATAGAGCAAGATTCTGTCTAAAAAAAAAGAAAA
AAGAAAAAAAAAAAAACAGTGAAAACATAACCTTCCCTTCATTACTGAAGATCTATCAAAACCAA
TTGTATATAATGCAAGCATCTATAGCAAAAGTTTACTTTTGTTTAGACATATCTAAACCAGTCTAA
TAAAAGTAGCAATGTTTTGTGATGGAGACAGATCACAAATTTAGATATTAGAAATTGTTGAGACT
ATTGGATAATTAATTAATTTAGGGACCCTAGCTATCTTATACATAAATTTTAATATGTATTATGATT
TATCAGTCATTTAAAAATGTCTACTTTTTCAACTCAAGATAATTTGTGAGCAGCATCAGAAAGGA
GGAAACTTCTTTGTCCTCTTCCTTTATAATAATTCCTTCCATTTGGTTTTCTACCTGTATTTACAAA
ACATTTATAAATCATCCATTGCTTCTTAATGGCTTACAGAAGCAATGATGAAACTGTGACAGGAT
AAAATTAACAAGATTTTCTCATAATATAAAACTAAGCTCCATTGTGTGCTAGATGGTTTTTGTCTC
AACGTTGTGATTTAACATAAATTTTAAACACAGAACACTATTTCAAGAATTCTTCTCTCATTCTGT
AAACAAATTTGAAGAGGAATGTATTAGTCCGTTCTCATGCTGCTAATAAAGACATACCTGAGACT
GAGTAATTTATTAAAAAAAAAGATGTAATGGACTTATAGTTCCCCATGGCTGGGAAGGCCTTAC
AATTATGGCAGAAGGTGAAGGAGGAGCAGAGGCATGTCTTACATGGCTGCAGGCAAGAGAGT
GTTGGCGGGGGAAACTGCCCTTTATAAAACCATCAGATCTCATGAGACTTACTCATTTTTATGAG
AACAGCATGGGAAAACCTGCCCCCATGATTCAATTACATCCCACCAGGTCCCTTCTATGATACA
AGGGGATTATGGCAGCTACAATCAAGATGACATTTGGGTGATGTCACAGCCAAACCATACCATT
GTCCTTACATTTCAAAACACAATCATGCCTTCTCAACAGTCCCCGAAAGTCTCAACTCATTCCAG
CATTAACTCAAAATTCCCAGTCCAAAATCTCATCTGAGACAAGGCAAGTCCCTTCTGCCTGTAAA
ATCAAAAGCAAGTTAGTTACTTCCTAGATACAATGGGGGTACAAGCACTGGGTAAATATACACA
TTTCAAATTGGAGAAATCAGTCAAAACCAAAGGGGCTATAGGCCCAATGCAAGTCTGAAATCCA
ATAGGGCAGTCACTAAACCTTAAACTTCCAAAACGATCCCCTTTGACTCCATGTCTCACATTCAG
GTCATGCTGATGCAAGAGGTAGTTTCCTATAGTCTTGAGCAGCTCTGCCTTTGTGGCTTTGCATG
GTACAGGACCCCTCCTGGTTGCCTTCACGGGCTGGCGTTGTGTCTGCAGCACTTCCTGGCACAT
GGTGCAAGCTGCTGTTGGATCTACCATTCTGGGGTCTGGAATATGGTGGCCTTCTTCTTACTGCT
CCACCAGGCAGTGCCCCAGCGGGGATCCAACCTCACATTTCCCTTCCACACTGCCCTAGCAGA
GGTTCTCCATGAGGGCTCTGCCCCTGTAGCAGACTTCTGCCTGGGCATCCAGGTGTTCCCATAC
ATCTTCTGAAATCTAGGCAGAGGTTCTCAAACCTCAATTCTTGACTTCTGTCCATCTACAGGCTC
AACACCACGTGGAAGCTGCCAAGGCATGGGGCTTGCACCCTCTGAAACAATGGCCTGAGCTGT
ATGTTGCCCACTTTTTGTCATGGCTGGAGCATGTAGGTCACAGGACACCAAGTCCCAAGACTGC
ATACATCAGGGAACCCCTGAACCTGGCCCAGGAAACTATTTTTCCCTCTTAGACCTCTGGACCT
GTGATGGAAGGGGCTGCCTCAAATGTCTCTGACATGCTCTGGAGACATTTTCTCCATGGTCTTGG
TGATTAACATTTGGCTCCTCATTCATTATGCAAATTTCTGCAGCCAGCTTGAATTTCTCCTCAAAA
AATGTGGTTTTCTTTTCTCTCATATCATCAGGCCATAGAGTTTTCAAAGTTTTATGCTCTGCTTCTT
GTTGAATGCTTTGCCAATTAGAAATTTCTTCCAGCAGATACTAAATCATCTCTCTCAAGTTCAAAG
TTCCACAGATCTCTAGGGCAGGGGCAAAATGCCACCAGTCTCTTTGCATAGCAAAAGTGACCTT
TACTCCAGTTTCCAACAAGTTCCTCATCTCCATCTGAGACCACTTCAGCCTGGACTTTATTGTCCA
TATCACTATCAGCCTTTTGGTCAAAGCCATTAAACGAGTCTCTAGGAAGTTCCAAACTTTCCCAC
ATTTTTCTACCTTCTTTGGAGCCCTCCAAATGGTTCCAGCCTCCACCTGTTACCCAGTTCCAAAGT
CACTTCCATATTCTTGAGTATCTTTACAGCAGTGCTGCATTCTCTGTGCAACCAATTGACTATATT
AGTCTATTCTCATGCTTCTAATAAAGACATACCTGAGACTGGGTGATTTATTGGAAAAAAAAAAA
GGTTTAATGGACTCACAGTTCCACATAGCTAGAGAGGCCTCACAATCATGGTGGAAGGTGAAG

GAAGAGCAAAGACATGTCTTACGTAGCAGAAGGCAAGAGAGCGTGTGCAGGGAAACTGCCCTT
TATAAAACCCATCAGTTCTCATGAGATTTATTCACTATCATGAGAAAAGCATGGAAAAATCCCAC
CCATGATTCAATTACCTCCCATCAGATCTCTCCCATGACACATGGGGATTATGGATAGCTACAAT
TCAAGATGAGATTTGGGTAGGGTCACAGTCAAACCTTATCAAGGAATTGAATACAAAGAGATAA
TATACTAGTTATCATACTTTTGTGTGTTTACATAGGCAATATGAATATTTTCTGTTTATCTTTTGCAT
TTAGGTGCTTATCTTTGATTTTTTTTACCAGTTTCTTAAAATTACATAAGTAATCCACAAAAATCAT
CTCCAGTTTATAAAAAAGATTCAAATTGGTTGACATAGAAATGTGTGTTTTCAAAGGCCAGTTCA
CCTTCTTTTTTTCGTCTGTAACACTCTCTTATCCCTAAATAAATTATATTGTAAGTTGTTGGTATAC
CTACATGGATCTCAAATGTGGACAATTTTTACCCCAGAATAATATTTGGCAATGTATAGAGACAT
TTTTGATTGCCTTATCTAAATAGAGGGAGTGAAGAAAACCTCTAGTGTGTATTAGCCAGAGATAA
TGCTCAATCATTCAGTCCTCAGGACAGCCCCATCACAAAGAATTATTAGTCCATATGCTGGTATA
TATCATGCCAGTCTTTCTCTGGATCAACTGCATTTACACATATGCACATGGATGTTTTGTTGACTG
TAGTATGTCAAAATTTAAAGACATATCAGGCACACAGTAGATAAAACTCTTCAAGCTATATCCAT
ATGAATGAAGGTCAATCATTCATATTGATAGAATTAATAAATTTGTTGACTAATAAAGCTCTTTAA
ATACATGTTATTTTAGATAAAAAATTAAGAGCTTCTCAATTTTGTAAAATAGTCATCTTAGTGTTTTA
GTTTTTGAATTATTAAAAAAAACACTGTTGAATTTTTTTTTAATAATAAAGCTCAGCTTTGCTACTA
CTTCTGGACTAGTCTATTTGCCAATTCTGAGATTTCTCAAATTTATGAGTCACATTAGATAATGTC
TGCTCTTTCAATTGTTCTAATTTGTTTTATTATCTGATCCAAATGCCTGAATCTCAATGGATTTATC
ATTAACTCTTTCTAACTCAGAAACTCTTGTGAGAGAGATGACAGACATATCACAAGATTATTGTG
CTGCACTGAACAAGGGCATGATACATGGTAAATAAATGAATGAAAAGAGAAGGAGAAGAGAG
CTATTTTGGGGTCTCATTTATACTCCACTTATATCATCTTTGATTTCTAATAATAAACTGATGAAAC
ATATTTTAAGTAATTTATAAATGTAAAATTCGTTTTTTATTTTACAATTTTAGCTACAGCACAGTAA
CTGTTATAATAGTTAAAACTTTTTTTCACATTAGAACCTTACTTTTTTAACAGTGAAAAGAATTATA
ATTATGTCTTTACATTTCTATCAGTATTTCATTGCAATATGTCCTGAATTTGACAGATGAACAATTA
TCTCTTTTTTTATTAATGGACTTTGCAGGAAACAAATTAAGGCCATTTGAGTAATTACAATAACTT
GATACATTTCTAATTTGCTATAGAATGTAACTCATGACTTGTATCTGAAAAAAAAGGAAGGAAGA
TAGAGAGACAAAAGATAGGAAGGGAGGGAAGAAGGAAACAAGGAAGGGAGGAAGGAAGGA
AGGGAGGGAGGGAGGAAAGAACGAAGGAAGGAAAGAGAAGAAGGAAGGAGGGAGGGAGGG
AAATAATCTAATGAGAAATGGGAAACAATAGTATCATTAATTTTTTTAAATTATACTTTATCTGGT
ACATGTGTAGAACGTGCGGGCTTGTTACATAGGTAAACACGTACCATGGTGGTTTGCTGCACCC
ATCAACCCGTCATCTACATTAGGTATTTCTCCTAATGCTATCACTCCCCCAGTCCCCCACCCCCC
AACAGGCCCCGGTGTCTGATGCCTGCCTCCCCGTGTCCATGTGTTCTCATTGTTCAGCTCCCATT
TATGAGTGAGAACATGCGGTGTTTGGTTTTCTGTTCTTGTGTTAGTTTGCTGAAAATGACTGGTAA
TGTTTAATAAGATGGGAGAGATGGATGTTACCTATTAAGATGCATATTGTTTTCCTCAAGGAATA
TTACCTTTTGAGCATTTTGCACTGTGGTCCAAAAATGGCTCAAATTCATCTGTTTCTTGCTGAATC
CCAGCTTTGCATGTGTGTCACTAAATCCACAAGGCAGCGATTCAGTGCTAATGAACTCTTCTGGT
GGTTACTAACTGCTCTATAAGCATTGTAGGTAGACCATAAATAAAATTTTACCAGTCAGTTATGA
AAGGAGAAAAATGAGAACAAAATAGGGAGTTTTCAATGAAGCTAATGTTAAAAGGAGTGATCTT
TATCTGCTCATATACATTTGCAGTTTTACAATATTAAAATAGCTGTGTATCATTGATATTTTATTAT
TTTGCTGTCCTTACTTTTCAAAACAATATATTGTAAACCTGTACTAGTTCTTACAAGTCTCTTGAAA
AATTACGGTTTGTGATACTGTATTTAATAGTCTATCAGGCCATGGTTTGCATTAATAAAATATGCC
GGGGACCCAGGGACACTACACTGCACCTCCTTTTTGTCTCAATTACATACCATGGTTAAGTCTTA
TTGCATTTTTATAAGTTTATTTAACAATTTAAGTTCGAAAGATATTACACAATTTCATAGTGCTCAA
TTTTAATACATTTCAAATGAAAGCAAAAAGGAAAAAAATGAATCCCATTAGAATTTTATTCTGTTCA

TATATGCATACTCACAATATAATTAATATTCTGAGCATTATTCATTAACTAGGTGGCCAGTATATT
ATGGATTCAGATTTCATTTAAACTTTGAGGAGAAAAGAAAGTAACTTTATAAATAATAGCATTAA
ATAATGTTCATAGGAAATATAACTTTATTGAAGTATTCATAAAATATAAATTGGACCTCTCTAAGA
CGTTTATTTCCTGTATAACTCTTACTACACTAATTTCTTACTTTCCTGTTGATTTTTTAAGCTATAGT
TAAAATACATAGGGAAGGGCTTCAAGATGGCTGACTAAAGGCACCTAGCACTCGCCTCCTCTG
GAAAAAAGGACCAAAACAGTGAGTAGATAATAATAAGTCAAATAGTAGATAATAACAAGTCAA
ATAGAGAGTATAACACAGAACAGTAAAATTCAGCAGGGAAGTGTCAGGGAACCTCTTAGGCAG
AGAAGTAAAAGGAAGTGAAGCATCCTCCCAGGCTGGGACCAGCTGTGAACCAGGAGAAACCTT
CCAGTGCAGAAAATATGTAAGTGACAGATCCCAGTAGTTCACATTCCCACTGCAGACTACTGTG
GTACTAGCCACAGAGATAGCCTCAGCCATCATGGTCTCTGGGACCAGGTTAGGGAACTGCCAC
ACTCCCTAGGGAGAGTCCACACAATAACATGGTTCGAGAGAATGAATTTGTACTGGGCCCCGC
ACACTTCCCTGAGACACAAACGACTGCAGCCTGGTGCCATTTAGAGAGCCCAGCCCCAACCAG
ACTACATCCTGCCCTGGGACCCAAAGGGCCCCTGTGTCTCCACATCCCTGGATCCTCATTGACA
TCTCCGTATGTCTACCCAGAAAACTGTAGCATCACTGCACTGGGTGGACCAGCGGTGCAGATGA
ATCTCCAGCAGTCTAGCCCATGCAGTTTCGTATAGCCAAGGGAATTTGTGGTGAAACTCAGTAA
GAAGACTGTCCCTGGGACAAAGGGAGCTGAAGCACATGCCTCCCAGAGCCTGAGGGCAGTGT
GCACAGGAATACTGCCCCTGACAGCAACCCTGCCCCCTCCCAGCAGTAGGGCTGCCACATATC
TGTACAAGCTTTCAGGGGGCCTAGGGACCTGAAGCTCAGGTACTTTCCGTGGACCTAAGAGCA
GACCTGTCCCACCCACCATTGCTCCCACTGAATCTCACCATCTGGGGGCCTGGAAATCAATACC
TCTTGCCAGCCACAGCTGGTGCTTGCCTGCATCATCAGTGAGCCTGAGGACAGGCCCCCTCTGA
CCAGTGCTGTCCCTACCAGTGCCCACAAGTGTCGTCTAGGAGCCAGGAGATTGACTTACCCCAC
GTACCACAGACTGTACCAACATATACCATTGGTGGCCTAAGGCCCAGGCCTTTTCACCACTGCG
GATGTGAGGCCTAGATATCAGCCTTCCCTGCCTATCACTGCTGATGCACAAAGGTGCACCATCA
GGAGACCTGGAAACAGATCCAGCCTGCCCGCTGTCAGCACCTGAGAACACTGGTAAGGGACA
AGAGAATTTACATGTCCTGCCTACCACCACTTGCATCCTGATAAACTATCATGGGGATGCAGAA
AAAGACCACTCCTTTTGATACCTTTCTTGCCAGTGCCTGTGTGCATTATCTGGGAGCTTGGGGTT
GATCTGACTCCCCCGACTTCCATTAGTTCCCATGCATGCCTTCTGGAGGCCAGTGGATTGCTCCA
CCCAGTCTGCTCATGCCTACACACACGAGCCACGGGGCTGGGGATTGACAAAAACATCTTCCA
CAGTCTGTGTGCACGGTAAGGGGCCTGAGGACAGGCCCAGCTCACCTGGTGCCACCCCTGCCA
GTACCGGCATGTGTCTTCTATGGTCCTAGAGGTCGACCTGTCCACCACCACTGTCATCTGTGCAC
CCCTAACGAGAGCCTGGGGATGGACCCGCCAAGCTTGCTGCCACCACTGCTGCTGGCATGTTTC
CCAACAAGCACTACCTGGAAGCCTGAGGATTGGCACACCACTGTTATTTTGATCACAGATGCTG
TATAAGCTGCCTAGAGCTTGTCCAGTCTACTGGTGCCTAGAGGATCTCTCCAGCCTACTGGTGC
CAATGCAAACGCTTAAGAAAGCCACCTGGAGGCCCTCAAAATGGTCTATCTCTAGCTGCTACCA
CCAGTACCCATGTATACTGCATACACAAGGGCTAACATGCTCAGCCCACTGCTGCCACCACTGG
GGCCTGAGGACTGGCCCAGCTTGTGTTGCCATCCTTAGCAATGTTCCACCACAGTCTCCACTAA
CAACTGCAGCTGAAGCCACTGAATAACTCACAGGAACCACTGATAGTGATTACAGTTAAAGAA
ATCATACAGAGACTGCACTACTGCATGCACTGAAAATCAAAGCCAAAGCATCGTATTCAATCAA
CGTAATAGATACATCTGTAGAAAGTCTTTCCCTATGAAAGCCATTCCATAAATTGGAACAAGCAA
CTGTTACATCGATGCAGATTTCAATGTAAGGACACAAGAAATAAGAAAAAGCAAGGAGACATG
ACACCTCCAAAGGAAAAAAAGAGTATCTATCTATTCAGCAACAGATTTCAACAAAAAGGAAGTC
TATGAAATCCTTAAATAATAATTCAGAATAATAACATTAAAATAACTCAGTGAGATACAAGAGCA
CACAGATAAAAAATACAAAGAAATCAGAAAAAAATTCATAATCTGAATAGGAAATTAAACAAATA
GATGTATATCATATAAAAGCAAACAGATATTCTGAACTTGAACAATTACATAAATAAAAATACAA

AAAGAGCTTCAATAAGACTAAATCAAGCAGAAGAAAAATTGTTGAACTTGAAAACAGGTATTTT
GAAATAATCTAGTCAGATCAAAAAAAAAAAAAGAATAAAAAATAATGAAGAAAGCCTACAGGAC
ATATGAGACTCCACAGAGAGAACAAGTGTTTGAGTTTGGGCATTCCAGAGAAAGAAGAAACAA
AGTCATAGAAACCTACTTAATGAAATAATAGCCAAAAACTTCCCAAGTCTTGCAAGAAATAAAA
GCATCCAAATGCAGAAATTTCAAATAATTCCCAAATAGAATCAACCCAAAAATGTCTTCTCCAAT
ACACATTATAGTCAAACCATCAAAAGTCAAAACAGAATTTTAAAAACAGGAACAGAAAAGCACT
AAGTCACATTTAAGTGAACCCCCATCAGACTTACAGGGTTTTTCTAACAGAAACCTTACAAGCCA
GCAAAGAATGGGATGATACACTCAAAGTCCTGAAAGGAAAAAAAAATCAGCCAAGAAGCAAA
GAACACTATATTAGAAAGCTATTCTTTAAAAATGAAGAAAAACTCCTTTCCAGACAAGCACAAA
CTGAGAGAATTTGTCACAAGACTGATCCTACAAGAAGGGAGTCTTGGTCCTTCGTAAGTGAATT
CTACATCTAGATGTGAGGGCCAGTATTTGCCATCATGAAATCACATGAAGTATAATACTCACTTA
CAGAGCACACACACAGATACAGAAATTTAAGAAAAAAATGGGCTTAAATATTACTATTACAGAA
AACCATCAAATTGCAATAAAAAACAAAGTGAAAGAAAGGAACAAAAGATACACAAAATAATCA
GAGAATAATTAACAAATTGACAGGAATAACTTTTTACATATCAATAATAAGCTGGAATGTAAATG
GATTCAGTTACCCCCTAAAAAGATATAGATTGTCTGAATGGATTAAGAAAAACATGACCCAACC
ATATGCTGTCCAGAAGAAACTCATCTCATCTATGAAGACAAATAAAATGAAAGTGAAAGGACGG
AAAAAGATATTCCATGCAAATGAAAACCAAAATTTAGCAGGAGTAGCTATATTTATACCAATAA
AAATGACTTTAGGTCAGAAAGAGTTAAAAGAGATGAAAAAGGTCATTATATAATAATAGAGGTA
TCAATTCAGCAAGACTATAACAATTGTAAATGTGTAGGCACCCAACACTACAACATCCAGATAG
ATACAACAAATATTATTAAATCTAAAGGCAGAGATAGACTCCAATACAATAATAGTTGAGGACT
TCAACACTCCACTCACATAATGTGTCAAATGCTGTGGGTGGAGTGTTGGAGTTTGAACTGAATTT
TAGACTAAGTGAACCTAACATTTACAGAATATTTTATCCAACAGTCACAGAATACACATTCTTCT
CAGGAGCATATGCAGCATTCTAGCAGGGTAACTCATATGTTAGGCTACAAAACAAGTCTCAACA
AATTTTAAAAAATCAAATTTTTTTCAAATATTTTCTCAGACTACGGTGCAACAAAACTAGATATCA
ATAGTAAGAGGAACTTTGAAAGCAATACAAATACATAGAAATTAACCAACAGGATCCTGAATGA
CCACTGGGTCAATGATGAAATTAAAAAGAAAATAATTCCAGAAACAAATAGAGAAAAAACACA
ACATATATACCTCTGGGATCCAACAAAACTAGTGCTAAGAGGGAAGTTTATAATAATGAATGCC
TACATAAAAAATACAAATATGTCAAATAGACAATCTAACAATGCACCTCAAGGAATTAAGAAAG
CAAGGAATTAGAAAACCAAATCCGGCCGGGCGCGGTGGCTCACGCCTGTAATCCCAGCACTTT
GGGAGGCCGAGGCGGGCGGATCACGAGGTCAGGAGATCCAGACCATCCTGGCTAAAACTGTG
AAACCCTGTCTCTACTAAAAATACAAAAAATTAGCTGGGCGTGGTGGCGGGCGTCTGTAGTCCC
AGCTACTCGGGAGGCTGAGGCAGGAGAATGGCGTGAACCCGGGAGGCGGAGCTTGCAGTGAG
CTGAGATTGCGCCACTGCACTCCAGCCTGGGCGACAGAGCGAGACTCCGTCTCAAAAAAAAAA
AAAAGAAAACCAAATCCAACATTAGTAGAATGAAAGTAATAACACATATCAGAGCAAAACTATA
CAAAATGGAGACTAAACACCAACAAAAATACACTGCAACCTCTGTCTCCTTGGTTCAAGCAATT
CTCCTGCCTCAGCCTCTTGAGTAGCTGGGATTACAGGCGCCACCACCACGCATGGCTAATTTTT
ATATTTTTAGTAGGGATGGGTTTTCACCATGTTGGCCAGGCTGTTCTCAAAACTCCTGACCTCAG
GTGATCCGCCTGCCTCGGCCTCCCAAAGTGCTGGGATTACAGGCATGAGCCACGGTGCCCAGC
CCAAAAATGAATAGTGTTTCTGTACATCAATAATAAACTAACTGAAAAATAAAGAAGACAGATC
TGTTTACAGTAGCTACAGAAAATAAAATATCTAGAAGTACATTTAACCGAGTTAGTGAAATTTTT
ATGCAAGGATAATGATGAAACATGAATGAAAGAAATTAAAGAGGACACAAACTGAAAGATATC
TAATACTCATGGGTTGAAAGAATTAGCATTGTTAAAATGACTGTAATACCGAAAGCAATCTACA
GATTCAATGCAATCCCTATCAAAATACCAATAATTATTTTTTTTCACAGAAACAGGAAAGCAGTC
CCAAAATTTGTATGAAACCACAAAAGACCCTAAATAGCCAAAACAATCCTGAGTTAGAAGAATA

AAGCTGGAGGCACCACAACACCTGACTTCAAACTATTCTACAAAGCTGTAATAACCAAATCAGC
ATAATATTAGTATAAAAACAGACATATAGACCATATAGACCAATGAAACAGAATAGAGAATCCA
GAAATAAATTTATATATTTACAGCCAATTGATTCTCAGAAAAGGCACCAAGAACATGCACTGAG
GAAAGGATGCCCTCTTTAATAAATCGTACTGGGAATACGTGATGTCCATATGCAGAACCATGGA
ACTATTCACCTATCTCTCATCATATACAAAAATCAACTCAAAAGAGATTAAAGACTTACATGTAA
CATCTGAAACTATAAAACTACTAGAAGTAAACACAGGGAGAAACTTTCAGGGCATAGATCTAGG
CAAAGATTTTATGGCTAAGATTTCAAAATCATAGGCAAAAAAAAAAAAATAGACAAATGTAACTTT
ATAAAACTAAAAGTCTTCTGCGAAGAAAAGGAAATCAACAGAGTGAAGTGACAACCTGTAGAA
TGGGAGAAAATATTTGCAAACTATATGACAAGAAATGAATAACCAAAATAATTAACTCAACTAA
ACAGCAATATCATGACAATAAATAATATAATAATAATAATTCCATTAAATAGTGGCTAAAAGGTC
TGAGTAGACATGCAAATGGCCAACAGGTATATGAAAAAAACAGTGAACATCACTAACCATCAG
AAAATGCAATTCAGGACCACAAAAAGTTATCATCTTACCCCAGCTAGAACGGCTATTATCAAAA
ACAAAACAAAAAAACAAAACAAAACAAAACAAAAAAAGAATCCCAGAAAGGATGCAGAGAAA
AGGGAACTCTTATACATTGTTATTGGGAATATAAATTAATATAAATTATTACAGCCTTTATGGAAA
ACAGTATGGATGTTTCTCAAATAACTGAAAATAGAATGACTACACGATCTGGCAATACCACGAC
TGGGTATTTATTCAAAGGAAAGGAAATCAGCACATAAAAGGGGTACATGCACCCTCATGTTTAT
TACAGCACTGTAGCTGAAGTGTAGAATAAACATGTGTCCATCAATGAATGAATGCATAAAGAAA
ATGTTATATGTATACATATGTGTCTGTATGCCTATACCCACAAATATATACAATGTATTTTATATAT
ACAATGTATTTTTTAATCCAGCAATAAAATATGGTAAAATACTATCATTGGAAGCAATATGTATG
GAACTAGAGGTTATTATGCTAACTGAAATAAACCAGGCACAGAAAGACAAATGTTGCATGTTCT
CACTAATAGGTGAAAGCCGAAACATTTGATCTCATGGGAACAGAGTGTAAAATGTTGGTTACCA
GAGGCTGGGGAGGGTGAGAATGAAGAGTTGTTTAATTGGTACAGACATGCAGAAGAAATAAGT
GCTGGTGTTCAATAGTACAGTAAGATAACTCTAGTTAACATATTATATTGTATATTTTAAAATAGC
TGAAAGAGAATATCTGAAATATTCCCAGTGAATTTTCTTTTCTTTTCTTTTCTTTCTTTCTTTTTTTTT
TTTTTAATGGAGTCTCGCTCTGTCTCCCAGGCTGGAGTGCAGTGGCGCCATCTGGGCTCACTGC
AAGCTCCACCTCCTGGGTTCAAGCCGTTCTCCCTCCTCAGCCTCCTGAGTAGCTGGGACTATAG
GCGCCCGCCACCACGCCAGGCTGATTTTGTTTTTGTATTAGTAGAGACAGGGTTTCACCGTGTTA
GCCAGGATGGTGGGCTGCACCTAATCAGTCTGCATCAAACCTTCCCTAATTAAAAAATCTTGGC
CGGGCGCGGTGGCTTGTGCCTGTAATCCCAGCACTTTGAGAGGCTGAGGCAGGCAGATTATTTG
AGGTCAGGAGTTCGAGACCAGCCTGGCCAACATGATGAAACCCCTTCTCTACTAAAATTACAAA
AATTAACCGGACGCAGTGGCTCCTGCCTGTGGCCTCAGCTACTCGGGAGGCTGAGGCTGGAGA
ATCACTTGAACCTGGGAGGCAGAGATTGCAATGAGCCGAGATCGCGCCACTGCACTCCAGTCT
TGGCAACAGAGAGAGACTCTATCTAAAAAAAAACCCAAAAAACAAAAAACAAACACAAGAAGTT
TTGGCTGCACCAAGATGCTAGCTGTGTGTGTTATTATTTTGACTACCATGTAGTTTAGTGAAGGT
GTTCAGTAAACAGATGACCAAGTTTAATTTTATAATTTTCAGGAGTATTATCCTGATTCTAGTATA
GAATTATCCTAATTCTAGTGTTAGAATTAGATTTTAGCAATATCTATTACTGTTCTTTGTTTGCCAT
TTTCCTATTGTACTATTGCTACATTTAGTGATATAAGGCCATCTGGATTCAAATTTCTAATTATTTT
CAACATGTATATTATTTTCAGTGTTGCTTTTGGTAATACATTTGAAATGATAATATTGAGCTACAT
GAAAAATAAATATATGATTATTCTATCTTTTATCAATTGCATGGATTAAATGTAGAGAAAGCTCGT
GAGTAATCCCATTATAAAAGATGAGAGAAATAAGAAATACATGCTTAGTGTATATAGAAGATAA
TGGGATATGGTGTTTATTTTAAAATATTTACGTACCTGTATTATTCACTACATATGATTACATTGAT
ACCTCAAACAAAATGTGTGAATATAATTGAATAAAATTATATCTAGAGGGAATTCATTTTCTAAA
GTGTGCATAGCAAAGGGTGCTATGTCAAAACATGTAAAATCATCACAGTGTACTTCAGAAACTT
CCAAGTATTGAATATACAAAATAAAAAACATGAATTTGTGCTGTTTTTCAGCCAAAGGCTCTTAT

CAACAAAGGTTTTCAGTTATTGTGGGAGAACCCTTTCTTGTTACCGTAGATCATCTTACTACATGA
TGGTATTTTATCTTTCTCTCGCTCACTTGACCTTATAGGGAAATCACCAGTAAGAATATACTTTTT
GTGACAATGTAAATATTATCCAGTCAAGATAGTAAATATTCATTCCTTAGCTATGATTGCCAGAA
TTTTCTTAAACTAAGATCAATGTGTGGAATGTCATCAGTATATAAAATCTTACTAATAACTTGGAA
AGACCATGATTTCTTTGTTTCTATATGTGATTTGTGGACGTTTACCTTCTCATGTGCAATCTTGGTA
AATCATAAAGTATGTTTAAATGTCAGTTTTCTCATCCATAAGCTGGGGATACCATTGTCTCCCTTG
CAGTATTGCTGTGGGTATGAAGACTGAAATTGAAGTGGCTGACATTTATAAGGCACATATGATA
GCTATTACATTTTTTATATTTCCATTTCCATTTGGGGAAATGTGGTTGATAGGGAGAATTCTTCTTT
TCTTTCTTCTTCTTCTTTTTTTTTTTTTTTGGAAGGAATCTCGCTCTGTCATCCAGGCTGGAGTGCA
GTGGTGTGATCTTGGTTCGCTGCAACCTCTGCCTCCTGGGTTCAAGCAATTCTCCTGCCCCAGCC
TCAAGAGTAGCTTGGACTACAGGTGTGTGCCACCACACCCAGCTAATTTTTGAATTTTTAGTGGT
GACAGGGTTTTACCATATTGGTCAGGCTGGTCTCGAACTCCTGACCTCATATCTGCCCACCTTGG
CCTCCCAAAGTGCTGGGATTACAGGTGTGAGCCACTGCGCCTGGCAGACAGGGAGAATTCTTC
TGTTAAGACTTCCCTGAAATTCAAGGTAGGCTAATTGTCCCTTATGTGCTCACTTACCACTGGTG
GAAATATCCCTCTTTAAGACCTAATATAATATACTTTATGTCTTTCTACTGGTAATCAGTAAGTGC
TTGGAGGATAGCAAATGCATTTTATTAAAACTTTTACTCCCAGTACATAGCAGATTGCTGAAGTC
ACAATTGTTAAATAAATGTTTGTTGCCCTGCACTAAAATGTGTAAGATACTTGATGAGAAAATTT
AGAAAATATTTTAAAAGACTTTTTATGTGGCTATAGAATATTTAACTTAAGATCCATGTGTTTGCT
TTATGTGTCAGAGTTTGTTATGTACTATGCTTTTGTATCTTCATGGCTTTTTTTTTAAATCTAGTTTT
TAAACATAAATAATTTTCTGATCTTTTTTAAGTGAAATATTTAAAATACATTAAAAATGTTTTGACA
TTATGATTATTTGTTTACACATGGTTAAATTATATCTATCTGAATTATGTGAATTATTCTTTGACAA
GTGATGATTCTTCATCTCAAAAGCCTAATGAAGATAGTATATTAATTGGTTCTCTGGTCTTAAATG
ATAATCTTTCAATAGTAAATTGCAAAGAAGCTTGCTGATATTTCTCTTGCATCGGTTAAATAATAA
TTTCCAGTAATCAAGCCATGGCTAATGATGATGGAAGTAACACATTTATGCAACAAAAATTTGCA
CATATTTCTTCCAGAAGCCAATAAGCAGTTACATGAAGCAACATAAAAGCAATTTTTTCCATCTT
TCAAGCAACCAACAAAATTAAATTTCATTGTTATTATTTAATAAAAATAGTAATATTTATGACTTT
ATATTACAGTTGGTATACTAATCTTTTAGTTAATAAATTTACAAGCTTAGTGGGAAAAACAAAAC
AAAAGCATAAACGAAATCTGTATGCAGTATGGAACTTATTAGGTACATATGACCCGATTCTAATT
TAAAACCTGTAATGCAATTGAAATAATACCCAACATAAAGCTTATGACAAAACAAATGCCTACA
ATTTACTTTAGAAAGTAAAGCGCATGCAGTTATTGAAAAAAATAGACTGTTTACACAGAATGTTA
ACTGTTCCTTTTGCATTGTTGTATCAAAATAGGTGATAAGCCCCAGTGTGTGTTGTTCCCCTCTAT
GCGTTTATGTGTTCTTATCATTTAGCTCCCACTTACATGTGAGAACATGCCGTATTTGGTTTTCTGT
CCCGTGTTAGTTTGCTAAGGATAATGGTCTCCAGCTCCATCCATGTCCCTGCAAAGGACATGAA
CAACACACACTGGGGCCTAACTGAGGGTGGAAGGTGTGATGAGGGAGAGGATCAGGAAAAAT
AACTAATGAATACTAGGCTTGATACCTGAGTGATGTAACAATCTGCTCAACAAACCTTCATGACA
CTGGTTTACCTAGGCAACAAACCTGCACATCCTGCAAATATACCCCTGAACTTAAAAGCTAAAA
AACAGGCCCGGGTGCAGTGGCTCACGCCTGTAATCCCAGCACTTTGGGAGGCCAAGGCGGGTG
GACCACAAGGTCAGGAGATCGAGGCCATCCTGGCTAACACGGTGAAACCCCATCTCTACTAAA
AATGCAAAAAATCAGCCAGGCGTGGTGGTAGGCACCTGTAGTCCCAGCTACTCAGGAGGCTGA
GGCAGGAGAATGGCGTGAACCTGGGAGGTGGAGCTTGCAGTGAGCCGAGATCACACCACTGC
ACTCCAGCCTGGGCGACAGAGCGAGACTCCTCAAAAAAAAAAAAAAAGCTAAAAAACAGAAAT
AATGACAAGGAAGAAAACACGTACATATTCAATAATAATAATAATAGCCAACAGGATTACTATG
AAAAAAGATAGGTGATAACTCTTCAGCTAACATTAATCAGGCTCTGTGTGCATGACTTGACTTGT
TATTTCTTATATATGTACAAACTCATTTAATACTCATAACCCTACATGTACATAGTTTTGATGACCT

ATTTTACAGATGGGAAAACTGAGACCCAGAGAAGTTAAACAACTCCCCCAAGGTTAGAATCTGA
TAGTTGCTAGAATCTGGCGAATGCAGATGTGTTTCAAATTATATTCGTTATTATAATTATATTTCTT
ATTTGTTAAATAAAAACACATGGGGGTCCAAGATGGCGGACTAGAAGCCACTAGTATGCCACG
GTGCTCATGGAGAGAAATAAAAGGGGTATGTAAATACTACATTTTCTTTTTTTTTTTTTTTTTTTT
ATTATACTCTAAGTTTTAGGGTACATGTGCACATTGTGCAGGTTAGTTACATATGTATACATGTGC
CATGCTGGTGTGCTGCACCCACTAATGTGTCATCTAGCATTAGGTATATCTCCCAGTGCTATCCC
TCCCCCCTCCCCCGACCCCACCACAGTCCCCAGAGTGTGATATTCCCCTTCCTGTGTCCATGTGA
TCTCATTGTTCAATTCCCACCTATGAGTGAGAATATGCGGTGTTTGGTTTTTTGTTCTTGCGATAG
TTTACTGAGAATGATGGTTTCCAATTTCATCCATGTCCCTACAAAGGATATGAACTCATCATTTTT
TATGGCTGCATAGTATTCCATGGTGTATATGTGCCACATTTTCTTAATCCAGTCTATCATTGTTGG
ACATTTGGGTTGGTTCCAAGTCTTTGCTATTGTGAATAGTGCCGCAATAAACATACGTGTGCATG
TGTCTTTATAGCAGCATGATTTATAGTCCTTTGGGTATATACCCAGTAATGGGATGGCTGGGTCA
AATGGTATTTCTAGTTCTAGATCCCTGAGGAATCGCCACACTGACTTCCACAATGGTTGAACTAG
TTTACAGTCCCACCAACAGTGTAAAAGTGTTCCTATTTCTCCACATCCTCTCCAGCACCTGTTGTT
TCCTGACTTTTTAATGATTGCCATTCTAACTGGTGTGAAATGATATCTCATAGTGGTTTTGATTTGC
ATTTCTCTGATGGCCAGTGATGATGAGCATTTCTTCATGTGTTTTTTGGCTGCATAAATGTCTTCTT
TTGAGAAGTGTCTGTTCATGTCCTTCGCCCACTTTTTGATGGGGTTGTTTGTTTTTTTCCTGTAAAT
TTGTTTGAGTTCATTGTAGATTCTGGATATTAGCCCTTTGTCAGATGAGTAGGTTGCGAAAATTTT
CTCCCATGTTGTAGGTTGCCTGTTCACTCTGATGGTAGTTTCTTTTGCTGTGCAGAAGCTCTTTAG
TTTAATTAGATCCCATTTGTCAATTTTGGCTTTTGTTGCCATTGCTTTTGGTGTTTTGGACATGAAG
TCCTTGCCCACGCCTATGTCCTGAATGGTAATGCCTAGGTTTTCTTCTAGGGTTTTTATGGTTTTA
GGTCTAATGTTTAAATCTTTAATCCATCTTGAATTGATTTTTGTATAAGGTGTAAGGAAGGGATCC
AGTTTCAGCTTTCTACATATGGCTAGCCAGTTTTCCCAGCACCATTTATTAAATAGGGAATCCTTT
CCCCATTGCTTGTTTTTCTCAGGTTTGTCAAAGATCAGATAGTTGTAGATATGCGGCATTATTTCT
GAGGGCTCTGTTCTGTTCCATTGATCTATATCTCTGTTTTGGTACCAGTACCATGCTGTTTTGGTT
ACTGTAGCCTTGTAGTATAGTTTGAAGTCAGGTAGTGTGATGCCTCCAGCTTTGTTCTTTTGGCTT
AGGATTGACTTGGCAATGCGGGCTCTTTTTTGGTTCCATATGAACTTTAAAGTAGTTTTTTCCAAT
TCTGTGAAGAAAGTCATTGGTAGCTTGATGGGGATGGCATTGAATCTGTAAATTACCTTGGGCA
GTATGGCCATTTTCACGATATTGATTCTTCCTACCCATGAGCATGGAATGTTCTTCCATTTGTTTG
TGTCCTCTTTTATTTCCTTGAGCAGTGGTTTGTAGTTCTCCTTGAAGAGGTCCTTCACATCCGTTGT
AAGAAATGGATACATTCCTCGACACATACACTCTCCCAAGACTAAACCAGGAAGAAGTTGAATC
TCTGAATAGACCAATAACAGGCTCTGAAATTGTGGCAATAATCAATAGTTTACCAACCAAAAAG
AGTCCAGGACCAGATGGATTCACAGCCGAATTCTACCAGAGGTACAAGGAGGAACTGGTACCA
TTCCTTCTGAAACTATTCCAATCAATAGAAAAAGAGGGAATCCTCCCTAACTCATTTTATGAGGC
CAGCATCATTCTGATACCAAAGCCGGGCAGAGACACAACCAAAAAAGAGAATTTTAAACCAAT
ATCCTTGATGAACATTGAGACAAAAATCCTCAATAAAATACTGGCAAACCGAATCCAGCAGCAC
ATCAAAAAGCTTATCCACCATGATCAAGTGGGCTTCATCCCTGGGATGCAAGGCTGGTTCAATA
TACGCAAATCAATAAATGTAATCCAGCATATAAACAGAGCCAAAGACAAAAACCACATGATTAT
CTCAATAGATGCAGAAAAAGCCTTTGACAAAATTCAACAACCCTTCATGCTAAAAATTCTCAATA
AATTAGGTATTGATGGGACGTATTTCAAAATAATAAGAGCTATCTATGACAAACCCACAGCCAA
TATCATACTGAATGGGCAAAAACTGGAAGCATTCCCTTTGAAAACTGGCACAAGACAGGGATG
CCCTCTCTCACCGCTCCTATTCAACATAGTGTTGGAAGTTCTGGCCAGGGCAATCAGGCAGGAG
AAGGAAATAAAGGGTATTCAATTAGGAAAAGAGGAAGTCAAATTGTCCCTGTTTGCAGACGAC
ATGATTGTTTATCTAGAAAACCCCATCGTCTCAGCCCAAAATCTCCTTAAGCTGATAAGCAACTT

CAGCAAAGTCTCAGGATACAAAATCAATGTACAAAAATCACAAGCATTCTTATACACCAACAAC
AGACAAACAGAGAGCCAAATCATGGGTGAACTCCCATTCACTACATTTTCAACTAAAGCATATA
GGTGCATGGACTGGGAATAATCAAGAAAACAACTCGAGCCACAGATAAAGCAGAGGAGCAAG
GCAAAATGACTGTCCACCCTGGAGTGACATGGAGCTAGGGGAGGCTCTCCTGCCCAGGGAAAC
AGAGAGTGAATGAGAGACCGCGGGGACTTATGCTTCTTCCACTGATCTTTGCAACCCGGGGGTC
AGGAGATCCCCTCACTTGACTTACTCCACCAGGACCTTCAGTCTGACATGCACAGCTACCTAGA
GTCTTAGCAGAGACACCTCTATGGTACATTTGGAGTCACCACACATCCTAGCAGTAGCAGCTGC
CGCTCTGGCAGAGGGGGTGGTTAAAGTCCTATACATGCCCACAGGAAAGAGGCTGAATCCAGG
GGGCTGAGCAGTGATGGTCTGCAGGTCTGGCTTCCAGGGCAGCTCACAGGATAAGACCCACTG
TCCTGGGACTTCAGCCAGACTCCAGTAGCAGCCCTGAGCTCCCTGAGACAGAGCTCCCAGAGG
GAAAGATGGGCTGCTATCTTTGCTGTTTCACAGCCTTGGCTGTTGTAGTCTTCAGCGCAGCTGCC
CTACGAAAAAGGAGTCAGCCTGCTTCTTTATGCGAGTCCCTGATGAAATTTCTCCTCACTGGGCA
GGACCAGGGTCTCCAGTCACCCTTTGCTGGTGTTTTCAGCAGCCCCATCCCCCCGTGGGTTGGA
GCTCCCAGAGGGAGGGGCAGGTCATCATCTTTGCTGTTTTGCAGCCTTAGCTGTTGTTTGCCTTC
AGCATTTAGGGAGTACGAGGTGACTAGGGACTGGAGCAGACCCCCAGCACAGCACTGCTGCCC
TTTGAAAATGCAGTCAGACTGCAAAAAAACAACAACAACAACATAAAAAAACAACTGTGGGTC
ACCTATCTTATTTCTCCTCACTGGGCAGGACCACTCGCCTGGGTCTCAAACCACCCCCTGCTGAG
GCTATCAAGCCAGTAGCAGCTCTGCACTTATCTAGGACAGAACTCCAAGTGGGAGGGACAAGC
TGCCATCCTTGCTGTCTAGCAGCCCTTGCCTTTGCCATCTCCAAGCTTGGGAGAGTCTGGGGGG
ACAAGGGGCTGTTCTGGGCCCTCAGCACAGGGCAGCCGCCTCACAGAAAAGTTAATGGACTGT
CCTCCACACAGGTCCTGGTCCTCACTTCACCTCACTGAACAGGGTCACCTGACCTGGACTGTCC
TCCACACAGGTCCTGGTCCTCACTTCACCTCACTGAACAGGGTCACCTGACCTGGGACTGGAGC
ACAAATACCCTGTCTCTGTCTGATTACTTCAGTGAGTGTCAGCTCAGAATTTCTGAGGAGAAAAT
TCCAAAATCAAGCCACAAACCCTCTGGCAGTACAGCCCTAACAGCCCTTGAGCTGAGGAAAGA
ACAAAGGGCCTAGTCTCTAAGCTGGCTCTGCCAGCACAAAGCAGGCACCATATGGAGAGGAGT
CCAGCCCCTCTACCCTGGGAACCTGCACCCTCACTCTTAAGACAGGACCCCTGGTGCAAAACCA
CAGAACAGCCACCTCTACCAGTGGCTGAACATGCCCAGCAGAAGTGGCTCTGAGATTTTCTATG
GTAAGGCTCCTAGAGCCAACTGACTATCCCTCTGCCACTGCCATAGCAGTGGTCCTTCCCCTGC
TGCCCTCAGCCTGGGGAAGAAACAAAAAGCCTGAAGGCTGCACTCAAGCTTACAGCATGCCAC
AGATACCATATGAAAAGGAGCTCAGTCTCTCTTCCCTGTGAGCCCTGGAACCCCTGCCACCCAA
CAAGCAGAGGCTCTAGTTCACACCAGCAGTGCAACTGCCCCAGTCCCCCGACTCAACACCGCC
AGTAACAGCAACTCCATGTTGGAGGTGGAGTCACTCGAGGGGCCACTGAAAACCCCTCTGCCA
CTGCCTCTGCAGTGGTACTGCCCCTACTATTCTCTTGAAAGAGCAAAGATCCTAAATGCTGTATC
TTAGCCCCAGCAAGCTGCAGTCAACACAAAGAGAGGAGGCCAGTCTGTCTCCCATGGGCTGCA
GCCCTGCTCCCCCTTGCTCATCAATAGGCAGGGCCTCCCTGGCTTGGACAGACAACAGAGAATC
CCATCCCAGGCTGATTGCACTGAGTGATTGAT

ID|30373|gene_id|ENSG00000251629|transcript_id|ENST00000514876 Homo sapiens lncRNA

TACTAGGCCACGTGGCAAGGGCCTGGTTGGGGACCTCCAGGGGTTGAGAGCATCCTCTGG
ACAACATGGAAAAAGAAAATGAAAATGGTGACCTCAGCCCTATAGCTAGAAGGAATTGAATCC
TGTCAACAACGATGTAAGTCTTAACGAAAACCCCAAGCTCTAGAAGAAAAGATAGCCCAGTTG
ACAGCTGAACTTTAACCTTGTGGGACCGTGAATGAAGGAGGAGCTAAAATGTGCTAGACGTCTG
ATGGATAGAAAGTGTGAGATAATAAATGTATGCTTTTTATAGCTGCAAGGTGTGATAATCTGTTA
CTCAACAGTATAAAACTAGTACATCTTACCTAGTAACTGGAAAACATAGGTTTATATGGAAAAAA

AATTTTTCACAATGTTTTTATCTATTCAGTGACAGTTTAAGACTAGATATCTTCTCAGGTAAGTTG
ATAAATAAATGCATAATTCTATTAGAAAAAAAGGAAAGATTGTATTTGAAAGTCTCCCATCATAA
ATGTGTGTTAGCATAATATATAGCACAGCAAAATTATTTTTACGCATTTTAATACAATTTGTAAAG
GCTCATTGACCAAAATATTTTAACTGGATGCTGCAATTAAAATAATTTGCAGATTTTGCTTGATTT
ACAGCTACACTAATGCAAGGTCATTAATTCTTGTAATTATGCTCATGCCATTCTTTATTGCAGGAG
AGTATACATATAGCACGTGATTGTATGCAATTATTATTTAAACTACTATAAAGAACATTTCACCAA
TGAAATACTGATGTTTTATATTTCTCAAAGAATAGGGTAACAAAAATAGTTTGCAACATGAGATA
AATCCAGAAGTTGTGAATATTAAATAGTTGTGGATTTAAATTTTGTTTTCTATGTTGCACTTTTTAT
ATATAATGCTAGAAGGTACAGACATAGGTTTAATTAGTGATAATGTGTCTGGAATTGGTTCCTTC
CAGTGGGTTCTCGGTCTCGCTGACTTCAAGAATGAAGCCGCAGACCCTCACGGTGAGCGTTACA
GTTCGTAAAGATGTTGTGTCAGGAGTTTGTTCCTTCACATGTTCAGATGTGTCCGGGGTTTCTTCC
TTCCGGTGGTTTCATGGTCTCCCCGACTTGACTTCAGGAGTGAAGCCGCAGACCTCTGCAGTGA
GTGCTACAGCTCATAAAGGTAGCACAGGCCTGAAGAGTGAGCAGCAGCAAGATTTACTGTGAA
AAGCAAAAGAATGAACCTTCCAAAGCTGGAAGGAGCCCCAAACAGGTTGCCGCGGCTGGCTCC
CCTGGCCAGCTATTATTCCCTTATTTGGCCCCACCCACGTCCTGCTGATTGGTCCATTTTACAGA
CCGCTGATTGGTCCGTTTTTACAGAGTGCTGACTGGTGTGTTTACAAACCTTTAGCTAGACACAG
AGCGCTGACTGGTGAGTTTTTACAGAGTGCTGATTGGTGCATTTACAAAGCTTTAGCTAGACACA
GAGCACTGATTGGTGCATTTACAATCCTTTAGCCAGACAGAAAAGTTCTCCAAGTCCCCACGTG
ACCCAGAAGCCCAGCTGGCTTCGCCTCTCAATAACATTGTTAAATTTATAACCTCAACTAGAAAG
GAGGATGAGCATAGTCCAGCTGAAGAAAGAGCTAAAATATCGATTTAGAACTAGTCTACATATT
TTGAGAAAAAAACATTTCATTCAATTTGCTGTTTTATCTAATAACTAAGAGTGATGCTAATACATG
CATGAAAATACTTCATTCTGAAATTATGAGCATTGTTTGAATAACTTATGAAAGGGGTTCCCCATT
CCTCAGAAAGATTTCCAAAAAGGAAAGATATAACAATACTCAGGCAAGTACTTTTTTCATTCTGT
AAAAATTTCATGCAAAGCAACATTTGTAACAGAAGTGTAAAGTGCTAATACAACATACCAAATA
CGTTTGTACACACTTTTTGTTTAATAAATATGCCATTCCTCAAAGCACGCTTCTAGCCATCCTCTT
ACTCACTATGTGTAAAATCTGTGCTGCTTTTTTTTTGTTTGTTTTTGAGATGTTTTCCCGCTTGTTAC
TCAGGCTGGAGTGTGTGGTGTGATCTCAGCTCACTACAACCTACACCTCCAGGTTCAAGTGATT
CTTGTGCGTCAGCCTCCCGAGTAGCTGGGACTACAGGGGCCCGCCACCACACCTGGCTGATTTT
TGTATTTTTAGTAGAGATGGGGTTTTGCCATTTGGCCAGGCTGCTGTGGAACTCCTGGCCTCAAG
TGAACTTCCTGCCTTGGCCTCCCAAAGTTCTGTGATTAAATGCATGAGCCACCACACCCAGCCT
GTGCTGCTTTTTTACAAAAAATAAATTTGCTATCTAAGGCTGTGTCCTTATGTAGTATCATGCTTA
CTTAAGCATATGCTTGAACTTAATATGAACGGGATAATAATACATTTTATACAAAAATTTTCCATC
AAAGTTGGTAAAATAACAAGGAACAAAAGCAAGCGGGAATTAGGTAAAATATTTAGCAAATTTA
TGACTAAGGATCCTTTCCACGGTAGCATTCTGCCTGTCTTGGGGGCAATGAGGTCAGATCCTTG
AAGCTCCACCTTTCTATTTTGGATAGAGTGGTGCTATTCAGTCAAAGTTTGCCTCTCTCTCTGCTT
CATCTGCTTTCCATTTATTTACAATTGACCAACTGGGGGAAATATTATTTCTTTATCAAGGGCAAT
AAAGTAATTAGGTGACTGCCAAGCTTGTTTGGGTGGTGCATTTCTATTCTGCTTTGAAGATAACT
ATTCATGATTCAATCTTGGGTTTTAATTTTCAGCTTACCTTTTAAAATGCAACCATATTTTCAAGTT
CATTGAAGTAAGCAAGAGGAAAGATCATTGCAATGTCACCATCCATATCACCTACAAATATGTT
AAAACTTTTATTATTTCTCTATAAGTATTATCTATGTGTAGACAATTGGCCAAATAGTTACATTATA
TTGACAAAAAGTCATGATTCTTGTCCTTGAGACATAAAATAGAGTATGAATTAATAAAGTAGGGC
CTCTGGGCCAAATCCAGCCCAGTGCTTGTTTTTATAAATAACGTTATTGGAACACATCCATGTTC
AATCAGTGACACATTATCCATGGCTGTTTTTATGCTACCATGACAAGTTCAGTGGCTAGGACAGA
GATCACATGGACTGCAAAGCCTAAAACATTTACTAACTGACCCTTTACAGAAAAAGTTTTCTGGC

CGGGCGCGGTGGCTCACGCCTGTAATCCCAGCACTTTGGGTGGCCGAGGCGGGTGGATCACGA
GGTCAGGGGACCCAGACCATCCTGGCTAACACGGTGAAACCCCGTCTCTACTAAAAATACAAA
AAATTAGTTGGGCGTGGTGGTGGGCGCCTGTAGTCCCAGCTGCTCAAGAGGCTCAGGCAGGAG
AATGGCATGAACCCGGGAGGTGGAGGTTGCAGTGAGCCGAGATCGCGCCACTGCACTCCAGC
CTGGGCGACAGAGCAAGACTCTGTCTCAAAAAAAAAAAAAAAGTTTTCTGACCCCTGATATAAAA
GAAAAGAAGGGTCAAATCAAGAATCAAAATTCAAATGATCTAATGGAGTATTTATATTTCTGGG
GGGTCTAACTTCTCCACACAACTATTATGATTCCCTAAATTACCTTCCAATAAACCACTGATACTT
GAATTCCCATCTCAAGGTTGATTTTGGGTAAATCAAAACAAAGACAAGTGGTATTAGAAGTGGA
TTCCTAGAAGGCAGATTCTCATAATGGGTTTCCAGTACAGAATTGTCCTCTGGCTATTTATGGTG
ATAAGTGAAGGAGTGAGAACTCTTAGCGTAATAAAACATCCGAAGTTCTAAGACCCCCCACATG
TTGTGAATTTGGTTGGAGTAAAGATCTAAGGGTCCATACTGGGCTATAAAAATAGTGTAGTACTT
GAGCAGTACTGTAAAAATGGCAATTATAAATAATTATGAGTGTTGAGGAGGTAATATGAACACT
CAAGTTCAAAGCAGTAAAATAAAAATTAAAAAAAAAAGACAGACTTAGGTAAGCCATCTATCAA
CTCGGAGAATGCTGATGCTTTGTTGAAAGAGAGCTTTGTCTTACAAATTAGGCACTTATAAAAGT
AAGTAAATCTCAGACTTGAGGAATATACTGAAGCAGGAAATTCCCCTGACCCCTTGGTGGGTGG
GAACTGCAGTGCAGGCACTGGAGCTAGCCAGCTGCTTCAGCATTGGCAGGGGCAAACTCCACT
CACTGGAACCTGTGGGCGCTCAACCCTTCCCGGGAAGGGGCACGCAGGTGAGCGCTTTTGGGT
GTCAGCTGGAACAAAACTCTGTGAGCCCCACGGAAGCATCTAGCGGGGAGTACCAATGACCTC
CAAAGCCCTAGACAGCAAGTGTTACAGTGGGAGGCACCTGGTGGAAGGTAATTGAATCATACG
GGCGGCTTTTTCCTGTGCTGTTCTCGTGATAGTGAGTAAGTATCACGAGATCTGACGGTTTTATA
AAGCGTAGTTTTCCCTGTACAAGCTCTCGCCTGCCGCCATGTAAACGTTCTTTGCTCCTGCTTCG
TCTTCTGCCGTGATTGTGAGGCCTCCCCAGCCATGTGGAACTGTGAGTCCAGTAAACCTCTTTTT
CTTTATAAATTACCCAGTTTCAGATACGTCTTTATTAGCGGCGTGAGAATGGACTAATACACTAA
CTTTCTCAGTGAGCATCTTATTCCAGTTATAAAACTACCTCTGGTTCTGAAGATACAAAGATAGG
ATAATGCTAACTATTTCTTTGCATAAGGCAAGTGAAAAAATACAGATGACCTGATATCTTCTATC
TGCCTACTCTCTCTACCTTTTGAAAGCTCTCTTTCTGTTGCTTTAATAGCATTGAATTGATACGCCA
TTATTGTGTCTATTCCTTTCACAATACCCTTAAATAAAGTCTTACTTTTAACTTTTTCCAGTGCCGT
TTTTGTTTTGCTGAACCTTAGAAAAATTAATAGTTTACAGTAAATTAGGTGGAGATGCTAGAACA
TCTGAGGCAAAATATTGAGAATGGGTTTTAAAGGCTACATAAAAATATGCTGGCCGGGCGCGGT
GGCTCACGCTTGTAATCCCAGCACTTTGGGAGGCCGAGGCGGGCGGATCACGAGGTCAGGAG
ATCGAGACCACAGTGAAACCCAGTCTCTACTAAAAATACAAAAAATTAGCCTGGCGTGGTGGTG
GGCGCCTGTAGTCCCAGCTACTCGGAGAGGCTGAGGCAGGAGAATGGCGTGAACCCGGGAGG
CGGAGCTTGCAGTGAGCCAGGATTGCACCACTGCACTCCAGCCTGGGTGACAGAGCGAGACTC
CGTCTTAAAAAAAAAAAAGAAAAAAAAAAATGCTAAAATGGATTATGTAAGAACAGATAATCCACG
ATTTAACTATGTCCCCGCAGAGAACTTAGGAGAGATTCCCTTCACTAAAGTGGTGAGATATATGT
CATGCCACTACCATCAAGATTTCAGATAAAACAGGAGAGGTAAATCTTCCCCAAAACCCAGTCT
ACTGCCATCAAAGGTGCAGAAAATATATATTGTCTATGAAATACAATTGATGTTCATCACATATA
ATCCTAATTTATTTTACCTTGCCTTTTGTGTATTCCAGTAGACTATTTAAATATTTCATAATGACAG
AAGCTATGAATAAAAACAAAATTAATTTTATTTTTCATATTTATATTCCATTTATTTCTAAATAGGA
TATTGCAACTTAATATTATTTTTTAAAGATGCAAACACAAACATCATTAAATTGTTTAAAAAAATTC
AGAAAAAAATTAAAATACATGGAATGAAAAAGGCACCTATATGAGGGATAAAGATCCTACTTAA
AATTAGTTGTGAGTATACTAGTGGACAACAAAAAAGAGATTGCAATTGGATATGTTGCTCCTGTT
ATCCAAAGTAGGAAGCCCATTCTTTCTGTGGATCAGTGTCTTAGTTTTCTATCACTGCTGTAGCA
AATAGTCACAAATTTCATTGTTTGGAACAACACAAACATATTTTCTTAAAATTCTGTAGGCCAAA

AGTCTTGGTAGGCTCAACTGCTTCCCCTTTTCCAAATCTCACAGGACTACAATCAAAGCATCGGT
TAGCCTGGGCTCTTATTTCAAAGCTTTGAGAGAGAATCTTCTTCCAAAATCATTCGTGTTTCTGGC
AAAATTCCTGTGCTATCCTGGCAGGCTATTGGCTGGGAGACCTAGCTGCTTTAGACCACCTGCA
TTCCTGATCGCTTCATCCTTTCATTCTTCAAATAAGCAATGTATGTTAAGTTCTTAAGCTTTAAGTC
TCTCTAACATCTCTTTTGCCAGATTTCTTCTTCTCCAGCTGAAGATAGCTCTCAGCTTTTAAAGGTT
CCTGTGACTAGATTGGACCCCCTCATAAATTTCAAATTAATCTTTTAATTTTAAGATCTTCAACCTT
AATTACATCTGCAAAATTCCTCTGTTCAAGAAATATTATGTATTCAAAGGTTCCAGGAATTAGGG
AGTAGATATCTTTAAGGGGAAATTCTGCCTTCCATGGGGAGAAACTTGGAATGTCAAGCTTTCCT
AGTACCAAAATATTGCCAAACACACAAGCACAGCCTTTTCGAATATGACTTAAGTTGGGTCATA
ATAATAAATGTAATAATATTAAGCAATAATGTCCTTAATACCTCTGAAATTATTTAGGCATACTTC
ATTTCCTTTGGCAGTTGTAAATTCCCAAGCAATCTTGTTTCATAATAATAAAATGTTTTACCCAGA
CAAGGGTAGTGTAGTTGCTTCTAGAAGTAAATTGTCTCAAGATGTGTTGAAATTGTACTTTCTTTA
GACAATGGTTCTGATATAATATTTTCTGACTTTTGATAGAGGCTAAAATTAGGTAGTTTTTTCTAA
AAGTATTTAATTAGGGCTTTGGATACTGGTAGTTTTATATTGTGAATTGGGAGATTTGTTCACTTT
GCAATTAGATAAACAGAAGATGAGTTTGAGCACCAATTGCATTTTTCTCTTCCTCTGTCAATAGA
GTTTGTTAGGTAAAATCCCTCAAATATCAGGTGAACTCAAAAGAATGTCTTTTGTATTTAAACTG
GTTGCATAGATTCATGTATATCAAGTGCTTGAAAATTAGCTAATATTTTCTTTAATATGATGAAGA
TGCCTCATTTTCATTCTTTCTAAATGTACTTCAGTTGCTGAGAGAATTTTAGTATGCTGAGTGATA
GTTCTGGCCTTTTGGCAAATCAGGCATATCCTGAATGTAATGATAATCCCCACCCAGCAGGTATT
TCTAACTGAATACAAACAAAATTAACTGTGTGAAAATGTTCATATAATTATGGTGTGACAGGATG
TGAGATAAGACACTTTATTTTCCTTATTGTCAATAGGCAGATTGCAGACTAATTTAGTGATTTAAA
AGACATAAACAACTAATTTTTTAGGCATAAAAAACCTAAATCAATGTTTGAGGCAACTAAATAAC
TAAATACTGTGAGGTAAACATTTTAAAATAAAATATTTTTATATTCTTATTTTCTTTTATGTACTATT
AACAGGCTTGGAAATATTGTTTCCTCTTCTTAAATATACACAATAATACATATTTTGGTAGTTTATT
CATCTTAAAAATAATGAAAGAATCCAGTATTTATTTTGAAGACATTGCATATGCATATACAGTTAT
ATAAATATGTATTCTTTTTCAATATTTTCCGATACTCCAAGTCTCATGACTCATAGAATAAAATTG
AAAATAAAAGCTGTTGACTAATATGATTTATTTCTGTTTGCAAATTTGTAAATAGGAAAGCCTCTC
AAATGCTTATCAGCTCCAAGAATACATAAATACAGAATATCGAGCTCCTATTGCTGCATATTCCA
ACCCCCCACAAGCTGCCATCTGTTACTCATAGGTACAGCTTTCTAACATCAGGTCTCTATGAACT
CGATGCTTATGTTTTACAAAATAAATGGTGGAAAGAAACCAAAAATGGTTAGAGTCTCATCTCTA
CCACTGTCAAATGGGCTTCATTTTTCCCTTTTCTCACAGCAAATAACAAAATATCCTCCAGAGAG
TTGTGCTAAATGCTGCCTCTGGTCATATGTTGCTTGGTGTGTAGGGAGCAGCCGCAATGGAGCA
ACAACATTATTTGCAGATGTTATTTTGTGTTCTGAAGTGTTATTTTCTTATTAATTTATGCTAAGTC
AAATTTGCACTGAAATACTAAAGTACTAAATACTGAATTTAGTATTTTGAAGAATATTGAGGAGT
ATAAGAAGGACATGTATCACAATTGCTTTTATTTATTAATAAAATAATAGTGTTTATCTTAGTTCTT
ATATATAACTTTTTTAAAGTAACATGGAAGCATGAGTAAAGTTAAAACTTTGAGTGACCATTTTC
AGAAATAAAACTGTATTTAGGTACCTGGAAACCAAAATTTAGACTTAAAGTGAAAGATAGGATT
TGGATTATCTAGTTTTGATGTGAAGAAAGCAAGCTGATCCAGGCTGCCATTTACTTGAACTACTC
TTTGTAGGCCTCAGCCTCACGTTCAAAAGTGTTCCACACAGCTACTGGGTACAGTTGTCTCTATT
TAACCATACATGAAGGAGGGTCATAAAAGCATGGGATTTAGTAAATCGGTATGCTGAGTCATAT
GTTGCAATAAACTGCATATAATAAATTAGTTGAAATATGCAATGCACAGGTGGGGATGTATATA
ATATACATAAAACTATGTAATATATACCTACATACATACATACGATATTACAGCAAAATTTGGAG
CTATATTCCAATTGTTTTCCCCTTATACATTTGAGGAACTCTTTGGTCAGCATATGGCTCTCTCATA
AGGTCTCCCTGCCTTTTTTTAATGTCCAACCCTCCTTTGCATTCCCACCTGGCCCAGAACGTGTG

CTCATCCTGTACTTAAGTAATTATTGCATTCATTCAATCTTTATCCCAGAGCTTTTTTTTTAATTGTG
CAGAGTTTTCACACTACCTACAATTTCTAGAATGTTCTGTCTACAAAGCATGAAGGATTCGTGTC
TATTTATCTTGATCTAGCAGTTTCTTTTTCTATGGCACTGAATAAACTTATAAACATGCAGGATTTA
AAAAATATGATTCAATTTTTCTAAAGATGGTGAAAGTGATATTTTCTTTAGAATTTTTTCCTGATGG
ATATTATGTATAAATCTCTCTGATTTCACATGGCAAAAATAAGGTTAAGTTAAAAATTACCTGAG
AGAATCCTTGAATTTATGACTTCTGGGCTTAAGTTTTATTGTAACATCTCCACGTTTCAATATTTAA
CTATCTCTGGGCCACATAAAGCATGAGGACAGGTACTTTTGATATATTTGCTGTGTTGTGGGAGA
AAAACAAATCTCACCATTTTCTTATAATTGCCCCAAAATTTTAGTTTAAGAAATACTTTTAATAAA
AGTAGTCTCCTTCCCAGATTAGTAAATGTATTCTAACTAAAGGGTTTTGTGTGTGTGTGTATGTGT
ATATGTATACACCTATATATATTCATGTATATATATACATCTATATATATACACACACACCTTTAGT
TAGTCTATGTGTGTGTGTGTATATATATATATATATATATATATAAAAGTTATAGTCAATTGAACTCA
ACGAATCAAATGATTGAATGTGCCCTATCTGCCAGGTATTCGATAATATAATTGAGAGTGAATAC
AAGAAAAAAATAAACTATACTTACTATCTCAGGAAGTCCATTTTAATTGGGAAGTAAAAATGTAGC
CACATAAACATGTTACATATTGATAGGATACATTTTGGTGGTGTTTGCCAGGTGCTGCAGTTGTG
GAATTAGCAGCCATTAATTTTATCCTAGGGGTTGAAGATACTGTTAGTGCTAGTTTTTGAAGAAC
AACTGGGGAATTTCCAGCTGAATTTGATAAGGACAATACAGGGAATAGAAATACTATTGTCAGA
GAGCTTTACAATGCAAAGTTTCTGTGTTCCAGTAATGTGTCTGTACAAGAACAGAAAAACTATCG
TGGATTCAAATTTGAAACTGTTTGTAAGCCTGAACAAATAATTTTAAGCAGAGGCATTCACAGTG
TGATTTATTTTATAAAGAATATTTGGGATCTCAAATTGCAGAATGGATAGTTTATATTAGAAAAGT
ATTTTAACAGTTCAACGTAGGAATAATTGCAAATTGAACTAGGGTAGAAATTGTGGGGAGAAGA
TAGATATGTATGCACTTATATGTACAGATGTGCATACCTCCTTGTTTGGATGATGGGACCCAACA
CATGGGCACTTGCATAGACGAAAAGCAAATTCTATGTTACTTTCAACCCCAAAGGAGAGAAGAC
TGCCTGGCAGGGTACACAAGGTGGCCCACCCAGAGACAGAGTAACCTCAGGATGGAGCTGTA
AGAGATAGGTAATGTATAGCAAGTGGGGAGAGATGAGTGAGGTTTGGTATCTGGCTCTGGGGA
AATTCAGGGAAGTGTTTGTGACTGAGCATGTGAGAGCCTGATAAGGGAGCTGGTTGAGGTGGG
CCTCTTCCAGCCAATTGGAGGTCTGAGTATAACAAAAAAGCAGAGTAAGAAGGAATTCCTCTGG
CTGGACTGCCTTGAACTGCAATATAGGAATTCTCCAGCCTGCAGACTTGAATTGAAACGTTGGC
TCTTTTGGGGTAGAGCTTTCTAGTCTTTAGATTAGAGCTTTTTGCATCAGCTCTTCTGGGTCTCCA
GCTTGCTGACTGCCCATCTTGGGACTTCTCTGTCTGCATCTCTCTTATGTGTATACATAAGATTTG
TTGTTAAATTTTACACACACACACACACACAATTGGAATCTGTTGGTTCTGTTTTTCTGAAGAATC
CTATTACAGGTCCACATGGGGAAGTACTAGATTTCGGCAGAAGGAGAAGTAAGGAAAAAGTAT
AGATTCCTCAGGGATCTAGAACTAGAAATACCATTTGAACCAGCCATCCCATTACTGAGTATATA
CCCAAAGGACTATAAATCATGCTGCTATAAAGACACATGCACACGTATGTTTATTGCAGCACTAT
TCACAATAGCAGACTTGGAACCAAGTCAAATGTCCAACAATGATAGACTGGATTAAGAAAATGT
GGCACATATACACCATGGAATACTAGGCAGCCTTAAAAAATGATGAGTTCATGTCCTTTGTAGG
CACATGGATGAAATTGGAAATCATCATTCTCAGTAAACTATCGCAAGAACAAAAAACCAAACAC
CGCATATTCTCACTCATAGGTGGGAATTGAACAATGAGAACACATGGACACAGGAAGGGGAAC
ATCACACTCTGGGGACTGTTGTGGGGTGGGGGGAGGGGGGAGGGATAGCTTTAGGAGATATAC
CTAATGCTAAATGACGAGTTAATGGGTGCAGCACACCAGCATGGCACATGTATACATATGTAAA
TACCCTGCACATTGTGCACATGTACCCGAAAACTTAAAGTATAAAAATAATAAAATAAAATAAA
ATAAAATAAAATAAATAATATTTAGGAAGAACCATTACTTAATAAAAGATAAACCATTTATTCAA
ATATTTTCTTTTATTTCTGAATCCATCACTGTGGGAAATCTGGAATTCTACATTTCACAAGACAAT
GAACTTTTGTTTGCCCTGGAGAAATATGTAACTTTTCACAGGCTTTTTCTATTTTAACATATATAGA
GATAGTGTTTGGAAATTTAAGAACAAGAGTGGACCTAAGTATGAAAAATACGTACAACTTTTTCT

TTAGTTACTCTTGATACTTTTCATTGTTCAGGTTTTATGTATAATAAAACCTTACTTTATTTCCTTTC
TTTTTAGTTTTCTTCCTTTGTTTTGCTATTGCTATAGCACAGGATAAAGAACTTACAGAATATACAT
TGGTTATGCAATTTAATTAATTTTTAGGAGTTATGATTATTATAATCATCAAAGCCTTGGGCTCAA
AATAAAAATAATGATTTTCATTTTTAAATATACTTTTATTTTAAGGAATCAGTGGCTATGATTAATT
TAATAGCACTCCTTCAATTCTAATAATGTTTTGTTACAAAGCTTACATGTCATTTCTTTAAGCCAG
CTCTTTTTATGTTATTAATGTTAGAACATTTTAAGTCAATGATTTCATTTGCTAATATTGGTTTATTT
CTAACAGCATCCATCATTGTTTAACAGTCTGTTGGAGTGTTGCAAATTTATGAATTTTTATTATGA
GGGCTCCTTTTGGCAACAGATAATTTTTTGTTAACTATCATCAAAAAAGATAGAGGCTTTCTTCTT
TCAAGAACAATTTGTGTTTTGATAAAAACAATGTGTTATAATAGATTACATTTATTTTATATAATGT
TATAACTATTAGGGATGAGGCTTTTATAGAGATTATTATAATAACTTTGCTAGTCATTGATTTTTAT
TCAGTAATATATGTTGTCTTGTGTGTTTGTGTGTGTGTATTCATTCCCATGGATACTTGTCCTATGG
TTGTAAAAACATTAATGTCAAAATGCATGAGTTGAATTTAAGACATGGAGAGACTGCAATTGTA
GTAGCAATTAATTTTGTTCACTATGTATTCAAATATGGTCACCTCACTGAATTCTGAAAATATAAT
AGAATTATCCTTCCTGATTCTGATGTAGGTGACTGCAGTCATAGGATTAGTTCTGAAAAATTATA
AGTGCATAAAACATCTCACTTATAGGAAAGACATTTACTTAATAAGAAGAGCTTCAAGAACATTC
TTTACTTACGCCACAATATCTGGCAGAATTTGTGATATAGTTAGTAAACAAGCCCAGATACCTAA
ATGACTACAGTGAGCACAGGAGCCACCTCCCATATACAAACTTACAACTGACATGTTCCTTGAG
CAAGTAATAAACTTTTATTGTATCAAGACATTGTTATTTTATTTGGAGAATATTATAGAATAATCTC
TGTTATCCTGATTTACATAACACAATTCACCTTTTTCATATGCATGTAAGAATACAGATCATTAAC
ATGGTTATTTTCTATGAAAATTATAGTTCAAGTTTTAATCTGATACTTGAAATAACAGTATGCCTTT
GCCCAACTAAATATAACTTGCTTTTTTATTGTCAGTGAGGATAATGATTTTTATCACTATATTTCTC
AAAATACAAAGCAAGAAAGAATTCACCGAGTCTGTAATTAGTTCAGGAAATATGTTGAAGAAAC
ATAAGGGGGCCAGGCGCAATGGCTCACACCTATAATCCCAGCACTTTGGGAAACTGTGACGGG
TGGATCAATAGAGCTCAGGAGTTTGAGACTAGCCTGGGAAACATGGCAAAACCCCTGCTCTAC
AAAAAAATACAATAATAAAAGTTATAATAATCTGGGTGTGGTGGTCTGCACCTGTAAGTCCCAG
CTATGTGGAAGGCTGAAGTGGGAGAATCCAGGCCCAGGAGGTTTAGGATGCAGTGAGCCGTAT
TCCTGACACTGCACTCCAGCCTAGGTGTCAGAGTGAGGCCCTGTCTCAAAACATAAAAAATAAA
AAAATAAGGATTACATGAAATAGGATACTGTTATTATTAAATGTAACACAAAATAAATCCCGATG
AGATTATAATATGCTGATATTGATAATAACAATTATAATACCAGCAAAATTATATTTATTAAATAT
TTAAAATGTGCCTGTCACTATACTAAGTGATTTGCATGTTGTATCCCATTTATTCTCATAGCAATC
TTGTCATATAAGTAATATTATCTCAATTTTTTCAGAAAATAAAGCCAAACTAAGGTTTAGTGAGGT
GTTTTGGGCTGAATTGTGTCACCCCAAAATTCAGAACCTCAGAATATAATGGTATTTAGAGATAA
GGTCTTTCAAGAAATGATTATGTCAAAATGAGTCTGTTAGGGTAGGGTCTTAACCCATATGACTG
GTGTCCTTATAAAAACAGGAGGAGACACCAGGGATGACACACACAGAGGAAAAGCCACGTGT
GGACACAGTAAGAAGGCAGCCATCTGAAAACCAACAAGAAAGATCTTATGGGAAACCAGCCCT
GCCTGCACCTTGATCTTGGATTTCTAATCTCCATAAATATGAGAAAAATATTTCTATCAAGTCACA
GATCTGTGGCATTTTGTTATGGTAGTCATATGGCAGTTATATTATTATCTTTAACTATAATAAGAT
AGAGGTAAAGATAACTTACGTGAAGTAACAAAAATAGTAAGTGACAGAGACAGCATTTACATC
AGGAGTGTTTAACATTAGTTTACACTTGTATCCCAAGCACACATTGCAGTTTGGATATTTCTTTGT
GGTTGCAACCTTTTTTGCTTAGCCTTTTTGTTGCTTCTGTAACAGTTATTGTTCAACGATAAAATTG
CCATAAGCCTATTTCACCATATTAGAATTTAGATCACAAATTCATTCCCAAACATCTGTTGAGAA
CACATTGGATGTCTGAATTAAAATGACTTAAGAAACACAGTTTTATGGTATGTGATCATCTGACT
TAGTCTTCCAAACGGTTAAAAATTCCTTTAAAAAATGGGAACTGTGCAGCTCATAAATCAACACA
ACATAGGTGAGGAATTTCCAAAGGTGATTAAATTTTTATGCACATTTATATAATTTAGCACATTAG

TATTCCTATGTCTGGGTAAAACTCTCCTGTGGGCTTTAAACTGCTTAGTTGGTATCATCAAATTAA
CGATGACCAAATTAGTACATATTAGCAGTTAGATTCTTATATTTAAGGTCAATGTCTTATTTCCTG
TATAATGTTAACAAACTTACAATAATTTGTAAGTCTTTGCTTGCATAAAATGCCACTCATAGTAAA
GAATATTCTATAGCTTAAATGATAATGAGAAATATTTTGGATTTATATTCCAATTTTATATTACCAA
AGGCTAGATGGTATAGATTCCAGTTATGTCATCTATACATGGTGCATCATTATTAACTTAAAGTA
GAGCTAAAGCAAAACAACAGTAACAGAGGCACTATTTATAACAGTGAAAACATGGCCAGGTAT
GGTGGTTTATGCCTGTAATCCCAGCACTTTGGGAGGCTGAGGCAGGCAGATCATGAGGTCAAG
AAGTCGAGACCATCCTGGCCAACATGGTGAAACGCCGTCTCTACTAAAATTACAAAACTTAGCT
GGGCATGGAGGCGTGTGCTTGTAGTCCCAGCTACTCAGGAGGCTGAGGCAGAAGAATTGCTTG
AACCCAGGAGGTGGAGGTTGCATTGAGCTGAGATCGCACCACTATACTCTAGCCCGGCAATAG
AGCAAGATTCTGTCTAAAAAAAAAAGAAAAAAGAAAAAAAAAAAAACAGTGAAAACATAACCTTC
CCTTCATTACTGAAGATCTATCAAAACCAATTGTATATAATGCAAGCATCTATAGCAAAAGTTTA
CTTTTGTTTAGACATATCTAAACCAGTCTAATAAAAGTAGCAATGTTTTGTGATGGAGACAGATC
ACAAATTTAGATATTAGAAATTGTTGAGACTATTGGATAATTAATTAATTTAGGGACCCTAGCTAT
CTTATACATAAATTTTAATATGTATTATGATTTATCAGTCATTTAAAAATGTCTACTTTTTCAACTC
AAGATAATTTGTGAGCAGCATCAGAAAGGAGGAAACTTCTTTGTCCTCTTCCTTTATAATAATTC
CTTCCATTTGGTTTTCTACCTGTATTTACAAAACATTTATAAATCATCCATTGCTTCTTAATGGCTT
ACAGAAGCAATGATGAAACTGTGACAGGATAAAATTAACAAGATTTTCTCATAATATAAAACTA
AGCTCCATTGTGTGCTAGATGGTTTTTGTCTCAACGTTGTGATTTAACATAAATTTTAAACACAGA
ACACTATTTCAAGAATTCTTCTCTCATTCTGTAAACAAATTTGAAGAGGAATGTATTAGTCCGTTC
TCATGCTGCTAATAAAGACATACCTGAGACTGAGTAATTTATTAAAAAAAAAAGATGTAATGGACT
TATAGTTCCCCATGGCTGGGAAGGCCTTACAATTATGGCAGAAGGTGAAGGAGGAGCAGAGGC
ATGTCTTACATGGCTGCAGGCAAGAGAGTGTTGGCGGGGGAAACTGCCCTTTATAAAACCATCA
GATCTCATGAGACTTACTCATTTTTATGAGAACAGCATGGGAAAACCTGCCCCCATGATTCAATT
ACATCCCACCAGGTCCCTTCTATGATACAAGGGGATTATGGCAGCTACAATCAAGATGACATTT
GGGTGATGTCACAGCCAAACCATACCATTGTCCTTACATTTCAAAACACAATCATGCCTTCTCAA
CAGTCCCCGAAAGTCTCAACTCATTCCAGCATTAACTCAAAATTCCCAGTCCAAAATCTCATCTG
AGACAAGGCAAGTCCCTTCTGCCTGTAAAATCAAAAGCAAGTTAGTTACTTCCTAGATACAATG
GGGGTACAAGCACTGGGTAAATATACACATTTCAAATTGGAGAAATCAGTCAAAACCAAAGGG
GCTATAGGCCCAATGCAAGTCTGAAATCCAATAGGGCAGTCACTAAACCTTAAACTTCCAAAAC
GATCCCCTTTGACTCCATGTCTCACATTCAGGTCATGCTGATGCAAGAGGTAGTTTCCTATAGTC
TTGAGCAGCTCTGCCTTTGTGGCTTTGCATGGTACAGGACCCCTCCTGGTTGCCTTCACGGGCTG
GCGTTGTGTCTGCAGCACTTCCTGGCACATGGTGCAAGCTGCTGTTGGATCTACCATTCTGGGGT
CTGGAATATGGTGGCCTTCTTCTTACTGCTCCACCAGGCAGTGCCCCAGCGGGGATCCAACCTC
ACATTTCCCTTCCACACTGCCCTAGCAGAGGTTCTCCATGAGGGCTCTGCCCCTGTAGCAGACTT
CTGCCTGGGCATCCAGGTGTTCCCATACATCTTCTGAAATCTAGGCAGAGGTTCTCAAACCTCA
ATTCTTGACTTCTGTCCATCTACAGGCTCAACACCACGTGGAAGCTGCCAAGGCATGGGGCTTG
CACCCTCTGAAACAATGGCCTGAGCTGTATGTTGCCCACTTTTTGTCATGGCTGGAGCATGTAGG
TCACAGGACACCAAGTCCCAAGACTGCATACATCAGGGAACCCCTGAACCTGGCCCAGGAAAC
TATTTTTCCCTCTTAGACCTCTGGACCTGTGATGGAAGGGGCTGCCTCAAATGTCTCTGACATGC
TCTGGAGACATTTTCTCCATGGTCTTGGTGATTAACATTTGGCTCCTCATTCATTATGCAAATTTCT
GCAGCCAGCTTGAATTTCTCCTCAAAAAATGTGGTTTTCTTTTCTCTCATATCATCAGGCCATAGA
GTTTTCAAAGTTTTATGCTCTGCTTCTTGTTGAATGCTTTGCCAATTAGAAATTTCTTCCAGCAGAT
ACTAAATCATCTCTCTCAAGTTCAAAGTTCCACAGATCTCTAGGGCAGGGGCAAAATGCCACCA

GTCTCTTTGCATAGCAAAAGTGACCTTTACTCCAGTTTCCAACAAGTTCCTCATCTCCATCTGAG
ACCACTTCAGCCTGGACTTTATTGTCCATATCACTATCAGCCTTTTGGTCAAAGCCATTAAACGA
GTCTCTAGGAAGTTCCAAACTTTCCCACATTTTTCTACCTTCTTTGGAGCCCTCCAAATGGTTCCA
GCCTCCACCTGTTACCCAGTTCCAAAGTCACTTCCATATTCTTGAGTATCTTTACAGCAGTGCTG
CATTCTCTGTGCAACCAATTGACTATATTAGTCTATTCTCATGCTTCTAATAAAGACATACCTGAG
ACTGGGTGATTTATTGGAAAAAAAAAAAAGGTTTAATGGACTCACAGTTCCACATAGCTAGAGAG
GCCTCACAATCATGGTGGAAGGTGAAGGAAGAGCAAAGACATGTCTTACGTAGCAGAAGGCAA
GAGAGCGTGTGCAGGGAAACTGCCCTTTATAAAACCCATCAGTTCTCATGAGATTTATTCACTAT
CATGAGAAAAGCATGGAAAAATCCCACCCATGATTCAATTACCTCCCATCAGATCTCTCCCATG
ACACATGGGGATTATGGATAGCTACAATTCAAGATGAGATTTGGGTAGGGTCACAGTCAAACCT
TATCAAGGAATTGAATACAAAGAGATAATATACTAGTTATCATACTTTTGTGTGTTTACATAGGC
AATATGAATATTTTCTGTTTATCTTTTGCATTTAGGTGCTTATCTTTGATTTTTTTTACCAGTTTCTTA
AAATTACATAAGTAATCCACAAAAATCATCTCCAGTTTATAAAAAAGATTCAAATTGGTTGACAT
AGAAATGTGTGTTTTCAAAGGCCAGTTCACCTTCTTTTTTTCGTCTGTAACACTCTCTTATCCCTAA
ATAAATTATATTGTAAGTTGTTGGTATACCTACATGGATCTCAAATGTGGACAATTTTTACCCCAG
AATAATATTTGGCAATGTATAGAGACATTTTTGATTGCCTTATCTAAATAGAGGGAGTGAAGAAA
ACCTCTAGTGTGTATTAGCCAGAGATAATGCTCAATCATTCAGTCCTCAGGACAGCCCCATCAC
AAAGAATTATTAGTCCATATGCTGGTATATATCATGCCAGTCTTTCTCTGGATCAACTGCATTTAC
ACATATGCACATGGATGTTTTGTTGACTGTAGTATGTCAAAATTTAAAGACATATCAGGCACACA
GTAGATAAAACTCTTCAAGCTATATCCATATGAATGAAGGTCAATCATTCATATTGATAGAATTA
ATAAATTTGTTGACTAATAAAGCTCTTTAAATACATGTTATTTTAGATAAAAATTAAGAGCTTCTC
AATTTTGTAAAATAGTCATCTTAGTGTTTTAGTTTTTGAATTATTAAAAAAAACACTGTTGAATTTT
TTTTTAATAATAAAGCTCAGCTTTGCTACTACTTCTGGACTAGTCTATTTGCCAATTCTGAGATTTC
TCAAATTTATGAGTCACATTAGATAATGTCTGCTCTTTCAATTGTTCTAATTTGTTTTATTATCTGAT
CCAAATGCCTGAATCTCAATGGATTTATCATTAACTCTTTCTAACTCAGAAACTCTTGTGAGAGA
GATGACAGACATATCACAAGATTATTGTGCTGCACTGAACAAGGGCATGATACATGGTAAATAA
ATGAATGAAAAGAGAAGGAGAAGAGAGCTATTTTGGGGTCTCATTTATACTCCACTTATATCATC
TTTGATTTCTAATAATAAACTGATGAAACATATTTTAAGTAATTTATAAATGTAAAATTCGTTTTTT
ATTTTACAATTTTAGCTACAGCACAGTAACTGTTATAATAGTTAAAACTTTTTTTCACATTAGAAC
CTTACTTTTTTAACAGTGAAAAGAATTATAATTATGTCTTTACATTTCTATCAGTATTTCATTGCAA
TATGTCCTGAATTTGACAGATGAACAATTATCTCTTTTTTTATTAATGGACTTTGCAGGAAACAAA
TTAAGGCCATTTGAGTAATTACAATAACTTGATACATTTCTAATTTGCTATAGAATGTAACTCATG
ACTTGTATCTGAAAAAAAAAGGAAGGAAGATAGAGAGACAAAAGATAGGAAGGGAGGGAAGAA
GGAAACAAGGAAGGGAGGAAGGAAGGAAGGGAGGGAGGGAGGAAAGAACGAAGGAAGGAA
AGAGAAGAAGGAAGGAGGGAGGGAGGGAAATAATCTAATGAGAAATGGGAAACAATAGTATC
ATTAATTTTTTTAAATTATACTTTATCTGGTACATGTGTAGAACGTGCGGGCTTGTTACATAGGTA
AACACGTACCATGGTGGTTTGCTGCACCCATCAACCCGTCATCTACATTAGGTATTTCTCCTAAT
GCTATCACTCCCCCAGTCCCCCACCCCCCAACAGGCCCCGGTGTCTGATGCCTGCCTCCCCGTG
TCCATGTGTTCTCATTGTTCAGCTCCCATTTATGAGTGAGAACATGCGGTGTTTGGTTTTCTGTTC
TTGTGTTAGTTTGCTGAAAATGACTGGTAATGTTTAATAAGATGGGAGAGATGGATGTTACCTAT
TAAGATGCATATTGTTTTCCTCAAGGAATATTACCTTTTGAGCATTTTGCACTGTGGTCCAAAAAT
GGCTCAAATTCATCTGTTTCTTGCTGAATCCCAGCTTTGCATGTGTGTCACTAAATCCACAAGGC
AGCGATTCAGTGCTAATGAACTCTTCTGGTGGTTACTAACTGCTCTATAAGCATTGTAGGTAGAC
CATAAATAAAATTTTACCAGTCAGTTATGAAAGGAGAAAAATGAGAACAAAATAGGGAGTTTTC

AATGAAGCTAATGTTAAAAGGAGTGATCTTTATCTGCTCATATACATTTGCAGTTTTACAATATTA
AAATAGCTGTGTATCATTGATATTTTATTATTTTGCTGTCCTTACTTTTCAAAACAATATATTGTAA
ACCTGTACTAGTTCTTACAAGTCTCTTGAAAAATTACGGTTTGTGATACTGTATTTAATAGTCTAT
CAGGCCATGGTTTGCATTAATAAAATATGCCGGGGACCCAGGGACACTACACTGCACCTCCTTT
TTGTCTCAATTACATACCATGGTTAAGTCTTATTGCATTTTTATAAGTTTATTTAACAATTTAAGTT
CGAAAGATATTACACAATTTCATAGTGCTCAATTTTAATACATTTCAAATGAAAGCAAAAAGGAA
AAAATGAATCCCATTAGAATTTTATTCTGTTCATATATGCATACTCACAATATAATTAATATTCTG
AGCATTATTCATTAACTAGGTGGCCAGTATATTATGGATTCAGATTTCATTTAAACTTTGAGGAGA
AAAGAAAGTAACTTTATAAATAATAGCATTAAATAATGTTCATAGGAAATATAACTTTATTGAAG
TATTCATAAAATATAAATTGGACCTCTCTAAGACGTTTATTTCCTGTATAACTCTTACTACACTAAT
TTCTTACTTTCCTGTTGATTTTTTAAGCTATAGTTAAAATACATAGGGAAGGGCTTCAAGATGGCT
GACTAAAGGCACCTAGCACTCGCCTCCTCTGGAAAAAAGGACCAAAACAGTGAGTAGATAATA
ATAAGTCAAATAGTAGATAATAACAAGTCAAATAGAGAGTATAACACAGAACAGTAAAATTCAG
CAGGGAAGTGTCAGGGAACCTCTTAGGCAGAGAAGTAAAAGGAAGTGAAGCATCCTCCCAGG
CTGGGACCAGCTGTGAACCAGGAGAAACCTTCCAGTGCAGAAAATATGTAAGTGACAGATCCC
AGTAGTTCACATTCCCACTGCAGACTACTGTGGTACTAGCCACAGAGATAGCCTCAGCCATCAT
GGTCTCTGGGACCAGGTTAGGGAACTGCCACACTCCCTAGGGAGAGTCCACACAATAACATGG
TTCGAGAGAATGAATTTGTACTGGGCCCCGCACACTTCCCTGAGACACAAACGACTGCAGCCTG
GTGCCATTTAGAGAGCCCAGCCCCAACCAGACTACATCCTGCCCTGGGACCCAAAGGGCCCCT
GTGTCTCCACATCCCTGGATCCTCATTGACATCTCCGTATGTCTACCCAGAAAACTGTAGCATCA
CTGCACTGGGTGGACCAGCGGTGCAGATGAATCTCCAGCAGTCTAGCCCATGCAGTTTCGTATA
GCCAAGGGAATTTGTGGTGAAACTCAGTAAGAAGACTGTCCCTGGGACAAAGGGAGCTGAAGC
ACATGCCTCCCAGAGCCTGAGGGCAGTGTGCACAGGAATACTGCCCCTGACAGCAACCCTGCC
CCCTCCCAGCAGTAGGGCTGCCACATATCTGTACAAGCTTTCAGGGGGCCTAGGGACCTGAAG
CTCAGGTACTTTCCGTGGACCTAAGAGCAGACCTGTCCCACCCACCATTGCTCCCACTGAATCT
CACCATCTGGGGGCCTGGAAATCAATACCTCTTGCCAGCCACAGCTGGTGCTTGCCTGCATCAT
CAGTGAGCCTGAGGACAGGCCCCCTCTGACCAGTGCTGTCCCTACCAGTGCCCACAAGTGTCG
TCTAGGAGCCAGGAGATTGACTTACCCCACGTACCACAGACTGTACCAACATATACCATTGGTG
GCCTAAGGCCCAGGCCTTTTCACCACTGCGGATGTGAGGCCTAGATATCAGCCTTCCCTGCCTA
TCACTGCTGATGCACAAAGGTGCACCATCAGGAGACCTGGAAACAGATCCAGCCTGCCCGCTG
TCAGCACCTGAGAACACTGGTAAGGGACAAGAGAATTTACATGTCCTGCCTACCACCACTTGCA
TCCTGATAAACTATCATGGGGATGCAGAAAAAGACCACTCCTTTTGATACCTTTCTTGCCAGTGC
CTGTGTGCATTATCTGGGAGCTTGGGGTTGATCTGACTCCCCCGACTTCCATTAGTTCCCATGCA
TGCCTTCTGGAGGCCAGTGGATTGCTCCACCCAGTCTGCTCATGCCTACACACACGAGCCACGG
GGCTGGGGATTGACAAAAACATCTTCCACAGTCTGTGTGCACGGTAAGGGGCCTGAGGACAGG
CCCAGCTCACCTGGTGCCACCCCTGCCAGTACCGGCATGTGTCTTCTATGGTCCTAGAGGTCGA
CCTGTCCACCACCACTGTCATCTGTGCACCCCTAACGAGAGCCTGGGGATGGACCCGCCAAGC
TTGCTGCCACCACTGCTGCTGGCATGTTTCCCAACAAGCACTACCTGGAAGCCTGAGGATTGGC
ACACCACTGTTATTTTGATCACAGATGCTGTATAAGCTGCCTAGAGCTTGTCCAGTCTACTGGTG
CCTAGAGGATCTCTCCAGCCTACTGGTGCCAATGCAAACGCTTAAGAAAGCCACCTGGAGGCC
CTCAAAATGGTCTATCTCTAGCTGCTACCACCAGTACCCATGTATACTGCATACACAAGGGCTA
ACATGCTCAGCCCACTGCTGCCACCACTGGGGCCTGAGGACTGGCCCAGCTTGTGTTGCCATCC
TTAGCAATGTTCCACCACAGTCTCCACTAACAACTGCAGCTGAAGCCACTGAATAACTCACAGG
AACCACTGATAGTGATTACAGTTAAAGAAATCATACAGAGACTGCACTACTGCATGCACTGAAA

ATCAAAGCCAAAGCATCGTATTCAATCAACGTAATAGATACATCTGTAGAAAGTCTTTCCCTATG
AAAGCCATTCCATAAATTGGAACAAGCAACTGTTACATCGATGCAGATTTCAATGTAAGGACAC
AAGAAATAAGAAAAAGCAAGGAGACATGACACCTCCAAAGGAAAAAAAGAGTATCTATCTATT
CAGCAACAGATTTCAACAAAAAGGAAGTCTATGAAATCCTTAAATAATAATTCAGAATAATAAC
ATTAAAATAACTCAGTGAGATACAAGAGCACACAGATAAAAAATACAAAGAAATCAGAAAAAA
TTCATAATCTGAATAGGAAATTAAACAAATAGATGTATATCATATAAAAGCAAACAGATATTCTG
AACTTGAACAATTACATAAATAAAAATACAAAAAGAGCTTCAATAAGACTAAATCAAGCAGAAG
AAAAATTGTTGAACTTGAAAACAGGTATTTTGAAATAATCTAGTCAGATCAAAAAAAAAAAAAGA
ATAAAAAATAATGAAGAAAGCCTACAGGACATATGAGACTCCACAGAGAGAACAAGTGTTTGA
GTTTGGGCATTCCAGAGAAAGAAGAAACAAAGTCATAGAAACCTACTTAATGAAATAATAGCCA
AAAACTTCCCAAGTCTTGCAAGAAATAAAAGCATCCAAATGCAGAAATTTCAAATAATTCCCAA
ATAGAATCAACCCAAAAATGTCTTCTCCAATACACATTATAGTCAAACCATCAAAAGTCAAAAC
AGAATTTTAAAAACAGGAACAGAAAAGCACTAAGTCACATTTAAGTGAACCCCCATCAGACTTA
CAGGGTTTTTCTAACAGAAACCTTACAAGCCAGCAAAGAATGGGATGATACACTCAAAGTCCTG
AAAGGAAAAAAAAATCAGCCAAGAAGCAAAGAACACTATATTAGAAAGCTATTCTTTAAAAATG
AAGAAAAACTCCTTTCCAGACAAGCACAAACTGAGAGAATTTGTCACAAGACTGATCCTACAAG
AAGGGAGTCTTGGTCCTTCGTAAGTGAATTCTACATCTAGATGTGAGGGCCAGTATTTGCCATCA
TGAAATCACATGAAGTATAATACTCACTTACAGAGCACACACACAGATACAGAAATTTAAGAAA
AAAATGGGCTTAAATATTACTATTACAGAAAACCATCAAATTGCAATAAAAAACAAAGTGAAAG
AAAGGAACAAAAGATACACAAAATAATCAGAGAATAATTAACAAATTGACAGGAATAACTTTTT
ACATATCAATAATAAGCTGGAATGTAAATGGATTCAGTTACCCCCTAAAAAGATATAGATTGTCT
GAATGGATTAAGAAAAACATGACCCAACCATATGCTGTCCAGAAGAAACTCATCTCATCTATGA
AGACAAATAAAATGAAAGTGAAAGGACGGAAAAAGATATTCCATGCAAATGAAAACCAAAATT
TAGCAGGAGTAGCTATATTTATACCAATAAAAATGACTTTAGGTCAGAAAGAGTTAAAAGAGAT
GAAAAAGGTCATTATATAATAATAGAGGTATCAATTCAGCAAGACTATAACAATTGTAAATGTGT
AGGCACCCAACACTACAACATCCAGATAGATACAACAAATATTATTAAATCTAAAGGCAGAGAT
AGACTCCAATACAATAATAGTTGAGGACTTCAACACTCCACTCACATAATGTGTCAAATGCTGTG
GGTGGAGTGTTGGAGTTTGAACTGAATTTTAGACTAAGTGAACCTAACATTTACAGAATATTTTA
TCCAACAGTCACAGAATACACATTCTTCTCAGGAGCATATGCAGCATTCTAGCAGGGTAACTCA
TATGTTAGGCTACAAAACAAGTCTCAACAAATTTTAAAAAATCAAATTTTTTTCAAATATTTTCTC
AGACTACGGTGCAACAAAACTAGATATCAATAGTAAGAGGAACTTTGAAAGCAATACAAATAC
ATAGAAATTAACCAACAGGATCCTGAATGACCACTGGGTCAATGATGAAATTAAAAAGAAAATA
ATTCCAGAAACAAATAGAGAAAAAACACAACATATATACCTCTGGGATCCAACAAAACTAGTGC
TAAGAGGGAAGTTTATAATAATGAATGCCTACATAAAAAATACAAATATGTCAAATAGACAATC
TAACAATGCACCTCAAGGAATTAAGAAAGCAAGGAATTAGAAAACCAAATCCGGCCGGGCGCG
GTGGCTCACGCCTGTAATCCCAGCACTTTGGGAGGCCGAGGCGGGCGGATCACGAGGTCAGG
AGATCCAGACCATCCTGGCTAAAACTGTGAAACCCTGTCTCTACTAAAAATACAAAAAATTAGC
TGGGCGTGGTGGCGGGCGTCTGTAGTCCCAGCTACTCGGGAGGCTGAGGCAGGAGAATGGCG
TGAACCCGGGAGGCGGAGCTTGCAGTGAGCTGAGATTGCGCCACTGCACTCCAGCCTGGGCGA
CAGAGCGAGACTCCGTCTCAAAAAAAAAAAAAAAGAAAACCAAATCCAACATTAGTAGAATGAA
AGTAATAACACATATCAGAGCAAAACTATACAAAATGGAGACTAAACACCAACAAAAATACAC
TGCAACCTCTGTCTCCTTGGTTCAAGCAATTCTCCTGCCTCAGCCTCTTGAGTAGCTGGGATTAC
AGGCGCCACCACCACGCATGGCTAATTTTTATATTTTTAGTAGGGATGGGTTTTCACCATGTTGG
CCAGGCTGTTCTCAAAACTCCTGACCTCAGGTGATCCGCCTGCCTCGGCCTCCCAAAGTGCTGG

GATTACAGGCATGAGCCACGGTGCCCAGCCCAAAAATGAATAGTGTTTCTGTACATCAATAATA
AACTAACTGAAAAATAAAGAAGACAGATCTGTTTACAGTAGCTACAGAAAATAAAATATCTAGA
AGTACATTTAACCGAGTTAGTGAAATTTTTATGCAAGGATAATGATGAAACATGAATGAAAGAA
ATTAAAGAGGACACAAACTGAAAGATATCTAATACTCATGGGTTGAAAGAATTAGCATTGTTAA
AATGACTGTAATACCGAAAGCAATCTACAGATTCAATGCAATCCCTATCAAAATACCAATAATTA
TTTTTTTTCACAGAAACAGGAAAGCAGTCCCAAAATTTGTATGAAACCACAAAAGACCCTAAAT
AGCCAAAACAATCCTGAGTTAGAAGAATAAAGCTGGAGGCACCACAACACCTGACTTCAAACT
ATTCTACAAAGCTGTAATAACCAAATCAGCATAATATTAGTATAAAAACAGACATATAGACCATA
TAGACCAATGAAACAGAATAGAGAATCCAGAAATAAATTTATATATTTACAGCCAATTGATTCTC
AGAAAAGGCACCAAGAACATGCACTGAGGAAAGGATGCCCTCTTTAATAAATCGTACTGGGAA
TACGTGATGTCCATATGCAGAACCATGGAACTATTCACCTATCTCTCATCATATACAAAAATCAA
CTCAAAAGAGATTAAAGACTTACATGTAACATCTGAAACTATAAAACTACTAGAAGTAAACACA
GGGAGAAACTTTCAGGGCATAGATCTAGGCAAAGATTTTATGGCTAAGATTTCAAAATCATAGG
CAAAAAAAAAAAATAGACAAATGTAACTTTATAAAACTAAAAGTCTTCTGCGAAGAAAAGGAAA
TCAACAGAGTGAAGTGACAACCTGTAGAATGGGAGAAAATATTTGCAAACTATATGACAAGAA
ATGAATAACCAAAATAATTAACTCAACTAAACAGCAATATCATGACAATAAATAATATAATAATA
ATAATTCCATTAAATAGTGGCTAAAAGGTCTGAGTAGACATGCAAATGGCCAACAGGTATATGA
AAAAAACAGTGAACATCACTAACCATCAGAAAATGCAATTCAGGACCACAAAAAGTTATCATCT
TACCCCAGCTAGAACGGCTATTATCAAAAACAAAACAAAAAAACAAAACAAAACAAAACAAAA
AAAGAATCCCAGAAAGGATGCAGAGAAAAGGGAACTCTTATACATTGTTATTGGGAATATAAAT
TAATATAAATTATTACAGCCTTTATGGAAAACAGTATGGATGTTTCTCAAATAACTGAAAATAGA
ATGACTACACGATCTGGCAATACCACGACTGGGTATTTATTCAAAGGAAAGGAAATCAGCACAT
AAAAGGGGTACATGCACCCTCATGTTTATTACAGCACTGTAGCTGAAGTGTAGAATAAACATGT
GTCCATCAATGAATGAATGCATAAAGAAAATGTTATATGTATACATATGTGTCTGTATGCCTATA
CCCACAAATATATACAATGTATTTTATATATACAATGTATTTTTTAATCCAGCAATAAAATATGGT
AAAATACTATCATTGGAAGCAATATGTATGGAACTAGAGGTTATTATGCTAACTGAAATAAACCA
GGCACAGAAAGACAAATGTTGCATGTTCTCACTAATAGGTGAAAGCCGAAACATTTGATCTCAT
GGGAACAGAGTGTAAAATGTTGGTTACCAGAGGCTGGGGAGGGTGAGAATGAAGAGTTGTTTA
ATTGGTACAGACATGCAGAAGAAATAAGTGCTGGTGTTCAATAGTACAGTAAGATAACTCTAGT
TAACATATTATATTGTATATTTTAAAATAGCTGAAAGAGAATATCTGAAATATTCCCAGTGAATTT
TCTTTTCTTTTCTTTTCTTTCTTTCTTTTTTTTTTTTTTTAATGGAGTCTCGCTCTGTCTCCCAGGCTGG
AGTGCAGTGGCGCCATCTGGGCTCACTGCAAGCTCCACCTCCTGGGTTCAAGCCGTTCTCCCTC
CTCAGCCTCCTGAGTAGCTGGGACTATAGGCGCCCGCCACCACGCCAGGCTGATTTTGTTTTTG
TATTAGTAGAGACAGGGTTTCACCGTGTTAGCCAGGATGGTGGGCTGCACCTAATCAGTCTGCA
TCAAACCTTCCCTAATTAAAAAATCTTGGCCGGGCGCGGTGGCTTGTGCCTGTAATCCCAGCAC
TTTGAGAGGCTGAGGCAGGCAGATTATTTGAGGTCAGGAGTTCGAGACCAGCCTGGCCAACAT
GATGAAACCCCTTCTCTACTAAAATTACAAAAATTAACCGGACGCAGTGGCTCCTGCCTGTGGC
CTCAGCTACTCGGGAGGCTGAGGCTGGAGAATCACTTGAACCTGGGAGGCAGAGATTGCAATG
AGCCGAGATCGCGCCACTGCACTCCAGTCTTGGCAACAGAGAGAGACTCTATCTAAAAAAAAC
CCAAAAAACAAAAAACAAACACAAGAAGTTTTGGCTGCACCAAGATGCTAGCTGTGTGTGTTAT
TATTTTGACTACCATGTAGTTTAGTGAAGGTGTTCAGTAAACAGATGACCAAGTTTAATTTTATAA
TTTTCAGGAGTATTATCCTGATTCTAGTATAGAATTATCCTAATTCTAGTGTTAGAATTAGATTTTA
GCAATATCTATTACTGTTCTTTGTTTGCCATTTTCCTATTGTACTATTGCTACATTTAGTGATATAA
GGCCATCTGGATTCAAATTTCTAATTATTTTCAACATGTATATTATTTTCAGTGTTGCTTTTGGTAA

TACATTTGAAATGATAATATTGAGCTACATGAAAAATAAATATATGATTATTCTATCTTTTATCAA
TTGCATGGATTAAATGTAGAGAAAGCTCGTGAGTAATCCCATTATAAAAGATGAGAGAAATAAG
AAATACATGCTTAGTGTATATAGAAGATAATGGGATATGGTGTTTATTTTAAAATATTTACGTACC
TGTATTATTCACTACATATGATTACATTGATACCTCAAACAAAATGTGTGAATATAATTGAATAAA
ATTATATCTAGAGGGAATTCATTTTCTAAAGTGTGCATAGCAAAGGGTGCTATGTCAAAACATGT
AAAATCATCACAGTGTACTTCAGAAACTTCCAAGTATTGAATATACAAAATAAAAAACATGAATT
TGTGCTGTTTTTCAGCCAAAGGCTCTTATCAACAAAGGTTTTCAGTTATTGTGGGAGAACCCTTTC
TTGTTACCGTAGATCATCTTACTACATGATGGTATTTTATCTTTCTCTCGCTCACTTGACCTTATAG
GGAAATCACCAGTAAGAATATACTTTTTGTGACAATGTAAATATTATCCAGTCAAGATAGTAAAT
ATTCATTCCTTAGCTATGATTGCCAGAATTTTCTTAAACTAAGATCAATGTGTGGAATGTCATCAG
TATATAAAATCTTACTAATAACTTGGAAAGACCATGATTTCTTTGTTTCTATATGTGATTTGTGGA
CGTTTACCTTCTCATGTGCAATCTTGGTAAATCATAAAGTATGTTTAAATGTCAGTTTTCTCATCC
ATAAGCTGGGGATACCATTGTCTCCCTTGCAGTATTGCTGTGGGTATGAAGACTGAAATTGAAGT
GGCTGACATTTATAAGGCACATATGATAGCTATTACATTTTTTATATTTCCATTTCCATTTGGGGA
AATGTGGTTGATAGGGAGAATTCTTCTTTTCTTTCTTCTTCTTCTTTTTTTTTTTTTTTGGAAGGAAT
CTCGCTCTGTCATCCAGGCTGGAGTGCAGTGGTGTGATCTTGGTTCGCTGCAACCTCTGCCTCCT
GGGTTCAAGCAATTCTCCTGCCCCAGCCTCAAGAGTAGCTTGGACTACAGGTGTGTGCCACCAC
ACCCAGCTAATTTTTGAATTTTTAGTGGTGACAGGGTTTTACCATATTGGTCAGGCTGGTCTCGA
ACTCCTGACCTCATATCTGCCCACCTTGGCCTCCCAAAGTGCTGGGATTACAGGTGTGAGCCAC
TGCGCCTGGCAGACAGGGAGAATTCTTCTGTTAAGACTTCCCTGAAATTCAAGGTAGGCTAATT
GTCCCTTATGTGCTCACTTACCACTGGTGGAAATATCCCTCTTTAAGACCTAATATAATATACTTT
ATGTCTTTCTACTGGTAATCAGTAAGTGCTTGGAGGATAGCAAATGCATTTTATTAAAACTTTTAC
TCCCAGTACATAGCAGATTGCTGAAGTCACAATTGTTAAATAAATGTTTGTTGCCCTGCACTAAA
ATGTGTAAGATACTTGATGAGAAAATTTAGAAAATATTTTAAAAGACTTTTTATGTGGCTATAGA
ATATTTAACTTAAGATCCATGTGTTTGCTTTATGTGTCAGAGTTTGTTATGTACTATGCTTTTGTAT
CTTCATGGCTTTTTTTTTAAATCTAGTTTTTAAACATAAATAATTTTCTGATCTTTTTTAAGTGAAAT
ATTTAAAATACATTAAAAATGTTTTGACATTATGATTATTTGTTTACACATGGTTAAATTATATCTA
TCTGAATTATGTGAATTATTCTTTGACAAGTGATGATTCTTCATCTCAAAAGCCTAATGAAGATAG
TATATTAATTGGTTCTCTGGTCTTAAATGATAATCTTTCAATAGTAAATTGCAAAGAAGCTTGCTG
ATATTTCTCTTGCATCGGTTAAATAATAATTTCCAGTAATCAAGCCATGGCTAATGATGATGGAA
GTAACACATTTATGCAACAAAAATTTGCACATATTTCTTCCAGAAGCCAATAAGCAGTTACATGA
AGCAACATAAAAGCAATTTTTTTCCATCTTTCAAGCAACCAACAAAATTAAATTTCATTGTTATTAT
TTAATAAAAATAGTAATATTTATGACTTTATATTACAGTTGGTATACTAATCTTTTAGTTAATAAAT
TTACAAGCTTAGTGGGAAAAACAAAACAAAAGCATAAACGAAATCTGTATGCAGTATGGAACTT
ATTAGGTACATATGACCCGATTCTAATTTAAAACCTGTAATGCAATTGAAATAATACCCAACATA
AAGCTTATGACAAAACAAATGCCTACAATTTACTTTAGAAAGTAAAGCGCATGCAGTTATTGAA
AAAAATAGACTGTTTACACAGAATGTTAACTGTTCCTTTTGCATTGTTGTATCAAAATAGGTGATA
AGCCCCAGTGTGTGTTGTTCCCCTCTATGCGTTTATGTGTTCTTATCATTTAGCTCCCACTTACAT
GTGAGAACATGCCGTATTTGGTTTTCTGTCCCGTGTTAGTTTGCTAAGGATAATGGTCTCCAGCT
CCATCCATGTCCCTGCAAAGGACATGAACAACACACACTGGGGCCTAACTGAGGGTGGAAGGT
GTGATGAGGGAGAGGATCAGGAAAAATAACTAATGAATACTAGGCTTGATACCTGAGTGATGT
AACAATCTGCTCAACAAACCTTCATGACACTGGTTTACCTAGGCAACAAACCTGCACATCCTGC
AAATATACCCCTGAACTTAAAAGCTAAAAAACAGGCCCGGGTGCAGTGGCTCACGCCTGTAATC
CCAGCACTTTGGGAGGCCAAGGCGGGTGGACCACAAGGTCAGGAGATCGAGGCCATCCTGGC

TAACACGGTGAAACCCCATCTCTACTAAAAATGCAAAAAATCAGCCAGGCGTGGTGGTAGGCA
CCTGTAGTCCCAGCTACTCAGGAGGCTGAGGCAGGAGAATGGCGTGAACCTGGGAGGTGGAG
CTTGCAGTGAGCCGAGATCACACCACTGCACTCCAGCCTGGGCGACAGAGCGAGACTCCTCAA
AAAAAAAAAAAAAGCTAAAAAACAGAAATAATGACAAGGAAGAAAACACGTACATATTCAATAA
TAATAATAATAGCCAACAGGATTACTATGAAAAAAGATAGGTGATAACTCTTCAGCTAACATTA
ATCAGGCTCTGTGTGCATGACTTGACTTGTTATTTCTTATATATGTACAAACTCATTTAATACTCAT
AACCCTACATGTACATAGTTTTGATGACCTATTTTACAGATGGGAAAACTGAGACCCAGAGAAG
TTAAACAACTCCCCCAAGGTTAGAATCTGATAGTTGCTAGAATCTGGCGAATGCAGATGTGTTTC
AAATTATATTCGTTATTATAATTATATTTCTTATTTGTTAAATAAAAACACATGGGGGTCCAAGAT
GGCGGACTAGAAGCCACTAGTATGCCACGGTGCTCATGGAGAGAAATAAAAGGGGTATGTAAA
TACTACATTTTCTTTTTTTTTTTTTTTTTTTTTTATTATACTCTAAGTTTTAGGGTACATGTGCACATTG
TGCAGGTTAGTTACATATGTATACATGTGCCATGCTGGTGTGCTGCACCCACTAATGTGTCATCT
AGCATTAGGTATATCTCCCAGTGCTATCCCTCCCCCCTCCCCCGACCCCACCACAGTCCCCAGA
GTGTGATATTCCCCTTCCTGTGTCCATGTGATCTCATTGTTCAATTCCCACCTATGAGTGAGAATA
TGCGGTGTTTGGTTTTTTGTTCTTGCGATAGTTTACTGAGAATGATGGTTTCCAATTTCATCCATGT
CCCTACAAAGGATATGAA

ID|30393|gene_id|ENSG00000253190|transcript_id|ENST00000517961 Homo sapiens lncRNA

AGTGCAATCTTCGGTTGCCTGGACTAGCGCTCCGGTTTTTCTGTGCTGAACCTCAGGGGAC
GCCGACACACGTACACGTCCCTTCGATAGCTCAGCTGGTAGAGCGGAGGACTGTAGCTACTTCC
TCAGCAGGAGACATCCTTAGGTCGCTGGTTCGATTCCGGCTCGAAGGAGACAAGTGCGGTTTTT
TTCTCCAGCTCCCGATGACTTATGGCACTTTCCTTGGGTGCCTTCAGTGACACATTGCATTCCAA
CGAGCAGTTTGAAAGTCTAGCGCTTTCTCCCCATTTTGGGCCTCCCAGCCTGCACGGTAATTCTT
TTTAGCCATTCGCCCTGCGGGAACGTGTCCGGGCAGGTTCCCAGCGCAGCTGTGGGTCTGCGCT
TGGCCGAGCGACTGCCGGGTCACGACTTCTGCGTCTTCTTAACCCGTCTTTGGCATTGCCCGGG
CCCCGAGTCACACAGGAGGCAGCGCCGGCTCCAGGGGGCCAGGCGGGGACCTTCTCCTCAGA
GCCCCGGGCAGCTTCTGCGACCCGAGGGCTCGCAACGGCTGCCGTGAGGAGGTGGGGGGTCC
GCGGGAAGAGGTATCTGGCGCTCCCGGAACCTGGGAATCAGAAAGAGAGAACACAATACTAA
AAACACGAAGCCTAAAAATGACACAATGTTATGGAGACAAGGCGGCACCCGGGAAGCTGTGC
CCGCTCCCTTCGATAGCTCAGCTGGTAGAGCGGAGGACTGTAGGCGCGCGCCCGTGGCCATCC
TTAGGTCGCTGGTTCGATTCCGGCTCGAAGGAGAGACACCCCCCCCCCCATTATTTTGTTGCTTT
GAACCAAAAAAGTCTGTCTTCAGCGCTCAATGTTCTGACCCTTCTCTAAAGGAACAGATAATAA
GCCGTGCCCAGCCGTGGGGGATTAGCTCAAATGGTAGAGCGCTCGCTTAGCATGCGAGAGGTA
GCGGGATCGATGCCCGCATCCTCCAGTTTTCCTTCCTGTCCCGTACGGTTTTTCTTTCGATTCTCA
GCCCAAACTAGAGCTGAAAAGTCAGACGAAGTCAGGTGAAGAGTAGGGCGAGCTCCAGCTTA
CCACTCTAAAACTTCCCAGACAATGAGTGGTGGGCGCTAGCAGTTTATCCTTCTAGCTTTAAATT
TTTAGACCCATTTAATTGGGGGGAAATTCACGAAACTGGTTATTTTTGCTTCAAAAATGGCGACA
GATTGCCGTCACATGTATTATCACTCAGAATCCTTTATGATTTGTGATAAGATGTCTGCATTTCCA
GGGACTCATCTGAGGCTAAGCTGCCCATAGTTCGGGGAGACCCACAGGGAAAACAAAACAAG
AAACAGAGAACTTGGAAACGGACGCTGATTACTTTGAACGTTTGCTCAACCGAGGAAGCAGGA
ACTGTTCGGCCCCATCTCAAGTCCGCAGGGCGGACTGCCTGGCTCTGGGCAGATCCGAGGTGG
ATAGGACAAGGCAAAAGGTAGCCCTGGAGAGGTGAGTGGATAAAGTCCCTGGACACAGGGGC
TGCCGTTGGAGGAGGTTGTAATGTCATTGTCCTCCTGTGGAAGTTAACTGTGGAGAATTAACTAG
AGACCAGCGCTGAGAGAGAATTAATTGGATAAAAGTTCTATCTAGGACAGTCCTTGAGCGTAAT

TTTGTGAAGCAAACACAAGACAACAACAAAACGAGTAAAACCTAAACACACATCCACACACTC
ACTGAAAGATTCAGAGATTGCACAGAACACAAAGACTTTTCTGGTAATAAAATCTCCCTATCCTA
ATTTTCTAGAAAAAAAGCTGTTGAAGCCACCGCGCAGTTCTTACAAGACATAACACAAATCTTC
CACACAAAGGCAGAAATACAGTATCAACCTTTGTGAGGAGTACAGCAAATAAATGATGTTTCTT
TAGGGTCCTAGG

ID|30394|gene_id|ENSG00000253190|transcript_id|ENST00000520930 Homo sapiens lncRNA

TCTGATGTCTTAGTAGATGCGGTGGAACCAGCTCTGTCTACAGAACCCCGGGCTTCAGTGG
CGTCTTCCTAACCCGGCTTGCCTGCCGGGGGCGTTCCGAGACCCTCGGGGCCTTCCCTTCACCC
CGCGGGAGTTGGACCGGTGGCGCTGGTAAGGCCTCCCGGGCTCAAAGTGCAACGGACACTGC
AGAAATCCAAACTGCTGGCATTCGCGGTTTGGGGACGCCAGAGGAGGTAATGATTTCTGGTTTG
TTAACCTCAAGTGACAATAATGCCGAGCCAGGCAGGAGCTGGACCTACAATCTTCTGATGCGTG
GTCAGACACGTTATCCCTTGCGCCACTGGCCTACCACGCTACTCCTTCAGTCGCCGTTGGATTAC
TGTGTGTTGAGAACACACTCGGCAACCACTTTAAAGGACAACGCAGGCTGGTAAAGGAAAAGT
ACGACAAGGGGGGGCGGTGGAATCGCAGGGTCTTGGCATCGCGGACCCCAGACACCTGGGTT
GAGGGCCTTTCCCGGGTCAGTCAGGCTAGCGAGCCGGAGCGTTCTGTCTTTCTGCGCACGCGTA
GAGCACACAGGCCGGCTCTGGGGCTCTGCGCTCCTCGGATTACGCATGCTCAGTGCAATCTTCG
GTTGCCTGGACTAGCGCTCCGGTTTTTCTGTGCTGAACCTCAGGGGACGCCGACACACGTACAC
GTCCCTTCGATAGCTCAGCTGGTAGAGCGGAGGACTGTAGCTACTTCCTCAGCAGGAGACATCC
TTAGGTCGCTGGTTCGATTCCGGCTCGAAGGAGACAAGTGCGGTTTTTTTCTCCAGCTCCCGATG
ACTTATGGCACTTTCCTTGGGTGCCTTCAGTGACACATTGCATTCCAACGAGCAGTTTGAAAGTC
TAGCGCTTTCTCCCCATTTTGGGCCTCCCAGCCTGCACGGTAATTCTTTTTAGCCATTCGCCCTGC
GGGAACGTGTCCGGGCAGGTTCCCAGCGCAGCTGTGGGTCTGCGCTTGGCCGAGCGACTGCCG
GGTCACGACTTCTGCGTCTTCTTAACCCGTCTTTGGCATTGCCCGGGCCCCGAGTCACACAGGA
GGCAGCGCCGGCTCCAGGGGGCCAGGCGGGGACCTTCTCCTCAGAGCCCCGGGCAGCTTCTG
CGACCCGAGGGCTCGCAACGGCTGCCGTGAGGAGGTGGGGGGTCCGCGGGAAGAGGTATCTG
GCGCTCCCGGAACCTGGGAATCAGAAAGAGAGAACACAATACTAAAAACACGAAGCCTAAAA
ATGACACAATGTTATGGAGACAAGGCGGCACCCGGGAAGCTGTGCCCGCTCCCTTCGATAGCT
CAGCTGGTAGAGCGGAGGACTGTAGGCGCGCGCCCGTGGCCATCCTTAGGTCGCTGGTTCGAT
TCCGGCTCGAAGGAGAGACACCCCCCCCCCATTATTTTGTTGCTTTGAACCAAAAAAGTCTGT
CTTCAGCGCTCAATGTTCTGACCCTTCTCTAAAGGAACAGATAATAAGCCGTGCCCAGCCGTGG
GGGATTAGCTCAAATGGTAGAGCGCTCGCTTAGCATGCGAGAGGTAGCGGGATCGATGCCCGC
ATCCTCCAGTTTTCCTTCCTGTCCCGTACGGTTTTTCTTTCGATTCTCAGCCCAAACTAGAGCTGA
AAAGTCAGACGAAGTCAGGTGAAGAGTAGGGCGAGCTCCAGCTTACCACTCTAAAACTTCCCA
GACAATGAGTGGTGGGCGCTAGCAGTTTATCCTTCTAGCTTTAAATTTTTAGACCCATTTAATTG
GGGGGAAATTCACGAAACTGGTTATTTTTGCTTCAAAAATGGCGACAGATTGCCGTCACATGTA
TTATCACTCAGAATCCTTTATGATTTGTGATAAGATGTCTGCATTTCCAGGGACTCATCTGAGGCT
AAGCTGCCCATAGTTCGGGGAGACCCACAGGGAAAACAAAACAAGAAACAGAGAACTTGGAA
ACGGACGCTGATTACTTTGAACGTTTGCTCAACCGAGGAAGCAGGAACTGTTCGGCCCCATCTC
AAGTCCGCAGGGCGGACTGCCTGGCTCTGGGCAGATCCGAGGTGGATAGGACAAGGCAAAAG
GTAGCCCTGGAGAG

ID|30396|gene_id|ENSG00000260464|transcript_id|ENST00000565336 Homo sapiens lncRNA

CCTGCAACTCTGGGGTCCCGGCCGGGCTGGAGCGGCCGCCGGAGAGACCTGGTAGGCGG

GGCGCTTGGGGCAGGAAGGGAGCCGGGGAGGGAAAAGCGCGACTGTGATTCGTACGGCTGG
GCCCGGACCGTGAAGGGGACCGGGTTGCGGCTGCCCTCACACCGACCAGGTGGGACCGTCGG
ACTGCGGCCGCCCTTGGCTCAACTGGGTCGCCGACCTTGTTGAGGGGCGACCACTGCGGGGAG
ACCGAGAGCCTGCGGCTGGCGTTTGAGGCGGATGGCAGTGCCCTGAGCGCGGCGGCTGGGTC
TCGGTGACACTGACGACGGGAGGCGCGGTCGGAAGAGCGCGGGGCCGTCGCCTCTGGCTTAA
CATAGCAGATGCGCTGAGACTCCAACAGGTGGCTCCGTGGCGCAATGGATAGCGCATTGGACT
TCTAGAGGCTGAAGGCATTCAAAGGTTCCGGGTTCGAGTCCCGGCGGAGTCGTAACGCTTTTTT
CCCTCCCCCCTACAATTTATTTTCTGCCTCCATCATGTCTCTTATTTTTTTTTTTTGCTAAACGGTTT
AACTTCTCTCTCATTCCCTGCTCTCGCAGTTCACTGCATTCGGTTCTTGCGGTCCTTTCTTAAGCG
GCTCGCAGGGTCCCGAGCCCCTCAGCTCCCCGGGCCTCGGTGGCCCAGGGCCCAGCTCAGCC
GACTGGGCAGTCGATGTAGGTCCTGAGAAGAGCGGCGGCGGCGGCGGCGGCGGCGGCGAAG
GAAAAGCGACACTGAAGCGAAGGCTCGCGGTTTCGGCCTAAAGGAAAGTGCAGGGGAGGCCC
GGGTCTCGGGTGACACCCCCACGCCTTGAGCGGGACTGTGGTTGCGAGGGAGGGACGAGAAA
ACACGCCTACGCCCTGCGTGCGCTGTGCTGACTACCTCTCCAGCATGGAGGCTTCCAGATGAGC
AGACACCCGAATCCCAGCGGGAGCCCGTCAAAAATGGAGATTTCTGCCGTCACTCTGGGACTC
TTAATTCGGTCCGTCTGGGGTGGGGCCCAGGAATCTGCATTTCTGATAAGGTCACCCGCTCCCC
TCCCCCATTATTCCAGTGCAAAGAGGTCCTAGGCCCAGTGCCCATCAGCTCCTCAAGACAGGAA
TTACTATATCTTTTTCTGACAACTGTTAACTTTGTACAAGGTTAGCAAATAAATCCAGGAATGAAT
GGAATCTTAAAACTCGTAAAACAACAATGAAAGGTAATTCACACAAAAGATACAAAATCCAAAA
TTGTCAAAAAAGATACAGGGAAAAGTAAACATCCATGTTATTCCTATAGTCCAGCCTTGCAATTC
TCTCCACAAGCCATTAGTGGTAATGGTTTCTTGGAAATATTTTCAGATTTATTTTATGCCCATATC
AGCATTTTTAACATCTTTTGTCATAAATATGTCGTAAATAACAATGTTATTGATACATATCCTTTTA
AAAACACAATGGTAGTATGCTATACTGGCTTGTTTTACTTATGAATATATGTAGAGCTGCCTCGTT
CCATTGTACGTATGTATGGTAATGATTTAACAGTCCCTAATAGCTGGACATTTAGATTGCCAGTA
TTTTCCTATTACAAACAATGCATCAGTGAACATCCTTGTTCTTTTATTATTGTACACAAGTACAGG
TACATCTATCTGTAGGATAGATTCCTAGAAAGTTGAATTGCTGGTTCAAAGGTTTGTGCGTTCTTA
ATCTGTACATTTTTTAATATATATTGGCAAATTGCCCCATAGA

ID|30404|gene_id|ENSG00000261519|transcript_id|ENST00000563151 Homo sapiens lncRNA

TAGATCTTCACAGACTGGATGTTAAGCAAGTGGAAAGGCAGAACAGCCTCCACTCGCGGT
GCACGGGGAAGAGTGGGCGGCGGACGTGGTTTTGGGCCTGGGACCCAGGTGTTCCTCTAGGCC
CAGGGCGGGCATCGCTGCCCATCCGTCCCACAGGTGGTTCTGAGGCGTCTCTCAACAGCCATAT
GGCGGACACCAGAACCCGGGTGTAACAGTGACCCGTTTCTGTGTGGGTCCCCACTCCCAGGGA
GGGCAGGAGAGCGCCGGGGGCGTAACCCCCCTCCCCCACGCTGGCACCAGAGTGTGAGCAGA
ATCGGCTTAAGGCAGCTCCCAGTCCCCATCGCCCCCAAGGTCGAAAGAATCGGGACAGGAAAT
GGGTCGGGGGACCGAGTTTGGGCCGGAATGTTCAGAGCGTGTCCGTCCTGCTCTTTAAAGGCC
CTCTCTCCATCCCAGAACCAAAGCCGAGCGCCCCCAGCGCTCGCTGCGGTCTCGCGCCCGGGT
AACGGCCGATGGCCGCCCCCGTCCCAGCCGCAGCCGGGGAGGAAATCCCGCGCCTCGGAAGA
GGCGCTTCGGGCTCGGGCGGGTTCCGGAAGCCGAGGGGCCGGGAGTAGGTGGGCGCATAGG
GGCTCCTGGGAGGCGGGACCAAGACGCGCGTTACCGGGAGGCCAGGCCCGGCGCTGCACCCT
CCGCGGCCCGGGGCCTGAGGCCGGGCTCCCGCGACTTCCCGGCCCAAAGCAGCGCAGCGCCC
GGGCGGCCCAGCCCCGCAGCGGCCACAGCAGCATAGCCGGGCGAGCGGATCCAGAGGCCCG
GGGTTCCTTCTTGTGCGAGTCGGCGCGGACGACTGCTAGTCCAGCCGTTGGGCGCCGCCTGGA
TCCCGGCGCCTGGCCCTTAAAGAGACCCGGTCGCATTTCATCTCAGCAAGAGGAGAAATACTC

GAAGGGAGAGGTTCGCCGCTGGCAGCCAGAAGCCTTACCACTGCTAGGACGCTGGCAGAACG
CACCCATAACTTGGCAACACTCGGAGGATGGATGGCAAATAATACCAGCGACTGAAGGGTTAA
TTAATGATTGCAGATTAATTGAACACCTTCTGTAATCAGACACCTGGCACACACTCATGATGTCA
CTTATTCCTCCCAACAACCCTGCAAATAGATAACTGTCTCTCCCATTCTCCGAGCTTGTCCTCAG
AGAATTAGGTAACTTGTTCAAAATGGTCAGAAGCAACAGATATAACTCCTTGCTCTACTCTTTCC
CCCCTTCTCACTACTGCACTTGACTAGTCTTTTTAAAACATAAATTTTTTGGCCAGGCACGGTGGC
TCACGCCTGTAATCCCAGCACTTTGGGAGGCCAAGGAAGGCAGATCACCTGAGGCCAGAAGTT
CGAGACCAGCCTTGGCCAACATGGCGAAACCCCATCGCTACTAAAAATGCAAAAATTAGCTGG
GTGTGGTGGGGGGCACCCGTATTCCCAGCTACTCAGGAGGCTGAGGCAGGAAAATCGCTTGAA
CCTGGGAGGCAGAGATTGCAGTGAGCCGAGATCACTGCACTGCACTCCAGCCTGAGCAACAGA
GGGACACTTCGTTCCCCCCGCCAAAAAAAAAATTTTTTTTAATGATCAGAAGCGGCTGGGCGC
GGTGGCTCGGGCCTGTAATCCCAGCACTTTCGGAGGCCGAGGAAGGTAGATCACTTGAGGTCA
TGAGTTCAAGACCAGCCTGACCAACATGGCCAAAAACTGTCTCTACTAAAAATACAAAAATTAG
CCGGGCGTGGTAGCGCACGCCTGTAATCCCAGCTACTCAGGAGGCTCAGGCACGAGAATCGCT
TGAGTCTGGGAGGCGGAGGTTGCAGTGAGCTGAGATCGTGGCCACTGCAATCCAGCCTGGGCG
ATAGAGTGAGACTACGTCTC

ID|30405|gene_id|ENSG00000261889|transcript_id|ENST00000570843 Homo sapiens lncRNA

ATAGCCGCCCGGCTAGCTCAGTCGGTAGAGCATGAGACCCTTAATCTCAGGGTCGTGGGT
TCGAGCCCCACGTTGGGCGTGAGATTTTGTCGGATGGATTAGACTTGCAACTTGGAAGAAAGTA
AACTAAATAAGAAAACGTGGAAGTGACTCCGTATACCTTGCAAATCGGGAATTGTTGGTCAGGC
AAACGCAAAACGCTCCACCAACCAACTGGCTTCAGGATCCCCTTTGTCTTGTTTCATAAAAAAA
AGAGGCCGAGCGTCGTGGTTCATGCCTATAATCCTCTCACTTTGACCCTAGGAGTTTGAGACCA
GCCTAGGCAACATGGTGAGACCCCCGGCCCTTAAAAAAAATACAAATTTTCGCCAGGCTTGGTG
GTGTGCGCCTGTAGTCCCAGCTATTCCAGAGACTGAGGCAGGAGAATCACTTGAACCCGGGAG
GTAGAGGTTACAGTAAGCCGAGACTGTGCCAGTGCACTCCAGCCTGGACAACACAGTAAGACT
CCGTCTCAAAATAATAATAAAAAGAAATTGGCCGGGCGTGGTGGCTCACGCCTGTAATCCCAGC
ACTTTGGGAGGTCGAGGCGGGCGGATCACGAGGTCAAGAGATCGAGACCATCCTGGCTAACAT
GGTGAAACCCCGTCTCTACTAAAAAAGAGAAAAAATTAGCCGGGCATGGTGGTGGGCGCCTGT
AGTCCCAGCTACTAGGGAGGCTGAGACAGGAGAATAGCGTGAACCCGGAAGG

ID|30407|gene_id|ENSG00000264727|transcript_id|ENST00000577346 Homo sapiens lncRNA

AATACCCAGCTAGTTGCAAAACCTTCTTTTATTGCTGCAATAGGACCTTTTCATGTGTCTAA
AGCATAATTTATTCAGTGAGTACCCTGCTGTTGGCCATGGAAGATAGTAGTCTTTTTCACTATTGC
AAACAATGTTTCAATAAATAACAAAATTTTTTTCACACCTAAAACAGCAGGTATTTTATATAGCAT
TTATAATTGTAAATAAGATCACTATTTTCTTCTCTCTTTCTCTCTTTTTCCTCCTTCTCTCACTAATT
ATTGCTGATATTAAAAGATTAATTCTTGTAGTCTGCCTGTGAATAGCCACTGCACTCCAGCCTGG
GCAACATAGCAAGCTATGCCTCTGTTTAAAAACAAAACTAAAAACTACTCATAATCTTACTCAAA
ATGACTAGTTTACATTTTGGTCTTTATTTGCATATATACCTACATTTTTAGAATAAGATTCTACTTT
ACACAGTATTCCTGTTGTGAATTCTGTCATTGCCAACCTTCTTGAAGATTCTTACTAGTTTCCATTT
TCTTCCACTGATTCTCTTGAGTTTTCTAGATCTATATTCTTATCATCTGATAACGAGGATGATCTTG
CCTCTTTCTTTTAAGTAGTCTTACTTTTTATTTCTTTTTCTTGTTTAACTGCACTGGCTGACCCGAAA
TGAGGCTAAATGATACTGAGGACAGTTGGTATCAGTGTCTCACTTCTTTCTAGAGTGGAAATGGT
TCTTTGTGTGGTTTATTTTTGCCTGATGGTGACTGTTGGTTTGTAATTGATCTTAACTGAGGAGTA

ACAAACATGGTAAAACATTTAACACAGAATCTTTCAAATAATAAATACACAACAAATGTTTTTGT
TATTATTATATTTTTGTCATGGTAAAGAGGTATACTTTAATTTCCTGTCATTAAGATTTATTTTTAAA
TTGAGAATGGATGTTGAATTTTGACTCCTGCCTTTCTTAAGGGGATATCTATTTAAATAATTACTT
ATATTTTTCTTTAAACTGTTAATAGAATGATATATTAGCATTGATATAGTTGTCTTTCTTGGACTGC
AAAACCAATTTTGTCAAAAGTTTGTTACCCTTTTGAAATAATGCTAGGTTTTATTTTCTAGTATTTT
ATTAGAGTATGTGCATCTATTTTCATGAGTTAAACTGTCCTCTATCATGATTTTTCAGAGATAGCT
TTGTTAAGATTTGAAATCAAGGTTATGCTAGCTTTGCAAAATGAATTGGAAGTTTTCTTTTTCCAT
CCTCCATCTCCCTTACCACCCCTTCCCCAGTATGTTCTGTTCCAAGAAATTTTGAAGGAGCTTAC
ATGTGAAATTGCCCTGGAAAAGAATCTTTTAAAATATAAATATACATACAAATATATATATATGT
ATATATATATACACACACACACAATATTTTCCATTTATTCTATAATTAGTACAACTTTTAGACTTTC
TTCTCAGTTTGAATTAATTTTGGCAATTGTACTTTCAAAGAAAATCATTCTGATTCCATTGAGTTTA
TTGGCATAGAGCTGCAAACAGCCTCTGATTCTGAAATAGCTCTTCTGGATCAGTGATTATATACC
TTTTTTGTTTTGTATCACTGAGTGCTTGTGCTCTCTCTCTTATTTCTGACTGAAGTTGCTAAAAATTT
AATTAGTTGATAGAACCAGTTAGTGTACAAATTAGTGTTGGATTACTAATTATTTTAATTTATTATT
TCCTTTTAAATATAAAAGAACTTGCAGCTGTAAGTTCTTCTCTAACAGTAGGTATAACTGAATCCT
AGATGTGTTCATATGTAATATTTTTGTTACTGGTGTGTTCTAAATATCTGTGGTGGTTGTTTTGAAT
TATGCTTTTGCCCAAAGGTTACTCGGTGAAGACTGTTTCTTAAATTTCAATTGGATTGGCTCTTTG
TTCATATTTTGTTATTAAAGTCTAATTTTATTGCATTTTAGGTAGATTTTGTGACCTAAATTATACCC
AATTTTGGAAATTTGTAGTTTTTGTGTGTGTATGCATGGCATATGTACAACTAATTTTTGTAACTAT
TATATGGGCACTTAAGAGTATCTTCACAATGTATAATTAATATATCATGATTAGGAGAAAGTGTG
TTTATGATGTTATTCAAATATTTTCTTTCTTTTTTTTTTTTTTTGAGATGGAATCTCACTCTGTTGCCC
AGGCTGGAGTGCAGTGGCGCAATCTCGGCTCACCACAACCTCCGCCTCCTGGGTTCATGCCATT
CTCCTGCCTCAGCCTCCCAAGTAGCTGGGACTACAGGCGCCCGCCACCACACCTGGCTAAGTTT
TTGTGTTTTTAGTAGAGACGGGGTTTCACCGTGTTAGCCAGGATGGTCTGGATCTCCTGACCTCG
TGATATGCCTGCCTCGGCCTCCTAAAGTGCTGGGATTACAGGCGTGAACCACTGTGCCGGGCCT
CAAATATTTTCTCTCTCTATATATTTATTTTTGTTCTCTTGATTAAAGTTGTTAGGAATGTGTTAATT
TCTTCCACTGTGATTGTGTTTTGTATTTTCTTGCATTATGCCATGGAAATTGCTGGTTTCCTAGGTC
AAAACAATTAAATATTGTCAGTTACA
ID|30408|gene_id|ENSG00000265091|transcript_id|ENST00000579933 Homo sapiens lncRNA

ACCCACGACGGCGAAGACCCGCTACAGCGCGTCCACGAAGCCCCAGTACTGAGCCAGGA
CGGGGCTCTCCCTCTCGATGTAGGCGACTACGAACTGCTCCGCCAGCCGGTGAGGCAGCTAAT
TAAGAAAGCCACCTTCAGGATGTCTTTCGAGAATCTAGCCTCGAAGAAAGTCATGTAAGAGAAC
GCCTGCATCAAAAAGTCGGGAGGCCGGGCGGAGTCGCCGTTAAACGTCCAGGGAAAGCCACC
AGGTGGGCCGGCGAACGCTCCCGGGCCAGCAGGTAGGCTTTCTCGCACAGCAGCCCCCGCAC
CCGGTCCGTAAGCTCGTTGTTCTCCCTGCGCCGGGCATGGTTCTAGGTCAGGAGGTCCTACATC
ATGACAGCCAACGCGTCCATCCTGCGCCTCCCCGCAGGCCTTGCTCCCCGCTCGGTCCCTCCCG
GCCGCGGCGCTGCCGGAAGTGCGTGTCGGCGCGGGGGCGGATCCCGGTGGCGGCGAGGGGC
GGACTCCGCGGACAAGGCCAGGCCTAGCCTTGAGCTCCGTCGGGCAGGGCCGCCGGGATTGG
TGTCCGGCAGAGCGTGAGCCCAGCGGGCTGGAGTCGGCGAGCGGGAGTGAAAGAAGAAAAG
CTAGAGAGCGAAGGCAAAGCCCGAGGAGAGAGGGCCGCGGTCGGCGGAGGAATCTGACTGC
ACGGTTGCGTGCGCTCACTCCCGGAGGGCTCCCTCAGGCGGGCGGCTCCGCTAGTGGCTAAAG
GCAAAGCATTCCGGGGCGCGGCGCATGAAGTTGAGCTTCGTCCCTGCTAGCCGCCGCTTTCTCC
CCAAAAATACATCCTAGCCTTAATGTTTATGCCTCCATTGCCCCAGTTCTTATCTGTTTTGCTCAA

TGTCTCATAGCTACAAGAAGGCAATTTCTGACGAAGCCCTCCGTCCCTTCCAAATGGATTATTTT
GGCGGGCTTCCACCCGGACAGTATGCCACCCGAATGACTGGACAAGTGCACGGGAGCGGCTG
TCATTTGCGGAGTGCGCCTTGCGATCTAGGCGCCTCACAGCGCAAATTATCCAGTAATTTCTCTG
AAATCGATGCTGGTTTGTTTTCCCAAGGCAAATCAGCAGCTTATACAGACATTGGGGCCACAAA
GCCGGTGGAACAACGGGAGACGGCTTCCTGAGTGTCAGGTCCTCCAAGATGAGCTTAAACTTC
GGGTGGTGGGCAGGCTCGGTAGGCGGGAAAGGCCCGTGTCCAGATGAATGCAGTCCATGTTTA
TATTGATGAAAGACACGTATACCATTTGGTCCAGCTCAACCATTCTTAACAAGTGCTGGCTGTGA
GCACTGGTGTGTGTGTGCCTGCGTGCAACCAGCAATGTAAGTGATGACCAGCTGCATGATGCCT
TAATTTGAGTGGACAAAGTCGGTCAAGAACCACTTTCAGGCTTCTGGGTCTCCAAGTCTTTGTCT
CCCTGTATCCAAACGAGGCATTCCAAGTTTGACAGCATGGAACATTGCATGTCCGAAATACCAA
CCGTGAAACTGGGTAAAAAGCTTCAGGAGGTGATATTGATGTGTATAGTTTAGGAAATAAAACC
CAATACTTACTTCAAAGAACGTAATTTGTGGCTTTTTAGAGAGAAGGATACTTTGACAGTTTTCAT
TTGTCGTCTACATGTATATGGTACGTTTCATTCTGCCTTTTAATTAATTTGGTATTTTACTATGAAA
ACTACTTTGTATTAATAAAATAATGTATTTCCAATGTTATAAGTTGTGTTATAACAGTTTCAAAAG
CCAAATAAACAAGATGCATTTTGATATCAATTGTTTAAAACGCGTATTTTGAGGAAGAACAAATT
CATACTCAAAAAAGTTATCTTTGGGTGGAGTATTGTGGAAAATTTTTACTTTATACATTTCTAGGT
TTCTGAAGTGACTGTATTCATTTTATAATTATAGAAACAAGACGTTAAATCCAGAAAACAAAACT
TAAGCTTAACCAACTAGAAACTGTCAACCAACTTCTCACTAGGGACTTCCCAATTTAACCAAATA
TTTTTATTTGTTTTGTGTCTGCAAACATCTTACAAAAGTTTTCCCTTTTTGCCTCCACAGTGGAGCA
CTGAACTGCTTGTGGTCTAGTGCTGACTGATTCATGAATCACTGGATGCTCAAATAAACTCTAAA
TTAAAAAAGACATTAAATGAAAGAAAAAGTTAAGAAAATAGAAATGCTTTCCATTTTTTAACAAA
TGCTTAAACGGAAGGAAGGATGCATGCAGAGGTTAAGTAAGACTGCGTTTCAGATCTGAGGCT
CGATCTGAAGAACATGTAGTATATGCCTATTCCTTTATAGCTAGACGTTATTTTCTTTGCTGAGGG
AAAAGGAACATGTCATTATATTCAACTCTTCTAAAAAAGGAAAGCCATCTACCGAGTCAGTATA
ATTTATGGAATTTATCTGGAGCTAAAGGTAGTATCAAAATGCCATTTAAAGTTGCAAAGCCAAAA
TGAACTCTTACTTTGTCCTTTATAATTTACATGAAATTTATGAAGCAAATAACGAGACTAGGCAGT
CATGCTCAAAACACTCAGGATGTGCCTACCACTGACTAATGATAGCTACATATATTCTTTTGTAG
GTATATATTAGAAAATAAAATTTTAAAACAACGTGGCCAGATATATGACAGATCTTCAATAGGTA
TGCAATGTCTGTTTTTAAGTAATGAAACAAAGCAAACACTCCTGAATCTATGGCCCATATGAAGA
CCTAGATTGTTGAATTTACTGTTATTCCCCTATCCCATCTTTACAGAGTTAACCACTAACCTGAGA
TTTGGGTTAATCATTATCATACCTGTTTTATTTTTTAAGTGTAGTTTTATATCTATCGATCAAGATCT
ATATATCTACATACTTTTAAAAGTATGGTTTACATATAAGTATAGTGCACACACATATATGTGTGC
TTAATGTTGCTTGTTTCTGAGATTTATACAATTAGTATGACTTTCAGATTTCTGCAACTTGCTTCAC
TAATTCAAGATTGTTTCTATCATTCCTCCAAGTTGTGCCATGTAGTATTAGTTCATTTCTTCTTGTT
GATACATAATATACTACTATGAATATACCATAGTTTATGTGCCGATTTTCCTGATGTTTGGACTGT
CTCTAACCTTTTGCTATTACAATTATATGATTATTCTTTATATGTCTTCTGGTGCACATGTTAAGAA
TATTTGTAGGGCATATATTTAGGAGTGGAATTGCTGGGTTAGAGGGCATGTGGTGATGCTACAG
TGTTTTCTAAAATAGTTGTTTCTACTATTGTAGTTGTTTAAAATACAATATTTTAAAAAGTTTATACT
TTTACCAACACTTGGTACTTTCAGAACTCTTAAATTTGCCAATGTAGTAGGAGTAAAGCTATAGC
TAATGAGTTGTTTTAATTTGTACTTTCTTGATTATTAGTGTGGCTGAGCATCTTTTCATATGTCTTC
AGGTACTGCTTGTTCTGTGCCATGCTTGTTCATGCATTTTATCTGTTTCATGGAGGTGTTTTTCCCC
CTAATCAATTTGCAAGAGTTCTTGGACATATCTTGGACATGAAACTTTTGTCAGTTATGTGGATTG
AATATCTCCAAGATTGTGGCTTTCCTTTATAATTTGTTTTTATATCTTTTGATGAGTAGAAATTACT
TTGATTTAGTTGAATTTATCTATCTTCTCTTGTGTTTAACATTTTTAAAATGGCTTTAAACTCTTCCA

ACCTCAAAGTAAAAAAAAAAATTCTGCACTTCTTTTTTAAAGCTTTAAAGTCTTGCCTTTTTATTTTA
AGTCTTGGAATTAACATTTTGTGAGATCAGATCCAATTATTTTGTCTTTCTTCTTATTTATTTATTTT
TGGACACAAGGTCTCACTCTGTTCCCTAGGCTGGAGGGCAATGGTGTGACCATGGCTTACTGCA
GCCTTGACCTCTCAGGCTCAAACGATCCTCCCACCTCAGACTCCCAAGTAGCTGGGAATACAAG
CATGTGCTACCACATCCAGATAATTTTTTCATTTGCAGAGACAGGGGTCTCACTCTGTTTCCATG
GCTGGTCTTGAATTCCTGGGCTCAAGTATTCCTCCCACCTTTGTCTTCCAAAGTGTTGGGATTAC
AAGCATAAGCCACTATGCCCCACCCAATTAGTTTTTCCTTGTGACTGAACATTTGTTCCAAGAAT
ACTTATTAAGTAGTCTCTTCTTTACTCACGGGTTTGCTATGCCATCTTTCTCATATATCAAGTTTCT
ATGTATGTGTGATTCTGAGGTTTCTATTCTGTTCTATTTATTTGCTTGTATTTCCATAAATACCAAG
TTGTCTTAATTTATAGAGCTTTATGATAATTTAAGAAGAACAGTCTTCTTCTGTCTTATTTGTCTTC
TTCAGGAGTGTCTTGGCTATTCTCAGGTCTTTGTTCTTCTATATACATTTTAGAATCAGCTTGTTGA
ACTATTAGTCTAATTGTTTTATCCCCTTGCCTGTAAAAAAATATTTTCCTTGCCTTTGGGGCTACG
GTTTCACTACAAACTGTCTAGGAGAGGATATATTTGTATTTAGCTTGTTTAATACATTTGTGTTTCC
TGTATCTGTGGGTTTATGACTTTGCTTCTGAAAATATCCCAGCTGTTATGTCTTCAAACATAGCTT
CTCTTTCATTCTTTCTATTTTCTCTGGCTGAAGCTCATATTAGCAGTACATTAGATCTTATTATTATT
CCAGTCTGTCTTTCTCTTAATCTCCTACAGTTACTAGATCCTTATCTCTCTGCTACTTCTGGGTAAA
TTATTTGAGCTATCTTTCATTTCAGTTATATTATCTACAGTTGCTTTAAGTTTGCTGCATAACCTAT
CGATTGCATTCTTAATTTCAATAGTTATGTCTTTTATTTCTAGAAGCTTATGTAGTTATTTTTAAAGT
AAGTCTATTTTGTTAATCCTATGTCTCTTGCTCATTTATTTTTTCATTTTATTAAACTTTTCACAGAT
ATTTTATATTCTGTATGTGATAATTCTGAATCCTTGAGAATCAATATCTGTTGTTTCTGATGGCTCT
CATAATGGCATGTTTCTTTATGTGTATCATGAGTTTATATATATGAACAATTGTCTACTAAGTCTTT
CACTAATATGGGATAAACAAGTTATAATAATAATAACTTATAATAACAATAAGTTATAATAATCA
GCCATCCTCAGTTGCCTGGTCCATGTCACAGTTATTAAGTGGTAGAGTTCAGAATTAAATTCAGG
TCTGTCTGACTTCAAAAACAACTTTTGCTCTCTCGGCCTTAGCGCCATCTTCTTGGAAACTTCTGC
ACCATGAGAGCCAAGCAGAGAAAGAAGTGAATGCGCAGGCTGAAACGCAAAAGAAGAAAGGT
GAGGCAGAGGGCCAAGTAAACTGCTAGCTTGTGCACCCGCGGAGGCCACAGCAGCAGAAACA
TGAAGTGCCAGAGGCTGGGGATGCTGGCACAAGTTGTTGGACTGCATGCTACTGTCTAGAGCTT
GTCTCAATGGATCTAGAACTTTCATTGCCATCTGATCGCTGAGACCACCTCTGAGACCCACCTTG
CTCATAATCAAAACTGTCCATCTTGGTCCTTTGCCCTGGAACTGTGACAT

ID|30409|gene_id|ENSG00000265091|transcript_id|ENST00000581170 Homo sapiens lncRNA

ACCCACGACGGCGAAGACCCGCTACAGCGCGTCCACGAAGCCCCAGTACTGAGCCAGGA
CGGGGCTCTCCCTCTCGATGTAGGCGACTACGAACTGCTCCGCCAGCCGGTGAGGCAGCTAAT
TAAGAAAGCCACCTTCAGGATGTCTTTCGAGAATCTAGCCTCGAAGAAAGTCATGTAAGAGAAC
GCCTGCATCAAAAAGTCGGGAGGCCGGGCGGAGTCGCCGTTAAACGTCCAGGGAAAGCCACC
AGGTGGGCCGGCGAACGCTCCCGGGCCAGCAGGTAGGCTTTCTCGCACAGCAGCCCCCGCAC
CCGGTCCGTAAGCTCGTTGTTCTCCCTGCGCCGGGCATGGTTCTAGGTCAGGAGGTCCTACATC
ATGACAGCCAACGCGTCCATCCTGCGCCTCCCCGCAGGCCTTGCTCCCCGCTCGGTCCCTCCCG
GCCGCGGCGCTGCCGGAAGTGCGTGTCGGCGCGGGGGCGGATCCCGGTGGCGGCGAGGGGC
GGACTCCGCGGACAAGGCCAGGCCTAGCCTTGAGCTCCGTCGGGCAGGGCCGCCGGGATTGG
TGTCCGGCAGAGCGTGAGCCCAGCGGGCTGGAGTCGGCGAGCGGGAGTGAAAGAAGAAAAG
CTAGAGAGCGAAGGCAAAGCCCGAGGAGAGAGGGCCGCGGTCGGCGGAGGAATCTGACTGC
ACGGTTGCGTGCGCTCACTCCCGGAGGGCTCCCTCAGGCGGGCGGCTCCGCTAGTGGCTAAAG
GCAAAGCATTCCGGGGCGCGGCGCATGAAGTTGAGCTTCGTCCCTGCTAGCCGCCGCTTTCTCC

CCAAAAATACATCCTAGCCTTAATGTTTATGCCTCCATTGCCCCAGTTCTTATCTGTTTTGCTCAA
TGTCTCATAGCTACAAGAAGGCAATTTCTGACGAAGCCCTCCGTCCCTTCCAAATGGATTATTTT
GGCGGGCTTCCACCCGGACAGTATGCCACCCGAATGACTGGACAAGTGCACGGGAGCGGCTG
TCATTTGCGGAGTGCGCCTTGCGATCTAGGCGCCTCACAGCGCAAATTATCCAGTAATTTCTCTG
AAATCGATGCTGGTTTGTTTTCCCAAGGCAAATCAGCAGCTTATACAGACATTGGGGCCACAAA
GCCGGTGGAACAACGGGAGACGGCTTCCTGAGTGTCAGGTCCTCCAAGATGAGCTTAAACTTC
GGGTGGTGGGCAGGCTCGGTAGGCGGGAAAGGCCCGTGTCCAGATGAATGCAGTCCATGTTTA
TATTGATGAAAGACACGTATACCATTTGGTCCAGCTCAACCATTCTTAACAAGTGCTGGCTGTGA
GCACTGGTGTGTGTGTGCCTGCGTGCAACCAGCAATGTAAGTGATGACCAGCTGCATGATGCCT
TAATTTGAGTGGACAAAGTCGGTCAAGAACCACTTTCAGGCTTCTGGGTCTCCAAGTCTTTGTCT
CCCTGTATCCAAACGAGGCATTCCAAGTTTGACAGCATGGAACATTGCATGTCCGAAATACCAA
CCGTGAAACTGGGTAAAAAGCTTCAGGAGGTGATATTGATGTGTATAGTTTAGGAAATAAAACC
CAATACTTACTTCAAAGAACGTAATTTGTGGCTTTTTAGAGAGAAGGATACTTTGACAGTTTTCAT
TTGTCGTCTACATGTATATGGTACGTTTCATTCTGCCTTTTAATTAATTTGGTATTTTACTATGAAA
ACTACTTTGTATTAATAAAATAATGTATTTCCAATGTTATAAGTTGTGTTATAACAGTTTCAAAAG
CCAAATAAACAAGATGCATTTTGATATCAATTGTTTAAAACGCGTATTTTGAGGAAGAACAAATT
CATACTCAAAAAAGTTATCTTTGGGTGGAGTATTGTGGAAAATTTTTACTTTATACATTTCTAGGT
TTCTGAAGTGACTGTATTCATTTTATAATTATAGAAACAAGACGTTAAATCCAGAAAACAAAACT
TAAGCTTAACCAACTAGAAACTGTCAACCAACTTCTCACTAGGGACTTCCCAATTTAACCAAATA
TTTTTATTTGTTTTGTGTCTGCAAACATCTTACAAAAGTTTTCCCTTTTTGCCTCCACAGTGGAGCA
CTGAACTGCTTGTGGTCTAGTGCTGACTGATTCATGAATCACTGGATGCTCAAATAAACTCTAAA
TTAAAAAAGACATTAAATGAAAGAAAAAGTTAAGAAAATAGAAATGCTTTCCATTTTTTAACAAA
TGCTTAAACGGAAGGAAGGATGCATGCAGAGGTTAAGTAAGACTGCGTTTCAGATCTGAGGCT
CGATCTGAAGAACATGTAGTATATGCCTATTCCTTTATAGCTAGACGTTATTTTCTTTGCTGAGGG
AAAAGGAACATGTCATTATATTCAACTCTTCTAAAAAAGGAAAGCCATCTACCGAGTCAGTATA
ATTTATGGAATTTATCTGGAGCTAAAGGTAGTATCAAAATGCCATTTAAAGTTGCAAAGCCAAAA
TGAACTCTTACTTTGTCCTTTATAATTTACATGAAATTTATGAAGCAAATAACGAGACTAGGCAGT
CATGCTCAAAACACTCAGGATGTGCCTACCACTGACTAATGATAGCTACATATATTCTTTTGTAG
GTATATATTAGAAAATAAAATTTTAAAACAACGTGGCCAGATATATGACAGATCTTCAATAGGTA
TGCAATGTCTGTTTTTAAGTAATGAAACAAAGCAAACACTCCTGAATCTATGGCCCATATGAAGA
CCTAGATTGTTGAATTTACTGTTATTCCCCTATCCCATCTTTACAGAGTTAACCACTAACCTGAGA
TTTGGGTTAATCATTATCATACCTGTTTTATTTTTTAAGTGTAGTTTTATATCTATCGATCAAGATCT
ATATATCTACATACTTTTAAAAGTATGGTTTACATATAAGTATAGTGCACACACATATATGTGTGC
TTAATGTTGCTTGTTTCTGAGATTTATACAATTAGTATGACTTTCAGATTTCTGCAACTTGCTTCAC
TAATTCAAGATTGTTTCTATCATTCCTCCAAGTTGTGCCATGTAGTATTAGTTCATTTCTTCTTGTT
GATACATAATATACTACTATGAATATACCATAGTTTATGTGCCGATTTTCCTGATGTTTGGACTGT
CTCTAACCTTTTGCTATTACAATTATATGATTATTCTTTATATGTCTTCTGGTGCACATGTTAAGAA
TATTTGTAGGGCATATATTTAGGAGTGGAATTGCTGGGTTAGAGGGCATGTGGTGATGCTACAG
TGTTTTCTAAAATAGTTGTTTCTACTATTGTAGTTGTTTAAAATACAATATTTTAAAAAGTTTATACT
TTTACCAACACTTGGTACTTTCAGAACTCTTAAATTTGCCAATGTAGTAGGAGTAAAGCTATAGC
TAATGAGTTGTTTTAATTTGTACTTTCTTGATTATTAGTGTGGCTGAGCATCTTTTCATATGTCTTC
AGGTACTGCTTGTTCTGTGCCATGCTTGTTCATGCATTTTATCTGTTTCATGGAGGTGTTTTTCCCC
CTAATCAATTTGCAAGAGTTCTTGGACATATCTTGGACATGAAACTTTTGTCAGTTATGTGGATTG
AATATCTCCAAGATTGTGGCTTTCCTTTATAATTTGTTTTTATATCTTTTGATGAGTAGAAATTACT

TTGATTTAGTTGAATTTATCTATCTTCTCTTGTGTTTAACATTTTTAAAATGGCTTTAAACTCTTCCA
ACCTCAAAGTAAAAAAAAAAATTCTGCACTTCTTTTTTAAAGCTTTAAAGTCTTGCCTTTTTATTTTA
AGTCTTGGAATTAACATTTTGTGAGATCAGATCCAATTATTTTGTCTTTCTTCTTATTTATTTATTTT
TGGACACAAGGTCTCACTCTGTTCCCTAGGCTGGAGGGCAATGGTGTGACCATGGCTTACTGCA
GCCTTGACCTCTCAGGCTCAAACGATCCTCCCACCTCAGACTCCCAAGTAGCTGGGAATACAAG
CATGTGCTACCACATCCAGATAATTTTTTCATTTGCAGAGACAGGGGTCTCACTCTGTTTCCATG
GCTGGTCTTGAATTCCTGGGCTCAAGTATTCCTCCCACCTTTGTCTTCCAAAGTGTTGGGATTAC
AAGCATAAGCCACTATGCCCCACCCAATTAGTTTTTCCTTGTGACTGAACATTTGTTCCAAGAAT
ACTTATTAAGTAGTCTCTTCTTTACTCACGGGTTTGCTATGCCATCTTTCTCATATATCAAGTTTCT
ATGTATGTGTGATTCTGAGGTTTCTATTCTGTTCTATTTATTTGCTTGTATTTCCATAAATACCAAG
TTGTCTTAATTTATAGAGCTTTATGATAATTTAAGAAGAACAGTCTTCTTCTGTCTTATTTGTCTTC
TTCAGGAGTGTCTTGGCTATTCTCAGGTCTTTGTTCTTCTATATACATTTTAGAATCAGCTTGTTGA
ACTATTAGTCTAATTGTTTTATCCCCTTGCCTGTAAAAAAATATTTTCCTTGCCTTTGGGGCTACG
GTTTCACTACAAACTGTCTAGGAGAGGATATATTTGTATTTAGCTTGTTTAATACATTTGTGTTTCC
TGTATCTGTGGGTTTATGACTTTGCTTCTGAAAATATCCCAGCTGTTATGTCTTCAAACATAGCTT
CTCTTTCATTCTTTCTATTTTCTCTGGCTGAAGCTCATATTAGCAGTACATTAGATCTTATTATTATT
CCAGTCTGTCTTTCTCTTAATCTCCTACAGTTACTAGATCCTTATCTCTCTGCTACTTCTGGGTAAA
TTATTTGAGCTATCTTTCATTTCAGTTATATTATCTACAGTTGCTTTAAGTTTGCTGCATAACCTAT
CGATTGCATTCTTAATTTCAATAGTTATGTCTTTTATTTCTAGAAGCTTATGTAGTTATTTTTAAAGT
AAGTCTATTTTGTTAATCCTATGTCTCTTGCTCATTTATTTTTTCATTTTATTAAACTTTTCACAGAT
ATTTTATATTCTGTATGTGATAATTCTGAATCCTTGAGAATCAATATCTGTTGTTTCTGATGGCTCT
CATAATGGCATGTTTCTTTATGTGTATCATGAGTTTATATATATGAACAATTGTCTACTAAGTCTTT
CACTAATATGGGATAAACAAGTTATAATAATAATAACTTATAATAACAATAAGTTATAATAATCA
GCCATCCTCAGTTGCCTGGTCCATGTCACAGTTATTAAGTGGTAGAGTTCAGAATTAAATTCAGG
TCTGTCTGACTTCAAAAACAACTTTTGCTCTCTCGGCCTTAGCGCCATCTTCTTGGAAACTTCTGC
ACCATGAGAGCCAAGCAGAGAAAGAAGTGAATGCGCAGGCTGAAACGCAAAAGAAGAAAGGT
GAGGCAGAGGGCCAAGTAAACTGCTAGCTTGTGCACCCGCGGAGGCCACAGCAGCAGAAACA
TGAAGTGCCAGAGGCTGGGGATGCTGGCACAAGTTGTTGGACTGCATGCTACTGTCTAGAGCTT
GTCTCAATGGATCTAGAACTTTCATTGCCATCTGATCGCTGAGACCACCTCTGAGACCCACCTTG
CTCATAATCAAAACTGTCCATCTTGGTCCTTTGCCCTGGAACTGTGACAT
ID|33358|gene_id|8123|transcript_id|NR_022008 Homo sapiens lncRNA
TTTCCTTCATGTAGATTGCTGTGTGTGTCTTTTTCTTCATAATTCCCCTTAATTCTGTGTGCTTT
GAATGACACTGTTGTTGTTACTGTTGTTTTATATCCATTGTCACATAAAACTCTTATCATTTATTCT
TCCAGGCCAGGGACTGTCCTTATGGGTAGAGATACACTATCTGCAGGAATTTATTTTTTTACGTA
TTTTTACTAGTTGATGGGAAAGACATTGGGAAAGACCTGGGCTGGATTTGTGATGAGCTGTGTTT
ACTGAGCATGATGAAGTAAAGCTCAACGTGATTACTCTGAAGTCCAGCCTTACTGTCAGTCTAA
GGACTCAGGATACAAATACTCAAAACATAAATTCTTAGACTCTAAATTTCACATGGTTTTTCCTAT
CAGTACAGGTTAAGGATAATGGCGTGTTGTAGGTTTTTCCAGTGCAATACAAGGAAGTTGGTTCT
AGATCCTTTAGTACAGTTAATGTTTTGTAGAGTGTGTTCTATGTTCACTGCATTGGTATGTCTCAT
CTTTGGCATGATCTTGGCTCACCGCAACCTCCGCCTCTTGCATTCAAGCACTTCTTGTCTCAGCC
TCCAGAGTAGCTGGGATTACAAGCATGTGCCAACATGCCCGGCTAATTTTTGTATGCTTTTGTGG
AGACAGGCTTTGCCATGTTTGCCAGGTTGGTCTCTAAGTGATCCACCCGCCTCAGCCCCACAAA
GTGCTGGGATTACATGCGTGAGCCACCGTACCCAGCCACACCTTTGCTTATTTCTACTATGCATT
TCAAGTGATCATTTCAAGAGAATAAGTACTTAGGAGCTCCTATGCTCTTTTTTTTTTTAACCCTGTT

GCTTGCATCACCCGGTTCCAACCCTATATGAAAGTTTCATGAGCATGTTTCTGTCACTGAGGCAG
CTTCTCCATTTGGCATATAATTGAGAAATTGCTAGTTTGTGGCTGAGACATTGGTTTGAGAGGAG
ACGTAATTTATATATTTAGCAGAATGCTTAAGTTACATTTCCAGAGATTAATTTAATTCTACTTTTA
ATTAAGTTAACCCTTTGCTGTATCTTTACAAGACGTAGGTGGAGATTTAATTTTGACTCTTAGATT
CAGTTAAGGTGATGAAATTGGCATTATATAGTTTTTGATGCTGAGCCCCACGGAGATTAAAAGAT
TTGCTAAACACCATGCAATTTTTGAATCACATCTCTAATCTCACATTATTCTGTACTCTATTTTTTG
CTTGTATGATTGATGGGTCTTTCATTATCTGTGATTGACATTCTATGAGTAGGTGCTTTTGCTTTGC
CTATAAGTCGTTATTATGAAGGAGGAATGGTGAATAAGAAGGTAATTTAGAAAAGCCTATATTA
AATATACCATGAACATTGAATATAGCAAGATCTTATTCTCTAGTTGTTATCTTAGTTGATAAATTC
TGTATGTGTTATGTGTTTGTGTATACATATGTACATATTAATGATGTGGGTGTTGATACTGTGATC
ACATGACAAATGACTGGTTTATAGTGAAAAGCTGTTGGGGTTCAGAGAAATGAACATTCTTCTA
AAAGTGAGGTAGATAAGTTATTTTATAGTTCTTGCCTTTGAGTCTCTGTTCTTCTGGTAGAAATGT
TAGTGTATTTATAACCTCTCAGATTTGTTTGGTTCTCTTAGTGTTCTTGATCCAGGCAGTTAGCGA
TATGTTGGATGAATTGAGAAAAATGAGTTTTTCCTTTCCTACTAACTGAATTAGATTAGAAATAAG
CAGTTAAGTGAGAAAAATAGGGAATTAGTGCCAGATATGTTGAGGTGTAGTGTTGACAGGGGG
TCTTTTACCTTCAGATGTTTGGTGCAACTTAGAGAATGCAGTGGTAGTTGCTGGCCCAGCCCATC
CAAACAACCTCATTTGTGGGGTGATACTTCACAGATGACTTAAAATAAATCCCCTGAAAATAAG
AATCTCTGGAAGAGATGACACGTGTATGTGCGTGTGTGCACGTGTGTGCGTGTGTGCACGTGTG
TGTGTGTGTGTGTTTTGGTGGAGTGGTAGGAGGAGGGTTGGCTTAATGATGAGAATCATTATTTC
TTGAATTGGATGACACTTTCCATTCCTGCAAAGGGAGCGTGAGGTCCAGCCTTGCTAATTCATGA
TCAAAGTAATTTGCTGAGAGTAGTGGAAGGAATGAGTTAGCAGAAGATGGCAAATTGCATTAAT
GAGTAGTAGGAGGAAAACAAATAAGCAAATTTTAATGTGGTTGGAACATGTTCCTGAGTTGTAT
GAAGGGAGACAGGTAAATTGAGAGTAATGTTATGATTGTTTCATTTGTTGGAAATAAGGAAAAC
TTGCATGGTTTGAATTGTGGAACAATAGGTGGTGTATGTTAGGAGTCAGAATTATCATTTAACAT
GTAAGTTTCTGTTTTACTTCAGATAAAGAATGTCAGCTACCTTTCCCATGTTAGCTTCTATACCTTT
TCTTGAGGTGACCATAAATAGGTAATATTGTCTTTCACTGAGATTGTTTTAATTAGCGAAGTGTGC
TGATTAAATGTCTTTTCTTGATAAATTATTAGAATGAATAAAGGATGGTATTTCTAGATAGCCTTG
GTTAGTCAGTGATTTGTTTAAATTTAGTGTGGATAAGAATCACCTGAGGGCTTTTTAAACACAGTT
TACTGGGTCCTGACCTTAGTGTTTCTGGCTTATTAGGTATAAGTATGAGAAGTTGATTTTTATCAA
GTTTCTAGATGCTACAGATCTAGGAACCATACTTAGAGTGCTATTATATGTCTTTTAATGTACACT
GATATGGAAAAAGCAATGATACATCATGTTATCGCATATTTTAAAATGTGATCATGACCAGGGA
ATCTTGAAAGTTGTCTTATGTAATAATGTAAATCAATGTAGAATACCATATATTTTTTTCTTGCAA
GGTTATTATTAATAGGAATGACTATTTGTAGCCAATGTGTTCAAATGCATGGGAGGACGTAAACA
CTTCATAATTTCCTGGAAATACAGACATATATCACTTAAAATAATACATTTCTAATATAAATTTAA
ATTATCTACGTGATATGAGGAAACGAGCTTATTTTTGATAATTTTCCTAGCAGTGAATCAACTATA
CTCATTTGAATCTAAATTAAATTAAATTTGAGTAAATTCAGCCATTTCTAAATTCAGTCCATATAA
ATTCACTTTGGGTAAAAGAAGACAAAATTCCTGAAAACAGTAGATTAAAAAAATAACATTTTAA
GAGCAGGTATGATCTCAATGAATATAGTGTGGTAATTTCTGCCTAATACCTACCCTTTTGGAGGA
AGGAAAGGTAATCCTGTTTGTGTTTAATGTTAATTACTGAAC

ID|33559|gene_id|8847|transcript_id|NR_002612 Homo sapiens lncRNA

GGAGCTTTGCTGAAACTGCACAAAAAATCGAGCCGGGGGGTTCCCTGGTCCCCGGCGATG
GGGCGGGGAGCGCTGCGCCGGGGGAGGGGGCGGGCGCGGCGGCGCGGGCCCCGCCGAGGG
GGGACACCTGGCTGAGGCACAGCTGCCGCCGTGCCTTTCCGCGCGAGCCCAGAGCTCCGATCC
CTGCGCGGGCTCGAGAGCTCGCCCCGAACGGGGGTCTTCCTCCTCCGCCGTTGACAGGTTTTG

ACCTGTAGCAGAGAACCAATTCTGGAGAACAGCCTCACTTCTTTGATTGAATACTTACATAATGC
ATTGGAACATGACATGAGATTAAGGTTTAATAATGATAGAATGAAGACCACAATAAAAGAGACC
TCTACTTAGCTCAGCAATTCTTACCTTTCTTACCTATTTGATGAAGATGTCTTTTGAAAGGTGTACT
GCAAGGAACAAAATGTTTGTAAATTCTCCTTTTACCAAGGTGGATAATTACTGTACCTTCCTCAT
GGAAAAAGTTTTATTTAAAGTGTTATTTCTCATTGAATACTATCAAAAAGGAAAAAAAAAATGACC
TAAACTTTTGAGATAGATTTGGCTCTAGGCCTACTCTAGGTGCGGTACTTCACTATAGTTTATAAA
AGATGGTCCCTGTCAGCAAAGAACTGTAACCTGTATGAGAATATTATACTAACATACAAGAAAC
AAGTAGAGAATAATGGAATGTAAACTCTGGCTTGCTAACTTAAGTGTGGTAGAAGCTTGAAGGA
AATGTGGACTTAAACTTATCAGAGAAGACTTATAAGAGGATATGAAAGGTGTAAATTTTGATAC
CACCATTGCCGCCTTCCCCTCAAAAAAGGTGAGAACTGACTAAACTACTGGTACTTAGACCATG
TGGAGAAGGAACTGAAAATGGAAACAGAAACACGTCTGTACAGAGCAGAGTGTCAGATAATAG
CTCATTTTAGTAGAGGGCAATAAATGCCACATGAGATGATCACCTCAGCAGAAAACTGGGATGT
GGAGACGCTGGTAGCGGGGCCAGGAAGGCTCCGGACGGAGCAGGTTTTCTGGACCACAGACA
CACTGCCCCGGCGCCCTCCGCAGGCCGCTCCTCCCCAGGCCCATGACAGTGCCCCGCTGGTTT
CTGCCGCGCTGCCGGACTGGAGCTCAGACGGCCTTCGCCAGCCCCCCAACCCACGCAGATCC
CTGCTGACCTGGGCAACTCCCCACCCTCGCTGAAGGTTCGAGGACCACCCCGCTTTCCCGAGA
GGAGCCGGGCGGCGGGTACTTATCTCCGACCTCCGGCTAGTGGGAAAGGCCGCGCGACCGCC
CGTCCTCCAACTACAGGGACCGCACGAACAGCGACTGGGAAACTGCCACTAGAAAAAGATGA
CTGTCCAACTAAAACCTGCATACACAGCTACATCCTTCCCTGGAAGAGCACAGTGGAACTAGAT
CCTAGTACAGATAGCACAGGGATTGTCAACATTCTTGTCACACTGAAATTTCCTTCTACTGATCT
CCAAACTTCTAAAAATTTGCTGTTGAGACCAGCTGAAATGTTTGAAAGAAGCAACTAAAGAAAG
AAACTTTGAAAGAAGCTTCCAAAAGAAGCAACCAAGTCTACAAAGGCACAGTTTGACTCAAATG
GATCCAACTGATTAGCAAATGGAAAATAACATGGAGGAATATATTTTGCATGACAAGCATAGCA
GCTCCAAGTCATCCCTAATGAAGCCAAAAATTTAAGTGCAACCCAGAGAATGGATGGTTCAGAA
TCTTTTTTTTTTTTGAGATGGAGTCTCGCTCTGTCACCTAGGCTGGAGTGCGGTGGCATGATCTCG
GCTCACTGCAAGCTCCGCCTCCTGGGTTCACGCCATTCTCCTGCCTCAGCCTTCCGGGTAGCTG
GGACTACAGGTGCCTGCCACCACGCCCGGCTAATTTTTTGTATTTTTAGTAGAGACGGGGTTTCA
CCGTGTTAGCTAGGATGGTCTCAATCTCCTGACCTCGTGATCTGCCCGCCTCAGCCTCCCAAAGT
GCTGGAATTACAGGCGTGAGCCACCGCGCCCGGCCCAGAATCTTTTTTAAATTTTTTATTATTATT
ATTTTTTCTGAGATGGAGTCCGGCTCTGTGGCCAGGCTGGAGTGCAGTGGCACAATCTCAGCTC
ACTGCAACCTCTGACTCCCAGGTACAAGTGATTCTCCTGCCTCAGCCTCCTGAGTAGCTGGGAT
AACAGGCACGTGCCACCACGCCCAGCTAATTTTTGTATTTTTAGTAGAGACGGGGTTTCACCAT
GTTGGCCAGGATGGTCTCGATCTGCCTACCTTGGCCTCCCAATGTACTGGGATTACAGGCGTGA
GCCACCGTACCCGGCCCAGAATCTTTGCCTTTTGTTTGCATAGTTTAATCTTCTAAACCAGTTGCT
TTCCCAGAAAAGGTGACTTAAAGTCATAGCCTTTGTGTTCATCACCATACTCTCCATAGTTTTACA
AATTATTCAGTTTATTTTTAAAAGGGGTAATTTTTAAAATTTTTATTTTTAATTCCGAAGCATTCCTT
TTAATACATTCTCTTGAGGGAAAAAAAAAATCACACATAGTGATTGTGTGTGGTAGTGATTATGT
GTGGCCTAAGTGGCAATTGATCAATACCCCAATCTCAAGCCTGTACATTGTTACTGGTTGACTGG
TTGGTTGCCTGGTGGGCTTTTTGTCCTTCCAGATGTTTTTCTACCTTCTGCCTTTTCCAATAAAGCT
TCCATTAT
ID|33993|gene_id|10230|transcript_id|NR_003108 Homo sapiens lncRNA
AGAGGAAGAATTCTACCTGAGTTTGCCATAAAGTGCCTGCCCTCTAGCCTCTACTCTTCCA
GTTGCGGCTTATTGCATCACAGTAATTGCTGTACGAAGGTCAGAATCGCTACCTATTGTCCAAAG
CAGTCGTAAGAAGAGGTCCCAATCCCCCACTCTTTCCGCCCTAATGGAGGTCTCCAGTTTCGGT

AAAAGTTTCATTTGATCTGAATAGTATTAAAATAAAATACCTGGATGAGGAAGATGAAGAGGTG
CTGGAAGCCCAGGAAGCACACATCAAGGCTCCCTTGCCAGCAGGGTGCTGCCAATAAAAGGTA
GTCACGTGGAATTTGGAATGTGGAAAGGAGGTAGAAGTCATCCTTTCCTCCCCTGTAGCAGCAG
GCGTGCAGGCTCTGGTGGTCAGCTGGACTCCATACTCCCCCACCAGTCACCAGCCTGGGGACC
GTGGGGCTGCAAGGACCTCAGCAGCGGTGTCCCAAGTTTCCTGACTTCTTCCATCCTCTGGAAA
TCAGCTGTGTTTGCTGAGGATAATGGCCTCAAGATCCATCTGTGTTCCTACAAAAGAGATGATCT
TGTTCTTTTTTATGATTGCACATCTTTTGTTCTAACGTTTGGTCCTTCACCTTGGTTCCTGACACAA
GGATTCCTAAATCCCTTGGAATTTTCTGCATGATAGGAGCGTCCTTTGTTCTCATGAGGTGACTCT
TGGTGGGCTCCTTATTTGGGGACTGGTCACCAAAAATACCTAACTATGGTTGGAAGCTTAGTGCT
TTCAGCCCCATTCCCCATCCTCTGGGGTGGGGAGCAGAGCTGGAGCTCGATCATGCCTGCGTGA
CAAAGCCTCCAGAAAAATCCTTGAAAGACAGGACATGGAGAGCTGCTGGGTTGGCGAACACAT
CCATGTGCCGGGAGGATGGTGCACCCCAACTCCACAAGGACCCTTCCAGACCTCACCCTGTGT
ATCTCTTCATCTGGCTGTTCATTTAGCAGCTCCGAGGGCAGGCATGGTGGCTCATGCCTGTAATC
CCAGCACTTTGGGAGGCCGAGGCAGGTGGTTCATCTAAGGTCTGGAGTTCGAGACCAGCCTGG
CCAACATAGCGAGAGCAGCTCCGGTGGCGGGAGGAGTGGCAGCGGCCAGGCAGCCCAGCTTC
GCGAAGGCTGTAGGCACACCGCGGCCAGCAGGCACCTGGCACCCACCTTCCCTGCTGCCAGG
ATGCCCAAGAAAAAGGTCAGCTCCACCGAAGGGGCTGCCATGGAAGAGCCCAAGAGGAGATC
AGCGCAATTGTCAGCTAAACCTCCTGCAAAAGTGGAAGCGAAGCCGAAAAAGGCAGCAGCGA
AGGATAAATTTTCAGACACAAAAGTGCAAAAAAA
ID|34013|gene_id|10301|transcript_id|NR_002605 Homo sapiens lncRNA
AGGCACATGCGCAGAATCATCGTGGTGCACGGCTCTCCCTTTGCTTCTTCGGTTGCAGTCC
TCTTGCTTCTTGCGCGTGCGTGTAGCGCTTTTGCAAAGCCGCGGAGGTGAAGTGAACTTAGAGG
TTGTGGGGCCGAGGGGTCGTCTTATAGCTACCAGCCCACAGGCATTTAGTCTACGTTGGAGGTA
AACAAATACGGGTCCTGCTTAGGAGAAAAGAAAAACGTCTTACAGCCAGTGTCTAAACTCCAA
ACAACGGAATGTATCAATGAGACCTTGTATATGGATACACGTGCATTTAAAACCGCCCTGCCGG
CTTGTAGAGCTTTTGCCGTTCTCCAGCGCTTTACAGGGGTTATCGCACTTAAGCCTCGGAACAAC
TTTACCAGTGATTCTACCAGAAAGGAATGAAGAACAGAACCTTCAGGAATTGAGTCACAATGCA
GACAAATATCAAATGGGAGATTGTTGCAAGGAAGAGATTGATGATAGTATTTTCTACTAGCCATT
GGGAAGATAAAAGGAGACAGAAGATTGAAGCCTTTGCCAGCCATTCTTTCCCTTTTTGCTTCCA
AACTCCTCAACTGGGAACCTTCATATGTGCAGTATTTATATTGGATCATACTGGTGATTATAAAA
GTTCCTAGGAGGCTAGAAGAGCCAACCAACAGAGAAGGGAAAGCAGTCTGTTCTGAACATAGG
GACATAAGTTCATTCATGCCAAGTATCTTTCCAGCATGTTTCTCCCATTTAGAATATCTAGCATGT
AAGGCCTTTCAATATTAATATAAGCCCAATATCAGCTCTTTCTCTTTGTATTTCATCTCTTTCTACT
CTCCTATTTGTATTTTGTGTTCCTATCAAAGTGTCGTATCTGGGAGATGACCTGCCTTATCCTGTT
CTATAACAGTTTTGTTTGCTGCTGTGTCTTTAGAACAGTGCCTGGCACACAGTAAGCACTCAATA
AATCTTTGATGAATGAATGAATGGTTATCTTTTCTCTCTGGCAATCCAAATTCACCACTCCTTAAA
AATTCTACATAAGATTTGCTGGCTGGGCGCAGTGACTCACGCCTGTAATCCCAGCACTTTGGGA
GACTGAGACAGGTGGATCACCTGAGGTCAGGAGTTTGAGACCAGTCTCAACATAGCGAAACCC
CATCTCTACTAAAAGTACAAAAATTAGCCGGGCGTGGTGGCAGGCGCCTGTAATCCCAGCTACT
TGGGAGACTGAGGCAGGAGAATCACTTGAGCCCAGGGGGGCAGAGGTTGCAGTGAGCTGAGA
TCACGCCACTTCACTCCAGCCTGGGCAAAAGAGCAAAACTCCGTCTCCAAAAAAAAAAAAAAA
AGATTTGCCTATTCCAGCAGACTTCTACCCAATATCAATCTAGATTTAAATCACTTGATTTTCCAC
ATCCCTTTAGCAATTATGTATAGTTTGCACTTCATTAACCTACCCTTATTTATGTTACTTCTCACAT
ACACCCATATCTCTTCTGTATTTTCAATCAAAAGATAATGCCTCAAATGTTATATATATTGTATGT

GCCACTCAACAGTGCCTCATATGCTGCAAAAAAAAAAAGACAGTAAACAATCGGGGTACCTTTTT
GTCTTTTAAGCCATATTTTCTGGGTCAGTGTGGTCTTTCAAGGGAGTTTAGACAGGCTCTATTTTT
TGTTACTTCACTCCTATAGTATGTGACTGTAAATTGTTCCTTCAGTGTTCAATTTAAGTACAAACT
AAAGAGAAACAGTTTCCTAAAGAAAAACATTTTAAAGTTACAGAGACACCTATGTGTTCGTAAC
ATTTCTCCAGAAATATTTGAAGTAACGTAAAACTTCTGGACTGGTCAGTTTAAATATTTTCTTGGA
ATTTCTATAGAAATTTCTGTGGAATGGTGTTATATTGGAAATATTAATTAACTATAAGAAGACTCC
TACTTCTTCACAGAGAACAAGCAGGGATATCCTGGATTTTCACAGTATTTAGGCCACTCCTTAAT
TGTCTCATGAAGGAATTAAGGTAGTGGAACACATTCAAGAATTGCTTAGGTGTCAAAATAAGAC
AAAGTGTAACAGTTTTTTTCTGTAGTAGATTTTTAAATAGTTAAAAGACTCTGCCCTCTTTATCTAT
ACTGTATTCTGGCAATAATAAAGTTGGGCAGCCTATACATAAAAGCAATTTCTAACAATGCCAAA
TTAACAGCAGTTTCTTGGCCCTTTCCTTTCCCTCCATGAAAGTTACAATATTAGGTTATTTCACAG
GTTTCCTCCATGCCCCCCATACCCAACCTAAAACTGTGTTAGGCTGTCAAAGAAAACAAATCAG
GTAAGATTAGTGTTTGCCTTTACGCAGTCATTCTAAGTACACATAAAATCATACATAATATGAAA
ATACAGCAGGAAGAGAAACATCAAACCATAGAACTGAGTTTTAGTTTTACTGACATTTATATGGC
ATACTTCCTGTTTTTGATTTCAGAAGTGCAACCATGCAGTGCTTCTATGAAAAACTGAATAGTTG
AGTACAGTTTGAGGATTATAACAACTTATCCAAAGGAAACAAACAGACCTAACTTACTGCTGTTT
TCATTTGTGGAACTGCTATCTTTGTTGTAAAAAGTTGAGTATTTTTCAAACATCATTTAATAATAG
GGTAGCCCCTAAAACAAGTAAGGAGCTATAAGTCTGAGTTGTGAATTTGACTAGTAATGGAATA
AGAAAACCAAGCCAAACTCCATCTAAATATGTATGTAAAGGTAATGCTTGTAAAAATTTGCTTTT
TCCTCATTTTGAGATTTCAGTTGCCAGTTGACTATTGGTATGCAAAATTAACAATTCTGCCTAAAG
GAGGTAAACGATAGAGGTTAATTCTTAAATAAAAGTCCCTTTGTTTCACTATT
ID|34014|gene_id|10301|transcript_id|NR_109973 Homo sapiens lncRNA
AGGCACATGCGCAGAATCATCGTGGTGCACGGCTCTCCCTTTGCTTCTTCGGTTGCAGTCC
TCTTGCTTCTTGCGCGTGCGTGTAGCGCTTTTGCAAAGCCGCGGAGGTGAAGTGAACTTAGAGG
TTGTGGGGCCGAGGGGTCGTCTTATAGCTACCAGCCCACAGGCATTTAGTCTACGTTGGAGGTA
AACAAATACGGGTCCTGCTTAGGAGAAAAGAAAAACGTCTTACAGCCAGTGTCTAAACTCCAA
ACAACGGAATGTATCAATGAGACCTTGTATATGGATACACGTGCATTTAAAACCGCCCTGCCGG
CTTGTAGAGCTTTTGCCGTTCTCCAGCGCTTTACAGGGGTTATCGCACTTAAGCCTCGGAACAAC
TTTACCAGATGAGGACACCTGAGGTTCAGATTAAGAAATCTGCCCCAAAGTCTTAGAACTGTGA
TTCTACCAGAAAGGAATGAAGAACAGAACCTTCAGGAATTGAGTCACAATGCAGACAAATATC
AAATGGGAGATTGTTGCAAGGAAGAGATTGATGATAGTATTTTCTACTAGCCATTGGGAAGATA
AAAGGAGACAGAAGATTGAAGCCTTTGCCAGCCATTCTTTCCCTTTTTGCTTCCAAACTCCTCAA
CTGGGAACCTTCATATGTGCAGTATTTATATTGGATCATACTGGTGATTATAAAAGTTCCTAGGA
GGCTAGAAGAGCCAACCAACAGAGAAGGGAAAGCAGTCTGTTCTGAACATAGGGACATAAGTT
CATTCATGCCAAGTATCTTTCCAGCATGTTTCTCCCATTTAGAATATCTAGCATGTAAGGCCTTTC
AATATTAATATAAGCCCAATATCAGCTCTTTCTCTTTGTATTTCATCTCTTTCTACTCTCCTATTTGT
ATTTTGTGTTCCTATCAAAGTGTCGTATCTGGGAGATGACCTGCCTTATCCTGTTCTATAACAGTT
TTGTTTGCTGCTGTGTCTTTAGAACAGTGCCTGGCACACAGTAAGCACTCAATAAATCTTTGATG
AATGAATGAATGGTTATCTTTTCTCTCTGGCAATCCAAATTCACCACTCCTTAAAAATTCTACATA
AGATTTGCTGGCTGGGCGCAGTGACTCACGCCTGTAATCCCAGCACTTTGGGAGACTGAGACA
GGTGGATCACCTGAGGTCAGGAGTTTGAGACCAGTCTCAACATAGCGAAACCCCATCTCTACTA
AAAGTACAAAAATTAGCCGGGCGTGGTGGCAGGCGCCTGTAATCCCAGCTACTTGGGAGACTG
AGGCAGGAGAATCACTTGAGCCCAGGGGGGCAGAGGTTGCAGTGAGCTGAGATCACGCCACT
TCACTCCAGCCTGGGCAAAAGAGCAAAACTCCGTCTCCAAAAAAAAAAAAAAAAAGATTTGCCT

ATTCCAGCAGACTTCTACCCAATATCAATCTAGATTTAAATCACTTGATTTTCCACATCCCTTTAG
CAATTATGTATAGTTTGCACTTCATTAACCTACCCTTATTTATGTTACTTCTCACATACACCCATAT
CTCTTCTGTATTTTCAATCAAAAGATAATGCCTCAAATGTTATATATATTGTATGTGCCACTCAAC
AGTGCCTCATATGCTGCAAAAAAAAAAGACAGTAAACAATCGGGGTACCTTTTTGTCTTTTAAGC
CATATTTTCTGGGTCAGTGTGGTCTTTCAAGGGAGTTTAGACAGGCTCTATTTTTTGTTACTTCAC
TCCTATAGTATGTGACTGTAAATTGTTCCTTCAGTGTTCAATTTAAGTACAAACTAAAGAGAAAC
AGTTTCCTAAAGAAAAACATTTTAAAGTTACAGAGACACCTATGTGTTCGTAACATTTCTCCAGA
AATATTTGAAGTAACGTAAAACTTCTGGACTGGTCAGTTTAAATATTTTCTTGGAATTTCTATAGA
AATTTCTGTGGAATGGTGTTATATTGGAAATATTAATTAACTATAAGAAGACTCCTACTTCTTCAC
AGAGAACAAGCAGGGATATCCTGGATTTTCACAGTATTTAGGCCACTCCTTAATTGTCTCATGAA
GGAATTAAGGTAGTGGAACACATTCAAGAATTGCTTAGGTGTCAAAATAAGACAAAGTGTAACA
GTTTTTTTCTGTAGTAGATTTTTAAATAGTTAAAAGACTCTGCCCTCTTTATCTATACTGTATTCTG
GCAATAATAAAGTTGGGCAGCCTATACATAAAAGCAATTTCTAACAATGCCAAATTAACAGCAG
TTTCTTGGCCCTTTCCTTTCCCTCCATGAAAGTTACAATATTAGGTTATTTCACAGGTTTCCTCCAT
GCCCCCCATACCCAACCTAAAACTGTGTTAGGCTGTCAAAGAAAACAAATCAGGTAAGATTAGT
GTTTGCCTTTACGCAGTCATTCTAAGTACACATAAAATCATACATAATATGAAAATACAGCAGGA
AGAGAAACATCAAACCATAGAACTGAGTTTTAGTTTTACTGACATTTATATGGCATACTTCCTGTT
TTTGATTTCAGAAGTGCAACCATGCAGTGCTTCTATGAAAAACTGAATAGTTGAGTACAGTTTGA
GGATTATAACAACTTATCCAAAGGAAACAAACAGACCTAACTTACTGCTGTTTTCATTTGTGGAA
CTGCTATCTTTGTTGTAAAAAGTTGAGTATTTTTCAAACATCATTTAATAATAGGGTAGCCCCTAA
AACAAGTAAGGAGCTATAAGTCTGAGTTGTGAATTTGACTAGTAATGGAATAAGAAAACCAAGC
CAAACTCCATCTAAATATGTATGTAAAGGTAATGCTTGTAAAAATTTGCTTTTTCCTCATTTTGAG
ATTTCAGTTGCCAGTTGACTATTGGTATGCAAAATTAACAATTCTGCCTAAAGGAGGTAAACGAT
AGAGGTTAATTCTTAAATAAAAGTCCCTTTGTTTCACTATT
ID|34015|gene_id|10301|transcript_id|NR_109974 Homo sapiens lncRNA
AGGCACATGCGCAGAATCATCGTGGTGCACGGCTCTCCCTTTGCTTCTTCGGTTGCAGTCC
TCTTGCTTCTTGCGCGTGCGTGTAGCGCTTTTGCAAAGCCGCGGAGGTGAAGTGAACTTAGAGG
TTGTGGGGCCGAGGGGTCGTCTTATAGCTACCAGCCCACAGGCATTTAGTCTACGTTGGAGGTA
AACAAATACGGGTCCTGCTTAGGAGAAAAGAAAAACGTCTTACAGCCAGTGTCTAAACTCCAA
ACAACGGAATGTATCAATGAGACCTTGTATATGGATACACGTGCATTTAAAACCGCCCTGCCGG
CTTGTAGAGCTTTTGCCGTTCTCCAGCGCTTTACAGGGGTTATCGCACTTAAGCCTCGGAACAAC
TTTACCAGATGAGGACACCTGAGGTTCAGATTAAGAAATCTGCCCCAAAGTCTTAGAACTGGTA
TTCTCCACTGGTTGAATGCAAATGGAAGCCAGAGGAAAAGGGAACCCTTGACACAGTTTTCATA
TAGATTGGCCTCCAGGGCAGACAGCAGTTGCTCTGGCTATATCAAATAAAAGTGTCAAGAGTGA
GCATCCTTGCCTTGTGCTGAATCACAAAGGAATACCTTTCAGTTTTTCTCCATTGATTATGATAGC
AGTGGGCTTTTCACAGTGGGCTTTACTGTGTTGAGGTACCCTAATGACAAGTTCACCCCAGTGTT
TTCTGAGGAGTCTACCTGGAAATGAGAACCATCTGTCCTTCCTATAGTAGTTCTTCTCAGAATGC
CGACTCTATGCTAATGGAGGAGGTCTGATGGCCGGGAGGACAGGAAGAAGGAACCCACAATG
AAGCAAAAGTTGCGTTTAACTCTGTCCTCACCATTCCTCTCCTGGACCTGTCCTAAACTGGAAGC
TGGGGGAGGATGGAGTGCTGGAATGCTGCAGAAGTCTCATGGGAGGGCGCCTGGTGTTCACCA
CATGTGTACACTGTTGCACGACCAGATGGAAATTTGAATGCTGGGGCTCAGTGGAACACCTGAA
GGAAGCTGGTGAGGCAGATGTCCCGCTATGCCTTCCAAGTGTCATGCAACCAGTTCATGCCTGT
CAGCATCTCTGCCGGTGGACTCTGTGAGTCAGCAAATCTACAGAGCAGAGAGCCACAAGTGGG
GACAGATGTGGTCAAATGCCATTTAAATCAGTGATTCTCAACCCAGGAGTTGTCATGGCACTGG

CCTTGAGTGAGCTGGACCAAGGGAAGTGGTAGAATTCCCCATGAAGGACCAAGTGAGCAGATA
GAAGTTTCTCCATTTTAACTTTTTCTGAGTCAATTTAAAAAACACCTAGTGCCTTTTTCTGTACATA
GTGCCTGTGGGTTTGAATTTAAGCCTACCCAAATGTGCTTTTATCCCACCTATTTTGAACCTTGCA
TGTCTCCGGGATAAGAATTAACATAAGCTATAAATATT

ID|36476|gene_id|84740|transcript_id|NR_026892 Homo sapiens lncRNA

GCTGCTGCCACGTAAGAAGTGTCTTTCGCCTCCCGCCATGATTCTGAGGCCTCCCCAGCCA
TGTGCAACTGCGTGTTTACTGCTCTGGGCCCAGTGCCTCCCTCGCTCAATGGAGTGACGGCATC
CAACTCACAAGACAGGAGACTCAACAGAATGACCAAGTGGAGAAGACGTCTAAGTTCTCAGCG
GTCTCAGCCGAATGACTGAAGAGGAACCAGGGACAGGGATGACTCACATGGGAAGAGGACCC
CACTTTGTTCTGTTTGATTCTAAGAGGACACAGACTGCTTCATTCATTTCAGTTTCCCCAGCACCT
GGCTTAACTCTCAGACATGTTAGACGGTTTGTAAGCACCGGCTCTACTGAACTGGCATCAAATC
ATGACCTGGTTCAGAAGAGACACGAGGACTGGATCTGTTCTAAACAGATTGTGCAAAGGGGAA
AGACACAGACTCAGCATTTCCACAGCTTTTAACATTTCAGCGAGAGGTGAGAAAGCATGTCAGG
AACACAGGCCCGGCCGATGAAAGTGTCTGATGCTAACACATGAACTGTCTTCTGCTGGGCACAC
TAAGGGGCCACAAGCAAGCTACGCCCCAGAGCCACTCCACATTCTCTCTGGGTGCACTGGCCC
TCGGCCACGCAAGGCCGCGCCAGCCTCCGAGGTGAGCCAGAAGGACACACACCTGTGGACTC
GATGTCCCTGACTTGGGCTCGATGGCCAGCCCCAGGGTGACTCCGCCCGCCAGCGCTTTCTTCA
GGCTCTCCTTGACCTCCGTCAGCTCCAGCTCCAGGCTGACACGCTCCGCCTCCTTCTGCCGGCA
CTCCTCCTCCAGCTGCTTCAGCTTCTCCTCCAGGATCGCCTGCGGCTTCCTGCCTGGAATTCCCA
GAAACGCCGTTACTCCCGCGGCAGGCACAGGTTCTCCAAACAACAGAGAAGGCACCTGGTCCC
CAAGAAGAACTTCCACAAAACGTCTGAGGTCGAGCTCCCCTGACTTAGGTCCTCGTTGTGAAAC
TTAAATTCAGCAGTTCTCTAAGGGATCAGAGTCCTTCTCCTACCATTTCCCTTTGCTGCAGCCGTT
GAGGACTCGGACCAGGAAAGAGGAGATGCTGATTCTGACGAAGGCCAGGAGCTCCTGGCTCT
GGGCCTCAATGTTCTCATTTTAAAACGTGGACCCTAAACTCCACAGTTCCCAAAGCTGCCACCTT
CTTGGAGTGTGAACCCTGTCCTCCCCTAACGCTCCGCCGATCCTTCAGTCATCTTATTTGCGCCC
GAGGCAATGAACAGTAGCTCAGCTTGCATCTCTACTGGACATGGCACACATTTCTTTAAGGCAC
ATTCACTTATTTTATTTGAGGTTTGCTGCAGTGACCTAAGATGCACCCACTGGGCAGATGAGAAC
ACCAATCCCAAGAGGTGAACGGACTTGTGCCCAGGGCCTGGGCCGGTGGTGGCCGGCAGGCG
AGGTTCTCTTTTTCAAAGCCAAAAGCACTCCTGGCCCTCCTGCATCTTCACTATCCTGCAAGCTG
GGAACACGGGAGAAATGCTACTTCTGTCTCATTAAACTCCATGCCCCGTCCCATTCTCTGGCAC
AGCCTCCTCCCGTAGGGTCAGCCTCAGACTGCAGGCCACATCCTGGGCCTCCTATAGGGTCAG
CCCGACCTGCCCTGCCAACACCTGCCTTCCCTCCTCTAAACTCCAAAAGCATTTCCCTTCATCAG
ATCATTGAGGCACAAAGTCCAGCAAGGCTGGAGCTGCTAACCACCTGCTGGGTGTGCGTCAGC
TCCTCCCCGCGCGGCGAGCCATGGGAAGGCCACAGCCTCTTCTCTGGACCACCCAGGGCTCTG
AGGCATCTGAAGCCCCCGTCCCAGCCCAGGCCAGTACAATCTCAATCTAAGGAGCCAGTCTTC
CGTAGTCTAAGCACCGGGCTGGAAGGGAGGCCATGCGTAGGTAAGCGGTGCCACCCCAGGCT
CAGGAGCTGAGCGCCAGCTCCCGCCAGCAGGAATTGCACGCGCCTGGCCCAGGGCTGCCACT
CTGGCTGCAAACTCAGTTTCTGTGACTTCAGTCCCTCTCTGGTGTCTGTATTCCATTAGCCTGGCC
TCTTCCCTGATGCATCCCAAGACAGATGCTGCCTTGCTCTCGCCTGTCCCTTCTCAACAAACGAG
GGGCTGCTTCTACCCTTGTCTGGACTTCTGAATATTACTGACCCCTGTGGGCTACCCTCTGTAGA
GAAATCCAGCTGATCCCTCCTACCCGGTGACCAGAATCTACTGCGTCCTGCGGATACAGGGGC
CATCCTCCAGACAATCTCTGTTGTCAGCGCCAGAAGCCGGAACAGGTCCCCCAGCACCTCCCCT
GCCTCGGTGAGCAGCGTGTGGGTCTGGCCCTGGCCTCTGCCTGCACGCCGCATGTTTGACCAC
ACAGGCACCTGCCTCGGGGTTACCAGCAATGTTTCCAAGCCCCAAGAAACACTGTGAGATAAC

CCACTCTTACTAAATAAAATGACAGCCTTGGTGAGCAATAGGTCCTTTGGGCAAAGCAGAATGA
AATCTCCAGTCTCTAATACAAACATGCACCCTGGGCAGTCACCCACACCCTTCATACCGGCGTT
CACTTCAATAGCCGCTCGAAGGTCTTTTCTTTCCTTGCGGAGCTGGGCCAGCCTATTCCGCAGG
GCCTCTTTCCTCTTCAGCAGCTCCTCCTCTTTGGTCTGTAGCCGCTTGGCATCTGCTTCTACCCGG
TTCTTGCCATACTTGTACTGGGCAGCATCTTGAGAAGAAAAAAAAGCAGCAATTAAAAATTAGG
TTTACTAGCCTGGGAAATATGGGACGATCCCTTCTCCACAAAAAATACAAAATTAGCCAGGCAT
GGCGGCACATGCCTATAGTCCCAGCTACTCAGGAAGCTGAGGTGGGAGAATCGCTTGAGCTTG
GGAGCTTGAGGCTGCAGTGAGCTGTGATCCTGCCACTGTGCTCCAGCCTGGATGACAGTGAGA
CCCCGTGTCAAAAAAAAATTAGGTTTATTCAAGCAGATAATGGGGTAACTCAAAAAGCCTGTTG
AATCAGCCAACTCAGTAAACAAACACAAAGCATTATTTTGCTAATTCAACAAGAAAAGGACAGG
GCTGGTATGAACCAAGCAGAAAAGTGACTAGTTCAAATTCTCCAATATTCTTTGAATCAAATTCT
TAAAAACGAAAACAGAATATTAGGAAATTCCTTTAATTCACTTCCCTTTGAGGCACACGGCTTAT
AATTAATCTGCAACAAGGTGGAGAATGAACATTCTATAATCGGAAAAGACAGGAGTAATATCAA
AAATAATGTCTGAAATTTGCTTCCTTCTCTACGTCTTCAAATCCTTCCCTAACACCGCAGTATCTG
TTCCTTATGGCCATGTCATCTGACTGGCTCTGAATTGCTGCTACACCCCCAGCACTGAGATTAGC
ACCCGGCACGGAGTGTGGCACAATAAATGCTTGTTTAGTGAATAAATGAACAACGGATACATGA
AGAGGCAACCAGGAGCGGTATCTGGAGCCTGACTGTCTAGGAAGGAGTCCCAGCTTACACTTG
TAGCTGCATGACCTGGAACAGGGCACGAATCCCTCTGTGCCTCAGTTTCCCTCATCCGTGAAAT
GGGGATAGTAAGAGCAGGCAATTCACTGAGTGGTTACGAGGATTAAATATTTATAAAGCACTTA
GAACAAACACAAATGGGCAACACATAAATGCTTATTTCATAAAATGAATAAATGAAGAGGAAA
CTAGCTGTAGGGAAAATTTACAGACTTGGCAACTTCACATCACCAGTGAAAACAGCTTTCAAAA
CCTGACCCCATGCTCTCTCCTATTATGTTGGCTTTTCAATAACAGTAATTTAGGACAACACCTCTT
TGTTAAGACACATGGAAGAGATGAAGGTTCTAACAAAACTCGGATGGAGGATCACCGATTTCAA
AGCTTCCGTGGTCTCCCAGTCGCTTCTAACAATCACTCACGCCTGAAGGCAACTCCCAGGCCTT
CCTGACTGCACACCCCAAGTGTCTGACTCCCTCACAAGGCTAGAAACTACTTCAGGTAGAAGCC
ACAGGGGTGGCATAATGATTAAGAATAAAAACACTGGACTCAGAGAGGTGGCTAGAAACCCAC
ACTCCACCCTCCCTTGCTCCATGACTCTGCAAGCAACCTCCGGAGAAGCTCAGCTTCACCCTCT
CTAAAGCAGAAACGAGAAGGAATCCTGTGTGTGTGTATGTGTGTGTGCGTGCGTGTGCATGCGT
GTTTTAAATTAGAACGTTATATCTGAAAAAAATCTAACAAAATGATACGACGCCTGATGTAGTGG
ACATTCACTGACAATTCTTACCATATCAGCGATAAGTGATTCCCAGAGCCTTGTCCACTCAGGGA
CAGCAGGGAGCCATTTCTAGCACCTGTCCTAAATGCTGGATTTGCCAATTCTTGCAACGTAAGTT
ATCATGATTTCAATGAAATACTGCATTTATGAAGGCATAAAAAGTGATCACTCCCCAGTCAATTT
TCCCCTAATCAAAAGAACCACATAGAATGACAGCAAAAGCGTCGGCAGCCTGAGGAGAGGCG
AAGCATTCGACAATCTGAAACAAAAGGATCGCTGTTTGCAGCTAAGGTTAACAACATATTTGGC
AAACAGGGCCAAGTCCAAACTCTACTCGCTTATTTCTAAACTAAGCTGTTTCCCCAATGAACTCA
ATGCAATCACCTATAAATTTCCACATCTTTTGGTCCCCATGTAATAAATCCCATCTAATCTATAAT
GCAACCGGCTGGATTCAAGCAAAAACACGGAATGCCATTATTTTTCTCTTTTGTCACCATTAGAC
GTCACCCACAGTTAATAAAAACTCCCTACAGAACATCCCATAAAATAAAGGAATAAATCATCTAG
GAAATAAAGTCAGCACAGGTACTGACAGAGAAACACCATGGCGATTGAGACTCCGTGGGACTC
CGATTTTGCTTGTAACAAAGCTTTCCAGGTGTGAGCTGCTCCTCTCCCCCATCTGCCCCTCCTCA
AAGAAACAACACTAAAAGAGGTGAAAGTAAATACACCTGATACATTCAGGAAATAAGATTTGC
CAGATAAGGTCAAAGGAAGTTAGTACATTAAACAAATGGTGGTAGGAGGGAGGATTTGGCCCA
ACTGGCTCCAACCTATCTGGTCAACACGTATGTTGGGTAGAACAGAGGTGGAGAAAAGCCTAG
ATCAGGGATGTATACGCTTCCTTGGGGCAGACGCAGCCTGGCCCTGCGGAGCGATTTCAAGCC

TCGTTCACGAACATGGCCAAGGACACTGCCAGCCTCTTCATTCCCCTGTGTGGATGCTCCAACT
CCAAAAGTGGAACCACAGGGCAAATGAATGTCGGCACGTGTCGGTATGGCAGCCTCGCTCTTC
GACAGCTGGTGTGGGGGTTACCACCTGGGGCTAGCTGGCCTCATCTTCGTTAGCACTGAGACTG
GTCTACCAGTTGCACAGATTCAGATTTGAAAATCTCAGGTGCAAATCCCAAGTGGCGATGGCAT
GTAACCACATCAAACTCAACATCTGCACCTAACTCCCACCTTCTTTCTCTGGGAAATCTTCCTGG
ACATGCCCAGATGGACTCAGGAGCCCATCACCTGTAAATCCCCACACTGCAGCCCTGCCACTTG
CTTGTCTGCATGTGTGGGTCCGCTGGACCACTTTGGTGTATCTTTGAGCCCCTTGAGTGAAAAAA
TGCTGCCTGGCTGGCTCCCTGATTAGACATCCAACATAATGAGGACTAACACCAATACAAACAC
TTAAGAGATGACATATCGCCCTAGCTGGATCTACTACAGGGAAGGGAAGAGGGTGCTGGGTCC
AGGCAGGCTGAGTGTCTCATGTCTTAATGCTTCTCTGCCCAATCTATTTCCGGCTGGATGTGGAG
TCTGAAGGCCTGGCACCCACTCTGGCTCTGTGATTTACCAGCTGTGAGCCTTGGGGGAGCTGCT
TACTCTCTTGGTGATTCTTTTCTCATTTCTATGATGGGGTAGAGGATAATGCCTATGCTTACAAAG
TGGCTGTGGGAAGTAAACCGGATGGGATAAGAATGGCTTGCTGTGGACCACAGGCACCGCAG
GATAACCATTCCTCAGAACTCCTCATACTGCTCTAGTGCTTGGAGGTCCGTGTATTACCTCAGCT
ATTCCAACCGCACCAACCACGGGAGCCACGTGTCTACGTCTGACAGATAAAGATGCTGAGTTTA
GAGTCTGCAAGGCTTGACAACCACAGATCAGGGACAGGAGCTGAGGTCTCCTGACCTGGAGCC
CAGGGCCACCCGGAGCTGCAAGAAACCTGCACTCACAACTGCCTCCTATTTTAAAATGCTGAGT
CGATCCCACAGGTGGCAAACCAGTTCTGGGCTTCAATTTACAAGCAGTCAGAAAAGCTGGGTTG
AAATCGCCACTGTCCTTCTATGTGGCTGATGAGGAAGGATGACGGTGCCCACCGCTCCATCTCT
CCAGCTGACCCCAAGCTGGCACTCACGGGTGGGCAGGCTCAGACAGGCCCAGCTCCACCAAG
TGCACTTGAAGCCGGAATGCAAGACATCCGATGGTATACTTACTCGAACCCGTCCTTTTCACAA
CAGCCGCGGGATCCGCTTTCTTTGGCTGACTGCTCAGAGTCTTCCCTTTTCCTGTGACCCCATTA
GACGCCACGGGGGGCTTTTTACCCTTGAGCTGTTGAAATAAACATATAAGAACATTAAAAATAA
ACACAAAGTCAAACAAAAAAAAAAAAAAAAAA
ID|36527|gene_id|85026|transcript_id|NR_122035 Homo sapiens lncRNA
TCGACCGGTGCGGCGCCTGCGCACTGGAACCAGGCGTGCGGCGCCCCAGGCGCCGGGCC
TGCGGCGGCCGGAACCGGAACGCGCGCCCGCGGGGTAGGAGACCTGGAGCTGGAGAACCCG
CCCGCCAGAGTCGTAGTAGTGGATGGGGAGTAGCCGTCCCATTTTCAACACATGTCTCATTCTA
AATTGACGTTTTGGTGGGCTGGCCGAAGAAGGAGAAAGACCCGCGGCTCCGACCTCTGACCTC
TAACCCCGGACGCACTTTGATTTCGCCGGCTTGCGCGGAAGGCTGACTCCCTGGGCCGCGGCC
TGGCGCGGGAGCCGCCTGTGGGGTGTCCCCGGCGCGGGAGGGTGCTCTGGTTCAGGCCCGCT
GCCCAGGTATGCACAAGTCAAGGCCTTTTCTGCAGACCAAGTAGCCTTCCCTGGAGAGCGCCC
CCAGCACCACGGAAACTACCTACATGCCCCGCGGTGGTCAGCCCGCCTTATCCCAGCCCCGAG
ACCCAAAACAAGAAGCATGGACTGAATTTGAGGTTTCTGGATGGTCGTGTGAACCGAGGGGAC
GGGTACTGGACTGACAAGAAATGAGCCTTGAAGGTCAATCTGGGTGACACCCGAGTCTCCTGA
TGGAGTTTCACTATGTTGCCCAGGCTGATCTGGAACTCCTGACCTCAAGCAATCCACCTGCCTCA
GCCTCCCAAAGTACTGGGATTACAGGAGGGAGCCACCGTGCCCGGCCTGGTCCTGTGCATTTTA
TAGATAAGGTAACTGACAAACCTAGCCACAGTCACCCCTTCGCCCTAAAGGAGAACTGGAATTT
GAACCCAGAGCCTTCTTCACCACCATCTCCGTTGTTTCTGGAAGCACCCTCCAGGCAGGCCAGC
CAGCATCACGGGGCCTCCCCTGGGGCTGGAACGTCTGCAGGATGCCCCTTTGAGAAATGCTGT
TCCACAGAACCCTGCCTTTCAGGCCTTGGAGACGTGGGCAGGGGAGAAGCAGCGTCCCTCAGA
GCCAGGCCTGGCAGTGGTGCTAGCAGGGGCCAAGGCCCAGGGAGCAGGGTCTCCTGTCGGAG
GGACCTGGGCAAGCCCCTCCACGCGCCAGCGGGTTTCTCAGCAGGGGAGGTCCACACCACAC
CGCTTGGGAACCTGGGTGCCTAAACGCAACAGGAGCCAAGGCACAAATTTAACCAAACACCAA

GGTTGCGTGAGGCCCCATTTCATGAGCCGGGCTCCAAGGACGTGTCCTTAGGCGGCTCTGGAA
GGCCCAGCGCCAGCCCCCGTCCTCTGTTAAAGGGAGCCAGCCCCGCGGCTCCGCCCAGGCAT
GGTAGCCTGAGCGCGCCCCCAGGGTAGTAGGGGGCACCTGAGGAGCAGGGTCTGCCCTGGCA
TGAGCAGAGCCCAGGATGGGGTCAGGGTCAGGCCCTGCCAGAGGCTGGAGGGTGCCCAAGGC
GAGGCCGGTGAGGCCCAGCTCTACAAATAAAGTGTTTTATTTACAGGAAAAAAAAAAAAAAAA
A
ID|36528|gene_id|85026|transcript_id|NR_122036 Homo sapiens lncRNA
CAGGTTCCCGTTGCGGCGGGGTTCCCTCGGTTGGGAGGAATGCCCCTGCCGAGGGCCCGC
TGCCCAGGTATGCACAAGTCAAGGCCTTTTCTGCAGACCAAGTAGCCTTCCCTGGAGAGCGCCC
CCAGCACCACGGAAACTACCTACATGCCCCGCGGTGGTCAGCCCGCCTTATCCCAGCCCCGAG
ACCCAAAACAAGAAGCATGGACTGAATTTGAGGTTTCTGGATGGTCGTGTGAACCGAGGGGAC
GGGTACTGGACTGACAAGAAATGAGCCTTGAAGGTCAATCTGGGTGACACCCGAGTCTCCTGA
TGGAGTTTCACTATGTTGCCCAGGCTGATCTGGAACTCCTGACCTCAAGCAATCCACCTGCCTCA
GCCTCCCAAAGTACTGGGATTACAGGAGGGAGCCACCGTGCCCGGCCTGGTCCTGTGCATTTTA
TAGATAAGGTAACTGACAAACCTAGCCACAGTCACCCCTTCGCCCTAAAGGAGAACTGGAATTT
GAACCCAGAGCCTTCTTCACCACCATCTCCGTTGTTTCTGGAAGCACCCTCCAGGCAGGCCAGC
CAGCATCACGGGGCCTCCCCTGGGGCTGGAACGTCTGCAGGATGCCCCTTTGAGAAATGCTGT
TCCACAGAACCCTGCCTTTCAGGCCTTGGAGACGTGGGCAGGGGAGAAGCAGCGTCCCTCAGA
GCCAGGCCTGGCAGTGGTGCTAGCAGGGGCCAAGGCCCAGGGAGCAGGGTCTCCTGTCGGAG
GGACCTGGGCAAGCCCCTCCACGCGCCAGCGGGTTTCTCAGCAGGGGAGGTCCACACCACAC
CGCTTGGGAACCTGGGTGCCTAAACGCAACAGGAGCCAAGGCACAAATTTAACCAAACACCAA
GGTTGCGTGAGGCCCCATTTCATGAGCCGGGCTCCAAGGACGTGTCCTTAGGCGGCTCTGGAA
GGCCCAGCGCCAGCCCCCGTCCTCTGTTAAAGGGAGCCAGCCCCGCGGCTCCGCCCAGGCAT
GGTAGCCTGAGCGCGCCCCCAGGGTAGTAGGGGGCACCTGAGGAGCAGGGTCTGCCCTGGCA
TGAGCAGAGCCCAGGATGGGGTCAGGGTCAGGCCCTGCCAGAGGCTGGAGGGTGCCCAAGGC
GAGGCCGGTGAGGCCCAGCTCTACAAATAAAGTGTTTTATTTACAGGAAAAAAAAAAAAAAAA
A
ID|36697|gene_id|114043|transcript_id|NR_026547 Homo sapiens lncRNA
GAACTTTCCTTATGGTCATCTTTGGGTGGAGCCACCACTTCCAGTTCTGATCTTCTCATTCTG
GTTGAGTACCTGGGCTTGCCAGTGCACCCCCCAGGAACACCGCCTAGTCCAGGGCTCCCACAG
GTCTCAGACTGACAGGAGACTGGGTCATATGACCAGCACTAGCCAATGAGCTGTGAGTACACG
TGACACAGGGACAAGCTGGGTCATGTGACTGGCACTAGCCAATGAGCTGTGAGGACGTGAGCA
CAGGTCCAGCTAGGCTGGGTCATATGACAAGCATTAGCCAATGAGCTGTGAGGACACGTGAGT
ACAGGTCCAGCTAGGCTGGGACATGTGACTGGCACTGGCCCATGGGCTGTGAGCACAGGTCAG
GTCAGCCCCTTCCTGCTGCTTCCCACCGTGTGTGCTCCTTGTCTGACTTGATATGAGAACTAACC
CAATATGGCAGGAAGCCACTGACATGTGGGAGCCGTGCATGGCAGTGGCCAGCGTCCTTGTCC
TGCGGGGGCTCTGTGGACATCACTCGCCTTCAGAACACTGAGCCCTCCTACCTCCTGATATCCA
GGGCTGCCGATGGGAAATCCCAGGCTGCCGCGGCTCCTGTGTGCTTTGAAGTTTTCAGGATTTT
TGTCAAACATCCGGGGACCCTTAGCTGGAGAGGATGGCATGGGTGACACGCAGCTGGCCAGG
GTGCGCGACTCTGCTCTCAAGACCCCCTGGAGGCCCGCCCCCTGCCCCCCACCAGCTCATTCGT
TAGATGATTGGAAGTGACTGCCTACGTCTTCTTTCAGGATAAAGCCTCAAGTCCTGCAACTATTC
CTTGTGTGATAACGTTTCAAATCCCTTCCTGGTTCTGGCAAAGCTCCTCAAACATGCTCCAGTGT
GCTGGGGCTCTGCATGTGTGAGCCTGGGGCCAGCAGGGCGCACGTTCATGTCAGGGCACCAGT
ATGATCCTGATGACACATGACTGGTGTGGAGCCCCGGGTGTTTCCACGGGAACTGCTGTCGGCC

GGATTGCTGTGCTCTGCAGCTGTGCCCA
ID|36698|gene_id|114043|transcript_id|NR_026548 Homo sapiens lncRNA

GAACTTTCCTTATGGTCATCTTTGGGTGGAGCCACCACTTCCAGTTCTGATCTTCTCATTCTG
GTTGAGTACCTGGGCTTGCCAGTGCACCCCCCAGGAACACCGCCTAGTCCAGGGCTCCCACAG
GTCTCAGACTGACAGGAGACTGGGTCATATGACCAGCACTAGCCAATGAGCTGTGAGTACACG
TGACACAGGGACAAGCTGGGTCATGTGACTGGCACTAGCCAATGAGCTGTGAGGACGTGAGCA
CAGGTCCAGCTAGGCTGGGTCATATGACAAGCATTAGCCAATGAGCTGTGAGGACACGTGAGT
ACAGGTCCAGCTAGGCTGGGACATGTGACTGGCACTGGCCCATGGGCTGTGAGCACAGGTCAG
GTCAGCCCCTTCCTGCTGCTTCCCACCGTGTGTGCTCCTTGTCTGACTTGATATGAGAACTAACC
CAATATGGCAGGAAGCCACTGACATGTGGGAGCCGTGCATGGCAGTGGCCAGCGTCCTTGTCC
TGCGGGGGCTCTGTGGACATCACTCGCCTTCAGAACACTGAGCCCTCCTACCTCCTGATATCCA
GGGCTGCCGATGGGAAATCCCAGGCTGCCGCGGCTCCTGTGTGCTTTGAAGGGTGCGCGACTC
TGCTCTCAAGACCCCCTGGAGGCCCGCCCCCTGCCCCCCACCAGCTCATTCGTTAGATGATTGG
AAGTGACTGCCTACGTCTTCTTTCAGGATAAAGCCTCAAGTCCTGCAACTATTCCTTGTGTGATA
ACGTTTCAAATCCCTTCCTGGTTCTGGCAAAGCTCCTCAAACATGCTCCAGTGTGCTGGGGCTCT
GCATGTGTGAGCCTGGGGCCAGCAGGGCGCACGTTCATGTCAGGGCACCAGTATGATCCTGAT
GACACATGACTGGTGTGGAGCCCCGGGTGTTTCCACGGGAACTGCTGTCGGCCGGATTGCTGT
GCTCTGCAGCTGTGCCCA
ID|37365|gene_id|285780|transcript_id|NR_026970 Homo sapiens lncRNA

GTGTGCAGGCATATGGCATCTGGTAGTTCTGTTTCATAACTGATTTCCATTTCAGCTTAGCT
ATGAGCTCACATTTGCCAATGTAGTGTTCCAGACAGTTTTATCATCCACATTGCTGACCTGGAAG
ATGCATCATTGTCTATGATATCTGCAATGGAAATGATCACTAGGCAATTTCAGCCTTGCAAATCT
AACTGTGGCGTGTGGAAAGAAGAGGAACCAGAAAAGAATGTTTGTGAAGTTCTGGCTTGAATCT
CGTGCCGGGAGTGTGAAGAAGGTCCTTGCTTCCCCTTCACCTTCTGCCATGATTCTAAGTTTCCT
GACGCCTCCCAGCCGTGCTTCCTGTTAAGCCTGTGAAACTCCTTTGGAATGAGTACACATCCAG
GAGTGAAGAAGACAAGCTCAACTGCCATTCTCTGAGCAGTTCAAGGCATACTGAGTTGCGGGA
GTTGTGTGGTGGTCCACTCTGACTGCAAGCACCCCTTTCTTGAACCGAACAGAGTCAAGTGGAA
TCAAAGGACATTACCTGTTGATTCTTGGGCAAGGCCCAGAATTTGGTGGACATACTTAAAGAAG
AAAGGAATCTTCAGCTAAAGTGTCTACGATCAATTCAGATTTGGAGGGCATGGATCTTCTGACA
GTAGGGGCCATTCTAGGATTTTTTGGTTGAAGAGATGAACTTCAACTTGGTTCCAGATGGGTGGT
CCAGCTATTCTGCTGACAACATTTTTCAGGGCAAGGCTTTTGCTACTGGAATGATTTAAGGAAGA
TCCCTCAGTGTTTCAGAGTAAAGATATTTCTAAAGAAGTACCTGTGTCATTGCCCATCTTGAAGA
TTGACTTTTATGGATTGCTCAAATACTGATATCAGCTCAATGTCAATATATACTCCTACATATAAA
ATCCATCTTCTAATCATAATCACTAGGTTTTAACCATTTAAGGCCATGAGGTGTGAGGTCTTTTGA
GAGTGAAAGAACTCATATTAATCCAGCTCCTGACTCCTAAAAAAACAGTAACTCTTGTAATTAAA
ATGGGCAGAGTCCATCAAGAAGCAGTGTCCAGAAGCACTGGGCCATGTGGCGAGCAGCAGTG
TCACCAGGAAACATGCCGAGCTGGGTCAGGAACTGCGCAAGGAGGAGGAGACACTGCTCCTG
TTGAAGGCAGCCCTCGCATGACCTGCTGAATCACATCAAGGTTGAAGCATTGGCCCTCCAATCA
ATGATTAGTTCTGGGAGAGGGGATGAGGTGCTGTCTTCTCCAACTTCTCCAACTGTGCCTGAAC
AGTCACACGTCATGTCAGTGCATATAGAGAATGAAGCATCAGTCAACCAAACTACGCTGGAGT
GGAGCATAGAGTCCTGTGACAGAAGAGGAGGAGGAAGAGAAAGAAGAATCAGATCCCTAAAG
GAGGGAAAAAGCCAGGATTCATTATACAAATTAAATTTCTAAGTCTCAGGCTCTTTCTCCCCAAG
GAAATCATCTTGCCATGAGCCCTAGGGCCTGCTTGAATGGAGAGCAAGAACTATAGGAGGATG
TGTAGCTGCAAAAATTTCTCATCAATAACTCAATCTTCAGAACGTTATGACATACAGAAAATCAA

AGATGCTGTGTCTTAGCATTGAGCAATATGGGTGGTGCTATGTCTGTAGTCTGGTCAAATTCAGT
AACTGTGTTCAAAACAAAATTTCCTCTGGAATACTATTGAGAAAATCTGGCATTTGCAAAAAGGG
CAGAGTTTAAATATGAGCCAGTCTTTCCAATCAACAATACCTCTTTTAAGAAAACCAACTAACAG
CTGTTTGCATGCCGTTGAATCCTTGTGAGCAGTTAGGCCACAGTCTAGGACAGAAAGATGACAC
GGGGAAGGACTGAGGGCCATCTGAGAACAAAAGTTGTTGCTACTTCTCAGAGGCATTGAGACA
CACTGCATGCTGTAGACTGCTTTGTGTAACTGTTGTCACATCTTGCTTGAGATGTCATTGGCAGA
ATGTTCTTAAAAATAAATTATAGATAAATAGCCAAAAAAAAAAAAAAA
ID|37412|gene_id|349114|transcript_id|NR_026999 Homo sapiens lncRNA
AGCGCAGATCCTGCCGGCTCTGGACTAGCCGCTCAGCACCAGGGCGGAAGGAAGACGAG
GCGAGGCAAGCGCCAAGCTTCCATCCGGACTTCAAGGCCAGTGGCTGGGTCTGGTTTTCTCAAC
ACGGCCTCCTAGTCTCCTTCCCGCTTGGGCATCTGTTTTTCTTATATAGACCCTGAAGCCGCCCG
TGATGTTCTGTAAGCAGGTTCCGCTTCAGAATCAGAGGTACTTAAGAGGGAACCTGGATGCTAG
ACCTGCCCTTGCTGCTGGCCTCAGTTTCTTTACCTGTAAAACAGGGATTTTGGAACTGTTAAATTA
AATCAAACTTAAAAGAATTACAACTTAAAGCTGCTGGAACTTTACATGATCCCGAGCCTTGAGA
GGAATGTGGCTATGCAGCCTGAGTCATGTGGCATGCAGATGCAACCTCTGCCTTTTTCCGCTGT
AAATAATTAAGAAGACCAAGTAGCATCCAAGAGTCACCTAAGCTGAAGATTCCCTTGAGAACAA
GTACTGTCCTGTGGTTTCATGGCCTTTCTTCCATTTGTGGTTCTTGCCAAGCGGAATTTAAATGAC
ATCTTATCAAGATGGATAAACCCTAGTTTCCCAGTGCTGGAATATAGAAAATGGATGGACAAGT
AAATCCCACTCAGCACCCATAGCCCAGGCATGGGGACCTCAACACATTTGAGCCGCAGAGATC
ACCTTTCATTGTGAATAGCTCTGAGATGATACTTCTGGTTGAATGTCTTTTTGTCACTTCCGGCTG
GATCTACCATGAAAGATTTCTGAATCCAGGAAGAGAGATTGACTGGGCAACATGTTATTCAGAA
ACAGGACCTTGCCCTGTCACTCAGGATGGAGTTCAGTGGTCCAATCATGGCTCATTGTAGCCTC
AAACTCCCAGGCTCAAGCAATCCTACCACGTCAGTCTTCCCAGTAGCTGACAGGGTCTCACTCT
GTCGCCCAGGCTGGAGTGCAGTGGCATGATCTTGGCTCACTGTAGTGTAGACTTCCCAGCCTCA
AGGGATCCTCCCACCCCAGACTCCCAAGTAGCTGGAATTACAGGTCTCATGCTCTCACTCAGGC
TGCAGTACAGTGGCACAACCTCTACTCACTGCAGCCTCTACCTGCCAGGCTCAAGCAATTCTCC
TGACTTAGCCTCCCGAGTAGCTGGGATTACAGTTGAAGCATATGTCCTGTGAGCTTAGGGGTGC
TACAAGTTGACCACTGCAGCAGTAAAGATGATTCTGAAGAATGGCATGGGATGGATCCTTTCGA
ATGCACTTGAGCAGCGGTCTCCAACCACAGGGCCACAGAGCTGGAGCTGGATCTACCATGAAA
GACTTCTGAATCCAGGAAGAGAGACTGACTGGGCAACATGTTATTCAGGAGATGGGGTCTCAC
CCTGTCGCCCAGGCTTGAGTACAGTAGTGCTATCACAGCTCACTGCAGCCTTGAACTCCTGGGC
TAAGGATCCTCCTACCTGAGCCTCCTGAGTAGCTGGGACTGTAGGGTCTCCCTCTGTTGTCCAA
GGCTGGAGTGTAGTAGTGCTATCACAGCTGACTGCAGCCTCAACCTTCCAGGCTGAAGCGATCC
TCCCATCTCAACCTCCCACGTGGCTGAGACTACAGGCGCTTGCCACTATGCCCAACTAATATTTG
GAATTTTCCTATACGTGGATTCCAGAGGGGTGACAGCGAAACGTGGGACCATCCAGTTGCAGG
AAAACGAGCTTAACACGCCCACTGATTCTACATTATGCTCCTACCTCCCGGCAGCCTCTCCAGG
CCCAGAACTTTCTCCAGTCGGCCTCCACAGACCAAGCTCATGACTCACAATGGCCTATTTAGGC
CCATACCCTACCTCACGGCAGTCTCCGCAGATGAGGCTACTGCCTCACAACAGCCTCCACAGG
CACAGCTCCATCGTTACAATGGCCTCTTTAGACCCAGCTCCTGCCTCCCAGCCTTCTCTCTAGGC
CCTGAACTTTCTCAAGTCGACGTCACCAGGCCCAGCTCATGCTTCTTGGCAGCCTCTCCAGGCC
CAGCTCTTCCTCCCGGCGGCCTCTGCAGGCCCAGACTGTCATCAAGTCGGCCTGTCCAGGGCC
AGCTCCTGCCTCCCGGCGCCCTCTGCAGGCCCAAGTCGTCCTCAAGTTGGCCTCCCCAGGCCCA
GCTCCAGCCTCTCGGCGGCCTCTCCGGGTGCAAAAGTTCCTCGAGTCAGCCTCTCCAGGCCCA
GCTCCTCCTGCCTCCCAGTGCCTCTTTCGGCCCAGCCCAGCTCATGCCTCTGGGCGGCCTTCCC

AGGCCCCGCTTTTGACTTTTGGCGGCCTCTGCAGGCCTCGACAAGGCCCGGCCTCCTGCCTCCC
GAAGCCTGCACAGGCCCAGCCTCTGCCTCACAGCAGACTCTCCACGCCCAGCTAGCTCTCGCC
TCACTGCGGCCTCCCCAGTCCAAAGCTCCTGCCTCTCGGCCGCTTCGGCAGGCCCAGCTCCCGC
CTGCCAGTGCCTCTTCAGGCCCATGGGGCTCATTCCTCACAACGGCCTTTCCAGGCCCCGTTTTT
CCCTTCCGGCGGCCTCTCCGGGCCCAGAACCTCCTCAAGTTGGCCTCTCCAGACCCACTTGCAC
CCTCCGGGCGTCCTCTCCAGGCCCAGCTCTTCCTCCCGGCTGCGTCTCCAGGCCCGACTCCTGC
CTCCCAACAACCTCTTTGGACTCAGTGCCTGCCCATCTCCTGGCGGCCTTGGTCGGCCCACAGC
TTCCTCAAGCCAAGCTCCCCAGGCCCAGGTCAGGCCTCACGGTGGCCTCTCCAGGATCAGCTC
CTGCCCTCCGATGGTGTCTCCAGGCCCCAAATGGTCTCCGGCCAGTGGGCTCCTCCACGCCCAG
CTTGGGCCTCCCGGCGACCTCTGCAGGCCCAAGTCGTCCTGAAGTCGGCCTCTCCCGGCCCTG
CCTCCCAGCAAGTAAGCAAGCTTTTTTGGCTTAGCTCCTGCCCAGCTCCCAACCGCCTTTGTAGG
CCCCGAACTTTCTCCAGCCAAGCTCTTCGGGCCCACCTCCTGCCTCCCGGTGGCCTGTACAGGC
CCAGCTCTGGCTGGAGAACAGCCTCTGCAGGCCCCGCTCTTGCCTCCCAGGGGCCTCTCCAGG
CCCAGCTCTCGCCCCCACAGCGGCCTCCCGGGGCCAAGTCCCTGCCTGCCTCCCGGCATCCCG
CATGCAGCCCAGCTCCTCCCTCACGGTGGCCTGTTGATGCCCAACTCATGCCTCTGGCACCCTG
CCCAGAGGCGTGAGCCCCTGCCTCACACCGGCTCCTCCCACGCTGACAGAGGTCAGCGTGAGC
CCCTGCCTCAACAGGCCACCGTGAGGGAGGAGCAGGGCCGCACGCGGGCTGCCGGAAGGCG
GACAGGGACTTGGCCCCGGGAGGCCGCGGTGGGGCAAGAGCTTGGCCTGGAGACGCCCCTGG
GAGGCAACAGCAGGGCCTGCAGACGCTCTTCTCCAGCCAGAGCTGGGACTGTACAGGCCACTG
GGAGGCAGGATGTGGGCCTGAAGAGCTTGGCTGCAGAAACTTTGGGGTCTACAAACGCCGGC
GAGAGCTGAGCCGAAAGAGCTTGCTTGCTGGGAGGCAGGAGCTGGGCCGGGAGATGCAGCCA
GGAGGAACAGCTGGGCCTGCAGAGGCCGCCATGCGGGAGGCAGAGGCTGGGCCTCCTCAAGT
TGGCCTCTCCAGACCCACTTGCAGCCTCCCGGCGTCCTCTCCGGGCCCAGCTCTTCCTCCCGGC
TGCGTCTCCAGGCCCAACTCCGGCCTCCCAACAAGGTCTTTGGACTCAGCTCCCCCGCAGCTTC
CAGCGGCCCTGGTAGGCCCACAACTTCCTGAAGCCAAGCTCCCCAGGCCCAGCTCAGGCCTCA
CGGTGGCCTCTCCAGGCTCAGCTCCTGCCCTCCGACAGCGTCTCCAGGCCCCAAACGGCCTCC
GGTCGGTGGGCTCCTCTAGGCCCAGCTTGGGCCTCCCGGCAGCCTCTGCAGGCCCAAATCGTC
CTGAAGTCGGCCTCTCCAGGCCCAGCTCCAGCCTCCCGGCGGCCTCTGCAGGCCTAAGTCGTC
CTCAAGTCGGCCTGGAAATGGGCCTGGAAGAGCTGCAAGTCGGCCTCCCCGGGCCCAGCTCCG
TCCTCTCGGCGGCCTCTCCAGGTGCAAAACTTCCTCGAGTCAGCCTCTCCAGGCCCAGCTCCTC
CTGCCTCAGTGGCCTCTTTCGGCCCAGCCCAGCTCATGGCTCTCGGCGGTCTTCCCAGGCCCCG
CTTTTGACTTTTGGTGGCCTCTTCAGGCCCAGAACTTGACCTCCAGTCGGCCTTTGCAGGCCCGG
CCTCCTGCCTCTCGAAGGTCTGCATGGGCCCGGCCTCGGCCTCACAGCGGACTCTCCACGCCC
AGCTAGCTCTCGCCTCACTGCACCCTCCCCAGTCCAAAGCTCCTGCCTTTCGGCCACTTCGGCA
GGCCCAGCTCCTGCCTGCCAGTGGCCTCTTTAGGCCCAGCTCATTCCTCACAACGGCCTTTCCA
GGCCCCGTTTTTCCCTTCCGGCAGCCTCTTGGCCTCTAATTTTTTTATCTTTTGTGTATAAATCCCA
AATTATGGAATTTTGGAATATTTCCACCATTATATAAATATTTTGGTAGGTAATTTAAAAAAAA
ID|37442|gene_id|387644|transcript_id|NR_026795 Homo sapiens lncRNA
GTCAGCCATATGCCTGTGCTCTCAAGTGCCAAGTTTGTGGGGATGCATGCAGGGGATTCTG
GACCTGATTGTTTCCTCTGAACCAGGATGTGGTCTGGTTGGCAGGGCAACTGGTCTTCACTTGGT
GGCCTTCAGTGGGTGTCCTCATTGGTTGCCTTCAGATAGTGCCCTCAGTTGGTACCATCAGTTGG
CGCTCTTCAGGTGGTGGTCCTCAGTTGGTGGTCTTCAGTTGTAGGGCAATGGTGGAAGGAGGAT
GAAACTGTGCTTTCTCCTCCCCCAGTCACAGCTCTCAGGCTTGCTGCCTCCGGGCCCTTCTAAAA
TAAACCAGACAGGTTGGCAGGTTTTTCTTAACAGACAAGTCAGCTCAGGGTACAGGGGTGACA

CAGGGTGCAGGCTGCTAGTGCAGTGCCACCCCATGGCCCAGGGGTGCCCGTGCCAGCCATGCC
TTTGCCAGGAAGAGGCGATAGGTGTTGAGCTCTGCAGACTTGGGGACAAGCGAACACCCAAGC
TGCCTGAGAAGTGCAGGGGTGCCCCATGGGGACCACACTGAACTGTCGCATGTGCACCAAAGC
CAGCCCCAGTCAGGGCCGGCACAAGGTTTCTGCAGAGCCAGCCTGTGGCTCTGACGTCTACAA
GTGTGCATACAACGAAGGCGCAGGCTGCGATATACAGGAGCAGAGGCACCCAGAACACGCTG
CCCCTGCTGCCAGTTGCAAGTGGGGTCTGCTCAGAATGCGGAGGGTGAAGCTGTCCTGCAGCT
TTGAGATCCACGAGGTGGTGGTGGCCCTAGCGCCAGACTCCACTGCTATGGAACCTGCCCAGTT
GAATTTGGGAGCCAGTGAGGGACCTGACAGCAGCACCAGGAGACGCCCAAAGGTAGGGAGGT
GACAGATACTGCTGAAGTTTAGGAACTCCTCTAGCCATTGCTGGCCCAAGAATCCTGCAGAGGC
ATCCAGAGTCTCAGTTTTCTCATCTGTAAACGATGTGCTTGTTATGAGGCTTCGGTAAGGTAAGT
GTGTAAAAACCATGGCAAAATGCTTGTAACTTAAAAGAGAGGATTTCTATGAGGCTTCTATAAA
GTAAGTGTATAAAAATCTGGACACAATACCTATGATTTTTTCAGTGTTCAGTATATGGAGAAGGA
GATTATATTCTCTCATCTAATTTTCAGAGCCAGTTTATAAAAATGGTGATACTCATATTTCTTAGA
CGATGAAATCAATGATCAGAAAGAATAAATAGATCATCAAAGTCACACAGCAGTTCACAATGCG
TTCCTTGTGTAATTTCTAGTTAAAAGTAGCAATGATGATCACATTGAAAATTTTGTATTATGTGGT
AGTTTTCACTTACATAATCCTTTTGGTGTTTTGAAACCATATATTCTAAGTTGCTCTACCAAAGCG
ATCATTAGAAAAATGTGATGTAGTTAGTGTGTAAATTTTAGAAAATTTCTTCCGTTAGTCTTGTTA
ATTTGATTGCAAATATGTGTTATTTTCAAAAACGTAAATGGAATATATATTGATTGGACTTGTATG
TGTATATATGAAATATGACTGGACATTATAGAGTAGGCTTAGTTCATGGCAATGTTTTTTTCTGAA
AGCACCTGAAGAAATCACTCTTATTTCCAGTTTGTTAGTGTTTCAGAGATTATGGGGGAAAGGG
GGTTCTATGAATGTCAGACACTGCGTGAGGCACATTTGGTTTCAGTAATTTTAGCATCATTCATT
GTAAAGTGATGATCCCTCGGAACTTCTTCACAATGAGAAACCTGAAAGGTCCCAGCAGCCAGC
AATGAATGAAAGGTGGGGTGGGGCCGCTGGCAGGGCGAGGCCTTGTGAGCCATGTGCCTGTG
CTCTCAAGTCCGAAGTTTGTGGGGATGTATGCAGGAGATTCTGGCCCTGATTGTTTCCCCAGAA
CCAGGATGCGTTCTGGTTGGCAGGACAACTGGCCTTCACTTGGTGGCCTTCAGTGGGTGTTCTC
ATTGGTTGCCTTCGTTTAGTGCCCTCAGTTGTTTCTCTTCAGTTGGCGGTCCTCAGTTGGTGGTCT
TCGGTTGTTGGGCAGTGGTGGGAGGAGGATGAATCCATTTGTGCTCTCTCCTCCCCCAGTCACA
GCTCTCAGGCTTGTTGCCTCCAGGCCCTTCTAAAATAAACTAGACAGGTGGCAGGATTTTTTTAG
CAGACAAGTCAGCTCAGGGTACAGGGGTGAAGCGGGTTGCGGGATGCTAGTGCAGTGTCGCCT
CATGGCCCAGGGCTGCCCGTGCCAGCCATGCCTTTGTCAGGAAGAGGCGATAGGTGTTGAGCT
CTGCAGTCGTGGGGGCCAGGGAAGGCCCAAGCTGCCTGAGAAGAGCAGGGGTGCCCCATGGG
GCCCACACTGAACTGTCGCATGTGCACCAAAGCCAGCCCCAGTCAGGGCTGGCAAAGGGGTTC
TGCAGAGCCAGCCTGTGGCTCTGACATCTACGAGTGTGCAGACAGCGAAGGCGCAAGCTGCGA
TATACAGGACCAGAAGCACCCGGAACACGCTTTCCCCCACTGCGAGCCGCAATTGGGCTAGCT
CAGGGTGCGGTGGGTGAAGCTTTCCTGCAGCTCTGAGATCCACGAAGTGGAGGTGGTAGTAGC
GCAGGACCCCACTGCTTTGGAGGCTGCCCAATCGAATTTGGGAGCCAGTGAGTGTCCTGACAG
CGGCACCAGAAGACCCCGAAAGCATGCAGACTGTGAAGATGCAGGCCGCGATATACGGGAGC
AGAGGTGCCCAGAACATGCTGTCCCCCAATGCCAGCCACAAGTGGGGCTATCTGAGGGTGTGA
TGGGTGAAGCTGTCCTGCAGCTCTGAGATCCACGAGGCAGAGGCAGCAGTAGCGCTGGACTCC
ACTGCTTTGGAGGCTGCCCAGTCGAATTTGGGAGCCAGCAAGAGTCCTGACAGCAGCACATTG
GCCACCAGAAGACACCCAAAGGGAGAGGAGCGGAAGACCTTCTGGTGTGGGCTGGGCAGCAA
TTCACTTGAAGGAGAAACAGCCCCGGTGTGGAGAGGCGGCCATCCTTGGCGGGATCCTTTCTA
AGGAGCCGAGAAATCAACGTAGAGCTTCCTCTGTCTGATTCTCTAACAACTGCAGACCTTCCAT
GAGTCAAGCTTTGTGTCAAAAGGACAAATAAAAAGGACCTATAAAAGGCATCACCAAGCCCAA

TGGACAGATGCCCCAGGCTGCACATTCTGTCAGTGCTGTTCTGGAAAAGGCCCAAACACATGCT
GAAACATCGAAGGATAAGAAGCCAGCCCTCGGGAACCGGCAGGAGCACTCTGGGCCCCGCAC
GGCCCCAAGTCCAGCCTGCCCCGCCTCCTCTGGTGCAGAGGTCCAGGGATACCAGCAGCAGCC
CCGCCACGCCCCTCCAAGGCCAAGAACACAGGCGGCAGGGGCTTCCCCTCCCTGCCAGACTAC
TTCCTGCCGCCACAGCCACCACCCTTGGACGACCCAGAGCTCCCGCCGCCCCTGGACTTCGTG
CTCCCTCCCCCCGCGGTCGCCAAGAGGCCTCCTAATGCCCCACCCCCGCAAGAGACACGAAGC
ATCAATGTTCAGAAGCAGCAAATAAATAAAATAAATGAATAAAAGTTAAACGTTCAAAAAAAAA
AAAAAAAAAAAAAAAAAAAAA

ID|37499|gene_id|642946|transcript_id|NR_027285 Homo sapiens lncRNA

GAGAACTTCGGGAGGCGGGGGAGGAAAGCGGCCGGCGAGCGCTGGCTGACATTTTCCTG
CCCGGAAGGATGCATGGCCCGGGGTCTCCTGCACCTGAGGGTGGGCGGGAGGCGGCCCCGCG
GGTTGTGTTGCTGGAAAAAGGGGTCTCGATCCAGACCCCAAGAGAGGGTTCTTGGGTCTACTTC
AGGGAAGAATTGGAGGCGAGTCACAGAGCGCAGTGAAGGAAGCAAGTTTATTGGAATCTACTC
CGTTAGAGAGTGCAAGTCCTCAGACTGCAGGAGGAGGAACTCCCGTCCTTCGGTAGTGTCACT
ACTTAGAGGCAGCTGTGAGGAGCTGTGATTAATCGTGGAATGTGCTGATGTGCCCACTAAAGAT
GCCTGGTAAGCAGTGGCTCCTCTAACAAGTGGGCAGCTTGGAACTGGGAACGAGAGAGCCACC
GAAACAGAATGAGGGAGACAAAGAAGTGGGCATCACCCTTAACTCCACAAGGGCTTCCCAAG
GGCGCTGTACCGTACGCAGGCCCAGGACGAACTTTATTTCTCGCCCCAGCATCGCTGTCCTTGT
CGGTGAGACCCTGGCTTTAGGGCAGACAGGACCACGTTTCATAAGTTCATGCTGTCCCAGCAGA
GGAATAACGCCAGAAAGTGTTCCAGTACAACCAGAGAAAGAGAGTCCATGAGAAATCTGCCCT
TGTGAAGTTGGAATCCCCTCAACCTCACCCCGCTGACTTGAATGAAGCGACTGAGACGGGCTAT
GATGGAGCAGATCCGGTTGCTCGACCTTCTCCCTTGCACCAACACATGTAGTTAATAGTTACTG
GACATGCATATTCAGTGGGTTCCAGGTACCAAACTTGTATTGAATGGTATGTGCCAGACACCGT
CTTGAGATCTGGAGAATAAAAAAATAAAAAAATAAAAACGAGACATCCAGAGCACAGTA

ID|37500|gene_id|642946|transcript_id|NR_027286 Homo sapiens lncRNA

GAGAACTTCGGGAGGCGGGGGAGGAAAGCGGCCGGCGAGCGCTGGCTGACATTTTCCTG
CCCGGAAGGATGCATGGCCCGGGGTCTCCTGCACCTGAGGGTGGGCGGGAGGCGGCCCCGCG
GGTTGTGTTGCTGGAAAAAGGGGTCTCGATCCAGACCCCAAGAGAGGGTTCTTGGGTCTACTTC
AGGGAAGAATTGGAGGCGAGTCACAGAGCGCAGTGAAGGAAGCAAGTTTATTGGAATCTACTC
CGTTAGAGAGTGCAAGTCCTCAGACTGCAGGAGGAGGAACTCCCGTCCTTCGGTAGTGTCACT
ACTTAGAGGCAGCTGTGAGGAGCTGTGATTAATCGTGGAATGTGCTGATGTGCCCACTAAAGAG
GAATAACGCCAGAAAGTGTTCCAGTACAACCAGAGAAAGAGAGTCCATGAGAAATCTGCCCTT
GTGAAGTTGGAATCCCCTCAACCTCACCCCGCTGACTTGAATGAAGCGACTGAGACGGGCTAT
GATGGAGCAGATCCGGTTGCTCGACCTTCTCCCTTGCACCAACACATGTAGTTAATAGTTACTG
GACATGCATATTCAGTGGGTTCCAGGTACCAAACTTGTATTGAATGGTATGTGCCAGACACCGT
CTTGAGATCTGGAGAATAAAAAAATAAAAAAATAAAAACGAGACATCCAGAGCACAGTA

ID|37528|gene_id|730101|transcript_id|NR_024403 Homo sapiens lncRNA

ATAAAGCGCGCCCGCTGCTCCCACAAAGCCGGGCTGTCCCAGGCTGAGGCGGTTGCTCCC
CTGCCGGGTGTGTTAGAAGCAAGTTAGAGGAAGAAGGTCTTGGGGACTACGGGCGCCACCGA
GAACCCATACGAGGCAGCCGCCGACGGCGGGTCGCCGGGGCGGGGCCAGAACGGCGCCAAG
GCCAACCAGAACGGCGGCAAGAACTAGGGGGACGCGGGGCAGGTGCTGTGGGAGTTGGCTG
GACCTGTGGGATCGACGTCTGCGCCGTCCAGGAGCCGCCCAGGGGAGAGAGCGGAGACTCGG
GAGGAGGATGTGCGCTCCGACCCGGGCAAGGCTTCCCATCCCTCCCTTCCTCTTCTCTCCTTCC
CTCCACCTGGGCTCGGAGTCAGGATCTGACCGCGGGCTCTGCCCAGGAAGACCGAGTTCCTTG

AGAGCGACACCTAAGCGCCGGGGAAGACAGGGATAGAAGAGACGACAGCCCTTTGATAAGAG
GGTTTTTAGACTAACTTCCACCTGTGACGAGTGCATGGGGCCTGCGCCCTGAGAGGTTGCCTTT
CCTTTTCCTCTCCCACCCTGGGGGTTTGTGGTGATGTTAAGTTCTGTTAGGACCAAGGTGGATCT
CAGTAAGGAGGTAGAGTAGATTTGTGAACAGCTGTCGGAGGTAGAATCTTTGCAGACCCAGAG
TGGGTGGGTGTAGACACTTTGCCATGGGTGGTAGCTTTATTTGTAATCCTCCCAGTGAAAGCTTT
GGGAAATCCAGCCCTCCCAAACTCTTGCCTTTCTTGTAAGACTTTCTAAGCGCATGGCTTTGTCG
TAAGACTTCTCGTGGCCATGTGTCTCACCCTACCGTTCGTCTCACCAATGAACCAGAGCTGGGA
TACCAGCAAAAAGGACCCAAAAGACTGTTTCACCACAATTGAAGAGGTCGTGGACCATCCCCC
AAAATGAATCCCAACACTGGAAGACCAAGAGGGTTGACGTTTGCTATCTTGAAAGCTGCAGCCC
CCGTAGAGAAGATCCTAGAGCAGAAGGAGCACAGGCTGGGCTGCCATAGCATGGACCCCCAA
AAAGCCACTGTCATGAAGAAATATCCTGTGAAGAAAATCTTTGCAGGGCACCTGAACTCCCAAA
GCCACTGAGGAGAAGGTAAGGGAGTACTTTGGTGAGTTTGGGGAGGTGAATTTCTCCCCAGGA
TATGGTTTATGCTCCCCACCAGCCACCAGTGGATGCTTTTTTCTGTTTTCCTTGGCTGGTGGAGTT
GTCAGTGGAGCTGCTCTCACCTCCTTTCTTGCCTTTGGAGGGCCGCCTTTTCTGAGTTCCCTTTAG
GGCCTGAGCCAGGGGAAGGCATTGGCCCTGAGCTTTGCCTGTGTTTCCAGATGGAGGCCATTG
AACTTTCAGTGGATCCAAAGTCTAACAAAAGACAATGTCTTGTTTTCGTCACCCCCCAAAGAAG
AGGAACCTGTGAGAAATATTCTGGAGAAATTTAACACCATCAGTGGAAGCAAGGTGAGGAGTC
TCTACCTCCATGTGCCTCCCCTCCTGCCTCTGGAGAGGTAGCCCTTTGGTTAATGGCAACCCAAT
AGGGTAACCAACCATCCCATTTTAGCATTGAGAGCATTGTGTCCTGGGAGACCCTTCCCTGGAA
TGTAAATAATCCTGGTTTTAGCAACAAAGTGTGGAAGGTTGGAGGAAATGCACAAAACCCGTAA
GTTTTAGAGGGTCTGAAAGCAACATTGTCTATCAAATGGATACGGCACAGATTATTCATCTTTAC
TTGTTGTCACCCATTTGACCACTGATACTGGCAACCATAATTTGTAGTTCAGAATGGTCATCCAT
CCATCGTTGCATCATTCAAAGTGATGTGGCTTAAGTGTATATAATGATTATTAATAGTTCATCTTC
GACTTTGCCTGTGGATTAATTGGTGTGCTTTGTAACAAGATAGTGCTAGTTTAATTTTTTAACATT
GTGAATATTGTGACCATGTTTTCTAAGAGGTCAGATAATGAGAATGGTTATAATACCAATGCAAC
CATGACCCCATCACTGAAGAAGAAGGCTAAACGACTATGTAATTTTGATGATGGATGGAAGGAC
ACATACTACTGAACTAGGAAGGTAAATAACCAAAACAGAGCATACTACATAATATACAGAAAA
AAATTTGAGATTGGACATGGTGGAGAAGGGGATGTGCAAGCACATATGGAGGGCAAATCTCAC
AAGTAAGATGGGACAGGTGAATGCTTCTAAACCAATCAAAAATACCTTTGTTTCCCCAAAAGAC
ACCAATGCTCAGTCAAAAATAGCAGCTGCTGAATTAGCATGGGCATACCAGGCAAATAAGCCT
GCATTGTCATAGCGTTCCCTTGATTGCTCTATGAAACTGAGTAAAGTTTCATTTCCTGATTCAAGA
ATTGCAGCTAAAATATCCTCTGGACAAAGAAGAAGGGAAATTTTTTGATAACAGATGTGTTGGC
TCCTTACAGTATAAAGCCAATTTCTGTCATATCTCACCAACAATCCTGGTTTCTACAGTACATCAA
TTTTAAGTAATATGCCAAATCATGGCAGCAAAAATATGTTCCCTCTAGCTGTTAGGTACTTTGACT
TGAAAACAGGAGTTTCAAATCATCTTCTTCATTTCTGTGAGGATTTTAATGAAACTGCTGAAATCA
TAAAATATTGTTGATATTTTGTCTAAATACAAACTAGACTTAGCTCATCTATCTGTGTATTCGTCA
GACAGTATAGATGTAAAATTTTGGCAAATTGCATTCAGTCTACAAACTTCTTACCAAAGAAAATG
AAAAGATCTTATCCATTCAATGCCTTGTACATCTTATATACAACACTGCTAAAAAGGGATGTGAG
TTGCTTAAATGTGACATTGAGACTTTCATAATGAAAGCTTTTGGTTACTTTTCAATTTCCTCAAAA
AGTGCAAAAGCACTTATGGAGATTTTTGACTTCACAGAAATGGAAGGTAACCTCTTTAGACGTGT
GCCTGCAAGATGGCTGTCATTGTAAGTGAGCCATAGAAACGATGCTAAAATGTTGGCCTGCCAT
AAGATCATATTTTCAAAGGACAAGAATAATGTCCTTTGACATTGTTTGAAATCTGAAAGGAGAAA
AAAATCAAGAATCTGAAGTGGGCCTTAAAAAAATCAGTAAGTCCCTTGTGACTCTGATTTTTATG
AATCTGTCTCTCAAAAGCCAGGGTGCCCCCAGAAGAACTGAGACAAACTGAGTTTCTGAACATT

TGAAAATGAGGAGCAGGCTCAGGATCCACAGGGTGTAACCATCCCGTGAGGCCCTGCCTGAGC
AGCCAGCCAGTCAGTGAGGCTCTTCACCTTACTTCCATTTCTCTGATTCCATCTATGTACAACTTA
CGAAAACTTGCATTATTATAGACTGGGAGTTCTTAAATTGGAATCTGTGAATCTAAAGGGGCCA
GAGGATAGAAATCAGGAAATCTGAAAACTTCGATGGAAAAAAGTTTTTATTTATTTCCATCAGGA
AAATAGGCAACAAACCACAGCAGTATTAGTACCTGTGTCTCTGTCATCACTAGGAATCACAGAT
ATCCACGTTACAGATATCTTAAAATATTTATGTACAGCACCACTCTGAAATTATGGTAATAATTAG
ATCCACCCCCTAGGTCTTGTAATCTTTAATATGTTAATAAAGAAGCACATCTATTATGTGACAGAT
TTAAATGTTTAAGAATTTGATTTCAATGAAATTGCATTTCAATAAAATTGGCTTCCTTTGC
ID|37529|gene_id|730101|transcript_id|NR_024405 Homo sapiens lncRNA
ATAAAGCGCGCCCGCTGCTCCCACAAAGCCGGGCTGTCCCAGGCTGAGGCGGTTGCTCCC
CTGCCGGGTGTGTTAGAAGCAAGTTAGAGGAAGAAGGTCTTGGGGACTACGGGCGCCACCGA
GAACCCATACGAGGCAGCCGCCGACGGCGGGTCGCCGGGGCGGGGCCAGAACGGCGCCAAG
GCCAACCAGAACGGCGGCAAGAACTAGGGGGACGCGGGGCAGGTGCTGTGGGAGTTGGCTG
GACCTGTGGGATCGACGTCTGCGCCGTCCAGGAGCCGCCCAGGGGAGAGAGCGGAGACTCGG
GAGGAGGATGTGCGCTCCGACCCGGGCAAGGCTTCCCATCCCTCCCTTCCTCTTCTCTCCTTCC
CTCCACCTGGGCTCGGAGTCAGGATCTGACCGCGGGCTCTGCCCAGGAAGACCGAGTTCCTTG
AGAGCGACACCTAAGCGCCGGGGAAGACAGGGATAGAAGAGACGACAGCCCTTTGATAAGAG
GGTTTTTAGACTAACTTCCACCTGTGACGAGTGCATGGGGCCTGCGCCCTGAGAGGTTGCCTTT
CCTTTTCCTCTCCCACCCTGGGGGTTTGTGGTGATGTTAAGTTCTGTTAGGACCAAGGTGGATCT
CAGTAAGGAGGTAGAGTAGATTTGTGAACAGCTGTCGGAGGTAGAATCTTTGCAGACCCAGAG
TGGGTGGGTGTAGACACTTTGCCATGGGTGGTAGCTTTATTTGTAATCCTCCCAGTGAAAGCTTT
GGGAAATCCAGCCCTCCCAAACTCTTGCCTTTCTTGTAAGACTTTCTAAGCGCATGGCTTTGTCG
TAAGACTTCTCGTGGCCATGTGTCTCACCCTACCGTTCGTCTCACCAATGAACCAGAGCTGGGA
TACCAGCAAAAAGGACCCAAAAGACTGTTTCACCACAATTGAAGAGGTCGTGGACCATCCCCC
AAAATGAATCCCAACACTGGAAGACCAAGAGGGTTGACGTTTGCTATCTTGAAAGCTGCAGCCC
CCGTAGAGAAGATCCTAGAGCAGAAGGAGCACAGGCTGGGCTGCCATAGCATGGACCCCCAA
AAAGCCACTGTCATGAAGAAATATCCTGTGAAGAAAATCTTTGCAGGGCACCTGAACTCCCAAA
GCCACTGAGGAGAAGATGGAGGCCATTGAACTTTCAGTGGATCCAAAGTCTAACAAAAGACAA
TGTCTTGTTTTCGTCACCCCCCAAAGAAGAGGAACCTGTGAGAAATATTCTGGAGAAATTTAACA
CCATCAGTGGAAGCAAGGTGAGGAGTCTCTACCTCCATGTGCCTCCCCTCCTGCCTCTGGAGAG
GTAGCCCTTTGGTTAATGGCAACCCAATAGGGTAACCAACCATCCCATTTTAGCATTGAGAGCA
TTGTGTCCTGGGAGACCCTTCCCTGGAATGTAAATAATCCTGGTTTTAGCAACAAAGTGTGGAA
GGTTGGAGGAAATGCACAAAACCCGTAAGTTTTAGAGGGTCTGAAAGCAACATTGTCTATCAAA
TGGATACGGCACAGATTATTCATCTTTACTTGTTGTCACCCATTTGACCACTGATACTGGCAACC
ATAATTTGTAGTTCAGAATGGTCATCCATCCATCGTTGCATCATTCAAAGTGATGTGGCTTAAGT
GTATATAATGATTATTAATAGTTCATCTTCGACTTTGCCTGTGGATTAATTGGTGTGCTTTGTAAC
AAGATAGTGCTAGTTTAATTTTTTAACATTGTGAATATTGTGACCATGTTTTCTAAGAGGTCAGAT
AATGAGAATGGTTATAATACCAATGCAACCATGACCCCATCACTGAAGAAGAAGGCTAAACGA
CTATGTAATTTTGATGATGGATGGAAGGACACATACTACTGAACTAGGAAGGTAAATAACCAAA
ACAGAGCATACTACATAATATACAGAAAAAAATTTGAGATTGGACATGGTGGAGAAGGGGATG
TGCAAGCACATATGGAGGGCAAATCTCACAAGTAAGATGGGACAGGTGAATGCTTCTAAACCA
ATCAAAAATACCTTTGTTTCCCCAAAAGACACCAATGCTCAGTCAAAAATAGCAGCTGCTGAATT
AGCATGGGCATACCAGGCAAATAAGCCTGCATTGTCATAGCGTTCCCTTGATTGCTCTATGAAA
CTGAGTAAAGTTTCATTTCCTGATTCAAGAATTGCAGCTAAAATATCCTCTGGACAAAGAAGAAG

GGAAATTTTTTGATAACAGATGTGTTGGCTCCTTACAGTATAAAGCCAATTTCTGTCATATCTCAC
CAACAATCCTGGTTTCTACAGTACATCAATTTTAAGTAATATGCCAAATCATGGCAGCAAAAATA
TGTTCCCTCTAGCTGTTAGGTACTTTGACTTGAAAACAGGAGTTTCAAATCATCTTCTTCATTTCT
GTGAGGATTTTAATGAAACTGCTGAAATCATAAAATATTGTTGATATTTTGTCTAAATACAAACTA
GACTTAGCTCATCTATCTGTGTATTCGTCAGACAGTATAGATGTAAAATTTTGGCAAATTGCATTC
AGTCTACAAACTTCTTACCAAAGAAAATGAAAAGATCTTATCCATTCAATGCCTTGTACATCTTAT
ATACAACACTGCTAAAAAGGGATGTGAGTTGCTTAAATGTGACATTGAGACTTTCATAATGAAA
GCTTTTGGTTACTTTTCAATTTCCTCAAAAAGTGCAAAAGCACTTATGGAGATTTTTGACTTCACA
GAAATGGAAGGTAACCTCTTTAGACGTGTGCCTGCAAGATGGCTGTCATTGTAAGTGAGCCATA
GAAACGATGCTAAAATGTTGGCCTGCCATAAGATCATATTTTCAAAGGACAAGAATAATGTCCTT
TGACATTGTTTGAAATCTGAAAGGAGAAAAAAATCAAGAATCTGAAGTGGGCCTTAAAAAAATC
AGTAAGTCCCTTGTGACTCTGATTTTTATGAATCTGTCTCTCAAAAGCCAGGGTGCCCCCAGAAG
AACTGAGACAAACTGAGTTTCTGAACATTTGAAAATGAGGAGCAGGCTCAGGATCCACAGGGT
GTAACCATCCCGTGAGGCCCTGCCTGAGCAGCCAGCCAGTCAGTGAGGCTCTTCACCTTACTTC
CATTTCTCTGATTCCATCTATGTACAACTTACGAAAACTTGCATTATTATAGACTGGGAGTTCTTA
AATTGGAATCTGTGAATCTAAAGGGGCCAGAGGATAGAAATCAGGAAATCTGAAAACTTCGAT
GGAAAAAAGTTTTTATTTATTTCCATCAGGAAAATAGGCAACAAACCACAGCAGTATTAGTACCT
GTGTCTCTGTCATCACTAGGAATCACAGATATCCACGTTACAGATATCTTAAAATATTTATGTACA
GCACCACTCTGAAATTATGGTAATAATTAGATCCACCCCCTAGGTCTTGTAATCTTTAATATGTTA
ATAAAGAAGCACATCTATTATGTGACAGATTTAAATGTTTAAGAATTTGATTTCAATGAAATTGC
ATTTCAATAAAATTGGCTTCCTTTGC
ID|37531|gene_id|100113386|transcript_id|NR_027287 Homo sapiens lncRNA

ACAAAACAAAACAAAACAAAAGAAATACACACAGGCAGCCGGGTGCAGTGGCTCACGCC
TGTAATCCCAGCACTTTCAGAGGCTGAGCGGGCAGATCATCTGAGGTCAGGAGTTCAAGACCA
GCCTGGCCAACATGGTGAAACCCCGTCTCTACTAAAAATACAAAAAATTAGCTGGGCGTGGTG
GCGGGCGCCTGTAGTCCCAGCTACTCGGGAGGCTGAGGCAGGAGAACTGCTTGAACCCGGGA
GGCGGAGGTTGCAGTGAGCCGAGATGGTGCCATTGCACTCCAGCCCAGGACTCTATCTCCAAA
AAAAAAAGAAAGGAAGAAAAATAAGCACAGGCATGTGTGGAGACCTGGAGGCAGGGCTGCCC
CTCACGAACCATCCACAATGCCAAGGAAACCAGAAGCAAACGTCCAGCAGCAGGGAGCGCAG
GGCATGTGAAACCGACACTGAGGAAACAGCCACGGGAGAGAGCACACAGGGAGAAAGGAAG
GCTCGGGCACTCCCTTGCTGTGGTTCAGGCCTGTTCTACCACCCCTGGGGTCACCATCGCCCAC
GGACCTTCCTGCTGGGTGCCTGTAGCACGGCACCTCCTGACAGGTCAGGTTTAAGGGAAGTTTG
CTGGATAACTAAAATGAGGCTGGGGGGAGGAGGGAGCAGGGAGGAGCTAGCCCACAGGAGC
CAATGCAGGCCTGACCCCACTGGTGCCGGACTGACTCAGGGAGAGTCATTAGGGCTGCTTTCTC
AGGCTGGATGAGCACTGGGGGTTGGGGGCTTGCCCTGGCCCACCCCACCACTCAGGAAACCTT
CCCTCAGCAGGACCTGCCCTGCACAGATCATGGGCAGCCTCCCTCTGCTCCCCACTTCCACAGC
CCCAGACTCCATCTCAACCGCAGCCAGGAGTCACTGCTAACACCACAAAGGACCTGTTCCTGC
ACCAGCACGGGCCAGCTCTGGGGGCAGGTGCCCAGGACAGAGTCACGAGTGGCAAGCAGACA
ATAAACAACAGGCAAGATGGACAGACTGATTGCCGGGAGGATGTGGCAGGAGGCACTGGAAC
GGTGGTGGAGGAACCCCAGCCCTGGGGACAGACCTCCACCCGTGCACAGACCTCCAAGCTGG
AGCCTGCTCTGGAACACTAAGGGCAGCAGCAGAGGTTGAAGAGGGGCAGACGCAGGAACGTT
GGGGACACGCTCGGGGAACAGTGAGAAAAGCCCTAGGCTGGGGCAGAGGAAGTCCTCGATGG
CACAAGCATCAGGACACAAAGTGCAAGAAATGTGCCTAAGACAAGAGGCAGTTCCAGGGCAG

ACCGGGGCCCCTGGAGAAGGGTGCTGCAATCTATCCTCACTGCCTGGAGCTTCCCTCCTGTCCC
TTGGTAACCACACCAAAGAAAGATAACAACAGTCTCTGCCTCGTGGGACACAGCGAGGGCCAC
CGTGCCCAGCCCACCGTCTGTGCACAGGGCAGGCCCAGATGGCTGCTGCCACCAACCTGTGTC
ATCCAGGAGTGCCTTTCTCAAGAGCTCTCAGTGCTTATCTAGCGTTGGCCTGCCCCACCTCGGGT
TAAATCAAAACCTCTTTGGTGGCTGGGCGCGGTGGCTCACGCCTGTAATCCCACGGAGGCCGA
GGCGGGCAGATCACCTGAGGTCAGGAGTTGGAGACCAGCCTGGACAACCTGACAAAATACCTC
TCTACTAAAAATACAAAAAAAAATTAGCCGGGCGTGGTGGCGCACACCTGTAATTCCAGCTACT
CGGGAGGCTGAGGCAGGAGAATTGCTTGAACTGAGCTCGGGAAAGGGAGGTTGCAGTGAGCC
GAGATCGAGCCACTGCACTCCAGCCTGGGCGACAGAGTGAGACTCCATCTCAAAACAAAAAAA
CAACGCCTCTTTGGAACCAGCAGAGGAACAGACTCAGGCCCTATTAGACTCCAGCCATGCAAA
GTTCCTAGTCACTGCCCACTGGGAGGGTGTGGACCTGAAGGGACGCAGGGCCAGGGGAAAGC
TCTTTCTGCTCAGCAGCTCCTGGAGAGGAGCGACCCTGTGCCGGGACACCCTTCCCCCTGCGGC
ATCCAGCGCAGCCCATCTCTGAGGGAGTCCTGGCTCACAGCGTTTCTGACAGGAGTCACAGCCT
GAACCCAGGCAAGAGGCGCCTGAGTGGGAACAACCCCTTCCTGGGGACCCCGCGGGCTCCCC
AAGGGACACTTCAGACCCGGAAGGGAGGGGCAGCGCCCAGAGCGCCCTCTTGCAGCCCCGGG
CACTGCGTGGCCACCGCCCCCTGACGGCCACGGCCCCAAAGGCGCCGCGCAGGGCCCGGCTC
CCAGAGGGGCCACTCGGGGTGACGAACCGGGGTGGGGGCGGGTTCCTCCCGCTGCACCCCCA
CAACTTCCCCACTTCATGCTCTTGCAAAGCCCCCGCAGAGGCTCGAAGGTGCCACGCGCGTGC
GAGGCAATCCGAAGGCACCGTGCGCCGCAGCACCTCCAAATCCTAAGCCCTGGCCTCGCGGGT
CGGGCAGGGCCACACCCCGAGCCGACAACATAGACCAACTCGCACAGTCGAGCAGGCACACA
GCCCGCCGGGCGCTCGAGGCCGAGGGCCCCACCCACCGGGGGCAGAGACCAAGGTCAACGC
TGGGGGTGCGCACCCGGACTCCTTCCCCGAGCCCCGAGGAGCCTGCCGGCTTGGGCCAGGTCC
GTCCTCCCGGCCGCTTCAAGCCCGCGGGGTTCCACCCGGCACGTGGGAGAACGGGGCGCCGC
CGCCTGGCCTCCGGGAGTGGGGGACTTGGGCTCGCGGCGGGGACGGACCACCCGCCCAGCTC
GGCCGGATCTGCAGCCCCTTCCCGCTCGCCAGTGGAGTGGAGGCGAGGCCCACGCCTACCGGT
CCTCGCACGCGGTCTCGCTTTTCTCAGCCTGCCGGCCTGGTGTGTCTCGGGCCGTAGGTGGCGA
CGTGGGCGAAGGATCAGCGTCCGCGCGGGCCGGGGGCGCAGCCATGGCGCTCGGAGGCCTCT
TTGCGGGCCTGGCCGGGCGGCGCGCATGGGCCGCCGGGAGCTGGCGGGTCCCTCTCTTGCCC
ACCGCGGACTACAAGCCCCACAAGGCCGCGCGTCGCGGACTACAAGCCCCACAAGGCCGCGC
GTCGCGGACTACAAGCCCCACAAGGCCGCGCGTCGCGGACTACAAGCCCCACAAGGCCGCGC
GGCGGGCATGGGGGACCCGTAGTTCGGCAGCCTCCGCCACGTGCCTGGAGGGCCCCGCCCCC
TGTTTTTCACCACAAACTGAGCCGCACAAAAACCCTGGAAAAAAATCAATAAACATTTATTTGAT
ACAGTCATTTTAATACATACAGTGATGGCCCGTCCCCACGCCACTCGGGACCAGGGCGCGGGG
CGGGGACGAGGACCGGGGACAGATGGTCACAAGGCGGGCGGGGCCCGAACTCACCGTTGAA
ACATCTCGCAAATAAACACTTTGTAGATAGAATATATATGTATATATATATGGTTACAAGTGATA
GCTGGAACCTCTTTCAAAATGCACTTATGTACTGAGAACTGGCATTAAAAAACCCAAACCAAG
ID|37550|gene_id|100287569|transcript_id|NR_027345 Homo sapiens lncRNA

AGCCTTCTGGGTCCGAGGCTCCCACCTGCTCTAAGCGCTTGACACCCTTTAAAAAAATGTA
TTTAAAGAGGCTGGTTCCTATCCATCCGACTGGAGGCATCTCAGTGCAAGAGCAAAGCTAAGTC
CTGCACACGCTCCTCCCCTCCTCCTCCTCCTTCTCCCCCCAGGTTTTCCCGAATGTATCTACTCCG
GTTACAACTAGACGCGGCCCCTCCCCCACCTGCCTCCCCCCTTCCTTCCCTCGATCGTGGAGGG
AGCGTTCTCTGTGCCTTCCCAAGTCCCCGTGGGGGACCTTCTATGTTGGAGTGGGGGGAGGGG
GGGAGGGTCATATAACGAAGGCCAGAAAGAACAAATTAGATAATCAAAAGAATTATAGTAATT

GCTTTCACTTTCCCCCGCCCGCTCAGCGGATTCCCTCCCCCGCCCCTCCCCTGGTTTTTCTGTCTG
TCGGGAATACTCGGTCTTTCCGACCCCCTCCCCTCCCCCAGGTTCCTCCTCTCCTCTCCCCTTGC
TCGCGCGTTCCCTCTCTTCCTCCGTTTTCTGGTGTGCTGGAACGTTCAGCGGAATATGATGAATG
ATCACCTGTCACAGCTTGTTTATTATAATGCAGGCAATCAATTACACATCCCCAATGCTGGCCGG
CCCGCAGGAAATTTATATGCTCAGCACAAACCAATGTGAAAATGGAATCTCATTTGCCAAATGT
CTTTCTCCCCGTACAGCACGATGATTACAGTCTGTGTTTGTTTCAACAGTCGTGTACAACTGACA
GTGCCATCATTTACTGCCTGGCTCAGGTCACGTTACTCTAAGGCTTTATTTATGGTGTTACGAAG
GGCAGCACAGGAAAAGGACAAGGGTGTCTGTCAGGGATGGCACTGTGTTAAAAAGTGGGCGT
GCAAGGGCCGCATTCCCGGGCAGCCGCTGCAACCTCAGCCCCTGGGCCCTTACCTCCGCAGCC
TCTCCCAGCATCCAGCTACCCAGACTCCAAGGCCCCAGGCGAGAGCCAGCTCTCGGTACCTGG
AGCTCCACAGGTCCCAGAATCGGGGTGGATCAGAGTTCAAATTCTGGTTCTGCTACTGTCTAATT
GCGTGCTGCAGGGACTCAATCTCTTCATCTGGGAAATGGGAGTAATAACCCTTGGCAGGAATGT
TGCGATCCTCTGGGATGTCAGAGGTGTTGATGAATGTTAGTTCCCGGGACTTCGGAAAGAGGTC
CCGTTGGAAGAGATGTGAATTGGAATTCACACCCTATATTAAAATCTCCTCCAATCTTCACCTCT
GAGACATGGCTGTCTCAAGACTGTTTTGTTTCCCTTCCTGGTGGAATTTTGCACTTTTATGTCCTG
TGTAGCAGCAGGTAGTGTGGCTTTGAGAAAATAAAATGGCCACCTTGCTCCGCTGTTCTTTCTTT
GTAAAAAAAAAAAAAAAAAAAAAAAAACGGCATAGCAATCTTGGCCTTTCTAGCTGTGTGACCC
CAGGCCGGTCAATCCCTCCTCCTCTCCAAGCCTCGGATTCCTCCCCTGAGAAGTAAAGAAAATA
ACTCCTAAACTGCCTCCCGAGGCTTGCTGGCAGGATCCAAGGTGTCCAGAGATGTT

ID|37551|gene_id|100287569|transcript_id|NR_027346 Homo sapiens lncRNA

CGAGGCTCCCACCTGCTCTAAGCGCTTGGTACCAGGGAGGGAATGGGAGGACGCTTGCG
GGATAGGGAGGCGGGGGCTGGAAATTGGCAGGGTGAGAGCTGGGGAAACTAGTCTGGGACGC
AGCCCTCCTTGTCCCCCTTCTTCATTCACCCCCTTGTTGAGGCCCTTGGCTTTTGCATAATTATGG
AAGCTCCAAAAGGAAAGATACCAGATTGATCTTGATCACCTCTGCCTCCCCAGCACCTAGTCTT
CCTTGGCACATCGTGAGTGCTCGGGTCAGGCTGTGCCACATCCACCCAGCCTTGCCTGGGGTCA
TTTATCCTACATGTACTCAGATCACGTGAACTGGTGATGGGCCACCCATGTGCCTGCCTGGAGA
CTCCTATTCACCCTTCAGATCAATATCCCCCAAATAAACTCCCAGTCACTCTGGCAAAAAAAAAA
AAAAAAAAAAAAAAA

ID|30369|gene_id|ENSG00000246350|transcript_id|ENST00000499525 Homo sapiens lncRNA

ACTGGTTGTGCAAACACCTACGGTACCAGTAATTCTGGGGAGGCAAGTACTCTGGAAACG
ACGGAGAATTAACTCCGGAATTACCTAGTTATCTGGCATCACGGTGAAAAGAGTTCCACAGTGT
GAGTAGTGCTGGAGTAGATGCTGCTGGTCTGTGTTTTCCTGTCACCGTGATAAACCATCGCTGG
CACTCAGACCCGTGGTGCTCACCGCAGCGACCTGAAACAAAATGGAGAGGAGCATCCCTAAGG
ACTACCACGTCAGCGAATCTGTAGGATCGTCCCCCGCCTTTCCTCGTTAATTTCGGGCCACTTTC
ACCTTTCCCTTTCACCCTAAATCAGACTTAAAAAGAAATGTAAGAGTTAGTCCATTGTTTCCATTT
TACGGATTAGAAAACCAAGACCACCGAGAATGAGCCTCAGTTGTTTAGTCACGGAGCACAGAG
CCGTGACTAAACAACTGTGGGAAACTGGTCCCAGAAATCTAACAAAATCTGGACGGCTGATCA
GCGCCTAGCGAATCACTGGAGTGGAGTAACCAGAACATTCTTTTCAAGCAGTCACTGGGTTCCT
CCATACCTCTGGTAGCGCAGAGATGCCCAAGGAATATTTGTTGAATTGCTGCCTGGTTCCCTTCT
CCATTCTCTGTTTCCTTCTGCTGTGGTCCTCATGTTGATTTCTCACATTTTGGCATTTCTGAGTAGA
GAGTCTTTGAATAATCAGAACATCTCTTTTAGAAATCTCTAAATAAAAAAGAAATCTTACGCTTA
GAGTCCGTGAATGGTCTTGAGTAAAATTCTGTATAAAATTTTTGTCTGTGTGCCTGTGTCATCCAA

CCCTGAAAAGATCATTTTCCCAGTCACTTTTAACGTGCTGAACTTATCTTTTGTTTCCCCCCCTAA
CTATCCTTTGCAATCAGGGATTCTACTACCTTTTTCTCATTCTGCAAGGCAGTGATCTCTGCCATG
CTGAGCACAGTAGGCAGTTAACATGGAGTAAGCATGACTTCTAAGTCCTTTACTGACGAGCCTT
ACTGTTTTGTTTGTTTTGTTTTGTAGATTTGCATTTGTTAATTACTACAACAGTGATAGACACTTAT
AAAAAAAGTCAAATATTTAAAAAGTCAACGTGGCAAGAAGTGAAGTTTCTTCATCCTCAGTTCC
ATTCCTCAGAAAAATTGATGCCGAGTTTGTTGTATGTACCTCCAACCTATTTTTAAAATGTACATA
CAAAATTGAGTACAATATTATTATGTACAAATATAACTGAGTCACAATTATAACTCATTATAATTT
GCAAAACTTAAATCATACTAGACTACAATTCACCAAGTTACTGGGCTTTTTTGGTTTGTTTTAAAC
TTAAATCCTCCCCCAGAAACTGCCAAGCCAACATGTGTTACATGTCTATCCCATTCTTTTTAGTG
GCTGTAGAAGAGACTATTTTGATAGATATTGCTAAAAGATCTGCACCCCAGAGGGATGTTTATC
GTGAGTCTCGCTAAAGTGTGTTAAGATCCTTTCCTTGCGCTGTGCTTTCCAAGGCCTTGGGAAGA
GGTTGCTCTGTATGAAACATATTTTGTAAGATTTGCAAAAGTATGCTAGTATAGTGAAAATTAGTT
AAGACAGCTTCCTCTCCCTACTCCAGCTTATCCTCAGATACATCATACAGGCCTTAGAGTACCCA
TAATTTTATGTTGATAACAGCTGTTCTTTTTCTTAAAGAGGACTTTCTAAATTGTTAAGTTTCAGAC
CTCACAAATCTGCCCCGGTTGATTTCTGGAAGCTAATTTATGCTCCCAACAAAAGCGCAGGAAA
GAGTGAAGAGATCTCCCTGAATTTTATTTTGACAGTTACTCCTTCTTCAGCAGCAAAGCGTCAGC
TTTCCTGTCAGCGTGAGTCTCACTGAAGCCTGACCTAACTCTTTCGGATCGGAACTAGAAAGTTT
ATTATCAAGGAGGTATTTAACCACGACTATACTTACCTACTGTACCCTTGTGGTTGGTCCCATGG
TGTAATGGTTAGCACTCTGGACTTTGAATCCAGCAATCCGAGTTCGAATCTCGGTGGGACCTTTC
AAAGGTGAACGTTTTACAGTTCCTGGCTTGGCCTCTGAATGTGGGAAAAATTCGTTCCTCCGCTT
CCCATCGAATTCTCGTCGAAAACAACTTTGTGGTCGCGTGCACGGTATTGAACTCTCCCCTTCTA
TCCAAGCATGAAAGTACCAAGCTCTTTCGCCTAGAAGAACGCTTTCAAAGCGTATCAAATTCAC
GGGTTTGGAGACAAAGAGAAAGATGCTGCTCTTATCGACTTACGAAAAGCTCCAGCAGCAACG
TGGAAACCCAATTCCTGTGATGGAGACAAACGCTTCCTCAAAATTTCCTAGGTTCGAAGCCCTC
CTCGCTGCCACAACCAAGTCCTGCGTGTAAAGTGGCTGAGCCTTGGTCTCGGGCGTGTTTCTTTT
GTTTGTTTTTTTGTTTGTTTTTTTTTTTTTTTTTCGCTGTGGACCTTGCGCCCCGGGTTACTTCTCGC
CCCTTTTCCCTATCCCCCATCTTGATGAGACAATTCACCCTCATCTGCTTTCTCTAGTCCCCTGTA
CTTCTTAGTCTCTCCGCTCACCCCGACTGCAACGTCTGGAAATCCCTCCAAAGCCAGCAGCCCC
GGAGGGCTCGGGTCCCTGGTATCCACGGCGTCGAGTCTTCCAGCCGGAGAGAAAAACTCACCT
AGGACCGGACCTGGGCCTCGCCGCGCTGGGAGCTGGCATTCCCAATTCAGGACCAGACACGGT
GGAGGGGCCCTACAACTTGTGTGGGAGACAAGGGAGATGGGGGTAAAAAGACCTCTGCCTTCA
GCCGTTCGCCTGGCATCTCGCCTCTTCAGCTCCTAGTACCAGGGTGAAAGGAGGGGGCTCCCTG
ACCGAGTCCTCCCCAGACACTACCAAACACTCGGTCCGAAGCATCCGGGAAACAGTCCCTTGG
AGCTGAAGGTCAAGAACAGAAAATGTAGCCGCTATCTTGCCTCCCCTGAACTGTGTGTGTAGAC
GAGGATTGAATTGGAGATAGAGTTGATGAAACCCGGAAGGTCTGGAAAATTCCTTATAGACACT
AATTTAGCTGTTAGAATGAGGTGGGAAATGTGAGGTGTCAAGGTGAGGGAAAGGTGTGCCCTA
GAGGACAGAGAGGACAAGAACTAAATTAGAAACAAGTATTTGTGTTTCGTACAAATTAATCTCT
TCTATGATGTAAGAGACAACCTTTAAATCCAATTATTTATTTTCAGATTTCTTACTTTGACATTTTG
ATTTTCGCATACATACCAAAAAACCAAATCGAATCCTACTTTCACCAAGTCCATTAAACCATTAA
TAATTCATTAGCTCATATTTTGAATTACCATTGGTTACACAGAGACTCTGTAACTAAGTTTACAAA
ATTCATGAAAGCATTATTTTGGCTCCCGTTATGTGTGCTTGATTGTACTTCATATGGTCTGTAATG
GTGCTAATTGCAGAGGGAAATAACATTTTCTTGTGGTGTTGCCTTAGCTAGTGTGGCCCCACCTT
CTTTCCACTTTGTTGATGTTAGCGTGATGAGATCTCACCCAAAGTGTTCTCCCTTTTCCTAATGGC
CTAACAGCCTCTTCTGCCTGTTTGGGATGCTTATAAGTCCAGCCATCCTTCCGACCAACACCATA

GCCTGCCTGTGTTACTGTAAGTAATCATTATGATCACATCCATCGTCAAGCATCCACTTATCCTA
GCTCAAACCAGTAACTCAGACTTTATCCCAAGCCTCTAAATTAAGGTAATGGACAAGACCTGAT
TGGAGATGGTGCCTGCACAACGTGACCCAGAACTGAACAGAAGGCCAAGGAGCAGCTGCTAT
GAGGAGGCGGAAGCAGCCACCAACACTGGGAGAGGCTGGGAGCATATGTAGATGCTCCCCCT
GATGCCTTACATGTTCTGTGGGTCTACCAATTCCTTTAGGAAGCTGAGGAAATTCCCGTGGGTAT
TTATAAAAACTCCACCCTTGAACAAACTTTAGTTAGGTTCCTCTGAGCCCTCCTCTCAACTAGGT
CCCATCCTTGGCTTGTCCAGCCCAATTTCAGCAAATAATCCAGTTGAGCCTAGTTTTAGCAAGAA
TCTTGCTAAGCCAATTCAGTAAGAATTCCTCACCTCAAAATCCTATCAAGTTTCTCTTTTTCCACC
CTTGATATCTAATCAAGTTCCTCGTAGTCACTTTCTATCAAATGGTTGTCTTACCCTTACTATTAGC
TATAGATCCCCAGCTGTCCCTGATGTATTTGGATCTGAGTTTATTTAATTTCTCTCCCCTATTGCA
ATTGTCTGGAATAAAGTCTTCCTTGCCTATTT

ID|30395|gene_id|ENSG00000254144|transcript_id|ENST00000517300 Homo sapiens lncRNA

GGATATGAGGGCGATCTGGCTGCGACACCTGTCATCCCATTGATCGCCAGGGTTGATTCG
GCTGATCTGGCTGGCTAGGCGGGTGTCCCCTTCCTTCCTCACTGCTTCATGTGCGTCCCTCCCGA
AGCTGCAGCTCAGTCGAAGAGAACGACCATACCCAATAGAAGAGCTGGGCTTCCCTGGTAGAA
CCTCCAAACAAGCTCTCAAGGTCCATTTGTAGGAGAAGGTAGGGTAGTCAAGATTCCAGACACA
TCCAAATGAGGCGCTGCATGTGGCAATCTGCCTTTCTAG

ID|30402|gene_id|ENSG00000261441|transcript_id|ENST00000562356 Homo sapiens lncRNA

AGTAACCACGCCCAGCAAGTCCCTGCGAGATTGCCCGCCTACGTGTCTCAGCGGAGGCAC
ATTTCTAAAATGTACCAGGTCTCTCGCACCCCGACGCAGGTGGAGGAAAGTGGAGAGGGAGGG
GCATCCTGTACAGAAATCTCAATTTATACTCAATTGATGGTTGGCAAGAGTTGGGGCTCGCAGC
CAAAGCGAGGCTCCCTCCCTCGCCCATCCGGTGCTGCCTCCTCGTCCAGAGCAATTCTGAGGG
GTAAAAATGTTGCCTTCCGTTGAACCTGAATTTGCCCGCAGATGCTAGCCTCCGGCCCACAGCG
CGGCTGGAGCTTGTCCCCTTCCCCAGCTGTTTCCAGTCTCCGCATCCGTCTTGTGTTGCTCCAGG
TGCTTCAACAGCTCTCAGCGCTCGGTGGTTGGTGAACTGTGGTAAAGTGCACGCAGAATAAGGT
CTACGTGATTAAGCACCGCCTGGGGGTTGGGGTCTGGAGGCCCAGCTGCACAGATGAAAGGGA
GGCTCAGAGACGTTTGGGACCAACCGAGATCACACAGCTGATTAGGAATAAAATTCAGGTCAA
GCTGAAAGTCGGCCGCCACCGTCCAGCGTGGATCCCAGGACACCGTGAACGGACTACAGAAG
TTGACTTTCGCGTTTTTAAGGAGTGGGGTGCAGGGAAACAATCCTCCGCCGGCCCTGCGGGCTG
ATGCCGACCCTCACCTGCTCCCGCCACGTGGAGAGGTAGAGAGAAGCCCCGGGCCAAGCAGC
ATTGAGCCCTGGTCCCTTCTATTTAAATATGAAATATTTTGCATACACTTTTCTAGATGTACTTAAT
ATACACAATGTTTTAATGTTTGTGATTTTTTTGTTTTGCCAAAAAAATACAATCAATCAGAAATTC
A

ID|30403|gene_id|ENSG00000261441|transcript_id|ENST00000569473 Homo sapiens lncRNA

AGAGACGGTAACAACCGACGGGCCGCGTGGCCTAATGGATAAGGCGTCTGACTTCGGAT
CAGAAGATTGCAGGTTCGAGTCCTGCCGCGGTCGAAGGGAGGTTATGATTAACTTTTAGTTTATT
CCTCCCTCAGGAACGAAGTATTGGGACAATGTGAACGTAGTCGCCGCCGATTCCCACCGCACTT
CAAAGATGTGGGAACGCCAAGATCCGCGGAAGTAACCACGCCCAGCAAGTCCCTGCGAGATT
GCCCGCCTACGTGTCTCAGCGGAGGCACATTTCTAAAATGTACCAGGTCTCTCGCACCCCGACG
CAGGTGGAGGAAAGTGGAGAGGGAGGGGCATCCTGTACAGAAATCTCAATTTATACTCAATTG
ATGGTTGGCAAGAGTTGGGGCTCGCAGCCAAAGCGAGGCTCCCTCCCTCGCCCATCCGGTGCT

GCCTCCTCGTCCAGAGCAATTCTGAGGGGTAAAAATGTTGCCTTCCGTTGAACCTGAATTTGCCC
GCAGATGCTAGCCTCCGGCCCACAGCGCGGCTGGAGCTTGTCCCCTTCCCCAGCTGTTTCCAGT
CTCCGCATCCGTCTTGTGTTGCTCCAGGTGCTTCAACAGCTCTCAGCGCTCGGTGGTTGGTGAAC
TGTGGTAAAGTGCACGCAGAATAAGGTCTACGTGATTAAGCACCGCCTGGGGGTTGGGGTCTG
GAGGCCCAGCTGCACAGATGAAAGGGAGGCTCAGAGACGTTTGGGACCAACCGAGATCACAC
AGCTGATTAGGAATAAAATTCAGGTCAAGCTGAAAGTCGGCCGCCACCGTCCAGCGTGGATCC
CAGGACACCGTGAACGGACTACAGAAGTTGACTTTCGCGTTTTTAAGGAGTGGGGTGCAGGGA
AACAATCCTCCGCCGGCCCTGCGGGCTGATGCCGACCCTCACCTGCTCCCGCCACGTGGAGAG
GTAGAGAGAAGCCCCGGGCCAAGCAGCATTGAGCCCTGGTCCCTTCTATTTAAATATGAAATAT
TTTGCATACACTTTTCTAGATGTACTTAATATACACAATGTTTTAATGTTTGTGATTTTTTTGTTTTG
CCAAAAAAAATACAATCAATCAGAAATTCA

ID|30406|gene_id|ENSG00000262899|transcript_id|ENST00000574245 Homo sapiens lncRNA

CCCAATTGGTACCACGTTTAACAAATGAGATCCTGTCTGGTCTTGTACAAAAGCAATAAAA
GGATCTTCTTCCTAAAAGCACTGAGGACAAGAAAAAGAAAAGTATGTATCAATAAGAAACTCAC
ATGAACCTCATCACTTGCGACTTATGAGAGAAAGAAGAACAGAAAAGGTGTCCTGTCTTCCCTT
CAAAATCAACTAGCAGGCCCTTTGTTTTTCTCCTTTTCTATTATTCAGCTGAAGCACAGCACAAAA
CACACAAATGTCAGTGGACAACTGTCTTTTTTATAAAAAAGAAAACAATTTAGGAAAGATTGGT
GGGTAAGAAAATTGGCACCCACTGGCTGAAAGGTATTTTGGGATTTTCCAAGGATTTAACTGGT
GTGCCTCCTATCCCATCTTCCCCATAAGAAAAGAATAGGAGAAAATAAGATTAGGAAAACATTC
TGAGATTCTCTTAAATTTCAACAGATCATCCCAGTCTGTCTCTAGTAAGGGGAGTACAACTTTTAT
GACCCTTCATCCTCTTGTGAAGATGGAAGCCTTCACTTTAAAAATATTTTAGGCCAGGTGCAGTG
GCTCATGCCTGCATTCCCAGCACTTTTGGAGACCGAGGTGGGAGGATCACTTGAGCCAGGAGTT
TCAGACCAGCCTGAGCAACATAGCGAGACCCCGTCTCTACAAAACACAAAATTTGAAGAAAAA
AAAAAAAAATAGCCAGGATGGTAGAGCACACCAGTAGTTCTAGCTCAGGAGGCTGAGGTGGGA
GAGTCGCCTGAGCCCAGGAATTCAAGGTTGCAGTGAGCCATGACCTCATCACT

Cytoplasm

ID|279|gene_id|100379571|transcript_id|NR_037803 Homo sapiens lncRNA

GGCTCACCGCAACCTCCACCGTCCTGAGTTAAAGTGATTCTCCTGTCTCAGCCCCCTGAGT
AGCTAGGATTACAGGCGTGCGCCACCACACCCAGCTAATTTTTGTACTTTTAGTAGAGATGGGA
TTTCACCCTGTTGGTCAGGCTGGTCTTGAACTCCTGACCTAGTGATCTGCCCACCTTGGCCTCCC
AAAGTGCTGGGATTACAGGCGTGAGCCACCACGCCTGGCTAGGGGAAGAGTGTTTTAAGAGCT
CTGAGTAGAAGGGTCTAAGTGCAGACATCTTGGCTGTTGCTGAAGAATGTGACTCTCACCGCCT
CCCTCTGACACTGTACCATCTCTTTTACCCCCATCCTTAGTCCACTCACGGAGGAGGCTGCCTTG
ATGGATTTGACTGCAGCTTCAAACACTTTCTTGGGCAAACGAAGGTTGGTGGTGCCACTGTCCA
CAATGCTCTTGTCATAGTTGTACTAAGAGGGAAAAGAGAGAGTTAAAAGAGTCAAAAGGTTTTT
GATGCTGGGCTCTGGGCAGTAGGGGGTTACTGCTGGGGCCCCAGCTGGGTTGGCATCTTGGCT
TTGGCACCTCCTAAGTGTACCTGCTTGGACAAGTTAACCTCTGTGCCTCAGTTCCTTCATCTCTAA
AGTGAGGATAAAAATAGCACCTACCTCAAAGGGTTATTGTAAGGATTAAATAAATCAGCAATGT
AAAGCACTTAGAATCGTGCCCAGCAGAGAGAAGGCACTTGGTAAATGTTTATTCTTGTTAATCTT
GGGTGGGCAGGTAGTCTCCAAACTTGAAAATAAAAATACCTTGTTTAGTGCTTTTAAAAAAAAA
AAAAAAAA

ID|490|gene_id|100885779|transcript_id|NR_048536 Homo sapiens lncRNA

GGTGAAATAAACAGCCATGTTGCTCACACAAAGCCTGTTTGGTGGTCTCTTCACACGGACG
CTCATGAAATTTGGTGATGTGACTCGGATAGGGGGACCTCCCTTGGGAGATCAGTACCCTGTCC
TCCTGCTCTTTGCTCCGTGAGAAAGATCCACCTACAACCTCAGGTCCTTAGACCAACCAGCCCA
AGAAACATCTCACCAATTTCAAATCCAGACCCCACTGGAAATCGGACTGTCCAACTCACCTGAC
AGCCACTCCCACAGCCGCTGGAACTCTGGCCCAAGGCTCTCTGACTCCTTCCCAGATCTTCTTG
GCTTAGCGGCTGAAGACTGACGCTGCCCGATCGCCTCGGAAGCCCCCTAGACCATCACGGACG
CCGAGCTTCGGGTAACTCTCACAGTGGAAGAAACACAACTACAGATTTCTACCTGGTGCATGGC
CATCGCTCATGAAGACTACAACTTCCAGCTTCCTTTGAAGAAAAAGAGGACTTGATGGCATTGT
CGCTAAGTAAGAAATAATGTGTGTGACTTCAGGGTTGCCCCCTTAAAGGGAGGGGACATTTTCC
ATCCTGCTGTTCAGAGTATGGATGTGATGAGAGACATCTTGGATGATGCAGAGGAGGTGAACAC
CCCAGGACAATGAAACCACAGGAGAGAAAGAGCCTGCGCTGGCCCATCTAGCACAGCCACTG
GACACAGGGACCACCTGCCTCTGCACTCTTATGGAAGGAGGAAATCTAACTTTCCCAGTTTAAG
GCACTGTTACTTTGGGCTCCTGTTACATAACTGTGGCAGAATGAAGGTTCAACATGGAAACTGG
CAATGTTGAAGAAACATAAAGTTCATTGCTTGAATATCTGAAGTTGTGGACTCAATCTCATACCT
GCTCCACTTATGAGTTATAGTTCTTCCAGGTCTCAGGAATGGGATCAGCAGGTCTCAGGGTTGTA
CTCTCCTGGATCTCTCACCAGCCACCTCAAACCAGCTGCCATAGCCTGTCCACTTCCACTCCAAT
CTTCTCTTCCTTCATCACCTCCCTTGCACACCCTGATAACCTCGAAAGAGAACTCTTCCCAAGGC
TCTGTTCCAAACACATCGCCACTCTGCTTAGAACCTTCAATGACTCCTCATGGCCTAGGAGGTTT
CTCTCCCATCTGGATCCAGCTGACGTTCCCAGCACCTTCTCCTGACTCCTGTCTTTTCTTGAACCA
GTTCTGCCCAACAAGGAGGAAAGGGCTGACAGAGTGAAAGTCCCAGGGCATGTGGGAATGTG
ACTCTTTTCACTTTAAATTCTATGACTGGAAAGTTTTGGGCAGAGTTGGACATGTGCACTTAGCTT
CCAGAAGACAGAATCCTTTTAAAAGAGTCAGAGAAAACACTGGCTTCCTGCCATGACATGAGAT
ACAGACAGGAGAGTTGGGAAGCTTTTTAAAGATGGCACTATGACTACAATCACAGAAACTCTCC
ATGAGGAAGTAAAAGAAAGCACCTGCAACACTCCAGCTATGCAGACCACTCTGTAATGGGCTC
AGATCTGGACAGGTGTGTGGAAAGGTGGGTCAACAGGTCAGGCGTCACAGACTTGGAACATTC
ATGGTGGAAAAGAAAAAGCCCCAAAGAAGAGACTTCAGGATAAATGAGAAAATACTCAAGAC
AGCAAAAGTCTCTTTTAGAAATGTTGGAGAAAGAACACTTAATGTCAGGAGTTACTGTTGATTGA
TGGCCTTACTGTGTAGCAGGTGAGAAACCCATTGTTCAGTTCCCTAAAGTCACCCTATTCTCCCA
ATCATCCTATGGAGGGGGAACCATGATGGTTATCCCCATCTTATAAATAAAGCAACAGAGGCTT
AGAAGGACGAACTCTTTTTCTCAAGGTTACCCAGATCATTTTGCAGAAGTCCCTAGATTTGAATC
ATGCTCTTGCTTTGAGGTTAAAGACACAGGGGAAGTCGAACTCCTTATCTCCTATATCCTGAATA
GGGAAAACCAAACATTGTCAAGAGGAGAGGAAGCCTGAGAGTTGGCATGAATCAGAGTGCTG
GGCAGTCTGGAGTCTTCCCCACTGAGTTGATGATGGAACAGTAGAGTGGGGCCTGAGCCCCGT
TAGGGCATGAGCTGCTGAATGATTCATGTGAACACCATGCACATGGGAGTGAGGTTTTGAGCAG
TGTGCCACAGGAGCCTACCCTCAGGCCCCACCATAAAATGTAGGGCCAGTCCTACATTTTATCA
ATGACTTGCGTGAACACAGAAAATGTGGATACAACCAAAAGGTAACAATCCAATTAAAAAATG
GACAAAAAACTTGAATAAACATTTCTCAAAAGAAGATATACAAATGATCAAAAAGCATATGAAA
AAATGCTCAACATCGCTAATTGTAAGAGAAATGCAAATCAAAACTATAATGAGATACCACCTTA
TATTGATTAGGAAGACTGCTATAAAAATAGTAAACAAACAAACAAACAAAGTAAGTCTTGGGGA
GGATGCAGAGAAATTAAAATTTTTGTGCACTGTTAGTGGGAATGTAAAATGGTGCAGCTGTTAC
GGGAAACGGTATGACAGTTCCTCAAAAAATAAAA

ID|10688|gene_id|285194|transcript_id|NR_015391 Homo sapiens lncRNA

GGGGTACCAAAGTCCACTCTGAGCTTTTCCTCTGGGAACAGATCCCAGATTATTCCAGACT
TAAGACATGGAGATATATCTTGCTAGAGGTAGGAAGATGATAAGATCAGTTTCTATAGGTAGAG

GATAATGTGTCCTAAAATGTAGGTAGAGATAAGTCTGACAGTGGTTTAGACAAAAAGGGCCTCT
GCTAATGGTGGAGAGATCAAGGAAGAAAAGAGCAACGTTAAAGTGAGGCTGTACCCACAGATC
ATGCTCAAGCAAATTCGAACCGTGAGCGCATTTCTCTTAAACAATGAACACTTAGAATAAAAATT
AAAATTTCCCACAAATTTCTAATTTTAAAATAGTTTCCCAAGATTTCAGTGGTATTGTATATATGC
CCATATAATTTTTTGGATTACTATTCCCACAAGTGTAAGATGAAAGAGGACCTGCCCTCCATTCT
ATCTACTCCCTCTCTGCAAAGGCCATTTTGAAGATCTCAGTTTTACTGAAAGGGGTCTGCCACAC
ATAACTTCCCTGGTGTACCAGCAACAAGTGTTCAGTGGACCATGGCAACACAAAGAATGACGG
AGGAGAGAAAATGCCATGCAGAAGTGATAGTCAAGATGAAGATGTGGGAGAGGAGCAGCCCC
CAGTATATATTCAGTGTGTCCTCTGAAGTAACAACCATCCCATCTGGCTGAGAAGTGATGGACA
CGGCTCCTTAACCACACTGTACCCTCTGTTCTTGGCACCTCTCTACCAGAAAGATATCAACAAAT
TGGATGGAATTCAGAGAACAGTAAGAAAAATGATTAAAGAGATGGAGGCAGTGACTTATGAGG
AAAGATGAAAAGGACTAAATCTATGTAGCTTGGCAAAGCAACAACTGAGGTGGCGTATGAAAA
CTGTCTACAAGCATTTGAAGAATATAAACACCAAGGGAAGAGAGGGAACTTTTTAGAATGGTCT
GGGGAATAAGCAGAGTAAGAGGACAGAATTGAAGAAAGGAGAGAGATATGCTAAGTTGGGGG
TGGGGGCGTTTTCTCATCAATTGTAAATTTTGATAAATAACAAAATTAAGTCTGGAGAGAAAGAG
TTGCATCTCATGCTTTTAGGTTACAATCAGTGTTGACCTGCACTGAGAAAATGTGACTTCCTATTA
TCCTTCTCAAAGTTATTGCTGCAGAGGAAAGAAGCATACATCTTTTACCCACCAGGAAACCCCC
AAAGCATCTATTACCATAATAGCCATGGGAAACAGAAGGCACCTCAAATAAAGGTGGGGAAAA
GAATGAAAGAAATGGCTTTGGCCTGTGCCTGTTTGACCTCTGAGAGATACTTTTTGCAAGAAATT
GTTAAGTTTTGGCCCAAAAAGTGGTCGGTCTTTTATCCTTCCTTGTGGAGGCCAAACTGCAAACC
AGGATGAAGAAACCATCAGTGGCAGTTTGGGGAGGTGGAGGAGGGACTATCTAGACTCAGTGA
ATCACCAAGGAAACATGAGGCCTATTTCTCTGTGAAGCTGAACTTCAAAAAACAATCTAAGAGA
TACAAAGGGAAAAGTGTGACTGCTCTGACAGCAAAAACAAGCAAATATCTCCAGAGGGGATTA
ATCCTTCTGCTTCAAAGTAACTAAAACAAAGAAAGCTGAGCCAGCTTCACTGGAAACAACACCT
GTCCATCAGACAAGGATGAGCTAATCAAAAGAAAACACTGCCTATGTGCACGACTCAGCAGCC
TGGACAGCTAATACCAAGGAACACGTTTTCACTATAAAGATTCAGCTTTCAAAATCCAGTTCCCT
CTTGTAGAGCCACAGGGTTAAAAGGGACCTTAGAGATCATCTACATACAGGCCAAACCCTCAAT
GAATGCCAGTGTTCGCCTTATGTTCTTCTTGCCGGACTCTTTTCTGGCCTCTGTTCTGTCAGGATG
TTTCCTGTTTTGCAAAACAGCTTTAATCCTTGAGTATATTTAATTGTTAGAAAACAGTTCCTCAAA
TTTGACCCCAAATTTAAGTCTAATTTATGAAGAAAGTTTTGCCTTTGGAGACAGGTTTAATTTTAC
TTCCATGTGGAAAACCTTCAAATAAAATGATGACTATTGTGTACTCACGAAAAA
ID|14351|gene_id|399959|transcript_id|NR_024430 Homo sapiens lncRNA
GAAGTTTTCTCCTCCTGTCCTGTAACTGGTTTTCACTCACTCAACTGAGAGATTTCTTTGTTG
CAATGATGGGGTCTAAGATAACACTTCTGGAGGCTGCAGGCGAGAAACACAGCTGATATCATCT
TTTATTGTGTGTTGTTATTTGCCTAGCATTATAAATTAAGGAGGAATAGTAACAAAGAGCTTGAA
GACATGCACAGCTCACAGGCCCCGGGTGGAGGCTGGCGACATCAGACAGACAGAACCAAGAC
ATCTGAGGGGCAACCAGGAGGTGCGTGTGGCTGCAGAGCACACAGACTTGTCTTTGGACAAAA
TTGAGAAGAACTCATAAACTTGGCTTCCTCGCTTCTGCTCCTCAGACGCGCGTGGATGATTTGAA
GATATTAGCCTAACAAGATGGAAGCAGGTTTAAAATTTTACATAAGAAGTTACCTTTTTCAGTTG
TGTTGATGTTCCTTTCAGCTGGTCCTATAGTACCCCTCCTCAGGAATGTCTCCCCAGTGCAAGGA
CAAAGACTGAAGAGACTGCTATATTGATGGACTCTCAAGCCAACTATGAAGTTGAAACAAAGAA
AGTGATCACCTGAAGACACCTCCTCTGCTAAGAAACACCCCCAAATTGTGCAGCTTCTGCCACT
AGAACTCTCAGAACAAGAGACAATCTTTTCAAGAAACAGAAAAACTCAATAATGACATCTAGAT
TTTCATGAGCCAAGAACTTTCCCTTCCTCATGTGTATTCCTCTGTTTGTACTTAAATTCATGTGACA

TTCATTTTTTTCCTAGTATGGATATGCTTATTAATGCACTTGTTTCAAAATCCCAAATTGCACAAAT
GTGTTAATATTTTAAGAAACAAAATGAATCCTACAAGGAGAATGATTTTTAGCCACACATAGGGT
TGGATCTTGAGAGTGACCTACAGAATAAAAGTACTTTTAAAATAAAGTAGTCAGAGGCTATTCA
AAGGGTAAAATAATCATAGTACCACATTGGTCCACTTGACACTAACCAATCGATCATTTTTTTTTA
ATCAAGAAAGCTAGATTCTATCAGATAAAATCACTGCTTCTAAAGAGTTTAAATCTAGTTAGAAA
AAGTTATAGAAATGTTTGCAAAGATAAGTAACAGATAGAGTCAGTAGAGGATAAGATCAAAAAC
AAAACCAAGCAAAAGATGAGTTCAGGGGAGTTTGCCATCAAGTTGGCAAAACTGACTTACTTAG
GGAAGAAAGTTATAAAACAGGAAAATATGAGATGAACCTTGAGTGATGTGGAAGATTTAGATA
AATGGAAAGGAAGGAGAAAATGGAGTTCTTTAGGTGGTTGTAATTGGAGGAGGAAATGAATAC
ACACATCTTGTTGACTTAAACCCAGACATTCAGCAGCTCTCTATACATATCTGGAAAAGACTGCA
CAGTCACCTCCTGTCTCTCACCCCAGGTATTACTTAGAATTATTATCATATTTCCCTTCCTTTAAA
GTAAGTAAGGGTGATTGGTGACAATATGGAGAACTATGATTTTTCCATTAACCTAATAATAATTG
GTATTTATTGAGTTCTGTTAAGCATTTTACATATTAACTCACTTAAGCCTTTCAACAGCCTTGCAA
AATAGGTATTATTATCCCCATTTTACAGGCAAGAAAACTGAGGTTTAAGTAACTTGCCGAAGTGC
CATATACAGGGCTCACATTCAGTATTGCAGTTGCAAAGCTCATGATCTATAGTGCCAAGTTGCAA
TATTGTAGTCAATGTCACAATTATTACCCCTTTTTATATTCCTTGATATTTTTCCATGGCAAACAAT
TAGCTATTTCATTTAATAATCACCTAAAACTTTTCAGTCTTCTGATTAAAATTACGCTGGAGTGAT
AGAATGTATTTTCATGATAGAAATTGGGAAAAAAAAATGGGGAATGAAGTTTATCAGCATTTCAG
ACTTGTTTTTTTTTTTTTTTTTTGCAAGACTTTGATGAGATTGTTCACTTTTGTCTATGTAAAATCCC
AAATCCTTGAGAATAAAAAAGGGGGAGGTTTAAGTCACTTGTTGCAATGCCCTTTTTAATAGAG
GCAATAAATCTAAAGGCCATAAATTTAGAGTGACTTACAGAAGATCGAACTTTGGAGTGTGGCA
GAGTAAGGGATGGAAACCGGGCCCTCCAGTTCACTATCAGTAGCTTTTGCACTGGTCTGCCCTT
CCTAAATTAAGTATGCACTTCAATTTGATGAGTGGAAACAGTCTATCTGGGCAGTAACCAGGGA
GCTTTGTGCCTAGTAGATTGCTTCTGTTCTGCACTTCTTTGGTTTCCCACCTCAATGTAAAAAATA
GCTAGCAATGAAGTCCAGAAGTTGTCAATGGTTCATCCCCAGAAGAATGCATAATGTCCAAAGT
TGTATGTGTATGATGTCTTCAATGGTATTAAGTTATTTCAAATTCTTAGTTCACCTACATAAATCAT
TTCTAACAAGCATCTTCTTAACCAACTTTATGCACAGTGTATGTTTGTAAGTGCTTCTGCACGAAT
GTTTATACATGACTGTTTCCATAGTACTTATGTTTTTAAAAATATTCAGTCATTTCCTACTATAATC
CTCATGTATCCATGTAACTGACTCAAAAATACTTCAGCCACAGAAAGCTAAAACTGAGCAAATC
TCATTCTTCTTTTCCATCCCCTTTGCATGTGGCTGGCATTTAGTAATGATTAATAATATGGCCAGC
TGAATAACAGAGGTTTGAGACACAATTCTTTCTCAAAGGAGTCAGCTAAGCTGGGTCTACTTATG
GACAAACATCTAAATGTGTGGAAGTATCTGATATTTGACAATGGTAAATTTCCACTTAGCTAGCT
AGCATTGTCAGACTTCAATCTCCTCATGGCTCTGGCCGTCCTGTTTTAAGCATGATAATTGTTGG
CCACATCTCACATAGTTCTCATTGAGTGAGTTCATAAATAAACAGGGTTTTTTTTTTTTTTTAAAGA
GCAGCCAAGCACAAAGTGTGACTTTGTTGACATTTTATGTGACTTTGTCATATGTTCCTAACCCC
CAATAAAAGCAATGTTGCATCAACTGTGAA
ID|24169|gene_id|728655|transcript_id|NR_004855 Homo sapiens lncRNA
ATGGGGGTGGAACTCATGATGGAATTGGAGCCTTTACAAGGGAATGAAGAGACAAGAGCT
CTCTTTATGCCACGTGAGGATACAGCAAGGCCCCAATCTGCAAGCCAGGAAGAGTCGTCACGA
GAACCAGACCATGCAGGAACTCTGATCGTGGACATTTCAACCTCCAGAACTGTGATCCAAAATG
CATATGTATCTTTGGAAGAAACTCTGAAGTAAAGGCCGGAATATTCTTTGTTTAAAACATTAAAA
ACAAAACAGACCAAAGCATCAAGCAAGAAGTTTCCTGGCAATAAACTAAGCACAGCATTATTTT
TTAAGGAACACAAATTAAGTGTTCAACCTGTGGCAAATTTGTACTTTCTCCCTGAATTATGTTGTT
ATCAAAGAAAAAAATTGGGAAGCATGGCAAAATATCATCAAAACTGAAACTAGAATTAAACAA

AACTAAATTAAAATGAAATAAAATGATGTCCATTCTTAAAAAAAAAAAAAAAAAA
ID|26750|gene_id|8420|transcript_id|NR_002909 Homo sapiens lncRNA
GATTCTCTAACTGCGCATGCTTCTGCGCACGCGCAATAGACATTCCAGGACTTCCGGGCAC
TTCGTAAGGTTTAAAAAGGATGCTTCGCGTTTTCTCTCTCCTTTTTGGAGACAGATTCGCAGTGGT
CGCTTCTTCTCCTTGGATTTGTTAAGGATTCCAAGTAACTCTTATTTGGAGAGAAGACGATCTGC
ACTTCGCATTTTGGCATTGACATTTAATTTTAGGGTCCTTTATATAGAAGGGAGAGTAGGTAAAC
TGATTTTTTTTTTTAACAGGGAGGGTTTGACAATCTTTGGCAGACTTGGAGCAAAAGATTGAGGT
GCATTTCATGCCTCCTTTTGAGAGTCTTGCTCTGTCGCCCAGGCTGTAGTGCAGTGGCGCAATCT
TGGCTGCAACCTCAGCCTCCCAAGTAGCTGGGATTACAAACATAAGCCACCACGCCCAGCCCT
CATACCTCTTTTAAAAGTCGACCTGTTTTGCAGAAAGTCTGCTGTTTTTGTACTAAAGGCTTTGGA
ATTTGGCATTTAGCTAGGAATGCACATTCTTTCACCTCATTCATACTTTAAGAACCACAGAAGTG
ACTCTGCTTGGCCAGAAGGCACACTGTGTTGGTGGTTATATTAAAAGTCCTTGAGTATTTTGCTTT
TCATGATCTTGCTCACTGCAACTTCCGCCTCCCAGGTTCAGGCGATTCTCCTGCCTCAGCCTCCC
AAGTAGCTGCGACTACAGGCGTGTAGCACCACACCTGGCTAATTTTTGTATTTTTAGTAGAGATG
AGGTTTCACCATATTGGCCAGGCTGTTCTCAACTCCTGACCTCGTGATCCGCCCACCTCAGCCTC
CTAAAGTGCTGGGATTACAGCTGTGAGCCACCCTGCCCGGCCACTTTTGTATGATTTCTAATGTA
TTTGTAATTTACCTAACAAATTGCCTAATCTGCTATGTTAATGTATTTATGAATTAAAATAAATAC
GACTGCATGTTTGTGGTTCATTTTTGTGGAGGTGGCTGTGGTGACATCAGCCAAGAATCTGAATG
GTACTGTTGAAGGAAACTAGCATGATAGCTTCAGTTCTAAAGGCCCTGAAACCTAGTCTCAGGT
GGGTCCCCCTTGGGTTCACTTTATATTGGCAGTTTATTGGGAAAATGGATATTAGGTCCTGACCA
ATAGGACCGTAAGTCTGGGTTGAGTGCAAGATGAGTTAGACCGATTCTTTAGCTTCCTGCAGTG
TAGTGGAGGAAAAATCGATGGTAGCAACGGGAGGTTGTATCCCTAGCTGATGAGTTGTATGAG
CCTCTACTACCTGGCGCACCTCCGCCTGAAGATTGCCAGAATTGCTTGCCTCATGACGTGAGTC
ACAATGGAAACTTTGTCAAGCCCCCTGCACTGGCTGCCAACATAAATGTTCAGTACCCTGAAGG
ATGGGACTGAAGGGGGATCATCTAGAAGGTAAAGTTACCTACTGGCATAGGGGAGGTGGGACA
GCCGTTAAGCCATTTGGAACTTGATGGAGACAGGTTTGAGGGAGGTGGGTGAGATTGGAGTTTG
GTGGACTGTAGAGCTTGCTTGCCAAGGTGTTGAGGTCAGGGTTGGTTTGAGAATGGAAGCTAGT
TACTAGCTATGATTGTGGGGGAACACAGCTTGATTTTTCTTACAAGCTAAGAGGAGTGAGGCAG
TGTTTAAGAGGGCATGTTAAATGCAGCCAGGCTTGGTGGCTCACACCCGTAATCCCAGCACTTA
GGCTAAGGCAGGCGGATCACAACATCTAGAGATCCTGGCCAACGCGGTGAAACCCTGTCTGTA
CTAAAAATACAAAATAACTGGGCATGGTGGTGTGCACCTGTGGGAGGCTGAGGCAGAATTGCT
GGAACCCGGGAGATGGAGGTTGTACTGAGCTGAGACCTTGCCACTGCGCTCCAGCCTGGTGAC
AGAGTTAAGTCTCAAAAAAAAAGGCATCTTCCTAAAGCAATTGTATTTGTGCTTACCTGTGCCAGG
CACTGTTCTAGGTAAGCACTAAGTGGGCTTTAATACAGCATATTCCAATGGGGAATCCCAGGAA
CCAAAAGACTAATTGTCCAAGTCCACAACTAGAAGTGGCACCTCTGCAGAAACAAGCATCAAAT
TCCCTGCTCAGGAAGAAGCCAGATGAGTCAGCCCCATTCGTCTGTATGCCCAGTCCCATCCGTG
TCCTGCTGTAACTACATAGATCTCACCTGAGTAAAGTGATTTTTTTCTGAA
ID|26751|gene_id|8420|transcript_id|NR_036473 Homo sapiens lncRNA
GATTCTCTAACTGCGCATGCTTCTGCGCACGCGCAATAGACATTCCAGGACTTCCGGGCAC
TTCGTAAGGTTTAAAAAGGATGCTTCGCGTTTTCTCTCTCCTTTTTGGAGACAGATTCGCAGTGGT
CGCTTCTTCTCCTTGACGGAGTCGGTTTGTCACTCAGGCTGGACTGCAGTGCTCGTTGCAACCTC
CGCCTGCCGGGTTCAAGCGATTCTCGTGCCTCAGCCTCTCCAGCAGCTGGGATTACAGGATTTG
TTAAGGATTCCAAGTAACTCTTATTTGGAGAGAAGACGATCTGCACTTCGCATTTTGGCATTGAC
ATTTAATTTTAGGGTCCTTTATATAGAAGGGAGAGTAGGTAAACTGATTTTTTTTTTTAACAGGGA

GGGTTTGACAATCTTTGGCAGACTTGGAGCAAAAGATTGAGGTGCATTTCATGCCTCCTTTTGAG
AGTCTTGCTCTGTCGCCCAGGCTGTAGTGCAGTGGCGCAATCTTGGCTGCAACCTCAGCCTCCC
AAGTAGCTGGGATTACAAACATAAGCCACCACGCCCAGCCCTCATACCTCTTTTAAAAGTCGAC
CTGTTTTGCAGAAAGTCTGCTGTTTTTGTACTAAAGGCTTTGGAATTTGGCATTTAGCTAGGAATG
CACATTCTTTCACCTCATTCATACTTTAAGAACCACAGAAGTGACTCTGCTTGGCCAGAAGGCAC
ACTGTGTTGGTGGTTATATTAAAAGTCCTTGAGTATTTTGCTTTTCATGATCTTGCTCACTGCAACT
TCCGCCTCCCAGGTTCAGGCGATTCTCCTGCCTCAGCCTCCCAAGTAGCTGCGACTACAGGCGT
GTAGCACCACACCTGGCTAATTTTTGTATTTTTAGTAGAGATGAGGTTTCACCATATTGGCCAGG
CTGTTCTCAACTCCTGACCTCGTGATCCGCCCACCTCAGCCTCCTAAAGTGCTGGGATTACAGCT
GTGAGCCACCCTGCCCGGCCACTTTTGTATGATTTCTAATGTATTTGTAATTTACCTAACAAATTG
CCTAATCTGCTATGTTAATGTATTTATGAATTAAAATAAATACGACTGCATGTTTGTGGTTCATTTT
TGTGGAGGTGGCTGTGGTGACATCAGCCAAGAATCTGAATGGTACTGTTGAAGGAAACTAGCAT
GATAGCTTCAGTTCTAAAGGCCCTGAAACCTAGTCTCAGGTGGGTCCCCCTTGGGTTCACTTTAT
ATTGGCAGTTTATTGGGAAAATGGATATTAGGTCCTGACCAATAGGACCGTAAGTCTGGGTTGA
GTGCAAGATGAGTTAGACCGATTCTTTAGCTTCCTGCAGTGTAGTGGAGGAAAAATCGATGGTA
GCAACGGGAGGTTGTATCCCTAGCTGATGAGTTGTATGAGCCTCTACTACCTGGCGCACCTCCG
CCTGAAGATTGCCAGAATTGCTTGCCTCATGACGTGAGTCACAATGGAAACTTTGTCAAGCCCC
CTGCACTGGCTGCCAACATAAATGTTCAGTACCCTGAAGGATGGGACTGAAGGGGGATCATCT
AGAAGGTAAAGTTACCTACTGGCATAGGGGAGGTGGGACAGCCGTTAAGCCATTTGGAACTTG
ATGGAGACAGGTTTGAGGGAGGTGGGTGAGATTGGAGTTTGGTGGACTGTAGAGCTTGCTTGC
CAAGGTGTTGAGGTCAGGGTTGGTTTGAGAATGGAAGCTAGTTACTAGCTATGATTGTGGGGGA
ACACAGCTTGATTTTTCTTACAAGCTAAGAGGAGTGAGGCAGTGTTTAAGAGGGCATGTTAAAT
GCAGCCAGGCTTGGTGGCTCACACCCGTAATCCCAGCACTTAGGCTAAGGCAGGCGGATCACA
ACATCTAGAGATCCTGGCCAACGCGGTGAAACCCTGTCTGTACTAAAAATACAAAATAACTGGG
CATGGTGGTGTGCACCTGTGGGAGGCTGAGGCAGAATTGCTGGAACCCGGGAGATGGAGGTTG
TACTGAGCTGAGACCTTGCCACTGCGCTCCAGCCTGGTGACAGAGTTAAGTCTCAAAAAAAAG
GCATCTTCCTAAAGCAATTGTATTTGTGCTTACCTGTGCCAGGCACTGTTCTAGGTAAGCACTAA
GTGGGCTTTAATACAGCATATTCCAATGGGGAATCCCAGGAACCAAAAGACTAATTGTCCAAGT
CCACAACTAGAAGTGGCACCTCTGCAGAAACAAGCATCAAATTCCCTGCTCAGGAAGAAGCCA
GATGAGTCAGCCCCATTCGTCTGTATGCCCAGTCCCATCCGTGTCCTGCTGTAACTACATAGATC
TCACCTGAGTAAAGTGATTTTTTTCTGAA

ID|55921|gene_id|ENSG00000226308|transcript_id|ENST00000412321 Homo sapiens lncRNA

GTTTACTGAGTGCCCACGGGGTGCCTAGAGCCTGGCGGGACGTCGCGGGGATGCGGCTG
GAGGAATCGGAGCCGGCCCTGCCCTCTCGGCACTCGCGGTCCGGACCAGGAAGGCGGCCCGA
GCAGGACGGGTGGGCTGCGGACCGCGGTGGGAAAAGTTGGGGGAGCGCGGAGGTGGCGCGG
AAATAGAAGGCTTCCTGGAAGCGGTGACCCTTGAGCCGGTCTTGCCCCTCAGTCCCTGTATCCT
TTTAAGCCAGCCACGCTGACCCCTCCGCGCCTCCCTGAAGCCGTTGCCAGCCTGTCTCTGCGCT
CGCCATTCCCTCCGCCTGGACCTCTCCCCACTCACCACCTCCCGCCCCCATCAGACGCTGCTTT
CTCCTCCCAGAGACCTCCAAGACCTGCCAAGCTGAGGTTGTTTCCTCGCCTCGCCCCTCGCACG
TTTGTTTTCTTTCCTGTAGTTTCTCTTGCTGTCTGGAATTGCTGTTTGAGTTACTCCGTGCCTTCTTT
ATTGCCTTCTTACCCCCGCTAGACACCCAGAGCACTGGGGCCCGGATCAGCGATCTTCTCCGCA
CCTCTGGTACCCGCAGTGCCTTGAACCCAGCGCCTGGTAGCCACTGTAGCTGCGCAGTCACGAT
TTGTTGAGTGAATAAGTGAACTGCGTGTGAGCTGGGGGGGTTCTTCCCACCTCCTCCTCTTCGG

GGCCCTGTATCTTTCCAGCCACCCCTCAGCCTGAGACCAGACACCCCAGCCCCACCACTAGGG
CTTCCCATCAGCCCTCCTCGTGGCAGTGCCTGGGGAGCCAGCCGCTGGTCTTCACATCCTCTTC
CCCTCCAAGGGTCTGTGCCTGAGACCTCCCCATCGGCCTCCTCTCCTGTCTCTCCTCATCCATTC
ATCCCTCAATCAATACCGAGCCATGCACGCTGTCACAGGGAGTCCCCAGACCACTCAGAACCC
ACCGGTCCCTCTGCCGGTGTGGGATCTCAAGGGAATTACTGCACCCAGAGTGGCAGTGAGGGC
AGCGGGGTAAAAGTTCATCCTGTCTCCTTCCCCTCCTGGGGCTGGGTGAGAGAGTGGGTCCCCA
GAGGGCCTTTGGGATGAACTGGGAAGGGCTGAGGTTGGGGAAGCGGGGGCGCTTGTCTGGGA
GGGGCTCTGGCTAAGCCTCACTTTGGGGCTGTCCTTCCTGGAGCAGGAGAGCTGGGGAAGAGA
ATTATGGTCTTCTACAGTTTTCTGGAGCAGAAGTAGTTTGGCCAAGTGGCCCTCTGGGTCTCCCT
TGGGGACTTTTGGACTAGATGGCAGCCTGAGGGGCCACATGGGTGGAGAAAGGGGAGGCCAG
AGGCCAGGCAAGGAGCAGAAAAATGCCAGCCTGCAGGTCCTGGCCATTTACACACAGGCCCC
AGGCTGTGGGATGGAACAGGTCTTGAGCCCCATGCCAGGGAGGGGCCTCTACCCACCCACCAG
GCAAGGCCCTCAAACCACCCCCACCACCTGCTCTTCATCCATTCACTCCTTCCTTCAACAGACAA
GCATGGAGCACAGGATATCAGGAGGCGAGCTGTGGGGCCTGCAACCTGGACAGTCGTACAGG
GCCCCCCAGCTCTTAAGGGCCCCACACTTGGTTTCCTACTCTGAGGTCACCATATGTGGTTCTGA
ATCACCTATGAACAAAGTACCCCCTGCCCCGTACTAATTTTGCACCTGGCCCAGATATTATGTGG
CCAGTCCCAGGGCCAAGCACCATGCTGGGCAGGAAGACATGGCCATGAGCGAACCCACATGG
CAGACTCCATCAAGGAGCCGGCCTCCAGGCCAGGGAAGCAAAGCAAGAGCCTTAAATAAGATT
CAGGGACTGAAGGAGAGGCAGATCTTGTCTATCACTCTCTCTGGAAACAATTCAGAAGCCAGG
CAGAGGAGGACATGCAGGAGGGAGAAGGAAAGGTGGGCATCCTGGCAAGCAGGAATCCTACC
CCAGGGAGACTGGGGAAATTGCAGGAGTGCCACAGTCGTCCAGGGTCCATCTGAGAGTGTACA
ACTGGAGAGCCTGGGTCACTTCAGCCTGCAATAAGTGACAGGCAGTGGGGGCGGGGGCGCAG
ATAAACAGGGTGAGGTGATGGAGGGTGGGAAGGGGAGTTTTTGGAGGAGATGCCACTGACCT
GAGTCCTGAAAGACAAGAAAGAGACAGGCTTTTCCAGGGAAAGGCATCCCAGGTGGAGGAAC
AGCAAGTGCTTGAGCCCTGCAGAGCAGGGAACAGAAACCAGGCTCGGTCCCCAGCCCTGCAC
ACGCCCTCCAGCGCTGGCTTGCTAGGGACAGGCTATGCCACCTCCAGTGCCAGTAGAGAAAAA
GGAGGTCATTTTCTTCCAAAGTCTGAAATAATAAGGAGCCAAAGCAGCCCTGTTCTTCAGTAGT
AGTCTCTGCTTTTTATCTACTCTCAAATCTTTTGGAGGGATCTTCACAAGCAGGTGAGGCTGGGG
GCAGGGGCAGCAGGTCTTGGTGCTACAATTTACATATGAAACAACTGATCCGTCAGTGTCTAAG
CAAGGATTCTAGTTTACCCTTCTTTGTGCATCTCACCTGAGCCTATTCTGAGCCTGGCACACAGC
AGGTGCTCAATTAGTACCTGTTGAATGAATAGTAATAAGCACAGCCAAAACCTAAAGAGACCGG
TTGACTGAGGGCTGACTGTGCAGGCGCCAAGCTAAGGCATCTCCCAGATCCCTCACCCCAGCA
ACCCTTCCTCTGAGACGCCATCTTTTCTCATGCAAGAGGAAGAGACAACATGCTCTTCATATCCA
AGACCTCAAGAGATAAGGTTTCCTCTGAAGTCCCACAAACCCCCATTTAGCCTTATCTTTTCTGT
GATCCATGCTTGGTGGGGGGGTCTTTCTTTTCCCTGTTTCCTCCCCGAGAGGCATCCCCAAGACT
GCACCAGGCCCTCAGTGAATGCCGAATGAATGAATGAATGAATGAATGAATGAATGAATGAAA
ACCGGCAAGCAAGAAATATCTGGCTGGGAGTGCATCCGATGCCAGTTTGGGAGCTGAGCCCAC
CACCAGAAAAGGCTTTGAGATTGCCGAGGGCAAGTCTTGTGTGGAGGAGGTAGCCTGGGCACT
GGAGCCTGCCCAGCCGCATTCACACTCCAGCTCTGTCTCTTCCAGCTGTGCACCATGGGCAAGG
GACTTAACCTCTCTACACCTCAGTTCCCCCATCTGTAAAATGGGGTGACTGATAGCGCCTGTCTT
CTGGGATCGCTGTGAGGTTGCAGAACTTAATTCATGGAAAGGGCTTTGCACAGTGCCTGGAACA
CAGAGAAAACCCTTGATAAATGTTATTGTGTTACCATCTGGATGAAAAGGAAAATATGGTAACA
GGAAAGCCTCCTGAGTTACAAAATAACAACAAAACTTTTCATGATGGTCTGCTTTTTGGCCAAGC
AGAGAAAAGCAGATGTATCCTGACTCTGAGAGCAAGAGCAAAAGTACCAGAGCAAAAGAGAG

AGTGAAGTGGAAGGGAACATGAGAGATGAAAAGGTCATCACTGCATTCCAATCCTCACATTGT
GCCTAGGCCCCCTGCAGGCCAGCTGGGGCCCAGGGACTTCCCTGGGTCCTATCTCATTTCCCCA
GCAGCTAGCCCCCAACCCACACACCCCCAGTTGTGGTTCCCCTGGCCAGGGCACCCAGCACTG
GGCTCCAGGACCGTAATCTGATCTGCCCCAGTCTGCTCCACACCTGGGGCCGCCTTTGCCTGGA
TGAAAGACGGCCTGGAATTGTAAGGGGTGTTATTGTGTCGGGTGGCCCGGTCCCAGTGTCCGC
GCCCCTCGCCTGCTGGCCTCCCAGTGCTGGGCCAGGAGTTTTCATTTCCTACATCATCAGTCACA
TCTTGCTGAAGTGATTTTGACCAAGTCAGCATGTCTCTCTAAGGCAGTAAAAAGAAGAAAAGAA
TTCTGCTGACACACACATTGTTCAGCGAGGTGGGGACCTCATCTTCATCCATCTGCCCTAGAGG
AGGCTCAGCTGGCCTGGAAGCTCCCTGGGGAGCACAGCCAAGGAAGCCAGTGGGCTGCTCTCT
CTCTGTCCACACCACCTGGCAAAGGCCAGATGACCTGGACCTCCAACCCCAACAAGGAATAGA
GGGCCAAGAGCTGCCTCCTGCACAGGCTTGAAAATCGTTGTGTGCAGAGGTGGACCCTGCAGA
GGAAATTATTAGAGCACACCTGTGGTGAAATTTTGTTTCCCACGCAGATCTCTCATTGCACGGGA
GGCTTGGACTCCTCACGCTTGCCCACTCTGAGATTTTCTTTGCACAACTTTAAAAGCAGCAGAAT
AAGAGATTAAACGTCTTCCATCAGTCTTAAGTACACATGGCAGTGGCCACAGTTCCAAATGGAA
AATACAGACGACACACACACTCTAGAGAAAGCTGATACCATGCAGGATTTCTAGGATCTGGAA
GCCAGCTAGGGGGAAGCAGTGGTTTTGTTTATTATTATCAGGGCTGTTATTTCTTCCTTGATTGGA
GGAAATCTGGTATGGTTTTGAGCACTAACTATCCTCGGTTCAGCACTAGAGGTAGCCCTTTCCTC
CTTCCAAAACGTGACAGGGGAGCGGCCGACCTGTCACCTGTTGGGAGCACTTGCTTCTGCTGG
GCACCTGCTAGGCCTGCACGGCCCAAGCCCACATCACCCGCAACTCCCCACTGCATGGAGAGG
CTAGTTCTGCTCCTCAGATTGCAGAGGAGGACCAACATCCCGAGAGCACCTTGTCCAGAGTCAG
ACAGCCAATCTAATCTGTAATGGAGCCAGGATCCAAAGCCACATCTGTCTACACAGCCCAGGC
CTTTAAGCATCAGTGCACGTTCAGTGACCTGGGAGCAGCCCCTTCCTTCCTTGCTTTAACCTAAA
GCAACACTTCTCCAACCCAACTGTGCGTGTGAATCCCCTGGGGGTCTGTTAGAACATAGATTCC
CACAGTCAGTCTGAGTGAGCTGAGATTCTGCACTTCTAACTAGCTCCTGGGGGCTCGGAGGCTG
TCGTCTTCAGGCTGTATGTTGACGATCGAGGCTCTAGATTTCTGAAGTTATAGCTGACAAATTGG
GGCTTCAAATTAAAACTTCAATATCTATGATTCCAGGTGTCTTTTAAAATTAACTCATTAATTCTC
CCAACTACCTTGTATGATTTTTATTATTATTATCCCCATTTTACAGATGAGGAAATTGAGGCTCAA
ATACTTGAGGGAACCTGCCCAAAGCCACACCAATGGTGAGTGAATGGTAAAATCAGAATTCTG
ACGCCGGCTATCTTGTTCCAGGACTTAAGTCCTTAAACATTGAGGTACACCAAAACACAGGGTT
TGTCTTAGTCCTGTAGGGACCTCCCAGTACCCATGGAGGCCTTGCACACAGTAGATGCCCTATC
AGTGTTTGAGGAACATACCTCTGAGCCCTTCTTCAATTCCTCCTTTATTGCAGAAGTCATACACTC
AAAAGCCAACGGGCACCAACCAGGTAACATAAATACAGAGATGCAACATGAAGCTCAAGGAA
TATTTCTCTTCCTCTTAATTTAATGGAAGGCAATCATGAGCACAGACATGGTGACAGGTGGCAG
GAGACACTGGCCTGGATAAATAAGGAGGGTGCAGACTGGGGTGAACTGGAAACCACATGCCC
ATGGGTGATGCCATCCAGAAATTTGATTCCAACATCACCAGAAAGTTCTGATTTTCCAAAGAAG
CTAGAAACTTGAGTTTGAATGTGACATCTCTCAATGTTTATCTGTTGGCAACTAATTCAACTCGTT
TTGGTTATTAGACCAAAGAAGACACATCTACCAGTCAGATTCGATGAATCTGGTTTGCCCCTGGC
CTCATAAAGGGCTCTGCTGTGTCTGGAGGATGTGCCCAGCCTCCCCCAACCCATCACCCAAACC
AAAGGACCACCAAGGATGGTGCATTTCAAGACAGCTCCATTGAATTCATGATTCGAGGGCTACT
GAGCCAGTGGTTGAAAAGGCTGGAACTGTCTGCCCCTTTAGGCAAAATCTGACCCTAACCTTCC
CCAGAGTCCCTTCCCAGCAAAACTCTGCTGTTTGGCCCTGGTGACTGATCTGATAAACTCCCCCT
CTCCCCAGAAACATCAAAAACCAGAATTCCATCTGCAAGAGCAGGAGTGAGTGTTCATTGCTCC
TGCTTTTCAGCATCTCCCTCCTCCCAGCTAGATCTGAGCCCTCTCTTATGGTTGAGGATGCCCCA
TCTGGTGAATATCCAGAGAGGCACTGTCTAGCCTGCTTTGCAGCCAGAGCCCTGACAGGTGACC

AAGTTTCAGCCAGTCAGAGGCACCCACCCCAGAAGCTATCAGTGCAGGCCCAGACTATTCTGC
AGAGTGATCTGGGGCCGTGTCCCAACTAGAGAGCCCCCAAGCCTGGTTCTCTGGCCTTCCCAG
GGGTTCTGTGAGCCCCCAAGATCCTCTAGTGGTCTCCTTTTCCGCACCAGCCTGCCCAAGCTGG
TTTCTGTTGCCTGCAGCCAGAAGCCCTGCTGGATACAGCACTGGGGAGCTGGAGAGGGCATGG
CAATCATGTCAGTTAAAAGGATACAGGCCTTCTCTTTTGTGCAGCGCTAAAACTTTTGGTCAAGG
AGTTTTTCACACATGCTCGACACAGAAAACAGAAACAGAGCTTCTTTGGTTACATAGAGCAGGG
GAGGGACTAGGGTTACCACATTTAGCAAAAAAAAAAAATAGGTGTCCAGTTCAATTTGAATTTCA
GATAAACAATGAATGAATTTTAGTATGACTATATCCCATTATATTGGAGTATATGTATTATATATA
CTCATACTGAAATTTACTCGTTGTTTATCTGAAGTTCAAATCGGACTGGACACCCTGCCTGTTATC
AGGTGATCCCAGCAGGGGCCCAGAGTCCCCAGGCCTGTTTCTTTTCACTACCATTTTCTCTGCTC
CCAGGGTTCCACAGAAAACACTTTGGAAGCTCACCTAATTTGAGACCCCTGCCTTTTCTCCTTCT
TCTGCAAATATTTCCTCTGTTTTCTTTTCTTTTCTTTTTTTTTTTAGACAGAGTCTCCCTCTGTCACC
CAGGCTGGAGTGCAATGGCACAATCTCAGCTCACTGTAACCTCTGCCTCCCGGTTTCAAGCAAT
TCTCCCTCCTCAGCCCCCCGAGTAGCTGGAATTACAGGTGTGCACCACCATGCCTGGCTAATTTT
TGTATTTTTAGTAGAAGACAGGGTTACACCACGCTGGCCAGACTGGTCTTGAACTCCGGGCCTC
AAACAATCCATCCATCTTGGCCTCTCAAAGTGCTGGGATTACAGGCATAAGCCACTGTGCCTGG
CCAAGATTTCCTCTGTTTTCCTCAGTAGAAATGCCCCTTCTTCACCATCAGTCCCTGTATTTCAGG
TTGAACTATCCTCTTCTCTAAAATTCAGACTGGGCACATGACCCAGCCTGGTCAACCAGAGATG
CCCATGCGATGGGTTCAGTGACAGTCATGTGACCTAATCCAGGCCAGTGAGATTCAACTTTTAG
AACTTTTGCTAGAACTACCGGTAAAGGGGTCTCTCCTCCCACTGGGGTTGCTAGGTCAGGGGCT
GCTGCTGGCTGTCATCTTCCCTCCCTTCAGGGAGAGCCTGCCCTGTGAGAGAAGCCACAAGACA
GTAAAACAAGAAAAGGCAGGAGACTGACAGCATCAGTGAGCCCCTGGAGCCCACTATCCCTGA
AGCCAGCCCCACCCAGGACTGTTCAGTTTAAGCCATTGTGTACGGAGTTTCCAGTCAGTTACAA
CAAAAGTCCTGCAGAAGCGTGGTTCAATTCCTTCATCTTGAAGATAGAGAAATTGACACCCAAA
GAGAAACTGCTGGGCCCAAAGTCTTACAGTGAATCAGGGATTAGACTGGGCTCCTCGCTCCCA
GAGTCCTGGGAACTCCTCACCACCCCACGTCGTGCTTCTCTCCATCTCTGTTGTTTTCTTCCTTCT
CTCCTCTCCTTCACTGTGTTTCTCCTCATTTAAAAAATTGTATTCTTTCTTTTACTTCGAGTTTTATC
TTTATTCCCTCCCCATTGGTAAACCATTACTCTAAATATAACAGGTTAGTCCCAACTCATGTGAAA
AGATCGTATGGGAAGATTAACAGATTTGAGCTCATCTTACATATGATGCAACAACTAATAAGAG
TTAGCATGAATGACACAGCTATCTGATGTCCAAGCTTTGCTGATTAGCTCTCGTCTTCTAATCGC
CCTGAAATTGGATGCAGTATCTCCCATTTCACAGATGAAGATCTGGAGGCCCAGGGGTCAAAG
GACTTGGCCAAGAAGCATGACTAACAAACGACAAAGCCAGCATTCAAGCCTGGATGACCCAAT
TCTGATCCCCTATTTCACCCAGCCAGTTGCCCCTGTAAAGCCAGCCTGAGATTTTTATTTCCAGA
GCTCACATGATAACAAACCACTGAGTTGAGTTGCCAGATGTGGCAAATAAAAAATATAGGCTAT
CCAGTTAAATCTGAATTTTAGATAAACAATGACTAATTTTTTTAGAATAAATATGTCCCAAATTGC
AAGGGACATACTTACACTAACAATGAGCCTTTGTTTATCTAAAATGCAAACTTAACCTTTCACGG
TGTATTCTGTTTGGCAATTCTACACTAAGGGGCTTTTACGCATAAAGCAAGAAGCATCAGCTGTG
TCCTTGGTTCTGCAAAAAGCCTCAGGGCCTTTGCACTTGCTGTGCCATTTGCCTGGAAGGCTCAT
ACTGATATTCACCTCCTTCACCTCCTCTGGGTCTCTGCTCAAATATTTCACCATTAAAGCTTTCCC
TTGCCCGTCTATCAGAAGCAACCCATGTTTCTTTTCCTCTTAACACCACCACCTGAAATACAATA
GTTACATGTTTATTTGTTCATTGTCTCTCTCCTGCCATGGAAATGTGGGCATGTGTTTTGGTCTGAT
TTGTTCACTACCGTGAATAGAGCCCAGAGCGGTGTGCCGTATGTAGTTGATACTCAATGAATCTT
TGCTGAATGAGTGAATGAACTCTCTGTGAGCACATCTTGGTTTCTGTCTGGAATACTGCCTGACT
TGAGATTGTCACTCACCTGCTGAGTGGCAGTGGGCATGCCCCTGAGCTCCCCACTCCTTGAGCT

GGACTCATCACACATCTCAGAGTCACTGGGAGATCAGGTGACGAAACAGCCTTGAGAGTCAGA
TGCAAACTTGCCGGAGCACGTATGCAAGCATTCCAGTGTGCTTTTCTCTTCTCTTCTCCTTCAAGC
ACTCGTGCCCCAGACAACTGAGCCTCGGAATACAAACCCTAAAAAACAATTTTTTCCAGTGTTC
AGAGCATATGCAAGCTCCTCCTACCCCTGGCATCACTGCAGACAGTGGCATCAAGCACTTCCAA
GTCGGAGGGAAGGACAGCACACCAACAGACATCAGTACCAACGACGGCTAATAGTCTAGACG
TCTGGTCCAGTTATCTGCATCACCTCCTTTCATCCTCACAACTCTTTTGAAATAGATATTTTTGTGC
CCATTTTACAGATGAGGAAATTGAGGCTCAGATTAATCAGTTCTACTGCCAGAAAGCAATGGAA
CCATGTTTTAATTCAGCCAGCCTGATAGCAGTCTCTGAGCCCATTTCCTCACCTGTAAAGTAGGT
ATAATTATAGTGCTACCTGGCCAGTCACAGTGGCTCACGCCTATAATCTCAGCACTTTGGGAGG
CCAAGGTGGGTGGATCACCTGAGGTCAGAAGTTCAAGACCACCCTGACCACTATGGTGAAACC
CGGTCTCTACTAAAAAATACAAAATTAGCTGGGTGTGGTGGTGCATGCCTGTAATCCCAGCTAC
TCGGGAGGCTGAGGCAGGAGAATCACTTGAACCCTGGAGGAGGAGGTTGCAGTGAGCTGAGA
TTGTGCCATTGCAGTCTAGCCTGGGCAACAAGAGCAAAACTCCATCTCAGATATAAATAGATAA
ATAAATAACTAAATGTGCTGCCTTGTTGGGATGTCGTGGGGTTAAAATGAGACATTGTGTGTGAA
GTATTTGCATGGGTCCAGGCAGGCTGCACACACAGGTGGCCACCACAGGCAACCTTGCCCTCC
CCCCAGCCAGAAATAATCAGGATAGAGGATGTCATGTGAAGGCCATTTTCCAAGCTGCCCAGA
GTGACTGATGACCTAGCTTGAGGCAAGACAACTATCCAGAGTTATCAGATTCACTTCCTTGGGT
ATTTGAAATAGGAAATCCAGAGAAAACGAGGTTACAGGAATGAGGCCAGGTCAAGAGGTAGA
AAGTGTGGCCATGATGGGTGGTGAGCAAGCTGGCCTTATAAAGGAGCTCAGGCCACTGGGGTC
CAGTAGTGTATGTTGTACACTGTGCACTCTGGGATCAACATTCCTATTCTTGCCTTTGAGAACGG
TGACCTCTGGAGTTGTGCAGTACACACTCTCAGCAACTGTACCCAGCAGCTCTGAAATTTTATTC
ATTCATTGATTCATTCACATATATATTAATTTATTCCCACCATGTGACAGTCTCTTTCAGACCATGA
GACGGCAGCAGTGAAAAGATATTCAAGACTTTTCACACAGGTATTTTAGTTAGGGGAGATGGAC
GAGAAGCAGGAGTGACTGACAAGTGCATGGAGAAAAAGAAAGCCCAGCGAGAGGCTGAGAG
CAGAGGGTGGGAAGGCCTCTCTGAGGAGGGGAGGTCTCCAAAGCCAGGCCTAGGAGGAGCAG
GGGGAGGGGACTGTGGGTAGAGGCAACGGTGGGTGCAAAGGCCCTGTGGTGACCCACAAGAG
CATCTGGGATGCTTGGCTCCAGAAGCCCCTCCTAACTCCGGCTTTTCCTGGAGTCGATGTTTCCC
TGTGCCCAGGTCCCCAGACACAAGTGTCTTTATGATCCCACCACATTCCTTGGGGTGGCTCGAG
TCAGTGCCTCTCACAGCCCAAAGAACTCTGACCCAACTTCCAGCTGGGGCCCCTGTCCCCGAAC
AGAGTCTCCAGACATGGGCATTGCTGGCCCTGGTCTGTTCTCCTCTTCCAAGTCTCAGTGACCCT
CCTCAGCCAGACTTCTGCCCCACCCATCTCTGCTTCCAACAAGCTCCCAGTGAGCTGCCTGCAT
CTGCAAAGCCGGGAAACATGATGTTCCCAGACATCAAAGAGTTTGCAAAAGATTGTGGTCACCT
TAGACCCAGACCATTTCCTGAGGCTGCTGTTTAGATACCAAGTCCAGGGTTTTTTCTTTAGGACT
CCAGTGAAGGGTAGAAGCTGTCCTACACCTCAAGCCACCACCACCTCCCTTCTCTGGACCCCAC
CGTAATCCATGGCTCCTTACAACCTCCACATCTCTGGCACTGGAGCCCTGATTTTGTTTTTATGG
GGTAATATTCTTTCATCCCCAAATGCCATGTAGGAATTTGCTGGCGGGGTGGGGTGGGAACCAG
AGAAACAAAAGGATGATTTACAGGGGTTCAAAAGCCTCATTATAACCAGAAAATTCAATTTACA
GACATTTGGTTCTTTTATTTCCATTTTTTTAAGGAGAGAAAAGAAATCAATCTAGTGTGGGCATTT
TGAGTCCCTTGCTTAGAGGTTCAAGGCATCTGTGACTTCAGAAAGCCTTCTTGGCATGAAGCAAT
AAACAGGCTGAGCAGATAAAACCACCCTGTGTTTAAAAAGCAGGCCTGTCTCCGAGGCAGAAT
TTTTATAGATAAAACAACAGAACGTGACTCAAAAAAGTCGTGTTGTGAAAATGGCCCCATGTAG
ACACAAAATTCTTTACTCAGGAGCTAAGTTCTACAGCAGCACAGGAAGGTTTCATCTAGATAATT
CTCTACGTCTGGCTTCAAGGAGCCACTCCAAAGCCCCGTTTTCCAGACGAGTTTCGTGTTGCAA
ATATCACCTCCATGCCTTCAATGTTTCACTGCATGGAGGACCCTCTCTCTTTCTCCGGTTCACCAA

AATGTTTGCACATGTTGTGAGGGAAAATGACTTTCTGTCAAGTAAGTTTAAGAAACGATGGGCTC
CAGAAAGGGAAACATGTTTCTTCACTGCAGGACTTCTCAATTTTTTTTTTTTTTTTTTTTTTTTTTGA
GACGGAGTCTTGCTCTGTCACCAGGCTGGAGTGTAGTGGCGCAATCTCAGCTCACTGCACCCTC
CGCCTCCTGAGTTCAAGCAATTCTCCTGCCTCAGCCTCCCAAGTAGCTGGGACTACAGGCACGC
ACCACTACGCCTAGCTAATTTTTGTATTTTTAGTAGAGATGGGTTTTCACCATGTTGGCCAGGAT
GGTCTTGATCTCTTGACCTTGTGATCCACCCGCCTCAGCCTCCCAAAGTGCTGGAATTACATTCT
CTGTCTTTAATTAGGGAGGAGAGGGTAGAGTGTTTCACAAGCTGTTATATTAAGTCAGAGATCTA
TCATGTGCAAGTGTCAGAAACCCAACTCAAAACGGCTCAATGCCCAACTCAGAGTGGTTTCCCT
AAGGGGGAAGTCCTATCAACGCTGTGCCCAGGGGCTCATGGAGTGACCTCTGTCTCTGGGGCT
CCATTTCTTTCTGTCTCCATCTTTCAACTCGGCATCTATCTGAATGCTGGCTTCATTATTTCCTACC
ACAGAGAGGTTTCCTCTGCATTGCCATATGTCAAGGCCAGCAAGCGACCCTGGGAAGAGAGAG
AGAGAGCCTCTTGCTCCCAGGAGAGAATGTCCAGGTCTTGGCTTACCTTGCGCACCAGTAATGG
CAAACGGGGAAGGGAGCACCTGGATTTCCCAGCCTTGTCTGCTCACCTCTGAGTCAGGGTGGA
GGTCAGGGCACTGGGTCAGCCCCACCAGAGACCCATATGGTAGGGCAGGCACCATTTCCCCAA
AGCAGGGGGACGCCATCACCTGAATCCAGGAAGGGGCACTGGGTCAGTAAAAAGGAAACCAA
ATTTTCCTGGGCATGGTGGCTCACACCTGTAACCCCAGCACTTTGGGAGGCAGAGGGGGGCAG
ATCACCTGAGGTCAGGAATTCAAGACCAGCCTGGCCAACATGGCGAAACCCCATCTCTACTAA
AAATACACAAATTAGCTGGGTGTGGTGGTGGGCCCCTGTAATCCCAGCTACTCAGAAGGCTGA
AGCAGGAGAATCACTTGAACCCTGGAGGCAGTGGTTGCAGTGAGCCGAGATGGCGCCATTGCA
CTCCAGCCTGGGCGACAAGAGCAAAATGCAATCTCAAAAAAAGGAAAGAATGAAAGAACGAA
AGAAAGAAAGAAAGGGAAAGAGAAGAGGAGAGAGAAAAGAACAGAAAAGAAAAGAAAATTT
GATCAAAAACCCCCCTTGATGGCCAAGCACCCAGCTGAACTGGGGTTCTGTAGACAGAGGCCC
TGGGGAGCAGATGCTTACCCCGGACAACGACCTCTCCAGCTCTGAATTTCTGTGCTGGCATGGG
TTTGCAGGTCATTAGCACTGGGTGCCACATGCCCGTTGGTTTGTGAGCTCCTAGAGGACATGCC
GCATATGCTGTTTTGTACTTTCTGTGTCTAACACGGGACTCTGCATACAGTCAGTGTTCAATGAA
CACTTGATCAATAGAGGAGTTCACAGATCCCCTGCTCTTTTTCAGCTGCGGCCCTAACTCAGTCC
AGTGTGGTTCCTGACTTCTCATCCACCTCCGTCATGAGACCATCTGCCACAGGCTCCTGCCTCCT
GGTTCAACACCTGGCAGGTAGCGGGGACTCGACACAGCTGCTCAATCAGGGAGCAGCCCTCGC
TTGCCGCTGGGTTTTGTTTGTGTGTTGGTTTTTGTGCCCGTCTTTGTTCCTACTTTGAATTTAAACT
CCTTTTGTCTGCTTTTTGAATAGATTCTTCTAGGGGTTCATTCTATCCTCTTGCATTAATTATTTAGT
GAGTATTACCATGTGCCAGACCTTATTCTGAGTGTGGTGCGTACAGGAGTGAACAACATGGACC
AAACTTCTCCGCACATGGGACTTTTACCCTAACAGGGCTGTTAGACTGGGCCCAGTAACACAGG
GCAGGGGTGCAGAGAGGGACAAGGATGCCACTTTAGCTATAATAATCAGGAAAAGCCCCGGAT
CTGAGCAGAGACCCCTGAGGACAGAGGGGAGGGAGCCGCTCACATCACTGGGGAAGAGCATT
CCTTTTTTTTTTTTTTTTAAGAGACGGAGTCTCGCTCTGTCGCCCAGGCTGGAGTGCAGTGGTGC
GATCTCGGCTCACTGCCACCTCCACCTCCCGGGTTCAAGCGATTCTCCTGCCTCAGCCTCCCGA
GTAGCTGGAACTACAGGCACCTGCCACCGTGCCTGGCTAATTTTTGTCTTTTTAGTAGAAATGGG
GTTTCAGCACCACCAGGAGCAGGACCTCAGCCCACCCAGGACTCCTGAATCAGAATCTGCCAT
TTGCTCACCATGCGATCCTGGACAATTTGCCAAACCTTTCTAGGTCTCTGTTTTCTCATTTGTAAA
ATGGGCTGAATTATTTCTCTCATCTATCTCCCTCAGATTGCTGGGAAGAACCAATGAGATAATCC
ATGGAAAGTGCCTAACACAAAATCAACTTAAGAAATGACAACTGGCCGGGCACAGTGTCTCAC
GCCTGTAATCCCAGCACTTGGGAGGCTGAGGCGGGCAGATCACTTAAAGTCAGGAGTTTAAGA
CCAGCCTGGTCAACATGGTGAAACCCTGTCTCTACTAAAAATACAAAAATTAGTCAGGTGTGGT
GGCGGGCGCCTGTAATCCCAGCTACTCGGGAGGCTGAGGCAGGAGAATTGCTTGAACCCAGG

AGGCAGAGGTTGCAATGAGCTGAGATCACCCCCACTGCAGTCCAGCCTGGGCAACAGAGCGA
GACTCTGTCTCAAAAAAAAAAAAAAAAAAAAGAAAGAAAAAGAAAGAAAGAAATGACTGGCAG
CCAGTACCACTCTTCATGTTGGCCTCGCAGTTGATATTTCCTCAGGAGGGCACTTCTGAATTCCT
CAAGGGAGAAAGAAAAACAGACTCCAGGAATATCCTGGTTGTTGGGCACAGGGAAAGCAACA
CTGCTGACGTCCATCTGATATAGAATTCTCAGAAATGCTGTGATACACCTGTTGGCCTGTAGCTT
TCTGGCTGGTTCTTGTTTGCACTTGGGGGTCATCTTCAGGGCCTTGCAGGGACGTGTTTCCTAGA
GTTGAGAATCCAGAACAGCAAAACGGGCTCCTGCGACTCCTCTGTTTCGACAAATTCATGACCC
GAATGAAAGTGGCTCTGAGCTGCACTAGGCCCTGCACACAGACACTAACTGGCAGCTTTGAAA
ATGACCATTCTGGAAATCTGCATGAGGTTTCCAGAGGTCAAACTAAATAAGCTACTGAAACTAG
TCATCTCTCGAAATTTCATCTTTGGTAATACAGCAAAAAAAAAAAATTCAAAAACTTTTTGTTTTGT
TTTGTTTTGAAACAGAGTCTCCCTAAGTCACTGAGGCTGGAGTACAGTGGCATGATCTCGGCTC
ACTGCAACCTCTGTCTCCTGAGTTCAAGCGATTCTCCTGCCTCAGCCTTCCAAGAAGCTGGGATT
ACAAGCGCCCACCACCACACCCGGCTAATTTTTTTGTATTTTTAGTGGAGACGGGGTTTTGCCAT
ATTGGCCAAGCTGGTTTCGAATTCCTGACCTCAGGTGATCCACTGGCCTCGGTTTCCCCAAGTGC
TGGGATTACAGGTGTGAGCCACCACAGCCAGCCTAAAAATAACTTTTTTAAAACAAACATAAAT
TTTAAAATAACTTCTTTTAGGCAGTCCATTCCATGGAATTGGTGGCACTGACCTCATCTGATGTCC
CAGAAAGCTGGCCACAAAAGTTTCCATCCCCCATGACAGACAAACAGTTCTACTCTGGGGCAAT
TCTCCCTGTGTCCAGACCACAAACGCCCCTGCCTGTTTGGTGCATCTCAGCAGCAGAAAATCAC
CAACCACAAAGCTTTCAGGACCGGCATTTAAAATCAACCCAAAGACACTCCCCTTCCACATGCT
CTGGGCTCCGTGCGATCTTTCATAGCGCAGCTCCACGCTCCTCCTCGCAGACGAGACTGGCATT
CAAACCAGGAATTCTTCTGAGGGGCTCTGTGTGCAGGAATACAATCACCTCAAGCTCGTGTAAG
AAGAAGAGAGCTCCAGTTACCAGGGCTTCCGAAGGCTTTTAATAGAAAACATTTGTGCCAAA
ID|55938|gene_id|ENSG00000205662|transcript_id|ENST00000381105 Homo sapiens lncRNA

GTGTCTTCTGTGGCTTCAGCGTTGCCTAGTGCAGGCTGCCATTCAACAAACGCATTGTCAA
CAGTCAACCAAAAGAAACCCATTGGCCACCATGCCCTGAGGACTAACCCTGACACAAATGCCC
TTCCAGATGCCCTCAATAGTCTGTTTCCATCACCCCAGCCTCGGAAGAGAAGCACTGCTGCCTA
TGCACTCCATTTACAGGTGAGACTGGGAGAGGTTTAGGGAGTGGCCAAGTCCCCTGCCTACAC
AGCTGTCTCCCAATCTATCTTCTCATCCTCCCCCTTCTCAGGCTAAAATCACACTCAGTGTTTCTG
TGGGATCATCTGATCTGGACATTGTCAGAAAAAATTGTACCAGACAAGTTAAACCTTCAAGGAA
GGACTCAGTCAGGACTACTGCAATACGGAAGAGAGAAGGAGCTTAACTTTGTTGAAACAAAAG
GCTGGAGAGTTTTTAAGTGCCGGGGTGAGATGCTGAGAAGGAACTGGAGACATCAGGAAGAG
GTTAGGAAATGCTGTAAAGCTATCTGCGTTGGCTCATTGGAATTTTTCAAAACTAGGAATCTGCC
CTTCTACAAAAACTGAGACACAGATGTATCATCTCCTTCAATAATTGCATTTTAAAGGGATGGCT
CTGAAGTCTTGAGAAAGGCATTCCTGGGTTGCAAAACTAGGAAGAGGCTAGAAGAAAATTTGG
GAGAAGATTTGCATCTCAAAGAGATAGAGAAAGAACTCACAATTGCAAGTTTCCTAAAATAAAT
CCTCCGTGAAAAAAGGAGGCCTGATGGATGGAACTGGAGGTCATTATGTTAAGTGAAATAAGC
CAGGCACAGAAGAAAAATACTGCATGTTCTCACTCACATGTGGGAGCTTAAAACCTGGATCTCA
TGGAGATAGAGAGTAGATTGGTGGCTACCAGAGGCTGGGAAGGGAGCAGAGAAGGGAGATAA
AAATAGTTTGATTAATGGATGCAAATATCTTAGGTTTTTTTGGTTTTTTGTTTTGTTTTTTTGGAGAC
AGGATTCTGCTCTGTTGCCAGACTGAAGTACAGTGGTGCAATCATAGCTCACGGTAACCTTGAA
CTCCTGGGCTCAAGCAATCCTCCTACCTCAGCCTCCCCAGTAGCTAGGACTACAGGTGCATGCC
ACCATGCCAGGCTGGCTTTTGTGTTTTTTTTTGTAGAGACAGGGTCTCACTAGGTTGCCCAGGCA
GGTCTCGAGCTCCTGAGCTCAAGAAATCCTCCCACTTTGCAAATATACACTTCGATAAGAGAAA

AAAGACATAGTGTTCAATAGATCAGCAAGTGACTGTAGTTTACAATAATCTATTGTACATGTCTT
TTCTTCTTCTTTTTTTTTTTTTATTTTTTTTTTTTGAGACAGAGTCTCGCTCAGTCACCCAGGCTGGA
GTGCAGTGGCATGATCTCCACTCACTGCAGCCTTGACTTCCCAGGCTCCAGAGATCCACCCACC
TTAGCCTCTTGAGTATGGGACTACAGGTACACAACACAACACCTGGCTAATTTTTGTATTTTTTGT
AGAGACAGGGTTTCGCCATGTTGGCCAGGCTAGTCTCAAACTCCTGGACTCAAGTGATCCACCC
ACCTTGTAGCAGGATGAGCCGCAGACAAAACCTCTCAGACCCCGAGTTGTAGAAGGAAGGGCT
TTATTCAGCTGGGAGCATCGGCAAGCTACTGCCTTAAAATCCGAGCTCCCCGAGTGCACAATTT
CTGTCCCTTTTAAGGGTTCACAACATTAAAGATTTCACATGAAAGGGTCGTGATTGATTTGAGCA
AGCAGGGGGTACGTGACAGGGGCTGTATGCACCGGTGGTCAGAGAGAAACAGAACAGGGCAG
GGAGTTTCACAGTGTTCTTCTATACAATGTCTGGAATCTATGAATAACACCGGTTTCTAAGTCAT
GAGTTGATTTTTAACTACTGGGTGTAGGCCAGGCAGGCCCAGGCCTGGTTTCAGGCCTGGCACC
GAGCTGCCTGTCTTTGGTTTTACTTCCTTGCTGTTTTTGCTTAAAACAGGTACTGAGTATAAAACA
ATATAAAATAATATGAGAGGGTCTTTCTCTTCCTTCAACCTCGGCCTCCCAAAGTGCTGGGATTA
CAGGCCTGAGCCACCGCACCCAGCTATCTTGGTGTTTTTAACCTTAATAACGACAAAGTGCATC
CTCGATATGGTCTCATCCCCCTTGCTGTAAGCACGTTAAAATGCTGTTATGACGTGCATTCAAGC
GATCAAATGGTGCTAACATCAATGCCTTTGCCTGCAGAATTAATTCAGGGGTTTATTTGAAACTC
AATAAAAAAGGCCTAATGTGCAACATTCGGCAGACAAGAAGCCATATGCAGATACTTTCTTTTA
AAATAACAGGTCATTATTTGGTTCTTTCTCATGTGAGAGTCAGATGACTCAGAGCCAACATAAAT
TCCAAGTGGTATGCAGAAAACCAGTGCATAGCTGGAGAGGGGAATGAAGGTTAGTATCTGCAG
TAAGGCTGAGTCGATGCAAACGGCCACACGGTCATGGATTCACAATGATCTGGGTCTCCATCAT
CCTGCAAACCAACACCTTGCACAGCCTTCAAACACCAGCTGGGAAATCAAAAGTAGCATGTGC
AGTAGCAGGAGATCTGTTCACCCTCTATTTCCTCTCATCTCCATGCAGCAATCTGTGAACGGAGA
GAAGCCTTTAAGCTCTGGGGTCTGCTGCTCTGCAGAGACAATTTGCATTTCTCTCAACACTCCTG
GAATTCTTACTGGGAAACCCAGGGTACCTGGGAGCTCAGGCCATCCTGTGTCCTTTGGGATGTT
AGGAGGAAGCAGAATAGCCAGCTCCCAAATGGAATACTGCAAAATGCTCACTTCTCAAGGGTC
TGTTTTGGGTTTTGTGTTGTTTTTTTTTTGAGACAGGGTCTCTGTCACACAGTCTGGAGTGCAGTG
GCATGATCCCGGCTCACTGTAACCTCCACCTCCCAGGTTCAAGGGATTCTCCCACCTCAACCTC
CCAAGTAGCTGGGACTACAGGCACGCACCACCACACTCAGTTAATTTTTGTATTTTCATTTTTGG
GGGTTTTTTGGTAGAGACAGAGTTTCACCATGTTGGCCAGGCTGGTCTCAAATGCCTGACCTCA
AGTGATCTGCCCACCTCAGCCTCCCAAAGTGCTGGGATTACAGGCATGAGCCACCGCGCCCGG
CCTCCTTCAAGGGTCTGAATCCAGCCAGTGAGCTTGCCCTACATCATGCCTGCTTCATCCTATGC
AATGGAGAGAGGCTGAGATTTTATTTTAATGAGAAAATCTATTGAATTGCATTACATTAAACTAA
AATGGAGAGACACAAACATAAAAAATGAAATAAAAATCCAGAAATTGCAATAGTAGGTAACCC
TCCAAAATGTGGCAAAGCTCTGTGTATATTAAAAGTGTACTGAGGTCGGGCGTGGTGGCTCACA
CCTGTAATCTCAGCACTTTGGGAGGCCAACGCAGGTGGATTGCCTGAGCTCAGGGGTTCAAGA
CTAGCCTGGGCAAAACAGTGAAACCCCATCTTTACTGAAGTACAAAAAATTAGCCAGGCATGG
CGGCATGCACCTGTAATCTCAGCTACTCGGGAGGCTGAGACAGGAGAATTGCTTGAACCCAGG
AGGCGGAGGTTGCAGTGAGCTGAGACCTCGCCATTGCACTCCAGCCTGGGCAACACAGCGAG
AGTCCATCTCAAAAACAAAACAAAACAAAAAATATACTGAAACATGCAACATCCACCTTTTCAT
AATAGTTCCCGTGGCCTACACAAGTGAGTATGAAATGCTTAATATTTTGCTTGCCCTACATTTTCT
TTTGCTGGAAGGGTGGTTCTGGATAAAAACTTTTTATTTTCCCATTCTTTGCTTTTTCATTAATGCT
ATTTTCTTTGCCCCAAAGTTATAGAGTGGAAAAACTCCCAATCTCTTTTTCTTCTCTCTTGTATTTT
CAGGTAGATGCATTCTATTACTATATTCTATCTATCCGTTCATTCCATCTATAATCTATCATCTATT
CCATATATCTATCCACCATCTATCTATCTATTCCATCTATCATCTATCTACCTACCTATTATTGTCT

ATCTGTCTATCTTTCTATCATCTATTCATTATCTATCTATTCTATCTACCTATCATTAATCTGTCATC
TATTCCATCTAACATCTATCTACCTATTGTCTATCTTTCTATCATCTATCGTCTATCTACATTTACCT
ATATTGTTCTCTCTGTCAATCATCTATCTATCCATCTATGTATATATGTATTTATGAATGTATGTGT
GTAATTTTCTATCTTCTAGTTATCCATCTGTCTATCTATCCATCCGTCCATCTATTTCTCTTATGTAT
ACAGATAAATATATAATCTATATATCTTACATCTGTAACATGTTCTTTTTACTTTTATACATCAGTG
ACATTAATTAAATCCCACCTAATAAAAATTGTATTCTAAAATTTGGTGTAGTAGGAGGCTGAATA
ACCTGACCCTAGATCAAGGTACACATTGTGTGGCTGAAAATCACCTGGCTTTGTTAGACTTCATC
TTTCTCCTACATAGGACTTGGTAGCCAGCCTAGAAAATCTCAAAGGTGCCTTCAACCTTTGAATG
CATTGGTTCCATGGAAGCAGGAGGCTTAAGCTCTAGACCCATGGCTGTTGTAATTACACATAAC
TCAGACATTGCCATAAGGGACCCTTAGGATGGCAGTACGACACTTGTTCCTCAGAATACCCTAC
ATGCAAATTTGCCCTCCCAAGAGCCATATTTTACAAAGTGATAATCTTGGGAACATGGTCCACCC
AAATGCCTCTGCCTGCAGTGGATTTCTGCAGACTTCAGACAATCTGGGCACCATCAAAGCCAAG
CACTCCCTGGTGTAGTATCTAATAGGGACCATGTAATTAGGGCATGTTTTATGTGAGATTCTGCT
CAAGAGATGTAAGTACAAGAAAAAGACAGCTTTTCACGGCAGAGATAAGATAATTGAGCTGTCT
GGCATAGTGACTCAAAGCCATTGAAGGTTCTAGTCAAATGATTCCTCTGGTTTTGACTTTTTAAAT
GGCTCTTTCACTGTTGTTGCCTCTGGCAACTACCATTATAATTGGAGGAAATTTTGTTCCGCTAAG
AGGCTACTCCATTTACGGACTCAAGCCAGACCAAAATAATGAAGCTCTCTTATAATCCCGGTAT
GTTTCCTGATCATTCCCATTTCCCTCTCTGGCTGTCCAGAAATAGACTCTCTTCCAAGGTATCACT
TTTATTTATAGCCAATATGAAGGTCTTGTAGAAAAACACTGACTTCCAAAAACAACAGACGCATG
TTGGCAAGAGTACAGAGACAAGGGAATGCTTATACACTGTTGGTGAGAAGTAAATTGTACAACC
TCTATAGGAAACAGTATGTAGATTTCCCAAAGAACTAAAAATAGATCTGCCCTTTGACCCAGCA
ACTCCACTAATGATTATCTATGCAAAGGAAAAGAAATTATCAAAAAAGATGCCTATGCTTACATT
TTTATTGCACCTCAATTCACAATAGCAAAATCGTGGAATGAACCTGAATGTTCATTGGATGAAGA
AAATGTGGTATACACACACCAAGGAATACTATGCATCTATAAAAAAGAATGAAACTGTATCACT
GTATCTTTTGCAGCAACATAAATGGAGCTGCAGGTCAATATCCTAAGTGAGATAACTCAGAAAC
AGAAAATCAAATACCACATGTTTTCACTTATAAGTGGGAGCTAAAAATGGGTACACATGGACCT
ATAGAATGGAATAACAGGCACTGGACACCTTTAAAAGGGGGAAGGTGGGAGAGGATGAGGGT
TGAAAAATTACCTATCTGGTATTATGTTCACTGTTTAGGGGACAGAAGGCCAATCCCCACCACTA
GGCAACATATCCATGTAACAAACCTGCACCCTTATGCCCTGAATCTATTTGTTTTTTCAACATTGA
CTTCCATGGTCACTATTTGTGAAATCACTCCTCATGAACCAGGACTTGCATGTTTTTTTGTTTCTC
AGGTGAACTGTCACCCCTACAACTCAGCTTGCAACCAGCCCTGGCCACCACCAGTTTCCCCACA
CTGAGCTGAATATTGGACATGCCCATCTTAGACATTCCAGCCCATTCTGAAATTCCACATCGATT
CACCCGACAAAGTCTGAAGTTCCAGGGCAATTTATCTGGAAAAGCTCACCTGGAATCATGTGTC
ATTTCAACCAACAACTGTCGAAGAGGACGTGGCATCAAAACCAAGGTTATCAATTATTTATAAG
GGTTGTGTGGTGGTTGGTCCAAGCATCTCTCCTTCATGCCATCACTCCTTTCAAGAGCTGCCTCT
ATTTTCTAATTTAGCACAGGAATTTAGATGCCCCTAAATAAGTAGGCATCCAGTATCTGCCCACA
CCAGTTTTAGTGAGAGTACATATGGAGACACTACCACCACCCCTGCTCTCCTACTGCATACCCC
ACCATGCCAAAGCATGGTGGCTCACACCTGTAATCCCAGTGCTTTGGGAGGCCAAAGAGGAGG
AACACTTGAGGCCAGAAGTATGAGACCAGCCTGGGCAACATAGTGAGACCTCATTTCTATAAAT
AAGTTAAAAAAACAAAAAAATAGGGCATAGTGGTGCACATGTGTGGTCCCAGTTACTCAGCAG
GCTGAGGCTGGAAGATCACTTGAGCCCAGGAGTTTGAGGCTGCAGTGAGCTAGGATTATGCCA
CTGCACTCTAGCCTGGGCAACAGAGAGAGACCCCATCTCTAAAAAGAGGGAGAAGAAGGAAG
GAGGAGGAAGAAGAGGAAGAGGAGGAGGAGAAATACTGCTCTTTCTCAGGCCCAAGGTAAAA
AGGTTTTTGGTCTCATGTTGACCAGAAAGACATTGATATTTAATTAAGCAATATAAAAAGGCTCA

TTGTTAGGATTAAAGAGTAAGTAAAATCAATATTGAGATAGGAGGATGGAGGACACCAACTGA
GTTCCGATCCCAATGAAGAGGAGAAAAAGCTACAACTATCTAGGTGAAATATCTACTTATCCTA
GAAGAAGCCTTTTGACAGCAACATAAAATTCATACCTATAGGTTTTTAATTCCTATGGATGCCGT
AGCAAATAACAAAAACTTGGATGGCGTAAAGTAACAGATGTTTACTCTTTTCCAGTATTGGAGA
CCAGGAATCTGAGATCAAGGTGTGGGCAGGGGTGCGCTCCCCCTGGAGGTTCTAGGAGAGGAT
CCTTCCTGCCTCTTCCAGCTCCTGGTGGCTCCAGGTGTCCCTGGGCTTGTGGCCACATCACTACA
TTCTCTGCCTCCATCTCCACGTGACCTTTTCCTCTGTGTCTGTGTCTCCTCTTCTGTCTCTTAGAAG
GACACTGGTCATTGGATTTAAAGGCCACCTGGGTAATTTATAGTGATCTAATCTCAAGAATCTTT
CCTTAATTACATGCAAATACTCTTTATCCAAATTAGTTTGCATTCACAAATTCTGGAGCTTAGTAC
TTGGACATATATTTTGGGGGGTTGATGGTTGGAGGGGCTTTTATTCAACTCAGTACATCTTAATA
AGGAATTAATGCCCCCCAACTTGCCTTACAAGTCATATATTAAAAACAATGTTGGCCTGGCACA
GTGGCTCATGCCTGTAATCTCAACACTTTGGGAAGCCAAGGGAGGAGGATCACTTGAGCCCAG
GAGTTGGAGACCAGCCTGGATAACAAAGGGAGACCCAGTTTCTACAAAATATTTAAAAATTAGC
CAGGCATGATGGTGCATGCCTGTGGTCCTAGCTATTCAGGAAACTGAGGTGGGAGGATCACTTG
AGCCTGGAAGTTCAAGACAGCAGTGAGCTATGCTCACACCACTGCACTCCATCCTGGGCAACA
GAGCTGGACTCTGTCTCAAAAAACATAACACTAAAACATCAAAATTAAAAAAAAAAAA
ID|56041|gene_id|ENSG00000236537|transcript_id|ENST00000432358 Homo sapiens lncRNA

TCCTGGGTCAAGCTGAGTATCTCCACGTTTAGGTGCACAAATACTATTTTGTGAAGATCTTA
AATTTGTGGTATAAGAACTTGAAGGAAATAGAACTGATTTGTTACTTCTAAAAGGTAAGCTTTTG
ATAAGTGAATATGAATTCCCTCTAAGAACTAGATGATATAAATCATTGCTGAACCCCAAACTAAT
GTTCATTATGTCCAGCTGTGATTATAACCTTTGACTGAAATGTGGGTTAAGGGGACATTTGGATC
ATTATCTTTTTTCCTGCTACCCGGTGTCTACTCTTTGATTTGAGGTAATGCTAGTTTCTGGGAAGA
ACGGCTACGAGATTTAAATCCTGCCATTAATTTTCTCATGTTATATTTGTGTACCTATAGGGCATG
GTAGTAGTCAAGAATCAACTGAATTTTCCCTGACAGTTTTTATTCTTGTTGGTGTTAATTGTAATA
ACACCTGTGTAAAGCAGGCATCTGGATCTTCCAGGACCACAGATGCTTTCTTGATGAACCCCAT
GTCATGGTAACCCTTTCTCTGTCTAGAATTATTTTAATTATTTCATATACAGAACGTTATATTATAT
GAAAATCTATGGGCTGGGTGCGGTGGCTCACACCTGTAATCCCAGTAATTTAGGAGGCCGAGG
CGGGCGGGTCACTTGAGGTCAGTTCGAGACCAGCCTGGCCAACATGGTGAAACCCCACCTCTA
AAAATACAAAAATTAGTTGAGCATGGTGGTGGCACACACTTGTAGTCCCAGCTACTCAGGAGGC
TGAGGCAGGAGAATCGCTTGAATCCAGGAGGCAGAGGTTGCAGTGAGCTGACATCCCACCACT
GCACTGCAGCCTGGGTGACAGAGCAAGACTCCGTCTCAAAAAAAAAAAAAACAAAAAAAAACC
CTATAAAGACGTTCATCTGTAAGAACCTAGATACAGTGCCGCCACCATGGCTCTATGGCTTCTTT
TAGCCTCATTTTGCCAACTTGTAACTATTAATATCTTCCATAAATGGTTATTGTTGTAATAAAAGA
AAATCATTTTTAGAAATTTTGAAACACTATGTCTAGCTACATTCAATAATAATTGAATCAGAATAA
TAAATGTTTCGAAGCCCCTGGTGGTAGTTCACTTATTTTCCACTCTGTATAACAAAATTCTTGTGT
AAAAGGTAAGGTTTTGGCTTGTGCTCTGAATCTTAGCACTTTACAGCAGGCAGAGCACAGCGTC
CCCTTCATTCATCTTAGTTCCTTGTACTTGGGGTTTCCTGTGTGCTGTTTCCTGCCTGAATTTGATT
CAACTTTGGATCTGTGTGCTTGTCCTTATAGAATTATATATGCAAGTGATACTACTGTGTTCTTAA
ACTGTCCTCTTTATTGGTGGAAGTACCAGTAAATAGAATCATTATATTCACTTACCTACCAGTCAT
CCTTCAGAACTAACTGTTAAAGTGCAAGTTGGTCTCCTTTCACTAGGAAATAAACTGCCTCCAGC
CTTCTGCTTCAGCAGGTCTGCTTCTTGCACCTCAGGCTCCTCCTCCACCCCAGCCAGCTTCAGCC
TCTCTCTTGTATTGGCTGCTTGTGTTTTTCTTCACCCTCTGGGAGTCCCCTGGGGTGTCCTCCTCT
CCTTTGACGGCTTATGTAGTTTCTGCGCCCATTTAACCTCAGGGAATGATTTCTAAACCACATCC

AAAGTGAAAAGTGGTAGATAATGGACCCTCCTATAAATGAGGCTGACCATATATTCTGGAAAAC
TCTCCTACTCTAAGCCCTTAGAAATACTGCAAAAGATACAGCAAACATCCGTTAAAAGTCTTTAT
CAAAAGAAAGTGAGAAAAATCCATAGGTGGGGAAAAAGAGAAAGAGGAGTGAGTGTTTTTGG
GAGACAGAAGGTCTAAAACTGAATTGGGAAGTGAGCACTGAAGGCCGGGGCCTGGGACTCATT
CAGGCCAGATGGAGACCAGGCTGGAAAACAAAACAAAAACATCCATCAGCAAATGGAGTTAA
AAAGAAAAAGTCTCTTTTGAGACTGTTTGGCTTGGGATTTGAATTAATGCTACCTGCATGGTCTG
GAAAACCCCAAGCTGAGAAACGAATCTAAATGCTTCTGAGTTGGAAGAGCCCAGGGCTGCTTG
GCAGAAAGAAAGAAATGCAAATCCTTTCAAGAAGAATGCTACCCTCTACCCGCTGCTGAAGAG
CTGGGGATATGCTGACGATAGAAACATTGCTAGACATATAAGGAAATGAGCCGCCGTGAATGA
GAGTCAGCTTAAAGTCAGTGATGACGTGCCATCTGCTTGATTCGTTCATTCCTCACTTTTTAGCTG
ACCACCCATTATATACTGAGTGCTGTTCTCAGTGCTGTGGTTATAATGGCAAAACATTATGTGGA
GAAATAGGTACTGAGCTGCTGACCAGAGGCTTTGGTACATTTGGCCTGCAGCCCTGCCCACTTC
AGGGCTCTCAGTCAATGCTAGAAAACTGGTGCTTGGCTGGTCCCAGTTTGAAAACTGCTGATTA
TGTTAATTCCTGGATTTTACAAATGAAAAAACTGAGACCACAGTGGTTAAGTAACTTGCCCGAG
GTGTGAGCCTCGGTTTCCTTGCCTGTGGAATGGGGATGATACCTACCTCACAGGGTTTCTGTGG
CTATGAAAGGTTAACCAGCATGTTTTCTGCCTCAGATATGTCTATTCTTCCTTTGTCTTCGTCTTTA
ATGATCACAGATAGTACAAGGACCCAGTTCTCCTGCTTTTCGGGTTCAACACTTTTCTGTTGCTG
GTATGATTCTCAAGGTCTTTGATTGTTCAAGCCCTACACAGAGCTTCACACCCTCACTCTTAATTG
TTCTCTGATTATTTCATGTATGTGAATTTCCCCTTCCTAACAGATTGTGAACAGTTTACGTTCAGG
GTCCAGATTTTGTATTTTTATGTTAGCTTTATGTTGTCTAGTCTTGGACTGGTCTGTTAGTAGATTT
TTTGTTGTTGTTAAGCAACAATAAACAATTCTTTATAACAAATAATATGTTGTTTATTAACAATAA
ACAACATATTATTTGTTTATTGTTGCTGGATTAATCACAGTAGTTCCATTGTAATGAGGAAACAAG
AATATTTCTGTCCAAAAAGTCATTATGCGAGAGTTTTGGCAACGTAAGGAAACACTTTTGCTGGC
CATTTATGATGTTGACATCTGTATTCTGCCCCTTCAGTCTCTGACTGATGATTCCTACCTGAATTC
CTGCTGTGGTTTGCCTTACAGTACAGGCCTGCTCATTCCTTCTGTACCAGGCCTGTTTGTGAGTA
CTGTTCACCACCCTGAGATTCTCTCTGTATGTTGGAAATCATTGAATGAATAGCTCACAGCCGCT
TCCATGTAGTTGTAAGCACATGTATTAGGATTGGAAGTGCATCCATTTGTATGGGCACGGGTCCA
TTGTTAGCTTTGTTCCCCTGTCGCCCTGCCCTGCCCTGCCCTGATCTCTGGCCTAGATGGACAAT
GTGGGTGTGCTCTCTGTGTCTTCCCTGTCCATCTGTTGGCCGAGGGTCAGGCACTTCTGCTTACC
ATCAGAACCAAGAAGGAATCACTTGGAATTCATCCCTCCCATAGAACCCAACTGTGGCTGGGCT
ATAGCCAGGGCTCTGTTCGGTTGCTGGCTGCGTTTCAGCTCTCCATTTGAGTCCTTTCTATATGAA
AAACATGAAAATGCCAGGGGATTATTTTTGCAAGTAAAAAAGATGGCAGCAGATGTGAAAACA
GATTTACTCATCATGATGTAAATTGGTGTTTTGTTTTCATTTTAGTTTAACAATATATAGGTATTCA
TGAAAATATTTGGCTGTAAAAAATTAGGAAAATTACAAACTGCCATTATCTGTTGCAATTCATTC
AATTCAGTTTTTTTTGGAAAGAACTTGAAATTGTAAGTTAAAAAATTTACTGAAATTTGTGCTAGT
TAAGCTTTAAGAATTCTAAGTAATAATCCAAAAGACATTCTAAAATTCAGATTTATCAAAATCAA
TTGTACCTTTCTTTGTACAGATATAATAATTTTCTGGAATATGGAATGTGGTGGTAGAAGTAATGC
AGAATTGTTTTGCCATAGCCTTTGTTTTCTCTCGCAATTATTTCTGTGTTTCTTCTGTTTTATAAAGG
AAATGGATTGTGTTTAGTAATTTCCTCTGTACCTGTAACCACGAGAGTAAACTTTTTATTACATCT
AAACTTGTTTATTCTTGTATACATGCTAGATCATAGACTAGTTGAAATTTTCTGTGTTACCATGGG
TCTGTGAAAAGATCTTGTCTTACTGGTCTTGTATTCCTAGTAAATTTCAGTATGATGATATCACTT
AAACACTGTAACTGTGGGACATTGGCAGGAGCTTGGGGATATTTTTAGCTACTTGGAACTCTTAT
TGTAATGGTATTTATTAGTAATTCAAGACAAATTTTATTGTTTAATTATGCTCTATGGGTTTATTGC
CATCCAAGACTGCAGTGTTTTAGATGACTAGAATTTAGCTTTTTAAGAACCCCAAATTTTTATTTC

AGCTCTTTCAGTGGAAAATTTTCTAAAATATGCTATTTCCTTCTTAATGAGAAGATAGTGAACATA
ATAGGACTTGTAATTTCCCACTTGAGTTCATCTCTTTATTCTTTTGTGAGGACGCACAGTAATGCT
TTTGGAAGGGGTCAGCATGTTGGGCTTCTTCCTACATTTAATGGTCATTTGCCAACAGATATTTA
CTGAGTTCTTTTCTAGGTCTGGAGATTCAGTGATGAAGGCATTATTTCTTGCCCTCAGAAGGTTC
ATATTGTAGTAGGGAAGATAAACCAGGTAGTAAATCTTAGAACACAATGGGATGTTTACAGTGA
AGAGGAGAGGCCAACCTGGAGTATATGGGTTTGAGATCCATACAGATCTGGGTTCAAATTACAG
GGTGAATGGAGTTGTTAAGCTCCATCTAGCATTTGCGTGGCCAGACATATGCATGTGTAGTGTTT
ATTTCTGTCGTGGCAGTAAAAAGACTTCTCCTGTAGTTTATTGGCCTATAAAATAATATAATTTAC
GACATCATTGGAAAAGTATTTTTATTCCTATGTAGGGTCATTTGTTTCTAGAGTAGAACAGTTTTA
ACACCGTATACAAATAAAAGCACTTTTATTCAACTGAACATGCATTTATTGAATACGTAATATAT
ACGTACATTTTGCTTGCTAGATGTCTTGATGAATAGACATTTAAAAGTCCTTACCCTTAAGTAATT
CCCAAGTTAGCACGGATGATAAGGGCTCAAATACTTATAATATGGTGTAGAATTGAATGAGCAA
CTTAAGATAGAAACATTTGCTGTAGAAGACCATACTTACTAGGGAGGAACTGGCAAGGATTTAT
GGAATCGGTGATGTCGAAGCTGCATCATGAAAGATGGCTAAAGTTGGGGGAATTTGGGATGAG
CATTGTACTTGGATGGATTAGCTTGGAGCTTAGTCCAGGCAGTAAATATATTCATACTTTGAAAG
GAGAGTAATTTTTATAGAGGACCCTGGTGGAAGACAAGACTGGCACAATAGGTAGGTGTATGG
CCCTTCTGTGAAGGCCCTTGAATGCCAGGCTGAGGAGGGTGTTCTTAGCCAGTGAATGTGACCA
AGTGACTGTTTTTAAAGTGGCAAATGATACTATTGCAGCTGTCCTTTAGCCCACTGGCAAAACGG
GTGAATATTAACTGAGGGGAGGTCATGTGGTCTACGGTGAAGAGCACTGAAATTATCAATTGGT
TTGAATTCTGGCTTCAGCACTTAATATGCTGGGGGGACTTCAACTTCAGGCAAGTTGCTCAAAAC
CTTTCTGAGCCTCTATTTCCTCATAGAGAAAATGGGAATAATGATTCCTTTTATATGGCTGTCTTA
AATGACATAACATTTATGAAATCACCTTTAATAATTTGCATGTCCAGTTTGTTTGGGATTTCAGCC
TAGGTGGTTTGTTTTCTTTCTTTCCAATCTTCCTATACCTTATATTGATTTTTGTTGTCTTCTGTGGT
AGCTCGGACCTCCAGTACTGTGTGGTAGTAGGGTTCTCATCTTTCTACTTTGTCTGGGATTTCTAG
CACAGCATGATACAGACATCTTTATCTTAATTCTTAAATATGATGTTTGCTATAGATTGTAGGTAA
ATATCCTTTATCAGAAAAAGGAAATTCTCTTCCTAATTTGGAATCTAAAAAAAAAAATGAGTGTTG
AATTTTTTTTTTTTTAAGAAGGAGTCTCACTCTGTCGCCCAGGCCGGAGTGCAGTGGCGTGATCTC
GGCTCACTGCAAGCTCCGCCTCCCGGGTTCACGCCATTCTCCTGCCTCAGACTCCGGAGTAGCT
GGGACTACAGGCACCCGCCACCACGCCCAGCTAATTTTTTGTATTTTTAGTAGAGACGGGGTTT
CACCGTGTTAGCCAGGATGGTCTCGATCTCCTGACCTCGTGATCCACCCTCCTCGACCTCCCAA
AGTGCTGGGATTACAGGCGTGAGCCACCGCGCCCAGCCATGAGTGTTGAATTTTAGGATACTTT
TTTGCTTGTATTGCAATAATCTTTTTCCCTGCTCTTTACTCTTTTAGTATGATGAATTATATCTAATA
GATTATATTTCTAATTAGTAGACAAACAGATTTTCTAATAACCTCTTAGAACTAGTTGTGGCTATT
ATCACTTTTTAAATATTCTGATTGAGTTTGTGTAATACTGCTGTAGGCTATTCCTGAATGTTTGGTA
GAACTCGTTTGTAAACTATATGGCCTGATTTCTTTGTGGGATGATTTTAAACTACAGATTCATTTT
CTTTAGTGGTTATAGAACAATTTAGACTGTCTTTCTTCTTGAGGTAGTTTTGTTAAATTGTGTTTCT
TAGGAATTTACTCATTTCATATGTTATCTAATTTTATAACATTAAGTTGTTCATGGTATTATTTTATC
TTAAGTTTCATGGTGTCTGTAGTTAGTTGTAGCCCTCTTTTCATTGTCAGTATTTATTTGTGCTGCT
CTGTCTTTATCTATCTTGCCAGAGCTTTGTGTCTTTGATTACATTTTGCAAAGAATCAGTGTTTGGC
TTTGTTTAGCTTGTCTAATTTTTTTTTCTTTTTATTTATTTAATTTCTGTTTTTACTATCTTCTTCCTTTA
CTCTCTTTTGGTTTATGCTGTACTTTTCTAACTTCTTTAAGTTGAATGTTTAGCCCCTTAATTTATAA
CATGTTTTCTTTTCTAACATAAGTTTTATAACATTTTATAACAGTTTCTTCTTTTATAACATAAGTTG
TTGTTGTTGTTTTGTTTTGTTTTGTTTTTTGAGACAGCGTCTCATTTTGTGGTCCAGGCTGGAGTAC
GGTGGTGCAATCATGGCTTACTTCAGCCTTGACCTCCTGGGCTCAAGTGATCCTCCTACTTCAGC

CTTCTGAGAAGCTGGGACCACAGGCATGTGCCACCATGCCTGGCTGATTTTTTAATTTTTTGTAG
AGACGGGGTCTCACTGTGTTGCCCAGGATGGTCTTGAACTCCTAGGTTCAAGCAGTCCTCCCAC
CCTGGCCTCCTAAAGTGTTGGGATTGCACATGTAATCCACCATGCCCAGACTAACATAAGCATTT
AAGGCAATGAATTTTCTTTCAAAATACCACCTTTGCTATAGCCAAGAAGTGTTTTGTTTTGTTTTG
AGACAGGGTCTCACCCTGTCACTGGAGTGTAGTGGCGTGATTAATGGCTCACTGCAGCCCCCAC
CTCCTAGACTTAAGCCATCCTCCCACCTCAGTCTCTTGTGTAGCTGGGACCATAGGCGCATGCC
ACCATGCTGGCTAATTTTAAAATTTTTTGTAGAAACGGGGTCCCACTGTGTTGCCCTGGCTGATC
TCGATCTCCTGGGCTCAAGCATCCACATCAGCATTTGTTATTTTCTGACTCTTTTAGTAATAGCCA
TTTTAACTGGGGTGAGAAGACATCTGCTTGTGGTTTTGATTTGCATTTCCCTGATGATAGTGATGT
GGAGCACTTTTTCATGTACCTGCTCTCCATTTGTTGTCTCCTTTTGAGAAATGTCTTTTCAGATCCT
TTGCCCATTTTCAAATCAGAATGTCTTTTGCTGTTGAGTTCCTTATATATTCTAGTTGTTAATCCTTT
GTTGGATGAATAATTTTGCAAATGTTTTCTCCCATTCTGTGGATTATGTCTACTTCGTTGATTGTTT
CCTTTGCTGTGCAGAAGCTTTGTACATTTTCATTTATTTTTCCTTTTGTATTGTTTTTCTCTTTCTAG
GGGATTGATTTTGGTCTCCCCTGCTCCCACCTCCCTTCTGTTTTTACCCCTCCAGTGATTTAGAAG
TTTTATACTTTATTTCTAGTTTTTTAGTGATTGCCCTTTAAATTTTACCATGCTTACCTAAGATAATT
AAGTATAAGGTTGAGTATTTTAACTATCCTTCTAAAACAATATAAAGGCCTTAGAGTACTTTTGG
CTCTGGTCATCCTTGTTTGATCAAATTGTCTTTTTTTTATTTTTTAGTTTTCTACCTTCCCCCCCACA
TTTGTCATAGACACAGTCTAGTGGTTAAGCACATAAACTAGAAACTGAAACCGGCAGCCTGGTG
TGACACCCAGCTCTGCTGCCTACCGGATAGTGTCGGGCAAGCATTTAACCTGCCTGTGCCTCAA
TTTCCTCACCTATAAAATGTGGACAACAAAAGGATCTTTCTCATAAGGCTAAGGCTTTTCAATGA
GAAATATAGTACCTATAAAGCATTAAGAGCTATACCTAGAAGTTGATAAGTACTGTGTATTAATA
TGAGTCTGCTGTTTCTGCTTTATTGTTCTGTTTTGAACAGACATTGTTTAATTTTACCTACATATTTA
ACAATTTCTTTTTTCGTTGTTTCTTAGACCTTCCTTCTGAGACATTTTCCTTCTACCTGAAGAATATT
GTTTAGATGTTATATTGCGAGGTCAGTTGGCCTTAAATTTCAGCTGTTATTCTTTTGAAATGGTCTT
CTTATTTCACTCTAATTCTTAAAAGACAATCTTTCTGGGAATATAATTGTGTTTTCTTTGAGTGCTT
TGAAGATACTATTTCATTGATTTTGGCAGCCAGTGCCGCTGTTGAGATGTCTCTGTCATCTAATTG
TCATTCTTTTGTAAGCACCTGTCTTTTCCTTTTGGCTACTTCTGAACTCTTCTCTTGGTGTTTTTATT
GTGCACTTTTACTAAAGTGTGCCCAGGTGTGGATTTCTTCTTTATTTCTCCTGCGTGGCATATATT
GTGCTGCTTCTTGTGTCTGTGCATTTTTTTTTATCAGTTTTGGCAAATTCTCAGTTGTTATGTCTTCA
GATATTTCTTTTCCATTTTCTCTGTTCTGTCCCTTTAGAATTTTAATTAGATTTGTCCTTTTTTAATCT
ATCTTTCATTTCTCTTGATCTCTTATATTTCTATCTCCTTGTGTCTCTGTGCTATAAAAAAATTGGGT
AATTTCTTGAGCTATTAGATTGAGTTCATTAATTCCTTTTTTAGCTGATGTCTTAATTGTAGACTGC
ATTTTATTGAAGGAATAGGTTTTTCATTTCTAAAAGATTTGGTTCTTTCTGGCATTTTAGATAGTGT
TTTGCTTTCTAATTCCTTTAAACATTTCAAACCTAATTATTTAAAGTTCTGTTTATTTCAGTTTCTGA
AGATTTGGGGATCTAAATTTGTTGTTTGTACTGAGAACTCATGGTGACTTATTTTGTGTGTTTAGT
AATTTTTAATTGTAAACAGATTTTTGGTTGAACCTTATCTGGGGGAATCCTTATTTCCTAAATTGG
GACAGTTTGCATGCAGCTAAGATTTGTATTTGCTCTGATATACAAGGATGTTAGAATTGTGGGAC
ACTTTAGCCTGTGAGTCCTGCTGAATGTGTGTGACTCAGGTTCAGCCTCTGTACCTAGAGGTTGG
ACCAAAGTCTCGATATCTGATAGAATGACCAACTGTCTGGTTAGTCCAGGACTGAGGGGTTTCC
TGGGATGTGGGACTTTCATTGCTAACACTGGGACAGTTCTGGGTAAACTGTGACTATTGGTCAC
CCTAATTCCGATTCCAGTGCTTGTTTTGTATCCGCTCTTGTGTCCCCTGTCAGATGACCCTGGCTT
TCCCTTATGTTTATGTGTATGTGCCTTCCATAGAGATTTTCTATACTTTCCTGCAAGTCCAGCAATT
AAAAATTACATTTGTTGTAATTTACTCAAGATCTAGTAGTGTGCAGAGGCCCTTTCAGAGCATTG
TGGAAGTGTAACATGTAATTTTACTTTTTACTTCCTGAGAACGTCTTTTCATGTTGTTTTGTAGTCT

TCTACCATATAATTATTAGTGGTTTTATAGTATTCTGTTTTATGTCTTTATCAACGTTTATTCAACCA
ATACCCTGTTTGGACATACTGATTATCTCATATTTTTCCCTTATACAAACGCTATAAGAAATCCTT
ATAGCCAAGTCTTTGCACAGTGTAAGATTATTTCCTTACGATAAACTTCCAGAAGTGGAATTGAT
GAATCAAAAGGTTTTGCAGCATTTTAAGTCTTTTTGAATTATATCACCAAAGTACTCTCTCATTTC
TGTGTACCCTGTGTGACATGTGACACAGGCTATTATTTCTTTCAGACTTCGTTGCTTTGATAAATA
AAATGCCCCCCTTTTTAATGAAGGCACCTGACTTTTGATACTGGTGAGCCCCAATATGGTACCAC
AGTATTTTAATATCTGAGTATCAGGTGTAGATTCACTCTAAGAAGTAGCTAACTTCCACTTTCTTT
TAAACTACTAATCAAGCAGTTTGTCTGCAAGCTCCATTCCCTCCTCTCTCTGTACAGATAATTACA
TGTTCATATTCTCAGAATCGGCTATTGAAATCAGCCATATAAAGTAAATAGGGAGTAGCTTTCCT
TGAAAATACACCTCTTTGTGATTCTCTGAACTTGGCAGTGGACCTGTGGCCCTTGTGGCTTTTGCT
CACAGGGCTGAAATGCCCACAGCCTTGGGCTTCCAAGTGGCTGGTGTTGACCTGGGGTTCACA
CACAGAAACTCATTCAGTTCATGTTTATTGTTTCCCACTTCCCTAAACTGAGGCAATGTGCCATG
CTCAGTACCTTATTGAACACTTAATTCAGTGCAGGGGTACTGGTGGGAATTGCAGGTAGCATAA
AAATTGGAGTAAAACATAGCCTCTGTTTATGAGTATATGATTTAGGTAGGGATAATTAATTAAAT
AAAAAATTACCTCTACCACAGGCAGAAAGACCCTTGCTATATGAGTCTGCTTATGTGCTCAGTG
CTTCTGTGTGCCACACCTGAAAGAGATGGTTGTGTTCTGAATAAGACAGAATTCTATAGACTCCA
CCTCTGTAAGTTACAATGCAAGTCAATGGGAAAATGATTTGAGACTACCACAGTTGTAGAAACA
CATCTTTTGAATAATCCTAGTCATACATTATTTATCTCACTAGCACGTTTTGAGAATTAGAGGTAG
AAAATGATAACACAATAAATATATAAAATCATTGTTAAATCCCATTACCTTTGCAGGGATGTTAG
TTTCTTCTGGATGTATGTGTTTCCCTGCGTTTGTCAATAGCCACATAACGAGTGGCTACTGAGAG
TATTCCAGCCTTCAAAGGCAGGCTCTGAGTGCAAATTTGAACCAGCCTCGGGGCAGCCATTAAA
ACTAATTAGGAAACAAAACTACCTCATGCTCAGTGAATATTTGTAGGTTCTGGAAAATTCTGAAA
GGAAAGGAGTGATTCTAATTAGAAGCAGGTCATCGGGCCAGGCGCAGTGGTTCACTCCTGTAA
TCCCAGCACTTTGGAAGACCGAGGCGGGTGGATCACCTGAGGTCAAGAGTTTGCAACCAGCCT
GGACAACATGGTGAAACCCTTTCTCTACTAAAAATACAAACATTAGCTGGGCGTGGTGGTGGGC
ACCTGTAGTCCAGCTATTTGGGAGGCTGAGGCAGGAGAATCGCATGAACCTGGGAGGCAGAGA
TTGCAATGAGCCGAGACCGTGCCACTGCACTCCAGCCTGGGCGACAGAGCAAGACTCCATCTC
AAAAAAAAAAAATAAAAAAAAAAATAAATAAAGCAGGTTGTCATAGTTACTTTATTTAAAAAGAA
ATGCAGAGTTCAGGACCTGCTTCCATCTGTGGACAGAAGGGCAACGAAGGGAGGCCTGGGTGT
GGAGAGGAGGAAAAGGGACAGAAAGCACAGGATCCCTGTTCCTAGGTAGTAGAGCTGTGAGG
ACGTCCTGTGTCCACATCCTCCATGGGTCCCTAAGGTAGCAGAGTCAGCCTCCCAGGAGGAGA
AACTGGGATGTGTTTGATTCAAATCATTAAACAGGAAATTCTCCTCCTCCTCCTCTACTTCCTTCT
CTTCCTCCTCCTTTCTTCCTCTTCCTTCTTCCTCTTTTTTCTTCTTTCTTCTTCTTCTTCTTTTTTTGAG
ACAGGGTCTGGCTCCTGTTTCCCAGGCCTGGAGCACAGTGGCGTGATCTCAGCTTACTGAAACC
TCCACCTCCCAAGCTCAAGCCATCCTCCTACCTCAGTCTCCTGAGTAGCCGGGACTACAGTCAT
ATGCCAGCATGCCTGGCTAATTTTTGTAGAGACTGGGTTTTGCTATGTTGCCCAGGTCCTGGGCT
CAAGTGATCTGCCTGTCTTGGCCTCCCAAAGTGCTGGGATTACAGGCATGAGCCACCTTGCCTG
GCTTCCTCTCTTTCTTTTCCTCAACCTCCAAGCAAACCTGTAGTCAAAAGCTGAAACATTAGGGT
GTTTGCATATTTTATTGAAAATTTTCTACTGTATATTTTTGTCTTGTTTTGTGTTCATCTGACACGTG
TATATTTTTTTCCAAATCTACTTTTCTGTTTAAGCACTGTCTTACATTGTTATCCTTTAGAAGAAATA
GAACTTTGTTCTGAAGTTTTATATCCATTCATTACTGTTAATGTGCATTTTCTTTGTAGACACCTTA
GCTTGAGAACTAAAATAGCATCTTTATGCTTTATTGGAATTTTCTAGATTGGGAGAGTACCCCTTA
TGGTTGGATACAATAATTTAAAAAAAACTTCCTTACTAATTTTTCTAAAAGTCCCCCAGGTCTGAT
GCCTAATGTAGTTCACAGCTTGTTAAACCTGTTTATATCAAGTTTCTCAAGTTAAAAAATTCTCAA

ATTAAAAAAAAAAACAAAACTTACTTGTTGTGAGATATACATACAGAAGCATATGTTATAAAGTGT
ATATATGTAATAAAGAATAAAGTGAACACCCCGTAACTACCACCCAGGTAAAGAAATATGGCCA
GCATTGGCCAGGCATGGTGGCTTTCGCCTGTAGTCCCAACACTTTGGGAGGCCAAGGTGGGCA
GATCACAAGGTTGAGAGATCGAGACCATGCTGGCTAACACAGTGAAACTCCATCTCTACTAAAA
AATACAAAAAAATTAGCCGGGCCTGGTGGCGGATGCCTGTAGTCCCAACTACTTGGGAGGCTG
AGGCAGGAGAATGGCACGAACCTGGGAGGCAGAGCTTGCAGTGAACCAAGATTGCACCACTG
CACTCCAGCCTGGGCAACAGAATGAGACTCCGTCTCAAAAGAAAAAAAGAAATATGGCCAGCA
TACAAGCAACACACCTTTGTGTGCCTCTCCCGGCTCACAATAGCCACCCCCCAAATAAGCAACA
TGCTGCTTTTTGTAATATTTTTTCTTTTTAATTTTTAGTCGTACCACCAATGTATTCTCTATTAACTG
ATAAAATTAGTTTGGCTTGTTTCTGAACTTCATATAATTGATTCATATTATAGGTATTCTTTCATAA
GATCCTTCCTTCCCCTTCCTTTTTTCTCTTTTTCCTTTCCTTTTCTTATTTTCTCCTTCCTTTCTTTCCT
TTCCCTTTCCCTTCCCATTCCTTTTTCTCTTCCCTTTTCCTCATGGCTCACTGTAGCCTCGATTTCCT
GGGCTCAAGCAATCCTCCCATATTGGGGGAACCCGCCCCCAATATTTCAACGTAGGTTCTATTTT
CCATAAGTGTCGGCCAGCTGAGAAATAAAGAGAGACAGTATAAAGAGAAGAATTTTACAGCTG
GGCCGCCGGAGGTGACATCACATATTGGTAGGACTGTGATGCCTGCCTGAGCCTCAAACCAGC
AAGTTTTTATTAAGGGTTTCAAAAGGGAAGGGGGTGTAAGAACAGGTAGTAGGTACAAAGATC
ACATGCTTCAAAGGGCAAAAAGCAGAACTACTGATAAGGGTCTAACAAAGATCACATGCTTCTG
AGGGAACAGGACAAAGGGCAAAAGCAGAACTACTGATAAGTATCGGGGGAACCCACCCCCAA
TATTTCAACGTAGGTTCTTTCTATTTTCCCTAAGTGTCAGCCGGTCTGAGAAATAAAGAGAAAGA
GTACAAAGAGAGGAATTTTACAGCTGGGCTGCCGGAGATGACATCACATATTGGTAGGTCCATG
ATGCCCCCTGAGCCTCAAAACCAACAAGTTTTTATTAGGGATTTCAAAAGGGGAGGGAGTGTAT
GAACAGGGAAAAGATTACATGCTTTAAGGGGCAAAAAAGCAGAGCAAAGATCACATGCTTCTG
AGGAAACAGGACAAGGCAAAATCAGAAACTCCTGATAAGGGTCTGTGTTCAGCAGTGCACGTA
TTATCTTGATAAACATCTTAACAGAAAACAGGGTTCGAGAGCAGAGAACCAGTCTGACCTGAAA
TTTACCAGGGCTGGTGTTTCCCAATCCTAGTAAGCCTGAGGGTACTGCAGGAGACCAGAGTGTA
TCTCAGTCCTTTTCTCAACCGCATAGGACAGACACTCCCAGAGCGGCCGTTTATAGACCTCCCC
CTAGGAATGCATTTCTTTTCCTAGGGTCTTAATATTTAATATTACTTGCTAGGAAAAGAATTTAGT
GGTATCTCTCCTACTTGGCACGTCCATTTATAGGCTCTCTGCAAGAAGAAAAATATGGCTGTATT
CTGCCTGACCCCGCAGGCAGTCAGACCTTATGGTTGTCTTGCATTGTTCCCTAAAATCGCTGTTA
TTCTGTTCTTTTTTAAGGTGCACTGATTTCATATTGTTCAAACACACATGTTTTACAATCAGTTTGT
ACAGTTAACGCAATCATCACAGGGTCCCGAGGTGATGTACATCCTCAGCTTACGAAGATAACGG
GATTAAAAGATTAAAGTAAGACAGGCATAAGAAATTATGAGAGTATTATTAGGGAAGTGATAAA
TGTCCATGAAATCTTCACAGTTTATGTTCAGAGATTGCAGTAAAGACAGGCTTAAGAAATTATAA
AAGTATTAATTTTAGGAACTGATATATGTCCACGAAATCTTCATAATTTATGTTCCTCTGCCACGG
CTCCAGCTGGTCCCTCTGTTCGGGGTGCCTGACTTCCCACAACAGATAAGGGTCCAACAAAGAT
CACAAGGCAAAGGGCAAAAGCAGAACTACTGATAAGGTTCTATGTTTAGCGGTGCACGTATTAT
CTTGATAAACATCTTAAACAACAGAAAACAGGGTTTGAGAGCAGAGAACTGGTCTGACCACAA
ATTTACCAGGGCGGAGTTTTTTCCCACTCTAGTAAGCCTGAGGGTACTGTAGGAGACCAGGGCG
TATCTCAGTCCTTATCTCAACCGCATAAGACAGACATTCCCAGAGCGGCCGTTTATAGACCTCCC
CCTAGGAATGCAATTCTTTTCCTAGGGTCTTAATATGTAATATTCCTTGCTAGGAAAATAATTTAG
CGATACCTCTCCTATTTGCACATCCATTTATAGGCTCTCTGCAAGAAGAAAAATATGGCTGTATT
CTGCCTGACCCCGCAGGCAGTCAGACCTTATGGTTGTCTTCCCTTGTTCCCTAAAAATCGCTGTT
ATTCTGTTCTTTTTCAAGGTGCACTGATTTCATATTGTTCAAACACACATGTTTTACAATCAATTTG
TACAGTTAACACAATTATCACAGTGGTCCTGAGGTGACGTACATCCTCAGCTTACAAAGATAAC

AGGATTAAGAGATTAAAGACAGGCATAAAAAATTATAAAAGTATTATTTGGGAACTGATAAATG
TCCATATTAAAATGAAATCTTCACAATTTATGTTCCTCTGCCGCAGCTCCAGCCGGTCCCTCCGTT
TGGGGTCCCTGACTTCCCGCAACATCTCTCCCTTTCTTTTTATATAAATGTGCCATGGCGATGAA
GGCTTGTTCGTTCTCCCAGTTTTGACACAGGATTCTTTGACTGGTCTGGCACATGGTATCCAGATT
CCTTCTTGATCTCGAACCAACATTATACTTTTCCTGGAGTCAAAACGGGAGTTAACACAAGTGTA
TAAATGACAATTAATGCATTGGACAGTTTGATTGTTCGTCCAAATTTTGATATTTTCCACTAACAG
CATGTAAGGAGGCTTAACACAACTCTGTATAGGAGTAGTCAGGTTGGCGGTAAACAAAGCAGA
ATGTTTGAGTCTACGTTGATACTGAGGGAGAGGAGCGGCAGTGGCAATGCCCAATGTTCTGCGC
CACAAAGAAGCAATCCTAGGTGCCCGGGGATGCCGAACAGGTAGAGGAGCATACCTGGGTCA
AGAAGAATTATCATAATGCCAATTGGAGTCCCATAAAGGAGGATCAGCATCAAAAAGAGGGAA
AAGGTTCAAAGGGGATTTATCATGGAGTTCAGAATCACGGATGCAAGGGGCGGTAGTGGGGAC
AACAGACAGAAACGTTTCCCCTTCCCATACTTGCAGTCTGGACATGGCAATAGCCAATTTCCAA
AGTTCTGGGTGTTCTGGGCTCGGAATGGGGAGTATCCATACTAGGCCTCGGTCGGGGTGGGGG
GTAATGCCCTTATCTTCCTGTTTTAAGGAAAAGGACGAGCTGAACCTCCTATGCCAAGTAGGAT
GATGATCCTCGTCATCCCAATAAGAAATATAAAATAAGTAGCCTCCAGGCATTCCCTTCTGCCA
GAGGAGCAATCGTTTTTTAAATAGCCCTTTGGTGCCCAGTCTATTACTAAACCATATGAGTCATTT
TTTTTAATACTACTGCATGTGAATTAACACAATCTTCCCAAATTAAAGTTTTAGATGGGCCCTCAA
AATTTTTAGGACATGGTTTTCCTACAGGTTTATATTGAAGGTATGGGGTATCTCCTATTACTCCCC
CTTTCATTTGTCTTAAAGGAGAAAGGGAGAGGCCGGAGACCAAATGTCCCCATTCCCTTGTGGC
TGATCTCTCCAGAAGGTAAGCAGCCCAGACTTGAGTTTCTAGATGGATACAACCAGGTGCATGT
CCAAAGCCCAGAGGAGGGCATTTATAACCCATAGTAACATTAAATGCAGTGCTTTCTTTTCCTGG
TTGAGCAGGGCAATGTTCATCTGTAGCTCCAGGCAGCCACACACGATATCGTTAGCATAGATTT
CCACAGGAGCATCCATCCAGGTGAGAGATTGAATAAGTGGGGGAAAAAGCACATAAGCCTAAT
AAGAATAATTTTGTGTAGCAGGTAAATCAGTTTGAGGGGAAACTGGTAAGACAAAGTGTAAGG
GGGAGAATTATTAAATAAAACCTATTATAAGCGAGATCCAGTGCTAAAGGAGGAAGAGAAGAA
CAGGGGGATGTTATTTTCAGGCTAATAGAAATGGTGAGATTTTTAGGTTCTTAAGGAGAAAAAG
GGAATTAGGAGAAGTGGGATTAGTTAGAGGGGTCTCTGTTGCCATTAGGGAGGATTGAACCAC
ACCCATTTTAATTTGGCGTGCCAGTTTCTGAGGAGTCGGCACAGATCTCACCAGGTATGAAGGT
GGTCTCTGACGCAGACGTCTCTTCCCTATGGTTTTTATTGTCAGTATTCACACGAAGTTTAAGTCT
CCTAGTGGGCACCCAGACAGGGGATTGATGATCTCCTGGTGAAACACAAGCATACCCTCTTCCC
CACGTTGTAATTGTTCCAGCTTCCCAGGTATTGGTCTGGGAGTTTTTCCCTAACACTGGCTTGCCT
TCATTTAGGGAGAATTTTTTGCCTGTTCAGCTGCAGTCAGAGTATGTCTTTAGGAACATTTAGAA
AGTTTAAAGTAAACAATGCTAAATGTAACTGGGAGTGGGGAGTGGTTAAATTATGCTTTTGTTGC
TCAGACTGTTTGGACAATTGAGTTTTTAAAGTGCAATTGGCCTGTTCCGCCACAGCTTGTCCCTG
AGGATTGTAAGTTACTCTGGTAATACGGGAAATTCCCCATTGTTGCATAAATAAATCAAAAGCCT
TACTAACATATCCAGGGGCATTGTCTGTCTTTATTTGATATGGAAGCCCCATAACTGCAAAGCAA
GAATACAGATGTCTTTTAATATTGGCTGTGCCTTCCCGTTTGGCAAGTAGCCCAAATAAAACCTG
AAAAGGTGTCTACAGAAACATGTACATGTGAAATTCTGCCAAAGGAGCTAACATGAGTCACATC
CATTTGCCATAAATCATTAGGAGTTAGGTCTCTGGGATTAATGCCAGGTTCCTGATTTGGAAGTA
CAAAAACTTGGCACTGAGGGCAGTGGTGGACAATAAGCTTAGCCTCCTTCCAGGTGAGAGCAA
ATTTATCTTTTAATCCAGCAGCATTGACATGAGTGAGATTATGGAACTCCTGAGCTTCTCGGATT
GCAATAGAGACCAGTCGACCTTATCGTTACCAGCAGACATGGGTCCCAGTAGAGTGGTATGAG
ATCTAATATGTGTAATATAGAAAGGGTGTCTACGTTGGCGAACCGCCTGTTGTAACCTTGAAAAT
AAAGAAGCCAATTCAGAATTACCAATATGTTTGATAGTAGCGGTTTCTATATTTTTAGTGGCATGT

ACAACATAAGCAGAATCAGAGACAATATTTAAAGGTTTGGGGAAATCCTGTAAGGCAATAATTA
CAGCAATTAACTCTACCTTTTGAGCAGAGGTATAAGGGTTAGAAATAAGCTTGTCTGTAGGACC
CACATAACCAGCATTTCTGTTACTGGAGCCATCAGTGAACACTGTAATGGCCTCAGGAATGGGC
TGATCTTTGGTCAATCAAGTGACCACCCAAGAAGTCATTTTTATAAAATCAAACAGTTTTTTTTTT
TTTGGATAATGATTGTCAACAACACTGATAAAATCAGCCAAGTGAATTTGCCACAGTATGGAAT
GTTGAAAAGTGGCCTGAACTTCGAGTTGATTTAAAGGAACCACAATTAAATTCGGATCAAATCT
GGAAAATTTAAGTATTCTACACCGAGCTTGTCCAATTAGGGTGGCCATTTGGTCCAGATAAACA
GACAAAGTTTTTGACACAGAATGAGGAAGAAAACACCACTCCACTAAGTCATTATGTTGAACTA
TTAGCCCAGTAGGGGAGTGCAATGAAGCGAAAACCAGAAGCTGAAAAGGCTGAGACGGCTGT
ACCCTAGACAACTGGGCAGTTTGGATTCTTTACTCCATGAATTCCAGTTCTAGTGAAGCCTCAGG
GGTGAAAGTCCTGGGACTGCAGAGATTGGAATCTCCACACAGCATAGAAAACAAGTTAGATGG
CGCGTATGTTGAAATGCCTAAAGTAGGTCTTAATTAATGTTATCTAAAAGTTTTTGGAATCATTTA
AGGTTTTCAAAGAATCTCTCCTAATCTGAACCTTTTGAGGTTGAATATATTCTTTATTGAACACCA
TTCCTAAATATTGAACAGGAGTGGTCTGTTGAATTTTATCCTGAGCGATGTGTAATCCAGCCTCTT
TAACACAATGGCTCAAAAATTGTTAACAGTCAATTCTTTATCAGTGGGGGCAGCGATTAATATAT
CATCAATATAATGAAGAATATAGGCCTGGGGAAATTGAGCTCAAAGTAGTGAAAGCACCTGTCC
AACATAAAGCTGGCAGATGGTAGGGCTATTCAGCACTCCCTGAGGAAGTACTTTCCATTGATAA
CGAGCTGCAGGCTCCTGATTATTGATAGATGGTACAGTAAAAGCAAATTTTTCACAATTCGATTT
ATGTAAAGCAATATGAAAGAAATAGTCTTTAAGATCAGTAACTATGAGAGGCCAATTCTTAGGT
ATTAAAGCAGGGGCAGGCATCCTGGGTTGGACAGCTCCCATAGGTTTAATTACAGCATTAATGG
CCCTTAAATCAGTTACCATCCTCCACTTGCCTGATTTCTTTTTTACTAGAAACACAGGAGACTTCC
AGGGGGAGAGAGAAGGTTCCACATTTCCAAGTTGTAACTGTTTAGCAACCAATTGAGTTAAAGC
CTCCAGTTTTTCTTGAGAGAGCGGCCGCTGCTCAATCCAAACAGGTGTTTCAGATTTTCATTGTA
GGGAGGCATAGCAGTGGCTGTCATTGAAAAGGATGACCTAAACCAGCCCTCTTCTACAGTAACT
TGAAGAGGTTTAGTAATTCCTTCATGTCTTGGACCGAGACCGAGTCCGGGAACAAACCCCATGT
TTTCCATTATATTTTGACTGGGAGCACTGTAAGAGTTATGTGGAATATTAATTTCAGCCCCCCACT
GTGTCAGCAAATCTCTACCCCAAATATTAGTGGAGATTGGCATGATATAAGGCTGAATTTTACCC
TTTTGACCCTCAGGGCCAGTGCAAGGCAAGATAAATGTGCTCTGGTGAACTTCATCAGCTTTTCC
AATCCCTGCTAGTTATATGTTAGTGGGATGTTTACGCCAGGAGGAAGGCCATAAATTAGAGGAA
ATAATAGAAACATCAGACCCAGTATCTAGTAGGCCCTTAAACTTTTTTCCTTGAATGTGTATGGT
GCAGGTGGGCTGTTGTTCAGAAATTACATTAATCCAATAAGTGGCTTTTTCACTGTCAGAGCCCA
TCCTAGGACCCCGTGTCTTATCTCCTTTGTTTAAAACAATATTAGGTAGTAAAAGTAATTGAGCA
ATTGACTCACCAGCCAGAGTGGAAACAGGAATTTCAATGTAGGTTCTATTTTCCGTAAGTGTTGG
CCGGCTGAGAAATAAAGGCAGCAGTATAAAGAGAGGAATTTTACAGCTGGGCCTCTGGGCATG
ACATCACATGTCGGTAGGACCGTGATGCCCGCCTGAGCCTCAAACCAGCAAGTTTTTATTAAGG
ATTTCAAAAGGGGAGGGGTTGTAAGAACAGGGAGTAGGTACAAAGATCACATGCTTCAAAGGG
TAAAAAGCGTAACTACTAATAAGGGTCTAATAAAGATCACATGCTTCTCAGGGAACACGACAAA
GGGCAAAAGCAGAACTACTGATAAGGGTCCAACAAAGATCACAAGGCAAAGGGCAAAAGCAG
AACTACTGGTAAGGGTCTCTGTTGTTTTCAGTTTGGAGCTGATGTAAACCCTGCTGCTGTGAATA
CTTTGTATGTGAATACCTTATATCTACCTCCCCTTGAGCACTCACAGACAATGTGTTGTGGTGCA
CACACTCAGGAGTGGGAATGCTATTTGCAGGGTATTCACGTCTTCAGTAATACTAGTAATACCA
AACTATTTTCCAGAGTGGTTGCATTCTTACAACTTGTAATGAATATGTTTCTGTTAAAATTGTGTA
AGTGGAAAATATCTTCCCTAATTTATCTCCAGTCTCTAACGTTAATTCAAACGGTTTAGAAAACA
AAACAAACCAAAAATGCTTTTTTACCTCTCCACTCCCGATGGTAAATTTTATCTGTGTTGGTGGA

AAATAGGAATCAGATAACACAAAACAAATTGTAGTGAAAATAAGTTAGAAAGTAGAATATCTAA
TTGGGGACAAGAGAGGGAGAGAACACAGCACAGGGGCCTTGTGTATAATATTAATAATATTAA
TAGCTGTTTAATGAAGATTTATTGGTTGATTAATCTATGGGTATCTAACATACCTGATACACCATC
CTGTCCCTAGGTTGAACTTTCCAAGCAAGTTCTGGGGCTCCTTCTTTGCCATTAGAACTGGATCG
TATGGAAGCTAGAGTGGGAAGCATCTTATGTGTTTCATTACTGGCATCTAGAACCCTAGCAGGT
CATTAATTCAATTAGACACATGTATTCTTTGGTTTGAGATTCTAATATCCTAAAAGCAGGTATGGG
TTTTGTACTGATATACAGGAATGTTCTGAAAATAAGCTGTAATTATTAGAAACAGGTTTGTTTCTA
ATAATATCTGTGGAGGTCAGCATTATTTATTCTTAATGAAAACACCCAGGATTGCCCTTGGGCCT
GTGTTAACAGTGCTCAGGGTAAAAAACATCATAATGACAAGCAGAGTAATATTTACTTTCAATCT
GTTATTGAAAGACTTTATATGAATAGTTGCTTTTTAACCAACTTATCTCATTTTTTAAATTGAGGGG
AAATATATATACCATTAAATTTACCAATTTTACCTTATTTTTTTTTTTGAGATGGGGTCTCACTCTG
TCACCCAGGCTGAAGTACAGTGGTGTGATCATGGCCCGCTGTAGCCTTGAGTTCCCGGGCTCAA
GCAATCCTCCCACCTCAGCCTTCCAAGTAGCTGGGACTACAGGTGTGCATCTCCATGTCTGGCT
GATTTTTTTATTTTTTGTAACGACGGGGTCTTGCTATATTGTCCATGCTGGTCTGAAACTCCTAGG
TTCAAGCCATCCTCCTGCCTCAGCCTCCCAATGTGTTGGGATTATGGGTGTGAGCCACTGTGCCC
AGCCCATTTTTACCATTTTTAAAGTGTACAGTTCAGTGATATTAAAGTACATTCACATTATTTTGC
AACCATTTGCCTTCAGAACTCTTTTCATCTTACAAAACTAAAACTCTATACCCATTAAACACTAAC
TCTTTATTTCCCCTCCCCTCAGCCCCTGGCACTCACCCTTCTACTTACTGTTTCTATGAATCCTACT
GCTATCTACTCTTGGTCCCTTATATCACTGGAGTCATAAGGTATTGGTCTTTTTGTGACTGGCTTA
TTTCACCTAGCATAATGTCCTCAGAGTTCATCCGTTTGGTAGCATGTACCAGAATTACTCTCCCTT
TTAAGGCTGAATGATATTTCATTCTGTGTGCGTGTGTGTGTGTGTGTGTGTGTGTGTGTGTGTGTG
TGTGTACATATACCACATTTTGTTTACCCGTTCATCCATCAGTGGACATTTGGGTTGTTGCTGGGT
GTGGTGGCTCACACCTGTAATTCCAGCCCTTTGGGAGGCTGAGGTGGGAAGATTGCCTGAGCC
CAGGAGTTTGAGACCAGCCTGGGCAATATAATAAGACCCTGTCTGTACCAAAAATTAAAAAAAA
AAAATTAGCTAGCCTGGTGGTACGCACCTGTAGTCTCAGCTATTTGGGAGGCTGAGGTGGGAGT
ATAGCTTGAGCCTAGGAGTTCAAGGCTGCAGTGAGTTGTGATCACGTCTCTGCACTCCAGCCCG
AAAGACAGAGGGAAACCCTGTCTCAAAAAAAAAAAAAAAAAGTCATTTATATTGGTTTCACCTTTT
GGCTACTGTGAATCATGCCGCCATGAACATTGGCATACAAATATCTGTTCAAGTTTCTGCTTTCA
ATCTGATAGTTGCTTTTTAATCATTTTATACCAACCCCCCTTACTAGTGTTGATTTGACAATATTCT
TATCATTCCTTGGAAACTTAATCAGTTTAGTTACCATTCTCGTTTTCTCATCTTTATGAGAAAAGGT
AGAAAGAAAGGAAGTGAAATGCCCAACACCACTCATTTTTGTTAATGGTATTGATAAAATTGGC
ACCAGCCTTTCAGGAATTCGGATGCTTTGATTGTTCTTTTTTGTTGTTAGATTTTTGTATCCATTAT
GCATTTTGAATCATAATAAAGTGGAATTAAAAAAAACTGCCATATTTGTCTAGCATATATGGCGT
TTTATGATTTTAAAGATATTTTTAAAGTTTGGTTAAAAACCTTAACAGTATATGTGAAATGGAACA
CTGTGCTTTTACTGTGGAAATTTATCCTATATACCTTAATAAAATAGTTATTTAAGATTTACATTTG
GCTGGGTGCTGTAGCTCATGCCTTTAATCCCAGTACTTTGGGAGGCTGAGGCAGGCAGATCACT
TGGGCCCAGGAGTTTTCGAGACTAACCTGGGCAACATTGGGAAACCCTGTCTCTTCAAAAAGTA
CAAAAAGTTAGCCGGGCATGGCGGCATGCGCCTGTAGTCCCAGCTCCTCAAGAGTCTGAGGTG
GAAGGATTGCTTGAGCCTAGTTCGAGGGAGGTTGCAGTGAGCCAAGATTGCGCCACTGCACTG
CAGTCTGGGTGAGAGTGAGACTCCTTTTCAAAATAAAGAAAGAAGGAAAGATAGATAGATTTAC
ATTTTAGAGAGTTAGTATATGTCTGTGATTTTTGAACTTTATTGTTTCAGGGCTCCTTTATATTCAT
AAAAATTATTGAAGGTCCAAAGGAACTTTTGTTTTTGTGGTTATCTGTTGATATTTACCATATTAG
AAATTAAAACTGGCTGGGTGCAGTGGCTGACACCTGTAATCCCAGCTACTTCAGGAGGCTGAGC
AGGGGTGGATCACTTGAGCCTCGGAGTTCGAGACCAGCCTGGCTAACGTGGCGAAACCCTGTC

TCTACACACAATACAAAAGTTAACTGGATGTGGTGGTGTGCACCTGTGGTCCTAGCTACTCAGG
AGGCTGAGGTGGGAGGATTTTTTTGAGCCCACGAATTTGAGGCTGCAGTGAGCTGTGATCACAC
CACTGTACTCCAGCCTGGGCGACAGAGTGAGGCTGTGTCTCAAAAAAATAAAATAAAACTGATA
AAAAATATTCCCTAATTTATTAAAAACATAAACCCATTAAATAAGTTATATATTTTTATGAAAACA
ACTGCTTGTCCTAAAACTAAAACATTTAATAATAAGAGTGTATTGTTTTATATTTTGGCAGCAATA
TCTAATGTCTGGCTTACTTAAAGATGGCTAGGTTCTCATATTTGCTTCTACATTTAATCTTGCAATA
TGTCGTTTTTGTTGAAGTATATGAAGAAAATTCAGCCTCACACACATATATAATTGGAAAAGAAA
GGAGGATTTTAATAGTCTTCAGATAATCATGGATGTTTTTCTTTGATAACCACACTAAAATTCAAC
AAGTGGTAGTTTCTCAAAGGTTAGTTGCAATGTAGAATTTGAATGAAATATATCAGTGAATTTTTC
ATCCTTGGTTACATTAAAATTCATTTGTCTTCTTTGCACTTTTTTTGTTTTGTTTTGTTTTTGAGACG
GAGTCTCACTGTCACCCAGACTAGAGTGCGGTGGTGTGATGTCGGCTCACTGCAACCTCCGCCT
CCTGGGTTCAATTGATTGTCCTGCCTCAGCCTCCTGAGTAGCTGGGACTACGGGCACCTGCCAC
CACACCCAGCTAATTTTTGTATTTTTTAGTAGAGATGGGGTTTCACCATGTTGGCCAGCCCGGTC
TCGAACTCCTAACCTCTGGTGACCCCCGCCTTGGCCTCCCAAAGTCTTGTACCTTGAATGGATCT
TTTACTCAAGTATAATTTCTATCATCTTTGTTGGTCATTTGGAAAATATTGGTTTACCAAGTATGTG
AGTCTTCCAAATATTGATATATAATGAAATATGGGATAAAAAATAAAAAAAATCACATTTATTAA
CATCACCCCCAGTTACGTGGGGAGCTCGCAAGCTCATAGTGATGAGTGCTGATTTTCGCAAACA
TCTAAATTTAACTTGAAAGCTCAGTTTTTCTTTTCTTTTTTTTTTCTTTTTTTATTTTTATTGATCATTC
TTGGGTGTTTCTCACAGAGGGGGATTTGGCAGGGTCATGGGACAATAGTGGAGGGAAGGTCAG
CAGATAAACAAGTGAACAAAGGTCTCTGGTTTTCCTAGGCAGAGGAACCTGCGGCCTTCCGCA
GTGTTTGTGTCCCTGGGTACTTGAGATTAGGGAGTGGTGATGACTCTTAACGAGCATGCTGCCTT
CAAGCATCTGTTTAACAAAGCACATCTTGCACCGCCCTTAATCCATTTAACCCTGAGTGGACACA
GCACATGTTTCAGAGAGCACAGGGTTGGGGGTAAGGTCACAGATCAACAGGATCCCAAGGCCA
AAGAATTTTTCTTAGTACAGAACAAAATGAAAAGTTTCCCATGTCTACTTCTTTCTACACAGACA
CGGCAACCATCCGATTTCTCAATCTTTTCCCCACCTTTCCCCCCTTTCTATTCCACAAAACCGCCA
TTGTCATCATGGCCCGTTCTCAATGAGCTGTTGGGTACACCTCCCAGACGGGGTGGCGGCCGGG
CAGAGGGGCTCTTCACTTCCCAGTAGGGGTGGCCGGGCAGAGGTGCCCCTCACCTCCCGGACG
GGGCGGCTGGTCGGGCGGGGGGCTGACCCCCCCACCTCCCTCTCGGACGGGGCGGCTGGCCG
GGCAGAGGGGCTCCTCACTTCCCAGTAGGGGCGGCCGGGCAGAGGCGCCCCTCACCTCCTGG
ACGGGGCGGCTGGCCGGGCGGGGGGCTGACCCCCCCACCTCCCTCCCGGACGGGGCAGCTGG
CCGGGCAGAGGGGCTCCTCACTTCCCAGTAGGGGCGGCCGGGCAGAGGCGCCCCTTACCTCC
CGGACGGGGCGGCTGGCCGGGTGGGGGGCTGACCCCCCCACCTCCCTCCCGGACGGGGCGG
CTGGCCGGGCGGGGGGCTGACCCACCCACCTCCCTCCCGGACGGGGCGGCTGGCCGGGCAGG
GGGCTGACCCCCCCACCTCCCTCCCGGACAGGGCGGCTGGCCTGGCGGGGGCTGACCCCCAC
CTCCCTCCCGGACGGGGTGGCTGCCGGGCGGAGACGCTCCTCACTTCCCAGACGGTGTGGTTG
CTGGGTGGAGGGGCTCCTCATTTCTCAGATGGGGCGGCTGCCGGGCAGAGGGGCTCCTCACTT
CTCAGATGGGGTGGTTGCCAGGCGGAGGGTCTCCTCACTTCTCAGACGGGACGGCCGGGCAGA
GACGCTCCTCACCTCCCAGACGGGGTCGCGGCCGGGCAGAGGCACTCCTCACATCCCAGACG
GGGCGGCGGGGCAGAGGCGCTCCCCACATCTCAGACCATGGGCAGCCGGGCAGAGACGCTCC
TCACTTCCTAGATGGGATGGCGGCCGGGCAGAGACGCTCCTCACTTTCCAGACTGGGCAGCCA
GGCAGAGGGGCTCCTCACGTCCCAGACGATGGGCGGCCAGGCAGAGACGCTCCTCACTTCCC
AGACGGGGTGGCGGCCGGGCAGAGGCTGCACTCTCGGCGCTTTGGGAGGCCAAGGCAGGCG
GCTGGGAGATGGAGGTTGTAGCAAGCCGAGATCATGCCACTGCACTCCAGCCTGGGCACCATT
GAGCACCGAGTGAACCAGACTCCGTCTGCAATCCCGGCACCTCGGGAGGCTGAGGCTGGCGG

ATCACTCGCGGTTAGGAGCTGGAGACCAGCCTGGCCAACACAGCGAAACCCCGTCTCCACCAA
AAAAATACGAAAACCAGTCAGGCGTGGCGGCGCCTGCAATCGCAGGCACTCGGCAGGCCG
AGGCAGGAGAATCAGGCAGGGAGGTTGCAGTGAGCCGAGATGGCAGAAGTACAGTCCAGCTT
CGGCTTGGCATCAGAGGGAGACCGTGGGAAGAGAGGGAGAGGGAGACCGTGGGGAGAGGGA
GAGGGGGAGGGGGAGGGGGAGAGGGAGAGGGAGCTCAGTTTTTATTTTCAACAACAAATTCTG
TTAGTTGTTTTCCTTAAAGTGGCAGGCTTACTTTGTTCATGTTCAAGAAAATGTCGGCCAAATATC
CCAGCTGGAACAACCATAATTTGTCATTCATACTTTTTAGTAAACTTGGGGTATTAGCCCATTTGC
ATTACTATAAGGAATACCTGAGACTGGGTAATTTATAAAGAAAATTGTTTTAATTGGCTCATGGC
TCTGCAGGCTTTACAGGAAGCATGGTGCTGGCATCTGTTTCTGGTGAGGCCTCAGGAAGCTTAC
AAACATGGCAGAAGGTGACAAGGAACTAGCATGTCACATAGCTAGAGCGGGAGCAGCAAGAG
AGGTGGGAGGTGCTACACTTTTTTTTTTTTTTTTTTTTTGAGACGGAGTTTTCTCTTTCGCCCAGG
CTGGAGTGCAGTGGTGCGATCTTGGCTCACCGCAACCTCTGCCTTTCGGTTTCAAGCAATTCTTC
TGCCTCAGCCTCCTGAGTAGTTGGGATTACAGGTGCCCACCACCATGCTCAGGTAATTTTTGTAT
TTTTAGTAGAGACAGGGTTTCACCATGTTGGCCAGGCTGGTCTCGAACTCCTGGTGCTACATTCT
TTTAAACAACCAGGTATCGTGAGAGTTCACTCACTGTCAGAAGACAGCACCAAGACATTCATGA
GGGACTGCCCCCATGATCCAAACACCTCCCACCAGGCCCCACCTCTAACACTGGGGGTTACGTT
TCAGCATGAGATTTGGAGGGGCCAAACATTCAAACCATGTCACATGGTATTCCATGAAAAAAGC
AAGCAGTTCATTTTGCAACTTAAACTGTATAATACATGCTTTTTCCTGGGACTACCATGGTGCTTC
AGTAGTCCACAGAAGTACTTTATGTGTACCTACTATTTTGGGACACAAGGGTATTAAAAACCATG
TACTCAAGGGCTGATATTTAATAAAATTAATAATTTTTACTGCCGTATCAAAGATGTTCTTAAGTG
AGGCTGGCTTTTTTTTTCTTTCTTTTTTTTTAATCTATGACTACTGGTGCAGTTGGTACCTCTACCTTG
ATTAGTGTTAAAGAGCTTGCAGTTTGACCCACTGTTGATTTTGCACCATGGGTACAAATGTGAAC
ACGGAAGAAGGCAAATTATGTCCTAGTATTATGATGAAAGTAGAGTCTTTTGCTGGGACTTGAT
GCTACAGGCCAGTAGTTCTCAAAGCCTGGTTCCCTGCCCAGCAGCATTAGCATCTCTTGGGAGC
TTATTAGAAATGCGAACACCTGGGCCCTTCCCTTCCCTTTCAGTGTCCAGTTCCTGTGGGATGGG
GCTGGGCCGCTTCAGCTGCCCCAGGAGACTGAGAGAGTCTGCTGAATTTGAGAAAGTCCACTC
TGCTTTCTGGAAGGACTGGCGGGGCGGGAGGGCAGGGAGGATGAGTAAGTTAGGGACAGAGT
GTTCTCGTGTTTGTAGAGGATGTGATGCAGCCTGGACTTCAGGCAGTAGGAAGAAGATGGAATG
GATTTGCTAGAGACAGATTCTCTAGAACTTTATTGCTGGTAGACCACATAAAAGCTTAGAAAACA
GCTGCGCTTTGCTCCGAGACCTGCCAGCCATCAGGGACGAATGAGTTTTAAAGGCCCTTACTGC
TTTGTGTCTTGAGGGAATATCTTTTATTAATCGTGAAAAAATCAATCCAGATAACTTGCCTGAGTC
ATTTCAGTCTCCTTTTTGAGGATGCCTGCTTAAACAGCGACCATGGTCTCTGGAGGGGTAGAGTC
TCACATTCGGAGTTCAGTCTCTTAGAAAGTACAGTTCAGTGTGGAAGAATGGCAGTTATGATTTC
ATTCAGATGTGCCCAGCTGCTGACAAATTGAATACAACGATCAGTGGCAGGCAGAACTCTCATT
TGTTGTGTGTTGTCTTGAGTCTGGTGAGGTGTATCCTTTCACTATTGGATTTTACCATTCAGTGCA
GTCAGTAGCTCCATTTGATGTAAGTGTAAAGATGGGTCTCTCATTTCAGTTTCAGCAATTATTCTT
AAAACCTTTATTTTCCAGTGTTTTAAAGTTTTTAAATTTTATATCTTTTTTTTTTTTTAAACAGATTTG
TAAGACTCCAGGGCCTCCCAGCCGTGAATAATCTGATGGTTCCTGAAATGACTGGGGAAGCAG
ACGCTTCGTATGGCAGTTGAAGAGTGTGTGTCTATGTGCATTTAAAACCTTCTTTCTGTACTTACA
CATTCACACGGGAAGACAGGCTCATTCTTGTGCACACTTGAGAGTTTTACAACTGATGAAAATTA
ATTTAAGAATCAGATGGAGCAACTTGACACCAGTGGGCTCAGGAGCCCGGGGAGAAAAATACA
TCACTAATGGCCAGTTTTCCATATGGTCTGCACGGGTAAAGAAAGT
ID|56108|gene_id|ENSG00000223812|transcript_id|ENST00000439804 Homo sapiens lncRNA

AAAAAGAATCTGGTTGAAACCTCAGTAACAGTTGGCCTGTCTACTGCCTCACACTAACAAG
GTAAGTCAGCTACCAGTGACAATGTGAGCAAGTTGCAACAGTGCCTAGGACCCCTGTATAACCA
CCCCACCCCCACCGCCGGATGTAACAAGCATATTATTGTTGCTTCATTTCCCTTTCTGAACTCAG
CCTCAGTACCAGACACAAGGACAGCAGCCTTACATGTGTTTTTAATCAGCTCCCACAATTGTATA
CCATCAAATATTTACAGTCAATCAAAGAGTGAGTGTTGGTGTGTGACTCCTAGTGCTGCAGTTTC
TTCTCTTGAATACTCATTGATTCATAATTCAAAAACCATTAGACTCCATAGGAGCAGGATTTGAT
GCCCTAATGGGAAACAAACTTTGCTTTCAAGCTGTGCTACTAATGATGATCATAACAATAGCTAA
AATTTACTGTGCTCTTACCATGTGACAGAAACTGTTCTAAATGTTCTATGTTAACTCTTTTTTTAAA
AAAAATAGGCTTGGAGGTAGGTACTATTACTATCCTCATTTTTATTTTACAAGTCAGGCAACTGA
CGTGCATGGAATTTAAGTCACCTCTCCAAGGTCACATAGTTGATGTAGGGAATCCACATCAATTA
CCAGCTATGACTTAAAGCCAGGGAGTCTGGCACCTTGAGAGCGTGTTCTAACCATTGTGTTTTTC
TACTCCCAGACCAATTCTCTAAATGAAGTTCATCCTGAACAATCTGATCTCAGATTTACTCCTATG
TCAGCTAATCCTCTGGAGGAAGCGGAAATGAGATAATGGGAATATCTGTGTAGTTATCTGTGGC
TTAGGAATTGTTAATTGGTAAATTGATGTTAAAAGGAAAGAGAAAGAATTAAAAGAAGAGAAAA
TGGGAATATTTTTTGCACCTTGAACATGCAGCCATCAGAGAATGAATAAGCCAGTTTGATAAGTC
TCAGTGAAAGTCAAAAACATTTTCTGCAGTGTGCAAAATATTTTATCAAGCAACACCTTGGCTTA
CTGTGCTAGACATCTGGCAAAGAGGATGTGTTCTAACTTTTTTTGTCCTTCATAGTGTCAGGAATT
TAGAATCATAACTTTTTATTCCATATTTAGTACATCAAAATAACCGCATTCTATATTTAAACATATC
AAATTGTTCTGTTTTTATATAATTTAATTTAAGAGAACCATAACTTAATTTTAAGTTCTGTGATCAA
ATTAACAAGATTCTTTTTATCTAATGTCTTCGATTTTATTAAGGTAAAAGTTATATAACAAAAAATT
AACTGTTTTAAAGTTAACAGTTCAGTGGCATCTAGTACACTCACAATATTGTCCAACAACCACCT
CTATCTAGTTCTAATATGTTTTTATTACCCCAGAAGGAAATGAAACAGAGTAACTGCTTATCATCT
TTGAATTTTAAAGAACATACGTATAATTTTGAGACAAGGTCTCACTCCATTGCCCAGGCTAGAGT
GCAGTGGTATGATCATGGCTTACTGCAGCCTCAACCTTCTGGGCTCAGGTGATCATCTCACCTCA
GCCTCCCAAGCAGCCAGGACTATGGGCGTGCACCACCATGCCTGGCTCTTTTTTTTTTTTTAAGA
GATGGGGTTTCACCATGTTGCCCAGGCTGGTCTCAAACTCCTGTGCTCAAGAGATCCTCCTGCCT
CAGCCTCCCAAAGTGCTAGGATTTATAGGCATGAGCCAATGTGCCTGGCCTAAAATAATTTTTAT
GTGGAAAATTCTAGTTGTCTTTTAAAGTCTACCAAAAATCTCCTATTCTGCATTTATTTTTTTGTCT
TTAACTTACTGATACTTGTATATTCCGCTCATCACTGACATTTTTTTCACTTTGTTGTGTATTTATCT
ATATAATTTATATATATATTATGGATAGCTATTACTAAATTTATGTATTAACATTTTATATATTTTAA
TTGTACTTTAAAGTTTATATATTGATATTTTAGTATTTCATACATTTAATTTTAAGATAGTCAAATCT
GATTATCTTTTTCTTTAAGTTTTGTACTTTCCAATCCCTGTTTTTAAATTTATTTTGCTATACTAAGG
TAATACAAAATGTTTTCCTCTAAAATTTTAAGGCTTTATTTTTCAAGATTTTACTTTGAAATTTATGT
TGTATACAGTGTAGAAGAGCAAATATTATTTGTTTTTTCTTAGGGGGAGTCAGTTGATTCAGAAT
CAGTATTTGATTAGAAAACTCTTTTCTGACTTGTTATTCTACCTCTTTCATTTATCAATTGCAATCT
ACATGTTGTTTTAGAAAGATCCATAAATTATCTACTTCATCCCCTTTATTTTACAGGGGGAGCTAA
TAAAACCGAGGTTAGAACACACAATGTGGAAATGAAAACCTTATTCTTGTCAATGGATGGAGGG
TGGGTAAGTGCCTGGGTAATAGCCTGATTCTTCCTATCATATCTGCTCCTATTGTGGCAGACAAG
AGAGCTTGTCCAGGGGAACTTCCATTGATAAAACCATCAGATCTTGTGAGACTTATTCACTACCA
GGAAAACATTATGGGGAAAACCGCCCCCATGATTCAGTTATCTCCACCTGGTCCTGCCCTTGAC
ATGTGGGGATTATTACAATTCAAGGTGAGATTTGGGTGGGGACACAGCCAAACCATATCACTTG
GATTAGCATTTTAGGTATCTTAGCTCTAGTCCTACTCTCTTTAATCACTATGCTATAGAGCTACTCT
ATCAATTTAAAAACAAATGTTATTCCTAAAACTGATATGTTAATTGTGTTTCATTTAAAGGTAGGA
ATAGATAAAAATTTCTTAATATTCTTAATACCAGAGCTTCTGTTAGCATCATTTCATCTTCTTAAAA

GTCTGAAAGGAAAAGATAAAGAAATAAGAGATACTAGGATTAGAAAAGAAGAAAACCTTTGCC
ACAGTTCATTTAGAGAGAAAGGATAGTGGAGTGGTTAAACAAATAACAGCATAGAACCATACC
CCTCAGTTTCAAACCTGACATTTTTCATTTCTGGCTGGGTGAATTTGAGCCAGTTGAGGGACTTTT
CCAGGTTTAAGTCAATGAAATGTGGTATTAATTATTCTTAACTTTTAGGGCTTTATGAAAATTAAA
TGATGCTTCTGCTCAGTAAACAGGAAATAATTTCCTTTAGATTAAGTAAAAGATAAAAATTTAAC
AATAATGTATGTAATTGGGAATAAACCCAAACAGATACTCATACCAAATACCAAGAATATGGGT
TTTATATCCTGGATCATTCTTTAGAATGAACATTTTTTGACATGATACTTGACTATTTAATTATCTG
TCTTTGTGATATGATTATTCATATCCTTAACTAAAACATCTTTAAGATTTAAAAACAATCTCAAAG
CAAATGGGTAAGGTTCATATCAGAGGATAAATAAGTGGTGTTATGGTTTGAATGTGTCCCCCAA
AGTTTATGTGCTAGAAGCTTGATTCCCCAATGCAGCATTGTTGGGAGATGGGACATTTAAGTGGT
GATAAGGCTATGAAGGCTTTTGTGTATTAGTCTGTTTTCACACTGTTATAAAGAAATACCCAAGA
CTGGGTAATTTATAAAGGAAAGAGGTTTAATTGACTCACAGCTCCACATGGCTGGGGAGGCCTC
AGGAAACTTACAATCATGGAGGAAGGGGAAGCAGGAACATTGTTAGGAAAGAGAGAAGAGTG
TGTGTAAGAGGAACTGTCAAACACTTACAAAACCATCAAGAACTTTCAAACACTTATAAAACCA
TCAAGAGAAGATAGTGAGAACTCACTCACCATCATGAGAACAGCATGGGGAAAACTGCCCCCA
TGATCCAATCACCTCCCACTGGGTCCCTCCCTTAACACATGGGGATTATGGGTATTACAATTCAA
GATGAGATTTGGGTGGGGACACAGCCAAACCATATCAGCTCTGCCCTTATGAATGGATTGTCAT
TATCATGAGAATGGAATTCTTATCTTGGTATTGGGTTTTTCATAAAAAGACAGATTTGGCACCCTT
TTGTCTCTCTCTCTCTCTCTCTCTCTTTCATTCTTGCCCTTTCTTTGCTCTTCTGCCATGGGATGATG
CAGCAAGAAGACCCTCACCAGATTCCAGCCCCTCGATCTTTGATTTTCCAGCCTCCAAAAACAT
GAGCCAGTAAATTTCTGTTTGTTTGTTATAAATTACCCAGTCTGTGGTGTTCTGTTATAGCAGCAC
AAAATGGAGACAAATGGTCAGGTAACACTGAAAGATGCTCATGCTTCCTAGGAAATAGGATCAT
TTATATTTTATAAAATAATACATCAACAATATGATACAAAAATTAAACACCTGGCAATAATATGTA
CAGTTAGAATGAAACCACTAATGATTATTCTTTGATGGAGCAGATAATAGTAAATACAATTAGAT
CAATTTGATAATATCCAGTAAATTTAAAGATGTGAAAACAATTTTCAGAGAAACATGCATGTGAT
TAAAAAGACATAAACAGAAGAGAACATTGCTATATTATGATAGAAGAAAATCTGAATGTTCATT
AAGTGAATAGATGAATAAATTATAATATACCCATAATGCGATATACTACAGGGTACTTCAAATGA
ATCAACTATATTTACACATATCAACAAAGATAAATATTAAAGAATGTAAGATCATGGCAAAAAG
CAAGTTGCAGAAAACTACGTATAGGATGTAGCACTTATGTAAAGTCTAAATCATACAAAATAAT
AATGTTCCAACTCAATAAAGGCAAATATAGGACGTAAATATATGCAACCATGAATGGTATTGAA
AAATGCAAAATTAAGCATATTTAGTATCTTTTCTGATGGGTCATGGAATGCAAGATGAGCATGAG
AGGCTTCAACCCCACCTATTTATTATTTATCACTTAAAAATTATTTTTTGATAACAAAATGATAAA
GTGGCTGAATTTCAAGAGAGACAGTGTCTACATAGGTGTTTGTTGTAATATTCCTTCTACAATTA
AAATTCACTGGGATCAAACATCCCCAGGTCTCTAGCCAGAACCAATAATTACTACGTTTTGAGTC
ATTTTTTAGTCTTTCTTCTGCAGGTAATTCTGATTTTCATTAAAAATAAGGAAGGCATGTTTTGTGC
TTAAGCTGAGACAGGTTTAAGTGACATTATTTTTCAATCCAATTAGGTGCCCTGATTCCATTTATT
CAGAAGGAGATTTATATCTATATATGCCACTTGCAAATATGACTGCCATTTTCTAATTAAGAAGA
AATTTTATCTTGACTAGATCTATTTCACCTATAGATGAATGCTTGATTTAACATTTACATAATTAAT
TGGGTGTGTCTGCTTGCTACTACATTCTCATGAAACTTTGCAGCTTCTCAACTACATCCAAACCTA
TTACCTTTTAAGAACCTAAGTATTTTCCCAGTATAAACAGTTTTCCATCGTATGAGCTTTAAATAA
TATTTTAACATAAAATATACAGTTTACACAGGGATATCTAAAGTGATGTCTATACAAATCCTAAT
GTGAATGTTTTTGTAAAGTGAAATATCTTCTTGTCGTGGGAATTAATCTTTGTAAATATAAATTAT
CTCTTTATTATCTTTCTAAAACTTTTCAATTCCTTAAATTCAGGTGAATGTTAACTCATTTTGTCAG
CAGAATATTTTTCAAAGTTATTTATTATCTATAAAATATATTTGCTTAAGGTTTAGCATTTTGGGGA

AATTATATAACAGCTATCAAATGAGGGTCTTCTGTAAGTGCTTATAAAATCAATAATCTAATGAT
ATAAATAGATCTATAAAGAGATGATAAAATTAAAGAACAATTACTAACATTCTAACTAGTCTAAC
ATTTTATGCCAATCAAGAACATATACCTGTTTAAGATATTCAGAGTATACTTTATGCTTTATTATTA
AACAGTATAAATAATATTTCAGAATTTTTTAGAATACCAAGTATATTGTACATGAACATGGCATC
AAATTTAGTGTCATGGGAAAGAATCTTTAGCTATAATTATGTAGTACTGGTGCAATACGTAATTT
GTTTTAGCGTGACAGACCCCCAGGTCTTCAGTAAGCTCCAAACTGCCAGGGAAAACAGATGAG
TAAGAAACTGGACAGAGAAGCGCAATTTTTAAAATGAAGAACTTGATCTAAATATTTTAGAGTGT
TTCCCATTTAAAAAAAAACAGAAATTGATTATATATATATATATATATATATATATATATATATATA
TAATTGTGTCAGTTCTCCAGATTGTTTCACATGGAATTTACATCTTTGCAACCCTGAAACCCTGTT
TCAGTCCTGTCTTTTTCACTGAGTGTTCCAAAATCTCTTTCCCATAGTTGCTGCATAATTTCTTAAA
TTGCTTAGAAGCTTTCAAGGGGAAAGGAGCTAAGTGAGTAGATTAACGAGGGGCTGTTTGTGAA
ATTCTTAAGAGTTCGTCAATGGCAGGTGTGATAAATTACAACATTGTTATGATGATGATCGTGTA
GCAAACCATTATTAGTGCTTAAGGTGGACTATCTCATTACACAAACACCACTTGAATTTGAACAT
ACGTCAGCAGAAAATGTAAACGGTTCACACAATTCCAACCAAACAGGCATTTCTCTAGTTGAAA
TTAGGCTTGGAAGTATTGGCCATGTTTACTATCAAATGGTTCAGTTACATTTTACTAATGTACAGG
AAAACCTAAACTCATGTACATCGAAAATTAACTCTTAAACATTTCTTTGCGTAATTATCTATTTTTC
TTGAAATTTTTGTATGTGACACGTTCCTTAAATAAAACCTCTGCTAAAATAACATAATTCAATGAG
TTTAAGAGCAGATAAATGACACAGTAAGATATTCAGAAGTTTTATAAAAAATAAATCATGTGAAT
AAACAATTGTGTTTGGTATAAATGGAATATTGTCAGTGTCCTTTTATGAATTAAAATGGCTTTATT
CCAGCCTGGGCAACATGGCAAAACTCTGTCCCTACAAATAATATAAAAATTAGCTGGGCGTGGT
GGCACACATTTGTAGTCCCAGCTGAGGTGGGAGAATCACCTGAGCCTGGGAAATCAAGGCTGC
AGTGAGCCGTGATTGTGCCACTGCACTCCAGGCTGGGCGACAGAGTAAGACCCTGTCTCAAAA
AATTAAATTAAATGGCTTAATAGCCTGGTTCTAAAAACCAATGTGTACCAAATAGAGAAATTCCA
GTTTTTAGCTTCATTCATTTAATCATTGTTAAAAATATCACCGATTATTCTTCACGATTTAAAAAAT
AAATTCTACACAACGTAGACTAATTGAATGTAAATTCAGTGACATAAACTCCGTGCTCATTTATA
CCCAAATCTGTTATTCTTCTACTCTGTATCCCAGCTATGAACACTTTCTATCATCTCCAATGCTCC
GGCCCAAGATTTCAATTGTTTCTTATCTGGAATTGTTAAAGTTGTTTAACTGATGTCCTTGCTTCA
ATCCCTGAACTCCTACTTTCATCATCTAGAGACATCAGTGTAAACTTTTTAAAATGTAAATTGGAT
CTGTTACTCTCAATTAAAACACTCCAATGAATTCCCATTGTAAATGGAATATTTTCTTCATGACCT
CCAAAGCAAGCCTGATCTTGTTTCTGCCGTCGTTTCCAGCTCCTCTTTCATTGTTTCTCTCATCTTC
TCTGGCTTCAGACACACTGGACTTTATTCTATCCATTGAAAATTCCAGGCTTGGTTTAGTTTCAGA
ATCTTCTCCCTTATTGTTAACTTTGTCTGGGATAGTCTCTTCCCTATTTTAAGTATGGCTTGTCTCC
TTATTTATTTCTCAAGTCAGATGTATCATCTTCTTAGCCAGACAGTACCTTGCTAAGATTCATTGTT
GTGCTAGATTATAATTAATTTAGATGTGCTTAGGGTCATTGCAGTTCCAAAATAAAAGATGCCAT
GAGTTTTAGCTGTATTCTAAGATAATGGAATTTATTCTCTATATATCCAAATCTTAGTAAATTAAC
ACCATGAAATACCCAGAATAACCACGTATCTCTAAAACCATATGAAAATAAGTGATGTGCTAAC
GACATTAACAAGTAAGAGAGTGCCAGGAATCCAAAGCATAATGGGGGAGATCCCCATATTGAT
ACCTAATCAATCATGTGCAGTTTAGTTTTAAAGATATATCCTAAAAATCAAAAACAAACACACAA
AAAAACTAAATAAACTAAGAAAGAAGTTTTTTCTCTTCACCATATATTATTACTAGCTGGCCTTTT
GATTTTTTCTGGTTTCCATTTTATAGGAAAACAGATGTTTCTGAACAGGTGTAAGTGGGTGGATTC
AAAAACCTAAAAACATTGAAAGCTTTTATTTCTCGTTTATTTTTAGAAAAGAAAGAAGAAAATAT
ATGATAGTATCCATTCTTTTAAAAGCCTTTATTGGCACTTGGTTTTATATTCTACTGCTATTTTAATT
TTTCTTCCCTTAAGTTAGTTGACCATATAAATTAATAATAATAAAATGAAATTTAGTATTTATTTTC
TTCTTCAAAATTAAACAAGCCCAGAGGGAAAATGCCCAGTTGTCCAGTGGTCTCTGTAATAACTT

CACACAACCTATCCCATCTGTCCTTTTATTTCCATCCCGTGTTCTAGTTCAGGCCCTCGTTATTTC
ATGTTTTTTTTTCTTTTTTTGCAGAAAATTCCTATATGAACTCGATAACTGGATTTTTTGTTTCACTA
AAGTATCTCATAAATAATGGTTCCAAAGAAATGTTTTAAATAGCAGATTTAATCAGGCAGGTGGA
CTGGAGTAAACCTGGAACCCAAAATTAGGATACGTATGTTTATTGTGGCACTATTCACAATAGC
AAAGATTTGGAACCAACCCAAATGTCCAACAATGATAGACTGGATTAAGAAAATGTGGCACATA
TACAGCATGGAATACTATGCAGCCATAAAAAATGATGAGTTCATGTCCTTTGTAGGGACATGGA
TGAAGCTGTTTTTAAACTATTTCTTGTACTTTCAAGCTGTTTTACCTTTTCATCTTATTTATCTTTTTT
GAAAATCTGTTTCCTCAATTTTACAATCTTATTCTCTGACTTATTGCAAGGTTTAAGCAAGGTTAT
GCATATTACATTTTAGCATGATGCCTGATAGTAAACAAACATTTTAAGATCGCTGTTTAATGTTTC
TCCCCCAAGCAAAACCATAAATAGCTTCCCATCACCAAGAGAATAAATTCCAAAAGTTTACCAA
TGGCATTCAAATCATGCCACAAACTGGAACCAACATGTTCATTCAGTGAACTTGCCCCTAATGTA
TTCAGTCCTTTAGAAAGTTGGACATTGTTTCTGTACATGCCTTGCATTCTCATCCCTTTATGTCATT
TTTTGCAGATTCTGTTATGTGGAATGCCTTTATACATCTTTGTCTACTGGTTGGGATGCTGCTCATT
TCCCTTTTCCTGAAAGCTTTCCAGACACTCAAGGTGCCATAAGGCAAAATATAATAGTTTATATTT
CTGTGTCCTCAAACATCGTAAACAGTCAATGTGTCGGATGAACTTTTCTCCAATTCATTATAACC
AGACAATAGGTTCTTTGAAGGCTATGACCAAATCGTGAGCCCATTTTCCTTCCTTAACATACTGC
ACCCAAATGAGCAGAACCTAGTGGAGATCTTGCGGCTTAGAGCTTGCTGAATTTGGTAAAGACA
AAAATTCTTGTGTTATGTGTGGTGGTTGCATGTGCATGTGTGTTAGTGTAGGTGCTGTGTGTGTGT
GTGTGTGTGTGTGTGTGTGTTTGCATGGGGCAATATGGAGATAGTGATGGAGTACAAGAAGGAG
CTTGACTTTCATGAGCTCCTGAGAAAACTCATCTATCCATCAATCCCCCATGCAATTACTAAGTG
CTTAATCCATGCCAGGCACTGTTAGAAGGCACCAGGAAGACAAAGTTCCTGCCATCGACACTTA
CATACTAGAGGAGGAAATAGGAAATAAACAGGTAAGGAAATACATGCAAAGTATGATATTAGG
ATGTGATAAGCATTATCAAGTGAGTAAAATGGAGAAAAGGAATAGAAAGTGACAGGGGTTGCA
ATTTTGGAATAGATGATTAGAGAAGTCCTACCAGCAAAGTGACATTTGTTGAGCAGGAACATAA
AAGAGGTGAGGTATTAAGCTAGGTACGTATCTAAAAAAAAAATGTATTTCCAGGCAGAGGAAAC
AGTAATGGCAAAGGTTTTAGAGAAAAAAAAAAAGTATAACCCAGGTTAACCTCTCTTGCAGAACT
AGTTTAGCCTGTCCCTTTTACTGGTGAAATCACTGTTAATGTTTCTGTAGTAAAGGTAGTGTTTTA
AATGATTTATATAATACAATAAAATTTGTAATCTCATTCAAGCTCCTTTCTTCATCTTTAAAATGAA
GTTAAACTCGGGAAAAAAGGATTGTTCTAGCTCTGAATTGCTGCACTACTGTGACTTAAGTGCTC
CTCTGCTCTCCTAGCAGCACTTTTATCTTACGAATTTATATTTATATATATATGATTAGAACATTAT
AAAGTAGAAAAAATTATGGTTGTTATTTTTATTAGTAGTAGTATTTTGTCTGTATTTTGTGATTTCT
GAAGCTGTGGCTACCTGGTTCTTTCTGTTTCAATTATGACTTTAATTGTGAGGCAACACTGTAAA
GCACTCAAATATCAGTGGTCAGGAATGTGCTGCAAGCAGATCCTCTCTCTCTTATTAATTCCAGA
ACACCAAATGTCTTAGTCCATTTTGCCTTACCATAAAGGAATACCTAAGGCTGGTAATTTATAAA
TAAAAGAGGTATATTTGGCTTATGCTTCCACAGCCTGTACAAGAAACATGGTGCCAGCATCTGC
TTCTGGTGAGGACTGCAGGAAGCTTCCAATCATGGTCCAAAAAATGCGGAGCCTGCGTGTCAC
GTGATAAGAGATGGAGCGTGAGAAAGAGGCAGGAGGAGAAAGGTGCCAAGCTATTTTTAACA
ATCAGATCTCGAGGGAACCAATAAGACAAGAACTCATGATCTCGGCACCAAGCCATTCTTAAG
GGATGTGCCCCATGACTCAAACACCTCTCATTGGCCCCACCTCTAATTCTGAGATCAAATTTCAA
CATGAGATTTTGAGAGGACAAATATGCAAACTCTATCACCTCGAAAGAGAATATTTCTGGGAAT
ATTAAGAAGGGGTCACAAGGCAGACTCAGGGGTTGGGTTTTGTGTGTAACCATGGTATGGAATC
CACACGCAGTCCATGTTGCTAGCTCCGGTTTTGGAGGCTGAGAGGTGACTAAGTCAGGCGTTCA
ACCTAGATTCTTCAACCTAGTCCTTCACTTAAGCACTTGGCTGAAAGATTGGCTTCAGAAGACAG
TACTCCAGCTGGGATGTTGCACATGGAACAGATTGATCTTGAAATCTGGTCCTGAAGTCAACTTC

ACAGCAGGAATTTCACACATGCAGGCACAAACCAGCACTTGTGAAAATCTCCATGAAATAGCTG
CTAATATTGTCTAAACTGGCTTTCAGCTCGGTAGCCAGTCTTCCAGATAAATGTCAAATTAACTG
GATAATTATACCTCAATATGAACTCAATGTCCAACATTAGCTTTTTGGGAGATTTATTTTTCTTTAA
ACTGTGCTGGCTTCGTAATCTTATAGAAACATTGTTTATCTCAGATTCTGAGAAGTATTCAAATAA
GTAAAAGAGTAAATGAAATGGATTATCATTTTCCTCTTTCCTCTTCCCTCCCTCCCTTCCTCCTTCT
TTCCCTGTACCTAGTGTGTGTGCAAAGAACTATGCTGGGTGTTGTGGAATAAAACAAGTTAACAT
ATAACCTTTCAGTAATCAACTAGCAACCTCATGGGGAAGACAGAAAAATGCTAAGCGTAAAATA
CCGTAAATATGCTCTAATAGAAATATCAACTATGTATTTTGAAAACATACAGATTCTAAGTCCCA
AAACAACCAGGACCTGCTGTCTTGGCTACTGCTTTTAACTATGTTGGTTAGCAAGATGATGACAC
ATAGTAGGTGTTCAAAAGATAGAACAGGGAATGTACTTTCTAATTTTGTGAAAAAGATGAGTAG
CAAAGAAATGAAAACAAAGATTATATTTGAGCTGGGATATAAAGGAATTTCTTTTTTCTTAACCT
GTTTTAATTTTGTTGTTTTTGTTTTGTGAAAAAAAAACACTGGATGCTTCAAACACTTTCTCTTTCT
ACAGTGACCCCCACCTTTACAAGTGTCTGTAATTCTCACTAGCAGTATATTAGAGGGCAAAAGA
TTTGAACAGACACATCAGAAAAGAAGACATACAAATGGCCGCTAAGCACTTGAAAGGATTCTG
AACACCATTAGTCATCAGTGAAATGCAAATTAAAGCCATAATGAATGGCGCTATACATTTTCAGA
ATGGCAAGGATTGAAGACCGAAAAAAATCAAGCGTTGGTGAGTATGTAAACTTATTGGGTTTAT
GTTTTACATATGCAAAATTTTGTCTCCGTAAGGGAGAGTTTTACTTCTTATTTCCTAACTTTGTATG
TTTTATTGCTTTTACTTTCCATATTTCGATTGACAGAACCTCTTATACAGTGTTTCCAGTTTTCATTT
TTGAATTTTAAGGGTTCTTTATATCATCTGGCATATGTGTTGTGAATATTATCTCGCAGTCTGTGG
GTTGCCTTTTGATTTTATTAACCGTGCTTCCAAGGAGAACAAATTGTGATTAAACAATTTATTAAT
GTTTTTATAGTGGTTAATAATATTCGTGTTCTAAGAAAACGTTTCTTAACCTCTCTAGAAATTTTAT
AATTTTAGGATTTACATCTAAGTCTGTAATCCATTTCCAGTGTATGGCATGAGATACGAATCTCTT
ATCTTACTTCCATTTAGAAATTTAATTGTTCCAGCCACATTTATTGAGAAGTATTTTCTTTCATTATT
GAATTGTCTTAGTGCCTTTGTAAAAAAAATCAATTAACTAAATATGTGTGGGTCTATTTCTGAACT
TGTGACTTTGTCTCATCCCTCTATGTCTTTAAACTTTTGCCACTCCTAAAGTGTCTTGATTTCTGTA
AGCTTTATTTTGTCTTGAAATCAAGTATGGTAATTATTCTAACTTTGTTCTTCTTTTAAAGAAAGTG
CTTAGCGAGGTAATGCTAAGTTATTTGAACTTCCATATAAATTGTAGAATCAGCTTGTAGAGTTT
GCCAAAAAAAACCTATGGGATTTTTATTGGAACTGCATGGAAAGCATAGATAATTTTTGAGTAAT
TGACATTTAACAATATTGAGACTTTCAATCCTTGAATGTAATATATCATCCCATTTATTTAGACCTT
CAATTTCTCACAAGAATATTTATAATATTTAGTGTGAGATCTTTGTTTATTCTATTGTATTTATCTAA
GTATTTTATGAATTTTAGTGCTATCATGAATGGATTTTGAGCAAATACTGTTTTCTAGTTATTTACT
GCCAACATATACAAGTAGAAAACATTTTTAATATTAACCCTTTATCCTGTAACCTTGTTAGACTCA
TTCATTAGTTCTAGCAGTTGTTTTGTAGACTTATTGAGGGTTTTTTGTACATAGATATTATTATGTA
TCTATATGTCTCAATAAATGCAAAATAGAAATTCCTGATTCTCTTTTCTACCAGGTTGTTTTATCTT
TATATATGGAAGTACCATCACTATCACATATTTGCTACAGACTAAAAACTAGACATTATCTTGAT
CACTCCTTTGTCTCATCTTCCAAATTTAATCCAGAAAATCCCCTGGTTATCTCTGCCATTCTAAAT
CTTTCCAGTTATATTTATTTTTATAGCCACCACCTTAACCCCCAGCTACAATCCTCCCATGTCTGA
TGGGTTAAAATCATTTACTTAATGATCTTCTTGCTTCTTCTATTGACCCTCCACACAACAAAGAGA
ATTATCTTTTTGAAATATTAATAGGATTATGCCTATACACTGTCAGAAGCACCTCATTAAGCTTCA
GTTGAGCCCCAGGTTACTTACTGCTGTCTTCAAGACTTGAAACACTGTGCTCCCTACTTAAGCTC
CATACTTGTACTTTTTCTTCTACTCATTATGGTGTGACTGCAGCTGTCTTTTCTGTTTCTTGAATATA
TCCAGTCCTTCTCACCCTCGGGTGCTTGAAGCTGTTCTTCCCTCTGCCTGGAGTTCTCCTCACCTT
TCAGGTTTCAGCTCAAAGATAAAATTCTCAGAGATGCCTTCCTTGAAACACTAGCTACAACAGTT
ATCCTACAATTAGTCTCCATGTCTGATTAACTTGCTTATTTCTTTCATTACATTATCATAACCTGCA

ATAACCTTTTTATTTTATTTTTGACTGGCTTTGTTCCTATTACTATAAGATATCTTGTCTTTTCAATTT
GTTGCTGTATTCATAATGCTTCAAATAATACCTAACACATAATAGGAATTCAACATGTGTATTGA
AGGAATAAGTCAATGAATAAATATTTCTAAAAGTCTAAGCTTTTGTTAAATTCCTAAAGGGGACT
TTTACTCAATTTAAGAACCACTGCTTTTAAAGTACAAAAAAAAACATCGATTTCTTCTGTTTTGTTCT
AAAGCATAAAAATCACAATAGCATTAATCAAAGGAGGACATAAAGTAGGAAAGCAGACATATA
GGTATAATAAAATAGACCTCTCTATATCAGTTGATTTCTATCAACCGACTAAATCTGAAATAGATT
ACACGTATTATGAGAGTTCCACTGGCAGCTTCTCAGAAATTTCCCTGAGATTTAGAATTTATTGG
CATCTTCGATTGCTTTAGCGTTGCACTAGTATGATATTTATGCGATTAAGACATTGTCAAGCACAC
CATTTGAGACAAATTTTGAAAGACAATATTTCCCTTGTTTAAAATACTAATTCATAATCCTTCCTAT
TTTTATATCGTGTCTTGCACTTACTTTAGTTATCTAGCTTTTGTTGCTTTTTTGTATCTCTAAAAACC
ATAATATCTTACCCTTGAATTTTTTGAATTGATATATTATTTATGTGACTGAAATCATTTTCCTTCTT
GTTTAGTTTTTTTAAGAGATACTAAGGGAAATGTTACTCTGTTCTATAAGATTAATTTATAAAGCA
TCTCCTTCTTTGTCACATGATCAGATACCACCAAAACATTAAATGTTTTCAAATAGATTTTAATAA
TGTCTGAACAAAACCATCATCTTCCCAAAAGTTATGTTGACGTTTACCTCATATGCTTAAATCAG
AGAGAAAAAGGGTCCAAACCACCACACAATTAAAATAATAAGCACACCAATATTATATTGCCCC
TAAAATGAAATCAATTCTTTTTAAAATCCAATGTGCTGTGTATCTCTCCTATATCATTTACTTGACA
GCTGCCGTTTATTGAATGCTTTCTCTGCTATTACAATATTTAATATACGTCTTTTCTGGTCCCTCTT
CCCACACTAGTAATATTGTAAGCCTCTTTAGTGTTGCATCAAAGATTCTTATTAGAAATGTCCCAT
GGTACCTGGAACACAATAGCTCCTTATTGAATTTAACTACTAAATTACCACAAATATTCATGACA
ACAAATTATAACCATATATTGGGAAAATGCCAACATAATCAAGCCAAAAATATCCATATCTATTA
ATCAAAGACTGTATTCTACAGATTTGCAAGGATTTTGTCTTTTTTAGCTTCAAGTTATTATAGAGT
GCTATTATTAACATAGAACTACATTTTTGTCCACTTACTTCAGTATGGCAGTGTTAGGCTGAAATA
TCTTACTCTAAATATTTATATGTTGAAGTCCTAGCCCCCAGTATCTCAAAATATGACTGTATTTGG
AGACAGGATCTTTACACATGTAACTAAGTTAAAATGAGGTGATTCCGGTAGGTCCTAATTCACTA
TGACTTATGTCCTTATTAGAGGAGAGTGACAAATGGGGAGACAGCCGTGGTGCATGATCGCCG
AATAAATACCATGTGAGGATACAGCAAGAAGGTGGCCATCTACAAGCCAAGGAGACAGGCCTC
AGAAGAAACCAACCCTTCTTACACCCTGGACTCAGACTTCTAATCTCCAGAACTGTAAGAAAAT
AAATTTCTGTTGCTTATGCTGCCCAGTCTGTGTTATTTTGTTATAACAGCCCTAGCAAACTAATAC
AGCCAGAGTACTGCTTCTTTAATGAAATTTCATTGATGTTTTATAGATAGGAACACTACTTCTTCA
GAGGGGGTGAGGAAGTTTAGGGCGATATTTAAAGACATGGCTTTGACATTCCATAAAAGTCACA
GACTGTCTAAGGGTTTCAGCTCTATGTCTTACCTGGCAGTTGTCATTTATCTGCTATTACAATTTTT
TACGTACATGTATGTCCCTGGAAAGTTCCTTAACTGTATTTAATTTCATTATGCATAAAATATTTTA
TTGGTTTGTAAAAATGGATGCAATATATATAAGGTACTTAGCATTAGTCAGACATATATTAATCA
ATTAATATATTTTAGTTTAAAAATAGATGTTTAAATTTTACATAGGATTTTAAAGAAAATAATTGCC
TCTAGGCCATATAGAATGTCTATGGAATTAAACATTTTGTTTATTGAACAAAATTGTTTAATTTAA
TTGTAGAACATTTTAAAGATGGAATAAATTTTAAAAAACGAACAACCTATACATTACCACCCATA
GATAGCTACTGTTAACTTGTAGTTTTATTACCTGTAACTTTTTTCTTTGGGTAGTTAGCAATTTTAA
AATATATTTATCATATGACATATTTAATTCTTAAAAAATATCTTCTTACTAAAGACTAATGCATACT
CACAGTTGAAAAATGAGGAAATGCATAAAAGTATTATAAATACACTTAGAGGTTATAGTAATTGT
TAATAATTTTCTATATTTTATTGAAATCATATAATGTAAATAAATATTAAAATAAATGAACTGGAA
ATACTTATAATTTGTATTTACTCAATAAATATTAATACATATCAGAAACTGTTTTATATGCACAAG
ATATAATGCTGATTAAGACAGGCCAAGTACCAGACTTTCCTTCTACCTCATTCCATTTGGGGAAA
GAAGTAATAAACAAGAAAAAATCAATTATTGATTCAGATGGTATGATAGACTTGATAACCGAAC
AAACTCGTTGAAATCAATTGCAATTGCTGATAAAATACTCTTACAAACCCATTCCTCTCAAAACA

CAGACAATCTTGCAAGAAAGATAGAAATTTTTAGATGCCAAAAAATAATTGAAAGTAGAAACTC
AGAGTTATAAGCAGAGAACTGAAGCAGGTTGTTACCTGAGGGTGTTTGCCAAACCAAAGAAAG
CTGAGCTTAGATTTTAATGACCTCAAGAAGTACAGAGGACAAGGGACAAATCTCATATCCAGAA
TAGATATGAGGAATTTCTGTCTCAACTTGACATTGGATAGAAAGAGAAAACATCTTCAATAATTG
ATAGACATAAAATCAAATTTAACGCAGTTTGAAATCATGAAGTAATATATGACAATGAAAATGA
CAAAATGTCTTTGAAAAAAATACTAAATAAGATTTCTGAAAGTGAAAAATAATAAAATAATTTAAA
TAATTCAATATGTGGAATTATCAGTAGTTTTAATTCAACTGAGGAAAGAATGTACTGGAAAATAT
ATTCAAGAACTTAATCAGACTATATAACAGAAAAACAAAGAAATAGAAAGCATGAAACATTAGG
AAACATAAAGAAGAGAATAAGAAAATCTAGCATAATTGAATTACCTGAATTTTAAAAATTGAAG
AAATGAAGACAGCTATATTTGAACAAATGATGCCTAAAAAATTTGCCATAAATTTTTACAAAACA
CAATCATTTGACTCAGGAAACCAAATGAATCATAGTAGGATACATAAAAAATAATTCCTATCGTC
ACATTGTAATAAAACTAAATAATTAAAAAGGCAATGTTCTTTTATTATTATTATTATTATACTTTAA
GTTTTAGGGTACATGTGCACAATGTGCAGGTTAGTTACATATGTATACATGTGCCATGCTGGTGT
GCTGCACCCATTAACTCGTCATTTTGCATTAGGTATATCTCCTAATGCTATCCCTCCCCTCTCCCC
CCACCCCACAACAGTCCCCAGAGTGTGATGTTCCCCTTCCTGTGTCCATGTGTTCTCATTGTTCA
ATTCCCACCTATGAGTGAGAATATGCGGTGTTTGGTTTTTTGTCCTTGCGATAGTTTACTGAGAAT
GATGATTTCCAGTTTCATCCATGTCCCTACAAAGGACATGAACTCATCATTTTTTATGGCTGCATA
GTATTCCATGGTGTATATGTGCCACATTTTCTTAATCCAGTCTATCGTTGGACATTTGGGTTGGTT
CCAAGTCTTTGCTATTGTGAATAATGCCGCAATAAACATACGTGTGCATGTGTCTTTACAGTAGC
ATGATTTATAGTCCTTTGGGTATATACCCAGTAATGGGATGGCTGGGTCAAATGGTATTTCTAGT
TCTAGATCCCTGAGGAATCGAAAAAGGCAATGTTCTTAAAAGTCTGATGAGAGAAAAAAAAAGAT
TGCTATTAACAAATAATAGATTGCTAGCTGACTTCTTAATAGCAACAATGGAAACCCAAACATAG
AATAACATTTTCAAAATGTTGAGAGAAAAAAAAAATCCAAAATGGTGTACCAAGACTAATTTTTAT
AAAAATATTCAGACCCTCCAGTAGCATTGTCTTAGGTCTGTAGTCCCAGCACTTTGGGAGGCTG
AGGCGGGTGGATCGCTTGAGGTTAGGAGTTCGTGACCAGCCTGGCCAACATGATGAAAATCCA
TCTCTACCAAAAATACAAAAATTAGCTGGACGTGGCGGCGGGTGCCTGTAATCCCAGCTACTCG
GGAGGCTGAGGCAGGAGAATCGCTTCACGACTGAGCAAGACTCCATCTCAAAAAAAAAAAAAA
AAAAAAAGTTTAGACAATTAAAAACAATGTGTTGTTTTGTTGTTGTTGTTTATTAGGTTTTGTT
TTTTAAGATGGAGTCTCACTCTGTTGCCCAGGCTGGAGTGTAGTGGCGCCATCTCAGCTCACTG
CAACCTCTGCCTTCTAGGTTCAAGTGATTCTCCTGCCTCAGCCTCCCGAGTAGCTGGGATTACAG
GTGCCTGCCACCATGCCCAGCTAATTCTTGTATTTTTTAGCAGAGATAGGGTTTCACCATATTGG
CAACAAAGTATTTTTATCAAAATACTCTGAGCACTCTAAATCATATTTCTGCTGAGTTGCAGTGAT
CCTACTACAAGGACAGGTAGGCAAGTATACCACTAAAATGGGGTCATTAATATGCCCTGCTTTT
ACAGTAATTGACCTTACTGACAACATTCAGTGAGAATGAGGATTTTATACCACCCAATGAACAA
GCTTGATTTAAGGGACATAGATATACACAATCATATTATTTGCACCTAATAATAGTTTACTTCCTT
TCTGTTACTCATATGTTTTATTTCTTTTTTCTGATCTTATTGTATTGGCTAAGATATTCAAATAAAAA
AAGTTAGTGAGAAGCAGTAATAGTGGATACCTTTATCAGTCAAGAATAACTATAGTGAACAGAA
AATTCTACAAGTACTTTAAGAAGGAAGGAATTTGATTAAAGGATATGGCAAGTCAGAAAATCAC
TAAAAGGACTGCCGGTCTAGGTTGCAGGTTAGGCCTTCAGGCCTGCCACTCAGAACAAGGCAA
AACTGCCTCCACATAGGAGTACTATTTCTAAAACCACAACTGATCAAGAAGATGCCACCGGCTG
CAGTAGTACTTTAAAGACACAGAAAAAAGAATTTACAGATTCCATTTTACATTCACATAAGTAGT
TGCAAGGAAATGTGGATTTGTAATTTTATGCCTCAGCATATCTAGCTAGCAAAAGGGTAAAAGA
CAGTTGTATGAGCTATAATTTTATGAGCAGTATCTTTTCTTTTTTTCTATTTTCATGAAATACTTTTT
AAAAATTATCATTAACTATGTTGTTCACAGTATGCTTTTTCTGCATATCTTTTTTAGGTTAACTGTTT

CTAAATTAATAATGGAGAACATTTATAGTTGCAAAACTATAATGAAAGACTTTTAATATTTTTTTT
AAGTCAGAGACAGGTTTTTTAGGGAGTCTTTAAATTAACTTCCTGTTTTCTGTTGGACTTTCTCTG
TACCTGTGAACTTTTATGCTTGGGCATCTCTGGGTTTCTGTCTGGAAAATGATCTCCTTTCTCCCA
TTACATTTTCCCTTATGTATAGTTTAGTCTGTTACTTCTGTGATTCTATCTGTCCTATCACTTTCGCA
TTCTGTCTTCCATAAATTCGTTAAATTACCTCATTCACTATAGGTAATGTCTCCCATTGTTTTCAGG
TGATAGGTTCATTCCATTTATTCCGTTACAATCATCGTAACTGGATTTCTAGAGGGAGTGGGGGA
AAAAAGACACAGAAAATTCTACTGAATTTTTCAAATCCTCCACTTATTAATTTAATCTCTTATTTTT
TCATTAATATCTGACAGAGATATGTTAAAACCTCTCATGATGATAAATTTTTCGTTTTCTTGGGAT
TCCATAAATTTTCTTTTTTATAAGTTTCAAAACTTTGTTGTTAGTTTCATTCAACTTTATGATCTTTCT
GGTGAATTGAATCTTTTCTCAATATCTGATTACTCTGTTTATTGGTAGTTTGATTTAAAGTACCTTT
TATCTGATATTAATATAGACACATCCAATATATTTATTTTGCTTGAATTTGCCTCATGTGTTTTTTCT
CTTCTGTATAGAAATACTTTTAAAAAATATATTCTTAAAATACAGTCTGACAATTTTTATTTTTATTT
TAATACAATATTTACTTTAGCCCTACATTGATTATGATTTGTTTATAAATTCTGTCTGTCCATGTGA
GGGTATTTTTTCTTTCTCTTTTTTTTCAGTCATTGTAATTTTTCCACCTACTGGTTTAGAGTTTATAT
CAAATCTATATTTTAGTAGTTTTTCTATAATTTTTTAAATTAATATATACATTTTAATAAATTCTAAA
GCTAACAACAAGAATTATATTCAGTTTATCCGTATAAGAATTCCTCTCACTTGACACTATTTTACT
TTTCTGGTCATGATTTATATATATGTTAATTAATTTTTAAACTGCATACTGTTACTGTTGTTTAATAT
AATTAATTTCTGCCTTAAGTTTGACTGATATAATATTTTCTTCATTCAGTATTTTTTCTCGAACCTTT
GACCTTCCTTTGGAATTACTTTTTTAAAATAAAGAATTTCTCTAGAAGTTCTTTTAGAGTTTATTGA
TAGAATATGTTTTTACTTTGTGTGTGCCTAAAAATACTTTTATTTTATCCTCATTACTGAAAGATAA
TTTCACTGGGTATAGATTTTCCAAGATTACTTTTTAAATATTTTTAGCTAATCACGGTAATGATATA
ATCACATTATCTTCTGAATTTTAGTGTTGCCAATATCTAATTATAGACCTTTTACAGTTAGTCTACA
TTTTGTCACTGACTGCTTTTAATTTTGTCTTTGGTATTTTACTATATTCTAATTTGGATTTTTAAAAA
ATATCTACTTTTTGAATTATTTATGTAACCAATTTGAGGCATGATTTTTTTCTGTCAGTCATCAAAC
TCTCTGCTATGTTTTTTAATTATTGCTTCTTCTCCCCACTCCCTCCACCTTTTTTTTCTTTTCCAAAG
ACAGGGCCTTGCTCTGTCACCCAGGCTGGAATGGTGCAGTGATGCAGTCACAGCCACTGCAAC
CTCAAATTTCTGGGCTCAAGCGATCCTCCAGCCTCGGCTTCTTGAGTAGCTGGAAATGTAGGTGT
GTGCCATGTGTTGCTAATTTTTTAATTTTTATTTTTGCAGACACAGGGTCTCTCTATATTTCTCAGG
CTGGTTTCACACTCCTGGCCTCAAGCAATTCTCCCTCCTAAGCCTCCTAAAGCATAAGGTACTCT
AAAAATAGATTATGTTAAATCTTCTTACCTTATTCAATATATCTTACAATCTCTCCTATTTTTCATGT
AGTTTCTGTTCTGCAGTTGGTTTAATTACTTTACTACTATTCACCATTTTACTCATGGAGGATACGT
CTTCTAATGTTTCTACTGTACTGTATTTTTTGTGCATTGAGTTTCTAAACCCTGTTATTAATGTCTG
CATTTTACATTTTATTATTTTTCAGATTTACTTGGCCTTATTTTTCAAAGCATTTTTTTAAAAAAAATA
ATATTCAACATATCAACATTATATTCTTGTTTGAAATTCTAATACTTGCAGACTTTATGGGTTTAAC
ATTTCATTATGTTTCTGCTAATTCATATGGAGAGACATGTTTAAATATTTTCCTCTTATTTATATATA
TATTAATTTTAGGCTTATCATATAACTTAGAATTTTATGTGAATTCTTTTGAGACTTGTTTTGAAAA
TACACATTCTTCTGGATTTTTTTTAACCTTTTCTAGGAGTCTGAGGGTAATACCAACCTGGAACCA
CTTTAAATGAAATTTTCAACTTGGAGATTTTTATCCCAAAGGTGATGGTAAATCTGTCCCCAAACT
CATACATGCAACCTTGCTGTTAAGAATTCTCAAAGAAATTCGCAGCAACATGAATGAAACTGGA
GGGCATTATGTTAAACGAAATAACCCAGGCCCGGATAGACAAAAACACTGCATGAAATTACTC
ATATATGGAATCTAAAAATGTTACTGTCATAGAAGTGTAGACAGTAGAGGCCAGGCACAGTGGC
TCACTCCTGCAATTCCAGCACTTTGGGAGGCTGAAGCGGGTGGATCACCTGAGGTCAGCAGTTG
GAGATCAGCCTGGCCAACATGATGACACCCCGTCTCTACTAAGAATACAAAAATTAGGTGGGC
CTGGTGTCGCATGCCTGAAATCCCAGCTACTTGGGAGGCTAAGGCAGGAGAATCACTTGAACC

CGGGACAGGGAGGTTGCAGTGAGTTGAGATCGCACCATTGCACTCCAGCTTGGGTGACTGAGC
AAGACTCTGTCTCAAAAAACAAAACAAAACAAAACAAAGTGTTAGAATAGAGTTTATCAGAGAT
AGAGGGTTGGGGGGAAGAGGGAGCGGTTGCTCAGTGTGTACAGAGTTGCAGTTAGTCAGGAA
GAATAAGTTATGGTGTTCTATTACACAGTAGAGTGAGTATAACTAATAGTGTATTGTATATTTCAA
GGTAACTAGAAGAGAGGATTTTAAATATTATCACCACAAAGAAATGATAAATTAAAATGACAGA
TATGCTAAATATCTTTATTATACAATGTATACATGCAATGAAACATCAAGCTGTACCACATAAAT
ATGTACAATTACCGTGTGTCAATTATAAACAAAATAATACTTAAAGAATTTTCATAGTAGACATTT
TATTTTTTGATTAGCTCTGCTTGGAATCAGAGACAGGTGAACACATCTCTGCTAACTCTCTCCAGT
CAGGTTTTAACCTACTTTGACTTATGATTGTTCAGAAGTCTTATTTTTCTCTGAATTTCTCTTAACT
GAGCTACCATTCTGCTAGCACCATAGTCACTGACCTCCTCTCTCCAGTGCAGTTCACTGATTATA
ATAGAGGCTTTTGGCTATCAGTGATGGACAGTTGACTCCAGGTTAATGCAAGCTCCAATACTCA
CAGAATTCTCGCCTATTCTGTACATAGGATTTTTGTTGTTTTCGCATTTTATTCATCAAAATTAGTT
GTATGGTTATGGGAAGTGATTCAATTAATATTTAGTTTGCCATATTGCAAAATAATGGAAGTCCTT
GTATTTTTCTATGTTTAGTTTTTAAGTTGTTGAGCATACTCTGTATGTGGTATTTAATGATAACCAA
CACAAATAGTAACAGTTAGATAAAAACATTTATACAATGGCACGTAATATCTGACTTTGGATAGG
TTGTTTTAACCAACCAAAGTCAGATATTATGTGCTATCATATAAATGTAAATTATATAATTTTTATA
TAATTATTAAAGAAATTAGTACAATAAAATGCAAGTTAGTATGTTTTACAGATGGAGAAAACCAT
TATTAAGTTTCTTCACAGTGACAATGGCCATTTTGTCCTTTATTCAACTTTAGTTTCAAAGTAAACT
AAAAAAATTAGAAATGTTCCTGGCATATTATCTTGAGAATTATCACTAATAATTATCCTGATTATT
AACACAACCAATGCTACAAATGCTAGGGCACAAAAGCTTCATATATAACTTTATTCATTAAATAT
AGTATAAGATTAATAATATCTTCATAGCAGAACTAAGGATGCTACTTATTTCTTATCAAAATTTCT
TAGGATATATCTAGACCTGGCTTATTTATTTTCCTGGAACATGATGATTCCTGAGGCAGAAACAA
TCACTATGTATGCTAAATGAAAACTATATTTTTTGTTTGTTTGTTTTGATGCAGAGTCTCGTTCTTG
TCACCTAGGCTAGGGTGCAATGGCGCAATCTCGGCTCACTGCAACCTCCGCCTCCCGGGTTCAA
GCGATTCTCCTGCCTCAGCCTCTCTAGTAGCTGGGATTACAGGTGCCTGTCACCTCCCCCAACTA
ATTTTTAAATTTTTTTAGTAGAGACAGGTTTTCACCATGTTGGCCAGGCTTGTCTCGAACTCCTGA
CCTCAGGTCATCTGCCTGCCTCAGCCTCCCAAAGTGCTGAGATTACAGGTGTGAGTGACTGCAC
CCAGCCAGTGAAAACTTTTGTGAGCTGACATTGGAGGTGCTCACCTTGATACAGTCACATCAAC
TAAGAAAAGCCTGAAAATGTTTTGTGTTAAAGCTGATAATCAGTCACGTTACGCTCAGCTAAACC
CTGTACAATTACTGCAGTCAAATTTTTTAGCACCCCTGTTGAAGACCATTTAGGTTCTACTTGTTC
TTTCTACTGTTTGTATTGCATTTTCCTTTTTAATAATCACAGTATTGTAGTATGTAGAAACCCACAA
AGCTGTTTCTCTCATGTTGTCCCAGTCGTATGTTCTATGAAAGAACAGAAAACATAAGTGTAATT
GCCATTCACTACATAAAAAAACCAAAAAAAAAAATAGATGAGAACAAGAAAAGTGAAGAAGTTT
ACCTAATGTCATACAAGAAATCACTTGCAGTACACAATTTCTAGTTCTGACAGCTTAACCTTCCG
CATTTATCTGGGAGAGATTTATAAGGACATCTTTCTCCCAATAACAAGAGCCGGGAAACTTACA
GAATGTCAAGGCCCTATATCTATCTAAAAGATTAAGAACAAAGTTAAGAGAAAATCTGTGGCCT
GAATAGAATAGTTGTAGGAGACTTTTCCCCGCAAGACCTCACCTGAGCTATCTGGATCTTTTATT
TTTTATTTTTTTTTTGAGACAGTCTCGCTCTGTTGCCCAGGCTGGAGTGCAGTGGTGCGATCTCGG
CTCACTGCAACCTCTTCCTCCTGGGTTCACGCCATTCTCCTGCCTCAGCCTCCCAAGTAGCTGGG
GCTACAGGCGCCCGCCACCACGCCCGGCTAATTTTTTGTATTTTTTAGTAGAGACAAGGTTTCAC
CATGTTAGCCAGGATGGTCTCGATTTCCTGACCTCGTGATCCACCCGTGATTTGGCAGAAACTTC
TAAGGAGCCACTGCATAGAAATAAATGCACAAAGGTTTTTATCCAACTCAAATAATGTATGGAC
TACTCTGGGTGATCTCTAAAGTTAACTTAGAGATCATCTAATTCTAAAAGTTTATACAGTAGTAAT
AAGATCAAAACAATAGGCCGGGCGCGGTGGCTCACGCTTGTAATCCCAGCACTTTGGGAGGCC

GAGGGGGGCAGATCACGAGGTCAGGAGATCGAGACCAGGGTGAAACCCCGTCTCTACTAAAA
ATACAAAAAATTAGCCGGGCGTGGTGGTGGGTGCCTGTAGTCCCAGCTACTTGGGAGGCTGAG
GCAGGAGAATGGCGTGAACCCGGGAGGCGGAGCTTGCAGTGAGCCGAGATCGCGCCACTGCA
CTCCAGCCTGGGCGACAGAACGAGACTCCATCTCAAAAAAAAAAAAAAAAAAAAAAAAAGATC
AAAACAATATTAGAGGATAAAGGTTAAATCTAATATTCTAATTTTACAGTTGTGCCAATTGAATG
TTAGATAATTTAAGTGTCTCCTTCATATTCAACAAGAGTATCCTTTTCCCATTTGGTGCTATTGCA
CTTCACTAATATACCTCTCATCTTTTCATTTGTTTTTTATATTTGAGCAAGCAGTGCCAACCCAAGC
CTAGCCCAACTCGCTAGAAAACATTTTTGGCTTTGAGTGCTGTATGACAGTGCTCACAACAAAA
ATGAGTCTGACAGAGAACGGAGAGAGATATGGAAAATCAAATCAATGGGATTAAAGGAAAGAT
CTAGGACAATCTTTAAAAATCCATTAGCCTGCATAATTGGAAGTTATATGCATTCTTAGGAACTG
GTCATGTTGTTCTTGTTCTCACTTTTCTCCGTTTCACTCAGAAATAGCCACCATTCTCATTAAATTG
AATTCTGATGTCATTGTCACTGTTGGGCTTAGGCATTCTATCCAAAAGTAACTGATTGTTAAGTGA
TGAATGAGATGATTCACACACAAACTGCAAATTGTAAGGCAACTTACATTGAGTTTTTAAAAAGT
ATGTATATGTGGTGTGTCCATTTGAGTTTTAGTCTAGTAGGATGTTATAGATCTATTTTGCATTTAA
TAAGATAAAAATTAGGCAGCTGAGTACAAGTTTCATATGCAAATAGCATTAAAGAGAGAATTTTT
TAAAAAACAGTTAAGCTTTTTTGAGAATACATCTGCTGTTCTTTCATGTAAATATTATTATATTTAA
AAGTTAATTAAAACCATTGTGTTTTCTTTTCTTTTTCTTTATTTTATTATTATTATACTTTAAGTTTTA
GGGTACATGTGCACAATGTGCAGGTTAGTTACATATGTATACATGTGCCATGCTGGTGAGATGG
AGTTTCACTCTTGTTGCCCAGGCTGGAGTGCAATGGCGTGATCTCGGCTGACTGAAACCTTCAC
CTTCCGAGTTCAAGTGATTCTCCTGCCTCAGCCTCCTGAGTAGCTGGGATTACAGACATGTGCCA
CCATACCTGGCTAATTTTTTGTATTTTTAGTAGAGACAGGGTTTTGCCATGTTGGGCAGGCTGGT
CTCAAACTCCTGACCTCAGGTGATCCCCCTGCCTCAGCCTCCCAAAGTGCTGGGATTACAGGTA
TGAGCCACCGCACCTGGCCTGTAATCATCCTTAATCTAGTAAAGATAAATACAAGGGTCTTAAA
GTTTCTTCTCTAATTCCTAAACAATACTTAGGGATTTGGTATGAAATATTCAGAAGTTGCATCAAC
AAATTTTTACATTGTGGTTTTAGATAAAAAGAAGGCTAACTTTCAAAAATGCTGAGTGATTGATA
GGAGAGTATGATATTTCAGATAGATTGATATCTTCTGTCAAGAAATCTTACAAATTCCACCCTTTC
TTTTGACCTCTTTAGAAATCATACCTGAATGCAAGTGAAGGATTGGGACATTATTTAAAGAAAAA
TAACTTGGATCATCTGTAGTTGTAAAATATTAAAGGCATCCAAATATGTGATATTTAAAATTTTCA
TTTAAACAAATTATATCTGGTATACTTCATATCTTACTGTGTTTTAGTTCCCTGAGTTTTCTCAGCT
ATTTTAAAAATTACTTTATTGAGATATGATTGACTGTACAAAAACGTTATACCCATTTAATCTATAT
GACTTGAAGATTTTGGAGATAAGTGTACACCCAGGAAATCATCACAATATATGCCATAAACATA
CCCATTACCTCCCAATGTTTCCTCCTGCCCTCTTTATTTATTGTTACATTTCTGTCACAGAAGGGAT
AACAGAAGATACACCCTTTAGCAAATTGGTAAGTATACAATGTAAAATAAACTACAGGCACTAT
ATTGTACAGTAAATCTCCAGGACTTATTTGTCTTGCATCACTGAAACTTTGTACCCTTTGACTAAT
TTTGTGTCCTTTGACCAACAACCTTCACACCATAAACAATGTTAAATATGTGAGGTAATATATATG
TTAAATAGCTTGATTTAATTATCCTGCACACACACATTGTACACAGACACATATTATGTTTTACAC
CATATATATAATTTTACTGTTATATGTTTATGCAAGTAATTATGCCCATAATTAAAAATAAGGAAA
CAATAAAAAAGTATTGGATTAGGAATCAATGCAGAATTAGGTTCAAGTCTTGATATCTCTTCAAT
TATTAATGGTCAAGATTTACTTCTCTGGATCTCTCCATCTGTAAATTTAGAGTTTGGGTTAGGTGA
GCTTTAAAGTTTCTTTTAGCTCTGAATTATACAAATCCTTTCATGAATAATTCTCAAATAATCACAA
CTATTCCCTAGAAAATTCTGAGAGAAGGATTTTTGTAAGTGAATATTTAAAAAATCACACAGGAC
AAGAGAATAAAATATGTAATTTCAAGTTTTAATAATTAAAATCAGATTTATTTCCTAAAGTATTAA
TATCTTGGATATTATGGGGAAGTACTTGCTCTGAGATTCTAAAAGAAGCCAGTATCTGTTTTTTCG
TGATGTTTTTGGAAAAGTCCACTAAAGGCCTCCTGATGCTACTCTTTCGTTTTGGTAAATGTTAAA

CCTCACTACATAATCTAAGACTATAACTGTGTGTTAGAAATTCCTAGTTTATGTTTTTTCTTGTATC
TTGGGTTCTCTTCCTTGACCTTGACATGTCCATTTAATGAAACCTGCTCAAATTTGAGGCTCATTT
AATTTGTTTCTCACAAACCTTCTGTATCTACTTTTACATATGTTCTTACGCCTGTGAATTCTCATGG
CCGCTTGTCCTAGGGCGCCTCAGCATAACCCTCCTTTATTAGTAATATATTATATATGTTACATTA
ATGCCTTAAGGCCTTGAACTATTCACTTATCCTGAAATACTTTCTTCAGATCTTTCTGATTGGCTTT
TTCTTGTTATCCAGGTCTTAGTTTATATTCTACCGCTGCAAAAAGCTCTCTGATCATCTAACGTAA
AGTAACTTCCCAGTTACTGTCTATTTTATTGTCATGTTTTACTTTCTTCATAGCACTATTTAAAGTT
ATTTTATGTATGTCTCTCATTAAAATGTAAGTTTCCTGGAGACAAAAGATGCTCAATAAATATGTA
ATGATGATAAATTTTTTGTATGTGTGCATTTTATTCATTTTGCTGGAGAAGGCATTCACCTTAGAA
TAGAGTTACTTCTGTTTCATTTCTTTTATCTTCTGCAATATCTGATGTATGGTCTTGCACACCAGAA
ATTCTCTGTAGATATTCTTAATGGAATTATGTTAATGTGGCAGAAAAAAAAACAGTAGAAAATTGG
AGCTGTTACTCTCCATTCTATTTGCTAAATGTAGTTTGTACTGTAACATCATTTCATTTCATGGGTT
CTTGACTTGATAAAAAATAAAGAAAAGAAAGAGAACCGCACAGTTTTAGCATGCTAACTTAAAA
ATCCATATAAAGGCCAAGCAACTGCATGCCCAAAGGAAACTTTGATTTAATTATTTTCTAAAAAG
ATGTCCATATCTATTTAACTGCCATTCAGAAAAATCATGAGTTTCAGAACTACAGTTCTTATAATA
AACCTACAAATATAATTATTTATTAATACAACTAGAAACATTTTTTATAGACCTGGTATAATCACA
GAATAATCAATCAGCCAGAAATGTAACATTTTTACTAATGATTTTGAAATGTCAAATGATATATA
ATGCTTTAATTGATCTAGGCTATTCAGAAACACCTAAGTCATTGTGACTATATAAAAGAAATAAA
AGCAGAAGATTTCTAAAGTGTCTAGTTCATTTTCATGTAAACAGGAGAATAAATGAAAGCAATGT
TTTATTTATTCAACATAAACTGGACTGGATTAAGATCAGACTTGCTACTTTCAGTTAAATTTACTA
TTAAATTGATATTCATTAGAATGTTAGAACAAGGAGGGATTCTAGCAGTCATTCAAATGAGGTTA
TTATAAGTGAGGAAACTGAACTAAAACAATCTATTCAATGTTGTAAAACTAGGAACTAAAACAA
TCTATTCAATGTTGTTAAACTAGCTACTCTAGATTAGCTCAGCTTTAAAGACTCGCTAGATGTCCA
GATGCAAGCACCTGCCTCTTAGTCCAGTGCTTACTCCACAACATAGCCTTTTGACTCCTATTTCA
AGGGACACCAAAGAGTAAAATTCTGTAACAGGTACTCAGCAGGGTTGGAAGCTAATAGGATTT
GGACTCATCTATTATATATCAAGAAAAGGATCTCCAGTTTATAATAATACATTGTAATCAAGTCA
ATACAGTAATATTAAAATTATATCAAGTCCTGAAAAATATCTTTCTTTGAATATTTGGGAGGCACT
TGTGGATCTAATACTGCTTTCTTTATGAAATTTCATAAGATTTACATTGTCTATGATGACTAAGATT
CTGGTTATCAGAATGCAAAGTAGAGAAGTATGTAAAGGTACTGCTTTAGTGTGTGTATTAGTAG
GAATTATTTCAGTTGCAAAAGGCAAGAACTCAGCTCAAATTAGTTTATACAAAAGAAGCTCACT
GGCTGGCAAGAAAATAGTAGCTTAAAGTTGAAAGCAAGAGATACAGAAAAACGAAGCACCTAG
GGCAGGAATTATGCACTCAGAATTGCTAAAATATTATCTCTCTTTTCTTATCTGTGCTTAGTTTCAT
TCTTCATATTTTAAATAAGATACGGCCCCTGGCCATTCCAGTATTATTTCCTCTCAGTTTAGCAAC
ACTGCCCCTTCCCTTAAAGGGGTAACAGCTTATTTATTCCAGGTTCTATAGATTAATATTAAGGA
AGGTCTTGAATTGGCACATCTTAGGGTATTTGTTGGCAACAGGGGAATAGGGAATTCCTGTAGC
AAGGTAACATGATTGAAATGTTCACAAGAACCACATGCCACTGGGGAATAGTGACTCTACCTCC
CAAAAGATGCTAAATTGTAAAAAGATGCAACACTAAAAAAAGTACCCCTTAGAATATTCATGAT
AATGGAATTTGCCTTGCTAATGTCCCATAAGTTAGTTGTTACAGAGGTGGCATTGCCAATTTCCA
GTATTTCCCTTATGCAGCTTACAAGTTTACAAAGATTAAAAAATGATAAAACCTAACATTGGTGG
AGGTGAAGTGAGATGGGCCTTCACTTAACACTGGAGGAAGTGTAAATTGGTAAAACCTTTTTGT
AAAGCAATTTGATAAAATGTCAAGAAAATTAAAATAGTTCACACTTGTAGATACAGTAATTCCAC
TTCTAAAAGGAAAATCTAATAAAATAATTAAAATGTGGGTACATACTTGTGTGTAATATTTTATGT
AATTTGAATATTAGAAACAAAATCAATGTTCAGTAAGGTTTACTCAGTTAAATATAAATATAGTC
ATGTACCACATAATGATGTTTCAGTCAAGACAGGTGACATATACAGGCCAATAAGATTATAACA

CTATTTTTACTGTTCATTCTCTATGATTAGATACACAGATGCTTACATTGTGCAACAGCTGCCTAT
AGTATTCAGCACAGTAATGTGCTATACAGTTTTGTAGCCTCTAGGCCATGCCATCTAGCCTAGTT
GCGTAGTAGACTATACTATCTACGTTTGTGTGAGTACCCTCTGTGATGCTCACATAATGAGGAAA
TCACCTAATGGCACATTTTGCAGAAGACACCCCTATTGTTAAATAATGCATGACTGTATGTAATA
GGCTACTATGCAGATTACACCTAATACCCTCACATGATTTTGATAATATATTGTCATTGCAAACTT
GAAAGGAAACAACTTCCTAAGTCATTGGCAACGATGTAAATTCATGCAAACTCGTTGGATAAAA
TTATATATAAGATGTATCATAAAAATATATATCAAAATTAGAAATGCAAATATGACCTAGAAATTT
TATTTTTAGGAATTTATCTCATATATATTCTAGTATGTACTCAAATAATGTTCACAGCAATACTGTT
TGCAATAGAAAATGTCTGGAAATAAGTCAAATTTCTGACAATGAGGGACATGTTGAATAAATTCT
GACATTAGAAAGGAATAATTTGTATATTAATCTGCAAAATACGCTAAATGAAAAAGCAAAAGTT
CAAACACTGTCTGTATAGTAGACCCTCATTTCTCTAGAAATAGATAACAGTTGTGTAGTGGACTT
GACTGAGTTCACTAATAGCTAGTTCTTCTCTCCTTGGACATACAGAAGATTATAGTTTCCATACTT
CTTGCAGTTAACTGAAGCCAGGAAACTAGATCTGGCAAATAAAATGAGTGGGACTGACTTTTGA
GCTGACAAAAATGAAGAGCCCTTTTTAAATTCTGCCATCTCTCTCTTTCCTTGCAGTGTTAATGCA
CAAGGTCTTGTGTTGAAAATGTAGAGCCACGAGATAAAAGCAGCTTGGATGGCTGACTCATATA
CAAGGACAACTGCCTGAGAGTCACCAGGGCTATCAAGAAGTTTGCAGAAACAAGAAATAAACT
TTTACTGTTTTAAACTTTTGTGAATTTTGGGTTTTGTTTATCA
ID|56111|gene_id|ENSG00000226308|transcript_id|ENST00000426580 Homo sapiens lncRNA

CTGAGTGCCCACGGGGTGCCTAGAGCCTGGCGGGACGTCGCGGGGATGCGGCTGGAGGA
ATCGGAGCCGGCCCTGCCCTCTCGGCACTCGCGGTCCGGACCAGGAAGGCGGCCCGAGCAGG
ACGGGTGGGCTGCGGACCGCGGTGGGAAAAGTTGGGGGAGCGCGGAGGTGGCGCGGAAATA
GAAGGCTTCCTGGAAGCGGTGACCCTTGAGCCGGTCTTGCCCCTCAGTCCCTGTATCCTTTTAA
GCCAGCCACGCTGACCCCTCCGCGCCTCCCTGAAGCCGTTGCCAGCCTGTCTCTGCGCTCGCCA
TTCCCTCCGCCTGGACCTCTCCCCACTCACCACCTCCCGCCCCCATCAGACGCTGCTTTCTCCTC
CCAGAGACCTCCAAGACCTGCCAAGCTGAGGTTGTTTCCTCGCCTCGCCCCTCGCACGTTTGTTT
TCTTTCCTGTAGTTTCTCTTGCTGTCTGGAATTGCTGTTTGAGTTACTCCGTGCCTTCTTTATTGCC
TTCTTACCCCCGCTAGACACCCAGAGCACTGGGGCCCGGATCAGCGATCTTCTCCGCACCTCTG
GTACCCGCAGTGCCTTGAACCCAGCGCCTGGTAGCCACTGTAGCTGCGCAGTCACGATTTGTTG
AGTGAATAAGTGAACTGCGTGTGAGCTGGGGGGGTTCTTCCCACCTCCTCCTCTTCGGGGCCCT
GTATCTTTCCAGCCACCCCTCAGCCTGAGACCAGACACCCCAGCCCCACCACTAGGGCTTCCCA
TCAGCCCTCCTCGTGGCAGTGCCTGGGGAGCCAGCCGCTGGTCTTCACATCCTCTTCCCCTCCA
AGGGTCTGTGCCTGAGACCTCCCCATCGGCCTCCTCTCCTGTCTCTCCTCATCCATTCATCCCTC
AATCAATACCGAGCCATGCACGCTGTCACAGGGAGTCCCCAGACCACTCAGAACCCACCGGTC
CCTCTGCCGGTGTGGGATCTCAAGGGAATTACTGCACCCAGAGTGGCAGTGAGGGCAGCGGGG
TAAAAGTTCATCCTGTCTCCTTCCCCTCCTGGGGCTGGGTGAGAGAGTGGGTCCCCAGAGGGCC
TTTGGGATGAACTGGGAAGGGCTGAGGTTGGGGAAGCGGGGGCGCTTGTCTGGGAGGGGCTC
TGGCTAAGCCTCACTTTGGGGCTGTCCTTCCTGGAGCAGGAGAGCTGGGGAAGAGAATTATGG
TCTTCTACAGTTTTCTGGAGCAGAAGTAGTTTGGCCAAGTGGCCCTCTGGGTCTCCCTTGGGGAC
TTTTGGACTAGATGGCAGCCTGAGGGGCCACATGGGTGGAGAAAGGGGAGGCCAGAGGCCAG
GCAAGGAGCAGAAAAATGCCAGCCTGCAGGTCCTGGCCATTTACACACAGGCCCCAGGCTGTG
GGATGGAACAGGTCTTGAGCCCCATGCCAGGGAGGGGCCTCTACCCACCCACCAGGCAAGGC
CCTCAAACCACCCCCACCACCTGCTCTTCATCCATTCACTCCTTCCTTCAACAGACAAGCATGGA
GCACAGGATATCAGGAGGCGAGCTGTGGGGCCTGCAACCTGGACAGTCGTACAGGGCCCCCC

AGCTCTTAAGGGCCCCACACTTGGTTTCCTACTCTGAGGTCACCATATGTGGTTCTGAATCACCT
ATGAACAAAGTACCCCCTGCCCCGTACTAATTTTGCACCTGGCCCAGATATTATGTGGCCAGTC
CCAGGGCCAAGCACCATGCTGGGCAGGAAGACATGGCCATGAGCGAACCCACATGGCAGACT
CCATCAAGGAGCCGGCCTCCAGGCCAGGGAAGCAAAGCAAGAGCCTTAAATAAGATTCAGGG
ACTGAAGGAGAGGCAGATCTTGTCTATCACTCTCTCTGGAAACAATTCAGAAGCCAGGCAGAG
GAGGACATGCAGGAGGGAGAAGGAAAGGTGGGCATCCTGGCAAGCAGGAATCCTACCCCAGG
GAGACTGGGGAAATTGCAGGAGTGCCACAGTCGTCCAGGGTCCATCTGAGAGTGTACAACTGG
AGAGCCTGGGTCACTTCAGCCTGCAATAAGTGACAGGCAGTGGGGGCGGGGGCGCAGATAAA
CAGGGTGAGGTGATGGAGGGTGGGAAGGGGAGTTTTTGGAGGAGATGCCACTGACCTGAGTC
CTGAAAGACAAGAAAGAGACAGGCTTTTCCAGGGAAAGGCATCCCAGGTGGAGGAACAGCAA
GTGCTTGAGCCCTGCAGAGCAGGGAACAGAAACCAGGCTCGGTCCCCAGCCCTGCACACGCC
CTCCAGCGCTGGCTTGCTAGGGACAGGCTATGCCACCTCCAGTGCCAGTAGAGAAAAAGGAGG
TCATTTTCTTCCAAAGTCTGAAATAATAAGGAGCCAAAGCAGCCCTGTTCTTCAGTAGTAGTCTC
TGCTTTTTATCTACTCTCAAATCTTTTGGAGGGATCTTCACAAGCAGGTGAGGCTGGGGGCAGG
GGCAGCAGGTCTTGGTGCTACAATTTACATATGAAACAACTGATCCGTCAGTGTCTAAGCAAGG
ATTCTAGTTTACCCTTCTTTGTGCATCTCACCTGAGCCTATTCTGAGCCTGGCACACAGCAGGTG
CTCAATTAGTACCTGTTGAATGAATAGTAATAAGCACAGCCAAAACCTAAAGAGACCGGTTGAC
TGAGGGCTGACTGTGCAGGCGCCAAGCTAAGGCATCTCCCAGATCCCTCACCCCAGCAACCCT
TCCTCTGAGACGCCATCTTTTCTCATGCAAGAGGAAGAGACAACATGCTCTTCATATCCAAGAC
CTCAAGAGATAAGGTTTCCTCTGAAGTCCCACAAACCCCCATTTAGCCTTATCTTTTCTGTGATCC
ATGCTTGGTGGGGGGGTCTTTCTTTTCCCTGTTTCCTCCCCGAGAGGCATCCCCAAGACTGCACC
AGGCCCTCAGTGAATGCCGAATGAATGAATGAATGAATGAATGAATGAATGAATGAAAACCGG
CAAGCAAGAAATATCTGGCTGGGAGTGCATCCGATGCCAGTTTGGGAGCTGAGCCCACCACCA
GAAAAGGCTTTGAGATTGCCGAGGGCAAGTCTTGTGTGGAGGAGGTAGCCTGGGCACTGGAGC
CTGCCCAGCCGCATTCACACTCCAGCTCTGTCTCTTCCAGCTGTGCACCATGGGCAAGGGACTT
AACCTCTCTACACCTCAGTTCCCCCATCTGTAAAATGGGGTGACTGATAGCGCCTGTCTTCTGGG
ATCGCTGTGAGGTTGCAGAACTTAATTCATGGAAAGGGCTTTGCACAGTGCCTGGAACACAGAG
AAAACCCTTGATAAATGTTATTGTGTTACCATCTGGATGAAAAGGAAAATATGGTAACAGGAAA
GCCTCCTGAGTTACAAAATAACAACAAAACTTTTCATGATGGTCTGCTTTTTGGCCAAGCAGAGA
AAAGCAGATGTATCCTGACTCTGAGAGCAAGAGCAAAAGTACCAGAGCAAAAGAGAGAGTGA
AGTGGAAGGGAACATGAGAGATGAAAAGGTCATCACTGCATTCCAATCCTCACATTGTGCCTAG
GCCCCCTGCAGGCCAGCTGGGGCCCAGGGACTTCCCTGGGTCCTATCTCATTTCCCCAGCAGCT
AGCCCCCAACCCACACACCCCCAGTTGTGGTTCCCCTGGCCAGGGCACCCAGCACTGGGCTCC
AGGACCGTAATCTGATCTGCCCCAGTCTGCTCCACACCTGGGGCCGCCTTTGCCTGGATGAAAG
ACGGCCTGGAATTGTAAGGGGTGTTATTGTGTCGGGTGGCCCGGTCCCAGTGTCCGCGCCCCTC
GCCTGCTGGCCTCCCAGTGCTGGGCCAGGAGTTTTCATTTCCTACATCATCAGTCACATCTTGCT
GAAGTGATTTTGACCAAGTCAGCATGTCTCTCTAAGGCAGTAAAAAGAAGAAAAGAATTCTGCT
GACACACACATTGTTCAGCGAGGTGGGGACCTCATCTTCATCCATCTGCCCTAGAGGAGGCTCA
GCTGGCCTGGAAGCTCCCTGGGGAGCACAGCCAAGGAAGCCAGTGGGCTGCTCTCTCTCTGTC
CACACCACCTGGCAAAGGCCAGATGACCTGGACCTCCAACCCCAACAAGGAATAGAGGGCCA
AGAGCTGCCTCCTGCACAGGCTTGAAAATCGTTGTGTGCAGAGGTGGACCCTGCAGAGGAAAT
TATTAGAGCACACCTGTGGTGAAATTTTGTTTCCCACGCAGATCTCTCATTGCACGGGAGGCTTG
GACTCCTCACGCTTGCCCACTCTGAGATTTTCTTTGCACAACTTTAAAAGCAGCAGAATAAGAGA
TTAAACGTCTTCCATCAGTCTTAAGTACACATGGCAGTGGCCACAGTTCCAAATGGAAAATACA

GACGACACACACACTCTAGAGAAAGCTGATACCATGCAGGATTTCTAGGATCTGGAAGCCAGC
TAGGGGGAAGCAGTGGTTTTGTTTATTATTATCAGGGCTGTTATTTCTTCCTTGATTGGAGGAAAT
CTGGTATGGTTTTGAGCACTAACTATCCTCGGTTCAGCACTAGAGGTAGCCCTTTCCTCCTTCCA
AAACGTGACAGGGGAGCGGCCGACCTGTCACCTGTTGGGAGCACTTGCTTCTGCTGGGCACCT
GCTAGGCCTGCACGGCCCAAGCCCACATCACCCGCAACTCCCCACTGCATGGAGAGGCTAGTT
CTGCTCCTCAGATTGCAGAGGAGGACCAACATCCCGAGAGCACCTTGTCCAGAGTCAGACAGC
CAATCTAATCTGTAATGGAGCCAGGATCCAAAGCCACATCTGTCTACACAGCCCAGGCCTTTAA
GCATCAGTGCACGTTCAGTGACCTGGGAGCAGCCCCTTCCTTCCTTGCTTTAACCTAAAGCAAC
ACTTCTCCAACCCAACTGTGCGTGTGAATCCCCTGGGGGTCTGTTAGAACATAGATTCCCACAG
TCAGTCTGAGTGAGCTGAGATTCTGCACTTCTAACTAGCTCCTGGGGGCTCGGAGGCTGTCGTC
TTCAGGCTGTATGTTGACGATCGAGGCTCTAGATTTCTGAAGTTATAGCTGACAAATTGGGGCTT
CAAATTAAAACTTCAATATCTATGATTCCAGGTGTCTTTTAAAATTAACTCATTAATTCTCCCAAC
TACCTTGTATGATTTTTATTATTATTATCCCCATTTTACAGATGAGGAAATTGAGGCTCAAATACTT
GAGGGAACCTGCCCAAAGCCACACCAATGGTGAGTGAATGGTAAAATCAGAATTCTGACGCCG
GCTATCTTGTTCCAGGACTTAAGTCCTTAAACATTGAGGTACACCAAAACACAGGGTTTGTCTTA
GTCCTGTAGGGACCTCCCAGTACCCATGGAGGCCTTGCACACAGTAGATGCCCTATCAGTGTTT
GAGGAACATACCTCTGAGCCCTTCTTCAATTCCTCCTTTATTGCAGAAGTCATACACTCAAAAGC
CAACGGGCACCAACCAGGTAACATAAATACAGAGATGCAACATGAAGCTCAAGGAATATTTCT
CTTCCTCTTAATTTAATGGAAGGCAATCATGAGCACAGACATGGTGACAGGTGGCAGGAGACAC
TGGCCTGGATAAATAAGGAGGGTGCAGACTGGGGTGAACTGGAAACCACATGCCCATGGGTGA
TGCCATCCAGAAATTTGATTCCAACATCACCAGAAAGTTCTGATTTTCCAAAGAAGCTAGAAACT
TGAGTTTGAATGTGACATCTCTCAATGTTTATCTGTTGGCAACTAATTCAACTCGTTTTGGTTATTA
GACCAAAGAAGACACATCTACCAGTCAGATTCGATGAATCTGGTTTGCCCCTGGCCTCATAAAG
GGCTCTGCTGTGTCTGGAGGATGTGCCCAGCCTCCCCCAACCCATCACCCAAACCAAAGGACC
ACCAAGGATGGTGCATTTCAAGACAGCTCCATTGAATTCATGATTCGAGGGCTACTGAGCCAGT
GGTTGAAAAGGCTGGAACTGTCTGCCCCTTTAGGCAAAATCTGACCCTAACCTTCCCCAGAGTC
CCTTCCCAGCAAAACTCTGCTGTTTGGCCCTGGTGACTGATCTGATAAACTCCCCCTCTCCCCAG
AAACATCAAAAACCAGAATTCCATCTGCAAGAGCAGGAGTGAGTGTTCATTGCTCCTGCTTTTC
AGCATCTCCCTCCTCCCAGCTAGATCTGAGCCCTCTCTTATGGTTGAGGATGCCCCATCTGGTGA
ATATCCAGAGAGGCACTGTCTAGCCTGCTTTGCAGCCAGAGCCCTGACAGGTGACCAAGTTTCA
GCCAGTCAGAGGCACCCACCCCAGAAGCTATCAGTGCAGGCCCAGACTATTCTGCAGAGTGAT
CTGGGGCCGTGTCCCAACTAGAGAGCCCCCAAGCCTGGTTCTCTGGCCTTCCCAGGGGTTCTGT
GAGCCCCCAAGATCCTCTAGTGGTCTCCTTTTCCGCACCAGCCTGCCCAAGCTGGTTTCTGTTGC
CTGCAGCCAGAAGCCCTGCTGGATACAGCACTGGGGAGCTGGAGAGGGCATGGCAATCATGTC
AGTTAAAAGGATACAGGCCTTCTCTTTTGTGCAGCGCTAAAACTTTTGGTCAAGGAGTTTTTCAC
ACATGCTCGACACAGAAAACAGAAACAGAGCTTCTTTGGTTACATAGAGCAGGGGAGGGACTA
GGGTTACCACATTTAGCAAAAAAAAAAAATAGGTGTCCAGTTCAATTTGAATTTCAGATAAACAAT
GAATGAATTTTAGTATGACTATATCCCATTATATTGGAGTATATGTATTATATATACTCATACTGA
AATTTACTCGTTGTTTATCTGAAGTTCAAATCGGACTGGACACCCTGCCTGTTATCAGGTGATCC
CAGCAGGGGCCCAGAGTCCCCAGGCCTGTTTCTTTTCACTACCATTTTCTCTGCTCCCAGGGTTC
CACAGAAAACACTTTGGAAGCTCACCTAATTTGAGACCCCTGCCTTTTCTCCTTCTTCTGCAAAT
ATTTCCTCTGTTTTCTTTTCTTTTCTTTTTTTTTTTAGACAGAGTCTCCCTCTGTCACCCAGGCTGGA
GTGCAATGGCACAATCTCAGCTCACTGTAACCTCTGCCTCCCGGTTTCAAGCAATTCTCCCTCCT
CAGCCCCCCGAGTAGCTGGAATTACAGGTGTGCACCACCATGCCTGGCTAATTTTTGTATTTTTA

GTAGAAGACAGGGTTACACCACGCTGGCCAGACTGGTCTTGAACTCCGGGCCTCAAACAATCC
ATCCATCTTGGCCTCTCAAAGTGCTGGGATTACAGGCATAAGCCACTGTGCCTGGCCAAGATTT
CCTCTGTTTTCCTCAGTAGAAATGCCCCTTCTTCACCATCAGTCCCTGTATTTCAGGTTGAACTAT
CCTCTTCTCTAAAATTCAGACTGGGCACATGACCCAGCCTGGTCAACCAGAGATGCCCATGCGA
TGGGTTCAGTGACAGTCATGTGACCTAATCCAGGCCAGTGAGATTCAACTTTTAGAACTTTTGCT
AGAACTACCGGTAAAGGGGTCTCTCCTCCCACTGGGGTTGCTAGGTCAGGGGCTGCTGCTGGC
TGTCATCTTCCCTCCCTTCAGGGAGAGCCTGCCCTGTGAGAGAAGCCACAAGACAGTAAAACA
AGAAAAGGCAGGAGACTGACAGCATCAGTGAGCCCCTGGAGCCCACTATCCCTGAAGCCAGC
CCCACCCAGGACTGTTCAGTTTAAGCCATTGTGTACGGAGTTTCCAGTCAGTTACAACAAAAGT
CCTGCAGAAGCGTGGTTCAATTCCTTCATCTTGAAGATAGAGAAATTGACACCCAAAGAGAAAC
TGCTGGGCCCAAAGTCTTACAGTGAATCAGGGATTAGACTGGGCTCCTCGCTCCCAGAGTCCTG
GGAACTCCTCACCACCCCACGTCGTGCTTCTCTCCATCTCTGTTGTTTTCTTCCTTCTCTCCTCTCC
TTCACTGTGTTTCTCCTCATTTAAAAAATTGTATTCTTTCTTTTACTTCGAGTTTTATCTTTATTCCCT
CCCCATTGGTAAACCATTACTCTAAATATAACAGGTTAGTCCCAACTCATGTGAAAAGATCGTAT
GGGAAGATTAACAGATTTGAGCTCATCTTACATATGATGCAACAACTAATAAGAGTTAGCATGA
ATGACACAGCTATCTGATGTCCAAGCTTTGCTGATTAGCTCTCGTCTTCTAATCGCCCTGAAATT
GGATGCAGTATCTCCCATTTCACAGATGAAGATCTGGAGGCCCAGGGGTCAAAGGACTTGGCC
AAGAAGCATGACTAACAAACGACAAAGCCAGCATTCAAGCCTGGATGACCCAATTCTGATCCC
CTATTTCACCCAGCCAGTTGCCCCTGTAAAGCCAGCCTGAGATTTTTATTTCCAGAGCTCACATG
ATAACAAACCACTGAGTTGAGTTGCCAGATGTGGCAAATAAAAAATATAGGCTATCCAGTTAAA
TCTGAATTTTAGATAAACAATGACTAATTTTTTTAGAATAAATATGTCCCAAATTGCAAGGGACAT
ACTTACACTAACAATGAGCCTTTGTTTATCTAAAATGCAAACTTAACCTTTCACGGTGTATTCTGT
TTGGCAATTCTACACTAAGGGGCTTTTACGCATAAAGCAAGAAGCATCAGCTGTGTCCTTGGTTC
TGCAAAAAGCCTCAGGGCCTTTGCACTTGCTGTGCCATTTGCCTGGAAGGCTCATACTGATATTC
ACCTCCTTCACCTCCTCTGGGTCTCTGCTCAAATATTTCACCATTAAAGCTTTCCCTTGCCCGTCT
ATCAGAAGCAACCCATGTTTCTTTTCCTCTTAACACCACCACCTGAAATACAATAGTTACATGTTT
ATTTGTTCATTGTCTCTCTCCTGCCATGGAAATGTGGGCATGTGTTTTGGTCTGATTTGTTCACTA
CCGTGAATAGAGCCCAGAGCGGTGTGCCGTATGTAGTTGATACTCAATGAATCTTTGCTGAATG
AGTGAATGAACTCTCTGTGAGCACATCTTGGTTTCTGTCTGGAATACTGCCTGACTTGAGATTGT
CACTCACCTGCTGAGTGGCAGTGGGCATGCCCCTGAGCTCCCCACTCCTTGAGCTGGACTCATC
ACACATCTCAGAGTCACTGGGAGATCAGGTGACGAAACAGCCTTGAGAGTCAGATGCAAACTT
GCCGGAGCACGTATGCAAGCATTCCAGTGTGCTTTTCTCTTCTCTTCTCCTTCAAGCACTCGTGC
CCCAGACAACTGAGCCTCGGAATACAAACCCTAAAAAACAATTTTTTCCAGTGTTCAGAGCATA
TGCAAGCTCCTCCTACCCCTGGCATCACTGCAGACAGTGGCATCAAGCACTTCCAAGTCGGAGG
GAAGGACAGCACACCAACAGACATCAGTACCAACGACGGCTAATAGTCTAGACGTCTGGTCCA
GTTATCTGCATCACCTCCTTTCATCCTCACAACTCTTTTGAAATAGATATTTTTGTGCCCATTTTAC
AGATGAGGAAATTGAGGCTCAGATTAATCAGTTCTACTGCCAGAAAGCAATGGAACCATGTTTT
AATTCAGCCAGCCTGATAGCAGTCTCTGAGCCCATTTCCTCACCTGTAAAGTAGGTATAATTATA
GTGCTACCTGGCCAGTCACAGTGGCTCACGCCTATAATCTCAGCACTTTGGGAGGCCAAGGTGG
GTGGATCACCTGAGGTCAGAAGTTCAAGACCACCCTGACCACTATGGTGAAACCCGGTCTCTAC
TAAAAAATACAAAATTAGCTGGGTGTGGTGGTGCATGCCTGTAATCCCAGCTACTCGGGAGGCT
GAGGCAGGAGAATCACTTGAACCCTGGAGGAGGAGGTTGCAGTGAGCTGAGATTGTGCCATTG
CAGTCTAGCCTGGGCAACAAGAGCAAAACTCCATCTCAGATATAAATAGATAAATAAATAACTA
AATGTGCTGCCTTGTTGGGATGTCGTGGGGTTAAAATGAGACATTGTGTGTGAAGTATTTGCATG

GGTCCAGGCAGGCTGCACACACAGGTGGCCACCACAGGCAACCTTGCCCTCCCCCCAGCCAG
AAATAATCAGGATAGAGGATGTCATGTGAAGGCCATTTTCCAAGCTGCCCAGAGTGACTGATGA
CCTAGCTTGAGGCAAGACAACTATCCAGAGTTATCAGATTCACTTCCTTGGGTATTTGAAATAGG
AAATCCAGAGAAAACGAGGTTACAGGAATGAGGCCAGGTCAAGAGGTAGAAAGTGTGGCCAT
GATGGGTGGTGAGCAAGCTGGCCTTATAAAGGAGCTCAGGCCACTGGGGTCCAGTAGTGTATG
TTGTACACTGTGCACTCTGGGATCAACATTCCTATTCTTGCCTTTGAGAACGGTGACCTCTGGAG
TTGTGCAGTACACACTCTCAGCAACTGTACCCAGCAGCTCTGAAATTTTATTCATTCATTGATTCA
TTCACATATATATTAATTTATTCCCACCATGTGACAGTCTCTTTCAGACCATGAGACGGCAGCAG
TGAAAAGATATTCAAGACTTTTCACACAGGTATTTTAGTTAGGGGAGATGGACGAGAAGCAGGA
GTGACTGACAAGTGCATGGAGAAAAAGAAAGCCCAGCGAGAGGCTGAGAGCAGAGGGTGGG
AAGGCCTCTCTGAGGAGGGGAGGTCTCCAAAGCCAGGCCTAGGAGGAGCAGGGGGAGGGGA
CTGTGGGTAGAGGCAACGGTGGGTGCAAAGGCCCTGTGGTGACCCACAAGAGCATCTGGGAT
GCTTGGCTCCAGAAGCCCCTCCTAACTCCGGCTTTTCCTGGAGTCGATGTTTCCCTGTGCCCAGG
TCCCCAGACACAAGTGTCTTTATGATCCCACCACATTCCTTGGGGTGGCTCGAGTCAGTGCCTCT
CACAGCCCAAAGAACTCTGACCCAACTTCCAGCTGGGGCCCCTGTCCCCGAACAGAGTCTCCA
GACATGGGCATTGCTGGCCCTGGTCTGTTCTCCTCTTCCAAGTCTCAGTGACCCTCCTCAGCCAG
ACTTCTGCCCCACCCATCTCTGCTTCCAACAAGCTCCCAGTGAGCTGCCTGCATCTGCAAAGCC
GGGAAACATGATGTTCCCAGACATCAAAGAGTTTGCAAAAGATTGTGGTCACCTTAGACCCAGA
CCATTTCCTGAGGCTGCTGTTTAGATACCAAGTCCAGGGTTTTTTCTTTAGGACTCCAGTGAAGG
GTAGAAGCTGTCCTACACCTCAAGCCACCACCACCTCCCTTCTCTGGACCCCACCGTAATCCAT
GGCTCCTTACAACCTCCACATCTCTGGCACTGGAGCCCTGATTTTGTTTTTATGGGGTAATATTCT
TTCATCCCCAAATGCCATGTAGGAATTTGCTGGCGGGGTGGGGTGGGAACCAGAGAAACAAAA
GGATGATTTACAGGGGTTCAAAAGCCTCATTATAACCAGAAAATTCAATTTACAGACATTTGGTT
CTTTTATTTCCATTTTTTTAAGGAGAGAAAAGAAATCAATCTAGTGTGGGCATTTTGAGTCCCTTG
CTTAGAGGTTCAAGGCATCTGTGACTTCAGAAAGCCTTCTTGGCATGAAGCAATAAACAGGCTG
AGCAGATAAAACCACCCTGTGTTTAAAAAGCAGGCCTGTCTCCGAGGCAGAATTTTTATAGATA
AAACAACAGAACGTGACTCAAAAAAGTCGTGTTGTGAAAATGGCCCCATGTAGACACAAAATT
CTTTACTCAGGAGCTAAGTTCTACAGCAGCACAGGAAGGTTTCATCTAGATAATTCTCTACGTCT
GGCTTCAAGGAGCCACTCCAAAGCCCCGTTTTCCAGACGAGTTTCGTGTTGCAAATATCACCTC
CATGCCTTCAATGTTTCACTGCATGGAGGACCCTCTCTCTTTCTCCGGTTCACCAAAATGTTTGCA
CATGTTGTGAGGGAAAATGACTTTCTGTCAAGTAAGTTTAAGAAACGATGGGCTCCAGAAAGGG
AAACATGTTTCTTCACTGCAGGACTTCTCAATTTTTTTTTTTTTTTTTTTTTTTTTTGAGACGGAGTC
TTGCTCTGTCACCAGGCTGGAGTGTAGTGGCGCAATCTCAGCTCACTGCACCCTCCGCCTCCTG
AGTTCAAGCAATTCTCCTGCCTCAGCCTCCCAAGTAGCTGGGACTACAGGCACGCACCACTACG
CCTAGCTAATTTTTGTATTTTTAGTAGAGATGGGTTTTCACCATGTTGGCCAGGATGGTCTTGATC
TCTTGACCTTGTGATCCACCCGCCTCAGCCTCCCAAAGTGCTGGAATTACATTCTCTGTCTTTAAT
TAGGGAGGAGAGGGTAGAGTGTTTCACAAGCTGTTATATTAAGTCAGAGATCTATCATGTGCAA
GTGTCAGAAACCCAACTCAAAACGGCTCAATGCCCAACTCAGAGTGGTTTCCCTAAGGGGGAA
GTCCTATCAACGCTGTGCCCAGGGGCTCATGGAGTGACCTCTGTCTCTGGGGCTCCATTTCTTTC
TGTCTCCATCTTTCAACTCGGCATCTATCTGAATGCTGGCTTCATTATTTCCTACCACAGAGAGGT
TTCCTCTGCATTGCCATATGTCAAGGCCAGCAAGCGACCCTGGGAAGAGAGAGAGAGAGCCTC
TTGCTCCCAGGAGAGAATGTCCAGGTCTTGGCTTACCTTGCGCACCAGTAATGGCAAACGGGG
AAGGGAGCACCTGGATTTCCCAGCCTTGTCTGCTCACCTCTGAGTCAGGGTGGAGGTCAGGGC
ACTGGGTCAGCCCCACCAGAGACCCATATGGTAGGGCAGGCACCATTTCCCCAAAGCAGGGG

GACGCCATCACCTGAATCCAGGAAGGGGCACTGGGTCAGTAAAAAGGAAACCAAATTTTCCTG
GGCATGGTGGCTCACACCTGTAACCCCAGCACTTTGGGAGGCAGAGGGGGGCAGATCACCTGA
GGTCAGGAATTCAAGACCAGCCTGGCCAACATGGCGAAACCCCATCTCTACTAAAAATACACA
AATTAGCTGGGTGTGGTGGTGGGCCCCTGTAATCCCAGCTACTCAGAAGGCTGAAGCAGGAGA
ATCACTTGAACCCTGGAGGCAGTGGTTGCAGTGAGCCGAGATGGCGCCATTGCACTCCAGCCT
GGGCGACAAGAGCAAAATGCAATCTCAAAAAAAGGAAAGAATGAAAGAACGAAAGAAAGAAA
GAAAGGGAAAGAGAAGAGGAGAGAGAAAAGAACAGAAAAGAAAAGAAAATTTGATCAAAAA
CCCCCCTTGATGGCCAAGCACCCAGCTGAACTGGGGTTCTGTAGACAGAGGCCCTGGGGAGCA
GATGCTTACCCCGGACAACGACCTCTCCAGCTCTGAATTTCTGTGCTGGCATGGGTTTGCAGGT
CATTAGCACTGGGTGCCACATGCCCGTTGGTTTGTGAGCTCCTAGAGGACATGCCGCATATGCT
GTTTTGTACTTTCTGTGTCTAACACGGGACTCTGCATACAGTCAGTGTTCAATGAACACTTGATC
AATAGAGGAGTTCACAGATCCCCTGCTCTTTTTCAGCTGCGGCCCTAACTCAGTCCAGTGTGGTT
CCTGACTTCTCATCCACCTCCGTCATGAGACCATCTGCCACAG

ID|56160|gene_id|ENSG00000253154|transcript_id|ENST00000518266 Homo sapiens lncRNA

GAGTCATTAAATCTCAAGGATATCTATTTGGTCATGTTTTCTTGTGGTATTTCAAGGCTACTG
GTGACTTCTTCAACAGTTGAGAAACTGAACTATGTGTTCATGAAAATACTCCATCATTAAAGTTT
ATGGTAAGTCTGATACCTATCTAAATAATAAGCAAACTTTAAAAGACACTATCTTTTAATAGTAT
GATTTATCATCATCCTCATCATTGATATCATCATTATTAAAAGAGAAATAATTATAAATGACCAAA
ACTTTTCTCATAATTACTATTCAGTTTATACTAATTCCAACCTCAAATTATATCTCTAAAAATCTGT
AGGATGTTGTAAGAAGTTAAAACAGTTGCCTATAGCACATAAATGCTCTTCTCTACTTTGCTTTTT
TCCTCTTTTATCTCTACATCCTTCCTCCTTGGAGTTTTTTCCTCTCAATTTATTCACTCATTTTTGTCT
CCAACATTGCTTTTTCTCTCCAATTTTATGAATGAAAATCCAAAGAAAAGCCTTAGTTTATATCTG
AAATATCTACATACAGTATAAAGAGAAACATATTTGCTTTTTGTCCTTAGAGCTCTTAAAATCCTT
GGAAATGAAGATTCAGGAAGCTTCCTGGTTGGTGAACACACCAAAGTTTAGGAGGGTGGTTCC
CCCAGAAATAACACAGAAACACTACACCCTACCCCCATACCTTGTTCTATGAATCTTTTTCATTTT
GCTATTCTTCAGTTGTATCCTTTCCTGAGGTCTGTCAGCCATTCTAATGAAGCATCAGACCTGAG
GAGGGAGTTATGGGAACCCCTGATTTAGAGCCAGTTGGTCAGAAGTACATGTGGCCCAAGACT
TGGAACTGCTGTTTGAAGGAGAGGCAGCCTTGTGAGACTGAGTCCTTTAACTTGTGTCTGACAC
TAAATCCAGGTAGATAGTATCAGCATTGAATTGAATTTGTAGACACCCAGTTGGTGTCAGAGAA
TTGGAGAACTGATTGATGTTAGAAAACACCATACTACAGATTTTTTTAAAATTAGCAGATGATTA
GATAACTACCTGTGATAATTATATCCTTGCTTATTAATAAGGCATTCAAAAGCTACTTACAATTGC
ATTTTGTATTCTAGGGCTGACTTTTGCTCACTCTTTAAATGACTTAAAAGTAGGACAGGTAGACA
ATTCTGATGTAATCTTCTTTCTTAAAGACCATTGGATTTGTAAATGCCAACCAAGCAATATTAAGA
CATTTTATGTAGGGCCAGGCATGGTGGTTCATGCCTTAATCCCAGCACTTTGGGAGGCCAAGGC
AGGTGGATCACCTGAGGTCAGGAGTTTGAGATCAGCCTGACCAACATGGAGAAACCCTGTCTCT
AAAATTAGCTGGGTATAGTGGTGCATTCCTGTAATCCCAGCTACTCAGGAGGCTGAGGCAGAAG
AATCGCTTGAACCTGGGAGACGGAGGTTGCACTGAGCCGAGATCACACCATTGCACTCCAGCT
TGAGCAACTAGAGCAAAACTCCATCTCAAAAAAAAAGACATTTTATGTAAAGTTTATTATAATAG
TTATTATATTACACATATGCAATATGTATTTATTATTGCCCTTGTGGAGAGAAAAATCTCAAATTA
CAAGTAAAGATTCAATTCTTTACTTGGGGGCAGCACCAGGTAGCAAACTGTATGTCTTAGATAA
AATTGAATTAACTGTAAGAGGACTCTGCTTGTTTAAAAAAGGAACATATATATATATATATATATA
TATATATATATATATATAAATTTTAAAACATCTATATGTGGGTGAGAATTACATTATGGATGATTT
ATTTACACACCAAAACTATATTGGCTTCTTTTATAAAACAATATAAATTTTTTTCCAATGGTCGAT

AAAACTCAAATGCCTTTAGGGGCCAGACAAATAACAGGCTACAATAGCAACAAGAGAGATAAA
ATCATAAAGAACAAACATTAAGAAAGTAGTATACATGAAGATTTCAATTATAAATTATCAGTCTA
TGAAAATAAGAACTGCAAACAAATCATGATCTCTTAGAAAATTTGTTTTCCATAATGTACAGTAA
TAGAAGTTATCGTTAATTTTGTTGTTGAATTGAGCAACTTAAAAGAGGGATTAAAAACAAGGAAA
AGCATTGTACATTGAGAACTACAAATCATTACTGAAAGAAATTAAGAAGTTCATAAATAAGTGG
AAATACATCTCATGTTCATGGGTTTGAAGACTTAATATCATTAAGATGTCAATACTACCAAAGCA
ATTGACAGATTCAGTGCAATCCCTATCAAAATCTCAATGATGTTTCTGCAGAAATAGAAAAATGT
AGCCTAAAATTCATATGGAATCACAAGGGACCTTGAATAGCCAAAACAATCTTGAAAAAGAAG
AAAAAAGTGAGAGGACTCATACTTCCTGATTTTGAAACATATTACAAGTCTACAGTAATCAAAAT
AGTGTGATACTAGCATAAGAGAGATATATAGATGAAACAAAATATAGGACCCAGAAATAAACA
CTCCTATATATGATCAAATTATTTTTCACAAGGGTGCCAAGACCAGTCAATGGGGAAAGGACAG
TCTTTTCACAAATGGTGCTGGGAAAACTGGGCATTCACCTGCAAAAGAATGAAGTTGCACCCTT
AACTGACATCACATACAAAAATTAATTCAAAATGGATCAAATACATAAATGTAAGACCTAAAAC
TATAACACTCTTAGAAGAAAACATAGGGGGAAATTGTCACAACATTGGATTTGGCAATAATTTCT
ATGACATCAATAGCATATGCAACAAAGAAAAATTAGACAGTTGGACTTTATGAAAATTTTAAAA
ATTGTGCATCAAAAGACAGTATCAACAGAGTAAAAAAGCAACCCACAATGGGAAAAACTGCAA
ATTACATATCTGATATGAGATTAATATTCAGAATATAGAGAGAACTCCAAAACTCAACAAGAAA
AAACAAACATCCCAATTCAAAAATAAGCAAAGGGCTTGAATAGACATTTCTCCATAGAAATGTA
CAAATGGCCAATAAGCACATGAAAAGATGCTCAGCATCACTAATCATTAGGGAACTGCAGATC
AAAACTACAATAGGATACCACATTATACCCATTAGAATGGCTACAATTTAAAAAAAAAGAAAAT
AACAAGTGTTGGCAAGAATGTAGAGAAAATGGAATGCTTGTGCATTGTTGGTGGGAATGTAAAG
TGGTGCAGTCCTTCTGAAAAACAATATGCTTACACCTGTAATCCCAGAGCTCTGGGGGCCGAGG
TAGGAGGATCACTGGAGCCCAGGAGTTCAAGACTAGCCCGAACTGAACTGTATACTCATAAGT
GGTCAAGATGGTAAATTTTATGTTATATGTATTTTATAATAATTTTTAAAATGGGAAAAAAGGAAA
ATTTTAAAAACAAACCTTTGAATAAAAATTAATCCATAAATTCATATTGATAAGAAAGAAAGAGA
GGGACAGTAAGGAAAGGAGGGAGGGACAGAGGAAAGAAGGAGGGAAGAAAGAAAGGAAGG
AAAGGAGAGAATAAGGGAGGGAGGGGGGTGAGAGGGAGGAAGGAAAGAAAGGTGGAAAGA
AAGAAGAAAGTTAATTTCTTTCTTACAGTATGGTGCTGATTAATACAGAGACAGAACAATGAACT
TAGAAAATAATCTGTTTACATCCATCATCAGAAAAATGATTTAGGCAAGAATCATTGATGGATGC
TAAATCAAAGTAGGGAAAGGCATTGAACAAGAATCAGGATGTCTACAGTCTCAAAGTATCTCTC
GCTAATTATTTAATAATGACAGAGGGAAAATAGCAACAATACAGTGGAGAAAATAGGCAAAAC
CTTCACCAAGTGATGAAAATTAACACCACCAAGGAAAGGCAGATGGGCATCTTGTGCTGCCAG
AAAGACACAACATCTGTTATAGAGTATTCCAGCCAAGAATGCATAACCTAAATCTGGTTATGAG
GAAACATTTGAAAAATCCAAACTAAATGACATTCTATAAAATAACTGGCCTGCATGCTTCAAATA
TGTCATCGATATAGCAAACAAACAAACAAAAACCAAAGTAAGGCTGAGGGTCTGGTTCGGATT
GAAGAAACTAAACAGTCATGACAACTAAATGCGAAATACGTTCTTGGACTAGGTTATGTATTGG
TGGAAATTGCTCTAAGAAATATTATTTGAACTAGGGGATGAAAGTAGAATATGGAATATAGATA
AGATAAATGTATCAATGTCAAATTCTCTAAATGTAAGTATTGTATTGAAGTTATGAATTTTAATTG
TTCTGGGAAATATGTAAAGACTGAAATATTAAGAAGTAAGGGGCAATAATGTATGCAATCTACT
GTCAAATAGTTTTAAAGAACCGTGTGTGTGTGTGTGTGTGTGTGTGTGTGTGTGCGCCTGGAGAG
ACAGAGAGAATGACAAAGCAAATGTGACAAAATGTGTAAACATTGGTGAGTCTAGGTAAAGCA
TGTATAGATGTTCTCATTTCAAATTTTCAAAATTAAAAATTAGATTTCAGAATTTTTTAATTCTGAA
GAAATTGTGTGGGGTAAATGGATGAAAACAATCTGTAAGTCTAATTCTATCCATGGGCTGCCAG
TTTGCAGCCTCAGGTTTAAAACATCATTTGACTTCCCTGAAATAGCTAAGGAAATCATTAAAAAA

CAAACCAAAAAACCCACCCATTTGCAATGAAACAGAAATCAATGAAAATAAAAAGCTTTTAAAG
TTTCCCACCTCAAGGCTGTTGTCTGGGGTGTGATTTCTTCCTAAAAATTAATTTTGAAAAGGATAA
AGGAGGGAAATTTCCAAACAACACAATATTGTTTTGTAAAAATTTCAACTTAAGTCAAAGGAGT
GAGGTTTGTGAAGGGTCCTGAGAGTCAGATTATCCTCCCATCCAGATCCTCTCCTCTTATCATGG
TGCAGGAGCATTGTTTTATCAGGTGGACTGGAGAGTAAGAAGTTACACCAGATGACAGTTTTCC
AGATAACAAGCTTTCACTTTAAATTCTCTTTCTAAAATTTTATTTTTTTCTAAAATACTTCCTATCAT
TAGGGTATACTTTCATCAATCTTCTTTCCATCATGTTTCAGCTAACGTTATTTGTTTTATTCGTCAG
TGCCATAACACAGGACTTATGTTCAATTTGCTGGTAGAATCTTGTTAAAAGGCAGGAACGTCATA
GGTTAAAAAAAAAAAAAACTATGGGCTTCCCATAGAAATATAGATGGATTCTCCAGACCAGGTTC
TTCCACCAATACACTAGGAAAGCTTTGGTTAATCATTTAGATTCACCGGGATCTAGTTCATCTGT
AGAAAAAATTTAGCTAAATTATATCACCTCTGAGGTCCTTCCAGATCTAGTAAGCATCATCCATTC
AGAAGCTGATTTGTTTTCATTCTCGAGACTGGACAAGAGGTAGGCATTTACAACAATGGTACTA
GGCTTTATTTTTTCAGGTACTTTTGTTTCATTAATTTTTTAAATTGACAAATAAAAATTATATATATT
TATGGTGTACTACATAATGTTTTGACCTATGTATACATTGCAGAATGGCCAAATCAAGCTAATTA
ACATAGCTATTATTTAACGTACTTATCAGTTTCTGTGGTGAGAATGTTTGAAATCTACTCTTAGTA
AGTTTCAGGTATATAATACATTGTTATTAACTATAGTCACCATGTTGTATAATAGACTTCTGAACT
GAAATTTCATATCTTTTGCAGTGGTCCCCAAGCTTTCGATTGTACTCTCCAACCCGGAAAAGGTG
GAACATGGATCCCCAATATATGCATATTAATTGGCACATGATATCCATGTAATGTTCCACTAATA
GGTAAATTGAGAAAGCACATTTAAAATAGACATTTAAAAAGAAAAATACAAAAAATAAATTATA
AAGGAGACCCTAATACTTTTTTCCAGCACACAATGCATCTCCTTGCCCACTCTAATTTGCATACC
ACTGTTTGTAGAAAAATGTCTAAACCCCTTCACATGTCATCTAAGGCCAGTCACATTTTGTCCAC
AATTTACCTCCTAGCTTTCCTTCCCACCACGTTCCTCAAGCACTCTTCACTCTGGCCACCTAGGAT
GGCTCACCAGCCTCTGAGCAAATCAAGCATTGTCACACCTCCTTGTTTCAGGCCCTGCTTTCCCC
TGTTTTTCTGTTGAGGGGGAAGACAAACATCACATATATCCTATGTAGTCCTTAAGACCTTCAAC
AAAGTTACTTCTTTTGTGAAGCCTTCCTCCCATTACAACTTATTCCTCCTCTCTCAAAATATGACA
GGATAACAATGCAATATAGTTCACCAAATTAATTTATATAAGTTGTATTGTATACACAAATTAATT
GAGTCTATGAACTCCTTCACAGCATTTTGGTGAGCGAAGATCAAAATTATCTTATTTTTATCTTTG
CCTCATTCCCAGCACCTTGTACATGGCAAGGGCTCAATAAAATTTAATTTCTTTAGGGAAGTTGT
ACCCTAGAGTTGAACTCTTGCTTTCAACCTATCCCATTTCTTGTAAAGCTGTGAAGGCAGCTATC
CACAGCAATGAAGATTACAGGTAAAACTAGTCCACAGACCTCTTATGTATCTAGAAGACTTGTT
GAAGATAATGATTATGATGAAATTGTAATTCTTGATTTCTTCTTTTAGCAACATCTTGCAAAGTGG
TGTTCAAAGAATGCTATTCCTGTGAGGTATAAAAGTAAATTAAATAAATAAATAAATAACTATAT
ATATATATGTATATATATATATATAAATAGTTATTCAATAGTTAACTATGCCTGCCTCATTTCATATTT
AAATGGATCAACTAATAATACATTTCAGGAATGAAATATTACCATGAACTTATTTTTTTCCTAAAC
CATTTTTAGCTTTCCTGATTCATGGTGAATGTGACAGTGTCTATAGCACTGTCAGGGGACCAGAT
TAACCCAGGCAGCGGGTGGTAAGTCAGCACCTTGGAGTTTGTGGTTTATGCAAACAACCTCACT
GACTTGAATGTGTAAAAATCAAAGAAATCCCACAGTCTTGGAGGTAATAGATGATGTTGTAGCA
GGACACACACCAGAAAGGAGGCATTGCTTTTGTGGCTAAGACTTATGACCTCCCTATATATAAA
AATAACCATGGTTCACCCTAGAGACTAATATAGTACTTGTTGGTTAACTGGGTACTAATCTAGTG
GTAAAAGTAAGGTTGATTACCATTCAAGCAGTGGAAAGCCTAGACCAGGGTTTTTTTAACCATTT
GTCTGCCAAGGACTTCTTTGAGAAGCTAAAGAGAAGAGCCATAAAATATTTTTAAAGATGAGTT
ATGCTCTTGGGAAAAATGTTGTCTCCTGGGATACCAGGCTAACAGGTTCTGGAGTGTTCTTTCTC
TCCTGTGGTTTTATGATTTTCATGTGCAATTCATTATTTGTGTGTGTTGTGTTCTTTCAAAATCACT
ATTGGAAAGTTGAAAAACTTCTTAGAATTCCGACCATTCAGATAAGGTCCCCAAAAGCATTTTAG

AGCAGAGATACTGAAGCCCAAGAATCCAAACTTCCTGGTAACTTTGGCAAAGCTTTTTTATATAT
AAATAATCAGAACATCTTGAATTAAATAAATTCAGGGTGAATTATATAAATTTCAACATATCTGTT
TTTTTCAGTAAAATGAAGAATGCATAAATAAGGTTTTAATGTGTACTAGAATTTGATCTACTTTCT
ATATGCTAAACCTGGGCCCTCCTAAGTCACCATTTGTCAATAAGAAATCAATCATAGAGATCCTA
ATTATACAGCCTAAAGCAATAGAAATATTTTATGAATGATTCTATGTTTCAAAAACTCTTCTATAG
AATAATATATTCACTTATTACTATATTTTCCTCTAAAAAGTAAGAAAGAAAGAAAAATTAAAAAA
ATAAAATAAATAAAAAGTAAGAAAGAAAAAGCTCAGGACACAAAATCTAGCAAAAGGATTAGA
TCAGCTGTAAGAATTCATCTGAGGGGACTATTAATAAGGAAGAAATATTTTTAAATTTTTGCTATT
AGACCTCCAAATAGAATGGCAGATCTACCAACACTTAAACCTATGTAGAGTTCTGATTATTCCTC
TTTTCTAAAGTACACACCTTAAATTATTAACTTTTAAGTTATTCATTATACCTTAAAAATTCAGTTG
AGCATGTTCTCATAAAGCAAAAGGAATATTTCAGTCTGGTTTCAGCAAGATGTGATTTAGGGAA
CCTCTCAAAAAATGGTGGGGAGAAAAGGGATTCTAAGATATATAACTGGCTTCTCCTGAACCCA
AAATACGGAGGAACTCTAACATCTCATCCAGTTTCTCTGTCTTCTTGTCTCCTTTGTGATATGCTG
AAGGCTTTATAAACACAAGGTTGGAGTGTAGTCAGCCAAGGTTTCAAAGGGCAGGCTACTTCTT
ACTCTATTTTTCTTAGGACTTTGATCTTATCTCTTCTGCTTATGCCAAATCAAGCTTCTGTTGCCCC
AACAGCTACCCCAGTTTAGGAGCTGAGGAGAACAACAACACTATTTTTATTGGAGAGACCCTGA
CTAGAGGGTGGGCATGGGAACAGGCAGGCTAAACTGCAGAGAGCAGGCTGAGTGAGCCACAG
AGTGATGTACAGTCATCCCGTTTCAGAGAACTCCAGGGACCATCAGAATGAAGTCAGATTTCAG
TGCAAAACTGTGTATTTGGCCACTGTTTCCATACAATAAGGTAAAAATTAATCATAGCAAATGGC
TGCTTATTAATAAAACCAAATTATTAAAATGGTCTAAATTGTAATGCCTTCTTAGTCATTCAAATT
GCCCTGGGTTAGACTTAAAATTGACACGCAGGTTTAGAAATGTAACTCTAGCTATCAGAAGGAA
ATGAGAAGAGATATTCTGTCATCTATTATTCTTTATAGGGGAAAACTGATTGATCCAAGGACAAA
CCTATCTCCATTACTTGGCCGGAATCTAAGAAGTAAACCTATGAGGATTATATATATTTATGTCCT
AATCTGTCATGCCAACATAATCATGATGTGCCTTTATGATATTCTTCAGTCTCTCTTCCTCAATCTT
CATATCTACTTGGTCACCAACATTTATTAATTCTACATTAGAAATTACTCTTGAATAAGGTTCCCG
TTCCATCATCCCCTGTGTCACTAACTTTAATTTACTCTTGTGGGTCATCCATCTACAAGCTGCCAA
GGTAATCATCCCAATAACCACAGGATAAATACACTCTCTTTGCACACAGGATAAAGCCCAACAC
CTTGGCAGCGTCTACAGGGCCCTTTGCAGTATAGCATCACTTAAACATCATCTAACCTCATTGTC
ACCAAGGCCCCCTGTATTATTTATACACATGAGGAGTCCTCATTCTTCCTCCAACATCCTTCTCA
GACTTCCAGCTTGAGAGAGACATTTTCTTGGTCTTTCCTTGGTGAATTCCTAGTAATCTTTCAAGC
ACCAGTTCAAATGTTATATTCTCTAAAGTTTTCTTTGACCCACCCATCTGCCATCCTTACTCCCAC
AGGTACAATATCTGGGCTTCCTCTTCCTGCAACATTTTGCTTGCATCTCTTTTGGAATACTTAACA
CGTTTTTCCGCCATGGTACAACATAGAGGCCAACTTCAAAGTTGAGTCTGTGACACGTCGTTGTG
TGACCTCATTCAAACCACTGTACTTCCCTGTACTTAATTTCTTCAACTGTAAAATGTAAATGAATA
TGGCTACTAAACAGAGCTGTTGTAACAGTTCAATAATGTATCAACACAGCTCCTGACCATGGTG
AAAATTCAGCAAATAGAACTATAATTATTATTATGAATAATTGTTATTAATTATATATGTAAATAC
CTATATAAGGCTTATTAAAACTCTGTGAAGGTAAGAACTGTGGGTTTTTAACACAGTCTTTATCG
CACATACACTTAAATTATGTTTTCTTGCTTTATCTCCTGGTTATTTGAATGGATTTTCTGTTTTCCAG
TGTAACCCAGATGTAATATCCTTACCAGATTCTTGTCTCTGGCAAGCATACCTAATCTGAAATGG
ATTCACCGAAGCCCAGCTAAACCATGACCAAATACAGGTCCTGATAAGGAATATACTATCCAGC
AACTACAGTGATTAAGTGTTGTGCTAAGAACATTTATTCTAAATATTTATAGGTCTTCTTTTTAAA
AATAGCCTAAAGAATTTTGGGGAAGATGATAACAGTGGGTAGAGTGAGTGTTCAAATGTGTACA
TATCAAGAATATACATTATCATAGAATCACTTAAATATTTAAATTGTGTTTATATTGCTCATTCTCA
GTAACTCTTTTAGATGCCATAAAACCTCTCTCAATCACCCAATGGAATTAAATTAATGGAGCTAT

TTTGCATACTTGGCTTGAACAGAATTTTAATGATAAATATCCCTTCTGTATTTTATATTTATATCAT
ATTCAGGCTCCTAAAGAAATATGCCATTTCAGGAACTTTAGTTCCAACTTCCTAACAGTTCGTTG
AAAGTGATATTAACTATATAACTGATGAATGACAATTAAAATTACACTGATAAAAGCAGAAAAA
ACCAGCAAGTCTTGCAGTTTTCCATTGATTTCTCTGTTGTGGCAGCATTTATTGACATGATAAAGC
CCTCTTCCTGCATCATCCAGAATTCTCCACAATATTTTAAAATCAAATATATATATATATTTTAAAT
TACATATATATATTTGTATACCTACACACACATGGCTTATCTTGGCTAATCAGGCAACATTTTTCT
AAATTCTAGCGCTTACAAGGTCATTACTGTCAGATTGGGGAGGTATGATAATAACTGATATGTAT
TAACTGCCCTGTACTATTAAAGGCACTTAGCATATACTCAAGCATTTTACACTTTAATGAAGGTC
ACAACTGCCCATTTTGTTTTCCTCAATAAGTTATATTTCTGGCCTCATACTGCCCAGTTCATTTAAC
GATTGTTGCCTCTAGCAAAAATTTCACTAGAAATATTTTTAGTAACTGGTGGCATCTGTCAATTAC
ATTTCTTAGAACATAGGGTTTAGATGGACAATAATTTTCTGATTAACTAGAAATGAGTCAGAAGA
CCCCGATAAGCATTTCTGGAAATGAGCACTCCTGGCTGTCTTTGTGGTACGTGTGTTACCTGGAT
GGATACACTGAGATTCCCATTCACTGGAATCCGTAGCTCCCTGGTCCTGGACCAGGGGCACCGA
TGTAGGAGGTACGAGACCTGAGGCATCATCTTCCCTTCACTGCTGACTAGCTTTGAAACCTCTTG
TCCATGTCTCTTCATATGGACTCAGTTCTCTCAGGTATAAATGTAAACTAAAAATGTCTGAAGGA
TACCATATTAGTCTCTAATATTTCAAGGTCTCTGATAATTCTAAAATCTAGGCTTTCTTTTATTTATT
TGAAATCCAGAAAGTACATACTAGAGTTTTATTCAAAAAAGCAAATTTGGAGATATGTCCTAGAT
CTGGCACATATATATATATATATATTCTAACATATCTTTCTGTTTCTGCACCTAAAGTTACGATAC
ATTTTTATTTATGTTCCCTTGCTCTTCCTGGAATATTTCATCTTCCACTCTTCTCCATTTTTATTCAG
ACCTTCCCCATTCTTCCTGATGATGTCCTATCTACCAGTCCTCATGTGGTCACAATGCAGTTATCA
TCTCTAAGCCAAAGAGTACTGATTACAATTTATTCTATAACATTAAATATGCAGTGTGTCATTGAC
TCTATTATATGCTGTGTGTCACTAATTCTAAGATGCATTTCTTATTGTAGTGTCTCTCAAATCAGG
ATGTATCTTAACAATCGATGACATATTGCAAATATAACTAACTGCTTTTCTTCTTAAGTGGCACAA
AAAGAAAGAGTGTATATCATAATTTCTGGCATCTTGCAGTCAATGGAAATTAGTCTTACTAGTAT
TTGAAATGCTATCTTATTCACTAGACAGATCTTGTCTGTATATTTTACTTCTTCAACTTATATATAA
GAATATATTTTTATATTTTCATACTCCTCAACCCCATATTAATAATGTTTTAGAAATTCATCAAGAA
TTTGATTAATAATTATAGAACTACCATGACTCAAGGAACCAGAGTCTGGACTTCTGAAGAAACAT
TCAATTATATTTGCCATAATGTATTTCTTGAGGTAGAGATATGGATCAATATATTTTAAGAATTTTC
AGTGACGATTAAATTAAAAGAATTCATTTATTCTTTATCACTGAGATGTAACTAGATGTTTTTCTTT
TTCTCATCATTTTTCAGGCTTTCCCAGAATCTTAAGGATGATAGGAATCTCTAACAGTGTTTTGGA
CATTCCATTTATCTGCACAGAACTTTATGCTAACCTGACGCAAAAAATAAGACAAATTTCTAAAA
AATACTTTTCTCTGTAGTTTATATTTTTGTACTTTTTGTCATAGTGAGATTTTATTCTGAGATATCCT
CCACGAGATGGTGGAAGTGTTGTTGTTCACTGAGTTTATAGCACTGCATAAACTGCCCGTCTCTC
AACCAAATAAAGTCTACAATTAACAATGCAGAAATAGCACTAAACATTCATTTTCTTAGGATCTG
GGCAATAATGGCATACCTTTAAAATCTCCACTTAAATGTATATCAAATAAATTTTAGAACCCAAT
ATTAATTTTCAAAATCACACAGAGGTCGGTCACAGTGGCTCATGCCTGTAATCCCAGCTCTTTGG
GAGGCCAAGGCGGGCAGATTACCTGAGGTCAGGAGTTTGAGACGAGCCTGGCCAACATGGCT
AGACCCCACTTCTACTAAAAAAAAAAAAACAAAAATTAGCTGAGTGTGGTGGCACATGCCTCTAA
TCTTAGCTACTTGGGAGGTTGAGGCACGAGAATCGCTTGAACCCAGGAGGCGGAGGTTGCAGT
GAGCCGAGACGGCACCACTTCACTCCAGCCTGGGTGACAAAGTGAGACTCCATCTCAAAAAAT
AAAAATAAAAAATAAAAATATATCACACAGAACTGCATTTTTCTATGATTATTGCTGCCTCTAGC
CTGACAGAATACAGCACTGTGTTTTACCTCTCTTCTGGGCCCGAAAAAGATTAATTTTATAAACA
ATTGTTAAGCACTGGCCACATGTGAGTACTCTGCCAGCACAGTTAAAATGCAAGAATAAATTAC
CTTCAAGACACCTACAGTCTAAAAAAGATGTACAAGAATGGGACCCCATCCACCTTCTGGGAAC

ATCACAGTTGTTTGGGAGCAGTGTCACTGGAAACTCTTTTTAAGCATAAATGTTTATATTGATGCT
GTGATGGGGAAGATATTATAGAAAACAGTATTTCATCCCCTTTACCTTAGGCAATAAAATAGCAT
AGATATTTTTAAAGGAACAAATGTCAGAAGAAATAAAGATATTAAAAAATCAATATCCATGTGG
GGCTAAAGGCAAGTTTTTACAGATCAGAAATAGATGAATTTTCTGAGCTCAGAAAAACCAGGCA
CTCAGGCATTAAGTTCCAAGAAAAAGAACTCACTTCAGAACTCTGAGAAATGTGTTAGAAGAGT
TGCAATCTGCTCTCTCCTTCATGCAGTCACAGAGATGCAGGAGGAGAATGTACATTTTTGACACA
AAGACACTGCAGCCACATCACAGTCCAACTCTGGCTGGGCAAGATCTCACTGCTATCTCTGGGT
GCAGGTGTCAGACTACCAGGCCGGTTACAATACCAGGGCACCTTTTGAACAGCTGGGACTGAC
AACACAGAGGGTTTCCAGGACACACACTTTTTTCTTTGGAAATGTTTAACTCGGGTACAGACTAA
TGAAAAGGAAATACACCTGGGAGTCATAAATAGTTTTAGAATGAGATCTAGTTGTTTAAATAATC
TCGTTTTGTTTTGGAATTTGTACATGTGAATACTAACTACAAGGATAAGCAAAGTCAAATACATT
CTGTATTACAGAATCTAGAAAAATAGGCCGCACAAATTTGGGCTGCAAAGATGTCCAGTGAGAT
ACAATAACATTAAGAAACTGTTAGGTTAAAAACAAGTAAATAAAAATATTGATCATTTTAAAATT
GCTATAAAGTTTATTCTAGTTCATCTGCTAACCAAACTATATTACCCTGAGTGTGTAAACTGGGTA
AAGAAAATGAACCATTTGGGAGCTAACTGCCTAAATAGTGATTTTATTTAAAATTTTTCTCTTTTT
AAAAATTCCTTTGATTAAAAAAATTTATGTAGCAATATAGCTACATTAATACAATAACATGGTTTC
ACAGGTGCAAACATAAGTTAAACTTATCAAACTGTACATTTTATGCTCAAGTATACATTAATAAA
TCTGCTAAAATGTCTATGTAGTTATCTATGATGTTATATTAATACAATGAGACAATATAGTGGATG
CATATTATTTAAGGGATAACATTTTAGATATTTTAGAGAGAGACTGATACTAATACTTTAATAAAA
ATCTTTTTTTCTTTTTCTGAGACAAGGTCTCACTCTGTTGCCCAGGCTGGAGTACAGTAGTGTGAT
CACGGCTTACTGAAGCCTTAACTTCCCCAGCTCAGGTGATTCTCCCACCTCAGCCTCCCCAGTA
GCTTGGACTACAGGCACGTGCCAACATACCTGTCTAATTTTTGTATTTTTGCAGAGACAGGGTTT
CACCATGTTGCCCAGGCTGGTCTCACACTCCTGAGCTCAAGGAATCCACCCACCTTGGCCTCCC
AAAGTTCTGGGATTACAGATGTGAGACGCTGTGCCTCGCCATTAATCATATATTTAAATCCATTT
ATTCAGGTATCCTTTTCTTCAGAAGTAGGAGAATTTATCGAATCAGCTGTGATGAAAGGGTAATA
ATATTAAGTGATCAGTCAAATTAAAGACAATTGTAATCCTGGTGAATAATGAAAATAACATTTTA
GCTGCAATTGCTATATATCTGCAAATATTGCAAATCTGTATCTTGCTACATATGGGTAGAAAATG
CTAGGCAGAAGTCAAAACCTGTAGATTAGAGAAGAAAATAATCTTCCTCCAAAAAACAGATACA
ATAAGTGGTAAGCATGGCACTTTATTTAAATCTTACCATATTTAATCCCAGCTCCCACATGTATCA
GCTATCTGATAGTTGTGGGAAGAAAAATTTCTCTTAAATTATGAAAGTTTAATTTGTTACCTTCAA
TGACTTTTTGTTGAAGAGATTAGGGAAATTCTTAGACTCCCTCTCTTCTGAATTAATTTCCATAAG
ATCTTTTAGAATAGATAAACTTGAGAAGAAAAGACCAGAGGAAAGAATGTATAAATTTAGTTTC
ATTCTAACAGGAAATGACATTTGGCTATGAAGGATTGTCTTTGCTGGAGCTCTCCATCCTTTTGA
AGTTTTGAATATCATAACAAACACCAGATAACCCAGTGTAACTAAGGGGGAATATACATGTAAA
CAGTTAATACATCTGTTTGAAATTTTGGACTGAACAGGAGATGAAAATAAAGCAGGAAAAAAGA
ATGAGAGTACCAGTGGGGGAAAACCACTTATTTCTGATTTTATGCACACACAAATACATATACAT
ATATAAACAAAAATAATACTTATCATGTGGTTGATATACATACAGAAAAAGCAACCATGAAAAT
GTATATTTTATCTAAAGAGGTTGCAAGGCCTTCCCGCCTTGTCAAATTTTGTATAAAGGCTCAGT
GAGACCACATTTGCTGAGGATAGTTCCTTGACAGCTGAGTAATAGAGCTTATTGGCAATGCCAA
GACTTAATGGCCTTCAGTGAATAGTGCATACGCTGGCATCTGGCAGATTTAGAGCTGTGGTTAT
GTAAAGGGTGGGTCTTTCAGGAGGCAATATTATAGATAGGCCCCTCCTTAGATAGTCATAGTCC
AAGAGAATAAAGGCAATGCAAACTCTTAATAGCAAAGTCTTTTAGTCTATAAAACTATTTAAGAG
TACAAATGGTTTCATTTAAAATTCAACATCAAAACACTTAATCCTTGCATGTGTAAAGATGAAAG
AAGTACCATTCTATTACAGTATTATATAATTAAAATAACTAACTGAGACATACTGTGGCTTGTAGT

TTTCATGAAAGGTCCCTTGTTCCCACCACCATGAAATGACATTTTTTTAAAAAATTTAATATAGGC
TGGGAGCAGTGGCTCATGCCTGTAATCCCAACACTTTGGGAGGCCAAGGCAGGCAAATTGCTT
GAGGCCAGGAGTTGGAAACCAGCCTGGGTAACATGGCAAAACCCTGTCTCTACTAAAAATACA
AAAATTAGCCAGGCATAGTGGTGCATGCCTGTAGTCCCAGCTACTGGGGAGGTTGAGGCGTGA
GAGTTGCTTGAACCTGGGAAGCAGACATTGCAGTGAGCCAAGATCCCGCCACACTGCACTCCA
TCCTGGGTGACAGAGTGAGATTCTGTCTCAAAAAAACAAAAAACAAACAAACAAAATAACACC
CAAAATAATAAAATGTATGGGCAATGTAGTTTGTGAAAATGACAGGTAAGGACACATACATAAT
AAGTTTAAAAATGATTTTGGATGATGACTACTCTAGCAGAAAGATACAACAATCGTGCTCTTTGA
AGAATTAGTTTTAAACTTGTCTTCATCTTGGGGATTATTACTAATCATGGTGGATTATCCCAGGTT
TTTTTGTCTCTCTTCATATACTTGGCAACTTGATTCTATCATAGGAAGAAATACGAGATGAAATTC
AAGTCAAGCAATTTTTTAACCTAAGATATAACTTGGTAGGAAACCAGTACTTTAATTTGAGTGTC
AATTATTGGTTATTATCTGAAGAGTCATTAAACTGCGGTGAAGCCAAGTATTGTCTCTGCAGAAT
CAGACCTATTTGCTCCAAATTATCACCTTCTTGCTTAATAATTATGTAACTTTGGGCCAATTGCTC
AATTTTGCTACATTTCATTTTTTCATTGGTATAATATAAGAAGAATGCCTATTATAGAATTTTTGTG
AGAATTAATATAGATAATATTTTCAAAACACAATCCCTGCCATATGTACGTACAACTCATGGTAC
TCACAGTTAGCTTTGCATTAGGAGCGGGTTAGTTTGCAACTGCATGGAGTGTAGTTTTCTTACCA
ACAACCATCAGGGTTGGTAAGAGAAGACTGTTGTACTGAAAAATAATATGAGGTAGCTAGAACT
AGAAGGAATATTCAAAAGTCTGAGAATCCCCACCATCCATCCCTGGGAAATAAAATCCCTAGGA
ATCAGAGACAATTTGTCTTCCTAAAATGAGAATTCATTCAATGTTAAAGTCTAAGAGTTCAAGAG
AAAAAAAAGCTTCAAAACAGACATCGTCCCTCTTCCCACTGTCTTCTCCTCCCAGGCCCCAGTG
CTTTCTATCACTATAGTTTAGTTCGCATTTTCCAGAATTATATGTACCTGGAATCATATAGTATGTA
CCCTTTTTTGGTGGGGAGGTGCAGACCTACCCAGAGTCTTTCCCTAACTGCTGCCAGATGCTTAA
AATTCCATTTTCAGTACATTACCCCTTGCAGTGGTTCTTAACCTTAGCTATGTCACAAATCCCCTC
GAGACTGATAAATTCTATGAGCCATCTTCCCAGAGAAATAAATGAAGAGAAATCCATTCCCACA
GAATGTGGCATATAATTTTAGGATATTCAGATGAGAGAATTTGGCTTACCTGAAAGCTTTTGTAG
CTGTCAAATACATAACAAGACTGAGGATCTATCACAATAAAGCTGGAAAGGCAAGAGCAACCC
AAGGCCCTGCAGAGCAGGGGGTTCATATGTTGGATGTTCACTAGAAGGATTGTATTTCTCCCTA
GATTTTTTCTTTCTGTTTCTTTTTAGGGAGTACTGCACACAATAGAACCAACTTTTATCTTTTCTGT
AATAGGAAAGTTGTCTTTTTTTTTTTTAATTTCTGTACCTGGTTTGTATGTATTTTGAGGGGGAAGC
ATTCTTGTTTTGCTTTTCTGCTTCCTTTTTTTTTTTTTTTCATTAGAGACAGGATCTCACTCTGTCAC
CCAGGCTGGAGAGCAGTGACATGATCACAGGTCACTGTAACCTAGAACTCCTAGGCTCAAGCA
GTCCTCCAAACTCAGCCTCCCAAAATGCTGACATTAAAAAGTGTGAGCCACTGTGCTCAGCCTT
GTGTTGCTTTTGAACACAGAAGCTAGCCTTGAATTAACTTTGGCTTTTAACTAAAATTCTGAGTTT
TTGATACTACGTTTTCTCTCACTCAGAATTTTTTCTTATCCATTAAAAAAATAAGAAATTGTGAGG
GCAATTTGATACTTACATCATTTTCAGTAGGTGAATTATACAGCCCTTAGTTCAATCATACTACTT
AAATAGATTGCTTAGAGGGTTAACTAGCTAGGGCTTGGCTTAGATCATTGACTCCAGAAATGCT
CCTTGTAACTATGGTCCAGGAGTATAATTTAAAGATCACATGTTAATATGATGTTAATGTGTGTAT
GTGTGTATGTGTATGTGTATTATATATAATATAAAGATGAACTCACCCAACCATTTATAGGCATA
AAATACCTTATAAAATGATCTCCATCCATATCCATATGCAAAATAATCTATCAGATCAAAACATT
ATATATCTCAAAACATTTCTCTGAAGAAGTCATTTTATGCTTAGATGACAACTTTTTTTTTCTATTTT
CCCTTTAGCAATACTCTCTCAACACTGTTTATCAAAGGAACCAATATGGAAGGAAAAACATTGCT
TTGAAGGGAAGATGGATGGATCCCCTAGTAAACTACAGCTTGAGGTGCAATTTCCTAAAGTTCA
AATGCCTGAAAAAGGAAACGAAAGAAAGAAACTCAACTCTTGAGACAGCTTCAATGCTAGCTTT
GATCTCAATTATAGAGTTATTTATGAAAAGTGCATTTTTACAACTAAAAATAAATTAGGATCAAA

AGCTTCTAGAGGCTATACTATGGAGTGCTTAATAAAAAGTTGAGTAAAGAGTAATGAATGGCTTT
GTACTATTAATTTGAAAGAATTACCTTCTCTTTTAAACAGCTGCCAAGATAGTGAAGATTGACAT
GTCACAGGAAATTAAAACAAATGGTTTCTAGTCCTGTATGCCTTGGTGGGTAAACAGTAGGATA
GAGCCCTTACCTGGGAAGCTGTGGGCTGGACTCTACCTCTAACTGTGTGCAACCATTAAAGGAG
CAACCTTCAGCAAGTTTATTTAACTCTCTGGGCCTCAAATTACCTACTCCTAAAGTGCACGAGAA
GAACTAGTTCTTCTCTGAAATTCTTCCTGTCTCCAATGGCCTGTTCCTTAGTTCCTGTTAACTAAG
CATACTCACACTCTAATAAATACATGTAAAATCTTACCCCTTATAGAAATAGTTTTTTTAAAAAAA
GAATAATCAGTTTTAAATTTAAATCATAGGTCTATTCACTCAATGGACATTGTTGGGATTCAGTGT
AGAAAAAGCACAAAGGATGAACTTCCAGTCAACTTTCAGTATTTGCATAAACACGACACAATTG
TAGATTATCAAGAAATAGTTTTTATCAGACTAAAATACTCTAATGAATGATTTCATAGTCTTTTGG
CTGTTTAATTAAATTCAGTGAATAAATCATTTCTTATTCTCTAGGTAACAATGAAAAAACATCTCA
GAATAGTATAGAACAGCAAAAGGAATTGAAAGATGGTGCTGAGGATGATAGATGATAGACAGA
CAGACAGACAGACAGACAGACAGATAGATAGATAGATAGATATTGATAGAGAGATAGATAAGA
CATATAGATAGAATCCACTATGTAGAAAATGTCTCCTGTGGATACTGGAAAGTAGCTACCTATCT
CAGGGAAAACAATGGCAAACCCATGAATAAGCTGATAGCTTTGACTAATAGGATATCTTAGTTG
CTGGGTTAAAATATGAGACTTGTGTTCAGATTACAGTATCTTTCATTTTTATAAGAGAACCTTTTTT
TTTGAGAAAACATATGTCACGTTCACACAATCTGGAAATGAATATTCTTATTCCTACCCCAATCTC
ATTTTCTTTTCTAGTTTTTTTTTTTTTTTTTTTCCTGTTCTTTGCAGCTCACAGGGATAAGGCAGGCAG
AGTAGGAGGTAAAGGAAGAGTACTCATGCCAATATGGCTCTTTGAGTAATTACCCTGCAGATGA
GTTCCTCCAAGCACTAAAGGAGTGAGTCAGATCCTCCCTTTGCCATTCAGGTTCCCTGAAGTAC
GTCTGAAGGAAAATAACAGGTCATCCAGTCTTTTTCCAATTTGGAGCTGTTGAATTGCCACTGTT
GTACTCCTTCCATATCTGAGTATCAACTGTAATATTATTTAGTAAATAATTTTTTAAATGGCTCAAT
TTTTTATTCAAATAATTTCTTTAAAAATGATATCTTACTAACCAGTTTATCAAAAATAGAGTTAAAT
AAAAATATGTAGAAGGAATTAAATAATATCATGTTATTTAATTACCACTAAATACTGTTGCCTACT
AAAACTGCTCTTTGATCCCTGCTCTCTGTTAACAAGATTAGTAAGATAATAAAGCCTTATTAATTT
GAAACTGAGACACCTTTCCTTGACATGATCAGAAGGAATGAAAGATAATCGAGAAGAAATAATT
TTCTCACTTTATGATTTAAGATTATGTAATGCCCTATCTATAAATTATTAATAATCTTTCCATACAA
AGCAGAGGTTAAAAACAAAATGGGAATAGGGTGTCTGTTGCTGATAGTGTACAAGGTAAAAAA
TACATTTGTTCTACCAGACTCATACATTTTAGATTGATACATTTGAAAGCAATCTAGTCCTCCTGT
TTACACTGCATTTGCTCTTGCTTCTGTAGTGAACAGCTCAAGTGGATTTAAAAAGAAAGAAGAGA
AAAAGAAAAAGAACCCAAATCTTTGTCTTTGATGTAAATTTTATAATTTGGTTTTGGAGAGAGAG
TAACCACATGAACAATAGAGAACAAAGCAATCTTAAAGCAAGTTGTTTAAGTGTCCGACAGCAC
AGTAATAGCTGACTTTTGTTGTCTTTTAATAATAATATTGTATACAAAGCATTTATTATGGGCCAG
ACGTGATATTAAGTACTTTAAAAACATTATCTCATTTAATCTTTCTGCTAATCCAATGAGGTAGGT
ACATCGTTATTCCCATTTTTGGATGGAGAAACCAAAGTTCAAAAAGAAAAAGAAACAAGCTCAG
GACAACACAATGGTAATGGGAAGACAGGATTCAAATCCAGCCTATCTCAGTTCCCATTATTTATT
GGATAAAGAATAGAGTCTAAGGGGGTAAGCCAGGAGAATTCAACTAACTTGCTTTTCATTTTAA
TTTGGGTTCTGACTACTACAATTTTTTTCTGTATAGAAACAGTAGCACATCCTCAAATCCTCCACT
TAGATAACAGTTTTAACCCCTGTCTATTGGCAGAATTAAAGAGCTTCTCCGAAGCCCCAACTCCA
CTGGAGAATATGTTGTGGGGGTGGAGCAAGTGCAGTGCCCAGTGCTTAGAGGTTTATCAAAAA
GGATTGTGTAAAAATCCTTTAATCTACCACAACTGGAACACAGGCTACAAAATCAGCCCATACT
CTTAGTTTTAAAAACTAAACCATGGCCACTACACGAACTATTCCCCAGCCTGTGTTGGGAAGGC
ATCAAAAGCCTATTGCTCTTAAGTATTGCCATGGGGCAGAGCATAAATTCTTGTCTTAAAAGCTC
AAGCTTGCACTGAAGTGTGACTCAAAATCAAAGGGCAAATTCAAATTAAAATCATGGGCTGGGA

TATGATGAAGGGAATGTTATCAAATATCTACCTGAACTCCTGGGGAGAAAGTTCAATTTGCTGTG
CAGACAGCTATAGCTGGAATGTAGCAAAGCTAAATCCCCATCCCCAGCTGGAATATTCAGACAC
AGGGCTGCTATGTTTTTGGAGCTCCAGCAGGTGTTGTCTGGAGTTTGACCAAAACAACAGTAGC
TCAGCTGCCAGCGAACTGAATTACACCTGAAGCCTTTCTAGGCATAAATGACTGTTCTTTGCACT
CTCTCCACCTCCTCTCCTCCAATCTGGTCCAACTTCCTAGATTCATATCACATAAAAATCCTTGTA
TTTAACAGCAATTAAAAGCAATATTAAGATGGAATGCCAATCAATATTAATATTCAATCTGTGCC
AATCAGCCTAACCTGTTTTCTAGGCTAATTTTTAGCCAGGAAAAATTAATCTCTCTTAGAAATTAA
AGTCCAAATAAGTCACAACTGTTTTCATGCTTAGAAAATGTATCTATCCATTTGTTTAAGGGGCTT
ACATAAAACTACTGAAGCTAAAGGAAGCCCTCAGGACTAACAGAATATTCCTGCAGCTCCTATG
CTTGCCAAAACTTGGCTGCTGAAAGGATATCACATGAAGCTAAAACAGCTGGGATGTCTAGGCC
TTAGAAACTTCCTTTAAATTTTCATGTTGTGATTTAACATCCTTTTGCAATTATTGTTTAAAATCACT
TATAGTTACTTAGTGATTGATCAGCAAAAATCAGTAATTTGATGTCTGGTGTAAGAGGAGCCTCT
CTCAGAATCAGTTTTCTACTTTCTTCAGTGTACAGAGCCAGATTCAGCAACTGGGGCTGTCAATA
TAGAGAAACAACTGGGGAGTTTTTTTAAATGTGAGTGCAGAACCCTTTCCCAGACAAATTAAAT
CAGAATCATTGGAGACAAGGTCCTGACTTTAGTGACTTTATAGGTTCCCAGATGATTCAAATATA
CAGGCACAGTTGAGGAAAGTTAGTTTAGGTTGTGAAATCCTACGCATGAAAAGTAACACATTGT
AATGGCTTAAAAATGGGAAAGATAATGCTGTATATTTTGCACATGGGCAGAAAGTATATATACG
CACACAGACATAAACACATGTATGTGTTTAATACACAATATGTAAAGGGCTTGGTCAAATTCTCT
ATAACAAATTTTGTGTAGATTCCTATTGTTATTTTAAAAGGGAGGAAATTATTGGTTTGGGAAGC
AAAGAAGACAAAATTAATTTTAGTTAAAAAATTGATTCAAAGGGCCAGCTTATCCTCATAATTAT
TTTTTAATGTTTCATTCTGGGAAGACTACTACATATTACACTAGTTTTTATGTCGTAGGTAGAAAC
TTAATTAAATAGCAGTCTCACATATTCAGAATCTGCTTAAAAACAATTAATTTCTTTGTATACATA
TTTTTTAAATTTACCTGGGTCCAAGCATTATTGAACTCTTGCAACAATAATTAAGCAACAAACTTT
AATAATTATTAAAATACCTTGGAACTAATGATAATTGTTCTTTTTAAGCTTTCGGAGCTCCCAAGC
CGGAAAGAATATATGTAATTGGCTAAAATACTTACAAGGCTCTGGAGGAATTCTTCTAATGAAA
AGCAAGATGGGAGACTTGCATCTATTGAATGGCTACCTTGTGTCAGACATTGTGTTAGGCACATT
ATCTCACTGAGTCCTAAACTAATCCCTGTAGAACAGGGGTCAGCAAACTATGGCCAAGGGCCA
AATTTCACATTTGTAGATAGTTTTATCGTTACATAGCCATACTCATTCATTTACCTATCATCTGTGG
CTTCTTTCTTGCTAAAAAGCAGGGTTTAGTAATTGCAACAGGGATCATATTGTCAGGTCCTTCAC
AGAAAAAGGTTACTAATCCTATTATAGAACATTTATGATTATCTGTTTTTGATGAAGGAATCAAC
GTTAAATTCAAAAGCACCAAAGCATTAATTTTTCTTCAGAAACTATTGTTGACTGAGAAAATGAC
CTCAAGAAGTGAACTAGCTTGAGAGATGACATGCTAATGATTAAGTAACTAATATTATATACAAC
TTGAATGTTTCTATAGTTTTGAAACAATAGGCAATTACCCTTGCGTTCAGATATCATATGAGTTTC
TCATTGCTTCTTGAATGAAACAGAATCAGGAAATAAAGATGTCATTTATTGAGTTTGGATAAATG
AACTAGAGCTGACATCTGTGAATACAACATTTATTTTTATTTTATTTATTTATTTATTTATTGAGAC
GGAGTCTCGCTCTGTTGCCCAGGCTGGAGTGCAGTAGCGCGATCTCGGTTCACTGCAACCTTTG
CCTCCCAGGTTCAAGCAATTCTCCTGCCTCAGCCTCCCGAGTAGCTGGGACTACAGGCATGCGC
CACCACGCCTGGCTAATTTTTTATGAATACAACATTATAATTTAGTCATGAAAATACTCCATTACT
TGAAATGTGGATATATGTCAAGAGACAGAAAATAATCACAAAAGAGATTTGTTCTAAGCAAAAT
TATATGCTGAACTTTTAATTTTTGTTGGTGAGAAGGACACTTCCTCCCTACTCCTGATGCTTCCTT
CCATTTGAAAGGAGCTGCACTAGCATAACACCTGGCCTACCTCTGTGCTTTCTCTTTCCTTGCAA
AAATCTAATTTTTTCCCACCCATCCTCTATCACTCTGATGCCATGAGTATCTGCTAAGATCTTTGG
CAACACTGGGTATGGTGGGTTTCCACTAAGTTGTAAACATATCTTTGTCTGAAATAGTTGGCCCA
GGAGCCTGGAGACTGGCTTGTTTTCTAAATTTGGTGGGTGAGTGGGGCCAGTTCTCAGATAGTC

TTTTGGGACTGTCAAGTGAGGCATGAGAGATTTGGCTTGAGACAGACTTGGCTGCAGCTGCTGA
AGAAATCATAGCACTCCTCAGTCATCGAGAACCATCTGGCTGGGACTGAGCAGAACAGGAAGT
TGAGGTGGTTGATGAATTTTGGACTTCAATTCCTGTGAAATTAGCCCACAAGGGCAGTAAGATT
GATTATGGATTTGAATTACTTTGGATTTTGAATTTTAGAAACTTTATATTTGGACTTTCATTCTCCTT
GACAATCTGATCCTGAGGTTGGGTTTTATGATCAAATGTGGGGCACCTTATTACTATCATGTTATT
TTTGGATTAAGTGGTAGATTTGACCATTGTTTCCAACTATTTGCCCAATGAGTTAAAAAAAAAATT
AAAACTGGGACTATTGAGAGATCTTTATTTTTCTTTTTTTTAATGTGAATGAAATGCCCTTTTTACA
ACTCTCCTAGTCTCTATGATTTAATCTGGCCAGTAACTTTTAGAAGCAAAACTGGACAAGTCAAG
TTTAATTCCCTCCTCTCCTCTTTCTCTTAGGTACAAGCTACCCATGTGAATTATGCGTTCTGTTCAG
CAATTCTATACTGTAAGCTTTTATGGAGTCAACCTTTGCCCCTGTGCAGGGAGGCTGCCCCAGAT
CTGCATTGCCCATTCCTTGGGGTTAAGATGGTATAAATTCACTTGTGCCCCTGGGATTTCTGAAG
CAGAATTATAAGATGAAAAATGTCCTGTGACTGTCACTGTTAAGCACTGGAAGAACAGTGTGAC
TGTGTGAAAGGCTGTGCTATTCAGTCACAAAGTATCCATCAGTCAATCTTTGTAGAGAACTGTGC
AGGGAAGTAGAATGTGGGGACTAGTCATGCAAAAAACAGGATGAGTTTTTCTTGGGATAAGCT
GAAGTCGAGGTATTATGAAAAATTCAATGCTAGCAGGGTTGCAGAGAAATAGGAATGCTTTTAC
ACTGTTGGTGGGAACGTAAATTAGTTCAACCATTGTGCAAGATGGTGTGGCAATTCCTCAAAGA
TCTAGAACCAGAAATATCATTTGACCCAGCAATCCCATTACTGGGTACATACCCCAAAGGAATA
TAAATCATTCAAATATAAATCATTCAAATATAAATCCATACAAAGATACATGCACATGTATGTTC
GTTGCAGCACTATTCACAATAGCAAAGACATGGAATCAACCCAAATGCCCATCAACGACAGACT
AGATAAAGAAAATGTGTTACATATACACCATGGAATACTATGCAGCCATTAAAAAAACGAGATC
ATGTCCTTTGCAGGGACATGGATGGAGCTGGAAGCCATTATCCTCAGCAAACTGAGGCAGGAA
CAGAAAACCAAACACTGCATGTTCTCACTTATAAGAGGGAGGAAAACAATGAGAACACATGGA
CACATGGAGGGGAATAACACACACTGAGGCCTGATTGGAGGAGGGAGAGCATTAGAAAAATA
GCTAATGCCTGCTGGGCTTAGTACCTAGATGACAAGTTGATAGGTGCAGCAAACCACCATGGCA
CATGTTTACCTATGTAACAAACCTGCACATCTTGCACAGGTACTCCAGAACTTAAAATTAAAATT
AAAATTAAAAAAGGCACATGCAATAAATGAGAGCGAGATGAAGAAAAAAGTCACAGTATGATG
GGGTCATCTGCATAACATAAGTGATATTGAAACCTCTAGGGTAGAATAAAAATAATTTAGAAAA
AAAGAACATCAAGAATATACACAGAATTGCCCACACACAATACCAAGAGCTTTCGTAACTGAG
GAGAAAAAAGCATCAGTGAAGCAGTTCTAGAAGGTTTAAGCAGAAGCAAATAGTAATGTTTCA
AAGAGAATTTTCAGGAGAGAGATCAATGGTATAAAAGTGATGTGAAGTCATCAAGCAGCATGA
ATTTAAAATACGTCGTCAGATTCAACAATCAACGTGGTAATTGTTGAAAAGAAAAGGTACATAG
AAATGTTGTCAGGGTCACATTGAGTTTTTCTTATTCAGGCTAGGAGATACCAGAAGATGTCTGAA
GATGGAGGAAATGCAAACTAGGAGGAGTTTCCTAGAAATGAGTGGAAATGTTTAGCAGGAGTT
GATATTATTAACTATTATCTTAACAAACCTTCAGGCGAACTGCTCTAAGTAATCACTTGGAGCAC
GTTAGTCGTATTCATTCCTTTTGTGTTCACTGCAGATTTCCAAATAGTAGAAGTTTCTGAAGCATA
TTAGAAGGGATTATAACCTTTACCATAATTATACAGCTGAGACATATAAAGTATAAACCTCAAAA
TAGATGTAAACGATCCACATAATCACAAGGCACAACCACTATGAAAATACCACAATATTTTGCA
GTTTATCTAAAAGTATCCACATTTCCTGCACTATCGTCTCATTGCACACTAGCAGCTGTTTAAATA
ATGCTATTTGCCACCTAGGGCACGCTCAATATATTCACAAGGCAAAGTTCTCATAAGTTCCATAA
GCAGAGCATTCTTTTTAGCTTTAAACTTAGCTTTAAAGCCTGTAGTTCCTAGGTGCACATATGTCC
CAATTTACCTAAACAATTCCAGTTCCTTCCTGTTGTCCTGATGTGATTGTTGATGGCACCCCTTTT
CACTCCTGAAAGTGTCCTTGCATGGATGATAGATTGTACCATCACCCTCCTCTTACCTCTTACTTT
CTCTCTGTTACACTCCAATGCAAGATCCACTCCACTCAGGGTTCTGTCTGACAAATATATTTTATA
CCCAATGACATAAAAATAAATATTACAGATTGATAAAAATATGTGGGCTCATCAGAACATTAGA

GCTTATTGCTTGAGAATCACTATTCAAGATAAATGTAAAAAATGGCAGAAAAGAGAGTGATGAC
AGAAACTGCAAAGATGCGGCAGTGGCCAGAAACATAATAAAAATCCTAAAACTGAGGTCCACA
ATGAAACGAAAAACAGAGTACCTATCAACATTAGAGATGAAAAATAACCTTTACAATGGATTAT
TATTCAAATTAACTTCTTTAAAAATGCTACCATATTGGGAAAACTGGCTAGCCATATTTAGCAAG
CTGAAACTGGATCCCTTCCTTACACCTTATACAAAAATTAATTTGAGATGGATTAAAGACTCAAA
TATTAGACCTAAAACCATAAAAACCCTAGAAGAAAATCTAGGCAATACCATTCAGGACATAGGC
ATGGGCAAGGACTTCATGTCTAAAACACCAAAAGCAATGGCAACAAAAGCCAAAATTGACAAG
TGGGATCTAATTAAACTAAAGAGCTTCTGCACAGCAAAAGAAACTCCCATCAGAGTGAACAGG
CAACCTACAGAATGGGAGAAAATTTTTGCAATCTACTTATCTGACAAAGGGCTAATATCCAGAA
TCTACTAAGAACTCAAACAAATGTACAAGAAAAAAACAAACAACCCCATCAATAAGTGGGCAC
AGGATATGAACAGACACTTCTCAAAAGAAGACATTTATGCAGCCAACACACACATGAAAAAAT
GCTCATCATCACTGGCCATCAGAGAAATGCAAATCAAAACCACAATGAGATACCATCTCACACC
AGTTAGAATGGTGATCATTAAAAAGTCAGGAAACAACAGGTGCTGGAGAGGAAGTGGAGAAAT
AGGAACACTTTTACACTGTTGGTGGGACTGTAAACTAGTTCAACCATGGTGGAAAACAGTATGG
CGATTCCTCAAGGATCTAGAACTAGAAATACCATTTGACCCAGCCATTCCATTACTGGGTATATA
CCCAAAGGATTATCAATCATGCTGCTATAAAGACACATGCACACATATGTTTATTGCGGCACTAT
TCACAATAGCAAAGACTTGGAACCAACCCAAATGTCCATCAATGATAGACTGGATTAAGAAAAT
GTGGCATATATACACCATGGAATACTATGCAGCCATAAAAAAGGATGAGTTCATGTTCTTTGTAG
GGACATGGATGAAGCTGGAAACCATCATTCTCAGCAAACTCTCGCAAGGACAAAAACCAAACA
CTGCGTGTTCTCACTCATAGGTGGGAATTGAACAATGAGAACACTTGGACACAGGAAGGGGAA
CATCACACACTGGGGCCTGTAATGGGGTAGGGGGAGGGGAGAGGGATAGCATTAAGAGATAT
ACCTAATGTAAATGACAAGTTAATGGGTGCAGCACAACAACATGGCACATGTATACATATGTAA
CAAACCTGCATGTTGTGCACATGTACCCTAGAAGTTAAAGTATAATAAAAAATAAATAAAAAAT
AAATAAAGAAAAATGCTAGCATATATAATTTTCACAAAATATCAACTTCATAATGTCCCATTCAG
GTTCGGATGCCCTCGGTGACTCTTGGTTACCTACACAGACTTAGCTAGACTTGCAGTTTGCACCA
TTCAGCTTCAACGTCCTTTTCCAAAATCCTCTCTCCCTAATTTGAACTCCTTGGAGAAATTTGAGC
TCCTTTTCCTTATTTATTATGGTCAATTGGCTGCATTGCTCATGAGGCAGTGATAAGGTTTCCTAG
CTTCTCTGGCCTATTCAACTGTATCTATTCTGCTTCGGGTAACATTCTGCTGCTCAGCGTGATTAC
TATTTTCTCCTGACTGTGAAGCACCTACAGTGGGGAATTCAAAATCTGGCTGGTCATCTAAGTGA
AATGGTACATTTTAAAAAGAAAGCTTCCCAGGCACCATTCTAGAGTTACAGGTTCAATTCTCTGG
GGAATAGGATTCAGGAATCTGTACGTTTTACAAGACCTTTACATTGTTCTCACATGCAGGGTCTC
TACCACACATTAGTCACTTAATCTGTGCTCCATTATTTTTGTTTTATATGTGCTTCTCAGGTTCACA
AATGACCTCTATGTTGCTAAATGCAGCAGTCAATTCAGTTTCTACAAATTGCATTTGATACAGTTT
ATATGACATATATACCACATATCCACCACACACACACACACACACACCCCTCCCTCTTCCATA
CCCCTACCCCAGGAATTTCTACACTTAGCTACATCCTTGTCTCAGTTTTCCTACTACCTCACAAAT
CACTCCTTCTCACTCACCTTTCCTGGTTTCTCCAGTTCTCCAGATCTTCAGTTTTAGAAGCGATCA
AGGGTGTACCCCTTGAATTTTTATCTTTTGTATCCAAATTTATTCCTAGATGATTCCATTCAATTTT
ATTGCTTTAAATACTACCTCTAGGCTGCCAACTTACAGACCAATATCTCCAAACTAGACCTCTCC
CTGCAAGTCCAGGCTTCCATATCCACTCACCTACTTGACACAGCCACTTGCCTAATGGGCACCT
CTAGCTCAGCGTGACTGAGGGTGATCTCACCACCCCACCCAGACCTGCTTGTCCATCAGTCTAC
TTCATCTCATCCTTTGCTCAGTCCGAAAGCTTCAGAGATTTTGGCATTCCTCCTTTCCAATCTAAC
CCACCAGAAAGTCCTTTCGGATATACTTTTTAAATGTACCCCAAGTCTAATCACTTATCACCCTCC
CTGACTTATAGCAAGTCACCATCATGGCTCATCTGGATTCCTGCAGTAGCCTCCCAAATGGTCTC
CCACTTCCATCTTTTTCCTTCCCTCTAATCACCACTCTCAGCCAAAGTGATGCTGTTCAGATGGAA

GTCTCATCTTTCTCTCATCTACAAAAATAAATAAATAAGAAAACCTAAACAAACAAAAAGCCCTC
TAATGCCTTTCTATCTTAGAGTGGAAGCCTGAGTCTTTCTAAGATGGACAAGGCTCTATGTGGCC
TGGTTCTCTGTCACCCTGTTATCTCATCTCCTGCTATTCTGCCCCTCATTCACCTGTTCCAAGCCA
TTCCATCTCCCAGCCATTCCTCCAACATGCCAGGCATGCTTCCTACCCAGGCCTCTTCCCCCAGA
TATCCACAGGGCTCCCTCTCTGGCCTCCTTGGGTCTTTATTCAAGTGACACCTTCTTCACGGGGC
CTTTCTGGGCCACCCTGTCTATAACTTCAACTCCTTCTCCCACCCTTCATTCCCTGTGCTATGTTTC
ATCCTCAGCACATACTCCTCATCACTATCTAATTTATCTACAATTTACTTATGTTGCTCATTGTCGT
CCCCAGTTAGCCAACTAGAATATAAGCCCCACGACAGCAGGAATTTTTGCTTGCTTTTTTTTTTT
TTTGTCTTTTTATTCAGTGCAGTAGCATATCCCATGACCTAATACAGGGTCTGGCATATAGTAAGT
ACTCAATATTTATTGAATAAATAAATGTGTGTTTGTTGTATTTCCCTAATACAAGAATTCAGCCTA
GCACAATATCCAATATACAGAAGGATCTGAAAAAATATCGGTTTTGAGACACTGGGTCCTACTT
GAGGGTGAAGGTTGGGAGGAGGATGAGGATCGAAAAATTACCTATTGGGTACTACGCTTAATT
ACCTGACTGATGAAATAATCTGTACACCAAACCTCTGTGTCACACAATTTACCTATAACAAACTT
GCATGTGTGCTCTTGAATCCCAAATAGTTACTAATTTAAAAAAAAAACTCCAAAATACATATATT
GAGTATTTTTTTTTTGAGATGGACTTTCACTCTTGTTGCCCAGGCTGGAGTGCAATGGTGCAATCT
TAGCTCATTGCAACCTCCGCCCACCCAGGTTCAAGCAATTCTCCTGCTTCAGCCTCCTGAGTAAC
TGGGACTACAGGTGCGTGCCACCATACCCTGCTAATTTTGTGTTTTTAGTAGAGACAGGGTTTCT
CCATGTTGGTCAGGCTGGTCTCGAACTCCTGACCTCAGGTGATCTGTCTGCCTTGGCCTCCCAAA
GTGCTGGGATTAAAGGCATGAGCCACTGCGCCTGGTCCTGTTTTGTTTGTTTGTTTGTTTGTTTGT
TTTAATGATTGAGTAAAGCAGAAAGGTTCTCTTCTCAAATTTGGCAAGAACCAATGTTCATGGTT
TCAAGAGTATCACTACTGGCAGCAATAAGGGCCCCACTGCAATGAAGGGGTTTTGAGGGAAGA
AAATATGTGTGGAAGACATATCTGCACAATTTTACACACATTTTCCAAAATTATTTTCTCTTAACG
ACAGACATAATTCACACCTATATCTTCTCAAAACCCACACTTCTAACAGGATCTGAAATACAAGA
CAAGCACTCTGCTGTGGTTTGGATATGTTTTGTTTGGCCCCACCAAGTCTCATGTTGAAATTTGAT
CCCCAGTGTGGGAGGTGGGAGGTGTTTGATCACAGTGGGGGATCCCTCATGAATGGCTTGGTG
CCATTGTCTTGGGAGTGAGCAGTCTCTCACTCTTAGTTCCCATGAGAACTGGTTGCTGAAAAGA
GCAAGAACTGGTTGTTGAAAGAGCACCTCCCCCTTCTCTCTCTTGTCTCCTCCTCTCCATGTGAT
CTCTGCACACAGTGGCTCCCGTTCACCTTCTGCCATGAGTGGAAACTTGCTGAGGCCCTCATCA
AAAGCAGATATTGGTGCCATGCTTGCTGTACAGCCTGCAGAACCATGAGCCAAATAAACCTCTT
TTCTTTATAAACTACCAAGCCTCAGCTATTCCTTTATAGCAACGCAAATGGACTAAGGCATAGTC
TTATGGAAAAGCTACTTGCAGGCCAGGCACGGGGGCTCAAGCCTTTAATCCCAACACTCTGGG
GGGCCAAGGTGGGCAGATCATTTAAGACTAGGCTGGCAATCTTGGCAAAACCTCGTCTCTACTA
ACAATACAAAAATTAGTCTGGCATGGTGACGCATGCCTGTAATCCCAGCTACTTGGAAGGCTGA
GGCACAAGAATTGCTTGAGCCTGGGAGGCAGAGGTTGCGGTGAGCTGAGATTAGGCCACTGCC
CTCCAACCTGGGAGACAGAGCAAGAATCTTTCAAAAACAAAAAACAAAACAAAACAAAACAAA
ACCGCAATCTACTTGCAGAAGTCTCAGGAAACATATATA

ID|56249|gene_id|ENSG00000228160|transcript_id|ENST00000454131 Homo sapiens lncRNA

GGGACTTACAGTATCATAAGAAAGATAGACATTTAAATCATCTCAACAAGGACAATGGACA
TCATGGAATCACACAGCAGGAGAACCTCGCCTAGTCTACCGATCAGGTAAGACTTCCCTGGATT
AAGTGGCATTTAAGGTGAGACTGTCAGAATGAGTAGGAAAGAAGAAGATGGGTATGGAGTGGA
GAAGATTTAAATGATTTAAGTCAAATGAATTCTGGAAAGAGACTCTCCAGATGGGAAGAAATGG
CTGTAGTAACAATCCCAATTCATCCTCTGGGGCTTCATTTTTCCTATGGCATTATTCCACACTAAT
ACCCAACACTTCTTTCTCTTTAGATACTCCATGCAGTTGTCACCTCCTCTATGAAGCCTCTCCTGC

CCCTTCCAAGTAGAACTGTCCATTCCATCTGTGGCAGAGTCTGGCTACATGTTCATCAAACCCTG
TTTCAAATATCCTCCTCTGCATGTCATTACACTGTATCTCCCAGCCCCCTTGCATCTACATGAGGC
CGCATGACCACATGCTGGGTCACTTCTGGGCCAAAATGGTATTGGAGAGTCCATCTTCTCCACT
CTGGCTCTCTTTCCATGCACCAGATAGGTGATTTTTAGTGATCTTTAGTGGAAGATGAAACAGGT
CTGTATCTCTAAATGACTTTGTGGAGTGGAGCTTCCCTTCCCAAG

ID|56262|gene_id|ENSG00000251387|transcript_id|ENST00000506892 Homo sapiens lncRNA

AAACCGAGAGTGCCGCAGATGGGCGCCCCTGACCTTCCCGCTGCTCCAAGCTCCCCACTC
GGCCTCGCGCGCCCTCCCTCCTCTCATAGCTCGGCGTCCTCGCCCGCCCCCGCGCGGCCCTCCC
AGGTACTGCAGCAGCCCCCGCCACTCCCGGAGACAGTGCCCCAACACTCCTGCGTCCCCTGCT
GCCTTGCAAAGATAGGGTGTGGAGTAATCTGACCTCAGTGCCTCAAGGCCTTGTCTCCAAACCT
TCGAAGTGTTGGCTGTTCTTCTTGGAGTCTAGCCTCACCTCCTGGGTATGGGTTCCAGATCCATC
TTGCTGATCCCAGACTGGTGGTGGGCGCCAGAAGCAACCTTGGCTGGGGCGGCCAATGGCACT
GCAGGCAGGAGGGCGGCGGCACAGTACGGTCTCGATAGAGGTCAAATCATGATCTGGGCTCCT
GGTGATGTCCAGGAACACACTCCACGCCTCCATGTGCACACATCCACAAGCACCACACTCCCAT
GGTGGACACAGCAACATCACTACCCAATCAGGCCCCGTCCCTCCCCTGTGCCCTGCGGACCCC
GAGGAAAGCGGTGCTTACTCTAAATTGCAAATAGAAAGCCTTTCCCGGGGCCGGGCGCAGTGG
CTCACGCCTGTAATCCTGGCACTTTGGGAGGCCGAGGTGGGCGGATCACGAGGTCAGGAGTTC
CAGACCAGCCTGACCAACATGGTGAAACCCCGTCTCTACTAAAAAATACAAAAATTAGCCGGG
CGTGGTGGTGGGCTCCTGTAATCCCAGCTACTCAGGAGGTTAAGGCAGGAGAATCGCTTGAAC
TCAGGAGGCAGACGTTGCAGTGAGCCGAGATCGTGCCACTGCACTCCAGCCTGGGTGACAGAG
CAAGACTCCATCTCCAGAAAAGAAAAGAAAAGCAAGAAAGCCTTTCCAGGGCCCACAGCCCTG
CATCGGCCAGCCCTGCCCACCTCTTAAACCACCTCTTCCCCCATTCCCCCTGCCCATTATTCACT
ATTTTCCAGCAAAAATACGCAGTGCTCACTCCACACTTCAGGTCCTGTGCACTTACTGTTCCCTC
TCCCTGAACCTCCACTCCTTATCCCAGATCCACCACTGGCTGCTTTCTTCCCTTCTTTTAGGCTTC
AGTTCAAATACCACCTCCCCAAAGAGTCCTTTGAGACCACTGAAGCTAAGAGGCTCCCCACCCT
CCATTCCTGAAAAACATGCTTCCTGCCTAATGTGTAATCTAATCATTAGGACAATGCACATCAAA
AAAAAAACGAGACCGAGTGCTGCGGCTCACGCCTCTAATCCCACACTTTAGGAGGCTGAGGTG
GGAGGATCACCTGAGTCTAGAAGTTCAAGGCTGCAGTGAGCTATGATGTAAACCCTTGGGACAT
TGAGGGACAATGTCCCTCAATGTCATTGGGACATTACATCAAGGGACATTGAAACCCTTGTCCC
TTGTGCACTGTTGGTAGGAATATAAAAGGGTTCAGCCAGTATGCAAAATAGTATGGTGGTTCCT
CAAACAATTAAAAATAAAATTATATGATCCACCAATTCCCCCATCTGGATATATTCCCAAAAGCA
TTGAAACAAGAACTCGAAGATATGCTTATACCCCTACATTCATAACAGCATCATTCACAACAGC
CAAGCGGTGGCAGCCACCCAAGAACCCATCAGCAGGTGAATGGTGGTCTATCCACACAGTGGA
GTACTACTCAGCCTTAAAAAGGAAAGAATTATGACATAGACAATATGAGTGAGCCTCAAAGGCA
CTATGCTGATGAAATAAGCCAGTCACAGAAGGACAAATATGGTATGATTCCATTTATATGATGTA
CCTAGGGTAGTCAAATTCATAGAGACAGAAAGTAAAATGGGAGGCCAAGATGGGAGAACTGCT
TGAGGTCAGGAGTTCAAGACCAGCCTGGGCAACATAGCAAGACTCTGTCTCTAGAAAAAAATG
AAAAAAAGCAGGGTGTGGTGGTGTGTGCCTGTAGTCCCAGCTATCAAGAGGCTGAGGCAGGAG
GATTCCTTGAGCCCAGGAATTCAAGGCTGCAGTGAGCTATGATTGTGCCACTGCACTCCAGCCC
GGGTGACAAAAGCGAGACCCAATCTAAAAAAAGGAAGAAAGGAAGGAAGGTAGGAAGAAAG
GAAGGTAGGTGGTTGCAGGGGTGGAGGGTGCTGGGAAAGGAGGTGGGGGAGTTACTGTTTTAA
TGAGTTCAGAGTTTCAGTTTTGCGAGATGAATGACTTCCAAAGATGGATGGTGGTGACGGTTGC
ACAATAATGTGAATGTATTTAATGTCACTGAACTGTATACTTAAAAAAGGTTAAGATGATAAATT

TTGTTATGTATATTTTACCGTAAATAAAAAAATATGTATCAAAACATTTCTTTGCTTTTTCAATCCT
GCCTCATTCATTAAACTGTGACCTCCATAAGAACAGTACCTGTATCTGCCTGGTGCACCTACTGT
GTGTTGCTAGTCACTGAAGACTTGGGGACCAAGACAGACAAGGTGCCCACTCACCTGGAGCTC
ACAGCCTGCGGGGAGGGGAAAAAGATACTGAACAAACAAATAAACAAGAATATTATCACCTAT
TACCTGCTGTGCAGAAAATTAAAAAGTGAAATGTAACAGAGTGGCTGCATGAGGAGGAGTTTCA
ACTGGAGCAGTCAGGCATACCATCCCAGAGGAGGTGACATTTGTGCAAAGACCCAAGGAATGA
TCAAGAGTCAGTCATCAAGCATCCAGGAGACCTGAGCTGCAGACAGAAGAAACAGCAAGTGCA
AAGGCCCTGTGCTGGGCATGAGTGTGGCATGCTAGAGGAACAGCAGAGAGAAAAGGAGGGGA
CGAGGAGGAGAGATG

ID|56265|gene_id|ENSG00000251061|transcript_id|ENST00000509548 Homo sapiens lncRNA

CTGCTCAAGATCGGCTGAAGGAAGCAAAGCTACATTTCTTGGTAAGAGTATTAAGATGCAT
CATTCCTATGGTTTTAGAGCTCAGCATATCTAATCCACAAAAGCACCTGAATAAGGAATGTTTGA
TTTTGCCTTTCTGTCCTCAATATTCTGTTATCTCAAGTTGTTTTCCTGATATGTTAAAATGCTCACG
CTGTTTTAACCTCCACTAATAGATATGTGAATTTGTAGAATATACAATTTTATCCTGATTTTCATGT
GGAGTGAATGCTTTATCTACCACATGAGAAATATGCCTGACAGAATTTTCTCGCTTCTGCTACTG
CATACTTATTCATACTTACTGACTTTTGTCTGGTAGACTTACTTGTTCCTGCTTATGAATTCAGTCT
GCCCAGAGTTTGAACCTCATGTTGCACTGGGCAGGGAATAAAGAGCCTTTGTCTTAGGGAAGAA
GAAAGTGCTCTTTTTATGCCTCAGTTCCTTTTTTCAGGTGTCCTGGGTGCTGCTACCTGGTACCTA
GTGGCAGAACCCCATGCCATGGTTCATGTGGGTCCCAGAACCCTGAGACTAATTGGAAACCCAT
CATCATTCTTTCTTTCCCTTCGGTTCTTACAGGCTTTGGATATCTGTAAGAATTCCTCACCCTACCA
CCCCATTCCCAACCTCATTAACTAGATTCATGGTCCAGATGCCTTCTGGATTCCACTACCCAAAT
GAACTTGCATCATACATGTTTTTCTCTGTATTACTGACCATCTTATCCAGACTCTTAATACCTGCA
AGGGCTCCCCTCCCCCGAAATACCCACCTTACTAACTGGATTCATAGTACTAGAGAACCCAAGG
CTGACCTGTAGGGAGACAAGTTTTCTGATAACTGTCAACTCTGGCATTTTTGTTGATAACATTTTC
AATGCTGAGGTGACATATGTTTACCTTGTAAAGTAGTTATAATCTCTGGCTTCTACCTACATAACA
TAAAGGGAATAGATTTCTAACAGCAGCAAAGTTATTATAAATTTTTTTATTAGAAATTCCTGATTA
TTTTGTAATTACCTTTTAGCCCAGTGTAATTAATTTTTTCATTAATTTCTTTGTTCCAAATCAAGAG
ATCAAAAGTAAAAGAAAAAGTAATATTAAGAGTGAAGCAACATGACACAAAAGATATATTAAA
GTGTAATCAAAGCATTTTAATTAAAAATGACTTTCTGAAGCCTAATTTTAGCTTTTTCATTTCACCA
GACCATGTTGTGATAATTATTTTAGAGGCCAAGTCACAGGTGAAGTATACCAAAAAATACCAAG
TGCTTATTTAATAATTCATTCAGAAGTATATTAATGTCAAACATAAAGCTCAAGTTTTTCTCTGTG
GTTCTCACCTCTTAAAAAATAAATTTATCCCAAATGTCTGTAAAATCTTTTCTTTCCACCTTATTCC
TTGATCCAACATAGAAAAATAAGTTGCATTTTTTTTTGGTAGAAAATACTGTGAAAAGCCAGGTC
ATATAATATAAACTGAAAGTTTTTGCCGCCCAAGTTTTTCTTAGTAATGGAATTTGGTTTCTGTGT
ATTGCTTTTCAGCACATTTTATGTTTACAACTTTTGGGGATTTGAAGGAAATAATACATTTATATTT
TCCATCATATTGTTTCTCATTTCCCCCCCTTTTCTGGGAAATTTCTATGATTTCATAAATTTTTAAAT
TATTATAATTATTATTTTGGAGAAGAGATGATTATTGTATGCTGACTTTTGAGTTCTTATTTTAGAG
ATACAATTTTTAATGCATCTGCAAAAATTAGTTAACAAATTTTAAAAGTAGTGAATGCTTTTTGCC
CTCTTGTTTTTCTGTTTTTGTTTTGGGGGCCAATTATTTTCTTTTGTTGGGTTGCTTGTCATCAAGAG
GGCCTTAAAGTTCCCCTCAGCTTGACTAAACTTTAGACATGCTTCTTGCCTTTAGGCCTCTAAACT
CACTTTTCTTACTGCATTTACTTTAGAAAACTGGCAATTATAAATTATTTCCCTGTCCCTTTGAGAT
GCAAATCTTTAAAAAAGCTTCTTAGGCCGGGAGCGGTGGCTCATGCCTGTAATTCCAGCACTTT
GGGAGGCCAAGGCAGGTGGATCACAAGGTCAGGAGTTCAAGACCAGCCTGGCTAACACGGTG

AAACCCCGTCTCTACTAAAAATACAAAAAAATTAGCTGGACGTGGTGGCGGGCACCTGTAGTCC
CAGCTACTCGGGAGGCTGAGGCAGGAGAATGGCATCAACCCGAGTGGCAGAGGTGGCAGTGA
GCCGAGATCACGTCACTGCATTCCAGCCTGGGTGACAGAGCAAGACTTCGTCTCAAAAAAAAA
AAAAAAAAAAAAGCTTCTTATCACTTTTGCAACCCAGGACTGTTCCTCTCCAGAACCTGGGAGCC
ATCCTTTTGAAATGTAATCATCAAGAAAGATAGTGCCCCTATCTCCCAGGCTCAGTATTTGGGGA
AGAGTCTAATTCTAATGGACACCTTGCTCCAAGTTATAAAACCACCTCTTGTCATGAAGATAGAA
CAGAATGTTTACCTTTTCTCTGGGTAAGGCCAATTAGCAAACACATCCCTCCATCCCAGCTCTCC
TGCACCCTTTGTTTTAGTAATGTCGAGTTCAGACTAAGCTGAGCTCTCTCCCTTATTGCAATATCC
TTGAATAAAGTCTTCCTTACTTGTTTAACATGATCTGGTGCTATTTTCGCATTGACACCGTTTTTTA
ATCAGTATATTGCTTTTTAAAACCAGCTTTAATTGAGGTATCACTTACATACCACAAATAGCATTT
ACCCACCTTCAGTGTCAAATTTAATGTTTTTAAATAATTCCCCAACTTTGTGCAGGCAGCCTGCA
CCACAATTCAGTTTTAGAACATTTTTGTCATCCCCAAACAGGTCCCTTTTTCCAATTTACAGTGAA
TCTATGATAGTCAATTATTTTTATGCTTTTGTTTTTCTTTATATGTCTGGGAATTCTTGGTTGTTATTT
TTTACTTAAATGGGAGGAGGTAATATTCCAGGTACCCGGTGTGGATTTCATTTGCCTTGCTTCCC
CTAAAGAACTCTCCCCTGACTGGGAAGTCTGATTGACAGTTCTCTGTAAGTGGGTAGGGCTTGT
CACCTGGCAGGGTTTTCTTCAGGTTGAGCAGGTGGGAAGCCAGCTATAAAGTTGTAGGACTCTA
AATGCTTTCTGTTGGTCATCAGGACTTTGTACTAGCAACATCTAGTTTTTCCCAGGAAAATGTTCA
CTTTTAACACCAATTGTTTTACCTGGGATATAGAAGGAAGGGTGTGTGTGTGTGTGTGTGTGTGT
GTGTGTGTGTGTGAGAGAGAGAGAGAGAGAGAGAGAGAAAACATGGGTAAGAACAGGTAAGAAC
TGGCACAATTGTTCTGAGGACATAATTTCAATTACTTCACCTGTTTTCAGCCTAACTTCATGCTCA
CAACTGATCAGTTGTTGCACCAGGAAAATCAGCTACCACCTCTATGCATCTCCCTCTCACATATA
TTTGATCTGAGACCTTCAGATCTTTCCCCCACTCATTTGTTTGTTTTTGTTTTATTTTATTAATATTA
ATTGAGCAACAAAAAAATTGTATTTATTTTTCATGGACAACATATTGCTTTGAAATATGTACACATT
TTAAATACATGTATACAATATAATGTATGCATTGTAAAATGGCTAAATTGAGCTAATATACATAA
GTATTACCTCACATACTTTCATTTTTTTTTTGTGGTGAGAACACTTAAAATCTACTTTTTTGGCAAC
TTTCAAAATTTTAAAAAAGTCATTCAATACTCTTTGTTTTACCACATCTTCCAGAAGTGTGTAAAA
ATCTCTAGCCTGCTGATGCTGCCCCCTGTTTTCTTTACTGTCATAATTTATTTTTTATGTTTCTTTAC
CATAACTTTAAAATTTTCTCAAGAAAGTCAGGAGGCAACTGCATGTGCTACTTAAAGAAAGAGG
AAAGAAATTAAAGAATTAAAGAAATTAAATTAAAGAAAGAATTAAAGAAAGAGGAAAGAAACA
TGAAAGGTGGTTTGCCAGTCAAGACAGGTTTATTTTAGAGAAAACGAACCAGAGAGGAGCTTCT
GGCCAAGTTAGGTCAGAGGCACACTCTTTTACAGACTGAGAATTTTTAAGGATTCAGGGTGGGA
GAGTTTATCAGAGGCTTGGACTGCTTCTGTGTCCCTTTGTTGTGCTTATTTGGGAGGGAGAGTTG
TGTGTCTGTTCCCATACACTTTTCTGCAGCTGCAGGCATACCCCCGAGTCTGCTTTTAGCTTTCCT
ATTTCAGTGCATCCTGAAAGGAAAGGAATGTGCTTATTAAGGCCCACTGTTTTGCTGGGGCCCA
TTGTATGAGGGAAGTTTGGCAGTTACTCAAGAGACTCTCCCCCACCTCCCTCTGTGCCCAAGCT
GTCTTATCTGTGTTTTACTGTCTGCTCTTTCTGGCTGCTTATAGTTAGAAGAGAAGTGATTTCCTTG
AAATGCATGAGGCTAGAAAGGGAGCTGGAACTTAAAGTAGTGGTGTTTGTCCGAGATGACGGT
GCTCCTGCTCTGTCAACTACCATCTTGAATTGCAGCTCAGTTTTCTTCTAAGAAATAATACCAAA
GTTGGATTGTAGTTATTTCAATAGAGCTATCTCTGTCCAGTAATATAGTGGCTAGGCAGTTTGCT
GAAAGAATGAATAAAATACAAATACATCTCCTTAAAAACATGGCAGAGATGACAAGATAATACA
AAATTTTAGGGCCAAAGTCAAATTGAAGACAGAAGCCCAGGGAGATAAGTATAGCAATAGAGT
TGTTTTTGTCAGAAGGGGACTTGCCAAAACCTTGATTTTTCTAATCTAGTGGAGTATGGGATCAA
AAGAACAGAATTCCCTCAAAAGACAAAGACCAGAGTTCTTCTAAGTTGAGGAGTCTGATAGGA
GAACTCCAGAAAGCTGAACTCAGATGGCATTTGCCTCATTCTGTGAGGAACAAGAGGAAAATCT

CCCTTTCCACCTTCCCTACCTTAAAGAGATTATAGGAATTTAGTCTTGATGCTTCAAAGAGCGGG
AGTGGGATGGGAAATTCAACTGAAGAGTTGTAACTCTAAGCCAGCATGGATTTGGTGTCCAAAG
ATGTATATTTTGGATTGTCCATAAAATCCCAAACCATAAATTTATTGTATTTCATTGATTCTGTCAT
GCACTTAATCCTCCCTGCCCCCACATTTTACATCAAAATGTACCCAAGGATCAACAATGTCTTCA
ATTCAATGAAATATTGAACTGTAAAATTTCCTGGTGGAAGCATAGACGAACCTTCTCTGGAGTAA
TACAACTTCATCTTCATCTCAGAGAATTCACACAAATAATTTTTCAGGAAAATGAGTAGCTCCCT
ATAAACAATTACTGACCATACAAGAAATGAAGCATAGTAAGTGTGGTAGCCAGCAGAACAAAA
CAGCCAGGAGGAACATATTCTATAAATTAATTAGGCTATTAGAAATTGACAGATTAAAAGAGGA
AGAAACTTTTGATAAGTATGTTTAAATAAATGATAGACAAGTCTGAGAGTATCTTCAGATAGGAA
ATTGTATGTATAAAGACCTAACAGTTTGAAAATGAGTCAAGTAGATAACCCATAAATGATGTGA
ATTTCTAAATTTACATAGTGTTTTTCATTTTTCCCTTAATGATGACAAAGTGAGATGGTTATGTTTC
CCATTCTGTATATTTGTGGCTTCCTAAATTTATGGTACAAGATCTGAGGATTTGCCTTTTGAAGAC
AACTTTTATACATTTCATGTATGTTAGTAATCGTAACGCAATCCCTCTCTGTGGAAAACTTTATTA
TTTCTTATTCTTCTGTGGAAAAAAATACTTTTTATATTTTAGGGTCATATCTTCACTATGGGCTGTT
TTGCTCATTTTTTCTGTATTCTTATCTACCTCCGAAGTGTGAAAAATGGCCTTGTGACTTGTTAGTT
GGGATTCTGGGAGCATTGTTTCTTCCATAAAGGAAATATTGTTGACTAAGCATAATATAGATTAT
ACTCTACCTATGTTATACATTGACCCTCAACCCACTAACCTAGAAATGTGCACACATTGCGTGGG
AGGTGATATTCATAGCTGTGCTTTGAGGATGGTGCTGGTTATAACATAAACCAGCACCACCCTG
GTTATAACATAAAAATAAAGGAGGCTTCTTTTCTTGGGTGTTGGTTTTCTTCATTTGAAACTCCAG
CCCTAGAAAACTCATGCCTTTGTTCAAAAATGTGAGTTGGTGACACACTGGCTGTTAGGTAGATT
CAGTATAGGACAATTATGCACCTGTTGTACTGCTGGTGTTTTAGTAGCAGCATTTTGCATATGAA
CTATAGCATTTCAAAGAATCATTTTGATTTACTTTCATTTCTGACTCCTTTTGTACTTGAAAATAAT
ACAAAAAATGAGAAATCTCTAAGAATTTGTTTTTGTCATTAAGGATATTTCTACGTATTATATTTT
GCAGAAATAACTTTTTACACAAGTCTGTTTCTGTAGAGGCAAACATCTTTGTAATCTTCTTTGAGT
TGAGGGATATGATTTGTTTTCATACCTTCCCTGTCAGTAACTGCTCCAGCATAACAGTATGAGTC
AAACTCATTCCCTGAAACTCTTTTTTTTTTCCTTAGGCGTTGATGGGTGAATGTGAAAATTGTACA
TCTCTGATGGCTGCTAAATCTCTTTGCCTTTCTAAATGACAAGGGGTAAATGGAATTTGTTTTTCC
CTTAATTTGACAGCATTTCCTTGTCAAAGGGTTCCTTCTTAGATAATTCGATAATTAGGTGACAAT
TACAGCAGATGTTTAAAAAATTTTCTTTGGGGGTTTCCAAAGTTTTTTAGACCTCAAAGTCATGTC
ATCCACCATAGCCACATAAAGACAAAAAAAGTGGCTGGGAGAATGTAGGATGGATGAGCTATA
GCTCATTAGTACTATGCTTTATATCCAATCAACTGAAGCCACAAGGCCAAAGATTGATACTGCTG
CTGACATGAATATCTGTGAAGATGGAATACCCTAAGTCAGGGTTTATCAACAGTGGTACTACTG
ATATTTTCGACTGGATGCGTCTTCATTTTGGGAAGCTATCTTGTCCACTGCCGGATGTTTAGCAG
CATTGCTGGCCTCTAACCAATAGATTCCTTCAATTGTGACACTCAAAAAGCTTCTGGACTTTGCC
AAATATTCCCAGGGGTGGGGCGAGGGGACAGAAATTGTGCCTGGTTGAGAACCACTGCTCTAT
GTCACTGTCATCAATCCTGGTCTTATGCAAGCTTGGGAAGAGAGATACAGAGAGGCATCAAGA
ATAATAGTCCATGTGATTTTAGTAGGCTAGGCTGAGATCGATGACTTCGTAAAGTGTCAGCTTCA
TCTTGTTTTATTTTTTATGCTAACAAATTTCTCGTTTCCTCCTGTAGTTTTCTGGTTCCTATGAACAG
AAGCAGAATTTTTTCACAACAGAAAGTGAAGCAACGAAGTTTTGAGGGTTGCTCCTCATTTAATG
GTGAGTCAAAGAGGCAGCATAGAGAGGATATATGAAGGTTAAGAGCTAAGGATGTGGATTCTG
GAGCCAGACTGCCTAGTTTGGAAGCCAGCTCCCCATGTTAGCTGGCTGATCTTGGCAAAGCACT
CACCTTCCGCTTGCCTCTACCAATAACATAGGGATAATGACAGAGTCAGTTTAAGGAGTTGTGA
GGACTAAATGAGATAATATGTATAAAATGCTTATAAGAGCAGTTAGTGCATAGAAATGGTAGTA
GATAAGTATTAAAGGAGATAAAAAGGTGGTTTTTTTTCCAGAAGAAAAAGGATACTTAAGTTGAT

TAGGAATTTTGTTGTCTGTAACTTCATTAACATAATCAACCCATTCCAAGTTAACAAGCTCTTTGA
CAACGACTGATTTAACGTTACCATCATAGCAGACGTGCATCACAAATGAAGTTTGATGTGCCTTA
AGTGAATCCGTGATTCTTAAAACACAAAAAAGTGATTACTTACAGTTTCCTGATATAAAATAGTT
GGCCCATAAATGTAAGTTAGTCCTATCCTCAAACACTATTAGATAAGGATTTGATTGAAAATTTG
TCTTCACTTGCTATAACCATCAAACCTGTACCGTATCTTAGTCTTAACCCAGAGATGACAAATAC
TTATTAGGCACTGATGCAGGCAGGTGAGCCCCGACATGGAGTTTAGCTCATGAGAGGGCTTGGT
TTTGCCCAGGAAAGAATTCAAGGACAAGACAGAGGTAGAAGAAAACAGCTACTGAAGAGGCA
GTGCTACAGCTCTAGCAGTGGGTTCTATGGCTGCTCTTGCAGGGCAGGGTTACCCCTTAGGCAG
AGACTAGCAGCTCAGGTCAGTTTTGCAGTCATATTTTTATCCACTCTTAATTGCATGCAGATTAAG
GAGTGACTTATGCAGAAATTTCTAGGGAGGGGGTAGTACCTTTAGGTCATTAGGTCACTGCCAC
AGAAGGGGGTGATAACCACGGGGTGTTGCCATGGCAAGAGTAAATTGACATGGCACACTATTG
GACATGTCTGATTGAAAGCTGCTTTGCCCAGGCCCTGTTTTAGCTAGTCCTCAATCTGTTCCGTG
TCCAAGCCCCACCTCTGGAGTCAAATCCCACCTCCTACCTCAGCCCCTTGCATGTATTAGGTTAG
TACAAAGGTTATTGTGTTTTTTGCCATTAATAGCAAAAACTGCAATTACTTTTGCACCAACCTATA
GTTATAAGTATCTGGCCAGGACATCCAAGCCAAAGGAGCACAGCACACATATATGAAAGCTAG
AGGGGGCCATCCTGCGGGAGGATCAAGTAAAGAACACACACAAAGAAGACTTAACTAACAGT
CACTCGAATCGTGAGTCCACTGAAGTTCTTTTTATTCAGGGTTAGAAACACATAGAGAAATTCAG
CTATAACACGAAGCACCATTTAACTGCAGCAATATAATATTTTTGCAGTTTAGGTTTAAAAAATA
GGTAGATATCAAGTTTTTATTATTTTTTACTATTGTAAGTACAGATACTTGGGATTCATAGAACTG
GCAACTATGCCTGGACTTCTGTTGGAAGCAGGAATTCATGCTGAGTTCCATTCCTTTTCTAAAAG
AAGTTATAAAAAGCCCAAGTGAAGTCAGATATTATGTGGATATTTTTTTTTATAGCAACAGTAAG
TAACTTCCACTTTAAGATCCTGATGGTGGATATGATTTTAAAGTATGAAGGAAATACAAAATGTC
TAAAATATTTCAGGGCTTAAGGGCATGAATGAAAAGAATTTGGTGTGTGGTGTGTATTGAGTCA
AGTTTAGGTAAAAAACAAAGGCATCAAGACCTCCGCAAGGCTCTTCCTGTGGCATGAATCACAC
AGTAGGGAGCTTACCTGGGCACCTTTCTTAAAGGTATGCCACTTGTCTGTGATAGGAGTGCTTGT
TGGGTTGGCTTTTGTTCAGTTAATCCTCTAAAACGATTTCAAAGGGGCTTCTGAATTTTTTGTTTTG
TTTTACTTTTTTTGAGACAGGTTCTCGCTCTGTTGCCCAGGCTGGAGCGCAGTGGCATGATCTCT
GCTCACTGCAACCTCCACATCCTGTGCTCAAGTGATGCTCCAACCTTGGCCTCCCAAGTAACTG
GAAACACAGGCGTGTGCCACCATGCCCAACTAATTTTTGTATTTTTTGTAGAGACAGGGTTTTTG
CATGCTGTCCATTCTATTCTCAAACTCCTGTGCTCAAGCGATTCTCCTCCCAAAGTACTGGGTTTA
CAGACCTGATATTTTTAAAGACAACAGAAAAGCATAGGTTTGGGAGAATTAAACGTGTGCTAAT
AGAAAAGTATTACACAATTACTAGTTTACTAAACAATTAGCTATATACACATTTGCTATGAGTTGT
ACTTTACAAAATTAGTTGAGTCTAAAAAACACACGCATGTATGTTTATTTTTCTCTAAATGAAACT
AAATATCCATGCTCCCCTTTCTTGCTGTCTTCAAATTAGTGGAAAATTCAACTTGAGTCATTTTAG
TGCACATTCTCTCTCTTATGATGGTTATGTATTAATCCACATATACCTCTCAGGAGGTGTGAGTTT
ACTGGGAAAACAGGTGAATGCCTCTAACTGTCATCCCCTCCTAAATGCCCTCTGTAGATTCTTGA
AGGTGAACAATTTCACTGCCGCTGTGCCAGGCAGGGCATATGGGCTCTGCTGAGACTGGGACC
CTGGTGTGCAAGGTCCAGCCCCAGGATCCGATATTACTCTTCTGTGTCCTACATTTTTCTCTTACG
ACATTTATCCTAACACAAGGGTGTTTTTATTTTCTCTGTATGCAATACCCTGCTGTTAATTATTACA
TAAGTCTTTATCAATCACACTTTATAGCTTTAACTCCCCAGGTTCCTAATTGGTTACTAAAGGTTT
GTTCCTCACTGCAGGCTCTGAAAATGCCATCCACACTGCCAGTTACCACCTATGGCTGTGCTCTC
TAAAATCTCTCTTAGCTTTGTCTACCCTTTTCTTTACACTAGCGGTAAGAGGTTGTTATAGATAGG
GTTGGGTTTTTGGCAGGAAAAGAACAGCTTCCAGGCTAAGAAATACCTGAAAAAATTCACCCTG
TGTGCCAAAGCCATTTCTTTTGGATGCCTCCAGTGTCAAGCCAGTGTCAGAGCTATTGCGGCGG

AAGCAGCCACATTCATTGCTTCATACGTTCTCTGGATGATATTCAAATGAACAGGACAAAGCTTC
ATGAAGAAAATCCAGGGTTCAGTTTTCCATTTACCAAAAAGGCTTGAATGACAGATGTCCACATT
TTCCTAATTTTTCTTTTTGCAGTATTTTATTATTTTCTCTCAACACGTTTTTTGTTTGTTTTTACTTAA
ATCTTCAATCTCCTATACATTTTTCTCAAGGGTATAGCCTAGTCTATTTCCTTCACTTAAAAGAGT
ATGAAGTTGGCCGGGTATGGTGGCTCACCCCTGTAATCCCAGCACTTTGGGAGGCCAAGGTGG
GCAGATCACCTGAAGTCAGGAGTTCGAGACCAGCCTGGCCAACATGGTGAAACCCCGTCTCTA
CTAAAAATACAAAAATTAGCCAGGCATGGTGGTGGGTGCCTGTAGTCCCAGCTACTCGGGAGG
CCAAAGCTGGAGAATCCTTTGAATCCGGGAGGCAGAGGTTGCAGTGAGCTGAGGTTGTGCCAC
TGCACTCCAGCCTGGGTGGCAGAGTGAGACCCTGTCTCAAAAAAAAAAAAAAAAACCCAAAAAG
TATGAAGTTGTATGTGTTTGATCTCTTGAGTGACTACATTGCAATGAATGTTGAAATAGGCTGCT
GTTTAAATCTTAATTCACATCCCTTGCAGGGGAATGGGGGTACTCATTGAATTAAAGATTAAACT
TATAATGAAAGACTATTCACTTAACAAGTATCACACCAGCACAAGATGAAGCAAAACTAGGCTA
TTTAGTTTGCAATGTGCTCGTCTTCGTATTAGAACACCTCTACTTAAACACAAATTATTATGCTGA
TAAATATCAATCATTAAAGAGGCCACCTTACTTTCTACTATATTAAGCTTATTATCTTACGTAGCA
CCTGAAATACTTGAGGTCATATTTTTATTGAAACACATATACATGGGTTTTTCTTTTTTTATTATTAT
ACTGTAAGTTCCAGGGTAAATGTGTACAACATCAGGTTTGATACATATGTATACATGTGCCATGT
TGGTGTGCTGCACCCATTAACTCCACATTTACATTAGGTATATCTCCTAATGCTATCCCTCCCCCT
TTCCCCCACCCCATGACAGGCCCCCATGTGTGATGTTCTCCACCCTGCATCCAAATGTTCTCATT
GTTCAATTCCCACCTATGAGTGAGAACATGTCGTGTTTGGTTTTCTGTCCTTGCAATAGTTTGCTG
AGAATGATGGTTTCTATCTTCATCCATGTCCCTACAAAGGACATGAACTCATCATTTTGTATGGCT
GCATAGTATTCCATGGTGTATATGTGCCACATTTTCTTAATCCAGTCTATCATTGATGGACATTTG
GGTTGGTTCCAAGTCTTTGCTATTGTGAACAGTGCTGCAATAAACATACAGTGCATGTGTCTTTA
TAGTAGCATGATTTATAATCCTTTGGGTATATGCCCAGTAATGGGATGGCTGGGTCAAGTGGTAT
TTCTAGTTCTAGATCCTTGAGGAATCACCACACTGACTTCCACAATGGTTGAACTAGTTTACAGT
CCCACCAACAGTGTACAAGTGTTCTTATTTCTCCACATCCTCTCCAGCACCTGTTGTGTCCTGATT
TTTTAATGATTGCCATTCTAACTGGTGTGAGATGGTATCTCATTGTGGTTTTGATTTGCATTTCTCT
GATGGCCAGTGATGATGAGCATTTTTTCATGTGTCTGTTGGCTGAATAAATGTCTTCTTTTGAGAA
GTGTCTGTTCATATCCTTTGCCCACTTTTTGATGGGGTTGTTTGATTTTTTCTTATAAAGTTTTTAAA
GTTTTTTGTAGATTGTGCATATTAGCGCTTTGTCAGATGGATAGATTGAAAAAATTTTCTCCCATT
CTGTAGGTTGCCTATTCACTCTGATGGTAGTTTCTTTTGCTGTGCAGAAGCTATTTAGTTTAATTA
GATCTCATTTGTCTATTTTGGCTTTTGTTGCCATTGCTTTTGGTGCTTTAGTCTTGAAGTCCTTGCC
CATGCCTATGTCCTGAATGGTATTGCCTAGGTTTTCTTCTAGGGTTTTTATGGTTTTATGTCTAACA
TTTAAATCTTTTTTTTATTATTATACTTTAAGTTTTAGGGTACATGTGCACATTGTGCAGGTTAGTT
ACATATGTATACATGTGCCATGCTGGTGCGCTGCACCCACTAACTCGTCATCTAGCATTAGGTAT
ATGTCCCAATGCTATCCCTCCCCCCTCCCCCCAACCCACAACAGTCCCCAGAGTGTGATATTCC
CCTTCCTGTGTCCATGTGATCTCATTGTTCAATCCCCACCTATGAGTGAGAATATGTGGTGTTTGG
TTTTTTGTTCTTGCGATAGTTTACTGAGAATGATGTTTTCTAATTTCATCCATGTCCCTACAAAGGA
CATGAACTCATCATTTTTTATGGCTGCATAGTATTCCATGGTGTATATGTGCCACATTTTCTTAATC
CAGTCTATCATTGTTGGACATTTGGGTTGGTTCCAAGTCTTTGCTATTGTGAATAATGCCGCAATA
AACATACGTGTGCATGTGTCTTTATAGCAGCATGATTTATAGTCCTTTGGGTATATACCCAGTAAT
GGGATGGCTGGGTCAAATGGTATTTCCAGTTCTAGATCCCTGAGGAATCGCCACACTGACTTCC
ACAATGGTTGAACTAGTTTACAGTCCCACCAACAGTGTACAAGTGTTCCTATTTCTCCACATCCT
CTCCAGCACCTGTTGTTTCCTGACTTTTTAATGATTGCCATTCTAACTGGTGTGAGATGGTATCTC
ATTGTGGTTTTGATTTGCATTTCTCTGATGGCCAGTGATGATGAGCATTTTTTCACATGTTTTTTGG

CTGCATAAATGTCTTCTTTTGAGAAGTGTCTGTTCATGCCCTTTGCCCACTTTTTGATGGGGTTGT
TTGTTTTTTTCTTGTAAATTTGTTTGAGTTCATTATAGATTCTGGATATTAGCCCTTTGTCAGATGA
GTAGGTTGTGAAAATTTTCTCCCATTTTGTAGGTTGCCTGTTCACTCTGATGGTAGTTTCTTTTGCT
GTGCAGAAGCTCTTTAGTTTAATTAGATCCCATTTGTCAATTTTGTCTTTTGTTGCCATTGCTTTTG
GTGTTTTAGACATGAAGTGCTTGCCCATGCCTATGTCCTGAATGATAATGCCTAGGTTTTCTTCTA
GGGTTTTTATGGTTTTAGGTCTAACGTTTAAGTCTTTAATCCATCTTGAATTGATTTTTGTATAAGG
TGTAAGGAAGGGATCCAGTTTCAGCTTGCTACATATGGCTAGCCAGTTTTCCCAGCACCATTTAT
TAAATAGGGAATCCTTTCCCCATTGCTTGTTTGTTTTTTTCAGGTTTGTCAAAGATCAGATAGTTGT
AGATATGTGGCATTATTTCTGAGGGCTCTGTTCTGTTCCATTGATCTATATCTCTGTTTTGGTACC
AGTACCATGCTGTTTTGGTTACTGTAGCCTTGTAATATAGTTTGAAGTCAGATAGTGTGATGCCTC
CAGCTTTGTTCTTTTGGCTTAGTATTGACTTGGTGATGCGGGCTCTTTTTTGGTTCCATATGAACTT
TAAAGTAGTTTTTTCCAGTTCTGTGAAGAAAGTCATTGGTAGCTTGATGGGGATGGCATTGAATC
TGTAAATTACCTTGGGCAGTATGGCCATTTTCACAATATTGATTCTTCCTACCCATGAGCATGGA
ATGTTCTTCCATTTGTTTGTATCCTCTTTTATTTCCTTGAGCAGTGGTTTGTAGTTCTCCTTGAAGA
AGTCCTTCACATCCCTTGTAAGTTGGATTCCTAGGTATTTTATTCTCTTTGAAGCAATTGTGAATG
GGAGTTCACTCATGATTTGGCTCTCTGTTTGTCTGTTGCTGGTGTATAAGAATGCTTGTGATTTTT
GTACATTGATTTTGTATCCTGAGACTTTGCTGAAGTTGCTTATCAGCTTAAGGAGATTTTGGGCTG
AGACAATGGGGTTTTCTAGATATACAATCATGTCGTCTGCAAACAGGGACAATTTGACTTCCTCT
TTTCCTAAATGAATACCCTTTATTTCCTTCTCCTGCCTAATTGCCCTGGCCAGAACTTCCAACACT
ATGTTGAATAGGAGTGGTGAGAGAGGGCATCCCTGTCTTGTGCCAGTTTTCAAAGGGAATGCTT
CCAGTTTTTGCCCATTCAGTATGATATTGGCTGTGGGTTTGTCATAAATATCTCTTATTATTTTGAG
ATATGTCCCATCAATACCTAATTTACTGAGAGTTTTTAGCATGAAGGGTTGTTGAATTTTGCCAAA
GGCTTTTTCTGCATCTATTGAGATAATCATGTGGTTTTTGTCTTTAGCTCTGTTTATATGCTGGATT
ACATTTATTGATTTGCGTATATTGAACCAGCCTTGCATCCCAGGGATGAAGCCCACGTGATCATG
GTGGATAAGCTTTTTGATGTGCTGCTGCATTTGTTTTGCTAGTATTTTATTGAGAATTTTTGCATCA
ATGTTCATCAAGGATATTGGTCTAAAATTCTCTTTTTTTGTTATGTCTCTGCCTGGCTTTGGTATCA
GAATGATGCTGGCCTCATAAAATGAGTTAGGGAGGATTCCCTCTTTTTCTATTGATTGGAATAGT
TTCAGAAGGAATGGTACCAGTTCCTCCTTGTACCTCTGGTAGAATTCGGCTGTGAATCCATCTGG
TCCTGGACTGTTTTTGGTTGGTAAGCTATTGATTATTGCCACAATTTCAGATCCTTTTATTGGTCTA
TTCAGAGATTCAACTTCTTCCTGGTTTAGTCTTGGGAGAGTGTATGTGTCAAGGAATTTATCCGTT
TCTTCTAGATTTTCTAGTTTATTTGCGTAGAGGTGTTTGTAGTATTCTCTGATGGTAGTTTGTATTT
CTGTGGGATCGGTGGTGATATCCCCTTTATCATTTTTTTATTGCGTCCATTCGATTCTTCTCTTTTT
CTTTATTAGTCTTGCTGGTGGTCTATCAATCTTGTTGATCCTTTCAAAAAACCAGCTCCTGGATTC
ATTAATTTTTTGAAGGGTTTTTTGTGTCTCTATTTCCTTCAGTTCTGCTCTGATTTTAGTTATTTCTT
GCCTTCTGCTAGCTTTTGAATGTGTTTGCTCTTGCATTTCTAGTTCTTTTAATTGTGATGTTAGGGT
GTCCATAACATTTAAGTCTTTAATCCATCTTGAATTAATTTTTGTATAAGGTGTAAGGAAGGGGTC
CAGTTTCAGCTTTCTACGTATGGCTAGCCAGTTTTCCCAGCACCATTTATTAAATAGGGAATCCTT
TCCCCATTTCTTGTTTTTGTCAGGTTTGTCTAAGATCAGATGGTTGTAGATGTGTGGTATTATTTCT
GAGGGCTCTGTTCTGTTCAATTGGTCTAGTTTGAAGTCAGGTAGCATGATGCCTCCAGCTTTGTT
CTTTTGGCTTATGATTGTCTTGGCAATGTGGGCTCTTTTTTGGTTCCACATGAACTTTAAAGTAGTT
TTTTCCAATTCTGTGAAGAAAGTCATTGGTAGCTTGATGGGGATGGCATTGAATCTATAAATTAT
CTTGGGCAGTATGGCCATTGTCGTGATATTGATTCTTCTTATCCATGAGCATGGAATGTTCTTCCA
TTTGTTTGTGTCCTCTTTTATTTCACTGAGCAGTGGTTTTTAGTTCTCCTTGAAGAGGTCCTTCACA
TCCCTTGTAAGTTGGATTGCTAGGTATTTTATTCTCTTTGAAGCAATTGTGAATGGGAGTTCACTC

ATGATTTGGCTCTTTGTTTGTCTATTATTGGTGTATAGGAATGCTTGTGATTTTTGCACATTGTGAT
TTTGTATCCTGCGACTTTGCTGAAGTTGCTTATCAGCTTTAGGAGATTTTGGGCTGAGACGTGAG
GTTTTCTAAATATAGAATCATGTCATCTGCAAACAGGGACAATTTGACTTCCTCTTTTCCTAACTG
AATACCCTTTATTTCTTTCTCCTGCCTGATTGCCCTGGCCAGAACTTCCAACACTATGTTGAATAG
GAGTGGTGAGAGAGGGCATTCCTGTCTTGCGCCAGTTTTCAAAGGGAATGCTTCCAGTTTTTGC
CCATTCAGTATGATATTGGCTGTGGGTTTGTCATAAATAGCTCTTATTATTTTGAGATACATCCCA
TCAATACCTAGTTTATTGAGAGTTTTTAGCATGAAGGGCTGTTGAATTTTGTCAAAGGCCTTTTCT
GCATCTATTGAGATAATCATGTGGTTTTTGTCTTTGGTTCTTTTTATATGCTGGGTTACATTTATTG
ATTTGCGTATGTTGAACCAGCCTTGCATCCCAGGGAAGAAGCCAACTAGATCGAGGTGGATAA
GCTTTTTGATGTGCTGCTGGATTTGGTTTGCCAGCATTTTATTGAGGATTTTCACATCAATGATCA
TCAGGGATATTGGTCTAAAATTCTCTTTTTTTGTTGTATCTCTATCAGGCTTTGCTATCAGGATGAT
GCTGGCCTCATAAAATGAGTTAGGGAGGATTCCCTCTTTTTCTATTGTTTGGAATAGTTTCAGAA
GGAATGGTACCAGCTCCTCTTTGTACCTCTGGTAGAATTCAGCTGTGAATCCATCTGGTCCTGGA
CTTTTTTTGGTTGGTAGGCTATTGATTTTTGCCTCAATTTCAGAGCCTGTTATTGGTCTATGCAGG
GATTCAACTTCTTCCTGGTTTAATTTTGGGAGGGTGTATGTGTCAAGGAATTTATCCATTTCTTCT
AGATTTTCTAGTTTATTTGCATAGAGGTGTTTATAGTATTCTCTGATGGTAGTTTGTATTTCTGTGG
GATCGGTGGTGATATCCCCTTTATCATTTTTTATTGTGTCTATTTTATTCTTCTCTCTTTTCTTCTTTG
TTAGTCTTGCTAGCGATCTATCAATTTTGTTGATCTTTTCAAAAAACCAGCTCCTGGATTCATTGA
TTTTTTGAAGGGTTTTTTGTGTCTCTATCTCCTTCAGTTCTGCTCTGATCTTAGTTATTTCTTGCCTT
CTGCTAGCTTTTCAATTTGTTTGCTCTTGCTTCTCTAGTTCTTTTAATTGTGATGTTAGGGTGTCAA
TTTTTGATCTTTCCTGCTTTCTCCTGTGGGCATTTAGTGCTGTAAATTTCTCTCTACACACTGTTTTA
AATGTGTCCCCAAGATTCTGGTATGTTGTGTCTTTGTTCTCGTTGGTTTCAGAGAACATCTTTATTT
CTGCCTTCATTTCGTTATTTACCCAGTGTCATTCAGGAACAGGTTGTTCAGTTTCCATGTAGTTGT
CCAGTTTTGAGTTTCTTAATCCTGAGTTCTAGTTTGATTGCACTGTGGTATGAGAGACAGTTTGTT
ATCATTTCTGTTCTTTTAGAATTGCTGAGGAGTGCTTTACTTCCAATTATGTGGTCAATTTTGGAAT
AAGTGCAATGTGGTACTGAGAAGAATGTATATTCTGTTGATTTGGGGTGGAGAGTTCTGTAGAA
GTCTATTAGGTCCATTTGGTGCAGAGATGAGTTCAAGTCCTGCATATCCTTGTTAACTTTCTGTCT
CATTGATCTGTCTAATGTTGACAGTGGGGTGTTAAAGTATCCCATTATTATTGTGTGGGAGTCTA
AGTCTCTTTGTAGGCCTCTAAGGACTTGCTTTATGAATCTGGGTGCTCCTGTAGTGGGTGCATAT
ATATTTAAGATGGTTAGCTCTTCCTGTTGAATTGATCCCTTTACCATTATGTAATGGCCTTCTTTGT
CTCTTTTGATCTTTGTTGGTTTAAAATCTGTCTTGTCAGAGACTAGGATTGCAACCCCTGCTTTTTT
TTGTTTTCCATTTGCTTGGTAGATCTTCCTCCATTCCTTTATTTTGAGCCTATGTGTGTCTCTGCAC
GTGAGATGGGTCTCCTGAATACAGCACACTGATGGGTCTTGACTGTTTATCCAATTTGCCAGTCT
GTGTCTTTTAATTGGGAGCATTTAGCCCATTTACATTTAAGGTTAATATTGTTATGTGTGAATTTGA
TCCTGTCATTATGATGTTAGCTGGTTATTTTGCTCAATAGTTGATGCAGTTTCTTCCTAGCATCGAT
GGTCTTTACAATTTGGTATGTTTTTGGAGTGGCTGGTACTGGTTGTTCTTTTCCATGTTTAGTGCTT
CCTTCAGGAGCTCTTGTAAGGCAGGCCTGGTGGTGACAAAATCTCTCAGCATTTGCTTGTCTGTA
AAGGATTTTATTTCTCCTTCAGTTATGAAGCTTAGTTTGGCTGGATATGAAATTCTGGGTTGAAAA
TTCTTTTCTTTAAGAATGTTGAATATTGGCCCCCACTCTCTTCTGGCTTGTAGAGTTTCTGCCGAG
AGATCCACTGTTAGTCTGATGGGCTTCCCTTTGTGGGTAACCTGACTTTTCTCTCTGGCTGCCCTT
AACATTTTTTCCTTCATTTCAACCTTGGTGAATCTGACAAGTATGTGTCTTGAGGTTGCTCTTCTTG
AGGAGTACCTTTGTGGCATTCTCTGAATTTCCTGAATTTGAATGTTGGCCTGCCTTTATAGGTCGG
GGAAGTAAGTTCTCCCGGATAATATCCTGAAGAGTGTTTTCCAACTTAGTTCCATTCTCCCTGTG
AATTTCAGGTACACCAATGACATGTAGATTTGGTCTTTTCACATAGTCCCATATTTCATGGAGGCT

TTGTTCATTTCTTTTTACTCTTTTTTCTCTAAACTTCTCTTCTAGCTTCATTTCATTCATTTGATCTTC
AATCACTGATACCCTTTCTTCAACTTGATCAAATTGGCTACTGAAGCTCGTGCATGCATCACATA
GTTTTCATGCCATTGTTTCCAGCTCCATGAGGAAGGTCTTCACTACTCTGTTTATTCTAGTTAGCC
ATTCGTCTAATCTTCTTTCAAGGTTTTTAGCTTCTTTCCGATGGGTTCGAACATCCTCCTTTAGCTT
GGATAAGTTTGTTATTACTGATCTTTTGAAGCCTACTTCTGTCAACTCGTCAAAGTCATTCACCAT
GCAGCTTGGTTTCATTGCTGGTGAGGAGCTGCATTCCTTTGGAGGAGAAGAGGTGCTCTGATTTT
TAGAATTTTTCAGTTTTTCTGCTCTGGTTTCTCCCCATCTTTGTGGTTTTATCTACCTTTGGTCTTTG
ATGATGGTGACCTATAGATAGGGTTTTGGTGTGGATGTCCTTTTTGTTGATGTTGATGCTATTCCT
TTCTGTTTGTTAGTTTTCCTTCTGACAGTCAGGACCCTCAGCTACAGGTCTGTTGGAGTTTGCTGG
AGGTCCACTCCAGACCCTGTTTGCCTGGGTATCACCAGCGGAGGCTGCAGAACAGCAAATATT
GCAGAACAGCAAATGTTGCTCTCTGATCCTTCCTCTGGAAGCTTTGTCTCAGAAGGGCACCTGG
CTGTATGAGGTGGCAGTTGGCCCATACTGGGAGGTATCTCCCAGTTAGGCTACTTGGTGGTCAG
GGACCCACTTGAGGAGGCAGTCTGTGTGTTCTCAGATCTCAAACTCCATAATGGAAGAAACACT
ACTCTCTTCAAAGCTGTCAGACAGGGACGTTTAAGTCTGCAGAAGTTTCTGCTGCCTTTTGTTCA
GCTATGCCCTGCCCCGAGAGGTGGAGTCTACAGAGGCAGGCAAGCCTCCTTGAGCTGCGGTGG
GCTTCAACTAGTTTGAGCTTCCTGGCCGCTTTGTTTACCTACTCAAGCCTCAGCAATGGCAGATT
CCCCTTCCGCAGCCTCACTGTTGCCTTGCAGTTTGATCTTGGACTGCTGTGGTAGCAGTGAGCAA
GGCTCCGTGGGCATGAGATCTGCCAAGCCAGCTGTGGGATATAATCTCCTGGTGTGCCATTTTC
TAAGACCATTGGAAAAGCACAGTATTAGGGTGGGAGTGTCCCGATTTTCCAGGTACCATCTGTC
ACGGCTTCCTTTGGCTAGGAAATGGAATTCTCCAACCCCTTGCACTTCCTGGGTGAGGCAATGC
CCTGCCCTGCTTCGGCTCACACTCCATGGGCTGCATCCACTGTCCAACAAGTCCCAGTGAGATG
AACCTGGTACCTCAGTTGGAAATGCGGAAATCACCCGTCTTCTGCGTGGCTCACTCTGGGAGCT
ATAGATTGGAGCTGTTCCTATTTGGCCATCTTGGAGGGATCTCAGGTTTTTCTGTTTAAAATAAAA
GGTGGTCATAGTCTTCAGAGTGTATATTTGGAATAAAATGTATTGAATCTGGAAAACATTATTTA
ATGAAAAATTAATATCATTTAAGCATAGAAACAAATTTTATATATACTTGTTGAATTAATGCCTTC
AGAATCTAAGAAAACCTCATTTAGTTGGTACATCCTCTTTAGTTTCTGTTTATCTTGAGTTTACTGA
TTTTGTCCTAAACAGAATGAAATATCATTATCTGAAGTATATAAAACCACATCTTTAAAAAATGA
AACTGAACTATCATTTCAAAATATTTTATAGTCTTCACTTAAACCATTGATTGAGAATCAACAGGT
TTTTTAAGGCAACAACTATGAATATTTTTAAATCTTTGGAGACTCAGTGGTTTGAGTTTTCATAGA
TAATATGATTAATCTGGATTCTGATTTAAGACAATCTTACTTTAAGGGCAAATTAAGAATCTTTCT
ACTGAAGACCACCTGACCATCTGGAATATTTTTACTGCTGTCAGGTAAATCACCTTCAAAATGAG
GACTGGATTTTGTTAAAAAGGGATGACTTCTGACTGCAAACCCTCTCTTAAGAACATTTTGTTTTC
TTTTTCCTGATAAAGTCCATTTTGTAACTCTCTTAAGGACATTTCATAAGCAATAGCACATGGCCT
CCTTTTGAGTACTGCTCTAAGGATGGTTCAATGAGACTTTTGATGACAATTAGGTTGATGCCTAG
AATTCACAGTGAGAGGAAGTTCTAATACATTTTGAAATTCCCAAGATATGTTCTTGACTGCCTTTA
GGCACCTGAAATTATTACATAAGATTTACTACTTCATCTAATATGAAGATATTTGGTGCATTTGTA
GAACTTTTGAAGGTGCTTTTGTCTCCAGTATCATTATTCATGGATCAAGGATTTTAAATAATGCGA
ATTGAGTGAATCAACAGTCATGCTGCTGTGTGTGCTTCGGGCAATCCTCTGTTTGGCAGATTTTT
AGATGTCATCCAAAGCAGCTCACCAGCAGCACCTTTTAATTGAACCCCATTGGCACCCACCATG
ACCCAAGAAAATGCCTTGGCTAATGAGATTACATCATACTGTGTTAGAGAACTTACAGTATCAA
ATTCCCATATAAATGCAAACTATAACATCTGTGTAAAAGTAGATAGTCAGAATTTAAATATTTCTT
CATATATGCTGGCCTTTGCACTGTCTGCAATTCTCTGTTCCCAGGCACAAAATCTATTAGAATTG
CATACAGGAACTATTGCCTCCTCTCTTGTATGATTTTTGTTGATTTTGATAAGGTTCGTATGATTTT
TCCAAACCATACAATGATTTGGAAGCTTGGGGGAGATCTCTCATGATATTCTTTCCCTCTCCACC

ATAAGACTTGGCACGTGGTTGCCATTCAGTAAGTAGTGACCAAAGAGTAGAATAAAGAGCTTTT
CCAAGGAAGCAAGTTTCACCAATTCAATACATTGGAAAGCAAGAATTGTATCTTTTGCTTCAAAT
TTTTAAAAAGTTTTAAATAATTTTCAACACCTACTCAAACTTTGGCTATCTCACAATACTATTATTT
CATTGAGCAGTCATTAGATAGTTTTTAAGCATCTACTATATTGCAAGCCCCTTGCCAGTTTCCCCA
AGTACGAAGATGAGTGGCATGTGATCTCTGAATGCAAAGCTCTCTGACCAATAGGGAAAAGAC
ACATGAAAACCATTATAGAGTGAGCTATGTGTGCTATGGTGCAATAGCACACACAGTGCTATAG
GTTTGCAGAGGAGAGTGTATTAAATGGTTCTTGGCCTTGTGGATAGGGATGAACCAGATGGTGA
GGGATAACTTAAGACAGATTCTTTGGTGAAGGATGTTGAAGAATGTTCCAGGTGGACAAGGGA
ATGATCCTCCAGGCAGATAAAGTGGCTTTTATAGAGGTACAGGGGATTGAAAGTCCTATTTGGA
ATTGAAGCATTTGAATGGGTTGTGAGAAGGTGCTGCTGGAAAGAACAGCTTCATCGTGATAGAT
TTTATATGTTGTAGTACATTTGGAGTTATTTTTACAGGCAGTGAACAAGGCTTCATATTAATTTTG
CAGGTCCATTATGTGCAAATTTCCTTGAAGGCTTAAGTGAAGATATTTATGTAGGTATGTATGTAT
GTATTGAGATGGAGTCTTGCTCTGTTGTCTAGGCTGGAGTGCAGTGGTGCGATCTTGGCTCACTG
CAACTTCCGCCTCCCAGGTTCAAGCAATTCTTCTGCCTCAGCCTCCTGAGTAGCTGGGATTACAG
GCACACATCACCATGCCCAGCTAATTTTTTGTATTTTTAGTAGAGACAGGGTTTCACTATGTTGGT
CAGGCTGGTCTCAAACTCCTGACCTCGTGATCTGCCCACCTCGGCCTCCAAAAGTGCTAGGATT
ACAGGCGTGAGCAAACATGCCCGGCCAATATGTTTATTTTTAACAATGTACTTCAGAATGAGAT
AATCTGGGATTATGTATTTAAGTTTATGTATTTTCAGACTACACAAATAATGGATAGATTACGAAT
TTCACAATATTTCTGGTCTTTCAGAGTGTACATGAAAAGACTTTCTTTAAATCTAAGAATTGCATG
CTTTTCTGAAAAATATAAGTTATGTGAGCCATGAACTTTTATATTATAACTACTGCAGATGTTGTG
AGCATATATGCTAGATGTTAGTGTGGTAATTGAAAAGTAATTTAGGTAAAAATAAACATTTCATT
CATTTACAAACTTTATGAGAACACTGATTTTATCTTATTTAGATCAAATATATGTTCTTTTCAATAT
GGTGAAAATACAACAGGGATCATAATCAAACTCCTTTTTGTACTTTTAAAGGCTGTAAATCAGAA
AAGGTTAAAGGAATTCATAGTTTTTCTCCTTTGTTTGCCAAGGTTGCTGTTGATATTTACTCCAAA
GTGGCCAATAGAACTAATGTCATATAAAGTTTCAGAGTTTACTGTCTTTTTGCCTTTGGACTCAAT
CAGAGTGTTGTACTTTCTGTTTATTTGCTTTGCTCTTATTTATTTTTAATAAGTTTATTGGGACGTAA
TCCTCATAACATTCAATTCACCAACTTAAATTGTATAATTTGATGTTTTAAAATATATTCATAGAGT
TGTACAATCATCACTAGAATAAATTTTAGAATATTCTGCCACCCTAAAAAGAAACCTCATATCCA
TTACCAGTCACTCCTCACTCACCTCTATTCTTACCCACTTCCTCATCCCTAGGCAACAACTAACCT
ATTTCCTGTATCTATAGATTTGATTCTGGATGTTTCATATAAAGACAGTCATACAACATATAGTCT
TTTGGCTCCTCTCACTTAGCATAATATTTTCAAGGTTCATTTATGTTGTGGTGCATATCAACACTTC
ATTCCTCTTTGTGGCTGAATAATATCCTATTATAAGACTATACCACTTTATTCGTTTGTCAGTTAAT
AGAGTGGCAAAGTGGATTGTTTTCACATTTTGGGTATTACAAATTATGCTACTATGAACATTTGTG
TATGAATTTTTGTGTGGATATATGTTTTGTTGATCTTGACTATACACTTAGGAATGGAACTTTTGG
GTCATATGGTAACTCCCTGTTTAGTATTTTGAGAAGCTGCCAAACTGTTTTCCAAAGTGGTTGGA
TTTGATTTATTTTAACATTATCTTACATCTACTTTGAATAAGAATTTGAGTCAACTTGATACAGATA
CTTGAGTTTGGCATTGTGTGTCCTCTCTGAGCCTTCAAGACAATCACATTTTTTCAGCTTACTCTG
TGTGTTCTGCATAAGACAGATGATTAGAATAATTGGAGGGCTTGTCTTGTCCTATGTGCTGGAAT
TTGGCTCTCTGTGACTTTCATTACAAGGTGGTGCTTTAGTTACTTGAGGTCTTGCCAGTCCTTATT
TCCTTAGTGTGTTCAGCTGCCTTCAGGTAGTGAGAGTTAGCTCTATCCAAGCAAGGATTTCAGGC
TGAGAAAATGAAGTTTGTTCATATAAGGCCACACATGCATTGATTCTTCCCTGTAAACTTACAAG
TTAAGGATGATTCATTGACAAGTGTGGGAATCCTGGGATAGTCTACGGCTTGATTTGTCTGTGTA
GTATGGTGCCATATGAATAGATGTACAAAAATTGGGGGTTATTGATAAGTGTGTCAAATACCTG
AGATTAGAGCTAAAGATAAGAGACCCATGTGCTGTAGATCACCACACCCTGAGCAGTGGAGAG

CATGCAGGAGAACTTTCTCTCGACTGAATAAAATCTGTGCAGCGATGAGGGAGGCGGACGGAA
CAGTGATGTGTGTTATGCACTGAAGTGTAACAGAGACTAGCTGCCTTGAGCAATCTGGAGAGCT
CTGAAACATTGGAAATAATTTTTCTTGATATACCACAAAGACCTCTGCAGGCTTTGATACCTAAA
AACCTCTGCAGGTCCAGTGTGGAAAACTTCTCCCTCTCATGCAATCCTCTGAACAACATGAGGC
ATTAGAGACCTGGTGATAGCTGTCCTACACAGCTAATAAAAGCCACGTGGGGCTACTACTATAA
TCAGGAGCTGCTGATGGGAACAACTAGCTTCTAGGGAGAACATTCTCTAAAACTGCCTCTAGTA
TCAATTTGGAAAGAACTAATTTTCTATGGTGTCTCCTAGCCTCTGACAACTGAGGATGAGTAGCT
TGAAGAAAAAAAGTGGCCAACTCCTTCTTGTAATTGCCAACCTCACATTTGACTGTTATTTTATTTC
TTGTCTTTTTTTTTGTATACTAAGAATAGTTTATAATTTCTTTCCAAAAAAATATGCATAGATTCTGG
TCCTCCCGGAGAGACCTGGTGTTATTAACCAACTCTCAGACGTCTGGTGATAAGTACAGTTCAG
AAGGAAAATACTATATAAAAATATAGTTTCTATCTCAAAACTCTAAAACTTATTTAAATCCTTTTA
GAAATTATAAAGTAACAATTCAAAAAACAAGGAATAGAGAAAAATTTCTCACACTTACAAGTAT
TTTTGTTCTACATTGATTGATATGTTTGTTATATTGCAGCTTCTTGTAACATTTTGTCTAGCCATGTT
GCTGCCAAAAATGGTCTTTCTTTATAATGAGGAAAAATAGCTTTCTATTATAGAGCATTATTGCCT
CGTATTATAATTAGCAGGGAGATTATGGGATTTGGAGTTTGTCAGATCTGAGTTGGAATTCCATT
CCAGCACAGATTAATTTTGGAGCTTGAACAAGATGCTTATCTTCTTTAAACCACACTTTCAACAG
TTTATAAACATGGATAATACCTATCTTGGGAAAATTTCATCTGATGGAGCATGTTACTCTGGTGG
CCAAGAGTTTTATTTGTTTTTCATCTAGAATCATCAGCTAGGAAGCCTAATTTTTAAAATCAATGT
TTGTTTAGAGCAGCGGTCCTCAATTTGGAATGACTTCATTTCTGAAGGTACAATTGGCAAAACCT
GGAGACATTTTTGGTTGTCACGTCTTGGTGGCGGGGGTGCTGCTGTGCCATTGAAATCTAGTGG
GGTAGAGGCCAGGGATGCTGCTACATATTCTATGATGCACAGGATCACTCCCTCCCTACCCTTA
ACAAAGACTTATCTGGCCCTATATTTCAATAGTGCTGAACTTGAGAAACCTGGTTTAGAGGAAG
GAAACAAAAATGTTCCCACTATGAGGACTCCTTCATAAAGGAAAAAATATTGATGTGGCAAATA
TCACTCTTAAGAGATTATGGTGAGGAACAAGAGAATTGCAACATGTAAATATTGGGTTTTAAGTA
TTTAAACCATTGGAGGAAGATAAGAATTGCTAATATTCAAGAGTATAAAAACTTCTGGAAAGAT
ATGTTCTGAAAATGTACTTATGTGAAGTGAAGCTTGACCCTATATATGTCGTTATGAGTGGAGAA
ACGCAAAGCAAAACAGCAGTTTAGAAGCCATGTAAGACTGCGTTTCAGTCCCATCTCCAATGCT
TGTTATCTGTGTGACCTTGGAGGGGTGACTAAACCTGTCTGAGCCTTGGTCCCTTCACTCTAAAG
TATAAGTAATAATACTTGCCTTCAAAGATGGTGAATTTTCTATTTCTTTTCTTTTTTTTCTTTTTAGA
CAGAGTCTCACTCTGTCGCACAGGCTGGAGCGCAGTGGCATGATTTCGGCTCACTGTAGCCTCT
GCCTCCCGGGTTCAAGCGATTCTCCCACCTCAGCCTCCCGAGTAGCAGGGATTACGTGTGCGTG
CCACGATACCCAGCTAATTTTTTATTTTTAGTAGAGACGGAGTTTTGCCATGTTAGCCAGGTCGG
TCTCGAACTCCTGACTTCAGGTGGTCCGCCTGCCTCGACCTCCCAAAGTGCTGGGATTATAGGC
GTGAGCCACCACACCCAACCTGAATTTTCTATTTCTATTAGGATTAACGAGATGATAAAGTGTCT
AAAACATTGTATGGGATCAATCAATTTCAGTTTTTGTTCCCATGATGAAGTTTGGGAATTAGAGTT
GTAGATATGAAAGTTATTTTTAACAATAACTTTTATAAATTATGGGAAGAAAATGGCAGAGGATA
GCAGAACCAGCTATTAAGATTCTGTATGAATTTTTAGATTTAAATAATTTTACTGGCTTATTTGTG
CACATGGCACACACAAAAAGAACAAATTACATTCAGTAAAGAGCAAGTCTACCTCCTACTCTTT
CCATTTGTCTCCTGATTCTTTTGCCGGTGGCAATTTCTGTTTCCAGCTTCTTATGTATTCTTCCATG
AGAGGAATCCTTGATATTCTGGCCCAAAATTGAGGGAAGTGTACAGTCTTTAGGAGGCCCTGTG
GACCAAGGGCAAGGAAACTCAGGTCAGAGAAAGACCAAATTTATAGGTCTGGATTTCAGGGAG
TAAAACTGACAGTCCTGAACAGGAACCAGGATAAAGACCAAATTTTAGGTCTGGATTTCAGGGA
GTAAAACTGACAGTCCTGAACAGGAACCAGGATAAAGACAAAATATAATGGAGGAAAGAGCAT
TTCTAGAAGACAGGCATTAGGTTATCAGCTTAGCTGTTGGAAGAGGGAGGGGAAAAACTTTAGA

GACAAAAAATTACATCCAGAAGCCAAAGAATTGTCTGTGGTTCAAAAAAGAATATAATATCAGCA
ACGGCTAGGCTGAAGATGGAGCATAATAGAACAAAAAGAAAAGGTTACTCTCAGGGCCTGGGT
TTGCATTAAAATGAAATAATACGTCAGGCGCAGTGGCTCATGCCTGTAATCCCAGCACTTTAGG
AGGCCGAGGTGGGTGGATCACCTGAGGTCAGGAGTTCAAGACCAGCCTGACCAACATGAAAC
GCCCTCTCTACTAAAAATACAAAAAATTAGCCAGCCATGGTGGCATGCACCCGTAATTCTAGCT
ACTTGGGAGGCTGAAGCAGGAAAATTGCTGAGCCCAGGAAGCAGGGGTTGCAGTGAGCCGAG
ATTGCACCATTGCACTCCAGCCTGGGCAACAAGAATGAAACTCCATCTCAAAGAAAAAAAAAA
AAATAATAATATTCATGAGTATGAAGGGAAAAATTAATGACTTTGTACATCTGCCAAGCCAGCT
GAGGAGTTTGAAAATGTATTTGGGATTTACCCTAAGAGTATATAATGAATATCCTGAAATGTCTG
TGGATGATACTGATATTGAACTATGGTTAGACAGCAGTTGCCAGAGCAGGGAAGGTGATAGAA
ACAGAGAAAATTTAAAAAGTCTTCCCCTCTATGCCCATAGCTGATAGCAGAGAAGAGTAATCCT
GAGACTCACCAAGGATGGAAATGATATGGCCCCCACCCTGCCAGCCTTAGCTTCACAAACTAG
AGGACCCTTGATAGCATAGGAGAACCTGTGTGGATTCACGAGAATGACTTACTATTGAGATTCT
GCAAGTGAAAGCATGAGATGGACTTGATTTTTTTTTTTTTTTAGATGTGGGATCTTGTTATGTTAC
CCAGGCTGGAGAGCAGTGGCTATTCACAGGTGTGATCGGCACACTATGACCTGGAACTCCTGG
CCTCAAGTGACCCTCCTGCCTCAGCCTCTCAAGCAGCTGGGACTACAGGCTCCTGACACCATAT
CCAGCTGAACCTGATTTTATATTCCAGTGGCTGACAAACTATTTAACTCCTCTGAACTACAGATT
CTACCCACCTAATTTTTGCCAGTTTGTATGCAAAAGAACAACTTATCACTTTTTATTTTCAAGCCA
CTTCCCTCCAGCCATGTACATTID|56267|gene_id|ENSG00000240562|transcript_id|ENST00000
479610 RP11-59J16.2 Homo sapiens
lncRNACCCGGTCTAGTAGGGAGGGCTTCCTGGAGTAGGTGAGGAAGTATGCTCCGGAGGACA
GAGGAAACGGCAGGAGCAAGAATTAGGAGATGACAACTGGAATAGCAAAATCCCTGGGTTTTT
GAACTCCACAGGGACAGCCACATTCATCCCGGCTGTGGACTGAACTGTGTCCCCTTCAGATGTC
ATATGTTGAAGCCCTTGCCTCCACTGTGACTGCATTTGGAGACTGGGCCTATCAGGAGGTAATG
AAGGTGAAATGAGAGCATGGGGTGGGCCCCTGATCCCATAAGATTCCTGTTCTCATAAGAAGA
GGCACCATGGTGGTATGCACCTGTGGTCCCAGCTACTCAGGAAGCTGAGGTGGGAGGATCCCT
TGAGCCCAGGAGCTCAAGGTTGCAGTGAGCTATGATGGTGCCACTGCACTCCAGCCTAGGCAA
CAGAGCAAGAGCTTGTCTCTAAACCAAAAAAGAATTAGAAGAAGAAACACCAGAAAACTCACT
CTCTTGCTCTCCAAACATGCACAGATAAGAGATCACATGGAGACACAGCAAGAAGGCAGCTGT
CTGCAGCCAGGAAGAGAGCCCTCAGCAGAACCTGCGCCTGCCAGCACCATGGTCTTGGACTCC
CAGTCTCCAGAACTATGAGAAAGTAAGTGTCTGTTGTTGAAGCCACCCAGTCTATGGCAGCCTG
AGCAGACTGACATCCTCACACCACCACCTAGAAAGAGCAGCTATTTGTATCCCCAGTTTACAGA
TGGGGAAACCAAGGCCCAGAGATAATACATGACTTGCCTGTGATCCCACAGCTGTAAGTCAGA
GTTAAGACTGGAGCCCAGGCCATCTCCCTCCACCACCCACACCTGACATCCGCACACTGCGCTT
CCTGCATGGTGGACAGTGCCTGCCTGCTCACAACTGGGACCACTGCCTCCTCTGCTTCTCTGTA
GCCCTGGCCCTAGCTCGTGGAAGGAGCAACAGGGCCATATTTGAGTTTCAGAGCACTGCTCAG
GGGGGCAGAAGGCAGGGTCAGATTCTTCTGGAAGAGGTCGGAGGTTTCCCAGCCAGAACAAA
ACTGGGATGAGAAGCTGGGGTTCCTCCCACCCCTCTCCAGGACCTGTGAATGTCCCTCCCCTGT
GGAGCTGGAAAGCCAGCCCGAAGGCACCTGCCCCGTGGGAGGACACACCCCTGAGCCTGGAT
GCCTCATCTCCTGCAGCTTTGGAATTCTAGCCCCTACCC
ID|56288|gene_id|ENSG00000253235|transcript_id|ENST00000522560 Homo sapiens
lncRNA
AACTCCAGGGCCATTGTTTTCCTGTTCTCCCCGCTCCAAGGCCTCTCCTCTCTCCTTGGCCC
AACTCTCTGACAGTGATGCAGGTACTTGGCAAATTTAAATGGGAGGGACTGCCTTTAAAAACCA

ATGAACCCCTTCCGAATGAACATATTCTATTGAATGCCATATGAAGAAAAATCTAGGGGTTTGTA
CTGGTTTCCTGAGGCTGCCAGTAACAAATTGCCACAAACTAGGTGGTTTACAACAACAGAAATG
TGTTCTCACAGTTCTGAAGGCCAGACGTCCAAAATCAGAATGGCATTTGCTTGAAGGATCCAGG
GGAGAATCCCTCCTTGCTTCTTCCAGCTTCTGGTGGCTCATGGCAATACCTTGACTTGTAGACAT
ATCCCTCCAATCTCTACTTATGCCTTCATATCACCTTCTCTTCTCTTATGAGAACAGTTGTCATTGG
ATTTAGGACCCACCCTAATCGAAGATGATCTCACATGTAGTTTAATTACATCTATAAAATATGCTT
TTCAGTAAG

ID|56321|gene_id|ENSG00000254002|transcript_id|ENST00000520375 Homo sapiens lncRNA

AGGAGAGAGGAACCTGCAGCTTGAGGAAGTTCAGAGAAAACCCAGCCCTTGTAGGTACA
GAGGCAGAGGTTATACCACATGCATGGAAGGCTCTGGGCTTCTTTAGCAACTTTTTTTTTTTTTTG
AGACGGGGTCTCACTTTGTCACCCAGGCAAGAGTGCAGTGGCACAATCACGACTCACTACAGC
CTCTGCGTCCCGGGCACAGGTGATCATCCACCTCAGCCTTGCAAGTAGCTCAGACCAAAGGTGT
GCACCGCCATGCCCAGCTATTTTTTAAATGTTTTGTAGAGGCAGAGGTCTCACTATATTGCTGAG
GCAGGTCTCAAACTCCTGGCCTCAAGCCATCCTCCTGCCTCAGCCTCCCAAAGTGCTGGGATTA
CAGGTATGAGCCACCATACCCAGCCTTTAACAACTCTCAATAACGATTTATCATTTTCACCACTA
GAGGTCTTATACATACTTTTAAAGATTTATTCTTATGTACTTCATTTCTTGGACTTATTATAGATGG
TATATATTTACTTAATTTCTGTTTGTTATTAATAATATAAATGTAATTGGCTTTTTTTTACCTTGGCT
TTATGGCCAGCACTCTTGGTAAGCTCTTATCAGTTCTAATTATTTATCTGTAGGATCATTTGAATTT
TTCATGTACACAGCCATATAATCTGTGAATGATGACTATTGTGTTTCTTCTTTCCAGTCCTTGTATT
TTGTGTGTCTTGTCCGTGCATTCCTACACTGGTTAGGACCTCCAGCCACAATGCTGAAAACACCT
GCTGGTAGTGAACATCTCGTCTTACTCTTGATATTGTAGGGAATGCTTTCTGTGTCTTGTCTTTTA
CCTCCACACTGTAGGTTTTAAAAGAATGAATACACATTATCTGATTATGAAAGCTTTCTTCTATTC
CTACTTTGCTGAGGTTTTCATTATAAATGTAGAATTATAATCAATATTGGCCAGGCGCAGTAGCT
CACGTCTGTAATCCCAGCACTTTGGGAGGCCACGGCAGGTGGATCACCTGAGGTCAGGAGTGC
AAGATCAGCCTGACCAACACGGTGAAACCCCATCTCAAAATACAAAAAAAATAGCTGGGCGTG
GTGGTGGGTGCCTGTAATCCCAGCTACTCGGGAGGCTGAGGCAAGAGAATCGCTTGAACCCAT
GAGGTGGATGTTGCAGTGAGCCGAGATAGAGCCATTGCACTCCAGCCTGGGCGACAAGTAAAA
AACTCCATCTCAAAAAAAAAAAAAAGAGAGAATTATAATTAATATTATTTCTGAATATATTGAAAT
GGCTCATGAACGTGACAAATGACATTCATTGATATTATAAAGTTAAACCAACCCTACATTTCTTG
GGTAAATACAAGATTCAGTGTTGACTGTCTTTGTATGCTCTGGAATAGTTTCTGTGGAATTGGAA
TTACTTATTCCTTGAATATTTAGAAGAACTTAGTGCAGTCAATAAGGACCACTGCTTTCTTGAAG
GAAAGATTTTTTAACTACCAATTAAGGACAAGGATAGTATACTGATTAAGAGCATAGACCTAAA
GCTCAACTGTCTGCATTTACATTCCAGCTTAGCCTCCTACAAGCATGCCTTTTAGCAAGTTATGTG
ATCTGTGACTTAATTTCCTCATCAGTAAACAAGAAGAATAAGATCACCTACTTTAAATGAGCTAA
CTTACCTAAAACATTTAAAATGATGCCCAGCTCATGGCAAGGACTATTCATTTGCTATTGTTATCA
CTGATAATGTCTTTGAAAATGGTAAAATTATTCAAGTTTTAAATTTTTATAAGTTATTCTAATTGTG
ATTAAATCTATATAACATAAAAAGTATATCTTAATCGTTTTTAAGTTTATAGTTCAGTGGTATTAAG
TATATTCATATTGTGCAACCATCACCACCATCCATCTCCAGAACTCTTTCATCTTGCAAAACTGAA
ACCCATTAAACAAGACTCCATTCTTCCCTCCCCATAGTGCCTGGAACTACTATTCTACTTGTTCTA
TCTATGAATTTGACTACTCTAGCTACTTCACGTAAGTGGAATCATGCAGCTATTTGTCTTTTTGTG
ACTGGCTTACTTCACTTAACAGAATGTCTTCAAGGTTCATCCTTGCAGCATGTATCAGAATCGCA
TTTCTTTTTCATGTTTTTTTGTTTGTTTGTTTGTTTGTTTTCTTTTTTTGAAACAGAGTCTCTCTCGTCT
CCCAGGCTGGAGTGCAATGGTACAATCTTGCCTCCTAAGTCCACGTGATTCTCCTGCCTCAGCCT

CCTGAGTAGCTGGGATTACAGGCGCCCACCACAAGGCCTGGCTAATTTTTTTGTATTTTTAGTAG
AGATGGGGTTTCACGATGTTGGCCGGGCTGGTCTCGAACTCCTGACCTCAGGTGATCCATCTGC
CTTGGCCTCCCAAAGTGCTGGGATTACAGGCGTGAGTGACCGCGCCCAGCTGCATTTCTTTTTA
AAGCTGAATAATATTGTTTTGTATGTGTGTATCATATTGTGCTTATCCATTTACCTGTTGTTGGGCA
TTTGTGTTGCATCCATATTTTGGCTGTTGTGAAAAATGCCACTGTGAACATGGGTGTACAAATAT
CTTCTCGAGGGCCAGCTGTATTAATCTGTTTTCATATTGCTATAAAGAACTGCCAGAGACTGAGT
AATTTATTATATATAAAGGAAAGAGGTTTAATTGACTCATAGTTCAGCCTGGCTGGAGAGGCCTCG
GGAAACTTAAAATCATGGCAGAAGGCAAAGGAGGAGCAAGCAATCTCCTTCACAAGGCGGCA
GGAGGGAGACGAGCTGAGGAAAGGCAAGGGGAAGAGGCCCTTATAAAACCATCAGATCTCGT
GAGACCTCTACTATCATGAGAACAGCATGGGAGAAAACGCCCCCAAGATTCAATTACCTTCACC
TGGTCTCTCCCTTGACACGTGAAAATTACAGGGATTACAATTAAGATGAGCTGTGGGTAGGGAC
GCAAAGCCTAACCGTATCACCTGCTTTCAATTATTTTGTTTATATACCCAGAAGTGGAATTGCTG
AATCATATAATTCTATGTTTAATTTCTTTTGGGAGCCAACATACTGTTTTCCACCACAGCTGTACC
ATTTTATATTCCCTCAGCAGTGCACAAGGATTTCAGTTTCTTCACATCATCACCAACACTTATTAT
TCTGGGATTTTTTATAGTAGCTGTCCTAATGGGTATGAAGGGGTATGATTTTGATTTGTATTTCCC
TAATGATTAGTGATGTTGAGCATCTTTTCATGTGCTTGTTGGATGCTTGTATATCTTCTTTGAAGA
AATGTCTCTCTAACTCATTTGCCCATATTTTAATCAGATTGTTTATCTTGTTGTTTTTGTTGTTGTTG
TTGAGTTTTAGGAGTTCTCTGTATACTGTGGATACTAATACCTTATTAGATATGTAATTTGCAAAT
ATTTTCTCACATTCTCTGGGTTGCCTGTTTTTCTGTTTTCTCTGTTGATAGTATCTTTTGATGCACAA
AAGTTTTTCATTTTCATGTGGTCTAGTTTGTCCACTCTTTCTTTTGCCTTTGCCTCTCACGTCCTACA
TTCTCCCTTTATTCTTTAATCAGTTTGGGTACGCTGTATTCTTCCAGGAATTTGACCATTTCATCTG
TTTCCAAATTTATAATATTATTCTATCATCTGATTAATGTCTGCAGCATATTTTATTATGATCCCTTT
TTTAATTCCTAGTATTGTTTTCTTGTGCCTTCTTCATTTCCTTAAACAATCTTGCCAGAGGTTTTTCA
ATTGTGTTCATCTTTACACACAGCATTTTGGCTTTGCTGAAGTTCTCTTTTGTATGTTTGTTTTCTAT
TCCATTAACTTCTACCTTTCTCTCAATTTCCACTTTCCCACTCTCTTTAGAATTCTCCTCCATCTTTT
TCCTAACTTCGTAAGGTAAATGCTTAGCTCACTCATTTTCAGCCTTTCTTCTTTTCTATTATAAACA
CCTTACAGCTATAAATTTTCTTCTATATTCCACAAATTCTGATATGTAGTATTTTTATGATAATTCA
GTCATAAATGATTTTTAATTTGTATTTTATGTTATTCTTTAACTCATGGGTTTATAAATTATGCTATT
ATTTATAAGTTATATACAATACCTGTGACTGAGTCTGTACATTAGGAACAATGGTGACAGAGAAC
TAATACTGTGGATTTAATTCAATACTGTGGCTCCAGATTTGAAAGCAAACTACATGGCAAATTAA
TAGTCTGGTTGCACTTATTTAAATAAGTCTAATCTCACAAAAGAAGCCTTATTTTGTGAACAAAA
ATAATCTTTCTTTGAGAGTATTATTATCACAAGGGGGAATATCATCATGAACAATGTTTGATTTTG
AAATGTGCAAACAAAAAAAAAAACGAGATTACTAAGTGTCCTGTGTACAGCATCATTCCAGGTTA
TACCTGCCTTTCATTGCCTTTGAACTATTTTATAACTCCGTAAGTTGAAAAAATCTGATAATGGTC
CAAATGTTTTTGTCTATGGAAGTACAAATTAATTAGGTCCAGTTGAATGCAATTGATTAGTGACC
CATTTTGCATCTATTTCAAATTCATTTTAGCATTCCTTATCTCTGTATGTGTGTGTGTCTGTGTATGT
GTGTGTGTGCGTGTGAGTGTGTTATTTTGAATTGTCCTTTGAATAAGTTATAACTTCTTACTGGTTC
CCTCAGTAATTAATGAGATAAATTAGAACTCTGGAACATGTTCATTTAAAGAAATCAATTATTTG
GCTGGACATGGTGGCTCCTGCCTGTAATCTCAGCACTTTGGGAGGCCAAGGCAAGAAGATTGCT
TGAGCCGAGGAGTTTGAGACCAGCCTGGGCAACATAATGAGACCCTGTCTCTACAAAAAAATCG
AAAAATTAGCAGGGCATAGTGGTGTGCATCTGTCTTCCCAGCAACTCAGGAGGCTGAGATGGTC
TGCACTCTAGCTTGGGTGACAGAGCTAGACCCTGTCTCAAGGAGGAAGAAGAAGAAGGGGAA
GGGGAAGGGGAAGGGGTGGGGAGGAGGAGGAGGAGGAGGAGGAGGAAGTCAATTGTTAAAA
GAAATATTTAAAAATTTTTTCACTCTGGACGTCTATTTATCTATTTCTCCTTACACATACAATAATT

TTTGCTCTATATGCTTTAAAACTATTTCATTAGATATATACAAGTCTAGAATATTGTCTCTTCTTGA
CAAATGTAATCTTCTCTCATTATAGGGCAACACTTTTTCTCTAAGAATGCCTCTTAACTCAAAGTC
TATTTTCTCACCATAATTGATAGTATTGAGTTAGTAGGTATGTGCTTTATCTTTTTTCATTTTTTTAA
TTTCAAGTATTTTTAAGTGAATATGTTTTTAGGTGTATTTCTTGGAAAAAATCTATTTTTTAACTGCA
GGATTTAGTCTGTTTGCATTTATTGTAATTGCTTGTATATTTGCACTTACTTTTTTTTTTTCTTTTATG
AAACAGGGTCTCACTCTGTTGCCCAGGCTGGAGTGCAGTGGTGCAATCTTGGCTCACTGCAACT
TCTGCTTCCCAGGTTCAAGTGATTCTCCTGCCTCAGCCTCCCAAGTAGCTGGGATTACAGGCACC
CACCACCAAGCCTGGCTGATTTTTGTATTTTTAATAGAGATAGGATTTTACCATGTTGGCCAGGC
TGTTCTCGAACTCCTAGTCTCAAGCAATCCAGCCACCTCAGCCTCCCAAAGTTCTGGGATTACAG
GCAGGACTACAGGTATGAGCCACTGAGCCAGGCCTGCACTTACTTTTATAATCTTGTTTTATATT
TCCTATCTGTCCTGTCCTGCCCTTTTTTTTTTTTTTTTTGCTTTTTTGTTTGTTTTGCTTCCTTTCTCTC
CTTTCTTGTCTTTTTTTATAATGAAGAAGTTTTTTTATCTCATTCCAATTTCCCTCCCTCTATTTCTGT
TCTTTTAGTAGCCATCTGTTATGAGTTATACTGTGTCCTCCCCCACCCCCTAAAAAATGTGATAAT
TCTAATCCTCAGTACCTCAGAACATGACATCATTTAAATTGGGTCATTACCAAAGTAATTAAGTT
AAATTAAGGTCATTGTACTGGGTAGGGTGGGCCCTTAACCTAATATGACTGATGTCCTTATGAAA
AGACAGAGACACAGAGGAAGAAAATCATGTAAAGATCAAGGCAGAAATTGGAGGGCTGCATC
TCCAAGCCAAGGAACACTATGGATTGCTAGCCAACAGCAGAAGATAGGAGAGAAGCATAGAA
GAGATTCTCCTTCTCAGCCCGCAGAACAAACCAACCCTGCCGGCACCTTGATTTTGGACTTCAA
GCCTCCAGAACCGGGAGAAAATCAATTTCTGTTGTTTTAAGCCTTCCAGTTTTTAGTACTTTGGG
ATGGCAGCCCTAGGGAATTAACACACTACCCCAGAAACTATAACATGCATACCCAATTTATCAA
AATCATTACCATTAATGAGGTCATTATCATCCTCATGAACAACGTAAGGACTATGGGATATTTTA
ACCATGGTTTTCCTTCATCTTAACGTGCTTTTTTTGTTCTTTTCTATTTTATGTTTTTCCCCAATCCC
ACAGAACATTATTTTTATGAATGTTATGTGCCTTTGTGATTTGTTTACTTTCAATTACTTTCAATTTG
TTTACTTTCAATTCACCATCTTCTTTTTTTACCATTCATTTTTTGTATCTCAAACTTCCCTTCTGGGA
TAACTTTCCTTGTGTCTAAAGTGCATTGATGAAGATTTGCTGGTGGAAATGCTGTTAGTCTAATCA
TGTCCTTCTTAGATACATAGTACTAGGTTGTCAGGTGTTCACTCTTGGCATTTGACACCCTTGGCA
TATCTTGGAGATATTATTCCACTGTCTTCTGGCTTTCACTGTTGCTCTTGAGAACTCTATTGATAA
GTCAGCCATCAGTCTAATCATTGCTCCTTTTTGAGTCATCTGTCTTTATGGCTTCCTTAACATTTTC
TCTGCATTTTGCATTCTTCAGCTTCCTCTGATGTACCTAGGGATAGATTTCTATTTATATTTCATGA
TTGGGATTTGTTGGGATTCTGGGATCTGAGAATCGATATCTTTATCATTTCAGAAAAATTGTCAGC
CCAGTCTACTCAAATATTGCCTTAGCCTCATGCTCACTCTCCTCGCCTTTGGGAACTTCTGGAAT
GTAGGTGACATATATGTTAGACCTTTTGCTCTCTCCTCTCTTTCAAAGCTCCTATCTGTTTGTCATT
CTATATGCATAATCAGTAAATTGTTTAGATGCATTTTCTTTCCAGTTCACTAATTCTCTCTCTAGTG
TTCAAGCTGCTGTTAAATGTGTCCAATGACTTTCTCACTTTATAATTTTATTTCTAGAAGTACTATT
TGATTATTTTTCAAATTGGCTTGATTTTTATATTATCAATTCTCTCCAGGCAAAGAACACCAGAAA
CACATCTGTAGTTGAACAAGTGGGTTCATTACTCATTGCAGCAAAGGCAAATGCATACCATAAG
GATCCATGAGGCATCTCAGTAAGAGGGTAGTCAAAGGACCCATTAAAAAGATTTGGACTTTGGC
TGGGTGATTTGAGGGAAGGTTTAAGAAACCAGAGTTTTGCTTTGTATTGGATGCTGTCAGGAAG
CAAAATTAATTCTATAACTGTAATTAATTCTATAAGAATAAATCTATAATTGTAACTATAATTCTAT
AACAATAAATCTTATCTAAAAAGAAGACAATTATGATCACTCATATTAGCCAAGATACGGGGAA
ATTTGGTCATTTTTGCTATTAGGACAATGTTCATATGTTGTCAGACATGACAGTGGAGTGGTCTTG
GTTTTGCCCAGGTCCACAATGGTCACAGAGTGGCCTTGTCTGATGTTGATTTTCATTTTTTTTTAG
ACAGTGTGTCACTCTGTTGCCCAGGCTGGACTACAGCGGCACAATCATGGCTCACTGCAACCTC
TGACTTCCAGGTTCAAGGAATCCTCCTACCTTAGCCTACCAAGTAGCTGGGATCAGAGGTACAT

GCCACCACTCCCAGCTAATGTTTTTATTTATTATAGAGATGGAGTCTCGCCATGTTTCCCAGGCT
GGTATCAGGCTCCTGGACTCAAGTGATCAGTCCACCTCGGCCTCCCAAAGTGTTGGGATTACAG
GTGTGAGCCACAGCACTCACCAATTTTCTGTCGTCTATGTTCAACACAAGAACAGCATGGACTA
GCAGTGAGCGTCAGGTCAGATATTAGATGTTAGAGGCTACTTTTTGCTTTCTCAATAGTCTCTTA
CATTTTAAATTTTCAATCCTTTCTTTTACATCATTACACACAATAAATATGCTTACTTTCGATTCTG
AGTCTGATCACTACAATATCTATAATTTTTGTGGACCTAATGTTGTCATGTGTTGTTTCCACTGGC
CCTAGCTTACCGTGCTTTGTTTGTGTGTATACGTGGGAGGTTTTTTATTTGTTTGTCTGTTTGTTTG
TTTTGAGATAGAGTTTCGCTCTTGTTGCCCAAGCTGGAGTGCGATGGCGTGATCTTGGCTCACTG
CAACCTCTGCCTCCCAGGTTCAAGTGATTCTCCTGCCTCAGCCTCCTGAGTAGCTGGAATTACAG
GCGCACAGCACCACGCCCTGGTAACTTTGTATTTTTAGTAGAAATGGGGTTTCACCATGTTAGCC
AGGCTGGTCTCAGCCGGACCTCAGGTGATCTGCCCACCTCGGCCTCCCAAAGTGCTGGGATTAC
AGGCATGAGCCACCATGCCCTGCCTACATGTGGTTTTTAACTCTGTCTTTGGTTTCCTACTTTGTG
ACATTTTACCTGTGTGAATTCTGAGTCTTGGAGTTGGGCTCCTGCAGTGAACATCTGCACGTGCT
TCCACCAGGACCCTGCGGGCATTACCAATCTATAATCATGCTTATAAATTTTTGGCTTGTAAATTA
TCAATGTGATGTTCTGACCAGCCTGATGATGTGACTGGTGCTGCAAACCTGTATGGGTGCCAGTT
TTAAGCTTGAGTTCTCAGGTAATATGTTTTTTACTTTCTGCTTTAGTAGGCAGATTAACTGGCTGT
ATCCTCTTTTCTAAATCATTCTTGCACAGAGAATGTCATCACATGAAGTCCCAGCTTTCTGTAGAA
ATTCTCTATTAGCTTATTCCCAGTGAGCCTTAGGCTCACTGTCTCTGGTGCCATAAAAACTGAAA
CTCAGCTGGGCATGGTGACTCACGCCTGTGATCCCAGCACTTTGGGAGGCCAAGGTGGGCAGA
TCACTTGGAGTCAGGGGGTTTGAGACCAGCCTGGCCAACATGGTGAAACCCTGTCTCTACTAAAA
ATACAAAAAAAAAAAAAAATTAGCTGAGCATGGTGATGGGCGCCTGTAGTCCCAGGTACTATG
GTGGCTGAAAGAGGCTGAGACAAGAGAATCACTTGAACCTGGGAGGCGGAGGTTCCAGTAAG
CTGAGATTGTGCCACTGCACTCCAGCCTGGGTGACAGAGCAAGACTCCGTCTCAAAAAAAAAAA
AAAAAAAAAAAAAAACAAACAAAAAACAAAGAGAAAAGAAATATATGATGCCAGTTCTTCCAG
TTTCTGAGAAATGCCAAAAATCAAGATTTGGTGAGTTTTTTTACCCTTTCTCATTTGAAAAACTTTT
AAACTTTGAAAAAGTTGCAAGAACAGTACAAAAATTCCCATGTGCCCTCTACCAAGTCCCCGGA
CATTAAAATTTTACACTTACTGCATCCTTTTTTTCTCTCTATATGTGCGTGTGTAAGTTATTTTGTAA
ACTGTGAAGTTTAAGACATAATGCCTCTTTCTACCTAAATACTTGGGTATATGCTTTCTAAATACA
TTCTCTTATATAACAAGAGTGAAGTTATCAAAATCAGGAAATTAGCACTAACATCTTACTCCGAT
TTCACCAACTTTCCAAATATTGCCTTTCATAGAAAAAAAAAAGATTCCTGGATCATGTTTTGCAT
TCAACGTCCAGGTCTCTTTAGTCTCATTGAATTAAGAAGAGTTATTCACTGTTTCCTTAACTTTTAT
GATCTTAACAATTTTGATGATTAAAGACCTGTAATAGATTGCACGTTCTAAAAATGGCCACAATA
TTTCTAGTTCCATATGCTCTTCCAGAATAGTCTTGCCACTCTCCATTGAGAGGTAGAGTCTATTTC
TTGAACATGAGTGGGTTGTTGTTGCTTTGACCAGTAGAGTACAGCAGAGGCGATGCTCTCTGATT
TCTGAAGCTAGGTTATGAAAAGAATGTGACTTTTGTCTGATTCTCTCTTTCTATCTCTATCTCTTTA
TCTCTCTTGCTGTCTCTCTCTTCACTTATCCTTGGAATCCAGTCACCAAGCTGTGAAGAAGCCAA
GCAGTTCTCATGGAAAAACCACATATATGTATTTCTGCCAAGAGGCCCAGCCAAGATCCCAGCA
ACAGCCAGCATCAACTACCAGACATGTAAGTGAATGAACATTAAGACACTTCCAATCCCCAGGC
TTTGAGTCTTCCAGCCAAGACCCACACATCATGGAGGAGGAACAAACCACTCCTTTTCACACTTT
CTGAATTCCCAGCCCAGAGGCCATAAACAAAACAAATGATTGTTTGACATAAATTTGTGTTGGG
GTTATTTGTCACACAGCCATAATAACCAAAATAAGACCTGTTATTTTTTAAGACATCCCTCAATTT
GTGTTTGTCCAATGTCAGATTTGTTTCTTAACATGCCTGCTTTTCTTCTTCTTTTTTTTTTTTTTTTTTT
GAGATGGAGTTTCACTCTTGTCACCCAGGCTGGAGTACAGTGGCACCATCTTGGCTCACTGCAA
CCCTCACCTCCCGGGTTCAAACAATTGTCTTGCCTCAACCTCCCAAGTAGCTGGAATTACAGATA

CCCACACCCACAGCCACGCCCAGCTAATTTTTATATTTTTAGTAGAGATGGGGTTTCACCACATT
GGCCAGGCTGGTCTCGAACTCCTGACCTCAAGTGATTCACCCGCCTCAGCCAAAGTGCTGGGAT
TACAGGAATATTTCTACTTTTAAAATATGGGCTTAAATTAAAACATTTTAACATGCAGTGTGGACC
AGGCAATGATTTTTTTTTTTAGATACAACATTGAAACACAATTCATGGAAGAAAAACTGATAAAT
TAGGCTTCATTAAAATTTAATACTGCTCTGCGAAAGATACTTATAAAAGAATGAAAAGATAAGCC
ATAGACTAGAAGAAAATGTTTGCAGAATACATATCTGAAAAAAGGCTTGTGTTCCAGATATACA
AAGAACTCAACTCTCAATACAAAAACAAATGACCTAATGTAAAAATGGGCAAAAGACCTCAAA
ACACACCTCACCAAAGAAGATACACAGATGGCAAAATAAGCACATAAAAGATGCTCCAGTCAT
TTCCCATTAATGAATTGCAAATTAAACCAGTGGTGAGATACCACTAGAATGACTAGTTGACTAGA
ATGAGGTATCATTCGAATGACTAATATCCAAAACACTAACAATTTTGAATGCTGGTGAGGATGTG
GAGCAGTAGGAACTCTCACTCATTGCTGATTGGACAGCAAAATTGTACAGCCACTTTGAAAGAC
TGTTTGGAAGTTTCTGACAAAACTAAACACACTCTTACTGGTCCAGCAATCAAACTCCTAGGTAT
TTACCCAGATGAGTTAAAAACCTCTGAGCACACAAAAATCTGCACACTAATGTTTATAGCTGCAT
TATTCATAATTGCCAAAGACTAGGAGTAAGGAAGATGACCTTCAATAGGTGAAAGGATAAGCAA
ACTGTGATACATCCAGATAATGGAATATTACTATGACTCATGTCCATAATCCTAGCACTTTGGGA
GGCCAAGACGGGAGGACAGATTGAACCCAAGAGTTTGAGACCAGCCTAGGCAACATAGGGAA
ACCTTGCCTCTACAAAAAATAAACAAAATTACCCAGGTGTGGTGGCACTCAACTGTGGTCCCAG
CTACTCAGGAGGCTGAGGTGGGAGGATCACTTGAGCCCAGGAGGATGAGGCTGCAGTGAGCC
ATAATCACACTACTGCACTCCAGCCTGGACAACAGACTGAGAGTCTCTTAATTTTTTTTTTTAAAG
AGCTATCAAGTCATGAAAAGACATGGAAGAACCTTAAACGCATATTGCTAAGTGAAAGAAACC
AATCTGAAAAGGCCACAGCCTATAATTCCAGCTATATGGCATTCTGGGAAATGCAAAGCTATAG
AAACATTTACAAAATCAGTGGTTGACAGGGATTCAAGGGGGAAGGACGGAGGGATCAAGGGA
ACACATAGGATTTTTAGGGCAGTGAAACTATTCTGTATGATATGGTGATAGTGGATACATGTCAT
TATACATTTGGGGAAACCTATAGAATGTGAAACACCAAGAGTGAACTCTACAGTAAACTATGGA
ATTTAGATTAAGAATAAGGTATCAAATATTGGTTCATCTATTATAACAAATATATCACACTAAGG
CAAGATTTTATTATCTAGTATGGGAAATTGTATAGGTGGGGTATATGGAAACACTGCTTTCTGCT
TAATCTTTCTATAAATCTAGGCCTAATAGGCCAAGGCGGGCGGATCACGAGGTCAGGAGATCG
AGACCAACCTGGCTAACACGGTGAAACCCCGTCTCTACTAAAAATACAAAAAAATTAGCCAGG
CATAGTGGCAGGTGCCTGTGGTCCCAGCTACTCAGGAGGCTGAGGCGGGAGAATGGCGTGAA
CCCGGGAGGCGGAACTTGCAGTGAGCCGAGATTGCGCCGCTGCACTCCAGCCTGGGCGACTG
AGCAAGACTCAGTCTCAACAACAACAAAAAAAAAGTCTATTAATTTGGCTGGGCATGGTGGCTC
ATGCCTATAATCCCAGCACTTTGGGAGGTGGAGGTGGGTGGATCATGAGGTCAGGAGATCAAG
ACCATCCTGACTAGCAGGGTGAAACCCAGTCTCTGCTAAAAATAAAAATGAAAAAATTAGCCAG
GCATGGTGGCAGGCACCTGTGGTCCCAGTTGCTTGGGGGGCTGAGGCAGGAGAATGGTGTGAA
CCCAGGAGGCGGAGGTTGCAGTGAGCCGGGATCATGCCACTGCACTCCAGCCTGGGTGACAG
AGCAAGACTCCGTCTCAAAAAAAAAAAGTCTATTAATTTTTAAAAAATACAGCATGGGCCAAACC
CACCATCTCTGCAGGATCAATAGTTGGTAACCCTTGCTTAAGAGAAGTGAAGCCATTAATATAC
AAATGAACCCTATTTTCAGAAAAAGGAACAGATGCCCATTAATGACTACAGTAGTTAGTTAATG
GCTAAGTCAAGATTAGAATTTAGGCTTCTTGTTCAATTCCAAGTTCTTGTCAATACACCTTACTGC
AACATTAGGGTCCTCCCCCTATCTAAGAACAGGCTCAGATAAAACTTATCTTTGCTCTAAGTGAG
CTGTAAGTTACCACAGTAAAAAGTCCACAGAATAGAAAAATGATCATCACTCCCAAAAATGAGT
TGGAATGAGGCCTGATAAAGTACCAAGGGCAGTGGTCAGTGTGGTCAAGGATGCCCAGCTCCA
GAGGGCACACTCTGCCCCTAAGGGTCCCAACCCCAATGCACATGCCCTCCTAAGATTGATGAG
AGGTTCAGAAACTAGTGTTGCCACAAACTGTCATTTTTCTTTCTATGCCTTTCCTTAAGAATTTTA

ATTTATAATAGGGAACCATGATCCTTGAGTCATTTCAGGGAGAGGAATGTAACTTTCACTTCTGA
ACAAAACAGCTCTGTGTGAATGCACATGTATTTAATATCCATTTTATTTGCGTGGTTAGTCACATC
AATCTACCTTTTGAGAACTTACTGCAAATCCATGGAGAAGAAAATATTAAACCTTTAAAGTCAGT
CAAGGCTTTACATCTAGAAAAAGCATGTTTATATAGGGGTTTTCCCCCCATAATTCTTGCATAAA
CCCAGTGATACAGATAATTATGCCCATTTTACAGATGAGGAAACAAAGTTCAGAGAAGCTGTGT
GACTTACCCAAATTCATGCAGTAAATGATGTAAGCCAAACTTAACAACTTGGCCTCCAAGAGGA
CTGCTCTTCATTACAAACCCCCTTGTCTTTTTTTTTTTTTTTTTTTTTGACCTTGTCTCACTCTGTCAC
CCAGGCTAGAGTGCAGTGGCATGATCTCGGCTTACAGCAGCAGCATCCACCTCCCATGTTCAAG
GGGTTCTCCTGCCTCAACCTCCCAAGTAGCTGGAATTACAGGGGCATACCACCATGCCCTGCTA
ATTTTTGTATTTTTAATAGAAATTGGGTTTCACCAAGTTGGCCAGGCCGGTCTCGAACTCCTGGC
CTCAGGTGATCCACCCACCTCTGCCTCCCAAAATGCTAGGATTACAGGCATGGGCCACCTCCCC
TGGCCAAACCTCCTTGCCTTTCTAATCAATTCATAAGCAAAGAGCCTGCTCAAGCAAAGGAAGC
TGGTGCCAACTGAGGGCTGTGGGGAAGGGCAACTACCCTAAGTTTTCTGTCCCTTGAGCTAGCC
CCAAACTCTGAATCCCATAGACCTTTGCTATTCAAAGTGTGGCCTGAGGACCAGCAGCTTGTGA
GAAGTGCGGAATCTTAGGTCCCACACTACTGAACCAGAACCTGCACTGTGACAAGGGCCAAGG
TAATTCCTAAGCCCTTTAAAGTCTGGGAAGCCCTGGTACAGACACACAGCCCTGTGGGATAAGG
CATTGTCTCTAGCAGAACTGTCCCTCATCAAGTGAACATGAAAAGTGGAGAAACAGATTGAGCC
AGACACCCAGTGTCATCAAATTTGCCAGTTAAGTTTTTGGTTTTTTCTTTCCCCAAAGGGGTATGT
CCTGGCAGTAAGCCTCAGTGGGCAGGTGAGCATAGGTACATGAAGGCTGTTTCAACAAGGCAG
CTCTAGCAGTCACAAAGTGGGCGAGCTCTTGAATGGCCCCAGAGTGGTCCTGGAGTGTAGGTG
AGGCACCACGGGAGAGCAAGCGGCAAGACTGTCAGCACTCCAGGAATCGCTTTCTAAGCCTAC
CTACAAAACCTCAGGTGCGGGCTGGCTGGTGCCAGCAGATGCCAGGTAGGTGGTCTCCTGCAC
CTCCCATCCCTTCCCAGGCTGGGAGAGCAGTGAATGTTCAGTCAAGGCCCCTGGGCTAGGAAG
CCAATGTCGACAGCCTCCTCCAGCCACCCAGGAGCTGAGCATCACCGTGCCAGGGATCCAAGC
GAGAAAGGCACGCGCAGCCGGGTGTGGGGCAGCTGCGGGGCAGATGTGGGGTAGGTGCGGG
GCAGGTGCAGGTCAGGTGTGGGGCGGGTGCGCGCTGCCATCCCCAAGAAAGGTCCCTACGCA
CCTCACCTCTTTCATCTGTAAAATGGGACTGGTTGTGGTGGGGAGAAGGGGTGTAAAATAGGAT
TAAAGAATCTCTCGAGTGTGGTGTGCAGGTCACAGACAGTGTGAAAGACGATGAGAAAAGACA
AACAGTTTTGCAAGTTATAGTTATAATGCAATAACAGCATCCGTTAAAACATAGTATCATATGCT
TTAACGAATGCTATTATCTAGATTTAAGTTAAAATAAATATTTTGTTTTACATTTTCTAAAAGGTAG
TAAAATCAACATAAACTATTAAGTAAATAATATTAACAGTACGGAGGGAAGTGAGTGCTAGAGG
AAAACAGGAGTAGGGTCCGGACGAAGGGTCTTGGCCACCATGCAGGTAAAGTGCTCAGGGTG
GGCGAGCGATCCCTGCCGGCTTAGCTCCTCCGGAGTCCGGGAAGAGTGGGAGGGCCCCTGGT
AGTTTCACCGCTCTGTGTGGGCTCGCGCCGCCCCTGACCCGCCCGGGGCCGCCGCCCGCCCCG
GCTCCTCTTGCGGTTCCCCCCGGCCTGCGCGGAGCTCCACCACCCGCCCTGGCGCCGGCGGCC
CCGCGTCGTCAACAGCGCGAGGTGTCGGGTGGCCCAGGGTGTTTGGGCCGCACCGGGAGGCT
GTTGGAATGACAAGGACGTCCCCGGGGCCAAACGAAAACAACCCCACCCCCTGCGGCGCCAC
CCGGCTGGGAGCGGAGAGAGACTGACGGCGGAGGCGGGGGTCAGGGCGCGGACCCGGACCC
CAGCCCCATGGGGGACAGAGTCCAAAAGTAAGCGGGACCACGGGCAGAGCGGGCACCACAG
CCGCGCCCTTCCAAGAAGCGCCTAGGAGGTGGCCGCGGTGTCAACGCCGTCCCTGAATTTGTTT
TATTTGTGGTTCCAGATAGCTCGCGTCCTTGGTGTTCGCCTTGTCGCAGGAGCCTCGCCATCGCG
CAATCTGCACTCGCCCCTCTGCAGATCTAAGGCCAGACCTGGGCCACCCTAACTTCTCGGTATC
TCCTATTTCTAGAAACCGGCCGGGTGGAGGATGCAGCCGGGCGTGGTGGCTACAAGGAAGTGC
CCTGGTGTTAAGAACTCAGCCTCTGAAACCCGCCGCCCAGCTTCGAGTCAGCGACGTACTTTTG

TTGTCCCGGCCTTAGTATCCCCGCTGCAATAAGAGGTACTCAGCTCAAACCATCTGGGTGAGGA
TTAAAGGAGTTCATATCCATAAAGGACTAGAACAGGTCCTGGCACCACGTAAACATTCTTATCA
TCAAATGTGAAAAAACAAAGTTCTCTAAGATTTCGAAA

ID|56419|gene_id|ENSG00000255135|transcript_id|ENST00000572035 Homo sapiens lncRNA

AGCGGTTGGAGCAGGGGCTCTGCTGCGACCTTTACGGTAGTGAGGTTAGGGACGATGGGA
GAGCAGCTTTGTTCCTGAAGTGCCGTCGAGGATTAGGATCGAGTTTCTTCGATCGTGAGGGCAA
TTATTAGTGGCCTCTAGCCCCCCAGATGGCATCTTGAGGAGAGAGAATCCACGCTACGAGAACT
AACATAGTGGACATCGCAGTTGTCCGTAGCCGATTTGCGGGTTACGGAGTTGTTGCAAGTAAAG
AAGGGGAAATCCGAGCGTTCTCGCGTTGGATTTCCTCCACGTGTGAAGTGGGAATGGTAGTGAT
ATCTCGTGCTATCTCTGTCAAGGGCAAGAAGGAGAAGGAATTTATTTGAGGCCCTACGACGTGG
TAGGTTCACCCTCAGCCTTTGTTCTTATTTCATCTTATTTGTGGAGAAGGTCTTTTCGTTCCTATTT
TACAGGTGAGAAAAGGGGCCCAATATATAGTTATTTGCGGCCATAAAATAGAAGCCAGTTGCCT
GATACTGAAGATTCAATGAAATCTTGTGATTGACTTCCCTCTTTAAATTTTGTACTTTGGGTAACA
TCGGATCCGGTAGAAACGCTCGTCAGAAAGAGTTTTTAATGTGCATTCTACAATTTCCAGACATA
GTTTCCTTCTTGAAAAATTGGGTTAATACTACCCTATAGGGATCTTGTGATGCACCACGTAAACG
TTGTAAGTTGTGAACCACTATACAAACGTATGAGACTCTTCATGTTCTTGAGTGGAACTGTAGGT
TAGGAAGAGAAGGTAAATAACAGCATTTAAGTTTCCTACTACTATTTGCCCATTAGCTAAAAATA
CCCTCCGAGGTCTAGGAAAGTCACTGTTGTTGAAACCCAACCTACACCTTCAGAAAATCGTAAA
GTGGAACATATTAATCACCAGGCCCTCCCTCCGCTGTCTCCCTGACCTTCCTATGCATCCAGTTG
CTTAAGAACTCTTGGAAAGATAGTAAGTGAAATCTTCATAGAGGATTTTTGTACTTTAACATATAT
TATTTGATTTGAGCCTCGCAACATTTCAGTGTGACAGGTACGATTAAGGTCTTTCATACTATGAAT
GAGAAAAACATAGATGTACCAAGGACTCACCAAATTTCACGGAGCTAGGAATTGGAAGAGAGC
TAGGCTTTGTAATCCCAGGTCTTCTGACCTCCAAATGTCCTTTCCTCTACAGTGAATGTCAAAAT
GACAATGAATGTCTACTCCTCTACAATGGAGTACAAATCGAAAGACAAGGGGCTTATAAAACTG
ACTCTGACCTTAAGAAGTAGCATATGAGTGATGAATTATTATAGTCAATCCTTGTGCTTACTAAG
TGCCACATACTGGTCTAAGCATTTTACATAGTAATTCAGTCCTTAGAACAACTTTATGAGGTGGA
AACTTTTATTATCTCTAATTACAAAGGAGGCAACTAAAGTCTAGGAAAGAAGCAAAATAGCTTG
CCTAAAGTCACACATAAGTAAATGGTGGCTCACGCCTGTAAACCCAGCACTTTGGGAGGCTGA
GGCAAGTGGATCATTTGAGGTCAGGAGTTCAAGACCAGCCTGGCCAACATGGTGAAAGCCCAC
CTCTACTAAAAATACAAAAATTAGCTGGGCATGGTGGCATGTGCCTGTAATCCCAGCTACTCGG
GAGGCTGAGACAGGAGAATTGCTTGAACCAGGGAGGTGGAGGTTGCAGTGAGCCAAGATCGC
TCTACTGCATTCCAGCGTGGGCAACAGAGCAAGACTCCATCTTAAAAAAAAAAAAAAAAAAAA
AAGCTAAAGCTGGGATTTAAACCTGATTATGTTGGCTCCAGAGCCTATGCTCTAGACCAGTGGTT
CTAAACTTTAATGGTCAATAGGAACCGCTTGAGGATCTTGTTAAAACGCAAATATTGGTAGTATA
GATATCAGGTAAGGCCTGGATCCTATAGTCTTAACAGGCCCCCAGGTGATGTTGATGGTGCTGG
TCATTACATACTACAGTTCACATCTTCCCTTTTCTCCTAACCGTATTTATTTTGCAGTCAGAAACTA
CCTTTCCCATGCTGCCTGTGACTGCTTTATAGTCTCAAATGTTCCCAACATGCTCTTCCTTAAGGA
TTAGACTCAAACTTTTTCCTCAAAAAGCTTATCCTGCCCCTGTCTTTCCCTCTAGGATAAGACAGA
CATCCCTGCCTCTGCTCCCCTACCACTCAGTGTACACCTTGTTACAGAGCTCAGAACACTGAATT
ATGGCCATTTATTTGAAAATCTTCCTGGCCGGGCATGGTGGCTCATGCCTGTATTCCCAGTACTT
TGGGAGGCCGAGGTGGGCTGATCACTTGAGGTCAGGAGTTCAAGACCAGCTTGGCCAACATGG
TGAAATCCCGTCTATACTAAAGATAGAAAAATTAGCCGGTCATGGTGTTGTGCATCTGTAGTCCT
AGGTACTCTACTCGAGAGGCTGAGGCAGGAGAATCTCTTGAACCCAGGAGGTGGAGGTTGCAG

TGAGCTGAGATTGCGCAACTGCACTTCAGCCTGGGCGACAGAGCAAGACTCTGTCTCAAAAAA
AAAGAAAAGAAAATCTTCCTGACTGGACAACGAGCTCTTTGAGGAGAGGAACCAATCAGGTCT
GTGTACTGTAGAGCCTAGCATATAATAAGAATAATAGCTAATACTTACATAGGACTTTCTGTATT
CCAGATCACGTTATACACATTAACTTGTTTAATCTCTACTATAACCTTACAGATACACTTTTATCC
CCATTTTATGAATGAAGAAACTAAGGCATAGAGAGGTTAAATAGCTGGCCCAAGGTAACAAGCT
TGTAAGTAACAGAGCCATGATTTTAAATAAAAGCACTTTGGCTCCAGGAAATGTGTGGTGAATT
AAATGAGAGCCACTCTACACTCAACCAAAGAAGGAAAACTGAGACTTACTATCCTATTAGACAT
GATTATAACTACCTCTAGTCTGGGAAAATACACAGAAATCTGTTCCCCTGGCCAGGTGCGGTGG
CTCACGCCTGTAATCCCAACACTTTGGGAGGCCGAGGCAGGCGGATCATCTGAGATCGAGAGT
TCGAGACCAGCTTGACCAACATGGAGAAACCCCGTCTCTACTAAAAGTACAAAATTAGCCAGG
CATGGTGGCACATGCCCGTAATCCCAGCTACTCGGGAGGCTGAGGCAGGAGAGTAGCTTGAGC
CCAGGAGGCGGAGGTTGTGGTGAGCCGAGATCGCGCCATTGCACAGCCTGGGCAACGAGCGA
AACTCCATCTCAAAAAAATAAATAAA

ID|56586|gene_id|ENSG00000254519|transcript_id|ENST00000530051 Homo sapiens lncRNA

ATCACAGGCAGGGGGAATGGGGAGGGGACGGTAGCGTCAGCCTACACCCACACGCAGGT
TAGAGCCTTGGACGGGGAAGCGGAAAAGGCTTAGCAGCTACTAATGGGTTCTATTAATAGTAGT
AGTGTTAGGTAGGCTGCACTGGCTGTATTCTGCCCCCATGCCTATCATTCCCTCACTCTGTGACC
CTGGGCGAGTCTCTTCCTCATCCTGGTGCTCAGTTGCCTTTTCATAAAGAAAGGGTTGATTCCCC
ATGATCTGTTGACTCCTTTGCTTTTGGATATTTTGGAAGACGCCCTGCGTCACGGGCTAGTTAATT
GTGAACGTCAGGGAAAGCACCAGAGTAGAGAAGCACTTCCAGGATCCAGAGCCAGGAGGATG
CTCAAAAGGAAGGGAGGAGAGATGCCCTTCGGCCAAAGGGAGAGCTTGGAGGGTGAGGGGA
AGTGGTTGCTTACCAGAAGGGCCTCGGAGTGTGAACTCAAAAGATAGAGGATGGCCGAGCGTG
GAGGCTCATGCCTGTAATTTCAGCACTTTGGGAGGCCGAGGCGGGCAGATCACTTGAGGACAG
GAGTTCGAGACCAGCCTGGCCAATATGACGAGACCCCATCTCTACTAAAAATACAAAAATTAGT
CAGGCGTCATAGCAGGCACCTGTAATCCCAGCTACTTGGCTACTTGGGAGGCTGAGGCATGAG
AATCACTTGAACCTGGGAGGTGGAGCTGCAGTGAGCCGAGATTGCACCACTGCACTCCAGCCT
GGGCGACACAGTGAGACCCTGTCTTAAAAAAATAATAATAATAGAGGAAGCGTTTTGGTCCACT
GTCCACTCACACAGGGTTAGGCGGAGAGGAAAAAGCTACTGAGACTGAAGAATGCCCTTTAAC
CCCACTTACTGGTTGTTTTGATTCTTTCCTGTAGTTTCCTGATAAAAGGTCCTTTGGAAACTTTGAT
TCTCTTCTCACCAAGTTGAACGGAGATGTGTTCCCATAGTAAGAGTGAGGCCCCTGCATTTGCTG
CCCTCGGTTTTCCCAACTAACACCAGGGCCTCCCCCCTGTACCCCATTCATATGCCTCAGAAGCT
GACAATGACTGTTGGGCTTTTCCAATGAATACCAGAACACCATTTCCTGCAGCCTGAAAAGAAG
ACAGTATAATTGTTTGGCAGCAAGAGGTGAGCATGGGTAAGGAGGGCGGAACTCTTTCACTCAT
ACCCACGCCTCCTCCTCTCTCCTTTCCTTTTCCCCATATTCTTTCTCCCCAGCCCTTCCCTCCTCCC
ACCTCCTGACTCTAGCCCAGGCCAGCGAAATTTTTTCCACTTTTCACTCCCGCCTGAGAATTTCC
GGGGGCCTGAAAGAAGACCTGTTCCCTAGCACTAGTTCATCGTCTGCTCCACTGAGAAATAACC
AGCTACCAGAAAGTTTGAGAAATTGATTTGCAGAAAGAGGGCACCAAATCAGTTTAGAGTCAG
GGTCTGTAGTCAGTAGAGTTGGCAAATGCCATTTCCCTGACCCACTGGGTATCTGAATACTTGTC
ATTGGCCCCTTGAGAGCTCATCAACTGTGGATGTGCAATAAGCTTGTCCAGGACCAAAAGTGTCT
CTTGCTGCTTAAATATCCACCAAAAAGATTCCACTTGTCAAAGCACCTGCCTCCCTGCACTGACT
TCTTTTACCTTCTGTCTCTTTTTTCTCACTTTCACACTGTTCTCTTTTTCTCTTTTCCTGTGCCCTTCT
CTCTCTCTGGTTTGATTTCTTTTTTCCAGGCCAAGCAGGAAACTAGAGATATGGTGACGGTAGTG
GTCCCAGGGAGGAGCTTTAACCTGAGCACCACCCACAGAAGGCAGACCACCCTCCCAGCCGC

AGTGCAAATGAATGTTGGCCCCCACCCAGGTCCCCAAGTGCTTTCCCAGTCACGCCCCAGCTGC
CAGGTTTTTTTCTCCAGGCCTACTACAGATGGGTTACCCTGACTCCACCCCCAACACCCTCCAAT
GATTAAAACCACTGACTCCTCCCTCCCATGCCTGCCTCCATGGCAGGCCCCTGCAGAAACCAAA
CATAGTCACCTTAGACTCTGGCATACTGAGACCAGGGCTTGATCCTGGCCCCCTTGGCCTCCCT
GGCAATTTATCATTTAATAAAAATATTGTTAAAAAA

ID|56614|gene_id|ENSG00000255135|transcript_id|ENST00000530759 Homo sapiens lncRNA

CCGTCGAGGATTAGGATCGAGTTTCTTCGATCGTGAGGGCAATTATTAGTGGCCTCTAGCC
CCCCAGATGGCATCTTGAGGAGAGAGAATCCACGCTACGAGAACTAACATAGTGGACATCGCA
GTTGTCCGTAGCCGATTTGCGGGTTACGGAGTTGTTGCAAGTAAAGAAGGGGAAATCCGAGCG
TTCTCGCGTTGGATTTCCTCCACGTGTGAAGTGGGAATGGTAGTGATATCTCGTGCTATCTCTGT
CAAGGGCAAGAAGGAGAAGGAATTTATTTGAGGCCCTACGACGTGGTAGGTTCACCCTCAGCC
TTTGTTCTTATTTCATCTTATTTGTGGAGAAGGTCTTTTCGTTCCTATTTTACAGGTGAGAAAAGGG
GCCCAATATATAGTTATTTGCGGCCATAAAATAGAAGCCAGTTGCCTGATACTGAAGATTCAAT
GAAATCTTGTGATTGACTTCCCTCTTTAAATTTTGTACTTTGGGTAACATCGGATCCGGTAGAAAC
GCTCGTCAGAAAGAGTTTTTAATGTGCATTCTACAATTTCCAGACATAGTTTCCTTCTTGAAAAAT
TGGGTTAATACTACCCTATAGGGATCTTGTGATGCACCACGTAAACGTTGTAAGTTGTGAACCAC
TATACAAACGTATGAGACTCTTCATGTTCTTGAGTGGAACTGTAGGTTAGGAAGAGAAGGTAAA
TAACAGCATTTAAGTTTCCTACTACTATTTGCCCATTAGCTAAAAATACCCTCCGAGGTCTAGGA
AAGTCACTGTTGTTGAAACCCAACCTACACCTTCAGAAAATCGTAAAGTGGAACATATTAATCA
CCAGGCCCTCCCTCCGCTGTCTCCCTGACCTTCCTATGCATCCAGTTGCTTAAGAACTCTTGGAA
AGATAGTAAGTGAAATCTTCATAGAGGATTTTTGTACTTTAACATATATTATTTGATTTGAGCCTC
GCAACATTTCAGTGTGACAGGTACGATTAAGGTCTTTCATACTATGAATGAGAAAAACATAGAT
GTACCAAGGACTCACCAAATTTCACGGAGCTAGGAATTGGAAGAGAGCTAGGCTTTGTAATCCC
AGGTCTTCTGACCTCCAAATGTCCTTTCCTCTACAGTGAATGTCAAAATGACAATGAATGTCTAC
TCCTCTACAATGGAGTACAAATCGAAAGACAAGGGGCTTATAAAACTGACTCTGACCTTAAGAA
GTAGCATATGAGTGATGAATTATTATAGTCAATCCTTGTGCTTACTAAGTGCCACATACTGGTCT
AAGCATTTTACATAGTAATTCAGTCCTTAGAACAACTTTATGAGGTGGAAACTTTTATTATCTCTA
ATTACAAAGGAGGCAACTAAAGTCTAGGAAAGAAGCAAAATAGCTTGCCTAAAGTCACACATA
AGTAAATGGTGGCTCACGCCTGTAAACCCAGCACTTTGGGAGGCTGAGGCAAGTGGATCATTTG
AGGTCAGGAGTTCAAGACCAGCCTGGCCAACATGGTGAAAGCCCACCTCTACTAAAAATACAA
AAATTAGCTGGGCATGGTGGCATGTGCCTGTAATCCCAGCTACTCGGGAGGCTGAGACAGGAG
AATTGCTTGAACCAGGGAGGTGGAGGTTGCAGTGAGCCAAGATCGCTCTACTGCATTCCAGCGT
GGGCAACAGAGCAAGACTCCATCTTAAAAAAAAAAAAAAAAAAAAAAGCTAAAGCTGGGATTT
AAACCTGATTATGTTGGCTCCAGAGCCTATGCTCTAGACCAGTGGTTCTAAACTTTAATGGTCAA
TAGGAACCGCTTGAGGATCTTGTTAAAACGCAAATATTGGTAGTATAGATATCAGGTAAGGCCT
GGATCCTATAGTCTTAACAGGCCCCCAGGTGATGTTGATGGTGCTGGTCATTACATACTACAGTT
CACATCTTCCCTTTTCTCCTAACCGTATTTATTTTGCAGTCAGAAACTACCTTTCCCATGCTGCCT
GTGACTGCTTTATAGTCTCAAATGTTCCCAACATGCTCTTCCTTAAGGATTAGACTCAAACTTTTT
CCTCAAAAAGCTTATCCTGCCCCTGTCTTTCCCTCTAGGATAAGACAGACATCCCTGCCTCTGCT
CCCCTACCACTCAGTGTACACCTTGTTACAGAGCTCAGAACACTGAATTATGGCCATTTATTTGA
AAATCTTCCTGGCCGGGCATGGTGGCTCATGCCTGTATTCCCAGTACTTTGGGAGGCCGAGGTG
GGCTGATCACTTGAGGTCAGGAGTTCAAGACCAGCTTGGCCAACATGGTGAAATCCCGTCTATA
CTAAAGATAGAAAAATTAGCCGGTCATGGTGTTGTGCATCTGTAGTCCTAGGTACTCTACTCGA

GAGGCTGAGGCAGGAGAATCTCTTGAACCCAGGAGGTGGAGGTTGCAGTGAGCTGAGATTGCG
CAACTGCACTTCAGCCTGGGCGACAGAGCAAGACTCTGTCTCAAAAAAAAAGAAAAGAAAATC
TTCCTGACTGGACAACGAGCTCTTTGAGGAGAGGAACCAATCAGGTCTGTGTACTGTAGAGCCT
AGCATATAATAAGAATAATAGCTAATACTTACATAGGACTTTCTGTATTCCAGATCACGTTATAC
ACATTAACTTGTTTAATCTCTACTATAACCTTACAGATACACTTTTATCCCCATTTTATGAATGAAG
AAACTAAGGCATAGAGAGGTTAAATAGCTGGCCCAAGGTAACAAGCTTGTAAGTAACAGAGCC
ATGATTTTAAATAAAAGCACTTTGGCTCCAGGAAATGTGTGGTGAATTAAATGAGAGCCACTCTA
CACTCAACCAAAGAAGGAAAACTGAGACTTACTATCCTATTAGACATGATTATAACTACCTCTAG
TCTGGGAAAATACACAGAAATCTGTTCCCCTGGCCAGGTGCGGTGGCTCACGCCTGTAATCCCA
ACACTTTGGGAGGCCGAGGCAGGCGGATCATCTGAGATCGAGAGTTCGAGACCAGCTTGACCA
ACATGGAGAAACCCCGTCTCTACTAAAAGTACAAAATTAGCCAGGCATGGTGGCACATGCCCG
TAATCCCAGCTACTCGGGAGGCTGAGGCAGGAGAGTAGCTTGAGCCCAGGAGGCGGAGGTTG
TGGTGAGCCGAGATCGCGCCATTGCACAGCCTGGGCAACGAGCGAAACTCCATCTCAAAAAAA
TAAATAAAAATTTTTTTAAAAAAG

ID|55977|gene_id|ENSG00000230918|transcript_id|ENST00000418335 Homo sapiens lncRNA

AACAACAACTGGGAAGCCTTCTCAGCTACACTTCGGGGCACTGGTCCAACCCCACGCAAA
ATCCCTCGTTTCCCTTAGCGTGGTAAGACGGAGCCTGACCTGAGCTCCAACTGTCCTATCTTTTT
CAAATGTTTCAAACTTACTGCCTTTGTTCAGCAGAACCACGGGCACGGTGATGATGGTGACAAG
CGCAGCAGCACCCAGCAGTCCCAGAAGAACCTTCCACGGTGTCTGCAAGCCGAGCAGATCAAG
TCCAATTAGAGGGAAGCGTGTGGCCCCAGTTTCCGTAGGAGGGTCGGGGCTGCTCCAGAGGCA
GCAGGATTTGCAGGTGGGAGTGCGTTAGAAGAGGGAGACCGCGGGCTGGGGGTGGGGGTGGC
GTCTGGAGTGCGCCAGTTGGAGTTCTCTAAGGCGGGTGCCCTTGAACTTGTGCCTTCAGAGCAC
ATTAGCGTTGGTTTCTCTACCCCTGCCCGGGTTCGGGCGTGCGTTCTGTGAGTGGCTCTCCGGG
ACATTCAAAGCTCGACGCCAGGGTCCTAGCAGAAGCCAGGGTCCGAAAGCTAAGCGAGAGCT
CTGGGACGTCCCTTCACCTGTCAGAGGGTGGCCTTGGGGCTTCCGCCTAAGGGGAGTCCCTGG
TCCGGTTTCGCCAGCTTTTGGGCCATTTGGGGAGTTTGGCGAAGAGGTCCCCACAGCTCGCCCC
GGGGACGTACGTGGCGCGGCACTCACCTTCATCGTCGGCGTCTCCTCGGAAGTGAGCGTTCAG
AGAAGGAGCGCAGGCAGAAGTCACCGCGGGCGGCGGAGACGCGCGTCCTGCACCGCTGCTCC
GGGCGGTGGAGTCACTCGCCGCTGGCAAGTTTCGGCCCCGAGTTAAACATTAGTGAGCGCCGA
GCCCGCTGGGTATAAAGGCGCCGCGGGCAGGCTGCAGGGCAGGCGGCGCGGGAGCAGGCGC
GCGTGGCGCGGGGCACTGGCATCCCGGCCGGGGGGAGCCCGCGAGGGCCCCCTGAGGGCGG
TGTAGGGCGCTGGGCGGCAGCCGGGGCGCAGAGTGCGGGGCCCGGAGGAGCCGTGGGGGAG
GGGAAAGGGCGCGCGGCCTCGGATGCGCAGACCCTGGGCCGGCGACTCGGGGACCCTGCTCC
CTCTTAGCTAAAAATGACGTCGGCGTTCAGCTCCTCCAACCTCACGTGGACAGGCGAGGGAAC
CGAGACCCAGAGAGGGCAGGGGACTTTGGCAAACTCACACAGCCCACCGCAGGCAACTGGAA
CTGAAACCCAGGACTCCGTCTCTTGCCAGTGAAAGTTATGTTAGGAAGCAGTGAGGGGTCTAAA
GCAGTATGAAAGGCAAAGAGAAAAGGTGATTGTTCCCTCTTGAATGGCCCTTGGAAGCTGAGTA
TCTGGATTCACCCTCCCTAGGGAATTTCCCGATTGTCTTGCAGGCTTACACACTCATCAAGATGA
CAAAAATAATGACAGTAACACTTATGTGGAACTTGACTTTTTCCCAGGTGCTGCTCTAAGCATTT
ACTGTGTTTGTTTTACAGGAAGGAAGACTGTACACAGAGAATAAATAACTTGGCCAAGCCATTC
AGCTAGGAAGTTGTAGATCCTAAATTAAGAGTTCAAGGTCTTAATGGCTACTCTATGCGGCCTCT
CATAGTCTTTTCAAGGGTTTTGGAGAAGAATAAAAGATCAGGTATGGCTTCTCCCTCCCCCAGCT
CTCTATTGTTCCCTAAAGGATTATTCATTCGTTCATTCATTCCTACATCCTCCCATTTATTCCAGTC

ATCCACAGATGTTTGAGTGGTTACAATGTGCCAGCCTCCCTTCTAGGTACTGGAGATAAAGTGG
GACAAGAGAGGTGAGGTCTGGACCCTTGGCACTTTCACGATAAAATACACAGGTAAACATTTGC
CTTTAGGGCCCCCTG

ID|55981|gene_id|ENSG00000228384|transcript_id|ENST00000416229 Homo sapiens lncRNA

GCCGGGCTGCCAGGCTCGACAGGAGGGGCAGGATCAGGCGGGAAGGGGTTGGGGCGGC
GAAGGCCGGGAGCGCCGGGCAGTGGTCCCCCGAGTGGCTCCGAAACCCCCGCGATGCGACCC
CCAACGAGCACCGATGCCGGGGCGGGAGCCCGCGCGGGCCGGGGTCGTGCAGTCTCACCGAC
AGCCAGCGTGATGAAGGCCACCTGGATGAAGAGCGACAGCCAGCTCAGCAGGTACATGAACC
ACATGGCGCCCCCACCCGCCCGCTCCCCGGCCACCGGGACGGGCCTGCGCGCTTCGGCTCCG
GCGACACACAGCCGCCACCGCCGCCGCCGCCGCGTCCCCGCCCCGCGGACGACAGGAC
GACGGTGCGCCGCCGCCGAGAGGAGCCCGGCCCTAGCGCTGCCCGGGGCCAGGCTCGCGCTA
GCGCCGCCCGCCGGAGAGGAGGGCCAACCGCCGCTGCCCCGCCAGGATCCCCCGCTCCACTG
AGGGGAATGAATGCAGACCGCTGCAGCCAGAGGGAGGCGGGACGGTGCCCAGGGGCGGAGA
CGACCAAGCGCCCATACTGGGGCTCGGAGTGCAGCCTCCAGTCCCTCCCTGTGCACTCCCGTA
CCAGCCTGGAGGTGGTCCAGGGCCGGACAGGGCCGCCCGACCGCGCCCCAGCAACCTCCCCA
GAACCTGCCTCCCGGCAGGTTGCGGACAAGTCTGTCTCTTGCTATCGCAGGTGATAGGGACAA
AATTATTGAATTCCTAATATGTGCCACGCTCTGTGATAAAGGCGAGGTGTGTTCTTGTCGGAAAT
TTGAGCAGATAATGTAAATCATTTTATAGACGGGGAAGCCGGGGCCAAAGGAAGTTGCCCTGG
GTCATGCACCTAGGAAGCGGCACAATCAGGAGTTGGGGTGATTTTAATCGGTCATCCTCCGGCC
TAATTCCCCTCTAGGCTGTTCTTTTGGGGTACATTCTGCACCTTTGTATCGCCTGCACCTCACCCC
TTGCCGGGCACGCAGGGCGTCCGCTAGGCGCTGGATGCATTTACTACACCTCTGGGAGGGTTC
CTGAGGGGCACCTGGAACCGGTTAGTCCTTGTCAACAAGTCTCCCATCTAGTGGCGGAGACAG
GCATGCAAAATAAAGTATGTAGAACAAGAGTCTAGTAGGCTTATTTCAGAATCATATTTTTCCTG
CATAAAAATACGTATTCCTAAAAAAAACAATTACCGTATGATTTAGCAATTCTACTTCTGGGTAT
ATACCCAAAAGAACTGAGAAAGCAGAGACATTTGTATACCAATATTCATAGCATTATTCACAAT
GGTCAGAAGAGGGAAGCAACCCAAATGTCCATCGGTTGACGAAAAGATAGACATAAAGTGCTA
TAAACATACAATGGAATATTATCTAGCCTTAAAATGAAGTGAAATTCTGACACATGCTACAACAT
CAATGAACCTTGAAGGCATGATGCTAAGTGAAATAAGCCAAATAGGGAAGGACACACATTGTA
TGATTCCCCTCATATGAGGTACCCAGAGTAGTCAAAATCATGGGCAGAAAGTTAAATGGTGCTT
GCCAGGGTCTGGGGGACAAAGAATGGGGAGTTACTTTTTAATGGATACAGGGTTTCAGTTTGGG
AAGATGGATGGTGCTGATGGCATCACAACAGTGTGAATGTGCTTAATGTCACTGAATTGTATAC
AGTGTTACAGAATTGTAAAATGGTTAAAACGGTCAATTTTATGTTATGCATAGCTTCCCCCTCCC
CCCAACAAAATACATAAGATTACAAAGCCCATCCTGGCTAACACGGTGAAACCCAGTCTCTACT
AAAAATACAAAAAATTAGCCGGGCTTGGTGGCGGGCGCCTGTAGTCCCAGCTACTCAAGAAAC
TGAGGCAGGAGAAGAGCGTGAACCCGGGAGGCAGAAGTTGCAGTGAGCCGAGATTGCGCCAC
TGCACTCCAGCCTGGGCGACAGAGCGAGACTCCGTCTCAAGAAAAAAAAAAAAAAAAAAGATTA
CAAAGCAGGCTGGGCACAGTGGCTCATGCCTGTAATCCCAGTACTTTGGGAGGCTGAGGTAGG
AGGATCACTTGAGGCCAGGAGTTCAAGACCAGCCTGGGCAAAGAAAAATTTTAAAATGGCCAG
GCATGGTGGTGCGTACCTGTAGTCGCAGTTACTCTGGGCTGAGGCAGGAGGATTACTTGAGTCC
AGGAGATGGCGGCTGCAGTGAGCTATGATTGGGCCACTGCATTCCAGCTGGGATGACAGAATG
AAAACTTGTCTCAAAAAAAAAAAAAAATTGCAGTTGCCGGGCGCGGTGGCTCACGCCTGTAATCC
CAGCACTTTGGGAGGCCGAGGCGGGAGGATCACGAGGTCAGGAGATTGAGACCATCCTGGCT
AACACGGTGAAACTCTGTCTCCACTAAAAAATACAAAAAAAAAAAATTAGCCGGGCATGGTGGC

GGGCGCCTGTAGTCCCAGCTACTCAGGAGGCTGAGGCAGGAGAATGGCGTGAACCCGGGAGG
CGGAGCTTGCAGTGAGCCGAGATCGCGTCACTGCACTCCAGCCTGGGGGACAGAGGGAGACT
CCGTCTCAAAACAAAAACAAAAACAAAAATAAAACAAAAAAGATTACAAAGCAAACCAATTAT
ATAGAAATGCAAGTATTAAACTATTTTTAGAAAACCAAATGTATTCTGTAATTTATGTGCTTCTCT
ATTAATATATGACATAAGCAGACCTATTGCAAAATCTAATAGCTACCTACCACAATGACAAAGTA
GGAGTGAGGGTAAATGATATTTCAAGATATCATTAATCATATGAGAGAGTTATAGATAAAAATA
GAAACTTTTCTCCTTCCAAGTTTCCAGGCGTCTTAAAATTGTCTCCCTAAATTCTATACCTGCTGA
CTTCTAAGGGGTCCCTGGACCCCCAGTTAAGAATACCTGAGAGATATGTATATGGGGCATGGTG
GGGTGGCTCACGCCTATAATCCTAGCACTTTGGGAGGCCTAGGCAGGAGAATTACTTGAGCCTA
GGAGTTCAAGATTGACCTAGACAACAAAGTGAGACCCCATCTCTACAAAAAATTTAGAAGTTAT
CCAGGTGCAATGGTGCCGGTAGTCCTGGCTACTCAGGAGTCTGAGGTGGGAGATCGCTTGAGC
CTGGGAGTTCAAGGTTGCAGTGAACCAGGATCACACCACTGCACTCCAGCCTGGGTGACAGAG
TGAGACCTTAAAGGGCTATATCCTTTTTTTTTTTTTTTTTGAGATGGAGTCTTGTTCTGTTGCCCAG
GCTGGAGTGCAGTGGCACGATTTCAGCTCACTGCAACCTCTGCCTCCTGGGTTCAACCGATTCT
CCTGCCCCAGCCTCCTGAGCAGTTGGGATTACAGGTGCCCACCACCACATCCAGCTAATTTTTG
TATTTTTGTAGAGATGGGATTTCGCCATGTTGGCGAGGCTGGTCTCAAACTCCTGACCTCAAGTA
ACCCACACACCTTGGCCTCCCAAAGTACTGGGATTACAGTTATGAGCCACTGCACCCGGCCTCA
CCATATGCTTATTTACAAGGCTTTTCATTGCAGCACTGTAAGAGACTGAAAACAACCTAAGTGTT
CATTGATAGGAAACTGGTTAAGTATATTTAGCCCTAAAAGCCAATGAGGGTGTTCTTTATGTACC
TATATAGGACTATTTTTGAAAAAAAGCAAAAGAATGGGGAAAAGAATCTATATACACATTTTTTG
CAGGGAGGGACAGGGTCTCACTCTGTCACCCAGGCTGGAGCACAGTGACAAGATCATGGCTCA
CTGCACCCTCAACCCACAGGGCTCAAGACATCCTCCCACTTCAGCCTCTCAAGTAGCTGGGACT
ACAAGTGCATGCCATAACACCTGGCTGATTTTTTTATTTTTATTTTTTGTACAGATGAGGTCTCATT
ATGCTGTCCAGGCTGGTCTTGAACTCCTGGGCTCAAGCAATCCTCCTGCCACAGCCTCCCAAAG
TAGTACTTTGGTAATACAGGCATGAGCCACGTCACCCAGGCTACACATTTCTTTCTATAAAATAT
CTCTGGAAGGATACGTTAGAAAACAGTAATATTGCTTGCTTCCTAGGAAGAGAAGGGGTGGGGT
TTCAGAGTAGGAAACAGCATTCCCACTGTATGTCTTTTTATATCCTTAAAATATTGAACTATGTAA
AGATATGGAAAGGTGTTACCTATTCATAAATGAAGTTTACAATTAAAATGTTCATAATATGATGG
GCCTGCTAGGATGTCTCCAATTTCCTGTTGTTACCCAGTCCCTATGTGAGGAAAAACCATTGAGT
TGTGCCTTACTGGTAAGTTTGCTAACCTTAGGTCATGGGACTTGGTCATCCCTGATTGTTCCAATA
GGGGAGCTTCATGTCCAGATCTTTTCTATTGAGGAGTTTGTATTGGAGACACGGAAGCATCAGT
GAGTAACGGCACAGTAAAGTATCTCTCTCTCACTCTCTCTCTCTCTCTGTCTCTCTCATTTATTT
ACTTATTTGACATGGAGTCTTGCTCTGTTGCCCAGGCTGGAGTGCAGTGGCACAATCTCAGCTCA
CTGCAACCTCCGCTTCCCGGGTTCAAGCAATTCTCCTGCCTCAGCCTCCCAAGCAGCTGGTATTA
CAGGCATTCACCACCATGCCCAGATAATTTTTATATTTTTGTAGAGATGGAGTTTTGTCATGATGG
CCAGGCTGGCCTCAAGTGATCCACCTGCCTCAGCCTCCCAAAGTGCTGGGATTACAAGCATGA
GACACCACACCCAACCAGTAAAGTCTCAATTGGTATTTTTTTTTCCCTAAACATCTTCCCAGGGA
AGGAGGAAACTATAAAAATATCCCCCATCCCTCTCCAGAATTGTAGATAAGGGAAACTGGAGG
CCCCAAAAGGTTAAGTGCTTTCACCTTTGAACCAGCCTTGTTTTTCCAGGGCTGGGCACTTTCAC
TTCCCACAGTTTTAAAGCCAAAAGTAGCCTGAGAGAGGTAAGGCTGAGGATTCAAATTTCCTCC
GATGGCAGGAAGAGAGAGGCCACCATTTCCTGAGAGTGGTGAGAAGTCAGGTCCACCTGACCC
AACAGGACAGGGCGGGGCTCAGGGAGGATCCTTAGGCTGTGTTTGGGGAAGGAGTTACAAGG
GGAGCAAACCCAGGCCTTGCCTGAAGGAGGGAAGGAAGGATAGCAGGTCCCCACACTCAATT
CAAGGGACTCATTTCAAGAGTGAAAGGGTTTGGAGGTTCAAAGGTGACTGACCAGTTTGTTGAT

TTAAATATTGACAGGTGGCCGGGCATGGTGGCTCACATCTGTAATCCCAGCAATTTGGGCACTG
AAGCAGGAGGATTGCTTGAGCCCAGGAGTTCAAGACTAGCCTGGGAAACATAGTGGGACCCCA
TCTCTACAAGAAATAAAAAAAATTAGCTGGGCATGGTGGCACACACTTGTGGTCCTAGCTACTT
GAGAGGTGCAGGTGGGAGGATCCCTTGAGCCCAGGGGGTTGAACCTGCAATAAGCCATGTTTG
CACCACTGCCCTCCAGCCTGAGTGACAGAGTGAGACCTTGTCTCCAAAAAACCCCACAAGGGC
CGGGCACGATAGCTCATTCCTGTAATACCAGCACTTTGAGAGGCCGAGGCGGGTGGATCACCT
GAGGTCGGGAGTTCGAGACCAGCCTGACCAACATGGAGAAACCCCCGTCTCTACTAAAAATTA
AAAAAATTAGCCGGCTTGGTGGCGCATGCCTGTAATCCCAGGTACTCGGGAGGCTGAGGCAGG
AGAATCACTTGAACCCAGGAGGTAGAGGTTGAGGTGAGCCAAGATCGCGCCATTGCACTACAG
CCTGGGCAACAAGAGCGAAACTCCATCTCAAGGGAAAAAAAAGAAAAAGAGAGAAAAAAAAA
CCACACACAAATGTTACCATTTAATGTTTGTAGGGTGAAGGACCAACTCCGTATATTTCATGAGA
GGGTTCTAGGGGAAACTAGTTATTTCAACATAATAGGTGTCTCTGCTTCAGAAAACAAAGACCA
AATTTCTTTTCAGGTTTTTGGCTGAGATGAAGTGCAGAAAGCAATAATTGCTAGCATTTATAGTGT
TCTTTCTCTGTGTCAGACACTGCTCTATGTGATTTGTATGTACTAACCTATTTAATACACACATCTA
GCACAGGCGATACTATTACTGTCTCCATCACAGCTAAGAAACCAAGGCACGGAGAGGTTCAAT
ATCTTGTCCAAAGTCATACCACTAATATTTTAAGCAGCCAGGGGTCAAACCGGGGCAGGCTGGC
TCCGGAGTCCACATGGTTTTCTCTGGTCTTTCTGAGTTGAGTCACCTGACTTTGCAGTTAGGGTG
ATTCTGCCTGCCTTGTCATCAGGTGCCATTTGGCCAGTGGGCTGGGTCCTGCCTCATCTCCCTGC
AGGCTGCCTGGTCCCCCGGGCTTTTCCTCTTCTCCCCTGCCTTTATCCTTGCAGGAACAAATGAA
TACACCTCAGGGTTCTGGCTTTGACCACCCCTGGTCCCCTCCACTGTCTTCACCCACTCCCCTTC
TCATTCTAACAGACTTACACATTCACTATGCCTGAGGCCAAGGACATCCTTAGTAGGATCTGTGG
TCCCAGTGGTCCACCCACACGCTGGGGACCTGCACATCCCCAGGGGCTAGGAAGGTGCGGGG
AGGTGGAAGTAGGAGGCGGTACGGCCGGAAGTCGGCCGCCAAAGTTCCCCTTGCCGGGCTGT
GTTGGCTGCTGCCCTCTAGTGGCGACCAGAGGAGCTGGCGAGGACCGAAGCCAGGTTCATCGC
TTATGGAAAGCAGTTTTGAGAGACAGTGGGGACCCCACTACCCGACTCATGAAGGCCAGGTGG
GCAGGCCCCGAGAGTACACACAGCCGACACCCACCCACCACACACACACAGTACACAAAATT
GTAGACAGGGTCCACCTCGGCATCAGGGCTCCTGCTGCCCCGGCATGGTCTGGCTACAGACGA
TGGTTGTTGTGTGAGAGACTGTGAGAGACACTGTGAAATCAGACATGGCAGAGACAGGCCTGG
AGGCCCAGGGCCCTCCCTCTGCCTAGTCTTCTCCTCCCCCGGTGTGGCTCTTACCTGGACCTGA
ACTCCTTCCCCACCAGCTCCCCAGGCCTCCCAGGAGCACAGCTTTGGGGTAGGAAGAGCTGAG
GAGAGAGGTTACCGCCCGCCCCACTGCACCCTAACACATGCCATCCACCACACGATGTCCCCT
CTTTAAGGTGCCACATGCTGGGGGAAATTTTTCCTGTGATGGCCCCTGTAGTTGGCAGGTGTGTT
GCTTGGCACCCCACCTCTAAGATCTTGGTTCTGCAGCCGGCTAGCAAGACCAAAATTCTAGTGA
GAGACAAACTACAATGAAAAACAATGACCCCTGCTATGTCCACATAATCTATGTGTCTAGGGAA
GCTAGGCCTCCCAACCTGGTTTCTACACGGTGCCTGTGCCTCACCCAAGCAGCTGCCACACAGT
CCTGGCACCCCCCCATCCCTGTCACATGCCTGCGGCCCCAACTCAGGTGGCTGGATTAAAGAG
CTTCCTAGTGACAAGTCCTCAAAGGGCACTATTAATTGCCTCAGCATTGTCTTCAGCTAAACCAC
TAGAGGATAAAGGGCAGGGGGTGGGGCGGGGGTGAAGGGGTGGGGCTGGGGAACGACAAGA
AGGAACGGGTGGGGAGGGAAGCACACACACACATGCACACACACACACACATGCATGCACAC
ACATGCAGCAGAGCCCCTGAGCAGGGCTGCTCTAGTCCCATAGCCTGGGGCGCAGGGAGGCG
GGCAGCTGGCTGATGAAAGTCCAGCCTCCCTGGGCTAGCAGCTCCTCTCCTGAGGCAGCCCCA
TTAGCCAGGACAGCAGAAAGCCATTCACGGTCACTCCTGTCATTAGCATAACGCGGATCACTGG
AATCTCGGCTGAAGTGTCTCTCTGTCTTTATCCAAGCAGCTCCAGGGGGTCCTCCCATGACAGG
AATTCCTGATGAGAAGAAAAGGTGCAGCTCTCTCTGACAAGCTGGCTCCTCTCCTCAGAAAAAA

GAAAGAAACAAAGAGAAGAGGATGACATTGAATGTATCAGAGAACTAAGAAACTTCTGCCAGC
CTGAGCAACTTCTCCAGCCAGGGCGACAGAGCAAGACCATGTCTCAAAAAAACAAACAAATGA
AAAAAGAAATTTCTGGATGAGGAGGATGC

ID|56695|gene_id|ENSG00000228384|transcript_id|ENST00000601923 Homo sapiens lncRNA

CTCGAGTCACCCTTTCTCGGGCAGTGCCGGAGCGGACTCTGTGACACCTGGAAGGAGCCA
CGGTCAGGCCGGGCTGCCAGGCTCGACAGGAGGGGCAGGATCAGGCGGGAAGGGGTTGGGG
CGGCGAAGGCCGGGAGCGCCGGGCAGTGGTCCCCCGAGTGGCTCCGAAACCCCCGCGATGCG
ACCCCCAACGAGCACCGATGCCGGGGCGGGAGCCCGCGCGGGCCGGGGTCGTGCAGTCTCAC
CGACAGCCAGCGTGATGAAGGCCACCTGGATGAAGAGCGACAGCCAGCTCAGCAGGTACATG
AACCACATGGCGCCCCCACCCGCCCGCTCCCCGGCCACCGGGACGGGCCTGCGCGCTTCGGC
TCCGGCGACACACAGCCGCCACCGCCGCCGCCGCCGCCGCGTCCCCGCCCCGCGGACGACAG
GACGACGGTGCGCCGCCGCCGAGAGGAGCCCGGCCCTAGCGCTGCCCGGGGCCAGGCTCGC
GCTAGCGCCGCCCGCCGGAGAGGAGGGCCAACCGCCGCTGCCCCGCCAGGATCCCCCGCTCC
ACTGAGGGGAATGAATGCAGACCGCTGCAGCCAGAGGGAGGCGGGACGGTGCCCAGGGGCG
GAGACGACCAAGCGCCCATACTGGGGCTCGGAGTGCAGCCTCCAGTCCCTCCCTGTGCACTCC
CGTACCAGCCTGGAGGTGGTCCAGGGCCGGACAGGGCCGCCCGACCGCGCCCCAGCAACCTC
CCCAGAACCTGCCTCCCGGCAGGTTGCGGACAAGTCTGTCTCTTGCTATCGCAGGTGATAGGGA
CAAAATTATTGAATTCCTAATATGTGCCACGCTCTGTGATAAAGGCGAGGTGTGTTCTTGTCGGA
AATTTGAGCAGATAATGTAAATCATTTTATAGACGGGGAAGCCGGGGCCAAAGGAAGTTGCCCT
GGGTCATGCACCTAGGAAGCGGCACAATCAGGAGTTGGGGTGATTTTAATCGGTCATCCTCCG
GCCTAATTCCCCTCTAGGCTGTTCTTTTGGGGTACATTCTGCACCTTTGTATCGCCTGCACCTCAC
CCCTTGCCGGGCACGCAGGGCGTCCGCTAGGCGCTGGATGCATTTACTACACCTCTGGGAGGG
TTCCTGAGGGGCACCTGGAACCGGTTAGTCCTTGTCAACAAGTCTCCCATCTAGTGGCGGAGAC
AGGCATGCAAAATAAAGTATGT

ID|56243|gene_id|ENSG00000248839|transcript_id|ENST00000502999 Homo sapiens lncRNA

GGTTCGTCCAAGCCGGACGCCTGCAGCAGCCACCTCCTGGGCCTCACGGCGGGGCCGGA
GAAGGCCAGGGCTGGGGCGGGGCAGCCGTGCTCGGCCCCGGGCCAGGGTCCCCCGGTACCC
GACCAGGCCCCGCCCTAGAGGGCTCGGCCCTGGGACAAATCCGCCGCTCGGAACCCGCCCAG
CCCGACCACGCCCGGGAAGGGGGCCCCTCTTCAAACCGCCGGGAACATGGAACCGCGACGCG
ACCCCTGCCCGGCGACGCAGGTCCCGCTCACTCACCGCCCATCCTCCTGCTCCGCCAGCTTCAC
CCTCTAGCTCAGACCACAGCACCCTACTCTCCCCGCGCCTCCTCTGACGCACTTCCCTGCAGAG
CCCGCCCCCTCCATAACTCCCGCGCCTCCGCCCGCCCCCACTCCGGGCCGGGCCGCGGCCCGG
GGCCGAGGGGCCTTGTGGGAGTTGTAGGAGGGACAGACTGAAAGCTGTCTGAGGCGGCGATA
GGCATTCCTGGCGCCGCGCGCGGCGCATTGTGGGAGATGTAGTTCAGCTCCTCAGGGCAAGCC
GGTACGCAGAAGGGAAGTCTAGGCTGTTGGATTCTTGCATTTTAGGGAGGAAGGGAGAATGGT
CCAGAAAGTAAAGCGGGCCTGTTTCCGGTCATCTCGAGTCAATGACCGTGACTTGTATAACTTA
GATCTGACTTTTAGGCCTGTGCTTTCCCTATCACACCACCCTGCCTCTAGATCTAAGTATAAATG
AACAAAAATGGGATTCTACTTGTGAAACGTTGACCCATTTTATTTCCCTCTCCCAGAATCCCTCC
CATTGGAGCAGAGCATCCTTCTGGTTTTATTGCATAAGTTTAGGCTAAGAAGTATGTGACCTGGC
AGACAGTATCCATCAAGAATGTAATGATATTATTTGGGTTTCATAAAATGTGTTTGTAGCTGCCTT
CTTTTTATGACAAGTGATACTAGTTTTCTATTGCAGCAATGTTGTTCCTTTTAAATTCTTTTCACTTA
TTTTAAAAACTATTAAGTTAATAATACTGAGGATACCAGATATGTCAAAAAATGAATAAAAAATT

GTGGGTATAGTAGGAAAAAGACTGAAATTTGGGGAACATCACTCAAGCAGATAAGGTCATGGA
CAGCATTGGAGATTGCAGATGATGTGTGGCACAGGGGCAACAATGATTCTGAACCCTCAGGTTT
AACACAGAAATTCATGGACAGGAAGCAGGGACATCTTAACAATAACATGAGTGGAACTATTAA
CGTGGCCTGGGACCAGCAAATGGTGGATTTCTCAACAGTAACCGACAGAGAAAGCTGACTCAA
GGACCAGAGGGGCTCTCAAACCCCCACTCTGTCCCCTAATGCCTTGTAATTTAGCATAGGAGAA
AGATGGGAGCTGCAAGTTGATATTAAATTTCTACCACTTTGACAGATTAAAGGCATGATATGTTT
CTCTATCTGTACTTACATTTCTTTGACCTGAGTGGGTGAAACTGATATTCATATGCAGCACATAAT
AAGGTAGTAGCCAAATATATTCAAACATGACTCTGGATTTTCATATACAGCATATAATCAAAAAA
AAATTTGTCAGAGGCACCAACTAGAGAAAGTGATGGAGAGGCACAGGCAAATTGTCAATGTGT
AGTAAGACACATAAATGCCCTTATACTCCAGGAAATGTCTTCATCCATTGGCCTTCATTAGAAAT
CCCCTATTTTCCGCCGGGCATGGTGGCTCACGCCTGTAATCCCAGCACTTTGGGAGGCCGAGGC
GGCAGATCACGAGGTCGGGAGATCGAGACCATCCTGGCTAACACGGTGAAACCCCGTCTCTAC
TAAAAATACAAAAAAATTAGCCAGGCGTGTTGGTAGGCGCTTGTGGTCCCAGCTACTTGGGAGG
CTGAGGCAGGAGAATGGCATGAACCCGGGAGGCGGAGCTTGCAGTGAGCCAAGATCACGCCA
CTGCACTCCAGCCTGGGCGACAGAACAAGACTCTGCCTCAAAAAAAGAAATTCCCCACTTTCAG
ATTTACCTCTCCACTGAGATCATGTGGTCAGTAAACCAGGCAAAAGAGAAGTTTAAAGGAGAGA
GAAGAAACAACTTCAGTCTACAGATAAAGGAACTAGAGAGAGTAACTGACTTGGTAGCAAACT
TGGATATTAGAGTAACTGACTACCATGGTAACACACTTGAATATACTCATTTTGTGACTTTGGAG
AATTAACCTTCTTTAAGTCTCGGTTGTCTTTTCTCCAAAATATGGATGTTGACAGTAGCATCTATC
CCACATGATCATTGTGAAGATTGAGAAAAACATATAAAACGCCCAGCACAGGACCTGAGGTTTA
GGAAAGAATCATTAAGTTATTAGTATTTGCAAATATTTGTTGAATATAATATTTGAGTAAGCTTAG
TGAGAACAGTCTTTTTTTTTTTTTTTTTTTGTATTCTAATACAGTTCAAATCAGTGTGGCTGGTTCAT
TTCAACTCCTTCACTACCAAACTAGGAGGCAGGGACTGGCTTCAGTTTTTCTGCCTTCTCTTTTTC
ACTGATGACCAGGGTATAAAGATATCTGCTGCATCGAACTTTAAACTTCACATTGTCCTTATTTTT
CTTGATCTTGACAGATTCAGCATCCTTTCATTGGGCTGTGAACAGAAAGTCCTTGATTTCCTCAAT
TTTCGGAGGCATGGCGACAAGGCACGCAGGGAGCAGGCGCGGACCGGGACAGCGTGCGGCG
ACCGGGAAAAA

ID|56247|gene_id|ENSG00000226969|transcript_id|ENST00000449154 Homo sapiens lncRNA

CCTGCGGCGCGCGCTGCCAGCCCGAGGGCCCCGCCGTCCAGGGGTCCCCTGACATCTCA
CACTTGCGGACTGAAGCTGCGAGGAGCGGAGAACCCGGCCAGGGTCACGGACGGGAGCGTCC
GGACTCGGGTTCTCTGGGCCCCAGCCCCCACTCCGCCCCCAGCGAATCCAAGGTCCCCTGTAG
GGAAGGGCCGTCCACACTCGTGAGTGCAACCGCTCACTTCGCCCAGGTCAAGGACGCGGCATT
GGCCCGAGTCGCCCATGACCTTCATTTCTGAAGCCACAGCCTCTCGGGCCTTACTCGAGGGGCT
TGATCTGGACAATCGCCCTCCAGGCCCCTCCACCGCCGCCCAGCTCCCTGCCTCCTGGGCTCCT
CCCCTGAAAATACGGTTTTCCCTGATCAGCCCATCCCTTACTGGCTGCATCCACTGAAGCCCAG
ACCTGCTGCCTGCAGGACCTCACCCAGGCACCAGGTGGACACCCAGACCCAGTTCCTGGCCTC
GGCCCCTCTCCCCTGAGAGTGGACCTGCTCAGGCCACGCCCTCACACCCCTCACCCAGACACA
GCCACCAGGACTTCAGCCACGTGCTTTCATCCCGCAGGTCGTGCCTGGCGCCTCCTTGATGCCA
GGCCCTGGGGAGACCTGTGGCCACAGAACCCATGTTCTCCCTGGAGATGGACAGGAGGCCCCT
CTCCTACGCAGAAGCTGTCGTTTTTTCTTCTTAAACACCGTTTGCCCCTCCCTCCACCCACCGAG
CCATCTCTGC

ID|56193|gene_id|ENSG00000250604|transcript_id|ENST00000513067 Homo sapiens lncRNA

GCGCAATCTCTAAGGAAAAGAGGTTTATTTGGCTCACTGCTCTGTGGGCTGTACAAGAAGC
ATGGCACCAGCATCTGCTCCTGGTGAGAGGCTCAGGCTGCTTCCACTCATGGCAGAAGGTGAG
GGGAGGTGTAATGTGCTAAGATCATACGGTGAGAGAGAAAGCAAGGAGGGAGGAGGGAGGC
GCCAGGCTCTATTTAACAACCAGCTCTCTTGAGAATGAATAAAGCAAGAACTCACTAACTGTTC
CCTACCCCCAGGGAGAGCATTAATCTATTCATGAGGGACCCTCCCTGATAACCCAAACGTCTCC
CATTAGGCCCCACCTCCGACACCTTGGTTTGAGGGAGACAAACATTCAAACCATAGCAGTCCTA
TTTTCTTGGAAATGAAGTCAAAATAGATGTCAAATGTTGTGTCCTGAGACCCATTCTCTGAAGCT
TTGCACAGGAGCTTAAATCAAGGCTTCACTGATCACAATGACTTAACTCCTCCAGATAAGAGTG
AGATGTTTCATTCTTATTGCAATAATTTTAAACAGCAATGCATCTCATAGTCTATGATTTCAGAGA
ATACAATTTCTCAGTAAGTAAGCAATAGTCCCTCTAAGAACTGGGTTTTTAGGGCATTTTATGGC
TGCAATATGTATTTGGGAAAAAATATTGTTTTCTGATAAATTTGCACCTAGCTTAATCTGCAGCTCT
GGTTCTAGATTGATGAATGTCAGGGACATTGCTTTCTTGGTCCAAGTAATTTTCTCAGACTATATT
CTATAACTTACTCTAATTCTAAACATTGATGAATAGGTCACTGTCCCTTCCTAGGTAATCTGAGTC
AAGACACATAAAGAGTTTGTATTTCATATTTTAAATTTATAATACAAGGTGGTTGGAAGAAGTCTT
CAACAGAGTATGTATATAGAATTAGATCGTTTTTAAATGTTAATGACTAAATTACCGTGCCTCCG
ACAGCATGTTTACCAGAACTCAGCATCCAAGTGTCCCACAGTTAGGTTTGCCAGTTTCCAACAG
GAGGCCTCAAGGTGCTCAGACTCCCACTGTGGCATCTTCATAGTTTTTCTTTCTGGTTCTAGCTTG
CTCTGGCTAACAACCACTATCATCAAAGTTGTCTGAGGAAGACCTCCTGTTGCTGTGACTGACG
GGACTCTTGGTGGAAGCCCTATTCCCATGTGCCCATGGCTGCAGCTCCCCAAGGAAGCAGGAC
ATTGTTGGATTCTCTAAGAAGCTCAGAGGAAGTTACTTCTCACTGTGGTGCCATATGGGATTTGA
AGTCGTTCTACTCATGCTCTGTTTTTCAATAAAGCTGCTTCTGCCTCTGT

ID|56205|gene_id|ENSG00000255237|transcript_id|ENST00000525893 Homo sapiens lncRNA

TTTTATAACTTTTTAACTTTAATAACATAAATATTAATTTTTAAAAATAAGGCTGGCCAGGCA
TGGTGGCTCATGCCTGTAATCCCAGCACTTTGGGAGGCTGAGGCGGGCGGATCACGAAGTCAG
GAGATCGAGACCATCCTGGCTAACACGGTGAAACCCCGTCTCTACTAAAAATACAAAAAATTAA
CCGGGCATGGTGGCGGGCACCTGTAGTCCCAGCTACTCGGGAGGCTGAGGCAGGAGAATGGC
GGGAACCCGGGAGGTGGAGCCTGCAGCGAACCAGGATTGTGCCACTGCACTCCAGCCTGGAC
AACAGAGCGAGAGTCTGTCTGAAAACAAAAAAATAAGGCTGGGAGCTGCAGTGGCTCAGGCTG
TTTGTTCTGGCGCTTGGGGAGGCGAGGCTACACGTTCGAGGCCAACCTGGTCAACATTGACCAA
AAAAAAAAAAAAAATTCCAACCTGGCTAACACGGTGAAACCCCGTCTCCACTAAAAATACAAA
AACTGTCTGGGCATGGTGGTGGGCCCTGTATTCCCAGCTACTCGGGAGGCTGAGGCAGGAGAA
TGGCGTGAACCCGGGAGACGGAGCTTGCGGTGAGCCGAGATCACACCACTGCACTCCAGCCTG
GGGTACAGAGCGAGACTCCATCTCAAAAAAAAATAAAATAAATAAAAAATAAAATTAAGCCTA
ATTTCTAGAAGGTATTTTCTTTTCTTTTCTTTTTTTTTTTTTGAGACGGAGTCTCGCTTTTTCGGCAG
GTGGAGTGCAGTAGTGCGATCTCAGCTCACTGCAAGCTCTACCTCCTAGGTTCATGCGATTGTC
CTGCCTCAGCCTCCCGAGTAGCTGGGACTACAGGCATGAGCCACCGCGCCCGGCTAATTTTTGT
ATTTTTAGTAGAGACAGGATTTCACCATGTTGGTTAGGTTGGTCTCGATCTCTTGACCTCGTGATC
TGCCCGCCTCAGCCT

ID|56210|gene_id|ENSG00000255206|transcript_id|ENST00000526901 Homo sapiens lncRNA

CACAGCTCGGTGCTCAGCACTCTTCCAGACATCGCCCTATTTATCTCTTCATCAGGCTTTTC
ACTTCTTATCCTTTATAATAAACCAGTGATTGTAAGTTTCTGGGTTCCCTGAGCTGTTCTAGCAAA
TAACTGAACCCAAGGAAGGGATGTGGGAACCCGCTGAATATATAGCCAGTTGGTCAGAAATAC

AGGTGAAAGCCTGGGACATGTGACAGGCGCCTGAAATGGGGGCAACCTTGTGGGGCTGATCCC
CTAACCTGTGTGTCTGTGCTAACACCTGGCTTTACAACTAAATTGAATTGTAGGACACCCAGTTG
GTGTTAGGAGATTTGGGGAATTGGTTGGTATGGGGGAAAACCCCACACATACAGGATGTCAGA
AGGGTTTTGAATGAAAACACCTCAGAATAGGGAATTATTCTCCAGCTGTGCTGGTTGGGAGGAA
CCTAATAGTGTAACAAAGATATCCAGAGACATCCACCAATAATATGAGTGGAAAGACACCAAG
AAAATATAAGAGGTGATTCATGGGCTTCTGTGAAAACGTTTTGAAACAATAATTTAAGGTATTTG
TCAATTGCAGACATAAATGTAATTGCCTCTGTCGCCCTTGGCAAAGAAGCCACTGAACAGAGCC
ATTTGCTGGTTTACTCCATTGAGGAAACAGAAATGATAACCGTCCTTATCAAAAGATCTCTCTCT
CCCTCTCTCTCTGTTTCTCTCTCATTTTCACACTACTAGTTTGCTCCTTTCCTTACTGTCAATGTTAG
AATACAGTAAAAGTAAAAAAGAATCTTCTAGAGCAGGGCTTCTCGAATTTTAATATGCATACAA
ATCAAATCTAGTTAAAATGAGAATTCTGACTCAGTAGATCTGGGGTACAGCTGAGATTCTGTATT
TCAGTCTAGCTCTCAGGTGATGTTGATACTGCTGCTCTGTGAGGCACACTGGAGGCCAGGTCTA
AACAACACTGAAACTCCAAACAAGGAAACACATTATAACCTGGGTTTGAATCCTCACTCTACTA
CTTAACTAGTTTGAGCAAGTCCCTTAACCTATATAGGCTTTAGTGTTTTCTCCACTCAAAAAAGAA
TGATGATATTCATTTCCATTTTAGGAGATTTTCTTTTTTGATGACTAAGTGAGATAGTCCACAAAA
ACACTTCATAAAATGCCTAACAAATAGAATTTGCCAGCTACTAGCTATTATTACTTATGCATTTGA
CAGACATCTGTTTGGTGATAACGACTTAAGAATGTGGCTTCAACATGTACAGGACCTTCAATATT
TGGGAGTAGGCACATGCACAGTGGAAAAGAATACTATAAGCACATGAAGAAGAAAATAAGAAA
CCTACCTTCTACTTTACAGTCAAAAGCATCCCCTGCTTCATCTCCAGGGGGCTTGGAGTGAGATT
TTTGAAGGTCTTCTTGAGCACTCTCAATGCTATTATAAACCCATTGTATGGAATCTTGCTGGAAAT
GAGGGGGGAAAGAAACCTCAGAGGTATTAGTGCTTCCTAAAATACATGCCCATTTGGAGACTTG
GCCTTATTTATTCCTCTAGATTCTTTAAAATTAGTAAAATTTGAAAGAGAGATACAATTAACATCA
GAAACTAATAAAAACTCAAGGACAATTATATTGTTTAACAAACACTCTTTCTTTCTTTTTTTTTTTT
TTTTTTTTTGAGATGGAGTTTCCCTCTTGTTGCCCAGGCTGGAGTGCAATGGTGCAGTCTCGACTC
ACCAAAACCTCCGCCTCCCTGATTCAAGCGATTCTCCTGCCTCAACCAACTGAGTAGCTGGCAT
TACAGGCATGCACTACCATACCTGGCTAATTTTGTATTTTTAGTAAAGACAGGGTTTCTCCATGTT
GGTCAGGCTGGTCTCGAACTCCCGACCTCAGGTGATCTGCCTGCCTCGGCCTCCCAAAGTGCTG
GGATTACAGGCAGGAGCTACTGCGCCTGGCCTAACAAACTGACTTTTTAAAGCTTCAAGTTTTAT
CTTCTAAGATATGTTCCAAAGATGTATGTTTTTAAATAGGATATATTCCAAAATATCTTACTTAAA
TGCTTCAAGTATATCGTAAGTAAGTTATAAATCACATTTTAAAATAATCAAATGTGGCTGGGCAT
GGTGACTCACACTTATAATCTCAGCACTTTGGGAGGCTGAGGTGGGAGGATCGCTTGAGCCCA
GAAGTTCAAGACCAACCTGAGCAACGTAGTGAGACCCCATCTCTTTTTAATAAGACAACAAAAT
AAAACAATTGTGAGAGGGACACTTGTCTGATGAATCAATTATCTATAATTGTACAAATCATTTAC
ATGTTCCCCTAATAGTCAGATATATTCTAATACATTTACTGCTATTTTAGAATCAAACAAATATGA
AAATTGGAAGGACATCATTCATCTCATTGGTCTCCCAAGTAATGTAACTATCTTTTAATCCCTCCA
AATAAAAAACATCCTATTTTTAAGGGCGATTTCCTTAAAAATTATCCTATATTTTAACAGCAGTTC
CAGTCTTATTTAGAGAAATGAGAGAGATGAGATGACTACCAAACAAATGCTTCAAGGGTTGCAT
TACTTTAAGGGTTATAGATTTTCTAGATAAGATGTTAAATCAAATTAAACTGCATTATTGAAGTAT
GATACAATATGTATTATCTATTTTTTGTATTCGATTCTCAAGTGTGTCTAATTAATATATTGCTACA
AATCATTAAAATGCTAATTAACATTTGATCATTCCATTACATCTTGGTGCCCTGTTGTGGCCACCA
ATGAGCCGCACCGCTTAGTTTCCCACATTGACCCTGGGCTTGGCCACGTGATTTGGTTTGTCCAA
AGGGATACTAGCAAGTACAATCTAAGTAGAACATTTATCACTTGTAATGTTCCCTCTTAAAATTC
AGACACACACTGAAAAGAAAACCCCGTTAGAGACTGGAAAGAGAGGCCATGCAAAGGTGCTC
TGGAGGGTGACCTGCCACATGGAAACAGAGGCCACGAACAGGGGAAGCAAAAGTCGGGTCCC

AGCAGAATGCAGCCACGTCAGTAACCTCATAGGAACCACCCCACGGCCGCCCCATAGAATCAA
GACAAATAGTCAATTATCATTGTTTTAAGCCACTAAGTTTTGCAGTGGTCTATTTTGTGGCAATGG
ATATCTGGTAGAGCATGTTAGTGGATTTTTCTGCCCATTTATTGCTTTTCTTTAATTATTTCACTGA
TCAACACCTCTATAAGAGGGTAGGCAGGGAAAATAAAAAGAAATGAAACTCAAATCGATTTCTC
TGCTTATTAACATTCTGGTAGAAGAGAAAAGATTTGATCATTTCCGATGAGACTTTAAACATTCA
TTCAATAATACAGTTCAACAAATATTTATCAAGCTTCTATCAACCAGAGTAACCTATGAGCCTAT
ATATAACTTCCAATTTGCTATGCTAGGTCTGACCTTCTCATTAAGTAAAACTGATCAGTTATTTTC
ATGTAGATTCAGCATTTTGCAGGCCATCAGCCCTGGCTTATGAGAAAATGGCCATTCATCTTCCA
GGTTTGGACATAGTTGCATCCTCAAATCCAATCCAGAAATGAGTACTTCATTATCCATGACAGGA
AAATACAAGGCTTCTCCTGTAAAGTTCTGCCTGCATCTAGCTAATTACATTGCAGGAGTTTTCTTT
TATATGCCAATACTCACATTTTCTTCATAATAGCTTTGCTTATGAATTATGATTCTAATTTTAGTTTT
TTGTGGCACTTGTAGATCAAAGGATAGTGTTCTACAATTTTAGATAACGCCGAGTACATTTTGGT
ATTTTCTTTTAAGACCTAAATGCAGTTACCTAAATACATTTTGGTATTTTCCTTTAAGAGCTAAATC
CAGTTACCTAAATAAATATACCTCAAAATCAATCATGGACCTTATAAAACCCACAATTTTTGCTTT
TTCACAGAAGTTTTCTAATAAAGAATATAGGTATTTATAGTTCATACATACAGACACAGACACAA
TGATCATTACATATTCTAAAGCAATCACAAGGGCCAAGAAAACAGTTCAAACCTTTGGATTTCAA
TTTGATAGAACTTGTATCTCAATGAAAATGAAAATACCCCAGATCACTTTTTAAACAGATGGCGG
TACTGTAATACTAAAATGTAACCATTTAAACACAATCAATTATAATTAATTATGCAGATAAAGCA
ATCATTATGAGATTTATAATGCTGTGTTTAATTTTAAACTTAAAATTGGAAACCACAAATATCTCT
GGCACTATCCCACCTCAACCCCTTAGGTCTTTATGCAAGTAGTCACTCAGATCCAGATTATCTGA
AGGGCCACTTACCTTCGTTGGCCGCCTGTACTTCCTGCAGGCTGTAACGTGTGCAATGACACTG
GCATGTGTCTCTTTGCTGACATTGATTCCATTCACCTTGATAATGCACTGTCCAGGGTGAAGACC
AGCTGCTGCAGCCACAGTTCCTATAAATACAAATGAGAAAATAACCAAAAACAAATTTCAGGAA
TAAGATTCATTTGTATGATATATACAATTATGGAAGCAATTTTAAAATATGATCAAAGTCTAGCTA
GTATAATCTTGATAAACTTGGTTCTGAGAGCAATGCCCACTCAACACTTTTCTGCTTTTTAACTTA
ACTTTCAAACTGTGATCAATAGAATATTTCTCACCTCCCTAAGAATTAGTTTGAAGGGATGTTGC
AAAGTGACCTTTATAATACTAATCAGTTCTTTTTCCTTCCAAATGTCTAGTCAGTCTTTCTAAATAT
GCATATATAATGGCAAAATGAGAAGACCAGAAACATATGCCTTACTTACCTTCCTTTATTCTACA
ACATTTCTAAATATTCAAATAGTATTCACTTCTGTTTTTTAAAATGACAACAATTTAACATGCACAT
AAAAAAGAAAATCCTTAAGAGAAAAATCAAGTCAATGAGCAACTATGATTGGACTGGTTTTTCC
AAGAAAAATAAGAGTTTGACATATATACAAAAAGTCAGAAAGATGGGAATTTGTAGAGAACAG
AATTAATGAGATAGCATCACTCATCCTGTCATTTTGTTTGCATTGATATAGATTTAAATATGAAAG
TAACATATCCTAACCAGTGAGCTAAGATTTCTCACAACCTTCCAATTATTGTTATTTGATAGTGTG
ATCAAATCAATATTAACAAAATACCATTAATAAATACATGTAATGAATACAATGCAAATAATTAA
TACAAATGATTATAAACTTGTAAATATATTAGAGGAGACTTCTTTTGTGACCAATGTCTCTCATAA
AGAAATTATTAAACATCTTTAAGAAAATAATTTACATTCAAGAATATATCTTTGCATTTCTTCTTAA
TCCCCATAAAATTCACTCCTAGAAATGTTAAGTGGCTTTCTGCATTGAATTGAGTCTTAGGGGAA
AATGGAAAATTATCAGCAGAAAGAACAGCCTGAAAAATGGCCTTGCTTCAAGAAATCACCTGG
CCACTTGGAAGCATCCAAGCTGTTCTGTTTGTGTGAGCTTAGAATGGGAGTGGATGGAAGAGAC
AGGATTGGAGAGTTGAATAGAGCCAACTGTGAAGTCTTTGCCTTGACTAAGAGTCTCAACCTTG
CTATAAATGGAAGACTCCGAGGGCTTCCTGTCTGAAAGGCTGAATTTGTCTGTAACGTAGGAAC
AAGTTCATCTTGGGATATAGGAAGCAATTTTGAGGAGCAAAAAAAACTGATCAAGGACACATTT
TTAAAAATGCTGAGTAGTAATACGGGCCCAGTAGAAGACAGTCAATTGGGAGACACTAATTCAA
TAACCATGACAGTCTTTTCCAGCTACAAGGGAAAAAGAGAGAAGATGTGGTTAGGTTGATCCAA

GGCTGAAGTGCAGTAGCTAGAATGCAGCTGAGGGTGGGAGGCAGGGGAGCCTGGGGATGCTG
GCAAGCGTGACTGCAGTGACCCACACGAGAAATCTCTTTTTCTTGTGGCAAAATTACAGTGCCA
AAAAATAACCAAATATCTTTCCATTTCATACACATCTGAGAGAGAAAGCACTTGAGCATTTCTGT
CTTTTACTCTTCAGAAGCAAAAAGCACAGAAACCAGACGTAGGCCAACCCCACCACTAAGCTCC
AAAGATAGAACCTTGCTCTTTAAATGACATTGTAATAAGGGAAGAGGTACAGCTTGATTCACTC
AGGCTTTAAGCTATTTTATAGCACTATTAATGTGAATTATTCCTTTTTGTCTTCATGAAAATGCAG
AAGATATTTGGTGCCTCAATATCCCATTTTAGTTTGACAATATTTAATGAGTTATTGGGAAGATTT
TAACTGTCTATCCATCTATATGTTTTAGAAATCTGAGAGAAACACAGCTTTCTGAAATCAGATGTT
TTTATCATACATACAATAATGAAAGTAATGTTATAATAAAGGTCTAGCTAGTATGTCCTTGATAAA
TTTGATTCTGATAAAAATGCCCTCTTAAAATTTTCTTGCCAAATTTTTGGGTGATCACATCAAGAT
ACTTTACATTCTTTAGTAATAAAAAGCATGTATGTCTCTTGTTAGATAAACACATGACTTCTTATG
CTAATAGTGACACTTCTTGCTATAGGCGCAAGTTACTTACTATGTTTAGTAATGTGGGATCTGAC
AGTTATTTCCTTCAATTGTTGCTAGTGTCAATTTAAAATTTAAAAAATACATATGATAAGAAGAGA
TGTCAATAATCAAACAAAAAAGAATAAGTGTAAACATAACTTTTTAGTCACTAAAATTATTGAAT
TTAAAATGCATCTTATGATGGGATTTAACCACGGTTTATAAAAGTACCCGAAGGAAGTATATAGC
AATAGGTTATAAAGTCCACAGATCAACTTTAAACCTAAAAACTCACAACTTTGGATTACTGACAA
AGAAATACAATTACTTGAAATCAAGAAAAATACAAATAGAAAATCTTAAATTTATTATATTCTTTG
GATAAACTGAAGGATGCAGAAAAGTGAGATTTAGGCATAATTCTTATATCAATTTTTTAACAATG
ATAAATATGGCTTAGAATTTTTTCGTTTTTTTAATGTTTCTGGAATGTGCTACCATTGAGTTAATAC
AGTGAAAATATAATTGAGAATCATCACAGCATGGTTCTGCCTATTTTAAACACATTCCTGTTCCAT
CGGGCACATCCCAAACCTGCTGGAAAGCTGTTTCCCTGCAAGACGAATTTCCTACCTCTTCCTAC
AGCATGCACAACAGAAGGGCCAAATCCCCGGATCTGGAAGCCAAGTCCATCAGCTGAATCTGG
AATTTTCACTGTCCTAAATCAATCAGAAAATAAGGTAAGATGAAGCGTAAGAATCAAAACTGGT
TGAAATAATCGTCGTGTACAGCTTTACACCTCTCAATAAATACATATTGAGGATCTACAGTGTGA
AAGATGTTGGGCTAGAAATCCTTGGAGACGAACATGGTCCCCAACCTCATGCAATACAGTGTTT
CTAAACTGAACACAGTCGTTACACAGTAAAGCCTGAAAAAGGAACATTGGAGTGTAGTGGCTTG
ATTACTGCTCACTGCAGCCTCAATCTCTCTGTCCCAGTGATCCTCACATCTCAGCCTCCCAAGTG
GCTGGGACTACAGGCACAAGGTGACACCATGCTTGGTTATTTTACCTTATTTTTTTCATAAACAC
AGGGTTTCCTACATTGCTCAAGCTCATCTTAAATTACTGAACTCAAGTGATCCTCCTACCTCAGT
CTCCCAAAGTGCTGGGATTACAGGTGTGAGCCACCATACCTGCTTAAACGTAGCCTTGAATCTT
CAAGTAGTCAGTTTGTAGGTGGATAACTTAGATAAGCTCCTTAACTACTCTGACACAGAAAGAT
AACATTGTTAAAAGTTATTTTGCAGGGTATCTGTGGCAATTAAAAAGAAATAACCTTGGTAAGTT
GCCCTGTACTGTGCTATGAGCTATACAGGTACTTAATAAAACAGAAACTTTTCTCCTTAGTTTACT
GTTACAAAGAAAACAATGAGGGACCTATGAAATTCTTTATAAAAATTAATGTAACAGAAATACA
GAGTTAATACATTTCTACCTATTGGGAGCTTTATCAAAGAACAACAAAGAGCTAGTTATAAAGAA
CAGATCTGTCAAACGTGGTATCAAAATATTTTAAGAAAATGATTCACTTTAGTATTTATGCAATGA
AGGTTTAAAAGAATAATACATCAGAAGGCAAACCTGAATAAGAGAAGAAATCATTCCAATGAAT
CTACATTAAAAAAAGAAAAAAGATTATGAAGAGATGGGAAACTTGAGCAAGCTGTCACGGCAA
ACCAGTCAGGGGTGTCCAATTTGGCACTCATAAATAAGATACTCCACTGAAGAAAGCCAGAAAT
CAGATAGCAACAATGAAAAGCAGAGAGTGCAAGATGCGAGGGAACATCATAACAATACAAGT
ATGATATCGCAGCCGGGAACAAAAACAAATGAATACAACGCAATAAATTGTGATAGATAGCCC
TTGACGCCCAAGAAACAAAAGTGTTTCCCAAAAGATGAGTTAATCACAAAGCAAATGTCAGCA
GCACTTCAAGGGCATATTAAACAGAGTCTGAAGGTAGATACAGCAGGACTTATCTGCAGTTTAT
GAAATTGCTGTGGGAGAGTTTGAGCAAAACTAAGGGCTCAAACACTTCAGAAAGCCATAGGCT

ACATTTAGGCCTAAGGCAGAGAGGCTGGCACTGCAATCTCAAATATAACCTAGGCAGGCATTCA
GGGATAGAAATGAAGATTTTCTGCTCTCGGAAAACTTGAAAGTATTATTATAAATTAGGTTATAC
ACTATGAAGTTATCTGTATGTATGTGGGATAGAGGTCACAGAATTAATGTATCCTTTATCCTTAAT
TAAACAGTTTTTGGTGTACAGGGCTGTATTCATTTCCTTTGAATAACACATAGTTATCAGTGTTTG
TCAAAGTTTCACTTGAAAATGAAAGATAACTGACCAAAGAAAGAAAAAGGGAATGAATACATAT
TTTGGTGAGCCTGAAACAAAATCAGAAGAAGTTCATGCTTCTATATATTACGAATGAGAGTTCAC
TTCTGCTGAAACAACATAGAACTTTCAGGCCATGGTGAATTATTGTTGCTTTTGGATTTTGAGTAA
TTCACAGTGGCCTCTCAATCTTACTGAGCAAGGCTTTTCTATCCTTCTAACACTGCTATGGAAGG
TTGACTCTAATCTTTAAGAGAATTATTTTTTCCTTAATCGAAATTGTACAAAACACCATGACATGC
ATTTTGGTCCAAATGTTGGAAGCCCCTTAGTAAAATGATCTTTAGAAAACATGTTTCCCTGGTGA
GAACATTATATTCCCTATTAAAAAAGAATTACGTTTATACAAGACGGATGTTCTATTTCCTACAGA
AAATAAGTGTACTAAATTATCTGCTACAAAGTCTTTTGAAAAATCCCTTGATGCTATCAAGTATG
AAATATAGCTAGAATCAAAAGAAAATTCTTTTGCATTTATCTCTCATATTTTTCCCTTTTCCTCAAA
ATGGGATACTACTTAAATTCCAAGAAGCTATTGTAAGAAAATATGACAGTGATTTATAGAACTCA
TACATTAGAATAAGGACATAAAGGAAAAGTCATTTATTTCAAACAAGTTTTTTAAAAATGAGTAT
GCCATCTTCAAAAAAAATGAGTATGCTATACAATGTAAAATATGCTCTTGATATAATAAAGTAGCA
ATGAAACATAATAAAATGAGAAGACAACAGCATCATATATAACATCACTGGGTACTTACTATGT
ACTGGATACTTCACTAAGTTCTTTACAAACCTAGTTTTATTCAACATTCTTTGCAGTTCCATGGCA
CAGGTACTCTTATCCCCATTTTATGGATGAGGACGTGAAGTTATACACTTAGCTAGCAAGTAGTG
GAAGAGAGGCTTGAACCTGTAATCTAGAGTTCTAGACACAGCGCAACGAACACTAAGAAGAGT
CAGAAGACACAGATTGCTATTCTGCTCATCTAGCAACTAAGTCTATGACTGAGGATTTGCTCCTA
TTTATTCAGTTTTGAAGTTGATGACCTCAAAGTCTCTAACAGTTGGAATGTCTCTGATCAGTAGAT
CAGTATTTGTAGGGTTAAATCAGGGGGCCCTAGGGAGGACGTAATGATTGCTTTCAATACTACT
CCCTTATTCCTTTCCAGTCTGTCTTCTTCCTTTTAAATGCCCTCAAAGTGTCAGGAGGTGGGCAAG
CAGGACAGTCTTCTCCTAGACAGAGTTGAATAAAGGGAGAGGAACAGACCATAATTAGGTTTA
GAGGGAAGACATTGCTGTGCTTCTTTCCTGCCCTCTAGCCCCGTCTTCCTACATTAGTAAAAAGT
GAGTGTCAGGCTCACTCTTTCAGACCTCTTGGTAAACTATTGGTAGAAGCTAAAGCATCTAATTT
AATGAATTCGCACTGCCTTGAAGAGACTCACTAGGGAGAACCCAGCCCACTGCACTGCACTTTG
ACTCTTAAGATACTTAACACTGCAGAAGTACATCCAGCTATTCCTGAATATATATAACATATAAA
CATATTACATATAAATATGTATTTTTACATATTAAAATATATTTAAGGCAGGGAAGAATACTGCAT
GTACTTTTAATAGGTAGTTAAAATTTAAAAAATCTTATTTTTGAAGTGCTAAATTTCATTTGCTTTA
AAACTAATGAAGTTGCTTATCATGATTGACAAATGATACAAGTAAAATTTAAACAATAATTTTGA
TATATTATGCTAAATAGTTTGTACAAATTATCTCAATTAATTAAGTGGTTAAACAATTCAAGGTAA
ATATGATAAGGAAATACTAAACCATTGATGGAAATGATTTGCACAAGAAAAGCCCATGTCTTGC
AAGTATATGCATGGTATGTTATTAAAAAGTAGCATACAGAGCATGCTCTCATTTTGTGGGTACCT
GATTGTGTATATGTGTACCAATACTTTAAAAACTTTAGTTAATACTTAATTTCAAAGGAATAACAA
TGGCTATTTATGGGTGATATTGCAAGAAAAAAAGTCTCCCTTTAGAAACATTTGTATTCTCTACAT
TTTCTATAATAAACATAACTTTTTTTCTAATCATAGGTAGATAGTTAATAGAATTTTTTAAAGAAAT
GGACAAATTACTAAGCACATGCAAGTATGGATTATCTTTCAGTCTTTTTTGCATCTCTTATGAGTG
TTAAATTATAAATGGTATCTGGTATCTAATCATCTGATAGCACCAGAAATTCTTATAGCCGAACA
TGGAGAAACACAGTTCCTTATTCTCAAATGTTCTACTAGTTTTCTTCACTCATAGTAAAACCCAAT
TCAACCCAGACCAGGAAAATGTGGGCAGCAAAAGAAAAGCATTGTTTCCACTAAAACTACATG
ATTCAAGTAACAAGTATGTCAGCATCACAAGCGATGTAGCTTCTCCCATCATCTGTAAACCAGTA
TTAATGATACCGGTGAGGTTCAAGCGTTCCAAGCAATGAGATGCTTAATTCTAAGCATTTACTTA

GTTATCTTTTCTGTGTTACTTATCTATGTATAACTTTTAAAAAATCACACATAAATCATACAACTTA
CTCTCTTGGCTTTGTGCTCACAAGAACTCTTAGAGGTTTTCTGCTGTTTAAACACGATTTCAGGAA
GCAATCCACTTCATTGAAAGGTCTCATAAAAACTAGGTCACCATTAATAGCAAAAATCTTTTTCC
CGACTTCCATGCCAGCCATCTAAGAGAGAAATTACAAAAAATAAGTCAAAGTATATTTTAATAA
GAAATTTTGAGTTTTTCACAAAATTGGAATATGAAAAGTTTCAGAGCTTATAGATATTGACATTTT
AACACACCTGCCGTGTTTTTATACAAATTCACAAAGCAGAACTGTAGATAACACAGTTATCATTC
TCTTTGGCATAGTTTTAAATTTGAACACAGAACTTTACCAAAATTATCCCCATTTCCCTCCCTGAC
TTAACATCCTACTCCCCTCTTCTGTTATATTTTACTGTTCTTTAGCTTATTTCATCCTTTTAACTATA
ATCAAATCCAAAGTTAATTTTTTTTACTGTGTTTTCCACCTATAGCCAATTAACTCAGAAAGTGTC
AACTTCTGTATCTACATCAATGACACCCAAATATGTATCTCTAGCCTGACATAGCTCAAGCGGCT
CCAGTGCAGGAGACAGAGGGAGCCTGGAATCCAAAGAAGAGAGCTCCAACCAGCATAGAGTT
GTCAACAGTGCAAGCTGCTGAAAAGAGGACAAGGAAGATATTATCCCTCCACGCCAACCAGCA
TGCACTTCTCAACCAGCCTTACTGCCTATAAACTACCGCTGTTTTGACTGTCAGCTAAATGATGA
AAGCCTGAGAGAGGGCATTTTATTAGTGGCATAGTCAGATGCAGCTGTGCTCTAAGACCATCCT
CTGAAAATGCCTGGGAGAAACTATTTCCTTCTCATTGACAATTACTAAAAATGGTCATTCGTTGG
CCAGGTCCACTAAACAGTGCATACGTTGATCAGAGTTATTTTGTTTGAAATTAGTGTGCTGTACA
GAAAGCTTTGTCTTGCATGAACCTGTGTTTTGATAAAACAGAGACCTTGGGCATTAGCAGATAG
GAGGATGGGTGACAGTTTACCACTAACTTTAAACAGGTTCAAAGGTAAATACCATATTTTGTTAA
TTTTCATATTCATTAAAGATCTAAATATTGAGAAGTTTGCTTGAGGATTAAGTATCTTCCAACCAG
GGGCACCAAGGTGAGG

ID|56153|gene_id|ENSG00000224505|transcript_id|ENST00000452741 Homo sapiens lncRNA

GACTAGGGTCCTCAATCCAAAACTGGAACCACTTACTCCTAACTGAAGCTTCAGTACACTT
GATGCCTACTGTGGTTTGATTTTTTACCTACATATGACATGTTCGCTCTACACTGATCAAGAGATG
TGAGTTTAAAAAAGCTAACTAACTTGGTAGGACTTAAATAGTCATAGGAACAGAAAAGGCTTTT
AAAGACGATCTTTGTGGGTCTTCTGTCAAGAATTTAATGGAGGATCCTAGACACTTGGGATTCAT
CCTCCATTATGAAGTAATCTAGTGAAATTTTTGGATGCTACTGAGTGGCAAATACTGTACCAGCA
GCAGAAATTTGTTACTCATTTGTAAATGAAACTTACTATCCTTAAGGTTGACCCACAAGCTTTTCC
ATTCTTTGCTAAGAAAAAATTGGGAATAGGCCGGGCACAGTGGCTCACGCCTGTAATCCCAGCAC
TTTGGGAGGCCAAGGCGGGCAGATCACGAGGTCAAGAGATTGAGACCGTCCTGGCAGACATG
GTGAAACCCCATCTCTACTAAAAATACAAAAATTAGCTGAGTGTGGTGGTGTGCACCTGTAGTC
ACAGCTACTTGGGAAGCTGAGGCAGGAGAATTGCTTGAACCCCGGGGCTGGAGGTTGCAGTGA
GCCGAGATCGTGCCACTGCACTCCAGCCTGGGTGACAGAGCAAGATTCTGTCTCAAAAAAAAA
AAAAGAAAAAATGGGAATAAAACCAGCTTATTAGTGGCCTCCTGGTTATCTAAGGAAAACACAG
GACTGTAGGACTGAAAATTAGGAACTCCGAGCTCTGTTTCTGTGTGTGCTGCCCTTAACTTGGTT
GACTTGGCCAGAGGCCAAATACTGATAATGAGGCCAAACTGGTCATTTTGCTGCTTAGTCTATTG
TTCATCTACTGTTCAACCAGACTTTCAACTTCCTTTTCAGCTCTCCCCTCTCAGATGAGTTGCATT
ATGCTTTTATGACAAATTCTACAAACTCTATATTCATTTGAAAATGGTATCAGCACTGTCTCCCTC
ATAAATACCTAGAGGAGTAACTAGTCTTAAGTCGCTATGTAAACACTTACTCCTTGTAACTTTGTT
ATATGAGAACTTCACAAGGAATGCTTCAAAGATCATTGGGGCAGTTGGAAAGCCTGCCTTACCA
AAGTGTGGCCTTAGTTGTCCATAGCATCACAAAAGAGCCATCCAAGTAGGCTGAATGAGGATCA
GTCTCTCAGGGTGCCCACTGGTTGTGGCTGTGAAGTCCTGGACACTCATTTTGCCTGATGGCCA
AATCCTAGGGAGCAGTGACTATGTGTGAGTAGGATTCGTTGACAGAGGTAGGAGGTTTTTTTTT
GTTTTTTTTTCCTGCTGCCTGTATAATTGCAGAAAAGGAGTGATTCTTTACAGTAATTCCCCCTCTT

CGGGATGAAGATGCTTGCTTCTGTTTGGTTTCCACAGTTAAATCAATTTTTTTTCCTTTTCTTTTGA
GATGGAATCTTGCTCTGTCGCCCAGGCTGGAGTGCAGTGGCGCGATTTCGCCCACTGCAACCTC
CACCTCCCAGGTTTAAGCGATTCTCCTGCCTCAGCCTCCCGCGTAGCTAGGATTACAGGCACTC
ACCACCATGCCTGGCTAATTTTTGCATTTTATTTTTTTCTGGAACGGAGTTTCACCTGGTTGCCAA
GGCTGGAGCGCGATGGTGTGATCTTGGCTCACCACAACCTCTGCCTCCTGGGTTCAAGCGATTC
TCCTGCCTCAGCCTCCCAAGGAGCTGGGACTATAGGCATGCGCCACCATGCACGGCTAGTTTTG
TATTTTTAGTAGAGATGGGGTTTCTCCATGTTGGTCAGGCTGGTCTTGAACTCCCAACCTCAGGT
GATCCTCCCACCTTGGCCTCCCAAAGTGCTGGGATTACAGTCAGTGAGCCACTGTGCCCGGCCT
GTATTTGTATTTTTAGTAGAGACAGGGTTTTACCATGTTGGCCAGGCTGGTCTCCAACTCCTGAC
CTCAGGTGGTCCGCCCACCTCGGCCTCCCAGTGTGCTAGGATTGCAGGCGTGAGCTACGGTGC
CTGGCCAGTTAATCAGTTTTTTACCCTCCTGTGTTTAGGCCCCAACATCCTGTTCTTGATCTTTTTT
GGCAAAAATTTGGCCAAAGATTGAAATTTTGCCTCCTGGACCATATATTTTGCTCCAGTTGCAAA
GTATGACTTTTATCCTTTGGTCAGCAGTTCCCATGGTGTTTAGGGCCTGAATTTGACATTCTTTTTT
TTTTTTCTTTTTGAGATGGAGTCTAGCTCTGTTGCCCAGGCTGGAGTGCAGTGGCACGATCTCGG
CTTGCCGCAACCTCTGCCTCCCGGCTTCAAGCAGTTCTCCCTGCCTCAGCCTCCCAAATATCTGG
GATTACAGGCGCCCACCACCACACCCGGCTAATTTTTGTAGCTTTTTTTGTTTGTTTGTTTGTTTTT
GAGAGTCTCACTCTGTCACCAGACTGGAGTGCAGTGGCGCAGTCTCGGCTCACTGCAACCTCTG
CCTCCTGGGTTCAAGCAATTCTCCTGCCTCAGCTTCCCAAGTAGCTGGGACTACGGGTGGGCAC
CACCATGCCTAGCTAATTTTTTTTCTGTATTTTTAGTAGAGACGGAGTTTTACCATATTGGCCAGG
ATGGTCTCGATCTCTTGACCTTGTGATGCGCCCGCCTCGGCCTCCCAAAGTGCTGGGATTACAG
GCGTGAGCCACCGTACCTGGCAACATTCTCTTTTTTTAAAAAAATAACTTTTTATTTCTAGCCTCA
ACCATTTACACTTTGCTTGAGAAAGAATAATAAAGTGGAATTGTTATTCCAGTGATGTTGTGCAA
ATGTGAGAAATAGTACTTCCCCCAAAAAAACAAACCTCGAAACCCCACAAACTCAAGTGTACTT
AAGTACTTAACGTTCAAGTACTTTTTAACTATAACCAACTTTTCCACCACACTGTTGTGAATATTT
GTTGCTAGCTGTTATGGGTATAAGTGATCAAGGGAAGAACTGATGCATTACCTAATGCTTGACTA
AATATAGCCAGCCCTTAAGTAGGTAGAGTATTTGGTTGTGGGTTTTGTTGTTTAAGTCTGTGGAG
TCCCTCTTTGGTTTCTTTAACTTTTGCAGTCATTAGGGCAGAGTATATTTATTTACATTTCTCCAAG
CAAAGTCTTGCTCTGTTGCCCAGGCTGGAGTACGGTGGCGCGATCTCGGCTCACTACAACCTCT
GCCTCCCAGGTTCAAGCAATTGTCCTGCCTCAGCCTCCCGAGTAGCTGGGATTACAGATACCCG
CCACGATGCCTGGCTAATTTTTGTATTTTTAATAGAGACGAGATTTTGCCATGTTGGCCAGGCTG
GTTTCGAACTTTTGACCTCAAGTGATCCATCTGCCTTGGCCTCCCAACGTGCTGGGATTACAGGC
GTGAGCCACCATGCCTGGCCTAAATTTACTTTTTAATAGAGACGAGGCTGGTTATGTTGCCTGTG
TTGGTAACTGTGTTGCCTAGGCTGGTGTCAAGTGCTTGAGCTCAAGTGACCCTCCTGCCTTGGCT
TCCCAAAGTGTTAGGATTACAGGCATGAAGCCACTGCGCCTGGCCTGTCTAATCTTTATTATGCT
TCCCCTCTTTAGAGATACTTAGTCTGTTTTTCAGAACTAAACCAATCTGGAACTCCCTGGCTCTCT
CTCCTAAAACTCTGAAGTCTAGAGGACCCTGTCTCCTGAGGGCTCTAAGAAAAGCTGTCCAGCT
AGCTGTGTCAGTTTGAGAAGCAGTTTTTCCTGATTGAATACCTAGGCCCCAGTAGGAAAAAGGG
AACAAACTGAAATTGCTAATTTGTAAGCCTGGTCTTTTGAGCAGCTCAGTTAAAATGCTTGACAG
AGGACTTAAGTGAAAGGCTAGAGTGGGCTTGGGGTTCATTTATCATCAAAGCCCCCTGTTCAAT
GTAGGTAAATCAGCGGGGTGTGTTGGTATCCACCTTTAGTCCCAGCTACTCAGGAACCTGAGGT
GGGAGGATCACTCAAGCCCAGGAATTTGAGGCTGCAGTGAGATATGATAGCGCCACTGCAGTC
TAGCTTGGGCAACTGAGGGAGACCCCATCTTTTTTTTTTTTTTTCAACTTTTTTTTTTTTTTTGAGAC
AGGGTTTCACTATGTTACCCAGGCTGGTCTCAAACTACTGAGCTCAAGGATTCTGCCCACCTTGG
CCTTCCAAAGTGCTAGGGTTACAGGTGTGAGTCACCGGGCCTGTTGAAACCCCAATTCTTTAAA

AAAAAAAAAAAAAGCTTTTTTTTTTTTTTTTTTTGAGACAGAGTCTTATTCTGCCACCTAGGCTGGA
GTGCAGTGTGGCGTGATGTTGGCTCACTGCAACCTTTGCCTCCCGGATTCAAGTGATTCTCCTGC
CTCAGCCTCCCAAGTAGCTGGGATTACAGGCACCCACCACCATGCCCAGCTAATTTTTGTGTTTT
TTTGTTGTTGTTTTTGTTTTTGAGACGGAGTCTCGCTCTGTTGCCCAGGCTGGAGTGCAGTGGTGT
GATCTCGGCCCACTGCAAGCTCTGCCTTCCGGGTTCACGCCATTCTCCTGTCTTAGCCTCCCGAG
TAGCTGGGACTAAAGGCGCCCGCCACCACACCCAGCTAATTTTTTGTATTTTTAGTAGAGACGG
GGTTTCACCATGTTAGCCAGGATGGTCTCGATCTCCTGACCTCGTGATCCGCCCGCCTTGGCCTC
CCAAAGTGCTGGGATTACAGGCGTGAGCCACCACACCTGGCCAATTTTTGTATTTTTAGTAGAG
ACAGGGTTTCACCATGTTGGCCAGGCTGGTCTGGAACTCTTGACCTCAGGTGATCTGCCTGCCT
CGGCCTCCCAAAGTGCTGGGATTACAGGCGTGAGCCACTGCGCCCGGCCTTTTTTTTTTTTTTTT
TTGAGGCTGAGTTTCACTCCTGTTGCCCAGGCCGGAGCACAATGGGGCGATCTCGGCTCACTGC
AAACTCCGCATCCCGGGTTCATGCCATTCTCCTGCCTCAGCCTCTCAAGTAGCTGGGACTACAG
GCACCTGCCACCATGCTCGGCTAATTTTGTATTTTGTATTTTTAGTAGAGATGAGGTTTCTCCATA
TTGGTCAGGCTGGTCTCTTAACTCCAGATCTCAAGAGATCCACCCACCTCAGCCTCCCAAAGTG
CTGAGATTATAGGCATGAACCACCACGCCCGGCCCAACCTTTTTTTTTTTAAATTAAAATATAGG
TAACTAATTGTTTTCTCTGCCAGGAGAGCAATATGCTAGTTAAATGTTTCAATAGAAAGTGAAGA
AATAGGAGGCCGGGTGTGGTGGCTCACACCCATCATCCCAGCACTTTGGGAGGCCGAGGCAG
GTGGATCACGAGGTCAGGAGATCGAGATCATCCTGGCTAACACGGTGAAACCCCGTCTCTACT
AAAACTACAAAAAAAAAAATTAGCTGAGCGTGGTGGCGGGTGCCTGTAGTCCCAGCTACTTGAG
AGGCTGAGGCAGGAGAATGGCATGAACCTGAGAGGCGGAACTTGCAGTGAGCCGAGATCGCG
CCACTGCACTCCAGCCTGGGCGACAGAGCAAGACTCCATCCCTCACCCCCCCCCCAAAAAAAA
GAAAAGTGTAGGAATAGGCTGGGCTTGGTGGCTCACAACTGTAATTTTGGGAGGCGGAGGAGG
GCGCATTGCTTGAGCCCAGGAGTTTGAGCCCAGGAGTTTGAGACCAGCCTGTGCAACAGGGTG
AAACCCTGTCTCTCCAAAAAAAAAAAAAAAAAAAAAAAAAAATAGCCAGGCGTGGTGGCAAGTAC
CAGCTACTCAGGAGGCTGCAGTGGGAGGATCGCTTTAGCCTGGGAAGTGGAGGTTGCCGTAAG
TCCAGATCGCGACACTGCACTCCAGCCTAGGCAACAGAGCAAGACCCATCTCAAAAAAAAAAA
AAAAAAAAAGCCAGGCGCGGTGGCTTATGCCTATATTGCCAACACTTTGGGAGGCTGAGACGG
GCGGATCACTTGAAGTCAGGAGTTCGAGACCAGCCTGGCAACCATGGCGAGACCCCGTCTCTA
CTAAAAATAGAAAAATTAGCCAGTTGCAGTGGCGTGCACCTGTAATCCCAGCTACTCGGGAGG
CTGAGGCAGGAGAATCGGTTGAACCCAGGAGGCTGAGGTTGCAGTGAGCCGAAATCACGCTA
CTGCACTCCAGCCTGGCGACAGAGTGAGACTCCGTCTCAAAAAAAAAAAAAAAAGTATAGAAAG
TGTTAAAACTGAAATTTTGAATAAACCTCATCATTGTGAGAATATAAATGCAAAAAAAAAAGGTAA
AATGCTAGTGCCTTTGAAAGCAAAATTCAGACATACTTGCATAGCTTAAGGTAAATTGATGTATG
AGAGTTATTTGTAATTAGATATTTGTGTATATATATATTTTTGAGACAGTTTCGCTCTTCTTGCCTA
GGCTGGAGTGCAATGGCGCGATCTCCTCTCACTGCAACCTCCACCTCCCGGGTTCAAACGATTC
TGCTGCCTCAGCCTCCCGAGTAGCTGGGATTACAGGCATGTGCCACCACACCCGGCTAATTTTT
GTATTTTTAGTAGAGACGGGGCTTCTGTATGTTGGTCAGGCTGGTCTCTTAACTCCTGACCTCAG
GTGATCTGCCCACCTCAGCCTCCCAAAGTGCTGGGATTACAGGCATGAGCCACTGTGCCCAGC
CTAGATATCTGTATATTTGAAGACTGGGCTGAATTTGTCTTCTAAAACATACTTTTTCTGATGATA
AATGCATTTATAGTCTTTCCAAGATTCATTTTCTAAATATCATTTTTCTAATACTAGGGGAAATAA
GATTTTCATAGCTGAATCCCTCTTTAAGAAGAGGGTCTGGGCTGGGCGCAGTGGCTCATGCCTG
TAATCCCAGCACTTTGGGAGGCCGAGGCGGGCGGATCACCTGAGGTCGTGAGTTCGAGAGCAC
CCTGACCAACATGGAGAAACTACGTCTCTACTAAAAATACAAAATTAGCTGGGGGTGGTGGCA
CATGCCTGTAATCCCAGCTACTAGGGAGGCTGAGGCAGGAGAATCGCTTGAACCTGGGAGGCA

GAGGTTGCGGTGAGCCAAGATTGCACCATTGCACTCCAGCCTGGGCAACAAGAGCGAAACTCC
GTCTCAAAAAAAAAAAAAAAATAAAGAAAAAGGGTCTAATATCAGAGATAATCACTGCTGCATCT
TACAGTTCAGTCAAAATGAGGCTTCAGCTCTGCACTGTAAAGCCAGAGGGCACTGTTGCTGCCT
TTCCAAGCAGCTGTTTTTCTGGGGCTGCTGCATGACGTCATAGCAGAAGAAAAAAACACGAAGA
AAACTATTTCAAAATAAATGTTTAATATAATTTTTAGACCAAAGAAAAACTCAATCATACATTGTC
TCCAGTGGACCATATGTGCCAGAACAGGACCAACTCAGTCCAGCTTTGAAGGCCCTGATTCAGT
CCTACTGGTATAATCTCCCTTGATTTATTGATTTGTGTATGCTTCTTATGCAATACAAGGTGCATTT
TACTCTTGAAGTCCCTATCACCTCCACCATCGCCATTTAACTGCAAAAGCATGCTTCCTAATGGG
AGGGTTTTCTTCCCCCTTCCCTGCAGAGGTTCTGAACCTTCAAGGAGTTTGACAATCTTATGAAT
CTCTTGAACCTTCTCCCCAGAAAAATGTACATACAAGTCTACAAATAATTTCATGGGGTTCATGG
ACCCCAAGGCCCACAATGGTTTCTAAGGCCCCAAATTAAGGAACCTCTGTGCTAGTGATTTGAG
CAACGCAATTGCCCTCAGAAGACACTGACACAGGGCAGGTGAAGGACTGGGTTAACCAAAATG
ACAGGAGGCAATCTCATAATTTCTGTTTTTATTTTGGGGCAAAATTTAGTTCTCAGGGCCTCCCTA
CGTTTAAGAGCAGAACTACTCAAGAACCTAAGCAGCTATCAGCATAGTTTAAGCGATTTAGAAT
AGGGCTGTGCAAGGGGATGTACAGGGTTCCTCATTCAGCTGAGGACCTCATTTAGCTCCTTAAC
TGGAAGCTTTACATTTGATCTGCAGTGAGGGGACATTTCTAATTTTAGTTTGACCCACATGGAAA
GGTGGATAGCTTTTTTTTTTTGTTTTGGTATAGAGAGCACGTAGAATTGGACAATTAGGTAAATCC
TTGGAATCCCCCAAACAGCCATTTGCTTTATAAATCCAGTATTTCTTACCTCTGAACGTCTTAAAG
AGCTGCCTCACAAATTAATCTAACCATTGCTTGCACTAAGAAGTTTGCCAAGAGCCTCTTCCAGC
CTTACTGGGGGGAAGGAAGAGATTACTGAAAACCAACTTGTAGGAATGAAATGAGGCTGTCAG
TTGTCTAACCTAACAAACTGCCAAATTAGGTTTACATTTTTTGTTATTAGACTTTTGAATGGCCGA
GGCAGAGTTAAATTTAAAATTTTGGATTGTAGCCACTTTCCACTCGCCTAAACACTCAATACCGG
TTTGTTTATTTTTCCAGCATCAAGAAAGAAAACCAAGGCACTAGAAAGCAAGCACACTTAAGTAT
TGAAGATAATTACATCTTAAATTTTGCCTGAACTGTATATTGTAGCATACTTAAACCCATTCAAGG
AAAAAATTGGCAAATTGAAAATTCTCCCATTAAAGATTGATATTTTTGCAAACTGACAAATTTAA
CGATTTCGGTACCCCTCCCCAAAAATGCAGTCACATAAAATATATCAATACTATTTTGAATTAGC
TCTAGAAATCACACTGAAACTATGTTACATTTACAAATATTAAATTTATTATAACTAA
ID|56552|gene_id|ENSG00000224505|transcript_id|ENST00000589950 Homo sapiens lncRNA

ATCGCCGCCATTTTGGGCTGGGAGGAGGCAGCGGAGGGACTCGCTGCGCAGTGAGAGCA
CCCCGTGGGGGGTGGTCCGGGCGCGGGGAGAGGCCGGGCCGAGTGGGCTACCTGAAGTGGC
GGCGCCGGGAGACGGCGGGAAGTGGGACCGGGGGCCAGGGAGGGTGAAGGTTGGAGTCTCC
TCGGCCGAGCCCGGTCCTCAAACCGTCCCGGCAGCCCCGCAGCCTCGCCTCTCACAGAGTGCG
GGGTTCTTCGGTAATTACCGCGTGTTTCCCGAGACCCCAGAAGGGGAGAGGCTGGTGAAACGG
CTTCCCTCTGCGGTCTTTCCACAGGGGCGCGGATCCTGAGGCCGAGCTCTGCCAGCTTCCCTCC
CCACCCCCATTCGGCCTGGGTATGGCGGCGAGGCCCCGCACAGAGGGACTCCCACTTCGACTG
GAGAGGTGGGGGCGACGCCAGATTCCAGAGGCTGGCGCCGCGGGGGAGGGCGGAGGTCGCA
GGTCTGGCCACAAGTCGGCGTGGCAGTCAGCTGGAGGCTCCGAGAGCGGAGATCCCAGTGCA
CCCACCTTACCTCGCTTACACCAAAGCGCCATCTCGCCGCCCGGGTTGCGAAAAACCACGACC
CTCTTAAACTACTGTAGCAACCACTAATTCCCCCTGGGAGCAGCTAGAGAGGGGCACTTGGAAA
CCTTCACGAGCTCGGTGCCTGAAGGTCTCTGAGGCTCTTGCCCTCAAGATCTCTCCCATTCAGCC
CCGATTCTCCGGACAACCTGACCTAGCATGTCGCCAGCCTCTTGGTGAAACGCCTAAAGTGTGC
TGCCCCACTTCTCTTGAAAGTTTACACCGAAGGGATGAGGTTTTGCCCTTTACAGGGTTGGGGC
ACAAAGTCGGAGTCGCTGCGGCTCCGAAATAGAAACGGAGTTGCCCATTTCGTATGCTTCATCA

ACATCTGCTTGCATTTTGAAATCCTAGTCTTAACTTTTACACAACTTTTAGAGAAATGGCTGCCCT
CCTAGAGGGAAAAACGAACTACAGTAATTCCAATTCCAATGTAATCTTTGTAAGACTACAAAAT
CTTTCCAGTTTAAAGATTTTTCTTTTCCCACAGACTAAACAAAGTCAGAGTATTTTGTGTTCTACT
GGTGCATTGTTACTAGGTAACCTCAATTTTTTGAAATTCCAAGTAATTGAAGGATCACTTTTGTAG
ATCCTAAATGCTACCAAAAAAGAAACCAGTTTTTATCTACTTCATATGTATAGCGTTTAAGAGAT
GAGGAGACCTGCTTTGAAAGAATAAACTCTTAACCTTACATTATAGCTGTAGCAGCTTCTTAAAG
ATTGAAGTTTGATGTGAAGTTCTTTTATTGAATTGCAGGTTAAACCATCTTACTTCCTACTGCTTG
AAATCTAAATCCAGAGAGATTTGTGGCTCAAGGATATCTTCAAATAAAAGTTTTATTGCTAAAAT
GTCAGTTAAAATTATGCCTAGGTAGGTGCTGAAGTAGGAGAGGCTTTGACGTAGCAGAGTGACT
GGCAGTGAATTTTGTGGGTACCTTGTGATGAGTCTTATTATCAGTTACAAATGGTCAAAACATGC
CTAAGCCTTCAGCCTTACTAAAAGTTTAACAGAACTTGGCCTGGCGCGGTGGCTCACACCTGTA
ATCCCAGCCCTTTGGGAGGTGGAGGCGGGTGGATCACCTGAGGTCAGGAGTTTGAGACCAACC
TGGCCAACATGGTGAAGCCCCTTCTCTACTAAAAATACAAAATTAGCCGGGTGTGGAGTTGCGT
GCCTGTAATCCCAGCTACTCGGGAGGCTGAGGCAGGAGAATCGCTTGAACCCAGGAGGCAGA
GTTTGCCATGAGCCTCTCACCGTTGCACTCCAGCCTGGGTGACAGAGTAAGACTCTGTCTCAAA
AAAAAAAAAAAGTTTTCCAGAACTGAAGTTCTGCTCCTCCTATTAATAATTTTTCTTCAGTTGGAG
TGACAACTCCATAGCATTAACTTTTTTCCCTCTGCTCCCCAGATGTTTTACAACTGGATTACAAAA
TAGGATCATCATTGACTAGGGTCCTCAATCCAAAACTGGAACCACTTACTCCTAACTGAAGCTTC
AGTACACTTGATGCCTACTGTGGTTTGATTTTTTACCTACATATGACATGTTCGCTCTACACTGAT
CAAGAGATGTGAGTTTAAAAAAGCTAACTAACTTGGTAGGACTTAAATAGTCATAGGAACAGAA
AAGGCTTTTAAAGACGATCTTTGTGGGTCTTCTGTCAAGAATTTAATGGAGGATCCTAGACACTT
GGGATTCATCCTCCATTATGAAGTAATCTAGTGAAATTTTTGGATGCTACTGAGTGGCAAATACT
GTACCAGCAGCAGAAATTTGTTACTCATTTGTAAATGAAACTTACTATCCTTAAGGTTGACCCAC
AAGCTTTTCCATTCTTTGCTAAGAAAAAATTGGGAATAGGCCGGGCACAGTGGCTCACGCCTGTA
ATCCCAGCACTTTGGGAGGCCAAGGCGGGCAGATCACGAGGTCAAGAGATTGAGACCGTCCTG
GCAGACATGGTGAAACCCCATCTCTACTAAAAATACAAAAATTAGCTGAGTGTGGTGGTGTGCA
CCTGTAGTCACAGCTACTTGGGAAGCTGAGGCAGGAGAATTGCTTGAACCCCGGGGCTGGAGG
TTGCAGTGAGCCGAGATCGTGCCACTGCACTCCAGCCTGGGTGACAGAGCAAGATTCTGTCTCA
AAAAAAAAAAAAGAAAAAATGGGAATAAAACCAGCTTATTAGTGGCCTCCTGGTTATCTAAGGA
AAACACAGGACTGTAGGACTGAAAATTAGGAACTCCGAGCTCTGTTTCTGTGTGTGCTGCCCTT
AACTTGGTTGACTTGGCCAGAGGCCAAATACTGATAATGAGGCCAAACTGGTCATTTTGCTGCTT
AGTCTATTGTTCATCTACTGTTCAACCAGACTTTCAACTTCCTTTTCAGCTCTCCCCTCTCAGATG
AGTTGCATTATGCTTTTATGACAAATTCTACAAACTCTATATTCATTTGAAAATGGTATCAGCACT
GTCTCCCTCATAAATACCTAGAGGAGTAACTAGTCTTAAGTCGCTATGTAAACACTTACTCCTTG
TAACTTTGTTATATGAGAACTTCACAAGGAATGCTTCAAAGATCATTGGGGCAGTTGGAAAGCCT
GCCTTACCAAAGTGTGGCCTTAGTTGTCCATAGCATCACAAAAGAGCCATCCAAGTAGGCTGAA
TGAGGATCAGTCTCTCAGGGTGCCCACTGGTTGTGGCTGTGAAGTCCTGGACACTCATTTTGCCT
GATGGCCAAATCCTAGGGAGCAGTGACTATGTGTGAGTAGGATTCGTTGACAGAGGTAGGAGG
TTTTTTTTTTGTTTTTTTTTCCTGCTGCCTGTATAATTGCAGAAAAGGAGTGATTCTTTACAGTAATT
CCCCCTCTTCGGGATGAAGATGCTTGCTTCTGTTTGGTTTCCACAGTTAAATCAATTTTTTTTCCTT
TTCTTTTGAGATGGAATCTTGCTCTGTCGCCCAGGCTGGAGTGCAGTGGCGCGATTTCGCCCACT
GCAACCTCCACCTCCCAGGTTTAAGCGATTCTCCTGCCTCAGCCTCCCGCGTAGCTAGGATTAC
AGGCACTCACCACCATGCCTGGCTAATTTTTGCATTTTATTTTTTTCTGGAACGGAGTTTCACCTG
GTTGCCAAGGCTGGAGCGCGATGGTGTGATCTTGGCTCACCACAACCTCTGCCTCCTGGGTTCA

AGCGATTCTCCTGCCTCAGCCTCCCAAGGAGCTGGGACTATAGGCATGCGCCACCATGCACGG
CTAGTTTTGTATTTTTAGTAGAGATGGGGTTTCTCCATGTTGGTCAGGCTGGTCTTGAACTCCCAA
CCTCAGGTGATCCTCCCACCTTGGCCTCCCAAAGTGCTGGGATTACAGTCAGTGAGCCACTGTG
CCCGGCCTGTATTTGTATTTTTAGTAGAGACAGGGTTTTACCATGTTGGCCAGGCTGGTCTCCAA
CTCCTGACCTCAGGTGGTCCGCCCACCTCGGCCTCCCAGTGTGCTAGGATTGCAGGCGTGAGCT
ACGGTGCCTGGCCAGTTAATCAGTTTTTTACCCTCCTGTGTTTAGGCCCCAACATCCTGTTCTTGA
TCTTTTTTGGCAAAAATTTGGCCAAAGATTGAAATTTTGCCTCCTGGACCATATATTTTGCTCCAG
TTGCAAAGTATGACTTTTATCCTTTGGTCAGCAGTTCCCATGGTGTTTAGGGCCTGAATTTGACAT
TCTTTTTTTTTTTTTCTTTTTGAGATGGAGTCTAGCTCTGTTGCCCAGGCTGGAGTGCAGTGGCACG
ATCTCGGCTTGCCGCAACCTCTGCCTCCCGGCTTCAAGCAGTTCTCCCTGCCTCAGCCTCCCAA
ATATCTGGGATTACAGGCGCCCACCACCACACCCGGCTAATTTTTGTAGCTTTTTTTGTTTGTTTG
TTTGTTTTTGAGAGTCTCACTCTGTCACCAGACTGGAGTGCAGTGGCGCAGTCTCGGCTCACTGC
AACCTCTGCCTCCTGGGTTCAAGCAATTCTCCTGCCTCAGCTTCCCAAGTAGCTGGGACTACGG
GTGGGCACCACCATGCCTAGCTAATTTTTTTTCTGTATTTTTAGTAGAGACGGAGTTTTACCATAT
TGGCCAGGATGGTCTCGATCTCTTGACCTTGTGATGCGCCCGCCTCGGCCTCCCAAAGTGCTGG
GATTACAGGCGTGAGCCACCGTACCTGGCAACATTCTCTTTTTTTAAAAAAATAACTTTTTATTTC
TAGCCTCAACCATTTACACTTTGCTTGAGAAAGAATAATAAAGTGGAATTGTTATTCCAGTGATG
TTGTGCAAATGTGAGAAATAGTACTTCCCCCAAAAAAACAAACCTCGAAACCCCACAAACTCAA
GTGTACTTAAGTACTTAACGTTCAAGTACTTTTTAACTATAACCAACTTTTCCACCACACTGTTGT
GAATATTTGTTGCTAGCTGTTATGGGTATAAGTGATCAAGGGAAGAACTGATGCATTACCTAATG
CTTGACTAAATATAGCCAGCCCTTAAGTAGGTAGAGTATTTGGTTGTGGGTTTTGTTGTTTAAGTC
TGTGGAGTCCCTCTTTGGTTTCTTTAACTTTTGCAGTCATTAGGGCAGAGTATATTTATTTACATTT
CTCCAAGCAAAGTCTTGCTCTGTTGCCCAGGCTGGAGTACGGTGGCGCGATCTCGGCTCACTAC
AACCTCTGCCTCCCAGGTTCAAGCAATTGTCCTGCCTCAGCCTCCCGAGTAGCTGGGATTACAG
ATACCCGCCACGATGCCTGGCTAATTTTTGTATTTTTAATAGAGACGAGATTTTGCCATGTTGGC
CAGGCTGGTTTCGAACTTTTGACCTCAAGTGATCCATCTGCCTTGGCCTCCCAACGTGCTGGGAT
TACAGGCGTGAGCCACCATGCCTGGCCTAAATTTACTTTTTAATAGAGACGAGGCTGGTTATGTT
GCCTGTGTTGGTAACTGTGTTGCCTAGGCTGGTGTCAAGTGCTTGAGCTCAAGTGACCCTCCTGC
CTTGGCTTCCCAAAGTGTTAGGATTACAGGCATGAAGCCACTGCGCCTGGCCTGTCTAATCTTTA
TTATGCTTCCCCTCTTTAGAGATACTTAGTCTGTTTTTCAGAACTAAACCAATCTGGAACTCCCTG
GCTCTCTCTCCTAAAACTCTGAAGTCTAGAGGACCCTGTCTCCTGAGGGCTCTAAGAAAAGCTG
TCCAGCTAGCTGTGTCAGTTTGAGAAGCAGTTTTTCCTGATTGAATACCTAGGCCCCAGTAGGAA
AAAGGGAACAAACTGAAATTGCTAATTTGTAAGCCTGGTCTTTTGAGCAGCTCAGTTAAAATGCT
TGACAGAGGACTTAAGTGAAAGGCTAGAGTGGGCTTGGGGTTCATTTATCATCAAAGCCCCCTG
TTCAATGTAGGTAAATCAGCGGGGTGTGTTGGTATCCACCTTTAGTCCCAGCTACTCAGGAACCT
GAGGTGGGAGGATCACTCAAGCCCAGGAATTTGAGGCTGCAGTGAGATATGATAGCGCCACTG
CAGTCTAGCTTGGGCAACTGAGGGAGACCCCATCTTTTTTTTTTTTTTTCAACTTTTTTTTTTTTTTT
GAGACAGGGTTTCACTATGTTACCCAGGCTGGTCTCAAACTACTGAGCTCAAGGATTCTGCCCA
CCTTGGCCTTCCAAAGTGCTAGGGTTACAGGTGTGAGTCACCGGGCCTGTTGAAACCCCAATTC
TTTAAAAAAAAAAAAAAAAGCTTTTTTTTTTTTTTTTTTTGAGACAGAGTCTTATTCTGCCACCTAGG
CTGGAGTGCAGTGTGGCGTGATGTTGGCTCACTGCAACCTTTGCCTCCCGGATTCAAGTGATTCT
CCTGCCTCAGCCTCCCAAGTAGCTGGGATTACAGGCACCCACCACCATGCCCAGCTAATTTTTG
TGTTTTTTTGTTGTTGTTTTTGTTTTTGAGACGGAGTCTCGCTCTGTTGCCCAGGCTGGAGTGCAG
TGGTGTGATCTCGGCCCACTGCAAGCTCTGCCTTCCGGGTTCACGCCATTCTCCTGTCTTAGCCT

CCCGAGTAGCTGGGACTAAAGGCGCCCGCCACCACACCCAGCTAATTTTTTGTATTTTTAGTAG
AGACGGGGTTTCACCATGTTAGCCAGGATGGTCTCGATCTCCTGACCTCGTGATCCGCCCGCCT
TGGCCTCCCAAAGTGCTGGGATTACAGGCGTGAGCCACCACACCTGGCCAATTTTTGTATTTTTA
GTAGAGACAGGGTTTCACCATGTTGGCCAGGCTGGTCTGGAACTCTTGACCTCAGGTGATCTGC
CTGCCTCGGCCTCCCAAAGTGCTGGGATTACAGGCGTGAGCCACTGCGCCCGGCCTTTTTTTTT
TTTTTTTTTGAGGCTGAGTTTCACTCCTGTTGCCCAGGCCGGAGCACAATGGGGCGATCTCGGCT
CACTGCAAACTCCGCATCCCGGGTTCATGCCATTCTCCTGCCTCAGCCTCTCAAGTAGCTGGGA
CTACAGGCACCTGCCACCATGCTCGGCTAATTTTGTATTTTGTATTTTTAGTAGAGATGAGGTTTC
TCCATATTGGTCAGGCTGGTCTCTTAACTCCAGATCTCAAGAGATCCACCCACCTCAGCCTCCCA
AAGTGCTGAGATTATAGGCATGAACCACCACGCCCGGCCCAACCTTTTTTTTTTTAAATTAAAAT
ATAGGTAACTAATTGTTTTCTCTGCCAGGAGAGCAATATGCTAGTTAAATGTTTCAATAGAAAGT
GAAGAAATAGGAGGCCGGGTGTGGTGGCTCACACCCATCATCCCAGCACTTTGGGAGGCCGAG
GCAGGTGGATCACGAGGTCAGGAGATCGAGATCATCCTGGCTAACACGGTGAAACCCCGTCTC
TACTAAAACTACAAAAAAAAAAATTAGCTGAGCGTGGTGGCGGGTGCCTGTAGTCCCAGCTACTT
GAGAGGCTGAGGCAGGAGAATGGCATGAACCTGAGAGGCGGAACTTGCAGTGAGCCGAGATC
GCGCCACTGCACTCCAGCCTGGGCGACAGAGCAAGACTCCATCCCTCACCCCCCCCCAAAAA
AAAGAAAAGTGTAGGAATAGGCTGGGCTTGGTGGCTCACAACTGTAATTTTGGGAGGCGGAGG
AGGGCGCATTGCTTGAGCCCAGGAGTTTGAGCCCAGGAGTTTGAGACCAGCCTGTGCAACAGG
GTGAAACCCTGTCTCTCCAAAAAAAAAAAAAAAAAAAAAAAAAATAGCCAGGCGTGGTGGCAAG
TACCAGCTACTCAGGAGGCTGCAGTGGGAGGATCGCTTTAGCCTGGGAAGTGGAGGTTGCCGT
AAGTCCAGATCGCGACACTGCACTCCAGCCTAGGCAACAGAGCAAGACCCATCTCAAAAAAAA
AAAAAAAAAAAAGCCAGGCGCGGTGGCTTATGCCTATATTGCCAACACTTTGGGAGGCTGAGA
CGGGCGGATCACTTGAAGTCAGGAGTTCGAGACCAGCCTGGCAACCATGGCGAGACCCCGTCT
CTACTAAAAATAGAAAAATTAGCCAGTTGCAGTGGCGTGCACCTGTAATCCCAGCTACTCGGGA
GGCTGAGGCAGGAGAATCGGTTGAACCCAGGAGGCTGAGGTTGCAGTGAGCCGAAATCACGC
TACTGCACTCCAGCCTGGCGACAGAGTGAGACTCCGTCTCAAAAAAAAAAAAAAAAGTATAGAA
AGTGTTAAAACTGAAATTTTGAATAAACCTCATCATTGTGAGAATATAAATGCAAAAAAAAAAGGT
AAAATGCTAGTGCCTTTGAAAGCAAAATTCAGACATACTTGCATAGCTTAAGGTAAATTGATGTA
TGAGAGTTATTTGTAATTAGATATTTGTGTATATATATATTTTTGAGACAGTTTCGCTCTTCTTGCC
TAGGCTGGAGTGCAATGGCGCGATCTCCTCTCACTGCAACCTCCACCTCCCGGGTTCAAACGAT
TCTGCTGCCTCAGCCTCCCGAGTAGCTGGGATTACAGGCATGTGCCACCACACCCGGCTAATTT
TTGTATTTTTAGTAGAGACGGGGCTTCTGTATGTTGGTCAGGCTGGTCTCTTAACTCCTGACCTCA
GGTGATCTGCCCACCTCAGCCTCCCAAAGTGCTGGGATTACAGGCATGAGCCACTGTGCCCAG
CCTAGATATCTGTATATTTGAAGACTGGGCTGAATTTGTCTTCTAAAACATACTTTTTCTGATGAT
AAATGCATTTATAGTCTTTCCAAGATTCATTTTCTAAATATCATTTTTCTAATACTAGGGGAAATA
AGATTTTCATAGCTGAATCCCTCTTTAAGAAGAGGGTCTGGGCTGGGCGCAGTGGCTCATGCCT
GTAATCCCAGCACTTTGGGAGGCCGAGGCGGGCGGATCACCTGAGGTCGTGAGTTCGAGAGCA
CCCTGACCAACATGGAGAAACTACGTCTCTACTAAAAATACAAAATTAGCTGGGGGTGGTGGC
ACATGCCTGTAATCCCAGCTACTAGGGAGGCTGAGGCAGGAGAATCGCTTGAACCTGGGAGGC
AGAGGTTGCGGTGAGCCAAGATTGCACCATTGCACTCCAGCCTGGGCAACAAGAGCGAAACTC
CGTCTCAAAAAAAAAAAAAAAATAAAGAAAAAGGGTCTAATATCAGAGATAATCACTGCTGCATC
TTACAGTTCAGTCAAAATGAGGCTTCAGCTCTGCACTGTAAAGCCAGAGGGCACTGTTGCTGCC
TTTCCAAGCAGCTGTTTTTCTGGGGCTGCTGCATGACGTCATAGCAGAAGAAAAAAACACGAAG
AAAACTATTTCAAAATAAATGTTTAATATAATTTTTAGACCAAAGAAAAACTCAATCATACATTGT

CTCCAGTGGACCATATGTGCCAGAACAGGACCAACTCAGTCCAGCTTTGAAGGCCCTGATTCAG
TCCTACTGGTATAATCTCCCTTGATTTATTGATTTGTGTATGCTTCTTATGCAATACAAGGTGCATT
TTACTCTTGAAGTCCCTATCACCTCCACCATCGCCATTTAACTGCAAAAGCATGCTTCCTAATGG
GAGGGTTTTCTTCCCCCTTCCCTGCAGAGGTTCTGAACCTTCAAGGAGTTTGACAATCTTATGAA
TCTCTTGAACCTTCTCCCCAGAAAAATGTACATACAAGTCTACAAATAATTTCATGGGGTTCATG
GACCCCAAGGCCCACAATGGTTTCTAAGGCCCCAAATTAAGGAACCTCTGTGCTAGTGATTTGA
GCAACGCAATTGCCCTCAGAAGACACTGACACAGGGCAGGTGAAGGACTGGGTTAACCAAAAT
GACAGGAGGCAATCTCATAATTTCTGTTTTTATTTTGGGGCAAAATTTAGTTCTCAGGGCCTCCCT
ACGTTTAAGAGCAGAACTACTCAAGAACCTAAGCAGCTATCAGCATAGTTTAAGCGATTTAGAA
TAGGGCTGTGCAAGGGGATGTACAGGGTTCCTCATTCAGCTGAGGACCTCATTTAGCTCCTTAA
CTGGAAGCTTTACATTTGATCTGCAGTGAGGGGACATTTCTAATTTTAGTTTGACCCACATGGAA
AGGTGGATAGCTTTTTTTTTTTGTTTTGGTATAGAGAGCACGTAGAATTGGACAATTAGGTAAATC
CTTGGAATCCCCCAAACAGCCATTTGCTTTATAAATCCAGTATTTCTTACCTCTGAACGTCTTAAA
GAGCTGCCTCACAAATTAATCTAACCATTGCTTGCACTAAGAAGTTTGCCAAGAGCCTCTTCCAG
CCTTACTGGGGGGAAGGAAGAGATTACTGAAAACCAACTTGTAGGAATGAAATGAGGCTGTCA
GTTGTCTAACCTAACAAACTGCCAAATTAGGTTTACATTTTTTGTTATTAGACTTTTGAATGGCCG
AGGCAGAGTTAAATTTAAAATTTTGGATTGTAGCCACTTTCCACTCGCCTAAACACTCAATACCG
GTTTGTTTATTTTTCCAGCATCAAGAAAGAAAACCAAGGCACTAGAAAGCAAGCACACTTAAGT
ATTGAAGATAATTACATCTTAAATTTTGCCTGAACTGTATATTGTAGCATACTTAAACCCATTCAA
GGAAAAAAATTGGCAAATTGAAAATTCTCCCATTAAAGATTGATATTTTTGCAAACTGACAAATTT
AACGATTTCGGTACCCCTCCCCAAAAAATGCAGTCACATAAAATATATCAATACTATTTTGAATTA
GCTCTAGAAATCACACTGAAACTATGTTACATTTACAAATATTAAATTTATTATAACTAAAATGAA
TTTAATTGTTCTCAGATTTGGCCACCTTATAGCTCCGTTTAAGGAGGGGATTTGTTAAAAACAAA
AATGCATTATAACTTGGTCAAATTACTTTCACATTAAGGAAAAAAACTTCTAAAAAGGAAAACAA
GAAAAGCAACTCTTCAGTTTCACATAATTAAAAGAACAGGAGAAAGCACGCAAGCTACATATAG
CTAAATTTACGAAACCAA

ID|56043|gene_id|ENSG00000234183|transcript_id|ENST00000443162 Homo sapiens lncRNA

TGAAGCGATTGCAGGCGCTGTGGCTTTCCCGCCCATTCGCGGCGTTGGTTTTCCCGCTTCT
GAGACTTGTTTGGGGAGGTAAAGAGTGTTGGACGTGCTTCTCCTGGTGGGTTTTCACAGACTGT
GAGGATTTATTCGTCTTAGAACGTGTGGATCAGGAGGGCGCTGGGTTTCAAGGTTAGGCAGGCC
TCATGCTCAGCCGTATTCTGGCGGACTGACTACCCTGACGTCCTCTCAGCGCGGACCCTGACAA
GGTCCGCGGCAGGCTGAGGCGGGAGAATCGCTTGAACCCCGGAGGCGGAGGTTGCAGTGAGC
CGAGATCGCGCCACTGCACTCCAGCTGGGCGACGCATCGTCTCAAAACAAACAAACAAAAACA
AAAGCTATCAATTGGCTACATATTGTTATTTGTATGACAAATTCTTTTTCCTTTTTTAGAGATGGAG
TCCTGGGACTACAGGCGCGCGCCCCAATGCCCAGCTAACTTTTGTATTTTTAATAGAGACGGGG
TTTCTCCGTGTTGGTCAGGCTGGTCTCGAACTCCTGACCTCAGGTGATCTGCCCCCCTTTGCCTC
CCAAAGTACTGGGATTACAGGCGTGAGCCACCGCGCCCGGCATGTATTACAAATTCTAAGAAC
ACGAAGATAATGGGGCCGGCTTGGTGGCGCGAACCTGTAGCCTCACCTACTGTCAAGACTGAG
ACAGGAGGATCTCTTGAGCCCAGGAGGTCGAGGCTGCAGTGAGTCATGATCGCACCACTGCAC
TCCAGCTTGGGAGACAGAATATACCCTGTCTAAAAAATAATAATAAAAAAAAAGGTAATGGATG
AGGCCGATAGTCAGGAATGAAATGACTTTAAACAATTGGGTATGCATGGGATTGGGTATGTGTG
TATGTGGGGGCAGAGGGTGGGCATAACCGAAGTCCCATACTCATGTCCCTCTAGGCCTAATACA
TTTTGCAGACCTCACAGAGCTCAGACTGTTGTGAGCCGTTTTTCTTTTCTCAACCTCTTCAGTGTG

TTTATTTTGCTTACATCATTAGGTTAAAATTTGGAGGATAACTCCCAGCTAGAAAGGAAGAAAAG
CTCTAAAGTGGAATCGCCTTCCCCCGGAAGCTGTTGTAAAAATTAAAGGATGTATGTGAGTGCA
CAG

ID|56324|gene_id|ENSG00000234183|transcript_id|ENST00000450016 Homo sapiens lncRNA

ATAATGGGGCCGGCTTGGTGGCGCGAACCTGTAGCCTCACCTACTGTCAAGACTGAGACA
GGAGGATCTCTTGAGCCCAGGAGGTCGAGGCTGCAGTGAGTCATGATCGCACCACTGCACTCC
AGCTTGGGAGACAGAATATACCCTGTCTAAAAAATAATAATAAAAAAAAAGGTAATGGATGAG
GCCGATAGTCAGGAATGAAATGACTTTAAACAATTGGGTATGCATGGGATTGGGTATGTGTGTA
TGTGGGGGCAGAGGGTGGGCATAACCGAAGTCCCATACTCATGTCCCTCTAGGCCTAATACATT
TTGCAGACCTCACAGAGCTCAGACTGTTGTGAGCCGTTTTTCTTTTCTCAACCTCTTCAGTGTGTT
TATTTTGCTTACATCATTAGGTTAAAATTTGGAGGATAACTCCCAGCTAGAAAGGAAGAAAAGCT
CTAAAGTGGAATCGCCTTCCCCCGGAAGCTGTTGTAAAAATTAAAGGATGTATGTGAGTGCA

ID|56057|gene_id|ENSG00000230113|transcript_id|ENST00000442757 Homo sapiens lncRNA

GGAACAGGAGAGAGGCAGACAGAAGGGAGGACTCAAAGAAAAGAAGCCACTAGAAATA
GCCTTCCCTCTGGAGTGGGGAGGAATCCAAAAATCCCAGGAGCTGCCCAGCGGGTGTGACCTT
AGCTAATAATAACAGGCCAGCGTTTTCTCACGAGAAAAAGGATCAGCATAGAGCACGGTGGAG
GGAAAGAGAAGCTGCTTAGCCCCCACACCGCAAGGGGTACAGACAGCACCCAGGGGTTCTGC
CAGGGCCAAACTCCTCTAAAAGCTCCTGGGGAAGGGCTCGGTTAGGGGAACCCAGAGTGGAC
AGAGGAGACCTTTTGGCAATAATGGCATGTCTGTGCTTTTCTTTTGCAGGAAGAGAAGACAATG
AAAAGAGCATAGTCAGGAATCAGGAATCTTTTGTGGTATCAAAGCTGAACCTAAGTGAAGAAG
CAATTTTCTGTCCTCTGCCCTTGTCCAAAAGAACACCCCTCAGTTCCTGCTTCTTACTAAGCCGGT
TGAGGGGCGTCAAGGGGCTGGACAGGACACCCCGCAAACTTTCCAGCCATTCCTGCTGTTCCTT
CTCACTGGGGCAAGTGAGGACAAATCTCCGCTCTGGGGTGACAATGGTGAGTCCGGCTTTCCA
GCGATTTCCTCGGATGCCCTTGGGCAGGTCTTCGTAGGCTTCATATCCCTGCTC

ID|56082|gene_id|ENSG00000230074|transcript_id|ENST00000434627 Homo sapiens lncRNA

GGGCTCCGAACGTCTCCTGGGGAGCGGCATGATTGAATCAGGGATTCCCTAGCCCCAGGT
CCCCTTCTCCGTCATTCTCAGGACGAGGCTGCCCAGTTCTCAGGTAATCTGAGGCTGATGCCCC
CGAGAAGCCTCAGTTGCCACCACGGGACAGGGTGGGCTTGGAGATTTGGGAGGCACTGGAAG
TTAAGGGGTGAGGAGGCCGGTCATCTCCGGGGCCTGGGGCATTTAGGGGGCCAGGGCGAGAG
GTTTGGCGGTCATGGGGCTGGGAGATTTGGGGCCCTGGGTGAGGATTTTCAGGGGCTGGGGTC
ACCTGGAGGGGGGGCTGAGAGGGGTCAGCGCAGCCTAAACTCCCGCCCTCCCTGGCTCCACC
CCGGAAACGGACCGTTACTATTACGTCACAGGGATGCCGTTGCGCACTAGGTTTGCCTGTCCCC
CGGAAGGGGTGGGGTGTTCATGTCTCCCAGAGGAGGCGGAGTCGAAACGTCATCTTACGGGGT
GAGGCCAGGGGCGGGGTCGGTTGATTAAGATGCGGGCAAAGGACCCTCTTTTATAGGTGTCAG
GTCAGGTTTTGGAGCTACACCTTGGGGGGCCGAGGCTTCCGCAGCAGGCAGCAAGAAAAGAG
AGTTGGGACTAAGTCCAGTACTGTTTTCTTAATCCCCACGTCTCCTCTACCCCTCCCTGGGCTTCT
GCCCTGCCGAGTGCCTCTCTTGCCTGCCTTCCTCCCCTTGTATGACCCTGGGCCGAGGAGGAAT
AGGTCCTCAGATAGAGTCCAGTCTAGAAAAGGCCAGGCTACGGAAATACCTAGTTGAGTGGCC
TCGGCTAAGTCACTGAATGGCCGGAGGGTCATTGCCCTTCCTCTCTGGTCTGAGACCTATCTTCT
TGTGATTTTTTTTTTTTTTTTTTTGCTACATTCATTCAATCAACAAATATTTGCTGAATGACCACCTGT
GTGTCAGGCACTGTGCTGGGCATGGGGATGTAGTAGTAAAGAAGACAGGTGGCCGGGCACGG

TGGCTCACGCCTTTAATCCCAGCACTTTGGGAGGCCGAGGTGGGTGGATCACGAGTTCAGGAG
TTCAAGACCAGCCTGGCCAAGATGGTGAAACCCCGTCTCTACTAAAAATACAAAAGTTAGCCGG
GCGTGGTGGCAGGCGCCTGTAATCCCAGCTACTCGGGAGGCTGAGGCAGAGAATTGCTTGAAA
CTGGGAGGCGGAGGTTGCTGTGAGCTGAGATCGCACCACTGCACTCCAGCCTGGGTGACAGAG
TGAGACTCCGTCTCAAACAAAACAAAACAAAACAACCAACAAACAAGACAGGCACCTTGTCTG
CCCTCTGAGAGGTAGGGGTAGAGCTGGGAGGATCAAAAGAGAATAGTGAGATTCTTACTATAA
ATGGAGCAGGCTGGCTGGAAGAAGTAGAAGGTGTCAAGAAAGGGCAATCAACCCAGTGTGGG
AGGTTGGAAATAGCTGCCCCAGAGCTCTGACATCCCAGTCAAGGCCCAGTACTGAGCTGTAGT
CGGTTTGAGTAAGGGGAAAGACAGTTTTGGGGCTGGGCTGACTGTATAGGAGGGAGTGACCAG
AAATAAGTGGGGTGAAAGTTTCCTCTGGTTGAAATGTGAGGAATGGATACCCCTCTTCTCTCCCT
CATCCCACCCTCTTTCTCCCTCTCATTCTTGTCTTCCCTTGTCTGAGCCACATACAGAGAGCAGAC
AGAGAGATCCATGGGTGCTAGGTGGTTTTATTGGAAGCACTGCAGTCTGGGAGTTCAGAAGCTA
TGCTTTCACCACGTTCACCCTTTCACTCACATTCACACTCATGTTGACACTCCAGGCCTGGCTGG
GTCAGGTCTGGTGCCGAGGAACTAGAGGCCTGGTCTTCCCTTCTGGTCCTTGGTTCCCCAGGCC
AGGGCTGTCTCTGGCACTGTCATGGGCTAACTGCTGTGGCTCTTGCTCTGGGAAAGGAAGGAGA
GGAAGGGCCATGGGCTGTAATCTTAGACAGGGCTCAGAGGGGGCAGACAGGACTGGGGAGGG
CCAGGCTTTCCTTGGCAGAGGTCTTCCGCAGTCTCCAGTTTGTGCAATCCATCCAGGGCTGGTCT
AGAGGCTGCCTTTCAGTGTGGTGAATCTGGAGTCAGAGAGAATGGAGCCATAGAACTCATCTTC
CTGAAAGGCCCTGGTGTGAGGGGCAGATGCTGGGGACCAGATCTGGGAACCTGTTTTAGGGGC
TTCCTTGTGGGAGGGATTGGGCAAGGAGAAGTGAAGGAAGTAACATGGTGAATGGAGACCTGG
ATGATGGAGGAAGTGATTGGGAGGGGAGGAGACTGGACATTCTCCACCCCGACTCATAGGACA
AAGTGCCCTGCAGCCATCCCTGAAGAGGAGGTAGCAGAAGGCACCAAAAATGTTCCCAGGAAT
TGGGTGTTGATTTGCTAAGAGGCAGAGTCCCCAGAGTTGTTGGTATCTCCCAGAGCTAGGGAGT
GAGTATTAGCTTTAGGGCTTAAGATGGGAAGGCCATAGCCCTGACTGGAACTTGGGATATCTGT
GTCCAGAGCTTTAGTGTTGCTCTCTACTCCACCCACATGCACTGTTCTCTTCTTCCTCTGGGAAGT
CCAGGCTAGAGAGACAGAGCCTATAAAGAGAAGAGCAGCCAGAGGCTCCAGGACCCATCCAG
GCACTGATACATACAGGAACAGCTGGGGTACAGGCACATCTACATCACACAGGTGCACACAAA
GGGCATAGGCATACCCACACCACACAGGCACACACATAGACTGCAAAGGAATACACACAATGC
ACAGGTATACCCATGTCACACAGGACGTAAAGTCATCACAAGGGCACACCCATTTGGCATATGT
ACAGTCTCAGGTGCATATCAGTCTCAGAACCTGGATGTTGATATTGTGAAAGGAGAATAAATCT
CAGAACCCCCAAATCATTCAACCAAGGGGAAAGTCAAGCTGGGAATTGTGTCAGGCAAACCTG
CCTCTTGTTTTATTCCTAAAAGAGATAGCTACAAAGAAAAAGCTACATACCTCCCTCACAATTTG
TCCACAGGGAAATTCCTTATGGGCCTCAAGATCTTTACCTTAAAACAGTTGTGCTGAATTTCACC
CTGGCAATGTAAACTGATAGCTTATCTTCACAGGTGCAGGACAATAGACAGAACTCAAAGTCAT
CCCTCTGCTCACCTGAGACAAATGTGTATCTGATTGCTGCTTCTGCCCTATTGTTTATGCAAAAAT
GCAGATCCACTGAGCCAGACTAAGGCATCAGTGTGACTATTCCTTTACTCCCCACTCCATGTAA
ATTGTGTATTCAGTGAAAGGCTGATCAAAGACCCCAAAAAATGCAGCCTTTTGTCTCTTATCTAC
CTATGACCTGGAAGCCCCCACTTCGAGTTGTCCCGCCTTTCCAGACTGAACCAGTGTACACCTT
ACACGTATTGATTGATGTCTCATGTCTCCCCAAAATGTATAAAACCAAGCTGTGCACCGACCAC
CTTTAGCATGTGTTGTCAGAACCTCCTGAGGCTGTATCATGTCATGAGTGTGTGCTCAACCTTGG
CAAAATAAACTTTCTAAATTGATTGAGACCCATCTCAGATACTTTTGGATTCACAACATGAACTC
AGCTTCCTTTACAGACCCCTGAGACCTGCACATTGATCCCTGCCATGCCCCTCCGTACCGTTTAA
CCCCTGCCCCATTTTACTCACTCATGGCAAGAAAACCAATATGCTGAGAAGAGGAGTGTTGCCA
CACACACAAGAGGCCATGGAGGATGAGCAGAGATGGACAAATGAGGAGAGTCCCTGGGAGCA

GCAGGTGTGCATGGGTGTGCAGGAGTCCTTCTGCTTGTGTGGAGTCTGTGAGACTGTGTGTGAG
GTTGGATGGGGAGAGCAAGCAGGAGAGGGATCATGATCATGAGGGCGAGGGAGTCCTTGGAA
GACTGAGGGTCCATGAGTGTGTGTGGATCTGTAGGAGGCTGTGGCTGTCTCCTATTTATGGCCC
CCTAAAACTTTCATTTTCTACGTTCAGAAACTTCCTTCCCTAATGGTGTGTAATAAAGAATTTGAC
TGCCTTTTGTCCCTGGCTCCTGAGAGGGAGACTAAACCTTTGGAATTTCCCAAGTAATAGGAGT
GTCTTTATTACTCATGAGCCCCTTGGAACACACCTGGGTTTATGCTAAGAGATGAGATGGCTCAG
GTTAGAGGTTGGTCACAGTAAAGACCAACCATGTGATTAGAGAGTTGAGACTTTGGGCCAGGCT
GACCTCTGGGGAGGAAGGGGGCGCTGGAGATTGAATTCAGGTATGTGGCCAATAATTCAATTA
TGCTGATGTAAGGAAATCTCAAAAAAAACTCTGGACACCAAAGCTCATAAAACTTTCTGGTTGA
TGAACACATTGATGTCCCAGGAGGCTGATGCACCTTATGTCTCCACAAGGAGAAACCACTGAGA
GAAGCTCTGCATTTGAGACCCTCCTAGACTTCATCCTGTGTGTCTTTTCATTTGGCTGTTTCTGAT
TTATACCCTTAATAAACTAATTAAACACAGCACTTTCCTGAGTTCTGTGAGTCACTCTAGCAAATT
ATCAAAACTGACGGGTTTTGGGAACTTCTCAGACTTGTAGCCAGTTGGCCAGAAATGTGGGTGG
CTTGGAGACCCTACTTGTGGCTGGTATCTAAACTGGGGACAGTCTTGTTGGGGACCTTTCCCTTA
AACCTGTGGAATCTGGTGCTAACTCTACGTGGCTAGTGTCAGAAGTGAATTGCAGTCCATGAGT
TGGTAGCAGAATACTTTCCCACTGGATAGCTCCCCATCGCCTCTCTCTCCCTTTTTGTGGAGGAA
ACTCTTTTCTCTTTCACAACTCTCCGCAGTAGACTGCACCTGTTTTTCACTTTTTGGCTTCAGCTCC
GAGAATTTCAGCAATAAGCAGTTTGGACAAGGGGAGGAGAGTTTGCACTATTACCATTAGGGG
AAGATTTGCAAGGTTTGTGATGTTGCTCCTTTGGAGGCATTTGAGCCATCAGTCTCAAGGCTGGG
AGTCAGGGTTTGGTTCAAGATTAGGGTCAGAGGTAGGAATGTGTCTCCTCCCTAGGTTCCTCACT
GCCAGCTCTTCTCTGTAGTACTAAGATACCTGGGAGTAGGAGGAGTGGGAAAGATGGGACATA
GCCACCCACTAGATTCCCTGAGGGACAATCACAAGAGCAGGGTAAGGATGAGAAGGTCCTCTC
CCAGATTCTCCAGACCTCCCTGGGCCTATCACAGTGCCTATATATCCACCATCTGAGATCCATTC
CTCCCTTGGAAAGTGAAGCTAAATGGAACATTCTTTGGAAGCAGGTTCAGCTCCCCTATCTCTCA
TCTCTGGACTCTAGGACTTCACCTTCCTGGCCCAGCCAGCACTGTCCCTCTCCCTTATTGACCCA
TTTTCCTATGCCCCAGAGCTGAGTTAGAATAGCTGGAGGAGAACTTGGCCTAGAACATGCCCAT
TCTCTACCGTGGCCTCCCTATCCTGGGAATTGAGAGGGATTTTTGAGGAGAGGGTTTGGTCTGCT
CACAGAGTAACAGGAAAAGAAAGGAATGAGGGAGTCCTGAGAAGCAGGCTTACTGTGTACTG
GTTGCCAATTTGTCTGAGTCCAGTGGGAGAACACATCCAACAAGTTATATGAAATGGATTTATTA
CTCTCAGGTAGGCAGCAAGGGAAACAGAATCCCAGGATTAATTGTGAGGCAGCCCCCAAGGCT
CAGGAAAGCTGCCCAGGGCAGATGGCTTCATCTGCCTATGCTGCACTTGCACTGCAGCCAAGG
ACTCCAGAAAGCAGCCTGCCTTGGGTTTTATACCCCAGGGCAATGTGAATTCCTGGGCTAAAAC
ATTGAAGGATATTGTTTCTGGGGTGGGACTAGAAGAGAGCCTGTTCCTTGTCTGTTTCTGTCACC
TCTTCCTTATCTCAAGATTTTGCATTCCAAGTGAGAAAGGGAGGAGAACTGGGTCAGTGTACGG
TCACCCAGAGAACTGTTCTGCCAGACCCAAGAGATAGACCTCTCAGAAGACACTATTGTGGAGT
GCTATTCTGTTTCCCATTCCAGATCTAGAGTATATAGTGGAGCAGATTTAATTGCCTGTATTTTTC
TGCCTCATCCATCCATCCATCCATCCATCCATCCATCCATCCATCCATCCATGTGTCTATATGTCT
ACTCATCTTTATCTCTTCATCAGCCCATTCTTCCTTCTTTCATCTTTCATTTATTTTTTCATATATTCA
TTGTGACCTGTTCATCTGTATGTTCAACCATTATTCATTTATTGTTTCCCTTCATTTGTTTGTCTCTC
AGTTATGGTAATAATTAACATTTATGATACTTAAGTGCCAGGCACCATGCAAAATGCTTTAAATT
CATTGTCTCATGACAAATCTATGAAGTGGATTGCTGTGGGCTGTTTTTGTTTATGCAGACCACTTT
TCTACTTTTCTATACCCCTTTTTGTGGTTCCTGTAGGAAACTCCTTTCCCACTTAACATGGTCTTGG
TGGGGCTGTCAGCTACAGTGCTCTGATCAGAGTACCCCTTTTCCTGGCCACTGTGATAGACTGA
AGAACTGAGTATGTATCACAAGTACAGTTAACCAGAGCTTTCTATGAGATTTGATGTTTGGATGC

TAGGAGAAAGAGAGTCTCTTTTAGATCAATCAATATAAAAATTTAGGCTTAAGGCTTCTAGTGGC
CATATTTCTTGCTACTAGAAGAGCACCTGTATCTTAGACCATTTTCTGTTTCTTAACAGAATACCT
AAATCTGGGTAATTTATAAAGAAAAATAATTTATTTCTTACAGTTATGGAGGCTGAGAAGTCCAA
GATTGAGGGGACATACCTGGTGAGATCCTTCTTGCTGGTGGGGACTCTCTGCAGGGTCGTGAGG
TGTGGCACAGGGCATCACATGGTGAGGGGGCTGAGTGTCCTAGCCCGTGTCTTTTCTTAGGAAG
CCTCTAGTCCCACACCCATAATAACCCATTACTCCATGGATGGATTCATCCATTCATCAAGGCAG
AGCCCTCATGACCCAATCACTACTTAAAGGCCCCACCTTTCAATATTGCTACATTTGGGATTAAA
TTTCTTTTATTTATTTTTAATTTGTTATATATTTTTAAGAGACGAAGTCTCACTCTGTCGCCCAGGCT
GGAGTGCAGTGGCATGATCATGGCTCACTGCAGCCTTGAACTCCTGGTCTCAGCCAGTTCTTCT
GCCTCAGCTTCCCAAGTAGCTGAGACTACAGCTACAGGTGTGCACCACCATGCCCAGCCAGGG
ATTAAATTTCAGCATGAGTTTTGGAGGGGACAAATATTCAAACCATAGCAACCTGTCTATGACA
GGAATGAGATCCCTACACAAAAAGATTAGTCAGAGCATGGTGGCTCATGCCTGTAAACCCAGC
ACTTTGGAAGGCCAAGGCGGACAGATCACCTGGGGTCAGAAGTTTGACACCAGCCTAGCCAAC
CTAGTGAAATCCTGTCTCTACTAAAAATACAAAAATTAGCCAGTCGTGGTGGCATGTACCTGCA
GTCCCAGCTACTCGAGAGATGAGGCAGGAGGATTCTCTAACCTGGGAGCGGGAGGTTGCAGTG
AGCCGAGATCGTGCCACTGCACTCCGGTCCGGGCTACAGAGCGAGACTCTATTTCAAAAAAAA
AAAAAAGAGAGGAATTGAGGTAAGAGAAGGAGATAACAACATTTCAACCCCTGGATCCAGCCA
GGGTTGCAGATAATCTTCTCCTTAATGAGAAGCTTTTTTGTTGTTGTTGTTTGTTTGTTTTTTTAAG
ATGGAGCCTCGCTCTGTTGCCTAGTCTGGAGTGCAGAGGTGTGATCTCGGCTCACTGCAACCTC
CACCTCTTGGGCTCAAGTGATTCTCCCACCTCAGCCTCCTGAGTAGGTGGGTGCCACAGCCTGG
CTAATTTTTTTTTTTTTTTTTGAGACGGAGTCTCGCTCTGTCGCCCAGGCTGGAGTGCAGTGGTGTG
ATCTCGGCTCACTTCAAGCTCTGCCTCCCAGGTTCACGCCATTCTCCTGCCTCAGCCTCCAGAGT
AGTTGGGACTACAGGCACCCGCTGCCAAACCTGGCTAATTTTTTGTATTTTTAGTAGAGACGGG
GTTTCACCATGTTAGCCAGGATGGTCTTGATCTCCTGACCTTGTGATCTGCCTGCCTCGGCCTCC
CAGAGTGCTGGGATTACAGGTGTGAGCCACCGCACCTGGCCAGCCTGGCTAATTTTTGTAGAGA
CAGAATTTCCCCATGTTGCCCAGGCAGGTGTCAAATTCCTGAGGTTAAGCAGGCCACCTGCCTC
AGCCTCCCAAAGTGCTGGGATTACAGGCGTGAGCCACTGCATCTGGCCTCATACCTAGTTTTGA
TATAACAAATTTAACTTTTTGCTCAAGGTGCTTTGAATTGGGTTTTTGCCGTTTGCAACCGAAAAA
GCCCTCAGTAAAAATGACTGTATTATCCCTATTATACAGATGGGAAACTAAGACCCAGAAAGGT
TAATTTTCCCAAGATTTCCCAGTTTATAATGAGTGGAACACTGGTATTCGGAGCAAAACTCTGCT
TTATTACCTGTATGTGTCCATCTGCCCATCTATCCATCCGTCCATCCCATCCATTTATTTCTCTGAC
CATCTTTCAATGCCAAGACACTGAAGACAATTAGTCTTGTTTCACTTGCCAACTTCAATTCTGAA
GCTGAGTCTGGATCAGGGATTGGTAAACTTTCTGTAAATGTCCAGATAGTAAATATTTAAGATTC
TGTGGGCCATACAGTCTTTGTAGCAACTACTCAGCTTTGCTGTTGCAGCACAAAAGCAGTCATA
GATATATTTAAATGAATAAGCATGGCTGTATTCCAATAAAACTCTATTTACAGAAACAGGTGGCA
GGCTGCATTTGGCCCATGGGCCATAGTTTGCCAATCCTAGTCTGGATCAATGGGGAAGACATAT
TATGTTTTATTATTGGGAACATGAACAGGGAGTGCCCTTGTGTTTGTACTGTGATTATATGCTTAA
ACGATTGTAGTGTGTCCTTGGTGTATGTGAGTGATTGGCTGTGTGATTGATTATGTGTCTGTGTAT
GTATGTGTGTGGGTAGAGACACGTAGGTGGGTAAGGGTGTCGGCAATTATGTGTTTGTGTATGG
TGTTTTTGTTGTTGTTGTTATTTTTGTTTTTTGAGACAGGGTCTCGCTCTGTCACTCAGGCTGGAAT
GCAGTGGCGTGATCTCGGCTCACTGTAACCACCACTGGGCTCAAGCTATCCTCCTACCTCCCAA
GTAGCTGGGACTACAGGTGCGTGCCACTACACCCCACTAATTTTTTGTATTTTTAGTAGAGATGG
GGTTTCACCGTGTTGCCCAGGCTGGTGTCAAACTCCCGGGCTCAAGTGATCCTCCCGCCTTGGC
CTCCTGCAGTGCTAGGAGTATAGGTGTGAGCCACTGCGCTGGGCAGGTTTGTTTTCTGGCTGCTT

TTTCAATCCATCTCTTTGTGGTTCTCAATCCGCAAGGGCCTGCAGGGGGCGTCCAGTGCCTAGG
GAAAAGGGGCTCCTGGCCTGGACTCTCAACCTCATGGGGCCTGCAGAGGGCGTCTAGCATACG
GGGAAAATGGAGTGTGTGGTTCCTGGCCTGTGGAAGAGGGGATCTGGCCTAAGTGGGCTGTCA
TGTCTTGTTCCAGAAAGGGGCTGGAAATGAGCAAGAAACCAAATTACTCAACTTCTGAAAGTCC
CTGATCCTGGGAAGGGGTCTGCTTCACTTCCTATCCTTTTGCAAATCCTCTATTCATGTCTGGAG
GGTCTCAGTCCAGCTGGAGGGGCAGAGCTTGGCTCTGCAGTCCCAGAGTGTGGGTGGAACATT
GCACAGAACTTCTGGAGGTGATGGGATATCAGGATAGGGGGAAGTCTTGAGCAATAGCATACA
CCCCTTCCCTGCGTCCTATCCCCTGGAGCTTGAAATAACGCCACTGCCAGGGTTTCCTCAATAGT
TATCTCCTCTATTCCCTGCAGTTTGGGTCACTCTGAACCTCAGGGTGCCTGGGCCTGGGCTGAGC
TTGCTTGGGAGAAGCTCAGGAAACAAGGGCCCCTGGATGTAAAGCTGAGGGTGACCTCTGCTA
TGTGGTTGGAGATGGGGAGCCCTGGGGTGTCAGAGGGAAGTAGGGAGGGGAGGCTGCCTGAA
GGAGATAGCTTTGCAAAAAGAACCTCAAGGATGGGTGGGAATGGAGAAAGGACATTCCAGAC
AGAATGAACAGCAAGCACAAAGGCTCAGAGGAGGGACATAAGAGTTTGTGATTAATCCCAATG
TAGGTGGGGCGGGGAAAGGGCTAGTGGTGGGAGAGGCTATATGTGTGTGGGAGGTGGAGTGG
GGGAGTTATTTCTGGTCATATATAATTTTGAAAAATTCCTGAGGAAGGAGCCATGGGGATGGCC
AAGCCCAGATGTTGCAACTGCCCTTAGGAAGGAGGCTGAAGTCAGGGCTGGGCTGTAGTAGCA
GGGCTGAAGAAGTGGCGCAGATACCAGAGACATTAAAGAGGTAGAAGTTGGGCTGGGCATGG
TGGCTCATGCCTGTAATCCCAGCACTTTGGAAGGCCAAGGCAGGTGGATCACTTGAGCCCAGG
AGTTCAAGACCAGCCTAGGCACAATGGGGAAACCACATCTCTACAAAAAATACAAAACTTTGCT
GGGCATGGTGGCGGGCCCCTGTAGTCCCAGCTTCTCAGGAGGCTGAGGTGGAGGATCTCTTGA
GCCTGGGAAGTTGAGGCTGAAGTGAGCCATGATTATGCCACTGCACTCCAGTCTGGGCAACAG
AGTGAGACCCTGGAAGAGCCAGGGATGGTGTACAGATCTACTTCTGGGTTCCAGGCTGCAGAG
CCAACTGCCTATTTGGGATTTCCTTGGCTTTCTTACAGATACTGGATTGGGCTGTTTTTGCATTGT
TATAAAGAAATACCTGAAACTGGGTAATTTATAAGAAAAAAGGTTTAGTTGGCTCACAGTTCTGC
AAGCAATACAGGAAGCATAACACTGGCATTTGCTTCTGAGGAGGACTCAGGAAGCTTACAATC
ACGGCAGAAGCTGAAGGGGGAGCAGGCATCTCATATGGCAGGGGAAGGAGCAAGAGAGAAA
CAGAGGGAGATGCCACACGCTCTTAAACAGCTAGATCACATGAGAACTCACTCATTTTCAGGAG
GACAGCCCCAAGGGGGTGGGGCTAAGCCCTCCATGAGAAATCCATTCCCGTGATCCAATCACC
TCCCGCCAGGCCCTATTTCCAATACTGGGGATGACGCTTCAACATGACATTTGGGCAGGGACAC
ATTTCCAAACTATATCAGATACCTTATTCCAAAACCAAACTCCTGATTCTCAACACCTCCCTCCTA
GCTTGTCTTCGTCTTCTCCCCAGTTATCCATCTCAGAAAAGGAAGCCACCATCCCTCCAGGGGCT
CAGGCCTGAAGCCTGGTGTCATCCCTGGTTTTTCCCTTGCCCTTATGCTTCAAATATAATCCATCA
GCTCAGCCTCATGTTCTACTTATAAGTCCATATTGGATTCCTCTTCTCTATTCCAACTGCCACCAC
CTAGGTTAGGCCAGCATCATCTCTTTCCTAGACTACTGGAAAAGCCCTCTGACTTCCCCATTCTT
TGTACAATTCATTTTTCCTGACTGCTGATAGGGTCTTTGTAAACTGTTTTTTTTTTTTTTTTTTTTTTT
TTGAGATGGAGTCTCGCTCTGTCGCCCAGGCTGGAGTGCAGTGGCACGATCTTGGCTCACTGTA
AGCTCCGCCTCCCGGGTTCACGCCATTCTCCTGCCTTAACCTCCCGAGTAGATGGGACTACAGG
TGCCCGCCACCACGCCTGGCTAATTTTTTGTATTTTTAGTAGAGACGGGGTTTCACCGTGTTAGA
AAGAGGATGTTCTCGATCTCCTGACCTCGTGATCCACCCGCCTGGGCCTCCCAAAATGCTGGGA
TTACAGGTGTGAGCCACTTCGCCCAGCCAGTAAACTGTTAATGAGATCATGTTACTCCTCAGATT
AAATCTTTCACAGCACTTATAGAACAAAATCCTCACTCCTTCTTGTGGCCTGCAAAGACTTGGAT
CCTATGATTCCAGCCTCTGTGTCCTACCTCTTCTTATGCCATCTCCTCCTCGTCCATTGTCCTCTG
GCCACACTGGTCATTCCTCCTTGAATGGCTGGCTTCTTCTCTTTTAGGTGTCCTGGTGGTTAATAC
TGAGTGTCAATTTGATTGGATTGAAGGATTCAAAGTATTGATCCTGGGTGTGTCTGTGAGGGTGT

TGCCAAAGGAGATTAACATTTGAGTCAGTGGGCTGGGAAAGGCAGACCCACCCTTAATGTGAG
TGGGCACAATCTAATTGCTGCCGGCAATATGTGCAGGCAGAAAAATGTGAAAAGAGAGACTGG
CCTAGCCTCCCAGCCTACATCTTTCTCCCATCCTGGATGTTTCCTGCCCTTGAATGTTGGACTCCA
AGTTCTTCAGTTTTGGAACTCGGACTGTCTCTCCTTGCTCCTCAGCCTGCAGACGGCCTATTGTG
GGACCTTGTGATCGTGTGAGGTAATACTTAATAAACTCCCCCCCTTTATATATTTTCTTTCTTTCCT
TCCTTCCTTCCTTTTTTTTTTTTTTTTTTTGGAATGACGTTTTGCTCTTGTTGCCCTGGCTGGAGCGC
AATGGCGCAGTATCGCTCACTCTGCAACCTCTGCCTCCTGGGTTCAAGCGATTCTCCTGCCTCAG
CCTCCTGAGTAGCTGGGATTACAGGCGCCTGCCACCACACCCAGCTAATTTTTGTATATTTAGTA
GAGACAGGGTTTCACCATTTTGACCAGGCTGGTCTTGAACTTCTGACCTCAAGTGATCCGCCCG
CCTAGGCATCCCAAAGTGCTGGGATTAAGAGCCACCATGCCTGGCTCTTTCTTTTTCTTTTTTTTT
GAGACAGAGTCTTGCTCTGTCACCCAGGCTGGAGTGCAGTGACACGATCTCGGCTCACTGCAA
CCTCCACCTCCCGGGTTCAGGCAATTCTTCCACCTCAGCCTCCTGAGTCTCTGGGACTACAGGC
ATGGGCTACCATGCCCGGCTAATTTTTGTATTTTTAGTAGAGATGGGGTTTCACCATGTTGGCCA
GCTGGTCTGGAACTCCTGACCTCAGGCGATCCGTCTGCCTTGGCCTCCCAAAGTGCTAGGATTA
CAGGCCTGAGCCATCACACCCGGCCCCTTTAGATATGTCTATTCCATTAGTTCTGTCCCTTTAGA
GTTTAAACTAACACCTGTCTGGTTTGAACCCCCTTCCTGCATAAAAGCCCTACAGGCCAGATACT
CACATTCCCAGCCTCTCTTGCATCTAAGAAAGTCCAACCAATCTGGATGTACCTTCTCAGGGCTG
TGAATCAAGTATTAGTGATGCAAAGAAGCAAGGACCATGGGGAATCCATTTTGGTGGTGGCAGT
AGAATTGGTGGCAGTGCCATTGTCTCTCACTTGGCCCTTTGACTCATCAGATCCAGTGGTGTTTG
AGGTGTCTGTGGCCAATTACCATGCAGTATGAAGCCCAAGAGCAGGTTGCTCACATTTCCAGCA
TGCTTGCTTCTGGGTCATTGTCACCTTTCCCTTAGTCTGCACCTGCAGCCTCATGGAGAGTTCTTG
ATGACCGTTGACTCATAAGCCAATTTTTCAGCTGGGTATATATGATTCTGCACAATTTTCTGGAAC
TAGTTTAGAGTGGATCACTATAGCATTAAGGCCCCATTCAGGGTGATCCTGAAAGACAGTGGTG
GGCGGATCACGAGGTCAGGAGATCGAGACCATCCTGGCCAACATGGTGAAACCCTGTCTCTAC
TAAAAATACAAAAATTAGCTGGGTGTGGTGGCGCGTGCCTGTAATTCCAGCTACTCAGGAGGCT
GAGGCATGAGAATCGCTTGAACCCAGGAGGCGGAGGTTGCAGTGAGCCGAGATCGCGGCTCT
GCACTCTAGCCTGGTGACAGAGCGAGACGCCTTCTCAAAAAACTTCCATATTGTGGAGAGGACC
ATGTGCCAGTGAATGGCAGGCAGTTCTAGGAGCTGGGAGCTTCAGTCCTACAAGCACAACTAC
CAAAAATAGCAGTGAGTTTGGAAGAGGGCTCTGAGCCTCAGATGAGATTGCAGTGCTGGATGA
CATCTTGAGTTCATTTCTTTTATATTTTTAATTTTTATTTTTGCCTGACAGTCCAAATATGTTTGTAT
GACACCTTGAGTTCAGCCTAGTGAGGCCAAGATCAGAGGACCCAGTCAACCTAGACCTACACT
GTTGGCTCACAGGAGCTGTGAGATAATAAATTTGAGTTATTTTAAGCTGCTAAATTTGTGGTGAT
TTATTTTACAGCAATAGAAAACCAATACAAGGCTGGGTGCAGTGGCTCATGCCTGTAATCTCAG
CACTTTAGAGGACAAGGTGGGCAGATCACCTGAGGTCAGGAGTTTGAGACCAGCCTGGCCAAC
ATGGTAAAACCCCATCTCTACTAAAAATACAAAAATTAGCCAAGCATGGTGGGGGGTGCCTGAA
GTCCCAGCTACTTGGGAGGCTGAGGCAGGAGAATCGCTTGAATCTGGGAGGCGGAGGTTGCAG
TGAGCCGAGATCGCACCACTGCACTCCAGCCTGGGCGACTGAGGGAGACCCTGTCTCAAAAAA
TAAAAAAATAAAAAATAAAATAAGAAGGCCGGGCGCGGTGGCTCACGCCTGTGGTCCCAGCAC
TTTGGGAGGCCGAGGCGGGTGGATCACGAGGTCAGGAGATCGAGACCATCCTGGCTAAAACG
GTGAAACCCCGTCTCTACTAAAAATACAAAAAATTAGCCGGGCGTAGTGGCGGGCGCCTGTAG
TCCCGGCTACTTGGGAGGCTGAGGCAGGAGAATGGCGTGAACCCGGGAGGCGGAGCTTGCAG
TGAGCCGAGATCCCGCCACTGCACTCCAGCCTGGGCGACAGAGCGAGACTCCGTCTCAAAAAA
AAAAATAAATAAATAAAAAAAAAAAATAAAATAAAATAAGAAAACCAATACAAGAAGAGAATAA
TAAATGTCAGCTAAGTCCCAGAACCAGTTGCAGAACTACTGACTGTAACTTTTATCCATATTCCC

TTCCATGTGAAATGTGTCTATTTTTCTGTTCTTTCACTGACTCCTCTTTTCTTTTATCCTCCTTTATTG
CATTCTTGAATATAGGTTGTTGTGGTGGTATTACTACTACTCAGCAAGATTCTGGTTTTACTTCTC
ATCTTGGGAGTTACCATCAAGACGTTAGGTATGGCTGTGTGACCCACTTTGGCCAATGAAATGT
GAACAGAAATGGCATATGTCACTTGTGGACAAAAACATTTAAGCAAGAATTTCCCTTGCTGTCTT
CCTTGACTGGAGCGACCATGGAAGCCTGTGTTGAGATGAAGAGGCTGCCAACAGATCAAAACA
CTTGAAAACTTGAGCAGCCACATGGACTACAACTGTGCTGGAGACTTGCCTGGACCCACAGCA
GGCTTGGTATGCATGGGGAATAGAATTTTGTTGTATTACACTCCTCAGATTTGGATGTCATTTGTT
ATTACAGCATTACCTAGCCTA

ID|56380|gene_id|ENSG00000234072|transcript_id|ENST00000453289 Homo sapiens lncRNA

CTTTCCGGCCGGACTGCCGCGCCTGTGGCCGGAAGAGCCAGCTAGACCAGAGAGTCTGTA
CCTGCAACCCTGGCGGCGTCCGAGCCCAAGGCGGAGTCTGGGAGACAAAGAAGTCCTCGCCG
CCGGCCGCTGGAGGATTCTGCCAGTAGTGAAGTCAGGGATGGGCCCGAGAGAGCTTGGAAAG
AGGACAAGGAAGGGAAGGAGTAGGAGTAGAAAAGCATGACTTTGGCTGGGTATGGTAGCTCCT
GTCTGTAATCCCAGCACTTTGGGAGGCCAAGGCGGGGGAGGATCACTTGAGCCCAGGAATTCG
ATACCAGCCTGGGCAACATAGTGAGACCCCGTTTTTATTTTTATTTTTATTTTTTCCTGAGATGGA
GTCTTGCTCTGTCCCCAGGCTGGAGTGCAGTGGCGCGATCTCGGCTCACTGCAAACTCCGCCTC
CCGGGTTCAAGCAATTGTCCTGCCTCAGCCTCCCGAGTAGCTGAGATTACAGGCGCCCGCCAC
CAAGCCCGGATAATTTTTGTCTTTTTAGTAGAGAAGGGGTTTCACTATGTTGGCCAGGCTGGTCT
CGAACTCCTGACCTTGTGATACGCCTGCCTCAGCCTCCCAAAGTGCTGGGATTACAGGCATGAG
CCACCGCGCCCGGCCTAATTTTTTAATTTTTTCTAGAGATGGGGTCTCGTTATGTTGACCAGGCT
GGTCTTGAACACCTGACCTGAAGTGATCCTCTTGCCTCAGCCTCCCATAGTGCTGCGATCACAG
GTGTAAGTCACTGAGTTTGGCCATTATTCCATTTCTGTACACAAAAACCTTTCTCATTCTGTGCTA
TAACTAGTTAGCATTTAATTGGGTGGATGTGGTATAACTAGTGCCTTGTTGATATTGATGCATATT
TACATGGTTTTGCTGTTATAAACAATGGTCCCTTGCTATTATAAATCACACTGCTATAAGTAACCT
TGCATCTAAATTACTTCAAATGTGTAACTATATGGGTAGGATAATTTCTAGAAGTGGAATTGTGA
ACCAAAGTTTTAGGTGCATTTGTAATT

ID|56383|gene_id|ENSG00000234072|transcript_id|ENST00000447070 Homo sapiens lncRNA

ACAACCCTCGGGGTTAAAGAGCCTCCTCGAGGAAGAGGCTGGGACACAGCTGAAAGAGA
TTGACCCACAACCACTCTAGGGGCCATCACCTGCCCTTCTTCAGTCCTGGGCGTGAAGGTAGAT
TTCTCCCCTAGCCTGTGCGGGAGAGGAGCCCTGGTTTTTCTAACAAGGACTACTGTAGGTTCGC
CGAGTTCGAGGGACGCTACGTATGCAGCGCCTACGCCCGGACACAGGCTTCCGCGTTACCGAC
ACCGCGTGGGGGAGGAGGGCAAGACCCGAGGCTTGCAGAGATGGGGGGTGCACGGTGGCCA
CAGTAGGGAAGGTGCGGAGCATGATGGTGCTCTGTAGGAGCGCAGACGCTGAAAGGCGCGCT
GCTGTGGCTGGGAGAAGAGCGGAGCCAGGAGTTTGTTCCCAATATCTTAGTGCTATGGCAAAA
GAAGGGGGAGGAGTAATTGTGGGGCCCCCCACACACCCGCTGCGGCGATACACTCACCCTCG
CCAAAGTCCTTCCGGAAGTCTAAGTTCGAAGATGGCGCCGCCTGTCTGGTCGCGGTCTTATGAC
GAAATCTTGTTAGCTGCTCTTCACTGACGCCCCCACCCCAGTGCACAAGGTAAACACGTGTGCT
TGGTCCGGAGTCGGTGCCGGGCTAGTTTTCTCTTAGACTCTGTATTCCATAATGCAGCGCGGCC
AGAGTCACCGAGCCCTCCGAAGCCAGCCGAGTTCCAGGGCGTCTTCAGCGGCGCCAGGGACG
CTGCAGGGCCTCATGGGAGTTGCAGTGTCAAAAAGGGGAGATCGACGCCTGCGCGGCCAACG
GGATCCCACGCGGCCTCGACGGGAGCCGGGAGTAGGCGGACTCCCAGGCCGGGCTCGCGCTC
TGGATCCCTGAATCCGGCCTTTCCGGCCGGACTGCCGCGCCTGTGGCCGGAAGAGCCAGCTAG

ACCAGAGAGTCTGTACCTGCAACCCTGGCGGCGTCCGAGCCCAAGGCGGAGTCTGGGAGACA
AAGAAGTCCTCGCCGCCGGCCGCTGGAGGATTCTGCCAGTAGTGAAGTCAGGGATGGGCCCG
AGAGAGCTTGGAAAGAGGACAAGGAAGGGAAGGAGTAGGAGTAGAAAAGCATGACTTTGGCT
GGGTATGGTAGCTCCTGTCTGTAATCCCAGCACTTTGGGAGGCCAAGGCGGGGGAGGATCACT
TGAGCCCAGGAATTCGATACCAGCCTGGGCAACATAGTGAGACCCCGTTTTTATTTTTATTTTTA
TTTTTTCCTGAGATGGAGTCTTGCTCTGTCCCCAGGCTGGAGTGCAGTGGCGCGATCTCGGCTCA
CTGCAAACTCCGCCTCCCGGGTTCAAGCAATTGTCCTGCCTCAGCCTCCCGAGTAGCTGAGATT
ACAGGCGCCCGCCACCAAGCCCGGATAATTTTTGTCTTTTTAGTAGAGAAGGGGTTTCACTATGT
TGGCCAGGCTGGTCTCGAACTCCTGACCTTGTGATACGCCTGCCTCAGCCTCCCAAAGTGCTGG
GATTACAGGCATGAGCCACCGCGCCCGGCCTAATTTTTTAATTTTTTCTAGAGATGGGGTCTCGT
TATGTTGACCAGGCTGGTCTTGAACACCTGACCTGAAGTGATCCTCTTGCCTCAGCCTCCCATAG
TGCTGCGATCACAGGTGTAAGTCACTGAGTTTGGCCATTATTCCATTTCTGTACACAAAAACCTT
TCTCATTCTGTGCTATAACTAGTTAGCATTTAATTGGGTGGATGTGGTATAACTAGTGCCTTGTTG
ATATTGATGCATATTTACATGGTTTTGCTGTTATAAACAATGGTCCCTTGCTATTATAAATCACAC
TGCTATAAGTAACCTTGCATCTAAATTACTTCAAATGTGTAACTATATGGGTAGGATAATTTCTAG
AAGTGGAATTGTGAACCAAAGTTTTAGGTGCATTTGTAATTTTGATTTATAACCAATCTTTTCACT
GATCTACTTCTCTGCCTCTAATTTCTACTAGGTGTTTGTTTGTTTGTTTGTTTGTTTTGAGACAGGG
TCTTGCTCTGTCACCCAGGCTGGAGGACAGTGGTGCAATCACGGCTCACTGCAGCCTCAACCTG
CCACCTCAGCCTCCTGAATAGTTGGGGTGTGTGGCAGGTGTATACCACCACTTCCAGGTTTTTTT
GTTGTTGTTGTTTAATAGAGATGAGGTCTTGCTATATTGCCCAGGCAGGTCTCACACTACTGAGC
GCAAGCGATCCTCCTGCCTATGCCTCCCAAAGTGCTGGGATTACAGGTGTGAGTTGCCATGCCT
GGCCCGGTTATCTTTCTAAAGCATAAAGCAACCCTGTTGATCCTCTGCTTAATTTTGATGATTAAG
TAGTATGTTCAAAATATAGAAATCCATGTATTGATAATGATCTTTTAAAAAAAGTAAAAGCCAAA
ATATCAGCTACTCATCCTACACCATTGAAAGTAGTGACCATTAAACTGCTCTACCTCTACCTCCA
CCTCTCTACCTCTCTACCTCTCTACCTCTACCTCTACCCACGGTCTCCCTCTCTTTCCATGGTCTCC
CTCTCATGTGGAGCCGAAGCTGGACTGTACTGCTGCCATCTCGGCTCACTGCAACCTCCCTGCC
TGATTCTCCTGCCTCAGCCTGCCGAGTGCCTGCGATTGCAGGCACGCGCTGCCACGCCTGACTG
GTTTTGGTGGAGACGGGGTTTCGCTGTGTTGGCCGGGCCGGTCTCCAGCCCCTAACCGCGAGT
GATCCGCCAGCCTCGGCCTCCCGAGGTGCCGGGATTGCAGACGGAGTCTCGTTCACTCAGTGC
TCAATGGTGCCCAGGCTGGAGTGCAGTGGCGTGATCTCGGCTCGCTACAACCTACACCTCCCA
GCCGCCTGCCTTGGCCTCCCAAAGTGCCGAGATTGCAGCCTCTGCCCAGCCGCCACCCCGTCT
GGGAAGTGAGGAGTGTCTCTGCCTGGCCGCCCATCGTCTGGGATGTGAGGAGCCCCTCTGCCT
GGCTGCCCAGTCTGGAAAGTGAGGAGCGTCTCCGCCCGGCCGCCATCCCATCTAGGAAGTGAG
GAGTGCCTCTTCCCAGCCGCCATCACATCTAGGAAGTGAGGAGCGTCTCTGCCCGGCCGCCCA
TCGTCTGAGATGTGGGGAGCGCCTCTGCCCCGCCGCCCCATCTGGGATGTGAGGAGCGCCTCT
GCCCGGCCGCGACCCCGTCTGGGAGGTGAGGAGCGTCTCTGCCCGGCCGCCCCGTCTGAGAA
GTGAGGAGCCACCCCATCTGGGAAGTGAGGAGCATCTCCGCCCGGCAGCCACCCCGTCCGGG
AGGGAGGTGGGGGGTCAGCCCCCCGCCCGGCCAGCCGCCCCGTCCGGGAGGGAGGTGGGGG
GGTCAGCCCCCTGCCCGGCCAGCCGCCCCGTCCGGGAGGTGAGGGGCGCCTCTGCCCGGCCG
CCCCTACTGGGAAGTGAGGAGCCCCTCTGCCCGGCCACCACCCCGTCTGGGAGGTGTGCCCAA
CAGCTCATTGAGAACGGGCCAGGATGACAATGGCGGCTTTGTGGAATAGAAAGGCGGGAAAG
GTGGGGAAAAGATTGAGAAATCGGATGGTTGCCGTGTCTCTGTAGAAAGAAGTAGACATGGGA
GACTTTTCATTTTGTTCTGCACTAAGAAAAATTCTTCTGCCTTGGGATCCTGTTGATCTGTGACCT
TACCCCCAACCCTGTGCTCTCTGAAACATGTGCTGTGTCCACTCAGGGTTAAATGGATTAAGGG

CGGTGCAAGATGTGCTTTGTTAAACAGATGCTTGAAGGCAGCATGCTCGTTAAGAGTCATCACC
AATCCCTAATCTCAAGTAATCAGGGACACAAACACTGCGGAAGGCCGCAGGGTCCTCTGCCTA
GGAAAACCAGAGACCTTTGTTCACTTGTTTATCTGCTGACCTTCCCTCCACTGTTGTCCCATGAC
CCTGCCAAATCCCCCTCTGTGAGAAACACCCAAGAATTAGCAATAAAAAAATAAATTAAAAAAT
ID|55892|gene_id|ENSG00000234072|transcript_id|ENST00000412749 Homo sapiens lncRNA

AGAGAGTCTGTACCTGCAACCCTGGCGGCGTCCGAGCCCAAGGCGGAGTCTGGGAGACA
AAGAAGTCCTCGCCGCCGGCCGCTGGAGGATTCTGCCAGTAGTGAAGTCAGGGATGGGCCCG
AGAGAGCTTGGAAAGAGGACAAGGAAGGGAAGGAGTAGGAGTAGAAAAGCATGACTTTGGCT
GGGTATGGTAGCTCCTGTCTGTAATCCCAGCACTTTGGGAGGCCAAGGCGGGGGAGGATCACT
TGAGCCCAGGAATTCGATACCAGCCTGGGCAACATAGTGAGACCCCGTTTTTATTTTTATTTTTA
TTTTTTCCTGAGATGGAGTCTTGCTCTGTCCCCAGGCTGGAGTGCAGTGGCGCGATCTCGGCTCA
CTGCAAACTCCGCCTCCCGGGTTCAAGCAATTGTCCTGCCTCAGCCTCCCGAGTAGCTGAGATT
ACAGGCGCCCGCCACCAAGCCCGGATAATTTTTGTCTTTTTAGTAGAGAAGGGGTTTCACTATGT
TGGCCAGGCTGGTCTCGAACTCCTGACCTTGTGATACGCCTGCCTCAGCCTCCCAAAGTGCTGG
GATTACAGGCATGAGCCACCGCGCCCGGCCTAATTTTTTAATTTTTTCTAGAGATGGGGTCTCGT
TATGTTGACCAGGCTGGTCTTGAACACCTGACCTGAAGTGATCCTCTTGCCTCAGCCTCCCATAG
TGCTGCGATCACAGGTGTAAGTCACTGAGTTTGGCCATTATTCCATTTCTGTACACAAAAACCTT
TCTCATTCTGTGCTATAACTAGTTAGCATTTAATTGGGTGGATGTGGTATAACTAGTGCCTTGTTG
ATATTGATGCATATTTACATGGTTTTGCTGTTATAAACAATGGTCCCTTGCTATTATAAATCACAC
TGCTATAAGTAACCTTGCATCTAAATTACTTCAAATGTGTAACTATATGGGTAGGATAATTTCTAG
AAGTGGAATTGTGAACCAAAGTTTTAGGTGCATTTGTAATTTTGATTTATAACCAATCTTTTCACT
GATCTACTTCTCTGCCTCTAATTTCTACTAGGTGTTTGTTTGTTTGTTTGTTTGTTTTGAGACAGGG
TCTTGCTCTGTCACCCAGGCTGGAGGACAGTGGTGCAATCACGGCTCACTGCAGCCTCAACCTG
CCACCTCAGCCTCCTGAATAGTTGGGGTGTGTGGCAGGTGTATACCACCACTTCCAGGTTTTTTT
GTTGTTGTTGTTTAATAGAGATGAGGTCTTGCTATATTGCCCAGGCAGGTCTCACACTACTGAGC
GCAAGCGATCCTCCTGCCTATGCCTCCCAAAGTGCTGGGATTACAGGTGTGAGTTGCCATGCCT
GGCCCGGTTATCTTTCTAAAGCATAAAGCAACCCTGTTGATCCTCTGCTTAATTTTGATGATTAAG
TAGTATGTTCAAAATATAGAAATCCATGTATTGATAATGATCTTTTAAAAAAAGTAAAAGCCAAA
ATATCAGCTACTCATCCTACACCATTGAAAGTAGTGACCATTAAACTGCTCTACCTCTACCTCCA
CCTCTCTACCTCTCTACCTCTCTACCTCTACCTCTACCCACGGTCTCCCTCTCTTTCCATGGTCTCC
CTCTCATGTGGAGCCGAAGCTGGACTGTACTGCTGCCATCTCGGCTCACTGCAACCTCCCTGCC
TGATTCTCCTGCCTCAGCCTGCCGAGTGCCTGCGATTGCAGGCACGCGCTGCCACGCCTGACTG
GTTTTGGTGGAGACGGGGTTTCGCTGTGTTGGCCGGGCCGGTCTCCAGCCCCTAACCGCGAGT
GATCCGCCAGCCTCGGCCTCCCGAGGTGCCGGGATTGCAGACGGAGTCTCGTTCACTCAGTGC
TCAATGGTGCCCAGGCTGGAGTGCAGTGGCGTGATCTCGGCTCGCTACAACCTACACCTCCCA
GCCGCCTGCCTTGGCCTCCCAAAGTGCCGAGATTGCAGCCTCTGCCCAGCCGCCACCCCGTCT
GGGAAGTGAGGAGTGTCTCTGCCTGGCCGCCCATCGTCTGGGATGTGAGGAGCCCCTCTGCCT
GGCTGCCCAGTCTGGAAAGTGAGGAGCGTCTCCGCCCGGCCGCCATCCCATCTAGGAAGTGAG
GAGTGCCTCTTCCCAGCCGCCATCACATCTAGGAAGTGAGGAGCGTCTCTGCCCGGCCGCCCA
TCGTCTGAGATGTGGGGAGCGCCTCTGCCCCGCCGCCCCATCTGGGATGTGAGGAGCGCCTCT
GCCCGGCCGCGACCCCGTCTGGGAGGTGAGGAGCGTCTCTGCCCGGCCGCCCCGTCTGAGAA
GTGAGGAGCCACCCCATCTGGGAAGTGAGGAGCATCTCCGCCCGGCAGCCACCCCGTCCGGG
AGGGAGGTGGGGGGTCAGCCCCCCGCCCGGCCAGCCGCCCCGTCCGGGAGGGAGGTGGGGG

GGTCAGCCCCCTGCCCGGCCAGCCGCCCCGTCCGGGAGGTGAGGGGCGCCTCTGCCCGGCCG
CCCCTACTGGGAAGTGAGGAGCCCCTCTGCCCGGCCACCACCCCGTCTGGGAGGTGTGCCCAA
CAGCTCATTGAGAACGGGCCAGGATGACAATGGCGGCTTTGTGGAATAGAAAGGCGGGAAAG
GTGGGGAAAAGATTGAGAAATCGGATGGTTGCCGTGTCTCTGTAGAAAGAAGTAGACATGGGA
GACTTTTCATTTTGTTCTGCACTAAGAAAAATTCTTCTGCCTTGGGATCCTGTTGATCTGTGACCT
TACCCCCAACCCTGTGCTCTCTGAAACATGTGCTGTGTCCACTCAGGGTTAAATGGATTAAGGG
CGGTGCAAGATGTGCTTTGTTAAACAGATGCTTGAAGGCAGCATGCTCGTTAAGAGTCATCACC
AATCCCTAATCTCAAGTAATCAGGGACACAAACACTGCGGAAGGCCGCAGGGTCCTCTGCCTA
GGAAAACCAGAGACCTTTGTTCACTTGTTTATCTGCTGACCTTCCCTCCACTGTTGTCCCATGAC
CCTGCCAAATCCCCCTCTGTGAGAAACACCCAAGAATTAGCAATAAAAAAATAAATTAAAAAAT
AAAAAAATAAAAAAATAAAAAAAACTCATTAAGCTCTAAAAAAAAAAAAAAAAAAGAAAGAAA
AAAAGAAAGTAGTGACCAATTCCTGAAAATTGGCAAAAAAAAAAAAAAAGGCAAGAGTAAGGCA
TTTATCTTGTCTTTCCAGTATGAACTCTAGTTCAAAATAACTGATGAGAGGAAGGTTTTGTTTGTT
TTTTGAGACTGAGTCTCACTCTGTCGCCCAGGCTGGAGTGCAGTGGCGCGATCTCGGCTCACTG
CAACCTCTGCCTCCCAGGTTCAAGCAATTCTTGTGCCTCAGCCTCCTGAGTAGCTGGGATTACA
GGCGCCCGCCACCACTCCCGGCTAATTTTTGTATTTTTTTAGTAGAGACGGGGTTTCACCATGTT
GGCGAGGCTGGTCTTGAACTCCTGACCTCAGGTGATCCGCACACCTAAGCCTCCCAACGTGCTG
GGATTACGGGTGTGAGCCATTGCCCAGCCCGGGATTTTTTTTTTTTTTTATAGATTAACAGCTAGT
AATTGTGCAAAGAATGATAGAAAAAAATCACTATTTTGCATGTCCCAGTGAAATAATTCAGGCA
ACAATCACCAGTGGATGAAATCCCTAAAGAGCTTTTAGGAAGCTTTAAAATGCCTGGACCCACT
CATCACTGAACATGAAACCACCAGTCATTGTTTGCCTCCTGATGTGAGGCAATATAAAGCACAC
AGAACCACTTATTAAACATTCTTGCCCAAAAAATTGAATAGGAATACAGGACTTAGGGGCCAGG
CGTGGTGGCTCACACCTGTAATCCCAGCACTTTGGGAGGCCAAGGAGGGCGGATAATCTGAGG
TCAGGAGTTCAAGACCAGCCTGGCCAATGTGGTGAAACCCTGTCTCTACTAAAAATACAAAAGA
AAATTAGCTGGATGTGGTGACGCACACCTGTAGTCCCAGCTACTCGGGAGGCTAAGGCAGGAG
AATCACTTGAACCTGGGAGGCAGAGGTTGCAGTGAGCTGAAATCGTGCCATTGCACTCCAGCCT
GGGCAACAGAGCAAGACTCCATCTCAAATAAAAAAAAAAGAAATACAGGACATAGGGAATGA
GTGAAATGACAACATAAGAAAGCAAACAGTCAAATCTAGAATGCAGAACATTTCATATGACAAT
CAACATGGCTTCTGTAAGCCAATGTCAATGTAAAAAGGGGATAGGAGACTGCTCTTTTTTTTTTTT
TTTTGTTTGTTTGTTTGTTTGTTTGTTTGAGACAGAGTCTCACTCTGTCGCCCAGGCTGGAGTGCA
GTGGCAAGATCTCTGCTCACTGCAAGGTCCACCTCCCGGGTTCACGCCATTCTCCTGCCTCAGC
CTCCCGAGTAGCTGGGACTACAGGCGCCCGCCACCACGCCTGGCTAATTTTTTGTATTTTTAGTA
GAGATGGGGTTCCACTGTGTTAGCCAGGATGGTCTCCATCTCCTGACCTCGTGAACTGCCCGCC
TCGGCCTCCCAAAGTGCTGGGATTAGGCGTGAGCCACCGCGCCCGGCTGAGGAGACTGCTTTT
TAAAAAATGTAACCAAATGTAATGTGTGGATTTGTTTGGATCTTGAGTCAAACTAAGTCATTTTTC
AGCCAAGGAAATTTGATTATGGATTGAATACTAAGAAAGTATGATTTAATAAGTGATATTATGGT
AAATTTCCATATTTCTTAAGAGACATTGAAGTTAAAGGAGTGAAATGATATAATGCTTGGATTTG
ATTTAAAGTAACTTCATCAAGGAAAAACAAGAAGGCTGAGTGCTGTGGCTCACGCCTGTAATCC
CAACACCTTGGGACGCTGAGGCGGGCGGATCACCTGAGGTCGGGAGTTCAAGACCAGCCTGA
CCAACATGGAGAAACCCCCATCTCTACTAAAAATACAAAATTAGCTGGGTATGGTGGCACATGC
CCATAATCCCAGCTACTCGAGAGGCTGAGGCAGGGGAATCGCTTGAATCCAGGAGGCGGAGGT
TGCAGTGAGCCAAGATTGCACCATTGCACTCCAGCCTGGGCAACAAGAGCGAAACTCTGTCTC
AAAAAAAAAAAAAAAAAAAAAGAAAAAAGAAAAACAACAAAAAGAGAAAGTAAATGTGGCAAA
TTTGATAACTGTTGCATCTGGTCATGGAGATTCTTTGTATAGTTCTACTTTTACGCTGCTTAAAAAT

GTTTGATAAAACCAACATGGCATATGTATACCTATGTAACAAACCTGCACATTGCGCACATGTAC
TGTAGAACTTAAAGTATAAAAAAAAAAATTTGATAAAATGGTTGGGTGCAGTGGCTCACGCCTGT
AATCCCAGCACTTTGGGAGGCCAAGGCAGGTGGATTGCTTGAGGTCAAGAGTTCAAGACCAGC
CTGACCCACATGGTGAAACCCCGTCTCTACTAAAAATACAAAAATTAGCTAAGTGTGGTGGCAG
GCACCTGTGATCCCAGCTACTTGGGAGGCTGAGGCAGGAGAATTGCTTGAACCCAGAAGGCAG
AGGTTGCAGTGAGCCGAGATAGTGCCATTGCACTCCAGCCTGGGAGACGAGTGACTCTTTCTCA
AAAAAAAAAAAAATTCAATAACACACATATATACCCAATAGATAACTCTTCATTTTAAGCTAACCA
GTGGTTGGACTCACAGTCTGGCCCCAACTTACCTTTGCAGTTTGATCACTCAACAATAAGACTCA
GTTCTAGCCTCACTATACTATGTGACACTTTTACATTGGCCACTCACTTTAAGGCCTATTCTTTGT
GTTCAAGCTGTTTCTTCTTTTTTTTTCTTCCCTAACTGGGCAATTACTTATCTTTCAAGGACTAGGT
TAGATGTGACTTGTAAGTTTTCACCAATCTTTTGTTCATACTACTACTGGGTTACCTCATACTGTA
GATACTATCTTCTTATGCCATGGTAAGCGTTCCTTAAGGGAATATAGCACAAAAGTTAAGGGCA
CAGTTTCTAGATCCAGACTGCCTGAGGTCACATCCTGGTTTAGCTACTGACTAGCTGTGTTACCT
AGGGCAAGTAACTTCATCTCTCTGTCTGCCTTGGGTAAGTAACTTAACCTTTCTGTGTCTGAGCT
GTGTTGCCTTGGGCAAGCAACTTAACCTTTCTATGCTTCCATTATCTCATCTGTAAAATCGCATTT
ACTTCATAGAGTTGTATTATTATTTTTATTTTTAGAGATGGAGCCTCGCTTTGTTGCCCAGGCTGG
AGTGCAATGGTGCCATCTCAGCTCACTGCAATCTCCATCTCCTGGGTTCAAGCGATTCTTCTGCC
TCAGCCTCTGGAGTAGCTGGGATTACAGGCATGCGCCACCACGCCCAGCTAATTTTTGTATTTA
GTGTAGAGACAGGGTTTCATCATGTTGGCCAGGCTGGTCTTGAACTCTTGACCTGAGGTGATCT
GCCTGCCTCGGCCTCCCAAATGAGCCACTGCGCCCAGCCTTCATAGAGTTGTATTAATCTTCCTA
AATGGGTAAGAAAGGGTTCAGAACAGTTTCTGACACACAGTTAAATATTCAACAAATGCTAATT
TATAAGGTTAGGACTCTCTTTTTGAGACAGGGTCTCACTCTGTCACCCAGGCTGGAGTGCAGCG
GCACAATCATGGCTTACTGCAGCCTCTACCTCCTGGACTCAAGCAATCCTCTGGCTTCAGCCTCC
TGAGTAGCTGGAACAACAGATGAGCACAACCATGCCCAGCTAATTTTTATTTTTTGTAGAGACC
GGATTGCCATGTTGCCCAGGCTGGTCTCAAACCTCTGGGCTCAAACAATCCTCCTGCCTTAGCCT
CCCAAAATGCTGAGATTACAGGAATGAGTCACTGCACTCAGCCCAGGACTATTTGTTTTCATATT
CCTCCTCACACCCAGTTTAGTGTCTGGCATGTAGATGCTCAGTGAATGATTGATTTGGGTAGTTT
TGGTTCTTTATACGGGTCTTCAGGTTAGTCCCAATGTACTGGCATGGAGCTAGTTACTTGAGCAG
GTCTTCACAAAGGTCTGAGTAGCAGGTGATAAAAGGGGTTGTGAAGTCTGGACTCCTAACTTAA
GTGGTGGAGATGCTAAAAGAGAAACAAAGGCAGCAGAGTTGCTGAGGGTAGGGAGTATGGCA
TTTATTAACCCTGTGTTTCCCCCGTCACTGGTCACTGCTCTTGACTCGTAGAACAACAGGTACAG
AACTGCAAGGGATCATCCCCAGCTCCGTGATCACCAGATCCACAAGCTCTGGGGGAGTCACAT
CATAGACTAGATTCAACAACCGTAGGGATGCGTGGTTCTGCCAGTTAGCCAGCGCAACATGTTC
TCCCCGCTTACATTGCAGATCATCAGGGTCATCTGCAATGGAAGGCGTACCCATTATGTTCTTTC
AGAAAAGAAGTTCTAGTTTATCCGCCCCTCTCCCTCCCTCTCAAGTCTTCACAGGTAGCTGGAGG
CTTCTCTTTTGCCTCCTTACCTAGCTCATTAGAGACAAAGGCATCAGTCTGCACACGCTCACAGA
ACTTGTATGTTTCACAGCAAACCAGCACTGGTACATTATGGGCTCGAGCCACCAGGGCTAACTG
TGCTGTCCCTACCCGTGACATCACAGACCCGTTGGCCAAGAGTGCATGAGCTCCCAATAGCACC
TTGGAAACCTGTTTCACAGGAGAAGAGGAAGAAAAAAATGTCATTGTTGTATCAATGCAAAATA
AGGTCCCCTACTTACAGGACAGCAACATTAAGACAAGTAATAAATCTCAACACCTGTGAAAAGA
AGGGAAAGAAACAAAAACAGACAACAAGTAATAAATCTTTTTTTTTTTTTTCTTTGAGATGAAGT
TCTGCTCTTGTTGCCCAGGCTGGAGTGCAATGGCGCGATCTCGGCTCACCACAACCTCTGCCTC
CTGGGTTCAAGTGATTCTCCTGCCTCAGCCTCCCGAGTAGCTGGGATTACAGGCATGCACCACC
ACTCCCAGCTGATTTGTTGTATTTTTATATTTTTAGTAGAGACGGGGTTTCTCCACGTTGGTCAGG

CTGGTCTCGAACTCCCAACCTCAGGTGATCTGCCCGCCTCAGCCACCCAAAGTGCCAGGATAAC
AGGCGTGAGCCACTGAGTCCGGCAATTAATAAATCCTTTTTTTTTTTTTTTTGAGATGGAGTCTC
GCTCTGTCGCCCAGGCTGGAGTGCAGTGGCATGATCTCGGCTCACTGCAAGCTCTGCCTCCTGG
GTTCACGCCATTCTCCTGACTCAGCCTCCAGATTAGCTGGGACTACAGGCGCCCACCACCACGC
CCGGCTAATTTTTTGTATTTTTAGTAGAGACGGGGTTTCACTGTGTTAGCCAGGATGATTGTGAA
CTCCTGACCTCGTGAACTGCCCTCCTCGGCCTCCCAAAGTGCTGGAATTACAGGCTTGAGCCAC
TGTGCCCGGCCTAATAAATCTTAATGATGGCTTTTCTTCTGTGAAAACACCTGACCACTGACCCA
CCAAATCTCCCACAGGAAAAGTAAACTTTGAAGTTACTTTTGTCTTGTTCCCTCTTCCTGGTTGCT
AGTTTAATGTAATGTTTTTGCAATAGCTAGACTGCTGTAATATACTGTCAGTGTTTTTCCAATTAA
ATAGAAACAGAGCTATATAATTATTTTTTTTGACACAGGGTTTCATTCTGTCACCCAGGCTGGAG
TGCAGTGGTGCAATGACAGCTCACTGTAGCCTCAACCTCCTGGGCTCAAGCAGTCCTCTCACCT
CAGCCTCCTAAGTAGCTGGAACTACATGCGCATGCCACCATGCCCGGGTGTGTGTGTGTGTGTG
TGTGTTCAAGCAGTCCTCTCACCTCAGCCTCCTAAGTAGCTGTGTGTGTGTGTGTGTGTGTGCGT
GCGCGCGCGCACGCACTCAAGCAGTCCTCTCACCTCAGCCTAAGTAGCTGGAACTACATGCGC
ATGCCACCATGCCCGGGGTGGGGGTGGGGTGGGGTGTGTGTGTGTGTGTGTGTGTGTGTGTGT
GTGTGTGTGTGTGTGTACAGGGTCTCATTATGTTGAGCAGGCTGGTCTTGAACTCCTGGGCTCAA
GTGATCCTCCTGCCTTAGTGCTGAGATTGCAGGCATTGAGCCATGGCACCAAGCCAGGGCTATA
TAATTCTAATATTTTTAAATAATTGTGGCTGGGCACAGTGGCTCATGCCTATGATCCCAGTACTTT
GGGAGGCCGAGACAGGGGGAACTGCTTGAGCCCAGGAGTTCCAGCCTGGGCAATGTAGCGAT
ATCCCATCTCTACAAGAAAAATAAAAAACTAGCTGGGCATGGTGGCACACACCAGTAGTCCCA
GCTACTCAGAGGCGGAGGTGGGAAGATCACCTGAGCCCAGGAGGTCAAGACTGCAGTGAGCT
GTGATCACGTCGCTGCACTCCATCCTTATCTCAAAACAAAAACAAAACTGTAACTTTGGGAAGG
TGAGATTATGTCTATACTACTACACCTTTTCACCGCCAACTTTGGAGTCCTTTTCCTCTGTACTTA
CCTCTGGGAGCACATAGGAGGCTGCAGGAATCAGCAGGTAGGAGGCTGGGACACCAGCATGG
ACTAGAGAACGTAGTGTGTGCCTTCCTTCCAGCCATGGCCGGCTGTCCACCACTACCACCCGAA
ACCGCCGGCCCTCTGTCCAAGCCTCCTGAAGAATTCGTGATACCAGAGATGAGCTAGAGTGAAT
GAAGAGGAGGATTCAGTTATAAATGTCAGTAACTGATGACTAACTTATTCCCAAAGGCAAGTGG
GTAGTCCCTTCTTCAGATCATTCCAACTCCCAATCTGCTCAGTCACAAGGTCTGGACCATACCAT
CCATATACCAGGATCACATCTCCATTACTGATCTTCTGGTAAGCAAAGCGTGAAATTGCCTGAGC
TGCTAGCACAATCTTCTCTTGCACATACCGATCAATGGCTGCTCGAAGTTCTGACTTGGCCTAAA
TGGAGTAAAATCCTTAGTGAACAAGAAATGGACACTTTTATCCCTCTGATGATCATGCCAGGTG
CTTGAATATGTGTATGATGACAAGCCTATAGAGGGCTCAGAGATGGCACAAGTCAGAATGGCC
AAACCATTCCTTAACCCTTGACTAGGGCAAAAAAAGGCTTACGTTGGCCTTCCCGGGAGTTTTTA
ACATGTTTCTTAATTTTACCCACAGGTGCATGCCTTCCTTATTTCTCATACTCATCACCTCCTCTTC
CCGCTTGGAACTGCCCACACTGGTGATTTCCTTGTTAAGGAACTTGATGGCGTTGTGCATGCTCG
CTGACAGGGGACGGCACTGAGTCAGGAAGCTATAATACAACAGCAATACCTTATATCTGCGCA
ACTCTTCACAACTTACAAAGCCTTCACAT

ID|56518|gene_id|ENSG00000254458|transcript_id|ENST00000534065 Homo sapiens lncRNA

GTGGGCGGGGCCTCTGGAGAGCTCCCGGGGGCTCTCTTCTGTTCTGCAGCGAGTAGGCGT
GGCTCGTAGCTCCCAGGTCCGGGCTGATACCGCTGGAGTATGCAGTTCGCCGCCGCGCCCGAA
TGAGGCGGGGCCTGCGAGTAAGTGGGCTGAGCCCGGGCTGGGCGCAGGCCGGGGCTCTGCCC
TGGGAGGCCGTCTGTTTAGGCCCGCACGCAGCTCCGCATCTGGTGCTCATTACCAGATGACAAA
GAGCCGGCTCTGGGCCCCCTTCCCCACTCCCATCCCCCGCCTCCGACTGCAGTGTATTTAACTG

TTTCCTGCCCTCGAGGCGCTCTAGATCCCTACCCGAGGTCACGGAGGGGACTCCAGACCATCCC
GTCTCTGCAGGCCTTGTCGCTCCCCAGGCTTCCTCACCTCCTTCCCAGACTCAGCCGTCTGGGC
CGCCTCGCAACCCTCATTGGCATCCAGCAAACCTGCCCGACGGCGTCTCCGAGGTTAAAGCTGT
GCAGGATCTGGCGGGGACGTCTGGTTTGAGTTAGGGGAACGTGAGAGCGGGGACCGGGGGCG
GTGCGTAGCGTTCCCTCCGCCCCCTCCGCGACCCCCTGGTTCATCCCCAGCTCCGGCCCCTCCT
TTCCTGGTTCCCTGGGGCCCAGCTCAGCCTTCCAGCTCAGGTCCAGGCCCGAAGGGGGCGCTG
GGCCACTCCTCCTCGCCTGCCCGGGTGGTCGTGGCGGCTCTGCCCCGTCCTAATTAGAGGCCCC
CAAGAGGCTGTAACTGAAAAATTACAGAGGCGGTGGGAACTGGCGCCTCCCCTCCCCAGGTCT
CACCCCCGCCCCCCAAGGCAGTGAGCACGAAATGAGGGAGGTCACTCCCACCAGGCTGTGAT
CTTTGACCAGGCCCTCCAGTTCCTGGGACACTTCAGTCCTGAGCAGCCGGAGCCCTCGCTCAAG
CTCAGGTCTGCTGAGCTCTGTCTGTCCCCCACCACCCCCAGCCAACATGGACAAGAGCAACGT
GCGGCCCTGGCAAAAGGAGGCCTTGCTCCTGCGGCTGTCCAAGCAGTTAGCATGGGATCTCTC
CCAGGTTCCCGGCCTTTCCCATCCCAGCCTCCTCCCCGGAGCAAGCGGAGGAATGCCTCCAAG
AGCACCCACAAACCAGGGGTTGGCAGTCCCCTTGAGTTACCCTGAGCCCCAGCCCCCAACTTC
CGCGAAGCTTACAGGGATGGGAAGGGAAGTGTCTGAAGAAAGGAGCAACTCACCCACTAGAC
CCCTCCTTCACTCCTCTGAATCACCAGCCCCAGAGCCTACTTTGACCCCTAAACCCCAGTCCGGT
TCAGCAGATGCCCTAATTCCACAACCGTCCCCCAGATTCAGCCCCACCTTCTTGGAGCTTTGGTA
AAATAAGGAATAAATATGAAATTCTCCCCATGCCAAACCTCCCACTGCTGGGCTCAACTGTGTG
ACCTTGGGCAAGGCTCTGCCCTCTCTGGGCTCCTGCTTCCCTGAGAATCTCTGAGGCATACCCT
CCCTTAAAGGGATCTTACCTTATCTCTTTGAGGACTCAGACAAACTGAAGGAAGATCCCCTCCTT
TTTGCTGAGTCCTCCCAGAACCTCCCTTAAGCACCAGTGGGGGTCAGAAGCCTTGGGTAAGGTT
GACACCCCATTTATTGGAGAAGACCCCAGCACCCGCCCCCTGAGGTCTTAAGGGCTTTGGTGTA
TCCTTGGTCACGAGCGCTGGGCCAGGAAGCAGAGTTCCTGAGAGCCAAGTCTAGTGGTTGAGA
GAGGACCCTGGCTGGGCCTGGGGAGCAGGAAGCCATCTGTCCAGCTGGGCAGCCCCCATGGG
TCCCTGGTGCAGCCCCGGCCATGTGTCCAGCGCCCCATACTCCATGAGGGGGGTCTGCACCCC
ATCACACGCTGGTTCTGCAGGTCTGCACCCCTGTGAGGCTGCCCCTGGGGGGCATGGGTTCTGT
TGGGCTCTTGCTCCCAGCATGGATGACCCAGCGATAGCAGTCAGTGATGCGCTTGTTGGGTGCA
TGGGGGCCACAGCGGGTGCAGTACACGATGCCCAGTGCAAGCAGGACCACCAAAAAGACACA
CGTTGGCACCAGGAGTGCCACCAGCAGCCACCGGTCATCCCTCTGGCTGTGCTCGGCAAGACC
AGCCTCCCCCAGGGCTGTTGGGGCTGCTGTGGGAGCTGGTGAGGGCAGCCACAGGGCCAACTT
GGGACTGGGGCCATCTTCCCTTGGGATTTGGGGGGCTTTGGAATGGGGATGTGTAGGGCTGAT
GGGTGAGGTCTGGTTAGTGGGGCTCTGAGAGGGCAGGAGGGTGGGGAGGGCTGCGGGCTGGG
TGGCAGCAGGCACAGAGATTTGATGGGCAGGAGACACAGGGGACCTGGAGGTGGTGGTCAGA
GAGGGCTGGGCAGTTGGGATAATGGGAAGCTGGGTGGCCTGGGTTCTGAGGACAAGGGCATC
TGGGGCTTGAGGGGGTAGCTGGGCACCGAGGGTGGTGACCAGAGGGGCATGGTTAGGTGGGA
TTCCAGGCAAATGAGTGGTGGTCTGGGTGCCAGCGACCCGGGTGTCTGGAAACATGGGGGACT
GGTGGGCAGGGAAGAGCTCCGGATATTTGGTTGAGATCATAGGGGGCTGGTGGGCAGGAGGC
TGTGCTGAATGAGAGACAGAGAGAATACCGGGTTGGTAGGCAGAAGGCAGATCTGGATAGTTG
GCTGCGATCACGGGGATCTGGTGGTCACGGGACAAAGCTGGGTGTGTGGCAGGGATCACAGG
AGGCTGGTGGGCAGAAGGCAGTGTGGGATGCGTGGCAGAGACCACCACAGGCCGGGTGACG
GAGAGCACTGAGGAGTGGTAGGGGACCCTGGGGGCACTGAGCGGGGGTGGCCAGGTGGGCT
CCGGGTAGGGTATCTGTGGCTCTCTGTCCTCTGGGAAGCTCGGTCTATAGGCCAGGGCAAAGTC
AGGCGGCTGCGTAGGCTCCATCCACAGGATCCCAGGCATCTCCGTCCAGCCACCGTTGAAGGC
CTTCCAGGCCTCGTCTTCATCTTCCTCATCCTCCCCGTCATCCAGCAACTCATCTCCGAGGTCCT

GGGAAGCCTGGGCACCCATGGCCCCTGCAGGGCTGCAGCTGATGCCATCAGCCTCCAGCTCAT
GTCCCTCGCTACAATAACACTCGAAGCCACCAACGTAGTTGACACACATCTGCTGGCACACACC
GGCAATCTGGCACTCATCTGTGTCCACACAGCGGTGCGGATCATCCTCCGCTGGCCGGAAACC
CAGGCGACAGTGGCAGCTGTAGCCTTGTGGCCCACCGGGCTCACACTGCTGCTCGCACGGAGC
CTGGGCACAGGGGTCCTCGCAACTGCGCCCGTCTGCTGCCAGCCGGAAGCCCTCAGTGCAGCG
GCAGGACACGTGACCATCCACCTCCTCCACACATTCGTGTTCGCAGCCCCCGTTGTCAGGGCTG
CAGCCAGTCCCCAGGCACAGGGGCCCAGCCCGTGACCAGCCCACACCTCCCTCAGGCTGCTTC
ACGCAGAGCAGAGAGGCTCCCCTGCCAGCCTGGCACTGCACAGCGGCCACAGAGCCGAAGGG
CAGCCACTCAAACTCTGTGGAGACCAGGTGGAAGGGCGTGGTATACACGGCTGGGCCGGCCT
GGCCCGCCTCATCTTGCAGCGCCGGGCAGGCGCCCTCGAAGCCAAACTGGCACAGGTAGCCG
TCGACAGCCAGCGTGCACGAGCCCTCCAGCCAGCGGTGCTCGCCACTTGCCTCCAGGGCCACA
CAGCGCTGGGCCGGGCAGGGGCCTCCAGAGGCTGGCTGGGCCCAGTTGGTGAAAGCCGTGTC
CTGGTCCCCTGTGGTCCACGTGAAGCCGCGCAGTGGGCGCTGCAGCTGGCATTGCCGGGCCTG
CCGCTGCAGCCCGATCCACAGCAGCCGGCTGGCTGGGCCCGCACCCACCAGGCTGTCCACAC
GCTGGGCCTCCTCGGGGGTCCGAGGAGTGGCCAGGTCGCCCCCCAGCTCGCGGCAGGCCCGC
CAGGCCTCCAGGAAGGTGCGGCGCCGTGGGAAGAGAGCGTAGCAGCTGCTGGGGCCGCAGG
CGGCACGGGGCTCAGCAGCCCAGGGGTCCTGGCCCAGTGTGGGCCCTGCGGCCGCCCAGGCC
AGCAACAGGCGCAGCAGCATCGCGATGCCCCCGGACTGGTCCGGCCCCGGGCTGGCGGCAGC
TCTTGACAGGGGGAGGGCCTGGGGCCTCCTGCGGGCGGGCCGGGGGCGGGCCTGGGGCCGG
GCTCAGGCCTTGTAAGGAGTGGGCTGGGGCGGCTGCCAGCTCCCTGCTCCCTCTTCCCCGGGG
GCCGCTAGAGCAGGAAGCAGTGGGGTGGGGGGCTCTAAGGTTGGGGGCGGAGGAGGGGAGA
AGGCGCAGTTGACGGCTGCCCCAGGCCCCATGGGGCTGAGAGTCAGGGGGTGGGAAAAGCCT
GCCTGCCGAGCTCTGTGACCCTCAAGTTGGGGCTCGGGGCTGTGGGGTGAGGTCGCTTCCCAG
AGACTCAGAGACCCAAATCTGGACACAGCAGCCCCATCACACCCGGGAGCAGTCTCAGCCCAG
CTGCTCGCCATGCACGGGAACACCCTCATGCCCACTCCTTTCTTGCCCCACAGACGCGCGCATG
CGCCTCCCCCACCCCCAACCCCATGCAAGAGTGCACTCTCTGCCAAGCTGTTTGTCCCTTCACC
TCACCGTGGACCTATGCTGGCTTGCAGATGACTCCCTTGCCACACGTACACACATTCTGACATG
CTGAGACCCTAGGTGCACATACAAAAATGGTGTGGCATTCTACATGGCCTGACATATGAAGACC
TCCGATTCGGCCTGAATGGAAACAGCTTGGTCACCCAGGCACAGGCATCCTCAGCCTCTCATTT
ATTTACTGAGCCCCTGCGTCAGCCTCTCCCCCTATACTCAAGCTTTCTGGTTCCAGCTGGGCCAG
CGTGTGGGGGTGTTGGGGGAGTGAGGGGGCCAGGGGCTGCCCTCCCCTCACCCCAGCTCAGC
CTGGGGCCCTGGGAACCTGGGCTTAGAGGGGGAGGAGGCAGCCCCTAAACAGGAAATGCCTC
TCTGGGCTCCTGCGTCAGGAAGGCAGGGTGCGTGGTTTAGGGGGCTGGGTGGGGGCTGGGCT
GCACGCAGGATGTGGAAGGCTGTGAGGGGACCCATGCAAGGCCATGTATGGGCATGGGGCAC
ACAGTGACATATATGTGTATATGTGACACAGGCATCACGTGTGTGGAGCCCTCCCTGCCCCCGG
GGCCTGCTGTTTCGGCCTGGCCAGGGTCCTCTTCCCGCCCTTCCTGGAGGCCTCTGGAGGCATG
TGCACTGAGCCCATCATCCAGGATGGCCTCTGGGGAGCTGGCTGGCGGATTAACCCATGCACA
CCCGCTGCACTCTTAGCAGAAGTTAGAAACAGTGTGGGGAGGTGGGGGAGAGGTTCCGCCCTT
CAGTAAACTGCCCTGATGCAGCTTCCCCCTCTGCCACAGATGGATCGACTTTGTGACTGCAGGG
GAGTCACTGGCCCTCTCTGGGTCTCCTTTTTTCTCAGTTGCAACATGAGGGCAATGAGTCCAGGA
TTTTTAGCTCAGCAGGGCTGAGATATTGCGTCACAGCGCTCAGTGTTTGCAAATGTGCTTTGTCA
GCTGTGGAGCCTGAAGCAAATGTTCCTGTCTTCTGAGCATATGTGGTGGGTGGAGACCAGGGTG
CGTTTTTGCAAACCACCTTACAGTGCCTAGGCTGGCCCTGCAGACCCAGCCAGCCCCTCGAGAA
CCAGGGCTTAGGAAGTTCTTGCCATATGCCTGGAAGTTCCTTCTTACAAGGTCTATACATGTGAA

GCACACAGAATGACTCCCCTTCTCAGGAGACAAAAGTCAAGATGGAGGAGTTCATCCTTCTGTT
GGCTCAGGCCCCAAACCTTGAGGTCATGCTTGACTCCTCTCTTTCTCCCACAGCCCACATCCAGT
CTCTCAGCAAATCCTGTTGGCTTCACTTTCAAAAGAGATCCAGAATCTGACCACTCCTGGACACC
TTCTTTGCCACCCACCCCCGGCCCAGGTGGCTAGCAACGCCAATCTAGATTTCCCCAACAGCCT
CTCCCGTGGTGTCTCTCTTGCCCTCTCCATCCTGCCAGAGGGATCCTTTCAAAATCCAAGTCAGA
TCTGATCACTTCTCCATTCAGAACCCTCTGAGCCCCCCATCTCGCTTAGAGGAAGGGCCACAGT
CCTTGCCATGGCCTCCTGGTCTATGTCATCTGCCTCCAAACTCTGGCCTCATTGCTGTTCCTTAAA
TGCTCCAGACACTCTGCCTCACAGCCTTGGTCTTGGCTGTTCCTGCTGCCTGGAATGCTCTTCCT
CCA

ID|56486|gene_id|ENSG00000259940|transcript_id|ENST00000567108 Homo sapiens lncRNA

TTTGCTGATGAACTCGTTGACCGTCATGAGGGTCTGGGACCATTCCTCATCTGTGTACCTCG
AACCAACTTCCACTGGGACAGTTCGGCAGCCAGCGATCTCCTGGATATACTCCAAACTAGCAGA
AAGACACACATTGGCATCGTCACTGCTAATATCTGTTGGGCATAGTACTAAGTGTGCTTTGCATG
TGGTCCCCTTTCATCCTCGGAAAACCTCACGTGCAGAGACAAGGACTGTCATCCCCAGCTTCAC
TGATGAGGAACCGGCATGGAGAGGTGGGGCTCCCTGCTCAAGGCTGTGTGGCTCATACACAGT
GCTGTGCAGCTTTGCAGACAGGGCCGGTAAGTCTGGTTGGCTGGGCTGCTCTAGTTCCCTGCTT
GCCAACAGCCTCCTCACTTCTTTCTTTGCTCATTTTGTCACTAAGCTGAGAAACCTTTGAACAAAT
AGCGGACAGCGTGGCAGGTGGCAGTCTCCTGGACTCTGCCCTCTAGCCTGGGACTGATTCCAG
GCCTCATCAGTTACAGGCCAGGGGATCTGAGGCAGGTTACTGAACCCGAGTCCTTGATCCCCAT
TTACAGAATGGGGGTGATGACAACACCTGTCCTAGTGCCAGGTACGTAGTAAAATGCTTAATGA
AGCCTTGCTGTGAGGAGAGCTCCTGCAATGAGAATTCCTCCACATGTGTCAGCGGGTACCATTC
CCTCCGCGAAACCACAAAACAGGCGGACTCCATCTACCTTCCTGGAGAGATGCCTCCTTATTTC
CAAACTCATGCAGAGCCGCGGCATGACAGCCACGCTCCAGCTGCCTGATCTGATCTCCCCATCA
TGATTCTGAGAAAGGACTAGAAACAAACACAAGAGACCACAGAATCCCTCAGAGAGAGAGGG
GCTGTCTCTTGGGTTATTTCTGCTCTTAGCTTCAGGGCCCTGAGATGGTCTGAAATTGCTAGGAT
CCTGTAAAATTGCATAACAGAAACATTCTAGGACTGCCACTTAGGGACTCATTAAAAAACCGCT
TAAGGCGAGCAAGGTAGAAGGTAAGAATCCCATAACCTGCGGCATAAATTCAGAGCACCTTAC
TTTTCACTCTTTTTTAACCCCTTGGCAATCAGTCAAGGGCTGCTAGTGAAGCATCTACAAGGTTG
CATTTCCCTGGGGATGGTCGGGAGGAATTCAGGGAGCCCTGGGGAACGCTAAAATGGGAAAA
GGGACCTCACCTCCCTCAGCGACCACCCACCTCCACTTCTGCATGCACGGCCAGTGGTCAGCC
ACGCCTTTCAGGATCACGGGCCTCCCTGGAACCAAAAACTGCTCCCTGAAATGCTGGAGGGAC
GGACGGTGCAGCCGGGGGACTGTTTTTTCTAACTTCACATCTGGAATCAAACCATGGTCCGCCC
TTGCTTTCTGTTTAAAATCAAAACAGAACAAAATAGCAACCACCTCAGGAAGTCGACATTTGAG
GTATTCCACCAGGAACCTGTGTCAGGGGTAGGAGAGCTTAGCTGTCAAGAGTACCAGTAACAA
CAATAGACAACGTTTGTTATTATTACTGCTACTATTACTATTTGCTCCTCATGAGATCAAGGTGGT
TTTTTTCTTAACCATTTCATCAAAAATGTTTGTTTCATTATAAACTGCTTTCACAGACATTCTGCAA
TAAAGGAAAACTTACTATCTCC

ID|56317|gene_id|ENSG00000224536|transcript_id|ENST00000454651 Homo sapiens lncRNA

CCCGGCGGTGCTGCCCTCCTAGGCCCCGGGAAGGCCGGCCGGCAGGGGCGGGGGCAGG
AGTCGCCGACTCTGGGGGTGCTCCGAGCTCGTGCTGCGGTCCCACTGTGCGCCCTGTCGCCGC
CACCGCGGAAAACGTCTTGCACGTGCGAGGGGCATTTCAGGAGTTTCCTGTCTGTCAGGTGAGA
AGGCGGAGATATGACCTTAGAGGTCAGACCCATGATAAACTTGAGCCCCTAACGAAAGGGTGT

CTCGAATTTACTTGTCCTGGTGGCAGAGGGTGAGGGACACATTTTGGGCGCCAGGGTAAGACA
GACCCTTCCAGGGCAGGTCTGATTCTGCCCATCCACTCACTAGCTGGGCCCTGGGCCTAGACAG
CCCTTCTGGGTTCAGGGTCATCTGTGAACGGCGTGTAATCTACAGGGATGTGAGGGGCTAAAAG
GAGATCCGGTAGGACCCGCAGACCTTCAGCAGCACCCGGCATGTAATAGACACTCAATAAATG
TAACTCCCCCCTCCAACAGCTCTTGCCCCTCAGTTTTTCCCAGTCTTCCCTGCTACCCTAAGGAC
AACACCTGGAAAAAGGTGTTCTCCCGCAAAAGCAGCAGTTCTGAAAAGTGGGTGCCTCCATAG
AGGCTTTTCTCTGGGGCTCCAACCTTCAGTAGGACAGACACAGGCTCAGGCCACTCAAGCTGCC
CCTGGGGCAGTTTCCAGGGGACATTCTGTCCACACCTCCTCACCCAGTCCCCAGCCCTTCCTGA
GTGCCCCGTGCAGGCATTTCAGGGAGCAGAGAAGCTCACAGCCTCCAGCTGGTCACCCTACAC
CCCAGGTGGCCCATAGATGCAGCTGCTATGTCTTCCACCCTGGGCAGGTGTCAAGGGTTCAGG
CCCCAACCGGCTTCACTTAAAGCCCTGGTTTGCTGAATCAACTTTTTGCCAGGAATAAAGTTAAG
CATCTTATGATCTAGAAAATGAAGTATTTGGCCAGGCACTGTGGCTCACGCCTGTAATCCCAAC
ACTTTGGGAGGCCGAGGCAGGCAGATTGCTTAAACCCAGGAGTTCGAGACCTGGTGAAACCCT
GTTTCTACAAAAAAATTAAAAAAATTAGCCAGGCGTGGTGGTGTGTGCCTGTAATCCAAGCTAC
TCGAGAGGCTGAGGTGGAAGGATCACTTGAACTCAGGAGGCAGAGGTTGCAGTGAGCCAAGA
TTGCACCACTGCACTCCAGCCTGGGCGACAGAGCAAGAGTGTTTCTCAAAAAAAAAAAGTGTTTA
TCTAGGTTTTTCTTGCTTTCTACCATAAGTAACATCTTTCCTTTGGCCAGGTCCATCCTATAAACA
AGGCCCTCCAAGATTAAAATGTCTAGAAGCACACAACCAGTGTTTCTTGCAGATTGGGCCATCT
TCATCTGACCCAACCAAGATGAAGGAGGAGAAACCAGAAGAATTTCTGGTCACGGCTGCACTG
ACTAAGCAATAGTTAAACAGGTCCATTAAACTGCAGAACCACCATCAGTGTCAGAGACCAAGG
GCTTCCGCCCAAAGATGGCGATCCCATGGGATTTCCCCCTCTCCTTTCAATTCTCAGTATCTTGG
AGAGATGAGCCTCGGAGTTTTGCCCAGGATTTTACAAAAGGGCAAACTCCTACATTACCTTCTTT
GGTCTGGGTTTGTCCTCTCTGGAGTCATGGGACTGTTGATAGATTTCCCTGTGAGGACATGCCAA
CCTGTGCTGTGAAATGGCTTTAGGGTAGATCCAGAAAAAGAAGAGCAGTCACTGCAGTCACACT
TGGATTTCTCCCAGATCTTAAAGCACCTTATAAACAGAATGGCTGTACAATGTGTCATCTAAACC
AGAATGCTTTGGATAGTAAGTGGTATGTGGTAGTATTCGTAACTACACTAAGGCAACAGGACTA
TCTCAGGCAGTCCAGGGCAGATGGTCACCCCTCTTTTCCACCCAAAGACTGTCAGGTCACTTGT
CTTAGGAGCTGTCTACGCAGAGCTACAGATGAAGCCAAGTAGGACCTGCCTTGTTTGGTGTTTTT
CCAGAACCTCTCACCTTCTGACTATATTATTCTCCCAGCCTGCTGCCAGTTACTTGGGCTTGGTCT
TAGATAGCACATTTGCAGATAAAGAAGACTTGAGGGATGAGGAAGGGCTTTGGGATTGAGTTG
AGTCAAAGGTGATGCTGGGGTTACCCACCATGCAAACAGGAACAAGCCTGATGTTGAAATGAC
AGAAAAATCAGTAAACATCATCAATACTTGGGACATCCTTAAGGCAGAGATTCTCATTCTGCAG
AGGTTCTCATTCTGGCTGACCTCATTTATATAACCATATCTAGACTGGCCCGAGCTGGTCAGCAG
CACCCACATCTGCAGAGACAATGCAGTCAGAGCTCATCACCAATAAATTATCTGCTAGGGGTTC
TAGAAGGAGGCTTGGCACACTTAGGGGCTTACCTGCTTCTCAGGACTCTCACAAATGTCTAACC
TTTTCAGTATCTTTATGTTCTCTCTGTGCACTCTATTTCCCAACACTTCTGGGTTGGTATTTGATGG
CTGCTAGGAATGAAGTGGGGCCTGTTACAGGCAGGAAGTAGGGCCCCTGTCCTCCAGCTTCCA
ACTTTGTCTGCTGCTTCCTCTGGCATCTTTTCTTCTCTTTCCTCCCTCATCAGCATCCTGATGCTAC
ATCTTCTCTCTCCTCTCCTGTGGTGAAACCCTTCTTTTCCGCATTGATCCCAAGTGAAGAGGACCT
GAGTGTGAGGCTGAGGAAAGAGTAAGGGAATGTGTCTGGCTTAGCTCTGCTCATCATCCCAGA
GAAATTAGAGATCCAGTCTGGGCTGGAGATCTTCTTGTCCATCCTTCCATCCATCTGTCCATCCA
TCCATTCATTCATTCACCCATCCATCCATTTGTTCATCCATCTGTCTCTCCATCCATCCATCCCTCC
CTCCATCCATCCATCCATCCATCCGTCCATCCACCTGTTCATCCATGTATCCATCCAACCATTCAC
TCAGAAGACATTTATTGGGGCCCCACTCTGTGCTGGTCTCTAGGTTCTGGGAATACAAGGAAGC

CTATAATTTGGATCCTGACCTCAAGAAGTTTACTCATCTAATGGTCTAAAAAAAGAAAAAAAAAA
TCCAGCCCTAAACTTGCAGTTTGAGTCTCAGCTCTGTCATCTATGATTTAAAATACTAATGATGG
CTGGCGCAGTGGCTCATGCCTTAATCCCAGCACTTTGGGAGGCCAAGGCCAGCAGATCACTTG
AGGTCAGGAGTTCAAGACCAGCCTGGTCAACATGGTGAAACCCCATTTCTACTAAAAATAGAAA
AATTAGCCGGGTATGGTGGCATGTGCCTGTAATCCCAACTACTGTGGAAGCTGAGACAAGAGA
ATCGCTTGAACCTGGGAGGTGGAGGTTGCAGTGAGGCGAGATCCAACCACTGCACTCCAGCCT
GGGTGACAGAGTGAAACTGTGTCTCAAAAAAACAAAAAACAAATAAAATACTGTTGATAATGA
GGAATAATTATAATTATTAAGCATTTACTACCTGTCAGGTACTTTACACAATTGTCCCCTTTAATC
TTCGCAGTAGCTGTATGAAGTAAGGACTATTTTTACCCCCTGCTTTGCATGTGAGGAGACCAGG
GCTTACAGCAGTAGCTAACTTAGTAGCTTTGTGACCATGGCCTAGGCCTCAGTTTTCCTCATGTG
TGAAATGGGAGGTTGGACTAACTCAGTATTCTCCAAAATTTCCACTGAGGTACCTCTGACAGCA
AAGAAGTAAATGTAAGGAACCCACAGTGTTGTCAGAATCAATCCACTCCCAGTCCTTGAAGTCT
TGGGGTTTATTGCAAAAATTTATTTGAATTTTGCATTCTCCTTGGCACATAACTGTATTTGTCTTCA
TAAAAAGATAATGTTTAAAATGACTCACTGGCCAGGCACGGTGGCTCACACCTGTAATCCCAAC
CCTTTGGAAGGCTGAGGCAGGTGGATCATTTGAGTCCAGAAGTTTGAGACCAAGTTTGGCAACA
TGGTAAAACCCAGTCTCTACAAAATATACAAAAATTAGCTGGACATGGTGGCATGCTTGTAGTC
CCAGCTACTAGGGACGCTGAGGTGGGAGGATCGCTTGAGCCCAGGAGGTAGAGGTTGCAGTG
AGCCGTACTATACTCCACTGTACTCCAGCCTGGGTGATAAAGTGAGACCCTGTCTCAAAAAATA
AATGAATAAATAAATAAAATTACTTACTGTTCAGAGAAATGGAGTTCTTAGGAAATGTATCGTGC
TTGGTGGGTAGTATCTCTGTGGGCCCCTGGGAATATGACTAGGCCCGCCTACCCACGTGGGTGA
TCTGCAAGGCTCTCCCTGGCTCTGGCACTGTTCGGCTGTGCCTAGGCTAGGATGAGGCAAGTCA
AAGGGGGCTTGTGGAATAAGGCCCAGCATTTGCTTTCAGTCCCTGTATTCATGTTTTCTAAGGAA
GATTCCTAGAATCTCTCAGGGTTTGCTGGAGAAATGAGCTTGGCAGCCAGAGGCAAATGAAAAT
CGGAAAAGGCAGTGTATACATGTTATAAGAATAAAATGCTTAGAGGCAAGAAAAGGAGAAATC
CATTTAGAAAGGAACAAAATAAAAACTCTAGGGGGTTCCTGATCCAGAAATCTCTTCTGATTTTC
AGGCTGAAATTTACACTGTGAAGCTCCAGGGGAGCTTGAAAGGTGATATTCAGAAATGCACTGG
GGTGGCTCAGGGTTGGGTCCTTAAGAACACTGTGAAACAGTCTAGATAGGTTTTTAAATGACCA
CTTCCTCTTCCCCTCCCCTTGGCCTGAAATTGATGTAAGGTCACTTCCCTCCTGCTCCCTCTGCAG
CCACTGCCTCTGTCCCAAACAGGCAAAACTGTGGAGTGGCTCTCACAGCCAAAGTGTGGGGAG
GGGAAAGCAGGAGGAGCTGGGGACTCAGGGCGGGAGCAGGCGGCAGGGAGCTCCCGGGCAG
CTGGCTGTGCATGGCTCCACTGTTCCGAGCCTGACAGGCTCTAGGGAAGTGTGGCCACAGGGA
GCTGGCTGAAGGCTCAGGAGAGGCTTTTCTGACAGGCCCAGTTCCAATCACGCCAGCTCCTAAT
CTACTGATCAGTTTGCTTTCCTCCTGCTTTAAAATCTCTTTCTCCCTGTTCTCCTGGTCCCTAGCCA
CACCACCTTCACTGCTCTGATCTTATCTATGATCACCTGGCCAATCTACTTAAAGCAGTGAAGTA
GACTGAAAATAACCAGACTTTCGGCAAAACTCTTTTTTTTTTTTTTTTTTTTTTTTGAGACGGAGTT
TCAGTCTGTTGCCCAGGCTGGAGTGCAGTGGGGCGATCTATGCTTACTGCAACCTCCACCTCCT
GGGGTCAAGCGATTCTCCTGCCTCAGCCTCCCAGCCTGGGACTACAGGCATGCGCCACCACAC
CCAGCTAATTTTTGTATTTTTAGTAGAAACCCTGCAACACGGCGTTTCACCATGTTGGCCAGGCT
GGTCTCAAACTCCTGGCCTCAGGTGATCCGTGCACTTCGGCCTCCCAAAGTGCTGGGATTACAG
GCGTCAGCCACTGCACCCGGCCTTGGCAAGACTCTTGACTCCTCTCCTCCATCAGGGTCGATTA
TATTAAATTACACTGACAGAAGAAAGGATTGGATGACTTTTGAAGAACTATTTCAACCCAAAGA
CAAGCTAAGGAAACATTGAGTTCACTCACCTGGAAGGTCTTGGAGGGCAGGAATTGTAGCTTAC
CCTCCTTTGTAGCCACACAGAGCCTTACATGGAGTAGGTGCTCAGTATTTTATGAATGTGTGCGT
GGATAAATCCATAGTTTGCAAGAAACCACGAATAAGCTTGCCACCTGCTGGTGAACTTGTGAAA

GACACCTCGCCGCACCTCGGCTCCCCCAAGTGGCCATACACTGTAATCCTAAGAAAACCCTCA
GCTTGGACAATCCTTTCTTTCCAAGGAGCCCTTTGTTACAGGAAGTCCCAATCCAGACCCCAAG
AGAGGGTTCTTGGATCTCACGCAAGAAGGAATTCAGGGCAAGTCCATAGAGTAAAGTGAAAGC
AAGTTTATTAGGAAAGTAAAGGAATGAAAGAATGGCTACTCCATAGACGGAGCAGCCCTGAGG
GCTGCTTTTTGCCCATGTTTATGCTTATTTCTTGATTATGTGCTAAACAAGGGGTGGATTATTCAT
GCCTCCCCTTTTAGACCATATAAGGTAACTTCCTGACGTTCCCATGGCATTTGTAAACTGTCATG
GCACTGGTGGGAGTGTAGCAGTGAGGACAGGTCACTCTCGTCGCCATCTTGGTTTTGGTGGGTT
TTGGCATCCTTCTTTACTGCAAGCTGTTTTATCAGCAAGGTCTTTATGATGTGTATCTGGTGCTGA
CCTCCTATCTCATCCTGTGACTTAGAATGCCTTAACCGTCTGGGAATGCAGCCCAGTAGGTCTCA
GCCTTATTTTACACATCCCCTACACAAGATGGAGTTGCTCCAGCTCAAACACCTCTGACAACTTG
ATACTTATTCATAAATCCTTTTGACTCTAACTCATATCCTACATTCTTAGCAGAGAAGCCAAAAAT
GTCCTAGGACACTAGTCAGTGAAAAAGGCCTTTGAAAGCCCTGGAAGCTGTTTTGCAACTCAGG
ACCCTCTCTTCTCCACTGGTAGATGTTCTCTCCCCAAGATCCCTTTCCCTATCCTTCACGCATCCA
GACAGAGAAAGCCTGTATTGAGCACCCACTATGTGCCAAGCACTGGGCTAGGTGCCGAGAGAT
ATGAATGGAGAATGGAGACTTTACAGGGAGGGGAGAGCTGAAGTGTGCACCCAGATGTGGGA
GAGGAGACTGGCACAGGAAGAGCACCGGGAGTTCAGACAGGGAGAGAACTCCATTGTGGGGA
TCAGAGAAGGCTTACATGGGAGATGACAGGCTAATGAGCTGGGCCATAAAGGGTGGTTAGGCT
CATAATGTATGGAGATGGACAGCATTCTGGTTACCTATTGCTCTGCAACAAACCACCCCAAACTT
GGTAGTGTAACAGAGCGGTCATTTGTTTTTCTCTCTCATGGTTTTAGGGTTTTCCCAAGCTCAGCC
GATTGTTCTTGCTGGGATCTCTCCTGTGATTACAGTCAAATGAGAGCTGGCTGGATTCTTCACTG
AGTCTCCCTTACTCCTGTGGCTGGAGGCTGATGCTGGCTGTCAGCTGGGACCTCAGCTGAGGCT
GTTAGCCAGAACCCCTACATGTGGTCTCTCCATGGGGCCTGGACTCTCTCACAGCATGGTGGCT
GGTTGCAAGGAGAAGCATCCCAAGAGATAGCAGGAGAAAGTTGTCTTGCCTTTTGAAAACTGG
CTTCGCAAGGCCCATAGCAGATCTTCCCCTGTGCTCCACTAGTTGAGACAGTCACGAAGCCCCA
CCCAGTTTCAAAGAAGGGGACAGAGACTCCATTTCCTGATGTGTGAGTGGCAAGGTTCTGGAA
GAGTATGTGGAATGGAAAGCATTATTGCAACCATTTGGGGAAAATACAACTTGCTGCTGGCAGG
AAGGTCATTTCAGGCAGAGATCTTCATTGATGGTACCTTCAGAAATGCATTTTTAGGAAAGAAA
GTGGTTTTTACAGACTTGGCATTGCTAAGGGAAAACATAAGGTTTTAGAAGTATTTACTGAGCCA
TCTTCACCAAGACTTATGAGAAACATTTCTTCGAAGAGAAGATGCTGAGGAAGAGGAAGATGAC
TTTTGAAGTGGACTCCAAGGGGCAGACAGGAGGCTGGGATGGATGGCAGCTCACAGCTGGCA
AAGGCAGTGGACTAGCAATCATGGGTGAAGGCCTACCAAGACTGATTCTTAGAGGGGGGAGAA
TTGACTTTGTGTTCTTATAGCAAGTGACCAGGAATAACCAGATGCTGTGTCCAAGCTGAGTCCTC
AAAAACAGTAGTAGGCAGGGGCCAAACCAGAAAGCAACAGTTAACAAAAACTGTATTGTAGCA
AGAGAGATAAGATAAGCACACAAGTGTAACCACCCAACGGGTTCACCTTGTCTGCTGCCTAGA
CAGAGCCGATTTATCAAGACAGGAGAATAGCAATGGAGAAAGAGTAATTCACGCAGACCCGGC
TGTGCAGGAGACTGGAGTTTTATTATTACTCAAATCAGTCTCTCCGAGCATTCGGGGATCAGAGT
TTTAATTTGGCAAGTAGGGGCTTGGGAAGTGGGGAGTGCTAATTGCTCAGGTTGGAGATGGAAT
CATAGAGGGTCGAAGTGAGTTTTTTGTGCTATTTTCTGTTCCTGGGTGGGATGGCAGAACTGGTT
GAGCCAGATTACAGTTCTGGGTTGTGTCAGCTGATCCACCCAGAGTGTAGGGTCTGCAAGATAT
CTCAAGCACTGATCTTAGGTTTTACAACAGTAGTGTTATCCCTAGGAACAATTTAAGGAGGTTCA
GATTCTTCAAGCCAGAGACTGCGTGACCCCTAAACTGTAATTTCGAATCTTGTAGCTAATTTGTT
AGCCCTGCAAAGGCAGACTGGTCCCCAGGCAGGAAGAGGGTCTTTTTGGGAAAGGTCTATTATT
AATTTTGTTTTAGAGTCAAATCATGAACTGAATTCCTTCAGGGCAAAGTTATTTCCACCTATGCCC
AGGAATGAACAAGGACAGCTTAAAGGTTAGAAGCAAAATGGTTAGGTAGGTCTGATTTCTTTAC

TGTCATAATTTCCTCAGTTATAATTTTGCAAAGGCAGTTTCACAAGCAACCACAAGGCATAACAG
CTATCATTTGGGGGTAATACAGGGAAGCAGGGAGCAGTCATCATGCATGAGCAAGAGCTGGGT
CAGAGATAAGACACAGCCCCAGGTTAGAAGGCAGCTTCACAGAGATGAATAGGGAGTGAGAT
CAAGGACCACAGCAGAGGTTGACAGCCCAAGGCCCTTGATGGCACTATGACCTGAATGTCCAG
GATCTTCCATGTCAACCACCAACTTTATAGGAAAGGGTGCACGAGCCAGGAATAATGGTGGTCA
AGCTTGGCTGTGTGGGAAATAGGGAGTTTGCCTGCCTGGCAGCTGCTGAGTGTCTCAGTAGTGA
GCTTCCAGCTACACAATATCTGGACCCTGAGACCCAAAGAGGAAGGAGACTTTGGCTTCCTTTA
CTATGGGCATTCTGTGACCCACCCTGGCAATGAGAAGGACTAAAGAACTACAAGATATTAAATC
TACCTTATGTTTTTCACTTGCTTATGAGCACTGGAGCTCCATCACCTGAGCCCCCACTAAGTGCA
GATGTATGTGACTTTAGAAAGGTTAGATTTACTCTTCATAGCTCAGGGTATAAAGAAGTGGTCCC
AATAAAAGTTTCCTTTGCTACAGGGGGCTGAGGACAATCATGAGGTTGTGAACTCTTTGAGGAC
AGGATTAGATCTTGGTCAATATCTGTATGCTCAGTGCCTAGGGTAGCATCTAGTTCATGCAAGGA
AGGAAAGGAGAGAGGAAGGAAGGAAGGAAGGAAGGAAGGAAGGAAGGAAGGAAGGAAGGA
AGGAAGGAAGGAAGGAAGGAAAGAAAGGAGGGAGGGGGGAGAGGGAGGGAGGGAGGAG
AGTCAGATAATACAGAGAGGCCATACCATGAATTCTTAGTCATACCTGACCTGGCCACTCCAGG
AGCGCCAGAAGAGTTGCTGGAAGCCCACATAGACATATGGTATATGATAACATTTTTTTTTAAAG
AGATATAATTCACATGTCATATGATTTTCCCTTTTAAAGTGTACATTTCAATGGTTTTTGGTATATT
TACAGTTTTGCAATCATCGCCACAGACCATTTTAGAACATTTTATTAACCTAATAAGAAAGCCTG
TACCCATTTGCAATCACTCCTGCTTTTTCCTGCAACATCCCTAGCAACTGAAACTGTCTTTGCAAA
AATTACAGCAATGAGAAAATTATCACAGTGAAAGAGATCCGACCTAATTGACTCCATCTTGCTTC
TAATCTCCAAGCTGCCCTTGTTCATTCCTGGGCAGAGGCTAAACTAACTTTGGGAGGAATTCAG
CTTATAACTTAACTTTGAAGCAAAGATGAAAACAGCCTTTTCCCTTAACACAGCCCCTTCTTGCTT
GGGGACCAGACTGCTTGTGTAGCACTAACAAATTAGCCTCAAGATTAATGCTGACAGTCCGGGC
GCACAGTGGCTCAGGCCTGTAATCCCAGCACTTTGGGAGGCCGAGGTGGGCAGATCACCTGAG
GTCAGGAGTTGGAGACCAGCCTGCCCAACATGGTGAAACCCTGTCTCTACTAAAATTACAAAAA
TTAGCTGGGAGTGGTGGCGGGGGCCTGTAGTCCCAGCTACTCGGGAGGCTGAGGCAGGAGAA
TCGCTTGAACCCAGGAGGCGGAGGCTGCAGTGAGCCAAGATCGTGCCACTGCATTCCAGCCTG
GGCGACAGAGTGAGACTCAGTCTCAAAAGATTAATGCAGACAGGCCACGAGATTTTGAACCTC
CCCCATTGCTCCGAAAGATAACATCACTATTGTAAAACCTAAGATCAGTGCTCCAGACATTTTTC
ACACTGCATTCTGATGCACCAGCTGGCCCCACCCAGATCGGTAATCTGGCTCAACCAGTTCTGG
GATCTCACCCAGGAACAGAGGACAGGAAGAAAAATCCCACTTTGACCCCCTATGATTTCATCGC
TGACCCAGCCAATCAGCATTCCCCACTTCCTGGCCCCCTACACACCAAATTATCCTTAAAAATTC
CAGTCTCCAAATTTTCCAGGAGACTGGTTTGAGTAATAATAAAGCTTCTATCTCTCTTTCACCAAG
TTTTTGTAAATTAAACTCTTCATTGCAATTCCCCTGTCTTGATAAATCTGCTCTGTCTGCGTATCAG
GCAAGGAGAAGCCATTGGGTGGTTACACAAGTACTTATCCACTTTATGTTTCTATAAATTTGCCT
GTTCCCTGTTCCGATTTTTTTTTTTTTTTTTTTTTTTTTGAGACAGGGCCTGGCTCTGTCACCCAGGC
TGGAGCACAGTGGCGCAATCACGGCTCACTGCAGCCTCCACTTCCTGGCCTCTAGCAATCCTCC
TGCCTCAGTACCCTCTACCCCCTTGCCCAGTAGCTGTGACTACTACAGGTGCAGGCCACCAAGT
CTGGCTAATTTTTATGTATTTTTTGTAGAGATGGGGTTTCGCCTTGCTGCCCAGGCTGGTCTCAAA
CTCCTGAACTCAAGTGATCAGCCCATCTAGGCCTCCCAAACTGCTGGGACTATAGGTGTGAGCC
ACCGCGCCTGGCCTGAACATTTCACATAAATGTAACCATACAATATGTGGTCTTTTGTGTCTAGT
TTATTTTATATAACTTAAGGGAAAGCTTCCTCTTTGCCCTCTGAAGGATCACTGAAAAAACAACT
GATGAAAGGCAGATTAATTGGAGACAAGGCATATAAATTTATCAACATGCACGCGGGGGAGTA
CTGCAGAGTTACCCTAACCTCCCAGTGGGGTATAGGAGCTTACATACCATCTTTTTTTGTTGTTTT

TTTGAGACAGGGTCTCACTCTGTCACCCAGGCTGGAGTGCAGTGATGCAATCTCGGCTCACTGC
AACCTCCACCTCCTAGATTCAAGCAATCCTCCTGCCTCAGCCTCCTGAGTAGCTAGGACTACAG
GTGTATGCCACCATGCCTGGCTACTTTTTGTAGTTTTTGGTAGAGATATGGGGTTTCACCCTGTTG
GCCAGGCTGGTCTCAAACTGCTGACCTCAAGTGATCCGCCCGCCTCCGCCTCCCAAAGTGCTGG
GATTACAGGCATGAGCCACCATTCTCGGCCCATACCATCTTGAGGTTACAAAATGAATGGGGAC
TCAGAGCATGGCCAAAAACAGATTATAGTGGTAAGCAGGTTTTAGTGGCAAGACACCTTATGGG
AGGGGGAAAAGATGAGGCCTGGCTAGCATAAGTGGTCTTGTTAGGTAGATGAAATCTCACAGG
TAGCAGCCCTCAGAAAGAATTGATGGTAAATACTTCTTTTCGACCTTTAAAGGTGTCAGAGCCTC
AGTTAATTGTTCCTAAATCTAAAGAGAAGCCCCACATAAGTGAAGATTTTATCCACTGATGCAAA
TCTCTCCCACCAAAGACAGCTTTGCAGGGCTACTTCTGTTTGCAAGGCCCTTTGAACAGCTGTCT
CAAAATATGTCAAAGAAGGATATTTTAAGGTGAAATATTTTTATTTCTTTCACTAACGTGTATAAA
TACTTCATTCCTTTTTTATTGCCAAATAATATGCACGGACATAGGACATTTTATTTATAAAGTCATC
GGTGCTGGGTAAGGTGTCAGAGCCCCGCGTCGAATAGTGGTTGACTCGCCGCTCGCAATGGCT
CACACCTGTAATCCCAGCACTTTGGGAGGCCAAGGCAGGTGGATCACCTGAGGTCAGGATTTC
CAAAGTTCCAGACCAGCCTGGCCAACATGGTGAAAACCCGTCTCTACTAAACATACAAAAATTA
CCCAGGTGTGGTGGTGCGTGGCTGTAATCCCAGCTACTCGGGAGGCGGAGACAGGAGAATCGC
TTGAACCCGGGAGGTGGAGGTTGCAGTGGGCCGAGATCGTGCCATTGCACTCCAGCCTGGGCC
ACAAGAGTGAAACTCAGTCTCAAAAAAAAAAAAAAAAAAAAAAGGGGGGGTTGACTCATGGGTT
GGTAGGAAGAATTTACTGACAACAGTATAGGTTTGAAAAAGGAAAGTTTTATTAGAAAGAAAGA
ACGTGCCTTTCCAGCCCCCCACCCGGACCCTACAGCCACCGAGATGTTGATGCCTAAGAAGAA
CTGGATTGCCATTTATGAACTCCTTTTTAAGGAGGGAGTCATGGTGGCCAAGAAGGATGTCCAC
ATGCCTAAGCACCCGGAGCTGGCAGACAAGAATGCGCCCAACCTTCGTGTCATGAAGGCCATG
CAGTCTCTCAAGTCCCGAGGCTACGTGAAGGAAGTTTGCCTGGAGACATTTCTACTGGTACCTT
ACCAATGAGGGTATCCAGTATCTCTGTGATTACCTTCATCTGCCCTCGGAGATTGTGCCTGCCAC
CTTACACCGCAGCTGTCCAGAGACTGGCAGGCCTTGGCCTAAAGTCTGGAGGGTGAGCGACCT
GCAAGACTCACAAGAGGGGAAGCCAACAGAGATACCTACAAACGGAGTGCTGTGCCCCCTGG
TGCCAACAAGAAAGCCGAGGCTGGGGCCGGGTCAGCAACCAAATTCCAGTTCAGAGATGGATT
TGGTTGTGGACGTGGTCAGCCACCTCAGTAAAATTGGAGAGGATTATTATGCGATGAATAAACT
TACAGTCAAAAAAAAAAAAGAAAGAAAGCTGCAGAAGAGTGCAGTGAGGTGCCTCAGCATGA
GAAAAGTAAGTGCGCCACGGCAGACTTTTCCTTAGGGGTATTTATGGACCTTAAAGCAGGAGCT
TAGGAGTAATGTGGACCATATTAGCCACATAGGTCATGATACGTGATTACATTTGTAGATATTTT
GGTGCCTTAATGTCAGCAAGGGCTGCACAACGAGTTTCAACATGCATTTATTCAGGAGATGTAC
AGAAATCCTAGTTACTTAAAAAATTTTTTAAAAGCCTGGTACCAGATGCTTGCTTTAGAATAATA
GGGACATCTAATTACTTCTAAATTCCTCAGATAAGAAATGTTGCCTCTGGATGGTCAGCTTGATG
GTCACCAGGTGATCTTTGCTCTCCTCAATCAGTTTTTTTGTTTTTTTGTTTTTTATTTTTTTTTGAGAT
GGAGTTTCATTCTTGTTGCCCAGGCTGGAGTGGGATGGCGCGATCTCGGCTCACCACAACCTCT
GCCTCCCGGGTTCAAGTGATTCTCCTGCCTCAGCCTCCCAAGTAGCTGGGACTATAGACACACA
CCACCACACCCGCTAATTTTTGTATTATTAGTAGAGATAGGGTTTCACCATTTTGGCCAGGCTGG
TCTTGAACTCCTGACCTCGTGATCTGCCTGCCTCGGCCTCCCAAAGTGCTGGGATTACAGGCGT
AAGCCACCGCGCCCAGCTATTTAAATCTTTTACTTATTTTATATTTAATTTTTTTCTCATGACGCAG
CCCTCAGGAGATCTTGAGAACATGTGCCCCTTTTTACCTATTTTTCTTTCTTTCCTTTTTTTTTTTTTT
TTTTTTTAAGAGACAGGGTCTCGCTCTGCCATCCAGGAGAGAGTGCAGCGGTGTGATCATAGCT
CACTGTAACTTGTAACTTCTGGACTCAAGCAATCCTCCCACCTCAGCCTTCCGAGTAGCTAGAAC
TACAGGTGTGTGCCCCTATGCCCAACTAATTTTTGAATTTTTTGTAGAGACGGGATCTCAATATGT

TGCCTAGGCTGCTCTCAAACTGCTGGCTTCAAGCAATTCTCCTACCTCAGCTTCCCAAAGTCCTG
GGATTACTATGCCTCGACTTTACCTATTTTTAAATTATTTGCCTTTTTATTATTGTATAAAATTTCTT
AAACTGATGTTCCACAAATGTAGTATCTGGAGACCTGTGTGTTCTCAGTCAGGTTTGTTTAATGT
GGCTCTTTTTTTCCTTCTTTCCTTCCTTCCTTCCTTTCTCTCTTTTCTTTTCTTCTCTTTTTTTCTTTTTT
TTTTTTTTGACAGTGCCTTGTTCTGATGCCCAGTCTGGAGTGCAGTGGTGTGATCTTGGCTCACTG
CAACCTCCACCCACTGGGTTCCAGCGATTCTTCCACCTCAGCCTCCTGAGTAGCTGGGATTACA
GGTGAGTGCCACCATGCCCAGCTAATGTTTGTATTTTTAGTAGAGACAGGGTTTTGCCATGTTGG
CCAGGCTGGTCTCCAATTCCTGACCTCAAGTGATCTGCCCCCATTGGCCTCCCAAGGTGCTGGG
ATTACAGGCGTGAGGCACTGCGCCCCCGGCCCTGTGGGACTTTCATTTTAATGGACATCTGCCT
CTGGCAAAGATGGAGTAACAGGTATCTTCCTGCTTAAAACAACAACAGAGGGCAAAATATATCA
AACAACAGTTTTCAGGACACTGGAAATAGGCAATAAAGGAGAGTGGTCTATGAGAGATGAGAA
ACAAATGAGGCGCCCTCTGATTGTCCCAGCTTTCCATGTGAAAGAGCTTCCAAGCTGTGGCGCA
GCAGAAGGAACCTAAGCAAAGCTCAGAGATTTCCTGAATTGAGAAGGAGCTGAGTCTGGAGAG
ATCAGAAAGTATAATAAATTAGCGGTTTCTTTCTACAGGGTCAGAAAGTAAGTTAGCAGTTGTTA
GGGCTGGGGTGGGGGCACAGAAGAGTGGAGAGTGACTTGTAATGGGTACCAGGTTTCTTTCTG
GGGTGATAAATGTTCTAAAATTAGACTGTGGTGATAGTTACAGAACTGTGTGAATATACTAAAAA
CTATTGAAATTCATTAAGTTGTATATACTAAATATGTACAGCTTTTCGCATGTCAGTTATATCCCA
ATTAAGTAGTTTTTTTAAATCATTGAGTTGTACATTTTCTTTTCTGTGGGGGTGGGGCAGGGCAGG
TCTCACTCTGTCACCCAGGTCGGAGTGCAGTGGCGCGATCTCAGCTTATTGAAGCCTCAACCTC
CTGGGCTCAAGCAATCCTCCCACCTCATTTTTTTGGATTTTTTGTAGAAATGAGGTCTCATCCAGC
CTGGCCAAAATGGTGAAACCCCATCTCTACTAAAAATACAAAAATTAGCCAGCCATGGTGGCAG
GTGCCTGTAATCCCAGCTACTTGGGAGGCTGAGACAGGAGAATCACTTGAACCTGGGAGGCAG
AGGTTGCAGTGAACCAAGACTGCGCCATGGCACTCCAGCCTGGGCAAGAAGAGCAAAACTCCA
TCTCAAAAAAAAAAAAAAAAAGGTCTCATTACATTGCCCAGACTGGTCTCGAACTTCTGGGCTCA
AGCAATTCACCTGTCTCAGCCCCGCAAAGTGCTAGGATTACAGGCACGAGCCACTGTGCCCAG
CTGAGTTGTATATTTTCAATAGGTGACTTGTTTGGTATGTAAAGTGTATATTAATAAAGTTGTTTTA
AAAATTAAAAGTGAAATAAAGACATGTTCAGACATGTGAAAGGGGAAATAATTCACAACAAGC
AGACCCCAATATAAGAAATGTTTAAAGTAAGTCCTTAAGGCGGAAGTAAAAAGAAGGAACAGA
GATAAAGATGATGTCTTATTTTTCATTGGAAACAATACAAGCAAGAAGACAGCAGAGTAACATT
TTTAATTTCTTTTAAAAAATTGTCAACAAAGAATTCTATATCTAGAAAAAATATCTTTTAGAAATA
AAGAGTAGAAGAAATGGTAGCTACATGGTTAAAAAATACGATTTTAAAAAAATTATTTAAAACT
GGCTGGGTGCAGTGGCTCATGCCTGTAATCCCAGCACATTCGGAGGCCAAGGCGGGCAGATCA
CTTGAGGTCAGGAGTTCAGGACCAGCCTGGCCAACATGGCGAAACCCCGTCTCTACTAAAAAT
ACAAAAATTAGCCAGGTGTGGTGGCGGGCGCCTGTAATCCCAGCTATTAGGGAGGCTGAAGCA
GGAGAATCGCTTGAACCCGGGAGGTGGAGGTTTCAGTGAGCCGAGATAGCACCACTGCACTCC
AGCCTGGGCAACAGAGAAAGATTCCGTCTCAAAAAAAAAAAAAAAAAAAAGGAAAGAAGAAAA
TTATTTAATACTTTTAAAAAGATAGTTGACTATTTAAACAAAAATAATAAGAATGTATTTCAGGGT
TTATAACATTTGGAAAAGTAAATGCATGGTTATAATAGTATAAAGGTCAGGAGGAGACAAATGG
AAGCACATTATTGCAAGGTTCTTATACTATGCATGAAATGGTATAATATCACTTGAAGGTAGATT
TGGGTAAGCTAAAGATAAATAGTATGAATCCTAAAGCAACCACCGAAATAACAAAACAAAGAG
TTATAGCTAATAAACCAGCAAAGGAGATAAATGGAATTGTGAAAATACTCAATTAATCTAAAGC
AGAAAAAGAGAAAAAATTGAACAAAGAACAGATGGGACAAATAGAAAATAAACAATGGAGCC
AGGTGTGGTGACTCAAGCCTGTAATCCCAGCACTTTGGGAGGCCAAGGTGGGCGGATCATTTG
CGGTCAGGAGTTTGAGACCAGCCTGGCCAACATGGTGAAACCCTGTCTCTACTAAAAATAAAAA

AATTAGCCTAGTGTGGTTGCACGCACCTGTAATCTCAGCTACTCTGGAGGCTGAGGCAGAAGAA
TCTCTTGAACCTGGGAGGCAGAGGTTGCAGTGGGCCAAGACTGCGCCACTGCACTCCAGCCTG
GGTGACAGAGTGAGACTCTGTCTCAAAAAAATTAAAAAATAAATAAATAAACAGCAAAGTGATA
GACTCAAATCCAACCACATCAATAATCACATTAAATCTAAATGGCCTAAACACTTCATTTTAAAG
GCAGAGACTATTAGATGGATTTTTTTTTAAAGTAAGACTCAATTATTTGCTATCTACAAGAAATGA
ACTTTAAATATAAAATCTTTAAAAGGATAGGAAGAATATACTATGCTAACATTAGTCAAAAGAAG
ATTGGAGTTGCTGTATTAACACCAGACAAAGTTGATTTCAGACCAGTCTGGGCAATATGGTGAA
AGCCTGTCTCTACAAAGAATACAAAAAATTAGCCAGGTGTGGTGGTGCAGGCCTATAGTCCCAG
CTACTTGGGAGGCTGAGATGGGAGGATCACCTGAGCCCAGGAGGCAGAGGTTGCAGTGAGCC
GTGGTCATGCCACTGCACTCCAGCCTAGGTGACAGAGTGAGACCCTGTCTCAAAAAACAAAAA
TGAAATGAAACAATAACAACAAGAGTTGTTTTCAGAGCAAAGAATATTACCAGAGATAAAGAAG
GACATTTCATAATGATTAGAGGTCATTTCATCAAGAGGATGTAATAATCCTATGTTTATGCACTTT
AACCACCAAACAGAAGGAAAAGCTTCCACTCCCTGAAAAATGGAAACAAAACACAAAAATTAT
ACTTAAATCTCTACTGCTAACCTCTACTGCTATTTCACACACAGGGTACAGAATACAAATTTTGT
GTGACATATGAAGAAGTAGAAAACTATGACTCATAATCACAAGACAACAAGACCATCAGGTGA
TCTAGTTATGAGAATCAGCAGACAAAGGCTTTAAAACAACAATTATAAGTATGCTGAAAATTTTA
AAGGAAAAAAAAACACACAATAGGTAAAGAGATAAGGTATTTCAAAAGAAATGGAAATTCAAG
AACGAACTAAAGGGAAATTACAAAATTGAAAAATACAGTGTATGAAGACTTACTGGATGAGCTT
AACAGCAAATTAGACACTGCAGAAGAAAAAAATAAGTGAATTGAATATAAGCTAATAAAAATTA
TCTAAATTGAAGTGGAGAGAGGAAAAAATGGTTGAAAAAAATGAACAGAGCATTAGCAAATTG
TGGGATAATAGTAAGCAGTCTAACATACATGCAATTAGAGTTCCATAAGGAAAAAAACAATAAG
GCAGAAAAATATTTAAAGAAACAGTGGCTTAAAAGTTGATGAAAAATAACAATCCACAGGTCCA
AAAAGTTCAACTAACCCCAAGCAGGATAAACTTTTTAAAAATCATACTTGGGGCCAGGCGCAGT
GGCTTATGCCTGTAATCCCAGCACTTTGGGAGACCAAGGCAGGCAGCTCATTTGAGGTCAGGA
ATTCAAAACCAGCCTGGACAATTTGGTGAAACCCAGTCTCTACTAAAAATATAAAATTAGCCAG
AGCTGGACGTGGTGGCTCACACCTGTAATCCCAGCACTTTGGGAGGCTGAAGTGGGCAGATCA
CCTGAGGTCAGGAGTTTGAGACTAGCATGGCCAACATGGTGAAACCCTGTCTCTACTAAAAATA
CAAAAATTAACCAGGAGTGGTGGTGGGTGCCTGTAATCTCAGTTACTGGGGAGGCTAAGGCAG
GAGAATCATTTGAACCCAGGAGGCAGAGGTTTCAGTGAGCCGAAATCACACCATTGCACTCCA
GCCTGGGCGACTGAGTGAGACTCTGTCTCAAAAAAAAAAAAAAACGAAAAAAGAAAAAAAATCA
CACTTGGTACATCAGAGTAAAATTGCTGAAAACCAAAACTAAAGAGAAAATCTTAAAAGCAGCC
ATAGGGGGAAAAGACACATTACATGCAGGGGGTAAAATATAAATGTGAGAGTTATTTCTCATCA
GAGATCATTAAGCCAAAAATCCATTAACTGGTAAATAAACAAAATGTGCTGTATTCATACAATGA
AATACTACTCAGCAATAAAAAGAAAAAAAAAACTACTATAGCTATACCGAAATGAACCTTAAAAA
CATAATTCAAAGGCCAGGCGCAGTGGCTCATGCCTGTAATCCCAGCACTTTGGGAGGCTGAGG
TGGGCAGATCATGAGGTCAGGAGATCAAGACCATCCTGGCTAACAAGGTGAAACCCCGTCTGT
ACTGAAGATACAAAAAATTAGCTGGGCATGGTGGCGGGCGCCTGTAGTACCAGCTACTCAGGA
GGCTGAGGCAGGAGAATGGCGTGAACCCGGGAGGCAGAGCTTGCAGTGAGCCCAGATCACAC
CACTGCACTCCAGCCTAGGCGACAGAGCGAGACTCTGTCTCAAAAAAAAAAAAAAAAAAAAAA
AAATTCTAAGTGAAAAGAGCCAGATGCAAAAGACCATAAATTAGATTATTTCATTTATATTAAAT
ATCTAGAAAAGAAAAATCTATAGAGATAGAAAGCACATTAGTGGTTGCCTGTGGCTGAGGGTG
AGATTGATTACAAATGGGTAGGAAGAATTGCTTTGGGATGTTATAAATGTTCTAAAACTAGGCTG
TGGTAATAGTTACACGACTCTTTAAATTTACTAAAAATCATTGAATCATACCTTTACCATGGGTGA
ATTTTAGGCTATGTTAATTATACCTTAATAAAGCTATTAAAAAGGTGCTGAGACAGAAGCCACCA

GTTTGGAAAGGATAAGAGAAAAACATGTAACAGAAAAAGGGCAATTGGCCACACTATAGAATT
ATTATATATCCAAGAGAAAATAACCAACAATATAATTGGAAAAAGATTAGGAAATAGGAATAGA
AGGCAATTCTAATCTGAACAAACTTATGAAAAGATGCTCAATCCTATTAATAGTTGGTGAAATAC
AAATTAAAATCATGATAATCTACAATTTCACATCCATCTGGTTGTCCAAAAAAAGGGTCTGACAA
TACCAGTGTTAGGATGAATATGGTAAAGTTAAAGTGTCCAGGCTCTGCGGCCATCAGTTTCACTC
CTGGGTAGACTACTGGAGAAACACAAATGTACACAATAAGATCTTTACAAGAATTTCCATAGCA
GCACGGTTTGTAATCGCAAAAAAATTAGAAACAATAAAATAAATAGGCCAGGTGCAATGGCTCA
TGCCTGTAATCCCAGCACTTTGGGAGGCTGAAGCAGGCAGATCACAAGGTCAGGAGTTCTAAC
CAGCCTGGCCAACAAGGTGAAATCCCATCTCTACTAAATATACAAAAATTAGCCAGGCGTGGTG
GTGGGCACCTGTAATCCCAGCTACTTGGGAGGCTGAGGCAGGAGAATCGCTTGAAACCGGAAG
GCAGGGTTGCAGTGAGCCGAGATTACGCCATTGCACTCCAGCCTGGGCAACAGAGTGAAATTC
TATCTCAAAGAAAAATAAAATAAAATAAATAAATTAGAATGTAGTTGTACAATGGAATATTATAC
AGCAGTGAAAATGAGTAAACTAAAGCTATATGCATTAACATGGATGAACTTTACAAATTTGTTGA
GTCAAAAAAGAAAATTGTAGAAGAATATACACAGCATGATGTTATTCATATAGTTAAAATGCAAT
GCATATAGAGTGTATAATATAGTAAAAAAAAAACATATGCATGGGAATAATTAACTCCAAGTTC
AGAATAACGTTTCCTCTGGGGGGAGAGAAAGGAATGCAATAGAGGAGGAGCTTAAACTATATT
TGTAATGCTTTATTTCTTTTTTTTTTTTTTTGAGACGGAGTCTCGCTCTGTCGCCCAGGCTGGATGG
AGTGCAGTGGCACGATCTCAGCTCACGGCAAGCTCCACCTCCCAGGTTCATGGCATTCTCCTGC
CTCAGCCTCTGGAGTAGCTGGGACTACAGGTGCCTGCCACAACGCCTGGTTAATTTTTTGTATTT
TTAGTAGAGATGGGGTTTCACCGTGTTAGCCAGGATGGTCTCGATCTCCTGACCTCATGATCTGC
CCGCCTTGGCCTCCCAAAGTGCTGGGATTATAGGCATGAGCCACCGCACCCGGCCGTAATGCTT
TATTTCTTAAACTCAGGGCATTTACAGATGTGATGGTTAAATTTTATGTGTCAACTTGTCTGGGCC
AAAAGATGCCCAGATATTTGGTAAAATATTATTCTGGATGTTTCTGTGAGGGTGTTTTTGGTTGA
GATTAACATTTAAATCAGTAGAATGAGTAAAGCAAATTGCCCTCTCTAATATAGGTGGGCCTTAT
CCAATCAGTTGATGGCCTAAATAGAACGAGAAAGGCTGACTCTCCCCTAAGTAAAAGCGAATTC
CTCTTGCCTAACTGCCTTCAAACTGGGACATTGGCTTTTTCCTGCCTTCAGACTTGAACTGAAAC
ACTGGTTCTTCCTGGGTCTTGAACTTGCTGGCTTTTGGATGGGAACTACATCATCAGCCCTCCTG
TTTCTAAGGCCTTCAGACTTTGGACTGGAACTAACATGCTATAGACTCTCCTAGGTCTCCAGCTT
GCTGACTCACCCTGCAGATCTTGGGACTTGTCAGTTTCTACAATTGCATGAGCCAATTCCTTATA
ATAAATCTCTTTCTCTCTCCCTCTCTCTATTTCTCCCTCTCCCTCCCGCCTCTCTTTCCCCCTCCAT
CCCCTCCCTGTCTATCTATAGTGTACATTCTATTGATTCTGCTTTTCTGGAGAACCCTGACTAATG
GAACAGATATGTTTGTTATATTATTCTGTATACCTTTTGCAGGCCTAAAATAGTTCATAATAAATTT
TAAAATAAATAATGAATGAATAGACGTTAAAATGTCAGATGCTATAGAGGGCAAGTAAAATGAG
AACTGGAATATGTACATTAGATTAGCTATTAAGAGGCCCATGATTGCCTTTGTAGGAGTGTTCAG
AGTGGAGGCCTAATGGTGATGGGTTGTAGAGTGATAGGGGGTCTACACACAATTCATGGAGTTT
AACAACAAAGTGAAAAAGAGCTCTGGGTCTAGAGTAGGGTGCAAAGGAGAGAAAGGGTTGTG
TTGTTGTTGTTGTTGTTGTTGTTGTTGTTTGAGACAGGGTCTCACTCTGTTGCCCAGGCTGGAGTG
CAGTGCAGTAGTGCAGTCTCGACTCACTGCAACCTCCGCCTCCTGGGCTCAGGTGATCCTCCCA
CCTCAGCCTCCCGGATAGCTGGAACCACAGACATGAACTAATTATTTTGTATTTCTTATAGAGAT
GGGGTTTTGTCACCTTGCCAAGGCTGGTCTCGAACTACTGGATGCAAGCAATCTGCCCGTCTTG
GCTTCCCAAAGTGCTGGGATTACAGGGTCAAGCCACCATGCCCAGACAGAGAAAAGAGTTTTA
ATAATGGAAACACTTCTCTGTGCTGTGGGGCTTCCATACTAATTCTGTTGGAATTATCAGAGTCC
TGGTCCTCCTTTGGTCGCTATGACTATTCCAGTAGAGATTCTGGAAGACTTGAACTTCGTCTCTGT
GGGTACAAAGTGATTTTTTTTTTTTTTTATGTTTCACTCTTGTCACCTAGGCTGGAGTGAAGTGGC

ACGATCTCGGCTCACTGCAACCTCTGTCTCCCGGGTTCAAGCAATTCTCCTGCCTCAGCCTCCCA
GGTAACTGGGATTACAAGCACCCACCACCACACCCGACTAATTTTTGTACTTTTAGTAGAGCCG
GGATTTCGCCATGTTGGCCAGGCTGGTCTCGAACTCCTGACCTCAGATGATCCACCCACCTAGG
CCTCCCAAAGTGCTGGGATTACAGTCGTGAGCCACCACACCTGGTCTTAGTAAAGATTTTTCAA
AGAGCACAGTGCTACTGTCTCTCATGGAAAGAAAAGTATTAAACCCATGATGATTACATCTTAGT
CTAAGGGGAATATTAAACCCACAATAAATTAAGCTAAAACTTCACAAAGACTCTGCAAGGCGTT
GGGGCAGGACAGGGGGTGGGTAAGGAAGGGGTTGATTGTAACTGGAAAATCTTGGGCTACTTG
TGGATGGTGTTTTTTTTCTTTTCTTTTCTTTTCTTTTTCTTTCTTTCTTACTTTTTTTTTTCCTCCTAAAG
GTTGAGCTTGGCTAAAGGAGTGTTGCTAATGGTTTCCCAATTTAGTTTCAAGGGGATTGGCAGCA
GGGTTTTCCAGGCCACAAAGGCTGAAGGGGGACATACTATTAGTCTGTCTCACTCCTCCCAGAA
ACTCAGTCTGGGTTTACTGTAATTTCATCTTAGTCTCGTCAAAGGCACTCCAGATGAGGAGATTC
TGAGCTGGGGTATTTCTTCCTACCCCTCCCAGACAGATTGCAAGGTTTTTCCAATACTGCATTCT
CCCTGGGGCTCTCCCTGGTTGGCGTGGAGAGAAAAGCGCCAGCAGGGGTCAGCAGTGTCCCAC
ATCTGGCCGGGAGCTGCTTGCCACATGTGCTGTTTCTTCTGGCACGGCACTGTCAGAGATACTG
GGGAAATTGGCGAGCACCGTTCCATTGAGGCGTTAGCATCATTATGAGCATCGCAGGCTCAAG
ATTCCTCTCCCTGAAAGTGGGGTGGGCTGGATGAGTGGAGGGTGTGTTCTTTATAAGCACTATG
TGGGGTGTGGGTGTAGAATCCAGACATCAGCAATGCCAACTCACCATCTGGTTTTCTATCGGGA
GGTTTTTATGTGTATTGGCGTGAGCAATATATTGTGTCGGTCTGGGTGTCAGAGGACGCGTGTCA
GAGTTGCCTTGTTGAAATTTTCCCAGGGACCTGGGATTTCTGGATTCACCCGGTATCCAGGCTCT
CCCAGAGCGGCACTTTAGCTGGCCAAGGCCTGGAGCAGAGTGTGTGCAGCGCTCCCACGGCC
GCCTGCCCGGGCCTGCCCCCGACACATGCTTAGTGGAATTCTCCCGTGGCAGCCCAGAGTCTC
CGGAACGAAGGTCTCCACAGCCAAAGCCACGCAAGAGGCTCTCCTGCTCTCATTTCCAGACGC
ACCCTCATCCTTGGCCTCTCCCTGCCTCAGTTCTCTTTGACTCCTCCGGTCCTTGTTGCTGCTCAG
CTCCAGGGCACCCCCACCTCCCAGGACAAGTCCCCTGACTTTACAGCTTACAGGCCCAGAATA
ACAGATGGCCAGAGGCATCCCATTCAAGACTCCCCGCCTCCTTCCTAGCTCCTCAGTCTCTCCC
ACCCCAATGTGGTGGTGCCCAGGGAGGGGGTCAGGAAACCACAGAGGGCCTGTGGGAGGCGT
ACAGCTGGGGACAAGCGTGGGCTGGAAGAGGAGCCCAGTCTGTAGCTGAGACTCCCAGGGCC
TCATGACCTTCCAGGCAGCCTCCACCGCTGCTATGACAGGCAGATGCCCGACTTTCCACCTGCT
TTCTGACCTGGCTTTGGCAAGGCCTGAGCACCTGGCCTGCTTTCCCAGGAGTACAGTTACAGCA
GCTGATCTAGGGAACAGGTGTGGGCTCCGTGTCACAGCTGCTGCTGAGCACATCTGTACCCACA
GTGAGGAGGCTGCAGCTGCAGCAGGGCCTGTCAGTCACTCATACTCTCCCCGGAGTTGGGCTG
GGGGCACTGAAAGGGCCAGGGGTGGAGAGAATCTGAGATAAGCATTCACAGAGCACCTTGCT
CCTAGAGATCCCAAAGTGTGACCCCTGACAAGGTTTTCCCACAGTCAGCCTCACTGGCACCAGC
AGCGTGGGAATTAGGGCTGGATTCTCCCCTTCTGCCTTGCTTCCCAGCCCTTGGGCCACAGTCA
GCTCCTCCTCCTTCCTGGCTAGCTCAGCTCAGCTTTTGCAGGATGGAACTCTGAATCCTGGCTTC
CAGTGGCTCTCCCAGAAAACTAGGAGGGAAGTGCTTGGAAGAATGGCTCTTTATTCATGCAAAT
GATTTCTTGCCATTTAAGAGCCTCAAACCACAAAATCAATTAAAGAGAAAAGGGGAAGATTTGG
GGGTCTGTGCCAGCCACTGGATGCAATGCTTTACCTCCATCATCTTATCAAATCTTCCCAGCAAC
CCTACTGCATAGCTATTATCAAGATCATTTCTGTTTCCCAGAGGAGAATTCAAGGTTACACAGTT
AGGATGCAGTGAATCTGGGATTTGATTGCCGTCCTGACTCCAAAGCTTGCACTCTGACCACTGG
GCCACACTGCCTCAAATCTCCCATTCTCTGTTAAATCCAACATCTGAGCCCCATTCTTTCTCCCTT
GCAGGGAATCACCTGCCTACCTCCACCTCATCCATTTCTCACCACAGCTGGATTCACCTCTACAG
CCTTTACCTCCAGTCGTATTTGGCAATTCATCCTTTTCCAGCTCCTATCATCCTCCCCTACCCTCA
CCAAAGTGGACATCGCAGCCTCTAAATCTGAACGGAGCTGACACCTAGCACCCACCCTCTCAC

CAGCTACCTCTGGGTTCCCCCACCCGGTATTCCCTTCCTCACATCCAAGCCTGTTCCCCTCAGGC
CCTGTCATGGCCTGAAATAGTCTTTGCCCCTGTCTACCACTCTCCAGGGTCCCTCTTGACACCTA
CTTCTGCTTTCCCCTCTCAAGACACCTTTTCTTGGCATTTTAAGACTATTTACTGACTATTTATTGA
TATAGGAAAATGCTTACAGAATGTATTAGGAGTATCTTACACTTTTTTAATAATTATGTATGTAAT
ACATGAATAGATTCTCCTTGTAAAAAACAAACTCAAACACACAGAAAAAGTTAATGACTCCCTT
GATGACTGCCAGTTTGAAGTGTTTTTCCAGACCTTAAAAAAATATACTTTCATACAAGCATATGT
ACATATAGAAAGATATACTTTAATGGGGCATCTTTCATCATAAATTGGATCATAATGTGCAAATT
GTTTCACAACTTTTATTCTCTGTTTGTTTTCAGACAGGGTCTCACTTTGTTGCCCAGGCTGGAGTG
CAATGGCACAATCATGGCTCACTGCGCCCTCCACCTCCCAGGCTCAAGCCATCCTCCTATCTCA
GCCTCCTGAGTAGTTGAGACAACAGGCATGTGCCACCATGTCTGGCTAATATTTTAATAAAATTT
TTTGTAGAGACAGGTTCTCCATA
ID|56119|gene_id|ENSG00000234913|transcript_id|ENST00000441167 Homo sapiens lncRNA

ATGACACATGTACACAGAAAAATACAAGAAAACACATACATCAAAATGGTAACAATGATA
TGCTGGTGAACGTAATGGGTATTTTTATTTTTTGTACTTTATTTAAAAAATTCTTTTACAATGAACA
TGTATTACTTTTGAAATCAGAAAATTGGCCGGGCGCGGTGGCTCACGCCTGTAATCCTAGCACTT
TGGGAGGCTGAGGAGGGCAGTTCACCTGAGGTCAGGAGTTCAAGACCAGCCTGGCCAACATG
GTGAAACCCTGTCTTTACTAAAATACAAAAATTAGCCGGGCATGATGGCGGGTGCCTGTAATCT
CAGCTACTCAGTTGGCTGAGACGAGAGAATCGCTTGAACCCAGAAGATGATGGTTGCTGTGAG
CAGAGATTGCACCACTGCACTCCAGTCCGGGTGGCTGAGAGGGACTCCGTCTCAAAACAAAAA
AAGAAATCAGAATTTTTTTGTGTGTGTGTGTGTGACAGAGTTTCGCTCTTGTTGCCCACGCTGGA
GTGCAATGGCACAATCACGGCTCATCACAACCTCCACCTCCCAGGTTCAAGCGATTCTCCTGCC
TCAGCCTCCCCAGTAGCTGGGATTATAGGCATACACCACCACGCCTGGCTAATTTTGTATTTTTA
GTAGAGACGGGGTTTCTCCATGTTGGTCAGGCTGGTCTCAAACTCCTGACCTCAGGTGATCTGC
CCTTCTCGACCTCCCAAACTGCTGAGATTAGAGGCATGAGCCACCGTCACCAGCCCAGAAAATA
TTTTTTAAATTGAAAATAAGATAGCATATTCCACAAATCTTCTACCGGCTCCATTGCGGATTGAAT
TAGATGATTCTCCCTATTGTTTACTAAGTGTCCAGTTATTTCTGGGCACTCTCCACCTCCGCAGGA
CACTGCCCATCTCCCCAACCCAGGGCCATTTCTCAACCTAGTGCTCCTTCTCCTACTAGGGGGG
CAGACTTCCCAACGTGAGCACCTACATTTCGCCGTAGATGGGATCCACTTGCACTCTCTGAGGT
TCTCAGGCAGATTCCTTCCTTACGGAAGGGGGTCTGATATTTAATCACCCCATGCACTAAGTACA
CACTTCATGTGCGGATCCATCAGGATAACCCCACACAAGCTGCCAGTTCTTTGCTTGTAGTGGGT
GCCCAATAAATGTTTGAATGCATAAAGAAAGCACTCAGATAAGCAGAGACATTTTAGAGCTCTC
TGGGGATGAGAAAAGAGAGAGCTATGGGAGTGAGAATTAACTTGATTGACTATGGCTGGACCT
GGCAAAGAACAACTCAGAAGTATTGGCCTCACAATCACCTTGTTCAGGCTGCTTAATCAATATTA
AAAGTTTGCTGCTCCCACACAATTTTGCTAGGCTCTCCAACCCTGTGAGGATGTCAACAGTTGAA
AAAGAAGAAATAATGAGCCCAGCCTGTATTTGCAGGGGAGTGTTAAAACTGCCGGTGGAGCAG
GATAGTTTGCTAAACAGCCAGTTGAGAACTTTCCCAAACTGGTCGGCAGCAGCATTGCACACAG
AAGTCCCTTCTTCATTGACATGGGGCTAATTCTAGCCACCAGCACTCCAGCGGTACGGGGCAGT
TTTGTGGCCTTTTGCTATTGAATCTGCCAGATGTGTCCAAGACACACTCTCATGGGCAGAATATA
AACAACAAACCAGTATAAAGAAGTCCACAGTGATCTCACCCTTCATAGAAGTCTTCTTTGACTTG
TTTTCTGGCCCCCTGTAAGCACTCCTTGTACCACACTCCCTAAACCCTGTGGCTTAGGTTTTGCTA
TTTTGTATTTGTTTCCTCCTTCTCTGGTTGTTACATGTAAAAAGGTTGTGTTCACGCAGGGGGCAG
GAATTGCAATGGAAAGAATGCAGTGTGGAAAGCCAGGCTCAGTTACTTACCAGCCTTGCTACCT
TAGCCAAACGGTCATGCTCTCTGAGCTGTAGTTTCCTTCCTCATCTGTGAATAGACTTCTACCAA

CTCTCCTCACATGACTGTTTTGAGACTGAAATGATATTATAGGTATAGAAATAAATGTAAACATA
ATATAAATGTATGAGGCCTGGCGTAGTGGCTCACGCCTGTAACCCCAGCACTTTAGGAAGTCGA
GGCAGGTGGATCACCTGAGGTCAGGAGTTCGAGACCAGCCTGGCCAAGATAGCGAAACCCCC
ATCTCTACTAAAAATATAAAAATTAGCCAAGCGTGGTGGCACGAGCCTGTAATCTCAGCTACTT
GGGAGGCTGAGGCATGAGAATCGCTTGAAATCAGGAGGCAGAGGTTGCAGTGAGCCGAGATT
GCGCCACTGTACTCCAGCCTGGGTGACAGAGTGTGACTCTGCCTCAAAAAAAATAAAAAAAAA
CAAAATAAAAATAAAAATAAATAAGTAAATATATGAGATTATCTTTCATCTGGCATGCAGTAGAT
TCTTAAGAAACTCAAGACAGTATTGCGTTGTGGAGTTATGGTGGGGGCACTAGATGTCAGACCT
CTGTGTGCACTCGGGCAGATCAGTTTCACTAAGCTCTTGTGTTTTCCTCAGGAAGGCAAAACGGT
ACTAAGGCGAATAATAAGGAACATCACAGAATTGATATGAGGGTGAAGTTAAATAATGCGGGC
AAAGCACTTCCCAACAGTACCTAGCACATAGTGAGCACTTAATAAATGTAAGCTGTGGCTGCTG
CTGCTGTGGCCATCAGGGAGATAGGGAAATATGTCAATAAGAAATGTATATTGGCCGGGCACG
GCGGCTCATGCCTATAATCCCAGCACTTTGGGAGGCCGAGGCGGGTGGATCACCTGAGGTTGG
GATTTCAAGACCAGCCTGACCAACATGGAGAAACCCCGTCCCTACTAAAAATACAAAATTAGCT
GGGCGTGTTGGCGCATGCCTGTAATCCCAGCTACTCGGGAGGCTGAGGCAGGAGAATCGCTTG
AACCCAGGAGGCGGAGTTTGTGGTGAGCCGAGATCATGCACTCCAGCCTGGGCAACAAGAAC
GAAACTCAGTCTCAAAAGAAAAAGAAAA

Ribosome

ID|195|gene_id|100128881|transcript_id|NR_036480 Homo sapiens lncRNA

AAAGCCTGGGCCCGTGGGGGACGCACATGGTCCTGTTCGTACTCCAGGATGGCGGCGTAA
AGGGCCCGCTGCTCCCGTTCCTCCGGGGTCAGGGCGACTTGGCTGCAAAACCTCCTGGGGAGA
AAGGCACGCGGTGGGTGCCGGGGCCAGGCCCTCCCCGCAGCGGCCCCAGGGGCGGGCAGGA
GTCCCACCTGTTGGCGGCCTCCTGGAGCCGCAGCCGGCGCTCCTCCATCTTTCTCTGGAGCTGG
GTGCAGAGTCAAGGGGCCGAGCGTGGGATCTGAGCGGGCCCTCGGTTCCCAACCCCGTCCCAC
ACCCGGAGAGGCCGGGACGTCCCAGGGACCTCCTCTCGGTGAGGGGCTGTCCCCAGGGAGGC
CTCCTCCAGTGGCGTCAGCTCTCTGGAAATGTGACAAGCTGCTGTCTAGACCCCAGGAAGCCCA
GTGGTGTCTGGACACCAGAGGAGTCTCTCTCATCCTCCCGGATTGCTCTGAGGCCAGGAGCTTT
GTGACTCTGCCCCCTGAAGCTTCTGGAAGATCTCGGGTGGCAGAAAAGGAGAGAGCTTTCCTC
CTTCATCGGAGTATACACGGCGGTGTCGGCCGGCAGGCTGGGGAATGGGAGCAGCTGCAGGC
ATGGTTGGCTTCAGGCGTGTTTTCCCTGCAAGCCATGGGTAACCAGGGGTCAAGCGGTGGGCC
ACATCCCACAGAGGAGATGCGGGCTTGGGGGATGCCCCAGGAGAGCCGCCTACTGCAGGAGA
CCCACCAAGCTTGGCGGCCGTCGACTGGGCCCTCTCCAGACACTGCTGTGCTAGCTTCAGCATC
TTGGAGGTGTCGGGGGGCACAGTTTCCCCAGCTTCTGGGGGAGGGACAAGAAGGCTGGTCACA
GTCGGCTCTACCACTGTTACTTACTGCTGAGATAAAATGAGGCAACGGAAAAGGCCTCTGGGAA
GTGGCCGTTTTACAGAGACAAGGACGTGCTGAGGGGATCGCCTCACCCATCTCCCACCGGCTG
TGTCCCCAAAGTCCTGCCTTCCCGTAGCTTGGCATGGAGCACCTCTGCGCGACCATGGACCTGC
TCACACAGCCCTTCTGCGCGCCGTGGGAGCTGCCTCTCTGTGACTCGCCCCTGGACCCACCGCC
CCAGCGCCTGACCCACCTCCCTGCTGCCCTTCACAGAAAACTTCTCATAGACTCCCATTTTCTCA
TCCCGCCCCGATTTAGCCCCCGACTCCCCCCGGAAACTGCCCTTACCCGTCCCCACCTTGCCAG
ACACGGTGGGCCACTGGAGTTCCTCTGTCTTCTGGGACTTTGCTCTCCCCAGGGGATCCTCTGC
CACTCCTCCACCCAACTCCTCAATCTTGGAGCGCCAGAGCTCAGCCGAGCTCACCGGCTCCCTA
GGGAGCTGGGGCAACTGTTAGCTCCCCCTGACCTGTGTGATCCTACGTCCAGTGGCTGCCCCGA
CCTCTGTGATCCCACGTCCAGGGGCTCCCCCGACCTCTGTGATCCCACGTCCAGTGGCTCCCCC

GACCTCTGTGCTCCCACGTCCAGTGGCTCCCCCGACCTCTGTGCTCCCACGTCCAGTGGCTCCC
CCGACCTCTGTGCTCCCACGTCCAGTGGGTCCCCCTGACCTCTGTGATCCCACGTCCAGTGGCT
CCCCCTGACCTCTGTGCTCTATGTCCAGTGGCTCCCCCTGACCTCTGTGATCCTTTGTGCAGCGG
CTGCTGGCCTCTCAAACTTAATGTGTTCATTGCGGAACTTGC

ID|277|gene_id|100306951|transcript_id|NR_028514 Homo sapiens lncRNA

AGTTGTCTCTTCGCCGCCGCCGCAGCCTCTGCCCCACTCGTGAGGGATCCGAGGGCTCCG
TCCTTGTGCTGTCTTCGCCCCGGCTCCCAGGGGTCAAACGAGGGTCGGGGGTGGGGACACGCA
GCCTAGCCACTGTCCTGCCTGGGATCCGGTCTCCCCCGCCCCCCGGCTGCAGGGTGGATAAAG
AGGAAGATGTGAAGAGGAGGCAGACAAAAGCAAAAGGACATCCTGTCAGTAGGAAACGGCCG
AATTACAATTCAGATGTGCAATGTTTGCATAAGTAAACTGACACGGGACTATTATATGCAGAAAT
CCACGTGCAAAAATTTTCAGACACACCTTTGTACCTCTTCCTAATCCTGCCCTGGTAAAATGGCG
GTTATCAGTGCAAGTTTGAGGATACATACAGCTGGAAAATGGACATCACCAAACTGGACTCCAG
AAAGTTCTGTCTAGGATTTTATTAGACTGCTTTTTTTCTCAATATACCTGTATTGGAAGATTGAGA
CAAAAAGGCACAAACTCACATTAAATAAACAAGGTAATCTAATCAAAAAAAAAAAAAAAAAA

ID|354|gene_id|100505761|transcript_id|NR_038937 Homo sapiens lncRNA

TTTTTTTTTTTTTTTTTTTTGTAGAGACAGGGTTTCGCCATGTTGCCTAGGCTGGTCTCCAACC
TGGTCTCCTGGGCTCAAGCGATCCGCCCGCCTCGGCCTCCCACAGTGCTGGGATTCCAGGCGT
GAGCTACCGCGCCCGGCCTATTTACTTTTCTTACTAAGCTGGGGATCACCGTCGCCCTCGGCTTG
GCAGGAAGGCGGGGGTGCAAGAAGAAAAGAGGTACAGAACACCCAGAGGTGCCCTCGATTCC
GTCTTGCACTTGCCCTTCTCCCACCGTCCAGCAATAAAGCGAGAGAAACAAGTGCAGGAAACT
GGCCGGCAGTCATGGGAGAAGCCAAAAAGACAGGTGAGCAGCGCGTCCGCCTCCCGCGCACA
GCCTTGGGGAGCCGGCGGGATCCAGAGCGGGGCTCCTCTCCGCACTTTGTAGCTGCGTTGCTC
CGCTCCATGCCCTGCCTCAGCCACTCCTCCTGGTTACCAGGCAAAAGGAAACACCTTGAGCTGG
CCAGGAGCTACCAGCGTCTGTCTACACCTGGGCTGGAGTGCAGCGGCATGATCTCGGCACACT
GCAGCCACCGTCTTCCAGGTTCAAGCGATTCTCCCACCTCAGCCTCCCGAGGAGCTGGGATTAC
AGGAGTTCAGTGGCATGACCAGGGCTCACTGCAACCTTGATCTGGGCTCAAGTGATCCTCCTAC
CTCAGCTTCCTGAGTAGCTAGGACCACAGGTGTGCACCAACCACACCCGACTAATTTTTGTAGA
GATGAGATCCCACTATGTTACCCAGGCTGGTCTTGAACTCCTGGGCTCAGGTGATCATCCTGCC
TTGGCTTCCCAAAGTACTGGGATTATAGGCTTGAGCCACCGTGCCTGGCCTGTGATCAGAATTCT
CATTTTTTTAGTCACTAAAAATGCTGGGGGGCACTCCATTCTCCATTATGTGATTAGTTCACATTG
CATGCTTGTATCAAAACATCATATATACCCCACAAATATATACAAAAAACTTTAAAATTTTAAGTA
TTAATTGCTCAGGAAAAAATTAAAATGCTGGGGTGCTGAAATCTCAAGGGCCCCATTACAAAAC
TCCTTAGGAACCTCGCCCTCTCTCTGCTGTAAGGACTGGTTCCAGAATGAGAGAATTTAAAAGA
CATTCCCCCCCCAAAATGTTCATAATGTCACCCCGGAAACCTGCGAATATGTTATATTACATGAC
CAGGGAGAAGTAAGGTTGCAGATGGCAGTAAGGTTGCTAATGGGCTGACCTTAAGATAAGGAG
ATGATCCTGGATTATCTGGGTGGACCCAATGTAATCACAAGGGTCCTTAACTGTGGAATAGTGA
GGTGGCTGAGTCAGAGGCAGAGTGATGCAATGACTGAAAGACTTAACCAGCCATCACCGGCTT
TGAATACGGAAGACGGTCATGAGCCAGGGAATGCAGGCAGGCTCTGGGAGCTGAAAAAAGCA
AGAAAATGGATTCTCCCCTGGAGCCTCCAGAAGGGATGCGGTCCTGCCAACCCCTTGTCAGTG
AGCCATTTCAGATTTCTGACTTCCAGGACTGTAAGAAAATAAACTTGTCTTGTTTTCAGCCAAAA
AAAAAAAAAAAAAAAAAAAAAAAAAA

ID|355|gene_id|100505761|transcript_id|NR_038938 Homo sapiens lncRNA

TTTTTTTTTTTTTTTTTTTTGTAGAGACAGGGTTTCGCCATGTTGCCTAGGCTGGTCTCCAACC
TGGTCTCCTGGGCTCAAGCGATCCGCCCGCCTCGGCCTCCCACAGTGCTGGGATTCCAGGCGT

GAGCTACCGCGCCCGGCCTATTTACTTTTCTTACTAAGCTGGGGATCACCGTCGCCCTCGGCTTG
GCAGGAAGGCGGGGGTGCAAGAAGAAAAGAGGTACAGAACACCCAGAGGTGCCCTCGATTCC
GTCTTGCACTTGCCCTTCTCCCACCGTCCAGCAATAAAGCGAGAGAAACAAGTGCAGGAAACT
GGCCGGCAGTCATGGGAGAAGCCAAAAAGACAGGAGTTCAGTGGCATGACCAGGGCTCACTG
CAACCTTGATCTGGGCTCAAGTGATCCTCCTACCTCAGCTTCCTGAGTAGCTAGGACCACAGGT
GTGCACCAACCACACCCGACTAATTTTTGTAGAGATGAGATCCCACTATGTTACCCAGGCTGGT
CTTGAACTCCTGGGCTCAGGTGATCATCCTGCCTTGGCTTCCCAAAGTACTGGGATTATAGGCTT
GAGCCACCGTGCCTGGCCTGTGATCAGAATTCTCATTTTTTTAGTCACTAAAAATGCTGGGGGGC
ACTCCATTCTCCATTATGTGATTAGTTCACATTGCATGCTTGTATCAAAACATCATATATACCCCA
CAAATATATACAAAAAACTTTAAAATTTTAAGTATTAATTGCTCAGGAAAAAATTAAAATGCTGG
GGTGCTGAAATCTCAAGGGCCCCATTACAAAACTCCTTAGGAACCTCGCCCTCTCTCTGCTGTA
AGGACTGGTTCCAGAATGAGAGAATTTAAAAGACATTCCCCCCCCAAAATGTTCATAATGTCAC
CCCGGAAACCTGCGAATATGTTATATTACATGACCAGGGAGAAGTAAGGTTGCAGATGGCAGT
AAGGTTGCTAATGGGCTGACCTTAAGATAAGGAGATGATCCTGGATTATCTGGGTGGACCCAAT
GTAATCACAAGGGTCCTTAACTGTGGAATAGTGAGGTGGCTGAGTCAGAGGCAGAGTGATGCA
ATGACTGAAAGACTTAACCAGCCATCACCGGCTTTGAATACGGAAGACGGTCATGAGCCAGGG
AATGCAGGCAGGCTCTGGGAGCTGAAAAAAGCAAGAAAATGGATTCTCCCCTGGAGCCTCCAG
AAGGGATGCGGTCCTGCCAACCCCTTGTCAGTGAGCCATTTCAGATTTCTGACTTCCAGGACTG
TAAGAAAATAAACTTGTCTTGTTTTCAGCCAAAAAAAAAAAAAAAAAAAAAAAAAAAAAAAA
ID|363|gene_id|100506054|transcript_id|NR_038429 Homo sapiens lncRNA
TGAGGGCCGGGGCCTAGGGAGGGGCGGTGTCGTAGGGCGGAGCCCGGAGCTCGGCCCC
TGACGAGCCCGAACTCGTCAACTTCCTGTCGGTACTTGAAGAAGCGGAGCCAGGGCTGAGATC
CCGAAGGCGGGCGAGGTCTGGGATGGGGCGGGGCCTATGGGAGCGGGGCTGAAGCCCTGGG
CCCGGCAGAGGAAGGTCGAGATGGACCATGTTGGGCCCCTTCTCTCCCCGCCCCCAGGCCGCA
GTTCGGGGGCCACGCCCCGGCGTGCTCGGGTCACCGCGGGAAGCCCTTGAACCCCCTGGCGC
CCGGCACCCACGTGCGGTAACCGCGGCTCCTCGAGAGCTCCAGGGATGCGGATCTACAGTAAG
GGCTGTGGCCAGATGAATGAATGCACATTTTTTAGTGGGCAGAAAGATGTTAGAATTCATGAAT
TAGAATAAGCACAAAGGAGGGCGAGAGGGGAGGCCGTGGAAGCCGCATTCCTCCCCTTGCCTT
GGGCGCCCACCCCCGAAGGGGCGCGTCCCCTCCCTGCTCCACTGCACGTGGGTGTTTAACCAG
GGGATATCCAGTGCCTCAAGCACCACGACCCGGGAGAAAACTGAGTTGAGAAGGCCTTGGGG
ATGAAGGTGAGAAATTCGAGAATCCCCGGAGATAACACATCAGCAAATAGTGCGCAGGCAGAA
CAAGCTGTTGGAGGTGAGAGACACAGAGCAGAGAAGCACCCGCACCTGGAGAGTCAGGAGGG
CCTCACATCAGAACGCCTGAGCAAATACAGTTTTGAAGGATACTAATTTTTTAAAAAGTTCATTC
ACATGAGTTAGACAAAGAGAAAGAAGCAGGACATTCTAGGAAGAAGGAACGGCCCAGGCAAA
AGCGACGCGTACATTGGCGTGGGTCCATTCAAGAATTGTGCCCACAATATACCAGGCACTTGTC
CAGACCTGCATCGTCCAATGCCCACCTCGACCTCCCAAAGTGTTGGGATTAGAGGCGTGAGCC
ACCGCGCCCTGCCTGCATGTGGCTATTGAGATGTGCTGTATGATACACACCGGACTTTGAAGAC
TCAGTGTGAAAAATAAAAAGAATTTTAAGAGGGGTGGGGGTCGAGGGGAGGCAGAGCAGTAG
GACAAATAGCTAATGCACGCAGGGCTTAAAACCTAGACAACAGGTTGATGGGTGCAGCAAACC
ACCATGACACATGTTTATCTATATAGCACCTACATGTTCTGCACTTGTATCCTGGAACTTAAAATT
AGAAAAAAAAAATAATAAAAGCCAACATGGCATTCATTACTGCCAAAAAAATTAAAACACATTG
TTAATTTTG
ID|364|gene_id|100506054|transcript_id|NR_038430 Homo sapiens lncRNA
GAGGGCCGGGGCCTAGGGAGGGGCGGTGTCGTAGGGCGGAGCCCGGAGCTCGGCCCCT

GACGAGCCCGAACTCGTCAACTTCCTGTCGGTACTTGAAGAAGCGGAGCCAGGGCTGAGATCC
CGAAGGCGGGCGAGGTCTGGGATGGGGCGGGGCCTATGGGAGCGGGGCTGAAGCCCTGGGC
CCGGCAGAGGAAGGTCGAGATGGACCATGTTGGGCCCCTTCTCTCCCCGCCCCCAGGCCGCAG
TTCGGGGGCCACGCCCCGGCGTGCTCGGGTCACCGCGGGAAGCCCTTGAACCCCCTGGCGCC
CGGCACCCACGTGCGGTAACCGCGGCTCCTCGAGAGCTCCAGGGATGCGGATCTACAGTAAGG
GCTGTGGCCAGATGAATGAATGCACATTTTTTAGTGGGCAGAAAGATGTTAGAATTCATGAATT
AGAATAAGCACAAAGGAGGGCGAGAGGGGAGGCCGTGGAAGCCGCATTCCTCCCCTTGCCTT
GGGCGCCCACCCCCGAAGGGGCGCGTCCCCTCCCTGCTCCACTGCACGTGGGTGTTTAACCAG
GGGATATCCAGTGCCTCAAGCACCACGACCCGGGAGAAAACTGAGTTGAGAAGGTCTTCTAAC
AAGCCTTTTGCACACCTGCCATGTATTTTGTCAGGCCTTGGGGATGAAGGTGAGAAATTCGAGA
ATCCCCGGAGATAACACATCAGCAAATAGTGCGCAGGCAGAACAAGCTGTTGGAGGTGAGAG
ACACAGAGCAGAGAAGCACCCGCACCTGGAGAGTCAGGAGGGCCTCACATCAGAACGCCTGA
GCAAATACAGTTTTGAAGGATACTAATTTTTTAAAAAGTTCATTCACATGAGTTAGACAAAGAGA
AAGAAGCAGGACATTCTAGGAAGAAGGAACGGCCCAGGCAAAAGCGACGCGTACATTGGCGT
GGGTCCATTCAAGAATTGTGCCCACAATATACCAGGCACTTGTCCAGACCTGCATCGTCCAATG
CCCACCTCGACCTCCCAAAGTGTTGGGATTAGAGGCGTGAGCCACCGCGCCCTGCCTGCATGT
GGCTATTGAGATGTGCTGTATGATACACACCGGACTTTGAAGACTCAGTGTGAAAAATAAAAAG
AATTTTAAGAGGGGTGGGGGTCGAGGGGAGGCAGAGCAGTAGGACAAATAGCTAATGCACGC
AGGGCTTAAAACCTAGACAACAGGTTGATGGGTGCAGCAAACCACCATGACACATGTTTATCTA
TATAGCACCTACATGTTCTGCACTTGTATCCTGGAACTTAAAATTAGAAAAAAAAAAATAATAAAA
GCCAACATGGCATTCATTACTGCCAAAAAAATTAAAACACATTGTTAATTTTG
ID|365|gene_id|100506054|transcript_id|NR_038431 Homo sapiens lncRNA
GAGGGCCGGGGCCTAGGGAGGGGCGGTGTCGTAGGGCGGAGCCCGGAGCTCGGCCCCT
GACGAGCCCGAACTCGTCAACTTCCTGTCGGTACTTGAAGAAGCGGAGCCAGGGCTGAGATCC
CGAAGGCGGGCGAGGTCTGGGATGGGGCGGGGCCTATGGGAGCGGGGCTGAAGCCCTGGGC
CCGGCAGAGGAAGGTCGAGATGGACCATGTTGGGCCCCTTCTCTCCCCGCCCCCAGGCCGCAG
TTCGGGGGCCACGCCCCGGCGTGCTCGGGTCACCGCGGGAAGCCCTTGAACCCCCTGGCGCC
CGGCACCCACGTGCGGTAACCGCGGCTCCTCGAGAGCTCCAGGGATGCGGATCTACAGTAAGG
GCTGTGGCCAGATGAATGAATGCACATTTTTTAGTGGGCAGAAAGATGTTAGAATTCATGAATT
AGAATAAGCACAAAGGAGGGCGAGAGGGGAGGCCGTGGAAGCCGCATTCCTCCCCTTGCCTT
GGGCGCCCACCCCCGAAGGGGCGCGTCCCCTCCCTGCTCCACTGCACGTGGGTGTTTAACCAG
GGGATATCCAGTGCCTCAAGCACCACGACCCGGGAGAAAACTGAGTTGAGAAGGGTCCATTCA
AGAATTGTGCCCACAATATACCAGGCACTTGTCCAGACCTGCATCGTCCAATGCCCACCTCGAC
CTCCCAAAGTGTTGGGATTAGAGGCGTGAGCCACCGCGCCCTGCCTGCATGTGGCTATTGAGAT
GTGCTGTATGATACACACCGGACTTTGAAGACTCAGTGTGAAAAATAAAAAGAATTTTAAGAGG
GGTGGGGGTCGAGGGGAGGCAGAGCAGTAGGACAAATAGCTAATGCACGCAGGGCTTAAAAC
CTAGACAACAGGTTGATGGGTGCAGCAAACCACCATGACACATGTTTATCTATATAGCACCTAC
ATGTTCTGCACTTGTATCCTGGAACTTAAAATTAGAAAAAAAAAAATAATAAAAGCCAACATGGC
ATTCATTACTGCCAAAAAAATTAAAACACATTGTTAATTTTG
ID|366|gene_id|100506190|transcript_id|NR_038955 Homo sapiens lncRNA
CCGGCCCGTCTCGGGGCCCTGAGTCATCGCCGCCCCCGGCCCCGACGCTGGCCTGCGGC
GTCTGTTTGTGCAGAGCGAGGCCCCGCCGGCCGGGCTTGACGTCAGGCTGGCAGGGCCTGGAA
CGGCCCCGCATCGCGGCCCCCTGGGGGCAGGACGACACCGCTGGGGGCCGAGGGCAGGGCA
CGCCTTCGGAATCCGGCTTCGAAGCCGGCCGCCTGACCTGGGGCAAGTGCTTTCAACTCTCTGA

ACTTCGGTTCCTCATCTGCCAGTTGGCAGACGCTCAGCAAATCTTCCTAGACTCACGGGGCGAA
TTGTACAGTTGGGTAAATCGAGGCCCAGAGATGGCAAAAGGACCGTCCAAGGTCGTACAGCCA
CCCGGAATTACAATTCTTTTCTGCCGGGTCACACGCTGTCATCCACGTGTTACCTAAGAGAGCTT
TCCAGCCTGTGCTAAGAAAACTGCCCCACATCATCTGCAGTAGGACGGGGGAGTTGGAGCCCT
GGTCAGGCCACTCTGCTACTGACCACAGTTTTCTCATCTCTAAAAAGGCGCAGTAACAATATAAT
TACCGTATGCAGTCCCCCAGGATACAGGGTCAAAGGAGAGCACAACCATCGCAGTTGGAAGCC
CATGGGGCAGCCCAGTGCAGATGCATCTGACTTACGAAACTTCAGTGACACCTGCTCTGTGCCA
GACACTGAAGATGGAGCAGTGAACAGCACTGACCCAGCCTGTCCTCCTGTTGCCTGCAGGCCA
GACGGAGTCTCACTCTGTCACCAGGCTGGAGTACAGTGGTGTGATCTCGGCTCACTGCAACCTC
CGCCTCCCAGGTTCAAGCGATTCTCCTGCCTCAGCCTCTCAAGTAGCTGAGATTACAGACGTGT
GTCACCATGCCCTGTTAATTTTTGTTTGTTTGTTTTGTTTTTTGTTTTGTTTTGTTTCGTTTTTTGTTTT
TTTTTTTTGAGACGGAGTCTTGCTCTGTCGCCCAGGCTGGAGTGCAGTGGCCCCGATCTCGGCTC
ACCGCAAGCTCCGCCTCGCGGGTTCATGCCATTCTCCGGCCTCAGCCTCCCGAGTAGCTGGGA
CTACAGGCATCCGCCACCACACCCAGCTAATTTTTTGTATTTTTTTTAGTAGAGACGGGGTTTCA
CTGTGTTAGCCAGGATGGTCTCGATCCCCTGACCTTGTGATCCACCCAAGGCCTCGGCCTCCCA
AAGTGCTGGGATTACAGGCATGAGCCACCGTGCCCGGTCAATTTTTATATTTTTTGTAGAGACAG
GGTTTCACCATGTTGGCCAGGCTGGTGTCGAACTCCCGACCTCAGGTGATTGTCGGCCTTGGCC
TCCCAAAGTGCTGGGATTACAGGCGTGAGCCACCGCGCCCAGCCGGTGGGCAGCCAGTTGTGT
TCCGGGAGGAGGAGGACACCCTGGAAAGACAGTGGGGCCAGACACTGAACTGCCTTTGGAAG
CAAGTAGGAGCAACAGCGAAGGTTTGCTGGCTCAGAAGCAAGGTCTCGCTGTGTTACCCTGGC
TGGAGTGCAGTGGCTACTTACAGGCATGATCATAGCATACTGCAGCCTCGAACTCCTGGACTAG
AGCAGTTCTCCTGCCTCAGCCTCCAGAGCAGCTGGGACTGCAGACACGCACCACAGAGCCCAG
CCTGAGTCATTTTGATTCTGTAACTGGCTTCCTCTCGGCGGGCAGGCTCACACCCTCCACCCCCA
GCTGGAGATGGAAGGAGGATGCTCACCGCCCATTCCTCATGCAGGCCATCGATGGAAATGTCA
GACAGTGCGAGTCCACGCCTGAACACTTCTGGGGTCCGCTCTGGGACTGACACTGGCAGATCC
ATCGATGCCGTTCCATTGTTTGCTGACTTTCATTGATTCTATGAGAAGTCTGCAGTCTTTTGCAGC
GTTGTTTTCCTATGTATAATTGTCTCCTCCCACCCCACTCACCCCAGCTGCTTTCCACATTCTCTCT
CTTTGATTTTCAGCAGTCTGCCCATGATGTGCCTAAATAGGACTTTCTTTGCATTCATCTTGGCTG
GGGCTCACTGAACTTTCTGAATCTGTAGATTAATTTTCTTTTTTCAATAAACCTTGGAATATTTTCA
GCATAAAAAA
ID|372|gene_id|100506668|transcript_id|NR_038854 Homo sapiens lncRNA
GCAGACCACAATGCACCGCTCACGGCGTCGGTTGCCCGGGCAATGGGCCGCACGCTACG
AGGCCCACACACCCAGAAGGTGGAGCCCCGGCCGGGTTACGAGGACCACCCAGCTGTCTGGA
GAGATGAAGAAAATGAGGTTCAAAGAGATGAAGTCTCTTGCCTAAAGTCAGTGACAGAAAGTG
ACAGAGCTGGGATGTGAATCCTGGTCTGACTCTACAGTCCCACATGGTAGATGGAACCTCCGAG
CAACACCTAAACAAAAGGAGTTGATGCCTCCGAACAGAGTAATGTCGCTGGAGACACTAAGCA
TCGTCGGCCCCTGGCAGGAGCTCCCTAAATATGTGTTGGATGGATGGAAGAAAGGATGGATAG
TTCAGAGTACTTCAGCCTTGATTCCTCCGCAACAGACACCTGAAACTTGACCCTACATTGGTTCT
TGGCCATCGTGATCTTCCTTGGACAGAATCACCTTTCAGCTCCGGTCATCAGACTTTCCCAGGGC
CCTCAGAAAGCCCTTCAGAGTTGTTACTCACAGGCAGGCTGAGGGATTCCTTACGGGGTCTGCA
GCTCTCCTCACCTCATCCACAAGTAGGACCGTGGCCTGTTCCTCACTACTGCCCCAGGATCACT
CTGTTCCCAGCCCAGTCCAGCAATCACTTGTCTAGCTTTCTGGAACCTTGAGTACTTTCTTGAAC
CATGAGTCCTGTGACCACCCTAGCAGCTCTAACCCTCCCTTATCTGAAAGGAAGTGTGAGGTGA
CCTTGCAGGTCCCAGAGTTGATTGAAGACCCCATCCAGAAAGAAGGCACCCTGTGGGAGAGAT

TGCAAGGCCTAGGTCTGAATCCGGAAGCTTCCACCCCATGGAGAAGGGCTGGCACCAGCCTGG
GGCTGGCAGTGGAGCTGAGCTTTGGAGCCAAGGACTGTACTGCAGTGCAGGGAGAGTGAGGC
CAGAAAGGCTGAGACAACTCAGGGAAAGAAAACCTCCCTTCTGGCTAATAGTCAAGCACCGCC
TGAGTAGACCAACACTCTCCTGTCCACAGGGGCAGCAGATGAAGACACAACCAGAGAGGACTA
ACAGGCCCCCTCAGCTCTCAGTCAGAGGGCAGAGCAACACAGAATAGACATTAAAGGAACAG
ACTTTGAGGCCAGGCAGCCTTGGGTGTGCATCTGTCCCTACTAAGCCATGTGACATTAAACAAG
TGAGTCCACCTCTCTGAGCCTCAGGTTCCTCATCTGTAAAATGGGGATTATAAGAGTTCTTGTTT
CTCAGGGACAATGTGAGGATTAAGTGAGATGATACACATAGAGAATGTGGTGCAGTACTGGGC
ACATGGCAAAGATCAGTGATGCTAGCTACCACTTATCATTAGTGTTCCTGTAGACCAGGACTTCT
CAACCTTGGCACTATTCACCTTTTGGGCCAGAAAGTTTTTTTTGTTGTTGTTGTGGGGAGCTGTCC
TATGCATTTGAGGATGTTTAACAGCACCCCTGGCCTCTACCCACTAGATGCCAATCACACCACA
CCCCCAGTTTCAACAACCAAAAATGTCTGCAGATGGTGCCGAATGTCTTCTGGAGGGTGAAATC
ACTCCCCAGCAGTTGAAAAGCACCACCCTAAACAATCTGGACTGAATTTGAATGTCACAGATCC
CAAGCTCACAGCTCCATGTAAAGGCCACAAGGCAGGCAGGCCTACTTGCAGATGAAGGAACG
GAAAGAGCAACAATTACAGATCAAGCGGCTAGCTCCGGTGTAGACTAAGAGGCGGGATCTGTA
GTCTGCTCTAGGAGCTAACAAGGCCTCTGTGACTCAGTAGGTGACCTCAGTGTCACTCTTTAATT
ATATGCAAAGTGGTCTTGTGTTAACTCTTAATTATTCTAGGGCTCCAAACAAGAA
ID|373|gene_id|100506714|transcript_id|NR_038956 Homo sapiens lncRNA
GCAGCCGCGCTTTGGGGCGTCGGCTCACGTGGGGCCGGCAACGCGTCTTCCAGGATGTTC
GCGCGCTAGTCGCCGCTGAGGGGCCGGCGATGCGCGAGGAGTTCCGGCGAGGAAGGTCGGTC
AGTGGACATGGCCGAGGCCCGCGGTGTCCGCGTGTGGGAGCTGCAGGGCTGAGGAGAGGCCG
CCCGCAACCCTCGGGAATCGTAGACGAGGTCTCACTATGTTGGCCAGGTTGGTCTCGAATTCCT
GGCCGCAAGCCGTTCTCCAGTCTGGGCCTCCCAAAGTGCTAGGATTACAGGAACAGAATTGCC
ATTGGGCCCAGGGAGAGACTACACTTCTTGTCTGCTTTGTTGCTAGTGCGGCCTTGTGGCCAAG
CACAGAACGTGTGCAGTGATGATGGTGCAACTTCTAGGTCGTCCCCAGCATGGAAAATGAGCC
CTGGGCATGGAAGATGAGCCCTGGTCCTGCAGCGGTGCTCTTTCCTCTTCTCACAAGCTGGAAT
GTGCATGTGCTCCCACCCAGCTTTGACCATGGGGCGAAAACAGTACTCTGCAGTCTGGCAGAG
CAACAAGGTGGAAAGGGCCTGGGTCCTTGAGTGACGTCATGAGCCCTCTCACTGTCCCGACTTT
GGGACTGTTATATGATAAGGACTTTCTGTGAGTCACTGTCTTTTAGAGTCTCTCTGTTTTAGCTGC
CTGGACTTTATCCTGAATAATATGGCTGTTATCATGGGTATAGTGTTACTATGAGCCTCATCTAAG
TAATTTCTACTCTGCCTTTGACCACTAAACTCATGTTGGACAAGAGTCACTTAGCATTATTTTTTC
CTCAATGAAGAGGTTAAGATGGAGGGAGCAGGAGAAATTGAGGCCTGTGTTTTACCAGAAGGT
GAAGGGCTGTCTTTGCCTTTCCTACATTTATGAAGGATAATTCTAGACAAAAACATTAAGTTCAT
ATCAAACCATCTGCTTTGTGAAGCCATGGGCTTTGCTAGAACTTTCTGTGACATGACATTTATTAA
CCCGAGTCTACCTGGAAGTTAGAGCTGAGGAAGTTTTCCTAGAGTGGAAAAAGTCCAATGCACT
TGTAAAAGTCAGACCAAGCTCAGCTCAGATTTCAGTTTTATTAGAAGTATGTCACCTTCCTGAGC
CCTTATTATTTCCCCATCTGTGAAATGAGGACACCGTTCTCTCTTTCTAAGCATTGCTGGGAAGA
CCACACACCACTGAGACAGAAGGTGGGGCTAGCGCAGTTCTGGGCATTTTGTACACCCCCAAC
AAATACTGATTTATTTCCTTCTTTTTCCTTGCTCAGAAAAAATTGGAGAAGGCTTTATTT
ID|374|gene_id|100506714|transcript_id|NR_038957 Homo sapiens lncRNA
CGAAACTCCAGTCTCTGCCCCCACCTTCACATCATCTTCTCCATGTCTCCTTACCTTCCTTTG
CCTCTGTCTTGAAAAGACACTGTGATGGCATTTAGGACCCACCCAGCTAATTCAGTGTGTTCTCA
TCTGCAGATCCTTGATCAAGTCACATTTGCAGAGACCTCTTTTTCAAATAAGAGACGAGGTCTCA
CTATGTTGGCCAGGTTGGTCTCGAATTCCTGGCCGCAAGCCGTTCTCCAGTCTGGGCCTCCCAA

AGTGCTAGGATTACAGGAACAGAATTGCCATTGGGCCCAGGGAGAGACTACACTTCTTGTCTGC
TTTGTTGCTAGTGCGGCCTTGTGGCCAAGCACAGAACGTGTGCAGTGATGATGGTGCAACTTCT
AGGTCGTCCCCAGCATGGAAAATGAGCCCTGGGCATGGAAGATGAGCCCTGGTCCTGCAGCGG
TGCTCTTTCCTCTTCTCACAAGCTGGAATGTGCATGTGCTCCCACCCAGCTTTGACCATGGGGCG
AAAACAGTACTCTGCAGTCTGGCAGAGCAACAAGGTGGAAAGGGCCTGGGTCCTTGAGTGACG
TCATGAGCCCTCTCACTGTCCCGACTTTGGGACTGTTATATGATAAGGACTTTCTGTGAGTCACT
GTCTTTTAGAGTCTCTCTGTTTTAGCTGCCTGGACTTTATCCTGAATAATATGGCTGTTATCATGG
GTATAGTGTTACTATGAGCCTCATCTAAGTAATTTCTACTCTGCCTTTGACCACTAAACTCATGTT
GGACAAGAGTCACTTAGCATTATTTTTTCCTCAATGAAGAGGTTAAGATGGAGGGAGCAGGAGA
AATTGAGGCCTGTGTTTTACCAGAAGGTGAAGGGCTGTCTTTGCCTTTCCTACATTTATGAAGGA
TAATTCTAGACAAAAACATTAAGTTCATATCAAACCATCTGCTTTGTGAAGCCATGGGCTTTGCT
AGAACTTTCTGTGACATGACATTTATTAACCCGAGTCTACCTGGAAGTTAGAGCTGAGGAAGTTT
TCCTAGAGTGGAAAAAGTCCAATGCACTTGTAAAAGTCAGACCAAGCTCAGCTCAGATTTCAGT
TTTATTAGAAGTATGTCACCTTCCTGAGCCCTTATTATTTCCCCATCTGTGAAATGAGGACACCGT
TCTCTCTTTCTAAGCATTGCTGGGAAGACCACACACCACTGAGACAGAAGGTGGGGCTAGCGC
AGTTCTGGGCATTTTGTACACCCCCAACAAATACTGATTTATTTCCTTCTTTTTCCTTGCTCAGAA
AAAATTGGAGAAGGCTTTATTT
ID|379|gene_id|100507316|transcript_id|NR_120682 Homo sapiens lncRNA
CGGTTTTTTCCTCAGGCTTCCGGTCTGTTTGGTGCCCTGGTCGCGTGTCTTCCGAACTCTGC
TGCTTCGCCCCGGCAAGAGCACTTCCTTCCGCGGCTGCTAAAACTGGTGCGCGGGTTCCCAACT
TCGCAGAAGAGCTTCATCGGCCCCCCGCTAGAGACCTCAGACCGCAGCCATCGACGCCTCGCC
TCGGGGATCTGTCCCCACCCTCGGGGCCAGCTCACCATGAGTCAGGCCCTGTGCTAGGTGCTG
GGATGAAGCAGTGACTGACACAGGCCTGGACCTGTCCTTGGGAAGAGTGCGTCTGTGACACAG
ATACTGTGCCATGTGGCAAAACTTGAATGGAGACATCAGCTCTTTGTGGGTCTCAAGTCTACTG
GCTTTCAATCAGCTCTTCTGCTTGACAGCTGCAGGTCTTGGGACTGCCCAGCCCCCATAACCCTG
TGAGTGCATTCCTGATAATAACCCTCTTTCTCTTGTCCTCCCCCACCCCACATCATTGGTTCTGTT
TCTCTGGAGAACCATAATACATCATTTACTAATCAGGAAATAGATTCAGAGACATTAATAACTCA
TCCAACCTCACAGTTAACTGTAGAGTTGGGCTTGAAATTCCAAGGTCGATTTTCTTAGCCAATAC
ACCATGCTACATATATGTATGTAAAAATAAAAACGCTTGTGAACTGAAAAGGGTCC
ID|380|gene_id|100507316|transcript_id|NR_120683 Homo sapiens lncRNA
CGGTTTTTTCCTCAGGCTTCCGGTCTGTTTGGTGCCCTGGTCGCGTGTCTTCCGAACTCTGC
TGCTTCGCCCCGGCAAGAGCACTTCCTTCCGCGGCTGCTAAAACTGGTGCGCGGGTTCCCAACT
TCGCAGAAGAGCTTCATCGGCCCCCCGCTAGAGACCTCAGACCGCAGCCATCGACGCCTCGCC
TCGGGGATCTGTCCCCACCCTCGGGGCCAGCTCACCATGAGTCAGGCCCTGTGCTAGGTGCTG
GGATGAAGCAGTGACTGACACAGGCCTGGACCTGTCCTTGGGAAGAGTGCGTCTGTGACACAG
ATACTGTGCCATGTGGCAAAGTAAGCGCGTGGGACAGCAGAGCCTTGTTTGCCATTAAAGTAAT
ATCACAAGTCTGTTAA
ID|489|gene_id|100874323|transcript_id|NR_046609 Homo sapiens lncRNA
AAAGAAAGAAGAGATAAAAGAGGAAGGGAAGACAGGGAAAAGAAGGAAGAAAAGAGA
GGCGACTCCTAGCAGCGGCCGAGCTTACAGAGAGAAGGGTGGAGGTGTGTGGTGGAAGGGGA
AAGCCGGCCGGCTGGCAAAGCGCTGCGGAGAAAGACACGAGGCTCCTGAGCAGGGAAAGCC
GAGGTTGCCACCGCAGGCCTGGCACGACCAGGGCCGTGATGCCCCGCCCGGCCCGACCCCCG
CGCGCAGAGGTACCTGGAGACGATTTCAACTGAAGTAATGAAGGCAGTGTCGTGCTGTCGAGA
GAAAGGTGGATCCCAACAACAGGAAACTACCTAAATCACCGACCAGTTCTGGTGCTGCCCGCG

AAGGGCTGCCTCGCCCGCCGCCGCCGCCGCCTCCGCCGCTGCCGCCGCCGCCAAGGAGAGAA
CCCTGCCATCGCGCCTGGCCCGGCCCAGCCCAGCCCCTAGGCAACCTGCGCCCGCCAGTGCAA
CAGAGTGCCCCAGGCGGCCGCAAATGCGTCAAGGAAGGGGAAGCCACAGGCCCCAGTAAGCC
AAAGTCAAGCCCGTTTGCCAACCTGCATGTCCATGCCTGTAAGCCCTTCTCTTGGCCAAGGAAG
AAAGGAAGAAAGAAAAAAGAAACCCAGGGGCCTGTATCCCCTGATTAAACACAGCACAGCACT
CCAGGCAGACATGCCCGGTGGCGGCTCCTTTGCACCATTGACCTCAGGCCAGACACCTCAGCG
CCAACAATGGGACCTCGGCCTTCCGGCTAGGTTTGCCCCAGGCTGGGCAGGAAACCAGCTCGG
CCGAAGACAGGGGCCATTTCGAGCAGTGGGACCCCAAGACAGCAAACCCAGCCCAGTCAGGA
CTTGACACTTAGGACAATATCTATCTCTATAGAATTTTAGCATGATATGGCTTTTTCCCCCAGAAA
ACAACAAATAAACCAGCACCAAGCAAACACAAAGAAACAAAAAGTCAGAACAAACCAGCCCT
GCACAGATGTAACGGCCCAGGAGATGGCGAGTGTGGGAGGGAGGAACAGGGCTCCAGCACA
GGTGCGAGTTCCTGGGCAGAGCCTGAAGACAGAGGGAGGGGACCAGCGCTCGGGAAGTGAAA
AAACCGCGTCGCCTGGAGATTCATCAGGAAAAATTAAAGT
ID|512|gene_id|100996579|transcript_id|NR_110163 Homo sapiens lncRNA
AGAGTGCCCCGGCGTCTTTCTAGCCTCCGCCTCCAGGCGGGTGAGGAGGTGACACCTGAG
AATTCCTCCCTCTCACCCCTGACCCTGCCGGAAAGCGAAACTGAGATGCGGGCATTGGGCCCT
GGGAAGCGTGGGGAAGGGGAAAGGAGAAAGCCAGGTTAACTACGTCAGGATTAATGTGTTCC
ACTTCTGCTGCCTGAGAAAATGGAGAAATTAAGTCCTTTCCATGGATCATTCCTTCTGTAAACAG
AGATCACAAAGCAAGAGCTTCAGCATCCTCGTGAAAAAGACATTTTGTTCTGGGTGTCATCATCT
CCTTCCACTACAGCTTGCAATTGGAACAAGCTTCACATCCTGGGGGTGCTGCCTATCTCTGTCTT
GATTTCTGTCTGTCTATTCCTCCCATTGACAGGAATGTCGTTTGTATCCTCCACGCAGCAGTTTGC
AGAAATCCAGCAAGGTTTATGGTTTATGTTGCCCAGCATGTATAAACACAACGCTCTCAAATGCT
TTTTAAAAATCTATGAATTTGTTAGATGTCTTCACATCCTGGTCCTCCTACCCCAATTCTCAAACA
CAAAGTACTCTGTTGCCCTATTATAGTCCTGCGATAGTTCTGGCTAGGATTAGGTTGACGGTCTA
AGCCCCAAGAAAGGCCTGGCATTTTCATAGCAGGCTTTCCCCAGTGCTTTGAGTCTTCCTCTCTC
ACTCCCTTTTTCTTTTTCCCTCGGAGGAGGGGGTGTTGGGGGTGGGAGGTGATTAGTAAATGTTT
CAGGTAATCTGTGAATTCTTAATAGTCATATTTCCGTGGTGGAAGATCTCCCTATTTCTCTGATGT
GACAGGTTACAGAAACCTTGGTGTTGGGGGTTTTACAGATATTTACAAGTTCTAGGAGTTTGAAC
TTTGGGTCAAGATGTTGATTCCTTTTAATGTGCGAGGGTGAAGGAGGTTTTATTTTGCTTTATTCT
TTTGGATATTATGAGATGAGATTGTATCCAGTCTTGGCTGTAACCTATACCATAAATGCAGAGCT
GCAACCATTATTTCTGTCTCTTTCTCCTTTTATCACCTACACTTCTAGAGATATTACTTTCAGTTTTA
TTGCAAATCATCTGTGTCTTTAGTTTAAACCATTATTTTTCATATACCCCAGGCACATTTCCCTTAT
AATGAGACAAATTTTAGATTATTAATGTTCCACCTAAAAGGATACTGTACACTTTTCATTGCCAAT
ATGGGCTTTGAAGCTTAAATATCAACATTTTTATAGGAAATTTACCCATGTCAAATTTAAATATAT
TTGCTTATATTTCTTGGAAAAAAGATTTTGGATAACTATCAATTAAGCAGCCAATTCCATTTGGGG
TGCAGAAAATTTTGAGCCCCTTGATAAGAAGGAACAAAATTTTTCCTCTAAATAACCAACTCTGG
ATCAGAAAAACTGAACTTTTCTAAATATTTATTATTAACAAAACTTTTTTTTGTCTGATTCCACTAG
AAGCATTCTATGTTGTGAACACTATAAAGTTATCCTATGTTGTGAACACCATAAAGTTATGAGGG
AAAAAATGTCAAATCTAAATTTTATTTAGAAGTAACATATTGTTTTTCTGGACTTGCAATCCAATT
CGAATGCATCGTTAGACTTTTCTGTTTTCTTATGATTAAACAACAATGGCTTAAAATGATAGCAAG
TCAACTGAAAAGTTGCTTTGGAATAAAACAAAAGTGGGAATGCCAACTTTCTTTTCTTCTCTATA
ACTTCATTAACATGGTTGTATAGACATTTGCATGTAAAATGTAGCTAACAAAAATTACACAGAAG
TAAATTTAGTCCTCTAGTGCCTACCTCTATGGTGAAATCAGACCATTTGGCATAGTTTGAAGTCTT
CCAAGAAGCCTGGTTTCCACCTGGAAAATAAATTACTAAATTTGAATGTGTTACTTATACTATGG

TGAGTTGTCTATCTGTTACATTTTATTTTAGTGCATATACTAGAAATTGTATTTAGGGTGTTCTAAA
ATTGTCATCTGTCAAGGTAGAGAATTATAATAATATAATTATACTCAGAAAGCGTTCTGATTTTAG
ATTATTTTGGCACTGTTACAAATATGATGAATTTTAAAAAAATGCAGAATGTTTATTACAAATTCA
TATGTTTAATTGTCAAATTCTGAGATTAATTTAGATAAGCTATAACCAAAGTTTATTTTCTTTGCAT
TTATTGGTACATAGTTAATGCTTATACACAGGCTTCAGTGTAACACCTATACACTATTACAATATT
TAGCTGTACTTATTTGCTTTATCTTTAAATGGTACTTTGTCCCAAAATTTTCTGAGTTTGATAAATT
ATTAAACATTTATCAAATCACAAAAAAAAAAAAAAA
ID|513|gene_id|100996579|transcript_id|NR_110164 Homo sapiens lncRNA
AGAGTGCCCCGGCGTCTTTCTAGCCTCCGCCTCCAGGCGGGTGAGGAGGTGACACCTGAG
AATTCCTCCCTCTCACCCCTGACCCTGCCGGAAAGCGAAACTGAGATGCGGGCATTGGGCCCT
GGGAAGCGTGGGGAAGGGGAAAGGAGAAAGCCAGAAAATGGAGAAATTAAGTCCTTTCCATG
GATCATTCCTTCTGTAAACAGAGATCACAAAGCAAGAGCTTCAGCATCCTCGTGAAAAAGACAT
TTTGTTCTGGGTGTCATCATCTCCTTCCACTACAGCTTGCAATTGGAACAAGCTTCACATCCTGG
GGGTGCTGCCTATCTCTGTCTTGATTTCTGTCTGTCTATTCCTCCCATTGACAGGAATGTCGTTTG
TATCCTCCACGCAGCAGTTTGCAGAAATCCAGCAAGGTTTATGGTTTATGTTGCCCAGCATGTAT
AAACACAACGCTCTCAAATGCTTTTTAAAAATCTATGAATTTGTTAGATGTCTTCACATCCTGGTC
CTCCTACCCCAATTCTCAAACACAAAGTACTCTGTTGCCCTATTATAGTCCTGCGATAGTTCTGG
CTAGGATTAGGTTGACGGTCTAAGCCCCAAGAAAGGCCTGGCATTTTCATAGCAGGCTTTCCCC
AGTGCTTTGAGTCTTCCTCTCTCACTCCCTTTTTCTTTTTCCCTCGGAGGAGGGGGTGTTGGGGGT
GGGAGGTGATTAGTAAATGTTTCAGGTAATCTGTGAATTCTTAATAGTCATATTTCCGTGGTGGA
AGATCTCCCTATTTCTCTGATGTGACAGGTTACAGAAACCTTGGTGTTGGGGGTTTTACAGATAT
TTACAAGTTCTAGGAGTTTGAACTTTGGGTCAAGATGTTGATTCCTTTTAATGTGCGAGGGTGAA
GGAGGTTTTATTTTGCTTTATTCTTTTGGATATTATGAGATGAGATTGTATCCAGTCTTGGCTGTAA
CCTATACCATAAATGCAGAGCTGCAACCATTATTTCTGTCTCTTTCTCCTTTTATCACCTACACTTC
TAGAGATATTACTTTCAGTTTTATTGCAAATCATCTGTGTCTTTAGTTTAAACCATTATTTTTCATAT
ACCCCAGGCACATTTCCCTTATAATGAGACAAATTTTAGATTATTAATGTTCCACCTAAAAGGAT
ACTGTACACTTTTCATTGCCAATATGGGCTTTGAAGCTTAAATATCAACATTTTTATAGGAAATTT
ACCCATGTCAAATTTAAATATATTTGCTTATATTTCTTGGAAAAAAGATTTTGGATAACTATCAAT
TAAGCAGCCAATTCCATTTGGGGTGCAGAAAATTTTGAGCCCCTTGATAAGAAGGAACAAAATT
TTTCCTCTAAATAACCAACTCTGGATCAGAAAAACTGAACTTTTCTAAATATTTATTATTAACAAA
ACTTTTTTTTGTCTGATTCCACTAGAAGCATTCTATGTTGTGAACACTATAAAGTTATCCTATGTTG
TGAACACCATAAAGTTATGAGGGAAAAAATGTCAAATCTAAATTTTATTTAGAAGTAACATATTG
TTTTTCTGGACTTGCAATCCAATTCGAATGCATCGTTAGACTTTTCTGTTTTCTTATGATTAAACAA
CAATGGCTTAAAATGATAGCAAGTCAACTGAAAAGTTGCTTTGGAATAAAACAAAAGTGGGAAT
GCCAACTTTCTTTTCTTCTCTATAACTTCATTAACATGGTTGTATAGACATTTGCATGTAAAATGTA
GCTAACAAAAATTACACAGAAGTAAATTTAGTCCTCTAGTGCCTACCTCTATGGTGAAATCAGAC
CATTTGGCATAGTTTGAAGTCTTCCAAGAAGCCTGGTTTCCACCTGGAAAATAAATTACTAAATT
TGAATGTGTTACTTATACTATGGTGAGTTGTCTATCTGTTACATTTTATTTTAGTGCATATACTAGA
AATTGTATTTAGGGTGTTCTAAAATTGTCATCTGTCAAGGTAGAGAATTATAATAATATAATTATA
CTCAGAAAGCGTTCTGATTTTAGATTATTTTGGCACTGTTACAAATATGATGAATTTTAAAAAAAT
GCAGAATGTTTATTACAAATTCATATGTTTAATTGTCAAATTCTGAGATTAATTTAGATAAGCTAT
AACCAAAGTTTATTTTCTTTGCATTTATTGGTACATAGTTAATGCTTATACACAGGCTTCAGTGTA
ACACCTATACACTATTACAATATTTAGCTGTACTTATTTGCTTTATCTTTAAATGGTACTTTGTCCC
AAAATTTTCTGAGTTTGATAAATTATTAAACATTTATCAAATCACAAAAAAAAAAAAAAA

ID|696|gene_id|101927746|transcript_id|NR_110115 Homo sapiens lncRNA

TGGTAGATGCAGCCGGCACGCACCTCTCCTCTGACGGGAACCGAAGGAGCCGGTAGGTTTTCACACTTGCAGCAGATCCGCTAAGAGAACGCGGGATTCAGCCGAGAAGCCACGGGGAGCCCGAGGAGCGGAGCAGAGGCACCCAGGCAGCCTGCGCGGAGAAATTGGATCGGCGGGGACGGCCTGCAGCTCCCGCGCGCGGGGAAAGGGAAGAAGTCCTCCCCTACAAAGCAAATTCACAAACTTGGAAGAAGCAATTTACACAGGATGTGCAGATCTCAATGGAAGGACACGGGAAACGTGAAAAAGCAAGGAAGTGGGACGCCTCCAAAGGAACCCAGTAATTCTCCAGCAACAGATCCCCATCCAAAAGAAATTCAAGAAATGTCATATAGAGAATTGTGGAAACTGATTTTAACCAAGATTAGAGGGATTCAAGAGACTTCTGAAAAAGAAAGTAAGGAAATGTCAACAGCAATTCTGGATATGGTTGAGGTATTTACCAACCAGATACAGAGTTTTCCAGAGCACATGGCAAATGTGGAACTGAAGAAATCACTGGATGAAATACAAAGTATACTCGAAAGCTTCAATGATAGACTAGATCAAGCAGAAAAAAAACTCTCAAAACTTAAAATCTGAAGGCTTTTACTCAATTCAAATATTTAATGGGTTGTACTCTGGCCATTCAGGTGAACAAAATCTCCTGGGTGACAGGGCGAGACTCTGTCTCAAAAAAA

ID|819|gene_id|102606465|transcript_id|NR_109849 Homo sapiens lncRNA

GACTAGCGGGAGGGGGCCGGGCTAGCGGGCTACTGGGCTGCGGAGACCAGAGCCCGGCTCCACTGAAGAAACCCGCCGGCTGCTGGGGCAGGGCGGCGAAAGGTCAGTCGGCCGAGAGGAGTCCGTGGAGCCACCGTTTCATAGCCTCAGGGAAAGGATCGGGCGTGCTCACGAGCCCTCTAACCCATGGCTTAAACACCCGCTTCCTAAGCCTGGGTTCTTATGCCTTTGTTTGAAAAACATGGTTCCCTGTGAAATTTTACATGTAGAAGAAAGCTTCACACTACGAGAGCTCTCAGGATATAATTATTTCAGACAGATACTGGGAACATAAGATGGCTTATCAATCTAAGATGGCCTTCCTGTCCTTCGGATTGAACTGAAACCATCCCTGATTCCAGGGATAGGTAGATGATCCAGGTTTGGACAATCAAAATATTGAACCACCCTGGACCTACAAGTGTTTCTGGGATGGGAACATGCAAACGTTGGTCCAAATTGTTGTTCTAGGCAGCCCAGTACATTTCTCCAATGCCTGATGGAATGATAGGGTATGCATAAATGTTCATGTGGGCTCTGGAGTATACCAGGAACTGAAGTAAGGTACATGGACGTCTTGAATATTGTAACTGAGATGGGCAAGCCTACACCTTTGCAAATAAAGGAGGATTGGGAAAGATTAAGCCTCTCAAGCACTGTGAAAAATCACTTTCTCTTGTTGTATTCTTGGAGCCCCTGCTAATTCATTATTGAAAAATACCTGCTGCCTGCATTTGGCCTTATGTTGTCATTTACTTGGTACTTCCTAAGCGTGGCTACTCCAATGAAGCTTTAGTATATGTACTGAAAGAAGATCCTCTCATACTGCCACAGCCAAGAGATAAGGTTGTAAATCTTTATTGGGAGAAGCCGTCAAGGACAGTTCTGTGAAATGAAAGGATCTCTGGATCACCACAAAGTCTGGCTCAGTGACTATGGCTTTGCAAACACTAAACATTCCTGTCTCAAGTCCTGTAGATGGCTTGAAATAGTATACATAAGCATGCCTTATAAACATCTTCAGTCTTTATCAACTCTTTGGAGATTCAGCCATTCTTTTTTGTACTTTAGGGAATTAAATCATTTTATAATGTATAAATCTGAGCATGGCCGATGGCAGAAGAAATTTTGTCATACGTTTTTAAAGAAATAAAAATGTATTATGTTTTAAAATTCCTTTAACTGTTTTTCTTACAAAATTCCCAACAACTTAGTTACCAAGTAAACTATCTCAGATATGATTTTTCAGTCCTTGCTAATCTATTTATAAAATCTTTTGCTATTTTAAAAAAAAAAAAAAAAAA

ID|1292|gene_id|104472717|transcript_id|NR_126448 Homo sapiens lncRNA

TTTGGCGGGGCTTTTGTCTCTCGCTGCAGCCAGAGCTTGGGATCGCTTTCTGCTATTCAACGTCCTCCACCTCTGCCCCCCTCTCGCCCCAGCCGGTGACAGGCTGTTGCCCTGTGATCTGCAGGTCCTGGGACGTGCACAGACAGCTAAGATGCCAGGACATTCCAGAAGGTGGGAAAGGCACCTGAGTAATTTGACTCTCCTGCCTGGACCCAGCGTACAGATGGGATTGTGCTTCATTGCTGGACCCAGCATTTAGGCCCACTACCTATGAGATTGCATGCTCCTGCCTGGGCTGTGCCCACAGTGACCTTGTGACATATATCTGCATCCATGACCTAAAAGATGTGACACTTCTTGCCTGCAACCTGCCCTGCACAGGAAAGATTGCGACTTCTCCCTGGAAGCAGACACCAGGGATCGTCACTCCCGTGGATGATCAAA

GGCGCCTGTAATTTCAGCTACTCCGGGGGCTGAGGCAGGAGAATCGCTTGAACTCGGGAAGCA
GAAGTTGCAGTGAGCCAAGGTCGCACCACTGCACTCCAGCCCAGGCGACATTGTGAGACTCCA
TCTCAAAAAAAAAAAAAAAGAAAAAAAGTGTACTAACAAGACCCAGCACACAGAGGAGACTTTT
ACCATTGTATGAACACCCATCAAACAGTACACATCATCATTGTGAGTTCTGAATCTCACACATAG
AGGAAGTCAAAGGTGGAAAACTTGACTCTCATATTTGGATCCAGTCCACAGGTGTGATTTTGAC
GCACACTTCTGCCCAGCACCTGAGTAATGTGATTCTTCAGAATTGGCCCGGCCCACAAATAGGA
TTGTGCCACACTGCTGGACCCAGTGCCTAAGTGATGTAACTCTATTCTTCTGCCTTGGTGCTACT
CGAAAAAGACATTGTAACATATCTCAGAGCCTTACACCCAGGTAATGTGAGTCTCCTCTTCTGCC
TTGCTGCTGCACACAGAAGCGGAATATGTACAGGATTGTTAATCTCATCTTGGATCTTCCTGCAC
TTGTGATTGTGACACATGCCTCTGTGTAGAACCTGAGTGAGTTTACTCTCCTGCCTGGATTTAAC
CCATAGATGGAATCGTGACATATCACTGTACCAGAAACTAGGTGATGTGACTCTATTTTCTTGCC
TCTGTGTTTTTATATGGGGCATCGTGACATATCACTGGGCCTTGCACCCAAGTGATGGGAGTTTT
CTCTCCTGCTTTGGTGCCGTCCACAATGAAAATTGTGAAATATTGCTGCCCTCCCCACTAGGTTA
TGTTACTCTCCTGCCTCTGCTTTGCCCACATGGGTCACTGTGACATATTGCTGGGTCTAAAACCC
AGGTAATGTCACTCTCTTGTTTGGGTTCTGCCTGCAGGAGTCATTGTCACATATCTCTATGCTCAT
CACCCAGGTAATATGATTCCCTTTTACTGCTTAGTTCCTTCTCACAAAACAACTTGTGACATATTG
CTAGGCCCAATACACCTAGCTGGTGTAACTCTTCTTTTCTTTTCTTTCCTTTTTTTTTTTTTTTTTTG
AGATGGAGTCTCACTCTGTTGCCCAGGCTGGAGTGCAGCGGCATGATTTCAGCTCACTACAGCC
TCTGCCTCCTAGGTTGAAGCGATTGTCCTGACTTGGCCTCCCAAGTAGCTGGGATTAAAGGCAT
GTGCCACCACGCCCGGCTAATTTTTGTATTTATAGTAGAGACAGGGTTTCGCCATGTTGGCTAGG
CTGGTTTCGAACTTCTAACCTCAGGTGATCCTCCTGCCTCGGCCTCCCAAAGTACTGGAATTACA
GGCATGAGCCACTGCACCTGGCCTGATTCTTCTCTTCTTTCTAGGTTTCAACTGCAGCAGAGATT
GTAACAAACCACTGGGCCCAGCCGCATAATGATGTTACTCTTTTGCCTTGGGCCTGCCCTCAGA
AGGCACTGTCACATATTCATGGGCCCAACGCCAAGGTGATATGTGTCTCCAGCCTGGACCCTGC
CCACAGGGGGCATTGTGACATATCTCTTGGCCCATCAGATGTTTGATTGTTATCCCCTCCCCTCT
TCTACCCTGTGTTTGCCTATAAAAGACACAGTCATATGTTCCTGGGCCCAGCACCTAGGTGTTGT
CTTACTGGAATGTGTCCACATGGGGATTGTGATATGTCACGTGGCCCAGTACTTAACTTGATGTG
ACTCTCCTCTCATGCCTAGGCCTTGTAGAGAATCCCCTTCCCCTTTTGTTTTCCTTTTTTTCTTTGC
AGATCCAGGAGATAATCAACTAAGAGCCAGGCACCCGTTTAGGTCTAATAAACATTTTACAAAC
TGCTCTCTCTTTTAATTCACCTATCTGAGAGATTCCTCTGTACAATAAAACTTGGTCTCCACAATC
CTTTACCTTAACCTAAACCTTCCCTTTCATTGATCCCAGGTCTTCAGATTCCACCACTTGTCAACC
ACAAAATATTTAAATTTACCTGGAAGCCGCCCGCTTTGAGTTGTCCCGCCTTTCTGAACCAAACC
AATATATTCCTGCAAAACCGGCCATGATTAAAACATCAAAAAA
ID|1293|gene_id|104472717|transcript_id|NR_126449 Homo sapiens lncRNA

TTTGGCGGGGCTTTTGTCTCTCGCTGCAGCCAGAGCTTGGGATCGCTTTCTGCTATTCAACG
TCCTCCACCTCTGCCCCCCTCTCGCCCCAGCCGGTGACAGGCTGTTGCCCTGTGATCTGCAGGT
CCTGGGACGTGCACAGACAGCTAAGATGCCAGGACATTCCAGAAGGTGGGAAAGGCACCTGA
GTAATTTGACTCTCCTGCCTGGACCCAGCGTACAGATGGGATTGTGCTTCATTGCTGGACCCAG
CATTTAGGGATCGTCACTCCCGTGGATGATCAAAGCTACTCCGGGGGCTGAGGCAGGAGAATC
GCTTGAACTCGGGAAGCAGAAGTTGCAGTGAGCCAAGGTCGCACCACTGCACTCCAGCCCAGG
CGACATTGTGAGACTCCATCTCAAAAAAAAAAAAAAAGAAAAAAAGTGTACTAACAAGACCCAG
CACACAGAGGAGACTTTTACCATTGTATGAACACCCATCAAACAGTACACATCATCATTGTGAG
TTCTGAATCTCACACATAGAGGAAGTCAAAGGTGGAAAACTTGACTCTCATATTTGGATCCAGTC

CACAGGTGTGATTTTGACGCACACTTCTGCCCAGCACCTGAGTAATGTGATTCTTCAGAATTGGC
CCGGCCCACAAATAGGATTGTGCCACACTGCTGGACCCAGTGCCTAAGTGATGTAACTCTATTC
TTCTGCCTTGGTGCTACTCGAAAAAGACATTGTAACATATCTCAGAGCCTTACACCCAGGTAATG
TGAGTCTCCTCTTCTGCCTTGCTGCTGCACACAGAAGCGGAATATGTACAGGATTGTTAATCTCA
TCTTGGATCTTCCTGCACTTGTGATTGTGACACATGCCTCTGTGTAGAACCTGAGTGAGTTTACTC
TCCTGCCTGGATTTAACCCATAGATGGAATCGTGACATATCACTGTACCAGAAACTAGGTGATG
TGACTCTATTTTCTTGCCTCTGTGTTTTTATATGGGGCATCGTGACATATCACTGGGCCTTGCACC
CAAGTGATGGGAGTTTTCTCTCCTGCTTTGGTGCCGTCCACAATGAAAATTGTGAAATATTGCTG
CCCTCCCCACTAGGTTATGTTACTCTCCTGCCTCTGCTTTGCCCACATGGGTCACTGTGACATATT
GCTGGGTCTAAAACCCAGGTAATGTCACTCTCTTGTTTGGGTTCTGCCTGCAGGAGTCATTGTCA
CATATCTCTATGCTCATCACCCAGGTAATATGATTCCCTTTTACTGCTTAGTTCCTTCTCACAAAA
CAACTTGTGACATATTGCTAGGCCCAATACACCTAGCTGGTGTAACTCTTCTTTTCTTTTCTTTCCT
TTTTTTTTTTTTTTTTTTGAGATGGAGTCTCACTCTGTTGCCCAGGCTGGAGTGCAGCGGCATGAT
TTCAGCTCACTACAGCCTCTGCCTCCTAGGTTGAAGCGATTGTCCTGACTTGGCCTCCCAAGTAG
CTGGGATTAAAGGCATGTGCCACCACGCCCGGCTAATTTTTGTATTTATAGTAGAGACAGGGTTT
CGCCATGTTGGCTAGGCTGGTTTCGAACTTCTAACCTCAGGTGATCCTCCTGCCTCGGCCTCCCA
AAGTACTGGAATTACAGGCATGAGCCACTGCACCTGGCCTGATTCTTCTCTTCTTTCTAGGTTTC
AACTGCAGCAGAGATTGTAACAAACCACTGGGCCCAGCCGCATAATGATGTTACTCTTTTGCCT
TGGGCCTGCCCTCAGAAGGCACTGTCACATATTCATGGGCCCAACGCCAAGGTGATATGTGTCT
CCAGCCTGGACCCTGCCCACAGGGGGCATTGTGACATATCTCTTGGCCCATCAGATGTTTGATT
GTTATCCCCTCCCCTCTTCTACCCTGTGTTTGCCTATAAAAGACACAGTCATATGTTCCTGGGCCC
AGCACCTAGGTGTTGTCTTACTGGAATGTGTCCACATGGGGATTGTGATATGTCACGTGGCCCA
GTACTTAACTTGATGTGACTCTCCTCTCATGCCTAGGCCTTGTAGAGAATCCCCTTCCCCTTTTGT
TTTCCTTTTTTTCTTTGCAGATCCAGGAGATAATCAACTAAGAGCCAGGCACCCGTTTAGGTCTA
ATAAACATTTTACAAACTGCTCTCTCTTTTAATTCACCTATCTGAGAGATTCCTCTGTACAATAAA
ACTTGGTCTCCACAATCCTTTACCTTAACCTAAACCTTCCCTTTCATTGATCCCAGGTCTTCAGAT
TCCACCACTTGTCAACCACAAAATATTTAAATTTACCTGGAAGCCGCCCGCTTTGAGTTGTCCCG
CCTTTCTGAACCAAACCAATATATTCCTGCAAAACCGGCCATGATTAAAACATCAAAAAA
ID|4218|gene_id|145200|transcript_id|NR_026774 Homo sapiens lncRNA

TGCCTTTCACCTTCTGCCATGATTGTAAGGCTTCCCCAGGCCCGTGGAACTGGGAGGAGCC
GGAAGAGAGCAGTGCTTCACAATAAAAGGAAGAATTGCTTGAACGGAGGCGTTTCAGGCATGA
GTCACAGGCCGGGTGTGGAAGAGGAAAGTGTGAAGCAAGGGACAGGTACCAGCTGGGATGTT
GGCAGCAGCCGCTCCCCTGCAAGGAGAACCTCCCACTTCCTGTCCCGCACACCTGCCTCATTGG
AAAGTGACGCACCCACTGTTTGGCCTCGGCAGCCTGATGTTTCTCATCACACAGGAAGACGGTC
ATGCTACAGGAAGACCTCGGACTTTGCATCCAGCAGATGGTTGTTCTAGAGATTGTCAGACCGA
GACCACGCCACACACCCAGAAGGCACCCAAGTCACAGAGCCTGATATTGTTCCTGGAGCGCCA
TTGCATCATCCTTGACCACAAGATCTGCGTCATTGGATTTGTCTCAAAAACTTGGGACTCTGGAG
ATAAAACCTCATTGCAAGGCTACCTTTGCCACTGTATCTTAGCAGCTTTTGAAGCAAAGCATTTC
AATATTTAGACACTGTGTTTCTAATGGGTACTGGGATCAAAATAATAAAAACTCAGTCTGCAAAG
GGAGAAAAAAAAAAAAAAAAAAAAAAA
ID|4351|gene_id|147727|transcript_id|NR_024333 Homo sapiens lncRNA

ATCTTACGCCCGTCGCCCTGAGTACGTTAGCGGAAATGGCTCCGAGAGGCTTTGACGCAT
GTGTACCTTTTTAGTGGCGGTATCTCGGCCGGGGATTTTCGGGCTCCAGTGGAGGTGGCTATGC
TCACCCCATCTCCCTTGAGAGGCACAGAAGGTTAGAGATGTGTGCGAGGAACGGCCAGGTCCA

GCGTCTCCTCGTTGCTGCCCCTTGAGCCCGGGACCGGCGCCAATGCGCGCCCGGGTTCTTGGG
TTCTTTCGTCCCGCAGACCTGCCTTGGTGTGGCGTCCCTGGGCCGTAGCTTCCGAAGGCTGCAG
CCGACCTGGTCGGGGTCCTCTAGCCCGCCTTCCCCTTCCCCCATTGACAGATGGAGAAACTGAG
GCCCAGGCCAAAAAATATCTCACTAGAGGGCTCACCGCCATTTCCTGGAAAAATTGGGGCTGG
GACTCAACGGACGCTCGTCCAGCAGCAGCGCGGAGCTCCGAAGTAAAGGCGCCCGTGACCGA
GTGCTTTGTCCTTACAAGCGTGGTGTTCTTGAATGCTTCCAGGCGCCTTATGAGGCCCCCGATGT
ACAATCTGTGCTTAACTAAACCCCACTGTCTTCCAGGGTCAGGTTGGCCATCCGCAGGCGTAGT
TGCTAGGAAGGTTTGACAGATGTCCGCCTGTTGATTCAGACGTTCCCAGGATTTGGAGCAAGAA
GAGCAAGGAAGGCCAGGCGCGGTGGCTCACGTTTGTAATCCCAGCACTTTGGGAGGCCGAGG
CGGGCGGATCACGAGGTCAGGAGTTCGAGACCAGCCTGGCCAGCATGGTGAAACCCCGTCTCT
ACTAAAAATACAAAAGAAAATTAGCCGGGTGTGGTGGCACATGCCTGTAGTCCCACCTACTTGG
GAGGCTGAGGCAGGAGAATTGTTTGACCTGGCAGGCGGAGGTTGCAGTGAGCCGAGATTGCG
CCACTGCACTCCAGTCTGGGCGACAGAGCGAGACTCTGTCTCAGAAAAAAAAAAAGAGCAAGGA
GGTGTTGAAAATGGGTTTCCTGGCTGGGCGTGGTGGCTCACGCTTGTAATCCCAGCACTTTGGG
AAGCTGAGGTGGGCGGATCACTTGAGGTCAGGAGTTTGAGACCAGCCTGGCCAACATGGTGAA
ACACCGTCTCCACTAAAAATAGAAAAATTAACTGGGTGTGGTGGAGGGCGCCTGTAATCCCAG
CTAATCAGGAAGCTGAGACAGGAGGATCGCTTGAACCCAGGAGGCGGAGGTTGCAGTGAGCC
GAGATTGCTCCATTGCACTCCAGCCTGGGCGATAGAGTGAGACTCAGCCTCAAAAAAAAAAAA
AAAAAAAAATTCGCCAGGTGTGGTGGTGTGCGCCTATAATCCCAGCTACTCAGGAGGCAGAGG
CACAAGAATGGCTTGAATCACTTGAACCTGGGAGGCAGAGGTTACAATGAGCCGAGATGGCAC
CACTGCACTCCAGCCTGGGCGACAGAGCGAGACTCCGACTTCCCATCTTAAAAAAAAAAAAAA
AAAAAAAAAAGAAGTGTAAAGATGTGATTATGTGGCAGGGGGTAAGGGTTAAAAAGAAAACAA
GAACAAGTTTTCCTCTGCTTAGCCAGCTTACTTCAAGGACAGTTATAACACTGAAAAGTCGAGG
CCAAAGGAATGGGCTCCAGACAGCCTCCTCCGGAGCAAAGTTGAAAAGAAAAATTCCTTTACT
GTCTCTCCTTTTCTGAACCATTAAATATGACTGTTTGCCAATGGTTGTATTTAGTAAGATTTGTAG
ACTCTGTTTTTCTTTTGACACAGCTGCAAGGCCAACAGCTGTGCAAAGCCACAAGTTATGCTAAG
TCAGCAGTTATGCTATAGATTACATGACCTGTGACTGTATCATTAACTGCTTTTGTTTTGCTTCTGT
AAGTTTGCCTATAAAAACCACACTCAGTCTTTGTTCAATGGTCAGCTTTTCAGATACAAATCCAC
TGAGCCGGTGTACATCTAAATAAATCCTCCTGTTTCCCGTGTCAAAAAAAAAAAAAAAAAAAAA
A
ID|14021|gene_id|389641|transcript_id|NR_033928 Homo sapiens lncRNA
CTCAGCTTCTGTTCCTCAACCACGGCCGCCGCGTGGATGCCGCTGCATTCGGAGTCGTTTT
GCCACTTGGTCCCAGCGCCAGGCTTCTCGGTCGGGAGTTGACCTCAGCCTTTCTGTGACCCAGT
TCCTGGCACCCAGCAGGCGCTCGGTGGACGGATGGGTGGCGGAATGAAGGACACAGAGGCCA
CTGCGGGGTCTGGCCTCTAAGAGGCTTTTACGGCCTCCTCCGTCACTACCGGGCGAGTGATTCA
GCCTGCCTTTTTTTTTTTTTTTTTTTTTTTGAGATGGAGTCAAGCTCTGTCGCCCAGGCTGGAGTGCA
GTGGCACGATCTCGGCTCACTGCAACCTCCGCCTCCCAGGTTCAAGCGATTTTCCTGCCTCAGC
CTCCTGAGTAACTGGGATTACAGGTGCCCGCCACCACGTCCGGCTAATTTTTTTTCTATTTTTAGT
AGAGACGGGGGTTTCACTGTTTTGGCCAGGCTGGTCTCGAGCTCCTGATCTCGTGATCCGCCCG
CTTCGGCCTTACAAAGTGCTGGGATTACAAGCGTGAGCCACCGTGCCCGGCTCATCCTGCTTTT
GGTTTCAGGTCTCCATGCCAGTGAAATGTCACCTCAACTCCACCGTCCCTGGTGTCAGGCAAGC
CCGAGGGACCCAATTCTGCCTTTCTTCTGGATTTTCCTAACCTGTTAAGAGTCTTGAAAGTCCTTA
AACCACGGGAGACTATCTGGCACATGACACGGGATGAGACTTGGTGGGGGTCCCACGGGGGA
CAGGTCCTTCCAGCGGTTCCTCTGCACCCAGCGCTGCACGGGCATGGGGCGTGGGTAATCAGC

CTCCCGAAAGCCACGCGAGTCACGGTCCTGCCTGCGAAGAACTCCCAGGGCGACCTTGGGGCT
GAGGAGTGGGCCTGCTTCGGCCTTCTGCTCTTCTGTTGTGTGGTGACCCTCACCAGAGGACAGC
TTCATGCTCTTGGACTTCCCAGCCTCAAGAACTTCTTGCTCTGTTGCCCAGGCTAGAATGCAGAG
GCACCATCTTGGCTCACTGCAACCTCCGCCTACCGGGTTCAAATGATTCTCCTGCCTCAGCCTCC
CGAGTAGCTGGGATTACAGGCACCTGCCACCATGCCCAGCTAATTTTTGTATTTTCAGCAGAGA
TGAGGTTTTACCATGTTGACCAGGCTGGTCTTGAACTCCTGACCTCAAGTGATCCGCCAACCTCG
GCCTCCCAAAGTGCTGAGATTACAGGCATGAGCCACCATGCCCGGCCTCCATCTTTTACTTTCA
ATCTATCTGAAGCTTTATATTTAAGGTGCATATATTTTAGACAGTTAATAATTTCAGCTTGCTTTTT
TTTTTCCTAGTTGGGGAAACTCTGCTTTTTAATTGGATTGTTTATTCTGGTTACATTTGTTGTTGTG
GTGAAATTTGGAAAGTTCAACCTGCCACAACGTCTTCACTTACTATTTTTAAAGGATATTCTCAAT
GAGTGTGAATAGTGAGTCAACAGCTTTCTTTCACCATTTCACACATCCTGAGCCTCCATTGTTTTG
AGTCAGCTGTCGTTCATATCTTTGTTACCTGTGAATGCACAAAATTTGCTCTGTATGATGACTATG
ATGTGTCCAGGTAGGGTTTTCTTTACATTTATTTTTATGAGGGGTTTATGAACTTTTTGAATCTATA
AGTTGATGTTTTTTCACTACATTTGAGACTATATCAACTTTTTTTTTCTTTCATCTTTTCCAGCCATT
TCTTCTTGGTTCTGGTTCCGTGTGTACTGCATGATATTGTACCACGTGTCACGGAGACTCGCTTCC
TTGTTTTCTTTCAGTTTCATTTTCCCTTGCACTTCAGATGGAATGATATCTATTGATCTCTTTTCAAG
TTCACTCATTCTTTCTTCTGTAATGTTCAATCTCTCTTAAGAAAGTCCAAATAAATTTTTCATTTCA
GAGAGTGGAATTTTCGGTTCT
ID|14378|gene_id|400619|transcript_id|NR_033876 Homo sapiens lncRNA
AAGGGGCGATGCGCCCCGGAGGGGGAGGGACGCAAGGGGCGACTACTGTTACCTCGCTT
GGAGATACCAAGGCCGGTCGGACCCCCCGCTGGCCGAATGTGGGGAGCGAGCAGATCCAGCC
TGTTTGGACGTGGTGAGGACCCAGCCGTGGGGTTTAAGGCCAAGTTAGCCTCTCCCTTCACTGA
TTACAAGAGCCATTGTTACAGGTGCTCACATGCCAGGAGAAATAAGCTGGTGATTTATGTGGCA
CAGCACCATCGATCGACCTACAAAGGAAGCTTCAGTGCAGCCGTGTTGCTATACAGACCTCTCT
CACCAAGGCGTCATGAACTGAAGGGCTGGGAGAGGCCTCGTGGAGGAGGAAGAGTTTCGCTC
AGCTGCTTGGGTGTGGGACTGAATGTGGTTCCAGAACTGGTCGCTGCCTTCACCCGCTGACTCT
GGTCTCGGCGATGACAAGCCCATGGAACAGACTTACGGCGTATGACCTCATCGGAGACTTGTA
CATGGGGCCAACCCCCATTTTCCCACAGGAAACCCACACTCTAATTAAGGGCTGACCCTGAACT
TGAATCTGAGTCTTCTCTTTCTTCAACAGAGAGCCATCTCTCCAGCCCAGCTGGCAAGGAGACA
GGTGATGTTACAGGAAAAAATACACAGCTTGTGCCCTTGGAATTACCAGAGTTCAAAGCCTGTT
CAGCATCTACCCACCATGTGACCCGGGTCGAATTCTTCAGTTTCTCTAAGCCCATTTTCTCATCTG
TAAGATGGGGAAGTGTAATCAGTTCTGAACCCACATATACCTCACTGGGCTTGAACAGGCCTGA
GCAGGCATGTGGGGCTTTACTAGCTAGCCATCCTTTCTGCCGCTCTCCCTCATTCTCCTTCAAGC
CCCCCAAAGTCCTTGGTGGAGGAGGAAGTGGAGACAGAACGTTTGGTGTTGGGTGAGGTACGT
GAGGAGGCTGATTTCAGAACCTCCAGGAAACAAAGCTTTAGTGTTCCGAGGGCAGTGCCCGCC
TGTGCCTCCTCCTCACAGGGGGTAGTAGGAGTGGGGTGGGGCCGAGGCATTTCCCAGCACAGC
TCAATCACATCTCCTGCGCCTGCCCTTGTGACCTGCTCACTGGAAAGGAAGAAATGACCGAGGG
AGAAGGGAGGGCATTTGAGGGGAGAAGAAGAGGATGGAGAAAAAAACTGGATCATCTCGTGA
CATGGAAAGAAAGGTCTGAGCTCTGACGATCCCCTCACGTGATTATAGGCATGAGGACTCACTC
TTCGAATTGTTTGAAAATTGAATGGACTGGCTCACACCTTCTGAAATTTCTTGGAAGCTCTGGAG
TAACTATTCCACTAAAGAACAGCGTGTTTTTGGCTGAAATCTGTGACTGTTTGTTTTTGAGTTATT
TTAATCTCTGTGTCAAGCATGAAAACCTCAGCAGCCTGGGATTTATAATTAGATCCCACTGGCAT
GCCCTCTCACCTCTCAAACATGCCACAGTGCTTTGGCTCTCTCCTTGTGCAAACCTCCCTTTGAT
ATTTTTGGATATCGATACCAAGTGTATCTGCATATTTTAACCAACTGATATGCATATCCTGTGATC

TGAGGCATTCGCTCAATGTGGGGCATCAGGCCAGCTGTGAACTTCCTTTAAAATTTATTTTGTGT
CAAATGATTCCCTAATGCTATAGCTCCCTTGAACTGCTAGATTTATGAAAATTCTGCACAACACA
TAGCTCAGAGAAAAAAAAAAAAAGAGTGTTGCAAGACCAGATATAGGATATCATTTGATTTGTTT
AAAACACTCTGTCCAGGGGGTTTTGACTTGCTAATTTAAAAATAATTCTAAAATTAGGTACAGTT
GACCCTTAGTATCCATGGGGGATTGGTTCCAGGATGCCCTGTGGATACCAGAATCCAAGGATGC
TCAAGTTCCTGACATAAATGGCTTAGTGTTTGCATATAACCTACTCCCCTCTTCCTATATACTTTA
AATAATCTCTAGATTACTTATAATGCCTAACACAACGCCTGTGCATCACTTCATTCATGTGGATTC
AATGTAGTACTTGGTGTGAAGAAAATTTAAGTCTTGCTTTTTGGAACTTTGTGGATTTTTTTTTCTG
GAATGTTTTTGATCTGCAGTTGGTTGAATCCACAGGTGCAGAACTATGGACACAGGGGGCTGAC
TATGTATATTTATATCCACTTGCAAAATTAAGAATAAAAATAATGTGCATCAGCAGGTGCAGATT
CTGATGCTCGGTGGACAGT
ID|14442|gene_id|401588|transcript_id|NR_015378 Homo sapiens lncRNA
AAAGCAATGCGCACGCGCAGTGGCTTTGAAGGCGGGGCAACAAAGCCTGTGGCCGATGT
GGCGCTCCTCGGCCCGCGCTTCAGTGGTTATGACAGTGTTCGCTCTAAGGGGAGAAGGAATGT
GTGACCATTTCCAACGGTTAACTTGCATTCCCCAGATAGTTTGGATGAGTTACAACCTAAATCTG
CAAACTCAATACAGCCAGACCCTGGGAAGTGACTCTCTCCAAGCCTCCTGTACAAGTCCCAGGA
AAAGAGCCTGTTTTCCCTTGGACATGTGAGCAGTGAATTACTGCTCATTCAGAATTTCATGTGTA
TGTGTTACTGATGGCCAGGTTTAGAGATACCAACCTATTTATTTCTAGAGGAGAGGTGATTTGAT
AACCTTTTCAGATATCACTATCAGTAGGCAGAAAAGGGTTAATAGTATGATAGGGTTAATATGTC
CTGAGGAGAGTAAAGAAATTGCAAACTGCAGAAGCACCTGATCTGTGGCCCTGGCCAGCATCT
CTCCATATCCTGATTCTGTAAGATCAAGTGTTTATGCAGAAAGAGAAGGGACTGATGATTTGAAT
GTGACCAGATGCAGGGCATGCCCTTATTGCCATGCCAGCATTTGCTATTCATGATGTTAGTATTC
AAAAATCAAGAAACACCCTTCCTGGGCCATTGGTGTCTACTAATCCATTGGAAGAAAAGACTGG
AATCCACCACTTACTAGCTTCTTGGGCCTCAGTCCCCTCTTTTCTGCCATAGCTAAGGACCACCT
AACCTAGATGGCTGTTGTTATTGTTCAAAGGGACATCTTTAGAGCCTCAAGCCCAGGAATAGCC
CGTGGCAGGCACCTGGTAAATGTTAGTTTTCATTGTCCTATCCCAACACTATGAAACATGCAGCA
CTCTCTGCAACTGCCCCTGTGGATTTCCATAGGCAGGAGAGTATCCAGTGTTGAGTGGAGCTGG
TATTCTCAGAAACGAAGCCCCTTTCCTCATTTTGCGCCAATCTCTTCAGTCATTTTTACCCTAGTA
GTATAATGCTTTGTCAAAGTCACTGTCATCTGATGTTAACAGTTGTTTTCGCCAGATGACTTTTCA
AAAGGTAACTTCACCATAATACCGGTTAATGTGATAAAAACACTGCATGTAGCCAATTTTTTTTTT
TTTTGAGATGGAGTTATTGGCTCTTGTTGCCCAGGCTAGAGTGCAGCGGCGTGATCTTGGCTCAC
TGCAACCTCCATCTCGTGGGTTCAGGTAATTCTCCTGCCTCAGCCTCCCGAGTGGCTGGGATTAC
AGGCACACGCCACCATGCCCAGCTAATTTTTTTGTATTTTTAGTAGAGTCGGGGTTTCACTATAT
GTTGGCCAGGCTGGTCTCGAACTCCTGACCTCAGGTGATCCGCCTGCCTCAGCCTTCCAAAGTG
CTGGGGTTACAGGTGTGAGCCACTGCACCCAGCCAAATATTTTCTTATTTATTTATTTATTTTAGA
GACAGGATGCCATTATGTTGCCTAGTTTGGAATGCAGTGTCTATTTAGAGGCGTGATCATGGCAT
ACTATAGCCTCGGCTCAAAGGACCCTGGTCTCAAAGGACCCTGCCACCTTAGCCTCCTGAGTAG
CTGGGACAAGTGTGCGCCACCACACCTGGCTAATTTTTTAAATTTTTTGTAGAGATAGTGTCTCG
CTATGTTGCCCACGCTGGTCTGGAATTCCTGGACTCAAGGCCTCTTCCTGCCCTCAGACTTCCAA
AGTGTTAGGATTACAGGGTGAGTCACCACGCCCAGCCAGTGTGAATTTTCTAATGCTGAGTAAG
TTGTGATCCAGAAGTAAATGTATCTGCAAGCTGATTTAAATTGTAGATTTTCTCTTCATTGCATAT
TTTCTGATGTTGAATAAAGTCTGAGTATTAACTAAAGTTTTTGCTTCTTAAATATGTAAGAATTGTC
CTCAAAATCTCTATTCTTGAGCACAGTAGTAAAAATATTTTTACATACATTGTATTTCAAGGGTTC
TGCATAAGCAACCTTGTTTTATTGTGACAATTTATTTGGGAATTGTATCAAATATATAATACTGAG

ATGTTCTCATTCCTTTTGTGAAAATACTTTTGTTTAAAAACCACTGCCTTCAAATGAAAACCACTA
GGTTTGTTAT

ID|17170|gene_id|51275|transcript_id|NR_015404 Homo sapiens lncRNA

ACGGAATCCGGGGGAGGCCGAAACCCGGTAGTCGATGGAACCTCTCACTGCAAAATCTCC
CTGCTTTTTGAGGCTAGAGCCGAGAAGAACGCCACAGGACAACGCGGGGGAAGAGGGGGCAC
CTCCCCCTTACCTTGATGGCTTTGTCCATGTCGCTCTCCTCCGACATACTCAGTGGGGCCCCCAC
AGCCGGGAGAGGCGGAGGCTGCTCAGCAGCCCTGTCCCCGCGCGGCCGAGGGAGCAAAGGG
CTCGGCACTCGGGCCCCTGGGCCTCGTGGCGACGGGCAGCAGCCGCCCCTCGGGACCGGTGG
GGATGAGGACCCCGGCGCCGGCTCTGCTTCGGCGGGCGGCTCTAGGCTGGCGGCAGCAGCAG
CGGCGGAGGCGGCGGCCCCTGGGGACAGGTCTTTGTGCTGGGCCCCACGCGGCCGCCTCCTA
GCCTCATCCCCAGCTGGGGAGGCAGGCTCCTGGGGTAGGGCGAGGCGGGGAGGGCCTCGACC
CGGGGCGCCGTGCAAGGGCCTCGCGGGGCCGCCGCTTAGACCTGGGCTCGCCAGGTCCTAAA
GAAGAGCCTGCGCCTCCGCCTCCGCCGCCTGCACCGCCGCGAAGCCGAAGCCGAAGCCGAAG
CAGAGCTCGGCGCCGCTGCTCCCGCCGCCGCCTCAGCGCCACACATCGCCGGGACACCCGCG
CCGGGCAGCCCCGCCGGCCGCCCTCACGCACGCGCTGTGCCCCGCGGGAGCTCCAGGCCGCC
CCTCCTGCCGCCCAGCTTCGTGACTCCACGGCCCCACGCGCCCCTTGCTGCCTTCCGCTGGGGT
TTCCGAGCCGCCCCCGAACCGCGCACCCGACTTGCAGGCGTCCGCCTGGGCCTCCCTAAGACA
CGCCGCATACAGGACAGGCCCCTGGGCCTTCCGCGGCGATGGCGCTGCGCTAATGACGTCATA
GGCCCGGACTCGCGTGAGTGCGCGTGCGTTGGGGCCTCAGCCTTTATCTCCACTCTGCGGAGAT
TCACGCCTGGAAAACGCTCTTCTGAGAGGATCTGTGGAGGTCAACCCAGGAGAGAGAAGACAG
GACTGAAGCACTGAAAGGGTCCTGCCGTTAAGGGCGCAGGATTGTATAGAATATATAATAGCA
GTAGCAGCTCTGTTTACGGAGCATTAACCTTACGTGGAGTTATTTCCTGCATTTCCTCCTTTCGTC
TTTACAAGGTAGCCGTTTGGCGTCGTGAGGTATGGATGTTCCCATCCCTAATTTGCAAAATAGGT
AACTGAGGCTCAGGAGAGCTAGACTAGTTTGTGCACAGCCGAGAAGTGGTCAAGCTAGAATGG
GACCCCAGGTTTGTGCTCCCTACTCTCCACCGATAACCTATCAAAGGGCTTTGCAAGAGCTTTTG
ACTAATCGTCTGTCAATGGTTCTTCATTCACGTATTAATTGCACATCAGTATGTGCCAAATCTACT
AGACATTGGAGAAACAGTAGGAACAACACATGGTACCGGCACATGGATCTTTCAGGAAAACGA
AGTAGGTAATAAACAGGAAAAAGCCCGAGTCTGATGCTAAGAAGAACATAAACAGGATGCTGA
GAGATTGGGTCTCTCCACATTGCCCCGGCTGCTCTCCAACCCCTGAGTTCAAGTGATTCACCTCC
CTTGGCCTCCCAAAGTACTGGGATTACAGGCGTGAGCCACCGTGCCTGGCTGAGAAGATGGAT
TTAAGACATATTTTGGAGGTAACATTGTCAGGACTTCCTGAAGGATTAGATGTGGAAGGGAAGG
ATAAGAAACAGACCAAGGATAACTTTCAAATGTATGGTTAAGCAACTGGATGGATAATGATGCC
ATTGAGTGAGTGAAAAACTTGATGGAAGTGGAAGATTCAGAGTTCATTTCTATCTAGGTTAATTT
GAGACATACCAGAGCATAAGTTAAGTAAGTAATTGAATATTGGAGTGGAGACTTATTTGTCTAC
CGAATTATTGTTTTCTTTGTCGGACATACACCTACACTGCATTCCTCAAAGTAAAATTTAAGTGTG
GCTCTGTGCCTATGCTCTCCCCAGCGGAAAGTGACCAGAAGAGGTGTGCAGTTTCCAGGCCTG
GCCCATACAGACCTCCAACAGGTGCTCCCCTGTGCTGTTACTCCTTCTGCCAACTGGAAGCAGA
TGGTGACCAGGCTCTGGAGAAGGCAAGGCCTGAAGATGGGAGATTCCTAAGTGGAGGAGAAC
TGTGCCTTACTGACCTAAATATCCACTCAGTATTGTTATGTGAGAATAAATAAACTTGTGTTGACC
GTTT

ID|21595|gene_id|645249|transcript_id|NR_038835 Homo sapiens lncRNA

GGGGACACCAACGGGGAGTGGATACTCCGAGGCTCAACCTGGGGCCTCTGCCCGCCCTC
CCCTGGGTGCGGAGCCGGACGGCGGGCACTGGCAACCCCCGGGCCCCTGGAGAGCCCCGCG
GCGGCCCAAGACCCGCTCCCGAGACACGTGGGCCCCTCCGGACTTGGTTGGTCCCTGGAGCCA

GCTGCGCGCGTCCTCTCCGGCCTCCTTGTCGGCGCTGGAGTGGGTCCACGTCTGCCTTGCGGAG
CGCGGTGGTTCCACGCTCCTGAGCGCCCACGCTGCGCAGACCTGGCCGCTCTGCGAGGCGACG
GCAGCGCACTGAAGACCGTGGTGTTTCCCAAGCCCTCCCTGCTCTCGGGCCTCCAGGGCGCAG
CAGGGGAGGGTTTTGTCCCCGTGGCCAAAGCTCTGCAGGTCGAACCTTATCTGCTATGGGAAG
GCCCCGCATTTTCAGATTCACGCAGGGCCCTCTGCAGACTGTTGGCAGAAAAAAGGTAGCCAC
CAAACACATGCATAAGCTGGACTTTTCCCAGAATGCTGCAGCAGAACTACCCTCCAGATAGACA
CTAGCCTGCGGGCCACAGTGCTACGTGAGTCTTGCAAAGAGGAGATCTTTATGGCGAGGCTGA
GAGCACTGCCTGCATGCTTTACCAGAGCCCAGCCTCCAGCCTCCACGGAAAATGTGTTTTGGAA
TCAACACTCTTTGCAAAGGCTCCACACTGCTTTCTGGTAGCATTGGCCTGGGCGCCCAGGCACT
CCTTTAGAACCCACCTGTTCCCCCCACCCACCCTAGTGGGAGGAGGAGAGGGTTACACTGACA
GATACCGGCAGCTCTGCAACCCGGGAACAACGCAGACAACATACAACTCGACAGAGTCACAG
AAGGTGGCGTCCATCGCGCCTGGATGGTGACTACTGCCCTGCGGGCTGCTGGGTGGGTGAAAC
ACACAGGGAAGAAGCACAAATACACACACAGATGTGGCTCAGGGAAAAAA
ID|24199|gene_id|729614|transcript_id|NR_024279 Homo sapiens lncRNA
TTGAAATGCTCGATGATCTTCTCTTCCCGCACGTTCTCGGGCCGGGCGTGGCGGCTCACAC
CTGTAATCTCAGCACTTTGGAAGGCCCTGGCGGAAGGATCTCTTGACGTCAGGACTTCCAAAAC
AGTTGGGCAACACAGCAAGACCCCGTTCCCAGGCTGGGGGCAAAGCCGGGCTCGGTGGCTCA
CGCCTGTAATTCCAGCACTTTGGGAGGCCGAGGTGGGCGGATCACCTGAGCTCAGGAGTTCGA
GACCAGCCTGACCAACATGGTGAAACCCATCTCTACTAAACATACAAAAATTACCCGAGCGTAT
TGGCGGGCGCCTGTAGTCCCAGCTACATGGGAGGCTGAGGCAGGAGAATCGCTTGAACCCGG
GAGGCGAAGGTTGCAGTGAGCCGAGACTGCGCCACTGCACTACAGCCTGGATGACAAGAGAC
TCCCGTCTCAAAAAAAAAAAAAAAAAGACACCTTTCTTTGGGCTTAGTGAGCAAACATTCTACG
CAGTGCCTAATATAAGCCGGCCCAGGCACCACCCAAGCCTTCGGCCAGGACTGTTTTCCCGTTT
CGAGAAGGCTCCATCACACTCCACACGGGATCCAGTCAACAGCCGTTCCAAACATGGCGCGGG
AAGGGGTGGAAGACAAGCCAGCGCGACGCCGTCCAGCCTCATCCCCGGGGCACAAAGGGGA
GACTAATCTCCAGGAAACTCGGGGTACAGAATCGATAACCCCACAAGAAACTAGTTTCCGCGA
GTGGGCCTTAAGGAAAAAGTAGGTCGGGCACAAGCGAGCTGTTCAGACGCACTTCACAGCAAA
GACTCGCGGACAACACAGCGAGACGAAAACGGCCCCGTGCGCAGGCGCGCGGAAACACGAC
TAGCGCGCTTCGGGACGACCCCTCCCCCTTCCCTCAAAGGCCAAGGAAGTGGCTCCGACGCGC
TTGCGCGAGGACGGAATGTTGAGGGGAGGGGGAATTCTTTCCCTTCATTGTCAGAGAGAACCG
CCCCCGCACGGCGAGCGCGCGCGCGCTCACGCACCACTCTCACACTCCGGCGCGCCAAAGGC
TTTCCGCTTGGCGTCTCGCGCATGCGCGGAGGGGGAAGCAGTGGCGAAATTGGATGCTTTTTGC
TGAGTTTCTCAGAGAAGTTTCTGGAAAGATGGAGCCAAAGGGACGCTGGGGGGTGGGGAGCG
CCAGGAAGCAAGCGTTTGAAAGAAAGCGAAAAAACCCAAGCGCACCGCGACACCTCCAACCT
TCAAGCCACGGCCCAAGCAGAGCCCAAGGCCACTGAGACCCTCACGCCAGAGAAAAAGCACA
GGGACCGCGGCAGCCGCTCCTCCCTCTGCCACTGCTCAGCCCCCGAGACTCCCCAGCACGGGG
AGAGGGCAGGGGGTAGCCATGAGAAACCTAGAGCGCTCACTGGACTTTTGCGAGGTGGAAGG
AAAGCCGAAACCCGCCCTTTGGAGCCGCCTGTGCGCACGGGCCCCGTCGAGTGGGCTCAGTAC
GTTAGGACTCAGCGTGGCCGCCAAAGCATTGGTTGGGGGCGTCTTTCACTAATTTTTCCTAGGTT
TTCTCTTCTATCGGGCTCTGTGGTCCTTTCACCACCCATCTTCACTTTGGTGGCCTGACGCTAACG
CTGCGAGTTGGGGAAACCGTAGTAACCCCGGGCCTGAGGGGCGTCCCGGGCGGCGGTGCTCC
GTTTCTTCCAGCGGTTGCCCCTTTAAGAAAAAGCCCCCCGGAGAGGGGCCCGTGTGGCTGAGT
AAAGGTGGCGTGCGCCCGCACAAAGGCGCCGCGTTGTTGGCGGGCTCCGGACCCTGTTGCCCC
TGGGCGGGGCGGCGTGGGAAGACCGGGGCGCCTGGGCGGGGAGAACGACGTTGGCGCTAGC

GGGGCAGGGCAGGGGGAGGGAGTCACGGGATTTAGCAGGTGCTTCTCCGCGACCGAGCGACT
GCCATTTTGTGGTGCGGCCGCCGCCATTTCGCGCGGCCGAGGGGCGGGGTGTGGGCGGAGCG
GCGGGCGCGCCCGGGCTGTCCCTCGGGGCGGCGGGCTAACGCGGCCGCTTTGTTCTTCGCTTC
TCGGCTCGAGGCCCCAGTCTCGACCGCCGACCGCCTCGGAACCGGGCTGCATCCGAGCTGCCA
CGCGGCGTGTGAACCTTGAAGCGCGGCCGGGGAATCCGCAAGGCGCCGACTCTCTTCCTCCTT
GCAAGCCCTTCGAAAGTGAGATTTCTCACCCCACCCTTGCAGGGTGGAGAGAACACGTTTCAAA
AAGGGGATGCCTAGAACTCAGCCGTGGGAAGTGCCTGTCTAAAGCCTTGTGCTGGTGCCAGTA
GCAAACCCGTGCGCGTGCTCGGCTTTGAGCGATCATGGAGCTCTGCAAATAGCTCAACTGCAG
AGCGTGCCCCTTAGAGCTCCTGTGCTGCTGGGAGGCCTCATAAAGGTGCCAAAACCAGGTGTC
CCCAGCGGTTGGCCTCTTTAAAAAGAAAAAAAAAAAAAAGGCGGAGTGGGATGTCCTTGACGTG
CTGAAAGACGGAGTAGAATGAGACGTTGGTTTTCATTGGCTGGCCATTAAGCACGCACTGCGCA
ACTGTTTTCCAGATACGGGTTTATAGCCCATTATTTATATCGGAGCCATAAACTTGAACAATAGA
TTGGAATATCTTATTTTTGCCTGTTGAGAATATCTCCCATTTAACTTCTTTTGAAAGTTCAGATGAG
TATATAAATAATACATTGACCCAG

ID|27042|gene_id|84791|transcript_id|NR_026761 Homo sapiens lncRNA

GTGGCGTAGGCCGGACATTTCTAGTCGACTGTTGCGCGTGCGCGCGCTGTGACGTTCCCTA
CGCGGTCGGGCGTTGGGTTTCGCGGCGCTCGCCGCACTGGTTGTTCAGCACCTTCGGTCCGGTT
GAGGTTGTCAAGTCGGACCAAACAGGTTGTTTCTCTGCAGTTTCCAACATGGCAGGGAGGTTTA
ATAGACATGGATAAGAAGTCCACTCACAGAAATCCTGAAGATGCCAGGGCTGGCAAATATGAA
GGTAAACACAAACGAAAGAAAAGAAGAAAGCAAAACCAAAACCAGCACCGATCCCGACATAG
ATCAGTGACGTCTTTTTCTTCAGATGATCCTATGTTTCCTTCTTCCTCATCATCGTCTTCAGGAAGC
CAGACAGATTCAAGTATTGAAGATGCTGCCAAGGGAAAAATTAAGAAGAAGAGAAGAGAGAA
AACAAATAAATGGGAAAAAAGAAAGGACAAAATATGAAACACCAAGAAAAAGAGAAGGAAAA
AAAGGAGGAAACAACCACATATGTCACCTTTCCAAGAGGGACTGAAACTGGGCTGACCCTTTTG
ATTTCCAAGCTCAGCGTTTTGGTGTAAGGCGGCCAAAGAAGGATGCGGAGCCCAGCACTGTGA
AGCCTACAAAAACATTGATGCGCTGGCTTGGGGATTTGAATTTGAACATCTTTCACACTAAGTTC
AGACTCATGAAACCAATCTTCAGATGCTCTGTAAACCACATAATAAAGAGTTTGGAAATTAAAA
AAAAAAAAAAAAAAAAAAAAAAAAAAAAAAAAAAAAA

ID|28725|gene_id|92659|transcript_id|NR_015454 Homo sapiens lncRNA

ATGCGCAACCCGCGCGCCGTCGTCCCTGGCAACACTGGCTGCTCTAGCGCCTGGCGGGGT
CTCACGGGCGGGCCTGGGCGCACGGCCAAAGCGCCAGGTCTCCACCCCCAGCCCCCGGCGTC
CTGCAGCCGGAGCCTGCCTCGCCTGGCTCCTGCGCCCGCCGTCCAGGGCCCCTGTCCAAGCTC
GCGGACGCCGGCGGCGTTTTCCAGCTCGAGTGCTCAGCCTAGGGCAGGCTCTGCGTGCGCCCC
CTCGAGTGCCGGCCTGGGCCCTGCCAGGCAGAGGCGCTCTTGCGTCAGGACGGCGCCGCCGG
GGCCCCGCGATTCCAACTCGGGGGTCCCGGAGCGGCGCGGGGCGGGGACGGCCTGGCAGCTC
CCAGCAGCGAGGGGCGATCCCCGCCCGCGCAGCAGACCCCGAGCTGCCCAGGCCCTTCCTGC
GCCACGTGTACCCAGAGCTCCGCCCGGGGGAGACTTCGCTGCTTGTTGGAGATGCCCAGCGGT
AGGGGGCGAGGACTCGGGAGGAAGATAAACGGGGGATCCCGTGTCTGGACTTTCTCCTCCACC
CTCTTCCCTTCGCAGCTGTGAATCCTGGGAGGGGAAGGGTGGCCGCCCCGCCAGCAGAGAGCT
GAAGGTGTTCCGTGGTCAGTGAGGGCTGGCACTGGCTCTGCACGAAGATCTCCTCACCTCCCCA
GCGGCTCCTGGCCAGCTCCCCACGGAGAGCCCAGGACACTTGTCCCTGGACACAGTGGTGCAG
CCATGCTTGGGCCAAACCATGAGGCCACCAGAACCGCGAAAGGCTACTGTACTGAGAGGGATA
CTGCGAGAGAGGCCCACAGGTTTCCAAGCATCCCTCTCACTCCGCACACTCATCTCCACGACCG
GCTTTAAGTGCGGCCTTGAGGCCTGGCTCCCTGTTTGGACTTCTCTGCACCTCCAGGAGCTCACT

TCCCGTCACAGCTGGATGCCTCCTCCCGCAGGCACCTCCCGGGCATCCTCCCGCCCCAGGCTG
CACCTTCTGTGGGACTGGCCCCCGCAGGCACCTCCCGGGCACCCTCCCGCCCCAGGCTGCAGC
TTCTGTGGGACTGAGCCCCGCAGGCACCTCCCCGGCATCCTCCCGCCCCAGGCTGCACCTTCTG
TGGGACTGAGCCCCGCAGGCACTTCCCCGGCATCCTCCCGCCCCAGGCTGCAGCTTCTGTGGG
ACTGACCTTAAGACCCCATCGGTTCCTCCTGAGAGCAGCAGATCTCAGCCAGATGCTGCCGGG
AGGGAGGTGCTAGCGTCCACACCCAGACGGGTCCAGTCCAGCCCTGCTCTCCCTGGTCTCCAG
GCAGGGCCCTCCCAGGGTGGACATTGCCCATCTCTGCCTGGCCAGGGGCTGCTGACCCACTGG
TTCCAGAGTCTGAGCCTGTCCTGCACAAGTACCATGGACAGGGGTCAGCCGGGCACTGAAGAG
GCGTGGCAGAATTGGGCAGGGCAAGGGCCCCAGCTGGCAAGCTGGAGTCAGGGCAATTCCAA
CCAAGAAACTGCGGCAGGCCGGGTGCAGTGTCTCAAGCCTATAATCCCAGCACTTTGGGAGGC
AGTGGGAGAATCGCTTGAGCCCGGGAGTTTGAGACCAGCATTGGTAACATAGAGACCCTATCG
CTACAAGAAAAAAAAAAAAAAAAAAGCTGGGCATGGTGGTGCACACCTGTTGTCCCAGCTATTC
AGGAGGCTGACGTGGGAGGATTGCTTGTGCCCAGGGGTTGCAGGCTGCAGTGAGCTGTGATCA
TACCACTGCACTCCAGCCTGGGCGACAGAGAACCTCTCTAAAAAAAAAGGAAAATGAAAAATA
AATGGGGGCAGAAGCTGAGCCAAGCAGAGGCGGGCAGCTCCTCCTCCAGGGCCCACCTGGCC
TGCTGGGGAAAAAAAAAAAAAAAAAAAA
ID|30352|gene_id|ENSG00000232019|transcript_id|ENST00000439105 Homo sapiens lncRNA

CCGGAGGCTGCCATGGCGGGTGGGCAGCTTGACTTCTCCTCCCGGCCAGTTGTCGAGCGC
CTCACCAGGCCTCGCCCTGCAGCCTCGCTCTCCCTGGCGCTGCGCGGCCTAGGGGACTGGGCT
GCTGGCCTCCGGGTGCGGGGTGGGGGCAGGCTCTGACCTGGGGCGTTCTGGCCGCGCGAGCC
GCGGGATGGGGCCCCCGGCCCCGGAGGAGGCGCCGCTGCTGTGTCCCTTGGTGGAGAGGGCG
CTGCCAGCCCTGCGCGGTTTCCAGCCAGGAAGCTTCGGGAAGCCTGGACGTCTGCTCACTGGA
GATGACACGTGCGTGGGGTGTTGGGAAGGAGGTGACTTCGCCTGTGATGGACTCCCAGTGTGA
GCTGTGATGGACTTCCAGTGTGAGCACTGGCCAGAGTGACCAGGCCGACCATCAACAGCCCTG
ATCCAGATGCAGAGGCCAGGATGTGGGCCCAGCCCTGTGCCAGAAGGCTGGCTGGAATAAAG
GTACAGATAGAGGCCTCACCCCCTCTGGGACCACTGGCACTCAGGGTGTTTGCAGCCTCAGAG
CCCACCTACCCCCAGGGCCACAGCTGCAGCTCCTGCCCTGCTGTCATTACAGGGATGGGCAGG
CTGGCATGGGGGCACCCGCTGCCCCTGCCTGGGTGTTGCTGTGTATTCCCGCCGGCCAGGGGC
CACTGCCAGGACCACCCCTCCCTTTTCATATCCCGATTCTTAAGTTCTGCTATTGTGGTATTGTGG
TCGAAAAAAAAGAACCGCTTGGCTGTTTTTGAACTGCCTGGAACCTAAGATCCTGAATTTTTTTT
CCCCCCCAAGGGGGAAAGTCTATATGGAAAACATTTATTTTAAAATACAGGATGAAGTGAATTA
AAAGATTTAAATGCA
ID|30370|gene_id|ENSG00000250920|transcript_id|ENST00000509399 Homo sapiens lncRNA

CAGCCCGCCTGCACCCAGGTGAAATAGACAGCCATGTTGCTCACACAAAGCCTGTTTGCT
GGTCTCTTCACACTGACTCGAGTGAAATTTGGTGCCGTGACTAGGATCGGGGGACCTCCCTTGG
GAGATCAATCCCCCGTCCTCCTACACTTTGCTCTGTGAGAAAGATCCACCTACAACCTCAGGTC
CTCAGACCAACCAGCCCAAGAAACATCTCACCAATTTCAAATCCGGTAAGCGGCCTCTTTTTACT
CTTTTCTCCAGCTTCCCTCACTGTCCCTCAACCTCTTTCTCCTTTCAATCTTGGTGCCACACTTCAA
TCTCTCCCTTCTCTTAATTTCAATTCCTTTCATTTTCTGGTAGAGACAAAGGAGACACGTTTTATCC
GTGGACCCAAAACTCCGGCGCCAGTCACGGACTGGGAAGGCAGCCTTCCCTTGGTGTTAAATC
ACTGCAGGGACGCCTCTCTGATTATTCACCCATGTTTCAAAGGTGTCAGACCACGCAGGGACGC
CTGCCTTGGTCCTTCACCCTTAGTGGCAAGTCCTGCTTTTCTGGGGAAGGGGCAAGTACCCCAA

CCCCTTCTCTCTGTGTCTCTACCCCTTCTCTGCTTTTCTGGGGGAGGGGCAAGTACCCCTCAACC
CCTTCTCCTTCACCCTTAGTGACAAGTCCTGCTTTTCTAGGAGGCAAGAACCCCCAATCCCTTAT
TTCCACACCCTGACCTCTTATCTCTGTGCCCCAATCCCTTATTTCCACACCCCAACCTCTTATCTC
TGTGCCCCAATCCCTTATTTCCGTGCCCCAACCCCCCTTCCCACTTTTCTGGAGGATAAGAACCC
CCGAACCCCTTCCCTCCGTGTCTCTATGCTCTCTTTTCTCTGGGTTTGCCTCCTTCACTATGGGCA
ACCTTCCACCTACCATTCCTCCTTCTTCTCCTTTAGCCTATATTCTTAAGAATTTAAAACCTCTTCA
ACTCTCACCTGACCTAAAATCTAAGCATCTTATTTCTTCTGCAATGCCACTTGACCCCAATACAA
ACTTGACAGTAGTTCCAAATAGCCAGAAAATGGCACTTCCAATTTTTCCATCCTACAAGATCTAA
ATAATTCTTTTCGTAAAATGGGCAAATGGTCTGAGGTGCCTGACATCCAGGCATTCTTTTACACA
ATAGTCCCTTCCTAGTCTCTGTGCCCAGTGCAACTTCTCCCAAATCTTCCTTCTTTCCCTCCTGCC
TGTCCCCTCAGTCCCAACCCCAAGCATCGCTGAGTCTTTCTAATCTTCCTTTTCTACAGACCCATC
TGACCTCTCCCCTCCTTGCCAGGCCAAGCTACGTCCCAATTCTTCCTCAGCCTCCGCTCCTCCAC
CCTGCAATCTTTTTATCACCTCCCCTCCTCACACCTGGTCCTGCTTACAGTTTCATTCTGTGACTA
GCCCTCCCCCACCTGCCCAGCAATTTACTCTTAAAAAAGTGGCTGGAGCCAAAGGCATAGTCAA
GGTTAATGCTCCTTTTTCTTTATGCCAAATCAGAAGTGTTTAGGCTCTTTTTCATCAAATATAAAA
ACCCAGCCCAGTTCATGGCTCGTTTGGCAGCAACCCTGAGACGCTTCACAGCCCTAGACCCTAA
AAGGTCAAAAGGCCATCTTATTCTCAATATACATTTTATTACCCAATCTGCTCCTAACATTAAATA
AAACCCCAAAAATTGGAATCTGGTCCTCAAACCCCACAACAGGACTTAATTAACCTCACCTTCA
AGGTGTACAATAATAAAAAAAAAGTTGCAATTCCTTGCCTCCACTGTGAGACAAAGCCCAGCCAC
ATCTCCAGCACACAAGAACTTCCAAACCCCTGAACTGCAGCAGTCAGGCATTCCTCCAGAACCT
CCTCCCCCAGGAGCTTGCTACAAGTGCCAGAAATCTGACCACCAGGCCAAGGACTGCCGGCAG
CCCAGGATTCCTCCTAAGCCACGTCCCATCTGTGTGGGACCCCACTGCAAATCGGACTGTTCAA
CTCACCTGGCAGCCACTCCCAGAGCCCCTGGAACTCTGGCCCAAGGCTCTCTGACTGACTCCTT
CCCAGATCTTCTCGGCTTAGCGGCTGAAGACTGACACTGCCCGATTGCCTCGGAAGCCCCCTAG
ACCATCATGGACGCCGAGCTTCAGGTAACTCTCACAGTGGAAGGTAAGCCCGTCCCCTTCTTAA
TCAATATGGAGGCTACCCACTCCACATTACCTTCTTTTTAAGGGCCTATTTCCGTTGCCTCCATAA
CTGTTGTGGGTATTGACGGCCAGGCTTCTAAACCTCTTAAAACTCCCCAACTCTGGTGCCAACTT
AGACAATACTCTTTTAAGCACTCCTTTTTAGTTATCCCCACCTGCCCAGTTCCCTTATTAGGCTGA
GACACTTTAACTAAATTATCTGCTTCCCTGACTATTCCTGGACTACAGCTATATCTCATTGCCGCC
CTTCTTCCCAATCCAAAGCCTCCTTTGCATCCTCCTCTTGTATCTCCCCAACTTAACCCACAAGTA
TAAGATACCTCTACTCCCTCCTTGGTGACCGATCATGCACCCCTTACCATCTCATTAAAACCTAA
TCACCCTTACCCCACTCAACGCCAATATCCCATCCCACAGCACGCTTTAAAAAGATTAAAGCCT
GTTATCACTGGCCTGCTACAGCACAGCCTTTTAAAGCCTATAAACTCTCCTTACAATTCACCCAT
TTTACCTGTCCTAAAACCAGACAAGCCTTACAACTTAGTTCAGGATCTATGCCTTATCAACCAAA
TTGTTTTGCCTATCCACCCCGTGGTGCCAAAACCATATACTCTCCTATCCTCAATCCCTGCCTCTA
CAACCCATTATTCTGTTCTAGATCTCAAACATGCTTTCTTTACTATTCCTTTGCACCCTTAATCCCA
GCCTCTCTTCGCTTTCACTTGGACTGACCCTGACACCCATCAAGCTCAGCAAATTACCTAGGCTG
TACTGCCGCAAACCTTCACAGACAGCCCCCATTATTTCAATCAAGCCCAAATTTCTTCCTCATCT
GTTACCTATCTTGGCATAATTCTCATAAAAACACACATGCTCTCCCTGCCATTCTTGTCAGACTGA
TCTCTCAAACCCCAGCACCTTCTACAAAACAACAACTCCCTTCCTTCCTAGGCATAGTTGGTGCA
GTCAGAATTCTTACACAAGAGCCAGGACCGCACCCTGTAGCCTTTCTGTCCAAACAACTTGACC
TTACTGTTTTAGCCTAGCCCTCATGTCTGCGTGCAGCGGCTGCCGCTGCTTTAATACCGTTAGAG
GCCCTAAAAATCACAAACTATGCTCAACTCACTCTCTACATTTCTCATAACTCCCAAAATCTATTT
TCTTCCTCATACCTGATGCATATACTTTCTGCTCCCTGGCTCCTTCAGCTGTACTCACTCTTTAAGT

CCTACAATTACCATTGTTCCTGGCCCGGACTTCAATAAGGCCTCTCACATTATTCCTGATACCAC
ACCTGACCCCCATGACTGTATCTCTCTGATCCACCTGATATTCACCATTTCCCCATATTTCCTTCT
TTCCTGTTCCCCACCCTGATCACGCTTGATTTATTGATGGCAGTTCCACCAGGCCTAATCGCCAC
ACACAAGCAAAGGCAGGCTATGCTGTAGTACAAGCCACTAGCCCGCCTCTCAGAACCTCTCATT
TCCTTTCCATCCTGGAAATCTATCCTCAAGGAAATAACTTCTCAGTGTTCCATCTGCTATTCTACT
ACTCCTCAGGGATTATTCAGGCCCCCTCCCTTCCCTACACATCAAGCTTGAGGATTTGCCCCCAC
CCAGGACTGGCAAATTAGCTTTACTCAACATTCCGGAGCCAGGAAACTAAAATACCTCTTAGGC
TGAATAGACACTTTCACTGAATAAGTAAAGGCCTTTCCTACAGGGTCTGAGAAGGCCACCGCAG
TCATTTCTTCCCTTCTGTCAGACATAATTCCTCAGTTTAGCCTTCCCACCTCTATACAGTCTGATA
ACAGACCAGCCTTTATTAGTCAAATCAGCCAAGCAGTTTTTCAGGCCCTTAGTATTCAGTGAAAC
CTTTACATCCTTTACGGTCCTCCGTCTTCAAGAAAAGTAGAATGGACTAAAGGTCTTTTAAAAAC
ACACCTCACCAAGCTCAGCCACCAACTTAAAAAGGACTGGACAATACTTTTACCACTTTCGCTTC
TCAGAATTCAGGCCTGTCCTCAGAATGTTACAGGATACAGCCCATTTAAGCTCCTGTATAGATGC
TCCTTTTTATTAGGCCCCAGTCTCATTCAACACCAGACCAACTTAGACTGTGCCCCAAAAAAACT
TGTCATCCCTACTATCTTCTGTCTAGTCATACTCCTATTCACCATTCTCAACTACTCATACATGCC
CTGCTCTTGTTTACACTGCCGGTTTACACTGTTTCTCCAAGCCATCACAGCTGATATCTCCTGGTG
CTATCCCCAAACTGCCACTCTAAACTCTTGAAGTAAATAAATAATCTTTGCTGGCAAGGCTAGGC
TGAACCTCCTTGGGCACTCTCTAATCAGATGTCCTAGGTCCTCCCAATTCTTAGACCTTTTATACC
TGTTTTTCTCCTTCTCTTATTCCATTTAGTTTTTCAATTCATACAAAACCGTATCCAGGCCATCACC
AATCATTCTATACGACAAATGTTTCTTCTAACAACCCCACAGTATCACCCCTTACCGCAAGATCT
TTCTTCAGCTTAATCTCTCCCACTCTAGGTTCCCACACCGCCCCTAATCCCACTCAAAGCACCCC
TGAGAAACATCGCCCATTCTCTCTCCATACCACCTGCAAAAATTTTCGCCGCCCCAACACTTCAA
CACTATTTTGTTTTATTTTTCTTATTAATAAAAGAAGGCAGGAATGTCGGGCCTCTGAGCCCAAG
CCAAGCCATCGCATCCCCTGTGACTTGCATGTATATGCCCAGATGGCCTGAAGTAACTGAAGAA
TCACAAAAGAAGTAAAAAGGCCCTGCCCTGCCTTAACAGATGACATTCCACCATTGTGATTTGTT
CCTGCCCCACCTTAGCTGAGTGATTAACCCTGTGAATTTCCTTCTCCTGGCTCAGAAGCTCCCCG
ACTGAGCACCTTGTGACCCCCGCCCCTGCCCACCAGAGAACAACCCCCTTTGACTGTAATTTTC
CATTACCTTCCCAAATCCTATAAAATGGCCCCACCCCATGTCCCTTCGCTGACTCTCTTTTTGGAC
TCAGCCCGCCTGCACCCAGGTGAAATAGACAGCCATGTTGCTCACACAAAGCCTGTTTGCTGGT
CTCTTCACACGGACTCGAGTGAAAATACACACGCACACACACACAAATGGACATTTACCCCACT
CCTGCTTTTGTGCTATTGTGGTCATGCATAGTATTTCTTTTTTGCTGTTGTTTTTCTTGTTGTTTTCA
CTGTCATACAGGTATTTATGATGGAAACAGAATCAGAGTCTGACCTTCCTGACTTGAAGTACAA
GGTTTTTGGGGTTTTTCATTCGTGTTTTATGTGTTTTTTAAAAAATTATTTGTGTTTTTAATCGACAA
ATAAAAACTGTATACACATATGCTTCCTGATTTATAATGGGGATACATCCCAAGAAACCTACTGT
AAGTTGAAAATAATGTAAGTCAAACATGCACTTAGTAAACCTAGCTTACCAAACATCATAGCTTA
ACCTAGCCTAGGTTAAACATTCGTGGGACACTTACATTAGTCTACAGTTAGGCAAAATCATCTAA
TACATAGCCTACTTTCTAATAAAGTTATTAGAAAGGATTTTTATTCAAAAATCAAAATATGGTTTC
TACTTAATGTTTATTGCTTTCACATCATCGTAAAGTGGAAAAATATAGGTCAAGCCATCGTAAGT
CAAGGAATGTTGGTATATCGTAATGGTGTACATGATATTTTGAAATATGTATACTTTAATAGCTAT
AGTGAGCTAATTAACATATGAATTGCCTCACATTCTTATCATTTATTTGTGGTGAGTAGATATAAA
ATCTACTCTTTTAGCAATTTTTAAGTATGCAATACACTATTGGAAGGGTCTCTGGCTAAGTTAGTT
TTCTTTCTGAACGGACATTTCTCACTAACAAGTGCAGAAATTCCCTGTGAACAGGGGAAGCGGT
AATGCTGACAACAAAACTCAGATGTAAATTTAGAATAAAAAAGGGCTCATGGTTGACTGCTTTG
GGCTATACTTCCATAATCTCTCAAAAAAAAAAGTAAGGTATTGTCCTCTGGAGTATTTTGATGGAA

AATTTTAGGAAGACTTGATAATCTGTAAAAGTTGTAGGCTTGAGATCCTAAAAGATCTGTATCTA
AATTCATGTATTCTACAGAAGTTTACTTAATTACTCCAGGTTTATTTTTCCTTACTTGTGAGGTAAA
TGTTGTAATACACATCTACCTCACAAAGGTGTTGTGAGAATTCAATGATATGTTATTTATAAAGA
AAGTATTGATTACCTTCCTTCTGTCCCAGTGATAGATCACAACAAGAGATCATGAAGAGGGCAT
GGCCGCCATGTCATTCTGGAGAGAGACAGAGTAAAACAAAGAAGGTGATGGGTAAAGCGCAG
TCGCCTGCTATATATTGTCTATTTTTGGTTTTTCACTTACCTTTTATATTTATGCCTTTTATGTACAA
CAGGATTATAAGCAGCTTGAGTCCAGTGAATATACCATTTCATTTTGCTATCCTTCACTGCACTTA
GCTTAGAGGAAATAATCACAGCTTATTATTGATTAATTAATTAATAGATGAATGGTGAACAGATG
ATTATCATCCCAAGAAATGGGATTTTTTTAATTCTGGGCAACTAGGAAAGTTTTTAGCTACAGAG
AAATATTGACATGGGCAACAACTTCATCTTACAGCAAAAAGTCCTTTTTTTTTTTTTTTTTTTCTTTT
TGGAGACAGAGTCTTGTTCTGCAACCCAGGCTGGAGTGGAGGGCAGTGGTGTGATCTCGGCTC
ACTTCAACCTCCGCCTCTTAGGTTCAAGTGATTCTCCTGCCTCAGCCTCCCGAGTAGCTGGGACT
ACAGGCATGCACCACCGCGCCCAGCTATTTTTTGTATTTTTAGTAGAAATGGGATTTCACCATGT
TAGCCAGGCTGGTCACGAACTCTTGACCTCAGGCAATCCACCTACCTCGGCCTCCCGGTGTGCT
GGGATTACAGGCATAAGCCACCATGCCCAGCCCAAAAAGTCATTTTATTTATTTTTTAAAAAGAT
ATTGCCAAGTTTGAGAGGGAAAAATGATTCATTTTACAACTGTGACTAGTACCAGATCACAGAT
AACATATGTATAAAAAACTTGCCTTTGTGGTAGTAACAGAGGCAGTTGGAAGAAAGCAAGCAG
AGAAAACGACCAACTGTTGAAGCTTTTATCACCACTTTGTTCAAACATGCACTAGAACAATTTGG
AATGGTATTAATTTTCAACTTAAACCTATAATGTAATATCTCAAGAATTGTTACAGAAAGACTGA
GTAAATATATACGAGAAAGAGCTCTATAATTTAATTATATTATATATTAGAGAAAGAGCTCTATAT
TCCACTATAACATGTGATTTAGGCATACCTCTCTCCTAGAAGAAAATGCAATTCAAGAGGCAAAT
TCATGATTTCTTTTTCAGTGGGTTGGGTGGGCTAGCTTACACTAACTTTCAAAAACTCAACTTATG
ACCAAAATTCTACAAAGAAGTTTTATGACTCAGGATGTGTGTGTTAATGTGCTTATTTTAACAGC
AGCCTCTCTACAAGTCTCCTAACTTAACCTGATGGGAAAACACTTCCTACCACTAACAGCATCTA
CTTGATTACTGATACTTTGATCATGGAGTTTGGGCATGCCACTTGATAGAAATTTGAAGAGCAAT
TATATTTTTCAAAAAGAGTTTTGAATAATGTTAAGATAGATTGCAACATGACTATCAATTCTTCCC
TTCCCATCAAAGGAGAGAGTCCGTTTATCCAGCCTTTGAATCTTGATTATTCATGTGACTTGCTTC
ACCCAATGTAACATTAATAAGCACAATACAAGCAGAGGCTTGCCAAGAACTTGGTTTGTTTCTA
ATGCTTAGAAGAAGAATGGTGTATGCCATATTTCTGCATTTAGAACTCACGTGGAGACATGTGT
ID|30412|gene_id|ENSG00000267321|transcript_id|ENST00000592381 Homo sapiens lncRNA

GGCGGAGAAGCAAAGGAGAGGGAAGCTGGAAGCACCTTTGGCCCGGGACAGAAATCTG
GAGAGCTTGGCTACCTCCATCCTCCTCAGGCCGGAGCAGGCTTCCTGAGAGAGTCCAGGTCGT
AGGAGTTTTACGACTTAGAAAAGCGGGCTGCAGATTCCTTCCTGGGTGTTTGGTTCAAGCCTTG
GCTCCAGCCTCACTCTCAGTCTTCCCGGGAGTTCGTGGGATTTGGACCTTAGATTATTAGTATTA
TTTTGAGGGCCTCCTGTGTGTAAGCACTGGTTGTGCGCAGATGGCTGTGCAGAGGGCCATGAGG
TAGAGGCTGGGGAAATGAGGGCTTGGAGGTGCTTGAGGTATGGTCTTTACCTACGTGAAATGTT
GGAGGTAAGTCGGATTTATAACTTGTGTTGATGCTTTTATAATCTAGGAGCTTAGGGTTTAGTAG
GAAGAGTCGCTCATTCAATGAATTAAACGCTCATTCCTATGTACTAGGCACCATTCCGAGTGCA
GTGGCTACAAAGACTAGTAGAACATAGTCCTGGTTCTTTAGGAACTCTGTGATTGGGCAAGTGG
GAGAGAGGGCGGAAAACGTGAACTGGTGAGTAGTTGGCGTGTGATGCGTGCCGTAGTGGAAG
AAAGTGCTGTGAGAGTTAGAAGAGGGGGATGATTGAAGGAATTAAGGACATTTAACAGAAAGT
GATATTTGAGTAAAGTTTATTTTTGTTTTGTTTTTTTTTTTTTTTTTGAGACGGGAGTCTCGCTCTGCC
TCCCAGGTTGGAGTCCAGTGGCGCTATCTCGGCTCACTGCAACCGCCGCCTCCCGGGTTCAAGC

AGTTCTCAGCCTCAGCCTCCCAAGTAGCTGGGATTACAGGCGCACACCACCACGCCCGGCTAA
TTTTTGCGTTTTTAGTAGAGATGGGGTTTCACCATGTTGGCCAGGCTGGTCTTGAACTCCTGACCT
CGTGATCCACCCGCCTCGGCCTCCCAAAGTGCTGGGATTACAGGCCAAAGCCACCGCTCCCGG
CCTTGAGTAAAGTTTAAAAAGTATACTTAGGGTGGGTATTTATCCCTCCATGAAAGCGCCTTTTA
CTCAGCCAGCACAGAGCCATCCCTTTGCCCTTCCAGATGCCTGACTGGGACCTTGCTTATGGAT
GTGTGAGCTTTTAATCTCTGGCTGTAATCTTACCCACTTCAACCTAAACTTGGAATGATTACTCTT
GACCCTTTTTTTGAGTGATAAGGCAAATCTCCCCACACCTGGGCATTTTAAAGTTATATACTGTAT
GTTTAAATATCCCCCTTAAAAAGCCTGAGCTATATAACAATCTAGATGTGTCTATCTTACTCTTTT
CCCACAAACTAAGAATCCAAATAATGTTAAAAATCTTTAAAAAAAAAAAAAAAAAGTATACTCAG
GTTTTCTCCCCATCGAAAGGGGTGGAGAAAGCACTCCAGGAACTAGGAAAATGTACCTGCCGC
ATGTGGCTGGAGCACATGGGAGGTTTGCCCAGCTGATGATGAAGCAGATAGGGAGTTCAACTT
GTAAAGATCTTGAATTACATGGTAGTCTGGCTGTCTGGATCTTAAAATACAAAGTGAAATAGGTA
CCACAAAACAGGTATTAAGGTGCTTTCATAATTCAGAAGGAGGACAGATACTTCCTGTTGGATT
GATGAAAAAAGGCTTCATGCAAAAGATGGCATTTTGAAGGGTTGCAAACTGCAGGGATAGGAT
TTTGAAGTACAGGAAGGATTTCTACACAGGGGGAAAGACTTTCTAGATGGAGGGCATAAGGTC
AAATAGAGCCAATATGAGGACTAAGGAATAATCTGGGTTGATTAGACTGTAAGATATGTTTGGG
GGCCCGGCACGGTGGCTCACACCTATAATCCCAGCACCTGGGAGGCCAAGGCAGGTGGATCA
CTTGAGGTCAGGAGTTTAAGACCAGTCTGGCCAACATGGCGAAACCCTGTCTCTACTAAAAATA
CAAAAATTAGTTGGGCATGGTGGCTCATGCCTGTAATCCCACCTACTTGGGAAGCTGAGGCAGG
AGAATCGCTTGAACCCAGGAGGCTGAGTTTGCAGTGAGCTGAGATTGCACCACTGCACTGCAG
CCTGGGCGACAGAGCGAGACTCTATCTCAAACAAACAAACAAACAAAAGACATGTTTGGGTTA
TTTATGGTGTATTTAAGGATAAATATTGGTCTGTTTTGAAACATCTTGATTGCTAAACAAAGTCGT
TAGGACTTTATTTCCTAGACTATGGGGAGCAACTGAATGCTTGTGAGTAATCACACCTCCATATA
TAAGGGTTAGTATGTGTGTAAGATTCAAAGTACATTGGATGGGAGAGAAACTGGAGGCAGGAG
GCCCATTGAGATAGCAGTATTGTCTGCCAGAAGGAACGTGGGTTTTAGAGCCAAGCACTCATTG
GTTCAGAGCTTGGCTTTGCTCACAAGTTTCATTATTTTTGGCAGATTTATTTAAGTTATTAAGCTCT
GATCTCTGTCTCAAAAGAGGGAAAGTAACTGATGAAGTGCCTGTTACACAATGGTTATGCTTGA
ATGCTTAAACAGAGGCTGAGGCCTCTGTTTTCTTTTTCTTTCTTTCTTTCTCTTTCTTTCTTTCTTTCT
TTCTTTCTTTCTTTCTTTCTCTTTCTTTTTTTCCTTCTTTCCTTCTTTCCTTCTTTCCTTCCTTCCTTCTT
TCCTTCCTTCCTTCTTTCCTTCTTTCTTTCTTTCTTTTTCTTTCTTTCTTTCTTTTTTTTTTTTTTTTTTGA
GACAGGATCTCACTCTTGTCGCCCAGGCTGGAGTGCAGTGGTGCAATCTCAGCTCACTGCAACC
TCCGCCTCCCAGGTTCAAGCTGTTGTCCTGCCTCAGCCTCTTGAGTAGCTGGCATTACAGGCGC
CTGCCATCATGCCCGGCTAATTTTTGTACTTTTAATAGAGACGGGGTTTCGCCACGTTGGCCAGG
CTGGTCTGGAACTCCTGGCCTCAGGGGACCTGCCTGCCTCGGCCTCCCAAAGTGCTGGGATTAG
AGGTGCGAACCACTGCGCCTGACCTAGTTTTCCTCTTTATGGAGCTTAGTTGCTGAATAGTGAAC
ACAGACCAATAGCTGTCAGTTTTAACTAGGGAATATCAGTCTATTGATGTTTATAGATGGCTGAA
GGCAGTATATGGTGGCCCCAGAATTTTAGATACTGACAATATCATTTTGAAAGTCTGGAGAGGC
CAGGTGCGGTGGCTCATGCCTGTAATCCCAGCACTTTCAGAGGCCGAGGTGGGCAGATCACTT
GAAGTCAGGAGTTCAAGACCAGCCTGGCCAACATGGCAAAACCCCATTTCTACTGAAAATGCA
AAAATTAGCTAGGCCTGGTTGTGCATGCCTGTAATCCCAGCTACCTGGGAGGCTGAGGTGGGA
GGACTGCTTGAACCCAGGAGGCAGAGGCTAAAGTGAGCTGAGATAGTGCCACTGCATTCCAGC
CTGGGTGACAGAGTGAGAATCTGTCTCAAAAAATAAAGAGGCCAGGCGCGGTGGCTCACGCCC
ATAATCCCAGCACTTTGGGAGGTTGAGGTGGGTGGATCACAAGGTCAAGAGTTCAAGACCAGC
CTGGCCAATATGGTGAAAGCCCATCTCTACTAAAAAGTGCAAAAATTAGCTGGGTGTGGTGGTG

CACGCCTGTAGTCTCAGCTACTCGGGAGGCTGAGGCAGGAGAATCACTTGAACCCGGGAGGTA
GAGGTTGCAGTGAGCCGAGATTGTGCCACCGCACTCTAGCCTGGGTGACAGAGTGAGGGAGTG
GAGTGGAGTGGAGGTCAGGTTTCTCATTCACAAATTGATTTTTCAGATTGTGAATTACCCATGCT
TTGCCCTGCTTTATCTTCCATGTCCTTCCTCATTAGTAAATCTAAAGGTAGTACCCTAGAGAAAAA
TGAAATTGAACCTTTTATTTAGTTGCTACTGAAAAAATATAAATCTGGTAATCATACCTCAAGTTC
TCTTTCCAGCTCTGCAGTTAATTTCCTATGGATCTTCGGCAAATCATAGCCTCTCTGGGTTCTTGG
ACTCTTCAGATAAAAGATTCTAGTTTAAAAACTTTTATGACTATAATTTTAATCTTTGTTGGCAGA
AGAATTATAAATCAAATTGCATATTAAATGAACTGGAGTTTATAAAGAAATTTAGTAGAAAATCT
TTTCTAGGCCCGGTGTGGTGGCTCACGCCTGTAATCCCAGCACTTTGGGAGGCCAAGGCTGGCA
GATCACTTGAGGTCAGGAGTTCAAGACCAGCCTGGCCAACATGGTGAAACCCCATCTCTACTAA
AAATACAAAAATTAGCTGGGCGTGGTGGCACATGCCTGTAGTCCCAGCTATTTGGGAGGCTGA
GGCTGCAGTGAGCCGAGATTGCGCCACTGCACTCCAGCCTGGGCGACACAGTGAGACTCCATT
TCAAAAAAAAAAAATATTTTCTAGGCCAGGTACAGTAGCTCACACCTGTAATCCAGCACTTTGGA
AGGCCAAAGAGGGAAGATCACTTGAGCCCAGCAGTTTGAGACCACCCTGGGCAACATGGCAA
GACTCTGGGCGTGGTGGTGCTCACCTGAGATGTCAGCTACTCAGAAGGCTGGGGTGGGAGGAT
TGCTTGAGCCCAGCAGTTTGAGGCCACAGTGAGCCATGATCGTGCCACTGCACTCCAGGCTAG
GTGACAGGGCAAGACCCTGTCTCCAGAAAAAATAAAGAAATTTTTAAAAAGAAAATATATTTTC
TAAAGCTTTTGCTTTTGAACTAGGCTAAGGGCAGGCTTATGGGCTCAAGCCTGTGATCCCAGGG
TTTTGGGAGGCCAAGGCAGGAGGATCACTTGAAGCCAGGAGTTTGAGACCAGCCTGGGAAACA
TACCGAGACTCTTGTCTCTACAAAAATTTAAAAAGAATTAGCCAGGGATGGTGGACATGCACCT
GTAGTCCTAGCTACCTGGTTGGATCACCTGAGCCCAGGAGTTTGAGGCTACAGTGAGCCATGAT
CACACCACTGCACTCCAGCCTGGTTGACAAGAGTGAAACCTTGTCTCAAAATAAAAATATATAT
ATATATTAATTGAAAACAGGCCAGGCATGGTGGCTCACGCCTGTAATCCGCGCACTTTGGGAGG
CTGGAGAAGGTGGATTGCTTGAGTCCAGGAGTTTGAGAACAGCCTGGACAACATGGTAAAACC
CTGTCTCTACAAAAAGTACAAAAATTAGCCAGATATGGTGGCCTGTGACTGTAGTCCCAGCTAC
TTGGGAGGCTGAGGTGGGATGATCGTCTGAGCCTGGGGAGGTTGAGACTGCAGTGAGCCATGA
TCAGGCCATTGCACTCCAGCCTGGGTGACAGAGTAAGACCCTGTCTTAAAAAAAAAAAGAAAG
AAAGAAATATTAATTAGAAACCCATGGTTGAAACTTACCTGAATAGAAGGCTTTATTTTTTTTCAT
AGGTAAGCCTATGTTTTCTTGGATGTGAAACTAGGGCACTGGGTTGCTCTTAGGTAATACAAGG
AGATGAAATGCAGCGATGCGATGATGAGTGAAGTAGAGCCTGACCTGGTATTGCCATTGCTTCA
CTGTTGGCTTTGACCAGGGTATGATCTCTTAATCTTCTCTCTGAGCTGATTCTGATTGTGGTTTTTC
CACACAGGTGTGTTGTATCCTGTTTGGACACTGGTTTTATTATGTTTAAGATGTTTCTTTTATTTTC
TTTTGAATTACTGATATTCTCATGTTTTCCTCCTTAGGTTGAGATGAAAACTCTTGCTTTGAATTCT
TCATGGAGGACTACATCATTTCAATCCTGAATCTGGCTCAATTCTATTATTCACTTATTACTGTATT
AAAAACGTTTAATTGGCCAGGCACAGTGGTTCACGCCTGTAATCCCAGCACTTTGGGAGGCCAA
GGCAGGCGGATCACTTGAGGTCAGGAGTTCGACACCAGCCTGGCCAACATGGCAAAACCCCG
CCTCTACTAAAAATACAAAAATTAGCCAGGCGTGGTGGCACGCACCTGTAATCACAGCTACTTA
GGAGGCTGAGGTGGCAGAATTGTTTGAACCCAGGAGGTGGAGGTTGCAGTGAGCCGAGATCAC
ACCACTGCACTCAAGCCTTGGCATCAGAGTGAGACTCTGTCTCAGTAAAAAAAAAAATAAATAAA
TAAAAATGTTTAATTAACCATGATATTTGCTTTGGTAAAACCTTTGAGGTTTAATGAGAATTTTAA
AATTCATTATACTTTTCTTATGGCATATAAAAGTTTTAATTTTTTTTATTGTCTCAACTAACATCATA
AATGTCTTAATGTTAAAATCCATGGGATATTCTATTTAAAATTTTAGCTTTATCACCTTTGTGCTAT
CAGAGAATGTGCTTTGAACTGGTAAAATGAGTTAAAGTATTGGAAAACACTTATGTCAGCAGAG
GCTCTTAAAAATCATTTTCATCCTTCCTCTTCTAGTGATGATTTTTTTTTAACCCATGTTTTCACCA

GTCTACCAATCCAATGGCATAAATGCTCCTACATAGGCAACTTAAGTGTTTCTTAAGTTGATGCA
AGGTATTTTGCATGAGGCTGAATTCTTGTTGTCGGAGCAGAGGATTTTTGTTCCAACTACTGAAT
TGGCAAAATGTTAAATATAAAAACACCTCAAAAAGTCAAA

ID|30414|gene_id|ENSG00000272086|transcript_id|ENST00000606994 Homo sapiens lncRNA

GCGCCCCCTGACCCGCGGTCCTGCAGTCCTGCTCCCGTGACGTGCCCTCCCTCCTTCCGTT
CCCCGTTTCCCCTTCACTACCCAGGGCTGGAGCCTGAACCTGGCGGGGCCCCCCGTGAATGGA
AGAGCCTGGGCCACGCGAAGGTGGACCCCACTCAGCCTCCAGCCGTGCCCTCCACGCCCCAG
ACACCGCCGTCTTCGGAGGCGACTCCGCGGTCGTGTGGACGGGGTCCCATTTGGACTCCGGGG
TCTGCAGGATCGGCAGGAAGGACTTTGCTCTCTGCTGCTGGGCGGGGAGGGGTACGCTATCTG
CCGGGACTCCCTAACCCTTTAGAAGGAGCAAGTGCGTCCAAATGGTTTGAAAGTGGACCTTTAT
TGTTTTCCAGGAGTAAGAAGATAGAACAGTTGACCAGTTTTGTTTGTAAAACTTCTTTCCTTGCCT
CGGACTTGTGCTATAAAGTGACCAGGGCGTAGTGAGTACAGTTAGTACCGCACACGTTAGCAC
GCTCGACCTTAACGATCTTTTTATAGAGACAATAAACCTGCAGCGTTTCATGCCACGTCTTTGTA
CGCATCGGATGTCTCTCCGTGTAGTTACTAGGTGAAATCCTCTTGGCGTGCGCAAATTAGGTTAA
AAAAATAGCTGTCAAACCTGAGACAGGAGTTGGAGGAGTAGAGGGTGGCGCTTGTTAAGTGAA
ACATTTAACCAGGAATGGTATTGTGTGCCATCCTGTATTAAACAATGTATGGACAGAGTGGAAC
AACAGCAAAGCAAAAAATAAAAATTTTTTAAAAAAAGTGA

ID|55893|gene_id|ENSG00000237253|transcript_id|ENST00000415104 Homo sapiens lncRNA

AGTTCTACTGGGCGCTATAGGAAGGGCGCACGCACGAAACTTCCTCCGTCCCTTGCAACC
TCAGGGCCCGCCCGGCAGGACGACTGAGCAAGGCCTTGGAAAACCGGAGAGATTGGAGCGGT
AAGTCGCGCTGGTCACGTTGGACACCTGCGCGTTGGGAGATTCTGGAGCCAGAAGGATAGCCG
AATGGCCTTCGCCCGCCCTGCCCCTCGCCTGCTTCAGAAACCCCCCGGAAACGTCCCGGTTGA
GACCCCGGCCCGAGCCGCCTGGGGGCCCTAGGGAGGCTGAACGCCCGGTGGCTCCCGGGATG
GCTCTTCCCGTTCTTCGCGCCGCCTTCACCCAGTGAGGGAGCCTGTGCCCTCCCTGCCCAGTCG
CTTTTGGGGCCGCTGCGGAGCTTCCGCTGCCATCTTCGGATCCTGTGTTCCGCACGGGGGCTCC
ACCAGGGCAGGGATCGTGGTGAGGGTGGCTCGTGGGTCCCCCTCGCGGGGAGCAGGGTCTGG
CACTCACCAGGGCGCAGGACTAGGACTTGTCGAATGAATCCATCCTTTTAGCTTTTAGTCCTTTG
AAGAGCCTTGAGAATGGAAATCATGAGAGATTTTTCCATGGGGAAGTTCCTTTTACAAAGCATTT
ATTTACGTGGACTTCTTGGCACCCCGCGGGGCGGCAACGGGCAGGGCCTCCAGTGCCCCTTCT
GCGCCGTGGAGCCGCGGGGGCTCAGCTGGGCGGTGGTCGGGTCCTGAGGCCGGAGGGCGGG
AGCAGGGGAGGGGAAAAGCAAAAGCGGGGAAAGAAGCCGGGGAGCGGTGGACCAGACGTCC
AGACCTCCTGAAAGGCTGGCGGGGAGGCACAGGCGGGATCTTCCGGAGGTGAGAATTTTTTTT
TATTATAGCAGAATGGGGAGGAATTGAGGGGAAAATGGAGATAGAACCTGAAAGAGCCCCAA
ACGCCAGAACCTGTAGCTCCCCAAGAATAAGATCTTCCAGAAGAACTAGACCCAAAACTAGCC
GTTGGGGAACACCGAAATCCTTGGAGGAGCAACATCCGCATGACCCTCTGTGTTCCTTTAGGCA
AAAGGACTTGCTTCCATTGTTTGTTCAATTGTTTGTGTTTGTTAAATAAATAAAACGATTTTCATGT

ID|55899|gene_id|ENSG00000230880|transcript_id|ENST00000400755 Homo sapiens lncRNA

ACTCCGCCATTTTCATCCGGAAGTGCGTCACCCAGAGGCGGTCTTGTAGCGGGGCCGGCT
TGGGGCTTGGTTCTATGTCCCTGCGGGTCGGTGCGAGGGCGAAGAGGAACCCGTGGGCCTCGG
GGGATCCCGGGGGGCCGGACCAGTGTCCCCTAGTTGTGGGAGCAGACGCGTGGGCGCATCGC
GGGCGGGCAGGGCCTGAAGTGCAGGTGCGGGCAGCGGACCCTGGCGGGGGCTGGGAGGACA

GGCGTGGGGTCCCGGCAGTGAAGCGGGTTCTAGAGGCGCAGGAGCGGGTAGGCGAGGCCGG
TGGCCCTGGGCCCGGAGTCTGCAGGCCGCGCTCCTGTCCTGCCGCTGAGGGACCCGGTTACCA
ACCCGCATGACGCTCAGTTTGCCCATCTCTCCCAGTGCTAACACACAGTTCTCGGGAGACGTTC
CCCATTCCCAGAGGAGTAGTGCGAAATGCGTGCGCCTCTAGTCTTAAACTTGGCGTTTGTATTAG
TTGGGTTTCCTGGTGTCTCTTTAGCAAGTGAAGTTTCTGGTTCCCTCCTTCACTGTGTGACCTGCC
TAGTCCTCCTGGGTTGCATTTACAGAAGTTTATACGAGACCTAGTTTCCAGGGAAGAACTCACTG
ATTCCGCGAGGGAGATGGCATAATGGATGATGGTCTTCAGCCTTAAGGGTACTTCAGTCTTAAC
TGTGTGTTACAAAGTTTGAAAGAGAGGGTTCCCTATGAATAAGAAGCGCACTTGAAAGAACAGC
TGTCTGGTCTAACCTCTCACTGGTGCTTCAGAGGAGGAAAAAAGGTCACAGGTGAGGATCCCA
GTTTTCCTCGCTCAGGAAATATTAATTCTACTCCCTAGAATGCACAAGATTTGCAAAGACTAGGT
GATAGTAGAAGGTTTGGACGAACTTTCAGAAGGTTGAGGTGTATTCCGCTGAGAAGAACAGGC
AAGGACCTAGGAAATATTCCTTATTTGAAGGGGTCTGAAAGTGTGGTCCGGGGTACAGGAGTG
ACCTGTCATACTTGAGAAGATTAAAATACTCTCCAAACACAGTCCCATTCCTTCAACCTTAGCTC
GTTTTTCCCAGCGTCTGAGATATATTAAACCTAGTCCATCCCCAAATTTAGCATTAGATTGCGAA
GTTCTATTGATTGTATTTGATTTGTAATTTAAGATTTTCTCCCCCTACGTAATTTTGTTAAAAACAC
AGAAGTGAATTCTGTTCACTTAGGTGTAACAGTTAATACTTGCTGTTTAAGGAACTAATTAAACC
TTACTGGCTTATAAAAAACAAACACCATTTTATTTGTTTGAAGTTCTGTGGATCTGCATTTTGGTG
TGGTGGGTTCAGCTGGGTAGTTGATATATTTGTGTTGCCTGGATCACAAAAAGTCCTTAGTCACC
TGGTGCCTTTACTGAGCCTGGTTGGTTTAAGATAGTTTCCTTCACAATCTGGTGGTTTGTGGTGAC
TCTTGGCTAGGCCCTGTGTCTCCAACAGGGTAGCTCCAGACCTCTTCACAATTTGACTGTGTCCA
AAATGGCAAGAACCAATGGATATTTGCATCACATTTTCCATTGTCCATTCACTGGACAAGTCAGA
TGGAAAAGCCCAATTTATTATCAGAGCATAATATGAGGGCTTGCATAGAAGGAAAGCTGTTATT
GGGAAACATGAGTACAATGGTGTACTGTAGGAAATACATATTATGTACATTTTAAAAAATGTAAT
TGTAGGCCAAAATTGCTGGTTTGCAAGATGCACTTTCCATGATGTTCAGATATAGAAAAGCAAG
ATGTACTGTCATGGGAACACTCATATGAAGTTATTTGTGGAATCTACATATAAATAGGAAAATAG
TTAATACAGCCTAGTATATTTCTATAACATTTATTTTAGTGAACTTATAATGTTTCTTTGCATTAAA
TTATTAGATTATATCTTTAGATCATATTGTTACTAAATTAATAGGTAATACATATTTTTATTCAAAA
ATAAATTGTGCATCTAATGTCTACCAATTAATGTACTTGTAGATGTATCTTATCTTAACTTGAGTCT
TTGATGCCCCTAATGAGGCGTGAAGGACTCTTCTCCCATGGGGAAGTTTTTCTTTTTCAGGAGGG
AGGAGAGCTTTCCCAGGTAATGTGTCTAGAGTGTTGGGCAGAAGAATCTGGGACTACACCACA
CCAGTTCTCTGCTTAATCCACGTCATTTGCCTTCTATCCCAGCTATGTTTCCAGTGTCCTCTGGGT
GTTTCCAAGAGCAACAGGAAACGAATAAATCTCTGGTGAGTTGTTTATTTGTTCTTCACTTTGTTT
TACATTGTATTTTCTGAGTTTTTGGGTGTCTGTGAATTAAAAAGGAAAAGTAGAAATAAGTAAAA
CTCAGGTTGAAGGAAATATACATAAATAAGATAAAGCTGACCTGTAGATATAGGCAGGTTATAA
GAGCTTAGAGTTGTCTAAGTTGGGTGCAAATTTTCCTCTGATCTTTCTGATGCCGAGACAAAAAA
GGCAGTCATATTTGTTACGTGATTGGAGTGGAACCCGAGAAGAGAACATGCTGTGTTCTTGTGG
GACAGGAAAGCTTGCGTGCACCAAGTCCGAACCACCACCTTCATTGGTGACATAGATTATGTGC
TGGAACATATTTCACACCGGCCTGGCAGTAACCACTTGTAGTGTTGTACAGTGGAAACGGTCAT
CTTCCGCTAAAGCACAGTGTGTTTTGCAGTGGAAATGGTCATCTGCCGCTAAAGCACAGCTTCC
ATCGTAAGATATGCTCCTTGCTCAAAGAGTGTGGTCCCAAACAGCTTTTGGGAGGTCCTCCTTGA
TTCATGGATGAAACCCAGAACATCTTGAGGACTGAGTTAACCATAGGTCCTTAAATAACTCTCCA
CACTTTTTCTTACTTTGTCTCTACATGCAGGGTGTGCAGCAGCCTGTTCAAAGTCATATTTTCTGG
GAAATATTTCCAGTGTTTATTTGCACTTTAGCCCACTCTGTGTAGTCTTAACTTATTTCTTCTAAAC
TCACCATTAACCTAAATAATAGTCAAATTTAGGGGGACTGTATTTGCCTTACTCGAGTCTTCTACC

ATAGTTGAAACTGTCGTACCTGAGTGAGAGAGAAACGCCACACTTTGAGACGAATTCAGGAGT
CCTTTATTAGCCGGTGACTGAGAGACGGCTAACGCACGAAATTCTCTCGGCCCCGAAGAAGGG
ACTAGATTTTCTTTTATAATTTGGTTTAGAGAGGGGAGGGGGGATTCTAGCTGCAGCAACTTTAC
AGAAGAAATAAACAGACAAAAAAGTTAAAAAGACAGATGGTTACAGGAAAACAAACTGTTCCA
GGTGCAGGGGCTTTAAATTCACCAAAAAGTGATAGGTGAGGGGGCTCTGGGCATTATCTGCCG
GACAAATGTGGGGGCTTTATGATACTATCTCTGAGTAATTTGCTGGGAACTGCGGACATCTCTTG
TCTCAGCACTTTATCAGTTAATTGCACTCTTTGATATGTTGAAAATCAGCTTGCACAAGTTAAAGT
CCTTGAGGAAAAGGGGTGGGTAAGAAGCCCTTGATGTTTTGTAAATGAAGGAGCCAAATGGAA
TTTGTCTGGTTTTCTCAGCTAAGGGAGAGTCTATTCATATTAAAAACAAGGTTAGCTATCTAAGG
AACATCTATTCATGTTAATACAACGTTGGGTATTACAAAACATCTGTTCATGATCTGGAAATTCTT
CTGTGTTAGTTCTGTTAAAAGAAAAACTTTAAAGGAGTTTAATTGAGCAATAAACGATTCACAAA
TCGGACAGTCCCCAGAATCACAGCAGATTCACAGAGACTCCAGCGCAGTCATGTGGTGGAAGA
AGATTTATAGACAAAAGGGAAATGGCATACCGAAATCGGAAGTGAGGTACAGAAACAACTCAG
CGTTTGCCTTGTTTGAACACAGTTTGAACATTTGGCAGTGCCTGAGTTGTTGAAGTTTGGCCATT
GGGATTGGCCAAGATGTAGCTGTTGTTCCAGGTGCATACTCTCAAGTTATTTTTTCATTCTTGTAT
ACCTATTAAGGTAGGTTGCAGTTCATCCACAAGTACTCATATATAGAATTATGGAGTCCTTCTCA
GGCCATACTTAGTTCACTTTAACAATGCCTTCCCTTTGGTTATTTTCTCAATTTTGAGAGATTGGC
CAAAACTTCAGTCACTGGTGTCACTATTACCATTGCAAATGTACTTACTTGGTTTAGAAACCCAC
TGGGAAATAGACCAGTGAGATTTGAAAAGGTGGAACAAGGACTTGAGTAGAAGGTATCTTCTTA
TGCTGGAACATCCTGTTTATAGGAGAAAAACAAAACCTGGTTTGTTCTAGGATTTATATGTTTCCT
TAAAGTCTTAGTTTGATTATGTTACATTTAGCATGAGTGACTCCATTTTGGTTTGGTTTGGTCTGTT
GGGACCTATTGCATGAGCTTAGTTCAAAACAATGGCCTCCCATAGTTTTGCTTAAAAAATTCCTC
CTTTTGGCTGGGCACGGTGGCTCACACCTGTAATCCTAGCACTTTGGGAGGCTGAGGTGGGCAG
ATCACGAGGTCAGGAGATTGAGACCATCCTTGCTAATATGGTGAAACCCTGTCTCTACTAAAAA
TACAAAAAATTAGCCAAGCGTGGTGGCAGGTGCCTGTATTCCCAGCTACTCAGGAGGCTGAGG
CAGGAGAATGGCCTGAACCCGGGAGGCGGAGCTTACAGTGAGCTGAGATCGTGCCACTGCACT
CCACTCTGTGGGACACACCAAGACTCTGTCTTAGAAAAAAAAAAATCCTCCTTTTCAGTCAAGTTC
TCACTTCGTTGAGAGTGTGACCAAAATATAGGGCCTTAGCATCACTCTTAGTTACCATTGTTTTG
GGTTCCGGTTTTAGCACATCATTCCCACTGTTTTGTGTTTCTGGTTTAGCACGTCACTCCCATTGT
TTTGGGTTCCGGTTTTAGCACATCACTCCCATGTTTTGGGTTCTGGTTTTAGCACGTCACTCCCAT
TGTTTTGGGTTCCTATTTAAGCACATCACTCCCATTGTTTTCATTTCCGGTTTTAGCACGTCACTCC
CATTGTTTTGGGTTCCAGTTTTAGCAAGTCACTCCCATTGTTTTGGGTTTCTGGTTTAGCATGTCA
CTCCCATTGTTTTGGGTTCCAGTTTAAGCACATCACTCCCATTGTTTTGGGTCTCTGGTTTCGCAG
GTCACTCATAGGTTACAGTGTCCTTATGGTTGCACATTTTTTTAAAATCTCTTGTCATTCCAGTTGA
AGAGATACCATTTGACATTTTAGAGATGCCTGCATGCAAACTCGTAAAACATCTGAGTAAGTAC
AGTGCACCAGGGAGACTCTTATGACTACTGGGATAATACCAAGAATTTGGTATATGCTCCTTACT
CAGGGTCCCCATAAATCAAACCACCTAAAATAAAATAGATTAAAGAATGAATTAGATAAAGAGT
TTACTTGCTTAACTAAGTGGGTTTTTTGTTAATTCCCTACAACCAAATTTTTATAATACCCCATGTT
TTCTCCACATGCTGTAAGTGTTAGCAGCTGCACAGATACTTAAGATAAGAGTCTCATGATAGTAG
AGAAGTCTTGATCTGTGATCTTGGGAAAAGCTGTTCACATTAAGGATGCCATCTTCTTCTGGGGG
GAACTGTCCTTGTTAGCTTTACCTTAAGGGTTCCAATGGGTATATGGTTCCAAGTGTGGAGGGAC
CCTTCTGAGTTGTGAAACTATGAACCCAAAGTTTAAGTTTTTAAAGTTTTGCTGTCATGTGGATGG
CAAGGGCAGTCCTTCTCTGATGTTCTCAGAAGATCCAGTCATCAGATTCTAGATTGTGAAGGGG
TTGACTGTCCCCAGTGAACCATAAAAGGCTTTCTTTACCTGGTGAAAATACACTTCAGGGTAATA

ATGTACTGTTTTAACATCAACTCTCTCGCATGGAAGAGCTTTTATACAATCAGAAAGCATGCACT
GAAAATGACAACTGAATGAAATCCCTTTATAAAATGTTTAAATGGCCCATCAGGTAATCAAATGT
ACCTGAAGTTTTGATTGTTTTCCTAGGAATATAGGTTTGACAAACCAAACATTGGTTATAAACTAT
TTTAGCAGTTTAGAAATCACCACACCAATATATTTAATTTGGATCATTTTCTCTTTCCATGATGAG
TTATGGAATGCAGAACTTTTAATAACAAAAGTTTTAAGGACTTAAGAAGGATAAGGTGGCCATC
CTGGTTCTTCATAAGTGTGTGCTTAATTAACATTAGACTTACATCCTCTTGAATACCAGCTGTTTC
TCCAAATTAGGTGCATGGCACTGGTAACTCATGAGTAGTTATAGGTAATTTGACTTAGACCATGG
AGTTTATTTAAATTATATATCTAAACAATTTCAATATTGGTGATTTAGCATGCAAATGTGGCAAAA
TATTTCCTTGGTATACAATTTTTGTTTTACTTGGGTTAGAAGTTTTATAAACCAGTTGGTCTTTTTAT
TAAAATTTTGGGATTTTTTTTTTTTTTGAGACAGAGTCTCACTGTGTTACCTAGGTTGGAGTGCAGT
GGCACAATCTTGGCTCACTGCAACCTCCACCTCCTGGGTTCAAGCAATTCTCTTGCCTCAGCCTC
CAGAGTAGCTGGGATTACAGGCACATACCACCACACCTGGCTAATTTCTGTATTTTTAGTAGAG
GTGGGCTTTCACCGTTGGCCAGGCTGGTCTCAAACTCCTGACCTCAAGTGATCCACTGGCCTTG
GCCTCCCAAAGTGCTGGGATTGCCGACGTGAGCTGCTGCACCCAGCCTAACTTTTGAGAATTCT
TAACCAGTCCAATTCTTGGGGTATCTTGGAACTTATGGGGAATTTTTACCCATGATATTAAAGTTA
TTAGAAATCTGTGTTCACAAGTGTTTTTTAGGGTCCTTTTCATTCTTTCATGAATCTTCTAAGAGAC
ACCATATTCTAGAATTTTGCATGCTTGTGAAGTTTTTAGAAACTGCATCACCATTAAGCAATTAAC
TGTGGAAATGACTTTAAATAGTTATAGTTAAAGACAATTGACAAGGAAATTTGGTTATTTTTGTG
GTCTACAATAACTTAATAACCATAATTAGGGTGGATGTGGTGGCTCATGCCTGTAATCCCAGCA
CTATGGGAGGCCGAGGTGGAAGGATCACGAGGTCAGGAGATGGAGATCATCCTGGCTAACAC
AGTGAAATCCGTCTTTACTAAAAATACAAAAATTAGCCTGGCATGGTGGTAGGTGCCTGTAGTC
CCAGCTACTTGGGAGGCTGAGGCAGGAGAATGGCATGAACCTGGGAGGTGGAGGTTGCAGTG
AGCCAAGATTGCACCACTGTACTCCAGCCTGGGTGACAGAGCAAGACTCCATCTCAGAAAAAA
AAAAAAAAAAAATCAAGAATTTTAGAAATCCTATACAATTTTAGAATGGATTGATGACATACACT
AAATATAACCTGAAGAAGGTTCAACATTATTTTTTATTTTGACAGTGCTACCCATGTGACTTAACA
TGTTAAATAGTCCTGTTTACCTCTCTTTTGGGTGCTTCAGGGGCCTCTGTAGTATCCCAAAGTTAG
AGGTCAGAAAAGACAATTTTGAAGTTGAAATTTGATTTTGGGAAGCCTATTAAATATATTAAAGG
TTTAAACACTTGATGTTATGAAATAGAATTCCACGTCACCATAAGTCATTCATTTACCTAAAATCA
TGACTTAAAAAATTTTTAAAGGGCAAAAATCTTTACTCATTGATAGGGGGAAGACTTATCTTCAC
AAACGATCTGCCTCTTGTTTTTCCTTTTTTTTTTTTTGGTAGTTTATTTACAAGGCAAACAAATTTTT
CATTTTTTTATTTTATTTTATTTATTATTATTATTATTATTATTATTATTATTATTATACTTTAAGCTTT
AGGGTTTATGTGCACAATGTGCCGGTTAGTTACATATGTATACATGTGCCAGTCTGGTGTGCTGC
ACCCATTAACTCGTCATTTAGCATTAGTTACATCTTCTAATGGTATCCCTCCCCCCTTCCCCCACC
TCACAACAGTCCCCAGAGTGTGATGTTCCCCTTCCTGTGTCCATGTGTTCTCATTGTTCAATTCCC
ATCTATGAGTGAGAACATGCAGTGTTTGGTTTTTTGTCCTTGTGATAGTTTACTGAGAATGATGAT
TTCCAATTTCATCCATGTCCCTACAAAGGACATGAACTCATCATTTTTTATGGCTGCATAGTATTC
CATGGTGTATATGTGCCACAATTTCTCAATCCAGTCTAACGTTGTTGGACATTTGGGTTGGTTCCC
AGTCTTTGCTATTGTGAATAGTGCCACAATAAACATACGTGTGCATGTGTCTTTATAGCAGCATG
ATTTATAGTCCTTTGGTTATATACCCACTAATGGGATGGCTGGGTCAAATGGTATTTGTAGTTCCA
GATACCTGAGGAATCACCACACTGACTTCCACTATCGTTGAACTAGTTTACAGTCCCACCAACA
GTGTTCCTATTTCTCCACATCCTCTCCAGCACCTGTTGTTTCCTGACTTTTTAATGATTGCCATTCT
AACTGGTGTGAGATGGTATCTCATTGTGGTTTTGATGTACATTTCTCTGATGGCCAGTGATGATG
AGCATTTTTTCATGTGTCCTTTGGCTGCATAAATGTCTTCTTTTGAGAAGTGTCTGTTCATATCCAT
TGCCCACTTTTTGATGGGGTTGTTTGTTTTTTTCTTGTAAATTTGTTTAAGTTCATTGTAGATTCTGG

ATATTATCCCGTTGTCAGATGAGTAGGTTGCGAAAACTTTTTCCCATTTTGTAGGTTGCCTGTTCA
CTCTGATGACAGTTTCTTTTGCTATGCAGAAGCTCTTTAGTTTAATTAGATCCCATTTGTCAATTTT
GGCTTTTGTTGCCATTGCTTCTGGTGTTTTAGACATGAAGTCCTTGCCCATGCCTATGTCCTGAAT
GGTAATGCCTAGGTTTTCTTCTAGGGTTTTTATGGTTTTAAGTCTAACATTTAAGTCTTTAATCCAT
CTTGAATTAATTTTTGTATAAGGTGTAAGGAAGGGATCCAGTTTCAGCTTTCTACATATGGCTAG
CCAGTTTTCCCAGCACCATTTATTAAATAGGGAATCCACTGCTTCTTTTTGTCAGGTTTGTCAAAG
ATCAGATAGTTGTACATATGTGGCATTATTTCGGAGGGCTCTGTTCTGTTCCATTGGTCTATATCT
CTGTTTTGGTACAAGTACCATGCTGTTTTGGTTACTGTAGCCTTGTAATATAGTTGGAAGTCAGGT
AGCGTGATGCCTCCAGCTTTGTTCTTTTGGCTTAGGATTGACTTGGTGATGCAGGCTCTTTTTTGG
TTCCATATGAACTTTAAAGTAGTTTTTTCCAATTCTGTGAAGAAAGTCATTGGTAGTTTGATGGGG
ATGGCATTGAATCTATAAATTACCTTGGGCAGTATGGCCATTTTCATGATATTGATTCTTCCTATC
CATGAGCATGGAATGTTCTTCCATTTGTTTGTATCCTCTTTTATTTCATTGAGCAGTGGTTTGTAGT
TCTCCTTGAAGAGGTCCTTCACGTCCCTTGTAAATTGGATTCCTAAGTATTTTATTCTCTTTGAAG
CTATTGCGAATGTGAGTTCACTCATGATTTGGTTCTCTGTTTGTCTGTTATTGGTGTATAAGAATG
CTTGTGATTTTTGTACATTGATTTTGCATCCTGAGACTTTGCTGAGGTTACTTAGCTTAAGGAGAT
TTTGGGCTGAGACAATGGGGTTTTCTAGATATACAATCATGTCATCTGCAAACAGGGACAATCT
GACTTCCTCTTTTCCTAATTGAATACCCTTTATTTCCTTCTCCTTCCTGATTGCCCTGGCCAGAACT
TCCAACACTATGTTGAATAGGAGTGGTGAGAGATGGCATCCCTATCTTGTACCCGTTTTCAAAGG
GAATGCTTCCAGTTTCTGCCCATTCAGTATGATATTGGCTGTGGGTTTGTCAAAGATAGTTCTTAT
TATTTTGAGATACGTCCCATCAATACCTAATTTATTGAGAGTTTTTAGCATGAAGCATTGTTGAAT
TTTGTCAAAGGCCTTTTCTGCATCTATTGAGATAATCATATGGTTTTTGTCTTTGGTTCTGTTTATA
TGCTGGATTACATTTATTGATTTGTGTATATTGAACCAGCCTTGCATCCCAGGGATGAAACCCAC
TTGATCATGGTGGATAAGCTTTTTGATGTGCTGCTGGATTCGGTTTGCCAGTATTTTATTGAGGAT
TTTTGCATCAATGTTCATGAAGGATATTCGTCTAAAATTCTCTTTTTTGGTTGTGTCTCTTCCCGGC
TTTGGTATCAGGATGATGCTGGCCTCATAAAATGAGTTAGGGAGGATTCCTTCTTCTTCTATTGAT
TGGAATAGTTTCAGAGGGAATAGTACCAGTTCCTCCTTGTACCTCTGGTAGAATTTGGTTGTGAA
TCCATCTGGTCCAGGACTTTTTTTGGTTGGTAGGCTATTGATTATTGCCACCATTTCAGCTCCTGT
TATTGGTCTATTCAGAGATTCAACTTCTTCCTGGTTTAGTCTTGGGAGAGTGTATGTGTCAAGGA
ATTTATCCATTTCTTCTAGATTTTCTAGTTTATTTGCGTAGAGGTGTTTGTAGTATTCTCTGATGGT
AGTTTGTATTTCTGAGGGATCAGTGGTGATATCCCCTTTATCATTTTTTATTGTGTCTATTTGATTC
TTCTCTCTTTTTTTCTTTATTAGTCCTGTTAGCAGTCTATCAATTTTGTTGATCCTTTCAAAAAACCA
CCTCCTGGATTCATTAATTTTTTGAAGGGTTTTTTTGTTGCTATTTCCTTCAGTTCTGCTCTGATTTT
AGTTATTTCTTGCCTCTGCTAGCTTTTGAACGTGTTTGCTCTTGCTTTTCTAGTTGTTTTAATTGTGA
TGTTAGGGTGTCAATTTTGGATCTTTCCTGCTTTCCCTTGTGGGCATTTAGTGCTATAAATTTCCCT
CTACACACTGCTTTGAATGTGTCCCAGAGATTCTGGTATGTTGTGTCTTTGTTTTCATTGGTTTCA
AAGAACATCTTTATTTCTGCCTTCATTTCGTTATGTACCCAGTAGTCATTCAGGAGCAGGTTGTTC
AGTTTCCATGTAGTTGAGCAGTTTTTAGTGAGTTTCTTAATCCCGAGTTCTAGTTTGATTTCACTGT
GGTCTGAGAGACAGTTTGTTATAATTTCTGTTCTTTTACTTTTGCTGAAGAGGCCTTTACTTCCAA
GTATGTGGTCAATTTTGGAATAGGTGTGGTGTTGTGCTGAAAAAAAGGTATATTCTGTTGATTTG
GGGTGGAGAGTTCTGTAGATGTCTATTAGGTCCGCTTGGTGCAGAGCTGAGTTCAATTCCTTGGT
ATCCTTGTGAACTTTCTGTCTCATTGATCTGTCTAATGTTGGCAGTGGGGTGTTAAAGTCTCCCAT
TATTATTGTGTGGGAGTCTAAGTCTCTTTGTAGGTCACTCAGGACTGGCTTTATGAATCTGGATG
CTCCTGTATTGGGTGCATATATATTTAGGATAGTTAGCTCATCTTGTTGAATTGATCCCTTTACCA
TTATGTAATGGTCTTCTTTGTCTCTTTTGATGTTTGTTGGTTTAAAGTCTGTTTCATCAGAGTCTAG

GATTGCAACCCCTGCCTTTTTTTGTTTTCCATTTGCTTGGTAGATCTTTCTCCATCCTTTTATTTTGA
GCCTATGTGTGTCTCTGCACGAGACATGGGTTTCCTGAATACAGCACACTGATGGGTCTTGACTC
TTTATCCAATTAGCCAGTGTGTGTCTTTTAATTGGAGCATTTAGTCCATTTACATTTAAAGTTAATA
TTGTTATGTGTGAATTTGATCCTGTCATTATGATGTTAGCTGGTTATTTTGCTCGTTAGTTGATGCA
GTTTCTTCCTAGTCTTGATGGTCTTTACAATTTGTCATGTTTTTGCAGTGGTTTGTACCGATTGTGC
CTTTGCATGTTTAGTGCTTCCTTCAGGAGCTCTTTTAGGGCAGGCCTGGTGATGACAAAATCTCT
CAGCATTTGGTTGTCTGTAAAGTATTTTATTTCTCCTTCACTTATGAAGCTTAGTTTGGCTGGATAT
GAAATTCTGGGTTGAAAATTCTTTTCTTTAAAAATGTTGAATATTTGCCCCCACTCTCTTCTGGCTT
GTAGAGTTTCTGCCAAGAGATCAGCAGTTAGTCTGATGGGCTTCCCTTTGTGGGTAACCCGACC
TTTCTCTCTGGCTGCCCTTAACATTTTTTCCTTCATTTCAACTTTGGTGAATCTGACAATGATGTGT
CTTGGAGTTGCTGTTCTCGAGGAGTATCTTTGTGGTGTTCTCTGTATTTCCTGAATCTGAATGTTG
CCCTGCTTTGCTAGATTGGGGAAGTTCTCTTGGATAATATCCTGCAGAGTGTTTTCCAACTTGGTT
CCATTCTCCCTGTCACTTTCAGGTACACCAATCAGATGCAGATTTGGTCTTTTCACATAGTCACAT
ATTTCTTGGAGGCTTTGTTCATTTCTTTTTATTCTTTTTTCTCTAAACTTCCCTTCTCACTTCATTTCA
TTCATTTCATCTTCCATCACTGATACCCTTTCTTCCAGTTGATCGCATCAGCTCCTGAGGCTTCTG
CATTCTTCATGTAGTTCTCCAGCCTTGGCTTTCAGCTCCATCAGCTCCTTTAAGCACTTCTCTATAT
TGGTTATTCTAGTTATACATTCGTCTAAAGTTTTTTCAAAGTTTTCAACTTCTTTGCCTTTGGTTTGA
ATTTTTCCCTGTAGCTCGGAGTAGTTTGATCGTCGGAAGCCTTCTTCTCTCAGCTCGTCAAAGTC
ATTCTCTGTCCAGCTTTGTTCCATTGCTGGTGAGGAACTGCATTCCTTTGGAGGAGGAGAGGTTT
TCTGCTTTTTAGAGTTTCCAGTTTTTCTGCTCTGTTTTCACCCCATCTTTGTGGTTTTATCTACTTTT
GGCCTTTGATGATGGTGATGTACAGATGGGTTTTTGGTGTGGATGTCCTTTCTGTTTGTTAGTTTT
CCTTCTAACAGACAGTACCCTCAGCTTAAGGTCTGTTGGAGTTTGCTAGAGACCCATTCCAGACT
CTGTTTGCCTGGGTATCAGCAGTGGTGTCTGCAAAACCATGGATTTTCGTGATCTGCGAATGCTG
CTGTCTGATCGTTCCTCTGGAAGTTTTGTCTCAGAGGAGTACCCGGTCGTGTGAGGTGTTAGTCT
GCCCCTACTGGGGGGGGGTTCCTCCCAGTTAGGCTGCTCAGGGGTCAGGGGTCTGGGACCCAC
TTGAGGAGGCAGTCTGCCCATTCTCAGATCTCCAGCTGCGTGCTGAGAGATCCACTGCTCTCCT
CAAAGCTGTCAGACAGGGACATTTAAGTCTGCAGAGGTTACTGCTGTCTTTTTGTTTGTCTGTGC
CCTGCCCCCAGAGGTGAAGCCTGCAGAGGCAGGCAGGCCTCCTTGAGCTGTGGTGGGCTCCAC
CCAGTTTGAGATTCCTGGCTGCTTTGTTTACCTAAGAGAGCCTGGGCAATGGCCGGTGCCCTTCC
CCCAGCCTCGCTGCCGCCTTGCAGTTTGATCTCAGACTGCTGTGTTAGCAATTAGCAAGATCCC
ATGGGCGTAGGACCCTCCGAGCCATGTGCAGGATGTAATCTCCTGGTGCGCCGTTTCCTAAGCC
CATCAGAAAAGCACAGTATTAGGGTGGGAGTGGCCCAATTTTCCAGGTGCCATCTCTCACCCCG
TTCCTTGACCAGGAAAGGGAACTAACTCCCTGACCTCTTGTGCTTCCCGAGTGAGGCAATGCCT
TGCCCTGCTTCGGCTAGTGCACAGTGCACTTCACCCACTGTCCTGCACCCACTGTCTGGCACTCC
CTAGTGAGATGAACCCAGTACCTCAAATGGAAATGCAGAAATCACCCATCTTCTGCGTCGTTCA
TGCTGGGAGCTGTAGAGTGGAGCTGTTCCTATTCGGCCATCTTGGCTCCTCCTCCCATTATTTTTT
AATATTTTCTGAAAATCTTCTTTAAAGAGAGAAAGCCAAATGTCACCCACTTTTTCATAAAACCTT
ATAGGCAAATCTATTATTCTTTTTTTTTTTTTTGAGATGGATTTTCCCTCTCGTTGCCCAGGCTGGA
GTGCAATGGTGCGATCTCGGTTTACTGCAATCCCCTGCCTCCCAGGTTCAAGCGATTCTCCCGC
CCCAGCCTCCTGAGTAGCTGGGATTAGAGGCATGCCCCACCATGCCCAGCTAATTTTGTGTTTTT
AGTAGAGACAGGGTTTTTCCTTGTTGGTCTGGCTGGCCCTGAACTCCTGACCTCAGGTGATCCA
CCTGTCTCGGCCTCCCCAAGTGTTGGGATTACAGGCGTGAGCCACTGCCCCTGGCCATGTTTTTT
TTTAAAGATAGCGTCTTGCTCTGTCACCCTCCTCACCACATTATAGCTCTGGGGGCCAAGCTGCA
TCACAATGGAAATCATGGAGCCACAGGAAGAATCCACTCAGCTTTGCAAGATGCTGCCCAAGG

GGTTGCTTGGAGTAACCAAATTAACATTTTTCATTCTGCTCAGAGCAAAATACATGTGACAAAAC
ATAGACACGAGCCACTTTGCTTAGCACACAGTGTCAAACTGGTAAGACTCAAACTTGCTCCCAG
ATAGGCCGTGCCATCTCTAAATCTTTTTAGAAGCTTCTGCATATTAATAGGCATCCCTAAGTGAG
ACTAATTTGGGAGCCATCATTTTTAAATGCACTTCATGGCATTATTCATTTGGAATGTTCCACTGT
AAGTTATCTTTAGTAAGATTTTGCCATTTCTGTAAGACTTTGCTGCTTCCCAGGCCTAATGAATTA
GCCAGAAGGAACTTAGTTTTCCAGAAATTAAGGATCCTATTTTTACCTAATATATTGGCTTTACTC
CCAGGTTCCCTTGATTGACTTAGCCAATGATTTTTTTTTCCTACCTAAGCATGCGAGGAAAATGA
AACAAAGGGGTAGAACACAAAAATCCCTGTGAATTTTGAAAAGCCAAATTTTACAACCCTCCAA
TATTATCATTTGCTACCACTTTCCTTCTGACCCATTCAGATGTAGGGGGCCTCTAACTGGAACTG
GATTCAAGCCAGTTAACTACTGGATCAAATCTGATCCTGGACCCGGTCCCGTTTCTGTCATAACT
TCTAAAACATCCAGCCAGTCATGGCTGGATAGCAGTTTGGAACAGAAATTTGCTCAAAGAAACT
CAGAGCTCAAAACACAAATCCATGGAGCTCTGAAATCCGAGAGAGAATTTACCACGATCCCCA
GCTGCTCTGAGAGGTCAAAGGGCACAAGTGTTACAGAATCCTGAGGCATCAATTTTCTGCCTGA
AACCTCTGGCTGGTGGCACATTTACCTGTGTTTTGCTCGAGCCCACTGGGTTCGTTCTGTCCACT
CGGCTCATGCTAGTGGTCTGGATCCCACACCTGCCAAGGGTGAGCTGGGTACAGAGCAGTGAA
GGGTGTGTGAGCAAGCGAGCATGGGATCTGGCCACTGCACACAGCCAAGCATGCCAGCTGCA
GTGGTGTGGGCAGCTCCAGGCACCGGCACAGGTGCCAGCTCCCTGTGAGGCTGCAGCTGGACC
AGGCCGACTGCAAACAGCTTCCACTGTGGGTATCAGGGAATGCAGTGGTGCCCAGAAGCTTAG
AGATGCAGGAACTGCAGAGCCCCAAAGAAGGTGTCACAGCCCTGGCTTGGGGAGCTCCTAGGT
CTGGGCTCCCTGAAGGGCCACAGCTCTTCTCTCCTTCTCTCTTCTCTTCTTCTTGCCTGCAATTTG
GCAAGCAAGGGGTGCGTTTCAGCCCTGTTTATGTTGCAACTCTTTCAGCCCTGCTAGTTGGCAG
GTCCCGAGTTCTTGTCCTGAGTCCAGGAAGAATGAGGTATGTGGGCAAGTAGAAGGTGAGCAA
GGTGAAGAGGTGCTTTATTGAGCAACAGTACAGCTCAGAGGAGACCTGCAGTGGGTAGCTCCT
TTCTGCAGGCAGGTCATCCCAACGTCTGTTCAGCTCTCAGCAGCTGAGAGAGACGCACGGTGGT
TAGCTGTGCACACGATGCCCAGGCTGTTCGAGCTGAGGAGTGCCTTCAGGCCAGTGCTGAGCC
ACCCTTAGCCCCACCTCAACCTCCCTCCTGTGCTCGTCAGTGCCCAAAGTCTGGAGGGGGCTGA
GGTGGCAGGGGGCTGGCATGTCAGCACTGCCCTGAGCTTGCACAAACTGGGCTGGGTTGCGAC
TGTACCCGGGTTCAGCCTCAACTTGGATCCGAAGTTGGAGTGGGCTCTGGGAGCGGAGACGCC
AGGTGGTGGGACCAGGTACAACTGAGCCTGCGGGGCAGGGGGGCTTGCTGGGCCTCTGAGAG
TGCAAAGATGCCCGGGTTTGCAGTCATGGCTGGATGGCTGCAGCTGTGCCTGGGAGGGCGGGG
CTCCTGCCTACCAATTTAGAAGGGGTGGGGCTCCCCCCCCCGTTCCTGGCTCCCACCAGCTTCG
AGGAGCGCACAGCCCCAGCCACTCCTCCCCACAGCAGCCAGTGTCTCCGTAGCAACTTCTCCA
CGTGGGCCACTGCTGCCATCACAGAGCAGTCCTTGCAGGTGCCTTTCTTGTACCTCAGCACTCC
TGGGGGTCATTAGAAGCCCTAGCAACACTGCTCACCACACTATAACTCCGGAGGCCCTAGCAG
TCCTGCTACCACAGATCCCACTTCTGATACCATCTATTAAAAGAAAATCTTCAGCTGAATTAAAT
TTAAAGGAACTTAATTGAGCAATGAATGATTCACGAATCAGGCAGCCCCCAGAATCACAGCAG
ATTTGGTGAGACTCCAGCACAGCTACATGGTGGAAGATTTATAGACAATAAAGGGAACGTGATG
TACAGAAGTCTGAAGTGAGGAGTGAGGTCCAGAAGCACCTGAGTCTGTTACAGTTCTCAGCAGT
GAGGTCCAGAAGCAACTGGACTGGTTACAGTGCTCAGCATTTGCCTTATTTGAACACAGCTGAA
CACTCAGCAGTGTGTGAGTGGCAGAAGTTTGGCTGTTGGGATTGGTCAGGACTCAGCTATAGTT
ACAGGCGCATACTCCTAAGTTAGGTTTTCAGTCTTTCTACCTATTAAGTTAGGTTGCAGTTTGTCC
ACAGGGACTCAAATCTAGAAGTACAGAGTCCTTCCCAGGCCATATTTAGTTCACTGTAACAGTT
CCTATTATGACCTCACTGACAGTTCTTTTTCTCTGAATTCTCCTTTCTTCTCAACAGCTTATCCAAA
TGTTCCGTTGGTCCCTGTTCATCCCGCCCTGCAGTTCTCCTTGACTGATTCAGCCCTTTGTGGTTT

GCAGTCCTGTTTCTCTACAGCTTGGACCCCTTCAGTCTTTCCATCATAAGTTTAGCTCTCTGTTGA
ATGCTTCTTTGTAGCTACACAAAAGTTACCTTAAGCTCAAAAAATTCAAAGTGAAAGCCACATCC
TCCTCTCTTCCCTTATGTATATGGTATTACTACCATGCAGCCAGTGACCCAAAATGGGATTTCTTC
TGGGCTTTTCTTGGTTAGATTCAGGCTCATCTGGTGTCAAGCTTTGTTACTTTTGTTTCCTTGTTCT
TTTATTTTTAATTTTTTTTCTTTTGAGACAGAGTTTCACTCTTGTTGCCCAGGCTAGAGCGCAGTGG
TGTGATCTTGGCTCGCTGCAGCCTCCACCTCCCGGGTTCAAGCAATTCTCCTGCCTCAGCTCCTG
AGTAGCTGGTATTACAGGCATTTGCCACCACGCCCGGCTAATATTGTATTTTTAGTAGAGATGGG
GTTTCTATGTTTTGATCAGGGTGGTCTCGAACTCCTGACCTCAGGTGATCCACCCGCCTCAGCCT
CCCAAAGTGCTGGGATTACAGGCGTGAGCCACCGTGCCTGGCCTGCTCGTTGTTTTCATCTCAT
CCTGATTTCTGAATACAGGAGAGGAGCTGAGTTGGTGTTCACTAACAAGCACGAAAGCTTTGTT
ATATTTACAGTGTCATTCTTGGCAAAACCTGAATGGTATGTTTGTGGGGTGATGAGATTCAGTCC
CCTGTGACCTGTACATCTGGCCAAAACTGTGGTGACATCCTTAGGAATCCATGGGGAGAGAGA
AAGCATTCAGGAGTTAGTGGGTCACGTTTGACAAGCGCCAATAAAGAAATATGCAAAGACAAA
AAACAAGAAGAACATTGTCATATTTTCTACCTTTTGTCTATATAAATTTATGTCAATGATTCTAGCT
TATGTTAATATGCAATGTATACAATATGCTAACATATACAATATGTTTTTATAGTTTAAACATTTCT
GTCATGTTTTCAGATTCTTTAAAGATTATATTACGCTTCCTATTTCAGATAGCTGTTTAAAATGAGT
AAGGAAAAACGGATGTGTATATCAGTTCTAACTGTTTATGGACTAAAACTAATTGATTTCTTGGT
TAAGAACAAAAAGTGACAACCTAATTACTGAAAATGTTAAGTAGGCAATTATAGTTTTAGCTTTA
ACGTAAAATATTAACTATGCTCCTTTCTTGCATTTTTAACCTAATACTCAATATAAATCGCCACAT
GCCATGTTTCAGATCAAGGTTCTACTTGTGATCTCTCATGAGTTTTTCAAGGTTTTAATTATCTGA
GATGTAACAATGTACCAGTAACCTTACTGGCTTAAACCAGGAATTTATTCTTTTTACATGTCACAA
TTTTCTTTGTCAAGACACTGGACAGGGTGGTGTGGGTTTGTTGCTTCATGATCTCCCTGGTCTCAT
CTGTAAGGACTCTAGTGGCTGGGGACGTGAAGCAGGCACCCAGAAGGACTCTAGTGGCTGGG
GACATGGAGCAGGCACCCAGCCCTCTCTTTGTGGCCAGCACGGACTTCCTCCCAGTCTGGCAGT
CAGGTAGTCAGGTTTGTCTGGCTTCTCCCAGGGTGTGTGTCCAAGAGGCCCAGGCAGAAGCTGT
AAGGTCTCTCATGATCATCCCTCAGAAGTCCCAGAGCATCTCTCCTGCCACACTGTCCAGTCGTA
CTCATCACTGAGATCAGCCATGATTCCAGGTGGGAAGGTGATTAGATTCCACCTCTTGATGAGA
AGCATAGTAGGAACCTGCAGCAGTCTTTCATAAACCACAGCTTGTCCTCTGGCCACAAACTATT
AACGTTTCTCCCACATGGAAATTATGCTTTGCCCCTCTCAAGAGCCCCAGAATGGTTTTCCTTAT
GGCACTGGCTGGTAGCCCAACTGAATCCTGAATCAGGTTGTGGTGGCCTGTCATCTGCACCCAC
ACACACAGACACAGTGAGGACTGAATCAGGTTGTGGTGGTCTGTCATTTGACCCCCGACACACA
GCCACAGTGGGGACTGAATCAGGTTGTGGTAGCCTGTCATCTGACCCCCCACACACAGCCACA
GTGGGGACTGAATCAGGTTGTGGTGGCCTGTCATCTGCGCCCGACACACACAGCCACAGTGAG
GGGACTGCTGTGAAAACAGTCGACATTTCCCTTTAGAAGCTGTTGGTGGGAGGCAGGAGGGAG
GTGCTGCCCTGCAGGCCCCGTTTAACAGTTGGTCATTCCCATGGGGCGCCTGTTACAGTTCTGT
GATTAGTGCCCAGTCCTGGTCCCTGAGAACGGCGCCCAGTCCCGGTCCCTGAGAATGGTGTTTG
TGTCCTTTTACTCCTCCCTCTGGGCTTTTGTCATTCTCCATGTTCTTTTTCCTTCAGTGTCTGGGTTG
CTGTTGACCAACTTTCCCTGCCTTTTTCTTATGGTCAATAGGGTATTCAATGGCTTCTTTTTCATTT
TTTTTTCCTTTTCTTTTCTTTTCTTTTTTTTTTTTTTTTTACTTTGGCCTTTTGAGACAAGAAATTATTTC
TTTATATTTTCTCTAAATTCTGTTTGAAAACTGAACCTCCTTCTTTAGATCATGTCCCTCTCCTGTC
ATATTTATTCAATGACAGTTAGGGGAGGCTGGTAGCACTTTCCATGTTCTTCCCAGATGTCTCCTT
AGGCAGATCCCTGAGATGATGCAGTGCCCTTTCAGTTTCCATGTTGTGGTCGTAGTTTTCCCACA
GTCCCTCAACACGTAACTCTCAGGCCTTTTCTCCAGTTTCCAATGACATTTTCTCACCGTCCTTCA
GGCCCTGACCAAGAGTCTTGATGCCCTTCCAGGTTGCATGAATGGTCTCCTTGAGGCCCAGTTA

CAGGTCAGCCTCACAGTCGTGGCACATATTGTAGCTTCTGATTACCACAGCAGCTCATTTCCAGC
TGCCATATTCTGTTCCAGTTATCTGTTCTGAAGAAACCATCCCCAAAAGTTGGCAGCTTAAAACA
ACTCATTATTACTTGTTGTTTGGCTTAGAGAGTCTTGGTGGCCAGCTCATCTCACACACATTTGCA
GCCAAGCTGGATTGTGTGAAAGCACAGTGGGGTGGTGTGCAGGGTGGCCCACTAGTGGTTGGG
AGTGGATGTTGCTGGAGGCTCACTAGTTGTTGGGAGTCGGTGTTGCTGGAGGCTCAGTGGGGG
ATGTCAATGCGTGTAGCTAGTCATGGACTGGCCATGTGGTTTCCATCATGAGGTCTCAGGGGAG
TGGGATTTCCTGCCTGGTGACTGGCTTTCTCCTGGATAAGTGTTCTGTTTTCTCAGCCTGGCTTCT
GAAGTCCCCAAATACCACCTTTGTCACCTTCTGTTGGCCAAACAAGTCAGTAGTCTGGGCAAGG
TTTAAGGGGAATTGGTTCTCACAGAGACAGGAGCAGGAAAGAATTTGTCACCTTTAGTCTACCA
GAAATGAGATTTTTATAACAAGTTTGTTCCAAATACATTCCAGTTCCCCTTGTGAATACTTTTTTG
ACTCACAGGGTATTTCAAAGTTTATTACTTGGCTTTCAGACATTTGAGGCTTTTCTGGATATCAAT
TTGTTGTTGGTTTCTAATTTAATTTCAAGTGTTCAGACAACATACTTTGTATACTATTTCAGGCTTG
AACCTTTTCTCAATCGATCGACATAGAGTCTATCTTGGTACTGCCAAGTACCATTTGGGTCAGGA
TTTTGTCATTTAGATCCGTATTTTTCCTATATTTTTATCTGGTTGTTCCATCAGTTACTGAGAGAGC
AGTATTAATTCACCAGCTATAATTTTGAATTGTCAATTTCCTGCTTTTGTTCTGTTGTTTTTGATTCA
CATACGTTGAGGCTCTGTGTGTGTGTGTGTGTGTGTACTTTGTGTGCACTTTGAGGCACAATTTATAA
TTGTAACATCATCGTCTCTGATTCTTTTATTTTTATTAAATTACCCTGTTTATTTCTGGTGATATATT
TTGTTCTGAAGCCTCTTTCATCTAGTGTTAACATCTCTGTTGAAGCTTTTTCTGATTAGTGTCTGAA
TAGCATATTTTTACGATTAGTGTCTGCATAGCATATTTTTTCTCATACTTTGTTTGTGTCTTTGTGTT
TAAATTGTGTCTCTGTGGATGCCATATTGTTGGGTCTTGCCTTCCTCTCAGGTCTGGCAGTCTCTG
TCTTAAGTAGAGTATTTGTCCAGTTACATTGTAACTAATCATTGCTAAGGTTGGATTTAGGTCTGC
CATTTTTCTAGTTATTTTCTATTTGTTTGTTTATTATTTTTAAGACAGGGTCTTGCTCTGTCACCCAG
ACTGTAGTGCAGTGGTGCAATCTTGGCTCACTGCAAACTCTGCCTCCCAGGTCCAACCAATCCT
CGCTTGAGCCCCCTGAGTAGCTGGGACTACACGTGCATGGCACCACACCTGGTTAATTTTTATAT
TTTTTGTAGAGATAGGGTTTTGCCATGTTGCACAGGCTGGTCTTGAACTCCTGAGCTCAAGCAAC
CTACCCACCTTGGCCTCCCAAATTGTTCAGATTACAGGCATGAGCCACCATGCCTGGCCTTCATC
TGTCTTTTGATCTTCTATATATTCTTTCCTAACTTCTTTTGGGTTAAATATTTCTAAATATTCCAGTT
TGATTAATCTTTTGGCTTTTTGAAATAATTTTTTATAGGCTGGGCATGTTGGCTTATGCTCGTAATC
TCAGCTCTGTGGGAGTCCAAGGGAGGTGGATTGCTTGAGCCCAGGAGTTTGAGACCAGCCTGG
GCAACATGGCAAAACCCTCTCTACAAAAAAACCAAACCAAAATTTAGACTGACATCTTGATGTG
CACCTGTAGTCCCAACTATTCGGGAGGCTGAGGTGGGAGGATTGCTTGAGCCTGGGAGGTTGA
GGCTGCAATGAGCTGTGATCATGCCATTGCACTCCTGCCAGGGCAACAGAGTAAGAACCTGTGT
CAAAAGGATCATTTTTTATAAATAATTTATTATTTCGAATTTTGGTAACAAACACATACCTTAAAA
TTTACCATCATAACCAGTTGTAAGTATAGAGTTTTGTAGAGTTAAGAATATTTGCAGTGTTGTGTA
GCAGATTTCTAGATTTTTTTTTTTTATCCTGGAAAAGTCTATACCCATTCAAGAACTATTTATTTCC
CCTTCCTTCCACCTCCTGGCAAGTACTATTCTACTTTGTGTTTCTAAAAATTTGGCTTATATACCTA
GGGTTATATAATATTTGTTGTTTTGTAAGTAGGTTCCATGTTATGTGTCAGATGTGTCAGGATTTT
CTTCCTTTCTATGGCTGAATAATATTTCTTCATATATATATATATATATATTTCTCTATCTATATATA
TATCTCCTTTTGTTTATCCATCTATTCCCGGATGGACGTTTTGGTTTCTTCCACCTTGTGGCTGTGT
AATGCTCCTGTGAACACATAGGTGTGAACATATCTGTTTGAGGTCCTGCTACTAGTTATTTTGTCT
CTGTAGAAGTTGGATGGCTGGATCATATGGTCATTTTATTTTATTTTTTTTGAGGAGCCAGTTCAT
ATTTCCACCAACAGTGTTCAAGGGTTTCAGTTTCACCTGCACTTGTTACTTTCTGTTGGGTTTGAA
GTGATGTCCCATTGTGGTTTCTATTTGCATTTCTCTAATGACTAGTGATGTTACACATCTTCTCATA
TATCTCATGTATCTGTTGGCTATTTGTATATCATCTTTGCATCTTTGGATAAATGTTCTTTGTCCATT

TTTTAATCAATTTATTTTGTTGTTGTGTTGTAGCGGAGTTTTTTGGTCATGACCATTCATTTATTTCA
CAGTTCATTCTTGTTACTTGGGCCAGGGTCATGATCATTCATTATCTCTCAGTTCATCCTCATTAC
GTTGGGCAAACAGTCATGCTGCAGGGTATAGATTATGTTATTCTGTTACTTTCAGGTAGAATTGG
GGTCTAGGTTCTAATTGTTTCTAAGTTTAGATTCTGAATGAGAATCAGCAGACGTAGACCACTGC
TGCTGAGGCCTGGGGATTGCTGGGAAAAAGGCAGGAAACAGATACGGACCTGACCATGGAGG
GTTTGTGTTTCACGGCTCCCATCTGGGTACCCAAGGAACCTACATGTAGCTCGTGTGTGGAGAG
CCTACATTGCCCACTCAAAGCAATTGAGGATGGAACGGTCTTGGGGCTGGAGCTCATTATTTGG
AATGATAACCACATCTGCACAGAGAGGAGCTGATAAGATGTTGTCCTTCCATGTATATCTGGGA
ATACTGTGTAGGGTCTCTCTGTAAGGACAGGGGCAGTATTGGCTCCTTGGCCTCTAGTTAACCTC
ACAAGTAGTCTAGTAAAGGCTTTGCAAACTTGTCACCATCTGTGGACATTCTGGCCAGCTCTTGT
TTTCACCCTACTGACTTCTTCAGACACTAGGCTTTTGCTTTAGACCATTCATGGTTTTTCTTCCTCT
TCAAATCAGTAATCAATAAATACTCTTCAAGTCAATAAATTTCCACTCCTTTAGGAAACCCTGAT
CTTCGGGTCGCACCACAAGGTTTAATTAACTCGTTTGATTGTTTTTCTGTTTTCTTGGGTTTTTTTTT
TTCCTTCTTCCTGGGGGTTTCTAGTAATTCTAGTTTGATGTCTCACTTTCTCCATTTTTTATTTCTTA
GTTTTCTTCTGTGATTATTTTCACTGCAGTTGCAGGGCCTAATCCTGGGTTGGCAGAGAACTAGC
ACTTACTCTGCCCTAATTAGAATCCAGGAGAGATAGGAGGTGCCCTAGTGTGAAAATGTGTTTG
CTCCTCTCTGCTTCTGGTAGTCTCTCTGTAGGAGTTCTTTACGTATTCTGAATGTTCACTTCTTATG
AGATACATGATGAGCTACTATAGGTTGAATGTCTCTGATCCAAAAATCTGAAATCCCAAATGCTC
CAAAGTCTGAAACTTTTTGAGTGCCAACATGACACTCAAAGGAAATGCTTATTGGAGCATCTCA
GACTCGGGTGTTTGAATTCGAGATGTTCAACCAGTAAGAATAATGCAAATATTACAAAATCTGA
AATACATCCCAAGCATTTCAAATAAGGGACACTCAACTGGTATTTTCTTTTATTCTACAGTTTGCC
TTTTACCCTGTTGGTTGTGACCTTTGAGGAACAGAAGTTTTTAGGTTTGATATATTTTTGCTTTTAC
TGCCTGAGATTTTAATGTCATATCCTAAAAATTATTGACAAATTCAACGTCATAAGGCATTTTCCA
AATTTGTTTTCCCTAGGAGTTTGATAGTTCTAGTTTTACATTTAGGTTTATAATTCACTTTGAATTA
ATTTTAACATGGTGTAAGGTAAGAGTCCAACTTCATTGTTTTGCATGTAGATATACAATTTTCCCA
ACACCATTTGTTGAAGAAAGTGTCCTTCGCCATTGAGTGGTCTTGGCATCCTTGTGGAAGATCAC
TGGACCATATATGCCAGGGTTGGTTTCTGAGGTCTCCGTTGTGTTGGTCCATAAGTGGGTCAAGA
GTGTCTTTTTGCCATGACCACATATTTTTTTTTTGGCTTATTGTAGTTTTGTAATTGTTTTGAGACCT
TTAATTTTGTTTTGTTTCAAGACTGATTTGCCTATTCATGGGCCCCATAGATTCCATATGAATTTTA
GGATAGGATTTTCTGTTTATCAAAAATGTCATTGGAATCTTTATAAGGATTGTATTGAATCTAGGT
CACTTCGAGTAGCGTTGACATCATTCCAAGATGAAATCTTTTAATCTGTAAACCCAGCTTTTCTTT
TCATTTATTTGTGTTTAATTTCTTTTAACAGTGTTTTGTAGTTTTCTGTGTTCAAATCTTTTGCCCTCT
TGGTTAAGCTTATTTCTAATTTTCATAATGTTGTTGTAAATGTAATTCTTTTTTTTTTTTTTTTTGAGA
TGGAGTCTTGCTCTGTCTCCCAGGCTGGAGTACAGTGGCACTATCTCAGGTCACTGCAACCTGC
ACCTTCCCTATTCAAGCGATTCTCCAACCTCAGCCTCTCAAGTACCTGGGATCACAGGTGCGCG
CCACCATGCCAGGCTAATTTTTTGATATCTTTAGTAGAGACAGGGTTTCTCCATGTTGACCAGGC
TAGTCTTGAACTTGTGACCTCAGGTGATCTGCCCACTTTGGCCTCCCAAACTGCTGGGATTGCAG
GCATGAACCACCGCACCTGGCCAAATATCATTCTTTTTAAAAATTTCTTTTCTTTTGTTTTCTCTTT
TCTTTTCTTTTCTTCCTCTCTCTTTCTTTCCTTTCTTTCTTTTTTTTTGAGACCGCATCTCACTCTGTTT
CCTAAGCTGGAGTGCAGTGGCACAATCTCAGCTGACTGCAACCTCCACCTTCCAAGTTCAAGCA
ATTCTCCTGCCTCAGCCTCCCAAGTAGCTGTGACTACAGGTGTCTGCCACTACGCCCAGCTAATT
TTTGTATTTTTAATAGAGATAGAGTTTTACTATTTATATTAGAGATGAGGTTGGCCCAGCTGGTCA
CGAACTCCTGACCTCAGGTGGTCCACCCACCTTGGCCTCCCAAAGTGCTGGGATTACAAGTGTG
AGCCACTGCACCTGGCCTCTTTTTAAAATTTTATTTGCAGATTGTTCATTGTTAGTTTATAGAAAT

GCAACTGACTTGTGTGTGTTACTGTATCCTGAAACTTTGTTGAATTTCATTATTCTACCAGTATTTT
GTGGAATTTCAGGATTTTTACACATTACATCCTGTTGTCTGTGAACAAAATTTTGTACTTTTTCCTT
TCCAATTTGCATCCTTTTTATTACTTTCTCTTGCCTAATTATTCTGAGTAGAAATTCCAGTACTGTG
ATGAATAGAAGTGGCAGGAAGAAATGTTGCTATCTTATTCCTGATCCTAGAGGAAAAGATTTTA
GTTTTTCACCATTGAGTATGATGTTAGCTGTGAGGCTTTCATGTATAATCTTTATTTATTGAGGAG
TTTCCATATATTACTAATTCTTTGAGTGTTTTTATTACAAAAAGTGTTCATCTGGCTCTGGAACCAG
ATAAACGTTGACCTGATAGAATGGATTGGAATGTCCCCTTCTGGTTTTTGAACATTTTTGGAATAT
TTTGCAGAGGATTGGCATTAATTCTTCTTGAAATGTTTGGTAAATTTTTCCAGTGAAGTTATCTGG
ACCTGGAATTTTCTTTTTTGGGGGGTTTTTGATTACTGGTTGAATCTTCTTACTAGTTACAGGTCTC
TTTGGATTTTTTATTTCTCCCTGATGCAGTATGGCGGTTTGTGTTTCTAGGAATTTATAAATTTATT
CTAGGTTGCCCAGTTTTGTGGCATATGGTTGCTCACATTAGTCTCTTGTAATCTTTTTCATTTTTGT
GACATCTGTTGTACTGTCACCTCTTTTATTTAAGATTTTAGCATTTGAGATTTCTCTTTTTTTCTTAA
TATAGCTGTGAGTTTTAAAATTTTTATTGATCTTTAAAAAAAAACAAACTCGGTGTTTTTTTTCCTTT
TTTTCTGGTCTTATTCTGCTTATCTCTGCTCTAATCTGTTATTTTCTTCCTTTTGCTTGGTTTGTCATT
AGTTTTTTTTTTTCCCCCCTTCAGGTGTAATGTTAGGTTATTGATTTGAGATCTTTCTTCTTTTTAAT
TTAAGCACCTGCAGCTATAAGCTTCCCTTTAGCATGGGTTTGAGATCTTTCTTATTTTTAATTTAA
GCATCTGCAGCTGTAAGCTTCCCTTTAGCACTGCCTTTGTTGCCTCCTCCTGAGTTTGGGTATGTC
ATGGTTTTCATTTGCTTAAACATTTTTTGTCCTATTGCAATATAATTGTGTTGTTTTTAATAAAGGTA
ATTAATGAAACCCATAATGAATTGTGCTTCTGTTTTTATAATATTTTAAGCATTCTTAACTCAGAA
ATGTAAATTTTAGAAAAAAAATTCCAGGCCAGGCACAGTGTCTCACACCTGTAATCCCAGCACTT
GAGGAGGCCGAGGCGGGAGGATCATCTGAGGTCAGGAGTTGGAGACCAGCCTGGCCAACATG
GTGAAACCCTGTCTTTACTAAAAATAGAAAAAAATATATAAAAGTTAGCTGGGTGTTGTGGTGG
GTGCCTGTAATCCCAGCTACTCTGGAGGCTGAGGCAGGAGAATCACCTGAATCTGGGAGGCGG
AGGTTGCAGTGAGCTGAGATTGCACCACTGCACTCCAGCCTGGGTGACAGAATGAGAGTCCGT
CTCAAAAAAAAAGAAAAAAGAAAAATTTCAGACATATTTATTTGTATTTCAATTTAGAAACTATG
ATCTCCTAAGTGTATTGACACAGGAACCTGACATAAAGATAAAGAATAATAAGCATATAACAAA
ACGGAAACTTGCAAATACCTGTTTTTTATTAATTTTTAATTATATATATTTAAAAATTGCCGGGTGC
AGTGGCTTACATCTGTAATCCCAGCACTTTGGGAGGCTGAGGTGGGCAGATCACATGAGGTCA
GGAGTTTGAGACCAGCCTGGCCAACATGGTGAAACCTCATCTCTATTAAAAATCAAAAAATTAG
CCAGGCATGATAGCATGCATCTGTATTCCCAGCTACTCGGGAGACTGAGGCAGGAGAATTGCTT
GAACATGGGAGGCAGAGGTTGCAGTGAGCCAAGATAGTGCCACTGCACTCCAGTCTGGGTGAC
AGAGTGAGACTCTGTCTCAAAAAAATAAAAATTGTCTGGGCGCGGTGGCTCACAACTATAATCG
CAGCACTTTGGGAAGCTGAGGCAGGCAGATCACGTCAGGAGATCGATACCATCCCGGCTAACA
CGGTGAAACGCCATCTCTACTAAAAATACAAAAAATTAGTTGGGCGTGGTGGTGGGTGCCTGTA
GTTCCAGCTACTCCGGAGGTTGAGGCAGGAGAATGGTGTGCACCTGGGAGGTGGAGCTTGCAG
TGAGCCGAGATTGCGCCACTGGACTCCAGCCTGGGTGACAGAGCGAGACTCTGTCTCAAAAAA
AAAAAATAAAACAAAATAAAACTAAGGTGTGGTTGACATACAAAAATTACACATATTTAATATAT
ACCGTGTGTGTGTGTGTGTGTGTGTGTGTGTGTGTGTGTGTGTGTGTGTGTTACGGAGGTTTTACT
TTTGTTGCCCAGGCTGGAGTGCAGTGACACGATCTCAGCTAGCTGCAACCTCCGCCTCCCGGGT
TCAAACAATTCTCCTGCCTCAGCCTCCTGAGTAGCTGGGATCACAGGCGTGCGCCCCCATGCCC
GGCTAATTATTGTATTTTTTTAGTAGAGACAGGATTTCATCATGTTGGCCAGGCTGATCTCGAGCT
CCTGACCTCAGATGATCCACCTGCCTCGGCCTCCCAAAGTGCTGGGATTACAGGTGTGTGACAC
CGAATATATACATCTTAATGAGTATAGAGATAAGTATTCGCCCCAGGTCTCATCACAACAAATAA
TGCCGTAAACTTGACCATCACTCCCCATATATTTCTCATTCTCACCCTTTTTAAAAAATGAGACCG

GGAGTGGTGGCTCACGCCTGTAATCCCAGCACTTTGGGAGGCCAAGGCAGGTGGATCATGAGG
TCAGGAGATCAAGACCATCCTGGCTAACACAGTGAAACCCCGTTTCTACTAAAAATACAGAAAA
TTAGCCGGGCATGATGGCGGGCACCTGTAGTCCCAGCTACTCGGGAGACAGAGGCAGGATAAT
GGTGTGAACTCGGGAGGCAGAGCTTGCAGTGAGCCTAGATCGTGCCACTGCACTCCAGCCTGG
GCAACAGAGTGAGAATCTATCTCAAAAAAAAAATGAGATGACCATTTCACCTAAAATATATCCT
CTTAAGTTTTTTTTTTAAGTGTACAATACAGGACGGCCATGCATCAGAGATATATCTGGGTTCGG
TTCCAGACCACTACAATAAAGTGAGTTATACAATCTCTTTTGGTTTCCCAGTGCATGTAAAAGTA
TGTTTATATTGTGCTGTATAAAGTGTGCAATAGCATATGTCTACAAAGTGTGCACACTTTAATTTA
CAAATACTTTATTGTTAACATGTGCTAACAGTCATCTGAGCCTTCAGAAAGCTGCAATCTTTTTTT
GTGTGTGTGACAGGGTTTTACTCTGTGGCTCAGGCTGGAGTAATTGCAGCCTCAACCTCATGCTC
AATCAAACCCCCACCTCAGACTCCTGACTAGCTGGGACTACAGGTGCATGCAACCATGTCCAGC
TAATTTTTGTATTTTTTTTTTGTAGAGATGGGGTTTTGCCATGTTGCTTTGATGTTCTGGGCTCAAG
CAATCCACCCACCTTGGCCTCCCAAGGAGTTGGGATGACAGTTGTGAGCCACTGCACCTGGCC
AAGTTTCAGTCTTCTTGCTGATGGAGGGTCTTGCCTTAATGTAAGGTGGTGGTTGCTGAGCGTTG
GGGTGGCTGTGGCTATTTCTTAAAATAAGACACCATTGAAGTTTGCTGTGTCAATTGACTCTCCC
TTTCACAAAAGAATTATCTGTAGCATACGATGTTGTTTGATAGCTTTTTACCCACAGTAGAACTTT
CAAAATTGGATTCAATGCTGTCAAACCTTCGTACTGCTGTACCAACTAAGTTTATGTATTATTGTA
AACCGTTGGGTTCAATTCTGTCAAGCCCTCCTTCTGCTGTACCAACTAAGTTTATTCTAAATCTGT
TGTCATCTCAACATTGTTTACACTGTCTTCACCACGAGTAGATTTCATCTCAAGAAACCACTTTCT
TTGCTCATCCGTGGAAGCAACTCATCCACTCACGTTTTCTCTGGAGACTGCTGCAGTCTCGCCAG
ATCTTCAGGCTCTGTCTCTGATTCTAGTGCTCTTGTTATTTCCACCATATCTGCAGTTACTTCCTCC
ACAGAAGTCGTGAACCCCTGTGTCATCTGTGAGGGTTGGAATAATCTTCCCAACTTCTCTCTCTC
TCTTTTTTTTTTTTTTTTTTGAGATGAAATCTTGCCTGGGCTGGAGTGCAGTGATGCGATCTCAGCT
CACTGCAACCTCCACCTCCCGGGTTCAAGCAATTCTCCTGCCTCAGCCTCCCAAGTGTTTGGGAT
TACAGTCACCCCCGACCAGACCCAGCTAATTTTTTTGTGTGTTTTTAGTACAGACAGGATTTCAC
TATGTTGGCCAGGCTGGTCTCAAATTCCTGACCTCGTGATCCACTTGCCTTGGCCTCCCAACGTG
CTGGGATTACAAGTGTGAGCCACCATGCCCGGCCCCAACTTCTCCTAATGTTGCTATTTTGATCT
TCTTTTTTAAATCATGAATGTTCTCAATGGCATCTAGAATGGTGAATCCTTTCCAGTAGGTTTTCA
ATTATTTTGCCCATATCCATCAAAGGAATCACTTTCTAGAGAAGTTATAGCTTTATGAAATATATT
TTTTAAGTGATAAGACTTGAAAGTTGAAATTATTCTTTGATCCAAGGGCACCAGAATGAATGTTG
GGTTAGTAGGCATGAAAACAATGTTCAGCTCTTTATACATCTCTGTAAAAGCCCTTGAGTCCAGG
GGCATTGTCAGTGAGTGGTAATACTTTGAAAGGAATCTTATTTCTTGAGCAGTAGGTGTCAACAG
TGGGCTTAAGATATTCAGTAAACCATATTTGTAAACCGATAGTCTGTCATCCAGGCTTTGTTCCC
ATTTGTAGAGTATAGGCAGAGCTGTGTTTTATCATAATTCTTCAGGGCCCTTGGATTTTCAGAATA
GTAAATCATCATTGGTTTCAAGTTAACATCACCAACTGCATTAGGCCTTAACAAAAGAGTCAGC
GTGTCCTTTGAAGCCTTAAATCCAGGCATCAACTCCACTCTAGCTGGGAACATCCTGGATGGCA
TCTCCTTCTAGTAGAGGGCTGTTTTGTCTCCATTGCAAATCTGTTTAGTGTAGCCATCTTAATCAG
TTACCTTCTAGATAGCTTTCTGCAGCTTTTCCATCAGTACTTGCTGCTTTATCTTGTGCTTTTATGTT
ATGGAGATGACTTTTTTCCTTAAACCTCAAGAAACAAGCTCTTCTAGCTTCAGACTTTTCTTCTGC
AGCTGCCTCACCTAAGTCTTCATAGAATTGAAGAGAGGCCGGGTGCGGTGGCTGTCACACCTGT
AATCCTAGCACTTTGGGAGGCCGAGGCAGGCAGATCACCTGAGGTCGGGAGTTCAACACCAGC
CTGACCAACGTGGAGAAACCCTGTCTCTACTAAAAATACAAAAAATTAGCCAGGCGTGGTGGT
GCATGACTGTAATCCCAGCTACTCGGGATGCTGAGGCAGGAGAATGGCTTGAAATTGGGAGGC
AGAGGTTGTGATGAGCCAAGATCACACCATTGCACTACAGCTTGGGCAAGAAGAATGAAACTC

TGTCTCAAAAACAAAGAAAAATAAGTAAAAAGAGAGTTAGGCTTGGGCTTAATGGAATGTTTTC
TGTTTTTTTTTTTTTATCTTCTATCTAGACCAATTAAACTTTCTTCATAACAGCAGCAAGATTGTTTA
GCTTTTTATCATTCATGTGTTCACTGGAGTAGTACTTTAAATTTCTTTCCAGAACACTTCCTTTGCA
TTCACAACTTGGCTAAGTGTTTGTTGCATGAGTTCTAGCTACTGACCTGTCTTGCTTACAGCATGC
CTTCCTCACTAAGCTTAATTATTTCTTCCTTTTGGTTTAAAGTGACAGACATGCAACTCTTCTTTCG
CTTGAACATATAGAGGCTATTGTAGGGTTATTAATTGGCCACATTTTAATATTAATAAAAAGAAG
CCTGAGAAAAAGTGAGAGAAAGAGAAATGTCCCATTGGTGGGGCAGTCAGAACAAACGCATTT
GTCAATTGTTTGCTGTCTTATCCTGGTGTGATTTGTGGTTCCCAAAACAATGACAACAGTAGCATT
AAGGATCACTCCTTACAGATCACCACAACAGATTCAATAATAAAAAGCTTAAAATACTGTGAGA
ATAACCGAAATGTGACACAGAGACATGAAGTGAGCACGTGCTGTAGGAACAATTGTGCCAGTG
AGACCTGCTTATTGCAGGGTGGCCACAAACCTTCAATATGTAAAACACATGGTCACAAAACACA
ATAAAGCAAAGTGCAGTGAAACAAGATGTGTCTGTCTTTTGATAGACTCTGACAATCTCTATCTT
TGAATTGGTACATTCATACCATTAACATTCAAAATGATTATTGATATCATTGGATTAATATCTACT
ATATTTGTTACTGTTTTCTATTCATTCTCCTCAGTCTTCATTCTTTTTTCTACCACTCTTTTTCTGCCT
TTTGCAGTTTTC

ID|55917|gene_id|ENSG00000230880|transcript_id|ENST00000412092 Homo sapiens lncRNA

GCGTCACCCAGAGGCGGTCTTGTAGCGGGGCCGGCTTGGGGCTTGGTTCTATGTCCCTGC
GGGTCGGTGCGAGGGCGAAGAGGAACCCGTGGGCCTCGGGGGATCCCGGGGGGCCGGACCA
GTGTCCCCTAGTTGTGGGAGCAGACGCGTGGGCGCATCGCGGGCGGGCAGGGCCTGAAGTGC
AGGTGCGGGCAGCGGACCCTGGCGGGGGCTGGGAGGACAGGCGTGGGGTCCCGGCAGTGAA
GCGGGTTCTAGAGGCGCAGGAGCGGGTAGGCGAGGCCGGTGGCCCTGGGCCCGGAGTCTGCA
GGCCGCGCTCCTGTCCTGCCGCTGAGGGACCCGGTTACCAACCCGCATGACGCTCAGTTTGCC
CATCTCTCCCAGTGCTAACACACAGTTCTCGGGAGACGTTCCCCATTCCCAGAGGAGTAGTGCG
AAATGCGTGCGCCTCTAGTCTTAAACTTGGCGTTTGTATTAGTTGGGTTTCCTGGTGTCTCTTTAG
CAAGTGAAGTTTCTGGTTCCCTCCTTCACTGTGTGACCTGCCTAGTCCTCCTGGGTTGCATTTACA
GAAGTTTATACGAGACCTAGTTTCCAGGGAAGAACTCACTGATTCCGCGAGGGAGATGGCATA
ATGGATGATGGTCTTCAGCCTTAAGGGTACTTCAGTCTTAACTGTGTGTTACAAAGTTTGAAAGA
GAGGGTTCCCTATGAATAAGAAGCGCACTTGAAAGAACAGCTGTCTGGTCTAACCTCTCACTGG
TGCTTCAGAGGAGGAAAAAAGGTCACAGGTGAGGATCCCAGTTTTCCTCGCTCAGGAAATATTA
ATTCTACTCCCTAGAATGCACAAGATTTGCAAAGACTAGGTGATAGTAGAAGGTTTGGACGAAC
TTTCAGAAGGTTGAGGTGTATTCCGCTGAGAAGAACAGGCAAGGACCTAGGAAATATTCCTTAT
TTGAAGGGGTCTGAAAGTGTGGTCCGGGGTACAGGAGTGACCTGTCATACTTGAGAAGATTAA
AATACTCTCCAAACACAGTCCCATTCCTTCAACCTTAGCTCGTTTTTCCCAGCGTCTGAGATATAT
TAAACCTAGTCCATCCCCAAATTTAGCATTAGATTGCGAAGTTCTATTGATTGTATTTGATTTGTA
ATTTAAGATTTTCTCCCCCTACGTAATTTTGTTAAAAACACAGAAGTGAATTCTGTTCACTTAGGT
GTAACAGTTAATACTTGCTGTTTAAGGAACTAATTAAACCTTACTGGCTTATAAAAAACAAACAC
CATTTTATTTGTTTGAAGTTCTGTGGATCTGCATTTTGGTGTGGTGGGTTCAGCTGGGTAGTTGAT
ATATTTGTGTTGCCTGGATCACAAAAAGTCCTTAGTCACCTGGTGCCTTTACTGAGCCTGGTTGG
TTTAAGATAGTTTCCTTCACAATCTGGTGGTTTGTGGTGACTCTTGGCTAGGCCCTGTGTCTCCAA
CAGGGTAGCTCCAGACCTCTTCACAATTTGACTGTGTCCAAAATGGCAAGAACCAATGGATATT
TGCATCACATTTTCCATTGTCCATTCACTGGACAAGTCAGATGGAAAAGCCCAATTTATTATCAG
AGCATAATATGAGGGCTTGCATAGAAGGAAAGCTGTTATTGGGAAACATGAGTACAATGGTGTA
CTGTAGGAAATACATATTATGTACATTTTAAAAAATGTAATTGTAGGCCAAAATTGCTGGTTTGC

AAGATGCACTTTCCATGATGTTCAGATATAGAAAAGCAAGATGTACTGTCATGGGAACACTCAT
ATGAAGTTATTTGTGGAATCTACATATAAATAGGAAAATAGTTAATACAGCCTAGTATATTTCTAT
AACATTTATTTTAGTGAACTTATAATGTTTCTTTGCATTAAATTATTAGATTATATCTTTAGATCATA
TTGTTACTAAATTAATAGGTAATACATATTTTTATTCAAAAATAAATTGTGCATCTAATGTCTACCA
ATTAATGTACTTGTAGATGTATCTTATCTTAACTTGAGTCTTTGATGCCCCTAATGAGGCGTGAAG
GACTCTTCTCCCATGGGGAAGTTTTTCTTTTTCAGGAGGGAGGAGAGCTTTCCCAGGTAATGTGT
CTAGAGTGTTGGGCAGAAGAATCTGGGACTACACCACACCAGTTCTCTGCTTAATCCACGTCAT
TTGCCTTCTATCCCAGCTATGTTTCCAGTGTCCTCTGGGTGTTTCCAAGAGCAACAGGAAACGAA
TAAATCTCTGGTGAGTTGTTTATTTGTTCTTCACTTTGTTTTACATTGTATTTTCTGAGTTTTTGGGT
GTCTGTGAATTAAAAAGGAAAAGTAGAAATAAGTAAAACTCAGGTTGAAGGAAATATACATAAA
TAAGATAAAGCTGACCTGTAGATATAGGCAGGTTATAAGAGCTTAGAGTTGTCTAAGTTGGGTG
CAAATTTTCCTCTGATCTTTCTGATGCCGAGACAAAAAAGGCAGTCATATTTGTTACGTGATTGG
AGTGGAACCCGAGAAGAGAACATGCTGTGTTCTTGTGGGACAGGAAAGCTTGCGTGCACCAAG
TCCGAACCACCACCTTCATTGGTGACATAGATTATGTGCTGGAACATATTTCACACCGGCCTGG
CAGTAACCACTTGTAGTGTTGTACAGTGGAAACGGTCATCTTCCGCTAAAGCACAGTGTGTTTTG
CAGTGGAAATGGTCATCTGCCGCTAAAGCACAGCTT

ID|55928|gene_id|ENSG00000225548|transcript_id|ENST00000414382 Homo sapiens lncRNA

AGTTCGAGCTTCCCAGCTGCATTGTTTACCTAAGCAAGCCTGGGCAATGGCGGGCGCCCCT
CCCCCAGCCTCGCTGCCGCCTTGCAGTTTGATCTCAGACTGCTGTGCTAGCAATCAGCGAGACT
CCGTGGGCATAGGACCCTCCGAGCCAGGTGCAGGATATAATCTTGTGGTGCGCCGTTTTTTAAG
CCTGTCCGAAAAGCGCAATATTCGCGTGGGAGTGACCCGATTTTCCAGGTGAGTCCGTCACCCC
TTTCTTTGACTCGGAAAGGGAACTCCCTGACCCCTTGCACTTCCCAAGTGAGGCAATGCCTCGC
CCTGCTTCGGCTCACGCATGGTGCGCGCACCCACTGACCTGGGCCCACTGTCTGGCACTCCCTA
GTGAGATGAACCCGGTACCTCAGATGGAAATGCAGAAATCACCCGTCTTCTGCGTGGCTCACG
CTGGGAGCTGTAGACCGGAGCTGTTCCTATTCGGCCATCTTGGCTGCTCCCCGGAAGTTTCTTAA
TGCATTTAAAATATTATTTGCTTGGCCATCATTTTTAAGAAACAACTATTGTAAAAATTAAGATGC
TCTTTTATCAATGAATGATGATTGTTTTCAACCAAGAGCTCTAGGCAGAAATTAAGTTGGAAATA
AGATCAACACTACCATTTGTATAGGGCATTTATGTTTTCTTGGGCTGAAGTTTTTAAAATCTTCTTT
AAAACCCTCAGAATAATGGGTAGCCTTTGTGATGCCCACCTTACCTTACTCTCAGCAGTGTTATG
GAGTAAAGTTAGGTTTTTTTTTTTTTTTTTTTTTTTTTTGTCAAATGTGTCTAATGGTCTAACCAATA
TGTACATTCTCCCTTGTAAAGACCCCAGCTGCCCAACTACATTTCCCATCCTGGGTTCCCTTATAT
CCCTGTAACTACTCCCCACCATGAAATGTGAGTGGAAGGGATGGGTATCACATATGGGTTGAAG
TTTTTAAGAAGCGGGTGTGCCTTCTGCATTCTTTCCTCTTCTGCCAAAGGCCCCAAAGTTCTACG
AGATGGTTAAGGTGACCAACTGTCCTGGTTTGCCCTGGACTGAGGGAGTTTCTGGAATGTGGGG
CTTTCAGTTTCAAAACTGGGACAGTCCAGGAAAACGGGGGATTTACTGGTCACCCTAAGAGACT
TTTATGGGTGTGAAACAGGACTTTTATGTCCCATATGGGACTTTTATGTAATTGTGAAATAAGCC
AGGAAAATTTTGAAGTTAATTCGTTGCTACTTACTAGCTTTATCCTAACTAATACAGATTGGAATT
ACTACTCAGGAAGTGGCCTTGTTCAGATAGACACATGTCAATGAGAGGATATCAAAAAGCAGA
AATGCTGGCTCTCGTCCTCAGCAGCTCCATGATCTAGAATGTATTGTTGGCCTAATCTTCTAGTT
GGAGTAGATTAATGGTTCTCAAAGCATGGTGTGAGAATCCACAGGGTTCCCTAAGATCTCAGGG
TGTCCATGAGGGCAAAACTATTTTCATAACAGTGGCAGATGTTATTACCTTTTCATTCTCATGCTA
TTAGGAGTGTGCAGTGGGATTTTCAAGAGGCTACACGAGGTGTGATGATGTCATGGATCTGGTG
GCTGATGGACTATGTGTTTGTGAATTATTGTGTTTTAAAATTGTCTTGGTCTTAATTTCTAATATGG

TCAATATTAATAGATGTAACTCACACTCCAAAAACTCTTCAGGGTCATTAATGATTTTTAAGAGT
GTAAGAACTCCTGAGACCAAAAAGTTTGAGAACTGTTGATGTGAATAATTTCTAAGGTTTTGTAT
ATTTCTCAAAGTTTGTGAATTAGTACTCATATATACCCAAGCCAGCTACAGAACTTTCTTTTTTTT
GAGATAAGGTCTTGCTGTGTTGCCCAGGCTGGAGTGCAATAATGGCACACTGCAGCCTAGAGA
GCCTGGGCTCAAGTAATCCTCCTACCTCAGCCTCCTGAGCAGCTAGAACTCCATGCATGCACCC
CCATGCCCAGCTAATTTTTTTTTATTTTTTGTAGAGATGGGGGTCTCATTATGTTGCCCAGGCTGG
TCTGGAACTCCTGGCCTCAAGAGGTCTTCCTGCCTCAGGCCCCCAAAGTACTTACAGGTGTGAG
CCACCATGCCTAGCCTCATTACAGAATTTTTAAGTTGATATGTCTTTCATTATTTTTCCATCTCTAT
CTCTATTTTTTTGTGTGTGTTCTATCTCTCTTTTTTTTTGAGACTGAATCTCACTCTGTCACCCAGGC
TGGAGTGCAGTAGCATGATCTTGGCTCACTGCAACCTCTGCCTCCCAGGTTCAAGCAATTCTCCT
GCCTCAGCCTCCTGAGTAGCTGGGATTACAGGCGTGTGCCACCATGCCAGGCTAATTTTTGTATT
TTTAGTGGAGGGGGGTTTCACCATGTTGGCCAGGCTGGTCTCGAACTCCTGACCTCAGGTGATCC
ACCTGCCTGTCTCAGCCTCCCAAAGTGTTGGGATTACAGGCATGAGCCACCACAGCCAGCCACT
TTTTTTTTTCCATCTTAAGCTGTGGTGGGGATAAAAAATGTTTTGGAATCTATTATTAAAAGATCT
CTTTCCAGAAAAGTATAGACACTTCCTGTTTTATATATTAGGAATTATACAGAGTTATTCCCCTTA
TGATATGGGACGGTTGCCTTAAACAAATCATAAAGTAGCAAAAATTAAAGTATATTTTTTCCCTT
CAGAGGAAGGACATTTTGCCGTTTCAGACAAGTAAATCTGACAATTATGTTTCCAGCAACCTTAG
AGAAATAGCTGTTGTGCTCACCCAGGGTTCTGGATAAAGATGCTTAGTGTACTTTCCTTGGAAGC
AAATCAAATGCAAATTTGGTTGTGTCTCTCTCTTGCTTAGACGCTTTCAGTAGCTTCCCTTGGGTC
CTTAGGATAAAGCCCCAACTTATTCAAAGTCCTTTCAGGTCTTCACCTGCACACTCCCTACCCTG
GTCTCCCTTCTGTGTGTTTTCTGTCTGGTTCTCTTTACTGAACCTCACAAGGGCTGACAGACGTGA
ACCCTGTTTCAGTTTCTGCTTAAAAGCCTTTTCTCCAGCCTGATTTATAATCTCAGTGCACCTATA
CTTCTAACACCTCAGAAACTCACTTGTAATTCTCTGTTCCAGGATGCCCACTGGATCCAAAGAGA
GAAACTCTATAGAATTATGATCTAGGTTTGACTGACTCCTGGCAAGAAGCCCTACCCGCCAGCC
AATTTGGATGAGGTGAGGGCTTTTTCTTATCCCGCAAAGGAGATGTGCAACATGCTGCTCAGGC
TTTTGAAGAAATGACTCAGAAAAGGGCTGTCCTCATAGCCAAATAACAAAATGTTTACTGAAAA
TGCAAAAGTGAAGTCATCAAATACCTGAATTCACTGGACATTGGTAAATTTAGAAAAAGCCAAG
ATCTGTCATCTATTCATACACACACACACACACACACAGACATACACACACAGACACACACACA
CAGACACACACATACACAGACATACACACACACAGACATACACACACACACACACACACACGC
ACAAACACGAACACCAGAGATCAATTCAAAATCCAGATTGTCATTTGAGGAATGTATAGTTGCT
AATTTATTTTGACAAATTCATGGAGTTGATTTAAATTATTTATGATAATGCTTCACAGTATATTTTA
TAAAAGTAATGAAAGTGTAATTTTAAAAACGTTCTTGGCTTTTACACTTTTCTTGGGGAGAAATG
AAATAATGCTATTTTTTTCTTTTAAAAAGCATTGAAACACCAGAACTTATGGAGTGAAAATATGA
AGGAAGTCTGTTTATATTTTAGGAAATATATATACTTCTACAAACAAACCTAGTAGAATATCTGA
CACAAAAACCGTACAGGCCTCTCCCAGCTCCTTATATGAACTCAGCTGCTGGTGAATGAGGAGA
TAATTCACCCATCTATATACATAAATTTCTGGATAATAATAATGATAATGGTAATTGGCATTTACT
AAGCTCTTACTGTGTTCTGAACACCTTATTTGCATGATATCTTTGAATGCTTATAACATTCTAGAA
GGTAGGTGCTGTATTGTTTCCGATTTATAAATAAGAAAATTGAGCCACAGAGAAGTTAAGTGACT
TACCCAAAGTCACACAGCAACAAAGATAAAATTCAAAATGATGTAGGCTTACTTCATAGCCTAG
TATTCTTTACTCCTAAGCCATACTGGGTTGTACAGTAATTGCTAAAGGAACAAATTTCAAAGTCTT
CCTTGTAGGTAACCTTTCTTTCTATTTGTACAAAGGATTATAAACTGTAAGAAGTTTTATGGCATG
TTGAATTAGCTCAGTAGTTCAATTGTAAGAGTTTCTCATTAATTATTTAAAGCTATTCATTTTTATT
AGGAGTACCAGAGCTAGACTCAGGACAAACCTCTATTTCATTCAATTCAATTTAGCTCAATTTAG
TTCATGTTATTGAATATACATTACATGCTGGGTTTGGGGAGGGAAGATTGTAACTGTAATTAGAA

AGGTATTATCTCTGCTTTCTAGGAACACAGAATCACTTGTGGAATTAAGAGATAAGGACAGCAA
GTAGACCAAACTATAAGCTGAGGTTCAAGGTGGCATAGGCTGGGTGCCACAGTGCTGTGTCTCA
GGTGGGCTAGAAGTGCGAAACGGACAGTACCAGAAGGAAAACTTGAGGGTAAACTTGGGTTTT
TTACCATTCCCAGCCTTAGGAGGCCAAAGGCTGAACAAATACTTGTCAAATGAATGATTAAGAT
CCCTTAAGGTACTAAGTAGGATATGAAAAAAATTCGTGGAGAAGAAAGCATTTGAAGTGAATAT
CTTTAAATTTTAGGGGAGAAGAGCATTACATTCTGAGATTGCAGGATATGCAGAGGGCAGATGC
TTAATATAAAAGTGCTTAAAATGATGATGGCAGAAGCCTGCTGAATGATGTGGAATAAAGAAGT
ATGTGATAATGATTATGACTTGACTAACATCTATTATACATCTCCATTGTGCCAGCTGTTTTCAAG
GGCTTAATCTTATTTGCTCTGCAGAAATTCAGTTTTGGTGGTAGACATAAAGGTGCAACCTTGTT
CTCCCTTCAAAGAGAAAGTGCTTCCCAGCTGTAGGGGGTGTGGTCAGCATACAGCCTCTGTTCA
GCTCTCAGCTCCTTCAGAGTTGGCCTAGGCAGCAGACAGGCACCTGGCCCATAATTGTGCCCTT
TTTAGGGAAAGCCCATGTCCCATAATTGAGGAAGAGATATAGAGGTCAGGTCATTCCCACCCAA
CATAGACAGACTCTGTAGGGTTTTGTTTGCTCCTGAGCTCCCTCCAGGATTGGCTATGGCACTGT
TAGGCTTTCATTGCTCCCTTCCCTTCCCATCACAGCTCAATTCCTAATCAATGTCTTGCTTAGTTTA
GAGTCTGCTTCCAGAAAACCTAATCTGCAAGACCGTTAATCTCCCACATTGACAGGCTGGAAAC
TGGAACACTGAGAGTCGATGCATTTCCCCAAGGCCACACAACTAGTGAATAGCAGAGCCAGGT
TTTAAATTAAGATTCATTTAACTTCTAATTGAGGTTCTCCTCAGAACCCCACACTGGCTACAGGA
CCAACCTGTATTTAGCCAAAGTTGCATTCTCGCACATGCACACATCAACAGGAATCTCTCCACTT
GTCGGCCCAGATAGATGTTTTCTGTCAGGAGAGCTCTGAGTCATCTGTGCCCCCTAGAGGAAGC
CAAAACTCTAGGTCATCTGTGCCCACTCTTGGCTTGATTACCCCTATCAATCTTTCAAGTCTTAAT
TCCTAAATCAAAATTAAGTTTTCTGGTTACAAGCTTTCGTAGTACCTGTCATAGCACTGGACACA
ATTTGCAATCACATATTTATTTTCTCCTGTTTGTTTGCTCCACTAGGCTATAACTTCAGGAGGGCA
TGGATTATGTTTGTTGAGTTTAGCACTAACTTCCTAGCACTTTCCCATACTTTTGGGTGTGATAGG
AGCTCAATGTACATTTATTAAAGGGCTGAGTTAAGGGTGTATGATAAATCCTGGGTACTTATCTC
AGTCAATATTATTTCTAGTTTTCTTCTCTTATGGAACTATATAAGATATTCACAAAAATATCATTAT
ATTATGGCAGATATTAATTATTATGATTATGATTGTTTTTAGGCTCACACCATACCTTAGACCATTT
TCAAATGACCAAGTTGGATGGTCAAAAATTAATAACTTACTCGGAAACTGTCCTTATATTTTTTTG
CTGAAGTCATGTTTAATTTTCTTCAGGAGAGATCTTGATCCTAAGACAATGCTGACATTTCCCAA
GAGTGGGAATTCCATGAGCTTTCTCAGATGGCTGCCCCAATGTCTGGAAACCTCTTGAAGGTGA
TCTCTATCATTTTGTCCACCTAGATATTTGATTCATAAACTCATTCTTTAAAACCAAAGTCATGGG
TGGCACCCTTTGACCTCGTTAAAGCTCATAAATCTCCCCTTTGTGAAGATTTTTCATCTTGAAATG
TGGCAAAGGTGTGAACTATTGAAGAGGTGTTCTCTTGAACATTTGCCCTACAAACTCACCCAATA
GAAAACCAGATTCAGGCATTTCCGACATGGGTCACTTCTCAGAAAGGCTATTTCAACTTCACTCT
ATATTATGAAGATTTTATAGACTTCAGTTATGCAGACTTTTTAGCTTTTTCATTTTCAGACTAGTCC
GACTTGTTTTTTTATAATTTTAAATGTATTCAAAAAAGTCATATTTTCAGACATCATATGTGTTTCC
ATTTGTGAGTTTTTCTGGTGTATAGAGATTATGAAATTCAAGTGGGAGGGAAAATGTTTGTTGAA
GGCTCATTCTTCTAGAGTTGCTCTAAAACCAAATTTAAACTAATCTCTAACAGTTGTGACTTAGAA
TTAGAAAACTCAGTCTTTAGGCTCTGTTGTCTAACAGCTGTGTGTGTTTCTACAATTTACTTAACT
GCTGAATCCAGTTTTTTGTTTTGTAAGATAGGAATAATAATTCCTGTTAGTGACCTAGTAGGAATG
TATTAGAGAATTAAGTGAGAAACGGCAAAATCCCTTCTGAACCCCAATAAACATAAGCTACTTTT
ATTACCGATCTTTTTTTGGATCTTTTTGTTAGATATGAGTTCTAAATTTCTTTTCAAAGAATTAATAT
GTCAGTATGTTCAATTCTTTGCCGTCTACTTTTAAACTTAACTTCCTCGTAAAGCAACCTTTTTCAA
TCACCTGCTCCACCCTACACTCCAATCACCTGCTCCACACTAACTCATTCCAATTACCTGCTACC
TGCTCTGCCCTGATTCCCGCCAAAGCACTCACCCCGTCATTCTCTTTAAATTAGCCAATCGGAAT

TAGTTTAGCCTGTGTGGTCTAACCCTAGCCAATAGGGGAAGGACACATTAGGGGCCACGTGCGT
CAGGGATAAGAACCCCTTCCCCTCCCTTGTCCAAGTGTGCACTCACCATTGTTTCATCTGTAAGG
GCGCAACTTTCTATATAGAAGTAACTTGCCTTGCTGAGAATTAAAAAGAAAATTTTATATTCGAG
TGCTCTTTCTTTTGTGGCACCGAAACTTTATATATAACATTTTCTGAACAATGTTGTAGCAATGCT
ATTGGTACCCACCCCCCTCATATTCCCTGGAGCTTTACTATTGTAGTGCCCACAGGCCCATCTCT
AATTACCAGCATTTGCCTCTCTTTGCGCAAGGACTTACTCTGACAGCCAGCTTCTGGTATATGCT
CACAAAAAGCAGGCTGAAACCAGAGAATTAGCATGCCCAGATCAGTCTACAACCAATGACTGA
AGGAAGTTGGTATATAAATACCTTGGCTGCCCTGTCCATCAGATGGGATTACACTGAGGCTTAT
AGTCTACACTGGATCTCAGTTTCCCTAGAGCTATTAAGCTGCAGGTGCCCACAGTGACAACCGG
CTCAATTATACACCATTTTTGGCTTCCTGAATCATTTATTGGCTTTATTCCATTCCACTTTCTGATTT
ACTTCTCCACTTTCTAACTGATATTTCCTGGAATTACCTTTCAAGTTAACTACTTGTACTTGAATTC
TTGTCTCAGGCTTTGCTTCTGAGGGATTCCCAAACTAAGACAGTGTTTATATGAATTACAATTGTT
TTGCATGGAAGTGATATACACTTACTTTAACTCAACAGAGCTTAGATCCGAACAAAGAAAAAAT
CAGTTCATTGACTTATATTAATGCAATGTCTAAAGATGACAGAATCTTATTTAAGAATGGCTAGA
TACAGGAAACTTATACAATGCCATTGACTTTTTTTTCTCTCACTCTCTGTCTCTCTGTTTTTCTCTCT
CCCTCACACACACACACACACACACACACACACCCCTACCTGTTTTCCTCTATGTACTTATTTTA
GGATAGAAAAGATGGTGCTGTAATGTCACTCTAAGCTTACATTGCCTCTACAGCATGAAATAGC
TGAGAAAGTAAAATTGTCTTTTGTTCATATCCATATCAATTCCAGAAAGAGTCTCAAATTGTCAGT
ATTTTCAATGTATGCTGACCTCTAGAAAAAAATGACTGGGTCCAGGTGGCTGAAATATGCTAGGT
GGTCATGCCAGGGAAGGTGACAACTCTAGCAGAGCTATATGAAATATGCGGGAGTGAGCTTGA
CTTTAAAATGATAAGTGCAGCAACAAGCATCCCACAAAGTAAAAAATACTATCTAGTGTTTCCAT
CCTTTTTTTTTTTTTTTTTTTTTTTTTTTTTTTTTTTTTTGAGACGGAATTTCACTCTTTTTGCCCAGGC
TGGAGTGCAATGGCATGATCTTGGCTCACAGCAACCTCCGCCTACTGGGTTCAGCGATTTTCCT
GCCTCAGCCTTCCAAGTAGCTGGGATTACAGGCATGTGCTACCACACCTGACTAATTTTGTATTT
TTAGTAGAGACGGCGTTTCTCCATGTTGGTCAGGCTGGTCTCGAACTCCCAAAATCAGGTGATTC
GCCCATCTCAGCCTCCCAAAGTGCTGGGATTACAGGTGTGAGCCACCACGCCCTGCTGTTTCCA
TCTTTTTTTTAATGATATCTTACATTTTTGGACCTTTTCATCTCAACAGAAGCAATTACTAATATAA
TATCACAGTTGGACCAGTGACTCCTTTCCCAGGTTCAGAATCGATGGATCAGATGTCATCATACT
ATAATGGAGGGACAGGCAAATAATATACTCATTAAATAGTAAAAATAGTGGTTACTGTTTGTCAA
ACACACATAGCATAAGTTCTAAATTCCATGCTAGGTATTTTACAAAGGTGATTCTACTTAAATCC
CAGAAACAAACCCTGCGACTAGTCATAGGTGTGACTAGGATTTTTATTTAATGAATGAAGAAAC
TAAAGCTTAGTGAAATTAGAGACTTGCTGAAGGTTTTCTTTTTTTTTGCTCGTTGATTGAAGAGGC
AATATTTGCATCCAGGTCTGTGTAGTTCAAAACCCATTTCTTAGCCACCTTGCTGCGTTGCCAGC
TTGAAGCACTGGTGCTTTGAGAACATTGCTTTTGGTGGACTTATTCTAATACTCATCTGCCCTTAG
GTTCCTTTGCATTCATTTTTTGGTGGAACATTCGGAGTCAATGAAAGCAATTTGAACTCTAGGAG
AAGATCAACTCTTTCCTTCAAATCTCATACTTCAGGTTGCAGACTTTTTGGTTCACACTAAATCCT
TGCGATCTGAGAACCTGAAAACCTTACACTCAGAATCAATGGGCCTAGAAAAACCTTTTTATCTA
GCTGTAGCACAACCAGAGCTAACTAAGTTACCATATTGGTTGTCCTTTTCCAGTTAGCCTAATGA
AGTCTCCTGATGGAGTAGGCAATAGATCAGACCGGATTTAATGAAAACAGAATTAATTCATGGA
GTTGTTTCATCAGCTTTCCAAGACCATCTCTCTCACTCGGACTGTAGGCAGCAGTTCTAAATATCT
GGCACTCTGTAAAAAATAACTCTATTTGCATTCTTTTTATACACTTTGAGATTTTGTTATTATTTAA
AGATATAAGAGGTAACTGAAGTGTGAATCTAATTATCTTCATGATCAGTTGACAAATGAATTACC
CACATAGTTGAAAGTGCTAAATTCTCTCATTGGTTTGCCAAATATTGATTACTCTCTTTTTTGTATG
TCAGACACTATCCTAGGTGCTGGGGACATAGCAGTGATCCAGATGGAAACAAGTTGCCCCACA

AATAAATATATAATTACATATTATGATAAGAATTAGAAGAGAGTTTAACAGGCAGACTTGTATCA
GTCAGGCTCCAGTGAAGAAAAGAGAAAAAACTCTAGGTATTTTAAGCAGGATATGGATTAATTC
AAGGAAGAGGATGTTTGTAAAACTCTTTTGAAGGGCTGAAGAGGCAGTTTAATCCTGGGTCTTC
AGGGGTATTTCTCAGAACACTGCAGAGCTGGCCCCCCTGGGGAGCTCCCATATGGAACTTTGCA
GGTCAAGAACACACTGCCACTTGCTTAAATTCTCTTATTGAAACTGTGTCTGAACACCTGGGTCG
CTACAAAATGGATATCCAAGGCTGTGATTCATGATTTAGCAAGCCAGTCAACATGCCACGATTG
CCACTAATGCCCCTGCACCCACTAAGTTCATTCTCAGGAATTTGAAAACTGGCACAGTACAGAG
AAACCTCATGTTTCCATGATAGTGCTTGCTAGTAGAAACTGCCAGGTGGAACCACATTAGCATC
CAGACCCTTAGCTGCAAGGGAGTCAGTAGTGCAGTTAGTAGCATTCTATCCTCTTCAAATAGGA
GGAGAGTGGAATGGAGAGTGAGTGAGATGATCTACAATGTCCAGCACAGGATGTTGATTGAGT
TTGAAGCTGGTGAAGACTTCTTTCAGGTAGTAATATTTCAGCTGAGACACAAAAAATGAGCTGA
AGGACAGCAGCCAAGGAGTTCAGTGTGCTCCACATGGAAGGAATAACATGAGAAAAGGCCTTG
CATTGGGAAGGTGTTTGGTGTGTTTGCACAGCTGGATGGGAGAGTGGTGGGGCAGAATTAGTC
AGAAGGAATGGTACACAGCTGGAGAGGTGGGAAGGACCTGGTGTGTAGGCTCTTCTTGATCAT
GTTAAAGACTTAGGATTTTTCTATAACTGCAATAGGACCATTTAAGTAGGGGAAGAAATCAATCT
GCTTTATATTTATAAAGGTTACTCTTGCTGATGTCTGACAACAGTTTAAAAGATGGTAGTTTAGAG
GAGGGTGTTTTCAGTGGAGGTATTGGAGAGGAATGGAGTAATAGGAATTACATTTTGGAGGCAG
AGCTAATAGGACTTGATGTTAGGCCAGTTGAAACAGAGGCAGGGGTAGGGAAGAGAAGCACC
CAAGCATGGCCCCTGAGTTTCCCATGTGAGTACGTGGATGGGATGTTCATACCACAAAGTGCAA
TTGGTAAGATGCATAGAACATGTTCTGTGGCAAGATCAATATTCTGTTGGACATATTATGCTTGA
GATGCCCCTGAAACATCCAAAAGGAGATTTCCAGCCTGTCTAGAAATGCATGCATGTCTAGATA
ATGATTGGTATGAACATGCAGGTGGACCTTGGGGAAGCCAGAGTGAAAAACAGGTATAACCTA
AATGAGTCAGTGCAGCAAGGATTATGCATTAATCTACTCCAAAAGGAAAAAAAAATCTAAAGGT
AGTACAGATGTAACTGACCACACAGTAGTAGTAGTAATAGTTCTTGTTTTTCCTCTTGCCTCTTTG
TTCTCCTCTTCCTGTTTCCCCCTTTTCCTTCCGTCTCTTCCTCCTCTTCTTATTTCTCCTTCTTTTTCT
CTCTTCCTTCAAAACATTCTTCTACGTTAATCTTCAGGCTGTTTCTTTTATTTTGAGCACAACCTCA
ACCTGTAAGAGCTTCCACAAAAAGAATTATTCTAAACTTTTAAATTGTGCTGATTCTTTGGAGGG
TTGTTTTGTCAGGTAAAAGTTACATTGTTATCTGTTTTATCTTCATTTCTTTTTCTGGTTTGAGAGAG
AACTGAGGAAAAGAACAAGATATTGTGTAGATTATTTTTCTTCTGGTCTTTGGGGCAAGCCAAGA
TCTTGGTTATAAAGAAGTACAGAAATCAGCAACAGTAATATTTGGAGTGAGGGATGTGTGAGCT
GACTTTACATACAGGCCTTTTTTAAATGGAAAGAGTCAACAAAATAGAGAGCAACTTGGAAGGG
GGAGAAAAAACAAACTGAAACAAAACAAAATTGCTAAGCAGAAGAGCCAAATATGATAGAAG
ATGAGTGACAGCTAATTAACACTTTCCATTGATTTTATAGAAGACAAAGTTTCTGTATAAAAAAA
TGTGGAAATTTGCAATTAAGTAGAATAGATTGGAGAGGAGAGAGAGAGAGAGAAATGGAGCAA
GAACATTGAAAAATAAATATCATCCTGAAACTTGCCTTTCAGTAAAATTGAAAGGGGCCTCTCCA
TAGGTTGTCTGGACTTCCTCACAGCATGGTGGCTGGGTTTCAAGAACAACCATCCCAAGAGAAC
CAGATAGAAGCTGCATTCCCTTTTGTGACCTTGCCTCTGAAAACACATAATGTCAATTCTCCCAT
AGTTACAGGTACACCCAGATTTAAGGCAAAGGAACAAAGACCTTACTTCACAATGAAAGGAGTT
TTAAGGCATCATTGAAAGAAGATCATGGGAGATAGGAAATACTGTTATGGCCATTCTGGAAAAT
ATAGTTTACCACACTATCCAGAAACAGGGTGCTCTTTTTATTTATTCAAGTCTTCTGTTGTGTCTCT
CTATAGATTTTTAGATTTTGTTCACAGAGATTGGGCAAATCTTATTTTCTTTTTTAATTTTTAAATTT
TTAAAAAACTTTTAAGTTCAGGGGTACATGTGCAGGATGTGCAGGTTTGTTACATAGGTAAACGT
GTCATGGAGGTTTGTTGTACAGATTAATCACTCAAGTATTAGGCCTAGTATCCATTAGTTATTTTT
CCTGATTCTCTCCCTCCCCACACCTTCTGCCCTTTGGTACGCCCCAGTGTGCATTGTTCCCCTCTA

TGTGTCCATGTGTTCTCATCATTTAGCTCCCACTTATGAGTGAGAACATACGGTATCTGGTTTTCT
GTTGCTGCATTAGTTTGCTAAGGATAATGGCCTCCAGCTCCATCCATGTCCCTGTAAAGGACATG
ATCTCATTCCTTTTTATGGCTGCATAGTATTCCATTGTGTAGGTGTACCACATTTTCGTTATCTGAT
CTGTTATTATTGGGCATTTAGGTTGATTCCATGTCTTTGCTATTGTGAATAGTGTTGCAAAAAAAT
ATGTGTGCATGTGTCTATATAATAGAACAATTTATATTCCTCTGTGTATATACCCAGTAATGAGAT
TGCTGGGTTGAATGGTATTTCTGTCTCTAGGTCTTTGAGGAATTGCCACAGTCTTCCACAATGGTT
TAACTAATTTACACTCCCACCATCAGTGTAAAAGTGTTCCTTTTTCTCCACAACCTTGCCGGCATC
AGTAGTTTTTTGACATTTTAATATATAATTATAGCCATTCTGATGGGTGTAAGATGATATCTCATTT
TGATTTTGATTTGTATTTCTCTAATAATCAGTGATGTTGAACTTTTTTACATATGATTGTTGGCTGC
ACAGATGTTTTCATTTGAGATGTGTCTGTTCATGTCCTTTGCCCACTTTTAAATGGGTTTGGTTTTT
TTCTTGTAAATTTGTTTAAGTTCCTTTTGGATATTGGATATTAGACCTTTGTCAGATGCATAGTTTG
CAAAAATTTTCTCCTATACTGTAGGTTGTCTGTTTACTCTGTTTAAATAGTTTCTTTAGCTATGCAG
AAGCTCTTTAGTTTAAGTAGATCCCATTTGTCAATTTTTGCTTCTGTTGCAATTGCTTTTAGTGTCT
TCATCATGAAATCTTTGCCCGTACCTGTGTCCAGAATGGTATTGGCTAGATTTTCTTCTAGGGTTT
TTATAGTTTTGGGTTTTACATTTAAGTCTTTAATCCATCTTGAGTTTATTTTTGTATATAGTTTAAGG
AATGGGTGCAGTTTCAATTTTCTGCATATAGCTAGCCGGTTCTCCCAGCACCATTTATTAAATAG
GAAATCCTTTCCCCATTGCTTGTATTTGTCAGGTTTGTCGAAGATCAGATAGTTGTAGGTGTGCA
GTTTTATTTCTGGGTTCTCTATTCTGTTCCGTTGGTCTATGTGTCTGTTTTGTACCAGTACCATGCT
GTTTTGGTTACTGTAGCCCTGTAGTATAGTTTGAAGTCAGGTAGTGTGATGCTTAGAGGGCACAT
TTTAAATAGGGTATTTAGAGTTATGATGTTTCATTTGGTGCTATTGTCATGAGAACATTTTCTTTAA
TTGCATTTCCTAACTGATTATTGCTTGTACAGAAAAGCTAAGGTATTCATGTGCTAATTTTGTAGC
CATTCACATTAGTAAATGTCCTGGGGAAGTACCAATTTGAAACATTCCTCAGTTACCAGGTCATA
ATGTGTTTGTGGTAAATTTTTACTGTCTTGTGACAAAGTTTCAATAGTTTACTCAAACCCAAAATA
CAACAGAAATAGAGAATCTTTCTTTTATTGTATCATTCACCAAAAGTGTCTCTGATTCAAACTTAG
TAAATCTACTTTATCTTTGTATAAAACAAAGATATATACTTTGTCATAGATATATGCTATGCTTTCA
CAGATATATGCTATGACATGATGGAAGAAATAATTCTGCTTATGGACCTTTTGGGGAGCTTGTGC
CTTTCAAGGTCTGCATTTTACAAAATATATGGAAGAGTTTGAAGTTGGCAATTTATTTTAATGTGG
TCATAAAGCTAGATTGTAGAACATTGCTGAAGTTTCACATTGAGCAGATTCCATAGACCAAGAC
CTTTTTGGAATTAAAGGACAGTTTGAGACTCAACTTGCTGACTTGTTCTGGCTCAAAAACTATAC
CTCTTTACTGATAAAACTACAATTTTTACTGAACAAAGTAAACCTGAAGGCAGAACACTATTCTC
AATCTGCAAAGAAGTAAGGGTGTTCAGATTCCAAATGAATCTATAAAGCAAATATCAATTTTTAT
TCTTCACTGGTAAAAAACTCTAAGCTCAATGGAATTACACTTACGTAAGGATTGCAGAGTGTATT
ACTGAGGGTTCTCCAGAGGGACAGAACGAATTGAATATATGTTTATATTGAAAGGAGTTTATTAA
GGAGAAGTGACTCACATGATCACAAGGTGAAGTCCCATGATAGGCCATCTGCAAACTGAAGAG
GTAGGAAGCCGTTAGTGGCTCAGTGCAAAAGCCTTAAAAGTGGGAAAACTTGACAATGCAGCC
TTCGGTCTGTGGCCAAATTCCTGAGATCCCTGGGCAGACCACTGGTGTAAGTCCAAAAGTCCAA
AGGCCGAAGAACCTGGAGTCTGATGTCCAAAGTAAGGAAGTATCCAGCATGGGAGAAAAATCA
AAGCCAGCAGACTCAGCAAGTCAACTTATCCCTTCTTCTGCCTGCTTTGTTCTAGCCGGGTTGTA
GCTGATTGGATGGTGCTGACCCACATTGACGATGGGTCTTCCTCTTGCAGTCCACCAACTCAAAT
GTTCATCTTCTCTGGCAACACCCTCACAGACACACCCAGAAACAATACTTTACCAGCTATCTAGG
CATCCTTCAATCTAATCAAGTTGACACTTAATATTAACCATTGCACAGAACTACTTACAGGAAAT
GAAACCACAAATTTACAAGTAAAAGTGTGTAAACAAATACAGTTATGGCTGCCTTTGGAGTGCA
AGCTTCAGGATGTTTCTGAAATATGTCTTGGCTTTGCAGTTAGCACTTACAACTAATATATTATAT
ATATTTTGCTTATTTATTTTGTTTATTACTATTTTCCATTTTAACATTAGCTCAATGATTTTCATCTGG

TTTTTAAAAATTCTATCTTCTCAGTACCAGATAATACCTAGCTTCCAGTAGGCATTCAATATGTAT
TTACTGACTGAATAATTCAATAGACAAATGAGATTGGAAGGTGTTTGAACTGAGACTGAGGTAT
CCACAACAGAAAGGCATATTATCTGTTGTTCTTTGGATGTGAGTATTGTGAGCTTTAAAGTTGTA
ATTCTACTAGATTCTGTGGAAGAACATTTTCTTTCACAAAATGAACTGAAGGTGAGAGTCACCAT
TAGTTGTGAAAATTTTGAGTTAGAATTGATGTATCTTAGTTGGAAGAAAAAAAAAAAACCTGGAAA
GAAATAGACCAACATTGACAGTAAAATGGATTGTGTGATTTTTTTATGCTCCTTCTTACTTTTCTG
TATTTTACAAATTTTCAACAACAAAGATGAACTACTTTTGAAGACAGAACAAAAACAAAAAAGA
AGTGGAAAACTATTGTGCCATTAAATTTGTTGTTGCTGTTGTTGTTTTGTGTTTTTTTGAGACAAG
GTCTTGCTCTGCCCCCTAAGCTGGAGTGCAGTGGTGCGATCATGGCTCACAATATCCTTGAACT
CCCAACTCAAGCGATCCTCCCACCTCAGCCTCCAGAGTACCTAGGACTATAGGCATGTGACACC
ACGCCCAGCTAATTTTCTTTTTTAGAGATGGGTTCTCACTGTGTTGCCTAGGCTGGTCTTGAATGC
CAGGCCTCAAGTGACCTCCTCCCTCAGCCTCCAAAAGTATTGGGATTACAGGCATGGGCCACCA
CACCTGGTTCCATTAAGTTTGTTAAAAAGCAGCATCTTACTAGTAATTAATATCAGAATATATGA
ATTAGAAGTAAAAGATGTATAACTGCACACTTAGGCACACATAAAATCAAACCACGTTTTCTTCT
GTTTTGAATATAACTACTGAAAAAATCTATATTTGAATAAATGACATATTCTTCTTTCCTTGTGTTA
TTAATAGTGGTTCCATGGCATCCCATCGACATATAGTTTTTTTTTCTTCTTTAAAAAAGTATAGAG
ATGGGATCTCACTATATTGCCCAGGCTCGTCCTGAACTCCTGATCTCAAGAGATCCTCCCACCTC
AGCCTTTCAAAGTGCTGGGATTATAGGGATGAGCCACTGCACCCGGCCCCACATATAGATTTTT
GAGTATAAGTTTAATTATTGAAATAGTTTTGACTATATGACCTTTAAGTTACCTTTGACTCTTATTC
TCTGCCACACTAAATTGGGCTTTATTTACATCAAAGGATTGTGAAAACTAAATAACGTGCAGATA
TATTTTCATACATGAGAAGACACTCTAAATTATAAAGGCCCACATACGTTAAATAATATTATTTAT
TAATTATCGGAAAATTGGTTGGAAGAATACCTCCCAGGACCTAGTCTAGTTCTCTCAGCCTCCCC
GTTGGTGGCTGTTTTCAACTTGGAGGGAGCTGGGTGGAATCTTTACTTGTCTGGGAATAAACGG
GAATAAGGGAACACTTTTTTCCTGTGCTGTGAGGCATCAGTCTGTAGGACACAGTTTAAACAGA
GGAGACTCCAGGCCCATAACATATGTAAATGCCTGTGCCAAGGGAGTAAGACCTGATGGAAAA
CTTTCCTGGAGTGTGGGGCTGCCCCAGCATGGGTCCAAAGGAGGCATTTCCCTACAAGTTTGCT
AACTTGGGAAAACTCCCAGCTAAGGGAACCCAAATCAAGTTTGTCTCTTCTGGAATGATACTGG
TTTGCGTTCTGTTCAGAGGCCTGCCACCCCAAATTATTGAGATCGTGGAGGGCATTCGATGGCC
TGTTTTATCGTGTAGGCTCCATATTAATAACAAGAGGAAGGTGGGTGTGTCCTAGCATCCAAAC
AAATTATCATTTGTTATTAAATTAGCTAATATTAAAATAAAATTTTAATAAGAATAAATTACTATTA
TATTATTACTAGTGCTTATTACTATTAAAGCTGCTTTTTATTCTTATTTATTTTTTCTTATACATGTTT
TTCAAGTTTCTATGTAATGCTTACATAATAAAATGAAATATTAATGCAAGTTACTTATAAGACATT
TAGAAAATGCAGGCAAGTAAAAGGAAGAACATTGAAATTATTTGGAATCCTGCTGCTTGGAGAT
AAGCATGGCAAACATTTTAGGATCAGATCTTCCAGATCAGGGAGGATTCCTTAAATTCTTTAAAC
AAAAATGGTCTTATGCTCCACGTACTATAGTCACCCTGTTCTTTTGCTTATAAAAAAATACTGTGA
GCATTTTCTTATACTAATAATGTTCTTCATATGTAACATATCAAAAACTGCATATCTAAGGCTTTTC
ATGCATATTAACTAGTTAGAAAAGTTGAATGTGTCCACACTCTGATCAGTGGTGTAATCCAGTGC
CCAGTCCCTCCCTCACTCTCATGTCAGCCTTCCCAACATCAGCAACTATAATAATTGGAAAATCT
TGGTCAGTAATGGTATCTAATTATTGTTTTAACTGGTATTTTGTTAATGACTAATAAGATTGGTCAT
TTTTCCCCAAAAGGTTTATTGAGCAATTGCCTTTGCCAGGATTGCTTCAAGATTCTATAGCAGTA
GAGTAGTGAGAGTGGCATCTATGTGGTGGAATGAGTTTTTATATGTAAATGTTTAATATTCAAGT
TGTACTATCTCTAAGCTCAGTCTCTTTTAACACATGTATACTCACTAAATCTGCCTTCTTATTCATT
AACACATTTACTGATGGTCTACTCTATGGCAAGCCTTGTTCTGGGTGCTGGTAATATAACCAAAC
AAAATTGACAAAGTGTCTGGTGCATACATTCTAACGGGGAGAGACAAACAACAACAAAGAAAG

AAAGAAAAAACAAGGTGAGATATTGCTGTAGTGGAACACAGAACAGAGTATGGGGGTTACAGA
GTGTCGGAGTGGGAAGGAGGTGCTGTATATTACTGGGGGATCAAGGAAGACCTCTCTGATGAG
ATGATATTTGGGCAGAGACCCAGATGAAGCAAAGAATGAGTGGACCATGCAGATGTCTGGAGA
AGACCATTCCAGATAGAAGGACCAGAAAGTGCAAAGTCCCTGAGGCAGTAAAGTAGTTGTTTT
GATGGGGGAGTGGCTAGAGCAAAATGAGAGGTCTTAGAGACAACAATGCAAGATGAGATCAG
AGAAATTGAGGGGTAGTGGAGGGCTGTTCACATAGAATCTTGTAGGCCGTATCCAGGGAGGAC
TTCAGATGTATTCTGAAGGAGCCCAGAGAGGAGACAAGGAGACCAGTAGGAAGCTACTGCAAT
GGTCCAGAGAGCAGTCAATAGTGTACATAGATATAAGCCATTTCTCCTTTATAAGCCTGCCCTTC
TCTGTCAAGCTGAAGCCATTGCCCACCTCTGGTGCCACCCAATCCCTCTCCTCCTGTGGCCAGAT
CATGGGTAGATATGAAAGAGGTGGAGTGGGTGCCTCCTATGAGGTCTGTTGGGTGCTAAGAGT
CAGTGTGACAGATAACCAAAATATCTAAGAGTGACAAGGCAGGATTAGTGGGTGTCACCCTCA
GAAGTCAATTTGACAATCTTGGTCTAGCACTTATGGGTCGAGAAGAACATGCTGGTAATACTAA
AGAAAAAGCAAAACAGAGGCACCATAGACTTCAGTAAGAAGCCTAAAAAAGAAGTGGCAGAC
ACAAACATGTAGTGTGTACCAAATCACTTCATATTTAATCTCTGTGATGAAGTACCTGGAAATCT
GTAAATGACAGCAAGAAGCAGCTTGACAGGCTGCTGTGTGTTCTACTTGGCAGTTACAAACTAA
GTGGAAGGTTTTCTCAAATCTGAGATGTCACTTTTGAATACAAAAGTAAGCAAAGCGGGTTCAA
AGTCCAAAGTCCAGGCCTCTATCTTATCCATAAACCAAATCAAAGAGCTATTAAACAAGAAGGG
CAGAGAGAATAAATGGCACCATTTATTTAGCACTTTATTGTTCTAGAGTGTAAACTTCATGAGGC
TAATGATATTTTACTCACTCCTATGCCCCCAATTTGGGAAACTGTGTGTGCAACACAGTAGGTAC
TCAATGAACAGTCTTTATATGGATGAATTTTAAATTCCTTTGGGTTATATGTATATTCTTGTAAGCC
TAGTGAGGATGAGATTACATTTTATTTCTTCCCATCTTTTTCTCCTCTCCTTATTTCTCTTCCATGTT
ACTGTAACAGATGCCATCAGCGACCTGCCCGTATCGCCTTGCCCCACCGAGAAGTCAGGCCAC
AGACAGTTCCTGTATATAGCAGTGCTTCTTTTGGCTTCTGTCTGTAGGTGTTTTCTCACCACAGAA
GAGTGCTCAGCCCTCTGGAAGTTCTCTAAAGAGTTAGATCTCCCAGGGGTGATCATCTATCAAT
GAAGGATGGGAATTGGTAAACAACTATTCCAGATTCCTTGCTTCTCAGTGGAATAATTCTGAGGT
GCATTCTACAGTTTCTCAGAAGGAATCGAGCCCTAGTTATTTGCAATGGTTTCTCACTCATTAATG
CATCTTTATGGCTTTACTACTTTCCGTATCTGAGGTCATCACTTTATCACTATTTCCTGGGACCACT
TCCTAAATAAACTATATACACCCAAATCCTTATATCAGGGGCTGCTTTGGGGGAAACCAAACTA
AGACAATTACCTACAATGCTTAGAAAAGCTGTCCATAGGCGGTTCTTGTGATGATTGAGGGAGA
TAATGCAAATCAAACATTTAGCACAGTGCTCAGGGAGTAAATGCTCAGCTCTCAGCCACCATTG
CTGCTGTTGGGGGTGCCCATTAAGTATTTATGCATGATTAATTTTGGGGGAGGAATTTTGAGGAG
GAATCAAAACAAAGACCAATGGGTAACCACAATAACAACAATAATGGTGGCAATTTATGAGGA
AAGACAGAGGCAAAGATCGATGACAAAGCTAGGAAAAATGGTGTCTATACTAGGTGTTAATCT
GAGTGGCACCAATAGCATAAGAAGGAAGACTTCACCCTGGGCACCAATGAGTAGAGCTTCCTG
TATTTTCAACAGAATTGATGAAAGCCTCATAGCTAGCTAGAGTATAAAGATATTTACATTCAAAT
GGCAAGGGCTCTATTAATACAATCCTGGAGAGAAGGGTCAAAAATTTGACTCCCTTTGAAGGAA
CAGAAAGATTCCATAGGTCTCATTAAAGTGGAGCAAGGTGGTCCAGTCTCAGGTAAGTGTAGAT
GGAGAACAGGAAAGCAAAGGGAGCTAGAATGCTGAGGTTGAGAAAATCAGAAAAAGGTGAAC
TGCAGAGCTCTGCACCCAGAAAGAAGCATTTAAATCTTTGCCTTTTTGATATTGTATATTAAATGC
TGTGTGAGTGACACTTGTGCTAACTGTATCTGAAAGAGGTCAGAGCTCATAGGGGAATTTTGCC
ATTAACTCTAAGCAGTGGCAGACCATTGGGATTATACTATAATGTCAACAAAGGTAAATGGTGC
TAGTAATTTTTTTCAGGACTGAGGCTTGTGCCTGACAGTGTTTGATTCTGTAGAATGCTCAAAATT
GGAAGTCTCTTTGAGGCACAGATAGTGTCCTATTCACTTTTAGATTTCCAGTGTCTCTCATTGTGC
CTAGCATGGAGTAGGTGGTCAGTAAACATACGTAGAAGAAGGGAAAAGAAAAACAAAATCAGT

GACATCGAGGTAGGAATGGAGAGAGAAGAAGAAATTAGTAAGTCCTCAATAAATGCCATAGTC
TATCGCTTCTCGGTCTTTTGGCTAAGATCAAGTGTAATGCCACAGTCTGGGTTGGGTGAGCAGGT
ACCAGTGGTACCAGGTTGGCTGCTTTTTCCTCCACTCTGCTGGTTGGGGCAAAAATAGACCAGA
GGTTCTCACTGATGTGGTTTCTTGTTAAACCACCACTATGATATACAGCTCATCCTGACCTGGTG
AAGTTATGTCTTCTCACCTACTTTTTATGAAAGATGGGATTCCACAATTGGAGAGAGGATGTGAA
GTAGGACAGGAAAGGAAATGTACATACTCTTGTCCATATGGAAAGGAGGCCTATGGCCAAATT
AATCAATAAAACATTTAGGTGTGGAGCCCAGGCTCGAAGGGGCATCCGGAAAACAGGACTATG
TGAGAGTTTGGATTCATTCTGACTCCTACGAGGAGACATTCTCCTACTCACTTTGGTCCCTTGGA
TTCCAAGTGTGTCTTACGGGTAATTACAGCTCATAAAGTGAGTCTCTTGTTTTATCAGTGAGATAC
TGTTGGTGAAAGTGCAGATACCTAACATGTTAATAAAGTTACCATCACATTTTATTTCTTGAATTT
AACTCAATGCCTGAAGCCCATATGAGTTATACTTTGATTCCTGTCATGACCAATCACCTTATGAT
ATTTGACCAAAAGCATGTATATTTTCCCCCATTTTGTTAGTTTTATTTATTTACTTTTTATTTCAATA
GTTTTTTGGGGTACAGGTGGTTTTTGGTTACATGGATAAGTTCTTTAGTGGTGATTTCTGAGATTT
TAGTGCACCTGTCACCTGAGCACTGTACACTGTACCCAATATATCATCCAAAAGCACAATATATC
TCTTGATTGGTGTCCCATCTGAACCAGTGAGCAGAAGGAGAAGAGTGGTTCCTGGACTTTCTTG
ATGTCTACAAAAGTTCATGAGCAAAAACAGAAACAAAAACCCAGTGATGTCAAGCTCATGTTCT
CCCAGTATCAAGGGTGTGGGCTGTGGTGATCACTTCTTCTCTGGGCTTCAGTGTCTTCCTCTTGA
AATGAGGTGCTTAGATTATGTAATGTTTGAGGTTCCATCTCAATCTGTTATTCTATGATTCCGATT
ATATACCAAAATTAGGAGAAAAGTTAGGTGTCAGGTGCTATGGGAGATTGCATTGTTAAGGCTT
TTGCCCCAGCTACCTTGCAGAGGAGAGGTTTCAGAAAGTTTTGCATTGTGTCTTTCTGAATCATT
CTACTTTTAGTACCTGTGGTAACTGAGTAATTCAATGATTTGTCAATTAACTTTGCTTATGCCCCTT
CAGCTGTAAAATCATTTTAATTTCTTTTTAAATTTTGTGTTCATAGGATCAAGAACAATACATTTAG
AAAGGCTTACTTACTCTAAGATTATACAAATATTCTCCTATGGAATTTTTCTATAGTTTTTGGATTT
ATTTTTAAATATCTAAATCCTTAATGCATCTGAAATTTATTTTTGCAAATGGTGTGAGTCAGGAAA
TGTAACAAATTTTCTTTCCAACTTGGCCCACTATTTCTTGGCTATTTGATAATTTATCCTTTTCCCC
ACTGATTTGAAATACTATTTTTATCCTATACAAATTCTCATAAAAATGTGACTCTTTTAATTTCTAT
TTTCTTAATATTAAATTTGTAGTGGATAATGTCGGTATACCTCCTAGATCTCTTAGATCCCTTTTAA
TGTTTGTTTTGGAGTACTTTGGACAGAGGTGTTTGTGAGTTTTGTACCTCTTAAGGTATAATCCCA
ATGGTCTGCCACTGCTTAGAGTTGTTGACACCTCTCTGTTTCTCTCTCTTCTTCCCTCTCCAAATTT
AACCAGTCCCAAAAGGGTTGGGCATTTAAATTGTTTTTAGTGTTTGTCTGTAAAAAAAAAATGCTG
CAATAAACATATTCACGGCATACTTTTGGATATTTCACTCACATATATTTCTGGAATAAATTCCTA
GCAATGGCACAGCCAAACTTCCTGCAGTTTTTTTTTTTACACTATCACATATTGTAATTAACAGTG
ACTATTTTTTTTTTTGGCAGCTTCTCCAACATTGGTTTTATAAAAACTAGCATTTTTTGACAAGTTGG
AGGATTTATAGTCTATTATAGCTTATTTATAGTTCTTTTAGTATATGCTGGTATCATGGAGTTAGTT
AATTCTCCAATGAAATTCATGCTTTCCTTTTCTTCTGAATTATTCCTTAGATGTGGCTGGCCAGGG
AGGCCTATTTTCTACCCCACTCCCTCACCCTTTTTGTGTCTAGATGCAGCCAGATGTCTAGTTCTT
GCTAGGGGATTATGAATTAAAGTGATGAATGACATTTACCAACTAAGACTTTCAAGAAATAGTG
TGCTTTTTCTCATTTCTTTCTCCTTCTGCTGGTAGGATGCAGAAGAAGATCAACCTAGGACTGGC
AGGGTCACAGGATGGAAAGGGCCTGGGTACCCAAATCACCACCTGGAGGAAAGCCACTTACC
AACCCAGAATACTCACCTTCTACTGTTACATAAATAAGAAACAAATATTATATTAAGCCACTGAA
ATTTGAAAGTTTATTTGCTTTAGGTGCCAACATTACTCTTATGAATATAGGTTACTTTGTAAATTAA
TATAACCTACTTAAAATTTGTTTCTTTTTTTTTTTTTTTTTGAGACGGAGTCTTGCTCTGTCACCCAG
GCTGGAGTGCAGTGGCGCGATCTCGGTTCACTGCAAGCTCCACCTCCCAGGTTCACGCCATTCT
CCTGCCTCAGCCTCCAAAGTAGCTGGGACTACAGGCACCCGCCACCACGCCCGGCTAATTTTTT

GTATTTTTAGTAGAGATGGGGTTTCACCGTGTTAGCCAGGATGGTCTCGATCTCCTGACCTCATG
ATTCACCCGTCTCAGCCTCCCAAAGTGCTGGGATTACAGGTGTGAGCCACCATGCCCGGCCTAA
AATTTGTTTCTTGATTTATTATTTTGAACATTTATTTTTCACCCCACTATGAATGATGAGTGAATTG
GTACACTTATGTTCCTCTTACCTCCCTAACCTTACCATCTCTCTATTTTTATCAGTATTCTTGTCATT
TTCAGGTCTACTATTGATTTATACTTATAGTAGATTTATATTCTTACCTTATACTTTAAAATAATAAT
ATATAGATTGATTTCTTGATTTTATTAGAGAGTATCACTTAACTCTGTGATATAAAATACTTGTTTT
ATACTTGTACTTCTTCTACCTCTATTTTCTTTTTTTGCTTTCTTATTTTAGCTAATTATATTTTACTTTT
ACTTTGCTAAAGCTTAAAACTTTTTTAAATTCTGTTCTATAATATTTATTCCCATAATGAGGTTTAT
CCTTCTTTTACTGGATTCAAAATTATGGCCAATTATTCAGTTTTTATTTTCCTAATTTTGTTAATTTA
ATTCAACCCTTTGTTATCTGAATTTTGTCATCAAATCGTTTTTGCACATTGGAGAAATTCTGTCTCT
TGCTGCTAATTTTGAATGACATCATTGTAATGAAGAATTCTGAAGGCAGTTTTATTTTTCTTTCTTA
TATTCCATCACTTTTTTTTATGCATGCATTTATGAACTTTTAAAAATATAAGTGACTTCAACAGCAG
GCCCAGTGGCTTACACCTCAGTACTTTGGGAGGCCAAGGTGGGTGGATTGCTTAAGCTCAGGA
ATTAAAGACCAGCCTGGATAACATGGCGAAATCCCATCTATACAAAAAATACAAAAATTAGCCA
GGTGTGGTGGTGTGCACCTGTAGTCCCAGCTACTAGGGAGGCTGATTTGGGAGGATTGCTTGAG
TCCAGGAAGTTGAGGCTGCAGTGAGCCATGAGCATGCCACTGCACTCCAACCTGGGTGACAGA
GGGAGACCCTGTCTCAAAAAAAAAAAAAAAAAAAAAAGTGACTTCAGCAGTCTATGTCTCTCCCTC
CCTCACTTTCTTTTTTTAATATTAAATTTTCTGAAATTTCTTGTCCCTTAATTTAAGTCTTCCATCATT
TTGGGAATAACTTCTTACATAAATATTTTTCAATTCCATTAGTTGTAGTCTCTTTTTCATGAAGCAA
ATTGTATGTACGGTTCACCCTTCATATCCATTTATGCAATTCAACCAACTGCAGATCAAAATATTT
GGAAAAAAACAACAATAAAACAATAAATAATAATACAAATAAAAACCAATACAGTATGACAAG
TATTAATATTTACATAGCATTTATGTTGCATTCAGTATTCTAAGTGATCTAGAGATGATTTAAAGT
ATACAGGATGATATGTGTGGGTTGCACATACTATGCACATACTATACCATTTTATATAAAGGACT
TGAGCATCTGTGGATTTGGGTATCCATGGAGGAAAATGGTCCCTGAAACCAATACCCTGCAGAT
ACTGAGGAAAAGAAACAAAAGTCAAATGGTTTTACTTTGCCATAATACCTTAGAAGTTGTTGACT
TCTTTCTTAATTTTTTTTAGAAAATCATTCTTGGTAAAGATTTTCCTCTGGAATGTTTTGTTCTTATC
TAAAAATGTCACCTTTAATAGCATAGCGCACACAAGTCATCAATCTTAATTATGGCTCCATTTCA
GAAAACCAAGAATGCAGCTGGGCGCAATAGCTGATGCCTGTAATCTCAACACTTTGGGAGGCC
AAGGCAGGTGGATTGTTTGGGTCCAGGAGTCCGCGACCAGCCTGGGCAACATGGCAAAACCCT
GTCTTTACAAAAAATACAAAAAGTTAGCTAGGCACGATGGTTGTGCCTGTAGTTCCAGATAGGA
GGCTGAAGTGGGAGCATTACCTGAGCCCGGGGCTCAAGGCTGTAGAGAGCTATGATCATGCCA
CTACACTCCAGCCTGGGGGATAGAGCGAGACCCTATCCCACCCCCCCCCAAAAAAAGAAAACC
AACAAAGCAAAGCAAAACTGAGGTTCCAGTCCATCATTCCTGAAGAAGGAGCCAAGGGCAGCT
TTGAATGCTGTAAATCATGCTGTAAAACTCATTGGCAGGTGTTGAAAAAATCCAGTGGGTTTTGG
ATCAAATTCTGCACTATGAGAGTTCATTATTCCCTCTGGTGAATAAGTGCATTCATTGATAAACCT
GGCAAAATTTGGAACAAAACCAATTTAAAGTGCCATATAAAAATTTGTTCATTTGATAACCTTAA
AGTGATTTCAGTTCTGTTTTACATTAGTCATCAAAGATAAGAGCAAAATTGTATTTATGATGGCA
GTGGCTTTCAGTATCTTGTATAAGAAAGGGGCTGTTAAGACTGCCACCAAGGAATGATGATTTC
AAAGTTCTCATTATGTACTTCAAACTTTCCAACACAGTTGTTATTATTGCTATAGTAAAGAGAAAC
AAGAATCCAAACATTTCCTCTTTTTCCTCCTGATCAACATCTTCATCCTTTCCTTCCTTGTCACCCT
CCAGGTCACAGGGGTCATCCATCTTTCCTTCTATGTGACTGGTTTGCTCTTGCATATCATGTTTTC
CGGTAAGATTGACTTGGAGGTTATTTCTGAGGGGATTTTTAATATTTGCAAGTGATATTTCCATAG
ATTAATAACAATCTGACTTTTAAAAAGTTTTAAAGAAATCTTAGCTAGATCTCCCCACTTCACCTC
CTCTTAACTTTTTCCCCTTTTTAAAAGCCTATATAAAAAAATGAATAGTGAGAACCTCCCACTAGC

AACCCACATATTTTCCCTCCAAATGGGCTGCTCATCTTTTGAGGCAGTTTTTCACATGGTTTTGGC
TTTCACACTTTTGGTCGTCTGTCATGTTTTGTTTACTAGAACAGGTGTAGATTTAGTTCTATCTTTA
CAAAGCATGTACAATGGAGAGGAAAATACAAGACTTTGCTTTATAGAAATGAAAGAGGAGGAA
TGAAGCCTTAGGCATACAAATAATAAACTGGAAGCCTGGAGTTAGTTTTCCTTATGCTGTCCTGA
CCTCTTTGATGAGTCCAGGTTTTTGTTTAGTGCCCCTGGGAAGGATCCATGTGATCGTAATTTTCG
GGGAGAAGATCATCCAGCGTCTAGTGTTGTGTCTTTACTTTAGCTGTGTCTCAGTGGGAACCTAG
TAATTTAAAAAAATCCATGTCAAGTCTCGGGAGGCTGAGCTGGGAGGATCACTTGAGCTGGCGA
GGTGGAGGCTGCAGTGAGCCAGATTGTGCCACTGTACTCCAGCCTGGGTGACAGAGCAACACC
TTTTCTCAAAATAAATAAATAAACAAATAAAAGAAAAAAATCCATGTCATTGGAGTTTTCTCCAA
GTTGTTCTCTTTGTGTAACATCTAAGATGGAAGCCTCAATCCTACCACTGCCACTCCTCCAATTCA
CAGCCGAGTATTCTGCAACTTCGAGCTCGTAAGGACCAGTGTACTAATGGCAGCAGAACATTCA
AACTCAGTAGAATGCTGCCCAGTACTGTGGCTATGGGGGCTGTGGTAGTCAACAGAAGAGGCA
GTGGCTCCCAGGGAGTGTGATGGTGCAAAAGAGGAGAGTAGTTGGGGAAAAGGCAGACTGCA
GATGAGCTTGCATGGGAGCCCTCTTCAAAGTACCATAGAGGAGGGATTTTTACAGGCACCTGGG
GTATGCATTAGCAGGAGCTAGTAGAGAAGGGAATGGAGAGCCAGCAAACATGTGTTATGATAT
CTTCTGAGAATGATATCTTACCAGATTTATTTAATATTTCCCTCTCTATTGTCAGTGAAAATGTAAT
GGTATAAGGGGAGAGTTGATGGAAGAGTGCTTACAGATTTTGGTACTTTGGTTATCTTTGTAGTA
AAATTTCCTAAACTCACAAGTAATTCATGTTCTCAACATAAAGATAACAATGAAAGAGTCTTACA
CTCCTGTGAGGCTCTCTCCTTTTCAGAGGGCCCCCTATAGATGCCCCTCATGCACAGTATTTCAG
TGAGGTTGACAGACCATGTATTTACTTCCACTTTGACAGGGGCAACCTGAACCTTCGAGATGTG
GAAGTATTTCCAAGAGTGAGGCAGCTAAGAAATGGAGGGACTAATAAACCCAGTTATTCTAACT
CCAAGATCATACTCTTTCTACCAAATCATGCTCATTATATTAATTTTTTAAAATAACAAAAATGGC
TTACATTAATTTTTTATAAATTTATATGAAATATACCACTGGGCTAAGCTGATTTCTATAGTTTCTA
TAATGTAAATAGAATAATGATACTATGTAGTAATGCATTATGCAATTTTCAAAGCACAGCAAATT
TTAAATAGCATAAGAAGTTAACAGGCAAGTTATTGTTACCTGTATTTGACATAGAAGGAAACTGA
GGAAAAAGGAAGTTAAATAACGTCTCAAATCTTCTGATTCCCTGTCATCATCTAAGAGTAGATTG
CTTCTTAAAATTCTTCACATCATCACATAAGTAACCTTTATAACTATGATATAGCCACATTTGTAT
ATTTACTGTCATGTTCCTAAATTTAACTCATGATTTTTCTTCCTTCATATGCTTCGTATATTGTCCTG
GGCTCTCTTTTCAGGGCATTTATGGTTAACTCTGAAGCAAAGTTGCTTCCATCTGTAATTAAATAG
TTCATTGATTTTCTTGGAAGCAAAATAAGATCATCTCAACAACCACATGCAAACATCCCTCTTTA
CCTCCCTCCCAAACCCAGCCTTTAAAAAAAAGCATGAAGCTTGTCAAAAATGTCACCTTTTATAT
TGGCTATGTATTAGAAAAACAAAAACAAAAATTGATTTGAAAATAATTGAGGTGTGTGGATGTC
AGAGGTGTTGCCTGCTGTGCTAACTGCCTTGCTGAGACTGATTCTTGGTATCTGTTGGCCTCCTT
GAGATAGCTACTGGGCTTGCACCTTCACCAAAGGAGACATAATAAAAAGTTTGCAGATATTTAA
GAAGGAAAAGAAAAACAGCCTTTTTTTTTCTTTCCTATAGCAGTCAGGAAGGACTATATATTGTA
TATATTGCTATATATATATATATAGCAAATCAACTGGGAGAAACCACCTTCAGCATTGAGTCTGA
AGCACATCCCTCCAGAATGCTAAGTTCCTTTCTCTTCAGCATCAAAAGCCAGAACACTCTTCTTG
GATCCCAATCACTGAAAGAAGCTTTTCTAAAAAGAAGTAATTATGTATCGAACTCTCTACTGAAG
AGAATAATTATGTAGAATAATTAAAGGGGATATTTTAGAAATTTTTCCCACTTTTTGATGTCTACT
GCTCCATGGAGAAAGTTAATGTAATTCAAAGATTAGCTAAAAATTTGGCAGGAAATAGGGGGA
GCTAAAACCATTGTGATGTGGATGACTGGGATGTAGACTCAGAGAGCCTTTCAATGTGTGGAGG
CTTCATCCCTAAAACTGTAAAATAGGAATATGGAGAGGACAGTAGTAGTTACCTGGGGATAATA
ATTCATATGTAAAATCTTTGAACATTCATTGAACTGTTGTCCTCCTTCCAGTTTCAAGGAGAGGA
GCCTTAGCATATGTAGCACCTGCCATATCAGGGAATGGCTATTTAGTATTGAGAATACATGGAG

GTGCATTTTAAAGTCAGATGGTATTTATTTCAACTCACAATCTCTACTTTTTACTTGTACTTGGATG
TGATTAGACTAGGGAGGAGGAGGAAGAAGGACTACTTAACTGAAGTTATCAAATATAAGGCTTT
CCCACAGAATAACTGTAGATGTGATGGTTATGTCAGTCATGACCATTTTGGTTGACCAGAACATC
AGAGACAAACCATATCATTCTGCACCAGCCCCTCCCAAATCTCATGTATTTTCATATTTCAAAAC
CAATCATGACTTCCCAACATTCCCCCAAAGTCTTAACTCATTTCAGCATTAATGCAAAAGTCCAT
AGTCCAAAGTCTCATCTGAGACAAGGCAAGTTTCCTCTGCCTGGGAACCAGTAAAATCAAAAAC
AAGTTAGTTACTTCCAAGATGCAATAGGGGTACAGTCATGGTATAAATACTCCCATTCTAAATGG
GAGAAATTGGCCCAAACAAAGGGGCTACAGGTCCTATGCAAATCCAAAATCTAGCAGGGCAGT
CATTAAATTTTAAAGCTCTAAAATGACCTCCTTTGACTCCCTGTCTCAGGGAGTCAGCTCCCAGT
GGATCTACCAGCTCTCAGTGGATCTACCATTCTGGAGTCTGGAGAATGGAGGCCCTCTTCTCAC
AGTTCCACTAGGCAATGGCCCAGTGAATACTCTGGGTGTGGGTTCCAAACACACACTTCTCTTC
CACACTGCCCTAGCAGAGGTTCTTCATGAGGGCTTCACCCCTGAAGACTTCTGCCTGGACATCC
AGGCATTTCCATACATCCTCTGAAATCTAGGTGGAGGTTCCCAAACCTCAGTTTTTGACTTCTGT
GCATCTTCAGGCCCAACACCACATGGAAACCGCCAAGGCTTGGGGCTTTCATCCTCCGAAGCA
ATGGCCCAAGCTGTACCTTGGCTCCTTTTAGCAATGGCTGGAGTTGGAGTGGCTGGGACACAGG
GCACCAAGTCCCTGGGCCTGGCTCACAAAATCATTTTTTACTCCTAGGCCTCTGGGCCTCTGATG
GGGTGAATGCTGTCAAGATCTCTGACATGCCCTGGAGACATTTTCCTCATTGGCTTGGTGATTAA
CATTGGCTTCTTGTTATTTATGTAAATTTTTGCACCTGGCTTGAATTTCTCCCCAGAAAATAGGTTT
TTTCTTTTCTTTTCTTTTCTTTTTTTTTTTTTTTTTAGATGGAATATCTCTCTGTTGCCCAGGCTGGAGT
GTAGTGGTGTGATCTCAGTTCACTGCAAGCTCTGCCTCCCAGGTTCACGCCATTCTCCTGCCTCA
GCCTCCCAGGTAGCTGGGACTACAGGCGCCCGCCACCATGCCCAGCTAACTTTTTGTATTTTTTT
AGTAGAGATGGGGTTTTACCATGTTAGCCAGGATGGTTTCAATCTCCTGACCTCGTGATCCACCC
GCCTCAGCCTCCCAAAGTGCTGGGATTACAGGCATGAGCCACTGCGCCTGGCTAGGTTTTTTCT
TTTCTATCACATTGTCAGGCTGCAAATTTTTCAAACTTTTATGCTCTACTTCCCTTTTAAACATAAA
TTCCAATTTAAAATCACCTCTCTCAAGTTCAAAGTTCCATTAGAGCAGGGGCAAAATGCCACCTG
TCTCTTTGCTAAAGCATAGCAAAAGTTACCTTTGCTCTAGTTCCCAAGAAGTTCCTCATCTCCATC
TGAGACCACCTCAGCCTGGTCTTCATTGTTCATATCATTATCAGCATTTTAATCAAAACCATTCAA
CAAATCTCTAGGAAGTTCCAAATTTTCCCACATCTTCTTATCTTCTTCTGAGCCCTCCAAATTGTT
CCAACCTCTGCCTGTTACCCAGTTCCAAAGTCACTTCCACATTTTCTGTATCTTAATGGCAGTGTC
CCACTCCCAGTGCCAATTAACCTCATTAGTCCATTTTCACACTGCTATAAAGAAATTTATCTGAG
ATTGGGTAATTTATAAAAAAAAATTTATAAAATTTACTCACAGTTCCACATGGCTGGGGAGACCT
CAATAAATTTACAATCATGGCAGAAAGGGAAGCAGGCACCTTCTTCACATGGAAGGAGGAGAG
AGAATGTGTGAAGGAGAAACTGTCAAACACTTATAAAACTATTAGATCTCAGGGGGACTATCAT
GAGAACAACATGGGGGAAACCTCTCCCCATGATCCACTCACCTTCTTCCCTTGGCACATGGGGA
TTACAGGTCCCTCTCTGGACATGTGGGGATTACAATTTGAGATAAGATTTGGGAGGGGACACAG
AGCCAAACCATATCACCCAGTAAGATACTCCATGAGAAGATCAACCCCAAGATACACAATCATC
AGATTCTCCAAGGTTGAAATGAAAGAAAAAATGTTAAGGGCAGTCAGAGAAAAAGGACAGGTA
ACCTACTAAAGGAAGCCCATCAGACTAACAGTG

ID|55978|gene_id|ENSG00000224698|transcript_id|ENST00000419814 Homo sapiens lncRNA

GAGTAGCTGGGATTACAGGCGCGCGCCACCACGCTCGGCTAATTTTTGTATTTGTTTAGTA
GAGACGGGGGTTTCACCATGTTGGCCAGGCTGGTCTCGAATGCCTGACCTCAGGCGATCCACC
CGCCTGGGCCTCCCAAAGTGCTGAGATTACAGGCGTGAGACACCGCGCCCGGCCGGAAAGTG
GCTATTTTAAGAAGATTGAGAACATGTTTCGTTGGGTCGCATTATTCTGCGTGACGAGGCTCACA

GAGGAGAACCCCACCCTTACTCCAAAGGCCTTTGAAAGCAGCTCTGGAAAGGGCAGGGTTTGC
TTCTCCCTCTGCCCAACGTCTCCCCAGGCTGCCTGTGCCACCTTCCGGTCTCACCTCCTCGGAGT
TGGGGTCAAACGCGGAGCTCCAGTTTCTCAGCCCCTCCTGGAGCTCAATGATGTCCACCACGCC
GTCCCCATTGCAATCCAGGTCTTCGAAGAGGATCCCGTAGCGTAAGTAGTCATCGTTATCCTGG
CAGGCCACCGCCTCCAGCACGAAGCCCTGCATCCAGAGCAGCATGGCTGGGTCCAGAGGCTG
CGGGGACCACTCGGCCACCCAGCCAGCACCGCAGCACTCGCCGCCGCCTCCTCGACGCCTGG
CTGCGCAAGGCAGGGGGTCCCGGCCGTCCGCCAGCTTCTGCAACTGTCTAGCGGAGCCGCCGC
AGTTCGGAGCCCACGCGGGCCAAGCCACCCGCGCCTGCGGGCTCTTGGGCGCCACGGAAAGG
CTGCGCCACCTTGTGGGACCTCTGGCCGAGGCGCCACGCGACAGTTGAGACGCCAGATGTGAG
TTGAGGTGCCAGACGTGAGTTGAGGTGCCAGACGTGGCGGCGTTGGGTACCCAGGACCCTCGG
ACGGGTAGGTCGACGGGGGAACCAAGGGACGTTTGGGGAAAGGGAGCGCATTCCCCAGCAGG
CTTGAAAGGGCACTCGCGGGTGTTTAAGATTCTCGCGGTACAGACTCAGTTCCTGCCGGAAGTC
ACTGCTGCGGGAGATGTGGCATGGAGTGAGCTGCTCAGACTCGCTCTTATGCCAGCATCCAGTA
CTCTGGTGCCACCCTTCCTCCCAGGACGACCTGCACCTGGGGGTCAGGACCCCGGCGACCCCC
AAAAGAGGTCTAAAGAGGGAAGGCCTCCTGAGGAGCCTCTGCCCTGTCCCTGTCTCTTCCCTCC
TTACCGGCCTAGCCCATGTTGGCAAACCTATTTTGGCCATACTGGGATTAGACTTTTCATGTTCTT
ATATGACCTTTTTCCAGTTAATTGGATTGGGTGCAGAAATACTTCTATCTCTATTAATAAAAGAAA
AATAAAAATAAATAATAACTAAAGGGAAGATACATACAGATTAAAATTAGAACATGTCTCCAAA
TCCAGAAATATGAAACACACATCATAACAGTTCCAATTTATATGCCACATTTCTATTCATAAATTT
TGTTCTAAAATAAAGAGAAAAAATAAATAGCAAAATACTTTAAAGTAGATCTGAAGCATTCAAA
GTATTTTTTGGTATGCATTTATTTTAAATCAATCTCTGATTTTTATTATCTATAGGGCTATTAAGGG
ATCCAGTTGCCGTTTATTTTTCCTCTATATTTTAGAAGTTCGTATAAACAAATTTAATTTCTAATTC
CTTGTTAATTATGAGGCATGATAACACATACTAATTTATCAAGATGTGCTTTATATTAGTCACATT
TATGTGAACTTTAGGAGTTGATTATATTTAAGTTTCAAATCTTTCTAGTAATCCTATTAAGAAAAA
AAGGGAGAATCTAACTAAAAATTGACTTTGGTTACTCAAATTTATGTTAATTGTGCATTTATATTC
ATTTCAATTTTTAGAAAATTTAACAACTTAATCCCATCATTAATCATTTATTAAGTACCCTATCCCA
ACCTGAGAAAATTAAATTTCTCTAATTTCACAGCAAATTCAAACATGGCATTTAACAAGAGGGAA
AAAATGGTATCAAATATTCTTCTGAATTTTAACACTTTACAGTGAGAGCAGATTTTAACTACATTT
TAATAACCTTGAGAAGTATGTTACAACCCCAAATATTAAAGTAACTCAAAATCTTTAATTTATAAA
ATGGCAGAAATATCTGTGTCCTGAAAAACTGGAAAAATGGACTGATCTAATGTATTACTAGTTGT
TTGGTATATTATAAATTATATAATTATAGTTACATTTATTAAATCACAATTTTAGAACCCATCTAAT
CTTTATAATGTAGCTATACATAGACATCTTAAAATATCTTTGGCCTAGTCTCTTTCAACATAAATT
GAAAAGATAGAGGCAAAAAAAGTAGGGATGGATGAGAATACTATTCATCCTTCATGTAAGTTTA
AGACTGTAAAGGGAAGTTCCAGTCAGCCTTTGTTCTAGTAGATCGGAGCTTGAAAGTAAAGTAT
AAAGTACATAGAGAAAAAAAGAAAAACTCAGTCTTTTTTAACACAGATTTAAGTATAAGAGCTC
ACACATTTTTCTGAATTACTTTGGTCACGTTGGTGATTTCGTGTAATGTTTAAATATTAAAAAAAA
AAGCAAGCGTAGCTAAAAATGTCTGTATCTCTAAATAAGAGTGCTCAGAAAAGGAATAAGAGGT
CACCATTATTCCATTGACAACACTGGTAGGAGACTGAGGTATTCTGATGCCATGAAATTCTGTTT
AGGACTTTCTATTTTGTTTTGTCTATTTTAGAGAAAGAAAAGGAGCCCAAACTTGACTGTTTTGCT
ATGAATTATAGTTTTAAATGCGTGAGTTTTCTATTAGTGCTTTCAAACATTTTAAATGTTCGCATAT
GGCAAGGAGAGGTGAAAAAATAAAAATAAATTTAAAATGCACATAAAGCACTAAAATGTTAGC
TTTTTAAAATACAAAGTAGAGAATTCAGGAAAACTTCAAAATATTCCAACTGTTTAATATGAAAT
ACTAGGTTAATTATATACTTAGGTTATATTCTGTATTATAAATTGCATTCTAATCCACTTAAATGCA
AATTTCTATGACTGTTCATTAAAATTTGGAGAAATATAAATAGATTTTATGAATGTTGAAGATTTT

AATTCACCAATAAATTAATAAACTGTTTCTTGTTTACTTAAAATTCAATATTTAATTTGTAAGTTTA
CCTCTTGGGAAAATGGATATAGCATCTTTTCATACAGACCTTTTGCAAATTAAGGAAAAAGCCAA
GACATTTTCATGTTTATAAACAAGTGTCCAGATAATGACGGTTCAAGATTTCATTTCTGCTTTTTAT
TTTTGAGACGGAGTTTCACTCTGGTTGCCCAGGCTGGAGTGCAGTGGCACAATCTCGGCTCACC
GCAACCTCTGCCTCCTGGGTTCAAGCGATTCTCCTGCCTCAGCCTCCTGAGTAGCTGAGATTACA
GGCGTGTGCCACCATGCCTGGCTAATTTTGTATTTTTAGTAGAGATGGGGTTTCTCCATGTTGGT
CAGGCTGGTCTTGAACTCCCGACCTCAGGTAATCCGCCCACCTCAGCCTTCCAAAGTGCCGAGA
TTACAGGCGTGAGCCACCATGCCCGGCCTCAAGATTTCGTTTGTAAAATATTCCCCAAGAGCGG
TTTTGCTGATTCATAAACCACACAGTTTACAACACACTGAAGTAAGAAACTTTATGAAATATGGA
GTCATGCCTCTGGAAAATCCTGTCACTCCACTCCTTTTATAAATTTAAGAAAATAAGCTGATCAT
ATTTAGCTGTGGGACTCATATCATGGCTAGAGAATTCAAGGAAAGGGAAAAGTCCAATTAATTT
GATATTTTAGCTTTCTTTTAAATAGTAAGCTAAAACTTGCTAAAACAGATAATAAAATAAAATTAA
TTAAATTACTTCACAAGGATCCCCAAGGGAAAATCTCTGGTTAATTACACTTTATAGTCGGAGGC
TCTCATGGAACTATGTGATGATCATCACTATTTAATTTTCATTCCTTTTAGGAACAAAGTCAGTCC
ACTAATGGTGAAATCTACTTATAATTATTGTGGAGTTACAATTTTAAAAATTCTCTAGGCATACAC
AGATACCTTCATTCAACCTCTCATGTCTTAAGAACATCTCAAATTCAGAAACAAAAAAAAATATG
TTTCTCATTCCATGTTTCACTTTTCAGGGAATGGCTGCACCATTCATATAGCTTCTGAAACTGAAA
ACCTGGAATGTATCCATGACATTTCCCTCTCCTTCATCTTACACATCTAAACCATCACCTACCAGT
TTCACTCTCCAAGTTTCTCTCAAATCCATCCACTTATCTTCATGTCCACTGCCACACCTTCTGTCTC
CAAACTACCATCATCTCTTGCCTAGACTGTTGTGATTACCCTCCTCTTTCCATTTTTTTCTCTATCC
TTTAACCAGAGTAAACTTCCAACAATGCAAATCTGATACTTTCCTTCTTCAGTAATTTCCCATTAT
GCTTAAAGACAGTATCATCTGACCTCTGTTTCCTCCTCCCAACTCACCTGTACCACTCTTGCTTTC
TGTACTCCAGCCATACAAGCTACTTTTCAGTTTCTCAAAGTGTCCCATTCTTCTTCCTTCTTCAGG
CTCATTACACATGCTTTCCCCTTTGCATGAAAGTCATTCCCTGTTCTTTGCTCATTTAACTCCACCT
CAGTCTTCAGCTCTCAGTTCAGTATTTTTCTCTGTTCATATATTTTTTAAGAAACTCTTTCCAGAAC
TTCCTCATTTCCCCTATTAATGGCCCTCAGAGAACCCTATTTTTCCTCTGTAGCTATAATCGCAGT
TTATAACTATATATTTGTATAAATGTTTAATTAATTTCTGTCTTCCCTACTAGACTATCAATTCTATA
ACAGCAGGGTCTCTGTTTGCTCACCCGAACCCCATGTGTTTGGCACATTGCACAGTACCTGGCT
ACCTTAAATTCTCAAATTACAATAGCTAGTATTTTTCTTTCTAATCTTTATATTTTGCTAGCATTTTT
CTCTCTAATCTTTATATTATATTCCCAAAATTGATGTAGCTAAATAGAGTGAAATCAATAGGCAAT
TGAAAAGTAAGTCTTTCACTGTTTTAGTCTAAACATTCTGTTCCCCCCTCCCACCCACCCCTACAA
GAAATTAAATAACTCCTTTAAAATGTAGAGAAGATCCCTTGAAAATTTAGAAAATATTTACTTGC
CACGGAATTTCTTGGAAAATGTTCTATTGCTATATGCGTCTTTTTGCTTTTTAATCAGAAACTAGT
AACATCCCCTCCAGTTTTTATAGTTTTGTAATTAAATTGGAAACTTTCTTACTTTTTTCTAATGTTG
AACTTACTTTGCCCATTGATTTTCTCCCAACTTATTATGAAAACTGTAAACTCTAAAAAGGATGGC
ACAATGAACATTTATATATTCACCACCTATAAAATTTAACATTTTGCCATAATTACATTTTGTCTCT
CTCCATTCTACTTAAACATATATATAAATGTGTGTGTGTCTATCTGAGCCATTTGAAAGTAAGTTA
CAGATATGATGACACTTAACCATAAAGTACTTGGACATATCCTAAAAACAAGGAGATTAGCTTA
CATAACCACAATGCCATAATTATATTTTAGAAATTTAAAAATAATTCTCTATTACTTGAGATGCTA
AGGCAGGAGGATCACTTGAGCTCCGGAGTTTGAAGCCAGCCTGGGCAACATAGCAAAGCCCTG
TCTCTTTAAAAATAAATAAATAAATAAATAAACAAACAAACAAATAACCTCTATACCATCAATAC
TTATTCTATATTCAAATTTTCCCAATTGTCCCCAAAATGCCATTTACAGATTTTTTCAAACTAATAT
CCAAATAAATTTACCCATTGCCCTTACTATGTTTATTCTCTTAATCAAGACTAGTAACAGTCCACC
ACCAACACTGTTTTTTCCACAACAATGACTTTTTGAAGAACCCAGCTCAACTGTCTTGTAGAATA

TTCCACAATCATGATTTGTTTGATTGTTTCTTAATGGTATTGTTTAACTTGTTCCTCCATCACCTGT
ATTTTCTACAGTTCTAAAGACATGGTCAGATTCTAGTAAAAAAATCTGGGCAAAAATACTTCATA
GGAGAGGCTATGAAATTTGCTGTCTCCTCAGTTTAATAACATCTTTGTTTTCTTTCTAAAATATTTA
CCATCTGTCCTGTAACTTGGACCCTATTCACCTAAGCTGAAATTTGTTAAATATCTAAGAAAAAA
AAACAGGGTGAATGAAGTTCTTATGTCAATGTACTTTTCTTCTCTCTCTTTGTTTTTCTTGCATTGT
TTGCTCTCCCATTGTTTGTTCTCCCATAACTTGAAGCAGTGACTTTCTTCCTTATTACTAGCAATAA
GGAAGGACATCTTATAATAATAAAAGGGTCAATCCTAAATTTATGTGCACATAATAATATAGCTT
CAAAATACATAATGCTAAAGTGGCAAACGTAGAGGAGTAATAGGGAAATCCACAATCATGGTT
AGAGATTTAAACATACCTTTTTCAGTAGCCAGTAAGACAAGCAGACAAAAAAATTAATAGGAAT
CTAAAACATTTGAATAACACATTTAACTAATTGATCTTATAGAACACATATATAGAACACAAAAT
ACAACTTCAAAATCCATTTATTTGAAAGAACAGAAATCATACAAAGCACATTCTGATGGCAGTG
CAATTAAAACAGGAGTTCACCGTCAGGAAGGTAATTGGAAAAATCCCCAAATGTTTGTAAATTC
AGCAATATATTTCTACATAACTGATGGGTGAAGAAGAAATCACAATGGAAACTGGACATTTTGA
AATAAACAATAATGAAAACATTACATATCAAAATTTATTGAGTGTAGGGAAAGCAGAACTTAGA
GGGAAATTAAAACTTTAAATTAATATACTAGAAAAGAAAAAAAAAGTCAATTATTTAAGACAGG
GATTGGCAACCTTTTTCTGTTACTGTCCAGATAATAAATATTTTAGGCTCCGCAGGCCATGTGAT
CTCTCGCAGTTACTCAACTCTGCCCTTGTAGCCATAGACAATACAAAAACAAATGGAAGTGGCT
GTGTTACAATTAAATTCAATTTACAGAAATAGGTGGCAGGTCAGATTTGGCCCTCAGGCCAAAG
TTTGCCAACCCATGACCTAAACTTCAAACCCAGGAAACTAAACAGGAAAATAAACAGAAAGAA
AGGAGGATGAAATAGTAAAGATAGGAGTAGAAACTAATGAAACAGGCCAGGTATGGTGGCTCA
CACATGTAGTCCCAGCAATTTGGGAGGCTGGGCAGGCAGATCACTTGAGGTCAGGAGTTCAAG
ACCAGTCTGGTCAACAAGGTAAAAAATCAGCCAGGCATGGTAGTGCATGCCTGTAGTCACAGC
TACTTGGGAGGCTGAGGTACATGAATCCCCCATATCATTTCTGATTGTGTTTATTTGAATTTTCTC
TCTTTTCATCTTTATTAGTCTTGCTAGTGGTCTATTTTATTGCCTTTTTTTTTTTTTTTCCAAAAAACC
AGCTCCTAGATTTGTTGATTTTTTGAAGGGTTTTTCATGTTTCTATCTCCTTCAGTTTCACTTTGAG
CTTCATTATTTCTTTTCTTTTTTTTTTAAATTATTATTATACTTTAAGTTTGAGGGTACATGTGCACA
ATGTGCAGGTTAGTTACATATGTATACATGTGCCATGCTGGTGTGCTGCACCCATTAACTCGTCA
TTTAGCATTAGGTATATCTGCTAAAACTATCCCTCCCCCCTTCCCCCACCCCACAACAGTCCCCA
GAGTGTGATGTTCCCCTTCCTGTGTCCATGTGTTCTAATTGTTCAATTCCCACCTATGAGTGAGAA
TATGCAATGTTTGGTTTTTTGTTCTTGTGATAGTTTACTGAGAATGATGATTTCCAATTTCATCCAT
GTCCCTACAAAGGACATGAACTCATCCTTTTTTATGGCTGCATAGTATTCCATGGTGTATATCTGC
CACATTTTCTTAATCCAGTCTATCATTGTTGGACATTTGGGTTGGTTCCAAGTCTTTGCTATTGTGA
ATAGTGCCGCAGTAAACATATGTGTGCAAGCGTCTTTATAGCAGCATGATTTATAGTCCTTTGGG
TATATACCCAGTAATGGCATGGCTGGGTCAAATGGTATTTCTAGTTCTAGATCCCTGAGGAATCG
CCACACTGACTTCCACAAGGGTTGAACTAGTTTACAGTCCCACCAACAGTGTAAAAGTGTTCCT
ATTTCTCCACATCCTCTCCAACACCTGTTGTTTCCTGACTTTTTAATGATTGCCATTCTAACTGGTG
TGAGATGGTATCTCATTGTGGTTTTGATTGAACTTCTCTGATGGCCAGTGATGGTGAGCATTTTTT
CATGTGTTTTTTGGCTGCATAAATGTCTTCTTTTGAGAAGTGTCTGTTCATGTCCTTCGCCCACTTT
TTGACAGTGTTGTTTGTTTTTTTCTTGTAAATTTGTTTGAGTTCATTGTGGATTCTGGATATTAGCC
CTTTGTCAGATGAGTAGGTTGCAAAAATTTTCTCCCATTTTGGAGGTTGCCTGTTCACTCTGACAG
TAGTTTCTTTTGCTGTGCAGAAGCTCTTTAGTTTAATTAGATCCGAGCTTCATTATTTCTTGTCTTC
TGCTAGCTCTGCGGTTTGTTTGCTCTTGGTTCTCTAGTTCTTTTAGTTGTACTGTTAGGCTGTCAAT
TTGAGATCTTTCTAGCTTTTTGATGTGGGCATTTAGTGCTATAAATGTCTCTCTTAACACTGCTTTT
GCTGCACCCCAGAGATTCTGGTACGTTGTCTCTTTGTTCCCATTGGTTTCAAAGAACTTCCTGATT

TCTGGCTTAATTTCATTATATACCCAGGAGTCATTCAGGAGCATGTTGTTCAATTTCCATGTAGTC
GTGTGGTTTTGAGTGGGCTTCTTAATCTTGAGTTCTAATTTGATTGCACTGTGGTCTTAGAGACTG
TTTGTTATTATTTCAGTTCTTTTTGCATTTGATGAGGAGTGTTTTACTTCCAATTATGTGATCAATTT
TCGATTAAGTGCCATGTGACACCAAAAAAAATGTATATTCTGTTGTTTTGGGTGGAGAGTTCTGT
AGATATCTATCAGGTCCACTTGGTCTAGAGCTGAGTTCAAGTCTTGAATATATTTGTAAATGTTCT
GTCTCAATGATCTGTGTAATATTGACAGTGGGGTATTAAAGTTTCCCACTATTATTGTGTGGGGG
TCTAAGTCTCTTTGTAGGTCTTTAAGAACTTGTTTTATGAATCTGGGTGCTCCTGTATTGGGTGCA
TGTATATTTAGGATAGTTAGCTCTTCCTGTTGAATAGATCCATTTACCATTATGTAATGCCCTTCTT
TGTCTTTTTTGACCTTTGTTGGTTTAAAGTTTTTTCTGTCAGAAACTAGGATTGCAACCCCTGTTTT
TTTTTTTCTGCTTTCCATTTGCTTGGTAAATTTTCCTCCATCCCTTAACTTTGAGTCTGTGTTCATCT
TTGCACATGAGATATGTCTCTTGAATACAGCATACCGATGAGTCTTGTCTTTGTATCCAACTTGCC
ATTCTGTGTCTTTTAATTGGGGGCATTTAGCTCATTTACACTTAAGGTTAATATTGTTATGTGTGAA
TTTGAACCTGTCATCATGATACTGGCTGGTTAATTTTGCAGACTTGTTAATGTAGTTGTTTCATAA
TGTCACTGATCTGTGTACTTCAGTGTGTTTTTGTGTGTGATTTTGCTATCCATGTTTAGTACTTCCT
TCAGGAGCTCTTGCAAGGCAGGCCTGGTGGTGATGAAATCCCTCAGCATTTTCTTGTCTGAAAA
AGATTTTATTTCTCCTTTGCTTATGAAGATTTGTTTGGTCTGATATGAAATTCTGGGTTGGCATTTC
TTTTCTTTAAGAATCTTGAATATTGGACCCCAATCTTGTCTGGCTGGTAGAGTTTCTGCTGAGAGG
TCCGCTGTTAGTCTGATGGGCTTCCCTTTGTACGTGACCTGGCCTTTCTTTCTGGCTGCCGTTAAC
ATTTTTTCCTTCATTTCAACCTTGGAGAACCTGATGATTATGTGCCTTGGGGTAGATCTTTTTGTG
GAGTATCTTACTGGGGTTCTCTGGATTTCCCTGATTTGAATATTGGCTTGTCTTGTTAGGTTGGGG
AAGTTCTCCTGGATGATATACTGAAGTGCGTTTTCCAACTCGGTTCCATTCTCCCCAACTCTTTCA
GGTACTCCAATCAGTCATAGGTTCCATCTTTTTACATACTCCCATAGTTCTCATAGGTTTTGTTTGT
TCCTTTTCGTTCTTTTTTCTCTAATTTTGTCTGCCTGCCTTATTTCAGCAAGATAGTCTTCAAGGTCT
GATATCTTCTCTTCCACTTGGTTGTTTTGGCTACAGATGCTTGTGTTTGCATCATAAAGTTCTTGTG
CTGTGTTTTTCAGCTTCATCAGGTCATTTATGTTCCTTTCTAAACTGGTTATTTTAGCTAATACCTC
CTGTAATCTTTTATCATGGTTCTTAGCTTCTTGCGTTGGGTTAGAACATAATCCTTTGGCTCAGTG
AAATTCATTATTACCCACTTTCAGAAACCTGCTTCTGTCAGTTCATCAGTCTCAGCTTCAGCCCTG
TTCTGTGCCCTTACTGGAGAAGTGTTGAGATCGTTTGGAACAAAAGAGGCATTCTGGCTTTTGAA
ATTTTCAGTGTTTTCGCATTGGTTTTTCCTCATCTTCATGGTTTTGTCTACCTGTGATCTTTGAAGCT
GTTGACCTTTGGATGTGGTTTTTGTGGGGTCTTTTGTGTTGATGTTGTTGTTGTTACTTTCTGTT
GGTTTTTCTTCTAACAGTCAGGCCTCTCTTCTGCAGGTCTGCTGCGGCTTGCTGGGGGTCCACTC
CAGACCCTGTTCGCCTGGGTATCACCAGTGGAGTTTGCAGAACAGCAAAGATTGCTGCTTGCTC
CTTCCTCCAGAAGCTTCTTCCCACAGTGGCATCAACCTGATTGATGTCAGCCGGAACTCTCCTGC
ATGAGGTGTCTGGCGACCCCTGATGGGAAGTCTCACCCAGTCAAGAAGCACGGGACTGGAAGG
GGGACCTCCTTACGGAGGCAGACTGACTGTCCCTTAGCAGAGCTGGTGCACTGTATTGGAGGA
ATAACCCTCCTTGGGATCAGCTGGTCTCTTCAGAGCCAGCAGGCAGGAAAGATTAAGTCTGCTG
AACCTCGGACCATGGCCGCCGCTCCCCCGAGGTGCTCTGTCCCAGGGAGATTAGAGTTCTTGTC
TGTAAGCCCCTGACTGTAGCTACTGGAATTCCTGCAGGGATGCCCTCCAGGTGAGGAGGGATG
GCTTCGGGTCCCACCTAGGAAGCAGTCTGGCCACGATCTGCAACAGCCACTTTGCTGCGTTGTG
GGGAATTCCACCCAGTCCAAACCTCCTAGTCTCCTTAGCACTGTCAGGGGAAAACCTCCAACTA
AAGCCTCAGTAATGACGGTCGCCCCTCCCCCTGGGAACTGAGTTGTCCCAGGCGGACTCCAGA
CTGCTGTGCCGGCAGCGGGGATTTCAAGCCAGTGGTTCTTAGCTTGCGGGGTTTCCGTGGGAGT
GGGACCCTCTGAGCGAGACCACTTGGCTCCCTGGCTACAGCCCCCTTTCCACAGAAGTGGACA
TTTCTCCTGCCTCACTGGAGTTCCAGGTGCTGCCGGAGTATGTAAAAATTCCTGCAGCTCAGTGC

CTGCCCAAACAGCCGCCGACGGGAGCAGCTGTCATTGGTCTGCCCAGTTTTGTATTTGAGACCC
AGGGCACTGGTGGTTGTAGGCTCCCGAGGGGATCTGCTGATCTGTGGATTGCAAAAATCTGTGG
GAAAAGCTTAGTACCCTTGGTGGGTAGCACTGTCCCTCACCACTTCCCTTGGCTGGGGAGGGAG
GTCCCCCTGCCCTGTGCAGTTCCTGGGTGAAGCAATACCCTGCTTCTTCTCGCTCTCTGTGGGTC
ATGCCGACCATCTAGTCAGTCCCAGTGAGATGAACTGGGTATCTCAGTTGGAAATGCAGAAATC
ACCCACATTCCTCGTTCCTCTCGCTGGCAGCTGCAGACCAGAGCTGCTTCTAGTCGGCAGTCTT
GGCCCCTCCCCGCCGAGGTAAGTTTAATAAGTCTTCTAAATTTGTCCTTGCCATAATGATCAGTG
ATGATCTGTCTTTTAATAAGTTAAATGCTTTCCAGACTTCAAGACTCTCACCCACATCAAAAATAA
AATTATATATCGGCCATATTACCAGGATTTTAGAATGTTGTATTTTATCCTCTAATTATAGGTCAA
ATAACTAAGGTAGAAAGGAGTAAAGCAACTTCCATGTATGTAGTGAGGATGTTGGGGGGTCTG
GGACTAAAATTCTGCCTTGGGATTCCCAAGAGGTTGGTGATTATGATTTTTGTACTCTTAGACAC
AAAATAAAATAACATTCTGTTTTGTTC

ID|55984|gene_id|ENSG00000227332|transcript_id|ENST00000418741 Homo sapiens lncRNA

CCAGGAGTAGGAGCCCTCTATATCCCTGCTCCTGAAAAACAAGCTAACTGGAGTCTCCATC
ACCTGCACCCAGCATAGACACACTACCAACTACCCAACTGAACTCCATGACTGATTTGCCAGCC
AATCATGCCCCTGACCCAGCCTACATGGACATGGGAAGGACATCAGTGAATCGGGAACAGAGG
CAGAGGTGAAGAGGCCACCTGTCCTGGGCCACACATCTATGTAGTCCAGCAATCTCCAGCCCCT
TCGTACTCCAGGGGCTCTAAGCCACCCCTCTGAAAGTCAGGACGGAAGTAGATGACACTACATT
TCTATCTGCTGTAGACACTCCTCCCAGTGTCTCAAAATGTTTTAGCATCTTTCAGTAAAATTCTTC
AAGTTTGTCAGTCCTTGATTTAAACAAAAGGAGCGAACTTTTTAGAGCTCCCTTGAACTCTTTATC
AAACTTCTCATAAACCCCAATATTTTGATCTCTTATTGAAGAGTCCATACTCCTATCCAATCCAGT
GTTGTTTCTCTTCAAACTTGGCCTTCCCCTGCTCATTCCATTTTTATCTACTTCCATTGGGTTCACC
AGCTAATTCCATTCTCATACTATCCACTGGGTGCACCAGACTCACTCCCATTTGTATTATTAAAGC
ACATGCAAATAGGATATAAAAAGAAGGAAGAGTACTGGGCCTCAAAATGAGTTCAAATCTCATT
TCTGCCAATTCCTATGTCTAAAAAAAAAGCATCCTAATCTCTTTGAGCCTCACATTCTCTGAGAAT
CACTTGACCAGAATGTTCAATACAGGTAAAAATACTAGAAAGTATTTTAATTAATTCTAAGATTC
CTTAAAATTCTGTAATTCTATGTCCTCTTGATTCTGTCTATAGGAAAACTGGGAATACATACCCAG
CAGAATTT

ID|55991|gene_id|ENSG00000234978|transcript_id|ENST00000423385 Homo sapiens lncRNA

GCTGCATAAGGGGAGTGAACACAAAGTACATTTGACCACAAAATGACTCTTTAAAAGTCA
GAACTACGGTAGCATGAAGCCAAACGAGGTAATCTACAATAAAATTTTCTATGCTTCTTTTCCTT
CTTTGCTCTCTTTCTACTCTAATAACTGCGATTCACACAGGTAATGAAGAGTGTAATTCCTTGATA
GAAACACAGCTCCAAGATTAATCCTTTCTTTAACTATGAAGTTCGCGTGTCCAAAATCTGTGGTA
GTTGCTGTCTGATTTTTGATCACTGATGGTGATACAGATATTTATCATCAACTCACAACTTCCCAA
ATCTTTGAAAAGTCTTACTATTGATGGTTCAACTAGTAGAAACATGATGTAAAATATCTGAAAAT
AAAGTTTTTATTTATTAGAATGTAAATAATAATACAAATTGTAATAAGGTGTAAAAGTTCTTTCTT
CACTGAAGCAGTACCATGTTGTCCTCTACCCCACAAATGCACTACTCCCCCGTGGTCTAATGTAT
TTTAAAAGTCTTGTAATTGCTATTAACTCAGACAAGTTTACTTAACTTGTTCTAAGCTTCTGGCATT
TACTACAGTTTACTTTCAATCACTCAACCATCTCTGTTATATATGTTGTTTTCCATGAGAAATTTGT
TTATTAGTAATTAAGATTCTTCAGGGATAAGAAAATATTTGAATAACTAAGTTTGTGCATAAACA
CATTAAGGTCAAATACCCATGACATTATTGTGTGTTTCTGTGTACTAGAGACAAAAACTTCAAAA
AAAATTTAATGAATATACGTTAAATTAAAAACTGCTTTCATTAAACTGAGATAATCTTCCCTCAAT

GCATGAATACCTTCAGAATTCACATAGACCAAAGAATTGTATAAAATATAATAGCCTTAAAAATC
TTATTTGTAGCTGGCACAGTGGCTCCCACCTGTAATCCCAGCACATTGGCAAGCCGAGGTGGGC
AGATCACCTGAGGTCAGGAGTTCAAGAGCAGCCTGGCCAAACTGGTGAAACCCCATCTCTACT
AAAAATAGAAAAATTAGCAGGGTATGGTAGCACGTGCATGTAGTATCAGCTACTCGAGGGGCT
GAGGCAGGAGAATTGCTTGAACCCGAGAGGCAGAGGTGGTAATGAACCAAGACTGAGCCACT
GCACTCCAGCCTTGGTGACAGAGCAAGACTCTGCCTCAAAAACACAAACAAACAAACAAAAAA
CCTAATTGTTCCCATATAAGTCTATGTTCATACAAGATCTGAAGAGTACACAACACCGTGAGACA
GGACAGACATATATTTTAAAAGTTATATTCCTGGTTTCTGTAAAAATAAAATAGTTGAATTTAAGC
TTTCAAGACAAGTCAACGAAAAGAGCAAAAAATGCAAAAGTGAAACTCGAAAGGTCATTTCCC
CATCAAGGGCTCATGATCACTGGACATTCACAAACTATACTGTTCAAAACATTAGATCTGAATTT
TGATCCGAGTATCCCTTTAGTAGCAGTTTCATTCAAGGATGTCCAAGAGGTAAAATAAGACAAT
ATCATTTGCTATTTTCAGTTTTCTTTTCTGAGAACAGCCCAGCATTCTTCTTCAGAGAAATGAATT
GTCCTAACTTCATAGGCTAAAGGCTCATGAGTCATAGTTCTAAGGGCATTAATAAAATATGGTGG
TGCATGCTTGTATTCTGAACTTTTCAGGTTTAAACTCTCATATAGTAAATGCTAATAGATACAAAC
CGATTAAAGAAAAGCCCTCTTAATCTGACATTATTTTTCTTTTTATTTCTTCATTTATCAGCAACAG
GAGAGTCTAACTAAATGTGGTAAAGTGGTATGAGGGAATACAATGAACAGTGTAAAATGAATTA
AACCAGAGATAATCACACCAATGTGGGTACAAGTGGAAAGTATAATACAAAACACACCAAAGA
AAGTGGCAGAAAGGTATATAAACTGTATAACCACTCACATACCATTTTAGGACACAAAAAATTC
TGCATATTATTTCTGAGCATCACAATGTAGTTAAAGATTTCAAAAGGGCATTGAAATGAAAAACA
ACCAACTTATGATGTTGGTAGCCTCTATGCAATCATGTTTTAAAAACTTTAACACCAAAAAGGCT
CAAAATCACCATTTTAAAAGGCTGTGTCTACCAGTCATAAATGAATCATTACTTTCATCATTTGTA
GTAGTCAAAGATGCCACAAGCGCACGCATACACACATCTATATATACACCTACACACACAGTCT
TACTCATTAGAACATCTGATAGGCTTCAGATCATCAGTGTAATAACACTAGCAGCAAGCCTCTGA
AGTTAAAACAGAAACTGACACTTTAATAAGTAAAGCTTTCCTCTAGGTAAAGATCAGAACTCCA
ACTAGCACTTAACTCACTGGAAATATCTTAAGAGTCTCAAAATTCACTGCTTTGAATCCCTGACA
AGTATAAAAATTTTATACTGAAAACTTCATGCTATTCAAAACATTAAAACAGAAACATCTGACCT
AAAGCTTACATTTTTAAAATCTTTTTTATGCTTCTAAATTTGTTTTTATTCAAATATGGATACCAAC
AATAACATTTATGTCAAAGCCTTCTGTTCAATATTGAACAAATAGAATTAGGAATAAGAATAATA
TGAGTACATCCAATCATTGAATGTACTTTATTTACAGTATTCCATTAAATGTACCTGCTCTCAATG
TCTGTACATTCTTTCTTTGTACTGCTCCTTTCACAGCAGGATCTTCCACTTCAGTGCTAGGCTGAA
TGGGTTTTAAAAGAAAACGATTCATAAATCATATATATTTTATACAACATGGAGTTAGTGATTCA
AAAATATACATAATTAATTACCTTCAAGGAAGGATGTTTTGCAGGAGGCCCTACAAAGCAAAGG
GGATATGTCATCAATTATATGTAAGTATGACAGGGCCAACCAAACATTCATGCAGTGTTACTGTC
GAGCTGAATTCTCAGGCCTGGCTATAAAAATAATTACTTAAGGTTTTGAGGGTTCTTCTTGGCTT
CTTCTTTTCATTGCCTAGGACAGCAACATGACAGAAACACAATGAGGAAAATAGGAATATAGGA
TTCCCAAAATGCACAGTTTACATTTCGGTAGTGAGATTATGTTTCAAATGCCTATACTTAAAATAG
AAAAGCATTGATATAACCGTGAACACGTGGACTGATAAGGAGAAAAGGGACCATTAAACAGAG
GGGCAAATTAAACCTGAGAGAATCAATGTCAAAGCTGATGGTGAATGTACAGAGTATTTTAACT
CCACACACCAGAGGCATTGCTGCCAGCACAGCACAAATAAATTCCCCTTGTCTTGTCACTGAGG
AAATACGCAGTTGGGATGACAGTTCAGATGAATGTGGGATTCACCTCTCATCAAAGAAAGGGTT
CTACATTGATCAGCTAGGATACACACTTATGAAATAACAGCTAATCAAACTACTCATTTTTCCCA
TGATCACATGGGCTACTGCAGCACCTACATTTCTCCTATCCCCTCATTTGGCCTTGAATTAGAGC
TCCTTGATCCACTCATGCAAGGTGGTCCATAAAACACATCAAATAAACCATGTCGAATAAGCTTC
TGATATCAAAATATTTATCAAAAAAGAAAACATTGAATGACCACAGACTTGCTGGATATTAATAC

ATATTTATATTTCAAAATCAGTGCAGTATTTATTGAAAATGATAATTTTGGTTTTCATGGAATGAAT
TTTATGATTACTTCTAAAATTAACTAAGTTTGGTATATTATCTTACACTGTAAAGGACTTTTATAAA
ACAGCTATCATATCAAAGAACTGGCTGTCTCAAAAAAATTTCACCAAAGCATCTATATGCAAGTT
AATCATATCTTATTCACTCATGTCAGTGAAACTTCTCTCCCTGAGGCCTGACAGTTATCAAGTGA
AATGAGCTGCTGTGGTTTACCCCAACTCTAGCACTCCCTCCTGTCTCCAGTACTCTCCACAGCAA
TAACCTCTTTTGTGAGACTGGGCATATGCTGAAGCAACTGGAAGTGAGTTGTCTCAAGTTTACTT
GGCTTTAACTCCCAAGACCCCAGCAAATGTCCTTCTTTCCTCCTTTTGTGTCCTTTCACCATCCCT
CTTCCTTTGAAAAAATGATTATCAGAACTGTCATCCTGATGCTTCCCTTCCTAACTGCTTTTTATG
GATGATTGTGACCACTTTTTTCATCTGTATTCAGCAGTAGTATACACCTGTAATCTCTCTTTTTTCA
TCTCATTTTCCTTCCCCTGTGGCTAGAATCATGCTCAGAAATAAAAGGAAATTAAAGCTTTCCCT
GGATTCTGTTATTTTTTAAATTGCTCTCCAGTGGTTCTTTTTCCAGATTTCTCTAAAGGAAAGCTAT
TCCCTTGCTATTCAGAGCTGTGTCCAAGGACCAGCACAAACATCACCTGAGTGCTCATGAGAAA
TGCAGACTCCAATACCTGCTGAGTCAGAATGTGCACTTTCCAGAAGCTCCTCAACGAATTCATG
ACAATTTGAATGCCCTGTTCCACACTGGTGTGCTTCCATATTGGTTTACCCTAACTGGCCTTTTTG
GCCTAGCCTCAACTTCTTTCTTATTATGTCCCTGAATTTAATACTACGTTATAAACCATAATGTTTC
TAATGAACTTTTAATCAGGCAAAGCTTCTCTAATTAATTTCTTCCCAATAAATCACCCAACACTAT
TCTTTTCATTTTCTATAAAAACAAATTATAGCCATACATGGCTGACCATTTACAGTGATGTTCATC
TATGGTAGATAAAACACAGGTCTGCATGGTAAAGTACCTCAATCCTTAATGCCTCCCCAGTAGC
GAGAATGACAGCAAGAGAAGGAAAATGTTACTGTAATTATATGACACATTTTGGTACTAGAAGC
TCACTTTATCTTCCTTCCTATTTCTAACACCCTGTTCCTCCTTCTTCTACAGATCAATTTGACTTTAC
TACCCTCCATTACATACATCCACTCTTTTTTATTTATTCCATGTACACTCTTCCCTCCTCATTCTTTC
TTTCTCTTTTATTCATTTCCTCTTCCCTCTCTCCTGACTTGCCTCAGGTCTTAGAGTATGTTAAAAT
GGAACTCATAACTCAGCTCCTTTAGTGGTACTTCCAATAGAATCAACTGCTGACCCTTGGTTAGA
GACACCACTTATCACCATTTCATTTCTCTTTTACTTATGATACAGTTAATAGGACATTTTCTTTAGC
TATTAAACTCTATTAGTGCTCATATTTTAAAAGAAACATTCCATCAATGCCTTTTTTTTTTTGAGAT
GGAGTCTCACTCTGTCACCCAGGCTGGAGTGCAGTGGCACGATCTCCGCTCACTGCAAGCTCCA
CCTCCTAGGTGCACACCATCCTCCTGCCTCAGCCTCCCGAGTTGCTGGGACTACAGGCACCCGC
CACCATGCCCGGCTAATTTTTTGTGTTTTTAGTAGAGATGGGGTTTCACCATGTTAGCCAGGATG
GTCTTGATCTCCTGACCTCATTACCCGCCCACCTTGGGCTCCCAAAGTGCTGGAATTACAGGCGT
GAGCCACCGCGCCTGGTCTCCATCAAATGACTTTTTAAATAAAATACGGTTCTCACCTTTTCCTTT
TCCATTGACTATTCTGTTTCCTTTTTCATGAGAAGGTCCACGTAAAGGCTCTGACACTTTCTCGGG
GACACACTGCTAAGGTAATATCAAGAATTAGTTTCCATTTAAAATTATAATGAGTTGCATCAAGA
GTTTCTTATCAATCTCTTTTTATGAAACTGGGTCTCACTCTGTCAACCCAGGGCTAGAATGCAGG
GGCCTGATTATGGCTCACTGTGGTCTCAAACTCCAGACCTCAAGCAATCTTCCCACCTCAACTTC
CTGGATAGCTGGAACTACAGGTGCATACCATCATGTCATGCTAATGTTTTTATTGTTATCTTTGTA
GAGACAAGGCCTCATTATACCTCCCAGGCTGGTCTCAAGCTCCTGGGCTCAAGCAAATCTTCCA
CTTCTGCCTCCCAAAGTGTTGAGATAAGCAGTGTGCACCACCACACCCAGCCCTAATCAATTTCT
TTAAATCAATCTCAATGTTGCCCAGGCATGGTGGCTCACACCTGTAATCTCAGCCCTTTGCAAGG
CCAAGGTGGGTGGATTGCTTGAGTTCAGGAGTTGGAGACCAGCCTGGGCAACATAATGAGAAC
ACATCTCTACACAAAAAATACCAAAAGGAGTCAGGCATGATGGTGTGTGCCTGTAGTCCCAGCT
GCTTGGGAAGCTGATGTGGGAGGATCACTTGAGCCTGAGAGGTGGATACAGCAGTGAGCCAAG
ATCATGCCACTACACTGCAGCATGGACAACAGAGCAAGACCCTGCCTCCCCAAAAATTTCAATT
TAAAATGTGAGAACAAAGAGAGATACAAACAAAAAACAAGCCTAATTAGTCAATGAAATATGA
GCTTAAGCCAAGAAAGAAAACGAAAAACATGAAGTATAATAAAGTACATGGGGAAATAGATCT

ATAACAGAGCCTTCGGTCTTTCATAACTCTGATAATACTAATTAATATTTATGCTGCAATTAGTTT
TTTGTAAGTACTTCTGTGATAGTGTTTCTTACTATAAGACATTCAATTAGCTAAATATGGTCATCT
ACCATTACCTGAAAGAACATTATTATAACAGAGAGAGAAAACTGGAACTTTCCATCAACTTTCC
ACCCAGAAAAAGAATTGGTCACCAGAATTCTAAAGAGTAATGTATGGCAGACACATGAAAAAA
TGCTCATCATCACTGGCCATCAGAGAAATGCAAATCGAAACTACTATGAGATATCATTTCACACC
AGTTAGAATGGCAATCATTAAAAAGTCAGGAATCAACAGGTGCTGGAGAGGATGTGGAGAAAT
AGGAACACTTTTACACTGTTGGTGGGACTGTAAACTAGTTCAACCATTGTGGAAGACAGTGTGG
AGATTCCTCAAGGATCAAGAATTAGAAATACCATTTGATCCAGCCATCCCATTACTGGATATATA
TGCAAAGGATTATAAATCATGCTGCTATAAAGACACATGCACATGTATGTTTATTGTGGCACTAT
TCACAATAGCAAAGACTTGGAACCAACCCATATGCCCATCAATGATAGACTGGATTAAGAAAAT
GTGGCACTTACACACCATGGAATACTATGCAGCATAAAAAAATGATGAGTTCATGTCCTTTGTA
GGGACATGGATGAAGCTGGAAACCATCATTCTGAGCAAACTGTTGCAAGGACAGAAAACCAAA
CACCACATGTTCTCACTCATAGGTGGGAACTGAACAATGAGAACACTTGGACACAGGATGGGG
AACATCACACACCAGGGCCTGTCATGGGGTGGGGGGAGAGGGGAGGAATAGCATTAGGAGAT
ATACCTAATATAAATGACGAGTTAATGGGTGCAGCACACCAACATGGCACATGTATACATATTT
AACAAACCTGCACGTTGTGCACATGTACCCTAGAACTTAAAGTATAATAATAATAATAAAGAGT
AATGTATGGCTTGAAACAGTATATTTAATGGAACATGAGTTGGGTCTAACAAAAAGCTTAAGAA
ATGTTAATCTAAAATCTCAATGTTAAGATTCCAGTTGAATGATACTAGAAAATATATTGTAACCCT
CTTTGCTACCGATGACCTATTTCTCTTTTATTTCCTTTTTAATTATGGCATAATTTCTCAACATAACA
TGTCAAAACTTATACACCCTTAAATATTAAAAAATAATACAATGTAAGCAATATTTTAAATACAAT
ATTTAATGATTAGATACATTAGGTTTATTATATTACTTATAACATTCCATTATATAAAAATTCATTT
GTTTATTTATTCAGATTAAACAACTATTAAGGCTGAATGTCTCATGTCTGTAACCCCAGCACTTTG
AGAGGCTGAGGCGAGCAGAACACCTGAGCCCAACAGTTAAAGAAGAGCCTGGGCAACAAGGC
AAAACCCTATCTCTACAAAACTCAGCCCAGCATGGTGACACAGGTCTATGGTGTCATAGCTCTA
TTGTTTCAACTACTTGGATGGCTGAGGTGTGAGGATCACCTGAGCCTAGGAAATGGAGATCGGA
GTGAGCCAAGATCTCACCAGTGCCCTCCAGCCTGGGTGACAGAGTGAAACCCCATCTCAAAAA
ACAACAAGTAAAATGCTTCTTACATGGAAGACTGTATTCTAGGTACTCCAGGATACACACAAAT
ATGTTTACTGACCTCCAGTAGCTTATGGTATGCAGGAGCTTCCAATGATCATTTAAAAAACTAAA
TAGAAAACCTTCTGACATTCAAACTTTCAGAATATGATATAAGGACTTTGAGTGGTTATTTTATTT
TATTTTATTTTTTAAGATGTAGTCTTGCTCTGTCACCCAGCCTACAGTGCAATGGTGCAATCTTGG
CTCACTGCAATCTTTGCCTCCTGGGTTCAAGCGATTCCCCTGCCTTGGCCTCCTGAGCAACTGGG
ATTACAGGCATGCACTATCATGCCTGGATAATTTTTGTGCTTTTTTTTTTTTTTTTTTTTTTTTTAGAG
ACAGGGTCTCACCATGTTGGCCAGGCTGATCTTGAACTCCTGACCTCAGGTGATCTACCCACCT
CGGCCTCCAAAAGTGCTGGGATTACAGGTGTGAGCCACCACGCCTGGCCAAGTAAATATTTTAA
ATAAACTACAATGACAAAATTATGATGATAAAGACTTACCATATTGGTATTAAGAGTCTCTGCTT
CTAGAACTGGTTATTTGCAGGAAAATACAAGTTATTCAATTAGATGAAGTGTTTTATATAAACTCT
TCATGGACAACTCATAAATCACATAAAAATCCCTTTGCAATACAAATCTTGAGAACATAAATTTA
AATTTCTACATTTCCACAATTTATATTTTTAAATCAGATACAATGTTGCCCAGGCTGTTCTCAAAC
TCCTGTGTTCAAGCAATCTTACTGTCTCAAACTCCCAAGTAGCAGAGACTACAGGTGTACACCA
CCAAACTCAGCTATTTTTCTACAATTTTTAATACTTTTTTAGTCTCACTACAGAACCAATAATATAA
GTAGAGAAACAATCTCTCCTAAAAACTATATGACACCAAAATGATAAGTTTCCAAGAACAAAAG
CTATATGCTATGTGCTGAACATTTCTGCCACTAAAAATTACCAGGAGGATTCCATGATTACTGCA
AACAATTTGATCCACTGAAGATTTATACAGGAATAAATATTAAGAAAGTCACACTCGTATGATTT
AAAAGTCAAAGTAGTAGTATTTATCCAAATAAACTTTAACCAAATTTCATATTTCCTCTATTGGAG

AAAGCATTTCCTAATGTGATTTTCCTGTGACTACTTGTTTTCCAGTTCATTTATTTTTCAGCTCCCA
CCCTGTCACAGTACTTACCAATCTTTATTAGTTACCAAAGTTAAACACATTTTTTGAATCAACTAG
CCATGTGTATGTTTTTCTCTGACCAGCTTTCCATTACCACCATAAAACAATGATAGGTAAACCACT
GCTAAATTTGAAAAGTAAATACTATGCAAAACTACACTCAGAGTGAGAAGATTAATTTTACAAG
AGACCACTTTACCTTAGTAGCAACACTCAAGTCTTCATCATCCAATGTAGGCAATGAATCCACAC
ACAGTTTATGCAAAATGCTTGAGAGCAAAAATACACAATGAAAATGAGCAAGTTGATTTCTTTAC
AATTTTTTTAACTGCCAGTTTATATCCAGCTTCCCCCTCAAAAAAAGGAAAACATAATCTGGGGA
AAGGTCAGTGATCTATATATTAAATTATGATTCTTGATATAATTAAAATATGTCCTCTGTTCTAAA
AATAGATTTTAGTTACCTATTTCTGCCTCCACCTGTCTAAATCTATAAAATATTCAATGAAAACTT
ACCTTGAGGTTTATAACAAATAGTGACAGTCAATATATTGGCAGAGCCTGACAATAATGTGCCC
TCACAAATTATCTGTCCTGAAGCTGAACTTAAAATTCAATTAATGGATGACATAAATTTTGTTCCC
TAAACTGGAATAAAACTGATGACCTAAAACAAGGTAGAAAGATCCACTGTCTCTTTTCTGTGATC
TGTCTCTGGATAAAAGACTAATCTACATCACTTCAAAATGGTAGTCTTGATTCCTCAGCATGGAT
CCAACTTAGGAAGGTCCTATTGCTTTCCTTTACCCTAAATTGGTACAGGAAAGCCCCCACAATAT
TTGAAATGTATGAAAGCTAAATGTACAGAAGTCAAATAGCAAAGGTGTATGTTCTTACTGAGAA
TACTTTTCCCAGAAATATTAAAATATTAACAATTATAAAATCCCATTATTTTCACTCTATAGGTCCT
ACCTTATTCAGGTCCACATAAACTAGCAAGCCCTTAAAAATTTTCATAGGCACTCAGACACCCA
AGGAGAGAGACTGCGAGGAAAGAAACAGAGTCATGATAGTTGTACCTCTATTTCCCTAAGTACT
ATCTAAGTATATTTCTTCCTATGGACACCCACTTCCAGATTCTACTTCTGCAGGGTTCCACAGAA
GTCTCCAATCTTCAAATCTTCAGTGTATGAAAGCACAGATTCCTGAAAGAATGGCCTCAAATGAC
CAGGAGTAGGAGCTCTCTATATCCCTGCTCCTGAAAAACAAGCTAACTGGAGTCTCCATCACCT
GCCACCAGCTATACACACTACCAACTACCCAACTGAACTCCATGACTGATTTGCCAGCTAATCA
TGCCCCTGACCCAGCCCACATGGACATGGGAAGGACATCAGTGAACCGTGAAAAGAGGCAGA
GGTGAGGAGACACCTGTCCTGTGCCACACATCTATGTAGTTCAGCAATTTCCAGCCCCTTAGTA
CTCCAGGGGCTCTAAGCCACCCCTTTGTAAGTCAGGATGGAAGTAGATGACACCACATTTTTAT
CTGCTGTAGACATTCTTCCCAGTGTCTCAAAATGTTTTGGCATCTTTCAGTAAAAATCTTCAAGTT
TGTCAGTCCTTGATTTAAAAAAAAAAAAGCAGCAAAATTTTTAGAGCTCCCTTGAACCTTCTATTTT
AATGTGCCTTTGTAGATAATTCCCAACATCTTGTGTCCTTCATTTTTATAATTTATCTTTATCAAAC
TTGTCATAAACCCCAATACTTTGATCTCTTGTAGAAGAGTCCGTACTCCTATCCAGTCCAGTGTT
GTTTATCTTCAAACTTGGACTTCCCCTGCTCATTCCATTCTTATCTACTTCCATTGGGTTCACCAGC
TAATTCCATTCTCATTCTATCCACAGGCTCACTCCCGTTTGTATTATTAAAACACACGCCAATAGG
ACATAAAAAGAAGCAAGAGTACTGGGCTTTACCATGAGTTCAAATCTCATTTCTGCCAATTCCTA
TGTCTAAAAAAAAAAAGCTTCCTAATCTCTTTGAGCCTCACATTCTCTATCTAGAGAATCACTTGAC
CAGAATGTTCAATGCAGATAAAAATACTAGAAGGTATTTTAATTCATTCCAAGATTCCTTAAAAT
TCTGTAATTCTATGTCCTCTTGATTCTGTCTATAGAAAAACTTGGAATACATAGGCAGCAGAGTTT
GAAAAAAATAATAGAACAAAAGAAACACCAAGAAAAACAGAGAAGAAAGTTTTAAAAAATGAA
GACAAGATTATATAAAAGTCATGGAAAAAGCAACAAGACTAAAAAATGTATTATGGAAGTAAG
CAGAAATACTTGCCTAAATGGAAAACCAAACTGGGAAGTCAAATAATTTGTCTCTAAGACTTGC
CTAAACTTGCTTTGGTAAAACTTACAGTCCTATGGCCAAAGCTAAGTCAGATCTGCCCTAGAGCC
TTTGAAGGTAAAAATAAGATACTGGTTACCACTGAAATCGTCAAATTTATTAGGACTAACCACAT
TCTAACAATAAACTTAAATGAGAGTTCAAGGTATTTAAACTCTCATTAATTTAGAAGTTAATCAAA
TTAATGAATCCGATTGATCTGATTCAGACCTATACTTTGATCCAAGGACTGCACAGATATCTATC
AATCTATGCTGAGGAGCAAGAGAAAGGATTTGGAAGGCAGGTAGGCTGATGGTCAAACTGTAG
GCCAGCTACTTTGTTAACTATGGGATCATGAGGCAATTAATCAGCTCTAATCCATAGTTGTTTATT

TAACAGTAGGTTATAGGAACACAGATGTTATGATGGCTTTATGAGATGATAAATGCATAGAACA
TATTAGGATGTCTAGCCCAGAATACAACACTCAACAGATATTAGTTTCTTCCATCTATATTTTCTT
AGTTAACATAATTTTTTAAATCTATGAAATCTTACCTGACTGCAGATTCATCAGGAATTCCAACAT
CTATTAAAGAAAAAGGTAAAATGCATTTTAAATCAATAATAAATGTACAGAATATTAAAATCATA
AGAATGCACAGTGATGCATGCCTCTAATCCAAACTACTTGAGAGGATGAGGCAGGAGGATCAC
TTGAGGAGCTCAGAAGTTTGAGACCAGCTTGGGAAACATAATAAGACTCTACCTTCATAAAAAA
ATTGTGCACACTTGTGTGTATGCTTTACATCCTGTTTGTTGTTGTTGTTGTTGTTGTTGTTTTGG
TTTGGTTTGGTTTTTTAAAGCATAAGACTGACGCTTTGTTACAAAGCA
ID|55994|gene_id|ENSG00000237643|transcript_id|ENST00000418403 Homo sapiens lncRNA
GCTGATGCCATTTTCAGCCTCAGCACGCCTGCACCCAGGCGCTCATTAAAACAGCATGTTG
CTCCCCACTGCCTCGTGTTGTCTGTTGGCGCGCTGTCGGGGTTCGAACCGATACAAGAACCTTC
CACCTACCTGGTGCTTTGGCCTCATCTATAAGGTAAAAGCAAAGCCATCTGTTGCTAAATATTGG
AAACTCTGTGTAGCAGTGAAGATTAGAAGTAGTTAATGATTGGAGGATGACCCCTCTCAAAAAA
AAAAAAAAGTTACATTGAGGGCCCCGCAGACATTGAATGAAAAATATGAACTCAAGTCAAAAA
ATTGTTTTCGCATTTTTTTCCAGCAGCTTTTCCACTGTCCTGAAACAAGATAGAGAATCTGAGGTA
AGATATGATCTGAAAAATGTTTGACATGGCTGCTTTTGCCATAAAAACAAAGTAGAAAATAATTA
AATGTAGTCCATTTCTAGAGTTACCTAAGTGTAGTGTTGGCTTATAAGACAGAATACCTGGACCC
TGCCTCATGTGTGCCTATTGTTTGCTGAAAGGATTTTAATCCTATAATAAAATCTCTCACAACATG
CCATGGAAAGTATTTCCTAGAATTCTTAAAAACTAATGAGAAGGAAGCTTGAATATGCTGATTAA
CATGGAAGCTTGTTACTGGAAAAGAAAAGCAAAACTGAAAGATATTGTTTTAATATCCTTTCAGC
TACTCTAAAAGATTACATAACTGCACATAGAACAAGCTATATAAAGTACAAGGCTTAGTCAAAG
AAACATCAGGCTAATACCTGAGTTTTCATACTTAATACAAAAGTCTAAAATATTTCTTACCCCCG
ACCTGAATATGGCTTCAACTTCTCATACAATGTACTACTAGCCTAATAGATATTTTAGAATTAATT
ATTGCAAGTAGTTCATGTATATAAATGCATTTTTATTCAACTTTAAGTAGTTTGTTTCATTGTTTTG
AGTATAAATAAGCAATCTCATCAATGATCACTTCAAAGATACAGTTTATTAAATATCTTAATCATT
ATTCACAAAGCATTATAGGATTATTAGTAAAGCTCCCTAATAGATAGTATTATTATTTTCACTCTTT
CCAAGGAGTCAGTTGTCAATATAAAAAAATACGTTTAGAGAATGGGTCAGTAAAGCAGCAATTT
GAATAATGAAGGATATTTGAACGATTGAAGGATAATGAATCAAACTGAGGCAAACAAAGGAAG
TGAGAATGAAGAATAAAGTGATGTAAAGAATTAATATAAGAATAGTAAGGAGCATTTTATAAAT
AAACCGTTAAAAGCAAGTTCTTCACAATAAACAAACTCTGAACAATGAGTCTATGAGGAGCTGC
CACTGAACTTTAAGTTGCTCAAACACACTAACTGTGGAAGAGTAGTACAAATGACAAGTAGGCT
TCCCACCAAGCAAAACTAGAAAATCAAAATTTGTTTCCAGCAGTAGCACATCTTCAAGTCCCTCC
TCCTCACTTCCTCCTTTCTTTTTTCTTTGCCAAAACAAAACAGATCTACTTAAAAGGCCAAAGGCC
ACTGGAGAAAGAGTTTATGATCAGACAACCCAGGTGTCCTATGTCTAAAAGGGAAATACAGGTC
TTGAAATGAGATAGACTGTGAAAATAATGAATTAGTTAGAATTCATAGGGAAAGAACTTCATTCC
TTAACACTTATCATCATGGTATTGTTTCTGCGGTGTGTTATTACTTCAGTAACTTCTTCCTGTAATG
ATCTAGGTTACATGTAGGTATGTATTTATCACAATAATCTGATGGCTGTCTAATAACTAGTCACAT
ACATGACATCTCTACTTCATAATAGTAGGAGCAACTTACTCTAATCTGACTAAAGTAAAATATGA
TACATAAGCCTTTTTAATTCTGCAATAACCCATTCATCTTTATTATTAATAGATTTTTAAAATTACC
AGTACGTATTTTTCTGAAATGTGTGCAATCAATCCTTTGTCAATTCTTTACTACTACGATCTGAAG
AAAGTTGCAATCCAAAGAGAATAAGGGTTACAATTATACTTGAAGATGAGAGTGCTAAAAATCT
GTGATATGTTTGAAAACCTAGCAAATGGGTGACTCATACTTTCATTACCTTACTTGCTGCCTAGTA
AACTAAACTGGGTGCCTAAAATTCCCTTTGTTTATTTTCAACTTCAATTATATATGCACAGTATTTT

AATTACTTAGCATCTATTGTTCCAGGTGCTTAGATTTTTCCACATCAAAATTCTCCAGTTTCCATTG
TGTTATTTCCTGTTATAAGATTATTCAAAGTAGTTTTTTTCTTTATTGTTGTTTTTCAAAGGGAGGC
AGACTGACCAGTCGTGTTTGGGGTTATTTCACAATGCTAGTTCCTTCCTGTATACAAATTCACAC
ACGCACCCATATTCATACAAATGCTGGCCTTGGGTAAAAAAAATAAGGTTGCCAAACATTTGAA
GTCTTCAGACCATTCTTTCTTTGTTCCTCCTTTGGGGAGCACCACCTCCCCTTCAATTATTCTGCA
TCCTGCATTTTCTCCTCTTCCTTACAAAGAGCCTGGTATCAGTCACAGCTCCTGGCACTCCTGAG
TTACATGGCCTACTTCTGAGGCACGCAAAGATTTGGTAGCAAAGCAAAGGGAAGGGAAGTGGC
AGACCTGTCTCTCAGCTGCCAGCTGCAATCTTTCTCGCACACCAGTGCCTCTCCTTCTTACAAGT
TACGTCCTGTCGGTAGACGGACTGGCCCAACAACAAGGTTATATCTATTGAGGTTAAACTTGCC
CCAAACAGTGTTTGTTGGAGCAGAGAGGCAGAAAGCGTACAAAATAGGAAGGTGAGTTCTCGA
AAGAGTTAAAATCCAGCCTTAAAAATTCCACATATGCTCAGTCAGGTAAATCATCATGAATTTTT
TACTGATATTTTGTCCTAGAAGATAGTGATATTAACATATTCTAGAAAAGAAAAACCTTGATTCA
GTAAAAACCTCCTTAAGTGAGAGATACATGATTAAATGTTGATGACAAAATATATTTTGAGGTTA
TCTCAAAGTTTAATGAAGTTCTAAATATATAGAATGATTGAATATACACTTTAAGAAAACATGCA
ATAGGTGACTGGATAGAGCTGAAAAAGAGGCATCCATATATTTTAGATTGTTCATTTCTTGTATA
TTTGATTTTATGTATGAGAGTGTTTGTTATCCTGATTTAGAATGAAATGACTTTGAGTGGTATTTGT
GGATTCAATTTTTTTCTAGCCTGAGGAATAATCTATTGTAGAAAAGTTGAAAAGTTGCTCTAATTC
GCTAATATTTCAAAGCATCATTTATTTTCTCAGGGAAAGGACCAGAAAACTCGGATCCAAACTTA
AAATGTCAATACTCATAGTTTAGCATTTCAAAATGAATAACTACATGGAGTTACCAGTGATACTT
TTAAAATTTATATTTTTGCTATGTGTACTGTTTCAACAAAGTCATGAGTATACCAAAGCGAGCTAA
CAGTCTTCATTCCAAAATGTTGCCCCAAAATAATTTCTGAACCACATGACAGATGTTTGATACAG
ATGACTAAATTTCCTGTCATGTTTGTTTATTGTTAATAGTAAAACCAACAGAGAGTGCTAGACTTT
AGACACTAATATCTTCAAAATATGGACTTACATGAAACTAGACAGCAATATCATCTTAAAGTACT
TTTTTCTCTTAAACAAACAAATCGAAAAAAGCAAAGATCAGAGATGAAAGGAAGAAAAGTATAT
CGAGTGACACCATGTGTCAGCCAGACACTGGGATAGATGCTTCATATACCTTAGGTCATTTAAC
GTTATAGTTAAGTAGAAAATGTTTTAAAATTGTGGAGGTGTTTAACTAGTTTTTCATTTTCTAAGC
CAGCACAGCTGTTGACTCACCTGTTCATAATGCAGAGATTATTTGTTCTGCCCTACGTTTCTGTTG
CACAACATTTTCCCTCTCTTAAAAGTCATTTATTGAATCATCATCTGTCTCAGGCTTGATACGGTA
TAGCCTCTGAAAGACTGGTTTAGTACTTAAACCCACCTTTCAAAATATTTCCAGTGCAGGCCATT
CAGTGTTCCAGGTACCTAATGAGTCATTTTCTTCTTCCCAGTCTACCAAGCAGGATCTCAAAGCC
TCCTTTATCTTTCTGGGTGTATACAGAGTCATTAAGCTCAAATATGTCCTTTGCCAATGGACTTGA
GGGTTGGGTTTAGTTATTAACTTGGTGAGTCTCCACCCAGCTAACTATTCTAGGAAATCATTTGG
TGAGTCAGGCATTCTTGTATTCACATTGCAGCGGCCAGTCATCTGCCCTAAGTGCTGCCGCCGA
AGACTGAATGTCCTGGAAAGTTTGCTGTCACATCTCCATTATGACAAAAGCATTGTGCCGAACA
GATGAAAAAATGCATTGTCAACGGAATCTTTTATGTTTGTTTGTCTTCCTTTAAGCAACATTGCCT
TACTTGTTATAAAAGATAAATAAATATTTGTTCATTTCAA

ID|56031|gene_id|ENSG00000227748|transcript_id|ENST00000436295 Homo sapiens lncRNA

CTCCTTTAAGGATCTTTCCAAAGGTCTTCAATTTTGATATAAGCAACAAAGAATAAGAGTGA
GTAACAGCTAAAGAAACCAGGGAACTGTAGCGGTAGAGTGTAGCAGTGAAAATATTTAAGACT
TGATGAAACAATGTCCCTTGCATAGACCGATGCTTTTATAGGCACTCCGGACCTGCAGTGGACT
GTTTCTGTTCTCCCAAAATCCATACGTTGAAATCTAATCTTCAATGTGATGGTATCAGGAGGTAA
GGCTGGGAGGTAATCAGGTCACAAGAGTGGAGCCCCCATGAATGGGATTAGTGCCCTTCTAAA
AGAGGCCCCCAAGTGCTCCCTTGCCAGCTACTGGCTGAGGACACAGTGAGAAGACAGCTGTCT

ATGAGCCATGAAGTGAGCCCTCACCTGACATCAATTCTGCCAGCACCTTCATTTTAAACTTTCCA
GCCTCCAGATCCGTGAGAAATAACTTTCTGTGTTTATAAGCCACCCAATGTATGTTTTGTTTTGTT
TTTACAGCGGCCTGAATAGATTTACAGCCAAGAAAATTAGAGAGGGGATCCAGGTTGGAATTAG
TCTTCAATCTGATTACTCAATTATGATGACTAAAAGCCCTACTGAGCACCAGAATTCTGGTGATT
TTTTTTTCCCAAGGTAGCCTAACATAAGGCAAAATTGTAGCATGAAGGAAGGGGATGACTTCTG
AGAAGGCTTTCTTCACCTTGTACAGAAACCCAGGAAGTGGGTTGTGGACACAGGTTTGCACATG
TCACCTTGTGGTGTGCATATGTGCGTGCTCTTGGGAATGTCATCCCTCCTTATTTTACCTGCATGT
GAAAGCAGCAGGAACCTGTGCTAGAAAACAGAGACAATTAGAAGATAGCTGTGCTTTGGGGAA
GCAAGTCAATTGACTGAAAAACAATTTGCCAAATGTCCAATTTGTGAGATTGTAAGGATGTTTAG
AAGATTTTCTTTTCCTCTTCACCCTATACTTTCCCTGTCCCTGTCTGTGAAGTTGGCCCAGACTATT
TTGTTACAGAGGCAGGGTCATGTTCAGAAGCAGGCGCAGGAATTAAACTAAAACATAAGCAAA
AACCCTCAATGAGTCAGTAAATTGGTCATTCATCAAGTTAATTTTAACAAATTGGCTTTCAACAA
ATTGGCCTGGAGCCCTACTTTGGCACCTTCTTCTGGAAATGTCTTCTTTTCCTCTTTTAGTCCTTTT
CCGTTCAATGTTGTACTTCACCTAGACCTTGAGATTATGCCATTCACAATTATAATCTCAACATTC
ATCACTTGCCTTGGGAGGTACAGACTTAACTGTTGGCTGGCTAAACGTTTATGTGGAATCTTAGA
GGACTTGTAACACTATTTAATGAAACACTCAAAACATTCAAAGTCTAAAGGGGGATGGTGATTT
GGAAATAACTTCTCTTACCTATTCCTACCTGCCCTCTGGCTTCATTCATGGTCATAGGGCCTCAGT
TTTTGGACAGAGAGCACCACCATTTTTGGCCATGCATTCTCTTTTTCCACAGCCCGTTTATATCCA
TCTGGTCACCACCTGCCAATGAAAAGTAAATAACAATGACTGCTCCCTCCACTGGCTTCTGTCCC
TGGGGTGAATGGCCAGGAGTTTGAGCCACTGCTGGACAAGAGCCGTTGATTGCTTGAAGGCTG
GCAAGGACAGCAGAGCCCTCATAGTGAACTGGCCTCTGCAGGAGAGAAAAAGGGATAAGCAG
ACATGCTGCACTGAGATTAGCTTCCTGCCAATTTCTCCTGAATTTTCCTAAAATAAAAGTGCATTT
TCTTCCCAGCCTGATTACATTTCAAAAGCCCTCTTCTACTAGCACAGCCTTGGTAGATGCTGGAC
TCTAGAGAGGATTAAAGGTGGTGGGAATGGTAAGGATGGTGAATCCGGGATTAAAATTGCCCT
GCCACACATTGGGTCAGAGCAAATATTTGTGAAGCTTGAGTTATTTGCAGTGCATCATTATGGTT
TCTGTATGGGAAATGCCAAGTTATACTTCGATGTGCAGGGAGATAAAAATATATTTACCAACAC
AGGGTGTTATGTGTTAAATGGTAAGGTGCCAGAATGCTCATAAACTTGACCAGTACAAGGATGT
TGTCAGAGGTGGTGTGGGAAGCAAAATTGTGGATCAGGTGCTGAAATGATTAAAGAAGATGGA
AGACTGAGCTGACGACAGTATGCATTAATTAATGGAAAGGTATGGCCTGAAATGTAAAAGAGG
AGATGTAGTTGAGTAAGAAGTGCAGCATCCTTTCATCTATCACACTGGAAATCCAGCAAAATCT
GAGACCAAGTGACTATTTAAAGTCAACTATATAGAACTCCAGAGCTGGGGATGATTTATCTGCA
GGAAGAAACCAGCTTTGCTCTGGGCTAAGAGTGAAAGCATATTAAATATGTAGCCTTTTAAAGA
ATCATATTTCTGTATCATCTATACATATATATATATACACACACACACACACTATTTCATATATATA
ATGGATTAAAGCAGATCATAACATAATAAATATCAAATTATTCATGTTCCTCTTTTGAGCCTCATT
CTGCAGAAGGAACACTTTCACGCTTTAAAAAAGACAGGATCACCTCTGATCTATGAACCATTTTA
TTTCAAATACATCTTACTGTTACAATTTGGCCTACTGCTTGGTGGGGAAAGGCCTTTCCACGTTTC
ACTCACTGCAGAGCAATCCAGCTTTCCTGTGATATTCATCTGATGTTTATATCCATCTTTTGAAAT
ATAGGGGCTATTAGATCAGAAATATTCCATGAAGAAGGGAGCAAAGATTCATGTCACTGACAAC
TGGAGAAGAAACAGAGAAAGGGTAATAATATCGTGGGACAGAATGGAGTCCCTTCCAGATCTT
TGGAGGGCTAGCACCATATCCTTCCCAATCATTTATTGCAAGGCTAACTAAGGTCCTGAAGAGT
GGGCCCCTTCAGTCTGAGGCCAAGAGGCCGCTGACCTACCCTGATATAGACCAAACTGTGCTTA
GTTTCCCTCTGGAAGCACCAGAATAGCTGCACTGGTTGTACTGTGTCATTTCAGAATGCTGACCT
CAACCCTTAGCCCATTTGTTTGCTGACACAATAGTTCTTTTTTTTTTTTTTTTTTTTTTTTTGAGAAA
AATAAATCTTTGTACTGCAGTTAGAAATAAAGCTTAAAGAAAAATCACTGGGATTGGTGACATGT

TGCTTTAAGTTAAAAAATGGCTCTGCAAGGTGGGTATCCAAACCTTTGCAGCTGTTCTCCATGCA
AGAAGAACAACTCAAGAGCAGCATGTCATGGGCAGCTCCCAGGAAAGGAGGAGAGAGGCTGG
AGGCACCAAACCCATCCAAGCACTCAGTACTTGCAAAGGCGCTGGTCTCCTTTACTATCCAAAC
ACCTTACACTTTCTAGGAATTCAATACACACGGATTAAAGCCCAACTTACTAAAACTAGTTATTT
AGCATTAATAATTTGAATCTTTAAGTTTTAGCTGGTGAAGGACTATATAAGTTTTTATTTGAAAAT
CAAGCGTGTTTTTCTTTCTTTCTTTCAAATCAGGTACTCATGATGTGTGACAAATGGCATGCCTCC
TGCAAGAGTTATGAGTTATGTGCCTAAACAGGAAGGATCATCTCATTGCAGAATTAGGGCATCA
TCCAGTGGGAAGGGAGTTTCAAGGTCATTTAGTCCAGCCTCCGAGTGATACAGAAATTCTCTTT
GCAGCATCCCTATCACGTGGCCATCAAACTTCTACTAGTGTATTCATCCACACCTGGTCTGTCTA
AAACCTTGAGAACAGCCCCTTGTATAGTTCAATAGCCCCAGCTGAAGGAAAAGTTTTTGTTATTT
TCAGTAAACATATTCTGTTGTGTAA

ID|56058|gene_id|ENSG00000228327|transcript_id|ENST00000428504 Homo sapiens lncRNA

GTTCGGCTACATCACTGGGGCGCCATGGTGCCTGGAGCTGGGCAGTTTTCTCATCAGAGTG
GGGACTGGTAAGAGTGACCTCCCCGCCAGGTTCTGTGTGTTGCCGGCTGAAGAAGGGTAGCTG
AAAAATTCAGACCCAGCACAGTGTTTATGTTGGTCAAAAATAGAAAACTATGTCTGGCGCGGCC
GAGGCGGGAGGACCCTTCAGGCCAAGAGCAGCCTAGCAACATGGCGCAACCCCATCTCTGTA
GTCCTACCTCAGCCCCCCAGCTACTTGAACCCAAAGGTTCAAGGCTCCAGTGAGCTATGATCCC
ACCACAGCATTCCAGCCTGCGAGATTGAGGTAAACCCTGTCTAAAAAAAATTTAAAAAACTATCC
AGGTGTGCAACAGGGAGGGACTGCTAAATAAAACATGAGGCTGGCTGGGCCCTACTGTAATCC
CAGCACTTTGGGAGGCCGAGGCGGGAGGATGGATGGCTTGGGCTCAGGAGTTCGAGACCAGC
CTGGGCAACATGACGAAACCCCGTCTCTACAAAAGATACAAAAATTAGCCGGGCGCGGTGGAC
ACCTGGCCTAATTTTTGTATTTTTTTCTAGAGATGGGGTCGGGGGGTGCCTCGCTATGTTGCCCG
GGCCAGTTTCGAACTCCTGAGTTGAAGCAATCTTCTCACCTTGGCCATCAGAGTTGTCGGGATTA
CAGGCGTGAGGGACAGCGACCCACCTGGGTTAGGCTACTTAATAACATAAAGTGCTCCGCCAG
GCTCAGTGGCTGACACCTGTAATCCCAACACTTTGGGAGGCCGAGGCGGGTGGATCACCTGAG
GTCAGGAGTTGGAGACCAGCCTGGCCATGGTGAAACCCCAGTCTCTACAAAAAATACAAAAATT
AGCTGGCCGTGGTGGCGCATGCCTGTAGTCCCAGCTACCTGGGAGGCTGATAAAGGAGAATTA
CTTGAACCCGGGAGGCAGAGGTTGCACTGGGCCAAGATCACACCACTGCACCCCAGCCTGGGC
GACACAGCGAGACTCCAAAGTTTGACACCAGCCTGGGCAATGTAGTGACACCCTGTCTCTACA
AAACAAACAAACAAAAACCCAGGCATAACTGTGTCCACCTGTGGCCCTAGCTAGTTAGGAGAC
TGAGGCAGGAGGATCACTTGAGGCCAGGAGCTCAAGGCTGCAGTGAGCTATGATAATCCCACT
GCTTCCCATCCTGAGCAATAGAATGAAAGCATGTCTCTAGATAGATAGCTAGCTAGATAATTGA
TACGTAGTTTTGTATCAAATTTTTTCCCTAGATTTGAGCATGTTTTTCTAAAGTAGCATTCAACACA
TCAGCATTTTACAGTGTTATTATTTGTTAATATGATTATGTTTTTCTGAAATACAGTGTCCTTTACA
AAAGCAGTTTTGTCTTTCAAAGCGCATAGATAAGGCCCTCAAGTAAATTTGTCTGATGTTGCCGA
CCTTGGTACCATTTTGTCCACTTGATTGAAAAGCCAGTCAATAATTTCAGGTCACTGTTGGCCTTA
GAAGAAGAGCCCAAAGGCAACAAGCAAAGGCGCTGGTGTCCAGTCGCCTTCTAGAAGCATTTT
CACTTTCCCTTAAGGTTTCCCTTGATGAACATAGAAGTACTGTATGTAGAATTGACCCAGTGCTG
CCCTGGCAACTTTGTATATTAGGCCAAATTTACATTTCTTAGCTTTATGAGAGGCACCCTGGTAG
GCTAGTGGAGTTACACACAAAGTCTGATCTCAGCTGCACTGTCCAGAAATGCAACACGGTCCAA
TCAAATAACATTCTCTGAGCCTGTTTCTTTAGCTGTGAAAGAAGAATAACATACCCATCTAAAAA
GGCAGCTTATTGTATTTGATTGGTCTTTTATTTTCTATGAAACTGTGTTTAACACAGTAATTATTTT
CATTTGTGTACTACATTTGTGTTGTGTTTTTGGTTTTAGTTTTGTTTTTGAAATGGAGTCTTTTTTTTA

GTGGTTTTTTGTTTTGTTTTGTTTTGTTTTGTTTTTGAGATGGAGTCTTTCTATTGTCACCCAGGCTA
GAGTGCAGTGGCGTGATCTCCGCTCACTGCAACCTCCACCTCCCAGGTTCAAGTGGTTCTCCTG
CCTCAGCCTCCTGAGAAGCTGGGATTACAGGTGCCCACCACCATGCCCAGCTAATTTTTAAAAT
ATATTTTTAGTAGAGATGGGGTTACAACATGTTGCCCAGGCTGGTCTCAAACTACTGACGTCAA
GTGATCCACCCGCTTTGGCTTCCCAAAGTGCTGGGATTATAGGCATGAGCCACCGCGCCTGGCT
TGTTTTAAAATAAGGGTTTCTTGGCTAGGCATGGTGGCTCACACCTGTAATCCCAGCACTTTGGG
AGGCCAAGGTCAGTGGATCACCTGAGGTCAGGAGTTCGAGACCAGCCTGACCAATATGGAGAA
ACCCTGTCTCAACTGAAAATACAAAATTAGCCAGGCGTGGTGGTGCATGCCTGTAATCCCAGCT
ACTCAGGAGGCTGAGGAAGGAGAATTGCTTGAACCCAGGGGGCAGAGATTGCAGTGAGCTGA
GATCGCACCATTGCACTCCAGCCTGGGCAATGAGCAAAACTCTGTCTCAAAATAAAAAAAAGAT
TTCTTAAAATGATATTTTCAGTATTTTATAGATGATGTGTAAGCAGCAAGCTTAATAGGATGTTAC
CCGACACTTTGCGAGACTGGCAGCTGATTTGATCCAGATGTCTCTAATTCTTTTTTCTTTTTCTTTT
TCTGTTTTTTTTTTTTTTTTTTTTGACAGAGCCTTGCTCCGTCCCCCATGCTGGAGTGCAGTGGCAA
GATCTCGGCTCACTGCAACCTCCACCTCCCGGGTTCAAGCGATTCTCCTGCCTCAGGCTCCCGA
GTAGCTGGGATTACAGGCGCGCGCCACCATGCCCAGCTAATTTTTTGTATTTTTGGTAGAGACA
GCATTTCACCATGTTGGCCAGGCTGGTCTCGAACTCCTGACCTTAGGTGATCTGCCTGCCTCGG
CTTCCCAAAGTGTTAGGATTACAGGCGTCAGCCACTGTGCCTGGCCCAGATGTCTCTAATTCTAA
CATGAGATGTATTGCAGGATCATAGCAGAGTGAGTTGCTGATGTATCCAGAAGGAAATGAGCAT
GGAACACTCACGACAGCTGTCCTGAGAAGTGTGTGTGTGCTGTGCTTGAATATCTCACTGCTCAT
TTATACACAGGCTTTCTGGTGACTGAGTTAACAGTACCTGTTTCATAAATAATGTAGCCCTCTTTC
TTTCTTTCTCTCTCTCTCTCTTTTTTTTTTTTTTTTGAGACAGGGTCTTGCTCTGCTACCCAGGCTGG
AGTGCAATGGTGCAGTCTCAGCTCACCGCAACTTCACGCCTGGCTAATTTTTTCTTTTTTTTTTTTT
TTGAGACGGAGTTTCGCTGTTTTTGCCCAGGCTGGAGTGCAATGGCACAATCTCGGCTCACCAC
AATCTTTGCCTTTTGGGTTCAAGGGATTCTCCTGCCTCAGCCTCCCGAGTAGCTGGGATTACAGG
CATGTGCCACCACACCTGGCTAATGTTGTAGTTTTAGTAGAGACGGGGTTTCCCTATGTTGGTTA
GGCTGGTCTCAAACTCCTGACCTCAGGTGATCTACCCACCTCGGCCTCTCAAAGTGCTGGGATC
ACAGGCATGAGCCATCACTCCTGGCCTAATTTTTGTATTTTTAGTAGAGAGAGGGTTTCACTCTG
TTGGCCAGGCTGGTCTTGAATTCCTGACCTCAAGTTATCTGCCTGCCTCGGCCTCCCAAACTGTT
GGAATTATAGGCGTGAACCACCATGCCTGGCCAGCTCTATTTCTTTAAGCCTACATGTTTTGCAA
GTATTTGAACATACAATTACTCAGCTTCCCTTGTTTACGCGTGAATTTTGTAGAATCTTAAATATTT
TTTCCAATCTAAGCTTTATTTTATCCCGTTTCTTCTATATTTGTATAACTTTAGGCGGCTATCTTCAT
TGAAAGTTTTTTCTCAAAAGCCTTAAGATAGAACATAGTTCTTGGCAGCAATTTGAAAGTTATTTG
AGGAGAAGGGGAGACTTACAATGATGATTCAAATGAAGGAAACTAAAAAGTAATGAAGCAAGG
CAGAGGAAAAAGCAGTACTCACTTGAGCACATCCCAAAAGAATAACATTTCAAATGTAACTAGA
AAAAAGTATGCTGAAGTTCGCAATACAGAAATAATTATTAATAAGATAGCTTTAAAGCCCTGCTC
AGCTTTTGAATGTTGGGAATTGACCCAGAGGTGGCTGTAACCTAAGATGGTTCCTTCAGTAATGA
CCATTTTTTCTTTTTCAAGATGATGATTATTCCCCACCTTCTAAGAGACAAAGACCAACGAGCCA
CCACAGCCACCAGTCCCAGAACCCGCCAATGCTGGGGAACGGAAAATGAGGGAGTTCAACTCT
GGTAAGTTCTCAGCGAAATCCATGACCTTTTCCTTTATCTTCTGGACTCTCAATGTGACTGATGAA
AGTTACCACATGCTCTGCAGGGGGAAATGGTTTAGCATGTGTTACTACATCTTAATCACATCTTT
GTAAAGCCAGGAGCATTTTACAAGTCACGTTACAGACATTGTTTAAACATAGTCTGTATTTACCA
AAGTATAGGACATTGTATCATCTCATATTAATTAGTTAGTTGGCTCAAAATTAGTGCTAATGACTT
AGTAATTCAGTGATTTCTGTTAGCTTTAAAACCTTTATTTCAGAACTATTTCACCTCTTGGTTTTCA
TTTTTGCGGTGTGTCACTGCCTGCCGGCTGCTAATTTATTAACTCCCAGTGAATCATGTCCTGTGA

AGGGACTGAATATTAGTGGCAATTTATGTTGATGATTTGTATTTTGAATAAACAGTTTGAATACAT
AGAACATTAAGCTTGTATACATTTTGAAAATAGTATTTTAATATTCTACTGTGTCATAGTTACAAT
GATTGGATATATATTGAATTTATATGTACTTTAAGTTGTTATATGTTTATGGTCTTTAGCATTCTAA
CGTGCAATTGTATATCTGTTAAGCCTTTTTTTTTTCGAGATTAGACTGATTTATTGAGGCGTCTGTT
TGATGCCACATTAAGTGGCCCAGGCTTTGTGTAGGGGTTGAGGTAAAAGCAGGAAGAAGGGTG
GTGAGAGGCGGGGCACCAGGGTTAGGTTGGAATACCTGGGGGTGCTCTGAGGCTCCCCAAGTT
TCCCTGGTCTTGGCCGGCTGTGCTGCTGGCCTGGGCATCTGATGGGCCTGCAAGGGTGGTCCAG
GGGCTAGGGCAGGGACTTTGGAGTCACGCCGTTGGCTTTGAATCCAGACTCCTACACTTGGTAG
CTGTGAACTCTCCATGCCTCAGGGACCTGCAGAACTGAGCTCTGTCTGAGCCAGGTTCCATCCA
GGCACTGCGCATCCATCCAGAGGGGCACTGCCTCAGGCTGCTCGCTGTTCACTGCCTTCTCAAG
CAGACCCTTGTCTCCTTCTAGGCCCTCACAATCCAGTGGAGGAGACGCAACTCATCTGCCTCTG
TCCCTCTGGGCACGCCTCATGCCAGGTGCATCTGTGGACAGGGGCCATGCTCCTGGGCTTCCAA
AGTTGGAGAAAGCTGCCAGGCTCAGGTGGGTACATCACAGCAGCTGCTGCCCTCTGAACACAG
TGACAAAAGAACACTCTGGGCCTGGAGCCCTGGTCTGGGGCATTGGGCAAGGCTGTTGCACTT
CTCTGATCCCATTTCCCCATCTGGAAAGTGCGCTGATTGTATCTCCCTGTGGGCACTGAGGGCTC
AGTGTTAGTTTGAGAGCCAGCATCTGGGGTTTGGGCTGTAATTCCCCGTCAGCCCCATAGCTGC
GGGGAACCAGGGACTTTGTTGGGATTACCCTAGGTATCAGTTTAGCTTCCTGCCCCTGGCTTGG
GCTCAGCACCTGAAGTAGTCTAGGGGATAGGTGGTGCTGGTGGGGGCTGGGGCTTTTACCCAG
ACTGAGGTCACACCCAGAGCCAGAAGTCTTGGTGCCTGCTCTGGGCAAAGGTGCCAGCCTGTG
TGACAAGAGCGAAACTCCGTCTCCAAAACAAAAACAAAAAACCTTGCATCGTTTCAAGGGGCT
CACACCTCCCTAAGGGCCTGGTAATTGGCTGGCTCTGGCCTGCATCTGGCCCCGAGGGTGTAG
GTAACACCCCACCTTACCTGGTTTCTTCCTGCCAGGGCCAATCTTCAGACCTCAGGACTTTACAG
CCTATCCCACCTCCCCTCTGGCCAGCCTAGAGCCCTTGTGGGTCCAGCACTTTTTCCAGGCTGTC
TCCTGGTTGTCCTTCTGCCTCGAGGCCTGGCTCATGCTGCTCCCCCTCCCACTCTCCAAGACCCA
CAAGGACCACTCCACACCCAGCTCAGCCCCATCCCCTCAGATAGTCCTTTCTCTTTCCTCAGGTG
GCCAGGTGCATATCTTGGTGTGAGGACCTTCGCTGTATCTGGGAATGCCTACTGGTTACCTTGGT
AACAGAGAACAAGGCATTTACCTGATATGAGTGTCTTGGTTCACTGTCTACATGGCTAGGGAGG
GAATCAATAATAGGCTTTTCACTTGCTGCAAGGGCCGGTTCTCCTGGCCCCATGGCTCTAGGGA
TGGAGGACGCTGCAGGAGATGCAGCGCTCACTTCCCAGCTGAGGACTGTGGGTCATCTCAGGG
CGATTTCACAGTCCCCACATGCCCCACCCCCTCAGCTCTGCAAATACCAAGCAGTGCAGCCTGC
CTAGGGGATGATGGGCTCGAGAGTGCCCAGGTAGTGCCCAGAGTGCCCTTGGCAGGCCCCTCA
CCTGGCTGCTTCCACAGCTCTGTAGCAAGAGTTCTAACCTTTTTTCACCGTGAAGTCTGCTGAGA
ATAAGCGCTGTGGACTGTTTTCCCAGAAAGGCATGTACATGCTCTCCACACAAAACCTTTCATCG
TGGCCAAGCACAGTGGCTCATGTGATCCCAGAACTTTGGGAGGCGGAGCCAGTCGGATCACCT
GAGGTCAGGAGTTCAAGACCAGCCTGCCCAACATGGCGAAACCCTGTCTCTACTAAAAATACA
AAAAATTAGCCAGGCGTGGTGGCAGCCACCTGTAATCCCAGCTACTCCAGAGGCTGAGGCAGG
AGAATCACTTGAACCTGGGAGGCGCAGGTTGTAGTGTGGTGAGATCACGCCACTGCACTCCAG
CCTGGGCGACAGGAGCGAAACTCTGTCTCAAAAAACAAAACAAAACAAAACCTTGCATCCTTTC
AGGGGGCTCACACCTCCCTAAGGGCCCAGTAATTAAACCCTTTGGGCCTGAGGGCGAGAAACT
TTGTCTCAGTTCTTCCCCAAGTGATCAGCCCAGGGGTAAGGAAGGAGAAGCCAGAAAGCAGGA
CCCATGAGAAGGGCCCCCTCCTGGAGTTTGAGGCCCACTCCCTCCTGCCCCTGCCTCTCCTCTG
TCCAGGACTCCTCCCTGCTCTGCCCCACTCCTGGGGCCATAACCATGGGGAGCTGTGGTTTTCT
ACAGGCCCCTGGGCACAAAGTGGGCAGGCTCACCTGGAGGCGATCAGAGTAACATGGCAGGA
AGTGAGGGGGAAAGCCACCCTGGAACTGCGCCTCTCTGCCCCCTGACGTCACTGGCGTGCACT

CCTCCCCCCCCTCAGGCAGTGGCATGAGTTCCATGTGAGCGCTGTCCTGCTCCCTCTGCTGCCT
CTTTTTTTTCTTGGGGCTGCCATAACACTTTCCCTTCCCCAGCCCTGCCAACCTGGTGGGACATT
GGGCTTCCCTCTCACAGGGTCCTGGGGACAGGCCCATCCTTTATCATACCCACAGAGAGACCGT
TTTTTTCTTCAGGACCTGGGGAGCAGCCAGGTTCCATGAGTTAAATGCAGATCTGAACCAAGCT
GGGATTGGGGTACACACTCTCCTCTACTGAAAAGTAGCTAGGGATTCCAACTAGGTGAGAAGG
AGAGTGGGGCAGAGCCAGACCAGACAAGGACTGATCACCTGGAAAAAGCCTGCCATCAAAGG
TCTTGGCAAATGCTGGGTGCAGTGGCTCACTCCTGTAATACCAGCACTTTGCGGGGCTGAGACA
GGTGGACTGGCAACGTGGCAAAACCGCATCTCTACTAGAAATACAAAGATTAGCTAGGCATGC
TCCACTCCTGTCATCCCAGCTACTCAGGAGACTGAGGCAGGAGAATCACTTGAACTGGGGAGG
CAGAGGTCGAAGTGAGCCGAGACTGTGCCCCTGCACTCCAGTCTGGGAGACAGAGTGAAACTG
GCCTCAAAAAAAAAAAAAAAGAATATGGCCTTGGCAGAGAGGGGCCAGCCCAGCAGTGCCTTCC
CTTGGGTTTCTCCTGGGTAGGCCTCTGCCATGAGGAGGTGCTTCCTTCTGCCTGTCCATGGCCCA
CAGCAATGGAATGTCTGTTTCTGGGGGTTGGGTGGGAGAGTGCTGGCAGAACTGGAAACCTTC
AGGTGGGGTTTTTTTGTTTTGTTTTGTTTTCGAGGTGGAGCGTCGCTCTGTCACCCAGGCTGGAG
TGCAGTGGTGGAATCTCAGTTCACTGCAACCTCCGCCCCCCTGGGTTCAAACAATTCTCCTGTCT
CAGCCTCCTGAGTAGCTGAGATTACAGGCATGTGCCACCATGCCCGGCTAGTTTTTGTATTTTTT
GTATAGATGGCATTTCACCATGTTGGCTGGGCTGGTCTCGAACTCCTGACCTCAAGTGATCCAC
CCATCTCGGCCTCCCAAAGTGCTGGGATTACAGGCATGAGCCACTGAGCCCAGCCCCTTCAGG
GGGGTTTTGAGGCTTCACTACAATACTAGTTTCCTGTGGCTGCTGCAACAAATTACCACACACTT
AGTGACTTAAAACAACCAAAATGTATTCCCTTACAGGTCTGAAGGCCAGAATTCTACAGTAAGT
CCTACTGAGTCAAGGTGGGAGCAGGGTCGGTAGCTTCCGAGGCTCTGCGGGAGAATCTGTTTC
CTGGCCGTAGAGGTGGCCTGCACTCCTCAGCCTGTGCTGCCCGTCTCGAATGACTGGAGTTTCC
TGCTTCTGTCACTACACCTCCCACCCTCTCCATCACCTGCTCTGCTCTTACAAGGATCCGAGTGA
GTACATCAACCCCAAAAGCCAAAGACCCTTAACTTCATTATATCTGCAAAGCTCCTTTTGCCATA
TAAGGTCATGTTCACCAGTTCCCGGGATTAGGATATGGGCATCTTGGGGGCATCAGCCTGCTAC
AGCTAGGCTGCAAAACTGTTACACCCTCCTGGTGTTTCAATGATTGGGAGAAAAAGGGTTGGCA
TTTTTTGCTTAGGGGTCCCTCTTAAACTTGCATCTGTAAGGTCGGGGGTCCCTCTTAACCTTGTGT
TTTTGTTTTTGTTTTTTTTGAGGTGGAGTCTTGCTCTGTCATCCAGGCTGGCAGTGGTGTGATCTTG
GCTCACTGCAATGTCTGCCTCCTGGGTTCAGGTGATTCTCCTGCCTCAGCCTCCTGAGTAGCTGG
GACTACAGGCGCCCGCCACCATGCCCTGCTGTTTTGTATTTTTGGTAGGGACGGGGTGGGGGTG
GGGCTGGGGAGGGGGGTTTTGGCTATGTTGCCCTGAGCTCAAAGTGATCCACCTGCCTCTGCTG
CCAAAGTGCTGGGATTACAGGCCTGCACCACTGCACCCGGCTGCTGTAAAGTCTATTTTCACAC
AGCTGAGACATGTTTTAGGAAGTTTGCTAAAAGACCCCTGGAGACCGCCTCATTGTGACCTCCC
TGTTATTGTGTTTAATTTGATTGAACTTTTCTGCCCTCCTGCTTTTCAGCTTCTCTAATAGTCTCCC
ATTAAACCAATTCTAAGAACCACCAAGAAGGGGAAATTTTTTCTTGAAAGCAGTAAAATGATAT
GGACTGTTAGAATGTAAAATATATGACATCAGTCATTATACGTTAGTGCTGCTCTGACATAGGGA
TGTGTTATTGAGAAGCAACTTTTGCTTGGTTTTCAGAGAAATGGAATCATCGTATCGCTGATCTA
CGTAAACAAACTGAAGAATTGTCTGAAAGAAAATATGGTATGTCTAAACTGGAAAAGTCTTGTA
ATCTTATGTTCATGGGCGTTTACACAGTGGAGTTACTGTTCATCATGGGGGTACCGTGGACAAG
CCCAGGGCTGCCGGCGAGTCATGCCATCCTTACATGTTTCTCCTTGTAAGGTGCTTTGTAGTGTC
TACACACTTTGTTTCTAGATTGCTGCAAAGCTGAGGAAAAGTTGTATTTCTTTAGTTATTAGTTAG
CATTTCTTTTAAACTTTCAGTATGGAGATTGGAAATTTATTTACATATTTATTGCAAAGCCCTGGA
TCTTAGGAATTTCATTGAATTATTTATTTATTTTTTTTGAGACGGAGCCTCACTCTGTCGCCCAGG
CTGGAGTGCAGTGGCACGATCTCGGCTCACTGCAACCTCCGCCTCCCGGGTTCAAGCAGTTCTC

TGCCTCAGCCTCCCGAGCAGCTAGGATTACAGGCACCAGCCACCACGCCTGGCTGATTTTTGTA
TTTTTAGTAGAGACGGGGTTTCATGATCTTGGCTAGGCTGGTCTTGAACTGCTGACCTCCTGATC
CACTCACCTCAGCCTCCCAAAGTGCTGGGATTATAGGTGTGAGCCACCATGCCTGGCCAAATAT
TATTTTTTTAAATGAATTGTTTCTCTTAGTCTGCTTTGTTAAATTTGGAATTCATCTGGGCGCGGTG
GCTCACACCTGTAATCCCAGCACTTTGGGAGGCCAAGGCAGGCAGATATCTAGGTCGGGAGTT
CGAGACCAGCCTGACCAACATGGAGAAACCCCGTCTCTACTAAAAATACAAAATTAGACGGGT
GTGGTGGCGCATGTCTGTAATCCCAGCTATTCGGGAGGCCAAGGCAGGAGAATCGCTTGAACC
CAGGAGGCAGAGGTTGCAGTGAGGCGAGGTTGGCACCATTGCACTCTAGCCTGGGCAAAAAG
AGCAAAACTCCATCTCAAAATAAATAAGTAAATAAATAAAATGTTCAGTACTCACCAAGGTGCC
CCTGTTGTCTCTGCTTTTATCTTGATGCATCACTGAATTGATGTTAGATTTCAAATTCATCATTGCC
CTGATACTATTCTATCCTGAAGCCACCTTTATATAGTGATGAAAGAAATTAGCGATTTGTTATTAT
CCTCTCTCTGTTGGTATACATCAAATACTCACCTAAAAAAGAGCAACAACCAGTGGAAAACATG
ATGTTTTTATTTGGGTGACTATTTACTTGTAACCTACTAGCAAACTATAAAATTGTATGATATGCA
GAATTTTAACTGAATTGCTTTAAGTGAACATTTAAACATGATAAACAATATTGATGGTATTTATGT
TAATATACTTAAAAGGAACATTTTTCTTCATCATGAGTAATATAACCTACTCCTCAATGAAAACCT
AGCACTAAATTTGCTAATGAATTCAATAACATTTCCATAATATTTTTAGTTACATGCTTAAGGTTCT
CTTAGTGTTTCTCCCACTTTTTAATAGCTTATGCCTTTTTCACCTTTGGTTTTTTTTTGGTTCATTTTA
AAGCAAAAATCTCACAACATGTGATATCTGGAAACACTGTAACCTAGTGGTAAGACCATAGGCC
CTGGGGACACAGGCTGGCCACGTCTCTTCTCCTGTCTGAGCTTTAGTATCCTCTTTTGTGGTCAT
GAGAACTGAAGACCTGTCCCGAAGATTTGATAAGATAGTAAAGTGCTTCACATAATACCAGACA
TATAAATACACAGTAAATGCTTCCTCCTTATATTTTTATTGATTGATTGATGGAGACAGAATCTTG
CTCTCTTGCCCAGGCTGGAATGCAGTGGCGTGATAATGGTTTCTGCAACCTCCACCTCCTGGGTT
CAGGCAATTCTCCTGCCTCAGCCTCCTGAGTCGCTGGGATTACAGGTGCCTGCCACCATGCCCA
GCTAATTATTGTACTTTTAGTACAGACGGGGTTTTACCATGTTGGCCAGGCTGGTCTCGAACTCC
TGACTTCATGATCTGCCTGCCTCGGCCTCCCAAACTGCTGGGATTACAGGTGTGAGCCACTGTG
CCCAGCCTGTCTTTTCTCTTCACACCCGCAGTTCATGATGAAATGTTAAATATGTACTAGTGGAT
ATTACTTTGCTGAAATTGCCTAGTGAATATTAAGTATTTATTCTCACCTTTCAGACATGAACTTAT
GAATTCAACAGGTGAAGATTTACAACTTGATAAATCAACTTTGTCAGGTACGTCTTCAGTCAAGT
CAGATTAGAAGATTATGTGAGGTAATTAACACTTAACATTGATTTAATGGTAGCTTCCACATGAA
ATAGTATGCCTCTAAGTATTAATTATGTCCTAGGACAGGAGAATTCATGTTGTCAAAATTCTCATA
CTCTCTAGAACAATAAACTCATTTTCTTTTTATTAGTAAATATTGCATTTATGGGTAGACAAAACT
GAAAGAACAATATTTGTTCTACTTTTGAGATGCAAGATTCATCTGGCATAATGCATTGAACAGGT
TATTATTGAAGTCTACACCAGTCAACTGAATAAGCATTCATCAAATGTCCATGATATGCAGGACA
TAAGTTTTCTTTTAGAGTATGGAACCATGCATATTATCTTTTAATTAGATGATTTAGTTAGATATGT
TTTTAAAGAACTAGAAATATAATTGATTTTCTTGTTTTGGCTCTGGAGTGGAGTGGGGACGAAAC
AGAATGGATTCACACTGTTTAGATTTACTAAAATGGAAGGATTGCAGCAAGATCATATCCCTAGT
CTCCCTATAGCAAATGTCACCTGCTAGCTGTTTTTTTTTTTTTTGGAGGTTGAAGTTTTGTTCTGTCA
CCCACGCTGGAGTGCAGTGGTATGATCTCAGCTCATGGCAAGCTCACCTCCTGGGTTCAAGCAA
TTCTCCCTGCCTCAGCCTCCTAAGTAGCTGGGATTACAGGCCTCTGCCACCACGCCTGCCTAATT
TTTGTATTTGTAGTAGAGTTGGGGTTTCACCATGTTGGCCAGGCTGGCCTTGAACTCCTGACTTC
AGGAGATTCACCCGCCTCAGCCTCCCAAAGTGCTTGGGATTATGGGTGTGTCACTGCACTTGGA
TTTAATGGGATATATCACTACAGACTTCGGTAAACAGAATATTAGCATTTTTGGTGTTCTTTTTATT
TTACTCATACTGTTTTTCTTTGGACTCAATCACAATAACAGAATTAAAGATCAAAGTGTAAAAGTT
AAAGACCAGTACAGATTCAATAATTATTCTTTTCTACATACTGTGTTTAAATGATATCCCTTTTTCT

TTTTGTTCTTATAGCTCGAGCTGTAAAAGCCAAAGGTCCGGTGATGATCCCATACCCTTTTTTCCA
GTCTCATGTTGAAGATTTTTATGTAGAAGGCCTTCCCAAAGGAATTTTTTTTTTTTTTTTTGAGAT
GGAGTTTTCACTCTTATCGCCCAGGCTGGGGTGCAATGGCGCAACCTTGCTGGTCACTGCAACC
TCTGCCTCCTGGGTTCAAGAAATTCTCCTGCCTTAGCCTCCCAAGTCACTGGGATTACAGGTGCC
CACCACCACACCAGGCTAATTTTTGTATTTTTAGTGGAGATGCGGTTTCACCATGTTGGCCGGGC
CAGTCTCGAACTCCTGACGTCAAGTGATCTTCCCGCCTCGACTCCTGATATCAAGTGATCTTCCC
GCCTCGGCCTCCC

ID|56062|gene_id|ENSG00000232019|transcript_id|ENST00000439105 Homo sapiens lncRNA

CCGGAGGCTGCCATGGCGGGTGGGCAGCTTGACTTCTCCTCCCGGCCAGTTGTCGAGCGC
CTCACCAGGCCTCGCCCTGCAGCCTCGCTCTCCCTGGCGCTGCGCGGCCTAGGGGACTGGGCT
GCTGGCCTCCGGGTGCGGGGTGGGGGCAGGCTCTGACCTGGGGCGTTCTGGCCGCGCGAGCC
GCGGGATGGGGCCCCCGGCCCCGGAGGAGGCGCCGCTGCTGTGTCCCTTGGTGGAGAGGGCG
CTGCCAGCCCTGCGCGGTTTCCAGCCAGGAAGCTTCGGGAAGCCTGGACGTCTGCTCACTGGA
GATGACACGTGCGTGGGGTGTTGGGAAGGAGGTGACTTCGCCTGTGATGGACTCCCAGTGTGA
GCTGTGATGGACTTCCAGTGTGAGCACTGGCCAGAGTGACCAGGCCGACCATCAACAGCCCTG
ATCCAGATGCAGAGGCCAGGATGTGGGCCCAGCCCTGTGCCAGAAGGCTGGCTGGAATAAAG
GTACAGATAGAGGCCTCACCCCCTCTGGGACCACTGGCACTCAGGGTGTTTGCAGCCTCAGAG
CCCACCTACCCCCAGGGCCACAGCTGCAGCTCCTGCCCTGCTGTCATTACAGGGATGGGCAGG
CTGGCATGGGGGCACCCGCTGCCCCTGCCTGGGTGTTGCTGTGTATTCCCGCCGGCCAGGGGC
CACTGCCAGGACCACCCCTCCCTTTTCATATCCCGATTCTTAAGTTCTGCTATTGTGGTATTGTGG
TCGAAAAAAAAGAACCGCTTGGCTGTTTTTGAACTGCCTGGAACCTAAGATCCTGAATTTTTTTT
CCCCCCCAAGGGGGAAAGTCTATATGGAAAACATTTATTTTAAAATACAGGATGAAGTGAATTA
AAAGATTTAAATGCA

ID|56063|gene_id|ENSG00000232053|transcript_id|ENST00000437569 Homo sapiens lncRNA

ATTTAAGTTCAGGAACTTTGCAGATCTCTTATTAACTCTTCAGTTAATAATGAGGTAAGTCTT
AAGTAAATATTCAAATTTTATCTAAGTTTTTTTTCACTTAATAGTGCCATCGTGAGTTCAGAGAAG
ACATTGAATAAGGATGAGAGGTGATTGATAAAGGGAGAAATGTGGAAGAATAATTTTTTATTCA
AATACATAAAATTTTACCTATTTAGAGATTTTTTTAAGTTTATCTTCTGCATTTTTAAAAACTTTTAA
TTGATTATTTTAATGGACACATTGTATTTGTATATATTTATGGGGTACAACTTGGTATCTTGATATA
TGTCGTATGATGATCAAATAAGGATAGTTATTGTATTCATCACCTCATGTACTTCTGATGAAGAC
ACCGAAAGCAATTGGGACAAAAGCAAAATTGGCAAATGGGATCTAATTAAACTAAAGAGCTTCT
ACACAGCAAAAGAAACTATCAACAGAGTAAGCGGACAACCTACAGAATGGGAGAAAATTTTTG
CAAACTGCATCCAACAAAGGTCTACTATCCAGCGGGAGGGATCACAGAAAATAACTTGTGGGT
ACTAGGTGTAATACCTGGGTGATGAAATAATTTGTACTGCAAATCCCCGTGACACGAGTTTACCT
ATATAACAAACCTGCACATGCACTCCTGAACTTAAAAGTTAAAAAAAAAAAAAGAGAGAGAGA
GAAAATGGTTTAGGCATTCAAAAAACTAATAGGTATTTTAAGAATGTAACTAATAATAGAAATTG
GTAGGTAGCAAGCACTTACATTTCACAAAGCATATGTGGATGCATTACTCCATTTAAAGAACAA
AGCCCGGCGTGGGGAGACACTCATATAATGGAAACACTGGAACTCTGAGATTGATCCGGACCC
ACAAAGCTCCCTAATGGCCAACCTAGGATACACTCAGACTTGGGACTGTTTAGCTCCACGTCTC
GTGGTCTTTATATGTGTCACAGTTAAGAATCAGACCGTGACAGACACAAATCATGTGAAACGGC
GTCCCTCTCTCTCTCACCCATTGATCCCCCGCAGTTGTAGATGTGATTCTGCTTCCTAGTAAAAG
AAACTTGCAATAAGAGTATGTGTCCACTAGAGGGCCGAACTCAGAATTCGCCGTTCTGCTGTAG

AGCCCAAGTAGACAATGCCATCAAATGCTCCAGGCTCTCTTTCATCAGCTTTGTACCACCTGATT
CAAACGCTAAAGCCCTGGGCATAGAGTGCAATGAAAAACACTCTTAATTCCAAGTAGAAAAAAT
AAAAGTTGTATTTTTCCCTGCTTGTCGATGCCTTCCGTGTGAAGTATGTTGTAAACTGTGACACCC
CATGGAGCGTGGTGTCTCACTATTAAGCGAAGCCTTTTCCTGTTTCTGATGTTTGCTGGGTTGGC
AGGAAGAAAAATATCAGTTCTATTTTTTAACCATACTATTTCTGTTGCCCTAACAGACTCCAGGTA
AATTAATTTACTTTGAATGAGTCATTAGTTCCCATTTGCTAGAGTTCATGAAAATGAAAGCATGA
AACAACATTTTATAGAAATTCCCTGAGGCACAGAACACCAAACCTGGGAAAGACGTTAGAGTCC
AAATTCCTTATTTTATAGACAATAGAACCAAGTTTGAGAGCAGGGTTGCCCTTAATTACCCAGCT
CACCTAAATCAGAGGCTGACCCAGAACCCAGGTCTCCTGTTTGCATGGTTCCATCCTACCCAGA
TTTTTTTTTTTTTAACACTGGTCCATAGAATCCTTTGGTGGCCTGACTCTCATGTTAATAAAGCCTG
ATCAGAGATTCTTGATTAAGTATATTGTTCACATATATTTTAATTTCCATTAGGAAATACTATTGTA
CCACATCAGTCAGTCCACTGCTAGAGAAGACTGGGGGGATCTTGCATCCTTATGCCAGTTAGGT
CTCCTTGCACACAATTCCTGTGAGCATCCTAGAGATCCATGCCTCCTCATTCCTGGATTTGCAGA
GATGGAGAGCTCACAGTTTTGAGGTTACCTAATTCTGTTGATTGAGTAATGTTGATTAAGGATTC
CCACCTTACTTTTGTTGGAGGAGAGAGAAAAGTTTGGGAAATGGGATTGGGGGTAGGAAGTAG
GGAGAATAATTTAAGATTGCTGTCCTTTCTTGTCTGATGATGAAGAAGTACAGAAGGACTGGGT
GATTCATCTGAGATTCTGAAATTAGATGTCAGAATAGGCATTATATAATGCGATAGAAGGCTCGT
TAGACTAGTAGTCAAATACGGTAAGTACTGACTCCATGATATAAGGGGCTGCTCAAATCTGAGA
GTCACCTAAACACCTTCTTTCTTCAAATCTCTAATTCCTCCCTAAGTATTGTCTAAATAGCCCCTC
AATATATTCCTCTCAACTCTGTCCTATCACTTTTGCTTTTTTCATCCAGATATGCCCAGCACCCTCC
AGTTTGTCCCCAGTATTGCAGTTTTCTGGACCTTCCCCAGAAGCAAATACAATCTTGCATCTTTTC
TGATTAAAATCCTTTACTGGCTCCCCCACTGTCCATACTGATAGCTATTCAATTGCACTTTGGAGA
TGAACAACTCCATTTTTGGAGCCAGAATATGTCCTTTTCCTTTCCCACTAATTTGCTTCCTCACCT
AGGAGACTCCCTGTCTAGCTTGTCCTTTCTTCCTTGCTGAAATTAAATTTCTATATGATAAAAGAG
GCTGGAAGCTGAGGAAGGTTTTGCAGGAATTGCCCCAGCCATAGCTACATGCGGCAACAAGAT
GCATGATATTTTAAGTTATGAGTAGCAGAAGCCTGTACATAATCAGAAAGATTCTAACATATTAT
ACCTCTAAGAAAAAAATAATATGCACACATGAATGTACATATATCTCTGGACAGAGCAGCCACT
AGGCTCCTGATGTGACTTGCTCTACATTCCTCTCCCCCTGGGGAACCACACAAGTGTTTTGCAAC
CTCCTTTCTCACCTCTGCCTACCATGCTGTGTTGCCTCTGAACATTTATATTCCACCTCCTTCTTTT
ATTTGTGGTAATTTTATACATGACAGCTACTAACTAACTAAGGGAAAATGATAGAGGGTCCTATT
TAAAAAACAAATGCAAGCCTGTTAGTAGAAGCATAATCCCTGTAAATAGTATAGCTTTCTCAGCT
AAAAATTTAAAGCAGGAGGGTTTTTTTAAAGTATAGGAAACTGGGGCTTCATTTCATCTCAGATT
CCTCATGAATACATCTTGGAGATTCATGTAGCTATATTTTTAAACTCATATCCAGAGGCACACCC
CCTTCACCAACATATATAAAAGATGATGAGGGGATTTTTCTTATCTTTACGTAAGAGCAGGAGTC
TGTACCAAGTTTGCGAGGTCATTCAGTAACAAATTTACTCAGCATGTATTCCTTCCATAGCTCCC
AGAGATCAGGTACCTTGCTAGCTCTTATGGTTGGAGATACACAGATGATGGAGTCTTGTCCTCC
ATCCCCTCACACTGAGAGGAGGCAGGGAATCTGACATGTAAACAAATGTGCACATGACTTGCTA
TATGCTATAATAGAAATGCTTGCCAAGTACTACATGAATAGAGCAAAGGAAATCCTAATGATTC
AGGTGGCCACTGAAGTCGACTTCCTGGAGAAGATGATACTACACTGACAGAGACTCAAATATG
AAAGAGGCAGAAATGTCACTCAGAAAGGGGCAGCATGAGCAGAAGTACTGGTTTACTTTATCC
TGGTATATGTAGTATTGTGGATCATATTAGTGTGGTCTGATCGATTATAGTTTCTTAAGGGGAGAT
TTTTAGCCAAGAACCACTTATGCACTTCAGAAAGTTGAGCAGTTATACAAACAATTTCTTTAACA
TTACCTTCTTCATATGCCCTTTGAAAAAATATTTATTAGCCTCTTTTAATTACAATATTAGTGTTTTA
TGTTAACTCCAAGTAAGTATAGGGTGTCAGGAGATATTGCTCTATCTAATTAGGTAATATATCATT

GTAATCATATCCATGGTATGACATCCAGATCATTTCCAGTTGAGAGAAGTGAGAGGAATCCTAC
CTTAAAATTATGAGGGGAAGTCTACCTTAATGGTATGGATGCTGGACAGCTGCCTTGTATAACCT
CTGGTCTAAGTTAGAAATATGAAAGGAAACAAGCCTAGTGAAACTTAAATATCCCATGGATTTA
TTTTCATCCTCATTGGTGATAAAACACCCAAACTAATGTTTCTCTGTGATAAGGATCTTGAGCATA
TCCTTATGCTCAAGTGTGAAGCCAAGAAAACAAGAAAACTCCGTAAGTCAGTCAATTCAAAAAG
CATTTCAGTGTTGAAACTCCCTAAAACGTGTTTATTCATGTCCAAGCAGAGGAAGACATATGCTT
TTGGAGAATTATTGGTGAAATACTGTGACCTAAACCTTGGTTTGTGACTTGCAACCCAGGAAGA
AGAAAAGGTGTATTTGGATGCTTGTCAAGTAGAAGTCTTCCTATGTGTTTTGTTTGTTTATTTTTG
GTTGTTTGTTTACTAAAGGGAGTTTACCACATGCTAGAGAATTTTTGAGGACCCAATAGCACTAT
GATGATACTTGATTTTTAACTTAATCATATTGATTGAAGAGGAAGTAACTGTTGCTATGTAAATCG
ATGCTATTTATTTATTCTCCATATGCTGTTTGAAAGCATTTAGAGAAGCTTGAGGCACATGCATCC
TGCTCTGGGAAACATGGGCCTGTATCTATTGCCATGTTTGCATTTTCTGCCAGTCCAACCTTGAA
TTCCTACAGAATCCTAATATCTATGCTAAACCCTTGCCTTAGGACCATGGTAATTTTATAAGGAA
GATTTTTATTTTTCAAATGTCAGTATCAGATTAATTATTTCATTGGTTCTGAAATGCCTTGTGCTGA
TTCTTGTTTCTATCTATAATCTATCTATCTATCTATCTACCTACCTACCTACCTACCTATCTATCTAT
CTATATCTATCATACAGTTTTGTGAGTTAGTAGGTGAACTGGCTTGCAAGATTTCCATTGTCTTTC
CAGATGTTGGTAGCCACTCACACTTCTTTCCATCTGTTTCTACTTATTATATTTATTGGCCATTGAC
AATTAAGGCTAGGTCTGCATGAATTTTAATTATTTTTAGCTAGTTTTTTATCTTTTGCTTAACATAT
GACATTGTATTGATGTTTCTTTCTAGACAGATTTAAAATCTCAGAAATTTAAGAGTATTTAAAGCA
GTTTAAAAGCTCTAGTCTTAAACAAATAATCTGTACAACAAACCCCCATGACAGAAGTTTACCTA
TGTAACAAACCTGCACCTGGACTACTGAATTTAAAAGTAAATAATAATAATAATAATAATAAAGT
AAATAAAATAAAAAATAAAGTTACAAAAAGGCAATAGAAGAAATAAAAATAATTATATAAACAT
TAAAGTTTGGAGGGTATTGTAAAATAACAAGAAAAGGTATAAACATGACTATCTAATTGGATCTT
TAAATGTCTCTAAATTCCCTTTATATTATTTTATTATTTGTACCCTCATAATCTTTTGCATAATTGAC
AATCTTTTAGTAATAATACTATAACATATGTGTTTGGCATTTGAAGAATTAACATTTAAGTAATGA
AGGTAATAGAATGCATAAAGTAGACTTAATGAGACTTTTAAATCATCATAAATGTTTATATTTGAT
AATTCTTATTTCGTGTTTTGTTGGGGGCCGATAATTGGTCATTGCCCCATAGGTAGGCATGACGA
CACATATTTTTCAGGGTTGAAACATGGGAGTGAAGACTGCAGGTTACAACTTGCCAATTAGTTCA
TTTGTTGGGTGGACATCCTATTGTCTTTTGACTATTGCTATCCTGGTCACACTGCACAAAATGCCA
GATAGAGGTAAAGTGCATTGCAAAAAGAGTAAAAAAAAAATTAAATGTCTGGACACTTCTCAAT
ATTTTCTATAAAAAAACATAATGCACACTTCATATTTTTAAATGGGCCTTGCTAAATGAAACCTAC
CTTTCTTTCTCTTTCTTTCTTTCTTTCTTTGTACATATGTGAGGCAACACTGCTTGGGTAAAGTCTCT
CTGTTCTATCTGTCTCTGTCTCCTCCTGTATCTCTTATGGTAAAATGGTTTATAAGCTGGTGAAAT
GGTAGTTCTGTAGAGACTTTTGCCAGTTAATTGAAAAAATACACACGTCTGTAGCAGATACATTC
AGGCTTTGTCTATGTGGCACACCTAAATAACTTTTCTGTTGTTACATTTTAATTCCGATATAAGAA
TAGGACAATGGCCAAATAATCAAGCAGATCACTGCCCAGTTTATTGGAATCTATTTTTACACTGA
CAGGAATAATTTTCTCAGATATGTCATGACTTGGCTATTTCAATAACTCCCAAGAACAAATTAAA
TCCTCCCATATTTCAATCAAATTGATATTGTAGGAGTCAGTGATTGTTTCAGTTAAACAATGAATT
GACTATGACTAAGGACTGTCACTCTCTACCCATTATTCTATCATAACTGTGTTTCTACAGATTATC
ACAAAATCAAAGAGTAGACAAGTGTAAGATTACGATTAAATGCAGAAGATTTGCAAAAAGAGTT
CACTGCTGGTTCAATTACTTTGGATCATGTTGATGCTACTTGGCTTCCTGCTGGACACAAGGGGT
AGTGAGTTTGACTTGTTTCGCTGAGGATTCTGGGAATGTTAGGGACGGTAAGACATCTGTCCATA
TTAAGGGATAGAATCCAAGTGAATGAAAAGGAGGTGCAGAGTGAGAATTCTTTGCAAGACTGT
CTCATTCATGTCCCTGACTCATGGCTTCAAGCAGTTCAGTGCTCTGCAGCCAAACTTATCCTTGT

GAGCAATTTGGTTGGATAGACAACTGATCTAAAATTATTGAGTTGCCTTTGCCCTTTTGAATATGT
AAAGAGAAACTGATAATCTAAATATATTTTTTCTTTCTACCTTTTAAATTTCTAATTGTGCTGACCT
GAGCTAGATATTGATTGAAGAAAGAAGTGATATCAAATGAGGATGACAAGTGGTATCTTCAATA
GACTAATTGAAAGTAATATGTCCACATATATAGACTGTGTCTCTCTGGGAGAGGGTGCACAAAA
AAAGAGGTAAATACTATCGGAAAAAACAATACCTCTAATTGGGGTGGTACTCCTACCTGACTCC
CGGTTAAACTACCACCCTAGGCTAAGTTCTTTTTCACATCTATCTGCAAGGAAAGAATGCTCACA
CTATTTTTTTTTTTTTTTTTTTTTTTTTTTTTTTTTTTTTGAGACAGAGTCTTGCTCTGTCGCCCAGGCT
GGAGTGCAGTGGTGTGATCTCGGCTCACTGCAAGCTCCGCCTTCCAGGTTCACGCCATTCTCCT
GCCTCAGCCTCCCGAGTAGCTGGGACTACAGGCACCCGCAACCACACCCAGCTAATTTTTTGTA
TTTTTAGTAGAGATGGGGTTTCACCGTGTTAGCCAGGATGGTCTCAATGCTCACACTAATTTTAC
GTCTAAACTAGCCGGAAGTAAGGACTGGAAGGGGGAGGTGTGGTGAAGGAGGAGGTGTGGGT
CAAGTTCACTGATGACGGTCTACACGGTGTCCTCAAGGGGTTATCTAAAAGGGGTAAAGGTGTT
ATGTGTTTTCTCTCAGTTCAGCATGACTACTGATCTCAGAAGGTAGGTTGTCTCACACATTTCTAT
GTGTGGCAAGTGTTAATGGGAGGTGAGAAGAAGGGGAGCTTGCTTTCAAGTGGTTGCAGCCCC
ATTTCTCCCCTGTACGCTTATCTGCCAATCCTCCCTACACATGGGGGTGCAGCTTAGTCATATGC
TGGCTCCTCCCAGGGCAGGCTTCTCTGTTTCCACCTGCACACGCTGAGTTTGGGGTGGTTTCTTG
TGGGCTGAGTTTGAATCTCTGACAGACTCTCGTGGGCCTCCTGGAAACTGAGGTGTGTTGGAGC
CCCACACTCTCCCTGCTGACCACCCATCTCACTGACCTCATTGCCAAGGCTGATAGCCTACTTAG
TACTACACTTACAAATTGTGTCTTGTAAGTCCACCCAGGGAGTCTGGAATGGACTTTGATTGCTA
GAGACATAATGTCCATCACAGGGCCAGCAATCTTTCTTTCCTAGTGCCCTCTCAGGAATGGCAG
GCCCCCTGCTCCCATGGCTTCAGGTCTCCTGTTCTCTAAAGGTTCTGGCTGTGGCAGATCCTGCC
CAATTAAACTCATGTACATAGTCTAAGTATCCATTAATGATTCTCTGTGAATCAAAATCCTCTCTT
AAAATGGGATATAAGTCCTCAGGGTTGTCTCAAGAAACTCTGACCCCTCCTAGGCACAAATATC
CCCTGTGGCAGGCAAATAGTCACAGTTTCGCAGATCAGTTTTTCTAGAATCTTATTTTCAACCAC
TTTTTTTCCCTAAAGATAGAGGAAATTAGTTTCCCCAGCCTTACGCACTCCCACCAGTGTGTGTTT
TCCCCAGCGTATAAGGCTGGGGACACAGGAATTATGCATACACAGGAATTATGAGGCAATAGA
CTGCTTCATAGATACAATAGGATACAGGTTTATGCATAGTTCCTGGCATCCTAGTTGTAACTCAA
CCTCTAATTCAGTTGAATATGGTTTAGTAGTTTGGAATATATGTTTTAGAGAAATTCTTAAACTTTA
ACAAGATTATGAATCAGCTGGACTTGTTAAAATGCAGATCCTGATTCAGTAGGTCAGAAATCGG
GTTTAACAGATTTTACACTTTCAGCAAGCTCAGGTAGTGCCAACGCTGATGGTCCATGAACCAC
ACCCTGAGTGCAAGGCTCTCAATGGTGATGTGTCTAGCCAGATGCATGATCTGGCCTCTCTAGC
TCTTTTTATTTATTTATTTTTATTATACTTTAAGTTTTAGGGTACATGTGCACATTGTGCAGGTTAGT
TACATATGTATACATGTGCCATGCTGGTGCGCTGCACCCACTAACTCGTCATCTAGCATTAGGTA
TATCTCCCAATGCTATCCCTCCCCCCTACCCCCACCCCACCACAGTCCCCAGAGTGTGATATTCC
CCTTCCTGTGTCCATGTGATCTCATTGTTCAATTCCTACCTATGAGTGAGAATATGTGGTGTTTGG
TTTTTTGTTCTTGCAATAGTTTACTGAGAATGATGGTTTCCAATTTCATCCATGTCCCTACAAAGG
ACATGAACTCATCATTTTTTTATGGCTGCATAGTATTCCATGGTGTATATGTGCCACATTTTCTTAAT
CCAGTCTATCATTCTTGGACATTTGGGTTGGTTCCAAGTCTTTGCTATTGTGAATAATGCCGCAAT
AAACATACGTGTGCATGTGTCTTTATAGCAGCATGATTTCTAGTCCTTTGGGTATATACCTGGTAA
TGGGATGGCTGGGTCAAATGGTATTTCTAGTTCTAGATCCCTGAGGAATCGCCACACTGACTTCC
ACAATGGTTGAACTAGTTTACAGTCCCACCAACAGTGTAAAAGTATTCCTATTTCTCCACATCCT
CTCCAGCACCTGTTGTTTCCTGACTTTTTAATGATTGCCATTCTAACTGGTGTGAGATGATATCTC
ATAGTGGTTTTGATTTGCATTTCTCTGATGGCCAGTGATGATGAGCATTTTTTCATGTGTTTTTTGG
CTGCATAAATGTCTTCTTTTGAGAAGTGTCTGTTCATGTCCTTCGCCCACTTTTTGATTGGGGTTG

TTTGTTTTTTTCTTGTAAATTTGTTTGAGTTCATTGTAGATTCTGGATATTAGCCCTTTGTCAGATGA
GTAGGTTGTGAAAATTTTCTCCCATGTTGTAGGTTGCCTGTTCACTCTGATGGTAGTTTCTTTTGCT
GTGCAGAAGCTCTTTAGTTTAATTAGATCCCATTTGTCAATTTTGGCTTTTGTTGCCATTGCTTTTG
GTGTTTTGGACATGAAGTCCTTGCCCACGCCTATGTCCTCAATGGTAATGCCTAGGTTTTCTTCTA
GGGTTTTTATGGTTTTAGGTCTAACGTTTAAATCTTTAATCCATCTTGAATTGATTTTTGTATAAGG
TGTAAGGAAGGGATCCAGTTTCAGCTTTCTACATATGGCTAGCCAGTTTTCCCAGCACCATTTAT
TAAATAGGGAATCCTTTCCCCATTGCTTGTTTTTCTCAGGTTTGTCAAAGATCAGATAGTTGTAGG
TATGCGGTGTTATTTCTGAGGGCTCTGTTCTGTTCCATTGATCTATATCTCTGTTTTGGTACCAGTA
CCATGCTGTTTTGGTTACTGTAGCCTTGTAGTATAGTTTGAAGTCAGGCAGTGTGATGCCTCCAG
CTTTGTTCTTTTGGCTTAGGATTGACTTGGCTATGTGGGCTCTTTTTTGGTTCCATATGAACTTTAA
AGTAGTTTTTTCCAATTCTGTGAAGAAAGTCATTGGTAGCTTGATGGGGATGGCATTGAATCTAT
AAATTACCTTGGGCAGTATGGCCATTTTCACGATATTGATTCTTCCTACCCATGAGCATGGAATG
TTCTTCCATTTGTTTGTATCCTCTTTTATTTCCTTGAGCAGTGGTTTGTAGTTCTCCTTGAAGAGGT
CCTTCACATCCCTTGTAAGTTGGATTCCTAGGTATTTTATTCTCTTTGAAGCAATTGTGAATGCGA
GTTCACTCATGATTTGGCTCTCTGTTTGTCTGTTGTTGGTGTATAAGAATGCTTGTGATTTTTGTAC
ATTGATTTTGTATCCTGAGACTTTGCTGAAGTTGCTTATCAGCTTAAGGAGATTTTGGGCTGAGA
CGATGGGGTTTTCTAGATAAACAATCATGTTGTCTGCAAACAGGGACAATTTGACTTCCTCTTTT
CCTAATTGAATACCCTTTATTTCCTTCTCCTGCCTGATTGCCCTGGCCAGAACTTCCAACACTATG
TTGAATAGGAGTGGTGAGAGAGGGCATCCCTGTCTTGTGCCAGTTTTCAAAGGGAATGCTTCCA
GTTTTTGCCCATTCAGTATGATATTGGCTGTGGGTTTGCCATAGATAGCTCTTATTATTTTGAAAT
ACGTCCCATCAATACCTAATTTATTGAGAGTTTTTAGCATGAAGGGTTGTTGAATTTTGTCAAAG
GCTTTTTCTGCATCTATTGAGATAATCATGTGGTTTTTGTCTTTGGCTCTGTTTATATGCTGGATTA
CATTTATTGATTTGCATATATTGAACCAGCCTTGCATCCCAGGGATGAAGCCCACTTGATCATGG
TGGATAAGCTTTTTGATGTGCTGCTGGATTCGGTTTGCCAGTATTTTATTGAGGATTTTTGCATCA
ATGTTCATCAAGGATATTGGTCTAAAATTCTCTTTTTTGGTTGTGTCTCTGCCCAGCTTTGGTATCA
GAATGATGCTGGCCTCATAAAATGAGTTAGGGAGGATTCCCTCTTTTTCTATTGATTGGAATAGT
TTCAGAAGGAATGGTACCAGTTCCTCCTTGTACCTCTGGTAAAATTCGGCTGTGAATCCATCTGG
TCCTGGACTCTTTTTGGTTGGTAAACTATTGATTATTGCCACAATTTCAGCTCCTGTTATTGGTCTA
TTCAGAGATTCAACTTCTTCCTGGTTTAGTCTTGGGAGAGTGTATGTGTCGAGGAATGTATCCAT
TTCTTCTAGATTTTCTAGTTTATTTGTGTAGAGGTGTTTGTAGTATTCTCTGATGGTAGTTTGTATTT
CTGTGGGATCGTTGGTGATATCCCCTTTATCATTTTTTATTGTGTCTATTTGATTCTTCTCTCTTTTT
TTCTTTATTAGTCTTGCTAGCGGTCTATCAATTTTGTTGATCCTTTCAAAAAACCAGCTCCTGGATT
CATTGATTTTTTGAAGGGTTTTTTGTGTCTCTATTTCCTTCAGTTCTGCTCTGATTTTAGGTATTTCT
TGCCTTCTGCTAGCTTTTGAATGTGTTTGCTCTTGCTTTTCTTGTTCTTTTAATTGTGATGTTAGGGT
GTCAATTTTGGATCTTTCCTGCTTTCTCTTGTGGGCATTTAGTGTTATAAATTTCCCTCTACACACT
GCTTTGAATGCGTCCCAGAGATTCTGGTATGTTGTGTCTTTGTTCTCGTTGGTTTCAAAGAACATC
TTTATTTCTGCCTTCATTTCGTTATGTACCCAGTAGTCATTCAGGAGCAGGTTGTTCAGTTTCCAT
GTAGTTGAGCGGCTTTGAGTGAGATTCTTAATCCTGAGTTCTAGTTTGATTGCACTGTGGTCTGA
GAGATAGTTTGTTATAATTTCTGTTCTTTTACATTTGCTGAGGAGAGCTTTACTTCCAACAATGTG
GTCAATTTTGGAATAGGTGTGGTGTGGTGCTGAAAAAAATGTATATTCTGTTGATTTGGGGTGGA
GAGTTCTGTAGATGTCTATTAGGTCCGCTTGGTGCAGAGCTGAGTTTAATTCCTGGGTATCCTTG
TTGACTTTCTGTCTCGTTGATCTGTCTAATGTTGACAGTGGGGTGTTAAAGTCTCCCATTATTAAT
GTGTGGGAGTCTAAGTCTCTTTGTAGGTCACTCAGGACTTGCTTTATGAATCTGGGTGCTCCTGT
ATTGGGTGCATATATATTTAGGATAGTTAGCTCCTCTTGTTGAATTGATCCCTTTACCATTATGTA

ATGGCCTTCTTTGTCTCTTTTGATCTTTGTTGGTTTAAAGTCTGTTTTATCAGAGACTAGGATTGCA
ACCCTTGCCTTTTTTTGTTTTCCATTTGCTTGGTAGATCTTCCTCCATCCTTTTATTTTGAGCCTATG
TATGTCTCTGCACGTGAGATGGGTTTCCTGAATACAGCACACTGATGGGTCTTGTCTCTTTATCC
AACTTGCCAGTCTGTGTCTTTTAATTGGAGAATTTAGTCCATTTACATTTAAAGTTAATATTGTTAT
GTGTGAATTTGATCCTGTCATTATGATGTTAGCTGGTGATTTTGCTCGTTAGTTGATGCAGTGTCT
TCCTAGTCTCGATGGTCTTTACATTTTGGCGTGATTTTGCAGCGGCTGGTACCGGTTGTTCCTTTC
CATGTTTAGCGTTTCCTTCAGGAGCTCTTTTAGGGCAGGCCTGGTGGTGACAAAATCTCTCAGCA
TTTGCTTGTCTGTAAAGTATTTTATTTCTCCTTCACTTATGAAGCTTAGTTTGGCTGGATATGAAAT
TCTGGGTTGAAAATTCTTTTCTTTAAGAATGTTGAATATTGGCCCCCACTCTCTTCTGGCTTGTAG
GGTTTCTGCCGAGAGATCCGCTATTAGTCTGATGGGCTTCCCTTTGAGGGTAACCTGACCTTTCT
CTCTGGCTGCCCTTAACATTTTTTCCTTCATTTCAACTTTGGTGAATCTGACAATTATGTGTCTTGG
AGTTGCTCTTCTCGAGGAGTATCTTTGTGGCGTTCTCTGTATTTCCTGAATCTGAACGTTGGCCTG
CCTTGCTAGGTTGGGGAAGTTCTCCTGGATAATATCCTGCAGAGTGTTTTCCAACTTGCTTCCATT
CTCCGCATCACTTTCAGGTACACCAATCAGACGTAGATTTGGTCTTTTCACATAGTCCCATATTTC
TTGGAGGCTTTGCGCATTTCTTTTTATTCTTTTTTCTCTAAACTTCCCTTCTCGCTTCATTTCATTCA
TTTCATCTTCCATTGCTGATACCCTTTCTTCCAGTTGATCGCATCGGCTCCTGAGGCTTCTGCATT
CTTCACGTAGTTCTCGAGCCTTGGTTTTCAGCTCCATCAGCTCCTTTAAGCACTTCTCTGTATTGG
TTATTCTAGTTATACATTCTTCTAAATTTTTTTCCAAGTTTTCAACTTCTTTGCCTTTGGTTTGAATG
TCCTCCCGTAGCTCAGAGTAATTTGATCGTCTGGAGCCTTCTTCTCTCAGCTCGTCAAAATCATTC
TCCATCCAGCTTTGTTCCGTTGCTGGTGAGGAACTGCGTTCCTTTGGAGGAGGAGAGGCGCTCT
GCGTTTTAGAGTTTCCAGTTTTTCTGTTCTGTTTTTTCCCCATCTTTGTGGTTTTATCTACTTTTGGT
CTTTGATGATGGTGATGTACAGATGGGTTTTCAGTGTGGATGTCCTTTCTGTTTGTTATTTTTCCTT
CTAGCAGACAGGACCCTCAGCTGCAGGTCTGTTGGAATACCCTGCCGTGTGAGGTGTCAGTGTG
CCCCTGCTGGGGGGTGCCTCCCAGTTAGGCTGCTCGGGGGTCAGGGGTCAGGGACCCACTTGA
GGAGGCAGTCTGCCCGTTCTCAGATCTCCAGCTGCATGCTGGGAGAACCACTGCTCTCTTCAAA
GCTGTCAGACAGGGACATTTAAGTCTGCAGAGGTTACTGCTGTCTTTTTGTTTGTCTGTGCCCTG
CCCCCAGAGGTGGAGCCTACAGAGGCAGGCAGGCCTCCTTGAGCTGTGGTGGGCTCCACCCA
GTTGGAGCTTCCAGGCTGCTTTGTTTACCTAAGCAAGCCTGGGCAATGGCGGGCGCCCCTCCCC
CAGCCTCGCTGCCGCCTTGCAGTTTGATCTCAGACTGCTGTGCTAGCAATCAGCGAGACTCCGT
GGGCGTAGGACCCTCTGAGCCAGGTGTGGGATATAGTCTCGTGGTGTGCCGTTTTTTAAGCTGG
TCTGAAAAGCGCAATATTCGGGTGGGAGTGACCCGATTTTCCAGGTGCGTCAGTCACCCCTTTC
TTTGACTCGGAAAGGGAACTCCCTGACCCCTTGCGCTTCCCAGGTGAGGCAATGCCTCACCCTG
CTTCGGCTCGTGCACGGTGTGCGCACCCACTGGCCTGCGCCCACTGTCTGGCACTCCCTAGTGA
GATGAACCCGGTACCTCAGATGGAAATGCAGAAATCACCCGTCTTCTGCATCGCTCTCGCTGGG
AGCTGTAGACCGGAGCTGTTCCTATTCGGCCATCTTGGCTCCTCCGACCCTCTCTAGCTCTTAAG
ATATATTTATACCTTGACCTTTCCATCTCTCTATATTCAATATTGTATATTTTCAAAACTGTCATTTA
TTATTGCACTTGTGCCTGTAACCTGCTAGGCTGCACTTCTGATAGAAACCTTGTTATCACCATCCT
TAGTCACCTTCCAGAAGAAAGGATGGACAAGCTGTCTTGTCATGAACTTTTATCCTCATTTTACT
CTCTTCTTCTCTCCTTTTTTTTCATCCAGTCCATACCTTTTCCCTTGTGATTTCTGTAAGCATTAAAT
TGTTACTCAAGAATCGGTGCAGCAACTGGCATGTGGTAGATTCACAATAAAAAGTTATATAAATT
GAAGCATCTTCCCTGCATGGCCCTTCCACCACCTTTTGCTGAACACTGGGTCTCCCCTGAGAGC
AAGACCTCTCTAGTAACCCTAACTTCGGAGGCTACTGGTTCCCTAAACCACAGGGTTTACAATTG
TGTGGATAGTCTCCCAGCTCCCTGTTTCTCCTTCCTGACTGTCACTTTTCCAAAGTCATGTCAAAA
GCCTTTGTCATTTGAGATCCATGCCACTCAATACTGCTTATGTCTTTTCTTGTTGTCTATCACTTTA

TAAATTGTTAAAGTTACAATAGGATTTGATCTTTTTTTACATTCAATCTCTTCTCATCATTATGTAT
GAGGATGACACATCCAATTATCCAGCCTCAAAGGTAATTAAACTCTTCGACTCCAACAACCTTCT
CTATATTCTACTTCAGCCACCTGTGTGTACAGCTACAACTTCACTTAGGGCATATTGAAGTCTATT
AGATCGAATTGTTCAATTGATTCTGCTGATCACAAAATAATACAAGCTTGTAATTCCAAAGTTAA
AGCCTACCTTTATGGAATAGAAGGTAAAAACATCTACATAATCCTAGCCAGATATAACAATTAA
AAACATACCATTTCAGACTTTTTCATAAAGAGATTAATTATCTACCTAAATTGAAACATACTAAAT
ATATAGGGTAGCATAGCATTTTGTATATATATTTCAATCTCATTTTTATCTAATATATAATGACATA
ATCATACATAATGATGAATTCTTCCTTTGAAGCAGCTCTCTACTATTTTACATTATCAATATGTTAT
AATTTATTCAATCCAATTCCCTCTTAGGAGGCATTTAAATTGTCTGCAGCATTGTAATATGCAATG
CTTCCTTTGAAAATTTGTATTTTCTTCTGTAGACAGATTCCTCATTTAGCTTAGAATATTTTCCTAG
AAGTAAAATTACTGAATCAAAAAATATGCACAGTATATTAGATTGTACTTTGGTAAGTTGAAAAG
TTTATTCTACGATATGTTCAGTTTGTCACTCTCCATTACCAATAAATATTATTCATCTTCTTAATTTT
TACCAATTTGTTAGATAGACAATGTCATTACTTTAGATTTATTTGACTAGTACTGAAATTTCACAT
ATGTTCAAGTGTGCATTGGCCATTTAAATATATAGATGTTTAACTAAAACTCTTATGTTTTTCTTTA
TATAAAAGGCAAAAGAAAGAGATTGGACTTTTTGGCCTTCTGTGTTAGGCTTTCAGTATTTAATTT
CGTTTGGAGGTAATTTCATCTTCTAGAGTTTTAAATGCATAGGAGATAATCTGTAGGTTAAAGTA
AAACTGTTGTATCTATTGTCTTTATGACTTCCAATCCATTGTGAGGATATTTAGAAGCCAAATTTC
TCTCTATATATAAATGCGTAATGAATCTCTCTTCTCACTTTCTGATTGTGTCTCTTAAAGCTGCATT
TAGATTTATACTTTAATCAGACCAAGTCAACATTTAATTTTCACTTGCTTTAACTACTGTGTTCAAT
GAAGCAGAGAATTCATCCATCTTAACAAATATAGAGAAACACAAATACAGCAAATAAAAGAAC
CTCAGGCATGTTTGTATACTGACTTTCTGAGAACAAATATCAGCTTGGAGTTTCTCATGTGCCTA
GGTCATTTTATGAGCCAGTTGGGCACATAGGATCAACACAAAAGATTGGCCTTTACTGAGATCA
TTTCCCTTTGTTATTCCCTTCCAGTTATTGTTCAGTGTTGATTTTCTACTGTCCTTTTCTTTAGTAGG
CTGTCACTGACCTCCACCTCTTAATTTTCTCTTACACATACAAAACCCATTCGATTCTTTCTTTTCT
CCCGCAACACTCTCCCCACTTTACCTTTCTTTCTTCTTCAAAGAACTGTCTTATTCTCTTAATCTCA
TGGGCAAGCTCTAATAATGTCTTCCCTTAATTACCTCCCTTTATTAACAGTGTGAACTAAGAAGG
AAGGCATTCTGCTCTAAAATGTATTTGAAAGTATCAGTTTCTAGCTTTCGGCTCAGTATAGTCAG
GTATTAAACCTCCTGTCCTAATATCAAAGCTTTTATTTTTCCTTTTTTTTTCTGAGACAGTCTTGCTC
TGTCTCCCAGGCTGGAGTGCAGTGGCATGATTTCAGCTCACTGTAAACTCCACCTCCCAGGTTC
AAGCAATTCTCCCACCTCAGCCTCCTGAGTAGCTGAAATTACAGGTGCATACCACCATGCCCGG
CCAATTTTTGTATTTTTTAGTAGGGACGGGGTTTCACTATGTTGGCCAGGCTGGTCTGGAAACTC
CTTACCTCAAGTGATCTGCCTGCCTTGGCCTCCCAAAGTGTTGGGATTACAGGCGTCAGCCACT
GTGCCCAGCCACAAGGCTATTCTTATGTTCCCAGGCTAATTTGGGTGCTCTTCCTTAACAGCACT
GCATTTTAGCCAGATTGGGTTCTTACGTGCTAGGCACATTTGACATTTTTTGCCCTAATTTTCACA
TCATTGTGTTATTATATATGCTGTTTCTTTCCCCAACCTACCAACCCCATCCCTGTCCTGTTTCTTT
GCCTTTCCAAATATTTCCCACTATTAAGACTGAGTCAAATTCCACCTTTACAATTCTATAGTTAGT
GACATCTATTCCCACTTCTCATCTAACAGTAGGATTCCCATATGACATCTCTGTAATTCGATCATG
CATACTTTGCTCAGTTTCAGTGAAAGGCTACCTTGTACATCAAGAGATCATTCATACCTTTATGA
ACTGATTGATGTATTCAATTTTTTCATTTCTACCTCTTTGTGATTTATATACATTAACCCCAATCTTT
AACACAGAGCACTGTTTCTCTTGTATGTTGCAGTGCTTTCAAAATTTGGAAACTACTATGACTTTC
TTCACTTTGGTATTCTAATCTCTGACATGTTTTTGAGAATCTTTCAACAGATCTGTCTCCTTTTCAG
GTATTCTCCTTATAATCTGCCATTAATAATTGAACAAAATATTTCACATGTGGTCTGACTAGTGCA
GAACAAGGTGAGGCTATTCTTTACAATGTTCTGGATGTCTACTTCTCTTCATGTAGTCTCATGTTG
AATGGGATTTTTAGTAATCATTTGACAATTGGCTCATTTACCTTGCAATAAGTTGTGTATATATAT

ATCACTGCAAAAGTTCTCTGATACCGCGTTTCAGTCAATAGATCCTCTACCCCTGACCCCAGCAA
ACCACTGATCTTTATGTCACTGTAGATTAGTTTGCATCTTCTAGAATTTTATATAGATGGAATCAT
ATATACACATATAGTCTATTAAAAAGAATTTTTCTCCTTCTGGAAATAGGTGTTTTTTTAAAAAAG
AAAACCAGATTTTTCCACAACAATATCTGCCCAATTGAATTTTTAAACCTAAATTCAAGAGATTTG
TCCTTTAAAATTTTATCTGGTTGGCTTCAGTTCAGTGTTCTTATATGTCAAAATGATTTTGAATCCT
GAATCTGTCATCCAAAGTATTAGCTAGCTATCATTTTTAGCTATATGCTTTCTGTAGGTTTGATGA
ACACATGTTAAAACATTATCACTGAACACAGAATCATAAAATATTAGACCAGAAAGAACCAAAG
TCATGGATAAAATTGTTGATCAGAACAACCACAGATAAAAACCTGCATGGTCAATGACCTGTCA
GGTTGATTCACAGCTAAGGGCCTGGCTTCCTCTTTTCCTTCCAACCTTCCAAAATGCTATGACAA
TTTTTGTTTTTTATATTTTGTAGCTTTTGAAAAATTTTGTTTATTTAAAAATTACTGTGATGCCATTTT
CACACATAACAAAATCTAAACAAGTCCAAAAATGCATTTGGTTAGGATTTCTCTTAACTTTTTGA
ACTTGTAATAGTTTCCCTGATTTTGTTTTGTTCACTCTTTTTGAACTTTTATTTTAAAATCCGGGGGT
ACACAGGTCAGTTACAAAGGTATGTTGTGTGATGCTGAGGTTCAGGTGGTAAGCATAGGACCCA
ACGGGTCGTTTTTCAGAATGACTTTTTTTTTTTTTTTTTTTTTTTGAGACAAAGTCTCGGTCTGTCAC
CCAGGCTGGAGTGCAGTGGTGCGATCTTGGCTCACTGCAAACTCCGCCTCCCCGGCTCAAGCA
ATTCTCCTACCTCAGCCTCCCGAGAAGCTGGGATTACAGGCACACACCACTATGCCTGGGTAAT
TTTTTTTTTGTATTTTTAATAAAGACGGGGTTTCACCATGTTGGCCAGGATGGTTTTGAACTCCTG
ACCTCGGAAGATCTGCCCACCTCAGCCTCCGAAAGTGCTGGGATTACAGGCGTGAGCCACCAC
GCCTGGCCCAGTATGACAATTTTTATATCTAGTACTAACTCTGACATATTTTCTAAGACTAAAAAA
GCTTGTTGTCTTCTAATTCCTTCACCATGTTTTTCATTCTTCTCAAGGTACCCTGTCTGTGTATGTG
TTTAAATTTTTTTTTTTTTCCTGAGGTAAAATTCACATTCACTGTAATTCAAATTATTTAAAATATAAT
CCATTTAAAATATAACATTGGATGATTTTGAACAAATGTAAACATTTATGTAACTGCTTCTCCAAT
CAAGATTCATAATATTTTCATCACTCCAAAAGTTTTCAGTCAATACCCCTTTCCCCAGTCCTAGGC
AACCACTGATCTACTTTATGTCTGTACAGATTAGTTTGCACTTTCTAGAGTTTCATATAAATGGAA
TCATGCAGTATCTACTGTTTTGTATCTGGCTTCCAGAATGTTTTGAGATATATCCATGATTTTGCTT
GTACCAGTAGTTCATTCCATTTCATTAATTAGTCATGTTCCATTGTTTGAATCAGTTTATTCATTGA
CTTTCTCATGGCCATTTGGGTTGTTCCCCATTCTAAATAATGTTGCTATGAACATTTCTGAAGTTG
TACAATTCGAGGTGTACAATTCTTTCTATGGATGTATGTTTTCATTTCTCTTAAGTGCATACCTATG
AGTGGAATTACTGAGTCCATGTGTCTAATTCCACAATAAACTTGAGAGTATTTTTCAAAGTCTAA
AGCTAACGACTACATCATTTTATATTCCTATCAGCAGCGTATAAGAAATCTAGTTGCTCCACATC
CTTACCAACATTCAATTTTTTCTTTTAAGCATCCTGTGGATACATTGTAGTAACTTATTTTGGTTTA
TATTGGCATTTCCCCGATGACTAATAATGGTGAGCATCTCTTGATGTTATTTTGTCATCCATATAT
CTTCCTTGGTAAAGTGTCTGTTCAAATCTCTTTTCAGGCCTTCATTCACTCTCTTTCTCCTTTTCTGT
CTTTCACCAAATTTCTATTCTCCTCATCCTTCTCTCAAGCCCTATTTTATTTGATTGGAATGATTTCT
CCTTGTAGTTCACCCAAGGTGTCCACCTTCTAATTTGCAAGCATCTCGGCATGTATCTGCTCAAA
AACATCTTCTCAGAGTATGAAAACAGTCCCATTTGTTTTCCCCAACAATAACTCCAAGCCAGGGA
TGCTACTGGGTGAATATAATAGCTATTGAGTAAACAGAAATGCTGCATGAAAATGTGTGAAGAA
TGGTTCTTTACTAAACTATCTTCATGGCATAATCTCTCTTATATAACAAAAAACCCAATTTTTAAG
AGACAATTGGAAATTTCTGCCCCATAAATATTGATGCTTTGAATCAAACAAAGTTAACAAAGTTG
AATTAAGAAAAAGATCTCTCCTGTGGTTTGCTAGGCTTATGTTTTAAACTTGTTAATAAAATGCAA
TGTTCTGTGTAGAAAATTTTCAGCGTTTCTTCATGAATTCTTCTTTTAGTTTCTAAAGGCTGTAACT
CATCTTGACAGTAAGCCGTATTCAACATTTTATTTGATCAGATTAGGAAGTTTCAATTTCTTAGGG
ATTTGTTTTGCCAACTATTTCAACAACTTGGAACAACTTGCCAAATATTTTTCTTGGGATCTGCGA
TATTAGAGCATAGAAGAAAGAGAGGGAGAGTAAATAGTGAGGAAAATCGTTGACCAGATAAAA

GTTACAGCATCACGCCCTACATCTAAAAGTGTTAAAACACAGAATAGAAATCTGCCCACTATGT
GCAACATGGCCATGACTGCACAATATGGGGGTTTGCAGAAGGTCTGCCTTTCACCCGTTCCCTT
CAGTGGCTTTTATCCCCTCATTTGGAGCCCTACTGCTTACCTGTGAATCTTTTTTCTTAATATTTCC
CCTTGGATACCTAGGGTTCCTACGAAAGTGAGATTTACATCCAGAGGTATCTTATCTATGATTCT
GTCCTAAATGACCACATTTCCAGATGTAAGAGTAAGGAGACGAATATCAGGCCCTCTTAGTAGG
CTTCAAATTCATTAAAATATATAATAAGCTCAGCTTCTCAGGGAATTTATTTCCATTGATATTTTAA
ATATAAGCCAGTTACTCTCTTTTTGATTCTTTTCATCATGTCCCATATGTAAAACCCTAAGTTGTCT
CAGTGTGTATACTAGTTTGGTTTGACACTTTTGGTATAGATATACAATATTAAAATGGTCTAAAGC
TAATATTATTAGTAAATATTTTCTCATACTTAGGGTATGAGAAAGTGAAGGTCATTGTTCAAAAAG
CAGATTGAACCTACTAGTATAATTTCATATCTTCAGATCTTTCATATTTTAATCTAATGATAAGTCA
GAGACCTTATCTCTGACTGCCCTGGCTTCATCTTTAAATATGTCAAAGTACCTAGTGCCAAAAAG
AACTGTGAGTTCCAGCAAACTGTAATACTTGTAACAACTCCTTACCCCTAGGCAGATCACTGAA
GCATCACTCCTGAATGTTCCTCTGTACCCTCAATGTGCATTTGCACTACCAGTGATCTCAAGAAT
TCTAGAAAATGAAAACTGGATTACCATCACCAACGTCTAGGAACTGTGGTTTGTACAATGCTAA
GTAAATGAGCTAACTGTTTTTAGTTTTCATTTTTCTTGATTTTTCTTGAACTTGATTCAGCGATTGC
CTAAGTTGATTCCCTCTCTGGGATTGGGCCTGACCTAACTTTTGGTTTATGAGAGCTTGGTTACAT
CTCTGGCTCTAAAAATATAGGAGGTTAAACTTTTTCTAGGAGGGGCTCCTAACATAGAATTTAAG
AGGGTTCAGTGGCCAGGTGCGGTGGTTCACGCCTGTAATCCTAGCACTTTGGGAGGCTGAGGT
GGGCGGATCACCTGAAGTCAGGAGTTTGAGACCAGCCTGGCCAACGTGACGAAACCCTGTCTC
TACTAAAAATACAAAAATTAGCTGGGCATATTGGTGGGTGCTTGTAATTGCAGTTCTCAGGAGG
CTAAGGCAGGGAGAATTGCTTGAACCCGGGAGGCAGAGGTTGCAGTGAGCCAAGATCGTGCC
ACCGCACTCCAGTCTGGGCAACAGAGCGAGACTGTGTCTCGAAAAACCCAAAAGCCAAAAAAC
AAAACAGACAAGCAACAACAACAACAAAAACAGAGGGTTTGGTAAACCACCTCCAATTATATA
TAAAATATAATGTAAATATCCTTTGTAATCCTTTTCTTCTTTTAAAATTATTATAATTTTAGACTTGT
AAACTATCTCATACATAAAAGTGAATATATTACATGTACAGAAGGTACACAATTACTTTCCAGAG
CCTTTGTTTGCATACTGCTCAACGTGAGAAATCACAACTAGCCTTCAAAGCTCTGTGCATCCCCT
TTGTGACTGAGTACTTCTGCAGCAGGTCACCACTATCCTGAGTTGTGGAGTTCTTTCACGTTCAT
GCACAAAGGTCATGTACCTTCCTTTTAATTTTTGTGGCTATATTGCAACACACATTCACATATTAT
TTATTTATCTGTGCTCCTGGTAAAGAACATTTAGAATGTTTTTATTTTGTATAATTATAGACAATAC
TGGTATAAACATTATCTTATGTATTTCCTGGTACACATATGCAACAATTATTGTAGACCATAACAT
CAGAATTATAATTGTTAAATTTACACATTTTCAACTCCCCACATAATCATTTTTAATCAACCATAT
GAAACATTTAAAATGCCATCTCAAATTTAAATTTTTATTCCACCTGGAATTGACTTTTGTGTCACG
AAAGATTTGATCCAATTTGTTTTTCCATGGGGATGATCAAGTGTATCAGTATCATTTGTTAAATTA
TCTCGAATTCCTCTACTGATTTACAATGTCAACTCTGTTGTATGTCAAGTTTCCCGACATATTTTTA
GGTTCTCAGTTCTGTTTCTTTTGTTTGCTTTCAATGTCATTCTTGTCAATGTCATACTCTGCTGTAAT
TTAACTAGAATTACATTGATTTTTAAAATTAATTTGAAAAATTTAACATTACAATACTAAATTTTCC
TATCTATGAACATAGTATCTCCTTTTCTTGTTTGATATTTTAAAAACATTTATCATTTTCTCCCTAAA
TGTCATTTTTTTAGATATGTACTACAAATGTTATATTTTGTTATTAGTTTGAATATTTTCTAACTCTG
TATTCCTATTGCTATGGTTTGAATGTGTGCATCCCTTCAAAATTCATATGTTGAAACCTAATCATC
AAAGTGATGGTATTAGAAAGTGTGACTTCTGGGAGGTGATTAGGTCGTGAGGGCTCCATTCTTA
TGAATGGGATTTATGCCCTTATAAAAGGTTTCAGAGAGCTAGCTGCCTCCCTCCTCTGTTCTGCT
ATGTGAGAACTCAGCAAGAAGGCACCAGAGAGCAAGCCCTTACTAGACACAAAATCTGCCAAA
AACTTGATGGTGGACTTCCTGGCCTCCAGAACTGTGAGCAATAAAGTCCTGTTATTTGTAAATTA
CCCAGTCTAAGGTATTCTGTTATAGCAGTACTAATGGACTAGGATAATCATGACAGAAATGCAA

TCACTATTTGTACATTGAATTTTTGTCTTGTAGCTCCGCTAAACTCTACTGCTAATTCCAATAACTT
GGGTACAGATTCTGTTTGCTTTCTAAGTGGACTTCTGAGACCAGTAGTTGCTTTTGCTTCTTTGTTT
TTCAATCCTGACCATTCTGGCTGTGGCCTCAGGTACAGTGTTGAAAAGGGGCTGTAACAGCTGG
AGTCCTTACTTCTTCCTGATTTAGAGAAAGTTTGTTGACTGATGTAAAGCTGTGGTAGATTACCTG
CAAAAGGGAAAATAAATTTTTTCCTATTATTAGTTTACCAAGAGGTATCTTTTGGGTATAAATAGG
TGGGAAATTATATTTAATTATATTATAATTTAATTATAAGTAGGTAAGTAGGTGTAAAATACTGTT
GAACCCTCTTTCTGCATCTTTTCTTTTCTTTTCTTTCTTTTTTTTTTTTTTTTTTTTTTTTTTGAGACAG
AGTCTCACTCTTTTGCCCAGGCTGGAGTGCAGCGGCATGATCTTGGCTCACTGCAACCTCTGTCT
CCTGGGTTCAAGTGATTTACCTGCCTCAGCCTCATAAGTAGCTGGGATTACAGGCACCTGCCAC
CATGTCCGGCTAATTTTTTTGTATTTTTAGTAGAGGGGAGGTTTTGCCATGTTGGTCAGGCTTGTC
TCAAACTCAATGATCCACCCACCTTGGCTTCCCAAAGTGCTAGGATTACAGGTGTGAGCCACTG
CACCTGGCCACTTTCTGCATCTTCTCGATGAATCATCTTGTCATCTTTATTTTATTTTTGTATATATT
TAAGGTATATCACATGATGTTTTGATATACATATACATAATGAAATTATTACACTACCGTGAAGC
AAATTAACCTATTCATTGCCTTGCATAATTACCCTTTTTGTGTGTGTGGTAAGAGCACATAAAGTC
TATTCTCTTAGCAAACTTTCAGTATACAGTGTTATTAAATATTCTCTTTATGCTGTACCACTTTGTC
ATCTTTAACTTGCTAAAGTGGTGATTTAAATTAATCTGTTTTTCTCACATTAACATTTCATTCCTGA
AATTAATGCACAGTTGGTCACACGTGTGTGCACATACACACATACACACACACACACACACACA
CTTCTGTATCTATGTTCATAAGTAAATATCCTGTTTTCCATATTTAATTTTGGGAAAAATTTTATAC
CTCCTTCCTAAAATTCCTTTGAGTGCATCTCCTCTACTTCTGTTTTCTGGAAAGATTTATATACAGT
TAGAATTGTTTCTTGGTAATTCAATAAAATTATCTGGACCTGATCTTTTATTTGTGAAAACATTAAT
TCCTGATTCATATCTTTGATGTTTATGGACCATTTTGATTTGCAGTTTCTCTTCAGCTTTGTTAAGTT
ATATTTTTTCTAGGAATTTTCCCATTTTATCTAACATTTAAAAATTTTTGGCAAAAGTACTTGCTTA
TTTCATGCACATACACACACGTACACACTCACACACATACATCTATAATCTCTGCTGTGTGTATCT
CTAGTTAATTTGTTGTTAATTTCTAGTATTGTTTATTTGTGCCTTTTATTATTTTTTTCACCCAGTTCA
TCTTGCCAGAGATAATTTGTTTCAAAGAACAAACTTTTCACTTTATCAAGCCTTTCTGTTGTCCTG
TGTGTTTTTATCTATCTCATTAATTCCTGTTATTTAAAAAAAATCCCTTATCTCCCCTGATTTGGGA
TTATTCTGTTACTTTGACTTTTTAAATTGGATGCATACCTCATCATTTTTTAGTTTTTTAAGTTTTCA
AATATAAGCTTGAAAACTAAATTTTTATTTTACACTTGATCTTAGCCAAAAGTCCGAGAAGACAA
CTTTAACTATGTTAATGAATTTTTAGTATACGGTATTTTTATTTTTATTCCCTTTTAAGTACTTTCTA
GTTTTCATTATAATTTCTTCCGTGAAGTGTTTTTTTTTTTAAGTGTAATTTAGGAAACCTAGGGTTTT
GTGTTTTTTTTTGTTATATTATAAGTGGTTGCATTGTGCTTAGAAAACATGGCCTATTTTTTTTTTTC
AGTTCTGTTGCTGTAGGTATGCGTATCATTTGTTTCATGGCTTAGTATATGGGCAATTTCCATAAA
TTTCCCTTTATGCAGAAGCGAGCCAGACTGCCTCTCTAGATTCCTCCTCTCTGGGCAGGTCATCT
CTGAAAGAAAGGCAGCAGCCTCAGTCAGGAGCTTATAGATAAAACTCCCATCTCCCTGGGACA
GAGCACCTGAGGGAAGGGGTGGCTGTGGGCACAGCTTCAGCAGACTTAAATGTTCCTGCTTGC
TGGCTCTGAAGAGAACAGCAGATCTCTCAGCACAGCACTCGATCTCTGCTAAGGGACAGACTG
CCTCCTCAAGTGGGTCCCAGACCCCAGTGCCTCCTGACTGGGAGACACCTTGAGCAAGGGTCA
ACAGACACCTCATACGGGAGAGCTCCAGTGGGCATCTGGCGGGTGCCCCTCTGGGATGAAGCT
TCCAGAGGAGGGAGCAGGCAGCAATCTTTGCTGTTCTGCAGCTTCTGCTGGTGATACCTAGGCA
AACAGGGTCTGAAGTGGACCTCCCACAAACTCCAACAGACCTGCAGACCTGACTGTTAGAAGG
AAAACTAACAAACAGGAAGGAATAGCATCAACATCAACAAAAAGGAAGTCCACACAAAAACC
CCAGCTGAGGGTCACCAACATTAAAGATCAAAGGTAGATTTATCTCAGGAAGATGAGGAAAAA
CCAGAACAATAAGACTGAAAATTCCAAAAATCAGAATATCTCTTCTACAAAAGATCACAACTCC
TCGCCAACAAGGGAACAAAACTGGACGGAGAATGAGTTTGACGAATTGACAGAAGTAGGCTTC

AGAAGGTGGGTAATAACAAACTTCTCTGAGCTAGAGGGGCATGTTCTAACCCAATGTGAGGAA
GCTAAGAACCTTGATAAAAGGTTACAGTAACTGCTAACTAGAATAAGCAGTTTAGAGAAGAATA
TAAATGACTTGATGGGGCTGAAAAACACAGCACAAGAAATTCATGAAGCATACACAAGTATCA
ATAGCTGAATTGATCAAGTGCTTCCACTTGATATCAGAAATTGAAGATCAACTTAATGAAATAAA
GCGTGAAGACAAGATTAGAGAAGAAAGAATGAAAAGGAACAAACAAAGCCTCCAAGAAATAT
GGGAATATGTGAAAAGACCAAACCTACATTTGATTGGTGTATCTGAAAGTGATGGGCAGAATGG
AACCAACTTGGAAAACAATGTCCCCAACCTAGCAAGACAGGCCAACATTCAAATTTAGGAAAT
GCAGGAAATACCCCTTCATGCTAAAAACACTAAATAAACTAGGTATTGATGGAATGTATCTCAA
AATAATAAGAGCTATTTATGACAAACCCACAGCCAATATCATACTGAATGGGCAAAAGCTGGAA
GCGTTCCCTTTGAAAACTGGCACAAGACAAGGATCCCTTCTCTCACCACTCCTATTCAAAATAGT
ATTGGATGTTCTGGCCATAGCATTTAGGCAAGAGAAAGACATAAAGGGTATTCAAATAGTAAGA
GAGGAAGTCAAATTGTCTGTTTGCAGATGACATGATTGTATATTTAGGAAACCCCATCGTCTCAT
TCCAAAATCTCCTTAGGCTGATAACTAACTTCAGCAAAGTCTCAGGATACAAAATCATTGTGCAA
AAATCACAAGCATTCCTATACACCAATAATAGACAGAGAGCCAAATCATGAGTGTACTCCCATT
CACAATTGCTACAAAGAGAACAAAATACCTAAGAATACAACTTACAAGGGATGGGAAGGACCT
CTTCAAGAAGAACTATAAACCACTGCTCAAGAAAATAAGAGAGGACACAAACAAATGGAAAAA
CATTCCATGCTCATGGATAGGAATAATCAATATTGTGAAAATGGCCTTACTACCCAAAGTAATTT
ATAGATTCAATGCTATCCCCATCAAGCTACCATTGACTTTCTTCACCGAATTAGAAAAAAACTAC
TTTAAACTTCATATGGCACCAAAAAAGAGCACGTGTAGCCAAGACAATACTAAGCAAAAAGAA
CAAAGCTGGAGGTATCACACTACCTGTCTTCAAACTATACTACAGGCTACAGTAACCAAAACAG
CATGGTACTGGAACCCAAACAGATACATAGACCAATGGAACAGAACAGAGGCCTCAGAAATAA
TGCCACACATCTACAACCATCTGATCTTTGACAAACCTGGCAAAAACAAGCAATGGGGAAAGG
ATTCCCTATTTAATACATGGTGTTGGGAAAACTGGCTAGCCATATGCAGAAAACTAAAACTGAA
CCCCTTTCTTACACCTTATACATAAATTAACTCACGATGGATTAAAGACTTAAACGTAAGATCTA
AAACCATAAAAACCCTAGAAGAAAACCTAGGAAATACCATTCAGGACATAGGCATGGGCAAAG
ACTTCATGACTAAAACACCAAAAGCAATGGCAACAAAAGCCAAAATTGACAGATGGGATGTAA
TTAAACTAAAGAGCTTCTGCACAGCAAAAAAAAACTATCATCAGAGTGAACAGGCAACCTACA
GAATGGGAGAAAATTTTTTCAATCTACTCATCTGACAAAGGGCTAATATCCAGAATCTACAAGG
AACTTAAACAAATTTACAAGAAACAAACAACCCCATCAAAAAGTGGGCAGAGGATATGAACAG
ACATTTTTCAAAAGAAGACATTTATGTGGCCAAAAAACATATGAAAAAAAGCTCATCATCACTA
GTCATTAGAGAAATGGAAATTGAAACCATAATGAGATGCCATCTCATGCCACTTAGAATGGTGA
TCATTAAAAAGTCAAGAAACAACAGATGCTGGAGAGGATGTGGAGAAATAGGAATGCTTTTAC
ACTGTTGGTGGGACTGCAAATTAGTTCAACTATTGTGGAAGGCAGTGTGGCAATTCCTCAAGGA
TCTAGAACTAGAAATATCATTTGACCCAGCAATCCCATTATTGGTTATACACCGAAAAGATTATA
AACCATTCTACTATAAAGTCCCATGCACATATATGTTTATTGCAGCAGTATTCACAATAGCAAAG
ACTTGAAACCAACCCAAATGTCTATCAATGATAGACTGGATAAAGAAAATGTGGCACATAAACA
CCATGGAACACTAGGCAGCCATAAAAAAGGATGAGTTCATTTCCTTTGCAGGGACATGGATGAA
GCTGGAAACCATCATTCTCAGCAAACTAACACAGGAACAGAAAACGAAACACTGCATGTTCTCA
CTCATAAGTGGTAGTTGAACAATGAGTACACATGGACACATGGAGGGGAATGTCATATACTGG
GGCCTGTCAGAGGTTGGAGGGCTAGAGAAGGGATAGCATTAGGAGAAATACCTAATGTAGATG
ACGGCTTGATGGGTGCAGCAAACCACCATGGCACATGTATACCTATGTAACAAACCTGCATGTT
CTGCCCATGTATCCCAGAACTTAAAGTATATAAAAAAATTCCCTTTATGCAGAAGAATATATTTTT
TAATGTTGTGTACATGATTCTGTAGAAGTCCATTGACCATGCTTGTTAATTTTGTGGTTCAGATCT
TTTCTGTCTCAGCTGATCTCTTTTTTATTTGATTGATGTACTAATTAGTGAGATTCATTATGGTGGT

GAATTTATCAATTTTGCTACATACTTCTTTCAACTTTCTCATTATGGATCTTGAAGCAATATTATTTT
GTGCCAAAAGATTTTAATATATCATTTTAGTAAATTGCATTCTTCATTAATGCCTTTTGCCCTAGTT
TTAATTTATCTAGAAGCAACAATAGCTTCACTTTCGTTAGTATTCACCTCTTATATAATTTCTCAGT
CTTTAGTTTTTAGCTTTTTCTGTTTTTATAATGTATATGTGTCTTTTTTTAAGCAACATAGAGACAGA
AATTTAAGTTTTTGGAATATGTTTTTCTTTTAATTTGTAAGCTTAATTCATTTGAATTATTTAGGCTC
CTTAATTTTATCTTATCTTCTTTGGACTCTATTTGTACTACATTTTCTGTTTCATTTTTCCTCTTTATT
GTTTGAGATTAAATAGTTGCTTTGATTCCATTTTTCTAGCTATTTGAAAATTATGTATTCTATTTTCA
TGCTATGTATTTTGTTTTATGCTTTTAGTTACTACTCCTTTTAATATGCACAGTTAATTCTAAAACTA
ATCAATATCTTTACCCTTCTTTTTATCAAAACAATTAGTTTAGAAGGCTTTAACTTAAAAAAGCAA
TTTCACAAATATCATTCTATTTTTCTCTGCTATTTTAGTATGATTTTGTTTTTATAGTCCTACAAATTT
TGTTACTATTATTTTTATACACATTCATATTGACATACTTACTTACATGTTTACACTTATTTTTTGCT
TGTCATTGCCCCTTTTACATTAGACCGTTTCTCTGGATCATTTTCTCTTTTCTCAAAGTACATTTTTT
AGACTTTATTCATGAGAGTCTGTTGGTGGTAAAATTTCTCAGGATTTCTTTTTTACTTGAAAATATT
TTTATTTTGCCTTCACTTTGGAAGGACAGTTTTTTCTGGGTGTACAGTTTTAGCTTGACAGTTATTT
TTTGCCATCTCTTTGAATACATTATTCACTTTGCTTCTAGTTTCCAGTATTGCTATTGGGAAGGCA
GCTGTCAGGATAAATTTGGTTCTTTTGTAGGTAATCTTCTTTTCTTTCAGCCTTTTTAAAGATCTTC
TTTGTTTTTGGTGTCTACAATTCTATTATAACGTACCTAGCAAGGACTTCTTTTAATTTAACTACAA
GTATTTGTGTACTAACTCCTATAATTTCTATCTTTCATCAGTTTTAAATATTTTCAGGCACAAACTC
TTCAAATACTGTTTTTCCCCCTATTCTTATCTCCTTCTTTAACTCCCATCACCAATGTCACTTTTTAA
CCTATTTTTTGTCCTTTCTTTCTTCTTTTCCATTTTTTTTGGCTCTTTGCTGCATTCTGGATAATTTTA
CAGATTTATATCTCAATTTACCAGTCTGTCTTCAAAAGCGTTTACTTAAGTCATAGCAATTTTTAAT
TTTAATTGGTTTGTGTCTTATTTCTATAAGTTCTATTTTATTCTTTATCAAAATAGCCATGTTAAAAA
AATGTAAAAAAGACATATGCACAACTCTTTTCTCTGAACCATTATTGTACTGGTGAAAGAATGGA
AACAGACCAAGTGCCATCAGTAAAGGAAAGTTTCCTAGTGTGGGAAGCCCATGATGACGTATT
GTGCAGCTTTATAAAAGAATGATGCATATTTCTATATATTGTTATCAAGTAATATTCAGACAATAG
TAAAAAATTTTTTAAAATTATGAAGTTAAGTGAGTATAATATATTGAGGATTATACATTGCTTTAT
AAAACATAAATACTTCCAAATTTAGTGTCTTAAAAGAATGATTTATTAATTTAATGTGACTGTGGG
TTGACTGGGTGGTTTGTCTCCTGTTCTTATCTGTGCAGAGTCCTATGGCTATTGATATCTAACATC
CTGGCTTCACTCACATGGATGAGCCCCACCTGGAATGCCTAGAAGACGTGGGACAGCTGGATC
TCTCTCCACACGGTTTTTCATTGTTGCCTCAGGTTCCAATAAAAGCAAAAGCTGCAAGGTCTTCT
GAGGCAAAGACTTAGAAATACCACAATGTTACTTCTGCTGCATTCTGTTGATAAAAGAATCCCA
CAATGCCAGCTTCATTTAAAGGGGTAAAGATATAGATTATTCACCTTCGATGGGAGGAACTGCA
AATGATGTAATTAGTGATAATTTTTAATATACTGTAATGTATTGACT

ID|56116|gene_id|ENSG00000213863|transcript_id|ENST00000438217 Homo sapiens lncRNA

ATCCCAGCTACTCGGGAGGCTGAGGCAGGAGAATCACTTGAACCCGGGAGGCAGAGGTT
GCAGTGAGCCGAGATCGCACCCCTGCACTCCAGCCTGGGTGACAGAGCAAGACTCCATCTCAA
AAAAAATAAAGGAAATATGCGTCGTTGGATGCTGTATGACAATCAAGCTACTTATAACAAACAA
AATTGAGAATGAAGGTAAATTAAAAATAATTTATAGTCTTTAAAAAGCAGGAACTACAAAATTTA
CATTTCTGAAAATGGCAGAAAAATCCCACATGGCTGTATATGTATGTGTGAGATTGTGTATGAGC
GCATGCATATATTTTTGTGTGTTTGGATTATATGTGTGACGGTGTGCATGAGTGTGTGTATGATTT
GGGGGTATGTGTGAGTGTGTATGACTGTGTGTATGAGTGTGTATGATTTGTGTGTGTGAGTGTGT
ACATGTATACACTAGTGTGCGTATGATTTGGGGGTATGAATGTGAATGTGTACATATGTATTTAT
GATCTGAGTGTGTATGAGTGTGAATGTGAACATGTGTGTGTGAGTGGGTATATGATTTGGGTGTG

TGCAAGTATGAATATGTATTGTGTGCATTAATGTGTATATTTTGGGCTGTGTATGAGTGAATGTGA
ACATGTGTGCACGAGTGTATATATGATCTGGGTGTGTATGAGTGAGCATGAGTGAACATGTGTG
CATGAATGTGAGTATGATTTGGGTGTGTATGAGTGTGAATGTGTGCACGAGTGTATATATGATCT
GGGTGTGTATGAGTGAGCATGAATGTGAACATGTGTGCATGAATATATGATTTGGGTGTGTATG
AGTGTGAATGTGTGCACGAGTGTACAATCGGTGTGTATGAGTGAGCATGAATGTGAACATGTGT
GCACGAGTGTATATATGATCTGGGTGTGTAGGAGTGAGCATGAATGTGTATGCACAAGTGTGTG
TATGTGTGTATGATCTGGGTGTGTAGGAGTGTGAATACGAACACGTATGCGCAAGTATGTATAT
GATCTGTGTATGTATGAGTGAGTGTGAATGTGTGCATAAGTGTGTGTATGATCTGGGTATGAGTG
ATTGTGAATGTGAGCTTGTGTGCACGAGTGTGTATATGATCTGGGTGTGTGGGAGTGAGCATGA
ATGTGTATGCACAAGTGTGTGTATGTGTGTATGATCTGGGTGTGTAGGAGTGTGAATATGAACA
CGTGTGCACAAATGTGTATATGATCCGAGTATGTAGGAGTGAGTGTGAATGTGAACATGTTTGC
ACGTGTGTATATGATATGGGTGCATGTGTGGGTGTGTTTGTGCAGGTGCACTGGGAGCTTTCAG
CATTGAGTCTGTGTGGCATCCAGGCTTTTCTGGATGATGTAGAATCAAACCCCATAATTAATTCC
AGTAACTTAGAAATTTTCACTTATGAATACTAAATTCTCCTCACCTAGCAACTGACTGAGCAGGT
TGAACAGCACCCTTCGCCGTTCTCCTGCAGAACCCCGTGGAATGGCCCTGCTTTCTCAGAGTGC
TCCTAGCGGTGATGAGGGAGCCCAGCTCATTGCGGTTCGTGCAGAAGGCTGTGTGCCATCTCC
GGGACTGCACGGAGAGGCGGAGGCTCAGGCGTGGGATCTGAGCTCAGTGGGCCCTTTAAGGA
CCGCTGCCTCCTTTCCTCCCTTTGGCATTCAGCATCGACTTACGATGGCTCCCAGTCACAGTCCT
TGTCACTGCCTGGCATTACACAGGGCCACAGTCCCTTAACTGAAACACTTGGGACCAGTTGCAT
TTTGGAATTCGGAATGTTTCTTGATTTCAGAGAGAGAAGAGGGGATGGACTGTTGGGCAGCCCT
GATTAACCCTGCAGCGTCTTGGCTCTAGAGGGGCCTACACGGCTGGGAGCGTCTCGGCTCTAG
AGAGGGGCCTGCACTGCCGGGAGCATCTCAGCTCTAGAGAGGGGCCTGCACTGCTGGGAAGC
CTACACGGCTGGGAGCGTCTCGGCTCTAGAGCCACAGCTGATGCATGGCAACGATGAGCTCTC
CTGCTTAGTGCTGATGTGCCTCAGCCCCTAACCCCACTGGTGTCCGGATGAGGAACTTGAGGCT
CAGAGAAGCTAGGTCAAGGTGAACACCAGTGTTGAGTTTCAGCCCCAGGTCTACCTGATGCTGC
CTCCACATTGACTGAGTTGGCCTGGTCCATTGTGAGGTCAGGTAGAGCCAGGGCCGAGGTGGG
GATGCCAAGGTCCCATGCCGCAAAGATGGGTGGGACGGCACAGGAGCCAGTCATCATCCCCA
AAGACACAGCTCCGAATACCTCATCCTGAAATCCCAAAGATCAAAATCCCAAAAGTATAAATCT
GGAAAAAATAATGTCGATGTTATTTATTTACATTTTTAAAAGGTATGTATTTGAGAAACATAAAAA
CACAACAGAACATTTCATAGGCCACTTTACACAATAAAATAGGCAATGATAACAGGTATTTTTGC
ATAAACACTGAGGTAACTAACGACGGCGGCATGGGTGTAGCAGTTATAAGAAGACAGACCATA
AAGAAATAAGTCAAAAGAGAAACGTGTACACGTATATCGCTACGGTTACTAATTGTGTGCACCT
AGCCCTGTAGCTGTGGTCATCTGAAATATCAGGGATTTTAGACTTGAGGGATTTTGATCTTTAGG
GATTTCAACATTCCACATTATGGTGCTTGGGATTGTGTCTTTCAGGATTATGATCCAAACTCAGCT
GGGCCTCCCCTCCCTGCCCCAGGATTGTGGAGTGAGAACGTTGCAGCAGGAGAGAACAACGCA
GCAAAGCACAGCAGGGGAACCGGAAATGCTCACCTTTTGACAGGGTACTTTTAGTTCTGGGGC
CTTATCTTAAGGATATTCCAACATATACAAAAAGATTCATGCACAGAGATATGTACTTTAGTATTA
TTTACCATAGGAAAAAAGTTGGAAACAATACATTTTATGTTCTGTAAAATGAAAGAACAGTTAAA
TAAATCATGGCTCTAAGACGACTCCAGGGCTGTGTACGGAAGTTCAGGGACAGAATCAGTTGA
GGCTGTTCCACCTGAGCCTAAGCTTCCTCTGCTGTATGTCGGGAATCAGGTGGGGGCCAAGCGA
GATCACCACAGTGCAGGCCAGTGTGCAGGGCACAGGTGGGCACCAGGGAGGGGACGCCGCA
CAGCCCCATGCTCTCGCCAAAAACGTGCAGCACAATTTGGAAGAAAACATTTCCATCTGTTAAT
AAAGAGCAAGGGCCTCTGGTCATAAGTG
ID|56128|gene_id|ENSG00000225135|transcript_id|ENST00000443556 Homo sapiens

lncRNA

GAGCTATTACTAACTTCTCCATTTGATTTTTTATGGATGTGAAAAATGCTGCTACTTGTCTAT
GTTATTATGTGTAAGTATATTACAATTTAATTAAGTACAATAGTTTAAAGAACGAAGCAATTTTTT
TTTTTTTGGCTTAGAACTTCTTAATTGTAGGTTCCTCTGAAGCGATTTCATGTAGATATGTGAGTGT
TTTAAACAAGTCTGAAAGTGTTACATACTTTTAGGTTACAGGGGTGCTGGGGAGACAGCTGAGG
AAAGGAAGAATATGTGGAAGACACCACGGAGTTCAAAGTTTTACCCTGAGTTCTATCTTCCATG
TATGTTTTGCTTAAGGCATTTCTCATGTGACATTAGAAAAGCTATATCCAAAGGTAAATTTTTTGT
GGCAAAGATTTATTTTACACTTTAACTTTTGGGATTTTATTTGTTTCAGCAAAATAAAGAGCACTG
AACTTTAAACTTGAATTTTTTCTGCACTTTTTTAGGTAATGAAAACTTTTTATTATCATTTAATCCAC
ATTGCTCAGTTTAAACCAAGTGATACATGTGTATAAAACATACCAAAATCATGAATATGCTGCTA
GCTGTACCTTAAATAAACTGATCAGTTTTAAAACCTTTAATAGGGTTTTATATAGATTTAAAAAAT
AGTAAAATAATCTGCTGTATGTTTCAGTGTTCTTGGTCTTAAATTATTGCAACACTTTCAGATTTGT
TTTAAGATCATACAGTAACATGTTATATTTATACATACTGCTAGAAAATATACTTTTAGTTTTAAA
ATGGAATTTTTATAAATGTACTTTAATTTTAAAATGGTGAACTTGTTAAGTTTAAGTCAAAGTACTT
AAAAAGTATGTATATTATCCATACAAAAAGTTATGTTTTACGCTTATAATAAGAAATACTGGTATC
TCATATCGAGATACAATTAATTTTAAAAAATCATACATCATTTAAAAATCTTCACACATGAACAG
GAAAGATATCATTTCTATGCTTTAAAAAACCCTCTATTGAGCATTTTCAAATATTAATTTTATTTTG
GGGGAAAATGCCCACAACAATAATTGCAAGATCTTTCTCAATAGCTTCAGTTTGCTGTTGGATGC
CTACCTTAACTATTGTTTTAAAATATAGATATATATATATATATTTAAAATAGTTTTCCAGGTATTT
TCTTCTACCTTTTTTAAAAATAAATTTCCAAAAATGAAAACAGAGTTTATGTATTTTTGTCAATGAA
GGTTTTTATTTTGTAGTTTATAGAATTTGTTCCTTATCAGTTTGATACTTGATCAACATTCTTAGACT
TTTTAATCTGTATTTACTTTGGGGGGAAAAAAGCACTCCTATATCTTAAATGTCCTTCTTTTTTCTT
TTCCCTTTTAGGGTATTTGTTCTCTTGGATTGAAATTTATGCCCACAGATTTTTCTTTTCATTTGAA
AATGAATGTGAAGATACTGTTTACAGCTTTTGTTAATTGCACTTTTCATCACTCTGATCAATTTCTT
GTAAAGTGCTATGGGAATTATTTTTCCTGTGAATATTAATGATTTTACTACTGCCTCCGAAGTTTT
AAAAAAGGTTCTGTAGACAGAAAACAGTTGTTGCAACCAAAAACAACCAAAAAAAGAAAAAAA
GGGAAAACTGGCTTACCTTTCTCAGATGAATTAAATGATTTTAATAACTTCCCAATTACCAGGGT
TATAACATTGCTTTCTTCAGTTATTAATACAGTCAAATTAATTTTAACAATATAGGCACAATTCATT
TACAAATTCAGTACAGTAGTCCAGGGCTACATGTAAGTGGTCTCTTTATTTTATTCTTTACATCAG
AAAAATCATGAATGTCAGTTTTGCCAGTATTTTACAATGAGGCTTATCGTTTAAAATTTTATAGTG
TATGACATGTAAATAAACCAGTATTTATTGTAGGTTGTTTGATTCAGGCATTTCAAATGGATATAG
GTTAATGGCAATGGAATCCTATTTATTTGGTAGTTGGTTTTGTTTGTTTTTGTTTATTTACTTTTGCT
ATTTGTTTTGTATTGTCTAAACTTTGCTTCAACTGAAGACAGTGTAAGGAGTTGAAAATACAATCA
ACATAGCAATGGAAAGCAATGCAAAGAGGGTAAATCTTGTTTTAATTTTTTTAACTTTTTTTTTTG
TAAATAAATAGTACTGTGATACTTTAGGTGTGACCTCTATTCCACATGATAGTTAGAACCAGAAA
AACACTTTTGCTTTCTAGTTATACTATTGGTTCATTGTAAATGCATTACCAAAAGACAATATGAAA
AAGAGCAAGATTAAGTTTTTCTAACATTGTCCACTTCAGGGGCAAGCATGCTTGGTATAGAGCTC
TAGCTTGTGTATACCTTAAACTGTAACTTGCAGGTGTTTTTCAGTTGCTGCACTTTTTATTTAATCT
TGCTCAGTCCCAGTAACTATCTCAAAGGTAATTGCTGGAATATGCATTTTGAAATACGGTTTAAA
AAAAATCTGTGTATTGTTTCATCTAAAAAGAAAAGCACTATTGGAAACACTAAAAACGTGTTAAA
CTTTGTAGTTATTTTGCATTCCTGTACTTTACAAACTGTCATCACAGCAAAAAGTTTAAAATTAGG
TAATGAAATATTTTTGTAAATAAATACCGTTAAGCTGGTATTTTAAAAAATGTATTATAAATTAATT
AATGTTTCTGGAAAATGTTCATATATATGTATATGAATGTCTCTTTATGCTGAAGGGCTCTGATTG
GGCAAAATAAAATACTCTAATCTCTCCTG

ID|56136|gene_id|ENSG00000236140|transcript_id|ENST00000427732 Homo sapiens lncRNA

AGTTCTACTGGGCGCTATAGGAAGGGCGCACGCACGAAACTTCCTCCGTCCCTTGCATCCTCAGGGCCCGCCCGGCAGGAGGACTGAGCAAGGCCTTGGAAAACTGGAGAGATTGGAGCGGTAAGTCGCGCTGGTCACGTTGGACACCTGCGCGTTGGGAGATTCTGGAGCCAGAAGGATAGCCGAATGGCCTTCGCCCGCCCTGCCCCTCGCCTGCTTCAGAAACCCCCGGAAACGCCCCGGTTGAGACCCCGGCCCGAGCCGCCTGGGGGCCCTAGGGAGGCTGAACGCCCGGTGGCTCCCGGGATGGCTCTTCCCATTCTTTGCGCCGCCTTCACCCAGTGAGGGAGCCTGTGCCCTCCCTGCCCAGTCGCTTTTGGGGCCGCTGCGGAGCTTCCGCTGCCATCTTCGGATCCTGTGTTCCGCACGGGGGCTCCACCAGGGCAGGGATCGTGGTGAGGGTGGCTCGTGGGTCCCCCTCACGGGGAGCAGGGTCTGGCACTCACCAGGGCGCAGGACTAGGACTTGTCGAATGAATCCATCCTTTTAGCTTTTAGTCCTTTGAAGAGCCTTGAGAATGGAAATCATGAGAGATTTTTCCATGGGGAAGTTCCTTTTACAAAGCATTTATTTACGTTGACTTCTTGACACCCCGCGGGGCGGCAACGGGCAGGGCCTCCAGTGCACCTTCTGCGCCGTGGAGCCGCGGGGGCTCAGCTGGGCGGTGGTCGGGTCCTGAGGCCGGAGGGCGGGAGCAGGGGAGGGGAAAAGCAAAAGCGGGGAAAGAAGCCGGGGAGCGGTGGACCAGACGTCCAGACCTCCTGAAAGGCTGGCGGGGAGGCACAGGCGGGATCTTCCGGAGGTGAGAATTTTTTTTTATTATAGCAGAATGGGGAGGAATTGAGGGGAAAATGGAGATAGAACCTGAAAGAGCCCCAAACGCCAGAACCTGTAGCTCCCCAAGAATAAGATCTTCCAGAAGAACTAGACCCAAAACTAGCCGTTGGGGAACACCGAAATCCTTGGAGGAGCAACATCCGCATGACCCTCTGTGTTTCTTTAGGCAAAAGGACTTGCTTCCATTGTTTGTTCAATTGTTTGTGTTTGTTAAATAAATAAAACGATTTTCATGTATCTTTGAAATTA

ID|56138|gene_id|ENSG00000229801|transcript_id|ENST00000428508 Homo sapiens lncRNA

CAGATCTAGGTTTCTCCACTCAGGAAAAGAGGAGAATGTACCCATACTTGGGAAAACAAGTTTCCGATGGCACAGCTTTGATCAAACAGCAAAATTCTATCCATCTATGTATTGCCATCTGACAGTATGACAAATGGTCCCATGTGCGATATTCACACTGCATTGCAGTCAAACCTGTAAGTCAAAGGATATGAAATAATAGTAACTATACATTAAGCACAGAAGAAAATGAAACAAACAAAAAGGTTTTAAACCAACCAAAAATATGTCTTATTTTGGATGTTCTATATGTTCTTACATTCTCTCAGGTCTTTTGTGTCATTATGAACACAATTCTAACAAGCTTGATTATTTTATTTCCATTCACATATTACAGGCAACAAGCTGAAAAAGTAGAACGGGGTGTAGAGAGACAGGACAAAGTACAGATTAGGGCTTGAAGTGCCCCTGACCAGTCGACAGCAACCACATGGAATAATGACTCATGTGCATTAATGATCACACTAAATGATATTTGTTTTTTACCTAGTCCTTCAACTGACAGCTTAAAGAACTTCAGGTTGTTCTGATTCTTGAGCCTCCTCTACAGCTTCAGAGAGGACTTTCATTTTATTTTGGATCAAATGCTCCACAACTAGTTGAAACTGGAATTAAATTTTATATGAAGTTCCTAGATGATTTAAAGCTGTAAGAAGAAGAATAATGAATCATAAGAAAACTTGCTGCTACAGATATCAAAAAGGAATGTTACCATCCCTCATGCTAATCCTTTTCATTTTAAATAAACAGGATCTAAAAAAAATAATGCTGGGAAGTCCTAACCACATCAAGAATGCCTCAGATCAGTGACCCAAGGAACCTTCCAGAATGGATGAAATAGACCCAAAGCTGAATTCACCTAATTTTAGGGCCAAAAACCCAAAAAACAAAACAAGACCAAAAAAATCTTCAGATACTGGGAGAACAAATCTCAATTGCTCAATTGTATCTTATGAAAACAATTTTTCAAAA

ID|56140|gene_id|ENSG00000250501|transcript_id|ENST00000513400 Homo sapiens lncRNA

TGATCTCTGAGAAGTGAGTAGCTGGAGGCACCAGGATACCAGCCAAGCCTAAAGGATAAAGCAAATCCCTGGACATGGCTTTTGATTCCAAGCTAAGAAGTGCAAGGTAAGCTACAAAAAGCATTCTAATCAGGGCCTAGATGTGTTTGATTACAATAAATCAACATATCTGTAAAATTTGTGAAAAAG

CTACCCAATGTTTTCATAGCCTTTATTTGACTTAAGAGCAATTAGGAAGCTTCATTTAAGTACAGT
AATCATTAAATCTATTTAAATAAGGTTTTCAATTGAAAAGCAAAAATAATGTACAAATTGGGGAT
TATACATCAGGAGCCAGATTCGTAATTAAACAAGGAGATTAAACCTATTCATTTATCTCTGCTTTT
TTTCTGAAATTTATCCAATATTAATATTAAAGGAATAAATAAATACATTAACTCTCAAGTTCTCAG
AGCATGAACGGGGCTTGACCACTGGCCCTAGACTGGGGAGAGAAAAATGGAAGTCTCGGCCA
GGCACAGTGGCTCATACCTGTAATCCCAGCACTTTGGGAGGCCAAGGCAGGCAGATCACTTGA
GGTCAGGAGTTCCAGACCAGCCCGGCCAACATGGTGAAACACCCCGTCTACTAAAAATACAAA
ATTTAGCCAGGTGTGGTGGTATGCACATGTAATTCCAGCTACTCAGGAGGCTGAGCCATGAGAA
TCGCTTGAACCTGGGAGGTGGAGGTTGCAGTGAGCCGAGATCACACCACTGCACTCCAGCCTG
GGCAACAGAGCAAGACTCTGTCTCACACACACACACACACAAAAAGTCTCTCAGAAAGCAAATCA
CAGCAAACAAGACAGCAGACAACAACAAAAGAGAAGAATCCACAAAATTCTGGAAGATGAAA
AGGAGGTGGACCCCTGGTAACTGAGTTAGTCAAAGAGAGAAGGCTGGAAACTATCATGCTGGA
GGAGAGAGGTAAGAAGACAATAGGAGCCAACTCTTTCACATCATAGAATCCCAGAAAGATACA
AGATTTGGAGGTCCTGGGAGCCAAATGAGACTGATAACTAACACCCTCCTTTTTCCTCAGTGGA
GTACTATGACATAGACACGGATGCCAGACAACGGAAGATCAAATGTCACCTTTAATCTGGAATC
CTACATTGGACACTGGACTAGAAAAACATTTGTGGAAAAAGTAATGAAATTCAAATACGGTTCC
ATGGATTAGCTAATTGTAATGTATTATGAATCACCTGCTTTCTATCTTTGTTCTGTGGTTATGTAAA
AAGTTAACATTAAAGAAAGCTGGGGGAGAACATACAGAAATTCTCTGTATTATTCGTGCAAATT
GTAGGTCTAAAATTATTTCAAAATTAAACTTTTTAAGTTAAACATCTCCTTGATTGCTGATAAAGT
CGATATTAGAAAGCATAGCTTATTCTGCCTGAATTGGATAAATTTGCTGACTTCAGATTTGGGCA
GATTTACCTAATATAACTGCAAGCAATACTGAGCCATTTCAAGTCTTACCACAGAATAGAGCCA
GCAGGCTCTAAGCCTACACAGGGCCGAGAGCTAAAGAGAGCACATACTCTGCTTCAGACAGGC
GAAACTTCAGTTTAAGATCTTGGGATTTCCTCAAAGAAAGGTGAGCAGAAAGGGCTGAGCTATG
CCTGCCCAGTTCCTTCCCAGCTCTCCCCAATTAAGTATGTCACTCCTTGTTGCCTGGGGGGGAGT
CAAGACGAGAAAAAAGAATCCAGAAGCATTGTTTTCAACGCAGTGCTCTCAGACTTTTTGGTCT
CAAGGCCCTTTTACACTATTAAAAATAATTGGAGACCTCAAAGAGCTTTTGTTCATGCTAGTTAT
ATATTTAAATATCTACTATAAAACTGATACTTTTTAGTATTTATTTATTTAAATACTATTACTAAACC
CAGTAGATGTTAACGTGAATAACATATTGTATGAAACATAATAATTTCTTCCAAACCAAAAAGTA
TAATGAGAAGAGTGTCCTTGTTGTTCATTTTTACAGAGTATCTGTAATGCCTGCCTTAACAGAAG
ACAGCTGGATTATCACAGCTGCTTCTGCATTCGATCTGTTGTGATATCACATGTCATGTTGCCTCT
GGAAAATTCCCCCTGCACACTTACAGGAGAATGAGAGTGAAAAGAGCAAATAATATCTAGTGTT
TTTAGAAAAAATATTTTGACCTTCCATCCCTCAGAGATCCCTGGGCCACACTCTGAGAACTGCTG
TTCTAATAAACATCTCTCTATGTGCAGAACAGACGAGGTAAATGTTCCCCAACAGAAGGCAGCA
CGAAGGCTGTAGAAGACAGCGATGAAGTCTAGAAGAAGGGCACCCCTGAGACAGAGAAGCTT
TGAAGAAAATCCCTCTATGAAGCAGTGAAACCCTTCCCCCAGTTCCTCTTTCGAGTAGCACTGTC
AGGTGACTGTCTCTCTGTGCTGGCAATTTACTGAGAGGCTGAACCCTGACAATCTCCAGATTCA
GGGAAGCCGGCAGAGCTGAGGAATACCCTACTAAAACAGGAAACTGGGAGGAATTTAGCACA
AGGTTCAATGAGATCCCTCATCCCTCTTGCCTTACTCAGCTCCTAGAATCCTGGCCTCTTCGTTTG
TTACCCACGCCCCAACACACACACACCCTAAGTGAGAGGTTGAGTAATTCTTCTCTGAGGAAACAG
AAACAAGCCAAGAAGGGGGAAAAGAAAAAAACCTATAAATACTGAGAGTTGGGATATCCATCA
ATGAAACAGCCCCAACCAATTACCCAAAATTAAAGCCCACGTCTTAGCAAGCCTCACCTCTGCA
CACACAGTTCCAATAGCTGCAGCTGTTGCACTTTTGTGTGCTAATAGACAGTCCAGAATGACTAA
AACATACATCCTCAGAAAGCATCCAATGGGGAAAAAAAAACAGACAAAATTATTAAAAAAACC
AATGAAAGGACAATGTGGTTTTTTTGTTTGTTTTTTGTTTTTTAAGAAAAAAAAGAACATTCATCA

ACAACAAAGGGCTTCTTGGATATTAAAAACATAAAAGCCGAAATTTAAAAATTAATTAAAGTTTT
GTAAGATAAAGCTGAGGAAGTCTTTCAGAAAGTAGATCAAAGAAAAATGAATAGAAAATAGAA
AAAAATAGTACTTAGAAGATCAGAGAATTGATACCAGTTAAACTTGCGACAAATACAACCCCAG
AAAGAGTTAACTGGGAAAATTACCAGATAAACAAAAATTAAATCTTCACAGAATTGAGGAGCCT
GAGTTTCCAAGTTAAAAGAGTCTATCACGTTTCCAGGAAAATATATAAATATGTGAAAAAGCAA
AAAAACACATAGATATGAAACTCCAGCAAACAAGGGATAAAGAGAAGATCCTAAAAGCTTCAA
GAGCACAAAAACAATGGAGATGCAGAATGGCATTAGACTTGAGAATAATAACTCTAAGAATTA
CACAATAATGGAGAAATGAAATGCCTTCAAAATGCTGAAGAAAAATGCTATCCACCTAGAATTC
TAAACTCAAATCATCATTCAAGATTGAGGATAAAGACATATGCACGAAGGCAACATCTAAAACA
GATTACTTCCCAGGAAAGCCTTTTTTCAAGATGCTCCTAGAAGATGTAATCCATTGAAAGAAAAG
AGTCAACCAAGGAAAGAAAACATGGAATCCAAGACAGAAGGTGTGGTGGTCAGTCTTCAAGAC
GGCCCTAAATGAGCCCCTCCTCCTGGGCTTCGCAGCCTTGTACAGTCCCCTCTGGTATTGTACA
GGGTCTGTGTGGACAGTGGAAGATAGCAGAAGTAACAAGCTATTCCTTCTGGCATTGGATTCCA
TCTTGCTCCCTTCTACTCTTTATCTCTCTTCAGTCACTAACTCTGGCAGAAGCCAATGGCCATGAT
GTGAGCAGTGCTATAGAGATGTCTGTTTGGTGAGAACTGAGTCCTCCTGCCCAGAGCTTCTAAT
CTGAGCTTAGAAACCGATTCTCCAGTGCCAGTCCAGCTTCAGATCATTTCAGCCTTGTGAGAAA
CCTGAACAAGAACCATCCAGCTAAGCTGTTCCTGGATATGACCCACAGAAACTGTGAAAAAATA
AATGTTTGTCAGTTGAAGCTGCTACGTTTTGAAGTGATACGTTACAAAGCAATAGACAACTAATA
CACAGTGGATCTAACATGGAAGAGAAGAGAAAGATATTTCTAAGACAAGAAAGCCCCAGGGCA
ACAGCCATGTAGCCATCAGTATCCTGTCCAGATTTGAGCAGAAGGACAGAGGTCTCTAGGAAA
GATGTCTCAAAAAAAAAAAAAAAAAAGTAGAACTGATAAATTATCTCATGTATTTTATGGTAGACA
GAAGTTGCATTCACTTACTTCAATGTGAATATACATTTAGTTACATTCACAAACACTTCAGTTTCA
GACTGAAGTATTTGCTAATGGTTACACGGAAAGCTAAGCAAAAGAAAAGTTGTAACAATTATTA
ACTCTAGAAAAAAAAAGGGTTGTTCAAGAAAGGAAAGGTAACTAAAGTACATATCTAGCCCATA
TGTGAATATTTGTATCATCATAGTAATGGAGATGCCAGACAGTGATATACCTCCCAGATTGTGGT
AATTCTACTGAGAGTATGAAGAAATGAAAGTATGTGTAGCAGGGAGGGAGTGGTGGTGGTACA
GTGTGTTGATTTAAGAAAGCTAAAATGTATCTTCTGTAATGAGAAGTCAGTGGACGATGTCTGAA
ACATGAAGATCAAAACAATTTAATCTGAGTATACTACTGACAAATGCAAAGGTAAATTCCACAA
TAACAGCTAAAATAATTGAAAGGAATTGACTTTGGGGAGCAGAAATTGAGGGTAGGAAGCAAT
GGCATCATGCAGTGCTCTGCCATAAGCTATGTTGTTCTCCTTGTCTCAAAAAATTTTGATCCAAAT
AAAATTTTATTATTATTATTGAGACGGAATCTCCCTCTGTTGCCCAGGCTGGAGTGCAGTGGCAT
GATCTCGGCTCACTGCAAACTCCGCCTCCCAGGTTCAAGTGATTCTCCTGGCTCAGCCTCCCAA
GTAGCTGGGATTACAGGCACCTGCCACCACGCTGGGCTAGTTTTTGTATTTTTAGTAGAGACGG
GGTTTCCCCAAGTTGGCCAGGCTGGTCTCAAACTCCTGACATCAGGTGATCCACCCACCTTGGC
CTCCCAAAGTGCTGGGATTACAGGCATGGGCCACCACACTCTAAAATAAAATTTTAACAGCATT
TACCTATCATGGGACAAAAATCAATAGGCAATGTCTATAATTAATAAACTACAAAATAGAAAAT
GTCTTCATATTTGTCAGTATCTATCAGAAGATACCAAAAATACAAATAGTTAAATATGTTTTCCTC
TGAAAGACAACACTGGGAATGGCAATATTTATTGCTGCAATACAAACCATCCCCAAACATAGTA
GCTGTGATGGTTAATATTAGATGTCAACTTGATCGCATTGAAAGATGCCTGGATGGCTGGTGGA
GTATTGTTTCTGAGTGTGTCTGTGAGAGTGTTGCCAGAGGAGATTGACATTTGAATTGCTGGAAA
AGCATTGGTTGTAGATGCTTTCCCCACCCTGAGATATCCTTCAATGCCTCAGGGAGAGGAAAAC
CACTGTTAGTGTGAGTGACCTTTAGTGTGAGTGGGCACCATCCAATCAGCAGCCAGTGCAGCTA
GAACAAAGCAGGTGGAAGAAGGTGGGATAAGTTTGCTTGCTGAATCTTCTGGCTCTTTTTCTTCT
TCCCATGCGAGACGCTTGCCTCCGCTCCTCGTGCCCTTGAACATCAGACCCCAGGTTCTTCCATC

TTTGAACCCTGGGACTTGCACCAGCGGCTTCCCAAAGGCTCTCAGGCCTTCGGCCACAGACGA
AGGCTGCACTGTCAGCTTCCCTGGTTTTGAGGCTTTCAGACTTGGACTGAGGGACTACTGGCTTC
TCTCTTTCCTCAGCTTGCAGACAGCCTATCGGGGGACTTCACCTTATAATTGTGCAAGCAATTTC
TCCCTAATAAACTCCCTTTTTTACTATTATTATACTTTAAGTTCTGGGATACATGTGCAGAATGTG
CAGGTTTGTTACATAGGTATACACGTACCATGGTGGTTTGCTGCACCTATCAACTCGTCATCTAC
ATTAGGTATTTCTCCTAATACTATCCCTCCCCTAGCCCCCGTCCCCCAAAAGACCCTGGTGGGTG
ATGTTACCCTCCCTGTATCCATGTGTTCTCATTGTTCAGCTCTCACTTATGAGTGAGAAAATGCAG
TGTTTGGTTTTCTGTACCTGAGTTAGTTTGCTGAGAATGATGGTTTCCAGCTTCATCCATGTCCCT
GCAGAGGACATGAACCTTTTGTATGGCTGCATAGTATTCCATGGTGTATATGTGCCACATTTTCT
TTATCCAGTCTATCCCTGATGGGCATTTGGGTTGGTTCCAAGTCTTTGCTATTGTGAATAGTGCTG
CAATAAACATATGTGTTCATTTGTCTTTACAGTAGAATAATTTACATACCCAGTAACGGGATTGCT
GGGTCAAATGGTATTTCTGGTTCTAGATCCTTGAGGAATCACCACACTGTCTTCCACAATGGTTG
AACTAATTTACACTCCCACTAACAGTGTAAAAGCATTCCTATTTCTCCACATCCTCTCCAGCATCT
GTTGTTTCCTGACTTTTTAATGATCGCCATTCTAACTGGCGTGAGATGGTATCTCATTGTGGTTTT
GACTTGCATTTCTCTAATGACCAATGATGATGAGCTTTTTTTCAAATGTTTGTTGGCTGCATAAAT
GTCTTCTTTTGAGAAGTGTCTGTTCATGCCCACTTTTTGATGGAGTTGTTTGTTGTTTTTTTTTTCTT
CTACATTTGTTTCAGTTCCCTGTAGATTCTGGACAATAGCCCTCTGTCAAATGGATAGATTGCAA
AAATTTTCTCCCATTCTGTAGGTTGCCTGTTCGTTCTGATGACAGTTTCTTTTGCCGCGCAGAAGC
TCTTTAGTTTAATTAGATCCCATTTGTCAATTTTGGCCTTTGTTGCCATTGCTTTTGGTGTTTTAGTC
ATGAAGTCCTTGCCCATGCCTATGTCCTGAATGGTATTGCCTAGGTTTTCTTCTAGGGTTTTTATG
GTTTTAGGTCTTGTGTTTAAGTCTTTAATCCATCTTGAGTTAATTTTTGTATAAGGTGTAAGGAAG
GGGTCCAGTTGCAGTTTTTTGTGTATGGCTAGTCTGTTTTCCCAACACCATTTATTAAATAGGGAA
TCCTTTCCCCCATTGCTTGTTTTAGTCAGGTTTGTCAAAGATCAGATGGTTGTAGATGTGTGGCAT
TATTTCTGAGGCCTCTGTTCTGTTCTATTGGTCTATATATCTTTTTTGGTACGAGTACCATGCTGTT
TTGGTTACTGTAGCCTTGCAGTATAGTTTGAAGTCAGGTAGCGTGATGCCTTCAACTTTGTTCTTT
TTGTTTAGGATTGTCTTGGCTATATGGGCTCTTTTTTGGCTCCATATAAAATTTAAAGTAGTTTTTT
CTAATTCTATGAAGAAAGTCAATGGTGGCTTGATGGGGATAGTGTTGAATCTATAAATTACTTTG
GCCAGTATGGCCATTTTCACGATGTTGATTCTTCCTATCCATGAGCATGAAATGTTTTTCCATTTG
TTTGTGTCCTCTTATTTCCTCAAGCAGTGGTTTGTAGTTCTCCTTGAAGAGGTCCTTCACAACCCT
TGTAAGTTGTATCCCTAGGTATTTTATTCTCTTTGTAGCAATTATGAATGGAAGTTCACTCATGAT
GTGGCTCTGTTTGTCTATTATTGGTATATAGGAATGCTTGTGATTTTTGAACACTGATTTTGTATCC
TGAGACTTTGCTGAATTTGCTTATCAGCTTCAGGAGATTTTGGGCTGAAACGATGGGGTTTTCTA
AATATACAATCATGTCATCTGCAAACAGAGAAAATTTGACTTCCTCTCTTTCTATTTGAATAGGCT
TTATTTCTTTCTCCTGCCTGATTGCTCTGGCCAGAACTTCCAACAATATGTTGAATAGGAGTGGTG
AGAGAGGGCATCCTTGTCTTGTGCTGGGTTTCAAAGGTAATGCTTCCAGCTTTTGCCCATTCAGT
ATCATACTGGCTGTGGGTTTGTCAGAAATAGCTGTTATTATTTTGAGATATGTTCCATCAATACAT
AGTTTATTGAGAGTTTTTAGCATGGAGGGGTGTTGAATTTTATCAAAGGCCTTTTCTGCATCTATT
CAGATAATCATGTGTTTTTTTTTTTCATTGGTTCTGTTTATGTCATGGATTACATTTATTGATTTGCA
TATGTTCAACCAGCCTTGTATCCCAGGGATGAGGCCTGCTTGATCATCGTGGATAAGCTTTTTGA
TGTGCTGCTGGATTCGGTTTGCCAGTATTTTACTGAGGATTTTCACAGCAATGTTCATCAGGGAT
ATTGGCCTGAAATTTTCCTTTCTTTTGTGTCTCTGCCAGGTTTTGGTATTAGGATGATGCTGGCCT
CATAAAGTGAGTTAGGGAGGAGTCCCTCTTTTTCTATTGTTTGGAATAGTTTCAGAAGGAATGGT
ACCAGCTCCTCTTTGTACCTCTGATAGAATTTGGCTGTGAATCCATCTGGTCCTGGGCTTTTTGTG
GTTGGTAGGCTATTAATTACTGCCTCAATTTCATAACTTGTTATTCGTCCATTCAGGGATTCAATT

TTTTCTTGGTTTAGTCTTGGGAGGGTGTATCTGTCCAGGAATTTACACATTTCTTCTAGATTTTCTA
GTTTATTTGTGTAGAGGTGTTTATAGCATTCTCTGATGGTAGTTTGTATTTCTGTGGGATCAGTGG
TGATATCCCCTTTGTCATTTTTTATTGTGTTTATTTGATTCTCCTCTCTTTTCTTCTTTATTAGTCTGG
TTAGCCGTCTATTTTGTTAATCTTTTCAAAAAATCACCTCCTGGATTCATTGATTTTTTGAGGGGTT
TTTCATGTCTCTATCTCCTTCAGTTCTGCTCTGATCTTAGTTATTTCTTGTCTTCTGCTAGCCTTTGA
ATTTGTTTGCTCTTGCTTCTCTAGTTCTTTTAATTGTGATTTTAGGATGTTGATTTTAGATCTTTCCC
GCTTTCTTCTATGGGCATGTAGTATATAAATTTCCATCTAAACACTGGCTTAACTGTGTCCCAGAG
ATTCCGGCACATTGTGTCTTTGTTCTCATTGGTTTCAAAGAACTTATTTATTTCTGCCTTGATTTCG
TTATTTACCCAGTAGTCACTCAGGAGCAGGTTGTTCAGTTTCCATGTAGTTGTGAGATTTTGAGT
GAGTTTCTCAATCCTGGGTTCTAATTTAATTGCACTGTGGTCTGAGAGACTGTTTGTTATGATTTC
CATTCTTTTGCATTTGCTGAGGAGTGTTATACTTCCAATTATGTGGTCAATTTTATAATAAGTGTG
ATGTGTTGCTGAGAAGAGTGTATATTCTGTTGATCTGGGATGGAGAGTTCTGTAGATGTCTATTA
GGTCTGCTTGGTCCAGAGCTGAGTTCAAGTCTTTACTATCCTTGTTAATTTTCTGTCTTGTTGATCT
GTCTAATATTGACACTGGGGTGTTAAAATCTCCCATTGTTACTGTGTGGGAGTCTAAGTCTCTTTG
TAAGTCTCTAAGAACTTGCTTTATGAATGTGGGTGCTCCTGCATTAGGTGCATGTATATTTAGGA
GAGTTAGCTCTTCTTGTTGTGTTGATCTCTTTACCATTATGTAATGTCCTTCTTTGTCTTTTTTGATC
TTTGTTGGTTTAAAGCCTGTTTTATCAGACACTAGGATTGAAACCCCTGCTTTTTTTTGCTTTCCAT
TTGCTTGGTAAATATTCCTCCATCCCTTTATTTTAAGCCTATGTGTGTCTTTGCACGTGAGGTGGG
TCTTCTGAATTCAGCACACTGATGGGTCTTGACTCTTTATCCAATTTGCTAGTCAGTGTCTTTCAA
TTGGGGCATTTAGCCCATTTACATTTAAGGTTAATATAGTTATGTGTCAATTTGATCCTGTCATTAT
TATGTTAGCTGGTTATTTTGCCTGTTAGTTGATGCAGTTTCTTCATAATGTCGATGGTCTTTACAAT
TTGGTATGTTTTTGCAGTGACTGGTACCAGTTTTTCCTTTCCATATTTAGTGCTTCCTTCAGGAGTT
CTTGTAAGGCAGGCCTTGTGGTGACAAAATCTCTCAGCATTTGCTTGTCTGTAAAGGATTTTATTT
CTTCTTCACTTATGAAGCTTAGTTTGGCTGGACAAGAAATTCTGGGTTGAAAATTCTTTTCTTTAA
GAATGTTGAATATTGGCCCCCACTCTCTTCTGGCTTGTAGGGTTTCTGCAGAGAGATCCACTGTT
AGTCTGATGGGCTTCCCTTTGTGGGTAACCCGACCTTGCTCTCTTGGCTGCTCTTAACATTTTTTT
CTTTCATTTCAACCTTGGTGAATCTGACGATTATGTGTCTTGTGGTTGACCTTCTCGAGGTGAATC
TTTGTTGTGTTCTCTGTATTTCCTGAATTTGAATGTTGGTCTGTCTTGCTAGGCTGGGGAAGTTCTC
CTGGATAATATCCTGAAGAGTGTTTTCCAACTTGGTTCCATTCTCTCTGTCACTTTCAGGTACACC
AATCAAATGTAGGTTTGGTCTTTTCACATAGACCCATATTTCTTGGAGGCTCTGTTCATTTCTTTTC
ATTCTTTTTTCTCTAATCTTGTCTTCTCACTTTATTTCATTAAGTTGATTTTCAATCTCTGATATCCTT
TCTTCTGCTTGATCAATTCCGTTATTGATACTTTCGTATGCTTCATGAAGTTCTTGTGCTGTGTTTTT
CAGCTCCATCAGGTTATTTTTGTTCTTGTCTAAAAGGGTTATTCTAGTTAGCAATTCCTCTAACCTT
TTTTCAAGGTTCTTAGCTTCCTTGAATTGGGCTAGAACATGCTCCTTTAGCTTGGAGGAGTTTGTT
ATTACCCACCGTCTGAAGGCTACTTCTGCCAATTCATCAAACTCATTCTCTATCCAGTTTTGTTCC
CTTGCTGGTGAGGAGTTGTGATCCTTCAAAGGAGAAGAGGCATTCTGGTTTTGGGAATTTTCAG
GCTTTTTGCAATGGTTTTTCCTCATCTTTGAGGATTTATCTACCTTTGGTCTTTGGTATTGGTAACC
TTCAGATGGGGTTTCTGTGGGTTTTTGTTGATGTTGATGCTATTCCTTTCTGTTTGTTAGTTTTCCTT
CTAACAATCAGGCCCCTCTGCTGTAGGTCTGCTGGAGTTCGCTGGGGGTCCACTCCAGATCCTG
TTTGCCTGGGTATCACCAGCAGAAGGGGCAGAACACCAAAGATTGCTTCCTGTTCCTTCCTCTG
GAAGCTTCTTCCCAGAAGGGCACTTGCCAGTTGCCACTCAGAGCTCCAACAGACCTGCCAGCA
GTGTCTGTCAACCCCTGCTGGGAAGTGTCTCTCAGTCAAGAGGCACGGGGGTCAGGGACCCAC
TTGAGGAGGCAGTCTGTCCCTTGGCAGAGCTCAAGCGCTGTGCTGGGAGATCCATTGCTCTCTT
CAGAGCCAGCAGGCAGGAACGTTTAAGTCTGCTGAAGCTGTGCCCACAGCCACCCCTTCCCCA

ACATGCTCTGTCCCAGGGAGATGGGAGTTATATGTACAAGCCCCTGGCTGGGGCTGCTGCCTTT
CTTTCAGAGATGTCCTGCCCAGAGAGAAGGAATCTAGAGAGGCAGTCTAGCTACAGTGGCTTTG
CCAAGCTGCAGTGGGCTCGGCCCAGTTTGAACTTCCCCCAGGACTTTGTTTACACTCTGAGGGG
AAAACTGCCTACTCAAGCCTCAGTAATGGTGGACGCCCCTCCCCCCACTAAGCTCGTGCATCCC
AGGTTGACTTCAGACTGCTGTCCTGGCAGCAAGAATTTCAAGCCAGTGGATCTTAGCTTGCTGA
GCTCCGTGGGAGTGGGATCCACTGAGCTAGACCACTTGGCTATCTGGCTTCAGCCCTCTTTCCC
GGGGAATGAATGGTTCTGCCTCACTGGCGTTCCAGGCGCCACTGGGGTATGAAAAAAAAAAAA
AAAAAAAAACCCCTAGTGAGCCGAGATCGTGCCATTGCACTCCAGCCTGGGAAACAGGGCAAA
CTCCATCTATAAAAAACAAACAAACAAACAAACAAACAAAAAAAAACTCCTGCAGCTAGCTCA
GTGTCTGCCCAAACGGCTGCCCAGTTTTGTGCTTGAAACCCAGGGCCCTGATGTTGTAGGCACC
CCAGGGAATCTCCTGGCCTGTGGGTTGTGAAGACCATGAGAAAAGCCTAATACCTAGGCCGGA
ATGCACAGTTCCTCACAGCACAGTCCCTCAAGGCTTCCCTTGGCTACGGGAGGGAGTTCCCTTA
CCACTTACACTTCCCAGGTTAGGTGACGCCCCACCCTGCTTCTGCTTGCCCTCCGTGTGCTGCAC
CCTTTGTCTAACCAGTCCCAGTGAGATGAGCCAGGCACCTCAGTTGGAAATGCAGAAATCACCC
GCCTTCTGCGTTGATCTCGCTAGGAGCTGCAGACCAGAGCTGTTCCTATTCGGCCATCTTTGTAC
CACATAAACTCCGTTTTAAATGTACATATATCCTATTGGTTCCATCCCTCTGCAGCACCCTAATAC
AGTATCTGAAAACAAAAAGAATCAAGACTTCAAACAGAAACAATGCAGAGCTTGGTGAGAAAA
GTTTGTCCCTGCTCCACACAGAGGCAGCCAGGATAGCTCCACTGGAGCTCACATGATATACAGC
CCAGATGGGCATGGGCATGAAGTGTCCAGTGCCATAGCTCCATAGTCTCTGTATGCCAGGCCTC
ACATGACTGTTCCTTGTGTTCTGCCTCAAGCCCACACTAAATTTCTAATGTGACTTTAGTCATGCA
TGCTGTTTCACAAACTTGCTGTCAGCCCCTAAGAATAACAGAAAATCATCAAAATTAAAGAGAG
ACTAGAGGGCACAGAACAGGTATAGAGCCCTAGCCTGAAGGGACTCCACTCTCCCCAAGGCTG
AAGGCTAGAGAGTGGCAGAGGATTTGAAAAGCATTCGTTTTCAAATGGTTTCCCTACCCTGAGG
TTTCCTTCAATGCTTGGGTTTCCTTTTGGAGCCAGCAATGTACACATTTCAGGATGACATCTTAAA
TGAGGCGGCCATTTCCTATGTTGTTCCTGCATGTTTTCAAATAAAAAGGACTGCATTCAGCCCTG
GCCTGCTACCGAACATCTCCACAAGACTTAAGTTACATGCTTGAACAGCCGAATGGGCAACAGC
AAAGCTGCCTCTCATGATACAGAGGCCTGCCAAGAGACAAGCAACTCTAAATCAAAAAAGGAA
GAAAAAAAATGCCATTCTCAGACCTCTCAGAAACTGGCCCAAAATGAAAACTCAGCTTCGCTGT
GTCTAAAGCCAAATCACTGAATCAGGTTAGTTGACTTTTAAGGTTAGAGGCAGATGACACTTGTA
AGGGTTGTGTCCTAGAACAGCCCACTGCTTTGACATGAGGTAGTGAAGAGGAAAACAGTACAG
CTCCAGGTGTAATAGGTCTTTCTTACTGAGAAAAAAAAGCCCATTTTCAAACAATTTTACTTCATG
TGACTTATGGTGACATTGAAAATTGGTTTTTTGTTGTTGATTTTTTGTTTTGTTTTGTTTTGCTTTGC
TTTTTGCTTTTGTCTAAAATGCTACTGTAGTAATAGTGTAAAACGTAAACAAGGCTAGAACTTTTC
TATTCTCTCTGAGTCATGATTGATTAGCAATTACTACTGGGAAGAAATACAGCAAGGATGAAGG
CAATCAACAGTTAATACTAGAACCTCAACCTCCCACATCAAAGCCTTTTTCTAGACTTAATATTAT
TCTCTCTGTTGAAGATGTTTATATATAATGGAATGATCTAAAATAAGAACAAAAGTTGTAAGCCT
AAATTCACCTTCCTTTACTCATATCTTGTGAGTAGTCCATATATATATTTAAAAATTCAAAGGGAA
CTGCTAACATGAAAATTTTAAATACACAAGGTGTTGTATAATATGTAAATCAATTAATCAAGTATT
GCCTTTTAATGAAGGAAGACTGGAAGAAGTCTTTTCCATCCAGAAACCAGTATATTTTTACTTCC
AGGGTTTTGCATCATTTGACTATTCAGAAAATATTTATTGAGGAAGCATGTGATAGGGTTTTGAT
GTCTGTCCCCTCCAAATCTCATGTTGAAATGTGACCTCCCGTGATGGAGGTGGGGCCTGGTAAG
AGGTGTTTGGGTCATGGGAGCAGATCCCTTATGAATGGCTTAGCACAAATCCCTCAGTGATAAG
TGAGTTCTCGCTCAGTTAGTTTATGCAAGATCTGGTTGTTTAAAAGAATCTGGGATCTCTCCATTC
TCTCTCTTGCTCCCTCTGCCATATGATACACCTGCTTCGTCTTCACCTTCTGCGATGATTGAAAGC

TTCCTGAGGCCCTCACCAGAAGCAGATGCCAGCACCATGCTTCCTGTACAGCCTGCAGAACCA
CAAGCCAATTAAACCTCTTTTCTTTATAACCTATCCAGCCTCAGTTATTTCTTTATAGCAATGCAA
AAATGGCCTAACACAGTATCTTTCAGGCACTAGTCTAGGCTTTAGAGAATATAGCCATGTTCTGG
ATATCGATTGTTGCCAAATGAATATCCCAAAATGTAGTGGCTTAAAAGATAGCAATCATTTTATT
ATTATGACTCATATTTTGACTGCCTAAGCTTATCTAAGCAGTTGTCTGTCAGGGTCTCTGAAATAG
TTGCAGTCAGATGATGACAGGGGCCTGGAGTCATTGCAAAGCCTTCTGCAATCCCATGTTTAGC
CATTGATGCTGGCTGTCAACTAGGGCCTCAGCTGTGGCAATTAAACATTGCCTCTTTGTGTAGCC
TGGATTTCCTCACAGCATGGTGACTGGGTTTCCAGACTGAGAGTCCCAAGAGAACCAGGAAGA
AACTATATTGCTTTTATGATTTATCCTTGGAAATCACATACCATCACTACTGCCAATAGTCACAGA
CCTGCCAAGATTCAAGAGAAAGAAGCATAGACCCCTCACATCTCTCAGTGGGAAATGTGTCACT
GTCACATTCTAATTAGACAATGTGGGTTGGGAGATCTTCTCATGCCCATCTTAGAAAAGCCAATC
TGCCGTGAGAAATAAAAATATTATACAAAGTATCTGCCCTCATACATATGTTTAGAGGCTGGGA
GAGCCTGGCAATAAATAAATCAAGAGAGGAATATCAGGCATGACAACTTCTATGCAGAAGAAT
TAGAGAAAGGAAGGGAAGAAATACAGATGGCGTTGAGCATACCATCTCTTTCATACAGTGGTC
ACAGAAGCCTTGTCTGATAAGTGGCCATGTGAGCAGAGATTCTGGAGGAACTGATCCATTGAGC
CACGTAGATAGTGGGGCAAGAGCATTCCAGGCAGGAGAAACAGCAAATACGAAGGTCACGTG
GGGAGACAGTGCTCCATGTGGTGAGGAAATGACCAGGAAGCCAATGTGGTTAGAGCAGTGACA
AAGGTTCAAGAAGAAGGCAGAGAGCTTGCTGGGGGCCAAGCTTGCAGAGCCTTTAGCCGTGGT
GAGGACCATGGTCTTTGCTCTGAGTGAGATGGGAAGCCATTGGATAGTTTTTAGAAGGAAAATG
GTAAAATTAACTTATGTTTTAACAGGACGACTCTGGCCACTACGTAGAAAATAGAAGCACCACA
TCCAGATACAGAGTGAAAGTGGGAGATGAGTATCGAGGCTCTTGCAATAAATCAGGTGTAAGA
TGATGATACCTTGGACAAGGTATTAGTAATGGAGTTGGTGAGGAGTCAGGTTCTAATGTTAAAT
AATGTCTTTTTTTAGCATTCAGATAATAAGCCATGTTTATGGATTTAAATATTAAAGCCTGAGCTT
AAATAATAAATGTTAAAGGATAAAAATGGATCAATTATCACTGGGCTGCATATCACAAGCAAAT
TTTCTTTTAACATAGCATTATGGGTTGTCTCTAAAACATTTTTGAAGCCTTTTCCTTTGGCAGCTTT
AATACTTTTCCCTCCTAGGCCACATATCCAAAGTGAAATGCAAATTAAGGAAATTATCACTTAGG
CCAAAAAAACCATTTGACTGAAGTATTCAGGTAATTGAAGAAAACATGTTTTAGGGAGTTTATATG
GCCTAGGTAATTCTCTCAAATTTCCACATCCCTTTACATATGCAGATAACAGTAGAAGCTGGGGT
GAAACTCATGGGTGAAAAAAAAATGTCACCCCTGTGAGGTAAACGGCTTCTTGTAGGACAAGAG
AGTAAATCATTCTCCAGGCCACTAGGTGCCCTAAAGGAGGTCATTCCCTCCTACGCATCAGATC
ATTTTGTTCCCAGGGAGTTTTACGTTCTTGCCGGTTCATTGACCACACAGCCAGGATGGTTAGGC
AGCTCTTAATAGATTACGGGTTATTTCCTTATTATGTTTGCTAAATGGTGGTGCATCTCATCATTTT
TATCATGTAGGGAAATTTCATGAGGTCTCTCTGGCCTAGCCAAATACCAAAACAGGATTCCCCC
ATTTATTCCCTTATGCCATAACTGAAAGTTAGCTGAACCAATTAGAGGGTACATGATAAGCCAAT
TAGTTGTTCCTTACTTTTCCTAATGAGTGTAAAATGTGTGCCTGCTCAAATTACAGCTCATTTGAC
AAGATTCTTTGCTATGCTACTATTACTACAACTTGGCATTATTTCATTTCTTTCAGTATTAACAGCA
TCAGTCACAGTAGCTGAGTATCTCCTGTGAGCTTCCATAGGAATTGAGCTTGTCTTTACAAGGTA
ATCCTTCACCCTCCAACTCCAGCATCATGACCAGCTGTCTGAATTGCTGGAAAATGGCCCCTCA
GTAAAACTAAGCTGAAGCCAAACCACATCCTGAAGATGAAACGCTTTTCTGGAGAATCCACTGA
CACTTCAGATATAAGAGGTTTCGGTCCCATATTTCCAAGCTTTATACATTATCTTGCTGCCACCAG
AGGTTAATCAATATGTTCTCTTAGGTTCTTTTTGTGATGATCCATCTACCAAGGTCAAGCATGAAA
AATGAGCAAAATCCATGCAAAGACTAAGATTTCAGCCATGGATTTTGAGGGAAGAGAAACTCTT
GGGTTATTGTAAAACAGCCAGCTCTAGTATCCAAAGGAGAACAACAACCAAAAAAAACAAGAA
TAGAGTCAGAAAATTTTGTAACTTTGTCAACATTTATTTTCACCAAACAACTCATTAAATCACTAT

AAAAGTCAACGCATATGGTCGGATATGACAGTTTAAATTCTAGAAAGAGTTATAAGTCAAGTTA
AACTCATAATACATAATGTAGTTTGGGTGTCTCATCCATGAATTATAACATTTTTATTACATCTCAT
CTGTAAATTACCAACTTGTTCCCTGTCCTGCCAATCTCTTGATTAAATGGGGGACTGTCAGGCAT
TGTGAAAATATATTATGGTAAAGGATAACTCCATCATTTGTCTCGCTTATTTCTACATGCTTATTTA
AAGCTTTATCACTAGAGCAGCTGGTCCAAACTGAATTGTTGATTTAGCAAAGTGACAGCAGAAA
TGAGGAAGTAGAAAAAAAATTGGCCAAAAAGACTCATGTTTTATTGTCATTATCTTACTGTATTT
CATCATATCTTTTTCTAATATTTGTGTATCTTGAATACTTTTGGTGTCCATTTTATATCACTTTAGTT
CCTATTCTTCAAAGCTATTCTTTCTCTACAGGCCTTATTCCCATGGGCCTAACTGAAGGGTGATTT
ATTCTGTATCTTACACAGATAATGGGACAATAACTTGTACCCATCAAATTTCCCCATAAAATGGT
TTAATATCTTGAATCAGTGCCAATAGGTCAGGTGAGTGAATCAAGTATACATCACTTCACATTTA
TCAGAGAGCCAAGAACTTACTTAAAAATTGACCTCCTTTATGAACTTTGATTCTAAAAGTAAAGG
ACACATTGATATATCAAAATATAAATAACAAGTACCTATATATTTGTCAATTTTTAATAATTTCCCT
TTATTTGTATTTGTTTTGATTAGTGTGCTTTATTTACTTTTTTCTAGTTGCAAAGTAACCACATAATT
ATTACAGAAAGTTTTGAAGATAAAAGAGTCCTAAAAAAAAGTCACCCACAATCTCAACACCCAA
ATATATGGAGTGGAAACATTTTATAGCTTTCATAAGTAATTCTGATTTATGTTCTCTGCTCATAGT
AAAATGAAAAAAAAAAATTGCATTACTAATAGAATTTTTCTCAAGGAGAAAACCATCTTTGCTTAG
CAACACACATAAGTAGACATGTTTGATTATAAACTTGAAGCCTGCAAGGCCTGACTTGAACCAT
CTAAGACAAAACAAAGGAATATCTGCATGGCTCTACCTCAAAAATGGACAGGAATCATAATCTA
GAAGGAAAAGAGTAGTTAAAGGAGAGAGAGTGTGCCTCTAGCATGGGAGAAGACTAGCATCTA
AGAAGAAGAGTTAGCCAGTAATTTTTAAGGCAGATCTGTCTGAAATGCCAAACAGGAATGTCTA
ATAGGCTTTTAAAAGAAGCAATGAACACAGATCCTCTGACCCCCCAGGTCACCCCCTGCCCCAA
ATATTCAGTAAGGTCTGCAGTACACTGCATCCTAAATTGTTGGGACTTTGAGGAGAAAGGACAT
TGGAGATGGGCGAATTCAGTGGAATAACATCATGCAGGAAAAGAAATGGCATTCAAGAGGGCA
AAATGCAGACTGGAAATTTATAAATGAAGCCAAAGTAAGAACTCCCAGCCATAAGTATTACCAA
AAATCTCACTTCCCTAGAGTTTCAGAGATAATGGAAGAAACACTGACTTTCCCACTGAAATGGA
GACTTACACCAAATTTTACTTAAATATTAAAGACAACTCCCAGCAAGTTGGAAATTTCAGGCCTG
ACATCTGGGTTACAGTTTTAAGAAAACACTAGAAGCTTTATATAATAATGATGATGATGATGATG
AAATAATAATGCAGTTTACATACTTACAGGTGATGTGATGCAGAAGTCTATGGAAGCATTCTAAA
AAGACTATTTAGTCAATCAACCAGCCAAATAAAATCCCAGTTTTGCCAACCACTTCCAGAGATA
GTTTGCTCAGATGAATAGAACACTATGCTAATTTCATCCAGGTTTCCTATCTGGTCCTATTAGCAG
CTTCTAACCCACCACCCCCATGCACAACTAGCTCTCTGGAGAATCTGTGGCACTGGTCATGTGTT
AGTTGGGGAGAGGTGCAGAAGACAGTAGAGAATGTTGTTAGCTAAGTATAAATCAGTTTGCATT
ATTAGAGTGTGCTCTGCCCTAAATCACAGGAAAAAAAAAAAAAGGACTATAATATGGTTTGACTC
TGTGTCCCCCGACAAATCTCATGTTAATTGTAATTCCCAGTGTTGGAGGAGGGGCCTGGTGGGA
GGTGACTGAATCATGGGGGTGGGCCTCCCCCTTGCTGTTCTCATCATAGAGTTCTCATGAGATCT
GGTTGTTTGAAAGTGTGTAGCACCTCCCCCTTCCCTCTCTCTTCCTCCTGCTGCAGCCATGTAGG
ATGTGCTGGCTTCCCCTTCACCTTCCACCATGACTGTAAGTTTCCTGAGGCCTTCTCAGCCATGC
TTCCTGTATAGCCCTCAGAACTGTGAGACAATTAAACCTCTTTTATTTATAAATTACCCAGTCTCA
CATAGTTCTTTATAGCAATGTGAGAATGGGCTAATACAGACTGCAAGAGTATAATATTCAATTCA
CAAATAAAATCACGTAGGAGATACCAACTACAGTTCAGTGGACAAGTAAGGGAAAGTCACCGG
AAATAAAAAGAGCCAGGAATGTCTGGTAACTCATTCTAGACTTTTAAATGTACAGAATGACAAT
GAAGATAGAAAAGAAAGAAAGATGGAAAGGAAGGGAGGGATGGGGGAGGGAGGGAAGGAG
GGAGGGAGGAAAGAAGAAGGAAGGAAGGAAGGAAAAAAAAGAAAGAGAGAGAAAGATGAA
AGAAAGAAAGAAAGAAAAAAGAAGACAGAAAGAAACAAAGAAAGAAAAAGAAAGAAAGAAA

AGAAAGAAAGAAAGAAAGAAAGAAAGGAGAAGAGGGGAGGGGAAGGGAAAAGTAGGGAAG
GGGAAGGGAAGAGAGGGGAAGTGGAAGGGAAAAAAGATAAGAGACGATCCTTCCAGGTGGG
ACAAAGAATTGTTACAGTATGGGAGATGCGAGATAGAGTAACCCAACCAAACAGAATGTCCAA
GCTTAAGTTGGAAAGCCAGAGCAGAATCATATTATGAAGGCTAAAAAGAAGACTCTGTTTCTTA
GCAAATGGAGAGCGAGTATTGATTCTTGATAAGAGACTGCCGTGATAAAAGTGTTGTATTCAAA
GATCAGTTTTGTCTAGTTAGAGCAATAAAATACAAGTAATATTTTTACCGTATAGTAGAGAACAC
AGAGAAGGTTGCTACAATAATCCAGGTTCAAGGTGATGAGGGATGTGACTAAACAAGCTCAGC
AGTGCCAGTTTCATGTGTGAAAATGACTGTATTTCTTAAGGCACCTGAGAGCAATTACACAAAG
GAACAGGAATTTAAGTGCCTATCCTATTTTATTGTCTAAAATTCCAAGGAATTAACAGGACTAAC
ATGGCCTCATGGCACAGGGTGGAGTTGGGAGAAGAGAGAGGTGGTGGGAGTTGTATGGAATA
AAGAGCTTAAGTAGAAAAGCAAGCAAAGCTAGTGTATTAGTTTCCAATTTCTGTTGTAACAAGTT
ATTACATACTTAATGGCTTGAAACAACACAAATGTATTATCTTATGGTTCTGGAAGTCAGAAGTC
CAACATGAGTTTCTCTTGGCTAAAATCAAAGTGTAGGTAGGGCTGTGTTCCTTCTAGAAGCTCCA
GGAGAGAACTGTTGTCTTCTCCAGTTTCTAGAAGCCACTTATGTTCCTTGGCTCGGCTCTTCTTCC
ATCTTCAAAGCCAATAGTGTAACATCTTCTAATTTCTCTCTCACTCTGACAATCCTGCCTCCCTCT
TCTAGAGAGGAGGCCCTTGTGATTACATTGGGCCGACCCAGATGATCCAGGAAAATCTTCCCAT
CTCAAGATTCTTATTTTAATCACATCTACAAAATTCCTTTTTGCCAAGTGAGGCAACATATTACAG
GGATTACAGGATTTGGGGGATAGGATGGGGACATCTTAGGGGTTCACTAGTCTACCTAACCACA
ACACCTGGCATTGCTTTTCCTAGCAGTACCAGGAAGAACTAGGGGTGGGCACCATACGGCTGA
CTAGGTAAGTGTGGGGCCAATTTCCTAAGTGAATGTCATTAACTACCTTTCAACTGATCAATTAT
TCTACCAATCTTTTACCAGAAGCTCTTCCAAATACAACTGCAACTGCCAAGAGATTGATGGACCA
GAGAATGGCCTCTTCATTCCAGGCACTTCTAAAGTCCAAGTGGTCAAATACCAGCCTCTTTTTAC
CAATGTTCCCACCTGTTTCATTCAGCCAAATTTCATTAGATGAAGGTAAAGCTAATGATTCCCTC
ATTCTCTGTAGTTTGACTCTCTCGAAGTGGCTAATGAGGAGAACAAATACAACTTAGACATCATG
CCACATTTAGGGCAAAGGCAGCTAATACTAATGTACAAAAGCAGTACTGATGTTTTTTAAGTCAA
TACAGGGGGTTGGGTCTTCATTTTGCTTTGAAAATATCTTTTTGTGGAAGATAGGTTTCACTTTAT
GTACACAGATGATTCGTATAAATAATAATAACTGTAATAATGAAAATAATGTCTACTTAGTGTAA
TTTAATCTTTAATTTGTCTTATGGAGATAAAAAATTTACAAATGGAAAAGGCAAGACCACGTTTG
AGAAAAATACAGAGGGAGAAAGAGTAGGATGCACAGAGATCAGTGTTGTCCAGGATTTTCATC
TTCTCTCTTCTCTCCTGTGGCTCAGGCTAAGAATGGCATGGGTCATGCCATCTCAAAGTAAACTA
GGCCTGTCTGTGGGCCTGCCTCTGAGATAGTCTAAAAGCCTTGCCTTTTTCCCAGAAGATTAGCA
TGGAACAGTGGCCCCTAGAAAATACCTTTAGCCCAATATTATGGGAATTAACCCAACCCAGTTT
ATGTAGAATATCCAGCTCTGGAATGAAGAATTGATGATAATCCAGGAGTTTAAAAAAGAAATAA
CTCAATTTTAGAAGATGAAAATAAATAGACACAAACAGGCAGGATACAGAAATCAATCTAGATG
TTGTGCAAACCTTAGTGTCATTGCAGGTGTTAGTGAAATCTACAGTCTGTAGCAAAGATTAGGAC
CAGCAGTGAGCCATGGTGATTGCATGGGTTAGAAGTAGACAAGGAGAACAATATTCCTGTCAG
CATCACCTTTGACCATGCAATCACATCTCTACATAGCCTAAATGAATAAAACAATTTTAACAGGG
AAAGGAAGACATACAATGACATCTATTATTGTGTACAGTTTCTAAATGACATGGTAATAAAAATT
TATCCCTTGTTTTTTTTTTTCTAATGAGCTACTTAGATGAAATTTTCAATGACAGCAACCAACAAT
GAGTAGAAGACCATTAAAATCAGTAGGATTGTGCTTCACTGTTCTTAATACAAAACCTGACAATG
ATTAAACAATTATGGGATTTGTTTGCCTCACACAACTAAGGATGGAAAGTGCTGGCCTGACAGT
GCCTCAGTGAAGCCATCAGCACCCAGCCTTTCCAGCTTTTGGGCTCTGCCATCCTTAGCATATAG
ACCTCTTTCTCCACACTTCCAACCTCCTGATTGCCAGATGGCTGCTGTATCCCCCAGTCCCACCT
CAGCGTCCCAGGGAAAAAGAAGGGGATTGGGCAAAAGGCCAAAAGGGTAGGTGAGCTAAACC

TATTCTCTTTTAAGAAGCTTTTTTTCTTTTCTTTGAGACGGAGTCTCACACTGTCGCCCCAGCTGG
AGTGCAATGGTGCGATCTCGGCTCACTGCAACCTCCGCCTCCCAGGTTCAAGCAATTCTCCTGC
CTCAGCCTCCTGAGTAGCTGGGATTGCAGGCACCTGCCACCACACCCGGCTAATTTTTGTATTTT
TAGCAGAGACAGGGTTTCACCATGTTGGTCAGGCAGGTCTTGGACTCCTGACCTCATGATCCAC
CTGCCTCAGCCTCCCAAAGGAAGCTTTTATAGAAGACTCACCCATATTGCAGACTCATTCTCTGC
TTGTAGTCATTGGCCAGAATTGTGTCACGTGGTCACGCTTAGCGGCGAGGTAAAGGTGCCTGGC
TCTGAGACGGGTCAGCTGAGATGAGTCAGTCGGAAGAAAGCATTCAACTGGAAATAAATCACC
GCTTCACAAGCACCAAGAACGAAGAAAAGAAGGGAGAGAAAAGAGAGAAAGAGGAGGAAAA
ACATTTCCTCGTGGAGACCATTTTCTTGGTATTTAATAGCATTTACGGGACACCAAAACTGTTTTC
TAGTGAGATGGTGGCATGTTTTTCCCTGGAGATTTTTAAGGGTTTTCCGTTGCAATCAAATGGAT
GAAAATGTTTTATAATTCATCTCTTATTGGAAAACAAGGAAAGGAACAAGATAGTCCCCTGAGTT
GCCATGTAATCTCTATTTAGGCTTCTGGAATACTAAGTTCCTTTCCTCTTCAAAATGTTTAGGAAA
GTGTTCCTGGCAATAATGCAGAGGCTGCAGCCCCAGGAATGAGGCCAAAGGGAAAGATGTTCA
GATAGTTTTTTAAATAAAAATATTGAAATATTCCCGGATATACATTAACTGCTGTGGATGGTTAAT
TTTGTGTTGGAATGATAAATTTAGGGTCAAGTTCTACACATAAGGGTCCCTCATAGACCCAAACA
TGTACATCCAAGATGGGAGCACAAAATTACAAAATCAAGAATCTAGTTGCTTAAGAGGGAATAT
TATCATTGCAAATATTCAGGGAAGACTTCAATTGAAAGAATATGTATATGTGTGAGTGTGTGTGT
GTGTGTGTGTGTGCGTGTGTGTGTGAGTGACAGAGGGAGGGAGGGAAACAGAGTTGGGAGAG
GGGAACAGAAAGAAAAGGCCATAGAAATGTGTTTTACAAATAAAACTCTGAAAACACTGTATTC
CACCTGCGCAGAACTTATTGAGAGCAGCCTTATTTTGCTGACCTAGCAAGATAGTTATCCAGCA
AATCTCCTAACTACATAAGAACACATTGTCAAATTTTTAAACTTGTTAATAAGTTTAAAAAACAAA
ACTTTTTTGCAAACTCTCTGCTCTGGAGGCATATCTCAGGTTTATAAATTCTCAGTCTCTGCCCTT
GCTGCAGCTACTACTCCCACTCCTCTCTGATCTGGCAGAGTCTTCCTCTCTCCACCCTTGGGTCC
AAATCCCAGTTTAGCATTTCTCTTTGTCCAAGAACCTGCATTATAGTCCTGCAATCTTGTGTGTAT
TTGCCCAGACTGAATATATTCAAGTGGTTCCCTCCAATAATCCTCTTGTCTAGACCCATATAATTT
ATGAAAAGGAATTTTACGAAGGCTTAAATGTAGTCTGTGCCATATTCAATTTAATTCAATAAATA
TTTATTGAGTGTCCACCTAGAATTGTGCTGTGCACTGTGTGAAATATAAAAGAAATATATAGCAC
AGTCCCTCCTGTGAAGGAGTTTGCAATATTCCTGGGAATAAAACTTGGGTGCTGAAGTAATTAC
AGGATGCAAGATAGTATAGAGCAATGCAATAAAATTATGTAACCAAGACCACAATATAAGGTAC
AGACAACATTATGTTCGGTCAGGGCAAGCAGGATAGAGCTATGGGGAATAGTTCACAGAGGAG
ACGGAACTTGAGCTTGTCCTTGAGAGATGTATGGGCTCAGCTGGGAGCAGCAGTGGAGGAGGG
CACTTCAGGCAGCGGAAAGAGTGTGAGTAAAATCAGAGAGCAGGAACAGTAGGCATGATATGT
AAGAGGAAATGTGAAGAGGCCAGCCTGACTGAAGAGAAGGGGGAATGGAGGTCACGAAGTTG
TGTTGGAAAAAGCTGGATAGAAAGAATATAATGAAACCACAGAAAGCCTTGAAGGACACAGTA
AAAAATTTGGACTTGAACCATATGCTTAATCTCTCTATATCCCTTCCTTGATGTTCTGTCATTGTCA
TTAGAGAATTCATTCATTGACAGCCAGCATGGCACTCTGAGAAGAGCATGGACTACAGAGTGA
GAAAGCACTGATTCTGCCTCATACACAACCTGAGAAAAGTCATTTTACTGCTCAACCTTCAGGTT
CACAACTGTAAAAATGAGAAAACCAATGCCTATAATTTAGGAGTGTTGTGAAAAATAGAGATTA
TGTGTATAACATATCTTGTGGATACAAACAGATGGAATCTTCTACGAGCTTCATTTATCCACTAA
CTATTTCTTGAATGTCTACTATGTGCCAAACACTGTTCAGCACTGGGAAAATGAAAACAAAAACA
CACAGTGCTTATTCTCAAGACTTGAACAGATACGGCTTAAAACAGTGGAAAGACAGTAGCTTGG
AATTGCATACACTTGCAACTAAAGCCCATTCCCACCTTGTACTAGTTGTATAACTTTGTGGAAAC
TATTCAATCACTCTGAATTTCAGTCTCATCATCTGTATAATTGGTCCAATGGTATCTACCTTATAG
GCTGACTTGAGGATTAAATACAAATATGTAAAGAATCTAATCTAGGGCCTGACAATTTTAAGGC

ATTTAACAAATGTAATTTGTATTATAGTGAAAAGGTAGGCATGAAAAATAATCTTATAACAAAAA
GTAAATGACTGGAAGGGAGTTAGAGAAGTCTTCACAGATGACACAGTCACTTCACTGAGACTTA
AATAACAAATAGGGTTGTAAGTGGGCATGTGTTAATAGGACATTCCTGGAAGAGGAAACAGCA
TATCAAAAGATACAGATATTAAAAGGACTGGCTTATTCTAGGACATGCAGAATTCAGTATTGAC
AGATTATAAAGTTGAAGGTGGAAAGTCCAGAAGAAGAAAATGATCAGATAGATAAAGGCCAGA
TTATCAAGAGCCTGGAGTGCCACAAAAAGGGGTTTGGATCCCTTCCAATAGGCAACGGGTAGT
CATTGATCAATGTTGAATAGTAAAGTGACTTGATCAGCTCTGTATGTTGGAGAACTCTCTCTAGT
ACGGAGATGGAAAGGCGGGGACAGCATTTAGAGGCTACTGAAAAAGTTCACATGAAACACGG
AGATAGACGAGATGGGATGATTACTAGTGATATTTAAGAGTTGGTTCCATCAGAATGCAGTTCT
GATTAGATTTTAGAGAAGGGTAAACATCAACAAAGAATCTAAAATGCCCCTCATTTTCTTGCAGG
AAGGTGGTGCATTCTTGAGATTGAGAATACAGATGAGAAGCAGGTTTGGAATAAAGATGATATT
TCCTGTCTGAGATACGATGAGCTTAAGGTGCCTGTGGCATATTCAAATAGAAATCTGGCCACAT
CGTTCTGGAGTACAGAATGGAGAACTAAGCTGGCTGGTGATGTAGAATTGGACATCATTTATGA
ATGAGGGTTAACCATGAGAATGGACTCACCCAGGGAAAAGGAAAAGAAAGGGGCAGGCTATT
CCTACTTAGCCTTAATATTGAGCTGCTCAAGGAGATGAAACTCAAATCCTTTAGGGTAAAAAGAT
AAACCCCAGATGTAGAGTCTGTTGAGGTATGCCCAAGGTCAAAGAAATCCATTTTCTGGCATAC
TCTACATGATGTAAGTTTCATTTGTCTGAAAGAAATGAGAGGGACCACATGGAAAGCCGATCTT
GGATATTGAGGGACTACATACAGGATGACAATTATAAACACACTGTCTCTAGAAAAACACTTCA
TTTACAAAGCAGATTTGACAGCTCAAGTTGCTTTTTGGCAGAGCAGTCCTGCCTGGCCCCTGTGC
GACCTGGTGAGCTTCGATTACAGCATTGATGCTGGCTCTACTACAGAGACCCCCTAAAAGGGGA
GTGATAAATGAGGTCCTGTCAGGCTTCATTCCCTGGGTGGCCTTGAATCTCTGCCGTTATCTGTT
ACCCTGCCAGTACTGACAGTGATGAACACAAGGCAGGCAGCTAATGACACCCCGATTCATTAC
CCAAAAAGAAGGGAAAGCAGAAATAAAGGGAAGGTGACTGCCATGTGGAAGCTTGTCTCACGT
CAGTGCCGTCTTCCTCCTACAGGTTTGAATGGTGAAGAAATGATGGACGCTGTTTGTCTCAAACA
AGGACTCTAATGATACAACAGAACAAGTCACAAGAAAGCTTGCCTGGAAAACCATACACTGGC
CTCTTCGTGGTGAAGTCCTTCACAATCCTTACAAACATAGAAAATATTCAGCAATTCGTTTGTTTG
TTTTCCAGTCCACTCTTACTTGAGAAAAATGCAAAACATCAAAGACACAGAAAAACCACCAGTC
TATACAGCTTGCCCATACTAAATTGTTATTCATTTCAGAAGAGTGTACAGTTTCTTTCTGTAAGAC
TCTATTAAACCAGTTACTCTACAATAGCAAACATTTTAGGCAAACCTAACCATTGCTTCCCATAA
AGATATTTATATCTTTAAGTGGTATCTGATTCATCCCTAAGAAAT

ID|56147|gene_id|ENSG00000227932|transcript_id|ENST00000446557 Homo sapiens lncRNA

TAACATGAATGACTCAGATTCTGAAGGAAGAAAAAGAATGACAAGTAAGTAATCCTTTTGA
TGTTCTCAACTTTAAGGATTATCACATAAAAGCAATGGTTTTTGAAAACCAAAAGCGATGACTTA
TGGTTTTGAGGCTGAGTTGCTTTGTTTTAAACTAGGATTTTCCTTCCTTCCTTCCTTCCTTCCTTCCT
TCCTTCCTTCCTTCCTTCCTTCCTTCCCTCCCTCCCTCCCTCCTTCCTTCCTTCCCTTCCTCCCTCCC
TCCTTCCCTTCTTTCTCTCTCTCTCTCTCTTTCTTTCTTGAGACGGAGTCTTGCTCTGTCGCCCAGG
CTGGAGTGCAGTGACGTGTTCTTGGCTCACTGCAAGCTCCGCCTCCCGTGTTCACGGCATTCTC
CTGCTTCAGCCTCTGGAGTAGCCGTGACTACAGGCACCCGCCACCGCGCCCGGCCTTAACTAG
GATTTTCTAGGTGGGTATATTTTAGTTGAGTTAAACTTTCTAATTTTAGCCAATGGCTTTAAGCTTG
GAGTATGACTACTCTGCTTTCAGTAAATTTGCATAGTAATGTTAAAATACTATTAACTTAAAAGAT
GGTGGACCGAGAAGGAAGGTGGTGTGGCATATGGTGGAAAGAACATGGGTTTTGGAATCTGGC
GGAAAATCTCACCTCTGCCAGTTTTTAGCTGTATGATCTGAAAGATATTTAACTCTCTGAATCTTC
AAGTGCTTCGCTGTAGTAAAATGGGGATGTTTTGCAAGCATCTTATTTATTTATATATTTTTTTGAG

ACAGAGTTTCGCTCTTGTCACCCAGCCTGGAGTGCAATGGCGGGATGTTGGCTCACTGCAAACT
CCGCCTCCCGGGCTCAATCGATTCTCCTGCCTCAGCTTCCTGAGTAGCTGGGATTACAGGCGCT
TGCCACCAAGCCTGTCTAATTTTTGTATTATTATTAGAGATGGGGTTTCACCATGTTGGCTAGGCT
GGTCTCAAACTGCTGACCTCAGGTGATCTGCCCGCCTCGGCCTCCCAAATTGCTGGGATTACAG
GCATGAGCCACTGCGCCCGGCCTTGCAAGGATCTTATAATGTGAGCAATGTATGTACAAAGCAT
CTTGTTATAGCCTGGGCCATAATGCTCATACAGTAAATTAGAGCTGTAGAAATCAATCACTGGG
CCGTAATACTCATACAGTAAATTAAAGCTGTAAAAATCAATCACTTCTATGTATTGATTTTCTTTT
CCTTTTTTTTTTTTTTTTTGTTTTTTGAGAGAGTTTCACTCTGTTTTCCAGGTTGGAGTGCAGTGGTG
AAATCACTTCACTGGGAGGGCCTTGACCTCCCAGGCCCAAGTGATCCTCTCATCTCAGCCTCTT
GAGTAGCTGGGACTACAGGCATGTGCCACTATGCCTGGCTAATTTTTTTGTACTTTTAGTAGAGA
TGAGGTCTCACTATGTTGCCCAGGCTGGCCTCGAATTCCTAAGCTCAAGCCGTTCTTCTACTTCC
ATCTCCAAAATGTTGGGATTGCGAGTGTGAGCCACTATACCTAGCCTTAATTTTCAGTTTACTGT
GTTGACTTCACACTTGGCTTGGAGAAGGGAAAAAACAGGGCTGGGCGTGGTGGCTCATGCCTA
TAATCCCAGCAATTTGGAGCCTGAGGCGGGAGGATTGCTTGAGCGCAGGGGTTCAAGACTAGC
CTGGGCAACATGGGGGATGCCTCTCTCTATAAAAAATTTAAAAATTAGTTGGGAGTCGTGTCAC
GTGCCTGTAGTTCCAGCTACTCGGGAGGCTGAGGTGGGAAGATCACTTGGGCCTGGGAGGTCG
AGGCTGCAGTGAACCATGATTGTGCCAGTGCACTCCAGTGTGGGCGACAGAGTGAGACCCTGT
CTCCAAACAAAGAAACAAACAAACAAAAACAACAAAAAAGGAAGGTAATTCTTTTTGCAGTTCA
GGTTCTAGCAAGATCAAAGAAGTTAAGGCAGAAGGATCTGAGCCTTTGAGTGCTTCTGCCTTAG
GACTTGTATAGAGAGTATGTGTGTGTATCGGGGTGGCTGGTTTCTGTCCATGAAAGAAGGATGA
CACTAACATTCCACATTAGGTTAGGGAATGAAATTGCTTCTTGAACTCGGGACATTATTGTAGAC
ATAAAAGCAAGTTTAGTTCTTACAGTGAAGTGGGTGAGCTCTTTGGCGTTCATTTCCTTGCCAGC
CATTTATGCCCGTTTGGGCAATCATACAGAACTTCCAGGCCAGCTCAGAATGTGAATAGTCCCA
GCAGCTGAGAGAAAGAAGTCTAAAATATTTTTAAATGCCATGTGGTGGCAGTCCTTAGTATTTGG
ATTTACTTGGTTAGGATAGATTGGATGAAATGGCAAAATGAAAGAAATGAAGTGGGTTCATGGC
TGCAGCTTTGCTTCTGTGATTTTAGAGAAATAGTAAATGCTAAACTATGTGCTCTCAAAGAAGGG
AAGAAATCAATTTCCATGAAGAACAATGGTATAATAAGAGTTTATAATGATAGTTCATTTACTTTT
TAAACGTACACTTCCATATATTCTTATATATTTAATTGATCTGTAAATGGAAATATATAAAATATT
GATTGCACATGTTTATGTAACAATGTATAGCCGGGATGTGGAGTTAAAGAATTATACAGACATG
AAGAAATAAACATCTAAGCAGGAGACAGCTGAGTAAAGTCTGATCCTCGTTTCCCTCATGGAAT
CTTTTTATTTCCGTGTAGCTCCTTTCTCTGGAGCAACCAGCTGTGCTGGTCCAGAGTTAGACCAG
CTATGCTTGAGTTCTGGTTGACCAAACATTTCCTAGATGACAGTAAGCCTGTCACTTGATGCCCC
ACTTCTTTCTTTCTCTCTGGTGTGTAATTTTTAACAGCTTTATTGACATATAATTTACACACCATAA
AGTTCATTCAAGTATAGAATTTTTTTTTACTAAAGAAATTGTGCAATCACGGAAGTCCAATTTTAA
AACATTTTTATCCACTTAACCTGTCCTAACTTTTATGCCCTCATTAATACTATACAGTGCATATGTA
TAATGGGTTGTTGCACAGACTCAATAAGATAGTGGTTGGCTGGCTCGCGCCTGTAATTCCAGCA
CTTTGGGAGGCTGAGGGGAGTGGATCACCAGGTCAGGAGTTTGAGACAAGCCTGACCAATGTG
GTGAAACCCTGTCTCTACTAAAAATACAAAAATTAGCTGGGCGTGGTGGTGCGCGCCTGTAATC
CCAGCTACTCAGGAGGTTGAGGCAGGAGAATCACTTGAACCCAGGAGGTGGAGGTTGCAGTGA
GCTGAGACTGTGCCACTGCACTCCAGCCTGGGCAACAGAGCGAGACTCCATCTCAAAAAAATA
AAAAAATAAAAAAAGATAGTGGTTGGCACATAGTAAGTACTCTATAAATACCAGCTATTGTATTA
TTATGGAGTGCATTAAATATCCTTGTCATATTGCAAACACTGGGATGGATATGTTTTTTAGAAACC
AGAAGATTAAAATATATATATATATATTTATATACATACAACATACATATATATACCATATTTATAT
TTATTTACATAAAACTATATATATGTTTTACATAGTTCCAAAACATGTTTTAAAAACTCAGGAGTA

GATTGGGGAAGATTTGCTATTAAAAATTTTTAGAAATGCTCTAGTTGCAAGCAATGGGAACATCA
AAGATGAGTGTTTTACAGGTCTTAGAAATATTTTTTAAATGCTCAATATTTATTATTCTTACACCTC
CAAATAACTCTAAGGAAGATCATAAACCACCAGTAGGCATTGGTCATTTCTTTTTCATGCTACAC
AGTGCCCATCCCAGAATAGTTAAAACTACATTTAAGAAATGCATCACGGCCGGGCATGGTGGCT
CACGCCTGTAATCCCAGCAGTTTGGGAGGCTGAGGCAGGCGGATCAGCTGAGGTCAGGAGTTT
GAGACCAGCCTGGTCAACATGGTGAAACCCCATCTCTACTAACAATAAAAAAATTAGCTGGGCT
TGGTGGCAGGTGTCTGTAATCCCGGCTACTTGGGAGGCTGAGGCAGGAGAATTGCTTGAACCC
GGGAGGTGGAGGTTTCAGTGAGCCGAGATCACGTCATTGCACCCAGCCTGGGCCACAAGAGTG
AAACTTCATCTCAAAAAAAAAAAGAAGAAGAAGAAGTAATGCATTTGATTTACTATGATACTCGG
GACAGGCATTGTTGATTTTGTATTTCAAGAAGATGATGTTTTGTATGGTGTTTAGCTCTGTAACTA
TAAAGATTGATAGGGAAGTAGTCATTTCTATCGTTTCCTTTTCTTCTTCTTTTTTTTTTTTTGAGATG
GAGCCTCGCCCTGTTTCCCAGGTTGGAGTGCAATGGTGGGATCTTGGCTCACTGCAACCTCCAC
CTGGTGGGTTCAAATGATTCTCCTGCCTCAGCCTCCCAAGTAGCTTGGATTACAGGCACCCATC
ACCACGCCCAGCTATTTTTTGTATTTTTAGTAGAGACGGGGTTTCTCCATGTTGGCCAGGCTGGT
CTGGAACTCCCGATCTCGTGATCTGCCCGCCTTGGCCTCCCAAAGTGCTGGGATTACAGGCATG
AGCCACTGTGCCTGGCCTACATTTCTATAGTTTCTTTGGAAAGAATAAGATCATCAGGCTCCTAT
GAGAAAACAGCTGATGTCCCAAAATGAAATATTTCCATGTATTTAAGTATGTTACCCTAGGTAGC
TCTAGCATTGTGTGATTGGGTTCATATTGCAAAGGAGAATTTTTTAAGCCCTGAAATATCTGTATC
TGCAATAAAATTAGGTTGGCTGTAATTCTTTTCCCAGCACTAAATTGAGTTATTTAGCAAACACAT
ACCAAGTCCCTATTATGTGTATCTTTGCATAGTCAAATTGTTAAAGCTAAAGCCACAAAGATGAA
TTTGATTCCCACCATTAAAAAGCCTGTGATCAGGCCAGGTGCAGTGGCTCATGCCTGTAATCCC
AGCACTTTGGGAGGCCAAGGCGGGAGGACACCTGAGGTCAGCAGCTGGAGACCAGCCTTACC
AACATGGCGAAACCCTGCATCTACTGAAAATACAAAAATTAGCTGAGCTTGGTAGCGCGCACCT
GTAGTCCCAGCTACTTAGGAGGCTGAGGCAGAAGAATCACTTGAACCCAGAAGGCGGAGGTTG
CAGTGAGCCAAGATTACGCCACTGCACTCCAGCCTGGGCAAGAGAGAGAGACTCAGTCTCAAA
AAAAAAAAAAAAAAAAAAGCCTATGATCAACTGGGCAAGGTGGCTCATGCCTGTAATTTCAGC
ACTTTGGGAGGCCGAGGCAGGCGGATCACATGAGGCCAGGAGTTTGAGACCAGCCTGGCCAA
CATGCCGAAATTCCATCTCTACTAAAAATACAAAAAAATTAGCTGGTCATGGTGGCACACACCT
GTAATCCCAGCTACTAGGGAGGCTGAGGCACGAGAATCTCTTGAACCCAGGAGGTGGAGGTTG
CAATGAGCCGAGGTCATGCCATTGCAATTCAATCTGACTGAGGGAGTGAGACTCTGTCTCAAAA
AAAATTTTTTTTTCATTAATAAAGCCTATGACCTAGTGCATCAGTGATTAACATATTATGTGGAAC
TTTGGTTGAACTTTTGTTTGGACATAGTCTTCCTTAACCACTGCTTAAAATTTTATTGCCAAAAGA
CATACAAACAGAAAATTTCGAACTGGATAAAAATGTACAGAATTAGATAATGCCTCAAAGAATC
TTATATACAGACCTAGAATGTGCTGATTTCCAAGTAGTTTATATTTTTACAATTTGATTCAAAGTG
CTTAGACTTACACCTGCAATTCCTAATGTATTTCAACAGTATTTTATTGCCAAGTTTTTATATAGCG
TTTGTCAAATTTGCCACAATTTTTAACTGGATGACTCTGTTCTTCTCTGCCTAAGTTGAGTCCATC
CACAGTTTCCTTTGCATGTCTGTCACCCATTTGTATGCATTTGTATGAAGACTCTTTATATTTTTGT
GTGTGTATACATTTTTATTTTAAAGGTGTTTGGTATGAATTTTTTTTTTTTTTTTTCTGAGACAGAGT
CATACACTGTTGCCCAGGCTGGAGTGCAGTGGCGCGATCTTGGCTCACTGCAACCTCTGCCTCC
TGGGCTCAAGCAATTCTCCTGTCTCCTCCTGAAGAGCTGGGATTACAGGCTCGTGCCACCATGC
CCAGCTAATTTTTTTATTTTTAGTAGAGACAGGGTTTCTCCATGTTGGCCAGGCTGTTCTTGAACT
CTCGACCTCAGGTTATCCGCCTGCCTCGGCCTCCCAAAGTGCTGGGATTACAGGCATGAGCCAC
TGCCCCTGGCCTGTATGAATTTTTTTAACTTGAAATATGCAAACATGGAAAGAATTTTAAAAGAT
CCCTCTTATTCCCATCACCCAAACACAATGACTATTCACTTTTTGATTCACTTTTGAGTAGACTTA

AACCTCTATTTTCTGTGTCTATTGATTAGGGGAAAATGTAGTAGGATAATAATTATACTATGGTAT
ACTTTTAGATTCTTCTTCTTTTCTTTTCTTCTTTCTTCCTTCCTTCCTTCCTTCCTTTCTCTCTCTCTCT
CTTTCTTTCTTTCTTTTTTTTTTTTTTTCAGAGACTCTCCGTTTCCCGGGCTGGAATGCAGTGGCATG
ATCATGGCTCACTGCAGCCTTGACCTCCTGGTCTCAAGCAATCCTTCGACCTTAGCCTCCCAAGT
AGCTGGGACTATAGACAGGCATCACCGTGCCCAGCTACTTAAAAAAATTTTTTTTGCAGAGATG
GGGTCGCACTATGTTGCCCAGTCTGGTCTTGAACTCCTGGGCTCAAGCAGTCCTCCTGCCTTGGT
TTCCCAAAGTGCTGAGATTATAGGCATGAGCCACCAGGCCTGGCCAGATTATTTTTTCACTAAAA
ATAAGCCAGGTGCCGTGGCTCACTTGAGGTCAGGAGTTTGAGACCAGCCTGGCCAACATGGTG
AAACCCCATCTCTGTGAAAAATACAAAAATTAGCCCGGCGTGGTGGTGCACATCTGTAATCCTA
GCTACTCAGGAGGCTAAGGGACCAGAATCGCTTGAACCTGGGAGGAGGAGGTTGTAGTGAGC
CAAGATTGTGCCACTGCACTCCAGCCTGAGTCACAGAGTGAGAAGCCATGTCAAAAAAAAAAAA
AAAGCGTTTTTTATTGCTACTTCATTCTTTTTTTTTTTTTTTTTTTGAGACAGAGTCTCGCTCTGTTG
CCCAGGCTGGAGTGCAATGGTGCAATCTCAGCTCACCACAACCTCCGCCTCTCGGGTTCAAGTG
ATTCTCCTGCCTCAGCTTCCCGAGTAGCTGGGATTACAGGGTCCTGCCACCATGCCCGGCTAAT
TTTTGTATTTTTGTTAGCGACAGGGTTTCACCATGCTGTCCAGGCTGGTCTCGAACTCCCGACCT
CAGGCGATCCGCCCGCCTCGGCCTCCCAAAGTGCTGGGATTACAGGCCTGAGCCACCATGCCC
CCAGCCTCTTCATTCTTTATAATGTAACATTTTTAATGGCTGTATAATATTTATTTGAAAGTATACG
TTTTACTTATTAAGTATCCATTTGTTTCTAGTTTTACCTTAATTTAAATAATGCTTTTTAATGTTTAC
ATTTGTTTCCTTAGACTATGTTCCAGGAGAAGTAGAATTACTGCATCACAGGGCACTTCATTTTTA
GATTACGTATCATGGCGGCAGACCTTCAATACCAAGGTGAGATGGAATATGTAGTCCATTCAAG
TAATGGGAACTTCAGATGAGAGTCATGTAACTGGGGTCCGCAGTCCTCTCAGAATTTCTGTTGTA
CCATTCAGGACAGCATCTCCCCTCCCTGACCAGTTGATAAATCATCTTGCAAAAGGCTCATCTCA
AATGAAGATGTAATTCCTGGAATTTTTTAAAATATGTCGTTTTTATAAGATTTTTAATAGGAAAAA
ATATATGGTTATTGTCAAAAATACAAATTTATAAAGGTTTTTAATTGCATGAGACCTTTCCTGTTT
GCTCTCAGGCAGCATCAGCATTAAGCAGTTCTACTGCGTCAGACCTTAATTCTGTGCCTTAAAAG
CAGCAACTGTTTTGTTTTGTTTCCTCCTCTCCACTTAGGCTGCAGGGCCCAATTCAGATGAATTAA
CTACATTGGCTTTATAAAATGCTTACTGAACTGAAAATGATGAAAGAAAAAACAAATTAGTGATG
AAAGAAAAGAAAAATAAAACATAACATTCATGAAAATGCTCACAAAAGGTTTTTTCTGCACCAA
AAGTTAATAAATGCTGTACATTGTTTATAAATAATTAAAATGGACTGATAGTGATAACGTGTTCTC
TTTTGACCCTTCCAGCAAACTTCTTAAAGGTTTTCAAATATAGTGGTGATTTCATATGGCCTGTGT
TGACTTCATTTATAGTTGTTGTTTGTAATTTATTAATTTGCTGTTTCCAGATGTGCTCTCTCCCGCC
CCTTGTTTAAGCTGTTAACCCTAGGTATGTGCTGAGGAACTGGATGGCAGAATCTGCAGTGCAG
AAAGCAGAAAGGAATGATTTCTCAGACCTAAGATGGTTGACTTCTCTCAGTTCATTTTTATTAATT
AAAAATCTATTATATTTTGAAGACGCTTGTTTGCAGACATGCTAAATTATACCATTGCCAACACC
AGCTTCCTAGATTTCTCCCTGCTTGGTCTCTCTTTGGTTTATCAGTTGAATTATTTTCTCATATTTTC
TATTTATGCTTTATAAACATTTTTAAATATTTGTTTTAAAACACACACATATACATTATTTAACCCTT
CAAGGGTCTGATTCTGCTGTTTATTGTTTGTGTTTTGGAATTGTGGATTTTAGTCTCTGTTTGAAAT
CTAGTGAAATGGAATTTGAGGGTATATGTCTCCAGAGGGCAGTAGCATTTGCTCCTTTCAGGCAT
GGATCCATGGTACTACCAACCTGGGTCCACTTTAATCCTGACAGCAATGAAGACGAATAAACTA
GAGCTACATGTATTAGCATCAATGACTCTCACAATTAAAATGAAAATTTTTAGACATGGGGTCTC
ACTCTGTCACCCAGAGTGGAGGGCAGTGGCATAATCATAGCTCACTGCAGCCTCCACCTCCCAG
GCTCAAGCGATCCTCCCCCATCAGCCTCTCAGAGACTACAGGCGAGCACCATCATACCTGGCTA
ATGTTTTTATTTATTGCAGAGACAGAGTCTCACTATGTTGCCCAGGTTGGTCTAGAACTCTCAGG
CTCAAGGGATCCTCCTGCCTTGGCCTCCCAAAGTGCTGGGATTACAGGCGAAGCTACTGTGCCC

AGCCAAATCTCACTTATAATGTTAAGCAAAACAAGCAAATGGCAAAGGAATTCACACAATGTGT
TACTACTTGAATATAGTATAAAAATAAGCAGAATCCTATCAATATCATACAATATGGCTTCAACA
GTATTGAAAGTATTTTATTGCTGAAGTTGAATGGAGGATACACAGGTGTCCATAATGTTATTCTG
TATATGTTTTCTGCCTCAAATATTTCATCATTAGCTGTTTAAAAATGCATTGCACGTTATGTATTTT
TTACAACAGGAATGGCCCTGGCTCTCATGCTGCCACTCTCCTGTCCCATGCCCGGAGGGTACGT
TATTGATAGTCATGACCCTCTTTCTGTCTCAAACCGGTCATAGCCTCAGAACCATTCTCATCACA
GCATGCCCTGACATCATCAGCCACTGATCAGAGTTGGCATGAGAGAGAAACCATTTTGCTCTTT
CTAATTTGGAGCTTGTATTAGCTTTTTATCTCAGTAGTTTAATTAGCCTGACAATTCTAGGTTTTCA
TTTCAGAAAACATAATATTTTTAAATAGAATCTTTTTATGATATGAAGGAGAAACAAAGAGAAGG
GAGATCCCTCAACATTTATCATTAGATAAATTTGTCTCCACAGGGGAAACTACACGGTGTTCAGT
AAGCTGTTTTGTTGGCCTGTGTCTCCTTTCTTCTATATCTTTTATTCCCCCCCTTCTTTCCATCCCTT
CCTCAAAATTATATATGAGTAATGAGGCAGCGCCCCTGGGGCAGGGTGGGTGGAGGTGTTTTCT
TAGGGTCCAGTAAACATTCAGATTCAAACGACTATCACAGGCCACACACCTGGCCCCAGCCAG
CTGAGAGAGACAGAGCACAGGAACAAAGCTTCCGGACAGGGCAGCACCAGGTTCTCCGTGTG
CAGGGGAACTTGGAGCAGTCCACACCAGCAGTAAGGCACAGCAGCTTCACTCAGGTGCAAGG
ATCTGAGAGTCCCACTGTGACCGTGCAGGGCAGCCCTGTCTTAGAAGCTTCAGGCTCTGCAGAA
AGCAGTGTCTCTTGTAAGAACTCCATAGGCATAATTTCCAGATCCTCACAAGAGACCTGCCTCAT
GGTAGGAGTCACCATGCTTAGGAAGGCCCAACGCATCCTATCAGGTATTTGCAGTTCTTCCAGT
CCAGAAGTTACTTGTCCTTTGTGGGCAATGCTGTATGGTGATAGAGTTGACCTACCGCCTTATCA
GGATACCAGGGAAACAGAACCAGAAAGGTAACTAAATGTCAACTTCTCATGCAGGAGAAGAAC
AACATTTTGTCAAACCTTTCTACATGCAATCCTTTACACATGGAACTGGTATTTATCTTCTTTCCTC
AAACATTTCTTGAGAATCCATTCAATGCAAGTTGCTGATTAATTAGAGGGGCTCTTAAACTAGTA
GGTAGGTGAGACCAATAGGTATACAGCCAACTTTAAAAAAAAGAAGTGTATAGAAAGTACCCA
GGGAGAAGTGCTAAGATTGGCTCCAAAGCCCTGTATTTATGATGATGCAATCTTCATATTTAAAA
TACTAGTGACACAATAATATGTTCTACTTGAACTCCCACCTTTAATTTCTTAGTATAGATAATCAC
CCACCTTTTCTATGATAGCTAAGCATTTCCTTCGTAATACCCAAGAAATAGCAAAAGCACTGAAA
ATATACATTTTGATATCAGATTGATTGTGGAAACAGGTGTGTTTTTCAGTGCCTCAGTTTATTACC
ATGGGGCCAAATTCCCAGTAAGAAGCAGCATGAGCTGGAATTCATGGCCTTCATTTGGAAAGAC
TAAATCATTGACATCCTTTTATTTCTGTAAATATTTTAGAAAATTACAATTGCCTAAATGCTGTACA
GGGATATTAAGATTAAAATATACATTTTCATTCACTTAGTTTAGTACAAAAAGTTAGAAAGTACTC
TTAAGGAACTTTCTGTGGAATAATAGAAACTCCAAGTAACAACTAGAGTTGAGGTTTTCCATAAA
GCATAAAATGTATAAACTTTTTTTTTGTTGATGTTGTTGTTGTTTTCTTTGAGAAGGAGTCTAGCTC
TGTCACCCAGGCTGGAGTGCAGTGGCACAATCCCGGCTCACTGAAACCACTGCCTCCCAGGTT
CAAGCGTTTCTCCTGCTTCAGCTTCCCGAGTAGCTGGGAGTACAGATGTGTGCCACAACGTCTG
GCAAATTTGTGTAATTTTTGTTGAGACGGGGTTTCACCATGTTGGCCAGGCTGGTCTCCAACTCC
TGACCTCCAGTGATCTGCCTGCCTCGGCCTCCCAAATTATAAACATTTTTAAACATTTTAAATTGA
CCTTGCCATTTAGAAATGGAGAAATTCATCAGGGTCCTGAGTAATTCTAATCTACATTTACCTGC
TCGCATTGTTTACTAGGTGCATCTTCTCCAGCAAGTTCTTCAGCATCCTTTCCAGAAGCATTCGG
CAGCCGAGAAAGCAGGCTACACCTTACCTACTCCTTCCTGGGCTAAAGATCTCAGACTTAGTTG
CTTATCTTAATTTGGGGAAAATAAATTAAGGAGTTCTTCTATCATTAAGCACAACATAATATAATC
TATTTAAT

ID|56195|gene_id|ENSG00000228742|transcript_id|ENST00000490162 Homo sapiens lncRNA

TATAATTAGTGTATAATGAGCAGTGAGACGACTGGAGGTCACTTTCATCGCCATCTTGGTTT

TGGTGGGTTTTGGACAGCTTCTTTACGCATGCTGTTTTATCAGCAAGGTCTTTGTGACCTGTATCT
TGTGCCGACCTCCTATCTCATCCTGTGACTAGGAATGCCTTAACCTCCTGGAATGCAGCTCAGTA
AGTCTCAGCCTTATTTTACCCACCCCCTATTCAAGATGGAATTGCTCTGGTTCAAAAGCCTCTGA
CAGGTAGAAATTCAGATGTTAGAATATTTTACCTCCTTAAAATAATTTAAAGGACCAATTACTGT
ATAGCTTATTTATTTGGATATTTTATATGATGTTTTCTAAACATATAGTATTTGTCAGTGTTTCCAGT
GACAAGGTTTTTTGTGAAGGGATTTAGACCAGTGGGGAGCCCAAGGATATTTATTTTGCTGTCGT
TCTTGAGTCACTATAAATATTTCAGTTAAGAGTAGTCATAGGAGAGCAAGCAATTGGAGCTATCT
GGATTTGAAAGTACTTCAGCTTTCTTCAGCATCCATGCTATCAGTAGCTGCCTAACCTTGCCTTCT
GATATTATGCAAGAATTGGACGCTTCTAGCTAACAAACCTTCACTCATGCTGGCTTTTCCCATCC
TTTGATCCCTTACCTTGATGCTGCTTTATGATGCCAAAACACCTCAAAGCTTGAACTTGGATAGC
ATCAAAACATAAAGACAGTACTGAATCAGCTCATGAATCCTCAAAATCAAAAGTCCTTCTCCCCT
GTCCGCATGTCCAGCATTTGTGACTTAGTGAATCATAGGGTGGGGAAGTGACTCACAGTCAGCC
CAACAGCACTCTTTTTTTCATTAGTTGTTTCTGAAGAATTGTAAAGTTCTGAATTCAAAGCGTGCT
TATAAGTTATCGAGATGGACTTTTTCTCCCCTGCAGCATGCCAGAAACTATACAATCCACCCCCT
CTGAAGGAGTTTGTTCATTTAAAAAAAAAAAAAAGGGTAAGCATGTTTTTAAAATTTAGTTGCAGC
AGACAGTACAGAAAACAGTTATTATGGCCACAGCACATGTTGCCTGGGTCTGCTGCTTTTTCTTC
TTTCCTATTTTCTCTCATCCCTTTGTTCCACTAAATCCTCCTGCTCCTACCCCCTTTTACCACCACC
CCCTCCTCTTCCTCCCTCCTCCACCCTGGATCCCTAGAGAGGGTTGACCTGGGGGCTAGGTAAT
AATCCCAGAAAGGGCTTGGGATTCCAAATTGCCAGAGATCAAGAAGGCCATCCAGGGAAAACC
TGGAAGGACACAGAGTGGTCAGTGTAAATGCATCCACGTGCTCCAAGTGGGCAAAGTAATTGG
TTAAAATGTGTAAATGTAAATGCTAAAAATCGAATCTCTATAGAGTGGCCAATGCTATAAAAGG
ATTACCTTATTCTAAACAATTTTTTCATTCTGTATTTTTCCTGCCAATCTATATATTGTTCATTATTTC
ACCTTCTCCTTGTTGTTTTCCTGAGCTTTGGGCAAACCTCATCACTTAATGCATGCAATAAATGGG
AGTAAGTTCAATTTTTCATCGAGGATAACCATGATAAAAAAAAGAAAGAAGAAATTCACTAATA
CTACAAACTGTGTTAACCTTGACATGAGAACTTTCAGAGGAGTAGTGGAGGCAAAAGCTTGATT
GGAAGGGACTGAAGGGAATGGGAGGAGAAGAATTGAAGGCAGTAAGTATAGACAACCCCAGA
AGAATCATTCAGTGAAGAGCAGCAGAGATAGTAGAGCTGGAGGGAGAGTTACAGGTGGATGTG
GGGTCAAGAAAGTTTTTAATATAATTAATGTGAGAGAAATAAGAGCATCTTTATATGGTGATAGT
AATGATGAGGCCAAGAGGGGAAAAACTGATGACATAGGGGAGGGAGTGTATTAGTCCATTCTC
ATACTGCTATGAAGAAATACCTGAGACTGGGTAATTTATAAAGAAAAAGAGGCTTAATGGACTC
ACAGTTCCACATGGCTGGGGAGGCCTCACAATTATGGCAGAAGACAAAGGAGGAACAAATGCA
CATCTTACATGGCGGCAGGCAAGAGAGCGTGTGCAGGGGAAGTGCCCTATATAAAACCGTCAG
ATCTTGTGAGACTTGTTCACTATCACAAGAACAGCACAGGAAAAACCTGTCTTCAAGATGCAAT
TACCTCCCACCAGATCCGTCCCACAACACATGGGGATTATGGTAGCTACAATTCAAGATGAGAT
TTGGGTGGGGACAGGGCCAAACCATATCAGGGAGTATGTTTGCCTTTGCCAAGCAGCATTAAG
GGTTTATGTTCATGAATTTAAGATGGGAGTGGAAGGACTGCTGGAGATGGAGCATGTTAGGGGT
TAAATTGTATGCCCCCCTCAAAAAAAAAATTTAAGTGTTGAAGTCCTAACCTCTGGTACCTCAGAA
TGTTACCTTACTTATCCTGACTAGTTAAGATGAGGGGGTGTCCTAATCCAGTGTGACTGGTGTCC
TTATAAAAATAGGAAATTTGGGTGCAGACAGGCACACAGGGAGAACACCATGTCAACACGAAG
GCAGAAGTTGGAGTGATGAGTCTACAAGCCAAGGATTGCCAGCAATTCATCAGAGGCCAGGAC
AGCAGCCTGGGGCAGCCCCCTACATGGTACCAACTCCATCAACACCTTGATCTCAGACTTCCAA
CCTCCAGAACTGTGTGAGAATTCATTTCTATTCTTTAAGCCACCTAATTTGTGGCACTTTGCTTTG
ACAGTTCTAGCACACAGGGTGATTGAGGAAGCGTGCTAGGAAGAGAGGAAGCAGGGGTCCTA
GAGTGGGCGGCATGCCTTGCAGCCGTGGAGGCCACACAGCTGTGGGAAATGACAGGGTCTGC

AATCACGGTGATGAAAGGGGAAGGACCAGATTGTGAGAGGAAAGGTCAGTAGGGAACTGAGA
GACCAGGATAATGGAGGGACAATCCATGTGTAAGTTGAACTTGCCCAGAGTTAAGACAGGAAG
AGGAATGCAATGGTGAGGCAGGAACACTGAGGAACCAGGGGGAATGACTTCAGGATGACAGA
TGTCACAAAATGAGAAGTCAGGTGACATAACCTGATGACCTGAGATTCAATGCAGGACACTTTT
AGGAAAGAGAAAGAGGTAATGATGCAGAAGTGGCAATGAGGAGTGTAAAAGAAACGCACCCC
ACCTCCAGGCCCCATGGCACAACTGGAGGGAGCAAACCACAGCCACCACGGGACAAGGAGAA
TAACGACAGAATCAGAAGGCAGTGTTCCCAGAGGACAGCCATGTTTTCATCAGAGTGAGATGG
TGCTGGAAGAGTCAGAGCCAAGGTTGACACTATTGGGTTTTGCTAATAATGGACTGTGAGTTCC
AGCAGGGCCCAAAAGGGGCTTCAGGATTGGGCAAGGGTAGATGTCCTCCTCTAGTCCATGACC
AATGCTGATCCACACTGGACTAATTACTTCCCTGATTCCAAGCTAGTAGATGGGTAGCAGTAAAT
GTTCCATCTCCAGCTGTACTCACCTTGTTGACTTTATTTTCTAACTGTAGCTAATTCTGTCCCAGG
ACCTGGCTATTACACAGGCTGAAGCAGCAGTTAGCTCCTCTTTTCTTTGGGACCCACAGAAATC
ACAGCAGCTGTGTTGTTGTGGGGTTTCTTGGCGTTTGTTTTGGTTTGGTTTGTTTTTTTTGCCAGTA
ATTTTTTTCCCAGAAAGGCATTCTTTCTCTTCACTGCAGTGGAAGTGAGAGAGGGAGAGAGGGT
GCAGAGAGGGAGAGGGCAAGTAATCAGATTCCAAGATGACCATCTCCCCAGAAGGCTTTCACT
ATTCCTCAACATATGGCATCATTTTAGCATCCTCAGCTCTGTTAGTTTGAGGGTAACCTCAGCAA
ACATGCCCCTCTCATGAGCAAGGTATGTGGTTTGACAAGCAGACTGTGACTTTTGTGATTAGAA
AAAAGCATCCTTCCTCCTGGTGAATGCAGGCCATGTCAGGGCGCTCAGCTTGACAAGGAGACA
GTAGGCTGGATTTTAGATCCTATTCATTTCTTGGGCTAAGTATTCTTACACAGTCAACAACCTGG
ACAACTGTACATAGCAGATCTAATGGGAGAAAAGTAATTGGGTGTTTTAAGGAATATTTTCATTA
AAGTATGTGGAATATGGTATCAAGTAGTGACTGTCATGTAACAATGCCTTTTACACATTCCAATG
TGTGTGAAATACAAAAAAAAAGTTTCCGTGTTAATCCCCTTATAAAATAATTGAAGAAGTCTTTTT
TATGTGCAATTGTTTCTGAAAATATGTTAAAGTTACCCAGAAAAGTGTTCACATTTTCAGACTATT
ATCTCATTCTAGGAAAAATTAGGACAACTTGACTCATGCTGGGGAGGCTAGAATCCTGGAGAGA
ATGAAATTAGAAAAACCTAGATTTTGGGTCCCCCAAGATAGCTCAAGTCTGTCAGTTCTGCAAA
ACAGAATTATGCCCCATTCCCATTCTCTCACTCCTTACCTGAGATGCAGACAGGGCTTAGAAAG
GACACGCCAAGATTTGCTGCCTTGCCACACAAGCTGGGTTTTCCCTCCCTGGGCCCTGCCAGCC
TGGAGGTCATATTTCCTTTACCTTTTGTCCATTTTTCCCTCCCTGGGGCCTGGCTTGAAGGACAAT
TCTTGTTTAGAAGAAATCAAGTCTAAGATGTTACTAATCTGGAAATGTTGATCTGGTCTGCCTCTA
ACATTTGTGGGGCCCAAAGCAAGAGTACAGACTAAGGTCCCCAAACCATGTGTCTAGTTATTTA
GAAGTTATAAATCAAAGCTAACAAACTGCTGAATAAAAGATCCTATTCTCCTATCTGATAAATAT
ACCCTCCAAACAACCCAGAAACCCAAGGTAGGAATGCGGTGGCACAGGGAGTGCTAACTCCTG
GCTCACAGCCCATGCTCCCTCTTTTCCCACAGCCAGCCCTCCATGAATGAGGAACTTCCTGGGG
CCACTAGTGCATGCTGCAGCTCACACAGTCAACTCCATCCACACCTCCACAAACAGCTGCCCTT
TGGCCACTACTCAGGCGTAAGGACACACCCAGCAGGGTATGCCCTCTGGAGGTCAGACCAGGG
AAGAAGCCCTTGCACAGTCAGGAGCCAGCTCAGCACCTGTGATGCTGAATTCTATGTGGCAACT
TGACTGGACGCAGGTGCTCGGATTAAACCTGATTTCTGGGTATGTCTGTGAGGGTATTTCCAGA
GGAGATTAGAATTTGAATCCGTGGATTGGCAAAGTTGGTGTCTGCCCCAGTGTGGGTGGTCACC
ATCTACTCTCCTGAGGGCCTGAAGAGAACAAAAGGCAAAGGAAGGAGGAATTCGCCCTTAACT
TGCTTGCCGCCTGCCTGCTTGAGCAGAGACATTTCATCACATCTTCTCTGGCCCTCGACTGAGAT
TCACACCATTGGCTTCCCTGGTTCTCAGGCATTTTGACTTGGACTGAACTACACCATTGGCTTTCC
TGGGCTCCAGCTTGCACATAGCAGATTGTGGGATTTCTCAGCCTCTATTATTACAGGCGCCAGTT
CTTCAAAATCAACCTCTCTCTCTCTGTCTCTCTCTCTCTCCCTAGATCACAGTCTGGAAGTCTGCT
CACAGTCTCAAGGTTGGCATATCCCCTAGAGCCCCCAGACTCCTCATCCCATGGAGACCAACAT

GGCCAGGGGAGGGCCAGAGCAGGGCCTTCTAAATCACAGACCCCAGGGCAGGAGCCCCTCCG
GTCTGGATCTAAGGATGGTATTTTCAACAATTCCTAAATTTTTATCCTAGGCTCTCCAAATGGGA
GTCTAAAGGTCTCAAAACATGGTCCAGAAGAAGTATGCAAAAATGTAAATTCCTAGGCCCCAAC
CCAGACCTATGAATCAGATGATCTGTATGGGGGTTCAAGGAATCTATATTATTAAACAATCTTCC
TAAATGACTTGTATTCATACTAAAGCTTGAGAATTGCTATCAAACAGCTAGAAGTTGGAGTGAAG
GGTCCTGCCTTTCCCATGAAATTCATCAACTCTATGCCCTCAAAAGCTGGGCTCTTGGATGAGCT
GCTCTCCATTCTAGAGACATTACCAGCTAGGTAGCCTGCTGGTCCTCAGGAAGTTAAGCCCAGA
CTCTTCAGCACAGAATCAGATAAACACTGATTACATTGTACTCTACAGTTCTGAGAACACCATTA
CTTAATTCCCGGCAAGTTGCAATGTAATTGAGTAGGTTGTAGTTTTTTGGGTTTGGGGTTTTTTTG
TTTTTTAAATATATCAGGCGTAGTTAGCCAGCTTCCTAGAAACACCATGATCTCTTGGTGAAAATT
CCAGAAATATGCATCACTTCCTAATTAAAGTAGGGAATTTTTAAAAATTATTAAACCACATGCTTT
CTAATTCCTATTGCCCTTTCTTTTCACCCACCATTCAGCAGTCAGCAGACACTTTCTGGGTACAAC
AGGGTAGGACCATCCAGGCAGAACAAAAGCCATTGATCATGAACTACAGAGGAGAGTTTGGTA
AATGGACACCAGAAACCATGGCCTACAGGGGCCTGGTTTTCTCCCATCTAAATACTAACTAGGC
CCAACGCTAGTTGGGCTTAGCTTCCTAGATCAGATGAAATTGGGCACATTTAGGGTTGGGGGTA
TGGCTCTCTATTGTAAGTAGCAAGAGTTTGAGAGAAGAAAAAAGGGATTTTCTTTTTTTTTCTTT
TAAATTTTAGAGCATTAACTTTAAACATCAAGATCCAAACCCCAAGGATTCAGAGAATCTTGTAC
TATAAACCATTTGTATTAGTCAGGGTTCTCCAGAGAAACAGAACCCATAGGAGGTATAGAGATA
GAGAAATATGGAATTGGCTCCCATGATTATGGAGGCTGAGAAGTCCCTCAATCTGCTGTCTGTG
AGCTGGAGACCTAGGAAAGCTTAGCTGGTGGTACAAACCAGCCCAAGTGCAAAGGCCCAAGA
ACTAAGAGCACCATTGTCAGAGAGCAGGAAAAGACAGATGTCCCAGCTCAAAAAGAGAGAGC
AAATTCACCTTCCTTCCACCATTTTATTCCGTCCTGTTCCATTCCTCAACAGGTTGGATGATGAGG
GAGATCTTTATCCATCTCAAGTTTTTTGTGCAAATATGGAATACAAATGAAAAATACTTAAAGCA
CATCATCATTGATTTCCAACTTAGACTAGTTTAACTTCCCTTTTTCTTCTATCCTGTCAGTTCCTTG
AGGGATCCTCTCTCCTTGTTCATCTCTGCATCTCTAGTAATTAGCACAGTGCCAGGCACATGGTA
ATATGGCTAAAACAGTGGATTATTGGTCTCCAACATAATTAAGCCCATCCAAAAGAAAAACCAA
CCTCATAAATCTGCTTTGCAAAGTTCATAAACAATGGATATGCTATGTCTCAAAGGTGCTGGCCA
AAAAAATTTAAGAACTTGGGAATTATGTGTTATTTTGTTTGTTTTTTTCACCAAGCCTACTTTTCTG
TCACAAGAGCCCATATCAACAACACACCCATGTATGTCCTTCTTTTTATCTACTTTTCTCCATGAA
GTTTGTCACCATCAACTGTTAAAACTGTAAAGATTTGATCAGCTAAATAAGTGGTCTTTGAACTA
ATTCCTCTCGTACTTCCTAAAGGAACTTTGAAAAACTATGTATCCCCTTGCACATTTTCAAGCAGA
CACCTAAGATTTTTCATCATAAATTTGAATAACTTCAAAATATGTACTTATTAGCATATTGTAAAT
ATTGATTTTTAAATAAAATATATCACACTTTGTATCCAATGAACTCTAAACACCATAGCAATTTGA
CACTTACTCTCCCACCCTCAGTCTTAAAAATTCACCAACAACCTCTTTTTTAACAACCGAAAAATT
TGTATTATTCCTTTTTAAAATTTTGAAGTTTTCTGGATTGAATTATTTTTATAATGATTTTTAGTATA
CAACTGACCAAAGCAACATAAATATATAGCATATTTTGATAAATAATTATAAAACATAGCAAGTT
ATGTGTTATTGGAAAATTAAACTAAATGAATGGATCTTTTTAAATTAGTTCGGGTATCTGTGGCTG
TTCCTAACTGTTAAAATGAGACAGAGTCAGCGTCAATTCTACTCTTATTTTTTATTTTTTTAGACGT
AAGCCCTGAGAAAACTTGTTCAATTAAATAAGTAATGAGAATGTTAGGCATTTTCTGATAAAAAT
GTCACTCTGTTATTTGAATGCCTTCTTGATTATAAGTCAAAAAGTACATAGTGATCTATTATCAAA
AACTATTGGCCTATCTGTTGACAACTTGGGTAGGTCCTCCTTTAATTTTATTGAAAGCAAAGAATT
GGAAACTACCTAATTTACAAAAAAAAGAAGAAGAAGAAGAAGAAGAAGAAATGTGTTACCTAA
TTATTAGAGCTCACTTTCTAAGTTTCTAGGAAGTATGACCCAAAAAAGCTTGGCCAAGACTTATC
ACATGGCTGTTGATTATACCATCACCCTTTTACTTACAGGCGTCTTGCTTATGGACCTAAAAAAG

ATGAAACTGGAGTTAGAAACAAAAACAGAAAATTGTAAATTGTGCATTTGGGGACTTTTCTCCTT
GACTGTGAACAGAGACCTATATACAATGCCAAATTTCATTTTTTAAGAATGTCTCAAAATGCAGA
GTAAAGAAATGACAGAAGGACAGACACTTGGGGATATTCATGTATATACTAATAACTTCTTCCC
AAATATGACTGAAGACAATACACTTAGCAGAACTGAAATCAATATATTAACATCTGAAAGGGAG
AAATTAATCCTAATTCAATCAAGCCAGTTTTCTAAAGTTCAGGTCATTGCATATTAGATTTACATT
TTGGAAAAATTTTTTTTGCTTTTGAACCAATAGATTTCTGCTTTTGACAGTATAGTTTAAATACTTT
ATGTAAGTTGACAGCAAGAGAAAGAACTTAGTCACCAATAAGTATAGTACAAGATTATGGAAAG
CACATTAAGGACCTCTAAGCACATTAGTTAGAGTATAATCTGCAATATATTCCTGTTAAATTACTT
AATGCTAATTCTGTGGACTGAATTTCATTCCCCCAAAATTTATATTTTGAAGGCTTAACCCTTAAT
GTGACTATATTTGGAAATAGTGCCTGTGAAGAGGTGATAAAGGTTAAATGAGGTCACAGGCTGG
GGTCCTACTTCACTAGGACTGGTGTCCTTTAAAGAAGAGGACAAGATGCCAGATGCTCTCGTTC
ATGTGCCCCCTTTCTCTCTCTCTCTCTCTCGCTGGTTCACTCTCTCTCTCTCTCCTCCTGCCCCCCA
TGTGAGGATATAAGGAGAAGGCAGCCATCTGCCAGCCAGGAAGAGAGCCCACACCAGAACTT
GACCATGCTGACACCCTAATCTTGGATTTCTAGCCTCCAGAGTTATGAGAAAATAAATTTCTGTT
ATTTAAACCACCTGGTGCATGGTATTTTGTTATGGCAACACAAGCTGACTTATATACCTAATAAC
TACATGGCAGGAATTAATACATGAACGGGAAAGTTGTTCATGAGTGACAGTGAGCAGGAGACC
TGTACCTTCATCAGAAATGAATATAGATTTCATATTTTGCTAACTTTTAGCAAAAATTAATGAAAG
TGTTTCTCCTATAAATCTGAGTAAATATTTAAGAAAATACAAAATACTAACTTACCTCTAATATGT
GTTTCTTTTGTTAGAAAATGTAGCCTTGATATGTTGGAAGAAACTGTAGTCTTTTCACCTTGAAGT
TATGCAAATTACACTCCTGAGACATAGAGTAAAATGCAGCTAAATGAGAGTATTCACAGAGCTT
GCTCTCATTACTGAGATGGTGAAATTGCAGAGAAAATGTGGTTCATAGCTCACTGGCATTTCCCA
GTACACTTCTTACAAATCTTGAATATTTCAAGATAGACAGAGACCTCCCATGAGTTTGCCACCTG
GATGAATAACTTACTGCCATCTTCAATATTTCTTTTTTCTTTTTCTTTTTTTTTTTTTTTTGAGATGGA
GTCTCGCTCTGTCACCCAGGCTGGAGTGCAGTGGCGCGGTCTCAACTCACTGCAACCTCCGTCT
CCCAGGTTCACGCCATTCTCCTGCCTCAGCCTCCTGAGCAGCTGGGACTACAGGCACTCGCCAC
CACACCCAGCTAATTTTTTGTATTTTTAGTAGAGATGGGGTTTCACCGTGTTAGCCAGGATGGTC
TCGATCTCCTGACCTCGTGATCCGCCCGTCTCGGCCTCCCAAAGTGCTGGGATTACAGGCGTGA
GCCACCGCGCCCGGCCAATATTTCTAAGAGATGTTCTCTTTGTTTTTTTCTTTGTTTTTTTTTTTTTT
TTTTTTTTTGAGATGGAGTCTTACTCTGTCACCGAGGTTGAAGTACAGTGGTGTGATCTCGGCTC
ACTGCAACCTCCACCTCCTGGGTTCAAGCGATTCTCCTGCTTCAGCCTCCTGAGTAGCTGGGATT
ATGGGGTCCCACCACTATGCCCGGCTAATTTTTGTATTTTTAGTAGAGACTGGGTTTCGCCATGT
TGGCCAGGCTGATCTCGAGCTCCTGACCTCAAGTGATCTGCCCGCCTTGGCCTGCCAAAGTGCT
GGGATTACAGGCGTGAGCCACTGTGCCCAGCCTGCTTTATTCTCAAGTCTCTTTCCTCAGGAACA
ATGCCTTCTTTGCCAGTAGTGAAGACAGGAGGAAAATGCCATCACTTCTCCTGCCTAATTCCATG
ATACTCCTGAAGTCCAAACACCCCGACATGGGTGAGCATACCTGCTTAAGGTATGCTTTGCCCC
TAAAAGAGATAAGCATAAGACAGAGAAAGATAATATGCCCTGCACCTTTGGTTTATGCTGGTTT
GATTAAAGGAAAATACCCATAGTATTTCCTTCAACAGAGTATTTCCTTTGAGAGTCTCTCTCTCTC
TCAAATACCCATAGTTTGACTTTCCTCAGCTTGGTGTCCCAGAGGTTTGTATACCCAGGGCAATT
AAGGTACCACAGCATAATTTGAGGTGGATGAAAAATATGTCACTTTTTTGTAGGGCAGAAGAAA
AGGAGCTATAAAAGAAAAGGTGGATAAGGAGAAGCAGCTTGATTCAGGGGCACTTTTGAGCAA
ATATAGAAATATATATATATAGAGAGAGAGAGAGAGAGAGAGGGAGAGAGAAAGAATTTGGA
AATTGATTCTGAGTCTGCATAGCTCTTGGGATGTCCTCATGTACCCCTAAAGCTATGCAAATCTC
AATTTGAATTAAATATTTCTACCATTGCTAATAATTATCATTGCAATGTACTGGGAACCTATTTTA
AACCAGGTAGTATATTAAGTTTCTCATATATATATATATATATATATATATATATATATATATACAC

ACATATATATATACACATGTATATATACATATATATATATGGAGAGACAACATGTGCTCATTTAAA
TTGTACTTCTCATATGATAGTCATTATTAACCCCATTTTAAAGGTGAATAAATTGGGACTCAGAGA
AAAGTAATTTGCCCAGGATCCCACGGCTAGTAAATGCCACAGCCAAATTCAACCCTAGCTCCAT
TTGATTGGAGAGCCTGTGCTCTGCAGTTCACCCTACGTGGCCTTGTTTCTGTGTTCTCAACTGGC
AGCAGCTGAATATCTCTGCCTTCTAGAGAACCAGTGCTTAAAAAGTAATAAGTCTCTAATGGTG
GATTTCAGGATTATGTGGCAGACTTTTGAAAGGTATTTTCATCAAGACACTCTTCCTAGTGCCTC
CTGAAATCATGAAAATACATTTTTTCTCTCTTTTTCTGATACACACATCACTCTGTTTCTTAATGAG
GTTACAGAGGTGGGGAGAATCAAGTATGAATAGCAAATAAGCATTCAAATTAAGAAAACTTGA
AATTATTCCAGGGTCTCTATTTAGTTCACTGTTAGAAATCCTAACTGTGTTTTCATACTTAATTTAA
AGACGTTGTGGTCACTAGTTCTTTTTTTTCCTTAAAATATCCCCCAAAGGATTCTTTAATTCTCAAT
TTTTGACCTAAGCCCATGAACCCAATCCCTTTAGTGATTAACCACACAGTTAAAAATTAAAGGTA
ATGATGATCATAAATGACCATTAATTCTTTAATTCTCAATTTTTGACCTAAGTCCATGAACCCAAT
CCCTTTAGTGAATAACCACATAGTTAAAAATTAAAGGGAATGATGACCATAATAGCAAACCATA
AAGAATATCCTACTATTCTCTGCAAAAATATAGACAAATTATGGTAATTAAAAAATAGCCCCCTT
TCAACCTATTTCACACATATGTACACACACAATTTTCAGTGGAACTGTGAGTAAAGTGAATCTAC
ACTTATATTTCATTATGGAGCTTAAACTAATATTTATCTATTCTTTAAGACAAATTAAAGCTTGTAT
TTTTGGGTGGGTTCAGCTAAATAGTAGTTTGGACAGTAGTCCTTGGTCTGGGGCTCATGTATTTTT
CAGACGTAAAGACACCTAAGAAGTTACAAGAGCACAGGGTGAATAAACAACATCAGTTTCCTA
GAGTGCAGATTTAGGAGAGGAGAGAAAGCTTAATGATCTAGACTAGAAAAGCAGCTCCCAAGA
CAAAAGAGCACCTCTGTTGGAGCCAGCCACACTGCATTTTGAGACGCAGCTCAAAAAGGTGGA
GAGGGGAACGGGAAAATCTAAGGAATTTTGTAAGGTGAGGGAGTCGGACAGAGAAATTAATTA
TGCTAGTTTTTAAGGGTATATTTGGTCCTCAATGAAATACTAATGTCATATGTTTATGTTTTGCTTA
AAGAACTTTTACATAAATGTATTATTCATAACGGCCTGTGGGGTTTGTGGGGAAGGCACTATTTT
TGTTAAGTGGCAGAAAAGAAAATGCAGGCACAGAGAGGCTGGGCGAATTGTCCATTGTCCCAT
AGCTGGTTCCTTGGCAGAGTAATGATCAGACAGACCCCAATCCAAGGGAGTCCCACCTCTTGCT
AGCAGTCAGAGGCACTCTTTTTTAATTTGCCAAATTGTTCTAAAACTAAATATACAAGTACCATAT
AATCCAATAATTGCACTCTCGAGTACTTATCTCAAAGCTTTGAAAACTTACGTTCACCAAAGACC
TGTATATGAATGTTCATGATGTCTTTATTCGTAATAAACAAAAGCAACCGAAATATTCTTCGGCA
GGTGAATGGTTAAACACGCTGTAGTCTATCCATACACCATACACTGGAATACTTTTCAGCAGTAA
AAAGGAACAAACTATTGATAAATGCAGCAACTTAGATAGCTCTTAAGGGAATTATGCTGAGTGA
AAAGAGCCAAACTGTTTTTATTTTATTTTTTTTTTCTGGAGAAAGGGTCTTGCTCTGGTGCCCAGG
CTGGAGTGCAGTGACGTGATCAGCCTCAAACTCCTGGGCTCAGGTGATCCTCCCACCTCAGCCT
CCCAAAGTGCTAGGATTATAGGAATAAGCCACCACACCTAGCCTAAAAGAGCCAATATTAAAA
AGTTATATACTATATGATTTCATTTATATAACATTCTTGAAATGAAAAATTCTAAAGATGGAGAAC
AGATTACTGATTGTTCATCAGGGTAGGAGTGGATGTAGGATAGCGACACAGCCTTAGTGATGGA
ACAGTTCTATATTTTGATTATAGTGGTGGTTACATGAACCTATACATTACAAAAGCTGCACAGAA
CTATACATACACACACATGCAAGAACACATCAAACAGGTGAAATGTGAATAAGCTCTGTGGATT
GGCCTAATGCCAATTTCCTGGTTTTGGTGTTGTGCTATAGTTAGGCAGGATGTTATCACTGGGGG
AAACAGAGTGAACATATATATATATATATAAATATATATATATATACACACACACACACACACACAC
ACATATATACATATATATATATACACATATATATATGTGTGTATATATAGATATACATTTTTTTTTTGA
GACAGAGTCTTGCTCTTTTGCCCAGGCTGTAGTGCAGTGGCACGATCTCGGTTCACCGCGAACT
CTGTCTCCCAGGTTCAAGCAATTCTCCTGCCTCAGCCTCCCAAGTAGCTGGGATTACAGGCACC
CAGCTAATTTTTGTATTTTTAGTAGAGGCAGGGTTTCACCATGTTAGCCAGGCTGTTCTAGAACT
CCTGACCTCGTGACCTATACATTTTTTATAACTTCCTATGGGTCTATAATTTTTTTCAAAATAAATC

ATGAAAAAACCATTTTCTAAGTAATCATAAACCACATTTAATAGTCCATTCTTGAAATTTGTCTGG
GATTAACACAAAGCTAAGGCCAGTTTTCCTGTACCTCCCCATTTCTTCTTACCTGTTGGTTTGTGA
TACAATTTGGATCTGTATCCCCACCAAATCTGATGTTGCATTGTAATTGCCCATGTTGGAGGTGG
GACCTGGTGGCAGGAGACTGGATCATCAGGGTGGATCCCTCATGGCTTAGTGCTGCCCCTGCA
ATAGTGAGTAGTAGTTTAAAAGTGCGTAGCACCCAGGTCGGGCACAGTGGCTCGCACAAGCAA
TCCCAGCACTTTGGGAGGCTGAGGCAGTTGGATCATTTGAGCCCAGGAGTTTGAGACCAGCCTG
GGCAACACGGCAAACCCCAGCTATATAAAAAATTAGCCTGCCATGGTGGTGCATGCCTGTAGTC
CCAGCTACCTGGCAAGCTAAGGTTGGGGGATCACCTGAGCCCAGGGCGGGGAGGAGGAGGGG
GTTGTCAAGGCTGCAGTGAGCCATGATCAAGCCACTGCACTGCAGTCTGGGCAACAGTGAGAC
CCCATCTCAAAAATAATAAATAAATAACTGAAAATGTTGTTAAGTATGTAGCACCTCTCTTCTTCC
TGCTTTGCCATGTGACATGCCTCCTCCCACTTTGCCTTTGGCCATGAGTAAAAGCTCCCTGAGGC
TTCCACAGAAGCCAAGCAGATACTGGCGTCATGCTTCCTGTACAGCCTGCAGAACTCGGAGCC
AGTTAAATCGCTTTTCTTTATAAATTACCCAGTGTCGTGTATTCCCTTATAGCGAGACCTAATACA
GTTTGTCTTATTGTCTTTCATTCTTTATTTGTTAATTCATTGCACACTTATTAAATGTTCATCATACA
CGCACTGCTGCTCCTGCAGGAGATCACTGGCCTTTATCCTATCCCTTGGCTCCATCTTTCTTCCCA
CCTTTCTGTGGAAACTGACAGGAGCAACTAATTCAGCCCTGAGGGTCATGAATATCCACAAAGG
GAGCATTTAAGTTGATTTTACCAGGATTTTCTTCTCCCTTTCCCAGAAAATTTGAGTGGAAAATCC
CTCTTGATCAAGTCAGCAAATCTCCTGCCAAACAAATTCTTCCCAGTCTTTGAAAGATCCAATCC
CCCTATTCTGGTAGGTTTGGCTATTATCCAGGAAACCTAATCTCACTCCTCTAAACCAGCAACAG
AAGCCAAGTCTTCCTTTCCTGCATCCTTTTCTTCCAAAGCCAACTGGAAAGAAAAAATAAACCAA
AAATTCTATCTAGACCCTAAAATTCTCTATTTTCTGTTCAAAACTCCAACAATGGAGATACAATTG
AAACTTCTTAACATCACAGAATTTGTTTTTAGCAGAAATCGATATAAGAGGCCAGCCTCCTCCTC
CTCCTAGAGATGAGGGAGAAATAGAGACTCAGAAGGGTATGCAATGTGCCAAGAGCCGGGCA
GGACAATGCACTTGTCAGGGCCTGAGTCTTAAGAAGTCAACATTTACCAAGTCCCCACAGCCTG
TCTTGTCATCAGGAACTGAAGGAAGCAAATGAGAGGGGAGCCCACCAACTCTCTGTGGTCCAG
GTGTAATCAGGAGGCCCCTCCCCATGTTCTCCTCCAGGACACCTTTGGCAGGAATGAAGAGGG
GACTCTGTATGCTCCTGACTGGAGCCCAACCTACCAATCCCCAGCCTTCACACTCCACACCACT
ATCCTCCTTGAGACCACCAAGATCAACCTCAGCATGCTCCCTAACCTTGTTTATGTCAACACAAG
AAGCCCAGGACTCCCAGGTCAGCCCTCAGACCCCACACGGCTCTATGAGAGCAAACAACCACC
TCCTCAAACACACACACACGATCCTAATTACCCCTCCTGCACTGCATTTTGTTTTTTTTTTTTTTT
ACTTAGCATTTATGATTATTTGATAATTCATAAGCTCTATTTACTTGTTTTTCCATCCTCTCCCAGA
ATAAAAGGTCTTTGAGAGCAGGAATTTTAGTTTGTTTTGTTCACTCTTCTATCTTCAGTGTTAGCA
CTTTGTAAGAGCTTAATAAACATTTGTTAAATCAATAAATCTCTAAGACCGTTTTTTGAAGTCTGA
AAATGCCAGGATGAAAGGATGTCGGAGAAGTTTAAGAGAAGAGCCATCATTTGTGGCAGCCAG
AACACTTGAAAAAGGGAAGGCTTGAATTCTAAAGATTTTCAGAACCGGCATGGATCAAGTGAAA
GAACAATTAGCAGTGGGGTGTTCAGAGCAGACACAGGGCCAGACATAAGCAAAATAGCGTTCC
TTCTGAAACAGGGAATAGAAATAACTTCACACCTACTATGTGCCAAGCACCGTGTCAACAAGCA
CTATTCAAGATGGAATTGTTATCCCCATATACAGATGAAGAAACTGAGGCAAAGAGGAGATAAG
TAACTGCCCAAGATCCACAGTTAGTAAGGAGGAGAGCCACTTTTCAAACCTGAAGTTTACCCAG
ATCCAAAATCCACCTCCCTTCCTCTCACTGAGTCTGCCTGGCCCATCCATCCCAGGCATCAGGC
CCCTTCCTCTGTGACCCCGACTGAATTCTTCAGTGCCTCCTTCTCCAAGTTCCAGATGACCCCGA
GGATCAATTAGTTCCCATGCAGTGCTAACAAATGCCACAGGCTCAGACCCTGGGTCCTGTCTTC
CTCTCTAAATCAACACTTGCAGGAAAAAAGTATGGACCCAACTAGAAAGTACTTTTAATGATTAA
GTTATTAAATAAACCCCAAAAAAGTACAGTGAAAGGGATTGGTAAGGGGGATAATTAGAATATA

GCCTACTGGTATATCTGGTAAAGTTTTTATGATAATAAATTAAAATATCATAAAGTTTTTATAATA
AATAGGATATTGATTAGTCTTTTCTAAACCTCAGCATACTTTTTTTTTGGTTTTGAAACAGAGTCTC
TGTCACCCAGGCTGGAATGTAATAGCACGATCTTGGCTCACTGCAACCTCCACCTCCTGGGCTC
AAGTGATCCTCCTGCCTCAGCCTCGGCATACTTTCTAACCTTGTTTATGTCACCACAAGAAACCC
AAGGCTCCTGGGTCAGCCCTTATCCCCCACACGGCTCTATAAGCAGTAAGCAACCACCTCCCCA
AACACACACACGCAATCCTTATCTTCCTTCCTGCCCTTTTGTAAAACTTAGCATTTATGATCATTT
GACAAATCATATACTTTATTTACTTGTTTGTCCATCCTTTCCTGGAATAAAAGGACTATTAGAGCT
GGAATTTGTTTGGACAGAAAAAAAAAAGTGTTTAAATAAAAGCAGAGAGAGAGAGACATGGTTT
ATAACAAAGTCTCATTAGGATGATGAAACACTTAAATAAATTTTTACTGGGGTCGTGATCCCTGG
AAAAACTTTCAAAATAAGAACTCTGACATTCACACTGAATGACTCTTCCTTCCATCACCGAGACA
CTCTGATCCAGATCAGATGGCCTCACAGTTCCGGCCAACTGGGTGAATCCAGGGTTAAAAATGG
CCACCTACTTTTCATGGGGCCCACTCGACAGCTCAGTTGTTTGGAGGTATAGTGCTTGCAGAAC
CAAGGTCACAGGTAATGAGCGGGATTATTGTTCATCATTAAGCATTAAGTGTTATCTATGAAATG
ATGTTGTTTAATGGTGCTATAGGCTTTTAGCAATAGAGTCATCAAGAAATATAAAATCCAGACTT
TAGGTTTAGTGGGAGAAGAGGGAAAGGAAAGCTTAAAATTAGGATAGTTGTAAGAAAGATGCT
TATTAAAGAAAGATGAATGGAGTTTCTCTGCTTCTAGTCCTAAAAGACACTAGTGTTACAGGTAC
CTGTTATTTTTCCACCTGCCCAGCAGGTGTCAGTGTAAGACAATGTTCCTAGAACTGACCAAGCT
TTTTTAAAAATTGAGAGTTATCAAATTATAAAATACAGAAGATACAATAAAGATGGGTTTGCAAA
AATTTTCACATGAGCCTTTAAATGAGGGAAGGAGATGACCAAAAATAAGTTTCCAGAAAGGTAA
GATGATCAGAGTTGGACTGGGGGGCCTCAGAAGGCCCCATCTGGCTCTCAGATTTCTTGTCAAT
TCAGCGGGAATTCCTGGAGCATCTCGGGGCTGCAGGAGGACTCAGCACTGTTGGAAGAGTTCG
TTGCTGGTAACTAGCACAGTAACAAATGAACACTCCCAGGGAAAATGGTAGTATTCCACCAGGC
ATACTCTCTTAAAAAACAGACACCGTCTCTGCTTCTCAGAGGCTTACAATAAGAACATAGAAATC
CTGTAATTCATGAAAATATATTATTATAAGAACTAAAAAAGTGAATCAAGGAAAAAGAGATGGT
CATCAATGGTTGCATGAATAATTTGCAGAATGCACTGAGTTAGTCAGGGTGAGCTTCCCGGAGG
AGCTGGCCATTGAGCTGTGACGAAAGAGAACAGAGCCACAAATCCAGGGTTCCACCCTAGCTC
CTGCCTTTCTGTTCTGCATTCAGTTTTTCTCCTCCAAATACAAATGTCCTATACACTGATGAAGGG
CAAGCAATACAGAAAAAGAAAATAAACACAACTTATTTTTTTAATTCACTATATAAAGGTTCCGT
GGGTATTTTAAAGGGAAAATGGTGGGGGGCGGGGGCTCTTATCTATTGCGCATGTAACAAGAC
ATACATTATGTCATACAAGTCTAACCAAAATCCTGAGATGTAAGTAGTATTGTTCTCATTTTTGCA
TAAAGGATCCAAGGCTCCCAGAAGCCATAAACTTGCCCAAGATTATGATATCTAATATTTATGA
GGGATTACTCCGCATACACAATAATCCTATGAAGTTACTATTATTCCCATTTATTGGATGAAGAC
ATTAAGCCTTACAAAAAATAAGTGATTGTCTCCCCAAAGCCACAGGCCTCAGGAGCCATTAAGA
TGAAATTCAAACTCATGTTTAGCAAGGTGGGTTCTCTGGGAACTCAGGTTGTAGGTGTTTGCTAG
GAAGTGCCCTTAGGATTAGTACCTATGGAGAGAAGGGGAGGGTGGAAGGATTGGCCAGCACTG
GGCCAAAGAGCCAGGCTTTTCTACACCTGTCTCCATCAGTCACTGGATATGGGCCTCACAGGAG
GGAATGGCCTTGGGGAAGGCAGCTCTCTGCAACTGAGGTGGACCTGAAGGGTTGGTACCTGAC
AACTGTCTGCTGACAGCACTCCCAGAGCTAGAACAAGTCCTTCCTAGGAGGGGCATCTGGCATC
ACCACGTTCATTATGCCATGCCTGTGTCTTACTTTGTTCCAGTTAATCATGATCTCTGCTACACCA
TGCTGGGAGAAGGCCAGAAGGGAGGCAGTGGTAGTCTTTGATAGAGTTGGAGGGTGGGGCAG
GGCTGGGGGTTGTGGAGTGGTGAGCCCAGACCCCAGACTTCCTGGGGAAATGAATACCAGGCT
CTCATCCAAAGAGACATTTCATCAAATAACATCAAGTGGAAATAAACAAAGGGGTTGAGGCTGA
TTGCACTGGCTCCAAGTACTGTCAGCCATTAGATGCTTGTTGTTCAGTTGATTGACAGCTTCCAG
GTGGCCAAGTAAGGAAGGATGCAACAGTTGGCCCAGACCTCGTGCTGCTTAGTAACATCTGATC

TCCTCCTGCTGTGTGCCGCTTTCTCACCCGGAGGCACCTGAGCTGCTCCATATGCCTGGTGTGCT
AAGCGCTGGCCTTTCTGTGCTGTGCGGTCCTGCTCTAAGGTGAGGGTAGCCTTGAGAGGGCTGT
TTGCCATTTGGAAATAGAGTCAGCTTGTGGCTCTCTCAAGAAGTGAGAACAGCAAAATTCAAGC
CTGAGTGGTAACCAGTTTCTCATACTTCTGCTTGAATTCAGAGAGCAGCTGACTTGACAGAAGG
CAAAAAGCCGGCCAGCTTAGCTCACTCAGTGACAAGTGAGCACAGAGCAAAACTAACTTTCCA
TGGTTCAATTCCTCTCCAGTGTCACCCTGCTTGCACATGGAAGGGAGGCCTGAGAGGCTGCAGA
ATGGCCCTGGAGAAGGCAAGAGACATCATCTGGTTGGCCTGGAGTGCACGTCTGTGAGAGACT
TTGGGGCAGTTCTCTCTTCTCTATGCTAGAGCCATTGATGCCCTGTGCACTTCATTGCCTTTGACC
ATCATTCCTTGCTCCTGGAATGCCACCTCCTCTTTGCCACAGTTAATAAAACCAGCTGAAGCCTC

ID|56209|gene_id|ENSG00000253476|transcript_id|ENST00000523840 Homo sapiens lncRNA

CTATAAAGGTGTCGCCAAAGGAGATTAACATTTGAGTCAGTGGGCTGGGGAAGGCAGACC
CACCCTTAATCTGGGTGGGCACCATCTAATCAGCTGCCAGCAAATATAAAGCAGGCAGAAAAA
CGTGAAAAGTCTAGATGGGCCTAACCTCCCAGCCTACATCCTTCTCCTGTGCTGGATGCTTCCTG
CCCTCGAACATTGGACTCAAAGTTCTTCAGCTTTGGGACTCAGACTGGCTTTCCTTGCTCCTCAG
CTTGCGGACGGCCTACTGTGGGACCTTGGGATCGTGTGAGTTAATACTTAATAAACTCTCCTATG
TGCGTGTGTTTGTGTGTGTGTGTGTGTGTGTATGTATTCTGTCCCTCTAGAGAATGCTGAGTAATA
CAATATTGCTGTGTTCATCTTAATAATTTCAAAACATTTTTCAAAAATTTAATCCCAACTGTTAGA
AATAACCTACAAGAAATCCAGTGAGGCCTACCTACCCAGAGATAAGTGATTCACCTGACAATAT
GCATCACAAGTCTTAACTCTCATTTCATTAGTCCTATCCAGAGACCACATTTTCTACTTAAGCAAT
ACTTAGAGGCACATCTCTCAGCAAACACAAACCAATTGGACAATTTAAACTAGCACTATTCAAC
CATAATGAACTCTGGTTGAAAAACAGTCTGTCCCTACTTGCATATTGTAGTGAGATGAGATTCCA
CTCTTCATGTCAGAATGGCACTTACATGAGTCAAAGTGTCTAAATGAAATAATTTAAATGGAACA
AAGTCAAATACTTACATTTTATTTTTAAATTCTCCTCTATTTGCAGTTATACTTTAAAGTGTACTCC
CTTCTCTATCCTATCATGAGGACTGGACAGCCTCCACAGAGATGGCTGGACAAAGCAAGGGAA
TTTACTTAGGTAAGACACCATTTCTCTTTACTCTGAAATCCCTGCAAATGTAGTTATTCCAGAGGA
GACTGTGCAATGACTGATGGGTTTAGCATCCTACATGAGGGGAAATTGCCATTCCCAGCTGTAA
AATGAACAAATATCCAGTTATTTTTT

ID|56220|gene_id|ENSG00000241732|transcript_id|ENST00000460407 Homo sapiens lncRNA

TGATGATTGAAGTATGTTTATTGTAAGGGCAGAAATGTGTTGGCATTTGGATAAAAAACTG
CTAACATTATAGAACTTATTACCTAACAAAATTTCACACCACAAAAAATATTTTAATGGCAAATTC
AAGGTGTTTTATTGCTTACAAATCAGCATCTTTGACTCTTTGAACATCAATTTGTGTTTACATTGAA
ATGACAAAAAGACAAACTAAGAAGAAATACAGCATGCAAGTTGGAATTCAGAGTTAAAACCAT
GATGTTGCCGCTCAGCCAGCTATGTGACTGTTGACCCTTTCAAGAACACACATGGATTTAAAAGT
TGGATGACATCCATTGTTGGGGCCTTGGGGGATATGGTAAAGCATGAAAACTAAACAGCCAGG
AGCCTGTGAAATCTGCTACTGTATTTTCCAGGACTTCATTCCACTCCTTGGCTAAAAAAATCTTG
GAAGTTTCACAGATTATGATGTGGACCTGTCACCTGTAAATTGTCTCAATCTACTCAGACAAGAC
ACTAAACTGTCTTTGGATACTATAGATGTCAGTGCTTATAGCAGCTGGAATTTGGCTAGTGACAA
TGTTTAAAGATGTAATACTAGTTAGTATCTATTGAAGCTTAAACTTTGCTGGTCAGGTTGTAGCTA
TTGTAAAAGTATTTATTGAAGAAGCTCACAGTCCTTCAGTTGTACAGACTGAAAAACTTTCATGA
AAGATCCAACATACTAATGTAAATTATATTTATTACAATGTATGATATTAATGTGTCAAACTGGTG
TATTTTACAAAATATATAATGCATACATAAATAAGAGTTGTATATTACAGTGCTTTTCAAATATCA
GTGTCTTGGAATATTTAAGTCTTCACATTTTTTGGTCTAAAATATGAAAATGTTTCATGATACAAG

TGATTAATTTTCCCTAGTAGTGCTTTTGCATGTTTGCCTTTTTATTTAAGTTTTTTTCTATATAGACA
CAATTTGGTGTCAGACTATCATAAGATCGATAGTGAATATAAAATATCTTAGCCAAATGGGGTCT
GTATTGTCTACATTTTATATATTAAATAAAAGTTTTTGTTGTCTTTTCAGGAGGTTTAGAGTATTGT
CACTAAATATGATCAAAGCTTCCCTTTCCAAATGCAAAAGTCTTGTCCTACATTTAAAGTTGATCT
GTCATGTTTTAGCAGTCAAGTGGGATGGGCATTATATAAACAACGTTACAATGTAAGGAAAATC
TTTAAGGAGATGGGGAGAGAAAAAGGCAGCTGGTATAATCGGTTACTGCTGCTTAGTTCTACTT
AATTTTTTGTGTTGCTTCTTCTTAAGGTGAGATAGCATAATCTTAACTGTTTTGAGATGGAATTTTA
AAGTAACACACTACCAGCGAGTTCAACACTGCTATTGATTTTAATCTGTTTTTTTTTGTTTAGTTGA
TAACTTAAATTCCAAGTTTCATAGTGATAATTGTATATTATTTGGCTGCTGAATTCTGTTAGAGTTT
TTTATTCTGTTGTACATTGTATTATACACATAATCACAAATTAATATGAAGGTGAATATATGTTAC
ATATCAAAATTTGTGAATTTGAATTATAGTATGTTTTAGTGCTATTGCAAAAAATGTTTATTTTTAT
ATTATCTGTGATTTTAATATAGATGATTGAACTAGATTTCTTTTTGAGTGATAGTGCCATTGAATG
AGCAGTATGGAAACAGTGTTACTTGATATTTTGAGCTTTCTCAGGTTTATCTAAATCAGTGGTAG
CTTAACAAAACCCAGACTAATTGTGTGTAATTGTATTTTTAATAAAAGGAAAGTACATTTCCTATA
ATAGCATAGTACTGTTTGCATGTAAGAGTATGCAAAACCTTGTGTGTGTGTGTGTGTGTGTGTGT
GTGTGTGTGTCTTAGTGTGTGTAAGGCATGGCAGCCAACTTTGTATCTGCTATTTTTAGTACGAG
CAGAGCTTCATAATTGTGGTCACTAGAACTGTACTTACCATGGACAGTTAAAACTGAAAAAGAC
TCAATAAAACTATGAAACATGG

ID|56242|gene_id|ENSG00000254298|transcript_id|ENST00000523781 Homo sapiens lncRNA

CCAGCAGTTTCCTCTGCCTGGAACACTTTCCGCAGGTATCTGTCCAATTCTTCTAACTTCCT
CATCTTCTCCTTACCAATCCCCTGGGGACAATAATTCTCAGTTTCCTCTGTATTCTCAGTTTATCA
ATTTTTCCTGTTCTAGCGCTGGATTACAACTGCTGGTCTCCCTGCCTGCCTTCCCTGCTTCCCAGA
GCACGAAGAAGCTTGCCTCCGCAACCCACTATTTGTGTGACCTTGGTCCAGGAACTGGACTGGG
CTCTCCTCCTGAAAGTGGGTAAATAATGTCACCGACCTAATGAGGCTGTTGAGGCATGGAATCT
GATCAGGCAGATAAAGTGCCCCTGGCAAACCTCAA

ID|56244|gene_id|ENSG00000251637|transcript_id|ENST00000529069 Homo sapiens lncRNA

GATTATGGGTTTTCTAATTACATAAGTCAGAAGTAGAAAAAGGGACACAAACTAAGAAGG
GTGCTGAGCGCTCGTCACCTGGAGGGACTTGTGCCTCTCTCAGTGGTAGTAGAGGGGCTACTTC
TTCCTGCCACGGTCATGATTGAGAGGCAACGGGTGGCGAGTCTACAGGGGACGTCGTCGAGCA
GACATGGGATAACTTTAAGGGAGAAGGTTGGTTGTAAGGCGGTGGGACTGGGTGAGGGAGACT
CTCCTCTTCTTCAGAGGGAGGCAGTACAGGGGGAGCTGAACTGGCTGAGGGTCGAGGCGAAAA
CTCGGTCTGGCTTAGGAGGACCTTGGAGGTAGAATTATGAATGGCTCATGAACGGAGCCATGG
AGAGTGGATCCTGACTAAACTTAGCTATTGATCAATGTAGAGAAACTGATCAGGGTGGCTAGGA
GTTTCAGTAACAACCTGCCACACAGCTTGAACAGTTGTGAGGTTCAATGACCCTTCAGGGGGCC
ACTTGACTTTAAACTTTGGCCATTTTATTTTGCAGAGTGTCTGGAGCTTACCTTTTTTAAGGCGGA
CTTTATAATCCTCTGAACTGAGAGAAAAATTCTGCAGCATACATTGGAGAGGGCTTTAACTTTAC
AAGGCTGGGAGGAAGTGTTTCTTATTTTTTTTTTTAAGGCAATTTAATAAGATTTGAGCATAGATA
TTAAACTTAGCATGGACAGAGAAACTTATTTCTTGGGGGACTGGCATAGTGAAAGAACAGAATC
AATATGACTAGAAAGAGCAGAAAAACTTAAAACAGCTAATACTACTTGCTACATTGCTGTAGCT
TTAAGATTGAGGGAGGAGGACTAGAGCCAGCCTGAGATCTTCTGGGTCAGTTTGATCTAGGCGT
TCTTCTTCTTCTTCTAGATCTGCGCTTTAAATACTTTTGGTGTCTTTATGACTTAAAGGCAAATAGC
TTAAACTTAGCTTTTTCTTTTAAGGGTTTAAGGAGTGAGAACAGAGCCAAGTCCTGGAGACGGT

GAACTTGCTGTCGCACCAGAAAACGAGATGTGCAGGATAGGGGGCAGGGACAAGGCGGAAAA
GGACTACTCGGATCATTTTTAAGATGGGAGAGTAGCCACAGAGGAGCAGAGTAAGAATCTAAA
CGAGGTAAAGCAGTACGGGCGTACATTTCTTTACACGGTGTTCTACTGAAGGGCACAGGAAAA
GTTACAGAATGACAAGAGAGGTGAGCAAGGAAATCTACAGGGTGGCTGTTTTGAATTCACTACT
GGTTTAGTTTAGAGGACGTCTAATCACTTGTACGTGGAATATGATGATCTAAATACTTACAACTTT
CATGGTGCTAGAAATCTTAATTAGGGAAATGTTTTTCACACTTGTTCTTGTAACAACACTTGATTT
GCTTCTGGCAGAAAAGACAGGACTGTGGTGACCAGCCTAAATGATTGATGAGAAATTTAACCTC
CTGTGACAAAAAATCAGCACTAAGGACTTTGAAGAAGTTTTTACTTAGACGTCTTGGGCAATATC
AACGTCTTAACATGCAAAACTTTGACAACTACTAACAAGACAATAGACACTGAACAGAACAATC
AACATAAAACAAACAATTGACTTTAGGGCATGTAAACAGTTATGACAGTTTCTTCCTTTTTTTTTT
TTTTTTCAGACAGACAAGGGGAGGGGCTCCTGTGATGGGATCAGTCAGATGCCTGCCTGGCCG
CTCCCCCTGAGGGGACTTGGGCTCCTCTTAACATTGGCAGGCTGGTATAAACTTCCGGCTCAGA
TCAAGCTATGCCTGATGCTGCCTTAAGCCTTATGAGGTCGCCACGGAACCGCAGGTGAGGGTCT
ACTTGAACTCCGTAGCTTTAGCCGTGGAGCTACAAACTGGAGGACAAGCGCAAGCCCTTGTCCT
CACTCATTCATTATTCACACAGAGTATATAACAGTTTTTTTTTTTTTCTTTCTTGGAGATTCTTCAA
GAAACTTGAACAAGAGAAAGATGAGAGATAGAAAGAGAGAGAGAGTGACCGGTCTGCCAGAA
ACCAGGACTCAGTCCTCCAGCATCCTGGGATGTGGACTGAGTCAAGGGAGGGCCGCTGTCAGA
GCCACTTCCCTCCTAGAAAGAGACACAGAGGTGCCTAACAGAAAACCAGGGCTCTACCTTCTA
GCGTCCTAGAGAAACAGGCAGAGTCAAAAGAGGGACACCCTCATCAGGGCCGCTTCCCTCTTA
CTAGAACTGAAGTCAAATCTGACCTACCTGACCTCAGGGTCAGAAGTCGAGGACTCAGATGTG
GAATTTTTATGGACACCCACACGGTAGTCGATCCGCTCTCCTCTGAAAGACGGTCACCTTTCGG
GGACCTAAAAATTTTTTTTCAGGTGGCACCCCCCCTACAAGCCGGCCGGCCTTCTGGGGGAGCC
CAGAGCGAGCCCGGCTCTTGCCTGGTGGCGTTTCTTGCTGGGGCCTCCGAATGTTGTACTTGAG
CGAGTTAGAGAAAATGTCACACTTTGACATGAATTAAGAGTCTGTTTATTTAGCTGACGGCCAAG
AAATGGCTAACTCTTAAAGTTCTCTTGGCCCCGAAGAAAGGGCTAGATTTTCTTTTATACTTCAGT
TTAGACAGGGGAAACGGGTCTAGTTAAAAGAATTTTACAGAAGTAAAGTAGGCAAAAAAGTTA
AAAGGATAAATTGTTACAGGAAAGTAAACAGTTCTAGGTGTAAGGGCTTTAAGACTATTACAAA
GTGATAGACGTGGGGCTTTAGGCATTATCAATCGGACAAATTCCTAGGAACTGCGGATATTGCT
CGCCACAGTATCTTATGAGTTAATTGCATTCTTAGATGTGCTAAGAGTCAGCTTGCACAAGTTAA
GTCCTTGAGGAAGGGGCTGTCAGTGAAAAGGCCAAGATAAAATCTATCTAGCTCTCTTAGCTAA
AAGAAAGTCAATTCAGGTAGAAACAAGGCTAAGTGATTAAAAGAAAAGGAAAGTCTAAGAACA
AAGTTAATAAAAACAAGGTTAGGCATTACAGATGGGGTCATCCTTATATTCAGCACAGCGTTTGT
CCTGAAACTTCAATGGAGTGCTCCTAGTTTGATAACTTGGACCATGCCAAATAAATTGAATTTCT
CTTTCTTAACAACAGATGTTTGAATACAGCCCCGTGACCCTTTCTGCTCCTTCCTGCCTTCCTCCT
CTCCTCTTCCTTCCCTTCCTTTTCAAGAGCAGGGTTTGCAAACTTTTTCTTGAAAGCATCAGATAG
TAAATATTTTCGAATTCGTGGGCCATACAGTCTCTTATAGTAGTTGCTCAACTCTGCCACCCATA
GCATGAAAGCAGCTGATATAGTTGAATATGAGTCCCCACCCAAAACTCATGTTGCAATCCCCAG
TGTTAGAGGTGGGGTCTGGTGGGAAGAGATTGGATCATGGGGGTGGATTTCTCATGAATGATTT
AGCCTCATCCCTTTGGTCCTGTCCTTGCAATAGTGAGTGAGTTCTTGCAAGATCTGGTTGTTTACG
AGTGTGTAGCACCTCCCTCCTTACTCTCTTGCTCCCACTTCACCTTCTGCCATCATTGTAAGTAAG
TTTCCTGAGGCCTCCACAGAAGCTCAGCAGATGTCAGTGTCATGCTCCCTGTATAGCCTACAAA
ACTGTGAGCCAATTAAAGCTCTTTATATATTGCCCAGTCTCAGGTATTTCTTTATAGCATGAGAG
AACAGCCAAATCCAGCAGCCATAGACAATATGTAGCAAATGGACATGACTGTGTTTCAATAAAA
CTTTATTGACAAACACATGCACAAGCAGGTCAGATTTGGCCCATGGGCATTAGTGTCCCAACCT

CTGCTCTGGAGTATTCTCTTCAGCAACCTCTGCCTCCTGGGTTCAAGCAATTCTCCTGCCTCAGC
CTCCCAAGTAGCTGGGATTACAGGCACCTGCCACCACACCTGGTTAATTTTTGTATTTTTTAATA
GAAATAGGGTTTCACCTTGTTGGCCAGGCTGGTCTCAAACTCCTGACATCAGGTGATCCAACCA
CCTCAGCCTCCCAAAATGCTGGGATTACAGCTGCCCACCACCACACCTGGCTAATTTTTGTATTT
TTAGTAGAGATGTGATTTCACTATGTTGCCTAGGCTGGTCTCGAACTCCTGATCTCAAGTGATCC
ACCTGCCTCGGCCTCCCAAAGTGCTGGGATTACAGGTGTGAGCCACTGCAGCTGGCCTCTAGAT
TTTTTTTTTTTTGATCGTTGCTTATTTGGTTTGACTTTCATTTTTTCCCCCATTGTGGTAGTCATTTTC
TGAACGCCTGTTAGTTTGTCCATCTCTCTCCTCTGTAGTCCCTAGAGTCAGATGAACTCCTCTGCA
GGTGCAATGGTGTAACACACTCTAGTGCTGAATTTTGAGCAGGAGAAAGAGAGCAAGAGTGAC
CAGTACCCTTGGAAACTCTGGCCTCCTGAGAATTTGGTGTCTTCCCTGCAAAGGTTGCAAACCTG
TTAACCCACAGGCCGGAGAGAGAGAGAAGTCAGAATCACGATCTGTTAAGCTTGAGTTTAATAT
TTGATGCACAGAGATCACTGAGTTTTTTGATAAGAATTAGAGGGGGAAGAAATAGCCAGAGAA
CAATTTCTATCTCCAGGTGAGGATTCAGGAGATAATTCTGCGAAAAGAACTTCCTGAGAACTAA
GATGGGGAAAATCACTGGTATTAGAAGAGTGAAAAGGTCAGTAATTAAGGCTACAACTGTGCTC
TTAGAGGCAAAAGAAGAAAATGAGACTGCCAGGCATGAATAATGAGAAATCTTTGATGGAATT
AGCCATGCAGAACAGATATTAAATGCATCCTCATTCTTCTCATAGTCAAAAGTATTTGCCTAAGC
TGGATGGGAAAAAGAGAATCCCATTTCACTAAGTATAAAAGAGGGGATTTTAGAGAAGGTCTCA
GAAGAAAGAGATGTGGCGGTTTGGTGAAACTCACCAGAGGCTGAACCCTCTCCAGCATAACAC
ACGGATTGGGAGGAGTGGGGTGGCATTAGGCCAGGTGCGTAGCCCAGTGCTGCTCTCTCTGGA
CTTGTTTGCTAGCTGAACTCATCCATTTGCATAATTTTCAGTGCCATTTCTCAGCTGATGAGTCCT
GAACTTTTGTCTTGAGGTTAGACTTCTCCTTCAAACAGCAGTCTTAAACACTCAACTTCTTTCTTG
TTTTTTCCACTTGTCAACTCATGAACACCTCAACCTTGTTAAATCCAAAGCCAAACTCATGGCTTG
GAGTGGTAGGTGATGGCTGTAATCCCAGTGCTATGGGAGGCTGAGGTGGGAGGATTACTTGAG
GTCAGGAGTTTGAAGCCAGCCTGGAAAACACAGTGAGACTCCCTATCTACAAAAAAAAAAAAA
AAAAAAAAAAAAAAAGCTAGGCATGGTGAAGTGCATCTGTAGGATCTGTAGTCCTGTTACATGG
CAGGCTGAGGCAGGAGGATCACTTGAGCCCAGGAGTTTGAGGCTGCAAAGAGCTATGATTGCA
CCAATGCACTCCAGCCTGGGTGACAGAGCCAAAGTCCCTGTCTTAAAAAAAAAAACAAAAGCAA
AACTCTTTTTCCTCCTCCTTCTCCATGGGCTCTGTCCGTGCCATCTCTGTTCTGTAAATGGCACCA
CCCCCTGCTGAGCTGCTCAAGGTGGTCATAACTCATGTGTTGTGCTAACTCTGCTTTTGCCCTCTT
CTCCAGTCAGAAGTCCTGTGATTCTAAACTTTATCCAACTTGTCCACTCTCTCTATCTTCACTGTC
ATTATCTTTGCCTAGGACACCGCTATCCCAGCTGGGCTACAGCAGCAGCCTCCTAACTGGTCTTA
ACTGGTCCTCTGCACCTGCTCTCCATGCTCAGCAATCCATTTCCTACCTAGCAGCTTCAGTGATC
TTAAGGTGTCCATTGAGTCTCATCCCTGCCTTTCCTGCCCATGGCACATAGAATAAAATCGAGAC
CCCGAGTCTTCTGCCTGTTCCTGCCACCTCTCCAGCTCTCTCCTATCTCCTCCCCTTGGCCTACTC
TATTTCAGCCACTCTGGCCTCCTTCTGTTTTCTTTGACTTTCAAACCTTTCTCCACAACAGGGCCTT
TGCACTTGCTGCTTCAGCCTGGAATGATTTTCCTCTGCACCTCCCCAAATTAGACCATCCTTTAG
GTCTCAGCTAAAATGGTGCTTCCAGACAGCTCTTTCCTGACCCCTTTATAAAGTGGACTTCCCTG
CTCTTCTCCACCTTAACCTCATTATTTCTTGTTGCGGGAAGTCAGGGACCCCAAATGGAGGGACT
GGCTGAAGCCATGGCAGAGGAACATAAATTGTGAAGATTTCATGGATATTTATCAGTTCCCAAA
TAATACTTTTATACTTTCTTATGCCTGTCTTTATTTTAATCTCTTAATCCTGTTATATTCATAAGCTG
AGGATGTACATCACCTCAGGACCACTGTGATAATTGTGTTAACTGTACAAATTGATTGTAAAACA
TGTGTGTTTCAACAATATGAAATCAGTGCACCTTGAAAAAGAAGAGAATAACAGCAATTTTTAG
GGAACAAGGGAAAACAACCATAAAGTCTGACTTACTGCAGGGTTGGGCAAAGGAGCCATATG
TTTCTTGCACGGAGTCTATAAATGGACGTGCAAGTAGGACAGATATTACTAAATTCTTTTCCTAG

CAAGGAATATTAATATTAATACCCTGGGAAAGGAACGCATTCCTGGGGGGAGGTCTATAAATGG
CCGCTCTGGGAATGTCTGTCTTATGCAGTTGAGATAAGGACTGAGATATGCCCTGGTCTCCTGC
AGTACCCAAAGGCTTACTAGGGCGAAAAACTCTGCCCTGGTAAATTTGTGGTCATACTGGTTCT
CTGCTCTCGAACCCTGTTTTCTGTTGTTTAAGATGTTTATTAAGACAATACGTGCACCACTGAACA
TAGACCCTTATCAGTGGTTCTGCTTTTGCCCTTTGCTTTGTGATCTTTGCTGGACCTTTATCAGTAG
TTCTGCTTTTGCCCTTTGTCCTGTTCCCTCAGAAGCATGTGATCTTTCTTAGACCCTTAGTAGTAGT
TCTGCTTTTTGCCCTTTGAAGCATGTGATCTTTGTACCTACTCCCTGTTCTTACAGCCCCTCCCATT
TTGAAACCCTTAATAAAAACTTGCTGGTCTGAGACTCAGCAGGCAGCACAGACTTACTGATATG
TACTGTCACCTCCAGCGGCCCAGCTGTAAAATTCCTCTCTTTGTAGTGTCTCTCTTTATTTCTCAG
CTGGCTGACACTTATGGAAAATGGAAAGAACCTATGTTGAAATATTGGGGGCAGGTTCCATCAA
TAGTTCTTACATGGATTTACTTATTTTTTGTCTCCCTCCATACCCTAGAAACTCCTGGAGGGCAGA
GCTGTGTCTGCCATCTTCATCATTGCATTACCACCACCAAGCACAGTGCCTGGCACAAAAGAGT
TGCTCAATAAATAAATCAGGATGAATGGATAAATACATGGATAGGCACTTTGAGCTACAGATGA
GTTTAAATACTTTGTGTTTTTCTTAGTCAAACATGTGCAATTAAGCATGTGATAAATGATATGATG
ACC

ID|56248|gene_id|ENSG00000249356|transcript_id|ENST00000514608 Homo sapiens lncRNA

TTTGCTAGAACACTGTGATAGATTAACTGCAGAATTCTTCCTGTAGGATTGGGGATATAACG
GGCTTCACTAACCTTCCCTAGGCAGTGAAACATCCCCCAAATCTGATGGATCTAGAGTCAGGGA
AGTCTGCTTTTGTACCTGCTGGGCCCCAAAGTTGGGCATTTTTCTCTCTGTTCCCTCTCTTTTGAA
AATGTAAAATAAAACCAAAAATAGACAACTTTTCCTTCAGCCATTCCAGCATAGAGAACAAAAC
CTTATGGAAACAGGAATGTCAATTGTGTAATCATTGTTCTAATTAGGTAAATAGAAGTCCCTATG
TATGTGTTACAAGAATTTCCCCCACAACATCCTTTATGACTTAAGTTCAATGACAGTTTGCCTTTG
GTAGTAAAGGATTTTCTCCATGGCCTGTATTAAGACCATTAGAAAGCACCAGGCCATGGGAGCA
GTGATCATCTGCTGACTGTTCTCGTGGATCTTGTGTCCAGGGACATGGGGCGATGTGCCTCGTAT
GTGTTAGAGGGTGGAATGGATGTGTTTGGCACTGCATGGGATCTGGTGCCCCTCTTCTCCTGGA
TTTGCATCCCCACCCAGGGCCCGCTTTTACTAAGTGTTCTGCCCTAGCTTGGTTCAAGGAGGTCA
TCCAACTGACTTTATCCAGTGGAATTGGGATATATTTGATATACTTTTGCCTAACAACATGGAAA
AGGGTTTTCCTTTCCCTGCATCCTACTGCTTTGAACTTCCAAGTATGTCTACTCACCTTTTAAAAT
GTAAACATTTTCAGAAAAATGAGGATTGCTTTCCTTGTATGCACTTTTTATCTTGACTACCTGAAT
TGCAAGGAATTTTTATATATTCATATGTTCCAAAGTCAGCAACTCTCCTGTTGGTTCATTATTGAA
TGTGCTGTAAATTAAGTCTTTTGCAATTAAAATGAGGTTTGCCCACATCCAAAAAAAAAAAAGAA
A

ID|56289|gene_id|ENSG00000247810|transcript_id|ENST00000501725 Homo sapiens lncRNA

GGAGCCAACAGAGTGAGATGAAGGGGACAACTTCAGATACTCAGGCAGAAGGTAGGCTA
CAGTGATGTAAGTCCAGCATCTTTATTTATTTTTATTTATTTTTTCATTTTTTAATTTCCATAGGTTTT
GAGGAATGGTAATATTTGGTTACATGAGTAAATTCTTTAGTGGTGAGTTGTAAGATTTTGGTGCA
CCTATAACCTGAGCAGTACACTGAACCCAATTTGTAGTCTTTTATCCCTCACCGCCTTCCCATCCT
TTCCCCTAGAGTCCCAAAGTCTATTGTATCATTCTTATGCCTTTGCATCCTCATAGCTTAGCTCCC
AGTTATGGGAAATCCAGCATCTTTAAATGATGCCTTGAGACCATATTCTCCTGTGCTTCCCCTGT
GAACAAGTAATGATGGATCTAGTAGTTACTACCATGATGTAATGCATGACAGTCCACCACAATC
ATGTTTCCTAGCCATGCATAACGACAGGAAAAATGACAGATAAGTAAATATACCAATCTAGGCC
TAAGGACAAGCATTTGGTTCTACTATTGCCTTTTTGTCCACCTGACCCAATTAGGCGGCTTCATAT

TTTTTAAAAGTTCAGTGTCTATCTGTAAGTCCCATGAATATATGATCCTAGAATTTAGAAATTATTT
TATAATTTGTATTTAATTTACATGGATTAGAATGTTATTAACTACCCTTGGTGCTCATGAAGCATC
TTTTCCCCTAATATATCTTCTGCAGTGCAACTTAATAGTGAGTCCCCATAGACAGAAAAATCTATT
TGAAGAAAATATTTTAGCAGTTTCTCCCATGAACAGTTTTTTGTTTGTTTGTTTGTTTTTGCCTCCA
GCTTCAGTAAAACAAGTTGTAAATAACTTTATAGGCAAAACTTTGGTCCCTGGTTAATCATGAGA
TTTTTTTTTTCTTTGAAGGCTAGTAATGATATTGATGAGGAAGAGCAGCTCGCTCTACAAAGTTAT
TTAGGGGCCCAGATGTGTTCCGTCTTGCTCTGCCATGCCCTCCTGTGCTATAACTCTTCTATTTAT
ATGGTAGTCCTTACACACATGGTTGCAGCTGTATTGTCAACACGACATATATACCTGAGAGGGG
AGAAAAAAATTCCATGGCAAGCATTTATCTTTTGAGACACTGCAATTTGAGACAGTTACAGTGATA
TTACATACATTACTCCCATTGTTCAGAACTAGTAACATCAGCTTGCCAGCTACAAAGGAGAGAG
GAAAATGTAGTCTCTAGTTTCTCATTCAAGGCCTAGGTAAAATGCTATTAAGATGGAAAAGGAA
AATAAAATGGGAAAAGACTACAAAGTCTACCGCATGCAATAAACAAGTATACAATTAAATACAT
AGTATGGCAGATAATGAACAGTACTATGGAAACTGTCTATATATACATATGTATATATATATGAA
AACGAGTGAGAGGTTAATATCATTTATTTTAAATAAGGTGTTCAGGAAAATGCCTTCTGGACATG
TTAGTAAAATGATGGCAGAAGGACCTCCAAAAACGATCTCCTCCACAAAAGTAATGAGAAAATT
GACCAAAAAAAAAAAAAGTCAGAATCATTTCTTCAAAATACTGGAAAAAATAACAAAAATCTTGA
AGTAACACAGGGAGGATTTATTCAAGAAAAATAAATGATCTCAGCAAGAACAGTGAGTGTCCTA
ATCTTATCTCCTGCTCTACAGCTCTGCAGTACCATTGAGAAAAAACAGCCCACATTTCCACTGTA
GCCCAGAAGCCATCAAAGGGAGTACAAAGGAGCTGCAACTTTTTAAAAAGCTTCATTCCAAAAT
AATTGTCATTATCTGAGCAGTCTGGTGGTTCTCTGAAATATATCAGTTACAAGGCCACCTGTACT
TGGCCTGTTTAGGAGCTCACCTAGTGCAAAAATTATTTTCCCTGAAGAATATTTATCAAAAACAA
TTACTGGAAAGTGTTTTAACTTTGTGGTTGTCTTAGGCAGTGGATAACAGGAGAAACAGTGACTA
AAAATTTAAAAGGAAAAGCTGAGAAATGACATGTCAATACAGTCTTTGAAAAAATCTCCAACAT
AATTTTTGTTATTTAGAAAGTCATATAGGGTTGTGCACATTCTCAGGGGTGTGGTAATGCTCAGA
AAATCCTTAAGAAGTCCCATATTACCACCTCTGGCTGACCATAGGGACCTGTGCAATGAGAAAG
TGAGAGCTAAGGCAGAGTTGTCAATTGCCTAACTGAATGTTGAGGGCCTGCTTTGCAACAAGCA
CACAAAGCCCCTCAGCAAAAGCTGGAAGATGTATTGGTTCCAGACTTTTAAGGAAATTATCCAA
TCATTAGCTGAGCACTAAGGTAATAGAGTAATCTAAAAGACCACATGTGACAATAAATATGGAC
TTTACCAAATTAACTCAGAAAATTACTAAACAAACATACAAGACTACTACAGCAAACAGCAGCA
TCAAATCCTGGAGAAGGGGGAGAATTTTATTTCTAGAATTGTCATATTAGATTATTTTAGATGTCC
AGTTTCAAACAAAAGATTATGAGATGGAAATACCTAAGAAAGTGTGACCCATGCATAAGAAAAA
AAGCAATAGCAACTATTCCCAAGGAAGCCCAAAAGTTGACTTACTAGATAAATATTTTTCTACCA
GCTATATGAAATATGTTCAAAGACCTCAAGGACATGATAGCTAAAGAACTAAAAAAAGGTATGA
AAACAATGACTTAACAAATATGGAATATCAATAGAGATAATCTTCGACAAACTTGACAAAAACA
AGCAATGAGGAAAGGATTCCCTATTTAATAAATGGTGCTGGGAAAACTGGCTAGCCATATGCAG
AAAACTGAAACTGGACCACTTCCTTACACCTTATACAAAAATTAATTCAAGATGGATTAAAGACT
TAAATGTATACCCTAAAATCTAAAAACCCTAGAAGAAAAAATCTAGGGGATACCATTCAGAACA
TAGGCATGGGCAAAGACTTCATGACTAAAACACCAAAAGCAACTGCAACAAAAGCCAAAATTG
AGAAATGGGATCTAATTAAACTAAAGAGCTTCTGGACAGCAAAAGAAACTATCATCAGAGTCAA
CAGGCAACCTACAGAATGGGAGAAAATTTTTGCAATCTATTCATCTGAAAAAGGTCTGATATCC
AGAATCTACAAGGAACTTAAACAAATTTACAAGGAAAAAAGAAACAACCCCATTAAAAGGTGG
GCAAAGGATATGAACAGATGCTTCCCAAAAGAAGACATTTACGTGGCCAACAAACATATGAAA
AAAAGCTCATCATCACTGTTCATTAGAGAAATGTAAATCAAAACAACAATGAGATACCATCTCAT
GTCAGTTAGAATGACGATCATTAAAAAGTCAGGAAACAACAGATACTGGAGAGGATGTGGAGA

AATAGGAATGCTTTTACACTGTTGGTGGGGGTGTAAATTAGTTCAACAATTGTGGAAGACAATAT
GGTGATTCCTCAAGGATCTAGAACCAGAAATACCATCTGACCCAGCAATCCCATTACTGGTTAT
ATACCCAAAGGATCATAAATAATTCTACTATAAAGACACATGCACACATATGTTTATTGCAGCAC
TATTCACAGTAGCAAAGACTTGGAACCAACCCAAATGCCCATCAATGTTAGGCTGGATAAAGAA
AATGTGGCACATACACACCATGGAACACTATGCAGCCATAAACAAGAATGAGTTCATGTCCTTT
GCAGGGACATGGATGAAGCTGGAAACCATCATTCTCAGCAAACTAACACAGGAACAGAAAATC
AAACACCACATGTTCTCACTCATAAGTGGGAGTCAAACAATGAGAACACACGGACACAGAATG
GGGAACATCACACACCAGAGCCTGTGGGGAGTGAAAGGCAAGGGGAGGGGGAGCATTAGGA
CAAATACCTAATGCATGAGGGGCTTAGAACCTAGATGATGGGTTGATGGGTGCAGCAAACCAC
CATGGCACTTGTATACCTATGTAACAAACCTGCATGTTCTGCACATGTATCCCAGAACTTAAAGT
ATAAAAAATAAAAAATATATATATTCTGGAATTGAAAATAAAACTAAAATAAAAATTTACTAGAG
TGGTTCAACAGCAAATTTGAGAGGGCAGAAGAAAGTATCAGCAAACATAGAGATAAGTGGATT
GAAATGATTCAGTCTCAGAAAGATAAAGAACAATAATAACAAATTAGCAGTCTTAGAGAGCTGT
TACAGATAACATCACCTGTACCAATATATGCATTATCGGAGTCCAGGAGGGGAGGAAAGAAAA
AAGGGACAGAAAGAAAACTTGAAAAAAAGGACAGGAAACATAACAAATTTTATTTCAAAAGTT
ACATTAATCTATACATCAAAGAAGCTCAATGAACTCCAGATAGTTTAAACTCAATAAGGATTATA
ATCAGATGAGTCATTAACTGTCAAAGACAAAGAAAGAAAGAATCTTCAAAGCAGCCAGAAAGA
AGTGATACATCATGTAAAAGGAATCCTCACTAAGATAAACAGCAAGTGTATAATTAGAAACTAT
TGAGGCCAGGGACAATGGAATGACATATTCAAGTTGCTAAAAGAAAAACAAAAATTTCACTCAA
TAATTCTAAATCCAGCAAAATTATCTTCCAAAAATTAAAACGAAATTAAGATATCGCCAAACAAA
AACTGAGATCATTGTCACTGACAAAACAGCTTTAAGAGAAATGTTAAATGTAGTACTTCAGACTG
ACCTGAAAAGACACTAGACAGTAACATGAATCTACATGAAGAAATAAAAGATACTGGTAAAAG
TAATTATACAGGTAGATGTAAAAGAGAGTATAAATGTATTTGAAATTTGTGATTACTCTTTTTTTC
GTATCTGATTTAAAAGACAACTACATAAAGCAATAATTATATATGTGTGTTCATTTATATTACATA
CAATGTACAAAGACGTAATATGAGTGACAATGATAGCACAAAGGAGAGCAAAAGAAGTGGTGC
AATATTGGAGAAAAGTTTTTGTACACTATGGAAATTAATTGGCATAAATCTGAAATAGACTCTTG
TAAATAATAATGTTAATCATAATTATCAGGGAACAATTAAGAAAATAACATAGTAAAAGGAATG
GTAAAGGAATTGCATGGTACACTAGAAAATATCTATGTAGCCCAAAGAAAGCAGGAATGGAAT
AATAGAGGAATGAAAAGACATTGAAAACAACTAGAAACATGACAGACATAAATCCTACCTTATC
AGTGATTATAGTAAATTTGAAGGGATTAAACATTTCAATCAAAAAGGAAGCAATTAGTAGAATG
GATAAAATGCTATGCTGTGACTATATTCCCTCTACAGAAGATACACTTTCTGTTAAAAGACACAA
TTGATTGAAACTAAACGGATGGAAAAAGACATACCATGTATACAGCAACCAAAGTGCTATAGTA
ACTTTCCTGATAGAAGACAAAATATACTTTGGAGGAAAACTATTACTAGAGACAAAAAGTACAA
TTTATAATGCTGAACAGGTCAGTCCATCAAGGAGATATAATTATAAAACTGTATGTCCCTAACAA
CAGAGACCCAAAATACATGAAGCAGAACTAGAAGAAGAAATAGACAATTCAACAATAATAGTT
GGAGATTTCAGCACCTGACTTTCAATGATGAATAAAATAAGAAGATAGAAGATTAGCAAGGAAA
TCGAATATACAAATATGAACTCAAGATAGGTAAGAGATGGCTGGGCACGGTGGCTCACGCCTG
TAATCCCAGCACCTTGGGAGGCCGAGGCGGGCAGATCACGAGGTCAGGAGATCGAAACCATC
CTGGCTAACATGGTGAAACCCTGTCTCTACTAAAAATACAAAAAATTAGCCGGGCGTGGTGGCG
GGTGCCTGTAGTCCCAGCTACTGGGGAGGCTGAGGCAGGAGAATGGTGCGAACCCAGGAGGT
GGAGCTTGCAGTGAGCTGAGATCGCGCCACTGCACTCCAGCCTGGGTGCAGAGCAAGACTCCA
CCTCAAAAAAAAAAAGATAGGTAAGAGACAAAAATGTAAGAGAAAAAAAGGATAAAACTCATAG
TAGAAAACATAGGTATAAATCTTTGTGGCCTCGGATTAGGCAAAAGTTTCTTAGACAAGATACCT
AAAGCATAAGCAACCAGAAAAAAAAAAAAAAATATATATATATATATACTGCACTTCATTGAATTA

AAAACATTTGTGCATCAAAGGACACTTTCAATAAAATTTAAAGGCAACGTACAGAATAGCCTAC
CTTACTTACAAGCCATGCATTTGATAAGATTTTAGATCCAGAACATATTTTTAAAACTCTTAAAGT
TCAATAATAAAAAGACAACTCAGTGAGAAAAAAAATGGGTCAAGTATTTGAATAGCTATTACTA
CACAGGAGATATATAAAGTTCATAAAACACATGAAAAGATTATCTAAATCATTAGTTATTAGGTA
AATGCAACCAAACCATAATGAGATACCCTTTCACACCAACTAAGATAGCTATAACAAAAAAGAT
GAACAATAACAAGTGTTGGCAAAGATGTGGAGAAATTGGAAACATACATTGTCAATATAAAATA
GCGCAGCTACTTTGGTGAACATTTTGAAGGTTCGTCAAAAAGTTAAACATAAATTTACTATATGA
CCCAGCCATGCCACTTTTAGGCACATACAAAAGAAATGAAAACATATATTCACTCAAAAACTTG
AACACAAATGTTAATAAGAGAATTATTCATACCATCTAAAAAGGTGGGAGCAACCTAAATGCCT
ATCAACTAATGAATGGATACACAAAGTATAGTATAACCACACAATGAAATATTATTCAGACATA
AAAGGGAATAAAGTTCTGATACATGCTACAACATGAATGAACTTTGAAAACATTATGTCAAGTG
AAAAAAAGCCAGACATAAAATCATACACATATTGTACAATTCTACTTGCACAAAAGGTCAAGAA
TATGCAAACTCTCAGAGACAGAAAGCTTAGTGGTGGCCAGGAGCCGGCAGGTGGCTGGGGAA
GAGAAGAGATTGAGGAGTGACTGATCTATGAGGATAGGGCTTCTTTGGGAAGGTTACAAAAAT
GTTCTAGAATTAGTGGTAATGGTTTCATAAGCTTTTAATACACTGAAACACACTGAATTGTATAAT
TTTAAATGGCGAAGTTTATTATATGTGAATTATATTTCAATACAATAAAAGAAGGGTAGAAAAGA
AATCCTTTTTTAGAAAGCAGCATTTGGAGAGAAATTTGAAGAAAGATAAGGAGTAATCTACCAG
ATATCCAGGAGAAAAAGAACACCAAATGATATTCAACAACAGTTTATTCAAAAGTTTAATAATTA
ATATTCCTCTTTTGATTTTTAAGTCTTTGTTACTGTCATGGAACAGTTTAATGTAGTCATATTTTGC
GGAAAGAATGAGGAGAGTAAATAAAAAGGTAGGAGACTTAGAAAGAGGAGATTTAATTTTTGA
ATCATGTAAAAAGGAAAGTGGGAATTTTTGAAAATATAAGAAAAATTAGAGTAAACTTAAAAGA
GCTGCAACGAGGAAAAAATATGAGAAGCATTGATATAGTGATAACAAAATTAAGCACTAAAATC
AGGCAGATACGGATTATATCTCACCTCTACATCAGCTAGCCCTTTCTCTAGAAAAGTTTCCTTAC
TCTGTGTTCTTCAGTTTCCCATGACCTATACAGAAAACTGAGGAATAATTTCAAGAAAATAATAT
GGGAATAAGGTGAGAAAAATAACTACCTTACTGGCTTGCTGTGAGAGATGAGCAAGATGACTTA
AGTAACCCACTGAATTTGACACCTGGTAGATTCTCAATATATAATAATTATTCACAATAATCATCT
GAATGGACGCTACATGAGTTCTGAGGGGTCTTAAGTTTTAAGAAGTAAAATTATCTATTTAGATA
TGTGTTTTTAAAATTTGTAAGATATATGGAACATATTCAAAAAGACTTCACATTTTAATTATTTAAA
TATTAATTTAATTTTGGACATTTTAATTTTAATATAACAGCATAGAAGACTAATGCAGAGGCTTTG
CCTATATTTTTAAGGTGATAATTGGTTACAAATTGACCTTTGTTGGAGAGATAAAAATGTATTTGG
AGACTAAATTAAAAAAAATGCTTTCCACATGAACTCAATATTTTTAGAAAACAAGCATGTTATCC
TTAAAACTATTTTGAGAATAACACTGTGGTGAAAAATGCTACCAAAAAAATTACTTTGCTAATTA
AAGCCTATGAAACCAAATAACTGTTTTTAGACATTGATTTTGCTGGTTCACATCCTAAAGAGAAG
TACTTCTCATGTTTCTTTCAAGTGAGATAAAGTTAGTGAAAAATCTGGCTAAGGGAGGATGAGAA
GGAGTTTACGTATTTGAACATTTTTGCTGGCATATGCCAGAATATTTTTATAAACAATGCTAGGG
AGATTGAAGTCTTCTGGGGATTAAAGGAAACCACGAAATAGAGAAAGAGCATGTCAGGGAAAG
GGGCCATGTCCGTCCTCTGAACAGTCTCCAGCCAAAAGAACTTGGCCAGTTAACACCTTTGTTTC
TCTTTTTCTAATTTTCTAAAATGAGAGGCTTTTACTTAATGGAATATGTAATCCCTCTTGATTCTAA
TTTTGTATTGTTCTGTGCAATATTTTCTCTTCTAGAAAAAAAAAAATGCCAAGGCTTAATTCAGACT
TTTCCTCTTTAGGTATTATTAAAGGTAGCTGTAAATTCCGGAGGTTGGGTCAAATTTGCCTTGGTG
GATTATATGATGTTTCTCTCTTCAGTTTCTTTCTTTCTTTTCTTTTTTCTTTGCTGTTGTGATTCCTTCT
GCCATTAAGAGAAGGCAACTAGAATTTCTGCTATAGGTCTCATTATACCTAATGCCAGAGGGAT
ATTCAAAACACACAGCAACCAGTACAGCAAAGGTATGAAACAGCAAAAGACATTGGCCATGTA
GACATACATCAGCCCTACCAAAGCTAGAGGCAAAACCTGATTGATTTAAGTGAAGCTCAGAAAC

ATTTGTGTATGTGTTGATGAACATATACGTATATCAACCTAAATAACAAGCATAGAGAGATTCTG
TAAAAGAAAATGATATTTATTCAGAAATAAGGCATTGCAATGGGACTACACATGCCATTGCAAA
CTATGTGCATATTCAGGAAGGTAAGAGAAGACAAAAATTTTTAAAGGAAAAATGCAGGTGGGTT
ACATAATTGTTTTCAGATAATTTCCCTTGGCCACAAGGCTCAATAGCAAAGGAGACACCAGTCT
GAGATTGGACAGGTAGTAGCTGGGCATATGTTCTTATGATAGTATTTTTTGTGTGAGGTTGCAAT
GACCTTTGTGCGAGGTAGTTTTTACCAAACCTTTTGTTATAGTTCTGATATCAGGCATACCAGGG
TAAGAAACCCTTTCTTTACAGCCTTCCCTGGCTCTATTTGCCTTTTTTTTCTTTTTTCACAAGTGATT
TCATTTTGATTCTGGTAACTCTTACACATACATTTGATAAATTAGAATTGTATCAATTTCAAAACAT
ATTTATTTTGTAAATGGAATTATTTTAATGTTTAAAATGCCCAAAGAGATAAATGAAGGAATAATA
TTCACTAAAACTGAACAGAAGTTATCTTTATCCAACCATCCATTGACCCTTAGGTTGATTATATGA
CTTTGTTATTGTGAGTGGTGCTGTGATAAACATACAAGTGCAGGTATCTTTTTAGCATAATGATTT
CTTTTCCCTTATGTAGGTAGTAGTGGGATTGTTGAATCAAATGCTAGTTCTATTTTTAGCTCTTTCA
GAAATCTCCATATTGTTATTCATAGACTTTGTCCTAATTTATATCCCAAACAACAGTGCATAAGCA
TTCCATTTTCTATGCATCCTCACCAACGTGTGTTGAACTTGACTTTTTAATAAAAGCCATTCTGGTT
GGTGTAAGATGGTATCCCATGTGGTTTTAAGTTGCATTTCTCTAATGACTAACGATGCTGAGCAG
TTTTATGTGTTTGTGGCCAGTTGTACATATTCTTTTGAAAAATGGCTGTTCATGTCCTTTGTCCGCT
TTTTAATAGGGTTATATGCTTTTTCTTGAGTTGTTTGACTTCCTTTTAGATTCTGGATATTAGTCCTT
TGTTGGATACATAATTTACAAATATTTTCTCCTTTTCTGTACATCATTTTGTCTACTCTGTTGATTAT
TTCTTTTGCTGCCCAGAAGCTTTTTTAGTTTACCTAGGTACCATTTGTATATTTTTGTTTTTGTTGCA
TTTGCTTTTGAGGACTTACTCATAAGTGCTTTGCTTTGCCCAATGTCCAGAAGAGTTTTTCCTAAT
TTTTCTTCTAGAATTTTTATAGTTTCATGTCTAACATGTAAGTCTTTAATTTATCCTTAGTTAATTGT
TGTATATGGTGGGAGATATGGGTCCAGTTTCACTCATAGCTATCCAATTTTCCCAGCATCATTTAT
TAACTATTGTGTCTTTTCTCCAGGGTAGTTTAAAAATTTTTTTTTAAGAATAAAACCTACTGATGCA
TTCATCCAGGGTAGGTTTTTTTGACTTTGTTGAAAATAATTCAACTGTAAATATAGAGGTTTTAAA
TCTGGATTCTCTACTCCGTTTCATTGATTTATGTGTCTATTTTTATACCAGTACCATGCTGTTTTTGT
TACTATAGCCTTGCAGTTTAATTTGAAATTGGATAAAATGACGCCTCCAGCTTTATTCACTTTGCT
TGTGATTACTTTGTCTATGTGGGCTATCTTTTGGTTTCACATGAATTTTAGGATTATTCTTTTCTAAT
TCTGTGAAAAATGACAGTGGCAATTTCATAGGAATTGCACTGAAGCTGTAGATTACATTGAGCA
ATATGGTCATTTTAATAATACTAAGTCTTCCAATCAATGAGTATGCCATGTTTTTTCATTTGTTTGT
GTCATCCACAATTTCTTTCATCAATGTTTTTTAGTTCTCCTTGCAGAGATCTTTCACCTCCTTGAGT
AAATGTATTCCAATAATTTTTTTTTGGTAGTTACTATACATGGGATTGAGTTCTTATTTGGTGCTCA
GCTGGATCATTACTAGTGTGTAAAAATGCTACTAATTTTTATACTTTAATTTTGTACCCTGAAACTT
TACTGACATTATTTATAAAATCTAGGAGTCTTTTGGAGGAGTCTTTAGGATTTTCTAGGTATAAGA
TCATATAATATGAAAATACAGATAATTTGACTTCCTCTTTTCCAATTTGAATGCCTTTTATTTCTTT
CTCTTGCCTGATTGCTCTAGGTAGGACTTCCAGTACCGTGTTGAATAGGGGTAGGGAAAGTGAG
CATTCTTGTCTTCTTAAAATCCTCAGGGGGAAATGCTTTTAACTTTTCCCCATTCAGTATGATGTT
GACTATGTGTTTCTTGTGTATGGTTTTTGTTATTTTGAGATTTTTTTCAATGTCTAGTTTGTATCCTG
AAATTGTACTGACATTATTTACTTATAAAATCTAGGGATCTTTTTTCATGAAGAGATGCCTAATTTT
ATCAAATGATTCAAATGTTTTTTCTGAATCTATTGAGATGATCAGATGTTTTTTTGCTTTTCTGTTTA
TGTTGTGAATCACATTTATTAATTTTTGTGTGTTGAACCATTCATGAATCCCTGGAATAAAACCCA
GTTGATAGTTGTTTATTTCTTTTAGATGAGTTGTTGGATTTGGTTTGCTAGCACCTTGTTGAGGATT
TTTGCATTATATTCACCAGGGATATTGGCCTGTAGCTTTTTGTTGTTCTTTCCTTCTCTGGCTTTGA
TATCAGGGTAATACTGGGTTCATGGAATGAGTTAGAGGGGATTTCTGGTTCCTCAATTATTTTGG
AGTAGTTTCTATAAGATTGGTAAAAGTTCTTCTGTGTATGTCTGGCATAATTCAGCTGTGAATCCG

TCTGGTCCTGAGCTTTTTTTGGTTGGGAGATGTTTCATTACTTATTTAATCTCACTACTCCTTATTG
GTCTATTCAGGATTTCTAGTCATTCCTGGTTCATGTTGAGAGGCTGTATGTTTCCAGGAATTTGCC
TGTTTCCTTTAGGTTTTCTAGCTTGTGAGTGTAGATATGTTCATTGAAATCTCTGATGATCATTTGT
ATTACTGTGGTATCAGTTGTAATGTCTCCTTTTCATTTCTGATTCTGCTTATTTGAATCTTTTCTCTT
TTTGTCTTAGTTAATCTAGCTAGTGGTCTATCAACTTTGTTTATTTTTCCAAAAGAGAAACTTTACA
TTTCATTGATCCTTTGCATTTTTTATCTCTATTTCATTTAGTTCTGTTCAGATCTTTGTTATTTCTTTT
CTTCTGCTAGCTTTGGGCTTGGTGTGTTCTTGTTTTTCTAGTTCCAAAGATGTGATGTTAGGTGGT
TAATTTGTAATCTTTCTATTGTTTGATGTAGGCCTTTCATTGCTCCCTCTTAGCACTGCTTTTGCTAT
ATGTTGCCTCTATTTTCATTCAATTCAAAAAAAGTGTTGACTTTCACTTTCATCTAATTATTGACCA
AATATAAGTCAAAACACATGGTTTGATTTCCACATATTTGCATAGTTTTGATTTCTAGTTTTCTTCC
ACTATGATCTGAGAATAATATACTTGATATAATTTTGATTTTTTTAAGTTTATCAAGACTTATTTTG
TGGCATAACATATGGCCTATTTTTAAAAAAGATGCATGCACTAATAAGAATGTATATTCTGCAGT
TGTTGGGTAGAATGTCTGTTAGGTTCATTTGGTCTAGAGTTCAATTTAAGTCTAGTGTTGCTTTGT
TGATCTTCTGCCTTGATGATCTATCTAGTGCTTTCAGTGGGGTGTTGAAATCTTCAACTATTATTAT
GTTGCTGTTTATCTCTTTCCTTAGGTCTCTTAGTATTTATTTTATAAATCTGGATACTCTGATGTTG
GGTGCATATATGTATGTACATATGTATATATGAATAATTATTATATTCTCATGTTGAATTGATCCCT
TTATCATTATATGACCTTCTTTGTCTTTTTTTTTACTATTGTTGATTTAAGGTCTGTTTTATCTGATAT
GAGCATAGCTACTCCTGCTTGCTTTTGGTTTCCATTGCATGGAATGTATTTTTCCACCCACTTATC
TTGAGTCCATAAGTGTCATTACCAGTTAGGTGAGTTTTCTTTCATCAGCATGTAATTTCATCCTGT
TTTTGTATCCATGTCATCAATGTATATCATTTAAGTGGAGCACTTAGTCAATTTACATTTGAGGTT
AATATTGGTGTGTGAGATTTTATTTCTGTTATAATGTTAATTGTTCCCTATTTACTTTGTAGTCTCA
GTTGTGTAATTGTTTTATAAGACCTGTGAGTTATATACCTCTGTGTCCTTTTGTGATGGTGATTATT
AACCTTTGGTTTCCATGTTTAGAATTTCTTTGAGTATTTCTCCCAGGACTAGTCTACAGGTGACAA
ATTCCCTTAATGTTTGCTTGTCTGGGGAAGACTTTATTTCTCTTTTATGTATGATACTTGGTTTAGT
AGGATAAAAAAAAAAATTCTTGGCTGACACTTTTTTCTTTAAGAAGACCAAAAATAGGACTCCAAT
CTATTCTAGCTTGCAGGATTTCTGCTGAGATCTGCTGTTAGTGTGATAAAATTTCCTTTATTGTTAA
TTACATGCTTTTCTCTTGCTGCCTTTCAGATTTTTTTCCTTCCTGATCACTTTAAATAGTCTGACGA
CTATATGCCTTGGTGGTCTGTCTTGAAAAAGTATCTTAAAGGAGTTTTCAAAGCTTCTTGTAGGTA
TAAATCTATAACTAGAATAGGAAAGCTTTCCTCAATTATTTCCTCAATTAGGTTTGTTAAACTTTCT
ATTTTTTCTTTTTCTCTCTCAGGAATACATATGACTTGTAGGTTTGAATGTTTTACATAATCTCATAT
TTTTCAAAGGGTTTGTTCCCTTTTTAATTTTTTTTATAAAATTTTTGTCTTACTATGTTAATTTGAAG
ATCTGTTCAAACTCTAAAATTCTTTTCTCTACTTGGTCTAGTCTACAGTTAAGGCTTTCAACTGTAT
TTTGTAATTCCTTCATGACTTTTTTGTTTCCAGAAGTTCAGAGTGTTTTTCTTTTTTAATCTATCTCT
CTAGTGAACTTTTCATTTGTACCCTCCTGGGGCTCATGACTCTCAATTCAGATAGACACTGCTGTT
TATCCCCTGGCCACAATGTAGCTAAGAGCCATAGAAAATAATTGTTCTATGGCACTCTGCCAAAT
GGCTTTGACTTGGAACCTCTTCCCTCAGTCTAAAACAGATAGCTTTGAGACCTGCCTGTTTTCCA
CTGCAAGAATGCTGCTGCTCCATGTGTAGAGGGAGAGGGGCTCTGCCTTTCACGCATTCCCAAT
CCAGTGGACACTCAACAGTGGAGGAGGTGCAGCTGCTCTTATTAGCCCTGGAATGGCCATCCA
CAGGTGTGTCCATGCCTCTTCCCTGTGGGATCAGCCACGTCTGTGTTTGCAGCAATGAGCAGGG
TGAGGAGAAGTTCCCTTCTCCACATCCACGTCCAAGCAATGGAGCTGCCTAGATTCTAGGACAG
AACACACTCCTCCCCCACAGGACTGAACACAGTGTCTGTGTATCCACTAGGAAAGGGGAAGTC
ACCTTCAGCCCACAAGTGTGGCACTCTGGAGGCACAGCAGAGCACATGTTCTTGTCTCCTTTGTT
CCAAGGGGGCTTTGTTGGTGTGCTACACTCTCTCTTCCCCTAGGAGCAGCATGGCCCAAGGGCC
AGATCACTGGGAATTCTGCAGCTTCCCTGGGTTCAGTCAGCCCTGTCTGGCTACCTTAATTTGAG

CAGGTGCTTTGGAATGTTTGCAGGGGATCCAATGATGTGGCAACAAAAGGGCTGAGATTCCCTA
GGCAGGACAGTCCCACAATGGGTATGCATCAACCCACCACTGCTCAGGTGTGGCAGAAGAGTG
AGCAACCTTGCATAAACTGGCAGCCCAGTGCAATGCCCTCAAGAAGTTCCCACATCACCACTCA
CACCAGTGCCTGGGCTCATGATGGCAGAGGGGCTCTCCAACAGTTCAGCAATCAGCAGCCTAC
TGCAAGGGTGACAGGAGCCAGAAAACCCCGCCTACCCTGTTCACCAGACACCAAGTCCCTTGA
AGCTCAATCTCTGCCAAACCCTTGCTTGTTTTTTTTTGTTGTTGTTGTTTTTTGTTGTTGTTGTT
CTGTGCTTTAGCTTGTCTGGTAGGCTCTGGCACTCTTCTCTTAATATTCTATTCAAGTTATGATTAT
TCATCTGTAAGTTTGGTTCTTCTTTCTGAGGAGAACTGGCCTCTGACATCTCTAGTCAGGCATCTT
GGAAAAAATCTCTATTTTTATTTTAAGGGGAAAAATATTAAGTAGCACATTTCAATTTAAAAATTC
TTAACCAATTTACAATTTTTATAAAATGAAACTAAGACACAGCAATATGTGTATATGCAAAAGTA
ACAAATTATAATAGTAAGATATGAAATGCTGAAAACATTGCTTCCTGATTATCAATTATTAAATAA
TATGGTTGTTAACAAACAATACCTTTATATCCAATGACATCATCTCGAATCTGCTCCTAATTAAGT
TTCCTCTGGTCCTGCTAGTTCTTGTTTCAGGAATAATATGTGAATTGAGGGGAATTCTCATGACCT
CTTGCTGAAGTTGGTGGAGGTTGAGTGGGGCTTTAAAATTCAAACCTTCTACTTCAACTACTAGG
TCACTTGCTCATATGCTCATTGTTTTAACAATGGCTGTGTGTTTCTTTTCCTAGCTGTGTGTTTCTTT
TCCTACTAGGGCATCGCACTCTGTTTCAGGAGCTGTTTGAGTACAGGCAAAGATAGTTGTTGTTT
AGCTATCAAGTGTGAAAAGAGGACAAAGGAGCAAAGGAGTATTGCAAGAGCATCTTTATAGTG
ACAAATATTAAGTCTTCTTTGGCAAAGAGGGAAGTGAACAAAAAAGAAGGTTGATGGACAGGA
ATGCACTGGGGAGACACAGACTCTGGGAAGGAGTAGTTTAAATGGTACTTTAAAAAATATTCAA
CCTTTCTTCTATGTCTCGGGAATTAAGGTATGGGTGACAGCATTGGAGGATAAGCTTACGCAAG
AATAAATTATTTGAATTGGTGCTATATTTGCTTTCAGTAGAAATGTTGCAGTTGATTAATCCATAA
ATCAATAAAATGTTAAGAAGGATGAAAATCACATTGGAAGTAAATTAATTAGAAGACTTTAGAT
AGTCTAAAAACTGAATCATGATGAGGTGGCAGGTTGTCATTTCTCATTAAGCCTTTTCACAATAA
TAAATAAAAGAGTGTATTTTTGGAGACAAGAATGTGCTGCTAGAATTCAATGCTTCAAGATATGA
GGGCATGAAAGAGAGCAGCCGAAAAGAAAATGAAATAAATAAATGTTTTCTCTCTTTAATCTGC
CCGAAATGCAGTCATGTAATGATGGATGGCTCAATTTATTTAGCGTACTCTGCACAGCAAAAGG
CAGGTTGGTTTCCATAGGTATTTGCATAGTTAGAAATCTTTGTTCCATTACTTGACTTAAAGAAGT
AACATGTCATCAAATATTTATTTCTTATACGGTAATGTAGCTTCTCAGTGGTGTCATATGATATGA
AAGGGAAAAATGAATATAGAATAAAGTAAAACAGATCCTAAAACTTTTCATAATAAAGATTTCT
AAACATAAGGCCCATGAATATCTGAACAATAGTTTTAAATATCAATGATTGCATCCCACACACTT
TCAATCTCTTTCCAAGGCATTTCCTCAGACCACCTGACCCTTCCTTATTCAAACTAGATTGGTATA
TTATTGCCACAGGAGAGAATATATAGCCAAACTCAGGTTACAAGGGAGTCCTCTCCATGAAAAT
CTAAAGTCTCTTGAAATTTATGGCAGAGGCATTAGCTTGAAGAAAAGATGTTTTGAGAATCAAAC
CAAAGGTCCTGAAGGATTCGAGACATAAAGTTTATGCCATAAAAAAATACATTTGAGAGAATGAT
GAAGGCCAGGATAACCTTGTCCCCTGAATAAGAATTCTATAAAGTAGCCAGGGAGCATGAGTT
GCCAAGGGAAGAAAGAGAAAACATACTTCAGTGCATCCCGGATGAGGAGCCTGTGTCTTACGC
CCTCACCCAAGGTCCAGAAGCAATTGAGGAGTTTTGCAAAAGTGGGTGTAAATTGCCTTTGATT
CTGATTATATGTTATATGCACTAGGAGAGGTAAATTTTTTAAAGTACTCATGCAGATTTGAAGAA
TTGAAAGCAGAGGTTCCAAATCTGTAGGTGAAGGAAAAAAAAAAAAAACATTGCTCCCCATGACT
AGGGAGCAGCCAAGGGAAAATGGCTATGACATGTAATTAGGTAGACACACCTCAAGCAGCCTC
AGAGAGTTCTCATGCTTTCCAGTGTGGCACAGGAAGCAGTAGAACTTTTCCTTATGTGGCATAG
AATGGTGAGGGCGTGACTCAGAGAGCTGAGATCCTGGAAAGTCCTAGTGGTTGAGATGATGGG
GACACTGAAGTCATCTGTATGAACAAAAAGCAGACAACAGAGAATGCTGAGGGTGTCTTAAAG
GAACATCAGCATGGATGCCTGAGACTAGATGTTCCCTACTTCCACCGTGCCACATCCTCATTCTC

ACACCTTGGCACTAGTTCAGACTCTGAAGAACTTAGGAAAAATATTGACATCGTTGGGAGTAAA
GCTAATGATTAACCAGTAAATTTGGGCTGGGCACAGTAGCTCACACTCTTAATCCCAGCACTTA
GGGAGGCCAAGGCAGGAGGATTGCTTGAGTTCAGGAGTTTGAGACCAGTCTGGGGAACATAAC
AACACCCCATTTTACAATACAATTGTTTTAAAAAGAAAATATTAGGTAAATGTGTTGGCACATGC
CTGTAATCCCAGCTACTCGGCAGACTAAGACAGGAGGACTGCTTGAGGCCAGGAATTCAGGGC
TACAGTGATTATAGCCTAGGAAACAGCAAAGCCCTATCTCCTAGGAAAAAAAAAGAAAATCGG
AAATTGACTAACAATACCCAGACGGAACTTCATCAAGAGGCATGGGGGAACATGAGAAAAAAA
ATTAAAATTTTTCTTAGCAAAAATATAAAGTTAGATGTTTTTGTTTTCCATTCAGGAGTTTGTGCTT
GGGAAGTCAACAGCATCTTTGAGTACACACAAAATCCAGATCAATAAACAGATACACTATGGG
GTGAATATTCCTTTTGTAAAAGTGTTCTTCACTATGGAATAATGTACCAGAGAATGCTTTTAAAAA
TCCAATACTCTTTTAGTGCATATAACACATAATTGGAATTACAGAAATGCAGTTTACATAAACTTG
TAAGAAGATAATCAAATGTCTAAAACAAAGCCATTTAAAACCTGCCATTTCTTTGGAGTGTTTAA
CAAAATTCGGGTATCCATCATTCTGTAGGGCTTAAGAAACTGGTGCCTGTTTTAGTCACAATCCA
GTAAGAAACAGATGATATTAACATGAATGAGCAAAGGGACAGGCCTATGGGTATCAGAAAGAG
GGGACAACCTGCTAAGATGTGACGGCAGGGCCTGAGCCCTCAGTTGTGGCATCTGGAAAGAAA
TTCAGGCCATTATCCTTAGCAAACTAACACAGGAACAGGAAACCAAATACCACGTATTCTCACT
TACAAGTCAGAGCTAAATGGTGAGAACACATGGACACATAAAGGGAAACAAAACACAAGAGA
ACCTTTTGGAGGGTGGAGAGGGGAGGAGGGAAAGGAAAATAACTAATAACTCATCGGTATTAG
GCATAATATCTGAGTAAGGAAATAATCTGTACGACAAACCCCCATGACATAAGTTTGCCTGTGT
AACAAACCTGCACTCGTACCCCTGAATTTAAATAAAAGTTAAAATAAAAAAGAAATTCAACTGA
TTCCTTTTTCCAATGAACATATGAGAAACACAATAATTAGGGACACATGCACACAACCTTGGCA
CTAGGACAAAAAAAAAACTAAACAAATAAATTCTGTAGGAAGTTGAAGAGGGCTGAATAGGAA
TCACTATAGGTTAGTGAATAGAATCAGCCCCTCTCAAGTCCATATTTATGGAATTCTTATTTCTCC
TACCTATAGCTTGAACACAGGAGTCAGAAAACTCTTCCATTTGATTACTCTGTAATTTGCAGAAG
ACAACATATGATCTGGGGAAAAATAATGTTTATGGCTGCCTTTGAACTCCACCAATTAGCCTCAT
TGTACTCCTCTTACAGTACTCCTGTAAGATGCTCAGGGCTTCACCTACTGATCTGGGTTCTTTTTC
CCAGGGCATAAAAGGTGCCAACAAATATTCAAAGATCATCCCATCCTGTAAGTCAGAAGCCAG
GTTAAAGCAGAATATCTGGGTTGGCAGAATCTGAAATGAAAAGAGGATTTCCTTTTTCTCTTGCT
TCCAGGCATGTACACTCACAGTTCTTATTCAGGACACCCCAAGGCTGCAGAATATACAGGGCAA
TGAAGTAATCACACTTGAGACTCTTACTATAGTATACTGAAAAATGCATTTATATAGCAGTATAC
GGCAAATTTTTGTTATTTATGGTAATAGTGTCCTAAAAGTCACCAAGAACACTGAATTAGTGAAT
ACTAGACTATTGCTTCTAGGGGAAAACGGTTAGGTTCCTATATGCCCCTAGTCATGCCATTTTCA
TCAACCAATCAATAAGTAACCTTGTTTTATGTATGTTTCTATTTAAAGACACCGTATTTAATATATA
TTGCTGATTAATATTGAACTTTAGGCTAATATCACTTTAACTCATGCCTGGATGGAGCTTATCTTA
TATAAGCTCTCTAAAGAACATCAAGGTTCCTTCCCCTTAAAAACACTAGATAGTACTTCAGCACT
GCCTTTGGAGCCAGTTAAAGCAGCAAAATAACCAACAAAAAAAGGAGAAACAATGCAAAAAAA
ATGGCACTAAATAGACCACAAAAAGGACACATTTACAGTATGAGAGCTGAAATAAGAAGGTGG
TACATCACCTTACCTGACCTTAGCTGGGAACTACACATTGGGAAGCTCAAATTTTTATCAGTCTTT
GCATGTCATAGTCTGCAAATTACCATCAAAGTGACATGAATATTGATTTGGGGGTTAGAGATAC
ATTTTAGTAAGAAGGCAAATTTTCCAATATGGAATCTGAATAATGAGGATCGACCATATTTAAGA
AGCATGAATGTTAGCTTGCTAGTGGGTACAAATCAGGCAGATGATTTTTCATCAATGAAAGAGT
GCATGGTTGAAAAGTTTTCTTTATTCTTATTTGTGTTTGGCTTTAGGAATTCTGCAGGTTCTTGCAG
GACTCCACATATTGTCTATCACAGCATATTGCAGACAGGTTCCCAGGAAGCAGATTCTGAGTTG
GTGATTTGCATGCAGGAAGTTTATGGAGAATTGTTTCTGAAATCAGCTCCTGTGGGGAAATGAA

GACAGCAGGACTGGGCAAAAGGAAACATTGAACTGGGATGAGATCACAAAGAAGGCATCAGG
GAGGGCTGGAGCCAGACTGGGACTTCAGAGTGGCCCCACATTGAAGAAGAGAAGCTTGGTTTT
TAAACTCACACTCTCTCCCCAACCAGGACAAGTTATGCAGGTGGTCCCCCAGAAGAAACTGTGA
CTTTGGAGTTGGTGGCGTTTTTCAGCAAATGGCAATTCCCAAAAAGGGATCCAGCTGCATGCAG
TTGCTGTCAATGCTCTCAGCAGCTGAGGGAATGAGTGCCTGCGTCATGGAGAGGAATGCAGGT
GGTACGCCCCGACACTCACTACACTGTCAAGCACAAGAGGGCATTTCTAGCAACTCAGTTGGA
AACGCTCTCAATGATGTTTAACAAAAGGATAGAATTACTTATGGTGGCAGAATAATGGCCCCTG
AAGATATCTATGCTGTGATTCCCAAAGCCTGTGACTATGTTACATTACATGGCAAAAGGCCTTTG
CAGATGTGATTAAGGTTAAGGACCTTGAGATGGGTTGATTATCCTGGATTATAGAGGTAAGTCC
AATGTAATCACATAAATCCTTGAAAGTGGAGGACCTATCCAGGCTTCAAAGAACCAGAGAGATT
TCAATGTGGGAAGGGCTCACCTAGCTGTTGCTGACTATGAAGGTGGAGGAAGGGGGGCCATGA
GGCAAGGAATACAGGCAGCCTCTAGAAACTGGAAAAGGCTAAGAACTGGATTCTCCCCTAGAG
CCTTCAGAAAGGAATGCAGCCTTCAACTTAACCCTAGTGAGACCCATCCTGGACTTCTGTCCAT
AGAATTAAAAGATAACAAATGCATATTATTTCAGCCATTAAGTTTATGGTAATTTGTTGCAGCAG
TCACAGTAAACTAATACACTGCCTGTCCAAATAGTGAACTTGGCTCCATGCATTGATGGAGAAG
AAAGACACCCGAGGGCTCCATTCTGCAGGATGAGTCTGCAGGGATCTCCTTGCCAGCTATGGA
AGTCAGTATGACAGCTTCAGCTACCTACAGTCCACTTGCCAGGTGATTATTATTATTATTATTATT
AATTTGCACTATCGCATCCCAGTGTGTGCCTCCAGAGCCATCTATTTTCTTTATTTTTTCACTGCCT
AAACTGCTTATAACTGAGAGTCACAATTGCAGAGCTATTATCAATTAATTTGCTAGCAGCAAAAG
TCGGTGTGACTCACAATCAGGCAGCCCTGCCCAACCTAACAGCTGTGGCTTCAGAAAGGCAGG
AGTGCCAAAACACCTTTCTTCTTGCCCTTCATACTATTGGTCTTCCCTGAAAGGAAACACTTCCC
AAAGCTCACTGATAAAATACTAAAGTACAATTTTGTTACTCGCCTAAGAGCACTCCTACCTACTC
TGTGCTGTGCAGGAGAAAAAATGGTGAACAAGAAAGACACAATCCTTACCTCTAAAGAACTTCA
GATTGGTGATGATTATAGACAAGATTTAGTTTTGATAGAGTGTCTGTGTTTTTTGATAGGAAAAA
AGCAGAGTTCTCTATGACCATGTTGGAGAAGCACCGAACCCAACTTTAGATGGTCAGGGAAGA
ATTCTCCTAAGAAACAACACTGAAACTGAGACTTAAGAATGCATAGAAATTGTCTAGACAAAAA
GAGTAATGGCAAAGACTGTTCCAAGCAAATGGAATGTGGACAACTGCAGAGGCAACAGTAATA
ATCACTAATATTTATAATATTTATTAAGTATTTACAATATGTTAGGCCCTGTGCTAAGTGCTGTAC
ACACACAGTCTTGTTTCATCCTCACAACAACCTGATGAGGTACGCACTGCTATTGATTCCATTTT
CCAGATAGGAAAGCTGAGCAACAGTGAGTTTGTACTTCTTGCCCAACATGACACAGCTTGTAAG
TAGTTGAGTATAGAAATGAACCCAAGCATTCTGGCTCTAGAATCCTCTTGCTTAACTGTTCTTCTG
CACTACCTCTTACTGAGTCTCAAGCAAGTGATCCAATGGCTCATCCAAGGAACTGAAGTAAATT
CCAGGTGCTTGGAGCTGAGACAAGTGGGGGCAGGGAATTAGATTCAGCAATAGCAAGAGATG
ATGCAAAAGTGAGTATTTATAACATATTATAATCATATCTATCCAGTACTCAAGATTACATCAATC
TTTAATGAGGACTTGGAATCTGCGGGATACAGTGGGAAGGGCAGAGGCTTTGGTGTCAGACAG
ACGTTAGTTCTGATCTTCCCACTAATGCGTAGAAGCTGAGGAATTTAACTTAAGCAAGTTCTTTA
AACTCCCCCTATACTCCCATCTGTGCCTTGGAGATTATAATAGCTACATCAAAGAGTTGTTGTGA
GGACTACATGGTAAAAAGTACCAAAGTGTCTGGCATATAATGAGTAGGCACTCAAATGTTCAGT
TTCTCCCCTTCCTTTCCCTTATTGAAAGCAGCAGAGCAGACTGTCTAAATATCTCTGCACACTTCA
ACCAACACAGAAAAAGAACTTGAACAAAACTGAACATTGATTCCCCCAATTTAAAAAAAAAATGC
ATTTGATTTGGGGCTCAGACCTCAGATATTGTTGAACCAAAAATTAAACACCAAAACCAGTGTG
GTTTTGAGGAAGGCCACATGGGTTAGGAATCAGGACATGCAAATGCTAGTGTCAGCTCCACCG
CAAGTAACTATGATGCTTTCTTCTCTGAGCATCAGCTTCCTCATCCAAGACAAGAGCTGGTTGAA
CTTGGTGGCCTCCAGGGACCACTGAGATCTAACTTTGAGCTCACTCAATGCCTTGAACATCTGAT

TAGAAACAGAACTGGAAGATCAAATTGATCTCCGAATTAAGACAAGATTCTGAGGTTAAACAAA
CAACAAAACAAAAGAGATCTAATAACCTTCTTGGGAGTTGCAGAGAATCTATGTAGGAAGATAA
GAGGTGGAGTCTTTGAAGGAGATAGACATTACAGTCTCATTTCCTCTTTGTTCTTTGCTAGATCTG
TCACCCAAAGTAAATCATTCTCTGTTTAAACTTGTTTCCTTACCTATCAAATGAAAATGATAACCT
TCTTTTATAGACTTGTATTCAAATTAAAGAATATGTATGGCCGGCCAGGCGTGGTGGCTCATGCC
TGTAATCCCAGAACTCTGGGAGGCTGAGGTGGATGAATCATGAGGTCAGGAGTATGAGACCAG
CCTGGCCAACATGGGAAAACCACGTCTCTACTAAAAATACAAAAATTAGCCGGGCGTGGTGGC
GAGCACCTGTAATCCCAACTACTCCGGAGGCTGAGACAGGATAATTGCTTAAACCTGGGGGGC
AGAGGGTGCAGTGAGCAGAGATTGCACCACTGTACTCCAGCCTGTGCGACACAGCGTGGCTCT
GTCTCTGGGACAAAAAAAAAAAGAAAGAATATTTACAATTTTCAGTGTACTCTTTATCCTCCAAGA
GAAAAAATAAAATCATTAATCACAGGCCTCCAAGAAAGGTTAAACTCTCCTCCTTTTTGCTCCCA
TCACTTAGGGTCCACCCTGTGCATCATCAGGACATTCACCTTGCTGCCTCCTATGTAAACATTTC
CTCTATTTTGAGCTCCCTCAGGGCAGATACTTTCATATTCACATTTATATCTAAATAGGGCCTAGA
ATTGCTCCCTGTGAATAGTAGTTTTTAATGAATGTTGGTTGTTGAAACTAATAAAAAAGAAAATA
AAAACAATGAGATTGCATTTTTATCTAGTAAATTAGTCAAGATTTCCAAAAACATACACATTTAAT
CCAGGCAAGTGCACATGAGCTGATGGAAGTTTTCTTGAAATCCATTTAGTAAATATGTTTCAAGA
CTTTAAAAAGTGTCTATACGATGGGGTTTTCTAGGTAAACAATCATGTCGTCTGCAAATAGGGAC
AATTTGACTTCCTCTTTTCCTAATTGAATACCCTTTATTTCCTTCTCCTGCCTGATTGCCCTGGCCA
GAACTTCCAACACTATGTTGAATAGGAGCGGTGAGAGAGGGCATCCCTGTCTTGTGCCACTTTT
CAAAGGGAATGCTTCCAGTTTTTGCCCATTCAGTATGATATTGGCTGTGGGTTTGTCATAGATAG
CTCTTATTATTTTGAAATACATCCCATCAATACCTAATTTATTAAGAGTTTTTAGCATGAAGGGTT
GTTGAATTTTGTCAAAGGCTTTTTCTGCATCTATTGGGATAATCATGTGGTTTTTGTCTTTGGCTCT
GTTTATATGCTGGATTACATTTATTGATTTGCGTATATTGAACCAGCCTTGCATCCCAGGGATGAA
GCCCACTTGATCACGGTGGATAAGCTTTTTGATGTGCTGCTGGATTCGGTTTGCCAGTATTTTATT
GAGGATTTTTGCATCAATGTTCATCAAGGATATTGGTCTAAAATTCTCTTTTTTGGTTGTGTCTCTG
CCAGGCTTTGGTATCAGAATGATGCTGGCCTCATAAAATGAGTTAGGGAGGATTCCCTCTTTTTC
TATTGATTGGAATAGTTTCAGAAGGAATGGTACCAGTTCCTCCTTGTACCTCTGGTAGAATTCGG
CTGTGAATCCATCTGGTCCTGGACTCTTTTTGGTTGGTAAACTATTGATTATTGCCACAATTTCAG
ATCCTGTTATTGGTCTATTCAGAGATTCAACTTCTTCCTGGTTTAGTCTTGGGAGAGTGTATGTGT
CGAGGAATGTATCCATTTCTTCTAGATTTTCTAGTTTATTTGTGTAGAGGTGTTTGTAGTATTCTCT
GATGGTAGTTTGTATTTCTGTGGGATCGGTGGTGATATCCCCTTTATCATTTTTTATTGTGTCCATT
TGATTCTTCTCTCTTTTTTTTCTTTATTAGTCTTGCTAGCGGTCTATCAATTTTGTTGATCCTTTCAAA
AAACCAGCTCCTGGATTCATTGATTTTTTTGAAGGGTTTTTTGTGTCTCTATTTCCTTCAGTTCTGCT
CTGATTTTAGTTATTTCTTGCCTTCTGCTAGCTTTTGAATGTGTTTGCTCTTGCTTTTCTAGTTCTTTT
AATTGTGATGTTAGGGTGTCAATTTTGAATCTTTCCTGCTTTCTCTTGTGGGCATTTAGTGCTATAA
ATTTCCCTCTACACACTTCTTTGAATGCGTCCCAGAGATTCTGGTATGTTGTGTCTTTGTTCTCGTC
AGTTTCAAAGAACATCTTTATTTCTGCCTTCTTAATAAATGGTGCTGGGAAAACTGGCTAGCCAT
ATGTAGAAAGCTGAAACCGGATCCCTTCCTTACACCTTATACAAAAATCAATTCAAGATGGATTA
AAGATTTAAACGTTAGACCTAAAACCATAAAAACCCTAGAAGAAAACCTAGGCATTACCATTCA
GGACATAGGCGTGGGCAAGGACTTCATGTCCAAAACACCAAAAGCAATGGCAACAAAAGCCA
AAATTGACAAATGGGATCTAATTAAACTAAAGAGCTTCTGCACAGCAAAAGAAACTACCGTCAG
AGTGAACAGGCAACCTACAACATGGGAGAAAATTTTCGCAACCTACTCATCTGACAAAGGGCT
AATATCCAGAATCTACAATGAACTCAAACAAATTTACAAGAAAAAAACAAACAACCCCATCAAA
AAGTGGGCGAAGGACATGAACAGACACTTCTCAAAAGAAGACATTTATGCAGCCAAAAAACAC

ATGAAAAAATGCTCATCATCACTGGCCATCAGAGAAATGCAAATCAAAACCACTATGAGATATC
ATCTCACACCAGTTAGAATGGCAATCATTAAAAAGTCAGGAAACAACAGGTGCTGGAGAGGAT
GTGGAGAAATAGGAACACTTTTACACTGTTGGTGGGACTGTAAACTAGTTCAACCATTGTGGAA
GTCAGTGTGGCGATTCCTCAGGGATCTAGAACTAGAAATACCATTTGACCCAGCCATCCCATTA
CTGGGTATATATACCCAAATGACTATAAATCATGCTGCTATAAAGACACATGCACATGTATGTTT
ATTGCGGCATTATTCACAATAGCAAAGACTTGGAACCAACCCAAATGTCCAACAATCATAGACT
GGATTAAGAAAATGTGGCACATATACACCATGGAATACTATGCAGCCATAAAAAATGATGAGTT
CATGTCCTTTGTAGGGACATGGATGAAATTGGAAACCATCATTCTCAGTAAACTATTGCAAGAA
CAAAAAACCAAACACCGCATATTCTCACTCATAGGTGGGAATTGAACAATGAGATCACATGGAC
ACAGGAAGGGGAATATCACACTCTGGGGACTGTGGTGTGGTGGGGGGAGGGGGGAGGGATAG
CATTGGGAGATATACCTAATGCTAGATGACGAGTTAGTGGGTGCAGCGCACCAGCACGGCACA
TGTATACATATGTAACTAACCTGCATGCACAATGTGCACATGTACCCTAAAACTTAAAGTGTAAT
AAAAAAAAAGTGCTTATAAAATATGAAAAAAAAAAAAGTGTCTATACACTCCCATCCAGTAACTC
CACTCGTAGGACTTCATCCAATGGAGATAATAGGTTATTTGCCATGTAATAATAGCAATGCATGG
GGAAAAAATTACTGTAAGTAAAAACACCAAAATATTAATAGTGGCCGTTTCTCAGTAGAGGGAT
TAAAGGTGATTTTCATTCTCTATTTAATTCCTTTCTGTATTTTCCAAAATTTATTTAGTGATCACGTA
AAATTTTTCCAGCAGGATTATATAGAACTTATTCATTACATGGAAGATTTAAAAAGTTTAAATGTA
ATAATTTAAACTTATACTAATTCTGAATATGTTTAAATAATTCTGAATATATTTAAAGCATGTTAAA
GTTTCTCAAATGCCTAAACTTTTAAAATTTATCCCAATACCCAATATAGTCCCTTGCATGTTGGAG
GCTCTCTGTAAATTCATATATACAGTTGGCCCTTCATATCAATGGGTTCCACATCTGTGGATTCAA
TCAAACATAAGTCAAAAATATTTGAAAAAAAGTGAATCTTTGTGTCTGTACTGAGCACGTACAGA
TTTCTTTTTTCATGTCATTATTCACTCTTCAATACAGTATGACAATTTCCATAGCATTTACATTATAT
TAGATATTATAAATAATCTAGAGATGATTTCAAGTATACAGGAGGATGTACATACATTATATGCA
AATAGTGCACCACCTTACATAAGGGACTTGCATCCGTGGATTCTGTCACCCTTGGGGATTCCTG
GAACCAATCCCCTACAGATACTGAGGAATGACTATACAATTTTACATGAGTGTATCTACTTGGGT
CTTTAATTTCTCTTTCTTTATTCTGTACATTTAGAAGTGTCACTTATATTTTTCTTCCAGTCATTTGA
TTTCCAGAGACATGCAAGAGTCAGCAGATTTATTTTTCTCCAAAACAGGTTAATGCTCCACTTAA
CTGCTGTCCAGATAGTTTTTATTAAAAAGTAGCACAGTTCTCCCTGTGAGATTTCAACATCACAT
GCTAGGAAGAACTGAGAAGTTAACTAGGTTATAAGGATTTGTTAGATTGTCTAATCATTGCTGTG
CGGTTACTCAGGTAAACACAAATGAAGTTCTTAAGTAAAAAATCATGATTGTGACTCTTTTTTGTT
AATGGAATTCTTTTAGGTTAAAAAAATGCTGTAATTCTTTTTTTCTGTAGAAAAGCCACTGTTAAC
TTGGTCAAATTTATTTTCACTGCATGAAAATTTTATCTTTCAAGAGAATTAGCCATCATTGCATCTT
TTAAATAGCATTAGCCATTATTGCATCTTTTTAAAGTTTTTAAACTTTTTCCCTGTCTCTTCAAGGC
TTCCTGCTTAGGTTTGTAACATCAAATACCAAACTTCTTGTATTTTCATGAGATTTCCACTATATTC
TTCCTCTCCTCTGGCCTCAACATGATAAAGTCTTCCCTCCTACATTAAAAATATGTCCTTCCATAT
TATTCCTTTAAGCTCTGAATCTGTTTCTCTCATTCCCTGAAGTATTAATGTTTTGAAAAGATAGTTC
ACACTCAGCTGAACAAATTTAAGTTCTTTAACCTTCTCTTACTCCAATTTCTTATTTCCACTCTTGC
TGAAATATTTTCTTGAAAGTCACCAATCAACTCTTATAACCACAGCAATGGCTCATTTCTTTAATG
TCCTCAACTTCTCTACATTTAATGTTACTTGTTCTTCTATCTTACTGAAACTCACCTCTGCTCACTT
AGACTCAAAGGGTAAGAATGTCAATGATCAAGGAACTGGGATTTCACCTTTGACTCAAAATTCT
CTACCAAGCAATAGTTTGAAAGTGGCTATAATTTTGTAAAACATTTCCACAAGTTAAACATAGGT
ATGTTTTTACTGTAACGTGAAGAATAAAACATGTGTCTATGGAATTCTGTCTTCATACATTGTTAT
CAACCTCAACTCATTCTACTCCTTCTGTGGTAACTATTTTTAATAACTTGGTATTTTTCAGCCCTTT
TTCTATGTAGCCATGTTAATATACTGGTATGTATACAAAAATAGAATTTTTACAAACATAAATATA

TACCTACAGTTTTAGGTAACAAATGAGATCATGTATGTATTTTTTCTTAAACCTGTTTATTGCTTTA
AAACTAAAAAGAAGAGATACTAAGTAAGGGTAAGAAGGAAAATAGCATGAAGAGTGGGGAGA
CACGAAGATGGGCAGATGGCTTGCGTTTATGCTTAAAATACCTTATGAATACATAGGCATATTTC
ATGTCCATGCCACACAAAGCTTCTTAAATTTAGCAGTGGAAGATTAAGTGTAGGATTTTGGATTT
TGATTTCTTTTTTATAGTGTTTTTGTTGCTAATATTAAAAATGAAACCACAGACAGCTTGGTTTGA
GGGGCATTGACTAAATTGTCTTTCCATTTTTATTAGGCAATTGACAAAAAGGACTTATTTCTTTGT
GACTTTATTATAATGGTTGGCTTTGATTTGCAAAAAGTGTGAAGTCGAAGATAGCTTTAGACAAG
GAATAGAAAGAAGGTATCTTAATTTTAGTATCCTTTTCACATTCTGTCTGTGCTGTCCAAGGTGAT
TAGATTTTACAGCCCACTCATGCTGTAAATATCTTTACATGTGGTAAGTCTTACCCTAGCACTTTA
GTAACATGCCTCAAACAAAGTATTAAAAGCTTTGAACAAGATGGCATCTAAGTATGATGAGGTT
TACTAGCTTTTTAAAAACATGAAACACCTTTTGAAGTGATTTTCAGAAGAAGAAAGATTTGTACT
ACACAGGAGAGGATTTCAGTGCTGAGTTTAAAAAGAAACTCAGGTTGGAAAGACTTTAGAGGA
TTTTTCTATGAACTAAGAGATTAGAGATTGATATCTTGTCAAGCACTTTGTAGAAAAAAGGAAAG
GTTATAGAAAGGCAGATAATAGACTGGGCAAGGGGATTAAAAGATGAGGCTAAGCAACATTTC
TTTTATTATACACATAGTCATTAACAAAGAACTAGAGTTCTTGTGAATTATGTTACATTGAAAAAA
ATTAACAGTTGATTAAGATAAGATCATTACCTATTACCTGTAGAGATCAGAAAAAAGATTTAAAT
AAAACATGTTACAAAAAATCCAAAATTTAAAAATATTTTGGCATTTAGAGACTATTAAAAAAGAG
ATAAAGGATACTTTAATACATGTTTAATGAAGGGTTGAGGAAATTGGTATCTTTTCCTAAGGACT
TTAAGACTTATGATGGCCTAACCCTCAGATGTGTGCAGCGAAAATATTACTTCAGCAGGACAGC
ATTGACTGATATTTTTCTCTCCTTCATTCTCATAAATGTGGCTAGAGAGACCTTAGTAATTGATAC
CCTCAACATCGACAGTTCTCATTTTACTTATTAATGTTGATCATCATACATACCAGGAGCATTAAA
AATGCAATAAACAATTGATTTGAGCTTTTTAAATAATTTCGACTTTTATTTTACATAAAGAGGATA
CATATGCAGGTTGTTACATGGGGGTATTGCATGATGCTGTGTTGTCTGCAAGGTTTTACAAAACA
AATACTTGGTGGCTGTTTATTTCTTCCAGCATAAGTCCCCACCTGCTGACCTGCTGCCTGCTATCT
TAACACGAATTCTTCATCTGAATGGATGAGCTTTTCACAGGTACGGTTACATAAATAAGGAATGT
GATACACATTTTTTTATTTAAAATGAAAGATAATATAATTTAAACCAAGAAGCTTTACATTTACTA
ATCATGTACTGCTGCTATTCAAATGCCTAAATACAGAGAGGTATCTTTTAAAACTCTAGACTCTA
CCCTTTTATTGTCTCACCCACTCCCATCCAAGATTCTTTATTCCATCCATGCAGGGCTTCATGCAT
GTGAGTGTACACATCCATTCTCCTCCAGGTGATTTTGACATGAATGCAGCCCATCAACACAACC
AAATGAAATCTACTGCTTCAATGCTTCAGTAAAGAGTAATCAAAAGAAAAGAGAAATCAAAATA
TCTGTTTACGATCTTAACTTAGTTCAAACCATTTTTTCACCCTTCTAAAGCAAGAGTCCTTCTTTCC
AGAGACAATATCTTTTTTGTACTTTGTAGGGGCTGAATCCTGTCTTTTATTTAATCTTCTTTCCTTG
GGCACACGAGTGGTTGGACATATGATCCAGCCCTATCAAATCTGCTTATCCTACCCCTCTGACC
ACAAACTCAAAATAAGCCAGTGAGAGTGAGTACTCCCAAGGATTTTCTGCCAGAGTTCTGAGGA
AAGAGAAAGTCTCCTTTTTCCACTGAGGATGTTAAGCTGATAAGATACATCTGGAGCTGAGCAG
TTCTATATCTGCCATATTTAGCCAGTTTGTTTGAGATCTACAGCTCCTAGGTTCAAACATTTCAGA
ATTTTCTGTTATGCTTATGCTGGTTTGAGTTGGGTTTCTATCATGGGCAAATGAAAAGGGGCAAC
AAATACATCACACTATATTGGGTAATTACTTAAACTCTGGCTCATTGTCCTCACCTGTAGAATTTG
GATAATGGCATCTACCATGCCTACGCACCAGAAAGGCAGTGATGGTCAGACCTAACAATTTAGG
TGCAAATGATATATTCATCCAGTAATAGGTTATTCAAATGATAAACTTTATATATATGCCAACAG
GATAAATATTTAATTCAGACAATAATATTAACCCAAACGGATTATTCAGTATTATGTAAAAATATC
TTGTGTTATATTCTCAATCCTTAGTTATTTTGTTGTCATATAAATATTGCTCATGAGCAGACAAAGC
TAAAACTTCGACCTATTCATATGCCAATAATTAATAGCAATGAATTATTCTTTGTTGGCTTACTTA
ATAGTTCAATGATTTAATAGTTATAATAGTTAATAGCTCTTTCCTCCTATATTGAAATTAAGTTTTA

GACTTCCAGTCTCCTAGCACAACATGTATCAATGCCTGTCAAGCTTGCCAAACACTTGGAGATC
ATGTTGCATGGCAGACACAACTGAAACCAATAACTTCACTTATTTGTGTTCAGAGTTCCAAACTA
AGGAATCCAGGAGTGGCCAATCCAGAGATTCATTCCTTATCTTTGACGAACATCTGAACTCCTG
GCTCATCCTGTGGAATTCAGGCCATACAGGAGATCAAGGTCATTTGCTTTGGGTTAAATGAATGT
TGCCAGGTGGAGGTTGCTAGAGGGAGAGTGCTATGTGAAAGTGCTGTATAAACTGCTTGCTTTT
CACAAACAGTAGTGATTCTTTATCCTGTCCAGCCTGCCACAACTGGACCATCCTGTATGTAAGTC
CCCTTAATAAATCCTGTGTCTCATTCACTGGC
ID|56303|gene_id|ENSG00000234147|transcript_id|ENST00000455011 Homo sapiens lncRNA

ACATTCTTTCAGATGCCAGAGGATGCTATAGAAAAATGCGTGCCCTCTTGTAATACTTTGTT
CAAGTTCTGATCAGAAGGATGAATGATGGAGCCCAAGAAGCCAGGTACAAATTCAAGCTTCTTC
CATTCATAAGCTAGGCAGTCCTTACTCAAATTTCTTATCGTATCTATTCTTTAGTTTCCTCATCTGA
AATGTAGGTGAGGCTAATTGTATATGCCAACTTGACTGGTTTCAAGACGCTTACATATTTGGTTA
GACATTATTTCCGAGTGTGTCAATAAGGGTGTTTCTGGAAGGACTGGGCATTTGAATTGGTGGA
CTGAGTAAAGCAGCTGGCCATCCCCAGTGTGAGTGGGCATCATCCAACTTGCTGAGGGTTTGAA
TAGAGCAAAACGGCAAAGGAAGGATGAATTCTCTCTCTCTGCCAGACTGTTTGAGCTGAGACAT
TGGTCTTTTCCTGCCCTTGAGCTAGAACTTACACCATCAGAACTCCTGGTTCTCAGGCCTTCAGC
TCCAAGCTGTAATTTATTCCACGACCTTTCCTGGGTCTCCAGCTTGCAGAGGGTGGACTGTAGGA
ATTTTCTGCCTCCATAATCACATAAGCCAATTCCTTATAATAAATCTCATTCTAGATATACATATCT
GTATATCTCCTGTTTGTTCTTGCTCTCTGGAGAACCCTGACTAATATAGTTATTCTGAGGATCAGT
GTTGTTTTGAAAACTGAATATTTCAGTGTGTGTGAAGTACTCAGAACAATGTCTGGCACAGGCAG
ATCCTGCACAAAGGTGGCTAATATCATTATTACCCCTTCTTTTCACCACACCTCTAGTATTTCTGT
AAGTTGCCCGTATACTAGCCCAGCAAATGAACCTTGATATTTCCATAACATGTAATTACCTAATT
CATATTGTTGCTACCATTCAACTAGAGATTATTACAAAATAATTTGAAAAACATCTTTATAATTGC
TCACTGATTAAAGATTTTAAGATGCTTTTCTTTCAAATATTTTTATTTCTCAGAGCACCTACAATGC
TTAGCGTAGGTCTTCATTTTCTGTCACTCATAATTACATTCTAATATTTTCATTATTTTAATATTTAT
CTATTTTCTCTACCTTGCATTACTGACAGGATTTTTGGCCATGTCTATGTTTTAATTTTCTGCTCCC
CACATACAATTTGAACAAAATATTATATTTTAAATTCTTTACAATTTTTCTCTTATTCTACATTTCTC
ATTTAAACTTAACCAGTTGTCTTTTCACCTAATTCTTATTTCTCTTTAGGGGTCTCTTCTTTTATTTC
ACAGTCTGGGTATATTTGTATTTTATTTATGATGCTAGTCCATCAATTTTTTTGATTTTTTAATAAAA
TAACGCACAGAACTACTTATCTTCTTCTGAGTCATTAGGATATTTTTATATATTTTTTATTCTATAGT
ATTTTTCATGCCAACTATTGGTACTTATAAGTTCTCAGGTGAGTTTGCTACACAACTACCTCTTTA
AATTGACTTTGTTGGCATGATATTAACTTCCAATGACATAGCTATATGATCAGTTCACATTGAAG
GCAAAACCCTGTAATAGACAAAATTACCTTGTCAGATTGACTCTGGTGACAAAAAAAAGGCCTTT
ATTTTCTTCAAACTATTATGAAATTATTTATATTCTATTAGAGGAAGAGGATTTAGGTAATTTAGC
AGGCCAAGTGTTTTGCAGGTGCACGGTCACAGTTACAGTGATTTGTAATTTGGGGACCCTAATA
GCACTAGAAGCTGAACTGCTCCCTGAATACTCTGCAGTGCAATAAATGGTGCCTATAACAGTGC
TGTGAAAAATCAACATACCCAAACTTACAGAGAAAATTAGCAAGTTCACTTGGGGGAAAGATAC
ATGGCCTGAGCAGATTTTTTTCCTCTCTCTTCAAGAGAAATCATTTAATCCATTATTTTGGGAATC
AAGACTGATAGTTATCTGAAACTGGATATTTGGAAAAAAAAAACAAGGAAAAAAGAAATATTAAC
CAATTTATGACAAAATAAAATAGAAATATACTAATAGATATTGAGTAGTATTTATACACTAGGTA
ATTTTCTAATCCCTGCAAAGTAGGATCTATCACCCACAATTTGCAGATGAAGAGAATGAGATAG
AAACATGTAAATAAACTGGTCTGAGATAACCATAAAGTAAAAGTCAGGAGTGGTTTTTAAATGC
ATGCAGTCTGACTCAGGTTCTCTGCTATTAATCTGATAGAAGTCTCTTAGGAAATTTGTCATCCTG

GTCAGATAGAAAAAACAGCCCAAGTAAAATAACCAGAATAAGCAGCTCAAACCCAGAGCTTGA
GATTCCCATGTCCCATCAGAAATATGTTTTGTCTATTCCTCAAGCTCCATAGTTTGTCTTTAGAAG
TACATGCTAGACACTTCTACAGTAAAAGTAGTGAACAAACGCCACTGATGTAAGCTACAAAAGA
CATTTGCCAATTGTGCATTCTAAAAATTTGAACATATTTGAAATACTTAAAAAATCCTGACAGTAT
CTTCAGATTTGACCATTCTTGTAAGCATTTTGACACTTAATAAGCAGAGAACCTCTTACAGAGAT
TCTTCTGGTCCCTTTCCATTCATCGTAAAAGATTTTAATGATTTCTTTATATATTGAAAATCTATTTT
TATAAAACTGACCATACTAATAAAATCATGTAACACTTTGTACACTTCTGGCTTTGACCTATAAAA
GATAATATTTTTTAAAAATCATATGTAATACTATATTGCTAACCATACATCAGTTTTATTAAAGAC
AAATAGACATTCCAAAAGGGGTTGCACGTCACATTTTTTGCTCTAGTAAGAAATAAGCTATTTTC
TTGTCATTCGCTTGGAATTCCAGTATTGACATTATATGCACTAATGATTTATTTGTAGGAATCTTTT
TAATTGAATTGTTGATGTTTCCAAGGTAAAATCATAATATAATTTAATTACAATAATATAAGAGGG
CAATTAATTATGTACCCTCTTGTATACCTGATTATGAGAACTGCATTCTGTGTTTCAACCCCTTAA
ACTGAGAACTGTTATAAAGTCCATAGTGTTAGGATATTTAGAAATAGAAATGTTAATGCAACCAA
GAGAGATTAAAGCCTCTTGAGTTCATATGGCATATTAATTTTGGCTTTTCTTCAGCTTTGAATTCC
CACTATTTAGTAACTTTGAAATTTCATTTCCTTTTTGGACATTTCTCAGCACCACCATGAGTCTAAT
ATAGTAAAATTGGGCTACCATCCAAGTGACACAAGCCCCATGCACTGGGGAAACGTAGGGATG
GTGGATTCTGCTCCAAGACTGAGATAGAATAGCTCTTTATTTTGCAGACCTTTCCTTTATTTTTACT
TAACTTTCCTATATTTGACATGAGAGCAACACAATCTCATGAGAGGTGTGAAACTAAAGGAACA
GGGCCAAAGAGGCCCATGCAGTCTCTTTTGAGCCAAAGTCCTGTAGATTTACAAGGTCTTCCTG
ATCTATACTTGATCTAATCCAGTGGTTCTCAAAATATAGTTCCTGAGTATCACTGTCACCCGGCTA
GTACAAATTTTCAAGCCCTAGGCTAGACCTGCGAAGAGGGCGTCACAGTCTGTGTTTTAATAAG
GCCTTCGGATGAAATCTGATGCATGCTAAAGTTTAAAAACCTGAGTTTTCAAATCACCTTGGCTA
AGCTCTACATAGGGGTTTAAGTCTATGCAAGTAGAGCAATACGTAGAGTGATTTTTGTTTTTCTG
CATTGTTGCTGAACCGTAAATAATCTATTTGTAAATCTTTGTTAGTAGTAATGAGTTTGAATTTAA
AAATTTGGAGGTTAAAAATTATGATCTTTTTTTTTTCTGCCTCTCTTGTATGTACAGGTTAATCCTC
ATTACTTACAAATTCCCTACTTGTGAAGTTGTCTACTTGGTAAAACTTTTTGTAATACAAAAATCA
ATTTTGTGGATATAAACAGATAAGTCCGGTGTAGTAAAAAATGTGAATTGTCCAACAAGCACATT
TCCAGCTGAGTTCAAAGTGATGTCTTGCCTTCTTTTTTCAGCTCACAAACAATAAACAAGTGTCCT
TTTGTGATCTATTTAGTGCCATACTTTAGGCATTTTTTTTTTTGCTTCTTCTTGGTGATTTCACTGCT
TAAAATGTCCCCCAAGGATATTGCTAAACTGTGGCCCTTTTTTCTAAACACAGGAAGACAAAAG
AATGTGCCTTATGGAGAAAATACATGTGTTAGCTTAACTTCTTTCTAGAATGAGCTACAGGGCTG
TAGACATAAGGTCAATGTTTATAAATCAACAATATATATTAAATAAGTTGTCTTTAGACAGAGAC
ATACAAAAAAATAAGGTCATGTATTGCATTAATCAGTTGATAAAAATATTGTAACCAGAGGCTTG
AGGGACCATAACCTGTATTTCCCATAGGAACAATGGTTCAATATTGACTGATTCTGTGTTTGCAG
CAAATTTATAGAATGTATCAGCCACTAATAATAAAAATCAGTTGTATATAATTTATATTTCTCAAT
TCTCTGCCAACTTGCCAATATAACAACTCCAACTAGTGCTATAGTATTTTTTTACATTGACCTAAT
CTTGCACTTCCCAACTCATTTTAAGATTGAGTTAATGTCTGACTTCCAAACCCTTCTAGGACAATT
TGTAATGGCTTGTTGAAATCCTGTTTTTCTATAGTGTAGTTGTAAATCATGACAGATTTTAAGTTC
ACTAAATATTTGACTTCAGCAGCATCCAAATATGTTTCTACTATTTGATAAAGAGGAACATGTTCT
AATTCTCATCACTGAGGTACATGGAACAAAGTTAATATACTCTGTTAAAACATGCCTTTCACTAA
ACTGGAAATTTAGAAACTGAAAATGTTAAGGAAATAGAATGGTGGGAAAGTAGATTTCATGAAT
TGACTCAAGCATAAGAAAACTTTTCACTGTCCTGTGTTTTAGATGCAGGCCGCTCCCCATCTTCT
GGCCATGTTAACACCTTTGGACCTATGGCTGTTTATCTCCATTAGATTACATAAAATGGTATAAA
GCACACTATACTACAGTGTTCTATACAAATTACATGCTTTTGGAATGCTTTTTTCTTATTAACTATT

AATATACAAATGTATTTTACAGTTTGATCCTTGGAATTTTTTTTCTGGCTTATTTATTTTATTCAGAT
GTATTCATTTAAAGCATTAGGAAAGATTTTCAAGAGATGGATACAGAGGTGAACAGCACAACTT
AAATAGCCAAAATATTTACTCATGTGAAATTATGAATTGTTAATTAACAGAAGCAACTGTCAAAA
TCATGAAACACTATTTCTACCATTTATTTTTATTGCTAGAGTACAGGAAAAACTCATGTCTGTTAA
TCATTCAGCTTTAGACAATAACCAAGCTTTCATATACTGTGTTTGATGATGAATCTACTAATGCCA
ACTTCCTTTTTTTCTTACAAACTTCCTAAAAGAAGCAAAGCAGTTTAGATAGCAAGTCGATTAAAT
CAATTAAATTAAAAATTGATATTTTTATTTTATTTAAACGTAAACTTTTTTGTGAACTATAAAATGA
AATATATTTAATAGTAAAAAGGAGGCATTAAATGGTTTTGGCTATTGTTAAATGACTCGTAGGTT
AAATTTCTATCAGAATTTTAGATAACTTACATTTTAATCTCAGAATTTACTTTGGCTTTTATGATTTT
ATGAGTCAAAGTGCAGATGCCCAGTTTATTTATTGGAATATTTGCTGCATTTGTAATATGAAAGG
TCATTAGGAATGACTCAACACCACAAAGCACAGTTAAGATTCTAAAACTGGGAAATGACAACTT
ATTATTTTTACACCAGCATTAAAGCCAATTCTCTTTTTATTTCATAATAGAAGTAAAATCCAATAAT
GAAGTCAATAGAAACAATGGTAGTCAGGGTTGATATTTATATTTTTATTCTAATGTGTGTTTTATC
AATTAATAACTTTTTCTCTTTCAAACAAGAACTCAAAAAATAAATTCCTAGCCTAAAGAAGTTCA
AAGATTTCTGATTTTAATATCATATATTTTATTTGACTCATCAATGTCCATGTAACCTCAGACAAG
AAGAATGTGGACAGCATAATAATTGTAAACCATTTGCTACTGCATGATCAGAAACAGACACATA
AACAAATTCTGGCCAAACGCATTAACGGAAGTGAGAGGGTTTCTCTAAAATCATCTCTTCATATC
TAAATAAGAACCATAGCAAAATTAAGCTCTTCACATCCTCTGAATCAGTTTTGCATTTTATGGACT
AGCTGAAATGGCTGTAGCCAAATTGCCACCATCTTTTAAGAAAAGCCAATGTCAAGGCTGGAGC
AGGAGAGAAGGGGACCTGCTGTTAATGACAATGCTGAGCCATTGACTCCACCCATGCTGAAGA
CTGCTCAAAGGCTAGAAGTTGTGCTATTATATATGCAAGATTCTTATTGTCTAGGTATTTAGGTAA
GTGTTTGTAAGTTTAATCAAAATCATTCCAATAGTTTTTGTTTGTTTGTTTGTTTGTTTGTTTTTGAG
ACAGAGTCTCATTGTGTTGCTCAGGCTGGAATCCAGTGGCATGATCTCGGCTCAGTGCAACCTC
CGCCTCCTGGGTTCAAGCAGTTCTCCTAGATCAGCATCAGCGTCCCGAGTAGCTGGGATTACAG
GCACCCGCCACCATGCCTGGCTAATGTTGGTCAGGCTGGCTCGAACTCCTGACCTCAAGAGATC
CACCTGCTTTGGCCTCCCAAAGTGCTCGGATTACAGGCATGAGCTGCCACACCCAGCCTCCAAA
TACTTTTTTTTTAAATCAAAATAATGATTTCAATGACTGAGATCTGTTATGTATTTCAACATAATCA
TGTTTCATAAAGATTTAGTCTTCTGAAA

ID|56338|gene_id|ENSG00000237343|transcript_id|ENST00000455105 Homo sapiens lncRNA

GGCCCGCCCGGCAGGACGACTGAGCAAGGCCTTGGAAAACCGGAGAGATTGGAGCGGTA
AGTCGCGCTGGTCACGTTGGACACCTGCGCGTTGGGAGATTCTGGAGCCAGAAGGATAGCCGA
ATGGCCTTCGCCCGCCCTGCCCCTCGCCTGCTTCAGAAACCCCCCGGAAACGCCCCGGTTGAG
ACCCCGGCCCGAGCCGCCTGGGGGCCCTAGGGAGGCTGAACGCCCGGTGGCTCCCGGGATGG
CTCTTCCCGTTCTTCGCGCCGCCTTCACCCAGTGAGGGAGCCTGTGCCCTCCCTGCCCAGTCGC
TTTTGGGGCCGCTGCGGAGCTTCCGCTGCCATCTTCGGATCCTGTGTTCCGCACGGGGGCTCCA
CCAGGGCAGGGATAGTGGTGAGGGTGGCTCGTGGGTCCCCCTCGCGGGGAGCAGGGTCTGGC
ACTCACCAGGGCGCAGGACTAGGACTTGTCGAATGAATCCATCCTTTTAGCTTTTAGTCCTTTGA
AGAGCCTTGAGAATGGAAATCATGAGAGATTTTTCCATGGGGAAGTTCCTTTTACAAAGCATTTA
TTTACGTGGACTTCTTGGCACCCCGCGGGGCGGCAACGGGCAGGGCCTCCAGTGCCCCTTCTG
CGCCGTGGAGCCGCGGGGGCTCAGCTGGGCGGTGGTCGGGTCCTGAGGCCGGAGGGCGGGA
GCAGGGGAGGGGAAAAGCAAAAGCGGGGAAAGAAGCCGGGGAGCGGTGGACCAGACGTCCA
GACCTCCTGAAAGGCTGGCGGGGAGGCACAGGCGGGATCTTCCGGAGGTGAGAATTTTTTTTT
ATTATAGCAGAATGGGGAGGAATTGAGGGGAAAATGGAGATAGAACCTGAAAGAGCCCCAAA

CGCCAGAACCTGTAGCTCCCCAAGAATAAGATCTTCCAGAAGAACTAGACCCAAAACTAGCCG
TTGGGGAACACCGAAATCCTTGGAGGAGCAACATCCGCATGACCCTCTGTGTTCCTTTAGGCAA
AAGGACTTGCTTCCATTGTTTGTTCAATTGTTTGTGTTTGTTAAATAAATAAAACGATTTTCATGTA
TCTTTGAAA

ID|56348|gene_id|ENSG00000225490|transcript_id|ENST00000454331 Homo sapiens lncRNA

CGCGGTCCCGGATCCTGCAACTTCTACCTCATTTTCTCCAGGTACAGTTTTCTGGTCGTTTA
ATTAGTTTGTTTTCTTGGCGGTGATGGGAATCACAGGCGTAGAGATAATAAGTTTGCTAGAGAG
GAAGCCACCCCACAGTTTCGGGTCTAACAAAATCCCTCCTTTAAAAACATTTGTACCCCCAATAA
AAATAAAATTAAAGAGGAATATAGAAGCACAAATTAGCCAACTTTTTTAAAAGAGAAAAAAGAA
AAGAAAATCATCGTAACAAATGAGCGTGAGAACTTCTAAAAAATTTGTCACGAACCCTTTCAGA
GGTCTGGATTCTTTTCCAGTAGCCAGTGACACATTTGGTCTGAAAAGAGAAATACACTATCCACT
GAGTCACTTATTTAAAGCCAGCACACACACACACACACACACACACACACACACACACACACA
CACACACAGACACGCATTCAGAAAGCTCTGGGTGCCCTCACATTTGAAAACAACACCTGGTGG
AGGACGCTGCGATACCTAATTGGACAAACAGCAATAAAATAAGACCAGTGGAAGTGCTGAGCA
CAGGGCGCAGGGCTAGGGCCCAGGTTTTGTGTTCAGGCCTGCGGCGAGGCAGCGGCCCTGGG
CGATGGGGCGCCGGGCGGTCAGGCACCGGCTGGCGACTCCGCTAGGCCTCCGAAGCCCGGCC
TGCAGACCCTTTCTCCCAACCTCTGTGCCTCGTGGGCTCTCTCCGCTCCGACGCCTGGTCGGGG
CTTCCCTGTTGAGAGGGTTTTAGGCAGAAGGGCCAAACGACTCAAGCCAGACTCTGCTTTTTCA
TTTTTCTCCCTTAGGCCTCTCCACTTGCCCGAGCTCTCAGCCACCAGGAGACAGACGGAAGGGG
CTTCTGGATTGCGTTGGGAGCGGGCACTGTCCCCTACCCACTCCTGCTCAGTTTCCCAAAGTCC
AGCGACTTCCGACCCTCCTTGGGGGGAAAAATACCAGTACCCAGACACCTTTGCACCCTGTTAT
ATGACTGGGCTTGCTTCAGGAATATCTTACAAAGAAGAAGAAGAAGAAAGAACGGCTTGGAGT
CATGTTTACATCTAGTTCTACTGATCCGTTCACATCTGGAGAGTTTATTTATAACTCCCTCTATAA
CTCTCTGTTTGGAGACACTGTGGCTCTATTGACAGACTGATATCTAGGAAATATTGCTCTATGTCT
AACCGTACCTAAATGTGCTGTGGTTGTATTTACAGATTTATGTTTGAGGGAAATATAACTATTTCC
ATTGTATTTGTGCATATACAGTAGTTATAGGAGCAGATTTATATATGGGAAAATATACGACTCTC
TTACAGCC

ID|56354|gene_id|ENSG00000247810|transcript_id|ENST00000508199 Homo sapiens lncRNA

CAGAAGGAGCCAACAGAGTGAGATGAAGGGGACAACTTCAGATACTCAGGCAGAAGGTA
GGCTACAGTGATGTAAGTCCAGCATCTTTATTTATTTTTATTTATTTTTTCATTTTTTAATTTCCATA
GGTTTTGAGGAATGGTAATATTTGGTTACATGAGTAAATTCTTTAGTGGTGAGTTGTAAGATTTTG
GTGCACCTATAACCTGAGCAGTACACTGAACCCAATTTGTAGTCTTTTATCCCTCACCGCCTTCC
CATCCTTTCCCCTAGAGTCCCAAAGTCTATTGTATCATTCTTATGCCTTTGCATCCTCATAGCTTA
GCTCCCAGTTATGGGAAATCCAGCATCTTTAAATGATGCCTTGAGACCATATTCTCCTGTGCTTC
CCCTGTGAACAAGTAATGATGGATCTAGTAGTTACTACCATGATGTAATGCATGACAGTCCACC
ACAATCATGTTTCCTAGCCATGCATAACGACAGGAAAAATGACAGATAAGTAAATATACCAATC
TAGGCCTAAGGACAAGCATTTGGTTCTACTATTGCCTTTTTGTCCACCTGACCCAATTAGGCGGC
TTCATATTTTTTAAAAGTTCAGTGTCTATCTGTAAGTCCCATGAATATATGATCCTAGAATTTAGA
AATTATTTTATAATTTGTATTTAATTTACATGGATTAGAATGTTATTAACTACCCTTGGTGCTCATG
AAGCATCTTTTCCCCTAATATATCTTCTGCAGTGCAACTTAATAGTGAGTCCCCATAGACAGAAA
AATCTATTTGAAGAAAATATTTTAGCAGTTTCTCCCATGAACAGTTTTTTGTTTGTTTGTTTGTTTTT
GCCTCCAGCTTCAGTAAAACAAGTTGTAAATAACTTTATAGGCAAAACTTTGGTCCCTGGTTAAT

CATGAGATTTTTTTTTTCTTTGAAGGCTAGTAATGATATTGATGAGGAAGAGCAGCTCGCTCTAC
AAAGTTATTTAGGGGCCCAGATGTGTTCCGTCTTGCTCTGCCATGCCCTCCTGTGCTATAACTCTT
CTATTTATATGGTAGTCCTTACACACATGGTTGCAGCTGTATTGTCAACACGACATATATACCTG
AGAGGGGAGAAAAAATTCCATGGCAAGCATTTATCTTTTGAGACACTGCAATTTGAGACAGTTA
CAGTGATATTACATACATTACTCCCATTGTTCAGAACTAGTAACATCAGCTTGCCAGCTACAAAG
GAGAGAGGAAAATGTAGTCTCTAGTTTCTCATTCAAGGCCTAGGTAAAATGCTATTAAGATGGA
AAAGGAAAATAAAATGGGAAAAGACTACAAAGTCTACCGCATGCAATAAACAAGTATACAATT
AAATACATAGTATGGCAGATAATGAACAGTACTATGGAAACTGTCTATATATACATATGTATATA
TATATGAAAACGAGTGAGAGGTTAATATCATTTATTTTAAATAAGGTGTTCAGGAAAATGCCTTC
TGGACATGTTAGTAAAATGATGGCAGAAGGACCTCCAAAAACGATCTCCTCCACAAAAGTAATG
AGAAAATTGACCAAAAAAAAAAAAAGTCAGAATCATTTCTTCAAAATACTGGAAAAAATAACAAA
AATCTTGAAGTAACACAGGGAGGATTTATTCAAGAAAAATAAATGATCTCAGCAAGAACAGTGA
GTGTCCTAATCTTATCTCCTGCTCTACAGCTCTGCAGTACCATTGAGAAAAAACAGCCCACATTT
CCACTGTAGCCCAGAAGCCATCAAAGGGAGTACAAAGGAGCTGCAACTTTTTAAAAAGCTTCAT
TCCAAAATAATTGTCATTATCTGAGCAGTCTGGTGGTTCTCTGAAATATATCAGTTACAAGGCCA
CCTGTACTTGGCCTGTTTAGGAGCTCACCTAGTGCAAAAATTATTTTCCCTGAAGAATATTTATCA
AAAACAATTACTGGAAAGTGTTTTAACTTTGTGGTTGTCTTAGGCAGTGGATAACAGGAGAAAC
AGTGACTAAAAATTTAAAAGGAAAAGCTGAGAAATGACATGTCAATACAGTCTTTGAAAAAATC
TCCAACATAATTTTTGTTATTTAGAAAGTCATATAGGGTTGTGCACATTCTCAGGGGTGTGGTAAT
GCTCAGAAAATCCTTAAGAAGTCCCATATTACCACCTCTGGCTGACCATAGGGACCTGTGCAAT
GAGAAAGTGAGAGCTAAGGCAGAGTTGTCAATTGCCTAACTGAATGTTGAGGGCCTGCTTTGCA
ACAAGCACACAAAGCCCCTCAGCAAAAGCTGGAAGATGTATTGGTTCCAGACTTTTAAGGAAAT
TATCCAATCATTAGCTGAGCACTAAGGTAATAGAGTAATCTAAAAGACCACATGTGACAATAAA
TATGGACTTTACCAAATTAACTCAGAAAATTACTAAACAAACATACAAGACTACTACAGCAAAC
AGCAGCATCAAATCCTGGAGAAGGGGGAGAATTTTATTTCTAGAATTGTCATATTAGATTATTTT
AGATGTCCAGTTTCAAACAAAAGATTATGAGATGGAAATACCTAAGAAAGTGTGACCCATGCAT
AAGAAAAAAAGCAATAGCAACTATTCCCAAGGAAGCCCAAAAGTTGACTTACTAGATAAATATT
TTTCTACCAGCTATATGAAATATGTTCAAAGACCTCAAGGACATGATAGCTAAAGAACTAAAAA
AAGGTATGAAAACAATGACTTAACAAATATGGAATATCAATAGAGATAATCTTCGACAAACTTG
ACAAAAACAAGCAATGAGGAAAGGATTCCCTATTTAATAAATGGTGCTGGGAAAACTGGCTAG
CCATATGCAGAAAACTGAAACTGGACCACTTCCTTACACCTTATACAAAAATTAATTCAAGATGG
ATTAAAGACTTAAATGTATACCCTAAAATCTAAAAACCCTAGAAGAAAAAATCTAGGGGATACC
ATTCAGAACATAGGCATGGGCAAAGACTTCATGACTAAAACACCAAAAGCAACTGCAACAAAA
GCCAAAATTGAGAAATGGGATCTAATTAAACTAAAGAGCTTCTGGACAGCAAAAGAAACTATCA
TCAGAGTCAACAGGCAACCTACAGAATGGGAGAAAATTTTTGCAATCTATTCATCTGAAAAAGG
TCTGATATCCAGAATCTACAAGGAACTTAAACAAATTTACAAGGAAAAAAGAAACAACCCCATT
AAAAGGTGGGCAAAGGATATGAACAGATGCTTCCCAAAAGAAGACATTTACGTGGCCAACAAA
CATATGAAAAAAAGCTCATCATCACTGTTCATTAGAGAAATGTAAATCAAAACAACAATGAGAT
ACCATCTCATGTCAGTTAGAATGACGATCATTAAAAAGTCAGGAAACAACAGATACTGGAGAG
GATGTGGAGAAATAGGAATGCTTTTACACTGTTGGTGGGGGTGTAAATTAGTTCAACAATTGTG
GAAGACAATATGGTGATTCCTCAAGGATCTAGAACCAGAAATACCATCTGACCCAGCAATCCCA
TTACTGGTTATATACCCAAAGGATCATAAATAATTCTACTATAAAGACACATGCACACATATGTT
TATTGCAGCACTATTCACAGTAGCAAAGACTTGGAACCAACCCAAATGCCCATCAATGTTAGGC
TGGATAAAGAAAATGTGGCACATACACACCATGGAACACTATGCAGCCATAAACAAGAATGAG

TTCATGTCCTTTGCAGGGACATGGATGAAGCTGGAAACCATCATTCTCAGCAAACTAACACAGG
AACAGAAAATCAAACACCACATGTTCTCACTCATAAGTGGGAGTCAAACAATGAGAACACACG
GACACAGAATGGGGAACATCACACACCAGAGCCTGTGGGGAGTGAAAGGCAAGGGGAGGGG
GAGCATTAGGACAAATACCTAATGCATGAGGGGCTTAGAACCTAGATGATGGGTTGATGGGTG
CAGCAAACCACCATGGCACTTGTATACCTATGTAACAAACCTGCATGTTCTGCACATGTATCCCA
GAACTTAAAGTATAAAAAATAAAAAATATATATATTCTGGAATTGAAAATAAAACTAAAATAAAA
ATTTACTAGAGTGGTTCAACAGCAAATTTGAGAGGGCAGAAGAAAGTATCAGCAAACATAGAG
ATAAGTGGATTGAAATGATTCAGTCTCAGAAAGATAAAGAACAATAATAACAAATTAGCAGTCT
TAGAGAGCTGTTACAGATAACATCACCTGTACCAATATATGCATTATCGGAGTCCAGGAGGGGA
GGAAAGAAAAAAGGGACAGAAAGAAAACTTGAAAAAAAGGACAGGAAACATAACAAATTTTA
TTTCAAAAGTTACATTAATCTATACATCAAAGAAGCTCAATGAACTCCAGATAGTTTAAACTCAA
TAAGGATTATAATCAGATGAGTCATTAACTGTCAAAGACAAAGAAAGAAAGAATCTTCAAAGCA
GCCAGAAAGAAGTGATACATCATGTAAAAGGAATCCTCACTAAGATAAACAGCAAGTGTATAAT
TAGAAACTATTGAGGCCAGGGACAATGGAATGACATATTCAAGTTGCTAAAAGAAAAACAAAA
ATTTCACTCAATAATTCTAAATCCAGCAAAATTATCTTCCAAAAATTAAAACGAAATTAAGATATC
GCCAAACAAAAACTGAGATCATTGTCACTGACAAAACAGCTTTAAGAGAAATGTTAAATGTAGT
ACTTCAGACTGACCTGAAAAGACACTAGACAGTAACATGAATCTACATGAAGAAATAAAAGATA
CTGGTAAAAGTAATTATACAGGTAGATGTAAAAGAGAGTATAAATGTATTTGAAATTTGTGATTA
CTCTTTTTTTCGTATCTGATTTAAAAGACAACTACATAAAGCAATAATTATATATGTGTGTTCATTT
ATATTACATACAATGTACAAAGACGTAATATGAGTGACAATGATAGCACAAAGGAGAGCAAAA
GAAGTGGTGCAATATTGGAGAAAAGTTTTTGTACACTATGGAAATTAATTGGCATAAATCTGAAA
TAGACTCTTGTAAATAATAATGTTAATCATAATTATCAGGGAACAATTAAGAAAATAACATAGTA
AAAGGAATGGTAAAGGAATTGCATGGTACACTAGAAAATATCTATGTAGCCCAAAGAAAGCAG
GAATGGAATAATAGAGGAATGAAAAGACATTGAAAACAACTAGAAACATGACAGACATAAATC
CTACCTTATCAGTGATTATAGTAAATTTGAAGGGATTAAACATTTCAATCAAAAAGGAAGCAATT
AGTAGAATGGATAAAATGCTATGCTGTGACTATATTCCCTCTACAGAAGATACACTTTCTGTTAA
AAGACACAATTGATTGAAACTAAACGGATGGAAAAAGACATACCATGTATACAGCAACCAAAG
TGCTATAGTAACTTTCCTGATAGAAGACAAAATATACTTTGGAGGAAAACTATTACTAGAGACAA
AAAGTACAATTTATAATGCTGAACAGGTCAGTCCATCAAGGAGATATAATTATAAAACTGTATGT
CCCTAACAACAGAGACCCAAAATACATGAAGCAGAACTAGAAGAAGAAATAGACAATTCAACA
ATAATAGTTGGAGATTTCAGCACCTGACTTTCAATGATGAATAAAATAAGAAGATAGAAGATTA
GCAAGGAAATCGAATATACAAATATGAACTCAAGATAGGTAAGAGATGGCTGGGCACGGTGGC
TCACGCCTGTAATCCCAGCACCTTGGGAGGCCGAGGCGGGCAGATCACGAGGTCAGGAGATC
GAAACCATCCTGGCTAACATGGTGAAACCCTGTCTCTACTAAAAATACAAAAAATTAGCCGGGC
GTGGTGGCGGGTGCCTGTAGTCCCAGCTACTGGGGAGGCTGAGGCAGGAGAATGGTGCGAAC
CCAGGAGGTGGAGCTTGCAGTGAGCTGAGATCGCGCCACTGCACTCCAGCCTGGGTGCAGAGC
AAGACTCCACCTCAAAAAAAAAAGATAGGTAAGAGACAAAAATGTAAGAGAAAAAAAGGATA
AAACTCATAGTAGAAAACATAGGTATAAATCTTTGTGGCCTCGGATTAGGCAAAAGTTTCTTAGA
CAAGATACCTAAAGCATAAGCAACCAGAAAAAAAAAAAAAATATATATATATATATACTGCACT
TCATTGAATTAAAAACATTTGTGCATCAAAGGACACTTTCAATAAAATTTAAAGGCAACGTACAG
AATAGCCTACCTTACTTACAAGCCATGCATTTGATAAGATTTTAGATCCAGAACATATTTTTAAAA
CTCTTAAAGTTCAATAATAAAAAGACAACTCAGTGAGAAAAAAAATGGGTCAAGTATTTGAATA
GCTATTACTACACAGGAGATATATAAAGTTCATAAAACACATGAAAAGATTATCTAAATCATTAG
TTATTAGGTAAATGCAACCAAACCATAATGAGATACCCTTTCACACCAACTAAGATAGCTATAAC

AAAAAAGATGAACAATAACAAGTGTTGGCAAAGATGTGGAGAAATTGGAAACATACATTGTCA
ATATAAAATAGCGCAGCTACTTTGGTGAACATTTTGAAGGTTCGTCAAAAAGTTAAACATAAATT
TACTATATGACCCAGCCATGCCACTTTTAGGCACATACAAAAGAAATGAAAACATATATTCACTC
AAAAACTTGAACACAAATGTTAATAAGAGAATTATTCATACCATCTAAAAAGGTGGGAGCAACC
TAAATGCCTATCAACTAATGAATGGATACACAAAGTATAGTATAACCACACAATGAAATATTATT
CAGACATAAAAGGGAATAAAGTTCTGATACATGCTACAACATGAATGAACTTTGAAAACATTAT
GTCAAGTGAAAAAAAGCCAGACATAAAATCATACACATATTGTACAATTCTACTTGCACAAAAG
GTCAAGAATATGCAAACTCTCAGAGACAGAAAGCTTAGTGGTGGCCAGGAGCCGGCAGGTGGC
TGGGGAAGAGAAGAGATTGAGGAGTGACTGATCTATGAGGATAGGGCTTCTTTGGGAAGGTTA
CAAAAATGTTCTAGAATTAGTGGTAATGGTTTCATAAGCTTTTAATACACTGAAACACACTGAAT
TGTATAATTTTAAATGGCGAAGTTTATTATATGTGAATTATATTTCAATACAATAAAAGAAGGGTA
GAAAAGAAATCCTTTTTTAGAAAGCAGCATTTGGAGAGAAATTTGAAGAAAGATAAGGAGTAAT
CTACCAGATATCCAGGAGAAAAAGAACACCAAATGATATTCAACAACAGTTTATTCAAAAGTTT
AATAATTAATATTCCTCTTTTGATTTTTAAGTCTTTGTTACTGTCATGGAACAGTTTAATGTAGTCA
TATTTTGCGGAAAGAATGAGGAGAGTAAATAAAAAGGTAGGAGACTTAGAAAGAGGAGATTTA
ATTTTTGAATCATGTAAAAAGGAAAGTGGGAATTTTTGAAAATATAAGAAAAAATTAGAGTAAACT
TAAAAGAGCTGCAACGAGGAAAAAATATGAGAAGCATTGATATAGTGATAACAAAATTAAGCA
CTAAAATCAGGCAGATACGGATTATATCTCACCTCTACATCAGCTAGCCCTTTCTCTAGAAAAGT
TTCCTTACTCTGTGTTCTTCAGTTTCCCATGACCTATACAGAAAACTGAGGAATAATTTCAAGAAA
ATAATATGGGAATAAGGTGAGAAAAATAACTACCTTACTGGCTTGCTGTGAGAGATGAGCAAGA
TGACTTAAGTAACCCACTGAATTTGACACCTGGTAGATTCTCAATATATAATAATTATTCACAATA
ATCATCTGAATGGACGCTACATGAGTTCTGAGGGGTCTTAAGTTTTAAGAAGTAAAATTATCTAT
TTAGATATGTGTTTTTAAAATTTGTAAGATATATGGAACATATTCAAAAAGACTTCACATTTTAATT
ATTTAAATATTAATTTAATTTTGGACATTTTAATTTTAATATAACAGCATAGAAGACTAATGCAGA
GGCTTTGCCTATATTTTTAAGGTGATAATTGGTTACAAATTGACCTTTGTTGGAGAGATAAAAATG
TATTTGGAGACTAAATTAAAAAAAATGCTTTCCACATGAACTCAATATTTTTAGAAAACAAGCAT
GTTATCCTTAAAACTATTTTGAGAATAACACTGTGGTGAAAAATGCTACCAAAAAAATTACTTTG
CTAATTAAAGCCTATGAAACCAAATAACTGTTTTTAGACATTGATTTTGCTGGTTCACATCCTAAA
GAGAAGTACTTCTCATGTTTCTTTCAAGTGAGATAAAGTTAGTGAAAAATCTGGCTAAGGGAGG
ATGAGAAGGAGTTTACGTATTTGAACATTTTTGCTGGCATATGCCAGAATATTTTTATAAACAAT
GCTAGGGAGATTGAAGTCTTCTGGGGATTAAAGGAAACCACGAAATAGAGAAAGAGCATGTCA
GGGAAAGGGGCCATGTCCGTCCTCTGAACAGTCTCCAGCCAAAAGAACTTGGCCAGTTAACAC
CTTTGTTTCTCTTTTTCTAATTTTCTAAAATGAGAGGCTTTTACTTAATGGAATATGTAATCCCTCTT
GATTCTAATTTTGTATTGTTCTGTGCAATATTTTCTCTTCTAGAAAAAAAAAAATGCCAAGGCTTAA
TTCAGACTTTTCCTCTTTAGGTATTATTAAAGGTAGCTGTAAATTCCGGAGGTTGGGTCAAATTTG
CCTTGGTGGATTATATGATGTTTCTCTCTTCAGTTTCTTTCTTTCTTTTCTTTTTTCTTTGCTGTTGTG
ATTCCTTCTGCCATTAAGAGAAGGCAACTAGAATTTCTGCTATAGGTCTCATTATACCTAATGCC
AGAGGGATATTCAAAACACACAGCAACCAGTACAGCAAAGGTATGAAACAGCAAAAGACATTG
GCCATGTAGACATACATCAGCCCTACCAAAGCTAGAGGCAAAACCTGATTGATTTAAGTGAAGC
TCAGAAACATTTGTGTATGTGTTGATGAACATATACGTATATCAACCTAAATAACAAGCATAGAG
AGATTCTGTAAAAGAAAATGATATTTATTCAGAAATAAGGCATTGCAATGGGACTACACATGCC
ATTGCAAACTATGTGCATATTCAGGAAGGTAAGAGAAGACAAAAATTTTTAAAGGAAAAATGCA
GGTGGGTTACATAATTGTTTTCAGATAATTTCCCTTGGCCACAAGGCTCAATAGCAAAGGAGAC
ACCAGTCTGAGATTGGACAGGTAGTAGCTGGGCATATGTTCTTATGATAGTATTTTTTGTGTGAG

GTTGCAATGACCTTTGTGCGAGGTAGTTTTTACCAAACCTTTTGTTATAGTTCTGATATCAGGCAT
ACCAGGGTAAGAAACCCTTTCTTTACAGCCTTCCCTGGCTCTATTTGCCTTTTTTTTCTTTTTTCAC
AAGTGATTTCATTTTGATTCTGGTAACTCTTACACATACATTTGATAAATTAGAATTGTATCAATTT
CAAAACATATTTATTTTGTAAATGGAATTATTTTAATGTTTAAAATGCCCAAAGAGATAAATGAAG
GAATAATATTCACTAAAACTGAACAGAAGTTATCTTTATCCAACCATCCATTGACCCTTAGGTTG
ATTATATGACTTTGTTATTGTGAGTGGTGCTGTGATAAACATACAAGTGCAGGTATCTTTTTAGCA
TAATGATTTCTTTTCCCTTATGTAGGTAGTAGTGGGATTGTTGAATCAAATGCTAGTTCTATTTTTA
GCTCTTTCAGAAATCTCCATATTGTTATTCATAGACTTTGTCCTAATTTATATCCCAAACAACAGT
GCATAAGCATTCCATTTTCTATGCATCCTCACCAACGTGTGTTGAACTTGACTTTTTAATAAAAGC
CATTCTGGTTGGTGTAAGATGGTATCCCATGTGGTTTTAAGTTGCATTTCTCTAATGACTAACGAT
GCTGAGCAGTTTTATGTGTTTGTGGCCAGTTGTACATATTCTTTTGAAAAATGGCTGTTCATGTCC
TTTGTCCGCTTTTTAATAGGGTTATATGCTTTTTCTTGAGTTGTTTGACTTCCTTTTAGATTCTGGAT
ATTAGTCCTTTGTTGGATACATAATTTACAAATATTTTCTCCTTTTCTGTACATCATTTTGTCTACTC
TGTTGATTATTTCTTTTGCTGCCCAGAAGCTTTTTTAGTTTACCTAGGTACCATTTGTATATTTTTGT
TTTTGTTGCATTTGCTTTTGAGGACTTACTCATAAGTGCTTTGCTTTGCCCAATGTCCAGAAGAGT
TTTTCCTAATTTTTCTTCTAGAATTTTTATAGTTTCATGTCTAACATGTAAGTCTTTAATTTATCCTTA
GTTAATTGTTGTATATGGTGGGAGATATGGGTCCAGTTTCACTCATAGCTATCCAATTTTCCCAG
CATCATTTATTAACTATTGTGTCTTTTCTCCAGGGTAGTTTAAAAATTTTTTTTTAAGAATAAAACC
TACTGATGCATTCATCCAGGGTAGGTTTTTTTGACTTTGTTGAAAATAATTCAACTGTAAATATAG
AGGTTTTAAATCTGGATTCTCTACTCCGTTTCATTGATTTATGTGTCTATTTTTATACCAGTACCAT
GCTGTTTTTGTTACTATAGCCTTGCAGTTTAATTTGAAATTGGATAAAATGACGCCTCCAGCTTTA
TTCACTTTGCTTGTGATTACTTTGTCTATGTGGGCTATCTTTTGGTTTCACATGAATTTTAGGATTA
TTCTTTTCTAATTCTGTGAAAAATGACAGTGGCAATTTCATAGGAATTGCACTGAAGCTGTAGATT
ACATTGAGCAATATGGTCATTTTAATAATACTAAGTCTTCCAATCAATGAGTATGCCATGTTTTTT
CATTTGTTTGTGTCATCCACAATTTCTTTCATCAATGTTTTTTAGTTCTCCTTGCAGAGATCTTTCAC
CTCCTTGAGTAAATGTATTCCAATAATTTTTTTTTGGTAGTTACTATACATGGGATTGAGTTCTTAT
TTGGTGCTCAGCTGGATCATTACTAGTGTGTAAAAATGCTACTAATTTTTATACTTTAATTTTGTAC
CCTGAAACTTTACTGACATTATTTATAAAATCTAGGAGTCTTTTGGAGGAGTCTTTAGGATTTTCT
AGGTATAAGATCATATAATATGAAAATACAGATAATTTGACTTCCTCTTTTCCAATTTGAATGCCT
TTTATTTCTTTCTCTTGCCTGATTGCTCTAGGTAGGACTTCCAGTACCGTGTTGAATAGGGGTAGG
GAAAGTGAGCATTCTTGTCTTCTTAAAATCCTCAGGGGGAAATGCTTTTAACTTTTCCCCATTCAG
TATGATGTTGACTATGTGTTTCTTGTGTATGGTTTTTGTTATTTTGAGATTTTTTTCAATGTCTAGTT
TGTATCCTGAAATTGTACTGACATTATTTACTTATAAAATCTAGGGATCTTTTTTCATGAAGAGAT
GCCTAATTTTATCAAATGATTCAAATGTTTTTTCTGAATCTATTGAGATGATCAGATGTTTTTTTGC
TTTTCTGTTTATGTTGTGAATCACATTTATTAATTTTTGTGTGTTGAACCATTCATGAATCCCTGGA
ATAAAACCCAGTTGATAGTTGTTTATTTCTTTTAGATGAGTTGTTGGATTTGGTTTGCTAGCACCTT
GTTGAGGATTTTTGCATTATATTCACCAGGGATATTGGCCTGTAGCTTTTTGTTGTTCTTTCCTTCT
CTGGCTTTGATATCAGGGTAATACTGGGTTCATGGAATGAGTTAGAGGGGATTTCTGGTTCCTCA
ATTATTTTGGAGTAGTTTCTATAAGATTGGTAAAAGTTCTTCTGTGTATGTCTGGCATAATTCAGC
TGTGAATCCGTCTGGTCCTGAGCTTTTTTTGGTTGGGAGATGTTTCATTACTTATTTAATCTCACTA
CTCCTTATTGGTCTATTCAGGATTTCTAGTCATTCCTGGTTCATGTTGAGAGGCTGTATGTTTCCA
GGAATTTGCCTGTTTCCTTTAGGTTTTCTAGCTTGTGAGTGTAGATATGTTCATTGAAATCTCTGAT
GATCATTTGTATTACTGTGGTATCAGTTGTAATGTCTCCTTTTCATTTCTGATTCTGCTTATTTGAAT
CTTTTCTCTTTTTGTCTTAGTTAATCTAGCTAGTGGTCTATCAACTTTGTTTATTTTTCCAAAAGAGA

AACTTTACATTTCATTGATCCTTTGCATTTTTTATCTCTATTTCATTTAGTTCTGTTCAGATCTTTGTT
ATTTCTTTTCTTCTGCTAGCTTTGGGCTTGGTGTGTTCTTGTTTTTCTAGTTCCAAAGATGTGATGT
TAGGTGGTTAATTTGTAATCTTTCTATTGTTTGATGTAGGCCTTTCATTGCTCCCTCTTAGCACTGC
TTTTGCTATATGTTGCCTCTATTTTCATTCAATTCAAAAAAAGTGTTGACTTTCACTTTCATCTAATT
ATTGACCAAATATAAGTCAAAACACATGGTTTGATTTCCACATATTTGCATAGTTTTGATTTCTAG
TTTTCTTCCACTATGATCTGAGAATAATATACTTGATATAATTTTGATTTTTTTAAGTTTATCAAGA
CTTATTTTGTGGCATAACATATGGCCTATTTTTAAAAAAGATGCATGCACTAATAAGAATGTATAT
TCTGCAGTTGTTGGGTAGAATGTCTGTTAGGTTCATTTGGTCTAGAGTTCAATTTAAGTCTAGTGT
TGCTTTGTTGATCTTCTGCCTTGATGATCTATCTAGTGCTTTCAGTGGGGTGTTGAAATCTTCAAC
TATTATTATGTTGCTGTTTATCTCTTTCCTTAGGTCTCTTAGTATTTATTTTATAAATCTGGATACTC
TGATGTTGGGTGCATATATGTATGTACATATGTATATATGAATAATTATTATATTCTCATGTTGAAT
TGATCCCTTTATCATTATATGACCTTCTTTGTCTTTTTTTTTACTATTGTTGATTTAAGGTCTGTTTTA
TCTGATATGAGCATAGCTACTCCTGCTTGCTTTTGGTTTCCATTGCATGGAATGTATTTTTCCACC
CACTTATCTTGAGTCCATAAGTGTCATTACCAGTTAGGTGAGTTTTCTTTCATCAGCATGTAATTT
CATCCTGTTTTTGTATCCATGTCATCAATGTATATCATTTAAGTGGAGCACTTAGTCAATTTACATT
TGAGGTTAATATTGGTGTGTGAGATTTTATTTCTGTTATAATGTTAATTGTTCCCTATTTACTTTGT
AGTCTCAGTTGTGTAATTGTTTTATAAGACCTGTGAGTTATATACCTCTGTGTCCTTTTGTGATGGT
GATTATTAACCTTTGGTTTCCATGTTTAGAATTTCTTTGAGTATTTCTCCCAGGACTAGTCTACAG
GTGACAAATTCCCTTAATGTTTGCTTGTCTGGGGAAGACTTTATTTCTCTTTTATGTATGATACTTG
GTTTAGTAGGATAAAAAAAAAAATTCTTGGCTGACACTTTTTTCTTTAAGAAGACCAAAAATAGGA
CTCCAATCTATTCTAGCTTGCAGGATTTCTGCTGAGATCTGCTGTTAGTGTGATAAAATTTCCTTT
ATTGTTAATTACATGCTTTTCTCTTGCTGCCTTTCAGATTTTTTTCCTTCCTGATCACTTTAAATAGT
CTGACGACTATATGCCTTGGTGGTCTGTCTTGAAAAAGTATCTTAAAGGAGTTTTCAAAGCTTCTT
GTAGGTATAAATCTATAACTAGAATAGGAAAGCTTTCCTCAATTATTTCCTCAATTAGGTTTGTTA
AACTTTCTATTTTTTTCTTTTTCTCTCTCAGGAATACATATGACTTGTAGGTTTGAATGTTTTACATAA
TCTCATATTTTTCAAAGGGTTTGTTCCCTTTTTAATTTTTTTTATAAAATTTTTGTCTTACTATGTTAA
TTTGAAGATCTGTTCAAACTCTAAAATTCTTTTCTCTACTTGGTCTAGTCTACAGTTAAGGCTTTCA
ACTGTATTTTGTAATTCCTTCATGACTTTTTTGTTTCCAGAAGTTCAGAGTGTTTTTCTTTTTTAATC
TATCTCTCTAGTGAACTTTTCATTTGTACCCTCCTGGGGCTCATGACTCTCAATTCAGATAGACAC
TGCTGTTTATCCCCTGGCCACAATGTAGCTAAGAGCCATAGAAAATAATTGTTCTATGGCACTCT
GCCAAATGGCTTTGACTTGGAACCTCTTCCCTCAGTCTAAAACAGATAGCTTTGAGACCTGCCTG
TTTTCCACTGCAAGAATGCTGCTGCTCCATGTGTAGAGGGAGAGGGGCTCTGCCTTTCACGCAT
TCCCAATCCAGTGGACACTCAACAGTGGAGGAGGTGCAGCTGCTCTTATTAGCCCTGGAATGGC
CATCCACAGGTGTGTCCATGCCTCTTCCCTGTGGGATCAGCCACGTCTGTGTTTGCAGCAATGA
GCAGGGTGAGGAGAAGTTCCCTTCTCCACATCCACGTCCAAGCAATGGAGCTGCCTAGATTCTA
GGACAGAACACACTCCTCCCCCACAGGACTGAACACAGTGTCTGTGTATCCACTAGGAAAGGG
GAAGTCACCTTCAGCCCACAAGTGTGGCACTCTGGAGGCACAGCAGAGCACATGTTCTTGTCTC
CTTTGTTCCAAGGGGGCTTTGTTGGTGTGCTACACTCTCTCTTCCCCTAGGAGCAGCATGGCCCA
AGGGCCAGATCACTGGGAATTCTGCAGCTTCCCTGGGTTCAGTCAGCCCTGTCTGGCTACCTTA
ATTTGAGCAGGTGCTTTGGAATGTTTGCAGGGGATCCAATGATGTGGCAACAAAAGGGCTGAG
ATTCCCTAGGCAGGACAGTCCCACAATGGGTATGCATCAACCCACCACTGCTCAGGTGTGGCA
GAAGAGTGAGCAACCTTGCATAAACTGGCAGCCCAGTGCAATGCCCTCAAGAAGTTCCCACAT
CACCACTCACACCAGTGCCTGGGCTCATGATGGCAGAGGGGCTCTCCAACAGTTCAGCAATCA
GCAGCCTACTGCAAGGGTGACAGGAGCCAGAAAACCCCGCCTACCCTGTTCACCAGACACCAA

GTCCCTTGAAGCTCAATCTCTGCCAAACCCTTGCTTGTTTTTTTTTGTTGTTGTTGTTTTTTGTTGTT
GTTGTTGTTCTGTGCTTTAGCTTGTCTGGTAGGCTCTGGCACTCTTCTCTTAATATTCTATTCAAGT
TATGATTATTCATCTGTAAGTTTGGTTCTTCTTTCTGAGGAGAACTGGCCTCTGACATCTCTAGTC
AGGCATCTTGGAAAAAATCTCTATTTTTATTTTAAGGGGAAAAATATTAAGTAGCACATTTCAATT
TAAAAATTCTTAACCAATTTACAATTTTTATAAAATGAAACTAAGACACAGCAATATGTGTATATG
CAAAAGTAACAAATTATAATAGTAAGATATGAAATGCTGAAAACATTGCTTCCTGATTATCAATT
ATTAAATAATATGGTTGTTAACAAACAATACCTTTATATCCAATGACATCATCTCGAATCTGCTCC
TAATTAAGTTTCCTCTGGTCCTGCTAGTTCTTGTTTCAGGAATAATATGTGAATTGAGGGGAATTC
TCATGACCTCTTGCTGAAGTTGGTGGAGGTTGAGTGGGGCTTTAAAATTCAAACCTTCTACTTCA
ACTACTAGGTCACTTGCTCATATGCTCATTGTTTTAACAATGGCTGTGTGTTTCTTTTCCTAGCTGT
GTGTTTCTTTTCCTACTAGGGCATCGCACTCTGTTTCAGGAGCTGTTTGAGTACAGGCAAAGATA
GTTGTTGTTTAGCTATCAAGTGTGAAAAGAGGACAAAGGAGCAAAGGAGTATTGCAAGAGCAT
CTTTATAGTGACAAATATTAAGTCTTCTTTGGCAAAGAGGGAAGTGAACAAAAAAGAAGGTTGA
TGGACAGGAATGCACTGGGGAGACACAGACTCTGGGAAGGAGTAGTTTAAATGGTACTTTAAA
AAATATTCAACCTTTCTTCTATGTCTCGGGAATTAAGGTATGGGTGACAGCATTGGAGGATAAGC
TTACGCAAGAATAAATTATTTGAATTGGTGCTATATTTGCTTTCAGTAGAAATGTTGCAGTTGATT
AATCCATAAATCAATAAAATGTTAAGAAGGATGAAAATCACATTGGAAGTAAATTAATTAGAAG
ACTTTAGATAGTCTAAAAACTGAATCATGATGAGGTGGCAGGTTGTCATTTCTCATTAAGCCTTTT
CACAATAATAAATAAAAGAGTGTATTTTTGGAGACAAGAATGTGCTGCTAGAATTCAATGCTTCA
AGATATGAGGGCATGAAAGAGAGCAGCCGAAAAGAAAATGAAATAAATAAATGTTTTCTCTCTT
TAATCTGCCCGAAATGCAGTCATGTAATGATGGATGGCTCAATTTATTTAGCGTACTCTGCACAG
CAAAAGGCAGGTTGGTTTCCATAGGTATTTGCATAGTTAGAAATCTTTGTTCCATTACTTGACTTA
AAGAAGTAACATGTCATCAAATATTTATTTCTTATACGGTAATGTAGCTTCTCAGTGGTGTCATAT
GATATGAAAGGGAAAAATGAATATAGAATAAAGTAAAACAGATCCTAAAACTTTTCATAATAAA
GATTTCTAAACATAAGGCCCATGAATATCTGAACAATAGTTTTAAATATCAATGATTGCATCCCA
CACACTTTCAATCTCTTTCCAAGGCATTTCCTCAGACCACCTGACCCTTCCTTATTCAAACTAGAT
TGGTATATTATTGCCACAGGAGAGAATATATAGCCAAACTCAGGTTACAAGGGAGTCCTCTCCA
TGAAAATCTAAAGTCTCTTGAAATTTATGGCAGAGGCATTAGCTTGAAGAAAAGATGTTTTGAGA
ATCAAACCAAAGGTCCTGAAGGATTCGAGACATAAAGTTTATGCCATAAAAAATACATTTGAGA
GAATGATGAAGGCCAGGATAACCTTGTCCCCTGAATAAGAATTCTATAAAGTAGCCAGGGAGC
ATGAGTTGCCAAGGGAAGAAAGAGAAAACATACTTCAGTGCATCCCGGATGAGGAGCCTGTGT
CTTACGCCCTCACCCAAGGTCCAGAAGCAATTGAGGAGTTTTGCAAAAGTGGGTGTAAATTGCC
TTTGATTCTGATTATATGTTATATGCACTAGGAGAGGTAAATTTTTTAAAGTACTCATGCAGATTT
GAAGAATTGAAAGCAGAGGTTCCAAATCTGTAGGTGAAGGAAAAAAAAAAAAAACATTGCTCCC
CATGACTAGGGAGCAGCCAAGGGAAAATGGCTATGACATGTAATTAGGTAGACACACCTCAAG
CAGCCTCAGAGAGTTCTCATGCTTTCCAGTGTGGCACAGGAAGCAGTAGAACTTTTCCTTATGTG
GCATAGAATGGTGAGGGCGTGACTCAGAGAGCTGAGATCCTGGAAAGTCCTAGTGGTTGAGAT
GATGGGGACACTGAAGTCATCTGTATGAACAAAAAGCAGACAACAGAGAATGCTGAGGGTGTC
TTAAAGGAACATCAGCATGGATGCCTGAGACTAGATGTTCCCTACTTCCACCGTGCCACATCCT
CATTCTCACACCTTGGCACTAGTTCAGACTCTGAAGAACTTAGGAAAAATATTGACATCGTTGGG
AGTAAAGCTAATGATTAACCAGTAAATTTGGGCTGGGCACAGTAGCTCACACTCTTAATCCCAG
CACTTAGGGAGGCCAAGGCAGGAGGATTGCTTGAGTTCAGGAGTTTGAGACCAGTCTGGGGAA
CATAACAACACCCCATTTTACAATACAATTGTTTTAAAAAGAAAATATTAGGTAAATGTGTTGGC
ACATGCCTGTAATCCCAGCTACTCGGCAGACTAAGACAGGAGGACTGCTTGAGGCCAGGAATT

CAGGGCTACAGTGATTATAGCCTAGGAAACAGCAAAGCCCTATCTCCTAGGAAAAAAAAAAGAA
AATCGGAAATTGACTAACAATACCCAGACGGAACTTCATCAAGAGGCATGGGGGAACATGAGA
AAAAAAATTAAAATTTTTCTTAGCAAAAATATAAAGTTAGATGTTTTTGTTTTCCATTCAGGAGTTT
GTGCTTGGGAAGTCAACAGCATCTTTGAGTACACACAAAATCCAGATCAATAAACAGATACACT
ATGGGGTGAATATTCCTTTTGTAAAAGTGTTCTTCACTATGGAATAATGTACCAGAGAATGCTTTT
AAAAATCCAATACTCTTTTAGTGCATATAACACATAATTGGAATTACAGAAATGCAGTTTACATA
AACTTGTAAGAAGATAATCAAATGTCTAAAACAAAGCCATTTAAAACCTGCCATTTCTTTGGAGT
GTTTAACAAAATTCGGGTATCCATCATTCTGTAGGGCTTAAGAAACTGGTGCCTGTTTTAGTCAC
AATCCAGTAAGAAACAGATGATATTAACATGAATGAGCAAAGGGACAGGCCTATGGGTATCAG
AAAGAGGGGACAACCTGCTAAGATGTGACGGCAGGGCCTGAGCCCTCAGTTGTGGCATCTGGA
AAGAAATTCAGGCCATTATCCTTAGCAAACTAACACAGGAACAGGAAACCAAATACCACGTATT
CTCACTTACAAGTCAGAGCTAAATGGTGAGAACACATGGACACATAAAGGGAAACAAAACACA
AGAGAACCTTTTGGAGGGTGGAGAGGGGAGGAGGGAAAGGAAAATAACTAATAACTCATCGG
TATTAGGCATAATATCTGAGTAAGGAAATAATCTGTACGACAAACCCCCATGACATAAGTTTGC
CTGTGTAACAAACCTGCACTCGTACCCCTGAATTTAAATAAAAGTTAAAATAAAAAAGAAATTCA
ACTGATTCCTTTTTCCAATGAACATATGAGAAACACAATAATTAGGGACACATGCACACAACCTT
GGCACTAGGACAAAAAAAAAACTAAACAAATAAATTCTGTAGGAAGTTGAAGAGGGCTGAATA
GGAATCACTATAGGTTAGTGAATAGAATCAGCCCCTCTCAAGTCCATATTTATGGAATTCTTATTT
CTCCTACCTATAGCTTGAACACAGGAGTCAGAAAACTCTTCCATTTGATTACTCTGTAATTTGCA
GAAGACAACATATGATCTGGGGAAAAATAATGTTTATGGCTGCCTTTGAACTCCACCAATTAGC
CTCATTGTACTCCTCTTACAGTACTCCTGTAAGATGCTCAGGGCTTCACCTACTGATCTGGGTTCT
TTTTCCCAGGGCATAAAAGGTGCCAACAAATATTCAAAGATCATCCCATCCTGTAAGTCAGAAG
CCAGGTTAAAGCAGAATATCTGGGTTGGCAGAATCTGAAATGAAAAGAGGATTTCCTTTTTCTCT
TGCTTCCAGGCATGTACACTCACAGTTCTTATTCAGGACACCCCAAGGCTGCAGAATATACAGG
GCAATGAAGTAATCACACTTGAGACTCTTACTATAGTATACTGAAAAATGCATTTATATAGCAGT
ATACGGCAAATTTTTGTTATTTATGGTAATAGTGTCCTAAAAGTCACCAAGAACACTGAATTAGT
GAATACTAGACTATTGCTTCTAGGGGAAAACGGTTAGGTTCCTATATGCCCCTAGTCATGCCATT
TTCATCAACCAATCAATAAGTAACCTTGTTTTATGTATGTTTCTATTTAAAGACACCGTATTTAATA
TATATTGCTGATTAATATTGAACTTTAGGCTAATATCACTTTAACTCATGCCTGGATGGAGCTTAT
CTTATATAAGCTCTCTAAAGAACATCAAGGTTCCTTCCCCTTAAAAACACTAGATAGTACTTCAG
CACTGCCTTTGGAGCCAGTTAAAGCAGCAAAATAACCAACAAAAAAAGGAGAAACAATGCAAA
AAAAATGGCACTAAATAGACCACAAAAAGGACACATTTACAGTATGAGAGCTGAAATAAGAAG
GTGGTACATCACCTTACCTGACCTTAGCTGGGAACTACACATTGGGAAGCTCAAATTTTTATCAG
TCTTTGCATGTCATAGTCTGCAAATTACCATCAAAGTGACATGAATATTGATTTGGGGGTTAGAG
ATACATTTTAGTAAGAAGGCAAATTTTCCAATATGGAATCTGAATAATGAGGATCGACCATATTT
AAGAAGCATGAATGTTAGCTTGCTAGTGGGTACAAATCAGGCAGATGATTTTTCATCAATGAAA
GAGTGCATGGTTGAAAAGTTTTCTTTATTCTTATTTGTGTTTGGCTTTAGGAATTCTGCAGGTTCTT
GCAGGACTCCACATATTGTCTATCACAGCATATTGCAGACAGGTTCCCAGGAAGCAGATTCTGA
GTTGGTGATTTGCATGCAGGAAGTTTATGGAGAATTGTTTCTGAAATCAGCTCCTGTGGGGAAAT
GAAGACAGCAGGACTGGGCAAAAGGAAACATTGAACTGGGATGAGATCACAAAGAAGGCATC
AGGGAGGGCTGGAGCCAGACTGGGACTTCAGAGTGGCCCCACATTGAAGAAGAGAAGCTTGG
TTTTTAAACTCACACTCTCTCCCCAACCAGGACAAGTTATGCAGGTGGTCCCCCAGAAGAAACT
GTGACTTTGGAGTTGGTGGCGTTTTTCAGCAAATGGCAATTCCCAAAAAGGGATCCAGCTGCAT
GCAGTTGCTGTCAATGCTCTCAGCAGCTGAGGGAATGAGTGCCTGCGTCATGGAGAGGAATGC

AGGTGGTACGCCCCGACACTCACTACACTGTCAAGCACAAGAGGGCATTTCTAGCAACTCAGTT
GGAAACGCTCTCAATGATGTTTAACAAAAGGATAGAATTACTTATGGTGGCAGAATAATGGCCC
CTGAAGATATCTATGCTGTGATTCCCAAAGCCTGTGACTATGTTACATTACATGGCAAAAGGCCT
TTGCAGATGTGATTAAGGTTAAGGACCTTGAGATGGGTTGATTATCCTGGATTATAGAGGTAAGT
CCAATGTAATCACATAAATCCTTGAAAGTGGAGGACCTATCCAGGCTTCAAAGAACCAGAGAG
ATTTCAATGTGGGAAGGGCTCACCTAGCTGTTGCTGACTATGAAGGTGGAGGAAGGGGGGCCA
TGAGGCAAGGAATACAGGCAGCCTCTAGAAACTGGAAAAGGCTAAGAACTGGATTCTCCCCTA
GAGCCTTCAGAAAGGAATGCAGCCTTCAACTTAACCCTAGTGAGACCCATCCTGGACTTCTGTC
CATAGAATTAAAAGATAACAAATGCATATTATTTCAGCCATTAAGTTTATGGTAATTTGTTGCAGC
AGTCACAGTAAACTAATACACTGCCTGTCCAAATAGTGAACTTGGCTCCATGCATTGATGGAGA
AGAAAGACACCCGAGGGCTCCATTCTGCAGGATGAGTCTGCAGGGATCTCCTTGCCAGCTATG
GAAGTCAGTATGACAGCTTCAGCTACCTACAGTCCACTTGCCAGGTGATTATTATTATTATTATTA
TTAATTTGCACTATCGCATCCCAGTGTGTGCCTCCAGAGCCATCTATTTTCTTTATTTTTTCACTGC
CTAAACTGCTTATAACTGAGAGTCACAATTGCAGAGCTATTATCAATTAATTTGCTAGCAGCAAA
AGTCGGTGTGACTCACAATCAGGCAGCCCTGCCCAACCTAACAGCTGTGGCTTCAGAAAGGCA
GGAGTGCCAAAACACCTTTCTTCTTGCCCTTCATACTATTGGTCTTCCCTGAAAGGAAACACTTC
CCAAAGCTCACTGATAAAATACTAAAGTACAATTTTGTTACTCGCCTAAGAGCACTCCTACCTAC
TCTGTGCTGTGCAGGAGAAAAAATGGTGAACAAGAAAGACACAATCCTTACCTCTAAAGAACTT
CAGATTGGTGATGATTATAGACAAGATTTAGTTTTGATAGAGTGTCTGTGTTTTTTGATAGGAAAA
AAGCAGAGTTCTCTATGACCATGTTGGAGAAGCACCGAACCCAACTTTAGATGGTCAGGGAAG
AATTCTCCTAAGAAACAACACTGAAACTGAGACTTAAGAATGCATAGAAATTGTCTAGACAAAA
AGAGTAATGGCAAAGACTGTTCCAAGCAAATGGAATGTGGACAACTGCAGAGGCAACAGTAAT
AATCACTAATATTTATAATATTTATTAAGTATTTACAATATGTTAGGCCCTGTGCTAAGTGCTGTA
CACACACAGTCTTGTTTCATCCTCACAACAACCTGATGAGGTACGCACTGCTATTGATTCCATTT
TCCAGATAGGAAAGCTGAGCAACAGTGAGTTTGTACTTCTTGCCCAACATGACACAGCTTGTAA
GTAGTTGAGTATAGAAATGAACCCAAGCATTCTGGCTCTAGAATCCTCTTGCTTAACTGTTCTTCT
GCACTACCTCTTACTGAGTCTCAAGCAAGTGATCCAATGGCTCATCCAAGGAACTGAAGTAAAT
TCCAGGTGCTTGGAGCTGAGACAAGTGGGGGCAGGGAATTAGATTCAGCAATAGCAAGAGATG
ATGCAAAAGTGAGTATTTATAACATATTATAATCATATCTATCCAGTACTCAAGATTACATCAATC
TTTAATGAGGACTTGGAATCTGCGGGATACAGTGGGAAGGGCAGAGGCTTTGGTGTCAGACAG
ACGTTAGTTCTGATCTTCCCACTAATGCGTAGAAGCTGAGGAATTTAACTTAAGCAAGTTCTTTA
AACTCCCCCTATACTCCCATCTGTGCCTTGGAGATTATAATAGCTACATCAAAGAGTTGTTGTGA
GGACTACATGGTAAAAAGTACCAAAGTGTCTGGCATATAATGAGTAGGCACTCAAATGTTCAGT
TTCTCCCCTTCCTTTCCCTTATTGAAAGCAGCAGAGCAGACTGTCTAAATATCTCTGCACACTTCA
ACCAACACAGAAAAAGAACTTGAACAAAACTGAACATTGATTCCCCCAATTTAAAAAAAAAATGC
ATTTGATTTGGGGCTCAGACCTCAGATATTGTTGAACCAAAAATTAAACACCAAAACCAGTGTG
GTTTTGAGGAAGGCCACATGGGTTAGGAATCAGGACATGCAAATGCTAGTGTCAGCTCCACCG
CAAGTAACTATGATGCTTTCTTCTCTGAGCATCAGCTTCCTCATCCAAGACAAGAGCTGGTTGAA
CTTGGTGGCCTCCAGGGACCACTGAGATCTAACTTTGAGCTCACTCAATGCCTTGAACATCTGAT
TAGAAACAGAACTGGAAGATCAAATTGATCTCCGAATTAAGACAAGATTCTGAGGTTAAACAAA
CAACAAAACAAAAGAGATCTAATAACCTTCTTGGGAGTTGCAGAGAATCTATGTAGGAAGATAA
GAGGTGGAGTCTTTGAAGGAGATAGACATTACAGTCTCATTTCCTCTTTGTTCTTTGCTAGATCTG
TCACCCAAAGTAAATCATTCTCTGTTTAAACTTGTTTCCTTACCTATCAAATGAAAATGATAACCT
TCTTTTATAGACTTGTATTCAAATTAAAGAATATGTATGGCCGGCCAGGCGTGGTGGCTCATGCC

TGTAATCCCAGAACTCTGGGAGGCTGAGGTGGATGAATCATGAGGTCAGGAGTATGAGACCAG
CCTGGCCAACATGGGAAAACCACGTCTCTACTAAAAATACAAAAATTAGCCGGGCGTGGTGGC
GAGCACCTGTAATCCCAACTACTCCGGAGGCTGAGACAGGATAATTGCTTAAACCTGGGGGGC
AGAGGGTGCAGTGAGCAGAGATTGCACCACTGTACTCCAGCCTGTGCGACACAGCGTGGCTCT
GTCTCTGGGACAAAAAAAAAAGAAAGAATATTTACAATTTTCAGTGTACTCTTTATCCTCCAAGA
GAAAAAATAAAATCATTAATCACAGGCCTCCAAGAAAGGTTAAACTCTCCTCCTTTTTGCTCCCA
TCACTTAGGGTCCACCCTGTGCATCATCAGGACATTCACCTTGCTGCCTCCTATGTAAACATTTC
CTCTATTTTGAGCTCCCTCAGGGCAGATACTTTCATATTCACATTTATATCTAAATAGGGCCTAGA
ATTGCTCCCTGTGAATAGTAGTTTTTAATGAATGTTGGTTGTTGAAACTAATAAAAAAGAAAATA
AAAACAATGAGATTGCATTTTTATCTAGTAAATTAGTCAAGATTTCCAAAAACATACACATTTAAT
CCAGGCAAGTGCACATGAGCTGATGGAAGTTTTCTTGAAATCCATTTAGTAAATATGTTTCAAGA
CTTTAAAAAGTGTCTATACGATGGGGTTTTCTAGGTAAACAATCATGTCGTCTGCAAATAGGGAC
AATTTGACTTCCTCTTTTCCTAATTGAATACCCTTTATTTCCTTCTCCTGCCTGATTGCCCTGGCCA
GAACTTCCAACACTATGTTGAATAGGAGCGGTGAGAGAGGGCATCCCTGTCTTGTGCCACTTTT
CAAAGGGAATGCTTCCAGTTTTTGCCCATTCAGTATGATATTGGCTGTGGGTTTGTCATAGATAG
CTCTTATTATTTTGAAATACATCCCATCAATACCTAATTTATTAAGAGTTTTTAGCATGAAGGGTT
GTTGAATTTTGTCAAAGGCTTTTTCTGCATCTATTGGGATAATCATGTGGTTTTTGTCTTTGGCTCT
GTTTATATGCTGGATTACATTTATTGATTTGCGTATATTGAACCAGCCTTGCATCCCAGGGATGAA
GCCCACTTGATCACGGTGGATAAGCTTTTTGATGTGCTGCTGGATTCGGTTTGCCAGTATTTTATT
GAGGATTTTTGCATCAATGTTCATCAAGGATATTGGTCTAAAATTCTCTTTTTTGGTTGTGTCTCTG
CCAGGCTTTGGTATCAGAATGATGCTGGCCTCATAAAATGAGTTAGGGAGGATTCCCTCTTTTTC
TATTGATTGGAATAGTTTCAGAAGGAATGGTACCAGTTCCTCCTTGTACCTCTGGTAGAATTCGG
CTGTGAATCCATCTGGTCCTGGACTCTTTTTGGTTGGTAAACTATTGATTATTGCCACAATTTCAG
ATCCTGTTATTGGTCTATTCAGAGATTCAACTTCTTCCTGGTTTAGTCTTGGGAGAGTGTATGTGT
CGAGGAATGTATCCATTTCTTCTAGATTTTCTAGTTTATTTGTGTAGAGGTGTTTGTAGTATTCTCT
GATGGTAGTTTGTATTTCTGTGGGATCGGTGGTGATATCCCCTTTATCATTTTTTATTGTGTCCATT
TGATTCTTCTCTCTTTTTTTCTTTATTAGTCTTGCTAGCGGTCTATCAATTTTGTTGATCCTTTCAAA
AAACCAGCTCCTGGATTCATTGATTTTTTGAAGGGTTTTTTGTGTCTCTATTTCCTTCAGTTCTGCT
CTGATTTTAGTTATTTCTTGCCTTCTGCTAGCTTTTGAATGTGTTTGCTCTTGCTTTTCTAGTTCTTTT
AATTGTGATGTTAGGGTGTCAATTTTGAATCTTTCCTGCTTTCTCTTGTGGGCATTTAGTGCTATAA
ATTTCCCTCTACACACTTCTTTGAATGCGTCCCAGAGATTCTGGTATGTTGTGTCTTTGTTCTCGTC
AGTTTCAAAGAACATCTTTATTTCTGCCTTCTTAATAAATGGTGCTGGGAAAACTGGCTAGCCAT
ATGTAGAAAGCTGAAACCGGATCCCTTCCTTACACCTTATACAAAAATCAATTCAAGATGGATTA
AAGATTTAAACGTTAGACCTAAAACCATAAAAACCCTAGAAGAAAACCTAGGCATTACCATTCA
GGACATAGGCGTGGGCAAGGACTTCATGTCCAAAACACCAAAAGCAATGGCAACAAAAGCCA
AAATTGACAAATGGGATCTAATTAAACTAAAGAGCTTCTGCACAGCAAAAGAAACTACCGTCAG
AGTGAACAGGCAACCTACAACATGGGAGAAAATTTTCGCAACCTACTCATCTGACAAAGGGCT
AATATCCAGAATCTACAATGAACTCAAACAAATTTACAAGAAAAAAACAAACAACCCCATCAAA
AAGTGGGCGAAGGACATGAACAGACACTTCTCAAAAGAAGACATTTATGCAGCCAAAAAACAC
ATGAAAAAATGCTCATCATCACTGGCCATCAGAGAAATGCAAATCAAAACCACTATGAGATATC
ATCTCACACCAGTTAGAATGGCAATCATTAAAAAGTCAGGAAACAACAGGTGCTGGAGAGGAT
GTGGAGAAATAGGAACACTTTTACACTGTTGGTGGGACTGTAAACTAGTTCAACCATTGTGGAA
GTCAGTGTGGCGATTCCTCAGGGATCTAGAACTAGAAATACCATTTGACCCAGCCATCCCATTA
CTGGGTATATATACCCAAATGACTATAAATCATGCTGCTATAAAGACACATGCACATGTATGTTT

ATTGCGGCATTATTCACAATAGCAAAGACTTGGAACCAACCCAAATGTCCAACAATCATAGACT
GGATTAAGAAAATGTGGCACATATACACCATGGAATACTATGCAGCCATAAAAAATGATGAGTT
CATGTCCTTTGTAGGGACATGGATGAAATTGGAAACCATCATTCTCAGTAAACTATTGCAAGAA
CAAAAAACCAAACACCGCATATTCTCACTCATAGGTGGGAATTGAACAATGAGATCACATGGAC
ACAGGAAGGGGAATATCACACTCTGGGGACTGTGGTGTGGTGGGGGGAGGGGGGAGGGATAG
CATTGGGAGATATACCTAATGCTAGATGACGAGTTAGTGGGTGCAGCGCACCAGCACGGCACA
TGTATACATATGTAACTAACCTGCATGCACAATGTGCACATGTACCCTAAAACTTAAAGTGTAAT
AAAAAAAAAGTGCTTATAAAATATGAAAAAAAAAAAAGTGTCTATACACTCCCATCCAGTAACTC
CACTCGTAGGACTTCATCCAATGGAGATAATAGGTTATTTGCCATGTAATAATAGCAATGCATGG
GGAAAAAATTACTGTAAGTAAAAACACCAAAATATTAATAGTGGCCGTTTCTCAGTAGAGGGAT
TAAAGGTGATTTTCATTCTCTATTTAATTCCTTTCTGTATTTTCCAAAATTTATTTAGTGATCACGTA
AAATTTTTCCAGCAGGATTATATAGAACTTATTCATTACATGGAAGATTTAAAAAGTTTAAATGTA
ATAATTTAAACTTATACTAATTCTGAATATGTTTAAATAATTCTGAATATATTTAAAGCATGTTAAA
GTTTCTCAAATGCCTAAACTTTTAAAATTTATCCCAATACCCAATATAGTCCCTTGCATGTTGGAG
GCTCTCTGTAAATTCATATATACAGTTGGCCCTTCATATCAATGGGTTCCACATCTGTGGATTCAA
TCAAACATAAGTCAAAAATATTTGAAAAAAAGTGAATCTTTGTGTCTGTACTGAGCACGTACAGA
TTTCTTTTTTCATGTCATTATTCACTCTTCAATACAGTATGACAATTTCCATAGCATTTACATTATAT
TAGATATTATAAATAATCTAGAGATGATTTCAAGTATACAGGAGGATGTACATACATTATATGCA
AATAGTGCACCACCTTACATAAGGGACTTGCATCCGTGGATTCTGTCACCCTTGGGGATTCCTG
GAACCAATCCCCTACAGATACTGAGGAATGACTATACAATTTTACATGAGTGTATCTACTTGGGT
CTTTAATTTCTCTTTCTTTATTCTGTACATTTAGAAGTGTCACTTATATTTTTCTTCCAGTCATTTGA
TTTCCAGAGACATGCAAGAGTCAGCAGATTTATTTTTCTCCAAAACAGGTTAATGCTCCACTTAA
CTGCTGTCCAGATAGTTTTTATTAAAAAGTAGCACAGTTCTCCCTGTGAGATTTCAACATCACAT
GCTAGGAAGAACTGAGAAGTTAACTAGGTTATAAGGATTTGTTAGATTGTCTAATCATTGCTGTG
CGGTTACTCAGGTAAACACAAATGAAGTTCTTAAGTAAAAAATCATGATTGTGACTCTTTTTTGTT
AATGGAATTCTTTTAGGTTAAAAAAATGCTGTAATTCTTTTTTTCTGTAGAAAAGCCACTGTTAAC
TTGGTCAAATTTATTTTCACTGCATGAAAATTTTATCTTTCAAGAGAATTAGCCATCATTGCATCTT
TTAAATAGCATTAGCCATTATTGCATCTTTTTAAAGTTTTTAAACTTTTTCCCTGTCTCTTCAAGGC
TTCCTGCTTAGGTTTGTAACATCAAATACCAAACTTCTTGTATTTTCATGAGATTTCCACTATATTC
TTCCTCTCCTCTGGCCTCAACATGATAAAGTCTTCCCTCCTACATTAAAAATATGTCCTTCCATAT
TATTCCTTTAAGCTCTGAATCTGTTTCTCTCATTCCCTGAAGTATTAATGTTTTGAAAAGATAGTTC
ACACTCAGCTGAACAAATTTAAGTTCTTTAACCTTCTCTTACTCCAATTTCTTATTTCCACTCTTGC
TGAAATATTTTCTTGAAAGTCACCAATCAACTCTTATAACCACAGCAATGGCTCATTTCTTTAATG
TCCTCAACTTCTCTACATTTAATGTTACTTGTTCTTCTATCTTACTGAAACTCACCTCTGCTCACTT
AGACTCAAAGGGTAAGAATGTCAATGATCAAGGAACTGGGATTTCACCTTTGACTCAAAATTCT
CTACCAAGCAATAGTTTGAAAGTGGCTATAATTTTGTAAAACATTTCCACAAGTTAAACATAGGT
ATGTTTTTACTGTAACGTGAAGAATAAAACATGTGTCTATGGAATTCTGTCTTCATACATTGTTAT
CAACCTCAACTCATTCTACTCCTTCTGTGGTAACTATTTTTAATAACTTGGTATTTTTCAGCCCTTT
TTCTATGTAGCCATGTTAATATACTGGTATGTATACAAAAATAGAATTTTTACAAACATAAATATA
TACCTACAGTTTTAGGTAACAAATGAGATCATGTATGTATTTTTTCTTAAACCTGTTTATTGCTTTA
AAACTAAAAAGAAGAGATACTAAGTAAGGGTAAGAAGGAAAATAGCATGAAGAGTGGGGAGA
CACGAAGATGGGCAGATGGCTTGCGTTTATGCTTAAAATACCTTATGAATACATAGGCATATTTC
ATGTCCATGCCACACAAAGCTTCTTAAATTTAGCAGTGGAAGATTAAGTGTAGGATTTTGGATTT
TGATTTCTTTTTTATAGTGTTTTTGTTGCTAATATTAAAAATGAAACCACAGACAGCTTGGTTTGA

GGGGCATTGACTAAATTGTCTTTCCATTTTTATTAGGCAATTGACAAAAAGGACTTATTTCTTTGT
GACTTTATTATAATGGTTGGCTTTGATTTGCAAAAAGTGTGAAGTCGAAGATAGCTTTAGACAAG
GAATAGAAAGAAGGTATCTTAATTTTAGTATCCTTTTCACATTCTGTCTGTGCTGTCCAAGGTGAT
TAGATTTTACAGCCCACTCATGCTGTAAATATCTTTACATGTGGTAAGTCTTACCCTAGCACTTTA
GTAACATGCCTCAAACAAAGTATTAAAAGCTTTGAACAAGATGGCATCTAAGTATGATGAGGTT
TACTAGCTTTTTAAAAACATGAAACACCTTTTGAAGTGATTTTCAGAAGAAGAAAGATTTGTACT
ACACAGGAGAGGATTTCAGTGCTGAGTTTAAAAAGAAACTCAGGTTGGAAAGACTTTAGAGGA
TTTTTCTATGAACTAAGAGATTAGAGATTGATATCTTGTCAAGCACTTTGTAGAAAAAAGGAAAG
GTTATAGAAAGGCAGATAATAGACTGGGCAAGGGGATTAAAAGATGAGGCTAAGCAACATTTC
TTTTATTATACACATAGTCATTAACAAAGAACTAGAGTTCTTGTGAATTATGTTACATTGAAAAAA
ATTAACAGTTGATTAAGATAAGATCATTACCTATTACCTGTAGAGATCAGAAAAAAGATTTAAAT
AAAACATGTTACAAAAAATCCAAAATTTAAAAATATTTTGGCATTTAGAGACTATTAAAAAAGAG
ATAAAGGATACTTTAATACATGTTTAATGAAGGGTTGAGGAAATTGGTATCTTTTCCTAAGGACT
TTAAGACTTATGATGGCCTAACCCTCAGATGTGTGCAGCGAAAATATTACTTCAGCAGGACAGC
ATTGACTGATATTTTTCTCTCCTTCATTCTCATAAATGTGGCTAGAGAGACCTTAGTAATTGATAC
CCTCAACATCGACAGTTCTCATTTTACTTATTAATGTTGATCATCATACATACCAGGAGCATTAAA
AATGCAATAAACAATTGATTTGAGCTTTTTAAATAATTTCGACTTTTATTTTACATAAAGAGGATA
CATATGCAGGTTGTTACATGGGGGTATTGCATGATGCTGTGTTGTCTGCAAGGTTTTACAAAACA
AATACTTGGTGGCTGTTTATTTCTTCCAGCATAAGTCCCCACCTGCTGACCTGCTGCCTGCTATCT
TAACACGAATTCTTCATCTGAATGGATGAGCTTTTCACAGGTACGGTTACATAAATAAGGAATGT
GATACACATTTTTTTATTTAAAATGAAAGATAATATAATTTAAACCAAGAAGCTTTACATTTACTA
ATCATGTACTGCTGCTATTCAAATGCCTAAATACAGAGAGGTATCTTTTAAAACTCTAGACTCTA
CCCTTTTATTGTCTCACCCACTCCCATCCAAGATTCTTTATTCCATCCATGCAGGGCTTCATGCAT
GTGAGTGTACACATCCATTCTCCTCCAGGTGATTTTGACATGAATGCAGCCCATCAACACAACC
AAATGAAATCTACTGCTTCAATGCTTCAGTAAAGAGTAATCAAAAGAAAAGAGAAATCAAAATA
TCTGTTTACGATCTTAACTTAGTTCAAACCATTTTTTCACCCTTCTAAAGCAAGAGTCCTTCTTTCC
AGAGACAATATCTTTTTTGTACTTTGTAGGGGCTGAATCCTGTCTTTTATTTAATCTTCTTTCCTTG
GGCACACGAGTGGTTGGACATATGATCCAGCCCTATCAAATCTGCTTATCCTACCCCTCTGACC
ACAAACTCAAAATAAGCCAGTGAGAGTGAGTACTCCCAAGGATTTTCTGCCAGAGTTCTGAGGA
AAGAGAAAGTCTCCTTTTTCCACTGAGGATGTTAAGCTGATAAGATACATCTGGAGCTGAGCAG
TTCTATATCTGCCATATTTAGCCAGTTTGTTTGAGATCTACAGCTCCTAGGTTCAAACATTTCAGA
ATTTTCTGTTATGCTTATGCTGGTTTGAGTTGGGTTTCTATCATGGGCAAATGAAAAGGGGCAAC
AAATACATCACACTATATTGGGTAATTACTTAAACTCTGGCTCATTGTCCTCACCTGTAGAATTTG
GATAATGGCATCTACCATGCCTACGCACCAGAAAGGCAGTGATGGTCAGACCTAACAATTTAGG
TGCAAATGATATATTCATCCAGTAATAGGTTATTCAAATGATAAACTTTATATATATGCCAACAG
GATAAATATTTAATTCAGACAATAATATTAACCCAAACGGATTATTCAGTATTATGTAAAAATATC
TTGTGTTATATTCTCAATCCTTAGTTATTTTGTTGTCATATAAATATTGCTCATGAGCAGACAAAGC
TAAAACTTCGACCTATTCATATGCCAATAATTAATAGCAATGAATTATTCTTTGTTGGCTTACTTA
ATAGTTCAATGATTTAATAGTTATAATAGTTAATAGCTCTTTCCTCCTATATTGAAATTAAGTTTTA
GACTTCCAGTCTCCTAGCACAACATGTATCAATGCCTGTCAAGCTTGCCAAACACTTGGAGATC
ATGTTGCATGGCAGACACAACTGAAACCAATAACTTCACTTATTTGTGTTCAGAGTTCCAAACTA
AGGAATCCAGGAGTGGCCAATCCAGAGATTCATTCCTTATCTTTGACGAACATCTGAACTCCTG
GCTCATCCTGTGGAATTCAGGCCATACAGGAGATCAAGGTCATTTGCTTTGGGTTAAATGAATGT
TGCCAGGTGGAGGTTGCTAGAGGGAGAGTGCTATGTGAAAGTGCTGTATAAACTGCTTGCTTTT

CACAAACAGTAGTGATTCTTTATCCTGTCCAGCCTGCCACAACTGGACCATCCTGTATGTAAGTC
CCCTTAATAAATCCTGTGTCTCATTCACTGGCTTCAAGTCTCTTCCTGGGCCTCTTGAACGTGGTG
CCACCTGTATTGAAGTCAACAGAAGTTCAGCACAACACAGGTCAAAATGAAGATTCTGACTCAG
TAGGTCAGGATGTTCTAACGAGCTCTAGCTGACATGATACGTCAGGGCTGCAGACTAGCTTTGA
GCAACAAGGTGCTAGAGAAATACCTATAGAACAACAGAGATGCAAGACAAATGAGTTGGAAGT
TAAAAGATTTAAATTCTTGTCTTAGTTTTCTCATTAAAGAAAAGCTGTGTGACTATGAAAAAAAAT
G

ID|56367|gene_id|ENSG00000225173|transcript_id|ENST00000457253 Homo sapiens lncRNA

GTCCGCGCCTCGCTGGGCTCAGCACCCCGGCCCCCGGCGGGACGGAGGCTCGGGTCCAA
AGCAGTGGCTGCACCGACACGAACCCGTCTGCGTTTCTCCACCTGTCTTCCCTCGCCACCTGCA
TCTACTATGGTCTCTAAATGCCTCGCTTTGTACTTTGTGGCTTTCTGGCAGCCCTTTAAAGCAGCG
GTGAAAGAAAGGAGAGAGTCAAGACACGGGAGAAAATGTTTCATCCCGGGTTCTTCTAGTGGT
GCCCGGTAAGTGACTCCGGACTGACGAGCAGAAAAATCCCAGGGCCATCAATAGAGGGCCTTC
GGTGTAGAATGCGGTTTGCTCAGCTGTCAGCAAAGAGGGACGCAAAACCTAAATTTGAGATATC
AGAAATAACTAAATAAAATAGACCTTGGCTGATTACAACTAATGGTTCTGAAACCTAGCAAAAG
GGTCAGATTCTGATCTTGAATATACTCTCGAGAGAACCGTTTCAGTACGGGGGTGTAGCTCAGT
GGTAGAGCGCGTGCTTAGCATGCACGAGGCCCCGGGTTCAATCCCCGGCACCTCCATTCCTTTT
GCTTTTAATTTTTTTCCCATTGCTTTACTTTATTTTCCTTGCTTAAACAGAAAGTAACCCTATTTCAC
AACGGTTTTGCCATTAATATTAGTGCTGTTCCATTTCTACTCCTCAGCCTTGACACGACTCGGGG
GACTGCCTAAGCAAAAGGTGGAAAAGATGGAGCTGGAAGACAAAGACGGCTCCGGATTCCAA
AACTTCAATGGCGTCGCCAAATTGACGAGCTCATTAACCTTTGGAGTATTGTTATTTAAAAAGTA
GGAAATAAAAGATAAATGTGGAAATATTTAATAAAAGAAAAACGTAAGCTCTCAATTTTCTTTAT
TACTTTTTTTTTTTTGAGACGGAGTCTCTCGCTCTGTCGCCCAGTGGCACGATCTTGGCTCACTGC
AAGCTCCGCCTCCCGGGTTTACGCCATTCTTCTGCCTCAGCCTCCCGAGTAGCCGGGACTACAG
GCGCCCGTCACCACGCCCGGCTAATTTTTTCTATTTTTTTGGTAGAGACAGGGTTTCACCGTGTT
AGCCAGGATGGTCGCGATCTCCTGACCTCGTGATCCTCCCGCCTCGG

ID|56372|gene_id|ENSG00000251637|transcript_id|ENST00000511677 Homo sapiens lncRNA

ACATAAGTCAGAAGTAGAAAAAGGGACACAAACTAAGAAGGGTGCTGAGCGCTCGTCAC
CTGGAGGGACTTGTGCCTCTCTCAGTGGTAGTAGAGGGGCTACTTCTTCCTGCCACGGTCATGA
TTGAGAGGCAACGGGTGGCGAGTCTACAGGGGACGTCGTCGAGCAGACATGGGATAACTTTAA
GGGAGAAGGTTGGTTGTAAGGCGGTGGGACTGGGTGAGGGAGACTCTCCTCTTCTTCAGAGGG
AGGCAGTACAGGGGGAGCTGAACTGGCTGAGGGTCGAGGCGAAAACTCGGTCTGGCTTAGGA
GGACCTTGGAGGTAGAATTATGAATGGCTCATGAACGGAGCCATGGAGAGTGGATCCTGACTA
AACTTAGCTATTGATCAATGTAGAGAAACTGATCAGGGTGGCTAGGAGTTTCAGTAACAACCTG
CCACACAGCTTGAACAGTTGTGAGGTTCAATGACCCTTCAGGGGGCCACTTGACTTTAAACTTT
GGCCATTTTATTTTGCAGAGTGTCTGGAGCTTACCTTTTTTAAGGCGGACTTTATAATCCTCTGAA
CTGAGAGAAAAATTCTGCAGCATACATTGGAGAGGGCTTTAACTTTACAAGGCTGGGAGGAAG
TGTTTCTTATTTTTTTTTTTTAAGGCAATTTAATAAGATTTGAGCATAGATATTAAACTTAGCATGGA
CAGAGAAACTTATTTCTTGGGGGACTGGCATAGTGAAAGAACAGAATCAATATGACTAGAAAG
AGCAGAAAAACTTAAAACAGCTAATACTACTTGCTACATTGCTGTAGCTTTAAGATTGAGGGAG
GAGGACTAGAGCCAGCCTGAGATCTTCTGGGTCAGTTTGATCTAGGCGTTCTTCTTCTTCTTCTA
GATCTGCGCTTTAAATACTTTTGGTGTCTTTATGACTTAAAGGCAAATAGCTTAAACTTAGCTTTT

TCTTTTAAGGGTTTAAGGAGTGAGAACAGAGCCAAGTCCTGGAGACGGTGAACTTGCTGTCGCA
CCAGAAAACGAGATGTGCAGGATAGGGGGCAGGGACAAGGCGGAAAAGGACTACTCGGATCA
TTTTTAAGATGGGAGAGTAGCCACAGAGGAGCAGAGTAAGAATCTAAACGAGGTAAAGCAGTA
CGGGCGTACATTTCTTTACACGGTGTTCTACTGAAGGGCACAGGAAAAGTTACAGAATGACAAG
AGAGGTGAGCAAGGAAATCTACAGGGTGGCTGTTTTGAATTCACTACTGGTTTAGTTTAGAGGA
CGTCTAATCACTTGTACGTGGAATATGATGATCTAAATACTTACAACTTTCATGGTGCTAGAAAT
CTTAATTAGGGAAATGTTTTTCACACTTGTTCTTGTAACAACACTTGATTTGCTTCTGGCAGAAAA
GACAGGACTGTGGTGACCAGCCTAAATGATTGATGAGAAATTTAACCTCCTGTGACAAAAAATC
AGCACTAAGGACTTTGAAGAAGTTTTTACTTAGACGTCTTGGGCAATATCAACGTCTTAACATGC
AAAACTTTGACAACTACTAACAAGACAATAGACACTGAACAGAACAATCAACATAAAACAAAC
AATTGACTTTAGGGCATGTAAACAGTTATGACAGTTTCTTCCTTTTTTTTTTTTTTTTCAGACAGAC
AAGGGGAGGGGCTCCTGTGATGGGATCAGTCAGATGCCTGCCTGGCCGCTCCCCCTGAGGGGA
CTTGGGCTCCTCTTAACATTGGCAGGCTGGTATAAACTTCCGGCTCAGATCAAGCTATGCCTGAT
GCTGCCTTAAGCCTTATGAGGTCGCCACGGAACCGCAGGTGAGGGTCTACTTGAACTCCGTAGC
TTTAGCCGTGGAGCTACAAACTGGAGGACAAGCGCAAGCCCTTGTCCTCACTCATTCATTATTC
ACACAGAGTATATAACAGTTTTTTTTTTTTTCTTTCTTGGAGATTCTTCAAGAAACTTGAACAAGA
GAAAGATGAGAGATAGAAAGAGAGAGAGAGTGACCGGTCTGCCAGAAACCAGGACTCAGTCC
TCCAGCATCCTGGGATGTGGACTGAGTCAAGGGAGGGCCGCTGTCAGAGCCACTTCCCTCCTA
GAAAGAGACACAGAGGTGCCTAACAGAAAACCAGGGCTCTACCTTCTAGCGTCCTAGAGAAAC
AGGCAGAGTCAAAAGAGGGACACCCTCATCAGGGCCGCTTCCCTCTTACTAGAACTGAAGTCA
AATCTGACCTACCTGACCTCAGGGTCAGAAGTCGAGGACTCAGATGTGGAATTTTTATGGACAC
CCACACGGTAGTCGATCCGCTCTCCTCTGAAAGACGGTCACCTTTCGGGGACCTAAAAATTTTTT
TTCAGGTGGCACCCCCCCTACAAGCCGGCCGGCCTTCTGGGGGAGCCCAGAGCGAGCCCGGC
TCTTGCCTGGTGGCGTTTCTTGCTGGGGCCTCCGAATGTTGTACTTGAGCGAGTTAGAGAAAATG
TCACACTTTGACATGAATTAAGAGTCTGTTTATTTAGCTGACGGCCAAGAAATGGCTAACTCTTA
AAGTTCTCTTGGCCCCGAAGAAAGGGCTAGATTTTCTTTTATACTTCAGTTTAGACAGGGGAAAC
GGGTCTAGTTAAAAGAATTTTACAGAAGTAAAGTAGGCAAAAAAGTTAAAAGGATAAATTGTTA
CAGGAAAGTAAACAGTTCTAGGTGTAAGGGCTTTAAGACTATTACAAAGTGATAGACGTGGGG
CTTTAGGCATTATCAATCGGACAAATTCCTAGGAACTGCGGATATTGCTCGCCACAGTATCTTAT
GAGTTAATTGCATTCTTAGATGTGCTAAGAGTCAGCTTGCACAAGTTAAGTCCTTGAGGAAGGG
GCTGTCAGTGAAAAGGCCAAGATAAAATCTATCTAGCTCTCTTAGCTAAAAGAAAGTCAATTCA
GGTAGAAACAAGGCTAAGTGATTAAAAGAAAAGGAAAGTCTAAGAACAAAGTTAATAAAAACA
AGGTTAGGCATTACAGATGGGGTCATCCTTATATTCAGCACAGCGTTTGTCCTGAAACTTCAATG
GAGTGCTCCTAGTTTGATAACTTGGACCATGCCAAATAAATTGAATTTCTCTTTCTTAACAACAG
ATGTTTGAATACAGCCCCGTGACCCTTTCTGCTCCTTCCTGCCTTCCTCCTCTCCTCTTCCTTCCCT
TCCTTTTCAAGAGCAGGGTTTGCAAACTTTTTCTTGAAAGCATCAGATAGTAAATATTTTCGAATT
CGTGGGCCATACAGTCTCTTATAGTAGTTGCTCAACTCTGCCACCCATAGCATGAAAGCAGCTG
ATATAGTTGAATATGAGTCCCCACCCAAAACTCATGTTGCAATCCCCAGTGTTAGAGGTGGGGT
CTGGTGGGAAGAGATTGGATCATGGGGGTGGATTTCTCATGAATGATTTAGCCTCATCCCTTTG
GTCCTGTCCTTGCAATAGTGAGTGAGTTCTTGCAAGATCTGGTTGTTTACGAGTGTGTAGCACCT
CCCTCCTTACTCTCTTGCTCCCACTTCACCTTCTGCCATCATTGTAAGTAAGTTTCCTGAGGCCTC
CACAGAAGCTCAGCAGATGTCAGTGTCATGCTCCCTGTATAGCCTACAAAACTGTGAGCCAATT
AAAGCTCTTTATATATTGCCCAGTCTCAGGTATTTCTTTATAGCATGAGAGAACAGCCAAATCCA
GCAGCCATAGACAATATGTAGCAAATGGACATGACTGTGTTTCAATAAAACTTTATTGACAAAC

ACATGCACAAGCAGGTCAGATTTGGCCCATGGGCATTAGTGTCCCAACCTCTGCTCTGGAGTAT
TCTCTTCAGCAACCTCTGCCTCCTGGGTTCAAGCAATTCTCCTGCCTCAGCCTCCCAAGTAGCTG
GGATTACAGGCACCTGCCACCACACCTGGTTAATTTTTGTATTTTTTAATAGAAATAGGGTTTCAC
CTTGTTGGCCAGGCTGGTCTCAAACTCCTGACATCAGGTGATCCAACCACCTCAGCCTCCCAAA
ATGCTGGGATTACAGCTGCCCACCACCACACCTGGCTAATTTTTGTATTTTTAGTAGAGATGTGA
TTTCACTATGTTGCCTAGGCTGGTCTCGAACTCCTGATCTCAAGTGATCCACCTGCCTCGGCCTC
CCAAAGTGCTGGGATTACAGGTGTGAGCCACTGCAGCTGGCCTCTAGATTTTTTTTTTTTTGATC
GTTGCTTATTTGGTTTGACTTTCATTTTTTCCCCCATTGTGGTAGTCATTTTCTGAACGCCTGTTAG
TTTGTCCATCTCTCTCCTCTGTAGTCCCTAGAGTCAGATGAACTCCTCTGCAGGTGCAATGGTGT
AACACACTCTAGTGCTGAATTTTGAGCAGGAGAAAGAGAGCAAGAGTGACCAGTACCCTTGGA
AACTCTGGCCTCCTGAGAATTTGGTGTCTTCCCTGCAAAGGTTGCAAACCTGTTAACCCACAGG
CCGGAGAGAGAGAGAAGTCAGAATCACGATCTGTTAAGCTTGAGTTTAATATTTGATGCACAGA
GATCACTGAGTTTTTTGATAAGAATTAGAGGGGGAAGAAATAGCCAGAGAACAATTTCTATCTC
CAGGTGAGGATTCAGGAGATAATTCTGCGAAAAGAACTTCCTGAGAACTAAGATGGGGAAAAT
CACTGGTATTAGAAGAGTGAAAAGGTCAGTAATTAAGGCTACAACTGTGCTCTTAGAGGCAAAA
GAAGAAAATGAGACTGCCAGGCATGAATAATGAGAAATCTTTGATGGAATTAGCCATGCAGAA
CAGATATTAAATGCATCCTCATTCTTCTCATAGTC

ID|56381|gene_id|ENSG00000234593|transcript_id|ENST00000447908 Homo sapiens lncRNA

CTTATGTATCAACAAAATCACACCAGACCACCCAAAGCTGGCCCTGCCAACTGACCACAC
ACACCCCTGCCTGAAAGGCATGTTTCAATGTGCAACAGACGCCTGCCACTCAGGGGACATCAA
ATGTTCATGAGAACTCGCTAACCTCGAAACAGGTGACTGTCAAGACTGGCTGAGGATTGAAACT
CTGGGGAAATTTGCTAGAATATGTGTGGATTGCTGGGCCAGCTTCCCGATGGGCTGATCTTTAG
GTGCCGGGCTGGGGATGCGCATTCTCAGCACTGCTGCTGGGTGGTTCTGTGAAGTAGACCCTTG
GTAACCACTGCATTAGACCACCTAGGTCTCTGCCACTTCCACACTGTGGACACTTCGATGGATC
ATTCTTTGTGGTGGGCACTGTCCAGTGCATTGCTGGATATTCAGCAGCATTCCTGAACTCTTCTTG
CCAGATACCAGTAGCACCCTTCCTGTCCCCTCCCATGTCACCATATGACAACCCAAAAGATCTC
CAGACGTTGCCACATGTTCCCAGGCAGCAAACTCCCTTCCATGGTAACTACTGTCCTAGGCTGTT
TACAAGGTTATTAGTGATGGTGACAGCCATAATGAGGAACCAGGCTCTGTGCCGAGCACTCACT
GCATCTCATTCTGTGTAATCCTCATGGTCATATTCTTAGGCAATCTTTTCATCATTGTTTATAAAAC
TCAGAGAATTTAAACAGCTTTCCCAAGGTCATGGGTAATCAGTGGCCACAGCTTGATGCCTACC
TCTGTTTGACTCCAAAGTGGACTCAGGGGTCAGATAGATCCAGAGGGTGACAGAAACCTCATCC
TGGAGACTGGGGGTTCTTCCAAGGCTTGTTCTTGTCACTTTTTTTTTTTTTTTTTTTGAGATGGAGT
CTTGCTCTGTTGCCCAGGCTGGAGTGCAGTGGCGCGATCTCAGCTCACTGCAAGCTCCGCCTCC
CAGGTTCACGCCTCCCAGGTTCACGCCTCCCGAGTAGCTGGGACTACAGGTGCCTGCCACCAT
ACCTGGCTAATTTTTTGTATTTTCAGTAGGGACGGGTTTTCGCCATGTTGGCCAGGATAGTCTCG
ATCTCCTGAACTCGTGATCCGCCCTCCTCCGCCTCCCAAAGTGCTGGGATTACAGGCGTGAGCC
ACCGCACCGGGCCTCTTGTCACTATTTAACAAAGCATAATGGCTCCTCTCTGCCTACTCTACCAG
ATCCATGCTCTTTAGCCTGCCAGGCCAGGCTGTCCCTACCTCACATCCCCTGATCAGCTACATTA
TAATCTAAGGCCTATCTCCTCTTTAACCCTGAACGTACCTTGCTCATTTTCACTGTAGGGCTGTTT
CTTTCATTCAGGGGTCTCAAACTCAAATGTCTATAGGGGCCTAGAGGGTAAATTAACAAACAAA
GTGGGCTCAGTGCAAGATGATAAATGGCAATGGATCTTTCAGCCTCATTTGGAGAGACCTTGGG
AATGGTGAGGACTGTGACCTCAGTGGAGCACCTACTTTACTGAGACAGGGCAGCTGCTTCTCCA
TCTTGCCCTGTGCTAGTCTTGCTGGATATTTTATTTTTCAAGAAATAAAATATGGAATGTGGAATA

ATATGTGTGACTTATCAATTTTTAAATTTTGGTAGGTTCTACAAATTCAAAAAAACCACCCGTGGC
CTGGCTCTGGGCTGAAGCTGAGTCCTGGCTTGAAATCCCCTGCCTTAATCTCTCTTCGAAGACTT
TTCTGCATTCATCTGAGTCCTCATCCTCCAAGGCAGGGCTCAGGCTTGGGCTCAGGCTCCCAGC
CATGTATCCCTCCTGGACCATTCCTGACTCCTTTCCAGTATCCTTATCACTTGTACCACATACGTT
GGCCTTTGATCCTGTGTACTGTTGGATTTGTCTTTACTTCACAGGTTCATCTTGTCTCCCCAAGAA
CAATGAGAGTTGCCTCAAGGGGTAGAACCATGCTTTTTGTTTTCTTTTGAATTCATTACAGCACA
GCCAAAAGCTCAACACACAAAACACACTCAGTAAATATCTGTTGAACAGAATAAAAGCCATTCC
GCA

ID|56630|gene_id|ENSG00000256101|transcript_id|ENST00000539795 Homo sapiens lncRNA

CCTGGAGGGACTTGTGCCTCTCTCAGTGGTAGTAGAGGGGCTACTTCTTCCTGCCACGGTC
ATGATTGAGAGGCAATGGGTGGCGAGCCTACAGGGGACGTCGTCGAGGAGACATGGGATAAC
TTTAAGGGAGAAGGTTGGTTGTAAGGCGGTGGGACTGGGTGAGGGAGACTCCCCTCTTCTTCA
GAGGGAGGCAGTACAGGGGAAGCTGAACCGGCTGAGGGTCGAGGCGAAAACGCGGTCTGGC
TTAGGAGGACCTTGGAGGTAGAATTATGAATGGCGCATGAATGGAGCCATGGAGGGGGGATCC
TGACTAATCTTAGCTATTGATCAATGTAGGGAAACTGATCAGGGTGGCTAGGAGTTTCAGTAAC
AACCTGCCACACAGCTTGAAGAATTGCAAGTTTCAATGACCCTTCAGGGGGCCACTTGACTTTA
AACTTTGGCCATTTTATTTTGCAGAGTGTCTGGAGCTTGCCTTTTTTAAGGCGGACTTTATAATCC
TCTGAGGAACTGAGAGGAAAATTCTGCAGAATACATTGGAGAGGGCTTTAACTTTTATAAGGCT
GGGAGGAAGTGTTTCTTTTTTTTTTTTTGAAGGCAATTTAATAAGATTTGAGCATAGATATTAAAC
TTAGCATGGACAGAGAAACATTTCTTGGGGGACTGGAGTAGTGAAAGAACAGAATCAACATGA
CTAGAAAGAGCAGAAAAACCACAACAGCTAATACTACTTGCTACATTACTGTAGCTTTAAGATT
GAGGGAGGAGGACTAGAGCCAGCCTGAGATCTTCTGGGTCAGTTTGATCTAGGCGTTCTTCTTC
TTCTTCTAGATCTGCACTTTAAGTACTTTTGGTGTCTTTATGACTTAAAGGCAAATAGCTTAAACTT
AGCTTTTTCTTTTAAGGGTTTAAGGAGTGAGAGCAGAGCCAAGTCCTGGAGACGCTGAACTTGC
TGTCGCACCGGAAAACGAGATGTGCGGGGTAGGGGGCAGGGACAAGGTGGAAAAGGACTACT
CGGATCATTTTTAAGATGGGAGAGTAGCCACAGAGGAACAGAGTAAGAATCTAAACGAAGTAA
AGCAGTACGGGCGTACATTTCTTTACATAGTGTTCTACTTCAGGGCACAGGAAAAGTTACAGAA
TGACAAAAGAGGTGAGTAAGGAAATCTGCAGGGTGGCTGTTTTGAACTCACTACTGGTTTAGTT
TAGAGGAGGTCTAATCACTTGGACGTGGAGTGTGACGATCTAAATACTTACAACTTTCATGGTG
CTAGAAATCTTAATCAGGCAAATGTTTTTCACACTTGTTCTTGTAACAACACTTGACTTGGTTCTG
GCAGAAAAGACAGGACTGTGGTGGCCAGCCTAAATGATTGATGAGAAATTTCACCTCCTGTGAC
AAAAAATCAGCACTAAGGACTTTGAAGAAGTTTTTACTTAGATGTCTTGGGCAATATCACCGTCC
TGACATGCAAAACTTTCACAACTACTAAGAAGACAATAGAAACTGAACAGAACAATCAATATAA
AACAAACAATTGACTTTAAGGCATGTAAACAGTTATGACAGTTTCTTCCTTTTTTTTTTTTTTAATA
GACAGACAAGGGGAGGGGTTCCTGTGATGGGATCAGTCAGATGCCTGCCTGGCCGCTCCCCGT
GAGGGGACTTGGGCTCCTCTTAGCATTGGCAGGCCGGTATAAACTTCCGGCTCAGATTGAGCTA
TGCCTGATGCTGCCTTAAGCCTTATGAGGTCGCCAAGGAACCGCAGGTGAGGGTCTACTTGAAC
TCCGTCGCTTTCGCCGTAGAGCTACAAACTGGAGGACAAGCGCAAGCCCTTGTCCTCCCTCATT
CATTCATTATTCACAAAGAGTTTATAACAGTTTTTTTTTTTCTTTCTTGGAGTTTCTTCAAGAAACTT
GAAGAAGAGAAAGAGGAGAGATAGAAAAAGACAGAGAGAGAGAGAGAGTGACCGGTCTGCC
AGAAACCAGGACTCAGTCCTCCAGCATCCTGGGATGTGGACTGAGTCAAGGGAGGACCCCTGT
CAGGGCCACTTCCCTCCTAGAAAGAGACACAGAGGTGCCTAACAGAAAACCAGGGCTCTACCT
TCTAGCTTCCTAGAGAAACGGGCAGAGTCAAAAGAGGGACGCCCTCATCAGGGCCGCTTCCCT

CTTACTAGAACTGAAATCAAATCTGACCTACCTGACCTCGGGGTCAGAAGTTGAGGACTCAGAG
GTGGAATTTTTGTGGGCATCCACACGGTAGTCGATCCGCTCTCCTCTGGAAGACGGTCACCTTTT
GGGGACCTGAAAATTTTTTTTCAGGTGGCACCCCCACTACAAGCCGGCCGTCCTTCCGGGGGAG
CCCGGAGCGAGCCCGGCTCTCGCCTGGTGGCGTTTCTCGCTCGGGCCTCCAAATGTTGTACTTG
AGCGAGTTAGAGAAAATGCCACACTTTGACATGAATTAAGTCTGTTTATTTAGCTGGCGGCCAA
GAGATGGCTAACTCTTAAAGTTCTCTTGGCCCCGAAGAAGGGGCTAGATTTTCTTTTATACTTCA
GTTTAGAAAGGGGAAAGGGGTGTAGTTAAAAGAATTTTACAGAAGTAAAGTAGGCAAAAAAGT
TAAAAGGATAAATTGTTACAGGAAAGTAAACAGTTCTAGGTCTAAGGGCTTGAAGACTATTACA
AAGTGATAGACGTGGGGCTTTAGGCATTATCAATCGGACAAATTCCTAGGAACTGCGGATATTG
CTCGCCACAGTATCTTATCAGTTAATTGCACTCTTAGATGTGCTAAGAGTCAGCTTGCACAAGTT
AAGTCCTTGAGGAAGGGGCTGCCAGTGAAAAAGCCAAGATAAAGTCTGTCTAGCTCTCTTAGCT
AAAAGAAAGTCAATTCAGGTAGAAACAAGGCTAAGTGATTAAAAGAAAAGGAAAGTCTAAGAA
CAAAGTTAATAAAAACAAGGTTAGGCATTACAGATGGGGTCATCCTGACATTCAGCACAGTGTT
TGTCCTGAAACTTCAATGGAGTGCTCCTAGTTTGATAACTTGAACCATGCCAAATAAATTGAATT
TCTCTTTCTTAACAACAGATGTTTGAATACAGCCCCGTGTCCCTCTCTGCTCCTTCCTGCCTTCCT
CCTCTCCTCTTCCTTCCCTTCCTTTTCAAGAGCAGGGTTTGCAAGCTTTTACTTGAAAGCATCAGA
TAGTAAATATTTTAGAATTCGTGGGCCATACAGTCTCTTGTAGTAGTTAATCAACTCTGCCACCC
ATAGCATGAAAGCAGCTGATATAGGTTGAATATGTGTCCCCACCCAAATCTCATGTTGCAATGTA
ATCCCCAGTGCTAGAGGTGGGGCCTTGTGGGAAGAGATTGGATCATGGGGGTGGATTTCTCAT
GAATGATTTAGCGTCATCCTTTTGGTCCTGTCCTTGCAATAGTGAGTGAGTTCCTGCAAGATCTG
GTTGTTTACGAGTGTGTAGCACCTCCCTCCTTACTCTCTTGCTCCCACTTCAGCTTCTGCCATCAT
TGTAAGTTTCCTGAGGCCTCCACAGAAGCTCAGCAGATGTCAGTGTCATGCTCCCTGTACAGCC
TACAAAACTGTGAGCCAATTAAAACTCTTTTCTTTATACATTACCCAGTCTCAGGTATTTCTTCAT
AGCATGAGAGAACAGCCTAATCCAGCAGCCATAGACAATATGTAGCAAATGGGCATGGCTGTG
TTTCAATAAAACTTTATTGACAAAAACATGTGCAGGCAGGTCAGATTTGGCCCATGGGCCATAG
TGTCCCAACCTCTGCTCTGGAATATTCTCTTTAGCCTGGATGGACATCTCTAGGGTGTTTTTTTTT
TTTTTTTTGAGACGAAGTCTCACTGTTGCCCAGGCTGGAGTGCAGTGGTGCAATCTTTGCTCGCC
ACAACCTCTGCCTCCTGGCTTCAAGCCATTCTCCTGCCTCAGCCTCCCAAGTAGCTGGATTACAG
GCACCTGCCACCACACCTGGCTAATTTTTGTATTTTTTAATAGAAATAGGGTTTCACCATGTTGGC
CAGGCTGGTCTCAAACTCCTGACATCAGGTGATCCACCCATCTCAGCCTCCCAAAATGCTGGGA
TTACAGGTGCCTGCCGCCACATCTGGCTAATTTTTTTGTATTTTTAGTGGAGATGTGATTTCACTA
TGTTGCCCAGGCTGGTCTCGAACTCCTGATCTCAAATGATCCACCTGCCTCAGCCTCCCAAAGT
GCTGGGATTACAGGTGTGAGCCACTGCAGCTGGCCTCTAATATATATATTTTTTTGACCATTGCTT
ATTTGGTTTGACTTTCATTTTCTTCCCCATTGTGGTAGTCATTTTCTGAATGCCTGTTAGTTTTTCCA
TCTCTCTCCTCTGTAGTCCCTAGAGTCAGATGAACTCCTCTGCAGGTGCAATGGTGTAACACACT
CTAGTGCTGAATTTTGAGCAGGAGAAAGAGAGCAAGAGTGACCAGTACCCTTGGAAACTCTGG
CCTCCTGAGAATTTGGTGTCTTCTCTGCAAAGGTTGCAAACCTGTTAACCCACAGGCCGGAGAG
AGAGAGAAGCCAGAATCATGATCTCTTAAGCTTGAGTTTAATATTTGATGCACAGAGATCACTG
AGTTTTTTGATAAGAATTAGAGGGGGAAGAAATGGCCACAGAGATCAATTTCTATCTCCAGGTG
AGGATTCAGGAGATAATTCTGCCAACAGAAATTCCTGAGAACTGAGATGGGGGAAATCGCTGG
TATTAGAAGGGTGAAAAGGTCAGTAATTAAGGCTACGACTGTGCTCTTAGAGGCAAAAGGAGA
AAATGAGACTGCCAGGCATGAATAATGAGAAATCTTTGATGGAATTAGCCATGCAGAACAGATA
CTAAATGCATCCTCATTCTTCTCATAGTCAAAAGTTTTTGCTTAAGCTGGATGGGAAAAAGAGAA
TCCCATTTCACTAAGCATAAAAGAGGGGATTTTAGAGAAGGTCTCAGAAGAAAGAGATGTGGC

GGTTTGGTGAAACTCACCAGAGGCTGAACCCTCTCCGGCATAACACAGGGATTGGGAGGAGCA
GGGAAGCATTAGGCCAGGTGCATAGCCCAGTGCTGCTCTCTCTGGACTTGTTTGCTAGCTGAGC
TCATCCATTTGCATAATTTTCAGCGCCATTTCTCAGCTGATGAGTCCTGAACTTTTGTCTTGAGGC
TAGACTTCTCCTTCAAACAGCAGTCTTAAACACTCAACTTCTTTCTTGTTTTTTCCACTTGTCAACT
CATGAACACCTCAACCTTCTTAAATCCAAAGCCAAACTCATGGCTTGGAGTGGTAGGTGATGGC
TGTAATCCCAGTGCTATGGGAGGCTGAGGTGGGAGGATTACTTGAGGTCAGGAGTTTGAAGCC
AGCCTGGAGAACACAGTGAGACTCCCTATCTACAAAAAAAAAAAAAAAAAAGCTAGGCATGGTGA
AGTGCATCTGTAGGATCTGTAGTCCTGTTACATGGCAGGCTGAGGCAGGAGGATCACTTGAGCC
CAGGAGTTTGAGGCTGCAAAGAGCTATGACTGCACCAATGCACTCCAGCCTGGTTGACAGAGC
CAGAGTCCCTGTCTTAAAAAAAAAAAAAAACAAAAGAAAAACTTTTTTTCCTCCTCCTCCATG
GGCTCTGTCCATGCCATCTCTGTTCTGTAAATGGCACCACCCCCTGCTGAGCTGCTCAAGGTGGT
CATAACTCATGTGTTGTGCTAACTCTGCTTTTGCCCTCTTCTCCAGTCAGAAGTCCTGTGATTCTA
AACTTTATCCAACTTGTCCACTATCTCTATCTTCACTGTCATTATCTTTGCCTAGGACACTGCTATC
CCAGCTGGGCTACAGCAGTAGCCTCCTAACTGGTCTTAACTGGTCCTCTGCACCTGCTCTCCATG
CTCAGCAATCCATTTCCTACCTAGCAGCTTCAGTGATCTTAAGGTGTCCATTGAGTCTCATTCCTG
CCTTTCCTGCCCATGGCACATAGAATAAAATAGAGACCCCGAGTCTTCTGCCTGTTCGTGCCAC
CTCTCCAGCTCTCTCCTATCTCCTCCCCTTGGCCTACTCTATTTCAGCCACTCTGGCCTCCTTTTGT
TTTCTTCGACTTTCAAACCTTTCTCCACAACAGGGCCTTTGCACTTGCTGCTTCAGCCTGGAATGA
TTTTCCTCTGCACCTCCCCAAATTAGACCATCCTTTGGGTCTCAGCTAAAATGATGCTTCCAGAC
AGCTCTTTCCTGACCCCTTTATCAAGTGGACTTCCCTGCTCTTCTCCACCTTAACCTCATTATTTCT
TGTTGCGGGAACTCAGGGACCCCAAATGGAGGGACTAGCTGAAGCCACAGCAGAGGAACATA
AATTGTGAAGATTTTGTGGATATTTATCATTTCCCAAATAATACTTTTATACTTTCTTATGCCTGTC
TTTATTTTAATCTCCTAATCCTGTTATATTCATAAGCTGAGGATGTACATCACCTCAGGACCACTG
TGATAATTGTGTTAACTGTACAAATTGATTGTAAAACATGTGTGTTTCAATAATATGAAATCAGTG
CACCTTGAAAAAGAAGAGAATAACAGCAATTTTTAGGGAACAAGGGAAGACAACCATAAGGTC
TGACTTCCTGCAGGTTTGGGCAAAAAGAGCCATATGTTTCTTCTTGCATGGAGTCTATAAATGGA
CATGCAAGTAGGACAGATATTGCTAAATTCTTTTCCTAGCAAGGAATATTAATATTAATACCCTG
GGAAAGGAATGCATTCCTGGGGGGAGGCCTATAAATGGCCACTCTGGGGATGTCTGTCTTATGC
AGTTGAGATAAGGACTGAGATATGCCCTGCTCTCCTGCAGTACCCAAAGGCTTACTAGGGTGGG
GAAAAACTCTGCCCTGGTGAATTTGTGGTCAGACTGGTTCTCTGCTCTCCAACCATGTTTTCTGTT
GTTTAAGATGTTTATTAAGACAATACGTGCACCACTGAACATAGACCCTTATCAGTGGTTCTGCT
TTTGCCCTTAGCTTTGTGATCTTTGCTGGACCTTTATCAGTAGTTCTGCTTTTGCCCTTTGTCTTGTT
CCTCAGAAGCATGTGATCTTTCTTAGACCCTTAGTAGTAGTTCTGCTTTTTGCCCTTTGAAGCACG
TGATCTTTGTACCTACTCCCTGTTCTTACAGCCTCCCCCCCTTTTGAAACCCTTAATAAAAACTTG
CTGGTCTGAGACTCAGCAGGCATCATGGACTTACTGATACGTAGTGTCACCCCCAGCGGCCCAG
CTGTAAAATTCCTCTCTTTGTAGTGTCTGTCTTTATTTCTCAGCTGGCTGACACTTACGGAAAATA
GAAAGAACCTATGTTGAAATATTGGGGGCAGGTTCCATCAATAGTTCTTTCATGGATTTACTTAT
TTTTTGTCTCCCTCCATACCCTAGAAACTCCTGGAGGGCAGAGCTGTGTCTGCCATCTTCATCATT
GCATTACCACCACCCAGCACAGTGCCTGGCACAAAAGAGTTGCTCAATAAATAAATCAGGATG
AATGAATAAACACATGGATAGGCACTTTGAACTACAGATGAGTTTAAATACTTTGTGTTTTTCTTA
GTCAAACATGTGCAATTAAGCATGTGATAAATGATATGATGACCACA
ID|30353|gene_id|ENSG00000232533|transcript_id|ENST00000429630 Homo sapiens lncRNA
GCTGCTCGTCTTTTACGGCCCTTCAACGCCCACCACGACCCACTCCTCTTGGAGACCCCGG

GCGACGGTGGGGCTCTTGGGCATTCTGAGACTGCGCTTGGTGGAGACCCCGGGCGACGGTGGA
GCTCTTGGGCATTCTGAGACTGCGCTTGGTGGAGCCCCCTACTGGCCAGACTGGATTTCTCAGC
CTGCGACTCAGCCCCAGGCTACACGAAAGAAGCCAGACCTGGGTAATTCTTCTAGTTCTTTTTTT
TTTTTTTTTTAATTGCACTGGGAAACTTCCCCAATCTCGGCCCCAGTTCTTTCTCCAAACTAAGGA
GTCATGGCCTTTCGCCCGCTAGTCCAGTATGCACCCGTAGGCGCTTCATTTTCTCTCCTCTTGTCA
GCTTTTACTGCCTCCTGAGGCCTTCGTCTTGTTCACACTGAGTGTCCAGTCCCTCCAAATCCGGC
TACACTCTACTGGCAAGGAGCACCTGGGCCATGTTTTAGAGATCATCCGAGGACTAACCCCAAA
AGTTTATGAAGAGAAAGCAGAGGCCGAGCTGAAGAGATGGACCCGGGTCACACCCAGGTAAA
GGCAGGATCTAAACTGAAACTGGTGTCAGATCTGGTTGCCTTGCACCCCTGATATCAGGTGAAG
CAACACTGGGCAGGATAGAGCAGAGTGAGGTCAGAGTGTGAAGATCCAGCCTGATGCCCAAA
CTGACGCCTCTTCATTCTCCCCTGCTCCATCTGTAAACGTCACGGTTAATCCATCTACTTTATTGC
ATTATATAGAGAAATAAATGAAATGTCATAGCTGTA

ID|30363|gene_id|ENSG00000236663|transcript_id|ENST00000424933 Homo sapiens lncRNA

CCCGCAGCGGACGCTCCCCACGAGGTAGAGCCCCGTTTCCACACCTGGAGCAGCTGCAC
CTGGAGGTGGGGCTCATCTCCAGACCTGGAGCAGCTGCCTGCCCCAGGGCAGAAGCCACTGCC
CGCAGCTAGGTGAGGCCATGTGCCCAGTCCTGGTCAATGGACTGAGCATGAGGGGATGTGTGC
CCAGCAGCAGGGACTACTGTGCACTGAAGGCCAGGGCCGCTGGATTGGGACAAATAAATACA
GCAGGTCCTCAAATCCCATCGTTTCATTCTAGGGAGCATTGAGGAGGTCTCCCCAGGTCCGCGC
GGGTTTTCTCCAGGTCCTCCAGTTTCCTCCCACATCCCAAGTATACACCTCAGCCTCACGGCATG
TCTACATGGGCCTAGTCCGAGTGAGTGTGGGTGTCCCATGGAGGCCTGTCCTATCCAGGGCGG
GTGCCCACCTTGGCACTGAGCCTCCAGGATGGGCCCTGGTCACCTGCCACTCTGAACTGGAGA
AAGCAAGTTGGAAAATGAATGAATACATGAATATAAATTATCAAATAAAAATTATAAAGCCTAC
GGTA

ID|30397|gene_id|ENSG00000261183|transcript_id|ENST00000563217 Homo sapiens lncRNA

AGTTTTAAAAATATTTTCCAAGATCCCTTCAGTGAACATGGGATGCTTCCATAATGAAGAGA
GAGACACACCGTGATGACCTCCACATACCATATTTGATGACAGTCTTCCTTGGTAATCACTCACA
TCCATCTCTTGTCTGTTCCCACTGCTGCCCTTAATCTAGGCTCTGTGCCAGCTTCCTGATGGAACT
CCAGGCCACCCTGCACACTGCCATGTGGTTCCCTCCTCCAGCCCTGCCTTCATCTGTCCCTCACC
TGTTCAGACCCTCAGTCATCCCCAAGAACTTCTCTGGCAGAGTGTACAGAGCCAACTGTTCCCT
GGACCGACTCATGAAGTTCAGTATTTGCTTAGGCAGAGAGTGTCAGAGCTGTCAGAGGAGAGT
CAGCTAAAACCCTTCCCCTGGCTGGGCACCGTGGCTTACGCTTGTAGTCCCAACACTTTGGGAG
GCCAAGGCAAGAGGATTGCTTGAGGTCAGGAGTTCGAGACCAGCCTGGGCAACATAGTGTCTG
AAAAAATAAATTTTAAAAAACAACCCAGCCAGGCACGGTGGCTCACGCCTGTAATCCCAGCACT
TTGAGAGGCCAAGGTGGGTGGATCACGAGGTCAAGAGATTGAGACCAGCTTGGTCAACATGGT
GAAACCCCGTCTCTACTAAAAATACAAAAATTAGCTGGCTATGGTGGCAGGCGCCTGTAGTCCC
AGCTACTCGGGAGGCTGAGTCAGGAGAATCGCTTGAATCCGGGAGGCGGAGATTGCAGTGAG
CCGAGATCGCACCACTGCACTCCAGCCTGGCGACAGTGCGAGACTCCAGCTCAAAAAATAAAA
TAAAATAAAATAAAAAATAAAAAAACTCTTCCCCTTACATACAGCAAACTGAGGCCCAGAGATT
TGGCCTTGGTTTGTTCATAGACACAGGGCCAGCTCCAGACCTCAGGTCTCTTGACTGCCAATCTT
AGGCCCTTTCCTTGATTTGAGCTGCCTTCCTGCAAAGTATTTGCAAATGATATTGTTTTTTAATTCT
ATACTGTGGGCCCAAGGGGAGTTCACTCTTCTCTTTTCTTTTCTTTTCAAGATGGAGTCTCACTCT
GTCACCCAGGCTGGAGTGCAGTGGCATGATCTCAGCTCACTGCAACCTCCGCCTCCCAGGTTCA

AGTGATTCTCTTGCCTCAGCCTCCCAAGTAGTTAGGACTGCAGGCACCTGCCACCATGTCTGGC
TATTTTTTTTATTTTTTGGTGGAGATGGGGTTTCACCATGTTGCCCAGGCTGGTCTTAAACTCCTG
ACCTCAAATGATCCACCCACCTCGGCCTCCCAGAGTATTGGGATTATAGGCATAAGCCACCGTG
CCCGGCCAAGGCTATGTAGCTCTTTTCTTAGGCCACGTAGCTCACTCTTTTCTTAGGCTGTGTAG
TTCACTTTCCCAGTGCAAACTTTGGGAAAAGCTCTGTGCCACAGGCTCCATTCCTGTTTGCCACA
TCACAAATACCAATCATTGCTCTAAGGAGTGGATGATTTGATGAGAATATTCAGTTCTTACACCA
CTACTAGAGGAGGAACACTGACTGCCTTGTCGTGGATGTGGTCTGGCAGTTTCTGTAGGTAGGA
GTGGTGGATTGTCTCCAAATGTGGCCACCGACAGTGCCTCCCATTCCTGTGTGTGCATGCTGCTT
CTCCCACTAGAAGGTGTCTTTTACTTTCCTTCTCTTTGCATCTGGAACTTGTTTTGACCAGTTGGA
CCTAGCAGAAGTGGGGCCCTGTGACTTCCAGGCCTTATGAAATCTTGTAGCATTGTTTTTCCCTT
TTAGAGCCAGCTGTGATGTAAAGAAGTTTGTGCTGCATGAGAGAGATAGCACCTGAAGAAAAT
GAGGCCCTGGAGGATGAGAGACTCTAAAGGAAAAGGGAAGGGCCTGCTGTCTTCCAGCCACG
CCAGCCCCCAACTGA

ID|30398|gene_id|ENSG00000261183|transcript_id|ENST00000564302 Homo sapiens lncRNA

GGAGCACCCCAGGATCGAAGAGGGCGCACGGGCCCGGCCCTCCCACGCGCCCTCCAGG
GACCCGGCAGCCAGACAGACGGACAGGCGGACCGCTAGGGAGCTCAAGTACCCGGACACAC
GCACGAGCGCAGAACAAAGGGCCGGACATGCTCCAGCCCGCTCCGCCTGCGCCTCGGCCTTG
CGCGCGGGGGCCCGGGTTACCTGCGCAGGTGAGTTGGGTGGGGCCGGGTCCGCCGAGGTTCC
GGTCCCAGCTTCCTGCGCGGCTCGGGTCGCGGCTTCCGACCCGGTGCTCGGAGAGCTGGGGCT
CGGCCGCCTTCTCCCCCTGGTTTCTGGTGAAACTGAGTCAGCGCCGTCGGGGCGGGGCCGACA
TTCACCCTTGCGCTGCCGGCCCCGCCCACGCCCGGTGCGCTGCGGCCGCCTCTAGCGCGGAGC
TCCAGGCCCGGCCCGCCCCGCGGCCCCGCAGCCCCGGTGCCTCCCCGGCAGCCAGCTCCCCG
GAGGGGCTCTCGGGCTCCACCCAGTCCAGCCTCGCGAGGGAGCCCCGCGCCCGAAGCGCATA
CCCAGCCGGGAGGGGATGCGGCCGGGACGCGAGGGTCCGGCCTCTGGGGCGGCGGCCTCCA
GTCTTCCTTTCCGGCCGGGGCCTGCTTTCCCAGCCCCAGACCTGAGGCCCGATGCAAATGATCT
TTCCGGCCCTCAGGAAATCATTTCCATTTCGTGGCGGGAAGGTAACCCAAAAGTTGCGTGGCTG
CACCTGTGATAACAGGGATCCGGGATGTCTGCCTCACTGTCAGCTCCGGGAAGCCCTCCCTGCT
GTCAGGGTGAGAAACGGAGGCGATTCCCCGCGTCCAGGCTCCCCTGCGTGCTCCACGGGGCTT
CCCTGTTGTTCGAGGCCCCTCCTCATCTTGTATGTACAGTTCGGTCCATTCCCTGCTATGGCTCAT
TGGACTGTTGCCCCCTTCCCTGGAGCCCAGGCTTCCTCTCTTTCACAAAGGGGACGCTGCTGGC
TACTTTCTTCCAGGGAAATGTCATCCTTGGAAGCCTGGAGGTTCCAGTTCACCAAGTAGCTGAGT
TAATGCCCTTCACGAAACAGTGGACTCCTTGAAGCCAAGCTCATTGCTCCTCCTGAGTAAAAAA
CTCTTAAGAGACCATTCTCTACGGCAAGGGTCTGAACAGGTGAAAGACAGATGAAGGCAGGGC
TGGAGGAAGGAATCACATAGCATGCTCTCTTAAAATACAGATTCCAAACCTTTTCCTGATGGCTT
AACAACCCCCAAACCAATGGGAACATTCTTCCCAGTTTCTCATCCAGAGCAGCTTTGCCTAGCA
GATCCTGTGGCCAACCCTTGGATTTTGCTGAACTGCAACATCCTCCAGCTGCTCACATGATGCGT
GTTGGGGTGGGAATCTGGGGCCTGTCTCCCCTTCTCTGGATGTTTAGGGTTTATCCACAGACCCT
GGCACACTGGTGGATATACAACAATGGTGGTATGCTGGGAATGAAGAAAATGATTGTGCAGTC
ACTGGGAAGAAAGGGTTTTGTTGTGATTTGATTTCCCCTTATGGCTAACACATTCAAGAATCTTTA
TTTATTTATTTATTTATTTTTTTTTTTTTTTTTTTTGAGACGGAGTCTTGCTCTGTCACCCAGGCTGGA
GTGCAATGGCACGATCTCGGCTCACTGCAACCTCTGCCTTCCAGATTCAGGAAATTCTGCCTCA
GCCTCCCAAGTAGCTGGGCTTACAGGTGGGCACCACCACGCCGAGCTAATTTTTGTATTTTTAGT
AGAAACGAGGTTTCACCTTTTTGGCCAGGCTGGTCTTGAACTCCTGACCTCAGGTGATCCACCC

ACCTCGGCCTCCCAAAGTGCTAGGATTATAGGCGTGAGCCACTGTGCCTGACCTCAAGAATCTT
AATACAGGAAAATATTACCTTATGGTAATATTACATCACCATAAATTTATCATTACATCCATTATA
CACACCATTATAAAAACCAACATGAATTCAGCACTGACAATGCCCAAACTCTGTTCCTAAGCAC
TGCCCACATATTACCCTATACTCAAAACAAACCTAGGGAGGTAGTATTTTTAGCCCCATTTTACA
AATGAGCAAACAGGCTCAGACAGGTAAACTGACTTGTCAGGTCATTCAGCATTGAAGCGGCAG
AGTAGGGATTGGAGCCCAGGCAGGCCAATTCCATGGCTTCAGTGCTTAAACCCTGGGCTTTATG
GCCTTTGCAATTCTGACTTCATCTGGAAGTTCTGCCCTCACCTCAGATTCAACTTGCTTAAACCCA
TAACTCACTTTCAAGTGGTGAGCACCCACCATTTGTTGAGATGCCCACTGTGTTCCCTCCAGGCA
CTTGACATAGGATTGCTAATGCTCAGGACAACTCTAAAAATTGCTTCCACTTTGCAGAAAGGTTC
ACTGACTCCATCAAGGTCAGGTAGTGAACGAAGAGTGGTGACAGGTTGTAGAGAAGTTGAACT
TGTGAGCTTCAGACTCAGACTACTGGATTTAGCTCTTGCATCTGCCACTTACTATTCGTGTGGCC
TTGTGCAAGTTATTTGATCTCTCTGCCTTAGTCTCCTTGAATGGGAAGTGGGGCTTCATAAGGTTT
TTTATGAGGATTAATTGAGTTAATGCAGACAAAGCATTTGACACAAAGAGTTCTCAATAAATGTT
ACTTGAAACGACAACAACAACAACAACAAAAACACATGGTGGTGAGGATGCAGAGAAAAGGG
AACGCTTATACATTGTTGGTGGGAATGTAAATCAGTTCAGCCACTGTGGAAAGCAGCTTGGAGA
TTTCTCAAAAACTTAGAACTACCATTCAACTCAGCAATCCTATTACTGGGTATATACCCAAAGGA
AAATATATTGTTTTTCCAAAAAGACACATGCACTTGTACGTTCATCACAGCACTATTCACAATAG
CAAAGACATGGAATCAACCTAGATGCCCATCAATGATGGATTGCATAAAGATAATGTACTTCAT
ACGTACCATGGAATACTATGCAGCCATAAAATTAACAAAATCATGTCCTTTCCAGCAATACGAAT
GCAGCTGGAGGCCATTATCCTAAGCAAATTAAGGCAGGAACAGAAAACCAAATACCACATATT
CTCACTTATAAATGGGAGCTAAGCATTGAGTACACATGGACATAATGATGGAACAATAGACACT
GAGGACTACCAGAAGAGAGAAGGAGGGAGGGGAGCGTGGGGAGCGTGGGCTGAAAAACAAC
CTTTTAGGTAATATGCTCACTACCTGGGTGACAGGCTCATCTGTACCCCAAACCCTATACCCATG
TAACAAACCTGCATGTGTACCCACTGAATCTAAAATAAAAGTTGAGGCCAGGTGCAATGGCTCA
TGTCTGTAATCCAAGCACTATGGGAGGCTGAGGCAGGAGGATTGCTTGAGCCCAGGAGTTCAA
GACCAGCCTGGGCAACATGGCAAGACCCCGTCTCTATAAAAAAAAATACAAAAAATTAGCCAG
GCATAGTGGCGCATGCCTGTAGTACCAGCTACTCAGGAGGTTGAGGTGGGGGGATTGCTTGAG
CCAGGGAGGTCAAGGTGGCAGTGAGCTATGATCATGCCACTGCACTCCAGCATGGGTGAAAGA
ACTAGACCCTGTCTCAAAAATAAGTAAACAAAATAAAAGTCGAAATGATTCTTTTAAAAAAGTG
ACAGAGCCAGGATTGTATCTGAAGTCTTTTTGACATTAAAAGCCATACTTTCCATACTACCTTAT
GCTGCTTCTTACATACTAATAAGAAAAACTACATGTGAAGAGAAGAAAGACTATAAGGTATGCC
ATCAAAGTAGGGAAAGTGATGGTATTTGAATGGTGAGACTGTAGGATAACATTTCTTCTTTCTAG
TTTTAAAAATATTTTCCAAGATCCCTTCAGTGAACATGGGATGCTTCCATAATGAAGAGAGAGAC
ACACCGTGATGACCTCCACATACCATATTTGATGACAGTCTTCCTTGGTAATCACTCACATCCAT
CTCTTGTCTGTTCCCACTGCTGCCCTTAATCTAGGCTCTGTGCCAGCTTCCTGATGGAACTCCAG
GCCACCCTGCACACTGCCATGTGGTTCCCTCCTCCAGCCCTGCCTTCATCTGTCCCTCACCTGTT
CAGACCCTCAGTCATCCCCAAGAACTTCTCTGGCAGAGTGTACAGAGCCAACTGTTCCCTGGAC
CGACTCATGAAGTTCAGTATTTGCTTAGGCAGAGAGTGTCAGAGCTGTCAGAGGAGAGTCAGCT
AAAACCCTTCCCCTGGCTGGGCACCGTGGCTTACGCTTGTAGTCCCAACACTTTGGGAGGCCAA
GGCAAGAGGATTGCTTGAGGTCAGGAGTTCGAGACCAGCCTGGGCAACATAGTGTCTGAAAAA
ATAAATTTTAAAAAACAACCCAGCCAGGCACGGTGGCTCACGCCTGTAATCCCAGCACTTTGAG
AGGCCAAGGTGGGTGGATCACGAGGTCAAGAGATTGAGACCAGCTTGGTCAACATGGTGAAAC
CCCGTCTCTACTAAAAATACAAAAATTAGCTGGCTATGGTGGCAGGCGCCTGTAGTCCCAGCTA
CTCGGGAGGCTGAGTCAGGAGAATCGCTTGAATCCGGGAGGCGGAGATTGCAGTGAGCCGAG

ATCGCACCACTGCACTCCAGCCTGGCGACAGTGCGAGACTCCAGCTCAAAAAATAAAATAAAA
TAAAATAAAAAATAAAAAAACTCTTCCCCTTACATACAGCAAACTGAGGCCCAGAGATTTGGCC
TTGGTTTGTTCATAGACACAGGGCCAGCTCCAGACCTCAGGTCTCTTGACTGCCAATCTTAGGCC
CTTTCCTTGATTTGAGCTGCCTTCCTGCAAAGTATTTGCAAATGATATTGTTTTTTAATTCTATACT
GTGGGCCCAAGGGGAGTTCACTCTTCTCTTTTCTTTTCTTTTCAAGATGGAGTCTCACTCTGTCAC
CCAGGCTGGAGTGCAGTGGCATGATCTCAGCTCACTGCAACCTCCGCCTCCCAGGTTCAAGTGA
TTCTCTTGCCTCAGCCTCCCAAGTAGTTAGGACTGCAGGCACCTGCCACCATGTCTGGCTATTTT
TTTTATTTTTTGGTGGAGATGGGGTTTCACCATGTTGCCCAGGCTGGTCTTAAACTCCTGACCTCA
AATGATCCACCCACCTCGGCCTCCCAGAGTATTGGGATTATAGGCATAAGCCACCGTGCCCGGC
CAAGGCTATGTAGCTCTTTTCTTAGGCCACGTAGCTCACTCTTTTCTTAGGCTGTGTAGTTCACTT
TCCCAGTGCAAACTTTGGGAAAAGCTCTGTGCCACAGGCTCCATTCCTGTTTGCCACATCACAA
ATACCAATCATTGCTCTAAGGAGTGGATGATTTGATGAGAATATTCAGTTCTTACACCACTACTA
GAGGAGGAACACTGACTGCCTTGTCGTGGATGTGGTCTGGCAGTTTCTGTAGGTAGGAGTGGTG
GATTGTCTCCAAATGTGGCCACCGACAGTGCCTCCCATTCCTGTGTGTGCATGCTGCTTCTCCCA
CTAGAAGGTGTCTTTTACTTTCCTTCTCTTTGCATCTGGAACTTGTTTTGACCAGTTGGACCTAGC
AGAAGTGGGGCCCTGTGACTTCCAGGCCTTATGAAATCTTGTAGCATTGTTTTTCCCTTTTAGAG
CCAGCTGTGATGTAAAGAAGTTTGTGCTGCATGAGAGAGATAGCACCTGAAGAAAATGAGGCC
CTGGAGGATGAGAGACTCTAAAGGAAAAGGGAAGGGCCTGCTGTCTTCCAGCCACGCCAGCCC
CCAACTGAGAAGCTGGGTGAAGCCATCTTGCATCTTTAGCCCCAGTCAAGTTACCTCAGCAAAG
ACTAGCTGACCCTGCCAAGCCCTGCCCAAGTTACAGAATCATGAGCAAATAAATGGCTGTTTCT
GTTTTAAGCTTTTAAATTTTGGGGGTGGTTTATGTGTCAATAATAACTGAAACAGATAATATATAC
AGAATAAACTTTAGTTTTAATAATCTAAGTAAAAGCCCACTAATTCATTATGCAGAAAAAAATGA
TTTTTTTGAGACGGGGTCTCGCTCTGTTGCCAGGCTGGAGTGCTGTGGCACAACCATAGCTCACT
GCAGCCTCCACCTCCCTGGTTCAAGCGATCTTCCCACCTCAGCCTCCCGAGTAGTTGAGACCAC
AGGTATGTGCCACAACACCTGACTAATTTTGTAAATTTTTTGTAGAGATGGGGTCCTGCCATATT
GCTGAGGCTGGTCTCTAACTTCTGGGCTCAAGCCATCCTCCTGCCTCAGCCTCCCAAAGTATTAG
GATTACAGGTGTGAGCCACTGTGCCCAGCCAATTTAACTTCTATTTTAAAAAGACAGCCCCTCAC
AGATACTGCTACAGCTCTGTTAAATCAAATTTAGCCTCCTTACATATTTTGAGTTTGACGTAAAGG
TTTCTCTGTACATAGTGAACTGTCACCTAAATGGAGGTGTAAACAGCCTGTAATCTACCCTTGTG
CCAAACACCAAGTTTTGGCCAATCAAAAGGGGCCAACTGTTCAAACCCTCTTCAAATAAGGCAA
ACACTGAGCTGTAACCAATCTGGCTGTTTCTGTCCCTCATTTCCATTTTCTGTACATCACTTTCCTT
ATGCTGCCCATAAATCTCTGAGCCTACTCTGGCTCAGAATGCTGCCCAATTTACCAATCGTACTT
TGCTAAATTGAACTCTTAAATTTAATTTGACTATGGATTTTTTTTTTTGAGACAGAAATTTGCTCTT
GTCGCCCAGGCTGGAGTGCAATGGTGCAATCTCAGCTCACCACAACCTCCATCTCCCGGGTTCA
AGTGATTCTCCTGCCTCAGCCTCCCGAGTAACTGGGATTACAGGCATGCACCACCATACCTGGC
TAATTTTGTATTTTTAGTAGAAACGGGGTTTCTCCATGTTGGTCAGGCTGGTCTCGAACTCCTAAC
CTCAGGTGATCTGCCCACCTCAGCCTCCCAAAGTGCTGGGATTACAGGCGTGAGCCATTGCGCC
TGGCCTAATTTGGCTATGGATTTTCTTTTAACAGCTCTTAAAGGGTTGTTTTTTGTTGTTGTTTTTTT
TTTTTTGAGATAGAGTTTTGCTCTTGTTGCCCAGGCTGGAATGCAATGGCTCGATCTCAGCTCAC
TGCAACCTCCGCCTCCCAGCTTCAAGTAATTCTCCCTCAGCCTCCTGAGTAGCTGGGATTACAG
GCATGCGCCACCATGCCCGGCTAATTTTGTATTTTTAGTAGAGATGGGGTTTCTCCATGTTGGTC
AGGCTAGTCTCGAACTCCCAACCTCAGGTGATCCACCCACCTCGGCCTCCCAAAGTGCTGGGAT
TACAGGCATGAGTCACTGCACCCATCCTGTTTTTATTTTATTTTATTTTATTTTATTAAGTTCTGGG
GTACATGTGCAGGATGTGCAGGTTTGTTGCATAGGTAAACATGTGCCATGGTGGTTTGCTGCAC

CTATCAGCCCATCACCTAGGTATTAAGCCCTGCATGCATTAACTATTTATCCTGATGCTCTCCCTC
CCTCCAACCTACCCCACAGGCCCCAGTGTGTGTTGTTCCCCTCCCTGTGTCCTAAAGGGTTATTT
TTGAGGAATTATTCTAATGTCAGAAATGCTCACATGAGGGTGGAGGGTACAGGGAGGTGATAAT
GAAAGGATACAATACCTCAGGAGGAAGTTTGATATTTTTGTTTGAGATCTATTGTACATGGCAAA
TATAACTAATAATTGAGTACTGTACATTTCAAAATTGCTGAGAGAATAAATTTCAAATGTTTTCAT
TATTAAAAATATTAAACATTTGAAAAAAAAATGTTCACAATATTCCATTGTGGTAGGTTGCTTCTA
AATGGCTCCCAGTGATCCATACCTCCTGGTACTCATGCCTTGTATAATTTCCTCTCCTTGAGTGTA
GTTGGACCTAGCGACTTGCTTCTAATGGATAGAATCAAGCAAAAGTCATAGGATGTCACTTCCT
GGATTTGGTTACAAAGACTGTAACTTTCTCTCTTTGTCTCTCTCTCTCTCTCTCTCTTTTAGTGCCC
TTGGTGTGGTGGAAGCAAGGTGCCATGTGATAAGTAAGCCTCCAGAGAGGCCTGCATGGCAGG
ATCCAATGTCTTTAGCCAACAGCATCTGGGAACCTGAGACCTGCCAACACCCTTGTGAGTGAGC
CTGGAAGTGGACCTCCTGCTCTTGAACCTTGAGGTGACTATAGTGCTGGCTGACACCTTGACTG
CAGTCCCTGAGCTGGGGGGCCCAGCTGATCTACTCCCAGAATCCCCCCTACTAAAACAGTGATA
TAATAAATGCTTATTGTTTCAAGCTGGTAA

ID|30399|gene_id|ENSG00000261183|transcript_id|ENST00000565315 Homo sapiens lncRNA

AGGGACCCGGCAGCCAGACAGACGGACAGGCGGACCGCTAGGGAGCTCAAGTACCCGG
ACACACGCACGAGCGCAGAACAAAGGGCCGGACATGCTCCAGCCCGCTCCGCCTGCGCCTCG
GCCTTGCGCGCGGGGGCCCGGGTTACCTGCGCAGGTGAGTTGGGTGGGGCCGGGTCCGCCGA
GGTTCCGGTCCCAGCTTCCTGCGCGGCTCGGGTCGCGGCTTCCGACCCGGTGCTCGGAGAGCT
GGGGCTCGGCCGCCTTCTCCCCCTGGTTTCTGGTGAAACTGAGTCAGCGCCGTCGGGGCGGGG
CCGACATTCACCCTTGCGCTGCCGGCCCCGCCCACGCCCGGTGCGCTGCGGCCGCCTCTAGCG
CGGAGCTCCAGGCCCGGCCCGCCCCGCGGCCCCGCAGCCCCGGTGCCTCCCCGGCAGCCAGC
TCCCCGGAGGGGCTCTCGGGCTCCACCCAGTCCAGCCTCGCGAGGGAGCCCCGCGCCCGAAG
CGCATACCCAGCCGGGAGGGGATGCGGCCGGGACGCGAGGGTCCGGCCTCTGGGGCGGCGG
CCTCCAGTCTTCCTTTCCGGCCGGGGCCTGCTTTCCCAGCCCCAGACCTGAGGCCCGATGCAAA
TGATCTTTCCGGCCCTCAGGAAATCATTTCCATTTCGTGGCGGGAAGGTAACCCAAAAGTTGCG
TGGCTGCACCTGTGATAACAGGGATCCGGGATGTCTGCCTCACTGTCAGCTCCGGGAAGCCCTC
CCTGCTGTCAGGGTGAGAAACGGAGGCGATTCCCCGCGTCCAGGCTCCCCTGCGTGCTCCACG
GGGCTTCCCTGTTGTTCGAGGCCCCTCCTCATCTTGTATGTACAGTTCGGTCCATTCCCTGCTATG
GCTCATTGGACTGTTGCCCCCTTCCCTGGAGCCCAGGCTTCCTCTCTTTCACAAAGGGGACGCT
GCTGGCTACTTTCTTCCAGGGAAATGTCATCCTTGGAAGCCTGGAGGTTCCAGTTCACCAAGTA
GCTGAGTTAATGCCCTTCACGAAACAGTGGACTCCTTGAAGCCAAGCTCATTGCTCCTCCTGAG
TAAAAAACTCTTAAGAGACCATTCTCTACGGCAAGGGTCTGAACAGGTGAAAGACAGATGAAG
GCAGGGCTGGAGGAAGGAATCACATAGCATGCTCTCTTAAAATACAGATTCCAAACCTTTTCCT
GATGGCTTAACAACCCCCAAACCAATGGGAACATTCTTCCCAGTTTCTCATCCAGAGCAGCTTT
GCCTAGCAGATCCTGTGGCCAACCCTTGGATTTTGCTGAACTGCAACATCCTCCAGCTGCTCAC
ATGATGCGTGTTGGGGTGGGAATCTGGGGCCTGTCTCCCCTTCTCTGGATGTTTAGGGTTTATCC
ACAGACCCTGGCACACTGGTGGATATACAACAATGGTGGTATGCTGGGAATGAAGAAAATGAT
TGTGCAGTCACTGGGAAGAAAGGGTTTTGTTGTGATTTGATTTCCCCTTATGGCTAACACATTCA
AGAATCTTTATTTATTTATTTATTTATTTTTTTTTTTTTTTTTTTGAGACGGAGTCTTGCTCTGTCACC
CAGGCTGGAGTGCAATGGCACGATCTCGGCTCACTGCAACCTCTGCCTTCCAGATTCAGGAAAT
TCTGCCTCAGCCTCCCAAGTAGCTGGGCTTACAGGTGGGCACCACCACGCCGAGCTAATTTTTG
TATTTTTAGTAGAAACGAGGTTTCACCTTTTTGGCCAGGCTGGTCTTGAACTCCTGACCTCAGGT

GATCCACCCACCTCGGCCTCCCAAAGTGCTAGGATTATAGGCGTGAGCCACTGTGCCTGACCTC
AAGAATCTTAATACAGGAAAATATTACCTTATGGTAATATTACATCACCATAAATTTATCATTACA
TCCATTATACACACCATTATAAAAACCAACATGAATTCAGCACTGACAATGCCCAAACTCTGTTC
CTAAGCACTGCCCACATATTACCCTATACTCAAAACAAACCTAGGGAGGTAGTATTTTTAGCCCC
ATTTTACAAATGAGCAAACAGGCTCAGACAGGTAAACTGACTTGTCAGGTCATTCAGCATTGAA
GCGGCAGAGTAGGGATTGGAGCCCAGGCAGGCCAATTCCATGGCTTCAGTGCTTAAACCCTGG
GCTTTATGGCCTTTGCAATTCTGACTTCATCTGGAAGTTCTGCCCTCACCTCAGATTCAACTTGCT
TAAACCCATAACTCACTTTCAAGTGGTGAGCACCCACCATTTGTTGAGATGCCCACTGTGTTCCC
TCCAGGCACTTGACATAGGATTGCTAATGCTCAGGACAACTCTAAAAATTGCTTCCACTTTGCAG
AAAGGTTCACTGACTCCATCAAGGTCAGGTAGTGAACGAAGAGTGGTGACAGGTTGTAGAGAA
GTTGAACTTGTGAGCTTCAGACTCAGACTACTGGATTTAGCTCTTGCATCTGCCACTTACTATTCG
TGTGGCCTTGTGCAAGTTATTTGATCTCTCTGCCTTAGTCTCCTTGAATGGGAAGTGGGGCTTCAT
AAGGTTTTTTATGAGGATTAATTGAGTTAATGCAGACAAAGCATTTGACACAAAGAGTTCTCAAT
AAATGTTACTTGAAACGACAACAACAACAACAACAAAAACACATGGTGGTGAGGATGCAGAGA
AAAGGGAACGCTTATACATTGTTGGTGGGAATGTAAATCAGTTCAGCCACTGTGGAAAGCAGCT
TGGAGATTTCTCAAAAACTTAGAACTACCATTCAACTCAGCAATCCTATTACTGGGTATATACCC
AAAGGAAAATATATTGTTTTTCCAAAAAGACACATGCACTTGTACGTTCATCACAGCACTATTCA
CAATAGCAAAGACATGGAATCAACCTAGATGCCCATCAATGATGGATTGCATAAAGATAATGTA
CTTCATACGTACCATGGAATACTATGCAGCCATAAAATTAACAAAATCATGTCCTTTCCAGCAAT
ACGAATGCAGCTGGAGGCCATTATCCTAAGCAAATTAAGGCAGGAACAGAAAACCAAATACCA
CATATTCTCACTTATAAATGGGAGCTAAGCATTGAGTACACATGGACATAATGATGGAACAATA
GACACTGAGGACTACCAGAAGAGAGAAGGAGGGAGGGGAGCGTGGGGAGCGTGGGCTGAAA
AACAACCTTTTAGGTAATATGCTCACTACCTGGGTGACAGGCTCATCTGTACCCCAAACCCTATA
CCCATGTAACAAACCTGCATGTGTACCCACTGAATCTAAAATAAAAGTTGAGGCCAGGTGCAAT
GGCTCATGTCTGTAATCCAAGCACTATGGGAGGCTGAGGCAGGAGGATTGCTTGAGCCCAGGA
GTTCAAGACCAGCCTGGGCAACATGGCAAGACCCCGTCTCTATAAAAAAAAAATACAAAAAATT
AGCCAGGCATAGTGGCGCATGCCTGTAGTACCAGCTACTCAGGAGGTTGAGGTGGGGGGATTG
CTTGAGCCAGGGAGGTCAAGGTGGCAGTGAGCTATGATCATGCCACTGCACTCCAGCATGGGT
GAAAGAACTAGACCCTGTCTCAAAAATAAGTAAACAAAATAAAAGTCGAAATGATTCTTTTAAA
AAAGTGACAGAGCCAGGATTGTATCTGAAGTCTTTTTGACATTAAAAGCCATACTTTCCATACTA
CCTTATGCTGCTTCTTACATACTAATAAGAAAAACTACATGTGAAGAGAAGAAAGACTATAAGG
TATGCCATCAAAGTAGGGAAAGTGATGGTATTTGAATGGTGAGACTGTAGGATAACATTTCTTCT
TTCTAGTTTTAAAAATATTTTCCAAGATCCCTTCAGTGAACATGGGATGCTTCCATAATGAAGAG
AGAGACACACCGTGATGACCTCCACATACCATATTTGATGACAGTCTTCCTTGGTAATCACTCAC
ATCCATCTCTTGTCTGTTCCCACTGCTGCCCTTAATCTAGGCTCTGTGCCAGCTTCCTGATGGAAC
TCCAGGCCACCCTGCACACTGCCATGTGGTTCCCTCCTCCAGCCCTGCCTTCATCTGTCCCTCAC
CTGTTCAGACCCTCAGTCATCCCCAAGAACTTCTCTGGCAGAGTGTACAGAGCCAACTGTTCCC
TGGACCGACTCATGAAGTTCAGTATTTGCTTAGGCAGAGAGTGTCAGAGCTGTCAGAGGAGAGT
CAGCTAAAACCCTTCCCCTGGCTGGGCACCGTGGCTTACGCTTGTAGTCCCAACACTTTGGGAG
GCCAAGGCAAGAGGATTGCTTGAGGTCAGGAGTTCGAGACCAGCCTGGGCAACATAGTGTCTG
AAAAAATAAATTTTAAAAAACAACCCAGCCAGGCACGGTGGCTCACGCCTGTAATCCCAGCACT
TTGAGAGGCCAAGGTGGGTGGATCACGAGGTCAAGAGATTGAGACCAGCTTGGTCAACATGGT
GAAACCCCGTCTCTACTAAAAATACAAAAATTAGCTGGCTATGGTGGCAGGCGCCTGTAGTCCC
AGCTACTCGGGAGGCTGAGTCAGGAGAATCGCTTGAATCCGGGAGGCGGAGATTGCAGTGAG

CCGAGATCGCACCACTGCACTCCAGCCTGGCGACAGTGCGAGACTCCAGCTCAAAAAATAAAA
TAAAATAAAATAAAAAATAAAAAAACTCTTCCCCTTACATACAGCAAACTGAGGCCCAGAGATT
TGGCCTTGGTTTGTTCATAGACACAGGGCCAGCTCCAGACCTCAGGTCTCTTGACTGCCAATCTT
AGGCCCTTTCCTTGATTTGAGCTGCCTTCCTGCAAAGTATTTGCAAATGATATTGTTTTTTAATTCT
ATACTGTGGGCCCAAGGGGAGTTCACTCTTCTCTTTTCTTTTCTTTTCAAGATGGAGTCTCACTCT
GTCACCCAGGCTGGAGTGCAGTGGCATGATCTCAGCTCACTGCAACCTCCGCCTCCCAGGTTCA
AGTGATTCTCTTGCCTCAGCCTCCCAAGTAGTTAGGACTGCAGGCACCTGCCACCATGTCTGGC
TATTTTTTTTATTTTTTGGTGGAGATGGGGTTTCACCATGTTGCCCAGGCTGGTCTTAAACTCCTG
ACCTCAAATGATCCACCCACCTCGGCCTCCCAGAGTATTGGGATTATAGGCATAAGCCACCGTG
CCCGGCCAAGGCTATGTAGCTCTTTTCTTAGGCCACGTAGCTCACTCTTTTCTTAGGCTGTGTAG
TTCACTTTCCCAGTGCAAACTTTGGGAAAAGCTCTGTGCCACAGGCTCCATTCCTGTTTGCCACA
TCACAAATACCAATCATTGCTCTAAGGAGTGGATGATTTGATGAGAATATTCAGTTCTTACACCA
CTACTAGAGGAGGAACACTGACTGCCTTGTCGTGGATGTGGTCTGGCAGTTTCTGTAGGTAGGA
GTGGTGGATTGTCTCCAAATGTGGCCACCGACAGTGCCTCCCATTCCTGTGTGTGCATGCTGCTT
CTCCCACTAGAAGGTGTCTTTTACTTTCCTTCTCTTTGCATCTGGAACTTGTTTTGACCAGTTGGA
CCTAGCAGAAGTGGGGCCCTGTGACTTCCAGGCCTTATGAAATCTTGTAGCATTGTTTTTCCCTT
TTAGAGCCAGCTGTGATGTAAAGAAGTTTGTGCTGCATGAGAGAGATAGCACCTGAAGAAAAT
GAGGCCCTGGAGGATGAGAGACTCTAAAGGAAAAGGGAAGGGCCTGCTGTCTTCCAGCCACG
CCAGCCCCCAACTGAGAAGCTGGGTGAAGCCATCTTGCATCTTTAGCCCCAGTCAAGTTACCTC
AGCAAAGACTAGCTGACCCTGCCAAGCCCTGCCCAAGTTACAGAATCATGAGCAAATAAATGG
CTGTTTCTGTTTTAAGCTTTTAAATTTTGGGGGTGGTTTATGTGTCAATAATAACTGAAACAGATA
ATATATACAGAATAAACTTTAGTTTTAATAATCTAAGTAAAAGCCCACTAATTCATTATGCAGAA
AAAAATGATTTTTTTGAGACGGGGTCTCGCTCTGTTGCCAGGCTGGAGTGCTGTGGCACAACCA
TAGCTCACTGCAGCCTCCACCTCCCTGGTTCAAGCGATCTTCCCACCTCAGCCTCCCGAGTAGTT
GAGACCACAGGTATGTGCCACAACACCTGACTAATTTTGTAAATTTTTTGTAGAGATGGGGTCCT
GCCATATTGCTGAGGCTGGTCTCTAACTTCTGGGCTCAAGCCATCCTCCTGCCTCAGCCTCCCAA
AGTATTAGGATTACAGGTGTGAGCCACTGTGCCCAGCCAATTTAACTTCTATTTTAAAAAGACAG
CCCCTCACAGATACTGCTACAGCTCTGTTAAATCAAATTTAGCCTCCTTACATATTTTGAGTTTGA
CGTAAAGGTTTCTCTGTACATAGTGAACTGTCACCTAAATGGAGGTGTAAACAGCCTGTAATCTA
CCCTTGTGCCAAACACCAAGTTTTGGCCAATCAAAAGGGGCCAACTGTTCAAACCCTCTTCAAA
TAAGGCAAACACTGAGCTGTAACCAATCTGGCTGTTTCTGTCCCTCATTTCCATTTTCTGTACATC
ACTTTCCTTATGCTGCCCATAAATCTCTGAGCCTACTCTGGCTCAGAATGCTGCCCAATTTACCA
ATCGTACTTTGCTAAATTGAACTCTTAAATTTAATTTGACTATGGATTTTTTTTTTTGAGACAGAAA
TTTGCTCTTGTCGCCCAGGCTGGAGTGCAATGGTGCAATCTCAGCTCACCACAACCTCCATCTCC
CGGGTTCAAGTGATTCTCCTGCCTCAGCCTCCCGAGTAACTGGGATTACAGGCATGCACCACCA
TACCTGGCTAATTTTGTATTTTTAGTAGAAACGGGGTTTCTCCATGTTGGTCAGGCTGGTCTCGAA
CTCCTAACCTCAGGTGATCTGCCCACCTCAGCCTCCCAAAGTGCTGGGATTACAGGCGTGAGCC
ATTGCGCCTGGCCTAATTTGGCTATGGATTTTCTTTTAACAGCTCTTAAAGGGTTGTTTTTTGTTGT
TGTTTTTTTTTTTTTGAGATAGAGTTTTGCTCTTGTTGCCCAGGCTGGAATGCAATGGCTCGATCTC
AGCTCACTGCAACCTCCGCCTCCCAGCTTCAAGTAATTCTCCCTCAGCCTCCTGAGTAGCTGGG
ATTACAGGCATGCGCCACCATGCCCGGCTAATTTTGTATTTTTAGTAGAGATGGGGTTTCTCCAT
GTTGGTCAGGCTAGTCTCGAACTCCCAACCTCAGGTGATCCACCCACCTCGGCCTCCCAAAGTG
CTGGGATTACAGGCATGAGTCACTGCACCCATCCTGTTTTTATTTTATTTTATTTTATTTTATTAAG
TTCTGGGGTACATGTGCAGGATGTGCAGGTTTGTTGCATAGGTAAACATGTGCCATGGTGGTTTG

CTGCACCTATCAGCCCATCACCTAGGTATTAAGCCCTGCATGCATTAACTATTTATCCTGATGCT
CTCCCTCCCTCCAACCTACCCCACAGGCCCCAGTGTGTGTTGTTCCCCTCCCTGTGTCCTAAAGG
GTTATTTTTGAGGAATTATTCTAATGTCAGAAATGCTCACATGAGGGTGGAGGGTACAGGGAGG
TGATAATGAAAGGATACAATACCTCAGGAGGAAGTTTGATATTTTTGTTTGAGATCTATTGTACA
TGGCAAATATAACTAATAATTGAGTACTGTACATTTCAAAATTGCTGAGAGAATAAATTTCAAAT
GTTTTCATTATTAAAAATATTAAACATTTGAAAAAAAAAATGTTCACAATATTCCATTGTGGTAGGT
TGCTTCTAAATGGCTCCCAGTGATCCATACCTCCTGGTACTCATGCCTTGTATAATTTCCTCTCCT
TGAGTGTAGTTGGACCTAGCGACTTGCTTCTAATGGATAGAATCAAGCAAAAGTCATAGGATGT
CACTTCCTGGATTTGGTTACAAAGACTGTAACTTTCTCTCTTTGTCTCTCTCTCTCTCTCTCTTTT
AGTGCCCTTGGTGTGGTGGAAGCAAGGTGCCATGTGATAAGTAAGCCTCCAGAGAGGCCTGCA
TGGCAGGATCCAATGTCTTTAGCCAACAGCATCTGGGAACCTGAGACCTGCCAACACCCTTGTG
AGTGAGCCTGGAAGTGGACCTCCTGCTCTTGAACCTTGAGGTGACTATAGTGCTGGCTGACACC
TTGACTGCAGTCCCTGAGCTGGGGGGCCCAGCTGATCTACTCCCAGAATCCCCCCTACTAAAAC
AGTGATATAATAAATGCTTATTGTTTCAAGCTGGTAACTTTTGGAATGCATTCACTGAAAAAAATT
AGAATATCAAACTATAAACCGTTTACTTAGTCAACTTTTTGGGGAACTAATATGGCCATAAATAT
CAAACCTCCTACAATTTTGCACATTCTTTGACTTTGTAGTTCTACATCTATAAACTTCTCTTAAGG
GAAATCATGGATGCCAACAAAGAAA

ID|30400|gene_id|ENSG00000261183|transcript_id|ENST00000568419 Homo sapiens lncRNA

AAGTACCCGGACACACGCACGAGCGCAGAACAAAGGGCCGGACATGCTCCAGCCCGCTC
CGCCTGCGCCTCGGCCTTGCGCGCGGGGGCCCGGGTTACCTGCGCAGGTGAGTTGGGTGGGG
CCGGGTCCGCCGAGGTTCCGGTCCCAGCTTCCTGCGCGGCTCGGGTCGCGGCTTCCGACCCGG
TGCTCGGAGAGCTGGGGCTCGGCCGCCTTCTCCCCCTGGTTTCTGGTGAAACTGAGTCAGCGCC
GTCGGGGCGGGGCCGACATTCACCCTTGCGCTGCCGGCCCCGCCCACGCCCGGTGCGCTGCG
GCCGCCTCTAGCGCGGAGCTCCAGGCCCGGCCCGCCCCGCGGCCCCGCAGCCCCGGTGCCTC
CCCGGCAGCCAGCTCCCCGGAGGGGCTCTCGGGCTCCACCCAGTCCAGCCTCGCGAGGGAGC
CCCGCGCCCGAAGCGCATACCCAGCCGGGAGGGGATGCGGCCGGGACGCGAGGGTCCGGCC
TCTGGGGCGGCGGCCTCCAGTCTTCCTTTCCGGCCGGGGCCTGCTTTCCCAGCCCCAGACCTGA
GGCCCGATGCAAATGATCTTTCCGGCCCTCAGGAAATCATTTCCATTTCGTGGCGGGAAGGTAA
CCCAAAAGTTGCGTGGCTGCACCTGTGATAACAGGGATCCGGGATGTCTGCCTCACTGTCAGCT
CCGGGAAGCCCTCCCTGCTGTCAGGGTGAGAAACGGAGGCGATTCCCCGCGTCCAGGCTCCCC
TGCGTGCTCCACGGGGCTTCCCTGTTGTTCGAGGCCCCTCCTCATCTTGTATGTACAGTTCGGTC
CATTCCCTGCTATGGCTCATTGGACTGTTGCCCCCTTCCCTGGAGCCCAGGCTTCCTCTCTTTCA
CAAAGGGGACGCTGCTGGCTACTTTCTTCCAGGGAAATGTCATCCTTGGAAGCCTGGAGGTTCC
AGTTCACCAAGTAGCTGAGTTAATGCCCTTCACGAAACAGTGGACTCCTTGAAGCCAAGCTCAT
TGCTCCTCCTGAGTAAAAAACTCTTAAGAGACCATTCTCTACGGCAAGGGTCTGAACAGGTGAA
AGACAGATGAAGGCAGGGCTGGAGGAAGGAATCACATAGCATGCTCTCTTAAAATACAGATTC
CAAACCTTTTCCTGATGGCTTAACAACCCCCAAACCAATGGGAACATTCTTCCCAGTTTCTCATC
CAGAGCAGCTTTGCCTAGCAGATCCTGTGGCCAACCCTTGGATTTTGCTGAACTGCAACATCCT
CCAGCTGCTCACATGATGCGTGTTGGGGTGGGAATCTGGGGCCTGTCTCCCCTTCTCTGGATGT
TTAGGGTTTATCCACAGACCCTGGCACACTGGTGGATATACAACAATGGTGGTATGCTGGGAAT
GAAGAAAATGATTGTGCAGTCACTGGGAAGAAAGGGTTTTGTTGTGATTTGATTTCCCCTTATGG
CTAACACATTCAAGAATCTTTATTTATTTATTTATTTATTTTTTTTTTTTTTTTTTTGAGACGGAGTCT
TGCTCTGTCACCCAGGCTGGAGTGCAATGGCACGATCTCGGCTCACTGCAACCTCTGCCTTCCA

GATTCAGGAAATTCTGCCTCAGCCTCCCAAGTAGCTGGGCTTACAGGTGGGCACCACCACGCC
GAGCTAATTTTTGTATTTTTAGTAGAAACGAGGTTTCACCTTTTTGGCCAGGCTGGTCTTGAACTC
CTGACCTCAGGTGATCCACCCACCTCGGCCTCCCAAAGTGCTAGGATTATAGGCGTGAGCCACT
GTGCCTGACCTCAAGAATCTTAATACAGGAAAATATTACCTTATGGTAATATTACATCACCATAA
ATTTATCATTACATCCATTATACACACCATTATAAAAACCAACATGAATTCAGCACTGACAATGC
CCAAACTCTGTTCCTAAGCACTGCCCACATATTACCCTATACTCAAAACAAACCTAGGGAGGTA
GTATTTTTAGCCCCATTTTACAAATGAGCAAACAGGCTCAGACAGGTAAACTGACTTGTCAGGTC
ATTCAGCATTGAAGCGGCAGAGTAGGGATTGGAGCCCAGGCAGGCCAATTCCATGGCTTCAGT
GCTTAAACCCTGGGCTTTATGGCCTTTGCAATTCTGACTTCATCTGGAAGTTCTGCCCTCACCTCA
GATTCAACTTGCTTAAACCCATAACTCACTTTCAAGTGGTGAGCACCCACCATTTGTTGAGATGC
CCACTGTGTTCCCTCCAGGCACTTGACATAGGATTGCTAATGCTCAGGACAACTCTAAAAATTGC
TTCCACTTTGCAGAAAGGTTCACTGACTCCATCAAGGTCAGGTAGTGAACGAAGAGTGGTGACA
GGTTGTAGAGAAGTTGAACTTGTGAGCTTCAGACTCAGACTACTGGATTTAGCTCTTGCATCTGC
CACTTACTATTCGTGTGGCCTTGTGCAAGTTATTTGATCTCTCTGCCTTAGTCTCCTTGAATGGGA
AGTGGGGCTTCATAAGGTTTTTATGAGGATTAATTGAGTTAATGCAGACAAAGCATTTGACACA
AAGAGTTCTCAATAAATGTTACTTGAAACGACAACAACAACAACAACAAAAACACATGGTGGTG
AGGATGCAGAGAAAAGGGAACGCTTATACATTGTTGGTGGGAATGTAAATCAGTTCAGCCACT
GTGGAAAGCAGCTTGGAGATTTCTCAAAAACTTAGAACTACCATTCAACTCAGCAATCCTATTAC
TGGGTATATACCCAAAGGAAAATATATTGTTTTTCCAAAAAGACACATGCACTTGTACGTTCATC
ACAGCACTATTCACAATAGCAAAGACATGGAATCAACCTAGATGCCCATCAATGATGGATTGCA
TAAAGATAATGTACTTCATACGTACCATGGAATACTATGCAGCCATAAAATTAACAAAATCATGT
CCTTTCCAGCAATACGAATGCAGCTGGAGGCCATTATCCTAAGCAAATTAAGGCAGGAACAGA
AAACCAAATACCACATATTCTCACTTATAAATGGGAGCTAAGCATTGAGTACACATGGACATAA
TGATGGAACAATAGACACTGAGGACTACCAGAAGAGAGAAGGAGGGAGGGGAGCGTGGGGA
GCGTGGGCTGAAAAACAACCTTTTAGGTAATATGCTCACTACCTGGGTGACAGGCTCATCTGTA
CCCCAAACCCTATACCCATGTAACAAACCTGCATGTGTACCCACTGAATCTAAAATAAAAGTTG
AGGCCAGGTGCAATGGCTCATGTCTGTAATCCAAGCACTATGGGAGGCTGAGGCAGGAGGATT
GCTTGAGCCCAGGAGTTCAAGACCAGCCTGGGCAACATGGCAAGACCCCGTCTCTATAAAAAA
AAATACAAAAAATTAGCCAGGCATAGTGGCGCATGCCTGTAGTACCAGCTACTCAGGAGGTTG
AGGTGGGGGGATTGCTTGAGCCAGGGAGGTCAAGGTGGCAGTGAGCTATGATCATGCCACTGC
ACTCCAGCATGGGTGAAAGAACTAGACCCTGTCTCAAAAATAAGTAAACAAAATAAAAGTCGA
AATGATTCTTTTAAAAAAGTGACAGAGCCAGGATTGTATCTGAAGTCTTTTTGACATTAAAAGCC
ATACTTTCCATACTACCTTATGCTGCTTCTTACATACTAATAAGAAAAACTACATGTGAAGAGAA
GAAAGACTATAAGGTATGCCATCAAAGTAGGGAAAGTGATGGTATTTGAATGGTGAGACTGTAG
GATAACATTTCTTCTTTCTAGTTTTAAAAATATTTTCCAAGATCCCTTCAGTGAACATGGGATGCT
TCCATAATGAAGAGAGAGACACACCGTGATGACCTCCACATACCATATTTGATGACAGTCTTCC
TTGGTAATCACTCACATCCATCTCTTGTCTGTTCCCACTGCTGCCCTTAATCTAGGCTCTGTGCCA
GCTTCCTGATGGAACTCCAGGCCACCCTGCACACTGCCATGTGGTTCCCTCCTCCAGCCCTGCC
TTCATCTGTCCCTCACCTGTTCAGACCCTCAGTCATCCCCAAGAACTTCTCTGGCAGAGTGTACA
GAGCCAACTGTTCCCTGGACCGACTCATGAAGTTCAGTATTTGCTTAGGCAGAGAGTGTCAGAG
CTGTCAGAGGAGAGTCAGCTAAAACCCTTCCCCTGGCTGGGCACCGTGGCTTACGCTTGTAGTC
CCAACACTTTGGGAGGCCAAGGCAAGAGGATTGCTTGAGGTCAGGAGTTCGAGACCAGCCTGG
GCAACATAGTGTCTGAAAAAATAAATTTTAAAAAACAACCCAGCCAGGCACGGTGGCTCACGC
CTGTAATCCCAGCACTTTGAGAGGCCAAGGTGGGTGGATCACGAGGTCAAGAGATTGAGACCA

GCTTGGTCAACATGGTGAAACCCCGTCTCTACTAAAAATACAAAAATTAGCTGGCTATGGTGGC
AGGCGCCTGTAGTCCCAGCTACTCGGGAGGCTGAGTCAGGAGAATCGCTTGAATCCGGGAGGC
GGAGATTGCAGTGAGCCGAGATCGCACCACTGCACTCCAGCCTGGCGACAGTGCGAGACTCCA
GCTCAAAAAATAAAATAAAATAAAATAAAAAATAAAAAAACTCTTCCCCTTACATACAGCAAAC
TGAGGCCCAGAGATTTGGCCTTGGTTTGTTCATAGACACAGGGCCAGCTCCAGACCTCAGGTCT
CTTGACTGCCAATCTTAGGCCCTTTCCTTGATTTGAGCTGCCTTCCTGCAAAGTATTTGCAAATGA
TATTGTTTTTTAATTCTATACTGTGGGCCCAAGGGGAGTTCACTCTTCTCTTTTCTTTTCTTTTCAA
GATGGAGTCTCACTCTGTCACCCAGGCTGGAGTGCAGTGGCATGATCTCAGCTCACTGCAACCT
CCGCCTCCCAGGTTCAAGTGATTCTCTTGCCTCAGCCTCCCAAGTAGTTAGGACTGCAGGCACC
TGCCACCATGTCTGGCTATTTTTTTTATTTTTTGGTGGAGATGGGGTTTCACCATGTTGCCCAGGC
TGGTCTTAAACTCCTGACCTCAAATGATCCACCCACCTCGGCCTCCCAGAGTATTGGGATTATAG
GCATAAGCCACCGTGCCCGGCCAAGGCTATGTAGCTCTTTTCTTAGGCCACGTAGCTCACTCTTT
TCTTAGGCTGTGTAGTTCACTTTCCCAGTGCAAACTTTGGGAAAAGCTCTGTGCCACAGGCTCCA
TTCCTGTTTGCCACATCACAAATACCAATCATTGCTCTAAGGAGTGGATGATTTGATGAGAATAT
TCAGTTCTTACACCACTACTAGAGGAGGAACACTGACTGCCTTGTCGTGGATGTGGTCTGGCAG
TTTCTGTAGGTAGGAGTGGTGGATTGTCTCCAAATGTGGCCACCGACAGTGCCTCCCATTCCTGT
GTGTGCATGCTGCTTCTCCCACTAGAAGGTGTCTTTTACTTTCCTTCTCTTTGCATCTGGAACTTG
TTTTGACCAGTTGGACCTAGCAGAAGTGGGGCCCTGTGACTTCCAGGCCTTATGAAATCTTGTA
GCATTGTTTTTCCCTTTTAGAGCCAGCTGTGATGTAAAGAAGTTTGTGCTGCATGAGAGAGATAG
CACCTGAAGAAAATGAGGCCCTGGAGGATGAGAGACTCTAAAGGAAAAGGGAAGGGCCTGCT
GTCTTCCAGCCACGCCAGCCCCCAACTGAGAAGCTGGGTGAAGCCATCTTGCATCTTTAGCCCC
AGTCAAGTTACCTCAGCAAAGACTAGCTGACCCTGCCAAGCCCTGCCCAAGTTACAGAATCATG
AGCAAATAAATGGCTGTTTCTGTTTTAAGCTTTTAAATTTTGGGGGTGGTTTATGTGTCAATAATA
ACTGAAACAGATAATATATACAGAATAAACTTTAGTTTTAATAATCTAAGTAAAAGCCCACTAAT
TCATTATGCAGAAAAAAAATGATTTTTTTGAGACGGGGTCTCGCTCTGTTGCCAGGCTGGAGTGCT
GTGGCACAACCATAGCTCACTGCAGCCTCCACCTCCCTGGTTCAAGCGATCTTCCCACCTCAGC
CTCCCGAGTAGTTGAGACCACAGGTATGTGCCACAACACCTGACTAATTTTGTAAATTTTTTGTA
GAGATGGGGTCCTGCCATATTGCTGAGGCTGGTCTCTAACTTCTGGGCTCAAGCCATCCTCCTG
CCTCAGCCTCCCAAAGTATTAGGATTACAGGTGTGAGCCACTGTGCCCAGCCAATTTAACTTCTA
TTTTAAAAAGACAGCCCCTCACAGATACTGCTACAGCTCTGTTAAATCAAATTTAGCCTCCTTAC
ATATTTTGAGTTTGACGTAAAGGTTTCTCTGTACATAGTGAACTGTCACCTAAATGGAGGTGTAA
ACAGCCTGTAATCTACCCTTGTGCCAAACACCAAGTTTTGGCCAATCAAAAGGGGCCAACTGTT
CAAACCCTCTTCAAATAAGGCAAACACTGAGCTGTAACCAATCTGGCTGTTTCTGTCCCTCATTT
CCATTTTCTGTACATCACTTTCCTTATGCTGCCCATAAATCTCTGAGCCTACTCTGGCTCAGAATG
CTGCCCAATTTACCAATCGTACTTTGCTAAATTGAACTCTTAAATTTAATTTGACTATGGATTTTTT
TTTTTGAGACAGAAATTTGCTCTTGTCGCCCAGGCTGGAGTGCAATGGTGCAATCTCAGCTCACC
ACAACCTCCATCTCCCGGGTTCAAGTGATTCTCCTGCCTCAGCCTCCCGAGTAACTGGGATTAC
AGGCATGCACCACCATACCTGGCTAATTTTGTATTTTTAGTAGAAACGGGGTTTCTCCATGTTGG
TCAGGCTGGTCTCGAACTCCTAACCTCAGGTGATCTGCCCACCTCAGCCTCCCAAAGTGCTGGG
ATTACAGGCGTGAGCCATTGCGCCTGGCCTAATTTGGCTATGGATTTTCTTTTAACAGCTCTTAA
AGGGTTGTTTTTTGTTGTTGTTTTTTTTTTTTTGAGATAGAGTTTTGCTCTTGTTGCCCAGGCTGGA
ATGCAATGGCTCGATCTCAGCTCACTGCAACCTCCGCCTCCCAGCTTCAAGTAATTCTCCCTCAG
CCTCCTGAGTAGCTGGGATTACAGGCATGCGCCACCATGCCCGGCTAATTTTGTATTTTTAGTAG
AGATGGGGTTTCTCCATGTTGGTCAGGCTAGTCTCGAACTCCCAACCTCAGGTGATCCACCCAC

CTCGGCCTCCCAAAGTGCTGGGATTACAGGCATGAGTCACTGCACCCATCCTGTTTTTATTTTAT
TTTATTTTATTTTATTAAGTTCTGGGGTACATGTGCAGGATGTGCAGGTTTGTTGCATAGGTAAAC
ATGTGCCATGGTGGTTTGCTGCACCTATCAGCCCATCACCTAGGTATTAAGCCCTGCATGCATTA
ACTATTTATCCTGATGCTCTCCCTCCCTCCAACCTACCCCACAGGCCCCAGTGTGTGTTGTTCCC
CTCCCTGTGTCCTAAAGGGTTATTTTTGAGGAATTATTCTAATGTCAGAAATGCTCACATGAGGG
TGGAGGGTACAGGGAGGTGATAATGAAAGGATACAATACCTCAGGAGGAAGTTTGATATTTTTG
TTTGAGATCTATTGTACATGGCAAATATAACTAATAATTGAGTACTGTACATTTCAAAATTGCTGA
GAGAATAAATTTCAAATGTTTTCATTATTAAAAATATTAAACATTTGAAAAAAAAATGTTCACAAT
ATTCCATTGTGGTAGGTTGCTTCTAAATGGCTCCCAGTGATCCATACCTCCTGGTACTCATGCCTT
GTATAATTTCCTCTCCTTGAGTGTAGTTGGACCTAGCGACTTGCTTCTAATGGATAGAATCAAGC
AAAAGTCATAGGATGTCACTTCCTGGATTTGGTTACAAAGACTGTAACTTTCTCTCTTTGTCTCTC
TCTCTCTCTCTCTTTTAGTGCCCTTGGTGTGGTGGAAGCAAGGTGCCATGTGATAAGTAAGCC
TCCAGAGAGGCCTGCATGGCAGGATCCAATGTCTTTAGCCAACAGCATCTGGGAACCTGAGAC
CTGCCAACACCCTTGTGAGTGAGCCTGGAAGTGGACCTCCTGCTCTTGAACCTTGAGGTGACTA
TAGTGCTGGCTGACACCTTGACTGCAGTCCCTGAGCTGGGGGGCCCAGCTGATCTACTCCCAGA
ATCCCCCCTACTAAAACAGTGATATAATAAATGCTTATTGTTTCAAGCTG
ID|30401|gene_id|ENSG00000261183|transcript_id|ENST00000568525 Homo sapiens lncRNA
GACCGCTAGGGAGCTCAAGTACCCGGACACACGCACGAGCGCAGAACAAAGGGCCGGA
CATGCTCCAGCCCGCTCCGCCTGCGCCTCGGCCTTGCGCGCGGGGGCCCGGGTTACCTGCGCA
GGTGAGTTGGGTGGGGCCGGGTCCGCCGAGGTTCCGGTCCCAGCTTCCTGCGCGGCTCGGGTC
GCGGCTTCCGACCCGGTGCTCGGAGAGCTGGGGCTCGGCCGCCTTCTCCCCCTGGTTTCTGGTG
AAACTGAGTCAGCGCCGTCGGGGCGGGGCCGACATTCACCCTTGCGCTGCCGGCCCCGCCCAC
GCCCGGTGCGCTGCGGCCGCCTCTAGCGCGGAGCTCCAGGCCCGGCCCGCCCCGCGGCCCCG
CAGCCCCGGTGCCTCCCCGGCAGCCAGCTCCCCGGAGGGGCTCTCGGGCTCCACCCAGTCCAG
CCTCGCGAGGGAGCCCCGCGCCCGAAGCGCATACCCAGCCGGGAGGGGATGCGGCCGGGAC
GCGAGGGTCCGGCCTCTGGGGCGGCGGCCTCCAGTCTTCCTTTCCGGCCGGGGCCTGCTTTCC
CAGCCCCAGACCTGAGGCCCGATGCAAATGATCTTTCCGGCCCTCAGGAAATCATTTCCATTTC
GTGGCGGGAAGGTAACCCAAAAGTTGCGTGGCTGCACCTGTGATAACAGGGATCCGGGATGTC
TGCCTCACTGTCAGCTCCGGGAAGCCCTCCCTGCTGTCAGGGTGAGAAACGGAGGCGATTCCC
CGCGTCCAGGCTCCCCTGCGTGCTCCACGGGGCTTCCCTGTTGTTCGAGGCCCCTCCTCATCTT
GTATGTACAGTTCGGTCCATTCCCTGCTATGGCTCATTGGACTGTTGCCCCCTTCCCTGGAGCCC
AGGCTTCCTCTCTTTCACAAAGGGGACGCTGCTGGCTACTTTCTTCCAGGGAAATGTCATCCTTG
GAAGCCTGGAGGTTCCAGTTCACCAAGTAGCTGAGTTAATGCCCTTCACGAAACAGTGGACTCC
TTGAAGCCAAGCTCATTGCTCCTCCTGAGTAAAAAACTCTTAAGAGACCATTCTCTACGGCAAG
GGTCTGAACAGGTGAAAGACAGATGAAGGCAGGGCTGGAGGAAGGAATCACATAGCATGCTC
TCTTAAAATACAGATTCCAAACCTTTTCCTGATGGCTTAACAACCCCCAAACCAATGGGAACATT
CTTCCCAGTTTCTCATCCAGAGCAGCTTTGCCTAGCAGATCCTGTGGCCAACCCTTGGATTTTGC
TGAACTGCAACATCCTCCAGCTGCTCACATGATGCGTGTTGGGGTGGGAATCTGGGGCCTGTCT
CCCCTTCTCTGGATGTTTAGGGTTTATCCACAGACCCTGGCACACTGGTGGATATACAACAATG
GTGGTATGCTGGGAATGAAGAAAATGATTGTGCAGTCACTGGGAAGAAAGGGTTTTGTTGTGAT
TTGATTTCCCCTTATGGCTAACACATTCAAGAATCTTTATTTATTTATTTATTTATTTTTTTTTTTTTT
TTTTTGAGACGGAGTCTTGCTCTGTCACCCAGGCTGGAGTGCAATGGCACGATCTCGGCTCACT
GCAACCTCTGCCTTCCAGATTCAGGAAATTCTGCCTCAGCCTCCCAAGTAGCTGGGCTTACAGG

TGGGCACCACCACGCCGAGCTAATTTTTGTATTTTTAGTAGAAACGAGGTTTCACCTTTTTGGCC
AGGCTGGTCTTGAACTCCTGACCTCAGGTGATCCACCCACCTCGGCCTCCCAAAGTGCTAGGAT
TATAGGCGTGAGCCACTGTGCCTGACCTCAAGAATCTTAATACAGGAAAATATTACCTTATGGTA
ATATTACATCACCATAAATTTATCATTACATCCATTATACACACCATTATAAAAACCAACATGAAT
TCAGCACTGACAATGCCCAAACTCTGTTCCTAAGCACTGCCCACATATTACCCTATACTCAAAAC
AAACCTAGGGAGGTAGTATTTTTAGCCCCATTTTACAAATGAGCAAACAGGCTCAGACAGGTAA
ACTGACTTGTCAGGTCATTCAGCATTGAAGCGGCAGAGTAGGGATTGGAGCCCAGGCAGGCCA
ATTCCATGGCTTCAGTGCTTAAACCCTGGGCTTTATGGCCTTTGCAATTCTGACTTCATCTGGAA
GTTCTGCCCTCACCTCAGATTCAACTTGCTTAAACCCATAACTCACTTTCAAGTGGTGAGCACCC
ACCATTTGTTGAGATGCCCACTGTGTTCCCTCCAGGCACTTGACATAGGATTGCTAATGCTCAGG
ACAACTCTAAAAATTGCTTCCACTTTGCAGAAAGGTTCACTGACTCCATCAAGGTCAGGTAGTG
AACGAAGAGTGGTGACAGGTTGTAGAGAAGTTGAACTTGTGAGCTTCAGACTCAGACTACTGG
ATTTAGCTCTTGCATCTGCCACTTACTATTCGTGTGGCCTTGTGCAAGTTATTTGATCTCTCTGCCT
TAGTCTCCTTGAATGGGAAGTGGGGCTTCATAAGGTTTTTTATGAGGATTAATTGAGTTAATGCA
GACAAAGCATTTGACACAAAGAGTTCTCAATAAATGTTACTTGAAACGACAACAACAACAACAA
CAAAAACACATGGTGGTGAGGATGCAGAGAAAAGGGAACGCTTATACATTGTTGGTGGGAATG
TAAATCAGTTCAGCCACTGTGGAAAGCAGCTTGGAGATTTCTCAAAAACTTAGAACTACCATTC
AACTCAGCAATCCTATTACTGGGTATATACCCAAAGGAAAATATATTGTTTTTCCAAAAAGACAC
ATGCACTTGTACGTTCATCACAGCACTATTCACAATAGCAAAGACATGGAATCAACCTAGATGC
CCATCAATGATGGATTGCATAAAGATAATGTACTTCATACGTACCATGGAATACTATGCAGCCAT
AAAATTAACAAAATCATGTCCTTTCCAGCAATACGAATGCAGCTGGAGGCCATTATCCTAAGCA
AATTAAGGCAGGAACAGAAAACCAAATACCACATATTCTCACTTATAAATGGGAGCTAAGCATT
GAGTACACATGGACATAATGATGGAACAATAGACACTGAGGACTACCAGAAGAGAGAAGGAG
GGAGGGGAGCGTGGGGAGCGTGGGCTGAAAAACAACCTTTTAGGTAATATGCTCACTACCTGG
GTGACAGGCTCATCTGTACCCCAAACCCTATACCCATGTAACAAACCTGCATGTGTACCCACTG
AATCTAAAATAAAAGTTGAGGCCAGGTGCAATGGCTCATGTCTGTAATCCAAGCACTATGGGAG
GCTGAGGCAGGAGGATTGCTTGAGCCCAGGAGTTCAAGACCAGCCTGGGCAACATGGCAAGA
CCCCGTCTCTATAAAAAAAAATACAAAAAATTAGCCAGGCATAGTGGCGCATGCCTGTAGTACC
AGCTACTCAGGAGGTTGAGGTGGGGGGATTGCTTGAGCCAGGGAGGTCAAGGTGGCAGTGAG
CTATGATCATGCCACTGCACTCCAGCATGGGTGAAAGAACTAGACCCTGTCTCAAAAATAAGTA
AACAAAATAAAAGTCGAAATGATTCTTTTAAAAAAGTGACAGAGCCAGGATTGTATCTGAAGTC
TTTTTGACATTAAAAGCCATACTTTCCATACTACCTTATGCTGCTTCTTACATACTAATAAGAAAA
ACTACATGTGAAGAGAAGAAAGACTATAAGGTATGCCATCAAAGTAGGGAAAGTGATGGTATTT
GAATGGTGAGACTGTAGGATAACATTTCTTCTTTCTAGTTTTAAAAATATTTTCCAAGATCCCTTC
AGTGAACATGGGATGCTTCCATAATGAAGAGAGAGACACACCGTGATGACCTCCACATACCAT
ATTTGATGACAGTCTTCCTTGGTAATCACTCACATCCATCTCTTGTCTGTTCCCACTGCTGCCCTT
AATCTAGGCTCTGTGCCAGCTTCCTGATGGAACTCCAGGCCACCCTGCACACTGCCATGTGGTT
CCCTCCTCCAGCCCTGCCTTCATCTGTCCCTCACCTGTTCAGACCCTCAGTCATCCCCAAGAACT
TCTCTGGCAGAGTGTACAGAGCCAACTGTTCCCTGGACCGACTCATGAAGTTCAGTATTTGCTTA
GGCAGAGAGTGTCAGAGCTGTCAGAGGAGAGTCAGCTAAAACCCTTCCCCTGGCTGGGCACCG
TGGCTTACGCTTGTAGTCCCAACACTTTGGGAGGCCAAGGCAAGAGGATTGCTTGAGGTCAGG
AGTTCGAGACCAGCCTGGGCAACATAGTGTCTGAAAAAATAAATTTTAAAAAACAACCCAGCCA
GGCACGGTGGCTCACGCCTGTAATCCCAGCACTTTGAGAGGCCAAGGTGGGTGGATCACGAGG
TCAAGAGATTGAGACCAGCTTGGTCAACATGGTGAAACCCCGTCTCTACTAAAAATACAAAAAT

TAGCTGGCTATGGTGGCAGGCGCCTGTAGTCCCAGCTACTCGGGAGGCTGAGTCAGGAGAATC
GCTTGAATCCGGGAGGCGGAGATTGCAGTGAGCCGAGATCGCACCACTGCACTCCAGCCTGGC
GACAGTGCGAGACTCCAGCTCAAAAAATAAAATAAAATAAAATAAAAAATAAAAAAACTCTTCC
CCTTACATACAGCAAACTGAGGCCCAGAGATTTGGCCTTGGTTTGTTCATAGACACAGGGCCAG
CTCCAGACCTCAGGTCTCTTGACTGCCAATCTTAGGCCCTTTCCTTGATTTGAGCTGCCTTCCTGC
AAAGTATTTGCAAATGATATTGTTTTTAATTCTATACTGTGGGCCCAAGGGGAGTTCACTCTTCT
CTTTTCTTTTCTTTTCAAGATGGAGTCTCACTCTGTCACCCAGGCTGGAGTGCAGTGGCATGATCT
CAGCTCACTGCAACCTCCGCCTCCCAGGTTCAAGTGATTCTCTTGCCTCAGCCTCCCAAGTAGTT
AGGACTGCAGGCACCTGCCACCATGTCTGGCTATTTTTTTTATTTTTTGGTGGAGATGGGGTTTC
ACCATGTTGCCCAGGCTGGTCTTAAACTCCTGACCTCAAATGATCCACCCACCTCGGCCTCCCA
GAGTATTGGGATTATAGGCATAAGCCACCGTGCCCGGCCAAGGCTATGTAGCTCTTTTCTTAGG
CCACGTAGCTCACTCTTTTCTTAGGCTGTGTAGTTCACTTTCCCAGTGCAAACTTTGGGAAAAGC
TCTGTGCCACAGGCTCCATTCCTGTTTGCCACATCACAAATACCAATCATTGCTCTAAGGAGTGG
ATGATTTGATGAGAATATTCAGTTCTTACACCACTACTAGAGGAGGAACACTGACTGCCTTGTCG
TGGATGTGGTCTGGCAGTTTCTGTAGGTAGGAGTGGTGGATTGTCTCCAAATGTGGCCACCGAC
AGTGCCTCCCATTCCTGTGTGTGCATGCTGCTTCTCCCACTAGAAGGTGTCTTTTACTTTCCTTCT
CTTTGCATCTGGAACTTGTTTTGACCAGTTGGACCTAGCAGAAGTGGGGCCCTGTGACTTCCAG
GCCTTATGAAATCTTGTAGCATTGTTTTTCCCTTTTAGAGCCAGCTGTGATGTAAAGAAGTTTGTG
CTGCATGAGAGAGATAGCACCTGAAGAAAATGAGGCCCTGGAGGATGAGAGACTCTAAAGGA
AAAGGGAAGGGCCTGCTGTCTTCCAGCCACGCCAGCCCCCAACTGAGAAGCTGGGTGAAGCC
ATCTTGCATCTTTAGCCCCAGTCAAGTTACCTCAGCAAAGACTAGCTGACCCTGCCAAGCCCTG
CCCAAGTTACAGAATCATGAGCAAATAAATGGCTGTTTCTGTTTTAAGCTTTTAAATTTTGGGGG
TGGTTTATGTGTCAATAATAACTGAAACAGATAATATATACAGAATAAACTTTAGTTTTAATAATC
T

ID|30415|gene_id|ENSG00000272189|transcript_id|ENST00000607749 Homo sapiens lncRNA

CTCAGTCCGGAGCTTCCGGTCGCCGCGGCCGACCAGCTGAGGGCTCCCGGAGATATGGA
GCGGGGTCAGCGAGGCCTGGTGGCGCCTCCTGGAGTCTGGGACGAGCGGCGGTCCGAGGAGC
AGCCCAGGTGGGGCGAGGGTACGGAGACGCTATTTCCCTCCGAACTCTGAAGTAGCCAGGTCA
GTGTCGGCTCCGCGAGGGCTGGAGTTCACGGTGACAGCCGGCAGAGCATCTGATGCCACGTGG
AGAGGCGAAGTACATGGGTCGGATTCCACGCTGGGAGCCGGCCTGGATCTGCGCTTTAGGAAG
CCACTGCGGGCGACCCTATCTTGTCGCCAGGACTTCGCATCTGATGAGCCTCAGGGCACACAA
GTTAGAGAGGCTTGTGCCGGCTGATGTGGCTTTTCTTTTTCTTAGTTTCCGTTTAGTAAACGACCT
TGTGACTTCTGGCTTGCTCAGGAAGACCAGAAAAAAAGGCAATAGGTAACGAATTTTTCTTTTTT
TAAAGAAACAGATTATTGGCCGAGGAAAAACCAATTTCGCTCTAAAGGATAAGCAACAAAATA
CTGAGGGGGGAAAAAGCCTACACAAATGGAAACATACGGCACAAAATCAAGGAGCGACCAGT
TCCGCATGGTTACACTAAAATGCACTGCACTCCCACTCTCTGGCTTGGCTGAAGATAACCAGCA
AGCTCTGAACAGTTTAAAAGCCTTCCAAGGACTGCAGCAGCACTGGACACACAAGAGAGTCGA
GAACAGTATTGGATTTATTGTTAGGGTTACAATTTTCACGATCTTTGCATGCAAAACTTGCCACTC
TGAGCCCAGTGAACTATTTTTATTTGGTTTACTTACTGGCTTTCTGGATGACTAGGTAGAGCTTTA
ACTAAAGAACACTTCTGCTGTGGTCTGTGATGATGAATTTTCCCGTAGTGTCATTTCTATAATGGA
ATAGGCAAACTGACCATTTAGAATGTGATATTCCTAAATCACATTCAAATCAGTCTTATCAGAGA
CTTCTAATCAATAAAAATAAAGCACCAGATGGCTAGAGGTAGTTTTTGACCTCAGCTTGGGCCTC
AGCAAAGATTTTTTGTGTTTTTTTTAACTACATTAAAACATCAGAGTTTAAGACACTTTTCTTTGAG

CATCAGAAATACTCTCAGCTGAATTTAGAGTTGAACTATCCGGAAGAACCCTGGAGAAGGAGG
GTGATTTATTTTCAACTTTCTGATTTACCACCGACTTAAATCAACCAATGAGTTCTTTCTTTGACCA
AAAAAAAAAAAAAAAAAAAAAAAAAAAAAAAAAAAAAATTCAAGACTTGTGATGTTTCTATTTGTT
TTGTTTTGTTTAGTTTGGTGTGTGCTTACTCATTGTCCCATTCTTTTGATAACCTGTATTTATCTGCC
ACAAGTTAAACAAAAATAAATGAAATAAGGAACACACCAAAATCCTGCACTTAGGCCCTGTATA
GACAGTGTGTTCCAAACCTAAGGAGGTGTCTGCAACAGGGGAGGCTGCTGAGTGACTCTGCAG
AGAACAGATGACATCTGTTAAATGTCAAACACCATCACGTCGTACTTCAAGTTGTACTTTACTTG
GAACATGGAAGCCATCTGTCTGGAGCTCCCACATCCATTTCAAATCTTCAGGGTTCCCCATTCCA
GGGGAGGAGCTGAACCTCCTGACACCGTCCACTTCCCTCCTGCCTCCTTCAAACATCATCCCTTT
TTTCTCCCGAATCCCTAAATGTTTCTGCACCCAAGGGCTCTACCCCAATAAACACAGCTAAGACT
CTTTGATTCTTAAAATGTCTTCCTTTAACGTCCCACCCTCCAGAATCTATTGTCGTATGTTTTGTTC
ACCAAAGGTAATAAAAATAAATGTGGA

ID|55918|gene_id|ENSG00000226465|transcript_id|ENST00000376445 Homo sapiens lncRNA

GGATCAACATTATCATGGCAACCCTGAGGAGGCTGTGAGAGTCTCTGGGGTCCTTGCCGG
CCTGCAGAAAATCAAGCTTTGAGCGGGAAGCTTCGAGGAGACACAGTGTCACAATTACAAGGT
GAGACCAGATGCCCTGGCCCCGCTGTCTCCTCGCAGGCAGGAGAGGTTTGGGAACACCCACGC
TTCCCTCAGCAAGGCAGAACTGCACTGGACGCATGGAAGTGTGATTTGGAAAGCGGGACCTTC
GTACGGAATGTTCGGAGTTGTAGTATCTGTTCTACTCCCAGCCTGTTGGTTCTAGGGCTGCGGAC
TATAATCCCAGCATGCACTGGGTTTGGGTAAGAGACTGAAGCATCCAGGTGAAGAACTGGCATT
GCTTCTACCTGTGGAGAGAGGACACCTGTAGGCTGCACGATTGACTGTGAATTTAGGAGGAGG
ATTAAAAGTTACCTGGCAAACAGTAGGCACCCTACAAATACTGATTTTTCCCCCTCCTTTAAAAA
GTTTGATCTCTTGTTAGATGGTGTTGAGTAATACAGAAATGGCAGGTATGCATGTGATTGGTGCT
TAAATGCACACAGTTCCCCATACCACAAGAGGAAACACACCACAGCCTGATCATTAGGACCAA
CAGCAAAATAAGTTTGCCTTTAATCTATTCACTGCATATCTAAACTTTGATCAAGCCCATGGACA
CACCTGAAGACATGGGGTTTCCTTCTTCTGGGCACAATCATCCTTTGATCAGTGTCTGCTCTGTA
AAACAGGATTCCCCCTAACTCTACCAACCAGCAGATGGGAAGTAGTAATCCACCCGTGGACAC
TTCCTGCTTGCAGCCCACTGCTCTCTCAGGTTACCATTTTCAGATGTTGCAAGGATGGGGTACTC
CCTTGCCTCTGAAAGGGAATGGAGGGTGGGGGGTGTGGTTCAGCACTCAGAGCTTCACTTCAAT
GTGGCAGCCACTGACCATTGGGACCACTGAGCCCCTGGAATGTGGCTGGTCTGTATTGAGATGT
GCTGGAAGTTTAAAATACAGAGTTTTATGGTTTTATTCTTTGTGTGTGTGTGTGTGTGTGTGTGTG
TGTATGTATGTGTTTAAAAATGGCTTTTATTTGTATGTTATTGCACATTCCTAATACAGTGATTTCT
GGAAATCACAGTATTTTGTAAACATAAGATTTATACTTTAACCATACCCAAAGTCTATTTTTATTT
CTTGACAGAATTGCTTTTCTTAGTACACATGGCTCTGCTGTGGTTTAAAACTGACTTTTTCATTTA
AGGCAATCTGGACTCTATTAGTGAGAAATGCATTGTTTTTCTTGGTTTTATTCTCTAAAGGTTCCA
GCATTTGGAGGCAAACATGAAGCTCCATCCTCTCCAATTCTGGGGCAACCGTGTGGAGATGATC
AAAATGCTTCACCTTCAAAACTTTCAAAGGAAGAGTTAATACAGAGTATGGATCGTGTAGATCG
AGAAATTGCAAAAGTAGAACAGCAGATCCTTAAACTGAAAAAGAAACAAGTAAAAGTCTTTGC
CT

ID|55954|gene_id|ENSG00000234869|transcript_id|ENST00000426112 Homo sapiens lncRNA

ATGTTTGTATTTTGCACTTGCCCGGAGGTGTTTCACGGGAAGTGAGCATTTTTTAAAACAAA
AATAGAATTTAGAAAAAAAAAAGGCAGACACGATGGATTTCTAGTGTTGTACGTGCTGTAGAAA
GCACTTGAAAATGCGTTTTGCCAGGGGCCGCAACCAGAAGCTACATGACTTCTGGAATCTGTTC

CTTACACAGTAAATTCCTGGGCCAGTTGGTAAACAGCAAGGAGCCACAAGAAATCACTCATAG
GCAAAAGCCGAGTGAGCCCAGGCCAAGCTCCACTTTGTCCACTTTGCCCTATTTTTGGAATTCA
GGGGGAAACATCTTGGGAGCAGGCCCATTGCACCCCTCTGCCTCCTCTCGATGCCTTACAGTTG
GTGGGGTTTGACACATGGAAAATTGTGAAAGTTAGAACACGGAACAAAAATGAAGCCTCAACA
TAGAGTTAATAGAGGCAAGGGGCTACAGAGAATTTAGATCCTAATCAAGCACGTCTTATGTACT
AGCGCTTCCCTGGAAGGCCAGGATAACTTTATGACTACAAAGAAAACATGTTTTCCTGCTTGGA
AGTTGCCAGAACTTGTCTAATATAAAAGCTGGGATTTCTTGCCCGTGGGCTGTCGCGTACGGCT
ACCCAGGCCGCCTCGTGAAGCCTCCTCCTGGGAGCTCGGCGCCCTCAGGTGCTCCCTGAGATG
CCAGCACCTGAACGACCTGCTCCAGGGAGGCTTTAGCCCCAAGAAGGTTCTGGAAAGCTCCAG
GAAGTTTAGCCGGCACGCACAAAAGCCAGAGACAAATCTCCAGCCTGCCTGGAAATGCTGTGG
GCTCAGGGCAATGGGCTGAGGTCTTGTGTCCAAAACTGCTCTTGCATCTCCCTGGGGAGCCCGG
GTGGGCAGGAACAACAAGGTCCTGTGGCCATGCCCTCGCCCCATGCTCACCCACACTGCCTGT
GCCTGCTGGGCCCATCTCAGGGCTGCACTGGCCCCTAGTGGCCAAGCATGGGCTCTACCCCTCT
GCGCCTGCCTGGCTGCCTGTGCAGGTGCCCACCACACTGTCCCTGAGCAGAGCAGAGACCAGC
CTGAGCCCCGGGCTCCGTGGCTACCATGCTGAGAGTGAGCCCTGTGCATGCACACACACTTAC
GATGCTGCTGGCCCCGAGCAGTGGCCGGCCACACGGGCTGCTAGTCATACTGCCCGCTGCTCC
TGGGACTCCTGAGCCCCTCACTCTCTATAGGGTGAGGATGCCCAGCTCTGCAAGACACACAGC
AAACGGAGCCTCAGAGTGCACAGCGTGGAGATGGGGCTCCCCCCACAGGCTGCACCCCCATCT
AGTCCCGGGGAACGAGAATGAAGGGCCTACCTGAGCACACAAGCCCCAGGAGGGCATGGGAG
CTGCATTTGGGGGCAGTGACTGGGAGGGCTGCTGACTCCAGGCTCAGGTAAGGGAGATGCACG
TGTTCCCGGAAGAGGGTCCACACCTGCTGAGTTCACGCTGCTGAATATCCTTGCTCCTATCACTA
TTATTATCATTGTCGTGGTCGATCTTGTTTCCTGGCCCCCACCTGATGCACATAAACGTTATCATG
GAGCCAATATGGGGGAATATTGTCTCACTTGATTACTGTTCTTTTTTTTTTTTTTTTTTTCTGAGATG
GAGTCTTGCTCTATCACCCATGCTGGAGTGCAGTGGCACAATCTCGGCTCACTGCAAGCTCCGC
CTCCCAGATTCACACCATTCTCCTGCCTCAGCCTCCTGAGTAGCTGGGACTACAGGCACCCGCC
ACCACGCCTGGCTAATTTTTTTGTATTTTTGGCAGAGACGGGGTTTCACCATGTTAGCCAGGATG
GTCTTGATCTCCTGACCTTGTGATCCGCCCGCCTCAGCCTCCCAAAGTGCTGGGATTACAGGCG
TGAGCCTGCGCCCGGCCCTGATTACTGTTTACTGATTACTGATTACTGTTCTTCTTTACATACCTC
CCTCCCTTTTTATTTCCCTGAAACTGGGTCTCACTCTGTCACCCAAGCTGGAGGGCAGTGGCATG
ACCACAGCTCACTGCAGCCTCAAACCCCTGGGCGCAAGTGATCCTTCTGCCTCAGCCTCCTGAG
TAGCTGGGACTACAGGCACATACCACTATGCTTGGCTAATTTTTTCTTTTTAATGTTTGTAGTGAC
AGGGTCTTGCTACATTGCCCAGACTGGTCTCAAACTCCCAACCTCAAGCTATCTTCCCTCCTCAG
CCTCCCAGAGTGCTGGCATTACAGGCATAAGCCTCTATGTCTGGCCTTATTTATTTATTTATTTAT
TTATTTTTGTTTCACTCTTGTCACCCAGGATGGAATGCAATGGCACGATCTTGGCTCACTGCAAC
CTCCAACTCCCAGGTTCAAGCGATTCTCCTGCCTCAGCCTCCCATGTAGCTGGGATTACAGGCA
TGCGCCACCACACCTGGCTAATTTTTTGTACTTTTAGTAGAGATGATGTTTCTCCATGTTGGTCAG
GGTGGTCACGAACTCCTGACCTCAGGTGATCCACCCACCTCAGCCTCCCAAAGTACTGGGATTA
CAGGCATGAGCCACTGCGCCCGGCAAAACCTGCATTTTCTTGCACCATCTCTATCTCTAAGAGA
AGTCTGAGTGACCTACATTTAAGTTCTCAAGTTAAACTATTACATTTCCCTGCAACCTTGGTGCG
GTGGCTCACGCCTGTAATCCCAGCACTTTGGGAGGCCGAGGCGGGCGGATCATGAGGTCAGGA
GATGGAGACCATCCTGGCTAACACGGCGAAACCCTGTCTCCACTAAAAACACAAAAAAATTAG
CCGGGCGTAGTGGCGGGTGTCTGTAGTCCCAGCTATTCGGAAGGCTGAGGCAGGAGAATGGCG
TGAACCCGGGAGGCGGAGCTTGCAGTGAGCCGAGATCGTGCCACTGCACTCCAGCCTGGGTGA
CAGAGTGAGACTCCGTCTCAAAAAAAAAAAAAATGAGATCAAGGGCCAGGTACGGTGGCACATG

CCGGCAATCCCAACACTTTGGGAGGCCAAGGTGGGTGGATCACTTGAGCCCAGGAGTTCAGGA
CCAGCCTGGGTAACATAATGAAACCCCATCTCTAAAAAAAAAAAAATTTTTTTTTTTTGAGACGG
ACTCTCACTCTGTCCCCAGGCTGGAGTGCAATGGCGCAATCTCAGCTCACTGCAACCTCCACCT
CCCGGGTTCAAGCGATTCTCCTTGATCTCCTCCTCAGCCTCCTGAGTGGCTGGGACTACAGGTG
TGCACCACCACGCCCAGCTAATTTTTGTATTTTTAGTAGAGATGGGGTTTCACCATGTTGGCCAG
GATGGTCTCAATCTCTTGACTTCGTGATCTGCCCACCTCAACCTCCCAAAGAGCTGGGATTACAG
GTGTGAGCCATTGCACCCGGCCCAAAAAATAAATGTAAAAAGAAAAAAAAAAAGGAGACACACT
TCACATATCTTAAAAATTACCCACTTTAAAGTGTACAATTCAATGCTTTCAGTATAGTCACAGAGT
TGTGTAGTCATCACCGCTATCTAATTCCAGAACATTGGATCACCCCAAAAAGAAGCCCCAAAGT
CATCATCAATCCCCTGCCAGCCCCCAGCAACTGATAACTGGCTTCCTGACTCTATGGATTTGCCT
GTCCTGGGTGGTTTGTGTCAGTGGAGTCACACACTGTGTGGCCTCTTGGGCCTGGCTGCTTTCTC
AGCATCACATTTCACTGTTCCTGCACATTGGGGCATAAATTGGTGCTTCCTTCCTTTTGAAGGCT
GAGTAACGTGTCACGCTGTGGATACACTACAGTTTGTTTATCTACTCATCAGCCAGTGGGGATTT
GGGCTGTTTCCACTTTTGGACTCTTTTGAATACCCTCCCTTCCTTTTGAGTGGAGAAAAAAGGGC
AAGACATTCCCTCTGCAAAATTGCACACAAAATCTTTTTTAAAATTATTCTTTTTATATTTTGGCAT
TGTAACTACTACTAACCATGCTGTCATCAACGGCTCAAAGAGGACTTTCCCTGGGGACTTTCCTC
TGCCCCTGCTGGGTATAGGGAGGAGCACTAAAGTTGCCCTGAGGGCCAGGCACGGTGGCTCAT
GACCGTAATCCCAGCACTTTGGGAGGCCAAGGTAGGTGGATCACCTGAGGTCAGGAGTTCGAG
ACCAACCTGGCCAACATGGTGAAACCCCATGTCTACTAAAAACACAAAAATTAGCTGGGCGTG
GTGGTGCGCACGTGTAGTCCCAGCTACTCAGGAACCTGAGGCAAGAGAATTGCTTGAACCGGG
GAGGCAGAGATTGCAGTGAGCTGAGATCAAGCCACTGCACTCCAGCCTGGGTGACAGAGCGA
GACTCCGTCTCAAAAAAAGAAAAAAACTGTTCTGAGGTAGGATTGGCAGATTGGGGGTGGGGG
AAGCCAGTGGCCCCTACTTTTCTGTCCCCTCCTAACACGCTCACCCACCCCCTGATGCTGTTGCT
GGACAGGAGGGCCACCCACCGCAGCAGCCGTGAGTTGATGACACTCTGCCAGCCTGGCCTTGT
CCTCCCTGGCCCCCCGCTTATGTCCTGATTGGAATGGGGAAGCAAGAACAACCACACTTGAACC
ACCCACTGTCTCCTTTCCTTCATGCACATTCAGCCATTCATTCAATATTCATTCAATACCGTGGTA
GACATGGACACCCCTCCCCCCACAGAGAGATTATTGTCATGGGAGAATACAGACAGTACATGT
CAGATTATTTTTTTCTCTTCCTGAACTTGCAAAACTTTTCTGGGGTGCAAGAGAGATGGGCAGGT
GATTGTGGGCAAGAACGATTCTACAGGAATGGGAGAGACCCAGAAAAGCTAGAGACAGTGTCA
GCATTCTAATTATTATTATTGGTTTTGTTTTTGTTGTTTTTTTTGAGACACAATCTTGCCCTGTTGCC
CAGGCTGGAGTGCAGTGGTGCGATCTCGGCTCACTGCAACCTCCGCCTCCCCGGTTCAAGCGA
TCCTCCTGCCTCAGCCTCCTGAGTAGCTGAGATCACTCCCGGCTAATTTTTTTGCATTTTTAGTAG
AGATGGGATTTCACCATGTTGGCCAGGCTGGTCGCAAACTCCTGACCTCAAGTGATCCGCCCCG
CCTTGGCCTCCCAGTGCTGAGATTGCCGCATCCTAATTATATCATTGTATCTTCTTTTTAAAGATT
AAAAGACAGACGGCAACACTGGGGTTGCAGAGCTCAGGAGCCAGTCCTGGCCCCCTGCCTCCT
CCCTCATGAGGTTCCCCTGATCCCCAGGACATCGGCTTCCACATCTGCACATCTGGGAGACAAA
TGCACCATCAGGGAGGGCAAATCATACCTGGGAAGGGCCTGACTCCAGCAGTTTCAGCAGTGG
CTGAAAGATCATGGCTATTTTTGCCATCATTACAATGGAATGTCCACTCCCTCCAGCCACCTCCA
CAGCAATCAACACCGGTGTCCAGCCACTCTACATGGACGTGTGGGGAACAGAGCACAGGCCTC
TTGGCTTGGCTGTGGTCACCGGGAGGGAGCAGGGAGGGACAGCTGCCAAGAGGATGGGGGAG
GGAGCCTGCTACTCAGGAACGCTGGGGCAAGACATGAGACAAAGGGCTGCCTTCACCCACCA
GTAGGAGCCTGACCCTGAGCCCAAAGAAACTGCCCACCTGTGTGCTGACCCCAGGGCCTGAGG
ACAGGTCCCCAAGTGGACGTCTCAAAGAGAGTCCCACATTCCAGGGCACATGGGAACTATGGC
CTTCTGGCAGCTGGAGGGCAGCAGGAAGGGCACAGCTGTCTGAGACCCGTGTGGCTTCCCAGA

AAGAGGTGCCACCCCTGAGGAGGCACCGGTGCTCACGGACTCCTGGTTGCCAGTCTTGCCAGG
GCAAAAAGGCAAGCGAAGGAAACCCTCTCTTCGTGGGGCACTCTGCCTTGCAGTGTCAGGTGA
GATCCCGGGGGGAAAGCTGGCTTGAGCTCCTCTGGAATGTGCAGGGAGGACTCCGTCAGGCCC
GGCTCTGCCTCCAAGCTGGGCAGCAGGCGAGCCCCAGAGCTCCACCAGCCCCACTGGCAGAG
GAGGGCCCTCACGAGGCAGGAGCCCATGATTCATGCCCTGCTGGAATTTGTCGAGCATGCCGA
GATTTGCAGCTCCTCCGAGATTGGGTCCTGCCCTGATCAAAAGAAAAGAAGATGGATGAGGAC
ACTATAAATAGGCCAGAGCCACCCAGCTGGCCTCGGGGATGCAGGCGGTATGTGTTCTGGGCT
CTCACGAAGCTAAGAAACTCTAAATCCAAGGTGACATCGTGGCACACCGCCACCTGCTTCAGG
GGGACACAGGGGACAAGGAGGAGCTATTGGCAGAGCGCTCGGTTCCTGCAAGTTCCAACTTCT
GCTAAAATTGGTTAATTGGTTCCTTTGCATGATTCAGGGCACACAGGCTTCTCTGTCCACAAAAT
TTGCTCGCCTGCACACCCAGAGAAATGGACTTGGCTTGGTTTTTCTGAACTTTTGCCTCTGCGGA
ATCAGCAGTGGACGTGTGAGCTGGGGCCTCTGTCCCTGTCATGTGAGGAGGTCAGGCAGGACA
AGCAGGAAGCACTGGGCAATGAGTCAGGAGGCCTGGTGGGGAGCTTGGGTGAGTCTGTTTTCC
TCTCTGGACCTCAGTCTTCTCCTCTGGTTGATGTAATAAAATCCTACAAACTG

ID|55972|gene_id|ENSG00000231025|transcript_id|ENST00000421806 Homo sapiens lncRNA

GTGGCCTTGAAACCTTCACTGTATTTGGACAGATGCAACAGAAGCACGTCTCCCCCACTGT
CACCCCATCCCACAACAAAGTGGGAATCCCATCATGGGTATGGCTGTGCCAGGCATGGCATCG
GGTGTGCCCCATGCAGCCTACTTCTTGCATGCACAGTTGGTACATTTAGCTGTATGGCATCGGGT
GTGCCCCACGCAGCCTACTTCTTGCATGCACAGTTGGTACATTCAGCTGTACACTTAACAGCCTG
TACAGGAAAATGAGCCTTAGTTGTAGCCCCAAGGAAATCCAAACCCATGCTTGTGAGATATGGA
GAAACCACTGGTTGCCTAGAGAAAGCATTAAGGTGTATTTCCAGTTCCCCTTTCATGTTGGTGAA
AGACACCTATTCACACTCTAATTAATCATGGGAGATAAGCATGTTACCTTCTGCACCTTGACTTT
GCTCGTGAGCACCTGGCTTTCCTCTTCCTCCTTCTCCCCTCTCTCCCTGTCTCAGTGTGCATCTGT
GCAGATGTGGGCCAGTCCCCCTGTAACCAGTGGTTACAGAATGATACCATCTGTTCTAAGATGT
GGTCAGCTTTACTTCTCAGGAGTCCTGTCCTACAATTCAGAGCACCCCTAGATGGGATGGAACC
AGTGCTTTATGATCATGTTCCTCTTCTCTGTGCCCTGTCCTTCGGCACCCCCATGTTAGTCATGAT
CCTCATTGTGCAGGGCTGGCTGGACTGAGAATTGCAACTCCAGGACAGCAGGGAGCTTTGGGA
CTAGGCACGCAGGGGTTTTGTCATCCATTTGAACCATAATTTGCATATTTTCTTACGGCCTCCCTT
TGTCTTTTCTAAACAAATGAGGCAGACACTTGAACAGTAATGCTGGAGAATGTTTTTTCTAGGAG
ACTTTGTAAGCAAATCTGATTTTAAGATGAAATGTGAATGAGAAACTAGGTGCCAGGGAATTTA
GCACTGAACTAGACAGTCAAAACTTTTTTAACTTTGCAGGAAGAAATAAATTTTGCTCTCGTCTA
AATGTTCACAAGAAGTAATGTTGTAAATAGTTTTCTAAAAATATTTATTACTTGTGCTGTATACAA
ATTCATGTTCTGAGTGATGTTGGTTTGCATTGTAGTATTATAGAATATATTTTGAGCAGTGGACTT
CCTTTCATAACACAGTTCACACATGGAGAAAGAACAAAAACCAGCAGGTTGAGAGCTGTTTGG
GGCCTTTGATCTGTAATGTTACATCAAATCTGAGCTTTAACTGCCCCCTCTTCTCATCTGGTTATG
TTATATTATATATATATATTATATATATATATATACAGGTGTATGATAAACTGGTCAGTGGTCAGT
ACTCAGATCTGCAGAGCAGATTTCCAGATGTTCTTTCTGATGTGTTGCCCATAAGCAATAAGATC
CTAGGTAATAACATTTATTCCAGGGCCCACATTGGCCCTTGTCTCAGAGGATAGGGTGGGGGTA
GAACAGCTCTTGCTAAGACCTCTTGCCTTTGCTGGAGAGGTTGCTGTACTTGAATTTTTGCTTCTG
TTTGTCTGCACGCTCCTTCATGTCATAGAACTCTAGCAATGGATGAGAAATCTGCCAGCACCAG
ACACATCAAAATTGGGCACATAGATGGAAAATTTTGCAATTTTTCATGAATTTATAGGGTTTCCA
GAATCTAGTTGAGGCAAAGCTCATTTGGCTATAGAGTAAATGTAAGACTTGTTACAACAGAAATT
TAAGTGGCCAGTTCAATGTCCTTTGGCTATATTTGACCTACCTTTAAAACCTAGCCCATTTCATAT

CAGCCTCTTCTGTGCCTGGGCTTGAAATGTCTAAAGCTGCCTTCGTGTCTGGGATTACACCATGT
AGGTCAGTATAAAGAGGGCAGTCACTCCTCCATTTCTCCCAGCGTGTCCAGTTCAGCAGATTTCT
AAAGCTGTTAAGCAGCCTCTCTTTTTGACCGTCCTAAACTTTGTGAAAATAAATACCCCACACCC
AGATAACCATTAATAGAAGTAATGTAGTTTTTTAGGCTTTTGGAATATGTCTTCTCTTTTGTATTTA
TGATGGTGTTTGGAATTTTTCACTAGTGTATTTTTAGACTGAAGTGGGTGCATTAGGATAAAACTT
TCCCTTGCTGAGCCTGAAGGCAAGCAAGAGGGAAGAAATTGACCTGTATTGTATCATTGGGGA
GGCGACTACTTCTTGTGGTGGTAGGGGGTGGGGGGAATGGGGCATTGTAGATTATGTGGGGGT
ACAATAGAATTCTGGCCTGAGGCTCCCCAAGTCTTATTACCTTGTATGAAAAATCCCCATATTAG
CCTCACTTAATCTCAATAGAAGCCTGCTGTTTACCCTCAGGGAACAAGTAGTTTTCAAAATTGAG
CTCCTCCAAGGTCATTGGCCAATGCTGTGTGCAGGCATTGGTCATCTTTGTAAACTCATGGCATT
ATTTCCTCATATGCATTATGATACTAAGAAATTCAACCCCAACCTGTATGAAAAGCACAGCCCAG
GGCCCTAATTGAGGCTAGCATGAATGCCTTTGAGGGGCCAGGGGGCCATAGGAGGCCCATGCA
ATTTAACTGAAGCTCAGGTGCCACGTTGGATTTTTATTCTTATTAGGCCGCAACCTTTCTGAATGA
GTTTAGGGCAGGGGCTGCTGATCCGGCTGTGGATTCAGGGAGCTCTAAAATGAATCTCTACCGT
TCCTTTAAAGGTCTTTCTGGTTCCTCCCAGTGTTGTCATTGCTGAGGTCGATCTCGTAGTTGTTCA
GTTGTGCTCTTTTTGCTGTCTTGTGAGTCTTAGCCAGTGATCCAGCTGCCCAAGGACAGGGCCTC
CAGAGGGCCTAGCGCCCTCCTGGTACCAAGCGTGTGCTCCTGGCCTGTGTGACAGTGTCTTTCC
CGAAGCAGTGAAATGAGAAATTCTGCAAGTGGCCACCCGGAAGATCAGTCTGTAGCTCTTGGG
CCTGGATTTCTACTATGTTAGTGAGTGTAGGAATATACTGTGTCTTTAATGGATGAAAATTCAATG
TATGCTGTGAATTTGTTGGTTTGAAACATTGAAATTTTTCATTAGACAATGTTTACATACCTCACCT
GAAGAACCTTTGAGAGTGTATATATAAAATTTTAAAATATATTAAGTTGCTTTAAAACAATATGTA
TAGCTATAAAAGTTGGAGTTATTTTAACTTTGAATATGTACCAAGTCTCTTAAATATTGAAATCTT
GTAGACTTCTTGTGCTTCTGTGTTTTGCTTTCCTATCAGGCCATGTAGGAAACTGTGTTCTTGTTAT
TGGTAGAGGGGCCCTCACTGAAATCCAAGCTTGGAAGGATGTCCTGTGTTGTTTCAGATTCTCTT
GTTTGGTTTTGAAGGATGCGGTGTGGAGCCAGAGGATGTGTGGGAGGGGTTTCTCTAACACTGA
CTTCTATTCCATGAGCTTTTTCAAGGCGCTTATTTTATGGCAGCGTATTAAACTTCTTTGATATTCA
AGTG

ID|55996|gene_id|ENSG00000237311|transcript_id|ENST00000424241 Homo sapiens lncRNA

GCTTTTATCTCAGGTTGCCCAATGGATAAGTACATTACTTTTTAACTCTATGCCTGATTGACC
AAGGTAAAGCTGAAAGAGTCTCATCTGAACTATAACCCAAGTGGGTAAGCCAGTCCCTTGGCAC
TGCCCAGGAAGAAGATATGGATGGGAGGACTACTCCTTCTTGAATGCAGAGGATAAAAGTTATC
TGGGAGCTCTTTTACGGTATGGTTGTGCCTTTGAATATGAGCAAAGCTTCTCCTAGCTGCAGATA
TTTTCAGGAAGAGCAGAAACTGCACCACAGTGTTTTGGGTAGATAAGGCTTACCCTGGTGGGGA
GAGAGGTCCTGGAAGGGGGTGAGCCCAAAATCTATATGCTTTCCTGGGAGGGGACCTGGTGTT
TTAAATCCATTTTTAGAATTTTTTTTTTATTGCTGCAAGAACTGGAGAGAGATCAAATCCATTTTCA
TTTGTTTCCAATAGCCCTTTTTTGCTGCTGCTGCTGCTGCTGCTGCTGCTGCTGTTTGTTCTGTTCC
TCCTCCTCCTCCTCTTTCTCGTCCTTTTTTTCCTTCTTCCTTCTCTTTTTCTTCTGGCTTTTGCAACTT
AGAATGCACATGGTGGCTAGATACAGTTAAAAACCCAGTTCCATGCTCAGGCTCATCCTAGCAC
TGGTTCTAATATCTATTCCAGATTGTTGGAGAAGATAGGAGAAAGTGAAGAAGGGAAGAAGAG
AAAAAAACAGAGAAGAAAGAAGGAAAAGAAGATAGAGGGACAAGTAGAAGAAGGAAAGGAA
AGGAAAAGCAAGGAAAAAGGATAACCTGTCCTCTTTGACTAGTAGGCTCACCAACTCTTCTTTA
CCATGGAAGGGATTTCAGTAGGACCAAAGGCTTTTCCCATGAAATAGTATTTTATCTTGATAAGA
ACCTAGCACATTGGTTACCAACCTCCTTAAATTGATTCCCTCAAAAGGAAGAAAGTGTATCTGGA

AATCCTCTGAAACTTCCAGAGTCCCCCAAGCCCAGTGCTATTTCTACCAGGGGAGAAAGCAGA
GCTCTTTGTATATATGGGTAGGAGCAAAGTAAGATTCTCCTCCCCCTTTCCCCACAACCCGAAGC
AGAAGTTTCTCCTGAGATTTCAACCAGTAAAATCACCATATAATAGATTAGGCATCCTGAGAAGT
TCTTAATGGAACGAGAGCTCTCACTAATCAAAGCAGGAAAGAACAGGGCCTTGAAGAAATCAG
AAATCTAATTAAGGTGGAATTTCAAGAGATCTGACTGAATGTTAAATAAGTTGTAGAAGCCTCAT
CAAAAGGATTAACTCAGCTGCAGAGACAACGC

ID|56237|gene_id|ENSG00000249639|transcript_id|ENST00000510230 Homo sapiens lncRNA

CTCACAATGGCCAGCTTTGTTCCTGCATGGGTTTGAGGAACACAAACTCTGCTATGTCCAC
CAGCACCCTGTGGCCCAGGACCTGCCATCATCTTCAACAGGAACCAAGGGTTGAGTGGATTTG
AATAACTAATTATACATTCTCTGGGTACCAGGCTTTTGCTCACATTATTTCTCTCCTAAGAATGCA
TCTTCCATCCCAAACTCTGTGTCCAAATACTGCCCACGGTTCAAGGCCCTGTTCAAGTGTTACCT
CTCCTGTGAAGCTCCCTTCCCTGGGGAAGGTGACCTTTCGCTGCCTTCTGACTCCCACAACAGG
GGGAATCCCCCAGGACACCTGGCCAACCTTACTCTCCATTCATTCCATCTTTGCATTTCATGCCC
AAGTTCTGTGGGCCCTTGTCCTGTCTTCCCTGAGTTCCTCTCTTCCTGCCCCTGAGGCCTCCCAA
GGGCTGGGTATGGTCTGGGCTGTGGTTCTTGCTGCCCCATGTTCCACTGCCCCATACAAATAGG
GCCTGGCCACCATAAATGTCCAGGGGCACATTTGCCGAGTGGCTAAATGAGAAGCCCACACAC
ACACATGGTGAAATCACCCAGAGAGACCACAAATGGGATCATGTCTCAAAGGCTGTGGGACAG
CGAGCCTGTTGCAGGTGGCTTGAACTCAATGTCCCTCCAGGTCTTGAACCTGCTGCCCCTCCTG
CCTTTTAGATGCAGTAGGTTAATGGCTGAGACCTTTTAGTCTGATAGTGAGGGCACCCTTTCACT
ATCAGAGGCCTGAGGGCACCCCCTTTCCCCTAAGGCACAGACACACATATGCCTCTACCCACA
CCCCATCTGGCCATAGTCTGCCGAAAGCCTTTCTTTTTTTTCTTTTTTTTTTTTTTCTGAGATGGAGT
CTCACACTGTCACCCAGGCTGGAGTATAGCGGCATGATCTTGGCTCATGGTAGCCTCCGTCTTC
CAGGTTCAAGGGATTCTCCTGCCTCAGCCTCCCCAGTAGCTGGGATTACAGGTGCCCGCCACCA
TGCCCAGCTAATTTTTGTATTTTCAGTAGAGACGGGGTTTCGCCATGTTGGCCAGGCTGGTCTCG
AACTCCTGACTTTAGGTGATCCGCCCGCCCCGACCTTCCAAAGTGCTGGGATTACAGGCGTAAG
CCACCACGCCCAGCCAGAAAGCCTTTTCTCGAGGTATGCGTTACAGGCTGAGCCCAGGACAGG
TGGGCTGGGCAACCCATGCTGTCAGGCCTGGCTGGAAACTCAAGAGTTCACTCCCAGAAACTA
ATATCACGGCACAGAGACCCTGAAAGAATCCCTCAGGAAGAAAGATTCACAGTTGCTGTGTGG
CCTTACTCTCTGCTCCAACCAGAGCTAACAGAAATCTCCCACTAAAGAGGAGCCCATCCACGAC
AGGCTGTCCCGTGTCTTTTGGATCCCAGGTGTTCTGGGGAAACAGAAACCCAGCCCTACAAATC
CAGCCTTTGGGACGGGGCAGGAGGAAGACACTCCAGAATGTGTGTCTCAGGGGAGCCCTCCTC
CCAGCCCCACCAGGCCCTCCCTTCTCCCCATGGGTTCAAGCCCCTCTGGCTTCAACTTACCTTAT
TGCCGGGGCACATCTGGCTCTAGTCCTGCAGCTGTGAGCTCTTGAGAGGGAGTAGGCTGGTTTG
GGTAGGGCTGGGAGGGCTCCTATGGCTGCAGTTCAGGAACGTGGCAGAACATACTTCAGTTTT
GTTGCATTCTTCCTTTTTCCCTTTAGGAAAGAAAATAGAAGTGTAATTTTTCATTGCAGAAGTGCA
ATTCCACTGCTGAGAGTTTGAGAAACCCCAGCGTGGGGCCCCTTCTGAGACAGATGGCCTGCCT
TCCTGGGTTTCAGTTCTGCCAGCCTCTCCTACCCCACCCCCAGGGACCTTAGGACCTGCACCCA
GGGTATCTGCAAAGTTCCTCTCCACCTGCCAGTAGGATGGGGTCAAAGCAGCCCAGGCATGTTA
GAAAAGAAGAGCTTTTTGGGACAGATGGCTACGGGACAGGAGAGTCCCCTGAGTGTGGGAGA
ATGAGGAGAGCGTTCTGGCTGGATAGAGGTTGGCCAAGGAAACTGTAGGCAACATGGCACTTC
CCTGAGGGTCTCAGGAGAGGGCAGGGCCTGGGGACGGGGAGGATGGAGAGGGCAGTAAATG
GCCACTCATAGAACATGATCTCTCCTGCAGAACAGTGAGAAGGTGGTCTCTCATGCCCACTCCC
CATCGCCCGAGCTCTGCCTATTTGCATGCAGTTTGGAAGGGGTGAAGATGGTCGCTTGTCCAGC

ACTGCGGGCTCTGGCTGCCCTGGAATGTGAAGGTGCTAACCCTTCACGTAGCCCAGCATTTCAT
AGTCCACCAGTGTGTTCAAGTCACCTGCGTTATCTCCTAGGAGTTTTATAGTTCTATACGTACATT
TTAATTTTTCTGACATGTGTAAGGTTTGTGTCTAGATTTTTTTTTTTTTTGGCTTATAGGTGTTCAGT
TGTCTCAGCACCATTTTCCGAAATGACCATGTTTTTTTTCCTTCAGTGTTTTCTCTTTACTCCTTTGT
CAAAGATTGGTTGACTGTATTCATGTGGGTTTATTTCTGGGCTCTCTACTTTGTATCATTGATCTAT
TTGTCTATTTTTTCACCAATACCTTACTGTCTTGATTGCTGCAGCTCTGTAGTAAGTCTTGAAGTTG
GGTACTGCCAGTCCTTCAATTCTGTTCTTCTCTTTCAATTTTGTCTTGGCTCTTTTGAGTTGTCTGT
CTATATAAATGTTAGAATCAGTTTGTTAATAGCCACAAAATAACTCGCTGGGATTTTGACGAGAC
TTCATCAAATCTATCAATAAAGGAGGGAAGAACTGACATCCTGACAGTATTGAATCTTCCTATTC
ATGGACATGGGATATCTCTCTATTTGTTCAGTTCTTTGATTTCCTTCATCATAGTTTCATGGGTTTC
CTCATATAGATCTTGTATATGTTTTGTTAGATTTATACAGAAATATTTCTTTTTTGAATATTAATGG
AAATGGCATTGTGTTTTTTATTTCAAATTCCACTTGTTCATTGCTGGCATATAGGAAAGTAATTGA
TTTTATGTATTAATCTTGTATCCTGCAATCGTCCGTTAATCACTTACTAGTTCCAGGAGCTTTTTTG
TCAATTCCTTTGGATTTTCTACATGGACAATTACGTCATCTGCAAATAAAGACAGTTTTTTTCTTCC
TTCCCAATATGTATACCTTTTTTTCCTTTTTAATCTTACTTCATTGGTTAAAACTTTTTGGTTAAACG
ATGTTGAAAAAGAATAGTGAGAGGAAATGTCCTTGTCTTGCTCCTGATCTTAGTGGGAAAATTTC
TGGTTTCTTATCAGTAAGTATGATGTTAACTGCAGGATTTTTGCAAATGTTCTTTTTCAAATTGAG
GATGTTTTCATTTATTCCTGGTTTGTTGAAGATATTTTGCTCTAAAGTAACCGCCAACCTAGAATT
CCATGCCCAGATAAAGAATCCTTCAAAAATAAAGATGACATAAAGTCATTTTCACACAAAAACT
GAAGAATTTGCCAACAGTACACCTACTCTAAAGTAAATTCTAGTGTTCTTCAGGCAGAAGAAAA
GTAATCCCAAATGAAAGCATAGAAATGTAGAAGAAAATGAAGAGCAATGGAAATGGATATATT
CATATAAATCAAAGTGAATATTAACCATATGAAAAACAATAATAATGACATTTTATTTTTGAAACT
TATTTTAGTTTCAGGGGTACATGTGAAGGTTTGTTACATATGTAAACTTATGTCATGGGGGTTTGT
TTTACAGATTATTTCATCACCCAGGTATTAAGCCCCGTACCCAATAGTAATCTTTTCTGCTCCTCT
CCCTCCTCCCACCCCCCACCCTCAAGTAGACCCCAGTGTCTGTCGTTTCCTTCTTTGTGTTCATGA
GTTCTCATCATTTAGCTCCCACTTATAAGTGAGAACATGTAGTGTTTGGTTTTCTGTTCCTGTGTTA
GTTTGCTAGTAGGATGATGACATTTTAAAATGTATTTAGAATATATGTATAATTAAAGTATATGAC
AACAATAACATCAAAAGTGGGAGGGGTAAAAATGTAATTATAGTGTCCCAAGGTCCTTGCATTG
TCCAGGATGCAATAAAACTACAATTTAAGGTAGACACTAATAAGTCAAAGGTGCATGATATAAT
CTCTAGGATAATCAGTAAAAGAATAATAATGTCTTAGTGACAAGCTAATAGATGGGAAGTGGAG
TAATACATTAGCAATGACAATGAAGGCTGAGAAGCCTTGGAAAGGCACAGTGGAACAGGGAG
GACATCCAAGTAGGGAGGATGAGGACAGCCCCAGACCAGGCCTGCACCTGGGTTGGGTAAAA
TTGAAGGGGAGAAGAAGGGCTCCAGCACTGGCTCTTCCTGGGCAGATACTGACCCATTCCAGT
AGCTCACCTCCTGCCTTCTTCTAGGCTGAGGCACTTGGGACCCTTCAGCATGGTGGCCTTGGAG
GCTGTTTCCTCTAAGGGTCTCTAAGTCTGCTCTTCTCCGGGCGGTGGGCTGCTACTCTGGAGGCC
TCACTGGTTACTCTTGGGCCTTTTCTGACCACCCTGAGATCCTCAGGGCCAGATGCTCAAATAAC
TGACCTGGAAGCTCAGCGAAGGGAAGTGACCAGCCAAGGTTACACAGCAAATCTGTAGCACAG
CTGGGTGAGGACTTAGGTGAGCTGACTTCCAGTCCCCTGGCCTAGCCACAATACACCTCCATGG
TCCAGATAGCTTGATAGCTGTGCCACATACTCCCTCTGGGTCATACTTGGGGCCAGTCATATGG
GTCTGGAAACAAATGTGGCTCCTGGTCCCTCGATAGCAAGCTAAGCAGAGAGGTGGGGGATCT
GGGGGGAGGGCAGTGAGGGGCAGGGCTAGCCCCCAGAACCACAAGATCTTTGGTGGCAGACC
AAGCAAGACAGGTGACTGAAGCTCAGTGAAGGCCCCATCATGGTCTTTACATCTCTCCAATTGC
CTTTTCTTGTATTCTTCCCCCATCTCCTGTACCTTGGCTCTTCCTGGTACTGGGGAAGGGTAGGGA
GCAAAGCTCAGATCACTGAGAAAATCCTCTCCTGACAATTCTTGGGGACTTGCTATGACTCCTTC

TCTAGAAACCTATGGAAGGCAGTAGTAACCTAAAGGAGTAGTTCTAAGAGCATGCCAGACCTG
CTGCTTGCTCTGTGCAGGAGAGAAGCCAAGTGCAGGCAAGGTAGCTAGAATGATCAAAAACCC
GAATGGCCACTCCAAGGTGGGAGAGAAGCCATGTGTAGGCTAGGCAGCTAAACTTGATCCCAG
GCCTCTTGCCGGCACCACACCCCTACTCAGGGCAGTGATAGACAGATGAATGACCGAAAACCC
AAATGGCTACATCAAGGCACTTCAAGGGCTGCAAAGGCACATAGATGGGGCTGAGCCTACTGT
GCGAAGGTGGCTGGCCATGCTCCAGGGCCTTTCCTCTCATCAGTCTCCCTGGTCCAAAGACCAC
CTGGCCTGCTGAGCATCCCTAAACCCTGGGCAAGGGCCTCATCTTCAGGGCTCCAGTCTGGGCA
AGACCTCAGTGATCCCCAGTTATTTCTGCCCACCAGCAGAGTAAGGTAGTGGGCAGAAGCCTG
GTAGGGAGCTTTGTCATCTCTAGATATGTGACCTCAGGCAAGTCCTATCTCTTCCCTGGGCCTAT
TTTCCTCCCACTGCCTTCTGTTGTAATCGTGAGTCATGAGGGGAGGGCTATGTCCGTGCTCACAG
CCCTGGCGTGGGAGGGTGACTGGCGAGAGGACATGTGTTCATGGTGCCTGTGTCCTTCATAACT
TGGGTCACAGCAGTTTCCAACAAGAAAGAGAACTGAAACAACCAGGGAATTCCCTGGCAAGGA
GAAGTCCTGCCCAGTCTGCATCTTTCAGAAGCCAGGAGGAATGCCCCAGGTTTGTGGGTAGGA
GTATGCATACACAGTGCTGGTTGTTGGCTTTCCGGAGGCAGATGGGAGCTGGAAGTCTCACGAA
GGGAGAGTAAGAGCCCGAAGGGTAAGAGCTGGGGAAGTGCCGGTTTCACTCAGCCTGGCTCC
CTTGGCTAGGACAGCCTCTGGTTGGGTGTTGGGCATCTGGTGTTGGGATCCTGGGACATATTAC
ATCAGTCAGCAAATGTCCCCCATCATGTGCTCAGGGAGGGCTCAGGGACAGAACAATTGCCTC
CCCAGGCAACCCCTGAAACTCCCACCTCCAGGGCTTTCTCCAACATTAGACCAGTGAGGCTTCT
GCAGCCAGACGGACTCAGGTCTGAGGCTTGGCTCCTACGTGCAACAACTCAAGCAAGTTATGC
AGCCTTTCTGCACCTCAGTTTTCTGCTCTGTAAAATGGGGACAAATTGCTGTTGTTGTGAGGACT
CAATGAGATATGTGGAAAGTGCCAAGCACGCGGTGAGAATTGATAAGTGAGAACTGCTCTTAG
TAGAACCCTTGATGGTAGAGCAAAAATGCACCCTGGGGATACGGGCTGTCAGAGGAGACTTAA
CAACTGCGCACACAAAACCTAAGTTTTCAGACAGCTCCAGTTTTTGCTTAAAATAAGCATGGCTT
TAGAGTTGAATTCCTAATGTGCATGAGTTTCCTATAAATTCCATTTTATATTATAATAAAGTAAAA
GGAAGTCTCCAAGTAATTGCTGGGACCTGTATCCTTGACGTCCAGGAAGGCTCTCCATCTCACA
AATGAGGAAGCTGGGGCTCAGAGCAGGTAGTTGATTTGCTCAAGGTCATCCTTCTGGGAAGTTT
CGGAGACAGGATTTGAACACAGTCTCTGAGACTCTTGGGTTTAACCATGAAGCTTTCAGGCCTC
AAAGGTAGGGTGTCTCTGGAGTCCAGCTGCCTGGCCTTCTCAAAATGTGTGCTCCAGCCCGCTG
GCCACCAGTGACACTCAGCGGAGAGTACCTGGTTCCAGCCCTAGCAAGCTAACGTGGCTGGCC
GCTGAGTCCCGATAGCCTGTGGCCACCAGGTGGCAGCATCCCTAAACCCACTGATCCTCTCTTG
GGGCCCAAGAGGGAGGATAGTTTGAAAGGTTGGGGAAGCCATGGCAGGAGGCTGCGCTGGAG
GAGGGAGAGGGGTCCTCCTTTCTCTGAGTCAAAATGCCCTGAATGGACCCCAGGTGAATCCAC
ACCCAATGCCCCTCCCACTCATAATGAGCATGGAGCTAAGAGTAGGAGTCAGAGCAGAAGCAG
GGGAAGCAGCCACTCAGCAGTGTGGATTGTTTGTAACAGTATAAATCTCAGGGAAGTGTTAATG
AGAGGATCGATGAATCTTCTATGCCATGAGCTCCCTGCCAGGCTTGGATTCTGCCATTTCCTGCC
CATGAACTGGATTCTGTGCCACTCGCTCACTGAGCTTTCCCAAGTATTCCTGGTGTCACAAATTT
TGGGGGCAAGGGTTATGTGATTATTATGTTTCTTTTTCCCCCACCATGGGCTTCTACGTAAAGCT
GTGGCCAGACCATCACCACCAAACACTGTTTCTAATGAGTTTGGGTCACAGACTCAAAGCCCAC
AGCAAAGTTCTGCGTTCCCAATATGGTAGGTGCACAAAACCCAGAGGGTCCACAGCATTTCTGG
ACTTTTGTATTGACTCCTCATTTAAAACTCTTTCCAGAAAGCCGATTCATTACAATGCTGGCTAAT
ACCCACAGGAACACTGAACTCCACTGTGTGGGAGCTTGAGGGGCCTGTGCTGCCCATGCCGAG
TGTCCACCATCTCACACTGCTTCTAACACCTTCAAGCCATCTCCTGAATCTGGAATAGTCATCAT
GAGCTGGAGGCTTCCTCAGAAGAACCCTAAAATCAAGGGGGATTCTGGTGCCAAGAAAAATAG
CCCAACCCAGCTTCACAGCTGTGAGCCTCCAATGCGCCCCAGGAAGCTGCCATCACCATGGGT

AGCGCGACCTGGCTTGATTTTGCCAAAACAAAGGAAAATTCCTCCAGCAATTTCTGTGAATTTGT
TTTGCAAAACTGAATTGTCTGCTTTTAATTCCCCTATACCAATTAA

ID|56319|gene_id|ENSG00000253982|transcript_id|ENST00000517959 Homo sapiens lncRNA

CTTGACAGCAGCTCCGACGGCACCTCTGTGGCTCTGCAGGAGCCGCCTCCCCGCCTCGTG
GGCACACAGGTGAGCTGACCGTACTTGCCCAGCCGGTCTGAGGGAAGCGAGGTCAGGGGCGA
CGGCGCCGCCTCCGCCTCCGCCTGTCCCCTCCCTCCGTCCGGCATCCGCGCCGCTCAAGGACT
CCAAAGTCAGCGTCGCGGTGAGGAGCCCCCCACGCCCAACCCGCGCAGGCAGGACCCCGGGC
TGCGGGGCTCGAAAGTCCCCCACAAGCTGCTGTCCGGGAACCGCACGGCCCGGGGTGCCGCA
CGCCCACCGGAAGGCAGGGCTGGGACCGCGGACGCCAGGAGCCACCCCGGCCCCTCACCCG
GGCTCCCCCTCACGTGGGCTCCCGCGCGCCCATCTGGGTTCCCCCTCAGCTGAGTTCCCGGCCC
CTCACCTGGGATCCCCGGTCCCCTCACCCGGGCTTCCCAGCCCCCTCGCCTGGGCTCCACGCG
CGCTCACCTGGGCTGCCCCTCACCTGGGTTCCCGGCCCCTAACCCGGGCACCCCGCGCGCTCA
CCTGGGCTGCCCCTCACCTGGGTTCCCGGCCCTCACCCGGGCTCCCTGAGCGCTCACCTGGGCT
GCCACTCAGCCGGGCTCCCTGCGCTTGGCGCCGCCCCGCCCCTGCAGGACTCCCGGCAGTCAC
TGACCTCATTGCCGCCCAGTCACGACTGGCCGGCCTCAAACACCGCCCGCGCACGCGCGTTCG
CACGCGCGTGCGCACGCGTTCTGCCCCCCTGGGGGCGTCGCTACGAGAGGCCCCGCCTAGGC
GCTACGACGGCGGCGGGCGCGGGAGGGGCGGGGCGGCGGGCGCGGGAGGGGCGCGGCGGG
GGGCGGGGCGCGGCGCGGGGGGCGGGGCGCGGCGGGGGGCGGGGCGCGGCGGGGGGCGG
GGCGCGGCGGGGGGCGGGGCGCGGCGGGGGGCGGGGCGCGGCGGGGGGCGGGGCGCGGC
GGGGGGCGGGGCGCGGCGGGGGGCGGGGCGCGGCGGGGGGCGGGGCGCGGCGGGGGGCG
GGGCGCGGCGGGGGGCGGGGCGCGGCGGGGGGCGGGGCGCGGCGGGGGGCGGGGCGCGG
CGCGGCGGGCGCTGTGGGGCGGCGGGGTGGACGCGCTGAACCACGGCCGGCGGGAGGGGCG
GGGCCGGGCTGAACCATGGCGGGCGGCGGCATGGGGGCGGTGGGGCTCCGCGGGCAGCCTG
GTTGCGCTGCACCCGGACGGGGCCGAGGGGACCTGAAAGCTGCGGCAGAGCCTGACCGCGCC
GTTCTCCAGAAGAGCCCCGGCCGCGGCTGAGTCGCGCTCCGGGTGTGGACGGAGCCGGAGCC
TCCCCGGGGCTCGGTGAGGACACAGGGCCCAAGCCCCGGACCTTCAAGTCTTGACCGAGCGC
ATCCCCGGCCCTTCTGCGCCCACACCTGAGTTTTTGTCTGTAGGAGTTTCCGCAGTTTGCTAAGG
TTGTTATTCGAGTTACTTATGTTTCTTCTCTCTAAGGTTGTTATTCGAGTTACTTATGTTTCTTCTGT
CTAATGTGTTTTGTTCCCTCTTTAGAATCGACATCTAGAGGAGTACTCACCACTTAATTGATGATC
AATACACGTTCCTTGAAAGAACCGATAGCATGCGATCGATACAGAGCATCTGCACTGGGATTGT
CCGCCCCGCCTGTCAGGGAGGCCTCGGGGGCTAATGGAGCTGAGGGTATAGTCGGCCTTGGAC
CCTCGCTTTTTTCCTTTCCTGTACTATGAAGATAACTGCCTTATCTCCCCCTCAGGACTGTTTTGG
GGATTAAATAGGACGTTTAAATTACCAACGCTCTTTGAAATAAACATTTCAAAATTATTATAACC
CTTTAGTACATGTGGGAAAACGTTGACATTCATGTTCAAGTTTATTTTTCTGCTGGCTCATTTAGG
TTTGTTGAAGCCTTGAGTCCCTAGGATTGTCTAAGAACATGGGTAAATGTGTGTGGAGGCCTCC
CTGGAGGCCAGAGAACTGCCAGCAGCCTTCATGATGTTAGGGGAACAGGAACCTAGGAGAGC
CAGGGTGACATCGTTTTAAAATCAACAACCCATCTTGGCGGGCGCGGTGGCTCAAGCCTGTAAT
CCCAGCAGTTTGGGAGAAAAAAATTAGCCGGGTGTGGTGGTGGGCACCTGTAATCTCAGCTACT
CAGGAGGCTGAAGCAGAATTGCTTGAACCCAGGAGGCAGAGGTTGCAGTGAGCTGAGATCAC
GCCATGGCACTCTAGCCTGGGCAACAAGAATGAAACTCTGTCTCAAAAAAATAAAAATAAAATA
AAATAAACTCCATCTTAAAACCAGTGAAACCACCATTGCAAAATTATAACTGAGACAGTGAGAG
ATCTGACCTAACCAACTCCATCCTACTTTTAACCTCCAA

ID|56339|gene_id|ENSG00000253982|transcript_id|ENST00000518481 Homo sapiens

lncRNA

GCGGTGGGGCTCCGCGGGCAGCCTGGTTGCGCTGCACCCGGACGGGGCCGAGGGGACCTGAAAGCTGCGGCAGAGCCTGACCGCGCCGTTCTCCAGAAGAGCCCCGGCCGCGGCTGAGTCGCGCTCCGGGTGTGGACGGAGCCGGAGCCTCCCCGGGGCTCGGTGAGGACACAGGGCCCAAGCCCCGGACCTTCAAGTCTTGACCGAGCGCATCCCCGGCCCTTCTGCGCCCACACCTGAGTTTTTGTCTGTAGGAGTTTCCGCAGTTTGCTAAGGTTGTTATTCGAGTTACTTATGTTTCTTCTCTCTAAGGTTGTTATTCGAGTTACTTATGTTTCTTCTGTCTAATGTGTTTTGTTCCCTCTTTAGAATCGACATCTAGAGGAGTACTCACCACTTAATTGATGATCAATACACGTTCCTTGAAAGAACCGATAGCATGCGATCGATACAGAGCATCTGCACTGGGATTGTCCGCCCCGCCTGTCAGGGAGGCCTCGGGGGCTAATGGAGCTGAGGGTATAGTCGGCCTTGGACCCTCGCTTTTTTCCTTTCCTGTACTATGAAGATAACTGCCTTATCTCCCCCTCAGGACTGTTTTGGGGATTAAATAGGACGTTTAAATTACCAACGCTCTTTGAAATAAACATTTCAAAATTATTATAACCCTTTA

ID|56258|gene_id|ENSG00000253982|transcript_id|ENST00000518822 Homo sapiens lncRNA

GCATCCGCGCCGCTCAAGGACTCCAAAGTCAGCGTCGCGGTGAGGAGCCCCCCACGCCCAACCCGCGCAGGCAGGACCCCGGGCTGCGGGGCTCGAAAGTCCCCCACAAGCTGCTGTCCGGGAACCGCACGGCCCGGGGTGCCGCACGCCCACCGGAAGGCAGGGCTGGGACCGCGGACGCCAGGAGCCACCCCGGCCCCTCACCCGGGCTCCCCCTCACGTGGGCTCCCGCGCGCCCATCTGGGTTCCCCCTCAGCTGAGTTCCCGGCCCCTCACCTGGGATCCCCGGTCCCCTCACCCGGGCTTCCCAGCCCCCTCGCCTGGGCTCCACGCGCGCTCACCTGGGCTGCCCCTCACCTGGGTTCCCGGCCCCTAACCCGGGCACCCCGCGCGCTCACCTGGGCTGCCCCTCACCTGGGTTCCCGGCCCTCACCCGGGCTCCCTGAGCGCTCACCTGGGCTGCCACTCAGCCGGGCTCCCTGCGCTTGGCGCCGCCCCGCCCCTGCAGGACTCCCGGCAGTCACTGACCTCATTGCCGCCCAGTCACGACTGGCCGGCCTCAAACACCGCCCGCGCACGCGCGTTCGCACGCGCGTGCGCACGCGTTCTGCCCCCCTGGGGGCGTCGCTACGAGAGGCCCCGCCTAGGCGCTACGACGGCGGCGGGCGCGGGAGGGGCGGGGCGGCGGGCGCGGGAGGGGCGCGGCGGGGGGCGGGGCGCGGCGCGGGGGGCGGGGCGCGGCGGGGGGCGGGGCGCGGCGGGGGGCGGGGCGCGGCGGGGGGCGGGGCGCGGCGGGGGGCGGGGCGCGGCGGCGCGGCGGGGGGCGGGGCGCGGCGGGGGGCGGGGCGCGGCGGGGGGCGGGGCGCGGCGGGGGGCGGGGCGCGGCGGGGGGCGGGGCGCGGCGGGGGGCGGGGCGCGGCGGGGGGCGGGGCGCGGCGCGGCGGGCGCTGTGGGGCGGCGGGGTGGACGCGCTGAACCACGGCCGGCGGGAGGGGCGGGGCCGGGCTGAACCATGGCGGGCGGCGGCATGGGGGCGGTGGGGCTCCGCGGGCAGCCTGGTTGCGCTGCACCCGGACGGGGCCGAGGGGACCTGAAAGCTGCGGCAGAGCCTGACCGCGCCGTTCTCCAGAAGAGCCCCGGCCGCGGCTGAGTCGCGCTCCGGGTGTGGACGGAGCCGGAGCCTCCCCGGGGCTCGGTGAGGACACAGGGCCCAAGCCCCGGACCTTCAAGTCTTGACCGAGCGCATCCCCGGCCCTTCTGCGCCCACACCTGAGTTTTTGTCTGTAGGAGTTTCCGCAGTTTGCTAAGGTTGTTATTCGAGTTACTTATGTTTCTTCTCTCTAAGGTTGTTATTCGAGTTACTTATGTTTCTTCTGTCTAATGTGTTTTGTTCCCTCTTTAGAATCGACATCTAGAGGAGTACTCACCACTTAATTGATGATCAATACACGTTCCTTGAAAGAACCGATAGCATGCGATCGATACAGAGCATCTGCACTGGGATTGTCCGCCCCGCCTGTCAGGGAGGCCTCGGGGGCTAATGGAGCTGAGGGTATAGTCGGCCTTGGACCCTCGCTTTTTTCCTTTCCTGTACTATGAAGATAACTGCCTTATCTCCCCCTCAGGACTGTTTTGGGGATTAAATAGGACGTTTAAATTACCAACGCTCTTTGAAATAAACATTTCAAAATTATTATAACCCTTTAGTACATGTGGGAAAACGTTGACATTCATGTTCAAGTTTATTTTTCTGCTGGCTCATTTAGGTTTGTTGAAGCCTTGAGTCCCTAGGATTGTCTAAGAACATGGGTAAATGTGTGTGGAGGCCTCCCTGGAGGCCAGAGAACTGCCAGCAGCCTTCATGATGT

TAGGGGAACAGGAACCTAGGAGAGCCAGGGTGACATCGTTTTAAAATCAACAACCCATCTTGG
CGGGCGCGGTGGCTCAAGCCTGTAATCCCAGCAGTTTGGGAGAAAAAAATTAGCCGGGTGTGG
TGGTGGGCACCTGTAATCTCAGCTACTCAGGAGGCTGAAGCAGAATTGCTTGAACCCAGGAGG
CAGAGGTTGCAGTGAGCTGAGATCACGCCATGGCACTCTAGCCTGGGCAACAAGAATGAAACT
CTGTCTCAAAAAAATAAAAATAAAATAAAATAAACTCCATCTTAAAACCAGTGAAACCACCATT
GCAAAATTATAACTGAGACAGTGAGAGATCTGACCTAACCAACTCCATCCTACTTTTAACCTCCA
AACTGTCCTTGTTCATTCCTGGGTGTAGGCCAAACTAACTTTGGAAGAAACTTAGTTTATGGTTTC
CCGAAACAAATCCTTTTCTTGTCTGG

ID|56270|gene_id|ENSG00000255224|transcript_id|ENST00000524499 Homo sapiens lncRNA

TGTAGGTGCACAAAACAAAGGGTTTATTGTAGGGCGAGAAGTACCGCGTGGATAATAAGG
TCGTCTGGGAAGGAGTGACGTCGGAGTAGAGCTGAAAGGAGAGGGAGCGAGTGGGGGAGTGC
CTAGCACGTTGAAGAGCCGCAAAGAGGAAGGAGGGCGCAGGGACAGAGGCCGATGAGAAAG
GCAGGACCACCGCGCAGCACACTAAGGACTTGCGTTTACTCCGAGTGAGATGCGGTGCTGAGC
ATCGGCTGTGTAGACTGAGGCGCGCCGGCCGTTGCCCCTCAAGTCCCCGAGCCAGCGCTCAGC
CCGGCTGCCCCGCAGCTCCCACGGCCACACGATTCCCCATCCCGCGCGCCCACTCGCCTCGTG
CGGGCCGTAGACCACAGCCAGTGCCTTGGTGTTGCCCTGCTCAATGTAGGCCGAGCCGTCAGC
CTGCGCGAACACGCCCATCCGCGCCTGGATCTTGCGCAGCTCCCCGGCGCGCCGCCCGTCCAC
CCGGTAGCCCTGGTCCGACAAGAGCTCCAGCCCCGCCATGCTGCCCGGCCGCCAGGTCCGCTC
TCTGCCTACTTCTCTGAGCCGCGGGAGAACTACAGCTCCCGGCGGCTCCGACCGCCCGGAATC
TACGGTTTCCGGAGGTCCGCCATCACTTCCGGTTCCGGGGAACTACAGCTCTCCGCGGCCCTGA
CCTCCGTGGATCTGCGGTTTCCGGCGTTCCGCTGTCACTTCCTGTCCTGGTGAACTGGAGTTCAC
CGCGGCCCCGACCTCCCCGGATCTACGGTTTCCGGCGGTCCGGCTCTACTTCTGTCTCCGGGGA
ACTGCGGCGGCGCTATCGGGGAAGCAGCGGCTGGGCCCGCAGCCGTTCCCGGAGCTGTGCCA
CGGGTGCCAGGAGGTGGGCGCCCGCCGAAGGAGGAATTGCCCAGTCACGCCCTCGTGACCAG
TGGTGCCTGCATTCAGCCCTTCCCCGCGCCCCAAGTGTGAAAGTGGAAGGATGAACCCAGTGG
GGTGCGACACTGACAGGAGAAGGCATTGAGCGGTCCTAGGAAAGTGACCAGGCTGGTGTCCC
AGGATGGTCCACACCTGGCACCTCTCCAGTCCTCTCATGCCCTCCCTTTACACCGAGCTTCCACA
CTGCAGTCAGGTGGGCCTCCCTTTTCCCTTTGAGGATGCAGATTTCCTCATCCTTTCCAGAGAGG
GGTTGTATGTCTGTTTATCTCTTGCCACTTAACCTCAGCGGCTTCAAATAATAAAAATCATTCTAT
TA

ID|56228|gene_id|ENSG00000239213|transcript_id|ENST00000461864 Homo sapiens lncRNA

GGCGGAGAGTAGAGAGCTAGAGGGTGAGAAGCGCTGGGCCGGACCAGAACTGTCTTAGC
TTTTCCCTCTGCGGCTGCTCTGGTTGGTTACTTTGAGCCTGCCTCCTCAGCGCCGCAGGAGAGAC
TTAACAGCAAAAATGACTGTGTCCTGAGTTCAGCCCCCACTGCTCTGGGAGCTTCCCCCTGGTTT
CTGCACCTGAAGAATGTCATCCCAGCCGAAGGTTCTTAGGAGCGGGGAGCGACAAAGAAAAAC
AAAGAAAAGCAGACCTGAAAATTGGGACCTGGGAGAAATGTAAGACATGTGCTTAATTTGAGG
CACAGACCAAAAGAAGCTGAGTCTGAGGGCCCTCTAAAAGTGTCCTCGTGCTTGGGCAGCTTA
CATTCTTGGCGGCAGTAGTGTTATGAAGAGGACCTTCGGAGCCAGGTCTGTCCATTCTTAGGGC
TCCAGCCACTGGCTGAGGCATCAGTAAGTTGGTTCTTCCCCTCGCCGGTGCTGCAGGCCCCCTT
CCCCGCCCCCGCGCCGTAACCTGCCTCTTTTTTTTTTTTTTTTTGAAACGAGTTTCGCTCTGTCGCC
AGGGTGGAGTGCAGTGGCACGATCTCGGCTCACTGCAACCTCCGCCTCCGGGTTCAAGCTATTC
TCCTGCCTCAGCCTCCCGAGTAGCTGGGATTACAGGCATGTACCACCACGCCCAGCTAATGTTT

TTGTATTTTTAGCAGTGACGGGGTTTCACCAGGATGGTCTCGATCTCTTGACCTCGTGATTCGCC
AGCCTCGGCCTCCCAAAGTGCTGGGATTACAGGCGTGAGCCACCACGCCCAGCCGCCTCATCT
CTTCTTAACCATCTTTGTGGCACAGTTTAGGATCGCTCCTCAGGGAAGCTTGCTCAGTTAGGTAC
CTTCCTTGGAGCACAGGTGTTTTCTCCATCCGAGCTATTGAAATGGTTTGCCTGTATGTCTACCTA
GATTGTAAACTCCGTTGAATCAGTAACCATGTCTGTTCAGTTTCTCCCTAGGGCCTTGCAGTAGG
GTAGATGGTCAGTAAATACTGTTTAAGGCATTAATACTTTTTATTTTAATCCTGGCCCTGGATATC
AGCCTCAAAAGTTTGCAGTTTTCTGCATTTAACTTCAAAATTATTTACGTGATTGGTAATGCATGC
ATGTGGTTGGCCTGTGCTGTAATTTAGATGTTTGTTCCCCTAAACCTCCTGTTGAAATTTGATCCC
CAATGTTGGAGGTGGGGACTAATGGGCAGTGTTGGGTCATGGAGGTGGTTCCTTCAGGAATAG
ATTAATGTCCTCCCTGGGGAGAGGGTAAATGAGTTCTCTTTATTTCCACCAGAGCTGGTTAAAAA
GAACGTCGCACTTCCTTTCTTCTTTTGCGTCCTCTCTGGCCATGTAATCTCTGCACACACCAGCTC
TCCTTTGCCTTCTGGCATGAGTAGAAGCAGTCTTAGGCTACCATCAGATGCAGATGCTCAGTCTT
GAACTTTTCCAGACATCAGAACCATAAGCCGAAAAACCTTTTCTATTTCTAAATTATCCAGCTTTG
GGTATTCCTTTCTAGCAACACTAAAGGGACTAAGACAGCCTGCCATATGTAGGATCACAAGGTC
AAGCACAAATTTACCTATTTATGCTGTACTATCCTCAGGTTGACTGGGACAAGTAGACCATGAAA
CTGGGCAACAGCTACTCTATGTCTACATTCTGTTATTAGCATCCAAAGTGAATAATGGCGGTGAA
TTTTGAGTTTATTCTAAGGGTTTAAATAACTGCATATTTTATCATGACTGGTATGGACTGAATTGC
ATCCCCCCCAAAAATTCATACTGAAGCCCTCACTCCTAATGTGACTATATAAATGTTCCTTAACTT
ACAAAGGGGTTATGTTCCAATAAACCCACCATAAACTGAAAATACCTTAAGTTGAAAAGATCTT
AAGTCAGAAATGCATTTAATGCTTCTAACCTACCAAACATCATAGCTTAGAGTAGCCTACCTAAA
ACATGCTCAGAATACTTACATTAGCTTTCAGTTAGGCAAAATCATCTAATACAAAGCTTATTTTTT
AATAAAGTATTGAGTATTTCATGTAATCTACTAAATACTGAATGAAAAACTGGATGGTTTTATGG
GTACTCAAAGTACAGATTCTAGAGTGCATATGGCTTTTGGACCATCATAAAGTCAAAAAAATCTT
AAGTCAGGGACCATCTCTAGTTGGAGCTAGGCCTTTAGGGAGGTAATAAAAATTAAATTAGGTC
ATAAGGGTGGGGCCCTAATCCCATAAGATTAATGTCTTTATAGAAAGTGGCACCAGAGAGCTCT
CTCCATTTGAGAACACAGCAAGCAGACAGACATCTGCAAGCCAGAGAGAGAGCCCTCACCCAA
AACTGACCATGCTGGCACCCTCTTTTGGACCTCCCAACCTACATAACTTTGAAAAAATAAACTTC
TGTTGTGTAAGCCACCCAATCTAGTATTTTATTATGGCAGCCCAAGCTAATACAATGGTTTTATTT
GTTGATACATTTTATAAAGTTTACAGACTGAGATATTATATTCATTTTTGAAGAAACTTGTCCAGA
GTTAAAACTGTTGCTGCAAAATCACCCATCAAACTGTTAGCACAGCGTGACTCAATTTTAATTCT
AATTACATATTTAACATACGATGGAGGCAGAAGGTGTGAATATAAATACAACAGGTAAAATACA
GAAATACAGTTCTTGCCTTCATGTAGCTTACCACCACCTTAAGTGGGGAAAATTAACCCAGCAA
AAAGTATGCTGAAGTCATACATTTAGGGTTTTTGTTTGTTTGTTTGTTTTTTGAGATGGAGTCTCGC
TTTTTTGCCCAGGTTGGAGTGCAGTGGCACCACCTCAGCTTATTGCAACCTCCACCTCCCGGGTT
CAAGCAATTCTCCTGCCTCAGCCTCCCAAGTTGCTGGGACTACAGGCACACACCACCACACCTG
GCTAATTTTTTGTATTTTTAGTAGAGACGGGGTTTCACAATGTTGGCCAGGCTTGTCTCAAACCCC
TGACCTCAGGTGATCCACCCACCTCAGCCTCCCAAAGTGTTGGGATTACAGGCGTGAGCCACA
GCACCCAGCTAGTTTATCCTCTTAATTTTCCTTCTTTAATCTTTCTCATCACTCATTCTTAACAGGA
ACTCTTTAAAAAAATTTAAGAAACCTCCAAAAATATATGCAAAAGAGGAGTCAAAGAAGAAAAA
TAAGCAAACAGCAATGAAAAAGGAAAGCATAGACTGGAAGGGCAGATAGGCAGAAAGGCATG
TATGTAATTGCACTTAGAAGGGATGCAGTACATGTTTTAATGGGGTGGGAGCTGTCCCAGAGAC
TTCCTTTCACACAGTCTGTCTCCCCAGCTTCCAAATATAAACATGCACATGGCTGCTGCTTCCTCT
CTTTCAGGGGAACTCAAACCAAAAACAGGAATCATCCTTGCTAATTAGTGCTGAGTGGATCAGG
GACTAACCAGAATCAGGCAACAAGAAAATGTAAGTCAAAACTTATGATCAGCCCTGGCAACAG

AGCTAGACCCTGTCTCTACAAAACAAAATTTTTTTTAATCAGCTGGGCATAGTGGCATGTGCCTG
TAGTCCCAGCCACTCTGGAGGCTGAGGTGGGAGGATGGCTTGAGCCCAGGAATTTGAGGCTGC
AATGAGCTATGATCACACCACATCTCAAAACAAAAACAAAACTTATCTAGGGACTACACGTGGA
GCTTTGGATATGGGCAGCTAATCTGTCTATGATTAAAAACACGCAGGTGGCTTACTTTATTAATC
CAAAATAAAAGAATTTGGGAACACAAGAAAGGGAAAAATGAGCCTCCATGTCAAGACCTTTCC
CAGAAACCTGATAGCTTATAGTTAGAAATTGTTATAGACAGTCCAGTGCAAGGAGGACATTAAG
CCACAGGGGAAAGGAGTTGTGTGGCCAGGTTAGTCAGGCAATTAACTAGAAAAAATCTGTCCA
CATAGAGGAGCCTGCATAATAAAAAGGTATTAAACGATAGTAGAATATTGCTAGAGCAGAAGA
TAGCTAGAAATATAATCAATAACCAGGTTATGGAAGTCAGGCACCTTAGAGTTTATCCTATAGG
CAATGAGAAGCCATTCAAAGGGTTTTTGTTTTTGTTTTCAGCACTTTAAAGATGTCATTCTCGTTTT
CCAGCCTCCATTATATCTGTGAGAAACTAGTTGTAATTCTTATCATTGTTTTCTTGTATGTCTTATG
CCTTTTTCTATGAATGCTTTTAAGATTTTGTTTATATTTATCAAAAGTTAACACTGATGTTTCTAGG
TGTCTTTTTCTATGAATGCTTATAAGATTTTCATTATATTTATCAAAAGTTACACTGTGATGTTTCAT
TACACTGTGATGTTTCATTACACTGTGATGTTTGTTTTGGTTTTGGTTTTGTATTTATCCTGCTTGG
GGTTCATTTCATTCCCTGGGTCTGTGGATTGCTGGTTTCTCATTTTCTTTTCTTTTCTTTTTTTTTTT
TATCAAATTTGGAAGACTTTTGTCCAGTATTTCTTCAAATTTTTTTTTCTGTCCCATTCTGTTTCTCC
TCTTCTTCTGGGACTACAATTACACTCATGCTAGACTTCTCAGTACTTAGTACAGGTCATTCAGG
CTCTGTTCATTCTTTTTTCAGTTTGTATAATTTCTATTGAGAGCCACTGTCTAGTAATCCCTGAAAA
AATCTAAATGTTGACATTTTCCCAAAGCACAGTACCCTGCTGATGGCTGTAGTTTCCTTTGGGGA
AGGTTTAGAGATATGTTAGTCAGGAAATCAGAAATTAGATGATATTACTACAGAGAGGATTTAG
TATTGAGTTTTCATTGACTGAAAAAGCAAAAACGGAACACTGAGGTTACCCAGAGGTGATAACT
GCAGGGAGCAGCTGTCAATGATAGATCTAGTGAAAATGGGGACTCAAGGTGGATTATCAGGTC
TCAGAAACTCAGACAAATGACCTGGCTGAGTATTGCTCAGACCCCTGAGAGGATGCTGTCTGGC
TGATCCTACTACTTCAGGGAAGGATATGATGAGGCTAGTTGTGGATGGGGAGGATGTCAAAAG
AAGCTCAAGGCTGAAATCAATTGCTGCTGCTGGAGCAAAGGGCTCTTGCTTGTTGCTGGGATAA
CAGTGACAGAAACTGCAAACAAATGGAAAGGAGGAAGTCCCTTCTTCCCTTCTGCCTTCCAGTC
TCCCTCTAATGCCCTTTATTGATGGAACCTAGTGAGACACCAGCTGACATAGAAAAAAATGTAG
TTTGCAGGGTTTGAGCCACAGCATTACAGAGAAATGTATAGAAGGGGGAATGTGGACCTGAGA
CAATAGTTTAATAAACAGTACCATATCATTTATTTGTGCTAAATTGGTAAGAATGGCCAAAATTA
GACTTAATGCCATCATATGTGCTTTTACAGACTTTTAGACCTTTTTGAAAAGAATCTCAGGATACC
ATGTCAATTTTCTGCTACATTATGATTACTGTTGGTGCCTTTTTCAGGAGTAAGGAGTACTTTTACT
GCAGCTCACACATACACTAGCCACAAAATCATAAACAAGGGCAGCCTTGGAAGAGATCTACAT
AACTGAGGATAAGCAGACCCTACCCCAACGTTAGGTCTCCAAACATCAGGGACAAAATGGCAT
GGGCTAGGTAGGACTGGCAAGAATCCACACTTCTCCCTATTTCTATTTCCTATGTATCGCCTAAT
GTGCTCATAATAAGGAGAAAACCAATTTCCTGTTCTAACTTTGGACAGCCACCCATTGTCATTAA
AAAACCACTTATTTTCCCTTCTTTCCTCTTTTCTTGCCTCAACCCAGGCTCCTTTACCCAGCCCTAT
GACTTCTAGTTTTCTAATGCAAGTAGATGACAAAACAGGGTTTGGTGTTCAACATTTCACCTTAT
AGTGTATTTTTCACAAATGAAGGAGTTTTGTTGAACATTTGTAGAACATTTCTAATTTTTGAACTTC
AGTTCCATCTGAAAGGGGTCATTTCTTTGAGTCTTCTATTATTTAGTAATAGCAGTTAACATTCTC
CTTCTACTACAATTTACCTGGAAAGACACCCATAAATACCAATAAAGATACCAGATTCATAACGT
ATTTCATATATTTAATATTTGTATTTGAATATATTAGACTAGAATAAATTTTCTGCATTTGTATATAT
TATATATTTTATAGCACTCTGTCAGTTATATAACACAAAAGACATTCATAGGTAAACATTAAAATG
GCAGTATACATAATAACTGAACATTAATGAGACAATAATAATCAGGTATTCATTTAATGGGAGA
GTCTAGTATTTCCTGGTAGTCCTGCTTTCTTAAATTCCTCCAGTAAAGTTTATAGCCAGCAAGGAC

AAAAACACTAATCATAATGATTACCCCTCCAAAAACATCATAGATGCTAGGAAATATGTGCAGC
ACGAGAAGCTGCAAGACCATAGCTACCACAATCTCCAAATGTTGTACTGTGCTAACCAAAGCTG
GATGGAATTTGTCCAAGGCATAATAAACTCCTAAGAATGCTGCAGTAGAACAGACACATATAGC
AATGAGATAACTCCAGGTTTCTCCATCTAATGGGATGATGGGTTCTTGAAGAATAAACATAGTA
GATATTCCCCAAATTGTCCCAGTCCAACCAAAAGTAAACAGTGCAGTCCACATGCTGATCTTCTC
CTTGATGGATCTGTATACTATCATTGAGAGAGCAGTGGTCAGTCCAGCCATCACAGTCATGGTG
TACCCAAAGGCTTCTTTCCAGGCATTTAACAAAGAATTGTCTTCATCAACAATGTTTGGGATCAT
GACAAGACAAACACCTAAGATGCTGCAAACAACTGTAGCCATGTCAACATAAGCCATTTTCTCA
TCTACGAGTAAAAAAGCCAAAATGGCACTGAAGACTGTAGTTGTGGCTCTCCACATAGTGGTCC
CATTGCTGGGAGGAACTATTGAAAATGATGTATAAGCACAAGTGATAGAAATGACATTGCATAC
ACCATAAAAGAAGAGTCGTAATCTGTATCCACTGGGTCCAAAGGGGGCCTCCTGATAGTAACAC
ACAACTAACACAGATAAGACCTGAAAAACAGAACGGATAAAAATCAGTTCTAGAGATGGAACT
TTAGACCGATCAGAAACAAGCCTAGTGATAAGAGCTACACATCCATGAGCCAAAGCAGATCCA
AACAGCACTATCCACATTTTTCGGGATTGAAAAATGTTTTTTTCTGCAAAGCTCTGGAATTGTCCA
ATCTCATTGATCATTGGGTCTTCTGTTGGTGGAGGTAGGGTATCCATGGTTCCAAAGAAAGCTCT
CCCTTTTTTTTTCATTTCACTCAGCAGACCTTTCTTTGGATTTTCCTCCATAAAATTTCCATAGCCTT
CATTGATTTCTTCATATCCATCATCGCCAGGCTGGGGATAATGAGAAGTATATTTCACCATCACT
GTGTTGGGATGTATTTTCACCCGTTTTTTAACTGGATATTTTCTGGAGGGAGAAGTATCCATTTCT
TCAGATAATCAATTCTATTTAAAGAAAAAAAATTGACAAAAAGACATTCATTTCATTGAAAATTTA
ATTTCTATTATAATCATATGCATCAATTCAATGATAGAAAGTAGAGGCTGTAACAGTAGTCCAGA
AAAAAAAGAAATTAGAGATGATCTGGCAATATGCCTTATCAGATGTATGGTACCTACTGGAATT
GTTTACCCTTTTTTCACTCTCATCTGAAGTCTGTCTCCATTTCTCTTTGCTTACTTGTTTAATTCAGC
TCTATCTTCTATCTTTATACCACAAACGGATTTTTCCATCTTTTCATTCCTACCTGTTTCTCCACCTA
TCCAACAAGCAAAACACAGAAAGATAAATAATAACTTAGGTCAAACCTTTCCTTCTCATTGGGTC
CATTTGCCTGTTATAAATTATTAGTTAAGTCCAAAGTATTTTGTATAATCAATTCTGTATAATACCA
GAATTCACCTTATAAATTATAGTGATTTTTAAACATTTATTCTGGACTCCCCATAAGTTTTGAGAT
ATAAAAATACACTGAAATTAGAACATAAATAACATGAATTTAGTAACACTCATGCATTGTTGGTA
GAAAGGTTAAATTGTACAACCTCTCTCGTGAACAGTTGGACCATATGTATTAAGATTTTAACTAT
CCTCTACTCAGCAATACCACTTTTAAGATTTTATGCTAAAGAGATGATCACAAGATGCTTTTTACA
TTCTGAATAAAAAAGGGGGGGAATAAGAATACGTATTTATATTTGTTTACATAACAAAGAAATTA
TATAAAGATGCCAAGAAAACTAATGGTGGTTCAGGTGACAGGAGGAACAAGAATGGAAGAAAA
CCTTTTTGTTATTTATTTTTTTACATTTTTATTTTTGAATTGTGATATTATCCAAAAATAGAATTAAA
ATGTTTATCTGTTACTTAAGAAAAAGAATATACCAAAAAATGTGGGGAAAATGTACATACAGGA
GAGTTCATTGTAGCTTACTACAATGAATGTTCATACAATGGAATACTATGCTATCTATTAAAAATG
ACAGTGTAGGCACGGTGGCTCACACCTGTAATCCCAGCATTTTGGGAGGCTGAGGCGGGCGGA
TCACTTGAGGCCAGAAGTTTGAGAGGAGTTGGCCAGCCTGGCCAACATGGTGAAATGCCATTTC
TACTAAAAATACTAAATTTAGCCGGGCCTGGTGGTATGTGTCTGTAATTCCAGCTACTCGGGAG
GCTGAGGCATGAGAATCACTTGAATTCAGGAAGCAGAGGTTGCAGTGAGCCAAGATTGTGCTA
CTGCACTCCAGCCTGGGTGACAGAGCGAGACTCTGTCTCAAAACAAAACAAAACAAAAACGAT
GTCCACTATATAATGTCAAGTGGGAAAAAAAGTTGAACTTACTGTAAGAATTAAATAAAATAAT
GCATATAAACAATTTGCCACAGTGCTGGCATATAGTAAACTCTCAATAAATGTTAGCAATAAATT
TGGCTTCCTTTCACCTATAACAACTTTATGAAACACTGGGTATCTTCTACAATATTATTATTTCTTT
TAGACCTTTATTTTTTCACTTGGTTCTTTCTTTCCTTCCTTTCAACAATTTCCTATTTCCCTCCCAAT
GATCCTCTCCTCCCGCTTAAAAAACAAAAAAGAGACCAGGCACGCTGGCTCATGCCTGTAATCC

CAGCACTTTGGGAGGCCGAGGTAGGCAGATCACCTGAGGTCAGGAGTTCGAGACCTGCCTGGC
CAACATGGTAAAATCCTGTCTCTACTAAAAATGCAAAAATTAGCTGGGAATGGTGGCAGGTGCC
TGTAATCCCAGCTACTCAGGAGGCTGAGGTAGGAGAACTGCTTGAACCCAGGAGACGGAGGTT
GCAGTGAGCCAAGATTGTGCCACTGCACTCCAGCATCAGCAACAGAGCAAGACTCCATCTCAA
AAAAAAAAAAAGAAAAAGAAAAAAAATCTTTTTTTTTCTTCCTCGTGACTCCCTGATTTCTTGCCA
AAAACTCATCGGTTCCAGGATCTTAAACAATGGCCCAGAAATAAAATCTCATTTTGTTCTTCGAC
ACCATCTGGGGAGCTAACTGTTCTATTCCTATTTACTCCTTTCTTTTCTAAAAGGCACCACCTTCA
GCTGGAAGAAGTCATGAAAAAAATATCACCTGTGCCTCCAAACCCTTCTATTTCCTAGCCTGGAT
GTCCCATTTGCAGTCCTTCTTCCTGCACACCACTATCAGGCTAATCTGACTGTTCTTTATTTCCAA
CTTTTGCCAGGACATGTCAAATCATAGCTTCAACAATCCCAAACTGAATAGTCTTGTTCTCCCAA
ATAGATTTTTTTCCCTAATGTCCTCATTTATGTCTGTGATACCACAATTTTTCCAGTTCCCCAAGCT
AGAAATCAGGGAATACTGTGAATTATCTTTTCTGCCATCTTACTTGCATTTGGTCACTAATCTTAC
TGTTTTTGTCTTTGAAATATTCCTGTTTTTCCCTTTCTCCTCCAATTCTATGTAACTTTTATAATTGT
GGTTTGGGTTATTGTGAATTCCCATCTAGAATATTGCAACAGCATCCTAACTAGCCTCCCTTGAA
TCCCAGTCCCTCTCTAAAATTTATATTATCCCACTGTCACATTGCTCTTCCTTTAAAAGACTCAAA
ACAAAAACAAAAACTTCTCAAGCTTTCCCCATTCAAGAACCTACAGTGATTTCCTATCTCTACCT
TATCAAATTTCAACTCCTCTGCCAGGCTCTCAATACCCTGGCTAATCTAGCTCCTATCAGTACTTA
GGTCTTCAACACGCCTACCTTGCTCTGAGCAGTCAGTCCAGTGTCTTCCCTGTCCTCATTCTCCA
TATATACCCTAAGCTTTTATTCTCACCAGTTTCCCTTCTGCTCCAACTTTGTAAACACTGTTCATCT
TTTTTTTTTTTTTTTTTTTTTTTTGAGACGGAGTCTCACTCTTCGCCCAGGCTGGAGCGCAGTGGCA
CAATCTCGGCTCACTGCAAGCTCCACCTCCCGGGTTCACACCATTCTCCTGCCTCAGCCTCCCG
AGTAGCTGGGAACTACAGGCTCCCGCCACCACGCCCGGCTAATTTTTTGTATTTTTTTTAGTGGA
GACAGGGTTTCACCGTATTAGCCAGGACGGTCTCGATCTCCTGACCTTGTGATCTGCCCACCTC
GGCCTCCCAAAGTGCTGGGATTACAGGCGTGAGCCACCGCGCCCGGCCAACATTGTTCATCTTT
AAAGGCCCAGTACAAATTCTGTCTTTTAGAGACAGGCCTTTCCTCACCTCTAGCCACTTGTGTAT
TTTTCTTCCTTGTTTAAACATCTGCAGTAGTACTTCTAACCTAATATTTTGTAATCTAATAATTTTGT
AACCTAGCTGTCTCAACTCAGTGGAATTTTGTTTCTAGTCACTGTGCCCAAATTTGGTATGTCAAA
TAATAATTATTTTGAGACAGGGTCTCACTCCGTTGCGCAGGCTGGAGTGCAGTGGCGTGATCAC
TGCTCACTGCAGCCTCGACCTCCAGGGCTCAAGCAATCCTCCCACCTCAGCCTCAGCTAAGACA
CTTGTACACCACCCCTCCTGGCTGTTTTTTACACTTTCTGTAGAGACAGGGTTTTGCCATGTTGCC
CACGATGGTCTCAAACTCCTGGGTTCAAGCAATAAGCATGCCTCAGCCTCTCAAAATGCTGGGA
TTACAGGCGTGAGCCACCACGCCCAGCTGCATTATTTTTTTTTTTTTAATTCAACAACTTTGTATATT
TTTGGATACAAATACAAAGTTATATCATGCTTTCAAGATATTCTTTTACTAATTCTCAGAACAATG
CAAGGCATAAATATTGTGCACACTCATGCCTACTTGTGTGATCCTGAGTTTAATAGAAGAAAGG
ATAGAAACCAGCTTGGCTTATACATTTTTTAAAATATTATTTGGCTCATTTTCCCTAAAAACATCA
AGAAAGGAATTGCCACATGCCTAATTGAAAAGATATGCTTAGTGGTAAGGATATATATACATCA
ACTTTTAAGAGGTTTAAAAAAAAAACTCCTCCCATTGTAACAGTTTATAGTTCACCACATACTACT
AAAACAACCCATAAACCATGAGTGCATTATCAGGAGTGACAGGAGGGTAAGTCGACCGATGTG
AAATTTACCTTGTAGGCAATTGACATTCTGCGGTGTATGGCAACTGGTCATGAGCAGTACAAAG
AATTTAAAAGGATCCAATATCTCAATATGTTGAAACAAACTGTATCACTTGGATCCATCAATATC
ATTTTCCATGGTTAGCTTTTGTTTGTTTTTAGTTATTTATTTATTTTTGAGACGGAGTCTCGCTCTGT
CCCCCAGGCTGGAGTGCAGTGGCATGATCTCAGCTCACTGCAACCTCCACCTCCCGGCTTCAAG
AGATTCTCCTGCCTCAGCCTCCACAGTAGCTGGGATCATAGGCATGTGCCACTACGCCTGGCTA
ATTTTTGTATTTTTTTGGTAGAGACGGGGTTTCACCATGTTGGTCAGGCTGGTCTCAAACCCCTGA

CCTCAAGTGATCCGCCCGCCGCGGCTTCCCGAAGTGCTGGGATCACAGGCATGAGCCACCACG
CCCAGCCTAAATCCTGTATTTTTAAAAACTAACAATGGGCCAGGTGCAGTGGCTCATGCCTGTA
ATTCCAGCACTTTGGGAGGCTGAGGCAGGCGGATCACTTGAGGTCAGGGGTTTGAGACCAGCC
TGGCCAACATGGTAAAACCTCGTCTCTACTAAAAATACAGAAAATTAGTTGGGAGTGGTGGCAC
ATGCCTGTAGTCCCAGCTACTTGGGAGGCTAAGACACCAGAATCACTTGAACCCAGGAGGCGG
AGGTTGCAGTGAGCCAAGATCGCACCACTGCACTTCAGCCTGGGAGACAGAGAACTTGTCTCA
AAAATAAAAAATAAAAATAAATAAAAGCACAGGATTTAAAGTAAAAAAAAAAAGTTGGGTTCA
ACTTTACTTATTAATCATGACTTTGGGTACATGATTTCGTTTTCATAAACTTTAGTTTCTACATGTA
TAAAAGATAATTATACTTATTACCTATGATTATTACAAAGTATAATGATAAAATATATATAAATTA
AATGGCATAACTTGTATATTTAGCACGCAATAAAAATTAGGTACTATTATAATTGCATAATTTTTT
TTTAGGTTCAGGGAGTACATGTGCAGATTCGTTACGTGTGTGTGTGTGTATATATAATCTTTTTTT
TTTTTGAGACGGAGTTTCGTTCTTGTTGCCCAGGCTGGAGTGTAATGACATGATCTTGGCTCACT
ACAACCTCCACCTCCGGATTCAAGCGACTCTCCTGCCTCAGCCTCCCAAATAGCTGGGATTACA
GGCATGTGCCACCACGCCCGGCTAATTTTGTATTTTTAGTAGAGACAGGGTTTCTCCATGCTGGT
CAGGCTGGTCTTGAACTCCCTACCTCAGGTGATCCGCCTGCCTCAGCCTCCCAAAGTGCTGAGA
TTACAGGCCACCGTGCCCGGTTCGTTACATGTATATATTATGTAATGGTAAGGTTTAGGATTCTG
GTGTATCCATCACCCAAATAGTGAGCATTGCACCCAATAGGTAATTTTTTAACCCTCTCTTCCCA
CCTACTCTAACCCGTTTCAGAGTCTCCAGGGTCTATTATTTCCATCTTTATGTGTAATTGTTTAGCT
CCCACTAATAAGAGCTTGTAAGACAGAACATGCAGTATTTGATTTTATGTTTCTGAGTTATTTTAC
TTAGGATAATAGCCTCTAGCTCTATCCATGTTGCTGCAAAGGACATAATTTCATTCTTTTTTATGA
CTATGATGGCTTTTTGTCACCCCAGATTCACTTTGTCACCCAGTGAATATGGCTGGCTCAAGTGA
TCCTCCCACCTCAGCCTCCTAAGTAGCTGGGGCTTCAGATGCCTGCCATCACGCCTACCTAATTT
TTGTATTTTTTGTAGAGATGGGTTTTCGCCATGTTGCCCAGGCTGATCTCAAACTCCTGGGCTCA
AGTGATCCACCAACCTCAGCCTCCCAAAGTGCTGGGATTATAAGCATAAGCCACCACACCCAG
CCTATGATATGCTGAACTACATTTTCCAATTTACTTAATCAAACACTAATCTATCTAAGTTGCTGT
TGTGAAGAGATTTTGCAGATGTGATAAAAGTCCCTTTTGTTTGCTGTAAATGTGTTGATAAATAAA
TAGCCCCTAATCTGTTGACTTTATCCTGGGTAGGCCTGACTTAATAAGGGGAGCCCTTTAAAGGT
GAGGGCTTAGGCCTTTCTTAAATTCAGAGACTCCAAACAGCTTATGCCCTTGGGATTCTAGCCTG
TTCATGATTTTTTCTTTACTGCCTGTGGACAACAAGCATTATCCTGTGCTCATGGGGTTCTAACCA
GTTCTTTCTCTTTTGTTTTGTTTTTGTTTGTTTTGAGACAGAGTCTTGCTCTGTCGCCCAGGCTGGA
GTGTAGTGATGCAATCCCGGCTCACTGCAACCTCTGCCTCCCAGGTTCAAGCAATTCTCCTGCCT
CAGCCTCCTGAGTACTTGGGATTACAGGCACCCACCACAGCCACCACCAAATCCAGCTAATTTT
TGTATTTTAGTAGAGGTGGGGTTTCGCCATGTTGGCCAGGCTGGTCTCGAACTCCTGACCTCAA
GTGATCCATCTGCCTCAGCCTTCCAAAGTGCTGGGATTACAGGCATGAACCACTGCTCCCGGCC
CCAGCTGGCCCTTAAATCTCCCCTTCCTGAATGACTGCCTGCAGACTTCAGACTTGCTTATCCAG
CCCCCACAAGAACATAAGCCAATTTTTTTTTTCTTTTTGAGATGGAGTTTCACTCTTGTTGCCCAG
GCTGGAGTGCAATGAAGCCAGTTCTTTATAATAAATCCCCTACTGGTTCTGTGTTTCAAATTAACT
CCTAATTCTGATATACGTTAGAATTCTCTCCTTGCTTGTTGTCTTCTGCTAACTCTAATAGGCCAC
CAAAATTCTCTTGGTTGCAAGCATCCAAATGTAAGTTATCTCAGGTAAATTCTGGAGGGCCTCCT
CAGTACACAAATCCCTTATGTGGGAGAGCCTTACATTTTGGGACTTAGAGTCCACAGATTTTTGC
TGTTTGGTCTTCTTGCCTAGTAGGCAGCTCTCCTTGGCAGGAACTAACAGTATTCACGTGGCCCC
AGGAAACTAGGAAATTTTATTTATTTATTTATTTATTATTTATTTATTTATTTATTTTTAGACAGTCTCACT
CTGTCGCCCAGGCTAGAATGTAGTGGCATGATCTTGGCTCACTGCAACCTCCACCTCCCAGGTT
CAAGTGATTCTCCTGCCTCAGCCTCCCTCAGCCTCCAGAGTAGCTGGGATTACAGGCATGCACC

ACCACGCCCAGCTAATTTTTGTATTTTTAGTAGAAATGGGGTTTCACCATGTTGGCTAGGTTTGTC
TCAAACTCCTGACCTCAGGTGATTCACTCGCCTTAGCGCAAAGTACTGGGATTACAGGTGTGAG
CCACCATGCCCAGCCGGAAATTTTATTTTAGAGATAAGAAATTTAAGACTCAGAAATTTTGAGTT
CTGGCTTGGCCACCAACTAGAGTAATAATTATTGTCTGTCTTAAGAGAAAGTGTTTTGGATGATA
ATTTTGGGGATCTTTTGACATAACCGTCTTCACAATCGGTTTGTCTTTTTAATGATTTCATACTTAC
AGTGTTTTGATATCTCTCCCAATGCAAGACCACTATGTAGTTTAGCCACTGTAACTTTCTACAATC
TGCTCTAAGTCATTCTGATCAAAGTGCCAACTCAACTTTTCCTCCTTCCTCAGAGACGAGTGCTC
TAAACCAGTATCATGGCTCCAGTTCCTCTGACAAGGATTCACATGGGGTAAGTGCCCACTAAGG
CAGAGGTGTCTACTTCAGCAGTGCAGAGCAACTCAGCTAAGTAGAGGGAATATGAAATAGAAC
CAGAAGTCTAGCCTGCTTAGCTACAAATGTTTCCACCAAAAATAGTGGATACAAATGAATTAAA
AGTATGCACAGTTTGGTGTCTGCTTGAACAATGGGCACATCCAGTGTGGAGAGCTTAAAATGAC
TATTTTTATCCAAAAGAGGAACTATTTCCCAAGCTAGATCCCAGAATATGAGGCCTGAGACCAC
CTCATTCCTATGGCTGAGAAAAGTAATACAGAATTATGGAGGCTGGCACGTCGGCATGTCCAAA
ATTTGCAGAGTGGGTAGCAGGCTAGTCCAGAGAAGTGCCAATATTGCGGTTTAAGTCCAAAGTC
TGTTGGCTAAAGAATTCCCTCTTGCTTGGAGGAGGTCAGTCTTTTGTGCTATTCAGGCTTTCAACT
GATTGGACGATGCCCACCCACATTATGGAAGGCAATTTGCTTTCTCCAAAGTCCACCCTTTCAAA
TGTAAATCTCATCCAAAAACACTCTCACAGAAACATCCAGAATAATGTTTGACCAGCCAAGTGG
ACACATAAAAGTAACCATAATTTTTTTTTTTTTTTTTACGGCCGGGTGCGGTGGCTCACGCCCGTC
ATCCCAGCACTTTGGGAGGCCGAGGCGGGCAGATCACAAGGTCAAGAGATCAAGACCATCCTG
GCCAACATGGTGAAACCCCGTCTCTACTAAAAATACAAAAATTAGCTAGACGTGATGGCGCGC
ACCTATAGTCCCACCTAATTGGAAGGCTGAGGCAGGAGAATTGCCTGAACCTGGGAGGCGGAT
ATTGCAGTGAGCCGAGATCGCGCCACTGACTCCAGCCTGGCGACAGAGCGAGACTCTGTCTCA
AAAAAAAAAAAATTAAATTTTTAGGGCATAACTGCAGCTGTTCCTTAAGTAATTTGTATCTGCTTT
AAACCACTAGAGAATTAAACTATTCTCAATCAATTCTAAAGGGTGCTGGAAATACATGTCTTTCA
GCATCTTCAGGCTGAGTTTCAAGTGCTAGTTTCCTGCTAGTTTCCCAGTGCATGCCTCACATTTTG
GCCCAGTCAAGTAAGTCCCATCACCAGCAAATCCACAGTGATTCCACATCAGGAAATTCCCAGT
GGTTCCATATCTGAGCTTGCTCAGAATAAACTTCTCACCCAAGAGCTTATTTACCACCATCAGAT
CTCTAAATAAATAACATTTTGTTTATTCCTATTACAAAAGCAATATATGTTCATTATAGAACATTTA
GAAATGTGGGAAAACAAAGAAAATAAAAATCACTCATAATCCTACCATTCAGAGACAACTACA
GACCCTTCCAGTAAGATTTTTCTTACAAAAATGATATCAAACATTATATCCTATTTTATAACTTCC
CCATTTTACAATACATTGTAAACATTTCCATGTCATTAAAAATTACATAATATCACTTTTAATGGCT
AGTTAATATGGAACCAACAAGGGCTTTGAACACATTAACTTCAGCTCTCTGAAAGCTGAAAGGC
AAATGGGATGACAAACTACCCTTATACAGGATTAGGTTCATAACAATCACAGGAAATGCCACAT
ATGTGAAAATAAACAGATAACAATTTTGAAGTTTTTCCATTGAATGTACATGAAAGAATGTTTTGT
CTTGTTTTCTTTTTTTTTTTTTTTTTTTTTTTTTTGAGACAGAGAGTCTTGCTGTCGCCCAGGCTGGAG
TGCAGTGGCCCAATCTCTGCTCACTACAACTTCCACCTCCCAGGTTCAAGCAATTCGTGCCTCAG
CCTCACGAGTAGCTGGGACTACAGGTGTTAGCCACCATGCCCAGCTAATTTTTGTATTTTCTGTA
GAGACAGGATTTTTGCCATGTTGGCCAGGCTGGTCTCAAACTCACGGCCTCATGCAATCAGTCA
GCCTGGGCCTCCCAAAGTGCTGGGATTACATGTGTGAGCCACCACGCCCAGTCAACAAATTTGT
TGTTTTTTTTTTTTTTTTGAGACGGAGTCTCGCTCTGTCTCCCAGGCTGGAGTACAGTGGCGCAATC
TCAGCTCACTGCAACCTCTGCCTCCCAGGTTCAAGCAATTCTCCTGCCTCAGCCTCCCGAGTAG
CTGGAACTACAGGCGCGTGCCACCACACCCAGCTAATTTTTTATATTTTTAGTAGAGACAGGGTT
TCACTGTGTTAGCCAGGATGGTCTCGATCTCCTTACCTCATGATCCGCCCGCCTCGGCCTCCCAA
AGTGCTGGGATTACCGGTGTGAGCCACCGCGCCTGGCTGTTTTTTTCTTCTTTTTGAGACGGAGT

CTCACTCTGTCGCCAGGCTGGAGTGCAGTGGCGAAATCTCGGCTCACTGCAACCTCCGCTTCCC
GGGTTCAAGCAATTCTCCTTGATTCTCTCCGTGCCCGGCCAGCAAATTTGTTTTCTTATAACTTGC
ACAGCCTGTCCACCTCCCAATCACTTCTACTCAAACCTTCATCTAGGCTCAGCTAGGCTAAGGG
CAAAGGGTAAGTAGTAAAATTCACTCAGAACTCTGATTTTTTTGTTTTGTTTTGAGACAGAGTCCC
ACTCTGCCACCCAGGTTAGAGTGCAGTGGGGCAATCTCAGCTCACCACAACCTCCACCTCCTGG
GTTCAAGGGATTCTCCTGCCTCAGCTTCCTCAGTAGCTGGGACTACAGGGGTGCGTCACCATGC
CCAGCTAATTTTTTTATTTTTAAGTAGAGATAGGGTTTTGCCATGTTGGCCAGGCTGGTCTCGAAC
TTGTGACCTCAGGTGATCCTCCCGCCTTGGCCTCCCAAAGTGCTGGGATTACAGCCTTGAGCCA
CTGCGCCCAGCCTTTTTGAAACAGGGTCTCACTCTGTCGCCCAGGCTGGAGTGCAATGGAGCAA
TCTCAGCTCACTGCAACCTCCACCTCCTGGGCTCAAGGGATTCTCCTGCCTCAACCTCCCACGTA
GCTGGGATTATAGGTGTGTGCCACCACACCCGGCTAATTTTTGTATTTTTTTAGTAGAGACAGGG
TTTTGCCACATTGGCCAAGCTGGTCTCGAACTCCTGGCCTCAAGTGAACTGCCCACCTCAGCCT
CCCTAAGTGCTGGGATTACACACCTGAGCTACCACACCCTGCCAGAACCCTGATTTTTTTTTTTTT
TTTTTTTTTTTTTTTTGAGACAGAGTCTCGCACTGTCACCCAGCCTGGAGTGCAGTGGCGCGATCT
CGGCTCACTGCAAGCTCCGCCTCCCGGGTTCACGCCATTCTCCTGCCTCAGCCTCCCGAGTAGC
TGGGACTACAGGCGGCCACCACCACACCTGGCTAATTTTTTGTATTTTTAGTAGAGACGGGGTTT
CACCGTGTTAGCCAGGATGGTCTTGATCTCCTGACCTCATGATCCGCCCACCTTGGCCTCCCAA
AGTGCTGGGATTACAGGCATGAGCCACCATGCCTGGCCCAGAACTCTGATTTTTAAAGTGCTCC
TTCTAAGAAGACTCAGAGCTACAGAGATGTCAGAAACAATCATAGAATGACTCTACATCCTCCC
ACAATGATACTAGACAGACACACTGGCACACCTCGAAATTTCTGGGTTGTGCACAGCCCAAGA
GGGCTCATTTAAGCTGCCATGTCCACTCCTCCAACAAATCCAGGCACCATCTAAAGGGAACATG
CCAAGTCACAGTCTCCTTTGTGAATTCCATAATGGCAAGAGAGCTCTCATTCATATTTGCGTTCC
TGGCACCCAGAGCACATTTAATAAAAGTTGATTAAACAAAAATAATGACTTGGTCCTGAACTGC
ATTCTACAACTGCTCCTTGAGTTTTGTCCCATCCTATAATAACCATCACAAGCTCTGCTATTTATT
CTACCATTTCTGTCCTGGCCCCACCTTTGAGATTGTTCTCACAGTTATCTTTGCCACTAGGTTGCA
ATTCTGACTGTGCTGGTGATGCTGGTTCCAGGTCCCTCAGGAACCCCTCCCCCAGTCTGGTCTG
CCCTTGAACTCTATACTGGCCCCAGGGTATCCCAGGATTGTCAGAAAGGCCTGGCCAGGATTCA
CCTGGGAGAGGGATCAGCATCTCTGCAAAGTTGTTGGATTGAAAGAACTTTCATTTTGAGCTAC
ATCATTGAAGAGCTTTAAATCTGAAAATAAGAAAGACGTTGAACCCTATTACTCACATCTCTTTG
TCATTTGAAGCAGATCCCGAACCACTCAAAAGGACAAAACAAGGAAATAGCAATGAAATTGGA
TTCAGTGAATGGGGTAAGGGTATTAAGTGTCTGAAATATAAGAGATAAAAAAATTAGTTTTTACC
TTTTTTTCTATTTTCTAAATGTTTTGGTGGCTAGAATAAGATATACTTTGAACTCAAGAGCAATAA
ATGTTAATATATTAAAAGTAGCTTTATGAAATATGGAAAGTATCTTTATTTCAAAATACTTCAAAG
TAATCATCTGAATATTTATCAGAAATATTAAGACAACTTATTAAATCCTGTATTAAAATGATTGCT
TATTACTTTTTGTTTATACTAGTCAGAAGTGATTCTCTTCCACATTTTAGATTTTTCAGAGTAAAAA
GGATATAATTTTAGACTTTCAATTGAATATAAACTGGGGTTTTTCCACACGATTTGTGGACATGG
CTGTCTGATGGTGACTCTTAGGTATTTATGCTAAAAATGTTATTTTGGGGATCCTTGGGAATGGA
CTCTGATATTTATTTATTTATTTATTTATTTATTTATTTATTTATTTATTTTGAGGCG
ID|56379|gene_id|ENSG00000239213|transcript_id|ENST00000474250 Homo sapiens lncRNA

CGGAGGCTGCGCGGCGGAGAGTAGAGAGCTAGAGGGTGAGAAGCGCTGGGCCGGACCA
GAACTGTCTTAGCTTTTCCCTCTGCGGCTGCTCTGGTTGGTTACTTTGAGCCTGCCTCCTCAGCG
CCGCAGGAGAGACTTAACAGCAAAAATGACTGTGTCCTGAGTTCAGCCCCCACTGCTCTGGGA
GCTTCCCCCTGGTTTCTGCACCTGAAGAATGTCATCCCAGCCGAAGGTTCTTAGGAGCGGGGAG

CGACAAAGAAAAACAAAGAAAAGCAGACCTGAAAATTGGGACCTGGGAGAAATGTAAGACAT
GTGCTTAATTTGAGGCACAGACCAAAAGAAGCTGAGTCTGAGGGCCCTCTAAAAGTGTCCTCGT
GCTTGGGCAGCTTACATTCTTGGCGGCAGTAGTGTTATGAAGAGGACCTTCGGAGCCAGGTCTG
TCCATTCTTAGGGCTCCAGCCACTGGCTGAGGCATCAGTAAGTTGGTTCTTCCCCTCGCCGGTG
CTGCAGGCCCCCTTCCCCGCCCCCGCGCCGTAACCTGCCTCTTTTTTTTTTTTTTTTGAAACGAGT
TTCGCTCTGTCGCCAGGGTGGAGTGCAGTGGCACGATCTCGGCTCACTGCAACCTCCGCCTCCG
GGTTCAAGCTATTCTCCTGCCTCAGCCTCCCGAGTAGCTGGGATTACAGGCATGTACCACCACG
CCCAGCTAATGTTTTTGTATTTTTAGCAGTGACGGGGTTTCACCAGGATGGTCTCGATCTCTTGA
CCTCGTGATTCGCCAGCCTCGGCCTCCCAAAGTGCTGGGATTACAGGCGTGAGCCACCACGCC
CAGCCGCCTCATCTCTTCTTAACCATCTTTGTGGCACAGTTTAGGATCGCTCCTCAGGGAAGCTT
GCTCAGTTAGGTACCTTCCTTGGAGCACAGGTGTTTTCTCCATCCGAGCTATTGAAATGGTTTGC
CTGTATGTCTACCTAGATTGTAAACTCCGTTGAATCAGTAACCATGTCTGTTCAGTTTCTCCCTAG
GGCCTTGCAGTAGGGTAGATGGTCAGTAAATACTGTTTAAGGCATTAATACTTTTTATTTTAATCC
TGGCCCTGGATATCAGCCTCAAAAGTTTGCAGTTTTCTGCATTTAACTTCAAAATTATTTACGTGA
TTGGTAATGCATGCATGTGGTTGGCCTGTGCTGTAATTTAGATGTTTGTTCCCCTAAACCTCCTGT
TGAAATTTGATCCCCAATGTTGGAGGTGGGGACTAATGGGCAGTGTTGGGTCATGGAGGTGGTT
CCTTCAGGAATAGATTAATGTCCTCCCTGGGGAGAGGGTAAATGAGTTCTCTTTATTTCCACCAG
AGCTGGTTAAAAAGAACGTCGCACTTCCTTTCTTCTTTTGCGTCCTCTCTGGCCATGTAATCTCTG
CACACACCAGCTCTCCTTTGCCTTCTGGCATGAGTAGAAGCAGTCTTAGGCTACCATCAGATGC
AGATGCTCAGTCTTGAACTTTTCCAGACATCAGAACCATAAGCCGAAAAACCTTTTCTATTTCTA
AATTATCCAGCTTTGGGTATTCCTTTCTAGCAACACTAAAGGGACTAAGACAGCCTGCCATATGT
AGGATCACAAGGTCAAGCACAAATTTACCTATTTATGCTGTACTATCCTCAGGTTGACTGGGACA
AGTAGACCATGAAACTGGGCAACAGCTACTCTATGTCTACATTCTGTTATTAGCATCCAAAGTGA
ATAATGGCGGTGAATTTTGAGTTTATTCTAAGGGTTTAAATAACTGCATATTTTATCATGACTGGT
ATGGACTGAATTGCATCCCCCCCAAAAATTCATACTGAAGCCCTCACTCCTAATGTGACTATATA
AATGTTCCTTAACTTACAAAGGGGTTATGTTCCAATAAACCCACCATAAACTGAAAATACCTTAA
GTTGAAAAGATCTTAAGTCAGAAATGCATTTAATGCTTCTAACCTACCAAACATCATAGCTTAGA
GTAGCCTACCTAAAACATGCTCAGAATACTTACATTAGCTTTCAGTTAGGCAAAATCATCTAATA
CAAAGCTTATTTTTTAATAAAGTATTGAGTATTTCATGTAATCTACTAAATACTGAATGAAAAACT
GGATGGTTTTATGGGTACTCAAAGTACAGATTCTAGAGTGCATATGGCTTTTGGACCATCATAAA
GTCAAAAAAATCTTAAGTCAGGGACCATCTCTAGTTGGAGCTAGGCCTTTAGGGAGGTAATAAA
AATTAAATTAGGTCATAAGGGTGGGGCCCTAATCCCATAAGATTAATGTCTTTATAGAAAGTGG
CACCAGAGAGCTCTCTCCATTTGAGAACACAGCAAGCAGACAGACATCTGCAAGCCAGAGAGA
GAGCCCTCACCCAAAACTGACCATGCTGGCACCCTCTTTTGGACCTCCCAACCTACATAACTTTG
AAAAAATAAACTTCTGTTGTGTAAGCCACCCAATCTAGTATTTTATTATGGCAGCCCAAGCTAAT
ACAATGGTTTTATTTGTTGATACATTTTATAAAGTTTACAGACTGAGATATTATATTCATTTTTGAA
GAAACTTGTCCAGAGTTAAAACTGTTGCTGCAAAATCACCCATCAAACTGTTAGCACAGCGTGA
CTCAATTTTAATTCTAATTACATATTTAACATACGATGGAGGCAGAAGGTGTGAATATAAATACA
ACAGGTAAAATACAGAAATACAGTTCTTGCCTTCATGTAGCTTACCACCACCTTAAGTGGGGAA
AATTAACCCAGCAAAAAGTATGCTGAAGTCATACATTTAGGGTTTTTGTTTGTTTGTTTGTTTTTTG
AGATGGAGTCTCGCTTTTTTGCCCAGGTTGGAGTGCAGTGGCACCACCTCAGCTTATTGCAACCT
CCACCTCCCGGGTTCAAGCAATTCTCCTGCCTCAGCCTCCCAAGTTGCTGGGACTACAGGCACA
CACCACCACACCTGGCTAATTTTTTGTATTTTTAGTAGAGACGGGGTTTCACAATGTTGGCCAGG
CTTGTCTCAAACCCCTGACCTCAGGTGATCCACCCACCTCAGCCTCCCAAAGTGTTGGGATTAC

AGGCGTGAGCCACAGCACCCAGCTAGTTTATCCTCTTAATTTTCCTTCTTTAATCTTTCTCATCAC
TCATTCTTAACAGGAACTCTTTAAAAAAATTTAAGAAACCTCCAAAAATATATGCAAAAGAGGA
GTCAAAGAAGAAAAATAAGCAAACAGCAATGAAAAAGGAAAGCATAGACTGGAAGGGCAGAT
AGGCAGAAAGGCATGTATGTAATTGCACTTAGAAGGGATGCAGTACATGTTTTAATGGGGTGGG
AGCTGTCCCAGAGACTTCCTTTCACACAGTCTGTCTCCCCAGCTTCCAAATATAAACATGCACAT
GGCTGCTGCTTCCTCTCTTTCAGGGGAACTCAAACCAAAAACAGGAATCATCCTTGCTAATTAGT
GCTGAGTGGATCAGGGACTAACCAGAATCAGGCAACAAGAAAATGTAAGTCAAAACTTATGAT
CAGCCCTGGCAACAGAGCTAGACCCTGTCTCTACAAAACAAAATTTTTTTTAATCAGCTGGGCAT
AGTGGCATGTGCCTGTAGTCCCAGCCACTCTGGAGGCTGAGGTGGGAGGATGGCTTGAGCCCA
GGAATTTGAGGCTGCAATGAGCTATGATCACACCACATCTCAAAACAAAAACAAAACTTATCTA
GGGACTACACGTGGAGCTTTGGATATGGGCAGCTAATCTGTCTATGATTAAAAACACGCAGGTG
GCTTACTTTATTAATCCAAAATAAAAGAATTTGGGAACACAAGAAAGGGAAAAATGAGCCTCCA
TGTCAAGACCTTTCCCAGAAACCTGATAGCTTATAGTTAGAAATTGTTATAGACAGTCCAGTGCA
AGGAGGACATTAAGCCACAGGGGAAAGGAGTTGTGTGGCCAGGTTAGTCAGGCAATTAACTAG
AAAAAATCTGTCCACATAGAGGAGCCTGCATAATAAAAAGGTATTAAACGATAGTAGAATATTG
CTAGAGCAGAAGATAGCTAGAAATATAATCAATAACCAGGTTATGGAAGTCAGGCACCTTAGA
GTTTATCCTATAGGCAATGAGAAGCCATTCAAAGGGTTTTTGTTTTTGTTTTCAGCACTTTAAAGA
TGTCATTCTCGTTTTCCAGCCTCCATTATATCTGTGAGAAACTAGTTGTAATTCTTATCATTGTTTT
CTTGTATGTCTTATGCCTTTTTCTATGAATGCTTTTAAGATTTTGTTTATATTTATCAAAAGTTAACA
CTGATGTTTCTAGGTGTCTTTTTCTATGAATGCTTATAAGATTTTCATTATATTTATCAAAAGTTAC
ACTGTGATGTTTCATTACACTGTGATGTTTCATTACACTGTGATGTTTGTTTTGGTTTTGGTTTTGT
ATTTATCCTGCTTGGGGTTCATTTCATTCCCTGGGTCTGTGGATTGCTGGTTTCTCATTTTCTTTTC
TTTTCTTTTTTTTTTTTTATCAAATTTGGAAGACTTTTGTCCAGTATTTCTTCAAATTTTTTTTTCTGTC
CCATTCTGTTTCTCCTCTTCTTCTGGGACTACAATTACACTCATGCTAGACTTCTCAGTACTTAGT
ACAGGTCATTCAGGCTCTGTTCATTCTTTTTTCAGTTTGTATAATTTCTATTGAGAGCCACTGTCTA
GTAATCCCTGAAAAAATCTAAATGTTGACATTTTCCCAAAGCACAGTACCCTGCTGATGGCTGTA
GTTTCCTTTGGGGAAGGTTTAGAGATATGTTAGTCAGGAAATCAGAAATTAGATGATATTACTAC
AGAGAGGATTTAGTATTGAGTTTTCATTGACTGAAAAAGCAAAAACGGAACACTGAGGTTACCC
AGAGGTGATAACTGCAGGGAGCAGCTGTCAATGATAGATCTAGTGAAAATGGGGACTCAAGGT
GGATTATCAGGTCTCAGAAACTCAGACAAATGACCTGGCTGAGTATTGCTCAGACCCCTGAGAG
GATGCTGTCTGGCTGATCCTACTACTTCAGGGAAGGATATGATGAGGCTAGTTGTGGATGGGGA
GGATGTCAAAAGAAGCTCAAGGCTGAAATCAATTGCTGCTGCTGGAGCAAAGGGCTCTTGCTTG
TTGCTGGGATAACAGTGACAGAAACTGCAAACAAATGGAAAGGAGGAAGTCCCTTCTTCCCTTC
TGCCTTCCAGTCTCCCTCTAATGCCCTTTATTGATGGAACCTAGTGAGACACCAGCTGACATAGA
AAAAAATGTAGTTTGCAGGGTTTGAGCCACAGCATTACAGAGAAATGTATAGAAGGGGGAATG
TGGACCTGAGACAATAGTTTAATAAACAGTACCATATCATTTATTTGTGCTAAATTGGTAAGAAT
GGCCAAAATTAGACTTAATGCCATCATATGTGCTTTTACAGACTTTTAGACCTTTTTGAAAAGAAT
CTCAGGATACCATGTCAATTTTCTGCTACATTATGATTACTGTTGGTGCCTTTTTCAGGAGTAAGG
AGTACTTTTACTGCAGCTCACACATACACTAGCCACAAAATCATAAACAAGGGCAGCCTTGGAA
GAGATCTACATAACTGAGGATAAGCAGACCCTACCCCAACGTTAGGTCTCCAAACATCAGGGA
CAAAATGGCATGGGCTAGGTAGGACTGGCAAGAATCCACACTTCTCCCTATTTCTATTTCCTATG
TATCGCCTAATGTGCTCATAATAAGGAGAAAACCAATTTCCTGTTCTAACTTTGGACAGCCACCC
ATTGTCATTAAAAAACCACTTATTTTCCCTTCTTTCCTCTTTTCTTGCCTCAACCCAGGCTCCTTTA
CCCAGCCCTATGACTTCTAGTTTTCTAATGCAAGTAGATGACAAAACAGGGTTTGGTGTTCAACA

TTTCACCTTATAGTGTATTTTTCACAAATGAAGGAGTTTTGTTGAACATTTGTAGAACATTTCTAAT
TTTTGAACTTCAGTTCCATCTGAAAGGGGTCATTTCTTTGAGTCTTCTATTATTTAGTAATAGCAGT
TAACATTCTCCTTCTACTACAATTTACCTGGAAAGACACCCATAAATACCAATAAAGATACCAGA
TTCATAACGTATTTCATATATTTAATATTTGTATTTGAATATATTAGACTAGAATAAATTTTCTGCA
TTTGTATATATTATATATTTTATAGCACTCTGTCAGTTATATAACACAAAAGACATTCATAGGTAA
ACATTAAAATGGCAGTATACATAATAACTGAACATTAATGAGACAATAATAATCAGGTATTCATT
TAATGGGAGAGTCTAGTATTTCCTGGTAGTCCTGCTTTCTTAAATTCCTCCAGTAAAGTTTATAGC
CAGCAAGGACAAAAACACTAATCATAATGATTACCCCTCCAAAAACATCATAGATGCTAGGAAA
TATGTGCAGCACGAGAAGCTGCAAGACCATAGCTACCACAATCTCCAAATGTTGTACTGTGCTA
ACCAAAGCTGGATGGAATTTGTCCAAGGCATAATAAACTCCTAAGAATGCTGCAGTAGAACAGA
CACATATAGCAATGAGATAACTCCAGGTTTCTCCATCTAATGGGATGATGGGTTCTTGAAGAATA
AACATAGTAGATATTCCCCAAATTGTCCCAGTCCAACCAAAAGTAAACAGTGCAGTCCACATGC
TGATCTTCTCCTTGATGGATCTGTATACTATCATTGAGAGAGCAGTGGTCAGTCCAGCCATCACA
GTCATGGTGTACCCAAAGGCTTCTTTCCAGGCATTTAACAAAGAATTGTCTTCATCAACAATGTT
TGGGATCATGACAAGACAAACACCTAAGATGCTGCAAACAACTGTAGCCATGTCAACATAAGC
CATTTTCTCATCTACGAGTAAAAAAGCCAAAATGGCACTGAAGACTGTAGTTGTGGCTCTCCAC
ATAGTGGTCCCATTGCTGGGAGGAACTATTGAAAATGATGTATAAGCACAAGTGATAGAAATGA
CATTGCATACACCATAAAAGAAGAGTCGTAATCTGTATCCACTGGGTCCAAAGGGGGCCTCCTG
ATAGTAACACACAACTAACACAGATAAGACCTGAAAAACAGAACGGATAAAAATCAGTTCTAG
AGATGGAACTTTAGACCGATCAGAAACAAGCCTAGTGATAAGAGCTACACATCCATGAGCCAA
AGCAGATCCAAACAGCACTATCCACATTTTTCGGGATTGAAAAATGTTTTTTTCTGCAAAGCTCT
GGAATTGTCCAATCTCATTGATCATTGGGTCTTCTGTTGGTGGAGGTAGGGTATCCATGGTTCCA
AAGAAAGCTCTCCCTTTTTTTTTCATTTCACTCAGCAGACCTTTCTTTGGATTTTCCTCCATAAAAT
TTCCATAGCCTTCATTGATTTCTTCATATCCATCATCGCCAGGCTGGGGATAATGAGAAGTATATT
TCACCATCACTGTGTTGGGATGTATTTTCACCCGTTTTTTAACTGGATATTTTCTGGAGGGAGAAG
TATCCATTTCTTCAGATAATCAATTCTATTTAAAGAAAAAAATTGACAAAAAGACATTCATTTCAT
TGAAAATTTAATTTCTATTATAATCATATGCATCAATTCAATGATAGAAAGTAGAGGCTGTAACA
GTAGTCCAGAAAAAAAAGAAATTAGAGATGATCTGGCAATATGCCTTATCAGATGTATGGTACC
TACTGGAATTGTTTACCCTTTTTTCACTCTCATCTGAAGTCTGTCTCCATTTCTCTTTGCTTACTTGT
TTAATTCAGCTCTATCTTCTATCTTTATACCACAAACGGATTTTTCCATCTTTTCATTCCTACCTGTT
TCTCCACCTATCCAACAAGCAAAACACAGAAAGATAAATAATAACTTAGGTCAAACCTTTCCTTC
TCATTGGGTCCATTTGCCTGTTATAAATTATTAGTTAAGTCCAAAGTATTTTGTATAATCAATTCTG
TATAATACCAGAATTCACCTTATAAATTATAGTGATTTTTAAACATTTATTCTGGACTCCCCATAA
GTTTTGAGATATAAAAATACACTGAAATTAGAACATAAATAACATGAATTTAGTAACACTCATGC
ATTGTTGGTAGAAAGGTTAAATTGTACAACCTCTCTCGTGAACAGTTGGACCATATGTATTAAGA
TTTTAACTATCCTCTACTCAGCAATACCACTTTTAAGATTTTATGCTAAAGAGATGATCACAAGAT
GCTTTTTACATTCTGAATAAAAAAGGGGGGGAATAAGAATACGTATTTATATTTGTTTACATAAC
AAAGAAATTATATAAAGATGCCAAGAAAACTAATGGTGGTTCAGGTGACAGGAGGAACAAGAA
TGGAAGAAAACCTTTTTGTTATTTATTTTTTTACATTTTTATTTTTGAATTGTGATATTATCCAAAAA
TAGAATTAAAATGTTTATCTGTTACTTAAGAAAAAGAATATACCAAAAAATGTGGGGAAAATGTA
CATACAGGAGAGTTCATTGTAGCTTACTACAATGAATGTTCATACAATGGAATACTATGCTATCT
ATTAAAAATGACAGTGTAGGCACGGTGGCTCACACCTGTAATCCCAGCATTTTGGGAGGCTGAG
GCGGGCGGATCACTTGAGGCCAGAAGTTTGAGAGGAGTTGGCCAGCCTGGCCAACATGGTGAA
ATGCCATTTCTACTAAAAATACTAAATTTAGCCGGGCCTGGTGGTATGTGTCTGTAATTCCAGCT

ACTCGGGAGGCTGAGGCATGAGAATCACTTGAATTCAGGAAGCAGAGGTTGCAGTGAGCCAAG
ATTGTGCTACTGCACTCCAGCCTGGGTGACAGAGCGAGACTCTGTCTCAAAACAAAACAAAACA
AAAACGATGTCCACTATATAATGTCAAGTGGGAAAAAAAGTTGAACTTACTGTAAGAATTAAAT
AAAATAATGCATATAAACAATTTGCCACAGTGCTGGCATATAGTAAACTCTCAATAAATGTTAGC
AATAAATTTGGCTTCCTTTCACCTATAACAACTTTATGAAACACTGGGTATCTTCTACAATATTAT
TATTTCTTTTAGACCTTTATTTTTTCACTTGGTTCTTTCTTTCCTTCCTTTCAACAATTTCCTATTTCC
CTCCCAATGATCCTCTCCTCCCGCTTAAAAAACAAAAAAGAGACCAGGCACGCTGGCTCATGCC
TGTAATCCCAGCACTTTGGGAGGCCGAGGTAGGCAGATCACCTGAGGTCAGGAGTTCGAGACC
TGCCTGGCCAACATGGTAAAATCCTGTCTCTACTAAAAATGCAAAAATTAGCTGGGAATGGTGG
CAGGTGCCTGTAATCCCAGCTACTCAGGAGGCTGAGGTAGGAGAACTGCTTGAACCCAGGAGA
CGGAGGTTGCAGTGAGCCAAGATTGTGCCACTGCACTCCAGCATCAGCAACAGAGCAAGACTC
CATCTCAAAAAAAAAAAAAGAAAAAGAAAAAAAATCTTTTTTTTTCTTCCTCGTGACTCCCTGAT
TTCTTGCCAAAAACTCATCGGTTCCAGGATCTTAAACAATGGCCCAGAAATAAAATCTCATTTTG
TTCTTCGACACCATCTGGGGAGCTAACTGTTCTATTCCTATTTACTCCTTTCTTTTCTAAAAGGCA
CCACCTTCAGCTGGAAGAAGTCATGAAAAAAATATCACCTGTGCCTCCAAACCCTTCTATTTCCT
AGCCTGGATGTCCCATTTGCAGTCCTTCTTCCTGCACACCACTATCAGGCTAATCTGACTGTTCTT
TATTTCCAACTTTTGCCAGGACATGTCAAATCATAGCTTCAACAATCCCAAACTGAATAGTCTTG
TTCTCCCAAATAGATTTTTTTCCCTAATGTCCTCATTTATGTCTGTGATACCACAATTTTTCCAGTT
CCCCAAGCTAGAAATCAGGGAATACTGTGAATTATCTTTTCTGCCATCTTACTTGCATTTGGTCA
CTAATCTTACTGTTTTTGTCTTTGAAATATTCCTGTTTTTCCCTTTCTCCTCCAATTCTATGTAACTT
TTATAATTGTGGTTTGGGTTATTGTGAATTCCCATCTAGAATATTGCAACAGCATCCTAACTAGCC
TCCCTTGAATCCCAGTCCCTCTCTAAAATTTATATTATCCCACTGTCACATTGCTCTTCCTTTAAAA
GACTCAAAACAAAAACAAAAACTTCTCAAGCTTTCCCCATTCAAGAACCTACAGTGATTTCCTAT
CTCTACCTTATCAAATTTCAACTCCTCTGCCAGGCTCTCAATACCCTGGCTAATCTAGCTCCTATC
AGTACTTAGGTCTTCAACACGCCTACCTTGCTCTGAGCAGTCAGTCCAGTGTCTTCCCTGTCCTC
ATTCTCCATATATACCCTAAGCTTTTATTCTCACCAGTTTCCCTTCTGCTCCAACTTTGTAAACACT
GTTCATCTTTTTTTTTTTTTTTTTTTTTTTTTTGAGACGGAGTCTCACTCTTCGCCCAGGCTGGAGCGC
AGTGGCACAATCTCGGCTCACTGCAAGCTCCACCTCCCGGGTTCACACCATTCTCCTGCCTCAG
CCTCCCGAGTAGCTGGGAACTACAGGCTCCCGCCACCACGCCCGGCTAATTTTTTGTATTTTTTT
TAGTGGAGACAGGGTTTCACCGTATTAGCCAGGACGGTCTCGATCTCCTGACCTTGTGATCTGC
CCACCTCGGCCTCCCAAAGTGCTGGGATTACAGGCGTGAGCCACCGCGCCCGGCCAACATTGT
TCATCTTTAAAGGCCCAGTACAAATTCTGTCTTTTAGAGACAGGCCTTTCCTCACCTCTAGCCACT
TGTGTATTTTTCTTCCTTGTTTAAACATCTGCAGTAGTACTTCTAACCTAATATTTTGTAATCTAAT
AATTTTGTAACCTAGCTGTCTCAACTCAGTGGAATTTTGTTTCTAGTCACTGTGCCCAAATTTGGT
ATGTCAAATAATAATTATTTTGAGACAGGGTCTCACTCCGTTGCGCAGGCTGGAGTGCAGTGGC
GTGATCACTGCTCACTGCAGCCTCGACCTCCAGGGCTCAAGCAATCCTCCCACCTCAGCCTCAG
CTAAGACACTTGTACACCACCCCTCCTGGCTGTTTTTTACACTTTCTGTAGAGACAGGGTTTTGC
CATGTTGCCCACGATGGTCTCAAACTCCTGGGTTCAAGCAATAAGCATGCCTCAGCCTCTCAAA
ATGCTGGGATTACAGGCGTGAGCCACCACGCCCAGCTGCATTATTTTTTTTTTTTAATTCAACAA
CTTTGTATATTTTTGGATACAAATACAAAGTTATATCATGCTTTCAAGATATTCTTTTACTAATTCT
CAGAACAATGCAAGGCATAAATATTGTGCACACTCATGCCTACTTGTGTGATCCTGAGTTTAATA
GAAGAAAGGATAGAAACCAGCTTGGCTTATACATTTTTTAAAATATTATTTGGCTCATTTTCCCTA
AAAACATCAAGAAAGGAATTGCCACATGCCTAATTGAAAAGATATGCTTAGTGGTAAGGATATA
TATACATCAACTTTTAAGAGGTTTAAAAAAAAAAACTCCTCCCATTGTAACAGTTTATAGTTCACCA

CATACTACTAAAACAACCCATAAACCATGAGTGCATTATCAGGAGTGACAGGAGGGTAAGTCG
ACCGATGTGAAATTTACCTTGTAGGCAATTGACATTCTGCGGTGTATGGCAACTGGTCATGAGC
AGTACAAAGAATTTAAAAGGATCCAATATCTCAATATGTTGAAACAAACTGTATCACTTGGATCC
ATCAATATCATTTTCCATGGTTAGCTTTTGTTTGTTTTTAGTTATTTATTTATTTTTGAGACGGAGTC
TCGCTCTGTCCCCCAGGCTGGAGTGCAGTGGCATGATCTCAGCTCACTGCAACCTCCACCTCCC
GGCTTCAAGAGATTCTCCTGCCTCAGCCTCCACAGTAGCTGGGATCATAGGCATGTGCCACTAC
GCCTGGCTAATTTTTGTATTTTTTTGGTAGAGACGGGGTTTCACCATGTTGGTCAGGCTGGTCTCA
AACCCCTGACCTCAAGTGATCCGCCCGCCGCGGCTTCCCGAAGTGCTGGGATCACAGGCATGA
GCCACCACGCCCAGCCTAAATCCTGTATTTTTAAAAACTAACAATGGGCCAGGTGCAGTGGCTC
ATGCCTGTAATTCCAGCACTTTGGGAGGCTGAGGCAGGCGGATCACTTGAGGTCAGGGGTTTG
AGACCAGCCTGGCCAACATGGTAAAACCTCGTCTCTACTAAAAATACAGAAAATTAGTTGGGAG
TGGTGGCACATGCCTGTAGTCCCAGCTACTTGGGAGGCTAAGACACCAGAATCACTTGAACCCA
GGAGGCGGAGGTTGCAGTGAGCCAAGATCGCACCACTGCACTTCAGCCTGGGAGACAGAGAA
CTTGTCTCAAAAATAAAAAATAAAAATAAATAAAAGCACAGGATTTAAAGTAAAAAAAAAAAGT
TGGGTTCAACTTTACTTATTAATCATGACTTTGGGTACATGATTTCGTTTTCATAAACTTTAGTTTC
TACATGTATAAAAGATAATTATACTTATTACCTATGATTATTACAAAGTATAATGATAAAATATAT
ATAAATTAAATGGCATAACTTGTATATTTAGCACGCAATAAAAATTAGGTACTATTATAATTGCAT
AATTTTTTTTTAGGTTCAGGGAGTACATGTGCAGATTCGTTACGTGTGTGTGTGTGTATATATAAT
CTTTTTTTTTTTTTGAGACGGAGTTTCGTTCTTGTTGCCCAGGCTGGAGTGTAATGACATGATCTT
GGCTCACTACAACCTCCACCTCCGGATTCAAGCGACTCTCCTGCCTCAGCCTCCCAAATAGCTG
GGATTACAGGCATGTGCCACCACGCCCGGCTAATTTTGTATTTTTAGTAGAGACAGGGTTTCTCC
ATGCTGGTCAGGCTGGTCTTGAACTCCCTACCTCAGGTGATCCGCCTGCCTCAGCCTCCCAAAG
TGCTGAGATTACAGGCCACCGTGCCCGGTTCGTTACATGTATATATTATGTAATGGTAAGGTTTA
GGATTCTGGTGTATCCATCACCCAAATAGTGAGCATTGCACCCAATAGGTAATTTTTTAACCCTC
TCTTCCCACCTACTCTAACCCGTTTCAGAGTCTCCAGGGTCTATTATTTCCATCTTTATGTGTAATT
GTTTAGCTCCCACTAATAAGAGCTTGTAAGACAGAACATGCAGTATTTGATTTTATGTTTCTGAGT
TATTTTACTTAGGATAATAGCCTCTAGCTCTATCCATGTTGCTGCAAAGGACATAATTTCATTCTT
TTTTATGACTATGATGGCTTTTTGTCACCCCAGATTCACTTTGTCACCCAGTGAATATGGCTGGCT
CAAGTGATCCTCCCACCTCAGCCTCCTAAGTAGCTGGGGCTTCAGATGCCTGCCATCACGCCTA
CCTAATTTTTGTATTTTTTGTAGAGATGGGTTTTCGCCATGTTGCCCAGGCTGATCTCAAACTCCT
GGGCTCAAGTGATCCACCAACCTCAGCCTCCCAAAGTGCTGGGATTATAAGCATAAGCCACCA
CACCCAGCCTATGATATGCTGAACTACATTTTCCAATTTACTTAATCAAACACTAATCTATCTAAG
TTGCTGTTGTGAAGAGATTTTGCAGATGTGATAAAAGTCCCTTTTGTTTGCTGTAAATGTGTTGAT
AAATAAATAGCCCCTAATCTGTTGACTTTATCCTGGGTAGGCCTGACTTAATAAGGGGAGCCCTT
TAAAGGTGAGGGCTTAGGCCTTTCTTAAATTCAGAGACTCCAAACAGCTTATGCCCTTGGGATTC
TAGCCTGTTCATGATTTTTTCTTTACTGCCTGTGGACAACAAGCATTATCCTGTGCTCATGGGGTT
CTAACCAGTTCTTTCTCTTTTGTTTTGTTTTTGTTTGTTTTGAGACAGAGTCTTGCTCTGTCGCCCA
GGCTGGAGTGTAGTGATGCAATCCCGGCTCACTGCAACCTCTGCCTCCCAGGTTCAAGCAATTC
TCCTGCCTCAGCCTCCTGAGTACTTGGGATTACAGGCACCCACCACAGCCACCACCAAATCCAG
CTAATTTTTGTATTTTAGTAGAGGTGGGGTTTCGCCATGTTGGCCAGGCTGGTCTCGAACTCCTG
ACCTCAAGTGATCCATCTGCCTCAGCCTTCCAAAGTGCTGGGATTACAGGCATGAACCACTGCT
CCCGGCCCCAGCTGGCCCTTAAATCTCCCCTTCCTGAATGACTGCCTGCAGACTTCAGACTTGCT
TATCCAGCCCCCACAAGAACATAAGCCAATTTTTTTTTTCTTTTTGAGATGGAGTTTCACTCTTGTT
GCCCAGGCTGGAGTGCAATGAAGCCAGTTCTTTATAATAAATCCCCTACTGGTTCTGTGTTTCAA

ATTAACTCCTAATTCTGATATACGTTAGAATTCTCTCCTTGCTTGTTGTCTTCTGCTAACTCTAATA
GGCCACCAAAATTCTCTTGGTTGCAAGCATCCAAATGTAAGTTATCTCAGGTAAATTCTGGAGG
GCCTCCTCAGTACACAAATCCCTTATGTGGGAGAGCCTTACATTTTGGGACTTAGAGTCCACAG
ATTTTTGCTGTTTGGTCTTCTTGCCTAGTAGGCAGCTCTCCTTGGCAGGAACTAACAGTATTCACG
TGGCCCCAGGAAACTAGGAAATTTTATTTATTTATTTATTTATTATTTATTTATTTATTTTTAGACAG
TCTCACTCTGTCGCCCAGGCTAGAATGTAGTGGCATGATCTTGGCTCACTGCAACCTCCACCTCC
CAGGTTCAAGTGATTCTCCTGCCTCAGCCTCCCTCAGCCTCCAGAGTAGCTGGGATTACAGGCA
TGCACCACCACGCCCAGCTAATTTTTGTATTTTTAGTAGAAATGGGGTTTCACCATGTTGGCTAG
GTTTGTCTCAAACTCCTGACCTCAGGTGATTCACTCGCCTTAGCGCAAAGTACTGGGATTACAG
GTGTGAGCCACCATGCCCAGCCGGAAATTTTATTTTAGAGATAAGAAATTTAAGACTCAGAAAT
TTTGAGTTCTGGCTTGGCCACCAACTAGAGTAATAATTATTGTCTGTCTTAAGAGAAAGTGTTTTG
GATGATAATTTTGGGGATCTTTTGACATAACCGTCTTCACAATCGGTTTGTCTTTTTAATGATTTCA
TACTTACAGTGTTTTGATATCTCTCCCAATGCAAGACCACTATGTAGTTTAGCCACTGTAACTTTC
TACAATCTGCTCTAAGTCATTCTGATCAAAGTGCCAACTCAACTTTTCCTCCTTCCTCAGAGACG
AGTGCTCTAAACCAGTATCATGGCTCCAGTTCCTCTGACAAGGATTCACATGGGGTAAGTGCCC
ACTAAGGCAGAGGTGTCTACTTCAGCAGTGCAGAGCAACTCAGCTAAGTAGAGGGAATATGAA
ATAGAACCAGAAGTCTAGCCTGCTTAGCTACAAATGTTTCCACCAAAAATAGTGGATACAAATG
AATTAAAAGTATGCACAGTTTGGTGTCTGCTTGAACAATGGGCACATCCAGTGTGGAGAGCTTA
AAATGACTATTTTTATCCAAAAGAGGAACTATTTCCCAAGCTAGATCCCAGAATATGAGGCCTG
AGACCACCTCATTCCTATGGCTGAGAAAAGTAATACAGAATTATGGAGGCTGGCACGTCGGCAT
GTCCAAAATTTGCAGAGTGGGTAGCAGGCTAGTCCAGAGAAGTGCCAATATTGCGGTTTAAGTC
CAAAGTCTGTTGGCTAAAGAATTCCCTCTTGCTTGGAGGAGGTCAGTCTTTTGTGCTATTCAGGC
TTTCAACTGATTGGACGATGCCCACCCACATTATGGAAGGCAATTTGCTTTCTCCAAAGTCCACC
CTTTCAAATGTAAATCTCATCCAAAAACACTCTCACAGAAACATCCAGAATAATGTTTGACCAGC
CAAGTGGACACATAAAAGTAACCATAATTTTTTTTTTTTTTTTTTACGGCCGGGTGCGGTGGCTCAC
GCCCGTCATCCCAGCACTTTGGGAGGCCGAGGCGGGCAGATCACAAGGTCAAGAGATCAAGA
CCATCCTGGCCAACATGGTGAAACCCCGTCTCTACTAAAAATACAAAAATTAGCTAGACGTGAT
GGCGCGCACCTATAGTCCCACCTAATTGGAAGGCTGAGGCAGGAGAATTGCCTGAACCTGGGA
GGCGGATATTGCAGTGAGCCGAGATCGCGCCACTGACTCCAGCCTGGCGACAGAGCGAGACT
CTGTCTCAAAAAAAAAAAAAATTAAATTTTTAGGGCATAACTGCAGCTGTTCCTTAAGTAATTTGT
ATCTGCTTTAAACCACTAGAGAATTAAACTATTCTCAATCAATTCTAAAGGGTGCTGGAAATACA
TGTCTTTCAGCATCTTCAGGCTGAGTTTCAAGTGCTAGTTTCCTGCTAGTTTCCCAGTGCATGCCT
CACATTTTGGCCCAGTCAAGTAAGTCCCATCACCAGCAAATCCACAGTGATTCCACATCAGGAA
ATTCCCAGTGGTTCCATATCTGAGCTTGCTCAGAATAAACTTCTCACCCAAGAGCTTATTTACCA
CCATCAGATCTCTAAATAAATAACATTTTGTTTATTCCTATTACAAAAGCAATATATGTTCATTATA
GAACATTTAGAAATGTGGGAAAACAAAGAAAATAAAAATCACTCATAATCCTACCATTCAGAGA
CAACTACAGACCCTTCCAGTAAGATTTTTCTTACAAAAATGATATCAAACATTATATCCTATTTTA
TAACTTCCCCATTTTACAATACATTGTAAACATTTCCATGTCATTAAAAATTACATAATATCACTTT
TAATGGCTAGTTAATATGGAACCAACAAGGGCTTTGAACACATTAACTTCAGCTCTCTGAAAGCT
GAAAGGCAAATGGGATGACAAACTACCCTTATACAGGATTAGGTTCATAACAATCACAGGAAAT
GCCACATATGTGAAAATAAACAGATAACAATTTTGAAGTTTTTCCATTGAATGTACATGAAAGAA
TGTTTTGTCTTGTTTTCTTTTTTTTTTTTTTTTTTTTTTTTTTGAGACAGAGAGTCTTGCTGTCGCCCAG
GCTGGAGTGCAGTGGCCCAATCTCTGCTCACTACAACTTCCACCTCCCAGGTTCAAGCAATTCG
TGCCTCAGCCTCACGAGTAGCTGGGACTACAGGTGTTAGCCACCATGCCCAGCTAATTTTTGTAT

TTTCTGTAGAGACAGGATTTTTGCCATGTTGGCCAGGCTGGTCTCAAACTCACGGCCTCATGCAA
TCAGTCAGCCTGGGCCTCCCAAAGTGCTGGGATTACATGTGTGAGCCACCACGCCCAGTCAAC
AAATTTGTTGTTTTTTTTTTTTTTTTGAGACGGAGTCTCGCTCTGTCTCCCAGGCTGGAGTACAGTG
GCGCAATCTCAGCTCACTGCAACCTCTGCCTCCCAGGTTCAAGCAATTCTCCTGCCTCAGCCTCC
CGAGTAGCTGGAACTACAGGCGCGTGCCACCACACCCAGCTAATTTTTTATATTTTTAGTAGAGA
CAGGGTTTCACTGTGTTAGCCAGGATGGTCTCGATCTCCTTACCTCATGATCCGCCCGCCTCGGC
CTCCCAAAGTGCTGGGATTACCGGTGTGAGCCACCGCGCCTGGCTGTTTTTTTCTTCTTTTTGAG
ACGGAGTCTCACTCTGTCGCCAGGCTGGAGTGCAGTGGCGAAATCTCGGCTCACTGCAACCTC
CGCTTCCCGGGTTCAAGCAATTCTCCTTGATTCTCTCCGTGCCCGGCCAGCAAATTTGTTTTCTTA
TAACTTGCACAGCCTGTCCACCTCCCAATCACTTCTACTCAAACCTTCATCTAGGCTCAGCTAGG
CTAAGGGCAAAGGGTAAGTAGTAAAATTCACTCAGAACTCTGATTTTTTTGTTTTGTTTTGAGAC
AGAGTCCCACTCTGCCACCCAGGTTAGAGTGCAGTGGGGCAATCTCAGCTCACCACAACCTCC
ACCTCCTGGGTTCAAGGGATTCTCCTGCCTCAGCTTCCTCAGTAGCTGGGACTACAGGGGTGCG
TCACCATGCCCAGCTAATTTTTTTATTTTTAAGTAGAGATAGGGTTTTGCCATGTTGGCCAGGCTG
GTCTCGAACTTGTGACCTCAGGTGATCCTCCCGCCTTGGCCTCCCAAAGTGCTGGGATTACAGC
CTTGAGCCACTGCGCCCAGCCTTTTTGAAACAGGGTCTCACTCTGTCGCCCAGGCTGGAGTGCA
ATGGAGCAATCTCAGCTCACTGCAACCTCCACCTCCTGGGCTCAAGGGATTCTCCTGCCTCAAC
CTCCCACGTAGCTGGGATTATAGGTGTGTGCCACCACACCCGGCTAATTTTTGTATTTTTTTAGTA
GAGACAGGGTTTTGCCACATTGGCCAAGCTGGTCTCGAACTCCTGGCCTCAAGTGAACTGCCCA
CCTCAGCCTCCCTAAGTGCTGGGATTACACACCTGAGCTACCACACCCTGCCAGAACCCTGATT
TTTTTTTTTTTTTTTTTTTTTTTTTTTGAGACAGAGTCTCGCACTGTCACCCAGCCTGGAGTGCAGT
GGCGCGATCTCGGCTCACTGCAAGCTCCGCCTCCCGGGTTCACGCCATTCTCCTGCCTCAGCCT
CCCGAGTAGCTGGGACTACAGGCGGCCACCACCACACCTGGCTAATTTTTTGTATTTTTAGTAG
AGACGGGGTTTCACCGTGTTAGCCAGGATGGTCTTGATCTCCTGACCTCATGATCCGCCCACCTT
GGCCTCCCAAAGTGCTGGGATTACAGGCATGAGCCACCATGCCTGGCCCAGAACTCTGATTTTT
AAAGTGCTCCTTCTAAGAAGACTCAGAGCTACAGAGATGTCAGAAACAATCATAGAATGACTCT
ACATCCTCCCACAATGATACTAGACAGACACACTGGCACACCTCGAAATTTCTGGGTTGTGCAC
AGCCCAAGAGGGCTCATTTAAGCTGCCATGTCCACTCCTCCAACAAATCCAGGCACCATCTAAA
GGGAACATGCCAAGTCACAGTCTCCTTTGTGAATTCCATAATGGCAAGAGAGCTCTCATTCATAT
TTGCGTTCCTGGCACCCAGAGCACATTTAATAAAAGTTGATTAAACAAAAATAATGACTTGGTCC
TGAACTGCATTCTACAACTGCTCCTTGAGTTTTGTCCCATCCTATAATAACCATCACAAGCTCTGC
TATTTATTCTACCATTTCTGTCCTGGCCCCACCTTTGAGATTGTTCTCACAGTTATCTTTGCCACTA
GGTTGCAATTCTGACTGTGCTGGTGATGCTGGTTCCAGGTCCCTCAGGAACCCCTCCCCCAGTC
TGGTCTGCCCTTGAACTCTATACTGGCCCCAGGGTATCCCAGGATTGTCAGAAAGGCCTGGCCA
GGATTCACCTGGGAGAGGGATCAGCATCTCTGCAAAGTTGTTGGATTGAAAGAACTTTCATTTTG
AGCTACATCATTGAAGAGCTTTAAATCTGAAAATAAGAAAGACGTTGAACCCTATTACTCACATC
TCTTTGTCATTTGAAGCAGATCCCGAACCACTCAAAAGGACAAAACAAGGAAATAGCAATGAAA
TTGGATTCAGTGAATGGGGTAAGGGTATTAAGTGTCTGAAATATAAGAGATAAAAAAA
ID|56158|gene_id|ENSG00000239213|transcript_id|ENST00000470236 Homo sapiens lncRNA

GGGAGCGGAGGCTGCGCGGCGGAGAGTAGAGAGCTAGAGGGTGAGAAGCGCTGGGCCG
GACCAGAACTGTCTTAGCTTTTCCCTCTGCGGCTGCTCTGGTTGGTTACTTTGAGCCTGCCTCCTC
AGCGCCGCAGGAGAGACTTAACAGCAAAAATGACTGTGTCCTGAGTTCAGCCCCCACTGCTCT
GGGAGCTTCCCCCTGGTTTCTGCACCTGAAGAATGTCATCCCAGCCGAAGGTTCTTAGGAGCGG

GGAGCGACAAAGAAAAACAAAGAAAAGCAGACCTGAAAATTGGGACCTGGGAGAAATGTAAG
ACATGTGCTTAATTTGAGGCACAGACCAAAAGAAGCTGAGTCTGAGGGCCCTCTAAAAGTGTCC
TCGTGCTTGGGCAGCTTACATTCTTGGCGGCAGTAGTGTTATGAAGAGGACCTTCGGAGCCAGG
TCTGTCCATTCTTAGGGCTCCAGCCACTGGCTGAGGCATCAGTAAGTTGGTTCTTCCCCTCGCCG
GTGCTGCAGGCCCCCTTCCCCGCCCCCGCGCCGTAACCTGCCTCTTTTTTTTTTTTTTTTGAAACG
AGTTTCGCTCTGTCGCCAGGGTGGAGTGCAGTGGCACGATCTCGGCTCACTGCAACCTCCGCCT
CCGGGTTCAAGCTATTCTCCTGCCTCAGCCTCCCGAGTAGCTGGGATTACAGGCATGTACCACC
ACGCCCAGCTAATGTTTTTGTATTTTTAGCAGTGACGGGGTTTCACCAGGATGGTCTCGATCTCT
TGACCTCGTGATTCGCCAGCCTCGGCCTCCCAAAGTGCTGGGATTACAGGCGTGAGCCACCAC
GCCCAGCCGCCTCATCTCTTCTTAACCATCTTTGTGGCACAGTTTAGGATCGCTCCTCAGGGAAG
CTTGCTCAGTTAGGTACCTTCCTTGGAGCACAGGTGTTTTCTCCATCCGAGCTATTGAAATGGTTT
GCCTGTATGTCTACCTAGATTGTAAACTCCGTTGAATCAGTAACCATGTCTGTTCAGTTTCTCCCT
AGGGCCTTGCAGTAGGGTAGATGGTCAGTAAATACTGTTTAAGGCATTAATACTTTTTATTTTAAT
CCTGGCCCTGGATATCAGCCTCAAAAGTTTGCAGTTTTCTGCATTTAACTTCAAAATTATTTACGT
GATTGGTAATGCATGCATGTGGTTGGCCTGTGCTGTAATTTAGATGTTTGTTCCCCTAAACCTCCT
GTTGAAATTTGATCCCCAATGTTGGAGGTGGGGACTAATGGGCAGTGTTGGGTCATGGAGGTGG
TTCCTTCAGGAATAGATTAATGTCCTCCCTGGGGAGAGGGTAAATGAGTTCTCTTTATTTCCACC
AGAGCTGGTTAAAAAGAACGTCGCACTTCCTTTCTTCTTTTGCGTCCTCTCTGGCCATGTAATCTC
TGCACACACCAGCTCTCCTTTGCCTTCTGGCATGAGTAGAAGCAGTCTTAGGCTACCATCAGAT
GCAGATGCTCAGTCTTGAACTTTTCCAGACATCAGAACCATAAGCCGAAAAACCTTTTCTATTTC
TAAATTATCCAGCTTTGGGTATTCCTTTCTAGCAACACTAAAGGGACTAAGACAGCCTGCCATAT
GTAGGATCACAAGGTCAAGCACAAATTTACCTATTTATGCTGTACTATCCTCAGGTTGACTGGGA
CAAGTAGACCATGAAACTGGGCAACAGCTACTCTATGTCTACATTCTGTTATTAGCATCCAAAGT
GAATAATGGCGGTGAATTTTGAGTTTATTCTAAGGGTTTAAATAACTGCATATTTTATCATGACTG
GTATGGACTGAATTGCATCCCCCCCAAAAATTCATACTGAAGCCCTCACTCCTAATGTGACTATA
TAAATGTTCCTTAACTTACAAAGGGGTTATGTTCCAATAAACCCACCATAAACTGAAAATACCTT
AAGTTGAAAAGATCTTAAGTCAGAAATGCATTTAATGCTTCTAACCTACCAAACATCATAGCTTA
GAGTAGCCTACCTAAAACATGCTCAGAATACTTACATTAGCTTTCAGTTAGGCAAAATCATCTAA
TACAAAGCTTATTTTTTAATAAAGTATTGAGTATTTCATGTAATCTACTAAATACTGAATGAAAAA
CTGGATGGTTTTATGGGTACTCAAAGTACAGATTCTAGAGTGCATATGGCTTTTGGACCATCATA
AAGTCAAAAAAATCTTAAGTCAGGGACCATCTCTAGTTGGAGCTAGGCCTTTAGGGAGGTAATA
AAAATTAAATTAGGTCATAAGGGTGGGGCCCTAATCCCATAAGATTAATGTCTTTATAGAAAGTG
GCACCAGAGAGCTCTCTCCATTTGAGAACACAGCAAGCAGACAGACATCTGCAAGCCAGAGAG
AGAGCCCTCACCCAAAACTGACCATGCTGGCACCCTCTTTTGGACCTCCCAACCTACATAACTTT
GAAAAAATAAACTTCTGTTGTGTAAGCCACCCAATCTAGTATTTTATTATGGCAGCCCAAGCTAA
TACAATGGTTTTATTTGTTGATACATTTTATAAAGTTTACAGACTGAGATATTATATTCATTTTTGA
AGAAACTTGTCCAGAGTTAAAACTGTTGCTGCAAAATCACCCATCAAACTGTTAGCACAGCGTG
ACTCAATTTTAATTCTAATTACATATTTAACATACGATGGAGGCAGAAGGTGTGAATATAAATAC
AACAGGTAAAATACAGAAATACAGTTCTTGCCTTCATGTAGCTTACCACCACCTTAAGTGGGGA
AAATTAACCCAGCAAAAAGTATGCTGAAGTCATACATTTAGGGTTTTTGTTTGTTTGTTTGTTTTTT
GAGATGGAGTCTCGCTTTTTTGCCCAGGTTGGAGTGCAGTGGCACCACCTCAGCTTATTGCAAC
CTCCACCTCCCGGGTTCAAGCAATTCTCCTGCCTCAGCCTCCCAAGTTGCTGGGACTACAGGCA
CACACCACCACACCTGGCTAATTTTTTGTATTTTTAGTAGAGACGGGGTTTCACAATGTTGGCCA
GGCTTGTCTCAAACCCCTGACCTCAGGTGATCCACCCACCTCAGCCTCCCAAAGTGTTGGGATT

ACAGGCGTGAGCCACAGCACCCAGCTAGTTTATCCTCTTAATTTTCCTTCTTTAATCTTTCTCATC
ACTCATTCTTAACAGGAACTCTTTAAAAAAATTTAAGAAACCTCCAAAAATATATGCAAAAGAG
GAGTCAAAGAAGAAAAATAAGCAAACAGCAATGAAAAAGGAAAGCATAGACTGGAAGGGCAG
ATAGGCAGAAAGGCATGTATGTAATTGCACTTAGAAGGGATGCAGTACATGTTTTAATGGGGTG
GGAGCTGTCCCAGAGACTTCCTTTCACACAGTCTGTCTCCCCAGCTTCCAAATATAAACATGCAC
ATGGCTGCTGCTTCCTCTCTTTCAGGGGAACTCAAACCAAAAACAGGAATCATCCTTGCTAATTA
GTGCTGAGTGGATCAGGGACTAACCAGAATCAGGCAACAAGAAAATGTAAGTCAAAACTTATG
ATCAGCCCTGGCAACAGAGCTAGACCCTGTCTCTACAAAACAAAATTTTTTTTAATCAGCTGGGC
ATAGTGGCATGTGCCTGTAGTCCCAGCCACTCTGGAGGCTGAGGTGGGAGGATGGCTTGAGCC
CAGGAATTTGAGGCTGCAATGAGCTATGATCACACCACATCTCAAAACAAAAACAAAACTTATC
TAGGGACTACACGTGGAGCTTTGGATATGGGCAGCTAATCTGTCTATGATTAAAAACACGCAGG
TGGCTTACTTTATTAATCCAAAATAAAAGAATTTGGGAACACAAGAAAGGGAAAAATGAGCCTC
CATGTCAAGACCTTTCCCAGAAACCTGATAGCTTATAGTTAGAAATTGTTATAGACAGTCCAGTG
CAAGGAGGACATTAAGCCACAGGGGAAAGGAGTTGTGTGGCCAGGTTAGTCAGGCAATTAACT
AGAAAAAATCTGTCCACATAGAGGAGCCTGCATAATAAAAAGGTATTAAACGATAGTAGAATAT
TGCTAGAGCAGAAGATAGCTAGAAATATAATCAATAACCAGGTTATGGAAGTCAGGCACCTTAG
AGTTTATCCTATAGGCAATGAGAAGCCATTCAAAGGGTTTTTGTTTTTGTTTTCAGCACTTTAAAG
ATGTCATTCTCGTTTTCCAGCCTCCATTATATCTGTGAGAAACTAGTTGTAATTCTTATCATTGTTT
TCTTGTATGTCTTATGCCTTTTTCTATGAATGCTTTTAAGATTTTGTTTATATTTATCAAAAGTTAAC
ACTGATGTTTCTAGGTGTCTTTTTCTATGAATGCTTATAAGATTTTCATTATATTTATCAAAAGTTA
CACTGTGATGTTTCATTACACTGTGATGTTTCATTACACTGTGATGTTTGTTTTGGTTTTGGTTTTG
TATTTATCCTGCTTGGGGTTCATTTCATTCCCTGGGTCTGTGGATTGCTGGTTTCTCATTTTCTTTT
CTTTTCTTTTTTTTTTTTTATCAAATTTGGAAGACTTTTGTCCAGTATTTCTTCAAATTTTTTTTTCTGT
CCCATTCTGTTTCTCCTCTTCTTCTGGGACTACAATTACACTCATGCTAGACTTCTCAGTACTTAGT
ACAGGTCATTCAGGCTCTGTTCATTCTTTTTTCAGTTTGTATAATTTCTATTGAGAGCCACTGTCTA
GTAATCCCTGAAAAAATCTAAATGTTGACATTTTCCCAAAGCACAGTACCCTGCTGATGGCTGTA
GTTTCCTTTGGGGAAGGTTTAGAGATATGTTAGTCAGGAAATCAGAAATTAGATGATATTACTAC
AGAGAGGATTTAGTATTGAGTTTTCATTGACTGAAAAAGCAAAAACGGAACACTGAGGTTACCC
AGAGGTGATAACTGCAGGGAGCAGCTGTCAATGATAGATCTAGTGAAAATGGGGACTCAAGGT
GGATTATCAGGTCTCAGAAACTCAGACAAATGACCTGGCTGAGTATTGCTCAGACCCCTGAGAG
GATGCTGTCTGGCTGATCCTACTACTTCAGGGAAGGATATGATGAGGCTAGTTGTGGATGGGGA
GGATGTCAAAAGAAGCTCAAGGCTGAAATCAATTGCTGCTGCTGGAGCAAAGGGCTCTTGCTTG
TTGCTGGGATAACAGTGACAGAAACTGCAAACAAATGGAAAGGAGGAAGTCCCTTCTTCCCTTC
TGCCTTCCAGTCTCCCTCTAATGCCCTTTATTGATGGAACCTAGTGAGACACCAGCTGACATAGA
AAAAAATGTAGTTTGCAGGGTTTGAGCCACAGCATTACAGAGAAATGTATAGAAGGGGGAATG
TGGACCTGAGACAATAGTTTAATAAACAGTACCATATCATTTATTTGTGCTAAATTGGTAAGAAT
GGCCAAAATTAGACTTAATGCCATCATATGTGCTTTTACAGACTTTTAGACCTTTTTGAAAAGAAT
CTCAGGATACCATGTCAATTTTCTGCTACATTATGATTACTGTTGGTGCCTTTTTCAGGAGTAAGG
AGTACTTTTACTGCAGCTCACACATACACTAGCCACAAAATCATAAACAAGGGCAGCCTTGGAA
GAGATCTACATAACTGAGGATAAGCAGACCCTACCCCAACGTTAGGTCTCCAAACATCAGGGA
CAAAATGGCATGGGCTAGGTAGGACTGGCAAGAATCCACACTTCTCCCTATTTCTATTTCCTATG
TATCGCCTAATGTGCTCATAATAAGGAGAAAACCAATTTCCTGTTCTAACTTTGGACAGCCACCC
ATTGTCATTAAAAAACCACTTATTTTCCCTTCTTTCCTCTTTTCTTGCCTCAACCCAGGCTCCTTTA
CCCAGCCCTATGACTTCTAGTTTTCTAATGCAAGTAGATGACAAAACAGGGTTTGGTGTTCAACA

TTTCACCTTATAGTGTATTTTTCACAAATGAAGGAGTTTTGTTGAACATTTGTAGAACATTTCTAAT
TTTTGAACTTCAGTTCCATCTGAAAGGGGTCATTTCTTTGAGTCTTCTATTATTTAGTAATAGCAGT
TAACATTCTCCTTCTACTACAATTTACCTGGAAAGACACCCATAAATACCAATAAAGATACCAGA
TTCATAACGTATTTCATATATTTAATATTTGTATTTGAATATATTAGACTAGAATAAATTTTCTGCA
TTTGTATATATTATATATTTTATAGCACTCTGTCAGTTATATAACACAAAAGACATTCATAGGTAA
ACATTAAAATGGCAGTATACATAATAACTGAACATTAATGAGACAATAATAATCAGGTATTCATT
TAATGGGAGAGTCTAGTATTTCCTGGTAGTCCTGCTTTCTTAAATTCCTCCAGTAAAGTTTATAGC
CAGCAAGGACAAAAACACTAATCATAATGATTACCCCTCCAAAAACATCATAGATGCTAGGAAA
TATGTGCAGCACGAGAAGCTGCAAGACCATAGCTACCACAATCTCCAAATGTTGTACTGTGCTA
ACCAAAGCTGGATGGAATTTGTCCAAGGCATAATAAACTCCTAAGAATGCTGCAGTAGAACAGA
CACATATAGCAATGAGATAACTCCAGGTTTCTCCATCTAATGGGATGATGGGTTCTTGAAGAATA
AACATAGTAGATATTCCCCAAATTGTCCCAGTCCAACCAAAAGTAAACAGTGCAGTCCACATGC
TGATCTTCTCCTTGATGGATCTGTATACTATCATTGAGAGAGCAGTGGTCAGTCCAGCCATCACA
GTCATGGTGTACCCAAAGGCTTCTTTCCAGGCATTTAACAAAGAATTGTCTTCATCAACAATGTT
TGGGATCATGACAAGACAAACACCTAAGATGCTGCAAACAACTGTAGCCATGTCAACATAAGC
CATTTTCTCATCTACGAGTAAAAAAGCCAAAATGGCACTGAAGACTGTAGTTGTGGCTCTCCAC
ATAGTGGTCCCATTGCTGGGAGGAACTATTGAAAATGATGTATAAGCACAAGTGATAGAAATGA
CATTGCATACACCATAAAAGAAGAGTCGTAATCTGTATCCACTGGGTCCAAAGGGGGCCTCCTG
ATAGTAACACACAACTAACACAGATAAGACCTGAAAAACAGAACGGATAAAAATCAGTTCTAG
AGATGGAACTTTAGACCGATCAGAAACAAGCCTAGTGATAAGAGCTACACATCCATGAGCCAA
AGCAGATCCAAACAGCACTATCCACATTTTTCGGGATTGAAAAATGTTTTTTTCTGCAAAGCTCT
GGAATTGTCCAATCTCATTGATCATTGGGTCTTCTGTTGGTGGAGGTAGGGTATCCATGGTTCCA
AAGAAAGCTCTCCCTTTTTTTTTCATTTCACTCAGCAGACCTTTCTTTGGATTTTCCTCCATAAAAT
TTCCATAGCCTTCATTGATTTCTTCATATCCATCATCGCCAGGCTGGGGATAATGAGAAGTATATT
TCACCATCACTGTGTTGGGATGTATTTTCACCCGTTTTTTAACTGGATATTTTCTGGAGGGAGAAG
TATCCATTTCTTCAGATAATCAATTCTATTTAAAGAAAAAAATTGACAAAAAGACATTCATTTCAT
TGAAAATTTAATTTCTATTATAATCATATGCATCAATTCAATGATAGAAAGTAGAGGCTGTAACA
GTAGTCCAGAAAAAAAAGAAATTAGAGATGATCTGGCAATATGCCTTATCAGATGTATGGTACC
TACTGGAATTGTTTACCCTTTTTTCACTCTCATCTGAAGTCTGTCTCCATTTCTCTTTGCTTACTTGT
TTAATTCAGCTCTATCTTCTATCTTTATACCACAAACGGATTTTTCCATCTTTTCATTCCTACCTGTT
TCTCCACCTATCCAACAAGCAAAACACAGAAAGATAAATAATAACTTAGGTCAAACCTTTCCTTC
TCATTGGGTCCATTTGCCTGTTATAAATTATTAGTTAAGTCCAAAGTATTTTGTATAATCAATTCTG
TATAATACCAGAATTCACCTTATAAATTATAGTGATTTTTAAACATTTATTCTGGACTCCCCATAA
GTTTTGAGATATAAAAATACACTGAAATTAGAACATAAATAACATGAATTTAGTAACACTCATGC
ATTGTTGGTAGAAAGGTTAAATTGTACAACCTCTCTCGTGAACAGTTGGACCATATGTATTAAGA
TTTTAACTATCCTCTACTCAGCAATACCACTTTTAAGATTTTATGCTAAAGAGATGATCACAAGAT
GCTTTTTACATTCTGAATAAAAAAGGGGGGGAATAAGAATACGTATTTATATTTGTTTACATAAC
AAAGAAATTATATAAAGATGCCAAGAAAACTAATGGTGGTTCAGGTGACAGGAGGAACAAGAA
TGGAAGAAAACCTTTTTGTTATTTATTTTTTTACATTTTTATTTTTGAATTGTGATATTATCCAAAAA
TAGAATTAAAATGTTTATCTGTTACTTAAGAAAAAGAATATACCAAAAAATGTGGGGAAAATGTA
CATACAGGAGAGTTCATTGTAGCTTACTACAATGAATGTTCATACAATGGAATACTATGCTATCT
ATTAAAAATGACAGTGTAGGCACGGTGGCTCACACCTGTAATCCCAGCATTTTGGGAGGCTGAG
GCGGGCGGATCACTTGAGGCCAGAAGTTTGAGAGGAGTTGGCCAGCCTGGCCAACATGGTGAA
ATGCCATTTCTACTAAAAATACTAAATTTAGCCGGGCCTGGTGGTATGTGTCTGTAATTCCAGCT

ACTCGGGAGGCTGAGGCATGAGAATCACTTGAATTCAGGAAGCAGAGGTTGCAGTGAGCCAAG
ATTGTGCTACTGCACTCCAGCCTGGGTGACAGAGCGAGACTCTGTCTCAAAACAAAACAAAACA
AAAACGATGTCCACTATATAATGTCAAGTGGGAAAAAAAGTTGAACTTACTGTAAGAATTAAAT
AAAATAATGCATATAAACAATTTGCCACAGTGCTGGCATATAGTAAACTCTCAATAAATGTTAGC
AATAAATTTGGCTTCCTTTCACCTATAACAACTTTATGAAACACTGGGTATCTTCTACAATATTAT
TATTTCTTTTAGACCTTTATTTTTTCACTTGGTTCTTTCTTTCCTTCCTTTCAACAATTTCCTATTTCC
CTCCCAATGATCCTCTCCTCCCGCTTAAAAAACAAAAAAGAGACCAGGCACGCTGGCTCATGCC
TGTAATCCCAGCACTTTGGGAGGCCGAGGTAGGCAGATCACCTGAGGTCAGGAGTTCGAGACC
TGCCTGGCCAACATGGTAAAATCCTGTCTCTACTAAAAATGCAAAAATTAGCTGGGAATGGTGG
CAGGTGCCTGTAATCCCAGCTACTCAGGAGGCTGAGGTAGGAGAACTGCTTGAACCCAGGAGA
CGGAGGTTGCAGTGAGCCAAGATTGTGCCACTGCACTCCAGCATCAGCAACAGAGCAAGACTC
CATCTCAAAAAAAAAAAAAAGAAAAAGAAAAAAAATCTTTTTTTTTCTTCCTCGTGACTCCCTGAT
TTCTTGCCAAAAACTCATCGGTTCCAGGATCTTAAACAATGGCCCAGAAATAAAATCTCATTTTG
TTCTTCGACACCATCTGGGGAGCTAACTGTTCTATTCCTATTTACTCCTTTCTTTTCTAAAAGGCA
CCACCTTCAGCTGGAAGAAGTCATGAAAAAAATATCACCTGTGCCTCCAAACCCTTCTATTTCCT
AGCCTGGATGTCCCATTTGCAGTCCTTCTTCCTGCACACCACTATCAGGCTAATCTGACTGTTCTT
TATTTCCAACTTTTGCCAGGACATGTCAAATCATAGCTTCAACAATCCCAAACTGAATAGTCTTG
TTCTCCCAAATAGATTTTTTTCCCTAATGTCCTCATTTATGTCTGTGATACCACAATTTTTCCAGTT
CCCCAAGCTAGAAATCAGGGAATACTGTGAATTATCTTTTCTGCCATCTTACTTGCATTTGGTCA
CTAATCTTACTGTTTTTGTCTTTGAAATATTCCTGTTTTTCCCTTTCTCCTCCAATTCTATGTAACTT
TTATAATTGTGGTTTGGGTTATTGTGAATTCCCATCTAGAATATTGCAACAGCATCCTAACTAGCC
TCCCTTGAATCCCAGTCCCTCTCTAAAATTTATATTATCCCACTGTCACATTGCTCTTCCTTTAAAA
GACTCAAAACAAAAACAAAAACTTCTCAAGCTTTCCCCATTCAAGAACCTACAGTGATTTCCTAT
CTCTACCTTATCAAATTTCAACTCCTCTGCCAGGCTCTCAATACCCTGGCTAATCTAGCTCCTATC
AGTACTTAGGTCTTCAACACGCCTACCTTGCTCTGAGCAGTCAGTCCAGTGTCTTCCCTGTCCTC
ATTCTCCATATATACCCTAAGCTTTTATTCTCACCAGTTTCCCTTCTGCTCCAACTTTGTAAACACT
GTTCATCTTTTTTTTTTTTTTTTTTTTTTTTTTGAGACGGAGTCTCACTCTTCGCCCAGGCTGGAGCGC
AGTGGCACAATCTCGGCTCACTGCAAGCTCCACCTCCCGGGTTCACACCATTCTCCTGCCTCAG
CCTCCCGAGTAGCTGGGAACTACAGGCTCCCGCCACCACGCCCGGCTAATTTTTTGTATTTTTTT
TAGTGGAGACAGGGTTTCACCGTATTAGCCAGGACGGTCTCGATCTCCTGACCTTGTGATCTGC
CCACCTCGGCCTCCCAAAGTGCTGGGATTACAGGCGTGAGCCACCGCGCCCGGCCAACATTGT
TCATCTTTAAAGGCCCAGTACAAATTCTGTCTTTTAGAGACAGGCCTTTCCTCACCTCTAGCCACT
TGTGTATTTTTCTTCCTTGTTTAAACATCTGCAGTAGTACTTCTAACCTAATATTTTGTAATCTAAT
AATTTTGTAACCTAGCTGTCTCAACTCAGTGGAATTTTGTTTCTAGTCACTGTGCCCAAATTTGGT
ATGTCAAATAATAATTATTTTGAGACAGGGTCTCACTCCGTTGCGCAGGCTGGAGTGCAGTGGC
GTGATCACTGCTCACTGCAGCCTCGACCTCCAGGGCTCAAGCAATCCTCCCACCTCAGCCTCAG
CTAAGACACTTGTACACCACCCCTCCTGGCTGTTTTTTACACTTTCTGTAGAGACAGGGTTTTGC
CATGTTGCCCACGATGGTCTCAAACTCCTGGGTTCAAGCAATAAGCATGCCTCAGCCTCTCAAA
ATGCTGGGATTACAGGCGTGAGCCACCACGCCCAGCTGCATTATTTTTTTTTTTTAATTCAACAA
CTTTGTATATTTTTGGATACAAATACAAAGTTATATCATGCTTTCAAGATATTCTTTTACTAATTCT
CAGAACAATGCAAGGCATAAATATTGTGCACACTCATGCCTACTTGTGTGATCCTGAGTTTAATA
GAAGAAAGGATAGAAACCAGCTTGGCTTATACATTTTTTAAAATATTATTTGGCTCATTTTCCCTA
AAAACATCAAGAAAGGAATTGCCACATGCCTAATTGAAAAGATATGCTTAGTGGTAAGGATATA
TATACATCAACTTTTAAGAGGTTTAAAAAAAAAAACTCCTCCCATTGTAACAGTTTATAGTTCACCA

CATACTACTAAAACAACCCATAAACCATGAGTGCATTATCAGGAGTGACAGGAGGGTAAGTCG
ACCGATGTGAAATTTACCTTGTAGGCAATTGACATTCTGCGGTGTATGGCAACTGGTCATGAGC
AGTACAAAGAATTTAAAAGGATCCAATATCTCAATATGTTGAAACAAACTGTATCACTTGGATCC
ATCAATATCATTTTCCATGGTTAGCTTTTGTTTGTTTTTAGTTATTTATTTATTTTTGAGACGGAGTC
TCGCTCTGTCCCCCAGGCTGGAGTGCAGTGGCATGATCTCAGCTCACTGCAACCTCCACCTCCC
GGCTTCAAGAGATTCTCCTGCCTCAGCCTCCACAGTAGCTGGGATCATAGGCATGTGCCACTAC
GCCTGGCTAATTTTTGTATTTTTTTGGTAGAGACGGGGTTTCACCATGTTGGTCAGGCTGGTCTCA
AACCCCTGACCTCAAGTGATCCGCCCGCCGCGGCTTCCCGAAGTGCTGGGATCACAGGCATGA
GCCACCACGCCCAGCCTAAATCCTGTATTTTTAAAAACTAACAATGGGCCAGGTGCAGTGGCTC
ATGCCTGTAATTCCAGCACTTTGGGAGGCTGAGGCAGGCGGATCACTTGAGGTCAGGGGTTTG
AGACCAGCCTGGCCAACATGGTAAAACCTCGTCTCTACTAAAAATACAGAAAATTAGTTGGGAG
TGGTGGCACATGCCTGTAGTCCCAGCTACTTGGGAGGCTAAGACACCAGAATCACTTGAACCCA
GGAGGCGGAGGTTGCAGTGAGCCAAGATCGCACCACTGCACTTCAGCCTGGGAGACAGAGAA
CTTGTCTCAAAAATAAAAAATAAAAATAAATAAAAGCACAGGATTTAAAGTAAAAAAAAAAAGT
TGGGTTCAACTTTACTTATTAATCATGACTTTGGGTACATGATTTCGTTTTCATAAACTTTAGTTTC
TACATGTATAAAAGATAATTATACTTATTACCTATGATTATTACAAAGTATAATGATAAAATATAT
ATAAATTAAATGGCATAACTTGTATATTTAGCACGCAATAAAAATTAGGTACTATTATAATTGCAT
AATTTTTTTTTAGGTTCAGGGAGTACATGTGCAGATTCGTTACGTGTGTGTGTGTGTATATATAAT
CTTTTTTTTTTTTTGAGACGGAGTTTCGTTCTTGTTGCCCAGGCTGGAGTGTAATGACATGATCTT
GGCTCACTACAACCTCCACCTCCGGATTCAAGCGACTCTCCTGCCTCAGCCTCCCAAATAGCTG
GGATTACAGGCATGTGCCACCACGCCCGGCTAATTTTGTATTTTTAGTAGAGACAGGGTTTCTCC
ATGCTGGTCAGGCTGGTCTTGAACTCCCTACCTCAGGTGATCCGCCTGCCTCAGCCTCCCAAAG
TGCTGAGATTACAGGCCACCGTGCCCGGTTCGTTACATGTATATATTATGTAATGGTAAGGTTTA
GGATTCTGGTGTATCCATCACCCAAATAGTGAGCATTGCACCCAATAGGTAATTTTTTAACCCTC
TCTTCCCACCTACTCTAACCCGTTTCAGAGTCTCCAGGGTCTATTATTTCCATCTTTATGTGTAATT
GTTTAGCTCCCACTAATAAGAGCTTGTAAGACAGAACATGCAGTATTTGATTTTATGTTTCTGAGT
TATTTTACTTAGGATAATAGCCTCTAGCTCTATCCATGTTGCTGCAAAGGACATAATTTCATTCTT
TTTTATGACTATGATGGCTTTTTGTCACCCCAGATTCACTTTGTCACCCAGTGAATATGGCTGGCT
CAAGTGATCCTCCCACCTCAGCCTCCTAAGTAGCTGGGGCTTCAGATGCCTGCCATCACGCCTA
CCTAATTTTTGTATTTTTTGTAGAGATGGGTTTTCGCCATGTTGCCCAGGCTGATCTCAAACTCCT
GGGCTCAAGTGATCCACCAACCTCAGCCTCCCAAAGTGCTGGGATTATAAGCATAAGCCACCA
CACCCAGCCTATGATATGCTGAACTACATTTTCCAATTTACTTAATCAAACACTAATCTATCTAAG
TTGCTGTTGTGAAGAGATTTTGCAGATGTGATAAAAGTCCCTTTTGTTTGCTGTAAATGTGTTGAT
AAATAAATAGCCCCTAATCTGTTGACTTTATCCTGGGTAGGCCTGACTTAATAAGGGGAGCCCTT
TAAAGGTGAGGGCTTAGGCCTTTCTTAAATTCAGAGACTCCAAACAGCTTATGCCCTTGGGATTC
TAGCCTGTTCATGATTTTTTCTTTACTGCCTGTGGACAACAAGCATTATCCTGTGCTCATGGGGTT
CTAACCAGTTCTTTCTCTTTTGTTTTGTTTTTGTTTGTTTTGAGACAGAGTCTTGCTCTGTCGCCCA
GGCTGGAGTGTAGTGATGCAATCCCGGCTCACTGCAACCTCTGCCTCCCAGGTTCAAGCAATTC
TCCTGCCTCAGCCTCCTGAGTACTTGGGATTACAGGCACCCACCACAGCCACCACCAAATCCAG
CTAATTTTTGTATTTTAGTAGAGGTGGGGTTTCGCCATGTTGGCCAGGCTGGTCTCGAACTCCTG
ACCTCAAGTGATCCATCTGCCTCAGCCTTCCAAAGTGCTGGGATTACAGGCATGAACCACTGCT
CCCGGCCCCAGCTGGCCCTTAAATCTCCCCTTCCTGAATGACTGCCTGCAGACTTCAGACTTGCT
TATCCAGCCCCCACAAGAACATAAGCCAATTTTTTTTTTCTTTTTGAGATGGAGTTTCACTCTTGTT
GCCCAGGCTGGAGTGCAATGAAGCCAGTTCTTTATAATAAATCCCCTACTGGTTCTGTGTTTCAA

ATTAACTCCTAATTCTGATATACGTTAGAATTCTCTCCTTGCTTGTTGTCTTCTGCTAACTCTAATA
GGCCACCAAAATTCTCTTGGTTGCAAGCATCCAAATGTAAGTTATCTCAGGTAAATTCTGGAGG
GCCTCCTCAGTACACAAATCCCTTATGTGGGAGAGCCTTACATTTTGGGACTTAGAGTCCACAG
ATTTTTGCTGTTTGGTCTTCTTGCCTAGTAGGCAGCTCTCCTTGGCAGGAACTAACAGTATTCACG
TGGCCCCAGGAAACTAGGAAATTTTATTTATTTATTTATTTATTATTTATTTATTTATTTTTAGACAG
TCTCACTCTGTCGCCCAGGCTAGAATGTAGTGGCATGATCTTGGCTCACTGCAACCTCCACCTCC
CAGGTTCAAGTGATTCTCCTGCCTCAGCCTCCCTCAGCCTCCAGAGTAGCTGGGATTACAGGCA
TGCACCACCACGCCCAGCTAATTTTTGTATTTTTAGTAGAAATGGGGTTTCACCATGTTGGCTAG
GTTTGTCTCAAACTCCTGACCTCAGGTGATTCACTCGCCTTAGCGCAAAGTACTGGGATTACAG
GTGTGAGCCACCATGCCCAGCCGGAAATTTTATTTTAGAGATAAGAAATTTAAGACTCAGAAAT
TTTGAGTTCTGGCTTGGCCACCAACTAGAGTAATAATTATTGTCTGTCTTAAGAGAAAGTGTTTTG
GATGATAATTTTGGGGATCTTTTGACATAACCGTCTTCACAATCGGTTTGTCTTTTTAATGATTTCA
TACTTACAGTGTTTTGATATCTCTCCCAATGCAAGACCACTATGTAGTTTAGCCACTGTAACTTTC
TACAATCTGCTCTAAGTCATTCTGATCAAAGTGCCAACTCAACTTTTCCTCCTTCCTCAGAGACG
AGTGCTCTAAACCAGTATCATGGCTCCAGTTCCTCTGACAAGGATTCACATGGGGTAAGTGCCC
ACTAAGGCAGAGGTGTCTACTTCAGCAGTGCAGAGCAACTCAGCTAAGTAGAGGGAATATGAA
ATAGAACCAGAAGTCTAGCCTGCTTAGCTACAAATGTTTCCACCAAAAATAGTGGATACAAATG
AATTAAAAGTATGCACAGTTTGGTGTCTGCTTGAACAATGGGCACATCCAGTGTGGAGAGCTTA
AAATGACTATTTTTATCCAAAAGAGGAACTATTTCCCAAGCTAGATCCCAGAATATGAGGCCTG
AGACCACCTCATTCCTATGGCTGAGAAAAGTAATACAGAATTATGGAGGCTGGCACGTCGGCAT
GTCCAAAATTTGCAGAGTGGGTAGCAGGCTAGTCCAGAGAAGTGCCAATATTGCGGTTTAAGTC
CAAAGTCTGTTGGCTAAAGAATTCCCTCTTGCTTGGAGGAGGTCAGTCTTTTGTGCTATTCAGGC
TTTCAACTGATTGGACGATGCCCACCCACATTATGGAAGGCAATTTGCTTTCTCCAAAGTCCACC
CTTTCAAATGTAAATCTCATCCAAAAACACTCTCACAGAAACATCCAGAATAATGTTTGACCAGC
CAAGTGGACACATAAAAGTAACCATAATTTTTTTTTTTTTTTTTACGGCCGGGTGCGGTGGCTCAC
GCCCGTCATCCCAGCACTTTGGGAGGCCGAGGCGGGCAGATCACAAGGTCAAGAGATCAAGA
CCATCCTGGCCAACATGGTGAAACCCCGTCTCTACTAAAAATACAAAAATTAGCTAGACGTGAT
GGCGCGCACCTATAGTCCCACCTAATTGGAAGGCTGAGGCAGGAGAATTGCCTGAACCTGGGA
GGCGGATATTGCAGTGAGCCGAGATCGCGCCACTGACTCCAGCCTGGCGACAGAGCGAGACT
CTGTCTCAAAAAAAAAAAAAATTAAATTTTTAGGGCATAACTGCAGCTGTTCCTTAAGTAATTTGT
ATCTGCTTTAAACCACTAGAGAATTAAACTATTCTCAATCAATTCTAAAGGGTGCTGGAAATACA
TGTCTTTCAGCATCTTCAGGCTGAGTTTCAAGTGCTAGTTTCCTGCTAGTTTCCCAGTGCATGCCT
CACATTTTGGCCCAGTCAAGTAAGTCCCATCACCAGCAAATCCACAGTGATTCCACATCAGGAA
ATTCCCAGTGGTTCCATATCTGAGCTTGCTCAGAATAAACTTCTCACCCAAGAGCTTATTTACCA
CCATCAGATCTCTAAATAAATAACATTTTGTTTATTCCTATTACAAAAGCAATATATGTTCATTATA
GAACATTTAGAAATGTGGGAAAACAAAGAAAATAAAAATCACTCATAATCCTACCATTCAGAGA
CAACTACAGACCCTTCCAGTAAGATTTTTCTTACAAAAATGATATCAAACATTATATCCTATTTTA
TAACTTCCCCATTTTACAATACATTGTAAACATTTCCATGTCATTAAAAATTACATAATATCACTTT
TAATGGCTAGTTAATATGGAACCAACAAGGGCTTTGAACACATTAACTTCAGCTCTCTGAAAGCT
GAAAGGCAAATGGGATGACAAACTACCCTTATACAGGATTAGGTTCATAACAATCACAGGAAAT
GCCACATATGTGAAAATAAACAGATAACAATTTTGAAGTTTTTCCATTGAATGTACATGAAAGAA
TGTTTTGTCTTGTTTTCTTTTTTTTTTTTTTTTTTTTTTTTTTGAGACAGAGAGTCTTGCTGTCGCCCAG
GCTGGAGTGCAGTGGCCCAATCTCTGCTCACTACAACTTCCACCTCCCAGGTTCAAGCAATTCG
TGCCTCAGCCTCACGAGTAGCTGGGACTACAGGTGTTAGCCACCATGCCCAGCTAATTTTTGTAT

TTTCTGTAGAGACAGGATTTTTGCCATGTTGGCCAGGCTGGTCTCAAACTCACGGCCTCATGCAA
TCAGTCAGCCTGGGCCTCCCAAAGTGCTGGGATTACATGTGTGAGCCACCACGCCCAGTCAAC
AAATTTGTTGTTTTTTTTTTTTTTTTGAGACGGAGTCTCGCTCTGTCTCCCAGGCTGGAGTACAGTG
GCGCAATCTCAGCTCACTGCAACCTCTGCCTCCCAGGTTCAAGCAATTCTCCTGCCTCAGCCTCC
CGAGTAGCTGGAACTACAGGCGCGTGCCACCACACCCAGCTAATTTTTTATATTTTTAGTAGAGA
CAGGGTTTCACTGTGTTAGCCAGGATGGTCTCGATCTCCTTACCTCATGATCCGCCCGCCTCGGC
CTCCCAAAGTGCTGGGATTACCGGTGTGAGCCACCGCGCCTGGCTGTTTTTTTCTTCTTTTTGAG
ACGGAGTCTCACTCTGTCGCCAGGCTGGAGTGCAGTGGCGAAATCTCGGCTCACTGCAACCTC
CGCTTCCCGGGTTCAAGCAATTCTCCTTGATTCTCTCCGTGCCCGGCCAGCAAATTTGTTTTCTTA
TAACTTGCACAGCCTGTCCACCTCCCAATCACTTCTACTCAAACCTTCATCTAGGCTCAGCTAGG
CTAAGGGCAAAGGGTAAGTAGTAAAATTCACTCAGAACTCTGATTTTTTTGTTTTGTTTTGAGAC
AGAGTCCCACTCTGCCACCCAGGTTAGAGTGCAGTGGGGCAATCTCAGCTCACCACAACCTCC
ACCTCCTGGGTTCAAGGGATTCTCCTGCCTCAGCTTCCTCAGTAGCTGGGACTACAGGGGTGCG
TCACCATGCCCAGCTAATTTTTTTATTTTTAAGTAGAGATAGGGTTTTGCCATGTTGGCCAGGCTG
GTCTCGAACTTGTGACCTCAGGTGATCCTCCCGCCTTGGCCTCCCAAAGTGCTGGGATTACAGC
CTTGAGCCACTGCGCCCAGCCTTTTTGAAACAGGGTCTCACTCTGTCGCCCAGGCTGGAGTGCA
ATGGAGCAATCTCAGCTCACTGCAACCTCCACCTCCTGGGCTCAAGGGATTCTCCTGCCTCAAC
CTCCCACGTAGCTGGGATTATAGGTGTGTGCCACCACACCCGGCTAATTTTTGTATTTTTTTAGTA
GAGACAGGGTTTTGCCACATTGGCCAAGCTGGTCTCGAACTCCTGGCCTCAAGTGAACTGCCCA
CCTCAGCCTCCCTAAGTGCTGGGATTACACACCTGAGCTACCACACCCTGCCAGAACCCTGATT
TTTTTTTTTTTTTTTTTTTTTTTTTTTGAGACAGAGTCTCGCACTGTCACCCAGCCTGGAGTGCAGT
GGCGCGATCTCGGCTCACTGCAAGCTCCGCCTCCCGGGTTCACGCCATTCTCCTGCCTCAGCCT
CCCGAGTAGCTGGGACTACAGGCGGCCACCACCACACCTGGCTAATTTTTTGTATTTTTAGTAG
AGACGGGGTTTCACCGTGTTAGCCAGGATGGTCTTGATCTCCTGACCTCATGATCCGCCCACCTT
GGCCTCCCAAAGTGCTGGGATTACAGGCATGAGCCACCATGCCTGGCCCAGAACTCTGATTTTT
AAAGTGCTCCTTCTAAGAAGACTCAGAGCTACAGAGATGTCAGAAACAATCATAGAATGACTCT
ACATCCTCCCACAATGATACTAGACAGACACACTGGCACACCTCGAAATTTCTGGGTTGTGCAC
AGCCCAAGAGGGCTCATTTAAGCTGCCATGTCCACTCCTCCAACAAATCCAGGCACCATCTAAA
GGGAACATGCCAAGTCACAGTCTCCTTTGTGAATTCCATAATGGCAAGAGAGCTCTCATTCATAT
TTGCGTTCCTGGCACCCAGAGCACATTTAATAAAAGTTGATTAAACAAAAATAATGACTTGGTCC
TGAACTGCATTCTACAACTGCTCCTTGAGTTTTGTCCCATCCTATAATAACCATCACAAGCTCTGC
TATTTATTCTACCATTTCTGTCCTGGCCCCACCTTTGAGATTGTTCTCACAGTTATCTTTGCCACTA
GGTTGCAATTCTGACTGTGCTGGTGATGCTGGTTCCAGGTCCCTCAGGAACCCCTCCCCCAGTC
TGGTCTGCCCTTGAACTCTATACTGGCCCCAGGGTATCCCAGGATTGTCA

ID|56173|gene_id|ENSG00000255471|transcript_id|ENST00000528660 Homo sapiens lncRNA

TTCCAGGTGACCAGTGGTCCTCCTTTCTGAAGGCTCTTGCTACAAAGGAAAGATGAGGATA
TTCTATTTCTTGATCCCTCTCAGCTACTGGGAGCTGCTTTGTGCCTTGGAAGCCTCAAGCTGAAA
TGGATGTCAGCCTCAAGTTTAACTCCAAGAGGGTTCACCTTGTTTCTTTGAACAAAATGTGCTGC
CTGTTGCTTCCCTTTGCCAGGAGTGGTGTTCCCTTTTCCACAGGTCTGGACCACAGTGTCTCTAG
CTCCCGCTCCTGGATACTTGGTTACTGTGTGTTTCGGTAAAAAAGCATCCACAACATGAATATCC
ACTAAGCTGTAACAGGGCCTGCTGCAACTATAGTATTTCCTGAAAAAGTATCAACTGCCACAAG
GGCCACAAATAAAGTCTGTGAACTTGAAATAACCATGGAGAATGAAATGTTCTGATTCAGATCA
TCGGTGGGATTTCACTGGGAAATCTCCCACTGGGCAAGAGTAGATGATGGAGGAGGAGAAACA

GTCACTAGAAAAAGGTGATACTTTGTATCAAGAAACAATTAAAAATTAGATCACATCCTGAAAC
ATTAAGCATATGCTCCTTTGTGAGGCTTGCCTGGCTGAACTTGAACTGGATTGAGTGAATAATTT
CTTAAAAACAATCAAAATCACAGATTTATTCTATTTTAAATACTTATATTTACTTATATTTCATTCA
GAAATTAGTTAACAAATAATTTTTGAGTATCCTGGGAACTGTTCTAATCATTAAACATGCAAGTTT
GAAGAAAGTAAGGATGAATCTGCCTGCGTGGGGCTCTCAATCTAGCGAGAAGCAGATAAGAAG
ACAAATGTCAGGGAAGATCACACAACCTTCAGGTAATATTTAAGCTGAGACCTCACTAGTTGCT
AAGACTGGATTTCAGAGGTCAGGTTTGGATATACAAAATTTAGGAGCTGGTACATGACATTTAA
AACCATGGGAATGAATGAGATTTCCTAGATAGAAAGAAGAAAAGAATATTGACATTTAATGTTG
AACGGAGTAGGAGGAGCTGAACAGGGAGACCAGGAAGGAGCAGACAGTGAACCTGGAGCAT
GAATAGGAGAGTGGGTGTCATGGAAGCCAAGAGAAACAAGAATTCAAGAAAGAAAGTTTCTTA
CTGTAGCAAATGGCGCTGAGAGGGAAAGTAAGGCCATGGAATGATGACAATGACAACAGTGAC
GAAGGATCATTGAGTATGGTATCATGCTGACATTTGTTTCTACCCAGGCTAGGTCGTTACTGAGG
GGTGGTAGCTAAGACAGGGTGGGTTGAACATGAGGAAATGAAGACAGAACATGAATTTTTGGA
AAGTTTGGCTACAATGGGAAGTAGAGAAATGGGGTAATATCTGGAAGGGGAGGGAATTGAGTC
AGTAGGGTTTTGTGTTTTGATTTTACGGTAGGGCATCTAGATAGATCATGTTTATGTACCTATTCC
CACTCTGTTCTCTCTTCCTGAAGAGTCTCCTCTGGCAAACTCCATTCTGTAAACGTCCGTATTTAA
GTGTCCTGTTCTCTGTCAAACATTCTTCCCAGGCCTAGTATGATGTTTGTCCCCAGGCTTCCATGC
CATTATGTGTACACATTATCTGCCATAAGCTTACCTTATTGCATGGCAATGGAAGTTTTTCCTCCT
TCTCTCTTCTTCATTAGACAAGAACAGGGTCTTGAAAAGTAGAGACTGTGTCTTCTCAAGTTCGG
ACTACTGGCACAGGTAGGTGCTCAGAACTTGGCACCAGGAAGATGCTTATTAAATATTTGAAGA
ACAAATAAGTAAATGAATGAATAGACTAACTGAGTTTGAAGGCAGTGCTATGGCAGATTTTTCTT
ATTTTATGAATGGAAAAACTATGGCATAGAACAATTTGGAATCTGATAATTTTACAAATTCGTAG
GAGTGCTGGAAAACAGTAAAATCTTCAGTCTTGCTCCTTGAGCTGTTGCCCAGGTCCCTCTCTGG
GACTGCACTAGCAGCCCTAAAAAACACCTGGGGCTGATACCCTCACATTCACACCAAACCGCA
GACCTCACCACAGAAGCACAGGCCTTCACGCTGGCGTCTGCAGTCTACTTGTAGTTTGATTTTAG
CAACATTGGCCAAGTTGAGGCTGGGAAGCAGCTGGTGAACAAAGGGTGGATGCACTTTTCTGA
ATCTAGGGGGAGACCAGGTTAGAGCCATGCTAAGTGAAGCTGCTAGGGTAGGTGGGCCAGGC
AGGGGAACAGGCAAAGATACCCTTGGCTCCCTTAAACTTGGGCTAGAAAAAACAAAGACATGG
CTCAAGGAGAACCACAAGGTGTCACTGTTCATCCTCAGTTAAACAAACACTGATTTGGGCAGGG
ACTTGAGAAACACTGCAAAGAACTTTATTTTGCAGATGAAGAAACGGAGGCCCGGGGAGGTAA
CATGATTTGACTAAGATCACGTAACAACTTAATGGCAGAACAATTATGAGAAACCAAGTCTCTTA
ACTTTCAGACTGGCAGGACCACTTTCTCCCATTGCTCCTATGAGATGGCATTTTGAGATGAGGAG
TAAGCCTTCTCTACAATCATTCACTCAAGACAATGGCTTTCAATTAGAAATAGAACAGAAAAAAA
ACAAGAGAGAGAGAGAAGAAAAGAATGAAAAAAGCGCAGCATTCACTCTCACATGTGCCTTATTC
AAAGTGCTGGGCTAAGATGCCAGTGCCCACAACGAGAGAGTGGCCCACAGGGCCTCCGTCTGT
GGCAGGCTTGAATACCCGAATAGAAAAGTATCAGGTAAAGGATTTCATTCTTTTTATGGCTCCAA
CTTAAAAAGAGTCTGGTTCCCAAGGAAAACAGGAAGCCAAGTTGCTCAGCAAAGAATTGGCTG
AAACCTAAGTAGGACCCAGATAAAAACATTTGCTGGATGGAAGGATGTGGGCAAATTTCTGGG
CCCCGCTGCTTGGGGATGGGAAGCTATGAGGCAGAAGACCCTTTGTGAAACAGTGACCCGTGC
ACCAGGCATTTTTGCGGATGTCTGGAAACTGCGAGGCAGTGGAGGAGTCATAAGTTAGTTGGTT
TTTTAATTCCAACTAACTTTACTCCACCAAGTATCTCAGGAGATTTCGCCTATGCTCTTTGGCCTG
ACTCCCACTAGGCCCTTACTCCTTCCATGGTGGCTTCTACTGGAGCCTCTTTCTTAGTTCCCAGGT
TATTAAAATAATAATAAATTCAAAGAGCTTTGTTGTTTTCTTAGGTAATATAATTAATATGCGCTC
ATTACAAAAATTCAGATACCAAGTAGCAGAATGAAGAAAATAAAAGACATCTGTAGTCTCTCCA

TTCAGAAATAACTGCTGTTAGCATAATGCTGTATACATTCAGGCTGTTGGCCTGTGGAACACCTG
GTATATGTCCTCGGCCCTGGGTTTACAGCTGTAAAGCTGGCACACACTAATCTCAGCTCTCACA
GAGCCTACATTCTAATTGTCTGGGAGAGACAGACAATAATATTTTGTAAAAAAGTAAAATATATA
GGATGTCAGATTGTGGTAAATGCTATGAAGACAGAGAAGGGGATGGGGATTTTAATGGTGGTG
AGGTGGCAATTTAAAAATAGGATATTCAGAAAGTGACATTTGAACAAAGATCTGAAGCAGATGT
GGATATCTGGGAGAAGACTTCTTTCTTTCTTTCTGTACATAGACAATAATGCAATTATAACATAAA
CAATATATGAATACATCTTTGATTTAAAATCTTCAAATAATACAGAAATATATGGGAAACAAGGG
AACAACCTTCTCACCACCCTCCTATTACCTTCTCCTCTCTCCCAATAAGCACTTTGGCAATGGTGT
GTTCCCTGCCCAAGCTTCATCTCTGCATTTACATTCATTTCTGTATATACTGACACAATAGCTTTAT
TTTATTTCCTACGTAAATGAGCTCATCCTTTGTGAAGGTGATATCTTTTTATTTTCAGCAAAAAGA
GTCGTCTTAACATTTTCTAAAATTCAAAGGTATGTGAGAGCTATAGGCCTCATTACTAAAAATGA
AATAGCTAAATGTGATTGCTAAGTGTGGGTAAATGTTTTACTTCTTCCTTTGGGCTAATAATCTAG
CATATACTGAATTCTATTGAAATAATTTGTTTCTGCCTCTGTCTCTGCTACTACACAGTGAATTTTT
TGTGGCCAGGGAATTAGGTCTTCGCATCTATTAATTTCCTGTGCTTGGTGCAATGCCCAGCATGT
GGTGTTTGGTAAATGAATAGGCAATCAATTATACTTTCATACATTACTGTGGAAGGCTTCCAATA
CTGGGTGAGATATTGGCTGAGATCAATCATCTGCCCTAATGTGGTCCAATCTTTTCTCCATTGAA
ATATCTATGTCACTATGGGACACAGCCACTGTGGGATGATAGCCAGGATGACTGTGGTCAGATC
CTACAAATCAGATAGTCCGATTTTAAGCATTAGAATTAAAAAATCAAGTAATGAGAGCTACCTTT
CATTCCTCTTGCTGCAAGAAAAGAGTTAGGACACAAATGTGCTAAGCACTTGACCTAACACTTCT
TGTTCAAGACACAAAAGTCTCAATGAAGTAGATATTATTTTGTGGACAAGGAAACTGAAAATCA
GTGATTAATATGGATATTAGTTAGATCCATTCATGTGTCTACCATACAGACTTATCAGATCTTAAC
ATTTCCCTATGTTTGCCTTAATTTTTTTCCCCAGAAATATTACACCAGAGGCCCACGATCACAAAT
TCATTGATTGATTGAGACGGAGTCTCACTCTGTCGCCCAGGCTGGAGTGCAATGGCACAATCTC
AGCTCACTGCACCCTCCGTCTCCCGGGTTCAAGCGATTCTCCTACCTCAGTCTCCCGAGTAGCT
GGGATTACAGGCTTGTGCCACCACGCCTGGGTCATTTTGTATTTTTAGTAGAGATGGGGTTTTGC
CATGTTGGTCAGGCTGGTCTTGAACTCCTGACCTCACGTGATCTGCCCGCCTTGGCCTCCCAAA
GTGCTGGGATTACAGGCGTGAGCGACCGCGCCCGGCTCCACAAATTTAATTGCAAACATTGGAT
GGCACATTTAAAAAGCCACTTAAAACAACTTTGTCATTTTATTCAATGGACATGAACTGAGCCTC
GGTGCTGGGCTAAGCTGTACGGATACAGAAAGGTATAAAGCTCTGTCCTTGTGCTCACAGGGAC
TCACTTTTTAGTGGGGGTGGCAGACGTATAAGTTTTACAGTTGAGAAACTGAGGCACAGAGAGG
GGTGGTTACTCTTTCAAGAGGCAAAGCCAGGTTTATACACCGGGGGTCAAACAAGCTGGCCTCT
TGGTGGACTGGGAGGCCCCTACACATTCCTGGTCTGTTGGAACAGTGTGGATTCTGAGTGGGGG
CACCTCTTCTCTGGGTGGGTCCAACTGGGAAGGGGAGCCAGTTTGACACAATGAAGCCGAATC
TGTGAGAAGCACTCATGAGTGTTTTTCTCTCTTTCCACTAACCATCTTTTTGCTGCAAAGAATTAA
ATTCTTTTCTGGTGACACACACTTCCTGTCAGTATGAGGCACACCCAGCCAGACAAAACCACCC
AAAGTAAATGAAGAAAAATCCTTTTAATTCCTCTAGGTCCACAGATTTGGTTTCTGCTGGATTGTT
GCCATTTTCCCAGGGGTAAAGCCAGAGGTGGATTTGGGGAACAGCAAATAAGAACAATACACT
GATAAGGGTAACACCAGTGACATGAAAACATTCACTATTTATTATCTCCAATTAAAATTCCATAT
TCTTATAAAAGCAATTCTCATCATAGAAAACTCAGAAAGCTACCAAAATTCGTGTTTATGGGTTT
TTTTTGTAAAAAGAAAACCAAAATCTCTCAGTCTAAACTACAATCACTTTAACATTTTGATGTATA
AACTTTCTTTTTAAATATTATATTTGTATGCATTATTTATTCTGGTCTGGTCATATAACTTTATATGA
CAAATACTTCCTTATATATTATGCACTTTGTTTTGTTTTGTTTTGTTTTGAGACAGAGTCCTGCTCT
GTCACCCAGGCTGGAATGCAGTGGTGTGATCTCGGCTCACTGTAACCTCTGCTTCCTGAGTTCA
AGCAATTCTTGTCCCTCAGCTTCCTGAGTAGCTGGGATTACAGGTGTGTGCCACCATGCCTGGCT

AACTTTTTTGTATTTTTTTAGTAGAGACAGGGTTTCACCATGTTGGGCAGGCTGGTCTTGAACTCC
TGGCCTCAAGTGATCCACCTGCCTTAGTCTCTCCCAAAGCGCTGGGATTACAGGCATGAGCCAC
CACACCTGACCCATTATGCTCTTTCAAATATAATTACAAATATAATATTTCATTATACAGTTGCTC
TATAATTTAGTTGGTCAATCTATTGTTGGACATTTAAATTTTCTATCTTTCAGTTACTATATTATTAC
CACTTTAGCCAGGCACAGTGGCTAAATATGTATTATATATAATTATATGTTATATATATATTACTT
ATATACATTATATATTAGTTTTATATATATAATATATATTATTATTACGACTCTAAGCAGATATTTG
CCTTATTGAGTGACATCACCGGCCTCTTTTCTTACATTATCTCATTTCCTTATAAGCCTCCAGAGT
AGACATTATTACTGTATTATTACTGTTTTATAAATGAAGGAACTAGAATTCAAAGAGGATAAAAA
ACTGTCCCAAGTTCAAAGCCAGGCTTTACACCTAAGTCTCTTTAACTCCTGAGCCAACACTACCT
ATTGCCACACTCTGGGTGTGCCTGGTGCTCCCTAACCCACCCTGAGTTTTCTGTCTTACCAGAAC
ATCATTACTTGTGATGTAAGAGTTTTCTTCTACTCTCCTTTGCTCAAAAAACTTAATCCTAGTCTCA
CTCCTTTCATGTTCCCATGAATCCCCTGGCCTCTTAGTAAAATTCGGGTTTTCCAATTAAACGCTC
TCCATTTTAAAGTTCCCAGGCAGCTGGACTGATGCGATTGATGGTTCCCAACTGCTGTGCCATGT
GCTCTGGTCCTCCTTCCTTCTCAGCTGTTCCCAGAAAGGCAGGCTATGAGGTTAATCACGTCTCT
ATGCTGTAAAACTGAATCCTGATACACTCACTAAAGCCCTCGTGGTTATTTGTGCCCCATTTGTA
TTCCCATTTATATTCTGAAACTTTTTCAAAATCTGAGATGCTCTCAGCAACTCAGTACATTCTAAA
CAGGATATTAAACTTTTAAAAAATGTATAAGGCTTAAAACTCTCTTCTAAATTCCAGACTTGGAA
CAGTTTAATTGACACCCACTCAGATGTTTTGAAACTTAACACATTCAAATCAGAATTATTGAAAG
TTTTTATATGTGCAATGATTACATGTCAACTAAAAAGGGGAAAAAAGGTTCTGCCACATTATAAT
CCTTTTTTCCAACTGTTTATTTCTGTTATTTTGAAAGAACAATCAGATGGTACTTTTTTCAAAGACA
GTCACATAAAAACTGAAACAAAATATAGATTAATTACATTTTTTGAAAATTGTGTGATACACGAT
TATGAAATTTGTAGAAATTTTATGTGGAAAAAGAACAAGGGAAATCGAGTGAAATGCTTTGAAG
GCATCTATGACATAGGATGCTGTGCTCTTCCTCACCTTCCATTCACTTTTTAACCCTCTCCAATGT
GCCTTTTCCACCATCTGAAATTGTTCTCACAAAGGTCACCAGTGACTTTCCAATTGCAAATCCAA
TGGGAGTTTTTTGGACCTCAGCTTACCTGATTAATTTGAGGCATTCAATGTTATTAATCAGTAACA
CTCCTTTTGCAACTTTCTTTCTTTTGTTGTTTTTTGTTTTTTGAGATGGAGTCTCACTCTGTCACCCA
GGCTGGAGTGTAGTGGCACGATCTCGGCTTGCTGCAACCTCTGTCTCCTGGGTTCAAGCAATTC
TCCTGCTTCAGCCTCCTGAGTAGCTGGGACTACAGGCACACGCCACCACGTCTGTGTCTGGCTA
ATCTTTGAGGTTTTTTTTTTTTTTTTTTTTTAGTAGAGATGGTGTTTCACCACATTCGTCAGGCCGGTC
TCAAACTCCTGACCTCAGGTGATCCACCCGCCTCAGCCTCCGAAAACATTTGGATTACAGGCAT
AAGCTACCGGGTCTGGCCACAACTTTCTAGTCTCAGTGTCATCACAACCAGTTTCCAGCTTCTCT
GACTTTTCTTAGCTACCCTGTTGTCCTATCACTCCCTGCCTCTTCTCCAGGGTTCAACCTCTGGTC
CACTTCCCTTCTCACACTTCATAGGAAAATACCACTCAAGATCATTGCCTCCCCTCTCACCACAA
CCTACATCTCTATCCAAAAACTCTTGATATTTTAGTTCAGACTGTTTTTAGCTATTTTACTGCAGAG
TTGGAATTTAAGAGTCTGGAGCTTGCAAATACAAACTTTCATTGCACTTGATTGCTGCTGTTCTGT
GAACTCTCATTACCTTTTCCCTGGGTGGCTGCTATATATCCTGACTGGTTTCTCTATCCCTAGTCT
CATTTCAGATCCATTCTCAACACTCCTTTAGAATACTTTCTAAAATATGGAAATCTGAGTTTCCAC
TTCTGCTACTGGCAGAATAGTTCCTATAAGACCAACCCTTCCACAAATAAAAACAGTAGACTATT
GAAAAAAAAAAAAACACCAATACAAACCTGAAGGCTATGGAGAAGAACCAAAAACAAACGCT
AGACAAGAGCCAGCATTTGGACAAAAAGACTATCATGTGTGAAATATGCATTTTTAATAGGTTTT
TTTTTTTTTTTTTGCCTGAGGGCAGACCCCAGTGAGTACTAAGCAGGGCAGCTAAAATGCAGATA
GATGCAGTCTTACCAAATAGAAGAATAAGAGGGAAGCATTCAGGGTAACTATAGCAGCTGTAA
ATTGGGATCAAGGGCAGGAGGATCCAAGAAAGGATAAAACAATGGAAAGGGAGCCCAAAATT
TTATGTATAAAACTCTACCTAAGTCTCTGGCAATTCTCTGAACTATGCATGTGTAAGACAGACGT

CAAGCAGCCCAACTAAGGCCAAAACAACTAAACAGAGATTTCACTTGGTGTCTGTTTTAGTGAG
AGATAGAGATTGAATTTAGCCATGTTAATTACTGGCTAAAACAAACAAACAAATAAAAATAACA
CTTTTTGGATGAATGTAACAAAAGACAGAGTCCTTACAACATATCACTCACAATGTCCAAGACAT
AATCCAAAATTATTATAAAGAAACAGGGAAACATGACTCATATTTAAGAGAAAGGGCTAATAGA
GACAAGCTCTGAGATGAACCATATGTTGGAATTAGTTGACGAGAATTTTAAAGTAGTTATTATAA
CCGTGGTTAAGGACATGAGAATAGACTTATAATGAATTAACAAATAGGAAATTACACTAGAGAA
ATAGAAATTAAAAAAAGAACCAAATGGAATTGAATCATATACAAAAAATGCAATATGAGAAATA
AAAAATTCCCTGATTGAACTTCACAGTAAATTGTAGATGACAGAAGAAAGGGTTAGTGAACTTG
AAGAGCAGTCAGTAGAAATCACTGACTTGCAGAACAGAGAGAAGAAAAATTGGAAAAAAAATG
AACAGAGCCTCAGGGAAAGTAGGCCAATATCAAAGGCCTAACGTGTGCAATTGGAGTCCCAAA
GACTAGAAGCTAGAAAATGGGGCAGAAGACATTTTTGAAGAAATTATATCTAAAAGCTTTCCAA
ATGTAATGAAATGCATATGTTCTCAGATTTAAGAAGTTTAATGAATCTCCAATAGGGTAAGTACA
AAGATAACCATCACATAATTCAGTAGTAAACAACAGTAACAACAATGACAACAAAGCCTATGGT
TATGTCTATAATCTTTTGGTGCAAAACCTTCTTGTCTTGTCTTTTTCTGCTTATCACCACCCTCTTTC
TTATTTTCACCAATCTGGAGATGCATATACAAAAACATTTTAAAATCCACTTTTTGGCCAGGTGCA
ATGGCTCATGCCTGTAATCCCAACACTTTGAGAGGCCGAGGCGGGAGGAATGCTTGAGCCCAG
GAGTTCAAGACCAGTCTGGGCAACATAGGGAAACCCTGTCTCTGCAAAAATAAAAAAATTAGCT
GGGCATGGTGGCACATGCTTGTGGTCTGAGCTACTGACCACTGAGCTACTGAAGTGGGAGGAT
TGCTTGGACCTAGGAGGTTGAGGCTGCAGTGAGCTGAGATCATGCCATTGCACTCCAGCCTGG
GTAACAGAGCAAGACCCTGTCACACTCACACACACACACACACACACACACTCCACTTCGTTTC
TAATCATGGTGTATAGATGCAAGCAATATTGTTGGTTCTATGACAATACTTTTAAAACTACAAAA
ACAAATTTGAAAGTGATGTGATGTGAAAAAGAATGAAATCATGTCATAGCCACATGGATGCAGC
TGGTGGCTATTATCCTAAGTGAAATAACTCACAAACAAAATCCGATACTGCATGTTCTCAATTAT
AAGTGGAAGCTAATCAATGGGTACATATGCACATAAAGATGGAAATATTAGATACTGGAGACTC
CAAAAGGGAGGAGGATGGAAGGAGGCCAGGGTTGAAAAACTACCTGTTGGAAGTTTTGGGGT
ACTATGTTCACTATTTGGGTGATGAGTTCACTTGAAGCCCAAACATCAGCATTATGCAATATACT
CACGTAACAAACCTGCACATGTACCCCATGTATTTATAATAATAAAAAGTAAATTAAAATATTTC
TATATAAAAATAATTTAAAAAGAAAGTGATGTGGTGGAAAGGGATAGGATTCCTTATAAGCTAA
TTATATATAAAATACTCTCCTGCTCTTTTTTATTTATAATTCAATCATTCCCTAATTAATTTATTCAT
CTAATCATGAAACTTGTTTAGTCACCTACTTGGGGGCAGGTACTGAGGACGTGATGACAAATAA
GGAAATCTTTACCCTTTAGAACTCATCATGTAATGATCCATACTGGTCACTTTGAGATTCTGGTAC
TTCTAAAAATAACTTATTATTTTTACTTCTTATTATAAAAATCATACATGGGACATTTAGAAGATAT
AGGAGAGCAAAGGTAAGAGAATTCACTGTGAATTAATTATTCATTGAACACCTATTTTGTGCCAT
TGACTATCCTTATTGCTGGGAGCACAGCGTTTAAAAAAAAACCAAAAACCTTTCTCCCACTCTAA
TAGATGAGTCACATAATGGGAAAACAAACAAATGTATCATCTATTAAAGTGGGAGAACTTGGTT
TTGTAGTTTTAAAAGTATTGTCATAGAACCAACGATACTGCTTGCATCTATACACCATGATTAGA
GAGGAAGTGGATGGAGAGTGTGTGTGTCTGAGAGAGAGAATATTTAGAATGCCAAAAGCGGGC
ACTTCAAGGAAGGCTTTTTTTATTTAAAAAAAAATTAGCTCATTCTACATACTTTAGAAATTACCTTT
GAGTAGCAAATGTTAAGGATATAAATACTTTGCACGTATATTCCTTGTTTATAGTTAACCATATGT
TATTTTTTACCTGCAAAATACTAAAACATATTCTACCTACGTGCAATCTAAGTTTTGTAGCAGAGT
CTTTTTACGGGGGCCAAACAATTCTGGACTAATTTTTAAAACCCTGAAATGGCTCACTGTTTTGA
GGTTATTTTGATGGAGTATGAATTTACTTCTTTGTCATTTGGAGAAGGTGCACAGTATTATTATGA
ACAGAGAAGAGCTGTATGAAAACTGATCTTCCAGCATCTATCATCTCCTTTTAGTCTAATGAGAT
ACTGCCATTATCTCCTGATGAAATATTGTTCTAGAAAACCTTACCCCATCATACGATTTCTGCAGA

TCTTACAGGAGCAATCTGAGCTCAAGCAATATTAATTACTGTTTCATTTGTCCATTTGTCTGTGTA
TAATCATGTATTTTAATGATCTTCTATTTTCTATGGTTTGTGTAGATTTGGAGACTAATTCAAAAGA
GTTGAAGTTTTTTGGACATGTTTAGTTGAATGTGCAGTTCTCTTTTGAAGAATCCTGTCTCCAAAT
CCTGTTTTGAAAAGAGTGTTGATGAGTAGCACAGCACCTGTCATATTGTGAGTGTGCAATAAAAT
CATGCCTTTTCAGCAGACCTTAACTAAAGAGTTAATTTTATTTATTTAAATCCACCTTTCAGATGA
CTCTCATTCTGCTTTGTCAGTGGTTGACGAGGGTAAAGGCACTTGAGGATGCACAACACACAGG
ACAGGGAGCAAGCCTTTCTCAGTGCGGAGAGCTAGGATAAACTTTTGCTTAGAAAGGGGTACAT
TAATGACAAAGGATCACAGCATTATGGGAGGATACTACACACCTGTTCTTCAGGGAGTACTCTG
GACATCCCTGTTCTTTCCCCACATGGCAGGTGTCTTTTCAGGTGCTCCCAACCACACTACCCTGA
ATTCCCATATCCGGGTTGCCCTCTATGCCAGCCTCTCCTCTTAGCCTTGAATTTAGCTTTGTTGCT
TAAGGCAAGTCACTTCCCTCTCCGGGTATGTTTCTTCTTCTGAAAATGGATGCAGTTGAACTAGA
TGAGAGCCAGGTGGCTTCAATTGATGTGCGGTGGCCCTCTGCCCTGCTTACTTTCATCTCCACCA
CTCCAAGGTGGCTCACAAACACGAATCCTCAGCCCCTTGGATTGGGTTCAGGGGAGTGCTATCA
GGAACCCATGGGAGAGACAGGAATGTTTTCAGAAACCTCAGCCTGTCCTCTTTTTACTGAAATA
GGGGCCCTTGTTGATGGTAGTCACTGCCCATGCTGTTTAGCCTAAGGAAGGTGACAGTGGGTGG
GCCACCTCAGGCCATATGGAGGAAGGCCTGGCACAGAAACACAAGGCCAGGCAGGCTGTGGC
TAGCCTGAATTTAGCTGTAGCCTCAGAATCAGGGGACTTGGGCTTGACAGCTGACATTACATGG
TGCACAACTCTGGTTAAGTCACTTAACTTAAATCTTATCCCCTCATCTGTAGATGGCTTGAAGAC
CAAATTAAGGAGCTCCTTCAACAAATACATTAAAAAAAAAAAAAAAGAAAAAGATAAGCCATAA
GGCACATACGAATCACTTGGGAATCTTGATAAAATGCAAATTTTGGTTTGGTAGATCTGGAGTG
GGGCTAAAAATTCTGCATTTCTAACGAGCTCCAGGGTGAACCTAGCTGATATTGCTGCTGCTTGA
GTGGGCCACACTCTGATGAACAAGGATAGAAACCATTACAATAGTTGAAAATGAAAATAACAAT
GAATTATATAAACCCCACCCCCCTCACACCACTTTCACGTCTGATATTTTTCAGTTAACCTAAAA
ATGTTTATTATTACCTAATAAACATTTTCCATGTTTCTACAGATTATATGGACCGCAAAGTTTCTGT
AATTACTATTATGGAGTAGCTACACAGTATCCCATAGTGTTGAACTAGCATCATTTATCCAATTG
CTTCCCTCACACTGGACCAGTGAGTCCTAGGAAGTGGCTGCAAGGAAGCAGCAGGTTTCTGGG
ACAGCCCAAGGGCATTTCTTATTTCCTCCTGTTCCTCCCTGGGATCAATCTGTAGGAGTAAACTG
ACTGAAGACTGTGAACACCTTCATGAACTCCTGTGACTTTGGACTTGTTTTCCAAAAGGCCAAAA
TTATAGATATGTACATATTTTGATTATTAAAAATATGTGTTTCCTTGTACATTGTTGGTGAGAATGC
AAAATGGTGAAGCTGCTATGGAAAGCAGTATGGAGTTTCCTTAAAAAATTAAAAATAGAATTAC
CATGTGATCCAATCAATAATCCCACTTCTGGGTATTTATTAAAAAGAATTGAAATCAGGATCTTG
CACTCCCATGTTCATTGCAGCATTATTCACAATAGCTGACATGTGGAAACAACCTAAATGTCTGT
AGATAAAGAAAATGTGTCATATACATACAATGGGATATTTTTCAGCCTTAAAAAGGAAGGAAAT
CCTGCCATATGCAACAACATGAATGAATCTTGAGGACATTATGCTAAGTGACATAAGCCAGTCA
CAGGACAACTACTGCGTGATTCCCACTGACGAGGAATCTGAAATAGTCAAACTTGTAGGAGCA
GAGAATAGATGGTGGTTGCAGGCACTGGGGGTAGGGAAAAATAGAGCTGCTATTCAACTGGGT
ATAAAGTTTCAGTTATGCAGCTGGATAAGTTCTAGAGAACTGCTCTACAACATTGCACTTACAGT
TAAAACGACGGCACTATGCACTTGAAAATTTGTTAAGGCCAGCGTGGTGGCTCACACCTGAAAT
CCCAGCATTTTGGGAGGTTAAGGCAGGCGGATCACTTCAGCCCAAGAGCTTGAGACCAGCCTG
GGCAACACAGAGGGACTCTGTCTCTACGAAAAATACAAAAATTATCTGGGCGTGGTGGTGTAC
ACCTGCAGTCTCAGCTACTCGGGAGGCTAAGATGGGGGGACTGCTAGAGCCCAAGAGGCTGCA
GGGAGCCATGATGGCACAGTGTGCTCTAGCCTGGGTGACAGAGTGATACCCTGTCTCAAAAAA
ACACAAAAAAATTGTTAAGAGGATAGATCTCATGTTAAGTGCTCTTACCACAATTTTAAAAAAGTT
CCCTATAGAAAAGTATTTTTAAAATGACAATTTCCCTAACACCACTGCTAACATTTTGATTGAAAA

AAAAATCATTAATTTGTTCAATAAACATTTATCATACACTTAGAATGTGCCAGGTTCAGTGTTATG
CATGAGGATGCTACAGCGAACAGTTTTTCTTTCTAGGCATATGTCCAACATTATCCATCAGTCAA
TCTATATAACAAAAAGTAGATACAATACAAAAATATACATGTACGTATATACACTACAAGATGTT
TTTATTCATGTTTTATGTGAATAGCAAGACCAAAAAAATCATTAATTGGGCCCAGTTTAGGCCAG
CAATGGTATTCTGTCCCCTGGATGTACACAAATAAAAACAACGCAGCTCATTCCTTTTAAGCATC
TTAAAGTATACCAAAGTTTGTTAACAGCCAGGTATGCACACGACAGAGAATGAGTTCCCTGACA
TCAGCCGGAATCTATCACCGGGTAAAATGGATGGAAACCTCTCCCTGCTACTTACTGCTTATTGC
TGTTCAGGCACTGTTTAATTTCTCGGTGCCTTAATTTGTACAGAGATACAAATGCCATCTCTGGTT
GTGGCTGAGAAGAATGAATGAAATAACGTACCTAAAATGTTCACAGGGCCTAGCATATGGCAA
GCGCTCCCTAAATAGACATTGCTTTTGTTGTCATTAGTGCTAGTGATAGAGGACCTTGGGTTTGG
GCCTCATTGTGCCACTAACCAGCAGTGTTATTTTGGGCAGCAGTGCTGTCCCTGGACCTCAGCTC
TACCTCTGGGAAATGCAGGGTATGGAGGAAACTGGTAGTTCAGCATTTGGAGATTCGTGGCAAA
CTATCAGGCACAACTCCTTGAAATCTTCCGGAGTTGGCGAGGCACTGCGGGGCTGATTAGATGG
GGGGCAACCTCGCGGTCCCAGCTACAACCTCGCCGAGCCAGCCTCCAGCCCCTAAGGTGCGCC
GGGGTGGCGCAGGGGCGGGCAGCTGGGCGAAAGCGAGTACCAGGCGGAGGGCGCAGCCTCA
GGGGGATCCTGGGCGAAGAGCTGAGGGTGGCGCTGGTGGGGGCCTTTGAGAGGCCGCAGCCG
AGCCCATGGCTTAGGGTTTCGATGAAAACAATAGCTGCGAGGTTTGGTTTCCTGGGCACAAGGG
TGGAGAAGGCCCGGGAACCCTTCCCTTTCCAATCCCGGTGTGGCCTTCTGACTGGCAGACAATA
TTAGAGGTATTAGCTTTCCTCTTGCCCCCGCCCCCCGGCCCCGCCAGGCCCTGGCCCTTCCTGC
AAGGCAATAGTGCGCCTGAGAAATCCCTGTGGTGCCTTGTTCTCTCTACAAAGGCAATGAATTC
TCAGCACAATTTGGCTTGGAGTAGGCTCTCAAGATAATTGTTGAATGAATGAATGAATGAGTGA
ATGAGTGAATGAATGTCATCAGTGGGGATGGGCCTAGGGCACCAGCAAGATCAGGAGATCTCA
TCTTTCATCCTCACCTTCTTTGTTCACTCCATTTGTTCACATCTGTACAGAATTTTCACCGTTGTGT
TGGTCACAGCGGGTTGGTGCAAAGGGGTCATGTTTTCAGGTAAGAGCAGCATTGCATAGTGAG
CAGAAACCCCAGATGAGTGGGACTTCTCTGAGTTCTGTGTCCACACCTTGGGGACTCAGACGTC
TATTCCTGCCTTCTATTAAGCTTGACAGCAAACTCAGCAGATGTAAATATTGACACCTTCTGGAA
AAGTGTGAAGGTATTTGTTAAAGAAAACCTTAAAGAAAGGGAGGCAGACATTGTGAGACTTTTT
ATGCCTTTCAGGGTAGCTTCAGGACTTCTGGATGGTTTAATATGCTTCAACTCAGCATATGACCT
AATGATTCATTTCTTAGAAGTTGAATCTGAAAGGAGTGAGGTTTTTAGCTGGCATTTTATTTTGTG
TCTGCTTGTGGCGGGAAATAACAGATTCATATATGACTCTCCACCTCCTGAAGAGGCTAGAGCA
AGAGTATCAATGATGTGCCGTAGGAAGAGCACCATACGTTTGAATGGTGCCTTATCACTGTCCT
GGGCTTTCACATGCTTTATCATGCTTAAAACCCATAGCCCTGTGGAATAGCCAGAAAGAGATTAT
TATTCCAATTTTCCAGCATTGTTACTTCAGTTATTGAATAGCCACTATGTGCTGAATATTAGAGGT
ACAAAGAGAAGGAAGGCCTATCTCTGCCTTTAGCGCACAGTCTAGTGAAAGAGAAAGATATGC
ACATGGATCATTTCGGTATGACATGTTACCTGATAGGGTAGATCAAGCCCAACATCTGTTTTTGA
AAATAAATGGGTTTTTTTGTTTTTGTTTTGTTGCTGTTTTGAGACAGGGTCTAACTCTGTCCCCCAG
ACTGAAGTACAGTGGTATAACCATAGCTCACTGCAGCCTCTAACACTCAGGCTCAAAGGATCTT
CCCATCTCAGCCTCCCAAGTAGCTGGGACTACAGGCATGCACCACCAGGCCCTGCTAATTAAAA
CTTTTTTTTTTTTTTTGGTGGAAATGGATCTCAATATGTTGCTCAGGCTAGTCTTAAACTTTTGGCC
TCAAGCAATCCTCCTGCCTCAGACTCCCAAGATTCTGGGATTACAGGCATGAGCCACCATATCT
GGCCCAGTGGTAAAGTTTTACTGGGACACAGCTTTACTCATTTATTTATATGTTGTCCACAGATG
CTTTTGTGCTACAATGGCAGAGTTTAGTGTAATAGTTGTGACAGAGGCTATCCGGCCTACAGAG
CCTAAAGTATTGACTATTGAACATTTATAGAAAAAGTTTGCTAGCCCATAGAATAGAGGAATAAT
GCTCAGGTGGGCGGGTATTTAGAGTTGGGGTACATAGCACCACCTGGGAGTTGAGAAAGGCTT

CCTGGGAAAGGCACTGCTTGAGCTAGAAGCTGAAGGGTAAGACTTGTGGGAGTTAGAAGGATA
AGATGGGTACTCCAGGCAAAGGAAAGCCAAGGGACTAAACTGCTAGAACCACAAGCAGTTCAT
GATTACTAGGATACAACATGCAAGATGGGGACGGAAGCAGATGAGGAAATCAGTCTCGACTTG
GGTGATTTGCTGGAGGTGATGTGTCTAGGTGGGGCAGGACTAAAATCCAGGTTTTCTAACTGGC
AGCCCTATGTGCTTATCACAACAGCAAGGACAGCAAATACACAGCACCCATGTTGTCATTCCTT
CAACCTGGGTCCACAGTGGCCAGTGCTAAGTGAGCACAGAAGTCTTTTCTGCAGCTTTATTTGTA
CTCCAGGTGACTGTTTGTAGAGTGAGTGTGCATGTTCAGACCTATATGCTATCCCTGATTTACCT
CATTCCAGCCATAGTGAGAAGGTTTTAGATTTATTTTTCTCTGAAGCTATTAGAAAAAGCCTTCA
GAAGCAAAAGCTATCAATTCAAGCAGCCCTATTTCAGCACATTCTAAATTATTGCTTACCCTGCC
TATGCCAGGTCATACACCAGGTGGTTTACACACTTTATCTATCTTAATCCTCACTTCAAGATCATG
AGGGAGGTAGAATAAATAAGTCACTTACCCAGGGGTCTGGAAGTAAGCATTTTAATTGTTCAAA
AAGATTTTAAAAGATTATATGTAGTCCGGGAGTGGTGGCTCATGTCTGTAATCCCAGCACTTTGG
GAGGCCGAGGCAGGTGGATCATGAGGTCAGGAGATCAAGACCATCCTGGCTAACACAGTGAA
ACCCCAACTCTACTAAAAATACAAAAAAATTAGCCAGGAGTGGTGGTGGGCACCTGTAGTCCC
AGCTACTCAGGAGGCTGAGGCAGGAGAATGGCATGAACCCAGGAGGTGGAGGTTGCAGTGAG
CCGAGATCACACCACTGCACTCGAGCCTGGGCAACAGAGCGAGACTCCGTCTCAAAAAAAAAA
AAAAAAAAAGATTGTATGTTAAAAATTACAATACTTAAGGAACAACTTGGTGTTGTGGTACTGTA
TAGGGCTGGGTTGTAAATGACCAGTGGGCATGCCAGAGGCTCAGCCCATACATCGCTAGTGGG
GTATGAACCATTATGCATGATTGCAGTGACCTTGTGACCTGTAGCTTGTAAAACAGAAGCTGGG
ATGATTATTGTTTGTTTATATTTTGCTGGATATCATATTTTTTATACCTTTTAACTGTGATTGTGGAA
ATTGACACTAGACATCCATCCCTTAATCCATGTAGTAGGTAAGAACATACAGATAAACACAGGG
CAGTGGGGAGGGGAAAGAAAAAAGAAAGGTAGGAAGGAAAGAAGGAAGAAATATATCCAGA
AGATGCAAGACCTGGATCAAATGGTAGCAGGAAAAGTTCTTTTGAAGATATCTAAGTGTTGGAA
ATATCATTCATCCAGGACAGGTAAGGTCCTGGACATTCCAGTGAGCAATTTCCCTCTGTGCTGTT
ATTTTTCATATTTTGGAGAGAACACTAAGGCCTGAAGAGGTTAGATAGCTTGCCCATGCTACTTA
GTGGTAGACTCAGAATTTCCACACTGGCCTTCCAGAGTCCACAATCCAAGATCTTAACTACGATT
ATATTCATCTTTGATCCTTGGGAAGAAGGGAAGACATTTTCAATTCAGGCTTGAAAACATAAAGT
AGCTGAATGAAGCAGTGTGGAATTAGCAGAGAGCAGAAAAAGTAAGCACCAGTCTGTGTCTTG
ATATCATGTTGTTTACATCTTGCTTTGGATTTTTTTAAACTGTCTTTCTAGAAAAAACATAGGTAGT
AGCATCATGTACACTAATCTTTTCTCCTCAGTTATCCACATCTGGAAGTAGTTTGATAGCTAGATG
TATGATATCATGCCTGTTAGCTGTTATCTGGTGGCAGCTCACCAATTATCCAATTTCTAGGGCATC
TCTCTTTAAAATCTGTTTCAGAAGTCTTGGTCTGACAGAAAGCCAGGTACACCTGAAGTGGCCG
AAGCTTGGGTGAGGACCCTTATCTAGGTCATCCAAAGATAAGAATGAGATAAGCTGAATAACAG
GTCTTAAGAAAGAAAAAAATTAAGTACCAATGCCTAGTCCAAGGATAGAGAAGACTCCAGCTG
GGAGATATTGCCAAAGGTCAGAAGACATGCTGAGTCTAAATAGAGCAGTAGAAATTGGGAGCA
AATAGATAAGGTTTTAGGGGAGGGGTTGTAGAGAATGTCCCTATAGTTTCTGAGCACAGGTGGC
TCAGTTTATAAGTACATAATCTTCCTAAATGAATATTCTAAGGGTCAACTTTATTTCAGTGAAAGA
AAACTCCACAAATTGCTTAGACACACTCCCATCTATCCATCATCTATGTAGACATTCAGTAGACA
TGAATTGACAATCTACTCTGTGCTCAGCACCATTCTAGGCTCCAGGTAAGCAAAGATGAGCAAC
ACCCAGTTTCTACCTTGAAGGATCACCCATGCCTGGTTCAGTAGAGACAGAGACATGAGTTTCC
TTTTTATCTATTTTCCCCCAGAAGTACCCTGGTTTTCATAAATGACCATGGCTTGACATGCCATTA
TGATAATAATGTATAATTAGTGGAAAACCAAAGGAATGACTGGAAATTGTCTTTAGTTATGGGAA
CAGCTGGGCTTCTCAGGATAACGCTGGAAAATACTATACTATGTGAGCAAGGTCTGTAGGGTCC
GGGTTTCTCTGGAGGTACAGCTAATTCCCTTCCAAATGCCAGCTTTAAATTCTGACATTTTGGCAT

TTATGTGAATGACTGTATGACTATATATGTAGGTGATCGGCCCTCTCAAGAGACTTTGAATTAGA
GGCTTTCTGGTTTTGATAAATGCACCTTCTTTTCCTCAAAGTCCTTTATAACTTTTTAGACCAGTGA
CCTCCCTTCTTAGCATCTCCTTGGCTAGAAGCAGAAGACAGTCAACCTACCATTTTGACTGTAAT
TCAATTTGGTTTCAGGCACTTTTAATAATTTGGTATTCTGTTATGAGATGAGGTATACAGGCCACC
TCTACTCTTTCAGTTCCGACAGCAGGAATAGAAAAGATGGTAGTTTTGTTCAAGCCAGCCATTCA
TTTATTCTCTAAACATTTATCATGCCCTCTGCTGGGACAAGAGCTGTTTTCGGTTCTTGGTATATA
ATGTAGATAAAATAGGTGCATATTGGTCAATGTCCCCATGAGGTTTACTCTCCATCAGGTGTAGG
GTGTCAGACATTTGGCAAATAACCTCACAAATAACAAAGTGTTAATAAGGAAAAGACTAGGCTG
TTATGAATGTACAATAGTGGTGGGCCTAATTTGGATTGGGAGACAGGGAAGACCTCTTTAAGAA
AGAGATGATAAGCCGGGTATGGTTGCTCATGCCTGTAATCCCAGCACTTTGGGAGGCTGAGGTG
GGCGGACCACCTGAGGTTAGGAGTTCGAGACCAGCCTGGCCAACATGGTGAAACCCCATCTCT
ACTAAAAATACAAAAGTTAGCTAGGTGTGGTGGTGGGTGCCTGTAGTCCTAGCTACTTGGGAGG
TTGAGGCAGGAGAATCGCTTGAACCTGGGAGGCAGAGGTTGCAGTGAGCCGAGATCGCATCAC
TGCACTCCAGCCTGGGCGACAGAGCGAGACTCCGTCTCAAAAAAAAAAAAAAAAAAAAAAGAG
AGAGAGATAATAATGCTGAAATTTGAAGGAGTGGGTACTAGCCAGGCAGAGTCTGAGAAAGAG
ATTCCAGGGAGAGGGAGCAGCATATGTGTAGACTCTGAGCTGAAAAGAAGAGGATGCCGCCTA
TATACTGTCAGAGGCAAACACAGGTGTGACATAGTCATGGTGGGGAAGAGTGGCACAAAATGA
GCGTGGTGAGGTGGCTCAGAGCCAGACCATGCAGGGCCCTGTAGCACCTGGTAAAGAATGTGG
ATTTCATTCTGAGTACAGTGGGAAGTCACAGGCAGGTGGCAAGCAGAAAAGTAGTATGATCTGC
TTACATTACAAAATATCTCTGAATTCTGTGTGCAGAATGGATCAGATATGCATTTAAGAAAAGCA
GTACACAGCCTGAGTCATCAAATGCATATTGCAAGGTGGGCACAGAGCCTGAGCATACGGGCA
CATGCAAGCCGCTTCCTCCTCCTGCCTCCCTGTACCAAATGTCCCGCCTGGGAGGCAGAATGCA
GTTTACAGACCTTGAAACCAAGTGCAAAGTGTCTACAATTAGAGGAGAGGAAAGCATTGAGGA
GGAAATAATAAATCAAGTATTGAAGATCTCAACAACTTAGAGAACAAGATACCAGTCTTAAGTG
AAAAGAACACATTTGGCATTAAGTATCTCAGGAAACATGTCCTTCAGGTCCATTGACTCAGAAA
CATCTCTATTTTCAAAGAGGCATTTAGTCAGCTCCTTCCCTCATCTAACTCAATTTCCTACTTCCCT
CCATCATAGACCATGCGTTCTTTGGTTATCAGATTTGCCTTTGGATTTTTTTTTTTAAAGACAACTA
AAAACATCTTTTAAAAATCTAGCCTTTTCAGTGTCACTTGTCACTTATGTGTTTCTCTTCCCAATTT
ACTTCATGGCTGAGGCCTCCCTTGGGGTAGTATTTTTTTTTTTTTTAAGAAATGGGGTCTCGCGGC
CGGACTTGGTGGCTCATGCCTGTAATCCCAGCACTTTGGGAGGCCGAGGCAGGTGGATCACGA
GGTCAGGCGATCGAGACCATCCTGGCTAACATGGTGAAACCCTGTCTCTACTGAAAAATAGAAA
AAAATTAGCCAGGCATGGTGGCGGGCATCTGTAGTCCCAGCTACTTGGGAGGCTTAGGCAAGA
GAATGGTGTGAACCTGGGAGGCAGAGCTTGCAGTGAGCCAAGATCGTGCCACTGCACGCCAGC
CTGGGTGACAGAGCGAGACTCTGTCTCAAAAAAAAAAAAAAAGAAACGGGGTCTCACTTTGTTG
CCCAGGCTGGAGTACAGTAGCATGATCTCAACTCACTGCTGCCTCAACCTCCCAGTCTCAGGTG
ATCCTCTCGCCTCTGCCCCCCAAGTAGCTGGGACTACAGGTGCGAGCTGCCATACCTGGCTAAT
TTTTGTATGTTTTGTAGAGATGGGGTTTTGCCATATTGCCCAGGCTGGTCTCAAACTCCTTAGCTT
ATGCAATCTGCCCCCTTCAGCCTCCCAAAGTTCTGGTATTACAGGTGTGAGAAGCCACGCCTGG
CCAGGGTAGTATTTTTTTCTTGACTTGTGAAGACTTAGCTTGATTCTCCCCTAGCAGTGCACCATG
AGTGGGGTGTGGGGTTGGGGAAGGGATCAGCACAGGAATGCATGCTGGCCAGGCCAGGGTTT
GTTGAAGCAGGTGGGGTTTGAAGGTTGTGGGACAAATACTTCCCAACTTTACCCTATGAACATG
GTGGCAGGTCAGCTGAGACTTGCACATACACTTTGTCTTCTGGGTGTGCCTGGAAGTAGAGTGA
TGCCCTGGAACCTGCATGACCTAGTGCTCAGAATACCTGGGCTTGGTTTCTGATCACTACCTAGT
CCAGTAACTCTGTGCCACAAACAAATTAACATAGATGAGTGCCCATGACTACCAGACAATATAT

GTAAGTGCTTCTGGGCACCCAGAGAGGTGTGCCTTGTTTTCTCCATGTAGAGGCCAGGAAATGA
AGGTTCAGAGAAGAGATATGCACACCGCATGCAATTCCCTCTGCCTGGAAAGCCTCTGCTCACC
CCATCACCCTCTGCTGATGGAGTCAGTTTCTTTGTGTGGTCTCAGCACACTTATCACATCTTCAGT
GTGACACATATTTTATTCACAAGACATATGTCCCAAGGACAGGGACCCATGAAGGAACAACAG
CAGAGAACTGTAGCTCTGAAAGGTGCTCCGCTTCATTTGTCCTTTCCCCAGACAATGAGGAAAG
TGACAAATAGCTATGGTCATAATGGAATTCTCAAAGAGACTCCTTCCTTATTGACTACATTCAAC
AGAAAATGAGTCCTTGGCATTCTGATCCTCAACAAAAAATATCAACATTAATATTTAGCATCCTT
ATATATCAAATAGACTCTAATGGGTTATGGGAGCTTTCTGAAATACTTTTTATACTATATTTATTG
AAGGAAATAAATAAGTTATAATAATTCACTTGTCCCATCTGGTTCAGTTTTAGGGGCCTTCTTCAC
TATGATTGTTTCTAAAATCCATGGAAATAGAGGTGAAAACCCTTCTCCTCCTCACTGCACTATTG
ACATTATAAACACTGACCTAGATTATAACCATTAGGTTCTCATCGAAATGAAATATCTGCCCTGG
GGTTTAAATTCCTAAACTTACTGTGTAAGATGAGCCTCCAATTAAAGATTTCTAATAAGAAAATC
AGAGATTTGGTGTTAAAACATCCTCAAGTAATCTGTGGGGAATGTTACTTTTAAAATGAGAGCAC
TTACTAGCATCTGATTCGAAGTAGAGTCTAGATCTATCTATCTTCTCCAGGGAGAGCTCCTTGAG
GACAAGGAATTTGCCTCTTTCATGTTATTTGGCACAGTTTTTAGCCCATAAGAGGCTCTCAATAA
ATACTGGTTAAACAGAAATAAGAAAAAGGAGATGCTGAGCGCAAAGAGCAAGGTAAAACCATT
CTGCTCTTTTTCTTCTTTGAAGATGATGATGGCAAGAGTTACCATAAACTGGTCAGGTGTGGTGG
CTCACACCATAATCCCAGCAACCAAAGCCCAGGGCAGGAGGATCACTTGAGGCCAGGAGTTTG
AAACTAGCCTGGGCAACAGCAAGACCCAGTATTTATAACAAACAAACAAAAAAAAAACTAGCCA
GGGGTGCATGCTTATAATTCTAGCCACTCAGGAGGCTGAGGCAGGAGGATTACTTGAGCCCAG
TAGTTTGAGGCTGCAGTGAGATATGATTATGCCACTGCACTCCAACCTGAGTGACAGAGCAAGA
CTTTGTATCTCAATTAAATAAGTAAATAAATAAAGAGTTACTATAAACTGAGTTGTTTACTATGTG
CCAGGCACTGTGTCAAGTACTTTATGTTGGCCAGGCATGGTGGCTCACTCCTGTAATTCCAGCA
CGTTGAGAGTAGGCCAAGTTGGGAGGATTGCTTGAGGCCAGGAGTTTCAGACCAGCCTGGGCA
ATATAGTGAGGCCCTGTCTCTGTCTAAGAAATTTAAAATAAAAAAAGCACTTTATGTGCAACAGT
TCTTTTAACTCTTTAATTCTATAAGGAATCCATTTTTCCTTTAAGTAGATGAGGGCCCAGGGCCAT
TAACTAAAGTATCCAAGGTTACCTGACTTAGAGGTGATTTTCCAAGATCTCAATTCAGGTCTCTC
TACATATTTTTAAACATTGCATTGCCTTTCCAAATGAACCAATATTTATTAGCCATTTTATGCAAAT
AATTGGGCCATGTATTTAAAAAATCATTTCTTATGTTCTTATTTCAGCTGGTTTGCAAAGCGGCCC
TGCTGGGGCTTGTATAGGGTAAATGATGGGCTGAAGTTAAAGATATACATATGTATAAGATGTA
TAGGATGGCGTTTTGTTAACCCTGGAAAGTTAAAGAGAATTAGAAGTTTTCAATGTAACATTTCC
AGGCAGGAAAGTGTTAGCATGTTATGAAGAATTTCCCTTCCTCTGAGCCATTGGGCTTTTGCCAA
GGCTTCAGGGTAGACGCTGCATTAGTGGAAGATGAATTTGATCCAAGAAGACATCTTCTCAATC
CTTGTGTAAGATTAGTCTGAGTAGGAATCACTTGGTAACAATTTGAAATGGAGTTCGTTAGATAA
ACAGAAAAACATAGCTTTGTCTGGGTTGAGCCCAGAAATGGGTGACAGATGTTAATGATGAGTG
ACTGAACAGATGTTTGGGGGAGAATTACAAAAAGGGTTTCTATCATGCAGGATCTAAACCTTAA
CCTTCCTGGCTGATGCTGAAAGAGAGCTTGTATGAAACACGTTCTTACAAAACTATTTGGTGGAT
CGTGACAAAGACGAATGACTGGGTACTATAATTTAAAATGGAAAATAAGGTAAATGGGAGTCCT
TGTTTATGTGCATGTGTATATGTGTGTTTTTTTTTTTTTACAAAGGTCAATGCTATATTAAAGAATT
GGAAAGTTGAATAAGTCATTCCATTCTGCTTCCCTATCTTTTCCACCCCTCCCCTCCAAACTGGTA
GGAATGAGCAGTATTTGATACTGTACCTTAAGGCTCAACAAATTATTAAAATACTATCACATATA
TACATCCTGAAATATCTGCCCTGTAATTACATTTGGAAAATAAAATTTCCTGTGCAAACTTAATTG
GTAGTTTTAGTTGGGGTCTCTGAGACTTACTTCAAGTTTTAAGGAGTTTAAAAAGACCCCCATTTC
AAAATAAATAAGAGACAAAGGCAGATTTTCAAAGTTGTACAGCTACTGCCATAAGCCTGTCCCC

ACTGTGAAGTGAGTACACTTCACAGAAAGATGAACAGTTAGTTGTGTTATACAAATTATATACTT
CCTGATAATTTGACTGAAATTTTGGATGGACTGATCAGATGCATTTGTTAATTCATCTTCATATCT
GGCCGTAATTGACATGAATTTGACTTACCTTCTGCTAATTATAGCTGTGGCTACACAATAGTGCA
AAACACCTGGTTAAAAAAGGTGAATACAGGCCAGGCACGGTGGTTCACACCTGTAGTCCCGGC
ACTTTGGGCAGCCAAAGTGGGCCGATCACTTGAGCCCAGGAGTTCGAGAGCAGCCTGGGCAAC
ATGGTGAAACCCCATCTCTATAGAAAATATAAAAATTAGCTGGGCATGGTAGTATGTGCTTGTG
GTCCCAGCTACTTAGGAGGCTGAGGTGGGAGGATCACTTGAGCCCAAGAGGTCAAGGCTGCAG
TGAGCCAAGATCACATCACTGTACTCCAGCCTGGGCAAGAGTGAGACCCTATCTCAAAAAAAA
AAAAAAAAAAAAAAAGGATAAAATAAAGGATAAATACAGTAAAAAATCCAGATATTCTCAGTTTA
GTATTCTAATATTAATGAATGCCACTGAATTTATGATAGACATTGTGTATACAATAGGCTCATATG
TCATCATAACCTTGCAAGCTGGATTTATTATCCTCATTTAGTGAAGGTGAAAGCAGAAGCCCAGA
GAAGTTAAGTGACTCACAGAAGTTCACATATGTCAGTGAGTAGCAGGACCAGGATCTGTACCTT
GGTCTGTGGTATTCAAAATTTGTCTCACTTTCTAATATGCCATACTGTCCTGGGTAGAATAGATGT
TTAATTATTTGATTAAATCCTTATATTTGATTAAATCTTTATATGTTACTGTATATCAAAATGGAAT
AGGACTTAAAAGCCAGAATTCTCAAACTCATGATCTGAAGTCTCACTGTAATGTCATCAAAGAA
AGAGTACTGGTAACTCTCTGTGTTATTGAAAGGGGAGGAAAAATACTAGATCCTTTCTTTGGCTT
TGTCCTGAAGACAGCTCAATAGAGAGGAAGGTAATAACTGAATAAAATGTCACCTTTGGGCAG
GGCAGTGTACTAGAAGCTATGAGCATTAAAAACATAAATGAGATACCTTAGAGCCTACAGAAG
AGATAAGACATATACACAAATAGTGATAAGGAAGAAGGGCATACATCAAGGGACATGAGAGTT
TAGGGGAGAAAGAGATTTTAGCTATGGTGATTAGGCAAGGCTGCATAGAGGGGATGGCATCTG
CACTGTGAGCTTTGAAGATAAAGACAACGACCGGTAAAGGTTAGTCCTTTCTTCAGCTTGATTTT
CCCTTCCTGTTCCTACTACCTCTCCTTCCCTACATTGCCAGACATGAAGGTGTCACCCTAGAAAT
GGAGAAGCAATAAGATGGAAGGCACTGGTCCCTTGACACTGGGAGCTGCCATGGGAGTTCTAG
AATACTTACTCTCATGTGATTATATAAGAGAGTGCCAGGAAAAATTGCAAAGGGTCTTGAAATG
GGCAACAAAAAGATTCTTGGGTGCCCTGAGTAGCTTACCCAGCCAGGTTACCTCTAACATTCAT
TGTCTTTGAAATATTTTACAACTCTGTAAGTTGTAGAGAACTGCTAGCAGGGCAAAGATGATTTC
TTTTCCATATGCAAACAAATTCTGGCTGGGCACAGTGGCTCATGCCTGTAATCTCAGCACTTTGA
GAGGCCAAGGCGGGCAGATCACTTGAGGTCAGGAGTTTGAGACCAGCTTGGACAACATGGTGA
AACCTCATCTCAAAAATACAAAAATTAGCCAGGCGTGGTGGCACATGTCTGTAATCCCAGCTAC
TTGGGACGCTGAGGCAGGAGAATTGCTTGAATCCAGGAGGCGGAGGTTGCAGTGAGCCAAGAT
TGTGCCACTGCACTCCAGCCTGGGCGACAGAGTGAGATTCCATCTCAAAAACAAAAACAAAAA
CAAACAAACAAAAACGGAAGACAAATTCTGTCCAGTGAATTGATTCCCCCCTTTACTCCCACTCT
ATTTCTGCCCCTTGGAAAAAGCCAGTTTTACATCTATTTGTAAATTCACTTCAGTATTCCTGATTG
ACCTCTATAAGTGGGGTACTTTAAATTCTCCTTTGAGCTGATAGTAATATCTTACTGGTTCCTTCA
TTAGTTAATGAGTTAAAATCTTTGACGTGTATTCATTTTATATTTGTATGAGAGTAAATAATTTACC
TTTATTTAAAAAAAATTTTTAACAAGAAGAATATACTTTTATTTAAGCCACTGCTATTTTGGGCAC
AAAATATACAATGCATGTATATATCTATATCTATCCATCTATTTCTATCTACCTATCTATAATGATC
ACATCTCTGTGCTAAGTAGATAGCTCCTGCCATCCTACCTTAATTATTTCCATGAGGACCAGCGT
TGACTCTTAAACCAAATCCAAAGATTTTTTTCTCAGTCATCATTCATCCTTAATGAGAGACATAGC
AGAACAATGTTTCTCCCAATCATGAGTCACAACAAATAAGTGGGTCGGAAATCAATTTAGTGGT
TTACAGCCAGCATTAATAGGATTTATCATAGTGCATAACACACAGTAAGTATAATTTGCGAAGCT
TTGGATTCAGATACTTATATGTATTAGTTTACTAGGGCTGCCATAACACATTACCACAAACTGGG
TGGCTTAGAACATTAGAAGTTTATTCTCTCATACTTCTGGAGGCCAGAAGTCTGAAATCAAGGTG
TGGCAGGACTAAGCTCTCTTTGAAAGCTCTAGGGGAGAATGCTTTCTAGACTCTTCTAACTTCTA

GTGAATTAGTGAATTAGCCTGGCATTCCCTGGCTGGTGTTAGCATAACTGCAATCTCTGTTTTCAT
CTTCACAGCATCTTCTCTCTGTGCCCTTTTCTGTCTAAGGACACAGCCATTGGATTTAGGCCCCA
CTCTAACCCATTGTGATCTTATCTTGATCTTTACCTAAATTATATCTGCAAAGACCCTATTTCCAG
ATAAGGCCACATTCCAAGGGTCAGTGTAGACATAAATTTTGGGGGACAATATTCAACCCACTAC
ATATATATATGTAAATTCTTGATCACGGTGTGAATTGTTTTTCTTATTATGAGAAGAGGTCTCAAA
ACTTTCAAACATAGGACTAGAAGACCCCTAGTACCTTTTTCACTCTGATTCCAGGAAGCATTTAA
CAAAGCTGGCTACATTTCCCTTTTTGGCAATAATAATATTAATAATTAATAAGTATAAAATTTATT
CAAGTAATTTTACATGCAACAAATAATTCAATCTTATAAAGCCTTAGGAGGTGGGTTTTAATAAT
ATGTCAGTTTTAAAGATTATCACACAGCTAGAGAGCTGAGCAGCCAGTATTCAATTCCAAACAC
ATGGTTTTCCATCATATCATGTGTATTTGGAACTTCATCTCCCTTTGCTTCCATATCGCTGCATTGA
TCTGGTGTATCTGACTTGGCTGCATTTTCTGGCCTCTATTTTCTTCTTTCCATCTCCTAATATTGTC
ATTCCATCAACTCTCAGCCATGGCCAGCTCCCGCCCAATCTCATTCAATTCTTCACTTTCTACTTA
CAGGGATGGAGAAGACTTAATCCTATCTGGAAGGAGCCAGTGCACAGCAAAGATGGCAGAGA
GAGAAAAGAGAGGGAGTGACAATGAAGTAAGGCTACAGAGAGATGACCAAGTGTCCCAGAGG
AGGAATTCTCTTTCGGAAAGAGATAGACACCTCTTCCT
ID|56678|gene_id|ENSG00000255471|transcript_id|ENST00000533672 Homo sapiens lncRNA

GCGGAAAAGGTAGCTTGGCGGCTTGTGCTAAAGAAGGGCCTTTGAAGGGTCAACTCCCCT
CGGTGAGTTAAAGTCTTCTTCAGAAAATGTTACAGAATGAAATACTGAGTCTCAGCAATCAGGA
TGTTGATTCTAAAAGTGTCACAAATCAGAAGCTGACCCCGGTTAGGTAGGGGTGAAGAGGCAG
AGGGTAGGAGGGAAGCTGTGGAAATAAGCTGGGCTCTTGGAGGAAAGGAAGCCATACTGAAC
CGTTCACTAAAGGTTTTGGGGAGCCCTTGGACCATTTTAAACAATGTAGTCTTTCCTACTCTTCAA
CTCAATATTGATTGCACCAGGACTCAGGAGCTTCCTCATTATCATGTAATCACTATAACAACTCTT
GGAGATAAGTTTCATTACTTTTCTTTTCCAGTTGAGTAAACTGAGAGTCACTGAATTGCCTAGGAT
TAAGTGGCTAGGTGGAGCCAAGAATTGAACCCAGGTCCAAGAGCCTTCAAGTCCAGTTCGCATT
TTCCAGTTTGGCTCCAATGCAAGCGCAGTATCTTATTTAACTCATTTTTGTTTTATCTATCCCTTAC
TTCTTGGTCCCAGGTCTCCAAAATTTAACAGAGTTTGGTGTGTTGTAAAAGTCAAACATGCCCTC
ATCATTTTGTTGTTGTTGTAGGCCCTAGAAATGGCTGTAGGAGTTGGCAAAATAAATGCATTCTG
AGGCCAGCAGCCCTTTCTTCTCTGAGCACATTCCCTGTTCCTTTCCCAGTCTCTGGGAGAGGAAT
TTTGCCCATGGGAGCTGTCTCAGGTGCCGACCTTTCTGTGCCTCCCTCACACATTCCTGCATCTA
AATTAGAACTGGTCAATGTTTCACTCCCCTGCTGTCCTCCCAGCCTTGTCTTCTGAAGCTGGTGA
ACAATTAACCACCAGCAAGCAGCCCCTGTTTTCCGGCTCTCGATCTTACCTCCTTAGAAGCCTCC
CTACATTTACTGCTAGGTGTCAATGATCTACCAAGCTTGAGAAGCACCGGGGCTTCCTCTCCATT
CTTTAAGGATCCTCTTGTATACCCTTTTACAATGATTCTGATGATATACGATTCTTCTCAAGGAAA
TATATCTTAGGGAATAACCAAGCCAAAGCACCCACATTGACTCTTGAATCGTGTTTTCTGTTGGG
AATTCTTACAAAGAACTGGAGAAAACGGGAAAAGATTCCAGGCCAGTTCTGTCACTTACAAGCC
AGGTGACATTAGCCAAGTTTCTTAACTTCTGTGGGTCTTCTTTTTACACTTATTAAATGAGGAGGG
TCATTCTTCCCTCTTAAGTGTGTTATACAGACTAAATAACAGTGTGTACAAAAGTGTTTGGCCCA
GACTAGCAACTTAATCAGTTTTCTTACATAAGAAACTGTAGTCTAAAGTTCATATGATCATAGAA
TACTGGAACTAGAAAATGCCTTGTAGAGCTTGTATAACTTTAACAAGAGAGGAGGTCCAGAGAA
AAAGTCTGAGATATACATATTCAATGACCTTTCAGCCAATTGGTGGCCTATAGTCTAGTTCTGCTT
TGTCCATTAGAAATATAATGTATAAGCCATACATGTAATTTAAAATTTTCTAGTAATCACATTAAA
GAAATAGAAGGTGGTTTATGCTGGTAATCCCAGCAATTTGGGAGGCTTAGGTGGGAGGATCACT
TGAGTTCGAGACCACCCTAGGCAACACGGCAAGACCCCATCTCTACAAAAATATTTTAAAAAAT

TAGCTGGGTGTGTTAATGTGTGCTTGTAGTTCCAGTTTTTTGAGAGGCAGAGGTGGAAGGCTCAC
TTGAGCCCAGGAGTTCGAGGTTGCAGTGAGCAATGATCACACCATTGCACTCCAGCCTGGGCG
ACAGAGACCCCGTCTCAAAAAAAAAAAAAAAAAAAGTAGACAGGAACAGGTGAAATTAATTTTAA
CATATATTATTGAACCCGATGTATGTGATATATCATTTTAACATGTAATGGATATAAAAATTAATA
ATGAAATATTTTACATTTAGTAGCCCCAAAAAAGGGCTACTAAATTTTCAAAACCTGCTGTGTGT
TTTGCATTCACAGCACAATCAATTCAGACTAACCACATTTCAAGTGCTTAATAGTCATATGAGGG
TAGGACCTACAGTGCAGGTGTAGTTTAATTCAAGCCAGCAAACATTTTGTGCACCTGTTATATGC
CAGGAAGTCTGTTAGATGCAGGAATATGAAGGTGAACAAACATACCAGGGTAATAGGCAGACA
GCTCTGGAAAGCACAAATTATAATTCAATGCTGGGTGTCCCTTCCTGTCTAGGCTGCTGTATTAT
TTATTCATATGTTTGAGCAAATGTAGAAAAAAATTCTCTAGAGAATATCTATATCTTTAGGGCATT
TTCTTCTGCTTAGATATCAAGGGATGGAAAGTATTTATTTTTACTGAGCTACTACTGTGTCCCAGG
TATTGTATCTGCACTTGACAGGTATTGTCTCATTTAATCTTCATAGTGATGGTGTGAGGACATTAT
TTTGCCCATTTTATTTTTATTTTTATTTGTTTATTTTTTTTAGAGATGGGGTCTTGCTATGTTGCCCA
GGTTGGTCTCTAACTCCTGGGCTCAAGCAGTCCTCCCGTCTCAGCCTCCCAAAGTTCTGGGATTA
CAGGCAAGAGCCACTGTACCTGGTCACATTTTGCCCATTTTAAAGAGGAGGAAATAAAAGTTGA
GAGTGGTTAAGTAATTGTCCATCTGATTTATCAGAGGTAGACCTGGGACTCAATCTCAGAAACA
AGGCATGTTTAACTTGGTCAGTATTGTCTCAGGGAGATTTGGAGAGAGGAATGTTCCTTGGCCAT
TATTATTTTTATCTATTTATAAACATTCCCAAAGTTTCCTGAATAATGTGACTGGAGCTTTATTTTA
ATAATAACTTTCCATTCCAGGTGACCAGTGGTCCTCCTTTCTGAAGGCTCTTGCTACAAAGGAAA
GATGAGGATATTCTATTTCTTGATCCCTCTCAGCTACTGGGAGCTGCTTTGTGCCTTGGAAGCCT
CAAGCTGAAATGGATGTCAGCCTCAAGTTTAACTCCAAGAGGGTTCACCTTGTTTCTTTGAACAA
AA

ID|56129|gene_id|ENSG00000237181|transcript_id|ENST00000429872 Homo sapiens lncRNA

TGGGCAGACACAGAAGAGGGAGAAGCATTCCACAGGGGAGGAAAGGCCTCAGTGGGGA
CCTATGGAGCTCCCAGGCCCACCGCAGGGAGGCAGGTCCTCGGTCATCTGCGGAGGTCAGTAT
CTATCAAAGCCCAGCCGCAGATAGATGAGCTGGGCCTGGCGGGGTCTTGTAGGATGTGTAGGA
GTTTGTGAGGCAGAGCAGGAATGAAAGCAGAGGGGACAGGAGGGGCAGAACCAGAGTCAGG
ACATTCATTGGCTAATTTCCTGTTCTTGGTCCCTCTCCCCCATTAAGCCACAACCTCCCCCACACC
AGAGCAGGCACCAGGTCCTCCTCGTCACAAGTGCCAGGCATAAAGCAGGCACTCAATAAACAT
CCAGGAAGGAGACGGCAGGGGAGGAGGCGACATGATGACTCCAGGACCCAGAGTGGAGGTG
ATGGGGGAGGAGGTGACACCAGGACCCAGAGTGGAGAGGGCAGGGGAGGAGGTGACACGGT
GACACCAGGACCCAGAGTGGAGGGGATGGGGGAGGAGGTGACACCAGGATCCAGAGTGGAG
AGGGTGGGGGAGGAGGTGACACCAGGACCCAGAGTGGAGGGGATGGGGGAGGAGGTGACAC
CAGGACCCAGAGTGGAGAGGGCGGGGGAGGAGGTGACATGGTGACACCAGGACCCAGAATG
GACAGGGCGGGGGAGGAGGTGACACGGTGACACCAGGACCCAGAGTGTGAACTAGAAGAGG
GGCTATAAGTAGTAGCATGAGATTTGGGGCTGGGATAAAGCGGTGGGTCCAGTCAGGAAGCTG
CCCATCCCATACCTCCCGCAGAGGTGGCTCTGCCTTCAGCACTGCTGACCACTGGGCAGGCCTG
GGCTGCCAGGGTCTCTCTGGGATCAGCCCTGCCCCATCTGCTGGGGGCTGAGCCCCATTCCCTG
CAGCCCCCCATCAGGCCCTGGTGTCTGGATACCAGGCACAACGGGGCCACCTCCTGGGACGG
GCCCAGGCCCCAAGACGATGCCCGGCTGGGATGCTGGCCGCATCCTCCTCTGTCTTCTCTATTT
CCTGGGCTAGGCTTTTTTTAAACTCGAAGCAAAGCCAGCAGGAGATGACTCTGCGGCCTGGCA
GCTGAGCCCAGGGTTGACCCGCCAGTGCCGCCCTCCAGCCTTCCCCCTCCACACTTCCTCTGAG
GCCGGGCAGTACCCGGGACCTTTGGCACCTGCCTCAGTTTCCATCTCTAGGGGAGACAGGAGC

ACCCCCACTCCCTGACACAAAGGTCTGCCCCAGAGGCCCTGTCTAGGGGCCCAGCCCGTGGGT
TCCGGCTCAGGACAGAGCACACCAGGGAGAAGAGAAACTGGCTCTTTCTCCGCCTGCGCGGGC
CAGGCCCGAGGCACGTGCCCCCCACGGGGAGGCGGGACCCCTGAGTCATTTCCTGTGCTATCA
GCTCCGGAAGCAGAGTGGGCCTGCTCAGGTTCCCAGAAACACAGCGCGATGGGGGACAGACT
CCGCGGCTGCCACGGAAACAAATGAGTAAATGCCCTCCCCAGAATAGCGTGGCCGGGACAGA
GGCACAGCCACCGGAGGCCAGACCTCGAAATAGCTCGTGCTGGGCAGCCGGGGGTGGCGTTG
CCTGCACGGAAGAGTGCGGAGGCCACCTCCTGGGGCAGGTGGACAAGGAGAAGGGCAGGCG
GACAAGGAGAAGGGCAGGCAGGCATCCCAGACCCAGAGAAGACTCAGCCAGCCTGGGAAGC
AGTGACCGTCTCGGGTAGCAGAATCCCCAAAGCGTCCTGCACTGTGGGGGCTTCCAGTCTGTGT
GCAGAAAGGCCGTAAAGAGACGGTCTCATGAGATGTCATCACCACGAGCCGCAGAGGGTCCTA
CGAGGGAGGCGTGGGACATGTCTGCTGATGCCCACTGGGAAGTGACATTTAGCAGAGAGCAG
AAGGAGAAGCAGGAGCTAACGGGGTTCCCGGCAGAGCAGGGGACACACACCAAGGCCCTGG
GGCCGCCAGTGCAGGCGGATTCCAGGAACCGAGGGAGGATGATGTCACTGAGGTCCTTCCCGC
TGACTCCGCAGGGGCTCGGGCCTGATTCAAAGGGAAACAAACTCCTAGCGGGCGTTTTCCCAG
AGGCTGCACCATCAGCTCCGCACCAGCAAGGTCAGGCTGCCGTGCGGGGGGCGGCCTGCAGA
ACAGGAGCCCAGCGGGGGATGAGGGGGCCTCAAAGCCTTCACACCAGAGCACCCAGGCTCTA
AACCGGACGGCAGAAGAGATGGGAGCCAGAGGCTCTGGGCAAGACTGGGCTGCGGGGCCAG
GGGTGATGTCATGTGCCGTGTGTATGTGTTTATTTACTTAGTTTTATACATATATGTATCTATTTTA
TACATACACATACACATGGCCTGGCCATCGGGCACGAGACGGGGTCTCCAGCGGAGGGGAAG
CTTCTCCTTCCAAAACAGTGGGAGCATCTCATAAAAGATGCTCTTGAGACAAGCGTGGGAAGAT
GACACAGTGAGACCCGACTCACGGCTGGGACGTTCCAAGCTGGGAGCCAAACGCAGGCGCAA
AAGGCAGCCGTTGTTTGATCGTGAAGTCGTCGGTCCTAGGGGGTCTCTAAGCACGTCGCTCTCC
CACCCACTTTGAGATGTTAACATCCCATGTGGTGGGTGAGCCTGGTGTATTCATCGGCCTCGGA
TCTGGAGACGGCGCTGGGTCAAACAGAACCGCCCTGGCCAGCCCCAGCAGTCGCAGCCCGGG
GGCAAGCGGAAGACAGCGGCCCTGGAAAACCTGAGGCCGCTGCCGGCACCTGCCAGCATCAC
CCTTGTTTCCCTCTGCACTTTCTCATATTCCCATATTCTCTTCCAAGAGATAGCAGTTGCATTTCTG
GATTAAAACACTAGGAATACGGCACTCAGCGCAGTGTTTGTTGGGGGCCTGGTGCTGCCTGGA
GGGCAAGTCTTCACACCGGACATGCAAAGACGCGCTATTTTCCAAAGACAAGTTAGCGACACT
CACATCCACTTCCCCTTGATCAACGACATAGAAGTTGTCTCCTTCATTCCCTGTAACAAAAGAAG
AAAGTCAACAGATACAAAGTACATCCAGACATACAAGTTACAGTTTCTCTTGCAAACGACTTTC
GCAACACTGTTGCAGAGACCCTCGAAGCAACATCCTGGGTGCTACCAATTCTTTTTAATGACAA
GATTATACAAACATTGAGATAACTTGTTAAAGAGCCTGTTCTTAAGTTTCATTAGTGAACTTTCTC
TAAACAATGAGAGGTGTTTTTCTTTTTGTTTGTTTTTTGTTTTTTTTGAGACGGAGTCAGGCTCTCG
GCTCACTGCAACCTCCACCTCCCAGGTTCAAGCGATTCTCCTGCCTCAGCCTCTGGAGTACCTG
GGATTACAGGCACCTGCCACCACGCCCAGCTAATTTTTTTGTAGTTTTAGTAGAGATGGAGTTTC
ACCATGTTGGCCAGGCTGGTTTCAAACTCCTGACCTCAGGTGATCCACCCGCCTCGGCCTCCCA
AAGTGCTGGGATTACAGGCGTGAGCCACCGCGCCCGGCCTCACAATGAGAATTTTATGCGTGT
GTGTGTGTGTGTGTGTGTGTGTGTATAATTTTATATTACTTTAGTTTTATACATATGTATATATTTTA
TATTTACTTAGTTTTATACATATATGTATCTATTTTATACACACACATATGTATATATGTGTACATAT
ATGTATACATGTACACACATATGTGTGCACATATACGTGCATATATATGTAGACATATATGCATA
TTATATGTGTGTATGTATGTACAAGTCTATATATATCCTTTTGAAATTTGAGACAGAGTCTTACTG
TATCACCTAGGCTAGAGTGCAATGGCGTGATCTCAGCCCACTGCAACCTCCGCCTCCCAGGTTC
AAGCGATTCTCCTGCCTCAGCCTCTGGAGCAGCTGGGATTACAGGCACCCGCCACCACACCCA
GCTAATTTTTATTTTTAGTAGAGACAGGGTTTCACCATGCTGGTCACCTGGACCACCAGGCTGGT

CTCGAACCCCTGACCTCAGGTGATCTGCCCACCTCAGCCTCCCAAAGTGCTGGGATTACAGGCG
TGGGCCATCGTGCCTGCCCTTATGCTATTTTTTTTTTAAGTGCATAGTCCATTTTTCCCCTCTGGAC
TTTGGCTCCGAGGAGCAGGCAGAACGTACCTTGCTGTATAACAGTCTCCCCAGCGATGTGAGTG
ACAGGGAACATGGCATCGAATATGTCACTGAAAAGCAAAACACGCCAAATTAGGGCTGCAGGC
AGCACAGGGACATTTAAACCCTTCCTCCCCTCATTTCACAGTCTTGTGTAAATCGCTTCACAGTC
ATTGGGGAAAATGAGAAAATCCATGAGAGTTCAAAGAAGAAAATTGAAATCCTTCATGACCCG
AAGGCCAGAGATGGCACCGCCAGCCCTGGGCACACAACCTCCCAGCCCCATTTCTGTTCTGTG
CACAGACGCAGTTTGCTGTTTTGGTTTTTACAAAAATGGGGTCATGCCATACATGCATTTTGAAC
TCCATTTAGAAAAACAAAAGTAACAATAGATTGTGAACATTCTCCCATCGGTAAATACAATTCTA
CTAGAATACAACCAGCAATCTTGATGACATGTTTTCTGCATTGTTTTCCTTTCAGGGAGAACACTT
TCAGTCGAAGGAGCCTGGGACCCTTGACACAGAAGTGACATGTGCATTCGGAACCTCTCTGTG
GAAAACACAGACCCTTCATGACACGCAAACTCGAAGCCGGTGAGGCCGAGTCTCAGGGCTGC
GCCAACCAGGAAGCCTGGCTGTCCAATGTTCCAAATCTGGATGTGAAATGGACCATGCAGACA
GGACCCAAGGGGCATGGGGGAGCTGGCAGAGGCACCCGGGACAAGTGGGGAAGCACCTGTC
GGCCAGGAAGGTAGAGAGGCACCTACCCCAGAACACGGTGCCAGCGTCACACTCTTATTAACA
GATGCAATAACCACCTTCGGAGACCAGACCCCTCAAAGAGCACTCAGCCCACAATGTGACGCA
GAGAAACCAGAGACCCAGGCAGGGAGTGCACCCACGGGGACACTCCGAGGGTGGCCTCCTGC
CTGCCCTGATTACTTAAAAAGGAGATTCTTGGAAACCTTTGCGGGCCCACAGGTGCACCCTGAC
TCCCAGGGATGGGGGCTGAGCCACCCAGGAAACAAATATGAAATCAAAGATCCAGACAGCCCT
GACCCTCCTGATTCCAGGGTCCATAAATAAGTCATTCTTAAAA

ID|56066|gene_id|ENSG00000237410|transcript_id|ENST00000433606 Homo sapiens lncRNA

CAGTGGCGACGGGCAGCAGCGCTTCGTGATCCTGATTTCTTCCACCCCAGGCAAGAAGTG
CCCACCATGGGTACCACCACCACCCCTCCCCCGAGGACTAAATGAGCAGCAGGGAGTGACAAA
CAGTCAGGTGCACCTAAGTTCCAGCCAAGCTCTACCATCGACACTCTATGACTTGGGGTGAGTA
ACTGCCTCTTACTGAGGCCTGGGTTTCCGTATCAGTTAAACACAAAGGCCACTGCCTTCTCTGAG
GATCAAATTAAAAGGGCCTTGTAGCAGCAAAACATGAAACTATTAGTGGGGAGTGTTACTATTA
AGCAACGAAGTTACCATCATCTCCAGATCTGTGCAAAACTGAGTTAGCTGGAGGGGAAGAGGT
CCAGCCACAGCCAGGTCTCCATGCCTTGGCAGTCAGGCTGGAACACCCTCAGCTGGCCATTGT
CTGGAGGCTTTGGAAGGCACAGACAGCAGCAGGTCAAGTAAGGAAAATCTTGGGAGACAGAG
TGGAGATGTGGTGGTACCCAAGGGCTGGACATGCCTAAGAACACGAGGAAAGCACTGAGGGA
GATGGGGTAGACATTTTTTTCAAAGGGGAAAGAAAAACTAACACCACCCAAGAGCTCCTGGAC
CCAGTAGGTTTCTCCTGTAAGAAACTCAGGACTCATGCCCAGGTGATGAAGGTCCCGCAGTGTG
GTATGGAGGCAAAGGCACTGACTGGAGTCTCACCCCAAATCTGCTATGAACTACCTCCAGAAAT
CGACTGTCCCTCTCCAGCCGTATTCCATGTCTGTAAAATGGAGACGCTTCGTTGTGTGTGTGTGT
GTGTGTGTGTGTGTGTGCATGTGTGTGTGCGTGCCTGTGTGTGTTTCTTCCCAGGCAGTTGACTTT
TCTGCTTGGTCTCTCAGCAGGACCCCAGGGAAGATGATTCAAGCAATGCTGAGGAGGCTGCAC
TCTGCCCACCCACTCCAGCCCCATCATCCAAGGGAAATGCCAAAGCTACCCCACAGAGTAGAA
GGTTGCCTCCCCGCCATTCCTGCCTCAGCTGCCCTCCCTCTGCAGGGCTCTAACCAGCTGCAGG
CCACCAACCACTAGGGACTGGGAGCTGCTGCTGACCCTGCCCTCTCTGGGGAGGGGCCTCAAA
CAGAATGCAAGTTGGCAATCAGATGTCCAGCCTAGGAGGAAAAGAACCAAGCGAAGACAGTA
GGGAGTGACCTTGTGGCTCAAGGGGACAAACTGCAGCCGGGATTATTGGGGAAGAAAGAGGA
GCCCATTCCAGGGAAACCAGCTGTCCAGCAGCCTGGCCTCCTGGAAGCCTCCAGAGCCTGTCC
TCTGGGGTGTGGTCCTGCTCTCAGCAGGAAATAACAGATTCCCACTTCATGCCCTGAGGGCACC

AAAGGGGGTGCAGATTGTTAACAGCCCCCCTCCCTGTGCAATAGTGTGTCTGTGTGAGGTACAA
GTACATGTAAGGCCATCACCAAAACTACACAGGACAGCAGCAAGTTCACCCCACTAAGGCCAA
CACCTTTGGTTTTGGGTGAAGGGAGCAGGGTCCCACCAGCTGAGAACAAGGAGGAGAGGCCAT
CTATCAGTGGGTCCCCCCAGAGCTTCAGGCAGCAGCAATCCTGCACAGCCCCAGGAGAAAGAC
CCTATGAGGGGACAGCAGGCCACAGCTCCCCTGGGACTCTGCAGACAAGGGATGAGAAGGAA
GAAGCAGCACAGCTCCTGAATGCTCAGAGGCCCTGTTTCCTCCCTGGCAGCCCCTATGGCTGGG
AATGGACCCTGGTCTCCCCAAACTGCCCCCAGCCCTCCCAGGCACTCACGATCATTGCAGACA
GGGCCTCCATAGGAAGTCATGGTGCAGTCGCAGGTGAAGCCATCCCACTGCTGCAAGCAGACG
CCCTGGTTGGCACAGGACTCTTCAGTGCAGGTGGTGCTGGGGCCTGGAAGGGGCAGGAGAAA
GGAACACCCCTGGCTCAGCCAAGCCCCCTCTTTCCCCAGGAGGTGTGGAAGCTTCAGAGCCAG
GCAGAGAGGTCCTACTCTGAGCTCTGCCTGGCTGAAAGGGCCAAGTACTGATGACAGGGATTG
GGGCAGGAGGGTCAGGTCTGCTAGGCTTGGCCTTGGACTGTGACAAGGGGCCAGGGTGTGAG
ACAACAGGGAGGGCAAGTGACCCTTCACACACCTGTATCCCTGCCCCAGAACTGTGGGGGCAA
GCTCCCATAAGGCCTGAGAAGGAAGGCCCCTTGGCAGAGCAAAGGCTACCTGCCCCGGTCTGC
CTCTGCAGCTGGCCATCCTGTAGCCAGTAGGGCCACACCTCACTCCTCCACCCTCCACTCACCA
TCACAGCCCCTCTCCACCTGCCCAATGCGGTGCAGGGCGTCGGCGATGAGGTCTGGGAGACGT
CCGTTGAGGTCCACTGAGGCCAGGCAGCCCTGAAAGCCATCCCGGGAGGCCACCAGCTTGGG
CAGGTTGCTGAACATATTCTTGCTCAGACCGCCAATGTACAACTCCCCTGCAAAGGGAGTGGGT
CAACCAAGGCATCCAGGTCCCCATTCCCATCCCAAGACAATGGCATCTGGCTGTCAGGTCCTCC
CAGCCTTCTCAGGTTCTCTGCGTTCCATCCCAGATTCCAGGTCCCTAACAGAGATCCAGAGACC
TGCACAACCCGCACCCCCCCAACACACTTCTTCCTTCTCCACTTTGCCATCTGCCTGCTCCCCTC
CCAGAGAAGCCCAGATGCCTGGGAGAGTGGGGGCTGGGGGACAGCTGGAGGAACCTGCTCTG
TGCTCTCTCCCACACATTTGATTCCAGTTCCGGCCCGTAATCAGGGCCTTTCATAGATGCGCAGC
CCAATCCCGTCCACCCCCATTCCCTCCCTCCCAAAGTTTCCCATTCCCAGAACTCCTTCAAGCCT
TTCCCAACTCTTGGGCTCTCTCCTAGGGAGTTCAAAGCCCAGCTCCCTTCCCCCAACAACTAGG
GCTTGGCCTTGGGACTCTGGGAAGTTCTCCTCCGCAACACCTGATCCCCTGCCACAGAGCCTAA
CTCCCTGGAATCCCTGGAAGCCCTAGCCAGCCCACAGCCGAGCTTCAGTCAAGACACTGGTCA
GTCTGCTGCCCTGGCCACGGGCTCAGTACAACACAAGACCCGCCAAGATAACCCCCGGGGTTT
GGGGCCACTTTGGATTCTCTGTGACACATCGCCCCCTTCTGAGAACAAGGGGAATTCACAGCAC
TATGTCAAGAGATCTGACAGATCTCAGATCTGAGGGAGTGGGGCAGTTAATTCCATGGTCTCTG
AGGACCCTCCCCAAGCTCTGACAGTATGAGGCAGGCTGATGCCCAGGGGTTCCTATGGGGCTG
AGGGCTGCTTTGGGCATGTGCAAGAAAGTAGATCACATTGCCCTCAGTAGGCTCTGGAGAAGCT
GCATCTTTCCTGTCCCAATGCTCAGGCCTTAGCTCACCCTCCTCCTCCCTGATCTCTCAGTCACC
CATTCTGGGGGCAGGCAATGACTGCTTCAATGACCACTGAAGAGGCCCTGGGGAATGTGGCAC
CCTGATGTGACCCTGAGCACACCAGGGAAGCCTAGGTCCAAAATGTCCCCAAGCTAGCCCAGC
CGATGCCCAAGACAGGTGACTCGGGGACTCTAGCTCCTGCTACGTCCTCTGCCCCATAGCGTCT
ACCTCACCCAGGAGGATGCTAAGCTCCCTATTCTCACTCCAGCCCTCAACCAACATAGGCTTCT
CAACCTTGGAGGCCCAGGAGGTCCTTGGAGGGCCTGGGTTACCTACTCCTAGCTCTGGAATGA
GGCCACCAAGCACCCTGTGAGACGCATCCTGCAACCAGGCTTTCAGTGGTGACATGCAGACTC
ACAAGAGAGCCAAGCAGAACTTGGACAGGAAGATGTCAGGCTGGACATAGTGGAGACAGGGA
AAGAGAGGTGGAAACTGGGGGCAGGGAACCGAGGGAAGCAGCGTCGCAGGAATGGGGTGAT
CTCGCTGGGGTGAGGGGTATCTGGGCTAGGGCCAGGCCTTCTCCAGAGGCCCCAGCCCCACCT
TTGAGATCGAGGTTTCGGGCGCCATTGGAGTGCTGCGTGACAGTGCGGGAGTCAATCTTGAGC
GTGTGCACGTTGCCTGGGTCCCTGGACACCACCACGTTGTGCCACTGGTTGTCATTGACTGGTTT

GTCTGAGTTCCCCTTCATCAAGGACGGGCCATTCCCCAGGTCAAACACGTAGTGGATGTACCTG
CCCCACAGTAGGGAGGAGACACTGGGTCAGGGCGGGGGTGATGCTGGGAAGGTGGGGTGAG
GAGGAGCTATGGCTGGCAGGGGTCCTAGGTGCCATGGGAAGAGGGATTGAAAGGGAGACCCC
AGAGGAGAGCGACCAAAACCAATGGCAAGAGGGCTATGGTGTGAGGTCCTGTCACGGGGAGA
ATGTAAGGAAGGAGGGCCACCAAGGAGCCGGCTCTTGTGACACAACCTCAGAAGAGGAGCTA
GAGGGGAGAGGTTTGTGGGTCAGCAGGGTCCTCTGGAGGCAGGGACTGGGCTGCTCTGGAGT
GGGGTCCCCTAAGGCAACGGAGGGGCAAGACTGACTTCTGCCTGTAGAGAAGGTGACAGTGG
ACAGAGAACAGATATAACAGGGAACTCTGGCAAGGAAGCAGCTGGGGAGTCAGCAGTGGAAG
AGGGTGTGCTTCTCCATCTTGGCTGAAGAGGGAGCCTCTCGACTTACGGTCGGGGACCTGAATC
TTGACTTGTGATCTGGGGGGATTGGACACCTCGGCCAGTAGCCAGGAGAGCTGTATGTGGTTCA
GCAGGGGGAGGGGGCCACCTCCTTGACAGCAGTGCAATCCCCAGCCCCTCACCCCTTGACCAG
CTCGATGACAATGAAGTCATTGCCGTTGCCCGAGTTGAACAGAAGAAGCCCATCAGGGGCCGT
GGTCTTGAACTGGAAGAAGAGGTGCATGGAAGCATAGGCTTGGAGCGTGGCGAGTGCCAGGT
AGCTGCTGCGACTCTTGAAGGTGACGGGATCGGCCACGATGGCACGCAGGCCAAAGCGAGCA
TTGAGCTCACAGTAGGTGATGTCACCATCCTTGCACTGGTCCATGTAGGGCTGGCCATTGAACA
CGAGCCCACTCAGATGCCCGATGAAGTTGGAGGGCACCACGGAGATAAACCGCCGCTCCGTCA
TGATGCCCGTCTCAATGTTGTGGAACTCCAGCCGCATATGGGCTCCTGCCATCTGTCCTGCTCAG
GACAGGAGACCAAGATGAGGGGGATGGCTTTGGGGCAGGGCTGGGGCTTGGGTTCCCAGGAG
CCTCCCCTCCACAGGAGGGCCACCCATGAGTGACATCTTTAGAGATGTCCTCGGCTAGGCACTA
GCAGCCAGCACAGCTCCAAGAGGAGTGGCAGGACTCGCCAGTCAAGAGCCAACAGGCTGCCA
GGGGAATGCTGCCTACAGGGAGTTGAAATCGTTCCTGGGGTCCAGATGCCCATAACATTCCACC
CCTGAAGGAGAAATGGCAGAGGCAGCTTGCCAGAACACTGCCCTAGGAATGGCAGCCCAGGC
AGTAGTCACCAAGTAGAACAGGGCCAAGCATAGGTCACTGGAGATGTGTCCACCTCCCTGGGC
CCAGACCACCTACCCTCCACAGTCACGTTGTCCACAGACAGCTGCAGGCTCTTGCCACGCCGG
ACCACCCTCACCGTGTGCCACTCATTGTCATTGAGCTTGTGCCCCGCAAACAGCGTTTCGGGGC
CTTTACCTGCGGCACCAAGGGGGGAATGAGGAGGGCACCTATACATGCTCATAGGTGACTTCC
TATATCACCTTGGATACACAGACAGCCTCCCCATCCCCGCACATGCCACACATGAACACATACA
TGACCAGTGGCTCATATTTGGCCACGCAACACACCCATGAATACAAGTACACACACTTATAGCC
TCCCATCACACCTGGAGTCCATGCACACGCAGAGCTTTTATACCCCAGTCATACATCCACTCTCA
GGACTGCCCTTTGTGTGACTTTAGGAGGTGCCATGCACATAGAATATTAAGTGAATGGTGCCTCT
GGAGTTGTACCCTGAGGCCCTGCACACAGACACCCACACCTATGACCAACATACTCTTCCCCCA
GATTCCTAAGGGCCTTCAGTGAGTGTGGCCAGAAGCTATATTCCCAGGGTGTAAACACAGACCT
AAAACTTTCAAAACTTCACACAGTACCCAAATGTAAATGCGAGCCCACAGCTTCGGCCTCTGTT
ACACAGACACCAGCTCCTCCATCTACAATGGGACCCGATGGGACCTTTCTACATACACATGCCT
GGTTAACGTCACACACCAAGTCACGCCACCACACGTACTGCCCATAAATATGCAGGATGGCCA
GAGAGGGGAAGGTTCTGTTTCTTAGGAACTATGGCCATTTACTCAAGACCAGGATTATCAGCTT
CCAGAGTCTAAAACAAAACATACTTATCTTTCAAATGCTGCTGGACTTTGCAGCACTCTCTGGTC
ATCCCTGCCCCCCCACCCCGAGGGCTCCAGCACGCACACAGCATGCCACACACACATGAAGAG
CAGGGCGCCCAGCACACACAGTCTCTCTGCCTGGGCCTACCACGTGCATGTGCTCCCCCATGTG
ACCCCAAGCCACACCATGCAGAAACACCCTGAGACACAGCATTGAGCCCAGGCGCACTGGGA
TGTCTGGAGATGTCCACCTGCTCACTCTCCATCTCACCCCTGCTGAGAGCCCTCACTGTCACCAT
AGAAGGAGCCCCCATTTGGCAGAAACTCACCCCAGCTAACACAGGAGCTGAAGAGGGCTCCCT
TCCCACCCTCATCCTCTTTCCTGCATCCCTCGCCTTCCCCCTTGTGGCCAATTCTGGAGATGCAG
CAGCGTATTCCAGCCCCTCTCAGGCTCCTCCCTGCCAGTCCATAGGACCACAGGTGCTACCCAA

GACTCTGCCCCAGCCCTTGATTTCTACCTCCTCCCCACCCTCCTGCTGACTTGGGTAAAGGGTGT
CCTTGTCTCTCCCTTCCTAGACCCAGTGAACATCCTGACTGCAGGCCCTCCCCCAGCTCTCCTTG
GCTCTGCTTCATTTGGTCCCTCCTGCCTTCCACAACCACCCAACCTCACCCTCCTGCCTGCACCC
CTCCCCACACTCAAGGCCCAACCCCCATTCGAGCCAGCAATCACGGCTTCTCTTCCTCCCAGCC
TCCCTCCCTAAATCCCAGCGTCCAGCATCCCAGTCCCCTTGGGTCTCTGCAGAAGAAGAGGGAA
GCCACTTACTGGGTGCGCAGCCGACGCGCAGGCAGTCTGGGGGGGCCATGGGGCGGGCAGAG
GGGAAGGGGAGACAAAAAGGTAAAACAAGTTTTTTTTTTTTTTACATCCTTCACAGGGAGGGGT
GTCTGCGGGCGGGGTTCCCCCCAGGGCCACTTTCCGGGGACCACGCGTGCCCAGGTGCCCTCT
TCCCTTCCTCAGTTGGACCCCTGTCCCTCCTGCCCCCAAGAGGGCTCCAACCCTCCCCAGAGCT
GGAAGGCAGGGCAGTCAGATGATGGGAAGAGGGCTTGTGGCCCCCTTCTTAGAGGGTACCAG
GCCTCTAGTGTCTGAGGGAGACGGGCACCTTGGGAATCCCAGTCGTACAGGGGATCAATGGGG
GCCGAGGGCTTGACCAACCCCACATCTCCCCTGTCTCTGGGAGGGAGATGGGTGGGGAGAGCC
AGAATCCCTGCATCCCCTGCTCTGGGACGACTCCCTCCTCAGAGCCCAGGTGTGACTTGTCACT
CCCTGTTCCCCTCTGGGCTCTGAGCTAAATGGAGGGGTCTGCCCCAGGGCCCCCTTCCCGAGGC
CATACACCTCCCAAAGCCCCAGTGGCTAGTACCACCCCTACAGGAACCTTCCTTCCACCCTTTC
CAATCCTGGGAAATGCCAGGCTGCTACTGGGGATAGATCATGGGAGTCCTTCACCCTGAAGAG
GACCTAGTATTAACCTGCTTTCCTAGTCAGAGGAGCCAAGGCCTCACAGATAGAGACAGTGCA
GCCCCCAAAATATGATTTCTCTGAGAACACAAAGCAGTTGGGGACAAGCAGCCACCATAAGCA
AAGTTTCCTGCCCCTGCCCCAGTCCGGTCCTGGCATTGCTATCCTCTGATTTCAGGACTGGGAAG
GCCCCGTGTGGCTACACAGAGCCAGCAGCCCAGGGCCCTGAAGCCTCTGGCCATCCCTCTGGT
TAGACACACCACCCAAAGGTGGGAGCCAGTCAGGTCTTGCAGAGGGAACAGTGGACCAGGGT
CCTATGAAGTCCAGATTTAAGGCCTTTAGGATGTCTGCACCACTCCATCAAACGCCAAGCCTGA
CGCAGCCCAAGGGAGGGGACTGGCTGGAGCTGCAGAAATCCTTTACTGGGAAGCAAGGGAGC
TGGGTCCTCATCTCCACCCTAACACTCACTAACTCACTGTGTGACCCTGGGCAAAGCACTTCTCT
CCATGCCTCAGCTTCCCTCCATGTGAGCTGAGGGAATGGATAACAGTAGGATCCTGAGCAGGA
AGTAGGCAGCAGGTAGACACTGGGTTGGCTTTGCCTTTCAGAGAGGGGCAGTCTCCCGGCCCT
CGATTGACAGGCCCAATACCCTGGCCTCCTGTCGCTCCACCCCAAATAGGCTCTGCCTACCCCA
CAGCCCAGGAAGCAAAGGGGGTGGGCAGCTGCATCCCCTGGGCCCCTGAAGGTTCCTGCAGT
GGAGGTAAACTGATGCCCCTGTGGCTTTGAGGGCCACGGCCCTTCGGCAGTACAGCTGAGCTC
CACACGCGTTGTCAACCGCAGCAGAAGAGTGGAGGCGAGCAGCCAGGACCCTGGGCCACAGC
CTGGCACCAAGGGAGGAGCAGAGGAGCAGAAAAAGGTCAAAAGGCAGACACAGAGGGAAGT
GAGAAGTCTCAGTGAGAGGAATGAGGTAGAGAGAGTGCAGAGAGAAAAGAGGAAGAAAACG
GAGCAGAGGAAGGAGCGGCTGCACAGAGGGGACAGGGGAGGCTGGACAGAAGAGGAAGGG
GAGGACTGTTAGGGTGAGGGTAGAAGGGTGCCAGGCCCTGGAGTCAGGTCACGGGGGCTCTC
TCGGGGCCAGCAGAGTCTTCACCAATGAAGGGTCCAAAAAAAGAAAAAAGGGGAGGCTGGAT
GGGCAAGCAGGTCCCCTACTGGGGAAAAGACCCAAAGAAGGAGCTTGATTCTTCTCCTTCCCC
AGCAAGGGAGCACCAGGAATGGGGGGTCCATCTGTCCAGAGAAGAGGTGTCAGCTCCAGGCC
CACCCCTGCAGAGACTCCACCCTCAGAAACACAGGCTCCAGTAGGGTTTGCAAGAGCAAACGA
CTTGAAGATGGGCACCCAGAGGGAAGCCAGCCCCATCCAGAAGAAAGCCAGAGACTGAGAGT
GAGCAGGAGAAACCCAGCCTCTTCAACATACACATTCACCAAAGTTCAGGGGGCACTAACTAG
TAACCTGTTAACTAGTGACCTGTGCCTCAAGTGCAGGGAATAGGCAGAAGTGAGTGGTCTTCGT
GGCACAGACCCACGAAGGCCCTGGCTGGACAGAGAGAGCAGGGTCGTAGTATGAACACGTGT
TGGGGAGGTAAGACAAAGCAGTCTGCATGGAAAGGAACTCAGGGGAAAGAACACTGGCTTCC
CAGCCCTGCCTCACCTGGGTTCCTTCCCCGCCCAGGCCCTGTGGGTTGGAGACAGTGGGCCCA

AGGCTCCCAGGCATCTGTCTCAGAAGTGACATCACAGAGTGGCAGCATGCACTCTGTCATAGCC
CTCCTTCAGAGCTGGCTCCCAGCAGCCCCCCTGATAATTACTGATCAGCCTCAGGTGGAAAGGC
AATGTACCCTCCCATGGCCCCAGGGATTAAAGGGCTTTGGGATACAATGTGTATTTAGCCTGATT
TAAAACACAAACGGGAGTTAGATTTCTCCGTTTGATTCCAAGGGGCAGAACTAACGGGAATTTC
ACAATAAGTGGGAATTTCACAATAAGTGGGAATTTCAGAAGGGCAGGGTGACACTAAGAAAGA
ACCACTCAATGATCAGAATGGATCTTGGAGGTGGGAGGTAGAAGGCCTCCCACTGGTAGGGAG
TGTATGGGTGATTCATGGGGTGGGGAGGGATGCTGAAGGAGGTAACCCCCTTGCAGCTCAGAG
ATTCTTTAATTACACCACACCCTTCAGGTCAGCTGCACTGACAGCTGGGACCCCAGGGGCACAC
CTAGTAGAGGACAGTGCCCAGCCTGGCCTGTCCTCTCCTCCCCTCCAGGGAGGGGCCCAGGCC
ATGCTGGGATAGGGCAAAGAATCCAGGGGACTCTACCATCCTCTAGGGACAGAGATCAACTCC
CCAACTCAGGGGCTGCAGACAGCAGAGCTCAAGGAACTTGCTCAGTGTTTGCAAACATAGTGG
GGGCTGGGAAGCTGCAGCAGGAGGCACGATAAGTTCCTTAGGATCCTCTGCCTGGAAAGAGGC
AGCAGTGCCTTTTTGTCCCTCTCTTATCTAGCAAATATCCCATCTCTTGAGGCTAGCACTAAGCG
ATCCCACCTAGTGGTCACCCGCAGCCTCCGCTCCCTCTTCAGGCCCAGCCTGGGGCCTGTTTCA
ATCCACTGAGTGCCTGTGGAGCTGCAGAGCTGCCTCCTGGAGCAATAGCTTCCCTCTACAGGAT
AAGATCATGAGGCCAGAGACCAAGCCTCACCAGGCTTCTCTCTCTTCCCCAGTAGTGAGTCCAG
CCCAGGCCTGGGGCCTTGGTGTTGCTGCCGGTCTGTGAGGCCCTGCAAGTCCTGGGGAGCAAG
CAATGGGGGACAGGAATGGGGCTCTTGAAGTGATTCACGTGGGCCAGGAGGCTGAAGGAGGT
GACGCCTTGTGGTTCAGAGATTCTTTACACTGCACTTTTCCCATCAGCTGCACTGACAGCTGGGA
CCCCATGGGCACACCTAGTAGAGGACAACTCCAGTCTGTCCATGCTATGTGGAAGTGGCCCCC
AGGCCTGGCTCCAAACTCAAGTCTCTATTCCAATATGGTTTTGTTTTCCTTCAAGAAGGCTGGGT
GGGTCTCTACTATGGGTCTTGCATCTACACAATGGTTTCAATGGTTTCATTCCCATTCTTCCATCT
GAGCCTCAAAACCACCACAGACGTAGGCAGAGTGAAGATTCTTATCTCCATTTTAAAGATTAAA
AAAAAGAGAGAGAGACTCAGAGATGGGAAATGACTTACCCAACAGTAAAAGATACCAAAGTTA
GGCCCCAGGTCTTCTGGCCCGGAGCCCAGAACTCTTCCCATCCCAACCCAAGGCTTGGTGGCT
GGGTAGGGGCCACCTGCGTGTCCACAGTGCCTACAGAGGAGGAATCTGTAGACGGATGCATTT
GCACCAAGCCTTTGGTCACAGATTGTCAGGAGTCATGCTAGAGATGGCATCTCAGCACACACAT
GTCTACAGATGGGGGCAGGGGCACAGCCAGGAGTAAGAAATGGGTGGGAGGAGAGTCCTGTT
ATCTGTCAGTCTGGGAAGCCTATTCTTAACATGATGTGTCTTCCAAGAGGCCACCTTGTTATCTG
ACTTCTGAGCACTTATGTGGCACAGACAGAAAAGTGTGCCCATGGAGAGGAGAGGGTGGGTCC
CAAGGGCCCACAGGATAGGTTTTCCTGACCAAGGACATTGTTTCCTGTCTAATCTTAAGAGACA
CAATCTTCTTCCCAGGGGCCTAGGACAGTCCAAATAAGCAAAATGCATCTGCCTTCCCTGCAGA
TCCAAGGCCCCAGATTTTTCCTGCTGTGTCCCCAGCAAGCACAGAGACATGTGTTTAGATGCAG
ACAACACACATATGCCCAGGCAAAGTAGGGGGGAACTCACACTGGATACACAAATCTGGGCAG
ACTTGGAAGTACAGAGCCACTTATGGGGTCCTTGGCTGGGGCTATCCTCTTGTGACAGTTTCAG
GGATGCAAGGGAGCATTGTGCAGACTTGTGGAGCATTGACTAGAGTGTCTCCCAGCCTGCTTTC
CCCTGCAGAGCTGCTGCACACCACCCGCTCTACAGGCTGTGATCGTGGTTAGAAACAGAGCTG
AGGGGAACCCTTCAAACAACTCTGAGGCCCTGGCCACCTGCATATCTCCCCACCTCCAGTCCCC
ACCTCTCTCAGCAAATCTTTGTCCTAGGAATACAAAGGCCAGGTGGCCAAAAGGGGAAAAGGA
AAGAACATAGTATAAATACATTTACAAGGCAATGGGTTCCCCAAAGGCCACAGTCTGTCCCACA
AATGCCCCGGAGACCAATGTTAGGATGAGACTCCAGATGAGTATTTAAAAGTTCAAAGTTCAGC
CAGGCACAGTGGCTCATGCCTGTAATCCCAGCACTTTGGGAGGCCGAGGCGGGTGGATCACGA
GGCCAGGAGATCAAGACCAGCGTGGCCAAGATGGTGAAACCCCATCTCTACTAAAAATACAAA
AATTAGCCAGGCGTGGTGGCGGGTGCCTGTAATCCCAGCTACTCGGGAGGCTGAGGCAGAGAA

TCACATGAACCCGGGAGGCAGAGGTTGCAGTGAGCCCAGATCACGCCACTGCACTCCCACCTG
GGTGAAAGAGCGAGACTCCATCTCAAAAAACAAAAAAAATGGTCAAAGCTCTGTATTCTCTCCA
GTCGAGGATTCTAACCTGCTCAGAAGAGCCCTGCTTCCCCTCTAGGTCCAGGATACAACAAGTG
ACCCTGATGACCTGCAAGCTTCCTATACGGCTGGTGCTACACCTTTATAAGCTTCTAGAGGCCA
GTCTGAAATGGACAGATTTGACCTCAGCCTGAACAGCTTAGGACCTCTCATTCCCAGGGTTCCC
CTGCTCTGAAAGCTCCAAAGGGGTGGGGACTGCTTATTCACTGCTGTAGCCCCTGGCTTGAACA
TACCTAGCATTTATATGGTGTCCCATAAATAGCTGAATGAGTGAACCTGCTGTTTTCTTTTTGCCC
TGACTAGGAGAGCTGAAGCCCGTGAGAAGAACCTAACAGGCTCATGGTCTGGCCCTCATGGAC
ACCCCGGGGACCACCAGGTGAAGTGGAGTCAAGGCCTGCTCTCAACCCTGGGCATGGGTGGA
GACATGAGTCAGCATGAGTGGCCTGAGGGGGAGGGTCAGCAGCCCTGTCCGAGGCAATGGGA
AAAGCACTGAATTTTAAGGCAAATGGCCCAGGTTGAGTCCTGGCTCCTCCCTCTGCCTTCACTA
GTTTTGTAATGTTGAACAAGTCAATACATGAATTTCATAATCTAT

ID|56067|gene_id|ENSG00000224699|transcript_id|ENST00000444238 Homo sapiens lncRNA

TAAGTTTTGTTAAATTTGAGTAAGAAGACCTGTTTGTTTCCTCAAGTTGCTTATAATCTAGGT
GAGGACACATCTGAGAGAATAACTAATAGTTCAGTGAAGGTAAGAGCCAAATGATTGGCACAG
ACCTCAAGTGGTCTGGCATTTACGGGAGAGGGGGATTGTGAAACAGGAATTGAAAGAAATTTA
AAAATGCATAAGCAAAAACTCAGTTTATGTTTTAAAAAAACCAATTCCCCCTGAGAAAGAGAAA
GAGGTGGAGTCCTTTAAAAATTATCTGCTTGTTTTTCTGTCTGTGGCTAGTGAGCCTTATCTCTCC
CTTTCCCAGGCATTGTGAAGACCCTGTTTCTCTAGCTGTGCAGCTGCAAGGTCACTAGGCAGAT
AAACTCAAGCCATAAAACATGTTTTTCCTTGAAAAGTAAGAAATAATGTAATGCATGTGTCAAAT
GAATAACTATTTCTCGCTTCTGTAATATGCTTCCCTTCGCACAGATCTCCCCCTGCCCCATGAAAT
GCTTTAAAGGTAACTTAACTCTTTGTTCGGGGCTCAGTCCTTTGGATGTTAATCTGACTGGCTTGG
TGCACCTAAATAATAAATAACATATATCCTCCTCAACCCCTCAGTCTCTCTGATTCCTAAATTATC
CCGCTGTAATTGCAGGGCTAGATTTGCCGACCTGCTTTATAAAGCTATGTTAAATCAGTATATTTT
ATTCTTTTTATTAGACTTTTCTATAGCATAAAAATGATAAAATACAGCAAGAAAAAAAGTCACCT
GAAACTCTAGTCCTAACCCTAATATGAATGTGAAGGAGCTGGGCTAGAATGGAATAGGGTAGA
ATTTGCATAAGGTGGAAATGGGGACAGGCCTGTCACAAGGATCACTAGAGTCTTGCTGGCAAA
AGCATCTTACTGCATTCATGCTGCAGCACAGGACCCAGATGCAGAACAGGTTGGGGACACAGC
CCTGCCTTCTAATAGTGGACACCATCTGAGAGCTGAGCCATAGATTCAAAGCCTAACTCCTAAT
AGCGATTGGTAGTAATATCATCAGCAGTTCATATACATGGAGTGACAACAGCGTGCCAGGCAAT
TCAAAGGGTCTCTCGGTCCTCACAGCATTTTTGAAGTAGCTAACATCACTATCCCCCTTTTACAG
TTAAAAACAACAACAACAACAACAAAAACACAAAACCCTGAGGCTCAGAAGATAAATATTTGCT
GAAGATCACTGTGCTTGTAAGTGGTGGAGCCTGACCATTAACTTCCCCACGACACTGCTTCAATT
TCTATCACTTCATCAACCACACAACCCGAGAACTGTGAGGGGATTTAGCAGGCCCAGTCCAGAT
CTCTTATTGTACAGAGAAGAAATCTGAGGCTCAGAGAGGGGAAGGGACTAATTTGGACCTTTAA
CTTCGGATTCTTAACTTTCTTTCTGGTGTTCTAACTATACTGTACTACAAGAGGCTGTCTTAGAGC
TTTAGTTCAACCAGCTTCAGAGATACTCATTGTTATGGGTTGAATTATGTCTCCCCAAAATTCATA
TGTCAAAGTTCTAACCCCCAGTACCTCAGAATGTGACCATATTTGGAGATAGGACCTTTACAGA
GGTAATCAGGTGAAAATGAGGTTGTTAGGGTGGGCCTTAGTCCAGTATGGCTGGTGTCCTCATA
AAAAGGGGAAATTTGGACACAGATGTACATAGAGGGAAGATGGTGTGAAGAGACATAAAGAA
CAGATAGTCATCTATAGCCAAGGCCTGGAACAGATCCTTCCCTAACAGCCCTCAGAAGGAAGC
AACCTCACTGACACCTTGATTTTGGACTGCTACCCTCTGGCACTGTGAGACAATAAATTCATGTT
GGTTAGGCCACCCAGTTTGTTATGGCAGCCCTAGGAAACCTTTGTGGATATATCTGTTCGTATAC

CCATAAAGTCTGCAAGTCAAAAATATCACAGACTTTTGGCAGCCCTTCATTCAAGCACTGGCTG
GGAGGGTCACCCTCCCCGAGGCAAAAGGATAGAGCAAATGATCTTTCCAGCTTGCTCCCAACC
TTGATCTTTGGCCAAGATATTTTTCTTTCACTGGACATTGGCCAACATTGGTGTCATAACTTAATT
TGACTGTGACCTTCTTGAGTCTTTTATGTCTCTGAGCTTGACCTGTAGAAGAAGCTCAATAAATGT
GGGTAGAGGGGATGAGTGGGAAAATCAGCTCCCAGCCATTCCAGTTCAAGCCTCTTCAAGCAG
AAGAGTCCCACCTGAAAGACCTCATTTTCACTTGATTACTCGATCTCCAAGCAATAGGTGGGGC
AGGAAGCAGGGTATGTAAGGCCTGTCAGTGGATGGAAGTTGAAGTTGGACACCATCTCCTAAA
GTTTAGAGCTGAGCCCAAATTCTGGAAAACACAAGCAAAATACATTTTATATGACTCAGGATGG
GCTAAAGAAGTTCCAGTACAGGAAACTGTTTTCCTGCTGCTTTATTAATTTTGCTACAGAGGATA
AATGAGATGCAGACTTTCATATCCAAGTCTTCATTAGATGCCCAGTTAATGCAGCCATGACAAAT
GGGAATGAAGCCCTTATGAATAAAAAAAAGATCAACCCAGTATAAAAAAATCATAGACCAGAG
AAATGAAAATTTAGAGGAGCTGATAAAATACCCTATCCTTAATTAGACTGTCAGCATACACATCA
AATCAGCCTTAATGCCAAGATTTATTGGCCTTGGAATTGGGTAAATGAAATAAGTGCTTACAAAT
GAATGGAAAAGAAATTACCTGTATTAAAACGTATTTGTGATGCAGGCCTGTGCACTGAGGGTTTT
AGTCAAACTCAGGCTTGCAGCTTCTTTTGCTCTGCATCCATTTCCACCCCATGTTCCCCAGTGGG
TTTCAGAGACCCTCTTTGTGGGGTAACAATATGTACCATTGTCAACTACCTCTGCTGTTGTCCTG
GAAAAATCCTTCTTGGACAGTAAAATATTTTTGTCTTTATTTCACCATCTCCAGATTAGATGGGTG
TTACTGATTCTTACCTCAGAGAAGAGAATGTCATGGAGACAAAATGACCTGCTTAGAGTTCTGC
ACCCCCCAGCATGGAGAGCAGAGCGCAAAGATTTCTTCCGGACTCCCACATTCCCCTTGGAATC
CAAGAACTGGTCTACTGTCACAACACTTTAGTACCCTCTTGTAAGCCTGTGGACAAATCCTCTGA
TGCCTTGATACCTCGGTTTTCCCCCACTATAAATGGGAAGATAAATTCACTAGAAACAGTACTTA
CCTTGCAGTGTCATTATGAGAATTTCATGAGACAATGCAATATAAAGCTCCTAGCACAGTTCCAG
GCACCATTGTAAGCACTGAACAAATGAAAGCTGTTGTCATTAGGATCTCAAGAAATGACAGCTG
AGGCAAATGTGGATTAGACTGCATTCAGCATTCTTAGCTGCAAGCAACAGAAGCTGACGCTGGT
TTAAAGAATAGGAAAGGAAGTTAATTAAAATGACATTGGGTAGCTTGTGGAACTGGGAGGACTA
GAGAGCTCAGTCTAAGTTCTAGTGACAAAGCTTCAAATCATACATGAGGAAAATGCCCCTCTGC
AGGAGGAACGTAATCCTGCAATCACTGCTTCCCCTGAAGCCAGATGCCTCTACTGCTGCCCTTC
CCAGCAGTGATGGAGCCTGGATCACATACCTGGCTTTAGTTGTAAAGGAGGCTGGAGAGAAAA
TGCTCTGCTCTATGTAACTGTCTTGAAAGTGGCCCACACATAGCAAAGCCATTCAAAAGCGGGT
GAGCAGCATGAAAGCATCCAGAAGGATTCTTGCTTTGCATGAGGGAGAACTCTTGCTCCCAGA
GTGTGAGACCAGCAGGGGCATTTTCCTCATGTGTGATTAGAAGCTTTGTCACTAGAAGCTTAGA
CTGAGTAGGACACCTCTGAGTGTGAATGTCCTACCTGAGTTCTGCCTCATGCAAAGCAAGAATC
CCTCTGGAGGGATTGACACCACAGCCTCTTCCACAATGTCTCCAAACAACTTTACTGGGCAGCA
TTCCACTCCTTGACAAACGGGAGAGGTGCATTCTACCTTCTCTCCTGCATGAGCCTAGACTAATA
TGGAATTTGGGAGTGTTGATCATTGTACTTTGTGCTCCTAGAGATAAGGGTCATCATAAAGCATC
TCAGAGTCTCACTGTAGATCCACAGTTCCATTTCTTTATGGATCCCTTACCCCTACTTTGCCAAAT
TCTATGCAGATAGCAATAATAATTCAGACAGGGCTTCCTCACTGAAGGAGTTTGTGCTCTATATA
GTTAATAAGCTAGAGATAATGAAAAAAAAAAAAAACTCAACTTGAACTTTGGGTAAGAGAAACTT
TTCAGACCAACGATCATTACCAATAACAGCTTCCACGCATGACCCTGCCCGGAGATTGGGGCCT
GCACTACTGTTTCCAGGAAACACATTCTTTTGAATTTTTTTATTTTCTTTGTAGAGATGGGGATCTC
ACTCTGTTGCCCAGGCTGGTCTCAAACTCCTGGCTTCAAGCAATCCTCCTGCTTCGGCCTCCCAA
ACTTCTGGGATTACAGATATGAGCCACCACGGCTGGCTGACAGGCACCAAATTCTAGGAGAGG
ACTTGAATAGAGTAGGCATCTGTCTCTCCCCATTCCTTTCAGGGACAGAGCTCTCCTGGTTGGGA
GAAAGGGAGAGGGAGAGGGAGGAGGATGAATAGTGAAGCACCAAACCTCAGAATTAAGGCCT

TTTTCTTTTCTTTCTTTCTTTCTTTTTTTTTTTTGAGATGGAGTTTCGCTCTTGTTGCCCAGGCTGGA
GTGCAATGGCACAATCTCGGCTCACCGCAACCTCTGCCTCCCAGGTTCAAGCGATTCTCCTGCC
TCAGCCTCCCGAGTAGCTGGGATTACAGGCATGCGCCACCACACCCAGTTAACTTTTTTGTATTT
TTAGAGACGGGGTTTCTCCATGTTGGTCAAGCTGGTCTCGAACTCCCGACCTCAGGTGCTCTGC
CCGACTTAGCCTCCCAAAGTGCTAGGATTACAGGCGTGAGCCACCATGCCCAGCCAGAATCAG
GGCCTTTTTAAAACTTCAGTGTCTTGTCTTTACCCTTCATGGTGCCTAGAAAAGCATCTGGCTCAT
AGGTGTTCAGTGAATGATTGTTTCATTAGTGAAGAAATTCCCTTTGGAAATCTCATAGGAAACTT
ACCCTCTCCAACCCTTCACCTGTGCATTATTCCAGCTCCCAAGGAGGCAGCAGAAACTGGGGTA
ATCACAACGGTCAACAGCAGCAAAGGCAGCACTTTCTGATTTACTTGACTGCTATGGATGCCAC
CTCTCTCCACGCCTCAGTTTAATATCTGTAAGTTTGGTCAGTATCTCATTCAAGAACATTGCATTT
ACTCTGGTACTTAAAGATGAAAAATCAACATTTACAAAGCAGATCCATTGTCAACCTAAATAACA
AACAAAGAGAGTCTCTATAAAAGAAAAGAATATTTATTTGGGAATAGAGCATTGCATAGGAATA
TGCCTGCCTTAGTAATGCATGTATTCAGGGAGGTAGAGGAAGACAAAGGTTTTTAACAGAAAAA
ATGTGGAGAATTACATAATTGTTTTGAAATAATTATCCTTGGCTATAAAGATCAATAACAAGGGT
GATGCGAGTCCAAGGTTGGACAGGCAGTTGCTGGGCAGATGTCTTTGCAGAAGTCTTTTTTGTG
TAAGGTTGTAATGGCTTTTGTAGGAGGTTGTGGCTTTTGCAATCTTTTGTGATAGCTTTTGTTACC
AGGCATACAAGCATGAGAATGCTCTCTTCAGGGCCTTCCCTAGCTCTATTTATCAGGGGTTTCTT
GTTAGTGATTCCATTTTGATTCTGACAACTTTCATGGACATTATCTCATTTAATCTCACAGTCATTC
CATGACTCTATCCCCATTTTGTGAATGAAGTAACTGAGGTCTGGAGAGGTCAACTAACTGGTCA
AGGTCATAGAACTTGAAAATTTTCATTGTCTTGGGTCTCTAATACTGGGGCTGACCATTCTCCCA
GGCAATGTTGAGTGAGTTGGCCCAGGTGAGTCGGCTCCTCTGCAATTGTGTCTCCTCAACTAGTT
TGAACAACACAGGCCTGTGAGCCAGTCCCATAAAGTGGCTAGGGGTTCCCTTCTCCCCATTTCC
CCTCATCAACAGGAGGACTTGTCTGTGTGCCTTCAGACACCACACAGTAAATCAACTGCCACAT
TCATTCTGGACCATCAGTATTTTGCCCCAGGAGAAAGTCAGAGGCAAGCACACTAATATCTCAT
TGATATTTTCAATTGCCAAAGGGGAAGTGAGATGAATCTAATCCCATCTGTCAAGGTGGAGAAG
AGCTGATTCGAAAGCAGCATAGATTACTTTAAGGAGATGACAGTTTATCACTCTCTGGACAAGG
GAATATGAAATGAGGACTAACCTCCTTTATGAGTTGGTAAAAACATTGATCAGCAAAGACAATG
TCTGGGGAAATGAACAGCAAGTTCTCTGGCCCTTTTTCCACTTTTCCTTTCCCCGAGACTTTATGT
TATATATACACACTCACACAAACAGGACAGAATCCACCTCCTGCCCCTCAAGAAATAGTTAACA
TTTGTTGAGGATTAATTATTGCCAGGCTCTGTACTCCATGTTTTATATTTACTATCCAGAAGCCTT
AATTGTTAAATGAGGTAAGTGTTATTATTAGAACCTTTGTACAGATGTGGAAACTGAGGTGTATA
GAGGTTGACTTGCCCAAGGTTACCCAGCTAGTGAAGAATGAAATCAGATGCAGTCCAGCCAAC
GCTTGGCCTGCCGTCAGTCACACGCAGTACACGTAGATCAGTCAGCCTGGAAAGCCACCTAGA
CAGCCCTGCTTCACTCCATCCTTGGTCAGCACCTTAGAGAGCCCTGCTGACCCCAAGAGGATGT
GCCTTTTACGGGTTGTAGAGGCTGAACCACAGCTGAGGGGCTGGAATGAGTGGGGGCCTCAGA
CAGGGTGTCTGGAGAGCTGAGGCTCAAGGAAGGGTGGAGGGGGTGCTGAAGCTGAAGGCCGT
GGGTCTTGCCAGCAAGTGGGGCTCAGTGAGCCGAGGTGCCTCTCAGTTACTTCCATGTGTGAAT
TTCCGGTGGAGGACAGTGATGGGCTCTGGGCCCTTGGAAACCGGGACATCCAGCGTGTCCAGC
CACAGTGCAGGCTGCCCTGGCTGCTGAGAGTCTCAGAAAATGCCACTGTCATGGGTTAGGAAG
GCCTGGGGACCACAACAATGTGACTTCAAAAAACCCAAGAAGGAATGTGCACACCTGAGATGC
TCAGAATGCATGTGTCAGTGTTCAGTTCTGACTCCTTGCTTCAGCCAGGGACAGAAGAACCTTCT
ACAGTAAAGAACCACAAGAGAGGGCTTTGCTGCCAGCCTCAGCCAGGGACACTTGGCGGCCA
AAGCAAAGGTCACTCCAGGGCTGCAGATTTCAGCCTCTGCGGCCAGAAACAAGCTTCCCAATT
AATAATACTCTGGATCGGACAGAGATGGCTGCTCTGTCTCAGGCGTGTTTGCCGTTGATCCCTGT

GATTGATGCTGATAACATTAAGAGTCTGTCAGGGTGATATTTCATGAATGTCTCATGTGATGGGA
TGATACAGTCGGGAAATGCAATTCCCTAGCTCAGGCAGAATCAGAGATGACCCAAAGTGCTGA
TGGAAAGGCTGAGGCCCTTTCCAGCCTCTGGCAGGGTAGGGAAAGCGGGTGAGGTGGGGGCT
TGGGAGGGGCTGGGCTGACCTAGAGCTGTAACAGGGGTCTCTCATGAAGGGCTGTGAAGAGAA
ACAGTATGTGAGAGAGGCAAGAAACTTTTCTCTGGGTTTCCTCATGTAGAATGATTCCATCTGTG
TAGGCCATGAAGGAGTCCAGGGAAGTTCACCCCAAAATGTTGAAACCACCTTTGCAAAGATTAT
GACAGTGAGAGAAGCCTGGCATGGCTGACTCTATCTTGCTTCTAGCCTCACAGGCTGGCTGTTC
TGTCTCATTCCTGGGCATAGGCCAAGCTAATCTTGGGAGGGATTTATGGTTTAACTTTGAAGCCA
GGATCATAATTGTCCCTCCCTAAAACTGACCCCTTTCTTGTTCAGGGACTGAAACTGCCTTTGTA
AGACTAATGAAAGGCCACAAAATTAGGATTATGGAAGGGGCCTGAATTCTGATAAGATGTAGG
CATAGCTTGCCTTTCTGTCATCGTTTATTGCACTGGCACTCACAAGATTTGTAACTTCCCCAATTG
GTCCCATAGATAACATCACTATTGTAGAACCTCAGATTGGTCTTTTGAGATCTTTTTCAGACTTTG
GTATTCTGGTGACTGACTCCACCCAGATCCCATGACTCATGACTCAGCTGGTCCTGTGGTTCTTA
CCCAGTCTGACACAGCACCAGAGGACCGTTTTCCACACTTCTATCTATGATTTCATCCCCAACCA
ATCAGCACCACCCATTCCCTAGCCCCCTGCCCACCAAATTGATATCCATAAAAACCCTAACCTCT
GAGTTCTCAGAGAAACTGATTGGAGTAATAAACTCCTGTCTTCTGTGTGGCTAGCCTTATGTTAA
TTAAACTCTTTCTTAGGGCAGTACTGCTGTCAGTGAATCAGCTTTATCTGTGCAGTGGGCAAGAA
GGACCCACTGGGTGACTACAATATGGCACTTTGGTATGTTGGTTATTTTAAGTTAGAGGCCTTTG
ATGCTGGAAGAAGCTTTACTCTGATATCTCCTTATCTACCACAAGACTGGATCTGCCAAAGAGA
ACACAATTGCGTTCCATTCCCTTCCTGAAATCTCATTATCTATGGCAGAAAAGAAGACTGGGGA
ATG

ID|56275|gene_id|ENSG00000224699|transcript_id|ENST00000457535 Homo sapiens lncRNA

GTTCCGCTCAAGTTCCTCCGCCGCGACCTCAGGACAGGCCGAAGAGGCGCGCACTACTCA
CGTGTCTTCCAAGTGCTGCTCCAAGGTCGCCTCCATCCCACCCACCGACCACTCCGGCTCAGAA
CCCAGCGGCACGGCACGTCCTTCTCCACCACAGGCCTCAGTCACTTGACGCGAGCGGGGCGTG
GACCGTAACGGCGCCTCCAAAGGGACAAAATTGATCACGGTGCAACGTCCGCGTTCTTTACTG
GAAAACATCACTGCGCTTCCGTCGCACAGAGCCTGACGAAGGCTTGGGCTCCCCGCGCGGTGA
CCGTCGAGGTGACCTGCACCTGGCTCCATGGCGGAACCGGCCGGCACGGATTATTCCCCTCTC
GGGAGAGGTCTGCAGCCGGGCCTCAGTGCTTCAGTTCGTGGCCTGGCATTTTGTGTCAGTGTTT
TTTAACCTTTGGACCCTAGTTCAACCGACTCCGAGACCTAGGGGACAGATCTTAAGTTTTACAAT
AACCCAGGGGTCAGACAGGGTCTGATCTAGCCTGTGGGGCAAGTACGGGGAAGACACGGGAC
CTCTTGGAGGGGGGTCAGTAGGCTTGTAGGGGGAAAGACGCACACAGAGACTAAAGGACGAGA
TGGAGCAAGGTTCAACCCCCTAGCTGAGTGTTGGCAGTGTCGGGATTTTAACGAAGACATTGGT
GCTTATTATGGTGAGGGGACTCAGGACAGTTTAGGGGAAGAGAGTGGCATTTGAGTCCTAACTC
GTAAGGAAGACTGGGTAGGACTACTTGAAAATAACTTTTAAAAATTAATTATAAAGCTGGTAAA
AATAGGAAAACATCTATGGCCCCACTGTCCAAAGACGACTTTTAACTTCTTGGAGAATATCCTTT
CAGATAGTTGTCTTTGTAAATGTATACTTTTAATGTTTTTTCGTTTGCCTTTTTAAAATAAATTTTTT
GGGGGACAGGGTCTTGCTCTGTTACCCAGGCTGTAGTGCAGTAGAACGATTATAGCTCATTGCA
ACCCGGACGTCCTGGGTTCAAGCTATCCTCACCTCTCAGCCTCTCGACTGGCTAGGACCACAGC
CTCGAGCCATCATGTCTGGCTAATTTTTACGTTTTTTTGTAGAGATGGGGGTCTCGCTGTGTTGTC
CAGGCTGGTCTTAAACTCCTTGCTTCAACAGATCCTCCCACTCCAGCCTCCCAAAATGTGATTAC
AGGCTGAGTCACCACGCCTAGCATGGTTTTTTTTGTTTTGTTTGTTTTGTAAGCAAAATTGACATA
CATACTGGAGGTACAATTCTGTAGCTGGCCTTCTTTACAGAGTTTAGAATAAATGTCTTTCCATGT

CAAGTTTTCACTTACATCCTTTGTAGTAGTTGCAAAATATTCCATTTTATAGCTGTATCTTAATTGT
GGATTAAGGTTGGAGTGCAGTGGCACTATCATGGCTTAGTCCAGCCTCAGCCTTCTAGGTTCAA
GCGATCTTCCCACCTCAGCCTCCCAAGAAGCTGGGACTACAGGGACCCGCCACCCCACCTAGC
TAATTAAAAAAAAAAATTGTAGAGATGGAGTCTCAGTGTGTAGCCCAGTCTCAAGTGACCCTCCC
TCGTTGGCTTCCCAAAGCGCTGAGATTGCAGGTGAGCCACTGTACTTGGACTTTTTTTTTTTTTT
TGCTTTTGTTTGTTTTTTGTTTTAGCTATTACAATGCTGGTTGAATATCCTCATAGCTAAATATCTGT
GCAGTTTTAAAATTTCCTTATAAGTTACTAGAAATGGAATGACTGGGTTAAAGGACATGATCATT
TTTTAAGACTCTTGATACATACTTACAAATTGCCTCCCGAAAAAGTGGTGCTAATTTACTCAAATT
GGTAAGCCTCACCCCCTCTGTGGATCATTCCTTGCACTAAGTTTCTTCCAACTCTGTCACCTCCA
GCTTTTTACTCACAGCCTACCTTCCAATCTGGGGCCCAGCTCCCAGATCGGAAGATACCAGACT
TTGGTATTGAACCAAAGTCCAATACACTGCACTGGCATTCAGAGTCCCTTTTTATTCCGCAGCTG
TCATAGTGAAGCTTGCAGCTTCCTTGAAATTCCAGTTTGTTTATTTCTTTATTTTTTTGAGACAGAG
TCTTGCTCTATCGCCCAGGCTGGAGTGCAGTGGCGCCGTCTCAGCTCACTGCAACCTCTGCCTC
CTGGTTCAAGCGATTCTTCTGCCTCAGCCTCCCGAGTAGCTGGGATTACAGGCATGCACCACCA
CACGTGGCTAATTTTTGTGCTTTTAGTAGAGATGGGGTTTCACCATGTTGGCCAGGCTGGTCTCA
AACTCCTGACCTCAAGCGATCCGCCCACCTCAGCCTCCCAAAGTGCTGGGATTATAGGCATGAG
CCATTGCACCCAGCCAAAATTCCAGTTTGAATGTTATAATTCAGGGGTCTTGGACTCCTGCTCTA
GAGAAATTATTCATGTTAAGTTAGGGGTAACACAGTACAGGCCATATAAGTTTTAGAAAAAGCA
GTTTAAAACTTAGCATTAGAAAACATAAGTACTGCCTGGTGAAATGGCACGTGCCTATAATCCC
AGCTACTCAGGAGGCTGAGGCAGGAGGATCCCTTGAGCCCAGGAGTTCAAGTCCAGCCTGGGC
AATATAGTCTAGTGGCCACATTAAAAAAAGTAAAACAGCCGGGCGCGGTGGCTCACGCCTGTA
ATCCCAGCACTTTGGGAGGCCGAGGCGGGTGGATCACGAGGTCAGGAGATTGAGACCATCCTG
GCTAACACGGTGAAACCCTGTCTCCACTAAAAATACAAAAAATTCTCCGGGTGTGGTGGTGGGC
GCCTGTGGTCCCAGCTACTCAGGAGGCTGAGGCAGGAGAATGGTGTGAGCCTGGGAGGCGGA
GCTTGCAGTGAGCCGAGATCGCGCCACTGCACTCCAGCCTGGGCGACAGAGCGAGACTCTGTC
TCAAAAAAAAAAAAAAAAAAAAAAAAAAAAGTAAAACAGGGCAGGGTGCAGTGGCTCACGCCT
GTAATCCTAGCACTTTGGGAGGCCGAGATGGGTGGATCAGTTGAGGTCAGGAGTTTGAGACCA
GCCTGGCCAACATGGCGAAACCCCATCTCTACTAAAAATACAAAAATTAGCTGGGTGTGATGGT
GCGTGCCTGTAGTCCCAGCTACTCAGGAGGCTGAGGCAGGAGGATCGTTTGAACTCGGGAGCT
GGAGGTTGCAGTGAGCCGAGATTGAGCCACAGCACTCCAGGCTGTGCTACAGTGTGAGACTGT
CTCAAAAAAAAGTAAAACAGATGAAACTAATTTTAATAACATTTTATTTATTCAAACAATATATTC
AAAATATTATTTCAACTTGTGATTGATAAAATACTAATGAGATATTTTATATTTTTTCTCCAGTACT
GTCTTCAAAATCCAGGGTATATTTTTCAGTTAGCAGCACATTTCAATTCAGACCAGCCGCATTTC
AAGTGCTCAGCAGCCACCTGTGGCTAGTGGCCACAGTATTGGCCAGTGTAGCCCAAGGATATCT
CAAATTATCTCAAAGCTAGATCAGTGATTAAAGGATTCTCAGGCCAGGCATGGTAGTTTTCAGG
CCTGGTATGGTGGTTTACGCCTGTAATCCCCGCACTTTGGGAGGCCTAGGTGGGCGGATGGCTT
GAGCCCAGGAGTTTGAGACCAGCCTAGCCAACGTGGGGAGACCCTGTCTCTACCAAAGCACAA
AAATTAGCTGGGCATGGTGATGCGCACCTGTGGTCTCAAGTACTTTGTGTGAGGTGGGAGGATC
ACTTGAGCCCGGGAGGTTGAGGCTGCAGTGAGCTGAGATTGCACAACTGCACTCCAGCCTGGG
TGACAGAATGAGACCCTGTCTCAAAAAAAAAAAAAGGATTCTCCGAAAGACTGGCGATTGCTTTT
TAAGCTATAAGGAAGGCCTTTGAAGGGAAATCAAGTAGGAAGAGGATGATAGCTGGCTCACAT
GTCCTTGCCACATGGGAGGTGAGAGAGCAGGCTACTCCTTACCACCCCCGCTTTCTCTGCCCTG
CTGCTGTCTTGCCCCTGTGCAAGTAGGCAATGGGAGAGGAATAGTATATATAAATTGGCAAGT
GCTAGGGGCTGGAGTGATGGTTGGGGAAAGTATCTGACCCTTGTTTTCTACTTTCTTTAGCTTGT

GGGGTATTTAACAATTCTTTGTTCTTAGTTTTGAGTGCTGGCAACCATTTCTGGGGTGACAGGAA
AAGTTCCATGACATCCGTATTAGAGCATTTGTTTCATTATATTGTAATTTTTTATGTTTACTTTTGA
AAAAGTTCATTCTTTTCTCTTTTTAATTCTTATTCTTTTTTTTTTCTTTTCTTTTTGAGGCAGGGTTTT
ACTCTATCACCCAGGCTGGAGTGCAGTGGCACAATCTTGGCTCACTGCAACCTCTGCCTCCTGA
GCTCGTGATTCATCCTCCTGAGTAGCTGGGACTACAGGTGTACACTACCATGTGTAGCTAAATTT
TTTGTATTTTTTGTAGAGATGGGGTTTTGCCATGTTGCCAGGCTGGTCTCGAACTCCTGCAATTTG
CTCTCCTTGGCCTCACGAAATGCTGGGATTACAGGCATAAGCTACAGCACCCAGCCCTTACGTT
TACTTTTCATTGACAGGAAGAGTGTCTTCATCTCTGCATCCCTAGTGCAACAGAGCATGACAAAA
ATTCAACAAATATTTATTGAAGAGTCTTGATGTCTTTTGGGAAAGGAACTCTATGGTTACCTTTAC
TCTAAGGTAAAGAAATTTAGGTCCTGAACTTTAACCACCTTCTCCTCTTCTTTCTTCCCTCTTTCAT
AAATGAGCTCTTGGAAAATTAAAATGCCAGGAGGGTCCAGCAGAGAATACCCAAGCCCACATG
TCCTGATGGGAAGTGGGGGATAAGTAGAGAGGGAAACACAGACCCTGTAGGCCATCTCACTGC
TGCTGGCCCAGGGGACCCTAACTCCTTTTGTTGACTCATCAGAAGTAGAATCCTCCTGAGGGTC
TGTTTAAGAAGCACAGCTCTGGTAGAAAGGGTAGCTTTCCTATGGGTACGGAGGGCTCTGCTAC
CCCAGGGCTATCCACACTGGCCTGTGAATCACTCTCAAATGCTTAGGGTCACGGGCTTTCAGTG
GAATGAGCCCTCATGTGCCACCAGGGGGCACCAAGGCCACAGAAAACCCTATTCAGAAGGTTC
TGGGGTGAGGGAAAGGAATCCACGGGTTTGTGGAGCTGGCGGAGCATGGGTGAGGACAGCAT
TATGCTAGGTTGGTGGCCTTGAAGTCAGATGTAAATTGGGATCCTGGCTTGGCCACTGACTATCC
ATGGGAACTTTGAATCTCAGGGGGCTCATTTGTGATGTGGGGGTACCATTATCTGCCTCATCAG
GTTGTGGGGAGGATTATATGAGATACCTTATGTAAGAGGGGCTGGTGCATTGTGGAAGTCAACA
AGTAAGTTTTGTTAAATTTGAGTAAGAAGACCTGTTTGTTTCCTCAAGTTGCTTATAATCTAGGTG
AGGACACATCTGAGAGAATAACTAATAGTTCAGTGAAGGTAAGAGCCAAATGATTGGCACAGA
CCTCAAGTGGTCTGGCATTTACGGGAGAGGGGGATTGTGAAACAGGAATTGAAAGAAATTTAA
AAATGCATAAGCAAAAACTCAGTTTATGTTTTAAAAAAACCAATTCCCCCTGAGAAAGAGAAAG
AGGTGGAGTCCTTTAAAAATTATCTGCTTGTTTTTCTGTCTGTGGCTAGTGAGCCTTATCTCTCCC
TTTCCCAGGCATTGTGAAGACCCTGTTTCTCTAGCTGTGCAGCTGCAAGGTCACTAGGCAGATA
AACTCAAGCCATAAAACATGTTTTTCCTTGAAAAGTAAGAAATAATGTAATGCATGTGTCAAATG
AATAACTATTTCTCGCTTCTGTAATATGCTTCCCTTCGCACAGATCTCCCCCTGCCCCATGAAATG
CTTTAAAGGTAACTTAACTCTTTGTTCGGGGCTCAGTCCTTTGGATGTTAATCTGACTGGCTTGGT
GCACCTAAATAATAAATAACATATATCCTCCTCAACCCCTCAGTCTCTCTGATTCCTAAATTATCC
CGCTGTAATTGCAGGGCTAGATTTGCCGACCTGCTTTATAAAGCTATGTTAAATCAGTATATTTTA
TTCTTTTTATTAGACTTTTCTATAGCATAAAAATGATAAAATACAGCAAGAAAAAAAGTCACCTG
AAACTCTAGTCCTAACCCTAATATGAATGTGAAGGAGCTGGGCTAGAATGGAATAGGGTAGAAT
TTGCATAAGGTGGAAATGGGGACAGGCCTGTCACAAGGATCACTAGAGTCTTGCTGGCAAAAG
CATCTTACTGCATTCATGCTGCAGCACAGGACCCAGATGCAGAACAGGTTGGGGACACAGCCC
TGCCTTCTAATAGTGGACACCATCTGAGAGCTGAGCCATAGATTCAAAGCCTAACTCCTAATAG
CGATTGGTAGTAATATCATCAGCAGTTCATATACATGGAGTGACAACAGCGTGCCAGGCAATTC
AAAGGGTCTCTCGGTCCTCACAGCATTTTTGAAGTAGCTAACATCACTATCCCCCTTTTACAGTT
AAAAACAACAACAACAACAACAAAAACACAAAACCCTGAGGCTCAGAAGATAAATATTTGCTG
AAGATCACTGTGCTTGTAAGTGGTGGAGCCTGACCATTAACTTCCCCACGACACTGCTTCAATTT
CTATCACTTCATCAACCACACAACCCGAGAACTGTGAGGGGATTTAGCAGGCCCAGTCCAGATC
TCTTATTGTACAGAGAAGAAATCTGAGGCTCAGAGAGGGGAAGGGACTAATTTGGACCTTTAAC
TTCGGATTCTTAACTTTCTTTCTGGTGTTCTAACTATACTGTACTACAAGAGGCTGTCTTAGAGCT
TTAGTTCAACCAGCTTCAGAGATACTCATTGTTATGGGTTGAATTATGTCTCCCCAAAATTCATAT

GTCAAAGTTCTAACCCCCAGTACCTCAGAATGTGACCATATTTGGAGATAGGACCTTTACAGAG
GTAATCAGGTGAAAATGAGGTTGTTAGGGTGGGCCTTAGTCCAGTATGGCTGGTGTCCTCATAA
AAAGGGGAAATTTGGACACAGATGTACATAGAGGGAAGATGGTGTGAAGAGACATAAAGAAC
AGATAGTCATCTATAGCCAAGGCCTGGAACAGATCCTTCCCTAACAGCCCTCAGAAGGAAGCA
ACCTCACTGACACCTTGATTTTGGACTGCTACCCTCTGGCACTGTGAGACAATAAATTCATGTTG
GTTAGGCCACCCAGTTTGTTATGGCAGCCCTAGGAAACCTTTGTGGATATATCTGTTCGTATACC
CATAAAGTCTGCAAGTCAAAAATATCACAGACTTTTGGCAGCCCTTCATTCAAGCACTGGCTGG
GAGGGTCACCCTCCCCGAGGCAAAAGGATAGAGCAAATGATCTTTCCAGCTTGCTCCCAACCTT
GATCTTTGGCCAAGATATTTTTCTTTCACTGGACATTGGCCAACATTGGTGTCATAACTTAATTTG
ACTGTGACCTTCTTGAGTCTTTTATGTCTCTGAGCTTGACCTGTAGAAGAAGCTCAATAAATGTG
GGTAGAGGGGATGAGTGGGAAAATCAGCTCCCAGCCATTCCAGTTCAAGCCTCTTCAAGCAGA
AGAGTCCCACCTGAAAGACCTCATTTTCACTTGATTACTCGATCTCCAAGCAATAGGTGGGGCA
GGAAGCAGGGTATGTAAGGCCTGTCAGTGGATGGAAGTTGAAGTTGGACACCATCTCCTAAAG
TTTAGAGCTGAGCCCAAATTCTGGAAAACACAAGCAAAATACATTTTATATGACTCAGGATGGG
CTAAAGAAGTTCCAGTACAGGAAACTGTTTTCCTGCTGCTTTATTAATTTTGCTACAGAGGATAA
ATGAGATGCAGACTTTCATATCCAAGTCTTCATTAGATGCCCAGTTAATGCAGCCATGACAAATG
GGAATGAAGCCCTTATGAATAAAAAAAAGATCAACCCAGTATAAAAAAATCATAGACCAGAGA
AATGAAAATTTAGAGGAGCTGATAAAATACCCTATCCTTAATTAGACTGTCAGCATACACATCAA
ATCAGCCTTAATGCCAAGATTTATTGGCCTTGGAATTGGGTAAATGAAATAAGTGCTTACAAATG
AATGGAAAAGAAATTACCTGTATTAAAACGTATTTGTGATGCAGGCCTGTGCACTGAGGGTTTTA
GTCAAACTCAGGCTTGCAGCTTCTTTTGCTCTGCATCCATTTCCACCCCATGTTCCCCAGTGGGTT
TCAGAGACCCTCTTTGTGGGGTAACAATATGTACCATTGTCAACTACCTCTGCTGTTGTCCTGGA
AAAATCCTTCTTGGACAGTAAAATATTTTTGTCTTTATTTCACCATCTCCAGATTAGATGGGTGTT
ACTGATTCTTACCTCAGAGAAGAGAATGTCATGGAGACAAAATGACCTGCTTAGAGTTCTGCAC
CCCCCAGCATGGAGAGCAGAGCGCAAAGATTTCTTCCGGACTCCCACATTCCCCTTGGAATCCA
AGAACTGGTCTACTGTCACAACACTTTAGTACCCTCTTGTAAGCCTGTGGACAAATCCTCTGATG
CCTTGATACCTCGGTTTTCCCCCACTATAAATGGGAAGATAAATTCACTAGAAACAGTA

ID|56405|gene_id|ENSG00000224699|transcript_id|ENST00000608602 Homo sapiens lncRNA

GGGGGTCTCGCTGTGTTGTCCAGGCTGGTCTTAAACTCCTTGCTTCAACAGATCCTCCCACT
CCAGCCTCCCAAAATGTGATTACAGGCTGAGTCACCACGCCTAGCATGGTTTTTTTTGTTTTGTTT
GTTTTGTAAGCAAAATTGACATACATACTGGAGGTACAATTCTGTAGCTGGCCTTCTTTACAGAG
TTTAGAATAAATGTCTTTCCATGTCAAGTTTTCACTTACATCCTTTGTAGTAGTTGCAAAATATTCC
ATTTTATAGCTGTATCTTAATTGTGGATTAAGGTTGGAGTGCAGTGGCACTATCATGGCTTAGTC
CAGCCTCAGCCTTCTAGGTTCAAGCGATCTTCCCACCTCAGCCTCCCAAGAAGCTGGGACTACA
GGGACCCGCCACCCCACCTAGCTAATTAAAAAAAAAAATTGTAGAGATGGAGTCTCAGTGTGTA
GCCCAGTCTCAAGTGACCCTCCCTCGTTGGCTTCCCAAAGCGCTGAGATTGCAGGTGAGCCACT
GTACTTGGACTTTTTTTTTTTTTTTTGCTTTTGTTTGTTTTTTGTTTTAGCTATTACAATGCTGGTTGA
ATATCCTCATAGCTAAATATCTGTGCAGTTTTAAAATTTCCTTATAAGTTACTAGAAATGGAATGA
CTGGGTTAAAGGACATGATCATTTTTTAAGACTCTTGATACATACTTACAAATTGCCTCCCGAAA
AAGTGGTGCTAATTTACTCAAATTGGTAAGCCTCACCCCCTCTGTGGATCATTCCTTGCACTAAG
TTTCTTCCAACTCTGTCACCTCCAGCTTTTTACTCACAGCCTACCTTCCAATCTGGGGCCCAGCTC
CCAGATCGGAAGATACCAGACTTTGGTATTGAACCAAAGTCCAATACACTGCACTGGCATTCAG
AGTCCCTTTTTATTCCGCAGCTGTCATAGTGAAGCTTGCAGCTTCCTTGAAATTCCAGTTTGTTTA

TTTCTTTATTTTTTTGAGACAGAGTCTTGCTCTATCGCCCAGGCTGGAGTGCAGTGGCGCCGTCT
CAGCTCACTGCAACCTCTGCCTCCTGGTTCAAGCGATTCTTCTGCCTCAGCCTCCCGAGTAGCTG
GGATTACAGGCATGCACCACCACACGTGGCTAATTTTTGTGCTTTTAGTAGAGATGGGGTTTCAC
CATGTTGGCCAGGCTGGTCTCAAACTCCTGACCTCAAGCGATCCGCCCACCTCAGCCTCCCAAA
GTGCTGGGATTATAGGCATGAGCCATTGCACCCAGCCAAAATTCCAGTTTGAATGTTATAATTCA
GGGGTCTTGGACTCCTGCTCTAGAGAAATTATTCATGTTAAGTTAGGGGTAACACAGTACAGGC
CATATAAGTTTTAGAAAAAGCAGTTTAAAACTTAGCATTAGAAAACATAAGTACTGCCTGGTGAA
ATGGCACGTGCCTATAATCCCAGCTACTCAGGAGGCTGAGGCAGGAGGATCCCTTGAGCCCAG
GAGTTCAAGTCCAGCCTGGGCAATATAGTCTAGTGGCCACATTAAAAAAAGTAAAACAGCCGG
GCGCGGTGGCTCACGCCTGTAATCCCAGCACTTTGGGAGGCCGAGGCGGGTGGATCACGAGGT
CAGGAGATTGAGACCATCCTGGCTAACACGGTGAAACCCTGTCTCCACTAAAAATACAAAAAAT
TCTCCGGGTGTGGTGGTGGGCGCCTGTGGTCCCAGCTACTCAGGAGGCTGAGGCAGGAGAATG
GTGTGAGCCTGGGAGGCGGAGCTTGCAGTGAGCCGAGATCGCGCCACTGCACTCCAGCCTGG
GCGACAGAGCGAGACTCTGTCTCAAAAAAAAAAAAAAAAAAAAAAAAAAAAAGTAAAACAGGGC
AGGGTGCAGTGGCTCACGCCTGTAATCCTAGCACTTTGGGAGGCCGAGATGGGTGGATCAGTT
GAGGTCAGGAGTTTGAGACCAGCCTGGCCAACATGGCGAAACCCCATCTCTACTAAAAATACA
AAAATTAGCTGGGTGTGATGGTGCGTGCCTGTAGTCCCAGCTACTCAGGAGGCTGAGGCAGGA
GGATCGTTTGAACTCGGGAGCTGGAGGTTGCAGTGAGCCGAGATTGAGCCACAGCACTCCAGG
CTGTGCTACAGTGTGAGACTGTCTCAAAAAAAAAGTAAAACAGATGAAACTAATTTTAATAACATT
TTATTTATTCAAACAATATATTCAAAATATTATTTCAACTTGTGATTGATAAAATACTAATGAGATA
TTTTATATTTTTTCTCCAGTACTGTCTTCAAAATCCAGGGTATATTTTTCAGTTAGCAGCACATTTC
AATTCAGACCAGCCGCATTTCAAGTGCTCAGCAGCCACCTGTGGCTAGTGGCCACAGTATTGGC
CAGTGTAGCCCAAGGATATCTCAAATTATCTCAAAGCTAGATCAGTGATTAAAGGATTCTCAGG
CCAGGCATGGTAGTTTTCAGGCCTGGTATGGTGGTTTACGCCTGTAATCCCCGCACTTTGGGAG
GCCTAGGTGGGCGGATGGCTTGAGCCCAGGAGTTTGAGACCAGCCTAGCCAACGTGGGGAGA
CCCTGTCTCTACCAAAGCACAAAAATTAGCTGGGCATGGTGATGCGCACCTGTGGTCTCAAGTA
CTTTGTGTGAGGTGGGAGGATCACTTGAGCCCGGGAGGTTGAGGCTGCAGTGAGCTGAGATTG
CACAACTGCACTCCAGCCTGGGTGACAGAATGAGACCCTGTCTCAAAAAAAAAAAAAGGATTCT
CCGAAAGACTGGCGATTGCTTTTTAAGCTATAAGGAAGGCCTTTGAAGGGAAATCAAGTAGGAA
GAGGATGATAGCTGGCTCACATGTCCTTGCCACATGGGAGGTGAGAGAGCAGGCTACTCCTTA
CCACCCCCGCTTTCTCTGCCCTGCTGCTGTCTTGCCCCTGTGCAAGTAGGCAATGGGAGAGGAA
TAGTATATATAAATTGGGCAAGTGCTAGGGGCTGGAGTGATGGTTGGGGAAAGTATCTGACCCT
TGTTTTCTACTTTCTTTAGCTTGTGGGGTATTTAACAATTCTTTGTTCTTAGTTTTGAGTGCTGGCA
ACCATTTCTGGGGTGACAGGAAAAGTTCCATGACATCCGTATTAGAGCATTTGTTTCATTATATT
GTAATTTTTTATGTTTACTTTTGAAAAAGTTCATTCTTTTCTCTTTTTAATTCTTATTCTTTTTTTTTTC
TTTTCTTTTTGAGGCAGGGTTTTACTCTATCACCCAGGCTGGAGTGCAGTGGCACAATCTTGGCT
CACTGCAACCTCTGCCTCCTGAGCTCGTGATTCATCCTCCTGAGTAGCTGGGACTACAGGTGTA
CACTACCATGTGTAGCTAAATTTTTTGTATTTTTTGTAGAGATGGGGTTTTGCCATGTTGCCAGGC
TGGTCTCGAACTCCTGCAATTTGCTCTCCTTGGCCTCACGAAATGCTGGGATTACAGGCATAAGC
TACAGCACCCAGCCCTTACGTTTACTTTTCATTGACAGGAAGAGTGTCTTCATCTCTGCATCCCT
AGTGCAACAGAGCATGACAAAAATTCAACAAATATTTATTGAAGAGTCTTGATGTCTTTTGGGAA
AGGAACTCTATGGTTACCTTTACTCTAAGGTAAAGAAATTTAGGTCCTGAACTTTAACCACCTTC
TCCTCTTCTTTCTTCCCTCTTTCATAAATGAGCTCTTGGAAAATTAAAATGCCAGGAGGGTCCAG
CAGAGAATACCCAAGCCCACATGTCCTGATGGGAAGTGGGGGATAAGTAGAGAGGGAAACAC

AGACCCTGTAGGCCATCTCACTGCTGCTGGCCCAGGGGACCCTAACTCCTTTTGTTGACTCATCA
GAAGTAGAATCCTCCTGAGGGTCTGTTTAAGAAGCACAGCTCTGGTAGAAAGGGTAGCTTTCCT
ATGGGTACGGAGGGCTCTGCTACCCCAGGGCTATCCACACTGGCCTGTGAATCACTCTCAAATG
CTTAGGGTCACGGGCTTTCAGTGGAATGAGCCCTCATGTGCCACCAGGGGGCACCAAGGCCAC
AGAAAACCCTATTCAGAAGGTTCTGGGGTGAGGGAAAGGAATCCACGGGTTTGTGGAGCTGGC
GGAGCATGGGTGAGGACAGCATTATGCTAGGTTGGTGGCCTTGAAGTCAGATGTAAATTGGGA
TCCTGGCTTGGCCACTGACTATCCATGGGAACTTTGAATCTCAGGGGGCTCATTTGTGATGTGG
GGGTACCATTATCTGCCTCATCAGGTTGTGGGGAGGATTATATGAGATACCTTATGTAAGAGGG
GCTGGTGCATTGTGGAAGTCAACAAGTAAGTTTTGTTAAATTTGAGTAAGAAGACCTGTTTGTTT
CCTCAAGTTGCTTATAATCTAGGTGAGGACACATCTGAGAGAATAACTAATAGTTCAGTGAAGG
TAAGAGCCAAATGATTGGCACAGACCTCAAGTGGTCTGGCATTTACGGGAGAGGGGGATTGTG
AAACAGGAATTGAAAGAAATTTAAAAATGCATAAGCAAAAACTCAGTTTATGTTTTAAAAAAAC
CAATTCCCCCTGAGAAAGAGAAAGAGGTGGAGTCCTTTAAAAATTATCTGCTTGTTTTTCTGTCT
GTGGCTAGTGAGCCTTATCTCTCCCTTTCCCAGGCATTGTGAAGACCCTGTTTCTCTAGCTGTGC
AGCTGCAAGGTCACTAGGCAGATAAACTCAAGCCATAAAACATGTTTTTCCTTGAAAAGTAAGA
AATAATGTAATGCATGTGTCAAATGAATAACTATTTCTCGCTTCTGTAATATGCTTCCCTTCGCAC
AGATCTCCCCCTGCCCCATGAAATGCTTTAAAGGTAACTTAACTCTTTGTTCGGGGCTCAGTCCT
TTGGATGTTAATCTGACTGGCTTGGTGCACCTAAATAATAAATAACATATATCCTCCTCAACCCC
TCAGTCTCTCTGATTCCTAAATTATCCCGCTGTAATTGCAGGGCTAGATTTGCCGACCTGCTTTAT
AAAGCTATGTTAAATCAGTATATTTTATTCTTTTTATTAGACTTTTCTATAGCATAAAAATGATAAA
ATACAGCAAGAAAAAAAGTCACCTGAAACTCTAGTCCTAACCCTAATATGAATGTGAAGGAGCT
GGGCTAGAATGGAATAGGGTAGAATTTGCATAAGGTGGAAATGGGGACAGGCCTGTCACAAGG
ATCACTAGAGTCTTGCTGGCAAAAGCATCTTACTGCATTCATGCTGCAGCACAGGACCCAGATG
CAGAACAGGTTGGGGACACAGCCCTGCCTTCTAATAGTGGACACCATCTGAGAGCTGAGCCAT
AGATTCAAAGCCTAACTCCTAATAGCGATTGGTAGTAATATCATCAGCAGTTCATATACATGGAG
TGACAACAGCGTGCCAGGCAATTCAAAGGGTCTCTCGGTCCTCACAGCATTTTTGAAGTAGCTA
ACATCACTATCCCCCTTTTACAGTTAAAAACAACAACAACAACAACAAAAACACAAAACCCTGA
GGCTCAGAAGATAAATATTTGCTGAAGATCACTGTGCTTGTAAGTGGTGGAGCCTGACCATTAA
CTTCCCCACGACACTGCTTCAATTTCTATCACTTCATCAACCACACAACCCGAGAACTGTGAGG
GGATTTAGCAGGCCCAGTCCAGATCTCTTATTGTACAGAGAAGAAATCTGAGGCTCAGAGAGG
GGAAGGGACTAATTTGGACCTTTAACTTCGGATTCTTAACTTTCTTTCTGGTGTTCTAACTATACT
GTACTACAAGAGGCTGTCTTAGAGCTTTAGTTCAACCAGCTTCAGAGATACTCATTGTTATGGGT
TGAATTATGTCTCCCCAAAATTCATATGTCAAAGTTCTAACCCCCAGTACCTCAGAATGTGACCA
TATTTGGAGATAGGACCTTTACAGAGGTAATCAGGTGAAAATGAGGTTGTTAGGGTGGGCCTTA
GTCCAGTATGGCTGGTGTCCTCATAAAAAGGGGAAATTTGGACACAGATGTACATAGAGGGAA
GATGGTGTGAAGAGACATAAAGAACAGATAGTCATCTATAGCCAAGGCCTGGAACAGATCCTT
CCCTAACAGCCCTCAGAAGGAAGCAACCTCACTGACACCTTGATTTTGGACTGCTACCCTCTGG
CACTGTGAGACAATAAATTCATGTTGGTTAGGCCACCCAGTTTGTTATGGCAGCCCTAGGAAAC
CTTTGTGGATATATCTGTTCGTATACCCATAAAGTCTGCAAGTCAAAAATATCACAGACTTTTGG
CAGCCCTTCATTCAAGCACTGGCTGGGAGGGTCACCCTCCCCGAGGCAAAAGGATAGAGCAAA
TGATCTTTCCAGCTTGCTCCCAACCTTGATCTTTGGCCAAGATATTTTTCTTTCACTGGACATTGG
CCAACATTGGTGTCATAACTTAATTTGACTGTGACCTTCTTGAGTCTTTTATGTCTCTGAGCTTGA
CCTGTAGAAGAAGCTCAATAAATGTGGGTAGAGGGGATGAGTGGGAAAATCAGCTCCCAGCCA
TTCCAGTTCAAGCCTCTTCAAGCAGAAGAGTCCCACCTGAAAGACCTCATTTTCACTTGATTACT

CGATCTCCAAGCAATAGGTGGGGCAGGAAGCAGGGTATGTAAGGCCTGTCAGTGGATGGAAGT
TGAAGTTGGACACCATCTCCTAAAGTTTAGAGCTGAGCCCAAATTCTGGAAAACACAAGCAAAA
TACATTTTATATGACTCAGGATGGGCTAAAGAAGTTCCAGTACAGGAAACTGTTTTCCTGCTGCT
TTATTAATTTTGCTACAGAGGATAAATGAGATGCAGACTTTCATATCCAAGTCTTCATTAGATGCC
CAGTTAATGCAGCCATGACAAATGGGAATGAAGCCCTTATGAATAAAAAAAAGATCAACCCAG
TATAAAAAAATCATAGACCAGAGAAATGAAAATTTAGAGGAGCTGATAAAATACCCTATCCTTA
ATTAGACTGTCAGCATACACATCAAATCAGCCTTAATGCCAAGATTTATTGGCCTTGGAATTGGG
TAAATGAAATAAGTGCTTACAAATGAATGGAAAAGAAATTACCTGTATTAAAACGTATTTGTGAT
GCAGGCCTGTGCACTGAGGGTTTTAGTCAAACTCAGGCTTGCAGCTTCTTTTGCTCTGCATCCAT
TTCCACCCCATGTTCCCCAGTGGGTTTCAGAGACCCTCTTTGTGGGGTAACAATATGTACCATTG
TCAACTACCTCTGCTGTTGTCCTGGAAAAATCCTTCTTGGACAGTAAAATATTTTTGTCTTTATTTC
ACCATCTCCAGATTAGATGGGTGTTACTGATTCTTACCTCAGAGAAGAGAATGTCATGGAGACA
AAATGACCTGCTTAGAGTTCTGCACCCCCCAGCATGGAGAGCAGAGCGCAAAGATTTCTTCCGG
ACTCCCACATTCCCCTTGGAATCCAAGAACTGGTCTACTGTCACAACACTTTAGTACCCTCTTGT
AAGCCTGTGGACAAATCCTCTGATGCCTTGATACCTCGGTTTTCCCCCACTATAAATGGGAAGAT
AAATTCACTAGAAACAGTACTTACCTTGCAGTGTCATTATGAGAATTTCATGAGACAATGCAATA
TAAAGCTCCTAGCACAGTTCCAGGCACCATTGTAAGCACTGAACAAATGAAAGCTGTTGTCATT
AGGATCTCAAGAAATGACAGCTGAGGCAAATGTGGATTAGACTGCATTCAGCATTCTTAGCTGC
AAGCAACAGAAGCTGACGCTGGTTTAAAGAATAGGAAAGGAAGTTAATTAAAATGACATTGGG
TAGCTTGTGGAACTGGGAGGACTAGAGAGCTCAGTCTAAGTTCTAGTGACAAAGCTTCAAATCA
TACATGAGGAAAATGCCCCTCTGCAGGAGGAACGTAATCCTGCAATCACTGCTTCCCCTGAAGC
CAGATGCCTCTACTGCTGCCCTTCCCAGCAGTGATGGAGCCTGGATCACATACCTGGCTTTAGTT
GTAAAGGAGGCTGGAGAGAAAATGCTCTGCTCTATGTAACTGTCTTGAAAGTGGCCCACACATA
GCAAAGCCATTCAAAAGCGGGTGAGCAGCATGAAAGCATCCAGAAGGATTCTTGCTTTGCATG
AGGGAGAACTCTTGCTCCCAGAGTGTGAGACCAGCAGGGGCATTTTCCTCATGTGTGATTAGAA
GCTTTGTCACTAGAAGCTTAGACTGAGTAGGACACCTCTGAGTGTGAATGTCCTACCTGAGTTCT
GCCTCATGCAAAGCAAGAATCCCTCTGGAGGGATTGACACCACAGCCTCTTCCACAATGTCTCC
AAACAACTTTACTGGGCAGCATTCCACTCCTTGACAAACGGGAGAGGTGCATTCTACCTTCTCTC
CTGCATGAGCCTAGACTAATATGGAATTTGGGAGTGTTGATCATTGTACTTTGTGCTCCTAGAGA
TAAGGGTCATCATAAAGCATCTCAGAGTCTCACTGTAGATCCACAGTTCCATTTCTTTATGGATC
CCTTACCCCTACTTTGCCAAATTCTATGCAGATAGCAATAATAATTCAGACAGGGCTTCCTCACT
GAAGGAGTTTGTGCTCTATATAGTTAATAAGCTAGAGATAATGAAAAAAAAAAAAAACTCAACTT
GAACTTTGGGTAAGAGAAACTTTTCAGACCAACGATCATTACCAATAACAGCTTCCACGCATGA
CCCTGCCCGGAGATTGGGGCCTGCACTACTGTTTCCAGGAAACACATTCTTTTGAATTTTTTTATT
TTCTTTGTAGAGATGGGGATCTCACTCTGTTGCCCAGGCTGGTCTCAAACTCCTGGCTTCAAGCA
ATCCTCCTGCTTCGGCCTCCCAAACTTCTGGGATTACAGATATGAGCCACCACGGCTGGCTGAC
AGGCACCAAATTCTAGGAGAGGACTTGAATAGAGTAGGCATCTGTCTCTCCCCATTCCTTTCAG
GGACAGAGCTCTCCTGGTTGGGAGAAAGGGAGAGGGAGAGGGAGGAGGATGAATAGTGAAG
CACCAAACCTCAGAATTAAGGCCTTTTTCTTTTCTTTCTTTCTTTCTTTTTTTTTTTTTGAGATGGAGT
TTCGCTCTTGTTGCCCAGGCTGGAGTGCAATGGCACAATCTCGGCTCACCGCAACCTCTGCCTC
CCAGGTTCAAGCGATTCTCCTGCCTCAGCCTCCCGAGTAGCTGGGATTACAGGCATGCGCCACC
ACACCCAGTTAACTTTTTTGTATTTTTAGAGACGGGGTTTCTCCATGTTGGTCAAGCTGGTCTCGA
ACTCCCGACCTCAGGTGCTCTGCCCGACTTAGCCTCCCAAAGTGCTAGGATTACAGGCGTGAGC
CACCATGCCCAGCCAGAATCAGGGCCTTTTTAAAACTTCAGTGTCTTGTCTTTACCCTTCATGGT

GCCTAGAAAAGCATCTGGCTCATAGGTGTTCAGTGAATGATTGTTTCATTAGTGAAGAAATTCCC
TTTGGAAATCTCATAGGAAACTTACCCTCTCCAACCCTTCACCTGTGCATTATTCCAGCTCCCAA
GGAGGCAGCAGAAACTGGGGTAATCACAACGGTCAACAGCAGCAAAGGCAGCACTTTCT
ID|56456|gene_id|ENSG00000224699|transcript_id|ENST00000608067 Homo sapiens lncRNA

ACTGCGCTTCCGTCGCACAGAGCCTGACGAAGGCTTGGGCTCCCCGCGCGGTGACCGTCG
AGGTGACCTGCACCTGGCTCCATGGCGGAACCGGCCGGCACGGATTATTCCCCTCTCGGGAGA
GGTCTGCAGCCGGGCCTCAGTGCTTCAGTTCGTGGCCTGGCATTTTGTGTCAGTGTTTTTTAACC
TTTGGACCCTAGTTCAACCGACTCCGAGACCTAGGGGACAGATCTTAAGTTTTACAATAACCCA
GGGGTCAGACAGGGTCTGATCTAGCCTGTGGGGCAAGTACGGGGAAGACACGGGACCTCTTG
GAGGGGGTCAGTAGGCTTGTAGGGGGAAAGACGCACACAGAGACTAAAGGACGAGATGGAGC
AAGGTTCAACCCCCTAGCTGAGTGTTGGCAGTGTCGGGATTTTAACGAAGACATTGGTGCTTATT
ATGGTGAGGGGACTCAGGACAGTTTAGGGGAAGAGAGTGGCATTTGAGTCCTAACTCGTAAGG
AAGACTGGGTAGGACTACTTGAAAATAACTTTTAAAAATTAATTATAAAGCTGGTAAAAATAGG
AAAACATCTATGGCCCCACTGTCCAAAGACGACTTTTAACTTCTTGGAGAATATCCTTTCAGATA
GTTGTCTTTGTAAATGTATACTTTTAATGTTTTTTCGTTTGCCTTTTTAAAATAAATTTTTTGGGGGA
CAGGGTCTTGCTCTGTTACCCAGGCTGTAGTGCAGTAGAACGATTATAGCTCATTGCAACCCGG
ACGTCCTGGGTTCAAGCTATCCTCACCTCTCAGCCTCTCGACTGGCTAGGACCACAGCCTCGAG
CCATCATGTCTGGCTAATTTTTACGTTTTTTTGTAGAGATGGGGGTCTCGCTGTGTTGTCCAGGCT
GGTCTTAAACTCCTTGCTTCAACAGATCCTCCCACTCCAGCCTCCCAAAATGTGATTACAGGCTG
AGTCACCACGCCTAGCATGGTTTTTTTTGTTTTGTTTGTTTTGTAAGCAAAATTGACATACATACT
GGAGGTACAATTCTGTAGCTGGCCTTCTTTACAGAGTTTAGAATAAATGTCTTTCCATGTCAAGTT
TTCACTTACATCCTTTGTAGTAGTTGCAAAATATTCCATTTTATAGCTGTATCTTAATTGTGGATTA
AGGTTGGAGTGCAGTGGCACTATCATGGCTTAGTCCAGCCTCAGCCTTCTAGGTTCAAGCGATC
TTCCCACCTCAGCCTCCCAAGAAGCTGGGACTACAGGGACCCGCCACCCCACCTAGCTAATTA
AAAAAAAAATTGTAGAGATGGAGTCTCAGTGTGTAGCCCAGTCTCAAGTGACCCTCCCTCGTTG
GCTTCCCAAAGCGCTGAGATTGCAGGTGAGCCACTGTACTTGGACTTTTTTTTTTTTTTTTGCTTTT
GTTTGTTTTTTGTTTTAGCTATTACAATGCTGGTTGAATATCCTCATAGCTAAATATCTGTGCAGTT
TTAAAATTTCCTTATAAGTTACTAGAAATGGAATGACTGGGTTAAAGGACATGATCATTTTTTAAG
ACTCTTGATACATACTTACAAATTGCCTCCCGAAAAAGTGGTGCTAATTTACTCAAATTGGTAAG
CCTCACCCCCTCTGTGGATCATTCCTTGCACTAAGTTTCTTCCAACTCTGTCACCTCCAGCTTTTT
ACTCACAGCCTACCTTCCAATCTGGGGCCCAGCTCCCAGATCGGAAGATACCAGACTTTGGTAT
TGAACCAAAGTCCAATACACTGCACTGGCATTCAGAGTCCCTTTTTATTCCGCAGCTGTCATAGT
GAAGCTTGCAGCTTCCTTGAAATTCCAGTTTGTTTATTTCTTTATTTTTTTGAGACAGAGTCTTGCT
CTATCGCCCAGGCTGGAGTGCAGTGGCGCCGTCTCAGCTCACTGCAACCTCTGCCTCCTGGTTC
AAGCGATTCTTCTGCCTCAGCCTCCCGAGTAGCTGGGATTACAGGCATGCACCACCACACGTGG
CTAATTTTTGTGCTTTTAGTAGAGATGGGGTTTCACCATGTTGGCCAGGCTGGTCTCAAACTCCT
GACCTCAAGCGATCCGCCCACCTCAGCCTCCCAAAGTGCTGGGATTATAGGCATGAGCCATTGC
ACCCAGCCAAAATTCCAGTTTGAATGTTATAATTCAGGGGTCTTGGACTCCTGCTCTAGAGAAAT
TATTCATGTTAAGTTAGGGGTAACACAGTACAGGCCATATAAGTTTTAGAAAAAGCAGTTTAAAA
CTTAGCATTAGAAAACATAAGTACTGCCTGGTGAAATGGCACGTGCCTATAATCCCAGCTACTC
AGGAGGCTGAGGCAGGAGGATCCCTTGAGCCCAGGAGTTCAAGTCCAGCCTGGGCAATATAGT
CTAGTGGCCACATTAAAAAAAGTAAAACAGCCGGGCGCGGTGGCTCACGCCTGTAATCCCAGC
ACTTTGGGAGGCCGAGGCGGGTGGATCACGAGGTCAGGAGATTGAGACCATCCTGGCTAACAC

GGTGAAACCCTGTCTCCACTAAAAATACAAAAAATTCTCCGGGTGTGGTGGTGGGCGCCTGTGG
TCCCAGCTACTCAGGAGGCTGAGGCAGGAGAATGGTGTGAGCCTGGGAGGCGGAGCTTGCAG
TGAGCCGAGATCGCGCCACTGCACTCCAGCCTGGGCGACAGAGCGAGACTCTGTCTCAAAAAA
AAAAAAAAAAAAAAAAAAAAAGTAAAACAGGGCAGGGTGCAGTGGCTCACGCCTGTAATCCTA
GCACTTTGGGAGGCCGAGATGGGTGGATCAGTTGAGGTCAGGAGTTTGAGACCAGCCTGGCCA
ACATGGCGAAACCCCATCTCTACTAAAAATACAAAAATTAGCTGGGTGTGATGGTGCGTGCCTG
TAGTCCCAGCTACTCAGGAGGCTGAGGCAGGAGGATCGTTTGAACTCGGGAGCTGGAGGTTGC
AGTGAGCCGAGATTGAGCCACAGCACTCCAGGCTGTGCTACAGTGTGAGACTGTCTCAAAAAA
AAGTAAAACAGATGAAACTAATTTTAATAACATTTTATTTATTCAAACAATATATTCAAAATATTA
TTTCAACTTGTGATTGATAAAATACTAATGAGATATTTTATATTTTTTCTCCAGTACTGTCTTCAAA
ATCCAGGGTATATTTTTCAGTTAGCAGCACATTTCAATTCAGACCAGCCGCATTTCAAGTGCTCA
GCAGCCACCTGTGGCTAGTGGCCACAGTATTGGCCAGTGTAGCCCAAGGATATCTCAAATTATC
TCAAAGCTAGATCAGTGATTAAAGGATTCTCAGGCCAGGCATGGTAGTTTTCAGGCCTGGTATG
GTGGTTTACGCCTGTAATCCCCGCACTTTGGGAGGCCTAGGTGGGCGGATGGCTTGAGCCCAG
GAGTTTGAGACCAGCCTAGCCAACGTGGGGAGACCCTGTCTCTACCAAAGCACAAAAATTAGC
TGGGCATGGTGATGCGCACCTGTGGTCTCAAGTACTTTGTGTGAGGTGGGAGGATCACTTGAGC
CCGGGAGGTTGAGGCTGCAGTGAGCTGAGATTGCACAACTGCACTCCAGCCTGGGTGACAGAA
TGAGACCCTGTCTCAAAAAAAAAAAAAGGATTCTCCGAAAGACTGGCGATTGCTTTTTAAGCTAT
AAGGAAGGCCTTTGAAGGGAAATCAAGTAGGAAGAGGATGATAGCTGGCTCACATGTCCTTGC
CACATGGGAGGTGAGAGAGCAGGCTACTCCTTACCACCCCCGCTTTCTCTGCCCTGCTGCTGTC
TTGCCCCTGTGCAAGTAGGCAATGGGAGAGGAATAGTATATATAAATTGGGCAAGTGCTAGGG
GCTGGAGTGATGGTTGGGGAAAGTATCTGACCCTTGTTTTCTACTTTCTTTAGCTTGTGGGGTATT
TAACAATTCTTTGTTCTTAGTTTTGAGTGCTGGCAACCATTTCTGGGGTGACAGGAAAAGTTCCA
TGACATCCGTATTAGAGCATTTGTTTCATTATATTGTAATTTTTTATGTTTACTTTTGAAAAAGTTC
ATTCTTTTCTCTTTTTAATTCTTATTCTTTTTTTTTTCTTTTCTTTTTGAGGCAGGGTTTTACTCTATCA
CCCAGGCTGGAGTGCAGTGGCACAATCTTGGCTCACTGCAACCTCTGCCTCCTGAGCTCGTGAT
TCATCCTCCTGAGTAGCTGGGACTACAGGTGTACACTACCATGTGTAGCTAAATTTTTTGTATTTT
TTGTAGAGATGGGGTTTTGCCATGTTGCCAGGCTGGTCTCGAACTCCTGCAATTTGCTCTCCTTG
GCCTCACGAAATGCTGGGATTACAGGCATAAGCTACAGCACCCAGCCCTTACGTTTACTTTTCAT
TGACAGGAAGAGTGTCTTCATCTCTGCATCCCTAGTGCAACAGAGCATGACAAAAATTCAACAA
ATATTTATTGAAGAGTCTTGATGTCTTTTGGGAAAGGAACTCTATGGTTACCTTTACTCTAAGGTA
AAGAAATTTAGGTCCTGAACTTTAACCACCTTCTCCTCTTCTTTCTTCCCTCTTTCATAAATGAGCT
CTTGGAAAATTAAAATGCCAGGAGGGTCCAGCAGAGAATACCCAAGCCCACATGTCCTGATGG
GAAGTGGGGGATAAGTAGAGAGGGAAACACAGACCCTGTAGGCCATCTCACTGCTGCTGGCCC
AGGGGACCCTAACTCCTTTTGTTGACTCATCAGAAGTAGAATCCTCCTGAGGGTCTGTTTAAGAA
GCACAGCTCTGGTAGAAAGGGTAGCTTTCCTATGGGTACGGAGGGCTCTGCTACCCCAGGGCT
ATCCACACTGGCCTGTGAATCACTCTCAAATGCTTAGGGTCACGGGCTTTCAGTGGAATGAGCC
CTCATGTGCCACCAGGGGGCACCAAGGCCACAGAAAACCCTATTCAGAAGGTTCTGGGGTGAG
GGAAAGGAATCCACGGGTTTGTGGAGCTGGCGGAGCATGGGTGAGGACAGCATTATGCTAGGT
TGGTGGCCTTGAAGTCAGATGTAAATTGGGATCCTGGCTTGGCCACTGACTATCCATGGGAACT
TTGAATCTCAGGGGGCTCATTTGTGATGTGGGGGTACCATTATCTGCCTCATCAGGTTGTGGGG
AGGATTATATGAGATACCTTATGTAAGAGGGGCTGGTGCATTGTGGAAGTCAACAAGTAAGTTT
TGTTAAATTTGAGTAAGAAGACCTGTTTGTTTCCTCAAGTTGCTTATAATCTAGGTGAGGACACA
TCTGAGAGAATAACTAATAGTTCAGTGAAGGTAAGAGCCAAATGATTGGCACAGACCTCAAGTG

GTCTGGCATTTACGGGAGAGGGGGATTGTGAAACAGGAATTGAAAGAAATTTAAAAATGCATA
AGCAAAAACTCAGTTTATGTTTTAAAAAAACCAATTCCCCCTGAGAAAGAGAAAGAGGTGGAGT
CCTTTAAAAATTATCTGCTTGTTTTTCTGTCTGTGGCTAGTGAGCCTTATCTCTCCCTTTCCCAGGC
ATTGTGAAGACCCTGTTTCTCTAGCTGTGCAGCTGCAAGGTCACTAGGCAGATAAACTCAAGCC
ATAAAACATGTTTTTCCTTGAAAAGTAAGAAATAATGTAATGCATGTGTCAAATGAATAACTATTT
CTCGCTTCTGTAATATGCTTCCCTTCGCACAGATCTCCCCCTGCCCCATGAAATGCTTTAAAGGT
AACTTAACTCTTTGTTCGGGGCTCAGTCCTTTGGATGTTAATCTGACTGGCTTGGTGCACCTAAAT
AATAAATAACATATATCCTCCTCAACCCCTCAGTCTCTCTGATTCCTAAATTATCCCGCTGTAATT
GCAGGGCTAGATTTGCCGACCTGCTTTATAAAGCTATGTTAAATCAGTATATTTTATTCTTTTTATT
AGACTTTTCTATAGCATAAAAATGATAAAATACAGCAAGAAAAAAAGTCACCTGAAACTCTAGT
CCTAACCCTAATATGAATGTGAAGGAGCTGGGCTAGAATGGAATAGGGTAGAATTTGCATAAG
GTGGAAATGGGGACAGGCCTGTCACAAGGATCACTAGAGTCTTGCTGGCAAAAGCATCTTACT
GCATTCATGCTGCAGCACAGGACCCAGATGCAGAACAGGTTGGGGACACAGCCCTGCCTTCTA
ATAGTGGACACCATCTGAGAGCTGAGCCATAGATTCAAAGCCTAACTCCTAATAGCGATTGGTA
GTAATATCATCAGCAGTTCATATACATGGAGTGACAACAGCGTGCCAGGCAATTCAAAGGGTCT
CTCGGTCCTCACAGCATTTTTGAAGTAGCTAACATCACTATCCCCCTTTTACAGTTAAAAACAAC
AACAACAACAACAAAAACACAAAACCCTGAGGCTCAGAAGATAAATATTTGCTGAAGATCACT
GTGCTTGTAAGTGGTGGAGCCTGACCATTAACTTCCCCACGACACTGCTTCAATTTCTATCACTT
CATCAACCACACAACCCGAGAACTGTGAGGGGATTTAGCAGGCCCAGTCCAGATCTCTTATTGT
ACAGAGAAGAAATCTGAGGCTCAGAGAGGGGAAGGGACTAATTTGGACCTTTAACTTCGGATT
CTTAACTTTCTTTCTGGTGTTCTAACTATACTGTACTACAAGAGGCTGTCTTAGAGCTTTAGTTCA
ACCAGCTTCAGAGATACTCATTGTTATGGGTTGAATTATGTCTCCCCAAAATTCATATGTCAAAG
TTCTAACCCCCAGTACCTCAGAATGTGACCATATTTGGAGATAGGACCTTTACAGAGGTAATCA
GGTGAAAATGAGGTTGTTAGGGTGGGCCTTAGTCCAGTATGGCTGGTGTCCTCATAAAAAGGGG
AAATTTGGACACAGATGTACATAGAGGGAAGATGGTGTGAAGAGACATAAAGAACAGATAGTC
ATCTATAGCCAAGGCCTGGAACAGATCCTTCCCTAACAGCCCTCAGAAGGAAGCAACCTCACT
GACACCTTGATTTTGGACTGCTACCCTCTGGCACTGTGAGACAATAAATTCATGTTGGTTAGGCC
ACCCAGTTTGTTATGGCAGCCCTAGGAAACCTTTGTGGATATATCTGTTCGTATACCCATAAAGT
CTGCAAGTCAAAAATATCACAGACTTTTGGCAGCCCTTCATTCAAGCACTGGCTGGGAGGGTCA
CCCTCCCCGAGGCAAAAGGATAGAGCAAATGATCTTTCCAGCTTGCTCCCAACCTTGATCTTTG
GCCAAGATATTTTTCTTTCACTGGACATTGGCCAACATTGGTGTCATAACTTAATTTGACTGTGAC
CTTCTTGAGTCTTTTATGTCTCTGAGCTTGACCTGTAGAAGAAGCTCAATAAATGTGGGTAGAGG
GGATGAGTGGGAAAATCAGCTCCCAGCCATTCCAGTTCAAGCCTCTTCAAGCAGAAGAGTCCC
ACCTGAAAGACCTCATTTTCACTTGATTACTCGATCTCCAAGCAATAGGTGGGGCAGGAAGCAG
GGTATGTAAGGCCTGTCAGTGGATGGAAGTTGAAGTTGGACACCATCTCCTAAAGTTTAGAGCT
GAGCCCAAATTCTGGAAAACACAAGCAAAATACATTTTATATGACTCAGGATGGGCTAAAGAAG
TTCCAGTACAGGAAACTGTTTTCCTGCTGCTTTATTAATTTTGCTACAGAGGATAAATGAGATGC
AGACTTTCATATCCAAGTCTTCATTAGATGCCCAGTTAATGCAGCCATGACAAATGGGAATGAA
GCCCTTATGAATAAAAAAAAGATCAACCCAGTATAAAAAAATCATAGACCAGAGAAATGAAAA
TTTAGAGGAGCTGATAAAATACCCTATCCTTAATTAGACTGTCAGCATACACATCAAATCAGCCT
TAATGCCAAGATTTATTGGCCTTGGAATTGGGTAAATGAAATAAGTGCTTACAAATGAATGGAA
AAGAAATTACCTGTATTAAAACGTATTTGTGATGCAGGCCTGTGCACTGAGGGTTTTAGTCAAAC
TCAGGCTTGCAGCTTCTTTTGCTCTGCATCCATTTCCACCCCATGTTCCCCAGTGGGTTTCAGAG
ACCCTCTTTGTGGGGTAACAATATGTACCATTGTCAACTACCTCTGCTGTTGTCCTGGAAAAATC

CTTCTTGGACAGTAAAATATTTTTGTCTTTATTTCACCATCTCCAGATTAGATGGGTGTTACTGATT
CTTACCTCAGAGAAGAGAATGTCATGGAGACAAAATGACCTGCTTAGAGTTCTGCACCCCCCAG
CATGGAGAGCAGAGCGCAAAGATTTCTTCCGGACTCCCACATTCCCCTTGGAATCCAAGAACTG
GTCTACTGTCACAACACTTTAGTACCCTCTTGTAAGCCTGTGGACAAATCCTCTGATGCCTTGAT
ACCTCGGTTTTCCCCCACTATAAATGGGAAGATAAATTCACTAGAAACAGTACTTACCTTGCAGT
GTCATTATGAGAATTTCATGAGACAATGCAATATAAAGCTCCTAGCACAGTTCCAGGCACCATT
GTAAGCACTGAACAAATGAAAGCTGTTGTCATTAGGATCTCAAGAAATGACAGCTGAGGCAAAT
GTGGATTAGACTGCATTCAGCATTCTTAGCTGCAAGCAACAGAAGCTGACGCTGGTTTAAAGAA
TAGGAAAGGAAGTTAATTAAAATGACATTGGGTAGCTTGTGGAACTGGGAGGACTAGAGAGCT
CAGTCTAAGTTCTAGTGACAAAGCTTCAAATCATACATGAGGAAAATGCCCCTCTGCAGGAGGA
ACGTAATCCTGCAATCACTGCTTCCCCTGAAGCCAGATGCCTCTACTGCTGCCCTTCCCAGCAGT
GATGGAGCCTGGATCACATACCTGGCTTTAGTTGTAAAGGAGGCTGGAGAGAAAATGCTCTGCT
CTATGTAACTGTCTTGAAAGTGGCCCACACATAGCAAAGCCATTCAAAAGCGGGTGAGCAGCAT
GAAAGCATCCAGAAGGATTCTTGCTTTGCATGAGGGAGAACTCTTGCTCCCAGAGTGTGAGACC
AGCAGGGGCATTTTCCTCATGTGTGATTAGAAGCTTTGTCACTAGAAGCTTAGACTGAGTAGGA
CACCTCTGAGTGTGAATGTCCTACCTGAGTTCTGCCTCATGCAAAGCAAGAATCCCTCTGGAGG
GATTGACACCACAGCCTCTTCCACAATGTCTCCAAACAACTTTACTGGGCAGCATTCCACTCCTT
GACAAACGGGAGAGGTGCATTCTACCTTCTCTCCTGCATGAGCCTAGACTAATATGGAATTTGG
GAGTGTTGATCATTGTACTTTGTGCTCCTAGAGATAAGGGTCATCATAAAGCATCTCAGAGTCTC
ACTGTAGATCCACAGTTCCATTTCTTTATGGATCCCTTACCCCTACTTTGCCAAATTCTATGCAGA
TAGCAATAATAATTCAGACAGGGCTTCCTCACTGAAGGAGTTTGTGCTCTATATAGTTAATAAGC
TAGAGATAATGAAAAAAAAAAAAAACTCAACTTGAACTTTGGGTAAGAGAAACTTTTCAGACCAA
CGATCATTACCAATAACAGCTTCCACGCATGACCCTGCCCGGAGATTGGGGCCTGCACTACTGT
TTCCAGGAAACACATTCTTTTGAATTTTTTTATTTTCTTTGTAGAGATGGGGATCTCACTCTGTTGC
CCAGGCTGGTCTCAAACTCCTGGCTTCAAGCAATCCTCCTGCTTCGGCCTCCCAAACTTCTGGG
ATTACAGATATGAGCCACCACGGCTGGCTGACAGGCACCAAATTCTAGGAGAGGACTTGAATA
GAGTAGGCATCTGTCTCTCCCCATTCCTTTCAGGGACAGAGCTCTCCTGGTTGGGAGAAAGGGA
GAGGGAGAGGGAGGAGGATGAATAGTGAAGCACCAAACCTCAGAATTAAGGCCTTTTTCTTTTC
TTTCTTTCTTTCTTTTTTTTTTTTTGAGATGGAGTTTCGCTCTTGTTGCCCAGGCTGGAGTGCAATGG
CACAATCTCGGCTCACCGCAACCTCTGCCTCCCAGGTTCAAGCGATTCTCCTGCCTCAGCCTCC
CGAGTAGCTGGGATTACAGGCATGCGCCACCACACCCAGTTAACTTTTTTGTATTTTTAGAGACG
GGGTTTCTCCATGTTGGTCAAGCTGGTCTCGAACTCCCGACCTCAGGTGCTCTGCCCGACTTAG
CCTCCCAAAGTGCTAGGATTACAGGCGTGAGCCACCATGCCCAGCCAGAATCAGGGCCTTTTTA
AAACTTCAGTGTCTTGTCTTTACCCTTCATGGTGCCTAGAAAAGCATCTGGCTCATAGGTGTTCA
GTGAATGATTGTTTCATTAGTGAAGAAATTCCCTTTGGAAATCTCATAGGAAACTTACCCTCTCC
AACCCTTCACCTGTGCATTATTCCAGCTCCCAAGGAGGCAGCAGAAACTGGGGTAATCACAAC
GGTCAACAGCAGCAAAGGCAGCACTTTCTG

ID|56470|gene_id|ENSG00000224699|transcript_id|ENST00000608499 Homo sapiens lncRNA

CCTGCACCTGGCTCCATGGCGGAACCGGCCGGCACGGATTATTCCCCTCTCGGGAGAGGT
CTGCAGCCGGGCCTCAGTGCTTCAGTTCGTGGCCTGGCATTTTGTGTCAGTGTTTTTTAACCTTTG
GACCCTAGTTCAACCGACTCCGAGACCTAGGGGACAGATCTTAAGTTTTACAATAACCCAGGGG
TCAGACAGGGTCTGATCTAGCCTGTGGGGCAAGTACGGGGAAGACACGGGACCTCTTGGAGG
GGGTCAGTAGGCTTGTAGGGGGAAAGACGCACACAGAGACTAAAGGACGAGATGGAGCAAGG

TTCAACCCCCTAGCTGAGTGTTGGCAGTGTCGGGATTTTAACGAAGACATTGGTGCTTATTATGG
TGAGGGGACTCAGGACAGTTTAGGGGAAGAGAGTGGCATTTGAGTCCTAACTCGTAAGGAAGA
CTGGGTAGGACTACTTGAAAATAACTTTTAAAAATTAATTATAAAGCTGGTAAAAATAGGAAAA
CATCTATGGCCCCACTGTCCAAAGACGACTTTTAACTTCTTGGAGAATATCCTTTCAGATAGTTG
TCTTTGTAAATGTATACTTTTAATGTTTTTTCGTTTGCCTTTTTAAAATAAATTTTTTGGGGGACAG
GGTCTTGCTCTGTTACCCAGGCTGTAGTGCAGTAGAACGATTATAGCTCATTGCAACCCGGACG
TCCTGGGTTCAAGCTATCCTCACCTCTCAGCCTCTCGACTGGCTAGGACCACAGCCTCGAGCCA
TCATGTCTGGCTAATTTTTACGTTTTTTTGTAGAGATGGGGGTCTCGCTGTGTTGTCCAGGCTGGT
CTTAAACTCCTTGCTTCAACAGATCCTCCCACTCCAGCCTCCCAAAATGTGATTACAGGCTGAGT
CACCACGCCTAGCATGGTTTTTTTTGTTTTGTTTGTTTTGTAAGCAAAATTGACATACATACTGGA
GGTACAATTCTGTAGCTGGCCTTCTTTACAGAGTTTAGAATAAATGTCTTTCCATGTCAAGTTTTC
ACTTACATCCTTTGTAGTAGTTGCAAAATATTCCATTTTATAGCTGTATCTTAATTGTGGATTAAG
GTTGGAGTGCAGTGGCACTATCATGGCTTAGTCCAGCCTCAGCCTTCTAGGTTCAAGCGATCTTC
CCACCTCAGCCTCCCAAGAAGCTGGGACTACAGGGACCCGCCACCCCACCTAGCTAATTAAAA
AAAAAATTGTAGAGATGGAGTCTCAGTGTGTAGCCCAGTCTCAAGTGACCCTCCCTCGTTGGCT
TCCCAAAGCGCTGAGATTGCAGGTGAGCCACTGTACTTGGACTTTTTTTTTTTTTTTTGCTTTTGTT
TGTTTTTTGTTTTAGCTATTACAATGCTGGTTGAATATCCTCATAGCTAAATATCTGTGCAGTTTTA
AAATTTCCTTATAAGTTACTAGAAATGGAATGACTGGGTTAAAGGACATGATCATTTTTTAAGAC
TCTTGATACATACTTACAAATTGCCTCCCGAAAAAGTGGTGCTAATTTACTCAAATTGGTAAGCC
TCACCCCCTCTGTGGATCATTCCTTGCACTAAGTTTCTTCCAACTCTGTCACCTCCAGCTTTTTAC
TCACAGCCTACCTTCCAATCTGGGGCCCAGCTCCCAGATCGGAAGATACCAGACTTTGGTATTG
AACCAAAGTCCAATACACTGCACTGGCATTCAGAGTCCCTTTTTATTCCGCAGCTGTCATAGTGA
AGCTTGCAGCTTCCTTGAAATTCCAGTTTGTTTATTTCTTTATTTTTTTGAGACAGAGTCTTGCTCT
ATCGCCCAGGCTGGAGTGCAGTGGCGCCGTCTCAGCTCACTGCAACCTCTGCCTCCTGGTTCAA
GCGATTCTTCTGCCTCAGCCTCCCGAGTAGCTGGGATTACAGGCATGCACCACCACACGTGGCT
AATTTTTGTGCTTTTAGTAGAGATGGGGTTTCACCATGTTGGCCAGGCTGGTCTCAAACTCCTGA
CCTCAAGCGATCCGCCCACCTCAGCCTCCCAAAGTGCTGGGATTATAGGCATGAGCCATTGCAC
CCAGCCAAAATTCCAGTTTGAATGTTATAATTCAGGGGTCTTGGACTCCTGCTCTAGAGAAATTA
TTCATGTTAAGTTAGGGGTAACACAGTACAGGCCATATAAGTTTTAGAAAAAGCAGTTTAAAACT
TAGCATTAGAAAACATAAGTACTGCCTGGTGAAATGGCACGTGCCTATAATCCCAGCTACTCAG
GAGGCTGAGGCAGGAGGATCCCTTGAGCCCAGGAGTTCAAGTCCAGCCTGGGCAATATAGTCT
AGTGGCCACATTAAAAAAAGTAAAACAGCCGGGCGCGGTGGCTCACGCCTGTAATCCCAGCAC
TTTGGGAGGCCGAGGCGGGTGGATCACGAGGTCAGGAGATTGAGACCATCCTGGCTAACACG
GTGAAACCCTGTCTCCACTAAAAATACAAAAAATTCTCCGGGTGTGGTGGTGGGCGCCTGTGGT
CCCAGCTACTCAGGAGGCTGAGGCAGGAGAATGGTGTGAGCCTGGGAGGCGGAGCTTGCAGT
GAGCCGAGATCGCGCCACTGCACTCCAGCCTGGGCGACAGAGCGAGACTCTGTCTCAAAAAAA
AAAAAAAAAAAAAAAAAAAAAGTAAAACAGGGCAGGGTGCAGTGGCTCACGCCTGTAATCCTAG
CACTTTGGGAGGCCGAGATGGGTGGATCAGTTGAGGTCAGGAGTTTGAGACCAGCCTGGCCAA
CATGGCGAAACCCCATCTCTACTAAAAATACAAAAATTAGCTGGGTGTGATGGTGCGTGCCTGT
AGTCCCAGCTACTCAGGAGGCTGAGGCAGGAGGATCGTTTGAACTCGGGAGCTGGAGGTTGCA
GTGAGCCGAGATTGAGCCACAGCACTCCAGGCTGTGCTACAGTGTGAGACTGTCTCAAAAAAA
AGTAAAACAGATGAAACTAATTTTAATAACATTTTATTTATTCAAACAATATATTCAAAATATTAT
TTCAACTTGTGATTGATAAAATACTAATGAGATATTTTATATTTTTTCTCCAGTACTGTCTTCAAAA
TCCAGGGTATATTTTTCAGTTAGCAGCACATTTCAATTCAGACCAGCCGCATTTCAAGTGCTCAG

CAGCCACCTGTGGCTAGTGGCCACAGTATTGGCCAGTGTAGCCCAAGGATATCTCAAATTATCT
CAAAGCTAGATCAGTGATTAAAGGATTCTCAGGCCAGGCATGGTAGTTTTCAGGCCTGGTATGG
TGGTTTACGCCTGTAATCCCCGCACTTTGGGAGGCCTAGGTGGGCGGATGGCTTGAGCCCAGG
AGTTTGAGACCAGCCTAGCCAACGTGGGGAGACCCTGTCTCTACCAAAGCACAAAAATTAGCT
GGGCATGGTGATGCGCACCTGTGGTCTCAAGTACTTTGTGTGAGGTGGGAGGATCACTTGAGCC
CGGGAGGTTGAGGCTGCAGTGAGCTGAGATTGCACAACTGCACTCCAGCCTGGGTGACAGAAT
GAGACCCTGTCTCAAAAAAAAAAAAAGGATTCTCCGAAAGACTGGCGATTGCTTTTTAAGCTATA
AGGAAGGCCTTTGAAGGGAAATCAAGTAGGAAGAGGATGATAGCTGGCTCACATGTCCTTGCC
ACATGGGAGGTGAGAGAGCAGGCTACTCCTTACCACCCCCGCTTTCTCTGCCCTGCTGCTGTCT
TGCCCCTGTGCAAGTAGGCAATGGGAGAGGAATAGTATATATAAATTGGGCAAGTGCTAGGGG
CTGGAGTGATGGTTGGGGAAAGTATCTGACCCTTGTTTTCTACTTTCTTTAGCTTGTGGGGTATTT
AACAATTCTTTGTTCTTAGTTTTGAGTGCTGGCAACCATTTCTGGGGTGACAGGAAAAGTTCCAT
GACATCCGTATTAGAGCATTTGTTTCATTATATTGTAATTTTTTATGTTTACTTTTGAAAAAGTTCA
TTCTTTTCTCTTTTTAATTCTTATTCTTTTTTTTTTCTTTTCTTTTTGAGGCAGGGTTTTACTCTATCAC
CCAGGCTGGAGTGCAGTGGCACAATCTTGGCTCACTGCAACCTCTGCCTCCTGAGCTCGTGATT
CATCCTCCTGAGTAGCTGGGACTACAGGTGTACACTACCATGTGTAGCTAAATTTTTTGTATTTTT
TGTAGAGATGGGGTTTTGCCATGTTGCCAGGCTGGTCTCGAACTCCTGCAATTTGCTCTCCTTGG
CCTCACGAAATGCTGGGATTACAGGCATAAGCTACAGCACCCAGCCCTTACGTTTACTTTTCATT
GACAGGAAGAGTGTCTTCATCTCTGCATCCCTAGTGCAACAGAGCATGACAAAAATTCAACAAA
TATTTATTGAAGAGTCTTGATGTCTTTTGGGAAAGGAACTCTATGGTTACCTTTACTCTAAGGTAA
AGAAATTTAGGTCCTGAACTTTAACCACCTTCTCCTCTTCTTTCTTCCCTCTTTCATAAATGAGCTC
TTGGAAAATTAAAATGCCAGGAGGGTCCAGCAGAGAATACCCAAGCCCACATGTCCTGATGGG
AAGTGGGGGATAAGTAGAGAGGGAAACACAGACCCTGTAGGCCATCTCACTGCTGCTGGCCCA
GGGGACCCTAACTCCTTTTGTTGACTCATCAGAAGTAGAATCCTCCTGAGGGTCTGTTTAAGAAG
CACAGCTCTGGTAGAAAGGGTAGCTTTCCTATGGGTACGGAGGGCTCTGCTACCCCAGGGCTAT
CCACACTGGCCTGTGAATCACTCTCAAATGCTTAGGGTCACGGGCTTTCAGTGGAATGAGCCCT
CATGTGCCACCAGGGGGCACCAAGGCCACAGAAAACCCTATTCAGAAGGTTCTGGGGTGAGG
GAAAGGAATCCACGGGTTTGTGGAGCTGGCGGAGCATGGGTGAGGACAGCATTATGCTAGGTT
GGTGGCCTTGAAGTCAGATGTAAATTGGGATCCTGGCTTGGCCACTGACTATCCATGGGAACTT
TGAATCTCAGGGGGCTCATTTGTGATGTGGGGGTACCATTATCTGCCTCATCAGGTTGTGGGGA
GGATTATATGAGATACCTTATGTAAGAGGGGCTGGTGCATTGTGGAAGTCAACAAGTAAGTTTT
GTTAAATTTGAGTAAGAAGACCTGTTTGTTTCCTCAAGTTGCTTATAATCTAGGTGAGGACACAT
CTGAGAGAATAACTAATAGTTCAGTGAAGGTAAGAGCCAAATGATTGGCACAGACCTCAAGTG
GTCTGGCATTTACGGGAGAGGGGGATTGTGAAACAGGAATTGAAAGAAATTTAAAAATGCATA
AGCAAAAACTCAGTTTATGTTTTAAAAAAACCAATTCCCCCTGAGAAAGAGAAAGAGGTGGAGT
CCTTTAAAAATTATCTGCTTGTTTTTCTGTCTGTGGCTAGTGAGCCTTATCTCTCCCTTTCCCAGGC
ATTGTGAAGACCCTGTTTCTCTAGCTGTGCAGCTGCAAGGTCACTAGGCAGATAAACTCAAGCC
ATAAAACATGTTTTTCCTTGAAAAGTAAGAAATAATGTAATGCATGTGTCAAATGAATAACTATTT
CTCGCTTCTGTAATATGCTTCCCTTCGCACAGATCTCCCCCTGCCCCATGAAATGCTTTAAAGGT
AACTTAACTCTTTGTTCGGGGCTCAGTCCTTTGGATGTTAATCTGACTGGCTTGGTGCACCTAAAT
AATAAATAACATATATCCTCCTCAACCCCTCAGTCTCTCTGATTCCTAAATTATCCCGCTGTAATT
GCAGGGCTAGATTTGCCGACCTGCTTTATAAAGCTATGTTAAATCAGTATATTTTATTCTTTTTATT
AGACTTTTCTATAGCATAAAAATGATAAAATACAGCAAGAAAAAAAGTCACCTGAAACTCTAGT
CCTAACCCTAATATGAATGTGAAGGAGCTGGGCTAGAATGGAATAGGGTAGAATTTGCATAAG

GTGGAAATGGGGACAGGCCTGTCACAAGGATCACTAGAGTCTTGCTGGCAAAAGCATCTTACT
GCATTCATGCTGCAGCACAGGACCCAGATGCAGAACAGGTTGGGGACACAGCCCTGCCTTCTA
ATAGTGGACACCATCTGAGAGCTGAGCCATAGATTCAAAGCCTAACTCCTAATAGCGATTGGTA
GTAATATCATCAGCAGTTCATATACATGGAGTGACAACAGCGTGCCAGGCAATTCAAAGGGTCT
CTCGGTCCTCACAGCATTTTTGAAGTAGCTAACATCACTATCCCCCTTTTACAGTTAAAAACAAC
AACAACAACAACAAAAACACAAAACCCTGAGGCTCAGAAGATAAATATTTGCTGAAGATCACT
GTGCTTGTAAGTGGTGGAGCCTGACCATTAACTTCCCCACGACACTGCTTCAATTTCTATCACTT
CATCAACCACACAACCCGAGAACTGTGAGGGGATTTAGCAGGCCCAGTCCAGATCTCTTATTGT
ACAGAGAAGAAATCTGAGGCTCAGAGAGGGGAAGGGACTAATTTGGACCTTTAACTTCGGATT
CTTAACTTTCTTTCTGGTGTTCTAACTATACTGTACTACAAGAGGCTGTCTTAGAGCTTTAGTTCA
ACCAGCTTCAGAGATACTCATTGTTATGGGTTGAATTATGTCTCCCCAAAATTCATATGTCAAAG
TTCTAACCCCCAGTACCTCAGAATGTGACCATATTTGGAGATAGGACCTTTACAGAGGTAATCA
GGTGAAAATGAGGTTGTTAGGGTGGGCCTTAGTCCAGTATGGCTGGTGTCCTCATAAAAAGGGG
AAATTTGGACACAGATGTACATAGAGGGAAGATGGTGTGAAGAGACATAAAGAACAGATAGTC
ATCTATAGCCAAGGCCTGGAACAGATCCTTCCCTAACAGCCCTCAGAAGGAAGCAACCTCACT
GACACCTTGATTTTGGACTGCTACCCTCTGGCACTGTGAGACAATAAATTCATGTTGGTTAGGCC
ACCCAGTTTGTTATGGCAGCCCTAGGAAACCTTTGTGGATATATCTGTTCGTATACCCATAAAGT
CTGCAAGTCAAAAATATCACAGACTTTTGGCAGCCCTTCATTCAAGCACTGGCTGGGAGGGTCA
CCCTCCCCGAGGCAAAAGGATAGAGCAAATGATCTTTCCAGCTTGCTCCCAACCTTGATCTTTG
GCCAAGATATTTTTCTTTCACTGGACATTGGCCAACATTGGTGTCATAACTTAATTTGACTGTGAC
CTTCTTGAGTCTTTTATGTCTCTGAGCTTGACCTGTAGAAGAAGCTCAATAAATGTGGGTAGAGG
GGATGAGTGGGAAAATCAGCTCCCAGCCATTCCAGTTCAAGCCTCTTCAAGCAGAAGAGTCCC
ACCTGAAAGACCTCATTTTCACTTGATTACTCGATCTCCAAGCAATAGGTGGGGCAGGAAGCAG
GGTATGTAAGGCCTGTCAGTGGATGGAAGTTGAAGTTGGACACCATCTCCTAAAGTTTAGAGCT
GAGCCCAAATTCTGGAAAACACAAGCAAAATACATTTTATATGACTCAGGATGGGCTAAAGAAG
TTCCAGTACAGGAAACTGTTTTCCTGCTGCTTTATTAATTTTGCTACAGAGGATAAATGAGATGC
AGACTTTCATATCCAAGTCTTCATTAGATGCCCAGTTAATGCAGCCATGACAAATGGGAATGAA
GCCCTTATGAATAAAAAAAAGATCAACCCAGTATAAAAAAATCATAGACCAGAGAAATGAAAA
TTTAGAGGAGCTGATAAAATACCCTATCCTTAATTAGACTGTCAGCATACACATCAAATCAGCCT
TAATGCCAAGATTTATTGGCCTTGGAATTGGGTAAATGAAATAAGTGCTTACAAATGAATGGAA
AAGAAATTACCTGTATTAAAACGTATTTGTGATGCAGGCCTGTGCACTGAGGGTTTTAGTCAAAC
TCAGGCTTGCAGCTTCTTTTGCTCTGCATCCATTTCCACCCCATGTTCCCCAGTGGGTTTCAGAG
ACCCTCTTTGTGGGGTAACAATATGTACCATTGTCAACTACCTCTGCTGTTGTCCTGGAAAAATC
CTTCTTGGACAGTAAAATATTTTTGTCTTTATTTCACCATCTCCAGATTAGATGGGTGTTACTGATT
CTTACCTCAGAGAAGAGAATGTCATGGAGACAAAATGACCTGCTTAGAGTTCTGCACCCCCCAG
CATGGAGAGCAGAGCGCAAAGATTTCTTCCGGACTCCCACATTCCCCTTGGAATCCAAGAACTG
GTCTACTGTCACAACACTTTAGTACCCTCTTGTAAGCCTGTGGACAAATCCTCTGATGCCTTGAT
ACCTCGGTTTTCCCCCACTATAAATGGGAAGATAAATTCACTAGAAACAGTACTTACCTTGCAGT
GTCATTATGAGAATTTCATGAGACAATGCAATATAAAGCTCCTAGCACAGTTCCAGGCACCATT
GTAAGCACTGAACAAATGAAAGCTGTTGTCATTAGGATCTCAAGAAATGACAGCTGAGGCAAAT
GTGGATTAGACTGCATTCAGCATTCTTAGCTGCAAGCAACAGAAGCTGACGCTGGTTTAAAGAA
TAGGAAAGGAAGTTAATTAAAATGACATTGGGTAGCTTGTGGAACTGGGAGGACTAGAGAGCT
CAGTCTAAGTTCTAGTGACAAAGCTTCAAATCATACATGAGGAAAATGCCCCTCTGCAGGAGGA
ACGTAATCCTGCAATCACTGCTTCCCCTGAAGCCAGATGCCTCTACTGCTGCCCTTCCCAGCAGT

GATGGAGCCTGGATCACATACCTGGCTTTAGTTGTAAAGGAGGCTGGAGAGAAAATGCTCTGCT
CTATGTAACTGTCTTGAAAGTGGCCCACACATAGCAAAGCCATTCAAAAGCGGGTGAGCAGCAT
GAAAGCATCCAGAAGGATTCTTGCTTTGCATGAGGGAGAACTCTTGCTCCCAGAGTGTGAGACC
AGCAGGGGCATTTTCCTCATGTGTGATTAGAAGCTTTGTCACTAGAAGCTTAGACTGAGTAGGA
CACCTCTGAGTGTGAATGTCCTACCTGAGTTCTGCCTCATGCAAAGCAAGAATCCCTCTGGAGG
GATTGACACCACAGCCTCTTCCACAATGTCTCCAAACAACTTTACTGGGCAGCATTCCACTCCTT
GACAAACGGGAGAGGTGCATTCTACCTTCTCTCCTGCATGAGCCTAGACTAATATGGAATTTGG
GAGTGTTGATCATTGTACTTTGTGCTCCTAGAGATAAGGGTCATCATAAAGCATCTCAGAGTCTC
ACTGTAGATCCACAGTTCCATTTCTTTATGGATCCCTTACCCCTACTTTGCCAAATTCTATGCAGA
TAGCAATAATAATTCAGACAGGGCTTCCTCACTGAAGGAGTTTGTGCTCTATATAGTTAATAAGC
TAGAGATAATGAAAAAAAAAAAAAACTCAACTTGAACTTTGGGTAAGAGAAACTTTTCAGACCAA
CGATCATTACCAATAACAGCTTCCACGCATGACCCTGCCCGGAGATTGGGGCCTGCACTACTGT
TTCCAGGAAACACATTCTTTTGAATTTTTTTATTTTCTTTGTAGAGATGGGGATCTCACTCTGTTGC
CCAGGCTGGTCTCAAACTCCTGGCTTCAAGCAATCCTCCTGCTTCGGCCTCCCAAACTTCTGGG
ATTACAGATATGAGCCACCACGGCTGGCTGACAGGCACCAAATTCTAGGAGAGGACTTGAATA
GAGTAGGCATCTGTCTCTCCCCATTCCTTTCAGGGACAGAGCTCTCCTGGTTGGGAGAAAGGGA
GAGGGAGAGGGAGGAGGATGAATAGTGAAGCACCAAACCTCAGAATTAAGGCCTTTTTCTTTTC
TTTCTTTCTTTCTTTTTTTTTTTTGAGATGGAGTTTCGCTCTTGTTGCCCAGGCTGGAGTGCAATGG
CACAATCTCGGCTCACCGCAACCTCTGCCTCCCAGGTTCAAGCGATTCTCCTGCCTCAGCCTCC
CGAGTAGCTGGGATTACAGGCATGCGCCACCACACCCAGTTAACTTTTTTGTATTTTTAGAGACG
GGGTTTCTCCATGTTGGTCAAGCTGGTCTCGAACTCCCGACCTCAGGTGCTCTGCCCGACTTAG
CCTCCCAAAGTGCTAGGATTACAGGCGTGAGCCACCATGCCCAGCCAGAATCAGGGCCTTTTTA
AAACTTCAGTGTCTTGTCTTTACCCTTCATGGTGCCTAGAAAAGCATCTGGCTCATAGGTGTTCA
GTGAATGATTGTTTCATTAGTGAAGAAATTCCCTTTGGAAATCTCATAGGAAACTTACCCTCTCC
AACCCTTCACCTGTGCATTATTCCAGCTCCCAAGGAGGCAGCAGAAACTGGGGTAATCACAAC
GGTCAACAGCAGCAAAGGCAGCACTTTCTGA

ID|56483|gene_id|ENSG00000224699|transcript_id|ENST00000596890 Homo sapiens lncRNA

AATGACCTGCTTAGAGTTCTGCACCCCCCAGCATGGAGAGCAGAGCGCAAAGATTTCTTCC
GGACTCCCACATTCCCCTTGGAATCCAAGAACTGGTCTACTGTCACAACACTTTAGTACCCTCTT
GTAAGCCTGTGGACAAATCCTCTGATGCCTTGATACCTCGGTTTTCCCCCACTATAAATGGGAAG
ATAAATTCACTAGAAACAGTACTTACCTTGCAGTGTCATTATGAGAATTTCATGAGACAATGCAA
TATAAAGCTCCTAGCACAGTTCCAGGCACCATTGTAAGCACTGAACAAATGAAAGCTGTTGTCA
TTAGGATCTCAAGAAATGACAGCTGAGGCAAATGTGGATTAGACTGCATTCAGCATTCTTAGCT
GCAAGCAACAGAAGCTGACGCTGGTTTAAAGAATAGGAAAGGAAGTTAATTAAAATGACATTG
GGTAGCTTGTGGAACTGGGAGGACTAGAGAGCTCAGTCTAAGTTCTAGTGACAAAGCTTCAAAT
CATACATGAGGAAAATGCCCCTCTGCAGGAGGAACGTAATCCTGCAATCACTGCTTCCCCTGAA
GCCAGATGCCTCTACTGCTGCCCTTCCCAGCAGTGATGGAGCCTGGATCACATACCTGGCTTTA
GTTGTAAAGGAGGCTGGAGAGAAAATGCTCTGCTCTATGTAACTGTCTTGAAAGTGGCCCACAC
ATAGCAAAGCCATTCAAAAGCGGGTGAGCAGCATGAAAGCATCCAGAAGGATTCTTGCTTTGC
ATGAGGGAGAACTCTTGCTCCCAGAGTGTGAGACCAGCAGGGGCATTTTCCTCATGTGTGATTA
GAAGCTTTGTCACTAGAAGCTTAGACTGAGTAGGACACCTCTGAGTGTGAATGTCCTACCTGAG
TTCTGCCTCATGCAAAGCAAGAATCCCTCTGGAGGGATTGACACCACAGCCTCTTCCACAATGT
CTCCAAACAACTTTACTGGGCAGCATTCCACTCCTTGACAAACGGGAGAGGTGCATTCTACCTT

CTCTCCTGCATGAGCCTAGACTAATATGGAATTTGGGAGTGTTGATCATTGTACTTTGTGCTCCTA
GAGATAAGGGTCATCATAAAGCATCTCAGAGTCTCACTGTAGATCCACAGTTCCATTTCTTTATG
GATCCCTTACCCCTACTTTGCCAAATTCTATGCAGATAGCAATAATAATTCAGACAGGGCTTCCT
CACTGAAGGAGTTTGTGCTCTATATAGTTAATAAGCTAGAGATAATGAAAAAAAAAAAAAACTCA
ACTTGAACTTTGGGTAAGAGAAACTTTTCAGACCAACGATCATTACCAATAACAGCTTCCACGC
ATGACCCTGCCCGGAGATTGGGGCCTGCACTACTGTTTCCAGGAAACACATTCTTTTGAATTTTT
TTATTTTCTTTGTAGAGATGGGGATCTCACTCTGTTGCCCAGGCTGGTCTCAAACTCCTGGCTTCA
AGCAATCCTCCTGCTTCGGCCTCCCAAACTTCTGGGATTACAGATATGAGCCACCACGGCTGGC
TGACAGGCACCAAATTCTAGGAGAGGACTTGAATAGAGTAGGCATCTGTCTCTCCCCATTCCTTT
CAGGGACAGAGCTCTCCTGGTTGGGAGAAAGGGAGAGGGAGAGGGAGGAGGATGAATAGTG
AAGCACCAAACCTCAGAATTAAGGCCTTTTTCTTTTCTTTCTTTCTTTCTTTTTTTTTTTTGAGATGG
AGTTTCGCTCTTGTTGCCCAGGCTGGAGTGCAATGGCACAATCTCGGCTCACCGCAACCTCTGC
CTCCCAGGTTCAAGCGATTCTCCTGCCTCAGCCTCCCGAGTAGCTGGGATTACAGGCATGCGCC
ACCACACCCAGTTAACTTTTTTGTATTTTTAGAGACGGGGTTTCTCCATGTTGGTCAAGCTGGTCT
CGAACTCCCGACCTCAGGTGCTCTGCCCGACTTAGCCTCCCAAAGTGCTAGGATTACAGGCGTG
AGCCACCATGCCCAGCCAGAATCAGGGCCTTTTTAAAACTTCAGTGTCTTGTCTTTACCCTTCAT
GGTGCCTAGAAAAGCATCTGGCTCATAGGTGTTCAGTGAATGATTGTTTCATTAGTGAAGAAATT
CCCTTTGGAAATCTCATAGGAAACTTACCCTCTCCAACCCTTCACCTGTGCATTATTCCAGCTCC
CAAGGAGGCAGCAGAAACTGGGGTAATCACAACGGTCAACAGCAGCAAAGGCAGCACTTTCT
GATTTACTTGACTGCTATGGATGCCACCTCTCTCCACGCCTCAGTTTAATATCTGTAAGTTTGGTC
AGTATCTCATTCAAGAACATTGCATTTACTCTGGTACTTAAAGATGAAAAATCAACATTTACAAA
GCAGATCCATTGTCAACCTAAATAACAAACAAAGAGAGTCTCTATAAAAGAAAAGAATATTTAT
TTGGGAATAGAGCATTGCATAGGAATATGCCTGCCTTAGTAATGCATGTATTCAGGGAGGTAGA
GGAAGACAAAGGTTTTTAACAGAAAAAAATGTGGAGAATTACATAATTGTTTTGAAATAATTATCC
TTGGCTATAAAGATCAATAACAAGGGTGATGCGAGTCCAAGGTTGGACAGGCAGTTGCTGGGC
AGATGTCTTTGCAGAAGTCTTTTTTGTGTAAGGTTGTAATGGCTTTTGTAGGAGGTTGTGGCTTTT
GCAATCTTTTGTGATAGCTTTTGTTACCAGGCATACAAGCATGAGAATGCTCTCTTCAGGGCCTT
CCCTAGCTCTATTTATCAGGGGTTTCTTGTTAGTGATTCCATTTTGATTCTGACAACTTTCATGGA
CATTATCTCATTTAATCTCACAGTCATTCCATGACTCTATCCCCATTTTGTGAATGAAGTAACTGA
GGTCTGGAGAGGTCAACTAACTGGTCAAGGTCATAGAACTTGAAAATTTTCATTGTCTTGGGTCT
CTAATACTGGGGCTGACCATTCTCCCAGGCAATGTTGAGTGAGTTGGCCCAGGTGAGTCGGCTC
CTCTGCAATTGTGTCTCCTCAACTAGTTTGAACAACACAGGCCTGTGAGCCAGTCCCATAAAGT
GGCTAGGGGTTCCCTTCTCCCCATTTCCCCTCATCAACAGGAGGACTTGTCTGTGTGCCTTCAGA
CACCACACAGTAAATCAACTGCCACATTCATTCTGGACCATCAGTATTTTGCCCCAGGAGAAAG
TCAGAGGCAAGCACACTAATATCTCATTGATATTTTCAATTGCCAAAGGGGAAGTGAGATGAAT
CTAATCCCATCTGTCAAGGTGGAGAAGAGCTGATTCGAAAGCAGCATAGATTACTTTAAGGAGA
TGACAGTTTATCACTCTCTGGACAAGGGAATATGAAATGAGGACTAACCTCCTTTATGAGTTGGT
AAAAACATTGATCAGCAAAGACAATGTCTGGGGAAATGAACAGCAAGTTCTCTGGCCCTTTTTC
CACTTTTCCTTTCCCCGAGACTTTATGTTATATATACACACTCACACAAACAGGACAGAATCCAC
CTCCTGCCCCTCAAGAAATAGTTAACATTTGTTGAGGATTAATTATTGCCAGGCTCTGTACTCCA
TGTTTTATATTTACTATCCAGAAGCCTTAATTGTTAAATGAGGTAAGTGTTATTATTAGAACCTTTG
TACAGATGTGGAAACTGAGGTGTATAGAGGTTGACTTGCCCAAGGTTACCCAGCTAGTGAAGAA
TGAAATCAGATGCAGTCCAGCCAACGCTTGGCCTGCCGTCAGTCACACGCAGTACACGTAGAT
CAGTCAGCCTGGAAAGCCACCTAGACAGCCCTGCTTCACTCCATCCTTGGTCAGCACCTTAGAG

AGCCCTGCTGACCCCAAGAGGATGTGCCTTTTACGGGTTGTAGAGGCTGAACCACAGCTGAGG
GGCTGGAATGAGTGGGGGCCTCAGACAGGGTGTCTGGAGAGCTGAGGCTCAAGGAAGGGTGG
AGGGGGTGCTGAAGCTGAAGGCCGTGGGTCTTGCCAGCAAGTGGGGCTCAGTGAGCCGAGGT
GCCTCTCAGTTACTTCCATGTGTGAATTTCCGGTGGAGGACAGTGATGGGCTCTGGGCCCTTGG
AAACCGGGACATCCAGCGTGTCCAGCCACAGTGCAGGCTGCCCTGGCTGCTGAGAGTCTCAGA
AAATGCCACTGTCATGGGTTAGGAAGGCCTGGGGACCACAACAATGTGACTTCAAAAAACCCA
AGAAGGAATGTGCACACCTGAGATGCTCAGAATGCATGTGTCAGTGTTCAGTTCTGACTCCTTG
CTTCAGCCAGGGACAGAAGAACCTTCTACAGTAAAGAACCACAAGAGAGGGCTTTGCTGCCAG
CCTCAGCCAGGGACACTTGGCGGCCAAAGCAAAGGTCACTCCAGGGCTGCAGATTTCAGCCTC
TGCGGCCAGAAACAAGCTTCCCAATTAATAATACTCTGGATCGGACAGAGATGGCTGCTCTGTC
TCAGGCGTGTTTGCCGTTGATCCCTGTGATTGATGCTGATAACATTAAGAGTCTGTCAGGGTGAT
ATTTCATGAATGTCTCATGTGATGGGATGATACAGTCGGGAAATGCAATTCCCTAGCTCAGGCA
GAATCAGAGATGACCCAAAGTGCTGATGGAAAGGCTGAGGCCCTTTCCAGCCTCTGGCAGGGT
AGGGAAAGCGGGTGAGGTGGGGGCTTGGGAGGGGCTGGGCTGACCTAGAGCTGTAACAGGG
GTCTCTCATGAAGGGCTGTGAAGAGAAACAGTATGTGAGAGAGGCAAGAAACTTTTCTCTGGGT
TTCCTCATGTAGAATGATTCCATCTGTGTAGGCCATGAAGGAGTCCAGGGAAGTTCACCCCAAA
ATGTTGAAACCACCTTTGCAAAGATTATGACAGTGAGAGAAGCCTGGCATGGCTGACTCTATCT
TGCTTCTAGCCTCACAGGCTGGCTGTTCTGTCTCATTCCTGGGCATAGGCCAAGCTAATCTTGGG
AGGGATTTATGGTTTAACTTTGAAGCCAGGATCATAATTGTCCCTCCCTAAAACTGACCCCTTTC
TTGTTCAGGGACTGAAACTGCCTTTGTAAGACTAATGAAAGGCCACAAAATTAGGATTATGGAA
GGGGCCTGAATTCTGATAAGATGTAGGCATAGCTTGCCTTTCTGTCATCGTTTATTGCACTGGCA
CTCACAAGATTTGTAACTTCCCCAATTGGTCCCATAGATAACATCACTATTGTAGAACCTCAGAT
TGGTCTTTTGAGATCTTTTTCAGACTTTGGTATTCTGGTGACTGACTCCACCCAGATCCCATGACT
CATGACTCAGCTGGTCCTGTGGTTCTTACCCAGTCTGACACAGCACCAGAGGACCGTTTTCCAC
ACTTCTATCTATGATTTCATCCCCAACCAATCAGCACCACCCATTCCCTAGCCCCCTGCCCACCA
AATTGATATCCATAAAAACCCTAACCTCTGAGTTCTCAGAGAAACTGATTGGAGTAATAAACTCC
TGTCTTCTGTGTGGCTAGCCTTATGTTAATTAAACTCTTTCTTAGGGCAGTACTGCTGTCAGTGAA
TCAGCTTTATCTGTGCAGTGGGCAAGAAGGACCCACTGGGTGACTACAATATGGCACTTTGGTA
TGTTGGTTATTTTAAGTTAGAGGCCTTTGATGCTGGAAGAAGCTTTACTCTGATATCTCCTTATCT
ACCACAAGACTGGATCTGCCAAAGAGAACACAATTGCGTTCCATTCCCTTCCTGAAATCTCATTA
TCTATGGCAGAAAAGAAGACTGGGGAATGTAATACACAGTATTACACAGTAATGTAACATACTA
CACAGTATTTACACAAAACATGTAACACAGAATGTACTACACAGTATTTTCACAAAACAATATCT
GTCTCTCCAACTCATTCAAATTCTAAAGAGTCATTTACACGTCAATTTGTGTCTTCTACGCCCATT
CATTCTCCCTAAGAATCATTTACTACTCCTCAAAAGAATTACCAACATTCTCCATCTCCCACTCCC
CTATGAGGAAGGGTATATAAGTTTCTGTCTGGATCTCATTAGATGACTGTGTGATCGTGCTTCTA
TGATCCCTACCCCACATGTTAAATGAATTTTGTATGCCTTTTTCTTTTATCAATCTGCCTTTTGTTA
GGGTATTTTCAGTGGACCTTCAGAAGGCAAAGGGGGAGCTTTCCTTCTTTGACCCTACAGCTATT
AGACTTGATTGAGTAAGACACACAATAATGAAAGTAAATAAGAAGAAAAACATTCATCCTGCTG
TCTCCAGAGTTTGAAATGTCTGCCTCAAACTTCGATTTCTATCTACCAAAAATTCATGCATTAATC
ATAAGGCCGATCTCAAAAGTGAATTTTTTTTTTTTTTTGAGAAGGAGTCTCACTCTGTTGCCTAGG
CTGGAGTGCAGTGGCACAATCTCAGCTCACTGCAACCTCTGCCTCCTGGGTTCAAGCGATTCTC
CTGCCTCAGCCTCCCAAGTAGCTGGGACTACAGGCACGTGCCGCCAAGCCTGGCTAATTTTTAT
ATTTTTAGTACAGACGGGGTTTCACCATATTGGCCAGGCTGGTCTCAAACTCCTGACCTCAAGTG
ATCCACCCACCTCTGCCTCCCAAAGTGCTGGGATTACAGGCGTGAGCCACCGCGCCTGGACGT

GAAAAGTTTTTTGAATTCCCCCATTAGAAATGATCTTCACCCTGACTCCCATAACACTTTGTACAT
TACTGTAAAAGCATAATATTCAAATATTTACAATATACCTCCTTCAAATATAAAATGGACACATG
CCGAAGACTTAGCAACTCATGAAGGAGGGAACCAATAATGTAGTTAAGCTAATTCAAAGAGCA
TTTTGAGGAAATGAATACTTATGGTCATTTAAAAGGAAATGTTAAAATTTCTAGGTTAAAAATAA
AACTTGCCATTGCATATATGGTCAAATGATTTTTGACAAGGGTGCCAAGACCATTCCATGGGGA
AAACACAGTCTTTTCAACAAATCGTGTTGGGAAAACTAGATATCCATGTTCAAAAGAATGAACC
CTTACCTTACCTTCCACCATATACAAAAATTAACTCAAAATGGATCAAAGACTTAAGCATAAGAG
CTAAAATTATAAAATTCTTAGAAGAAAACATAGGGAAAAAGGTCCATGGTATTATATTTGACAAT
GACTTTTTTTTTTTTTTTTGAGGCAAGATTTCACTCTGTCACCCTGGCTGGAGTTCAATGCAGTGG
CACAATCTCAGCTCACTGCAACCTCCACCTTTCAGGCTCAAGCAATCCCCCTACCTCAGCCCCT
AAGTAGCTGAGACTACAGGCATGCACCACCAAGCACAGCTAATTTTTGTATTTTTGTACAGAGTT
TCGCCATGTTGCCCAGGCTGGTCTCGAACTCCTGAGCTCAAGCTATTCACCTGCCTCAGCCTCC
CAAAGTGTTGGGATTACAGGTGTGAGCCACTGTGCCCAGCCCTGACCCCCACATTTTTAAACAA
AGCTTCTCTTCCATAATCAATTGCAAATCAGAAGATCTTTGAATCTATCTGCAGCCTGCAAGCCC
CCTATTCAAGACATCCCACCCTTTTAAACTTAAGCCAATGTGTAACCTCCTGGCAATCAATTTTG
CAATTTATGATTTTGCCTGTAGCTTCTGCTGTTCTGAAATTTATTCCTGCCTCTAAAAATCCTTGCC
GGCAAGCCACTGGGGAGGCTAGGTTTTGAGCACTTAGCTGTCTGGTCTTCCTTACTTAGTGCTCT
GCAATAAACACCTTCCTTTCTATCACCACAAAATCCTGGGTGTGGGTATCTGGTTTTACTGTGTTG
GGTGAGCAGACCTCAGTTTGGTTCTGTTACAAGTCCCTGCTCCATCTCACTTGCTACAACATGAA
TGAAACTTGAGGACATTATGCAAAATGAAATAAGCCAGTCACAAAAAGACAAATACTGTATTAT
TCCACTTATATGAGGTACTTATGATAGACAAAATCATCAAGACAGAAGGTATGATGGTTGCCAG
GGGTTGAGGGAAGACAAAATAGGGAGTTATTGTTTAATGGTTGAGAAGTTTCAGTTTTGCAAGA
TGAAAAGTGTTCTGGAGATGGATGGTGGTGATGGTTGCATGACATAAATGTGCTTAATACCACT
GAACTGTTCTTAACTCCGCTGAAATATGGTTAAGATGGCCCGGGTGCAGTGGCTCATGCCTGTA
ATCCTAACACTTTGGAACGCCGAGGCGGGCAGATCACCTGAGATCAGGAGTTCGAGACCAGCC
TGACCAATATGGCGAAACCCAGTCTCCACTAAAAATACAAAAATTAGCGAGGTGTGGTGGTGC
GCACCTGTAATTCAAGCCACTTGGGAGGCTGAGGCAGGAGAATTTTCTGAATCCAGGATGCAG
AGGTTGCAGTGTACTGAGATCGCACCACTGCACTCCAGCCTGAGCAACAGAGCAAGACTCCAT
CTCAAAAAAAAAAAAAAAGGTTAAGATGGTAAACTTTGTACCAATTAAAAAACAAAGCTGGCTAA
CACCCTACAGGGCAATTAAAACATCATAATCTTTGGTGTTATCTTTTTTATTAGATAGAAATTATT
TATACTTACTGGATAAGATCTGTAAGTTAACACATAACTTCACAGAGTCTGAGCCCTTAATGAAT
AATAAATAGCCAAGTCACAGGGAGATACTTTCCCTGAGGTAATTAAGCAATCCTTGGGTAGGCT
TGTGAAGAAATGCCCAGAATGACGTTGGAAGGACATGCTGTTCTCTCTTGGCCTTATTTCCAAG
GTTTGGACTCAAAGTATTATTTTTTAACATCACAGAAATTTTTTATGCCCTGGTCTCTTGAGGGTA
GGGACTGTGTCTTTTTCATCTTTGCCCCTTTCCACATGCTCTGGTTTGTAGAACAGAATTGAAGAA
GGAGCCATGTGCTAGATAGAGCGGGTTGGGAGCAGCAGACCTGCCTGCTCATTGTTTCTGTGAC
CTTGACCCATCCTGTAGTCTCCCCACGCCTTGCTATTCTCATCCATCAACGATGAGATTGCACCT
AAATCCTGGGTGCTGTGCTGGGCCTGGGGATGCTGTCTAATCCCTGGGTTTCCTTCTAGTTTGCT
CTAATCACCCATATGAAAAAGAAGCTACTCAAGGCAAACATATAAATGAAACGGATAATGAATG
AATAGTATTTTTTCTTGCTTTCTTTGGAAGAGGCATCCTGGATTCTACTTGAAGCCTGCAGCCCTG
ACTCCTGGGCTGGGAGGTGGGCGAGGACTTGTATTTTGTAGCTCTGCTAGAGAGGGAACAGCT
GTCCACTGGTGGCAGGACTGACGCCTCCCAGAATACAGGGGCTTTGGATCAGCAGGCGTCAGA
GGGAAAGGAAACTGGCATCCGAGTCTGCCTTTGAGAAAGGAGTAAATTACTATCTTATTATAAA
TTCATGATCCACTCATTGCTGTTTTCAATTAATTTCTTGACAAATCTGGCTGTTCCTTTTCTTATTTT

CCTTAGGACAGGAGGTGTCCTGGTGATCAGCTGGAGCTCGGGGGAGCTGTTGCTGAAATTAAC
TTGAGTGCTTTGGAGAGAGATTTACATGGCAGAGTGGAGCCAGGGCTGCTCCTTTGGCAGGCA
CTGCTGTGCCACTGTGGAGGCGCCCCTGCTTTTCCCATGCTTTGCAGTGTCTTTAAAGGCCTTTG
GAGCCATGCCCAGGCTCAGCCCCATCCTGGTGTCCCCCTGTGTCCGTTTTGCCACTGCCCGGCT
ACTTCACTTGGCCATGGGCCTTACCAGCTTGCAGACAAACCCGTCCATCTACGCCTCCCAGCGC
AGCTTGGCCCACTTGCAAGCACTTACAGAGGGTTTCAGAAGGAACATGTCTGCCATCTGTGTTT
GGAGACTGTGTCCCAGACTTTTGAGCTCACATTAGTCATGGCCACAGACGGGACTGGAGTCCTG
CCCACTGGAGTCCCAAAGGTCTCTGGTGTTTGTTTTCCACTCTGCATGCCTGGGTTGGGG

ID|56489|gene_id|ENSG00000224699|transcript_id|ENST00000608253 Homo sapiens lncRNA

AAGACGACTTTTAACTTCTTGGAGAATATCCTTTCAGATAGTTGTCTTTGTAAATGTATACTT
TTAATGTTTTTTCGTTTGCCTTTTTAAAATAAATTTTTTGGGGGACAGGGTCTTGCTCTGTTACCCA
GGCTGTAGTGCAGTAGAACGATTATAGCTCATTGCAACCCGGACGTCCTGGGTTCAAGCTATCC
TCACCTCTCAGCCTCTCGACTGGCTAGGACCACAGCCTCGAGCCATCATGTCTGGCTAATTTTTA
CGTTTTTTTGTAGAGATGGGGGTCTCGCTGTGTTGTCCAGGCTGGTCTTAAACTCCTTGCTTCAAC
AGATCCTCCCACTCCAGCCTCCCAAAATGTGATTACAGGCTGAGTCACCACGCCTAGCATGGTT
TTTTTTGTTTTGTTTGTTTTGTAAGCAAAATTGACATACATACTGGAGGTACAATTCTGTAGCTGG
CCTTCTTTACAGAGTTTAGAATAAATGTCTTTCCATGTCAAGTTTTCACTTACATCCTTTGTAGTAG
TTGCAAAATATTCCATTTTATAGCTGTATCTTAATTGTGGATTAAGGTTGGAGTGCAGTGGCACTA
TCATGGCTTAGTCCAGCCTCAGCCTTCTAGGTTCAAGCGATCTTCCCACCTCAGCCTCCCAAGAA
GCTGGGACTACAGGGACCCGCCACCCCACCTAGCTAATTAAAAAAAAAATTGTAGAGATGGAG
TCTCAGTGTGTAGCCCAGTCTCAAGTGACCCTCCCTCGTTGGCTTCCCAAAGCGCTGAGATTGC
AGGTGAGCCACTGTACTTGGACTTTTTTTTTTTTTTTTGCTTTTGTTTGTTTTTTGTTTTAGCTATTAC
AATGCTGGTTGAATATCCTCATAGCTAAATATCTGTGCAGTTTTAAAATTTCCTTATAAGTTACTA
GAAATGGAATGACTGGGTTAAAGGACATGATCATTTTTTAAGACTCTTGATACATACTTACAAAT
TGCCTCCCGAAAAAGTGGTGCTAATTTACTCAAATTGGTAAGCCTCACCCCCTCTGTGGATCATT
CCTTGCACTAAGTTTCTTCCAACTCTGTCACCTCCAGCTTTTTACTCACAGCCTACCTTCCAATCT
GGGGCCCAGCTCCCAGATCGGAAGATACCAGACTTTGGTATTGAACCAAAGTCCAATACACTG
CACTGGCATTCAGAGTCCCTTTTTATTCCGCAGCTGTCATAGTGAAGCTTGCAGCTTCCTTGAAA
TTCCAGTTTGTTTATTTCTTTATTTTTTTGAGACAGAGTCTTGCTCTATCGCCCAGGCTGGAGTGCA
GTGGCGCCGTCTCAGCTCACTGCAACCTCTGCCTCCTGGTTCAAGCGATTCTTCTGCCTCAGCCT
CCCGAGTAGCTGGGATTACAGGCATGCACCACCACACGTGGCTAATTTTTGTGCTTTTAGTAGA
GATGGGGTTTCACCATGTTGGCCAGGCTGGTCTCAAACTCCTGACCTCAAGCGATCCGCCCACC
TCAGCCTCCCAAAGTGCTGGGATTATAGGCATGAGCCATTGCACCCAGCCAAAATTCCAGTTTG
AATGTTATAATTCAGGGGTCTTGGACTCCTGCTCTAGAGAAATTATTCATGTTAAGTTAGGGGTA
ACACAGTACAGGCCATATAAGTTTTAGAAAAAGCAGTTTAAAACTTAGCATTAGAAAACATAAG
TACTGCCTGGTGAAATGGCACGTGCCTATAATCCCAGCTACTCAGGAGGCTGAGGCAGGAGGA
TCCCTTGAGCCCAGGAGTTCAAGTCCAGCCTGGGCAATATAGTCTAGTGGCCACATTAAAAAAA
GTAAAACAGCCGGGCGCGGTGGCTCACGCCTGTAATCCCAGCACTTTGGGAGGCCGAGGCGG
GTGGATCACGAGGTCAGGAGATTGAGACCATCCTGGCTAACACGGTGAAACCCTGTCTCCACT
AAAAATACAAAAAATTCTCCGGGTGTGGTGGTGGGCGCCTGTGGTCCCAGCTACTCAGGAGGC
TGAGGCAGGAGAATGGTGTGAGCCTGGGAGGCGGAGCTTGCAGTGAGCCGAGATCGCGCCAC
TGCACTCCAGCCTGGGCGACAGAGCGAGACTCTGTCTCAAAAAAAAAAAAAAAAAAAAAAAAAA
AAGTAAAACAGGGCAGGGTGCAGTGGCTCACGCCTGTAATCCTAGCACTTTGGGAGGCCGAGA

TGGGTGGATCAGTTGAGGTCAGGAGTTTGAGACCAGCCTGGCCAACATGGCGAAACCCCATCT
CTACTAAAAATACAAAAATTAGCTGGGTGTGATGGTGCGTGCCTGTAGTCCCAGCTACTCAGGA
GGCTGAGGCAGGAGGATCGTTTGAACTCGGGAGCTGGAGGTTGCAGTGAGCCGAGATTGAGC
CACAGCACTCCAGGCTGTGCTACAGTGTGAGACTGTCTCAAAAAAAAGTAAAACAGATGAAAC
TAATTTTAATAACATTTTATTTATTCAAACAATATATTCAAAATATTATTTCAACTTGTGATTGATA
AAATACTAATGAGATATTTTATATTTTTTCTCCAGTACTGTCTTCAAAATCCAGGGTATATTTTTCA
GTTAGCAGCACATTTCAATTCAGACCAGCCGCATTTCAAGTGCTCAGCAGCCACCTGTGGCTAG
TGGCCACAGTATTGGCCAGTGTAGCCCAAGGATATCTCAAATTATCTCAAAGCTAGATCAGTGA
TTAAAGGATTCTCAGGCCAGGCATGGTAGTTTTCAGGCCTGGTATGGTGGTTTACGCCTGTAATC
CCCGCACTTTGGGAGGCCTAGGTGGGCGGATGGCTTGAGCCCAGGAGTTTGAGACCAGCCTAG
CCAACGTGGGGAGACCCTGTCTCTACCAAAGCACAAAAATTAGCTGGGCATGGTGATGCGCAC
CTGTGGTCTCAAGTACTTTGTGTGAGGTGGGAGGATCACTTGAGCCCGGGAGGTTGAGGCTGCA
GTGAGCTGAGATTGCACAACTGCACTCCAGCCTGGGTGACAGAATGAGACCCTGTCTCAAAAA
AAAAAAAGGATTCTCCGAAAGACTGGCGATTGCTTTTTAAGCTATAAGGAAGGCCTTTGAAGGG
AAATCAAGTAGGAAGAGGATGATAGCTGGCTCACATGTCCTTGCCACATGGGAGGTGAGAGAG
CAGGCTACTCCTTACCACCCCCGCTTTCTCTGCCCTGCTGCTGTCTTGCCCCTGTGCAAGTAGGC
AATGGGAGAGGAATAGTATATATAAATTGGGCAAGTGCTAGGGGCTGGAGTGATGGTTGGGGA
AAGTATCTGACCCTTGTTTTCTACTTTCTTTAGCTTGTGGGGTATTTAACAATTCTTTGTTCTTAGTT
TTGAGTGCTGGCAACCATTTCTGGGGTGACAGGAAAAGTTCCATGACATCCGTATTAGAGCATT
TGTTTCATTATATTGTAATTTTTTATGTTTACTTTTGAAAAAGTTCATTCTTTTCTCTTTTTAATTCTT
ATTCTTTTTTTTTTCTTTTCTTTTTGAGGCAGGGTTTTACTCTATCACCCAGGCTGGAGTGCAGTGG
CACAATCTTGGCTCACTGCAACCTCTGCCTCCTGAGCTCGTGATTCATCCTCCTGAGTAGCTGGG
ACTACAGGTGTACACTACCATGTGTAGCTAAATTTTTTGTATTTTTTGTAGAGATGGGGTTTTGCC
ATGTTGCCAGGCTGGTCTCGAACTCCTGCAATTTGCTCTCCTTGGCCTCACGAAATGCTGGGATT
ACAGGCATAAGCTACAGCACCCAGCCCTTACGTTTACTTTTCATTGACAGGAAGAGTGTCTTCAT
CTCTGCATCCCTAGTGCAACAGAGCATGACAAAAATTCAACAAATATTTATTGAAGAGTCTTGAT
GTCTTTTGGGAAAGGAACTCTATGGTTACCTTTACTCTAAGGTAAAGAAATTTAGGTCCTGAACT
TTAACCACCTTCTCCTCTTCTTTCTTCCCTCTTTCATAAATGAGCTCTTGGAAAATTAAAATGCCA
GGAGGGTCCAGCAGAGAATACCCAAGCCCACATGTCCTGATGGGAAGTGGGGGATAAGTAGA
GAGGGAAACACAGACCCTGTAGGCCATCTCACTGCTGCTGGCCCAGGGGACCCTAACTCCTTTT
GTTGACTCATCAGAAGTAGAATCCTCCTGAGGGTCTGTTTAAGAAGCACAGCTCTGGTAGAAAG
GGTAGCTTTCCTATGGGTACGGAGGGCTCTGCTACCCCAGGGCTATCCACACTGGCCTGTGAAT
CACTCTCAAATGCTTAGGGTCACGGGCTTTCAGTGGAATGAGCCCTCATGTGCCACCAGGGGGC
ACCAAGGCCACAGAAAACCCTATTCAGAAGGTTCTGGGGTGAGGGAAAGGAATCCACGGGTTT
GTGGAGCTGGCGGAGCATGGGTGAGGACAGCATTATGCTAGGTTGGTGGCCTTGAAGTCAGAT
GTAAATTGGGATCCTGGCTTGGCCACTGACTATCCATGGGAACTTTGAATCTCAGGGGGCTCAT
TTGTGATGTGGGGGTACCATTATCTGCCTCATCAGGTTGTGGGGAGGATTATATGAGATACCTTA
TGTAAGAGGGGCTGGTGCATTGTGGAAGTCAACAAGTAAGTTTTGTTAAATTTGAGTAAGAAGA
CCTGTTTGTTTCCTCAAGTTGCTTATAATCTAGGTGAGGACACATCTGAGAGAATAACTAATAGT
TCAGTGAAGGTAAGAGCCAAATGATTGGCACAGACCTCAAGTGGTCTGGCATTTACGGGAGAG
GGGGATTGTGAAACAGGAATTGAAAGAAATTTAAAAATGCATAAGCAAAAACTCAGTTTATGTT
TTAAAAAAACCAATTCCCCCTGAGAAAGAGAAAGAGGTGGAGTCCTTTAAAAATTATCTGCTTG
TTTTTCTGTCTGTGGCTAGTGAGCCTTATCTCTCCCTTTCCCAGGCATTGTGAAGACCCTGTTTCT
CTAGCTGTGCAGCTGCAAGGTCACTAGGCAGATAAACTCAAGCCATAAAACATGTTTTTCCTTG

AAAAGTAAGAAATAATGTAATGCATGTGTCAAATGAATAACTATTTCTCGCTTCTGTAATATGCTT
CCCTTCGCACAGATCTCCCCCTGCCCCATGAAATGCTTTAAAGGTAACTTAACTCTTTGTTCGGG
GCTCAGTCCTTTGGATGTTAATCTGACTGGCTTGGTGCACCTAAATAATAAATAACATATATCCT
CCTCAACCCCTCAGTCTCTCTGATTCCTAAATTATCCCGCTGTAATTGCAGGGCTAGATTTGCCG
ACCTGCTTTATAAAGCTATGTTAAATCAGTATATTTTATTCTTTTTATTAGACTTTTCTATAGCATA
AAAATGATAAAATACAGCAAGAAAAAAAGTCACCTGAAACTCTAGTCCTAACCCTAATATGAAT
GTGAAGGAGCTGGGCTAGAATGGAATAGGGTAGAATTTGCATAAGGTGGAAATGGGGACAGG
CCTGTCACAAGGATCACTAGAGTCTTGCTGGCAAAAGCATCTTACTGCATTCATGCTGCAGCAC
AGGACCCAGATGCAGAACAGGTTGGGGACACAGCCCTGCCTTCTAATAGTGGACACCATCTGA
GAGCTGAGCCATAGATTCAAAGCCTAACTCCTAATAGCGATTGGTAGTAATATCATCAGCAGTT
CATATACATGGAGTGACAACAGCGTGCCAGGCAATTCAAAGGGTCTCTCGGTCCTCACAGCATT
TTTGAAGTAGCTAACATCACTATCCCCCTTTTACAGTTAAAAACAACAACAACAACAACAAAAA
CACAAAACCCTGAGGCTCAGAAGATAAATATTTGCTGAAGATCACTGTGCTTGTAAGTGGTGGA
GCCTGACCATTAACTTCCCCACGACACTGCTTCAATTTCTATCACTTCATCAACCACACAACCCG
AGAACTGTGAGGGGATTTAGCAGGCCCAGTCCAGATCTCTTATTGTACAGAGAAGAAATCTGAG
GCTCAGAGAGGGGAAGGGACTAATTTGGACCTTTAACTTCGGATTCTTAACTTTCTTTCTGGTGT
TCTAACTATACTGTACTACAAGAGGCTGTCTTAGAGCTTTAGTTCAACCAGCTTCAGAGATACTC
ATTGTTATGGGTTGAATTATGTCTCCCCAAAATTCATATGTCAAAGTTCTAACCCCCAGTACCTCA
GAATGTGACCATATTTGGAGATAGGACCTTTACAGAGGTAATCAGGTGAAAATGAGGTTGTTAG
GGTGGGCCTTAGTCCAGTATGGCTGGTGTCCTCATAAAAAGGGGAAATTTGGACACAGATGTAC
ATAGAGGGAAGATGGTGTGAAGAGACATAAAGAACAGATAGTCATCTATAGCCAAGGCCTGGA
ACAGATCCTTCCCTAACAGCCCTCAGAAGGAAGCAACCTCACTGACACCTTGATTTTGGACTGC
TACCCTCTGGCACTGTGAGACAATAAATTCATGTTGGTTAGGCCACCCAGTTTGTTATGGCAGCC
CTAGGAAACCTTTGTGGATATATCTGTTCGTATACCCATAAAGTCTGCAAGTCAAAAATATCACA
GACTTTTGGCAGCCCTTCATTCAAGCACTGGCTGGGAGGGTCACCCTCCCCGAGGCAAAAGGA
TAGAGCAAATGATCTTTCCAGCTTGCTCCCAACCTTGATCTTTGGCCAAGATATTTTTCTTTCACT
GGACATTGGCCAACATTGGTGTCATAACTTAATTTGACTGTGACCTTCTTGAGTCTTTTATGTCTC
TGAGCTTGACCTGTAGAAGAAGCTCAATAAATGTGGGTAGAGGGGATGAGTGGGAAAATCAGC
TCCCAGCCATTCCAGTTCAAGCCTCTTCAAGCAGAAGAGTCCCACCTGAAAGACCTCATTTTCA
CTTGATTACTCGATCTCCAAGCAATAGGTGGGGCAGGAAGCAGGGTATGTAAGGCCTGTCAGT
GGATGGAAGTTGAAGTTGGACACCATCTCCTAAAGTTTAGAGCTGAGCCCAAATTCTGGAAAAC
ACAAGCAAAATACATTTTATATGACTCAGGATGGGCTAAAGAAGTTCCAGTACAGGAAACTGTT
TTCCTGCTGCTTTATTAATTTTGCTACAGAGGATAAATGAGATGCAGACTTTCATATCCAAGTCTT
CATTAGATGCCCAGTTAATGCAGCCATGACAAATGGGAATGAAGCCCTTATGAATAAAAAAAAG
ATCAACCCAGTATAAAAAAATCATAGACCAGAGAAATGAAAATTTAGAGGAGCTGATAAAATA
CCCTATCCTTAATTAGACTGTCAGCATACACATCAAATCAGCCTTAATGCCAAGATTTATTGGCC
TTGGAATTGGGTAAATGAAATAAGTGCTTACAAATGAATGGAAAAGAAATTACCTGTATTAAAA
CGTATTTGTGATGCAGGCCTGTGCACTGAGGGTTTTAGTCAAACTCAGGCTTGCAGCTTCTTTTG
CTCTGCATCCATTTCCACCCCATGTTCCCCAGTGGGTTTCAGAGACCCTCTTTGTGGGGTAACAA
TATGTACCATTGTCAACTACCTCTGCTGTTGTCCTGGAAAAATCCTTCTTGGACAGTAAAATATTT
TTGTCTTTATTTCACCATCTCCAGATTAGATGGGTGTTACTGATTCTTACCTCAGAGAAGAGAATG
TCATGGAGACAAAATGACCTGCTTAGAGTTCTGCACCCCCCAGCATGGAGAGCAGAGCGCAAA
GATTTCTTCCGGACTCCCACATTCCCCTTGGAATCCAAGAACTGGTCTACTGTCACAACACTTTA
GTACCCTCTTGTAAGCCTGTGGACAAATCCTCTGATGCCTTGATACCTCGGTTTTCCCCCACTAT

AAATGGGAAGATAAATTCACTAGAAACAGTACTTACCTTGCAGTGTCATTATGAGAATTTCATGA
GACAATGCAATATAAAGCTCCTAGCACAGTTCCAGGCACCATTGTAAGCACTGAACAAATGAAA
GCTGTTGTCATTAGGATCTCAAGAAATGACAGCTGAGGCAAATGTGGATTAGACTGCATTCAGC
ATTCTTAGCTGCAAGCAACAGAAGCTGACGCTGGTTTAAAGAATAGGAAAGGAAGTTAATTAAA
ATGACATTGGGTAGCTTGTGGAACTGGGAGGACTAGAGAGCTCAGTCTAAGTTCTAGTGACAAA
GCTTCAAATCATACATGAGGAAAATGCCCCTCTGCAGGAGGAACGTAATCCTGCAATCACTGCT
TCCCCTGAAGCCAGATGCCTCTACTGCTGCCCTTCCCAGCAGTGATGGAGCCTGGATCACATAC
CTGGCTTTAGTTGTAAAGGAGGCTGGAGAGAAAATGCTCTGCTCTATGTAACTGTCTTGAAAGT
GGCCCACACATAGCAAAGCCATTCAAAAGCGGGTGAGCAGCATGAAAGCATCCAGAAGGATTC
TTGCTTTGCATGAGGGAGAACTCTTGCTCCCAGAGTGTGAGACCAGCAGGGGCATTTTCCTCAT
GTGTGATTAGAAGCTTTGTCACTAGAAGCTTAGACTGAGTAGGACACCTCTGAGTGTGAATGTC
CTACCTGAGTTCTGCCTCATGCAAAGCAAGAATCCCTCTGGAGGGATTGACACCACAGCCTCTT
CCACAATGTCTCCAAACAACTTTACTGGGCAGCATTCCACTCCTTGACAAACGGGAGAGGTGCA
TTCTACCTTCTCTCCTGCATGAGCCTAGACTAATATGGAATTTGGGAGTGTTGATCATTGTACTTT
GTGCTCCTAGAGATAAGGGTCATCATAAAGCATCTCAGAGTCTCACTGTAGATCCACAGTTCCA
TTTCTTTATGGATCCCTTACCCCTACTTTGCCAAATTCTATGCAGATAGCAATAATAATTCAGACA
GGGCTTCCTCACTGAAGGAGTTTGTGCTCTATATAGTTAATAAGCTAGAGATAATGAAAAAAAA
AAAAACTCAACTTGAACTTTGGGTAAGAGAAACTTTTCAGACCAACGATCATTACCAATAACAG
CTTCCACGCATGACCCTGCCCGGAGATTGGGGCCTGCACTACTGTTTCCAGGAAACACATTCTT
TTGAATTTTTTTATTTTCTTTGTAGAGATGGGGATCTCACTCTGTTGCCCAGGCTGGTCTCAAACT
CCTGGCTTCAAGCAATCCTCCTGCTTCGGCCTCCCAAACTTCTGGGATTACAGATATGAGCCAC
CACGGCTGGCTGACAGGCACCAAATTCTAGGAGAGGACTTGAATAGAGTAGGCATCTGTCTCTC
CCCATTCCTTTCAGGGACAGAGCTCTCCTGGTTGGGAGAAAGGGAGAGGGAGAGGGAGGAGG
ATGAATAGTGAAGCACCAAACCTCAGAATTAAGGCCTTTTTCTTTTCTTTCTTTCTTTCTTTTTTTTT
TTTGAGATGGAGTTTCGCTCTTGTTGCCCAGGCTGGAGTGCAATGGCACAATCTCGGCTCACCG
CAACCTCTGCCTCCCAGGTTCAAGCGATTCTCCTGCCTCAGCCTCCCGAGTAGCTGGGATTACA
GGCATGCGCCACCACACCCAGTTAACTTTTTTGTATTTTTAGAGACGGGGTTTCTCCATGTTGGT
CAAGCTGGTCTCGAACTCCCGACCTCAGGTGCTCTGCCCGACTTAGCCTCCCAAAGTGCTAGGA
TTACAGGCGTGAGCCACCATGCCCAGCCAGAATCAGGGCCTTTTTAAAACTTCAGTGTCTTGTCT
TTACCCTTCATGGTGCCTAGAAAAGCATCTGGCTCATAGGTGTTCAGTGAATGATTGTTTCATTA
GTGAAGAAATTCCCTTTGGAAATCTCATAGGAAACTTACCCTCTCCAACCCTTCACCTGTGCATT
ATTCCAGCTCCCAAGGAGGCAGCAGAAACTGGGGTAATCACAACGGTCAACAGCAGCAAAGG
CAGCACTTTCTGA

ID|56494|gene_id|ENSG00000224699|transcript_id|ENST00000609709 Homo sapiens lncRNA

CGCGCGGTGACCGTCGAGGTGACCTGCACCTGGCTCCATGGCGGAACCGGCCGGCACGG
ATTATTCCCCTCTCGGGAGAGGTCTGCAGCCGGGCCTCAGTGCTTCAGTTCGTGGCCTGGCATT
TTGTGTCAGTGTTTTTTAACCTTTGGACCCTAGTTCAACCGACTCCGAGACCTAGGGGACAGATC
TTAAGTTTTACAATAACCCAGGGGTCAGACAGGGTCTGATCTAGCCTGTGGGGCAAGTACGGG
GAAGACACGGGACCTCTTGGAGGGGGTCAGTAGGCTTGTAGGGGGAAAGACGCACACAGAGA
CTAAAGGACGAGATGGAGCAAGGTTCAACCCCCTAGCTGAGTGTTGGCAGTGTCGGGATTTTA
ACGAAGACATTGGTGCTTATTATGGTGAGGGGACTCAGGACAGTTTAGGGGAAGAGAGTGGCA
TTTGAGTCCTAACTCGTAAGGAAGACTGGGTAGGACTACTTGAAAATAACTTTTAAAAATTAATT
ATAAAGCTGGTAAAAATAGGAAAACATCTATGGCCCCACTGTCCAAAGACGACTTTTAACTTCTT

GGAGAATATCCTTTCAGATAGTTGTCTTTGTAAATGTATACTTTTAATGTTTTTTCGTTTGCCTTTTT
AAAATAAATTTTTTGGGGGACAGGGTCTTGCTCTGTTACCCAGGCTGTAGTGCAGTAGAACGATT
ATAGCTCATTGCAACCCGGACGTCCTGGGTTCAAGCTATCCTCACCTCTCAGCCTCTCGACTGG
CTAGGACCACAGCCTCGAGCCATCATGTCTGGCTAATTTTTACGTTTTTTTGTAGAGATGGGGGT
CTCGCTGTGTTGTCCAGGCTGGTCTTAAACTCCTTGCTTCAACAGATCCTCCCACTCCAGCCTCC
CAAAATGTGATTACAGGCTGAGTCACCACGCCTAGCATGGTTTTTTTTGTTTTGTTTGTTTTGTAA
GCAAAATTGACATACATACTGGAGGTACAATTCTGTAGCTGGCCTTCTTTACAGAGTTTAGAATA
AATGTCTTTCCATGTCAAGTTTTCACTTACATCCTTTGTAGTAGTTGCAAAATATTCCATTTTATAG
CTGTATCTTAATTGTGGATTAAGGTTGGAGTGCAGTGGCACTATCATGGCTTAGTCCAGCCTCAG
CCTTCTAGGTTCAAGCGATCTTCCCACCTCAGCCTCCCAAGAAGCTGGGACTACAGGGACCCGC
CACCCCACCTAGCTAATTAAAAAAAAAAATTGTAGAGATGGAGTCTCAGTGTGTAGCCCAGTCTC
AAGTGACCCTCCCTCGTTGGCTTCCCAAAGCGCTGAGATTGCAGGTGAGCCACTGTACTTGGAC
TTTTTTTTTTTTTTTTTGCTTTTGTTTGTTTTTTGTTTTAGCTATTACAATGCTGGTTGAATATCCTCAT
AGCTAAATATCTGTGCAGTTTTAAAATTTCCTTATAAGTTACTAGAAATGGAATGACTGGGTTAA
AGGACATGATCATTTTTTAAGACTCTTGATACATACTTACAAATTGCCTCCCGAAAAAGTGGTGC
TAATTTACTCAAATTGGTAAGCCTCACCCCCTCTGTGGATCATTCCTTGCACTAAGTTTCTTCCAA
CTCTGTCACCTCCAGCTTTTTACTCACAGCCTACCTTCCAATCTGGGGCCCAGCTCCCAGATCGG
AAGATACCAGACTTTGGTATTGAACCAAAGTCCAATACACTGCACTGGCATTCAGAGTCCCTTTT
TATTCCGCAGCTGTCATAGTGAAGCTTGCAGCTTCCTTGAAATTCCAGTTTGTTTATTTCTTTATTT
TTTTGAGACAGAGTCTTGCTCTATCGCCCAGGCTGGAGTGCAGTGGCGCCGTCTCAGCTCACTG
CAACCTCTGCCTCCTGGTTCAAGCGATTCTTCTGCCTCAGCCTCCCGAGTAGCTGGGATTACAG
GCATGCACCACCACACGTGGCTAATTTTTGTGCTTTTAGTAGAGATGGGGTTTCACCATGTTGGC
CAGGCTGGTCTCAAACTCCTGACCTCAAGCGATCCGCCCACCTCAGCCTCCCAAAGTGCTGGG
ATTATAGGCATGAGCCATTGCACCCAGCCAAAATTCCAGTTTGAATGTTATAATTCAGGGGTCTT
GGACTCCTGCTCTAGAGAAATTATTCATGTTAAGTTAGGGGTAACACAGTACAGGCCATATAAG
TTTTAGAAAAAGCAGTTTAAAACTTAGCATTAGAAAACATAAGTACTGCCTGGTGAAATGGCAC
GTGCCTATAATCCCAGCTACTCAGGAGGCTGAGGCAGGAGGATCCCTTGAGCCCAGGAGTTCA
AGTCCAGCCTGGGCAATATAGTCTAGTGGCCACATTAAAAAAAGTAAAACAGCCGGGCGCGGT
GGCTCACGCCTGTAATCCCAGCACTTTGGGAGGCCGAGGCGGGTGGATCACGAGGTCAGGAG
ATTGAGACCATCCTGGCTAACACGGTGAAACCCTGTCTCCACTAAAAATACAAAAAATTCTCCG
GGTGTGGTGGTGGGCGCCTGTGGTCCCAGCTACTCAGGAGGCTGAGGCAGGAGAATGGTGTGA
GCCTGGGAGGCGGAGCTTGCAGTGAGCCGAGATCGCGCCACTGCACTCCAGCCTGGGCGACA
GAGCGAGACTCTGTCTCAAAAAAAAAAAAAAAAAAAAAAAAAAAAAGTAAAACAGGGCAGGGTG
CAGTGGCTCACGCCTGTAATCCTAGCACTTTGGGAGGCCGAGATGGGTGGATCAGTTGAGGTC
AGGAGTTTGAGACCAGCCTGGCCAACATGGCGAAACCCCATCTCTACTAAAAATACAAAAATTA
GCTGGGTGTGATGGTGCGTGCCTGTAGTCCCAGCTACTCAGGAGGCTGAGGCAGGAGGATCGT
TTGAACTCGGGAGCTGGAGGTTGCAGTGAGCCGAGATTGAGCCACAGCACTCCAGGCTGTGCT
ACAGTGTGAGACTGTCTCAAAAAAAAAGTAAAACAGATGAAACTAATTTTAATAACATTTTATTTA
TTCAAACAATATATTCAAAATATTATTTCAACTTGTGATTGATAAAATACTAATGAGATATTTTATA
TTTTTTCTCCAGTACTGTCTTCAAAATCCAGGGTATATTTTTCAGTTAGCAGCACATTTCAATTCA
GACCAGCCGCATTTCAAGTGCTCAGCAGCCACCTGTGGCTAGTGGCCACAGTATTGGCCAGTGT
AGCCCAAGGATATCTCAAATTATCTCAAAGCTAGATCAGTGATTAAAGGATTCTCAGGCCAGGC
ATGGTAGTTTTCAGGCCTGGTATGGTGGTTTACGCCTGTAATCCCCGCACTTTGGGAGGCCTAG
GTGGGCGGATGGCTTGAGCCCAGGAGTTTGAGACCAGCCTAGCCAACGTGGGGAGACCCTGTC

TCTACCAAAGCACAAAAATTAGCTGGGCATGGTGATGCGCACCTGTGGTCTCAAGTACTTTGTG
TGAGGTGGGAGGATCACTTGAGCCCGGGAGGTTGAGGCTGCAGTGAGCTGAGATTGCACAACT
GCACTCCAGCCTGGGTGACAGAATGAGACCCTGTCTCAAAAAAAAAAAAAGGATTCTCCGAAAG
ACTGGCGATTGCTTTTTAAGCTATAAGGAAGGCCTTTGAAGGGAAATCAAGTAGGAAGAGGATG
ATAGCTGGCTCACATGTCCTTGCCACATGGGAGGTGAGAGAGCAGGCTACTCCTTACCACCCCC
GCTTTCTCTGCCCTGCTGCTGTCTTGCCCCTGTGCAAGTAGGCAATGGGAGAGGAATAGTATAT
ATAAATTGGGCAAGTGCTAGGGGCTGGAGTGATGGTTGGGGAAAGTATCTGACCCTTGTTTTCT
ACTTTCTTTAGCTTGTGGGGTATTTAACAATTCTTTGTTCTTAGTTTTGAGTGCTGGCAACCATTTC
TGGGGTGACAGGAAAAGTTCCATGACATCCGTATTAGAGCATTTGTTTCATTATATTGTAATTTTT
TATGTTTACTTTTGAAAAAGTTCATTCTTTTCTCTTTTTAATTCTTATTCTTTTTTTTTTCTTTTCTTTTT
GAGGCAGGGTTTTACTCTATCACCCAGGCTGGAGTGCAGTGGCACAATCTTGGCTCACTGCAAC
CTCTGCCTCCTGAGCTCGTGATTCATCCTCCTGAGTAGCTGGGACTACAGGTGTACACTACCATG
TGTAGCTAAATTTTTTGTATTTTTTGTAGAGATGGGGTTTTGCCATGTTGCCAGGCTGGTCTCGAA
CTCCTGCAATTTGCTCTCCTTGGCCTCACGAAATGCTGGGATTACAGGCATAAGCTACAGCACC
CAGCCCTTACGTTTACTTTTCATTGACAGGAAGAGTGTCTTCATCTCTGCATCCCTAGTGCAACA
GAGCATGACAAAAATTCAACAAATATTTATTGAAGAGTCTTGATGTCTTTTGGGAAAGGAACTCT
ATGGTTACCTTTACTCTAAGGTAAAGAAATTTAGGTCCTGAACTTTAACCACCTTCTCCTCTTCTT
TCTTCCCTCTTTCATAAATGAGCTCTTGGAAAATTAAAATGCCAGGAGGGTCCAGCAGAGAATA
CCCAAGCCCACATGTCCTGATGGGAAGTGGGGGATAAGTAGAGAGGGAAACACAGACCCTGT
AGGCCATCTCACTGCTGCTGGCCCAGGGGACCCTAACTCCTTTTGTTGACTCATCAGAAGTAGA
ATCCTCCTGAGGGTCTGTTTAAGAAGCACAGCTCTGGTAGAAAGGGTAGCTTTCCTATGGGTAC
GGAGGGCTCTGCTACCCCAGGGCTATCCACACTGGCCTGTGAATCACTCTCAAATGCTTAGGGT
CACGGGCTTTCAGTGGAATGAGCCCTCATGTGCCACCAGGGGGCACCAAGGCCACAGAAAACC
CTATTCAGAAGGTTCTGGGGTGAGGGAAAGGAATCCACGGGTTTGTGGAGCTGGCGGAGCATG
GGTGAGGACAGCATTATGCTAGGTTGGTGGCCTTGAAGTCAGATGTAAATTGGGATCCTGGCTT
GGCCACTGACTATCCATGGGAACTTTGAATCTCAGGGGGCTCATTTGTGATGTGGGGGTACCAT
TATCTGCCTCATCAGGTTGTGGGGAGGATTATATGAGATACCTTATGTAAGAGGGGCTGGTGCA
TTGTGGAAGTCAACAAGTAAGTTTTGTTAAATTTGAGTAAGAAGACCTGTTTGTTTCCTCAAGTTG
CTTATAATCTAGGTGAGGACACATCTGAGAGAATAACTAATAGTTCAGTGAAGGTAAGAGCCAA
ATGATTGGCACAGACCTCAAGTGGTCTGGCATTTACGGGAGAGGGGGATTGTGAAACAGGAAT
TGAAAGAAATTTAAAAATGCATAAGCAAAAACTCAGTTTATGTTTTAAAAAAACCAATTCCCCCT
GAGAAAGAGAAAGAGGTGGAGTCCTTTAAAAATTATCTGCTTGTTTTTCTGTCTGTGGCTAGTGA
GCCTTATCTCTCCCTTTCCCAGGCATTGTGAAGACCCTGTTTCTCTAGCTGTGCAGCTGCAAGGT
CACTAGGCAGATAAACTCAAGCCATAAAACATGTTTTTCCTTGAAAAGTAAGAAATAATGTAAT
GCATGTGTCAAATGAATAACTATTTCTCGCTTCTGTAATATGCTTCCCTTCGCACAGATCTCCCCC
TGCCCCATGAAATGCTTTAAAGGTAACTTAACTCTTTGTTCGGGGCTCAGTCCTTTGGATGTTAAT
CTGACTGGCTTGGTGCACCTAAATAATAAATAACATATATCCTCCTCAACCCCTCAGTCTCTCTG
ATTCCTAAATTATCCCGCTGTAATTGCAGGGCTAGATTTGCCGACCTGCTTTATAAAGCTATGTTA
AATCAGTATATTTTATTCTTTTTATTAGACTTTTCTATAGCATAAAAATGATAAAATACAGCAAGA
AAAAAAGTCACCTGAAACTCTAGTCCTAACCCTAATATGAATGTGAAGGAGCTGGGCTAGAATG
GAATAGGGTAGAATTTGCATAAGGTGGAAATGGGGACAGGCCTGTCACAAGGATCACTAGAGT
CTTGCTGGCAAAAGCATCTTACTGCATTCATGCTGCAGCACAGGACCCAGATGCAGAACAGGTT
GGGGACACAGCCCTGCCTTCTAATAGTGGACACCATCTGAGAGCTGAGCCATAGATTCAAAGC
CTAACTCCTAATAGCGATTGGTAGTAATATCATCAGCAGTTCATATACATGGAGTGACAACAGC

GTGCCAGGCAATTCAAAGGGTCTCTCGGTCCTCACAGCATTTTTGAAGTAGCTAACATCACTATC
CCCCTTTTACAGTTAAAAACAACAACAACAACAACAAAAACACAAAACCCTGAGGCTCAGAAG
ATAAATATTTGCTGAAGATCACTGTGCTTGTAAGTGGTGGAGCCTGACCATTAACTTCCCCACGA
CACTGCTTCAATTTCTATCACTTCATCAACCACACAACCCGAGAACTGTGAGGGGATTTAGCAG
GCCCAGTCCAGATCTCTTATTGTACAGAGAAGAAATCTGAGGCTCAGAGAGGGGAAGGGACTA
ATTTGGACCTTTAACTTCGGATTCTTAACTTTCTTTCTGGTGTTCTAACTATACTGTACTACAAGAG
GCTGTCTTAGAGCTTTAGTTCAACCAGCTTCAGAGATACTCATTGTTATGGGTTGAATTATGTCTC
CCCAAAATTCATATGTCAAAGTTCTAACCCCCAGTACCTCAGAATGTGACCATATTTGGAGATAG
GACCTTTACAGAGGTAATCAGGTGAAAATGAGGTTGTTAGGGTGGGCCTTAGTCCAGTATGGCT
GGTGTCCTCATAAAAAGGGGAAATTTGGACACAGATGTACATAGAGGGAAGATGGTGTGAAGA
GACATAAAGAACAGATAGTCATCTATAGCCAAGGCCTGGAACAGATCCTTCCCTAACAGCCCTC
AGAAGGAAGCAACCTCACTGACACCTTGATTTTGGACTGCTACCCTCTGGCACTGTGAGACAAT
AAATTCATGTTGGTTAGGCCACCCAGTTTGTTATGGCAGCCCTAGGAAACCTTTGTGGATATATC
TGTTCGTATACCCATAAAGTCTGCAAGTCAAAAATATCACAGACTTTTGGCAGCCCTTCATTCAA
GCACTGGCTGGGAGGGTCACCCTCCCCGAGGCAAAAGGATAGAGCAAATGATCTTTCCAGCTT
GCTCCCAACCTTGATCTTTGGCCAAGATATTTTTCTTTCACTGGACATTGGCCAACATTGGTGTCA
TAACTTAATTTGACTGTGACCTTCTTGAGTCTTTTATGTCTCTGAGCTTGACCTGTAGAAGAAGCT
CAATAAATGTGGGTAGAGGGGATGAGTGGGAAAATCAGCTCCCAGCCATTCCAGTTCAAGCCT
CTTCAAGCAGAAGAGTCCCACCTGAAAGACCTCATTTTCACTTGATTACTCGATCTCCAAGCAAT
AGGTGGGGCAGGAAGCAGGGTATGTAAGGCCTGTCAGTGGATGGAAGTTGAAGTTGGACACC
ATCTCCTAAAGTTTAGAGCTGAGCCCAAATTCTGGAAAACACAAGCAAAATACATTTTATATGAC
TCAGGATGGGCTAAAGAAGTTCCAGTACAGGAAACTGTTTTCCTGCTGCTTTATTAATTTTGCTA
CAGAGGATAAATGAGATGCAGACTTTCATATCCAAGTCTTCATTAGATGCCCAGTTAATGCAGC
CATGACAAATGGGAATGAAGCCCTTATGAATAAAAAAAAGATCAACCCAGTATAAAAAAATCAT
AGACCAGAGAAATGAAAATTTAGAGGAGCTGATAAAATACCCTATCCTTAATTAGACTGTCAGC
ATACACATCAAATCAGCCTTAATGCCAAGATTTATTGGCCTTGGAATTGGGTAAATGAAATAAGT
GCTTACAAATGAATGGAAAAGAAATTACCTGTATTAAAACGTATTTGTGATGCAGGCCTGTGCA
CTGAGGGTTTTAGTCAAACTCAGGCTTGCAGCTTCTTTTGCTCTGCATCCATTTCCACCCCATGTT
CCCCAGTGGGTTTCAGAGACCCTCTTTGTGGGGTAACAATATGTACCATTGTCAACTACCTCTGC
TGTTGTCCTGGAAAAATCCTTCTTGGACAGTAAAATATTTTTGTCTTTATTTCACCATCTCCAGATT
AGATGGGTGTTACTGATTCTTACCTCAGAGAAGAGAATGTCATGGAGACAAAATGACCTGCTTA
GAGTTCTGCACCCCCCAGCATGGAGAGCAGAGCGCAAAGATTTCTTCCGGACTCCCACATTCCC
CTTGGAATCCAAGAACTGGTCTACTGTCACAACACTTTAGTACCCTCTTGTAAGCCTGTGGACAA
ATCCTCTGATGCCTTGATACCTCGGTTTTCCCCCACTATAAATGGGAAGATAAATTCACTAGAAA
CAGTACTTACCTTGCAGTGTCATTATGAGAATTTCATGAGACAATGCAATATAAAGCTCCTAGCA
CAGTTCCAGGCACCATTGTAAGCACTGAACAAATGAAAGCTGTTGTCATTAGGATCTCAAGAAA
TGACAGCTGAGGCAAATGTGGATTAGACTGCATTCAGCATTCTTAGCTGCAAGCAACAGAAGCT
GACGCTGGTTTAAAGAATAGGAAAGGAAGTTAATTAAAATGACATTGGGTAGCTTGTGGAACTG
GGAGGACTAGAGAGCTCAGTCTAAGTTCTAGTGACAAAGCTTCAAATCATACATGAGGAAAATG
CCCCTCTGCAGGAGGAACGTAATCCTGCAATCACTGCTTCCCCTGAAGCCAGATGCCTCTACTG
CTGCCCTTCCCAGCAGTGATGGAGCCTGGATCACATACCTGGCTTTAGTTGTAAAGGAGGCTGG
AGAGAAAATGCTCTGCTCTATGTAACTGTCTTGAAAGTGGCCCACACATAGCAAAGCCATTCAA
AAGCGGGTGAGCAGCATGAAAGCATCCAGAAGGATTCTTGCTTTGCATGAGGGAGAACTCTTG
CTCCCAGAGTGTGAGACCAGCAGGGGCATTTTCCTCATGTGTGATTAGAAGCTTTGTCACTAGA

AGCTTAGACTGAGTAGGACACCTCTGAGTGTGAATGTCCTACCTGAGTTCTGCCTCATGCAAAG
CAAGAATCCCTCTGGAGGGATTGACACCACAGCCTCTTCCACAATGTCTCCAAACAACTTTACT
GGGCAGCATTCCACTCCTTGACAAACGGGAGAGGTGCATTCTACCTTCTCTCCTGCATGAGCCT
AGACTAATATGGAATTTGGGAGTGTTGATCATTGTACTTTGTGCTCCTAGAGATAAGGGTCATCA
TAAAGCATCTCAGAGTCTCACTGTAGATCCACAGTTCCATTTCTTTATGGATCCCTTACCCCTACT
TTGCCAAATTCTATGCAGATAGCAATAATAATTCAGACAGGGCTTCCTCACTGAAGGAGTTTGTG
CTCTATATAGTTAATAAGCTAGAGATAATGAAAAAAAAAAAAAACTCAACTTGAACTTTGGGTAA
GAGAAACTTTTCAGACCAACGATCATTACCAATAACAGCTTCCACGCATGACCCTGCCCGGAGA
TTGGGGCCTGCACTACTGTTTCCAGGAAACACATTCTTTTGAATTTTTTTATTTTCTTTGTAGAGAT
GGGGATCTCACTCTGTTGCCCAGGCTGGTCTCAAACTCCTGGCTTCAAGCAATCCTCCTGCTTCG
GCCTCCCAAACTTCTGGGATTACAGATATGAGCCACCACGGCTGGCTGACAGGCACCAAATTCT
AGGAGAGGACTTGAATAGAGTAGGCATCTGTCTCTCCCCATTCCTTTCAGGGACAGAGCTCTCC
TGGTTGGGAGAAAGGGAGAGGGAGAGGGAGGAGGATGAATAGTGAAGCACCAAACCTCAGA
ATTAAGGCCTTTTTCTTTTCTTTCTTTCTTTCTTTTTTTTTTTTGAGATGGAGTTTCGCTCTTGTTGCC
CAGGCTGGAGTGCAATGGCACAATCTCGGCTCACCGCAACCTCTGCCTCCCAGGTTCAAGCGA
TTCTCCTGCCTCAGCCTCCCGAGTAGCTGGGATTACAGGCATGCGCCACCACACCCAGTTAACT
TTTTTGTATTTTTAGAGACGGGGTTTCTCCATGTTGGTCAAGCTGGTCTCGAACTCCCGACCTCAG
GTGCTCTGCCCGACTTAGCCTCCCAAAGTGCTAGGATTACAGGCGTGAGCCACCATGCCCAGC
CAGAATCAGGGCCTTTTTAAAACTTCAGTGTCTTGTCTTTACCCTTCATGGTGCCTAGAAAAGCA
TCTGGCTCATAGGTGTTCAGTGAATGATTGTTTCATTAGTGAAGAAATTCCCTTTGGAAATCTCAT
AGGAAACTTACCCTCTCCAACCCTTCACCTGTGCATTATTCCAGCTCCCAAGGAGGCAGCAGAA
ACTGGGGTAATCACAACGGTCAACAGCAGCAAAGGCAGCACTTTCTGA

ID|56496|gene_id|ENSG00000224699|transcript_id|ENST00000598158 Homo sapiens lncRNA

AATGACCTGCTTAGAGTTCTGCACCCCCCAGCATGGAGAGCAGAGCGCAAAGATTTCTTCC
GGACTCCCACATTCCCCTTGGAATCCAAGAACTGGTCTACTGTCACAACACTTTAGTACCCTCTT
GTAAGCCTGTGGACAAATCCTCTGATGCCTTGATACCTCGGTTTTCCCCCACTATAAATGGGAAG
ATAAATTCACTAGAAACAGTACTTACCTTGCAGTGTCATTATGAGAATTTCATGAGACAATGCAA
TATAAAGCTCCTAGCACAGTTCCAGGCACCATTGTAAGCACTGAACAAATGAAAGCTGTTGTCA
TTAGGATCTCAAGAAATGACAGCTGAGGCAAATGTGGATTAGACTGCATTCAGCATTCTTAGCT
GCAAGCAACAGAAGCTGACGCTGGTTTAAAGAATAGGAAAGGAAGTTAATTAAAATGACATTG
GGTAGCTTGTGGAACTGGGAGGACTAGAGAGCTCAGTCTAAGTTCTAGTGACAAAGCTTCAAAT
CATACATGAGGAAAATGCCCCTCTGCAGGAGGAACGTAATCCTGCAATCACTGCTTCCCCTGAA
GCCAGATGCCTCTACTGCTGCCCTTCCCAGCAGTGATGGAGCCTGGATCACATACCTGGCTTTA
GTTGTAAAGGAGGCTGGAGAGAAAATGCTCTGCTCTATGTAACTGTCTTGAAAGTGGCCCACAC
ATAGCAAAGCCATTCAAAAGCGGGTGAGCAGCATGAAAGCATCCAGAAGGATTCTTGCTTTGC
ATGAGGGAGAACTCTTGCTCCCAGAGTGTGAGACCAGCAGGGGCATTTTCCTCATGTGTGATTA
GAAGCTTTGTCACTAGAAGCTTAGACTGAGTAGGACACCTCTGAGTGTGAATGTCCTACCTGAG
TTCTGCCTCATGCAAAGCAAGAATCCCTCTGGAGGGATTGACACCACAGCCTCTTCCACAATGT
CTCCAAACAACTTTACTGGGCAGCATTCCACTCCTTGACAAACGGGAGAGGTGCATTCTACCTT
CTCTCCTGCATGAGCCTAGACTAATATGGAATTTGGGAGTGTTGATCATTGTACTTTGTGCTCCTA
GAGATAAGGGTCATCATAAAGCATCTCAGAGTCTCACTGTAGATCCACAGTTCCATTTCTTTATG
GATCCCTTACCCCTACTTTGCCAAATTCTATGCAGATAGCAATAATAATTCAGACAGGGCTTCCT
CACTGAAGGAGTTTGTGCTCTATATAGTTAATAAGCTAGAGATAATGAAAAAAAAAAAAAACTCA

ACTTGAACTTTGGGTAAGAGAAACTTTTCAGACCAACGATCATTACCAATAACAGCTTCCACGC
ATGACCCTGCCCGGAGATTGGGGCCTGCACTACTGTTTCCAGGAAACACATTCTTTTGAATTTTT
TTATTTTCTTTGTAGAGATGGGGATCTCACTCTGTTGCCCAGGCTGGTCTCAAACTCCTGGCTTCA
AGCAATCCTCCTGCTTCGGCCTCCCAAACTTCTGGGATTACAGATATGAGCCACCACGGCTGGC
TGACAGGCACCAAATTCTAGGAGAGGACTTGAATAGAGTAGGCATCTGTCTCTCCCCATTCCTTT
CAGGGACAGAGCTCTCCTGGTTGGGAGAAAGGGAGAGGGAGAGGGAGGAGGATGAATAGTG
AAGCACCAAACCTCAGAATTAAGGCCTTTTTCTTTTCTTTCTTTCTTTCTTTTTTTTTTTTGAGATGG
AGTTTCGCTCTTGTTGCCCAGGCTGGAGTGCAATGGCACAATCTCGGCTCACCGCAACCTCTGC
CTCCCAGGTTCAAGCGATTCTCCTGCCTCAGCCTCCCGAGTAGCTGGGATTACAGGCATGCGCC
ACCACACCCAGTTAACTTTTTTGTATTTTTAGAGACGGGGTTTCTCCATGTTGGTCAAGCTGGTCT
CGAACTCCCGACCTCAGGTGCTCTGCCCGACTTAGCCTCCCAAAGTGCTAGGATTACAGGCGTG
AGCCACCATGCCCAGCCAGAATCAGGGCCTTTTTAAAACTTCAGTGTCTTGTCTTTACCCTTCAT
GGTGCCTAGAAAAGCATCTGGCTCATAGGTGTTCAGTGAATGATTGTTTCATTAGTGAAGAAATT
CCCTTTGGAAATCTCATAGGAAACTTACCCTCTCCAACCCTTCACCTGTGCATTATTCCAGCTCC
CAAGGAGGCAGCAGAAACTGGGGTAATCACAACGGTCAACAGCAGCAAAGGCAGCACTTTCT
GATTTACTTGACTGCTATGGATGCCACCTCTCTCCACGCCTCAGTTTAATATCTGTAAGTTTGGTC
AGTATCTCATTCAAGAACATTGCATTTACTCTGGTACTTAAAGATGAAAAATCAACATTTACAAA
GCAGATCCATTGTCAACCTAAATAACAAACAAAGAGAGTCTCTATAAAAGAAAAGAATATTTAT
TTGGGAATAGAGCATTGCATAGGAATATGCCTGCCTTAGTAATGCATGTATTCAGGGAGGTAGA
GGAAGACAAAGGTTTTTAACAGAAAAAATGTGGAGAATTACATAATTGTTTTGAAATAATTATCC
TTGGCTATAAAGATCAATAACAAGGGTGATGCGAGTCCAAGGTTGGACAGGCAGTTGCTGGGC
AGATGTCTTTGCAGAAGTCTTTTTTGTGTAAGGTTGTAATGGCTTTTGTAGGAGGTTGTGGCTTTT
GCAATCTTTTGTGATAGCTTTTGTTACCAGGCATACAAGCATGAGAATGCTCTCTTCAGGGCCTT
CCCTAGCTCTATTTATCAGGGGTTTCTTGTTAGTGATTCCATTTTGATTCTGACAACTTTCATGGA
CATTATCTCATTTAATCTCACAGTCATTCCATGACTCTATCCCCATTTTGTGAATGAAGTAACTGA
GGTCTGGAGAGGTCAACTAACTGGTCAAGGTCATAGAACTTGAAAATTTTCATTGTCTTGGGTCT
CTAATACTGGGGCTGACCATTCTCCCAGGCAATGTTGAGTGAGTTGGCCCAGGTGAGTCGGCTC
CTCTGCAATTGTGTCTCCTCAACTAGTTTGAACAACACAGGCCTGTGAGCCAGTCCCATAAAGT
GGCTAGGGGTTCCCTTCTCCCCATTTCCCCTCATCAACAGGAGGACTTGTCTGTGTGCCTTCAGA
CACCACACAGTAAATCAACTGCCACATTCATTCTGGACCATCAGTATTTTGCCCCAGGAGAAAG
TCAGAGGCAAGCACACTAATATCTCATTGATATTTTCAATTGCCAAAGGGGAAGTGAGATGAAT
CTAATCCCATCTGTCAAGGTGGAGAAGAGCTGATTCGAAAGCAGCATAGATTACTTTAAGGAGA
TGACAGTTTATCACTCTCTGGACAAGGGAATATGAAATGAGGACTAACCTCCTTTATGAGTTGGT
AAAAACATTGATCAGCAAAGACAATGTCTGGGGAAATGAACAGCAAGTTCTCTGGCCCTTTTTC
CACTTTTCCTTTCCCCGAGACTTTATGTTATATATACACACTCACACAAACAGGACAGAATCCAC
CTCCTGCCCCTCAAGAAATAGTTAACATTTGTTGAGGATTAATTATTGCCAGGCTCTGTACTCCA
TGTTTTATATTTACTATCCAGAAGCCTTAATTGTTAAATGAGGTAAGTGTTATTATTAGAACCTTTG
TACAGATGTGGAAACTGAGGTGTATAGAGGTTGACTTGCCCAAGGTTACCCAGCTAGTGAAGAA
TGAAATCAGATGCAGTCCAGCCAACGCTTGGCCTGCCGTCAGTCACACGCAGTACACGTAGAT
CAGTCAGCCTGGAAAGCCACCTAGACAGCCCTGCTTCACTCCATCCTTGGTCAGCACCTTAGAG
AGCCCTGCTGACCCCAAGAGGATGTGCCTTTTACGGGTTGTAGAGGCTGAACCACAGCTGAGG
GGCTGGAATGAGTGGGGGCCTCAGACAGGGTGTCTGGAGAGCTGAGGCTCAAGGAAGGGTGG
AGGGGGTGCTGAAGCTGAAGGCCGTGGGTCTTGCCAGCAAGTGGGGCTCAGTGAGCCGAGGT
GCCTCTCAGTTACTTCCATGTGTGAATTTCCGGTGGAGGACAGTGATGGGCTCTGGGCCCTTGG

AAACCGGGACATCCAGCGTGTCCAGCCACAGTGCAGGCTGCCCTGGCTGCTGAGAGTCTCAGA
AAATGCCACTGTCATGGGTTAGGAAGGCCTGGGGACCACAACAATGTGACTTCAAAAAACCCA
AGAAGGAATGTGCACACCTGAGATGCTCAGAATGCATGTGTCAGTGTTCAGTTCTGACTCCTTG
CTTCAGCCAGGGACAGAAGAACCTTCTACAGTAAAGAACCACAAGAGAGGGCTTTGCTGCCAG
CCTCAGCCAGGGACACTTGGCGGCCAAAGCAAAGGTCACTCCAGGGCTGCAGATTTCAGCCTC
TGCGGCCAGAAACAAGCTTCCCAATTAATAATACTCTGGATCGGACAGAGATGGCTGCTCTGTC
TCAGGCGTGTTTGCCGTTGATCCCTGTGATTGATGCTGATAACATTAAGAGTCTGTCAGGGTGAT
ATTTCATGAATGTCTCATGTGATGGGATGATACAGTCGGGAAATGCAATTCCCTAGCTCAGGCA
GAATCAGAGATGACCCAAAGTGCTGATGGAAAGGCTGAGGCCCTTTCCAGCCTCTGGCAGGGT
AGGGAAAGCGGGTGAGGTGGGGGCTTGGGAGGGGCTGGGCTGACCTAGAGCTGTAACAGGG
GTCTCTCATGAAGGGCTGTGAAGAGAAACAGTATGTGAGAGAGGCAAGAAACTTTTCTCTGGGT
TTCCTCATGTAGAATGATTCCATCTGTGTAGGCCATGAAGGAGTCCAGGGAAGTTCACCCCAAA
ATGTTGAAACCACCTTTGCAAAGATTATGACAGTGAGAGAAGCCTGGCATGGCTGACTCTATCT
TGCTTCTAGCCTCACAGGCTGGCTGTTCTGTCTCATTCCTGGGCATAGGCCAAGCTAATCTTGGG
AGGGATTTATGGTTTAACTTTGAAGCCAGGATCATAATTGTCCCTCCCTAAAACTGACCCCTTTC
TTGTTCAGGGACTGAAACTGCCTTTGTAAGACTAATGAAAGGCCACAAAATTAGGATTATGGAA
GGGGCCTGAATTCTGATAAGATGTAGGCATAGCTTGCCTTTCTGTCATCGTTTATTGCACTGGCA
CTCACAAGATTTGTAACTTCCCCAATTGGTCCCATAGATAACATCACTATTGTAGAACCTCAGAT
TGGTCTTTTGAGATCTTTTTCAGACTTTGGTATTCTGGTGACTGACTCCACCCAGATCCCATGACT
CATGACTCAGCTGGTCCTGTGGTTCTTACCCAGTCTGACACAGCACCAGAGGACCGTTTTCCAC
ACTTCTATCTATGATTTCATCCCCAACCAATCAGCACCACCCATTCCCTAGCCCCCTGCCCACCA
AATTGATATCCATAAAAACCCTAACCTCTGAGTTCTCAGAGAAACTGATTGGAGTAATAAACTCC
TGTCTTCTGTGTGGCTAGCCTTATGTTAATTAAACTCTTTCTTAGGGCAGTACTGCTGTCAGTGAA
TCAGCTTTATCTGTGCAGTGGGCAAGAAGGACCCACTGGGTGACTACAATATGGCACTTTGGTA
TGTTGGTTATTTTAAGTTAGAGGCCTTTGATGCTGGAAGAAGCTTTACTCTGATATCTCCTTATCT
ACCACAAGACTGGATCTGCCAAAGAGAACACAATTGCGTTCCATTCCCTTCCTGAAATCTCATTA
TCTATGGCAGAAAAGAAGACTGGGGAATGTAATACACAGTATTACACAGTAATGTAACATACTA
CACAGTATTTACACAAAACATGTAACACAGAATGTACTACACAGTATTTTCACAAAACAATATCT
GTCTCTCCAACTCATTCAAATTCTAAAGAGTCATTTACACGTCAATTTGTGTCTTCTACGCCCATT
CATTCTCCCTAAGAATCATTTACTACTCCTCAAAAGAATTACCAACATTCTCCATCTCCCACTCCC
CTATGAGGAAGGGTATATAAGTTTCTGTCTGGATCTCATTAGATGACTGTGTGATCGTGCTTCTA
TGATCCCTACCCCACATGTTAAATGAATTTTGTATGCCTTTTTCTTTTATCAATCTGCCTTTTGTTA
GGGTATTTTCAGTGGACCTTCAGAAGGCAAAGGGGGAGCTTTCCTTCTTTGACCCTACAGCTATT
AGACTTGATTGAGTAAGACACACAATAATGAAAGTAAATAAGAAGAAAAACATTCATCCTGCTG
TCTCCAGAGTTTGAAATGTCTGCCTCAAACTTCGATTTCTATCTACCAAAAATTCATGCATTAATC
ATAAGGCCGATCTCAAAAGTGAATTTTTTTTTTTTTTTTGAGAAGGAGTCTCACTCTGTTGCCTAGG
CTGGAGTGCAGTGGCACAATCTCAGCTCACTGCAACCTCTGCCTCCTGGGTTCAAGCGATTCTC
CTGCCTCAGCCTCCCAAGTAGCTGGGACTACAGGCACGTGCCGCCAAGCCTGGCTAATTTTTAT
ATTTTTAGTACAGACGGGGTTTCACCATATTGGCCAGGCTGGTCTCAAACTCCTGACCTCAAGTG
ATCCACCCACCTCTGCCTCCCAAAGTGCTGGGATTACAGGCGTGAGCCACCGCGCCTGGACGT
GAAAAGTTTTTTGAATTCCCCCATTAGAAATGATCTTCACCCTGACTCCCATAACACTTTGTACAT
TACTGTAAAAGCATAATATTCAAATATTTACAATATACCTCCTTCAAATATAAAATGGACACATG
CCGAAGACTTAGCAACTCATGAAGGAGGGAACCAATAATGTAGTTAAGCTAATTCAAAGAGCA
TTTTGAGGAAATGAATACTTATGGTCATTTAAAAGGAAATGTTAAAATTTCTAGGTTAAAAATAA

AACTTGCCATTGCATATATGGTCAAATGATTTTTGACAAGGGTGCCAAGACCATTCCATGGGGA
AAACACAGTCTTTTCAACAAATCGTGTTGGGAAAACTAGATATCCATGTTCAAAAGAATGAACC
CTTACCTTACCTTCCACCATATACAAAAATTAACTCAAAATGGATCAAAGACTTAAGCATAAGAG
CTAAAATTATAAAATTCTTAGAAGAAAACATAGGGAAAAAGGTCCATGGTATTATATTTGACAAT
GACTTTTTTTTTTTTTTTTGAGGCAAGATTTCACTCTGTCACCCTGGCTGGAGTTCAATGCAGTGG
CACAATCTCAGCTCACTGCAACCTCCACCTTTCAGGCTCAAGCAATCCCCCTACCTCAGCCCCT
AAGTAGCTGAGACTACAGGCATGCACCACCAAGCACAGCTAATTTTTGTATTTTTGTACAGAGTT
TCGCCATGTTGCCCAGGCTGGTCTCGAACTCCTGAGCTCAAGCTATTCACCTGCCTCAGCCTCC
CAAAGTGTTGGGATTACAGGTGTGAGCCACTGTGCCCAGCCCTGACCCCCACATTTTTAAACAA
AGCTTCTCTTCCATAATCAATTGCAAATCAGAAGATCTTTGAATCTATCTGCAGCCTGCAAGCCC
CCTATTCAAGACATCCCACCCTTTTAAACTTAAGCCAATGTGTAACCTCCTGGCAATCAATTTTG
CAATTTATGATTTTGCCTGTAGCTTCTGCTGTTCTGAAATTTATTCCTGCCTCTAAAAATCCTTGCC
GGCAAGCCACTGGGGAGGCTAGGTTTTGAGCACTTAGCTGTCTGGTCTTCCTTACTTAGTGCTCT
GCAATAAACACCTTCCTTTCTATCACCACAAAATCCTGGGTGTGGGTATCTGGTTTTACTGTGTTG
GGTGAGCAGACCTCAGTTTGGTTCTGTTACAAGTCCCTGCTCCATCTCACTTGCTACAACATGAA
TGAAACTTGAGGACATTATGCAAAATGAAATAAGCCAGTCACAAAAAGACAAATACTGTATTAT
TCCACTTATATGAGGTACTTATGATAGACAAAATCATCAAGACAGAAGGTATGATGGTTGCCAG
GGGTTGAGGGAAGACAAAATAGGGAGTTATTGTTTAATGGTTGAGAAGTTTCAGTTTTGCAAGA
TGAAAAGTGTTCTGGAGATGGATGGTGGTGATGGTTGCATGACATAAATGTGCTTAATACCACT
GAACTGTTCTTAACTCCGCTGAAATATGGTTAAGATGGCCCGGGTGCAGTGGCTCATGCCTGTA
ATCCTAACACTTTGGAACGCCGAGGCGGGCAGATCACCTGAGATCAGGAGTTCGAGACCAGCC
TGACCAATATGGCGAAACCCAGTCTCCACTAAAAATACAAAAATTAGCGAGGTGTGGTGGTGC
GCACCTGTAATTCAAGCCACTTGGGAGGCTGAGGCAGGAGAATTTTCTGAATCCAGGATGCAG
AGGTTGCAGTGTACTGAGATCGCACCACTGCACTCCAGCCTGAGCAACAGAGCAAGACTCCAT
CTCAAAAAAAAAAAAAAAGGTTAAGATGGTAAACTTTGTACCAATTAAAAAACAAAGCTGGCTAA
CACCCTACAGGGCAATTAAAACATCATAATCTTTGGTGTTATCTTTTTTATTAGATAGAAATTATT
TATACTTACTGGATAAGATCTGTAAGTTAACACATAACTTCACAGAGTCTGAGCCCTTAATGAAT
AATAAATAGCCAAGTCACAGGGAGATACTTTCCCTGAGGTAATTAAGCAATCCTTGGGTAGGCT
TGTGAAGAAATGCCCAGAATGACGTTGGAAGGACATGCTGTTCTCTCTTGGCCTTATTTCCAAG
GTTTGGACTCAAAGTATTATTTTTTAACATCACAGAAATTTTTTATGCCCTGGTCTCTTGAGGGTA
GGGACTGTGTCTTTTTCATCTTTGCCCCTTTCCACATGCTCTGGTTTGTAGAACAGAATTGAAGAA
GGAGCCATGTGCTAGATAGAGCGGGTTGGGAGCAGCAGACCTGCCTGCTCATTGTTTCTGTGAC
CTTGACCCATCCTGTAGTCTCCCCACGCCTTGCTATTCTCATCCATCAACGATGAGATTGCACCT
AAATCCTGGGTGCTGTGCTGGGCCTGGGGATGCTGTCTAATCCCTGGGTTTCCTTCTAGTTTGCT
CTAATCACCCATATGAAAAAGAAGCTACTCAAGGCAAACATATAAATGAAACGGATAATGAATG
AATAGTATTTTTTCTTGCTTTCTTTGGAAGAGGCATCCTGGATTCTACTTGAAGCCTGCAGCCCTG
ACTCCTGGGCTGGGAGGTGGGCGAGGACTTGTATTTTGTAGCTCTGCTAGAGAGGGAACAGCT
GTCCACTGGTGGCAGGACTGACGCCTCCCAGAATACAGGGGCTTTGGATCAGCAGGCGTCAGA
GGGAAAGGAAACTGGCATCCGAGTCTGCCTTTGAGAAAGGAGTAAATTACTATCTTATTATAAA
TTCATGATCCACTCATTGCTGTTTTCAATTAATTTCTTGACAAATCTGGCTGTTCCTTTTCTTATTTT
CCTTAGGACAGGAGGTGTCCTGGTGATCAGCTGGAGCTCGGGGGAGCTGTTGCTGAAATTAAC
TTGAGTGCTTTGGAGAGAGATTTACATGGCAGAGTGGAGCCAGGGCTGCTCCTTTGGCAGGCA
CTGCTGTGCCACTGTGGAGGCGCCCCTGCTTTTCCCATGCTTTGCAGTGTCTTTAAAGGCCTTTG
GAGCCATGCCCAGGCTCAGCCCCATCCTGGTGTCCCCCTGTGTCCGTTTTGCCACTGCCCGGCT

ACTTCACTTGGCCATGGGCCTTACCAGCTTGCAGACAAACCCGTCCATCTACGCCTCCCAGCGC
AGCTTGGCCCACTTGCAAGCACTTACAGAGGGTTTCAGAAGGAACATGTCTGCCATCTGTGTTT
GGAGACTGTGTCCC

ID|56511|gene_id|ENSG00000224699|transcript_id|ENST00000598350 Homo sapiens lncRNA

AGATGGGGATCTCACTCTGTTGCCCAGGCTGGTCTCAAACTCCTGGCTTCAAGCAATCCTC
CTGCTTCGGCCTCCCAAACTTCTGGGATTACAGATATGAGCCACCACGGCTGGCTGACAGGCAC
CAAATTCTAGGAGAGGACTTGAATAGAGTAGGCATCTGTCTCTCCCCATTCCTTTCAGGGACAG
AGCTCTCCTGGTTGGGAGAAAGGGAGAGGGAGAGGGAGGAGGATGAATAGTGAAGCACCAAA
CCTCAGAATTAAGGCCTTTTTCTTTTCTTTCTTTCTTTCTTTTTTTTTTTTGAGATGGAGTTTCGCTCT
TGTTGCCCAGGCTGGAGTGCAATGGCACAATCTCGGCTCACCGCAACCTCTGCCTCCCAGGTTC
AAGCGATTCTCCTGCCTCAGCCTCCCGAGTAGCTGGGATTACAGGCATGCGCCACCACACCCA
GTTAACTTTTTTGTATTTTTAGAGACGGGGTTTCTCCATGTTGGTCAAGCTGGTCTCGAACTCCCG
ACCTCAGGTGCTCTGCCCGACTTAGCCTCCCAAAGTGCTAGGATTACAGGCGTGAGCCACCATG
CCCAGCCAGAATCAGGGCCTTTTTAAAACTTCAGTGTCTTGTCTTTACCCTTCATGGTGCCTAGA
AAAGCATCTGGCTCATAGGTGTTCAGTGAATGATTGTTTCATTAGTGAAGAAATTCCCTTTGGAA
ATCTCATAGGAAACTTACCCTCTCCAACCCTTCACCTGTGCATTATTCCAGCTCCCAAGGAGGCA
GCAGAAACTGGGGTAATCACAACGGTCAACAGCAGCAAAGGCAGCACTTTCTGATTTACTTGAC
TGCTATGGATGCCACCTCTCTCCACGCCTCAGTTTAATATCTGTAAGTTTGGTCAGTATCTCATTC
AAGAACATTGCATTTACTCTGGTACTTAAAGATGAAAAATCAACATTTACAAAGCAGATCCATTG
TCAACCTAAATAACAAACAAAGAGAGTCTCTATAAAAGAAAAGAATATTTATTTGGGAATAGAG
CATTGCATAGGAATATGCCTGCCTTAGTAATGCATGTATTCAGGGAGGTAGAGGAAGACAAAG
GTTTTTAACAGAAAAAATGTGGAGAATTACATAATTGTTTTGAAATAATTATCCTTGGCTATAAAG
ATCAATAACAAGGGTGATGCGAGTCCAAGGTTGGACAGGCAGTTGCTGGGCAGATGTCTTTGC
AGAAGTCTTTTTTGTGTAAGGTTGTAATGGCTTTTGTAGGAGGTTGTGGCTTTTGCAATCTTTTGT
GATAGCTTTTGTTACCAGGCATACAAGCATGAGAATGCTCTCTTCAGGGCCTTCCCTAGCTCTAT
TTATCAGGGGTTTCTTGTTAGTGATTCCATTTTGATTCTGACAACTTTCATGGACATTATCTCATTT
AATCTCACAGTCATTCCATGACTCTATCCCCATTTTGTGAATGAAGTAACTGAGGTCTGGAGAGG
TCAACTAACTGGTCAAGGTCATAGAACTTGAAAATTTTCATTGTCTTGGGTCTCTAATACTGGGG
CTGACCATTCTCCCAGGCAATGTTGAGTGAGTTGGCCCAGGTGAGTCGGCTCCTCTGCAATTGT
GTCTCCTCAACTAGTTTGAACAACACAGGCCTGTGAGCCAGTCCCATAAAGTGGCTAGGGGTTC
CCTTCTCCCCATTTCCCCTCATCAACAGGAGGACTTGTCTGTGTGCCTTCAGACACCACACAGTA
AATCAACTGCCACATTCATTCTGGACCATCAGTATTTTGCCCCAGGAGAAAGTCAGAGGCAAGC
ACACTAATATCTCATTGATATTTTCAATTGCCAAAGGGGAAGTGAGATGAATCTAATCCCATCTG
TCAAGGTGGAGAAGAGCTGATTCGAAAGCAGCATAGATTACTTTAAGGAGATGACAGTTTATCA
CTCTCTGGACAAGGGAATATGAAATGAGGACTAACCTCCTTTATGAGTTGGTAAAAACATTGAT
CAGCAAAGACAATGTCTGGGGAAATGAACAGCAAGTTCTCTGGCCCTTTTTCCACTTTTCCTTTC
CCCGAGACTTTATGTTATATATACACACTCACACAAACAGGACAGAATCCACCTCCTGCCCCTC
AAGAAATAGTTAACATTTGTTGAGGATTAATTATTGCCAGGCTCTGTACTCCATGTTTTATATTTA
CTATCCAGAAGCCTTAATTGTTAAATGAGGTAAGTGTTATTATTAGAACCTTTGTACAGATGTGG
AAACTGAGGTGTATAGAGGTTGACTTGCCCAAGGTTACCCAGCTAGTGAAGAATGAAATCAGAT
GCAGTCCAGCCAACGCTTGGCCTGCCGTCAGTCACACGCAGTACACGTAGATCAGTCAGCCTG
GAAAGCCACCTAGACAGCCCTGCTTCACTCCATCCTTGGTCAGCACCTTAGAGAGCCCTGCTGA
CCCCAAGAGGATGTGCCTTTTACGGGTTGTAGAGGCTGAACCACAGCTGAGGGGCTGGAATGA

GTGGGGGCCTCAGACAGGGTGTCTGGAGAGCTGAGGCTCAAGGAAGGGTGGAGGGGGTGCTG
AAGCTGAAGGCCGTGGGTCTTGCCAGCAAGTGGGGCTCAGTGAGCCGAGGTGCCTCTCAGTTA
CTTCCATGTGTGAATTTCCGGTGGAGGACAGTGATGGGCTCTGGGCCCTTGGAAACCGGACAT
CCAGCGTGTCCAGCCACAGTGCAGGCTGCCCTGGCTGCTGAGAGTCTCAGAAAATGCCACTGT
CATGGGTTAGGAAGGCCTGGGGACCACAACAATGTGACTTCAAAAAACCCAAGAAGGAATGTG
CACACCTGAGATGCTCAGAATGCATGTGTCAGTGTTCAGTTCTGACTCCTTGCTTCAGCCAGGG
ACAGAAGAACCTTCTACAGTAAAGAACCACAAGAGAGGGCTTTGCTGCCAGCCTCAGCCAGGG
ACACTTGGCGGCCAAAGCAAAGGTCACTCCAGGGCTGCAGATTTCAGCCTCTGCGGCCAGAAA
CAAGCTTCCCAATTAATAATACTCTGGATCGGACAGAGATGGCTGCTCTGTCTCAGGCGTGTTTG
CCGTTGATCCCTGTGATTGATGCTGATAACATTAAGAGTCTGTCAGGGTGATATTTCATGAATGT
CTCATGTGATGGGATGATACAGTCGGGAAATGCAATTCCCTAGCTCAGGCAGAATCAGAGATG
ACCCAAAGTGCTGATGGAAAGGCTGAGGCCCTTTCCAGCCTCTGGCAGGGTAGGGAAAGCGG
GTGAGGTGGGGGCTTGGGAGGGGCTGGGCTGACCTAGAGCTGTAACAGGGGTCTCTCATGAA
GGGCTGTGAAGAGAAACAGTATGTGAGAGAGGCAAGAAACTTTTCTCTGGGTTTCCTCATGTAG
AATGATTCCATCTGTGTAGGCCATGAAGGAGTCCAGGGAAGTTCACCCCAAAATGTTGAAACCA
CCTTTGCAAAGATTATGACAGTGAGAGAAGCCTGGCATGGCTGACTCTATCTTGCTTCTAGCCTC
ACAGGCTGGCTGTTCTGTCTCATTCCTGGGCATAGGCCAAGCTAATCTTGGGAGGGATTTATGG
TTTAACTTTGAAGCCAGGATCATAATTGTCCCTCCCTAAAACTGACCCCTTTCTTGTTCAGGGACT
GAAACTGCCTTTGTAAGACTAATGAAAGGCCACAAAATTAGGATTATGGAAGGGGCCTGAATTC
TGATAAGATGTAGGCATAGCTTGCCTTTCTGTCATCGTTTATTGCACTGGCACTCACAAGATTTGT
AACTTCCCCAATTGGTCCCATAGATAACATCACTATTGTAGAACCTCAGATTGGTCTTTTGAGAT
CTTTTTCAGACTTTGGTATTCTGGTGACTGACTCCACCCAGATCCCATGACTCATGACTCAGCTG
GTCCTGTGGTTCTTACCCAGTCTGACACAGCACCAGAGGACCGTTTTCCACACTTCTATCTATGA
TTTCATCCCCAACCAATCAGCACCACCCATTCCCTAGCCCCCTGCCCACCAAATTGATATCCATA
AAAACCCTAACCTCTGAGTTCTCAGAGAAACTGATTGGAGTAATAAACTCCTGTCTTCTGTGTGG
CTAGCCTTATGTTAATTAAACTCTTTCTTAGGGCAGTACTGCTGTCAGTGAATCAGCTTTATCTGT
GCAGTGGGCAAGAAGGACCCACTGGGTGACTACAATATGGCACTTTGGTATGTTGGTTATTTTA
AGTTAGAGGCCTTTGATGCTGGAAGAAGCTTTACTCTGATATCTCCTTATCTACCACAAGACTGG
ATCTGCCAAAGAGAACACAATTGCGTTCCATTCCCTTCCTGAAATCTCATTATCTATGGCAGAAA
AGAAGACTGGGGAATGTAATACACAGTATTACACAGTAATGTAACATACTACACAGTATTTACA
CAAAACATGTAACACAGAATGTACTACACAGTATTTTCACAAAACAATATCTGTCTCTCCAACTC
ATTCAAATTCTAAAGAGTCATTTACACGTCAATTTGTGTCTTCTACGCCCATTCATTCTCCCTAAG
AATCATTTACTACTCCTCAAAAGAATTACCAACATTCTCCATCTCCCACTCCCCTATGAGGAAGG
GTATATAAGTTTCTGTCTGGATCTCATTAGATGACTGTGTGATCGTGCTTCTATGATCCCTACCCC
ACATGTTAAATGAATTTTGTATGCCTTTTTCTTTTATCAATCTGCCTTTTGTTAGGGTATTTTCAGTG
GACCTTCAGAAGGCAAAGGGGGAGCTTTCCTTCTTTGACCCTACAGCTATTAGACTTGATTGAGT
AAGACACACAATAATGAAAGTAAATAAGAAGAAAAACATTCATCCTGCTGTCTCCAGAGTTTGA
AATGTCTGCCTCAAACTTCGATTTCTATCTACCAAAAATTCATGCATTAATCATAAGGCCGATCTC
AAAAGTGAATTTTTTTTTTTTTTTGAGAAGGAGTCTCACTCTGTTGCCTAGGCTGGAGTGCAGTGG
CACAATCTCAGCTCACTGCAACCTCTGCCTCCTGGGTTCAAGCGATTCTCCTGCCTCAGCCTCCC
AAGTAGCTGGGACTACAGGCACGTGCCGCCAAGCCTGGCTAATTTTTATATTTTTAGTACAGAC
GGGGTTTCACCATATTGGCCAGGCTGGTCTCAAACTCCTGACCTCAAGTGATCCACCCACCTCT
GCCTCCCAAAGTGCTGGGATTACAGGCGTGAGCCACCGCGCCTGGACGTGAAAAGTTTTTTGA
ATTCCCCCATTAGAAATGATCTTCACCCTGACTCCCATAACACTTTGTACATTACTGTAAAAGCA

TAATATTCAAATATTTACAATATACCTCCTTCAAATATAAAATGGACACATGCCGAAGACTTAGC
AACTCATGAAGGAGGGAACCAATAATGTAGTTAAGCTAATTCAAAGAGCATTTTGAGGAAATGA
ATACTTATGGTCATTTAAAAGGAAATGTTAAAATTTCTAGGTTAAAAATAAAACTTGCCATTGCAT
ATATGGTCAAATGATTTTTGACAAGGGTGCCAAGACCATTCCATGGGGAAAACACAGTCTTTTC
AACAAATCGTGTTGGGAAAACTAGATATCCATGTTCAAAAGAATGAACCCTTACCTTACCTTCCA
CCATATACAAAAATTAACTCAAAATGGATCAAAGACTTAAGCATAAGAGCTAAAATTATAAAAT
TCTTAGAAGAAAACATAGGGAAAAAGGTCCATGGTATTATATTTGACAATGACTTTTTTTTTTTT
TTTGAGGCAAGATTTCACTCTGTCACCCTGGCTGGAGTTCAATGCAGTGGCACAATCTCAGCTC
ACTGCAACCTCCACCTTTCAGGCTCAAGCAATCCCCCTACCTCAGCCCCTAAGTAGCTGAGACT
ACAGGCATGCACCACCAAGCACAGCTAATTTTTGTATTTTTGTACAGAGTTTCGCCATGTTGCCC
AGGCTGGTCTCGAACTCCTGAGCTCAAGCTATTCACCTGCCTCAGCCTCCCAAAGTGTTGGGAT
TACAGGTGTGAGCCACTGTGCCCAGCCCTGACCCCCACATTTTTAAACAAAGCTTCTCTTCCATA
ATCAATTGCAAATCAGAAGATCTTTGAATCTATCTGCAGCCTGCAAGCCCCCTATTCAAGACATC
CCACCCTTTTAAACTTAAGCCAATGTGTAACCTCCTGGCAATCAATTTTGCAATTTATGATTTTGC
CTGTAGCTTCTGCTGTTCTGAAATTTATTCCTGCCTCTAAAAATCCTTGCCGGCAAGCCACTGGG
GAGGCTAGGTTTTGAGCACTTAGCTGTCTGGTCTTCCTTACTTAGTGCTCTGCAATAAACACCTT
CCTTTCTATCACCACAAAATCCTGGGTGTGGGTATCTGGTTTTACTGTGTTGGGTGAGCAGACCT
CAGTTTGGTTCTGTTACAAGTCCCTGCTCCATCTCACTTGCTACAACATGAATGAAACTTGAGGA
CATTATGCAAAATGAAATAAGCCAGTCACAAAAAGACAAATACTGTATTATTCCACTTATATGAG
GTACTTATGATAGACAAAATCATCAAGACAGAAGGTATGATGGTTGCCAGGGGTTGAGGGAAG
ACAAAATAGGGAGTTATTGTTTAATGGTTGAGAAGTTTCAGTTTTGCAAGATGAAAAGTGTTCTG
GAGATGGATGGTGGTGATGGTTGCATGACATAAATGTGCTTAATACCACTGAACTGTTCTTAACT
CCGCTGAAATATGGTTAAGATGGCCCGGGTGCAGTGGCTCATGCCTGTAATCCTAACACTTTGG
AACGCCGAGGCGGGCAGATCACCTGAGATCAGGAGTTCGAGACCAGCCTGACCAATATGGCG
AAACCCAGTCTCCACTAAAAATACAAAAATTAGCGAGGTGTGGTGGTGCGCACCTGTAATTCAA
GCCACTTGGGAGGCTGAGGCAGGAGAATTTTCTGAATCCAGGATGCAGAGGTTGCAGTGTACT
GAGATCGCACCACTGCACTCCAGCCTGAGCAACAGAGCAAGACTCCATCTCAAAAAAAAAAAA
AAGGTTAAGATGGTAAACTTTGTACCAATTAAAAAACAAAGCTGGCTAACACCCTACAGGGCAA
TTAAAACATCATAATCTTTGGTGTTATCTTTTTTATTAGATAGAAATTATTTATACTTACTGGATAA
GATCTGTAAGTTAACACATAACTTCACAGAGTCTGAGCCCTTAATGAATAATAAATAGCCAAGTC
ACAGGGAGATACTTTCCCTGAGGTAATTAAGCAATCCTTGGGTAGGCTTGTGAAGAAATGCCCA
GAATGACGTTGGAAGGACATGCTGTTCTCTCTTGGCCTTATTTCCAAGGTTTGGACTCAAAGTAT
TATTTTTTAACATCACAGAAATTTTTTATGCCCTGGTCTCTTGAGGGTAGGGACTGTGTCTTTTTCA
TCTTTGCCCCTTTCCACATGCTCTGGTTTGTAGAACAGAATTGAAGAAGGAGCCATGTGCTAGAT
AGAGCGGGTTGGGAGCAGCAGACCTGCCTGCTCATTGTTTCTGTGACCTTGACCCATCCTGTAG
TCTCCCCACGCCTTGCTATTCTCATCCATCAACGATGAGATTGCACCTAAATCCTGGGTGCTGTG
CTGGGCCTGGGGATGCTGTCTAATCCCTGGGTTTCCTTCTAGTTTGCTCTAATCACCCATATGAA
AAAGAAGCTACTCAAGGCAAACATATAAATGAAACGGATAATGAATGAATAGTATTTTTTCTTGC
TTTCTTTGGAAGAGGCATCCTGGATTCTACTTGAAGCCTGCAGCCCTGACTCCTGGGCTGGGAG
GTGGGCGAGGACTTGTATTTTGTAGCTCTGCTAGAGAGGGAACAGCTGTCCACTGGTGGCAGG
ACTGACGCCTCCCAGAATACAGGGGCTTTGGATCAGCAGGCGTCAGAGGGAAAGGAAACTGG
CATCCGAGTCTGCCTTTGAGAAAGGAGTAAATTACTATCTTATTATAAATTCATGATCCACTCATT
GCTGTTTTCAATTAATTTCTTGACAAATCTGGCTGTTCCTTTTCTTATTTTCCTTAGGACAGGAGGT
GTCCTGGTGATCAGCTGGAGCTCGGGGGAGCTGTTGCTGAAATTAACTTGAGTGCTTTGGAGAG

AGATTTACATGGCAGAGTGGAGCCAGGGCTGCTCCTTTGGCAGGCACTGCTGTGCCACTGTGG
AGGCGCCCCTGCTTTTCCCATGCTTTGCAGTGTCTTTAAAGGCCTTTGGAGCCATGCCCAGGCTC
AGCCCCATCCTGGTGTCCCCCTGTGTCCGTTTTGCCACTGCCCGGCTACTTCACTTGGCCATGGG
CCTTACCAGCTTGCAGACAAACCCGTCCATCTACGCCTCCCAGCGCAGCTTGGCCCACTTGCAA
GCACTTACAGAGGGTTTCAGAAGGAACATGTCTGCCATCTGTGTTTGGAGACTGTGTCCCAGAC
TTTTGAGCTCACATTAGTCATGGCCACAGACGGGACTGGAGTCCTGCCCACTGGAGTCCCAAAG
GTCTCTGGTGTTTGTTTT

ID|56528|gene_id|ENSG00000224699|transcript_id|ENST00000609653 Homo sapiens lncRNA

GTAGCTAAATTTTTTGTATTTTTTGTAGAGATGGGGTTTTGCCATGTTGCCAGGCTGGTCTCG
AACTCCTGCAATTTGCTCTCCTTGGCCTCACGAAATGCTGGGATTACAGGCATAAGCTACAGCA
CCCAGCCCTTACGTTTACTTTTCATTGACAGGAAGAGTGTCTTCATCTCTGCATCCCTAGTGCAA
CAGAGCATGACAAAAATTCAACAAATATTTATTGAAGAGTCTTGATGTCTTTTGGGAAAGGAACT
CTATGGTTACCTTTACTCTAAGGTAAAGAAATTTAGGTCCTGAACTTTAACCACCTTCTCCTCTTC
TTTCTTCCCTCTTTCATAAATGAGCTCTTGGAAAATTAAAATGCCAGGAGGGTCCAGCAGAGAAT
ACCCAAGCCCACATGTCCTGATGGGAAGTGGGGGATAAGTAGAGAGGGAAACACAGACCCTG
TAGGCCATCTCACTGCTGCTGGCCCAGGGGACCCTAACTCCTTTTGTTGACTCATCAGAAGTAG
AATCCTCCTGAGGGTCTGTTTAAGAAGCACAGCTCTGGTAGAAAGGGTAGCTTTCCTATGGGTA
CGGAGGGCTCTGCTACCCCAGGGCTATCCACACTGGCCTGTGAATCACTCTCAAATGCTTAGGG
TCACGGGCTTTCAGTGGAATGAGCCCTCATGTGCCACCAGGGGGCACCAAGGCCACAGAAAAC
CCTATTCAGAAGGTTCTGGGGTGAGGGAAAGGAATCCACGGGTTTGTGGAGCTGGCGGAGCAT
GGGTGAGGACAGCATTATGCTAGGTTGGTGGCCTTGAAGTCAGATGTAAATTGGGATCCTGGCT
TGGCCACTGACTATCCATGGGAACTTTGAATCTCAGGGGGCTCATTTGTGATGTGGGGGTACCA
TTATCTGCCTCATCAGGTTGTGGGGAGGATTATATGAGATACCTTATGTAAGAGGGGCTGGTGC
ATTGTGGAAGTCAACAAGTAAGTTTTGTTAAATTTGAGTAAGAAGACCTGTTTGTTTCCTCAAGTT
GCTTATAATCTAGGTGAGGACACATCTGAGAGAATAACTAATAGTTCAGTGAAGGTAAGAGCCA
AATGATTGGCACAGACCTCAAGTGGTCTGGCATTTACGGGAGAGGGGGATTGTGAAACAGGAA
TTGAAAGAAATTTAAAAATGCATAAGCAAAAACTCAGTTTATGTTTTAAAAAAACCAATTCCCCC
TGAGAAAGAGAAAGAGGTGGAGTCCTTTAAAAATTATCTGCTTGTTTTTCTGTCTGTGGCTAGTG
AGCCTTATCTCTCCCTTTCCCAGGCATTGTGAAGACCCTGTTTCTCTAGCTGTGCAGCTGCAAGG
TCACTAGGCAGATAAACTCAAGCCATAAAACATGTTTTTCCTTGAAAAGTAAGAAATAATGTAAT
GCATGTGTCAAATGAATAACTATTTCTCGCTTCTGTAATATGCTTCCCTTCGCACAGATCTCCCCC
TGCCCCATGAAATGCTTTAAAGGTAACTTAACTCTTTGTTCGGGGCTCAGTCCTTTGGATGTTAAT
CTGACTGGCTTGGTGCACCTAAATAATAAATAACATATATCCTCCTCAACCCCTCAGTCTCTCTG
ATTCCTAAATTATCCCGCTGTAATTGCAGGGCTAGATTTGCCGACCTGCTTTATAAAGCTATGTTA
AATCAGTATATTTTATTCTTTTTATTAGACTTTTCTATAGCATAAAAATGATAAAATACAGCAAGA
AAAAAAGTCACCTGAAACTCTAGTCCTAACCCTAATATGAATGTGAAGGAGCTGGGCTAGAATG
GAATAGGGTAGAATTTGCATAAGGTGGAAATGGGGACAGGCCTGTCACAAGGATCACTAGAGT
CTTGCTGGCAAAAGCATCTTACTGCATTCATGCTGCAGCACAGGACCCAGATGCAGAACAGGTT
GGGGACACAGCCCTGCCTTCTAATAGTGGACACCATCTGAGAGCTGAGCCATAGATTCAAAGC
CTAACTCCTAATAGCGATTGGTAGTAATATCATCAGCAGTTCATATACATGGAGTGACAACAGC
GTGCCAGGCAATTCAAAGGGTCTCTCGGTCCTCACAGCATTTTTGAAGTAGCTAACATCACTATC
CCCCTTTTACAGTTAAAAACAACAACAACAACAACAAAAACACAAAACCCTGAGGCTCAGAAG
ATAAATATTTGCTGAAGATCACTGTGCTTGTAAGTGGTGGAGCCTGACCATTAACTTCCCCACGA

CACTGCTTCAATTTCTATCACTTCATCAACCACACAACCCGAGAACTGTGAGGGGATTTAGCAG
GCCCAGTCCAGATCTCTTATTGTACAGAGAAGAAATCTGAGGCTCAGAGAGGGGAAGGGACTA
ATTTGGACCTTTAACTTCGGATTCTTAACTTTCTTTCTGGTGTTCTAACTATACTGTACTACAAGAG
GCTGTCTTAGAGCTTTAGTTCAACCAGCTTCAGAGATACTCATTGTTATGGGTTGAATTATGTCTC
CCCAAAATTCATATGTCAAAGTTCTAACCCCCAGTACCTCAGAATGTGACCATATTTGGAGATAG
GACCTTTACAGAGGTAATCAGGTGAAAATGAGGTTGTTAGGGTGGGCCTTAGTCCAGTATGGCT
GGTGTCCTCATAAAAAGGGGAAATTTGGACACAGATGTACATAGAGGGAAGATGGTGTGAAGA
GACATAAAGAACAGATAGTCATCTATAGCCAAGGCCTGGAACAGATCCTTCCCTAACAGCCCTC
AGAAGGAAGCAACCTCACTGACACCTTGATTTTGGACTGCTACCCTCTGGCACTGTGAGACAAT
AAATTCATGTTGGTTAGGCCACCCAGTTTGTTATGGCAGCCCTAGGAAACCTTTGTGGATATATC
TGTTCGTATACCCATAAAGTCTGCAAGTCAAAAATATCACAGACTTTTGGCAGCCCTTCATTCAA
GCACTGGCTGGGAGGGTCACCCTCCCCGAGGCAAAAGGATAGAGCAAATGATCTTTCCAGCTT
GCTCCCAACCTTGATCTTTGGCCAAGATATTTTTCTTTCACTGGACATTGGCCAACATTGGTGTCA
TAACTTAATTTGACTGTGACCTTCTTGAGTCTTTTATGTCTCTGAGCTTGACCTGTAGAAGAAGCT
CAATAAATGTGGGTAGAGGGGATGAGTGGGAAAATCAGCTCCCAGCCATTCCAGTTCAAGCCT
CTTCAAGCAGAAGAGTCCCACCTGAAAGACCTCATTTTCACTTGATTACTCGATCTCCAAGCAAT
AGGTGGGGCAGGAAGCAGGGTATGTAAGGCCTGTCAGTGGATGGAAGTTGAAGTTGGACACC
ATCTCCTAAAGTTTAGAGCTGAGCCCAAATTCTGGAAAACACAAGCAAAATACATTTTATATGAC
TCAGGATGGGCTAAAGAAGTTCCAGTACAGGAAACTGTTTTCCTGCTGCTTTATTAATTTTGCTA
CAGAGGATAAATGAGATGCAGACTTTCATATCCAAGTCTTCATTAGATGCCCAGTTAATGCAGC
CATGACAAATGGGAATGAAGCCCTTATGAATAAAAAAAAGATCAACCCAGTATAAAAAAATCAT
AGACCAGAGAAATGAAAATTTAGAGGAGCTGATAAAATACCCTATCCTTAATTAGACTGTCAGC
ATACACATCAAATCAGCCTTAATGCCAAGATTTATTGGCCTTGGAATTGGGTAAATGAAATAAGT
GCTTACAAATGAATGGAAAAGAAATTACCTGTATTAAAACGTATTTGTGATGCAGGCCTGTGCA
CTGAGGGTTTTAGTCAAACTCAGGCTTGCAGCTTCTTTTGCTCTGCATCCATTTCCACCCCATGTT
CCCCAGTGGGTTTCAGAGACCCTCTTTGTGGGGTAACAATATGTACCATTGTCAACTACCTCTGC
TGTTGTCCTGGAAAAATCCTTCTTGGACAGTAAAATATTTTTGTCTTTATTTCACCATCTCCAGATT
AGATGGGTGTTACTGATTCTTACCTCAGAGAAGAGAATGTCATGGAGACAAAATGACCTGCTTA
GAGTTCTGCACCCCCCAGCATGGAGAGCAGAGCGCAAAGATTTCTTCCGGACTCCCACATTCCC
CTTGGAATCCAAGAACTGGTCTACTGTCACAACACTTTAGTACCCTC

ID|56555|gene_id|ENSG00000224699|transcript_id|ENST00000590826 Homo sapiens lncRNA

GCTTAGTCCAGCCTCAGCCTTCTAGGTTCAAGCGATCTTCCCACCTCAGCCTCCCAAGAAG
CTGGGACTACAGGGACCCGCCACCCCACCTAGCTAATTAAAAAAAAAATTGTAGAGATGGAGT
CTCAGTGTGTAGCCCAGTCTCAAGTGACCCTCCCTCGTTGGCTTCCCAAAGCGCTGAGATTGCA
GGTGAGCCACTGTACTTGGACTTTTTTTTTTTTTTTTGCTTTTGTTTGTTTTTTGTTTTAGCTATTACA
ATGCTGGTTGAATATCCTCATAGCTAAATATCTGTGCAGTTTTAAAATTTCCTTATAAGTTACTAG
AAATGGAATGACTGGGTTAAAGGACATGATCATTTTTTAAGACTCTTGATACATACTTACAAATT
GCCTCCCGAAAAAGTGGTGCTAATTTACTCAAATTGGTAAGCCTCACCCCCTCTGTGGATCATTC
CTTGCACTAAGTTTCTTCCAACTCTGTCACCTCCAGCTTTTTACTCACAGCCTACCTTCCAATCTG
GGGCCCAGCTCCCAGATCGGAAGATACCAGACTTTGGTATTGAACCAAAGTCCAATACACTGC
ACTGGCATTCAGAGTCCCTTTTTATTCCGCAGCTGTCATAGTGAAGCTTGCAGCTTCCTTGAAATT
CCAGTTTGTTTATTTCTTTATTTTTTTGAGACAGAGTCTTGCTCTATCGCCCAGGCTGGAGTGCAG
TGGCGCCGTCTCAGCTCACTGCAACCTCTGCCTCCTGGTTCAAGCGATTCTTCTGCCTCAGCCTC

CCGAGTAGCTGGGATTACAGGCATGCACCACCACACGTGGCTAATTTTTGTGCTTTTAGTAGAG
ATGGGGTTTCACCATGTTGGCCAGGCTGGTCTCAAACTCCTGACCTCAAGCGATCCGCCCACCT
CAGCCTCCCAAAGTGCTGGGATTATAGGCATGAGCCATTGCACCCAGCCAAAATTCCAGTTTGA
ATGTTATAATTCAGGGGTCTTGGACTCCTGCTCTAGAGAAATTATTCATGTTAAGTTAGGGGTAA
CACAGTACAGGCCATATAAGTTTTAGAAAAAGCAGTTTAAAACTTAGCATTAGAAAACATAAGT
ACTGCCTGGTGAAATGGCACGTGCCTATAATCCCAGCTACTCAGGAGGCTGAGGCAGGAGGAT
CCCTTGAGCCCAGGAGTTCAAGTCCAGCCTGGGCAATATAGTCTAGTGGCCACATTAAAAAAAG
TAAAACAGCCGGGCGCGGTGGCTCACGCCTGTAATCCCAGCACTTTGGGAGGCCGAGGCGGGT
GGATCACGAGGTCAGGAGATTGAGACCATCCTGGCTAACACGGTGAAACCCTGTCTCCACTAA
AAATACAAAAAATTCTCCGGGTGTGGTGGTGGGCGCCTGTGGTCCCAGCTACTCAGGAGGCTG
AGGCAGGAGAATGGTGTGAGCCTGGGAGGCGGAGCTTGCAGTGAGCCGAGATCGCGCCACTG
CACTCCAGCCTGGGCGACAGAGCGAGACTCTGTCTCAAAAAAAAAAAAAAAAAAAAAAAAAA
AGTAAAACAGGGCAGGGTGCAGTGGCTCACGCCTGTAATCCTAGCACTTTGGGAGGCCGAGAT
GGGTGGATCAGTTGAGGTCAGGAGTTTGAGACCAGCCTGGCCAACATGGCGAAACCCCATCTC
TACTAAAAATACAAAAATTAGCTGGGTGTGATGGTGCGTGCCTGTAGTCCCAGCTACTCAGGAG
GCTGAGGCAGGAGGATCGTTTGAACTCGGGAGCTGGAGGTTGCAGTGAGCCGAGATTGAGCCA
CAGCACTCCAGGCTGTGCTACAGTGTGAGACTGTCTCAAAAAAAAGTAAAACAGATGAAACTA
ATTTTAATAACATTTTATTTATTCAAACAATATATTCAAAATATTATTTCAACTTGTGATTGATAAA
ATACTAATGAGATATTTTATATTTTTTCTCCAGTACTGTCTTCAAAATCCAGGGTATATTTTTCAGT
TAGCAGCACATTTCAATTCAGACCAGCCGCATTTCAAGTGCTCAGCAGCCACCTGTGGCTAGTG
GCCACAGTATTGGCCAGTGTAGCCCAAGGATATCTCAAATTATCTCAAAGCTAGATCAGTGATT
AAAGGATTCTCAGGCCAGGCATGGTAGTTTTCAGGCCTGGTATGGTGGTTTACGCCTGTAATCC
CCGCACTTTGGGAGGCCTAGGTGGGCGGATGGCTTGAGCCCAGGAGTTTGAGACCAGCCTAGC
CAACGTGGGGAGACCCTGTCTCTACCAAAGCACAAAAATTAGCTGGGCATGGTGATGCGCACC
TGTGGTCTCAAGTACTTTGTGTGAGGTGGGAGGATCACTTGAGCCCGGGAGGTTGAGGCTGCA
GTGAGCTGAGATTGCACAACTGCACTCCAGCCTGGGTGACAGAATGAGACCCTGTCTCAAAAA
AAAAAAAGGATTCTCCGAAAGACTGGCGATTGCTTTTTAAGCTATAAGGAAGGCCTTTGAAGGG
AAATCAAGTAGGAAGAGGATGATAGCTGGCTCACATGTCCTTGCCACATGGGAGGTGAGAGAG
CAGGCTACTCCTTACCACCCCCGCTTTCTCTGCCCTGCTGCTGTCTTGCCCCTGTGCAAGTAGGC
AATGGGAGAGGAATAGTATATATAAATTGGGCAAGTGCTAGGGGCTGGAGTGATGGTTGGGGA
AAGTATCTGACCCTTGTTTTCTACTTTCTTTAGCTTGTGGGGTATTTAACAATTCTTTGTTCTTAGTT
TTGAGTGCTGGCAACCATTTCTGGGGTGACAGGAAAAGTTCCATGACATCCGTATTAGAGCATT
TGTTTCATTATATTGTAATTTTTTATGTTTACTTTTGAAAAAGTTCATTCTTTTCTCTTTTTAATTCTT
ATTCTTTTTTTTTTCTTTTCTTTTTGAGGCAGGGTTTTACTCTATCACCCAGGCTGGAGTGCAGTGG
CACAATCTTGGCTCACTGCAACCTCTGCCTCCTGAGCTCGTGATTCATCCTCCTGAGTAGCTGGG
ACTACAGGTGTACACTACCATGTGTAGCTAAATTTTTTGTATTTTTTGTAGAGATGGGGTTTTGCC
ATGTTGCCAGGCTGGTCTCGAACTCCTGCAATTTGCTCTCCTTGGCCTCACGAAATGCTGGGATT
ACAGGCATAAGCTACAGCACCCAGCCCTTACGTTTACTTTTCATTGACAGGAAGAGTGTCTTCAT
CTCTGCATCCCTAGTGCAACAGAGCATGACAAAAATTCAACAAATATTTATTGAAGAGTCTTGAT
GTCTTTTGGGAAAGGAACTCTATGGTTACCTTTACTCTAAGGTAAAGAAATTTAGGTCCTGAACT
TTAACCACCTTCTCCTCTTCTTTCTTCCCTCTTTCATAAATGAGCTCTTGGAAAATTAAAATGCCA
GGAGGGTCCAGCAGAGAATACCCAAGCCCACATGTCCTGATGGGAAGTGGGGGATAAGTAGA
GAGGGAAACACAGACCCTGTAGGCCATCTCACTGCTGCTGGCCCAGGGGACCCTAACTCCTTTT
GTTGACTCATCAGAAGTAGAATCCTCCTGAGGGTCTGTTTAAGAAGCACAGCTCTGGTAGAAAG

GGTAGCTTTCCTATGGGTACGGAGGGCTCTGCTACCCCAGGGCTATCCACACTGGCCTGTGAAT
CACTCTCAAATGCTTAGGGTCACGGGCTTTCAGTGGAATGAGCCCTCATGTGCCACCAGGGGGC
ACCAAGGCCACAGAAAACCCTATTCAGAAGGTTCTGGGGTGAGGGAAAGGAATCCACGGGTTT
GTGGAGCTGGCGGAGCATGGGTGAGGACAGCATTATGCTAGGTTGGTGGCCTTGAAGTCAGAT
GTAAATTGGGATCCTGGCTTGGCCACTGACTATCCATGGGAACTTTGAATCTCAGGGGGCTCAT
TTGTGATGTGGGGGTACCATTATCTGCCTCATCAGGTTGTGGGGAGGATTATATGAGATACCTTA
TGTAAGAGGGGCTGGTGCATTGTGGAAGTCAACAAGTAAGTTTTGTTAAATTTGAGTAAGAAGA
CCTGTTTGTTTCCTCAAGTTGCTTATAATCTAGGTGAGGACACATCTGAGAGAATAACTAATAGT
TCAGTGAAGGTAAGAGCCAAATGATTGGCACAGACCTCAAGTGGTCTGGCATTTACGGGAGAG
GGGGATTGTGAAACAGGAATTGAAAGAAATTTAAAAATGCATAAGCAAAAACTCAGTTTATGTT
TTAAAAAAACCAATTCCCCCTGAGAAAGAGAAAGAGGTGGAGTCCTTTAAAAATTATCTGCTTG
TTTTTCTGTCTGTGGCTAGTGAGCCTTATCTCTCCCTTTCCCAGGCATTGTGAAGACCCTGTTTCT
CTAGCTGTGCAGCTGCAAGGTCACTAGGCAGATAAACTCAAGCCATAAAACATGTTTTTCCTTG
AAAAGTAAGAAATAATGTAATGCATGTGTCAAATGAATAACTATTTCTCGCTTCTGTAATATGCTT
CCCTTCGCACAGATCTCCCCCTGCCCCATGAAATGCTTTAAAGGTAACTTAACTCTTTGTTCGGG
GCTCAGTCCTTTGGATGTTAATCTGACTGGCTTGGTGCACCTAAATAATAAATAACATATATCCT
CCTCAACCCCTCAGTCTCTCTGATTCCTAAATTATCCCGCTGTAATTGCAGGGCTAGATTTGCCG
ACCTGCTTTATAAAGCTATGTTAAATCAGTATATTTTATTCTTTTTATTAGACTTTTCTATAGCATA
AAAATGATAAAATACAGCAAGAAAAAAAGTCACCTGAAACTCTAGTCCTAACCCTAATATGAAT
GTGAAGGAGCTGGGCTAGAATGGAATAGGGTAGAATTTGCATAAGGTGGAAATGGGGACAGG
CCTGTCACAAGGATCACTAGAGTCTTGCTGGCAAAAGCATCTTACTGCATTCATGCTGCAGCAC
AGGACCCAGATGCAGAACAGGTTGGGGACACAGCCCTGCCTTCTAATAGTGGACACCATCTGA
GAGCTGAGCCATAGATTCAAAGCCTAACTCCTAATAGCGATTGGTAGTAATATCATCAGCAGTT
CATATACATGGAGTGACAACAGCGTGCCAGGCAATTCAAAGGGTCTCTCGGTCCTCACAGCATT
TTTGAAGTAGCTAACATCACTATCCCCCTTTTACAGTTAAAAACAACAACAACAACAACAAAAA
CACAAAACCCTGAGGCTCAGAAGATAAATATTTGCTGAAGATCACTGTGCTTGTAAGTGGTGGA
GCCTGACCATTAACTTCCCCACGACACTGCTTCAATTTCTATCACTTCATCAACCACACAACCCG
AGAACTGTGAGGGGATTTAGCAGGCCCAGTCCAGATCTCTTATTGTACAGAGAAGAAATCTGAG
GCTCAGAGAGGGGAAGGGACTAATTTGGACCTTTAACTTCGGATTCTTAACTTTCTTTCTGGTGT
TCTAACTATACTGTACTACAAGAGGCTGTCTTAGAGCTTTAGTTCAACCAGCTTCAGAGATACTC
ATTGTTATGGGTTGAATTATGTCTCCCCAAAATTCATATGTCAAAGTTCTAACCCCCAGTACCTCA
GAATGTGACCATATTTGGAGATAGGACCTTTACAGAGGTAATCAGGTGAAAATGAGGTTGTTAG
GGTGGGCCTTAGTCCAGTATGGCTGGTGTCCTCATAAAAAGGGGAAATTTGGACACAGATGTAC
ATAGAGGGAAGATGGTGTGAAGAGACATAAAGAACAGATAGTCATCTATAGCCAAGGCCTGGA
ACAGATCCTTCCCTAACAGCCCTCAGAAGGAAGCAACCTCACTGACACCTTGATTTTGGACTGC
TACCCTCTGGCACTGTGAGACAATAAATTCATGTTGGTTAGGCCACCCAGTTTGTTATGGCAGCC
CTAGGAAACCTTTGTGGATATATCTGTTCGTATACCCATAAAGTCTGCAAGTCAAAAATATCACA
GACTTTTGGCAGCCCTTCATTCAAGCACTGGCTGGGAGGGTCACCCTCCCCGAGGCAAAAGGA
TAGAGCAAATGATCTTTCCAGCTTGCTCCCAACCTTGATCTTTGGCCAAGATATTTTTCTTTCACT
GGACATTGGCCAACATTGGTGTCATAACTTAATTTGACTGTGACCTTCTTGAGTCTTTTATGTCTC
TGAGCTTGACCTGTAGAAGAAGCTCAATAAATGTGGGTAGAGGGGATGAGTGGGAAAATCAGC
TCCCAGCCATTCCAGTTCAAGCCTCTTCAAGCAGAAGAGTCCCACCTGAAAGACCTCATTTTCA
CTTGATTACTCGATCTCCAAGCAATAGGTGGGGCAGGAAGCAGGGTATGTAAGGCCTGTCAGT
GGATGGAAGTTGAAGTTGGACACCATCTCCTAAAGTTTAGAGCTGAGCCCAAATTCTGGAAAAC

ACAAGCAAAATACATTTTATATGACTCAGGATGGGCTAAAGAAGTTCCAGTACAGGAAACTGTT
TTCCTGCTGCTTTATTAATTTTGCTACAGAGGATAAATGAGATGCAGACTTTCATATCCAAGTCTT
CATTAGATGCCCAGTTAATGCAGCCATGACAAATGGGAATGAAGCCCTTATGAATAAAAAAAAG
ATCAACCCAGTATAAAAAAATCATAGACCAGAGAAATGAAAATTTAGAGGAGCTGATAAAATA
CCCTATCCTTAATTAGACTGTCAGCATACACATCAAATCAGCCTTAATGCCAAGATTTATTGGCC
TTGGAATTGGGTAAATGAAATAAGTGCTTACAAATGAATGGAAAAGAAATTACCTGTATTAAAA
CGTATTTGTGATGCAGGCCTGTGCACTGAGGGTTTTAGTCAAACTCAGGCTTGCAGCTTCTTTTG
CTCTGCATCCATTTCCACCCCATGTTCCCCAGTGGGTTTCAGAGACCCTCTTTGTGGGGTAACAA
TATGTACCATTGTCAACTACCTCTGCTGTTGTCCTGGAAAAATCCTTCTTGGACAGTAAAATATTT
TTGTCTTTATTTCACCATCTCCAGATTAGATGGGTGTTACTGATTCTTACCTCAGAGAAGAGAATG
TCATGGAGACAAAATGACCTGCTTAGAGTTCTGCACCCCCCAGCATGGAGAGCAGAGCGCAAA
GATTTCTTCCGGACTCCCACATTCCCCTTGGAATCCAAGAACTGGTCTACTGTCACAACACTTTA
GTACCCTCTTGTAAGCCTGTGGACAAATCCTCTGATGCCTTGATACCTCGGTTTTCCCCCACTAT
AAATGGGAAGATAAATTCACTAGAAACAG

ID|56556|gene_id|ENSG00000224699|transcript_id|ENST00000608719 Homo sapiens lncRNA

GGTGCATTGTGGAAGTCAACAAGTAAGTTTTGTTAAATTTGAGTAAGAAGACCTGTTTGTTT
CCTCAAGTTGCTTATAATCTAGGTGAGGACACATCTGAGAGAATAACTAATAGTTCAGTGAAGG
TAAGAGCCAAATGATTGGCACAGACCTCAAGTGGTCTGGCATTTACGGGAGAGGGGGATTGTG
AAACAGGAATTGAAAGAAATTTAAAAATGCATAAGCAAAAACTCAGTTTATGTTTTAAAAAAAC
CAATTCCCCCTGAGAAAGAGAAAGAGGTGGAGTCCTTTAAAAATTATCTGCTTGTTTTTCTGTCT
GTGGCTAGTGAGCCTTATCTCTCCCTTTCCCAGGCATTGTGAAGACCCTGTTTCTCTAGCTGTGC
AGCTGCAAGGTCACTAGGCAGATAAACTCAAGCCATAAAACATGTTTTTCCTTGAAAAGTAAGA
AATAATGTAATGCATGTGTCAAATGAATAACTATTTCTCGCTTCTGTAATATGCTTCCCTTCGCAC
AGATCTCCCCCTGCCCCATGAAATGCTTTAAAGGTAACTTAACTCTTTGTTCGGGGCTCAGTCCT
TTGGATGTTAATCTGACTGGCTTGGTGCACCTAAATAATAAATAACATATATCCTCCTCAACCCC
TCAGTCTCTCTGATTCCTAAATTATCCCGCTGTAATTGCAGGGCTAGATTTGCCGACCTGCTTTAT
AAAGCTATGTTAAATCAGTATATTTTATTCTTTTTATTAGACTTTTCTATAGCATAAAAATGATAAA
ATACAGCAAGAAAAAAAGTCACCTGAAACTCTAGTCCTAACCCTAATATGAATGTGAAGGAGCT
GGGCTAGAATGGAATAGGGTAGAATTTGCATAAGGTGGAAATGGGGACAGGCCTGTCACAAGG
ATCACTAGAGTCTTGCTGGCAAAAGCATCTTACTGCATTCATGCTGCAGCACAGGACCCAGATG
CAGAACAGGTTGGGGACACAGCCCTGCCTTCTAATAGTGGACACCATCTGAGAGCTGAGCCAT
AGATTCAAAGCCTAACTCCTAATAGCGATTGGTAGTAATATCATCAGCAGTTCATATACATGGAG
TGACAACAGCGTGCCAGGCAATTCAAAGGGTCTCTCGGTCCTCACAGCATTTTTGAAGTAGCTA
ACATCACTATCCCCCTTTTACAGTTAAAAACAACAACAACAACAACAAAAACACAAAACCCTGA
GGCTCAGAAGATAAATATTTGCTGAAGATCACTGTGCTTGTAAGTGGTGGAGCCTGACCATTAA
CTTCCCCACGACACTGCTTCAATTTCTATCACTTCATCAACCACACAACCCGAGAACTGTGAGG
GGATTTAGCAGGCCCAGTCCAGATCTCTTATTGTACAGAGAAGAAATCTGAGGCTCAGAGAGG
GGAAGGGACTAATTTGGACCTTTAACTTCGGATTCTTAACTTTCTTTCTGGTGTTCTAACTATACT
GTACTACAAGAGGCTGTCTTAGAGCTTTAGTTCAACCAGCTTCAGAGATACTCATTGTTATGGGT
TGAATTATGTCTCCCCAAAATTCATATGTCAAAGTTCTAACCCCCAGTACCTCAGAATGTGACCA
TATTTGGAGATAGGACCTTTACAGAGGTAATCAGGTGAAAATGAGGTTGTTAGGGTGGGCCTTA
GTCCAGTATGGCTGGTGTCCTCATAAAAAGGGGAAATTTGGACACAGATGTACATAGAGGGAA
GATGGTGTGAAGAGACATAAAGAACAGATAGTCATCTATAGCCAAGGCCTGGAACAGATCCTT

CCCTAACAGCCCTCAGAAGGAAGCAACCTCACTGACACCTTGATTTTGGACTGCTACCCTCTGG
CACTGTGAGACAATAAATTCATGTTGGTTAGGCCACCCAGTTTGTTATGGCAGCCCTAGGAAAC
CTTTGTGGATATATCTGTTCGTATACCCATAAAGTCTGCAAGTCAAAAATATCACAGACTTTTGG
CAGCCCTTCATTCAAGCACTGGCTGGGAGGGTCACCCTCCCCGAGGCAAAAGGATAGAGCAAA
TGATCTTTCCAGCTTGCTCCCAACCTTGATCTTTGGCCAAGATATTTTTCTTTCACTGGACATTGG
CCAACATTGGTGTCATAACTTAATTTGACTGTGACCTTCTTGAGTCTTTTATGTCTCTGAGCTTGA
CCTGTAGAAGAAGCTCAATAAATGTGGGTAGAGGGGATGAGTGGGAAAATCAGCTCCCAGCCA
TTCCAGTTCAAGCCTCTTCAAGCAGAAGAGTCCCACCTGAAAGACCTCATTTTCACTTGATTACT
CGATCTCCAAGCAATAGGTGGGGCAGGAAGCAGGGTATGTAAGGCCTGTCAGTGGATGGAAGT
TGAAGTTGGACACCATCTCCTAAAGTTTAGAGCTGAGCCCAAATTCTGGAAAACACAAGCAAAA
TACATTTTATATGACTCAGGATGGGCTAAAGAAGTTCCAGTACAGGAAACTGTTTTCCTGCTGCT
TTATTAATTTTGCTACAGAGGATAAATGAGATGCAGACTTTCATATCCAAGTCTTCATTAGATGCC
CAGTTAATGCAGCCATGACAAATGGGAATGAAGCCCTTATGAATAAAAAAAAAGATCAACCCAG
TATAAAAAAAATCATAGACCAGAGAAATGAAAATTTAGAGGAGCTGATAAAATACCCTATCCTTA
ATTAGACTGTCAGCATACACATCAAATCAGCCTTAATGCCAAGATTTATTGGCCTTGGAATTGGG
TAAATGAAATAAGTGCTTACAAATGAATGGAAAAGAAATTACCTGTATTAAAACGTATTTGTGAT
GCAGGCCTGTGCACTGAGGGTTTTAGTCAAACTCAGGCTTGCAGCTTCTTTTGCTCTGCATCCAT
TTCCACCCCATGTTCCCCAGTGGGTTTCAGAGACCCTCTTTGTGGGGTAACAATATGTACCATTG
TCAACTACCTCTGCTGTTGTCCTGGAAAAATCCTTCTTGGACAGTAAAATATTTTTGTCTTTATTTC
ACCATCTCCAGATTAGATGGGTGTTACTGATTCTTACCTCAGAGAAGAGAATGTCATGGAGACA
AAATGACCTGCTTAGAGTTCTGCACCCCCCAGCATGGAGAGCAGAGCGCAAAGATTTCTTCCGG
ACTCCCACATTCCCCTTGGAATCCAAGAACTGGTCTACTGTCACAACACTTTAGTACCCTCTTGT
AAGCCTGTGGACAAATCCTCTGATGCCTTGATACCTCGGTTTTCCCCCACTATAAATGGGAAGAT
AAATTCACTAGAAACAGTACTTACCTTGCAGTGTCATTATGAGAATTTCATGAGACAATGCAATA
TAAAGCTCCTAGCACAGTTCCAGGCACCATTGTAAGCACTGAACAAATGAAAGCTGTTGTCATT
AGGATCTCAAGAAATGACAGCTGAGGCAAATGTGGATTAGACTGCATTCAGCATTCTTAGCTGC
AAGCAACAGAAGCTGACGCTGGTTTAAAGAATAGGAAAGGAAGTTAATTAAAATGACATTGGG
TAGCTTGTGGAACTGGGAGGACTAGAGAGCTCAGTCTAAGTTCTAGTGACAAAGCTTCAAATCA
TACATGAGGAAAATGCCCCTCTGCAGGAGGAACGTAATCCTGCAATCACTGCTTCCCCTGAAGC
CAGATGCCTCTACTGCTGCCCTTCCCAGCAGTGATGGAGCCTGGATCACATACCTGGCTTTAGTT
GTAAAGGAGGCTGGAGAGAAAATGCTCTGCTCTATGTAACTGTCTTGAAAGTGGCCCACACATA
GCAAAGCCATTCAAAAGCGGGTGAGCAGCATGAAAGCATCCAGAAGGATTCTTGCTTTGCATG
AGGGAGAACTCTTGCTCCCAGAGTGTGAGACCAGCAGGGGCATTTTCCTCATGTGTGATTAGAA
GCTTTGTCACTAGAAGCTTAGACTGAGTAGGACACCTCTGAGTGTGAATGTCCTACCTGAGTTCT
GCCTCATGCAAAGCAAGAATCCCTCTGGAGGGATTGACACCACAGCCTCTTCCACAATGTCTCC
AAACAACTTTACTGGGCAGCATTCCACTCCTTGACAAACGGGAGAGGTGCATTCTACCTTCTCTC
CTGCATGAGCCTAGACTAATATGGAATTTGGGAGTGTTGATCATTGTACTTTGTGCTCCTAGAGA
TAAGGGTCATCATAAAGCATCTCAGAGTCTCACTGTAGATCCACAGTTCCATTTCTTTATGGATC
CCTTACCCCTACTTTGCCAAATTCTATGCAGATAGCAATAATAATTCAGACAGGGCTTCCTCACT
GAAGGAGTTTGTGCTCTATATAGTTAATAAGCTAGAGATAATGAAAAAAAAAAAAAACTCAACTT
GAACTTTGGGTAAGAGAAACTTTTCAGACCAACGATCATTACCAATAACAGCTTCCACGCATGA
CCCTGCCCGGAGATTGGGGCCTGCACTACTGTTTCCAGGAAACACATTCTTTTGAATTTTTTTATT
TTCTTTGTAGAGATGGGGATCTCACTCTGTTGCCCAGGCTGGTCTCAAACTCCTGGCTTCAAGCA
ATCCTCCTGCTTCGGCCTCCCAAACTTCTGGGATTACAGATATGAGCCACCACGGCTGGCTGAC

AGGCACCAAATTCTAGGAGAGGACTTGAATAGAGTAGGCATCTGTCTCTCCCCATTCCTTTCAG
GGACAGAGCTCTCCTGGTTGGGAGAAAGGGAGAGGGAGAGGGAGGAGGATGAATAGTGAAG
CACCAAACCTCAGAATTAAGGCCTTTTTCTTTTCTTTCTTTCTTTCTTTTTTTTTTTTGAGATGGAGT
TTCGCTCTTGTTGCCCAGGCTGGAGTGCAATGGCACAATCTCGGCTCACCGCAACCTCTGCCTC
CCAGGTTCAAGCGATTCTCCTGCCTCAGCCTCCCGAGTAGCTGGGATTACAGGCATGCGCCACC
ACACCCAGTTAACTTTTTTGTATTTTTAGAGACGGGGTTTCTCCATGTTGGTCAAGCTGGTCTCGA
ACTCCCGACCTCAGGTGCTCTGCCCGACTTAGCCTCCCAAAGTGCTAGGATTACAGGCGTGAGC
CACCATGCCCAGCCAGAATCAGGGCCTTTTTAAAACTTCAGTGTCTTGTCTTTACCCTTCATGGT
GCCTAGAAAAGCATCTGGCTCATAGGTGTTCAGTGAATGATTGTTTCATTAGTGAAGAAATTCCC
TTTGGAAATCTCATAGGAAACTTACCCTCTCCAACCCTTCACCTGTGCATTATTCCAGCTCCCAA
GGAGGCAGCAGAAACTGGGGTAATCACAACGGTCAACAGCAGCAAAGGCAGCACTTTCTG
ID|56557|gene_id|ENSG00000224699|transcript_id|ENST00000608152 Homo sapiens lncRNA

ATGTAAGAGGGGCTGGTGCATTGTGGAAGTCAACAAGTAAGTTTTGTTAAATTTGAGTAAG
AAGACCTGTTTGTTTCCTCAAGTTGCTTATAATCTAGGTGAGGACACATCTGAGAGAATAACTAA
TAGTTCAGTGAAGGTAAGAGCCAAATGATTGGCACAGACCTCAAGTGGTCTGGCATTTACGGGA
GAGGGGGATTGTGAAACAGGAATTGAAAGAAATTTAAAAATGCATAAGCAAAAACTCAGTTTAT
GTTTTAAAAAAACCAATTCCCCCTGAGAAAGAGAAAGAGGTGGAGTCCTTTAAAAATTATCTGC
TTGTTTTTCTGTCTGTGGCTAGTGAGCCTTATCTCTCCCTTTCCCAGGCATTGTGAAGACCCTGTT
TCTCTAGCTGTGCAGCTGCAAGGTCACTAGGCAGATAAACTCAAGCCATAAAACATGTTTTTCCT
TGAAAAGTAAGAAATAATGTAATGCATGTGTCAAATGAATAACTATTTCTCGCTTCTGTAATATG
CTTCCCTTCGCACAGATCTCCCCCTGCCCCATGAAATGCTTTAAAGGTAACTTAACTCTTTGTTCG
GGGCTCAGTCCTTTGGATGTTAATCTGACTGGCTTGGTGCACCTAAATAATAAATAACATATATC
CTCCTCAACCCCTCAGTCTCTCTGATTCCTAAATTATCCCGCTGTAATTGCAGGGCTAGATTTGCC
GACCTGCTTTATAAAGCTATGTTAAATCAGTATATTTTATTCTTTTTATTAGACTTTTCTATAGCAT
AAAAATGATAAAATACAGCAAGAAAAAAAGTCACCTGAAACTCTAGTCCTAACCCTAATATGAA
TGTGAAGGAGCTGGGCTAGAATGGAATAGGGTAGAATTTGCATAAGGTGGAAATGGGGACAGG
CCTGTCACAAGGATCACTAGAGTCTTGCTGGCAAAAGCATCTTACTGCATTCATGCTGCAGCAC
AGGACCCAGATGCAGAACAGGTTGGGGACACAGCCCTGCCTTCTAATAGTGGACACCATCTGA
GAGCTGAGCCATAGATTCAAAGCCTAACTCCTAATAGCGATTGGTAGTAATATCATCAGCAGTT
CATATACATGGAGTGACAACAGCGTGCCAGGCAATTCAAAGGGTCTCTCGGTCCTCACAGCATT
TTTGAAGTAGCTAACATCACTATCCCCCTTTTACAGTTAAAAACAACAACAACAACAACAAAAA
CACAAAACCCTGAGGCTCAGAAGATAAATATTTGCTGAAGATCACTGTGCTTGTAAGTGGTGGA
GCCTGACCATTAACTTCCCCACGACACTGCTTCAATTTCTATCACTTCATCAACCACACAACCCG
AGAACTGTGAGGGGATTTAGCAGGCCCAGTCCAGATCTCTTATTGTACAGAGAAGAAATCTGAG
GCTCAGAGAGGGGAAGGGACTAATTTGGACCTTTAACTTCGGATTCTTAACTTTCTTTCTGGTGT
TCTAACTATACTGTACTACAAGAGGCTGTCTTAGAGCTTTAGTTCAACCAGCTTCAGAGATACTC
ATTGTTATGGGTTGAATTATGTCTCCCCAAAATTCATATGTCAAAGTTCTAACCCCCAGTACCTCA
GAATGTGACCATATTTGGAGATAGGACCTTTACAGAGGTAATCAGGTGAAAATGAGGTTGTTAG
GGTGGGCCTTAGTCCAGTATGGCTGGTGTCCTCATAAAAAGGGGAAATTTGGACACAGATGTAC
ATAGAGGGAAGATGGTGTGAAGAGACATAAAGAACAGATAGTCATCTATAGCCAAGGCCTGGA
ACAGATCCTTCCCTAACAGCCCTCAGAAGGAAGCAACCTCACTGACACCTTGATTTTGGACTGC
TACCCTCTGGCACTGTGAGACAATAAATTCATGTTGGTTAGGCCACCCAGTTTGTTATGGCAGCC
CTAGGAAACCTTTGTGGATATATCTGTTCGTATACCCATAAAGTCTGCAAGTCAAAAATATCACA

GACTTTTGGCAGCCCTTCATTCAAGCACTGGCTGGGAGGGTCACCCTCCCCGAGGCAAAAGGA
TAGAGCAAATGATCTTTCCAGCTTGCTCCCAACCTTGATCTTTGGCCAAGATATTTTTCTTTCACT
GGACATTGGCCAACATTGGTGTCATAACTTAATTTGACTGTGACCTTCTTGAGTCTTTTATGTCTC
TGAGCTTGACCTGTAGAAGAAGCTCAATAAATGTGGGTAGAGGGGATGAGTGGGAAAATCAGC
TCCCAGCCATTCCAGTTCAAGCCTCTTCAAGCAGAAGAGTCCCACCTGAAAGACCTCATTTTCA
CTTGATTACTCGATCTCCAAGCAATAGGTGGGGCAGGAAGCAGGGTATGTAAGGCCTGTCAGT
GGATGGAAGTTGAAGTTGGACACCATCTCCTAAAGTTTAGAGCTGAGCCCAAATTCTGGAAAAC
ACAAGCAAAATACATTTTATATGACTCAGGATGGGCTAAAGAAGTTCCAGTACAGGAAACTGTT
TTCCTGCTGCTTTATTAATTTTGCTACAGAGGATAAATGAGATGCAGACTTTCATATCCAAGTCTT
CATTAGATGCCCAGTTAATGCAGCCATGACAAATGGGAATGAAGCCCTTATGAATAAAAAAAAG
ATCAACCCAGTATAAAAAAATCATAGACCAGAGAAATGAAAATTTAGAGGAGCTGATAAAATA
CCCTATCCTTAATTAGACTGTCAGCATACACATCAAATCAGCCTTAATGCCAAGATTTATTGGCC
TTGGAATTGGGTAAATGAAATAAGTGCTTACAAATGAATGGAAAAGAAATTACCTGTATTAAAA
CGTATTTGTGATGCAGGCCTGTGCACTGAGGGTTTTAGTCAAACTCAGGCTTGCAGCTTCTTTTG
CTCTGCATCCATTTCCACCCCATGTTCCCCAGTGGGTTTCAGAGACCCTCTTTGTGGGGTAACAA
TATGTACCATTGTCAACTACCTCTGCTGTTGTCCTGGAAAAATCCTTCTTGGACAGTAAAATATTT
TTGTCTTTATTTCACCATCTCCAGATTAGATGGGTGTTACTGATTCTTACCTCAGAGAAGAGAATG
TCATGGAGACAAAATGACCTGCTTAGAGTTCTGCACCCCCCAGCATGGAGAGCAGAGCGCAAA
GATTTCTTCCGGACTCCCACATTCCCCTTGGAATCCAAGAACTGGTCTACTGTCACAACACTTTA
GTACCCTC

ID|56561|gene_id|ENSG00000224699|transcript_id|ENST00000608077 Homo sapiens lncRNA

AATGGGGACAGGCCTGTCACAAGGATCACTAGAGTCTTGCTGGCAAAAGCATCTTACTGC
ATTCATGCTGCAGCACAGGACCCAGATGCAGAACAGGTTGGGGACACAGCCCTGCCTTCTAAT
AGTGGACACCATCTGAGAGCTGAGCCATAGATTCAAAGCCTAACTCCTAATAGCGATTGGTAGT
AATATCATCAGCAGTTCATATACATGGAGTGACAACAGCGTGCCAGGCAATTCAAAGGGTCTCT
CGGTCCTCACAGCATTTTTGAAGTAGCTAACATCACTATCCCCCTTTTACAGTTAAAAACAACAA
CAACAACAACAAAAACACAAAACCCTGAGGCTCAGAAGATAAATATTTGCTGAAGATCACTGT
GCTTGTAAGTGGTGGAGCCTGACCATTAACTTCCCCACGACACTGCTTCAATTTCTATCACTTCA
TCAACCACACAACCCGAGAACTGTGAGGGGATTTAGCAGGCCCAGTCCAGATCTCTTATTGTAC
AGAGAAGAAATCTGAGGCTCAGAGAGGGGAAGGGACTAATTTGGACCTTTAACTTCGGATTCTT
AACTTTCTTTCTGGTGTTCTAACTATACTGTACTACAAGAGGCTGTCTTAGAGCTTTAGTTCAACC
AGCTTCAGAGATACTCATTGTTATGGGTTGAATTATGTCTCCCCAAAATTCATATGTCAAAGTTCT
AACCCCCAGTACCTCAGAATGTGACCATATTTGGAGATAGGACCTTTACAGAGGTAATCAGGTG
AAAATGAGGTTGTTAGGGTGGGCCTTAGTCCAGTATGGCTGGTGTCCTCATAAAAAGGGGAAAT
TTGGACACAGATGTACATAGAGGGAAGATGGTGTGAAGAGACATAAAGAACAGATAGTCATCT
ATAGCCAAGGCCTGGAACAGATCCTTCCCTAACAGCCCTCAGAAGGAAGCAACCTCACTGACA
CCTTGATTTTGGACTGCTACCCTCTGGCACTGTGAGACAATAAATTCATGTTGGTTAGGCCACCC
AGTTTGTTATGGCAGCCCTAGGAAACCTTTGTGGATATATCTGTTCGTATACCCATAAAGTCTGC
AAGTCAAAAATATCACAGACTTTTGGCAGCCCTTCATTCAAGCACTGGCTGGGAGGGTCACCCT
CCCCGAGGCAAAAGGATAGAGCAAATGATCTTTCCAGCTTGCTCCCAACCTTGATCTTTGGCCA
AGATATTTTTCTTTCACTGGACATTGGCCAACATTGGTGTCATAACTTAATTTGACTGTGACCTTC
TTGAGTCTTTTATGTCTCTGAGCTTGACCTGTAGAAGAAGCTCAATAAATGTGGGTAGAGGGGAT
GAGTGGGAAAATCAGCTCCCAGCCATTCCAGTTCAAGCCTCTTCAAGCAGAAGAGTCCCACCTG

AAAGACCTCATTTTCACTTGATTACTCGATCTCCAAGCAATAGGTGGGGCAGGAAGCAGGGTAT
GTAAGGCCTGTCAGTGGATGGAAGTTGAAGTTGGACACCATCTCCTAAAGTTTAGAGCTGAGCC
CAAATTCTGGAAAACACAAGCAAAATACATTTTATATGACTCAGGATGGGCTAAAGAAGTTCCA
GTACAGGAAACTGTTTTCCTGCTGCTTTATTAATTTTGCTACAGAGGATAAATGAGATGCAGACT
TTCATATCCAAGTCTTCATTAGATGCCCAGTTAATGCAGCCATGACAAATGGGAATGAAGCCCTT
ATGAATAAAAAAAAGATCAACCCAGTATAAAAAAATCATAGACCAGAGAAATGAAAATTTAGA
GGAGCTGATAAAATACCCTATCCTTAATTAGACTGTCAGCATACACATCAAATCAGCCTTAATGC
CAAGATTTATTGGCCTTGGAATTGGGTAAATGAAATAAGTGCTTACAAATGAATGGAAAAGAAA
TTACCTGTATTAAAACGTATTTGTGATGCAGGCCTGTGCACTGAGGGTTTTAGTCAAACTCAGGC
TTGCAGCTTCTTTTGCTCTGCATCCATTTCCACCCCATGTTCCCCAGTGGGTTTCAGAGACCCTCT
TTGTGGGGTAACAATATGTACCATTGTCAACTACCTCTGCTGTTGTCCTGGAAAAATCCTTCTTG
GACAGTAAAATATTTTTGTCTTTATTTCACCATCTCCAGATTAGATGGGTGTTACTGATTCTTACCT
CAGAGAAGAGAATGTCATGGAGACAAAATGACCTGCTTAGAGTTCTGCACCCCCCAGCATGGA
GAGCAGAGCGCAAAGATTTCTTCCGGACTCCCACATTCCCCTTGGAATCCAAGAACTGGTCTAC
TGTCACAACACTTTAGTACCCTCTTGTAAGCCTGTGGACAAATCCTCTGATGCCTTGATACCTCG
GTTTTCCCCCACTATAAATGGGAAGATAAATTCACTAGAAACAGTACTTACCTTGCAGTGTCATT
ATGAGAATTTCATGAGACAATGCAATATAAAGCTCCTAGCACAGTTCCAGGCACCATTGTAAGC
ACTGAACAAATGAAAGCTGTTGTCATTAGGATCTCAAGAAATGACAGCTGAGGCAAATGTGGAT
TAGACTGCATTCAGCATTCTTAGCTGCAAGCAACAGAAGCTGACGCTGGTTTAAAGAATAGGAA
AGGAAGTTAATTAAAATGACATTGGGTAGCTTGTGGAACTGGGAGGACTAGAGAGCTCAGTCTA
AGTTCTAGTGACAAAGCTTCAAATCATACATGAGGAAAATGCCCCTCTGCAGGAGGAACGTAAT
CCTGCAATCACTGCTTCCCCTGAAGCCAGATGCCTCTACTGCTGCCCTTCCCAGCAGTGATGGA
GCCTGGATCACATACCTGGCTTTAGTTGTAAAGGAGGCTGGAGAGAAAATGCTCTGCTCTATGT
AACTGTCTTGAAAGTGGCCCACACATAGCAAAGCCATTCAAAAGCGGGTGAGCAGCATGAAAG
CATCCAGAAGGATTCTTGCTTTGCATGAGGGAGAACTCTTGCTCCCAGAGTGTGAGACCAGCAG
GGGCATTTTCCTCATGTGTGATTAGAAGCTTTGTCACTAGAAGCTTAGACTGAGTAGGACACCTC
TGAGTGTGAATGTCCTACCTGAGTTCTGCCTCATGCAAAGCAAGAATCCCTCTGGAGGGATTGA
CACCACAGCCTCTTCCACAATGTCTCCAAACAACTTTACTGGGCAGCATTCCACTCCTTGACAAA
CGGGAGAGGTGCATTCTACCTTCTCTCCTGCATGAGCCTAGACTAATATGGAATTTGGGAGTGTT
GATCATTGTACTTTGTGCTCCTAGAGATAAGGGTCATCATAAAGCATCTCAGAGTCTCACTGTAG
ATCCACAGTTCCATTTCTTTATGGATCCCTTACCCCTACTTTGCCAAATTCTATGCAGATAGCAAT
AATAATTCAGACAGGGCTTCCTCACTGAAGGAGTTTGTGCTCTATATAGTTAATAAGCTAGAGAT
AATGAAAAAAAAAAAAACTCAACTTGAACTTTGGGTAAGAGAAACTTTTCAGACCAACGATCAT
TACCAATAACAGCTTCCACGCATGACCCTGCCCGGAGATTGGGGCCTGCACTACTGTTTCCAGG
AAACACATTCTTTTGAATTTTTTTATTTTCTTTGTAGAGATGGGGATCTCACTCTGTTGCCCAGGCT
GGTCTCAAACTCCTGGCTTCAAGCAATCCTCCTGCTTCGGCCTCCCAAACTTCTGGGATTACAGA
TATGAGCCACCACGGCTGGCTGACAGGCACCAAATTCTAGGAGAGGACTTGAATAGAGTAGGC
ATCTGTCTCTCCCCATTCCTTTCAGGGACAGAGCTCTCCTGGTTGGGAGAAAGGGAGAGGGAGA
GGGAGGAGGATGAATAGTGAAGCACCAAACCTCAGAATTAAGGCCTTTTTCTTTTCTTTCTTTCT
TTCTTTTTTTTTTTTGAGATGGAGTTTCGCTCTTGTTGCCCAGGCTGGAGTGCAATGGCACAATCT
CGGCTCACCGCAACCTCTGCCTCCCAGGTTCAAGCGATTCTCCTGCCTCAGCCTCCCGAGTAGC
TGGGATTACAGGCATGCGCCACCACACCCAGTTAACTTTTTTGTATTTTTAGAGACGGGGTTTCT
CCATGTTGGTCAAGCTGGTCTCGAACTCCCGACCTCAGGTGCTCTGCCCGACTTAGCCTCCCAA
AGTGCTAGGATTACAGGCGTGAGCCACCATGCCCAGCCAGAATCAGGGCCTTTTTAAAACTTCA

GTGTCTTGTCTTTACCCTTCATGGTGCCTAGAAAAGCATCTGGCTCATAGGTGTTCAGTGAATGA
TTGTTTCATTAGTGAAGAAATTCCCTTTGGAAATCTCATAGGAAACTTACCCTCTCCAACCCTTCA
CCTGTGCATTATTCCAGCTCCCAAGGAGGCAGCAGAAACTGGGGTAATCACAACGGTCAACAG
CAGCAAAGGCA

ID|56573|gene_id|ENSG00000224699|transcript_id|ENST00000585330 Homo sapiens lncRNA

AGTGTCTTCATCTCTGCATCCCTAGTGCAACAGAGCATGACAAAAATTCAACAAATATTTAT
TGAAGAGTCTTGATGTCTTTTGGGAAAGGAACTCTATGGTTACCTTTACTCTAAGGTAAAGAAAT
TTAGGTCCTGAACTTTAACCACCTTCTCCTCTTCTTTCTTCCCTCTTTCATAAATGAGCTCTTGGAA
AATTAAAATGCCAGGAGGGTCCAGCAGAGAATACCCAAGCCCACATGTCCTGATGGGAAGTGG
GGGATAAGTAGAGAGGGAAACACAGACCCTGTAGGCCATCTCACTGCTGCTGGCCCAGGGGA
CCCTAACTCCTTTTGTTGACTCATCAGAAGTAGAATCCTCCTGAGGGTCTGTTTAAGAAGCACAG
CTCTGGTAGAAAGGGTAGCTTTCCTATGGGTACGGAGGGCTCTGCTACCCCAGGGCTATCCACA
CTGGCCTGTGAATCACTCTCAAATGCTTAGGGTCACGGGCTTTCAGTGGAATGAGCCCTCATGT
GCCACCAGGGGGCACCAAGGCCACAGAAAACCCTATTCAGAAGGTTCTGGGGTGAGGGAAAG
GAATCCACGGGTTTGTGGAGCTGGCGGAGCATGGGTGAGGACAGCATTATGCTAGGTTGGTGG
CCTTGAAGTCAGATGTAAATTGGGATCCTGGCTTGGCCACTGACTATCCATGGGAACTTTGAATC
TCAGGGGGCTCATTTGTGATGTGGGGGTACCATTATCTGCCTCATCAGGTTGTGGGGAGGATTA
TATGAGATACCTTATGTAAGAGGGGCTGGTGCATTGTGGAAGTCAACAAGTAAGTTTTGTTAAAT
TTGAGTAAGAAGACCTGTTTGTTTCCTCAAGTTGCTTATAATCTAGGTGAGGACACATCTGAGAG
AATAACTAATAGTTCAGTGAAGGTAAGAGCCAAATGATTGGCACAGACCTCAAGTGGTCTGGCA
TTTACGGGAGAGGGGGATTGTGAAACAGGAATTGAAAGAAATTTAAAAATGCATAAGCAAAAA
CTCAGTTTATGTTTTAAAAAAACCAATTCCCCCTGAGAAAGAGAAAGAGGTGGAGTCCTTTAAA
AATTATCTGCTTGTTTTTCTGTCTGTGGCTAGTGAGCCTTATCTCTCCCTTTCCCAGGCATTGTGA
AGACCCTGTTTCTCTAGCTGTGCAGCTGCAAGGTCACTAGGCAGATAAACTCAAGCCATAAAAC
ATGTTTTTCCTTGAAAAGTAAGAAATAATGTAATGCATGTGTCAAATGAATAACTATTTCTCGCTT
CTGTAATATGCTTCCCTTCGCACAGATCTCCCCCTGCCCCATGAAATGCTTTAAAGGTAACTTAA
CTCTTTGTTCGGGGCTCAGTCCTTTGGATGTTAATCTGACTGGCTTGGTGCACCTAAATAATAAAT
AACATATATCCTCCTCAACCCCTCAGTCTCTCTGATTCCTAAATTATCCCGCTGTAATTGCAGGG
CTAGATTTGCCGACCTGCTTTATAAAGCTATGTTAAATCAGTATATTTTATTCTTTTTATTAGACTT
TTCTATAGCATAAAAATGATAAAATACAGCAAGAAAAAAAGTCACCTGAAACTCTAGTCCTAAC
CCTAATATGAATGTGAAGGAGCTGGGCTAGAATGGAATAGGGTAGAATTTGCATAAGGTGGAA
ATGGGGACAGGCCTGTCACAAGGATCACTAGAGTCTTGCTGGCAAAAGCATCTTACTGCATTCA
TGCTGCAGCACAGGACCCAGATGCAGAACAGGTTGGGGACACAGCCCTGCCTTCTAATAGTGG
ACACCATCTGAGAGCTGAGCCATAGATTCAAAGCCTAACTCCTAATAGCGATTGGTAGTAATAT
CATCAGCAGTTCATATACATGGAGTGACAACAGCGTGCCAGGCAATTCAAAGGGTCTCTCGGTC
CTCACAGCATTTTTGAAGTAGCTAACATCACTATCCCCCTTTTACAGTTAAAAACAACAACAACA
ACAACAAAAACACAAAACCCTGAGGCTCAGAAGATAAATATTTGCTGAAGATCACTGTGCTTGT
AAGTGGTGGAGCCTGACCATTAACTTCCCCACGACACTGCTTCAATTTCTATCACTTCATCAACC
ACACAACCCGAGAACTGTGAGGGGATTTAGCAGGCCCAGTCCAGATCTCTTATTGTACAGAGA
AGAAATCTGAGGCTCAGAGAGGGGAAGGGACTAATTTGGACCTTTAACTTCGGATTCTTAACTT
TCTTTCTGGTGTTCTAACTATACTGTACTACAAGAGGCTGTCTTAGAGCTTTAGTTCAACCAGCTT
CAGAGATACTCATTGTTATGGGTTGAATTATGTCTCCCCAAAATTCATATGTCAAAGTTCTAACC
CCCAGTACCTCAGAATGTGACCATATTTGGAGATAGGACCTTTACAGAGGTAATCAGGTGAAAA

TGAGGTTGTTAGGGTGGGCCTTAGTCCAGTATGGCTGGTGTCCTCATAAAAAGGGGAAATTTGG
ACACAGATGTACATAGAGGGAAGATGGTGTGAAGAGACATAAAGAACAGATAGTCATCTATAG
CCAAGGCCTGGAACAGATCCTTCCCTAACAGCCCTCAGAAGGAAGCAACCTCACTGACACCTT
GATTTTGGACTGCTACCCTCTGGCACTGTGAGACAATAAATTCATGTTGGTTAGGCCACCCAGTT
TGTTATGGCAGCCCTAGGAAACCTTTGTGGATATATCTGTTCGTATACCCATAAAGTCTGCAAGT
CAAAAATATCACAGACTTTTGGCAGCCCTTCATTCAAGCACTGGCTGGGAGGGTCACCCTCCCC
GAGGCAAAAGGATAGAGCAAATGATCTTTCCAGCTTGCTCCCAACCTTGATCTTTGGCCAAGAT
ATTTTTCTTTCACTGGACATTGGCCAACATTGGTGTCATAACTTAATTTGACTGTGACCTTCTTGA
GTCTTTTATGTCTCTGAGCTTGACCTGTAGAAGAAGCTCAATAAATGTGGGTAGAGGGGATGAG
TGGGAAAATCAGCTCCCAGCCATTCCAGTTCAAGCCTCTTCAAGCAGAAGAGTCCCACCTGAAA
GACCTCATTTTCACTTGATTACTCGATCTCCAAGCAATAGGTGGGGCAGGAAGCAGGGTATGTA
AGGCCTGTCAGTGGATGGAAGTTGAAGTTGGACACCATCTCCTAAAGTTTAGAGCTGAGCCCAA
ATTCTGGAAAACACAAGCAAAATACATTTTATATGACTCAGGATGGGCTAAAGAAGTTCCAGTA
CAGGAAACTGTTTTCCTGCTGCTTTATTAATTTTGCTACAGAGGATAAATGAGATGCAGACTTTC
ATATCCAAGTCTTCATTAGATGCCCAGTTAATGCAGCCATGACAAATGGGAATGAAGCCCTTAT
GAATAAAAAAAAGATCAACCCAGTATAAAAAAATCATAGACCAGAGAAATGAAAATTTAGAGG
AGCTGATAAAATACCCTATCCTTAATTAGACTGTCAGCATACACATCAAATCAGCCTTAATGCCA
AGATTTATTGGCCTTGGAATTGGGTAAATGAAATAAGTGCTTACAAATGAATGGAAAAGAAATT
ACCTGTATTAAAACGTATTTGTGATGCAGGCCTGTGCACTGAGGGTTTTAGTCAAACTCAGGCTT
GCAGCTTCTTTTGCTCTGCATCCATTTCCACCCCATGTTCCCCAGTGGGTTTCAGAGACCCTCTTT
GTGGGGTAACAATATGTACCATTGTCAACTACCTCTGCTGTTGTCCTGGAAAAATCCTTCTTGGA
CAGTAAAATATTTTTGTCTTTATTTCACCATCTCCAGATTAGATGGGTGTTACTGATTCTTACCTCA
GAGAAGAGAATGTCATGGAGACAAAATGACCTGCTTAGAGTTCTGCACCCCCCAGCATGGAGA
GCAGAGCGCAAAGATTTCTTCCGGACTCCCACATTCCCCTTGGAATCCAAGAACTGGTCTACTG
TCACAACACTTTAGTACCCTCTTGTAAGCCTGTGGACAAATCCTCTGATGCCTTGATACCTCGGT
TTTCCCCCACTATAAATGGGAAGATAAATTCACTAGAAACAGTA
ID|56576|gene_id|ENSG00000224699|transcript_id|ENST00000608111 Homo sapiens lncRNA
GAGGATTATATGAGATACCTTATGTAAGAGGGGCTGGTGCATTGTGGAAGTCAACAAGTA
AGTTTTGTTAAATTTGAGTAAGAAGACCTGTTTGTTTCCTCAAGTTGCTTATAATCTAGGTGAGGA
CACATCTGAGAGAATAACTAATAGTTCAGTGAAGGTAAGAGCCAAATGATTGGCACAGACCTCA
AGTGGTCTGGCATTTACGGGAGAGGGGGATTGTGAAACAGGAATTGAAAGAAATTTAAAAATG
CATAAGCAAAAACTCAGTTTATGTTTTAAAAAAACCAATTCCCCCTGAGAAAGAGAAAGAGGTG
GAGTCCTTTAAAAATTATCTGCTTGTTTTTCTGTCTGTGGCTAGTGAGCCTTATCTCTCCCTTTCCC
AGGCATTGTGAAGACCCTGTTTCTCTAGCTGTGCAGCTGCAAGGTCACTAGGCAGATAAACTCA
AGCCATAAAACATGTTTTTCCTTGAAAAGTAAGAAATAATGTAATGCATGTGTCAAATGAATAAC
TATTTCTCGCTTCTGTAATATGCTTCCCTTCGCACAGATCTCCCCCTGCCCCATGAAATGCTTTAA
AGGTAACTTAACTCTTTGTTCGGGGCTCAGTCCTTTGGATGTTAATCTGACTGGCTTGGTGCACC
TAAATAATAAATAACATATATCCTCCTCAACCCCTCAGTCTCTCTGATTCCTAAATTATCCCGCTG
TAATTGCAGGGCTAGATTTGCCGACCTGCTTTATAAAGCTATGTTAAATCAGTATATTTTATTCTT
TTTATTAGACTTTTCTATAGCATAAAAATGATAAAATACAGCAAGAAAAAAAGTCACCTGAAACT
CTAGTCCTAACCCTAATATGAATGTGAAGGAGCTGGGCTAGAATGGAATAGGGTAGAATTTGCA
TAAGGTGGAAATGGGGACAGGCCTGTCACAAGGATCACTAGAGTCTTGCTGGCAAAAGCATCT
TACTGCATTCATGCTGCAGCACAGGACCCAGATGCAGAACAGGTTGGGGACACAGCCCTGCCT

TCTAATAGTGGACACCATCTGAGAGCTGAGCCATAGATTCAAAGCCTAACTCCTAATAGCGATT
GGTAGTAATATCATCAGCAGTTCATATACATGGAGTGACAACAGCGTGCCAGGCAATTCAAAGG
GTCTCTCGGTCCTCACAGCATTTTTGAAGTAGCTAACATCACTATCCCCCTTTTACAGTTAAAAAC
AACAACAACAACAACAAAAACACAAAACCCTGAGGCTCAGAAGATAAATATTTGCTGAAGATC
ACTGTGCTTGTAAGTGGTGGAGCCTGACCATTAACTTCCCCACGACACTGCTTCAATTTCTATCA
CTTCATCAACCACACAACCCGAGAACTGTGAGGGGATTTAGCAGGCCCAGTCCAGATCTCTTAT
TGTACAGAGAAGAAATCTGAGGCTCAGAGAGGGGAAGGGACTAATTTGGACCTTTAACTTCGG
ATTCTTAACTTTCTTTCTGGTGTTCTAACTATACTGTACTACAAGAGGCTGTCTTAGAGCTTTAGTT
CAACCAGCTTCAGAGATACTCATTGTTATGGGTTGAATTATGTCTCCCCAAAATTCATATGTCAA
AGTTCTAACCCCCAGTACCTCAGAATGTGACCATATTTGGAGATAGGACCTTTACAGAGGTAAT
CAGGTGAAAATGAGGTTGTTAGGGTGGGCCTTAGTCCAGTATGGCTGGTGTCCTCATAAAAAGG
GGAAATTTGGACACAGATGTACATAGAGGGAAGATGGTGTGAAGAGACATAAAGAACAGATAG
TCATCTATAGCCAAGGCCTGGAACAGATCCTTCCCTAACAGCCCTCAGAAGGAAGCAACCTCAC
TGACACCTTGATTTTGGACTGCTACCCTCTGGCACTGTGAGACAATAAATTCATGTTGGTTAGGC
CACCCAGTTTGTTATGGCAGCCCTAGGAAACCTTTGTGGATATATCTGTTCGTATACCCATAAAG
TCTGCAAGTCAAAAATATCACAGACTTTTGGCAGCCCTTCATTCAAGCACTGGCTGGGAGGGTC
ACCCTCCCCGAGGCAAAAGGATAGAGCAAATGATCTTTCCAGCTTGCTCCCAACCTTGATCTTT
GGCCAAGATATTTTTCTTTCACTGGACATTGGCCAACATTGGTGTCATAACTTAATTTGACTGTGA
CCTTCTTGAGTCTTTTATGTCTCTGAGCTTGACCTGTAGAAGAAGCTCAATAAATGTGGGTAGAG
GGGATGAGTGGGAAAATCAGCTCCCAGCCATTCCAGTTCAAGCCTCTTCAAGCAGAAGAGTCC
CACCTGAAAGACCTCATTTTCACTTGATTACTCGATCTCCAAGCAATAGGTGGGGCAGGAAGCA
GGGTATGTAAGGCCTGTCAGTGGATGGAAGTTGAAGTTGGACACCATCTCCTAAAGTTTAGAGC
TGAGCCCAAATTCTGGAAAACACAAGCAAAATACATTTTATATGACTCAGGATGGGCTAAAGAA
GTTCCAGTACAGGAAACTGTTTTCCTGCTGCTTTATTAATTTTGCTACAGAGGATAAATGAGATG
CAGACTTTCATATCCAAGTCTTCATTAGATGCCCAGTTAATGCAGCCATGACAAATGGGAATGA
AGCCCTTATGAATAAAAAAAAGATCAACCCAGTATAAAAAAATCATAGACCAGAGAAATGAAA
ATTTAGAGGAGCTGATAAAATACCCTATCCTTAATTAGACTGTCAGCATACACATCAAATCAGCC
TTAATGCCAAGATTTATTGGCCTTGGAATTGGGTAAATGAAATAAGTGCTTACAAATGAATGGAA
AAGAAATTACCTGTATTAAAACGTATTTGTGATGCAGGCCTGTGCACTGAGGGTTTTAGTCAAAC
TCAGGCTTGCAGCTTCTTTTGCTCTGCATCCATTTCCACCCCATGTTCCCCAGTGGGTTTCAGAG
ACCCTCTTTGTGGGGTAACAATATGTACCATTGTCAACTACCTCTGCTGTTGTCCTGGAAAAATC
CTTCTTGGACAGTAAAATATTTTTGTCTTTATTTCACCATCTCCAGATTAGATGGGTGTTACTGATT
CTTACCTCAGAGAAGAGAATGTCATGGAGACAAAATGACCTGCTTAGAGTTCTGCACCCCCCAG
CATGGAGAGCAGAGCGCAAAGATTTCTTCCGGACTCCCACATTCCCCTTGGAATCCAAGAACTG
GTCTACTGTCACAACACTTTAGTACCCTCTTGTAAGCCTGTGGACAAATCCTCTGATGCCTTGAT
ACCTCGGTTTTCCCCCACTATAAATGGGAAGATAAATTCACTAGAAACAGTACTTACCTTGCAGT
GTCATTATGAGAATTTCATGAGACAATGCAATATAAAGCTCCTAGCACAGTTCCAGGCACCATT
GTAAGCACTGAACAAATGAAAGCTGTTGTCATTAGGATCTCAAGAAATGACAGCTGAGGCAAAT
GTGGATTAGACTGCATTCAGCATTCTTAGCTGCAAGCAACAGAAGCTGACGCTGGTTTAAAGAA
TAGGAAAGGAAGTTAATTAAAATGACATTGGGTAGCTTGTGGAACTGGGAGGACTAGAGAGCT
CAGTCTAAGTTCTAGTGACAAAGCTTCAAATCATACATGAGGAAAATGCCCCTCTGCAGGAGGA
ACGTAATCCTGCAATCACTGCTTCCCCTGAAGCCAGATGCCTCTACTGCTGCCCTTCCCAGCAGT
GATGGAGCCTGGATCACATACCTGGCTTTAGTTGTAAAGGAGGCTGGAGAGAAAATGCTCTGCT
CTATGTAACTGTCTTGAAAGTGGCCCACACATAGCAAAGCCATTCAAAAGCGGGTGAGCAGCAT

GAAAGCATCCAGAAGGATTCTTGCTTTGCATGAGGGAGAACTCTTGCTCCCAGAGTGTGAGACC
AGCAGGGGCATTTTCCTCATGTGTGATTAGAAGCTTTGTCACTAGAAGCTTAGACTGAGTAGGA
CACCTCTGAGTGTGAATGTCCTACCTGAGTTCTGCCTCATGCAAAGCAAGAATCCCTCTGGAGG
GATTGACACCACAGCCTCTTCCACAATGTCTCCAAACAACTTTACTGGGCAGCATTCCACTCCTT
GACAAACGGGAGAGGTGCATTCTACCTTCTCTCCTGCATGAGCCTAGACTAATATGGAATTTGG
GAGTGTTGATCATTGTACTTTGTGCTCCTAGAGATAAGGGTCATCATAAAGCATCTCAGAGTCTC
ACTGTAGATCCACAGTTCCATTTCTTTATGGATCCCTTACCCCTACTTTGCCAAATTCTATGCAGA
TAGCAATAATAATTCAGACAGGGCTTCCTCACTGAAGGAGTTTGTGCTCTATATAGTTAATAAGC
TAGAGATAATGAAAAAAAAAAAAAACTCAACTTGAACTTTGGGTAAGAGAAACTTTTCAGACCAA
CGATCATTACCAATAACAGCTTCCACGCATGACCCTGCCCGGAGATTGGGGCCTGCACTACTGT
TTCCAGGAAACACATTCTTTTGAATTTTTTTATTTTCTTTGTAGAGATGGGGATCTCACTCTGTTGC
CCAGGCTGGTCTCAAACTCCTGGCTTCAAGCAATCCTCCTGCTTCGGCCTCCCAAACTTCTGGG
ATTACAGATATGAGCCACCACGGCTGGCTGACAGGCACCAAATTCTAGGAGAGGACTTGAATA
GAGTAGGCATCTGTCTCTCCCCATTCCTTTCAGGGACAGAGCTCTCCTGGTTGGGAGAAAGGGA
GAGGGAGAGGGAGGAGGATGAATAGTGAAGCACCAAACCTCAGAATTAAGGCCTTTTTCTTTTC
TTTCTTTCTTTCTTTTTTTTTTTTGAGATGGAGTTTCGCTCTTGTTGCCCAGGCTGGAGTGCAATGG
CACAATCTCGGCTCACCGCAACCTCTGCCTCCCAGGTTCAAGCGATTCTCCTGCCTCAGCCTCC
CGAGTAGCTGGGATTACAGGCATGCGCCACCACACCCAGTTAACTTTTTTGTATTTTTAGAGACG
GGGTTTCTCCATGTTGGTCAAGCTGGTCTCGAACTCCCGACCTCAGGTGCTCTGCCCGACTTAG
CCTCCCAAAGTGCTAGGATTACAGGCGTGAGCCACCATGCCCAGCCAGAATCAGGGCCTTTTTA
AAACTTCAGTGTCTTGTCTTTACCCTTCATGGTGCCTAGAAAAGCATCTGGCTCATAGGTGTTCA
GTGAATGATTGTTTCATTAGTGAAGAAATTCCCTTTGGAAATCTCATAGGAAACTTACCCTCTCC
AACCCTTCACCTGTGCATTATTCCAGCTCCCAAGGAGGCAGCAGAAACTGGGGT
ID|56578|gene_id|ENSG00000224699|transcript_id|ENST00000585433 Homo sapiens lncRNA

CTCCCACATTCCCCTTGGAATCCAAGAACTGGTCTACTGTCACAACACTTTAGTACCCTCTT
GTAAGCCTGTGGACAAATCCTCTGATGCCTTGATACCTCGGTTTTCCCCCACTATAAATGGGAAG
ATAAATTCACTAGAAACAGTACTTACCTTGCAGTGTCATTATGAGAATTTCATGAGACAATGCAA
TATAAAGCTCCTAGCACAGTTCCAGGCACCATTGTAAGCACTGAACAAATGAAAGCTGTTGTCA
TTAGGATCTCAAGAAATGACAGCTGAGGCAAATGTGGATTAGACTGCATTCAGCATTCTTAGCT
GCAAGCAACAGAAGCTGACGCTGGTTTAAAGAATAGGAAAGGAAGTTAATTAAAATGACATTG
GGTAGCTTGTGGAACTGGGAGGACTAGAGAGCTCAGTCTAAGTTCTAGTGACAAAGCTTCAAAT
CATACATGAGGAAAATGCCCCTCTGCAGGAGGAACGTAATCCTGCAATCACTGCTTCCCCTGAA
GCCAGATGCCTCTACTGCTGCCCTTCCCAGCAGTGATGGAGCCTGGATCACATACCTGGCTTTA
GTTGTAAAGGAGGCTGGAGAGAAAATGCTCTGCTCTATGTAACTGTCTTGAAAGTGGCCCACAC
ATAGCAAAGCCATTCAAAAGCGGGTGAGCAGCATGAAAGCATCCAGAAGGATTCTTGCTTTGC
ATGAGGGAGAACTCTTGCTCCCAGAGTGTGAGACCAGCAGGGGCATTTTCCTCATGTGTGATTA
GAAGCTTTGTCACTAGAAGCTTAGACTGAGTAGGACACCTCTGAGTGTGAATGTCCTACCTGAG
TTCTGCCTCATGCAAAGCAAGAATCCCTCTGGAGGGATTGACACCACAGCCTCTTCCACAATGT
CTCCAAACAACTTTACTGGGCAGCATTCCACTCCTTGACAAACGGGAGAGGTGCATTCTACCTT
CTCTCCTGCATGAGCCTAGACTAATATGGAATTTGGGAGTGTTGATCATTGTACTTTGTGCTCCTA
GAGATAAGGGTCATCATAAAGCATCTCAGAGTCTCACTGTAGATCCACAGTTCCATTTCTTTATG
GATCCCTTACCCCTACTTTGCCAAATTCTATGCAGATAGCAATAATAATTCAGACAGGGCTTCCT
CACTGAAGGAGTTTGTGCTCTATATAGTTAATAAGCTAGAGATAATGAAAAAAAAAAAAAACTCA

ACTTGAACTTTGGGTAAGAGAAACTTTTCAGACCAACGATCATTACCAATAACAGCTTCCACGC
ATGACCCTGCCCGGAGATTGGGGCCTGCACTACTGTTTCCAGGAAACACATTCTTTTGAATTTTT
TTATTTTCTTTGTAGAGATGGGGATCTCACTCTGTTGCCCAGGCTGGTCTCAAACTCCTGGCTTCA
AGCAATCCTCCTGCTTCGGCCTCCCAAACTTCTGGGATTACAGATATGAGCCACCACGGCTGGC
TGACAGGCACCAAATTCTAGGAGAGGACTTGAATAGAGTAGGCATCTGTCTCTCCCCATTCCTTT
CAGGGACAGAGCTCTCCTGGTTGGGAGAAAGGGAGAGGGAGAGGGAGGAGGATGAATAGTG
AAGCACCAAACCTCAGAATTAAGGCCTTTTTCTTTTCTTTCTTTCTTTCTTTTTTTTTTTTGAGATGG
AGTTTCGCTCTTGTTGCCCAGGCTGGAGTGCAATGGCACAATCTCGGCTCACCGCAACCTCTGC
CTCCCAGGTTCAAGCGATTCTCCTGCCTCAGCCTCCCGAGTAGCTGGGATTACAGGCATGCGCC
ACCACACCCAGTTAACTTTTTTGTATTTTTAGAGACGGGGTTTCTCCATGTTGGTCAAGCTGGTCT
CGAACTCCCGACCTCAGGTGCTCTGCCCGACTTAGCCTCCCAAAGTGCTAGGATTACAGGCGTG
AGCCACCATGCCCAGCCAGAATCAGGGCCTTTTTAAAACTTCAGTGTCTTGTCTTTACCCTTCAT
GGTGCCTAGAAAAGCATCTGGCTCATAGGTGTTCAGTGAATGATTGTTTCATTAGTGAAGAAATT
CCCTTTGGAAATCTCATAGGAAACTTACCCTCTCCAACCCTTCACCTGTGCATTATTCCAGCTCC
CAAGGAGGCAGCAGAAACTGGGGTAATCACAACGGTCAACAGCAGCAAAGGCAGCACTTTCT
GATTTACTTGACTGCTATGGATGCCACCTCTCTCCACGCCTCAGTTTAATATCTGTAAGTTTGGTC
AGTATCTCATTCAAGAACATTGCATTTACTCTGGTACTTAAAGATGAAAAATCAACATTTACAAA
GCAGATCCATTGTCAACCTAAATAACAAACAAAGAGAGTCTCTATAAAAGAAAAGAATATTTAT
TTGGGAATAGAGCATTGCATAGGAATATGCCTGCCTTAGTAATGCATGTATTCAGGGAGGTAGA
GGAAGACAAAGGTTTTTAACAGAAAAAAATGTGGAGAATTACATAATTGTTTTGAAATAATTATCC
TTGGCTATAAAGATCAATAACAAGGGTGATGCGAGTCCAAGGTTGGACAGGCAGTTGCTGGGC
AGATGTCTTTGCAGAAGTCTTTTTTGTGTAAGGTTGTAATGGCTTTTGTAGGAGGTTGTGGCTTTT
GCAATCTTTTGTGATAGCTTTTGTTACCAGGCATACAAGCATGAGAATGCTCTCTTCAGGGCCTT
CCCTAGCTCTATTTATCAGGGGTTTCTTGTTAGTGATTCCATTTTGATTCTGACAACTTTCATGGA
CATTATCTCATTTAATCTCACAGTCATTCCATGACTCTATCCCCATTTTGTGAATGAAGTAACTGA
GGTCTGGAGAGGTCAACTAACTGGTCAAGGTCATAGAACTTGAAAATTTTCATTGTCTTGGGTCT
CTAATACTGGGGCTGACCATTCTCCCAGGCAATGTTGAGTGAGTTGGCCCAGGTGAGTCGGCTC
CTCTGCAATTGTGTCTCCTCAACTAGTTTGAACAACACAGGCCTGTGAGCCAGTCCCATAAAGT
GGCTAGGGGTTCCCTTCTCCCCATTTCCCCTCATCAACAGGAGGACTTGTCTGTGTGCCTTCAGA
CACCACACAGTAAATCAACTGCCACATTCATTCTGGACCATCAGTATTTTGCCCCAGGAGAAAG
TCAGAGGCAAGCACACTAATATCTCATTGATATTTTCAATTGCCAAAGGGGAAGTGAGATGAAT
CTAATCCCATCTGTCAAGGTGGAGAAGAGCTGATTCGAAAGCAGCATAGATTACTTTAAGGAGA
TGACAGTTTATCACTCTCTGGACAAGGGAATATGAAATGAGGACTAACCTCCTTTATGAGTTGGT
AAAAACATTGATCAGCAAAGACAATGTCTGGGGAAATGAACAGCAAGTTCTCTGGCCCTTTTTC
CACTTTTCCTTTCCCCGAGACTTTATGTTATATATACACACTCACACAAACAGGACAGAATCCAC
CTCCTGCCCCTCAAGAAATAGTTAACATTTGTTGAGGATTAATTATTGCCAGGCTCTGTACTCCA
TGTTTTATATTTACTATCCAGAAGCCTTAATTGTTAAATGAGGTAAGTGTTATTATTAGAACCTTTG
TACAGATGTGGAAACTGAGGTGTATAGAGGTTGACTTGCCCAAGGTTACCCAGCTAGTGAAGAA
TGAAATCAGATGCAGTCCAGCCAACGCTTGGCCTGCCGTCAGTCACACGCAGTACACGTAGAT
CAGTCAGCCTGGAAAGCCACCTAGACAGCCCTGCTTCACTCCATCCTTGGTCAGCACCTTAGAG
AGCCCTGCTGACCCCAAGAGGATGTGCCTTTTACGGGTTGTAGAGGCTGAACCACAGCTGAGG
GGCTGGAATGAGTGGGGGCCTCAGACAGGGTGTCTGGAGAGCTGAGGCTCAAGGAAGGGTGG
AGGGGGTGCTGAAGCTGAAGGCCGTGGGTCTTGCCAGCAAGTGGGGCTCAGTGAGCCGAGGT
GCCTCTCAGTTACTTCCATGTGTGAATTTCCGGTGGAGGACAGTGATGGGCTCTGGGCCCTTGG

AAACCGGGACATCCAGCGTGTCCAGCCACAGTGCAGGCTGCCCTGGCTGCTGAGAGTCTCAGA
AAATGCCACTGTCATGGGTTAGGAAGGCCTGGGGACCACAACAATGTGACTTCAAAAAACCCA
AGAAGGAATGTGCACACCTGAGATGCTCAGAATGCATGTGTCAGTGTTCAGTTCTGACTCCTTG
CTTCAGCCAGGGACAGAAGAACCTTCTACAGTAAAGAACCACAAGAGAGGGCTTTGCTGCCAG
CCTCAGCCAGGGACACTTGGCGGCCAAAGCAAAGGTCACTCCAGGGCTGCAGATTTCAGCCTC
TGCGGCCAGAAACAAGCTTCCCAATTAATAATACTCTGGATCGGACAGAGATGGCTGCTCTGTC
TCAGGCGTGTTTGCCGTTGATCCCTGTGATTGATGCTGATAACATTAAGAGTCTGTCAGGGTGAT
ATTTCATGAATGTCTCATGTGATGGGATGATACAGTCGGGAAATGCAATTCCCTAGCTCAGGCA
GAATCAGAGATGACCCAAAGTGCTGATGGAAAGGCTGAGGCCCTTTCCAGCCTCTGGCAGGGT
AGGGAAAGCGGGTGAGGTGGGGGCTTGGGAGGGGCTGGGCTGACCTAGAGCTGTAACAGGG
GTCTCTCATGAAGGGCTGTGAAGAGAAACAGTATGTGAGAGAGGCAAGAAACTTTTCTCTGGGT
TTCCTCATGTAGAATGATTCCATCTGTGTAGGCCATGAAGGAGTCCAGGGAAGTTCACCCCAAA
ATGTTGAAACCACCTTTGCAAAGATTATGACAGTGAGAGAAGCCTGGCATGGCTGACTCTATCT
TGCTTCTAGCCTCACAGGCTGGCTGTTCTGTCTCATTCCTGGGCATAGGCCAAGCTAATCTTGGG
AGGGATTTATGGTTTAACTTTGAAGCCAGGATCATAATTGTCCCTCCCTAAAACTGACCCCTTTC
TTGTTCAGGGACTGAAACTGCCTTTGTAAGACTAATGAAAGGCCACAAAATTAGGATTATGGAA
GGGGCCTGAATTCTGATAAGATGTAGGCATAGCTTGCCTTTCTGTCATCGTTTATTGCACTGGCA
CTCACAAGATTTGTAACTTCCCCAATTGGTCCCATAGATAACATCACTATTGTAGAACCTCAGAT
TGGTCTTTTGAGATCTTTTTCAGACTTTGGTATTCTGGTGACTGACTCCACCCAGATCCCATGACT
CATGACTCAGCTGGTCCTGTGGTTCTTACCCAGTCTGACACAGCACCAGAGGACCGTTTTCCAC
ACTTCTATCTATGATTTCATCCCCAACCAATCAGCACCACCCATTCCCTAGCCCCCTGCCCACCA
AATTGATATCCATAAAAACCCTAACCTCTGAGTTCTCAGAGAAACTGATTGGAGTAATAAACTCC
TGTCTTCTGTGTGGCTAGCCTTATGTTAATTAAACTCTTTCTTAGGGCAGTACTGCTGTCAGTGAA
TCAGCTTTATCTGTGCAGTGGGCAAGAAGGACCCACTGGGTGACTACAATATGGCACTTTGGTA
TGTTGGTTATTTTAAGTTAGAGGCCTTTGATGCTGGAAGAAGCTTTACTCTGATATCTCCTTATCT
ACCACAAGACTGGATCTGCCAAAGAGAACACAATTGCGTTCCATTCCCTTCCTGAAATCTCATTA
TCTATGGCAGAAAAGAAGACTGGGGAATGTAATACACAGTATTACACAGTAATGTAACATACTA
CACAGTATTTACACAAAACATGTAACACAGAATGTACTACACAGTATTTTCACAAAACAATATCT
GTCTCTCCAACTCATTCAAATTCTAAAGAGTCATTTACACGTCAATTTGTGTCTTCTACGCCCATT
CATTCTCCCTAAGAATCATTTACTACTCCTCAAAAGAATTACCAACATTCTCCATCTCCCACTCCC
CTATGAGGAAGGGTATATAAGTTTCTGTCTGGATCTCATTAGATGACTGTGTGATCGTGCTTCTA
TGATCCCTACCCCACATGTTAAATGAATTTTGTATGCCTTTTTCTTTTATCAATCTGCCTTTTGTTA
GGGTATTTTCAGTGGACCTTCAGAAGGCAAAGGGGGAGCTTTCCTTCTTTGACCCTACAGCTATT
AGACTTGATTGAGTAAGACACACAATAATGAAAGTAAATAAGAAGAAAAACATTCATCCTGCTG
TCTCCAGAGTTTGAAATGTCTGCCTCAAACTTCGATTTCTATCTACCAAAAATTCATGCATTAATC
ATAAGGCCGATCTCAAAAGTGAATTTTTTTTTTTTTTTGAGAAGGAGTCTCACTCTGTTGCCTAGG
CTGGAGTGCAGTGGCACAATCTCAGCTCACTGCAACCTCTGCCTCCTGGGTTCAAGCGATTCTC
CTGCCTCAGCCTCCCAAGTAGCTGGGACTACAGGCACGTGCCGCCAAGCCTGGCTAATTTTTAT
ATTTTTAGTACAGACGGGGTTTCACCATATTGGCCAGGCTGGTCTCAAACTCCTGACCTCAAGTG
ATCCACCCACCTCTGCCTCCCAAAGTGCTGGGATTACAGGCGTGAGCCACCGCGCCTGGACGT
GAAAAGTTTTTTGAATTCCCCCATTAGAAATGATCTTCACCCTGACTCCCATAACACTTTGTACAT
TACTGTAAAAGCATAATATTCAAATATTTACAATATACCTCCTTCAAATATAAAATGGACACATG
CCGAAGACTTAGCAACTCATGAAGGAGGGAACCAATAATGTAGTTAAGCTAATTCAAAGAGCA
TTTTGAGGAAATGAATACTTATGGTCATTTAAAAGGAAATGTTAAAATTTCTAGGTTAAAAATAA

AACTTGCCATTGCATATATGGTCAAATGATTTTTGACAAGGGTGCCAAGACCATTCCATGGGGA
AAACACAGTCTTTTCAACAAATCGTGTTGGGAAAACTAGATATCCATGTTCAAAAGAATGAACC
CTTACCTTACCTTCCACCATATACAAAAATTAACTCAAAATGGATCAAAGACTTAAGCATAAGAG
CTAAAATTATAAAATTCTTAGAAGAAAACATAGGGAAAAAGGTCCATGGTATTATATTTGACAAT
GACTTTTTTTTTTTTTTTTGAGGCAAGATTTCACTCTGTCACCCTGGCTGGAGTTCAATGCAGTGG
CACAATCTCAGCTCACTGCAACCTCCACCTTTCAGGCTCAAGCAATCCCCCTACCTCAGCCCCT
AAGTAGCTGAGACTACAGGCATGCACCACCAAGCACAGCTAATTTTTGTATTTTTGTACAGAGTT
TCGCCATGTTGCCCAGGCTGGTCTCGAACTCCTGAGCTCAAGCTATTCACCTGCCTCAGCCTCC
CAAAGTGTTGGGATTACAGGTGTGAGCCACTGTGCCCAGCCCTGACCCCCACATTTTTAAACAA
AGCTTCTCTTCCATAATCAATTGCAAATCAGAAGATCTTTGAATCTATCTGCAGCCTGCAAGCCC
CCTATTCAAGACATCCCACCCTTTTAAACTTAAGCCAATGTGTAACCTCCTGGCAATCAATTTTG
CAATTTATGATTTTGCCTGTAGCTTCTGCTGTTCTGAAATTTATTCCTGCCTCTAAAAATCCTTGCC
GGCAAGCCACTGGGGAGGCTAGGTTTTGAGCACTTAGCTGTCTGGTCTTCCTTACTTAGTGCTCT
GCAATAAACACCTTCCTTTCTATCACCACAAAATCCTGGGTGTGGGTATCTGGTTTTACTGTGTTG
GGTGAGCAGACCTCAGTTTGGTTCTGTTACAAGTCCCTGCTCCATCTCACTTGCTACAACATGAA
TGAAACTTGAGGACATTATGCAAAATGAAATAAGCCAGTCACAAAAAGACAAATACTGTATTAT
TCCACTTATATGAGGTACTTATGATAGACAAAATCATCAAGACAGAAGGTATGATGGTTGCCAG
GGGTTGAGGGAAGACAAAATAGGGAGTTATTGTTTAATGGTTGAGAAGTTTCAGTTTTGCAAGA
TGAAAAGTGTTCTGGAGATGGATGGTGGTGATGGTTGCATGACATAAATGTGCTTAATACCACT
GAACTGTTCTTAACTCCGCTGAAATATGGTTAAGATGGCCCGGGTGCAGTGGCTCATGCCTGTA
ATCCTAACACTTTGGAACGCCGAGGCGGGCAGATCACCTGAGATCAGGAGTTCGAGACCAGCC
TGACCAATATGGCGAAACCCAGTCTCCACTAAAAATACAAAAATTAGCGAGGTGTGGTGGTGC
GCACCTGTAATTCAAGCCACTTGGGAGGCTGAGGCAGGAGAATTTTCTGAATCCAGGATGCAG
AGGTTGCAGTGTACTGAGATCGCACCACTGCACTCCAGCCTGAGCAACAGAGCAAGACTCCAT
CTCAAAAAAAAAAAAAAAGGTTAAGATGGTAAACTTTGTACCAATTAAAAAACAAAGCTGGCTAA
CACCCTACAGGGCAATTAAAACATCATAATCTTTGGTGTTATCTTTTTTATTAGATAGAAATTATT
TATACTTACTGGATAAGATCTGTAAGTTAACACATAACTTCACAGAGTCTGAGCCCTTAATGAAT
AATAAATAGCCAAGTCACAGGGAGATACTTTCCCTGAGGTAATTAAGCAATCCTTGGGTAGGCT
TGTGAAGAAATGCCCAGAATGACGTTGGAAGGACATGCTGTTCTCTCTTGGCCTTATTTCCAAG
GTTTGGACTCAAAGTATTATTTTTTAACATCACAGAAATTTTTTATGCCCTGGTCTCTTGAGGGTA
GGGACTGTGTCTTTTTCATCTTTGCCCCTTTCCACATGCTCTGGTTTGTAGAACAGAATTGAAGAA
GGAGCCATGTGCTAGATAGAGCGGGTTGGGAGCAGCAGACCTGCCTGCTCATTGTTTCTGTGAC
CTTGACCCATCCTGTAGTCTCCCCACGCCTTGCTATTCTCATCCATCAACGATGAGATTGCACCT
AAATCCTGGGTGCTGTGCTGGGCCTGGGGATGCTGTCTAATCCCTGGGTTTCCTTCTAGTTTGCT
CTAATCACCCATATGAAAAAGAAGCTACTCAAGGCAAACATATAAATGAAACGGATAATGAATG
AATAGTATTTTTTCTTGCTTTCTTTGGAAGAGGCATCCTGGATTCTACTTGAAGCCTGCAGCCCTG
ACTCCTGGGCTGGGAGGTGGGCGAGGACTTGTATTTTGTAGCTCTGCTAGAGAGGGAACAGCT
GTCCACTGGTGGCAGGACTGACGCCTCCCAGAATACAGGGGCTTTGGATCAGCAGGCGTCAGA
GGGAAAGGAAACTGGCATCCGAGTCTGCCTTTGAGAAAGGAGTAAATTACTATCTTATTATAAA
TTCATGATCCACTCATTGCTGTTTTCAATTAATTTCTTGACAAATCTGGCTGTTCCTTTTCTTATTTT
CCTTAGGACAGGAGGTGTCCTGGTGATCAGCTGGAGCTCGGGGGAGCTGTTGCTGAAATTAAC
TTGAGTGCTTTGGAGAGAGATTTACATGGCAGAGTGGAGCCAGGGCTGCTCCTTTGGCAGGCA
CTGCTGTGCCACTGTGGAGGCGCCCCTGCTTTTCCCATGCTTTGCAGTGTCTTTAAAGGCCTTTG
GAGCCATGCCCAGGCTCAGCCCCATCCTGGTGTCCCCCTGTGTCCGTTTTGCCACTGCCCGGCT

ACTTCACTTGGCCATGGGCCTTACCAGCTTGCAGACAAACCCGTCCATCTACGCCTCCCAGCGC
AGCTTGGCCCACTTGCAAGCACTTACAGAGGGTTTCAGAAGGAACATGTCTGCCATCTGTGTTT
GGAGACTGTGTCCCAGACTTTTGAGCTCACATTAGTCATGGCCACAGACGGGACTGGAGTCCTG
CCCACTGGAGTCCCAAAGGTCTCTGGTGTTTGTTTTCCACTCTGCATGCCTGGGTTGGGGGATG
GCAGGGGGATACAGGTTGTTGGAGTACCTCCCAGCCGGGCCGTTTCCTTGGTTCCCTGGGGCTG
CCTTGCTTCCTCTCTAGATCATCAGTCTCAGCAGAGGAAACCAAAAATCTCCTCCTCGCCCCGCA
ACCACCTTTTGCTGACTGCTAAGCATCCCTGCTCTGAAACCATAACCACCATAAAAGCAATAGG
CTTGTCTTCTCTGTTCTAAGCATGTACTATTCAGGTGATTTAGGGGGAGTAAACAGGCCCAGAGA
AATTAAATAATTTGTCTAAGGGTACCGGCTAATGAGGATCTGAGCTCAGTTGTAAATGACTCCAT
CACCCATGCTCTTTTTTAGTCCCCTTTATAATTATTTTAAAATAACATTTAATTAATTTTCTGTTATA
AAAGGAACACATGCTCAAACAAAAAACTTAGAAAATATAAAAATGTATGAAGACAAAACAAAA
TAACCCCCAGTATTAGCAATGCCAACTTGCCAAGACAAAAATGTTAACATTTTTATTTCTTTTTCT
TTTCAGTCTTTACTCTCTGTGTGCACACACACACACAGGCAAAGTGAAATCACATTGTATATTCTT
TTTTGCTTCTAGCATTTTCCACTTAGCTATCTAATATTACTATTTCCTGATATCAATAAATGATCTT
CAATCCCATGCTTAACTCTTCTGTCCAAGGCATGTTCTGCCCAATTTTGTTTTCTCCTGTGTCTGTT
TGAATGCCTCCTTTCTGGCCTGCATCTTAGGGGGACACTGAAGTCTCCTTTCCAACAGAAGTTCT
ATTCAGGGGGAATCTGTTATCTTGTTTAAAGCCCATCCTGGGCTGGGGAGCAAGCAGGAGAGA
GAGAGAGAGACACCACCCATACGTGACTAGAGTGCCATTTGTAGCAGGATGCCCCAGAGGCAG
CGTGGCCCTTCACCACCACAGTTATCTGGAACGAGCAGGAACTGTGTAGAGGAAGAGGCACAT
CCCCTTTTGAATGGCTGTGCCCTTGCAGCCACCTCAGTCCCTGCTTCCTGAAGGGAATCAGTTAC
TGGGCTCTGGGAGACAATTTTCAAAGTGAACTTCCACGGATGTGAAGGGAACCATGACCTAGA
AATCTGAAGGTGCTAGGTCTTATTTTGCTCAGAGCTGAAAAGAGCCACTTTAAGAGGGCAAACA
TTGAGGAAAAAAATGAAGAATATTTCATTTTTTAGGGAAATCCTACGAGCCTATAGGAGTCATCTT
AGCCCATGTGCATGGTCACATGGCCTCTGTCCAGGATGGCCAGGACGGGGTGACTTCCTTAGA
AGGGACTGGCATCTTGGGAGGCAGGCCTTTTCAGGGCTCAGCCCCAGGACTCCTTATCAAAAT
GTTGAAAAAAAAAGTTTATTCCATGGACTTCTAGAAATGGGGAAAGCATTGATGGGTTATAACT
CAATTCTCAACACCAACAATCCATAACTCTCAAAGAAGATCTATGACTCCAAAAGGGTGACCTG
AATCAGGAGGCTTATAATTGCATTGCCAAAGTGCAATGTGATCAGAGGCAGAAACATAACTAGG
ACCTCCCACAAGGAGTTGAAACTCCCTTTATCCTGGGCCTGTGGTTATCTCATTGATATTTTCCAC
TGTAGGTGAATTATTTCATTGTAGAGTCCCCTATTTAGAAAATACATGGCATGTGTGCCAGCATTT
TCCTATCCTGCACCCATGGCAGGCATCACCAATCAACATTGGCACCCTCCCCACTGAGCCTAGA
AGCAGCTTCAGACTCAGTCTCAACATACTTCTCCAGGCAGCCATTACGAATCAGTCAGCACTGA
CCTGGAATGAAATCCTTTGACATTCCTGAATTAGTTTATAATCACAAGCAGGAATTGTATATCAA
GTTCTTATTAGATAGAATACTTAGAAGGCCCTTAGGTTGGTTCATTTGCCTGTTTCCTCATAAGAA
TGAGGGCAGAGCTGGGCATGGTGGCTCATGCCTATAATCCCAGCACTTTGGGAGGCTGAGATG
GGAGGATTGCTTGAGGCCAGGAGTTTGAGACCAGCCTGGGCAACATGGCAAGATCCCATCTCT
ACAAAATATATATTAAAAAAATAAAATGAAGAATGAGGGCAGGAGGTGGCGGGAGAAGTGGA
AGCAGATGGAGGAAGTTAGGTTGAAGAGAGCCAAGGATTCTTATTCTGATCCTATGATTTTCTTT
CTTTCTTCACTCTGTCACCCAGGCTGGAGTGCAGTGACACTATCCCAGCTCACTGCAATCTCTGC
CTCCCGGGCTCAAGTGATTCTCGTGCCTCAGCCTCCCACGTAGCTGGGATTACAGGTGTGTGCC
ACCATGCCTGCTAAATTTTGTATTTTTAGTAGAGAAAGAGTTGCGCCATGTTAGCCAGGCTGGTT
TCAAACTCCTGGCCTCAAGCATTCCATCCACCTTAGCCTCCCAAAGTGCTGGGTTTACAGGCAT
GAGCTACCATATCCAGCCCCATTTTCTTATTTAAGACTGAATATGTTTCTCCTGACATTGCCACAG
GTAGCCTCCTTTCCTGCCTGTTCCTAGTCCAGCCCTGGGACTCTTCTCACCTCTTGTCAGGGTCC

CTTGAAGCAGGGGCTGGGTTGCCCTTCTCCCTGAGGCAGGCAAGAGGCAGAGCCGGGTTCAAG
CAATTCTAGTGCCTCAGCCTCCCAAGTAGCTGAGATTACAGGCATGTGCCACCATGCCCGGCTA
AGTTTTGTATTTTTAGTAGAGGTGGGGTTTCACCATGTTGTCCAGGCTGGTCTCAAACTCCTTACT
TCAGGTGAGCCACCCACCTTGGCCTCCCAAAGTGCTGGGATTACAGGAATGAGCCACTGCATCT
GGCCTATTCTTTAATGAGAAGAATAGAGCAATGAAGTCCCTGAACTGGGCCGGGGTTCCCAGG
GGCTTCCCAGAAGCATGTGGCTGACTTATTGTAGTGAAAAATTAGCCAGCACAGATTTATCACC
CAATATTTAGCTTTGGCCATCAGTATTCTCATAGCAGCTTCTGCTGATTGCCTCATTCAATCAACA
CAGGGTGGAGGTAATATTCCATTCACGTTGCGAGATCCTGGCTTTGTGTCCTGATGGGCCATGT
GGGCAGGCTGCTCTCTGCTCTAACACTCCCTCAACACTATGTTCACACCATCCTGATTTTTTCCC
CCAAGTGTTTGCACGACTCAGCCTATGCAAGGGATTATGGAGCTCAGTGGCTCTGTGGCTTCAG
GTTCTGGCAAATGACTATTTGCTTTTTCTTCTTCTTCTTTTTTTTTTTTCTTTTTTTTTTTTGAGATGAA
GTTTTGCTCTTCTTGCCCAGGCTGGAGTGCAATGGTATGATCTCAGCTCACCGCAACCTCCGCCT
CCTGGGTTCAAGTGATTCTTCTGCCTCAGCCTCCCAAGTAGCTGGGATTACAGGCATGTGCCAC
CATGCCCAGCTAATTTTGTATTTTTGGTAGAGACGGGGTTTTTGCATGTTGGTCAGGCTGGTCTC
GAACTCCCGACCTCAGGTGATCCGCCCATCTTGGCCTCCCAAAGTGCTGGGATTACAGGTGTGA
GCCACTGCGCCCAGCCGATTATTTGCTTCTTCATGGCAACTCACAGACCATGATCCCCACTCAAT
CTTGTGGGTTTGTAATAGCAGCACAGAAAAGCTGTGTTGAACCGTGGCACATCTGTTAGCTTTTC
CAGGTGCTCAGGAGGAGAAGGTGAACTGGCTGTGTGTAGTATGGTAAATAAAGGGGCCGGGCT
ACCTTTCAGAGGTTTGGCACACTGAATGACTTATGCCCTGACCAGAGACCTGGGCTAGGAACTT
AGCTCCATGGCCTGGATGCCTGGGACCTTGGTCCGATTTGCAAACACAACAGCTCCACAGGAA
GAAGCTTTGTCACAATAGTTATTATTATAGCAACCATCCAGAGGATGCTGCATCCTAAATATCCC
TCAGCAGTAGTCCAGGTAAGAAGCACACCTGACTATGTATGCTCTTCTCTGCTTCAGAACCCTTC
AGGGAGCTCCACTGCCTATAAGGAAAAGTCCACATCTGGGCACTTGGGTCTCCATGAGCTTCTG
CCTGCTTATGGCTCCAGTCTTACCTCTCACTGCTTCCTGCATTAAACGTTTGCCCTAAACATTGAA
CTGGGTTCATTGTTGCATGTTCCCCTTCACCCCCAAATCAGACTTTGCTCATCCCCAGGATACCTT
TCAACTTCCCCCACCTCCCCACCTGCCTAACACCACCACTCACTAACAAGGAGCTGTGCTCCGA
AGCTTGTGGACAGCTGTGTCATGGAGGAGCTGCCGTGCTCTGTCCAGAGGGCTGTCTCTTGAGC
ACAGAGACTGAGCCTTCCCCACCTCTCTAGCCTCTAGTCCCAAGCACAGCCTGCCCCCTGGTAG
CCCCTAGGAAATTTCCACCAAATGCATGCAAGGTCTTGCAGCTGCCCCACAGCTTGACATCCCA
CTTCCTCCTGAGCCTCCTCCTCCACTGTCTTCTATCCTCTGCTCAAAGCCCTTAACCGATGCCCC
GGTTTTTCAAGGGCAGCTTCTTCAGTGTTGTTTGTTCATTCACTCAACTAACAAGGGCTGACCAT
GCATCGGCCTTGAGGGACTGAGGAACAAAGGTGCTCACACTCTGATGGGAGGCACACGGAAG
CAGTGGCAATAAAATCTGTAAAGTCTTCTGTGAGAAGTTAAAGAGAAAATGCTAGAGGATCCCA
GAGGAGGAAGCTACTGCTGACTGTGTGGTGGCTCCTCCCCAGCCTCGTACCCTGACTCTCACTC
TTCAGCCTGCCCCAGTTCCCCTCTAAGCTACACCTTCTCCAGGAAATCGCAGCACACCCATGGC
ATCCACCTTTCCACTGAAGACTCCTGAGTATTCAGCCCCAACCTCTCTCTGAGGATTAGAGAAGT
TAGGTAACCTGGCCAAGGCCACACAGCTAGGGGCAGCAGGGATGGCGCTCTCCTAGGACCAT
GCTGCACTCCTCCAAATCTGCTCCTTGCTCCCCTCCCATCCATCTCCACCTTCTTTGCCCTGCTGT
GTGTCCTGGGAGGCTGATCTGTGAGGATGTCTTGGTGGCTGCCTTCGGTGGATTTGGCTAGTAA
CCCTCAGAGGAGTGGAGGAAAGGAGGAACATGAAACCGGGGAATTAACTCCTCTGTCATGCTC
CCAGCAAGACCACCTAGGTTGGCTGTCCCTTGGCAGAAGGGGATCCTCTCAAGGGAGCTTCTCT
ACAAGACCCTCTCCTTCTGGTCCAGGTAGCTGTTCTCCTCCTGGTCTTCTGGCTGGGTGATGGGA
GAAGCTCCTTCACTCCACTGCTACTGGCTCTAGGTTACTGCATTAGTCCTGCCTTTATCCGACAC
CACGCTCACACCTTTGTGAAAACACCCTCTGTGCATTATCCTAGTTTGAGTGGCTCCCTCTTTCCT

GCGGGCCCGGGGCTGATGCATGCTCCTTCTGCATGTCCTGGATTGGCGAATAGCATCAATATCT
GTACAGCTCTCCAGGCCCCAACCTTAACCGTCTCCTATTCTTCTTTCTCTCTCATCCTACTCTAGG
ACCTGAAAGCCTCTGAAGTTCACTCCTTCGGCCCCACTCCCTTCCTGCTGCCTTTGGGCTCTCCT
CATGTGACTCTGCCTGTTGAAACTGTCTGCTAGCTGGCCCAGCATCTCCAGGCATGGCCTCACT
CATCCACCTCTCCCTTGCTGAAGGTCTCCTCATGTAACACACGATTCCTTTAGATGGTCCTTCATC
ACCAGCAAGATGAAACCTAAATTCATTGGCATGGTAGACAAAACTCTAGCCTTGCCTCCCAGAG
GCCAGTCTCTGTCTCCGCCACTCCCCAGGTGGACTGCATTGTGTTTCTGCTGCCATAAACATAAT
TTATTCTGACTGGAACATCTTCTGTGCATGTCTGCAGAGTGCACTTCACTCCCCCTGCAGTTCAA
GCATCCTTCCTCCACAAAGCCTTTTCTTTTGATCACTGCAGGACCTGTAAAAGCTATCCTGGGAT
AGCCTGTATTTACTGGACTGAAATGATCTGTTTGCCTATCAATTTCCCCAAATGATATATGGCAA
GGAGTAGTTGAATCTAAGAGCAGATCTCAAAGCCTATGTCCTTTCCATTGCCTTGTACCCTGCTC
CCCTCAAGAATACCTATTTTTTGGGGTTGGTGGGAGGATCATGTGAGATAATGCATGGGCAGTG
CCTGGCACACAGTAAGTGCTCAATAAATACTGTTATAAATGGGCGACCAG

ID|56602|gene_id|ENSG00000224699|transcript_id|ENST00000609245 Homo sapiens lncRNA

ATATGAGATACCTTATGTAAGAGGGGCTGGTGCATTGTGGAAGTCAACAAGTAAGTTTTGT
TAAATTTGAGTAAGAAGACCTGTTTGTTTCCTCAAGTTGCTTATAATCTAGGTGAGGACACATCT
GAGAGAATAACTAATAGTTCAGTGAAGGTAAGAGCCAAATGATTGGCACAGACCTCAAGTGGT
CTGGCATTTACGGGAGAGGGGGATTGTGAAACAGGAATTGAAAGAAATTTAAAAATGCATAAG
CAAAAACTCAGTTTATGTTTTAAAAAAACCAATTCCCCCTGAGAAAGAGAAAGAGGTGGAGTCC
TTTAAAAATTATCTGCTTGTTTTTCTGTCTGTGGCTAGTGAGCCTTATCTCTCCCTTTCCCAGGCAT
TGTGAAGACCCTGTTTCTCTAGCTGTGCAGCTGCAAGGTCACTAGGCAGATAAACTCAAGCCAT
AAAACATGTTTTTCCTTGAAAAGTAAGAAATAATGTAATGCATGTGTCAAATGAATAACTATTTCT
CGCTTCTGTAATATGCTTCCCTTCGCACAGATCTCCCCCTGCCCCATGAAATGCTTTAAAGGTAA
CTTAACTCTTTGTTCGGGGCTCAGTCCTTTGGATGTTAATCTGACTGGCTTGGTGCACCTAAATAA
TAAATAACATATATCCTCCTCAACCCCTCAGTCTCTCTGATTCCTAAATTATCCCGCTGTAATTGC
AGGGCTAGATTTGCCGACCTGCTTTATAAAGCTATGTTAAATCAGTATATTTTATTCTTTTTATTAG
ACTTTTCTATAGCATAAAAATGATAAAATACAGCAAGAAAAAAAGTCACCTGAAACTCTAGTCCT
AACCCTAATATGAATGTGAAGGAGCTGGGCTAGAATGGAATAGGGTAGAATTTGCATAAGGTG
GAAATGGGGACAGGCCTGTCACAAGGATCACTAGAGTCTTGCTGGCAAAAGCATCTTACTGCAT
TCATGCTGCAGCACAGGACCCAGATGCAGAACAGGTTGGGGACACAGCCCTGCCTTCTAATAG
TGGACACCATCTGAGAGCTGAGCCATAGATTCAAAGCCTAACTCCTAATAGCGATTGGTAGTAA
TATCATCAGCAGTTCATATACATGGAGTGACAACAGCGTGCCAGGCAATTCAAAGGGTCTCTCG
GTCCTCACAGCATTTTTGAAGTAGCTAACATCACTATCCCCCTTTTACAGTTAAAAACAACAACA
ACAACAACAAAAACACAAAACCCTGAGGCTCAGAAGATAAATATTTGCTGAAGATCACTGTGCT
TGTAAGTGGTGGAGCCTGACCATTAACTTCCCCACGACACTGCTTCAATTTCTATCACTTCATCA
ACCACACAACCCGAGAACTGTGAGGGGATTTAGCAGGCCCAGTCCAGATCTCTTATTGTACAGA
GAAGAAATCTGAGGCTCAGAGAGGGGAAGGGACTAATTTGGACCTTTAACTTCGGATTCTTAAC
TTTCTTTCTGGTGTTCTAACTATACTGTACTACAAGAGGCTGTCTTAGAGCTTTAGTTCAACCAGC
TTCAGAGATACTCATTGTTATGGGTTGAATTATGTCTCCCCAAAATTCATATGTCAAAGTTCTAAC
CCCCAGTACCTCAGAATGTGACCATATTTGGAGATAGGACCTTTACAGAGGTAATCAGGTGAAA
ATGAGGTTGTTAGGGTGGGCCTTAGTCCAGTATGGCTGGTGTCCTCATAAAAAGGGGAAATTTG
GACACAGATGTACATAGAGGGAAGATGGTGTGAAGAGACATAAAGAACAGATAGTCATCTATA
GCCAAGGCCTGGAACAGATCCTTCCCTAACAGCCCTCAGAAGGAAGCAACCTCACTGACACCT

TGATTTTGGACTGCTACCCTCTGGCACTGTGAGACAATAAATTCATGTTGGTTAGGCCACCCAGT
TTGTTATGGCAGCCCTAGGAAACCTTTGTGGATATATCTGTTCGTATACCCATAAAGTCTGCAAG
TCAAAAATATCACAGACTTTTGGCAGCCCTTCATTCAAGCACTGGCTGGGAGGGTCACCCTCCC
CGAGGCAAAAGGATAGAGCAAATGATCTTTCCAGCTTGCTCCCAACCTTGATCTTTGGCCAAGA
TATTTTTCTTTCACTGGACATTGGCCAACATTGGTGTCATAACTTAATTTGACTGTGACCTTCTTGA
GTCTTTTATGTCTCTGAGCTTGACCTGTAGAAGAAGCTCAATAAATGTGGGTAGAGGGGATGAG
TGGGAAAATCAGCTCCCAGCCATTCCAGTTCAAGCCTCTTCAAGCAGAAGAGTCCCACCTGAAA
GACCTCATTTTCACTTGATTACTCGATCTCCAAGCAATAGGTGGGGCAGGAAGCAGGGTATGTA
AGGCCTGTCAGTGGATGGAAGTTGAAGTTGGACACCATCTCCTAAAGTTTAGAGCTGAGCCCAA
ATTCTGGAAAACACAAGCAAAATACATTTTATATGACTCAGGATGGGCTAAAGAAGTTCCAGTA
CAGGAAACTGTTTTCCTGCTGCTTTATTAATTTTGCTACAGAGGATAAATGAGATGCAGACTTTC
ATATCCAAGTCTTCATTAGATGCCCAGTTAATGCAGCCATGACAAATGGGAATGAAGCCCTTAT
GAATAAAAAAAAAGATCAACCCAGTATAAAAAAATCATAGACCAGAGAAATGAAAATTTAGAGG
AGCTGATAAAATACCCTATCCTTAATTAGACTGTCAGCATACACATCAAATCAGCCTTAATGCCA
AGATTTATTGGCCTTGGAATTGGGTAAATGAAATAAGTGCTTACAAATGAATGGAAAAGAAATT
ACCTGTATTAAAACGTATTTGTGATGCAGGCCTGTGCACTGAGGGTTTTAGTCAAACTCAGGCTT
GCAGCTTCTTTTGCTCTGCATCCATTTCCACCCCATGTTCCCCAGTGGGTTTCAGAGACCCTCTTT
GTGGGGTAACAATATGTACCATTGTCAACTACCTCTGCTGTTGTCCTGGAAAAATCCTTCTTGGA
CAGTAAAATATTTTTGTCTTTATTTCACCATCTCCAGATTAGATGGGTGTTACTGATTCTTACCTCA
GAGAAGAGAATGTCATGGAGACAAAATGACCTGCTTAGAGTTCTGCACCCCCCAGCATGGAGA
GCAGAGCGCAAAGATTTCTTCCGGACTCCCACATTCCCCTTGGAATCCAAGAACTGGTCTACTG
TCACAACACTTTAGTACCCTCTTGTAAGCCTGTGGACAAATCCTCTGATGCCTTGATACCTCGGT
TTTCCCCCACTATAAATGGGAAGATAAATTCACTAGAAACAGTACTTACCTTGCAGTGTCATTAT
GAGAATTTCATGAGACAATGCAATATAAAGCTCCTAGCACAGTTCCAGGCACCATTGTAAGCAC
TGAACAAATGAAAGCTGTTGTCATTAGGATCTCAAGAAATGACAGCTGAGGCAAATGTGGATTA
GACTGCATTCAGCATTCTTAGCTGCAAGCAACAGAAGCTGACGCTGGTTTAAAGAATAGGAAAG
GAAGTTAATTAAAATGACATTGGGTAGCTTGTGGAACTGGGAGGACTAGAGAGCTCAGTCTAAG
TTCTAGTGACAAAGCTTCAAATCATACATGAGGAAAATGCCCCTCTGCAGGAGGAACGTAATCC
TGCAATCACTGCTTCCCCTGAAGCCAGATGCCTCTACTGCTGCCCTTCCCAGCAGTGATGGAGC
CTGGATCACATACCTGGCTTTAGTTGTAAAGGAGGCTGGAGAGAAAATGCTCTGCTCTATGTAA
CTGTCTTGAAAGTGGCCCACACATAGCAAAGCCATTCAAAAGCGGGTGAGCAGCATGAAAGCA
TCCAGAAGGATTCTTGCTTTGCATGAGGGAGAACTCTTGCTCCCAGAGTGTGAGACCAGCAGGG
GCATTTTCCTCATGTGTGATTAGAAGCTTTGTCACTAGAAGCTTAGACTGAGTAGGACACCTCTG
AGTGTGAATGTCCTACCTGAGTTCTGCCTCATGCAAAGCAAGAATCCCTCTGGAGGGATTGACA
CCACAGCCTCTTCCACAATGTCTCCAAACAACTTTACTGGGCAGCATTCCACTCCTTGACAAACG
GGAGAGGTGCATTCTACCTTCTCTCCTGCATGAGCCTAGACTAATATGGAATTTGGGAGTGTTGA
TCATTGTACTTTGTGCTCCTAGAGATAAGGGTCATCATAAAGCATCTCAGAGTCTCACTGTAGAT
CCACAGTTCCATTTCTTTATGGATCCCTTACCCCTACTTTGCCAAATTCTATGCAGATAGCAATAA
TAATTCAGACAGGGCTTCCTCACTGAAGGAGTTTGTGCTCTATATAGTTAATAAGCTAGAGATAA
TGAAAAAAAAAAAAAACTCAACTTGAACTTTGGGTAAGAGAAACTTTTCAGACCAACGATCATTA
CCAATAACAGCTTCCACGCATGACCCTGCCCGGAGATTGGGGCCTGCACTACTGTTTCCAGGAA
ACACATTCTTTTGAATTTTTTTATTTTCTTTGTAGAGATGGGGATCTCACTCTGTTGCCCAGGCTG
GTCTCAAACTCCTGGCTTCAAGCAATCCTCCTGCTTCGGCCTCCCAAACTTCTGGGATTACAGAT
ATGAGCCACCACGGCTGGCTGACAGGCACCAAATTCTAGGAGAGGACTTGAATAGAGTAGGCA

TCTGTCTCTCCCCATTCCTTTCAGGGACAGAGCTCTCCTGGTTGGGAGAAAGGGAGAGGGAGAG
GGAGGAGGATGAATAGTGAAGCACCAAACCTCAGAATTAAGGCCTTTTTCTTTTCTTTCTTTCTTT
CTTTTTTTTTTTTGAGATGGAGTTTCGCTCTTGTTGCCCAGGCTGGAGTGCAATGGCACAATCTCG
GCTCACCGCAACCTCTGCCTCCCAGGTTCAAGCGATTCTCCTGCCTCAGCCTCCCGAGTAGCTG
GGATTACAGGCATGCGCCACCACACCCAGTTAACTTTTTTGTATTTTTAGAGACGGGGTTTCTCC
ATGTTGGTCAAGCTGGTCTCGAACTCCCGACCTCAGGTGCTCTGCCCGACTTAGCCTCCCAAAG
TGCTAGGATTACAGGCGTGAGCCACCATGCCCAGCCAGAATCAGGGCCTTTTTAAAACTTCAGT
GTCTTGTCTTTACCCTTCATGGTGCCTAGAAAAGCATCTGGCTCATAGGTGTTCAGTGAATGATT
GTTTCATTAGTGAAGAAATTCCCTTTGGAAATCTCATAGGAAACTTACCCTCTCCAACCCTTCAC
CTGTGCATTATTCCAGCTCCCAAGGAGGCAGCAGAAACTGGGGTAATCACAACGGTCAACAGC
ID|56616|gene_id|ENSG00000224699|transcript_id|ENST00000609512 Homo sapiens lncRNA

GCCTCGAGCCATCATGTCTGGCTAATTTTTACGTTTTTTTGTAGAGATGGGGGTCTCGCTGT
GTTGTCCAGGCTGGTCTTAAACTCCTTGCTTCAACAGATCCTCCCACTCCAGCCTCCCAAAATGT
GATTACAGGCTGAGTCACCACGCCTAGCATGGTTTTTTTTGTTTTGTTTGTTTTGTAAGCAAAATT
GACATACATACTGGAGGTACAATTCTGTAGCTGGCCTTCTTTACAGAGTTTAGAATAAATGTCTT
TCCATGTCAAGTTTTCACTTACATCCTTTGTAGTAGTTGCAAAATATTCCATTTTATAGCTGTATCT
TAATTGTGGATTAAGGTTGGAGTGCAGTGGCACTATCATGGCTTAGTCCAGCCTCAGCCTTCTAG
GTTCAAGCGATCTTCCCACCTCAGCCTCCCAAGAAGCTGGGACTACAGGGACCCGCCACCCCA
CCTAGCTAATTAAAAAAAAAAATTGTAGAGATGGAGTCTCAGTGTGTAGCCCAGTCTCAAGTGAC
CCTCCCTCGTTGGCTTCCCAAAGCGCTGAGATTGCAGGTGAGCCACTGTACTTGGACTTTTTTTT
TTTTTTTTGCTTTTGTTTGTTTTTTGTTTTAGCTATTACAATGCTGGTTGAATATCCTCATAGCTAAA
TATCTGTGCAGTTTTAAAATTTCCTTATAAGTTACTAGAAATGGAATGACTGGGTTAAAGGACAT
GATCATTTTTTAAGACTCTTGATACATACTTACAAATTGCCTCCCGAAAAAGTGGTGCTAATTTAC
TCAAATTGGTAAGCCTCACCCCCTCTGTGGATCATTCCTTGCACTAAGTTTCTTCCAACTCTGTCA
CCTCCAGCTTTTTACTCACAGCCTACCTTCCAATCTGGGGCCCAGCTCCCAGATCGGAAGATAC
CAGACTTTGGTATTGAACCAAAGTCCAATACACTGCACTGGCATTCAGAGTCCCTTTTTATTCCG
CAGCTGTCATAGTGAAGCTTGCAGCTTCCTTGAAATTCCAGTTTGTTTATTTCTTTATTTTTTTGAG
ACAGAGTCTTGCTCTATCGCCCAGGCTGGAGTGCAGTGGCGCCGTCTCAGCTCACTGCAACCTC
TGCCTCCTGGTTCAAGCGATTCTTCTGCCTCAGCCTCCCGAGTAGCTGGGATTACAGGCATGCA
CCACCACACGTGGCTAATTTTTGTGCTTTTAGTAGAGATGGGGTTTCACCATGTTGGCCAGGCTG
GTCTCAAACTCCTGACCTCAAGCGATCCGCCCACCTCAGCCTCCCAAAGTGCTGGGATTATAGG
CATGAGCCATTGCACCCAGCCAAAATTCCAGTTTGAATGTTATAATTCAGGGGTCTTGGACTCCT
GCTCTAGAGAAATTATTCATGTTAAGTTAGGGGTAACACAGTACAGGCCATATAAGTTTTAGAAA
AAGCAGTTTAAAACTTAGCATTAGAAAACATAAGTACTGCCTGGTGAAATGGCACGTGCCTATA
ATCCCAGCTACTCAGGAGGCTGAGGCAGGAGGATCCCTTGAGCCCAGGAGTTCAAGTCCAGCC
TGGGCAATATAGTCTAGTGGCCACATTAAAAAAAGTAAAACAGCCGGGCGCGGTGGCTCACGC
CTGTAATCCCAGCACTTTGGGAGGCCGAGGCGGGTGGATCACGAGGTCAGGAGATTGAGACCA
TCCTGGCTAACACGGTGAAACCCTGTCTCCACTAAAAATACAAAAAATTCTCCGGGTGTGGTGG
TGGGCGCCTGTGGTCCCAGCTACTCAGGAGGCTGAGGCAGGAGAATGGTGTGAGCCTGGGAG
GCGGAGCTTGCAGTGAGCCGAGATCGCGCCACTGCACTCCAGCCTGGGCGACAGAGCGAGAC
TCTGTCTCAAAAAAAAAAAAAAAAAAAAAAAAAAAAAGTAAAACAGGGCAGGGTGCAGTGGCTCA
CGCCTGTAATCCTAGCACTTTGGGAGGCCGAGATGGGTGGATCAGTTGAGGTCAGGAGTTTGA
GACCAGCCTGGCCAACATGGCGAAACCCCATCTCTACTAAAAATACAAAAATTAGCTGGGTGTG

ATGGTGCGTGCCTGTAGTCCCAGCTACTCAGGAGGCTGAGGCAGGAGGATCGTTTGAACTCGG
GAGCTGGAGGTTGCAGTGAGCCGAGATTGAGCCACAGCACTCCAGGCTGTGCTACAGTGTGAG
ACTGTCTCAAAAAAAAGTAAAACAGATGAAACTAATTTTAATAACATTTTATTTATTCAAACAATA
TATTCAAAATATTATTTCAACTTGTGATTGATAAAATACTAATGAGATATTTTATATTTTTTCTCCA
GTACTGTCTTCAAAATCCAGGGTATATTTTTCAGTTAGCAGCACATTTCAATTCAGACCAGCCGC
ATTTCAAGTGCTCAGCAGCCACCTGTGGCTAGTGGCCACAGTATTGGCCAGTGTAGCCCAAGGA
TATCTCAAATTATCTCAAAGCTAGATCAGTGATTAAAGGATTCTCAGGCCAGGCATGGTAGTTTT
CAGGCCTGGTATGGTGGTTTACGCCTGTAATCCCCGCACTTTGGGAGGCCTAGGTGGGCGGATG
GCTTGAGCCCAGGAGTTTGAGACCAGCCTAGCCAACGTGGGGAGACCCTGTCTCTACCAAAGC
ACAAAAATTAGCTGGGCATGGTGATGCGCACCTGTGGTCTCAAGTACTTTGTGTGAGGTGGGAG
GATCACTTGAGCCCGGGAGGTTGAGGCTGCAGTGAGCTGAGATTGCACAACTGCACTCCAGCC
TGGGTGACAGAATGAGACCCTGTCTCAAAAAAAAAAAAAGGATTCTCCGAAAGACTGGCGATTG
CTTTTTAAGCTATAAGGAAGGCCTTTGAAGGGAAATCAAGTAGGAAGAGGATGATAGCTGGCTC
ACATGTCCTTGCCACATGGGAGGTGAGAGAGCAGGCTACTCCTTACCACCCCCGCTTTCTCTGC
CCTGCTGCTGTCTTGCCCCTGTGCAAGTAGGCAATGGGAGAGGAATAGTATATATAAATTGGGC
AAGTGCTAGGGGCTGGAGTGATGGTTGGGGAAAGTATCTGACCCTTGTTTTCTACTTTCTTTAGC
TTGTGGGGTATTTAACAATTCTTTGTTCTTAGTTTTGAGTGCTGGCAACCATTTCTGGGGTGACAG
GAAAAGTTCCATGACATCCGTATTAGAGCATTTGTTTCATTATATTGTAATTTTTTATGTTTACTTT
TGAAAAAGTTCATTCTTTTCTCTTTTTAATTCTTATTCTTTTTTTTTTCTTTTCTTTTTGAGGCAGGGT
TTTACTCTATCACCCAGGCTGGAGTGCAGTGGCACAATCTTGGCTCACTGCAACCTCTGCCTCCT
GAGCTCGTGATTCATCCTCCTGAGTAGCTGGGACTACAGGTGTACACTACCATGTGTAGCTAAA
TTTTTTGTATTTTTTGTAGAGATGGGGTTTTGCCATGTTGCCAGGCTGGTCTCGAACTCCTGCAAT
TTGCTCTCCTTGGCCTCACGAAATGCTGGGATTACAGGCATAAGCTACAGCACCCAGCCCTTAC
GTTTACTTTTCATTGACAGGAAGAGTGTCTTCATCTCTGCATCCCTAGTGCAACAGAGCATGACA
AAAATTCAACAAATATTTATTGAAGAGTCTTGATGTCTTTTGGGAAAGGAACTCTATGGTTACCTT
TACTCTAAGGTAAAGAAATTTAGGTCCTGAACTTTAACCACCTTCTCCTCTTCTTTCTTCCCTCTTT
CATAAATGAGCTCTTGGAAAATTAAAATGCCAGGAGGGTCCAGCAGAGAATACCCAAGCCCAC
ATGTCCTGATGGGAAGTGGGGGATAAGTAGAGAGGGAAACACAGACCCTGTAGGCCATCTCAC
TGCTGCTGGCCCAGGGGACCCTAACTCCTTTTGTTGACTCATCAGAAGTAGAATCCTCCTGAGG
GTCTGTTTAAGAAGCACAGCTCTGGTAGAAAGGGTAGCTTTCCTATGGGTACGGAGGGCTCTGC
TACCCCAGGGCTATCCACACTGGCCTGTGAATCACTCTCAAATGCTTAGGGTCACGGGCTTTCA
GTGGAATGAGCCCTCATGTGCCACCAGGGGGCACCAAGGCCACAGAAAACCCTATTCAGAAG
GTTCTGGGGTGAGGGAAAGGAATCCACGGGTTTGTGGAGCTGGCGGAGCATGGGTGAGGACA
GCATTATGCTAGGTTGGTGGCCTTGAAGTCAGATGTAAATTGGGATCCTGGCTTGGCCACTGAC
TATCCATGGGAACTTTGAATCTCAGGGGGCTCATTTGTGATGTGGGGGTACCATTATCTGCCTCA
TCAGGTTGTGGGGAGGATTATATGAGATACCTTATGTAAGAGGGGCTGGTGCATTGTGGAAGTC
AACAAGTAAGTTTTGTTAAATTTGAGTAAGAAGACCTGTTTGTTTCCTCAAGTTGCTTATAATCTA
GGTGAGGACACATCTGAGAGAATAACTAATAGTTCAGTGAAGGTAAGAGCCAAATGATTGGCA
CAGACCTCAAGTGGTCTGGCATTTACGGGAGAGGGGGATTGTGAAACAGGAATTGAAAGAAAT
TTAAAAATGCATAAGCAAAAACTCAGTTTATGTTTTAAAAAAACCAATTCCCCCTGAGAAAGAG
AAAGAGGTGGAGTCCTTTAAAAATTATCTGCTTGTTTTTCTGTCTGTGGCTAGTGAGCCTTATCTC
TCCCTTTCCCAGGCATTGTGAAGACCCTGTTTCTCTAGCTGTGCAGCTGCAAGGTCACTAGGCA
GATAAACTCAAGCCATAAAACATGTTTTTCCTTGAAAAGTAAGAAATAATGTAATGCATGTGTCA
AATGAATAACTATTTCTCGCTTCTGTAATATGCTTCCCTTCGCACAGATCTCCCCCTGCCCCATGA

AATGCTTTAAAGGTAACTTAACTCTTTGTTCGGGGCTCAGTCCTTTGGATGTTAATCTGACTGGCT
TGGTGCACCTAAATAATAAATAACATATATCCTCCTCAACCCCTCAGTCTCTCTGATTCCTAAATT
ATCCCGCTGTAATTGCAGGGCTAGATTTGCCGACCTGCTTTATAAAGCTATGTTAAATCAGTATA
TTTTATTCTTTTTATTAGACTTTTCTATAGCATAAAAATGATAAAATACAGCAAGAAAAAAAGTCA
CCTGAAACTCTAGTCCTAACCCTAATATGAATGTGAAGGAGCTGGGCTAGAATGGAATAGGGTA
GAATTTGCATAAGGTGGAAATGGGGACAGGCCTGTCACAAGGATCACTAGAGTCTTGCTGGCA
AAAGCATCTTACTGCATTCATGCTGCAGCACAGGACCCAGATGCAGAACAGGTTGGGGACACA
GCCCTGCCTTCTAATAGTGGACACCATCTGAGAGCTGAGCCATAGATTCAAAGCCTAACTCCTA
ATAGCGATTGGTAGTAATATCATCAGCAGTTCATATACATGGAGTGACAACAGCGTGCCAGGCA
ATTCAAAGGGTCTCTCGGTCCTCACAGCATTTTTGAAGTAGCTAACATCACTATCCCCCTTTTAC
AGTTAAAAACAACAACAACAACAACAAAAACACAAAACCCTGAGGCTCAGAAGATAAATATTT
GCTGAAGATCACTGTGCTTGTAAGTGGTGGAGCCTGACCATTAACTTCCCCACGACACTGCTTC
AATTTCTATCACTTCATCAACCACACAACCCGAGAACTGTGAGGGGATTTAGCAGGCCCAGTCC
AGATCTCTTATTGTACAGAGAAGAAATCTGAGGCTCAGAGAGGGGAAGGGACTAATTTGGACC
TTTAACTTCGGATTCTTAACTTTCTTTCTGGTGTTCTAACTATACTGTACTACAAGAGGCTGTCTTA
GAGCTTTAGTTCAACCAGCTTCAGAGATACTCATTGTTATGGGTTGAATTATGTCTCCCCAAAATT
CATATGTCAAAGTTCTAACCCCCAGTACCTCAGAATGTGACCATATTTGGAGATAGGACCTTTAC
AGAGGTAATCAGGTGAAAATGAGGTTGTTAGGGTGGGCCTTAGTCCAGTATGGCTGGTGTCCTC
ATAAAAAGGGGAAATTTGGACACAGATGTACATAGAGGGAAGATGGTGTGAAGAGACATAAAG
AACAGATAGTCATCTATAGCCAAGGCCTGGAACAGATCCTTCCCTAACAGCCCTCAGAAGGAA
GCAACCTCACTGACACCTTGATTTTGGACTGCTACCCTCTGGCACTGTGAGACAATAAATTCATG
TTGGTTAGGCCACCCAGTTTGTTATGGCAGCCCTAGGAAACCTTTGTGGATATATCTGTTCGTAT
ACCCATAAAGTCTGCAAGTCAAAAATATCACAGACTTTTGGCAGCCCTTCATTCAAGCACTGGC
TGGGAGGGTCACCCTCCCCGAGGCAAAAGGATAGAGCAAATGATCTTTCCAGCTTGCTCCCAA
CCTTGATCTTTGGCCAAGATATTTTTCTTTCACTGGACATTGGCCAACATTGGTGTCATAACTTAA
TTTGACTGTGACCTTCTTGAGTCTTTTATGTCTCTGAGCTTGACCTGTAGAAGAAGCTCAATAAAT
GTGGGTAGAGGGGATGAGTGGGAAAATCAGCTCCCAGCCATTCCAGTTCAAGCCTCTTCAAGC
AGAAGAGTCCCACCTGAAAGACCTCATTTTCACTTGATTACTCGATCTCCAAGCAATAGGTGGG
GCAGGAAGCAGGGTATGTAAGGCCTGTCAGTGGATGGAAGTTGAAGTTGGACACCATCTCCTA
AAGTTTAGAGCTGAGCCCAAATTCTGGAAAACACAAGCAAAATACATTTTATATGACTCAGGAT
GGGCTAAAGAAGTTCCAGTACAGGAAACTGTTTTCCTGCTGCTTTATTAATTTTGCTACAGAGGA
TAAATGAGATGCAGACTTTCATATCCAAGTCTTCATTAGATGCCCAGTTAATGCAGCCATGACAA
ATGGGAATGAAGCCCTTATGAATAAAAAAAAGATCAACCCAGTATAAAAAAATCATAGACCAG
AGAAATGAAAATTTAGAGGAGCTGATAAAATACCCTATCCTTAATTAGACTGTCAGCATACACA
TCAAATCAGCCTTAATGCCAAGATTTATTGGCCTTGGAATTGGGTAAATGAAATAAGTGCTTACA
AATGAATGGAAAAGAAATTACCTGTATTAAAACGTATTTGTGATGCAGGCCTGTGCACTGAGGG
TTTTAGTCAAACTCAGGCTTGCAGCTTCTTTTGCTCTGCATCCATTTCCACCCCATGTTCCCCAGT
GGGTTTCAGAGACCCTCTTTGTGGGGTAACAATATGTACCATTGTCAACTACCTCTGCTGTTGTC
CTGGAAAAAATCCTTCTTGGACAGTAAAATATTTTTGTCTTTATTTCACCATCTCCAGATTAGATGG
GTGTTACTGATTCTTACCTCAGAGAAGAGAATGTCATGGAGACAAAATGACCTGCTTAGAGTTCT
GCACCCCCCAGCATGGAGAGCAGAGCGCAAAGATTTCTTCCGGACTCCCACATTCCCCTTGGA
ATCCAAGAACTGGTCTACTGTCACAACACTTTAGTACCCTCTTGTAAGCCTGTGGACAAATCCTC
TGATGCCTTGATACCTCGGTTTTCCCCCACTATAAATGGGAAGATAAATTCACTAGAAACAGTAC
TTACCTTGCAGTGTCATTATGAGAATTTCATGAGACAATGCAATATAAAGCTCCTAGCACAGTTC

CAGGCACCATTGTAAGCACTGAACAAATGAAAGCTGTTGTCATTAGGATCTCAAGAAATGACAG
CTGAGGCAAATGTGGATTAGACTGCATTCAGCATTCTTAGCTGCAAGCAACAGAAGCTGACGCT
GGTTTAAAGAATAGGAAAGGAAGTTAATTAAAATGACATTGGGTAGCTTGTGGAACTGGGAGG
ACTAGAGAGCTCAGTCTAAGTTCTAGTGACAAAGCTTCAAATCATACATGAGGAAAATGCCCCT
CTGCAGGAGGAACGTAATCCTGCAATCACTGCTTCCCCTGAAGCCAGATGCCTCTACTGCTGCC
CTTCCCAGCAGTGATGGAGCCTGGATCACATACCTGGCTTTAGTTGTAAAGGAGGCTGGAGAGA
AAATGCTCTGCTCTATGTAACTGTCTTGAAAGTGGCCCACACATAGCAAAGCCATTCAAAAGCG
GGTGAGCAGCATGAAAGCATCCAGAAGGATTCTTGCTTTGCATGAGGGAGAACTCTTGCTCCCA
GAGTGTGAGACCAGCAGGGGCATTTTCCTCATGTGTGATTAGAAGCTTTGTCACTAGAAGCTTA
GACTGAGTAGGACACCTCTGAGTGTGAATGTCCTACCTGAGTTCTGCCTCATGCAAAGCAAGAA
TCCCTCTGGAGGGATTGACACCACAGCCTCTTCCACAATGTCTCCAAACAACTTTACTGGGCAG
CATTCCACTCCTTGACAAACGGGAGAGGTGCATTCTACCTTCTCTCCTGCATGAGCCTAGACTAA
TATGGAATTTGGGAGTGTTGATCATTGTACTTTGTGCTCCTAGAGATAAGGGTCATCATAAAGCA
TCTCAGAGTCTCACTGTAGATCCACAGTTCCATTTCTTTATGGATCCCTTACCCCTACTTTGCCAA
ATTCTATGCAGATAGCAATAATAATTCAGACAGGGCTTCCTCACTGAAGGAGTTTGTGCTCTATA
TAGTTAATAAGCTAGAGATAATGAAAAAAAAAAAAAACTCAACTTGAACTTTGGGTAAGAGAAAC
TTTTCAGACCAACGATCATTACCAATAACAGCTTCCACGCATGACCCTGCCCGGAGATTGGGGC
CTGCACTACTGTTTCCAGGAAACACATTCTTTTGAATTTTTTTATTTTCTTTGTAGAGATGGGGATC
TCACTCTGTTGCCCAGGCTGGTCTCAAACTCCTGGCTTCAAGCAATCCTCCTGCTTCGGCCTCCC
AAACTTCTGGGATTACAGATATGAGCCACCACGGCTGGCTGACAGGCACCAAATTCTAGGAGA
GGACTTGAATAGAGTAGGCATCTGTCTCTCCCCATTCCTTTCAGGGACAGAGCTCTCCTGGTTGG
GAGAAAGGGAGAGGGAGAGGGAGGAGGATGAATAGTGAAGCACCAAACCTCAGAATTAAGG
CCTTTTTCTTTTCTTTCTTTCTTTCTTTTTTTTTTTTGAGATGGAGTTTCGCTCTTGTTGCCCAGGCTG
GAGTGCAATGGCACAATCTCGGCTCACCGCAACCTCTGCCTCCCAGGTTCAAGCGATTCTCCTG
CCTCAGCCTCCCGAGTAGCTGGGATTACAGGCATGCGCCACCACACCCAGTTAACTTTTTTGTAT
TTTTAGAGACGGGGTTTCTCCATGTTGGTCAAGCTGGTCTCGAACTCCCGACCTCAGGTGCTCTG
CCCGACTTAGCCTCCCAAAGTGCTAGGATTACAGGCGTGAGCCACCATGCCCAGCCAGAATCA
GGGCCTTTTTAAAACTTCAGTGTCTTGTCTTTACCCTTCATGGTGCCTAGAAAAGCATCTGGCTCA
TAGGTGTTCAGTGAATGATTGTTTCATTAGTGAAGAAATTCCCTTTGGAAATCTCATAGGAAACT
TACCCTCTCCAACCCTTCACCTGTGCATTATTCCAGCTCCCAAGGAGGCAGCAGAAACTGGGGT
AATCACAACGGTCAACAGCAGCAAAGGCAGCACTTTC

ID|56621|gene_id|ENSG00000224699|transcript_id|ENST00000597455 Homo sapiens lncRNA

CTGCCATCTGTGTTTGGAGACTGTGTCCCAGACTTTTGAGCTCACATTAGTCATGGCCACAG
ACGGGACTGGAGTCCTGCCCACTGGAGTCCCAAAGGTCTCTGGTGTTTGTTTTCCACTCTGCATG
CCTGGGTTGGGGGATGGCAGGGGGATACAGGTTGTTGGAGTACCTCCCAGCCGGGCCGTTTCC
TTGGTTCCCTGGGGCTGCCTTGCTTCCTCTCTAGATCATCAGTCTCAGCAGAGGAAACCAAAAAT
CTCCTCCTCGCCCCGCAACCACCTTTTGCTGACTGCTAAGCATCCCTGCTCTGAAACCATAACCA
CCATAAAAGCAATAGGCTTGTCTTCTCTGTTCTAAGCATGTACTATTCAGGTGATTTAGGGGGAG
TAAACAGGCCCAGAGAAATTAAATAATTTGTCTAAGGGTACCGGCTAATGAGGATCTGAGCTCA
GTTGTAAATGACTCCATCACCCATGCTCTTTTTTAGTCCCCTTTATAATTATTTTAAAATAACATTT
AATTAATTTTCTGTTATAAAAGGAACACATGCTCAAACAAAAAACTTAGAAAATATAAAAATGTA
TGAAGACAAAACAAAATAACCCCCAGTATTAGCAATGCCAACTTGCCAAGACAAAAATGTTAAC
ATTTTTATTTCTTTTTCTTTTCAGTCTTTACTCTCTGTGTGCACACACACACACAGGCAAAGTGAAA

TCACATTGTATATTCTTTTTTGCTTCTAGCATTTTCCACTTAGCTATCTAATATTACTATTTCCTGAT
ATCAATAAATGATCTTCAAT

ID|56634|gene_id|ENSG00000224699|transcript_id|ENST00000608635 Homo sapiens lncRNA

TCCTCAAGTTGCTTATAATCTAGGTGAGGACACATCTGAGAGAATAACTAATAGTTCAGTG
AAGGTAAGAGCCAAATGATTGGCACAGACCTCAAGTGGTCTGGCATTTACGGGAGAGGGGGAT
TGTGAAACAGGAATTGAAAGAAATTTAAAAATGCATAAGCAAAAACTCAGTTTATGTTTTAAAAA
AACCAATTCCCCCTGAGAAAGAGAAAGAGGTGGAGTCCTTTAAAAATTATCTGCTTGTTTTTCTG
TCTGTGGCTAGTGAGCCTTATCTCTCCCTTTCCCAGGCATTGTGAAGACCCTGTTTCTCTAGCTGT
GCAGCTGCAAGGTCACTAGGCAGATAAACTCAAGCCATAAAACATGTTTTTCCTTGAAAAGTAA
GAAATAATGTAATGCATGTGTCAAATGAATAACTATTTCTCGCTTCTGTAATATGCTTCCCTTCGC
ACAGATCTCCCCCTGCCCCATGAAATGCTTTAAAGGTAACTTAACTCTTTGTTCGGGGCTCAGTC
CTTTGGATGTTAATCTGACTGGCTTGGTGCACCTAAATAATAAATAACATATATCCTCCTCAACC
CCTCAGTCTCTCTGATTCCTAAATTATCCCGCTGTAATTGCAGGGCTAGATTTGCCGACCTGCTTT
ATAAAGCTATGTTAAATCAGTATATTTTATTCTTTTTATTAGACTTTTCTATAGCATAAAAATGATA
AAATACAGCAAGAAAAAAAGTCACCTGAAACTCTAGTCCTAACCCTAATATGAATGTGAAGGAG
CTGGGCTAGAATGGAATAGGGTAGAATTTGCATAAGGTGGAAATGGGGACAGGCCTGTCACAA
GGATCACTAGAGTCTTGCTGGCAAAAGCATCTTACTGCATTCATGCTGCAGCACAGGACCCAGA
TGCAGAACAGGTTGGGGACACAGCCCTGCCTTCTAATAGTGGACACCATCTGAGAGCTGAGCC
ATAGATTCAAAGCCTAACTCCTAATAGCGATTGGTAGTAATATCATCAGCAGTTCATATACATGG
AGTGACAACAGCGTGCCAGGCAATTCAAAGGGTCTCTCGGTCCTCACAGCATTTTTGAAGTAGC
TAACATCACTATCCCCCTTTTACAGTTAAAAACAACAACAACAACAACAAAAACACAAAACCCT
GAGGCTCAGAAGATAAATATTTGCTGAAGATCACTGTGCTTGTAAGTGGTGGAGCCTGACCATT
AACTTCCCCACGACACTGCTTCAATTTCTATCACTTCATCAACCACACAACCCGAGAACTGTGAG
GGGATTTAGCAGGCCCAGTCCAGATCTCTTATTGTACAGAGAAGAAATCTGAGGCTCAGAGAG
GGGAAGGGACTAATTTGGACCTTTAACTTCGGATTCTTAACTTTCTTTCTGGTGTTCTAACTATAC
TGTACTACAAGAGGCTGTCTTAGAGCTTTAGTTCAACCAGCTTCAGAGATACTCATTGTTATGGG
TTGAATTATGTCTCCCCAAAATTCATATGTCAAAGTTCTAACCCCCAGTACCTCAGAATGTGACC
ATATTTGGAGATAGGACCTTTACAGAGGTAATCAGGTGAAAATGAGGTTGTTAGGGTGGGCCTT
AGTCCAGTATGGCTGGTGTCCTCATAAAAAGGGGAAATTTGGACACAGATGTACATAGAGGGA
AGATGGTGTGAAGAGACATAAAGAACAGATAGTCATCTATAGCCAAGGCCTGGAACAGATCCT
TCCCTAACAGCCCTCAGAAGGAAGCAACCTCACTGACACCTTGATTTTGGACTGCTACCCTCTG
GCACTGTGAGACAATAAATTCATGTTGGTTAGGCCACCCAGTTTGTTATGGCAGCCCTAGGAAA
CCTTTGTGGATATATCTGTTCGTATACCCATAAAGTCTGCAAGTCAAAAATATCACAGACTTTTG
GCAGCCCTTCATTCAAGCACTGGCTGGGAGGGTCACCCTCCCCGAGGCAAAAGGATAGAGCAA
ATGATCTTTCCAGCTTGCTCCCAACCTTGATCTTTGGCCAAGATATTTTTCTTTCACTGGACATTG
GCCAACATTGGTGTCATAACTTAATTTGACTGTGACCTTCTTGAGTCTTTTATGTCTCTGAGCTTG
ACCTGTAGAAGAAGCTCAATAAATGTGGGTAGAGGGGATGAGTGGGAAAATCAGCTCCCAGCC
ATTCCAGTTCAAGCCTCTTCAAGCAGAAGAGTCCCACCTGAAAGACCTCATTTTCACTTGATTAC
TCGATCTCCAAGCAATAGGTGGGGCAGGAAGCAGGGTATGTAAGGCCTGTCAGTGGATGGAAG
TTGAAGTTGGACACCATCTCCTAAAGTTTAGAGCTGAGCCCAAATTCTGGAAAACACAAGCAAA
ATACATTTTATATGACTCAGGATGGGCTAAAGAAGTTCCAGTACAGGAAACTGTTTTCCTGCTGC
TTTATTAATTTTGCTACAGAGGATAAATGAGATGCAGACTTTCATATCCAAGTCTTCATTAGATGC
CCAGTTAATGCAGCCATGACAAATGGGAATGAAGCCCTTATGAATAAAAAAAAGATCAACCCA

GTATAAAAAAATCATAGACCAGAGAAATGAAAATTTAGAGGAGCTGATAAAATACCCTATCCTT
AATTAGACTGTCAGCATACACATCAAATCAGCCTTAATGCCAAGATTTATTGGCCTTGGAATTGG
GTAAATGAAATAAGTGCTTACAAATGAATGGAAAAGAAATTACCTGTATTAAAACGTATTTGTGA
TGCAGGCCTGTGCACTGAGGGTTTTAGTCAAACTCAGGCTTGCAGCTTCTTTTGCTCTGCATCCA
TTTCCACCCCATGTTCCCCAGTGGGTTTCAGAGACCCTCTTTGTGGGGTAACAATATGTACCATT
GTCAACTACCTCTGCTGTTGTCCTGGAAAAATCCTTCTTGGACAGTAAAATATTTTTGTCTTTATTT
CACCATCTCCAGATTAGATGGGTGTTACTGATTCTTACCTCAGAGAAGAGAATGTCATGGAGAC
AAAATGACCTGCTTAGAGTTCTGCACCCCCCAGCATGGAGAGCAGAGCGCAAAGATTTCTTCCG
GACTCCCACATTCCCCTTGGAATCCAAGAACTGGTCTACTGTCACAACACTTTAGTACCCTCTTG
TAAGCCTGTGGACAAATCCTCTGATGCCTTGATACCTCGGTTTTCCCCCACTATAAATGGGAAGA
TAAATTCACTAGAAACAGTACTTACCTTGCAGTGTCATTATGAGAATTTCATGAGACAATGCAAT
ATAAAGCTCCTAGCACAGTTCCAGGCACCATTGTAAGCACTGAACAAATGAAAGCTGTTGTCAT
TAGGATCTCAAGAAATGACAGCTGAGGCAAATGTGGATTAGACTGCATTCAGCATTCTTAGCTG
CAAGCAACAGAAGCTGACGCTGGTTTAAAGAATAGGAAAGGAAGTTAATTAAAATGACATTGG
GTAGCTTGTGGAACTGGGAGGACTAGAGAGCTCAGTCTAAGTTCTAGTGACAAAGCTTCAAATC
ATACATGAGGAAAATGCCCCTCTGCAGGAGGAACGTAATCCTGCAATCACTGCTTCCCCTGAAG
CCAGATGCCTCTACTGCTGCCCTTCCCAGCAGTGATGGAGCCTGGATCACATACCTGGCTTTAG
TTGTAAAGGAGGCTGGAGAGAAAATGCTCTGCTCTATGTAACTGTCTTGAAAGTGGCCCACACA
TAGCAAAGCCATTCAAAAGCGGGTGAGCAGCATGAAAGCATCCAGAAGGATTCTTGCTTTGCAT
GAGGGAGAACTCTTGCTCCCAGAGTGTGAGACCAGCAGGGGCATTTTCCTCATGTGTGATTAGA
AGCTTTGTCACTAGAAGCTTAGACTGAGTAGGACACCTCTGAGTGTGAATGTCCTACCTGAGTTC
TGCCTCATGCAAAGCAAGAATCCCTCTGGAGGGATTGACACCACAGCCTCTTCCACAATGTCTC
CAAACAACTTTACTGGGCAGCATTCCACTCCTTGACAAACGGGAGAGGTGCATTCTACCTTCTCT
CCTGCATGAGCCTAGACTAATATGGAATTTGGGAGTGTTGATCATTGTACTTTGTGCTCCTAGAG
ATAAGGGTCATCATAAAGCATCTCAGAGTCTCACTGTAGATCCACAGTTCCATTTCTTTATGGAT
CCCTTACCCCTACTTTGCCAAATTCTATGCAGATAGCAATAATAATTCAGACAGGGCTTCCTCAC
TGAAGGAGTTTGTGCTCTATATAGTTAATAAGCTAGAGATAATGAAAAAAAAAAAAAACTCAACT
TGAACTTTGGGTAAGAGAAACTTTTCAGACCAACGATCATTACCAATAACAGCTTCCACGCATG
ACCCTGCCCGGAGATTGGGGCCTGCACTACTGTTTCCAGGAAACACATTCTTTTGAATTTTTTTAT
TTTCTTTGTAGAGATGGGGATCTCACTCTGTTGCCCAGGCTGGTCTCAAACTCCTGGCTTCAAGC
AATCCTCCTGCTTCGGCCTCCCAAACTTCTGGGATTACAGATATGAGCCACCACGGCTGGCTGA
CAGGCACCAAATTCTAGGAGAGGACTTGAATAGAGTAGGCATCTGTCTCTCCCCATTCCTTTCA
GGGACAGAGCTCTCCTGGTTGGGAGAAAGGGAGAGGGAGAGGGAGGAGGATGAATAGTGAA
GCACCAAACCTCAGAATTAAGGCCTTTTTCTTTTCTTTCTTTCTTTCTTTTTTTTTTTTGAGATGGAG
TTTCGCTCTTGTTGCCCAGGCTGGAGTGCAATGGCACAATCTCGGCTCACCGCAACCTCTGCCT
CCCAGGTTCAAGCGATTCTCCTGCCTCAGCCTCCCGAGTAGCTGGGATTACAGGCATGCGCCAC
CACACCCAGTTAACTTTTTTGTATTTTTAGAGACGGGGTTTCTCCATGTTGGTCAAGCTGGTCTCG
AACTCCCGACCTCAGGTGCTCTGCCCGACTTAGCCTCCCAAAGTGCTAGGATTACAGGCGTGAG
CCACCATGCCCAGCCAGAATCAGGGCCTTTTTAAAACTTCAGTGTCTTGTCTTTACCCTTCATGG
TGCCTAGAAAAGCATCTGGCTCATAGGTGTTCAGTGAATGATTGTTTCATTAGTGAAGAAATTCC
CTTTGGAAATCTCATAGGAAACTTACCCTCTCCAACCCTTCACCTGTGCATTATTCCAGCTCCCA
AGGAGGCAGCAGAAACTGGGGTAATCACAACGGTCAACAGCAGCAAAGGCAGCACTTTC
ID|56663|gene_id|ENSG00000224699|transcript_id|ENST00000610148 Homo sapiens lncRNA

GCGCGGTGACCGTCGAGGTGACCTGCACCTGGCTCCATGGCGGAACCGGCCGGCACGGA
TTATTCCCCTCTCGGGAGAGGTCTGCAGCCGGGCCTCAGTGCTTCAGTTCGTGGCCTGGCATTTT
GTGTCAGTGTTTTTTAACCTTTGGACCCTAGTTCAACCGACTCCGAGACCTAGGGGACAGATCTT
AAGTTTTACAATAACCCAGGGGTCAGACAGGGTCTGATCTAGCCTGTGGGGCAAGTACGGGGA
AGACACGGGACCTCTTGGAGGGGGTCAGTAGGCTTGTAGGGGGAAAGACGCACACAGAGACT
AAAGGACGAGATGGAGCAAGGTTCAACCCCCTAGCTGAGTGTTGGCAGTGTCGGGATTTTAAC
GAAGACATTGGTGCTTATTATGGTGAGGGGACTCAGGACAGTTTAGGGGAAGAGAGTGGCATT
TGAGTCCTAACTCGTAAGGAAGACTGGGTAGGACTACTTGAAAATAACTTTTAAAAATTAATTAT
AAAGCTGGTAAAAATAGGAAAACATCTATGGCCCCACTGTCCAAAGACGACTTTTAACTTCTTG
GAGAATATCCTTTCAGATAGTTGTCTTTGTAAATGTATACTTTTAATGTTTTTTCGTTTGCCTTTTTA
AAATAAATTTTTTGGGGGACAGGGTCTTGCTCTGTTACCCAGGCTGTAGTGCAGTAGAACGATTA
TAGCTCATTGCAACCCGGACGTCCTGGGTTCAAGCTATCCTCACCTCTCAGCCTCTCGACTGGCT
AGGACCACAGCCTCGAGCCATCATGTCTGGCTAATTTTTACGTTTTTTTGTAGAGATGGGGGTCT
CGCTGTGTTGTCCAGGCTGGTCTTAAACTCCTTGCTTCAACAGATCCTCCCACTCCAGCCTCCCA
AAATGTGATTACAGGCTGAGTCACCACGCCTAGCATGGTTTTTTTTGTTTTGTTTGTTTTGTAAGC
AAAATTGACATACATACTGGAGGTACAATTCTGTAGCTGGCCTTCTTTACAGAGTTTAGAATAAA
TGTCTTTCCATGTCAAGTTTTCACTTACATCCTTTGTAGTAGTTGCAAAATATTCCATTTTATAGCT
GTATCTTAATTGTGGATTAAGGTTGGAGTGCAGTGGCACTATCATGGCTTAGTCCAGCCTCAGCC
TTCTAGGTTCAAGCGATCTTCCCACCTCAGCCTCCCAAGAAGCTGGGACTACAGGGACCCGCCA
CCCCACCTAGCTAATTAAAAAAAAAAATTGTAGAGATGGAGTCTCAGTGTGTAGCCCAGTCTCAA
GTGACCCTCCCTCGTTGGCTTCCCAAAGCGCTGAGATTGCAGGTGAGCCACTGTACTTGGACTT
TTTTTTTTTTTTTTGCTTTTGTTTGTTTTTTGTTTTAGCTATTACAATGCTGGTTGAATATCCTCATAG
CTAAATATCTGTGCAGTTTTAAAATTTCCTTATAAGTTACTAGAAATGGAATGACTGGGTTAAAG
GACATGATCATTTTTTAAGACTCTTGATACATACTTACAAATTGCCTCCCGAAAAAGTGGTGCTA
ATTTACTCAAATTGGTAAGCCTCACCCCCTCTGTGGATCATTCCTTGCACTAAGTTTCTTCCAACT
CTGTCACCTCCAGCTTTTTACTCACAGCCTACCTTCCAATCTGGGGCCCAGCTCCCAGATCGGAA
GATACCAGACTTTGGTATTGAACCAAAGTCCAATACACTGCACTGGCATTCAGAGTCCCTTTTTA
TTCCGCAGCTGTCATAGTGAAGCTTGCAGCTTCCTTGAAATTCCAGTTTGTTTATTTCTTTATTTTT
TTGAGACAGAGTCTTGCTCTATCGCCCAGGCTGGAGTGCAGTGGCGCCGTCTCAGCTCACTGCA
ACCTCTGCCTCCTGGTTCAAGCGATTCTTCTGCCTCAGCCTCCCGAGTAGCTGGGATTACAGGC
ATGCACCACCACACGTGGCTAATTTTTGTGCTTTTAGTAGAGATGGGGTTTCACCATGTTGGCCA
GGCTGGTCTCAAACTCCTGACCTCAAGCGATCCGCCCACCTCAGCCTCCCAAAGTGCTGGGATT
ATAGGCATGAGCCATTGCACCCAGCCAAAATTCCAGTTTGAATGTTATAATTCAGGGGTCTTGG
ACTCCTGCTCTAGAGAAATTATTCATGTTAAGTTAGGGGTAACACAGTACAGGCCATATAAGTTT
TAGAAAAAGCAGTTTAAAACTTAGCATTAGAAAACATAAGTACTGCCTGGTGAAATGGCACGTG
CCTATAATCCCAGCTACTCAGGAGGCTGAGGCAGGAGGATCCCTTGAGCCCAGGAGTTCAAGT
CCAGCCTGGGCAATATAGTCTAGTGGCCACATTAAAAAAAGTAAAACAGCCGGGCGCGGTGGC
TCACGCCTGTAATCCCAGCACTTTGGGAGGCCGAGGCGGGTGGATCACGAGGTCAGGAGATTG
AGACCATCCTGGCTAACACGGTGAAACCCTGTCTCCACTAAAAATACAAAAAATTCTCCGGGTG
TGGTGGTGGGCGCCTGTGGTCCCAGCTACTCAGGAGGCTGAGGCAGGAGAATGGTGTGAGCCT
GGGAGGCGGAGCTTGCAGTGAGCCGAGATCGCGCCACTGCACTCCAGCCTGGGCGACAGAGC
GAGACTCTGTCTCAAAAAAAAAAAAAAAAAAAAAAAAAAAAAGTAAAACAGGGCAGGGTGCAGT
GGCTCACGCCTGTAATCCTAGCACTTTGGGAGGCCGAGATGGGTGGATCAGTTGAGGTCAGGA
GTTTGAGACCAGCCTGGCCAACATGGCGAAACCCCATCTCTACTAAAAATACAAAAATTAGCTG

GGTGTGATGGTGCGTGCCTGTAGTCCCAGCTACTCAGGAGGCTGAGGCAGGAGGATCGTTTGA
ACTCGGGAGCTGGAGGTTGCAGTGAGCCGAGATTGAGCCACAGCACTCCAGGCTGTGCTACAG
TGTGAGACTGTCTCAAAAAAAAGTAAAACAGATGAAACTAATTTTAATAACATTTTATTTATTCAA
ACAATATATTCAAAATATTATTTCAACTTGTGATTGATAAAATACTAATGAGATATTTTATATTTTT
TCTCCAGTACTGTCTTCAAAATCCAGGGTATATTTTTCAGTTAGCAGCACATTTCAATTCAGACCA
GCCGCATTTCAAGTGCTCAGCAGCCACCTGTGGCTAGTGGCCACAGTATTGGCCAGTGTAGCCC
AAGGATATCTCAAATTATCTCAAAGCTAGATCAGTGATTAAAGGATTCTCAGGCCAGGCATGGT
AGTTTTCAGGCCTGGTATGGTGGTTTACGCCTGTAATCCCCGCACTTTGGGAGGCCTAGGTGGG
CGGATGGCTTGAGCCCAGGAGTTTGAGACCAGCCTAGCCAACGTGGGGAGACCCTGTCTCTAC
CAAAGCACAAAAATTAGCTGGGCATGGTGATGCGCACCTGTGGTCTCAAGTACTTTGTGTGAGG
TGGGAGGATCACTTGAGCCCGGGAGGTTGAGGCTGCAGTGAGCTGAGATTGCACAACTGCACT
CCAGCCTGGGTGACAGAATGAGACCCTGTCTCAAAAAAAAAAAAAGGATTCTCCGAAAGACTGG
CGATTGCTTTTTAAGCTATAAGGAAGGCCTTTGAAGGGAAATCAAGTAGGAAGAGGATGATAGC
TGGCTCACATGTCCTTGCCACATGGGAGGTGAGAGAGCAGGCTACTCCTTACCACCCCCGCTTT
CTCTGCCCTGCTGCTGTCTTGCCCCTGTGCAAGTAGGCAATGGGAGAGGAATAGTATATATAAA
TTGGGCAAGTGCTAGGGGCTGGAGTGATGGTTGGGGAAAGTATCTGACCCTTGTTTTCTACTTTC
TTTAGCTTGTGGGGTATTTAACAATTCTTTGTTCTTAGTTTTGAGTGCTGGCAACCATTTCTGGGG
TGACAGGAAAAGTTCCATGACATCCGTATTAGAGCATTTGTTTCATTATATTGTAATTTTTTATGTT
TACTTTTGAAAAAGTTCATTCTTTTCTCTTTTTAATTCTTATTCTTTTTTTTTTCTTTTCTTTTTGAGGC
AGGGTTTTACTCTATCACCCAGGCTGGAGTGCAGTGGCACAATCTTGGCTCACTGCAACCTCTG
CCTCCTGAGCTCGTGATTCATCCTCCTGAGTAGCTGGGACTACAGGTGTACACTACCATGTGTA
GCTAAATTTTTTGTATTTTTTGTAGAGATGGGGTTTTGCCATGTTGCCAGGCTGGTCTCGAACTCC
TGCAATTTGCTCTCCTTGGCCTCACGAAATGCTGGGATTACAGGCATAAGCTACAGCACCCAGC
CCTTACGTTTACTTTTCATTGACAGGAAGAGTGTCTTCATCTCTGCATCCCTAGTGCAACAGAGC
ATGACAAAAATTCAACAAATATTTATTGAAGAGTCTTGATGTCTTTTGGGAAAGGAACTCTATGG
TTACCTTTACTCTAAGGTAAAGAAATTTAGGTCCTGAACTTTAACCACCTTCTCCTCTTCTTTCTTC
CCTCTTTCATAAATGAGCTCTTGGAAAATTAAAATGCCAGGAGGGTCCAGCAGAGAATACCCAA
GCCCACATGTCCTGATGGGAAGTGGGGGATAAGTAGAGAGGGAAACACAGACCCTGTAGGCC
ATCTCACTGCTGCTGGCCCAGGGGACCCTAACTCCTTTTGTTGACTCATCAGAAGTAGAATCCTC
CTGAGGGTCTGTTTAAGAAGCACAGCTCTGGTAGAAAGGGTAGCTTTCCTATGGGTACGGAGG
GCTCTGCTACCCCAGGGCTATCCACACTGGCCTGTGAATCACTCTCAAATGCTTAGGGTCACGG
GCTTTCAGTGGAATGAGCCCTCATGTGCCACCAGGGGGCACCAAGGCCACAGAAAACCCTATT
CAGAAGGTTCTGGGGTGAGGGAAAGGAATCCACGGGTTTGTGGAGCTGGCGGAGCATGGGTG
AGGACAGCATTATGCTAGGTTGGTGGCCTTGAAGTCAGATGTAAATTGGGATCCTGGCTTGGCC
ACTGACTATCCATGGGAACTTTGAATCTCAGGGGGCTCATTTGTGATGTGGGGGTACCATTATCT
GCCTCATCAGGTTGTGGGGAGGATTATATGAGATACCTTATGTAAGAGGGGCTGGTGCATTGTG
GAAGTCAACAAGTAAGTTTTGTTAAATTTGAGTAAGAAGACCTGTTTGTTTCCTCAAGTTGCTTAT
AATCTAGGTGAGGACACATCTGAGAGAATAACTAATAGTTCAGTGAAGGTAAGAGCCAAATGA
TTGGCACAGACCTCAAGTGGTCTGGCATTTACGGGAGAGGGGGATTGTGAAACAGGAATTGAA
AGAAATTTAAAAATGCATAAGCAAAAACTCAGTTTATGTTTTAAAAAAACCAATTCCCCCTGAGA
AAGAGAAAGAGGTGGAGTCCTTTAAAAATTATCTGCTTGTTTTTCTGTCTGTGGCTAGTGAGCCT
TATCTCTCCCTTTCCCAGGCATTGTGAAGACCCTGTTTCTCTAGCTGTGCAGCTGCAAGGTCACT
AGGCAGATAAACTCAAGCCATAAAACATGTTTTTCCTTGAAAAGTAAGAAATAATGTAATGCAT
GTGTCAAATGAATAACTATTTCTCGCTTCTGTAATATGCTTCCCTTCGCACAGATCTCCCCCTGCC

CCATGAAATGCTTTAAAGGTAACTTAACTCTTTGTTCGGGGCTCAGTCCTTTGGATGTTAATCTGA
CTGGCTTGGTGCACCTAAATAATAAATAACATATATCCTCCTCAACCCCTCAGTCTCTCTGATTC
CTAAATTATCCCGCTGTAATTGCAGGGCTAGATTTGCCGACCTGCTTTATAAAGCTATGTTAAAT
CAGTATATTTTATTCTTTTTATTAGACTTTTCTATAGCATAAAAATGATAAAATACAGCAAGAAAA
AAAGTCACCTGAAACTCTAGTCCTAACCCTAATATGAATGTGAAGGAGCTGGGCTAGAATGGAA
TAGGGTAGAATTTGCATAAGGTGGAAATGGGGACAGGCCTGTCACAAGGATCACTAGAGTCTT
GCTGGCAAAAGCATCTTACTGCATTCATGCTGCAGCACAGGACCCAGATGCAGAACAGGTTGG
GGACACAGCCCTGCCTTCTAATAGTGGACACCATCTGAGAGCTGAGCCATAGATTCAAAGCCTA
ACTCCTAATAGCGATTGGTAGTAATATCATCAGCAGTTCATATACATGGAGTGACAACAGCGTG
CCAGGCAATTCAAAGGGTCTCTCGGTCCTCACAGCATTTTTGAAGTAGCTAACATCACTATCCCC
CTTTTACAGTTAAAAACAACAACAACAACAACAAAAACACAAAACCCTGAGGCTCAGAAGATA
AATATTTGCTGAAGATCACTGTGCTTGTAAGTGGTGGAGCCTGACCATTAACTTCCCCACGACAC
TGCTTCAATTTCTATCACTTCATCAACCACACAACCCGAGAACTGTGAGGGGATTTAGCAGGCC
CAGTCCAGATCTCTTATTGTACAGAGAAGAAATCTGAGGCTCAGAGAGGGGAAGGGACTAATTT
GGACCTTTAACTTCGGATTCTTAACTTTCTTTCTGGTGTTCTAACTATACTGTACTACAAGAGGCT
GTCTTAGAGCTTTAGTTCAACCAGCTTCAGAGATACTCATTGTTATGGGTTGAATTATGTCTCCCC
AAAATTCATATGTCAAAGTTCTAACCCCCAGTACCTCAGAATGTGACCATATTTGGAGATAGGA
CCTTTACAGAGGTAATCAGGTGAAAATGAGGTTGTTAGGGTGGGCCTTAGTCCAGTATGGCTGG
TGTCCTCATAAAAAGGGGAAATTTGGACACAGATGTACATAGAGGGAAGATGGTGTGAAGAGA
CATAAAGAACAGATAGTCATCTATAGCCAAGGCCTGGAACAGATCCTTCCCTAACAGCCCTCAG
AAGGAAGCAACCTCACTGACACCTTGATTTTGGACTGCTACCCTCTGGCACTGTGAGACAATAA
ATTCATGTTGGTTAGGCCACCCAGTTTGTTATGGCAGCCCTAGGAAACCTTTGTGGATATATCTG
TTCGTATACCCATAAAGTCTGCAAGTCAAAAATATCACAGACTTTTGGCAGCCCTTCATTCAAGC
ACTGGCTGGGAGGGTCACCCTCCCCGAGGCAAAAGGATAGAGCAAATGATCTTTCCAGCTTGC
TCCCAACCTTGATCTTTGGCCAAGATATTTTTCTTTCACTGGACATTGGCCAACATTGGTGTCATA
ACTTAATTTGACTGTGACCTTCTTGAGTCTTTTATGTCTCTGAGCTTGACCTGTAGAAGAAGCTCA
ATAAATGTGGGTAGAGGGGATGAGTGGGAAAATCAGCTCCCAGCCATTCCAGTTCAAGCCTCTT
CAAGCAGAAGAGTCCCACCTGAAAGACCTCATTTTCACTTGATTACTCGATCTCCAAGCAATAG
GTGGGGCAGGAAGCAGGGTATGTAAGGCCTGTCAGTGGATGGAAGTTGAAGTTGGACACCATC
TCCTAAAGTTTAGAGCTGAGCCCAAATTCTGGAAAACACAAGCAAAATACATTTTATATGACTCA
GGATGGGCTAAAGAAGTTCCAGTACAGGAAACTGTTTTCCTGCTGCTTTATTAATTTTGCTACAG
AGGATAAATGAGATGCAGACTTTCATATCCAAGTCTTCATTAGATGCCCAGTTAATGCAGCCATG
ACAAATGGGAATGAAGCCCTTATGAATAAAAAAAAAGATCAACCCAGTATAAAAAAAATCATAGA
CCAGAGAAATGAAAATTTAGAGGAGCTGATAAAATACCCTATCCTTAATTAGACTGTCAGCATA
CACATCAAATCAGCCTTAATGCCAAGATTTATTGGCCTTGGAATTGGGTAAATGAAATAAGTGCT
TACAAATGAATGGAAAAGAAATTACCTGTATTAAAACGTATTTGTGATGCAGGCCTGTGCACTG
AGGGTTTTAGTCAAACTCAGGCTTGCAGCTTCTTTTGCTCTGCATCCATTTCCACCCCATGTTCCC
CAGTGGGTTTCAGAGACCCTCTTTGTGGGGTAACAATATGTACCATTGTCAACTACCTCTGCTGT
TGTCCTGGAAAAATCCTTCTTGGACAGTAAAATATTTTTGTCTTTATTTCACCATCTCCAGATTAG
ATGGGTGTTACTGATTCTTACCTCAGAGAAGAGAATGTCATGGAGACAAAATGACCTGCTTAGA
GTTCTGCACCCCCCAGCATGGAGAGCAGAGCGCAAAGATTTCTTCCGGACTCCCACATTCCCCT
TGGAATCCAAGAACTGGTCTACTGTCACAACACTTTAGTACCCTCTTGTAAGCCTGTGGACAAAT
CCTCTGATGCCTTGATACCTCGGTTTTCCCCCACTATAAATGGGAAGATAAATTCACTAGAAACA
GTACTTACCTTGCAGTGTCATTATGAGAATTTCATGAGACAATGCAATATAAAGCTCCTAGCACA

GTTCCAGGCACCATTGTAAGCACTGAACAAATGAAAGCTGTTGTCATTAGGATCTCAAGAAATG
ACAGCTGAGGCAAATGTGGATTAGACTGCATTCAGCATTCTTAGCTGCAAGCAACAGAAGCTGA
CGCTGGTTTAAAGAATAGGAAAGGAAGTTAATTAAAATGACATTGGGTAGCTTGTGGAACTGGG
AGGACTAGAGAGCTCAGTCTAAGTTCTAGTGACAAAGCTTCAAATCATACATGAGGAAAATGCC
CCTCTGCAGGAGGAACGTAATCCTGCAATCACTGCTTCCCCTGAAGCCAGATGCCTCTACTGCT
GCCCTTCCCAGCAGTGATGGAGCCTGGATCACATACCTGGCTTTAGTTGTAAAGGAGGCTGGAG
AGAAAATGCTCTGCTCTATGTAACTGTCTTGAAAGTGGCCCACACATAGCAAAGCCATTCAAAA
GCGGGTGAGCAGCATGAAAGCATCCAGAAGGATTCTTGCTTTGCATGAGGGAGAACTCTTGCTC
CCAGAGTGTGAGACCAGCAGGGGCATTTTCCTCATGTGTGATTAGAAGCTTTGTCACTAGAAGC
TTAGACTGAGTAGGACACCTCTGAGTGTGAATGTCCTACCTGAGTTCTGCCTCATGCAAAGCAA
GAATCCCTCTGGAGGGATTGACACCACAGCCTCTTCCACAATGTCTCCAAACAACTTTACTGGG
CAGCATTCCACTCCTTGACAAACGGGAGAGGTGCATTCTACCTTCTCTCCTGCATGAGCCTAGA
CTAATATGGAATTTGGGAGTGTTGATCATTGTACTTTGTGCTCCTAGAGATAAGGGTCATCATAA
AGCATCTCAGAGTCTCACTGTAGATCCACAGTTCCATTTCTTTATGGATCCCTTACCCCTACTTTG
CCAAATTCTATGCAGATAGCAATAATAATTCAGACAGGGCTTCCTCACTGAAGGAGTTTGTGCTC
TATATAGTTAATAAGCTAGAGATAATGAAAAAAAAAAAAAACTCAACTTGAACTTTGGGTAAGAG
AAACTTTTCAGACCAACGATCATTACCAATAACAGCTTCCACGCATGACCCTGCCCGGAGATTG
GGGCCTGCACTACTGTTTCCAGGAAACACATTCTTTTGAATTTTTTTATTTTCTTTGTAGAGATGG
GGATCTCACTCTGTTGCCCAGGCTGGTCTCAAACTCCTGGCTTCAAGCAATCCTCCTGCTTCGGC
CTCCCAAACTTCTGGGATTACAGATATGAGCCACCACGGCTGGCTGACAGGCACCAAATTCTAG
GAGAGGACTTGAATAGAGTAGGCATCTGTCTCTCCCCATTCCTTTCAGGGACAGAGCTCTCCTG
GTTGGGAGAAAGGGAGAGGGAGAGGGAGGAGGATGAATAGTGAAGCACCAAACCTCAGAATT
AAGGCCTTTTTCTTTTCTTTCTTTCTTTCTTTTTTTTTTTTGAGATGGAGTTTCGCTCTTGTTGCCCAG
GCTGGAGTGCAATGGCACAATCTCGGCTCACCGCAACCTCTGCCTCCCAGGTTCAAGCGATTCT
CCTGCCTCAGCCTCCCGAGTAGCTGGGATTACAGGCATGCGCCACCACACCCAGTTAACTTTTT
TGTATTTTTAGAGACGGGGTTTCTCCATGTTGGTCAAGCTGGTCTCGAACTCCCGACCTCAGGTG
CTCTGCCCGACTTAGCCTCCCAAAGTGCTAGGATTACAGGCGTGAGCCACCATGCCCAGCCAG
AATCAGGGCCTTTTTAAAACTTCAGTGTCTTGTCTTTACCCTTCATGGTGCCTAGAAAAGCATCTG
GCTCATAGGTGTTCAGTGAATGATTGTTTCATTAGTGAAGAAATTCCCTTTGGAAATCTCATAGG
AAACTTACCCTCTCCAACCCTTCACCTGTGCATTATTCCAGCTCCCAAGGAGGCAGCAGAAACT
GGGGTAATCACAACGGTCAACAGCAGCAAAGGCAGCAC

ID|56671|gene_id|ENSG00000224699|transcript_id|ENST00000590413 Homo sapiens lncRNA

TTGCCAGCTTTATACCTGCACAAAAACGAAGAGATGACATGATGGATCCAATCCAACCAAT
TTGCTCTGAGGTGCCTTCAAACTCTTCTTGAAAGACCACAAAGAAAATTGCAAAAGTCTTGGTCA
TCCCCATCACAAACACATTCACCTAAATCAAAGCAGACCAGAGTCCAACTCAGGTGGTAAGTGA
GCCATTACAGAGGCAATTACGGCTACTAGAAGTTATGATTCCCTCAGCTTACTACTATTTACCAG
TTTGCCTTATTGGTTGAGCCTCATTATACATCTTATATTTTATGGGGGAAGTCATTCCTTTTAAAGT
AGCTAAGCATTTTAAATGGAAACATCTCATTTAATCCTCATTTCATGGCTTTGGAATAGGCATGC
CAATGTTGTGTACCAAATAATTTATACCAAATAAACCACCCAGTGGGTTCATCTTGCCTGCTGCC
TAGATACAGTTGATTTATCAAGACAGGAATTGCAATAGAGAAAGAGTTATTCCCACAGAGCCCA
TTGTGCAAGAGACTGGAGTTTATTAGACCAATCAGTTTCCCCGAGCTTTTGGGGATCAGAGTTTT
TAAGGATAATTTGGTGGGTGGGAGGCCAGTGAGTTGGGAGTGCTGATTGGTTGGGTCAGAGAT
GAAATTCTATCAAAGCTGCCCTTTTGCGCTGAGTCACTTCTGGGTGGAAGGGTGGAGGGGGACC

CACAACCAGATGAGCCAGTTTATTAATCTGGGTGGTGCCAGCTGATCCATCAAATGCAGGGCCT
GCAAATTATCTCAAGCACTGATCTTAGGTTTCACAATAGTGATGTTATCCCCCGAGCAATTTGGG
GAGGGTCAGTCTTGTGGCCTCCAGCTGTATGACTCCTAAACCATAATTTCTAATCTTATGGCTAA
TGTGTTAGTCCTACAAAGGCAATCTAGTCCCCAGGCAGGAAGGAAGTGTTTTTTTTTTTTTTTGGA
AAGGGCTGTTAAAAGGAGGTTTTTTTTTTTTTTTTTTTTTTTTTTTGGAAACGGCTGTTAGTTTCTCT
TTCAAAGTTAAGCTATCAACTAAGTTTCCCCCAAAGTTAGTTCAGCCTACGCATGGGAATGAACA
AGGACAGCTTGGAAGTTAGAAGCAGGATGGATGGAGTTGGTTAGGTCAGATCTCTGTCACTGTA
ATAATTTTCTCAGTTATAACTGCAACAGCAGTTCCAAGATCAGGAAATTGAAGTTTAGAGAGGTA
AAAAGTTGCTTAAAATAGGGGTACTGGAACTGAGATTTGAACCCAGATTAGACTGACTCTAGAC
CTGGCACTCTTTTACTGCCCTCAGTAGCATCTGTTAAAGACAGCTAATTCCAGTTACCTTTGTGG
GAGAAAAGTGTTTGTCCAAAGAGGAATAATTATTCCTGTCAGTTCTTAGATTAGTTTTGATTTAAC
TCTGCTGCTGAGCTGAGTACCTTCCTTCACTAGATATATAAATAGGTTGACCATAAAATGTATCA
TCCAAATTGAGACACTCAAGAGTGAAAAGGGGTGCTTAATTTTGGATTAACTGGGATTTGTGGC
CATCTTAAGGTTGGGCCAGGGGCCCCATCCTTTTCTTTCTGCCCCACCTAATCTTCCTTACCTGTA
CTCCACTAGAATTGCTGCTTTGGAGATGAGGTTTTCTTTCTGTGGAGTATTAACTTTTATTTCTCA
GAATTCTAGTTAGCCGGGGGGCTGTTAAGTGCTATTCCTTCTTTCCGTTTTTAAGTAACAGTCATG
CTTTATCCCACAGGGGTCTAGAGGGGAACCTAGCCAGGGTTTGCATTCACTCGTAAAGCCCAGG
TGTACTCTCTGTCTCCAGGGGTTTCGTAGCGTTGGCTTTTCAAGGCCACTTCTGCTTTCAACCTGG
CCATGCATTGCTTATGAACCACAAAACCAGCTGGGCATGGAGGCTCCTGCCTATAATCCTAGTA
CTTTGGGAGGCCGAGGTGGGTGGATCGCTTGAGCCCAGGAGTTCAAGACCAGCCTGGGCAACA
TGGCGAAACCCCGTCTCTACAAAAAGTACAAAAAATTAGCTGGGCGTAGTGGTATAACACCTGT
AGTCCCAGCTACCTGGGAGGGTGAGGTGGGAGGAACACCTGAACCTGGGAGGTCGAGGGTAC
GGTGAGTTGTGATCACACTACTGCACTCCAGCCTGGGCGACAGTGAGACCTTGTCTCAAAATTA
ATAATAATAAAAGAACCACAAAGCCTTAGGTTCCCCTTCATTAATTCCACTTTCTCGGCCCGCCT
GTTACAGTTCTGTACTCATCTCGCTTGATATCTGCTCCCTGGACAATCATTTACTCTCAAGGTTTT
AATTACCACCTGCATACTTATGGTGGTGTAGCTGAACAGGGTTCTGCAATCAGTCTGCCTTAACT
TGCATCCAGGCTGTTCTTCCTACTGGGGCGTGACCTTGGGCAAACAACTTCGCACGTCACATGG
GTTAAGCTTCCGCTTTTATGATGGGGGAGGCGGCATTGGGGATGAAAATATTACCTAGCTCATA
AGACTGTGAAGATTAATTGAGAAAATTCTGATAAAGTACTTAATATTTGGCACTTAACACTCAAA
CAATTAACTGTGGTTTCCATTCTGCTTTCCCAAGAGCTTCAGACTCCTAATTGTCTACCATATCTT
TATAACTTCTGGTCAACTTGTCCAAACATAAACTCACATTTCCCTTATCCTATGCCTACTCTTTTCA
GTTTTTTCTATTGGTACCATCCACCCATTCATTTGAGTGACAGATGTGCTTTCTCTTAGACACCTC
CCTCCCTCAGCCCCTGCTTTTAAATAATGGTCAAGACCTGCCCCCTTCTTTTCACCCCATCACTAC
TGGCGATGCTCAGAAATTGCAAAAGTGGTAGTGGTGAGGAAGAAGTCTCTTAATATACTTTTTCC
CATTCTGATTTGATAAAAGGAACAAAACTTATTTAGAATGAAGAGTGCTATAAACGCCAGGACT
ATATATTCAAAAGTTAAAAAGACATTTTATCAGGCCCCCATTGAGGCAATTTATAACCATAATTTT
CCTCTAAGGCACTTCTGTCAGGCTAGAAATTAGGTTAGAAAATATACACAGATCTCAAATGACTT
AGACTTTCTTCTCTCAGTTCATAATAATGTGATGATTGTGTCCTCTCATGAGCAAGGAATAATACA
GTGCATCATTCTGTAAAGTAAACTTCCTTTTGTTCTATGGACGTGGGTTCTGTGCTGGTGTCTAGA
GGATGTTGACCTCTATATTCTTAGCTACCTAAATTTAGCTAGCTACTGGTCCCCCAGTTCAGAGC
TTTTCATCTCTGGTCTGTCCTTGGTGTCTGCGTGTAGCAGCAGTGAATGGTTAGGACTTGTCTCTA
ATAACTGAGTCCGTGTCCACTATGTATCAAGAAGACCAAGGGCAGAGCCTTTGCACCTACCATG
CTTCCCGTCCCTTAACTTGAAGTCTTCCAACTTTGTTTCAGTTTTGACTCAAAGATATTAAAAATC
TCTCCCAAGGTCTAGGTTTTTCCTTAGTTAATTTAGCAGACTACATAAATCTGCATTATTCTACAT

AATCCCATGTCTTAGACCAAGCTTGGTGGGTTTTAGTTCTGTTACCAGAGTCCACGTCATACATT
GTAAATGGCTCACTCCAAAATAAAGCAAAAACCCTAGAACTTCTCCAGTCCCCTCATTTTCTTTT
TGTTTTTAAGAGCAGTTAATTGGGTTGAACCTGGCAGGAACACCTTAAGCAGGACTTCCCCCAC
ACCCCACCTCCAATAAGAGCTTCCTCACTAGATCATTTTAATTATTGTCTGGGAAAGGAGTTGGA
TCAGCCTCAGTGACTTATTTTCAATACAGGCACCAGGCTTTGTCAGAGTTAGTTTTATCTAATAAC
AACTTCTTATCCTGCGTCCTTTTCTAGTATCACCCTAGCTCCTCCTGACCTTCACAGGCAAACTTC
TGTAAAAAATGGTCTATTAGGTATCTCCACTTTCTGTCATTCTGCTACTCCACTGAAAACTAAAGT
TCACTAACTTTGGCCCCCACTGCTCCACTGTACTAGCTCTTATCAAGGTTATTAGATTTCTTTGTT
GCTAAACCAATAGAGTCTTGTTATTTCTCTCCTATTTGACCTCTCAGTAGCATCTGCTCGTCACAT
TTCTCTGAAATGCAAATCTATCACATCCCTGCTTAATTTTTCTCATTAGCTTCTCATAGCCTCAAG
AGCCATTCTCATCTCTTCAGTTTCATCAACTATCCCCTTCATCCTTATTTGTTTATTCATAAATGTG
TGCAGCTATCAATTCATCCATCCATCTATCCATCCACCAACAGTATTTTACTGAGTGCCTACTATG
TTCCAGGCTGTATACTGGCACTGGGCTTACGGTAATGAGCATATACTCATGAGCAATGCAGACA
TAGACCCTGCCCCCATGGAACATTCAGTCTAGTAAGACAGACTGAATACAGAAAACAGTCATAC
AAATATATAAAGTGTGGTAAGTTGGTTAGAATGAATCTCTCTCTCTTTTTTTAAGTAGGAAGCTCC
CAAAGAACATTGTTATTTTTCACTTACTAGGAGCTCATCTGTTATAGAAAAGAACAGTTGTAAGT
CATACAGAAACAAAAGCACAGTCATTCAAGTATCCACATTACAACTGTGGGGGATTAGCCCCTG
AATTTATTGTTAACATTTTGAGGATCCAGTGCATTTTTTGTGCTTAGTAAGCAGTATCACACCCTA
AAAGTGGGACAGTGTTTATTAATCTGGTTATTAGATGGTTTTTCCTATCAGCAACTAAATTGATCA
AAAACCATTCTATCCTCAGAGGATAATGTAGAAAAAGTCAGGGTGGTTACTGGGGAGAAAGGC
AGGAGAATTAGAGATCCTCACCAACAGTGCCTATTCTGCAAAGCCTATGATTTAGGGGGGTGGC
CCAGCCCCAGGAAGGGCTCTCAGACTTAGGGCATCCTTTAGTTTCCCTATTAGTAACATTTCTAG
GGGGAAAATGAGATTGTTTAGTACTGAAGCTAATGTTTAGCAAGTACTACTATTATGTGTTAAGA
GTTTTGTATTACATAAATCCTGGCTGGGGACCTCTGCCCCTATCTTGTCTAGCACGAGGGAGGA
GGCCTGATGTTTTCCCACCATCCTATTTGTAGAATTCTTAGATATCTGCATGTGAACTATAGGATT
ATTAGAAGGCACCAAGACCTGTGCTACCTTTCCCCGTGAGCTGCTTACTGTAGGGCTGAGTAGG
GGGGAAGTGTCGGCAAGAAAAGGGTGTTTTTTTTTGAGTGCATCTCAAAGTTAATAGAACTCTG
GAAACACTTAAGAATTGTGCAGCTGGTGCCACTCTCTGGAGAGCTGTGTGGGGCATAGCTGGG
GTTCACCAACATTCTGGAATGGGCATGCTGATGATGCAGATCACCACTCTCACACCTCAACCCC
TGGGAGGGTGAGGGAAAGAAGTTAATTTAAAAAATGGAATGCCTCGTCTAGTTGCTTATGGGTA
AAGGAGAAGAATCAGGCTCCCCCTTCAAAATCCTTGTCCTCCCTTTCCAAACACATCGGAAATA
TAGATAGCATAAACTATAGTTAGTGGGGATGGATTGGGAAGTAGACAGCAGGCCAAACAGAGG
AAATGTCCCCACCCCTTATCGGTAGAAGGGGCTGTGCCCACAGTGTTCTATCTATGCTGGGCTG
AACTGGGGAGATCACCCCCAGAGCACTTTTTAGCACCTCCCAGTGCTACTTTTGGGATGGTTAA
AAGTATGATTGTCCCCAAACCCTAATTGCCATGCAGCCAGTACAGAACTGCTGATTGTGACTGA
GCATTCTCAGGTTTAAGAGATGATAGGAAGAGTAGATGACTGGATTCAGGAACACTTCTGTTTG
TTTGTTTTGAGAGAGTCTCTCATGGTTGCCTAGGCTGCAGTGCAGTGGCACGATCTTGGCTCACT
TCAGTCTCCACCTCCTGATTTCAAGCAATTCTCCTGCCTCAGCCTCCCAAGTAGCTGGGACTACA
GGTGCATGCCACTGCACTCAGCTAATTTTTGTATTTTTGTAGTGATCGGGTTTCACCATGTTAGCT
AGGCTGGTCTCGAACTCCTGACTTCAAGTGATCCACACACCTAGGCCTCCCAAAGTGCTGGGAT
TATAGGTGTGAGCCCCCACACCCAGCCCAGGAACATTTCTGCTTTTGCTCTAGGGGCATTTTCAA
CTTTCTCCTGGGTTCGGCATGGGCCTCTGACTTTAGCAGTTTTTGGCAGCTCCTGGCCTTGTTCCT
GCCCACCCCTCCACTCAGATGCCAAAGATTTCCTAAGTGATCCTATTCGTAGGCAGCTCTGAGC
CTTTCTCCAGAAAGCCTGCTTTTAGGCAATAGGAAAGGGGGAAAAAAGTGAATTCTTACCAGGA

AAAAATGAATCACAATCATCCATCCCCATCCTCCATCCAGGGTTTTAGTGTAAGGTTGGACCTTC
CCCTCCCTCTTCAGCATGATGCCTCTTCTATTTTAAATGCAGAAGATCCCTGTGATAAATCCAAG
ACAGTTGATTCAGTGGACCAGAAACTAATCTAAACTTTTAAACTTTTTATCTGAAAGGGAACGTT
CATACATTGTTGGTGGGAATGCAAATTAGTTCAGCCACTGTGCAACGCAGTTTGGAGATTTCTGA
AAGAACCAAAAATTGAACTACCATTTGACCCAGTAATCCCATTCCTGGGTATATACCCAAAGGA
AGATAAATTGTTCTACCAAAAAGCCACATGCACTTTTATGTTCACTGCAGCATTATTCACAATAG
CAAAGACATGGAATCAACCTACGTGCTCATCAACAGTGGGTTGGATAAAGAAAATGTGATACAT
AGACACCATGGAATACTACACAGCCACCATAAAAAAGAACAAAATCATATCCTTTCCAGCAATA
TGGATGCAGCTGGACCATTATCTTGAGCAAATTGATGCAGAAACAACCAAATACTGCATGTTCT
CACTTATAAGTGGGAGCTAAAAATTGGGTACATATGGACATAAAGAGGGGAACCACAGACACT
GGGGACTACAAGATAGGGAGGGAGGAGGGTTGAAAAACTCCCTATTGGATGCTGTGCTCACTA
TCTGGGTGAGGGGTTCAATTATACTCCAAATCTCAGCATCAGACAATATGCCTTTGTAACAAACC
TACACATGTACCCCCTGAATCTAAAATAAAAGTTGAAATGTAATACAAAACAGAAAAAAAACCT
CTTTCCCTATGATATAAAACCACTTGTAATTTTTTTTTAACCCCTCAGTTACAGATGAAGTCTGCC
TGATGCAACTAAGGACTTAGTTACCTGGAAAGCCTGATCTCAGTGACTGTGGGGTGGCCACTCC
TAGAACTGTACTCAGTGTCCCTCCCCCCAGGTCTGCAAGCCAGTTTCTTGGAGAAGCTGTGCGT
CACCCTGCTGGTCCTCCGGGTGAACTCTTAAGAGAAATAGAAAGGGAAGGCGAGCCTTGAGTT
TAGGAATTCTCCTTAGAAAACACTTGCTAAATGTAAATATTTTTGGAGCTAAACAGACATGACAA
CGAAATGCGATACCCGCTTATTGAATATATCCTAGATCAGAAAAAAAAAGTTGTAAAAGACAGTT
TGTGGGGACAGTTGGGGAAATTTGAATATGGACTGTATAATACAGAATACTGTACCAAGGTTAA
CCTCTTGGAACCTCTTCCACAAGAGAATATTCTTAGGAGATACATGCTAAAGTATTCAGGAGTAA
CATGACACTATGCTACCTTCTCCCAATCGGTTCCGTAAAATAAACAAGTGTATATAAAGAATGAT
AGCAAAAAGAAAATGTAAATGTTCTGAGGAAGGGGGAACAGTACTCATTATTTCCCCTGTCGGG
TCGAAGGCTGACTGTTTCTAACTGGAAAGGAAAGTCAGCTGGCAGGGTATGCGGAATGGCCAT
CTGGTGAGCAGGCTGGGTGGGGAGAGATGCCAGTCGTTGTTAGAAAACCAACCCAGCACACA
GTCCCATTCCCATCTTACCTTTGATGTGGTGTTCAGCAAAGAAATCCTCCACAGATGGGGCAATT
AAGGAAGAAAAATAAATTACACCGTTTTCTGGAATGTTACTCTTTTTCTAAGGCATTTCATTTTGC
TGCATTGTGAAAACAAGTGAGCATTGTAAGAGCCAAGGAGGGCTCAGACGATTTAGGCCCTCC
TGCTAGTGGACTTTACTTCCTGAGGGGAGGAGGAAAGACTGTGATTTGATGTCATCTCAGGCCC
TCCCTCCACCCACAGTCACTCAGAAGGTAGAATTTTTTGCCGCAGGCTCAAAAGCAAGCATGAA
GTAAACTAAGTGGAAAGTCCTTTTGGCGACTATGCTCAGGGCAGGGGGATGGGGCTCCAGAGC
TCTCATACACTTGTCTTCAAATTTCCTCAGGGAACTTCTCACAACATGCTTATTTTACAGGAGAAT
ATAAAACTCCTGGATGGGTGGTGAGAGAGGCATTTCCTGGGCCACAAACAGAAATCTTCAGTA
AGCTCAACCCATTCGTGTGGCCTTTTGTATATTGACTAAAGATTCTGGTTACTAATTTGGGGTTTC
AGACCCCATTTTAATTGCATTTTTGAGATCTGATTACCTCAACCACCTCCTGCAAAAGACGTATG
CTCGTAGTAATTTTAATGAGATTTTTTTTATTATATTTTGGAAATGGTTAAGGCTGAGACCCCTAG
AAAATTACTGGGATAATTCAACTTTCTCTTCCCATTTCCTCCCTCTACCCTCCACATAAAACAAAA
CAAAACAAACAAGTCAAATGGGAAATTGTCTTTCGTCTCCCACACCAATATTCCAGATTTTAAAC
CAGAGTATATTTTAGACGAAAGGATGTGTTTTTAGTTTTATCTGGAAAATCTGCACATATTTATGC
ATGTTCCTTTTTAAGAAGCAAGAGGCTTCTCAGATTCTTAAAATCAAACTCTATAATACTTTTTGA
TTAAAGTGCTTGCAGAAGGACAGCTTAGTGGTTTGGGAGGGTGCCCTGTAAGTGTAAGCATGGA
AGGATTATGCAGTTACATGATGCTGGTGGCAAGGATCAAATAAACATTCAACAGAAGGAGAAA
ACCCAAAGAGAGAAGGCAAATAAATGCTTTTCAGAGAGCTGTTTGTAATTATGAATACACTCTA
GACAACTTTTTTTTTTTTGAGACAGAGTCTCACTCTGTCGCCCAGGCTGGAGTGCAGTGGTGCAA

TTTCGGTTCACTGCAACCTCTGCCTCCCGTGTTCAAGTGATTCTCCTGCCTCAGTCTCCCAAGTA
CCTGGTACTACAGGCGCATACCACCACGCCCAGCTAATTTTTTGTATTTTAGTAGAAACGGGGTT
TCACCGTGTTGCCCGGGCTGGTCTCAAACTCCTGAGCTCAGGCAATCCGCCTGCCTTAGCCTGC
CAAAGTGCTGGGATTACAGACGTAAGCCACCGTGGCCAGCCTGAAAACATTTTTTAGTAAACAA
GACTGATTTATTTATTTTTTTGGTGGCACGCTTGTTGCTCTTTTAAAGCAGGGCTATCTATGATTTC
TTTTTTTCTTGAGACAGGGCCTGCTCTATTGCCCAGGCTGGAGTCAGTGGCATAATCATGGTTTA
CTGCAGCCTTGACCTCCCCAGCTCAAGCAATCCTCCTACCTCAGCCTCCTGAGTAGCTGGGACT
ACAGGCATGCACCACCATGCTCAGCTAATTAATTTTTTTTTTTTTTTAAGAGATGGGGGTTTCACTC
TGTTGCCCAGGCTGTTTGCAATGTCCTGGGCTCAAGCGATCCTCCCATCCTGACCTCCCAAAGTA
TTAGGATTACAGGCATAAACCACTGTAAGGAGCCAGCTATCCATAATTCTAATCACAGGATGAA
ATGATGCTAGGCTATATATATATATTTTTCTTACACACACACACACAGAGGAATATATATATACACA
CATATACATATAGGAACATATACAACTGGGAAGTTAGATTTCAGGAGGGTTCCTACCATTCCTTA
AATGATAATAGTGAATCATTGTGCCTAACCTATGTAAACAATACATTATATATGTGTGTATATATT
CCTATTCATCTTTGTCTTATAATAGGCTTTATATTGATAGAGAACTCTGATGCTAATGCCTGGGGT
CAGATTTGAAATGCGGCAGAACAGCAAGTCATTCATAGAGGCCAAGAGGACAGCAAAGTAATC
AGAGATGAACTTCAAACATATGTTTCATAGAGGCACATGAACAGAAATGCAGGCTTGGTGATAA
TCTCAATTTTGTCAGTTTTTAAGCCCGTAGACTCTGGGCAATTTACTCTCTTTGAGTCTGTTTCTAA
AACTCTAAAGCGTAGCTAATATTTTGCAAGGTTGTTGTGAGGATTAACAATATATAATGTAAGAT
GACTGCACATTGGACCTACTATTGGTACTTGGTTCATGAAAAATAATTTTTGTTAGTCAAATGAG
GCCTAATAGCAAGAAGAAAGAATTCTTAAGCCACCTTTGGGATATTAGTTAACATTTTGGCATCT
GTACCCAATTGTCTTAATAAAGCTTGTAGGGTCTTTAACGAAACATAAACAATTCCCTCAACTTC
TAACCTTTTAACTCTTGGCCCTAAAGCTATTCAGCAAGAATGCAGCATGATTTGGCAATCACCAC
TATTACCCCCATCCCAGTGTCAAACATAGCCAGCCCAGGAATAATTTTTAACACTTATGGATTTA
GACTTTAATAAGCTTTACCAACTAGAATTAAGATACTTTATTTTTAGTTGACTCTCTCCTTGCCCTC
ATTCAATCCTTGTTCAGAAAAGAGTTAACATCGCTGGCCTGAGATTCCTATTCTTTGAAAGGCCT
GTTTACATGGTTGGCCTCTGGCTGGCAACTGGGAACTTAGATTTCAGGAGGGTTCCTACCATTCC
TTAAATGATAATAGTGAATCATTGTGCTTAAACTATGTAAACAATACAATTTTTATGCTGAACATT
TGCTTTTTTCGGGAGCTTGGAATTTTGATATGTGCTAGGCAGAGAGGGTACTTTGGTGACCAGCC
CCCTTGGGCACTGAGTCTCTACTAAGCTTTCTGGATTGGCAACATTTCACACATGTTGTTACAAC
TCATTGCTGGAGGAATTAACTGCATCCTGTGTATCTTCACTGATAGAAGAATTCTTGCAAGCTTC
TGCCTGGTTTCTTCTGGACTTTGCCCCATCCTCCTTTTGCCTTTGTTGAGTCAGCTTTGTATCCTTT
CACTTTAATAAACCATGTAAGTACAACAACTAAATGCTCAGTCCTGTGAGTCATCCTAGTGAATT
ATCAAACCTGAGGGGTTGAATGAGTCTGGGTTCTCGAGAAAGAATAGAATATTCTCCAGAGCAA
GAACCAGTGATGGGGGAGGAGATATTAAGGTTGATTTACTATGAGAATTGACTCACATGATTAT
GGAGACTGAAAAGCCTGCACTCTGGTCAGCAAGCGGGACACTCAAGAGAGTTGATGGTGTAGT
TCCAGTCTGAGTCCAGAAGCCTGAAAACCAGAAGTGCTGATGGTGTAAGTTTTAGTCTGAGAGG
GCCTGAGAACCAGGAGAGCCATTTGTGTAAGTTCCATTCTGAGTTTGAGTCCAAAGTCAGGAGA
AGACTGATGTCCCGACTCCAAGACAGTCAGGCAGTGAGAAATTCTTTCTTAGCCTTTTATTCTAT
TCAGGCCTTTAATGGATTGCATGAGTTCCGCTCACATTGGGGAGGGCAGTCTTCTTCAGTCTACT
GATTCAAATGCTGATCTCATCCAGAAACACCTTCACGGTTACACCCCAAAATAATGTCTAGCCA
AATACCTGAGCATCCCATAGCCCAGCCAAGCTGACACATAAAATTAACTATTATAGAAGTGTCC
TGGGACTCCCTCATACAAGCATACTTCAGCTTTGGCTCTGTACAGACTAGAAAATGAGCCTTTTC
CAGCCTCAAGCTCTTTCCAGCACATCTTATGTTTCATTGATAAATTGGATACATTATATCCTACCT
CAAAGGGTGCTGTGAAGAATAAATGAGAATGTATGTTCATTTATTTCATTCAATAAAAACAATTG

CTTCCTATGTGCCAGACGCTGTGCCAGAGGGGGATATGTGGTAGACACAGGGTGGCATGAGAT
GTGGCCCAAGGGGTTAGCAGGGCTTGAATTATGCAGGGGTTGGGAGGTCATGGTTAGAACTTA
TTTTTTTTTTTGGATACAGGGTCTCGCTCAGTCACAGCCCAGGCTGGAGTGCAATGGCACAATCT
CATCTCACTGCAACCTCCGCCTCCTGGGTTCAGGTGATTCTTGTGCCTCATCCTCCTGAGTAGCT
GGGATCATGTGCACCACCACACCCAGCTAATTTTTTTGGATTTTTAGTAGAAATGGGATTTCACC
ATATTGGCCAGGCTGGTCTCGAACTCTGGACCTCAAGTGATCTGCCCCTCTCGGCCTCCCAGAG
TGCTAGGATTACAGGCATGAACCGCTGTGCCCAGCCATAACTTTTTAATTCTAAATGCAATGGG
AGGCTGTTCGTGGTGGCTCATACCTATAATCCCAGTGTGCCCGGAATTGGTGGGTTCTTGGTCTC
ACTCACTTCAAGAATGAAGCCGCAGACCCTGGCCGTGAGTGTTACAGTTCTTAAAGGTGGCGTG
TCTGGAGTTTGTTCCTTCTGATGTTCAGATGTGTTCAGAGTTTCTTCCTTCTGGTGGGTTCGTGGT
CTCGCTGGCTCAGGAGTGAAGCTGCAGACCTTCGCCGTCAGTGTTACAGCTCATAAAGACAATG
TAGACCCAAAGAGTGAGCAGTAGCCAGATTCATTGCAAAGAGTGAAAGAACAAAGCTTCCACA
GTCTGGAAAGAAACCACACGAGTTTGCCACTCACTGCTGGCTCAGGCAGCCTGCTTTTACTCTC
TTATCTGGCCCCACCCACATCCTGCTGATTGGTCCATTTTACAGAGAGCCGATTGGTCTGTTTTA
CAGAGAGGTGATTGGTCCATTTTGACAGGGTGCTGATTGGTGCGTTTACAATCCCTGAGCTAGA
CACAAAAGTTCTCCACATCCCCACTAGATTAGCTAGATACAGGGTGTAGATTGGTGTATTTACAA
ACCCTGAGCTAGACACAGAGTGCTGATTGGTGCATTTACAAACCCTGAGCTAGACACAGAGTG
CCCATTGGTGCATTCACAATCCCTTAGCTAGACATAAAGATTCTCCAAGTCCCCACCAGATTAGC
TAGATACAGAGTGCTGATTGGTGCATCCACAAACCCTGAGCTAGACACAGGGTGCTGATTGGTG
TGTTCACAAACCTTGAGCTAGACACAGAGTGCTGATTGGTGCACTTACAATCCCTTAGCTAGAC
ATAAAGGTTCTCCAAGTCTCCACTAGACTCAGGAGGCCAGCTGGCCTCACCCAGTGGATCTCGC
ACTGGGGCCGCAGGTGGAGCTGCCCGCCAGTCCAGTGTGGTGCGCGCAGTCCTCAGCCCTTGG
GCGGTGGATGGGACCGGGCGCCGTGGAGCAGGGGGCGGTGCTCGTCAGGGAGGTTCAGGCC
GTGCAGGAGCCCACAGCCGGCCGTGGGGGTAGACTCAGACATGGCGGGCTGCAGGTTCCAAG
CCCTGCCCTGCGGGGAGGCAGCTAAGGCCTGGCGAGAAATCGAGCGCAGCGCCGGTGGGCCA
GCACTGCTGGGGGACCCCGCGCACCCTCCGCAGCTGCTGGCCTGGGTGCTAAGCCCCTCACTG
CCCGGGGCCGGCAGGGCTGGCGGGCCAGCCTCTCCGAGTGCGGGGCCCGCCAAGCCCACACC
CACCCGGAACTCTAGCTGGCCCGCAAGCGCCGCGCACAGTTCCGGGTCCCGCCCCTGCCTCTC
CCTCCACGCCTCCCCGCAAGCCGAGGGAGCCGGCTCCAGCCTTGGCCAGCCCAGAGAAGGGC
TCCCACGGTGCAGCGGCGGGCTGAAGGGCTCCTCAAGCACTGCCAGAATGGGGGCCGAGGCC
AAGGAGGCACCGAGAGCAAGCGAGGGCTGCATGGGCTGCCAGCACGCTGTCACCTCTCACAG
GCACTTTGAGAGGCCAAGGTGAGTGGATCAGTTGAGGCCAGGAGTTTGAAACCAACCTGGCCA
ACATGGCGAAACCCTGTCTCTGTGCAAAAATTAGCCAGGTGTTGTGGTGCATGCCTGTAATCCC
AGCTACTTGGGACGCTTGAGGCACGAGAAGCACTTCGACCTGGGAGGAAGAGGGTTGCAGAG
AGCCGAGATGGTGCCACTACGCCCCAGCCTGGGTGACAGAGTGACACTCTATTTCAAATAAATA
GATAGATAAATAAATAATGTAAATGCAATGAGAGGCTCCTTAGGGGAGGTTTAGCATTGTGCCT
GGTAAACAGCAAGCACTCAATACATGCTTGCTATTAAGGATTACCACCAAGATGCCCTGTCAAC
ATCTAAAATCAAACCCAGTCATCTTTTCGCTGTTATTGAGTTCACTGCTTTTTTCTAAGACACCCA
GTTCTATCGCCTAGACTAGAAAATTCAATCATCTTTAATTCTTCATTCTCCTTTATTCCCTTTCTGC
CAAATTTTTTTATTTTATTTTATTTTTTTGAGATCGCATTTTGCTTTTGTTGCCCAGGCTGGAGTGCA
ATGGCGCGATCTCACTGCAACCTCCCTCTCCTGGGTTCAAGGAATTCTCCTGCCTCAGCCTCCC
GAGTAGCTGGGATTACAGGCATGTGCCACCATGCCCAGCTAATTTTGTATTTTTAGTAGAGATGG
GGTTTCTCCATGTTGGTCAGACTGGTCTTGAATTGCCGACCTCAAGTGATCCACCCGCCTCAGCC
TCCCAAAGTGCTGGGATTACAGGCGTGAGCCACCACGCCCGGCCTAATTTGCTTTTTTTTGTCTC

TTACATTTGATCCTGAGCTAATGATCTTGTGGAGCCTATTTCTTCTCTAAAATGGGGATAATACCT
TGTAAGATTGAGGATCAAATGAGGTAAATGAATGTAAATTGCTTAGCATGTGGCAAAAACTCAC
TAAAGATTCACTGTTATTACTTTGGGCTAGGAACTGGAGATACATAGATGAACAAAAGGAAGCT
CACCTTGAAAAAAATGAGCTCTTTCTACAAATTAATTTTGTTTTTCTTTGAGATGGAGTCTCACTC
TGTCACCCAGGCTGGAGTGCAGTGGTGCAATCTTGGCTCACTACAACCTCCGCCTCCCGGGCTC
AAGTGATTCTCCTGCCTCAGCCTCTCAAAGTAGCTGGGATTGTAGGTGCTCGCCACCACACCCA
GCTAATTTTTATATTTTTAGTGGAGATGGGGTTTCACCAGGTTGACCAGGCTGGTCTTGAACTCCT
GACCTCAGATGATCCAGCTGCCTCGGCCTCCCAAAGTGCTGGGATTATAGGCGCGAGCCACGG
CGCCTGGCCACTACAAATTAATCTTAAAACTCCACCTTTACTCTCACCTGCTCCAAAATCCAATC
TGCTAATAAGATAAAGCCTAGATTTCTTAAAAAGCAGATATTCAGTTTCCTTGAGTTAGCTGCCC
TACCTTTCAGCTTTTTCACTTCCTACCTCACAGACCAAACTAAGGCATTTGCTATTACATATTCCTT
TTGGTTTATCCACTCTTCACACTGCCATTTCCTCTTGCTGGAAACCCCATATTCCTTTTACTGCAA
GCATCCATTCATTCTATCTTTGGAGGCTCAGTTCATACTACAAAACTCTTTTGTGGGGCCAGGCA
TGGTTGCTCACACCTGTAATCCCAGCACCTTGGGAGGCCGAGGCGGGAGGATTGCTTGAGGTC
AGGAGTTCAAGACCAGCCTGGGCAACACAGCAAAACCCTGTCTCTACTAAAAGTACAAAAATT
AGCCAGACGTGGTGGTGTGCGCCTGTAGCCCCAGCTACTCGGGAGGCTGAGGCACAAGAATCT
CTTGCACCTGGGAAAGCAGAGGTTGCAGTGAGTCAAGATTGTGTCCCTGCACTCCAGCCTGGG
CGATAGAGCAGGACCCTGTCTCAAAAACAAAAAAATAAAGAAAACCTCTTGTACTAGTTATATA
CTCATCTATTCAGCAATTATTCAGTGCCTACTCTGTTCTAGGAACTGTTCTAGGTGCTTAAGATAT
ATATGTAAATAAGACTAAGGGGCTGGGTGCGGTGGCTCACGCCTGAAATCCTAGCACTTGGGA
AGCCGAGGCAGGTGGATCGTTTGAGGCCAAGAGTTTGAGACCAGCTGGCCCAACATGGCAAAA
CACTGTCTCTACTAAAAATACAAAAATTAGCTGGGCGTGGTGGCGTGTACCTGTAGTCCCAGCT
ACTCGGGAGGCTGAGGCACAAAAATCACTTGAACCCGGGAGGCGGAGGTTGCAGTGAGCCGA
GATTGTGCCACTGCACTCCAGCCTGGGTGACAGAGCGAGACTGTCTCAAAAAAAAAAAAAAAA
AAAGACAAAGCTTTTGCCCTCAAAGTGTGTACATTATTGGGGAAATCAGACATAAAACAACATG
TATGTCAGGTATTAATAAGTATGGTGAAGACCATACATATATATCCCATTTATTAAAGTACAAGC
TCAAGGGCTGGAGCTATGTCTTACTAAAAATCTTTGCTTTCTCACATAGCACCTTATATTTGTAGA
TGCTTAATATTTTTGGATAAATAATATCCTGGAGGTTTTTATACTGTCAAATGTTAAATCGCTAGA
GCAACAGAACTACATTCAAGTTCAACAAATCAGTCTTTTGTGCCTGCTTGTGCACAATTCCGTTG
AGAACTATAAAGGTTGAAGCGAACCTTTTTTTTTTTTTGAGACAGTGGCTTGCTCTGTCACCCAG
GCTAGGGTGCAGTGGCACATCAACGTCAACCTTCTGGGCTCAAGTGATTCTCCTGCCTCAGCCT
CCCAAGTAGCTGGGACTACAGGCCCATGCCACCATGCCTGGTTAATTAAAAAAAAAACATTTTT
TGTAGAGATGGGGTCACACTATGTTGCCCAGGCTGGTCTTGGCTTAAGCCATCCTCCTGCCTTG
ACCTCCCAAAGTGTTGGGATTACAGGCATGAGTCACTGTGCCCAGCTAGACCTTTTCAAATGTA
AAAATTTATTTTCTAGCCAACATGTCCTATTTGGTGGTCTGGGTGTTAAAGAGTACAGACTCAGC
TGGGTGCGGTGGCTCATGCCTATAATCCCAGCACTTTGGGAGGCTGAGGCGGGTGGATCATGA
GGTCAGGAGATTGAGGCCATCCTGGCTAACACAGTGAAACCCCATCTCTACTAAAAGACAAAA
AATTAGCCGGGTGTGGTGGAGGGCACCTGTAGTCCCAGCTACTCGGGAGGCTGAGGCAGGAG
AATGGCGTGAACCCAGGAGGCGGAGCTTGCAGTGAGCTGAGATCGTGCCACTACACTCCAGCC
TGGGCAACAGAGCGAAACTCCGTCTCAAAAAAAAAAAAAAAAAAAAAAAAAAAAAGTACAGACT
GAATTGGAGTCCCGAGTTCAAATGATGACTGGTAATTTCATAGCCTTAGTTACACAAACCTAAG
ATAGATAGGGTAAGTAAGAGTACCTACTGTATAGGGTTGTTAAGGATAATTAAATGAGTTCCTG
GCATATAGTATACTTGATCCTTGAACAACACGGGTCCACTTACACATGAATTTTCTTTCACCTCTG
TCACCCCTGAGACAGTGAGACAAACCCTCCTTTTCAGCCTACTCAATGTGAAGATGACAAATAT

AGACCTTTTATAATGATCCACTTCTACTTAATGAATATTGAATATATTTTATCTTCATAGTTTTAAT
AACATTTTTTCTAGCTTACTTTACTCTAAGAATATAGTATATAATACATAAAACAGCCGGGTGCG
GTGGCTCATTGCCTATAATCCCAGCACTTTCGGAGGCTGAGGCGGGTGGATCACGAGGTCAGG
AGATCGAGACGATCCTGGCCAACATGGCGAAACCCCATCTCTACTAAAAATACAAAAATCAGC
CAGGTGTGGTGTCGTGCGCCTGTAGTCCCAGCTACTCTGGAGGCTGAGGCAGGAGAATCGCTT
GAACCCAGGAGGCGGAGCTTGCAGTGAGCTGAGATCGCACCACTGCACTCCAGCCTGGCGAC
AGAGCAGGACTCTGTCTACTGAAAAAAACAAAACAAAACAAAACAAAAAACGAAAACGTAAAA
CATAGAAAACTCGTGTTACTGTTTAAGTCAGTAGGCTACTGAGGGGTCAAAAGTTACATGTAGA
TTTCTGACTGCACAGGGGTGGGATCCATGGACATACCTGTGAGAATGTAGGTATTTCAGGAAGC
TTCTTAAACAAAAGTAATTTAGAACAAAGACCTTTTAAAAAAATTACAGTTTGATAACTGAACAA
AAGGAACATTTGGTTAAGTTTAAAGAATCTAGAAAAGATCCATTTTCCTCCAAACAGATTTATTG
AATACAGCAAAATTCTATATACAAAGTGACCTGGACCTGCTGCTTCAAAACATGATCCTTTCTTA
CTAATATCTTGATAGTCGGTCCATAGAGCATTAGAAAGCAATTGACTCTTAAATAAACAGAAAA
GTGCCTAATGCACATTAAATGAATGGCCTAACTACTGGAACTTTAGTAGTTCTATAAGGTAATTA
ACATAGGTAGGATCCAGTTCCTATGACAGGCTGCTGAAGAACAGATATGAGCATCAAGAGGCC
ATTTTGTGCACTGCCACCGTGATGCCATCGTGTTTCTGGATCATAATGTTCCTGAAATGCAGGAA
GAAAATATTCAGTAAAACGTAATCAGTGGGACTTCACTGCAAGATTAAAGATGTTGTTTGCTTTA
GGATACAATATTTAAAATAATGTAATTGTTTCTACGTATAAAAGCAATATTTAAACATAACAAAA
CATTTGGGAAATACATAAACATATAGCATGTGAACACGAAATGAATCCTTAATCCTACTACACA
CAGACAACCACTGTTAATATTTCCCTGGTTCTTTGTTTCGGTTTTTTAGGCACTGTCTTGCTATGTT
GCCCAAGCTGGCCTCAAACTCCTGGGCTCAAGCAATCCTGCTTCAGCCACCTCAGTAGCTGGAA
CTACAAAAAAAAAGGCTTTACTGGTCTTTGTTTTCTTTGCATGTATATGATACAGTCATGTGCTGCA
TAACAATGCTTCAGTCAACAGTGGACCCTACATATGATGGTGGTCCCGTAAGATTATAATGGAA
CTGAAAAAATTCCTATTGCCTAGTGACCTTTTAACCGTCCCAGCATCACATAGCACAACACACTAC
TCACATGTTTGTGGTAATGCTGGTGTAAACAAATTGTGCTGCCAGTCATAAAAAGTATAGCACAT
ACAATTATGTACAGTACGTAACGCTTGATAATAATAAATGACTATGCTATGGTTTATGTATTTACT
ATACTTTTTTTCTTTTTTTTGAGACGGAGTCTTGCTCTGTTGCCCAGGCTGGAGGGCAGTGGCGTG
ATCTCAGCTCACTGCAACCTCTGCCTCCCGGGTTCAAGCAATTCTCCTGCCTCAGCCTCCTGAGT
AGCTAGGATTACAGGTGCCTGCCACCATGCCTAATTTTTGTATTTTTAGTAGAGACAGGGTTTCA
CCATGTTGGTTAGGCTGTCTCACACTCCTTACCTCAAGTGATCTGCCCGCCTTGGCCTCCCAAAG
TGCTGGGATTACAAGTGTGAGCCACCATGCCGGGCCTACTTTTTATCATTATTTTAGAGTATACT
ACTCCTACTTATTAAGACAAGGTTAACTATAAAACAGCCTCAGACAGGTCCTTTAGGAGGTATTC
CAGAAGGTATCATCATTACAGGAGATGACAACTCCATGCATGTTACTGCCCTGGATGACCTTCC
AGTGGGAGAAGATGTGGAGATAGAAGCCAGTGATATTGATGATCCTGACCCTGTGCAGGCCTA
GGCTAATGTGTGTTTCTTCTTTGCTTTTAACAAAAAAGTCTAAAAAGTAAAAAAGTTTAAAAAAA
GAATTTTTGTACAGCTGTACAACTGTTTTAAGTGTTATTATAAAGTCAAAAAGTTTTATTGACTCA
CTCAGAGCAACTTCCAGTGTTGTAAGCTCCATTATGGTAAGTGCCCTATTCAGATATACCTTTTA
AAAAATTTCCCTATACCATACTTTTACTGTATCTTCTCTACGTTTAGATACATAAATACCACTGTTA
TAACTGCCTACATTATTTAGGCTACAAATCTTTGTAGTCTAAGAGCAATACAGCCTAGGTGTGTA
ATAGGGTGTACCACCTAGGTTTAAGTACACTCTATGATGTTCATACAACAAAACTGCCTGACACA
TTTCTCAGAACATATCCCCATTGTTAAGCAATGCATGACTGTATGTTTTTTCTTTTTACAAGGCTGT
GATAGTAGCAGTTGTATATATATACTTTTTTTCACATATTATTCTGAGGACCATCACTTGGAATAT
CTTTAGTAATTTTATTGCTTTAGAAGTAATTTTTGGCTGGGTGCAGTGACTCATTCCTGTAATCCC
AGCACTTTGGGAGGCTGAGGCGGGCAAATTACTGGAGCCCAGGAGTTCGAGACCAGCCTGGG

AAACATGGTGAAACCTCATCTCTACAAAAAAATACAAAAATTAGCTGGGTGTGGTAGTGTACAC
CTGTAGTCTCAGCTACAACTCGGGAGGCTGAGTTGGGTGAATCGCTTGAGCCCGGTGGGGTTG
AAGTCACAGTGACCCATGATCACACCACTGTACTCCAGCCTGGTGCCAGAGCAACACCCTGCCT
CAAAAAGAAAAAAAAAAAAAAGTAATTTTTATTCTCTGATTAGTTTTCCTTTGTGCTATTTGTTACTT
TCTTTTTGTTGGTAAACTGATAGCTTCTGCCAATTCTTATTCTTCATTTAAATAGCATTGTGGCCTG
AGAGCACATTAGCTTTAGGGATCAACTTGTCTGTTCCTGTGATTCAAAAGATTAAGAACTTTTCC
CAAAGTAGTCTGAATAAGGAATCACTGATGAACACAGGGCCGGCCCCCGCCAATTTTACCAGG
AATTTTTCTGAACAAAAAGGAAAGGATCTGCTGAAATCTACTCACCCATTATCTGATTCTAGACA
CACCACAGGAATATCAGTGGGGTCAGAGGTTAGCTTAGCTGCTTGCTGGGCTAGAACAGATATC
ACTCCAGCATGCTCATCTGACAGGGTCCCGCGGCCTGGAAAATAGAGATGATATATGTCACCTG
ATTGCTCATAGAGTATTTCTGCTAAAATAAGCTAGCTGGCTGTTTTAAAATGTCTACAGAATTGTC
TTTTTATTAATGTCTGTATTAGTCTGTATATATTATAGTAACTAAATCTCGGGTACAAAAGTACCTA
TAAATTCTAGGTACGAAAATTAATTACTAAGTGTACTTTACAATATATTTCTGTATATAAAGGCAA
CACAAAGTTAACTGTACCTGAAACCAAATCAGGTAATAGACATGCCACTATGGCAGAGCATTTG
GATGTACTATTACTATACAAAACATTTTGACCAGATCCGATGCCGACGTTTGAGCCTTGAAGGTT
TCTTGAAGGAACTCATTTAGTTTTAGATTTCCATACCTGAATTTCAGTCTTTCCATTAGTAAGCCT
AGCAAATGCTTCCAAAAATTTACCCTTTTTCTCCCAAATGACTGATCTGCATGTTAAACATAACTG
AGTTACACCGATTACCTCTTTCAGTTACCCTTCCAGCAAAAATTCAGAGCTTTAAACATTCCTGC
ATTAGGAGCAACAAGTCTTGGGTTTACTAGTTGTCACTAATTAACTGAATGCTTTACTTATTTACC
TTTCCAAACTTATTTCCTCATGTGTAATTGCTACCATTTATTAAGTATTATGTCTCAGTTCTATGTA
AGCATTTTATACATATTATATAACCTTAAAATATCGTGATTAGGCCGGGTGTAGTGGCTCATTCCT
GTAATCCCGCACTTTGGGAGGCGGAGGTGGGTGGATCACCTGAGATAAGGAGTTCGAGACCAG
CCTGGCCAACATAGTGAAACCCTGTATCTACTAAAAATACAAAAATTATCTGGGCGTGGTGGCA
GGCGCCTGTAATCCCAGCTACTCAGGAGGCTGAGGCAGGAGAATTGCCTGAACCTGGGAAATG
GAGGTTGCAGTGAGCCAAGATCGTGCCACTGCACTCTAGCCTGGGCGACAGAGCAAGATTTCG
TCTCAAAAAAAAAAAAAAAAAAAAGTGATTAAGAGGCCTCAGGGCCAGACAAAAATCCTAGCTTA
ACGACTTACTAGCTGCATGACCTCAGGCAAGTTATTTAACCTCTGTGTCTCAGTTTCTTTTTTTATT
TTTTGAGACAGAGTCTCGCTCTTGTCATCCAGGCTGGAGTGCAATGGTGCAATCTCGGCTTACTG
CAACCTCTGCCTCCCAGGTTCAAGCGATTCTCCTGCCTCAGCCTCCCGAGTAGCTGGGATTACT
AGCACCTGCTACCATGCCTGGCTAATTTTTTTTTGCATTTTTAGTAGAGATGTGGTTTCACCATGT
TGGCCAGGCTAGTCTCGAACTCCTGACCTCAGGTGATCCACCCGCCTCCGCCTCCCAAAGTGCT
GGGATCACAGGTGTGAGCCACCGCATCCGGCCTCTTTTTTTAAATTTTAAAAACATATATATATG
TTTATTGTAAAAATAGAGATGGGGGGTCTCACTGTGTTGCACAGGCTGGTCTTGAACTCCTGGG
CTCAAGTGATCTGCCCACCTTGGTCTCCCAAAGTGCTGGGATTACAGGCGTGAGACACTGCACC
TGGTCCCTCAATTTCTTTTTCTACCTCACTGGGTTGTTATGAGGAATAAAAGAGTATATGTAAAGC
ACTTACAATGGTGCTTGGCACATAACAGCTCAATAAATATAATTCTATGATTATTACCATTATCAA
ATAACAACCCAGTGAAGAAAATGAGGGCCTAAAAGGTAGTTTTTTGGTCCACGTTCACACTACT
AACAAATGAAAGAGCAAGGGATTGAGACCAGGTATGTCCAACACTGAAATCCCATTTTACTGCC
TATTCACTTCTTGTTTAGTGTAGTTGAGCTTCAAAATTGTACACCACCCACCTTGCCGGGTTGTTG
TGAAAATTAAAGATAATGTATTTAGAGCCTAGCAGTCTCAGCATATGGCATACATTCAATAAATG
GTTAACAGCTGTTATTATCTGAAGTCCTTTCTAGACTCAATTTAAAATGATTCAAATATTATTTTTA
GTAGAGACAACTATTTATTGTTATAGGACTGCAAGGAACATCTTCCTTCATAAATACCAACTGAA
AAGTTTCAATTTTCTGATACTGTTATTTGGAGATAATCTCTACTCTCAAACTCAGAAAGACCTGGC
AAGGTAACAGGAAATAAAAGCTAGAAAATAATAGCACGCTAGGCTCCAAGTCAACATCAGATG

CTATGCAATTCATCAAATAGTTGGTATGAGAGATACTTACAACCCAGATTAAGTCCTTGTGAATC
TGTGCACAGGACTCCAACAATGGAGGGATTCTTCATTCTAATTCCAGGAACACAAGAGAGTTGA
AGTACATAATGGGAGAAAAAGGATTTAAATAAGTACAAACATACAGGTGTTCTATGTACCAGGC
ATTAAGTGCTTTAAATCAATATTTCTCAATCTTTTTTCATTGTCACACCCTAAGAAAAAAATTAAG
TTCTAGGGCCGGGCGCGGTTGCTCACGCCTGTAATCCCAGCACTTTGGGAGGCCCAGGTGGGC
GGATCACCTGAGGTCGGGAGTTCAAGACCAGCCTGACCAACATGGAGAAACCCCGTCTCTACT
AAAAATACAAAATTAGCCAGGCGTGGTGGCGCATGACTGTAATCCCAGCTACTCGGGAGGCTG
AGGCAAGAGAATCGCTTGAACCTGGGAGGCGGAGGTTGCAGTGAGCCGAGATCACGCCATTG
CACTCCAGCCTGGAAGACGAGCGAAATTCCGTCTCAAAAAAAGAACACAAAAAATTTAAGTTCT
AGTTAATTAATTGAAATAAACCAGTTTCTCTCTGATGAGAGAAAAAAATGTGCTAAGTACTGCTG
AGTTCTGGAGGGCCACAAACACTGCGTGAAAAACATCTAGGATGTTTTCACTCTTGTTAAAAAC
GCATGTTTTAGTACTAACTCATTTAAAACAACTGTTGAAAAACTCAAGTTACCTCCTGGAGGGTA
AGTGGAAGCTGAGGGACATCGGTGGGAGGCAGGTGTCTCTCTGTACCCTTTACACTTTTCCAGT
TTTGAATTTTACATAGATACTGCTTGTTCGAAAAATTTAAAGAATGATTTTTATCTACTTAAGCAC
CAAGTAACTGATAGGTTACACACAGGAGATACGTGTTTGTTTACACTGCAGGGCTCGACACTGC
ACAACTGGCACAATCTTCCCAAAAGTCAACCCGTTTTCTAAAACTTCCGCAGAACCACCCTGAC
CGTAAAGACGATTTCAACAACGAGATAGGCAGTTTTGGCCCGTGGCCTGGGCCCGGGAGACTC
GCTCAAGAGGGAATGTGCGGTGCCCGGCAGGATTTTAATTCCGGGTCACCCGCCCCGCCCTGC
GGCCTTGGGTAGAGTTTAGCCCCCTCATCCCGTGTCCCGGGGGTCGCGCGACGTCCCCGAGAC
GCCCTGGCCGGTACTCGCAGCTCGCGCCTTTCGTTCCGCTCAAGTTCCTCCGCCGCGACCTCAG
GACAGGCCGAAGAGGCGCGCACTACTCACGTGTCTTCCAAGTGCTGCTCCAAGGTCGCCTCCA
TCCCACCCACCGACCACTCCGGCTCAGAACCCAGCGGCACGGCACGTCCTTCTCCACCACAGG
CCTCAGTCACTTGACGCGAGCGGGGCGTGGACCGTAACGGCGCCTCCAAAGGGACAAAATTGA
TCACGGTGCAACGTCCGCGTTCTTTACTGGAAAACATCACTGCGCTTCCGTCGCACAGAGCCTG
ACGAAGGCTTGGGCTCCCCGCGCGGTGACCGTCGAGGTGACCTGCACCTGGCTCCATGGCGGA
ACCGGCCGGCACGGATTATTCCCCTCTCGGGAGAGGTCTGCAGCCGGGCCTCAGTGCTTCAGTT
CGTGGCCTGGCATTTTGTGTCAGTGTTTTTTAACCTTTGGACCCTAGTTCAACCGACTCCGAGAC
CTAGGGGACAGATCTTAAGTTTTACAATAACCCAGGGGTCAGACAGGGTCTGATCTAGCCTGTG
GGGCAAGTACGGGGAAGACACGGGACCTCTTGGAGGGGGTCAGTAGGCTTGTAGGGGGAAAG
ACGCACACAGAGACTAAAGGACGAGATGGAGCAAGGTTCAACCCCCTAGCTGAGTGTTGGCAG
TGTCGGGATTTTAACGAAGACATTGGTGCTTATTATGGTGAGGGGACTCAGGACAGTTTAGGGG
AAGAGAGTGGCATTTGAGTCCTAACTCGTAAGGAAGACTGGGTAGGACTACTTGAAAATAACTT
TTAAAAATTAATTATAAAGCTGGTAAAAATAGGAAAACATCTATGGCCCCACTGTCCAAAGACG
ACTTTTAACTTCTTGGAGAATATCCTTTCAGATAGTTGTCTTTGTAAATGTATACTTTTAATGTTTTT
TCGTTTGCCTTTTTAAAATAAATTTTTTGGGGGACAGGGTCTTGCTCTGTTACCCAGGCTGTAGTG
CAGTAGAACGATTATAGCTCATTGCAACCCGGACGTCCTGGGTTCAAGCTATCCTCACCTCTCA
GCCTCTCGACTGGCTAGGACCACAGCCTCGAGCCATCATGTCTGGCTAATTTTTACGTTTTTTTGT
AGAGATGGGGGTCTCGCTGTGTTGTCCAGGCTGGTCTTAAACTCCTTGCTTCAACAGATCCTCCC
ACTCCAGCCTCCCAAAATGTGATTACAGGCTGAGTCACCACGCCTAGCATGGTTTTTTTTGTTTT
GTTTGTTTTGTAAGCAAAATTGACATACATACTGGAGGTACAATTCTGTAGCTGGCCTTCTTTACA
GAGTTTAGAATAAATGTCTTTCCATGTCAAGTTTTCACTTACATCCTTTGTAGTAGTTGCAAAATA
TTCCATTTTATAGCTGTATCTTAATTGTGGATTAAGGTTGGAGTGCAGTGGCACTATCATGGCTTA
GTCCAGCCTCAGCCTTCTAGGTTCAAGCGATCTTCCCACCTCAGCCTCCCAAGAAGCTGGGACT
ACAGGGACCCGCCACCCCACCTAGCTAATTAAAAAAAAAAATTGTAGAGATGGAGTCTCAGTGT

GTAGCCCAGTCTCAAGTGACCCTCCCTCGTTGGCTTCCCAAAGCGCTGAGATTGCAGGTGAGCC
ACTGTACTTGGACTTTTTTTTTTTTTTTTTGCTTTTGTTTGTTTTTTGTTTTAGCTATTACAATGCTGGT
TGAATATCCTCATAGCTAAATATCTGTGCAGTTTTAAAATTTCCTTATAAGTTACTAGAAATGGAA
TGACTGGGTTAAAGGACATGATCATTTTTTAAGACTCTTGATACATACTTACAAATTGCCTCCCG
AAAAAGTGGTGCTAATTTACTCAAATTGGTAAGCCTCACCCCCTCTGTGGATCATTCCTTGCACT
AAGTTTCTTCCAACTCTGTCACCTCCAGCTTTTTACTCACAGCCTACCTTCCAATCTGGGGCCCA
GCTCCCAGATCGGAAGATACCAGACTTTGGTATTGAACCAAAGTCCAATACACTGCACTGGCAT
TCAGAGTCCCTTTTTATTCCGCAGCTGTCATAGTGAAGCTTGCAGCTTCCTTGAAATTCCAGTTTG
TTTATTTCTTTATTTTTTTGAGACAGAGTCTTGCTCTATCGCCCAGGCTGGAGTGCAGTGGCGCCG
TCTCAGCTCACTGCAACCTCTGCCTCCTGGTTCAAGCGATTCTTCTGCCTCAGCCTCCCGAGTAG
CTGGGATTACAGGCATGCACCACCACACGTGGCTAATTTTTGTGCTTTTAGTAGAGATGGGGTTT
CACCATGTTGGCCAGGCTGGTCTCAAACTCCTGACCTCAAGCGATCCGCCCACCTCAGCCTCCC
AAAGTGCTGGGATTATAGGCATGAGCCATTGCACCCAGCCAAAATTCCAGTTTGAATGTTATAA
TTCAGGGGTCTTGGACTCCTGCTCTAGAGAAATTATTCATGTTAAGTTAGGGGTAACACAGTACA
GGCCATATAAGTTTTAGAAAAAGCAGTTTAAAACTTAGCATTAGAAAACATAAGTACTGCCTGG
TGAAATGGCACGTGCCTATAATCCCAGCTACTCAGGAGGCTGAGGCAGGAGGATCCCTTGAGC
CCAGGAGTTCAAGTCCAGCCTGGGCAATATAGTCTAGTGGCCACATTAAAAAAAGTAAAACAG
CCGGGCGCGGTGGCTCACGCCTGTAATCCCAGCACTTTGGGAGGCCGAGGCGGGTGGATCAC
GAGGTCAGGAGATTGAGACCATCCTGGCTAACACGGTGAAACCCTGTCTCCACTAAAAATACA
AAAAATTCTCCGGGTGTGGTGGTGGGCGCCTGTGGTCCCAGCTACTCAGGAGGCTGAGGCAGG
AGAATGGTGTGAGCCTGGGAGGCGGAGCTTGCAGTGAGCCGAGATCGCGCCACTGCACTCCA
GCCTGGGCGACAGAGCGAGACTCTGTCTCAAAAAAAAAAAAAAAAAAAAAAAAAAAAAGTAAAA
CAGGGCAGGGTGCAGTGGCTCACGCCTGTAATCCTAGCACTTTGGGAGGCCGAGATGGGTGGA
TCAGTTGAGGTCAGGAGTTTGAGACCAGCCTGGCCAACATGGCGAAACCCCATCTCTACTAAAA
ATACAAAAATTAGCTGGGTGTGATGGTGCGTGCCTGTAGTCCCAGCTACTCAGGAGGCTGAGG
CAGGAGGATCGTTTGAACTCGGGAGCTGGAGGTTGCAGTGAGCCGAGATTGAGCCACAGCACT
CCAGGCTGTGCTACAGTGTGAGACTGTCTCAAAAAAAAAGTAAAACAGATGAAACTAATTTTAAT
AACATTTTATTTATTCAAACAATATATTCAAAATATTATTTCAACTTGTGATTGATAAAATACTAAT
GAGATATTTTATATTTTTTCTCCAGTACTGTCTTCAAAATCCAGGGTATATTTTTCAGTTAGCAGCA
CATTTCAATTCAGACCAGCCGCATTTCAAGTGCTCAGCAGCCACCTGTGGCTAGTGGCCACAGT
ATTGGCCAGTGTAGCCCAAGGATATCTCAAATTATCTCAAAGCTAGATCAGTGATTAAAGGATT
CTCAGGCCAGGCATGGTAGTTTTCAGGCCTGGTATGGTGGTTTACGCCTGTAATCCCCGCACTTT
GGGAGGCCTAGGTGGGCGGATGGCTTGAGCCCAGGAGTTTGAGACCAGCCTAGCCAACGTGG
GGAGACCCTGTCTCTACCAAAGCACAAAAATTAGCTGGGCATGGTGATGCGCACCTGTGGTCTC
AAGTACTTTGTGTGAGGTGGGAGGATCACTTGAGCCCGGGAGGTTGAGGCTGCAGTGAGCTGA
GATTGCACAACTGCACTCCAGCCTGGGTGACAGAATGAGACCCTGTCTCAAAAAAAAAAAAAGG
ATTCTCCGAAAGACTGGCGATTGCTTTTTAAGCTATAAGGAAGGCCTTTGAAGGGAAATCAAGT
AGGAAGAGGATGATAGCTGGCTCACATGTCCTTGCCACATGGGAGGTGAGAGAGCAGGCTACT
CCTTACCACCCCCGCTTTCTCTGCCCTGCTGCTGTCTTGCCCCTGTGCAAGTAGGCAATGGGAG
AGGAATAGTATATATAAATTGGGCAAGTGCTAGGGGCTGGAGTGATGGTTGGGGAAAGTATCT
GACCCTTGTTTTCTACTTTCTTTAGCTTGTGGGGTATTTAACAATTCTTTGTTCTTAGTTTTGAGTG
CTGGCAACCATTTCTGGGGTGACAGGAAAAGTTCCATGACATCCGTATTAGAGCATTTGTTTCAT
TATATTGTAATTTTTTATGTTTACTTTTGAAAAAGTTCATTCTTTTCTCTTTTTAATTCTTATTCTTTTT
TTTTTCTTTTCTTTTTGAGGCAGGGTTTTACTCTATCACCCAGGCTGGAGTGCAGTGGCACAATCT

TGGCTCACTGCAACCTCTGCCTCCTGAGCTCGTGATTCATCCTCCTGAGTAGCTGGGACTACAG
GTGTACACTACCATGTGTAGCTAAATTTTTTGTATTTTTTGTAGAGATGGGGTTTTGCCATGTTGC
CAGGCTGGTCTCGAACTCCTGCAATTTGCTCTCCTTGGCCTCACGAAATGCTGGGATTACAGGC
ATAAGCTACAGCACCCAGCCCTTACGTTTACTTTTCATTGACAGGAAGAGTGTCTTCATCTCTGC
ATCCCTAGTGCAACAGAGCATGACAAAAATTCAACAAATATTTATTGAAGAGTCTTGATGTCTTT
TGGGAAAGGAACTCTATGGTTACCTTTACTCTAAGGTAAAGAAATTTAGGTCCTGAACTTTAACC
ACCTTCTCCTCTTCTTTCTTCCCTCTTTCATAAATGAGCTCTTGGAAAATTAAAATGCCAGGAGGG
TCCAGCAGAGAATACCCAAGCCCACATGTCCTGATGGGAAGTGGGGGATAAGTAGAGAGGGA
AACACAGACCCTGTAGGCCATCTCACTGCTGCTGGCCCAGGGGACCCTAACTCCTTTTGTTGAC
TCATCAGAAGTAGAATCCTCCTGAGGGTCTGTTTAAGAAGCACAGCTCTGGTAGAAAGGGTAGC
TTTCCTATGGGTACGGAGGGCTCTGCTACCCCAGGGCTATCCACACTGGCCTGTGAATCACTCT
CAAATGCTTAGGGTCACGGGCTTTCAGTGGAATGAGCCCTCATGTGCCACCAGGGGGCACCAA
GGCCACAGAAAACCCTATTCAGAAGGTTCTGGGGTGAGGGAAAGGAATCCACGGGTTTGTGGA
GCTGGCGGAGCATGGGTGAGGACAGCATTATGCTAGGTTGGTGGCCTTGAAGTCAGATGTAAA
TTGGGATCCTGGCTTGGCCACTGACTATCCATGGGAACTTTGAATCTCAGGGGGCTCATTTGTGA
TGTGGGGGTACCATTATCTGCCTCATCAGGTTGTGGGGAGGATTATATGAGATACCTTATGTAAG
AGGGGCTGGTGCATTGTGGAAGTCAACAAGTAAGTTTTGTTAAATTTGAGTAAGAAGACCTGTTT
GTTTCCTCAAGTTGCTTATAATCTAGGTGAGGACACATCTGAGAGAATAACTAATAGTTCAGTGA
AGGTAAGAGCCAAATGATTGGCACAGACCTCAAGTGGTCTGGCATTTACGGGAGAGGGGGATT
GTGAAACAGGAATTGAAAGAAATTTAAAAATGCATAAGCAAAAACTCAGTTTATGTTTTAAAAA
AACCAATTCCCCCTGAGAAAGAGAAAGAGGTGGAGTCCTTTAAAAATTATCTGCTTGTTTTTCTG
TCTGTGGCTAGTGAGCCTTATCTCTCCCTTTCCCAGGCATTGTGAAGACCCTGTTTCTCTAGCTGT
GCAGCTGCAAGGTCACTAGGCAGATAAACTCAAGCCATAAAACATGTTTTTCCTTGAAAAGTAA
GAAATAATGTAATGCATGTGTCAAATGAATAACTATTTCTCGCTTCTGTAATATGCTTCCCTTCGC
ACAGATCTCCCCCTGCCCCATGAAATGCTTTAAAGGTAACTTAACTCTTTGTTCGGGGCTCAGTC
CTTTGGATGTTAATCTGACTGGCTTGGTGCACCTAAATAATAAATAACATATATCCTCCTCAACC
CCTCAGTCTCTCTGATTCCTAAATTATCCCGCTGTAATTGCAGGGCTAGATTTGCCGACCTGCTTT
ATAAAGCTATGTTAAATCAGTATATTTTATTCTTTTTATTAGACTTTTCTATAGCATAAAAATGATA
AAATACAGCAAGAAAAAAAGTCACCTGAAACTCTAGTCCTAACCCTAATATGAATGTGAAGGAG
CTGGGCTAGAATGGAATAGGGTAGAATTTGCATAAGGTGGAAATGGGGACAGGCCTGTCACAA
GGATCACTAGAGTCTTGCTGGCAAAAGCATCTTACTGCATTCATGCTGCAGCACAGGACCCAGA
TGCAGAACAGGTTGGGGACACAGCCCTGCCTTCTAATAGTGGACACCATCTGAGAGCTGAGCC
ATAGATTCAAAGCCTAACTCCTAATAGCGATTGGTAGTAATATCATCAGCAGTTCATATACATGG
AGTGACAACAGCGTGCCAGGCAATTCAAAGGGTCTCTCGGTCCTCACAGCATTTTTGAAGTAGC
TAACATCACTATCCCCCTTTTACAGTTAAAAACAACAACAACAACAACAAAAACACAAAACCCT
GAGGCTCAGAAGATAAATATTTGCTGAAGATCACTGTGCTTGTAAGTGGTGGAGCCTGACCATT
AACTTCCCCACGACACTGCTTCAATTTCTATCACTTCATCAACCACACAACCCGAGAACTGTGAG
GGGATTTAGCAGGCCCAGTCCAGATCTCTTATTGTACAGAGAAGAAATCTGAGGCTCAGAGAG
GGGAAGGGACTAATTTGGACCTTTAACTTCGGATTCTTAACTTTCTTTCTGGTGTTCTAACTATAC
TGTACTACAAGAGGCTGTCTTAGAGCTTTAGTTCAACCAGCTTCAGAGATACTCATTGTTATGGG
TTGAATTATGTCTCCCCAAAATTCATATGTCAAAGTTCTAACCCCCAGTACCTCAGAATGTGACC
ATATTTGGAGATAGGACCTTTACAGAGGTAATCAGGTGAAAATGAGGTTGTTAGGGTGGGCCTT
AGTCCAGTATGGCTGGTGTCCTCATAAAAAGGGGAAATTTGGACACAGATGTACATAGAGGGA
AGATGGTGTGAAGAGACATAAAGAACAGATAGTCATCTATAGCCAAGGCCTGGAACAGATCCT

TCCCTAACAGCCCTCAGAAGGAAGCAACCTCACTGACACCTTGATTTTGGACTGCTACCCTCTG
GCACTGTGAGACAATAAATTCATGTTGGTTAGGCCACCCAGTTTGTTATGGCAGCCCTAGGAAA
CCTTTGTGGATATATCTGTTCGTATACCCATAAAGTCTGCAAGTCAAAAATATCACAGACTTTTG
GCAGCCCTTCATTCAAGCACTGGCTGGGAGGGTCACCCTCCCCGAGGCAAAAGGATAGAGCAA
ATGATCTTTCCAGCTTGCTCCCAACCTTGATCTTTGGCCAAGATATTTTTCTTTCACTGGACATTG
GCCAACATTGGTGTCATAACTTAATTTGACTGTGACCTTCTTGAGTCTTTTATGTCTCTGAGCTTG
ACCTGTAGAAGAAGCTCAATAAATGTGGGTAGAGGGGATGAGTGGGAAAATCAGCTCCCAGCC
ATTCCAGTTCAAGCCTCTTCAAGCAGAAGAGTCCCACCTGAAAGACCTCATTTTCACTTGATTAC
TCGATCTCCAAGCAATAGGTGGGGCAGGAAGCAGGGTATGTAAGGCCTGTCAGTGGATGGAAG
TTGAAGTTGGACACCATCTCCTAAAGTTTAGAGCTGAGCCCAAATTCTGGAAAACACAAGCAAA
ATACATTTTATATGACTCAGGATGGGCTAAAGAAGTTCCAGTACAGGAAACTGTTTTCCTGCTGC
TTTATTAATTTTGCTACAGAGGATAAATGAGATGCAGACTTTCATATCCAAGTCTTCATTAGATGC
CCAGTTAATGCAGCCATGACAAATGGGAATGAAGC

ID|56676|gene_id|ENSG00000224699|transcript_id|ENST00000587691 Homo sapiens lncRNA

GTCGAGGTGACCTGCACCTGGCTCCATGGCGGAACCGGCCGGCACGGATTATTCCCCTCT
CGGGAGAGGTCTGCAGCCGGGCCTCAGTGCTTCAGTTCGTGGCCTGGCATTTTGTGTCAGTGTT
TTTTAACCTTTGGACCCTAGTTCAACCGACTCCGAGACCTAGGGGACAGATCTTAAGTTTTACAA
TAACCCAGGGGTCAGACAGGGTCTGATCTAGCCTGTGGGGCAAGTACGGGGAAGACACGGGA
CCTCTTGGAGGGGGTCAGTAGGCTTGTAGGGGGAAAGACGCACACAGAGACTAAAGGACGAG
ATGGAGCAAGGTTCAACCCCCTAGCTGAGTGTTGGCAGTGTCGGGATTTTAACGAAGACATTGG
TGCTTATTATGGTGAGGGGACTCAGGACAGTTTAGGGGAAGAGAGTGGCATTTGAGTCCTAACT
CGTAAGGAAGACTGGGTAGGACTACTTGAAAATAACTTTTAAAAATTAATTATAAAGCTGGTAA
AAATAGGAAAACATCTATGGCCCCACTGTCCAAAGACGACTTTTAACTTCTTGGAGAATATCCTT
TCAGATAGTTGTCTTTGTAAATGTATACTTTTAATGTTTTTTCGTTTGCCTTTTTAAAATAAATTTTT
TGGGGGACAGGGTCTTGCTCTGTTACCCAGGCTGTAGTGCAGTAGAACGATTATAGCTCATTGC
AACCCGGACGTCCTGGGTTCAAGCTATCCTCACCTCTCAGCCTCTCGACTGGCTAGGACCACAG
CCTCGAGCCATCATGTCTGGCTAATTTTTACGTTTTTTTGTAGAGATGGGGGTCTCGCTGTGTTGT
CCAGGCTGGTCTTAAACTCCTTGCTTCAACAGATCCTCCCACTCCAGCCTCCCAAAATGTGATTA
CAGGCTGAGTCACCACGCCTAGCATGGTTTTTTTTGTTTTGTTTGTTTTGTAAGCAAAATTGACAT
ACATACTGGAGGTACAATTCTGTAGCTGGCCTTCTTTACAGAGTTTAGAATAAATGTCTTTCCAT
GTCAAGTTTTCACTTACATCCTTTGTAGTAGTTGCAAAATATTCCATTTTATAGCTGTATCTTAATT
GTGGATTAAGGTTGGAGTGCAGTGGCACTATCATGGCTTAGTCCAGCCTCAGCCTTCTAGGTTC
AAGCGATCTTCCCACCTCAGCCTCCCAAGAAGCTGGGACTACAGGGACCCGCCACCCCACCTA
GCTAATTAAAAAAAAAAATTGTAGAGATGGAGTCTCAGTGTGTAGCCCAGTCTCAAGTGACCCTC
CCTCGTTGGCTTCCCAAAGCGCTGAGATTGCAGGTGAGCCACTGTACTTGGACTTTTTTTTTTTT
TTTGCTTTTGTTTGTTTTTTGTTTTAGCTATTACAATGCTGGTTGAATATCCTCATAGCTAAATATCT
GTGCAGTTTTAAAATTTCCTTATAAGTTACTAGAAATGGAATGACTGGGTTAAAGGACATGATCA
TTTTTTAAGACTCTTGATACATACTTACAAATTGCCTCCCGAAAAAGTGGTGCTAATTTACTCAAA
TTGGTAAGCCTCACCCCCTCTGTGGATCATTCCTTGCACTAAGTTTCTTCCAACTCTGTCACCTCC
AGCTTTTTACTCACAGCCTACCTTCCAATCTGGGGCCCAGCTCCCAGATCGGAAGATACCAGAC
TTTGGTATTGAACCAAAGTCCAATACACTGCACTGGCATTCAGAGTCCCTTTTTATTCCGCAGCT
GTCATAGTGAAGCTTGCAGCTTCCTTGAAATTCCAGTTTGTTTATTTCTTTATTTTTTTGAGACAGA
GTCTTGCTCTATCGCCCAGGCTGGAGTGCAGTGGCGCCGTCTCAGCTCACTGCAACCTCTGCCT

CCTGGTTCAAGCGATTCTTCTGCCTCAGCCTCCCGAGTAGCTGGGATTACAGGCATGCACCACC
ACACGTGGCTAATTTTTGTGCTTTTAGTAGAGATGGGGTTTCACCATGTTGGCCAGGCTGGTCTC
AAACTCCTGACCTCAAGCGATCCGCCCACCTCAGCCTCCCAAAGTGCTGGGATTATAGGCATGA
GCCATTGCACCCAGCCAAAATTCCAGTTTGAATGTTATAATTCAGGGGTCTTGGACTCCTGCTCT
AGAGAAATTATTCATGTTAAGTTAGGGGTAACACAGTACAGGCCATATAAGTTTTAGAAAAAGC
AGTTTAAAACTTAGCATTAGAAAACATAAGTACTGCCTGGTGAAATGGCACGTGCCTATAATCC
CAGCTACTCAGGAGGCTGAGGCAGGAGGATCCCTTGAGCCCAGGAGTTCAAGTCCAGCCTGGG
CAATATAGTCTAGTGGCCACATTAAAAAAAGTAAAACAGCCGGGCGCGGTGGCTCACGCCTGT
AATCCCAGCACTTTGGGAGGCCGAGGCGGGTGGATCACGAGGTCAGGAGATTGAGACCATCCT
GGCTAACACGGTGAAACCCTGTCTCCACTAAAAATACAAAAAATTCTCCGGGTGTGGTGGTGGG
CGCCTGTGGTCCCAGCTACTCAGGAGGCTGAGGCAGGAGAATGGTGTGAGCCTGGGAGGCGG
AGCTTGCAGTGAGCCGAGATCGCGCCACTGCACTCCAGCCTGGGCGACAGAGCGAGACTCTGT
CTCAAAAAAAAAAAAAAAAAAAAAAAAAAAAGTAAAACAGGGCAGGGTGCAGTGGCTCACGCC
TGTAATCCTAGCACTTTGGGAGGCCGAGATGGGTGGATCAGTTGAGGTCAGGAGTTTGAGACC
AGCCTGGCCAACATGGCGAAACCCCATCTCTACTAAAAATACAAAAATTAGCTGGGTGTGATGG
TGCGTGCCTGTAGTCCCAGCTACTCAGGAGGCTGAGGCAGGAGGATCGTTTGAACTCGGGAGC
TGGAGGTTGCAGTGAGCCGAGATTGAGCCACAGCACTCCAGGCTGTGCTACAGTGTGAGACTG
TCTCAAAAAAAAGTAAAACAGATGAAACTAATTTTAATAACATTTTATTTATTCAAACAATATATT
CAAAATATTATTTCAACTTGTGATTGATAAAATACTAATGAGATATTTTATATTTTTTCTCCAGTAC
TGTCTTCAAAATCCAGGGTATATTTTTCAGTTAGCAGCACATTTCAATTCAGACCAGCCGCATTTC
AAGTGCTCAGCAGCCACCTGTGGCTAGTGGCCACAGTATTGGCCAGTGTAGCCCAAGGATATCT
CAAATTATCTCAAAGCTAGATCAGTGATTAAAGGATTCTCAGGCCAGGCATGGTAGTTTTCAGG
CCTGGTATGGTGGTTTACGCCTGTAATCCCCGCACTTTGGGAGGCCTAGGTGGGCGGATGGCTT
GAGCCCAGGAGTTTGAGACCAGCCTAGCCAACGTGGGGAGACCCTGTCTCTACCAAAGCACAA
AAATTAGCTGGGCATGGTGATGCGCACCTGTGGTCTCAAGTACTTTGTGTGAGGTGGGAGGATC
ACTTGAGCCCGGGAGGTTGAGGCTGCAGTGAGCTGAGATTGCACAACTGCACTCCAGCCTGGG
TGACAGAATGAGACCCTGTCTCAAAAAAAAAAAAAGGATTCTCCGAAAGACTGGCGATTGCTTTT
TAAGCTATAAGGAAGGCCTTTGAAGGGAAATCAAGTAGGAAGAGGATGATAGCTGGCTCACAT
GTCCTTGCCACATGGGAGGTGAGAGAGCAGGCTACTCCTTACCACCCCCGCTTTCTCTGCCCTG
CTGCTGTCTTGCCCCTGTGCAAGTAGGCAATGGGAGAGGAATAGTATATATAAATTGGGCAAGT
GCTAGGGGCTGGAGTGATGGTTGGGGAAAGTATCTGACCCTTGTTTTCTACTTTCTTTAGCTTGT
GGGGTATTTAACAATTCTTTGTTCTTAGTTTTGAGTGCTGGCAACCATTTCTGGGGTGACAGGAA
AAGTTCCATGACATCCGTATTAGAGCATTTGTTTCATTATATTGTAATTTTTTATGTTTACTTTTGA
AAAAGTTCATTCTTTTCTCTTTTTAATTCTTATTCTTTTTTTTTTCTTTTCTTTTTGAGGCAGGGTTTT
ACTCTATCACCCAGGCTGGAGTGCAGTGGCACAATCTTGGCTCACTGCAACCTCTGCCTCCTGA
GCTCGTGATTCATCCTCCTGAGTAGCTGGGACTACAGGTGTACACTACCATGTGTAGCTAAATTT
TTTGTATTTTTTGTAGAGATGGGGTTTTGCCATGTTGCCAGGCTGGTCTCGAACTCCTGCAATTTG
CTCTCCTTGGCCTCACGAAATGCTGGGATTACAGGCATAAGCTACAGCACCCAGCCCTTACGTT
TACTTTTCATTGACAGGAAGAGTGTCTTCATCTCTGCATCCCTAGTGCAACAGAGCATGACAAAA
ATTCAACAAATATTTATTGAAGAGTCTTGATGTCTTTTGGGAAAGGAACTCTATGGTTACCTTTAC
TCTAAGGTAAAGAAATTTAGGTCCTGAACTTTAACCACCTTCTCCTCTTCTTTCTTCCCTCTTTCAT
AAATGAGCTCTTGGAAAATTAAAATGCCAGGAGGGTCCAGCAGAGAATACCCAAGCCCACATG
TCCTGATGGGAAGTGGGGGATAAGTAGAGAGGGAAACACAGACCCTGTAGGCCATCTCACTGC
TGCTGGCCCAGGGGACCC

ID|56679|gene_id|ENSG00000224699|transcript_id|ENST00000599202 Homo sapiens lncRNA

AATGACCTGCTTAGAGTTCTGCACCCCCCAGCATGGAGAGCAGAGCGCAAAGATTTCTTCC GGACTCCCACATTCCCCTTGGAATCCAAGAACTGGTCTACTGTCACAACACTTTAGTACCCTCTT GTAAGCCTGTGGACAAATCCTCTGATGCCTTGATACCTCGGTTTTCCCCCACTATAAATGGGAAG ATAAATTCACTAGAAACAGTACTTACCTTGCAGTGTCATTATGAGAATTTCATGAGACAATGCAA TATAAAGCTCCTAGCACAGTTCCAGGCACCATTGTAAGCACTGAACAAATGAAAGCTGTTGTCA TTAGGATCTCAAGAAATGACAGCTGAGGCAAATGTGGATTAGACTGCATTCAGCATTCTTAGCT GCAAGCAACAGAAGCTGACGCTGGTTTAAAGAATAGGAAAGGAAGTTAATTAAAATGACATTG GGTAGCTTGTGGAACTGGGAGGACTAGAGAGCTCAGTCTAAGTTCTAGTGACAAAGCTTCAAAT CATACATGAGGAAAATGCCCCTCTGCAGGAGGAACGTAATCCTGCAATCACTGCTTCCCCTGAA GCCAGATGCCTCTACTGCTGCCCTTCCCAGCAGTGATGGAGCCTGGATCACATACCTGGCTTTA GTTGTAAAGGAGGCTGGAGAGAAAATGCTCTGCTCTATGTAACTGTCTTGAAAGTGGCCCACAC ATAGCAAAGCCATTCAAAAGCGGGTGAGCAGCATGAAAGCATCCAGAAGGATTCTTGCTTTGC ATGAGGGAGAACTCTTGCTCCCAGAGTGTGAGACCAGCAGGGGCATTTTCCTCATGTGTGATTA GAAGCTTTGTCACTAGAAGCTTAGACTGAGTAGGACACCTCTGAGTGTGAATGTCCTACCTGAG TTCTGCCTCATGCAAAGCAAGAATCCCTCTGGAGGGATTGACACCACAGCCTCTTCCACAATGT CTCCAAACAACTTTACTGGGCAGCATTCCACTCCTTGACAAACGGGAGAGGTGCATTCTACCTT CTCTCCTGCATGAGCCTAGACTAATATGGAATTTGGGAGTGTTGATCATTGTACTTTGTGCTCCTA GAGATAAGGGTCATCATAAAGCATCTCAGAGTCTCACTGTAGATCCACAGTTCCATTTCTTTATG GATCCCTTACCCCTACTTTGCCAAATTCTATGCAGATAGCAATAATAATTCAGACAGGGCTTCCT CACTGAAGGAGTTTGTGCTCTATATAGTTAATAAGCTAGAGATAATGAAAAAAAAAAAAAACTCA ACTTGAACTTTGGGTAAGAGAAACTTTTCAGACCAACGATCATTACCAATAACAGCTTCCACGC ATGACCCTGCCCGGAGATTGGGGCCTGCACTACTGTTTCCAGGAAACACATTCTTTTGAATTTTT TTATTTTCTTTGTAGAGATGGGGATCTCACTCTGTTGCCCAGGCTGGTCTCAAACTCCTGGCTTCA AGCAATCCTCCTGCTTCGGCCTCCCAAACTTCTGGGATTACAGATATGAGCCACCACGGCTGGC TGACAGGCACCAAATTCTAGGAGAGGACTTGAATAGAGTAGGCATCTGTCTCTCCCCATTCCTTT CAGGGACAGAGCTCTCCTGGTTGGGAGAAAGGGAGAGGGAGAGGGAGGAGGATGAATAGTG AAGCACCAAACCTCAGAATTAAGGCCTTTTTCTTTTCTTTCTTTCTTTCTTTTTTTTTTTTGAGATGG AGTTTCGCTCTTGTTGCCCAGGCTGGAGTGCAATGGCACAATCTCGGCTCACCGCAACCTCTGC CTCCCAGGTTCAAGCGATTCTCCTGCCTCAGCCTCCCGAGTAGCTGGGATTACAGGCATGCGCC ACCACACCCAGTTAACTTTTTTGTATTTTTAGAGACGGGGTTTCTCCATGTTGGTCAAGCTGGTCT CGAACTCCCGACCTCAGGTGCTCTGCCCGACTTAGCCTCCCAAAGTGCTAGGATTACAGGCGTG AGCCACCATGCCCAGCCAGAATCAGGGCCTTTTTAAAACTTCAGTGTCTTGTCTTTACCCTTCAT GGTGCCTAGAAAAGCATCTGGCTCATAGGTGTTCAGTGAATGATTGTTTCATTAGTGAAGAAATT CCCTTTGGAAATCTCATAGGAAACTTACCCTCTCCAACCCTTCACCTGTGCATTATTCCAGCTCC CAAGGAGGCAGCAGAAACTGGGGTAATCACAACGGTCAACAGCAGCAAAGGCAGCACTTTCT GATTTACTTGACTGCTATGGATGCCACCTCTCTCCACGCCTCAGTTTAATATCTGTAAGTTTGGTC AGTATCTCATTCAAGAACATTGCATTTACTCTGGTACTTAAAGATGAAAAATCAACATTTACAAA GCAGATCCATTGTCAACCTAAATAACAAACAAAGAGAGTCTCTATAAAAGAAAAGAATATTTAT TTGGGAATAGAGCATTGCATAGGAATATGCCTGCCTTAGTAATGCATGTATTCAGGGAGGTAGA GGAAGACAAAGGTTTTTAACAGAAAAAATGTGGAGAATTACATAATTGTTTTGAAATAATTATCC TTGGCTATAAAGATCAATAACAAGGGTGATGCGAGTCCAAGGTTGGACAGGCAGTTGCTGGGC AGATGTCTTTGCAGAAGTCTTTTTTGTGTAAGGTTGTAATGGCTTTTGTAGGAGGTTGTGGCTTTT

GCAATCTTTTGTGATAGCTTTTGTTACCAGGCATACAAGCATGAGAATGCTCTCTTCAGGGCCTT
CCCTAGCTCTATTTATCAGGGGTTTCTTGTTAGTGATTCCATTTTGATTCTGACAACTTTCATGGA
CATTATCTCATTTAATCTCACAGTCATTCCATGACTCTATCCCCATTTTGTGAATGAAGTAACTGA
GGTCTGGAGAGGTCAACTAACTGGTCAAGGTCATAGAACTTGAAAATTTTCATTGTCTTGGGTCT
CTAATACTGGGGCTGACCATTCTCCCAGGCAATGTTGAGTGAGTTGGCCCAGGTGAGTCGGCTC
CTCTGCAATTGTGTCTCCTCAACTAGTTTGAACAACACAGGCCTGTGAGCCAGTCCCATAAAGT
GGCTAGGGGTTCCCTTCTCCCCATTTCCCCTCATCAACAGGAGGACTTGTCTGTGTGCCTTCAGA
CACCACACAGTAAATCAACTGCCACATTCATTCTGGACCATCAGTATTTTGCCCCAGGAGAAAG
TCAGAGGCAAGCACACTAATATCTCATTGATATTTTCAATTGCCAAAGGGGAAGTGAGATGAAT
CTAATCCCATCTGTCAAGGTGGAGAAGAGCTGATTCGAAAGCAGCATAGATTACTTTAAGGAGA
TGACAGTTTATCACTCTCTGGACAAGGGAATATGAAATGAGGACTAACCTCCTTTATGAGTTGGT
AAAAACATTGATCAGCAAAGACAATGTCTGGGGAAATGAACAGCAAGTTCTCTGGCCCTTTTTC
CACTTTTCCTTTCCCCGAGACTTTATGTTATATATACACACTCACACAAACAGGACAGAATCCAC
CTCCTGCCCCTCAAGAAATAGTTAACATTTGTTGAGGATTAATTATTGCCAGGCTCTGTACTCCA
TGTTTTATATTTACTATCCAGAAGCCTTAATTGTTAAATGAGGTAAGTGTTATTATTAGAACCTTTG
TACAGATGTGGAAACTGAGGTGTATAGAGGTTGACTTGCCCAAGGTTACCCAGCTAGTGAAGAA
TGAAATCAGATGCAGTCCAGCCAACGCTTGGCCTGCCGTCAGTCACACGCAGTACACGTAGAT
CAGTCAGCCTGGAAAGCCACCTAGACAGCCCTGCTTCACTCCATCCTTGGTCAGCACCTTAGAG
AGCCCTGCTGACCCCAAGAGGATGTGCCTTTTACGGGTTGTAGAGGCTGAACCACAGCTGAGG
GGCTGGAATGAGTGGGGGCCTCAGACAGGGTGTCTGGAGAGCTGAGGCTCAAGGAAGGGTGG
AGGGGGTGCTGAAGCTGAAGGCCGTGGGTCTTGCCAGCAAGTGGGGCTCAGTGAGCCGAGGT
GCCTCTCAGTTACTTCCATGTGTGAATTTCCGGTGGAGGACAGTGATGGGCTCTGGGCCCTTGG
AAACCGGGACATCCAGCGTGTCCAGCCACAGTGCAGGCTGCCCTGGCTGCTGAGAGTCTCAGA
AAATGCCACTGTCATGGGTTAGGAAGGCCTGGGGACCACAACAATGTGACTTCAAAAAACCCA
AGAAGGAATGTGCACACCTGAGATGCTCAGAATGCATGTGTCAGTGTTCAGTTCTGACTCCTTG
CTTCAGCCAGGGACAGAAGAACCTTCTACAGTAAAGAACCACAAGAGAGGGCTTTGCTGCCAG
CCTCAGCCAGGGACACTTGGCGGCCAAAGCAAAGGTCACTCCAGGGCTGCAGATTTCAGCCTC
TGCGGCCAGAAACAAGCTTCCCAATTAATAATACTCTGGATCGGACAGAGATGGCTGCTCTGTC
TCAGGCGTGTTTGCCGTTGATCCCTGTGATTGATGCTGATAACATTAAGAGTCTGTCAGGGTGAT
ATTTCATGAATGTCTCATGTGATGGGATGATACAGTCGGGAAATGCAATTCCCTAGCTCAGGCA
GAATCAGAGATGACCCAAAGTGCTGATGGAAAGGCTGAGGCCCTTTCCAGCCTCTGGCAGGGT
AGGGAAAGCGGGTGAGGTGGGGGCTTGGGAGGGGCTGGGCTGACCTAGAGCTGTAACAGGG
GTCTCTCATGAAGGGCTGTGAAGAGAAACAGTATGTGAGAGAGGCAAGAAACTTTTCTCTGGGT
TTCCTCATGTAGAATGATTCCATCTGTGTAGGCCATGAAGGAGTCCAGGGAAGTTCACCCCAAA
ATGTTGAAACCACCTTTGCAAAGATTATGACAGTGAGAGAAGCCTGGCATGGCTGACTCTATCT
TGCTTCTAGCCTCACAGGCTGGCTGTTCTGTCTCATTCCTGGGCATAGGCCAAGCTAATCTTGGG
AGGGATTTATGGTTTAACTTTGAAGCCAGGATCATAATTGTCCCTCCCTAAAACTGACCCCTTTC
TTGTTCAGGGACTGAAACTGCCTTTGTAAGACTAATGAAAGGCCACAAAATTAGGATTATGGAA
GGGGCCTGAATTCTGATAAGATGTAGGCATAGCTTGCCTTTCTGTCATCGTTTATTGCACTGGCA
CTCACAAGATTTGTAACTTCCCCAATTGGTCCCATAGATAACATCACTATTGTAGAACCTCAGAT
TGGTCTTTTGAGATCTTTTTCAGACTTTGGTATTCTGGTGACTGACTCCACCCAGATCCCATGACT
CATGACTCAGCTGGTCCTGTGGTTCTTACCCAGTCTGACACAGCACCAGAGGACCGTTTTCCAC
ACTTCTATCTATGATTTCATCCCCAACCAATCAGCACCACCCATTCCCTAGCCCCCTGCCCACCA
AATTGATATCCATAAAAACCCTAACCTCTGAGTTCTCAGAGAAACTGATTGGAGTAATAAACTCC

TGTCTTCTGTGTGGCTAGCCTTATGTTAATTAAACTCTTTCTTAGGGCAGTACTGCTGTCAGTGAA
TCAGCTTTATCTGTGCAGTGGGCAAGAAGGACCCACTGGGTGACTACAATATGGCACTTTGGTA
TGTTGGTTATTTTAAGTTAGAGGCCTTTGATGCTGGAAGAAGCTTTACTCTGATATCTCCTTATCT
ACCACAAGACTGGATCTGCCAAAGAGAACACAATTGCGTTCCATTCCCTTCCTGAAATCTCATTA
TCTATGGCAGAAAAGAAGACTGGGGAATGTAATACACAGTATTACACAGTAATGTAACATACTA
CACAGTATTTACACAAAACATGTAACACAGAATGTACTACACAGTATTTTCACAAAACAATATCT
GTCTCTCCAACTCATTCAAATTCTAAAGAGTCATTTACACGTCAATTTGTGTCTTCTACGCCCATT
CATTCTCCCTAAGAATCATTTACTACTCCTCAAAAGAATTACCAACATTCTCCATCTCCCACTCCC
CTATGAGGAAGGGTATATAAGTTTCTGTCTGGATCTCATTAGATGACTGTGTGATCGTGCTTCTA
TGATCCCTACCCCACATGTTAAATGAATTTTGTATGCCTTTTTCTTTTATCAATCTGCCTTTTGTTA
GGGTATTTTCAGTGGACCTTCAGAAGGCAAAGGGGGAGCTTTCCTTCTTTGACCCTACAGCTATT
AGACTTGATTGAGTAAGACACACAATAATGAAAGTAAATAAGAAGAAAAACATTCATCCTGCTG
TCTCCAGAGTTTGAAATGTCTGCCTCAAACTTCGATTTCTATCTACCAAAAATTCATGCATTAATC
ATAAGGCCGATCTCAAAAGTGAATTTTTTTTTTTTTTTGAGAAGGAGTCTCACTCTGTTGCCTAGG
CTGGAGTGCAGTGGCACAATCTCAGCTCACTGCAACCTCTGCCTCCTGGGTTCAAGCGATTCTC
CTGCCTCAGCCTCCCAAGTAGCTGGGACTACAGGCACGTGCCGCCAAGCCTGGCTAATTTTTAT
ATTTTTAGTACAGACGGGGTTTCACCATATTGGCCAGGCTGGTCTCAAACTCCTGACCTCAAGTG
ATCCACCCACCTCTGCCTCCCAAAGTGCTGGGATTACAGGCGTGAGCCACCGCGCCTGGACGT
GAAAAGTTTTTTGAATTCCCCCATTAGAAATGATCTTCACCCTGACTCCCATAACACTTTGTACAT
TACTGTAAAAGCATAATATTCAAATATTTACAATATACCTCCTTCAAATATAAAATGGACACATG
CCGAAGACTTAGCAACTCATGAAGGAGGGAACCAATAATGTAGTTAAGCTAATTCAAAGAGCA
TTTTGAGGAAATGAATACTTATGGTCATTTAAAAGGAAATGTTAAAATTTCTAGGTTAAAAATAA
AACTTGCCATTGCATATATGGTCAAATGATTTTTGACAAGGGTGCCAAGACCATTCCATGGGGA
AAACACAGTCTTTTCAACAAATCGTGTTGGGAAAACTAGATATCCATGTTCAAAAGAATGAACC
CTTACCTTACCTTCCACCATATACAAAAATTAACTCAAAATGGATCAAAGACTTAAGCATAAGAG
CTAAAATTATAAAATTCTTAGAAGAAAACATAGGGAAAAAGGTCCATGGTATTATATTTGACAAT
GACTTTTTTTTTTTTTTTTTGAGGCAAGATTTCACTCTGTCACCCTGGCTGGAGTTCAATGCAGTGG
CACAATCTCAGCTCACTGCAACCTCCACCTTTCAGGCTCAAGCAATCCCCCTACCTCAGCCCCT
AAGTAGCTGAGACTACAGGCATGCACCACCAAGCACAGCTAATTTTTGTATTTTTGTACAGAGTT
TCGCCATGTTGCCCAGGCTGGTCTCGAACTCCTGAGCTCAAGCTATTCACCTGCCTCAGCCTCC
CAAAGTGTTGGGATTACAGGTGTGAGCCACTGTGCCCAGCCCTGACCCCCACATTTTTAAACAA
AGCTTCTCTTCCATAATCAATTGCAAATCAGAAGATCTTTGAATCTATCTGCAGCCTGCAAGCCC
CCTATTCAAGACATCCCACCCTTTTAAACTTAAGCCAATGTGTAACCTCCTGGCAATCAATTTTG
CAATTTATGATTTTGCCTGTAGCTTCTGCTGTTCTGAAATTTATTCCTGCCTCTAAAAATCCTTGCC
GGCAAGCCACTGGGGAGGCTAGGTTTTGAGCACTTAGCTGTCTGGTCTTCCTTACTTAGTGCTCT
GCAATAAACACCTTCCTTTCTATCACCACAAAATCCTGGGTGTGGGTATCTGGTTTTACTGTGTTG
GGTGAGCAGACCTCAGTTTGGTTCTGTTACAAGTCCCTGCTCCATCTCACTTGCTACAACATGAA
TGAAACTTGAGGACATTATGCAAAATGAAATAAGCCAGTCACAAAAAGACAAATACTGTATTAT
TCCACTTATATGAGGTACTTATGATAGACAAAATCATCAAGACAGAAGGTATGATGGTTGCCAG
GGGTTGAGGGAAGACAAAATAGGGAGTTATTGTTTAATGGTTGAGAAGTTTCAGTTTTGCAAGA
TGAAAAGTGTTCTGGAGATGGATGGTGGTGATGGTTGCATGACATAAATGTGCTTAATACCACT
GAACTGTTCTTAACTCCGCTGAAATATGGTTAAGATGGCCCGGGTGCAGTGGCTCATGCCTGTA
ATCCTAACACTTTGGAACGCCGAGGCGGGCAGATCACCTGAGATCAGGAGTTCGAGACCAGCC
TGACCAATATGGCGAAACCCAGTCTCCACTAAAAATACAAAAATTAGCGAGGTGTGGTGGTGC

GCACCTGTAATTCAAGCCACTTGGGAGGCTGAGGCAGGAGAATTTTCTGAATCCAGGATGCAG
AGGTTGCAGTGTACTGAGATCGCACCACTGCACTCCAGCCTGAGCAACAGAGCAAGACTCCAT
CTCAAAAAAAAAAAAAAAGGTTAAGATGGTAAACTTTGTACCAATTAAAAAACAAAGCTGGCTAA
CACCCTACAGGGCAATTAAAACATCATAATCTTTGGTGTTATCTTTTTTATTAGATAGAAATTATT
TATACTTACTGGATAAGATCTGTAAGTTAACACATAACTTCACAGAGTCTGAGCCCTTAATGAAT
AATAAATAGCCAAGTCACAGGGAGATACTTTCCCTGAGGTAATTAAGCAATCCTTGGGTAGGCT
TGTGAAGAAATGCCCAGAATGACGTTGGAAGGACATGCTGTTCTCTCTTGGCCTTATTTCCAAG
GTTTGGACTCAAAGTATTATTTTTTAACATCACAGAAATTTTTTATGCCCTGGTCTCTTGAGGGTA
GGGACTGTGTCTTTTTCATCTTTGCCCCTTTCCACATGCTCTGGTTTGTAGAACAGAATTGAAGAA
GGAGCCATGTGCTAGATAGAGCGGGTTGGGAGCAGCAGACCTGCCTGCTCATTGTTTCTGTGAC
CTTGACCCATCCTGTAGTCTCCCCACGCCTTGCTATTCTCATCCATCAACGATGAGATTGCACCT
AAATCCTGGGTGCTGTGCTGGGCCTGGGGATGCTGTCTAATCCCTGGGTTTCCTTCTAGTTTGCT
CTAATCACCCATATGAAAAAGAAGCTACTCAAGGCAAACATATAAATGAAACGGATAATGAATG
AATAGTATTTTTTCTTGCTTTCTTTGGAAGAGGCATCCTGGATTCTACTTGAAGCCTGCAGCCCTG
ACTCCTGGGCTGGGAGGTGGGCGAGGACTTGTATTTTGTAGCTCTGCTAGAGAGGGAACAGCT
GTCCACTGGTGGCAGGACTGACGCCTCCCAGAATACAGGGGCTTTGGATCAGCAGGCGTCAGA
GGGAAAGGAAACTGGCATCCGAGTCTGCCTTTGAGAAAGGAGTAAATTACTATCTTATTATAAA
TTCATGATCCACTCATTGCTGTTTTCAATTAATTTCTTGACAAATCTGGCTGTTCCTTTTCTTATTTT
CCTTAGGACAGGAGGTGTCCTGGTGATCAGCTGGAGCTCGGGGGAGCTGTTGCTGAAATTAAC
TTGAGTGCTTTGGAGAGAGATTTACATGGCAGAGTGGAGCCAGGGCTGCTCCTTTGGCAGGCA
CTGCTGTGCCACTGTGGAGGCGCCCCTGCTTTTCCCATGCTTTGCAGTGTCTTTAAA
ID|56681|gene_id|ENSG00000224699|transcript_id|ENST00000609244 Homo sapiens lncRNA

CGGCCGGCACGGATTATTCCCCTCTCGGGAGAGGTCTGCAGCCGGGCCTCAGTGCTTCAG
TTCGTGGCCTGGCATTTTGTGTCAGTGTTTTTTAACCTTTGGACCCTAGTTCAACCGACTCCGAGA
CCTAGGGGACAGATCTTAAGTTTTACAATAACCCAGGGGTCAGACAGGGTCTGATCTAGCCTGT
GGGGCAAGTACGGGGAAGACACGGGACCTCTTGGAGGGGGTCAGTAGGCTTGTAGGGGGAAA
GACGCACACAGAGACTAAAGGACGAGATGGAGCAAGGTTCAACCCCCTAGCTGAGTGTTGGCA
GTGTCGGGATTTTAACGAAGACATTGGTGCTTATTATGGTGAGGGGACTCAGGACAGTTTAGGG
GAAGAGAGTGGCATTTGAGTCCTAACTCGTAAGGAAGACTGGGTAGGACTACTTGAAAATAACT
TTTAAAAATTAATTATAAAGCTGGTAAAAATAGGAAAACATCTATGGCCCCACTGTCCAAAGAC
GACTTTTAACTTCTTGGAGAATATCCTTTCAGATAGTTGTCTTTGTAAATGTATACTTTTAATGTTT
TTTCGTTTGCCTTTTTAAAATAAATTTTTTGGGGGACAGGGTCTTGCTCTGTTACCCAGGCTGTAG
TGCAGTAGAACGATTATAGCTCATTGCAACCCGGACGTCCTGGGTTCAAGCTATCCTCACCTCTC
AGCCTCTCGACTGGCTAGGACCACAGCCTCGAGCCATCATGTCTGGCTAATTTTTACGTTTTTTT
GTAGAGATGGGGGTCTCGCTGTGTTGTCCAGGCTGGTCTTAAACTCCTTGCTTCAACAGATCCTC
CCACTCCAGCCTCCCAAAATGTGATTACAGGCTGAGTCACCACGCCTAGCATGGTTTTTTTTGTT
TTGTTTGTTTTGTAAGCAAAATTGACATACATACTGGAGGTACAATTCTGTAGCTGGCCTTCTTTA
CAGAGTTTAGAATAAATGTCTTTCCATGTCAAGTTTTCACTTACATCCTTTGTAGTAGTTGCAAAA
TATTCCATTTTATAGCTGTATCTTAATTGTGGATTAAGGTTGGAGTGCAGTGGCACTATCATGGCT
TAGTCCAGCCTCAGCCTTCTAGGTTCAAGCGATCTTCCCACCTCAGCCTCCCAAGAAGCTGGGA
CTACAGGGACCCGCCACCCCACCTAGCTAATTAAAAAAAAAAATTGTAGAGATGGAGTCTCAGT
GTGTAGCCCAGTCTCAAGTGACCCTCCCTCGTTGGCTTCCCAAAGCGCTGAGATTGCAGGTGAG
CCACTGTACTTGGACTTTTTTTTTTTTTTTTGCTTTTGTTTGTTTTTTGTTTTAGCTATTACAATGCTG

GTTGAATATCCTCATAGCTAAATATCTGTGCAGTTTTAAAATTTCCTTATAAGTTACTAGAAATGG
AATGACTGGGTTAAAGGACATGATCATTTTTTAAGACTCTTGATACATACTTACAAATTGCCTCC
CGAAAAAGTGGTGCTAATTTACTCAAATTGGTAAGCCTCACCCCCTCTGTGGATCATTCCTTGCA
CTAAGTTTCTTCCAACTCTGTCACCTCCAGCTTTTTACTCACAGCCTACCTTCCAATCTGGGGCCC
AGCTCCCAGATCGGAAGATACCAGACTTTGGTATTGAACCAAAGTCCAATACACTGCACTGGCA
TTCAGAGTCCCTTTTTATTCCGCAGCTGTCATAGTGAAGCTTGCAGCTTCCTTGAAATTCCAGTTT
GTTTATTTCTTTATTTTTTTGAGACAGAGTCTTGCTCTATCGCCCAGGCTGGAGTGCAGTGGCGCC
GTCTCAGCTCACTGCAACCTCTGCCTCCTGGTTCAAGCGATTCTTCTGCCTCAGCCTCCCGAGTA
GCTGGGATTACAGGCATGCACCACCACACGTGGCTAATTTTTGTGCTTTTAGTAGAGATGGGGT
TTCACCATGTTGGCCAGGCTGGTCTCAAACTCCTGACCTCAAGCGATCCGCCCACCTCAGCCTC
CCAAAGTGCTGGGATTATAGGCATGAGCCATTGCACCCAGCCAAAATTCCAGTTTGAATGTTAT
AATTCAGGGGTCTTGGACTCCTGCTCTAGAGAAATTATTCATGTTAAGTTAGGGGTAACACAGTA
CAGGCCATATAAGTTTTAGAAAAAGCAGTTTAAAACTTAGCATTAGAAAACATAAGTACTGCCT
GGTGAAATGGCACGTGCCTATAATCCCAGCTACTCAGGAGGCTGAGGCAGGAGGATCCCTTGA
GCCCAGGAGTTCAAGTCCAGCCTGGGCAATATAGTCTAGTGGCCACATTAAAAAAAGTAAAAC
AGCCGGGCGCGGTGGCTCACGCCTGTAATCCCAGCACTTTGGGAGGCCGAGGCGGGTGGATC
ACGAGGTCAGGAGATTGAGACCATCCTGGCTAACACGGTGAAACCCTGTCTCCACTAAAAATA
CAAAAAATTCTCCGGGTGTGGTGGTGGGCGCCTGTGGTCCCAGCTACTCAGGAGGCTGAGGCA
GGAGAATGGTGTGAGCCTGGGAGGCGGAGCTTGCAGTGAGCCGAGATCGCGCCACTGCACTC
CAGCCTGGGCGACAGAGCGAGACTCTGTCTCAAAAAAAAAAAAAAAAAAAAAAAAAAAAAGTAA
AACAGGGCAGGGTGCAGTGGCTCACGCCTGTAATCCTAGCACTTTGGGAGGCCGAGATGGGTG
GATCAGTTGAGGTCAGGAGTTTGAGACCAGCCTGGCCAACATGGCGAAACCCCATCTCTACTAA
AAATACAAAAATTAGCTGGGTGTGATGGTGCGTGCCTGTAGTCCCAGCTACTCAGGAGGCTGA
GGCAGGAGGATCGTTTGAACTCGGGAGCTGGAGGTTGCAGTGAGCCGAGATTGAGCCACAGC
ACTCCAGGCTGTGCTACAGTGTGAGACTGTCTCAAAAAAAAAGTAAAACAGATGAAACTAATTTT
AATAACATTTTATTTATTCAAACAATATATTCAAAATATTATTTCAACTTGTGATTGATAAAATACT
AATGAGATATTTTATATTTTTTCTCCAGTACTGTCTTCAAAATCCAGGGTATATTTTTCAGTTAGCA
GCACATTTCAATTCAGACCAGCCGCATTTCAAGTGCTCAGCAGCCACCTGTGGCTAGTGGCCAC
AGTATTGGCCAGTGTAGCCCAAGGATATCTCAAATTATCTCAAAGCTAGATCAGTGATTAAAGG
ATTCTCAGGCCAGGCATGGTAGTTTTCAGGCCTGGTATGGTGGTTTACGCCTGTAATCCCCGCA
CTTTGGGAGGCCTAGGTGGGCGGATGGCTTGAGCCCAGGAGTTTGAGACCAGCCTAGCCAACG
TGGGGAGACCCTGTCTCTACCAAAGCACAAAAATTAGCTGGGCATGGTGATGCGCACCTGTGG
TCTCAAGTACTTTGTGTGAGGTGGGAGGATCACTTGAGCCCGGGAGGTTGAGGCTGCAGTGAG
CTGAGATTGCACAACTGCACTCCAGCCTGGGTGACAGAATGAGACCCTGTCTCAAAAAAAAAA
AAGGATTCTCCGAAAGACTGGCGATTGCTTTTTAAGCTATAAGGAAGGCCTTTGAAGGGAAATC
AAGTAGGAAGAGGATGATAGCTGGCTCACATGTCCTTGCCACATGGGAGGTGAGAGAGCAGGC
TACTCCTTACCACCCCCGCTTTCTCTGCCCTGCTGCTGTCTTGCCCCTGTGCAAGTAGGCAATGG
GAGAGGAATAGTATATATAAATTGGGCAAGTGCTAGGGGCTGGAGTGATGGTTGGGGAAAGTA
TCTGACCCTTGTTTTCTACTTTCTTTAGCTTGTGGGGTATTTAACAATTCTTTGTTCTTAGTTTTGAG
TGCTGGCAACCATTTCTGGGGTGACAGGAAAAGTTCCATGACATCCGTATTAGAGCATTTGTTTC
ATTATATTGTAATTTTTTATGTTTACTTTTGAAAAAGTTCATTCTTTTCTCTTTTTAATTCTTATTCTTT
TTTTTTTCTTTTCTTTTTGAGGCAGGGTTTTACTCTATCACCCAGGCTGGAGTGCAGTGGCACAAT
CTTGGCTCACTGCAACCTCTGCCTCCTGAGCTCGTGATTCATCCTCCTGAGTAGCTGGGACTACA
GGTGTACACTACCATGTGTAGCTAAATTTTTTGTATTTTTTGTAGAGATGGGGTTTTGCCATGTTG

CCAGGCTGGTCTCGAACTCCTGCAATTTGCTCTCCTTGGCCTCACGAAATGCTGGGATTACAGG
CATAAGCTACAGCACCCAGCCCTTACGTTTACTTTTCATTGACAGGAAGAGTGTCTTCATCTCTG
CATCCCTAGTGCAACAGAGCATGACAAAAATTCAACAAATATTTATTGAAGAGTCTTGATGTCTT
TTGGGAAAGGAACTCTATGGTTACCTTTACTCTAAGGTAAAGAAATTTAGGTCCTGAACTTTAAC
CACCTTCTCCTCTTCTTTCTTCCCTCTTTCATAAATGAGCTCTTGGAAAATTAAAATGCCAGGAGG
GTCCAGCAGAGAATACCCAAGCCCACATGTCCTGATGGGAAGTGGGGGATAAGTAGAGAGGG
AAACACAGACCCTGTAGGCCATCTCACTGCTGCTGGCCCAGGGGACCCTAACTCCTTTTGTTGA
CTCATCAGAAGTAGAATCCTCCTGAGGGTCTGTTTAAGAAGCACAGCTCTGGTAGAAAGGGTAG
CTTTCCTATGGGTACGGAGGGCTCTGCTACCCCAGGGCTATCCACACTGGCCTGTGAATCACTC
TCAAATGCTTAGGGTCACGGGCTTTCAGTGGAATGAGCCCTCATGTGCCACCAGGGGGCACCA
AGGCCACAGAAAACCCTATTCAGAAGGTTCTGGGGTGAGGGAAAGGAATCCACGGGTTTGTGG
AGCTGGCGGAGCATGGGTGAGGACAGCATTATGCTAGGTTGGTGGCCTTGAAGTCAGATGTAA
ATTGGGATCCTGGCTTGGCCACTGACTATCCATGGGAACTTTGAATCTCAGGGGGCTCATTTGTG
ATGTGGGGGTACCATTATCTGCCTCATCAGGTTGTGGGGAGGATTATATGAGATACCTTATGTAA
GAGGGGCTGGTGCATTGTGGAAGTCAACAAGTAAGTTTTGTTAAATTTGAGTAAGAAGACCTGT
TTGTTTCCTCAAGTTGCTTATAATCTAGGTGAGGACACATCTGAGAGAATAACTAATAGTTCAGT
GAAGGTAAGAGCCAAATGATTGGCACAGACCTCAAGTGGTCTGGCATTTACGGGAGAGGGGGA
TTGTGAAACAGGAATTGAAAGAAATTTAAAAATGCATAAGCAAAAACTCAGTTTATGTTTTAAAA
AAACCAATTCCCCCTGAGAAAGAGAAAGAGGTGGAGTCCTTTAAAAATTATCTGCTTGTTTTTCT
GTCTGTGGCTAGTGAGCCTTATCTCTCCCTTTCCCAGGCATTGTGAAGACCCTGTTTCTCTAGCT
GTGCAGCTGCAAGGTCACTAGGCAGATAAACTCAAGCCATAAAACATGTTTTTCCTTGAAAAGT
AAGAAATAATGTAATGCATGTGTCAAATGAATAACTATTTCTCGCTTCTGTAATATGCTTCCCTTC
GCACAGATCTCCCCCTGCCCCATGAAATGCTTTAAAGGTAACTTAACTCTTTGTTCGGGGCTCAG
TCCTTTGGATGTTAATCTGACTGGCTTGGTGCACCTAAATAATAAATAACATATATCCTCCTCAAC
CCCTCAGTCTCTCTGATTCCTAAATTATCCCGCTGTAATTGCAGGGCTAGATTTGCCGACCTGCTT
TATAAAGCTATGTTAAATCAGTATATTTTATTCTTTTTATTAGACTTTTCTATAGCATAAAAATGAT
AAAATACAGCAAGAAAAAAAGTCACCTGAAACTCTAGTCCTAACCCTAATATGAATGTGAAGGA
GCTGGGCTAGAATGGAATAGGGTAGAATTTGCATAAGGTGGAAATGGGGACAGGCCTGTCACA
AGGATCACTAGAGTCTTGCTGGCAAAAGCATCTTACTGCATTCATGCTGCAGCACAGGACCCAG
ATGCAGAACAGGTTGGGGACACAGCCCTGCCTTCTAATAGTGGACACCATCTGAGAGCTGAGC
CATAGATTCAAAGCCTAACTCCTAATAGCGATTGGTAGTAATATCATCAGCAGTTCATATACATG
GAGTGACAACAGCGTGCCAGGCAATTCAAAGGGTCTCTCGGTCCTCACAGCATTTTTGAAGTAG
CTAACATCACTATCCCCCTTTTACAGTTAAAAACAACAACAACAACAACAAAAACACAAAACCC
TGAGGCTCAGAAGATAAATATTTGCTGAAGATCACTGTGCTTGTAAGTGGTGGAGCCTGACCAT
TAACTTCCCCACGACACTGCTTCAATTTCTATCACTTCATCAACCACACAACCCGAGAACTGTGA
GGGGATTTAGCAGGCCCAGTCCAGATCTCTTATTGTACAGAGAAGAAATCTGAGGCTCAGAGA
GGGGAAGGGACTAATTTGGACCTTTAACTTCGGATTCTTAACTTTCTTTCTGGTGTTCTAACTATA
CTGTACTACAAGAGGCTGTCTTAGAGCTTTAGTTCAACCAGCTTCAGAGATACTCATTGTTATGG
GTTGAATTATGTCTCCCCAAAATTCATATGTCAAAGTTCTAACCCCCAGTACCTCAGAATGTGAC
CATATTTGGAGATAGGACCTTTACAGAGGTAATCAGGTGAAAATGAGGTTGTTAGGGTGGGCCT
TAGTCCAGTATGGCTGGTGTCCTCATAAAAAGGGGAAATTTGGACACAGATGTACATAGAGGG
AAGATGGTGTGAAGAGACATAAAGAACAGATAGTCATCTATAGCCAAGGCCTGGAACAGATCC
TTCCCTAACAGCCCTCAGAAGGAAGCAACCTCACTGACACCTTGATTTTGGACTGCTACCCTCTG
GCACTGTGAGACAATAAATTCATGTTGGTTAGGCCACCCAGTTTGTTATGGCAGCCCTAGGAAA

CCTTTGTGGATATATCTGTTCGTATACCCATAAAGTCTGCAAGTCAAAAATATCACAGACTTTTG
GCAGCCCTTCATTCAAGCACTGGCTGGGAGGGTCACCCTCCCCGAGGCAAAAGGATAGAGCAA
ATGATCTTTCCAGCTTGCTCCCAACCTTGATCTTTGGCCAAGATATTTTTCTTTCACTGGACATTG
GCCAACATTGGTGTCATAACTTAATTTGACTGTGACCTTCTTGAGTCTTTTATGTCTCTGAGCTTG
ACCTGTAGAAGAAGCTCAATAAATGTGGGTAGAGGGGATGAGTGGGAAAATCAGCTCCCAGCC
ATTCCAGTTCAAGCCTCTTCAAGCAGAAGAGTCCCACCTGAAAGACCTCATTTTCACTTGATTAC
TCGATCTCCAAGCAATAGGTGGGGCAGGAAGCAGGGTATGTAAGGCCTGTCAGTGGATGGAAG
TTGAAGTTGGACACCATCTCCTAAAGTTTAGAGCTGAGCCCAAATTCTGGAAAACACAAGCAAA
ATACATTTTATATGACTCAGGATGGGCTAAAGAAGTTCCAGTACAGGAAACTGTTTTCCTGCTGC
TTTATTAATTTTGCTACAGAGGATAAATGAGATGCAGACTTTCATATCCAAGTCTTCATTAGATGC
CCAGTTAATGCAGCCATGACAAATGGGAATGAAGCCCTTATGAATAAAAAAAAGATCAACCCA
GTATAAAAAAATCATAGACCAGAGAAATGAAAATTTAGAGGAGCTGATAAAATACCCTATCCTT
AATTAGACTGTCAGCATACACATCAAATCAGCCTTAATGCCAAGATTTATTGGCCTTGGAATTGG
GTAAATGAAATAAGTGCTTACAAATGAATGGAAAAGAAATTACCTGTATTAAAACGTATTTGTGA
TGCAGGCCTGTGCACTGAGGGTTTTAGTCAAACTCAGGCTTGCAGCTTCTTTTGCTCTGCATCCA
TTTCCACCCCATGTTCCCCAGTGGGTTTCAGAGACCCTCTTTGTGGGGTAACAATATGTACCATT
GTCAACTACCTCTGCTGTTGTCCTGGAAAAAATCCTTCTTGGACAGTAAAATATTTTTGTCTTTATTT
CACCATCTCCAGATTAGATGGGTGTTACTGATTCTTACCTCAGAGAAGAGAATGTCATGGAGAC
AAAATGACCTGCTTAGAGTTCTGCACCCCCCAGCATGGAGAGCAGAGCGCAAAGATTTCTTCCG
GACTCCCACATTCCCCTTGGAATCCAAGAACTGGTCTACTGTCACAACACTTTAGTACCCTCTTG
TAAGCCTGTGGACAAATCCTCTGATGCCTTGATACCTCGGTTTTCCCCCACTATAAATGGGAAGA
TAAATTCACTAGAAACAGTACTTACCTTGCAGTGTCATTATGAGAATTTCATGAGACAATGCAAT
ATAAAGCTCCTAGCACAGTTCCAGGCACCATTGTAAGCACTGAACAAATGAAAGCTGTTGTCAT
TAGGATCTCAAGAAATGACAGCTGAGGCAAATGTGGATTAGACTGCATTCAGCATTCTTAGCTG
CAAGCAACAGAAGCTGACGCTGGTTTAAAGAATAGGAAAGGAAGTTAATTAAAATGACATTGG
GTAGCTTGTGGAACTGGGAGGACTAGAGAGCTCAGTCTAAGTTCTAGTGACAAAGCTTCAAATC
ATACATGAGGAAAATGCCCCTCTGCAGGAGGAACGTAATCCTGCAATCACTGCTTCCCCTGAAG
CCAGATGCCTCTACTGCTGCCCTTCCCAGCAGTGATGGAGCCTGGATCACATACCTGGCTTTAG
TTGTAAAGGAGGCTGGAGAGAAAATGCTCTGCTCTATGTAACTGTCTTGAAAGTGGCCCACACA
TAGCAAAGCCATTCAAAAGCGGGTGAGCAGCATGAAAGCATCCAGAAGGATTCTTGCTTTGCAT
GAGGGAGAACTCTTGCTCCCAGAGTGTGAGACCAGCAGGGGCATTTTCCTCATGTGTGATTAGA
AGCTTTGTCACTAGAAGCTTAGACTGAGTAGGACACCTCTGAGTGTGAATGTCCTACCTGAGTTC
TGCCTCATGCAAAGCAAGAATCCCTCTGGAGGGATTGACACCACAGCCTCTTCCACAATGTCTC
CAAACAACTTTACTGGGCAGCATTCCACTCCTTGACAAACGGGAGAGGTGCATTCTACCTTCTCT
CCTGCATGAGCCTAGACTAATATGGAATTTGGGAGTGTTGATCATTGTACTTTGTGCTCCTAGAG
ATAAGGGTCATCATAAAGCATCTCAGAGTCTCACTGTAGATCCACAGTTCCATTTCTTTATGGAT
CCCTTACCCCTACTTTGCCAAATTCTATGCAGATAGCAATAATAATTCAGACAGGGCTTCCTCAC
TGAAGGAGTTTGTGCTCTATATAGTTAATAAGCTAGAGATAATGAAAAAAAAAAAAAACTCAACT
TGAACTTTGGGTAAGAGAAACTTTTCAGACCAACGATCATTACCAATAACAGCTTCCACGCATG
ACCCTGCCCGGAGATTGGGGCCTGCACTACTGTTTCCAGGAAACACATTCTTTTGAATTTTTTTAT
TTTCTTTGTAGAGATGGGGATCTCACTCTGTTGCCCAGGCTGGTCTCAAACTCCTGGCTTCAAGC
AATCCTCCTGCTTCGGCCTCCCAAACTTCTGGGATTACAGATATGAGCCACCACGGCTGGCTGA
CAGGCACCAAATTCTAGGAGAGGACTTGAATAGAGTAGGCATCTGTCTCTCCCCATTCCTTTCA
GGGACAGAGCTCTCCTGGTTGGGAGAAAGGGAGAGGGAGAGGGAGGAGGATGAATAGTGAA

GCACCAAACCTCAGAATTAAGGCCTTTTTCTTTTCTTTCTTTCTTTCTTTTTTTTTTTTGAGATGGAG
TTTCGCTCTTGTTGCCCAGGCTGGAGTGCAATGGCACAATCTCGGCTCACCGCAACCTCTGCCT
CCCAGGTTCAAGCGATTCTCCTGCCTCAGCCTCCCGAGTAGCTGGGATTACAGGCATGCGCCAC
CACACCCAGTTAACTTTTTTGTATTTTTAGAGACGGGGTTTCTCCATGTTGGTCAAGCTGGTCTCG
AACTCCCGACCTCAGGTGCTCTGCCCGACTTAGCCTCCCAAAGTGCTAGGATTACAGGCGTGAG
CCACCATGCCCAGCCAGAATCAGGGCCTTTTTAAAACTTCAGTGTCTTGTCTTTACCCTTCATGG
TGCCTAGAAAAGCATCTGGCTCATAGGTGTTCAGTGAATGATTGTTTCATTAGTGAAGAAATTCC
CTTTGGAAATCTCATAGGAAACTTACCCTCTCCAACCCTTCACCTGTGCATTATTCCAGCTCCCA
AGGAGGCAGCAGAAACTGGGGTAATCACAACGGTCAACAGCAGCAAAGGCAGCAC
ID|56688|gene_id|ENSG00000224699|transcript_id|ENST00000608486 Homo sapiens lncRNA

GGGCTCCCCGCGCGGTGACCGTCGAGGTGACCTGCACCTGGCTCCATGGCGGAACCGGC
CGGCACGGATTATTCCCCTCTCGGGAGAGGTCTGCAGCCGGGCCTCAGTGCTTCAGTTCGTGGC
CTGGCATTTTGTGTCAGTGTTTTTTAACCTTTGGACCCTAGTTCAACCGACTCCGAGACCTAGGG
GACAGATCTTAAGTTTTACAATAACCCAGGGGTCAGACAGGGTCTGATCTAGCCTGTGGGGCAA
GTACGGGGAAGACACGGGACCTCTTGGAGGGGGTCAGTAGGCTTGTAGGGGGAAAGACGCAC
ACAGAGACTAAAGGACGAGATGGAGCAAGGTTCAACCCCCTAGCTGAGTGTTGGCAGTGTCGG
GATTTTAACGAAGACATTGGTGCTTATTATGGTGAGGGGACTCAGGACAGTTTAGGGGAAGAGA
GTGGCATTTGAGTCCTAACTCGTAAGGAAGACTGGGTAGGACTACTTGAAAATAACTTTTAAAA
ATTAATTATAAAGCTGGTAAAAATAGGAAAACATCTATGGCCCCACTGTCCAAAGACGACTTTTA
ACTTCTTGGAGAATATCCTTTCAGATAGTTGTCTTTGTAAATGTATACTTTTAATGTTTTTTCGTTTG
CCTTTTTAAAATAAATTTTTTGGGGGACAGGGTCTTGCTCTGTTACCCAGGCTGTAGTGCAGTAG
AACGATTATAGCTCATTGCAACCCGGACGTCCTGGGTTCAAGCTATCCTCACCTCTCAGCCTCTC
GACTGGCTAGGACCACAGCCTCGAGCCATCATGTCTGGCTAATTTTTACGTTTTTTTGTAGAGAT
GGGGGTCTCGCTGTGTTGTCCAGGCTGGTCTTAAACTCCTTGCTTCAACAGATCCTCCCACTCCA
GCCTCCCAAAATGTGATTACAGGCTGAGTCACCACGCCTAGCATGGTTTTTTTTGTTTTGTTTGTT
TTGTAAGCAAAATTGACATACATACTGGAGGTACAATTCTGTAGCTGGCCTTCTTTACAGAGTTT
AGAATAAATGTCTTTCCATGTCAAGTTTTCACTTACATCCTTTGTAGTAGTTGCAAAATATTCCAT
TTTATAGCTGTATCTTAATTGTGGATTAAGGTTGGAGTGCAGTGGCACTATCATGGCTTAGTCCA
GCCTCAGCCTTCTAGGTTCAAGCGATCTTCCCACCTCAGCCTCCCAAGAAGCTGGGACTACAGG
GACCCGCCACCCCACCTAGCTAATTAAAAAAAAAAATTGTAGAGATGGAGTCTCAGTGTGTAGCC
CAGTCTCAAGTGACCCTCCCTCGTTGGCTTCCCAAAGCGCTGAGATTGCAGGTGAGCCACTGTA
CTTGGACTTTTTTTTTTTTTTTTTGCTTTTGTTTGTTTTTTGTTTTAGCTATTACAATGCTGGTTGAATA
TCCTCATAGCTAAATATCTGTGCAGTTTTAAAATTTCCTTATAAGTTACTAGAAATGGAATGACTG
GGTTAAAGGACATGATCATTTTTTAAGACTCTTGATACATACTTACAAATTGCCTCCCGAAAAAG
TGGTGCTAATTTACTCAAATTGGTAAGCCTCACCCCCTCTGTGGATCATTCCTTGCACTAAGTTTC
TTCCAACTCTGTCACCTCCAGCTTTTTACTCACAGCCTACCTTCCAATCTGGGGCCCAGCTCCCA
GATCGGAAGATACCAGACTTTGGTATTGAACCAAAGTCCAATACACTGCACTGGCATTCAGAGT
CCCTTTTTATTCCGCAGCTGTCATAGTGAAGCTTGCAGCTTCCTTGAAATTCCAGTTTGTTTATTTC
TTTATTTTTTTGAGACAGAGTCTTGCTCTATCGCCCAGGCTGGAGTGCAGTGGCGCCGTCTCAGC
TCACTGCAACCTCTGCCTCCTGGTTCAAGCGATTCTTCTGCCTCAGCCTCCCGAGTAGCTGGGAT
TACAGGCATGCACCACCACACGTGGCTAATTTTTGTGCTTTTAGTAGAGATGGGGTTTCACCATG
TTGGCCAGGCTGGTCTCAAACTCCTGACCTCAAGCGATCCGCCCACCTCAGCCTCCCAAAGTGC
TGGGATTATAGGCATGAGCCATTGCACCCAGCCAAAATTCCAGTTTGAATGTTATAATTCAGGG

GTCTTGGACTCCTGCTCTAGAGAAATTATTCATGTTAAGTTAGGGGTAACACAGTACAGGCCATA
TAAGTTTTAGAAAAAGCAGTTTAAAACTTAGCATTAGAAAACATAAGTACTGCCTGGTGAAATG
GCACGTGCCTATAATCCCAGCTACTCAGGAGGCTGAGGCAGGAGGATCCCTTGAGCCCAGGAG
TTCAAGTCCAGCCTGGGCAATATAGTCTAGTGGCCACATTAAAAAAAGTAAAACAGCCGGGCG
CGGTGGCTCACGCCTGTAATCCCAGCACTTTGGGAGGCCGAGGCGGGTGGATCACGAGGTCAG
GAGATTGAGACCATCCTGGCTAACACGGTGAAACCCTGTCTCCACTAAAAATACAAAAAATTCT
CCGGGTGTGGTGGTGGGCGCCTGTGGTCCCAGCTACTCAGGAGGCTGAGGCAGGAGAATGGT
GTGAGCCTGGGAGGCGGAGCTTGCAGTGAGCCGAGATCGCGCCACTGCACTCCAGCCTGGGC
GACAGAGCGAGACTCTGTCTCAAAAAAAAAAAAAAAAAAAAAAAAAAAAAGTAAAACAGGGCAG
GGTGCAGTGGCTCACGCCTGTAATCCTAGCACTTTGGGAGGCCGAGATGGGTGGATCAGTTGA
GGTCAGGAGTTTGAGACCAGCCTGGCCAACATGGCGAAACCCCATCTCTACTAAAAATACAAA
AATTAGCTGGGTGTGATGGTGCGTGCCTGTAGTCCCAGCTACTCAGGAGGCTGAGGCAGGAGG
ATCGTTTGAACTCGGGAGCTGGAGGTTGCAGTGAGCCGAGATTGAGCCACAGCACTCCAGGCT
GTGCTACAGTGTGAGACTGTCTCAAAAAAAAGTAAAACAGATGAAACTAATTTTAATAACATTTT
ATTTATTCAAACAATATATTCAAAATATTATTTCAACTTGTGATTGATAAAATACTAATGAGATATT
TTATATTTTTTCTCCAGTACTGTCTTCAAAATCCAGGGTATATTTTTCAGTTAGCAGCACATTTCAA
TTCAGACCAGCCGCATTTCAAGTGCTCAGCAGCCACCTGTGGCTAGTGGCCACAGTATTGGCCA
GTGTAGCCCAAGGATATCTCAAATTATCTCAAAGCTAGATCAGTGATTAAAGGATTCTCAGGCC
AGGCATGGTAGTTTTCAGGCCTGGTATGGTGGTTTACGCCTGTAATCCCCGCACTTTGGGAGGC
CTAGGTGGGCGGATGGCTTGAGCCCAGGAGTTTGAGACCAGCCTAGCCAACGTGGGGAGACC
CTGTCTCTACCAAAGCACAAAAATTAGCTGGGCATGGTGATGCGCACCTGTGGTCTCAAGTACT
TTGTGTGAGGTGGGAGGATCACTTGAGCCCGGGAGGTTGAGGCTGCAGTGAGCTGAGATTGCA
CAACTGCACTCCAGCCTGGGTGACAGAATGAGACCCTGTCTCAAAAAAAAAAAAAGGATTCTCC
GAAAGACTGGCGATTGCTTTTTAAGCTATAAGGAAGGCCTTTGAAGGGAAATCAAGTAGGAAG
AGGATGATAGCTGGCTCACATGTCCTTGCCACATGGGAGGTGAGAGAGCAGGCTACTCCTTAC
CACCCCCGCTTTCTCTGCCCTGCTGCTGTCTTGCCCCTGTGCAAGTAGGCAATGGGAGAGGAAT
AGTATATATAAATTGGGCAAGTGCTAGGGGCTGGAGTGATGGTTGGGGAAAGTATCTGACCCTT
GTTTTCTACTTTCTTTAGCTTGTGGGGTATTTAACAATTCTTTGTTCTTAGTTTTGAGTGCTGGCAA
CCATTTCTGGGGTGACAGGAAAAGTTCCATGACATCCGTATTAGAGCATTTGTTTCATTATATTGT
AATTTTTTATGTTTACTTTTGAAAAAGTTCATTCTTTTCTCTTTTTAATTCTTATTCTTTTTTTTTTCTT
TTCTTTTTGAGGCAGGGTTTTACTCTATCACCCAGGCTGGAGTGCAGTGGCACAATCTTGGCTCA
CTGCAACCTCTGCCTCCTGAGCTCGTGATTCATCCTCCTGAGTAGCTGGGACTACAGGTGTACA
CTACCATGTGTAGCTAAATTTTTTGTATTTTTTGTAGAGATGGGGTTTTGCCATGTTGCCAGGCTG
GTCTCGAACTCCTGCAATTTGCTCTCCTTGGCCTCACGAAATGCTGGGATTACAGGCATAAGCTA
CAGCACCCAGCCCTTACGTTTACTTTTCATTGACAGGAAGAGTGTCTTCATCTCTGCATCCCTAG
TGCAACAGAGCATGACAAAAATTCAACAAATATTTATTGAAGAGTCTTGATGTCTTTTGGGAAAG
GAACTCTATGGTTACCTTTACTCTAAGGTAAAGAAATTTAGGTCCTGAACTTTAACCACCTTCTCC
TCTTCTTTCTTCCCTCTTTCATAAATGAGCTCTTGGAAAATTAAAATGCCAGGAGGGTCCAGCAG
AGAATACCCAAGCCCACATGTCCTGATGGGAAGTGGGGGATAAGTAGAGAGGGAAACACAGA
CCCTGTAGGCCATCTCACTGCTGCTGGCCCAGGGGACCCTAACTCCTTTTGTTGACTCATCAGAA
GTAGAATCCTCCTGAGGGTCTGTTTAAGAAGCACAGCTCTGGTAGAAAGGGTAGCTTTCCTATG
GGTACGGAGGGCTCTGCTACCCCAGGGCTATCCACACTGGCCTGTGAATCACTCTCAAATGCTT
AGGGTCACGGGCTTTCAGTGGAATGAGCCCTCATGTGCCACCAGGGGGCACCAAGGCCACAG
AAAACCCTATTCAGAAGGTTCTGGGGTGAGGGAAAGGAATCCACGGGTTTGTGGAGCTGGCGG

AGCATGGGTGAGGACAGCATTATGCTAGGTTGGTGGCCTTGAAGTCAGATGTAAATTGGGATCC
TGGCTTGGCCACTGACTATCCATGGGAACTTTGAATCTCAGGGGGCTCATTTGTGATGTGGGGG
TACCATTATCTGCCTCATCAGGTTGTGGGGAGGATTATATGAGATACCTTATGTAAGAGGGGCT
GGTGCATTGTGGAAGTCAACAAGTAAGTTTTGTTAAATTTGAGTAAGAAGACCTGTTTGTTTCCT
CAAGTTGCTTATAATCTAGGTGAGGACACATCTGAGAGAATAACTAATAGTTCAGTGAAGGTAA
GAGCCAAATGATTGGCACAGACCTCAAGTGGTCTGGCATTTACGGGAGAGGGGGATTGTGAAA
CAGGAATTGAAAGAAATTTAAAAATGCATAAGCAAAAACTCAGTTTATGTTTTAAAAAAACCAAT
TCCCCCTGAGAAAGAGAAAGAGGTGGAGTCCTTTAAAAATTATCTGCTTGTTTTTCTGTCTGTGG
CTAGTGAGCCTTATCTCTCCCTTTCCCAGGCATTGTGAAGACCCTGTTTCTCTAGCTGTGCAGCT
GCAAGGTCACTAGGCAGATAAACTCAAGCCATAAAACATGTTTTTCCTTGAAAAGTAAGAAATA
ATGTAATGCATGTGTCAAATGAATAACTATTTCTCGCTTCTGTAATATGCTTCCCTTCGCACAGAT
CTCCCCCTGCCCCATGAAATGCTTTAAAGGTAACTTAACTCTTTGTTCGGGGCTCAGTCCTTTGG
ATGTTAATCTGACTGGCTTGGTGCACCTAAATAATAAATAACATATATCCTCCTCAACCCCTCAG
TCTCTCTGATTCCTAAATTATCCCGCTGTAATTGCAGGGCTAGATTTGCCGACCTGCTTTATAAAG
CTATGTTAAATCAGTATATTTTATTCTTTTTATTAGACTTTTCTATAGCATAAAAATGATAAAATAC
AGCAAGAAAAAAAGTCACCTGAAACTCTAGTCCTAACCCTAATATGAATGTGAAGGAGCTGGG
CTAGAATGGAATAGGGTAGAATTTGCATAAGGTGGAAATGGGGACAGGCCTGTCACAAGGATC
ACTAGAGTCTTGCTGGCAAAAGCATCTTACTGCATTCATGCTGCAGCACAGGACCCAGATGCAG
AACAGGTTGGGGACACAGCCCTGCCTTCTAATAGTGGACACCATCTGAGAGCTGAGCCATAGA
TTCAAAGCCTAACTCCTAATAGCGATTGGTAGTAATATCATCAGCAGTTCATATACATGGAGTGA
CAACAGCGTGCCAGGCAATTCAAAGGGTCTCTCGGTCCTCACAGCATTTTTGAAGTAGCTAACA
TCACTATCCCCCTTTTACAGTTAAAAACAACAACAACAACAACAAAAACACAAAACCCTGAGGC
TCAGAAGATAAATATTTGCTGAAGATCACTGTGCTTGTAAGTGGTGGAGCCTGACCATTAACTTC
CCCACGACACTGCTTCAATTTCTATCACTTCATCAACCACACAACCCGAGAACTGTGAGGGGAT
TTAGCAGGCCCAGTCCAGATCTCTTATTGTACAGAGAAGAAATCTGAGGCTCAGAGAGGGGAA
GGGACTAATTTGGACCTTTAACTTCGGATTCTTAACTTTCTTTCTGGTGTTCTAACTATACTGTACT
ACAAGAGGCTGTCTTAGAGCTTTAGTTCAACCAGCTTCAGAGATACTCATTGTTATGGGTTGAAT
TATGTCTCCCCAAAATTCATATGTCAAAGTTCTAACCCCCAGTACCTCAGAATGTGACCATATTT
GGAGATAGGACCTTTACAGAGGTAATCAGGTGAAAATGAGGTTGTTAGGGTGGGCCTTAGTCC
AGTATGGCTGGTGTCCTCATAAAAAGGGGAAATTTGGACACAGATGTACATAGAGGGAAGATG
GTGTGAAGAGACATAAAGAACAGATAGTCATCTATAGCCAAGGCCTGGAACAGATCCTTCCCTA
ACAGCCCTCAGAAGGAAGCAACCTCACTGACACCTTGATTTTGGACTGCTACCCTCTGGCACTG
TGAGACAATAAATTCATGTTGGTTAGGCCACCCAGTTTGTTATGGCAGCCCTAGGAAACCTTTGT
GGATATATCTGTTCGTATACCCATAAAGTCTGCAAGTCAAAAATATCACAGACTTTTGGCAGCCC
TTCATTCAAGCACTGGCTGGGAGGGTCACCCTCCCCGAGGCAAAAGGATAGAGCAAATGATCT
TTCCAGCTTGCTCCCAACCTTGATCTTTGGCCAAGATATTTTTCTTTCACTGGACATTGGCCAACA
TTGGTGTCATAACTTAATTTGACTGTGACCTTCTTGAGTCTTTTATGTCTCTGAGCTTGACCTGTA
GAAGAAGCTCAATAAATGTGGGTAGAGGGGATGAGTGGGAAAATCAGCTCCCAGCCATTCCAG
TTCAAGCCTCTTCAAGCAGAAGAGTCCCACCTGAAAGACCTCATTTTCACTTGATTACTCGATCT
CCAAGCAATAGGTGGGGCAGGAAGCAGGGTATGTAAGGCCTGTCAGTGGATGGAAGTTGAAGT
TGGACACCATCTCCTAAAGTTTAGAGCTGAGCCCAAATTCTGGAAAACACAAGCAAAATACATT
TTATATGACTCAGGATGGGCTAAAGAAGTTCCAGTACAGGAAACTGTTTTCCTGCTGCTTTATTA
ATTTTGCTACAGAGGATAAATGAGATGCAGACTTTCATATCCAAGTCTTCATTAGATGCCCAGTT
AATGCAGCCATGACAAATGGGAATGAAGCCCTTATGAATAAAAAAAAGATCAACCCAGTATAA

AAAAATCATAGACCAGAGAAATGAAAATTTAGAGGAGCTGATAAAATACCCTATCCTTAATTAG
ACTGTCAGCATACACATCAAATCAGCCTTAATGCCAAGATTTATTGGCCTTGGAATTGGGTAAAT
GAAATAAGTGCTTACAAATGAATGGAAAAGAAATTACCTGTATTAAAACGTATTTGTGATGCAG
GCCTGTGCACTGAGGGTTTTAGTCAAACTCAGGCTTGCAGCTTCTTTTGCTCTGCATCCATTTCCA
CCCCATGTTCCCCAGTGGGTTTCAGAGACCCTCTTTGTGGGGTAACAATATGTACCATTGTCAAC
TACCTCTGCTGTTGTCCTGGAAAAATCCTTCTTGGACAGTAAAATATTTTTGTCTTTATTTCACCAT
CTCCAGATTAGATGGGTGTTACTGATTCTTACCTCAGAGAAGAGAATGTCATGGAGACAAAATG
ACCTGCTTAGAGTTCTGCACCCCCCAGCATGGAGAGCAGAGCGCAAAGATTTCTTCCGGACTCC
CACATTCCCCTTGGAATCCAAGAACTGGTCTACTGTCACAACACTTTAGTACCCTCTTGTAAGCC
TGTGGACAAATCCTCTGATGCCTTGATACCTCGGTTTTCCCCCACTATAAATGGGAAGATAAATT
CACTAGAAACAGTACTTACCTTGCAGTGTCATTATGAGAATTTCATGAGACAATGCAATATAAAG
CTCCTAGCACAGTTCCAGGCACCATTGTAAGCACTGAACAAATGAAAGCTGTTGTCATTAGGAT
CTCAAGAAATGACAGCTGAGGCAAATGTGGATTAGACTGCATTCAGCATTCTTAGCTGCAAGCA
ACAGAAGCTGACGCTGGTTTAAAGAATAGGAAAGGAAGTTAATTAAAATGACATTGGGTAGCTT
GTGGAACTGGGAGGACTAGAGAGCTCAGTCTAAGTTCTAGTGACAAAGCTTCAAATCATACATG
AGGAAAATGCCCCTCTGCAGGAGGAACGTAATCCTGCAATCACTGCTTCCCCTGAAGCCAGAT
GCCTCTACTGCTGCCCTTCCCAGCAGTGATGGAGCCTGGATCACATACCTGGCTTTAGTTGTAAA
GGAGGCTGGAGAGAAAATGCTCTGCTCTATGTAACTGTCTTGAAAGTGGCCCACACATAGCAAA
GCCATTCAAAAGCGGGTGAGCAGCATGAAAGCATCCAGAAGGATTCTTGCTTTGCATGAGGGA
GAACTCTTGCTCCCAGAGTGTGAGACCAGCAGGGGCATTTTCCTCATGTGTGATTAGAAGCTTT
GTCACTAGAAGCTTAGACTGAGTAGGACACCTCTGAGTGTGAATGTCCTACCTGAGTTCTGCCT
CATGCAAAGCAAGAATCCCTCTGGAGGGATTGACACCACAGCCTCTTCCACAATGTCTCCAAAC
AACTTTACTGGGCAGCATTCCACTCCTTGACAAACGGGAGAGGTGCATTCTACCTTCTCTCCTGC
ATGAGCCTAGACTAATATGGAATTTGGGAGTGTTGATCATTGTACTTTGTGCTCCTAGAGATAAG
GGTCATCATAAAGCATCTCAGAGTCTCACTGTAGATCCACAGTTCCATTTCTTTATGGATCCCTTA
CCCCTACTTTGCCAAATTCTATGCAGATAGCAATAATAATTCAGACAGGGCTTCCTCACTGAAGG
AGTTTGTGCTCTATATAGTTAATAAGCTAGAGATAATGAAAAAAAAAAAAAACTCAACTTGAACTT
TGGGTAAGAGAAACTTTTCAGACCAACGATCATTACCAATAACAGCTTCCACGCATGACCCTGC
CCGGAGATTGGGGCCTGCACTACTGTTTCCAGGAAACACATTCTTTTGAATTTTTTTATTTTCTTT
GTAGAGATGGGGATCTCACTCTGTTGCCCAGGCTGGTCTCAAACTCCTGGCTTCAAGCAATCCT
CCTGCTTCGGCCTCCCAAACTTCTGGGATTACAGATATGAGCCACCACGGCTGGCTGACAGGCA
CCAAATTCTAGGAGAGGACTTGAATAGAGTAGGCATCTGTCTCTCCCCATTCCTTTCAGGGACA
GAGCTCTCCTGGTTGGGAGAAAGGGAGAGGGAGAGGGAGGAGGATGAATAGTGAAGCACCAA
ACCTCAGAATTAAGGCCTTTTTCTTTTCTTTCTTTCTTTCTTTTTTTTTTTTGAGATGGAGTTTCGCTC
TTGTTGCCCAGGCTGGAGTGCAATGGCACAATCTCGGCTCACCGCAACCTCTGCCTCCCAGGTT
CAAGCGATTCTCCTGCCTCAGCCTCCCGAGTAGCTGGGATTACAGGCATGCGCCACCACACCC
AGTTAACTTTTTTGTATTTTTAGAGACGGGGTTTCTCCATGTTGGTCAAGCTGGTCTCGAACTCCC
GACCTCAGGTGCTCTGCCCGACTTAGCCTCCCAAAGTGCTAGGATTACAGGCGTGAGCCACCAT
GCCCAGCCAGAATCAGGGCCTTTTTAAAACTTCAGTGTCTTGTCTTTACCCTTCATGGTGCCTAG
AAAAGCATCTGGCTCATAGGTGTTCAGTGAATGATTGTTTCATTAGTGAAGAAATTCCCTTTGGA
AATCTCATAGGAAACTTACCCTCTCCAACCCTTCACCTGTGCATTATTCCAGCTCCCAAGGAGGC
AGCAGAAACTGGGGTAATCACAACGGTCAACAGCAGCAAAGGCAGCACTTTCTG

ID|56704|gene_id|ENSG00000224699|transcript_id|ENST00000598454 Homo sapiens lncRNA

CCCCGCGCGGTGACCGTCGAGGTGACCTGCACCTGGCTCCATGGCGGAACCGGCCGGCA
CGGATTATTCCCCTCTCGGGAGAGGTCTGCAGCCGGGCCTCAGTGCTTCAGTTCGTGGCCTGGC
ATTTTGTGTCAGTGTTTTTTAACCTTTGGACCCTAGTTCAACCGACTCCGAGACCTAGGGGACAG
ATCTTAAGTTTTACAATAACCCAGGGGTCAGACAGGGTCTGATCTAGCCTGTGGGGCAAGTACG
GGGAAGACACGGGACCTCTTGGAGGGGGTCAGTAGGCTTGTAGGGGGAAAGACGCACACAGA
GACTAAAGGACGAGATGGAGCAAGGTTCAACCCCCTAGCTGAGTGTTGGCAGTGTCGGGATTT
TAACGAAGACATTGGTGCTTATTATGGTGAGGGGACTCAGGACAGTTTAGGGGAAGAGAGTGG
CATTTGAGTCCTAACTCGTAAGGAAGACTGGGTAGGACTACTTGAAAATAACTTTTAAAAATTAA
TTATAAAGCTGGTAAAAATAGGAAAACATCTATGGCCCCACTGTCCAAAGACGACTTTTAACTTC
TTGGAGAATATCCTTTCAGATAGTTGTCTTTGTAAATGTATACTTTTAATGTTTTTTCGTTTGCCTTT
TTAAAATAAATTTTTTGGGGGACAGGGTCTTGCTCTGTTACCCAGGCTGTAGTGCAGTAGAACGA
TTATAGCTCATTGCAACCCGGACGTCCTGGGTTCAAGCTATCCTCACCTCTCAGCCTCTCGACTG
GCTAGGACCACAGCCTCGAGCCATCATGTCTGGCTAATTTTTACGTTTTTTTGTAGAGATGGGGG
TCTCGCTGTGTTGTCCAGGCTGGTCTTAAACTCCTTGCTTCAACAGATCCTCCCACTCCAGCCTC
CCAAAATGTGATTACAGGCTGAGTCACCACGCCTAGCATGGTTTTTTTTGTTTTGTTTGTTTTGTA
AGCAAAATTGACATACATACTGGAGGTACAATTCTGTAGCTGGCCTTCTTTACAGAGTTTAGAAT
AAATGTCTTTCCATGTCAAGTTTTCACTTACATCCTTTGTAGTAGTTGCAAAATATTCCATTTTATA
GCTGTATCTTAATTGTGGATTAAGGTTGGAGTGCAGTGGCACTATCATGGCTTAGTCCAGCCTCA
GCCTTCTAGGTTCAAGCGATCTTCCCACCTCAGCCTCCCAAGAAGCTGGGACTACAGGGACCCG
CCACCCCACCTAGCTAATTAAAAAAAAAAATTGTAGAGATGGAGTCTCAGTGTGTAGCCCAGTCT
CAAGTGACCCTCCCTCGTTGGCTTCCCAAAGCGCTGAGATTGCAGGTGAGCCACTGTACTTGGA
CTTTTTTTTTTTTTTTTTGCTTTTGTTTGTTTTTTGTTTTAGCTATTACAATGCTGGTTGAATATCCTCAT
AGCTAAATATCTGTGCAGTTTTAAAATTTCCTTATAAGTTACTAGAAATGGAATGACTGGGTTAA
AGGACATGATCATTTTTTAAGACTCTTGATACATACTTACAAATTGCCTCCCGAAAAAGTGGTGC
TAATTTACTCAAATTGGTAAGCCTCACCCCCTCTGTGGATCATTCCTTGCACTAAGTTTCTTCCAA
CTCTGTCACCTCCAGCTTTTTACTCACAGCCTACCTTCCAATCTGGGGCCCAGCTCCCAGATCGG
AAGATACCAGACTTTGGTATTGAACCAAAGTCCAATACACTGCACTGGCATTCAGAGTCCCTTTT
TATTCCGCAGCTGTCATAGTGAAGCTTGCAGCTTCCTTGAAATTCCAGTTTGTTTATTTCTTTATTT
TTTTGAGACAGAGTCTTGCTCTATCGCCCAGGCTGGAGTGCAGTGGCGCCGTCTCAGCTCACTG
CAACCTCTGCCTCCTGGTTCAAGCGATTCTTCTGCCTCAGCCTCCCGAGTAGCTGGGATTACAG
GCATGCACCACCACACGTGGCTAATTTTTGTGCTTTTAGTAGAGATGGGGTTTCACCATGTTGGC
CAGGCTGGTCTCAAACTCCTGACCTCAAGCGATCCGCCCACCTCAGCCTCCCAAAGTGCTGGG
ATTATAGGCATGAGCCATTGCACCCAGCCAAAATTCCAGTTTGAATGTTATAATTCAGGGGTCTT
GGACTCCTGCTCTAGAGAAATTATTCATGTTAAGTTAGGGGTAACACAGTACAGGCCATATAAG
TTTTAGAAAAAGCAGTTTAAAACTTAGCATTAGAAAACATAAGTACTGCCTGGTGAAATGGCAC
GTGCCTATAATCCCAGCTACTCAGGAGGCTGAGGCAGGAGGATCCCTTGAGCCCAGGAGTTCA
AGTCCAGCCTGGGCAATATAGTCTAGTGGCCACATTAAAAAAAGTAAAACAGCCGGGCGCGGT
GGCTCACGCCTGTAATCCCAGCACTTTGGGAGGCCGAGGCGGGTGGATCACGAGGTCAGGAG
ATTGAGACCATCCTGGCTAACACGGTGAAACCCTGTCTCCACTAAAAATACAAAAAATTCTCCG
GGTGTGGTGGTGGGCGCCTGTGGTCCCAGCTACTCAGGAGGCTGAGGCAGGAGAATGGTGTGA
GCCTGGGAGGCGGAGCTTGCAGTGAGCCGAGATCGCGCCACTGCACTCCAGCCTGGGCGACA
GAGCGAGACTCTGTCTCAAAAAAAAAAAAAAAAAAAAAAAAAAAAGTAAAACAGGGCAGGGTG
CAGTGGCTCACGCCTGTAATCCTAGCACTTTGGGAGGCCGAGATGGGTGGATCAGTTGAGGTC
AGGAGTTTGAGACCAGCCTGGCCAACATGGCGAAACCCCATCTCTACTAAAAATACAAAAATTA

GCTGGGTGTGATGGTGCGTGCCTGTAGTCCCAGCTACTCAGGAGGCTGAGGCAGGAGGATCGT
TTGAACTCGGGAGCTGGAGGTTGCAGTGAGCCGAGATTGAGCCACAGCACTCCAGGCTGTGCT
ACAGTGTGAGACTGTCTCAAAAAAAAGTAAAACAGATGAAACTAATTTTAATAACATTTTATTTA
TTCAAACAATATATTCAAAATATTATTTCAACTTGTGATTGATAAAATACTAATGAGATATTTTATA
TTTTTTCTCCAGTACTGTCTTCAAAATCCAGGGTATATTTTTCAGTTAGCAGCACATTTCAATTCA
GACCAGCCGCATTTCAAGTGCTCAGCAGCCACCTGTGGCTAGTGGCCACAGTATTGGCCAGTGT
AGCCCAAGGATATCTCAAATTATCTCAAAGCTAGATCAGTGATTAAAGGATTCTCAGGCCAGGC
ATGGTAGTTTTCAGGCCTGGTATGGTGGTTTACGCCTGTAATCCCCGCACTTTGGGAGGCCTAG
GTGGGCGGATGGCTTGAGCCCAGGAGTTTGAGACCAGCCTAGCCAACGTGGGGAGACCCTGTC
TCTACCAAAGCACAAAAATTAGCTGGGCATGGTGATGCGCACCTGTGGTCTCAAGTACTTTGTG
TGAGGTGGGAGGATCACTTGAGCCCGGGAGGTTGAGGCTGCAGTGAGCTGAGATTGCACAACT
GCACTCCAGCCTGGGTGACAGAATGAGACCCTGTCTCAAAAAAAAAAAAAGGATTCTCCGAAAG
ACTGGCGATTGCTTTTTAAGCTATAAGGAAGGCCTTTGAAGGGAAATCAAGTAGGAAGAGGATG
ATAGCTGGCTCACATGTCCTTGCCACATGGGAGGTGAGAGAGCAGGCTACTCCTTACCACCCCC
GCTTTCTCTGCCCTGCTGCTGTCTTGCCCCTGTGCAAGTAGGCAATGGGAGAGGAATAGTATAT
ATAAATTGGGCAAGTGCTAGGGGCTGGAGTGATGGTTGGGGAAAGTATCTGACCCTTGTTTTCT
ACTTTCTTTAGCTTGTGGGGTATTTAACAATTCTTTGTTCTTAGTTTTGAGTGCTGGCAACCATTTC
TGGGGTGACAGGAAAAGTTCCATGACATCCGTATTAGAGCATTTGTTTCATTATATTGTAATTTTT
TATGTTTACTTTTGAAAAAGTTCATTCTTTTCTCTTTTTAATTCTTATTCTTTTTTTTTTCTTTTCTTTTT
GAGGCAGGGTTTTACTCTATCACCCAGGCTGGAGTGCAGTGGCACAATCTTGGCTCACTGCAAC
CTCTGCCTCCTGAGCTCGTGATTCATCCTCCTGAGTAGCTGGGACTACAGGTGTACACTACCATG
TGTAGCTAAATTTTTTGTATTTTTTGTAGAGATGGGGTTTTGCCATGTTGCCAGGCTGGTCTCGAA
CTCCTGCAATTTGCTCTCCTTGGCCTCACGAAATGCTGGGATTACAGGCATAAGCTACAGCACC
CAGCCCTTACGTTTACTTTTCATTGACAGGAAGAGTGTCTTCATCTCTGCATCCCTAGTGCAACA
GAGCATGACAAAAATTCAACAAATATTTATTGAAGAGTCTTGATGTCTTTTGGGAAAGGAACTCT
ATGGTTACCTTTACTCTAAGGTAAAGAAATTTAGGTCCTGAACTTTAACCACCTTCTCCTCTTCTT
TCTTCCCTCTTTCATAAATGAGCTCTTGGAAAATTAAAATGCCAGGAGGGTCCAGCAGAGAATA
CCCAAGCCCACATGTCCTGATGGGAAGTGGGGGATAAGTAGAGAGGGAAACACAGACCCTGT
AGGCCATCTCACTGCTGCTGGCCCAGGGGACCCTAACTCCTTTTGTTGACTCATCAGAAGTAGA
ATCCTCCTGAGGGTCTGTTTAAGAAGCACAGCTCTGGTAGAAAGGGTAGCTTTCCTATGGGTAC
GGAGGGCTCTGCTACCCCAGGGCTATCCACACTGGCCTGTGAATCACTCTCAAATGCTTAGGGT
CACGGGCTTTCAGTGGAATGAGCCCTCATGTGCCACCAGGGGGCACCAAGGCCACAGAAAACC
CTATTCAGAAGGTTCTGGGGTGAGGGAAAGGAATCCACGGGTTTGTGGAGCTGGCGGAGCATG
GGTGAGGACAGCATTATGCTAGGTTGGTGGCCTTGAAGTCAGATGTAAATTGGGATCCTGGCTT
GGCCACTGACTATCCATGGGAACTTTGAATCTCAGGGGGCTCATTTGTGATGTGGGGGTACCAT
TATCTGCCTCATCAGGTTGTGGGGAGGATTATATGAGATACCTTATGTAAGAGGGGCTGGTGCA
TTGTGGAAGTCAACAAGTAAGTTTTGTTAAATTTGAGTAAGAAGACCTGTTTGTTTCCTCAAGTTG
CTTATAATCTAGGTGAGGACACATCTGAGAGAATAACTAATAGTTCAGTGAAGGTAAGAGCCAA
ATGATTGGCACAGACCTCAAGTGGTCTGGCATTTACGGGAGAGGGGGATTGTGAAACAGGAAT
TGAAAGAAATTTAAAAATGCATAAGCAAAAACTCAGTTTATGTTTTAAAAAAACCAATTCCCCCT
GAGAAAGAGAAAGAGGTGGAGTCCTTTAAAAATTATCTGCTTGTTTTTCTGTCTGTGGCTAGTGA
GCCTTATCTCTCCCTTTCCCAGGCATTGTGAAGACCCTGTTTCTCTAGCTGTGCAGCTGCAAGGT
CACTAGGCAGATAAACTCAAGCCATAAAACATGTTTTTCCTTGAAAAGTAAGAAATAATGTAAT
GCATGTGTCAAATGAATAACTATTTCTCGCTTCTGTAATATGCTTCCCTTCGCACAGATCTCCCCC

TGCCCCATGAAATGCTTTAAAGGTAACTTAACTCTTTGTTCGGGGCTCAGTCCTTTGGATGTTAAT
CTGACTGGCTTGGTGCACCTAAATAATAAATAACATATATCCTCCTCAACCCCTCAGTCTCTCTG
ATTCCTAAATTATCCCGCTGTAATTGCAGGGCTAGATTTGCCGACCTGCTTTATAAAGCTATGTTA
AATCAGTATATTTTATTCTTTTTATTAGACTTTTCTATAGCATAAAAATGATAAAATACAGCAAGA
AAAAAAGTCACCTGAAACTCTAGTCCTAACCCTAATATGAATGTGAAGGAGCTGGGCTAGAATG
GAATAGGGTAGAATTTGCATAAGGTGGAAATGGGGACAGGCCTGTCACAAGGATCACTAGAGT
CTTGCTGGCAAAAGCATCTTACTGCATTCATGCTGCAGCACAGGACCCAGATGCAGAACAGGTT
GGGGACACAGCCCTGCCTTCTAATAGTGGACACCATCTGAGAGCTGAGCCATAGATTCAAAGC
CTAACTCCTAATAGCGATTGGTAGTAATATCATCAGCAGTTCATATACATGGAGTGACAACAGC
GTGCCAGGCAATTCAAAGGGTCTCTCGGTCCTCACAGCATTTTTGAAGTAGCTAACATCACTATC
CCCCTTTTACAGTTAAAAACAACAACAACAACAACAAAAACACAAAACCCTGAGGCTCAGAAG
ATAAATATTTGCTGAAGATCACTGTGCTTGTAAGTGGTGGAGCCTGACCATTAACTTCCCCACGA
CACTGCTTCAATTTCTATCACTTCATCAACCACACAACCCGAGAACTGTGAGGGGATTTAGCAG
GCCCAGTCCAGATCTCTTATTGTACAGAGAAGAAATCTGAGGCTCAGAGAGGGGAAGGGACTA
ATTTGGACCTTTAACTTCGGATTCTTAACTTTCTTTCTGGTGTTCTAACTATACTGTACTACAAGAG
GCTGTCTTAGAGCTTTAGTTCAACCAGCTTCAGAGATACTCATTGTTATGGGTTGAATTATGTCTC
CCCAAAATTCATATGTCAAAGTTCTAACCCCCAGTACCTCAGAATGTGACCATATTTGGAGATAG
GACCTTTACAGAGGTAATCAGGTGAAAATGAGGTTGTTAGGGTGGGCCTTAGTCCAGTATGGCT
GGTGTCCTCATAAAAAGGGGAAATTTGGACACAGATGTACATAGAGGGAAGATGGTGTGAAGA
GACATAAAGAACAGATAGTCATCTATAGCCAAGGCCTGGAACAGATCCTTCCCTAACAGCCCTC
AGAAGGAAGCAACCTCACTGACACCTTGATTTTGGACTGCTACCCTCTGGCACTGTGAGACAAT
AAATTCATGTTGGTTAGGCCACCCAGTTTGTTATGGCAGCCCTAGGAAACCTTTGTGGATATATC
TGTTCGTATACCCATAAAGTCTGCAAGTCAAAAATATCACAGACTTTTGGCAGCCCTTCATTCAA
GCACTGGCTGGGAGGGTCACCCTCCCCGAGGCAAAAGGATAGAGCAAATGATCTTTCCAGCTT
GCTCCCAACCTTGATCTTTGGCCAAGATATTTTTCTTTCACTGGACATTGGCCAACATTGGTGTCA
TAACTTAATTTGACTGTGACCTTCTTGAGTCTTTTATGTCTCTGAGCTTGACCTGTAGAAGAAGCT
CAATAAATGTGGGTAGAGGGGATGAGTGGGAAAATCAGCTCCCAGCCATTCCAGTTCAAGCCT
CTTCAAGCAGAAGAGTCCCACCTGAAAGACCTCATTTTCACTTGATTACTCGATCTCCAAGCAAT
AGGTGGGGCAGGAAGCAGGGTATGTAAGGCCTGTCAGTGGATGGAAGTTGAAGTTGGACACC
ATCTCCTAAAGTTTAGAGCTGAGCCCAAATTCTGGAAAACACAAGCAAAATACATTTTATATGAC
TCAGGATGGGCTAAAGAAGTTCCAGTACAGGAAACTGTTTTCCTGCTGCTTTATTAATTTTGCTA
CAGAGGATAAATGAGATGCAGACTTTCATATCCAAGTCTTCATTAGATGCCCAGTTAATGCAGC
CATGACAAATGGGAATGAAGCCCTTATGAATAAAAAAAAGATCAACCCAGTATAAAAAAATCAT
AGACCAGAGAAATGAAAATTTAGAGGAGCTGATAAAATACCCTATCCTTAATTAGACTGTCAGC
ATACACATCAAATCAGCCTTAATGCCAAGATTTATTGGCCTTGGAATTGGGTAAATGAAATAAGT
GCTTACAAATGAATGGAAAAGAAATTACCTGTATTAAAACGTATTTGTGATGCAGGCCTGTGCA
CTGAGGGTTTTAGTCAAACTCAGGCTTGCAGCTTCTTTTGCTCTGCATCCATTTCCACCCCATGTT
CCCCAGTGGGTTTCAGAGACCCTCTTTGTGGGGTAACAATATGTACCATTGTCAACTACCTCTGC
TGTTGTCCTGGAAAAATCCTTCTTGGACAGTAAAATATTTTTGTCTTTATTTCACCATCTCCAGATT
AGATGGGTGTTACTGATTCTTACCTCAGAGAAGAGAATGTCATGGAGACAAAATGACCTGCTTA
GAGTTCTGCACCCCCCAGCATGGAGAGCAGAGCGCAAAGATTTCTTCCGGACTCCCACATTCCC
CTTGGAATCCAAGAACTGGTCTACTGTCACAACACTTTAGTACCCTCTTGTAAGCCTGTGGACAA
ATCCTCTGATGCCTTGATACCTCGGTTTTCCCCCACTATAAATGGGAAGATAAATTCACTAGAAA
CAGTACTTACCTTGCAGTGTCATTATGAGAATTTCATGAGACAATGCAATATAAAGCTCCTAGCA

CAGTTCCAGGCACCATTGTAAGCACTGAACAAATGAAAGCTGTTGTCATTAGGATCTCAAGAAA
TGACAGCTGAGGCAAATGTGGATTAGACTGCATTCAGCATTCTTAGCTGCAAGCAACAGAAGCT
GACGCTGGTTTAAAGAATAGGAAAGGAAGTTAATTAAAATGACATTGGGTAGCTTGTGGAACTG
GGAGGACTAGAGAGCTCAGTCTAAGTTCTAGTGACAAAGCTTCAAATCATACATGAGGAAAATG
CCCCTCTGCAGGAGGAACGTAATCCTGCAATCACTGCTTCCCCTGAAGCCAGATGCCTCTACTG
CTGCCCTTCCCAGCAGTGATGGAGCCTGGATCACATACCTGGCTTTAGTTGTAAAGGAGGCTGG
AGAGAAAATGCTCTGCTCTATGTAACTGTCTTGAAAGTGGCCCACACATAGCAAAGCCATTCAA
AAGCGGGTGAGCAGCATGAAAGCATCCAGAAGGATTCTTGCTTTGCATGAGGGAGAACTCTTG
CTCCCAGAGTGTGAGACCAGCAGGGGCATTTTCCTCATGTGTGATTAGAAGCTTTGTCACTAGA
AGCTTAGACTGAGTAGGACACCTCTGAGTGTGAATGTCCTACCTGAGTTCTGCCTCATGCAAAG
CAAGAATCCCTCTGGAGGGATTGACACCACAGCCTCTTCCACAATGTCTCCAAACAACTTTACT
GGGCAGCATTCCACTCCTTGACAAACGGGAGAGGTGCATTCTACCTTCTCTCCTGCATGAGCCT
AGACTAATATGGAATTTGGGAGTGTTGATCATTGTACTTTGTGCTCCTAGAGATAAGGGTCATCA
TAAAGCATCTCAGAGTCTCACTGTAGATCCACAGTTCCATTTCTTTATGGATCCCTTACCCCTACT
TTGCCAAATTCTATGCAGATAGCAATAATAATTCAGACAGGGCTTCCTCACTGAAGGAGTTTGTG
CTCTATATAGTTAATAAGCTAGAGATAATGAAAAAAAAAAAAAACTCAACTTGAACTTTGGGTAA
GAGAAACTTTTCAGACCAACGATCATTACCAATAACAGCTTCCACGCATGACCCTGCCCGGAGA
TTGGGGCCTGCACTACTGTTTCCAGGAAACACATTCTTTTGAATTTTTTTATTTTCTTTGTAGAGAT
GGGGATCTCACTCTGTTGCCCAGGCTGGTCTCAAACTCCTGGCTTCAAGCAATCCTCCTGCTTCG
GCCTCCCAAACTTCTGGGATTACAGATATGAGCCACCACGGCTGGCTGACAGGCACCAAATTCT
AGGAGAGGACTTGAATAGAGTAGGCATCTGTCTCTCCCCATTCCTTTCAGGGACAGAGCTCTCC
TGGTTGGGAGAAAGGGAGAGGGAGAGGGAGGAGGATGAATAGTGAAGCACCAAACCTCAGA
ATTAAGGCCTTTTTCTTTTCTTTCTTTCTTTCTTTTTTTTTTTTGAGATGGAGTTTCGCTCTTGTTGCC
CAGGCTGGAGTGCAATGGCACAATCTCGGCTCACCGCAACCTCTGCCTCCCAGGTTCAAGCGA
TTCTCCTGCCTCAGCCTCCCGAGTAGCTGGGATTACAGGCATGCGCCACCACACCCAGTTAACT
TTTTTGTATTTTTAGAGACGGGGTTTCTCCATGTTGGTCAAGCTGGTCTCGAACTCCCGACCTCAG
GTGCTCTGCCCGACTTAGCCTCCCAAAGTGCTAGGATTACAGGCGTGAGCCACCATGCCCAGC
CAGAATCAGGGCCTTTTTAAAACTTCAGTGTCTTGTCTTTACCCTTCATGGTGCCTAGAAAAGCA
TCTGGCTCATAGGTGTTCAGTGAATGATTGTTTCATTAGTGAAGAAATTCCCTTTGGAAATCTCAT
AGGAAACTTACCCTCTCCAACCCTTCACCTGTGCATTATTCCAGCTCCCAAGGAGGCAGCAGAA
ACTGGGGTAATCACAACGGTCAACAGCAGCAAAGGCAGCACTTTCTGATTTACTTGACTGCTAT
GGATGCCACCTCTCTCCACGCCTCAGTTTAAT

ID|56080|gene_id|ENSG00000237436|transcript_id|ENST00000442889 Homo sapiens lncRNA

CGCGGGGGGCCTCGGGGCTGAGGTCCGCAGGGGTGCCGCGCCCTGGAGTCCGACTGCCT
AGGAGTCCGGGCGTCGCTGCCGGCCGTGGGGGAGGGGCGAGCCTCCCGCCAGGCCCCGCCCC
CTCGGCGCCGCGTCCCCACGGCCCCGGCGCCGCCCGCCTGCGCCCGCCTCCTGTGCCCGCCCC
CGGTGAGCTCCGTTGTCCCCTTTTCTGGCGGCTTAAAATACCGAAACTTTATTTTGGTCACGTTG
CCCATGTGACCCGCGGAGCGGATGACTCATGGTTGCCTGTCCCAGGATGAGGCCGCCGGCCGA
AAGCAGAGAAGCGCCAGCCCCGGCCGCCCGGGTGGAGCGCTGGGCAGCCGAGTTTCCCACCC
TCCTCAATCCGGAGAGTCCGCGCGGGGCTTTTCCTAATAAATAGCCAGGCACCCGCTGCCCTCG
CGCTGCGTACGGGAGCACGTGCCCCCCGGGAGGTGGGCGCCCGCCAGGTGCCCCGGGAACGA
GCCTAGGAACCGGGTCCGCCGAGTGCGGCTCTCGGGGCGGCTAGGCGTGCGGGTGGCTGGGC
AGCGCTCCCAGGGACCGGCCTCTCTCCGGAGAAAGTAAGGAATTCTGCTCCTGGGCAGGTAAT

ACCGGAAACAAAAGGAGAGGCTGCAATCGCAAATTGTTTAAAAAGAAGAAAAAGGAAAGAAA
AGTTGAATGTAAGAGCTCCAGCCCCTCAATGTGATCTTGTACGGTTTGCAGTAAGAACATTTCAT
TCTTCCGCATTTTATTTTTCCTTTTGCTACTGTTGGTGACAACTAGCAAAATAAGTGAAAAATCCT
CGTTCAAGTGTTCTGGCATTAAGATTGACTGTCACACTGAATTCTGTGCCAATCCTAACAACATG
TGTTCTTTTTCTGGTCTTCACAAGTAAGAATGTCTTAGGTTTTCCAGAAATAAAGATGTCCCAAAA
GAA

ID|56088|gene_id|ENSG00000237429|transcript_id|ENST00000443579 Homo sapiens lncRNA

CCCAGGAGCTGTGTGTGTCTGTGTGTCTGTCTACCTGGCAAGGAGATATGGCCCCCATCAG
ACTGGAGCTCCACAGAGCAGGTCTGTGCCTCTTCTGCCCATCCAGGGACTCCAGGGGACAGAG
CTCTGTCTCCTTCAGGCCAGGGGCTCCCAGGGAAAAGGCTGGTGCTGTCTCCTCCCTAAGCCTG
AGGGTTACGGATGCAGCCCAGGCCAGACAGGGCTTCACTGGGCTCTGGTTCTGGGGCCACTAG
GGCCCTCTGACCCCAGGGCAGATCTGCTGTGATGATGAGCTTGTGGGTAGGGAGGAGTCTCAG
CTCTGCCCACACCACACCCTAGGCAGGCACGTGTTTGTGCTATGGGTACTTGTGTGGGTGCAGG
TGGGACATGTCAGGGCCCGTGGTCCTCTGGGGACCTGTGCCTCTCCACATGTCGGGCTCCTGCC
TGCATCAGTGGTGTCAGCGACTGCCTGCCTTGGGCACTGTGGTCAATCGGTACAAGTTGAGTGT
ATTGTATGTTTCTCTGTGTTCTTTTTTTCTTTGGAGGGGGGGACGGAGTCTCGCTCTGACACCTCC
CGAGTAGCTGGGACTACAGGCGTGTGCCACCACACCCGGCTAATTTTTTGTATTTTTAGTAGCAA
GAGGGTTTCATCATGTTAGCCAGGATGGTCTCGATCTCCTGACCTCAGGTGATCTGCCCGCCTC
GGCCTCCCAAAGTGCTGGGATTACAGGCGTGAGCCACCACACCCGACCTTCTCTGTGTTCTTGT
GTGTGCACATGGATGACTGTGTATGTGGGTTTCTGCCTTTCATTGTGCAGGCCTGCAGATACTAG
CAGGGATGGACTCTGCATTCATGTGGGAGTTGCTGATGTCACCATGGGGGGTTTCTGTTTGTGT
GTCAATGTGCTCGGGTGTGTGTCTCTGTCAGAAGGTCAGTGCCTGTTTGTTCTGTGTATGGAGGT
GTATGTAGGTGCATCTGTGTGTCAGTGTTTCAATTACCATGTTTCTGTGTGTCTGTGTGTCTGTCTT
CCTGGTTGTCATTATGTCTGCGGTACCTCCGTGCCTGTGTTGCGTGCCTAGGTCAGTTTGATTTTG
CAGGTCTGTAGGGCCACGTGTCCCTCTGTATGTTGGTGTTTTCCATGTGGGTTGGAATCTGCCTC
TGCTTTTCCTAATCTCCCACCATTTCTGTGTGGAAGGCCTCAGGCATTGTGTGTGTGTGTGTGTGT
GTGTGTGTGTGTGTATGTGTGTGTGTGTTCAGCAACTTTCCCCAGCCCCACAGCCTGCAACCC
TGAGATCTGCTCTCCCACTCTTTCTCCGTTGGACTTCAGGTGGGAGCTGAAGCAAAGAGGGCAG
GCAGGCAGGGAGCCCACGACAGCCGGGGGCCGGGGCCAGGCTGCCACGAGGGCCCTACCTC
AGCTTTCCTCCAAGCAAGCGCTAAGCCAGAGCCTCGGTTACATGTAAACACCAGCTGTGTCAAG
ACGGCGCAGCTTCTTCTAGGTCAACAGACAGCTGTGGGGTTGCTAGGCAACCGCCTGGCTCCC
CCCTCCATTAGGCCCCCTCCCACTGCCTCTACCTGCAAACCCTCTGCTGGCTCCCCCCACGCCA
GGATAGGCCTCACCCCTCCGCGCCAATTAACACAGCGGGACTGAGGGGGTAGGGGGTCAGGG
CAGCTCCGGGACTGGAATCAAGGTATGAGGACGTATTAACCCTTCACGTGCCACCACCCCCTCC
ACCTAGTCCTGGGGCACTTCACACACCCCTAACTTCACCAGTCACTTTAGGGCCTGGAGTGATG
AGGAGCACGTCCTGAGGGATGGGGACCCTCAGTGCAGCCACAACCAGGACTGGAGAGACGCT
GGAGGCAGATGGCGCAGGGCTTGGTCAAGGCCTCTCCCCTCCCCAGCCAAGCCTGGGGGAAG
GAAGGGTTAAATTCCCCCTTTATTTCCCCTGTCCCGCCCCCTCCCAGGACAACCACCCCATTTCC
AGGGCAGCACCTGCGCTCCCAGCTGCCGTGCCCGCTTGGTGCCTGAGTCCGGGCCAGGCAGCC
CCCGCAGCGCCCAGGCCCTCGCCCTCGCCGTCGCCCTCGCCCTCGCCGGCCCCAGCGCCGCCC
TTTGCCCGGCCTGGGACTTCTCTGCCGGGAAAGGGGAGGCGAGGGCCCCTAGGCCTTAGGCAA
GGGGCTCAGGCCCACCGGGTGGGGTGCGTGGGCCAGGGCCGCCAGCCCCGGGGTGACTTGGC
TGGAGCCGGAGGAAGGAGTTGGAGGAGGGAGCCCGGCCGGCAGGGGGGCAGCTGTGGGGGC

AGCGCCCGTGGCCGCCCCATTAGTATGCGGCCGCGCTCTGCGCGCCATGGCAACAGCTCCCCC
CGCGCAGCGCTAGGCGGGCAGCCCCGGGCGCAGCGCGCACCTCTCCTCCCACAGGTGGCGGC
GCGCACGGGCACGCACGCACACTCGGCTCGGGCGCCTAGGGGAACGCCGGGGGCGCCAGGT
GCGCTGGGGCGTCCTGCCGCCCGTGCAGCCGCCCATGGGCGTGTCCCCAGGTGCTCCAGAGTG
AGGCCCGAGGGAGGAGTGTGTCCTTGAGGACGTTCTGCCCGCGCCTGGGTGTGCCCCTCAGTG
GGTCCCTCGGCCGGCCTGCTGTGCTTTTATGTCGGTCTCCGATTGTCCTGGAGGGTCTGTGCCTG
TCTGTGCTCATCTGCCTCCTTAGCCCCGAGTGCCTGAGTGACTGCCAGTTAACCGAGCGTACCT
GTGCCTCTGTATCTGTGTCTGTACGAGCCCAAGGGGTGTGTGTGTGTCCGTGTGCGCGTCCATG
TCTCTGGGTTTTCCCCAGGAGGAACGCCCTGGGTGTGTCGGCGACTCTGGATGTCCGTGTGTGC
CAGAATGAGTACCTGTGTGTGCGCCTTTGTGTCTGTGTGTGGCCCGTGGATATCTGTCTGGCATC
CCTGAGTGTTTCGGGTGTGGCTGTGTGTGGACTTTTGTACGTCTATGTCCCTGTAAGTGCCCTCA
GGAGCCCCTCTCTCCATGCATATCACTGTGTGTTCCTGTGAGTGCCCCACGTCTCCTGGAGTGTG
GCTGTGTGTCCAGGGGCTGCTGGCTCTTTGCCCTCCTCGGCTTCACTCCCTGCTGCCTGAGTGA
GCCGACACCTGCTCCCTGAGCACTGGGCCCCCTTCCCCCCACCTGGCAAGGACAGGGAAGGAC
ACGGAGGCAGAGGCAGAGGCAGACATGGAGGCAGAGACAGAGACATGGAGGGAGACACAGC
CAAGTAGGCTGGAGAGGTGGAGATCCAGTCACGTGATCAGCCTCAAAGCCCAAGACGGAGAA
GAGAGGCACAGAGATGCCAAGATCCGGACATTAGAGAGAGGGAGACTCAGAGGCACACAGAG
ACCCAGAGCACGGCGTCCGGAACTGCAGAAACAGAAGAGATCAAGGATGCGGCTCCTCAGCC
TCCCTTCCTCTCTCCCTCCCTCCCACTCTAGAGTTTCCAGCCCCAGCTGTGCCTCCCCCACATAC
CCAAAGCAGAGTTCTCCTGGCCCTGGGACTGGAAGCCAGCCTCCTCCTCACAGCTGGCTGTGTG
TGTGTGGACACGTGTACATGCATGTGCGCATCTGTGTGACGCACTGGGCTAGTGCCTACCTTGT
ACACAGCCCCCCTCCATTCAAAACCTCCTCTCCCCACCACCACCGTCCACATTTTCACAGGCCC
AAATCATGCACACCTTTACACACATGCCACACAGCCAGCCACACTCATTCAAGCCCAGAAACAT
GCCCTGGGCCTCCCTCTCTCAATCTCATCCACACAGGGCCAGAAGACCCAGCATACACAGCCA
CACCAGAAAGTCTCTCCCCGTCCTAAGGTGTGAGGGTCAGATGTCCACCCCTCTTTCCCCCTCTC
AGCAACCCTCGAGGGGCTCGAACCCCTGCTACTCACAGGGAAAAACGCATGACTAAATTTGTC
AATTCCATCAGATTAGACACTCAAAGGCAGGGCCCCTGACTGTCCTGCTCACTGTTGACTCCCC
AAGTGCCTTCGACGGTGCCTGGAAGGTGCTTAAGAAATGTTTGAATAAATATGTTCATACCAGG
TCTCTGGTGTGGGGGAGAACCCATAGTAACTGACAGCCACTCAGAGTGAAAAACGCTAATGGA
TATCACAGTGTACACACACCTAGGACAGTACACTAAATACAGGTGTGTGTGACACTCAGCTCAA
CAGCTCAATTAGAGGTTATCCAGTGGGCTGTGTGTTGGAACCACCCAAGAAGCTTTCAAAAATC
CCCATGCCCAGGCCCCACCCCAGACCCATAAAATCGGAATATTTTGGGGTAGGACTTGGGCAT
CGGCATTTTGAAGCTCTCCAGGCAATTCCTTTCTAGGATCTAGAACCCCTTATGTACATGAATAG
ACTAACAAGTGTTGACACAGGGGTTATAGCCAGGGATGGGAGTAGAGAGGATTTTCACTTTTTT
TGTTGTTTGTTTTTTTGTTTTTGAGATGGAGTCTGGATCTGTCACGCAGGCTGGAGTGCAGTCGCG
CGATCTCAGCTCACTGCAACCTATGCCTCCCAGGTCAAGCGATTCTCCTGCCTCGGCCTCCCAA
GGAACTGGAACCACAGGCGCGCGCCACTGCGCCCAAGGATTTTCACTTTTAACTGTACAGTGAA
GCACAGACTTCTGAGCCAGACTGCTTGGTCTGAACTCTGGTGTCACCACTTAGTAAGTTCTGGTT
TGAATGTACTCACTGAGTGGTCTTCATAAATTACCTCTCTGC
ID|56110|gene_id|ENSG00000237950|transcript_id|ENST00000446167 Homo sapiens lncRNA

GGGACCACCCAATAAGCAACCGGGAAACCACATACCATGGACAGTGCTCGGGGAATTTC
GTAAGGTTTTTGGAAGTAACTTTATGTGTAACTAGAGTAAGAGTAGCTTACATTTAAGTGCTAGG
AGCAGGACTTTGCTTACCTTAGGTTCAGTTTTCTTAATAGCTCTGAGGTGGATATTTTTATTCATC

ATTTTCAGATGAGATGAGGAAATCGAGCTTGGGGAAAGTTAGGCTTATCTTGCCCCTGGCACAC
AGACAGGGTGGCAGACCATGCCATAGATCTGGTCTGCCACAGGCCCCGGTAGTTCCTGCCTGT
CACCCCTCCAGTTGTATTTTATTGTTTATTAACTCCTGCCAGCTACCCTAGCAGGGAAGAGGAAA
AAATTAAAAGTGCAAAGAGCTCCGTAGTTTTGTTCAAATGGAGGAAGCTGGCATTGGAAGCTGG
CATTGGCAGCAGCTGCTGAGGCCTGAGAAGGTCCATGAAGGCTTCCCTGAGAAGAGAAACTTG
ATCTGAGCTTGAAGAATAGAGAGCCTGGGAAGGAGAGGGGCCCATTTAGCTGGTAAGCAAAAT
CCAGTAGGACTCTAAGTCATTCAGCAAGTCTTCATCAATAAAGAATAGCAAGTAGGCCGGGCGC
GGTGGCTCACGCCTGTAATCCCAGCACTATGGGAGGCTGAGGCGGGCGGATCACGAGGTCAG
GAGATCGAGACCATCCTGGCTAACACGGTGAAACCCCGTCTCTACTAAAAATACAAAAAATTAG
CCGGGCATGGTGGCAGGCACCTGTAGTCCCAGCTATTCGGGAGGCTGAGGCAGGAGAATGGCT
TGAACACGGGAGGTGGAGCTTGCAGTGAGCTGAGATCACGCCACTGCACTCCAGCCTGGGCGA
CAGAGTGAGACTCCGTCTAAAAAAAAAAAAGAAAAGAAAAGAAAGAATAGTAAGTAATTTATA
TTCATTTTAAGAGAATATATGTGGGGTGGGGAAGGGGAAGGGTATGAGTAACAGAGATGAGGT
TGGAAAGACAAGTTGGTGGCAATAGATTCTTGTAGGAGTTTGGACATTATCCTAAGTGCAATAT
GAAACCATGATGGGGTTTCAAGTGAGCAATGATGATTACATTTGCATTTTAGAAAAATCTGCAAC
ATGAAAGACAAATTGAAGGAAGAAAGAGAGGCAGGAGACATTTGGAAGGAGGAAATAGAAAG
CCAAAATAAAGTAACTGATTCCAGGCCTGTGCCTAGGGTGTGAAATACCTTGGGCCTGGTGCTG
GCTGGCGGTTTCACTGGTGGAGGTGGTGGTGGCAGGGGAGATGGTGTTGGGAGTGGGGTTTA
AATGCACAAATTCTGGACTTAGTCCTGAGTTCAAAACCTGTTTATGCCTCTTTCTAGCAATGAGA
CCTTAGCTTCTTCTGAAACTCATTTTAGTTAAGGGGTTTTAGGGGTGGGGGGCTCAGCCCAGAG
ACCACAACTTAGTCAGTATGTGTTTTCTTCCTGCTTCCCATCCAGGGCTCCTCCTCATCCCTGGA
GGTTCCAGGGATCTGGCAGAGAAAGAAGCCCAGTCTCACAGCAATGACTCCTGAACATCCTTTG
CAAACACTATTTAGGACAGATGGACTGAAGTAGGCCCTGGGGAGAGTTGGGGGCATTTCCGGA
GGCCAGACCAAAGGTGATCTCCAGGAGATTTGGATGTTAGTGCAGGATAGCTCAGAGCCAAGT
GGCAGATAAGTCTTTGGGAGCCAGAAGGGCTCTTTCTTCTCTAAGGAACAAGTGTAGACCTATA
GGGGCAAGAACCTGTGGCAACCCTGGGAGAGGTCTGCAGATTCAGGAGGGAACATCCCAAAA
CTCAGCAGAGGAGGAGAGGGAGCTTTTGTTGGTCACCTGGAACCACTACCAATCCATGTTCATG
TCAAATTAAATGATCACTTTGAAGTTT

ID|55926|gene_id|ENSG00000237950|transcript_id|ENST00000412378 Homo sapiens lncRNA

CAGACCGGCGCGGGGCGCTGCGGCCCAACTCCTTAGTAGGACGACGTGACTCGAGGGGC
CGGAGGACGGAGGTGGGAGCCCGGGCTAGAGGGTGGAGGCTGGGGAGGTTGCCTGGTCACTC
TGGCGCTCCGCTGCCGGGGGCATCCTCCGGCCCAGCAAAGGTGCAGCCTGGGAAGAAGGCGT
CAGCACCACCCAGAGAAGAGGAGAGGGGACTTGGCACGCCTCAGGAAGGAAAAAAGGAAGG
CTCTCGGGACCACCCAATAAGCAACCGGGAAACCACATACCATGGACAGTGCTCGGGGAATTT
CGTAAGGTTTTTGGAAGTAACTTTATGTGTAACTAGAGTAAGAGTAGCTTACATTTAAGTGCTAG
GAGCAGGACTTTGCTTACCTTAGGTTCAGTTTTCTTAATAGCTCTGAGGTGGATATTTTTATTCAT
CATTTTCAGATGAGATGAGGAAATCGAGCTTGGGGAAAGTTAGGCTTATCTTGCCCCTGGCACA
CAGACAGGGTGGCAGACCATGCCATAGATCTGGTCTGCCACAGGCCCCGGTAGTTCCTGCCTG
TCACCCCTCCAGTTGTATTTTATTGTTTATTAACTCCTGCCAGCTACCCTAGCAGGGAAGAGGAA
AAAATTAAAAGTGCAAAGAGCTCCGTAGTTTTGTTCAAATGGAGGAAGCTGGCATTGGAAGCTG
GCATTGGCAGCAGCTGCTGAGGCCTGAGAAGGTCCATGAAGGCTTCCCTGAGAAGAGAAACTT
GATCTGAGCTTGAAGAATAGAGAGCCTGGGAAGGAGAGGGGCCCATTTAGCTGGTAAGCAAAA
TCCAGTAGGACTCTAAGTCATTCAGCAAGTCTTCATCAATAAAGAATAGCAAGTAGGCCGGGCG

CGGTGGCTCACGCCTGTAATCCCAGCACTATGGGAGGCTGAGGCGGGCGGATCACGAGGTCA
GGAGATCGAGACCATCCTGGCTAACACGGTGAAACCCCGTCTCTACTAAAAATACAAAAAATTA
GCCGGGCATGGTGGCAGGCACCTGTAGTCCCAGCTATTCGGGAGGCTGAGGCAGGAGAATGG
CTTGAACACGGGAGGTGGAGCTTGCAGTGAGCTGAGATCACGCCACTGCACTCCAGCCTGGGC
GACAGAGTGAGACTCCGTCTAAAAAAAAAAAAAGAAAAGAAAAGAAAGAATAGTAAGTAATTTA
TATTCATTTTAAGAGAATATATGTGGGGTGGGGAAGGGGAAGGGTATGAGTAACAGAGATGAG
GTTGGAAAGACAAGTTGGTGGCAATAGATTCTTGTAGGAGTTTGGACATTATCCTAAGTGCAAT
ATGAAACCATGATGGGGTTTCAAGTGAGCAATGATGATTACATTTGCATTTTAGAAAAATCTGCA
ACATGAAAGACAAATTGAAGGAAGAAAGAGAGGCAGGAGACATTTGGAAGGAGGAAATAGAA
AGCCAAAATAAAGTAACTGATTCCAGGCCTGTGCCTAGGGTGTGAAATACCTTGGGCCTGGTGC
TGGCTGGCGGTTTCACTGGTGGAGGTGGTGGTGGGCAGGGGAGATGGTGTTGGGAGTGGGGTT
TAAATGCACAAATTCTGGACTTAGTCCTGAGTTCAAAACCTGTTTATGCCTCTTTCTAGCAATGA
GACCTTAGCTTCTTCTGAAACTCATTTTAGTTAAGGGGTTTTAGGGGTGGGGGGCTCAGCCCAG
AGACCACAACTTAGTCAGTATGTGTTTTCTTCCTGCTTCCCATCCAGGGCTCCTCCTCATCCCTG
GAGGTTCCAGGGATCTGGCAGAGAAAGAAGCCCAGTCTCACAGCAATGACTCCTGAACATCCT
TTGCAAACACTATTTAGGACAGATGGACTGAAGTAGGCCCTGGGGAGAGTTGGGGGCATTTCC
GGAGGCCAGACCAAAGGTGATCTCCAGGAGATTTGGATGTTAGTGCAGGATAGCTCAGAGCCA
AGTGGCAGATAAGTCTTTGGGAGCCAGAAGGGCTCTTTCTTCTCTAAGGAACAAGTGTAGACCT
ATAGGGGCAAGAACCTGTGGCAACCCTGGGAGAGGTCTGCAGATTCAGGAGGGAACATCCCA
AAACTCAGCAGAGGAGGAGAGGGAGCTTTTGTTGGTCACCTGGAACCACTACCAATCCATGTTC
ATGTCAAATTAAATGATCACTTTGAA

ID|56083|gene_id|ENSG00000237950|transcript_id|ENST00000445226 Homo sapiens lncRNA

CAACCGGGAAACCACATACCATGGACAGTGCTCGGGGAATTTCGTAAGGTTTTTGGAAGT
AACTTTATGTGTAACTAGAGTAAGAGTAGCTTACATTTAAGTGCTAGGAGCAGGACTTTGCTTAC
CTTAGGTTCAGTTTTCTTAATAGCTCTGAGGTGGATATTTTTATTCATCATTTTCAGATGAGATGA
GGAAATCGAGCTTGGGGAAAGTTAGGCTTATCTTGCCCCTGGCACACAGACAGGGTGGCAGAC
CATGCCATAGATCTGGTCTGCCACAGGCCCCGGTAGTTCCTGCCTGTCACCCCTCCAGTTGTATT
TTATTGTTTATTAACTCCTGCCAGCTACCCTAGCAGGGAAGAGGAAAAAATTAAAAGTGCAAAG
AGCTCCGTAGTTTTGTTCAAATGGAGGAAGCTGGCATTGGAAGCTGGCATTGGCAGCAGCTGCT
GAGGCCTGAGAAGGTCCATGAAGGCTTCCCTGAGAAGAGAAACTTGATCTGAGCTTGAAGAAT
AGAGAGCCTGGGAAGGAGAGGGGCCCATTTAGCTGGTAAGCAAAATCCAGTAGGACTCTAAGT
CATTCAGCAAGTCTTCATCAATAAAGAATAGCAAGTAGGCCGGGCGCGGTGGCTCACGCCTGT
AATCCCAGCACTATGGGAGGCTGAGGCGGGCGGATCACGAGGTCAGGAGATCGAGACCATCC
TGGCTAACACGGTGAAACCCCGTCTCTACTAAAAATACAAAAAATTAGCCGGGCATGGTGGCA
GGCACCTGTAGTCCCAGCTATTCGGGAGGCTGAGGCAGGAGAATGGCTTGAACACGGGAGGT
GGAGCTTGCAGTGAGCTGAGATCACGCCACTGCACTCCAGCCTGGGCGACAGAGTGAGACTCC
GTCTAAAAAAAAAAAAAGAAAAGAAAAGAAAGAATAGTAAGTAATTTATATTCATTTTAAGAGAA
TATATGTGGGGTGGGGAAGGGGAAGGGTATGAGTAACAGAGATGAGGTTGGAAAGACAAGTT
GGTGGCAATAGATTCTTGTAGGAGTTTGGACATTATCCTAAGTGCAATATGAAACCATGATGGG
GTTTCAAGTGAGCAATGATGATTACATTTGCATTTTAGAAAAATCTGCAACATGAAAGACAAATT
GAAGGAAGAAAGAGAGGCAGGAGACATTTGGAAGGAGGAAATAGAAAGCCAAAATAAAGTAA
CTGATTCCAGGCCTGTGCCTAGGGTGTGAAATACCTTGGGCCTGGTGCTGGCTGGCGGTTTCAC
TGGTGGAGGTGGTGGTGGGCAGGGGAGATGGTGTTGGGAGTGGGGTTTAAATGCACAAATTCT

GGACTTAGTCCTGAGTTCAAAACCTGTTTATGCCTCTTTCTAGCAATGAGACCTTAGCTTCTTCTG
AAACTCATTTTAGTTAAGGGGTTTTAGGGGTGGGGGGCTCAGCCCAGAGACCACAACTTAGTCA
GTATGTGTTTTCTTCCTGCTTCCCATCCAGGGCTCCTCCTCATCCCTGGAGGTTCCAGGGATCTG
GCAGAGAAAGAAGCCCAGTCTCACAGCAATGACTCCTGAACATCCTTTGCAAACACTATTTAGG
ACAGATGGACTGAAGTAGGCCCTGGGGAGAGTTGGGGGCATTTCCGGAGGCCAGACCAAAGG
TGATCTCCAGGAGATTTGGATGTTAGTGCAGGATAGCTCAGAGCCAAGTGGCAGATAAGTCTTT
GGGAGCCAGAAGGGCTCTTTCTTCTCTAAGGAACAAGTGTAGACCTATAGGGGCAAGAACCTG
TGGCAACCCTGGGAGAGGTCTGCAGATTCAGGAGGGAACATCCCAAAACTCAGCAGAGGAGG
AGAGGGAGCTTTTGTTGGTCACCTGGAACCACTACCAATCCATGTTCATGTCAAATTAAATGATC
ACTTTGAAGT

ID|55922|gene_id|ENSG00000227070|transcript_id|ENST00000415031 Homo sapiens lncRNA

AGGCAAGAGGGAAGCTCGAGAACAGGTTTTGCTGAGGATTCCCCCCAAGAAAAGTGCTCC
CCGGGAGGCCGTTCCTGGCACCAAGACTGGCGGAGCCACCACACCCGTTAAAGGGATCCACCT
GGACAGCGATCAGGCGTGAGCCCCCCGAGGGGAAGTTGAAGTGGAAGCAGACGTGGGTGGCA
AAAGGCTGCATTGGGGGTCTGACCCAGAAGGAAACCTGATCAGGAGGGGGGAGAGGCTGAAC
GGAGGAAGGGGCCGGCAGAGTGGGTAGTTGAGTAGGATGAGACTTCTTTGGAGGCGAGGCTA
ACTACATTAAACTCTGGGAAGGGAGGGCTTCCGTCGGACCCTGGGAGAAAGGCAGGTCTGGAG
AGGAAAGACTGGCTTGGGGGGCCTGCGCGTCCGGCTCTGGAAGGCAACTCCTACTGGGGGCG
GCGCGCAGCCGGCCTGGCGGGCGAGCCTCGTGCGGCCCGGGGGTGCGGCCGGCCCGCGCGA
GCTCAACAGGAGCTCACCTGGGATGCGTGCGGCCCTGTGGGAGGAAACTTCCTGGGTAGGTGA
AAGCTGCCCCGCGGGAGCCGGCCCGGCACCGGCAGAGCGCGGCCGGGTCACAGTCCACTCGG
CAGCGGCGCGGAGGGCGGCGAGGGCCGCGCGGGGCGGGGCAGGAGGCGCCCGACGACCCT
GGGCGAGGTCGCGGCGGCCGCAGGGGGGCTCCGGCTCCGGAGGGCGCCCTCCCGCAGTCGC
ACCCGCTTTCCCTCACACACACCGGCCGCCCGGCCCACAAGCCAAGAAGCTGCCTGCTTGGCC
CACAAGCCAAGAAGTCGGCGAAGCTGAGGGCGGTGGGGGAGGGGGCACCGGCCGGCCAAGC
CCGGCGGACGGGCGTGTGGCGTTACCTGTGTCAGAGAGAGCTGGGCCGCCGCAGCCATGGTG
TTTCCATCATGCAAGGGAGGGGAAGGAAGACGGGCTGACTGGGGCTCGGGGCGGGGAGGAG
GCGGAGGGAGCGGCCGCAGGCGAGGCCGGGGCGGGCGGGCGGGCGTCTCCCCTAACTTCCA
CAACTTCCCTCCCCACGCCCGCCCCGCCCCGCCTGGCCCCGCCCGCCTGCCCGCCCGCCGGAG
CCGGCCGCGGCGCCGCCCAAACCCTGGCGCCGGAGTCTCGGCCACCGGACCAGAGCCTGCCC
CCGGGGCCGCCTTGGAAAACCCGGCGCGGACATAAAATTAAAGCGGACGGGCTGCGGAGGCG
ACTGCAGATTGACCTGTGAACTTCAGACAGGCCACCTACCTACGCTGAGCTCCTGCTTCTGAAA
AATGGGGGAAATTGTAACTAGAGGCCCAGTTACTTACAAAGACTAAATGAGATAATGATAGAA
AATAATAATGGCACTTTATATATGCCAGGCTCTGTACTAGACTCTTATTTTTTGAATTGTATGAAT
GAAGGAACTGAAACACAAATAAGTGTCTTACTAAGGTCATACAGCTAATTATATAGCTGAATTA
GAATCCAAGTCTAACTGTAAATATTTAACCATCTGCTAGTATATTGGAAAAGAAGACATTAAGTC
ATTCCCTCCCACAGCCAAAATCCATTTGGAAAGGGGAAGCAAAAATGTATTTGCCTGGTGCCTG
TCACATTTAGATTGTTTAACAAGGGAACGCTGGTGCCTGTAGCACTCCTAGCTTCTCCCATCATC
CACTTAGCAAACTCCTCCTTCTTTCTCAGGACATAATTCAAGTTTCACCTCCTTTGCAAAGTATCT
TTTTCTCTTCCTTATGTCAGAGTTACACACTCTCCTTTATAACCTTTCTTCTCTACCTTGTGAAGTC
ATCACCCTAATGACAGTTTACACCTATCTATCCCTCTAGATTAAGAGCTCCTCAATGGCAAGAAC
TGTTTTCAATATATTTCTATATCGTCAGACCCTAGCATTACAGTGCATGCTCAGTAAAGGCCTTCG
TTCCTGCCTATATTAAACATAACACATCCTACACCTTCTGATGCTGCCAACCTTCAGTTGGAATA

CCCAGTTACTTGAAATTCATCTTCAAAGTCTCAGAGTATATACCACCTACTTCAAGAAGCCTTCT
AGGAACTCCACACCCCACCTCTAAGTTGTTCTGATGTGACCGTTTTTATACTCTGCCTTATCTGTA
AGCGTTAGTTGAGGGTTTGTCTCCACCTCACTGCACTGTAAAATCCTTGAGGACAAGGGGCTGA
CTCACTGCTTTATCTCCCTCATAGTTTAGCCTACCACAGAGGCCTCAATAAATGTATTTGCCCGA
ATTGGTCTTAATTTCTTAGTCACGATGGCTTCCCATTACAACAAACCACAACTCTATCAGGCCTA
CACCCATTACCATTATCATAGCAGTCCCCGTGGTAGGAGGGACAACTGAACAAGACCCAGGAT
GGAGTTAGCCCCACCTAAGTTTTTAGACTATTGCCTCCACAAGCAAACCATTCCAGAAATTTCCC
AAGGAACTCCTAATTCTACTCAGACTCCTTCTGGCAAAGGCCAGCTAACAAGAGCAAGCCATAT
AGGTCTGGTGAGTAACCACTTATTCAAGAGCATCTTCCTTGGCTTCTTTTTTGCTGCTAATCTCTTT
AACTGAAGAGAGAATAAGAGATACTGTTTTACATTACTTCACTTTGGTACACTTATCAGCCATAA
CCACAACCGGTGTTTCTACCGGTGTTTTCTACTTTATATTAGCACATTTGAGCTTCACAACTCTAA
AAGGAAGTAATCGTCATACTCACTTTACAAATGAGGTAATGCAGGGTCACAGAGGCTAAGTGAT
TTGCCCAGGGTTATACAACTGATAGACAAAGCAGCATAGTATAGTGAAATAAATAGGACTCAGA
CATTAGGAATATCCAGGTTCCATTCCTGGCTCTCCCATTCACTAGTTGTATGCCCTTAGGGTTGTT
GTGAGGATGAATGAGATGACATATACAAAAGTGCCTAAACTTGTAGCTACACAAATTATGAAAT
CTGAAAATCATGGAGCAAATCGCTTTGGGGAGTATAATTTCACTTGCTATTCAAGGGGACTCCTC
CAAACCATATGAGATGTAAGATATTCCTCTCAGATGAATAAGGTGGAAACCCTGCCATGTAGTT
TCCGGAAAGGCAGTGCAGAGTAGCAAAGAGAATATTAAGAATCAGAGTGTGACCGCAGGCAA
ACCATTTAACCTCTTTGAGCTTCAGTTTCTCATACAGGAAAAGTAAGGAATTATTACTGTTTTAAC
TTTTATATGATTTATATTAAGTCAAAAGCACACCTTACCCAGGCATGGTGGCACATGCCTATAGT
CCCAGCTACACAGGAGACTGAGGCAGTTGCACAGAGCACGAATTCAGCCTGAGCAACATAGAG
AGACCCCATCTCTAATAAATAAATAACACCTTGTATGTATACAGTATTGCTTTTCACCGTTTATAA
ATGTTTTTATTTTATTTTTATTTTTTTGAGACAAGGTCTCACTCTGTCACCCAGGCTGGAGTGCAAC
GGTGCAATCTCGGCTCACTGCAACCTCCACCTCCTGGGTTCAAGCGATTCTTGTGCCTCAGCCTC
CTGAGTAGCTAGGATTACAGGCACCCGCCACCACACCAGCTAATTTTTTTTTTTCTAGTAGAGAC
AGGGTTTCACCGTGTTGGCCAGGCTGGTCTCGAACTCCTGACCTCAAGTGATCTGCCTGCCTCG
GCTTCCCAAAGTGCTGGGATTACAGGCGTGAGCCACCGTGCCCGGCCAAATGTTTAGAAGCAC
AACTTCTAATGTTCAGTAAGTACATTATAAAAGTTTGTTGAATTGAACTGAATACAATCTCATATA
TAATTGTTATTTGGATCTTCACTTTATCCCCATAAGAAAATCAAGGAACAAATATTTTATAAATTT
ATTTTATAAATAGCTTCTATAAGTAAGAGCCATTGACAGCAGGCTTGGGTCTTGGTTAACAAAAA
ATAAAAAGAATCAGATGCCCTAGCAAGGGAAGACTTGCTTAAAATCTTTATATGGGAATTGCCC
CACGTCCCCTTCCAGACCACTAGTAATTTGTAGTGGCTTCTTTCTTCATTCGTAGGTTCTGGTTTA
AATTAATGACATGTAATGGGAGATGGGAGCATGTCTATAGGGAAGAGAATAATGGGCCAAATT
CCCAAAAAAGGCCTAGAACTTCTGAGAGAGGTGTTTTTGTTTGTTTGTTTGTTTGTTTGTTTTAAT
GACAAACCAATGACAATTTGCAAATGTGTGGCCCAGATCTCGTCAGAGCACTGGGTCATATCCC
AAATCTATTTTAGGAGAGGAAAAATGTAGCCAATCGAATATATGTTATAATCTAATTCATTATAA
CAAGAATGAGTTGCATGGAGCAGACAAGAAGAATTTATCTGTTTTTTCTAGGACAACTTAATGTC
TCTGGGTCACTAAGCCTCATTTTCCTCATGATTACATTGAAGATAATAATATCTACTTTGTATGGA
TGGTTTAAGGATTAGAGCTCATGTATAATGGCCTATCAGAGAGACTGGCCTGGAATAGATGTTC
AATAAGTAGCAGCTATAAGATACCATTCTTCAAGATCACCATTTAACTCTCTACCCTTCAGGCAA
CCCTCCAACAACTAAGTTGGTTTGACAGATATATTAGTTACATGAATGGGTCTGCCAGACCAGA
CCAGGTGGCTCACATTTGTAATTTTAGCACTCTGGAAGGCCGAGGCAGGAGGATTGCATGAGCC
CAGGAGTTCAAGACCAGCCTGGGCAACATAGTGAGACCTCGTCTCTATTTTTTTAAAATCAATCA
AATAAAAATAAATAAATAAATAAGAATAAGGGTGCTTTGAACAACTGACATATCCCTGTCATTG

AAAAAGTAGATGTTCCCTAGGTAAACAAAGTTGTATCTGATGACTTAAATCTGAATACTAATAAC
CAACAATTGCTGTTTTCTTTTTTTTGTTTTATTTATTTATTTATTTTATTTGAGACAGTGTCTTGCTAT
GTTGCCTAGGCTGGTCTTCAACTCCTGGGCTCAAGTCATCCTCCTGCCTCAGCCTCCCAAAGTGC
TGGGATTATAGGTGTGAGCCACCGCACCCAGACAAATTGCTGCTTTAATGTGCATTATTTCATTT
ATTCCTCAAAACAGCTCTACAAGGGTTATTTTTAATCTTTTTTTTTTAACAGAAGAAGAATTTGTC
CAAATTCATAATGACAAAGCTGGGAATTATACACAATTATACACGGGTCTTCTGATTCAAATGCG
CTTTTTCCTGGGGTGTGTTGCTGGTGTCCTGCAGACATTAATCACCACCCAGGAAGACTTGTTAA
AATTTTGCCCTTGAACATGGGAAGCTGAAGATGAGGCTCTGGATTTACGATAGCCCTAAATCCA
GGATGAGCCTAAAGAGAAGACCTTAAATCATAAACAGCTTTGTCACCAATCATCTTTCTATCCTA
CTCTAACTCCAACCCCCCTCCCCAACCCATCCTTAAAGGAAATCAAAACTACTTTTATTTTCTTAT
TTTCTAGCTTTTAGAGGCAGTTGTCATATCAACGCTTCATGAACACTGCGTGTCTGTGAAGTCAG
CATCTAGAAAGTTGAGAAATGTGAAGTGATTGAAACTATGGAAAAAAAAACTGAGGAACTCTCT
GAATACACAAAGGGCCAGTTTATTTGCATGAGACTTTGAGAGATGGGCTTTCCATATAGATGGT
GCTAAACTGTATTTTAAAATCTTACTACAATTCTCATGGTAATCTGAAGCTCGTGCTTCTAAGCTG
CTGGTTTCATAAATATAGTCCATCATTGTTCGCGTTTGCCCAGGGCAGAAAGAATATGTGAGGC
AGCACAATAACAACAATTGTGCTATGCAAATAACCATCCCTGTGCCAGGGATTGAGCTATGAAA
AGAACAAGCAGTAAGAGATTTGTAAATGCCATACACACGCAGGGTGTGATAGAACAGTTCAAA
ATTGTCATCATCACTAACTTTTCAGGGCAGTGGGATACCCTGGGAAGAAGCTGAGTTTAGGACT
GGGAAGGCCCAGGTTGGTCCAGTTTTGGTCCTGCAGGTTCTTTGGTGACTTTGCAGAAGTGAGG
AACTATACCAAGCCACGGTTTCCTCTCCTGTAAACTAAAAGTAATAATAATACCTACCTGCTCAT
GCTATTACAATGATTAAATGAAGGTTTATGTGAAAACCTGGGACACAGCAGAAACTAGATAAGC
ATTAGCTCCTTTTTCTTCCTACTGGAGAACATGTAGGCTTTAAGTAACCAGAACACAAAAATAGA
AAATATTTTTAAAGGTTTTCAGTGGCCTTCCAAAGCCCACTTAATGGTTTTTTACATAAAATTTATT
TAATCCATTAGTAATATTTATTGAGTACTATACAGTAAGAGAGATTATTATATTTATGTCCCCAAA
GGCCATTGGCTTCTAGTGAGAAAGCAAACACTGTTTTGCCCTTGCCCTATTTCCTTCCTTTCCTCT
CTGTCCTTTATGAAGAGTGCTGGAAGCCATAAGAAACCAAAAGGAACACCATAGGAAAGAAAC
CTTGCACTCATGGCTGCAGGTTCTTTCATCTGCAGGTTTCAAAGCATTTTCTCCATAGGTAATTAC
TAGCCCTACTTACCATGTGTGAATGCTGAGACATCATCAAATTAAGAAGCTTGCCCTAAATCACA
AGGCAAGTCAGAGGATGGGCTGCAGACAGAACAAAGCTGTCCTGTTTGCCAGACTTGGGCTCT
AAAGTAGGGCACATGAGTGTCAACAGAAGACTTCAAGTGATGAGGAAAAAAGTTTGAGGAATC
CTGATTCTGAATCAGCGCAATAACAAGAGCATGGGCTTCAGAGTCAAATTTGATTTCAAATCCC
AGCTCAGCCCCATTTGACTGGCTGCCTGACCTTCATCAAATCATAAACTAAGTCTAGTTTCCCTTT
TTGTAAAATGAGGATATTCTTACTTCTAAGGGTTTTGGGGAAAGTTAAATTATGTAACTAGTGTG
AGAGAGCCTGGTTCAGTATCTGTCACTTAGCAGGTGCTGATAATGTTGGTTTATGTCATTGGGAA
AAGTTAATATATCTCCCCCACAAACACAAAATAACAAATAGACAACTCTTTCAGTCAAAGATTTT
CAAAGCCATAATCACAGTTGTGTCTCAGTTTAAACCCATGACATCTCACATTATAGCACAAATAG
AGGATGCCTTGTTCAGGCCTTCATCACTTTTCTTCCTTGATTCTGTGCTTTCTCTCATAGCCCAAG
TCCAATCTACCAGAAAGTTCTGTCGGCTCGATCTCCAATATATATCCAAGATCCTAATTTCCAGG
TCTGACCACTTTTCACCACCTCCACTACCATGGCCTTTCTCTTGGACCACTGCTAAAGTTTCTTAA
CCAACATCCCTACTTCCATGATTTTGCCTAAGTTCATTCTCCATATCATAAACTGAATGATCTATT
TTTATTTATTTACATCACCCAGCTGCAGTGCAGTGGCACGATCATGGCTCACTGTAGCTTTGAAC
TCCTGGGCTTAAGCGATCCTCTTGCCTCAGCCTGAATGATTTTTTTTGTTCTTTTTGAGACAGCAT
CTCACTCTGTCACCTAGACCGGAGTGCAGTGGTCCAATTTCAGCTCACAGCAGCCTCCATCTCC
CAGGCTCAGTTGATCCTCCTACCTCAGCCTCCAGAGAAACTGGGACTACAGGTTCACACCACCA

CACCCAGCTAATTTTTGTATTTTTTGTAGAGACAGGGTTTTGCCACATTGCCCAGGTTGGTCTTAA
ACTCTTGGGCTTAAGCAATCCACCTGCCTTGGCCTCCCAAAACGCTGGGATTACAGGGGTGAAC
AACTGCACTCTGCCTGAATGTTCTTTTTAAAACATAAACCAGATCTTATTATTTTCCTGGTTAAAA
CCCTACAGGGACTTCTCATCAAGCACCTGAATATGGCCTACCGTGGGTAAAGTATGTGTCAGCA
AAGCCCAGAAGAGCGAGATGTCTGGTTAAATGGAGCATGGTAGAGATGAGAGAGAAAGAAAC
TAGCAGAGAAGGGTGGAAGGGGTGTTAGAAAATACCCAGAAGGACTCATGGCTGGACTACAA
GAGGCTGAGAGAAATGTTTCTCTCTGGAGATTCTCTTTTTTGTAAAATGAGGATATTCTTACTTCT
GAGGATTTCGGGGAAAGTTAAATGATGCAACTGGTGTGAGAGAACCTAGTTCAGTATCTGTCAC
TTAGCAGGTGGTGGTAATGTTTGTTTATGTCATTGGGGAAAGTAGCTCAAAAGAGTCCCAAAAA
TAGCTCAAGAGATTCACAATCAACACTGGAAGCAAAGGAGATTCTTACTGTTTTCCATTGATGGC
ATTTTTAAATCTAGTGGCCTGGAATTTTTAAGCACCCAATCAGGAATCTATTTTACCCTTTTTTTTT
TAACTTTTATTTTTATTTTTTGAGATGGAGTCTCGCTCTGTCACCAGGCTGGAGTGCAGTGGCGCA
ATCTCGGCTCACTGCAACCTCTGCCTCCCAGGTTCAAGCGATTCTCATGCCTCAGCCTCCTGAGT
AGCTGGGATTAGAGGCAAGCGCCACCACACCTGGCTAATTTTTTGTATTTTTAGTAGAGACGGG
GTTTCACCAAGTTGGCTAGGATACTCTTGATCTCCTGACCTCGTGATCTGCCCACCTAGGCCTCC
TAAAGTGCTGGGATTACAGGCATGAGCCATCGCGCCTGGCCTATTTACCCTTTTAAAAGTAAGA
ATTATACGCTAGGACCCCTAACATCAGGACAATGGTTAAGATGATGGACTTTGGAATCAAATTT
GACATTGAATCAAGGTCCTCCACTTATAAGCTACAACACCATGGTCAAGTTATTAAAACTCTTTG
ACCCTCAGTTTCATGCAGACTACCATACAGAATGAGTAAAAGAATTAAATAGACAATGATTATA
AAGCACTTTGCATAGTATCTGAGACATGGGGAGTAAGTGCTCAGCAAATGGGGACTATCCAATG
ATGATGGTGATGATGATGATGATGATGATGATGATGATGATGATGATGATGGTGATTAAGAATTGGG
TTATCTTTTTTAAAAAATTTTTTTAGATACAGGGACTCACCCTGTTGCCCAGGATGGAGTGCAGTG
GTGCAGTCATAGCTCACTGTAGACTCAAACTACTGGGCTCAGGCAGTTACCCACCTCAGCCTCC
TGAGTAGCTGGGACTACAGACACATGTCCCCATACCTAGATAATGTTTTTATTTTTTATAGAGAC
TGGGTCTCACTATGTTGCCCAGGCTGGTCTTGAACTCCTGGACTCAAGCAATCCTCCTGTCTCAG
CCTCCCAAAGTGCTGGGATTACAGATGTGAGTCACTGTGGCTGGGTGCCTGCCTGGGGTGAGAT
TTAAATTGGCCTTGTAAGCTAATAAAAAATGAAGTCTATTCTGAGGGCAATGTGGAGTCATTGAA
AGGTTCCCAGGAAGGAAAATAAAAATCCAAAATCATGTTATAGAAAGGTAACTCAGCCGGGCA
CCGTGGCTCATGCCTGTAGTCCCAGCACTTTGGGAGACCGAGGCAAGCAGAGCACTTGAGGTC
AGGAGTTCGAGACCAGCCTGGCCAACATGGCAAAACCCCATCTCTACTAAAAATACAAAAAAA
AAAAAAAAATTAGCCAGGGGCTGTGGCACACACCTGTAGGCCCAGCTACTTGGGAGGCTGAGG
CACGAGAATCTCTTGAACCTAGGAGGCAGAGGCTGCAGTAAGCCAAGATCACACCACTGAACT
CTAGCCTGGGGGTGACAGAGCAAGACTCTGTCCCAAAAAAAAACCAAGAAAGGTAACTCTATTC
AGCATTATACTGGAGTTTCCAGCCAGAGGAATTAGGCAAGAAAAAAATAAAAAACATCCATATT
GAAACAGAAGTAAAACTATATTTGCAGATGAGATGATCTTGTATATATAGAAAATCCTAAAGAA
ACAAACAAAAATCTATTGGAATAAATGAGTTCAGCAAAGTTGCAAGATACAACATTAATATACA
AAAATCAATTGTATTTCTATACACTAGCAATGAATAATCTGAAAATGATAGCCAGGTATGGTGGC
TCATGCCTGTAAGTCCCAGCGACCTGGGAGGCTGAGGCAGGAGAATTGCTTGAGCCCAGGAGG
CGGAGGTTGCAGTGAGCCAAGATCCTGCCACTGCACTTCAGCCTGGGTGACAGAGTGAGACTC
TGTCTCGGGGAAAAAAAAAAACAAACTGAAAATGAAATTAAAAAATCAATTCCAGCCCAGCACA
GTGGCTCACCTCTGTAACCCCAGCACTTTGGGAGGTCAAGTTGGGTTGATCGCTTGAGGTCAGG
AGTTTGAGACCAGCCTGGGCAACATGGTGAAACCCTGTCTCTGCAAAAAAATGCAAAAATTCGC
CAGGCATGGTGGAGTACACCCATAGTCCCAGCTCCTCAGGGGGCTAACGTGGGGAAATCACTT
CAGCCCTCCAGGGCAGAGGTTACAGTGACCCCAGATTCCGCCACTGCACTCCAGCCTGGGAGA

CAGAACGAGACTGTCTCAAAAATAAAGAATAAAAAATATCAATTCCGTTTACAATAACATCAAA
AAGAAAAAAATAGTTAAGAATAAATTTAATAGAAGTACAAGATTTGCAAATAAAAACTACAAAAT
ATCATTGAAAGAAAGTAAAGAAGACCTAAACAAATGGAAAGACTTCTTATGTTCATGAATTGGA
AGACTTAAGATTGTTAATATGGTAATACTCCCCAAATTGATCTACTGATTCAGTGCAATCCTTATT
AGAATACCAGTTAGCTTCTTTGTATAAATTGACAAGCTGATTCTAAAATTCATATGGAATTTCCAG
TAACTCAGAATAGCCAAATAATCTTAACAATGAAGAACAAATTTGGAAGACTCATGCTTCCTGAT
TTTTTTTTTTTTTGAGACAAGAGTCTTGCTCTGTTGCCCAGGCTGGAGTGCAGTGGCACGATCTCG
GTTCACTGCAAGCTCTGCCTCCCAGGTTTGCCATTCTCCTGCCTCAGCCTCCCGAGTAGCTGGG
ACTACAGATGCCCGCCACCATGCCTGGCTAATTTTTTGTTTTTGTATTTTTAGTAGAGACAGGGTT
TCACCGTGTTAGCCAGGATGGTCTTGATCTCCTGACCTCATGATCCACCTGCCTTGGCCTCCCGA
AGTGCTGGGATTACAGGCTCCTGATTTTAAACTTACTACAAAGGTATAGTAGTCAAGATAGCATG
GTACTAGTATAAAGAGAGACATATAAACCAATGGAATGTAATTGAGAGTTCAAACTAACTCTAT
ACATTAATAGTCAATTGATTTTCAGCAAGGGTATGAAGATAATTCAATGGCGGAAAGAATAGTC
TTTTCAACAGATGGTACTGTTGAACTACCATATTCACATGCAACTACATATTCACATGCAAAAGG
ATGAAGTTGAACCCCTACGTCATACCAGATGCCAAAACTAGCTCAGAATGGATCCTTGACATAA
ATGTAAAACTGTAAAATTCTTAGAAGAATCTAAGAGTTGCTTAAGTATAAAACTCTGAGAAGAG
GCTGGGTGCGGTGGCTCACGCCTGTAAACCCAGCACTTTGGGAGGTGGAGGTGGGTGAATCCC
TAGGTCAGGAGATCGAGACCATCCTGGCTAACACGGTGAAACCCGTGTCTACTAAAAATACAG
AAAAATTAGCCAGGTGTGGTGGCGGGCGCCTGTAGTCCCAGCTACTTGGGAGGCTGAGGCAGG
AGAATGGCATGAACCTGGGAGGCGGAGCTTGCAGTGAGCTGAGATCGTGCCACTGCATTCCAG
CCTGGGTGACAGAGTGAGACTCTGTCTCAAAAAAAAAAAAAAAAAAAAAAACAAAAAAAAAAAA
CCTCTAAGAAGAAAACATAGATGCAAACCATCTTTATTAGAGGAGGCAATGATTTCTTAGATATA
ACACCAAAGCAGAGCAACAAAAGAAAAATTAGATAAACTGGATGTCATCAGAACTTCTGTACTT
CGAAGGACACCATCCAGAAAGTGAAAAGACAATCCAAGGAATGGGAGAAAATATTTGCAAATC
ATATACCTGACAAGTGAATTCTAACTAGAATATATAAAGAACCCTTACAACTCAACAAGAAAAA
GACAAATAAACCAGGCATGGTGGCTCACACCTGTAATCCCAGCACTTCGGGAGGCTGAGGCTG
GAGAATACCTTGAAGCCAAGGAGTTCAAGACCAGCCTGGACAACATATCAAGACCGTCTCTTA
AACAAAAAAAAAATTAGCCAGGCATAGTGAGCCCAAGAGTTTGAGGCTGCAGTGAGCTATGAT
TGCGTCACTGCACTCCAGCCTGGGTGACAGACTGAGACCCTGTCTGTAAAAAAAATTAAAATTA
TGGCCTGGCACAGTGGCTCACACCTGTAGTCCCAGTACTTTGGGAGGCCGAGGCAGGCAGATC
ACTTGAGGTCAGTTTGAGACCAGCCTCTATTAAAGGGTGAAACCCTGCCTCTACTAAAAATATC
AAAATGCCTGGAATCCCAGCTACTCGGGAGGCTGAGGCAGGAGAATCGCTTGAACCCGGGAG
GCCGAGGCTGCAGTGAGCCGAGATCAGGCCACCGTACTCCAGCCTGGGTGACAGAGCGAGAC
TTTGTTGTAAAAAAAGAAAAAAAATAGGGCCAGGTGCGGTGGCTCATGCCTGTAATCCCAGCACT
TTGGGAGGCCGAGACAGGCAGATCACCTGAGGTCAGGAGTTAGAGACCAGCCTGGCCAACAT
GATGAAAACCTGCCTCTACTATAAATACAAAAGAACTAGCTGAGTGTGGTGGTGCATGTGCCTG
TAATCCCAGCTACTCGGGAGGCTGAGGCAGGAGAATCACTGGAACCTGCGAGGCAGAGGTTGC
AGTGAGCCAAGATCGTGCCACTGCACTCCAGCCTGGTGACTGAGCAAGACTCTGTCTAAAAATA
AATAAATAAATAAAAACATTTAAAGAAAAATATTATTTGGACTATTGTGGGGAAATAAACTGTAG
ATCAACAAGGGCAGAAACTGGTAAAACAAATATAAGATTATTGCAATAATCCAGATATGATGAT
GGCTTGGTGGTGAGAGGTGGTAAGAAGTGGTCAGATTCTGTACATATTGAAGGTAGAGCACAC
CAGATTTGCTAAAGGATTAAGGATGAGGTATGAGAGGAAGAAAAGGATCACGGATGATGCCAG
GGATTTTGCCTGAGCAACTGGATTGATGAAATTGTTTTTTACTGAGATGAGTAAGACTATGGAAG
AAGCCAATTTAGAGAGAATATCGGGAATTTGGCCTTTGACTAAATTTGATACACTTTTAGATATC

CAAGTTGAAATGTCAAGGATGCGGTTGGAATAAGAGTCTGGAGTTCAGGGGAGAGGCCTGGGT
TGGTGATATAAATTTGGGTGTCATTAGCATATGGAGGCATTTAAAGCCATGAGGTTGGGTGAGA
CTGTCCAAGTGTCTATAATAAAACCCAAGAAGTCCAACTACCAAGCCCTGGGCCACTCCAATGT
TAGGACTCAGGGAAATGAAGAGGAACTAGTAAAGGAGACCAAAGAAAGAGCAGCTGAGCTTA
GAGGAAAACCAGGAGAGTATGATGTCTGTCCTTGAGCCAAATGAGCAATGTGTTTCAGGAAGG
AGCAAGGAAGGAAGGAGTGAGAAAGCATGTCAAATCTTATCAAAGAGCTAGTTAGATGAGGAT
TGCAATTAATATCCAGAGTTAGCAATGTGGAAGTCTTTGGTGACCTTGGTAATCGTTTCAGGGGA
AGGTGAAAGAAAAGTATAATTAGAGGAGGCTCGGGAGAGAATGAAAAGAGAGGACTTGGAGT
CAAGAAAAACAGATAATTCTCTAGAAATGTCTAGCTGAAATGAAGAGCAGAGAAATGGTGTGA
ATACCTAGTAAGGGATACAGAGTCAAGAGAGGTTTCTTTTCTTAAGGTGAGCAATAATGCAGCA
TTTTTTAGAAAGTGAACGGAATGATCTGGTAGAAGAGGAATCACTGATGATACAAGCAAGAGA
GGGAATTGCTGGAGTGCAGGGGAAAGAATTGGCTTTGAGAATACCGAAGAGGGCTGAGTATGT
GGGCATGGATGCAGGTGGATGGGTTATCTGCTGAGAGGGAGGAAAAAGGAGAGGATATGAAA
AGTTTGAAAAAAGTTACGAAATAAGCATTTAGGAAAAGTGGATGGACTTGAAAATGTAGTATGA
TTCCCAGGTAGCACTAAAAGCCCACTTGAGGTCAGTGGCGATGAATTTTAAGAGTCCAGTTGAC
ACAGTTGTGGGTTTTTCTTCATCCACTTTCCCAAAGGCAGAGAGTAGGTAGAGTTAGATTGTGCA
GGGTAGGGATCTTGTGAAGCAAAATGAGAATGCAAGAGGAGGCAAGAGAGAAGAGGACCTAT
CAAGGGAGTGATTTTAACATTGGACCTTGGAATTTAGGTGGGTAAAAAGGGAAGTGAGGACAC
AAGATAGTTGAGGAATGGTGAAGAGATATTAGGATCACTGGATTATAAGGCCCAGGAAGGGAT
CAGAGAAGTGTTGGAGTCAGGGTGTTGGAAGGAGTGAGCTGAGAAACAGGAGATTTAGAAAG
ATAAGTAAAATTATTGTTAGATTATTATTAATAATTGGTAATTATTAAATGGTAATTGGTAATTAAA
TGGTAATTGGTAATTAATTGGTAATTGGTAATAATTGGTAATTATTAATTGGTAATTAATAAATGG
TAGATATTTATTAATAACTGGAAGCAACCTCTAAAAAAATCATAGTATTAAGACCCAGTTCATGC
CCTCTAAGTGCTCACTCTGTGATGGGAAAGACAAGACAGAATGAAATAAATATTAAAGAGAAGT
ACATGCTGCTGGGTGTAGTGGCTCACGCCTGTAATCCTTGTACTTTGGAAGGCTGAGGTGGGTG
GATTGGCTGAGCTCAGGAGTTCGAGACCAGCCTGGGCAACATGGTGAAACCCCATCTCTACTA
AAAAAAATACAAAAAATTAGCTGGTCGTGGTGGTGCATGCCTGTAGTCCCAGCTACTCTGGAGG
CTGAGGCAGGAGAATCACTTGAATCCAGGAGGCGGAGGTTTCGGTGAGTGGAAATTGCACCAC
TGCACTCCAGCCTGGGCAACAGAGCAAGACTCTGTCTCAAAAAAAAAAAAAAAAAAGAAAGAAA
GAGAAAGAAAAGAAAAAAAAAGAGGGAAGTAGATGAAATGGCTGTGGGAGTCAAAAGGAATC
TGCGAGTAAACGTAAGGGTCAGATCAAGGAAAGCCTCCAAAGCAGGTGGCATTTGAATCGAGC
CTCAAAATGGAGCAAATGCAGCACATGGCCTGAATAAATGTTCACAGTTACACCATTGCAATGA
GTGCTCAGAAAACAGAGGTTGGAGTGCAGGGTGGGAGCTATGGCAGGACATGAGACTAGAAA
ATTATCTGGAACAGGCAAGGGATTTTTGAATGCCAGGCTAATGCCTGGGCATTTTTTTTTCTTTTG
TTTTTTCTTTTTTCTTTCTTTTTTTTTTTTTTTTTGCAGAGGAATAGATAATACTGAGGTTAGGGGTT
AGAGGTTATTATTCTAAATAATTGATGCTGACTGGGCGCAGTGGCTCACACGTGTAATCCCAGC
ACTTTGGGAGGCCGAGGCAGGTGGATCATGAGGTCAGGAGTTCGAGACTAGCCTGACCAACAT
GGTGAAACCCCATCTCTACTAAAAATATAAAAACTAGCGGGGAGTGGTGGCACATGCTGTAGTC
CCAGCTACTCAGGAGGCTGAGGCACAAGAATCACTTGAACCCGGGAGGAGGAGTTTGCAGTGA
GCTGAGATCGTGCCACTGCACTCCAGCCTGGGCGATAGAGTGAGACTACATCTCAAAAAAAAA
AAAATGCTCCCAGCTGTTTATCATGTTATGAAGTCATACTGGTTTTTTTGTTTTGAACAGTCTAGT
AATTTTATTCTCCATCATGTCCTTAAGTCATATTAGGGAAGTTTTATGCTTTGGATTTTCTGATTGT
CTTTTTAAGATCATCTCCCCTCTATGGCAATGATAATGTTGTTTCTTTCCAAATAATGTTCTGTCAC
ATGCTCATTCACTTTTTTTTCCAAATGGCCTTCAACTTCATCCCCTGATGTTGGTAGGAAAGTCTA

AGATCCCTCAGCAGTCTGAAGCTCCTCTCAGAAAGGGGTGAGGAGTGGGTTGGATAGGATCAG
AACTATGCTGTCCAGGGCATTCAGACCCGTTGAAGTGGATAAGAATTAAAGGTGAACAGTCAG
ATCCTCCCAGGGAGAGGAAGCCACAAGAGACACAGCCTCACAACTGTGTTGTCACAGTGTTTTC
AGAACTCAGTACCAATACCAGTGATGGGGCCACGCTTTTTGCAAGTGACGTTTATTATGTCATTT
GAAAATATCAGAATCTCTCTCTTCACAGGCTAGCCAGATAAGTATTGAAACCATTAATTTCTCTA
CCATGTCACTGTGTCTTTGGGCAAGCCCCTTCTCTCCTCTGGACCCAGGGTCCTGCCTATAAAAC
GAAGGGGCTCTATTCATCCTCCTCTAGGATCCCTTTCAATTCTGACAGGCTCTAATTTTTCTGTTA
TCAGTTATTCTTTATTAAACTTACAGTATGTGCCAGCTTTTTTCAAGATGTGAGACCCTCTCACCC
TCTTACTCCTCAACCCCCTGTTATAACTTATCTTGGGCTTTTCTTGTTTAAAACATTTGGAAAAAGT
GGTGTGTTCACGGGACCTTTATTTGCCCTAAGACATTTGAGGGTAAATCATGTGAAGGCCTTTGT
TTTCTTCCAGCAGCCAAAACAATAAAACCACCAAATTGCGTGCAGCATCATTTTCTACTGCCCTC
AAGTGGTGGTGGCCCAAATGTCTTTTCCTGGTTGCAGTTTAAGGAATAAAACATGTTTTCAGAAG
AACTCTAGGCATCTAGGAGAGGGTAGGAGAGTCATAGAGTCACAAAGGGCCTGAGGTGGAAA
GGCCCTTACAGGATGTCTGATCTAAGCCCCTCGTCTTGAAAGGGAGAGACTGAAGTCCACCTTG
GTTCTCTGATCAGTGAACAAACCTAGTTTCTTCTGTCTTTAAAGAGCTATCTTATGACCCTGACTC
TATTTTTGGCTCCATTTCTCTGCTCCCATTCACAGTGAAATTTTCTAAAGAGTTATCTCCATACTGT
TTCTATTTCTTCATCTATGCTTTATTATTATTATTACTTTTTATTTTTTTTTAAAAACAAAATAATAAT
CTCACTCTGTCGCCCAGGCTGGAGTGCATTGGGCTGATGTTGGCTCATTTCAGCCTCTGCCTATG
GGTTCAAGTGATTCTCCTGCCTCAGCCTCCCAAGTAGCTGGGACTATAGGAGGATGCCACCACA
CCTGGCTAATTTTTATATTTTTAGTAGAGACAGAGTTTCACCATGTTGGCCAGGCTGGTCTCGAA
CTCTTGACCTCAAATGTTAACCCCCCACCCCGCCGCCCTTGTCTACCAAAGTGCTGGGATTACA
GGCATGAACCACCGTGCCCACCTTTACACTTTATTTTTAACTTTTTACTTTGAAATAATCTCAAA
CTTGCAGAAGAGCTGCAGAAATATTACAAAGAATTCCCAGATGCCCCAGAAGTTCTCATGTGTG
GGCATATGCCTACATGCATATACATGGATATGTATATTTTTTCCTGAGCCATTTGAATTGCAGTAG
TTACCCCTAATACCTTAAATATTTCAATGTGTATTTTCTGAGAAAAGGACATTCTCTTATATACCT
ACATACCCACTCAGTATGACAATCAAATCCAGAAATTTTAAATTGACATAGTGCTTATATACAGT
CCATACTCAAATTTCACTAATTGGCCTGATAATGTCCTATATAGCAGTATTTTCCCCCATCAGGTT
CTGATCCAGTGCCTGATCCAGGTAATGCATTATACTTAACTGTCATGACTTTTAAAAAGGTTTTTT
TTCTTTTAATTATTATTTTTTAGAAACAGGATCGCGCTCTGTCACCCAGGCTGGAGGGCAGTGGT
TCAATTACAGCTCACTTACAACCTTGAATTCCTGGGCTCAAGCGATCCTCCCACCCCTGCTTCCA
GACTAGCTAGCTAGTACCGCAGGCACGCACCACTATGCCTGGCTAATTTTTTAAAAAATTATTTT
TGTAGAGGTGGGGTCTCGTTATGTTGCCCAGACTGGCCTATAATTCTCAGCCTCAAAGTGATCTT
CCTGTCTCTGCTTCCCAAAGTGCTGGAATTTCAGGTGTGAGCCACCACACCTAGCCTGTTATAAC
TCTTTAGTGTCCTTTAATCTAGAATAGTTCCCCAGTCTTTCACTATTATTCACGATATTCACATTTT
TAAAGAGTACTGGCCAGGTGTTCTGTAGTGTCTCCCCCTGTTTGGCTTTGTCTCATGTTTTCTCAT
GAAGGGATTTACATATTTTTCCAGGAAGAAATCCTAGTTATATGATGTTGTGTCCTTCTCAGTGCA
TCATATGGGGAGAAACGTGGTGTCACTTGGTCCCATTATTGATGGTGTTCACTTTGATCAGCGGA
TTAAGGTGGTATCTACCAGATTTCTTCACTGTAGAGTATTTTTTCCTGTGTAAGTAATGGTAATCT
GTAGAGAGGGACTTTGAAACTATGTAAATATCCTGTTCCTCTTCAAACTTTTGCCCAATGGTTTCA
ACACTCATTGATAATTCTTGTCTGAATCAATTACTGTGATAGTTGCAAACTGGTGATTTTCTACTT
CTATCATTCCATCTACATTTTTTAGTTGGCATTCTACTGTAAGCAAGAAGGTTTCCTTCTCTCTAAT
TATTATCATGAGTATAAACTAATGTATTCTTAATTTATTTAATGAGTTATAAACATTACCCTTATTA
TTTTTTAAATGTTCAACTTGTCCCAGGTTTGGCCAATGAGAGCCTCTTCAAACTAGCTCCTGTGTC
CTTTTGACATGTCTTCATCACTTTTTTTTAACACCTCCTTGCTTTCTGGCATAATAAGATGTTCCAG

GCTTATCTTGTACTTTCACTGCCTAGTCCTGAAAATCAGCCATTTCTCCAAAGAGCTACTCTTTAG
TGGGGAATGGTATTTAGAAACCAAGATTTGGGCATCAGATGTGCTCATTCCTATTGGAGTGTAAT
TGCTTCTAGGCCATTTTAGTAAACAGAGCCAAAGGGGAAAAATAGTAACCTAATTTTGTTTAATT
ATTATTATAATTATTTTTAACAGAGACAGGGTCTCACTCTGTTGCCCAGACTGGAGTGCAGTGAT
GCTATCATAGCTCACTGCAGATATGAACTCCTGAGATCACCTCAGCCCCTGGAATAGGCTAGGA
CTACACGCCACTACACTCAGCTAATATTTTTAAATTCTTTATAGAGATGGGGTCTCACTATGTTGC
CCAGGCTGGTCTCAATCTCCTGGCCTCAAGTGAACCTCCTACTTTGGCCTCCCAAAGTGCTGGG
ATTACAAGCATGAGCCACTGCACCCAGCTCTGGTCAACCATTTTAAATTAATAATTCATTACTGA
TACTTTAATTCCAATTTAACACCACAGGGTTCTTTCTCCTATTCCTTATTTGCATCTCCCTTTTCCC
ACAGTAAGAACTCTGGCTCTCAACAACATCAATGTCTTTATTCATTTGTTTAAAATTATGATATAG
GCTGGGCATGGTGGCTCATGCCTGTAATCTCAGCACTTTGGGAGGCCAAGACGGGCAAATCAC
TTGAGGTCAGGAGTTCGAGGCCAGCCTGGCCAACATGGCGAAACCCCATCTCTACTAAAAAAT
ACAAAATTTAGCCGGGCATGGTAGTGCATGCCTGTAATCCCAGCTACTAGGGAGACTGAGGCA
GGAGAATCACTTGAACCTTGGAGGCAGAGGTTGCAGTGAGCCAAGATCACGCCACTGCACTCC
AGCCTGGGCAACAGAGCACGACTCCATCTCAAAAATAAATAAATAAATAAATAAACAAGATAG
ACACAAAATAGAATTGTTACATCCATTCTGCTAACAAAAACAATCTACTAAACAGGGTTCAAGAT
TTGTTTGCAGTTTTGTTTTGTTTTTAGACAAAAGCTGTATATTCAAGTACTATGTTCAAAAGTATTT
AGATTCATTTTTTTTCCTTCAGAGTGATTATATTACTTATTTAAATATAGTTTGGTTCACTTGTTTCT
GTTTATATTCAGTTTTAGGATCCCTTCCATTCTTGTTTATTTAATTCCGTTTTTAAATATGTAAAGGT
AAACATTGTCACAAAATTAAAATCTATACAAAAGGCATATAGAATGCTTCACAGATGTGTCATCC
TTGTGTAGGAGTCAAGCCAATTTTCTCTGTATTGTTCCTTTTTTTTTGTAAATATGCTGTCAAAGCA
AGCACACCTCTAATTCATTCTTCAACTTATTTAATTCTTACTTAGACCACCCACTTCACTGACCCT
ACTACTGCCAAGATCATCAGTGTCCAATATTTTCTTTAGTCTTATTTGACCTTTCCACTCAGCTGA
CCTCTCCAATTTTCTTGAAACTTTCTCGTCTCTTGGTTTTTGTGATACCACATTCTGCTGATTTACT
CTCTCTTCGCAGGTCTGTCCTCACTCTCTTTGTCTATTCCACCACCCTAAAATCCTCTGGACTCTT
CTTGGCTCTCTTCACTTCTCTCTGCAGACTCTCTTCCTAGATGATCTCAACTATTTCTTTGTCTTCA
AATGCTGATCATGCTAATGACTTCCAAATTTACATCTTTTTCCCAGACCTCTCTTCTGGTCTCCAA
AATGTGTAACCAACTGTCTAATAGACATCCACTTGGATGTCTTAAGACATCTCAGGTTTAACATA
TCTAAAATAGAACTCCTAATTTCCCTGCCAAACCTGCTTCGCCTCGAGTCCTAATCTCTGTAAAT
GTCACTTCCATCCCCCGGTTACTCTAGCCAGAAACTTAGAAGTTATTCTTGATTCTTCCATTTTCC
TCAACCCCCATGTCCAGTCCATCTGCAAAATATGAAAATTTTACCTCCAAAATTATGTGAAATTA
GTCCATTTCTCTCTGTGTCTACCACCATCACTTCACTCGTCATCTCTTACCCAGGCTACTCATAAG
CCTCATAACTGGCCTCCCCTCACCTATTCTTTACCCTCTTCCTTTTATGCCCCCACATTCATTCTCC
ATACACCAGCTACAATGACCTTTCTAAAAACAAAATAATAATACGAACTTACATTTATTAGTGTC
TATTATGTGCCAGGAACTGTTTTACATGTATCTAATCTATACAACAACTCCATGAAGTAAAACCC
AAATCTACCTCAATTTTACAGATAAGGAAACTAGGGCAGAGAGAGATTGAGAAATTTGCCCAAG
GTCACACAGCCTTGGGCACACAGCCAGTAAATGGTAGAACTTAGGTTTGAACCCAGGTTGACTT
CTTAGCCTTCAAGCTTATCTGCCACACTATGCTAGATCATGTCACTACCTAGCATCAAATATTTGA
TGGCTTCTCACTACACTGGAACTAAATTCCCAACTCCTTACCAAGGCCTAGAGAGGCCTGTGTG
ATCTATCGTCTGCCTGTATCTCCAAACTCATCTCATGCTTCCTTTTCTTTTCCTTTGCTCCATCCAC
ATGGCTTTCACACCGCAACCCCCATCATCTTCCTTGAACATGATAAGCTCTTTCCTGCCAGCAGG
CCTTAAACATCCTCTATCTCAAGTATTCTTCTTTTTTTCTTTTAAATCCCCTATTCTGAGAAATGTC
AAATATTGTTTTCTCTGATCTTCACACAGCTGGATCCTTCTCATTCTTTAGGTCTCATTTTAATTTTC
TGTCCAGAGAGACTCTCCCTCCCATTGCTTTTTTTAATTATTATTTTAAATTTTTTTTTTAAAGCTGG

ACACCATTAATTAGAATCATGCTATTTCTTAAGTAGGTCCCTCCCTACTCCTGCCAAGATCATTAG
TGACCCTCCCTGATATGTTCCATCTTGGCCCCATATTTGTTTTCTTCTTAGCCTTTAAAACAATTTG
TTAGCCGGGCGTGGTGGTTCATGCCTGTAATCCCAGCACTTTAGGAGGCCGAGGTGGGCAGAT
CACGAGGTCAAGAGAGCAAGACCATCCTGGCCAACATTGTGAAACCCCATCTCTACTAAAAAA
AAAAAATTAGCTGGGCGTGGTGGCGCACGCCTGCAGTCCCAGCTACTCAGGAGGCTGAGGCAG
GAGAATCACTTGAACCTGGGAGGTGGAGGTTGCAGTGGGCCAAGATAGCACCACTGCACTCCA
GCCTGGCCGCAGAGCAAGACTCTGTCTCAAAAAAAAAAAAAAATTTTTTTTGTTGGCCAGGTGTG
GTGGCTCACGCCTGTAATCCAGCACTTTGGGAGGTCAAGGTGGGCAAATCACTTGAGGTCAGG
AATTCCAGACCAGCCTGGCCAACATGGTGAAACCCCGTCTCTACTAAAAATACAAAAATTAGCC
AGGTGGGGTGGTGGGCGTCTGTAATCCCAGCTACTTGGGAGGCTGAGGCAGGAGAATTGCTTG
AACCTGGGAGGTGGAGGCTGCAGTCAGCCAAGATAGCGCCACTGCACTCCAGCCTAGGCAAC
AGAATGAGACTCCATCTCAAAAAAAAAAAAACAAAAAAAAAAATTACTCAGGCATGGTGGCGCGC
ACCTGCAATCCCAGCTATTCAGGAGGCTGAGGCAGGAGAATCGCTTGAACCCGAGAGGCAGA
GGTTGCAGTGAGCCAAGATCATGCTATTGCACTCCAGCCTAGGCAACAGAGTGAGACTCTGTCT
CAAAAAAAAATTGTAATTATTTATTTGTTTGTTTGCTTGTTTGTCTCTCTTCCCCACTAAAATTAAG
TGTTGTGAAGGCAGAGGCTGAGTCTTAGTCACTTTCATTATGTTCAGAGCCAAACACAGTTTCCA
GCTCATAGAAGGAACTCTAGAAATATCTGTTGTCTATCCATATATTATCTTTTCTACTTCATAGAT
CTGTTCATTCATAATTTCTGCTTCTTTATCACTTTGGTTTGTCATCATCTACACTCCATCTCCTCCC
ACACATACCCCTACTCAAACTCATGGCCTCTCTCTTTACATCCTGTTAGTCATAGTTTTTATTGCT
GCTAATTGCCTCAATCACCTCATGCTGCCTGATTCAAATTCCTGACAGAGAAATATTGATTTGTC
CAACTCATATTTTAGGAGCCAAGACACATTTGTAGGTCATTGGCAAGCCCATGGGTTGCTTGCCT
TTGGGTCATATTCCCAGTCTTAGTCCAGAAGCTGTGTTAGAGAAGGCTGTGGGTGGGGATGGTC
AGCATAGTGCTGCCTTTCTGCAAGGATGAGGATGTAGCAGGCTTGGTGACTGATATTTGAGCAA
AAGTTGTTGCAGCAGAAGCAACAAATAGTTAACAAGGCCTCACTTAAGGGCTGGGCTTCCCAGT
AGTTGCTCTCTGGGAGGAAGGTCTCAGGTTATGGTCGAGTCCAGTTTACTCTCCCTCTGGGGAG
AATAGTAAACACAAATTGAAAGCACTCACATTTACCCAGCCTTTTTTTTTTTATTTTTTGAGACAG
GGTCTGGCTCTGTCACCCAGGCTGAAGTGCAGTGGCACGATCTTAGGTCACTGCAACCTCTACC
TCCCAGGCTCAAGCCATCCTCCCACCTCAGCCTCTTGTGTATCTAGGACTACAGGTGTATGTCAC
CATGCCCAGCTAGTTTTTGAATTTTTAGTAGAGATGGGGTTTCACCACGTTGCCCAGGCTGGTCT
CAAACTGCTGAGCTCAAAGCAGTCCATCCACCCTGGCCTCCCAAAGTGCTGGGATTACAGGCTT
AAACCACTGCACCTGGCATATACAGCCTTTCTAAAATTGCAAAAAGAGCATTTTTATCCATCTGC
ATTTATACCTCCAGTGAGGTAAAGAGTAGTTTCCATTTAACAAATAATAAAACTGAGGCTGGGA
AGATAGAGTGCTATGCCTAAGGTCCCACACACACTGAGTAAGAGTCATCATTACATCCCAGATC
TCTAGACTCCCACGCAAGGTCTCCTTTCACTATACATATCTTGTTGCCCTCATTTCCTACCTGGGA
CTTTAATCTTGAATCACAGCTCAAGGGATTTTTAAGGGCAACCATTTGAACGGCCAGTGAAAAG
TTCTAAAACAGCTTGATGATAATTGAAGTTTCCTATAAGGCAGCACTCGATAGGTAGGTTCTATG
GGCTCTGGGCTCCCAGTATAATTATCAAGTGTCTAAGAATTTATGGAATAATTACCATGTGCCAG
GCACTCTACTTTATACATACTCTCTCAATTCAACCTCACAACATGCATGTAAGGTATATGTTATTA
TAATCCCAACTTTACAGATGAGGACACTGAAACTTAAGAAACTAGGTCACTGGACCAAAGTTCA
AAATCTAGTAAATACAGTGCTGGAATCCTAACTCAGGTCTGTCTGACTCCAAAGCCCACCTCCCT
TTTCATGATCAGCTTGATTTTGTCACTTGATTTATGTGAACTTCCACTTCCTTATCGGTAAAATGG
GGATATCAGGGCCTAATTCATGGGATTATTGTAGGTTTATGAACAATCACATAGAGGGCAAACC
TATTTCAGTAAACAGAGCAGATATACACTATCTTTCAGTATAGTCTGAAACTAAATCCTTCATGTG
TAAAAAGAAGCAGAATTATCAACTCCAGGTGCCTATAAGAAACAGGATTTTTCTTGTGAGACTG

TAATCCTGGGGCAAGGATTCCTGGTCCAAATTCTGAAAACATAAGGCCTGGAGAGAAATAACC
ATTTCTCCAAGGAAACAGATCAATTTATTGTATCTCAGAGATGAGAAAGAACGTGCACTTAAACT
AGTCTTAATGCATCAGGATGGAAATTAGTAAATGCCCAATAAAGAGATTTGAATTTGTGTCTGAT
AAACTTGAGATCTTCTCCTAGGCCAAGGCCTTTTGCATATAATCTTCTTAATCCTACCACAACTCT
AAGAGGCAGGTCTTCTTACAACCTCCATTTTTCAGGCAAGTACTTTGAGACTCAGAGAGGTTTTT
TTGTTTTTTTTGAGACGGAGTCTCACTCTGGCCCCCGGACTGGAGTGCAGTGGCGCCATCTCAG
CTCACTGCAACCTTCACCTCCTGGGTTCAATCGATTCTCCTGCCTCAGCCTCCTGGGTAGCTGGG
ACTACAGGCACGTGCCACCATGCCCGGCTAATTTTTTGTATCATTAGTAGAGACGGGATTTCACC
GTGTTAGCCAGGGTGGTCTTGATCTCCTGACCTCGTGATCCACCCGCCTCAGCCTCCCAAAGTG
CTGGGATTACAGGCGTGAGCCACTGCGCCTGGCCTCAACTTTGAGGCTCAGAGAGTTTAAGTGA
CCTGGCAAAAGTCACACATCTAACTCAAAGCAGAGAAGAAACTGGAGCCAAGCTTTCAGCTGT
GTGCAGTCTGGCCAGTCTGCTAGTAAGTTAGCTGCCTCAGCACTGTAGACCTCTTTGGAAGGAA
ACTGACCCCTAGGTCAAACATAGTGCTGACATCATGCCCACTCATTATTCTCAAGAAATGAAAAT
CAATTCTACGTCCATCAGCACACTTTAGGGAGAATGCTGGCTAGGACAATACTTCCCTTGCAGC
ATTATAGATAAATACCAACACCTAAACAAATGTCAATAATACCATGTGATGAATGCAAGAGTGA
GAATAATGACTAAACTTTGCGTAGCACTTGATGATTCACAAGGAACATTCATATCTGTTACCCAA
TTTGCTGTTCATATCACCCCTTGGAGATAGGTATTATCATTGCCATTTCACAGATGAAGAAACTG
AGGCTCAAAGGAGTTTATTTCATATGAGAGAAGATGTGAAGCTAGCTGTGGTGGCACACACCTG
TAACCCCAGCTACTTGGGAGGTTGAGGCAGAAGGATTACTTGAACCAGGAGCTTGACAGTTCA
AAAGTTTGAGTCCAGCCTGGGCAACATAGTAAGACTGAATCTCTGAAGGAAAAAAAAAGAAAG
ATGTGGACATATACCATTTTACGATCTCTTAGCCTCCATTTCCCTTCTTTTGGGACTGGTACTCTG
TGTTTCCTTTGAAGATCAAATAACCCTTGCTTCAGCAACATTGATTCGATCAGCGTGAACACATA
ACACTTGACTCTTTGAGAATCAAGCTTGGAACTTTCGTGTAGGCTTTTGTTCAAACTGTTGAGAG
AGAGAAGCTCTCTTAAGCCCATGGGTGTGAGTCTGAAGTTAAGGGCTACCACCATAAGGAAAA
AAATTATGTATAAATTTTAAACCAGCAGAAGATAATTCAGCCTATTCAATGGAAAGCAGAAATG
GAGATTAAAAATAAAAAAAGAATGTAGAGTAAATGGAGGATAGAACTAAATCTTAATATATAAT
AATAACATTAAATGTAAAAAAGTGAATCTCACCAATGGGATTTTTAAAAATTACATCCAACAGTA
AACTAACTTTAAGAGACATGCTTTTTTTTTTTTTTTTTTTTTTTTGAGATGGAGTTTCACTCTGTCAC
CCAGGCTGGAGTGCAGTGGCTTGATCTCGGCTCACTGCAACATCCACCTCCCAGGCTCAAGCA
ATTCTCCTGCCTCAGCCTCCCAAGTAGCTGGGATTACAGGTGCCCACTACCACTCCTGGCTAATT
TTTGTATTTTTAGTAGAGACAGGGTTTCACCATGTTGGCCAGGCTTGTCTCAAACTCTTGACCTCA
AGTGATCTGCCTGCCTTGGCCTCCCAATGTGCTGGGATTACAGGCGTGAGCCACCACATCCGAC
CCAGAGACATACTTAAAGAGATAGATTAAAAATAAAAAGAAAGATAAACCAGGCAAATGTGAA
CTGGGTTGCGATCTTATTATCTGTCAAAGAGGAATTTTAGTTTCAAAAGATGAGATGGAAAGTCA
TTATTTTTTCTTAAAAGGAACAATAGACAAATACATAGATACAACAGGCAAAACACACAGATGC
ACCTAATCATACAGTCTTAAAATATACAGAACTCAACCTGGACAAACATGGCGAAACTCTGTCA
CTACTAAAAATAAAAAATAAAAAAATTATCCAGGCGTGGTGGCATACACCTGTAGTCCCAGCTA
CAGTCCCACCTGTTTCAAAAAATAAACATAACTGAGCCAGGTGCAGTGGCTCACACCTATAATC
CCAGTGCTTTGGGAGGCTGAGGTGAGAGGATAACTTAAGGCCAGGAGTTCAAGACAAGCACCA
TAGCAAGACCACATCTCTATAAAAAATAAAATAAAAATTTACCAGCATGGTGGAACAATTCTGT
AGTCCTAGCTACTCAGGAGGCTGAGGTGAGAGGATCGCTTGAGTGCAGGAGTTTGAGGCCAGA
GTGAGCCATGATCATGCCACTGTATTCCAGCTACACACAAACAAACACACACACAAAACAACTG
CAGAGAAAAATCAAGAGATGCTTAATTATAGCTGAAGACTTTAACATCCCTCTCTCAGAAACTG
ATATATCAAGGCTTGAATAGTATAATTACCATATGTCAACAATTCTACATCGTATAAGAGTAGAC

TCTAGGAGGGTTAAAAACATAATAAATGTGAAAAACTAACAAAGAAAAATATGAGATAATTATA
GTTTGTGACTGAAAAGGAAAGGCAAAACTTCAAATGCACAGACTGTAAGACAATTTTTTTTGAG
ACAGGGTCTCACTTTGTCACCCAGACTGGAGTGCAGTGGCGCAATCTCAGCTCACTGCAGCCTC
AACCTCCTGGGCTCAAGCGATCCTCTCACCTCAGCCCCCAAGTAGCTGGGACTACAGGTATCAG
CCACCATGCTCGGCTAATTTGTTGTGTTTATTGTAGAGATGGGGTTTCACCATGTTGCCCAGGCT
GGTCTCGAATTCCTGAGCGCAAGCGATCTGCCTGCCTTGGACTCCCAAAGTGCTAGGATTACAG
GTGTGAGCCACCACACCAGGCTATAAGACAAAAATATTGATGAATATAATGACATTAAAATTAA
GAATTTCTGTTCAACAAAGAAGATTGTTGAACAAAATAACTAGATAAAAGACAACATGGCTGGG
CGCGGTGGCTCACGCCTGTAATCCCAGCACTTTGGGATGCCGAGGCGGGTGGATCACGAGGTC
AGGAGATCGAGACCATCCTGGCTAACACGGTGAAAACCTGTCTCTACTAAAAATACAAAAAATT
AGCTAGGCATGATGAAGGGCACCTATAGTCCCAGCTACTCAGGAGGCTGAGACAGGAGAATGG
CATGAACCCGGGAGGCGGAGCTTGCAGTGAGCCGAGATCGTGCCACTGCACTCCAGCCTGGG
CGACAGAGCCAGACTCTGTCTCAAAAAAAAAAAAACAAAAAACACAACATAACTAGATAAGAGA
ATGGGAGAATATGTTCAAAATACGCAACACACAAAACTGGTAAATAAATAAGAGGGCAGCAAA
CCTTAGGAAAAAATAAAAGCAAAACATACAAATAGTTGGCATGAACATTATCACCAAAAAAAG
AGAGAAAAAGATATAAACAGAGAGGAATTCCAAAAATCAAAAGGCATATGAAGAGATGCCCAA
AGTACCACAGAAATGCTAATTAAATCAGCAACGTACTATCCTTTTACATCTACTAAGCTACAGAA
ATTAGAATGCTTGATAATGCCAGCTGGTGGAGTTGTGGGGATGCTGGAACTCTGGTGGGTGTAT
GGACTGGTACAGCCACTGTGGATCACAATCTGACATGAAGTATGCAAATTCAGAACTCATACAC
TCTATGAGCTTGCAGTTAGACTCCTGGGATATATCCCAAAGAAATTCTTATGCAGGCACATAGG
GGTTCTGCATTAAAATATTTATTGTGTGCTATTTATGGTCATAGGGATTCAAAGACAACCTGGTTA
TCATATGGAGAGGAGATAGCTAAAATGTGGTAGATGTTGTATTGTAAAATATTATGCAACAATCA
AAAGCAATGGATTAGATGCATACACAGCAAGATGAAGGCATTTTAAAACAGTGCTCAGTGAAA
AATATGAAGAAAAAATGCGATTAAAACATGATGTTATTTGCAGAAATTAAAAATGTATTAATACA
AAACAAAAAATTCACTTTTTATAAGAACAGATACAAATAAAAATAACAGACACTTTAAATAAAACT
TAATTATCTAAGTGGGAAACAGAAAAGGAATAAAAGAGAAAAAAATGGACAAATAGGTAGGGC
ACGGTGGTTCACCTGTGAATCCCAGCACTTTGGGAGGCCAAGGCAGGTGGATCACTTGAGCCG
AGGAGTTTGAAACCAGCCTGAGCAACATGGCGAAACCCCATCTCTACTAAAAATACAAAAATTA
GCTGGGCATGGTGGTGTGCAGCTGTAGCTGTAGTCCCAGCTACTTGGGAGGCTGAGGTAGGAG
GATTGCCTTGAACCCAGGAGGTCGAAGCTGCAGGGAGCCAAGATTGCACTACTACACTCCAGC
TTGGATGACAGAGTGAGATCCTATCTCAAATATATATATATAAATAAAATAAAATAAAATTTAGA
AGTGACATTGTCACCAACCAAAGATGATAAACTACCATGTACTAAGTAGTGTGATTAATTAAATC
CTCAAAACTGGAAGGAAATATAATAATATTGTATATTATACAATAATAATATTAACTTTCTTCCTA
ATACATATGTTGACTCCAGATTTCTACACTGAATACCTATTAATTTTCAAAATTAAATATACAATC
ATAATTTTTAAAAAAATCATCTGGAGGAGTATGGTTCAGTTGCAGAATGGTGGTGTTCAAAAGAT
GTGTCCTGGAAAGAAACACAAAAAATTTCAGTCTATGTGCAGAATGGGTAGAATTATGCAGGCA
TTTTTTTCTTGTTAAAATTACAATTTCTTTTTGTTTGGTTTGGATTTTTTTTATTTTGATTTTTTATAGA
GACAGAGTCTTGCTGTGTTGCCCAGGTTGGTCTTGAACTCCTGGGCTCAAGTGATCCTTCTGCCT
TGGCCTCCTAAAGTGCTGAGATTACATGTGTGAGCCACCACACCTAGCTTGGTTTTTATTTTTTTG
AAACAAGGTCTTGCTCTGTTGCATAGGCTGGAATGCAGTGGCATCATCTCAGCTCACTGAAGCC
TCTATCTCCTGGGCTCAAGCGATCCTCTAACCTCAGGGTAGCTGGGACTACAAACATGCTCCAC
GATGCCCAGCTGATTTTTAATTTTTTCGTGGGGATGGAGTTCCACTATGTTGCCCAGGCTGGTCT
AGAACTCCTGGGCTCAAGTGATCCTCCTGCCTCGACCTCCCTCAGTCCTGGGATTACAGGTGTG
GGCCACCATGTCTGGCATACCATAATTTCTGAAACAGTGTATGCACAGTCAATCAAAATAAAAG

GCAAAAGAAAATTTATCAAAGGTGAGAGCAGCAGCTTTAAAAAATATTCAAATGAATCCTTACT
GTAAACAAGGATTACAAAATCCTGCCTGAAAATATCTTCATCTGGTGATACTGAAAATATGTAAC
AATTGGTAGAGCATACATCTGAGCAATCAGAGCAGACATTTATAGTAAACAATGCTCATATCAG
ACTGGCATCCCTAAAATATAAATCACCATCCACACCAGTCCTCCAGCCCCAAAACAAAATATTT
AGCCTCAGCCCCTGAAGCTTAGCTCTTTTTCACAACAGGAAGACTCTCAGGCTGTAGAAATTGG
TATAAAGTCAAGTTTTCCAAACCAGAATATTCCAAAGGTTGTAAGGTCTAGAAACCACACCAGG
CACCAGTCTACAGGGAGGAAGGAAGAAGTTCTCACTGCTGCTCCCTGAACTCACCACCCTACTT
CCTGTCATCTGGTGCTCTGCTCTTTGACTCTGAAATCTGGTCCACACATCCCCTTCTCCTTTGGGC
AGACTTGTGCATTCTTTTTTGTTTTTAACCTTCCCCCAAAGATGCACACTCATGCATTCTTTAAGA
GCCGCCTCAAAACTCACTTCCTTCATGAAGTCTTTTCTCAAACCACCAAAACTACCAGGCAGAA
AATTGTTCATTTATTCAACTAATTTTTACTGAGCACCTACTATGTTCCAGATCCTGCACCTGCTAT
GTTCCAGAGCCCTGGCTTATCATTTACTGAACTGTATGACAGCCATGTCTGTGGGTCTGACTCCG
TGGCTGTTGGATATAGTCATTTAAAAATACATTTGAAGGGCAGGTACACTGGCCTACGCCTGTA
ATCCCAGCAGTTTGGGAGGCTGAGATGGGCAGATCACTTGAGGTCAGGAGTTAGGGACCAGCC
TGGCCAACATGACAAAACACTGTCTCTACTAAAAATACAAAAATTAGCCAGATGTGGTGGCACA
CGCCTGTACTCCCAAGCTACTCGGGAGGCTGAGGCAGGAGGATGGCTTAAACCCTGGAGGTGG
AGGTTACAGTAAGCTGAGATTGTGCCACTGCACTCCAGCCTGGGCAACAGAGCAAGACTCTGT
CTCAAAACAAAACAAAACAAAAAGCACTTAAAAAGGCATTTTTTTTCCATGTGGAATGTCGGTT
GCCTACTTTATGTATTCAATAGTAATAGATATTCACTCACTCATTCCACAGCCTGGTGCAGTGGA
AGGATAAACTGTTTTCTTTTATAGTGCTTTGTTAGAGTTATTTATAGGGTTATTGTTTTATGGAAGT
ATAAACTTCGAAACCAGACAAGCTTGAGAGACTGGTCCTGTCCCTTACTAGCTGTTTAACCTTGA
GCAAGTGACTTACCCACTCTGAGCAGTTTCCGCATCTGTAAAATGAAGATAAGAATGGTATCCT
CTACAATGGCTGCTATGAGGATTAGATGAGCACATTGTAGAAGCTCCACAAATTTATGGCAAAG
TGCTTTCTAATCTATAAAGAGCTACAAGAATGTGCATGATGACACGTGAGCAGGTAGTACAGAA
AAAAATAAATATGAGACTAGCAAACTTGCAAACAAGTGACAATTATTTAAAAGTTAGGGCACAA
AACAGCAGAAAAGCAAATAGATTTTCACAAAGTATTTTTTCTTATGTTAGGGGGAAATCTAGTTG
TTAAGTGCTTGATACATCTGCAAGAGGAAAACATCATCTGTCCACAAGTATAACCATCAGCACT
GCTCAGTAAATACTAATACATACGTTTTTATTTTATTTTATTTTATTTATTTTTTGAGACGGAGTCTC
GCTCTGTTACCCAGGCTGGAGTGCAGTTGTGCGATCTCGGCCCACTGCAACTTTCGCCTCCCAG
GTTCAAGCAATGCTCCTGCCTCAGCCTCCCAAGTAGCTGGAATTACAGGTGCCCGCCACCAAGC
CCAGCTAATTTTTTGTATTTTTAGTAGAGACAGGGTTTCACCACATTAGCCAGGCTGGTCTTAAA
CTCCTGACCTCAGGTGATCGACCCACCTTAGCCTCCCAAAGTGCTGGGATTACAGGCAAGAGCC
ACTGTGCCTGGCCCCATACATTTAAACAAAATAAAATCCTTGGGTGTCCTGGGATAAATTGAAA
GTGAGGATTAAGTTTATACAGAAGTTTATTTACTGATAACTAATTTCATAAACACCATAATAATGA
TAGTAGTGATAGCAACCATTTTTTGACTACCTATTTGTATCAGGCACTTTACATACATTAGTTCAT
TTAATCCCTACAACCTCATAAAACAGAAATGATCACTCCCTTTCTACTAATAAGGAAACAGAAGC
CCCAAG

ID|55975|gene_id|ENSG00000227070|transcript_id|ENST00000426017 Homo sapiens lncRNA

CTGGCACCAAGACTGGCGGAGCCACCACACCCGTTAAAGGGATCCACCTGGACAGCGAT
CAGGCGTGAGCCCCCCGAGGGGAAGTTGAAGTGGAAGCAGACGTGGGTGGCAAAAGGCTGCA
TTGGGGGTCTGACCCAGAAGGAAACCTGATCAGGAGGGGGGAGAGGCTGAACGGAGGAAGG
GGCCGGCAGAGTGGGTAGTTGAGTAGGATGAGACTTCTTTGGAGGCGAGGCTAACTACATTAA
ACTCTGGGAAGGGAGGGCTTCCGTCGGACCCTGGGAGAAAGGCAGGTCTGGAGAGGAAAGAC

TGGCTTGGGGGGCCTGCGCGTCCGGCTCTGGAAGGCAACTCCTACTGGGGGCGGCGCGCAGC
CGGCCTGGCGGGCGAGCCTCGTGCGGCCCGGGGGTGCGGCCGGCCCGCGCGAGCTCAACAG
GAGCTCACCTGGGATGCGTGCGGCCCTGTGGGAGGAAACTTCCTGGGTAGGTGAAAGCTGCCC
CGCGGGAGCCGGCCCGGCACCGGCAGAGCGCGGCCGGGTCACAGTCCACTCGGCAGCGGCG
CGGAGGGCGGCGAGGGCCGCGCGGGGCGGGGCAGGAGGCGCCCGACGACCCTGGGCGAGG
TCGCGGCGGCCGCAGGGGGGCTCCGGCTCCGGAGGGCGCCCTCCCGCAGTCGCACCCGCTTT
CCCTCACACACACCGGCCGCCCGGCCCACAAGCCAAGAAGCTGCCTGCTTGGCCCACAAGCCA
AGAAGTCGGCGAAGCTGAGGGCGGTGGGGGAGGGGGCACCGGCCGGCCAAGCCCGGCGGAC
GGGCGTGTGGCGTTACCTGTGTCAGAGAGAGCTGGGCCGCCGCAGCCATGGTGTTTCCATCAT
GCAAGGGAGGGGAAGGAAGACGGGCTGACTGGGGCTCGGGGCGGGGAGGAGGCGGAGGGA
GCGGCCGCAGGCGAGGCCGGGGCGGGCGGGCGGGCGTCTCCCCTAACTTCCACAACTTCCCT
CCCCACGCCCGCCCCGCCCCGCCTGGCCCCGCCCGCCTGCCCGCCCGCCGGAGCCGGCCGCG
GCGCCGCCCAAACCCTGGCGCCGGAGTCTCGGCCACCGGACCAGAGCCTGCCCCCGGGGCCG
CCTTGGAAAACCCGGCGCGGACATAAAATTAAAGCGGACGGGCTGCGGAGGCGACTGCAGAT
TGACCTGTGAACTTCAGACAGGCCACCTACCTACGCTGAGCTCCTGCTTCTGAAAAATGGGGGA
AATTGTAACTAGAGGCCCAGTTACTTACAAAGACTAAATGAGATAATGATAGAAAATAATAATG
GCACTTTATATATGCCAGGCTCTGTACTAGACTCTTATTTTTTGAATTGTATGAATGAAGGAACTG
AAACACAAATAAGTGTCTTACTAAGGTCATACAGCTAATTATATAGCTGAATTAGAATCCAAGTC
TAACTGTAAATATTTAACCATCTGCTAGTATATTGGAAAAGAAGACATTAAGTCATTCCCTCCCA
CAGCCAAAATCCATTTGGAAAGGGGAAGCAAAAATGTATTTGCCTGGTGCCTGTCACATTTAGA
TTGTTTAACAAGGGAACGCTGGTGCCTGTAGCACTCCTAGCTTCTCCCATCATCCACTTAGCAAA
CTCCTCCTTCTTTCTCAGGACATAATTCAAGTTTCACCTCCTTTGCAAAGTATCTTTTTCTCTTCCT
TATGTCAGAGTTACACACTCTCCTTTATAACCTTTCTTCTCTACCTTGTGAAGTCATCACCCTAAT
GACAGTTTACACCTATCTATCCCTCTAGATTAAGAGCTCCTCAATGGCAAGAACTGTTTTCAATA
TATTTCTATATCGTCAGACCCTAGCATTACAGTGCATGCTCAGTAAAGGCCTTCGTTCCTGCCTAT
ATTAAACATAACACATCCTACACCTTCTGATGCTGCCAACCTTCAGTTGGAATACCCAGTTACTT
GAAATTCATCTTCAAAGTCTCAGAGTATATACCACCTACTTCAAGAAGCCTTCTAGGAACTCCAC
ACCCCACCTCTAAGTTGTTCTGATGTGACCGTTTTTATACTCTGCCTTATCTGTAAGCGTTAGTTG
AGGGTTTGTCTCCACCTCACTGCACTGTAAAATCCTTGAGGACAAGGGGCTGACTCACTGCTTT
ATCTCCCTCATAGTTTAGCCTACCACAGAGGCCTCAATAAATGTATTTGCCCGAATTGGTCTTAA
TTTCTTAGTCACGATGGCTTCCCATTACAACAAACCACAACTCTATCAGGCCTACACCCATTACC
ATTATCATAGCAGTCCCCGTGGTAGGAGGGACAACTGAACAAGACCCAGGATGGAGTTAGCCC
CACCTAAGTTTTTAGACTATTGCCTCCACAAGCAAACCATTCCAGAAATTTCCCAAGGAACTCCT
AATTCTACTCAGACTCCTTCTGGCAAAGGCCAGCTAACAAGAGCAAGCCATATAGGTCTGGTGA
GTAACCACTTATTCAAGAGCATCTTCCTTGGCTTCTTTTTTGCTGCTAATCTCTTTAACTGAAGAG
AGAATAAGAGATACTGTTTTACATTACTTCACTTTGGTACACTTATCAGCCATAACCACAACCGG
TGTTTCTACCGGTGTTTTCTACTTTATATTAGCACATTTGAGCTTCACAACTCTAAAAGGAAGTAA
TCGTCATACTCACTTTACAAATGAGGTAATGCAGGGTCACAGAGGCTAAGTGATTTGCCCAGGG
TTATACAACTGATAGACAAAGCAGCATAGTATAGTGAAATAAATAGGACTCAGACATTAGGAAT
ATCCAGGTTCCATTCCTGGCTCTCCCATTCACTAGTTGTATGCCCTTAGGGTTGTTGTGAGGATG
AATGAGATGACATATACAAAAGTGCCTAAACTTGTAGCTACACAAATTATGAAATCTGAAAATC
ATGGAGCAAATCGCTTTGGGGAGTATAATTTCACTTGCTATTCAAGGGGACTCCTCCAAACCAT
ATGAGATGTAAGATATTCCTCTCAGATGAATAAGGTGGAAACCCTGCCATGTAGTTTCCGGAAA
GGCAGTGCAGAGTAGCAAAGAGAATATTAAGAATCAGAGTGTGACCGCAGGCAAACCATTTAA

CCTCTTTGAGCTTCAGTTTCTCATACAGGAAAAGTAAGGAATTATTACTGTTTTAACTTTTATATG
ATTTATATTAAGTCAAAAGCACACCTTACCCAGGCATGGTGGCACATGCCTATAGTCCCAGCTA
CACAGGAGACTGAGGCAGTTGCACAGAGCACGAATTCAGCCTGAGCAACATAGAGAGACCCC
ATCTCTAATAAATAAATAACACCTTGTATGTATACAGTATTGCTTTTCACCGTTTATAAATGTTTTT
ATTTTATTTTTATTTTTTTGAGACAAGGTCTCACTCTGTCACCCAGGCTGGAGTGCAACGGTGCAA
TCTCGGCTCACTGCAACCTCCACCTCCTGGGTTCAAGCGATTCTTGTGCCTCAGCCTCCTGAGTA
GCTAGGATTACAGGCACCCGCCACCACACCAGCTAATTTTTTTTTTTCTAGTAGAGACAGGGTTT
CACCGTGTTGGCCAGGCTGGTCTCGAACTCCTGACCTCAAGTGATCTGCCTGCCTCGGCTTCCC
AAAGTGCTGGGATTACAGGCGTGAGCCACCGTGCCCGGCCAAATGTTTAGAAGCACAACTTCT
AATGTTCAGTAAGTACATTATAAAAGTTTGTTGAATTGAACTGAATACAATCTCATATATAATTGT
TATTTGGATCTTCACTTTATCCCCATAAGAAAATCAAGGAACAAATATTTTATAAATTTATTTTATA
AATAGCTTCTATAAGTAAGAGCCATTGACAGCAGGCTTGGGTCTTGGTTAACAAAAAATAAAAA
GAATCAGATGCCCTAGCAAGGGAAGACTTGCTTAAAATCTTTATATGGGAATTGCCCCACGTCC
CCTTCCAGACCACTAGTAATTTGTAGTGGCTTCTTTCTTCATTCGTAGGTTCTGGTTTAAATTAAT
GACATGTAATGGGAGATGGGAGCATGTCTATAGGGAAGAGAATAATGGGCCAAATTCCCAAAA
AAGGCCTAGAACTTCTGAGAGAGGTGTTTTTGTTTGTTTGTTTGTTTGTTTGTTTTAATGACAAAC
CAATGACAATTTGCAAATGTGTGGCCCAGATCTCGTCAGAGCACTGGGTCATATCCCAAATCTA
TTTTAGGAGAGGAAAAATGTAGCCAATCGAATATATGTTATAATCTAATTCATTATAACAAGAAT
GAGTTGCATGGAGCAGACAAGAAGAATTTATCTGTTTTTTCTAGGACAACTTAATGTCTCTGGGT
CACTAAGCCTCATTTTCCTCATGATTACATTGAAGATAATAATATCTACTTTGTATGGATGGTTTA
AGGATTAGAGCTCATGTATAATGGCCTATCAGAGAGACTGGCCTGGAATAGATGTTCAATAAGT
AGCAGCTATAAGATACCATTCTTCAAGATCACCATTTAACTCTCTACCCTTCAGGCAACCCTCCA
ACAACTAAGTTGGTTTGACAGATATATTAGTTACATGAATGGGTCTGCCAGACCAGACCAGGTG
GCTCACATTTGTAATTTTAGCACTCTGGAAGGCCGAGGCAGGAGGATTGCATGAGCCCAGGAGT
TCAAGACCAGCCTGGGCAACATAGTGAGACCTCGTCTCTATTTTTTTAAAATCAATCAAATAAAA
ATAAATAAATAAATAAGAATAAGGGTGCTTTGAACAACTGACATATCCCTGTCATTGAAAAAGT
AGATGTTCCCTAGGTAAACAAAGTTGTATCTGATGACTTAAATCTGAATACTAATAACCAACAAT
TGCTGTTTTCTTTTTTTTGTTTTATTTATTTATTTATTTTATTTGAGACAGTGTCTTGCTATGTTGCCT
AGGCTGGTCTTCAACTCCTGGGCTCAAGTCATCCTCCTGCCTCAGCCTCCCAAAGTGCTGGGAT
TATAGGTGTGAGCCACCGCACCCAGACAAATTGCTGCTTTAATGTGCATTATTTCATTTATTCCTC
AAAACAGCTCTACAAGGGTTATTTTTAATCTTTTTTTTTTAACAGAAGAAGAATTTGTCCAAATTC
ATAATGACAAAGCTGGGAATTATACACAATTATACACGGGTCTTCTGATTCAAATGCGCTTTTTC
CTGGGGTGTGTTGCTGGTGTCCTGCAGACATTAATCACCACCCAGGAAGACTTGTTAAAATTTTG
CCCTTGAACATGGGAAGCTGAAGATGAGGCTCTGGATTTACGATAGCCCTAAATCCAGGATGA
GCCTAAAGAGAAGACCTTAAATCATAAACAGCTTTGTCACCAATCATCTTTCTATCCTACTCTAA
CTCCAACCCCCCTCCCCAACCCATCCTTAAAGGAAATCAAAACTACTTTTATTTTCTTATTTTCTA
GCTTTTAGAGGCAGTTGTCATATCAACGCTTCATGAACACTGCGTGTCTGTGAAGTCAGCATCTA
GAAAGTTGAGAAATGTGAAGTGATTGAAACTATGGAAAAAAAAACTGAGGAACTCTCTGAATA
CACAAAGGGCCAGTTTATTTGCATGAGACTTTGAGAGATGGGCTTTCCATATAGATGGTGCTAA
ACTGTATTTTAAAATCTTACTACAATTCTCATGGTAATCTGAAGCTCGTGCTTCTAAGCTGCTGGT
TTCATAAATATAGTCCATCATTGTTCGCGTTTGCCCAGGGCAGAAAGAATATGTGAGGCAGCAC
AATAACAACAATTGTGCTATGCAAATAACCATCCCTGTGCCAGGGATTGAGCTATGAAAAGAAC
AAGCAGTAAGAGATTTGTAAATGCCATACACACGCAGGGTGTGATAGAACAGTTCAAAATTGTC
ATCATCACTAACTTTTCAGGGCAGTGGGATACCCTGGGAAGAAGCTGAGTTTAGGACTGGGAA

GGCCCAGGTTGGTCCAGTTTTGGTCCTGCAGGTTCTTTGGTGACTTTGCAGAAGTGAGGAACTAT
ACCAAGCCACGGTTTCCTCTCCTGTAAACTAAAAGTAATAATAATACCTACCTGCTCATGCTATT
ACAATGATTAAATGAAGGTTTATGTGAAAACCTGGGACACAGCAGAAACTAGATAAGCATTAGC
TCCTTTTTCTTCCTACTGGAGAACATGTAGGCTTTAAGTAACCAGAACACAAAAATAGAAAATAT
TTTTAAAGGTTTTCAGTGGCCTTCCAAAGCCCACTTAATGGTTTTTTACATAAAATTTATTTAATCC
ATTAGTAATATTTATTGAGTACTATACAGTAAGAGAGATTATTATATTTATGTCCCCAAAGGCCAT
TGGCTTCTAGTGAGAAAGCAAACACTGTTTTGCCCTTGCCCTATTTCCTTCCTTTCCTCTCTGTCC
TTTATGAAGAGTGCTGGAAGCCATAAGAAACCAAAAGGAACACCATAGGAAAGAAACCTTGCA
CTCATGGCTGCAGGTTCTTTCATCTGCAGGTTTCAAAGCATTTTCTCCATAGGTAATTACTAGCCC
TACTTACCATGTGTGAATGCTGAGACATCATCAAATTAAGAAGCTTGCCCTAAATCACAAGGCA
AGTCAGAGGATGGGCTGCAGACAGAACAAAGCTGTCCTGTTTGCCAGACTTGGGCTCTAAAGT
AGGGCACATGAGTGTCAACAGAAGACTTCAAGTGATGAGGAAAAAAGTTTGAGGAATCCTGAT
TCTGAATCAGCGCAATAACAAGAGCATGGGCTTCAGAGTCAAATTTGATTTCAAATCCCAGCTC
AGCCCCATTTGACTGGCTGCCTGACCTTCATCAAATCATAAACTAAGTCTAGTTTCCCTTTTTGTA
AAATGAGGATATTCTTACTTCTAAGGGTTTTGGGGAAAGTTAAATTATGTAACTAGTGTGAGAGA
GCCTGGTTCAGTATCTGTCACTTAGCAGGTGCTGATAATGTTGGTTTATGTCATTGGGAAAAGTT
AATATATCTCCCCCACAAACACAAAATAACAAATAGACAACTCTTTCAGTCAAAGATTTTCAAAG
CCATAATCACAGTTGTGTCTCAGTTTAAACCCATGACATCTCACATTATAGCACAAATAGAGGAT
GCCTTGTTCAGGCCTTCATCACTTTTCTTCCTTGATTCTGTGCTTTCTCTCATAGCCCAAGTCCAAT
CTACCAGAAAGTTCTGTCGGCTCGATCTCCAATATATATCCAAGATCCTAATTTCCAGGTCTGAC
CACTTTTCACCACCTCCACTACCATGGCCTTTCTCTTGGACCACTGCTAAAGTTTCTTAACCAACA
TCCCTACTTCCATGATTTTGCCTAAGTTCATTCTCCATATCATAAACTGAATGATCTATTTTTATTT
ATTTACATCACCCAGCTGCAGTGCAGTGGCACGATCATGGCTCACTGTAGCTTTGAACTCCTGG
GCTTAAGCGATCCTCTTGCCTCAGCCTGAATGATTTTTTTTGTTCTTTTTGAGACAGCATCTCACT
CTGTCACCTAGACCGGAGTGCAGTGGTCCAATTTCAGCTCACAGCAGCCTCCATCTCCCAGGCT
CAGTTGATCCTCCTACCTCAGCCTCCAGAGAAACTGGGACTACAGGTTCACACCACCACACCCA
GCTAATTTTTGTATTTTTTGTAGAGACAGGGTTTTGCCACATTGCCCAGGTTGGTCTTAAACTCTT
GGGCTTAAGCAATCCACCTGCCTTGGCCTCCCAAAACGCTGGGATTACAGGGGTGAACAACTG
CACTCTGCCTGAATGTTCTTTTTAAAACATAAACCAGATCTTATTATTTTCCTGGTTAAAACCCTA
CAGGGACTTCTCATCAAGCACCTGAATATGGCCTACCGTGGGTAAAGTATGTGTCAGCAAAGCC
CAGAAGAGCGAGATGTCTGGTTAAATGGAGCATGGTAGAGATGAGAGAGAAAGAAACTAGCA
GAGAAGGGTGGAAGGGGTGTTAGAAAATACCCAGAAGGACTCATGGCTGGACTACAAGAGGC
TGAGAGAAATGTTTCTCTCTGGAGATTCTCTTTTTTGTAAAATGAGGATATTCTTACTTCTGAGGA
TTTCGGGGAAAGTTAAATGATGCAACTGGTGTGAGAGAACCTAGTTCAGTATCTGTCACTTAGC
AGGTGGTGGTAATGTTTGTTTATGTCATTGGGGAAAGTAGCTCAAAAGAGTCCCAAAAATAGCT
CAAGAGATTCACAATCAACACTGGAAGCAAAGGAGATTCTTACTGTTTTCCATTGATGGCATTTT
TAAATCTAGTGGCCTGGAATTTTTAAGCACCCAATCAGGAATCTATTTTACCCTTTTTTTTTTAACT
TTTATTTTTATTTTTTGAGATGGAGTCTCGCTCTGTCACCAGGCTGGAGTGCAGTGGCGCAATCTC
GGCTCACTGCAACCTCTGCCTCCCAGGTTCAAGCGATTCTCATGCCTCAGCCTCCTGAGTAGCT
GGGATTAGAGGCAAGCGCCACCACACCTGGCTAATTTTTTGTATTTTTAGTAGAGACGGGGTTTC
ACCAAGTTGGCTAGGATACTCTTGATCTCCTGACCTCGTGATCTGCCCACCTAGGCCTCCTAAAG
TGCTGGGATTACAGGCATGAGCCATCGCGCCTGGCCTATTTACCCTTTTAAAAGTAAGAATTATA
CGCTAGGACCCCTAACATCAGGACAATGGTTAAGATGATGGACTTTGGAATCAAATTTGACATT
GAATCAAGGTCCTCCACTTATAAGCTACAACACCATGGTCAAGTTATTAAAACTCTTTGACCCTC

AGTTTCATGCAGACTACCATACAGAATGAGTAAAAGAATTAAATAGACAATGATTATAAAGCAC
TTTGCATAGTATCTGAGACATGGGGAGTAAGTGCTCAGCAAATGGGGACTATCCAATGATGATG
GTGATGATGATGATGATGATGATGATGATGATGATGATGATGGTGATTAAGAATTGGGTTATCTT
TTTTAAAAAATTTTTTTAGATACAGGGACTCACCCTGTTGCCCAGGATGGAGTGCAGTGGTGCAG
TCATAGCTCACTGTAGACTCAAACTACTGGGCTCAGGCAGTTACCCACCTCAGCCTCCTGAGTA
GCTGGGACTACAGACACATGTCCCCATACCTAGATAATGTTTTTATTTTTTATAGAGACTGGGTC
TCACTATGTTGCCCAGGCTGGTCTTGAACTCCTGGACTCAAGCAATCCTCCTGTCTCAGCCTCCC
AAAGTGCTGGGATTACAGATGTGAGTCACTGTGGCTGGGTGCCTGCCTGGGGTGAGATTTAAAT
TGGCCTTGTAAGCTAATAAAAAATGAAGTCTATTCTGAGGGCAATGTGGAGTCATTGAAAGGTT
CCCAGGAAGGAAAATAAAAATCCAAAATCATGTTATAGAAAGGTAACTCAGCCGGGCACCGTG
GCTCATGCCTGTAGTCCCAGCACTTTGGGAGACCGAGGCAAGCAGAGCACTTGAGGTCAGGAG
TTCGAGACCAGCCTGGCCAACATGGCAAAACCCCATCTCTACTAAAAATACAAAAAAAAAAAA
AAAATTAGCCAGGGGCTGTGGCACACACCTGTAGGCCCAGCTACTTGGGAGGCTGAGGCACGA
GAATCTCTTGAACCTAGGAGGCAGAGGCTGCAGTAAGCCAAGATCACACCACTGAACTCTAGC
CTGGGGGTGACAGAGCAAGACTCTGTCCCAAAAAAAACCAAGAAAGGTAACTCTATTCAGCAT
TATACTGGAGTTTCCAGCCAGAGGAATTAGGCAAGAAAAAAATAAAAAACATCCATATTGAAAC
AGAAGTAAAACTATATTTGCAGATGAGATGATCTTGTATATATAGAAAATCCTAAAGAAACAAA
CAAAAATCTATTGGAATAAATGAGTTCAGCAAAGTTGCAAGATACAACATTAATATACAAAAAT
CAATTGTATTTCTATACACTAGCAATGAATAATCTGAAAATGATAGCCAGGTATGGTGGCTCATG
CCTGTAAGTCCCAGCGACCTGGGAGGCTGAGGCAGGAGAATTGCTTGAGCCCAGGAGGCGGA
GGTTGCAGTGAGCCAAGATCCTGCCACTGCACTTCAGCCTGGGTGACAGAGTGAGACTCTGTCT
CGGGGAAAAAAAAAAACAAACTGAAAATGAAATTAAAAAATCAATTCCAGCCCAGCACAGTGGC
TCACCTCTGTAACCCCAGCACTTTGGGAGGTCAAGTTGGGTTGATCGCTTGAGGTCAGGAGTTT
GAGACCAGCCTGGGCAACATGGTGAAACCCTGTCTCTGCAAAAAAATGCAAAAATTCGCCAGG
CATGGTGGAGTACACCCATAGTCCCAGCTCCTCAGGGGGCTAACGTGGGGAAATCACTTCAGC
CCTCCAGGGCAGAGGTTACAGTGACCCCAGATTCCGCCACTGCACTCCAGCCTGGGAGACAGA
ACGAGACTGTCTCAAAAATAAAGAATAAAAAATATCAATTCCGTTTACAATAACATCAAAAAGA
AAAAATAGTTAAGAATAAATTTAATAGAAGTACAAGATTTGCAAATAAAAACTACAAAATATCAT
TGAAAGAAAGTAAAGAAGACCTAAACAAATGGAAAGACTTCTTATGTTCATGAATTGGAAGACT
TAAGATTGTTAATATGGTAATACTCCCCAAATTGATCTACTGATTCAGTGCAATCCTTATTAGAAT
ACCAGTTAGCTTCTTTGTATAAATTGACAAGCTGATTCTAAAATTCATATGGAATTTCCAGTAACT
CAGAATAGCCAAATAATCTTAACAATGAAGAACAAATTTGGAAGACTCATGCTTCCTGATTTTTT
TTTTTTTTGAGACAAGAGTCTTGCTCTGTTGCCCAGGCTGGAGTGCAGTGGCACGATCTCGGTTC
ACTGCAAGCTCTGCCTCCCAGGTTTGCCATTCTCCTGCCTCAGCCTCCCGAGTAGCTGGGACTA
CAGATGCCCGCCACCATGCCTGGCTAATTTTTTGTTTTTGTATTTTTAGTAGAGACAGGGTTTCAC
CGTGTTAGCCAGGATGGTCTTGATCTCCTGACCTCATGATCCACCTGCCTTGGCCTCCCGAAGTG
CTGGGATTACAGGCTCCTGATTTTAAACTTACTACAAAGGTATAGTAGTCAAGATAGCATGGTAC
TAGTATAAAGAGAGACATATAAACCAATGGAATGTAATTGAGAGTTCAAACTAACTCTATACATT
AATAGTCAATTGATTTTCAGCAAGGGTATGAAGATAATTCAATGGCGGAAAGAATAGTCTTTTCA
ACAGATGGTACTGTTGAACTACCATATTCACATGCAACTACATATTCACATGCAAAAGGATGAA
GTTGAACCCCTACGTCATACCAGATGCCAAAACTAGCTCAGAATGGATCCTTGACATAAATGTA
AAACTGTAAAATTCTTAGAAGAATCTAAGAGTTGCTTAAGTATAAAACTCTGAGAAGAGGCTGG
GTGCGGTGGCTCACGCCTGTAAACCCAGCACTTTGGGAGGTGGAGGTGGGTGAATCCCTAGGT
CAGGAGATCGAGACCATCCTGGCTAACACGGTGAAACCCGTGTCTACTAAAAATACAGAAAAA

TTAGCCAGGTGTGGTGGCGGGCGCCTGTAGTCCCAGCTACTTGGGAGGCTGAGGCAGGAGAAT
GGCATGAACCTGGGAGGCGGAGCTTGCAGTGAGCTGAGATCGTGCCACTGCATTCCAGCCTGG
GTGACAGAGTGAGACTCTGTCTCAAAAAAAAAAAAAAAAAAAAAACAAAAAAAAAAAAACCTCT
AAGAAGAAAACATAGATGCAAACCATCTTTATTAGAGGAGGCAATGATTTCTTAGATATAACAC
CAAAGCAGAGCAACAAAAGAAAAATTAGATAAACTGGATGTCATCAGAACTTCTGTACTTCGAA
GGACACCATCCAGAAAGTGAAAAGACAATCCAAGGAATGGGAGAAAATATTTGCAAATCATAT
ACCTGACAAGTGAATTCTAACTAGAATATATAAAGAACCCTTACAACTCAACAAGAAAAAGACA
AATAAACCAGGCATGGTGGCTCACACCTGTAATCCCAGCACTTCGGGAGGCTGAGGCTGGAGA
ATACCTTGAAGCCAAGGAGTTCAAGACCAGCCTGGACAACATATCAAGACCGTCTCTTAAACAA
AAAAAAAATTAGCCAGGCATAGTGAGCCCAAGAGTTTGAGGCTGCAGTGAGCTATGATTGCGT
CACTGCACTCCAGCCTGGGTGACAGACTGAGACCCTGTCTGTAAAAAAAATTAAAATTATGGCC
TGGCACAGTGGCTCACACCTGTAGTCCCAGTACTTTGGGAGGCCGAGGCAGGCAGATCACTTG
AGGTCAGTTTGAGACCAGCCTCTATTAAAGGGTGAAACCCTGCCTCTACTAAAAATATCAAAAT
GCCTGGAATCCCAGCTACTCGGGAGGCTGAGGCAGGAGAATCGCTTGAACCCGGGAGGCCGA
GGCTGCAGTGAGCCGAGATCAGGCCACCGTACTCCAGCCTGGGTGACAGAGCGAGACTTTGTT
GTAAAAAAAGAAAAAAATAGGGCCAGGTGCGGTGGCTCATGCCTGTAATCCCAGCACTTTGGG
AGGCCGAGACAGGCAGATCACCTGAGGTCAGGAGTTAGAGACCAGCCTGGCCAACATGATGA
AAACCTGCCTCTACTATAAATACAAAAGAACTAGCTGAGTGTGGTGGTGCATGTGCCTGTAATC
CCAGCTACTCGGGAGGCTGAGGCAGGAGAATCACTGGAACCTGCGAGGCAGAGGTTGCAGTG
AGCCAAGATCGTGCCACTGCACTCCAGCCTGGTGACTGAGCAAGACTCTGTCTAAAAATAAATA
AATAAATAAAAACATTTAAAGAAAAATATTATTTGGACTATTGTGGGGAAATAAACTGTAGATCA
ACAAGGGCAGAAACTGGTAAAACAAATATAAGATTATTGCAATAATCCAGATATGATGATGGCT
TGGTGGTGAGAGGTGGTAAGAAGTGGTCAGATTCTGTACATATTGAAGGTAGAGCACACCAGA
TTTGCTAAAGGATTAAGGATGAGGTATGAGAGGAAGAAAAGGATCACGGATGATGCCAGGGAT
TTTGCCTGAGCAACTGGATTGATGAAATTGTTTTTTACTGAGATGAGTAAGACTATGGAAGAAGC
CAATTTAGAGAGAATATCGGGAATTTGGCCTTTGACTAAATTTGATACACTTTTAGATATCCAAG
TTGAAATGTCAAGGATGCGGTTGGAATAAGAGTCTGGAGTTCAGGGGAGAGGCCTGGGTTGGT
GATATAAATTTGGGTGTCATTAGCATATGGAGGCATTTAAAGCCATGAGGTTGGGTGAGACTGT
CCAAGTGTCTATAATAAAACCCAAGAAGTCCAACTACCAAGCCCTGGGCCACTCCAATGTTAGG
ACTCAGGGAAATGAAGAGGAACTAGTAAAGGAGACCAAAGAAAGAGCAGCTGAGCTTAGAGG
AAAACCAGGAGAGTATGATGTCTGTCCTTGAGCCAAATGAGCAATGTGTTTCAGGAAGGAGCA
AGGAAGGAAGGAGTGAGAAAGCATGTCAAATCTTATCAAAGAGCTAGTTAGATGAGGATTGCA
ATTAATATCCAGAGTTAGCAATGTGGAAGTCTTTGGTGACCTTGGTAATCGTTTCAGGGGAAGGT
GAAAGAAAAGTATAATTAGAGGAGGCTCGGGAGAGAATGAAAAGAGAGGACTTGGAGTCAAG
AAAAACAGATAATTCTCTAGAAATGTCTAGCTGAAATGAAGAGCAGAGAAATGGTGTGAATACC
TAGTAAGGGATACAGAGTCAAGAGAGGTTTCTTTTCTTAAGGTGAGCAATAATGCAGCATTTTTT
AGAAAGTGAACGGAATGATCTGGTAGAAGAGGAATCACTGATGATACAAGCAAGAGAGGGAA
TTGCTGGAGTGCAGGGGAAAGAATTGGCTTTGAGAATACCGAAGAGGGCTGAGTATGTGGGCA
TGGATGCAGGTGGATGGGTTATCTGCTGAGAGGGAGGAAAAAGGAGAGGATATGAAAAGTTTG
AAAAAAGTTACGAAATAAGCATTTAGGAAAAGTGGATGGACTTGAAAATGTAGTATGATTCCCA
GGTAGCACTAAAAGCCCACTTGAGGTCAGTGGCGATGAATTTTAAGAGTCCAGTTGACACAGTT
GTGGGTTTTTCTTCATCCACTTTCCCAAAGGCAGAGAGTAGGTAGAGTTAGATTGTGCAGGGTA
GGGATCTTGTGAAGCAAAATGAGAATGCAAGAGGAGGCAAGAGAGAAGAGGACCTATCAAGG
GAGTGATTTTAACATTGGACCTTGGAATTTAGGTGGGTAAAAAGGGAAGTGAGGACACAAGATA

GTTGAGGAATGGTGAAGAGATATTAGGATCACTGGATTATAAGGCCCAGGAAGGGATCAGAGA
AGTGTTGGAGTCAGGGTGTTGGAAGGAGTGAGCTGAGAAACAGGAGATTTAGAAAGATAAGTA
AAATTATTGTTAGATTATTATTAATAATTGGTAATTATTAAATGGTAATTGGTAATTAAATGGTAAT
TGGTAATTAATTGGTAATTGGTAATAATTGGTAATTATTAATTGGTAATTAATAAATGGTAGATAT
TTATTAATAACTGGAAGCAACCTCTAAAAAAATCATAGTATTAAGACCCAGTTCATGCCCTCTAA
GTGCTCACTCTGTGATGGGAAAGACAAGACAGAATGAAATAAATATTAAAGAGAAGTACATGCT
GCTGGGTGTAGTGGCTCACGCCTGTAATCCTTGTACTTTGGAAGGCTGAGGTGGGTGGATTGGC
TGAGCTCAGGAGTTCGAGACCAGCCTGGGCAACATGGTGAAACCCCATCTCTACTAAAAAAAA
TACAAAAAATTAGCTGGTCGTGGTGGTGCATGCCTGTAGTCCCAGCTACTCTGGAGGCTGAGGC
AGGAGAATCACTTGAATCCAGGAGGCGGAGGTTTCGGTGAGTGGAAATTGCACCACTGCACTC
CAGCCTGGGCAACAGAGCAAGACTCTGTCTCAAAAAAAAAAAAAAAAAAGAAAGAAAGAGAAA
GAAAAGAAAAAAAAAAGAGGGAAGTAGATGAAATGGCTGTGGGAGTCAAAAGGAATCTGCGAG
TAAACGTAAGGGTCAGATCAAGGAAAGCCTCCAAAGCAGGTGGCATTTGAATCGAGCCTCAAA
ATGGAGCAAATGCAGCACATGGCCTGAATAAATGTTCACAGTTACACCATTGCAATGAGTGCTC
AGAAAACAGAGGTTGGAGTGCAGGGTGGGAGCTATGGCAGGACATGAGACTAGAAAATTATCT
GGAACAGGCAAGGGATTTTTGAATGCCAGGCTAATGCCTGGGCATTTTTTTTTCTTTTGTTTTTTC
TTTTTTCTTTCTTTTTTTTTTTTTTTTTTGCAGAGGAATAGATAATACTGAGGTTAGGGGTTAGAGGTT
ATTATTCTAAATAATTGATGCTGACTGGGCGCAGTGGCTCACACGTGTAATCCCAGCACTTTGGG
AGGCCGAGGCAGGTGGATCATGAGGTCAGGAGTTCGAGACTAGCCTGACCAACATGGTGAAA
CCCCATCTCTACTAAAAATATAAAAACTAGCGGGGAGTGGTGGCACATGCTGTAGTCCCAGCTA
CTCAGGAGGCTGAGGCACAAGAATCACTTGAACCCGGGAGGAGGAGTTTGCAGTGAGCTGAG
ATCGTGCCACTGCACTCCAGCCTGGGCGATAGAGTGAGACTACATCTCAAAAAAAAAAAAAATG
CTCCCAGCTGTTTATCATGTTATGAAGTCATACTGGTTTTTTTGTTTTGAACAGTCTAGTAATTTTA
TTCTCCATCATGTCCTTAAGTCATATTAGGGAAGTTTTATGCTTTGGATTTTCTGATTGTCTTTTTA
AGATCATCTCCCCTCTATGGCAATGATAATGTTGTTTCTTTCCAAATAATGTTCTGTCACATGCTC
ATTCACTTTTTTTTCCAAATGGCCTTCAACTTCATCCCCTGATGTTGGTAGGAAAGTCTAAGATCC
CTCAGCAGTCTGAAGCTCCTCTCAGAAAGGGGTGAGGAGTGGGTTGGATAGGATCAGAACTAT
GCTGTCCAGGGCATTCAGACCCGTTGAAGTGGATAAGAATTAAAGGTGAACAGTCAGATCCTCC
CAGGGAGAGGAAGCCACAAGAGACACAGCCTCACAACTGTGTTGTCACAGTGTTTTCAGAACT
CAGTACCAATACCAGTGATGGGGCCACGCTTTTTGCAAGTGACGTTTATTATGTCATTTGAAAAT
ATCAGAATCTCTCTCTTCACAGGCTAGCCAGATAAGTATTGAAACCATTAATTTCTCTACCATGTC
ACTGTGTCTTTGGGCAAGCCCCTTCTCTCCTCTGGACCCAGGGTCCTGCCTATAAAACGAAGGG
GCTCTATTCATCCTCCTCTAGGATCCCTTTCAATTCTGACAGGCTCTAATTTTTCTGTTATCAGTTA
TTCTTTATTAAACTTACAGTATGTGCCAGCTTTTTTCAAGATGTGAGACCCTCTCACCCTCTTACT
CCTCAACCCCCTGTTATAACTTATCTTGGGCTTTTCTTGTTTAAAACATTTGGAAAAAGTGGTGTG
TTCACGGGACCTTTATTTGCCCTAAGACATTTGAGGGTAAATCATGTGAAGGCCTTTGTTTTCTTC
CAGCAGCCAAAACAATAAAACCACCAAATTGCGTGCAGCATCATTTTCTACTGCCCTCAAGTGG
TGGTGGCCCAAATGTCTTTTCCTGGTTGCAGTTTAAGGAATAAAACATGTTTTCAGAAGAACTCT
AGGCATCTAGGAGAGGGTAGGAGAGTCATAGAGTCACAAAGGGCCTGAGGTGGAAAGGCCCT
TACAGGATGTCTGATCTAAGCCCCTCGTCTTGAAAGGGAGAGACTGAAGTCCACCTTGGTTCTC
TGATCAGTGAACAAACCTAGTTTCTTCTGTCTTTAAAGAGCTATCTTATGACCCTGACTCTATTTT
TGGCTCCATTTCTCTGCTCCCATTCACAGTGAAATTTTCTAAAGAGTTATCTCCATACTGTTTCTAT
TTCTTCATCTATGCTTTATTATTATTATTACTTTTTATTTTTTTTTAAAAACAAAATAATAATCTCACT
CTGTCGCCCAGGCTGGAGTGCATTGGGCTGATGTTGGCTCATTTCAGCCTCTGCCTATGGGTTCA

AGTGATTCTCCTGCCTCAGCCTCCCAAGTAGCTGGGACTATAGGAGGATGCCACCACACCTGGC
TAATTTTTATATTTTTAGTAGAGACAGAGTTTCACCATGTTGGCCAGGCTGGTCTCGAACTCTTGA
CCTCAAATGTTAACCCCCCACCCCGCCGCCCTTGTCTACCAAAGTGCTGGGATTACAGGCATGA
ACCACCGTGCCCACCTTTACACTTTATTTTTTAACTTTTTACTTTGAAATAATCTCAAACTTGCAGA
AGAGCTGCAGAAATATTACAAAGAATTCCCAGATGCCCCAGAAGTTCTCATGTGTGGGCATATG
CCTACATGCATATACATGGATATGTATATTTTTTCCTGAGCCATTTGAATTGCAGTAGTTACCCCT
AATACCTTAAATATTTCAATGTGTATTTTCTGAGAAAAGGACATTCTCTTATATACCTACATACCC
ACTCAGTATGACAATCAAATCCAGAAATTTTAAATTGACATAGTGCTTATATACAGTCCATACTC
AAATTTCACTAATTGGCCTGATAATGTCCTATATAGCAGTATTTTCCCCCATCAGGTTCTGATCCA
GTGCCTGATCCAGGTAATGCATTATACTTAACTGTCATGACTTTTAAAAAGGTTTTTTTTCTTTTAA
TTATTATTTTTTAGAAACAGGATCGCGCTCTGTCACCCAGGCTGGAGGGCAGTGGTTCAATTACA
GCTCACTTACAACCTTGAATTCCTGGGCTCAAGCGATCCTCCCACCCCTGCTTCCAGACTAGCTA
GCTAGTACCGCAGGCACGCACCACTATGCCTGGCTAATTTTTTAAAAAATTATTTTTGTAGAGGT
GGGGTCTCGTTATGTTGCCCAGACTGGCCTATAATTCTCAGCCTCAAAGTGATCTTCCTGTCTCT
GCTTCCCAAAGTGCTGGAATTTCAGGTGTGAGCCACCACACCTAGCCTGTTATAACTCTTTAGTG
TCCTTTAATCTAGAATAGTTCCCCAGTCTTTCACTATTATTCACGATATTCACATTTTTAAAGAGTA
CTGGCCAGGTGTTCTGTAGTGTCTCCCCCTGTTTGGCTTTGTCTCATGTTTTCTCATGAAGGGATT
TACATATTTTTCCAGGAAGAAATCCTAGTTATATGATGTTGTGTCCTTCTCAGTGCATCATATGGG
GAGAAACGTGGTGTCACTTGGTCCCATTATTGATGGTGTTCACTTTGATCAGCGGATTAAGGTGG
TATCTACCAGATTTCTTCACTGTAGAGTATTTTTTCCTGTGTAAGTAATGGTAATCTGTAGAGAGG
GACTTTGAAACTATGTAAATATCCTGTTCCTCTTCAAACTTTTGCCCAATGGTTTCAACACTCATT
GATAATTCTTGTCTGAATCAATTACTGTGATAGTTGCAAACTGGTGATTTTCTACTTCTATCATTCC
ATCTACATTTTTTAGTTGGCATTCTACTGTAAGCAAGAAGGTTTCCTTCTCTCTAATTATTATCATG
AGTATAAACTAATGTATTCTTAATTTATTTAATGAGTTATAAACATTACCCTTATTATTTTTTAAAT
GTTCAACTTGTCCCAGGTTTGGCCAATGAGAGCCTCTTCAAACTAGCTCCTGTGTCCTTTTGACA
TGTCTTCATCACTTTTTTTTAACACCTCCTTGCTTTCTGGCATAATAAGATGTTCCAGGCTTATCTT
GTACTTTCACTGCCTAGTCCTGAAAATCAGCCATTTCTCCAAAGAGCTACTCTTTAGTGGGGAAT
GGTATTTAGAAACCAAGATTTGGGCATCAGATGTGCTCATTCCTATTGGAGTGTAATTGCTTCTA
GGCCATTTTAGTAAACAGAGCCAAAGGGGAAAAATAGTAACCTAATTTTGTTTAATTATTATTAT
AATTATTTTTAACAGAGACAGGGTCTCACTCTGTTGCCCAGACTGGAGTGCAGTGATGCTATCAT
AGCTCACTGCAGATATGAACTCCTGAGATCACCTCAGCCCCTGGAATAGGCTAGGACTACACGC
CACTACACTCAGCTAATATTTTTAAATTCTTTATAGAGATGGGGTCTCACTATGTTGCCCAGGCTG
GTCTCAATCTCCTGGCCTCAAGTGAACCTCCTACTTTGGCCTCCCAAAGTGCTGGGATTACAAGC
ATGAGCCACTGCACCCAGCTCTGGTCAACCATTTTAAATTAATAATTCATTACTGATACTTTAATT
CCAATTTAACACCACAGGGTTCTTTCTCCTATTCCTTATTTGCATCTCCCTTTTCCCACAGTAAGA
ACTCTGGCTCTCAACAACATCAATGTCTTTATTCATTTGTTTAAAATTATGATATAGGCTGGGCAT
GGTGGCTCATGCCTGTAATCTCAGCACTTTGGGAGGCCAAGACGGGCAAATCACTTGAGGTCA
GGAGTTCGAGGCCAGCCTGGCCAACATGGCGAAACCCCATCTCTACTAAAAAATACAAAATTTA
GCCGGGCATGGTAGTGCATGCCTGTAATCCCAGCTACTAGGGAGACTGAGGCAGGAGAATCAC
TTGAACCTTGGAGGCAGAGGTTGCAGTGAGCCAAGATCACGCCACTGCACTCCAGCCTGGGCA
ACAGAGCACGACTCCATCTCAAAAATAAATAAATAAATAAATAAACAAGATAGACACAAAATA
GAATTGTTACATCCATTCTGCTAACAAAAACAATCTACTAAACAGGGTTCAAGATTTGTTTGCAG
TTTTGTTTTGTTTTTAGACAAAAGCTGTATATTCAAGTACTATGTTCAAAAGTATTTAGATTCATTT
TTTTTCCTTCAGAGTGATTATATTACTTATTTAAATATAGTTTGGTTCACTTGTTTCTGTTTATATTC

AGTTTTAGGATCCCTTCCATTCTTGTTTATTTAATTCCGTTTTTAAATATGTAAAGGTAAACATTGT
CACAAAATTAAAATCTATACAAAAGGCATATAGAATGCTTCACAGATGTGTCATCCTTGTGTAGG
AGTCAAGCCAATTTTCTCTGTATTGTTCCTTTTTTTTTGTAAATATGCTGTCAAAGCAAGCACACC
TCTAATTCATTCTTCAACTTATTTAATTCTTACTTAGACCACCCACTTCACTGACCCTACTACTGCC
AAGATCATCAGTGTCCAATATTTTCTTTAGTCTTATTTGACCTTTCCACTCAGCTGACCTCTCCAAT
TTTCTTGAAACTTTCTCGTCTCTTGGTTTTTGTGATACCACATTCTGCTGATTTACTCTCTCTTCGC
AGGTCTGTCCTCACTCTCTTTGTCTATTCCACCACCCTAAAATCCTCTGGACTCTTCTTGGCTCTC
TTCACTTCTCTCTGCAGACTCTCTTCCTAGATGATCTCAACTATTTCTTTGTCTTCAAATGCTGATC
ATGCTAATGACTTCCAAATTTACATCTTTTTCCCAGACCTCTCTTCTGGTCTCCAAAATGTGTAAC
CAACTGTCTAATAGACATCCACTTGGATGTCTTAAGACATCTCAGGTTTAACATATCTAAAATAG
AACTCCTAATTTCCCTGCCAAACCTGCTTCGCCTCGAGTCCTAATCTCTGTAAATGTCACTTCCAT
CCCCCGGTTACTCTAGCCAGAAACTTAGAAGTTATTCTTGATTCTTCCATTTTCCTCAACCCCCAT
GTCCAGTCCATCTGCAAAATATGAAAATTTTACCTCCAAAATTATGTGAAATTAGTCCATTTCTCT
CTGTGTCTACCACCATCACTTCACTCGTCATCTCTTACCCAGGCTACTCATAAGCCTCATAACTG
GCCTCCCCTCACCTATTCTTTACCCTCTTCCTTTTATGCCCCCACATTCATTCTCCATACACCAGCT
ACAATGACCTTTCTAAAAACAAAATAATAATACGAACTTACATTTATTAGTGTCTATTATGTGCCA
GGAACTGTTTTACATGTATCTAATCTATACAACAACTCCATGAAGTAAAACCCAAATCTACCTCA
ATTTTACAGATAAGGAAACTAGGGCAGAGAGAGATTGAGAAATTTGCCCAAGGTCACACAGCC
TTGGGCACACAGCCAGTAAATGGTAGAACTTAGGTTTGAACCCAGGTTGACTTCTTAGCCTTCA
AGCTTATCTGCCACACTATGCTAGATCATGTCACTACCTAGCATCAAATATTTGATGGCTTCTCA
CTACACTGGAACTAAATTCCCAACTCCTTACCAAGGCCTAGAGAGGCCTGTGTGATCTATCGTCT
GCCTGTATCTCCAAACTCATCTCATGCTTCCTTTTCTTTTCCTTTGCTCCATCCACATGGCTTTCAC
ACCGCAACCCCCATCATCTTCCTTGAACATGATAAGCTCTTTCCTGCCAGCAGGCCTTAAACATC
CTCTATCTCAAGTATTCTTCTTTTTTTCTTTTAAATCCCCTATTCTGAGAAATGTCAAATATTGTTTT
CTCTGATCTTCACACAGCTGGATCCTTCTCATTCTTTAGGTCTCATTTTAATTTTCTGTCCAGAGA
GACTCTCCCTCCCATTGCTTTTTTTAATTATTATTTTAAATTTTTTTTTTAAAGCTGGACACCATTAA
TTAGAATCATGCTATTTCTTAAGTAGGTCCCTCCCTACTCCTGCCAAGATCATTAGTGACCCTCCC
TGATATGTTCCATCTTGGCCCCATATTTGTTTTCTTCTTAGCCTTTAAAACAATTTGTTAGCCGGGC
GTGGTGGTTCATGCCTGTAATCCCAGCACTTTAGGAGGCCGAGGTGGGCAGATCACGAGGTCA
AGAGAGCAAGACCATCCTGGCCAACATTGTGAAACCCCATCTCTACTAAAAAAAAAAAAATTAGC
TGGGCGTGGTGGCGCACGCCTGCAGTCCCAGCTACTCAGGAGGCTGAGGCAGGAGAATCACTT
GAACCTGGGAGGTGGAGGTTGCAGTGGGCCAAGATAGCACCACTGCACTCCAGCCTGGCCGC
AGAGCAAGACTCTGTCTCAAAAAAAAAAAAAAATTTTTTTTGTTGGCCAGGTGTGGTGGCTCACG
CCTGTAATCCAGCACTTTGGGAGGTCAAGGTGGGCAAATCACTTGAGGTCAGGAATTCCAGACC
AGCCTGGCCAACATGGTGAAACCCCGTCTCTACTAAAAATACAAAAATTAGCCAGGTGGGGTG
GTGGGCGTCTGTAATCCCAGCTACTTGGGAGGCTGAGGCAGGAGAATTGCTTGAACCTGGGAG
GTGGAGGCTGCAGTCAGCCAAGATAGCGCCACTGCACTCCAGCCTAGGCAACAGAATGAGACT
CCATCTCAAAAAAAAAAAAACAAAAAAAAAAATTACTCAGGCATGGTGGCGCGCACCTGCAATCC
CAGCTATTCAGGAGGCTGAGGCAGGAGAATCGCTTGAACCCGAGAGGCAGAGGTTGCAGTGA
GCCAAGATCATGCTATTGCACTCCAGCCTAGGCAACAGAGTGAGACTCTGTCTCAAAAAAAAAT
TGTAATTATTTATTTGTTTGTTTGCTTGTTTGTCTCTCTTCCCCACTAAAATTAAGTGTTGTGAAGG
CAGAGGCTGAGTCTTAGTCACTTTCATTATGTTCAGAGCCAAACACAGTTTCCAGCTCATAGAAG
GAACTCTAGAAATATCTGTTGTCTATCCATATATTATCTTTTCTACTTCATAGATCTGTTCATTCAT
AATTTCTGCTTCTTTATCACTTTGGTTTGTCATCATCTACACTCCATCTCCTCCCACACATACCCCT

ACTCAAACTCATGGCCTCTCTCTTTACATCCTGTTAGTCATAGTTTTTATTGCTGCTAATTGCCTCA
ATCACCTCATGCTGCCTGATTCAAATTCCTGACAGAGAAATATTGATTTGTCCAACTCATATTTTA
GGAGCCAAGACACATTTGTAGGTCATTGGCAAGCCCATGGGTTGCTTGCCTTTGGGTCATATTC
CCAGTCTTAGTCCAGAAGCTGTGTTAGAGAAGGCTGTGGGTGGGGATGGTCAGCATAGTGCTG
CCTTTCTGCAAGGATGAGGATGTAGCAGGCTTGGTGACTGATATTTGAGCAAAAGTTGTTGCAG
CAGAAGCAACAAATAGTTAACAAGGCCTCACTTAAGGGCTGGGCTTCCCAGTAGTTGCTCTCTG
GGAGGAAGGTCTCAGGTTATGGTCGAGTCCAGTTTACTCTCCCTCTGGGGAGAATAGTAAACAC
AAATTGAAAGCACTCACATTTACCCAGCCTTTTTTTTTTTATTTTTTGAGACAGGGTCTGGCTCTGT
CACCCAGGCTGAAGTGCAGTGGCACGATCTTAGGTCACTGCAACCTCTACCTCCCAGGCTCAAG
CCATCCTCCCACCTCAGCCTCTTGTGTATCTAGGACTACAGGTGTATGTCACCATGCCCAGCTAG
TTTTTGAATTTTTAGTAGAGATGGGGTTTCACCACGTTGCCCAGGCTGGTCTCAAACTGCTGAGC
TCAAAGCAGTCCATCCACCCTGGCCTCCCAAAGTGCTGGGATTACAGGCTTAAACCACTGCACC
TGGCATATACAGCCTTTCTAAAATTGCAAAAAGAGCATTTTTATCCATCTGCATTTATACCTCCAG
TGAGGTAAAGAGTAGTTTCCATTTAACAAATAATAAAACTGAGGCTGGGAAGATAGAGTGCTAT
GCCTAAGGTCCCACACACACTGAGTAAGAGTCATCATTACATCCCAGATCTCTAGACTCCCACG
CAAGGTCTCCTTTCACTATACATATCTTGTTGCCCTCATTTCCTACCTGGGACTTTAATCTTGAAT
CACAGCTCAAGGGATTTTTAAGGGCAACCATTTGAACGGCCAGTGAAAAGTTCTAAAACAGCTT
GATGATAATTGAAGTTTCCTATAAGGCAGCACTCGATAGGTAGGTTCTATGGGCTCTGGGCTCC
CAGTATAATTATCAAGTGTCTAAGAATTTATGGAATAATTACCATGTGCCAGGCACTCTACTTTAT
ACATACTCTCTCAATTCAACCTCACAACATGCATGTAAGGTATATGTTATTATAATCCCAACTTTA
CAGATGAGGACACTGAAACTTAAGAAACTAGGTCACTGGACCAAAGTTCAAAATCTAGTAAATA
CAGTGCTGGAATCCTAACTCAGGTCTGTCTGACTCCAAAGCCCACCTCCCTTTTCATGATCAGCT
TGATTTTGTCACTTGATTTATGTGAACTTCCACTTCCTTATCGGTAAAATGGGGATATCAGGGCCT
AATTCATGGGATTATTGTAGGTTTATGAACAATCACATAGAGGGCAAACCTATTTCAGTAAACAG
AGCAGATATACACTATCTTTCAGTATAGTCTGAAACTAAATCCTTCATGTGTAAAAAGAAGCAGA
ATTATCAACTCCAGGTGCCTATAAGAAACAGGATTTTTCTTGTGAGACTGTAATCCTGGGGCAAG
GATTCCTGGTCCAAATTCTGAAAACATAAGGCCTGGAGAGAAATAACCATTTCTCCAAGGAAAC
AGATCAATTTATTGTATCTCAGAGATGAGAAAGAACGTGCACTTAAACTAGTCTTAATGCATCAG
GATGGAAATTAGTAAATGCCCAATAAAGAGATTTGAATTTGTGTCTGATAAACTTGAGATCTTCT
CCTAGGCCAAGGCCTTTTGCATATAATCTTCTTAATCCTACCACAACTCTAAGAGGCAGGTCTTC
TTACAACCTCCATTTTTCAGGCAAGTACTTTGAGACTCAGAGAGGTTTTTTTGTTTTTTTTGAGAC
GGAGTCTCACTCTGGCCCCCGGACTGGAGTGCAGTGGCGCCATCTCAGCTCACTGCAACCTTCA
CCTCCTGGGTTCAATCGATTCTCCTGCCTCAGCCTCCTGGGTAGCTGGGACTACAGGCACGTGC
CACCATGCCCGGCTAATTTTTTGTATCATTAGTAGAGACGGGATTTCACCGTGTTAGCCAGGGTG
GTCTTGATCTCCTGACCTCGTGATCCACCCGCCTCAGCCTCCCAAAGTGCTGGGATTACAGGCG
TGAGCCACTGCGCCTGGCCTCAACTTTGAGGCTCAGAGAGTTTAAGTGACCTGGCAAAAGTCAC
ACATCTAACTCAAAGCAGAGAAGAAACTGGAGCCAAGCTTTCAGCTGTGTGCAGTCTGGCCAG
TCTGCTAGTAAGTTAGCTGCCTCAGCACTGTAGACCTCTTTGGAAGGAAACTGACCCCTAGGTC
AAACATAGTGCTGACATCATGCCCACTCATTATTCTCAAGAAATGAAAATCAATTCTACGTCCAT
CAGCACACTTTAGGGAGAATGCTGGCTAGGACAATACTTCCCTTGCAGCATTATAGATAAATAC
CAACACCTAAACAAATGTCAATAATACCATGTGATGAATGCAAGAGTGAGAATAATGACTAAAC
TTTGCGTAGCACTTGATGATTCACAAGGAACATTCATATCTGTTACCCAATTTGCTGTTCATATCA
CCCCTTGGAGATAGGTATTATCATTGCCATTTCACAGATGAAGAAACTGAGGCTCAAAGGAGTT
TATTTCATATGAGAGAAGATGTGAAGCTAGCTGTGGTGGCACACACCTGTAACCCCAGCTACTT

GGGAGGTTGAGGCAGAAGGATTACTTGAACCAGGAGCTTGACAGTTCAAAAGTTTGAGTCCAG
CCTGGGCAACATAGTAAGACTGAATCTCTGAAGGAAAAAAAAAGAAAGATGTGGACATATACC
ATTTTACGATCTCTTAGCCTCCATTTCCCTTCTTTTGGGACTGGTACTCTGTGTTTCCTTTGAAGAT
CAAATAACCCTTGCTTCAGCAACATTGATTCGATCAGCGTGAACACATAACACTTGACTCTTTGA
GAATCAAGCTTGGAACTTTCGTGTAGGCTTTTGTTCAAACTGTTGAGAGAGAGAAGCTCTCTTAA
GCCCATGGGTGTGAGTCTGAAGTTAAGGGCTACCACCATAAGGAAAAAAATTATGTATAAATTT
TAAACCAGCAGAAGATAATTCAGCCTATTCAATGGAAAGCAGAAATGGAGATTAAAAATAAAA
AAAGAATGTAGAGTAAATGGAGGATAGAACTAAATCTTAATATATAATAATAACATTAAATGTA
AAAAAGTGAATCTCACCAATGGGATTTTTAAAAATTACATCCAACAGTAAACTAACTTTAAGAGA
CATGCTTTTTTTTTTTTTTTTTTTTTTTTGAGATGGAGTTTCACTCTGTCACCCAGGCTGGAGTGCA
GTGGCTTGATCTCGGCTCACTGCAACATCCACCTCCCAGGCTCAAGCAATTCTCCTGCCTCAGC
CTCCCAAGTAGCTGGGATTACAGGTGCCCACTACCACTCCTGGCTAATTTTTGTATTTTTAGTAG
AGACAGGGTTTCACCATGTTGGCCAGGCTTGTCTCAAACTCTTGACCTCAAGTGATCTGCCTGCC
TTGGCCTCCCAATGTGCTGGGATTACAGGCGTGAGCCACCACATCCGACCCAGAGACATACTTA
AAGAGATAGATTAAAAATAAAAAGAAAGATAAACCAGGCAAATGTGAACTGGGTTGCGATCTT
ATTATCTGTCAAAGAGGAATTTTAGTTTCAAAAGATGAGATGGAAAGTCATTATTTTTTCTTAAAA
GGAACAATAGACAAATACATAGATACAACAGGCAAAACACACAGATGCACCTAATCATACAGT
CTTAAAATATACAGAACTCAACCTGGACAAACATGGCGAAACTCTGTCACTACTAAAAATAAAA
AATAAAAAAATTATCCAGGCGTGGTGGCATACACCTGTAGTCCCAGCTACAGTCCCACCTGTTT
CAAAAAATAAACATAACTGAGCCAGGTGCAGTGGCTCACACCTATAATCCCAGTGCTTTGGGAG
GCTGAGGTGAGAGGATAACTTAAGGCCAGGAGTTCAAGACAAGCACCATAGCAAGACCACATC
TCTATAAAAAATAAAATAAAAATTTACCAGCATGGTGGAACAATTCTGTAGTCCTAGCTACTCAG
GAGGCTGAGGTGAGAGGATCGCTTGAGTGCAGGAGTTTGAGGCCAGAGTGAGCCATGATCATG
CCACTGTATTCCAGCTACACACAAACAAACACACACACAAAACAACTGCAGAGAAAAATCAAG
AGATGCTTAATTATAGCTGAAGACTTTAACATCCCTCTCTCAGAAACTGATATATCAAGGCTTGA
ATAGTATAATTACCATATGTCAACAATTCTACATCGTATAAGAGTAGACTCTAGGAGGGTTAAAA
ACATAATAAATGTGAAAAACTAACAAAGAAAAATATGAGATAATTATAGTTTGTGACTGAAAAG
GAAAGGCAAAACTTCAAATGCACAGACTGTAAGACAATTTTTTTTGAGACAGGGTCTCACTTTGT
CACCCAGACTGGAGTGCAGTGGCGCAATCTCAGCTCACTGCAGCCTCAACCTCCTGGGCTCAA
GCGATCCTCTCACCTCAGCCCCCAAGTAGCTGGGACTACAGGTATCAGCCACCATGCTCGGCTA
ATTTGTTGTGTTTATTGTAGAGATGGGGTTTCACCATGTTGCCCAGGCTGGTCTCGAATTCCTGA
GCGCAAGCGATCTGCCTGCCTTGGACTCCCAAAGTGCTAGGATTACAGGTGTGAGCCACCACA
CCAGGCTATAAGACAAAAATATTGATGAATATAATGACATTAAAATTAAGAATTTCTGTTCAACA
AAGAAGATTGTTGAACAAAATAACTAGATAAAAGACAACATGGCTGGGCGCGGTGGCTCACGC
CTGTAATCCCAGCACTTTGGGATGCCGAGGCGGGTGGATCACGAGGTCAGGAGATCGAGACCA
TCCTGGCTAACACGGTGAAAACCTGTCTCTACTAAAAATACAAAAAATTAGCTAGGCATGATGA
AGGGCACCTATAGTCCCAGCTACTCAGGAGGCTGAGACAGGAGAATGGCATGAACCCGGGAG
GCGGAGCTTGCAGTGAGCCGAGATCGTGCCACTGCACTCCAGCCTGGGCGACAGAGCCAGACT
CTGTCTCAAAAAAAAAAAAACAAAAAACACAACATAACTAGATAAGAGAATGGGAGAATATGTT
CAAAATACGCAACACACAAAACTGGTAAATAAATAAGAGGGCAGCAAACCTTAGGAAAAAATA
AAAGCAAAACATACAAATAGTTGGCATGAACATTATCACCAAAAAAAGAGAGAAAAAGATATA
AACAGAGAGGAATTCCAAAAATCAAAAGGCATATGAAGAGATGCCCAAAGTACCACAGAAATG
CTAATTAAATCAGCAACGTACTATCCTTTTACATCTACTAAGCTACAGAAATTAGAATGCTTGAT
AATGCCAGCTGGTGGAGTTGTGGGGATGCTGGAACTCTGGTGGGTGTATGGACTGGTACAGCC

ACTGTGGATCACAATCTGACATGAAGTATGCAAATTCAGAACTCATACACTCTATGAGCTTGCA
GTTAGACTCCTGGGATATATCCCAAAGAAATTCTTATGCAGGCACATAGGGGTTCTGCATTAAA
ATATTTATTGTGTGCTATTTATGGTCATAGGGATTCAAAGACAACCTGGTTATCATATGGAGAGG
AGATAGCTAAAATGTGGTAGATGTTGTATTGTAAAATATTATGCAACAATCAAAAGCAATGGATT
AGATGCATACACAGCAAGATGAAGGCATTTTAAAACAGTGCTCAGTGAAAAATATGAAGAAAA
AATGCGATTAAAACATGATGTTATTTGCAGAAATTAAAAATGTATTAATACAAAACAAAAATTCA
CTTTTTATAAGAACAGATACAAATAAAAATAACAGACACTTTAAATAAAACTTAATTATCTAAGT
GGGAAACAGAAAAGGAATAAAAGAGAAAAAAATGGACAAATAGGTAGGGCACGGTGGTTCAC
CTGTGAATCCCAGCACTTTGGGAGGCCAAGGCAGGTGGATCACTTGAGCCGAGGAGTTTGAAA
CCAGCCTGAGCAACATGGCGAAACCCCATCTCTACTAAAAATACAAAAATTAGCTGGGCATGGT
GGTGTGCAGCTGTAGCTGTAGTCCCAGCTACTTGGGAGGCTGAGGTAGGAGGATTGCCTTGAA
CCCAGGAGGTCGAAGCTGCAGGGAGCCAAGATTGCACTACTACACTCCAGCTTGGATGACAGA
GTGAGATCCTATCTCAAATATATATATATAAATAAAATAAAATAAAATTTAGAAGTGACATTGTC
ACCAACCAAAGATGATAAACTACCATGTACTAAGTAGTGTGATTAATTAAATCCTCAAAACTGG
AAGGAAATATAATAATATTGTATATTATACAATAATAATATTAACTTTCTTCCTAATACATATGTTG
ACTCCAGATTTCTACACTGAATACCTATTAATTTTCAAAATTAAATATACAATCATAATTTTTAAA
AAAATCATCTGGAGGAGTATGGTTCAGTTGCAGAATGGTGGTGTTCAAAAGATGTGTCCTGGAA
AGAAACACAAAAAAATTTCAGTCTATGTGCAGAATGGGTAGAATTATGCAGGCATTTTTTTCTTGT
TAAAATTACAATTTCTTTTTGTTTGGTTTGGATTTTTTTTATTTTGATTTTTTATAGAGACAGAGTCT
TGCTGTGTTGCCCAGGTTGGTCTTGAACTCCTGGGCTCAAGTGATCCTTCTGCCTTGGCCTCCTA
AAGTGCTGAGATTACATGTGTGAGCCACCACACCTAGCTTGGTTTTTATTTTTTTGAAACAAGGT
CTTGCTCTGTTGCATAGGCTGGAATGCAGTGGCATCATCTCAGCTCACTGAAGCCTCTATCTCCT
GGGCTCAAGCGATCCTCTAACCTCAGGGTAGCTGGGACTACAAACATGCTCCACGATGCCCAG
CTGATTTTTAATTTTTTCGTGGGGATGGAGTTCCACTATGTTGCCCAGGCTGGTCTAGAACTCCTG
GGCTCAAGTGATCCTCCTGCCTCGACCTCCCTCAGTCCTGGGATTACAGGTGTGGGCCACCATG
TCTGGCATACCATAATTTCTGAAACAGTGTATGCACAGTCAATCAAAATAAAAGGCAAAAGAAA
ATTTATCAAAGGTGAGAGCAGCAGCTTTAAAAAATATTCAAATGAATCCTTACTGTAAACAAGG
ATTACAAAATCCTGCCTGAAAATATCTTCATCTGGTGATACTGAAAATATGTAACAATTGGTAGA
GCATACATCTGAGCAATCAGAGCAGACATTTATAGTAAACAATGCTCATATCAGACTGGCATCC
CTAAAATATAAATCACCATCCACACCAGTCCTCCAGCCCCAAAACAAAATATTTAGCCTCAGCC
CCTGAAGCTTAGCTCTTTTTCACAACAGGAAGACTCTCAGGCTGTAGAAATTGGTATAAAGTCAA
GTTTTCCAAACCAGAATATTCCAAAGGTTGTAAGGTCTAGAAACCACACCAGGCACCAGTCTAC
AGGGAGGAAGGAAGAAGTTCTCACTGCTGCTCCCTGAACTCACCACCCTACTTCCTGTCATCTG
GTGCTCTGCTCTTTGACTCTGAAATCTGGTCCACACATCCCCTTCTCCTTTGGGCAGACTTGTGC
ATTCTTTTTTGTTTTTAACCTTCCCCCAAAGATGCACACTCATGCATTCTTTAAGAGCCGCCTCAA
AACTCACTTCCTTCATGAAGTCTTTTCTCAAACCACCAAAACTACCAGGCAGAAAATTGTTCATT
TATTCAACTAATTTTTACTGAGCACCTACTATGTTCCAGATCCTGCACCTGCTATGTTCCAGAGCC
CTGGCTTATCATTTACTGAACTGTATGACAGCCATGTCTGTGGGTCTGACTCCGTGGCTGTTGGA
TATAGTCATTTAAAAATACATTTGAAGGGCAGGTACACTGGCCTACGCCTGTAATCCCAGCAGTT
TGGGAGGCTGAGATGGGCAGATCACTTGAGGTCAGGAGTTAGGGACCAGCCTGGCCAACATG
ACAAAACACTGTCTCTACTAAAAATACAAAAATTAGCCAGATGTGGTGGCACACGCCTGTACTC
CCAAGCTACTCGGGAGGCTGAGGCAGGAGGATGGCTTAAACCCTGGAGGTGGAGGTTACAGT
AAGCTGAGATTGTGCCACTGCACTCCAGCCTGGGCAACAGAGCAAGACTCTGTCTCAAAACAA
AACAAAACAAAAAGCACTTAAAAAGGCATTTTTTTTCCATGTGGAATGTCGGTTGCCTACTTTAT

GTATTCAATAGTAATAGATATTCACTCACTCATTCCACAGCCTGGTGCAGTGGAAGGATAAACTG
TTTTCTTTTATAGTGCTTTGTTAGAGTTATTTATAGGGTTATTGTTTTATGGAAGTATAAACTTCGA
AACCAGACAAGCTTGAGAGACTGGTCCTGTCCCTTACTAGCTGTTTAACCTTGAGCAAGTGACTT
ACCCACTCTGAGCAGTTTCCGCATCTGTAAAATGAAGATAAGAATGGTATCCTCTACAATGGCT
GCTATGAGGATTAGATGAGCACATTGTAGAAGCTCCACAAATTTATGGCAAAGTGCTTTCTAATC
TATAAAGAGCTACAAGAATGTGCATGATGACACGTGAGCAGGTAGTACAGAAAAAAATAAATA
TGAGACTAGCAAACTTGCAAACAAGTGACAATTATTTAAAAGTTAGGGCACAAAACAGCAGAA
AAGCAAATAGATTTTCACAAAGTATTTTTTCTTATGTTAGGGGGAAATCTAGTTGTTAAGTGCTTG
ATACATCTGCAAGAGGAAAACATCATCTGTCCACAAGTATAACCATCAGCACTGCTCAGTAAAT
ACTAATACATACGTTTTTATTTTATTTTATTTTATTTATTTTTTGAGACGGAGTCTCGCTCTGTTACC
CAGGCTGGAGTGCAGTTGTGCGATCTCGGCCCACTGCAACTTTCGCCTCCCAGGTTCAAGCAAT
GCTCCTGCCTCAGCCTCCCAAGTAGCTGGAATTACAGGTGCCCGCCACCAAGCCCAGCTAATTT
TTTGTATTTTTAGTAGAGACAGGGTTTCACCACATTAGCCAGGCTGGTCTTAAACTCCTGACCTC
AGGTGATCGACCCACCTTAGCCTCCCAAAGTGCTGGGATTACAGGCAAGAGCCACTGTGCCTG
GCCCCATACATTTAAACAAAATAAAATCCTTGGGTGTCCTGGGATAAATTGAAAGTGAGGATTA
AGTTTATACAGAAGTTTATTTACTGATAACTAATTTCATAAACACCATAATAATGATAGTAGTGAT
AGCAACCATTTTTTGACTACCTATTTGTATCAGGCACTTTACATACATTAGTTCATTTAATCCCTAC
AACCTCATAAAACAGAAATGATCACTCCCTTTCTACTAATAAGGAAACAGAAGCCCCAAGAGGC
AGTGTGACTTTCCCCAGGTCACACAGAGCAAAGGGAGCGGGATGGAAATACAAATCCAAAGTT
TATTTCA

ID|56038|gene_id|ENSG00000237587|transcript_id|ENST00000439245 Homo sapiens lncRNA

GGCTGCATAAAGGGAGTAAACACAAAGTACATTTGACCACAGAATGACTTTTTAAAAGCC
AGAACTATGGTAGCATGAAGCCAACTGAGGTAATCTAGAATAAAATTTTCTATGTTTGTTTCCCT
TCTTTGCTCTCTTTCTACTCTAATAACTGCGATTCACAGAGGTAATGAAGAGTATAATTCCCTGAT
AAAAACACAGCTCCAAGATTAATCCTTTCTTTAACTATGAAGTTCGTGTGTCCAAAGTCTTGTAG
TTGCTGATTTTTGATCACGAATGGTGATACAGATATTTATCATCAACTCACAACTTCCCAAATCTT
TGAAAAGTCTTACTATTGATGGTTCAACTAGTAGAAACATAATCTAAAATATCTGAAAATAAAGT
TTTTATTAGAATGTAAATAGTAATACAAATTGTAATAAGGTGTAAAAGTTCTTTCTTCACTGAAGC
AGGACCATGATGTCCTCTACCCCACAAACACACTACTCCCTCATGGTCTAATGTATCTTAAAAGT
CCTGTAATTGCTATTAACTCAGACAAGTTTACTTAACTTGTTCTAAGCTTCTGTTATTTACTACAAT
TTACTTTATCACTCAACAATCTCTATTATATATGTTGTTTTCCATGAGAAATTTTTTTATTAATAATT
AGGATTCTTCAGGGATAAGAAAATATTTGAATAACTAAGTTTGTGCATAAACACATTAAGGTCAA
ATACCCATGACAATATTGTGTGTTTCTCTGTACTAGAGACAAAAACTTCAAAAAAATTTTTAATGA
ATATACATTAAAAACTGCTTTCATTAAACTGAGATGATCTTCCCTCAATGCATGAATACCTTCAG
AATTCACATAGACCAAAGAATTGTATAAAATATAATAGCCTTAAAAATCTTATTTGTACCTGGCA
CAGTGGCTCCTGCCTGTAATCCCAGCATACTGGCAAGCTGAGGCAGGCAGATCACCTGAGATC
AGGAGTTTGAGAGCAGCCTGGCCAACATGGTGAAACCCCATCTCTACTAAAAATAGAGAATTTA
GCAGGGTATGGTAGCACATGCAGGTAGTATCAGCTACTCGAGGGCCTGAGGCAGGAGAATTGC
TTGAACCCGAGAGGCAGAGGTGGTAATGAGTCAAGACCGAGCCACTGCACTGCAGCCTTGGTG
ACAGAGCAAGACTCTGTCTCAAAAACACAAACAAACAAACAAAACACCTAATTGTTCCCACATA
AGTCTATATTCACACAAGATCTGAAGAGTACACAACACCGTGAGACAGGACAGACATAAATTTT
AAAAGTTATATTCCCAGTTTCTGTAAAAATAAAACGGTTGAATTTAAGCTTTTAAGACAAGTCAA
GGAAAAGAGCAAAAAATGCAAAAGTGAAACTTGAAAGGTCATTTTCCCATCAAGGGCTCATGA

TCCACTGGACATTCACAAACTATATTGTTCAAAACATTAGTTCTGAATTTTGATCTGAGTATCCCT
GGAGTTGCAGTTTCATTCAAAGATGTCCAAGAGGTCAAATAAGACAATATCATTTGCTATTTTCA
GTTTTCTTTTCTGAGAACAGCACAACAAACTTCTTCAGAGAAATGAATTGTCCTAACTTCATAGG
CTAAAGGCTCATGAGTCACAGTTCTAAGGGCATTTATAAAATATGGTGGTGCATGCTTGTATTCT
GAACTTTTCAACTTTAAACTCTCATATAGTAAATATTAATAGATACAAACTGATTAAAGAAAAGC
CCACTTAATCTGACATTATTTTTATTTTTCTTTCTTTCTTCATTTATCAGCAACAGGAGAGTCTAAC
TAAATGTGGTAAAGTGGTATAAGGGAATACAATGAAAAGTGTAAAATGAATTAAACCAGAGATA
ATCATATCAATGTGGATACATCTGGAAAATATAATACAAAATACACCAAAGAAAGTGGCAGAAA
GGTATGTAAAGTGTATAACCACTCACATACCATTTTGGGACACAAATAAAAAATTCTGCATATTA
TTTCTGAGCATCACAATATAGTTAAAGATTTCAAAAGGGCATTGAAATGAAAAACAACCAACTTA
TGATGTCGGTAGCCTCTATGCAATCATGTTTTAAAAACCTTAACACCAAAAAGTCTCAAAATCAC
CATTTTAAAAGACTGTGTCTACCAGTTATAAATGAATCATTACTTTCCTCATTTTTAATAGTCAAA
GATGCCACAAACACACACATACACACAGCTATATATACACCTACACACACAGTCTTGCTCATTA
GAACATCTGATTGGCTTCAGATCATCAGTGTAATAACACTAGCAGCAAGCCTCTAAAGTTAAAA
CAGAATCTGACATGTTAATAAGTAAAGCTTTCCTCTAGGTAAAGTTCAGAACTCCAAGTAGCACT
TAACTCATTGGAAATATCTTAGAGTCTCAAAATTCACTGCTTTGAATCCCTGACAGGTATAAAAA
TTTTATACTGAAAACTTCATGCTATTCAAAACATTAAAAGAGAAACATCTGAGTTAAAGCTTACA
TTTTTAAAATCTTTTTTATGCTTCTAAATTTATTTTTATTCAAATATGGATACCAACAATAACATTTA
TGTCAATGCCTTCCATTCAATTTTGAACAAATAGAATTAGGAATAAGAATAATGTGAGTACTTCC
AATCATTGAATGTACTTATTTCCAGTATTCCATTAAATGTACCTGCTCTCAATGTCTGTACATTCTT
TCTTTGTACTGCTCCTTTCACAGCAGGATCTTCTACTTCAGTGCTAGGCTGAATGGGTTTTAAAAG
AAAATGATTCATAAATCATATATATTTTATACAACATGGAGTTAGTGATTCAAAAATATACATAAT
TAATTACCTTCAAGGAAGGATGTTTTGCAGGAGGCCCTACAAAGCAAAGGGGATATGTCATCAA
TTATATGTAAGTATGACAGGGCCAACCAAACATTCATGCAGTGGTACTATCGAGCTGAATTCTCA
TGCCTGGCTATAAAAATAATTACTTAAGGTTTTGAGGGTTCTTCTTGGCATCTTTTCATTGCCTAG
GACGGCAACATGACAGAAACATAATGAGGAAAATAGGAATATAGGATTCCTAAAATGCACAGT
TTACATTTCAGTAGTGAGATTATGTTTCAAATGCCTATACCTAAAATAGAAAAGCATGGATATCA
CCGTGAACATGTGGACTGATGAGGAGAAAAGGGACCATTAAACAGAGGAGCAAATCAAACCT
GAGGGAATCGACGTCAAAGCTGATGGTGAATGTACAGAGTATTTTAACTCAACACAGCAGAGG
CATTGCTGCCAGCATGCCACAAACAAATTCCCCTTGTCTTGTCACTGAGGAAATACACAGTTGG
GATGACAGTTCAGGTGAATGTGTGATTCACCTCTCATCAAAGAAAGTGTTCTACATTGATCAGCT
AGTATACACACTTATGAAATGACAGCTAATCAAACTACTCATTTTTCCCATGATCACATGGGCTA
CTGCAGCACCTACATTTCTCCTATCCCCTCATTTGGCCTTGAGTTAGAGCTCCTTGATCCACTCAT
GCAAGGATCACATAAAATAAACCATGTCGAATAAGCTTCCGATATCAAAATATTTATCAAAAAA
GAAAACACTGAATTACCACAGACTTGCTGGATATGAATACATATTTATATTTCAAAATCAGTGCA
GTATTTATTGAAAAAGAGAATTTTGGTATTCACAGAATGAATTTTGTAATATCAAAAAAAATTAG
CCAAAGCATCTATATGCAACTTAATCACATCTTATTCACTCATGTCAGTGAAACTTCTCTCTCTGA
GGCCTGACAGTTATCAAGTGAAATGAGCTGCTGTGGTTTACCCCAACTCTAGCACTCCCTCCTG
CCTCCAGTACTCTCCACAGCAATAACCTCTTTTGTGAGACTGGGCATATGCTGAAGCAACTGGA
AGTGAGTTGTCTCAAGTTTACTTGGCTTCAACTCCCAAGACCCCAGCAAATGTCTTTCTTTCCTCC
CTCCGTGTCCTTTCACAATCCCTCTTCCTTTGAAAAAGTGATTTTTAGAACTGTCATCCTGATGCT
TCCCTTCCTAACTGCTTTTTATGGATAATTGTGACCACTTTTTTCATCTGTATTCAGCAGTAGTATA
CACCTGTAATCTCTCTGTTTTCATCTCATTCTCCTTCCCCTGTGGCTAGAATCATGCTCAGAAATA
AAAGGAAATTAATGCTTTCCCTGGATTCTGTTATTTTTTAAATTGCTCTCCAATGGTTCTTTTTCCA

GAGTTCTCTAAAGGAAGGCTATTCCCTTGCTATTCAGAGCTGTGTCCAAGGACCAGCACCAAAA
ATCACCTGAGTACTCATGAGAAATGCAGACTCCCATACCTGCTGAATCAGAATGTGCACCTTCC
AGAAGCTCTTCAACTCATTCATGACAATTTGAATGCCCTGTTCTACACTGATGTGCTTCCATATTG
GTTTCCCCTAATTGCCCTTTTGGCCTAGCCTCAATTTCTTCCCTATTATGTCCCTCAATTTAATACT
ACATTATAAGCCATAATGTTTCTAATGAACTTTTAATCAGGCAATAACTTCTTGAATTAATTTCTTC
TCCATAAATCACCCAACACTATTCAATTATGTTAATTTGGCCTATATGATACTATCCTATGAGGTT
ACAACATTTTCTATAAAAACAAATTATAGCCATACATGGCTGACCATCTTTGGTGATGTTCATCTA
TGGTAGATAAAACACAGGTCTGTGTGGTGAGGTGCCTCAATCCTTAATGCCTCCCCAGTAGCGA
GGATGACAGCAAGAGAAGGAAAATGTTACTGTAATTCTCTGACACATTTTGGTACTGGAAGCTC
ACTTTATCTTCTTTCCTGTTTCTAACACCATGTTCTTCCTTCTTCTACAGATCAATTTGCCTTTACTA
TCCTCTCTTACTTACCTCTGCATATGCTTTTTTATTTATTCCATGTACAATCCGCTCTCCTCATTCTT
TCTTTCTCTTTATTCATTTCCTCTTCCCTCTCTCCTGACTTGCCTCAGGTCTTAGAGTATCTTAAAAT
GGAACTCACAATTCAGCTCCTTTAGTGGTACTCCCAATAGAATCAACTGCTGACCCTTGGTTAGA
GACACAACTTATCACCATTTCACTTCTCCTTTACTTATTATACAGTTAATAGGACACTTTCTTTAGC
TATTACACTCTATTAGTGCTCATATTTTCAAAGAAACATTCCATCAAATGACTTTTTTTGTTTTGTTT
TTTCTGAGACGGAGTCTCGCTCTGTCGCCCAGGGTGGAATGCAGTGGTGGTATCTCCGCTCACT
GCAAGCTCCGCCTCCCGGGTTCAAGCCATTCTCCTGCCTCAGCCTCCTGAGTAGCTGGGACTAC
AGGCGCCCACAACACCACCTGGCTAGTTTTTTGTATTTTTAGTAGAGATGGGGTTTCACCATGTT
ATCCAGGATGGTCTGGATCTCCTGACCTCGTGATCCGCCCGCCTCGGCCTCCCAAAGTGCTGGG
ATTACAGGCGTGAGCCACCGCACCCGGCCTCCATCAAAAGACTTTTTAAATAAAATACGGTTCT
CACCTTCTCCTTGTCCATTGACTATTCTGTTTCCTTTTTCATGCGAAGATCCAGGTAAAGGCTCTG
ACACTTTCTCGGGGACACACTGCTAAGGTAATATCAAGAATTAGTTTCCATTTTAAAATTATAAT
GAGTTGCATCAAGAGTTTCTTATCAATCTCTTTTTATGAAACTGGGTCTCACTCTGTCAACCCAGG
GCTAGAATGCAGGGGCCTGATTATGGCTCACTGTTGTCTCAAACTCCTGACCTCAAGCAATCTTC
CCACCTCAACTTCCTGAATAGCTGGAACTACAGGTGTGTCCCTTCATGCCATGCTAATGTTTTTAT
TGTTATCTTTGCAGAGACAAGGCCTCATTATACTGCCCAGGCTGGTCTCAAACTCCTGAGCTCAA
GTAAATCTTCCACTTCTGCCCCCCAAAGTGTTGAGATAAGCAGTGTGCACCACCACACCCAGCC
CTAATCAATTTCTTTAAATCAATCTCAATGTTGCCCAGGCATGGTGGCTCACACCTGTAATCTCA
GCCCTTTGCAAGGCCAAGGTGGGTGGATTGCTTGAGTTCAGGAGTTTGAGACCAGCCTGGGCA
ACATAATGAGAACACATCTCTACTCAAAAAATACCAAAAGGAGTCAGGCATGATGGTGTGCGC
CTGTAGTCCCAGCTGCTTGGGAAGCTGATGTGGGAGGATCACTTGAGCCTGAGAGGTGGATAC
TGCAGTGAGCCAAGATCATGCCACTACACTGCAGCATGGGCAACAGAGCAAGACCCTGACTCC
CCAAAAATTTCAATTTAAAATATGAGAATGAAGAGAGATACAAACAAAAAACAAGCCTAATTGG
TCAATGAAATATGAGCTTAAGCCAAGAAAGAAAAGAGACAACATGAAGTACAATAAAGTACAT
GGGGAAATAGATCTATAACAGAGCCTTTTGCTCTTTCATATCCCTGATAATACTAATTAATATTTA
TGCTACAATTAGTTTTTTGTAAGTACTTCTGTGATATAGCTTATTACTATAAGACATTCAATTAGCT
AAATATGGTCATCGACCACTACCTGAAAGAACATTATTATAACACAGAGAGAAAACTAGAACTT
TCAATCAACTTTCCACCCAGAAAAAAATTGGTCACCAGAATTCTAAAGAGTAACGTATGGCAGA
CACATGAAAAAATGCTCATCATCACTGGCCATCAGAGAAATGCAAATCAAAACCACAATGAGAT
ACCATCTCACACCAGTTACAATGACGATCATTAAAAAGTCAGGAACCCACAGGTGCTGGAGAG
GATGTGGAGAAATAGGAGTACTTTTACACTGTTGGTGGGACTGTAAACTAGTTCATCCTTTGAGG
AAGACAGTATGGTGATTCCTCAAGGATCTAGAACTAGAAATACCATTTGACCCAGCCATCCCAT
TACTGGGTATATACCCAAAGGATTATAAGTCATGCTGCTATAAAGACACATGCACACGTATGTTT
ATTGCGGCACTATTTACAATAGCAAAGACTTGGAACCAACCCAAATGTCCATCAATGATAGACT

GGATTAAGACAATGTGGCACATATACACCATGGAATACTATGCAGCCATAAAAAAGGATGAGTT
CATGTCCTTTGTAGGGACATGGATGAAGCTGGAAACCATCATTCTGAGCAAACTATCGCAAGGA
CAAAAAACCAAACACTGCATGTTCTCACTCATAGGTGGAAATTGAACAATGAGAACACTTGGAC
ACAGGATGGGGAACATCACACACTGGGGCCTGTCGTGGGGCGGACAAGGGGGGAGGGATAG
CATTAGGAGATATACCTAATGTAAATGATGAGTTAATGGGTGCAGCACACCAACATGGCACGTG
TATACACATGTAACAAACCCACACATTGTGCACATGTACCCTAGAACTTACAGTATAAGAATAAT
AAAGAGTAATGTATGGCTTGAAAACGTATATTTAATAGAACATGAGTTAGAGCTAATAAAAAGC
TTAAGAAATGTTAATCTAAAATCTCAATGTTAAGATTCCAGTTGAATGATACTAGAAAATATATTG
TAACCCTATTTGCTACTGATGACCTATTTCTATTTTATTTCCTTTTTAATTATGGCATAATTTCTCAA
CATAACATATCAAAACTTATACACCCTTAAATATTAAAAAATACAATGTAAGCAATATTTTAAATA
CAATATTTAATTATTAGATACATTAGGTTTATTATATTACTTATAACATTCCATTATATAAAAATTC
ATTTGTCTATTTATTCAGATTAAACAACTATTAAGGCTGAATGTCTTATGCCTGTAACCCAGCACT
TTGAGAGGCTGAGGCAGGCAGAACACTTGAGCCCAACAGTTAAAGACCAGCCTGGGCAACAA
GGCAAAACCCTGTCTCTACAAAACTCAGCTGAGCATGGTGACACAGGTCTATGGTGACATAGCT
CTATTGTTTAAACTACTTGGATGGCTGAGGTGTGAGGATCACCTGAGCCCAGGAAATGGAGATT
GGAGTGAGCCAAGATCTCACCAGTGCCCTCCAGCCTGGGGGACAGAGTGAAACCCCATCTCAA
AAAACAACAACTAAAATGCTTCTTACATGGAAGACTGTATTCTAGGTACTCCAGGATACACACA
AATATGTTTACTGACCTCCAGTAGCTTACGGTATGCAGGAGCTTCCAATGATTATTTAAACAACT
AAATAGAAAACCTTCTGAAATTCAAAATTTCAGAATATGATATGAGGACTTTGAGTGGCTATTTT
ATTTTTTAAGATGTAGTCTTGCTCTGTCACCCAGGCTGGAGTGCAATGGTGCGATCTTGGCTCAC
TGCAACCTCCGCCTCCCGGGTTCAAGCGATTCTCCCACCTTGGCCTCCTGAGCAGCTGGGGTTA
CAGGCATGCACCATCACGCCTGGTTAATTTTTCTGCTTTTGTTTTGTTTTGTTTTGTTTTGGTTTTGG
TTTTTTTTAGAGACAGGGTTTCACCACGTTGGCCAGGCTGATCTTGAACTCCTGACCTCAGGTGA
TCTACCGACCTCGGCCTCCAAAACTGCTGGGATTACAGGTGAGTCTCCACACCTGGTGAAGTAA
ATATTTTAAATAAACTACAATGACAAAATTATGAGGATAAAGTCTTACCATCCATTGGTATTAAG
AGTCTCTGCTTCTAGAACTGGTTATTTGCAGCAAAATACATGTTATTCAATTAGATGAAGTGCCTT
ATATAAACTCTTCATGGACAACTCATAAACCACACAAAAAATTCCTTTGCAATACACATTTTGAGA
ACATAAATTTAAATTTCTATATTTCCACAATTTATATTTTTAAATCAGATACGATGTTGCCCAGGCT
GTTCTCAAACTCCTGGGTTCAAGCAATCTTCCTGCCTCAAATTCCCAAGTAGCAGGGACTACAG
GTGTACACCACCACACTCAGTTATTTTTCTACAATTTTTATTTTTTTTTGTCTCACTACAGAACCAA
TAATATAAGTAGAAAAACAATCTCTCGTAAAAACAATATGATACCAAAATAATAAGTTTCCAAG
AACAAAAGCTATATGCTATGTGCTGAATATTCTTGCCACTAAAAATCACCAGGAGGATCCCATG
ATTACTGCAAATAATTTGATCCACTGAAGATTTATACAGGCATAAATATTAAGAAAGTCACACTC
GTATGATTTAAAAGTCAAAGTATTAGTATTTATCCAAATAAACCTTAAGCAAATTTCATATTTCCT
CTATTGGAGAAAGCATTTCCTAATGTGATTTTCCTGTCACTACTAATTTTCCAGTTCATTTTTTTTC
AGCTCCCACCCTGTCACAGTACTTATCAATCTTTGTTAGTTACCAAAGTTAAACACATTTTTTGAA
TCAACTAGCCATATGTATGTTTTTCTCTGACCAACTTTCCATTACCACCATAAAACAATGATAGGT
AAACCACTGCTAAATTTGAAAAGTAAATACTATGCAAAAGTACATTCAGAGTGAGAAAATTAAT
TTTACAAGAGACCACTTTACCTTAGTAGCAACATTCAAGTCTTTGTCATCCGATGCAGGCAATGA
ATCCACACGCAGTTCATGGAAAATGCTTGAGAGCAAAAACACACAACGAAAATGAGCAAGTTG
ATTTCTTTACAATTTTTTTAACTGCCAGTTTATATCCAGCTTCCCCCTCAAAAAAGGAAACCATAA
TCTAGGGAAAGGTCAGCAATCTATATATTAAATAGTGATTCTTGATATAATTAAAATATGTCCTCT
TCTAAAAATAGATTTTAGTTACCTATTTCTGCCTCCACTTGTCTAAATCTATAAAATATTCAATGAA
AACTAACCTTGAGCTTTATAACAAATAGTGACAGTCAATAAATTGGCAGAGCCTGACAATGATTT

GCCCTCACAAATTATCTGTCCTGAAGCTGAACTTAAAATTCAATTAATGGATGACATAAATTTTGT
TACCTAAACTGGAAGAAAACTAACAACCTAAAACAAGGTAGAAAGATCCACTGTCTCTTTTCCA
TGATCTGTCTCTGGATAAAAGACTAATCTGCATCACTTCAATATGGCAGTCTTGATTCCTCAGCA
TGGAACCCACTTAGGAAGGTCCTATTGCTTTCCTTTGCCCTAAATCAGTACAGGAAAGCCCCTAC
AATATTTGAAATGTATGAAAGCTAAATGTACAGAAGTCAAATAACAAAGGTGTATGTTCTTATTG
AGAATACTTTTCCCAGAAAGATTGAAATATTAACAATTATAAAATCCCATTATTTTCACCCTATAG
GTCCTACCTTATTCAGGTCCACATAAACTAGCAAGCCCTTAAAACTTTTCATAGGCACTCAGACA
CCCAAGGAGAGAGACTGTCAGAAAAAAACAGAGTCCTGGTAGGTGTACCTCTATTTCCCTAAGT
ACTCTCTAAGTATCTGTCTTCCTATGGGCTCCCACTTCCAGATTCCACTTCTGCAGGGCTCCACA
GAAGTCTCCAATCTTTAAATCTTCAGTCTATGAAAGCACAGATTCCTGAAAGGATGGCCTCAAAT
GACCAGGACTAGGAGCTCTCTATATCCCTGCTCCTGAAAAACAAGCTA

ID|56320|gene_id|ENSG00000237587|transcript_id|ENST00000453946 Homo sapiens lncRNA

GGCTGCATAAAGGGAGTAAACACAAAGTACATTTGACCACAGAATGACTTTTTAAAAGCC
AGAACTATGGTAGCATGAAGCCAACTGAGGTAATCTAGAATAAAATTTTCTATGTTTGTTTCCCT
TCTTTGCTCTCTTTCTACTCTAATAACTGCGATTCACAGAGGTAATGAAGAGTATAATTCCCTGAT
AAAAACACAGCTCCAAGATTAATCCTTTCTTTAACTATGAAGTTCGTGTGTCCAAAGTCTTGTAG
TTGCTGATTTTTGATCACGAATGGTGATACAGATATTTATCATCAACTCACAACTTCCCAAATCTT
TGAAAAGTCTTACTATTGATGGTTCAACTAGTAGAAACATAATCTAAAATATCTGAAAATAAAGT
TTTTATTAGAATGTAAATAGTAATACAAATTGTAATAAGGTGTAAAAGTTCTTTCTTCACTGAAGC
AGGACCATGATGTCCTCTACCCCACAAACACACTACTCCCTCATGGTCTAATGTATCTTAAAAGT
CCTGTAATTGCTATTAACTCAGACAAGTTTACTTAACTTGTTCTAAGCTTCTGTTATTTACTACAAT
TTACTTTATCACTCAACAATCTCTATTATATATGTTGTTTTCCATGAGAAATTTTTTTATTAATAATT
AGGATTCTTCAGGGATAAGAAAATATTTGAATAACTAAGTTTGTGCATAAACACATTAAGGTCAA
ATACCCATGACAATATTGTGTGTTTCTCTGTACTAGAGACAAAAACTTCAAAAAAATTTTTAATGA
ATATACATTAAAAACTGCTTTCATTAAACTGAGATGATCTTCCCTCAATGCATGAATACCTTCAG
AATTCACATAGACCAAAGAATTGTATAAAATATAATAGCCTTAAAAATCTTATTTGTACCTGGCA
CAGTGGCTCCTGCCTGTAATCCCAGCATACTGGCAAGCTGAGGCAGGCAGATCACCTGAGATC
AGGAGTTTGAGAGCAGCCTGGCCAACATGGTGAAACCCCATCTCTACTAAAAATAGAGAATTTA
GCAGGGTATGGTAGCACATGCAGGTAGTATCAGCTACTCGAGGGCCTGAGGCAGGAGAATTGC
TTGAACCCGAGAGGCAGAGGTGGTAATGAGTCAAGACCGAGCCACTGCACTGCAGCCTTGGTG
ACAGAGCAAGACTCTGTCTCAAAAACACAAACAAACAAACAAAACACCTAATTGTTCCCACATA
AGTCTATATTCACACAAGATCTGAAGAGTACACAACACCGTGAGACAGGACAGACATAAATTTT
AAAAGTTATATTCCCAGTTTCTGTAAAAATAAAACGGTTGAATTTAAGCTTTTAAGACAAGTCAA
GGAAAAGAGCAAAAAATGCAAAAGTGAAACTTGAAAGGTCATTTTCCCATCAAGGGCTCATGA
TCCACTGGACATTCACAAACTATATTGTTCAAAACATTAGTTCTGAATTTTGATCTGAGTATCCCT
GGAGTTGCAGTTTCATTCAAAGATGTCCAAGAGGTCAAATAAGACAATATCATTTGCTATTTTCA
GTTTTCTTTTCTGAGAACAGCACAACAAACTTCTTCAGAGAAATGAATTGTCCTAACTTCATAGG
CTAAAGGCTCATGAGTCACAGTTCTAAGGGCATTTATAAAATATGGTGGTGCATGCTTGTATTCT
GAACTTTTCAACTTTAAACTCTCATATAGTAAATATTAATAGATACAAACTGATTAAAGAAAAGC
CCACTTAATCTGACATTATTTTTATTTTTCTTTCTTTCTTCATTTATCAGCAACAGGAGAGTCTAAC
TAAATGTGGTAAAGTGGTATAAGGGAATACAATGAAAAGTGTAAAATGAATTAAACCAGAGATA
ATCATATCAATGTGGATACATCTGGAAAATATAATACAAAATACACCAAAGAAAGTGGCAGAAA
GGTATGTAAAGTGTATAACCACTCACATACCATTTTGGGACACAAATAAAAAATTCTGCATATTA

TTTCTGAGCATCACAATATAGTTAAAGATTTCAAAAGGGCATTGAAATGAAAAACAACCAACTTA
TGATGTCGGTAGCCTCTATGCAATCATGTTTTAAAAACCTTAACACCAAAAAGTCTCAAAATCAC
CATTTTAAAAGACTGTGTCTACCAGTTATAAATGAATCATTACTTTCCTCATTTTTAATAGTCAAA
GATGCCACAAACACACACATACACACAGCTATATATACACCTACACACACAGTCTTGCTCATTA
GAACATCTGATTGGCTTCAGATCATCAGTGTAATAACACTAGCAGCAAGCCTCTAAAGTTAAAA
CAGAATCTGACATGTTAATAAGTAAAGCTTTCCTCTAGGTAAAGTTCAGAACTCCAAGTAGCACT
TAACTCATTGGAAATATCTTAGAGTCTCAAAATTCACTGCTTTGAATCCCTGACAGGTATAAAAA
TTTTATACTGAAAACTTCATGCTATTCAAAACATTAAAAGAGAAACATCTGAGTTAAAGCTTACA
TTTTTAAAATCTTTTTTATGCTTCTAAATTTATTTTTATTCAAATATGGATACCAACAATAACATTTA
TGTCAATGCCTTCCATTCAATTTTGAACAAATAGAATTAGGAATAAGAATAATGTGAGTACTTCC
AATCATTGAATGTACTTATTTCCAGTATTCCATTAAATGTACCTGCTCTCAATGTCTGTACATTCTT
TCTTTGTACTGCTCCTTTCACAGCAGGATCTTCTACTTCAGTGCTAGGCTGAATGGGTTTTAAAAG
AAAATGATTCATAAATCATATATATTTTATACAACATGGAGTTAGTGATTCAAAAATATACATAAT
TAATTACCTTCAAGGAAGGATGTTTTGCAGGAGGCCCTACAAAGCAAAGGGGATATGTCATCAA
TTATATGTAAGTATGACAGGGCCAACCAAACATTCATGCAGTGGTACTATCGAGCTGAATTCTCA
TGCCTGGCTATAAAAATAATTACTTAAGGTTTTGAGGGTTCTTCTTGGCATCTTTTCATTGCCTAG
GACGGCAACATGACAGAAACATAATGAGGAAAATAGGAATATAGGATTCCTAAAATGCACAGT
TTACATTTCAGTAGTGAGATTATGTTTCAAATGCCTATACCTAAAATAGAAAAGCATGGATATCA
CCGTGAACATGTGGACTGATGAGGAGAAAAGGGACCATTAAACAGAGGAGCAAATCAAACCT
GAGGGAATCGACGTCAAAGCTGATGGTGAATGTACAGAGTATTTTAACTCAACACAGCAGAGG
CATTGCTGCCAGCATGCCACAAACAAATTCCCCTTGTCTTGTCACTGAGGAAATACACAGTTGG
GATGACAGTTCAGGTGAATGTGTGATTCACCTCTCATCAAAGAAAGTGTTCTACATTGATCAGCT
AGTATACACACTTATGAAATGACAGCTAATCAAACTACTCATTTTTCCCATGATCACATGGGCTA
CTGCAGCACCTACATTTCTCCTATCCCCTCATTTGGCCTTGAGTTAGAGCTCCTTGATCCACTCAT
GCAAGGATCACATAAAATAAACCATGTCGAATAAGCTTCCGATATCAAAATATTTATCAAAAAA
GAAAACACTGAATTACCACAGACTTGCTGGATATGAATACATATTTATATTTCAAAATCAGTGCA
GTATTTATTGAAAAAGAGAATTTTGGTATTCACAGAATGAATTTTGTAATATCAAAAAAAAATTAG
CCAAAGCATCTATATGCAACTTAATCACATCTTATTCACTCATGTCAGTGAAACTTCTCTCTCTGA
GGCCTGACAGTTATCAAGTGAAATGAGCTGCTGTGGTTTACCCCAACTCTAGCACTCCCTCCTG
CCTCCAGTACTCTCCACAGCAATAACCTCTTTTGTGAGACTGGGCATATGCTGAAGCAACTGGA
AGTGAGTTGTCTCAAGTTTACTTGGCTTCAACTCCCAAGACCCCAGCAAATGTCTTTCTTTCCTCC
CTCCGTGTCCTTTCACAATCCCTCTTCCTTTGAAAAAGTGATTTTTAGAACTGTCATCCTGATGCT
TCCCTTCCTAACTGCTTTTTATGGATAATTGTGACCACTTTTTTCATCTGTATTCAGCAGTAGTATA
CACCTGTAATCTCTCTGTTTTCATCTCATTCTCCTTCCCCTGTGGCTAGAATCATGCTCAGAAATA
AAAGGAAATTAATGCTTTCCCTGGATTCTGTTATTTTTTAAATTGCTCTCCAATGGTTCTTTTTCCA
GAGTTCTCTAAAGGAAGGCTATTCCCTTGCTATTCAGAGCTGTGTCCAAGGACCAGCACCAAAA
ATCACCTGAGTACTCATGAGAAATGCAGACTCCCATACCTGCTGAATCAGAATGTGCACCTTCC
AGAAGCTCTTCAACTCATTCATGACAATTTGAATGCCCTGTTCTACACTGATGTGCTTCCATATTG
GTTTCCCCTAATTGCCCTTTTGGCCTAGCCTCAATTTCTTCCCTATTATGTCCCTCAATTTAATACT
ACATTATAAGCCATAATGTTTCTAATGAACTTTTAATCAGGCAATAACTTCTTGAATTAATTTCTTC
TCCATAAATCACCCAACACTATTCAATTATGTTAATTTGGCCTATATGATACTATCCTATGAGGTT
ACAACATTTTCTATAAAAACAAATTATAGCCATACATGGCTGACCATCTTTGGTGATGTTCATCTA
TGGTAGATAAAACACAGGTCTGTGTGGTGAGGTGCCTCAATCCTTAATGCCTCCCCAGTAGCGA
GGATGACAGCAAGAGAAGGAAAATGTTACTGTAATTCTCTGACACATTTTGGTACTGGAAGCTC

ACTTTATCTTCTTTCCTGTTTCTAACACCATGTTCTTCCTTCTTCTACAGATCAATTTGCCTTTACTA
TCCTCTCTTACTTACCTCTGCATATGCTTTTTTATTTATTCCATGTACAATCCGCTCTCCTCATTCTT
TCTTTCTCTTTATTCATTTCCTCTTCCCTCTCTCCTGACTTGCCTCAGGTCTTAGAGTATCTTAAAAT
GGAACTCACAATTCAGCTCCTTTAGTGGTACTCCCAATAGAATCAACTGCTGACCCTTGGTTAGA
GACACAACTTATCACCATTTCACTTCTCCTTTACTTATTATACAGTTAATAGGACACTTTCTTTAGC
TATTACACTCTATTAGTGCTCATATTTTCAAAGAAACATTCCATCAAATGACTTTTTTTGTTTTGTTT
TTTCTGAGACGGAGTCTCGCTCTGTCGCCCAGGGTGGAATGCAGTGGTGGTATCTCCGCTCACT
GCAAGCTCCGCCTCCCGGGTTCAAGCCATTCTCCTGCCTCAGCCTCCTGAGTAGCTGGGACTAC
AGGCGCCCACAACACCACCTGGCTAGTTTTTTGTATTTTTAGTAGAGATGGGGTTTCACCATGTT
ATCCAGGATGGTCTGGATCTCCTGACCTCGTGATCCGCCCGCCTCGGCCTCCCAAAGTGCTGGG
ATTACAGGCGTGAGCCACCGCACCCGGCCTCCATCAAAAGACTTTTTAAATAAAATACGGTTCT
CACCTTCTCCTTGTCCATTGACTATTCTGTTTCCTTTTTCATGCGAAGATCCAGGTAAAGGCTCTG
ACACTTTCTCGGGGACACACTGCTAAGGTAATATCAAGAATTAGTTTCCATTTTAAAATTATAAT
GAGTTGCATCAAGAGTTTCTTATCAATCTCTTTTTATGAAACTGGGTCTCACTCTGTCAACCCAGG
GCTAGAATGCAGGGGCCTGATTATGGCTCACTGTTGTCTCAAACTCCTGACCTCAAGCAATCTTC
CCACCTCAACTTCCTGAATAGCTGGAACTACAGGTGTGTCCCTTCATGCCATGCTAATGTTTTTAT
TGTTATCTTTGCAGAGACAAGGCCTCATTATACTGCCCAGGCTGGTCTCAAACTCCTGAGCTCAA
GTAAATCTTCCACTTCTGCCCCCCAAAGTGTTGAGATAAGCAGTGTGCACCACCACACCCAGCC
CTAATCAATTTCTTTAAATCAATCTCAATGTTGCCCAGGCATGGTGGCTCACACCTGTAATCTCA
GCCCTTTGCAAGGCCAAGGTGGGTGGATTGCTTGAGTTCAGGAGTTTGAGACCAGCCTGGGCA
ACATAATGAGAACACATCTCTACTCAAAAAATACCAAAAGGAGTCAGGCATGATGGTGTGCGC
CTGTAGTCCCAGCTGCTTGGGAAGCTGATGTGGGAGGATCACTTGAGCCTGAGAGGTGGATAC
TGCAGTGAGCCAAGATCATGCCACTACACTGCAGCATGGGCAACAGAGCAAGACCCTGACTCC
CCAAAAAATTTCAATTTAAAATATGAGAATGAAGAGAGATACAAACAAAAAACAAGCCTAATTGG
TCAATGAAATATGAGCTTAAGCCAAGAAAGAAAAGAGACAACATGAAGTACAATAAAGTACAT
GGGGAAATAGATCTATAACAGAGCCTTTTGCTCTTTCATATCCCTGATAATACTAATTAATATTTA
TGCTACAATTAGTTTTTTGTAAGTACTTCTGTGATATAGCTTATTACTATAAGACATTCAATTAGCT
AAATATGGTCATCGACCACTACCTGAAAGAACATTATTATAACACAGAGAGAAAACTAGAACTT
TCAATCAACTTTCCACCCAGAAAAAAAATTGGTCACCAGAATTCTAAAGAGTAACGTATGGCAGA
CACATGAAAAAATGCTCATCATCACTGGCCATCAGAGAAATGCAAATCAAAACCACAATGAGAT
ACCATCTCACACCAGTTACAATGACGATCATTAAAAAGTCAGGAACCCACAGGTGCTGGAGAG
GATGTGGAGAAATAGGAGTACTTTTACACTGTTGGTGGGACTGTAAACTAGTTCATCCTTTGAGG
AAGACAGTATGGTGATTCCTCAAGGATCTAGAACTAGAAATACCATTTGACCCAGCCATCCCAT
TACTGGGTATATACCCAAAGGATTATAAGTCATGCTGCTATAAAGACACATGCACACGTATGTTT
ATTGCGGCACTATTTACAATAGCAAAGACTTGGAACCAACCCAAATGTCCATCAATGATAGACT
GGATTAAGACAATGTGGCACATATACACCATGGAATACTATGCAGCCATAAAAAAGGATGAGTT
CATGTCCTTTGTAGGGACATGGATGAAGCTGGAAACCATCATTCTGAGCAAACTATCGCAAGGA
CAAAAAACCAAACACTGCATGTTCTCACTCATAGGTGGAAATTGAACAATGAGAACACTTGGAC
ACAGGATGGGGAACATCACACACTGGGGCCTGTCGTGGGGCGGACAAGGGGGGAGGGATAG
CATTAGGAGATATACCTAATGTAAATGATGAGTTAATGGGTGCAGCACACCAACATGGCACGTG
TATACACATGTAACAAACCCACACATTGTGCACATGTACCCTAGAACTTACAGTATAAGAATAAT
AAAGAGTAATGTATGGCTTGAAAACGTATATTTAATAGAACATGAGTTAGAGCTAATAAAAAGC
TTAAGAAATGTTAATCTAAAATCTCAATGTTAAGATTCCAGTTGAATGATACTAGAAAATATATTG
TAACCCTATTTGCTACTGATGACCTATTTCTATTTTATTTCCTTTTTAATTATGGCATAATTTCTCAA

CATAACATATCAAAACTTATACACCCTTAAATATTAAAAAATACAATGTAAGCAATATTTTAAATA
CAATATTTAATTATTAGATACATTAGGTTTATTATATTACTTATAACATTCCATTATATAAAAATTC
ATTTGTCTATTTATTCAGATTAAACAACTATTAAGGCTGAATGTCTTATGCCTGTAACCCAGCACT
TTGAGAGGCTGAGGCAGGCAGAACACTTGAGCCCAACAGTTAAAGACCAGCCTGGGCAACAA
GGCAAAACCCTGTCTCTACAAAACTCAGCTGAGCATGGTGACACAGGTCTATGGTGACATAGCT
CTATTGTTTAAACTACTTGGATGGCTGAGGTGTGAGGATCACCTGAGCCCAGGAAATGGAGATT
GGAGTGAGCCAAGATCTCACCAGTGCCCTCCAGCCTGGGGGACAGAGTGAAACCCCATCTCAA
AAAACAACAACTAAAATGCTTCTTACATGGAAGACTGTATTCTAGGTACTCCAGGATACACACA
AATATGTTTACTGACCTCCAGTAGCTTACGGTATGCAGGAGCTTCCAATGATTATTTAAACAACT
AAATAGAAAACCTTCTGAAATTCAAAATTTCAGAATATGATATGAGGACTTTGAGTGGCTATTTT
ATTTTTTAAGATGTAGTCTTGCTCTGTCACCCAGGCTGGAGTGCAATGGTGCGATCTTGGCTCAC
TGCAACCTCCGCCTCCCGGGTTCAAGCGATTCTCCCACCTTGGCCTCCTGAGCAGCTGGGGTTA
CAGGCATGCACCATCACGCCTGGTTAATTTTTCTGCTTTTGTTTTGTTTTGTTTTGTTTTGGTTTTGG
TTTTTTTTAGAGACAGGGTTTCACCACGTTGGCCAGGCTGATCTTGAACTCCTGACCTCAGGTGA
TCTACCGACCTCGGCCTCCAAAACTGCTGGGATTACAGGTGAGTCTCCACACCTGGTGAAGTAA
ATATTTTAAATAAACTACAATGACAAAATTATGAGGATAAAGTCTTACCATCCATTGGTATTAAG
AGTCTCTGCTTCTAGAACTGGTTATTTGCAGCAAAATACATGTTATTCAATTAGATGAAGTGCCTT
ATATAAACTCTTCATGGACAACTCATAAACCACACAAAAATTCCTTTGCAATACACATTTTGAGA
ACATAAATTTAAATTTCTATATTTCCACAATTTATATTTTTAAATCAGATACGATGTTGCCCAGGCT
GTTCTCAAACTCCTGGGTTCAAGCAATCTTCCTGCCTCAAATTCCCAAGTAGCAGGGACTACAG
GTGTACACCACCACACTCAGTTATTTTTCTACAATTTTTATTTTTTTTTGTCTCACTACAGAACCAA
TAATATAAGTAGAAAAACAATCTCTCGTAAAAACAATATGATACCAAAATAATAAGTTTCCAAG
AACAAAAGCTATATGCTATGTGCTGAATATTCTTGCCACTAAAAATCACCAGGAGGATCCCATG
ATTACTGCAAATAATTTGATCCACTGAAGATTTATACAGGCATAAATATTAAGAAAGTCACACTC
GTATGATTTAAAAGTCAAAGTATTAGTATTTATCCAAATAAACCTTAAGCAAATTTCATATTTCCT
CTATTGGAGAAAGCATTTCCTAATGTGATTTTCCTGTCACTACTAATTTTCCAGTTCATTTTTTTTC
AGCTCCCACCCTGTCACAGTACTTATCAATCTTTGTTAGTTACCAAAGTTAAACACATTTTTTGAA
TCAACTAGCCATATGTATGTTTTTCTCTGACCAACTTTCCATTACCACCATAAAACAATGATAGGT
AAACCACTGCTAAATTTGAAAAGTAAATACTATGCAAAAGTACATTCAGAGTGAGAAAATTAAT
TTTACAAGAGACCACTTTACCTTAGTAGCAACATTCAAGTCTTTGTCATCCGATGCAGGCAATGA
ATCCACACGCAGTTCATGGAAAATGCTTGAGAGCAAAAACACACAACGAAAATGAGCAAGTTG
ATTTCTTTACAATTTTTTTAACTGCCAGTTTATATCCAGCTTCCCCCTCAAAAAAGGAAACCATAA
TCTAGGGAAAGGTCAGCAATCTATATATTAAATAGTGATTCTTGATATAATTAAAATATGTCCTCT
TCTAAAAATAGATTTTAGTTACCTATTTCTGCCTCCACTTGTCTAAATCTATAAAATATTCAATGAA
AACTAACCTTGAGCTTTATAACAAATAGTGACAGTCAATAAATTGGCAGAGCCTGACAATGATTT
GCCCTCACAAATTATCTGTCCTGAAGCTGAACTTAAAATTCAATTAATGGATGACATAAATTTTGT
TACCTAAACTGGAAGAAAACTAACAACCTAAAACAAGGTAGAAAGATCCACTGTCTCTTTTCCA
TGATCTGTCTCTGGATAAAAGACTAATCTGCATCACTTCAATATGGCAGTCTTGATTCCTCAGCA
TGGAACCCACTTAGGAAGGTCCTATTGCTTTCCTTTGCCCTAAATCAGTACAGGAAAGCCCCTAC
AATATTTGAAATGTATGAAAGCTAAATGTACAGAAGTCAAATAACAAAGGTGTATGTTCTTATTG
AGAATACTTTTCCCAGAAAGATTGAAATATTAACAATTATAAAATCCCATTATTTTCACCCTATAG
GTCCTACCTTATTCAGGTCCACATAAACTAGCAAGCCCTTAAAACTTTTCATAGGCACTCAGACA
CCCAAGGAGAGAGACTGTCAGAAAAAAACAGAGTCCTGGTAGGTGTACCTCTATTTCCCTAAGT
ACTCTCTAAGTATCTGTCTTCCTATGGGCTCCCACTTCCAGATTCCACTTCTGCAGGGCTCCACA

GAAGTCTCCAATCTTTAAATCTTCAGTCTATGAAAGCACAGATTCCTGAAAGGATGGCCTCAAAT
GACCAGGACTAGGAGCTCTCTATATCCCTGCTCCTGAAAAACAAGCTAACTGGAGTCTCCATCA
CCTGCCCCCAGCTAGACACACTACCAACTACCCAACTGAACTCCATGACTGATTTGCCAGCCAA
TCATGCCCCTGAACCAGCCCACATGGACATGGGAAGGACATCAGTGAATCGGGAAAAGAGGC
AGAGGTGAG

ID|56336|gene_id|ENSG00000244239|transcript_id|ENST00000458624 Homo sapiens lncRNA

CACCAGCCGGAATAGCAACCGGCGGAGCCCGTGCGCACCGCAGCGCAGCGCAGCGGCC
GGGGGGTGCGGGCGCCCTCGCCGCCCGGCTACCTACCGCCGCCTGGGCGCCGCCGCTGCCTC
CCTTCCCCGCTTCTACCCTCCAGCAGACGCCCGCCGGAGCGACCCCTCCCAGGGTGACCGGGG
GAAGGTGGGTGGCGTTGACCTCACCTTGCTTGGGGAGGGTCGGGGACTCGGGGAGTGAGTTTC
TCTCTCTCTACTCCCCGGGGTCTCTCCTCGACCCCCATCTCCTGGGGTATCCCCCGCTCGGATCC
GCCGCCCAGAGTGACCCCGCCCCTGCTCTCCGGCAGGTGTTCCACGTGGCCATAGCGTTTGCGT
CACAATGACCTGGCGGACAGGTGGGAGCCACAGCGTACACCCCAGGGTTCCCTCTGTGCTGTG
CGTAGTGCCTACTGCTTGCTTCCAGGTTAAATTTCTGTAGCTTTAGAGGACAGGAGACTTCATGA
CTTCACTGCCAAGAAAAGTTACAGAGGCCTGCTGTACCTGGAAATCAAGTTTGAGCTTCGAGAA
TAAAGGTCATGTTTTAAAAACA

ID|56355|gene_id|ENSG00000229953|transcript_id|ENST00000448869 Homo sapiens lncRNA

TGTCAGGGACAGGGCAGTATTCCAGCTTCAGCCTGTCTACCTCTGGAGCTCTCCTGATCAA
TACATCAGAGAAGATGATGGAGCCCTCCTGCCGCTCTTGGGGAGAGTTGGTGAGAATGCCTTG
ATGGGAAACCAAAGCCCCTTCCAGCTCACCACCATGGGCTCTCTGGGGAATCCAATATACTATC
TAAACTCCACCTCAGGGAACCTCCCGTAAGAGCCCAGGGACAGACACTGCCCCTCCCCAGCCT
TCTCCTCTTCAGGCCCTACTTTCAAAGGCCCACCTTGATCTTCATCCACTCACAGTTCCACCTAA
ATGCCCCCGAGAGCCCTAAAAATGCATTGACTGGACAATCACAGGGTAATACAGGATACAGTG
AGGATCTACTCTGGCCCCAGAATCATCCTCTTCCCTATAGCTCCTTAAGAAACAGAAGCCCCTG
CTCCCTAGCCACGCAGGGCAGAGTTGTGTGGGGGGCAGAAGGTGGGGGAGGTGAGGCCTGGG
GTGAGGGAGGAATGTGGTGGTAGGTGGGTGTGGGAGGTCACACTGGTATATCAGAGACCAGCT
GGTTTCAAGTTTAGAAATAGGGTGGGGAAGGCCCAGGAGCTGGGCAGGGATGGCCTAGGAGA
GCCCCAGCTCCCACCAACACAGAGCTGTATTGAACTTGTGTCTGCCTGTCTTGCCCACTATCTCT
GGGTCTCCCCGTATTACTATCAGCCTTTGCTCCTCCGGATCTGTCTCCCTGTCTCTGGATTTTACT
GGCTTCAGAGAAGGAATAAGTATTGCCCATGTGGGTGATGTGCTGTAACTAAGTGGGCACATGC
AGGAGTGTGTACACACGACAGTGTGCACGTGTGTGCCTATGTGTGACCGTGTGTGCCTGAGGAT
CTGCAGCAATGATTCATCCTCATCATGAGGACAGACTGCATCCTGACATGCACTTCACCAACAA
TTCTGTGCACTGGGAGAAGGGTGCACACTCACACACACGCATGCGCGTACACACACGCACGCG
TACACACACACACACACACAGGCACACGCGGGCGCTGGGTCACTCTCCAGTTCTCTGAGTGAC
CTCATCCTCTCAACCTCCGCTGACTCACAGCCCCCAGGGCCAGATCAGCCTGGCCGGAAGTCTA
ACCCCCCCACATAGCCAGCCACCCCCCTTCCCCTCCCGCCTCCTCAGGACGCAAGTCCGCGGC
GCCCCCTAGCGGGATCCTGGGGCTCCTCGCACCCCTCTGCCCGTCTCCACCGGCGCACGCGTA
CACGGCACTCTCAGAGCACCGTGCTGGGTCCGCGGCGCCCTCTGCTGGCCGCTGCCTGTGCGG
GCCCCCAGTTCGTTCCCCGAAGCCCAGTCCACCGCAGGGCTGGGAACCTCCCCTTGGCCCTTA
GTTTGTTTCCCTATTTCTTGCATCTCTGCCTCCTCTGCTGGACGTAAACCTTCCTGCGGCCTAACC
CCCAGACATGCTGCAGTGCCAGCGGAGCTCTCCCATCACCCTCACTCTCACACCCCCATGAGG
GAAGGCCGGCCCTTCCGGAAGCTCAGAACTGGGACACCAAGGCACCAACCTCTGCCTTCCTCT

CTGGGACCCCAGCCCTTCTCCCTCCCAGGACAGAACTCATTGACGTTCACTTGACTCTTAGGCA
CTCAGGGCACTCCATCTCTTTGCAAACATGCTGCATCACTCCAGCTGTCCCCCAACCCCTGTGCC
TTCCTTCATTCCATAAGTACTTCCTGGCAGCTATTCTGGCACTGTTCTACTTGCTGACATCTTGGG
GACGTCTCTGACCTTTGGAAGCGAATAAACCCCGAAGCTCTATATATCCTATGATGCCCTGCCC
AACCACACCTCTCTCTGTCTGTCACGGACATACACACAGAAGCATCCAACTTGCCTGGGCTCAT
ACACAGTTTTATTTCCTGTTGATTTTACAGACACTCCATCCTGCAAGCCCATTCCCTTGGAAAACC
CAGAAAGAGTGGGCACAGTGCTCCCTAGAGGAATAGAGGGGACAAGATGGCTGCCAGGGAGA
GGGCAGTTGAGGCACTTAGGGATTTACTCCGGCCCTGATGGAAGATCTGGTGCCCAGGGTAGG
GGGAGAGGGCCTGGGCTGGGCTGGAGCCTCCTAGGTATTTCCCAGAAGCCCCTTCAGGAACTG
TCACCTGGACTCCAGTACCACCCCTCGTCATGTTGTCACTTCCTGTGGTGGCGGGAGCGCAGGG
TGTGAGGGAAAATTTGGGTGACAGGGCAGTGCTGTGGCCGGCAGTGTTCAAGGCCTTGCCCCC
TAGGGACCTGGCATGGGGCTGGAAGGGGGGATCAACAGCGCCTTCCAGCGTCCCAGTAGCCTC
CCCTGACGTCCTTCTGGGTCCTCCTCTGGGTGCAGAGCTCGGGCCTGGGGAAGACACACACTC
ACCCTCCACCCTGGCTCTTCCTTCTCCTCTCCTCAGAGCCTGCCCTCTGATGTTCTTGCTCAGCCC
GTAAGAGCCCAGAATGGGGCTGCATGTTATCAGACATCAGCACTGAAGGAACCCCAGAGCCTC
CAGCCCACCTCTTCCCCTTTGCCTCAGAGAGGGGCAGTGATGGCTCCAAGGTCACATGGCCAGT
GACAGGCAGGACTTTAAGTGGAGTCCAAGGCTACTGTTGGCTACTGTCTCCCACTCCCACCTGC
CTCTTCCTGGCACTCACAGGTCTTCTGGGCACACAGGAGATCTGGGGGGCCTCCCAACGACCGT
TCTCTTGGCATCGGATCAGCGGCAGATTGCGCTGGGCCAGTCCTTCCCGGCACCGGTAGCGAA
GCACAGTGTCCACCTCATAGCGCAGCCGTGGGCGGCCGAACACTTGAGCCAGGGGCAGCTCC
GGTGGCGGCCCACAGGACACTGCAAAGGAAGACAGTTAGGAACTGAGCCTGGCTCTGTGGGT
GACAGTTCCCAGAATCTCCATGGGGAAGAGAAAAATGGGCCACAACATGACAAAAAAAGATCA
GGGATTCCGAGTGGACCCACGTAACTTGTTAGCTCTCCCATGGTCTGGAACACGTTCTGACTCC
TCTATGTTGCTTTGCACTCTCTGTCTACAAACATCAGTCCCTCTGGTCCCCTAGCCCACCCCTCCA
CTTCCTTGCTGGGACCCTCCTTTGCCTGCCCTCACCCAGCCCCATCTTGCAGGTGTAGGACAGGT
GGTAGTTGCAGGGCACGTCACTCCATTGTCCCTGATCATGCCACACCATGACCACGCAGTTCTC
TCCAGACAGGAAGTAGCTGTCAGGCTGCCCAGGGTTCCAGTTCTCATAGAGCTGGGGGAACGG
AGGAGGTGGTGAGGAGCTCCTACACCAGGAGCACCGGGAGAGGGCCTAGAGGATCTGGGGA
GGAGGGCTGGTGAGCGGGGCCCCAGGAGAGGGAAGGTTAGGGCTAGGGGGTAGGGGATCTC
CCGGGGAAGGGGAAAGGAGAGGGCAGGGACTTTAGGGGAGGGAGAATGGGAGCCAGAAGG
GAAGAAAGAGGACACAACGAAGGGAGGAGAGCCCAGGGAGAAGAGGCACAGGGCACGAGA
AAGGGAAGGAGGCCGGAAAAAGAAGGGGACAGGGTCGGGAGGGGTGGGGGTGGTAGGGAA
CAGAAGTGCTTAGAGGAAGTGAGCTAGACGGCGGGGTTCGACTGGGGCCTCTCACCAGGGGG
ACGCCATCCGACCACAAGAAGTCGCCTTCGATGGTCCTGTCGTTGAGTCCGATCCACTGGTACT
CCCGGTACCGGTCTAGGGAGACGAAGGCGTGCAGTGAGCGCAGCCGCCAGCAGATGGCGGCC
GTTCCCCGCTCCCGGGCGGTCTGCGGTGACTGCCTTGGAAACCCTTCCCCGCGATCCAGCACCC
GCCCTTGTCGGGTTCTTCTACCCCAGCCTCTGCTCAAGGGCCAGATGGAAAGCAGGGGAATGG
GGGTGGGAGAATAAGGAAGATATCAACCCAGGCTAGAAAAGGGGAGTTTTGGAAAAAGAGTC
AAAACTTTCAACAAGATTGGAGTTGGAGTTGGAGGAGGCCGACGGAGGTCGGAGAGCAGGGA
GGTTGGAGGGATTGGAGGCGGCCCCCTGCACGGCGGCCCGGCCCACACAGGCGCACTCTACT
GCGCGAACCTCCACTCTCCGTTCCCGAAGGCCACTGCCTAAGCGTAGGGTGGGAAGGGAGAG
AGAATGAGAAGGGGAGTGAACGAATGAGTGAGGGAATGCACGCTTGAGAAAACCAAGGAATA
AGTGCATGGGTTAATGAGGAAGGCGGTTAGGCGAGTGAGAGAGAGTGAGAGCATATGGCAGG
GGCCCCTCCCGCCCTGTCTCCCAGCCCACTGTTGATGAAGTCCTGTTCCTCGGGTGTGCTGATG

CTGGCCAGATGCGCGCCGTACATCCGGCACTGGGTCTCTGCCTCCTCCCAGCTCCTTCGTGTGG
AAAAGTGCTTGTAGCAGGCGCCCTGGAAGGCGTCCCAGCCGGGGTTGCAGAAGCGGAGGCCT
GCGGCACATAAGGGCGGGCATCTTAGGGCCCCAAAACCCAGAGGGCCAGGTCCCCTGGAAGG
GGCTGGTCTCCTTCCACTACCGCAGGCCGAGAGGGGCGGGACTAAGGGGGACAGGAGGGCGG
TGCAAAAGAGCTAGTAGAGAATGAGGGGGTAGCAGGAGCCTGGAGGGGCTCTTGGGCAGGGG
CGCTGGTGCCTTTTCTAAGAGACAGGAACCAGGGTGAGGGATGGAGTTCCGGCCTGGGTCTCC
ATCCTAACGAGCAGTGGCTCTCCTCTCCAGGAGAATGGAGCCTGTCATGTATTGACCACCGATT
AAATGCCAGGAGTATGCTTTGCACTGGTCTCAAATGGACCCCCTATGAGAGGCATTGTGGTTTC
ACTTAGATAAGAAAATTGAGGGTAGGACTCAAAGAAGTTATGCAACTTGCCTAAAACAGGGACT
CATTAATGACAAGGCCAAAACCAATGTTCTCCCTCCCCCCTCCAGAAGTAGCACGTTGTGGCAG
GGTCCAGGCTTCAAACCTGCCCCCCGTCCCTCAACACTCACCAACATCGCACAGGTCCCCCCCA
TAGCCAGGCAGACATAGGCAGCGGACCCCTTCCTCCTCCTCCAAGCATGTCCCACCATTGTGGC
AGGGGCTGGGGACACAGTCACCTGAGAGGGGGAAAGAAGTGAGGCGGGGGGAGCGAGGCAG
CCGCACTCTGCAGCCTGAGCCCTCCAGGAGAGGGTGCAGAGGGTCCCCCAGCCCTGGTCTCTC
TGTCCTGGAAGCTATAACAGCACCCTAAAAGTTCTCCCTAGAGCCTGTCTCTGGTCACAACCCA
GCCCTGAGTTCTGGGTTTCTTTCCTCTTCCCGCTCCCGACTTCAGCATTGGCTCAGCCTTCCATTG
TTATGGCCACCAGTGTGAGCCCAGCCCAAAAGGGGCTGGGGAGAAGAGGCTAGGAGAGCCCT
GAGAACCCAGCCCCACAATGGAGAAAGATACTGGGCACAGAAAGTTATAGCTCTAAACTCTTA
TCTGGCTTCTGTGTGATATTGGGCAGGTCATTTTCCTTCTCTAGGCTTGGAGTTTAGTGCCACACC
CCCTTGTCTTTGTGCCAGGTCCCAGCACAGTTCTGTATAGTCCAGGTGGAATGAATGGAAGTGA
AGGAGGGGACAGAAGGATTACCGCAGCAACAGTGGAGGGTGGTATCCCTTGACTGCTCCTGAG
ACCTGATATTGGTGTGGTCCAGGTGAATACTAACCATTCCTCCTGCTGTCCACTGGGCAGTCAG
CCAGTCCCAGAAGTGGTTAGTGGAGATCCAGCCCACTTTCCTCACTCTAGGCCCTGCCCCTTCT
CCCCCAGCTTAGGAACTAAGTCGCAGCAGCTCCCATTGTCCTGCAGTGCTGACTGGCCTCACAC
ATGCAGCTTTCTCTCAATATCACCCAACCTCTCACTTCCCCTTCTTATATCCACAGACTCTTCCAC
TTACCAGGCTCCCTCTCCCAACTCCACTTTGGGCCTTCTGCAGGTTCTCCAACCTAGAATCCATC
TCAATCCCTCCTTCTGGCTCCCTCTCCTCCAGGCAGTCCTCTTAGACTAACACACTGCCCTGAAG
GTCTGCAATCTGTTGCCTGGATCTAGCTCCATCTGGCTAGGCCATCTGCTTTACTGGACTTTTTCT
CTCTGTATCCTGGGGTAGGAGGTGGCTGTCCTTAGGATGTGCTTGGGTGCTACAGGAGCTGTCA
GTTTCCAATCCTTTCTGGTTCAGATGACCCAAAGGAAGATGGCTCCACAGACCAACTGTGTAGG
TCATGGCCCTCAGCCATGGTAAGGTCAAGGCCCTCAGCCATGAGAGGCAAAACCACTCCAGGG
CAGAACCCTTCAGACTTTACCATCCACCTCTAGAAGCCATGGCCAAGGTCCACAGTGCTTCCCG
CTGGCCTGGCCACAGGTCCCACTGCCTGGTGGGAATCAGTAGGGGTGGAGAGAGGAGAAGCT
GGAGTGGATTCAAAGAATTTTGCTGGGATGACCTAGCTGTTTTGGGTGAAATGAATCAGCTTTTC
CAGCAGCACTCCTTCTGTCATCTTCCCTCCTGCCAATAGTTTCTTCCCAGCTTGTGGTGCTGCCCA
CCTCCCAACCCCAGAGGAAGAGGTATTTCATTTAACCCATTAAGCACCAGGCCCCACAGAGGG
TAGAATTCTGAACTCCCCTCCCACTCCTCACACTTTCCTGTCCCTCCTGTTGTCCTTTCTGCTCCTT
CCCATTCAACCCTCCAACCCTGGGGGACAGAGGCTCTCAAACTTGGTCTTTGGTCCCCTACCCA
AGCCATAGGCCCCTCCCCCTGGCTGTGGGCTACAGAACCTGAACCCAGCGCAGGCAGAGTCGG
GTGAAGAACTCTTCTTCTTGTCATTTTCCCTCTCCCTTTTTGGGGACAACTATTCTACACAACCAG
AAAGTTTTCCCTTTCTTTCCATTGAAACAGCAGCCAAGGAGACCTCTCACTTACCCTCCCGGTGG
GGAAACTAAGACACTGTAGATACCTCCAATTGTGCTCCTCTTCCCACCAAACCCCCTCCCTATCC
CCCACAGGGCTGAACTCCAAGGCCAGTTGTGCAGGGACCTAACAGTTGACATGGGTAAGCCTG
GGTGGGACTCAGGATGTGATCCATCTAACATGGGGGTCCTAGGGCTAAGGGAATCCCAGCCTT

CTCCAGGAACCTCCACTGGGAATCTTAGCCTTCCTGAGTAGAGAGGGCTTCTCTCAGACCCCAG
AACCTGATTCCTTCCTTCACACTCTCCATCACTTGCTTTTGATCAGTGTCTTCTCCAGCATTAGGT
ACTTTGAGACCCTTTGGGTCTGGGGAGGGTGAAGCCATTCCTTCTGGAGAGTAGGGAATGATCA
TTAGTGCAGGAGTTAGTGGAGAAGACGGAAGTTGTAGGTAGTGGGATGAGAGTTAGATACCAC
CCCCTGCTGAGATGCAGGGCTAGGGGATGGGGGTGAGGGCAACTGAATGCCAGGGAACTGCC
TCTATGCTGAGAGACCCTCAGTTCCCTCAGTTACTATGACCCTTGGCACCACCAAGATGGATTG
GAAGTGAATATAAATGCACAACACATGCACTCCGGGCTGCAGGGGTTTCTCCTCATCAGGAGTG
AGGGACTACAACTCCCAGCACTGCCTCAGACTATGGATGATGACTTCTCCAGGGGTAATGTGTA
CAGTCAAATTTTGGGATCTTAGCAGCCAGGAAGGAAAGTGTAGACAGGCCTGAGAAGGAGAGG
CTGGGGGAACAATGAGGGGCTGTTAGACTGCCAGGACAGACAAGGGGAGAAGCCGAAAATGA
AAAAGGAAGAGAGGCCAAGACACACACAGAGACAGGGAGAGACATGGAGCCAGAGAAGAAG
CATCCAAGTGCTCCCCATGACACAGACCCATGGAATGAGAGAGGTGGACAGCAGGAGATGGA
GACAGAGGGTACCAGAGCTTGGTTACAAAGCGGTTTAATAGAGATTTATTTCCAAGGCTTGTTTT
CCATGAAACAGTCAAAAAATACTTCACAGAAGTAGTTGCTTAAGGCTCTGTGGGGGTACGGGTT
CTCCTGGACTCCGGAGGTTGAGGGGTGAGAAGGGACTCCCAGGCTTCCCTGAGATCAATAGGA
GGCAGCTAAAGGGGACGGCACACAGGCGGATGAAGGGAGTAGTGGAAAATAGGCCGAGCCC
TGAAGGCTGCAATAGGTGATGAGGACCCTTGGAGAGGCCTGAGGATGCTCTGTGGGGCTGGCG
AGATGTGAGCCACAGGTGGAGGGCCCAATGGCAGGGAAGGGTCACAGATGGTCAGTGGCGGG
GCTGGGATCACAGGGTAAAGACGCGGAGAGCTGAGATACCCACAAGGTGGATATCCGAGATT
GGAGACCCATAGGTAGAGGTGGGTAAGAGAATGAAGGCAAAGGACAGGAGAGTTTGGGATGA
TGGTGGGTTAAAGGACTACAGGTCAGGTGACCCAGAGCTGCAGGGGGAAGAAAAGGAGTAGG
ATAGAGAGGGCAGTTGAGCCTTGGGCAGAATTACCTGATGCGGGGACCACGGCCACTCCACCT
CGGCTGGCGCTGTCAGTGGGCAGCACTGGCTGGGCCTGCACTGAGGTCCCTGCTGGGGCAGTT
CTTCCAGAATTATCTTCAGAGGGGGCCTCCAGCTCCCTGGTACCCTCAGGGGCCCGTGTGGCTG
GAAGCAGGGAAGGGGCACCCTCGGAGCTTCCTGTCTCCTCGCTCTCTCCTCGAGGGACCCCAG
ATAGCTCAGGACCACCAGTTGCCTCCCCCACCTCTCTTGCCTCAACCAGAGTGGAAGGTGATGG
GGATGCTAGGTTCCTCTCCCTGGGAGTGGGCAGAGTCTCAGTAGGTGGTCCATGGACCCTTGGA
GGCCTGGAAGCTTCTGACTCTCCATCAGGAAGTGGTGATGCACCAGGCTGCAGGACTGCCCTT
GCTGGCGCCTGGGAGAGTGACTCCTCCTGGGCTGCTGGCTCAGTGGGGAGAGAGGCCTCAGG
GCCCGGGCTGCTGAGCTCGCTGGGCCATGCCCACAGAGCCTCATCCTCCACCTCCTCCTCTTCT
TCTTCCTCCTCTTTCTCTTCTTCATCTTCATATTTCTCTTCTTCCTCCAATGCCTTACCTTCCTCTTCT
GAGAACCCCGTGGGCGGTACCATGGATTGTGTTTCAAATTCTAGGCAAAGCACAAACGGTTCA
GCACCAGGGACAGCGTCTGTCCAAGGACCGAAAGGAAAAAGGGAAGGGGAGAAAAGTAGGG
CTCAGGAGGGTGAGCCCTGGGGAATAGGGAAGAGACCGCCCTGAGGACAGGTGGTCCTTGCG
CCACACTGGAAGAACTTGACCCCAGCCATATTGTTGAGGCAACACTTGAGAAGGCTCTGTGAGC
CATCATTAGCCCCGGAGAACATGGTCATTTGTCTCCCTCCTACTCTGTCAAAACTCTAGTCTGTTT
TGGGTGTGTGAGGCGGAAGAGGGACCAAAGAAGGCAGGTTCCACCTTTCCAGGGCGCAGCTC
CTTAGTGCAAGCATCTCTAGCTCCATAGAGTTGGGGGTGGAATTTACTGGAAAAGCAAAGGTTG
GGAGAGGGACAGGAAAGGTGCCCTGCACCCCTGAGCTACTGAGAAAGGACAGGGCAGAGTCA
TGTCATCCATCACCCTAGCATCAACCTGGGCTTGCCTCTTCTGCCCCCAACTTCTTTCAATCAAG
ACATCCTTAGGATAAGGGATCCGACTTACCTAGGAGCGTCCTAGGGGCCTCTGCTGGGTCTTCT
GGAGTGGAGCTTCCACCTCCTCCGTCCTCCATGATGGGGATGGAGTAGATGGCCCCACGGGAT
TCACTCTCTGTGGCTTCCTGAGGCAGCTGCAGTTCCTCCAGGGTCTCTGTCACTGTGACGATAGC
CTCTAGTCCATCAGAGGCTGGGTTGGAGGCTGGGTTGGAGGCCTCAGGGATGGCAGAAGGCTG

GGCCGAGTCTGTGGCAGGAGGAAAGTATTGATGCGAGCCTGAATAGGTAGCTCTGCCCTCCCA
GGTCACAGCTTGCACCATGGAATTCTCTCAATTCTCTCACATCTTCCCTGTGGAGCACCTACTGG
GGCCCAGGGAGCATCCCAGAACTTCGTCACTCATTTCTCAAGCATTCATCAAGTGCCTACAGTG
TGTCATGCCAAGCATTCCCCTGGGCACTGAGGATACAGTGCAGAACAAAATAGCAAACCTCCTA
CCCTCAAGGGAAATTATATTCTAGAACAGATGACAAACAAACATATATTATAATCTTACAAGATA
AAGAACACTTGAGCAGAGTTAGAAGATGGAGTGATGGAAAATACTACTTTGGAGTGGTCAGGA
AAGGCTCAGGTGAGAGATATTCCCTAGGGGGAGGGGCTGGAGCCAGCAGGATGGATGAAATA
GCTTTACCACCTTGTGATCTTGGGCTTGGGGCAAGGTGTGAGGTGTCCCAGGTATCCCCAAATT
CTTTTATTTTTTAGACAGAGTCTCCTTCTGTCACCCAGGCTGGAGTGCAGTGGCACGATCCCAGC
TCATTGCAACCTTCGCCTCCTGGATTCAAGCGGTTCTCCCGTCTCAGCCTCCCAAGTAGCTGGGA
CTACAGGCATGTGCCACCGTAACTGGCTAATTTTTGTATTTTTAGTAGAGACAGGGTTTCCCCGT
GTTTGCCAGGCTGATCTCGAACTCCTGGCCTCAAGTGGTCTTGGCCTCCCAAAGTGCTGGGATT
ACAGGCATGAGCCACTGCACCGAGCCTCCAAAATTCTTGAAATAGAAGGGGAGAGGCTGTCAT
CCCCTCAGGATAGCTGGAGGGCAGAGTGCAGGAATAACTTCAGGCATCACCTGAGGATCTCAG
TGGTGGGGCAAGTAGGCAAGGGAAAAGGCCACCTCCTTCTGGGACCTACTCCTTTCAAATAAC
CAGAAGTTTAGAATGGTTTACTTGTCCTCTGGGGAAGGGAGCCAAGAGCAGAAAACCAATGAA
GTGAAGGTGGAATTGGGCTAAGAAAAGTCCTGAGGGCTGTCTGTGCTCAGCCAGGCAAGACAG
GACAGTGAGTCCCTGCCCTCATCACCTGCTCCAGTGAGCCTTACTCCTCACCAGACCTTGTGTCC
TTGCTGCCTGTGTCCTCCACCAATCGTAGTCCCAACTGTAGTCATATTCCCCCTCCCCTGCCCCT
CCTCATGATCCAGGCCATTTCTTTCCAAAACAGTGCTTGGCTTGGCACACAATCATAGCCAGCA
CACAGAAGTTTTAGCTATTACTATTATTATTATTACTACTACTACTACTACTACTACCCCTGTT
CCAGGAGGTCTTTTCAGATGGACCTGTCTTCCTATGGACTTTTACTTCCCCACCCCTCCCCAACC
CCAGCCAAATATGTCCACAGATCTGTCCAAATGGAAGTAATGCTTTTCTCTCAGTCTCTCCTCAT
CCTGGGAGTGAGGCATCTTGTCTTCCCTTTGCTGGGGGTTCCCATGGCAAAAGCTGTGTCTTCAT
CCTTCAGACCAAGTGCTCCCTGAGGTTCAAGGCTGTGTCTGCTCCTTCCATTTCTGCTTCCAGCT
CTTGTAACAGTATTTCAGGCTTAAAACTGGGGAAGGACCGAGACACCGCATGGCAATGCCTCTC
TTATTGTCGGTCACCCTTGGCCTGGGCCTTTTGCATCTGAGAGTCCCAGAAACTGCCTCCCGCTC
CAGCTCCCAACATAAGATATGGGCAGGTTTGGGTGCAGTGGCTCACACCTATAAATCCCAGCAC
TTCGGGAGTCCAAGATAGGTAGATTGCTTGAGCTCAGGAGTTCAAGACCAGCCTGGGCATGTG
GTGAAACCCCATCTCTACAAAAATTACAAAAATTTGCTAGACATGGTGGCATAAGCCTGTGGTC
CCACCTACTCCGGAGGTTGAAGTGGAAGGATCCCCTGAGCCCAGGGAGGTTGAGGCTACAATG
AACCATGATCACACCACTGCACTCCAGCCTGGGGGACGGAGTGAGACCCTGTCTCAAAAAAAG
AAACAAGCCATAAGATGTGGGCAGAAAGCTATCTCTATTCCCAACTGTACTTAAGGTCTTGCTC
CCTTTTGGGGGAATTGTATCCATCCACCAGTATTTGGTAGTTAAAGATCTGACACCTTCTTAAGC
TGTAGTTTCTGGAAATCCTCCAGAGGGCTCCTGAGGGCATTTGGTTGTTGACTGGAAGAACTTG
GGCAGATTACTCATTGAATTCACAGAATGTTGGTCGTCTTTGCTCTGCCAATAAGCTGACAGGAG
ATGGGAGCTTATTCAGCAGCCCAGCTGGAGCAAACAAGGCATGGACTACTGGTGGGAAGGCA
GATAAGGAGAGGCCAAAAGGTGACTACTCCTTGGGCCAAACAGCTGACAGACACCACACCAG
AGGGCATGCCCAGAATTAGAACCCTAGATGACAAGCTGGACTTCACCCACGACCACTTCAGGC
AAATGGCAAAGCCCTGAGCTAAACTGATCAGACTGGGGTAGGACTTTTAGGACTCAACTCTGCC
TTTCTGAGATTGGTCTCAGCTTCTGCAGGGAGGTGGGCTCACCTCGGAAGCAGTAGACGTTGAA
GCGGCTGTGCTTATTGGGGAAGCCAGTCTGGTTGGGGAAGAGGAAGAGAGTCTTGACACCAGG
CAAGCCCCCACCACAGCGCTGGCTGGGTGTGACGATGGGGTAGCGCACACTGCCATCAGCTAG
CCACCCTGGGCTGCAGTGGTCCAGGCCACCATCCCAGGCTGCATACAGTTGGCCCGTGGTGGC

AATCTCTGCACCCCGCTCCTGGCAGTACGCCCGTGCTTCCTCCAATGTCAGCTTCTCTGGAGGG
TCACCCAGGAACAGTTCTCCTGGGTGGAGAAGAGAAGGCTGGGTTATCAGGCAGCTGGTAGCT
CCTTCCACTCTGGTCTCCCATGGGAACCCTGGGCAAGCTTCTCCAGTTCCCTCATCTGTGGCAG
GGAGTCAACTACTCTACTTCATAGGATCATAGTCTGAATAGAACCTCAAGATGGATATTAGAGC
TCAAAGAGATGGGGCCCAAACTTCTTGTTCTGCAGAGTCATGCACAGAGTCCACCTGGCAGGTG
GTAGATGAGTTAAACTCGGTATCCTGCTGCCTGGCTGAGACTTTGGTCATGAGCTTGCCTCTGTG
ACAGTGTTTGGAGAGAGGGACACCAATTGTCTCCTTACTCCTGCTTGCCCCCTTAAACTCAGGA
GGGATAGTAAGGTCCTGCAGAGGCCCCGGGAGGACCTATGGGCAGCATGGGAGTAGGAAATT
GTCCCCTGGGAACCTCTCACTCCTAATCACCATTTAGGTCTTCAGCATAACAGTACACATCATAG
AGGTCATCCGGGTCCACCACACCATAGTTCCGGACCCCGGGGAAGCCATCCATGTCTCCGTAA
CAGGCCTCTCGTGGGGTCTGGATGGGATACCTAGGGAGGAAGGACAGAATCACTTAGTACAGA
TGCTTTCACCAGGGAGCCAGCCCATATTGATTCTATTGAACCCCACTGCCACCACAGTGCAGAC
ACTTGCTGACAGCTGGCTGTCCTTCTAGCACTGCAGGGATGCACATGCTCAGGGTCCTCACCTC
CCCTCCTCAGACCAGCCTCATCCCCCAGAGGCAAAAAGCCCTGCATGTCCAGCCTGACACAGC
AGGCAGCACCCAGCAGAAGGGGGGCCATGGCCACCTCAGGGGCCAATAGAAGCCCCAATCCA
CAGCCCCTGCCCACCTCACGGTCTGATCCGACAGCCAGCCAGCATCACATTGCTCATAGCCCCC
AAGGTAGGCGGCATAGAGCTGCTCCGGGGTGGCGATGTGGGCTCCAATGCGGGCACAGGCCT
CCTGGGCCCCAGAAAAGGAGAAAGCATAGCGGGCAGAGCCCTCTCGGTAGAGAAAGACGACC
CCTGTGGGGCAGAGAGGACCCCTGAGCCAGGTGAGCACCCAGCACGCTGGCCTCCCCTCACC
CTCCTTGTCACTCCTCTGTACACAAGGGTAAATAAAGTTCAATTGTGCTCACTCTACAGCATGAG
GGACTTAAGTTAGATTGTGGGTAGAATGTCCTCCACCTGAAGAGAGAAAGGAGACAACAAAGG
CCCGTGTCTCCTCACTCTTTTTCCAACCCAGGCTGCGCCTTCCAGCTACCTTTGCTTCCCTCTCAC
ACTGTGGGATCCCCGAAGGCAAGAGTCCTCCCTTCCCTCCCTCCCTCCCTCCCTCTGGAACCTC
CCTGCCCTCTCACCTTTGACCTTGACCTCCACAGCGTCGCTGCTGTCATCGATGCCGTGCTGGAC
CTCACAGCGATAGATACCTGAGTCGTTGGGGCGCAGCTCGCTCAGCGCCAGGGAGACGTCGGT
GAGCGACGCTGGGTACGCAGGCAGTGCCACGCGGAACCGGTAGGCCTCGTTCACCTTGACGC
GCACTCCCCGCGCCACCAGCACCTCTGCCTCCCGGCCCCGGGACAGGAAAGTCCACTTGACCC
GCGGAGAGCCCAGCACAGCCCGGCGGCTCGGCGGTGGCCGCAGGTAGTGGACGTGGCAAGG
GATGGTGAGGGCGCCGCCGAGCACGCCCTGCAGTGGCGCGTCGCCCGCGATGCGCACGCGAA
AAGCGCGGTCCTCTGTGAACGGGTGGAGCCGGCTGGAACTTAACGCTGTCGACCCTCAGAGTC
CCACCCGGTGGCCTCCAAGCCCCGCTCCAGGATCCCTCCTTCCGGCCCCAGGATTCCCCGGCC
CCCCTAGGACCAGGGGCCAGGGGCCAGGGGCCAGGGTCCTGCAGCCCTACCTCTAGGTCCTC
CAGGACCACCCCGACGGTCTCCAGACCCATCCCCCAAAGTCTCCCAGTCAATTCAGCCCCCAG
GATTCCTTGCCCTGCTCTCAGAGTCTTCCACTTCCTCTCCAAAATTCCTAGACCA
ID|56356|gene_id|ENSG00000236782|transcript_id|ENST00000448923 Homo sapiens lncRNA

GGCAGGCGCGGGTGAGCATCCTCTGAGAGCTTGGGTCTTGCGTATGTGGAAGGTCAGAGA
CCCCTCTACCCTAGTTTCCACTTCTTGGTGTCATGAAGACCACTGGTCTTCACGTCCGCTCCCAC
CCTACAGGCCGCCAGGGAGACCAGTGCCGCCCACCATGCGATTTCGACGCTTGACCCCTGGTT
ACTTCCGGGTGTTACAGGTAAGTACCCCTGATCAACGGTTTCTGGCACGACTGACCCCTTCCTCC
CCCTACCATAGCTGGGTTGACACCCCCAACAGCCAGGCTGTCACGTTCCCTGCCGCCTGAGCA
GGCAGGCCCAGATGGGCAGTGAGGCGAAAGGGATTGGGGTGGAATCTCCCTGGGGCAGGACC
GGCGTCCATGTCTCCTTCCCCTAGCTACATCTACTCAGCCCTTTCCCACTCCCCACCTTCCTGGC
AGTCTCAACTGACCCCTGAGGCACACCCTCTGTGCACCTCCAGATGCAGGTAGCTGGTGAGCTG

AAGGCAGAGCCCCGGAGTCTGCTGGCAGGAGTTGTGGCTACAGTGCTAGCTGTCCTCGGCCTG
GGTGGCTCCTGCTATGCCGTCTGGAAGATGGTGGGGCAGCGGCGGGTGCCACGGGCCCCGTA
ACGAAGAAGAGAGACTCTCACTTGGATGCTGGGGGTAGGTGACTCGGGAGGGGGGTGCAATCT
CAGAAGCCTTCTTTTCTGGGGATCAGTCTCTGTCAGAAGCAGGACAGAGTAATCTGATCCCACT
CCCGCCACCCCACACTGCATCAGGCCTCCATCTGGGTGCTCCAGTTCAGATGCTAGGACCTCAG
AGGGAAGGCAGAGATAGACCAAGTGGGGAGTAGACACCCTAGGCCTGGTGTACAGAAAGTAG
TAGAGAAGCCCTTCCAGGGCAGGAGTTTGAGACAGCGGCCTGGAAGTTAGAGTTGGAGGCAA
GTTCTCTTTGTCCAGTTCCTTTATTTGGGGCAGGGCACCAAGAAGAGGCCCTCCGCTCCCCAAA
CCCAGAGGCAAAAGGGGTTGGCACGCTCCCTCCCAGCCTAGTCCTTGCGTCACTGTCCATGGG
CAATTCCTCTGCCCTGCATCTTCAGGCCATGTCAGGTAGAGGTATCCATCTCAGGGACCTCAGT
GGACACTTCCGTGGGCACTGCCAGCCGCCTGGGGGGCACATAGGATCCCATACCCGCTGCCCT
CTCCGCCTCTTCCTGACTGTAGGGCTCGACGCTCAGCTGCTTCAGCCTGGGAGAAAGAAAAACA
GGAGTTGGGAGCTGACCAGGTGAGAGCCCTATCTGCTGAGCACCCATCCATCAATCCTCTCCTT
A

ID|56377|gene_id|ENSG00000232093|transcript_id|ENST00000452962 Homo sapiens lncRNA

AACAAATGTCCACACAGTTAGGAAGCCCAAGGGCCCTCTGCCCTTTCCTCTCTGCCTTCCT
GGAGCATGAACCCACACAGGGCACACAGCAGCAAGGCATCCCCGGGCAGTGCCGTGCCCACT
CACCAGCTTCTTCCTGCGGTCATCGATCTGGCAGAAGTAGGTACTGATGTAGATGTTGTCAAAA
CGAATGTCATGGTTATAGCTGTCCATGTAGGAGAAAGACCTGTGGGTGGATGGGCAGGGATGT
CTCCATCTCTTCCAGGTTCTCCTCATCTATCCCCAAACATGGGCTTCCTTGAGGCACAGTCATTC
AACCAACCAGCCAGCATTCATTGAGCACCATCTATGTCCTGGGCACTGCTAGGGGATGGTGATA
ACAGGGAGAAGACTCTGTCCCTGCCTTCCAATTGTGTAGAGGAAGACATCCCCCTACATGATGG
GTGAGACATAGCAGAAGTGAGTAGGGGATGAGGTGGGGGCTCAGAGGAGGGCATGGTCAGCC
TGTCTGGGAGGGAGTTGCATGTGTGCATCTGAGGTAGGGACAGGCATGCATCTTACAGGATGA
ATATCGAGCAGAGTTACAGAGAGGGGGAAACTCCTTGAGGTTTCAGGAATCACCTAATCCACTG
TGACTCACAAATTCCTGCCTCTTGGCTTTGCCTGCAGCATATCTCCTGGAAGTGTGCTGGGGCAA
AACTCATCCCAGACCACCATCTCCATCCTCCCCCAATACACCCTGGCTCTCCCTGGCTACCCTTG
AGCACGGTGCACGTGTGCATGGGTGCATGCCTGCATATATAGCTATCCCCCATGTATTTCCCAA
AGCCCTACATAATGCTTCAGTTTGCTAAGGAAAAAATGTTAATTACTGCAAATGTGTTTAAAACT
GTAAAAGTACATTAAACAAACTCTGTAAAGTGTGAAA

ID|56389|gene_id|ENSG00000250222|transcript_id|ENST00000508877 Homo sapiens lncRNA

AGGATTGGGGAGCACGTTTTGCGTCTTGCTGTGCGTGAGGAAGCGGGGATCCAGGTCAAG
GCTATGGAAAGGTCAGGGAAGGGTGTCAGTGCGCCCGTCCTGCAGGGAGTCCAGATCCGAGA
GGTTAGGGCGGGACCTGGGGCGAGCTGGGCGGTGGGGGAACAGTGGGGTCATCAGGAGGTG
GAGGCCCAGCGGGAGTGTGGCGGGGGAGGCGGGGAATCAGGAGGGCCAGGTGTTTGGAGGC
GCTGGGGTGGGAAGGGTCCAGGCGAGGTTGCGGGTGGGTGCCAGCCCCGTCTCACATTTGGAT
GAAGTAGGAAAAGGCAGCTTTTAGGGTACACCCTGACATGTTCTGTCAGGGCCCTTAAAGGGC
CCACGGGCCGGAGGAGAGGGGAAAAGGATGAGGGCTCAGCCTGGCAAAGGACAGAAAGTGG
TGGGTGTAGGAGGTGGACCTTGGTCTGGTGGCCTAGCCTGCCCTGTGCTCCCTGCAATGCCGG
GTGACTTGGGTCACTGGGCGGCCAGGCCAGAGGTGGGCCTCTAGGTGCCCCCCATTCTGGATG
CTGAGGGTCCCTGTGAGGGTCTGGCGGCAGAGGCCTGCGCTTATGGGTCAGCAGCTAGGACTG
ATGGGTTGAATAAGAAGGGCCCAAGTTAGGCCAGTTGTTCTCACAGACCCAGAGGTCCCTAGA

GTTTATGATGCCCAGAGTCTAGGAGAGGGAAATGCACACACATGCCCTCGAGGACACCAGCAT
CCTGACATCCAAGTTACTGTACTGCCCTGGTTAGCAAGAAAGAACATCTGTTCCCTTCTCTATGC
AATTGTTACAGTAACTGTTGTTACATTTCTTAAGATACATAGCTATATATGTAGTCACTCTGCAAA
CACACACACTCTGATAGACACACGATTGCACTCATGGACACAAAAACGGAAAATAAACATGCG
GACAGCTACTCCCAGTCCCCAGACCTGACCCTCATGTGACACCTTCACCAGACATGGTACTTTC
TGGGACCTGTAATGTGTGTTGTGATGCCCAGCACATTTGTTGGATGGTACAAAGGGAATTATGAT
ACTTAATCAAAAAGCCATACAAGCCAGATAACATTAAGGCTTAACGGTAACAATAGGTGATTCA
GTTAGGAAATTAATGTTAGAGATCGGTTTTCCTTGTGGAAATCAGTGGTATTCAAATGCCTGAAT
TGGCTACCCGGTTTTAGAGCTGCTCTCCATGTATGAGCAGGACATAGGATTCACTCTCTGGAATG
CTTCTGTAGGCAGGACTTTGAAATGACCCTGTCCTCAGCCTGGAGGATGCGGTATTCTGATTGTC
TGGTGCTGTGTCAACTGGGCCTGCCTCTGGGATAGTGTTGTGGCTGCCCCACGAGAACCCTATA
TGCCAGGTGAGCTTGAAGCACCTCAGAGACAGAGCACTGAAGCTCCATGACACAGGGCTATTC
AGCTATCATTGCCCCTGTTTGCCAGCACCTCTGTCTAAATAACTTGTGTGGGACAGAATTAGGTC
CGAGTCTGGTATTCTGTTCATCTAGCCTGGTAGCCAGGTAGTGAGAGAAGAAACCTGTGGCCCT
TGTCTAAATATGAGGTGGTCAGTATTTAACAACAAATCAACAATAGGGTATGACCAGACTGACT
TGGGTCTGTTTGCAACACCTGAACCTTATGCACATGCTCATAAATGCCCTCTCCAAAATACACAC
ACACACCCCCAAGAAAGGTGAGATACAAATACACACTCCTCCCACACAGAAACATGCATGCTC
ATTCACTCTGGCAGAAACCCTGATCACACTTACGCTCCTGCACTCAGATCTAGGGAGTAGAAAA
TGGGCCAAGCCAACTCCAAAGACAGCTCATAGTTTCATTTTCATCTCCTCACCCCCTTCCCAGGG
GAGCGCTGAGCAGGGAGTAGCCCACTGCCACCCTTCCAGAAGCCCAGCTACCCAGAAGCTGG
AGAACAAGTAGACAAGGGGCTGAGGCTGGCCAGGGGAATGCCCTCCAGGGCATGGGCTGAGA
AGGCCTTGGCACCAGGGTGCTCTGCTGTTGGCATTTTCCTGAGTCCGTCAGGAGCATATTTGTGC
TCTGTGTGTTGTTTAGAGCTGGCTGCATCTTTAAAATTGTAAGTGCCAACTTGTGTTTTGTGTTTC
AAAGTTCCAGAAGCAAAAACAAAAAAAGTTCGGGTACAGCGGCTCACACCTGTAATCCCAGCT
CTTAGGGAGATGGAAACTGGAGGATCACTTGAGGCCAGGAGTTCAAGACCAGCCTGGGCAACA
TAGCAAGACCCAGTCTCTTAAAAGAAAATTAAAAAGAAAGAGTAAATAAAGTTCCAGAAGCTTT
GCTCTCCTT

ID|56278|gene_id|ENSG00000250222|transcript_id|ENST00000514487 Homo sapiens lncRNA

CCCGGCAGAGGGCTCCGCGAAGACAGTGCCGTCGTTGGGCGCGCGCGGGCGGGAGC
TGTCGGGGCGGGGCGGCACCGCCGAGCTCCGAGCTGTGAGGACCGCCCCGGGCCTTGGGCGG
GGCGGGGGCCAGGGAAGGGCGTGCGCAGGATTGGGGAGCACGTTTTGCGTCTTGCTGTGCGT
GAGGAAGCGGGGATCCAGGTCAAGGCTATGGAAAGGTCAGGGAAGGGTGTCAGTGCGCCCGT
CCTGCAGGGAGTCCAGATCCGAGAGGTTAGGGCGGGACCTGGGGCGAGCTGGGCGGTGGGG
GAACAGTGGGGTCATCAGGAGGTGGAGGCCCAGCGGGAGTGTGGCGGGGGAGGCGGGGAAT
CAGGAGGGCCAGGTGTTTGGAGGCGCTGGGGTGGGAAGGGTCCAGGCGAGGTTGCGGGTGGG
TGCCAGCCCCGTCTCACATTTGGATGAAGTAGGAAAAGGCAGCTTTTAGGGTACACCCTGACAT
GTTCTGTCAGGGCCCTTAAAGGGCCCACGGGCCGGAGGAGAGGGGAAAAGGATGAGGGCTCA
GCCTGGCAAAGGACAGAAAGTGGTGGGTGTAGGAGGTGGACCTTGGTCTGGTGGCCTAGCCTG
CCCTGTGCTCCCTGCAATGCCGGGTGACTTGGGTCACTGGGCGGCCAGGCCAGAGGTGGGCCT
CTAGGTGCCCCCCATTCTGGATGCTGAGGGTCCCTGTGAGGGTCTGGCGGCAGAGGCCTGCGC
TTATGGGTCAGCAGCTAGGACTGATGGGTTGAATAAGAAGGGCCCAAGTTAGGCCAGTTGTTCT
CACAGACCCAGAGGTCCCTAGAGTTTATGATGCCCAGAGTCTAGGAGAGGGAAATGCACACAC
ATGCCCTCGAGGACACCAGCATCCTGACATCCAAGTTACTGTACTGCCCTGGTTAGCAAGAAAG

AACATCTGTTCCCTTCTCTATGCAATTGTTACAGTAACTGTTGTTACATTTCTTAAGATACATAGCT
ATATATGTAGTCACTCTGCAAACACACACACTCTGATAGACACACGATTGCACTCATGGACACA
AAAACGGAAAATAAACATGCGGACAGCTACTCCCAGTCCCCAGACCTGACCCTCATGTGACAC
CTTCACCAGACATGGTACTTTCTGGGACCTGTAATGTGTGTTGTGATGCCCAGCACATTTGTTGG
ATGGTACAAAGGGAATTATGATACTTAATCAAAAAGCCATACAAGCCAGATAACATTAAGGCTT
AACGGTAACAATAGGTGATTCAGTTAGGAAATTAATGTTAGAGATCGGTTTTCCTTGTGGAAATC
AGTGGTATTCAAATGCCTGAATTGGCTACCCGGTTTTAGAGCTGCTCTCCATGTATGAGCAGGA
CATAGGATTCACTCTCTGGAATGCTTCTGTAGGCAGGACTTTGAAATGACCCTGTCCTCAGCCTG
GAGGATGCGGTATTCTGATTGTCTGGTGCTGTGTCAACTGGGCCTGCCTCTGGGATAGTGTTGTG
GCTGCCCCACGAGAACCCTATATGCCAGGTGAGCTTGAAGCACCTCAGAGACAGAGCACTGAA
GCTCCATGACACAGGGCTATTCAGCTATCATTGCCCCTGTTTGCCAGCACCTCTGTCTAAATAAC
TTGTGTGGGACAGAATTAGGTCCGAGTCTGGTATTCTGTTCATCTAGCCTGGTAGCCAGGTAGTG
AGAGAAGAAACCTGTGGCCCTTGTCTAAATATGAGGTGGTCAGTATTTAACAACAAATCAACAA
TAGGGTATGACCAGACTGACTTGGGTCTGTTTGCAACACCTGAACCTTATGCACATGCTCATAAA
TGCCCTCTCCAAAATACACACACACACCCCCAAGAAAGGTGAGATACAAATACACACTCCTCCC
ACACAGAAACATGCATGCTCATTCACTCTGGCAGAAACCCTGATCACACTTACGCTCCTGCACT
CAGATCTAGGGAGTAGAAAATGGGCCAAGCCAACTCCAAAGACAGCTCATAGTTTCATTTTCAT
CTCCTCACCCCCTTCCCAGGGGAGCGCTGAGCAGGGAGTAGCCCACTGCCACCCTTCCAGAAG
CCCAGCTACCCAGAAGCTGGAGAACAAGTAGACAAGGGGCTGAGGCTGGCCAGGGGAATGCC
CTCCAGGGCATGGGCTGAGAAGGCCTTGGCACCAGGGTGCTCTGCTGTTGGCATTTTCCTGAGT
CCGTCAGGAGCATATTTGTGCTCTGTGTGTTGTTTAGAGCTGGCTGCATCTTTAAAATTGTAAGT
GCCAACTTGTGTTTTGTGTTTCAAAGTTCCAGAAGCAAAAACAAAAAAAGTTCGGGTACAGCGG
CTCACACCTGTAATCCCAGCTCTTAGGGAGATGGAAACTGGAGGATCACTTGAGGCCAGGAGTT
CAAGACCAGCCTGGGCAACATAGCAAGACCCAGTCTCTTAAAAGAAAATTAAAAAGAAAGAGT
AAATAAAGTTCCAGAAGCTTTGCTCTCCTTA
ID|56173|gene_id|ENSG00000255471|transcript_id|ENST00000528660 Homo sapiens lncRNA

TTCCAGGTGACCAGTGGTCCTCCTTTCTGAAGGCTCTTGCTACAAAGGAAAGATGAGGATA
TTCTATTTCTTGATCCCTCTCAGCTACTGGGAGCTGCTTTGTGCCTTGGAAGCCTCAAGCTGAAA
TGGATGTCAGCCTCAAGTTTAACTCCAAGAGGGTTCACCTTGTTTCTTTGAACAAAATGTGCTGC
CTGTTGCTTCCCTTTGCCAGGAGTGGTGTTCCCTTTTCCACAGGTCTGGACCACAGTGTCTCTAG
CTCCCGCTCCTGGATACTTGGTTACTGTGTGTTTCGGTAAAAAAGCATCCACAACATGAATATCC
ACTAAGCTGTAACAGGGCCTGCTGCAACTATAGTATTTCCTGAAAAAGTATCAACTGCCACAAG
GGCCACAAATAAAGTCTGTGAACTTGAAATAACCATGGAGAATGAAATGTTCTGATTCAGATCA
TCGGTGGGATTTCACTGGGAAATCTCCCACTGGGCAAGAGTAGATGATGGAGGAGGAGAAACA
GTCACTAGAAAAAGGTGATACTTTGTATCAAGAAACAATTAAAAATTAGATCACATCCTGAAAC
ATTAAGCATATGCTCCTTTGTGAGGCTTGCCTGGCTGAACTTGAACTGGATTGAGTGAATAATTT
CTTAAAAACAATCAAAATCACAGATTTATTCTATTTTAAATACTTATATTTACTTATATTTCATTCA
GAAATTAGTTAACAAATAATTTTTGAGTATCCTGGGAACTGTTCTAATCATTAAACATGCAAGTTT
GAAGAAAGTAAGGATGAATCTGCCTGCGTGGGGCTCTCAATCTAGCGAGAAGCAGATAAGAAG
ACAAATGTCAGGGAAGATCACACAACCTTCAGGTAATATTTAAGCTGAGACCTCACTAGTTGCT
AAGACTGGATTTCAGAGGTCAGGTTTGGATATACAAAATTTAGGAGCTGGTACATGACATTTAA
AACCATGGGAATGAATGAGATTTCCTAGATAGAAAGAAGAAAAGAATATTGACATTTAATGTTG
AACGGAGTAGGAGGAGCTGAACAGGGAGACCAGGAAGGAGCAGACAGTGAACCTGGAGCAT

GAATAGGAGAGTGGGTGTCATGGAAGCCAAGAGAAACAAGAATTCAAGAAAGAAAGTTTCTTA
CTGTAGCAAATGGCGCTGAGAGGGAAAGTAAGGCCATGGAATGATGACAATGACAACAGTGAC
GAAGGATCATTGAGTATGGTATCATGCTGACATTTGTTTCTACCCAGGCTAGGTCGTTACTGAGG
GGTGGTAGCTAAGACAGGGTGGGTTGAACATGAGGAAATGAAGACAGAACATGAATTTTTGGA
AAGTTTGGCTACAATGGGAAGTAGAGAAATGGGGTAATATCTGGAAGGGGAGGGAATTGAGTC
AGTAGGGTTTTGTGTTTTGATTTTACGGTAGGGCATCTAGATAGATCATGTTTATGTACCTATTCC
CACTCTGTTCTCTCTTCCTGAAGAGTCTCCTCTGGCAAACTCCATTCTGTAAACGTCCGTATTTAA
GTGTCCTGTTCTCTGTCAAACATTCTTCCCAGGCCTAGTATGATGTTTGTCCCCAGGCTTCCATGC
CATTATGTGTACACATTATCTGCCATAAGCTTACCTTATTGCATGGCAATGGAAGTTTTTCCTCCT
TCTCTCTTCTTCATTAGACAAGAACAGGGTCTTGAAAAGTAGAGACTGTGTCTTCTCAAGTTCGG
ACTACTGGCACAGGTAGGTGCTCAGAACTTGGCACCAGGAAGATGCTTATTAAATATTTGAAGA
ACAAATAAGTAAATGAATGAATAGACTAACTGAGTTTGAAGGCAGTGCTATGGCAGATTTTTCTT
ATTTTATGAATGGAAAAACTATGGCATAGAACAATTTGGAATCTGATAATTTTACAAATTCGTAG
GAGTGCTGGAAAACAGTAAAATCTTCAGTCTTGCTCCTTGAGCTGTTGCCCAGGTCCCTCTCTGG
GACTGCACTAGCAGCCCTAAAAAACACCTGGGGCTGATACCCTCACATTCACACCAAACCGCA
GACCTCACCACAGAAGCACAGGCCTTCACGCTGGCGTCTGCAGTCTACTTGTAGTTTGATTTTAG
CAACATTGGCCAAGTTGAGGCTGGGAAGCAGCTGGTGAACAAAGGGTGGATGCACTTTTCTGA
ATCTAGGGGGAGACCAGGTTAGAGCCATGCTAAGTGAAGCTGCTAGGGTAGGTGGGCCAGGC
AGGGGAACAGGCAAAGATACCCTTGGCTCCCTTAAACTTGGGCTAGAAAAAACAAAGACATGG
CTCAAGGAGAACCACAAGGTGTCACTGTTCATCCTCAGTTAAACAAACACTGATTTGGGCAGGG
ACTTGAGAAACACTGCAAAGAACTTTATTTTGCAGATGAAGAAACGGAGGCCCGGGGAGGTAA
CATGATTTGACTAAGATCACGTAACAACTTAATGGCAGAACAATTATGAGAAACCAAGTCTCTTA
ACTTTCAGACTGGCAGGACCACTTTCTCCCATTGCTCCTATGAGATGGCATTTTGAGATGAGGAG
TAAGCCTTCTCTACAATCATTCACTCAAGACAATGGCTTTCAATTAGAAATAGAACAGAAAAAAA
ACAAGAGAGAGAGAGAAGAAAAGAATGAAAAAAGCGCAGCATTCACTCTCACATGTGCCTTATTC
AAAGTGCTGGGCTAAGATGCCAGTGCCCACAACGAGAGAGTGGCCCACAGGGCCTCCGTCTGT
GGCAGGCTTGAATACCCGAATAGAAAAGTATCAGGTAAAGGATTTCATTCTTTTTATGGCTCCAA
CTTAAAAAGAGTCTGGTTCCCAAGGAAAACAGGAAGCCAAGTTGCTCAGCAAAGAATTGGCTG
AAACCTAAGTAGGACCCAGATAAAAACATTTGCTGGATGGAAGGATGTGGGCAAATTTCTGGG
CCCCGCTGCTTGGGGATGGGAAGCTATGAGGCAGAAGACCCTTTGTGAAACAGTGACCCGTGC
ACCAGGCATTTTTGCGGATGTCTGGAAACTGCGAGGCAGTGGAGGAGTCATAAGTTAGTTGGTT
TTTTAATTCCAACTAACTTTACTCCACCAAGTATCTCAGGAGATTTCGCCTATGCTCTTTGGCCTG
ACTCCCACTAGGCCCTTACTCCTTCCATGGTGGCTTCTACTGGAGCCTCTTTCTTAGTTCCCAGGT
TATTAAAATAATAATAAATTCAAAGAGCTTTGTTGTTTTCTTAGGTAATATAATTAATATGCGCTC
ATTACAAAAATTCAGATACCAAGTAGCAGAATGAAGAAAATAAAAGACATCTGTAGTCTCTCCA
TTCAGAAATAACTGCTGTTAGCATAATGCTGTATACATTCAGGCTGTTGGCCTGTGGAACACCTG
GTATATGTCCTCGGCCCTGGGTTTACAGCTGTAAAGCTGGCACACACTAATCTCAGCTCTCACA
GAGCCTACATTCTAATTGTCTGGGAGAGACAGACAATAATATTTTGTAAAAAAGTAAAATATATA
GGATGTCAGATTGTGGTAAATGCTATGAAGACAGAGAAGGGGATGGGGATTTTAATGGTGGTG
AGGTGGCAATTTAAAAATAGGATATTCAGAAAGTGACATTTGAACAAAGATCTGAAGCAGATGT
GGATATCTGGGAGAAGACTTCTTTCTTTCTTTCTGTACATAGACAATAATGCAATTATAACATAAA
CAATATATGAATACATCTTTGATTTAAAATCTTCAAATAATACAGAAATATATGGGAAACAAGGG
AACAACCTTCTCACCACCCTCCTATTACCTTCTCCTCTCTCCCAATAAGCACTTTGGCAATGGTGT
GTTCCCTGCCCAAGCTTCATCTCTGCATTTACATTCATTTCTGTATATACTGACACAATAGCTTTAT

TTTATTTCCTACGTAAATGAGCTCATCCTTTGTGAAGGTGATATCTTTTTATTTTCAGCAAAAAGA
GTCGTCTTAACATTTTCTAAAATTCAAAGGTATGTGAGAGCTATAGGCCTCATTACTAAAAATGA
AATAGCTAAATGTGATTGCTAAGTGTGGGTAAATGTTTTACTTCTTCCTTTGGGCTAATAATCTAG
CATATACTGAATTCTATTGAAATAATTTGTTTCTGCCTCTGTCTCTGCTACTACACAGTGAATTTTT
TGTGGCCAGGGAATTAGGTCTTCGCATCTATTAATTTCCTGTGCTTGGTGCAATGCCCAGCATGT
GGTGTTTGGTAAATGAATAGGCAATCAATTATACTTTCATACATTACTGTGGAAGGCTTCCAATA
CTGGGTGAGATATTGGCTGAGATCAATCATCTGCCCTAATGTGGTCCAATCTTTTCTCCATTGAA
ATATCTATGTCACTATGGGACACAGCCACTGTGGGATGATAGCCAGGATGACTGTGGTCAGATC
CTACAAATCAGATAGTCCGATTTTAAGCATTAGAATTAAAAAATCAAGTAATGAGAGCTACCTTT
CATTCCTCTTGCTGCAAGAAAAGAGTTAGGACACAAATGTGCTAAGCACTTGACCTAACACTTCT
TGTTCAAGACACAAAAGTCTCAATGAAGTAGATATTATTTTGTGGACAAGGAAACTGAAAATCA
GTGATTAATATGGATATTAGTTAGATCCATTCATGTGTCTACCATACAGACTTATCAGATCTTAAC
ATTTCCCTATGTTTGCCTTAATTTTTTTCCCCAGAAATATTACACCAGAGGCCCACGATCACAAAT
TCATTGATTGATTGAGACGGAGTCTCACTCTGTCGCCCAGGCTGGAGTGCAATGGCACAATCTC
AGCTCACTGCACCCTCCGTCTCCCGGGTTCAAGCGATTCTCCTACCTCAGTCTCCCGAGTAGCT
GGGATTACAGGCTTGTGCCACCACGCCTGGGTCATTTTGTATTTTTAGTAGAGATGGGGTTTTGC
CATGTTGGTCAGGCTGGTCTTGAACTCCTGACCTCACGTGATCTGCCCGCCTTGGCCTCCCAAA
GTGCTGGGATTACAGGCGTGAGCGACCGCGCCCGGCTCCACAAATTTAATTGCAAACATTGGAT
GGCACATTTAAAAAGCCACTTAAAACAACTTTGTCATTTTATTCAATGGACATGAACTGAGCCTC
GGTGCTGGGCTAAGCTGTACGGATACAGAAAGGTATAAAGCTCTGTCCTTGTGCTCACAGGGAC
TCACTTTTTAGTGGGGGTGGCAGACGTATAAGTTTTACAGTTGAGAAACTGAGGCACAGAGAGG
GGTGGTTACTCTTTCAAGAGGCAAAGCCAGGTTTATACACCGGGGGTCAAACAAGCTGGCCTCT
TGGTGGACTGGGAGGCCCCTACACATTCCTGGTCTGTTGGAACAGTGTGGATTCTGAGTGGGGG
CACCTCTTCTCTGGGTGGGTCCAACTGGGAAGGGGAGCCAGTTTGACACAATGAAGCCGAATC
TGTGAGAAGCACTCATGAGTGTTTTTCTCTCTTTCCACTAACCATCTTTTTGCTGCAAAGAATTAA
ATTCTTTTCTGGTGACACACACTTCCTGTCAGTATGAGGCACACCCAGCCAGACAAAACCACCC
AAAGTAAATGAAGAAAAATCCTTTTAATTCCTCTAGGTCCACAGATTTGGTTTCTGCTGGATTGTT
GCCATTTTCCCAGGGGTAAAGCCAGAGGTGGATTTGGGGAACAGCAAATAAGAACAATACACT
GATAAGGGTAACACCAGTGACATGAAAACATTCACTATTTATTATCTCCAATTAAAATTCCATAT
TCTTATAAAAGCAATTCTCATCATAGAAAACTCAGAAAGCTACCAAAATTCGTGTTTATGGGTTT
TTTTTGTAAAAAGAAAACCAAAATCTCTCAGTCTAAACTACAATCACTTTAACATTTTGATGTATA
AACTTTCTTTTTAAATATTATATTTGTATGCATTATTTATTCTGGTCTGGTCATATAACTTTATATGA
CAAATACTTCCTTATATATTATGCACTTTGTTTTGTTTTGTTTTGTTTTGAGACAGAGTCCTGCTCT
GTCACCCAGGCTGGAATGCAGTGGTGTGATCTCGGCTCACTGTAACCTCTGCTTCCTGAGTTCA
AGCAATTCTTGTCCCTCAGCTTCCTGAGTAGCTGGGATTACAGGTGTGTGCCACCATGCCTGGCT
AACTTTTTTGTATTTTTTTAGTAGAGACAGGGTTTCACCATGTTGGGCAGGCTGGTCTTGAACTCC
TGGCCTCAAGTGATCCACCTGCCTTAGTCTCTCCCAAAGCGCTGGGATTACAGGCATGAGCCAC
CACACCTGACCCATTATGCTCTTTCAAATATAATTACAAATATAATATTTCATTATACAGTTGCTC
TATAATTTAGTTGGTCAATCTATTGTTGGACATTTAAATTTTCTATCTTTCAGTTACTATATTATTAC
CACTTTAGCCAGGCACAGTGGCTAAATATGTATTATATATAATTATATGTTATATATATATTACTT
ATATACATTATATATTAGTTTTATATATATAATATATATTATTATTACGACTCTAAGCAGATATTTG
CCTTATTGAGTGACATCACCGGCCTCTTTTCTTACATTATCTCATTTCCTTATAAGCCTCCAGAGT
AGACATTATTACTGTATTATTACTGTTTTATAAATGAAGGAACTAGAATTCAAAGAGGATAAAAA
ACTGTCCCAAGTTCAAAGCCAGGCTTTACACCTAAGTCTCTTTAACTCCTGAGCCAACACTACCT

ATTGCCACACTCTGGGTGTGCCTGGTGCTCCCTAACCCACCCTGAGTTTTCTGTCTTACCAGAAC
ATCATTACTTGTGATGTAAGAGTTTTCTTCTACTCTCCTTTGCTCAAAAAACTTAATCCTAGTCTCA
CTCCTTTCATGTTCCCATGAATCCCCTGGCCTCTTAGTAAAATTCGGGTTTTCCAATTAAACGCTC
TCCATTTTAAAGTTCCCAGGCAGCTGGACTGATGCGATTGATGGTTCCCAACTGCTGTGCCATGT
GCTCTGGTCCTCCTTCCTTCTCAGCTGTTCCCAGAAAGGCAGGCTATGAGGTTAATCACGTCTCT
ATGCTGTAAAACTGAATCCTGATACACTCACTAAAGCCCTCGTGGTTATTTGTGCCCCATTTGTA
TTCCCATTTATATTCTGAAACTTTTTCAAAATCTGAGATGCTCTCAGCAACTCAGTACATTCTAAA
CAGGATATTAAACTTTTAAAAAATGTATAAGGCTTAAAACTCTCTTCTAAATTCCAGACTTGGAA
CAGTTTAATTGACACCCACTCAGATGTTTTGAAACTTAACACATTCAAATCAGAATTATTGAAAG
TTTTTATATGTGCAATGATTACATGTCAACTAAAAAGGGGAAAAAAGGTTCTGCCACATTATAAT
CCTTTTTTCCAACTGTTTATTTCTGTTATTTTGAAAGAACAATCAGATGGTACTTTTTTCAAAGACA
GTCACATAAAAACTGAAACAAAATATAGATTAATTACATTTTTTGAAAATTGTGTGATACACGAT
TATGAAATTTGTAGAAATTTTATGTGGAAAAAGAACAAGGGAAATCGAGTGAAATGCTTTGAAG
GCATCTATGACATAGGATGCTGTGCTCTTCCTCACCTTCCATTCACTTTTTAACCCTCTCCAATGT
GCCTTTTCCACCATCTGAAATTGTTCTCACAAAGGTCACCAGTGACTTTCCAATTGCAAATCCAA
TGGGAGTTTTTTGGACCTCAGCTTACCTGATTAATTTGAGGCATTCAATGTTATTAATCAGTAACA
CTCCTTTTGCAACTTTCTTTCTTTTGTTGTTTTTTGTTTTTTGAGATGGAGTCTCACTCTGTCACCCA
GGCTGGAGTGTAGTGGCACGATCTCGGCTTGCTGCAACCTCTGTCTCCTGGGTTCAAGCAATTC
TCCTGCTTCAGCCTCCTGAGTAGCTGGGACTACAGGCACACGCCACCACGTCTGTGTCTGGCTA
ATCTTTGAGGTTTTTTTTTTTTTTTTTTAGTAGAGATGGTGTTTCACCACATTCGTCAGGCCGGTC
TCAAACTCCTGACCTCAGGTGATCCACCCGCCTCAGCCTCCGAAAACATTTGGATTACAGGCAT
AAGCTACCGGGTCTGGCCACAACTTTCTAGTCTCAGTGTCATCACAACCAGTTTCCAGCTTCTCT
GACTTTTCTTAGCTACCCTGTTGTCCTATCACTCCCTGCCTCTTCTCCAGGGTTCAACCTCTGGTC
CACTTCCCTTCTCACACTTCATAGGAAAATACCACTCAAGATCATTGCCTCCCCTCTCACCACAA
CCTACATCTCTATCCAAAAACTCTTGATATTTTAGTTCAGACTGTTTTTAGCTATTTTACTGCAGAG
TTGGAATTTAAGAGTCTGGAGCTTGCAAATACAAACTTTCATTGCACTTGATTGCTGCTGTTCTGT
GAACTCTCATTACCTTTTCCCTGGGTGGCTGCTATATATCCTGACTGGTTTCTCTATCCCTAGTCT
CATTTCAGATCCATTCTCAACACTCCTTTAGAATACTTTCTAAAATATGGAAATCTGAGTTTCCAC
TTCTGCTACTGGCAGAATAGTTCCTATAAGACCAACCCTTCCACAAATAAAAACAGTAGACTATT
GAAAAAAAAAAAAACACCAATACAAACCTGAAGGCTATGGAGAAGAACCAAAAACAAACGCT
AGACAAGAGCCAGCATTTGGACAAAAAGACTATCATGTGTGAAATATGCATTTTTAATAGGTTTT
TTTTTTTTTTTTTGCCTGAGGGCAGACCCCAGTGAGTACTAAGCAGGGCAGCTAAAATGCAGATA
GATGCAGTCTTACCAAATAGAAGAATAAGAGGGAAGCATTCAGGGTAACTATAGCAGCTGTAA
ATTGGGATCAAGGGCAGGAGGATCCAAGAAAGGATAAAACAATGGAAAGGGAGCCCAAAATT
TTATGTATAAAACTCTACCTAAGTCTCTGGCAATTCTCTGAACTATGCATGTGTAAGACAGACGT
CAAGCAGCCCAACTAAGGCCAAAACAACTAAACAGAGATTTCACTTGGTGTCTGTTTTAGTGAG
AGATAGAGATTGAATTTAGCCATGTTAATTACTGGCTAAAACAAACAAACAAATAAAAATAACA
CTTTTTGGATGAATGTAACAAAAGACAGAGTCCTTACAACATATCACTCACAATGTCCAAGACAT
AATCCAAAATTATTATAAAGAAACAGGGAAACATGACTCATATTTAAGAGAAAGGGCTAATAGA
GACAAGCTCTGAGATGAACCATATGTTGGAATTAGTTGACGAGAATTTTAAAGTAGTTATTATAA
CCGTGGTTAAGGACATGAGAATAGACTTATAATGAATTAACAAATAGGAAATTACACTAGAGAA
ATAGAAATTAAAAAAAGAACCAAATGGAATTGAATCATATACAAAAAATGCAATATGAGAAATA
AAAAATTCCCTGATTGAACTTCACAGTAAATTGTAGATGACAGAAGAAAGGGTTAGTGAACTTG
AAGAGCAGTCAGTAGAAATCACTGACTTGCAGAACAGAGAGAAGAAAAATTGGAAAAAAAATG

AACAGAGCCTCAGGGAAAGTAGGCCAATATCAAAGGCCTAACGTGTGCAATTGGAGTCCCAAA
GACTAGAAGCTAGAAAATGGGGCAGAAGACATTTTTGAAGAAATTATATCTAAAAGCTTTCCAA
ATGTAATGAAATGCATATGTTCTCAGATTTAAGAAGTTTAATGAATCTCCAATAGGGTAAGTACA
AAGATAACCATCACATAATTCAGTAGTAAACAACAGTAACAACAATGACAACAAAGCCTATGGT
TATGTCTATAATCTTTTGGTGCAAAACCTTCTTGTCTTGTCTTTTTCTGCTTATCACCACCCTCTTTC
TTATTTTCACCAATCTGGAGATGCATATACAAAAACATTTTAAAATCCACTTTTTGGCCAGGTGCA
ATGGCTCATGCCTGTAATCCCAACACTTTGAGAGGCCGAGGCGGGAGGAATGCTTGAGCCCAG
GAGTTCAAGACCAGTCTGGGCAACATAGGGAAACCCTGTCTCTGCAAAAATAAAAAAATTAGCT
GGGCATGGTGGCACATGCTTGTGGTCTGAGCTACTGACCACTGAGCTACTGAAGTGGGAGGAT
TGCTTGGACCTAGGAGGTTGAGGCTGCAGTGAGCTGAGATCATGCCATTGCACTCCAGCCTGG
GTAACAGAGCAAGACCCTGTCACACTCACACACACACACACACACACACTCCACTTCGTTTC
TAATCATGGTGTATAGATGCAAGCAATATTGTTGGTTCTATGACAATACTTTTAAAACTACAAAA
ACAAATTTGAAAGTGATGTGATGTGAAAAAGAATGAAATCATGTCATAGCCACATGGATGCAGC
TGGTGGCTATTATCCTAAGTGAAATAACTCACAAACAAAATCCGATACTGCATGTTCTCAATTAT
AAGTGGAAGCTAATCAATGGGTACATATGCACATAAAGATGGAAATATTAGATACTGGAGACTC
CAAAAGGGAGGAGGATGGAAGGAGGCCAGGGTTGAAAAACTACCTGTTGGAAGTTTTGGGGT
ACTATGTTCACTATTTGGGTGATGAGTTCACTTGAAGCCCAAACATCAGCATTATGCAATATACT
CACGTAACAAACCTGCACATGTACCCCATGTATTTATAATAATAAAAAGTAAATTAAAATATTTC
TATATAAAAATAATTTAAAAAGAAAGTGATGTGGTGGAAAGGGATAGGATTCCTTATAAGCTAA
TTATATATAAAATACTCTCCTGCTCTTTTTTATTTATAATTCAATCATTCCCTAATTAATTTATTCAT
CTAATCATGAAACTTGTTTAGTCACCTACTTGGGGGCAGGTACTGAGGACGTGATGACAAATAA
GGAAATCTTTACCCTTTAGAACTCATCATGTAATGATCCATACTGGTCACTTTGAGATTCTGGTAC
TTCTAAAAATAACTTATTATTTTTACTTCTTATTATAAAAATCATACATGGGACATTTAGAAGATAT
AGGAGAGCAAAGGTAAGAGAATTCACTGTGAATTAATTATTCATTGAACACCTATTTTGTGCCAT
TGACTATCCTTATTGCTGGGAGCACAGCGTTTAAAAAAAAACCAAAAACCTTTCTCCCACTCTAA
TAGATGAGTCACATAATGGGAAAACAAACAAATGTATCATCTATTAAAGTGGGAGAACTTGGTT
TTGTAGTTTTAAAAGTATTGTCATAGAACCAACGATACTGCTTGCATCTATACACCATGATTAGA
GAGGAAGTGGATGGAGAGTGTGTGTGTCTGAGAGAGAGAATATTTAGAATGCCAAAAGCGGGC
ACTTCAAGGAAGGCTTTTTTTATTTAAAAAAAAATTAGCTCATTCTACATACTTTAGAAATTACCTTT
GAGTAGCAAATGTTAAGGATATAAATACTTTGCACGTATATTCCTTGTTTATAGTTAACCATATGT
TATTTTTTACCTGCAAAATACTAAAACATATTCTACCTACGTGCAATCTAAGTTTTGTAGCAGAGT
CTTTTTACGGGGGCCAAACAATTCTGGACTAATTTTTAAAACCCTGAAATGGCTCACTGTTTTGA
GGTTATTTTGATGGAGTATGAATTTACTTCTTTGTCATTTGGAGAAGGTGCACAGTATTATTATGA
ACAGAGAAGAGCTGTATGAAAACTGATCTTCCAGCATCTATCATCTCCTTTTAGTCTAATGAGAT
ACTGCCATTATCTCCTGATGAAATATTGTTCTAGAAAACCTTACCCCATCATACGATTTCTGCAGA
TCTTACAGGAGCAATCTGAGCTCAAGCAATATTAATTACTGTTTCATTTGTCCATTTGTCTGTGTA
TAATCATGTATTTTAATGATCTTCTATTTTCTATGGTTTGTGTAGATTTGGAGACTAATTCAAAAGA
GTTGAAGTTTTTTGGACATGTTTAGTTGAATGTGCAGTTCTCTTTTGAAGAATCCTGTCTCCAAAT
CCTGTTTTGAAAAGAGTGTTGATGAGTAGCACAGCACCTGTCATATTGTGAGTGTGCAATAAAAT
CATGCCTTTTCAGCAGACCTTAACTAAAGAGTTAATTTTATTTATTTAAATCCACCTTTCAGATGA
CTCTCATTCTGCTTTGTCAGTGGTTGACGAGGGTAAAGGCACTTGAGGATGCACAACACACAGG
ACAGGGAGCAAGCCTTTCTCAGTGCGGAGAGCTAGGATAAACTTTTGCTTAGAAAGGGGTACAT
TAATGACAAAGGATCACAGCATTATGGGAGGATACTACACACCTGTTCTTCAGGGAGTACTCTG
GACATCCCTGTTCTTTCCCCACATGGCAGGTGTCTTTTCAGGTGCTCCCAACCACACTACCCTGA

ATTCCCATATCCGGGTTGCCCTCTATGCCAGCCTCTCCTCTTAGCCTTGAATTTAGCTTTGTTGCT
TAAGGCAAGTCACTTCCCTCTCCGGGTATGTTTCTTCTTCTGAAAATGGATGCAGTTGAACTAGA
TGAGAGCCAGGTGGCTTCAATTGATGTGCGGTGGCCCTCTGCCCTGCTTACTTTCATCTCCACCA
CTCCAAGGTGGCTCACAAACACGAATCCTCAGCCCCTTGGATTGGGTTCAGGGGAGTGCTATCA
GGAACCCATGGGAGAGACAGGAATGTTTTCAGAAACCTCAGCCTGTCCTCTTTTTACTGAAATA
GGGGCCCTTGTTGATGGTAGTCACTGCCCATGCTGTTTAGCCTAAGGAAGGTGACAGTGGGTGG
GCCACCTCAGGCCATATGGAGGAAGGCCTGGCACAGAAACACAAGGCCAGGCAGGCTGTGGC
TAGCCTGAATTTAGCTGTAGCCTCAGAATCAGGGGACTTGGGCTTGACAGCTGACATTACATGG
TGCACAACTCTGGTTAAGTCACTTAACTTAAATCTTATCCCCTCATCTGTAGATGGCTTGAAGAC
CAAATTAAGGAGCTCCTTCAACAAATACATTAAAAAAAAAAAAAAAGAAAAAGATAAGCCATAA
GGCACATACGAATCACTTGGGAATCTTGATAAAATGCAAATTTTGGTTTGGTAGATCTGGAGTG
GGGCTAAAAATTCTGCATTTCTAACGAGCTCCAGGGTGAACCTAGCTGATATTGCTGCTGCTTGA
GTGGGCCACACTCTGATGAACAAGGATAGAAACCATTACAATAGTTGAAAATGAAAATAACAAT
GAATTATATAAACCCCACCCCCCTCACACCACTTTCACGTCTGATATTTTTCAGTTAACCTAAAA
ATGTTTATTATTACCTAATAAACATTTTCCATGTTTCTACAGATTATATGGACCGCAAAGTTTCTGT
AATTACTATTATGGAGTAGCTACACAGTATCCCATAGTGTTGAACTAGCATCATTTATCCAATTG
CTTCCCTCACACTGGACCAGTGAGTCCTAGGAAGTGGCTGCAAGGAAGCAGCAGGTTTCTGGG
ACAGCCCAAGGGCATTTCTTATTTCCTCCTGTTCCTCCCTGGGATCAATCTGTAGGAGTAAACTG
ACTGAAGACTGTGAACACCTTCATGAACTCCTGTGACTTTGGACTTGTTTTCCAAAAGGCCAAAA
TTATAGATATGTACATATTTTGATTATTAAAAATATGTGTTTCCTTGTACATTGTTGGTGAGAATGC
AAAATGGTGAAGCTGCTATGGAAAGCAGTATGGAGTTTCCTTAAAAAATTAAAAATAGAATTAC
CATGTGATCCAATCAATAATCCCACTTCTGGGTATTTATTAAAAAGAATTGAAATCAGGATCTTG
CACTCCCATGTTCATTGCAGCATTATTCACAATAGCTGACATGTGGAAACAACCTAAATGTCTGT
AGATAAAGAAAATGTGTCATATACATACAATGGGATATTTTTCAGCCTTAAAAAGGAAGGAAAT
CCTGCCATATGCAACAACATGAATGAATCTTGAGGACATTATGCTAAGTGACATAAGCCAGTCA
CAGGACAACTACTGCGTGATTCCCACTGACGAGGAATCTGAAATAGTCAAACTTGTAGGAGCA
GAGAATAGATGGTGGTTGCAGGCACTGGGGGTAGGGAAAAATAGAGCTGCTATTCAACTGGGT
ATAAAGTTTCAGTTATGCAGCTGGATAAGTTCTAGAGAACTGCTCTACAACATTGCACTTACAGT
TAAAACGACGGCACTATGCACTTGAAAATTTGTTAAGGCCAGCGTGGTGGCTCACACCTGAAAT
CCCAGCATTTTGGGAGGTTAAGGCAGGCGGATCACTTCAGCCCAAGAGCTTGAGACCAGCCTG
GGCAACACAGAGGGACTCTGTCTCTACGAAAAATACAAAAATTATCTGGGCGTGGTGGTGTAC
ACCTGCAGTCTCAGCTACTCGGGAGGCTAAGATGGGGGGACTGCTAGAGCCCAAGAGGCTGCA
GGGAGCCATGATGGCACAGTGTGCTCTAGCCTGGGTGACAGAGTGATACCCTGTCTCAAAAAA
ACACAAAAAAATTGTTAAGAGGATAGATCTCATGTTAAGTGCTCTTACCACAATTTTAAAAAAGTT
CCCTATAGAAAAGTATTTTTAAAATGACAATTTCCCTAACACCACTGCTAACATTTTGATTGAAAA
AAAAATCATTAATTTGTTCAATAAACATTTATCATACACTTAGAATGTGCCAGGTTCAGTGTTATG
CATGAGGATGCTACAGCGAACAGTTTTTCTTTCTAGGCATATGTCCAACATTATCCATCAGTCAA
TCTATATAACAAAAAGTAGATACAATACAAAAATATACATGTACGTATATACACTACAAGATGTT
TTTATTCATGTTTTATGTGAATAGCAAGACCAAAAAAATCATTAATTGGGCCCAGTTTAGGCCAG
CAATGGTATTCTGTCCCCTGGATGTACACAAATAAAAACAACGCAGCTCATTCCTTTTAAGCATC
TTAAAGTATACCAAAGTTTGTTAACAGCCAGGTATGCACACGACAGAGAATGAGTTCCCTGACA
TCAGCCGGAATCTATCACCGGGTAAAATGGATGGAAACCTCTCCCTGCTACTTACTGCTTATTGC
TGTTCAGGCACTGTTTAATTTCTCGGTGCCTTAATTTGTACAGAGATACAAATGCCATCTCTGGTT
GTGGCTGAGAAGAATGAATGAAATAACGTACCTAAAATGTTCACAGGGCCTAGCATATGGCAA

GCGCTCCCTAAATAGACATTGCTTTTGTTGTCATTAGTGCTAGTGATAGAGGACCTTGGGTTTGG
GCCTCATTGTGCCACTAACCAGCAGTGTTATTTTGGGCAGCAGTGCTGTCCCTGGACCTCAGCTC
TACCTCTGGGAAATGCAGGGTATGGAGGAAACTGGTAGTTCAGCATTTGGAGATTCGTGGCAAA
CTATCAGGCACAACTCCTTGAAATCTTCCGGAGTTGGCGAGGCACTGCGGGGCTGATTAGATGG
GGGGCAACCTCGCGGTCCCAGCTACAACCTCGCCGAGCCAGCCTCCAGCCCCTAAGGTGCGCC
GGGGTGGCGCAGGGGCGGGCAGCTGGGCGAAAGCGAGTACCAGGCGGAGGGCGCAGCCTCA
GGGGGATCCTGGGCGAAGAGCTGAGGGTGGCGCTGGTGGGGGCCTTTGAGAGGCCGCAGCCG
AGCCCATGGCTTAGGGTTTCGATGAAAACAATAGCTGCGAGGTTTGGTTTCCTGGGCACAAGGG
TGGAGAAGGCCCGGGAACCCTTCCCTTTCCAATCCCGGTGTGGCCTTCTGACTGGCAGACAATA
TTAGAGGTATTAGCTTTCCTCTTGCCCCCGCCCCCCGGCCCCGCCAGGCCCTGGCCCTTCCTGC
AAGGCAATAGTGCGCCTGAGAAATCCCTGTGGTGCCTTGTTCTCTCTACAAAGGCAATGAATTC
TCAGCACAATTTGGCTTGGAGTAGGCTCTCAAGATAATTGTTGAATGAATGAATGAATGAGTGA
ATGAGTGAATGAATGTCATCAGTGGGGATGGGCCTAGGGCACCAGCAAGATCAGGAGATCTCA
TCTTTCATCCTCACCTTCTTTGTTCACTCCATTTGTTCACATCTGTACAGAATTTTCACCGTTGTGT
TGGTCACAGCGGGTTGGTGCAAAGGGGTCATGTTTTCAGGTAAGAGCAGCATTGCATAGTGAG
CAGAAACCCCAGATGAGTGGGACTTCTCTGAGTTCTGTGTCCACACCTTGGGGACTCAGACGTC
TATTCCTGCCTTCTATTAAGCTTGACAGCAAACTCAGCAGATGTAAATATTGACACCTTCTGGAA
AAGTGTGAAGGTATTTGTTAAAGAAAACCTTAAAGAAAGGGAGGCAGACATTGTGAGACTTTTT
ATGCCTTTCAGGGTAGCTTCAGGACTTCTGGATGGTTTAATATGCTTCAACTCAGCATATGACCT
AATGATTCATTTCTTAGAAGTTGAATCTGAAAGGAGTGAGGTTTTTAGCTGGCATTTTATTTTGTG
TCTGCTTGTGGCGGGAAATAACAGATTCATATATGACTCTCCACCTCCTGAAGAGGCTAGAGCA
AGAGTATCAATGATGTGCCGTAGGAAGAGCACCATACGTTTGAATGGTGCCTTATCACTGTCCT
GGGCTTTCACATGCTTTATCATGCTTAAAACCCATAGCCCTGTGGAATAGCCAGAAAGAGATTAT
TATTCCAATTTTCCAGCATTGTTACTTCAGTTATTGAATAGCCACTATGTGCTGAATATTAGAGGT
ACAAAGAGAAGGAAGGCCTATCTCTGCCTTTAGCGCACAGTCTAGTGAAAGAGAAAGATATGC
ACATGGATCATTTCGGTATGACATGTTACCTGATAGGGTAGATCAAGCCCAACATCTGTTTTTGA
AAATAAATGGGTTTTTTTGTTTTTGTTTTGTTGCTGTTTTGAGACAGGGTCTAACTCTGTCCCCCAG
ACTGAAGTACAGTGGTATAACCATAGCTCACTGCAGCCTCTAACACTCAGGCTCAAAGGATCTT
CCCATCTCAGCCTCCCAAGTAGCTGGGACTACAGGCATGCACCACCAGGCCCTGCTAATTAAAA
CTTTTTTTTTTTTTTTGGTGGAAATGGATCTCAATATGTTGCTCAGGCTAGTCTTAAACTTTTGGCC
TCAAGCAATCCTCCTGCCTCAGACTCCCAAGATTCTGGGATTACAGGCATGAGCCACCATATCT
GGCCCAGTGGTAAAGTTTTACTGGGACACAGCTTTACTCATTTATTTATATGTTGTCCACAGATG
CTTTTGTGCTACAATGGCAGAGTTTAGTGTAATAGTTGTGACAGAGGCTATCCGGCCTACAGAG
CCTAAAGTATTGACTATTGAACATTTATAGAAAAAGTTTGCTAGCCCATAGAATAGAGGAATAAT
GCTCAGGTGGGCGGGTATTTAGAGTTGGGGTACATAGCACCACCTGGGAGTTGAGAAAGGCTT
CCTGGGAAAGGCACTGCTTGAGCTAGAAGCTGAAGGGTAAGACTTGTGGGAGTTAGAAGGATA
AGATGGGTACTCCAGGCAAAGGAAAGCCAAGGGACTAAACTGCTAGAACCACAAGCAGTTCAT
GATTACTAGGATACAACATGCAAGATGGGGACGGAAGCAGATGAGGAAATCAGTCTCGACTTG
GGTGATTTGCTGGAGGTGATGTGTCTAGGTGGGGCAGGACTAAAATCCAGGTTTTCTAACTGGC
AGCCCTATGTGCTTATCACAACAGCAAGGACAGCAAATACACAGCACCCATGTTGTCATTCCTT
CAACCTGGGTCCACAGTGGCCAGTGCTAAGTGAGCACAGAAGTCTTTTCTGCAGCTTTATTTGTA
CTCCAGGTGACTGTTTGTAGAGTGAGTGTGCATGTTCAGACCTATATGCTATCCCTGATTTACCT
CATTCCAGCCATAGTGAGAAGGTTTTAGATTTATTTTTCTCTGAAGCTATTAGAAAAAGCCTTCA
GAAGCAAAAGCTATCAATTCAAGCAGCCCTATTTCAGCACATTCTAAATTATTGCTTACCCTGCC

TATGCCAGGTCATACACCAGGTGGTTTACACACTTTATCTATCTTAATCCTCACTTCAAGATCATG
AGGGAGGTAGAATAAATAAGTCACTTACCCAGGGGTCTGGAAGTAAGCATTTTAATTGTTCAAA
AAGATTTTAAAAGATTATATGTAGTCCGGGAGTGGTGGCTCATGTCTGTAATCCCAGCACTTTGG
GAGGCCGAGGCAGGTGGATCATGAGGTCAGGAGATCAAGACCATCCTGGCTAACACAGTGAA
ACCCCAACTCTACTAAAAATACAAAAAAATTAGCCAGGAGTGGTGGTGGGCACCTGTAGTCCC
AGCTACTCAGGAGGCTGAGGCAGGAGAATGGCATGAACCCAGGAGGTGGAGGTTGCAGTGAG
CCGAGATCACACCACTGCACTCGAGCCTGGGCAACAGAGCGAGACTCCGTCTCAAAAAAAAAA
AAAAAAAAAGATTGTATGTTAAAAATTACAATACTTAAGGAACAACTTGGTGTTGTGGTACTGTA
TAGGGCTGGGTTGTAAATGACCAGTGGGCATGCCAGAGGCTCAGCCCATACATCGCTAGTGGG
GTATGAACCATTATGCATGATTGCAGTGACCTTGTGACCTGTAGCTTGTAAAACAGAAGCTGGG
ATGATTATTGTTTGTTTATATTTTGCTGGATATCATATTTTTTATACCTTTTAACTGTGATTGTGGAA
ATTGACACTAGACATCCATCCCTTAATCCATGTAGTAGGTAAGAACATACAGATAAACACAGGG
CAGTGGGGAGGGGAAAGAAAAAAGAAAGGTAGGAAGGAAAGAAGGAAGAAATATATCCAGA
AGATGCAAGACCTGGATCAAATGGTAGCAGGAAAAGTTCTTTTGAAGATATCTAAGTGTTGGAA
ATATCATTCATCCAGGACAGGTAAGGTCCTGGACATTCCAGTGAGCAATTTCCCTCTGTGCTGTT
ATTTTTCATATTTTGGAGAGAACACTAAGGCCTGAAGAGGTTAGATAGCTTGCCCATGCTACTTA
GTGGTAGACTCAGAATTTCCACACTGGCCTTCCAGAGTCCACAATCCAAGATCTTAACTACGATT
ATATTCATCTTTGATCCTTGGGAAGAAGGGAAGACATTTTCAATTCAGGCTTGAAAACATAAAGT
AGCTGAATGAAGCAGTGTGGAATTAGCAGAGAGCAGAAAAAGTAAGCACCAGTCTGTGTCTTG
ATATCATGTTGTTTACATCTTGCTTTGGATTTTTTTAAACTGTCTTTCTAGAAAAAAACATAGGTAGT
AGCATCATGTACACTAATCTTTTCTCCTCAGTTATCCACATCTGGAAGTAGTTTGATAGCTAGATG
TATGATATCATGCCTGTTAGCTGTTATCTGGTGGCAGCTCACCAATTATCCAATTTCTAGGGCATC
TCTCTTTAAAATCTGTTTCAGAAGTCTTGGTCTGACAGAAAGCCAGGTACACCTGAAGTGGCCG
AAGCTTGGGTGAGGACCCTTATCTAGGTCATCCAAAGATAAGAATGAGATAAGCTGAATAACAG
GTCTTAAGAAAGAAAAAAATTAAGTACCAATGCCTAGTCCAAGGATAGAGAAGACTCCAGCTG
GGAGATATTGCCAAAGGTCAGAAGACATGCTGAGTCTAAATAGAGCAGTAGAAATTGGGAGCA
AATAGATAAGGTTTTAGGGGAGGGGTTGTAGAGAATGTCCCTATAGTTTCTGAGCACAGGTGGC
TCAGTTTATAAGTACATAATCTTCCTAAATGAATATTCTAAGGGTCAACTTTATTTCAGTGAAAGA
AAACTCCACAAATTGCTTAGACACACTCCCATCTATCCATCATCTATGTAGACATTCAGTAGACA
TGAATTGACAATCTACTCTGTGCTCAGCACCATTCTAGGCTCCAGGTAAGCAAAGATGAGCAAC
ACCCAGTTTCTACCTTGAAGGATCACCCATGCCTGGTTCAGTAGAGACAGAGACATGAGTTTCC
TTTTTATCTATTTTCCCCCAGAAGTACCCTGGTTTTCATAAATGACCATGGCTTGACATGCCATTA
TGATAATAATGTATAATTAGTGGAAAACCAAAGGAATGACTGGAAATTGTCTTTAGTTATGGGAA
CAGCTGGGCTTCTCAGGATAACGCTGGAAAATACTATACTATGTGAGCAAGGTCTGTAGGGTCC
GGGTTTCTCTGGAGGTACAGCTAATTCCCTTCCAAATGCCAGCTTTAAATTCTGACATTTTGGCAT
TTATGTGAATGACTGTATGACTATATATGTAGGTGATCGGCCCTCTCAAGAGACTTTGAATTAGA
GGCTTTCTGGTTTTGATAAATGCACCTTCTTTTCCTCAAAGTCCTTTATAACTTTTTAGACCAGTGA
CCTCCCTTCTTAGCATCTCCTTGGCTAGAAGCAGAAGACAGTCAACCTACCATTTTGACTGTAAT
TCAATTTGGTTTCAGGCACTTTTAATAATTTGGTATTCTGTTATGAGATGAGGTATACAGGCCACC
TCTACTCTTTCAGTTCCGACAGCAGGAATAGAAAAGATGGTAGTTTTGTTCAAGCCAGCCATTCA
TTTATTCTCTAAACATTTATCATGCCCTCTGCTGGGACAAGAGCTGTTTTCGGTTCTTGGTATATA
ATGTAGATAAAATAGGTGCATATTGGTCAATGTCCCCATGAGGTTTACTCTCCATCAGGTGTAGG
GTGTCAGACATTTGGCAAATAACCTCACAAATAACAAAGTGTTAATAAGGAAAAGACTAGGCTG
TTATGAATGTACAATAGTGGTGGGCCTAATTTGGATTGGGAGACAGGGAAGACCTCTTTAAGAA

AGAGATGATAAGCCGGGTATGGTTGCTCATGCCTGTAATCCCAGCACTTTGGGAGGCTGAGGTG
GGCGGACCACCTGAGGTTAGGAGTTCGAGACCAGCCTGGCCAACATGGTGAAACCCCATCTCT
ACTAAAAATACAAAAGTTAGCTAGGTGTGGTGGTGGGTGCCTGTAGTCCTAGCTACTTGGGAGG
TTGAGGCAGGAGAATCGCTTGAACCTGGGAGGCAGAGGTTGCAGTGAGCCGAGATCGCATCAC
TGCACTCCAGCCTGGGCGACAGAGCGAGACTCCGTCTCAAAAAAAAAAAAAAAAAAAAAAGAG
AGAGAGATAATAATGCTGAAATTTGAAGGAGTGGGTACTAGCCAGGCAGAGTCTGAGAAAGAG
ATTCCAGGGAGAGGGAGCAGCATATGTGTAGACTCTGAGCTGAAAAGAAGAGGATGCCGCCTA
TATACTGTCAGAGGCAAACACAGGTGTGACATAGTCATGGTGGGGAAGAGTGGCACAAAATGA
GCGTGGTGAGGTGGCTCAGAGCCAGACCATGCAGGGCCCTGTAGCACCTGGTAAAGAATGTGG
ATTTCATTCTGAGTACAGTGGGAAGTCACAGGCAGGTGGCAAGCAGAAAAGTAGTATGATCTGC
TTACATTACAAAATATCTCTGAATTCTGTGTGCAGAATGGATCAGATATGCATTTAAGAAAAGCA
GTACACAGCCTGAGTCATCAAATGCATATTGCAAGGTGGGCACAGAGCCTGAGCATACGGGCA
CATGCAAGCCGCTTCCTCCTCCTGCCTCCCTGTACCAAATGTCCCGCCTGGGAGGCAGAATGCA
GTTTACAGACCTTGAAACCAAGTGCAAAGTGTCTACAATTAGAGGAGAGGAAAGCATTGAGGA
GGAAATAATAAATCAAGTATTGAAGATCTCAACAACTTAGAGAACAAGATACCAGTCTTAAGTG
AAAAGAACACATTTGGCATTAAGTATCTCAGGAAACATGTCCTTCAGGTCCATTGACTCAGAAA
CATCTCTATTTTCAAAGAGGCATTTAGTCAGCTCCTTCCCTCATCTAACTCAATTTCCTACTTCCCT
CCATCATAGACCATGCGTTCTTTGGTTATCAGATTTGCCTTTGGATTTTTTTTTTTAAAGACAACTA
AAAACATCTTTTAAAAATCTAGCCTTTTCAGTGTCACTTGTCACTTATGTGTTTCTCTTCCCAATTT
ACTTCATGGCTGAGGCCTCCCTTGGGGTAGTATTTTTTTTTTTTTTAAGAAATGGGGTCTCGCGGC
CGGACTTGGTGGCTCATGCCTGTAATCCCAGCACTTTGGGAGGCCGAGGCAGGTGGATCACGA
GGTCAGGCGATCGAGACCATCCTGGCTAACATGGTGAAACCCTGTCTCTACTGAAAAATAGAAA
AAAATTAGCCAGGCATGGTGGCGGGCATCTGTAGTCCCAGCTACTTGGGAGGCTTAGGCAAGA
GAATGGTGTGAACCTGGGAGGCAGAGCTTGCAGTGAGCCAAGATCGTGCCACTGCACGCCAGC
CTGGGTGACAGAGCGAGACTCTGTCTCAAAAAAAAAAAAAAAGAAACGGGGTCTCACTTTGTTG
CCCAGGCTGGAGTACAGTAGCATGATCTCAACTCACTGCTGCCTCAACCTCCCAGTCTCAGGTG
ATCCTCTCGCCTCTGCCCCCCAAGTAGCTGGGACTACAGGTGCGAGCTGCCATACCTGGCTAAT
TTTTGTATGTTTTGTAGAGATGGGGTTTTGCCATATTGCCCAGGCTGGTCTCAAACTCCTTAGCTT
ATGCAATCTGCCCCCTTCAGCCTCCCAAAGTTCTGGTATTACAGGTGTGAGAAGCCACGCCTGG
CCAGGGTAGTATTTTTTTCTTGACTTGTGAAGACTTAGCTTGATTCTCCCCTAGCAGTGCACCATG
AGTGGGGTGTGGGGTTGGGGAAGGGATCAGCACAGGAATGCATGCTGGCCAGGCCAGGGTTT
GTTGAAGCAGGTGGGGTTTGAAGGTTGTGGGACAAATACTTCCCAACTTTACCCTATGAACATG
GTGGCAGGTCAGCTGAGACTTGCACATACACTTTGTCTTCTGGGTGTGCCTGGAAGTAGAGTGA
TGCCCTGGAACCTGCATGACCTAGTGCTCAGAATACCTGGGCTTGGTTTCTGATCACTACCTAGT
CCAGTAACTCTGTGCCACAAACAAATTAACATAGATGAGTGCCCATGACTACCAGACAATATAT
GTAAGTGCTTCTGGGCACCCAGAGAGGTGTGCCTTGTTTTCTCCATGTAGAGGCCAGGAAATGA
AGGTTCAGAGAAGAGATATGCACACCGCATGCAATTCCCTCTGCCTGGAAAGCCTCTGCTCACC
CCATCACCCTCTGCTGATGGAGTCAGTTTCTTTGTGTGGTCTCAGCACACTTATCACATCTTCAGT
GTGACACATATTTTATTCACAAGACATATGTCCCAAGGACAGGGACCCATGAAGGAACAACAG
CAGAGAACTGTAGCTCTGAAAGGTGCTCCGCTTCATTTGTCCTTTCCCCAGACAATGAGGAAAG
TGACAAATAGCTATGGTCATAATGGAATTCTCAAAGAGACTCCTTCCTTATTGACTACATTCAAC
AGAAAATGAGTCCTTGGCATTCTGATCCTCAACAAAAAATATCAACATTAATATTTAGCATCCTT
ATATATCAAATAGACTCTAATGGGTTATGGGAGCTTTCTGAAATACTTTTTATACTATATTTATTG
AAGGAAATAAATAAGTTATAATAATTCACTTGTCCCATCTGGTTCAGTTTTAGGGGCCTTCTTCAC

TATGATTGTTTCTAAAATCCATGGAAATAGAGGTGAAAACCCTTCTCCTCCTCACTGCACTATTG
ACATTATAAACACTGACCTAGATTATAACCATTAGGTTCTCATCGAAATGAAATATCTGCCCTGG
GGTTTAAATTCCTAAACTTACTGTGTAAGATGAGCCTCCAATTAAAGATTTCTAATAAGAAAATC
AGAGATTTGGTGTTAAAACATCCTCAAGTAATCTGTGGGGAATGTTACTTTTAAAATGAGAGCAC
TTACTAGCATCTGATTCGAAGTAGAGTCTAGATCTATCTATCTTCTCCAGGGAGAGCTCCTTGAG
GACAAGGAATTTGCCTCTTTCATGTTATTTGGCACAGTTTTTAGCCCATAAGAGGCTCTCAATAA
ATACTGGTTAAACAGAAATAAGAAAAAGGAGATGCTGAGCGCAAAGAGCAAGGTAAAACCATT
CTGCTCTTTTTCTTCTTTGAAGATGATGATGGCAAGAGTTACCATAAACTGGTCAGGTGTGGTGG
CTCACACCATAATCCCAGCAACCAAAGCCCAGGGCAGGAGGATCACTTGAGGCCAGGAGTTTG
AAACTAGCCTGGGCAACAGCAAGACCCAGTATTTATAACAAACAAACAAAAAAAAACTAGCCA
GGGGTGCATGCTTATAATTCTAGCCACTCAGGAGGCTGAGGCAGGAGGATTACTTGAGCCCAG
TAGTTTGAGGCTGCAGTGAGATATGATTATGCCACTGCACTCCAACCTGAGTGACAGAGCAAGA
CTTTGTATCTCAATTAAATAAGTAAATAAATAAAGAGTTACTATAAACTGAGTTGTTTACTATGTG
CCAGGCACTGTGTCAAGTACTTTATGTTGGCCAGGCATGGTGGCTCACTCCTGTAATTCCAGCA
CGTTGAGAGTAGGCCAAGTTGGGAGGATTGCTTGAGGCCAGGAGTTTCAGACCAGCCTGGGCA
ATATAGTGAGGCCCTGTCTCTGTCTAAGAAATTTAAAATAAAAAAAGCACTTTATGTGCAACAGT
TCTTTTAACTCTTTAATTCTATAAGGAATCCATTTTTCCTTTAAGTAGATGAGGGCCCAGGGCCAT
TAACTAAAGTATCCAAGGTTACCTGACTTAGAGGTGATTTTCCAAGATCTCAATTCAGGTCTCTC
TACATATTTTTAAACATTGCATTGCCTTTCCAAATGAACCAATATTTATTAGCCATTTTATGCAAAT
AATTGGGCCATGTATTTAAAAAATCATTTCTTATGTTCTTATTTCAGCTGGTTTGCAAAGCGGCCC
TGCTGGGGCTTGTATAGGGTAAATGATGGGCTGAAGTTAAAGATATACATATGTATAAGATGTA
TAGGATGGCGTTTTGTTAACCCTGGAAAGTTAAAGAGAATTAGAAGTTTTCAATGTAACATTTCC
AGGCAGGAAAGTGTTAGCATGTTATGAAGAATTTCCCTTCCTCTGAGCCATTGGGCTTTTGCCAA
GGCTTCAGGGTAGACGCTGCATTAGTGGAAGATGAATTTGATCCAAGAAGACATCTTCTCAATC
CTTGTGTAAGATTAGTCTGAGTAGGAATCACTTGGTAACAATTTGAAATGGAGTTCGTTAGATAA
ACAGAAAAACATAGCTTTGTCTGGGTTGAGCCCAGAAATGGGTGACAGATGTTAATGATGAGTG
ACTGAACAGATGTTTGGGGGAGAATTACAAAAAGGGTTTCTATCATGCAGGATCTAAACCTTAA
CCTTCCTGGCTGATGCTGAAAGAGAGCTTGTATGAAACACGTTCTTACAAAACTATTTGGTGGAT
CGTGACAAAGACGAATGACTGGGTACTATAATTTAAAATGGAAAATAAGGTAAATGGGAGTCCT
TGTTTATGTGCATGTGTATATGTGTGTTTTTTTTTTTTTTACAAAGGTCAATGCTATATTAAAGAATT
GGAAAGTTGAATAAGTCATTCCATTCTGCTTCCCTATCTTTTCCACCCCTCCCCTCCAAACTGGTA
GGAATGAGCAGTATTTGATACTGTACCTTAAGGCTCAACAAATTATTAAAATACTATCACATATA
TACATCCTGAAATATCTGCCCTGTAATTACATTTGGAAAATAAAATTTCCTGTGCAAACTTAATTG
GTAGTTTTAGTTGGGGTCTCTGAGACTTACTTCAAGTTTTAAGGAGTTTAAAAAGACCCCCATTTC
AAAATAAATAAGAGACAAAGGCAGATTTTCAAAGTTGTACAGCTACTGCCATAAGCCTGTCCCC
ACTGTGAAGTGAGTACACTTCACAGAAAGATGAACAGTTAGTTGTGTTATACAAATTATATACTT
CCTGATAATTTGACTGAAATTTTGGATGGACTGATCAGATGCATTTGTTAATTCATCTTCATATCT
GGCCGTAATTGACATGAATTTGACTTACCTTCTGCTAATTATAGCTGTGGCTACACAATAGTGCA
AAACACCTGGTTAAAAAAGGTGAATACAGGCCAGGCACGGTGGTTCACACCTGTAGTCCCGGC
ACTTTGGGCAGCCAAAGTGGGCCGATCACTTGAGCCCAGGAGTTCGAGAGCAGCCTGGGCAAC
ATGGTGAAACCCCATCTCTATAGAAAATATAAAAATTAGCTGGGCATGGTAGTATGTGCTTGTG
GTCCCAGCTACTTAGGAGGCTGAGGTGGGAGGATCACTTGAGCCCAAGAGGTCAAGGCTGCAG
TGAGCCAAGATCACATCACTGTACTCCAGCCTGGGCAAGAGTGAGACCCTATCTCAAAAAAAA
AAAAAAAAAAAAAAGGATAAAATAAAGGATAAATACAGTAAAAAATCCAGATATTCTCAGTTTA

GTATTCTAATATTAATGAATGCCACTGAATTTATGATAGACATTGTGTATACAATAGGCTCATATG
TCATCATAACCTTGCAAGCTGGATTTATTATCCTCATTTAGTGAAGGTGAAAGCAGAAGCCCAGA
GAAGTTAAGTGACTCACAGAAGTTCACATATGTCAGTGAGTAGCAGGACCAGGATCTGTACCTT
GGTCTGTGGTATTCAAAATTTGTCTCACTTTCTAATATGCCATACTGTCCTGGGTAGAATAGATGT
TTAATTATTTGATTAAATCCTTATATTTGATTAAATCTTTATATGTTACTGTATATCAAAATGGAAT
AGGACTTAAAAGCCAGAATTCTCAAACTCATGATCTGAAGTCTCACTGTAATGTCATCAAAGAA
AGAGTACTGGTAACTCTCTGTGTTATTGAAAGGGGAGGAAAAATACTAGATCCTTTCTTTGGCTT
TGTCCTGAAGACAGCTCAATAGAGAGGAAGGTAATAACTGAATAAAATGTCACCTTTGGGCAG
GGCAGTGTACTAGAAGCTATGAGCATTAAAAACATAAATGAGATACCTTAGAGCCTACAGAAG
AGATAAGACATATACACAAATAGTGATAAGGAAGAAGGGCATACATCAAGGGACATGAGAGTT
TAGGGGAGAAAGAGATTTTAGCTATGGTGATTAGGCAAGGCTGCATAGAGGGGATGGCATCTG
CACTGTGAGCTTTGAAGATAAAGACAACGACCGGTAAAGGTTAGTCCTTTCTTCAGCTTGATTTT
CCCTTCCTGTTCCTACTACCTCTCCTTCCCTACATTGCCAGACATGAAGGTGTCACCCTAGAAAT
GGAGAAGCAATAAGATGGAAGGCACTGGTCCCTTGACACTGGGAGCTGCCATGGGAGTTCTAG
AATACTTACTCTCATGTGATTATATAAGAGAGTGCCAGGAAAAATTGCAAAGGGTCTTGAAATG
GGCAACAAAAAGATTCTTGGGTGCCCTGAGTAGCTTACCCAGCCAGGTTACCTCTAACATTCAT
TGTCTTTGAAATATTTTACAACTCTGTAAGTTGTAGAGAACTGCTAGCAGGGCAAAGATGATTTC
TTTTCCATATGCAAACAAATTCTGGCTGGGCACAGTGGCTCATGCCTGTAATCTCAGCACTTTGA
GAGGCCAAGGCGGGCAGATCACTTGAGGTCAGGAGTTTGAGACCAGCTTGGACAACATGGTGA
AACCTCATCTCAAAAATACAAAAATTAGCCAGGCGTGGTGGCACATGTCTGTAATCCCAGCTAC
TTGGGACGCTGAGGCAGGAGAATTGCTTGAATCCAGGAGGCGGAGGTTGCAGTGAGCCAAGAT
TGTGCCACTGCACTCCAGCCTGGGCGACAGAGTGAGATTCCATCTCAAAAACAAAAACAAAAA
CAAACAAACAAAAACGGAAGACAAATTCTGTCCAGTGAATTGATTCCCCCCTTTACTCCCACTCT
ATTTCTGCCCCTTGGAAAAAGCCAGTTTTACATCTATTTGTAAATTCACTTCAGTATTCCTGATTG
ACCTCTATAAGTGGGGTACTTTAAATTCTCCTTTGAGCTGATAGTAATATCTTACTGGTTCCTTCA
TTAGTTAATGAGTTAAAATCTTTGACGTGTATTCATTTTATATTTGTATGAGAGTAAATAATTTACC
TTTATTTAAAAAAAAATTTTTAACAAGAAGAATATACTTTTATTTAAGCCACTGCTATTTTGGGCAC
AAAATATACAATGCATGTATATATCTATATCTATCCATCTATTTCTATCTACCTATCTATAATGATC
ACATCTCTGTGCTAAGTAGATAGCTCCTGCCATCCTACCTTAATTATTTCCATGAGGACCAGCGT
TGACTCTTAAACCAAATCCAAAGATTTTTTTCTCAGTCATCATTCATCCTTAATGAGAGACATAGC
AGAACAATGTTTCTCCCAATCATGAGTCACAACAAATAAGTGGGTCGGAAATCAATTTAGTGGT
TTACAGCCAGCATTAATAGGATTTATCATAGTGCATAACACACAGTAAGTATAATTTGCGAAGCT
TTGGATTCAGATACTTATATGTATTAGTTTACTAGGGCTGCCATAACACATTACCACAAACTGGG
TGGCTTAGAACATTAGAAGTTTATTCTCTCATACTTCTGGAGGCCAGAAGTCTGAAATCAAGGTG
TGGCAGGACTAAGCTCTCTTTGAAAGCTCTAGGGGAGAATGCTTTCTAGACTCTTCTAACTTCTA
GTGAATTAGTGAATTAGCCTGGCATTCCCTGGCTGGTGTTAGCATAACTGCAATCTCTGTTTTCAT
CTTCACAGCATCTTCTCTCTGTGCCCTTTTCTGTCTAAGGACACAGCCATTGGATTTAGGCCCCA
CTCTAACCCATTGTGATCTTATCTTGATCTTTACCTAAATTATATCTGCAAAGACCCTATTTCCAG
ATAAGGCCACATTCCAAGGGTCAGTGTAGACATAAATTTTGGGGGACAATATTCAACCCACTAC
ATATATATATGTAAATTCTTGATCACGGTGTGAATTGTTTTTCTTATTATGAGAAGAGGTCTCAAA
ACTTTCAAACATAGGACTAGAAGACCCCTAGTACCTTTTTCACTCTGATTCCAGGAAGCATTTAA
CAAAGCTGGCTACATTTCCCTTTTTGGCAATAATAATATTAATAATTAATAAGTATAAAATTTATT
CAAGTAATTTTACATGCAACAAATAATTCAATCTTATAAAGCCTTAGGAGGTGGGTTTTAATAAT
ATGTCAGTTTTAAAGATTATCACACAGCTAGAGAGCTGAGCAGCCAGTATTCAATTCCAAACAC

ATGGTTTTCCATCATATCATGTGTATTTGGAACTTCATCTCCCTTTGCTTCCATATCGCTGCATTGA
TCTGGTGTATCTGACTTGGCTGCATTTTCTGGCCTCTATTTTCTTCTTTCCATCTCCTAATATTGTC
ATTCCATCAACTCTCAGCCATGGCCAGCTCCCGCCCAATCTCATTCAATTCTTCACTTTCTACTTA
CAGGGATGGAGAAGACTTAATCCTATCTGGAAGGAGCCAGTGCACAGCAAAGATGGCAGAGA
GAGAAAAGAGAGGGAGTGACAATGAAGTAAGGCTACAGAGAGATGACCAAGTGTCCCAGAGG
AGGAATTCTCTTTCGGAAAGAGATAGACACCTCTTCCT

ID|56500|gene_id|ENSG00000256940|transcript_id|ENST00000538355 Homo sapiens lncRNA

GGTGGCCTAGCTGTCCGTACTCCCCAAACGCAACCTTCCAGTCCCAAACCCAGGCGTTCTA
AGCGCTCAGGACGACGATTTGCGCTCACGCCAGGACCCTAGTCTTCATTGGACCCTTCCTCATT
CTGTCCTCATTGAGGCCATCTGAGAATAGGGGCTATCTGAGAATGCAGGGGAGTGGACATCGT
GTGCAGACAGGGAGTCTGGTGAGGAGACGCTGCGGTCCCCGATGTCCAGGTCCTCCAGCCCTC
GGCCAGCGAGGAGCGAACGGGCTCCTCCGCACGGACCGACACGTGGACTGGGCTCCTGGGAA
AGGAAGAGTGGAGAAGGCGGGCAGGAGCTGTCCGGGTCCCTAGACTCTCCGAGTAGCAGGTC
CCACGCCCCCAGCGGAAGAAGCCGGGGCTGGGGGTGAGCGGTGGCACCCGGCCGGCCCAAA
CGGCCAACCCCGGGCAGCCACCGCCCACCGGCAAGATGGCGCGAGGCTCGGGCTTGTGGGAA
ACGGAGTCCGCTCCCGGCGCCGCCCCTCCCCCGCCCTGCGGCCAGGCCGGGAAGCGCAAAGT
TGTTTGGGGCGTCCTCTGCGCCTCGCGACCCGAGTGCGCACAGAGGGGGAAACTAAGGCGCAA
AGAGCTGGGCAGTGCGGGGGCCGCGACTTATAATAATAAAGGCGGTAATTGCGGCTGACCCTA
GGAGCGTGCGTCGGGACCGCCGCCCAGTCGGCTCCGCAGAGAGGGCGCCGTGCCGGGGCTCC
TGCACCCCCGCCCTGCGGCTCCTCGGTCCTTCACATAGCCCTGGCCCACCTCCAATAAGCCGAC
GGCGCATCCCATTCCCTCCCTCTATTTTATTTTCTGCATCTTTAAATGGTCTCATTCATTCGTGTAT
TGTCTGTCTCCCACTTCTAGATTGTGAGTGCCACGGGGCTGAGGCCTGCGCCGACCTGGTCTGC
TGGTGCTACCAGGCTTGAACAGTCTTCAAATCCACTGCTATTAGGCAAATTACCTGGCTCCCGCT
GAACTCCAGCACCTAGAACTATGTCACACTCGTAGTAGGCCGCTGCATTGGTTGAACAAATGAT
TTTGAAAGAATGAATGTCTTCCTCTGTGCCTGCATTTCCTCAGAAGGCTGTA

ID|56558|gene_id|ENSG00000256940|transcript_id|ENST00000544553 Homo sapiens lncRNA

GAAAGCGGGCTAGCCTGCACGCACGCCAAGATGGAGCTCCAGGCTAGCCCACAGAACAG
CCCAGCCGCAGCCGTCCTACCAGACCAGCACCTTGTAACCACAGTCTAACCCAGCGGCACCA
GGCGGTGAGACCTCCTGCCGCTGCCAGCCCAGGATAGCCCCCTTGCCTCTTGCCCAAGGCTCA
GGCTACCCCTTGAGGCGTCTGGAGGACACTAGGCTTGACCTGGGGAGTGGCATGATGGGGGGC
AGGGTCCGAGGCAACGGAGAAGGCAGAAGTGACTTAGGTCAGGCAAGAAAGAGTATATATTG
GGGGCGGGAGTGCAGAGGGGAGGGGAGCGCGCGGGGAAGCAAAGCGCCCTTTGACTCCAGT
GACAGGCGAGCTCACAGTGGAGCTGCCAGGCGGACCGGCGCGAGACCGATTTTGGGGTGTCG
GCGCGGGGTGGGAACACACCTGGCAGTCGTAGAGGCGCGTGAGCTGCTCCAGGATCCACTCCT
CTAGGTTGAGGCGCTTCCGTAGCTCCTTGCGGTCATACTTGACGGTGACCTTCCCTTGGCGCCTC
ACTGGGCCCTCATCGTCCGCCCCGCCCGGGCCCTCTCCTGCGGCCCCGGGGGGGCTCTGAAAG
TAGACGCGTGGTCCTGGGCCGCCACTGCCCGGCCCGGGGGCCGGGGCCGCCAACGCCGCGCC
CCCCGCGGTGCCGCTGTCCGCCATGGCGGCCGCCGGGGCCACGTGCGAGCGTCGGGCCCCTC
CCTGCGCCACCGCCTCCGGGACGCCCGCCGGCTGGCTCGGGTTAGCTCGCCGGCTCCGCTCCG
CGCGGCTCCGCGGCGAGGGCGGCGGCGGGGGCGCCCGGGGGCAGCTGCAGCATGCGGAGCC
CCCGGGCTTGGCCTGGCCGCGCCCCGCCCCCTCCCCGCCGATCCGCCCGCCCTTTGTCCCCCG
CGGCCGCCGCCCGAGCTGCAGCCGCCGAAGCGCTTTCTCTGTCTCGCTCTTCCTCCGCCCCCCG

ACCACACCCCCTTAAAGGGCCAGCCCCTCTACAGGCCCCTCTCCCCTACTCCGCCCCCGGGCA
GGGGTAGGGGAGGGTCTATTCGGCAGGGGGTGGCCTAGCTGTCCGTACTCCCCAAACGCAACC
TTCCAGTCCCAAACCCAGGCGTTCTAAGCGCTCAGGACGACGATTTGCGCTCACGCCAGGACC
CTAGTCTTCATTGGACCCTTCCTCATTCTGTCCTCATTGAGGCCATCTGAGAATAGGGGCTATCT
GAGAATGCAGGGGAGTGGACATCGTGTGCAGACAGGGAGTCTGGTGAGGAGACGCTGCGGTC
CCCGATGTCCAGGTCCTCCAGCCCTCGGCCAGCGAGGAGCGAACGGGCTCCTCCGCACGGAC
CGACACGTGGACTGGGCTCCTGGGAAAGGAAGAGTGGAGAAGGCGGGCAGGAGCTGTCCGG
GTCCCTAGACTCTCCGAGTAGCAGGTCCCACGCCCCCAGCGGAAGAAGCCGGGGCTGGGGGT
GAGCGGTGGCACCCGGCCGGCCCAAACGGCCAACCCCGGGCAGCCACCGCCCACCGGCAAG
ATGGCGCGAGGCTCGGGCTTGTGGGAAACGGAGTCCGCTCCCGGCGCCGCCCCTCCCCCGCC
CTGCGGCCAGGCCGGGAAGCGCAAAGTTGTTTGGGGCGTCCTCTGCGCCTCGCGACCCGAGTG
CGCACAGAGGGGGAAACTAAGGCGCAAAGAGCTGGGCAGTGCGGGGGCCGCGACTTATAATA
ATAAAGGCGGTAATTGCGGCTGACCCTAGGAGCGTGCGTCGGGACCGCCGCCCAGTCGGCTCC
GCAGAGAGGGCGCCGTGCCGGGGCTCCTGCACCCCCGCCCTGCGGCTCCTCGGTCCTTCACAT
AGCCCTGGCCCACCTCCAATAAGCCGACGGCGCATCCCATTCCCTCCCTCTATTTTATTTTCTGC
ATCTTTAAATGGTCTCATTCATTCGTGTATTGTCTGTCTCCCACTTCTAGATTGTGAGTGCCACGG
GGCTGAGGCCTGCGCCGACCTGGTCTGCTGGTGCTACCAGGCTTGAACAGTCTTCAAATCCACT
GCTATTAGGCAAATTACCTGGCTCCCGCTGAACTCCAGCACCTAGAACTATGTCACACTCGTAG
TAGGCCGCTGCATTGGTTGAACAAATGATTTTGAAAGAATGAATGTCTTCCTCTGTGCCTGCATT
TCCTCAGAAGGCTGTAACAAAGATTAAATAGGAAAATTCGTGGAAAGTTCAATTAC

ID|56471|gene_id|ENSG00000232006|transcript_id|ENST00000585996 Homo sapiens lncRNA

CTGAAGAGTTAGGTATTGTAGTCTTCACAGTCTGAGTTTGTTTGTACCTGTCCTTCTTGGAA
AGGCTCTCCAGGTATTTGAAGGTACTTGGGTGTTGTGATCTACGTTTTTGGTCACTGCAGTCATA
TCTGCATTAAGGGGCACCCCAAGCCCAGTAATGCTGTGGGTCTTTTAGACTCATAGGAGTACTG
CCTTAGTGGTCTTGGATAAGATCCAGAGGAATTCTCTGGATGCCCAGGCAAAGACTCTCATTCT
CTTCCCTTACTTTGTCTCAAATAGATGGGGTCTCTCTTTCTGTGCTGAGCTGCCTGGAGCTAGGG
GAATGGTGACACAAGCACCCCTGTGGCCACCACTGCTGGAACTGCATTGGTTTGGACCTGAAA
CCAGCATAGCACTGGGTCTCACACAAGGCCCACTGTAACCACTACCTCACTCCTGCCTATGTTT
GCTCAAGGCCCTAGGGCTCTACAGTCAGCTTCAGGTAAAGCCAGCCAGGCTTGCATCCTTTCCT
TTGGGGTGGTGAGTTCCTCCTAGTCCTGGGCAGGTTTAGAGATGCCATCTGGGAACAAGGGCCT
GGAGTCGGAAACCTTAGGAATCTACCTGATGCTTTATTTTACTGTGGCTGAGCTGGCACCAAAA
CCACAAGACAAAGTCCTTCCCACTCTTCCCTTCCCTTGCGACAAGCAGAGGAGTATCTTCCCAT
GGCCACCACCACCACACACCCATGAGGAGTCCTGCTAGGCTACTACCAGTGTTCACTCAAGGC
CCAAGGGCTCTTCAGTCAGCTTGTGGTGAATGCTTCCAGGCCTGGGACTCACCTTTCAGAATAG
TGGACTCCCTTTTGGCCCAGGGCAGTCCAAAAATGCCACCCAAGAGCCAAGGACTAGAATCAA
TGATTCCAAGAGCCTGCTTAGTACTCTACCTTACTGTGACCAAACTGGTACCTAAACTGCAAGAA
AAAGTCCCCTTTACTCTTCCCTCTTCTTTTCTCTAGAAGAAGGAGTATTTCTCCATAGCCATCACA
ACTAGGAATGTACTGGGTCACATCTGAAGCCAGCACGTCTCAGAGTCTCTCTCAAGGCCCATGG
TGTGTACTATCTCATTATTGCTGCTGATTATTTAGGGTCCAAGGGCTCTTCAGTCAGCAGGTTATG
AGTTCTGTCAGTACTGGGTTCTTTCCTTCAAGGCAGAAGGCTCCCTTCTGGCCTGGGGAATGTCT
AGAAATGTATCTGGGAGTTAGGGCCTGGAATGAGTGCCTCAGGTCCCTGTCCAGTGCCAGATCC
TACTGTGGCTGAGCTGGAATCCAAGTTGCAAGACAAAGCCCTCTTTACTCTTCCTTCTCATTAAG
CAGAAGGAAGGAGTGTCTTTTGAAGCTGCAAGCTGTGCTACCTGGGGTTGGGGGTGGGGAGGC

ACAAGCACTCCCTTGGCCACCCCGGCTTGGTGTCTCACTAGGTCACATGCCCCACAAGTTCACT
GGGTCTGAGCCCAGCACAGCACTAGGACCTGCCTAGGAGTTGCAGTCCTCGTGGCCTAGACTG
CCTTTCAAATTTATTTATGTCCCCACAGCACTTTAGCCTGTGGTGGCGAGGCTCACCGAAACTCA
AGTTCAGACTGTTGGGATGGACTATTCCCCTTTGGTTGGGGCTGGTCTAAATGCTACCTCCGAGA
GTGTTGGCTGAGTTCTGCCTGGTGTTGTTTTCTGCTGTGACTGGGCAGCACTGAGTTCCAAAGCA
AAGTCCCACAATCACTGTGCTGTCACTTCCCCAAGCACACAGATTCTGTTTCTGTACCATGCAGC
CACTGCTGGGGGATGGGGAGGGGTAGTGCCAGTGATTCAAGACTGTCTTTCCTACCCTCTTCAA
TGCCTCTTTCTGTGATATGAAATTAAAACCAGGTACTGTGATCACTCACCTGATTTTTGGTTCTTA
TGAAGGTACTTTTTTTGTGTGTGAATAGTTGATCAATTTGGTGTTCCTGTGGGGGGAATGACTAG
CGAAGGCTTCTATTCAGCCATCTCCCATCCTAGAGGTATATCTTATCCTCCATGAATAAAATGTC
ATAAAAAAGGAAGATCACAAATATGTAAAATTTGGTGTGTGATTGGAAGCTACATATTTCACAC
ATTTTTTACTATGATATTCATTGATAAAATGAATATCCTTTTTTTACCAATTCGATTAAGTACTATG
TAAAAGGTACTAAATTAAGCTCTAAAACTTCAGAAATAAATACAAAATTCAAGAAAATAAGTTAT
CACAGTTTTTACTTTTGTTTGATGTGAATGTATGTCTTTTGAGAAGCAGACACCAAGAAAGGATT
AAACATGCAAGGATTTTATTGAGTTATAAAATAGGAAGTGGAGAGAGAGCTGGGAAAAGCTGA
AAGAGCTGTCAGACTGCATTGCAATCCTGGCCCTGAATGAAGGAGAGAGAGAAGAATGACTGG
ATGGGAGACATCCTAGACCCCATGTGGCAATAATGAAGGTTCAGCAAGACTCAAGGGGTCCTT
GAGGCACAGTTGGCTGTCAAAGAGTGTTATGTCTCCCCAGAATGGGGCTGTCCCTGCTGTACTC
AGCTGCTGGCTGGGAACAGCCCATGGGAAGTGTTGCTTTGGGTCAAACATGGCAATGAATTTAA
AAATGAGTGGCTGGGCTCTTGCTCAAATGTGTTCCATGTACTTAGAGGCTGCAAAATGAATTTTC
ATGGCTGCCATATACTTTGTTCACTTTTTAATTGAAACATTACCCAGTTTAATTCATCCTCCTTGCT
AATAATAAAAATTAAACCTTATTTATGCCCAAATCATAAGATAAAATGTTGATTTTCTGTTTTATA
AAATTAGATTCGGGGAATGTGACTGTGAAATCCCTGAAATATCAAATTCTTCAGTCCAACGTCTA
AACATATCTGTGGTTTGTATATTTGGTGCTTGATTCCAACCTGAAGGCAATAAAATGTGTTTGTAA
AACAAGAGCAAAGCTTTAAAATCATTCACGGGAAAATAACCTGCAAAATACAGGCTACAGAGC
AAAACTTTGGTGATATCCTATTACCTTTATTTCTTTTACATTCTGAGTCGGTATCTTTGAATTGGTA
ACAAAGTTGGTGTCTTTCCATTGTTGAAATCAAGGTTATTGTGAATGCAAAGCATTTTCAAGCAT
CTTTAGTATCTTTTACTTTGTGTATCAGTCTAGTTTGAGGCCATTGAGTTTTATTGACTTCTTGACA
GTTTGGTGCTCAATTTTTTTGTTGTTGTTCTTTGCTTGCAAGAATTCTCAAGTCTCAGATGCCTTTG
CTAGCTTAAGTATTTGTTTATGTTTTAAATACTTTTGAAATAGAAGTTTTGAAGGCTGTGTCATTTG
GTGGAAAGACCACGGTCTTTGAGGTCAAACAGATTGTATTGGGTTCTCATTTCAACAACTTATTT
TTTTTTCTTTTTAAAGATCAGATTTATTTGGAGAAGACAAAAAACCCACAACCCAAAGGATGGGA
TTTTACATCTCAAGACATCTCCTAGGTAATAAGTCTACAGATTACAAATCATTTTCACAGAAGAT
ATTTTTGTACAAATTTTACATGTATTCAGGAGTAGGATATTAATCCCAATTTCTATTTTAACAGGA
GGGCAAGGTAGGGGAGAGGGAAAAACGGTTTTGGATATAACTATTTGGAAGGCAAGTAGACAT
GAAGAGGGCAAACCCTAGCTTTTCATTATCTACAATCAATATTTTTTTCTTTCTTAAAAAAATTTTT
TTCAATCTTTAGTATCTCTTTAAAAACCCACTTCTTAGCTACTAGTCAATCCACACCAATTATTTAA
ATTCACTTGGTACACACCTTTGTCTACTGGGTAAATTATATTCATTATGCCCACTGCTGCAGCATG
CATAAACCAACACCCCTGCATGGCCTAATCTGGGACTGATGGGAGAAGTGCTTGCAAACCAAG
ATCAAGGTGTCATTTCTCTGCTAATACTGTCTAACAAGCTGACCCTTACAAAAACGCACGTAAAA
TCAGGCAGGTTTAGCTACTGTGTTGCAAGAGAAACCAGGACCTTTCTAAATAGTTCTCTCCATTA
CCATTTATTCTCTCAAGGGAAACTTAAAAAAAAAAAAAAGAAGAAAAAGAAAAAACAACACATT
AGTCTGGCCACCTCATAAATCGAACAAGCATTGCTGTGGCATTTCAGTGGAAAAGGAAACTTGA
GGGGGAAAAAAACCATCAACGTTGTAAGAAAGGCTTCCAATTTAACCGTCCCTGTCCCTATTTA

TCCACTTTAACTCACCACCCAAGACCATCCATTATTCTAGAGCACCCTGATCTATAAAAGGGGTC
AAAGCATCAGGAACAGGCAAGGAGTGAGAACCAAAAGACATCAAGAAACCGATTTGCTTGAG
AAAAGCAGTGATTCTTCCTCTTATAGCCTCCATGGCTGAGAGAGAAAATGCCCAAGAGATTATC
CATGTGACTTGGAGACTGCTTATTTATTGCATTACTTTAGACAAGCTGACAAATCTTTCTGAACTT
GCTTGCTTCATCCTCTGTAATTTAGGGACAGCAATTCCTATCTTGTAGGTTGCTGTGGGACAGAG
GCGAGGCAGCATACACAAGTTAATCCTGAGGCTCCCCATCTGGACTGTTGTGTTACCCAGTGTT
TTTGCCTTCCTCTCACTTGGGGTCACTGGAAACATGCAGGTTCCAAGCTTGGCCAGAAGAATTG
CCCAAGCGCCAAGCACAGCCCCTTAGCAGCTTGCCGCACAATGCCTGAGCTGAGAGGGCCATA
GCCCATTGGCCTCCTGGGGTCTTGCTGTGCTCTTGTAATCCCTCCTTCAGCCCATGTGTCAAATG
CTACCCAATGCTCTCATTTCCACCATGATCCAATAATTATAGTGGTGGGAGGGAATATTGTCCAG
AAAGGACCAGGAGGGTGGGAAGCAAGGTCCTCAATGACTGCATTCACCTGGACCTTTGGCAGT
CAGTGGTCTACTGTCAGTGGGTTCCAGCCATCAGGTGTCTACCACGAGGCATGCCCCTCTTCTT
GTCTTACTGCCAATTCATCTCCTCCTATAAGTGCTCCCTCAGTCAAGGCTCTCATATCCATCAAAT
TCTGCTCTTACAATCTTGCCCACTCAGCTCTATTAACCATGCATTCTGAAATTTCCACTTCCCCCA
TTCCCTCAATTACATACCCTCAGAGGCAGAGGGTCCTGACAGTGCTGGACACATGCAGCTCAGA
AGCTGAGTAAATGCCCCTTCTCTTGTCCCCTTGTGTCCCAGAAGCTAGGTGGTCTATGTGGCTGG
AGCTCCATGTCAGCTCTGTGGTGTTCATCACACTGCCCTGTGTGAGATTCTCCTGCAAGTTGGTC
TCTGAGAGACTAGCTGAGTTGGATATTCCTTCAAGACTCTAAAAAATAGGACATTTACTGAAATC
ATTTTTTAATCCTTTTTACTGAATATGTAGAGGTGCAGTATTTGTTTGTGTGCTTAGTCCTCAATCT
GTTTGCATTTTCATGAATAAAGTCGTCGAAAGAAATACATAGCTGAGCCATGCAATGCTTCCGG
GTTGTGGGAATTATGCATTCTTTAGACCATGGGGAATCTGAGGGGAGAGGCATTTGTTACTTGTC
TGCTGCTGGTTTGGCTCCCTCAGTGTCTGTGTCTAACAGGACACAGTTTTAGTTGCAACAAGGGG
CCCTGGTGGGTAGAGGGGCTCTCAAAGGGCTGTTAACATATCCAAGTACATGATGATCTGAAGA
ACCCAAAGGTTATTTCTGATGTAAGTGTCCAGGGATGGAGATTGTGTGTTGAGAACTAGGGCCA
GGACGACGGTGAGGTGAGTAGGCACCTGGCTTGGGTGCAAAATTTGTAAATAATATTTTAATGC
AATATTTTTAAAAAACTCAAATAAAAAAATTCATGTTGGACAAAATATAAAAATTCTAAATAAAG
AGAGGATCAGTAATGGTGCTGTGTTGAGCCATATTTGAGCTTGTAGTAAAAGGAAAAATCAGTA
ACCTATCCAGTTTTCATTTAAAATTTTTATATTTGATTCATCATGAATTTTTTGCATTCATTTGATTT
TCCAGATCAGATTAAAATATTATTTTTCTTGAAAATAAAAAAATAAAATTAAAATTAAAAAACCA
AAAAAAAGCAAATAAAAAAAGGAAAATATATAATAATAATATTAAATTATTAGTTCTGACCCTGC
TGAGAGCCATTGGATTTAAAAAGCATTCCTTTTGCTTCCTTTAAATCTTTTCTTTAGAAAAGATTTT
CTTTAGAAAAGATTTAAGACTAAAGAAAGACTAAAGAAAATTTTGTTTTCTTTAGTCTCCTGCTGA
CTGGTAAAAGGCATTTATGGCCCAAGGGCCACAAAACTTCTATATATCAGAAACCATACTGACA
ATATTTAATATTAATTTGCATGTGATTTATTTTTGGTTTTGTAAATCAATCTTTTTCTGTACCAGAAA
TCCTTCAGGGGAAATTCGAATACTACTAGAGTAGATTAATAATGAAAACAATAAATGATTTTTAA
AAATAGAATTTTAAGTACTGTAAACCTTCAGGGTATTTTTAAATAATGGCCTTATTTTTCACTTCA
ACACATTTTCCTCAACACATTTTCCAAGTGGAGTGGTACTCACCTTGTCTATGAGATTATTGCCTG
GCATATTTCAAACTCCTACCATTGGAAGCCATCATGAGTCTGTGACCTATGGGGGTGTGGCAGC
TGTTAGGAAGAGCTATCTTCACCCCACGCCTCGTATCTTAAGGAGGGGGTGATATGTTGGGAAG
TGCACAGGATTCATGAGCAGAAGACATCAATTCCTCCCTCATTTTTAACTCATTACCTGGCTGGG
CTTCAACTTCTCTAAGCTTGATTAGCCACATCCTTGCAATGGGAATAACAATAGATTTTTTTCCAG
GGTTGTTGGAAGGGGAAAAAAAGGAATCATAGGAGAAAGAGCTTTGCAAACTGCCAAAAGGCT
CTCCAAATTAAGCAAGCTCTTTCTTGATCAATGCCCACCCACAGGGATGGCTCCTCTTGGCTGGA
AACTAAAGTTGACTTCTCTGCTAGAGAGATTATCTGTCATCTAGGTTGCATTATCAGCTGTAGTAT

GTTATTCTAAAACACACTAAACATATAAAGGAAAGAAAAGGAAATCCTGAGAGGTCTTCATGGA
AAAGTTCTTGCTTATAAAGTGCAAAGTAATCATAGAGTGTGGCCTCCAACAAAGCTTCCTGAGA
AAGGTAAAATGACTTCAAATGAACTCTAGTTAATTCCACAAAACTTTCCTTGATCAGCAGTGTGA
AAAAATGAACAAAGAAGTGGATTATGCCCCATGAAGAAGATAATTGCTTCTTCAGGAAATGCAA
AAGTTGTACCAATTCCTGGAACTAAGCCAATGCATCTTCAACTAATTGGACTTGCAGTTGGTCAT
CTCAACTTTAAGTGTCCTAGCTTTTGGATACTGAAATAATCCCTTAATATGTCTAACTGAAGCACA
AAAAGAGGTAAAATTATCCTTTAAGATTTTCATCTGTCATAGTCTGGACATGATGGCTCACTCTTA
TAATCCCAATACTTTGGGAGGCCAAGGCAGAAAGATCGCTTGAGGCCAGGAGTTTGAGACCAG
CCTGGGCAATATAGAGACACTGTCTCTACATAAATCATTTATTTTTTAAAAAAAGATTTTTATCTG
TCATACATATAGACATCATTTTTGAAGAGTGATTATTTTTCAAAACAAAAACAGCTGAATTCAGA
GACCGCCAGTTTCATTGATTTCACAGCCCTTACCTGGTCAGTGAGCAGGTGGTATAAGAAGATC
TGGTCTTTGGTTAAGCTGATGGCAATGCCATGTGGCAAGGGAAAAATATTCATTCTTCACTGACT
GCACTATAGGACGTGCTATGTTTGAATCTGAAGCTAGAGGTGGATCAACTCCTATCTGACAATG
CTGTGGATTGTCATTAGTTTGATCTTTGGAAAACCTTGAAGCCTTTGAGGAACTCTCTAAGGAAT
ATACCTCTACCACTAAGGAGAAAATAGCCAAGGGGCTTATTTCCACTATACACTTAACCCAGAT
CCCAAAAATGCAGTTCCCTGAAGTGAAGGATTTACTTGGTCTTACATAGTATAGTAATCAGGTCT
CCTAAAATACTTGCTGATAGTAAACTAAGATATCTTGGTGGGATGAGGCTATTTTTCAGTAAACA
CCAATGGCATTTGGCATCCCACCAAATGCCATTGCTTATCCAACAACATTTAAAAACTTGAGTTT
TGTGTAGAACTTATTAAAGGAAAGTAACAAACATCTGTTACTGATTTAAATGATTTTTATTATACT
GTTATTTAAAATATGTCAAACACAGTGATACACCAAGTAGAATCATCATGCTTGCATGCTGTGTT
GCCTGGGCAATGAGCAAACAGGGACCAGGGATCCCCCTTTCCCTTGGGACCAGCAACTTCCTT
CTTTTGATGGCAGGGCCTGAACTATCCTTTCCAGACCACTCCTGCAAATAGCTGGCATCCATATC
CCAGCTGCTGGCACCCTGCTGGGGTTATCTCCCCATTGACAAGACAACATTTGCATACTTGCTCT
TTAAGGAGGAACTTCCGCATGGGAAAGGGCCCCTGCACTGGGACACAAGCCCCATCGTGTATC
CATCACGTTCTGGCTGGCCCTGGGCTTCCTCTCTATGGAGTGATGGGAACATGGGTGATCTCTTT
GCCTTTTCTTTTCTTAATCCAACCTTGTTACTTTTTTTTTCTTCTCAGTAATGCCTATTCCTTATGAC
TTTCCTTGTGGAATTTGTCCCAAATTCTGGTAAATAACAGATAGAGACCTGCTAGGACTCAGTTT
TTATGACAAACATGAAACTAGGTATAAATGCTCTTATAAGCATGGCTCTTAACCACAAAACTAAA
ACAAAACTTAAATATAAGTATAAAAAAATCACATTAATGACCATAATGTAATATAATAATTGATG
TTATATTGGTGAAATTAAATCATTCAATTTGGGCTTAATGAAATAAATGAGATAGCCTTAGTTCAA
CTCTTTATTTAATCCATTTCTATATTTTAACTTGAACGAAATTTTTAGAGAATGCTTGTTTGGATAA
GTTTGATGGGCAAAGTGATATGAATAACTACAAGGCTTTTTCAGTTCAGGATAAACAGACTTCAT
ATTTAACCCAAATTCTGCTAGCAAGCAATCATTGAATGCCTGTTTTGAAGAGGCGTCATATAGTA
ACTTGTAATGACTAGTTCATCTGAAAATAAGATCGGCATTGTGATATTATTAAATCTATAATAAAT
GGGTATTTAATCCAATTATTTCTGGAATTGAAAACAAAAAGAAATCTTCATTGTAATTCCAATAGT
TGCTGCTTTTGAATTTTTGCAAGTGACTACGGAGCAGGAGGGGCTGGCTGGACAGGGTGCCATG
TGCCTGCCTGAAATGGAAACCTGGCTCGATTGTTGTCTGGTGCAGACCTTTGAGTTTTATAAACC
TTGGCTCCATGCACTGTGTGAGAACTCACATGGCCCACAACTGTTATCTATTTAAAGCATGATGA
AAGTTTCCTTGAAGCAGAAAGCTATGCATGATTCATTCTATTCTGCATTCTATTCAGTCGCTCACT
CACTTGCACACTCATTCAAACATGTGCCATGTGCCAGACAGGCTCTAGGCAATTGCATACACTG
GTGGACAAAATGAAAAACCTAATCTTCATGAAATTCCTATTCCAGCAGGAAGAGACAGAAAACA
AGTTATTTTAATAAGTAAATTTTATAGTGTGTTTAAAAACAATTTTTTTAAAAAGGGAAAAAGTAG
GACAGGGCTTTAGGGACTGCTGGGGCTGGAGTGGGGCAGGTTGCAATTTGAAATAGGGTTCAG
GGTAGGCTTCACTGGAAAAGTGTGATGAGAGAAAACATTTGTGAAAGGTGAGGGTGTGAGCCA

AGGGGCTGTTGAGGAGAAGTGAGCAGTTCAGGCTGAAGGACCGGGTAGGGCAGATTCTTGAG
GTGGGGATATACCTGATGTGTTCAGGAAAGAGCTGAGAAGTCAATGTGGCTGTACATCAGTGA
GCTGGGAGTGGAGGAGTAAGAGAGGAGGTTGAAGAAGTAAAGGTTATCTTAGTCTGTTTTCTGT
TGCTTATAACAGAATACCTAAAACTAGGTAATTTATAAAGGAAAGAAAGTTATTTCTTATAGTTCT
GGAGGCTGGGACCAGCTCCAAGGTCGAGGCAGCATCTGGTGCGGGCCTTCTTGCTGTTGGCGA
CTCTGCAGAGTCCCAGGGTTGCTCTAGGCAACACATGGTGAGGGAGCTGAGCGTGCTAGCTCA
TTTCTCTCTTTGTCTTCTTATAAAGCCACCAGCCCCACTCTTGTGATAACCCATTAATCCATTAATC
CATGAAAGCTTTCATGACCCAATCCCTTCTTAAAGGCCCCACCTCCCAATACTGCCACATTGGA
GCTAAGTTTCTACATGATTTTCAGAAGGGACAAAACATTCAAAGCATAGCAGCGGGGAAGAATG
TGAAAACTCCAAGAAATCACGGTAAGCACTTTGGCTTTTTCTTTGAGTGAAATGGGGAGCTATTT
CAGTGCTTTGAGCAGAGTTGTGGCATGATCTCACTCACAGTTTAGAAGTGTCACTCAGGTGGCT
GGGCTCAGTGGCTCACCCCTGTAATCCTTTGGGAAGCTGAGTCAGGTGGATCACCTGAGGTCAG
GAGTTGGAGACCAGCCTGGCCAACATGGCGAAACCCCGTCTCTACTAAAAATACAAAAATTAG
CCAGGCATGGTGGAGGATGCCTATAATCCCAGCTACTCAGGAGGCTGAGGCAGGATAATTGCT
TGAACCTGGGGGGTGGAGGTTGCAGTGAGCCGAGATCGCACCACTTCACTCCAGCCTGGGTGA
AAGAGTGAAACTCCATCTCAAAAAAAAAAAAAAAGGAAATGTCACTCAGGAAGGATGGAGCTGC
CTTCACCAGTGTTGGGGAAGCTGCAGGTAGAGCAGGTTCCAAAGTGGAAAGAAATAACAAGCT
CAGTTCTGACTCGATTAACTTTGGCACATCTGGTAGGCATCCAAGTGGGGTATAAGTTGGGTTCT
AGCATCTGAAGTTTGGGAGAGAGGTCTGAGCTGGAGATTTATAGTTAAAAGTCAGGGAGTAGAT
GGATGGCATTGAAGCCAAGGCACTGAATGAAAGATGCAAGGGAGTAAATGTGGCTGAAGAAA
CAAAGAGGACCAAGGATTCAGGCCTGGGGCCCCCATAACACAGAGGATACCAAAATCCTACA
GGAGGTCTCGAGATAGTTTTCATCTGGATGCTCCAGTCTACACTATAGTTACATTGTTTCCAAGTT
ATCATTACAGTGAAAGCATCACTTATAGGTTATGTGGAGAACTCACAGACCTTGGTGATGCATCT
GGAACCCTCCTTTGAGAAATCCTGCTTCACAGGCCTCAATCATAGGCAAGATAGTTCTTCAAGTT
GAGACAGAATGATATGATTCTAAAATCATGTTCATCACTGTCTTGCTCCACAGGGGAGATTTTCA
CATTTACACCCTTGTTCTACTTTATCAATGTATTAGAAATTATACCCAAAATATTCTTCACCACAG
AAAAGGTAGAAAATTTAGAAGTGAGTGTTTAAAAAGTAAAGTTTTAATGGTTTCATTTCACCCCA
CACTGGGATATACCCCACTTGCCCTTAGAGACTGTTTTCCTTGAGCAGTGATGTACATTGAAGTT
CACTTCCCTGGGCTTGGACAAAGGCTTCCTAGGGCAATTGGTTTTCAGGCCCTCAAGGTTCATAT
GTACTCTTTTCTCACACGGAACCACATGGGAACTCATCTGCCACATTAATGCTTTCTCAAATTTCC
TACCAGTACTTCCTTTCATGTACCTTACAAAAGATTCACCCAACTGAAATCCAACTCTGGTGCAG
TGACTCAAGTTGCAAAAGTGTCTGGTGCTCTAAGCAGAAGAACAATTGTCAATATCACTCAGGA
AAGTCACCTTCAGCAAAGACCTCATAGAATTCCTCCCCTAGTCTATTTTAGTAACAGCTTCATTTC
CAATAAAATTTTCTTTTTATGGTTAAAAATAATTTATCAAAATTTATTATATGTTTAAGGTGTTTTG
ATTTATAATAATTAAACTAAAATGAAAATTTTAAGTTTAGAGAGTTTTAACCAGAAAAAAAATAAA
AAATAAATTTTAATTTATGGACATGTTTTTCTTTCAAAGAAATTTGACAGCATAAAGAATAAAAGT
TGCACTAAGACAACTCGATGGAGGAAGTGTGATGGAAATGTGATTTAAGTCAGAAGTGAATGAT
GTAAATTTTCTCCCTGTTAAATAAGAAATGTTCATATATTTAAAAAATGGATGTTGCCTATCAAAT
TGCAATAGTGTTTATATTCCATTTTCAGAACACATTTTATAATTATCAGAGCAATGTAACCGTTTC
ATTTGAAAATGTTAACATTTACAATATGCCAGAAATCACATATTTTGCCTCTATTTTAGAGAAAAA
AGTTTTAGATATCATCTTTAAAATATAGAAGGGAATACCTCATTTTCCAAACTCCATTCAATGAGC
AACATAGGTTTCAAATTCCACAATGACTTAAACTACACCAAAGACAATCTCAGCAAGATATGTAT
CAAAAGATCTCAACGGCAATGGAAAACTTCACTTTTCCGAAGCAGACCAATCAATTTTGAGCCA
TCAATTTCTTTCTTTTGTAAGATTCATTCCTACTGCACTGACCCCAAAACCTTATCTTATTTCTTGA

CCATGGATTATACCAGTACATATTGATCATGTTAAACTGTTAACACCCACAATGATATGCCCCAG
ACACATTTCTTACAATGAGCAGAGGGTTCTGTGTATTGGATATAGGTCAATCACTGAAGGTAGC
AGTAACTCAGTGGCTCTCTGCACTTTACCTTCTTAAGGCCACCACACTCACGTACTAGGTGTTGT
CCCAGTGGAGGACAGTTGGATACAAAGTCTCTTCCATCTTGACATCTTGGTACTGCAGTGGCCT
CCTGTTCCATTTCTCACTTCTCCCACCCTTAGTTTTCTAGCTTTGGGGAACATGGCTGCCTCAGCA
ACCATCTTATCTGCTCCCTCTACCCCACCGTTTTGCAAGGAGGAACGGGCATTGGTTTCTTTCTC
ACACTGTCACTACCTTCTCCCCTTTCCTTTACCATTTTTTCATTTGCTCTTCACTTCCAGTCTCCCTT
GCGATCTTCTTCCTTTCTTTCTGGCCAGCCTCTGACTTCTGCAGAGTGGTTCCCTCCACCCAGAC
AAAACCCTGGACTGATGCCAGGCACAGGGTATGTGACCTATGCTTCCATCTCCTCTTCCGTCCCT
GGGACTGTGGCCATAGATATTCACCTCCATCAGGTTCCACTGATAAAGGAGAAGGAGAGGACA
CAGTTCATAGAAAAGGAAGCTCTGGCCAAACTGGAAGCCACCAAACCCAGGGTGTGGCTCCTA
GAGTATTGCCATCTCTCAGGAGCCTTCCAAGGCTTATCCCGTAACACCTTCCACTCTTCTTAGCA
TCTCTCTTCTTACCCTTTATTATTTTTTATGTGAAAAAATATACATAACCATAAAATTTACTATTTTA
ATCATTTTTAAGTGTACAGTTCAGTGGCATGAATTACATTTATATGTTGTACAAACATTACCACTA
TCAATTTCCAGAACTTTCTCATCATCCCAAACAGAAATTCTTTACCCATTAAACATTATTTCCTTTT
CCTTTCTCACTCCAGCCCTTGGCAACTGCCATTCTACTTTCTGTCTCTATGAACTTGACAATTCTA
GGTACCTCATATAAATACAAATAAAAATACAGCATTTGTCCTTTTGTGACTGGTTTATTTCATTTG
ACATAATGTCTTCAGGGTCCATCCATGTTGTGGCATGTGACAAAATTGCCTTCCTTTTTAAGGCT
GAATGATATTCCATTGTGTATATATCCACATTTTGTGTATATATTTATCCCTGCGTGGGCACATGG
GTTTCTTCCTACTCTCAGCTATTGTGAATAATGCTGCTAAGAACATGAGTTTATAAATATATGTTC
AAGTCCCTTCTTTTACTTCTTTTCAGTATAAACCCAGAAGTGAAATTGCTGTATCATATAGTAATT
CTATATTTAATTTTTTGAGGAACCACCATACTGTTTTTTATAGTGGCTACACCATTTTACATTTCTA
CCAGGAATGCACAAGAGTTCCAATTTCTTTACATTCTCACTGATGTAGTTTGAATACATGAGCCC
GCCAAATCTCATGTGGAATTGTAATCCTCAATGTTAGAGGTGGGGCCTGGTGGGAGGTGATTGG
GCCATGGGGGCAGATCCCTCATGGCTTGGTGCTGTCATCAAGATACTGAGTGAGTTCTCACAAG
ATCTGGTTGTTTAAGTATATGGCACTTCCCCTCTACTCTCTCTCTCTTGCTCTCCCTCCCACCATGT
GAGATGTCTGCTCCCCCTTTGCCTTCTTTCATGATTGTAAGCTCCTCGAGGCTTCCTAGAGGCAG
AAGAGCAGGTATGCTTCCTGTACAGCCTGCAGAAGCACAAGCCAATTAAACCTCTTTTCTTTATA
AATTACCCAGCCTCAGGTATTTCTTTATAGCAATGCAAGGATGGACTACCACACTCACCAACACT
TGTTATTTGTTTATTTATTGATTTTCATAGTAGCCATCCTAATGGGTGAGAAGGGATATCTTCTTGT
GGTTTTGCTTTGCATTTTGCTAGTGACCAGTGACACTAAACACACTTTCATGTGTTTATTGGCCAT
TTGTGTATCTTCTTCAGAGAAATACCTATTCGAGTCATTTGGTCATTTTAAATTGGGTTGTTGGTTT
TGTCGTTGTTGTTATTGAGTTTATTTTTATTAATTGTAATTATTTTTTCTCTTAAAAATTCTAATGCTT
ATTTTAGAAAATTAAATTAAGAATTGAAAAAACATTGAGACAAAAATGATTCATATTACAAATAA
AGAATATATTTTCTCAATTTATTTTAAGTGTTTATAAATTTTATTTAGGCCACATTCTTTGAGTGCA
ATATGAAGAAGCTAGGATTCATTACTTAGGAATTAAAAGTAGACATTACTAAATAAGTCTTGGGT
AAAAGAGAAAATTTAAATTTAAAGCAGGTCACAGATTATAAGACTAGTAGGTAACACACTACTA
CCCAAACTAATGAAATATGTTCAGAGCCATACTTAAAAGAGAATTCCTGACTTTAAATATTCTTA
TTACAAATCCAAAAGAATTTTAGTTAAGCATTCAATAAAATACAAATTCTTGAAAATACAAATGC
ATAAAAGAGGCTATAATAGCTGGTAAAGAATTATTTCTGGATGTGTCTGTCACGGTGGTCTGGA
AGAGATTAGCGTTTGAATCTGTAGACTGAGAGAAAATCACCACCTCCGGTGTAAGTAGGCATCA
TCCATTCTCTCAAGGGTTCTGAATAGGGCAAAAAAGCAAAGGAAGGGGGAATTTGCTCCCTCTG
AACTAGTGTATCTGTCTTCTTCTTCTTCTTCTTCTTCTTCTTCTTCTTTTTTTTTTTTTTTTTTTTTTTTTGA
GACAAAGTCTTGCTCTGTTGCCCAGGCTGCAGCACAGTGGCGTGATCTCAGTGCACTGCAACCT

CTGCCTCCCAGGTTTAAGCAATTCCCGTCTCAGCCTCCCCAGTAGCTGGGACTACAGGTGCAAA
CCACCACACCCAGCTAATTTTTGTATTTTTAGTAGAGACAGGGTTTCACCATATTGGTCAGGCTG
GTCTCAAACTCCTGAGCTCAGGTGATCCACCTGCCTCAGCCTCCCAAAGTGCTGGGATTACAGG
CATGAAGCCACCACACCCAGCCCACATCTGTCTTCTGATGTTAGACGTGGGCACTCCTGGTTCT
CAGGCCATTGGACTGAATCATACCACTGGCTTTTCATGGTTCACCAGCTTGCAGACAGATCATG
CAACTTAGCCTCCATGAGCCAATTTCTATAATACATCAATCTCTTTCTCTAAGTATATGTAAATGT
AGAGAAAGCGATATATATGTTCCATATGTATACTGTTTCTGCGAAGAACCCTAATACAAACATTA
ATAAATTGTTTTAAAACTCACAAAATAATATAATTAAAGAGCAGGTAACCCCCAACTGTGTTTTC
AGTTTGCATATTCTAAGATAATTACTAAGAACATTGGTTTCATATCTCTGGATGCTGTGCTTCTGT
TAGTCCCGTAATTATTCATGACCATTATATCTTCATTTTGGATTATGTATTTTATTTATAAAGTCTC
CATTGTTCAAGTGATTTTTCCTTAATTCTATTTGGTCTATATTAATATTCTTATTTGTTTTCTATTTGC
ATTCATCTGGTGTGTCTTTGTCCACCTCTTCAAAAAATATTTCTGCTTCAATTTGTTTTACATATTTT
TTTGAAAATAATGTTTAGCTAGATTTTGTTTTGCTGACCCAATTTAAAAATTATCTTTTAAAGATGA
AATTTGATCAACTTACATTTATCTTAGTTGATGCAGTTGTGTTTCATGCTTTTCTATTGCTTTGTTCT
TTACTTTTGCTGGTTTTTTGTTTGTTTATTTTAGTATATTGTATTTTATATTTCTACTGATTTGGAAAA
TATACATTCTACTCATAGTCTGTTAGAGAACATCTTTCATTAAAAGCATGTAAGCCTTTATTTCTCT
GTTATAACAATAAAACCTCAATGCCTCAAATAAGATGAGGAATTCAGCTTGCTTTTAAAAGTTTC
CTCTTTTTTCTCTATCTCCCAGATTACATTAGAATTATCTCAGATTTTAAAACCAGAATATTACAAG
TAGGTTTTTTACTCTGCAATTTCTCTTTAAAAACCATTTGGACATTTTAAGTGTATCTCATAACCAC
AGAAACAGCAATTATTCACTGGGATACTTTGTTTCATTGGTATCACTATTGTTTACCTCTTTCACTT
TCTTCCTGGATATTTTTACTGTAACATGGATAATTCCCTCTTGGAATACTTGCCTATCTCAAACTA
CGTTTGTCATTTTCTGATGCTATGATGATAAATTGTCCTGGTTTACAAGTCCTGAGCCACATCCTT
TCCCCTCTAAACTCTGTGGATCTTATTCCATGATCTTTGGCATGACTATATTGCAGAGAAGCCTG
GAGACAAATCCTATAGTTAACCTGGTTTTACTGTCTATGTCCTTTGGGAATTTTCCTTTATCTTTGA
AAATCTGAAATTTCATTGGAATAAATACAGGTGTGTCTTCTCATTTTCTAAGGTAAAAAGACAAA
ACAAAACACAACCTAATACACTGAGAGCATCTTCAACCTGAAGTGTCAAGCATTTCTGTGATTTA
TTTAATTTTGTGTTTGTTCCTCCACTTGCTCTTTTTCTTCACTCTTAATCTTCATCTTCCATGACCTC
AACCTTACTCTTCATGGGTATTCTAGTTTCCATCAAAATCTCTTTTTCCAGCACATTGGAGAAGAT
AAAAAGGCTGGGAAGGAAGTGAGGAGAATTGTGCGTGCCATTAGCTGCCTTGCAAATTATCTCT
TCCCTGGGGCTGGTGTGGCTTCTTGGCCCTGCAGGGTGCTAAGATGGTGCTCAGTGGCTCCAGT
GGAATCTGTCTTCATCACTGGCACACGTGGGCCGCCTGCAGACTCCCCAGACAGATTTCTCTGT
GGCACCTGAGCTCCTCTCTCATGGATAAGATCTCCTTTGTGACTGAGATTAAGCTGCTGAAATTT
GCCTGCTGGAAACAGTCCTCTCATCACAGCCAAGCCTTTTTCTTTCTGCAGCCCATGAAGAAGTC
TATTCCGACTTTCTTTCCATCCAGGTTCCAGGGACTTCTCCTGCCTGCATGTTCCTTCTCCACCAA
TGGCTTCTGCCATTCTAGCTTTCACTTTCTCTTGGAGGTGTCAGCCAGAACATTTCACTTGGAAG
CTTTTGGTATGGACATTTCCCTCCCTACTTCCCACCAAACATCCCAGTGAAATATTAATACTTGTA
GTTTCTTGACCAGAGATTCCAACGGGTGATAGGAAAATTGGCAGAGGTCATGTTGTTGATTTTAG
GTTTGTTGTTGTTGTTTATTTTAATCATTTAGCTAAGAACTCCAGAAATGGCCTTTGGATATAGCC
TCCAAAATACTCCAAATAGCTTCAAGAAACATGAATCGTAACCGCACGCATAGCCTGTGATCAG
AAATAGTCCCAGCAGGAAAGAAAATGAGTTACAGAATGAGTTTGCATTCCCTTTGGCTGGGAAC
CTCAGCACCGGCCCTGGGCTCCTTCATGAAGCAAGAGCCTTTCCCAGGTCCAGGAGTCCTTGAT
TCTTGTGGTTCTCTGTTCTTCTTCTTCTTTCTTCTTCTTTTTTTTTTTTTTTGAGAGGGAGTCTAGCTCT
GTAGCCAGGCTGGAGTACAGTGGTGTGATCTCAGCTCACTGCAACCTCCGCCTCCTGGGTTCAA
GTGATTCTCCTGCCTCAGCCTCCTAAGTAGCTGGGAACACAGGCATGCACCACCACACCCAGCT

AAGTTTTGTATTTTTAGTAGAGGCAGGATTTCACCATGTTGGTCAGGATGGTCTTGATCTCTTGAC
TTTCCGATCCACCCATCTCAGCCTTCAAAAGTGCTGGGATTACAGGTGTGAGCCACCGCACCCG
GCCAGTTGTCTGTTCTTTTAGACACCTCTGGGATCACACTGTGAGAAGCAAAATCTTAAGAGACT
ATCATGCATTCATTAATTCACATGGGGATGAAAGGGCTGGTGCCCAAGCTCCAGCTCTCAAATC
CCGCTTTTGATTCCCCCACCTCATTCTCCACCTCAGCCACACTTTCTTTGAAGGATTTGACAAGTT
TGGCCCCCAATCCATGTAGTGAAGTGCCTCTCTTCTGCCCATCTACTGAGCTTTCTGCTGTTGAT
GGATAATTTCTCTGGAATGTCTTGATTGAGCCCCTTCTCCTTTTTGCCTGTGTTTTACACATGGGTT
TCCAACCATTGCCCCAAGAGATGAAGATTCACCTGGGATGACTAAATTCCACTGTAGAATCTAG
AAACCCTCTCACAGCTCCTGGCCTTCCAACAGGTGTTACAGTTTACAGCTGTGTCCTACCTCCTA
GACTTGCAAAAGTGGTGTCTGGTGTTTTTTTTTGGAGATGGAGTCTTGCTCTGTTGCCAGGCTGG
AGTGCAGTGGCACCATCTTGGCTCACTGCAACCTCCACCTCCCAGGTTTAAGTGATTCCCCTGC
CTCAGCCTCCTGAGTAGCTAAGACTGCAGGTACACGCCACCATGCCCAGATAATTTTTTGTATTT
TGGTAGAGTTGGGGTTTCACCATGTTGGCCAGGATTGTCTCGATCTCCTGACCTCATGATCCCTG
GACACCCACCCCGGCCTCCCAAAGTGCTGGGATTACAGGCATGAGCCACCGCACCCGGCCTGG
TGTCTGGTTTATATGAACGAAAGATCTGGCTGGAATAGCAAATGTCTGCAAGGGAGGCTGGGCT
AGATATCACCAGGGCCTGGAACACTTCTCACATTCTTTCCTCCACTAACCTCCAGTTATCTAAGA
GGATTATGACTCTTCTTTCTCTCTTGTCCTCAGAATGTCTCTCTGGCTTCCTCTCCCTCTCAGAGA
CCCAATTTGGAACTGAAACCCTGAGGTGAGATAACTGAGGTGAGATAATTGCAATAATGGAATA
ATTCTTTACCAAGTCACTGCGGTAACTCAGTAACTCCCCCTTTTGTGTTGGTGCTACACAAGGCT
AAGTTCTGAATCTACAGCTATTCGTGATGCTATTTCTTTAAAAACCTAGATATCAACAAACGAAA
CTACATTAATCCAATGTGGCAATCAGGCGCCGTGAACTAATCCTAGTACTTTAATTCTAAAAGCT
GAACCCTCTTGGCAAAAGAAAATGATAGTCAGAATTTCTCTTCCCAAGACATTTCATTTAGCCAC
CTCTATTTGTCTTCTATTTTCTTCCAAGATTTCCCACTACCAAGCATAGTAGTTTTTCTTCAAACTA
CTCTCAATAGCCTAAGGTAGTCTAATTAAAGCAATGGATCATTCACTCTGCTTTTCAGTAATCCTA
CAGTGACTTAATGAACAGAGTGCAGCAATTCAACACATGGATTCTTACTCATTGTATCCTAATTG
TATGTAAGTACCACACAATTCTATTGAGACTCACAGACATTTCTTTCTTCTCCTCTTCAATGAAAA
AGCAAACATTAATTGAACCATACCACGATTTGGGAAATAAACATTTCATGTAAATTGAAAAAAAT
ACTTCTCTACTTGTAACATGCACCATTCTTGTTTATCTGAGTTTTCTTAATTTTCTACAATGAGCAT
GTACTAGTTTTGTTAAGGGAAAAAAATACGTTTTTTTTATCTTTAAGTCAGGAGTCTGCCTATTGA
AAATAGCAGTGTGGTACCTTTATCTTTAGAAATAAGTGTCTTTACTTTTGATAGAAAAGATTAGAA
GTGTTTCAACAAAAGAAATAGTACATTATGGTTTCAAACCTCAAAGCATGGAGACAACCTGGGA
TGTTAATGAAGGAAACCTGAATTCCGAGATCCATCTGCAAAAGGGAGGCTGGAAGTCTTGGAA
GAAGATTCTGATTGTGTAATCAAGTTATTAATATCAGAAATGCTTTTCCATAGGCCTTTACTTTGA
ATTCATTTTTCGAATGAGATAGCTGGTCTTTAAAAACAACTTACTTTTTACTGTTACAGCACTACA
CATGTATGGTGAAAATAATTCAAATGGTAAAAAGAAAGAGGTGCTGTGAAAAAGATGAAAAAC
TCCTGACATCCCAACTCTGCAGTCCTCAGAGATAATGATCATTTCTTGTGCCCCTTTTTAGAAATT
TTCTCTGGGCATACACTCCCTCTATGCGTCTATTCCTTTAAATCTATATTCTATTGCTTTATATCTC
CATATCTATGTATTAATATGTAGATATTACAGTTGCTATATACTTATATCATTAAAACAGTTATTTG
ACTCTTTGTATTTCACATTCTGGGGCTTCCAGCTGGCACCATGACTCCAAGCACCCCTCTGCTAG
GACATGAGAGAGAAGGCATCAGTCAGGATGATAGAGCTTGACTGATGCACGTGCCCGAGCCAC
ACTAGTGATCTTTGAGTGCAGGCTGGAATCCAGGTAATCTGGCCGGGCCTCCTCATTCATGGCA
ATCTCTGGGCAGAGATCTTAGGAAATTCTCCTCCCCTCTCAAGACTGAGCCATCTCATTGGTAGG
ATGAAGGGCTGGGAATACCCGAGATACAGTCTAATGTTCTATGTCTCTTGTCTGTGATGTCATAA
CTGTGGCACCTAGGGACACTTCCAAATGACCAATATTCCGCAAAGATCCACATCACGTTCTTTA

AGACCCACGCCTGTCAGAGCTGTCAGACACTGGCCAGGGCCCTGCAGACGTGCACATTTTCTC
ATAGCCTCGCCTGGTGCTTTGAGTTCCTGCCACACTCAGTGGGTAGCTGTAGCTCACTGATGTGC
ATTTAACTGGCAGGAAGTGGGAGAGAGAAACAGAGGAGTGGGAGAGTGGAGTTCTTAAGGTTC
AGACAGAAGCTTCATTTTCCGCCTCACTCCTGGCCTAAGTGCTACCCTGTCCTCATGCCACTTTG
TCCTCTACTCTCCAGGGGACTGCTATCACCACAGAATATCTATATTTCCTGACTTGGCCTTGTGA
GAATCAAATGATTTACTAAATACTGTCTATACTCCTGCAATTTGCCACACAAATGCAGGGTGAAA
ATCTGAGGATAGCAAAATGAGTCCTTAAACTGTGTGCTAAAAGAGGTCTAAGTTCAGGTGGGGA
AACAGAAATGTACATAGATAAACTGGAACGCAGCACTGACAATGTCAGATGAAAACGGGGAAG
CTCTGCAGAGAAGAGGGACCTTGGGCAAAGAAATGGAGGCCAAAGTGGGGTCTGCCAGGTTC
AGGAGGTAGAAAGCACTCCAGGAAGTAGCGAAGTGGAGTAGCAAGGCACAGAGACATATATG
AGAGTGGCATGTTCAGGGGATGACAAGCTATTCTGGATGCCTTGTGTGTAGGGTGCACATGGG
GAAGGACCAGGTGAATGAGCCTAGAAAAGTAGGCCTCTGCCTGACCATACAGGGTTTGGATAA
TGAGCTAAAAAGTTTAGAATTTGTTCTGAATAGCAGCAGCTCCCAACCCTACCACATCCAATGG
CCCTTTTTATAACAAGTATTTTGTAATGTCGTCTATATGTCCTGAAATGAAATTCATATTGCTATAT
ATCCTACCTACATATACAGTGAAAAATTAAATGAAAAATGAAAATCACTATAATGGCTTGACATG
AAGAACACCAAAGGAAAATATGTATTTCATGTAAATGCTTGAATCTGACTAAAAAAAAAACAGA
AAACATACTGTAGTGGTTGGTTGTTTATGACTATACACAGAAGCACCAAGATGTGACAGCTACA
AATACAAACAATACAGGGGCATGGAACTAGCGAGTCATGGACCTTGTCATTTGATGTGATTTTCT
GAAATGTTAACTTTGGAGAAATGCTGAACACAAAAAAGTACAATCATCTCTTGATTCACTTAGTG
TTTTCACTTGTAGATCATTGAATATATTAAAATGATGCAAAAATACTTTGTGTGTCTATGCAAATT
GGAATTCATTCTAGGTTTATACAATTATAAAAAGCATTTTCAATTATTGGCATGTTTAATAAAACA
TTCAGAGTCACATTAAAAGTGGGACCGTTTCTCCTTTGAAAGGATAGTTTTGTGTGTGTCTGGAT
GACCTAGACATATATGTTTGGTTGCAAAGGTCAGGGACGTGGAAGTGGCTGTGAAGATGAGGC
ACTGCCACATGGCCATGGGAGTTGCAGCTGCAGGTGCCCAACAAGGGAATTCCTGAGAAGCCC
ATAGAAGTGCCCTAGAGAAAGAGTCAGTCTGAAGCCACTGCTGTGGTCAGAGGAAACCAACAA
CAGATTATGTCAATTGTATCTTGTATCAGAACCAGCCACGTAAGGGTTCTCTTGACTCTTAACTCT
CACTTAAGGCCCTGCTTCAACTCTGGGAAGCCAGCAGCAACACAGTGAGTAGGGAAAAAGAAA
TAGAAATTTTAGTTGAAGTGTTAGAAAAATTCATTGTTCACTCGGGGAAAAAAACCTAAACTGTT
AGATCACTTCATAACACTGTACATAAAATTAACTACAAGAGGATTAACCATCTAACAATAAAAA
ATAAAATTGTAAAGAACCAATAGAATATATTGTTAAACATCTTTAGAATCATGAGTTAGGTAAGA
CCCTCTTAGATGGAGAAAATCAATTTCTTAGAAAAATCTGAAAAAATGAATGGGCAATCCCCAG
AGAAGAAATACAAGCAGTCCAAAATCATGAGCAGATACTCGACTCAACTTCACCAGTAACTGG
GGAAATGCAAATCACACCAAGTATCTATTACCAGAGTGGCAGCATACCAAATTGATAATATTCA
GTGTTGTCAAGGAATATGGTAGTATCTATCAACATTTTAAAAGTGTGTACCATAAGTCTGGCTAG
TCTATGCATAGAGATCTATCCTTGGGAATAAGTATATACAAAGTGTGTGCAAAAATTTTCACTAT
GGCATTGTTTTAATTGTGAAAACATGAAACATTACATATGTTTATCAATAGGAGTATGCTTGAAT
GGATTATGGTGCCTCTATACTGTAGGATAATCTGTACAAGTTAAAGAAAAAAGACATGAATTTAT
ACATATTGACATGAAGAAAAACCACTCCATGACACATTCTTAAGTATAGAAAGAAATTTCGCAC
ACTATGAACAAATGATCCCATTTATATAAAGAGTAAAAATTATGCACACACAAATATATCTATCT
ATCTATCTATATATATACACAAAAATACATGAATATGTATGCATCCCCCCATACACACAAACAAA
CACATGCACATAAAATTGAGGAGATTGACTGGAAGATATATACAATGAATAGTTAAAAAAGGTT
TACTTCTATGCTAGAAAATAAGATTGGGCCATGATGAGGACCTTTACTTTGTGCTCTTTCTGTATG
TTTATTTTTTAAGTGTTATGTGAGAACATACTGATGTATTTCTGATGCAGACTTTTACCAAATGTTT
AAAACTTGGCAATCACAGATTTTATATGGAAGCTATACTGACTTCAGGAACACCACACTCTTGGT

TTTGCTGTCCTCATTGGCTGCCTCTTCTTTGGGTTGTACAGTGTCCTTCTCATCTCGCTGACCTCTA
AATATTAGAGTGCCCCAGGGCCCAGTCTTGCAAGCTATGCTTTCTCAGCCAACAATTATTTCCTA
CACATTGACAACCAATTCCATGACTTTTTGGCAGGTTAAATTTTAGCTCCAACCTCCAGCTTTGC
CGCTTTGCCCTGACTTGCGGAGTCTTATAAACAACTGTGTACTCAATATCTGCAATGGGATATCT
GAGAATATCTACACCTTAACTTGTCAAAACCAAAACTGCCTGTTTAACTTTTTTTCTTCAAACCTG
CTCCTCCCACAGTCTTCCCCACTCAGGAAGTGGCATCTCCATTCAAGTTGCTTGGACAAAAGTCC
TTAGGGCCATTTTTTAACCTTATGACTAGACCCAAATAGAGCTGTTTCTCACTGCTCCAATCGCTT
TGACTCTAGTCCAAGCCACATCATCTCTCACCTTGACTATTAGAATAGCTTCCAAAAACCCATCT
TTGTGCTTCCATCCCTGCCCAGTCTGCAGTCCATTTTTCACTCTGCAGTCAAGGGATCCTTCTAAA
ATCTGAATTTCAGCCTGGCACATGCCTGTAATCCCAGTGCTTTGGGAGCTGGAGGTGGGAGCAT
GGCTTGAGGCCAGGAGTTTAAGGCCAGCCCGGACAACATAGTGAGACCACATCTCTAAAAAAA
ATTAAAAATAAATAAATAAAAGCTGAATTAGGGCCCCGGCACAGTGGCTCATGCCTGTAATCCC
AGCACTTTGGGAGGCTGAGGCGGGCAGATCACGAGGTCAGGAGATCGAAATCATCCTGGCTAA
CATGGTGAAACCCCATCTCTACTAAAAATACAAAAAATTAGCCAGGCGTGGTGGCACATGCCTG
TAATCCCAGCTACTTGGGAGGCTGCGGCAGGAGAATCACTTGAACTGGGGAGGCAGAGGTTGC
AGTGAGCCGAGATGGTGCTACTGCACTCCAGCCTGTGCGACAGAGCGAGACTCCATCTCAAAA
AAAAAAAAAAAAAAGCTGAACTAGGTCATGTATTATGCCACTTTTCTACTCAAAACCCTGCAATTA
CTTTACATCACAATAACAATTTCACATCACACTAACAATTTCAAATCTCTTATCACAGCTGATCAC
ATCTACCAAGGTCCATTCCCCCCAAGGGGCCTCCCCCATTGACCTCAGCTTCCACCACTCTCCTC
TTACTTATTACCTGTGAATCCACCTGCATTGGCAAGCTAGCTGGTCCTCATGTACCCTAGACAGT
ACGCTGTCTTAGCACACCTGTTCTGCTCTTGGCCTGACGCTCTTCCCCCAGATATTTGCATGCAT
CCTTTTCTTGCTTCATTCAAGAGTTTGCTCTAAACTCAACTCCTCAGAGTGCATTTTAACACCCTC
CTAACTACGAAACTATCCCCCATCCTTCTGTTTGCCTGTCCTCCACATAAGGGGCTTTCCTCATAT
GTATGGCCATTCTTGATGGCATGGTCAAGATAAAGAGTGGGAGGAAAGAAAGCCACGTGGAAA
TTCTAAACCCATGCTGGTGTGGTGGGGCTTGTCAACTCTGAGCTTCACTTTAAGATATTTTGTGTG
GGCCACTGGTCGGGCTGCCTTGACGTCAGTATTTTCAGATCTGTTCGTTTGGTCTGGGCACATTC
CCCAGAGAGGCATCTTGCCTGTTAGGTTAAGGTCTGACTGCTGGTTTTCCGAGAGCAAAGAAAA
GGCGTGAGGCTGGGAGGAGGTGTCTTGGGAGTCAGCATTCTGTATGCCGGCAGTCTACTTAATT
CCCCGGATTTTGATAAGATCCAAAGGCTCTCAACTGTACTCCCAGTCCAGAGAACCTGTTTTTAC
AGTCTCTAGGGGAAAAAAACCTTCAGAAATCTAGGAGAGAGGATGAGGGAGCAGCAGTGAGG
AGCAGCTGCCTCATGTATGGAGTAAGGAGTCGGGAGATGTAGGTGCTTCCCAAACAACTTATGT
GATTCTCCTTAATTTAGCAGGTTTCTATGCCCCATGAGCCAGAGATTTAGAGATTTAGTGCTGAC
AGTTCTCAAGCATTTTGAGGATTCTGAGTGTATACTGGGTTGAACTCTCAGGAGAGCCCCGGCT
GAGGTATATGAGTATGTGGATAAGAATGCTCTTCTAGACAAAGGCAGTTGCTGAGCCCACGATG
GCTTTGAATGAATTAGCAAAGGCACCCCTTCTTCAGGGCAGACATAGCCCCATGTCAGGGTGCA
TGCTTGGCTGGAGAAATGTGTGGCCAGGATTTCAGCTCCAACTCAGCCTCCAGAGTGGGAGCCT
TTTTACCATGACCAAGCCACACACAGCTGCGGGCTCTCAGCTTCCTCCATGACTGCCCTGTTTAC
ACCTTGTCTGTTTCCTTGCATCCATTTTCAAGAATGTGTTGCTGTTGTCTTCTCCCATGTTCTCAAT
GTTCTTGTGGATTTATGTGCTCTTTGTAAATGCCTTTTTTTATGGGATTTTAGCAGGGTTTTGGGAA
ATAATAAAATTAAATGAATGAATTTAATCTATTAACTCAGAACGGTGCTCCCTCTTCTGAACTCCT
GCCTTTTCCCATGTTGTCCTATTTCCTGTTTGTTCATCATTATGTGGAAGACATTTGTGTTTGTTCC
ATACACAATTTGTATGAATTTTGTTTGTTCCATATACAATTTGACAGAATGGTCAAAAAATGGTCT
CATCTTGGTCCCTTACTTTAAGCCAAGATGAAGGAATAGGTGGAAATTTATGCTTCTAGCTATTA
AGAGGAATGCTCCAATGACCATCTTTGAAGCTGTCTATTTTAGTCCACTTTAGCCTTCACCTAATC

TTTTAATAAGAGAATTAAATATGTTTATACTTTTTTGGTGTAAATTGCACATCCAACTTAATGTTGT
TACCTTAGATTTGCTTTCCCTTTTTTTATTCCCTTCCCCCCTTTTTATTGTGTTATATATTCTGTATAC
TTTAATTTCCCTAAAGACTTTTTACTTAGAATGATTTTTGATATGTGCACTAGTTTTAAATTCTACTT
TTAGGTGTTAACGTTAACCTGCAATTTATTTATATGATAATTTTCTTTTTCTTTTTATTAATTAACTG
GAAATGCTTTTGATACATTTTATAGAAAGACTGTATATGAGGTTTATTTTGGAATTATTTAATATG
GAATAGTATTTCTTTTGCCTTTATGATAATATATCGTTTAATATTGTGAAGGTGAGATCTGAAGTC
TGATCTTTTTTTCTTTAGAATTAACTTAGGTGAAATTTTTCCTATGGTTGAGACAGAACAGGGAGC
CCTCTTAGCAACCTGTGACCAACCCCCAACCCCTGGCAAGCATGAAAATAAAGAAAAATCTCG
AGTTCTGTCAAAGGAAATTCCAGGCACCTAGGTAGGCCTGAGAAGTAAATAAGCAACTTGATAA
ATAAGAAGGTAATAGCAACCTAAAACAATAGCCAAGGAAGCTACAGTCAGGAGAATCATTCCC
TATAGAAACTAAAGATAACATCTTAACAGGTGTTTCATATCTTAAGATATACATACATTCTTCAGA
AACCCAGACCCCCACCAAACGAATCTGCTGGCACAGACCTCAAATGAGGGGGGAGCTGAGGA
CTGAACCTTGACTGCCATGTTTTGTTCTAAATTTCTTCCTGGGGGCCTAGAGGAGGTCACGTTCA
TGAGCCAGAGCTAGCATTCTTTTCTGATAACCCAAAATTTTTAGACAAAGCTTCTCCTCCTTAACC
AATCACAAATCAGAAAAGCTTTGAATCCACCTATGACTCATGGCCCCTGCTTTGAGATGTCCTAC
CTTTTAAAGTCAAACCAATGTAGAGCCTCCATTACTGATTTATGACTTTGCCTGCAGCTTCCACCT
TCCCACCTTTAAAAACCCTTACCTGTAAGCCATTGGAGAAGTCAGGGCTGAAGCAAGAGCTGCC
CAAGTCTCCATGTTTGGTGCCCTGCAAATAAACACCCTCGTTTCTCCTGCTGCAAATCTCAGTGT
GGTTGTTTGCCTTATTGCACCAGCCAGTGGACCCCAATTTGGTTTGATAACATGGTGAAGTAGAC
TTTGTACTCTATTTATATGTAAGATATGTGTGTGTGTGTGCGTGTGTGTGTCTGTGTGTATGCCTTG
CTGAATTCTTTAACTTTGAAAATCTAAATTTTACTATGATAGGTTGAAGTCTCTTTTCATCTATGTA
TGGAATGGAAATACTGATGACCAACAAAATAAAAAAGATGCACAGACACAGAAGTAATCAGGG
AAACTCAAAGTATCTGTAACAAGATACTTTATTTGAATTGTCAAGCATTTTTTATTGAAGTCTAGT
ATGTATACAGAGAAAAACACATATCATAATTGTATAGCTTGATGAATTTCCACAAACTGAACCCC
TGTAACCAGCAGCCAGACCAAGAGCAGAACTGTGCCAGCTCCCAGGAATCCTCCGCTGGGCTT
CTGTCCAGCTATTGCCTACCCCCAGAGCAACCGTGATCCTGACTTCTAAAAGCCTAGTTTAATTT
CGAGTTTGGCAAACAATTTAAAGTTTTGATGATATGCAAGTCATGACCAGATTGAGGGAAGAGT
ATATAAATACTCTTTATATTTATATATAAATTATATATAAATTTTGCAGGTGTTTGGAGAACAGCTA
GGCAATGCCCATATTCTTATTTTTTTTTTTTTTTAAGATGGAGTCTCACTGTGTTGCCCAGGCTGAA
GTGCAGTGGCATGGTTTTGGCTCACAGCAACCTCTGCCTCCCAGGTTCAAGCGATTCTCGTGCC
TCAGCCTCCTGAGTGGCAGGGATTACAGGCATGTGCTACCACGCCCGGCTAATTTTTGTATTTTT
AGTAGAGACCAGGTTTCGCCATGTTAGCCAGGCTGGTCTCAAACTCTTGACCTCAGGTGATCCA
CCCACCTCGGCCTCCCAAAGTGCTCGGATTACAGGCGTGAGCCACCGTGCCCACTCATATTCTT
AATTTCTCAGTTTTCCTTTCAGCTCATTCATTGCTTACTCTCCTTGTCCTCTTTAATACAGGTTTAAT
TGTTTCCTCTTACCTAATTATGATTCATCCAACCAACATTTGCCATTGTACTTCCTCAGAAAACAG
CCTTGCTCTTAGAGATGACTAAGAATGTTTTGGCCTATGGTGTCTCTCAGACTCCTCAATTCCATG
CTGCCTGCCAAATGTACCTTGGCTTAAAATATCTCTTTCAATGCCACCTTGTCCTTCCAGTCATCC
TCAATTCCTTTTATTATTTCCCTAATGAAAATTGAAATTTCTGCCAATTGCAAATATAATTAAAGCT
TCTCATAAATGAAATAAAATGGAAATAATGTCTATGAAGCATCTACTACAGCAACTAACACGTA
GTATGTTGTTTGAAATGTTAGTTTCCTTCCTTTTCTTCCTTGTCCTTTTTTAGAAAGCCTCATTTACC
CTCAAAACAGCTGTATACAGCATGAAAACCACTCTGAAGCACTTGCACCGTGTATAAAGAATCC
TTTTCTCTTTCAGAGAAATGTTCATTCATGCGTCAAGTTTTTTAAAATAATATTTTTGCTTATTCTTT
TAACAGTGATAAATGCCATCTGTAAACTTGGAGTTTCCTACAGACCAATTATATTGGGGTGTTTT
CCAATTAGTAAAATCCTCATGGTGTTTTATACTCTGGGACAAGAATAGTATAGATGAGAAATGAA

CCTGTAGGGACTGGAAGCAGGCAACATGAAGCATTTAGACACAGGACACAGCGTACAGCACTC
ACGTGCATGGAGCACACAATTATATACATAGAAGTCAGAACAGGCTTCCCGTGCAGAGTCTCCT
GGGCTACACCCAGCATTTAAATGTAGTGGCATTTAGCCTCATTCTTCCCATTTCCGTATTCCTCTG
ATATGGCTTCCCTGCGGGATGCTGTGACTTAATTTGTCTGACAACCAAAATTTTGCCACGGTGCC
CCCAAAAATATTTACTCTGTTAGTGGCCTAATTTTCTGGGATCTAGAGAAGTAGGCAAGAAGACT
AAAAAACTGACATAATTCTTCAAAACTGAGCTGCAAAAGGATTTAAAGAAATAGTTGTGGATTC
CAGGAAGATCATTAGTAGCTTAGACACCAAAATAAAATACCATCTTGCACTCAGAGCAGGCTCT
TTACTCTCTGTAGTTTAAGGAGACATGCTGAATATCCATGCACAGACATACACGTTGAGGTGTCT
TCTTATTTTCTTATTGAAATGGATTTATACTAAACTCCTTCATCTACCTCTCGCTGTAGTATTCCAC
ATGACTTTTAATTTGTGTATTTGTTTATTTATTTATTACCTAAAGCTTGGAAAATAATCTACAAATA
AATATCCCTTCCGAGATCAATTCCCTAACTAGGGTTTCATTAGTACCTCTGCCCCCCATTTTCTTC
GCAGGAATGCAAGGCTTTATATGATAAAATTAGTAACTGACAAGCTCTTGGTCTTCCAGTTTCAA
CAGCCTTTATTTGTTTTACCATCATCACTAATGTCAATTGAACGCCTTCTTGGCTCTTGATCTTCTG
AGCCTCTCAGACACTTTGTGACTTTAGAGATAGTAGTAGTACTCTATGACTATTTAGAAGGTAAG
GGAAATTAGAAGCTCAGCTTGGAAACAACTATTTTTGTTTTTCCAACTTCTCTCCTGTGTTTTCCTT
TCTTTCCAATTACCTAACCACAGTCCTGATCTGCTGCTCTATTCCAGGGCATCTGTTTGTCTATCA
CTACACTTGGCTGCTCAGGAACCTTGTTCATTTCCTCGTTTTCTTTGATCACTCTATCCTGATATAG
TACATACACACTTGTGATCTCCAAACTTCTATTTTCAGCTGTTTCTGGGATATTACCATTCAAATG
TTCCCACTCTCTCACTTCAAAATAAGCACACCTGGAACTCAACTGCTTATCTCCTCCTGTCTCCTA
AAACCAGCTTCTTGTTCCCATTTCCAGCATTATTCTGGTTAAATTTCCCAAAAGACTGGTGATCTG
ACCTGGAATCAGAAAAGGACTGAGTATTAATTTTCTTTTTCATTGGTTAGAACAACAGATTATTA
GTTTCTTGAAGGTGGGGATTACTTTTTATGATGTGTATCCCTACAGTGCCTTGTAAAGTATCTCAG
TGAATATCTGTTAAGCGGGTGATACATTAAAATGCTGTGCTAAGTGAGGCAAAATATAACGCTC
CCCGATTGCAATCATCTGTTAGCTAACAATCCATAAGATAATGTGAATATATGAAGACCTACCCA
TGCCAGGTGCGGTGCCTCATACCTGTAATACCAGCACTTTGGGAGGCTGAGGCGGGCAGATCA
TGAGATCAGGAGATTGAGACCATCCTGGCCAAGATGGTGAAATCTCATCTCTACTAAAAGTACA
AAAATTAGCTTGGCATGGTGGCGCACCCCTGTAATCCCAGCTACGCAGGAGGCTAACACAGGA
TAATCGATTGAACCAGGGAGTCAGAGGTTACAGTGAGCCAAGATAGAGCCACTGCACTCCAGC
CTGGTGACAGAGTGAGACTCTGTCTCAAAAAAAAAAAAAAAAAGATGTACCCATTTCTGCTACTA
CCAAAATAATTCCATGTACCCGCCTTACTAATGGTGGGTGGGTAGCAGAATTCCTGGTTTCTAAT
TACTAGCTTTGGTCCTATTTAACCAAACTTGTTGGGTGGACAGAGATGGAAATAAATTAGGCTTT
ACTAACCTAATTGTAACTGGCTCTTTGGGAGTAAAAAATTAAAAAATTAACCCTAAGACTATCTT
CAATCATGGGATGAACATAATAGTACCGGAAGCTACCAAGAACAAAATAAAACAAAGCAAAAC
AAAAACCAACAGCCCTCATTTCAAAAGTGTTCAAACTTCCTTATGAATATAATGATTCTGTGTGC
ATAATTACTTTGTGCAATACACCATTAATGGGACTATGTATCACCTAAATTCTTCAAAGGCATTTT
TTTCTGAGATGGAATAGATCTTCAGAGACTGTACCCTATAATGATACATGAACTTTTTACTTTGAT
GCAAGAAAAACATCAGCAGAGGCTTGCTGAGGGGTGCCTGGGAATTAACACTCTCTCCTTTAAG
AATTACCCTGGAGTCCTAAATAATTAGTCAACTATGCATGTTTTTCTCTAGCCTTACAAACTAACT
ATATTGCATATAAGATTAGAAGGTAGGCGGGCAGGATTAAGGGCTTTGCTATTAGATGACAATG
GCTTGAATACCTCCGGTGCCTTTGTTAGAAAGATACCATGGTTCCCTTGTGTGAAGTCCTTAATG
GAGGTGATCAAATGTGAACTGCTTGGGAAAATGACTGCCTTTTCAAATGATTGTTGTAAAATGTA
TGGCGATATTCCCGTTTACTGTATCTTCTTCTTCTAAGCCCAGAATTAACAATTAAATGTGTT
ID|56399|gene_id|ENSG00000232006|transcript_id|ENST00000609382 Homo sapiens lncRNA

GGGCAATGGCGGGCGCCCCTCCCCCAGCCTCGCTGCCGCCTTGCAGTTTGATCTCAGACT
GCTGTGCTAGCAATCAGCAAGTCTCCGTGGGCGTAGGACCCTCCGAGCCAGGTGCAGGATTTA
ATCTCGTGGTGCGCCGTTTTTTTAAGCCGGTCGGAAAAGCGCAATATTCGGGTGGGAGTGACCC
GATTTTCCAGGTGCGTCCGTCACCCCTTTCTTTGACTTGGAAAGGGAACTCCCTGACCCCTTGTG
CTTCCCAAGTGAGGCAATGCCTTGCCCTGCTTCGGCTCGCGCACGGTGCGGGCACCCACTGAC
CTGCGCCCACTGTCTGGCACTCCCTAGTGAGGTGAACCCGGTACCTCAGATAGAAATGCAGAA
ATCACCCGTCTTCTGCGTCGCTCACGCTGGGAGCTGTAGACCGGAGCTGTTCCTATTCGGCCAT
CTTGGCTCCTCCGGGTAAATCATTTTATTGTTATAAAAATATATAAGGTAGTTGTCATTATTATTCC
AATTTTTAGATTAGTAAACAGAGGCACAAAAAAAGTAAAATAACTGACTCAAAGGTGATATGGT
GAATAAGTAGTGGAGTTGGCACTTGAACCCAGGCAGCCTTCTTCTGGAGTCCTTGTTCTTAACA
GTGTTCCTTGATTTTTGTCACCTGGTACCTCTGACCCAGGCACTTGTGGCACCTGAGGCCAATGT
GCTCAAAAAGGGTTGAGAATTGCTTGCAGTGATCACATGTTCATAAACCATATTCTTGGGCAAA
CGTGTGTATACTCTCAGTCAGAAATGGATTTTCACTGTTTCCAGGAGTCTGTGTTGGAGAAAGGG
AAGGGCGGGTTACTCTGCTGTATGTTCAGTACATCTGTAAATGGCCCATCTGCAGATGAGCTCA
AGGTGAATGGGGGTTTCAGAGGACCATTTGCATCAAAGAATGTAAGACAGAAGTGAAACTCTA
CTGAGAGCTCAACTCTTGAAATCTCCCTGTAGAACAGACTAAGGAGTGGTAAGGAATGCCCACA
CACTGAGTCCCAAAAGAACCCCACGTGTCACTAAAATGCTGGCTACTCCAATGGAGCCAATAAC
CTTCCAGGGTTCTTCAGGGGCACAGCAGTCTGCATAGGAGCCTGAAGGTCAACATGGGTGGGC
TGCCAAGTTTCCTTCCCACAGCCAGCTATAAGGTAGGCGGATTAACCATGAGACGAGGTCTATG
ACTTCATGAGGTGCTAGAAGGGGTTGGATGTCTTGTAATTCTTATCTGAAATGTTTTGGGAGGGG
CAGAGGGAGGAGACAGATGAGATGTCGGGAAAGTGTAAGGATAACACCCAGTTGTTAAGGTTC
TAGTCTTTTAGGAACTCTGGGACCATTCATATACAGACCTTCTTGAGAAACAACTTTTTCCTAGTC
TCACATGTCCCCTCCCTTTAAATATGGAGAGAAATAGCTGGGATAGCTGGTCTGGCCTAAACAG
ACTCCTATCTACAAGAATGTGGAGATGTGTGCACACACACATGCACACGCACACACTTACTGCT
TTCCTTACTTTTAGCCAGGGAGGAGTCACTGAGGGCACTCCATTGACACTCCTTCCTTCTATTAA
ACACCTAAGTCTTAGGCCAGGTGTGGTGGCTCACACCTGTAAGCCCAGCACTTTGGGAGGCTG
AGGCGGGTGGATAACCTGAGGTCAGGAGTTTGAGACCAGCCTGGCCAACAATGGTGAAACCTC
ATCTCTACTAAAAATACAAAAATTAGCCGGGCGTGGTGGCATGTGCCTATAATCCCAGCTATTTG
GGTGGCTGAGGCAGGAGAATTGCTTGAACCCAAGAGGTGGAGGTTGCAGGGAGCCAAGATCG
AGCCACTGCACTCCAGCCTGGGCAACAAGAGCGAGACTCTGTCTCAAAACAAAACACAAAAAC
AAAAAAGTAAGTCTTCGGGTTGAGATTTTTATATAAGTAGGACAGGTGAGGTAGCATGGCTCAT
GCCTGTTACCACAGAGCTTTGGGAAGTCGAAGTGGGAGGATCACTTGAGGCAGGAATTCAGGA
CCATCCTGCACAACATAATGAGACCCCCATCTGTACCAAAAAAAAAAAAATTTATCTGGATGAAG
TTTGTGTGTGCTTGTAGTTCCAGCTACTCGGGAGGCCAAGGTGGTAGGATTGCTTGAGCTCAGT
AGGTCAAGGCTGCAGTGAGCCAAGATTGCACCATGCACTCCAGCCTGGGTGACAGAGTGAGGC
CCTGTGCCAAGACAACAACAAAAAAGACAAATAGAAAGCAGGGAAAAGTGGTATTGCAGAAG
CCAAGGAAATATAGAACTTTATAATTTATAAGAGGCTGTCCGAAATTCAATGTAGCCAAGTTTCT
GCTGGATGGGACCTGAAAACGAGGTGCTGATTTTGGGGGAGAACATTGCTGGTAACTTCTGACC
CAGGGATACTAACAGCAAAACCAAATGGTAATGGATTGAAGAGCAAAACCAATGGATCTACTG
AATGTAGATTTTGTTTAGTTCGGTCAAAACCTTGAGTAAGATTTTAATAGGAAACCAATAAGAAA
GAATGGAATTCCGGCACATGAACAAAGACAGGCATAAAGGCATAGGAAGGGCTTTTTCATCTTT
TCATTTTTTAAAGTGAAGTTTTATGTCATGTACTCAATGTGAATGTAATATTCTTTCTCTTTTGAAA
ATAATTGTTTTTTAAACTTTGTCTTTAATTGTGGAATGAAGGGTAATAGTACAGGAAAAGAATTAG
AGCCCCTGAAGTTAATCCTTCATTGCCAGTCACTTATTTGGCTAACCCTGACCTTAGGGGGCACA

GTTATGAATTATTTTAGCATGGCATAGAGAAACAACATTTTGCAGTAAAGAAATGCTTGAATCAT
TAACATTTAATATAAATAAGTGCACATTCTACATAGTCCTTTTTGCTTTCGAGAGCATTTGGGGTT
AGGCTGCACTCGTGAGCTATAGTCCATCTTTGTCCAAATAGCAATAAAGAGGATAAAAAAGAAA
AATGGGATGAAGCATTAACTCCCTTCCCCTGGCCACCTGTTCTAAACCCTTCTGGAAGCAGCAA
AGGACAGAAGGAGTTATCAAAGTATCAGGCTTTGTAAATTGCTAGCAAAGACAGTTCACAACAT
CATCTCACCAGAAGAGCTCCAGCATCTTCCCATCCTAGGTTTTCTGCAGTGAAGCAGCAACCAC
CAGGGGAAGCATCTGGAGCATGGCTGTGGGGTACACAGCAGGTCTGCGTTTTTCAAGGCTTGCT
CCACTGTTTTTATTTATTTATTTATTTTAAACAGGGTCTCACTCTGTCACCCAGGCTGGAGTGCAG
TGGCATGTTCTGGGCTCACTGCAACCTCCACCTCCCAGGATCAAGTGATTCTCATGCCTCAGCCT
CCTGAGTAGCTGGGAACACAGGCTTGCACCACCATGCTCAGCTAATGTTTGTATTTTTATTAGGT
TGGTGCAAAAGCAATTGCATTTTTTTGCCATTACTTTAATGGCAAAAAAAACGCAATTGCTTTTGC
ACCAACCTAATAGTGGAGACAGGGTTTTGCCATGCTGGCCAGGCTGGTCTTGAACTCCTGGCCT
CAAGCGATCTGCCCACCCCGGCCTCCCAAAGTGCTGGGATTACAGGCATGAGCCACCGCGCCC
AGCCCATTGTTTTTATTTATAACCCTAAGACTATCCCTCAAACCCTTCCCAAGCTCTGCCACTATC
CTATTTAGAATCTCTTTATTTCTGTGTGGCTTTCTTAATGAGGCTAGAATCTGTAACGAAGACATT
TGTGAGGGAAACAGGCAGAGCTTAAATATTATGGAGACTTTCCATCATTTGAATGCATTCTAGCC
ATATCTTTAATTACTTGTAAATATAATTGGGAGTTATTTTTTTCCCCTGATTCTGTCAGTCAGCCAT
ATAGCTCCTATTTATCTTGTTAACCAAGATGCTTAAAACTTTTCGGGTATGGCTGGGCGCGGTGG
CTCATGCCTGTAATCCCAGCACTTTGGGAGGCCAAGGTGGGTGGATCACCTGAGTTCAGGAGTT
CGTGACCAGCCTGGCAAACATGGTGAAACCCTGTCTCTACTGAAAATACAAAAATTAGCCGGGT
GTGGTGGCATGCGCCTGTAGTCCCAGCTACTTGGGAGGCTGAGGCAGGAGAATCACTTGAACC
CGGGAGGTGGAGGTTGCAGTGAGCCGAGATTGCGCCATTGCACTCCAGCCTGGTGACAGAGCA
AGACTCTATCTCAAACAACAATAACAAAAAGAAAACCAACCAACCAAAAACAAAAAAAGCGTT
TTGAGTATGTATGGCATAGACCTTCTTCCTGTTTTTAAAGCTTCTTAATACATACAGGTGTAGCTT
TCATGAGTATATATACATTTCGCTACAAATGACTATTGGTTTTATTCTAACACAATAGATTGCCTT
TTAAAAGACGCCTACCTTTTCATTTCCTTACAGTCTTTCCTCATTTTTCAGACCCAGAATCTCCAC
AAAATGCCTTACTCTACTATTCAGTGGGAAAGTAGTAATGTTTAACCCTTATGAAACATCTCTCG
GTCAGGTTTCAGTCCCAGTGATACCAGAACCAGGGAGCGAAGGTGGTTACAACTGAGTGATGT
AATTCCCTAGAGACATGGGTTTCCTTTGTCTCAAGCCTGGCAGCCTAATTAATATGCTGAAAACA
CCTTAACTCTAACTGGGCTTGAGACATTTGCAGAAGATGACTTTCAGCTGCCTGCAGGAGCGGC
CAAAGGAGCCTGGGCCTTGCTTTTTACCAATCTTAACATCATCTCTCAGTCTGCAGACCAGCTTT
CCATTTTACCACATTGTTGACATCCTCCTTCAAGAGCTTCACTTCACAAAAAAATGTGGTGCTCTC
TTTTGTCTTCTTAGTCTCATGAAGCATTGGGATAAAAGCCTGAAAGCTTTGCATCTAAAGAGTTA
AAATAATTTGCATGGCAGAATCTCAAATCTCGCTGCTTCTCCCTGCCAAAGAGGGAGAAAAAAA
GAAAGCAATAAGGAGGAAGGGAAAGCGGGAACATCACTATTAGAAAATCAATCAGTTCCTGGA
AAATAATCCTCTTAAATCCTTTCCTAAAGGTGGGGAGAGCCCTCTTATCAGGGTTTTCAGTGAAA
TCTATAGCAAGGTCTGGCAACTCTGTCATGGTTGATAGTCTGCATTTAGTCCTATTTATCTGGATT
TCAGGATCATCCTATGGAAATGATCCACGAGTACAATTATAGGAGTACTTAGTAATTTTATCCTTT
TACTCATTAATATTAATTAGTGCTTGGAGGGGAAAGAGGACAGGGAACAGCTTCTCATATCCAA
ACTAATTAACTTCGTCTAGCTTGTATGAAAGTGAGAAAGCACTTTATTAAAAACCATAAGGTAAG
CTCTCAAGGGACTTTTATTTTCTGCTTAGGCTAAACTATGATAACATTTGGCTCTTAGTGAGCTCC
CTAAAATTGCCAGAAAGAATAAAAAAAATTGTGGCCAGGAGATTAAAGACTCCTGCATGGTTTC
CAAGACACCCACTGTATTGGTGGTAACGTACGTTTGATGTTTTTATGCTATTCTGGAAAATGAAG
GGTAGTTTCCAAGCCACCTATTTAGGACATAATAATGCTAGAAATGAAAAGGAAGATTAAGGCA

ATGGTAGTCATTGTTACTTCTTTTTAGTAATATATGTTGATACGTCATCTATTGACACTGAAGCGG
TGAGTTAAAATCCTTTTTAATCTAATGCCTAAAAAATGTATTCGTCTCCCAGTTAGAATTGGGAAC
ATTTTAAATTATTTATTTATTTTTTGAGACTGAGTCTCACTCTGTCACCCAGGCTGGAGTGCAGTG
GTACAATCTCAGCTCACTGCAACTTCTGCCTCCCAGGTTCAAGCGATTCTCCTGCCTCAGCCTCC
CTAGTAGCTGGGATTACAGTTGCGGGCTACCATGCCCGGCTAATTTTGTATTTTTAGTAGAGACG
GAGTTTCACCATGTTGGCCAGGCTGGTCTCAAACTCCTGACCTCAGGTGATCCACCCACCTCGT
CCTCCCAAAGTGCTGAGATTACAGGCATGAGCCACCACACCTGGCTGGAACATTTTTTTAAACC
AAAGATTTCATTAGACATATATTCCCCCTGCTGAATCCCACATTCTTTGACTTTTTTCCAGTTTTTC
TTTTTTTTTTTTTTTTGAGACAGGGCCTCATCTGTCACCCAGGCTGAAGTGCAGTGGTGCAATCAT
GGGCTCACTGCAGCCTCGACTTCCCAGGCTCCAGTGATTCTCCCACCTCAGCCTCCCAAGTATC
TGGGACCACAGGCATGCACCACCATTAGCCTGGCTAATTTTTTTTTTTTTTTTTTTAAGATGGAGT
TTCCCTCTTGTTGCCTAGGCTGGAATGCAATGGTGCGATCTTGGCTCACTGCAACCTCTGCCTCC
TGAGTTCAAGTGATGCTCCTGCCTCAGCCTCCTGAGTAGCTGGGATTACAGGCATGTGCCATCA
TGCCTGGCTAATTTTTTGTATTTTTAGTAGAGATGGGGTTTCACCATGTTGGCCAGGCTGGTTGCA
AACTCCTGACCTCAGGTGGTCCACCCACCTTGGCCTCCCAAAGTGCTGGGATTACAGGTGTGAG
CCACCACACCTGGCCATGCCTGGCTAATTTTTGCATTTTGGGTAGAGATGGGGTTTCACCATGTA
TCCCAGGCTGGTCTCGAACTCCTGAGCTCAAGTGATACACCTGTCTTGGCCTTCCAAAGTGCTG
GCATTACAGGCATGAACCACTGTGTCTGCCCAATTTTTAATTTTTGTGCGTACATAGTAGGTGTAT
ATATTTATGGGGTATGTGTTAGGCCATTCTTGCATTGCTATAAAGGAATACCTGAGACTGGCTAA
TTTATAAAGAAAAAATATTCAATTGGCTCATGCTTCTGCAGGCTAACAGGAAGCATAGCACCAA
CATCTGCTTCTGGTAGGGCCTTAGGTAGCTTACAATCATGGTGGAAGGCCAAGGGGAAACAAG
TATTTCACATGGCAGGAGTGGGAACGAGAGAGAGTTGTGGAGATAGGACACACTTTAAACAAC
CAAATCTCCCGAGAACTCACTCACTCAGTACTTTGAGGACAGCACCAAGCCATCAGGGATCCAC
CCTCATGACCCAGACACATCCCACCAGGGACCACCTCCAACACTGGGGATTATATTCCAACATG
AGATTTGGGCAGGGACATATATCCAAACTATATCGGGGTACATGAGATATTTTGATACAGGTGT
ACAATGCATAATAATCACATCAGGGTAAATGGGGTATTTATCTACCTCAAGCATTTATCCTTTCTT
TGTATTACAAACAATGCAATTATACTCTTTCAGTTATTTTTAAATGTATAATAAATTATTGTAGACT
ATAGTCACTCTGTTGTACTATCAAATACTATATCTTATTCATCTATTTATATTTTTGTGCCCATTAA
CCATCCTCCCTTCACCCCACTACACTCTCCAGCCTTTGATAACCATCATTCTACTCTCTATCTCCA
TGAGTTCAATTGTTTTAATTTTTAGCTCCTACAAATGAGTAAGGACATATGAAGTTTGTCTTTCTGT
ACCTGGCTTATTTTACTTAACATAATGACCTTCAGTTCCATCCACGTTATTGCAAATGACAAGTTC
TCATTGTTTTCTATGGCTGAATAGTGCTCCATTGTGTGTATGTAACACATTTTCGTTATCCACTCAT
CTGTTGATGGACACTTAGGTTGCTTCCAAATCTTGGATATTGTGAATAGTGCTGCAATAAACATG
AAAGTGCAAGTATCTCTTTGATATACTGATCTCCTTTCTTTTGGGTATATACCTAGCAATGGGATT
GCTGGATAGCTGGTAGCTCTATTTTTAGGTTTTTGAGGAACCTCCAAACTGGTCTCCTAGGGTTG
TACTAATTTACATTCCCACCAACAGTGTATGAGGGTTCCCTTTTCTCCATATCCTCAGCAGTGTTT
GTTATTGCCTGTCTTTTGGATAAAGACCATTTTAACTGGGATGTGATAATATCTCATTGTAGTTTTT
ATTTGTATTTCTCTGATGATCAGTGATGTTATGCACCTTTTCATATGTCTGTTTGCTATTTGTATGT
CTTCTTTTGAGAAATGTCTATTCAGATCTTTTGCCCATTTTTAAATCAGATTATTAGATTTTTTCCTA
TGGAGTTGTTTGAGCTCCTTATATATTCTGTTCATTAATCCCTTGTCAGATGAATAGTTTGCAAAT
ATTTTCTTCCATTCTGTGGGTTGTCTCTTCACTTTGCTCATTGTTTACTTTGCTGTGCAGAAGGTGT
TTAATTTGATGTAATCCTATTTATCCATTTTTCCTTTGGTTTCCCGTGCTTATGGGGTATTATTCAA
GAAATCTTTGCCCTATCCAATGTCCTGGAGAGTTTCCCTGATGTTTTCATAGTTTGAATTTAGTAG
TTTCATAGTTTGTAGTCCTAGATTTAAGTCTTTAATCCATTTTGATTTGATCTTTGTATATGGTGAG

AGACAGACGTCTAGTTTCATTCTCTTGCCTGTGGATGTCTAATTTTCCCAGCACCATTTATTGAAG
AAAGAGTCCTTTCCCTATTGTATGCCCTTGGCGCCTTTGTTGAAAATGAATTCACTGTAGATGTAT
GGATTTATTTTGGTGTTCTCTTTTCTGTTCTGTTGGTCTATGTGTCTGTTTTTATGCCAGTACCAGG
CTGTTTTGCTTATGATAGCTCTGTAGTATAATTTGAAGTCAGGCAATGTGATTCCTCCCGTTTTTTT
TTTTTTTTTTTTTCCTCAATATAGCTTTGATTATTCTGGGTCTTTTGTGTCTCCATATAAATTTTAGG
ATTGTTTTTTATATTTCTGTGAAGAATGTCATTTTTTTGATAGGGGTTGCATTGAATCTATAGATTG
CTTTGGACATTTTAACAATCTTTCAAACCATGAAAATGGAATACCTTTCAAATTTTTTGTGTGTCC
TTTTTAATTTCTTTCATCAATGTTTTATAGTTTTCATTGTAGAGCTCTTTCACTTCTTTGGTTAATTTC
TAGGTATTTAATTTTATTTGTAGCAATTGTAAATGGAATTACTTTTTAAGTTTCTTTTTCAGATTGTT
TGCTGTTGACATACAGAAATGCTACAGATTTTTCTTTGTTAATTGTGTATCCTGCAAATTTACTTAA
TTTATCAGTTCTAATAGTTTTTTGGTGGATCCTTTAGGTTTTTCCAAATATAAGATCATATCATCAG
CAAACAAGGATAATTTGACTTTTTCCTTTCCAAGTGAGATAAATGCCCTTTATCTCTTTCTCCTGT
CTGATTGCTCTAGCTAGGACTTTCAGTACCATGTTAAATAACAGTGATGAAAGTGGGCATCCTTG
TAATGGTCCAGACCTTAGAGGAAAGACTTGTTTTTCCCCACTCAGTATGATACTAACTGTGGGTC
TGTTGCAAATGGCTTTTTTTATGTTGAGGTATACTCCTTCTATAACCAGTTTTTTGAGGGCTTTTAT
CATGAAGCGTTACTGAATTTTAATCAAACACTTCTTTGCGTCAATTGAAATGATCATATGTTTTTT
GCCATTCATTCTGTTGATATGATGTGATCATTGATTGATCTGTATATGTTGAACCATCCTTGCATC
CCACAAATAAATCCCAGTTGCCCATGATGAATGATCTTTCTAATGTGTTGTTAAATTTGGTTTGCT
AGTATTTTGTTGAGGATTTTTGCATTAGCCCTCATCAGGGGTATTGGCCTGTAGTATTTTGTTTTTT
GTTTTTGTTTTTTTGATGTGTCTTTAGTTTTGATATCAGGGTAAAACTGGCCTCATAGAATGAGTTT
GGAAGTATTCCCTCCTCCTCTATTTTTCAGAACAGTTTGAGTAGGATTGGTATTAGTTCTTCCTTA
AATGTTTGGTAAAATTCAGCAGTGAAGCTATCAGGTCCTAGGCTTTTCTTTGCTGGGAATCTTTAT
TGTGGCTTCCATCTCATTTCTTGTTATTGGTCTGTTCAGGTTTTGGATTTCTTCATGGTTTAATCTTG
GTAGGTTGCATATATCTAGGAACTTATCCATTTCTTCTAGTTTTCCAATTTATTAGCATATAGTTGC
TTGTAGTAACCTCTAATGATCCTTTGAATTTCTGCAGCATTGGTTGTAATGTCTCCTTTTTCATATC
TGATTTTATTTGGGTGTTCTCTATTTTTTTCCTTAGTCTGGCTAAATATTTGTCAATTGTGTTTATCT
TTTCAAAAACCAACTTTTCACTTTATTGATCTTTTGAATTGTTTTCTTTGTTTCAATTTCATGTATTTC
TGCTCTAATCATTATTATTTCTTTTCTTTTATTAATTTTGGCCCATTTGGTCTATAGTGCAGATTAAG
TCCAAAGTTTCTTTGTTGATTTTCTGTCTGGATGATCTGTCCAATGCTGAAAGTAGGGTGTTGAAG
TCTTTAGCTATTATTGTATTGGGGTCTAGAGCTCTCTTCAGCTCTAATAATATTTGCTTAATATGTC
TGGGTCCTCCAGTGTTGAGTGCATATATACTTATAATTGTTATATTCTCTTCCTGAATTGACCTCTT
TACTATTATAAAGAGACCTCCTTGTCTCTTTTTATGGTTTTTGTCTTGAGATCTATTTTGTCTGATGT
AAGTATAACTACTACTGTTGTTTTTTGGTTTCTATTTGCATGGAATATGTTTTTCCATCCCTTTATTT
TCAGTCTATGTGTGTCTTTATAGGTGAAGTATGTTTCTTGTAGGCAACAGATCATTGGGTTTTTTTT
TTTAATCCATTCAGAAACTGTGTCTTTTGAATGGAGAGTTTAGTCCATTTACATACAATGTTATTA
TTAATAAGTAAGAATTTACTCCTGCCATTGTGTTGTTTTCTAGTTGTTTTGTGGTCTTCTCTTACTTC
TTTCCTCCCTTCCTGTCTTCCTTTTAATGAAAGTAATTTTCTCTGGTGATGTATTTTAATTTCTTTTTT
TTTAGTTTTTGTGTATTCATTGTATGTTTTTTTAAATTTGAGGTTACTATGAGGCTTGCAAATAATAT
CTTATAACCCATTATTTTAAACTGATGACAATTTAACTGTGTATTTTCAAATAACCTATCTTCAAGC
TCACTAATTCTTTCTTCTGCTTGATCAGTTCTCTGATGCATTCTTCAGTATATCAATTGCATTTTTCA
ACCCCAGAATTTTTGCTTGATTCTTTTAAATTATTTCAATCTCTTTATTAAATTTATCAATAGGATTT
TGAATTCCTTCTCTGTGTTATCTTAAATTTCATTGAGTTTCCTTAAAACAGCTATTTCGAATTCTCT
GTCTAAAAGGTCACATATCACTGTCTCTCCAGGATTGGTTTCTGGTGCCTTATTTAGTTTATTTGG
TGAGATTTTGTTTTCCCAGATGGTTTTGATGCTTGTGGATATTTATCAGTGTCTGAGTACTGAAGA

GTTAGGTATTGTAGTCTTCACAGTCTGAGTTTGTTTGTACCTGTCCTTCTTGGAAAGGCTCTCCAG
GTATTTGAAGGTACTTGGGTGTTGTGATCTACGTTTTTGGTCACTGCAGTCATATCTGCATTAAGG
GGCACCCCAAGCCCAGTAATGCTGTGGGTCTTTTAGACTCATAGGAGTACTGCCTTAGTGGTCTT
GGATAAGATCCAGAGGAATTCTCTGGATGCCCAGGCAAAGACTCTCATTCTCTTCCCTTACTTTG
TCTCAAATAGATGGGGTCTCTCTTTCTGTGCTGAGCTGCCTGGAGCTAGGGGAATGGTGACACA
AGCACCCCTGTGGCCACCACTGCTGGAACTGCATTGGTTTGGACCTGAAACCAGCATAGCACTG
GGTCTCACACAAGGCCCACTGTAACCACTACCTCACTCCTGCCTATGTTTGCTCAAGGCCCTAG
GGCTCTACAGTCAGCTTCAGGTAAAGCCAGCCAGGCTTGCATCCTTTCCTTTGGGGTGGTGAGT
TCCTCCTAGTCCTGGGCAGGTTTAGAGATGCCATCTGGGAACAAGGGCCTGGAGTCGGAAACC
TTAGGAATCTACCTGATGCTTTATTTTACTGTGGCTGAGCTGGCACCAAAACCACAAGACAAAGT
CCTTCCCACTCTTCCCTTCCCTTGCGACAAGCAGAGGAGTATCTTCCCATGGCCACCACCACCA
CACACCCATGAGGAGTCCTGCTAGGCTACTACCAGTGTTCACTCAAGGCCCAAGGGCTCTTCAG
TCAGCTTGTGGTGAATGCTTCCAGGCCTGGGACTCACCTTTCAGAATAGTGGACTCCCTTTTGGC
CCAGGGCAGTCCAAAAATGCCACCCAAGAGCCAAGGACTAGAATCAATGATTCCAAGAGCCTG
CTTAGTACTCTACCTTACTGTGACCAAACTGGTACCTAAACTGCAAGAAAAAGTCCCCTTTACTC
TTCCCTCTTCTTTTCTCTAGAAGAAGGAGTATTTCTCCATAGCCATCACAACTAGGAATGTACTG
GGTCACATCTGAAGCCAGCACGTCTCAGAGTCTCTCTCAAGGCCCATGGTGTGTACTATCTCATT
ATTGCTGCTGATTATTTAGGGTCCAAGGGCTCTTCAGTCAGCAGGTTATGAGTTCTGTCAGTACT
GGGTTCTTTCCTTCAAGGCAGAAGGCTCCCTTCTGGCCTGGGGAATGTCTAGAAATGTATCTGG
GAGTTAGGGCCTGGAATGAGTGCCTCAGGTCCCTGTCCAGTGCCAGATCCTACTGTGGCTGAGC
TGGAATCCAAGTTGCAAGACAAAGCCCTCTTTACTCTTCCTTCTCATTAAGCAGAAGGAAGGAG
TGTCTTTTGAAGCTGCAAGCTGTGCTACCTGGGGTTGGGGGTGGGGAGGCACAAGCACTCCCTT
GGCCACCCCGGCTTGGTGTCTCACTAGGTCACATGCCCCACAAGTTCACTGGGTCTGAGCCCAG
CACAGCACTAGGACCTGCCTAGGAGTTGCAGTCCTCGTGGCCTAGACTGCCTTTCAAATTTATTT
ATGTCCCCACAGCACTTTAGCCTGTGGTGGCGAGGCTCACCGAAACTCAAGTTCAGACTGTTGG
GATGGACTATTCCCCTTTGGTTGGGGCTGGTCTAAATGCTACCTCCGAGAGTGTTGGCTGAGTTC
TGCCTGGTGTTGTTTTCTGCTGTGACTGGGCAGCACTGAGTTCCAAAGCAAAGTCCCACAATCA
CTGTGCTGTCACTTCCCCAAGCACACAGATTCTGTTTCTGTACCATGCAGCCACTGCTGGGGGAT
GGGGAGGGGTAGTGCCAGTGATTCAAGACTGTCTTTCCTACCCTCTTCAATGCCTCTTTCTGTGA
TATGAAATTAAAACCAGGTACTGTGATCACTCACCTGATTTTTGGTTCTTATGAAGGTACTTTTTT
TGTGTGTGAATAGTTGATCAATTTGGTGTTCCTGTGGGGGGAATGACTAGCGAAGGCTTCTATTC
AGCCATCTCCCATCCTAGAGGTATATCTTATCCTCCATGAATAAAATGTCATAAAAAAGGAAGAT
CACAAATATGTAAAATTTGGTGTGTGATTGGAAGCTACATATTTCACACATTTTTTACTATGATAT
TCATTGATAAAATGAATATCCTTTTTTTACCAATTCGATTAAGTACTATGTAAAAGGTACTAAATT
AAGCTCTAAAACTTCAGAAATAAATACAAAATTCAAGAAAATAAGTTATCACAGTTTTTACTTTT
GTTTGATGTGAATGTATGTCTTTTGAGAAGCAGACACCAAGAAAGGATTAAACATGCAAGGATT
TTATTGAGTTATAAAATAGGAAGTGGAGAGAGAGCTGGGAAAAGCTGAAAGAGCTGTCAGACT
GCATTGCAATCCTGGCCCTGAATGAAGGAGAGAGAGAAGAATGACTGGATGGGAGACATCCTA
GACCCCATGTGGCAATAATGAAGGTTCAGCAAGACTCAAGGGGTCCTTGAGGCACAGTTGGCT
GTCAAAGAGTGTTATGTCTCCCCAGAATGGGGCTGTCCCTGCTGTACTCAGCTGCTGGCTGGGA
ACAGCCCATGGGAAGTGTTGCTTTGGGTCAAACATGGCAATGAATTTAAAAATGAGTGGCTGGG
CTCTTGCTCAAATGTGTTCCATGTACTTAGAGGCTGCAAAATGAATTTTCATGGCTGCCATATACT
TTGTTCACTTTTTAATTGAAACATTACCCAGTTTAATTCATCCTCCTTGCTAATAATAAAAATTAAA
CCTTATTTATGCCCAAATCATAAGATAAAATGTTGATTTTCTGTTTTATAAAATTAGATTCGGGGA

ATGTGACTGTGAAATCCCTGAAATATCAAATTCTTCAGTCCAACGTCTAAACATATCTGTGGTTT
GTATATTTGGTGCTTGATTCCAACCTGAAGGCAATAAAATGTGTTTGTAAAACAAGAGCAAAGCT
TTAAAATCATTCACGGGAAAATAACCTGCAAAATACAGGCTACAGAGCAAAACTTTGGTGATAT
CCTATTACCTTTATTTCTTTTACATTCTGAGTCGGTATCTTTGAATTGGTAACAAAGTTGGTGTCTT
TCCATTGTTGAAATCAAGGTTATTGTGAATGCAAAGCATTTTCAAGCATCTTTAGTATCTTTTACTT
TGTGTATCAGTCTAGTTTGAGGCCATTGAGTTTTATTGACTTCTTGACAGTTTGGTGCTCAATTTTT
TTGTTGTTGTTCTTTGCTTGCAAGAATTCTCAAGTCTCAGATGCCTTTGCTAGCTTAAGTATTTGTT
TATGTTTTAAATACTTTTGAAATAGAAGTTTTGAAGGCTGTGTCATTTGGTGGAAAGACCACGGT
CTTTGAGGTCAAACAGATTGTATTGGGTTCTCATTTCAACAACTTATTTTTTTTTCTTTTTAAAGAT
CAGATTTATTTGGAGAAGACAAAAAACCCACAACCCAAAGGATGGGATTTTACATCTCAAGACA
TCTCCTAGGTAATAAGTCTACAGATTACAAATCATTTTCACAGAAGATATTTTTGTACAAATTTTA
CATGTATTCAGGAGTAGGATATTAATCCCAATTTCTATTTTAACAGGAGGGCAAGGTAGGGGAG
AGGGAAAAAACGGTTTTGGATATAACTATTTGGAAGGCAAGTAGACATGAAGAGGGCAAACCCT
AGCTTTTCATTATCTACAATCAATATTTTTTTCTTTCTTAAAAAAATTTTTTTCAATCTTTAGTATCTC
TTTAAAAACCCACTTCTTAGCTACTAGTCAATCCACACCAATTATTTAAATTCACTTGGTACACAC
CTTTGTCTACTGGGTAAATTATATTCATTATGCCCACTGCTGCAGCATGCATAAACCAACACCCC
TGCATGGCCTAATCTGGGACTGATGGGAGAAGTGCTTGCAAACCAAGATCAAGGTGTCATTTCT
CTGCTAATACTGTCTAACAAGCTGACCCTTACAAAAACGCACGTAAAATCAGGCAGGTTTAGCT
ACTGTGTTGCAAGAGAAACCAGGACCTTTCTAAATAGTTCTCTCCATTACCATTTATTCTCTCAAG
GGAAACTTAAAAAAAAAAAAAAAGAAGAAAAAGAAAAAACAACACATTAGTCTGGCCACCTCAT
AAATCGAACAAGCATTGCTGTGGCATTTCAGTGGAAAAGGAAACTTGAGGGGGAAAAAAACCA
TCAACGTTGTAAGAAAGGCTTCCAATTTAACCGTCCCTGTCCCTATTTATCCACTTTAACTCACCA
CCCAAGACCATCCATTATTCTAGAGCACCCTGATCTATAAAAGGGGTCAAAGCATCAGGAACAG
GCAAGGAGTGAGAACCAAAAGACATCAAGAAACCGATTTGCTTGAGAAAAGCAGTGATTCTTC
CTCTTATAGCCTCCATGGCTGAGAGAGAAAATGCCCAAGAGATTATCCATGTGACTTGGAGACT
GCTTATTTATTGCATTACTTTAGACAAGCTGACAAATCTTTCTGAACTTGCTTGCTTCATCCTCTGT
AATTTAGGGACAGCAATTCCTATCTTGTAGGTTGCTGTGGGACAGAGGCGAGGCAGCATACACA
AGTTAATCCTGAGGCTCCCCATCTGGACTGTTGTGTTACCCAGTGTTTTTGCCTTCCTCTCACTTG
GGGTCACTGGAAACATGCAGGTTCCAAGCTTGGCCAGAAGAATTGCCCAAGCGCCAAGCACAG
CCCCTTAGCAGCTTGCCGCACAATGCCTGAGCTGAGAGGGCCATAGCCCATTGGCCTCCTGGG
GTCTTGCTGTGCTCTTGTAATCCCTCCTTCAGCCCATGTGTCAAATGCTACCCAATGCTCTCATTT
CCACCATGATCCAATAATTATAGTGGTGGGAGGGAATATTGTCCAGAAAGGACCAGGAGGGTG
GGAAGCAAGGTCCTCAATGACTGCATTCACCTGGACCTTTGGCAGTCAGTGGTCTACTGTCAGT
GGGTTCCAGCCATCAGGTGTCTACCACGAGGCATGCCCCTCTTCTTGTCTTACTGCCAATTCATC
TCCTCCTATAAGTGCTCCCTCAGTCAAGGCTCTCATATCCATCAAATTCTGCTCTTACAATCTTGC
CCACTCAGCTCTATTAACCATGCATTCTGAAATTTCCACTTCCCCCATTCCCTCAATTACATACCC
TCAGAGGCAGAGGGTCCTGACAGTGCTGGACACATGCAGCTCAGAAGCTGAGTAAATGCCCCT
TCTCTTGTCCCCTTGTGTCCCAGAAGCTAGGTGGTCTATGTGGCTGGAGCTCCATGTCAGCTCTG
TGGTGTTCATCACACTGCCCTGTGTGAGATTCTCCTGCAAGTTGGTCTCTGAGAGACTAGCTGAG
TTGGATATTCCTTCAAGACTCTAAAAAATAGGACATTTACTGAAATCATTTTTTAATCCTTTTTACT
GAATATGTAGAGGTGCAGTATTTGTTTGTGTGCTTAGTCCTCAATCTGTTTGCATTTTCATGAATA
AAGTCGTCGAAAGAAATACATAGCTGAGCCATGCAATGCTTCCGGGTTGTGGGAATTATGCATT
CTTTAGACCATGGGGAATCTGAGGGGAGAGGCATTTGTTACTTGTCTGCTGCTGGTTTGGCTCCC
TCAGTGTCTGTGTCTAACAGGACACAGTTTTAGTTGCAACAAGGGGCCCTGGTGGGTAGAGGG

GCTCTCAAAGGGCTGTTAACATATCCAAGTACATGATGATCTGAAGAACCCAAAGGTTATTTCTG
ATGTAAGTGTCCAGGGATGGAGATTGTGTGTTGAGAACTAGGGCCAGGACGACGGTGAGGTGA
GTAGGCACCTGGCTTGGGTGCAAAATTTGTAAATAATATTTTAATGCAATATTTTTAAAAAACTC
AAATAAAAAAATTCATGTTGGACAAAATATAAAAATTCTAAATAAAGAGAGGATCAGTAATGGT
GCTGTGTTGAGCCATATTTGAGCTTGTAGTAAAAGGAAAAATCAGTAACCTATCCAGTTTTCATT
TAAAATTTTTATATTTGATTCATCATGAATTTTTTGCATTCATTTGATTTTCCAGATCAGATTAAAAT
ATTATTTTTCTTGAAAATAAAAAAATAAAATTAAAATTAAAAAACCAAAAAAAAGCAAATAAAAA
AAGGAAAATATATAATAATAATATTAAATTATTAGTTCTGACCCTGCTGAGAGCCATTGGATTTA
AAAAGCATTCCTTTTGCTTCCTTTAAATCTTTTCTTTAGAAAAGATTTTCTTTAGAAAAGATTTAAG
ACTAAAGAAAGACTAAAGAAAATTTTGTTTTCTTTAGTCTCCTGCTGACTGGTAAAAGGCATTTA
TGGCCCAAGGGCCACAAAACTTCTATATATCAGAAACCATACTGACAATATTTAATATTAATTTG
CATGTGATTTATTTTTGGTTTTGTAAATCAATCTTTTTCTGTACCAGAAATCCTTCAGGGGAAATTC
GAATACTACTAGAGTAGATTAATAATGAAAACAATAAATGATTTTTAAAAATAGAATTTTAAGTA
CTGTAAACCTTCAGGGTATTTTTAAATAATGGCCTTATTTTTCACTTCAACACATTTTCCTCAACAC
ATTTTCCAAGTGGAGTGGTACTCACCTTGTCTATGAGATTATTGCCTGGCATATTTCAAACTCCTA
CCATTGGAAGCCATCATGAGTCTGTGACCTATGGGGGTGTGGCAGCTGTTAGGAAGAGCTATCT
TCACCCCACGCCTCGTATCTTAAGGAGGGGGTGATATGTTGGGAAGTGCACAGGATTCATGAGC
AGAAGACATCAATTCCTCCCTCATTTTTAACTCATTACCTGGCTGGGCTTCAACTTCTCTAAGCTT
GATTAGCCACATCCTTGCAATGGGAATAACAATAGATTTTTTTCCAGGGTTGTTGGAAGGGGAA
AAAAAGGAATCATAGGAGAAAGAGCTTTGCAAACTGCCAAAAGGCTCTCCAAATTAAGCAAGC
TCTTTCTTGATCAATGCCCACCCACAGGGATGGCTCCTCTTGGCTGGAAACTAAAGTTGACTTCT
CTGCTAGAGAGATTATCTGTCATCTAGGTTGCATTATCAGCTGTAGTATGTTATTCTAAAACACA
CTAAACATATAAAGGAAAGAAAAGGAAATCCTGAGAGGTCTTCATGGAAAAGTTCTTGCTTATA
AAGTGCAAAGTAATCATAGAGTGTGGCCTCCAACAAAGCTTCCTGAGAAAGGTAAAATGACTTC
AAATGAACTCTAGTTAATTCCACAAAACTTTCCTTGATCAGCAGTGTGAAAAAATGAACAAAGA
AGTGGATTATGCCCCATGAAGAAGATAATTGCTTCTTCAGGAAATGCAAAAGTTGTACCAATTC
CTGGAACTAAGCCAATGCATCTTCAACTAATTGGACTTGCAGTTGGTCATCTCAACTTTAAGTGT
CCTAGCTTTTGGATACTGAAATAATCCCTTAATATGTCTAACTGAAGCACAAAAAGAGGTAAAAT
TATCCTTTAAGATTTTCATCTGTCATAGTCTGGACATGATGGCTCACTCTTATAATCCCAATACTTT
GGGAGGCCAAGGCAGAAAGATCGCTTGAGGCCAGGAGTTTGAGACCAGCCTGGGCAATATAG
AGACACTGTCTCTACATAAATCATTTATTTTTTAAAAAAAGATTTTTATCTGTCATACATATAGAC
ATCATTTTTGAAGAGTGATTATTTTTCAAAACAAAAACAGCTGAATTCAGAGACCGCCAGTTTCA
TTGATTTCACAGCCCTTACCTGGTCAGTGAGCAGGTGGTATAAGAAGATCTGGTCTTTGGTTAAG
CTGATGGCAATGCCATGTGGCAAGGGAAAAATATTCATTCTTCACTGACTGCACTATAGGACGT
GCTATGTTTGAATCTGAAGCTAGAGGTGGATCAACTCCTATCTGACAATGCTGTGGATTGTCATT
AGTTTGATCTTTGGAAAACCTTGAAGCCTTTGAGGAACTCTCTAAGGAATATACCTCTACCACTA
AGGAGAAAATAGCCAAGGGGCTTATTTCCACTATACACTTAACCCAGATCCCAAAAATGCAGTT
CCCTGAAGTGAAGGATTTACTTGGTCTTACATAGTATAGTAATCAGGTCTCCTAAAATACTTGCT
GATAGTAAACTAAGATATCTTGGTGGGATGAGGCTATTTTTCAGTAAACACCAATGGCATTTGGC
ATCCCACCAAATGCCATTGCTTATCCAACAACATTTAAAAACTTGAGTTTTGTGTAGAACTTATTA
AAGGAAAGTAACAAACATCTGTTACTGATTTAAATGATTTTTATTATACTGTTATTTAAAATATGT
CAAACACAGTGATACACCAAGTAGAATCATCATGCTTGCATGCTGTGTTGCCTGGGCAATGAGC
AAACAGGGACCAGGGATCCCCCTTTCCCTTGGGACCAGCAACTTCCTTCTTTTGATGGCAGGGC
CTGAACTATCCTTTCCAGACCACTCCTGCAAATAGCTGGCATCCATATCCCAGCTGCTGGCACC

CTGCTGGGGTTATCTCCCCATTGACAAGACAACATTTGCATACTTGCTCTTTAAGGAGGAACTTC
CGCATGGGAAAGGGCCCCTGCACTGGGACACAAGCCCCATCGTGTATCCATCACGTTCTGGCT
GGCCCTGGGCTTCCTCTCTATGGAGTGATGGGAACATGGGTGATCTCTTTGCCTTTTCTTTTCTTA
ATCCAACCTTGTTACTTTTTTTTTCTTCTCAGTAATGCCTATTCCTTATGACTTTCCTTGTGGAATTT
GTCCCAAATTCTGGTAAATAACAGATAGAGACCTGCTAGGACTCAGTTTTTATGACAAACATGA
AACTAGGTATAAATGCTCTTATAAGCATGGCTCTTAACCACAAAACTAAAACAAAACTTAAATAT
AAGTATAAAAAAATCACATTAATGACCATAATGTAATATAATAATTGATGTTATATTGGTGAAATT
AAATCATTCAATTTGGGCTTAATGAAATAAATGAGATAGCCTTAGTTCAACTCTTTATTTAATCCA
TTTCTATATTTTAACTTGAACGAAATTTTTAGAGAATGCTTGTTTGGATAAGTTTGATGGGCAAAG
TGATATGAATAACTACAAGGCTTTTTCAGTTCAGGATAAACAGACTTCATATTTAACCCAAATTCT
GCTAGCAAGCAATCATTGAATGCCTGTTTTGAAGAGGCGTCATATAGTAACTTGTAATGACTAGT
TCATCTGAAAATAAGATCGGCATTGTGATATTATTAAATCTATAATAAATGGGTATTTAATCCAAT
TATTTCTGGAATTGAAAACAAAAAGAAATCTTCATTGTAATTCCAATAGTTGCTGCTTTTGAATTT
TTGCAAGTGACTACGGAGCAGGAGGGGCTGGCTGGACAGGGTGCCATGTGCCTGCCTGAAATG
GAAACCTGGCTCGATTGTTGTCTGGTGCAGACCTTTGAGTTTTATAAACCTTGGCTCCATGCACT
GTGTGAGAACTCACATGGCCCACAACTGTTATCTATTTAAAGCATGATGAAAGTTTCCTTGAAGC
AGAAAGCTATGCATGATTCATTCTATTCTGCATTCTATTCAGTCGCTCACTCACTTGCACACTCAT
TCAAACATGTGCCATGTGCCAGACAGGCTCTAGGCAATTGCATACACTGGTGGACAAAATGAA
AAACCTAATCTTCATGAAATTCCTATTCCAGCAGGAAGAGACAGAAAACAAGTTATTTTAATAAG
TAAATTTTATAGTGTGTTTAAAAACAATTTTTTTAAAAAGGGAAAAAGTAGGACAGGGCTTTAGG
GACTGCTGGGGCTGGAGTGGGGCAGGTTGCAATTTGAAATAGGGTTCAGGGTAGGCTTCACTG
GAAAAGTGTGATGAGAGAAAACATTTGTGAAAGGTGAGGGTGTGAGCCAAGGGGCTGTTGAGG
AGAAGTGAGCAGTTCAGGCTGAAGGACCGGGTAGGGCAGATTCTTGAGGTGGGGATATACCTG
ATGTGTTCAGGAAAGAGCTGAGAAGTCAATGTGGCTGTACATCAGTGAGCTGGGAGTGGAGGA
GTAAGAGAGGAGGTTGAAGAAGTAAAGGTTATCTTAGTCTGTTTTCTGTTGCTTATAACAGAATA
CCTAAAACTAGGTAATTTATAAAGGAAAGAAAGTTATTTCTTATAGTTCTGGAGGCTGGGACCA
GCTCCAAGGTCGAGGCAGCATCTGGTGCGGGCCTTCTTGCTGTTGGCGACTCTGCAGAGTCCCA
GGGTTGCTCTAGGCAACACATGGTGAGGGAGCTGAGCGTGCTAGCTCATTTCTCTCTTTGTCTTC
TTATAAAGCCACCAGCCCCACTCTTGTGATAACCCATTAATCCATTAATCCATGAAAGCTTTCAT
GACCCAATCCCTTCTTAAAGGCCCCACCTCCCAATACTGCCACATTGGAGCTAAGTTTCTACATG
ATTTTCAGAAGGGACAAAACATTCAAAGCATAGCAGCGGGGAAGAATGTGAAAACTCCAAGAA
ATCACGGTAAGCACTTTGGCTTTTTCTTTGAGTGAAATGGGGAGCTATTTCAGTGCTTTGAGCAG
AGTTGTGGCATGATCTCACTCACAGTTTAGAAGTGTCACTCAGGTGGCTGGGCTCAGTGGCTCA
CCCCTGTAATCCTTTGGGAAGCTGAGTCAGGTGGATCACCTGAGGTCAGGAGTTGGAGACCAG
CCTGGCCAACATGGCGAAACCCCGTCTCTACTAAAAATACAAAAATTAGCCAGGCATGGTGGA
GGATGCCTATAATCCCAGCTACTCAGGAGGCTGAGGCAGGATAATTGCTTGAACCTGGGGGGT
GGAGGTTGCAGTGAGCCGAGATCGCACCACTTCACTCCAGCCTGGGTGAAAGAGTGAAACTCC
ATCTCAAAAAAAAAAAAAAAGGAAATGTCACTCAGGAAGGATGGAGCTGCCTTCACCAGTGTTG
GGGAAGCTGCAGGTAGAGCAGGTTCCAAAGTGGAAAGAAATAACAAGCTCAGTTCTGACTCGA
TTAACTTTGGCACATCTGGTAGGCATCCAAGTGGGGTATAAGTTGGGTTCTAGCATCTGAAGTTT
GGGAGAGAGGTCTGAGCTGGAGATTTATAGTTAAAAGTCAGGGAGTAGATGGATGGCATTGAA
GCCAAGGCACTGAATGAAAGATGCAAGGGAGTAAATGTGGCTGAAGAAACAAAGAGGACCAA
GGATTCAGGCCTGGGGCCCCCATAACACAGAGGATACCAAAATCCTACAGGAGGTCTCGAGAT
AGTTTTCATCTGGATGCTCCAGTCTACACTATAGTTACATTGTTTCCAAGTTATCATTACAGTGAA

AGCATCACTTATAGGTTATGTGGAGAACTCACAGACCTTGGTGATGCATCTGGAACCCTCCTTTG
AGAAATCCTGCTTCACAGGCCTCAATCATAGGCAAGATAGTTCTTCAAGTTGAGACAGAATGAT
ATGATTCTAAAATCATGTTCATCACTGTCTTGCTCCACAGGGGAGATTTTCACATTTACACCCTTG
TTCTACTTTATCAATGTATTAGAAATTATACCCAAAATATTCTTCACCACAGAAAAGGTAGAAAA
TTTAGAAGTGAGTGTTTAAAAAGTAAAGTTTTAATGGTTTCATTTCACCCCACACTGGGATATAC
CCCACTTGCCCTTAGAGACTGTTTTCCTTGAGCAGTGATGTACATTGAAGTTCACTTCCCTGGGC
TTGGACAAAGGCTTCCTAGGGCAATTGGTTTTCAGGCCCTCAAGGTTCATATGTACTCTTTTCTC
ACACGGAACCACATGGGAACTCATCTGCCACATTAATGCTTTCTCAAATTTCCTACCAGTACTTC
CTTTCATGTACCTTACAAAAGATTCACCCAACTGAAATCCAACTCTGGTGCAGTGACTCAAGTTG
CAAAAGTGTCTGGTGCTCTAAGCAGAAGAACAATTGTCAATATCACTCAGGAAAGTCACCTTCA
GCAAAGACCTCATAGAATTCCTCCCCTAGTCTATTTTAGTAACAGCTTCATTTCCAATAAAATTTT
CTTTTTATGGTTAAAAATAATTTATCAAAATTTATTATATGTTTAAGGTGTTTTGATTTATAATAATT
AAACTAAAATGAAAATTTTAAGTTTAGAGAGTTTTAACCAGAAAAAAATAAAAAATAAATTTTAA
TTTATGGACATGTTTTTCTTTCAAAGAAATTTGACAGCATAAAGAATAAAAGTTGCACTAAGACA
ACTCGATGGAGGAAGTGTGATGGAAATGTGATTTAAGTCAGAAGTGAATGATGTAAATTTTCTC
CCTGTTAAATAAGAAATGTTCATATATTTAAAAAATGGATGTTGCCTATCAAATTGCAATAGTGTT
TATATTCCATTTTCAGAACACATTTTATAATTATCAGAGCAATGTAACCGTTTCATTTGAAAATGTT
AACATTTACAATATGCCAGAAATCACATATTTTGCCTCTATTTTAGAGAAAAAAGTTTTAGATATC
ATCTTTAAAATATAGAAGGGAATACCTCATTTTCCAAACTCCATTCAATGAGCAACATAGGTTTC
AAATTCCACAATGACTTAAACTACACCAAAGACAATCTCAGCAAGATATGTATCAAAAGATCTC
AACGGCAATGGAAAACTTCACTTTTCCGAAGCAGACCAATCAATTTTGAGCCATCAATTTCTTTC
TTTTGTAAGATTCATTCCTACTGCACTGACCCCAAAACCTTATCTTATTTCTTGACCATGGATTAT
ACCAGTACATATTGATCATGTTAAACTGTTAACACCCACAATGATATGCCCCAGACACATTTCTT
ACAATGAGCAGAGGGTTCTGTGTATTGGATATAGGTCAATCACTGAAGGTAGCAGTAACTCAGT
GGCTCTCTGCACTTTACCTTCTTAAGGCCACCACACTCACGTACTAGGTGTTGTCCCAGTGGAGG
ACAGTTGGATACAAAGTCTCTTCCATCTTGACATCTTGGTACTGCAGTGGCCTCCTGTTCCATTTC
TCACTTCTCCCACCCTTAGTTTTCTAGCTTTGGGGAACATGGCTGCCTCAGCAACCATCTTATCTG
CTCCCTCTACCCCACCGTTTTGCAAGGAGGAACGGGCATTGGTTTCTTTCTCACACTGTCACTAC
CTTCTCCCCTTTCCTTTACCATTTTTTCATTTGCTCTTCACTTCCAGTCTCCCTTGCGATCTTCTTCC
TTTCTTTCTGGCCAGCCTCTGACTTCTGCAGAGTGGTTCCCTCCACCCAGACAAAACCCTGGACT
GATGCCAGGCACAGGGTATGTGACCTATGCTTCCATCTCCTCTTCCGTCCCTGGGACTGTGGCC
ATAGATATTCACCTCCATCAGGTTCCACTGATAAAGGAGAAGGAGAGGACACAGTTCATAGAAA
AGGAAGCTCTGGCCAAACTGGAAGCCACCAAACCCAGGGTGTGGCTCCTAGAGTATTGCCATC
TCTCAGGAGCCTTCCAAGGCTTATCCCGTAACACCTTCCACTCTTCTTAGCATCTCTCTTCTTACC
CTTTATTATTTTTTATGTGAAAAAATATACATAACCATAAAATTTACTATTTTAATCATTTTTAAGTG
TACAGTTCAGTGGCATGAATTACATTTATATGTTGTACAAACATTACCACTATCAATTTCCAGAAC
TTTCTCATCATCCCAAACAGAAATTCTTTACCCATTAAACATTATTTCCTTTTCCTTTCTCACTCCA
GCCCTTGGCAACTGCCATTCTACTTTCTGTCTCTATGAACTTGACAATTCTAGGTACCTCATATAA
ATACAAATAAAAATACAGCATTTGTCCTTTTGTGACTGGTTTATTTCATTTGACATAATGTCTTCA
GGGTCCATCCATGTTGTGGCATGTGACAAAATTGCCTTCCTTTTTAAGGCTGAATGATATTCCATT
GTGTATATATCCACATTTTGTGTATATATTTATCCCTGCGTGGGCACATGGGTTTCTTCCTACTCTC
AGCTATTGTGAATAATGCTGCTAAGAACATGAGTTTATAAATATATGTTCAAGTCCCTTCTTTTAC
TTCTTTTCAGTATAAACCCAGAAGTGAAATTGCTGTATCATATAGTAATTCTATATTTAATTTTTTG
AGGAACCACCATACTGTTTTTTATAGTGGCTACACCATTTTACATTTCTACCAGGAATGCACAAG

AGTTCCAATTTCTTTACATTCTCACTGATGTAGTTTGAATACATGAGCCCGCCAAATCTCATGTGG
AATTGTAATCCTCAATGTTAGAGGTGGGGCCTGGTGGGAGGTGATTGGGCCATGGGGGCAGAT
CCCTCATGGCTTGGTGCTGTCATCAAGATACTGAGTGAGTTCTCACAAGATCTGGTTGTTTAAGT
ATATGGCACTTCCCCTCTACTCTCTCTCTCTTGCTCTCCCTCCCACCATGTGAGATGTCTGCTCCC
CCTTTGCCTTCTTTCATGATTGTAAGCTCCTCGAGGCTTCCTAGAGGCAGAAGAGCAGGTATGCT
TCCTGTACAGCCTGCAGAAGCACAAGCCAATTAAACCTCTTTTCTTTATAAATTACCCAGCCTCA
GGTATTTCTTTATAGCAATGCAAGGATGGACTACCACACTCACCAACACTTGTTATTTGTTTATTT
ATTGATTTTCATAGTAGCCATCCTAATGGGTGAGAAGGGATATCTTCTTGTGGTTTTGCTTTGCAT
TTTGCTAGTGACCAGTGACACTAAACACACTTTCATGTGTTTATTGGCCATTTGTGTATCTTCTTC
AGAGAAATACCTATTCGAGTCATTTGGTCATTTTAAATTGGGTTGTTGGTTTTGTCGTTGTTGTTAT
TGAGTTTATTTTTATTAATTGTAATTATTTTTTCTCTTAAAAATTCTAATGCTTATTTTAGAAAATTA
AATTAAGAATTGAAAAAACATTGAGACAAAAATGATTCATATTACAAATAAAGAATATATTTTCT
CAATTTATTTTAAGTGTTTATAAATTTTATTTAGGCCACATTCTTTGAGTGCAATATGAAGAAGCT
AGGATTCATTACTTAGGAATTAAAAGTAGACATTACTAAATAAGTCTTGGGTAAAAGAGAAAATT
TAAATTTAAAGCAGGTCACAGATTATAAGACTAGTAGGTAACACACTACTACCCAAACTAATGA
AATATGTTCAGAGCCATACTTAAAAGAGAATTCCTGACTTTAAATATTCTTATTACAAATCCAAA
AGAATTTTAGTTAAGCATTCAATAAAATACAAATTCTTGAAAATACAAATGCATAAAAGAGGCTA
TAATAGCTGGTAAAGAATTATTTCTGGATGTGTCTGTCACGGTGGTCTGGAAGAGATTAGCGTTT
GAATCTGTAGACTGAGAGAAAATCACCACCTCCGGTGTAAGTAGGCATCATCCATTCTCTCAAG
GGTTCTGAATAGGGCAAAAAAGCAAAGGAAGGGGGAATTTGCTCCCTCTGAACTAGTGTATCTG
TCTTCTTCTTCTTCTTCTTCTTCTTCTTCTTCTTTTTTTTTTTTTTTTTTTTTTTTGAGACAAAGTCTTGCT
CTGTTGCCCAGGCTGCAGCACAGTGGCGTGATCTCAGTGCACTGCAACCTCTGCCTCCCAGGTT
TAAGCAATTCCCGTCTCAGCCTCCCCAGTAGCTGGGACTACAGGTGCAAACCACCACACCCAG
CTAATTTTTGTATTTTTAGTAGAGACAGGGTTTCACCATATTGGTCAGGCTGGTCTCAAACTCCTG
AGCTCAGGTGATCCACCTGCCTCAGCCTCCCAAAGTGCTGGGATTACAGGCATGAAGCCACCA
CACCCAGCCCACATCTGTCTTCTGATGTTAGACGTGGGCACTCCTGGTTCTCAGGCCATTGGACT
GAATCATACCACTGGCTTTTCATGGTTCACCAGCTTGCAGACAGATCATGCAACTTAGCCTCCAT
GAGCCAATTTCTATAATACATCAATCTCTTTCTCTAAGTATATGTAAATGTAGAGAAAGCGATAT
ATATGTTCCATATGTATACTGTTTCTGCGAAGAACCCTAATACAAACATTAATAAATTGTTTTAAA
ACTCACAAAATAATATAATTAAAGAGCAGGTAACCCCCAACTGTGTTTTCAGTTTGCATATTCTA
AGATAATTACTAAGAACATTGGTTTCATATCTCTGGATGCTGTGCTTCTGTTAGTCCCGTAATTAT
TCATGACCATTATATCTTCATTTTGGATTATGTATTTTATTTATAAAGTCTCCATTGTTCAAGTGATT
TTTCCTTAATTCTATTTGGTCTATATTAATATTCTTATTTGTTTTCTATTTGCATTCATCTGGTGTGTC
TTTGTCCACCTCTTCAAAAAATATTTCTGCTTCAATTTGTTTTACATATTTTTTTGAAAATAATGTTT
AGCTAGATTTTGTTTTGCTGACCCAATTTAAAAATTATCTTTTAAAGATGAAATTTGATCAACTTA
CATTTATCTTAGTTGATGCAGTTGTGTTTCATGCTTTTCTATTGCTTTGTTCTTTACTTTTGCTGGTT
TTTTGTTTGTTTATTTTAGTATATTGTATTTTATATTTCTACTGATTTGGAAAATATACATTCTACTC
ATAGTCTGTTAGAGAACATCTTTCATTAAAAGCATGTAAGCCTTTATTTCTCTGTTATAACAATAA
AACCTCAATGCCTCAAATAAGATGAGGAATTCAGCTTGCTTTTAAAAGTTTCCTCTTTTTTCTCTA
TCTCCCAGATTACATTAGAATTATCTCAGATTTTAAAACCAGAATATTACAAGTAGGTTTTTTACT
CTGCAATTTCTCTTTAAAAACCATTTGGACATTTTAAGTGTATCTCATAACCACAGAAACAGCAA
TTATTCACTGGGATACTTTGTTTCATTGGTATCACTATTGTTTACCTCTTTCACTTTCTTCCTGGATA
TTTTTACTGTAACATGGATAATTCCCTCTTGGAATACTTGCCTATCTCAAACTACGTTTGTCATTTT
CTGATGCTATGATGATAAATTGTCCTGGTTTACAAGTCCTGAGCCACATCCTTTCCCCTCTAAACT

CTGTGGATCTTATTCCATGATCTTTGGCATGACTATATTGCAGAGAAGCCTGGAGACAAATCCTA
TAGTTAACCTGGTTTTACTGTCTATGTCCTTTGGGAATTTTCCTTTATCTTTGAAAATCTGAAATTT
CATTGGAATAAATACAGGTGTGTCTTCTCATTTTCTAAGGTAAAAAGACAAAACAAAACACAAC
CTAATACACTGAGAGCATCTTCAACCTGAAGTGTCAAGCATTTCTGTGATTTATTTAATTTTGTGT
TTGTTCCTCCACTTGCTCTTTTTCTTCACTCTTAATCTTCATCTTCCATGACCTCAACCTTACTCTTC
ATGGGTATTCTAGTTTCCATCAAAATCTCTTTTTCCAGCACATTGGAGAAGATAAAAAGGCTGGG
AAGGAAGTGAGGAGAATTGTGCGTGCCATTAGCTGCCTTGCAAATTATCTCTTCCCTGGGGCTG
GTGTGGCTTCTTGGCCCTGCAGGGTGCTAAGATGGTGCTCAGTGGCTCCAGTGGAATCTGTCTT
CATCACTGGCACACGTGGGCCGCCTGCAGACTCCCCAGACAGATTTCTCTGTGGCACCTGAGCT
CCTCTCTCATGGATAAGATCTCCTTTGTGACTGAGATTAAGCTGCTGAAATTTGCCTGCTGGAAA
CAGTCCTCTCATCACAGCCAAGCCTTTTTCTTTCTGCAGCCCATGAAGAAGTCTATTCCGACTTTC
TTTCCATCCAGGTTCCAGGGACTTCTCCTGCCTGCATGTTCCTTCTCCACCAATGGCTTCTGCCAT
TCTAGCTTTCACTTTCTCTTGGAGGTGTCAGCCAGAACATTTCACTTGGAAGCTTTTGGTATGGAC
ATTTCCCTCCCTACTTCCCACCAAACATCCCAGTGAAATATTAATACTTGTAGTTTCTTGACCAGA
GATTCCAACGGGTGATAGGAAAATTGGCAGAGGTCATGTTGTTGATTTTAGGTTTGTTGTTGTTG
TTTATTTTAATCATTTAGCTAAGAACTCCAGAAATGGCCTTTGGATATAGCCTCCAAAATACTCCA
AATAGCTTCAAGAAACATGAATCGTAACCGCACGCATAGCCTGTGATCAGAAATAGTCCCAGCA
GGAAAGAAAATGAGTTACAGAATGAGTTTGCATTCCCTTTGGCTGGGAACCTCAGCACCGGCCC
TGGGCTCCTTCATGAAGCAAGAGCCTTTCCCAGGTCCAGGAGTCCTTGATTCTTGTGGTTCTCTG
TTCTTCTTCTTCTTTCTTCTTCTTTTTTTTTTTTTTTGAGAGGGAGTCTAGCTCTGTAGCCAGGCTGGA
GTACAGTGGTGTGATCTCAGCTCACTGCAACCTCCGCCTCCTGGGTTCAAGTGATTCTCCTGCCT
CAGCCTCCTAAGTAGCTGGGAACACAGGCATGCACCACCACACCCAGCTAAGTTTTGTATTTTT
AGTAGAGGCAGGATTTCACCATGTTGGTCAGGATGGTCTTGATCTCTTGACTTTCCGATCCACCC
ATCTCAGCCTTCAAAAGTGCTGGGATTACAGGTGTGAGCCACCGCACCCGGCCAGTTGTCTGTT
CTTTTAGACACCTCTGGGATCACACTGTGAGAAGCAAAATCTTAAGAGACTATCATGCATTCATT
AATTCACATGGGGATGAAAGGGCTGGTGCCCAAGCTCCAGCTCTCAAATCCCGCTTTTGATTCC
CCCACCTCATTCTCCACCTCAGCCACACTTTCTTTGAAGGATTTGACAAGTTTGGCCCCCAATCC
ATGTAGTGAAGTGCCTCTCTTCTGCCCATCTACTGAGCTTTCTGCTGTTGATGGATAATTTCTCTG
GAATGTCTTGATTGAGCCCCTTCTCCTTTTTGCCTGTGTTTTACACATGGGTTTCCAACCATTGCC
CCAAGAGATGAAGATTCACCTGGGATGACTAAATTCCACTGTAGAATCTAGAAACCCTCTCACA
GCTCCTGGCCTTCCAACAGGTGTTACAGTTTACAGCTGTGTCCTACCTCCTAGACTTGCAAAAGT
GGTGTCTGGTGTTTTTTTTTGGAGATGGAGTCTTGCTCTGTTGCCAGGCTGGAGTGCAGTGGCAC
CATCTTGGCTCACTGCAACCTCCACCTCCCAGGTTTAAGTGATTCCCCTGCCTCAGCCTCCTGAG
TAGCTAAGACTGCAGGTACACGCCACCATGCCCAGATAATTTTTTGTATTTTGGTAGAGTTGGGG
TTTCACCATGTTGGCCAGGATTGTCTCGATCTCCTGACCTCATGATCCCTGGACACCCACCCCGG
CCTCCCAAAGTGCTGGGATTACAGGCATGAGCCACCGCACCCGGCCTGGTGTCTGGTTTATATG
AACGAAAGATCTGGCTGGAATAGCAAATGTCTGCAAGGGAGGCTGGGCTAGATATCACCAGGG
CCTGGAACACTTCTCACATTCTTTCCTCCACTAACCTCCAGTTATCTAAGAGGATTATGACTCTTC
TTTCTCTCTTGTCCTCAGAATGTCTCTCTGGCTTCCTCTCCCTCTCAGAGACCCAATTTGGAACTG
AAACCCTGAGGTGAGATAACTGAGGTGAGATAATTGCAATAATGGAATAATTCTTTACCAAGTC
ACTGCGGTAACTCAGTAACTCCCCCTTTTGTGTTGGTGCTACACAAGGCTAAGTTCTGAATCTAC
AGCTATTCGTGATGCTATTTCTTTAAAAACCTAGATATCAACAAACGAAACTACATTAATCCAAT
GTGGCAATCAGGCGCCGTGAACTAATCCTAGTACTTTAATTCTAAAAGCTGAACCCTCTTGGCA
AAAGAAAATGATAGTCAGAATTTCTCTTCCCAAGACATTTCATTTAGCCACCTCTATTTGTCTTCT

ATTTTCTTCCAAGATTTCCCACTACCAAGCATAGTAGTTTTTCTTCAAACTACTCTCAATAGCCTA
AGGTAGTCTAATTAAAGCAATGGATCATTCACTCTGCTTTTCAGTAATCCTACAGTGACTTAATG
AACAGAGTGCAGCAATTCAACACATGGATTCTTACTCATTGTATCCTAATTGTATGTAAGTACCA
CACAATTCTATTGAGACTCACAGACATTTCTTTCTTCTCCTCTTCAATGAAAAAGCAAACATTAAT
TGAACCATACCACGATTTGGGAAATAAACATTTCATGTAAATTGAAAAAAATACTTCTCTACTTG
TAACATGCACCATTCTTGTTTATCTGAGTTTTCTTAATTTTCTACAATGAGCATGTACTAGTTTTGT
TAAGGGAAAAAAATACGTTTTTTTTATCTTTAAGTCAGGAGTCTGCCTATTGAAAATAGCAGTGT
GGTACCTTTATCTTTAGAAATAAGTGTCTTTACTTTTGATAGAAAAGATTAGAAGTGTTTCAACAA
AAGAAATAGTACATTATGGTTTCAAACCTCAAAGCATGGAGACAACCTGGGATGTTAATGAAGG
AAACCTGAATTCCGAGATCCATCTGCAAAAGGGAGGCTGGAAGTCTTGGAAGAAGATTCTGATT
GTGTAATCAAGTTATTAATATCAGAAATGCTTTTCCATAGGCCTTTACTTTGAATTCATTTTTCGAA
TGAGATAGCTGGTCTTTAAAAACAACTTACTTTTTACTGTTACAGCACTACACATGTATGGTGAA
AATAATTCAAATGGTAAAAAGAAAGAGGTGCTGTGAAAAAGATGAAAAACTCCTGACATCCCA
ACTCTGCAGTCCTCAGAGATAATGATCATTTCTTGTGCCCCTTTTTAGAAATTTTCTCTGGGCATA
CACTCCCTCTATGCGTCTATTCCTTTAAATCTATATTCTATTGCTTTATATCTCCATATCTATGTATT
AATATGTAGATATTACAGTTGCTATATACTTATATCATTAAAACAGTTATTTGACTCTTTGTATTTC
ACATTCTGGGGCTTCCAGCTGGCACCATGACTCCAAGCACCCCTCTGCTAGGACATGAGAGAG
AAGGCATCAGTCAGGATGATAGAGCTTGACTGATGCACGTGCCCGAGCCACACTAGTGATCTTT
GAGTGCAGGCTGGAATCCAGGTAATCTGGCCGGGCCTCCTCATTCATGGCAATCTC
ID|56458|gene_id|ENSG00000232006|transcript_id|ENST00000588324 Homo sapiens lncRNA

CACAGATTTCAGAGAAATAGGATCAGTGCTCTGAAGTTAAAAGTTAAGTTTTTGCTTATATA
GGAAGAAGACAAAGAAATTTAACAGGATTATATTTCCTATATACTGGTTTAAGAGTTATAACAAA
TTAATTAGTTAGAGTTTGTTTTCTTTTCTGTATGGCTTGTTTTATTCATAGCTGATTTTCATTTCCTTT
CCAATTTAAAAGAGTATGTTTAACATTCCATCTTAAGACAATGTGGTAGTCATGAAGTCTTTGTGT
GAGAAAGGTGAGAGGGAGGTTAATCTACAATGAAGATCAACAGAGGAGAAGGAAGGGGTCTT
CCTTGGTGCCCTTCTGTCATTCATGTCATTTTATTAACACAAAACAATACAGGTAAGGAAGGAGG
CTTAATCTATAATCAGAGAAACAAAGGTTACAGCTGTCTGTTATGTGATGCAGGCCCATAATCAC
ATTCCTTTAAGGTTCAAAATAATTTTAGAGTTCCAATACCTTCAATTTGGATTACTTATTTTTACAT
TTTTCTTCTTTTTGGAAGAAAGTTCTTCAAGAACTTTTTGAAGAAACCATCATAGATGAATTTAAT
GGTTGGACTCCTTTTATGTTGAGAAATTTTGATCCCATGTCACTAGGAAGGCACACTCCTAGGAT
GTCATGTCCCCTAGCAAAGGATGATTAAACCAGCTGTAAGCTGATGTAGTATAGGCTGAATTTA
AGTCAGCATAAAGAAAGTGATTTTGTGTGTTAGGCTAAATAGCTATATCTTCAATTTATGCAACT
ATTTGGGGGACCATTATTTTAGTCTTTTCAGTCACAAGTTGACTCCATTTTAAACAAATGCTGTAA
CAACAGTGTAGACAAAACCAGAACAAAACACAATATGCAATTACAATTCCTAGAATAAGTAACA
GCTTTTGCTGTTAAGTTACCCAAGATCCAAACCATCTACTTAGCTAGTCACTTAGAGAAGATTTG
GGATCTGAAAAGTTGGTTATTGAGGTTTTCATATCAGCCATTCATTTATTGATGTTATTAGACTCA
TCTAGTATAGAGACACAACATTCAGTTTTTATGATGGTACCTTGGGCTGCAGTGGGTGTGTCTAA
AACTTGTTGTGCCATACAGCCTTTCTTGTAATAGCAACTTCATTGTTTAATAACAAAGTACTGATG
TGGCTATCATTTAGAGCCTGTAGTGTATAATTGATCGGGGCCTCTACATGCCAAATAACATTCTC
AAATACCCAGTTGTGGGATGAAGATGGAAGCTAAATGATCATACCAATGGAACACAGAAAAAG
CCCAGCAGGATTATAAATGAGGAAAATTTGCAGGTTTTGATAGGGAATGAACTATTTGACCTTGT
GCCCAAGCATAACCCAAGGAACATTGTCCTAGCCATCCTGGGAGTAGCCATGTCCATAGATTAG
TGCCACATGACCAAAACTTTCCATTTGAAGCTAACCAAGAAATACTCAGTTGTTGTATCTATTCA

GTGGCAGGCCAATCAGTATTTTGTAATGTAATGGTGTTGAAACACTGTTCTTTGGGTATCCATCC
CATATTTCTGGTGCAATTAGGCCAGTTATCTTTAATGTAATTTCTCTGGTCCCAACATAGACAACG
TAGCTGAGCTGACGGGAGGGAATAAGCCAGATAAACCCATCTTAAATTTGTGTTATATGATACT
GAAGTTGAGTTTTCTTATAACTGAGTACCCTATTTTTAAGGTTTTTTTCCTCTTCCTTCTTCAGTCCT
TTTCTATTAACTTTTTAGTAATAAAATAATATCCTTTACTCTCCTTCTTTCCACAAGGCACTCCCTT
GCATTGCTAGCTTATCTAATTATGCTTGCTTAGAAGTTCCAGAGATTGAATCTTGAGACAATCCA
GGTGCCTATGGAATTATCCCCCACCAGGAGATTACTTCAAGCCTGCAGCTAATTTACAACCCAG
TCAAGCCCGAGATGGCACCAGCTCATTCACAAGTTGGAGCAATAACTCAAGATAAGTCATCAG
AACAAGTCATATAGACCCACACCACCTTGCCACCCCCAACTCCCGCATTTCCTCCATACCAAAC
TTCCCCTTCTCAAGCTCTAGTGTTTTGCCTGAGAATTTTAAAGTGGTTTCATCAAGGCAGGAGCC
TGAACTACTTCCCACTGTCAATATTGGAACATAAAGTCACTTCACTTTCTTTCTATCACACCTTGT
CCTTGTTACTTGATTCTGCAAGTGACAAACGGCCAAATCTGCATTCAGTTACAATTTTTGGTGTCC
TGTACAGGGAAGCTGTGCATTCTGGGTGATCCTAGTCTGTCAGTCTGGCTTCATGCTACCAAATG
GGCCAGGGGGCTGCCTGTGAGCACCAGCTGCTTATAGCTAGCTGACATTATGGCCAAAACCCT
GGGGAATTTCCCTGAAACTGACAGTTTTGTCCTGGGGAACCTCCCTGCTCTTTCTGCCATGGCAC
TGGCTGGCTTCTACACTTCACTGGTACAAAGAAAGTGACCTCTGAGTAAGTCGACACACTTTGG
AAATTGGATGAGGCAGCTGGTGTGCACCCAGCTTAGCTCTGTCTTTTTGGGTGGTATTGCTTGGA
CTCTGCTCTGCTTAGAATTAGGTGCTCACAACACAGTTTGGGCATGCACACCATTTGTGTTTGTG
ATGCCACTGGAGCTTTGCTCTGTTTGGACTCAGCTGCTCTTGGAGCCATTTGTGATCAGGATGGA
GGAGGTTTCCAGACTCTTTACCCAGCCCCTTGAGCGGGGACTTTGTTTGGGAGAGCGCCATTTG
AAATTGTGACTGTGTGAGTGTGTAGCATTTATATTTGGACCCTTATCTTTCTTTATTTCTTTTCTTTT
CTGCCTACTTTGTATCTGCTATTATTAAGTTACTAGTACTGAGATTTTGTTTTTCTTATACAGTTCA
GCCACTTCTAACTAAAATGTAAACAGTGAAAATTCATTCAAAATTGAAGGAAAAAAAATTCAAA
TGCCAGAAATATTGGCTGCTTTTCGTAGCTAAAATCTGGTAATAAAAGATTTGAAAGGATTTTAT
TTTGAGAGCTTTATAGTTAAAAGTCAACTTACTTAAAACTGATATTTAAGACATATATTGTTTTAA
AGCCCCTGCTTTCTCTCTGTAAACACTTCTTAGCAGAATTCCGTCTGACTCTCCATATACTCCTGT
CTGTTGTTCCTTCCTCTTTTATCCAGTCTTTTTTACTTTGTAGGGGGAGACCAGAAATTACTTGGC
ATTATGAGAGAATTTTGACCTTGATATGTAATAGCTAGATTTAAAAATATACTTTGTAAAATGGCT
GATGGCAGTTATAATAAATAATTCTTCTTACAGGGCCATACAACTTTCATTGGGCATTTAAATAA
GCAGAGTATGCTCCTGGACAACTGGAAGGTATGGAACGGGGGATGGGCTGATTACAGAAAGG
GCTGATTGGCTTTGGGTTGCCCACCAGCCTCACAGAAATGTCCTTTTGATGGTATACACTGTGAA
AGCATTGCACTATCTCATCCTGTAGCACTTCCCTCTTTGGGGCACCCAGGATTCGGTGGAAAAAT
GAGATCTTTGATTTGGGGAGGTCTGTTTGCCTTCCAGCTGTGCCTGCTTGTTAGCCCTACAAAAT
GCATGTTTCCTGGCCCTATTCCTTAAAAGTCTCCACCTTAAAGCCAGTAATCTTAAAGCCAGATC
TAAGTTCCAATTAAGAAACTCAGATCTTTTAGAAAATTTCCATTTGTAAGAGTGTCTGCCTTTGCT
GGCTGTCTTGACTGAACTTTTACCCACACCATTTTTCCTCACTCTAAATAGAATATGAATTCTCTA
TTTTGTTTCACCTAATAAATATTTCTACGAAAATGTAAATTTGAAGCTGTCTGGCTGGCAATTGTT
TGGGAAAGGGAACAGGTAATCAAGAGACCGATCGTCTAAAACAAGAAAAAGAAACTTAAAACT
GGCAAATGAAAAATAATATCACCTGCTTCTGTCTGTCTCTCTGTGTATTTATATGTGTTGTGTGTG
TGATGTTTCACTACCAAAATATAGAAAAGGGCTCTAATTAATTGGCTGAAAGAAAAATAAGCAG
CTAAACACAATATTTTATTAGAAAAATAGAAAAACTTTAAAATGTCATGTGACTTTAGTAATCTTT
GGGAAATAAAGACAGTGGTAAAGATTATTGGTAAAATAAAATAAAATTGTCTTCAGAATTTAGA
CACTTACTCTGAATTAAGCAAGCCAGATACTGTCTTTGCTAGATGTTTTAAGATCACAAACTGCT
TCTGTGACATTTTAATAATTGTTGGACTCGCCAGCTTTGGAGCCATTAAATTCTAAGTAAAGCCA

GGGCACACATGGACTTAGCTGTGCTCCCTGGCTATGCTGGAAAAAGTCAAACATTATCTGAAGT
TCTATCCTGTCTCCTAGGTTCTACACCTGGTATATAATTAAAATTGCTTACTTCCTTTATCTTTCAT
TAAGAATAAAAGTTGCTAGGAGTTTACATTATAACATATGTAATTGGGACTACTAGAAAAACAGT
TTTACATGCAAGTTGTCTAAAGAAAGTAAAATATGTCTTGGTAAAAAGTTATAAAAAGGCATGGA
AACATGGTTTTTTATTAAAGATAAAGTAATTTTGCCTAGTTTAGATGGTTTAAAGATTATTTTAAGT
TAAAAAAACGAAGTTTAAGTAAGTTATAAAAGGTTTATAGAAGATTAATTTTTTTTTTTTTTTTGA
GATGGAGTCTCACTCTGTCACAGAGACTGAAGTGAAGTGGTACGATCTTGGCTCACTGCAACTT
CTGCCTGCCAGGTTCAAGCCTCCCTGGTAGCTGGGATCACAGGCACATGCTATCATGTCCGGCT
AATTTTTTTATTTTTTGTAGAGATGGGGTTTCACCATGTTGGCCAGGCTGGTCTTGTACTCCTGAC
CTCAAATGATCCACACACCTTAGCCTCCCAAAGTGTTAGGAATACAAGTGTGAGCCACTGTGCC
CAGCCTGATTAATCTTATAAAATAAATTCTGCATGTGAAGAAGCTGGCTAAAATTAAAGGATATT
ATTTTGTATATCCATAAATTGAGTATTAAAATAAAAAGACACTGATATAGGGCCAGAATCTGGGC
CCATGTATCAAAATAACAGAGGTTTCTTAGAGCATTGATCTGCTCTTTAACAAAAAATTATAGAG
TTATAAATGGTTTATAAAAGTCTTACCTTACGGTCAAACTAATAGACTGGCTTACAAGGTTTTATA
AAAAATTAACTTTAGGTCAAGCATGGTGGCTCAAGCCTATAATCCCAGCACTTTGGGAGGCCAA
GGCAGGTGGATCACATGAGGCCAGGAGTTCAAGACCAGTCTGGCCAACATGGTAAAACCCTGT
CTCTACTAAGAATACAAAAATTAGCCAGGAGTGGTGGCATGCACCTGTGATCCCAGCTACTGGG
GAGGCTGAGGCATAAGAATCATTTGAACCTGGGAGGTGGAGGTTGCACTGAGTTGAGATCATG
CCACTGCACTCCAGCCTGGGTGACAGAGGGAGACTATGTCTCAAAAAAGAAAAAAAGAAAAGA
AAAAAAATAACTTCACTGTTAGTAATGCAACAATACAAAGGTGAAATTTGTTTTTCTTTTTTAAAC
AAAATTTTCATATAATATTGAGAGATAATGAAAGGGTTTTCTTTGACTTTTGAGTAAGCTACAGG
AAAACAAAGAAAACAAAGAAAAAAAAAGACAGAAAGAGAGAAGAGACAGATTTAGTTGGCCTG
GTGTTTTCTTTATTGGGTCTTTGTATTAGGCTTTTCTTGCTATGAAGAAATACCTGAGACTGGATA
ATTTATAAAGAAAAGAGGTTTAATTGGCTCACGATTCTGCAAGCTTTATAAAAAGTATGGTGCTT
GCATCAGCTGGGCTTCTAGGGAGGCCTCAGGAAGCTGACAATCATGGCAGAAGGCAAAGGGG
GAGGAGGCATCTCACAAAGCAAGAGCAAGAGAGAGAGAGAATATGGGTGCCACACGCTTTTC
AACAGCCAGATCTCATGAGAACTTACTCACTATTGTGAGAACAGCACCAAGTGGGTGGTGCTAA
GCCATTCATGAAAAATCCACCTCCATAATCTAATCACCTCCCACCAGGCCCCACCTCCAATGCT
GGGAATTACAATTCAACATGAGATTTGGGTGGGGACAAATATACAAACTATATCAGTTTGTTGTT
TAGAAAACTGAGTCTCCCCTCTATTAGTGAGTAAAGGTTTTTGTCTTTTGATATTTTTGAGTTGTC
ATTTCAGCTAAATGAATAATCTGTGATTCCATTTTGTGTTATCAAATGTTTTAAGCCGTTGATATTT
GACAAACCTTCCAAAATTGAAATTCCAAGTTCTAAATTAAGCCTTTTTAGATCTCATTAACCTTTT
TAAATATTACATCTCCTGAAGTCTAAAAGAGACATATTTGACTTATTTGGATATTAAAATCATACA
GGAAGCATTGTGAAATATAAAATAATGTTTAACTTTCTTGGATTCATTATACATTCATATAAATGT
ATTACTAATATCTGTTCCAAAACTGTGTAAGATTCCTATAATTCTGATATATCTCAGTATATGCTAT
CAGTAATAATTAATGGTTGCTATGTTAAATTATTGTGTGCCACAGAGATAATGAAATTTCCTTGTC
AATTGTGTCTTCAACCAAGGCTGCTCTGAAACTGTTGTCATCTATAATTGTTGTTTTATTTTGATTC
TTTTCAAAATGCGGATTTATAATCAGCTATAGGACTCTGATCCTATAGGTAGGTGCTCTTGAATG
CAGGTTTCTGATAACTTTTGAGAGTATGACATTAGAATAAAGTAAAAAACTTCCAAGACTCCCAT
GAAGAGCTAATGTGTTCATGAATATCAAGCAGAAAAAGAGTTGATTACACAGACTGAACTAATG
GAAGACCAAAATAATCTTTTTATGACTTTTTGTTTAAAGTATTGGTAATTCCTTTGTTTTTCAAAGT
CCAGAAAATCTTTCTTTTTCTTTTGAGTGATTTACAGAGTATATTCTTGTGAGAAAAATTTGAAAC
ATTTCTGTCCTTTACCTGATTTCTCCAGAATTTGAAAACTATTTGTAAGTATACTTAATTTATGGCA
GTATGTTTATTTCTATAAGTTCAATAAAAATCTGTTTTCTTTTTTTTTAAACAGGACACCATTGAAG

GCACTGGTTATTTTACCAAGGCTTTGACTGGAATGGCATGGTTTCAGTTATAAACAGACTGCTTT
AAGGAATCAAAGTTGACTTACAGAGCTGATAAAAGGCCCCTAAGAAAAACTGGCCTCACCTCTT
GTCTATATAGTCCCTATATAGGTTTCCTGACCTGTGGTAAGTAAAGAATGTCACTTTCTGACAGG
CCTAGGAGCTCCAAGTTTTTATGGGATCTTGAAAGGAGAAGAATTTACCCAATTCATACAGATAT
TTGCAGGCAGATAAATCCATGGCTGAACTCAGGGCTTTAAACAGTCTAATCTGAGATTTCTTATA
AAAAAATAGCATTCCAGCAAAGTCAATTTTTTTAAAAGGGCCTATATGGCAAATAATTATTCTTG
CTGCACTTTATACAAATAATGAGGCTAATCAGGCCAAATATAATAATACTAAAACTTGTTTTACA
AATAGTTCCATGATTTGTCTTTAGTAAAAATGGGGACTGAAAAAAGAAAATTTGTGTTTCAAAAA
AAAAAACTATAGTACACCTATTGTTAGAGTGTAGTCTTGTCCATTGTTTTTGGATCTGTATTATTTT
CTGCAATTTGGAATGAATCTTTATTTTTTCCAGGCAACAATTCCCCAGACTAATGCTTTTAAATTT
CTTTTTCCAGTTTTTTGACTTGGACTCAATAAAATTGCTGCTACCTTTTTACTGAGGCCTTACAAG
CTAAAGCTTATTCCTTGAGACACAGGTGAGAAAAATGAGTCAGATTGCCACTGCCTTCTTCTTTT
GAAACTGAAGATAGATTCTGCCCAACATCAACTTTGTTTTTCTTCTGTTTCCATAAAAATACCTCC
TATTCACCTTCATCACATATTGAGACCTAGCCCATCTGCAATGGCACCTCCTGGAATGGGAAAC
AGCCATTTAACTAAACTGATCCATTCTTAAGAACTAGACAGGGTTTATAACCATCTTCATGAATA
TTCATAGCTCCTCCTGTAATCTGTTAAATATACATATATACATACACACACACACGTATATATACA
TATATACATACACATACACGTATATATACATACATACATAAGACATGCAAGAAATTCAAGCCATG
TTAAACCCAGACCCTACATTGCCATTAGATGGTTTACATCCTAGTTTGGGTCAGGACCATCCTGA
TGGGAACAGTTACTGGTAATTCTCACTATAATTGTGGGAAATAGGGATACTTCTGTGCTGTGGGT
TTTACTGCTGCAGCGCCTTTTGCATGGGATACTAGATAGACTTTCTCAGGGACTTGCAAGGCTCT
CCACCATAATGCTGCAACAGGTTTCATCTGAGCCCAGGTACCTGAGAACACTTTCACCTCCAAG
TAGATCAGTTCCATTCCAATACCTTTTAACTACACCCCTTTGCAATAGGAAGTAGCTAGAATGAA
TATGTCACTTGAGTTCCATAGAAATAGAATGGCATTTGATAGTGAGGTGTTGTAACCAAGGACC
CTATTTTTGTTATTTTTTTCTTTTTCTTTCTGCAGTCCTTTCTACAACTTATTAGTAATGAAATAATAA
GCTTTACTCTCCTTCTTTCTACCAGGTACTTCCTTGCATTGCTAACTTATCTAATTATGTTTGCTTA
GAAGTTCCAGGGATTGAATCTTGAGACAATCTGGGTGCCTATGGAATTATCTCCCACCAGAAGA
TTACTTCAAGGCAGCAGCTAATTTACAACCTGGTCAAGCCTGAGATGGTGTCAACCCATTCTCAA
GATGGGACAATAACTCAAGATAAGTCATCAAAACAAGCCACGTAGACCAGCACCACCTCTCAC
GCCCCCACCCACCACCCCCCACCCACCACATATCCTCCATACCAAACTTCCCCTTCTTAAACCCT
AGCATTTTGCCCAAGAATTTTGAAGCAGTTTCATTAAGGCAGGAGCCTGAACCACTTCCCCACT
GTTAGCTTTGGAAAATGAAGTCAATTTCCTT

Nucleus

ID|128|gene_id|100048912|transcript_id|NR_003529 Homo sapiens lncRNA

AGCTACATCCGTCACCTGACACGGCCCTACCAGGAACAGCCGCGCTCCCGCGGATTCTGG
TGCTGCTCGCGTCCCCGCTCCCCTATTCCCCTTATTTTATTCCTGGCTCCCCTCGTCGAAAGTCTT
CCATTCTTCAAACTAGATTATTTAAAAATGAAAAAGGAAGAAAGGAAAGCGAGGTCATCTCATT
GCTCTATCCGCCAATCAGGAGGCTGAATGTCAGTTTTGAACTAAAAGCCGCTCCGCTCCTCTTCT
AGATTTGGAAAACAAGCGAAATTAAACTAAACCGCTGCACGCCTCTGACGCGACATCTGGACA
CGGCGCGGCGCTGGCGCTGCCGGAGCTGTCGACCCGGCCTGGCGCCGGACTAGGACTATTTG
CCACGACATTTCAAAGGATTCCAAGAGAGAATATTGGTGTCCATGCTGTGATGATTCCTCAGCTC
CTCTCATCTGATCTCCGTCCTGGCCCCCATGACTTTCTTTGTGGTAGTTAGGGTGTGGTATGTGCC
ACTGAGGCCCACACCTATTGCTGCAATTTATAGCACTGATCTGTCATCAATACCACTTGCTGTCT
TGGATGTGAAGATGATTTTTCCTGCAGGGATTCCCTCTACAAAATTAAAAACACTGGGCATGTG

GAAATAATATTCATGCTTTAAATTGTCTTTTCTCTTCACTACACCAGGGGTCCCCAACCCCTAGG
CCACAGACTGTGGCCCTAGTGTAGTGAATAGAAAAGACAATTTAAAGCGTGAATATTATTTCCT
CATGCCCAGTGTTTTTAATTTTGTACTGGTCTGTGGCTTGTTAGAAACCAGGCTGCACAGCAGAA
GGTGGGCAGCAGATATTGAGAAACCACAGAAAGAGAGAAGTTAAATAATTTTCCTGTCAAAGA
CCACATCAATGATGAAGCCAGAATTTGAGCTCATGTACTTAACCACTGGACTACCTGCCTGCCC
TGTCGAGGAACAGCTAAGTGTCCCTTTTGATGAGAAGAATAAGCCTCATTCTGATTCAACAGCA
GAGATCAAAGAAAAGACTTCTGTTTTCTGGCCACCAGATATATGTTATCTGTGCTTAAAGAATTG
AAAAACACACATCAAAGGAGAATTTTCTTGGAAAGAGAGGGTTCAAGCATCACTGTTAGGTGTG
CTGGAATCCTTTCCCGAGTCAGTACTGCTTTCTAGAAGAAAACCGGGGAGATCTATTTGGAATGT
ATCTAACTCCAAAGAAACCATCAGAGGTAACAGTAGAGACGGGGTTTCACCATGTTGGCCAGA
CTGGTCTTGAACTCTCGACCTCGTGATTCGCCCGCCTCGGCCTCCCAAAGTGCTGGGATTACAG
GTGTGAGACACCACACCCGGCGGATAGAGAGAATTTTGACAGTCTCTCCAATGAACGCCTTCAC
TGATATCCAAAGCATGAAGGACACACCAGGGAAAAACATAGACCTAACACAGGACAAATGGA
ATTATTAGAAACATTTTCTAGCAGAAGAACACTATTCTGTTGCCATTTGAATCTTTGCTTCTTTCTA
GGTTTGACAATGAGCCTATCATATAAGCCCAAATGTAAACAGAAAGAGGTTGAATCAGTCACGA
TAAGCCCAATTATGCTGTGGTAACAAACAACCTCAAAATCTCATTGGCTTAAAATATACAGAATT
ATTCTTACTCATGGCACATATCCATCTATCATCTGCAGGGGATCTGCTCACTGAAGTCACTTAGG
AACTTGGACTGATGGAACGGCCACTTTTTGGTCACTATATGTATTAATCTGTTTTAATCCTGCTGA
TAAAGACCCAAAATTGGGAACAAAAAGAAGTTTAACTAGACTTACAGTTCCGCATGGCTGAGG
AGGCCTCAGAATCATGGTGGGAGGCGAAAGGCACTTCTTACATGGTGGCAGCAAGAGAAAAAT
GAGGAAGAAGCAAAAGCGGAAACCTCTGATAAACCCATCAGATCTTATGAGACTTATTCCACTA
TCAAGAGAATAGCATGGGAAAGACTGGCTCCCATAATTTACCTCCCTCTGGGTCCCTCCCTCAA
CATGTGGGAATTCTGGGAGAAACAATTCAAGATATGACACATTCATAATTTAAACAGAAGCCTA
CGAAGAACTCATAAATTAAAAGAAGATAATCTTTTCACAAGGTGATGGAGGCTTTTTATTTTGCC
ACAAAACCACTGGTGACGTTGCCTGTGGCCACCTTGGAGAAGACACTGGAGGCCTGGGACATG
GAGACTGCTTTTCTGCAGAAACCACATCCCTTGGAGTAATGAGCTACACCTACCTCAATTATTCA
GTGCAGTACAACACTCCAGACAGGGTCTCACTCTGTCACCCAGGCTGGAGTGTATTGGCATGAT
TACAGCTCACTGCAACCTTGAACTCCCAGGCTCAAACCTGAGCAGCTGGGACTACAGATGCAC
CACCATGCATGGTACCAGAGATATAATAATGAGAAACAGACATGCTCCCTCCCCTCATTGAGGT
TACAGCTTAGTGTGGAGACACACAGATGCCTAACGCACTATGGTATGGAAGGTGCTATGGACA
CAGTGCTCAAATCCATGATCTACATAGGTGGAGAACTTCAGTAGAGGAAGTGGCAGGAATTTGG
GAATGAGGAGCACAGTGATTAAACTGGGGCCATTCATATGAGAGTTTAAGAACTCAGACCAGT
GACTTAGATTGGCTTCTCTCACATGGCAAGAAACATTGCTGCTAGCACTTCCCGAGTTCTACGTT
CTACAACATCCACCACTGGATCTTAACATAGACGTAAGATCAAATGCAATAGCATGTCAAACAA
TGTGTAACTCCAGTTATACAAACATTACTGTATCTCATTGGGGATACGAAGCTCTACACACTTGA
AGATGGTGAAGGAATATAAAAATCTATGTCTCACAGTCCAGACTTGGAGTACAAGTAATAAGAA
GAATAAAACTTAATCCCTTAAGTAGATTCACCATAAGTTAGCTCAGAGCAATTCCAGTGCAAGTA
TGGTCTGTGATCCAGTAGTATCTTACAGACAGCAAGTTGAACATTGTGGGATGCATGAGCTATTG
AGGCCTTTGCAGCTTTCTGCTACATGGAGGCTAGGGCCAGAGTCAAGATTTATGCTTTGCAGCA
CACTGGTCAGCTGTTTTTGCAAATCAGATTAAATGATTTTTAAATGAGGCTGAGAGCATGGGAGA
TACTAATGTGTGTTTCCTTGTGAGCTACTGCATAAGTTAGGAAATTGAAATACAGAAAGATGAAA
AGTGATTTGCCCAAGCATATAGATCAAAGCTGTGGCAGAACCAGGACTGGAACCTATATCTCTC
TACTAATGGTTTTTTTAAAAAAATAACCTTGTTTCAAAAATATTAAAAAGTCACAAGAAAGGTAA
ACATGTGGATAAACAAAATGAAGAAAATAAAAATTATCCAGTAATAACATATTGGCATATGTCTT

TCTGGTATATTTTCCTGTGTTGTCATCATTATCATCTCCATCATCATTATATCCATCATTATCATCAT
CATCATCATCATCATCATCATTATCATCACCATAGTGAACATGTAATGCTTACCTAGTGCCAGAT
GCTGTCTAGGCATTTTACATGTGTTACTGGTAACTCATGTAATCCTCATAACAACCTTATAAGGTG
GTTGCTATTATCCCCATGTTACATATGAAGAGACAGAAGCATAAAGAAGTTGCACCGCTGGTAA
TTGGCTGGGATTTGAACTTAAGCAGTCTAACCTTAGAGTAATGATTTTAACAACTATGCTATATA
CATACAAATTTACAAAATAAAACTGGGCTCAGACAATAAAAAAAAAAAAAAAAAAAAAAAAAA
ID|129|gene_id|100048912|transcript_id|NR_047532 Homo sapiens lncRNA
AGCTACATCCGTCACCTGACACGGCCCTACCAGGAACAGCCGCGCTCCCGCGGATTCTGG
TGCTGCTCGCGTCCCCGCTCCCCTATTCCCCTTATTTTATTCCTGGCTCCCCTCGTCGAAAGTCTT
CCATTCTTCAAACTAGATTATTTAAAAATGAAAAAGGAAGAAAGGAAAGCGAGGTCATCTCATT
GCTCTATCCGCCAATCAGGAGGCTGAATGTCAGTTTTGAACTAAAAGCCGCTCCGCTCCTCTTCT
AGATTTGGAAAACAAGCGAAATTAAACTAAACCGCTGCACGCCTCTGACGCGACATCTGGACA
CGGCGCGGCGCTGGCGCTGCCGGAGCTGTCGACCCGGCCTGGCGCCGGACTAGGACTATTTG
CCACGACATTTCAAAGGATTCCAAGAGAGAATATTGGTGTCCATGCTGTGATGATTCCTCAGCTC
CTCTCATCTGATCTCCGTCCTGGCCCCCATGACTTTCTTTGTGGTAGTTAGGGTGTGGTATGTGCC
ACTGAGGCCCACACCTATTGCTATATTGAGAAACCACAGAAAGAGAGAAGTTAAATAATTTTCC
TGTCAAAGACCACATCAATGATGAAGCCAGAATTTGAGCTCATGTACTTAACCACTGGACTACC
TGCCTGCCCTGTCGAGGAACAGCTAAGTGTCCCTTTTGATGAGAAGAATAAGCCTCATTCTGATT
CAACAGCAGAGATCAAAGAAAAGACTTCTGTTTTCTGGCCACCAGATATATGTTATCTGTGCTTA
AAGAATTGAAAAACACACATCAAAGGAGAATTTTCTTGGAAAGAGAGGGTTCAAGCATCACTGT
TAGGTGTGCTGGAATCCTTTCCCGAGTCAGTACTGCTTTCTAGAAGAAAACCGGGGAGATCTAT
TTGGAATGTATCTAACTCCAAAGAAACCATCAGAGGTAACAGTAGAGACGGGGTTTCACCATGT
TGGCCAGACTGGTCTTGAACTCTCGACCTCGTGATTCGCCCGCCTCGGCCTCCCAAAGTGCTGG
GATTACAGGTGTGAGACACCACACCCGGCGGATAGAGAGAATTTTGACAGGCTTTGGTATCAG
GATGATTCTGGCCTCATAAAATGAGTTGGGGAGAATTCCCTCTTTTTCTATTGAATGGAATAGTTT
CAGAAGGAATGGTACCAGCTCCTCTTTGTACCTCTGGTAGAATTCGGCTGTGAATCCATCTGGTC
CTGGACTTTTTTTGGTTGGTACCAGAGATATAATAATGAGAAACAGACATGCTCCCTCCCCTCAT
TGAGGTTACAGCTTAGTGTGGAGACACACAGATGCCTAACGCACTATGGTATGGAAGGTGCTAT
GGACACAGTGCTCAAATCCATGATCTACATAGGTGGAGAACTTCAGTAGAGGAAGTGGCAGGA
ATTTGGGAATGAGGAGCACAGTGATTAAACTGGGGCCATTCATATGAGAGTTTAAGAACTCAGA
CCAGTGACTTAGATTGGCTTCTCTCACATGGCAAGAAACATTGCTGCTAGCACTTCCCGAGTTCT
ACGTTCTACAACATCCACCACTGGATCTTAACATAGACGTAAGATCAAATGCAATAGCATGTCA
AACAATGTGTAACTCCAGTTATACAAACATTACTGTATCTCATTGGGGATACGAAGCTCTACACA
CTTGAAGATGGTGAAGGAATATAAAAATCTATGTCTCACAGTCCAGACTTGGAGTACAAGTAAT
AAGAAGAATAAAACTTAATCCCTTAAGTAGATTCACCATAAGTTAGCTCAGAGCAATTCCAGTG
CAAGTATGGTCTGTGATCCAGTAGTATCTTACAGACAGCAAGTTGAACATTGTGGGATGCATGA
GCTATTGAGGCCTTTGCAGCTTTCTGCTACATGGAGGCTAGGGCCAGAGTCAAGATTTATGCTTT
GCAGCACACTGGTCAGCTGTTTTTGCAAATCAGATTAAATGATTTTTAAATGAGGCTGAGAGCAT
GGGAGATACTAATGTGTGTTTCCTTGTGAGCTACTGCATAAGTTAGGAAATTGAAATACAGAAA
GATGAAAAGTGATTTGCCCAAGCATATAGATCAAAGCTGTGGCAGAACCAGGACTGGAACCTA
TATCTCTCTACTAATGGTTTTTTTAAAAAAATAACCTTGTTTCAAAAATATTAAAAAGTCACAAGA
AAGGTAAACATGTGGATAAACAAAATGAAGAAAATAAAAATTATCCAGTAATAACATATTGGCA
TATGTCTTTCTGGTATATTTTCCTGTGTTGTCATCATTATCATCTCCATCATCATTATATCCATCATT
ATCATCATCATCATCATCATCATCATCATTATCATCACCATAGTGAACATGTAATGCTTACCTAGT

GCCAGATGCTGTCTAGGCATTTTACATGTGTTACTGGTAACTCATGTAATCCTCATAACAACCTT
ATAAGGTGGTTGCTATTATCCCCATGTTACATATGAAGAGACAGAAGCATAAAGAAGTTGCACC
GCTGGTAATTGGCTGGGATTTGAACTTAAGCAGTCTAACCTTAGAGTAATGATTTTAACAACTAT
GCTATATACATACAAATTTACAAAATAAAACTGGGCTCAGACAATAAAAAAAAAAAAAAAAAAA
AAAAAA

ID|130|gene_id|100048912|transcript_id|NR_047533 Homo sapiens lncRNA

AGCTACATCCGTCACCTGACACGGCCCTACCAGGAACAGCCGCGCTCCCGCGGATTCTGG
TGCTGCTCGCGTCCCCGCTCCCCTATTCCCCTTATTTTATTCCTGGCTCCCCTCGTCGAAAGTCTT
CCATTCTTCAAACTAGATTATTTAAAAATGAAAAAGGAAGAAAGGAAAGCGAGGTCATCTCATT
GCTCTATCCGCCAATCAGGAGGCTGAATGTCAGTTTTGAACTAAAAGCCGCTCCGCTCCTCTTCT
AGATTTGGAAAACAAGCGAAATTAAACTAAACCGCTGCACGCCTCTGACGCGACATCTGGACA
CGGCGCGGCGCTGGCGCTGCCGGAGCTGTCGACCCGGCCTGGCGCCGGACTAGTGTCCCTTTT
GATGAGAAGAATAAGCCTCATTCTGATTCAACAGCAGAGATCAAAGAAAAGACTTCTGTTTTCT
GGCCACCAGATATATGTTATCTGTGCTTAAAGAATTGAAAAACACACATCAAAGGAGAATTTTCT
TGGAAAGAGAGGGTTCAAGCATCACTGTTAGGTGTGCTGGAATCCTTTCCCGAGTCAGTACTGC
TTTCTAGAAGAAAACCGGGGAGATCTATTTGGAATGTATCTAACTCCAAAGAAACCATCAGAGG
TAACAGGATGATTACGGAGCTAGGAATTTCCTACGAAGCTGGGTGATAAAAGGACATTGGACA
AAAACACAGATAGCTTCATTCTATACCAGGATCCATCACTGATTAGCTGTATTATATTTGTCTCTA
CTGTAATTTGAGGAGAATGATACCTGCTTCTCTATCTTGTTGGGTTTTACAGATGATGTATTGAAA
GTTCAATAAAATCTATAAACAAT

ID|131|gene_id|100048912|transcript_id|NR_047534 Homo sapiens lncRNA

AGCTACATCCGTCACCTGACACGGCCCTACCAGGAACAGCCGCGCTCCCGCGGATTCTGG
TGCTGCTCGCGTCCCCGCTCCCCTATTCCCCTTATTTTATTCCTGGCTCCCCTCGTCGAAAGTCTT
CCATTCTTCAAACTAGATTATTTAAAAATGAAAAAGGAAGAAAGGAAAGCGAGGTCATCTCATT
GCTCTATCCGCCAATCAGGAGGCTGAATGTCAGTTTTGAACTAAAAGCCGCTCCGCTCCTCTTCT
AGATTTGGAAAACAAGCGAAATTAAACTAAACCGCTGCACGCCTCTGACGCGACATCTGGACA
CGGCGCGGCGCTGGCGCTGCCGGAGCTGTCGACCCGGCCTGGCGCCGGACTAGTGTCCCTTTT
GATGAGAAGAATAAGCCTCATTCTGATTCAACAGCAGAGATCAAAGAAAAGACTTCTGTTTTCT
GGCCACCAGATATATGTTATCTGTGCTTAAAGAATTGAAAAACACACATCAAAGGAGAATTTTCT
TGGAAAGAGAGGGTTCAAGCATCACTGTTAGGTGTGCTGGAATCCTTTCCCGAGTCAGTACTGC
TTTCTAGAAGAAAACCGGGGAGATCTATTTGGAATGTATCTAACTCCAAAGAAACCATCAGAGG
TAACAGGTGGAGAACTTCAGTAGAGGAAGTGGCAGGAATTTGGGAATGAGGAGCACAGTGATT
AAACTGGGGCCATTCATATGAGAGTTTAAGAACTCAGACCAGTGACTTAGATTGGCTTCTCTCA
CATGGCAAGAAACATTGCTGCTAGCACTTCCCGAGTTCTACGTTCTACAACATCCACCACTGGA
TCTTAACATAGACGTAAGATCAAATGCAATAGCATGTCAAACAATGTGTAACTCCAGTTATACAA
ACATTACTGTATCTCATTGGGGATACGAAGCTCTACACACTTGAAGATGGTGAAGGAATATAAA
AATCTATGTCTCACAGTCCAGACTTGGAGTACAAGTAATAAGAAGAATAAAACTTAATCCCTTAA
GTAGATTCACCATAAGTTAGCTCAGAGCAATTCCAGTGCAAGTATGGTCTGTGATCCAGTAGTAT
CTTACAGACAGCAAGTTGAACATTGTGGGATGCATGAGCTATTGAGGCCTTTGCAGCTTTCTGCT
ACATGGAGGCTAGGGCCAGAGTCAAGATTTATGCTTTGCAGCACACTGGTCAGCTGTTTTTGCA
AATCAGATTAAATGATTTTTAAATGAGGCTGAGAGCATGGGAGATACTAATGTGTGTTTCCTTGT
GAGCTACTGCATAAGTTAGGAAATTGAAATACAGAAAGATGAAAAGTGATTTGCCCAAGCATAT
AGATCAAAGCTGTGGCAGAACCAGGACTGGAACCTATATCTCTCTACTAATGGTTTTTTTAAAAA
AATAACCTTGTTTCAAAAATATTAAAAAGTCACAAGAAAGGTAAACATGTGGATAAACAAAATG

AAGAAAATAAAAATTATCCAGTAATAACATATTGGCATATGTCTTTCTGGTATATTTTCCTGTGTT
GTCATCATTATCATCTCCATCATCATTATATCCATCATTATCATCATCATCATCATCATCATCATCA
TTATCATCACCATAGTGAACATGTAATGCTTACCTAGTGCCAGATGCTGTCTAGGCATTTTACAT
GTGTTACTGGTAACTCATGTAATCCTCATAACAACCTTATAAGGTGGTTGCTATTATCCCCATGTT
ACATATGAAGAGACAGAAGCATAAAGAAGTTGCACCGCTGGTAATTGGCTGGGATTTGAACTTA
AGCAGTCTAACCTTAGAGTAATGATTTTAACAACTATGCTATATACATACAAATTTACAAAATAA
AACTGGGCTCAGACAATAAAAAAAAAAAAAAAAAAAAAAAAAA
ID|132|gene_id|100048912|transcript_id|NR_047535 Homo sapiens lncRNA
AGCTACATCCGTCACCTGACACGGCCCTACCAGGAACAGCCGCGCTCCCGCGGATTCTGG
TGCTGCTCGCGTCCCCGCTCCCCTATTCCCCTTATTTTATTCCTGGCTCCCCTCGTCGAAAGTCTT
CCATTCTTCAAACTAGATTATTTAAAAATGAAAAAGGAAGAAAGGAAAGCGAGGTCATCTCATT
GCTCTATCCGCCAATCAGGAGGCTGAATGTCAGTTTTGAACTAAAAGCCGCTCCGCTCCTCTTCT
AGATTTGGAAAACAAGCGAAATTAAACTAAACCGCTGCACGCCTCTGACGCGACATCTGGACA
CGGCGCGGCGCTGGCGCTGCCGGAGCTGTCGACCCGGCCTGGCGCCGGACTAGTGTCCCTTTT
GATGAGAAGAATAAGCCTCATTCTGATTCAACAGCAGAGATCAAAGAAAAGACTTCTGTTTTCT
GGCCACCAGATATATGTTATCTGTGCTTAAAGAATTGAAAAACACACATCAAAGGAGAATTTTCT
TGGAAAGAGAGGGTTCAAGCATCACTGTTAGGTGTGCTGGAATCCTTTCCCGAGTCAGTACTGC
TTTCTAGAAGAAAACCGGGGAGATCTATTTGGAATGTATCTAACTCCAAAGAAACCATCAGAGG
TAACAGTAGAGACGGGGTTTCACCATGTTGGCCAGACTGGTCTTGAACTCTCGACCTCGTGATT
CGCCCGCCTCGGCCTCCCAAAGTGCTGGGATTACAGGTGTGAGACACCACACCCGGCGGATAG
AGAGAATTTTGACAGATTGGCTTCTCTCACATGGCAAGAAACATTGCTGCTAGCACTTCCCGAGT
TCTACGTTCTACAACATCCACCACTGCTATGTCTCACAGTCCAGACTTGGAGTACAAGTAATAAG
AAGAATAAAACTTAATCCCTTAAGTAGATTCACCATAAGTTAGCTCAGAGCAATTCCAGTGCAA
GTATGGTCTGTGATCCAGTAGTATCTTACAGACAGCAAGTTGAACATTGTGGGATGCATGAGCT
ATTGAGGCCTTTGCAGCTTTCTGCTACATGGAGGCTAGGGCCAGAGTCAAGATTTATGCTTTGCA
GCACACTGGTCAGCTGTTTTTGCAAATCAGATTAAATGATTTTTAAATGAGGCTGAGAGCATGGG
AGATACTAATGTGTGTTTCCTTGTGAGCTACTGCATAAGTTAGGAAATTGAAATACAGAAAGATG
AAAAGTGATTTGCCCAAGCATATAGATCAAAGCTGTGGCAGAACCAGGACTGGAACCTATATCT
CTCTACTAATGGTTTTTTTAAAAAAATAACCTTGTTTCAAAAATATTAAAAAGTCACAAGAAAGGT
AAACATGTGGATAAACAAAATGAAGAAAATAAAAATTATCCAGTAATAACATATTGGCATATGT
CTTTCTGGTATATTTTCCTGTGTTGTCATCATTATCATCTCCATCATCATTATATCCATCATTATCAT
CATCATCATCATCATCATCATCATTATCATCACCATAGTGAACATGTAATGCTTACCTAGTGCCA
GATGCTGTCTAGGCATTTTACATGTGTTACTGGTAACTCATGTAATCCTCATAACAACCTTATAAG
GTGGTTGCTATTATCCCCATGTTACATATGAAGAGACAGAAGCATAAAGAAGTTGCACCGCTGG
TAATTGGCTGGGATTTGAACTTAAGCAGTCTAACCTTAGAGTAATGATTTTAACAACTATGCTAT
ATACATACAAATTTACAAAATAAAACTGGGCTCAGACAATAAAAAAAAAAAAAAAAAAAAAAAA
AA
ID|133|gene_id|100048912|transcript_id|NR_047536 Homo sapiens lncRNA
AGCTACATCCGTCACCTGACACGGCCCTACCAGGAACAGCCGCGCTCCCGCGGATTCTGG
TGCTGCTCGCGTCCCCGCTCCCCTATTCCCCTTATTTTATTCCTGGCTCCCCTCGTCGAAAGTCTT
CCATTCTTCAAACTAGATTATTTAAAAATGAAAAAGGAAGAAAGGAAAGCGAGGTCATCTCATT
GCTCTATCCGCCAATCAGGAGGCTGAATGTCAGTTTTGAACTAAAAGCCGCTCCGCTCCTCTTCT
AGATTTGGAAAACAAGCGAAATTAAACTAAACCGCTGCACGCCTCTGACGCGACATCTGGACA
CGGCGCGGCGCTGGCGCTGCCGGAGCTGTCGACCCGGCCTGGCGCCGGACTAGTGTCCCTTTT

GATGAGAAGAATAAGCCTCATTCTGATTCAACAGCAGAGATCAAAGAAAAGACTTCTGTTTTCT
GGCCACCAGATATATGTTATCTGTGCTTAAAGAATTGAAAAACACACATCAAAGGAGAATTTTCT
TGGAAAGAGAGGGTTCAAGCATCACTGTTAGGTGTGCTGGAATCCTTTCCCGAGTCAGTACTGC
TTTCTAGAAGAAAACCGGGGAGATCTATTTGGAATGTATCTAACTCCAAAGAAACCATCAGAGG
TAACAGATTGGCTTCTCTCACATGGCAAGAAACATTGCTGCTAGCACTTCCCGAGTTCTACGTTC
TACAACATCCACCACTGCTATGTCTCACAGTCCAGACTTGGAGTACAAGTAATAAGAAGAATAA
AACTTAATCCCTTAAGTAGATTCACCATAAGTTAGCTCAGAGCAATTCCAGTGCAAGTATGGTCT
GTGATCCAGTAGTATCTTACAGACAGCAAGTTGAACATTGTGGGATGCATGAGCTATTGAGGCC
TTTGCAGCTTTCTGCTACATGGAGGCTAGGGCCAGAGTCAAGATTTATGCTTTGCAGCACACTG
GTCAGCTGTTTTTGCAAATCAGATTAAATGATTTTTAAATGAGGCTGAGAGCATGGGAGATACTA
ATGTGTGTTTCCTTGTGAGCTACTGCATAAGTTAGGAAATTGAAATACAGAAAGATGAAAAGTG
ATTTGCCCAAGCATATAGATCAAAGCTGTGGCAGAACCAGGACTGGAACCTATATCTCTCTACT
AATGGTTTTTTTAAAAAAATAACCTTGTTTCAAAAATATTAAAAAGTCACAAGAAAGGTAAACAT
GTGGATAAACAAAATGAAGAAAATAAAAATTATCCAGTAATAACATATTGGCATATGTCTTTCTG
GTATATTTTCCTGTGTTGTCATCATTATCATCTCCATCATCATTATATCCATCATTATCATCATCAT
CATCATCATCATCATCATTATCATCACCATAGTGAACATGTAATGCTTACCTAGTGCCAGATGCT
GTCTAGGCATTTTACATGTGTTACTGGTAACTCATGTAATCCTCATAACAACCTTATAAGGTGGTT
GCTATTATCCCCATGTTACATATGAAGAGACAGAAGCATAAAGAAGTTGCACCGCTGGTAATTG
GCTGGGATTTGAACTTAAGCAGTCTAACCTTAGAGTAATGATTTTAACAACTATGCTATATACAT
ACAAATTTACAAAATAAAACTGGGCTCAGACAATAAAAAAAAAAAAAAAAAAAAAAAAAA
ID|134|gene_id|100048912|transcript_id|NR_047537 Homo sapiens lncRNA
AGCTACATCCGTCACCTGACACGGCCCTACCAGGAACAGCCGCGCTCCCGCGGATTCTGG
TGCTGCTCGCGTCCCCGCTCCCCTATTCCCCTTATTTTATTCCTGGCTCCCCTCGTCGAAAGTCTT
CCATTCTTCAAACTAGATTATTTAAAAATGAAAAAGGAAGAAAGGAAAGCGAGGTCATCTCATT
GCTCTATCCGCCAATCAGGAGGCTGAATGTCAGTTTTGAACTAAAAGCCGCTCCGCTCCTCTTCT
AGATTTGGAAAACAAGCGAAATTAAACTAAACCGCTGCACGCCTCTGACGCGACATCTGGACA
CGGCGCGGCGCTGGCGCTGCCGGAGCTGTCGACCCGGCCTGGCGCCGGACTAGTGTCCCTTTT
GATGAGAAGAATAAGCCTCATTCTGATTCAACAGCAGAGATCAAAGAAAAGACTTCTGTTTTCT
GGCCACCAGATATATGTTATCTGTGCTTAAAGAATTGAAAAACACACATCAAAGGAGAATTTTCT
TGGAAAGAGAGGGTTCAAGCATCACTGTTAGGTGTGCTGGAATCCTTTCCCGAGTCAGTACTGC
TTTCTAGAAGAAAACCGGGGAGATCTATTTGGAATGTATCTAACTCCAAAGAAACCATCAGAGG
TAACAGTAGAGACGGGGTTTCACCATGTTGGCCAGACTGGTCTTGAACTCTCGACCTCGTGATT
CGCCCGCCTCGGCCTCCCAAAGTGCTGGGATTACAGGTGTGAGACACCACACCCGGCGGATAG
AGAGAATTTTGACAGTATCTTACAGACAGCAAGTTGAACATTGTGGGATGCATGAGCTATTGAG
GCCTTTGCAGCTTTCTGCTACATGGAGGCTAGGGCCAGAGTCAAGATTTATGCTTTGCAGCACA
CTGGTCAGCTGTTTTTGCAAATCAGATTAAATGATTTTTAAATGAGGCTGAGAGCATGGGAGATA
CTAATGTGTGTTTCCTTGTGAGCTACTGCATAAGTTAGGAAATTGAAATACAGAAAGATGAAAA
GTGATTTGCCCAAGCATATAGATCAAAGCTGTGGCAGAACCAGGACTGGAACCTATATCTCTCT
ACTAATGGTTTTTTTAAAAAAATAACCTTGTTTCAAAAATATTAAAAAGTCACAAGAAAGGTAAA
CATGTGGATAAACAAAATGAAGAAAATAAAAATTATCCAGTAATAACATATTGGCATATGTCTTT
CTGGTATATTTTCCTGTGTTGTCATCATTATCATCTCCATCATCATTATATCCATCATTATCATCAT
CATCATCATCATCATCATCATTATCATCACCATAGTGAACATGTAATGCTTACCTAGTGCCAGAT
GCTGTCTAGGCATTTTACATGTGTTACTGGTAACTCATGTAATCCTCATAACAACCTTATAAGGTG
GTTGCTATTATCCCCATGTTACATATGAAGAGACAGAAGCATAAAGAAGTTGCACCGCTGGTAA

TTGGCTGGGATTTGAACTTAAGCAGTCTAACCTTAGAGTAATGATTTTAACAACTATGCTATATA
CATACAAATTTACAAAATAAAACTGGGCTCAGACAATAAAAAAAAAAAAAAAAAAAAAAAAAA
ID|135|gene_id|100048912|transcript_id|NR_047538 Homo sapiens lncRNA
AGCTACATCCGTCACCTGACACGGCCCTACCAGGAACAGCCGCGCTCCCGCGGATTCTGG
TGCTGCTCGCGTCCCCGCTCCCCTATTCCCCTTATTTTATTCCTGGCTCCCCTCGTCGAAAGTCTT
CCATTCTTCAAACTAGATTATTTAAAAATGAAAAAGGAAGAAAGGAAAGCGAGGTCATCTCATT
GCTCTATCCGCCAATCAGGAGGCTGAATGTCAGTTTTGAACTAAAAGCCGCTCCGCTCCTCTTCT
AGATTTGGAAAACAAGCGAAATTAAACTAAACCGCTGCACGCCTCTGACGCGACATCTGGACA
CGGCGCGGCGCTGGCGCTGCCGGAGCTGTCGACCCGGCCTGGCGCCGGACTAGTGTCCCTTTT
GATGAGAAGAATAAGCCTCATTCTGATTCAACAGCAGAGATCAAAGAAAAGACTTCTGTTTTCT
GGCCACCAGATATATGTTATCTGTGCTTAAAGAATTGAAAAACACACATCAAAGGAGAATTTTCT
TGGAAAGAGAGGGTTCAAGCATCACTGTTAGGTGTGCTGGAATCCTTTCCCGAGTCAGTACTGC
TTTCTAGAAGAAAACCGGGGAGATCTATTTGGAATGTATCTAACTCCAAAGAAACCATCAGAGG
TAACAGTATCTTACAGACAGCAAGTTGAACATTGTGGGATGCATGAGCTATTGAGGCCTTTGCA
GCTTTCTGCTACATGGAGGCTAGGGCCAGAGTCAAGATTTATGCTTTGCAGCACACTGGTCAGC
TGTTTTTGCAAATCAGATTAAATGATTTTTAAATGAGGCTGAGAGCATGGGAGATACTAATGTGT
GTTTCCTTGTGAGCTACTGCATAAGTTAGGAAATTGAAATACAGAAAGATGAAAAGTGATTTGC
CCAAGCATATAGATCAAAGCTGTGGCAGAACCAGGACTGGAACCTATATCTCTCTACTAATGGT
TTTTTTAAAAAAATAACCTTGTTTCAAAAATATTAAAAAGTCACAAGAAAGGTAAACATGTGGAT
AAACAAAATGAAGAAAATAAAAATTATCCAGTAATAACATATTGGCATATGTCTTTCTGGTATAT
TTTCCTGTGTTGTCATCATTATCATCTCCATCATCATTATATCCATCATTATCATCATCATCATCAT
CATCATCATCATTATCATCACCATAGTGAACATGTAATGCTTACCTAGTGCCAGATGCTGTCTAG
GCATTTTACATGTGTTACTGGTAACTCATGTAATCCTCATAACAACCTTATAAGGTGGTTGCTATT
ATCCCCATGTTACATATGAAGAGACAGAAGCATAAAGAAGTTGCACCGCTGGTAATTGGCTGG
GATTTGAACTTAAGCAGTCTAACCTTAGAGTAATGATTTTAACAACTATGCTATATACATACAAAT
TTACAAAATAAAACTGGGCTCAGACAATAAAAAAAAAAAAAAAAAAAAAAAAAA
ID|136|gene_id|100048912|transcript_id|NR_047539 Homo sapiens lncRNA
AGCTACATCCGTCACCTGACACGGCCCTACCAGGAACAGCCGCGCTCCCGCGGATTCTGG
TGCTGCTCGCGTCCCCGCTCCCCTATTCCCCTTATTTTATTCCTGGCTCCCCTCGTCGAAAGTCTT
CCATTCTTCAAACTAGATTATTTAAAAATGAAAAAGGAAGAAAGGAAAGCGAGGTCATCTCATT
GCTCTATCCGCCAATCAGGAGGCTGAATGTCAGTTTTGAACTAAAAGCCGCTCCGCTCCTCTTCT
AGATTTGGAAAACAAGCGAAATTAAACTAAACCGCTGCACGCCTCTGACGCGACATCTGGACA
CGGCGCGGCGCTGGCGCTGCCGGAGCTGTCGACCCGGCCTGGCGCCGGACTAGGACTATTTG
CCACGACATTTCAAAGGATTCCAAGAGAGAATATTGGTGTCCATGCTGTGATGATTCCTCAGCTC
CTCTCATCTGATCTCCGTCCTGGCCCCCATGACTTTCTTTGTGGTAGTTAGGGTGTGGTATGTGCC
ACTGAGGCCCACACCTATTGCTGCAATTTATAGCACTGATCTGTCATCAATACCACTTGCTGTCT
TGGATGTGAAGATGATTTTTCCTGCAGGGATTCCCTCTACAAAATTAAAAACACTGGGCATGTG
GAAATAATATTCATGCTTTAAATTGTCTTTTCTCTTCACTACACCAGGGGTCCCCAACCCCTAGG
CCACAGACTGTGGCCCTAGTGTAGTGAATAGAAAAGACAATTTAAAGCGTGAATATTATTTCCT
CATGCCCAGTGTTTTTAATTTTGTACTGGTCTGTGGCTTGTTAGAAACCAGGCTGCACAGCAGAA
GGTGGGCAGCAGATATTGAGAAACCACAGAAAGAGAGAAGTTAAATAATTTTCCTGTCAAAGA
CCACATCAATGATGAAGCCAGAATTTGAGCTCATGTACTTAACCACTGGACTACCTGCCTGCCC
TGTCGAGGAACAGCTAAGTGTCCCTTTTGATGAGAAGAATAAGCCTCATTCTGATTCAACAGCA
GAGATCAAAGAAAAGACTTCTGTTTTCTGGCCACCAGATATATGTTATCTGTGCTTAAAGAATTG

AAAAACACACATCAAAGGAGAATTTTCTTGGAAAGAGAGGGTTCAAGCATCACTGTTAGGTGTG
CTGGAATCCTTTCCCGAGTCAGTACTGCTTTCTAGAAGAAAACCGGGGAGATCTATTTGGAATGT
ATCTAACTCCAAAGAAACCATCAGAGGTAACAGTAGAGACGGGGTTTCACCATGTTGGCCAGA
CTGGTCTTGAACTCTCGACCTCGTGATTCGCCCGCCTCGGCCTCCCAAAGTGCTGGGATTACAG
GTGTGAGACACCACACCCGGCGGATAGAGAGAATTTTGACAGTCTCTCCAATGAACGCCTTCAC
TGATATCCAAAGCATGAAGGACACACCAGGGAAAAACATAGACCTAACACAGGACAAATGGA
ATTATTAGAAACATTTTCTAGCAGAAGAACACTATTCTGTTGCCATTTGAATCTTTGCTTCTTTCTA
GGTTTGACAATGAGCCTATCATATAAGCCCAAATGTAAACAGAAAGAGGTTGAATCAGTCACGA
TAAGCCCAATTATGCTGTGGTAACAAACAACCTCAAAATCTCATTGGCTTAAAATATACAGAATT
ATTCTTACTCATGGCACATATCCATCTATCATCTGCAGGGGATCTGCTCACTGAAGTCACTTAGG
AACTTGGACTGATGGAACGGCCACTTTTTGGTCACTATATGTATTAATCTGTTTTAATCCTGCTGA
TAAAGACCCAAAATTGGGAACAAAAAGAAGTTTAACTAGACTTACAGTTCCGCATGGCTGAGG
AGGCCTCAGAATCATGGTGGGAGGCGAAAGGCACTTCTTACATGGTGGCAGCAAGAGAAAAAT
GAGGAAGAAGCAAAAGCGGAAACCTCTGATAAACCCATCAGATCTTATGAGACTTATTCCACTA
TCAAGAGAATAGCATGGGAAAGACTGGCTCCCATAATTTACCTCCCTCTGGGTCCCTCCCTCAA
CATGTGGGAATTCTGGGAGAAACAATTCAAGATATGACACATTCATAATTTAAACAGAAGCCTA
CGAAGAACTCATAAATTAAAAGAAGATAATCTTTTCACAAGGTGATGGAGGCTTTTTATTTTGCC
ACAAAACCACTGGTGACGTTGCCTGTGGCCACCTTGGAGAAGACACTGGAGGCCTGGGACATG
GAGACTGCTTTTCTGCAGAAACCACATCCCTTGGAGTAATGAGCTACACCTACCTCAATTATTCA
GTGCAGTACAACACTCCAGACAGGGTCTCACTCTGTCACCCAGGCTGGAGTGTATTGGCATGAT
TACAGCTCACTGCAACCTTGAACTCCCAGGCTCAAACCTGAGCAGCTGGGACTACAGATGCAC
CACCATGCATGGATGATTACGGAGCTAGGAATTTCCTACGAAGCTGGGTGATAAAAGGACATTG
GACAAAAACACAGATAGCTTCATTCTATACCAGGATCCATCACTGATTAGCTGTATTATATTTGT
CTCTACTGTAATTTGAGGAGAATGATACCTGCTTCTCTATCTTGTTGGGTTTTACAGATGATGTAT
TGAAAGTTCAATAAAATCTATAAACAAT
ID|137|gene_id|100048912|transcript_id|NR_047540 Homo sapiens lncRNA
AGCTACATCCGTCACCTGACACGGCCCTACCAGGAACAGCCGCGCTCCCGCGGATTCTGG
TGCTGCTCGCGTCCCCGCTCCCCTATTCCCCTTATTTTATTCCTGGCTCCCCTCGTCGAAAGTCTT
CCATTCTTCAAACTAGATTATTTAAAAATGAAAAAGGAAGAAAGGAAAGCGAGGTCATCTCATT
GCTCTATCCGCCAATCAGGAGGCTGAATGTCAGTTTTGAACTAAAAGCCGCTCCGCTCCTCTTCT
AGATTTGGAAAACAAGCGAAATTAAACTAAACCGCTGCACGCCTCTGACGCGACATCTGGACA
CGGCGCGGCGCTGGCGCTGCCGGAGCTGTCGACCCGGCCTGGCGCCGGACTAGTGTCCCTTTT
GATGAGAAGAATAAGCCTCATTCTGATTCAACAGCAGAGATCAAAGAAAAGACTTCTGTTTTCT
GGCCACCAGATATATGTTATCTGTGCTTAAAGAATTGAAAAACACACATCAAAGGAGAATTTTCT
TGGAAAGAGAGGGTTCAAGCATCACTGTTAGGTGTGCTGGAATCCTTTCCCGAGTCAGTACTGC
TTTCTAGAAGAAAACCGGGGAGATCTATTTGGAATGTATCTAACTCCAAAGAAACCATCAGAGG
TAACAGTAGAGACGGGGTTTCACCATGTTGGCCAGACTGGTCTTGAACTCTCGACCTCGTGATT
CGCCCGCCTCGGCCTCCCAAAGTGCTGGGATTACAGGTGTGAGACACCACACCCGGCGGATAG
AGAGAATTTTGACAGATATGACACATTCATAATTTAAACAGAAGCCTACGAAGAACTCATAAATT
AAAAGAAGATAATCTTTTCACAAGGTGATGGAGGCTTTTTATTTTGCCACAAAACCACTGGTGAC
GTTGCCTGTGGCCACCTTGGAGAAGACACTGGAGACAGGGTCTCACTCTGTCACCCAGGCTGG
AGTGTATTGGCATGATTACAGCTCACTGCAACCTTGAACTCCCAGGCTCAAACCTGAGCAGCTG
GGACTACAGATGCACCACCATGCATGGATGATTACGGAGCTAGGAATTTCCTACGAAGCTGGG
TGATAAAAGGACATTGGACAAAAACACAGATAGCTTCATTCTATACCAGGATCCATCACTGATT

AGCTGTATTATATTTGTCTCTACTGTAATTTGAGGAGAATGATACCTGCTTCTCTATCTTGTTGGG TTTTACAGATGATGTATTGAAAGTTCAATAAAATCTATAAACAAT

ID|138|gene_id|100048912|transcript_id|NR_047541 Homo sapiens lncRNA

AGCTACATCCGTCACCTGACACGGCCCTACCAGGAACAGCCGCGCTCCCGCGGATTCTGG TGCTGCTCGCGTCCCCGCTCCCCTATTCCCCTTATTTTATTCCTGGCTCCCCTCGTCGAAAGTCTT CCATTCTTCAAACTAGATTATTTAAAAATGAAAAAGGAAGAAAGGAAAGCGAGGTCATCTCATT GCTCTATCCGCCAATCAGGAGGCTGAATGTCAGTTTTGAACTAAAAGCCGCTCCGCTCCTCTTCT AGATTTGGAAAACAAGCGAAATTAAACTAAACCGCTGCACGCCTCTGACGCGACATCTGGACA CGGCGCGGCGCTGGCGCTGCCGGAGCTGTCGACCCGGCCTGGCGCCGGACTAGTGTCCCTTTT GATGAGAAGAATAAGCCTCATTCTGATTCAACAGCAGAGATCAAAGAAAAGACTTCTGTTTTCT GGCCACCAGATATATGTTATCTGTGCTTAAAGAATTGAAAAACACACATCAAAGGAGAATTTTCT TGGAAAGAGAGGGTTCAAGCATCACTGTTAGGTGTGCTGGAATCCTTTCCCGAGTCAGTACTGC TTTCTAGAAGAAAACCGGGGAGATCTATTTGGAATGTATCTAACTCCAAAGAAACCATCAGAGG TAACAGTAGAGACGGGGTTTCACCATGTTGGCCAGACTGGTCTTGAACTCTCGACCTCGTGATT CGCCCGCCTCGGCCTCCCAAAGTGCTGGGATTACAGGTGTGAGACACCACACCCGGCGGATAG AGAGAATTTTGACAGGTGATGGAGGCTTTTTATTTTGCCACAAAACCACTGGTGACGTTGCCTGT GGCCACCTTGGAGAAGACACTGGAGGATGATTACGGAGCTAGGAATTTCCTACGAAGCTGGGT GATAAAAGGACATTGGACAAAAACACAGATAGCTTCATTCTATACCAGGATCCATCACTGATTA GCTGTATTATATTTGTCTCTACTGTAATTTGAGGAGAATGATACCTGCTTCTCTATCTTGTTGGGTT TTACAGATGATGTATTGAAAGTTCAATAAAATCTATAAACAAT

ID|139|gene_id|100048912|transcript_id|NR_047542 Homo sapiens lncRNA

AGCTACATCCGTCACCTGACACGGCCCTACCAGGAACAGCCGCGCTCCCGCGGATTCTGG TGCTGCTCGCGTCCCCGCTCCCCTATTCCCCTTATTTTATTCCTGGCTCCCCTCGTCGAAAGTCTT CCATTCTTCAAACTAGATTATTTAAAAATGAAAAAGGAAGAAAGGAAAGCGAGGTCATCTCATT GCTCTATCCGCCAATCAGGAGGCTGAATGTCAGTTTTGAACTAAAAGCCGCTCCGCTCCTCTTCT AGATTTGGAAAACAAGCGAAATTAAACTAAACCGCTGCACGCCTCTGACGCGACATCTGGACA CGGCGCGGCGCTGGCGCTGCCGGAGCTGTCGACCCGGCCTGGCGCCGGACTAGTGTCCCTTTT GATGAGAAGAATAAGCCTCATTCTGATTCAACAGCAGAGATCAAAGAAAAGACTTCTGTTTTCT GGCCACCAGATATATGTTATCTGTGCTTAAAGAATTGAAAAACACACATCAAAGGAGAATTTTCT TGGAAAGAGAGGGTTCAAGCATCACTGTTAGGTGTGCTGGAATCCTTTCCCGAGTCAGTACTGC TTTCTAGAAGAAAACCGGGGAGATCTATTTGGAATGTATCTAACTCCAAAGAAACCATCAGAGG TAACAGTAGAGACGGGGTTTCACCATGTTGGCCAGACTGGTCTTGAACTCTCGACCTCGTGATT CGCCCGCCTCGGCCTCCCAAAGTGCTGGGATTACAGGTGTGAGACACCACACCCGGCGGATAG AGAGAATTTTGACAGGATGATTACGGAGCTAGGAATTTCCTACGAAGCTGGGTGATAAAAGGA CATTGGACAAAAACACAGATAGCTTCATTCTATACCAGGATCCATCACTGATTAGCTGTATTATA TTTGTCTCTACTGTAATTTGAGGAGAATGATACCTGCTTCTCTATCTTGTTGGGTTTTACAGATGA TGTATTGAAAGTTCAATAAAATCTATAAACAAT

ID|140|gene_id|100048912|transcript_id|NR_047543 Homo sapiens lncRNA

AGCTACATCCGTCACCTGACACGGCCCTACCAGGAACAGCCGCGCTCCCGCGGATTCTGG TGCTGCTCGCGTCCCCGCTCCCCTATTCCCCTTATTTTATTCCTGGCTCCCCTCGTCGAAAGTCTT CCATTCTTCAAACTAGATTATTTAAAAATGAAAAAGGAAGAAAGGAAAGCGAGGTCATCTCATT GCTCTATCCGCCAATCAGGAGGCTGAATGTCAGTTTTGAACTAAAAGCCGCTCCGCTCCTCTTCT AGATTTGGAAAACAAGCGAAATTAAACTAAACCGCTGCACGCCTCTGACGCGACATCTGGACA CGGCGCGGCGCTGGCGCTGCCGGAGCTGTCGACCCGGCCTGGCGCCGGACTAGTGTCCCTTTT

GATGAGAAGAATAAGCCTCATTCTGATTCAACAGCAGAGATCAAAGAAAAGACTTCTGTTTTCT
GGCCACCAGATATATGTTATCTGTGCTTAAAGAATTGAAAAACACACATCAAAGGAGAATTTTCT
TGGAAAGAGAGGGTTCAAGCATCACTGTTAGGTGTGCTGGAATCCTTTCCCGAGTCAGTACTGC
TTTCTAGAAGAAAACCGGGGAGATCTATTTGGAATGTATCTAACTCCAAAGAAACCATCAGAGG
TAACAGTAGAGACGGGGTTTCACCATGTTGGCCAGACTGGTCTTGAACTCTCGACCTCGTGATT
CGCCCGCCTCGGCCTCCCAAAGTGCTGGGATTACAGGTGTGAGACACCACACCCGGCGGATAG
AGAGAATTTTGACAGATTGGCTTCTCTCACATGGCAAGAAACATTGCTGCTAGCACTTCCCGAGT
TCTACGTTCTACAACATCCACCACTGGATCTTAACATAGACGTAAGATCAAATGCAATAGCATGT
CAAACAATGTGTAACTCCAGTTATACAAACATTACTGTATCTCATTGGGGATACGAAGCTCTACA
CACTTGAAGATGGTGAAGGAATATAAAAATCTATGTCTCACAGTCCAGACTTGGAGTACAAGTA
ATAAGAAGAATAAAACTTAATCCCTTAAGTAGATTCACCATAAGTTAGCTCAGAGCAATTCCAGT
GCAAGTATGGTCTGTGATCCAGTAGTATCTTACAGACAGCAAGTTGAACATTGTGGGATGCATG
AGCTATTGAGGCCTTTGCAGCTTTCTGCTACATGGAGGCTAGGGCCAGAGTCAAGATTTATGCTT
TGCAGCACACTGGTCAGCTGTTTTTGCAAATCAGATTAAATGATTTTTAAATGAGGCTGAGAGCA
TGGGAGATACTAATGTGTGTTTCCTTGTGAGCTACTGCATAAGTTAGGAAATTGAAATACAGAAA
GATGAAAAGTGATTTGCCCAAGCATATAGATCAAAGCTGTGGCAGAACCAGGACTGGAACCTA
TATCTCTCTACTAATGGTTTTTTTAAAAAAATAACCTTGTTTCAAAAATATTAAAAAGTCACAAGA
AAGGTAAACATGTGGATAAACAAAATGAAGAAAATAAAAATTATCCAGTAATAACATATTGGCA
TATGTCTTTCTGGTATATTTTCCTGTGTTGTCATCATTATCATCTCCATCATCATTATATCCATCATT
ATCATCATCATCATCATCATCATCATCATTATCATCACCATAGTGAACATGTAATGCTTACCTAGT
GCCAGATGCTGTCTAGGCATTTTACATGTGTTACTGGTAACTCATGTAATCCTCATAACAACCTT
ATAAGGTGGTTGCTATTATCCCCATGTTACATATGAAGAGACAGAAGCATAAAGAAGTTGCACC
GCTGGTAATTGGCTGGGATTTGAACTTAAGCAGTCTAACCTTAGAGTAATGATTTTAACAACTAT
GCTATATACATACAAATTTACAAAATAAAACTGGGCTCAGACAATAAAAAAAAAAAAAAAAAAAA
AAAAAA
ID|141|gene_id|100048912|transcript_id|NR_120536 Homo sapiens lncRNA
AGCTACATCCGTCACCTGACACGGCCCTACCAGGAACAGCCGCGCTCCCGCGGATTCTGG
TGCTGCTCGCGTCCCCGCTCCCCTATTCCCCTTATTTATTCCTGGCTCCCCTCGTCGAAAGTCTT
CCATTCTTCAAACTAGATTATTTAAAAATGAAAAAGGAAGAAAGGAAAGCGAGGTCATCTCATT
GCTCTATCCGCCAATCAGGAGGCTGAATGTCAGTTTTGAACTAAAAGCCGCTCCGCTCCTCTTCT
AGATTTGGAAAACAAGCGAAATTAAACTAAACCGCTGCACGCCTCTGACGCGACATCTGGACA
CGGCGCGGCGCTGGCGCTGCCGGAGCTGTCGACCCGGCCTGGCGCCGGACTAGTGTCCCTTTT
GATGAGAAGAATAAGCCTCATTCTGATTCAACAGCAGAGATCAAAGAAAAGACTTCTGTTTTCT
GGCCACCAGATATATGTTATCTGTGCTTAAAGAATTGAAAAACACACATCAAAGGAGAATTTTCT
TGGAAAGAGAGGGTTCAAGCATCACTGTTAGGTGTGCTGGAATCCTTTCCCGAGTCAGTACTGC
TTTCTAGAAGAAAACCGGGGAGATCTATTTGGAATGTATCTAACTCCAAAGAAACCATCAGAGG
TAACAGGTTAGGAAATTGAAATACAGAAAGATGAAAAGTGATTTGCCCAAGCATATAGATCAAA
GCTGTGGCAGAACCAGGACTGGAACCTATATCTCTCTACTAATGGTTTTTTTAAAAAAATAACCT
TGTTTCAAAAATATTAAAAAGTCACAAGAAAGGTAAACATGTGGATAAACAAAATGAAGAAAAT
AAAAATTATCCAGTAATAACATATTGGCATATGTCTTTCTGGTATATTTTCCTGTGTTGTCATCATT
ATCATCTCCATCATCATTATATCCATCATTATCATCATCATCATCATCATCATCATCATTATCATCA
CCATAGTGAACATGTAATGCTTACCTAGTGCCAGATGCTGTCTAGGCATTTTACATGTGTTACTG
GTAACTCATGTAATCCTCATAACAACCTTATAAGGTGGTTGCTATTATCCCCATGTTACATATGAA
GAGACAGAAGCATAAAGAAGTTGCACCGCTGGTAATTGGCTGGGATTTGAACTTAAGCAGTCTA

ACCTTAGAGTAATGATTTTAACAACTATGCTATATACATACAAATTTACAAAATAAAACTGGGCT
CAGACAATAAAAAAAAAAAAAAAAAAAAAAAAAA
ID|161|gene_id|100093630|transcript_id|NR_003584 Homo sapiens lncRNA
CACATTCGGGAAGCGTCGGGATTAGGTGAAAGTACGTAGTTGTCTTTCGTAAGTTAAAATG
ATAATTGGGCCGAAACTTACTGCCTTACCTAAAAGGCAGCGCAGTCAGGATATTGGTAGGTCGG
GGGCGGCTTTGGAAACCCTTAAGTTTACAAGCATGCGCGGACTTGAGTGCTCATTAGGTCGCCG
GGCGTCCACGTGCAGCCCTGGACCCTGAACCCCGGCGTGCGTGGGCCGTGGGCCCTCGGGGA
AAGGTTCCGTGCACTCGGGGACTCCGGTGAAGCCTGTTCAGCCGTCTGTGTCATGTGGCCATCT
TGAGTCTACTCTGTCGCTCTTGTGCCCTAGCACCCCGAGAACCGTCAGTTTGAGCCAGATGGAA
GCTGAGCTGAACACATTACGATGGATGATGGAAACATAAGACTATCAAGAAATCCAAGTGGTA
ATGGGCGAAGTTTATTCAGCATCCGGCAATGGACTTATCGTAGTTGGGGAAACGGGTGTTCCGA
ATAATATCCTGGAAGTTATCAGGACACCTATTTTAAATATAGGCCTGAATTTTGTAAAGTAATATT
TAAGGTGGTCCGTGATAATTAAATAAAATGCTTAATTCATGTGGCTAAAAAAAAAAAAAAAAAA
AAAAAAAAAAAAAAAAA
ID|162|gene_id|100093630|transcript_id|NR_034010 Homo sapiens lncRNA
CACATTCGGGAAGCGTCGGGATTAGGTGAAAGTCGCCGGGCGTCCACGTGCAGCCCTGG
ACCCTGAACCCCGGCGTGCGTGGGCCGTGGGCCCTCGGGGAAAGGTTCCGTGCACTCGGGGA
CTCCGGTGAAGCCTGTTCAGCCGTCTGTGTCATGTGGCCATCTTGAGTCTACTCTGTCGCTCTTG
TGCCCTAGCACCCCGAGAACCGTCAGTTTGAGCCAGATGGAAGCTGAGCTGAACACATTACGA
TGGATGATGGAAACATAAGACTATCAAGAAATCCAAGTGGTAATGGGCGAAGTTTATTCAGCAT
CCGGCAATGGACTTATCGTAGTTGGGGAAACGGGTGTTCCGAATAATATCCTGGAAGTTATCAG
GACACCTATTTTAAATATAGGCCTGAATTTTGTAAAGTAATATTTAAGGTGGTCCGTGATAATTAA
ATAAAATGCTTAATTCATGTGGCTAAAAAAAAAAAAAAAAAAAAAAAAAAAAAAAAAAAA
ID|163|gene_id|100093630|transcript_id|NR_034011 Homo sapiens lncRNA
CACATTCGGGAAGCGTCGGGATTAGGTGAAAGAAGCTGAGCTGAACACATTACGATGGAT
GATGGAAACATAAGACTATCAAGAAATCCAAGTGGTAATGGGCGAAGTTTATTCAGCATCCGGC
AATGGACTTATCGTAGTTGGGGAAACGGGTGTTCCGAATAATATCCTGGAAGTTATCAGGACAC
CTATTTTAAATATAGGCCTGAATTTTGTAAAGTAATATTTAAGGTGGTCCGTGATAATTAAATAAA
ATGCTTAATTCATGTGGCTAAAAAAAAAAAAAAAAAAAAAAAAAAAAAAAAAAAA
ID|370|gene_id|100506385|transcript_id|NR_038885 Homo sapiens lncRNA
AAGCAGAGCTCCATCAGTCTCTTTTGGAGAGCTGTGAACGTGACCAGACCTATTAATTACT
TAACTGAGGTGCTGAACTTTGAAAATGAAAGTTCCGGTGGGGAGTTCTGTCCTTTCCAGCCTGG
GGAGCTCTGTCAGGGCTCTCCTGAGCGTCTCTTTCAGCTTATCAAGCTCGCTCGGGGACCGTCT
CTGCCGCCATGTAGGAAGTGCCTTTAGCCTTCTGCCATGATTGTGAGGCCTCCCCAGCCATGTG
CAACTACTGGGAAAGTGAGGTGCAGAGGTATTTTGCAACGTGCCCACAGAACCCCAGCGAGCC
CGTGTTGGAGTTACAACTGTGCCTCAGGGAGCATTCTCAGGCAGCATTCTCTTATGCTTCACCGT
CTCACAGATGATAAGGTTTCATTCAAGGACTTACAACAGCAACCCGGAGGGAGCATTCTCAGTG
GCTGGTAGATGCCATTTGTACTGAGCAGGACAATTAAGCCCAATTCTGGAAAAATTCTGCTTTCT
CTATTCAATGCATTTTATAAATGTCTTCACTCAGCACATTTACCCTACTAATTATATTTTGCTTTGT
GATCATGGTATGTCATTTGCATTCCTTCAGCACACACTGACTGATGGGGAAGAGATGTTGGGTG
ACCCAGGCAAGCCAAACAGGATTAAAGCCTGCTGTCTCACCAGGTGGATAATCCACTTTGAACA
CTTGAGAGGGGAGTGTTGAGATCACACCCCTTCCCATGGGACATTTTTCTGGGAAATAAAATCG
GTGACAAACACCACGCTTGAACACTCACATTGACTCTAGGGTGCAGGCTGAGCTCATCTGGTGC
AGGGGAGTAATGCAGAGGCGAGGCCTGAGTCAGCGCCAGGCACAGGAAGGGGATGCAGGAT

GCACCACAGCCCATGCCTCTTAGGAGAAAATCTGGGCCTTCTCCATGGCTGTGTCTCTATTAATT
TGCTAAGGCTGCCCAAATAAAGCACCACACATGGGCAGCTTACATAACAGGAATTTATTCTCAG
TTCTGGAGGCCGGCAGTCTGAGATCAAGGTGTCAGCACAGCTGGTTCCTTCTGAGGACTGTGCA
GGAGAATCTGTTCCAGGCCTCTCCCTGAACTTCTGGGGGTCACTGGCCATATTTGGCATTCCTTG
GCTTATAGAAGCAATACCCCAATTTCTGCCTTCATCCGCATGTGGCATTCTCCCTGTGTGCGTGT
TTCTGTGTCCACATTTTTCCTTTTTATAAGGATACCAGTCATGTTGGAGGTCTACCCTACTCCTTG
ATGACCTTATCTTAACTAGTTATATCTGCAACAAACCTATTTCCAAATTAGGTCACATTCTGAGGT
CCTGGGGGTTAGGACTTTAACATATGAATTTTAAGAGGACACAGTCCAACCCATAATAGTAACT
TTGTCCCTCTCTCTCTTTTGACACAATAATGACCCTATGCAGGGATATTTCTAGGTGGCTACTGTC
TGTGTGTGTGTGGTAGGGGGACTTCTAGTACTGTTTTGGATTACTGTTGACAGACAGCAGCCTGA
GGCTAGGAAGCACTTTGGGGCCATGACTTAGAGCAAAGCTAGTAAGTTTTCTCAGGAATTTGAA
CCCAGAATTTCATCCCCATGGCACTTTAATCCAGTACATTTAATGGCTCAGATGACCCTGGCCTT
ATCAGCCTGGTGCTCTAAGCAGCTGACCTAACAGTGTGTGCATGCGTGTATGCACATGTGTATG
TTATGTGCATATATAAGTGCATACACATAATGTTGGACACAGAAATATCTCTGAGAACTGTCCCT
TTATCACCCTTCAGATAAAATTATTCTCAGCTTGCTTAGTTGCAGTGTTTCTTACTTTGCATCTCCT
AACACTTTCATACATTTCTCCCGAAGTGCATAGGCAAGGAGCTTCAACGGAATTCAATGTCAAC
CTGGTCATCTTACCATGATTTCTAGGAAAAAATGTGTGTAATTGGTTCATGCCCTCCTTCTGTACA
GATTACAATTCTCCTTTTTTGGCATGAAAACCTCCATCTGTCTTTTGAAAGCACATGTCACTTATTT
ATTAAAGTCCTCCCCAACTTGTTCAGGTTGAGTTTCACTAGTACCTTTCTGGAGTATTTTATACCT
ATGTTATAGCCCCATATGTTATACTTTGGATGTCTGTTTACCTGTCTGTAAGACTGTCTGCATAAT
TCATGGGCCTTGTACAAAATAAGAATTCAGGGACTCTTGTTACAAGAAACTATTAAGAATTTCAA
GATGGGTCAGGCGCGGTGGCTCACGCCTGTAATCCCAACACTTTGGGAGGCTGAGGCGGGCAG
ATCACGAGGTCAGGAGTTCGAGACCAGCCTGGCCAACATGGCGAAACCCTGTCTCTACTAAAA
ATACAAAAATGAGCTGGGCGTGGTGGTGTCTGCCTGTAATTCCAGCTACTCAGGAGGCTGAGG
CAGGAGAATCACTTGAACCTGGGAGGTGGAGGTTGCAGTGAGCCAAGATCGCACGGTTGCACT
TCAGCCTGGGAAATGGAATGAGACTCCATCTCAAAAAAAAAAAAAAAAAA
ID|371|gene_id|100506385|transcript_id|NR_038886 Homo sapiens lncRNA
AAGCAGAGCTCCATCAGTCTCTTTTGGAGAGCTGTGAACGTGACCAGACCTATTAATTACT
TAACTGAGGTGCTGAACTTTGAAAATGAAAGTTCCGGTGGGGAGTTCTGTCCTTTCCAGCCTGG
GGAGCTCTGTCAGGGCTCTCCTGAGCGTCTCTTTCAGCTTATCAAGCTCGCTCGGGGACCGTCT
CTGAGAGAGAGTCTCACTCTGTCACCCAGGCTGGAGTGCAGTGGCATGATCTCGGCTCACTGCA
ACCTCCGCCTCCTGGGTTCAAGTGATTCTCCTGCCACAGCCCCTTGAGTAGTTGACGTTACAGG
CACCCACCACCGCACCCAGATACTTTTCGTATTTTTAGTGGAGACGCTGTTTCACCATGTTGGCC
AGTTGGTCTCGAACTCCTGACCTCAAGTGATCTGCCTGCCTTGGCCTCCCTATGTGCTGGGTTAT
AGGCGTAAGCCACTGTGCCCTGCTTCATGTCATTTTTAAAGAGCCCACACAACTCCTGTAGCTGC
ATTACGACAACCTCAACGGCAAGACCTCGACGTGATATTACTCCACGTGAAAGTCTAAAAGGCG
CTGAGCACAGTGCTCTGCCAATGGTGTGTCTTCCAGCCTCGCTTGCTGAGAGAACAGATGTGCC
GGGGAAGAAATGGACAAAGCTGGGAAAGGAGTCTAACTTCTTCAAAGCGGTTGGGCACCGGTG
AGGCTCTTTTGGTTACAAACCACAGAAATGGATTCTGGTTTATCAGTTTAATCTCGGTGTAACTA
AAAGTGGGAAG
ID|381|gene_id|100507428|transcript_id|NR_108062 Homo sapiens lncRNA
CCCTTGGCTGACTCTCTTTTCGGACTCAGCCCGCCTGCACCCAGGTGAAATAAACAGCCTT
GTTGCTCACACAAAGCCTGTTTGGTGGTCTCTTCACACAAACGCGCATGAAATTTGGTGCCATG
ACTCGGATCGGGGTACCTCCCTTGGGAGATCAATCCCCAGTCCTCCTGCTCTTTGCTCCGTGAG

AAAGATCTACCTAGGACCTCAGGTCCTCAGACTGACCAGCCCAAGGAACATCTCACCAATTTCA
AATCTGGACCCCACTGAAAATCGGACTGTTCAACTCATCTGGCAGCCACTCCCAGAGCCCCTGG
AACTCTGGCCCAAGCCTCTCTGACTGACTCCTTCCCAGATCTTCTCGGCTTAGCAGCTGAAGACT
GACACTGCCCAATCGCCTTGGAAGCCCCCTAGACCATCACGGATGCCGAGCTTCGAGTAACTCT
CACAGTGGAGGGAACGCGCATGAAAAAACCAAACAAACAAAAAAATTTCTTTTGGTAGCAGAA
TAAAAAAACAAAAAAAAGGACTTTTTCTTCTGGACTGAACTATATTTAAATCTCAAAGGATGGAC
ATCTCACAACCTTCCTACAGCAAGTACTGTGAGAACTCAAAGGGGGAGAGAAAAAGACGAAAA
ATATCTTCAAAGAGGAGAACAAATCTGATCTTTCTCTACGTCTGCTTAGTTGGCGTAACTTTAGG
ATGTCCATAAGTTGTAGCTTTCCTGGGAAAATTTGATTTTACACTGGTTATCCCAACATAATATTA
ATGGTATCCTCTTAGACTCTCAATAGTGTCCGGTTTGGATGACAAATTCTGTGGTCTTTTTAGCCA
ACTGCTACTGAAGTGGTCAGATTTATTGCACAAAATATATTTTTAAAAATAAGTAGCACAAGATG
AAATATGGCCTCAAATTTCTTGGTTAGTTTCCACTAGAAAGTTTTAAACATTAAAAAGTACAAAA
TTCATTACATTTATTGAATGATAAAGTATAACATTGTATGAAGTTGATGACTATTAAAACTATTTG
AGTGTTTAAAAAAAAAAAAAAAAAAAAAAA
ID|382|gene_id|100507428|transcript_id|NR_108063 Homo sapiens lncRNA
CCCTTGGCTGACTCTCTTTTCGGACTCAGCCCGCCTGCACCCAGGTGAAATAAACAGCCTT
GTTGCTCACACAAAGCCTGTTTGGTGGTCTCTTCACACAAACGCGCATGAAATTTGGTGCCATG
ACTCGGATCGGGGTACCTCCCTTGGGAGATCAATCCCCAGTCCTCCTGCTCTTTGCTCCGTGAG
AAAGATCTACCTAGGACCTCAGGTCCTCAGACTGACCAGCCCAAGGAACATCTCACCAATTTCA
AATCTGGACCCCACTGAAAATCGGACTGTTCAACTCATCTGGCAGCCACTCCCAGAGCCCCTGG
AACTCTGGCCCAAGCCTCTCTGACTGACTCCTTCCCAGATCTTCTCGGCTTAGCAGCTGAAGACT
GACACTGCCCAATCGCCTTGGAAGCCCCCTAGACCATCACGGATGCCGAGCTTCGAGTAACTCT
CACAGTGGAGGGAACGCGCATGAAAAAACCAAACAAACAAAAAAATTTCTTTTGGTAGCAGAA
TAAAAAAACAAAAAAAAGGACTTTTTCTTCTGGACTGAACTATATTTAAATCTCAAAGGATGGAC
ATCTCACAACCTTCCTACAGCAAGTACTGTGAGGTAATAATTGATATATTAATTATTAAAATTATT
GAAGCTGGAACTCAGATTTCCATTTTATATTTACATATATAATTATTCTATTAATGGATTCTAGCTT
AAATAAAGAGAAGCAACATGAGTCATTGGGGCAAGGGGTGAGATCGATGACCTCTGGAGATTG
ATCCCAGTGTTCTGACAGAGTTAGCTTCTGTTATCAGGTGCTATAGTTTTTCATAGTGATGCTGAT
AGAGCCTACTCAAGGATTGGACTCATTCTTTTGTTCCATGAATGCCAATTCTGCAGAAGCCACTG
TGGTAGAAATTCAAATGTAATGAAAAACAAACATTCACTCATTTATTATCACTATTTGCCTGCCA
CAAAGACAGATTTTGAGGAAGATAAACAAATAAAAACGTGTTTGCCATTTGAAGGGAAA
ID|488|gene_id|100874054|transcript_id|NR_051960 Homo sapiens lncRNA
GCGCATCTCCTACGGCCTCCAGGACAGAGGAACCGGGGGAGGCAGGGGGAAAAGGCCG
GCCCAGCAATTCCCCTACCCCCCGGTCCCACGTGTACCCTCCTGGCCTGGGTCGCCCCAGCCCA
CGGGGAGCGGGCGGAGTCCTGGCCCACGAAGCCTTGTCACCTGGCGGGCGAATCCGCAAGCG
GAGACTTGTCTTTAAAGGGCTTTGGGCCGGGCGCGGTGGCTCATGCCTGGAATCCCAGCACTTT
GGGAGGCCGAGGCGGTGGATCACGAGGTCAGGAGTTCAAGACCAGCCTGGCCAAGAAGCTCA
TACTGACTAAGGCAGCAGAACATACAGGAGGAAGAGGAGCAGGTTTCACAGAGGGAAGACAT
GAGTTCAATTTTGGACTTCTCAGTAGTCGGTGTCCTCAGTGGTAGCAACTTCAAACGGAAGGTGT
CAAAAGTCAAATTCTGGAGAGTTGAGTATGAATGGGAGATGAAGAAAAGGAGGCAGCACTTGT
AGCCTGCTCTTAATGTATTTCTGCACTCTACACTAGCAGCCTATTACACAGGACACTTGGATGTC
T
ID|689|gene_id|101805488|transcript_id|NR_109834 Homo sapiens lncRNA
CCGAGGTGATCAGGTGGACTTTCCTGGATGTTCTGGGTCTTGACCTGATTGCTGAAAAATG

AATACAAATTCAGAGAAGAAGAAAGCTAGTATGAGACTACCAAATGATCATCAGACATTTCTTG
AACACCAATTAAATTGCTAGGTATGCTAAAGTTTGCAAAACTGGTATAGACACCAAGAGGGAGG
TATCAACAGAGACTCCCCAAGAGCTAAGAGGAAACCACCTTGGACTGGAAGTCAAGAGCCAAA
ATTCTAGACTCTACTCTGTAATTAACTACCTATTTGAATTTGGAAAAATCACCATCAACTTTCCCA
GCCTCGTTCTCTGCATCTGGGAAATGAAGGCGTCGTCCAAATGATTACAAGCTTCTTTTCCTGCT
CTTATTGCATGATTCCACTTCCACAGCCCTCCAGCATTTTTTAGCAGCTGCATCGCTCCATAGAG
CCTGCAGAGGGCACTAGACTGGGAATTAGAAAACCTGATTTCCCTTCCAGCTCCACCTCTGACC
AATTGCCTGACCCTGGTCAAATTGCTTAACCTCTTCCTATCTCAGCTCCCTATCCATAAAACAGA
GGGACGAATAAACTCTCCTCCTACCACTAAGAGGTGTAGCCAGAGTTAATACCCTCATCGTCCT
TTGAGCTCAGCAGATGAAAGGCACTGAGAAAAGTACAAAGAATTTTTATGTGCTATTGACTTTAT
TTTATTTTATGTGGGGGAGGGAGCCGGCCCCAGCTGGAAAGCTGCTTTCTCTGAATCAAAGGGC
AGGAACCCAGCAAGTTTCTCAGGATTGGGGCCTTAGACTGGGCTGTGTATACAGACAGTGCCA
GCCAACCCCACAGTTCAGTTTCCTTTAACCTGGTGCTCCAGGCAATAACTGTGCAACTCTGCAAT
TTAACAATGTGTTCTTTGTCCCACAACTGTTCTCGTTTCTCAACTGCCCAGGTAATATGTTTGGGC
CTGTAGGAAGAGTCAAATAGTTAATAAGGGAAGGGTTTGGCATGCCCTACGTAAGTTCTACCAG
CAAGTCCCAACAAGAAGGCATTCTGTGTCTCCTGATTCCTGACCTACCCCCAAAATGTACAAAT
GTACAAGGAATGAGCCCACTTTCCCAGCAGGCTGTAATACCAGTTTGGCCTATATCAATGCATT
GGTGAGCTGTGTTTTGTTTATGGTTTTATGCCATCTATTTTCCCATGGATATTATGTTTTCTAAAGA
GCCCTTAAGTTTACGTCAGCTTTTAAAGCTACCAGCAGCACCATTTCAGTTCATATTAAGCCCTT
AATATGGTATGAATAGGAGAGCTATTAGACTAAAGAGCCATAATCATCCCTGAGGAAAACATCC
ATCACCAACATTTATGTGGTCCCTGAACTTCTAAAAGGTGTCATCTCTCTGGGGTGTATCTGGTG
AGAGCTTTCTCTGGGAGATGCCAAAAAGCCAATGCATTAGATGAAGCTTAGAAGGGCATTTTCT
AACCATTACAAATTGCCTAGTCTAGCATCTCAATTTCATCTACGTGAAGAGCCTTAATTAAATTTG
TTGGGGTTTGATCCTTTATCCCCAGATGTGGCGCTGACAGAGATTGCTTACATAAATAATGTGTG
CTCCAAGTGCTTGCCAGGCTCCTGGCTCAGCTGGGACAGCTGTAGCTTTTTGAATGTCATTCCCA
AGATATCCTGCAGGTGTTCAGCTTCCCCTGTTCTACTCTGGGAAGAGAGCCGTGGGCAACATCA
GCCCAGAAGAC
ID|754|gene_id|102216342|transcript_id|NR_108096 Homo sapiens lncRNA
GGAGTTGTATATTTCCCTAAAGGGTCAAAAATAGAACATTTATTAAACGACGAGTTATTCAA
AAACCGGCACAGGCCCCAGTTGTCTTTCAAAAGCTCTGATTTACTGCATATTTCTCCCTTGAGAA
AGGCCTTTTTTAAATGTTCATTTCCATTTTAAGAGACTGTGGGCTTTGATCTGTTGGGAGCATTCT
TTTTCTTTCCCTGACTGGGGCCTGTCGGCTACTCAGGCCTGTGTTTTTCCATGGCCTTCCCCAGCA
TTCACACGGCATCTCCTGCATCTGCGTTCACCCTGGCTAATTTGGAGGTCAGAACTGGTGAGTC
CCTGTAGCAGGTGACACCAAGAACCAATGAGCCATATTTGCTCTTCACACCTCAACAAAGATGC
TGCTTCCAGGAGTGAGTGTTTGTGTAAGTTTCAAATCCAGCAACCTGAAGATTCGCCTCCAATGG
CACAGGGCTGGACCGCACTCAGAGGTGGCTGCCACCCAATCGTCACTCAGCGTTTTATCGCTG
GGCTATTCTGTTCGAGTGCTAAGCAATTGTTCACAGAAATAAGATAATGGAAAGAGCAGAGACA
GAATGAGCAATGGAGAGAGTCGGGCCTACCATCGCGCTTACTGCTGTTCGTCACAGATCTGCAA
GAACTCTGAACTCATAGATTATGTCCAAACTGAGAAGCACTGGACAGTCATTTTGCTTATCTGCG
GTTTCTCATTTCTGAGAAGAATCTCCATATGGACATTATCACTTTTGTTATAATCTCGAGGATGGT
TAAGGTAGCTTTGATCGCCACTTTCATATCTCCTGTGATAATCAGCACCAATGGAATGTGCAGAA
ATCCATCCCTATTCCTAAGGACTCCTGATGTCATCTCACCAGGTGGGACGGTTTGTCCTGCTCGA
GTCTGCACGCCCAGCTCCAACAGCTTCGCCCAAGTTTGGGGACAGGCTTCACCCAACACCCAG
AATCTGTGCAGTTATTTGGCCCCTCATTGAAAAGCCTCTCACGGTTACAGACAAAATTAACTTGG

TCATCCCTGCAGCGCCGGAGGGATACTTCTGGACTCTCTGTTTGGGGAGTGTACGCTCAAACCG
TTTCAAGAAGAGATTTAAACCTCTTCTTGGAGAAGACTGAGGCTGGCACAAGGCTAACAAATCA
AATAACTGCACCGAGGCAAAGCTGCCGTCTCTCCCGGGCTGTACCCTGCCTGCTTCTCCCATCC
CTGCCTTCTGGCCGATTTGTCTTTAAGGTTTTAGCATTATCCCTCTGTTGACAAATGAATGCTTTTC
TGGAAGATGAGGTATTTCCAGAAGCAGGCAGACACTGGCTGGAGCGCCGTAAGAGCTCTGCCT
CCCTCGATTCGTTATGTGAACGGGAGAGGCCTGGAGGTTTTGCGCGAGCATTACGTTCAAGGTG
ATGATAAATGGAGTCTGGCCGGGGTGGTAATAAGGGAATTTCTAGCCAGATCCTGTCTAGACCA
CGGTTTGTTATGGAGGGAAAGCCACAAAAGGGGGCTATAGTACCCCGTTCCTCAGAGAAATTGT
CCTCAGGGAGAGCCAAGCACATTTCCAGTCCGAACCTTCGTCCGGCCCCTCACTGGGGCCGCT
GTAAATAACATTAGCCTGTCTTTCAGCAAGCTCTGGGAAGCTTATAAACCGAACACGGTTTTTGT
TGTCTGTAGTTGTGCATTAAGCTCAGTACTGTCAAGTCTAATTATTCTCATTAAGGCCTGAAAGTT
TTAGGGATTAATTAAGCAGAGAGCCAAAGCTTATGCAGAACAGCAATATTTTCCCCCTGAAGGA
TTAGCCCGTCATTCTGATGGGGTTTTCCTTGAGCGTTGCCCATTACTTTATAAGATAGCAAAGTGT
GGAGCTCATATAATATGGATTAGAAATCGCCTACATTTCACATGTGCTATTCAGCCATGAAAATA
AGTCCTGGCCCTTGTGCCTGTAACCCGGAAGGCTCACTAATGAGAACCAGGCAGTGGGGCTAA
TGAGAAATTCAATACGTGCCGTCTCTCCTTCCCGTTTCCTTTCAATTTACTTTCTACAGGAGCCAA
TATGTGTAGGAAAAGTCTGAGTGGATGCTGGTATTCTCCTCTTTACCTGGTAGGGATCTTAGAAA
TTATCTGATTCTAGTCCTTCTTGCTATAGAAGTGAAAACTGGGATTCAGCAACCTGCTTAAAGGG
ACCCAGTTAATGGCAAAGCTACTTGAAGAGAAGGTGACAGGAAACTTCAGGAGGTGTAGACAA
TCATTAGGTACATTAGGATGGTGAGGGTACTTAGTCTTAGGTTTTGGTGTGATGGAAAATGCCGC
AGAAATGAGAAGGTCCCACAATGAAAGGAGGATGCTGGTGAGGACCTAATTTGCAGACGAGCC
CCAGGAACTCTGGTGCCCTGCTTGGCAAAGGATTTCAAGGCTTCCTGGAGAAATGGGGGTAGG
GGGAGGGATCGTTAAGCCCTGGCACCCCAGATATAAAAAGGAAAGTCAGACGTGGGTAATCAC
CCTGTCTTCACCCAGCAGCTCCAGTTTTCAGCAAGGCAGAGGGGACACTTCTGCCTTCAGTCTC
CCCTACTTCACATCAAGGCGCCGTTGCATTTGATGGCTCGGGACGCGGCCTTCCTGCCTCCCAC
ACTCCCAAATCCATGCCTTGTTTATCTTTTTAGAATGGATCTCTGATGCCTGCCCACCACGCGGC
TGGAGTGGCGCCAGCATCCCCAGGGCACGTCTGTGATGACCGATGAGGTCCACCACCTCTACC
CCAGACCCTCCCATACCCAAAGGCTCCCTCTGCCCAGTGAAGTCCCCTTCGCCCCAGCATTCCT
GCACCAGGCACATCCCAAGGGTGGCCCCGTGTCCTGCAGGTGGCAGGAGAGTGCACCCTCCTC
AGACACCAGTCTGATATGTCACCAGGAGAGAGCGGGCGGTCCTCAGTGTATGCTCTTTTCTTGT
CTCTGGGGAGCTTGTTTTGGCTCCTCAAGAAGACCTTGGAAAAAGCACAGGTTGTAGAGGCGG
CATTCGCCGGGCAGAACCATGTCCCAAGACAACTGGTTAGTGAGGCCAGGCGTGACAATCATA
ATCGGAGCAGCAACTGTGCAATGGCATAGGCAAAGGGGGCCGAAGGCGCAGCAGCCACCACC
ATTATAGTCAGACTGGGCACCGTTTCCTAAATGTCACACATCCTTCCAGCCCCTTGATCTTTGCTT
ATGAAAAGGGTCATTCCCTCTACAATCCTCTAGAACTCTTTGTAGGTTTTTGTTTGTTTTCCAAAC
ACTGTACTTGCTTCTTTATCTGTCTCTCTATGAACCGTGAGTCCCAGAGGACCTCGTGGTCCTCA
ATTATCCTTGCTTCTCTGTGGCCTGTGCCATGGGGCTGAATGTTTGTAGCCCCCAGAATCCATAT
GTGGAAGCCCTAATCCCCACTGTGCTGGTATTAGGAGGTGGGGCCTTTGGGATGTGATTAGGGT
TAGATGAGGGCATGAGGGTGGGGCCCTCATAATGAGATTAGTGACCTTATCAAGAGGATGAGA
CACTAGATCCCTTGCTCCCTCTCTGCCTGCAATCACCAGTGAAAGGCCAAGTGAGGACATAATA
GAAGCCATCCCTTCCCAGAACCCAGCCATGCTGGCACCCTGATCTCTAGCTTCTCAGCCTCCAG
AACTGTGAGAAATAAATGTCTGTTGTCTACTCCA

ID|755|gene_id|102216342|transcript_id|NR_108097 Homo sapiens lncRNA

GGAGTTGTATATTTCCCTAAAGGGTCAAAAATAGAACATTTATTAAACGACGAGTTATTCAA

AAACCGGCACAGGCCCCAGTTGTCTTTCAAAAGCTCTGATTTACTGCATATTTCTCCCTTGAGAA
AGGCCTTTTTTAAATGTTCATTTCCATTTTAAGAGACTGTGGGCTTTGATCTGTTGGGAGCATTCT
TTTTCTTTCCCTGACTGGGGCCTGTCGGCTACTCAGGCCTGTGTTTTTCCATGGCCTTCCCCAGCA
TTCACACGGCATCTCCTGCATCTGCGTTCACCCTGGCTAATTTGGAGGTCAGAACTGGTGAGTC
CCTGTAGCAGGTGACACCAAGAACCAATGAGCCATATTTGCTCTTCACACCTCAACAAAGATGC
TGCTTCCAGGAGATCTGCAAGAACTCTGAACTCATAGATTATGTCCAAACTGAGAAGCACTGGA
CAGTCATTTTGCTTATCTGCGGTTTCTCATTTCTGAGAAGAATCTCCATATGGACATTATCACTTTT
GTTATAATCTCGAGGATGGTTAAGGTAGCTTTGATCGCCACTTTCATATCTCCTGTGATAATCAG
CACCAATGGAATGTGCAGAAATCCATCCCTATTCCTAAGGACTCCTGATGTCATCTCACCAGGT
GGGACGGTTTGTCCTGCTCGAGTCTGCACGCCCAGCTCCAACAGCTTCGCCCAAGTTTGGGGAC
AGGCTTCACCCAACACCCAGAATCTGTGCAGTTATTTGGCCCCTCATTGAAAAGCCTCTCACGG
TTACAGACAAAATTAACTTGGTCATCCCTGCAGCGCCGGAGGGATACTTCTGGACTCTCTGTTTG
GGGAGTGTACGCTCAAACCGTTTCAAGAAGAGATTTAAACCTCTTCTTGGAGAAGACTGAGGCT
GGCACAAGGCTAACAAATCAAATAACTGCACCGAGGCAAAGCTGCCGTCTCTCCCGGGCTGTA
CCCTGCCTGCTTCTCCCATCCCTGCCTTCTGGCCGATTTGTCTTTAAGGTTTTAGCATTATCCCTC
TGTTGACAAATGAATGCTTTTCTGGAAGATGAGGTATTTCCAGAAGCAGGCAGACACTGGCTGG
AGCGCCGTAAGAGCTCTGCCTCCCTCGATTCGTTATGTGAACGGGAGAGGCCTGGAGGTTTTGC
GCGAGCATTACGTTCAAGGTGATGATAAATGGAGTCTGGCCGGGGTGGTAATAAGGGAATTTCT
AGCCAGATCCTGTCTAGACCACGGTTTGTTATGGAGGGAAAGCCACAAAAGGGGGCTATAGTA
CCCCGTTCCTCAGAGAAATTGTCCTCAGGGAGAGCCAAGCACATTTCCAGTCCGAACCTTCGTC
CGGCCCCTCACTGGGGCCGCTGTAAATAACATTAGCCTGTCTTTCAGCAAGCTCTGGGAAGCTT
ATAAACCGAACACGGTTTTTGTTGTCTGTAGTTGTGCATTAAGCTCAGTACTGTCAAGTCTAATTA
TTCTCATTAAGGCCTGAAAGTTTTAGGGATTAATTAAGCAGAGAGCCAAAGCTTATGCAGAACA
GCAATATTTTCCCCCTGAAGGATTAGCCCGTCATTCTGATGGGGTTTTCCTTGAGCGTTGCCCAT
TACTTTATAAGATAGCAAAGTGTGGAGCTCATATAATATGGATTAGAAATCGCCTACATTTCACA
TGTGCTATTCAGCCATGAAAATAAGTCCTGGCCCTTGTGCCTGTAACCCGGAAGGCTCACTAAT
GAGAACCAGGCAGTGGGGCTAATGAGAAATTCAATACGTGCCGTCTCTCCTTCCCGTTTCCTTTC
AATTTACTTTCTACAGGAGCCAATATGTGTAGGAAAAGTCTGAGTGGATGCTGGTATTCTCCTCT
TTACCTGGTAGGGATCTTAGAAATTATCTGATTCTAGTCCTTCTTGCTATAGAAGTGAAAACTGG
GATTCAGCAACCTGCTTAAAGGGACCCAGTTAATGGCAAAGCTACTTGAAGAGAAGGTGACAG
GAAACTTCAGGAGGTGTAGACAATCATTAGGTACATTAGGATGGTGAGGGTACTTAGTCTTAGG
TTTTGGTGTGATGGAAAATGCCGCAGAAATGAGAAGGTCCCACAATGAAAGGAGGATGCTGGT
GAGGACCTAATTTGCAGACGAGCCCCAGGAACTCTGGTGCCCTGCTTGGCAAAGGATTTCAAG
GCTTCCTGGAGAAATGGGGGTAGGGGGAGGGATCGTTAAGCCCTGGCACCCCAGATATAAAAA
GGAAAGTCAGACGTGGGTAATCACCCTGTCTTCACCCAGCAGCTCCAGTTTTCAGCAAGGCAGA
GGGGACACTTCTGCCTTCAGTCTCCCCTACTTCACATCAAGGCGCCGTTGCATTTGATGGCTCGG
GACGCGGCCTTCCTGCCTCCCACACTCCCAAATCCATGCCTTGTTTATCTTTTTAGAATGGATCTC
TGATGCCTGCCCACCACGCGGCTGGAGTGGCGCCAGCATCCCCAGGGCACGTCTGTGATGACC
GATGAGGTCCACCACCTCTACCCCAGACCCTCCCATACCCAAAGGCTCCCTCTGCCCAGTGAAG
TCCCCTTCGCCCCAGCATTCCTGCACCAGGCACATCCCAAGGGTGGCCCCGTGTCCTGCAGGTG
GCAGGAGAGTGCACCCTCCTCAGACACCAGTCTGATATGTCACCAGGAGAGAGCGGGCGGTCC
TCAGTGTATGCTCTTTTCTTGTCTCTGGGGAGCTTGTTTTGGCTCCTCAAGAAGACCTTGGAAAA
AGCACAGGTTGTAGAGGCGGCATTCGCCGGGCAGAACCATGTCCCAAGACAACTGGTTAGTGA
GGCCAGGCGTGACAATCATAATCGGAGCAGCAACTGTGCAATGGCATAGGCAAAGGGGGCCG

AAGGCGCAGCAGCCACCACCATTATAGTCAGACTGGGCACCGTTTCCTAAATGTCACACATCCT
TCCAGCCCCTTGATCTTTGCTTATGAAAAGGGTCATTCCCTCTACAATCCTCTAGAACTCTTTGTA
GGTTTTTGTTTGTTTTCCAAACACTGTACTTGCTTCTTTATCTGTCTCTCTATGAACCGTGAGTCCC
AGAGGACCTCGTGGTCCTCAATTATCCTTGCTTCTCTGTGGCCTGTGCCATGGGGCTGAATGTTT
GTAGCCCCCAGAATCCATATGTGGAAGCCCTAATCCCCACTGTGCTGGTATTAGGAGGTGGGG
CCTTTGGGATGTGATTAGGGTTAGATGAGGGCATGAGGGTGGGGCCCTCATAATGAGATTAGTG
ACCTTATCAAGAGGATGAGACACTAGATCCCTTGCTCCCTCTCTGCCTGCAATCACCAGTGAAA
GGCCAAGTGAGGACATAATAGAAGCCATCCCTTCCCAGAACCCAGCCATGCTGGCACCCTGAT
CTCTAGCTTCTCAGCCTCCAGAACTGTGAGAAATAAATGTCTGTTGTCTACTCCA
ID|2079|gene_id|10984|transcript_id|NR_002728 Homo sapiens lncRNA
AGGCAGAACGGTCGCCGCGTCGCCTCAGCACGGACCTCCAGGGAGCTCCTCAGCAAGAT
CCTGCCAGGGCGCCCCTCAGCGCGATTCTGCCGGGGTGCCTCTCAGCGTGGTCCTCCCCGGGG
CTCCTCAGCACGATTCTCCCGGTGCGCCCCTCAGCGCGGTCCTCCTCGGTGCGTCAGTCATCGT
GGTTCTCCCCGGCGCGCCCCTCGGCGCGGTTCTCCTCGGGGCTCCTCAGCGCGGCGCTCTTCTG
GGGGCTCCTCGGCGCAGTTCTCCCCGGGGACTCCTCGGCGCCGTTCTCCTCGGGGCACCCGGG
GCTTTTCGGCGCGGTTCTCCCCGGGGGTTCTTCGGCGCGGTTGTCCCCGGGGGTTCTTCGGCGC
GGTTCTCTCCGGGGGCCTCTCGCCGCGGTTCTGTTCTCCCCGGGGGCTCCTCAGCATGGTTCTC
CTCCGCGCGGTCCTCCCTGGGCCTCCTCAGCGCGGCACTCTCCTGGGGGCTCCTCAGCGCGGC
ACTCTCCCCGGGGGCTCCTCAGCGCGGCACTCTCCCGGCGGCTCCTCAGTGCGGTTCTCCCAG
ACTCTCCTCAGCGCGGCCCTCCCCATCTCTCTGGGAGGGTTTGAACACGGTCAGCACGGACCTG
GGCGGACGGCGCGGGACGGGTGATCACTGGCGTTGCTGAGGTGAGCTGTGTGCCCCGCGGCC
GTCCCAGATCACAGGCGTCAGCAGTGCAGCCTGGCCTGGGCAGTGCGCTCCCATCTGCACCTT
ATGGACAGCGTGGCCAGGGTCGAGGTCCGAGTTCCTGGCCGCGTCCCAAGGATCGGATTCCGG
GTCTATAGTTCTCATGGTGGTTCAGAGTGGGCTGAATTGGGATTGGAGTCTGGAATCCGCATCG
TGGTTCTGAGTCCGCGCTATTGGGATGGAAGTTGGGAATCCATGTTGTGGTTTTGAGTCAAAGC
CCGAATTGGGACCGGAGTCGGAAATCCACTTAGTGGTTCTGAGTCAGGGCCCGAATTAGGATC
GGAGGTGGGAATCCCCGTTGTGGTTCTGAGTTAGGTATCCTAGTTGGGATCAGAATTCGGGTCT
AGGGTCCACATCCTGGCTGGGAGTTTGCCTGAGTTGGGATCTGAGTAGTTGGGGTCCCAGTCAG
TCCGGGTCTGGAGTCCGCATGGTGGTTTTAGGTAGGGCCTCATTAGGGACAGAGGATCGGGAG
TCTGAGTTGGGGACCTGAATCCCGGTCTGGAGTCTGCATCATGGCTCTGAGTTGAGATCCAAGG
GAGGTCCGAGTTGGCGACTGGAGTCAGGGTCTGGAGTCTGTCTTTGGGTAGAGGTTCTGAATGG
GGGGCTCTGAGTTAAGAGATTGGGATTGGGGTTAGAGGTCTCAGTGGGGTATGGGAGTCAGGA
ATTGAAGTCGAAGTTCTGCCTCTAGGTGGGGATTCTGAGTCAGGAATTAGGGGGCGAAGTAAA
GGTTGCAGTCTGAGTTGGGGGTGGTGATCACCTGCCAGGTGAGAGGTAGTGGTAGAAGTCTCTT
GGTCATATTGGAGATGAGAGTTGATCAGATGCAGGTCTCAGTCTGGGTAGTAGGCTTGAAGGGC
CACCACTATGTTCAGATCCCAGTACCCCACTGTGGTCCCTGAGCAGGTCAAATAGCCTTTTATAT
AAAATTGAGAGTAAGAGTGCCTTCCCACAGCACTGTTCTGGTTATGAAAGAGAATAATTCATCC
AGGTAGAATAGTTCTGTCTTAGATTATCACAAAGTATAAAAACTTTCCTGATAATATCCACATTTG
ATGTGCATTTACCAGTTCTTTCTTTACTGTGGTGCCCCTTTTGCTAGACCAAAAGCTCCCAACGA
GGAACTCTGTTTTGTTTTCTGCTGCCTGCTCTGTACCCAGCCAAGAGCCTGATACACAGCCTTAC
TCAGTCAGTACTTGAAGGAAAGCAGGCAGGCAGGATTAACCAGATTTCTATGGTGTCTCAAATG
ATCCATTTCTGCACCTGTTTTCCAGATACTCAGTAGGAGAAAAATTTAAGGATTGAGTTGCTTGA
TTATGGGTGTATTTTAATGTGTTTCCTAACATGCTGAGGAGAAAAGTGGGTCTGTTTCAGGATTTA
TGCTCCAGCTAGAGGGATTTTTTAAAATTGCGAATCAGAGCATGTCCAAATTCAGATAGAAAGC

ACAAGAAAACTATAAAGAATTTCCCTTATGAATGTAGATGCAGAAGTTCCTTGTACAATGCTAGC
TAACTGAATCCAATAATATGTATTAAACAACAATTCTTAAACAAGTGGGATTGCTCTTAGGAATG
CAAAGATGATCCAATATCAGAAACTTTATCCATGTAATTCACATTAAGAGGCTACAGGAGAAAA
ACCATATGGTTCCCTCAATAGAGGCAGAAAAAAATGATGTGATAAAGTTTAACGCCTATGTGTG
ATTTTGGGGTAGTGGAGTACACCACTTAGCAAGCCAGGACTAGAGAGGAACTTCTTCAACTTGA
TACAAGTTATAAACCAAAACTTAACTTTTCAGTAAACTTGATAGAGAAATTAAAAGTGTATCATT
CTCTTTAAGATAAGGAGTAGGCCAAGGATGTCATCCTGGCCATACCCTAATACAAGAAAAAGAA
ATGGGGATGTGAGGATCAGGTAGGAAGTGATAAAACTTAGTGTAGCTCATAGGGTTGATGGTCT
CCATAAAGATTCTCAAACATTTTGGTGTTACGACTTGTTGTATTCTTAAAAGTTATTGAAACCCCC
AAAGGGCTTTTCTTGATAGAGATTCTATCTGTTGATAGTTACCATCTTAGAAGTTAAAACTAAGA
AATGTTTAAAGCAGAACTATACAAGCACATATTCCACTGGGGTCATGGTGTGCTGCCTCTGGAA
AACTCTTTATCTTTGTGAGAATGAAAGAAAAGGCAAATAATGTCGAGTATTATTATGAAAATGTT
TTTGACCTTTCCTGAAAGGGTCTGGGGGGCTCTCTGGAATCCCCAGACTACACTTTGAAAATTAC
TGGGCTACATGGAAAAAAAAAATTAAGGAACTTAACAACTAAAGAGAGATTTCAGATTTCAGAG
AGGTTTTGGATATAAGATCAGTGTACTCAAAACAAAAAACAAAAACAAACCACATTTCTTTATGC
CAATAGCAACTGACTAAAAAAAAGCATAATAGAGATTAGGACAGCAATCAGAATAGCAGCAAA
ATTTATACCAGTCTAGGAATTAACAAAGACGACGACTTTTATGGAAAAAATTTTAAAACTCTATT
GGAGGATATAGAGGGAGATCTTTCCATATGCAGAGACATTTTATTTTCTTGAACAGGGCAACTTG
GCATTAGAATGACGTCAGGATTTTCCAAATCTGTTAAGTTCAACAGGACTCCACTCAAACTTCCA
GTTGGAGGGCTTGAGTAATTTAACAAACATATTCTAAAATGTATGTGGAAGAAAAATATTTGTAA
ATGCCAACTGAACTTTTGAAAAGGAGAGCAGAGGACTTCTGCTGGCAGATACCAGGGCACACG
CCAACTTCAGCAATCAGAGCGGTGTGGTGACAGAGGCCCAGAAGAGAGCTCAGAGGTAACTCC
AAGTCTAGAGGAAGGGGACATGTGATAAAGGTGGCACCACATATCAGTGGGGACTGTTCAGAA
GATGATGTTGGAAAGCCAGCCCTAAAATGGAGGAATAGGAATATGGATTCCTAACTGAGCCCC
TAGGAAAAGTAGACTCCAAATGGACTAAATATTTGAAAGCAAAAGGTAGTTGCCATTTGTTTAAT
AGGAGAAAAAATGAATAATCTCCAGGGCCAGGGTGGCCGTGGGGTTCTTAAACAGGATCCAGA
AAGCACAAACCTCAAGACTAAAATGTATGAACTTGAATGCATTAATGATTATGGATTAAGGATTT
CTGTTAAATAAATCCATCATGGCAAGGGTCAAGACTGTTTTCATAATAATACTAAGGAAACAATT
TGCGTTTTTCACTTTTACACTCATGAATGTAACTTTGCCATGACTCATTACAAGATATTTGAAACA
ATTAAAGCTGAGGAGGGAACAACAGCTAAAATATACCAGAAAGCACCACAAATCATTAAGAAA
AAGGTAGGAAGATTGCCAGTTTGCAGGAGGGTTAACAAACAGATGAAAATTTGCCCAAGCTAA
TCAGTTATCAGAGAAATGTAAATGAAAGCAACAGCTATTCTTCATAGGAGAAGCATTAGAAAGT
AGGATAAGGCCAAGTGTTGGCAGAAGGCAAGGAACAAGCCTCCCTGTGTGCTGCAGGTGGGG
ACGGAGACAGGCACAGACGTTCTGAAGAGCTGGCCAACACTTCTTCCTCAAATAAAATATTTTA
TACCTGTGGCCCAGCAGTTTCCCTCCTAAGTAAACATGCTGGAGCAGTTCTCACCCAGAACAAC
GAGAGGCTGTGTGTGTGCATGCTCAGGGCAGCATGGCCCATAGGGGTGGGCAGTTGGAGCTGG
TCACTGTGGTTAGGAGCAGGGGACTAGATGTACACTTCACAAGATAGAGACGCGTGGAAACAC
AGTGCTGAGTGACACAAATGCCAAACAGAAAGAGGGCCTCAGTACATGACTGTGTATTTAAATT
AAAATTACACACCACCCAGACACGCAGTACTAGGGAGTGCTCGTGAGTGCACACACACACACA
CAATGATATAAATAAATTACCCTGGAGAAGCTGTTTGTGTAGGGAGGAGAGCAAGGATAGAGG
CTAGAGATGAAGTGGAAAAGCATGGAGAGGAAGAGAGCGGTGACTTTATGCAGACCCTTACAG
GGTGACAGTGTTTCATAGGCTGAGGGCTGTGATCAACTCTGTGCAGAGTCCCAAAAGCCAAGA
ACCAACGAATGAATGTGCCTCCATGACTCTCCTGCCAGATACCCTCTAGTGGCTTCTATTTCTAG
GAAATAAAATTCAAATTCCATCAGGTTTTTACAAGATCTGCCCTGCTTACCTCTCTGACCACTGTC

CTCCCTCACTGAGCTTTGGCTCTGCCAGCCTTCTTCTTCTTTGTTAAATCTGCCAAGTATCCCTCC
TGCCCCAAGGCCTTCACCACCTCTGCCTGGCTTGTCTTTTCTGGTGATCTTCCCTTGACAATTCCT
TCTTCTTATTTAAATCTCACAGGAAATTTTGCTCTGACCTGCCATATCTGTCATCCCCTTCAGTCA
CTCACTAGCTGACGACTACCTTTCGAGTCTCTGCAAAGCACAGGCTACGCTTTTCTAGTTCCTTA
GACATCTACTTATTTGTTGGCTGCCTATCTCTTCCACCGGAAGAGAGGCCCCAAGAGAGCATAT
CTGTCTTCCGTATAGGGTCCGCACATGGTGGGTGTGTCACAGATTTCTGTTGAATGGTTTCATTC
ATTTATTGAACGAAGGAAGAGGCCAGGCCAGGAAAGTGTGCCTCCATTTTCACAACTCAGTGAG
AAAGCAAATAGCATGTCAAAATCTGACCCCTTGCAACCAGGGTTTCTTAGCCAGCCACACTGGT
AATGCTTGGGACAGGATGTATCTTTGTTGTGAGGGGCTGTCATTTATATTGTAAGATGTTTAGCA
CCCTGCCTGGTCTCTACCTAGTTGATATTAGTAATTCTCCCATAGTCATGATAGCCAAAAATATCT
CCAGACATTGCCATATGTCCCCTGGGGGGCACCGCAGATGACAACCACTTCCTTAGAACAGCTC
TTGGGACTGGGATGGTGTCTCTGCTGTGGCTCTTTAAAGGCAGCAGGCCCTGGCTTGCATCCTG
CCACATCTAACACCTATAGCCTCACCACCTCGGTGAGAAAGACCCTGGCCCAGGCCTGGCCAA
TCAGAGTACCCTCTTCCCTGGTCAGCAGTATAATTTGTTTGGCAAGCACTTCCAGTCCCACAACC
TATGGGCTGCCACCCAGAGGTTTAGTTGGAACTACGTGTCAGCTGCCGTCCCTCAGCCATCTAT
GGCATGGGCACCTGTGGCTTGACCAAGTTGGAGGTGGCCTAGAGAGGCCGGGCTGGGCCTGCT
CACCTTCTCCAGTTAGTGCAGACTGATGAATGAGTGGTTCTCAGCGGATACCGGATCCAGGTTT
GCAGTACACTCCAGGAGGAGGACGAGTAGGGTTTCCCTCAGAGCAGGCCTGGATGGGAGGGC
AAGGGGTGGCCTCTAATACTGCCTTTCCTTCCGGTGCCTTTATCAGCCTGTGCCATGCTCCTCTG
AACTGGCCGGCCCCAGCATGTCCAGGTTACTGGCAGGAGCCTGTATAACCCAGGGTTCCAGTC
ACACCAGCCAGGAAGGCCCATGAGGCTGGAGCGCACCCAGTCTCTGGCTGCTCTGTCTGCGGA
ACTAAAAGCCTTTCTCCCAGAGCCTTTCCTACGGCTGGGCGAAGCTCTTGTTTGCAATTTAAATG
TGGCCTCATTTATGGAAGAATCCCTGCTCCTCCGAAAAGCCTTGGGCTCAACCTCCCTAGCAGC
CCGGGCCTCAGCCAGGAGAAGAAATGGATCTAAAAGGCCTTTCTCCTGCTAATTGTAGCAATTA
GCATTAGAGAATGGGGACTCGATGGGGGGCCAGCGCCTGGCAAAACAAGCTTATTCCCAGCTT
AGGCCCAGGCCCAGCTCCACACACTGCTGCTTGGCCAGACTCGGCCCCCACCCAGGGTCTGGT
TGGCTTCAGGGCAGGGAGAGAGGGCCCAGGCCTGTGGGTGCTAAGAGCAGTTGTGGAGGGTG
TGCATCTGAGTTGTTAAGCCTGGTAGCTGTACAGGTAGCCCTGGGCTATGACTTGTGGTCCTCTC
AGAAAAGGGTCTGACTGGAACGCAGAGCCAGACCAGATATTCATGAGTGGATCACAGAGTGTA
GACTGAGGGCTATCTGGTGATTGACAGAAAGTAGTCTTGGGGCAAATGACTCTTAAAGAGTAAG
GAGCCATTCCCCTCTATGGCTAACTGGATGCCTTTCTCTCCCCCTGCTCCAGAAAGGACTTCCTG
TTCCTTCACTGAGCGTGCTTGCTTATGAGGCTTCTGGAAACCAGGAGAGTTCAGTGGGCCTCATT
ATTGAGCGTATGGGCAGGACAACCTGCTTGGGACATAGGCCATGCTACAGTTGATTTTAGCCAG
CTTGCTGCATCTACACAGCACAGTGCCTTCCCAGGCAGTGAATTATGTCTAACAGGCAGGGGAA
TGATCATTGCCTCATGTTAGGGATGCAGTCATTCAAGAAATATTTGTCAAACACTCAGCAGGTAC
TAGACCCTGGCCCAGAGGGAGGGATTTGATAGGAGCAGGGACCTGCCTACCCTCCCAGGGTTA
GTGGACAAAGGGGAGAAAGACTAACTAATGTTGAAGGACCGTTTGCACCCTGAGGGGAGTGCT
GAGGCCTGGGAGCACAGCAGGTGTATAGCAGGAACCCACTGGAACTAGCCTGGGGTCTGAAA
AACTCCCTTGGGGAAAGTGGCAGGGGCAGGCCAAGGGCCCTCTGGTGGTAGGGAGCCTGTGA
GCACCTGGGATGGAAAGAAGCCAAGCATCTGAACTACCAGAAAGGCAGCTGCAGGATTAAGC
AAGACCCACAGGTCTCTAAACTTTGAAAGGATGTCTGTCCACTGAGTAGATAATTGATTGGAAT
GGGGCAGGGGATGTGAGAATGCCAGTTAGGAGATGCCTGCAGTACAGAGAAATGGTGGCCTAT
CAGGACAGTGGCAATAGGGATGGAGGGAAGCAGGGAACCTCTAGAACTATGGATGGGTGAAG
TAAGCCTGGCTTGGTGATGGGCTTGAGGTGATGGGCAGGTGGTGAGGAAGCGGGTGTATCGGA

AATGACCCCAGAACTTCTGACTTGTGCCACTGAAAGGATGGGTGACACTGAAAGGACAGGGGC
CTTCAGTAGAGTGGGCTATAAAATGATGGAGGCTGTGGAGAGGAGGTGGGGGCAGGTGACTG
GAATGTGGGTCTGGCTGGGTTGGAGAGACATTTGGGGGGTCAGGGCCTCACAGGTGGTCTGGA
AGCCCTTGGTGCTGAGGGAACAGCTTAGGGAAATGGTTTGCATAGAGAAAGGCACTGGCATTT
AGTGGCCATGCCAGAAGGGAAAACCTGCCAAGGAGACTGAGGATAGAGCCAGAAAGATGGCA
AGAAAGCCAGGAGAACTGGAGGGATGGAAAAGAGGACCTAGGGGTCATGGTCCTGGCAGAGG
TCACATTGGATCCAGTGCTTGTGATTCTCTGATTGCTGGCAGTGGATGGCCACTGGCCTGCAAAT
GGCCCTAGTGGAATGTCCCCTGCCTGTGTGGCTCCCTGTGGGCTGCCTCTCCCCTGGACAGATG
GGGAGAGACCCCTTCCCCACCTGGTTCTCTGTTTCACCCTTGGAACTGTGGCGGTAAAGAGGAC
CTCCCCACAGTACGTGCAGGGAACAAGCTAGGTTGACTCCCTCAGGGAGGCCTCGTCCTTGCT
GAGGTATTTCATAACCTCAGGATAGAGAAATCCCCCCAACTTTAATGCAAAGCCAAGCAAGCTG
AAAAATGGCCTTGAATAGGCTGAGATGAAAAAACAAAGAGGCTGTCAACATCAGAGACTCAGT
GGGGGCAGGGAGCTTCAGTGGACTCCCCACAGACAGCAGTTCCTGGGCTTCTATGCCCAGACC
CAGAGGCCTGAGCCCAAGATGTCAGGATGGGTGGTCACTGTAAGTGCAGTGGATTGTAGCTCA
GGGGTGAGACATCGCCTTTTTCTTGTCAGCTCTCCTTCCTGATTAGTGGCTCAAACAGCCTTCCG
AGACTCAAGGGGTTAACTGCAGATCCTCTAACTCCTCCACTTGCTAATTTTTAAACACAGCTGAG
CTCTGAGCACCCCTCCCAAGGCTGTGAGAGGCCAGAGGCCAGCACAGACTCTGCACCAGGTCT
GCACAGCCTCCACCCCTACTGTTGTCAGGAATCTGCAGGCATCTAGAACAAAGGTGCCTCCATT
ATTTCCCTCACCCAGCATATCCTGATACCTGAGCCAAGAGTTCACCCAGAGCTTTTGCCCACTTC
ATTGATGGTCTGGTGGGCTTTTGTTGACACATACCCGTGACTCCCAAATGAACCTTTCAGGTTGG
CTTAAAGCCGCTGGGACCTACACATGACAAAACCCAGACGGAAACAGTGGCTTACACTCCTGT
GTCCCTAAAGGTACCTGTTCCTAACAGTGGGAGTGCCAGAGCCTGGAAGCTGGAAGCCAAGGA
GACAGGGACCTCCCTCCTGCTGACCCCCTGCCAGGCCACATCCACCTAAGGGATTTGGGATCAT
ACTCCATCTTCAACCACTGGGATCAAATGGAAAGCCCTCAGGAATTCTGAGAGACAGCCCCTAT
ACCTATGTGCACTGACACACATACACTGACAAATACGCATTCTGACACATATATGAACATGCAC
ATATGTGTGCCCCACTCCCTGCCAGGAGGTGGGTTCTGTCCCATCTTTGACATGTTACTTCTTGT
CCTTCCCTAGGCCCAGTGGCACTTTCTTTGTGATAGGTGCTGAGACCCCCCCGACTGGGCCCAG
TGGCTGTTCCCAGGACCCAGTCTCTGCTCTTATCTTAACCCAGCTCTAGCTCCCAGGACCCCAGC
CTTTCGCAGGGAGAGAGATAGCTCTTTTGGTCTGGCACATGTTCCATGCCAAGTAGTGGAAACA
GCAGCTGCAGGGCAGCCTCAGACTTGACATCATTTAATCCTTGTGACAATCCTACAGGGAGGAA
GACACTACACATATGTGCAGATGCAGCAAGGAGCAGCTCAGCCACATGGAATGACTTGCTTGA
GGTCACCAGGCACTTGACACAGCAGAGTTTAGCCCAGTCTCTGGAGAGACCAGCCTTCCACAT
CCTGTCATGCTGGGGAGGGCTGCCCGCTTTTTGTCCCTGCTCCCCCAGTTCTAACCCCGGAAGA
GCGATTGTCCCTGCTGTGCCTTCAGCCCAGACAGAAGCAGGCTAGGAGAGGGGAGGAGAGGC
AGGGCTGGCCCTCGGAAGGCTCACCTTCCTCAACAGTTCTCTGCTTGGGCAAAGGTCTTCCAAG
CAGCCAGGCTCTGAGGCTAGTCACTGTCATCGTTGGGTGAGGCAGCCTGGGAGTACCAGAAGT
TGGAGCAGGTGGGAACCTTCCCCAGGGATGGGGCTGGATAGGCAGGGGTGGGTGCTAAGTGG
CAGGCCACAACCAGGCACCTGGGAGGGCCAAGCACCACACTGAGAACGCAGTGCTTCCCCTC
CCCTCCTAGGCTGCTCAGGAACCCATCAGTCTCCTGTAAACAAATGAGACTAAGTCTCTGGCAC
TCTAGGCCCAGGTGCAAAGGACAGCTGCCCCTGCAGCTACTCACTCTGCCCTGGCCCTCTCAGG
AGGCTTGTGACCTCTGCACCTGCTGGCTTGTTTTGTTCCTAAAATACCCGGATCCTTGAGAATCT
AGAAGACACCACTGCTAACAGATTTCCACATCATTCATTCTGTTCTTCCTGTGCAAGCTTCATGC
TGGGACAGGGACCATAAAACAAGGTGACCCAAAACCTTCAGCAGAATGCAGGTAGGCAGAGC
AGAGGTCTCTGGGAGTGTCATTCCAAGGCAGTGTCTTCCTCCTCCCCTATGCTCCCCATTTCCCA

AGAGTGGGCTTCTTCCACCCCACTCAAGCACCTCTTCCCAAGGCTGACCTAAAGGCCTCAAGCT
CTGCGGATAGGGGCAGTCCTGAGGTGGCATTGAGTCTTAGGGTTGGAGTGGGAGATATCTGTG
CAAAGGCTGATAGGAACGTCCTCCAAGGGCTGACTTCAAAGCTTGGCCTAAACCACTGCATCA
AAGTCAGCAAGCTGAGCTGTTTGCACAGAAAAGGAGGAGGAGCAGGCCCTTGAGCCACCTGTA
GCTCTCCTTAGGGTCTCAGGCTTTCACACCAGGGCAGGCCTTGGGCCAGACTACCCAACTTCCA
GGCCGGATGTGCCACCGGGGAGGAGGAGGTAACTGCCCCTCAAAACCCTGCAGCCCAGGGCT
CCAGGATTCCAACCCAGGGACCTGCAGGCTCCCCTGCTCTCCAGATCAGACCCCTACTCTCAGA
GCCCTAGGCCCAGTGGGTGACAGCCTGTGAGACAGGCCTGTGTGGGGTGGCCCTAGCCCCACC
TCAGCTTCCAACCTGACCTTGCTGCATGACCTCTGGCCCAGCCCTCTCCACCTGGAGTGCCATCT
CTCTTTGTGGGATGGGCCAGGTGGGCTGTGTGTGGGGGAGGGCAAGTATATAGGTGTGGTATA
GGTGGAAACTGCCACAGTAACCAGCAGCAGCTCAGAGCCGGGCCACCCAATTTGTATGCCAGC
CCCAGCTGGGCCTAGGCACGTGGTGTGTGGAAATGAACGAAGCAGGCAGCCCTATGTTTAGGA
GGCAGGTCCAGAGCTCACCCAGGCACAGCACAAGATGAGTGCCTGGCCTCAGTGAGCGAGCA
CTGGCTGCTATGAGGCTGTGGTGTGGAACCTTGACACTAGGGCCACTGGCCTGGCTGTGTGACC
TCTACCTCATCACCCAGAGCAGGGCAACCAGAGGCAGAGCCTCAGTGTGGTGTTCTCCTGTGA
AATAGTGATGGCACAGGCAGGGGCTGGGGCCCAGTGCCTACACAGAGGATCAGCAAAGGATG
GCAGTTATTGAAACCTCTACGGGGAAGTTCCCCAGACACGAGAGGAAGACCCCCATGGAAGCT
GTGTGTGTGACGTGCTTAGATTTTCCAGAGTCTTCATTTTCCTTGTCTAAACAGAGGTCATGGCG
CCTCCCTCTCAGATCAAACAGCCCTGTATGAGTGGCTACAACCTTTGGTGGGCTCAGTGCAGCC
CCTCCTGCCCCCTTGCTTGGGAGCTGTCTGCAGAGGCCTAGGGAGCTGCGTGGGGTCTCTCCAC
CTCAGCCTGTATCCCCACCCCCACCAGGTCCCCTGCCTGACTCCAGCATACCCAGGCCCTAGAA
GCACCAACTCCATGGCTGCAGCTGAGTGGAGTGTGTGCCTGCCCAAGCCTAGAGGGGTGCTGA
CCCCTCCCTCGCACGAGGAGACATCAAAGGGAAGCAGGAAGCAGCGGCCTGGCTGACCCACA
GCGGGTGGGAGAAGTGCAGCCTCACAACTGCTGGGGATTTTCCATCCCACCAGGATTTCCGCG
GGACACCCCAAGTCCAGCCTTTGAGGCTCCTCAGGAACCAGAGTTTGGCCTGGAGCCAGTTGG
GGCAGAGGAGGGGGGCCAGGCTGGGTGGCTTAGGGGTGTGGTTAGGCCTGCTAAGGGGCTCC
TCTTCTCAGTCTCCCCATGATTTGCTGGTTACTGGCTTGAAATCTGGGCCCAGACCCATTCCAAC
ACTGAACCCTGAGCTCCCTGAGACCTGGCTGGCAAGGACAGGTCCCAATTTTTACAGGGGCCT
GAGAGGAATGGAGAAGGGATCTCTGCCCCTTCCTTTCTCAGTCACACCTCCTTGACAGGGGGTC
ATCAGAGTGGGCTGTGGTAGGTGCACCAGGGAAGGGACCATACAGAGGAGGCTGGTCTCAAG
TCCTAGGGTGCCTGGGATGGCCCTATTGAAATGCTGCAGCAGCTCTTGTGCAGGCAGGGATCTG
TATGGACAGCTGGGTGGACACAGGTGCCTACTGACCTCCCAGATCCTTCAGCAGAGCTCAGCC
CTGAGTGACAGCCCAAGGCAGACCTGGCTACCTCAGCCTCTGGCTCACAGCCCTCAGCCAGAG
CCCTGAGAGGCCTGGGATCTCAAAGCTGCAAGGTGGGAGGCCAGAGGAGAGAAGCCCGGAGC
TGGTAGTGATGCTGGCTGAGTGAAAATTGCACATTTCCCAACAGTGTGATGCTCCCTATCCAGC
CTGGGCTTTTGGCAGACTTCTGTGCTTTGTGGAGGAAGCTGGCTCAGATTCCTCCCCTCCCTGTG
CAGTTTGTGTGTCAAGGTGTACTTGTCACACCCAACTCAGGATATCTTGCATCACTTTGTGGGTC
CTTGTCCCCCAGAGCTGGGACTGCTGAGTTTTAGCCCTAAGCTCTTGCTCCCATCTTAGCAAGTC
AGGGCCCAGTGTTGTCCTTGCCTCTGCATCAGGATGGTAAGCTGGGCATGCTCCGCATCACGGA
GACCTGCATGGACCTGGATTGGGTTGCCTCTATCTTCAGGCTGGTCACTGGGCAGACACAAACC
AGGGCCTCTGGCCAGAGGGGACAGTGGCTCTGAGTTACTTGTGCTGCTCTTTAGGGGTGAGCA
GGGGCTGCTTGTGCATCATAGTGTATCACAGGGGATGCACATTTACAATTGAAAGCTACTGGTC
CTGGGTAGAAGTGGGGCCGAGTAGGACTTCCCAGCCTTGAACATGGGTTTGTCATCCAACTGG
GACCCACACTTGATCCAGCGGCCATGCTCTGTGCAGCCTGGGCCTGAGGGCAGCCACGCTCAT

GTGAAAAGTGCTAGTAGCCAAGCTGCCATTCTAGTCACCACAGCTGTCCTTGCAGGAGCACATT
GCCCAGGGCTGGGAGCTGTCATTTCCGTGACGGGAGTGAGACTCAACACTGGGTGAGGGTGGG
GGTATCTGTTGGGAATTATTTCAAAACAATTGTGGAAACTTGGCAGTGGAGGGGCTCTGGGCAT
AGGTATCCACAATATCCTAGTCAGGGGTTGGGGTACCCTGGCTCTGCTGATAAGCCCAAGTGAG
CATACTCCTCCCCCAGCTTGGAACAGGTGGGACGGGCCAGGTGACACTAGTGTGGTCTGTGGT
CTGGCCTCCCAAGCCAGTGGCAGTACTGGGGTGGCAGACATTTGCTGTGTGCTATCCCAAGGC
CTAAAGGTCACACAGCTAGGTTGGAGCAGGGTTTTGGGCAGGATGAGTAGGCATTGCTGTGCT
CTCAGAAAGGCGAAGCAAACCCCAGAAGCAAGGGGGCGAGACAGGGCAAAGGCCTACACTTT
GCCATCAACTCAGGCCTACTCAATGCTGGCCCTGTCCTTCCCCTGCCCCTGCCCCATCAGATGG
TGCGAGCTGGCGGGGCTTAGAGTGAGCCCCCCTGACTCTTCTTGGTGTGGAGTCTGAGGCCCA
GAGTGTACAGGCCTCATCCGTGTGACCCAGCAGGAGGATGGGAGAGTGGGCCCAGAACTTGGT
CAGGCTCATCCGTGGAGAACAAGTTCTTCCCTAGTCTAGTTGTGGTCTGAGTCCTGGGTGAAGC
AGGAGGCAGCCTGGGAAACAGCCTCGTTCTAGTAGGAATCGCATTCTCAGGAACGCTGACTGT
GCAGGGCCCTTTCTCACTGCCACCTGGGAGGATGCCTGCCTGCTGGCCATTTCGGCGGACTGTC
AGAGCACCCTCCCTGGGGTCCCAGACCGAAGGCCTGAAGTCCACATGGGACCCCTGAACTTCT
GGTACCTGTGTGGGCTTGTCCCCTCAACCCTGAGTGGAATGGCAACAGCCAGCTGCAACCCTCC
TAGCTTCCTGTCATGTGCTGTATGGAAGGGCCAAGAGCTGTAGTCATCTGCAGATCCTCCCACT
GCACCTGTAGGACTAGCGTCTCCTGTCTGGATGTGAGCAACTTGCTGGTGGCCTGTGAGATGGG
AGTTGCACTGGGCTTCTGGATAAGGGCTTCTCGTTCAGGTGACAATTCTATGTGAAGGAGACCC
ATGGAAACACATCTATCCCCTGCCCATGTACCAGGCAGAGATGGTACCTGCCCATGTACCCTCC
ATGACATGGCTGGGTCCCTGAAACATCAGACAGATCGATACTCTGTGTGCATGTTTGTGGGCAA
GACACAGTGGCACGTGTGCCCTGGAGTTTGGTAATGGGAGGACTGAAGTAGTGCCTCCGTGTG
TGAGCTGTGCCAGCCATCCCCACGAAGGGGGCATTCCATGCAGATGGGGATCCTCTCCAGGCA
GCTTCTTCCACACCTGATTAGCTCTGACCATCAGACCCCCCGTGCCAAAAGGGCCCCTTCCCTG
GAATCTGTAATTCTCACAGGCTGTCCCATACATTTTAAATCCAGGAAATGTGCTCACCCATGAGA
CAGGGATGGCAAGAAGATATGAATGAAAAGCAACCAGTCCGTAAAATTGGAAGCCATTTCACT
CCTTACCAGGTACAAAGATAACATGCTGCATGGATCAGGGACACAAACTTACCTACCTTATGAT
GTAGTGAAAGAATCTAAAACTTTTGTCTCAGCAGACTCCCTGGAGGAAGCCAATAACAGGAGG
CCTTGACTAGGAGACCTCAAGCCTTGGAATGTGCCCAGACCAGTGGGGAGGGAAGGCTGGGA
GGCAGTCCTCCCTTACCCCTTTTCTCCCAGGCAGACCTACCCTGGGTTCTCTCCAGGCCCCTCCG
CCTGAGCAAGTGCTCAGTTGGCCACTAGCTCCCTGCACATCCAACAAGCCTTTCTTTCCTGTTCC
TTCTTCAAGGAAGGCTTAGCCTCAAATCCAGGGCCACAGACAAAGGACAGTCACTCTCCTCTGC
CAGAAAGTCATTTATGGGACATGGAGAAAGCATCAAATATATTGCTTTTCCAACACCTAACAGT
GGAATAGGAGGAATAGAAGGAGAACTCAGCCAGAAAGTTCTGGAGCATAGTCTTTTTGCCAAA
AGAACACGTTTTCAAATGAAAGAGGTGGTGCTGGGGCACTGAGGAATCAGGGGGATGGACCCC
ACTGGAGCTTGTCTCATGCTGGCAGCAAGTCCCAGGCCTCAAACACATACCTGGGCCACCTCAG
GAGAGAGACTGCAGGGAAGAAATGAGTTGCAATGCTCCAGCCTAACCAACTCCCCTTTTGTTAA
TCCAATCCCAGAAGGTACTGGGTAGGGTCAGTTAACAGACAGCTGCCTGTCTCCTGGCAGAGTC
TGACCAAGCCCCAGTGGGCGCTTCTCTTCCTAAGTCCTCTGGTGTACCCCATTCTTCAAGGGAA
ACGGTCCCCTTAAGAATCTATGTGAAAGGGTTCTGAAATTGCTGTACCAGTTAATGAATTATTTA
CTCTAAACAATGTCTTGATAAAGGGGGAGGATGAAGTTAGCTGATGTTGACTAAGGAGAGACG
GAGTTGCCAAGGAAATTCTTCTAAATGAAAAAAAAAAAAAAAGTAAAAGCAGTCAGCTGGTTTTG
TCTGACTATGAAACGTGTCACAGAGCATGCACCTTTGGCCATCTTTTGCATTTATGATTTGGAAG
AGAGATGTAGCAGTGAGTGTGGGGCCAGGGAGGGGAATCCAACAAAGCCATTCTGCAAGCTA

GTGGCTGGGATTTTGGGGTATAGAGTAAGAGCAGCCAGCCTTAGAGGCCCATGTCATCTGGGC
TGCCTCAGAGCTAAAGCTAGGTAACCAGAGGGAGTAAGGAAAGGCCTGAGTCCCAGGCTAGG
CTTTTCACAGTTGTATTGAGATGGGAAAGTAAGGGATGTTCCTTGCAGCAGATGCAGGGCCCAG
TTGTCTAGAGCAGTATTTGAAATGAGTTGGCATTTCAGGGCAGTGTTTGTGATGCCAGTCAGTAG
GTAGGGGCTTCTACCTCTTTTTTAGAAATGTTTACTCTGAACTAAGCTCTATGCTTTAACAAGTTA
TGTTTTAATAGGAAAACTGTCATTGATGGAATCATATGTAATTCCTACATTTTTCCAGTTACCCAT
TCCTGGGGAAAGCTTAGCCATTGGACAAACCACCCAGAGCCCCAGGAACCTGCATTTGCAGAC
AGCAGTGGCAGGCACAGTGGCCTAGTGTTGAGCGGGGGAAACCTGGCCTCTATGTGTCTGGTT
TTGCAAACTAAAACGCCATTGCCCATACTCAGGCTGACAGGTTCTGGAGGTGCCATGGACTCCT
GCTCTGTATCTCACCTCTAGTTCCATCACAATGCATGAGCTGTCAGTGGGAGCCTTTTAATGCAG
ATGCTAATACCCATTCCTAATGCTAGCCCATCCCCACACCCTCCTGAGTTGGTGCGATATATCCT
GGGAACAAGAAAATCTGAAGGCTCCTGGTCCTCAGGCTAGCAAGAGCCTTGCCTGTAAGAAGT
GGGAGAAGGTCACCTCTCTCAGGAGGCAGAACTAAGGCTGCACAGCACAAGGGAGATGTCTG
AATGCACAGTCTGGGTGGCAGAAGGACTCCTGTCCTTTCTTCCACACAGACCTGAGCACCTCTC
CAGGTGCCACCATTTTCTGTCTGGGAACATCTGCTATCCCCAAGCCTCAGTTTCCTGGTCTACAA
AACTGAGATGGTCTCTTCTATACAAGGCCCCAGGAGGATCACTGCAAGTCACTGCATTTCATGG
TGCCCAGATTTCCCTGCATTTCTAGAATGTCAACAGAGATGAGTGGGCTCAAGGGGAAGAGATT
CTTTATGACTAGAGATATTCTGGTCAACTTTGGACCCCAGCTTCTATGGACAATAGCATGTGGAC
ATGAGCCCTGGGGAAGCAGGGATGACTGAGCCTGAAGAAGAGCTCTCAAGCCTTACACTCTCC
AGACCAGCCTTGGGTTGCCTGAACCCCAGCATAGCCTTATGGAACCCCTGCCAGGGTCCTAGC
ACCTGGTTTGACCTGGGCACAAGTACTCACTCACTCACTCACTCACTCACTCACTCGTCAAACAT
TCACTGAGGGTCCGTGACAGGCTCCATATGGATTAAGCACTGAAGATAAAACACTGAATAGAA
ACAGCCAGCCTATACCCTCATGCTCCTTGAATGTGATAGAAGGAGCCAGCCAGTAAACCCTTTA
CCACAAATAAACACACAGTTACACATTAAGAATGGGAGGGGAGGTATGTGATTTTTTACAGCCC
AGTCTTAAGCTTTAGAAATTGGCTCCTCTGAGGAAGGGACATTTCAGCTAAGAACCACAGGATA
AGACAGTCCTGGCCTGGGGAAGGGGAGAGAAGGTCAGGAGAGAGCTTGATATGTTTAATACTG
AAAGAATATTTGATGAGCTGGGACATCTCCTGCAGATCCACGGCTCCCTAGCTTTGTGACCCTG
GGCCCTAGACTCAATCCCTCAACCCTTAGGAGATTATCTATAAAACAGGAGTGATAATATTCTTG
TGTTAAAAGCTGCTGTGAGGATTAGTGAAATGATGTCTATATTTGCCTGGCAAACAGGAAGTGT
AAGTAGTAGTATCATTTGAAGATTATTCTTCAGCCTAAACGTGTGTTCATTATGGCCGGTGTGTTT
TCTCCCCACCTAGATGGTAGTGCTGGGCCTCCTTTGGAGTACCTTGATGTTTGGGGAGCATGGAT
GATATGACCATAACCTGAGAAGGCAGGGACTGTACCCATTCTGGTCCTCCCAGGTCCCAGGGC
CCCCACAGTGCCCTGGTGTGTGATCAGTGCCCACAATACTGAATAGAGGAACAGACACATGGG
AAGCAACGGCAGCCTGGTCTTTAAGGGTGAGGGAGCACCTTCTCCTGAGAGAGGGAGAGATAG
GTTGGGGGAGGTTGGGTGGGGGTGGGGAGAGCGAGTGAGCAAGCAAGCTAGAAACTTCCTGA
AAAGGGAAGTTACTCTAGAAAAGGGTAAGTTCCTTCCAGGCATCATGGGCTGGCTTAACCTGGA
CCAGTTTGACTTTACTAAGTCCCATTTCTCATCTAGGGAGCCTGAGGCCATTAAGGTTATCTGGT
CCCAAAATCCCCTAGAGCTGTCACAGGGATCCCCATTCTTTGCCTTTAGTCACCCCAGCCCCAA
GAGCCATCCTCCACATGGAGTGAGCAGGGAGCTACAAGGAAAAGACGGTCACAGTTGGAGAG
AGACAGAGAACCAGGTGGGCTGGTAGGACAGGAAGGTATAGGGAGAAAGTGAAGCTCTCTCC
TGGGGAGGCAGGAATACTCTCCTGCTTCCTTCTACCCACTCTTCATAGAACACAAACACTGCAA
AAATGACTTTTTCCAAACCCTCTTAGCATTCAGAAGTAGAATTGCTATGTAAGAATTTTTTCATAG
GTGCTTTTTTGGGGGGCATCTATTTAAAAAATAACCACATCATTAGTGAAATCTTCCAAAGTCTCT
TTTTGGAGCCCACATTTCACTAATGTCCACATCTGAGGAGGCTTTCATAATAAAAGAACACATAC

CTATTTCACCTATGATGGTCAGTGCAATCTTGAAAACACTAGTAAATAGTGTCCATCAGCTTTAA
AAATACAAACCCAAATTGGGTCATACCAAAATGCAAGGATACACACAATAACCTGGGTGAATCT
TCAGGGAATTGTGCTGAGTGAAAAGAGCCGATCCCAAAAAGGTGATTTGCTATGTGGTTCCATT
TATATAATTTTCTTGGAATGATAAAGAAATGAAGAATAGGTTAGTAGTTGTCAGGGGCTAAGGA
CAGGGTAGGGTGGGAGGGAATTGGGGGTAGCTATAAAAGGGGGACATGAGGGATTCTTGAAG
AGTTAGAAATGTCCTGTAGTGAGACTATCAATGCAATATCCTGGTCAGAATATTGTACTACAGTT
TTGCAAGATGTTACTATCGGGGGAGACTAGGTAAAGGAGTATGTAAGATTTCTCTGTGTTGTTTC
TTAGAACTGCATGAGAAATGTACAATTATCTCAAAATAAAAATTTATTTTAAAAAATGCCCAAAT
TAAAAAAAAATCAGTAGTCCTCCTAAAAATACCCAGAAATGAATTTTAGAATGTCCAAAATCCAT
ACAAAGATCTAAAAAAAATATGGAAGGATGTTGCAATGGATTTGAACATGTGGGTAAACTGCTTC
TATTTGTGGCTGGAAAGACTTTTTTTTTGAGGTGGGGAAGGCAATTCTTCATCAGTTAATCTAAAT
TGAATGAAGTTTCAATAACAAATGCCAATGGGATATGAATATACACACAGATAGACAACTTTTTT
TTTTTTTTTTTTTTTAATTAGGCAAGCTGAGGCCAGGTGCATTGGCTCACGCCCGTAATCCCAGCA
CTTTAGGAGGCTGAGGCGGGCAGATCACTTGAGGTCAGGAGTTTGAGACCAGCCTGGCCAACA
TGGTGAAACCCTGTCTCTAATAAAAAAAAAAAATTAGCCAGTCATGGTGGCAGATGTCTGTAGTC
CCAGCTACTAGGGAGGCTAAGGCAGGAGAATTGCTTGAACCCAGGAGGTAGAGGTTGCAGTAA
GCCGAGACTGTGCCACTGCATTCCCGCCTAGGTGACAAAGTGAGACTCTGCCTCAAAAAAAAT
AAAAATAAAAAAAAAAATTAGGCGTGCTAGTTCTAAGTTTATATGAAAAAAATGCATGTCCTATAA
AATTCAGAAGTAGCAGCAGTCTGGCCCCTTGTGCTCCTCTAGCCCTCAACTGGCCTAACAGGCC
CTAGTGTGCAGCAGGGACTATTTTCCCATCATCACTGTCCACAGCATCTGGACAAGAATACAGA
GAACGGCTGATTTAAAAAAAATCTAATCTGGGATCCTATGCATCCTAAATAATCTTATGAGGATG
CACTTGTATTTTTTCTGATGCTGATACTATTCATTCCAACTCGCCTCCCCATCAGCTTTGTCAGGC
ACTGTGAATTGCATGGTACACATGATTACAATGGATGGAATGGCCATCATGAGGTATAGCAATA
CATTTAAGTAACACTGTGTCACTACTATAGGACAGGCACTTTTCTGCATACTTTATATTATTAATT
TGTGTAATCCTCCTAACAACCCTCTGAGGAAGGTACTCTTATGATTCCCATTTTACAAATAAAGA
AACTGAGACTAAGTGACTCACTCAAGGTGTAGCAGGTAGAAAGTAGTGGAATTAGGATATGAG
TTCAGACTCCAGACCCTGTTATTAGCCCCTGTCTTTGGCTATGGATTAGTAAAGGAGAGCACAG
CCAAGGGTGGGACAGGCAGCTGTAGTCCTCTGGACCAGCTTCACATGGATGGGGCTGCATTGT
TACAGGTGGCCTAACTCAGACATGGAAACTGAGTCGCTGCTGCCCAACTCATGCCATGAAACC
ATTTTGGCCTGCACAGCTTATAACAGCACTCTAGCTCTAGCTATGACACTTTATGGTGTTAGTGG
GTTTTTTTGGTGGTTGGTATGGAGGAGGGGCTGGGCTACAAGTTTTAGATTATGTTTCTTATTGCG
GGTTTTTAAAAATTCTTGAATTAGGCCTATTTGCTCTTTTGTTCTCTATTGAGTTAGCAAATTCAGT
TTTTCCAAGATGCCCTGAAAGTATGATCATACATGTATACGAAATGGTCCTTGGGTCCAGCTGGC
AACTCCTGTGCACCCCCGACCTGGATTCTGCCTTCCTTGAAGTGAGCCTTGGAAGAAACAAGTG
CTCATGCTCCCTGACATTATTAGCTGCCACGATGAAGCCTCAGTCCCAAACACCCCAGCTTCCT
GTCTAACTCAAGAGCTAATCCTGTGTTTAAATCTTAAGAGTGTTTAGATCTTAGGCCATGCCATG
AATTCCCCTGGTTGACTGCATTTGTGAAAATGAGCTTCCCATTATTGGCTCTTAAGTAATGGAGA
TGAGGGGGAGTCCTGCTACACAGATAAGTGCATCATAAAGTTACATAATTAAAATAACTGAGCT
CTGGCCTATCAACAGGCCCATCAGCAGTCCAGAATAGAGTCTAGAAATGGACACAAATTAAAT
GAGAGTTTGAAATATGATAAAGGTAGCAGTTGAAATTAGTAGAGAGATGATGATGTACTCAAAT
AAATAGTGTTGGACAACTGGACAAATATATGGGAAACCAAATTGGATTTAATTACTCTGTCAAA
ATCCCGATAAAGCAAGGATTTAAATGTAAAAAATGAAATAAAAAATGATAGAAGAAAACATGG
AAGAATTTATTAAAACAATTTCAGGATGAGAGGTTATTTCAGAATTTAAGATAAGGACCTCAAAC
CATAAAAGACTGACACAGCTGACTATGTGAAATTAAAAAAATTCTGCCACCAGAAAAATATTAT

AGAAAAAGCCAAAACACCTAAGGAAAATGAGGATAAAATATTTTCAATGAATATGACAAAGCA
AGTTTCTACAAATCAGTAAGACAAGGTATTTTACAAAGTCCAAAGGGTATGAAGAAATAGTTCA
CAAGAAAGAAAAACAAATGATTTTAAGCATTTGGAAAGATAAAAACCTCATTTCCAACAAAAGA
AATGTAAATTAAAGCTCCAGTGTACCATTTCTCATCTATGAAATGAATGAAGATAAAAACCTGAG
TATACACTTGGTAAGGGTGGGGGAAGAAAGTGCATTTGTGCAGTCAAAGTGATGTAAATGGTAC
AGCCCTTATTTGGCAGTATCTATCACAACATAAATGAACATTTTAATCCTGCTGGAAATGGGGGG
AAAGGGAGGATCTCTTAAAGTAAATCCACTGAAAACGGAGACCAAAATTTTACAATAGAGATAT
TTGATTGGGAAGTTAATTTGAAAAATAACAGTAGTTAAAATATTATTTTATCTCATATGCAATAAC
AGTTGTTAGAGTTACAATTATGTACACGTTTTAGAAAAAAACAAATCCAGAGGGGATTTTGGTTT
GTCTTTTGTAAAGCATTATATGGTAACATCTGAGGGCATCTTTTATACTTGGCAGAGGATGTTGG
CAGCACTTCCACAGAGAGGATCATTTTTTGTAGAAGTATGGCATGGTTTCACTGCTTGACCAAGA
GGCCACCTGCATCAATTATCATCTCCCCTGCTGTCCTCTGAAATGCTTATCTAGGCATCGATCTT
GGTTTCCTCTTCAACTGATTAACTAACTGGTCTACTCTGCCCAGTCTCTATTTGAGAAATCATTTG
ACATCATTTTCTTTAACTTGATGAAATCTCCTATTTTGACAAGATTTACAGTTCCTCTTTATAAGTT
CCTCTTTACTTTGTGTGTGCTTTATATTGTCTTTGGTTGACAAGAGACAGCCAAAGAGGTCAGTC
AGCAGAGATACAAGTAGGGATAAGATGGATAGGGAGAAACAACAGTGTTCAATAGGGAGGGA
TGTTTCAGGGAAACTAATTTCATAAGTGGTCATTTCAGTAGTGAATATATTTGAATGGTTAATGGC
TGCTGTTTTTTAGCTGCCCTACATATCCATCAACAGAGGATCTGTTCCATAAATCATGGCACAAT
GAAACTCTGCAGCTGTTAATGAAAAGGAGATTTGCTAGATGTCTTAATACAGATCCATCTCCAG
AACATACCAGTGTCTTCCTTTTAAGTGGTGCACACTGGGGCTGAACACTTACTAAGAAATGAAA
GGGCTCCCCAGTCAAAGCTGACAGGTGTCATTACAACATGACCCCTGTGGTAGATCCCAGCAC
AATACACTGGCCATCACTCCATTCTCTTCAAATGTCCTAGGGTTGCTTCATACAGCAGACCATCG
TACCCAAAGCCACTATGCATATGACTTGGTTATCTCCTGCCAGGCTGTAGTCCAGTTAGTGCTCC
CTTGGAAATACTCAAGACCTTAATGAACTATCTATCTGTGAATCTGTGAAGCTACCCATTTCTTA
GAGGCCTCTTTGGAGAGCTGGCTGTTGAATACCCAGTATGATGGTCCTTAGCCTTCTTAGACCTT
TCCTGTACCACTACCTCTATGCCTTTTGCCAAGTCCAAGTGTTCACCAGAAATGGAACTGATGCC
AGAAAGACTACAGCTAACAATCTGAGCAGAACAGTGAGCATGTCCTATCTGGGAAAAGCCCAG
CCCATTGGGCTAAATCTCTAGCTGAACACAGAGACTATGCTCCTTCCAATTATACTGGAAGCCC
AACCATGAGAAAACAGCACTTCAACTACACTGTTCAGAGCCATCACCTGTTCACACGTGTGTCT
GTATACATGTAAACACACATTTTGCAATAAAAATGCTTCTCAGATGTACTGCACCATAATAAATC
AGCATTGTATGTACAGCTCAAAAAGAAGTATGGCATACCTATGGAAGAAGTCAAAGGCAAAGT
GTAGAAAAATACATACAGTATGCTACCATTTGTATAAAAAGGAGCCGTATCTACTAAGTACATG
CACACACAGACACACGTATATTTGTTTATATAAAATACGTCTGGAAAGATGCAATAGGAAAGTG
TTGAGTGGTAACCTTTAGGGAAGGAAACTTGGGAATTAGGGTTGGGTGGGGAGAGTGTTATTTT
TCACCCCTTTTGTACTCTGAATTGGTTTTACCATGTGTGTAAAGACTGTTGTATGATTTTTGTAGCA
CAACCCTTGAATACATAAAGCACGATTGGCTCCCCAGAAGGCAGGTGTTGGCCAGGCAGTGGA
TGCACGGTAGGGCAGTGTGCAGCCCTGCATGCACGTGGCTGGTATGCCCTGCGGGCAGCAGCT
CCCCGCACACAGTGCTGTGAGTCTCTGGTTCATGTCACTCTGTGGAGCAGGGGCTGCTCCAGCA
CAAAGAGGTTTTTGACAGGGAAAGGCAGAGCCAGAGTCAGGGCAGGCCCTGGGCGGGCTCCT
AGAGAGCCCAACCACCCTCTAATAAGGGGTGCTCTCCACCCTATCTGAGGAGCAATTGGGATCT
CCTAGGAAGTGAAACTGTTACGTATGTCTTATTACAGAATTTCCAGTGGAGTGATTCTGGAAGAA
ATGTGTATGGCATGTTGTGACTGCTATGTCTCATGAAATATTTACGTTTTTATGTAACTTTTTTCCA
TTTTCAGATCATCTTTTATTTTAACGCAAAATGACAATATATTTTTCAATTAATTTGCCCAATATCT
GTGTTTAAATGTTTTCTAATGCACACACGTGTTTTAAACTCATTAAAAAAATATGACTTAGATCCC

ATCCCACTTTCATGTTTTATATTGCCAAAACTCTGCTTTTGGGAGAGGTGAGTTATCTTCACTTCT
GAAACAGTACTGCTTGGGAGCTGGTTTGGCCCTGGCTATTCTGGGGAAATGACACAATATTGAC
CTAGAGGGGCCCTAATCTTTCCCCGCTAGAGCACTACCTCTAATAGGGTGAAGGATGGGAGAA
AGAGGCTAGTGAACCCACCTTCAGGTCTGTCCACTCAGGACATGCCCACACTAGCCTCTCCAGG
GGCTCTGCCCTTGGCATCCCCGTCCCCCACCTGGGCTCTGGCAGAGAAGTCAGGCGGAAGAA
GCCGGCTGAGTGCCCGCCACTGTAAGAGGAAACGAAGAGAAACAGCTCCAGTCAGCCCCAGA
AGCGAGGCCTGGAAAAACTCTGCAGCTGGAGCTAACACAAAGGACTTTTCAGACCCTGCAGCC
AAGGTGCCCAGTGAGCTTTTTTTTTTTTTTTTTTTTTTTTTTTTAAAAAACAGCTATTAGGGTGACA
AGCCTTTCCAAAGGCCAGCAGTTGGCGAGAGTTTCCAGGAAGCTGGTCTGGCACTGGGCTGGG
ATTGGAGCAACTTTAACGAGTTAAGTTCAGAAGGAAGGAGTCATCAGGAAAGGTTTAAGGTTCA
ACAAACTTTTCAAATTATTTTGCAAAAAGTTCTGGCTTTCCAATTCAAAGGCATGTTTCGCTGTGA
CTGTTTTAAGCCAGAGCCTATTTAAAAGGCAACACAACTAGCATGCTTCAGATTCACTCACTCGA
GGTATTTGTCTAAATCCTCCGAATGGGCTTTGCAAGTGCCATCTAGTGCCTAGTAACACTGGGGC
CATCTTACACATCTCGACAGGCAGCCCTCCTCCCCACGTGCCTCCCCCCAGCCTCTGTGGTCGT
GTGCGCGCGGGCGCGCACACACACACGCACGCATGCACCTTCCCTAAGCAGGAAGCTGCTCTT
TGCCAAGAGCGAATGGAATTCAGATTTCCAGAGGGTGTCCTTGGCCTCCAGCTCTACGGCTGCA
TGATGAAGAAAGGTCCTAGTGTGTGTGCCGGGGGCTGGGAGAGAGGGGCCTCCAGCTCTGCTC
ACATCTATCTGGGCCCCAAGAAAAACCCACAACTGGCTGTGCTCTCCCCAGCCACACCCAGCCT
TGGTCACATTCCTTCCCTTCCTGAGCCCACACTGGGTGGCCCCGGCAAACCAGCTTCCACACCT
CCCTCCCCACCGGCAGGGAGACCTAGCCCAAGCCCCCATGGGGCAGCCAGCAAGTACAAATG
AGGGGCTGCACCCCAGGCCCAAAGAGCCAGAAAGCACGGAGGGCCCTCCCCCCACCCCACCC
CCAATGCTGGGCATCCCCTTGTGGCTGCTGTCACTCATGGGGAAAGAGACACACTCCCTTGTCT
AGCACCCGGTGAGCTGAGCTCCCATCCCTTCCTGTGCCTGCTTTCAGGAAGTCCCTCTTCATATC
CAACCTTGACTCTCCTGGCTGTGGTGCTTGTGGGCCTGGGATTGAAGGGCCATCACTGCCCTTT
GCACCAGAGCTTCCCAACATACCAGCAAGAAGGGACTGTACCCCTTCCCCTGTCCAGTCTGGG
CCTTCCAAGCCCTGAGATTCAGCAACCTCAGGAATTAAACATTTATAGGAAAACACCTATGCAG
ACAAGGGACATGAGTCAGGGTGGTCATCAGCAGTAGCAAGGTGGGTAGGCTGGTCACGTTTAG
CTTCTCTTTAGCCCAAGTAATAGGGTCTTCCACCTGCATGGCCAGGGAGGGGATAGGCTGAGAG
CAATCTTTCTACAGGCTGTCAGCATCAACAAGTGGTTGTGATGAGCAGGAGTCTGTATGGCTTTT
CAGGTGTTCCGGCTCAAGGAGGCCCAACACCAAGTTGGAACCTCCAGGCCTGGTGCCTAGGCA
GAAGGTGGGCTAAACAAAGGCCAAGGGGAGCTCTGCTCTGTCACTTCCCTTTGTTTGCTAATGA
GACTGTTTCCAAAAGCTGGCCTGGTAGCCTGTGGGATTGTTTCTCTCAAGATTTCTTCTAGGGGA
GAAGGTGGCTGAGGCTTTGCAGTCTGCATCACCCAGTCCCCAGAATCTACAGGGGACTCAGCTT
GCCTAGTCACAGTTCGGCTTTTGGCCTTGGCTCCAGGGCCCATTCTGATCCTCGTGCTTGGGTCA
GGGATGGTCAGCACCCCTGCCCTGATGCTGCCTGCTGGGGTCTCCTGCTGAGTACCGTGAAGG
CCTACTGTGGCCTGAGAAGCCAACTTCACTGTCCCTTTTGGCCAGCCCTAGACCCTATATTTGTT
CAAGTAACACATTGATTCACTGGCTTCTCCTTTAGGCAAGGCCTGCAGGGGACTCAGCTGGGGT
TCATGATTCCTTGCCACCTGTGGTGGGCCTGCATGGCCAGCCCAAGGCCTCAAGTGCCACATAC
TGCTCCCCTGACAGTCAGGTGGCTAGGGTATGACACTCAAGAAAATGCCCAGGCAGAACCCAG
AGTTGGGGTCACTTGGTCCAGCCCTTCTGAAGGGGACTGGTTGGATCTGTTGCGGGGAGGAGA
CAGGCCATGCTGGACCAGCAAAAAAGCATAGAACTTGGAGTCTGGAACCTGACATCTGGCCTT
GGCTCTGCCATCAGCTGGCTGTGTCCACTGGGCAAGGTCCTATCATATCTGGTCTCAGTGCTTCC
ATTTACCCAGAGTCCTTCCATCACCTGACACCTACAGGGATGCTTTCCCAATCAAAATTCAGGCC
ATGTGGTTTGATAAGGCCACCTTCCAATGTGGAAGACAGGCTGGTAATGGAGAGGCCTGGGAG

CACAGAGCTCCTAGGCACTGCCATGGAATGTGGGGACCTTAGGGAAGGATATCTCAATTGAAG
CTCTTCAGAGCCCCTGGGGTGGACTGTTCTGTACTCTCATCCCCTAACCCATACCAGCCTGAGCT
CAATCATATCAGCCTGTGAAGGCTGCCCCACCACACTGCTTCTGCACCCACCACACCAGGCCCC
CTTGAGCTCCCACCTGGAGCCCACACACCCAGCTGGTGGGGTTCGTGGGGCAGTTCAAAAAGA
AAAGCAGAAAAAAGCATTTGAGTATTTTGGACTGGAAGGAATTCAATTTAACTTAACAAATCAG
GACATGAACCTAAAGAAAACACTGAAAAACAAGCCCAACTTTGAAAGGCCCAAAGAGGGAGG
ATTAGGTGGGAGAAGAAAGGTCCCCTGTGGGCAGAAGACAGCTGCTCAGTCCAGACCAGGGA
CCAGGGGAGGATACTGGGCTTGAGTGCAGACCGGGCCAGGGTAGGGAGACAAGCCCAACATA
CACTCGAGGACTTGAAGTCAGGGGTGCTAGCTGACTTTTTAGCCCACTCTTTCTACCCCAGATAC
AGAGACTAGCTGCCTGAGGAAGCATAGCGGGGCCCACAGGCTGGCTGCCTGGTGCTGCCCAC
CTCCTCTCCAGGGCTTGGCTCCCTGGGGCAGAAGGCTCAGACCTATAACCACAGTGGGGTTGC
AGGGATTTATTTGGGGGAAGAACCCTGACTGGGTCAGAATGATTATGTGCATGCATGTATGTGT
ATGTAGCTCCTTGTTCTCTGAAAAGCCTTGCCTACCACCCTACTTCCCAAGTACCCTCTATTGCCT
TGCAAAGTCACCCCTGCTCTCTGCCTCAGTTTCCCCAGCAGGGCACCACAGGACACTGCTGTGG
CAAGGTTTATACTTAGGGTGCAGTGCAGTCTGATCTTGTATAAATGCTCAGTGCCAAAGACTTAG
GAGATAAGTCAGGCAGAGTCAAAATTAACCTTTGAAAAATCCCTTGAATTGGCCCTAAAAATAA
TCCCTTTCTCAGATCTTTGTGTATATCCCCTGCTGGGTTGCTCTTCTGCCTGCTAAAGTGGGAGAA
TCTCTGGGTGCACATCCAGAGTACCCTGGGAGAGGCATGCCAGGTGTTCCTGTACCTTTTTCAG
GATATTCCCAGTGTCATCTTCAGAGGGAGCCTCTGAGGCTGCAGCCACACATGGGAAAAGATTA
CTTATGGAGAAGATCAGAGGCTAAGGGGCTGGGGATGGTGGGGACATCGGATTTTGAGGTCAG
ACCTGGACTCCTGTCTACCTCTGTCTCTCTACCTTTTATTTTTCTTTCCTTTTCTCCTAACAAGGCC
CTATTTCTAAGATGGCACCTCAGGCCTCCCATGGGACCAACCAGGCCTGACAGACTAACTGCTC
AGACTAACTGCCAGACTTCCTCCTGCTAGCCAGTCACTACCAAGGATGTGTGCCTCAGATACAG
CCCCTGCCCAGGACACCTACGTTGTCCCTAACAGGGCCCACATATCTAAAGCATGAATGCCTAG
CCAGGGGCTCTGGGGCACCATAAGAAGGCATGAAGCTGGGCCTGCCAATTTGCATCCTGGGAG
GGCAGGTCAGCCTTGTCCAGGCAATCCAGGAGGCTTCCCAGTGGCCCACTGGCCAGTGAGGAC
CTTTGACCTCCTTGGCTGGTGGCACCAGGTTTATGATGCACATAGAGGCTGAACTTGAAGAATT
CTTATTGCAGAGGGCAGCCACAAGAGCCACTGGCCAGTGCCCAGATAATATCTGCAGACTGTTC
CCTCCTGCTCCATGGGCCACAGAATCCCTTTGTCTTGTTACTGCTACACTGCCTTCCTACCAATCT
TCAGGGTTCATCCTGGCCTCTGCCATGCATATCCAGGCTCCCCCAGAGATTGTGTGTCTGGGAA
TGAGCCCTGGCTTCTGCTCCTATCTCCCAGTGATACTGGCAAGTCCCTATCCTTCTTGAGGCTTC
AATTTCCCCTTCTGTGAGCTGGGAGGACAGGCCCACTAGATGCTCTCTGAGGGACCTACCAAGG
ATGTGGGCTGTGTGTCTCAGCCAACAGCAGTATCTCCTAACCCCAACACAAGAAATACACATAG
TAGGTGTCTGGTTAATGCTCTTGGAATTTCAGCCTATACTAACCATCTGAAAGCCACTGAGTCTG
AACTCTGCTACCATATTTGAGATTATCTCTCCATTGGGCACCTATGGGTTGCCTAGAGACAAGCT
ACCATGGTCCTCTTCCCTCGCCCGTCATGTGCAGGGTCAAAGGCAGGTGAGCTACACAGCTCGC
CCTGCTCTTCACTCTGCTTTAATGTGGGTGCTTGCCTTGGCAGATGGGCCCGACAGTGATAAGA
ACAAATGACAGATGCATACTGGGGCACCTGTGACTGCCATGAGACACAGTCCACTGATTCTGCC
TCCAACCCCCTCCCCAGAGCAGAATAGTGAGTGCTAGGTTTCCGCCTGTACCTCCACGGGACCC
CAACAGCTCCTGAACCCCATTCCGAGATTTGAGGTTGTGACTTGGGGACCTCTAGACTTCCCTTC
CCTCCACCTGACTCCAGACTGTCCTGGAGCTTGGGACTCTCAGGCCTCTAGGCACATCTTATTAA
GGACAGCAGCTGACAGCCATATGTTGTGACCCCATTTGTATCCCATGGTAACTCATGACCTGGC
AGTCTCAAAAGCTCTCAGGCCAAATAAGGAATGTCCCCACAGGATCTCCCATCACAAAGAGAC
ATTCTATGCTGGCTAATATTTTCAGGCATTTCCCCGAGTCAGGTATGTGGCTGGATGCCTAACAC

CATCTCCTCAAGGCCTTACACTAATACTGTGAGATATACTCCCAATGTATACAAGAGGAAACTG
AGGCTGATAGAGATTAGGTGACTGCCCCAGTGTCCTGTGGGCAGGATGAAATGACCCAAGCTG
GCCAGGGCTCCCTGACAGACCTTGTCCCTGGATTCAGCGAACATACATCAAACCATTAGCTGAC
AAGGAATGAATCTGACAAGCTGTTCATGACCACCACACTGAAAACTACAGGGCAAGGTAAAAG
ATATGAAAGTCAACCTAAATAAACAAGGGACACATGATGCCATGTTTCAGGAGCCCCAAAACTA
TAAGGATACCCATTCTCCCATGCCCATCTAGAGGCTGCTGCGCCTAGAGGAGGCTTTCCATGGC
AACTGGCATATGTATGGTGAGGAGAAACCCTGCAAAGACAGCAAAGCCAGTGATGGGGAAGG
AGAAGCAGCCAGAGCCAGCAGGACCAGCTGTCAAAACCTGTTAGCAAACTACAGTGAGAAGG
AAGCAAGGTCCTGGCCACAAAGAACAACTTGAATCTGACCAGTGGCACCCAATAGGGTCCAGA
AACAGAGCCACACGTAGCCAGTGAGCTGATTGTAAAGACACTGATACGGCAGGGTAGTTGGGA
GAGGACGGGCCTTCCCATGCCGGGTGCTGAGCTAGACTGGATATCCAAGTAGGGGAAGAGTG
GACCTTGACCTCGGCCTCACATTGGACACAAAAATCAATCCCAGATGGAGTGCAGATCTACATG
GGAAAGGTGAGCTAATAAAGCTTTAGAATAAACCAGAATATCTTTATGACCTTGGAGTAGTCAA
AGTTTCTTAAATAGGACACAAACAGCCCTATCCAGAGAGGAAATGTTTGATTAACTGGACCACA
TGAAAATTAAGACCTTCTGTTCATCAAAAGACACCATCAAGAGAGGAAGAAGGCAGCCTGCAG
AGGGGGAGGCGATATTTGCAATGTGTGTGTCTGTGAGAGAGTGTGAGTGTGTGTATCTCTCTCT
CTGACAAATGACTCCAGAATATCTAAAGATATATAGGACCATAAATAAACATAAAACAGGCAGA
AGATGTAGGCAATTTATACAGAATGATGTATCTGACTAAATGATAACATGGAAAGGGACTCAAC
TTCATTAGTCAACAGGTTACTGCAAATTAAAATCACCATTTGATGCTGCTAGACATTCACCATCA
TGGCTGAAAAGGAAACGACGGAAAATGCCAAGTGTTAGGATGTGGAGCAGCCAGAATGCTGC
CTGTGGGAATGAAAATTGGAATGACTCCTTTGGTAGACTGCTGGGTAGTATTCACTAAGAGTAA
ACATATGCCCACCCAGGACCCTGCAGCTCCACACATAGGCATATATTCCCCAGAAACGCAACCT
GATGCTCACCAAAAGCATGTACAGAATATTCTTAAACAGCAACCTACACCAGAAACTGCCTGCT
GGCCACAGGTGGTAGAATGGATGAATGATGTAGGCCATGCACACAATGGAGCACCAGTCAGCA
AGGAGCATGAGGGAGCACTCTCCCTTCAGCCCCATAGAGAGGCTGCCAGACATCAAATTGAGT
GAGGGACATTGGACACAAAGGTGTATGAGTTGCATCATTCCCACCGCATAAAGTTCAAATGTGG
GCAAAGCTGGTCCTTGCTGCTAGCAGTTAGGCCTGTGGTTAATCTCAAGGTCAGTGACAGGAAA
TGGGAGCCTGAGAGGCTTTCTGGGGGTTCTGTTGCTTCCTCCGGGTGCTGCACACATGGGTCCC
AGGGACTGAAGATGCCTCAAGCTGCGCACTTGAGACCTGTGTACTAATCTGTGTGCATGCACAT
TACCCTTTAACTAAAAGTTTAACACCACCACCCTATACCAGGGTAGCCCAGGTGAGCCTAATTA
GGTGGATAGCCCTGGCCTTCAGGGCCGCTAGGCCCAGCCCCATCCCACTCCCTCACTTAAGGG
GACACGGCTTCCAGACTTCCTGACTAGGTCACCTCCGTGCTTCAGCCTCCAGAGGCTCTGCAGT
TCAATCTCCCCTTTCAGGTATCAGCAGAGCACACAAGAGATCTATATGGGATGTGCCCCCCGCC
CAACCCCGCCCCGACCCCCTGCCCCAACTGCCAGAAGTGTCTCCATGACAATATCAGTGCAGTT
GTGAAGTCCTTTGCCCTCCAGCCACCTCTGTGGCGTGAATGTTCTCTCTGTCACATCCTCCAACA
CCATCTGCTTGGCTGAGGACCTGAGCAGCATCATGGCTGGGAGTTCACAAGTGGAGAGGGCTG
AGCCTGGCCAGAGTGCACTGGGGCAGGGTGATTTGGATGGCCTTTCCCTTGCAGAGGAAATGA
GAAGCCTCACTGCTTTCTTCTGGGCCATCCACCTAGACAGTGCGGCCCTCTCCCCATAGCAGCA
GCCCTAGGCCAGCCGCTTCTTCCTCACGTGCTCAG
ID|3505|gene_id|128439|transcript_id|NR_003239 Homo sapiens lncRNA
GCAGCCGCGCCCCCAGCCGTATTTCCGCGGGCGCTGAGCACTAGAGAGAGCGTCTTGTGG
CTGCGGCCTGCCCCTCAGCCTCCTCCGCGCGGTTACCCCTGTACCCGCCGCCATCCGTCCTGGC
GCTCCGGATGAGTCAATGAGGGGCAGGGCCCGAGGAGTGGTCTTCCCAAGAACCCCTGGTGG
CCTCCCAAGGCCGGTGCTGTGTACCTCCTCCCCGACAAAAGGGGAAACTGAGGCCCCGAGGG

GAGTGGGAAGAGCCGGCTGGACGTCAGGCCCAGCCGCTGGTGCAGTGGTCCGTCCCCTCTGCC
GGGGTGGGCCCCTCGGGTTTCGCGTGTCCTCGGGAAAGAGACTGGCGGGCCTCGTGGGCTGTG
CGGCTATCCTGGAGACAGATGACAGCTCTCCCTTGGATGGCTTTGCTGGTTCCGCACCAGCCAG
CGCCCCCATTTTTCCTGCAGCACCCTGATCTGCACTCCCTGAGGGGCTCCCACTGTCCGCGGTG
TGAGGATGTCCCTGGATAGTCCACTGTGTGCAGAGGCATGGGAGTTGTCATGTTGGGAACATGC
TAGACCTCAGTATCCTTGAGGGATGCTGCCTTGGGTCTGGAAACTGTTAGAGGAAACCCCAAGA
GGTGCAGGCACTGAGCCTCTCAGGACAATGACCTGGGGTCCCAGCTCCCCTGGAGGGGCCTCC
TCATGATTGTTTGGGGGTTGATCACAGACCAAGAGTGACGAGTGATGTCACCCTGTGACTCATG
GCTGGACCTTCTTGCCCCTATTGTCTCAGCACAACATTATTCGACTTTTCCCTCAGCGTGGGTGG
GCAGAGGAAAAGCCCTGTGGCTCTGGGGACTTGGGATCCAGAGTTGAAGACCCTTCAGCTGGC
TCTGCCCTGCCAGTGCCACAGAGTGCCATGGCCCAGGAAGACAGGTTTTCTTCCATCTAGGCCA
GGCCATCCAGTGGCCATCCTCCGTGTCCTCCCGCCTCCTCCTGGTGTGACTTCTGAAAACCAAG
AATTTGTTCCTGTTGACTTTTTCTGTGCTATGGACCATTGTCCTCTCACCCACTCAATAAATCTTGA
AACATGCAAAAAAAAAAAAAAA
ID|5943|gene_id|196047|transcript_id|NR_002791 Homo sapiens lncRNA
GTGCTTGGCTACAGCCGCTGCTGCCTCTCGCGAACTGGGCTCCGGGGCTCCCGGCTCCCG
AGAACTAGAAGAGAAACGCGAGCGAAGGGATCGAAACCCGGGGGGTTACCGACTTGCAGACA
CCGCCAGGACAGTCTGTAACGCAGGAAGATCCCAGCGGCTCCGGGTCTGGTGAGGGGACCAT
AAGCATGACTGATAGCGAATGAGGAAGGGCAGCCCTAAACTTTTCAAGCAAAGCCTCAGAGTT
TTGGGTTCACTCATTAGCATAGGAAATCGATTCACCGAAAACCCAAACAAAGAAAAACAAGCC
GACAGTCCAGGCAGGATGCAGGCAAATCCAGTTCGGGATTAAGGGTAAAAGGCTTTTTGGGTTT
TTTTTCCTTTGGTTTGATTTTTTAAAATATGGGGAGGGGGGTGACATCTACCCGATTCTAGGCTCC
GGCAGGAACGCAATGGGTTAATGAATGGACAAGCCGCGGAGTATTGATCGGCTGCCGCCGGA
GAAAGAAAGAAAAACAAAAACCAGACCGAACCTGCCTTCCCGCTGTGGCTGCTCGGCGCCCCA
ATTAAGCAGGGTCATCTCAGGCTGGCTGCATGCCTCAGCTGAAGATCCCAGCTCCTGTCAATGC
CACCTCTCTGCTTGACTGTCTCCTTCCAGATTCGAGCAGGTATGAGCTGGGAAGAATGAAGGCA
GGGCATGCCCGTGTGCCAGCTCTGCACAGCTGGATAGCTGAGGAAAGATGTGGAGGAGAAGC
CGGGGATTGTGTGGAAGTCTAAGGGTGTTGTTTGCCCTTTGGGTTCCAGAAGATGCATGCCAGG
ACCCTGGGTGGCACTGCCAGGAAGCAACAGAGAGGAGATAAAACTCACAGCAGACAGACTTG
CCTTAACAACAACTCCCTTGAATTAAAACACGCTTTTCAAGAAAACAAATTATCAGTTCGATCAG
CAAACAGCAGAGAAGTTTCTCTCATAATGGCAAAGAAGGGCCGGGTTGCTGACCAGTGAAAGA
GCTTCAGAAAAGGAGAGGGGAGATGAGATGGCCAGAAGGAGCAAGAGCACCGTACATCCCTG
GACAACCTCATTCTAATGGGTCAGGGGCTGGGACGTGCATTTTGGAGTGCAGGAGAAGTGGCA
ACTCACAAATGCTAGATTTTCTTCTAGAGATGACCAAGCTGTAGTTCTTAAAGCAGTGGCACTAG
GGCAGAAAACTCTCACACTTTGATGTGCACACACAGCCCCTGGGGATCTTGTTGACATGTAGAT
TCTGATTCCGTAAGTCGGGCTGAGATTCTGCATTTCCAACAAGCTCCTAGATGAGGTCCATTTTG
CTGGTCCATGAAACACACTTAGAATAAGTAGCAAGGTATAGGAGGATACTGACTTTGCTCAGTG
ATGCTTGGGCTTCCGTCCAAACTAAAATAAAACAAAAGCAGACATAAATGGCCCAATTCAACAG
CCTGAGAAGTTTGGTGATAATGACCCAAGCCCTGGCCTGGTGACCAAGTGGCTGCTCAGAGAG
CTCTATCTCCAAACTCCTTCCCTTCCTGCCTCCCCTCCAAGAGTGAGTAATGTGCCAGAGTTCAT
TGGGGTCTGCTGTCCCCAGGGAAAGGTGGCCCAGTTGCCAAGTGCAAGTGTATAGAACGGCTT
ACCTGTGCAAAGCTATCAGCCCTGGACCTATGGGCCTAGAAACATTACTTGCCACTCACTCACT
CACTATCATTCATTCATTTCTGCAACAAACACTTCCTGAGCATATGCTATACACCAGGCCCTGAG
CTCAGCTCTGGGGATACAGGATGAATGGCAGATTGTGCCATCAGGAAGCCGACTGCACAGTCA

GCCCGCTGGAGCACATATGAAAAACAATACGGATGTTTTGATTTATTATCATATTGATCATTATG
ATGGTAGCGTATACTTTTGGAAGGCTTGCCATACATTAGGCATTGTGCTAAGCACATTATATGGT
TAATCTCATCAATCTTCACAACCTATGTGGCAGGTACTATTATTAAACCCACTTCAAAGAGGAGG
GAACTGAACCTCAGAGAGGGTAAGTGACTTGCACAAGGACACAAGCTAGTGAGGAGAGGAAC
TGAGAGGAATGGCCAGACAGGCCATCAGCCTCTGTCCCCACATGCTGGGTCTGAGTGTCTGAC
GGTGGACACATTCCCTTCCTGGAGGGAGAGCTCCTAGGGAGCTGATGGCAGCACTGAGTCTGG
AATGTGATGGGAGTGCATTAGATGACGTGGGTGATCTGATGAGTACAGAGGCGTCACACTGCA
ATGACTTCATGCACATATCTCCCCTTGAATGCACAGAGATTCAGCAGAGGCCAGGAGCTCAAGT
TTCTGGCCCAGTGTCCCCCTCTTCCCCTGGGGAAAGCCAGGGACACACTGCCTGGCTTTGCTAC
ACCTTTCTAGGCAAAGGCAGTATGCTCTCCCTCTCCTTGGCCTCCCTGGCTGTCCACACATCCAC
ACCCACTCACACACACATGCACATGATCATACACATTACACAAGCCCACATGCCCACAGACATG
CTCACACTCACACTCACATGCATACACTGCCATACACAACACACATACATACATTCACACACAC
TTGCACACACACACACACACACAGTTCTGATGGCCAATCTTTTGGGGCTATGTTGACACTGTGA
AGCAGGTGAAACAAAGTCTTTGCCACTCCCTGCTTTGGGCCTATTATGTTTGTTTTATAGTTTAAC
TCTCAAAAGACAACTTTATTTTCCAAAATTATTCAGGTTCCCAGCCAGGTGCAGTGGCTCACACC
TGTAATCCCAGTACTTTGGGAGGCCAAGGCGGGCAGATCACCTGAGGTCAGGAGTTTGAGACC
AGCTTGGCCAACATGGTGAAACCCAGTCTCTACTAAAAATACAAAAATTAGCCAGACATGGTGG
CACACACATGTAATCCCAGCTATTCGGGAGGCTGAGGCAGGAGAATCGCTTGAACCCGGGAGG
CGGAGGTTGCAGTGGGCCAAGATCGTGCCACTGCACTCCAGCCTGGGCAACAGAGCAAGACTC
CATCTCAAAAAACAAAAACAAAATCATTCAGGTTCTGTAGGGGACCCTCACTGTGTCCTGGATT
CCATTCCACTTTTAAAGTCCCAAACAGCTTGTATGGCCACTTCCCTTGACACCCAAACCAGATAG
GAGTCTTAAATGACACAGAGTATTGATGGTTTCTTTAAAAGGAAATCCACTCTCAATGGGAAGCT
GGATAACTTCGCATCCAGATTTCATCCATCTTGGGCTTTGGTGGCTGCTGTTTGTTTTTGTGCAGG
AGAATCTCTCCCAGCCAACGTCGATTTCACTAACACAGTGGTTAACCTTTGCTCTCTACTCCTGC
ATCAATCTTCCGCCTACCGCATGTTGAGCCATGCTGCTTTCTGGCATTCCTTTTGGATTTTCAGTT
TACTACCCAACTACCAGTCCTAATAACATCAGATAAAAGGGCTTCCGCTGTGCTGCACTTGTTTT
CAAAAACTATAAATGCCATCGACCCAGAGTTAAGTCCAGCCTCAGAGAGACAGAATATAAATA
CCCAAGTGGCCCTGGGCCACTGAGTTCCTCAGACATTATGTGCTATGGGAGTTCCAGAAAGGG
GGATTGCCTGGTTTTGAGTGCAGAAGGCTTCCTGGAGGAGGCGAGATTTCTGATGGGCCTCTGT
TATGAGCTAAATTGAGTCCACCCCAAAATTCAGATGTTCAAATCCTAACCTGTAGTACCTAAGCA
TGTGGCCTTATTTGGAAGCAGAAGCATAACAGAGGTAATTCGTTAAGATGAGGTCACAGTGGAA
GAGGGTGGACCCCAATCTGATACAACTCGTGTCCTTATAAAAAGCAGAAATTTGAAGACAGACA
CACAAGAAGATGAAGGCAGAGACCTGGCTGATGCGGCTATAAGCCAAGGAACGCCAAAGATT
GCCAGCAAAGCACCAGCAGCCAGGGAGAGGCCTCGAACAAATTCTCCCTCCCAGCAAAAAGG
AGGAAGGAAGGAACCTCCAAAAGAAGCAGGCCAGGCACAGTGGCTCACGCCTGTAATCCCAG
TACTTTGGGAGGTTAAGGCAGGCGGATCACCTGAGATCAAGAGTTCAAGACCAGCCTGGCCAA
CATGGTGAAACCTCATCTCTACAGAAAAATACAAAAATTAGTCAGGTGTAGTGGCGTGCACCTG
TAGTCCCAGTTACTCGGGAGGCTGAGGCAAGAGAATCGCTTGAACCTGGGAGGTGGAGGTTGC
AGTGAGCCAAGATCGTGCCATTGCACTCCAGCCTGGGTGACAGAGCGAGACTCCATCTCAGAA
AAACAAAAAAGAACCCTACTGACACCTGGATTTCAGACTTCCAGCCTCCAGAACTGTGAGATGT
TACATTTCTGTCATTTAAACACCCAGTTTGTAGTACTTTGTTGCAACAGCTCTAGTGAGCTAATAC
AGCCTTGTAGGACAGGTAGGAATTTGTAATGCACAGAGAAAGCCAACTGGCCACAGTTGTTGA
AAATAATAGAATCGAAGCGCCCCTATCACTACCACCCCAGGCCATCACTTTCAAAGCTGTTCAA
AATGATGCAGCCGCGTGAGAAGGCCGGAAGGCCAGGGCGGGAGATGGGCTCCAGGTGGGCC

CAGGCTGCCTGCAGAACCAGCTTGCCTCACTGCCTGGTTGCCAAGGAGCACCTGTGGCTAGGA
GGAGATGCCCAGGAAGAAAATTTGGCCACATCCCTGCCTGCCTCCCTGGCAATGGGTGATGCT
CTCTGCCAGCTGGCCCAGCTCCCCCACCACATACCTGGCTAGGAGTCAGTAGGAAAACAGTGT
GTGTTTGTAGGGCTCTGCACTGAAGGGCATTAGATCCATGCAAAGGCTAATAAATTAGAGATAA
GTGGGATTTGTAGATTTGCTGGAGGTTAGAGCTGCAGGGTCTGGCATCAGCTCTGGGTCTTTTCC
ATGGCCCCAGGCTGGCTCAGGTGTGAGCTGAAGAGGCAAAACTGTCTGTTTAGGTTGCATTGG
GTTCCCCTGAAAGATCTGTTGAAGTCCTAACCTCGGGTACCTCTGTTTGTGCTATTTGCAAACAG
GCTCTTTGCAGATGTAATCAAGGAAAGATGAGGCAACGCTGATTTAGTCAGGCCTTAATCCAGT
GACTGGTGTCTTCATAAGACAAGGAAATCTGGACACAGACACACAAAGAGGAAAAAGCCAAAG
ACACAGAGACACATGTACACAGGGAGAACTCCATGGGACAACGCAGGCACAGATCGGAGTGA
CGCGGCTGCAAGCCAAGGAGCGCCAAGATTGCCAGCAACCCCCTGGGGCTGCAAGAGGTGAG
AAGGAGCCTTCGGAGGGAACATGCACTGCTGATTCCTTGATTCCAGATTTCTGACCTCCAGAAG
CATGGGAGAACAGATTTCCCTTGTTTGAAGGCACCCAGTCTGTGGTCCTTCATCATGGCAGCCC
TCAGGAATGGATACAGGCCCTTCCCTGCCTGCCTGGTGGAGCAGCCTCCAAGGGGAGCCTTTG
CTAGCAGGAAGAGGCATCGCATGGAGCTCAGACCCGCCTCAGAGGAACTTCTCTCAGCCTCCT
CTCTCCGCACACCCGACCAAGGTGTGAAATCCCTCCCCTTTACCTCTGCCACTTGCAGGTGGCA
GGATGCTGGAGCTGTCCCTGCCTCTTTGTAAAGTGAGGGAAGGGCAGCCCTCTCTGCTCTTTTTT
TTTTTTTTTTTGAGACAGAGTCTCACTCTGTCTCCCAGGCTGGAGTGCAATAGCACGATCTCGGC
TCACTGCAACCTCCGCCTCCCAGGTTCAAGCGATTCTCCTGCCTCAACCTCCTGAGTAGCTGGA
ATTACAGGTGCCTGCCACCACGCCCAGCTAATTTTGTATTGTTAGTAGAGCCAGGGTTTCACCAT
GTCGGCCAGGCTAGTCTCAAACTCCTGACCTCAGGTGATCCACCTGCCTCGGCCTCCCAAAGTG
CTGCGATTACAGACATGAGCCACCACGCCCGGCCTGCTCCACTTCTAAGGCTTCTTGTGACAAT
GTAAGAGAAAGGAGATGACAGAGCTTTGCAACGGGAGGAGGGCTATGTGTTCTGGTGACCAAT
TCACTGTCTTGTGTCGGGACAGGAAGAAGCCCTTCATACGGGCAGCAGGCTGGGAGCCAGGGA
GGAGGAAAGATCACGATCCACTCCCTGGTACATGGCCCTTCTGCACCCCGCAGTCTCCTTCCAG
GTGCCACAACGAGAAGGCACACATCCTTGGCACAGCACTTGAGGCTTTTCACCACTGGCTGCA
CTCACCCCTCCAGACTCACTGCCTTGCACCAACCCTTTTCCGCCCACCCCACTCTATGCTGTCCA
CAGCCTCCACCCCAGCCACCTGATTCTGCAGGCCAATGTCACATTCTTCCAGTCCAGGTTCTATT
CTGGCATTTCTTGTCATTATTTTGCTGAGAATGTGTCTCTCTTGACTTTGAACTTATTGAGAGCAG
GAATCATGACTCAGCCATATATCCCAGCACTTGGCCCAGGGCCTGTCGTTTCCAGGGTAGGTGG
TCTAGGCTGATTGAAGGAATGGCATTTAGTCTTTAAAATGAAAGCATGTTGCCTAGCTTGGTTAT
TTTTGAACTCTATAATCAAGGACTACGTTTACCTGAATAGCCTCTGCAGAACACCAATTCCGTAA
GGTGCTTCACACACACACACACCAATTCTATCATTTAATACATTTTGGAAAGGCTACATACTACTAC
AGCCTCTTTTACAGATTAGCAATGTCCATGAGCGCACTAAAGGTTGAGACATTCTGCAGTGAAG
AAGCCTATTTCATTTTGTTTAACCAAGTATTTCTCAAATTTATTTGATCATATGTGGCAGAAAATG
CTGTGTCTGGCATTCTTCATTTCCCTCTTCCTCCTTTAACATGGAACCCCTGATGTCTTTAGCTTGG
CACATCGCCACCCAGAATAAAAACTACCTTTCCCAGCTCTTCCTGCAGCTAGGGGCAGCCCTGG
GATAAATTCTGGACAATGAAATACAGGCAGAAGTAAATCATATACGATTTCCATGAAGGGACCT
TAAACAGAAGTGTGCCCTTCTCTTCCCCACACATTCCTCCTCCTGTCTGAAATGTAGATGCAACT
GCTGGCATTTGAGCAGCCATCTTGGGCCATGTGGTAGCTTCTTATGGATGATCTAGGACTAATTC
AAGGGTCTAGATTTACCTCCAAACTTTGTTTATCTACAAAAAATAAAACTCTATCTTCTTAAAGCT
A
ID|5953|gene_id|196475|transcript_id|NR_024037 Homo sapiens lncRNA
TTTCATTTGTGATTCGGATGATGCAGCTCTAGGTGGATTGACTATGAAAGGCGCTGAATATC

TTCAGGAAAATGGGTTCCATGAGAAGTGACATCCTCCTCGAATCAAAAGATTCTCAGGATGATG
GAGTGAGTGATGGAATAGGTTGCCAACTGTAGTTACTCCACTCAGCACAGAATGGCTGCGGGG
AAATATAATCAGACATGCCCAAAGAAGTCTGCTTAGGGTGAAACAAATACCTTTGAGGTTTAAA
TCAGGAAGGAGAGACTGTTTCTACAGGTGTCATACAAAAATCAATACTGAGCCAAGGATTATTG
GATCCCATAGAACCACGGAGACTTGGTAGTGAAATTCTTCCAAAGCATTCCATCCGTGCAGGCA
GATGGTGTTCCTAGGTTTGGAAATATAAATCCCATGGGAAGTCAGCTGAAGACCATTTTATGCAT
CACTCATCCTGTTAGAGCCCTGTGCTGTTCTGTCTCTCTATAAGACTGGCTGAAACCACCACGGT
TGTTTACAATTTATGCTCTAACTTTGGATCACCTAGTTAATACTCTAACTCCGATTATTACCAAAG
ACAATGTTCTATAAAAGATATTGTCTATTAGAAGGATGCTTCTGCATTATTAATAAAATCACAGG
GAAAATGCAACATTTTGGATGTCACATCTGATAAGTTGGCAGTTAAAGAACAGAAACAGCAATA
AGACTGCGTGGCCACATTTTCCCAGTGCCACCAACTGGGATTTAAGCTGACTAACATTTTGAAA
GCCCAGCCCATTTCATTCACAGCAGGATGGCAGTGGGTGACTGATCGTACCCGCCAGCTGGCT
AATGTCATAATTAACAAAGTTGCTACTACATCTGCAAAGTTTAATCGAAAAATTTGCCAGTGATA
TGATGGCCTTGTCACAGAGATCACAAGGGACACGGAGTTGACTTTTGCTCAAGGTGGAACGAC
AGAGCTGTGTAGGCAAGATGAGAAATTGCGAACTTGTTCGGCCAAAGGATTATCTCCTGAGATG
TGTAGAAATGAACTCTTGTCAGAGTTTCAAAGGAATTAACGTTTTTTGGTAAAATGTGCTAATGT
AAAGAGTGAGAAGATTAGTGATACATAATGAAAGCAATGCATTCTTTCACATGGAAATGGGAAT
ATAATAAATGTCCTGCAGGTGATTGGGGCTGTAGCAATTTTATTGAAGATTAAAGTTTGATTGAC
CTTTGTTAGGGTGATTAACCCTTCTGAAATTAAAAGCCAGAGCTGAATTATTCTGCTCAATTTAAA
TGCAAACATCGAGCAGGTCGTATGTCATTCAAATAATAATAAGAAGAAAATCTTCCTATTTTAAT
TTTCATTTTTGCACTTCTGAGTGGTATGCTGCTTTAGAAGGACCATAGCTCTCGCAAAAGCATTCT
GGTGGCAGAGAATATACAATAACGTTACAGATAATTGGGAGCATATAGGAAGAGTCCCAAGCA
CTACTGCCTGCATTCGAAACATCAAAACCACCATCCCAACAAAAGCACGGAGCATGAAGCCAA
TCTCTGTACACTGAAAATGCATGCTCCCAATAGAAAAACAACCTAGGGATGTCCTGAAGGGGTT
AGGAATAAATAACAATGACATCTCTATTCTAGTTCAGTGCCAACCACCGAAATTGCATATCATAT
CCATGTTAAATCTACATGATGTCTCCTGGAAATGCGTGCTTTTTGGTACAGTTGCTTATGAAGCA
GTTTCTAAAAGTTGATTATTTCTGATGTCTGGAATTTCATTCTCCATGATCTCTGGCAGTTTCTGAA
TATATTCAGTATTTTCATTAAGTTCAAAAATGCTGTAGGAGAAGCTAACGAATTATTTGCAATGC
CATTTAATGGTTTAGTTTTATTTTTCTCTGTTAAATTTTCTAAGCCTTGGTGAAAAGTTGTGTATTC
ACATTTCTTCAAAAAACTGTTTCCATTTTTCATATCATGTTAATTTTGTTTAGTTCTAAGTCAGTGA
ATATTTTTAGTCTTAGGTCCCACCACATAGTGAATGTGTCTGCCTTTTATGTATCTGTGAACTATT
AAAATGATAATAAAGGTTCTCTTCCTTCCAT
ID|10680|gene_id|284801|transcript_id|NR_040095 Homo sapiens lncRNA
GGGCTTGGGCTGGTGACGCGTGCGCCGGCCGGCCGCCGAGGGGCTACCGTTCTGCCTCC
GACCGGTCGTGTGTGGGTTTACTCGGAGGTGCTTTGCCTCTGAAGAAAAGAGAAATACTAGGCT
TTAGAATTTCACCATATGAATTTTGGGAGAGAGGAGCACAGTTCAGCCCATAACTAACACACGA
CGGAATGAAGTTGATAATAGTGAGACCTTGTTGGTACAAGACTATGTAACATAACCTGCGCTTCT
CAACAAAGAATTGCTTTTCTGACTTCTGCACTCAATAGCAAGACAAATCTCTCCTCTGCCTTCTC
CTTATCAGACTACTCAACATGAAGGCAATAAGAATGAAGACCTCTATGTATAATAATTGACTTCC
ACTTAATAAATAAGGTTACTTGGTACACTGGTCGACAAAACCTTGAAATGTTGATGTTTAAGGGC
TGTTGATTGCTGGCAGCAAAGAAGTCCAACTGTGCTGGTACCCGAGATAGCGTCTCCACATTTT
CTTCCAGGATCATCACCTTTGTTCTAAATAGCTCCCACATAGTGTGAACAGAAGTCATATGTTTTA
GATTTTCTGCCTTGGCACCCAGGAAACCAGTATGATTGAGTAAACATATTAAACTTCCTCTGCAT
TTCCAGCCTGAACAGATGTTGAGTACAAGCTGATCCACCTACGTTCAACCTGAGAGAGTCCACA

TCCACAGACTGTTTCATTTCCCTTTTTCAATTTACATAGAAGCATAGATTCTTGCTGGCATATACA
AATGCGCCATTTAAGGCAATCAAAAATGGTTCCACATCTCATCTGATTTCCACAATTGTTGCTGT
AACGATGGACTACTGAGGAGAGAGACAATTTCTCTGGCACATCTTCCCACATTTTAGCAACATG
TTCATGGAGAGCCAATATTCAGTAATTACAGCCCAATTCCTCACAATTTCACAATTCGCTAACAA
TATGCATTTTCACAATGTAAAGCTGATTTTCCTTTAACCAAATCAAACATGAAAAGATAGCTGCA
TGCAATGGGCAATCTAGATCATTAGAAGATTGATGTAAAATTAGAGGACACTGAGACTGGAATA
GATTGCAAACTTCAGGGAGCTCAGAAAACTTGTAAACTCTACATTTTGAGGAAATATGTTGTCGA
TGCTTTTGCAACTCTATGCAGTAACTTAACAAATAAAGCCTTAAAGTAACTTAAAAAAAAAAAA
ID|13631|gene_id|378805|transcript_id|NR_015431 Homo sapiens lncRNA

TTGGCCCCACTGTGCACCACACACTCCTTTCCCAGCCCAGGGGCACGCGAACAAAATGAG
GCTCAAGCTGACCAGGCCGAGCCGGAGGAACGCTGGGGCTTGGCAGCAGAAGGGATGGGACC
AGAGAGAAGGGTGTGGAGGAGACCCCAGTGAGGGCCAGGACATTTCAGGTAAAGAGAGGTCA
TATCTCCGTACCTCACTTCCTGACACAAACAAGTTTTCACTGTTGTCAGCAACAAAGCCCTAATA
TAGCTGCGGAAGAGAAAAACTGCATTGCATTTTGCCTCCTGCAAGCATCATCAACAGTTACTGG
AGGAACGTAATTCCAGAAAGCTTGAAAGCCGTGGTGATGGTAATTATGTATCAAATGCCTGGTT
CTATTTCTGTTATTATTGTTTTGTCATTTCTGTTTTCCCAGCGATCTGACTGAACTCGCAGAGGGA
CAAATCCAGTTTTTCTTTTTGACTTTTGTCAAACTAAATCAGGCCTGATAGAAAACTCATTGCTCT
CCGGGGAAACAAAGTAGGAGCCACGAAATGTCATTTTAACAGAGCGTGGGTTTGGTGACTGTA
GGAAAGGATTTGAGGACGCTCCTTCTGTTCGGCTTCCTATGTCATGAGCACAGGCTCCACGCAC
GCACAGACACCACGGCTCCCGGATGCTGTGGCTCCCCGATCGGGGCTCCTGCAGCGCCAGAA
GCCCCTCCGGGATGCTTCGAGGGGCTCCCGGTGGGTGGAGGTACGGACGCCGCTGCGGCCGC
CGCCGCCAGTCCTGCTGCTGTTGTTGCTGCTGCAGTCACGTGGGAGCCCCTTTAAGTTTCCATAG
AGAGGCCTCTCTGGTGTCACATGATGGACATGATATAATGAAACAACATTGTGGAGAGGAAAG
CATTAGGGGAGCCCACGGCTACAAAAACAAGTGAGTGAGAAGAGGTGGGAGGAAGAGAAACT
ACGCCACCTCCCCTGCAGCCGAGTGCACGCAGCAGCCTGGCGTGACAAGTGGGCGACGCCGG
GGGGCAGGGAGCCGGGGTCCTTGGCCCTGGCCGGGGACCCCACCGCCCACCGCGCGGAGGA
CAACTTTTAGCCGGCAGCCCAGACCAGCGCGGCACCTGTCTCCGGAGTCTCCACCGCTCCTCCC
GATTCATCCCAGGGAAATTCTCAAGAATACGCTCTACAAATCTACGTGCGCATCATTTTCACCTC
GCGTCGCGCCCGGGAGGAAGGAACGAGGCAAGGAGCTAAAGCAGCGTGCGTTCAGCCCTGGG
GCATTTTATTAATGCTTTTACGAGTTAGAAGAGTTGGGATAATTTGCCATCTGGAGTTTCTCTGCC
TTGCTGATCTGAGCTCAGACCTGCCAATTTACCAGAGATAATTGATAACACCCTGTAACAGCTG
AGAGGAAAATGGAAGAAACGGAGATACTTTTAGTGAAGCAGAATAAACCACTGAACAGGAAAA
ATGAGGAAGCTGTGAGTACCAGTGGAAGGAACGAGCCAGGAAGAGGGACTGGAACCATCTCA
GAAGCCATGCCCCTCAGGCTGGAACTTGCCCTGTCTCCTCGCAGATGAGGTAGGAGGCTCAGC
ACGGGCTGGTGGGAGCAGCCCACAGCAGCAGTGAGGGTCAGTGGGCCTGTGAGTTAAGAAGA
AGGTGGCACAGAGCGAGGGTCTCTGGATCCTGACTGTTTGACTTTTCCATGATTAGGATAAGTA
GCCAGGGCTTGGCTAGTTGGAGAGTTACTCGAACCTCAGGTGACAGTTGTAAGGCAGCACATA
GTGAAAAAGAGTCCTAGCCTGGGAAAGTCCAAAACCTTAGGTCTGGTTTCAGTTCACTCACCTA
TCTCTGTGACATAGCAAAGTCTGTTAATTTCTCTAATTTTCTGATTAGTAGTATAGTTGCAGGAAT
GAAATAAAAATGGTCCTGATTACTCAGAGATTCATTTACTGATTACTCTTTTTGTGACCTGAATAA
AGAGTACAACATCTCTCAAAAGGTAACAATATGATGTTTCAGGAAATTAGAGAAGTTAAGAGAC
TTTGCTGTATTTACTATACAGATAGTTGACTGACAGCTGAGCCCGGACCCAAGATTCATTTATAA
AATGAAGGAGTTGAGAAGTACATACTCTGAGGTTTATCCTAAATAAACTGAAGTCTAGGCAAGT
GGCTGAGCTAGGTTTGCCATGAATCAATCTGCTGAGTATATTTCTTGGTAACTAGTTCATCTTTCC

TTAATTCAGTCAACAAATATGATATCAAGTTTAGTAAGATGATGACAGTTGTCATGTAATACTCC
ATCTGGAAGATTGTATGCTTCTCAGCTTCCCTAACTTTTCTTAATCTACCTTAAGCTTCCTTTCTGA
AAGGTTTCTGTCTTTCCTAACAGTCTCTTCTTTCCGCCAGCTACCTCTTGTGTTTAAGAATTAAAG
GAAGATGAGCTAGGAATCAAGAAATACGCAGAACTGATTCCTGTCACTGGCGCCACCCTGCCA
CTTGCCCAGAGCTGTGGTATAAATTTTTTTAATAGGCTGAAATAAAAATATGAATAGCATTTTGG
TATTAAGCATTAAATTGATAAAGGCTATGAGATACACCTGCTCTCAACCATGTTAATTTTTTATTA
TTGGTATTAATAAATTATTACTTACTAACATATTAACAAGAATTGCATTGAGAAACAAAGCATCC
ACAGGCCAAATCTTCTGAATTTCAAATGTTTATATTAATGCGTTGTATTCTAGAAAAGTAGAATTG
TCTTAAGTAGCTTTGTAATATAAAGTCATCTATCAGCCCATTACACCTATTAGAATGTTTTAACCT
TTATTTCCCACTTTTTTGTTTCTAGACTGAGTGTACTTGGTCTGGTATTTGTGGCATAACTTACGGA
ACACATAAGAATGATACAGATACTATTTAATGATGACCTAATACAAGCTTAGATAGCTAAGGTG
AAAGCTTCTATGGCCTTAACATTTTCCTCTTGAAGAATGTATTTTCTGTAATAAAATACAGTGGCT
ACTTGAAATCTATAAACTTATGTGAGGTCTGGATAAATCTGAGCAACTTTCTTCTTTGTGCTCCAG
GAACCTACGCACTATATATATAAATAAAGCTTAAGTAAACATCACTGCAAAAAAAAAAAAAAAA
AA
ID|13632|gene_id|378805|transcript_id|NR_024153 Homo sapiens lncRNA
ACTGGAATCAGCAACTCCCTGGAACTCGGGCCTCTGGCCCCGTCCCTCTTTTCTCCGGCAG
GGACGCAGGATGCCTTGGTGCACTCTAGAGACGGGGACCGGAGGGTGCAGCGCGCCTGGGTG
GGGCGAAGGGCGCCGGTCTCCTCACGCCCTCTGACTTTAGGATACCAGACAGTGTCTCTTCAAC
CCCACCCCAGGCGCAGGTTTCCAGAAGGCGGGGTGGGTGGGGCCCCCGATTTCCAGAAGGCG
AGTTACCTAGCAGGTGTGTGGGGTGGGCATTGCTATTCGGAGAAAAATCAAGACGTTCCCTTCA
GTCCTCCCTCGGTGTTAACGTCTGCTCCAGCTCGCAGCCCGCTGCTGGGAGGAAAATGGAAGA
AACGGAGATACTTTTAGTGAAGCAGAATAAACCACTGAACAGGAAAAATGAGGAAGCTGTGAG
TACCAGTGGAAGGAACGAGCCAGGAAGAGGGACTGGAACCATCTCAGAAGCCATGCCCCTCA
GGCTGGAACTTGCCCTGTCTCCTCGCAGATGAGCTACCTCTTGTGTTTAAGAATTAAAGGAAGAT
GAGCTAGGAATCAAGAAATACGCAGAACTGATTCCTGTCACTGGCGCCACCCTGCCACTTGCCC
AGAGCTGTGGTATAAATTTTTTTAATAGGCTGAAATAAAAATATGAATAGCATTTTGGTATTAAG
CATTAAATTGATAAAGGCTATGAGATACACCTGCTCTCAACCATGTTAATTTTTTATTATTGGTAT
TAATAAATTATTACTTACTAACATATTAACAAGAATTGCATTGAGAAACAAAGCATCCACAGGCC
AAATCTTCTGAATTTCAAATGTTTATATTAATGCGTTGTATTCTAGAAAAGTAGAATTGTCTTAAG
TAGCTTTGTAATATAAAGTCATCTATCAGCCCATTACACCTATTAGAATGTTTTAACCTTTATTTCC
CACTTTTTTGTTTCTAGACTGAGTGTACTTGGTCTGGTATTTGTGGCATAACTTACGGAACACATA
AGAATGATACAGATACTATTTAATGATGACCTAATACAAGCTTAGATAGCTAAGGTGAAAGCTT
CTATGGCCTTAACATTTTCCTCTTGAAGAATGTATTTTCTGTAATAAAATACAGTGGCTACTTGAA
ATCTATAAACTTATGTGAGGTCTGGATAAATCTGAGCAACTTTCTTCTTTGTGCTCCAGGAACCTA
CGCACTATATATATAAATAAAGCTTAAGTAAACATCACTGCAAAAAAAAAAAAAAAAAA
ID|13633|gene_id|378805|transcript_id|NR_034120 Homo sapiens lncRNA
AAACAGCGATTCTTCTGCAGTGAATCACTTCCAGAAATGAATAGCCACAGTTTTTTGGAAT
GAACGTTGTGAAATCCCTCCTTTATAATGGCAGGTTTTCAGTTGGTGGTTTTATCAGAATTTCTCA
AGATCAAAACGAAACCTTCTCTTTAAAAAGGAAAGAAAGTACTATCGATACAGAAAGCAAAAGT
ATTTCCAGTCTCCTACTGAACTGTCACGGCAGACCTCTCTGTATCTATATTTAGAGCTGTATGTCC
ATATATTTGCCTGAATGTGTGAGTTCTTGGAAGTATGGATTCATTGCCAGGAGCTGGTGATTTCC
TAAGCAGAGGTCGCTAACTACAAGAAATGTTACACTCGGACAAGTCCTGCGCTTGGGGATCCTC
TGTACGCCCGTTCACTGGTGCAGGTCTTCTGAACAAATGCCTCCTGAGGATTTCTGTGCCTGGAA

AATATCTGTTAACAGACTGCTTTTACACCTACCTATTTGGTGCTACTTCTACTCCTCATGACCAAA
GCTGGCTTTCATGGTTTACTCATGTACTTACTGATGAAACCCTGAGACTTGGGGCCAGTGACTGT
TCAGAGGTCCATGTGAAGATGCGCTGAATGGTATAAAGGACAGAGCAGTAAAGAGTGCTGGCT
GAATGCCTGGGCTGGCAATCAGGCATGTTGGAGTTTGAGTCCCAGTCTGCCACTCCAGCTCTGT
GACCAAGAGGAAGTGAAGTCATGTACCCTTTCTAAGTCTCTAGCCCCTCTTCTGAAAGACGAAG
ATGCCACCACCAACCATGTTAGTGGTTGTGAATGCCAAGAAAGTCCATGTGAAGCACATAGCAT
AGTGTCTGCCATGCGTTAAGTGTTCCAAAAATGGAAGCTATGACTATTTCACAGAGTACACTTCC
CATCACTTTTCAGTTCATTTTGGCCTTCTCCCTCAAAAGCACTCCAGTGTTCTGATCCAGCTGATA
AAACACCAAACATTCACATAGTGCTTTAAAAGTGACCCACTGTTTTTACAGACTGCATTACATAT
GACCCTAACAACCACTGCAATATAGAAATTCGCATCTCCATTCTCCAGATCAGGATACCGAAGC
TCAGAAAAATTAAATGATTTGCCCAGGATCACATAGTTAGTAAGCAGCCCAGCCAGATCCTGTG
TTAACTTGTTCATCCGCTTCCTATAAGAAAACATCCATTCAAGGCACATGGGGCAACAAAGAAG
GACTTAGAGCTGTGTGGCCCCTCGTCAGGGCAGTACCAGTTGCACCCATAGTCCGGCCTGAATA
CTCAGCTTAGGACTATTGTGTCCAATGTCCCAAACATTTTGAAGCTACATCTTAAAGAAGTGTCC
TGCTCAGATGCAGTTCTCCTTGTGAAATCCGCAGGCCCCAAGGAAAGTGACTTTAATCCAGTTT
GCTCATGCAAGAACAGACTGGCATTTCACATCCAGGAAAACAATGGATTTGTACTGTTCAGCTTT
TGCCATGCAAGGAGTTCCTGGTGTGGTTTATTAATTTACATCTCAGGCTCCAGCTTCACTATCAAT
CAGATTTGGAAGAAAAAAGTTACAAAGAAAGGCAGCTTGCTGAGAAACAGCGGAAGCATGAC
GGTACAGACTGGGTTTTGAAATGGAGACACATTCTCATGGCTTGTGGGCTGAGAGACACTGAGA
AAGGATTTTGTTCTTGAAAGATGATTGTTTTAATGCTGGAGATGGAGAGTTTTGACAATTTTAAGT
GCATGTTCTGTGTGTAAGAAAGAGTCATTACGTTTTTTTTTTCTTTCAGATGTTGCCATGCCTTAAA
ATTGCTGATGATTAAAATAGAACATCCTGAGTTACAGAAATTCAGCCCTAGTGTATCCTGGCCTA
AAAATACAGAACAATCAAGTTGATTGTTGGAAATGAGAGGCTAGGCAGGGTTGGAAACATGCT
AATGTTTACTGAGTGAAATCTTTCCTTCTCAGTAGAGTTGCCCTTGCAGCTGAAAGTCACTGAAA
GACTCAACAAAATAAGCAGATCCCACTTGTCTCACTCTGTTCTCCTATGCATGCCCTGGAAAGA
GAATGAATGAGCCCTTTTTAATTTATCAACTGGTTTTCTTCCATCTCTTTACTAGTGAGCCATGGG
GGTTTTTTGCCGTTAACTGGGTAGCCAGTCTCTTCATGGAGACTTATTTTCAGGAAACTAGCCTCT
TGCTTTATGTGAAAACAAGGACCCAACTCAA
ID|13634|gene_id|378805|transcript_id|NR_109850 Homo sapiens lncRNA
TTGGCCCCACTGTGCACCACACACTCCTTTCCCAGCCCAGGGGCACGCGAACAAAATGAG
GCTCAAGCTGACCAGGCCGAGCCGGAGGAACGCTGGGGCTTGGCAGCAGAAGGGATGGGACC
AGAGAGAAGGGTGTGGAGGAGACCCCAGTGAGGGCCAGGACATTTCAGGTAAAGAGAGGTCA
TATCTCCGTACCTCACTTCCTGACACAAACAAGTTTTCACTGTTGTCAGCAACAAAGCCCTAATA
TAGCTGCGGAAGAGAAAAACTGCATTGCATTTTGCCTCCTGCAAGCATCATCAACAGTTACTGG
AGGAACGTAATTCCAGAAAGCTTGAAAGCCGTGGTGATGGTAATTATGTATCAAATGCCTGGTT
CTATTTCTGTTATTATTGTTTTGTCATTTCTGTTTTCCCAGCGATCTGACTGAACTCGCAGAGGGA
CAAATCCAGTTTTTCTTTTTGACTTTTGTCAAACTAAATCAGGCCTGATAGAAAACTCATTGCTCT
CCGGGGAAACAAAGTAGGAGCCACGAAATGTCATTTTAACAGAGCGTGGGTTTGGTGACTGTA
GGAAAGGATTTGAGGACGCTCCTTCTGTTCGGCTTCCTATGTCATGAGCACAGGCTCCACGCAC
GCACAGACACCACGGCTCCCGGATGCTGTGGCTCCCCGATCGGGGCTCCTGCAGCGCCAGAA
GCCCCTCCGGGATGCTTCGAGGGGCTCCCGGTGGGTGGAGGTACGGACGCCGCTGCGGCCGC
CGCCGCCAGTCCTGCTGCTGTTGTTGCTGCTGCAGTCACGTGGGAGCCCCTTTAAGTTTCCATAG
AGAGGCCTCTCTGGTGTCACATGATGGACATGATATAATGAAACAACATTGTGGAGAGGAAAG
CATTAGGGGAGCCCACGGCTACAAAAACAAGTGAGTGAGAAGAGGTGGGAGGAAGAGAAACT

ACGCCACCTCCCCTGCAGCCGAGTGCACGCAGCAGCCTGGCGTGACAAGTGGGCGACGCCGG
GGGGCAGGGAGCCGGGGTCCTTGGCCCTGGCCGGGGACCCCACCGCCCACCGCGCGGAGGA
CAACTTTTAGCCGGCAGCCCAGACCAGCGCGGCACCTGTCTCCGGAGTCTCCACCGCTCCTCCC
GATTCATCCCAGGGAAATTCTCAAGAATACGCTCTACAAATCTACGTGCGCATCATTTTCACCTC
GCGTCGCGCCCGGGAGGAAGGAACGAGGCAAGGAGCTAAAGCAGCGTGCGTTCAGCCCTGGG
GCATTTTATTAATGCTTTTACGAGTTAGAAGAGTTGGGATAATTTGCCATCTGGAGTTTCTCTGCC
TTGCTGATCTGAGCTCAGACCTGCCAATTTACCAGAGATAATTGATAACACCCTGTAACAGCTG
AAGACAGAGTCTTACCCTGTCACCCAGGCTGGAGTACAATGGTGCGATCTCGGCTCACTGCAAC
CTCTGCCTCCTGGGTTCAAGTGATTCTTCTGCCTCAGCCTCCTGAGTAGCTGGGACTACAGGAG
GAAAATGGAAGAAACGGAGATACTTTTAGTGAAGCAGAATAAACCACTGAACAGGAAAAATGA
GGAAGCTGTGAGTACCAGTGGAAGGAACGAGCCAGGAAGAGGGACTGGAACCATCTCAGAAG
CCATGCCCCTCAGGCTGGAACTTGCCCTGTCTCCTCGCAGATGAGGTAGGAGGCTCAGCACGG
GCTGGTGGGAGCAGCCCACAGCAGCAGTGAGGGTCAGTGGGCCTGTGAGTTAAGAAGAAGGT
GGCACAGAGCGAGGGTCTCTGGATCCTGACTGTTTGACTTTTCCATGATTAGGATAAGTAGCCA
GGGCTTGGCTAGTTGGAGAGTTACTCGAACCTCAGGTGACAGTTGTAAGGCAGCACATAGTGA
AAAAGAGTCCTAGCCTGGGAAAGTCCAAAACCTTAGGTCTGGTTTCAGTTCACTCACCTATCTCT
GTGACATAGCAAAGTCTGTTAATTTCTCTAATTTTCTGATTAGTAGTATAGTTGCAGGAATGAAAT
AAAAATGGTCCTGATTACTCAGAGATTCATTTACTGATTACTCTTTTTGTGACCTGAATAAAGAGT
ACAACATCTCTCAAAAGGTAACAATATGATGTTTCAGGAAATTAGAGAAGTTAAGAGACTTTGC
TGTATTTACTATACAGATAGTTGACTGACAGCTGAGCCCGGACCCAAGATTCATTTATAAAATGA
AGGAGTTGAGAAGTACATACTCTGAGGTTTATCCTAAATAAACTGAAGTCTAGGCAAGTGGCTG
AGCTAGGTTTGCCATGAATCAATCTGCTGAGTATATTTCTTGGTAACTAGTTCATCTTTCCTTAAT
TCAGTCAACAAATATGATATCAAGTTTAGTAAGATGATGACAGTTGTCATGTAATACTCCATCTG
GAAGATTGTATGCTTCTCAGCTTCCCTAACTTTTCTTAATCTACCTTAAGCTTCCTTTCTGAAAGGT
TTCTGTCTTTCCTAACAGTCTCTTCTTTCCGCCAGCTACCTCTTGTGTTTAAGAATTAAAGGAAGA
TGAGCTAGGAATCAAGAAATACGCAGAACTGATTCCTGTCACTGGCGCCACCCTGCCACTTGCC
CAGAGCTGTGGTATAAATTTTTTTAATAGGCTGAAATAAAAATATGAATAGCATTTTGGTATTAA
GCATTAAATTGATAAAGGCTATGAGATACACCTGCTCTCAACCATGTTAATTTTTTATTATTGGTA
TTAATAAATTATTACTTACTAACATATTAACAAGAATTGCATTGAGAAACAAAGCATCCACAGGC
CAAATCTTCTGAATTTCAAATGTTTATATTAATGCGTTGTATTCTAGAAAAGTAGAATTGTCTTAA
GTAGCTTTGTAATATAAAGTCATCTATCAGCCCATTACACCTATTAGAATGTTTTAACCTTTATTTC
CCACTTTTTTGTTTCTAGACTGAGTGTACTTGGTCTGGTATTTGTGGCATAACTTACGGAACACAT
AAGAATGATACAGATACTATTTAATGATGACCTAATACAAGCTTAGATAGCTAAGGTGAAAGCT
TCTATGGCCTTAACATTTTCCTCTTGAAGAATGTATTTTCTGTAATAAAATACAGTGGCTACTTGA
AATCTATAAACTTATGTGAGGTCTGGATAAATCTGAGCAACTTTCTTCTTTGTGCTCCAGGAACCT
ACGCACTATATATATATAAATAAAGCTTAAGTAAACATCACTGCAAAAAAAAAAAAAAAAAA
ID|13635|gene_id|378805|transcript_id|NR_109851 Homo sapiens lncRNA
TTGGCCCCACTGTGCACCACACACTCCTTTCCCAGCCCAGGGGCACGCGAACAAAATGAG
GCTCAAGCTGACCAGGCCGAGCCGGAGGAACGCTGGGGCTTGGCAGCAGAAGGGATGGGACC
AGAGAGAAGGGTGTGGAGGAGACCCCAGTGAGGGCCAGGACATTTCAGGTAAAGAGAGGTCA
TATCTCCGTACCTCACTTCCTGACACAAACAAGTTTTCACTGTTGTCAGCAACAAAGCCCTAATA
TAGCTGCGGAAGAGAAAAACTGCATTGCATTTTGCCTCCTGCAAGCATCATCAACAGTTACTGG
AGGAACGTAATTCCAGAAAGCTTGAAAGCCGTGGTGATGGTAATTATGTATCAAATGCCTGGTT
CTATTTCTGTTATTATTGTTTTGTCATTTCTGTTTTCCCAGCGATCTGACTGAACTCGCAGAGGGA

CAAATCCAGTTTTTCTTTTTGACTTTTGTCAAACTAAATCAGGCCTGATAGAAAACTCATTGCTCT
CCGGGGAAACAAAGTAGGAGCCACGAAATGTCATTTTAACAGAGCGTGGGTTTGGTGACTGTA
GGAAAGGATTTGAGGACGCTCCTTCTGTTCGGCTTCCTATGTCATGAGCACAGGCTCCACGCAC
GCACAGACACCACGGCTCCCGGATGCTGTGGCTCCCCGATCGGGGCTCCTGCAGCGCCAGAA
GCCCCTCCGGGATGCTTCGAGGGGCTCCCGGTGGGTGGAGGTACGGACGCCGCTGCGGCCGC
CGCCGCCAGTCCTGCTGCTGTTGTTGCTGCTGCAGTCACGTGGGAGCCCCTTTAAGTTTCCATAG
AGAGGCCTCTCTGGTGTCACATGATGGACATGATATAATGAAACAACATTGTGGAGAGGAAAG
CATTAGGGGAGCCCACGGCTACAAAAACAAGTGAGTGAGAAGAGGTGGGAGGAAGAGAAACT
ACGCCACCTCCCCTGCAGCCGAGTGCACGCAGCAGCCTGGCGTGACAAGTGGGCGACGCCGG
GGGGCAGGGAGCCGGGGTCCTTGGCCCTGGCCGGGGACCCCACCGCCCACCGCGCGGAGGA
CAACTTTTAGCCGGCAGCCCAGACCAGCGCGGCACCTGTCTCCGGAGTCTCCACCGCTCCTCCC
GATTCATCCCAGGGAAATTCTCAAGAATACGCTCTACAAATCTACGTGCGCATCATTTTCACCTC
GCGTCGCGCCCGGGAGGAAGGAACGAGGCAAGGAGCTAAAGCAGCGTGCGTTCAGCCCTGGG
GCATTTTATTAATGCTTTTACGAGTTAGAAGAGTTGGGATAATTTGCCATCTGGAGTTTCTCTGCC
TTGCTGATCTGAGCTCAGACCTGCCAATTTACCAGAGATAATTGATAACACCCTGTAACAGCTG
AGAGGAAAATGGAAGAAACGGAGATACTTTTAGTGAAGCAGAATAAACCACTGAACAGTCTGT
TTCATCTTCATAAAAACGCTGCTTTCAGTGCACTGGATTTATTTTACAACCCACTAATGGATTACC
CTACAGTTTGAAAAACATTGCGTTAGACCAGTTTGACTGTTGTCAAGAACCAAAAAAGGAAAAA
TGAGGAAGCTGTGAGTACCAGTGGAAGGAACGAGCCAGGAAGAGGGACTGGAACCATCTCAG
AAGCCATGCCCCTCAGGCTGGAACTTGCCCTGTCTCCTCGCAGATGAGGTAGGAGGCTCAGCA
CGGGCTGGTGGGAGCAGCCCACAGCAGCAGTGAGGGTCAGTGGGCCTGTGAGTTAAGAAGAA
GGTGGCACAGAGCGAGGGTCTCTGGATCCTGACTGTTTGACTTTTCCATGATTAGGATAAGTAG
CCAGGGCTTGGCTAGTTGGAGAGTTACTCGAACCTCAGGTGACAGTTGTAAGGCAGCACATAGT
GAAAAAGAGTCCTAGCCTGGGAAAGTCCAAAACCTTAGGTCTGGTTTCAGTTCACTCACCTATC
TCTGTGACATAGCAAAGTCTGTTAATTTCTCTAATTTTCTGATTAGTAGTATAGTTGCAGGAATGA
AATAAAAATGGTCCTGATTACTCAGAGATTCATTTACTGATTACTCTTTTTGTGACCTGAATAAAG
AGTACAACATCTCTCAAAAGGTAACAATATGATGTTTCAGGAAATTAGAGAAGTTAAGAGACTT
TGCTGTATTTACTATACAGATAGTTGACTGACAGCTGAGCCCGGACCCAAGATTCATTTATAAAA
TGAAGGAGTTGAGAAGTACATACTCTGAGGTTTATCCTAAATAAACTGAAGTCTAGGCAAGTGG
CTGAGCTAGGTTTGCCATGAATCAATCTGCTGAGTATATTTCTTGGTAACTAGTTCATCTTTCCTT
AATTCAGTCAACAAATATGATATCAAGTTTAGTAAGATGATGACAGTTGTCATGTAATACTCCAT
CTGGAAGATTGTATGCTTCTCAGCTTCCCTAACTTTTCTTAATCTACCTTAAGCTTCCTTTCTGAAA
GGTTTCTGTCTTTCCTAACAGTCTCTTCTTTCCGCCAGCTACCTCTTGTGTTTAAGAATTAAAGGA
AGATGAGCTAGGAATCAAGAAATACGCAGAACTGATTCCTGTCACTGGCGCCACCCTGCCACTT
GCCCAGAGCTGTGGTATAAATTTTTTTAATAGGCTGAAATAAAAATATGAATAGCATTTTGGTAT
TAAGCATTAAATTGATAAAGGCTATGAGATACACCTGCTCTCAACCATGTTAATTTTTTATTATTG
GTATTAATAAATTATTACTTACTAACATATTAACAAGAATTGCATTGAGAAACAAAGCATCCACA
GGCCAAATCTTCTGAATTTCAAATGTTTATATTAATGCGTTGTATTCTAGAAAAGTAGAATTGTCT
TAAGTAGCTTTGTAATATAAAGTCATCTATCAGCCCATTACACCTATTAGAATGTTTTAACCTTTA
TTTCCCACTTTTTTGTTTCTAGACTGAGTGTACTTGGTCTGGTATTTGTGGCATAACTTACGGAAC
ACATAAGAATGATACAGATACTATTTAATGATGACCTAATACAAGCTTAGATAGCTAAGGTGAA
AGCTTCTATGGCCTTAACATTTTCCTCTTGAAGAATGTATTTTCTGTAATAAAATACAGTGGCTAC
TTGAAATCTATAAACTTATGTGAGGTCTGGATAAATCTGAGCAACTTTCTTCTTTGTGCTCCAGGA
ACCTACGCACTATATATATAAATAAAGCTTAAGTAAACATCACTGCAAAAAAAAAAAAAAAAAA

ID|13636|gene_id|378805|transcript_id|NR_109852 Homo sapiens lncRNA

TTGGCCCCACTGTGCACCACACACTCCTTTCCCAGCCCAGGGGCACGCGAACAAAATGAG
GCTCAAGCTGACCAGGCCGAGCCGGAGGAACGCTGGGGCTTGGCAGCAGAAGGGATGGGACC
AGAGAGAAGGGTGTGGAGGAGACCCCAGTGAGGGCCAGGACATTTCAGGTAAAGAGAGGTCA
TATCTCCGTACCTCACTTCCTGACACAAACAAGTTTTCACTGTTGTCAGCAACAAAGCCCTAATA
TAGCTGCGGAAGAGAAAAACTGCATTGCATTTTGCCTCCTGCAAGCATCATCAACAGTTACTGG
AGGAACGTAATTCCAGAAAGCTTGAAAGCCGTGGTGATGGTAATTATGTATCAAATGCCTGGTT
CTATTTCTGTTATTATTGTTTTGTCATTTCTGTTTTCCCAGCGATCTGACTGAACTCGCAGAGGGA
CAAATCCAGTTTTTCTTTTTGACTTTTGTCAAACTAAATCAGGCCTGATAGAAAACTCATTGCTCT
CCGGGGAAACAAAGTAGGAGCCACGAAATGTCATTTTAACAGAGCGTGGGTTTGGTGACTGTA
GGAAAGGATTTGAGGACGCTCCTTCTGTTCGGCTTCCTATGTCATGAGCACAGGCTCCACGCAC
GCACAGACACCACGGCTCCCGGATGCTGTGGCTCCCCGATCGGGGCTCCTGCAGCGCCAGAA
GCCCCTCCGGGATGCTTCGAGGGGCTCCCGGTGGGTGGAGGTACGGACGCCGCTGCGGCCGC
CGCCGCCAGTCCTGCTGCTGTTGTTGCTGCTGCAGTCACGTGGGAGCCCCTTTAAGTTTCCATAG
AGAGGCCTCTCTGGTGTCACATGATGGACATGATATAATGAAACAACATTGTGGAGAGGAAAG
CATTAGGGGAGCCCACGGCTACAAAAACAAGTGAGTGAGAAGAGGTGGGAGGAAGAGAAACT
ACGCCACCTCCCCTGCAGCCGAGTGCACGCAGCAGCCTGGCGTGACAAGTGGGCGACGCCGG
GGGGCAGGGAGCCGGGGTCCTTGGCCCTGGCCGGGGACCCCACCGCCCACCGCGCGGAGGA
CAACTTTTAGCCGGCAGCCCAGACCAGCGCGGCACCTGTCTCCGGAGTCTCCACCGCTCCTCCC
GATTCATCCCAGGGAAATTCTCAAGAATACGCTCTACAAATCTACGTGCGCATCATTTTCACCTC
GCGTCGCGCCCGGGAGGAAGGAACGAGGCAAGGAGCTAAAGCAGCGTGCGTTCAGCCCTGGG
GCATTTTATTAATGCTTTTACGAGTTAGAAGAGTTGGGATAATTTGCCATCTGGAGTTTCTCTGCC
TTGCTGATCTGAGCTCAGACCTGCCAATTTACCAGAGATAATTGATAACACCCTGTAACAGCTG
AGAGGAAAATGGAAGAAACGGAGATACTTTTAGTGAAGCAGAATAAACCACTGAACAGATGGA
GTCTCACTCTGTTGCCCAGACTGGAATGCAGTGGTGTGATCTCAACTGACTGCAACCTCCGTATC
CTGGGTTCAAGCAACTCTCCTGCTGCAGCCACGAAAAATGAGGAAGCTGTGAGTACCAGTGGA
AGGAACGAGCCAGGAAGAGGGACTGGAACCATCTCAGAAGCCATGCCCCTCAGGCTGGAACT
TGCCCTGTCTCCTCGCAGATGAGGTAGGAGGCTCAGCACGGGCTGGTGGGAGCAGCCCACAGC
AGCAGTGAGGGTCAGTGGGCCTGTGAGTTAAGAAGAAGGTGGCACAGAGCGAGGGTCTCTGG
ATCCTGACTGTTTGACTTTTCCATGATTAGGATAAGTAGCCAGGGCTTGGCTAGTTGGAGAGTTA
CTCGAACCTCAGGTGACAGTTGTAAGGCAGCACATAGTGAAAAAGAGTCCTAGCCTGGGAAAG
TCCAAAACCTTAGGTCTGGTTTCAGTTCACTCACCTATCTCTGTGACATAGCAAAGTCTGTTAATT
TCTCTAATTTTCTGATTAGTAGTATAGTTGCAGGAATGAAATAAAAATGGTCCTGATTACTCAGA
GATTCATTTACTGATTACTCTTTTTGTGACCTGAATAAAGAGTACAACATCTCTCAAAAGGTAACA
ATATGATGTTTCAGGAAATTAGAGAAGTTAAGAGACTTTGCTGTATTTACTATACAGATAGTTGA
CTGACAGCTGAGCCCGGACCCAAGATTCATTTATAAAATGAAGGAGTTGAGAAGTACATACTCT
GAGGTTTATCCTAAATAAACTGAAGTCTAGGCAAGTGGCTGAGCTAGGTTTGCCATGAATCAAT
CTGCTGAGTATATTTCTTGGTAACTAGTTCATCTTTCCTTAATTCAGTCAACAAATATGATATCAA
GTTTAGTAAGATGATGACAGTTGTCATGTAATACTCCATCTGGAAGATTGTATGCTTCTCAGCTTC
CCTAACTTTTCTTAATCTACCTTAAGCTTCCTTTCTGAAAGGTTTCTGTCTTTCCTAACAGTCTCTT
CTTTCCGCCAGCTACCTCTTGTGTTTAAGAATTAAAGGAAGATGAGCTAGGAATCAAGAAATAC
GCAGAACTGATTCCTGTCACTGGCGCCACCCTGCCACTTGCCCAGAGCTGTGGTATAAATTTTTT
TAATAGGCTGAAATAAAAATATGAATAGCATTTTGGTATTAAGCATTAAATTGATAAAGGCTATG
AGATACACCTGCTCTCAACCATGTTAATTTTTTATTATTGGTATTAATAAATTATTACTTACTAACA

TATTAACAAGAATTGCATTGAGAAACAAAGCATCCACAGGCCAAATCTTCTGAATTTCAAATGTT
TATATTAATGCGTTGTATTCTAGAAAAGTAGAATTGTCTTAAGTAGCTTTGTAATATAAAGTCATC
TATCAGCCCATTACACCTATTAGAATGTTTTAACCTTTATTTCCCACTTTTTTGTTTCTAGACTGAG
TGTACTTGGTCTGGTATTTGTGGCATAACTTACGGAACACATAAGAATGATACAGATACTATTTA
ATGATGACCTAATACAAGCTTAGATAGCTAAGGTGAAAGCTTCTATGGCCTTAACATTTTCCTCT
TGAAGAATGTATTTTCTGTAATAAAATACAGTGGCTACTTGAAATCTATAAACTTATGTGAGGTCT
GGATAAATCTGAGCAACTTTCTTCTTTGTGCTCCAGGAACCTACGCACTATATATATATAAATAAAG
CTTAAGTAAACATCACTGCAAAAAAAAAAAAAAAAAA

ID|13637|gene_id|378805|transcript_id|NR_109853 Homo sapiens lncRNA

TTGGCCCCACTGTGCACCACACACTCCTTTCCCAGCCCAGGGGCACGCGAACAAAATGAG
GCTCAAGCTGACCAGGCCGAGCCGGAGGAACGCTGGGGCTTGGCAGCAGAAGGGATGGGACC
AGAGAGAAGGGTGTGGAGGAGACCCCAGTGAGGGCCAGGACATTTCAGGTAAAGAGAGGTCA
TATCTCCGTACCTCACTTCCTGACACAAACAAGTTTTCACTGTTGTCAGCAACAAAGCCCTAATA
TAGCTGCGGAAGAGAAAAACTGCATTGCATTTTGCCTCCTGCAAGCATCATCAACAGTTACTGG
AGGAACGTAATTCCAGAAAGCTTGAAAGCCGTGGTGATGGTAATTATGTATCAAATGCCTGGTT
CTATTTCTGTTATTATTGTTTTGTCATTTCTGTTTTCCCAGCGATCTGACTGAACTCGCAGAGGGA
CAAATCCAGTTTTTCTTTTTGACTTTTGTCAAACTAAATCAGGCCTGATAGAAAACTCATTGCTCT
CCGGGGAAACAAAGTAGGAGCCACGAAATGTCATTTTAACAGAGCGTGGGTTTGGTGACTGTA
GGAAAGGATTTGAGGACGCTCCTTCTGTTCGGCTTCCTATGTCATGAGCACAGGCTCCACGCAC
GCACAGACACCACGGCTCCCGGATGCTGTGGCTCCCCGATCGGGGCTCCTGCAGCGCCAGAA
GCCCCTCCGGGATGCTTCGAGGGGCTCCCGGTGGGTGGAGGTACGGACGCCGCTGCGGCCGC
CGCCGCCAGTCCTGCTGCTGTTGTTGCTGCTGCAGTCACGTGGGAGCCCCTTTAAGTTTCCATAG
AGAGGCCTCTCTGGTGTCACATGATGGACATGATATAATGAAACAACATTGTGGAGAGGAAAG
CATTAGGGGAGCCCACGGCTACAAAAACAAGTGAGTGAGAAGAGGTGGGAGGAAGAGAAACT
ACGCCACCTCCCCTGCAGCCGAGTGCACGCAGCAGCCTGGCGTGACAAGTGGGCGACGCCGG
GGGGCAGGGAGCCGGGGTCCTTGGCCCTGGCCGGGGACCCCACCGCCCACCGCGCGGAGGA
CAACTTTTAGCCGGCAGCCCAGACCAGCGCGGCACCTGTCTCCGGAGTCTCCACCGCTCCTCCC
GATTCATCCCAGGGAAATTCTCAAGAATACGCTCTACAAATCTACGTGCGCATCATTTTCACCTC
GCGTCGCGCCCGGGAGGAAGGAACGAGGCAAGGAGCTAAAGCAGCGTGCGTTCAGCCCTGGG
GCATTTTATTAATGCTTTTACGAGTTAGAAGAGTTGGGATAATTTGCCATCTGGAGTTTCTCTGCC
TTGCTGATCTGAGCTCAGACCTGCCAATTTACCAGAGATAATTGATAACACCCTGTAACAGCTG
AGAGGAAAATGGAAGAAACGGAGATACTTTTAGTGAAGCAGAATAAACCACTGAACAGGAAAA
ATGAGGAAGCTGTGAGTACCAGTGGAAGGAACGAGCCAGGAAGAGGGACTGGAACCATCTCA
GAAGCCATGCCCCTCAGGCTGGAACTTGCCCTGTCTCCTCGCAGATGAGCTACCTCTTGTGTTTA
AGAATTAAAGGAAGATGAGCTAGGAATCAAGAAATACGCAGAACTGATTCCTGTCACTGGCGC
CACCCTGCCACTTGCCCAGAGCTGTGGTATAAATTTTTTTAATAGGCTGAAATAAAAATATGAAT
AGCATTTTGGTATTAAGCATTAAATTGATAAAGGCTATGAGATACACCTGCTCTCAACCATGTTA
ATTTTTTATTATTGGTATTAATAAATTATTACTTACTAACATATTAACAAGAATTGCATTGAGAAAC
AAAGCATCCACAGGCCAAATCTTCTGAATTTCAAATGTTTATATTAATGCGTTGTATTCTAGAAA
AGTAGAATTGTCTTAAGTAGCTTTGTAATATAAAGTCATCTATCAGCCCATTACACCTATTAGAAT
GTTTTAACCTTTATTTCCCACTTTTTTGTTTCTAGACTGAGTGTACTTGGTCTGGTATTTGTGGCAT
AACTTACGGAACACATAAGAATGATACAGATACTATTTAATGATGACCTAATACAAGCTTAGATA
GCTAAGGTGAAAGCTTCTATGGCCTTAACATTTTCCTCTTGAAGAATGTATTTTCTGTAATAAAAT
ACAGTGGCTACTTGAAATCTATAAACTTATGTGAGGTCTGGATAAATCTGAGCAACTTTCTTCTTT

GTGCTCCAGGAACCTACGCACTATATATATAAATAAAGCTTAAGTAAACATCACTGCAAAAAAAAAAAAAAAAAA

ID|13638|gene_id|378805|transcript_id|NR_109854 Homo sapiens lncRNA

TTGGCCCCACTGTGCACCACACACTCCTTTCCCAGCCCAGGGGCACGCGAACAAAATGAGGCTCAAGCTGACCAGGCCGAGCCGGAGGAACGCTGGGGCTTGGCAGCAGAAGGGATGGGACCAGAGAGAAGGGTGTGGAGGAGACCCCAGTGAGGGCCAGGACATTTCAGGTAAAGAGAGGTCATATCTCCGTACCTCACTTCCTGACACAAACAAGTTTTCACTGTTGTCAGCAACAAAGCCCTAATATAGCTGCGGAAGAGAAAAACTGCATTGCATTTTGCCTCCTGCAAGCATCATCAACAGTTACTGGAGGAACGTAATTCCAGAAAGCTTGAAAGCCGTGGTGATGGTAATTATGTATCAAATGCCTGGTTCTATTTCTGTTATTATTGTTTTGTCATTTCTGTTTTCCCAGCGATCTGACTGAACTCGCAGAGGGACAAATCCAGTTTTTCTTTTTGACTTTTGTCAAACTAAATCAGGCCTGATAGAAAACTCATTGCTCTCCGGGGAAACAAAGTAGGAGCCACGAAATGTCATTTTAACAGAGCGTGGGTTTGGTGACTGTAGGAAAGGATTTGAGGACGCTCCTTCTGTTCGGCTTCCTATGTCATGAGCACAGGCTCCACGCACGCACAGACACCACGGCTCCCGGATGCTGTGGCTCCCCGATCGGGGCTCCTGCAGCGCCAGAAGCCCCTCCGGGATGCTTCGAGGGGCTCCCGGTGGGTGGAGGTACGGACGCCGCTGCGGCCGCCGCCGCCAGTCCTGCTGCTGTTGTTGCTGCTGCAGTCACGTGGGAGCCCCTTTAAGTTTCCATAGAGAGGCCTCTCTGGTGTCACATGATGGACATGATATAATGAAACAACATTGTGGAGAGGAAAGCATTAGGGGAGCCCACGGCTACAAAAACAAGTGAGTGAGAAGAGGTGGGAGGAAGAGAAACTACGCCACCTCCCCTGCAGCCGAGTGCACGCAGCAGCCTGGCGTGACAAGTGGGCGACGCCGGGGGGCAGGGAGCCGGGGTCCTTGGCCCTGGCCGGGGACCCCACCGCCCACCGCGCGGAGGACAACTTTTAGCCGGCAGCCCAGACCAGCGCGGCACCTGTCTCCGGAGTCTCCACCGCTCCTCCCGATTCATCCCAGGGAAATTCTCAAGAATACGCTCTACAAATCTACGTGCGCATCATTTTCACCTCGCGTCGCGCCCGGGAGGAAGGAACGAGGCAAGGAGCTAAAGCAGCGTGCGTTCAGCCCTGGGGCATTTTATTAATGCTTTTACGAGTTAGAAGAGTTGGGATAATTTGCCATCTGGAGTTTCTCTGCCTTGCTGATCTGAGCTCAGACCTGCCAATTTACCAGAGATAATTGATAACACCCTGTAACAGCTGAGAGGAAAATGGAAGAAACGGAGATACTTTTAGTGAAGCAGAATAAACCACTGAACAGGAAGAGGGACTGGAACCATCTCAGAAGCCATGCCCCTCAGGCTGGAACTTGCCCTGTCTCCTCGCAGATGAGCTACCTCTTGTGTTTAAGAATTAAAGGAAGATGAGCTAGGAATCAAGAAATACGCAGAACTGATTCCTGTCACTGGCGCCACCCTGCCACTTGCCCAGAGCTGTGGTATAAATTTTTTTAATAGGCTGAAATAAAAATATGAATAGCATTTTGGTATTAAGCATTAAATTGATAAAGGCTATGAGATACACCTGCTCTCAACCATGTTAATTTTTTATTATTGGTATTAATAAATTATTACTTACTAACATATTAACAAGAATTGCATTGAGAAACAAAGCATCCACAGGCCAAATCTTCTGAATTTCAAATGTTTATATTAATGCGTTGTATTCTAGAAAAGTAGAATTGTCTTAAGTAGCTTTGTAATATAAAGTCATCTATCAGCCCATTACACCTATTAGAATGTTTTAACCTTTATTTCCCACTTTTTTGTTTCTAGACTGAGTGTACTTGGTCTGGTATTTGTGGCATAACTTACGGAACACATAAGAATGATACAGATACTATTTAATGATGACCTAATACAAGCTTAGATAGCTAAGGTGAAAGCTTCTATGGCCTTAACATTTTCCTCTTGAAGAATGTATTTTCTGTAATAAAATACAGTGGCTACTTGAAATCTATAAACTTATGTGAGGTCTGGATAAATCTGAGCAACTTTCTTCTTTGTGCTCCAGGAACCTACGCACTATATATATAAATAAAGCTTAAGTAAACATCACTGCAAAAAAAAAAAAAAAAAA

ID|13639|gene_id|378805|transcript_id|NR_109855 Homo sapiens lncRNA

TTGGCCCCACTGTGCACCACACACTCCTTTCCCAGCCCAGGGGCACGCGAACAAAATGAGGCTCAAGCTGACCAGGCCGAGCCGGAGGAACGCTGGGGCTTGGCAGCAGAAGGGATGGGACCAGAGAGAAGGGTGTGGAGGAGACCCCAGTGAGGGCCAGGACATTTCAGGTAAAGAGAGGTCATATCTCCGTACCTCACTTCCTGACACAAACAAGTTTTCACTGTTGTCAGCAACAAAGCCCTAATA

TAGCTGCGGAAGAGAAAAACTGCATTGCATTTTGCCTCCTGCAAGCATCATCAACAGTTACTGG
AGGAACGTAATTCCAGAAAGCTTGAAAGCCGTGGTGATGGTAATTATGTATCAAATGCCTGGTT
CTATTTCTGTTATTATTGTTTTGTCATTTCTGTTTTCCCAGCGATCTGACTGAACTCGCAGAGGGA
CAAATCCAGTTTTTCTTTTTGACTTTTGTCAAACTAAATCAGGCCTGATAGAAAACTCATTGCTCT
CCGGGGAAACAAAGTAGGAGCCACGAAATGTCATTTTAACAGAGCGTGGGTTTGGTGACTGTA
GGAAAGGATTTGAGGACGCTCCTTCTGTTCGGCTTCCTATGTCATGAGCACAGGCTCCACGCAC
GCACAGACACCACGGCTCCCGGATGCTGTGGCTCCCCGATCGGGGCTCCTGCAGCGCCAGAA
GCCCCTCCGGGATGCTTCGAGGGGCTCCCGGTGGGTGGAGGTACGGACGCCGCTGCGGCCGC
CGCCGCCAGTCCTGCTGCTGTTGTTGCTGCTGCAGTCACGTGGGAGCCCCTTTAAGTTTCCATAG
AGAGGCCTCTCTGGTGTCACATGATGGACATGATATAATGAAACAACATTGTGGAGAGGAAAG
CATTAGGGGAGCCCACGGCTACAAAAACAAGTGAGTGAGAAGAGGTGGGAGGAAGAGAAACT
ACGCCACCTCCCCTGCAGCCGAGTGCACGCAGCAGCCTGGCGTGACAAGTGGGCGACGCCGG
GGGGCAGGGAGCCGGGGTCCTTGGCCCTGGCCGGGGACCCCACCGCCCACCGCGCGGAGGA
CAACTTTTAGCCGGCAGCCCAGACCAGCGCGGCACCTGTCTCCGGAGTCTCCACCGCTCCTCCC
GATTCATCCCAGGGAAATTCTCAAGAATACGCTCTACAAATCTACGTGCGCATCATTTTCACCTC
GCGTCGCGCCCGGGAGGAAGGAACGAGGCAAGGAGCTAAAGCAGCGTGCGTTCAGCCCTGGG
GAGGAAAATGGAAGAAACGGAGATACTTTTAGTGAAGCAGAATAAACCACTGAACAGGAAAAA
TGAGGAAGCTGTGAGTACCAGTGGAAGGAACGAGCCAGGAAGAGGGACTGGAACCATCTCAG
AAGCCATGCCCCTCAGGCTGGAACTTGCCCTGTCTCCTCGCAGATGAGACAGACGTTTAAGTAC
GAGAAAGCTAATGGCGCAGATTCTGCTGACTGTGTGGCTCTCGTCTTAATGCGTTTTCCAACCTT
GAACCTGAGAATGCCAGA
ID|13640|gene_id|378805|transcript_id|NR_110472 Homo sapiens lncRNA
AAACAGCGATTCTTCTGCAGTGAATCACTTCCAGAAATGAATAGCCACAGTTTTTTGGAAT
GAACGTTGTGAAATCCCTCCTTTATAATGGCAGGTTTTCAGTTGGTGGTTTTATCAGAATTTCTCA
AGATCAAAACGAAACCTTCTCTTTAAAAAGGAAAGAAAGTACTATCGATACAGAAAGCAAAAGT
ATTTCCAGTCTCCTACTGAACTGTCACGGCAGACCTCTCTGTATCTATATTTAGAGCTGTATGTCC
ATATATTTGCCTGAATGTGTGAGTTCTTGGAAGTATGGATTCATTGCCAGGAGCTGGTGATTTCC
TAAGCAGAGGTCGCTAACTACAAGAAATGTTACACTCGGACAAGTCCTGCGCTTGGGGATCCTC
TGTACGCCCGTTCACTGGTGCAGGTCTTCTGAACAAATGCCTCCTGAGGATTTCTGTGCCTGGAA
AATATCTGTTAACAGACTGCTTTTACACCTACATCAGGATACCGAAGCTCAGAAAAATTAAATGA
TTTGCCCAGGATCACATAGTTAGTAAGCAGCCCAGCCAGATCCTGTGTTAACTTGTTCATCCGCT
TCCTATAAGAAAACATCCATTCAAGGCACATGGGGCAACAAAGAAGGACTTAGAGCTGTGTGG
CCCCTCGTCAGGGCAGTACCAGTTGCACCCATAGTCCGGCCTGAATACTCAGCTTAGGACTATT
GTGTCCAATGTCCCAAACATTTTGAAGCTACATCTTAAAGAAGTGTCCTGCTCAGATGCAGTTCT
CCTTGTGAAATCCGCAGGCCCCAAGGAAAGTGACTTTAATCCAGTTTGCTCATGCAAGAACAGA
CTGGCATTTCACATCCAGGAAAACAATGGATTTGTACTGTTCAGCTTTTGCCATGCAAGGAGTTC
CTGGTGTGGTTTATTAATTTACATCTCAGGCTCCAGCTTCACTATCAATCAGATTTGGAAGAAAA
AAGTTACAAAGAAAGGCAGCTTGCTGAGAAACAGCGGAAGCATGACGGTACAGACTGGGTTTT
GAAATGGAGACACATTCTCATGGCTTGTGGGCTGAGAGACACTGAGAAAGGATTTTGTTCTTGA
AAGATGATTGTTTTAATGCTGGAGATGGAGAGTTTTGACAATTTTAAGTGCATGTTCTGTGTGTA
AGAAAGAGTCATTACGTTTTTTTTTTCTTTCAGATGTTGCCATGCCTTAAAATTGCTGATGATTAA
AATAGAACATCCTGAGTTACAGAAATTCAGCCCTAGTGTATCCTGGCCTAAAAATACAGAACAA
TCAAGTTGATTGTTGGAAATGAGAGGCTAGGCAGGGTTGGAAACATGCTAATGTTTACTGAGTG
AAATCTTTCCTTCTCAGTAGAGTTGCCCTTGCAGCTGAAAGTCACTGAAAGACTCAACAAAATAA

GCAGATCCCACTTGTCTCACTCTGTTCTCCTATGCATGCCCTGGAAAGAGAATGAATGAGCCCTT
TTTAATTTATCAACTGGTTTTCTTCCATCTCTTTACTAGTGAGCCATGGGGGTTTTTTGCCGTTAAC
TGGGTAGCCAGTCTCTTCATGGAGACTTATTTTCAGGAAACTAGCCTCTTGCTTTATGTGAAAAC
AAGGACCCAACTCAA
ID|13641|gene_id|378805|transcript_id|NR_110473 Homo sapiens lncRNA
AAACAGCGATTCTTCTGCAGTGAATCACTTCCAGAAATGAATAGCCACAGTTTTTTGGAAT
GAACGTTGTGAAATCCCTCCTTTATAATGGCAGGTTTTCAGTTGGTGGTTTTATCAGAATTTCTCA
AGATCAAAACGAAACCTTCTCTTTAAAAAGGAAAGAAAGTACTATCGATACAGAAAGCAAAAGT
ATTTCCAGTCTCCTACTGAACTGTCACGGCAGACCTCTCTGTATCTATATTTAGAGCTGTATGTCC
ATATATTTGCCTGAATGTGTGAGTTCTTGGAAGTATGGATTCATTGCCAGGAGCTGGTGATTTCC
TAAGCAGAGGTCGCTAACTACAAGAAATGTTACACTCGGACAAGTCCTGCGCTTGGGGATCCTC
TGTACGCCCGTTCACTGGTGCAGGTCTTCTGAACAAATGCCTCCTGAGGATTTCTGTGCCTGGAA
AATATCTGTTAACAGACTGCTTTTACACCTACGTTGTCTTGGGTTTATTGTAAGAGAGCATTATGA
AG
ID|13643|gene_id|378825|transcript_id|NR_024089 Homo sapiens lncRNA
TGAAGTGCTGACGGGTGGAGCGACACGGAGGAGCCTCAGAAACACCCACCATGCTGCTG
CCCGGAAGAGGCAGGCACGGAAGGCCACGCGCTCTATGACCTCATCCATGGGAAACGCACAG
AGGCGAGGCCTGCCCGGGGCTGGGAGGAGGGGGCACAGGAGCGCCGGCTGTGGGGATGGTG
TTTCTCCCGCCGGGGGTGGCTGGCTCACCCTGGCACTGCACCGCAGTGGGGGTGGACCGGATG
CCGCTGAACTGCCTGGACTTTCAAGAGGTAAATTCCGAGCTCTGCTCTGAGGCCTGACCGTCCC
TGGCCGCCAAGTGTCTGCTGACTGAGAGCTCCAGCTCCGGAGAGGTGCCCAGTACCTGGTGAG
CAGAGGGACCTGAAGGAGCAGGTGCCTCTTCCTCAGACATCTGCAGTCACTTCACAGTGAATG
AGGCTCCAGCCGGGTGCCCTGAGAAGTGGCCAACACCCTACCCAAGAGGAGACCGCACAGCC
ACGGCCAACGTGGAGCTCTAACTCAGGGCTCCAGCCTCCAGCCCAGTCCTTTTCCTTGAGTAGG
GACGACCCCCACAGCGGCCTCTCTGCATTGCTTAGGTGGGGAGCACATGTGGTGAAGATGGGC
AGCCTGCAGGTGCCTGCTACACGTGGGGGCCACGGCCGACGGAAAGCTCAGTCATGAGTGAA
AACAGAGGAAATGTTTCTGAAAATATAACAAGTCAGTGGGAAATCAAAAAAAAAAAAAAAAAA
ID|13654|gene_id|379025|transcript_id|NR_029434 Homo sapiens lncRNA
ACCAGCTTGTCGGCGCCATTTTGTCTCGGCAGCGGTGGCCGTAGCTCCATCGCATTTTATG
TTTCTGGCGAGAAGGGAACGGAGTTTTCATCAGGTAGATTGGTTTTTGTGCGGCCGTCCTCCAC
CGTTTCCTCCAGGACAGCACCTAGTCGTGGCCGGAGGAGTCTCAGAGCTGTCAGAAAGAATAA
GACTGATTTTATGGGAAAATTAAGCAGATGCTCCAGTTTGAGAAACCTGGATCTGCGATCTGTTT
GTGAGTGGTATTCAGTTAAGAGAACAATTATTTCGTATAAGCCACATCAGAGACGACTGAAGAT
GAAAAAACTACCATCCCCGTATATAACTAATTTGTGCTGTGCACCAACAAGAACCTGCTTTAAAT
TTCCATGGCAATTTACAACCCCCATACTGTACCAGGCAAGGTACCAGCATCAAGATGATTTATG
GTAATAAGATATAAAACCAAGGAAAATAACCTAAAGTCTGAAAAAGACCAGAATCGAAGTTTCC
TGATTCATATTTTAATGTTTTGAAATTTATACTCCAGGCTGGGTGCAGTGGCTTGTGCCTGTAATC
CCAGCACTTTGGGAGGCCGAGGCGGCCGGATTGCCTGAAGTCAGAAGTTCGAGACCAGCCTGA
CCAACATGGTGAAACCCTGTCTCTACTTAAAAATACAAAAGAGCTGGGCATGGAGGTGGTGCG
CGCCTGTAACCCTAGCTACTCGGGAGGCTGAGGCCTAAGAATCTCTTGAACCCAGGAGGCAGA
GGTTGTAGTGAGCCGGGATCACGCCACTGCACTCTAGCCTGGGCGACAGAGAGAGAGACTGTC
TAAAAAAGGAAAAGAAAAAAATTTATACGCCAAAAAAGATATTCTGAGATAACCTGTAGTTACC
ACTAACTTTGTGACAAAATTATAAAAATCCACAGCCATCTATGAATCTGTAGGCAGACCTGAAGT
TTGAACGACTGGTGAAGACATCTGATTTTCTTTATAGCCAAGTTAGGATAACAAAATGCAAACAA

GTCATTAATATTTACTATATGCAAGATACAGAAAGATGAAGGAAGGAGTAAGAAGTTATCCTTC
GTGGAACTATTCAAAGCAAAAATGCAAAATACCAGGGAAAGCTTTGAAGAACCAAGATTTGAG
CAGGTTCTTAGAATATAGATTGGATTGGATTTGATTTCAAGAAAGATGAACGTGTAAAGCAGAA
GTGGGAAGCCAAAAACTTTTTTCCAAATTAGAATTAAGCATTCTTTCAATGAACATTTGGAAATC
AAGGTAGAAAAATATCCCTAGGGCCTAGCTGAGGAGTCTGTATGTTGTTGGGTTTTGATAGTAA
CCAGTCCAAGATTTTGTTATTAGACGTGGGTTTCTGAATAGACAGGCATTTCTGAACAGACATGA
CAGGATCAAAACAATGTTTGAGCAAACTGCCCTTCATCTACTGTGGATATGTTGGGGGATGATG
GAATATAGTGAAAGATAATGGGTGCTCATACAGCAGTCTAGACTTAAGGTGATTCAACTACTAT
ATATTAAACTAGATTATCTTTTATTTTTTAATTTTGAAATCTGGATGCTCAAGCTCTGCCTGCACAA
CCACATGAGGAAGAAGGAACAATGACAACAAAAATAACACTAAATTTAAATTTAAGAGTACTAC
TTTTAGGAAATAGACAAACCATTATTTGGGTACAACTAAAGGCAACTGGCATGGACTCAAATATT
TTGGGGAAGAAAAAGACTAAAAGTTCTAAGGAAGAAAATGCGAACCTTGATAGTTTGAAATAGT
TAAAAAGACAGTGTAGAAACTGCTTAGGCAGTTTGATTATGGACTATTAGATGATACTTGGGTCT
GATAATGGTATAAGGAGAATAAAGTATTTAGGGATCCAATATTACGCCTGCAGCTTTTTCCAAAT
AGTTCATGGGGGAGGGGGATGTGTAAGTGGTTAACTGAAGTCTAACTAGATAGGTTTGTTGTAA
GCTTAGGATGTTTACAGTTCTTCATGTTAAGTTGAGCGTGATGGGAAGGGAAAGAATGCTGATC
TTTAAATTTTTGTCCTTAGTTAAGTTCTGTATTTAGTGAATTAATTGCATCCTAAAAAGTCAAACTT
GAAAAGCACATTTTAAATGGCAAATCTATTTTTTACATGTTTGTGAAGTTTTTATTTTTTAGTAAAC
AGACCATCAGAAGAGAACAATGGTACAGAGCAGGGGTCAGCAGATGGATTTTGTAAAGCATCA
ACTTGTAAATATTTTCAAGTTTATTAGCTGTATGGCTCTGGTTTCTGTTCCCTGTTCCAAATGTTAA
AGTCTACTGTTGTATTCTAAAAGCAGCCATGGACTGAATGTAGCTGTGTTCCAATAAAACTTACA
CAAAAGCAGGCAGTGGGCCATAATTTGCAACACCTGATTCACAGCATAATTTTGTCACAAACTG
AAAGTGTTCCTCAATTAAAGTGATTTTTTTTTCTTGAAAAAAAAAAAAAAAAA
ID|13655|gene_id|379025|transcript_id|NR_029435 Homo sapiens lncRNA
ACCAGCTTGTCGGCGCCATTTTGTCTCGGCAGCGGTGGCCGTAGCTCCATCGCATTTTATG
TTTCTGGCGAGAAGGGAACGGAGTTTTCATCAGGTAGATTGGTTTTTGTGCGGCCGTCCTCCAC
CGTTTCCTCCAGGACAGCACCTAGTCGTGGCCGGAGGAGTCTCAGAGCTGTCAGAAAGAATAA
GACTGATTTTATGGGAAAATTAAGCAGATGCTCCAGTTTGAGAAACCTGGATCTGCGATCTGTTT
GTGGTCTCTATGAATAAAGAAAAGCAAATAGAATCTTATCAGATAAGTGCTTTTATAAGTCTAAA
TATATTCTTTGGAATCTCTAAAGCAAAACTTTTAAACTTTTTTTTTTTTTTTTTTTTTTTTTAGGTACCA
GCATCAAGATGATTTATGGTAATAAGATATAAAACCAAGGAAAATAACCTAAAGTCTGAAAAAG
ACCAGAATCGAAGTTTCCTGATTCATATTTTAATGTTTTGAAATTTATACTCCAGGCTGGGTGCAG
TGGCTTGTGCCTGTAATCCCAGCACTTTGGGAGGCCGAGGCGGCCGGATTGCCTGAAGTCAGA
AGTTCGAGACCAGCCTGACCAACATGGTGAAACCCTGTCTCTACTTAAAAATACAAAAGAGCTG
GGCATGGAGGTGGTGCGCGCCTGTAACCCTAGCTACTCGGGAGGCTGAGGCCTAAGAATCTCT
TGAACCCAGGAGGCAGAGGTTGTAGTGAGCCGGGATCACGCCACTGCACTCTAGCCTGGGCGA
CAGAGAGAGAGACTGTCTAAAAAAGGAAAAGAAAAAAATTTATACGCCAAAAAAGATATTCTG
AGATAACCTGTAGTTACCACTAACTTTGTGACAAAATTATAAAAATCCACAGCCATCTATGAATC
TGTAGGCAGACCTGAAGTTTGAACGACTGGTGAAGACATCTGATTTTCTTTATAGCCAAGTTAGG
ATAACAAAATGCAAACAAGTCATTAATATTTACTATATGCAAGATACAGAAAGATGAAGGAAGG
AGTAAGAAGTTATCCTTCGTGGAACTATTCAAAGCAAAAATGCAAAATACCAGGGAAAGCTTTG
AAGAACCAAGATTTGAGCAGGTTCTTAGAATATAGATTGGATTGGATTTGATTTCAAGAAAGATG
AACGTGTAAAGCAGAAGTGGGAAGCCAAAAACTTTTTTCCAAATTAGAATTAAGCATTCTTTCAA
TGAACATTTGGAAATCAAGGTAGAAAAATATCCCTAGGGCCTAGCTGAGGAGTCTGTATGTTGT

TGGGTTTTGATAGTAACCAGTCCAAGATTTTGTTATTAGACGTGGGTTTCTGAATAGACAGGCAT
TTCTGAACAGACATGACAGGATCAAAACAATGTTTGAGCAAACTGCCCTTCATCTACTGTGGAT
ATGTTGGGGGATGATGGAATATAGTGAAAGATAATGGGTGCTCATACAGCAGTCTAGACTTAAG
GTGATTCAACTACTATATATTAAACTAGATTATCTTTTATTTTTTAATTTTGAAATCTGGATGCTCA
AGCTCTGCCTGCACAACCACATGAGGAAGAAGGAACAATGACAACAAAAATAACACTAAATTT
AAATTTAAGAGTACTACTTTTAGGAAATAGACAAACCATTATTTGGGTACAACTAAAGGCAACTG
GCATGGACTCAAATATTTTGGGGAAGAAAAAGACTAAAAGTTCTAAGGAAGAAAATGCGAACC
TTGATAGTTTGAAATAGTTAAAAAGACAGTGTAGAAACTGCTTAGGCAGTTTGATTATGGACTAT
TAGATGATACTTGGGTCTGATAATGGTATAAGGAGAATAAAGTATTTAGGGATCCAATATTACGC
CTGCAGCTTTTTCCAAATAGTTCATGGGGGAGGGGGATGTGTAAGTGGTTAACTGAAGTCTAAC
TAGATAGGTTTGTTGTAAGCTTAGGATGTTTACAGTTCTTCATGTTAAGTTGAGCGTGATGGGAA
GGGAAAGAATGCTGATCTTTAAATTTTTGTCCTTAGTTAAGTTCTGTATTTAGTGAATTAATTGCA
TCCTAAAAAGTCAAACTTGAAAAGCACATTTTAAATGGCAAATCTATTTTTTACATGTTTGTGAAG
TTTTTATTTTTTAGTAAACAGACCATCAGAAGAGAACAATGGTACAGAGCAGGGGTCAGCAGAT
GGATTTTGTAAAGCATCAACTTGTAAATATTTTCAAGTTTATTAGCTGTATGGCTCTGGTTTCTGTT
CCCTGTTCCAAATGTTAAAGTCTACTGTTGTATTCTAAAAGCAGCCATGGACTGAATGTAGCTGT
GTTCCAATAAAACTTACACAAAAGCAGGCAGTGGGCCATAATTTGCAACACCTGATTCACAGCA
TAATTTTGTCACAAACTGAAAGTGTTCCTCAATTAAAGTGATTTTTTTTTCTTGAAAAAAAAAAAA
AAAA
ID|15575|gene_id|440823|transcript_id|NR_003491 Homo sapiens lncRNA
CAGCTACAAAGACGACGCCGGCTGCGCTCGCGGGACCAGAGGGAGGGAGGCTGCGGAC
GAGTGCGGGGAGGAGTTTGAGATGGGAGGCGCTTGCAGGGGCTCCCCCAGTTCTGCGCTGTGA
GCCGGGGCACAAAGAGCCCTCTGCACTAGCGCCGCAGGACCGCGGACCCGAGTTGGAGGCAT
CTGTCCACCCATGTGGTTCCAGACACGTTCATGTGGCCACCATGACCCCGTCGGCATCACAGGG
GTAACCAAGGTCCACAGAACGAGGGGAAAGGGTCACTGTGGATCAAGCCAGGCTCCTCTGAG
CAGTCCAGGGTCTATTTACAGAGAGCAGCAAGTTCCTGGGACAGAGGAGACAGAAGGGAATTT
CCAGGTTCTTCAGGACGTTCACAACCACACTGAGAAGCATCTTTGCAGATAAGTATTTAAACTTA
CCAGCCCATAGCCTACCAGCCTGCCCTCTTCTGGTCTGTGCAGGAAAGTGTAGTATCCTAGAAT
GCCAAAGTGGGAGGGGAAATGGGTGATGTAGCTCATTCTCTTTTCTACCTCTATGGAAGAAAGA
AAGAGCCTGTCCCCATTTTGTGGGGTCCCAGAAAGGGTGATTTTCACACTTCACATTTGGCGTTA
GGGCTAGTATTTCACAAACATTACCGTCTGGAACTTTTGAAGGCTGAGTTAAATGACTTCATCTT
GTGTACTTGTAGAACTGCTATTAGAAGGAGGCTGGGGACTCCCTGATGATGTGAACCAGCCCTT
CTCTTCTCAGTTTCAGCACTTTGTGATCATATTTATAAACCTTGGACAGGGCTCCTTGGCCTTAGA
GTTAAGTGAAGAGTTAACACTGTGACTTTAGATGCATTTTTCTCAGTGTAAGTGTCTGAGCTCAT
CTCCAGTTCTGGACTCGTGCCCCCACTCCCGGGTGCTGACAGCAAGCTCTGAAGATATGCTGAG
AGAGCTCCCTGACTCTCCTGACTCAGGTCTGCCAGTTGATGAAGACAGCACAGTTGTTACCAAA
AACAAAACCTGGCAGATGGTCCTAGGTCAGGATTAGTGGTCATTCTCTGGTCTTCTCCAGACTG
GTGACACAGGGTGGTCTTAAAAGAGTCCTAGTCCAGCCTCTGGCTCCCCGAATTTCCTCTGGTG
CATGGGGCATCAGGAAAGGGCAGGGGAGAAGGAGGGGGAGCAGAAAAATAACTGAGGCTGG
GCAGAAGGGTCCCTTCCATTTCCCATCCATTGCTTCAAACAATGGGTGATTACCGTGCACCTTGA
GTGAATGTCAAGGCAGGAAGGGTTCCAGGGCCACCCAGTGTGGCAGGAGCAGAGCTGGGATC
AGAATTCTGGATTCCTGACTCTCCGTTCAGCTGCTATTCAGCTATCACCAAGCTGCTGAGTTTGT
CCTACTTTCTCCTGGGTCACATTCTGCTGTTGTTGGGAAACAGGGAGTGAGTGAAGGGAACCTG
GGAACCCTTCTCCCCTCACCTTTTAAGGATGTTCTGGGGTGGAATAAAGAGAGGTGGAGGGTGG

GGTGCCTGGGCCCAGCCTCCCTGGCTCCTGGTCCTTCTTTCTTCAGCAGGGAGTTCTGGCAAGG
CCTGGGCCTGGGGAGGGGGCCTGGGTGCAGTTTTCTCCTGCGGTGTGGTTGGCTCTTTTGTCTT
CCAGGATGGGCCTGTGGGGTCTGTGTGGGGAAGGAAGGAGCTGGGAGCCTGGGCTGCCTGTG
TGTCGGCTGGATGCTGCCAGCACCGGCATGGACCAAGCAACTTCTCTGAGCCAGATGCTGGGG
ACGCAGAGGTGAACCCAGAGACATGATCCCATCTGCTCAGATCTTCATGTCAGAACACGCTTTA
TTACAGTCTCGGCAAGGGGGTGGCTCCATCTGGGCCGCATCTCCAGGACTCCTGGCCTGGACTT
CTCTTTGGACCTCGGGTGACCTTGGATTCTGGCCACAGTGACTTAGCGGTCAGACTCCGGTGGT
TTTCTAGCCCGCTGGGTGACTCCCTGAAGATCTCATCCTCTTCACACCTGTTGGGATGGAGCCTC
CCATGGATAGGAACTCTTCCTGGTTGATGCTCAGCAGATGACTGATCTGGCTCTGGAGGACAGC
TCCAGGGGTATGAGGGAGGCCTGGCAGCCCACTACTCCCTCCTCACCTGCTCGGGCCCTGCCT
GCTGGGCCCTATTGTCTGGGTCTGGGGGCCCTTTCCTGCCCCATGTGAACTTGATTCCTCCTCGC
GTTTCTGCCGGTGACCAGACAGAGCCTGGCCTGGGCCAAGTCCCCACTGACCACACTTTCCCCA
AGGCTGGGTCTCAAAGGACCCTACAGGATGGGGACAGGAACAAGGATGGGAGTCGGGGAGG
GGCTGTCAGGCAGAGGGCAGATGCAGCTCCTCGGGTGCCGCTGGCTGGAGTCCTTTGTCTCCA
TTTGCTCAGTGCGTAATTGTCATTTTGTGGGGGACTTCCTTGGTGGGGCTTTTCCATGCTTTTCCT
CTCCCTGATGGTTACAAAGAGGAGACAGTCAGGATCTGGGAGGAGCTGGAGCCTTCCCCCTGT
GCTGTTGAACTCTGGGAGGGGCAGCCAGAGGGGCTGAGAGTGCACCATCCTGAAAAGAGAGG
GTCTTTAAGAGATAAGATTTAGGCTAGGGAAATTCACTAGAAAGAGACAGGTGGCCCTGGGTTT
TTTTTCCTGCCATCCAAAGGGAACCAGTCTTACCCAAAGGCTTAAGGGGTCACCTTAGCCCAGT
CTCAGGACACTCTTCTCCAATCAGATCGCTGCTTCTAGTTGCACTGCTCCTGGATTCTGTTCTTGG
GGAACAGAAGGTGGCAGGACAGGCCCCACAATCTGCAATTCCAGCTCCCTCCAGAGGAGACA
GTCCCAGCGTTTGCAAGAGAGCAGCTTGTGGCCTCCCTTAGGCAAGTTTAAAAGCCAGATGTCC
TTTTTCCCAGAATGCGGAGTGGTGTGTACTATTCATCAGCTTGGGCTGCCACAGCACCATGCCA
GACTGGATGGCTTAAACATCAGGAATTTATTTTCTGCCAGTTCTGGAGGCTGGAAAGTCCAGAT
CAAGGTGTTAAGACTTGGTTTCTGGTGAGGCCTCTCTCCTTGGCTTGCAGGTGGCACCTTCTCAC
TATGTCCTCACATGGCCTTTTCTCTGTGGAGAGGGACAGAGAGCATGAGCAGGCTCTGGTGTCT
CCTCCTCTTCTTATAAAGACACTAATATCACCATATTAGGGCTTAAACCTATGACCTCATTTAACC
TTAACCCCTTAAAGGTCCCATCTCCAAAAACAGTCACATAGCAGGCTACTGCTTCAACATATGC
ATTTGGGGGAGGGGACACCATTCAGTTCTTAACAGGGTGGTCACCGCAAACATGGAAAGTCAG
AGCCTTCTCCCCTTCAGAATTCCCGCCCCCACCCAGGGATGGGGAGGAGGAGCAGAGAGGTAT
GGGAAGCAGACACGGAGAGTGGCAGGTACCATGCTGGGGTGGCTCAGGAGTGCTTCGGAGGA
CATATGGAACTGGCAGGGCTCAGTGCAGGGAGGCGGAGGCCCTGGGAGAGCCGTGTCCTGAG
AAGGGCCTGGGCTACAACCCTGGGCAAGTTACTTCACCTCTGAGCCTCCGATGCTCTGTGAAAT
GGAAGGAATGTGCTTGCCTGTCAGGTGCAGGGAGAATTCAGTGAGATTGTGTGCCTAGCACAG
TGCCTGACTCCCAGTAGGTGCTCAGCAAATGCTCCCATCCATCTGGGAGTATAGACTTAGGGTT
TATCTATTTTTTTTTTTTTTTTGGCTCTCTGGACTTTAAAACTCAGCATCTTCTGAACCAGAGGCATT
TCTGATTAGCCCTTCCCTACCTATTTTCCTAGTATCACTCTTTAATCAGCTTGGGGAGGTGGCAGC
ATTTCATGGCCTCCGTAGTAACTCACAATGCTTCCTGGGGTATTTAAATTCTACTCTCTCATCAGC
ACTGAGCACCTACTTTGGGCCCTTTCCCGTGCTAGCCATTTGGGGGAAATATAGGTGAAGAGTG
GCTGGGGTTTGAACCTTTAGGATTTCACAGCCATGCTGGATGGGATAAAACCAGCTCACATGGA
GAATCAGAGAATGGGACAGTGCCACAAAACAGTCTACTGAGGCACAAGTTCTGAGTGCCTGGG
AAGTGGTAGAGAGACCCCAGAGAGGGCTGCTGCAGTCTGGAACAGCTTCCTAGCAAGCTGTAC
CAAACAGGGCCTTGAAGGATGAGAAAGATCTGGCTGAGATGATACCCGACCCTCTAGGGAAAT
TCTTAAAGTAACTTCTAGGAAATGTCATTGCTCCTTAAAAAAAAAAAAAAAAAAAAAAAAAAGCAG

GTTCTAATTCAAATTCCGCTGCACTACTGAAGTAGTGCAGGACTTATCAGAACCTTTAAAATGCT
AATGGCAATTTCAAATACTCAGGGACAGGGCATAGGATGAAGTGTTCCTTGACTTTTATGGCCG
TAGAATCTTTTTCTCATGGAACATCGTTTAGAACTGTGGGAGATGCTAGGCTGGGGTTTCTATGG
GAAAAGTACAGCAGTGATAGAAGATGGCATAATTAGGGTACACTTAGGTGCTTTGTAGCATTGG
CTGAAAAGGAAGCAGACCGGGTGGGCGTGGGGGAGTCTCAACTTGGTTCCAGGAGCCAGGCC
AGCTTGATGCGTTTTTTTTTTTTCTAATGGTGGGAGGGCAGGCCAATAGTTGGAGAAACTAGAGG
CCTGACAGTCGGTGATGGAAGAGATGTCTACTAAGCACGGTTGTCAGATCTTTTGCGGAGAGAT
GCGGGTGTGCGTGTGTCTGTCAGAGTGTAACAGGATCCAGCCCTCTGGGAAGGGGCTGACCAG
AACCAGGCAGGGGGTTTCTGTCTAGGACAAGTAGGGTCCATTTTCAGGGGAGCTGAAATATTTG
CAGGGGGTGCTCTGAGACCCACAGCCAATAAATAGGGAAGCAACATGCTTTTGGGCAGGGTCC
ATGTGGTTAGGGTTGATTTTGATGGGGCTGGGGGGCTTAGAGTTAGACCCCCTGCCCTGGTCAG
GCCCCACTCCCTGGCTGGTGGGGGCGGGAGGCCTCTGAAGCTGCAGCTGGAGGGGCCGGGGC
AGTGTTGCATGGTGTGATTAACCTACTAACCCAGGGCTCAGCCGGGACGCTGTGGCGTGTGAC
GAGGTGCCTGGCCTTCTGCCAACCAACTAACCCACTAACCAGGCCCGAGCATGCCCTACTAAC
CACCCCCCCGCAGCCAGATCCTACTAACTGTGCAGCTCATGGGCCTTTCAAGGGGCTGGGATAT
GGGGGCAGCCAAGGCTCCAGAAGGCTCTGCCTATGGGCTGGGATCTTTGTGCAGACCCACGTT
CCAGGACAGAGAATGCAAATAAGACTCACCAACTCTGCCACTTCCTGTTGAGTGAGTTTGAGTA
AGTCACCTCACTTCTCTCAACCTCAGTTTCCTCAGTGGTAGAATGCGAGTTCTGATACCTTACAG
GTTGCAGTGAGGATTCCCGTCAATATAAGGGTCTCAGCACATGGCAGACACCTAAAAAAAAAA
AAAGTATGAGGATGGAGGAAGGCCCCTCAAACCTGGTTGAACACGTCTGCTGCCTAGGCCCTG
GCCTCTCTGGGTGTTCTGCACACAGATCCTTCTGCCTGGGGCAAGACACTTGGCTTTGAATCCAT
GTCACTTCCCCACATTCTTCCTCCGTCTCAGCTTTCTTTTCTGTAGGCAGCTCTTTTGTTCGATTAC
TAACCCGTCTAACCCCTGGGGTGCCTCAACTGCACCCCGATCATCCCTTAACAAACTAACGGAC
CTGCGTGCGTTCTTCCTTTTCTCTTAGCGACTCCTGTGTGTGTCTGCTGAGGTGCCCTGTCCGCTG
GTGCTGTGCTCTGACTTACTAACCCAGCCCCTACTAACCCTGTTTTCTCTTCTTACTAACCCCAGC
CCTGCCGAGCTCTGGGCTCCCCCCGGGGGCTGGTCCCCCTCCTTTTGGCAAGCAGATGACCTG
GGGCTACTGGCCCTGTAGACAGATGTCCCACTTTGCTGCCCCATATTGGCTGTAAGATCAGAGT
CCACTGGGCCAGGTCTAAGGCAGGGGATGGCCCTATTAACAAGACTCAGAGGAGGAAGAGGT
GGTCCTGTGGATGTGGGAGGCTGGACTCTGAGTATGACATCTCTCCTATGTGCAGAAGTCTGGT
TGCCACTGGGAGTAGGTGGGACCAGGGAAATCTCTGGGACGTGAGTGTGGAGGCCTGTTGGTC
TAGACTCTAGACTGTGGAGCTCTGAGCTTTTGTGTCCTCTGGAAGGAAGCTGGGGAAGAATCCT
CTCCATTGTTAAGTGACGGGGATAGAAGCTGTCCTGCACAGGAAGTCACGAGGGGGGCGTATC
CCACGAGGAAGGCAGGAGGGGGCGTGCCCCTCACCGGAAATTAGCAGAGGGGCGTGTCCCAC
ACCGGAAGTCAGAAAGCGGAGCCTTTCTTACACCGGAAGTCAATGAAGCGGGTCTTTCCTACG
CTAAAAACCACTGAGTGGAGTATTTAGTACACAGGAAGTCGGCCAGAGAAACATTTCTCATATT
TGAAGGCCGGAAAGAGGGACATTTCTGACACCGGAAGTCAGTGAGAGGACTCTTTCCCACACA
GGAAGTCAGCTAGAGAGCCGTCTCCCCTCTCTGGAGCCGAGAGAGGCCGGTTTCCCCCACCGT
AAGTAGACGTGGGGCCGTGACCGGAAGTCCTTGGGAAAGATCCGTCCCATTCCCGGAAGCTAG
AGGGCGTTAGTTGTCGGGTTGAAAAGGGGTGTGGGGAGGGGAAGCAGCTTTACCCCGGGCTC
GGAGTTTGCAGGAGAGAGAAGTGGGGAGCAAGAAGTGAACCTCAGGGGCTCACAGGGTTCCC
GCAGATGCTCAGGCCGGCCAGGAATGCATCTCTGGCTCTCTGTTCCCACGGACGTCACTGCCTC
AGCCAGCCTCCCCCAGAGCCCGCCAGCCGCTAAGCCGGGGCCACACCTGGGGGTGATTTCATG
CCTCACCTCCAGTAGGCACCTTGGTTTCTTTGGGCTAATCTCTGGCTCCCTTGCGCTAACTCTTGC
TCTCACCCAGCTAATCCCTGCCTCACCCTGACTGCCCCAGGGGCTGACCACTAACAACCAACCT

GGCCCTGTCTGGGGGTTCCAGGCTCCTGGCCTGGCCCTGACCGGTTCTTAATTAACCTTTCCTTC
ACCTTGACTAACTCCTGCCTTCCTGGTCTGTTCCTTTCAGCAGAAACTAATGGTTTGTGGATTTTT
TTCTGACTAACAACAGGTCTAACATTCCTCGTTACTGTTAACAGCTTGGATGTCGGCATGGCTGG
GAAGGGGCTAACACAGCTTTGAACTTGGCTAACACAGGTTTGAACTTGGCTAACACAGGTTTGA
ACTTGACTAACACAGGGAAAAGCATAGCTAACAATTTTGGGCGTGGTGGCTGCTCTGAGTCAGA
ACAATCAGAAGTCGGTAAAGATGGTAGTTTTCTAAAGGAGGTGCCAGGGCTCTGGTGTGGACC
AGGCCTGATGGAGCAGTGGTACCCACCAAGGTGGGGTCAGAAGTATAGCCAGTCTTGCAAGGT
TTTGGCCATTGGGCATATCTTCACTCCTCATAGTCTGCATTTGGTTTCAGTTCTTAAAAAAATATA
GCCTTATAGCTACAGTAGTTTGCACAAGTAGATGCAGCTCTTATAAACCTTAAAATACCTGTCTG
GTGTCTACAGAGCATATGCCATTTTGTATAGAGTCACCCTTCCCCAGGCCAGGGCCTAGAGTCTT
CATTTTGGGGACTTTGTGTTTTGGAAGTTCTAGGACATAAAGCTTTAGATCAGAGTATTCAGAAG
GGTTATAACTCCGTCAGTCATTTACATGTTAGTAGTATAAATTATCTGAGCTTCCTGTTCTAACTTT
TAGCTCTTCTAGCATAAACTCTTCTGTGCAATTTAGCTGCACCGAGGAAACGGGAGTTTTTCTGG
AAGGGACTTTTGATCTCTTTAGACTGAGGGAACGTCCTTTGGGAGTAGAGGGGCAGGGAGCAT
ACGCAAGGGATTCCAGGTGCAGGTAAAAGGTGGCACTAGTTCAAGGTTTTGCTGACTCAGTCTG
GTAGTCAGAGTCTGCAGGAGAAGACAGTTCAAGGCAGGGCCTGGAGGATTGGATCAGTTTAGG
GACAGGTCAAAGGCTGGCTTACAGACCTTAGAGGCAGGTTGCTTGGGTCGTTGAATGCTAGTCT
GGTGCTGAGAGCCCTTTTCTCTGGCAACTGTGGACTCAGAGCTAACCAATTGTAGTTGGCAGTG
GGGGTGAAGGGTGATCCAGAGGCCTGAGCTGCAGAGGGCACAAGAGAGAAAAGATGTCTTAG
AAAGAGCTTTGAGAACATGCCTTGGCTGCTGGCAGGGACCTTGGATGGGGTAGTCTACACCCG
GAAGTGCCTGCCTGCCATCCTCTAGTGGCTGCCTTGCTCCATTTCACTCAAAGCAGGAAGCTCA
CACCTCCTATTCCTGAAACTCCTCTTTGTTTAACTGCAAAGACTTGATGCTGCTAAGGATCTACTA
TGTGCCAGGCACTGCTCTGGGCGCTGGGACCTGCACCTGGGCTTTTTCGTCATGGTGCTTTTATA
GCCTAGTGGGAGAGTTGGTGAAGTAGATAGTGATTCAGTGAGATGGGTGTTATGATTGGTCAGG
GGTCTGTGGGAGCACCAAGGAGACAGACAAGATTGATGTGCACCTACTCTGTGCCAGGCGTGT
GCCAGGCATTGGGGATGTAGTGGTAGTTAAACACCATTTGGTCTTCAGGAGCTTTAATTCTAGTG
TGTTGGGTGCAGGGGGGTGGAATGGGGACAGAGAGACACCTAATCCACCCTGTGGTGGCTTTC
TGGAGAGGGAGGCATCTAAGCTGAGCTGTGGCTGGGTGGAGTGTGGGTGGGGATGAGTTCCG
GGCAGCGAGAGTGGTGGACACCAGTTTCTGGGGATCAGAGAGGATCCAAAGAGGTTCTGGAA
GGTTCATGTGGAATGTAGCAAGAGATAGGAGACATGGACATGGTGCCGGGTCTGGTTGCCAAG
AAGTTTAGATTTTATCCTTAGGCCTTGGGGAGCGACGGATATGATCTGAGAAAGGGAGTTAGTG
GATTTGAGTTTTAGGCTGGCCATTTGGCTTTTCCAGCCCAGGTGGAACTCAGAGGAGTTTGCAAT
GGCCTCTGGCCACATTTTAGACAACTGAGCAGAACTTTTTGAAACTAGGAAGACCCTTTGGTCC
ATCTTTTGATAAACAGAATCCATACATGTCTACCCCAGTTGGAAGTATCTCTGCAATGACTGGAA
AGTAAAGAGGACCAAGGTGAAAATAAAGGCTCGGAAGGGGAGCAATCTTGAAAACATGTCATC
CCATGGTGGTGGGAAGTCCCTGGAGAAGATCAGGGGAAACACAGTCATAGGCTGCAAGTCTAT
AAGATAATTCCATTGGGGAGGGAGCCCATTTGTCATGCATGGCTGCAAGGGGCAGATACAAGT
GTGGAGTAAGCTTGCAAGAGCTGATCCTGGTCCCAGAGAGGGAAAAATATGCCTTGGTGGGTA
ATGAACCTTTTGTTCCCAGAGGCAGAAGGATTGGGACTAGGCCAACATAGAGATTGGCGATGG
TTGTGAGATTCTAAGAGTGTGTGTGCATCTTGACAATATTAGAGGAGGCTGAGCCCAAGCAGGC
ACATTCTCTTCGACCCCTCCCTCATTCAGTCTGCTTTGGAGTCTACTGAACATCAAGCTTGCTATG
AGCAGGATCTTAGAGCTGAGGAATTGGCCTCCCAATCCGAACAGGTGTTATAATCCTTTCTTAAT
AGGTTGTGCTGTGGACCCAATGTGAGGGCTGTGCTGGTGTAAATGGTGACATGTTGAGCTGGGG
GGATGCTTTCGGGGTGGGGGGACTGGTTCCATTCCATCAAAGGCCCTCTTGAGAGTCTATCCAG

GGACCCATTGTTTTACTTTAACAGACCAGAAAAGATGTTTGTTTTCCATGTCATTACCCCCAGGG
GATACCGAATGTGTGGGTAGAAATTTCTCTGTAGATTAAAAATCAGATTTTTACATGGATTCAAC
AAAGGAGCGTCACTTGGATTTTTGTTTTCATCCATGAATGTAGCTGCTTCTGTGTAAAATGCCATT
TTGCTATTAAAAATCAATTCACGCTGGAAAAAAAAAAAAA
ID|15576|gene_id|440823|transcript_id|NR_033319 Homo sapiens lncRNA
CAGCTACAAAGACGACGCCGGCTGCGCTCGCGGGACCAGAGGGAGGGAGGCTGCGGAC
GAGTGCGGGGAGGAGTTTGAGATGGGAGGCGCTTGCAGGGGCTCCCCCAGTTCTGCGCTGTGA
GCCGGGGCACAAAGAGCCCTCTGCACTAGCGCCGCAGGACCGCGGACCCGAGTTGGAGGCAT
CTGTCCACCCATGTGGTTCCAGACACGTTCATGTGGCCACCATGACCCCGTCGGCATCACAGGG
GTAACCAAGGTCCACAGAACGAGGGGAAAGGGTCACTGTGGATCAAGCCAGGCTCCTCTGAG
CAGTCCAGGGTCTATTTACAGAGAGCAGCAAGTTCCTGGGACAGAGGAGACAGAAGGGAATTT
CCAGGTTCTTCAGGACGTTCACAACCACACTGAGAAGCATCTTTGCAGATAAGTATTTAAACTTA
CCAGCCCATAGCCTACCAGCCTGCCCTCTTCTGGTCTGTGCAGGAAAGTGTAGTATCCTAGAAT
GCCAAAGTGGGAGGGGAAATGGGTGATGTAGCTCATTCTCTTTTCTACCTCTATGGAAGAAAGA
AAGAGCCTGTCCCCATTTTGTGGGGTCCCAGAAAGGGTGATTTTCACACTTCACATTTGGCGTTA
GGGCTAGTATTTCACAAACATTACCGTCTGGAACTTTTGAAGGCTGAGTTAAATGACTTCATCTT
GTGTACTTGTAGAACTGCTATTAGAAGGAGGCTGGGGACTCCCTGATGATGTGAACCAGCCCTT
CTCTTCTCAGTTTCAGCACTTTGTGATCATATTTATAAACCTTGGACAGGGCTCCTTGGCCTTAGA
GTTAAGTGAAGAGTTAACACTGTGACTTTAGATGCATTTTTCTCAGTGTAAGTGTCTGAGCTCAT
CTCCAGTTCTGGACTCGTGCCCCCACTCCCGGGTGCTGACAGCAAGCTCTGAAGATATGCTGAG
AGAGCTCCCTGACTCTCCTGACTCAGGTCTGCCAGTTGATGAAGACAGCACAGTTGTTACCAAA
AACAAAACCTGGCAGATGGTCCTAGGTCAGGATTAGTGGTCATTCTCTGGTCTTCTCCAGACTG
GTGACACAGGGTGGTCTTAAAAGAGTCCTAGTCCAGCCTCTGGCTCCCCGAATTTCCTCTGGTG
CATGGGGCATCAGGAAAGGGCAGGGGAGAAGGAGGGGGAGCAGAAAAATAACTGAGGCTGG
GCAGAAGGGTCCCTTCCATTTCCCATCCATTGCTTCAAACAATGGGTGATTACCGTGCACCTTGA
GTGAATGTCAAGGCAGGAAGGGTTCCAGGGCCACCCAGTGTGGCAGGAGCAGAGCTGGGATC
AGAATTCTGGATTCCTGACTCTCCGTTCAGCTGCTATTCAGCTATCACCAAGCTGCTGAGTTTGT
CCTACTTTCTCCTGGGTCACATTCTGCTGTTGTTGGGAAACAGGGAGTGAGTGAAGGGAACCTG
GGAACCCTTCTCCCCTCACCTTTTAAGGATGTTCTGGGGTGGAATAAAGAGAGGTGGAGGGTGG
GGTGCCTGGGCCCAGCCTCCCTGGCTCCTGGTCCTTCTTTCTTCAGCAGGGAGTTCTGGCAAGG
CCTGGGCCTGGGGAGGGGGCCTGGGTGCAGTTTTCTCCTGCGGTGTGGTTGGCTCTTTTGTCTT
CCAGGATGGGCCTGTGGGGTCTGTGTGGGGAAGGAAGGAGCTGGGAGCCTGGGCTGCCTGTG
TGTCGGCTGGATGCTGCCAGGTGGCTCCATCTGGGCCGCATCTCCAGGACTCCTGGCCTGGACT
TCTCTTTGGACCTCGGGTGACCTTGGATTCTGGCCACAGTGACTTAGCGGTCAGACTCCGGTGG
TTTTCTAGCCCGCTGGGTGACTCCCTGAAGATCTCATCCTCTTCACACCTGTTGGGATGGAGCCT
CCCATGGATAGGAACTCTTCCTGGTTGATGCTCAGCAGATGACTGATCTGGCTCTGGAGGACAG
CTCCAGGGGTATGAGGGAGGCCTGGCAGCCCACTACTCCCTCCTCACCTGCTCGGGCCCTGCC
TGCTGGGCCCTATTGTCTGGGTCTGGGGGCCCTTTCCTGCCCCATGTGAACTTGATTCCTCCTCG
CGTTTCTGCCGGTGACCAGACAGAGCCTGGCCTGGGCCAAGTCCCCACTGACCACACTTTCCCC
AAGGCTGGGTCTCAAAGGACCCTACAGGATGGGGACAGGAACAAGGATGGGAGTCGGGGAGG
GGCTGTCAGGCAGAGGGCAGATGCAGCTCCTCGGGTGCCGCTGGCTGGAGTCCTTTGTCTCCA
TTTGCTCAGTGCGTAATTGTCATTTTGTGGGGGACTTCCTTGGTGGGGCTTTTCCATGCTTTTCCT
CTCCCTGATGGTTACAAAGAGGAGACAGTCAGGATCTGGGAGGAGCTGGAGCCTTCCCCCTGT
GCTGTTGAACTCTGGGAGGGGCAGCCAGAGGGGCTGAGAGTGCACCATCCTGAAAAGAGAGG

GTCTTTAAGAGATAAGATTTAGGCTAGGGAAATTCACTAGAAAGAGACAGGTGGCCCTGGGTTT
TTTTTCCTGCCATCCAAAGGGAACCAGTCTTACCCAAAGGCTTAAGGGGTCACCTTAGCCCAGT
CTCAGGACACTCTTCTCCAATCAGATCGCTGCTTCTAGTTGCACTGCTCCTGGATTCTGTTCTTGG
GGAACAGAAGGTGGCAGGACAGGCCCCACAATCTGCAATTCCAGCTCCCTCCAGAGGAGACA
GTCCCAGCGTTTGCAAGAGAGCAGCTTGTGGCCTCCCTTAGGCAAGTTTAAAAGCCAGATGTCC
TTTTTCCCAGAATGCGGAGTGGTGTGTACTATTCATCAGCTTGGGCTGCCACAGCACCATGCCA
GACTGGATGGCTTAAACATCAGGAATTTATTTTCTGCCAGTTCTGGAGGCTGGAAAGTCCAGAT
CAAGGTGTTAAGACTTGGTTTCTGGTGAGGCCTCTCTCCTTGGCTTGCAGGTGGCACCTTCTCAC
TATGTCCTCACATGGCCTTTTCTCTGTGGAGAGGGACAGAGAGCATGAGCAGGCTCTGGTGTCT
CCTCCTCTTCTTATAAAGACACTAATATCACCATATTAGGGCTTAAACCTATGACCTCATTTAACC
TTAACCCCTTAAAGGTCCCATCTCCAAAAACAGTCACATAGCAGGCTACTGCTTCAACATATGC
ATTTGGGGGAGGGGACACCATTCAGTTCTTAACAGGGTGGTCACCGCAAACATGGAAAGTCAG
AGCCTTCTCCCCTTCAGAATTCCCGCCCCCACCCAGGGATGGGGAGGAGGAGCAGAGAGGTAT
GGGAAGCAGACACGGAGAGTGGCAGGTACCATGCTGGGGTGGCTCAGGAGTGCTTCGGAGGA
CATATGGAACTGGCAGGGCTCAGTGCAGGGAGGCGGAGGCCCTGGGAGAGCCGTGTCCTGAG
AAGGGCCTGGGCTACAACCCTGGGCAAGTTACTTCACCTCTGAGCCTCCGATGCTCTGTGAAAT
GGAAGGAATGTGCTTGCCTGTCAGGTGCAGGGAGAATTCAGTGAGATTGTGTGCCTAGCACAG
TGCCTGACTCCCAGTAGGTGCTCAGCAAATGCTCCCATCCATCTGGGAGTATAGACTTAGGGTT
TATCTATTTTTTTTTTTTTTTGGCTCTCTGGACTTTAAAACTCAGCATCTTCTGAACCAGAGGCATT
TCTGATTAGCCCTTCCCTACCTATTTTCCTAGTATCACTCTTTAATCAGCTTGGGGAGGTGGCAGC
ATTTCATGGCCTCCGTAGTAACTCACAATGCTTCCTGGGGTATTTAAATTCTACTCTCTCATCAGC
ACTGAGCACCTACTTTGGGCCCTTTCCCGTGCTAGCCATTTGGGGGAAATATAGGTGAAGAGTG
GCTGGGGTTTGAACCTTTAGGATTTCACAGCCATGCTGGATGGGATAAAACCAGCTCACATGGA
GAATCAGAGAATGGGACAGTGCCACAAAACAGTCTACTGAGGCACAAGTTCTGAGTGCCTGGG
AAGTGGTAGAGAGACCCCAGAGAGGGCTGCTGCAGTCTGGAACAGCTTCCTAGCAAGCTGTAC
CAAACAGGGCCTTGAAGGATGAGAAAGATCTGGCTGAGATGATACCCGACCCTCTAGGGAAAT
TCTTAAAGTAACTTCTAGGAAATGTCATTGCTCCTTAAAAAAAAAAAAAAAAAAAAAAAAGCAG
GTTCTAATTCAAATTCCGCTGCACTACTGAAGTAGTGCAGGACTTATCAGAACCTTTAAAATGCT
AATGGCAATTTCAAATACTCAGGGACAGGGCATAGGATGAAGTGTTCCTTGACTTTTATGGCCG
TAGAATCTTTTTCTCATGGAACATCGTTTAGAACTGTGGGAGATGCTAGGCTGGGGTTTCTATGG
GAAAAGTACAGCAGTGATAGAAGATGGCATAATTAGGGTACACTTAGGTGCTTTGTAGCATTGG
CTGAAAAGGAAGCAGACCGGGTGGGCGTGGGGGAGTCTCAACTTGGTTCCAGGAGCCAGGCC
AGCTTGATGCGTTTTTTTTTTTTCTAATGGTGGGAGGGCAGGCCAATAGTTGGAGAAACTAGAGG
CCTGACAGTCGGTGATGGAAGAGATGTCTACTAAGCACGGTTGTCAGATCTTTTGCGGAGAGAT
GCGGGTGTGCGTGTGTCTGTCAGAGTGTAACAGGATCCAGCCCTCTGGGAAGGGGCTGACCAG
AACCAGGCAGGGGGTTTCTGTCTAGGACAAGTAGGGTCCATTTTCAGGGGAGCTGAAATATTTG
CAGGGGGTGCTCTGAGACCCACAGCCAATAAATAGGGAAGCAACATGCTTTTGGGCAGGGTCC
ATGTGGTTAGGGTTGATTTTGATGGGGCTGGGGGGCTTAGAGTTAGACCCCCTGCCCTGGTCAG
GCCCCACTCCCTGGCTGGTGGGGGCGGGAGGCCTCTGAAGCTGCAGCTGGAGGGGCCGGGGC
AGTGTTGCATGGTGTGATTAACCTACTAACCCAGGGCTCAGCCGGGACGCTGTGGCGTGTGAC
GAGGTGCCTGGCCTTCTGCCAACCAACTAACCCACTAACCAGGCCCGAGCATGCCCTACTAAC
CACCCCCCCGCAGCCAGATCCTACTAACTGTGCAGCTCATGGGCCTTTCAAGGGGCTGGGATAT
GGGGGCAGCCAAGGCTCCAGAAGGCTCTGCCTATGGGCTGGGATCTTTGTGCAGACCCACGTT
CCAGGACAGAGAATGCAAATAAGACTCACCAACTCTGCCACTTCCTGTTGAGTGAGTTTGAGTA

AGTCACCTCACTTCTCTCAACCTCAGTTTCCTCAGTGGTAGAATGCGAGTTCTGATACCTTACAG
GTTGCAGTGAGGATTCCCGTCAATATAAGGGTCTCAGCACATGGCAGACACCTAAAAAAAAAA
AAAGTATGAGGATGGAGGAAGGCCCCTCAAACCTGGTTGAACACGTCTGCTGCCTAGGCCCTG
GCCTCTCTGGGTGTTCTGCACACAGATCCTTCTGCCTGGGGCAAGACACTTGGCTTTGAATCCAT
GTCACTTCCCCACATTCTTCCTCCGTCTCAGCTTTCTTTTCTGTAGGCAGCTCTTTTGTTCGATTAC
TAACCCGTCTAACCCCTGGGGTGCCTCAACTGCACCCCGATCATCCCTTAACAAACTAACGGAC
CTGCGTGCGTTCTTCCTTTTCTCTTAGCGACTCCTGTGTGTGTCTGCTGAGGTGCCCTGTCCGCTG
GTGCTGTGCTCTGACTTACTAACCCAGCCCCTACTAACCCTGTTTTCTCTTCTTACTAACCCCAGC
CCTGCCGAGCTCTGGGCTCCCCCCGGGGGCTGGTCCCCCTCCTTTTGGCAAGCAGATGACCTG
GGGCTACTGGCCCTGTAGACAGATGTCCCACTTTGCTGCCCCATATTGGCTGTAAGATCAGAGT
CCACTGGGCCAGGTCTAAGGCAGGGGATGGCCCTATTAACAAGACTCAGAGGAGGAAGAGGT
GGTCCTGTGGATGTGGGAGGCTGGACTCTGAGTATGACATCTCTCCTATGTGCAGAAGTCTGGT
TGCCACTGGGAGTAGGTGGGACCAGGGAAATCTCTGGGACGTGAGTGTGGAGGCCTGTTGGTC
TAGACTCTAGACTGTGGAGCTCTGAGCTTTTGTGTCCTCTGGAAGGAAGCTGGGGAAGAATCCT
CTCCATTGTTAAGTGACGGGGATAGAAGCTGTCCTGCACAGGAAGTCACGAGGGGGGCGTATC
CCACGAGGAAGGCAGGAGGGGGCGTGCCCCTCACCGGAAATTAGCAGAGGGGCGTGTCCCAC
ACCGGAAGTCAGAAAGCGGAGCCTTTCTTACACCGGAAGTCAATGAAGCGGGTCTTTCCTACG
CTAAAAACCACTGAGTGGAGTATTTAGTACACAGGAAGTCGGCCAGAGAAACATTTCTCATATT
TGAAGGCCGGAAAGAGGGACATTTCTGACACCGGAAGTCAGTGAGAGGACTCTTTCCCACACA
GGAAGTCAGCTAGAGAGCCGTCTCCCCTCTCTGGAGCCGAGAGAGGCCGGTTTCCCCCACCGT
AAGTAGACGTGGGGCCGTGACCGGAAGTCCTTGGGAAAGATCCGTCCCATTCCCGGAAGCTAG
AGGGCGTTAGTTGTCGGGTTGAAAAGGGGTGTGGGGAGGGGAAGCAGCTTTACCCCGGGCTC
GGAGTTTGCAGGAGAGAGAAGTGGGGAGCAAGAAGTGAACCTCAGGGGCTCACAGGGTTCCC
GCAGATGCTCAGGCCGGCCAGGAATGCATCTCTGGCTCTCTGTTCCCACGGACGTCACTGCCTC
AGCCAGCCTCCCCCAGAGCCCGCCAGCCGCTAAGCCGGGGCCACACCTGGGGGTGATTTCATG
CCTCACCTCCAGTAGGCACCTTGGTTTCTTTGGGCTAATCTCTGGCTCCCTTGCGCTAACTCTTGC
TCTCACCCAGCTAATCCCTGCCTCACCCTGACTGCCCCAGGGGCTGACCACTAACAACCAACCT
GGCCCTGTCTGGGGGTTCCAGGCTCCTGGCCTGGCCCTGACCGGTTCTTAATTAACCTTTCCTTC
ACCTTGACTAACTCCTGCCTTCCTGGTCTGTTCCTTTCAGCAGAAACTAATGGTTTGTGGATTTTT
TTCTGACTAACAACAGGTCTAACATTCCTCGTTACTGTTAACAGCTTGGATGTCGGCATGGCTGG
GAAGGGGCTAACACAGCTTTGAACTTGGCTAACACAGGTTTGAACTTGGCTAACACAGGTTTGA
ACTTGACTAACACAGGGAAAAGCATAGCTAACAATTTTGGGCGTGGTGGCTGCTCTGAGTCAGA
ACAATCAGAAGTCGGTAAAGATGGTAGTTTTCTAAAGGAGGTGCCAGGGCTCTGGTGTGGACC
AGGCCTGATGGAGCAGTGGTACCCACCAAGGTGGGGTCAGAAGTATAGCCAGTCTTGCAAGGT
TTTGGCCATTGGGCATATCTTCACTCCTCATAGTCTGCATTTGGTTTCAGTTCTTAAAAAAATATA
GCCTTATAGCTACAGTAGTTTGCACAAGTAGATGCAGCTCTTATAAACCTTAAAATACCTGTCTG
GTGTCTACAGAGCATATGCCATTTTGTATAGAGTCACCCTTCCCCAGGCCAGGGCCTAGAGTCTT
CATTTTGGGGACTTTGTGTTTTGGAAGTTCTAGGACATAAAGCTTTAGATCAGAGTATTCAGAAG
GGTTATAACTCCGTCAGTCATTTACATGTTAGTAGTATAAATTATCTGAGCTTCCTGTTCTAACTTT
TAGCTCTTCTAGCATAAACTCTTCTGTGCAATTTAGCTGCACCGAGGAAACGGGAGTTTTTCTGG
AAGGGACTTTTGATCTCTTTAGACTGAGGGAACGTCCTTTGGGAGTAGAGGGGCAGGGAGCAT
ACGCAAGGGATTCCAGGTGCAGGTAAAAGGTGGCACTAGTTCAAGGTTTTGCTGACTCAGTCTG
GTAGTCAGAGTCTGCAGGAGAAGACAGTTCAAGGCAGGGCCTGGAGGATTGGATCAGTTTAGG
GACAGGTCAAAGGCTGGCTTACAGACCTTAGAGGCAGGTTGCTTGGGTCGTTGAATGCTAGTCT

GGTGCTGAGAGCCCTTTTCTCTGGCAACTGTGGACTCAGAGCTAACCAATTGTAGTTGGCAGTG
GGGGTGAAGGGTGATCCAGAGGCCTGAGCTGCAGAGGGCACAAGAGAGAAAAGATGTCTTAG
AAAGAGCTTTGAGAACATGCCTTGGCTGCTGGCAGGGACCTTGGATGGGGTAGTCTACACCCG
GAAGTGCCTGCCTGCCATCCTCTAGTGGCTGCCTTGCTCCATTTCACTCAAAGCAGGAAGCTCA
CACCTCCTATTCCTGAAACTCCTCTTTGTTTAACTGCAAAGACTTGATGCTGCTAAGGATCTACTA
TGTGCCAGGCACTGCTCTGGGCGCTGGGACCTGCACCTGGGCTTTTTCGTCATGGTGCTTTTATA
GCCTAGTGGGAGAGTTGGTGAAGTAGATAGTGATTCAGTGAGATGGGTGTTATGATTGGTCAGG
GGTCTGTGGGAGCACCAAGGAGACAGACAAGATTGATGTGCACCTACTCTGTGCCAGGCGTGT
GCCAGGCATTGGGGATGTAGTGGTAGTTAAACACCATTTGGTCTTCAGGAGCTTTAATTCTAGTG
TGTTGGGTGCAGGGGGGTGGAATGGGGACAGAGAGACACCTAATCCACCCTGTGGTGGCTTTC
TGGAGAGGGAGGCATCTAAGCTGAGCTGTGGCTGGGTGGAGTGTGGGTGGGGATGAGTTCCG
GGCAGCGAGAGTGGTGGACACCAGTTTCTGGGGATCAGAGAGGATCCAAAGAGGTTCTGGAA
GGTTCATGTGGAATGTAGCAAGAGATAGGAGACATGGACATGGTGCCGGGTCTGGTTGCCAAG
AAGTTTAGATTTTATCCTTAGGCCTTGGGGAGCGACGGATATGATCTGAGAAAGGGAGTTAGTG
GATTTGAGTTTTAGGCTGGCCATTTGGCTTTTCCAGCCCAGGTGGAACTCAGAGGAGTTTGCAAT
GGCCTCTGGCCACATTTTAGACAACTGAGCAGAACTTTTTGAAACTAGGAAGACCCTTTGGTCC
ATCTTTTGATAAACAGAATCCATACATGTCTACCCCAGTTGGAAGTATCTCTGCAATGACTGGAA
AGTAAAGAGGACCAAGGTGAAAATAAAGGCTCGGAAGGGGAGCAATCTTGAAAACATGTCATC
CCATGGTGGTGGGAAGTCCCTGGAGAAGATCAGGGGAAACACAGTCATAGGCTGCAAGTCTAT
AAGATAATTCCATTGGGGAGGGAGCCCATTTGTCATGCATGGCTGCAAGGGGCAGATACAAGT
GTGGAGTAAGCTTGCAAGAGCTGATCCTGGTCCCAGAGAGGGAAAAATATGCCTTGGTGGGTA
ATGAACCTTTTGTTCCCAGAGGCAGAAGGATTGGGACTAGGCCAACATAGAGATTGGCGATGG
TTGTGAGATTCTAAGAGTGTGTGTGCATCTTGACAATATTAGAGGAGGCTGAGCCCAAGCAGGC
ACATTCTCTTCGACCCCTCCCTCATTCAGTCTGCTTTGGAGTCTACTGAACATCAAGCTTGCTATG
AGCAGGATCTTAGAGCTGAGGAATTGGCCTCCCAATCCGAACAGGTGTTATAATCCTTTCTTAAT
AGGTTGTGCTGTGGACCCAATGTGAGGGCTGTGCTGGTGTAAATGGTGACATGTTGAGCTGGGG
GGATGCTTTCGGGGTGGGGGGACTGGTTCCATTCCATCAAAGGCCCTCTTGAGAGTCTATCCAG
GGACCCATTGTTTTACTTTAACAGACCAGAAAAGATGTTTGTTTTCCATGTCATTACCCCCAGGG
GATACCGAATGTGTGGGTAGAAATTTCTCTGTAGATTAAAAATCAGATTTTTACATGGATTCAAC
AAAGGAGCGTCACTTGGATTTTTGTTTTCATCCATGAATGTAGCTGCTTCTGTGTAAAATGCCATT
TTGCTATTAAAAATCAATTCACGCTGGAAAAAAAAAAAAA
ID|15577|gene_id|440823|transcript_id|NR_033320 Homo sapiens lncRNA
CAGCTACAAAGACGACGCCGGCTGCGCTCGCGGGACCAGAGGGAGGGAGGCTGCGGAC
GAGTGCGGGGAGGAGTTTGAGATGGGAGGCGCTTGCAGGGGCTCCCCCAGTTCTGCGCTGTGA
GCCGGGGCACAAAGAGCCCTCTGCACTAGCGCCGCAGGACCGCGGACCCGAGTTGGAGGCAT
CTGTCCACCCATGTGGTTCCAGACACGTTCATGTGGCCACCATGACCCCGTCGGCATCACAGGG
GTAACCAAGAGAGCAGCAAGTTCCTGGGACAGAGGAGACAGAAGGGAATTTCCAGGTTCTTCA
GGACGTTCACAACCACACTGAGAAGCATCTTTGCAGATAAGTATTTAAACTTACCAGCCCATAG
CCTACCAGCCTGCCCTCTTCTGGTCTGTGCAGGAAAGTGTAGTATCCTAGAATGCCAAAGTGGG
AGGGGAAATGGGTGATGTAGCTCATTCTCTTTTCTACCTCTATGGAAGAAAGAAAGAGCCTGTC
CCCATTTTGTGGGGTCCCAGAAAGGGTGATTTTCACACTTCACATTTGGCGTTAGGGCTAGTATT
TCACAAACATTACCGTCTGGAACTTTTGAAGGCTGAGTTAAATGACTTCATCTTGTGTACTTGTA
GAACTGCTATTAGAAGGAGGCTGGGGACTCCCTGATGATGTGAACCAGCCCTTCTCTTCTCAGT
TTCAGCACTTTGTGATCATATTTATAAACCTTGGACAGGGCTCCTTGGCCTTAGAGTTAAGTGAA

GAGTTAACACTGTGACTTTAGATGCATTTTTCTCAGTGTAAGTGTCTGAGCTCATCTCCAGTTCTG
GACTCGTGCCCCCACTCCCGGGTGCTGACAGCAAGCTCTGAAGATATGCTGAGAGAGCTCCCT
GACTCTCCTGACTCAGGTCTGCCAGTTGATGAAGACAGCACAGTTGTTACCAAAAACAAAACCT
GGCAGATGGTCCTAGGTCAGGATTAGTGGTCATTCTCTGGTCTTCTCCAGACTGGTGACACAGG
GTGGTCTTAAAAGAGTCCTAGTCCAGCCTCTGGCTCCCCGAATTTCCTCTGGTGCATGGGGCAT
CAGGAAAGGGCAGGGGAGAAGGAGGGGGAGCAGAAAAATAACTGAGGCTGGGCAGAAGGGT
CCCTTCCATTTCCCATCCATTGCTTCAAACAATGGGTGATTACCGTGCACCTTGAGTGAATGTCA
AGGCAGGAAGGGTTCCAGGGCCACCCAGTGTGGCAGGAGCAGAGCTGGGATCAGAATTCTGG
ATTCCTGACTCTCCGTTCAGCTGCTATTCAGCTATCACCAAGCTGCTGAGTTTGTCCTACTTTCTC
CTGGGTCACATTCTGCTGTTGTTGGGAAACAGGGAGTGAGTGAAGGGAACCTGGGAACCCTTCT
CCCCTCACCTTTTAAGGATGTTCTGGGGTGGAATAAAGAGAGGTGGAGGGTGGGGTGCCTGGG
CCCAGCCTCCCTGGCTCCTGGTCCTTCTTTCTTCAGCAGGGAGTTCTGGCAAGGCCTGGGCCTG
GGGAGGGGGCCTGGGTGCAGTTTTCTCCTGCGGTGTGGTTGGCTCTTTTGTCTTCCAGGATGGG
CCTGTGGGGTCTGTGTGGGGAAGGAAGGAGCTGGGAGCCTGGGCTGCCTGTGTGTCGGCTGGA
TGCTGCCAGCACCGGCATGGACCAAGCAACTTCTCTGAGCCAGATGCTGGGGACGCAGAGGTG
AACCCAGAGACATGATCCCATCTGCTCAGATCTTCATGTCAGAACACGCTTTATTACAGTCTCGG
CAAGGGGGTGGCTCCATCTGGGCCGCATCTCCAGGACTCCTGGCCTGGACTTCTCTTTGGACCT
CGGGTGACCTTGGATTCTGGCCACAGTGACTTAGCGGTCAGACTCCGGTGGTTTTCTAGCCCGC
TGGGTGACTCCCTGAAGATCTCATCCTCTTCACACCTGTTGGGATGGAGCCTCCCATGGATAGG
AACTCTTCCTGGTTGATGCTCAGCAGATGACTGATCTGGCTCTGGAGGACAGCTCCAGGGGTAT
GAGGGAGGCCTGGCAGCCCACTACTCCCTCCTCACCTGCTCGGGCCCTGCCTGCTGGGCCCTA
TTGTCTGGGTCTGGGGGCCCTTTCCTGCCCCATGTGAACTTGATTCCTCCTCGCGTTTCTGCCGG
TGACCAGACAGAGCCTGGCCTGGGCCAAGTCCCCACTGACCACACTTTCCCCAAGGCTGGGTC
TCAAAGGACCCTACAGGATGGGGACAGGAACAAGGATGGGAGTCGGGGAGGGGCTGTCAGGC
AGAGGGCAGATGCAGCTCCTCGGGTGCCGCTGGCTGGAGTCCTTTGTCTCCATTTGCTCAGTGC
GTAATTGTCATTTTGTGGGGGACTTCCTTGGTGGGGCTTTTCCATGCTTTTCCTCTCCCTGATGGT
TACAAAGAGGAGACAGTCAGGATCTGGGAGGAGCTGGAGCCTTCCCCCTGTGCTGTTGAACTC
TGGGAGGGGCAGCCAGAGGGGCTGAGAGTGCACCATCCTGAAAAGAGAGGGTCTTTAAGAGA
TAAGATTTAGGCTAGGGAAATTCACTAGAAAGAGACAGGTGGCCCTGGGTTTTTTTTCCTGCCAT
CCAAAGGGAACCAGTCTTACCCAAAGGCTTAAGGGGTCACCTTAGCCCAGTCTCAGGACACTCT
TCTCCAATCAGATCGCTGCTTCTAGTTGCACTGCTCCTGGATTCTGTTCTTGGGGAACAGAAGGT
GGCAGGACAGGCCCCACAATCTGCAATTCCAGCTCCCTCCAGAGGAGACAGTCCCAGCGTTTG
CAAGAGAGCAGCTTGTGGCCTCCCTTAGGCAAGTTTAAAAGCCAGATGTCCTTTTTCCCAGAAT
GCGGAGTGGTGTGTACTATTCATCAGCTTGGGCTGCCACAGCACCATGCCAGACTGGATGGCTT
AAACATCAGGAATTTATTTTCTGCCAGTTCTGGAGGCTGGAAAGTCCAGATCAAGGTGTTAAGA
CTTGGTTTCTGGTGAGGCCTCTCTCCTTGGCTTGCAGGTGGCACCTTCTCACTATGTCCTCACAT
GGCCTTTTCTCTGTGGAGAGGGACAGAGAGCATGAGCAGGCTCTGGTGTCTCCTCCTCTTCTTAT
AAAGACACTAATATCACCATATTAGGGCTTAAACCTATGACCTCATTTAACCTTAACCCCTTAAA
GGTCCCATCTCCAAAAACAGTCACATAGCAGGCTACTGCTTCAACATATGCATTTGGGGGAGGG
GACACCATTCAGTTCTTAACAGGGTGGTCACCGCAAACATGGAAAGTCAGAGCCTTCTCCCCTT
CAGAATTCCCGCCCCCACCCAGGGATGGGGAGGAGGAGCAGAGAGGTATGGAAGCAGACAC
GGAGAGTGGCAGGTACCATGCTGGGGTGGCTCAGGAGTGCTTCGGAGGACATATGGAACTGG
CAGGGCTCAGTGCAGGGAGGCGGAGGCCCTGGGAGAGCCGTGTCCTGAGAAGGGCCTGGGCT
ACAACCCTGGGCAAGTTACTTCACCTCTGAGCCTCCGATGCTCTGTGAAATGGAAGGAATGTGC

TTGCCTGTCAGGTGCAGGGAGAATTCAGTGAGATTGTGTGCCTAGCACAGTGCCTGACTCCCAG
TAGGTGCTCAGCAAATGCTCCCATCCATCTGGGAGTATAGACTTAGGGTTTATCTATTTTTTTTTT
TTTTTGGCTCTCTGGACTTTAAAACTCAGCATCTTCTGAACCAGAGGCATTTCTGATTAGCCCTTC
CCTACCTATTTTCCTAGTATCACTCTTTAATCAGCTTGGGGAGGTGGCAGCATTTCATGGCCTCC
GTAGTAACTCACAATGCTTCCTGGGGTATTTAAATTCTACTCTCTCATCAGCACTGAGCACCTAC
TTTGGGCCCTTTCCCGTGCTAGCCATTTGGGGGAAATATAGGTGAAGAGTGGCTGGGGTTTGAA
CCTTTAGGATTTCACAGCCATGCTGGATGGGATAAAACCAGCTCACATGGAGAATCAGAGAATG
GGACAGTGCCACAAAACAGTCTACTGAGGCACAAGTTCTGAGTGCCTGGGAAGTGGTAGAGAG
ACCCCAGAGAGGGCTGCTGCAGTCTGGAACAGCTTCCTAGCAAGCTGTACCAAACAGGGCCTT
GAAGGATGAGAAAGATCTGGCTGAGATGATACCCGACCCTCTAGGGAAATTCTTAAAGTAACTT
CTAGGAAATGTCATTGCTCCTTAAAAAAAAAAAAAAAAAAAAAAAAAGCAGGTTCTAATTCAAAT
TCCGCTGCACTACTGAAGTAGTGCAGGACTTATCAGAACCTTTAAAATGCTAATGGCAATTTCAA
ATACTCAGGGACAGGGCATAGGATGAAGTGTTCCTTGACTTTTATGGCCGTAGAATCTTTTTCTC
ATGGAACATCGTTTAGAACTGTGGGAGATGCTAGGCTGGGGTTTCTATGGGAAAAGTACAGCA
GTGATAGAAGATGGCATAATTAGGGTACACTTAGGTGCTTTGTAGCATTGGCTGAAAAGGAAGC
AGACCGGGTGGGCGTGGGGGAGTCTCAACTTGGTTCCAGGAGCCAGGCCAGCTTGATGCGTTT
TTTTTTTTTCTAATGGTGGGAGGGCAGGCCAATAGTTGGAGAAACTAGAGGCCTGACAGTCGGT
GATGGAAGAGATGTCTACTAAGCACGGTTGTCAGATCTTTTGCGGAGAGATGCGGGTGTGCGTG
TGTCTGTCAGAGTGTAACAGGATCCAGCCCTCTGGGAAGGGGCTGACCAGAACCAGGCAGGG
GGTTTCTGTCTAGGACAAGTAGGGTCCATTTTCAGGGGAGCTGAAATATTTGCAGGGGGTGCTC
TGAGACCCACAGCCAATAAATAGGGAAGCAACATGCTTTTGGGCAGGGTCCATGTGGTTAGGG
TTGATTTTGATGGGGCTGGGGGGCTTAGAGTTAGACCCCCTGCCCTGGTCAGGCCCCACTCCCT
GGCTGGTGGGGGCGGGAGGCCTCTGAAGCTGCAGCTGGAGGGGCCGGGGCAGTGTTGCATGG
TGTGATTAACCTACTAACCCAGGGCTCAGCCGGGACGCTGTGGCGTGTGACGAGGTGCCTGGC
CTTCTGCCAACCAACTAACCCACTAACCAGGCCCGAGCATGCCCTACTAACCACCCCCCCGCA
GCCAGATCCTACTAACTGTGCAGCTCATGGGCCTTTCAAGGGGCTGGGATATGGGGGCAGCCA
AGGCTCCAGAAGGCTCTGCCTATGGGCTGGGATCTTTGTGCAGACCCACGTTCCAGGACAGAG
AATGCAAATAAGACTCACCAACTCTGCCACTTCCTGTTGAGTGAGTTTGAGTAAGTCACCTCACT
TCTCTCAACCTCAGTTTCCTCAGTGGTAGAATGCGAGTTCTGATACCTTACAGGTTGCAGTGAGG
ATTCCCGTCAATATAAGGGTCTCAGCACATGGCAGACACCTAAAAAAAAAAAAAAGTATGAGGA
TGGAGGAAGGCCCCTCAAACCTGGTTGAACACGTCTGCTGCCTAGGCCCTGGCCTCTCTGGGTG
TTCTGCACACAGATCCTTCTGCCTGGGGCAAGACACTTGGCTTTGAATCCATGTCACTTCCCCAC
ATTCTTCCTCCGTCTCAGCTTTCTTTTCTGTAGGCAGCTCTTTTGTTCGATTACTAACCCGTCTAAC
CCCTGGGGTGCCTCAACTGCACCCCGATCATCCCTTAACAAACTAACGGACCTGCGTGCGTTCT
TCCTTTTCTCTTAGCGACTCCTGTGTGTGTCTGCTGAGGTGCCCTGTCCGCTGGTGCTGTGCTCTG
ACTTACTAACCCAGCCCCTACTAACCCTGTTTTCTCTTCTTACTAACCCCAGCCCTGCCGAGCTCT
GGGCTCCCCCCGGGGGCTGGTCCCCCTCCTTTTGGCAAGCAGATGACCTGGGGCTACTGGCCC
TGTAGACAGATGTCCCACTTTGCTGCCCCATATTGGCTGTAAGATCAGAGTCCACTGGGCCAGG
TCTAAGGCAGGGGATGGCCCTATTAACAAGACTCAGAGGAGGAAGAGGTGGTCCTGTGGATGT
GGGAGGCTGGACTCTGAGTATGACATCTCTCCTATGTGCAGAAGTCTGGTTGCCACTGGGAGTA
GGTGGGACCAGGGAAATCTCTGGGACGTGAGTGTGGAGGCCTGTTGGTCTAGACTCTAGACTG
TGGAGCTCTGAGCTTTTGTGTCCTCTGGAAGGAAGCTGGGGAAGAATCCTCTCCATTGTTAAGT
GACGGGGATAGAAGCTGTCCTGCACAGGAAGTCACGAGGGGGGCGTATCCCACGAGGAAGGC
AGGAGGGGGCGTGCCCCTCACCGGAAATTAGCAGAGGGGCGTGTCCCACACCGGAAGTCAGA

AAGCGGAGCCTTTCTTACACCGGAAGTCAATGAAGCGGGTCTTTCCTACGCTAAAAACCACTGA
GTGGAGTATTTAGTACACAGGAAGTCGGCCAGAGAAACATTTCTCATATTTGAAGGCCGGAAAG
AGGGACATTTCTGACACCGGAAGTCAGTGAGAGGACTCTTTCCCACACAGGAAGTCAGCTAGA
GAGCCGTCTCCCCTCTCTGGAGCCGAGAGAGGCCGGTTTCCCCCACCGTAAGTAGACGTGGGG
CCGTGACCGGAAGTCCTTGGGAAAGATCCGTCCCATTCCCGGAAGCTAGAGGGCGTTAGTTGT
CGGGTTGAAAAGGGGTGTGGGGAGGGGAAGCAGCTTTACCCCGGGCTCGGAGTTTGCAGGAG
AGAGAAGTGGGGAGCAAGAAGTGAACCTCAGGGGCTCACAGGGTTCCCGCAGATGCTCAGGC
CGGCCAGGAATGCATCTCTGGCTCTCTGTTCCCACGGACGTCACTGCCTCAGCCAGCCTCCCCC
AGAGCCCGCCAGCCGCTAAGCCGGGGCCACACCTGGGGGTGATTTCATGCCTCACCTCCAGTA
GGCACCTTGGTTTCTTTGGGCTAATCTCTGGCTCCCTTGCGCTAACTCTTGCTCTCACCCAGCTA
ATCCCTGCCTCACCCTGACTGCCCCAGGGGCTGACCACTAACAACCAACCTGGCCCTGTCTGGG
GGTTCCAGGCTCCTGGCCTGGCCCTGACCGGTTCTTAATTAACCTTTCCTTCACCTTGACTAACT
CCTGCCTTCCTGGTCTGTTCCTTTCAGCAGAAACTAATGGTTTGTGGATTTTTTTCTGACTAACAA
CAGGTCTAACATTCCTCGTTACTGTTAACAGCTTGGATGTCGGCATGGCTGGGAAGGGGCTAAC
ACAGCTTTGAACTTGGCTAACACAGGTTTGAACTTGGCTAACACAGGTTTGAACTTGACTAACAC
AGGGAAAAGCATAGCTAACAATTTTGGGCGTGGTGGCTGCTCTGAGTCAGAACAATCAGAAGT
CGGTAAAGATGGTAGTTTTCTAAAGGAGGTGCCAGGGCTCTGGTGTGGACCAGGCCTGATGGA
GCAGTGGTACCCACCAAGGTGGGGTCAGAAGTATAGCCAGTCTTGCAAGGTTTTGGCCATTGG
GCATATCTTCACTCCTCATAGTCTGCATTTGGTTTCAGTTCTTAAAAAAATATAGCCTTATAGCTA
CAGTAGTTTGCACAAGTAGATGCAGCTCTTATAAACCTTAAAATACCTGTCTGGTGTCTACAGAG
CATATGCCATTTTGTATAGAGTCACCCTTCCCCAGGCCAGGGCCTAGAGTCTTCATTTTGGGGAC
TTTGTGTTTTGGAAGTTCTAGGACATAAAGCTTTAGATCAGAGTATTCAGAAGGGTTATAACTCC
GTCAGTCATTTACATGTTAGTAGTATAAATTATCTGAGCTTCCTGTTCTAACTTTTAGCTCTTCTAG
CATAAACTCTTCTGTGCAATTTAGCTGCACCGAGGAAACGGGAGTTTTTCTGGAAGGGACTTTTG
ATCTCTTTAGACTGAGGGAACGTCCTTTGGGAGTAGAGGGGCAGGGAGCATACGCAAGGGATT
CCAGGTGCAGGTAAAAGGTGGCACTAGTTCAAGGTTTTGCTGACTCAGTCTGGTAGTCAGAGTC
TGCAGGAGAAGACAGTTCAAGGCAGGGCCTGGAGGATTGGATCAGTTTAGGGACAGGTCAAA
GGCTGGCTTACAGACCTTAGAGGCAGGTTGCTTGGGTCGTTGAATGCTAGTCTGGTGCTGAGAG
CCCTTTTCTCTGGCAACTGTGGACTCAGAGCTAACCAATTGTAGTTGGCAGTGGGGGTGAAGGG
TGATCCAGAGGCCTGAGCTGCAGAGGGCACAAGAGAGAAAAGATGTCTTAGAAAGAGCTTTGA
GAACATGCCTTGGCTGCTGGCAGGGACCTTGGATGGGGTAGTCTACACCCGGAAGTGCCTGCC
TGCCATCCTCTAGTGGCTGCCTTGCTCCATTTCACTCAAAGCAGGAAGCTCACACCTCCTATTCC
TGAAACTCCTCTTTGTTTAACTGCAAAGACTTGATGCTGCTAAGGATCTACTATGTGCCAGGCAC
TGCTCTGGGCGCTGGGACCTGCACCTGGGCTTTTTCGTCATGGTGCTTTTATAGCCTAGTGGGAG
AGTTGGTGAAGTAGATAGTGATTCAGTGAGATGGGTGTTATGATTGGTCAGGGGTCTGTGGGAG
CACCAAGGAGACAGACAAGATTGATGTGCACCTACTCTGTGCCAGGCGTGTGCCAGGCATTGG
GGATGTAGTGGTAGTTAAACACCATTTGGTCTTCAGGAGCTTTAATTCTAGTGTGTTGGGTGCAG
GGGGGTGGAATGGGGACAGAGAGACACCTAATCCACCCTGTGGTGGCTTTCTGGAGAGGGAG
GCATCTAAGCTGAGCTGTGGCTGGGTGGAGTGTGGGTGGGGATGAGTTCCGGGCAGCGAGAGT
GGTGGACACCAGTTTCTGGGGATCAGAGAGGATCCAAAGAGGTTCTGGAAGGTTCATGTGGAA
TGTAGCAAGAGATAGGAGACATGGACATGGTGCCGGGTCTGGTTGCCAAGAAGTTTAGATTTTA
TCCTTAGGCCTTGGGGAGCGACGGATATGATCTGAGAAAGGGAGTTAGTGGATTTGAGTTTTAG
GCTGGCCATTTGGCTTTTCCAGCCCAGGTGGAACTCAGAGGAGTTTGCAATGGCCTCTGGCCAC
ATTTTAGACAACTGAGCAGAACTTTTTGAAACTAGGAAGACCCTTTGGTCCATCTTTTGATAAAC

AGAATCCATACATGTCTACCCCAGTTGGAAGTATCTCTGCAATGACTGGAAAGTAAAGAGGACC
AAGGTGAAAATAAAGGCTCGGAAGGGGAGCAATCTTGAAAACATGTCATCCCATGGTGGTGGG
AAGTCCCTGGAGAAGATCAGGGGAAACACAGTCATAGGCTGCAAGTCTATAAGATAATTCCATT
GGGGAGGGAGCCCATTTGTCATGCATGGCTGCAAGGGGCAGATACAAGTGTGGAGTAAGCTTG
CAAGAGCTGATCCTGGTCCCAGAGAGGGAAAAATATGCCTTGGTGGGTAATGAACCTTTTGTTC
CCAGAGGCAGAAGGATTGGGACTAGGCCAACATAGAGATTGGCGATGGTTGTGAGATTCTAAG
AGTGTGTGTGCATCTTGACAATATTAGAGGAGGCTGAGCCCAAGCAGGCACATTCTCTTCGACC
CCTCCCTCATTCAGTCTGCTTTGGAGTCTACTGAACATCAAGCTTGCTATGAGCAGGATCTTAGA
GCTGAGGAATTGGCCTCCCAATCCGAACAGGTGTTATAATCCTTTCTTAATAGGTTGTGCTGTGG
ACCCAATGTGAGGGCTGTGCTGGTGTAAATGGTGACATGTTGAGCTGGGGGGATGCTTTCGGG
GTGGGGGGACTGGTTCCATTCCATCAAAGGCCCTCTTGAGAGTCTATCCAGGGACCCATTGTTT
TACTTTAACAGACCAGAAAAGATGTTTGTTTTCCATGTCATTACCCCCAGGGGATACCGAATGTG
TGGGTAGAAATTTCTCTGTAGATTAAAAATCAGATTTTTACATGGATTCAACAAAGGAGCGTCAC
TTGGATTTTTGTTTTCATCCATGAATGTAGCTGCTTCTGTGTAAAATGCCATTTTGCTATTAAAAT
CAATTCACGCTGGAAAAAAAAAAAAA

ID|15578|gene_id|440823|transcript_id|NR_033321 Homo sapiens lncRNA

CAGCTACAAAGACGACGCCGGCTGCGCTCGCGGGACCAGAGGGAGGGAGGCTGCGGAC
GAGTGCGGGGAGGAGTTTGAGATGGGAGGCGCTTGCAGGGGCTCCCCCAGTTCTGCGCTGTGA
GCCGGGGCACAAAGAGCCCTCTGCACTAGCGCCGCAGGACCGCGGACCCGAGTTGGAGGCAT
CTGTCCACCCATGTGGTTCCAGACACGTTCATGTGGCCACCATGACCCCGTCGGCATCACAGGG
GTAACCAAGAGAGCAGCAAGTTCCTGGGACAGAGGAGACAGAAGGGAATTTCCAGGTTCTTCA
GGACGTTCACAACCACACTGAGAAGCATCTTTGCAGATAAGTATTTAAACTTACCAGCCCATAG
CCTACCAGCCTGCCCTCTTCTGGTCTGTGCAGGAAAGTGTAGTATCCTAGAATGCCAAAGTGGG
AGGGGAAATGGGTGATGTAGCTCATTCTCTTTTCTACCTCTATGGAAGAAAGAAAGAGCCTGTC
CCCATTTTGTGGGGTCCCAGAAAGGGTGATTTTCACACTTCACATTTGGCGTTAGGGCTAGTATT
TCACAAACATTACCGTCTGGAACTTTTGAAGGCTGAGTTAAATGACTTCATCTTGTGTACTTGTA
GAACTGCTATTAGAAGGAGGCTGGGGACTCCCTGATGATGTGAACCAGCCCTTCTCTTCTCAGT
TTCAGCACTTTGTGATCATATTTATAAACCTTGGACAGGGCTCCTTGGCCTTAGAGTTAAGTGAA
GAGTTAACACTGTGACTTTAGATGCATTTTTCTCAGTGTAAGTGTCTGAGCTCATCTCCAGTTCTG
GACTCGTGCCCCCACTCCCGGGTGCTGACAGCAAGCTCTGAAGATATGCTGAGAGAGCTCCCT
GACTCTCCTGACTCAGGTCTGCCAGTTGATGAAGACAGCACAGTTGTTACCAAAAACAAAACCT
GGCAGATGGTCCTAGGTCAGGATTAGTGGTCATTCTCTGGTCTTCTCCAGACTGGTGACACAGG
GTGGTCTTAAAAGAGTCCTAGTCCAGCCTCTGGCTCCCCGAATTTCCTCTGGTGCATGGGGCAT
CAGGAAAGGGCAGGGGAGAAGGAGGGGGAGCAGAAAAATAACTGAGGCTGGGCAGAAGGGT
CCCTTCCATTTCCCATCCATTGCTTCAAACAATGGGTGATTACCGTGCACCTTGAGTGAATGTCA
AGGCAGGAAGGGTTCCAGGGCCACCCAGTGTGGCAGGAGCAGAGCTGGGATCAGAATTCTGG
ATTCCTGACTCTCCGTTCAGCTGCTATTCAGCTATCACCAAGCTGCTGAGTTTGTCCTACTTTCTC
CTGGGTCACATTCTGCTGTTGTTGGGAAACAGGGAGTGAGTGAAGGGAACCTGGGAACCCTTCT
CCCCTCACCTTTTAAGGATGTTCTGGGGTGGAATAAAGAGAGGTGGAGGGTGGGGTGCCTGGG
CCCAGCCTCCCTGGCTCCTGGTCCTTCTTTCTTCAGCAGGGAGTTCTGGCAAGGCCTGGGCCTG
GGGAGGGGGCCTGGGTGCAGTTTTCTCCTGCGGTGTGGTTGGCTCTTTTGTCTTCCAGGATGGG
CCTGTGGGGTCTGTGTGGGGAAGGAAGGAGCTGGGAGCCTGGGCTGCCTGTGTGTCGGCTGGA
TGCTGCCAGGTGGCTCCATCTGGGCCGCATCTCCAGGACTCCTGGCCTGGACTTCTCTTTGGAC
CTCGGGTGACCTTGGATTCTGGCCACAGTGACTTAGCGGTCAGACTCCGGTGGTTTTCTAGCCC

GCTGGGTGACTCCCTGAAGATCTCATCCTCTTCACACCTGTTGGGATGGAGCCTCCCATGGATA
GGAACTCTTCCTGGTTGATGCTCAGCAGATGACTGATCTGGCTCTGGAGGACAGCTCCAGGGGT
ATGAGGGAGGCCTGGCAGCCCACTACTCCCTCCTCACCTGCTCGGGCCCTGCCTGCTGGGCCC
TATTGTCTGGGTCTGGGGGCCCTTTCCTGCCCCATGTGAACTTGATTCCTCCTCGCGTTTCTGCC
GGTGACCAGACAGAGCCTGGCCTGGGCCAAGTCCCCACTGACCACACTTTCCCCAAGGCTGGG
TCTCAAAGGACCCTACAGGATGGGGACAGGAACAAGGATGGGAGTCGGGGAGGGGCTGTCAG
GCAGAGGGCAGATGCAGCTCCTCGGGTGCCGCTGGCTGGAGTCCTTTGTCTCCATTTGCTCAGT
GCGTAATTGTCATTTTGTGGGGGACTTCCTTGGTGGGGCTTTTCCATGCTTTTCCTCTCCCTGATG
GTTACAAAGAGGAGACAGTCAGGATCTGGGAGGAGCTGGAGCCTTCCCCCTGTGCTGTTGAAC
TCTGGGAGGGGCAGCCAGAGGGGCTGAGAGTGCACCATCCTGAAAAGAGAGGGTCTTTAAGA
GATAAGATTTAGGCTAGGGAAATTCACTAGAAAGAGACAGGTGGCCCTGGGTTTTTTTTCCTGC
CATCCAAAGGGAACCAGTCTTACCCAAAGGCTTAAGGGGTCACCTTAGCCCAGTCTCAGGACA
CTCTTCTCCAATCAGATCGCTGCTTCTAGTTGCACTGCTCCTGGATTCTGTTCTTGGGGAACAGA
AGGTGGCAGGACAGGCCCCACAATCTGCAATTCCAGCTCCCTCCAGAGGAGACAGTCCCAGCG
TTTGCAAGAGAGCAGCTTGTGGCCTCCCTTAGGCAAGTTTAAAAGCCAGATGTCCTTTTTCCCAG
AATGCGGAGTGGTGTGTACTATTCATCAGCTTGGGCTGCCACAGCACCATGCCAGACTGGATGG
CTTAAACATCAGGAATTTATTTTCTGCCAGTTCTGGAGGCTGGAAAGTCCAGATCAAGGTGTTAA
GACTTGGTTTCTGGTGAGGCCTCTCTCCTTGGCTTGCAGGTGGCACCTTCTCACTATGTCCTCAC
ATGGCCTTTTCTCTGTGGAGAGGGACAGAGAGCATGAGCAGGCTCTGGTGTCTCCTCCTCTTCTT
ATAAAGACACTAATATCACCATATTAGGGCTTAAACCTATGACCTCATTTAACCTTAACCCCTTA
AAGGTCCCATCTCCAAAAACAGTCACATAGCAGGCTACTGCTTCAACATATGCATTTGGGGGAG
GGGACACCATTCAGTTCTTAACAGGGTGGTCACCGCAAACATGGAAAGTCAGAGCCTTCTCCCC
TTCAGAATTCCCGCCCCCACCCAGGGATGGGGAGGAGGAGCAGAGAGGTATGGGAAGCAGAC
ACGGAGAGTGGCAGGTACCATGCTGGGGTGGCTCAGGAGTGCTTCGGAGGACATATGGAACTG
GCAGGGCTCAGTGCAGGGAGGCGGAGGCCCTGGGAGAGCCGTGTCCTGAGAAGGGCCTGGG
CTACAACCCTGGGCAAGTTACTTCACCTCTGAGCCTCCGATGCTCTGTGAAATGGAAGGAATGT
GCTTGCCTGTCAGGTGCAGGGAGAATTCAGTGAGATTGTGTGCCTAGCACAGTGCCTGACTCCC
AGTAGGTGCTCAGCAAATGCTCCCATCCATCTGGGAGTATAGACTTAGGGTTTATCTATTTTTTTT
TTTTTTTGGCTCTCTGGACTTTAAAACTCAGCATCTTCTGAACCAGAGGCATTTCTGATTAGCCCT
TCCCTACCTATTTTCCTAGTATCACTCTTTAATCAGCTTGGGGAGGTGGCAGCATTTCATGGCCTC
CGTAGTAACTCACAATGCTTCCTGGGGTATTTAAATTCTACTCTCTCATCAGCACTGAGCACCTA
CTTTGGGCCCTTTCCCGTGCTAGCCATTTGGGGGAAATATAGGTGAAGAGTGGCTGGGGTTTGA
ACCTTTAGGATTTCACAGCCATGCTGGATGGGATAAAACCAGCTCACATGGAGAATCAGAGAAT
GGGACAGTGCCACAAAACAGTCTACTGAGGCACAAGTTCTGAGTGCCTGGGAAGTGGTAGAGA
GACCCCAGAGAGGGCTGCTGCAGTCTGGAACAGCTTCCTAGCAAGCTGTACCAAACAGGGCCT
TGAAGGATGAGAAAGATCTGGCTGAGATGATACCCGACCCTCTAGGGAAATTCTTAAAGTAACT
TCTAGGAAATGTCATTGCTCCTTAAAAAAAAAAAAAAAAAAAAAAAAAAGCAGGTTCTAATTCAAA
TTCCGCTGCACTACTGAAGTAGTGCAGGACTTATCAGAACCTTTAAAATGCTAATGGCAATTTCA
AATACTCAGGGACAGGGCATAGGATGAAGTGTTCCTTGACTTTTATGGCCGTAGAATCTTTTTCT
CATGGAACATCGTTTAGAACTGTGGGAGATGCTAGGCTGGGGTTTCTATGGGAAAAGTACAGCA
GTGATAGAAGATGGCATAATTAGGGTACACTTAGGTGCTTTGTAGCATTGGCTGAAAAGGAAGC
AGACCGGGTGGGCGTGGGGGAGTCTCAACTTGGTTCCAGGAGCCAGGCCAGCTTGATGCGTTT
TTTTTTTTTCTAATGGTGGGAGGGCAGGCCAATAGTTGGAGAAACTAGAGGCCTGACAGTCGGT
GATGGAAGAGATGTCTACTAAGCACGGTTGTCAGATCTTTTGCGGAGAGATGCGGGTGTGCGTG

TGTCTGTCAGAGTGTAACAGGATCCAGCCCTCTGGGAAGGGGCTGACCAGAACCAGGCAGGG
GGTTTCTGTCTAGGACAAGTAGGGTCCATTTTCAGGGGAGCTGAAATATTTGCAGGGGGTGCTC
TGAGACCCACAGCCAATAAATAGGGAAGCAACATGCTTTTGGGCAGGGTCCATGTGGTTAGGG
TTGATTTTGATGGGGCTGGGGGGCTTAGAGTTAGACCCCCTGCCCTGGTCAGGCCCCACTCCCT
GGCTGGTGGGGGCGGGAGGCCTCTGAAGCTGCAGCTGGAGGGGCCGGGGCAGTGTTGCATGG
TGTGATTAACCTACTAACCCAGGGCTCAGCCGGGACGCTGTGGCGTGTGACGAGGTGCCTGGC
CTTCTGCCAACCAACTAACCCACTAACCAGGCCCGAGCATGCCCTACTAACCACCCCCCCGCA
GCCAGATCCTACTAACTGTGCAGCTCATGGGCCTTTCAAGGGGCTGGGATATGGGGGCAGCCA
AGGCTCCAGAAGGCTCTGCCTATGGGCTGGGATCTTTGTGCAGACCCACGTTCCAGGACAGAG
AATGCAAATAAGACTCACCAACTCTGCCACTTCCTGTTGAGTGAGTTTGAGTAAGTCACCTCACT
TCTCTCAACCTCAGTTTCCTCAGTGGTAGAATGCGAGTTCTGATACCTTACAGGTTGCAGTGAGG
ATTCCCGTCAATATAAGGGTCTCAGCACATGGCAGACACCTAAAAAAAAAAAAAAGTATGAGGA
TGGAGGAAGGCCCCTCAAACCTGGTTGAACACGTCTGCTGCCTAGGCCCTGGCCTCTCTGGGTG
TTCTGCACACAGATCCTTCTGCCTGGGGCAAGACACTTGGCTTTGAATCCATGTCACTTCCCCAC
ATTCTTCCTCCGTCTCAGCTTTCTTTTCTGTAGGCAGCTCTTTTGTTCGATTACTAACCCGTCTAAC
CCCTGGGGTGCCTCAACTGCACCCCGATCATCCCTTAACAAACTAACGGACCTGCGTGCGTTCT
TCCTTTTCTCTTAGCGACTCCTGTGTGTGTCTGCTGAGGTGCCCTGTCCGCTGGTGCTGTGCTCTG
ACTTACTAACCCAGCCCCTACTAACCCTGTTTTCTCTTCTTACTAACCCCAGCCCTGCCGAGCTCT
GGGCTCCCCCCGGGGGCTGGTCCCCCTCCTTTTGGCAAGCAGATGACCTGGGGCTACTGGCCC
TGTAGACAGATGTCCCACTTTGCTGCCCCATATTGGCTGTAAGATCAGAGTCCACTGGGCCAGG
TCTAAGGCAGGGGATGGCCCTATTAACAAGACTCAGAGGAGGAAGAGGTGGTCCTGTGGATGT
GGGAGGCTGGACTCTGAGTATGACATCTCTCCTATGTGCAGAAGTCTGGTTGCCACTGGGAGTA
GGTGGGACCAGGGAAATCTCTGGGACGTGAGTGTGGAGGCCTGTTGGTCTAGACTCTAGACTG
TGGAGCTCTGAGCTTTTGTGTCCTCTGGAAGGAAGCTGGGGAAGAATCCTCTCCATTGTTAAGT
GACGGGGATAGAAGCTGTCCTGCACAGGAAGTCACGAGGGGGGCGTATCCCACGAGGAAGGC
AGGAGGGGGCGTGCCCCTCACCGGAAATTAGCAGAGGGGCGTGTCCCACACCGGAAGTCAGA
AAGCGGAGCCTTTCTTACACCGGAAGTCAATGAAGCGGGTCTTTCCTACGCTAAAAACCACTGA
GTGGAGTATTTAGTACACAGGAAGTCGGCCAGAGAAACATTTCTCATATTTGAAGGCCGGAAAG
AGGGACATTTCTGACACCGGAAGTCAGTGAGAGGACTCTTTCCCACACAGGAAGTCAGCTAGA
GAGCCGTCTCCCCTCTCTGGAGCCGAGAGAGGCCGGTTTCCCCCACCGTAAGTAGACGTGGGG
CCGTGACCGGAAGTCCTTGGGAAAGATCCGTCCCATTCCCGGAAGCTAGAGGGCGTTAGTTGT
CGGGTTGAAAAGGGGTGTGGGGAGGGGAAGCAGCTTTACCCCGGGCTCGGAGTTTGCAGGAG
AGAGAAGTGGGGAGCAAGAAGTGAACCTCAGGGGCTCACAGGGTTCCCGCAGATGCTCAGGC
CGGCCAGGAATGCATCTCTGGCTCTCTGTTCCCACGGACGTCACTGCCTCAGCCAGCCTCCCCC
AGAGCCCGCCAGCCGCTAAGCCGGGGCCACACCTGGGGGTGATTTCATGCCTCACCTCCAGTA
GGCACCTTGGTTTCTTTGGGCTAATCTCTGGCTCCCTTGCGCTAACTCTTGCTCTCACCCAGCTA
ATCCCTGCCTCACCCTGACTGCCCCAGGGGCTGACCACTAACAACCAACCTGGCCCTGTCTGGG
GGTTCCAGGCTCCTGGCCTGGCCCTGACCGGTTCTTAATTAACCTTTCCTTCACCTTGACTAACT
CCTGCCTTCCTGGTCTGTTCCTTTCAGCAGAAACTAATGGTTTGTGGATTTTTTTCTGACTAACAA
CAGGTCTAACATTCCTCGTTACTGTTAACAGCTTGGATGTCGGCATGGCTGGGAAGGGGCTAAC
ACAGCTTTGAACTTGGCTAACACAGGTTTGAACTTGGCTAACACAGGTTTGAACTTGACTAACAC
AGGGAAAAGCATAGCTAACAATTTTGGGCGTGGTGGCTGCTCTGAGTCAGAACAATCAGAAGT
CGGTAAAGATGGTAGTTTTCTAAAGGAGGTGCCAGGGCTCTGGTGTGGACCAGGCCTGATGGA
GCAGTGGTACCCACCAAGGTGGGGTCAGAAGTATAGCCAGTCTTGCAAGGTTTTGGCCATTGG

GCATATCTTCACTCCTCATAGTCTGCATTTGGTTTCAGTTCTTAAAAAAATATAGCCTTATAGCTA
CAGTAGTTTGCACAAGTAGATGCAGCTCTTATAAACCTTAAAATACCTGTCTGGTGTCTACAGAG
CATATGCCATTTTGTATAGAGTCACCCTTCCCCAGGCCAGGGCCTAGAGTCTTCATTTTGGGGAC
TTTGTGTTTTGGAAGTTCTAGGACATAAAGCTTTAGATCAGAGTATTCAGAAGGGTTATAACTCC
GTCAGTCATTTACATGTTAGTAGTATAAATTATCTGAGCTTCCTGTTCTAACTTTTAGCTCTTCTAG
CATAAACTCTTCTGTGCAATTTAGCTGCACCGAGGAAACGGGAGTTTTTCTGGAAGGGACTTTTG
ATCTCTTTAGACTGAGGGAACGTCCTTTGGGAGTAGAGGGGCAGGGAGCATACGCAAGGGATT
CCAGGTGCAGGTAAAAGGTGGCACTAGTTCAAGGTTTTGCTGACTCAGTCTGGTAGTCAGAGTC
TGCAGGAGAAGACAGTTCAAGGCAGGGCCTGGAGGATTGGATCAGTTTAGGGACAGGTCAAA
GGCTGGCTTACAGACCTTAGAGGCAGGTTGCTTGGGTCGTTGAATGCTAGTCTGGTGCTGAGAG
CCCTTTTCTCTGGCAACTGTGGACTCAGAGCTAACCAATTGTAGTTGGCAGTGGGGGTGAAGGG
TGATCCAGAGGCCTGAGCTGCAGAGGGCACAAGAGAGAAAAGATGTCTTAGAAAGAGCTTTGA
GAACATGCCTTGGCTGCTGGCAGGGACCTTGGATGGGGTAGTCTACACCCGGAAGTGCCTGCC
TGCCATCCTCTAGTGGCTGCCTTGCTCCATTTCACTCAAAGCAGGAAGCTCACACCTCCTATTCC
TGAAACTCCTCTTTGTTTAACTGCAAAGACTTGATGCTGCTAAGGATCTACTATGTGCCAGGCAC
TGCTCTGGGCGCTGGGACCTGCACCTGGGCTTTTTCGTCATGGTGCTTTTATAGCCTAGTGGGAG
AGTTGGTGAAGTAGATAGTGATTCAGTGAGATGGGTGTTATGATTGGTCAGGGGTCTGTGGGAG
CACCAAGGAGACAGACAAGATTGATGTGCACCTACTCTGTGCCAGGCGTGTGCCAGGCATTGG
GGATGTAGTGGTAGTTAAACACCATTTGGTCTTCAGGAGCTTTAATTCTAGTGTGTTGGGTGCAG
GGGGGTGGAATGGGGACAGAGAGACACCTAATCCACCCTGTGGTGGCTTTCTGGAGAGGGAG
GCATCTAAGCTGAGCTGTGGCTGGGTGGAGTGTGGGTGGGGATGAGTTCCGGGCAGCGAGAGT
GGTGGACACCAGTTTCTGGGGATCAGAGAGGATCCAAAGAGGTTCTGGAAGGTTCATGTGGAA
TGTAGCAAGAGATAGGAGACATGGACATGGTGCCGGGTCTGGTTGCCAAGAAGTTTAGATTTTA
TCCTTAGGCCTTGGGGAGCGACGGATATGATCTGAGAAAGGGAGTTAGTGGATTTGAGTTTTAG
GCTGGCCATTTGGCTTTTCCAGCCCAGGTGGAACTCAGAGGAGTTTGCAATGGCCTCTGGCCAC
ATTTTAGACAACTGAGCAGAACTTTTTGAAACTAGGAAGACCCTTTGGTCCATCTTTTGATAAAC
AGAATCCATACATGTCTACCCCAGTTGGAAGTATCTCTGCAATGACTGGAAAGTAAAGAGGACC
AAGGTGAAAATAAAGGCTCGGAAGGGGAGCAATCTTGAAAACATGTCATCCCATGGTGGTGGG
AAGTCCCTGGAGAAGATCAGGGGAAACACAGTCATAGGCTGCAAGTCTATAAGATAATTCCATT
GGGGAGGGAGCCCATTTGTCATGCATGGCTGCAAGGGGCAGATACAAGTGTGGAGTAAGCTTG
CAAGAGCTGATCCTGGTCCCAGAGAGGGAAAAATATGCCTTGGTGGGTAATGAACCTTTTGTTC
CCAGAGGCAGAAGGATTGGGACTAGGCCAACATAGAGATTGGCGATGGTTGTGAGATTCTAAG
AGTGTGTGTGCATCTTGACAATATTAGAGGAGGCTGAGCCCAAGCAGGCACATTCTCTTCGACC
CCTCCCTCATTCAGTCTGCTTTGGAGTCTACTGAACATCAAGCTTGCTATGAGCAGGATCTTAGA
GCTGAGGAATTGGCCTCCCAATCCGAACAGGTGTTATAATCCTTTCTTAATAGGTTGTGCTGTGG
ACCCAATGTGAGGGCTGTGCTGGTGTAAATGGTGACATGTTGAGCTGGGGGGATGCTTTCGGG
GTGGGGGGACTGGTTCCATTCCATCAAAGGCCCTCTTGAGAGTCTATCCAGGGACCCATTGTTT
TACTTTAACAGACCAGAAAAGATGTTTGTTTTCCATGTCATTACCCCCAGGGGATACCGAATGTG
TGGGTAGAAATTTCTCTGTAGATTAAAAATCAGATTTTTACATGGATTCAACAAAGGAGCGTCAC
TTGGATTTTTGTTTTCATCCATGAATGTAGCTGCTTCTGTGTAAAATGCCATTTTGCTATTAAAAAT
CAATTCACGCTGGAAAAAAAAAAAAA
ID|22804|gene_id|677766|transcript_id|NR_003023 Homo sapiens lncRNA
GTTTTAGGGAGGGAGAGCGGCCTGGGTCCTGGGTGTTGTGTGCGGAGCTGTGGCGTCGCG
TGTGAGGCGCGTGCAGGGTGAGTGTGAGTGGACGCGTGAGTGTGTGAGTGTGCGCGCTTGGAG

CGTGTTAGGCGAGTGCGTGCGCCCACCCCTGCGCCCCTCCTCCCGCTTACACTTTGATCTTATTT
GATCGGATCGTGACCCCAGCCCCGCCGGGCCGACCCGAAATGAAAAGCTCTCCTCCTGCGAAG
CCCCCTCGGGGCGCTGTGCAGCGAGGCCCCTTAGGCGGCGGCCACGCTGTGGTCCCCGAGGT
CCCGGAGCTGGCCCTGCGGGGCCCGGCGCTCAGAAGTGATGAATTGATCAGATAGACGAGGC
CGGGCTTGTCCCCGGCCACTGATTATCGAGGCGATTCTGATCTGG
ID|25794|gene_id|79940|transcript_id|NR_026807 Homo sapiens lncRNA
ATGCGAGGCTGGGGCCGGTTGCCTACCGGCCGCTTCTCGCCGAGGCAGTCCAGACTTTTC
CCCCGGCGGTGCCCGCTCCAAGACAGCATCTGTCAACGCTCCTCTTCTCCCCTCCTCCTCCTGC
CGGGCCGGGCTCCGCCGGCTGCGGCCGAGAGGACGCGGACCCGGCGCGGTGAGCCCATCA
GCTGTCAGGCGAGCGGCGAAGCGGCTGGAGGGCGGCGAGAGACACACAAAGAACGCGGTGG
GCGGCGGCGGCGAAAGGGGACGGCAACTCCTCCCCGCGCCCGCCGGTGCCACCGCCGGCCGT
GCTTGTTCCGAGGCCGCGCAGACAATGCGGCCGGGCTCGTCCCCGCGTGCCCCAGAGTGCGG
AGCGCCCGCGCTCCCCCGACCCCAACTTGACCGTCTCCCGGCTCGCCCAGCCCCCTCCCGGGG
TAGGGGCGCCCCCTCCCTCCGGTGGCCGGCGAAGGAAGTCGGTCCGCGGCCGCAGATCCCGG
CAACTTGCGAACCGGGAAAAGTTTGCGGCGCCTCCGCGGGGCGGCGCGACGCGGCCCGCCCC
TCGCGTCCGCGGTCATCGCGGGTGACTTTCTCGACTCGTCGTCAGCCGGGGCCGCAGCGCGGC
CGGTGGGGACTGCGGGGCGGGCCGGAGTCCGTCCGAGGGCTCCCGCACCTCGGGCTGCGGAT
TTCAGGTACTCCACTGGGCATTTTCTCTTCATGGAGCTTATAGCAACAAAAGTGTTTTACAAAAC
ACCAGACATGTTGGCATCATCTCTTAGTTGGGCCATGTGGCCAGGGAACCCTTCATGAAACCAC
CTCTGCAGTCCTGGAGAGTTGCTGTAGAAGAAGGAAAAGAGAGGAAGACAACACAACACAGG
AGGGGATAAGTACCTCTGGACACAATGGAAGTAGTAGTTGTTGAGCAGAGGGAAAGGGATAGG
AGAAGGATGTGGACTGCAGTGCTGCAGTGCCAGGTAATAACTTTGATCATGTACCTCCTTGGCT
CAGGTGCTGTCTCTTCTACTCTCCTCCTTACTTGTACTACCTTATTTAGCACTTAATTATATACTTT
CTTGACTCACTTTGAGACACACTGATTATGTGGTCTGCCTCAAAGACAGAATCCTAGTTTTGGAT
TTTAGCATTACTGATCTATAGCCTTGCAGGGGCTTAAGGATGGCAGCTGTCTCTCTCCCTTGGGA
TCTAGCTTTTGCAAATATTGAAGGGAAGAGATTTCTTTGATCTTATAGGAATAGATCCACAGTTC
CTCTCCAGATTTGCTTTTATAGAGAAGTGTGTGTCACCAAAACAATGGAGATACGGTCAGAGAG
GCCCCTACCCCCAGAATTTTAATATAATTTTACTTTCTGGGGCATTTATTCTTGACAAATACTTTCT
CTCCTATCAGCTCACTTGTTCCTCACAAAAACCCTATAAGATATTAATATTATTAAACAGACCCCT
ATGAAGCACCTCAAGTCTTTCTCTCTTCCCACTTCCATTCTTGCACACCTTTTGCTAGACTAGTCT
CTGCGTTCATCACATCAGGTTTCTAAAATAAAAGTCCCCTAATAACTTTCTATTTGTTCTCTCATC
ATATTGAACCTTTTTTGCCTGGCTTATAAGGTGGCCTTAAAACCCCATTTTCCTCTAAACATTGTC
TCTGTAGTTCAGGAATATGGCAGGCTCAACACTATGCTTGAGCCCATATTGCTTCCCTCATTAGT
GATGCCTCCTCTCAGACCACCCAGATTCTACTCATTCTTCAATTTCCAGCTTCTCCTTGGAGCCTT
CCCAGATCATTAGCTTCTTAAAAATCCCAGCTTTCTCTGAATTCTGATGGTAGCCCCCGTTATTTG
GACAATTATGTCCTGGTCTTAAAGTCCTAATGGGAAAAAAAAGTACATGATGGACTTCATGTAG
AGAAGAAAGCACTGGACTGAGTTTTAAGACAGCTGCTAAGGAGCTGTGTGAGCCAAACCAGGT
TACTTTACCTCTCTGGATCTATATTTGCTGATCTGTTAAGTGGAGACAATTGGGTCAGATAATCTT
GGTGGTCTTTGCTTATTCTGAGGTTCTTGGACAACTTGGCCTAGTTCACGTGGGATGGGATTGAA
GATCTTACTGAGTTCTTGGATGGAAATGTCAGTAATGGGCACTTAGTGCCTTCAGGACTTGTTCT
ATACTATCCCAGAAATTTCTGATGCTAAACTTGGCAATTTTTAAATGTCATATTCCTTTTCTCCAA
ACTCTTTAACCACAAGAATTCAGGGAAGGTTATGGGGGGTGACTTAGTTATCTGGATAATACAT
ATTGTGGACAATATAGCATTATCCTTGGGAATGATTTCATATTGTTGAATAAATAGGATCAACTTC
TTGATAAACCTTAGTCCCAAGCATTTCATCTTCATTGGATAGTGTTACATAGTAATATATTTATGTT

TTCTTTTAATCATTTCATAACTTGGAAAATACTAACATAGTCAAAACTCTAGGGTAGGTGATACAT
GAGTTTCTGTAGTAATCTGGTTGGAGACATGTTGTAATTCTGTATATATATGTACATTTATCCCAT
GCATGTTATGCCTAAACTAAGACGGATACCCCTGAATTAAGAGGTGCTGTTATACATTGACCAG
GCTTAAGAATATCTCTTTAAAGTGTGTCGACATTTAATTGACCTTTGGAAGTTCATTCTGTTAATC
ATACTCAAAGTGCTAAAGCTATGGTTGACTGCTCTGGTGTTTTTATATTCATTCGTGCTTTAGCAT
ATAAATTCTTCAGCATAATTGCTACTTATTTAGCAAGAGTTTCCTTTATTTGAAAATGTGAGTTGT
GCTTGTATTTTTGTGTCTTTCTTTCTTTCTTTCTTTTTTTAAACTTTGCTTCAGGCTGGGTAGTGGTA
GAGGTTTGAATTAAAATGTTTTCCTGTCAGTAGTTGTATGAGGTGTGGCTTCTTAATCTAGAAGTA
ATGGAAATTTCTTGCTTAAATGAAGTGCACTTTTCTTCCTGCAGAAGTTAGAACTCTTTTAGACAA
GCTTAACACTTCAAAACTGTATATCTTTAACAGAAACTTTTGGAAATTTTTAAATTCCATTTGTGTA
GATTAAAAATATCAATGCTAATACTTTCTATTAAATAAGGATTTATCTATTTTCATAAAACCTCTA
ATTATATTCCTTGAATTGCTATGAGTAGAATTTTGTTGTAAACACAATATTTTTAAAAAGTCAAAT
ATATTTTAAAAATCTTAACATTATATTACTATAACTTAGAGAACAGGTGTTTACATGTTTAAAATTT
TCTAGGCCTTAAATTTGTGAAATCAGTCTTTCAGAAGTTATTTTTAATATAGTTATCTGTGTTACAG
GTTTATAAGTTGACATTTCAATTTAAAATTTTTTCAATTTATATTTCAATGACCAGGAAAAAAATAGT
TAAACTTATTCCTAAATTTTAATAGAAAAATAAAAAACCACATAGTTGTTAATTTCTGGTAATTTA
TCTTGAGACTCCAGGCATGTTTTTAGAACATTTTTACCTTAAAAGCAATTTTGAAATAGGGAAATT
AAAGTTTAAAGCAGAGTTTTCACTTTAATGAGGTAATTTTATACTTCCCAGATAGGTAGGCCCAT
ATTCAAGAACACTCTGATCTCTGTTTATAGTAAGTTTTGATGCCATTATATCATTTACTTATTCACG
CATTATAAGCATAGTAGATAGCCCCATTGTCACGAAGCTGGCATTATTTTCTTGTCTGTCATTCAA
TATTGTCCCCTTTTTTTTTTCTGTTTTCATCATAGCCGTCCTTTTCATTTTATTGTAGCCCTGACTCG
AAATGAAAGTAAGTTTGAACTCTTAAAAAAGAGTTCCTCAAACTCTTTTTTAAGAATATACAGTG
AAATGTCTTAAATAATATTATCTAAATAATACCTAAAGAATGCAAATGACTTGCAATGCTGAATA
GTCAGACACAGGATGTTCCATGCTAAGGGTAGACTCATATTTCCACTTTATAAAATGTGGTTTCA
GAAAACAAATAGATGTGTTTCTTCCTTAATAATTAGTGTTTCAGGACCAAAGTTGACTAAATATG
GAAGTTTAAGATAAAACCCGGTTTCTTAGGGAATCTTTCTCAGTCCTTAGTAATTCTGTAGAATTG
ATGATAGAGTGGCAACTTCAATACACTCAGTATTCTTTTCTGTTTGAAGTCTGTTTTGCCATTGGA
AAAAACTTATTGTTGTTTGAATTTGGGTATATGTATTCACAGATGGTTTACTTTATTTTTATTTTGTT
TAAACAAATACTTTCATGTCCCAAACACTGAGACATGTTTCATGTCCTAAACACTTTATAAATATT
AGAATATTTAATCCCCTTAACTCTGTAGTTATAATTATCTTCATTTTACAATTGAGAAAACTAAGA
TACGACGTTTTATTTAATGGTAACGCCTTAAGTAAGGGGAGAGTGATAAGTGTAAGCAGAGTGG
AAAACTTAGTAGTGCTAAGGAGCAACAGAAGTATGTGCAAGAGTTTGGGAAGATGTGTCTGCTT
GGAAAGGATCAGGGACTCAGCTGGGATCAGTCTTAAAGAGCAACATGTCCTCATTCCTCAATTT
TAAGCCTTTGGGCTCTAGAAGTAAAGGGCAAACTTAGAGGAGGCACTCCTCAGGTTTCAGCTGC
CTGTTCCGGCCTGGTGAGGTGGCTGTACTGGCTCATGTGTTTCTCATAGGGCTACATGAGCCTTT
ATGTCTTACCTCCTCCTCATTTCTTGGCTGACTCCTGCTTTTTTGGAGGGTTTAACTTCTCTCTCAG
CTAGAAGGTTAGGCATTGCAAATACCAGTGGATAATTTTTTTCTTAGCTTTAACCCCAGCCCATTT
CAACCCCCTCTTTGCCCTTTGTATATTCTTTTGAAAATATGATCCAGTAGTGTTTATGAATGTGTGT
TGTGTAAAATTTAGAGATTGATGTTAAACAACAGAATTAAAGGACAAAGCTGTCTTTTTTGTTGG
AATTGGGGATGGGAGAGCAGCTCAAAGTGGGAAATATGGAGAAAGGAGAGACATTGTGGAAA
AGAAGAGATAAATTGAGGGGAAAAAAAGAGGATGAAAAAATGAAGAGAGAGAATTACTGATG
TGCTTCACACCCACACCTCCTCTCCAACTTCACAGTAACAGAGGCAATGTCACTATCTTTTACCT
GACAAGTGCTTTACCAGGGAGTTGTGCCTGGCATCGTGGGAGTGGAAAATTTTTCCCTTTTGCAT
TTTGAGACTTGGGTGTGCTGCTTTAAGATGCCACTGGTATCTAAGTAATGTTTGTTTATACTGGAT

ATTTTTCTCTGTAGCCTTTGTGGATTGGACTTATAATATAAACAATATTGTTAGCAGAACTGCAGG
TACTCTATTGCTATGTTTATTTTATTAGTTAAAAAACTACCAGAGCCCTAGGACTTCTGAGCACAT
TTAGAAAATACCAGAGGCAATTTTAAGACTAGCATTAGAAAGAAAGAAAAGAATAAGAATAAA
ACTAAGACTTGCATACCTTAGAATCCTAACCAATGTGGTGATTTTTAAAAATTAATGTTTCAGAG
ACTCTAAATTAGGGTAGTTGGTTTCATCAATTACATTTTTTAAGTTAATATAAAGTGCTTTTGTATG
TAAATTCTCTTAATAGATAATTTAGGTAAAATGAAAGTGTGGCATTTTTTGTTCCATAGAATGTAA
AACTAACGTCAGAATAAGAATTCTTTTGGGAATTTAGGATTTTTTTGATGCTTCTCAAATCATCTG
TAATGACTTTTATTTATAGAAAAGGTAGATTATTCATTAGTTTTTATGAAAAACACTCAGTTTAAAT
AGCCGGGTATGGTGGCGCATGGCTGTAGTCCCAGCTACTCAGGAGGCTGGGCACGAGAATTGC
TTGAACCTGTGAGGCAGAGGTTGCAGTGAGCCAAGATCGCACCACTGCACTCCAGCCTGGTCC
AGAGTGAGATTCCGTCTCGAACAAAACAAAACAAAAATATTGAGCTAATCTAAATGGTAAAAAT
CTAGATTTGAAGAAATTTATTTTAAAATAGTTGTTCATTTGTCAAATAGAAAAGTATGATCTTAGC
AGGTTTGGTTAGATCCCTATCTGAAACAAAGTAAACTAAAGATGTTTTTCTTGATCAATGCATAG
CAATAACTGCTGTTCAAACATGTGAGGAATCACCAGTAAATTGGGGGATAGAAAGCAGGAAAA
GGAGCTTTTTTCTCTTATTTTTTTTCATTTATCAGTTTTTCCCATTTGTCCATCCTTTCCTATAATCAT
GGAAATTTTTATTTTTTACACTCCTCTCCCCATTTTTTTATCCTAGATTGCCACCACACCAAGGTTT
TCTAGAGTTAGGCAGAAAGAGAACCTGGGCCTAGATGCTAATGGAAACTATCCCATGCTTTCTC
AGACTCTCTTTCCTCCCCAGAGACAAGAGGAGCAAAACAGGAGTTGATGGCTCATTGCTGTTGC
TTCTGTGGCTGCCCTGTTTGGTTGACCTGAGCTTTTTTTAAAAACCTCCACTCTTGACGCTTAACT
CTATGAATAAAATTAGGGCCAGTCACACTGTATTTTCATCATCAGTTATCCCTAGTGATTCAAATT
TAGCACAAACCTGCTGTGAGTACATATTAATTAGGTGAATACAGTAGAATGCAATCATAACATG
AGTCTTTTAATAACTATTCTTTGTTTGATTTTTATTTTAGAAGTTTGAAGTTATTTTGTTGAAAATCT
AGAAAATCTTTATGTGAGCAACTGAGGTTCGGGTAGCCCACAACAGTTTAAATCAAACATTAAC
TTGCAGTAAAACACTACTGAAAGTGTCTTTGATTTTGAGGTGCAGAGAGCTGTTTCAAGTTAAGT
TGCTATATTAGTCTGTTCTCATGCTGCTAATAAAGACATACCTGCAACCGGGCAATTTATTAAAA
AAAAAAAGAGGTTTAATTGACTGACAGTTCCACATGGCTGGGGAGGCCTCACCGTCATGGTGA
AAGAGCAAGGGATGTCTTACATGGTGGCAGGCAAGAAGTTTGTGCAGAGGAACTCCTGTTTATA
AAACCACCAGATCTCATGAGACTTACTCACTACCAGGAGAACAGGATGCGGGAAACCACCTCG
GTGATTCAGTTATCTGCACCTGGCCCCACCCTTGACACATGTTGATTATTACAATTCAAGGTGAG
ATTTGGGTAGGTACACAGCCAAACCATATCAGCTGCCCATTCTCCTGTGCAGTGACATAAAGGT
CTGATAACCTGATTTAAAGCTTGGACTCTAAGATTTTTTTTTTTCTTTTTTTGAGTCAGGTTATCTCT
GTCGCTCAAGCTGGAGTTCAGTGGCATGAACAGGGCTCATTGCAGCCTCAACCTTCCAGGTTCA
AGTGATTCTCTTGCCTCAACCTCCTGAGTATCTGGGGCTACAGGCATGTGCCACCGTGCCAGGC
TAATATTTTTATTTTTATTTTTGTAAAGATGGGGTCTCACTTCTGGGCTCAAGCAATCTTTCCTCAG
CCTCCCAAAGTGCTGGGATTATAGGTGCGAGCCACTGCACCCTGCTGTAAAGAATTTCATATCC
CTGTTTTCGGTGTGCTGTGTCACTGTTTGCCATGGGTAAAGGTATATGAATCTCTTTTATTTTGCT
GCTTGCAACCCAATTCCCATGCCAAACCCAATTTGTATCCAAACAAACAATATTGGAGACAAGT
TCTTCTATAGAAAGCTTCATAAGCCAGTTACTGACTACTGTGTCTGTAAATATACTCAAACTATTT
TGTAGTCTTGCTATTTTAAAAAATGTGTGGGTGTGGGAAGTGATGTTGAATGAATTCAGGGTAAT
TGGTGGTTCTTTGTATTAGGAAAACTCACTAGTTAAGGGAAGTCTGTTTTCATGATTCATGTATGT
TTAACTTAAAAAAAAAATTCTTCCATCCTTCTCTCCAGTTTCCACTCTACCTCATAAATTCAAGTAA
TGAAGGAATTTAAATAATAGAATTTTATAACTGAAAGGATTTTGGAGAGAATCTATTAACCCTCC
TACTACAGTTGAGGAAATAGATGTCCAGTGAGGTCAAATGAGATCATGTAGCTAGTTTAAAAAC
CCAAATAACATTTTAGTAGTTTGTTTCCATTAGTAAACACTGTTCTGTTTGATTTGCCCTAACATGT

TCATAATCAGAGTCATAATAAGGAAAAATCACTAATATGTGTGTGGTACTTTCTTGTGCATACCT
CAGTCACTTCTCAAAAGAGGTCTGTAAGGTGTCGGTATCCTGGTTTCCATTCAGGGAGAGAACA
GTCTATGAGCCTAAGAGACCTGTCTGTGCCAGTCACTATCAGAACAGTCTAATGCTATGTGTCTT
CCTACCTTGGCAAAAGCATTGGCTGGTCTCTGGACAGTTCTCATAGACCACAGGATTGGGCAGG
GGGGAGCCACATCTTCATGTTAGGGCTCTAACCTGTACCAATTATTAACTGTTGTCTCTATTTCAT
CATTTCTTAAAGGGAAAAAAAAGCACTTTTTAAAGAGCATGAGATTTTAAATTTTTCAAAAGAAG
GATGCTGGATCATTATAAGTAAGGCATGTTTTAAAGATCTTCTTATCAACAGCTTAAAAATTAGC
CAGTATATACTGAACATCCACTGTATAAAAAGCACATTATGGGGTGCTCCAGTAGGTACAGCAG
TGATCGAGACAGAATCATAGCCTTCAGGGAAGAGGTATATGCAATGTTCTCTCCTTCCAAAGAT
TACCTAGATAGAGAACTGTTCATAAGAAATATCTTGGTACTGTCTACATTACTCTTGAGATATAA
AATATATATTATTTCTTACGTCTGAAATGAGTCAGTTGTTTCTAAAACAATTTGTATTAAAGTATTC
ACATTAAAAATTTGTAAAATACAGAAAAGTAGAGAGAAAAAAATCATGAATTTTTAACATGGTAAT
GATTATATCAGTTTCGTTCTTGGCGTTTCTTTAACTTGATGGGATAGCTAATGTTTTTTGTCATTGC
ATAGCTTTGTAAACTTGTCCTAAGGATTATATAATTTCAGCATGAGTTAGTTTCTAAGTATGTCAG
AAATCTAGCTTTTTTGTGGTAATTGTTGTTTTACACAAAATGATTAATAAAAGACTCTAAACTAAC
CTTGGCTTTAGAAATGCTTTTTAAATTTTAAAATAGTGAATGAGTAGCAGATTACTTTCTAGTATTT
CTTTAAGGTCACCAAAATCACTTAACTTGCTTAAAATATTGATGGAAATATCAACTACGTACAGT
TGACCTAAATTAGAAATGGGTGTTTTTTAAATAGTGAAATTTATAAATATAATATGTATATTTTAA
AATACAGTTTATATTATAGAGTTATGTTCCTCTGTAATAATTTCTCTTTACACTGAATCATGTTCAG
TTTTTATTATGCAGATAATTTAATCTTACAGCTTTGATCTTTTTTAAAAAATAAAACAGCCATTTGG
AGTCTAAA
ID|25795|gene_id|79940|transcript_id|NR_121612 Homo sapiens lncRNA
ATGCGAGGCTGGGGCCGGTTGCCTACCGGCCGCTTCTCGCCGAGGCAGTCCAGACTTTTC
CCCCGGCGGTGCCCGCTCCAAGACAGCATCTGTCAACGCTCCTCTTCTCCCCTCCTCCTCCTGC
CGGGCCGGGCTCCGCCGGCTGCGGCCGAGAGGACGCGGGACCCGGCGCGGTGAGCCCATCA
GCTGTCAGGCGAGCGGCGAAGCGGCTGGAGGGCGGCGAGAGACACACAAAGAACGCGGTGG
GCGGCGGCGGCGAAAGGGGACGGCAACTCCTCCCCGCGCCCGCCGGTGCCACCGCCGGCCGT
GCTTGTTCCGAGGCCGCGCAGACAATGCGGCCGGGCTCGTCCCCGCGTGCCCCAGAGTGCGG
AGCGCCCGCGCTCCCCCGACCCCAACTTGACCGTCTCCCGGCTCGCCCAGCCCCCTCCCGGGG
TAGGGGCGCCCCCTCCCTCCGGTGGCCGGCGAAGGAAGTCGGTCCGCGGCCGCAGATCCCGG
CAACTTGCGAACCGGGAAAAGTTTGCGGCGCCTCCGCGGGGCGGCGCGACGCGGCCCGCCCC
TCGCGTCCGCGGTCATCGCGGGTGACTTTCTCGACTCGTCGTCAGCCGGGGCCGCAGCGCGGC
CGGTGGGGACTGCGGGGCGGGCCGGAGTCCGTCCGAGGGCTCCCGCACCTCGGGCTGCGGAT
TTCAGGTACTCCACTGGGCATTTTCTCTTCATGGAGCTTATAGCAACAAAAGTGTTTTACAAAAG
AACATTAAAATCTCCCCAGATGCTCACTGCTCTGATCATTTCCCCTACAGCACCAGACATGTTGG
CATCATCTCTTAGTTGGGCCATGTGGCCAGGGAACCCTTCATGAAACCACCTCTGCAGTCCTGG
AGAGTTGCTGTAGAAGAAGGAAAAGAGAGGAAGACAACACAACACAGGAGGGGATAAGTACC
TCTGGACACAATGGAAGTAGTAGTTGTTGAGCAGAGGGAAAGGGATAGGAGAAGGATGTGGA
CTGCAGTGCTGCAGTGCCAGGTAATAACTTTGATCATGTACCTCCTTGGCTCAGGTGCTGTCTCT
TCTACTCTCCTCCTTACTTGTACTACCTTATTTAGCACTTAATTATATACTTTCTTGACTCACTTTGA
GACACACTGATTATGTGGTCTGCCTCAAAGACAGAATCCTAGTTTTGGATTTTAGCATTACTGAT
CTATAGCCTTGCAGGGGCTTAAGGATGGCAGCTGTCTCTCTCCCTTGGGATCTAGCTTTTGCAAA
TATTGAAGGGAAGAGATTTCTTTGATCTTATAGGAATAGATCCACAGTTCCTCTCCAGATTTGCTT
TTATAGAGAAGTGTGTGTCACCAAAACAATGGAGATACGGTCAGAGAGGCCCCTACCCCCAGA

ATTTTAATATAATTTTACTTTCTGGGGCATTTATTCTTGACAAATACTTTCTCTCCTATCAGCTCAC
TTGTTCCTCACAAAAACCCTATAAGATATTAATATTATTAAACAGACCCCTATGAAGCACCTCAA
GTCTTTCTCTCTTCCCACTTCCATTCTTGCACACCTTTTGCTAGACTAGTCTCTGCGTTCATCACAT
CAGGTTTCTAAAATAAAAGTCCCCTAATAACTTTCTATTTGTTCTCTCATCATATTGAACCTTTTTT
GCCTGGCTTATAAGGTGGCCTTAAAACCCCATTTTCCTCTAAACATTGTCTCTGTAGTTCAGGAA
TATGGCAGGCTCAACACTATGCTTGAGCCCATATTGCTTCCCTCATTAGTGATGCCTCCTCTCAG
ACCACCCAGATTCTACTCATTCTTCAATTTCCAGCTTCTCCTTGGAGCCTTCCCAGATCATTAGCT
TCTTAAAAATCCCAGCTTTCTCTGAATTCTGATGGTAGCCCCCGTTATTTGGACAATTATGTCCTG
GTCTTAAAGTCCTAATGGGAAAAAAAAAGTACATGATGGACTTCATGTAGAGAAGAAAGCACTG
GACTGAGTTTTAAGACAGCTGCTAAGGAGCTGTGTGAGCCAAACCAGGTTACTTTACCTCTCTG
GATCTATATTTGCTGATCTGTTAAGTGGAGACAATTGGGTCAGATAATCTTGGTGGTCTTTGCTTA
TTCTGAGGTTCTTGGACAACTTGGCCTAGTTCACGTGGGATGGGATTGAAGATCTTACTGAGTTC
TTGGATGGAAATGTCAGTAATGGGCACTTAGTGCCTTCAGGACTTGTTCTATACTATCCCAGAAA
TTTCTGATGCTAAACTTGGCAATTTTTAAATGTCATATTCCTTTTCTCCAAACTCTTTAACCACAAG
AATTCAGGGAAGGTTATGGGGGGTGACTTAGTTATCTGGATAATACATATTGTGGACAATATAG
CATTATCCTTGGGAATGATTTCATATTGTTGAATAAATAGGATCAACTTCTTGATAAACCTTAGTC
CCAAGCATTTCATCTTCATTGGATAGTGTTACATAGTAATATATTTATGTTTTCTTTTAATCATTTC
ATAACTTGGAAAATACTAACATAGTCAAAACTCTAGGGTAGGTGATACATGAGTTTCTGTAGTAA
TCTGGTTGGAGACATGTTGTAATTCTGTATATATATGTACATTTATCCCATGCATGTTATGCCTAA
ACTAAGACGGATACCCCTGAATTAAGAGGTGCTGTTATACATTGACCAGGCTTAAGAATATCTCT
TTAAAGTGTGTCGACATTTAATTGACCTTTGGAAGTTCATTCTGTTAATCATACTCAAAGTGCTAA
AGCTATGGTTGACTGCTCTGGTGTTTTTATATTCATTCGTGCTTTAGCATATAAATTCTTCAGCATA
ATTGCTACTTATTTAGCAAGAGTTTCCTTTATTTGAAAATGTGAGTTGTGCTTGTATTTTTGTGTCT
TTCTTTCTTTCTTTCTTTTTTTAAACTTTGCTTCAGGCTGGGTAGTGGTAGAGGTTTGAATTAAAAT
GTTTTCCTGTCAGTAGTTGTATGAGGTGTGGCTTCTTAATCTAGAAGTAATGGAAATTTCTTGCTT
AAATGAAGTGCACTTTTCTTCCTGCAGAAGTTAGAACTCTTTTAGACAAGCTTAACACTTCAAAA
CTGTATATCTTTAACAGAAACTTTTGGAAATTTTTAAATTCCATTTGTGTAGATTAAAAATATCAAT
GCTAATACTTTCTATTAAATAAGGATTTATCTATTTTCATAAAACCTCTAATTATATTCCTTGAATT
GCTATGAGTAGAATTTTGTTGTAAACACAATATTTTTAAAAAGTCAAATATATTTTAAAAATCTTA
ACATTATATTACTATAACTTAGAGAACAGGTGTTTACATGTTTAAAATTTTCTAGGCCTTAAATTT
GTGAAATCAGTCTTTCAGAAGTTATTTTTAATATAGTTATCTGTGTTACAGGTTTATAAGTTGACA
TTTCAATTTAAAATTTTTTCAATTTATATTTCAATGACCAGGAAAAAATAGTTAAACTTATTCCTAA
ATTTTAATAGAAAAATAAAAAACCACATAGTTGTTAATTTCTGGTAATTTATCTTGAGACTCCAGG
CATGTTTTTAGAACATTTTTACCTTAAAAGCAATTTTGAAATAGGGAAATTAAAGTTTAAAGCAG
AGTTTTCACTTTAATGAGGTAATTTTATACTTCCCAGATAGGTAGGCCCATATTCAAGAACACTCT
GATCTCTGTTTATAGTAAGTTTTGATGCCATTATATCATTTACTTATTCACGCATTATAAGCATAGT
AGATAGCCCCATTGTCACGAAGCTGGCATTATTTTCTTGTCTGTCATTCAATATTGTCCCCTTTTTT
TTTTCTGTTTTCATCATAGCCGTCCTTTTCATTTTATTGTAGCCCTGACTCGAAATGAAAGTAAGTT
TGAACTCTTAAAAAAGAGTTCCTCAAACTCTTTTTTAAGAATATACAGTGAAATGTCTTAAATAAT
ATTATCTAAATAATACCTAAAGAATGCAAATGACTTGCAATGCTGAATAGTCAGACACAGGATG
TTCCATGCTAAGGGTAGACTCATATTTCCACTTTATAAAATGTGGTTTCAGAAAACAAATAGATG
TGTTTCTTCCTTAATAATTAGTGTTTCAGGACCAAAGTTGACTAAATATGGAAGTTTAAGATAAAA
CCCGGTTTCTTAGGGAATCTTTCTCAGTCCTTAGTAATTCTGTAGAATTGATGATAGAGTGGCAA
CTTCAATACACTCAGTATTCTTTTCTGTTTGAAGTCTGTTTTGCCATTGGAAAAAACTTATTGTTGT

TTGAATTTGGGTATATGTATTCACAGATGGTTTACTTTATTTTTATTTTGTTTAAACAAATACTTTCA
TGTCCCAAACACTGAGACATGTTTCATGTCCTAAACACTTTATAAATATTAGAATATTTAATCCCC
TTAACTCTGTAGTTATAATTATCTTCATTTTACAATTGAGAAAACTAAGATACGACGTTTTATTTAA
TGGTAACGCCTTAAGTAAGGGGAGAGTGATAAGTGTAAGCAGAGTGGAAAACTTAGTAGTGCT
AAGGAGCAACAGAAGTATGTGCAAGAGTTTGGGAAGATGTGTCTGCTTGGAAAGGATCAGGGA
CTCAGCTGGGATCAGTCTTAAAGAGCAACATGTCCTCATTCCTCAATTTTAAGCCTTTGGGCTCT
AGAAGTAAAGGGCAAACTTAGAGGAGGCACTCCTCAGGTTTCAGCTGCCTGTTCCGGCCTGGT
GAGGTGGCTGTACTGGCTCATGTGTTTCTCATAGGGCTACATGAGCCTTTATGTCTTACCTCCTC
CTCATTTCTTGGCTGACTCCTGCTTTTTTGGAGGGTTTAACTTCTCTCTCAGCTAGAAGGTTAGGC
ATTGCAAATACCAGTGGATAATTTTTTTCTTAGCTTTAACCCCAGCCCATTTCAACCCCCTCTTTG
CCCTTTGTATATTCTTTTGAAAATATGATCCAGTAGTGTTTATGAATGTGTGTTGTGTAAAATTTAG
AGATTGATGTTAAACAACAGAATTAAAGGACAAAGCTGTCTTTTTTGTTGGAATTGGGGATGGG
AGAGCAGCTCAAAGTGGGAAATATGGAGAAAGGAGAGACATTGTGGAAAAGAAGAGATAAAT
TGAGGGGAAAAAAAGAGGATGAAAAAATGAAGAGAGAGAATTACTGATGTGCTTCACACCCAC
ACCTCCTCTCCAACTTCACAGTAACAGAGGCAATGTCACTATCTTTTACCTGACAAGTGCTTTAC
CAGGGAGTTGTGCCTGGCATCGTGGGAGTGGAAAATTTTTCCCTTTTGCATTTTGAGACTTGGGT
GTGCTGCTTTAAGATGCCACTGGTATCTAAGTAATGTTTGTTTATACTGGATATTTTTCTCTGTAG
CCTTTGTGGATTGGACTTATAATATAAACAATATTGTTAGCAGAACTGCAGGTACTCTATTGCTAT
GTTTATTTTATTAGTTAAAAAACTACCAGAGCCCTAGGACTTCTGAGCACATTTAGAAAATACCA
GAGGCAATTTTAAGACTAGCATTAGAAAGAAAGAAAAGAATAAGAATAAAACTAAGACTTGCA
TACCTTAGAATCCTAACCAATGTGGTGATTTTTAAAAATTAATGTTTCAGAGACTCTAAATTAGG
GTAGTTGGTTTCATCAATTACATTTTTTAAGTTAATATAAAGTGCTTTTGTATGTAAATTCTCTTAA
TAGATAATTTAGGTAAAATGAAAGTGTGGCATTTTTTGTTCCATAGAATGTAAAACTAACGTCAG
AATAAGAATTCTTTTGGGAATTTAGGATTTTTTTGATGCTTCTCAAATCATCTGTAATGACTTTTAT
TTATAGAAAAGGTAGATTATTCATTAGTTTTTATGAAAAACACTCAGTTTAAATAGCCGGGTATG
GTGGCGCATGGCTGTAGTCCCAGCTACTCAGGAGGCTGGGCACGAGAATTGCTTGAACCTGTG
AGGCAGAGGTTGCAGTGAGCCAAGATCGCACCACTGCACTCCAGCCTGGTCCAGAGTGAGATT
CCGTCTCGAACAAAACAAAACAAAAATATTGAGCTAATCTAAATGGTAAAAATCTAGATTTGAA
GAAATTTATTTTAAAATAGTTGTTCATTTGTCAAATAGAAAAGTATGATCTTAGCAGGTTTGGTTA
GATCCCTATCTGAAACAAAGTAAACTAAAGATGTTTTTCTTGATCAATGCATAGCAATAACTGCT
GTTCAAACATGTGAGGAATCACCAGTAAATTGGGGGATAGAAAGCAGGAAAAGGAGCTTTTTTC
TCTTATTTTTTTTCATTTATCAGTTTTTCCCATTTGTCCATCCTTTCCTATAATCATGGAAATTTTTAT
TTTTTACACTCCTCTCCCCATTTTTTTATCCTAGATTGCCACCACACCAAGGTTTTCTAGAGTTAG
GCAGAAAGAGAACCTGGGCCTAGATGCTAATGGAAACTATCCCATGCTTTCTCAGACTCTCTTT
CCTCCCCAGAGACAAGAGGAGCAAAACAGGAGTTGATGGCTCATTGCTGTTGCTTCTGTGGCTG
CCCTGTTTGGTTGACCTGAGCTTTTTTTAAAAACCTCCACTCTTGACGCTTAACTCTATGAATAAA
ATTAGGGCCAGTCACACTGTATTTTCATCATCAGTTATCCCTAGTGATTCAAATTTAGCACAAAC
CTGCTGTGAGTACATATTAATTAGGTGAATACAGTAGAATGCAATCATAACATGAGTCTTTTAAT
AACTATTCTTTGTTTGATTTTTATTTTAGAAGTTTGAAGTTATTTTGTTGAAAATCTAGAAAATCTTT
ATGTGAGCAACTGAGGTTCGGGTAGCCCACAACAGTTTAAATCAAACATTAACTTGCAGTAAAA
CACTACTGAAAGTGTCTTTGATTTTGAGGTGCAGAGAGCTGTTTCAAGTTAAGTTGCTATATTAGT
CTGTTCTCATGCTGCTAATAAAGACATACCTGCAACCGGGCAATTTATTAAAAAAAAAAAAGAGG
TTTAATTGACTGACAGTTCCACATGGCTGGGGAGGCCTCACCGTCATGGTGAAAGAGCAAGGG
ATGTCTTACATGGTGGCAGGCAAGAAGTTTGTGCAGAGGAACTCCTGTTTATAAAACCACCAGA

TCTCATGAGACTTACTCACTACCAGGAGAACAGGATGCGGGAAACCACCTCGGTGATTCAGTTA
TCTGCACCTGGCCCCACCCTTGACACATGTTGATTATTACAATTCAAGGTGAGATTTGGGTAGGT
ACACAGCCAAACCATATCAGCTGCCCATTCTCCTGTGCAGTGACATAAAGGTCTGATAACCTGA
TTTAAAGCTTGGACTCTAAGATTTTTTTTTTTCTTTTTTTGAGTCAGGTTATCTCTGTCGCTCAAGCT
GGAGTTCAGTGGCATGAACAGGGCTCATTGCAGCCTCAACCTTCCAGGTTCAAGTGATTCTCTT
GCCTCAACCTCCTGAGTATCTGGGGCTACAGGCATGTGCCACCGTGCCAGGCTAATATTTTTATT
TTTATTTTTGTAAAGATGGGGTCTCACTTCTGGGCTCAAGCAATCTTTCCTCAGCCTCCCAAAGTG
CTGGGATTATAGGTGCGAGCCACTGCACCCTGCTGTAAAGAATTTCATATCCCTGTTTTCGGTGT
GCTGTGTCACTGTTTGCCATGGGTAAAGGTATATGAATCTCTTTTATTTTGCTGCTTGCAACCCAA
TTCCCATGCCAAACCCAATTTGTATCCAAACAAACAATATTGGAGACAAGTTCTTCTATAGAAAG
CTTCATAAGCCAGTTACTGACTACTGTGTCTGTAAATATACTCAAACTATTTTGTAGTCTTGCTAT
TTTAAAAAATGTGTGGGTGTGGGAAGTGATGTTGAATGAATTCAGGGTAATTGGTGGTTCTTTGT
ATTAGGAAAACTCACTAGTTAAGGGAAGTCTGTTTTCATGATTCATGTATGTTTAACTTAAAAAA
AAATTCTTCCATCCTTCTCTCCAGTTTCCACTCTACCTCATAAATTCAAGTAATGAAGGAATTTAA
ATAATAGAATTTTATAACTGAAAGGATTTTGGAGAGAATCTATTAACCCTCCTACTACAGTTGAG
GAAATAGATGTCCAGTGAGGTCAAATGAGATCATGTAGCTAGTTTAAAAACCCAAATAACATTT
TAGTAGTTTGTTTCCATTAGTAAACACTGTTCTGTTTGATTTGCCCTAACATGTTCATAATCAGAG
TCATAATAAGGAAAAATCACTAATATGTGTGTGGTACTTTCTTGTGCATACCTCAGTCACTTCTCA
AAAGAGGTCTGTAAGGTGTCGGTATCCTGGTTTCCATTCAGGGAGAGAACAGTCTATGAGCCTA
AGAGACCTGTCTGTGCCAGTCACTATCAGAACAGTCTAATGCTATGTGTCTTCCTACCTTGGCAA
AAGCATTGGCTGGTCTCTGGACAGTTCTCATAGACCACAGGATTGGGCAGGGGGGAGCCACAT
CTTCATGTTAGGGCTCTAACCTGTACCAATTATTAACTGTTGTCTCTATTTCATCATTTCTTAAAGG
GAAAAAAAAGCACTTTTTAAAGAGCATGAGATTTTAAATTTTTCAAAAGAAGGATGCTGGATCAT
TATAAGTAAGGCATGTTTTAAAGATCTTCTTATCAACAGCTTAAAAATTAGCCAGTATATACTGA
ACATCCACTGTATAAAAAGCACATTATGGGGTGCTCCAGTAGGTACAGCAGTGATCGAGACAG
AATCATAGCCTTCAGGGAAGAGGTATATGCAATGTTCTCTCCTTCCAAAGATTACCTAGATAGAG
AACTGTTCATAAGAAATATCTTGGTACTGTCTACATTACTCTTGAGATATAAAATATATATTATTT
CTTACGTCTGAAATGAGTCAGTTGTTTCTAAAACAATTTGTATTAAAGTATTCACATTAAAAATTT
GTAAAATACAGAAAAGTAGAGAGAAAAAATCATGAATTTTTAACATGGTAATGATTATATCAGTT
TCGTTCTTGGCGTTTCTTTAACTTGATGGGATAGCTAATGTTTTTTGTCATTGCATAGCTTTGTAAA
CTTGTCCTAAGGATTATATAATTTCAGCATGAGTTAGTTTCTAAGTATGTCAGAAATCTAGCTTTT
TTGTGGTAATTGTTGTTTTACACAAAATGATTAATAAAAGACTCTAAACTAACCTTGGCTTTAGAA
ATGCTTTTTAAATTTTAAAATAGTGAATGAGTAGCAGATTACTTTCTAGTATTTCTTTAAGGTCAC
CAAAATCACTTAACTTGCTTAAAATATTGATGGAAATATCAACTACGTACAGTTGACCTAAATTA
GAAATGGGTGTTTTTTAAATAGTGAAATTTATAAATATAATATGTATATTTTAAAATACAGTTTATA
TTATAGAGTTATGTTCCTCTGTAATAATTTCTCTTTACACTGAATCATGTTCAGTTTTTATTATGCA
GATAATTTAATCTTACAGCTTTGATCTTTTTTAAAAAATAAAACAGCCATTTGGAGTCTAAA
ID|25796|gene_id|79940|transcript_id|NR_121613 Homo sapiens lncRNA
ATGCGAGGCTGGGGCCGGTTGCCTACCGGCCGCTTCTCGCCGAGGCAGTCCAGACTTTTC
CCCCGGCGGTGCCCGCTCCAAGACAGCATCTGTCAACGCTCCTCTTCTCCCCTCCTCCTCCTGC
CGGGCCGGGCTCCGCCGGCTGCGGCCGAGAGGACGCGGGACCCGGCGCGGTGAGCCCATCA
GCTGTCAGGCGAGCGGCGAAGCGGCTGGAGGGCGGCGAGAGACACACAAAGAACGCGGTGG
GCGGCGGCGGCGAAAGGGGACGGCAACTCCTCCCCGCGCCCGCCGGTGCCACCGCCGGCCGT
GCTTGTTCCGAGGCCGCGCAGACAATGCGGCCGGGCTCGTCCCCGCGTGCCCCAGAGTGCGG

AGCGCCCGCGCTCCCCCGACCCCAACTTGACCGTCTCCCGGCTCGCCCAGCCCCCTCCCGGGG
TAGGGGCGCCCCCTCCCTCCGGTGGCCGGCGAAGGAAGTCGGTCCGCGGCCGCAGATCCCGG
CAACTTGCGAACCGGGAAAAGTTTGCGGCGCCTCCGCGGGGCGGCGCGACGCGGCCCGCCCC
TCGCGTCCGCGGTCATCGCGGGTGACTTTCTCGACTCGTCGTCAGCCGGGGCCGCAGCGCGGC
CGGTGGGGACTGCGGGGCGGGCCGGAGTCCGTCCGAGGGCTCCCGCACCTCGGGCTGCGGGA
ACATTAAAATCTCCCCAGATGCTCACTGCTCTGATCATTTCCCCTACAGCACCAGACATGTTGGC
ATCATCTCTTAGTTGGGCCATGTGGCCAGGGAACCCTTCATGAAACCACCTCTGCAGTCCTGGA
GAGTTGCTGTAGAAGAAGGAAAAGAGAGGAAGACAACACAACACAGGAGGGGATAAGTACCT
CTGGACACAATGGAAGTAGTAGTTGTTGAGCAGAGGGAAAGGGATAGGAGAAGGATGTGGAC
TGCAGTGCTGCAGTGCCAGGTAATAACTTTGATCATGTACCTCCTTGGCTCAGGTGCTGTCTCTT
CTACTCTCCTCCTTACTTGTACTACCTTATTTAGCACTTAATTATATACTTTCTTGACTCACTTTGA
GACACACTGATTATGTGGTCTGCCTCAAAGACAGAATCCTAGTTTTGGATTTTAGCATTACTGAT
CTATAGCCTTGCAGGGGCTTAAGGATGGCAGCTGTCTCTCTCCCTTGGGATCTAGCTTTTGCAAA
TATTGAAGGGAAGAGATTTCTTTGATCTTATAGGAATAGATCCACAGTTCCTCTCCAGATTTGCTT
TTATAGAGAAGTGTGTGTCACCAAAACAATGGAGATACGGTCAGAGAGGCCCCTACCCCCAGA
ATTTTAATATAATTTTACTTTCTGGGGCATTTATTCTTGACAAATACTTTCTCTCCTATCAGCTCAC
TTGTTCCTCACAAAAACCCTATAAGATATTAATATTATTAAACAGACCCCTATGAAGCACCTCAA
GTCTTTCTCTCTTCCCACTTCCATTCTTGCACACCTTTTGCTAGACTAGTCTCTGCGTTCATCACAT
CAGGTTTCTAAAATAAAAGTCCCCTAATAACTTTCTATTTGTTCTCTCATCATATTGAACCTTTTTT
GCCTGGCTTATAAGGTGGCCTTAAAACCCCATTTTCCTCTAAACATTGTCTCTGTAGTTCAGGAA
TATGGCAGGCTCAACACTATGCTTGAGCCCATATTGCTTCCCTCATTAGTGATGCCTCCTCTCAG
ACCACCCAGATTCTACTCATTCTTCAATTTCCAGCTTCTCCTTGGAGCCTTCCCAGATCATTAGCT
TCTTAAAAATCCCAGCTTTCTCTGAATTCTGATGGTAGCCCCCGTTATTTGGACAATTATGTCCTG
GTCTTAAAGTCCTAATGGGAAAAAAAAGTACATGATGGACTTCATGTAGAGAAGAAAGCACTG
GACTGAGTTTTAAGACAGCTGCTAAGGAGCTGTGTGAGCCAAACCAGGTTACTTTACCTCTCTG
GATCTATATTTGCTGATCTGTTAAGTGGAGACAATTGGGTCAGATAATCTTGGTGGTCTTTGCTTA
TTCTGAGGTTCTTGGACAACTTGGCCTAGTTCACGTGGGATGGGATTGAAGATCTTACTGAGTTC
TTGGATGGAAATGTCAGTAATGGGCACTTAGTGCCTTCAGGACTTGTTCTATACTATCCCAGAAA
TTTCTGATGCTAAACTTGGCAATTTTTAAATGTCATATTCCTTTTCTCCAAACTCTTTAACCACAAG
AATTCAGGGAAGGTTATGGGGGGTGACTTAGTTATCTGGATAATACATATTGTGGACAATATAG
CATTATCCTTGGGAATGATTTCATATTGTTGAATAAATAGGATCAACTTCTTGATAAACCTTAGTC
CCAAGCATTTCATCTTCATTGGATAGTGTTACATAGTAATATATTTATGTTTTCTTTTAATCATTTC
ATAACTTGGAAAATACTAACATAGTCAAAACTCTAGGGTAGGTGATACATGAGTTTCTGTAGTAA
TCTGGTTGGAGACATGTTGTAATTCTGTATATATATGTACATTTATCCCATGCATGTTATGCCTAA
ACTAAGACGGATACCCCTGAATTAAGAGGTGCTGTTATACATTGACCAGGCTTAAGAATATCTCT
TTAAAGTGTGTCGACATTTAATTGACCTTTGGAAGTTCATTCTGTTAATCATACTCAAAGTGCTAA
AGCTATGGTTGACTGCTCTGGTGTTTTTATATTCATTCGTGCTTTAGCATATAAATTCTTCAGCATA
ATTGCTACTTATTTAGCAAGAGTTTCCTTTATTTGAAAATGTGAGTTGTGCTTGTATTTTTGTGTCT
TTCTTTCTTTCTTTCTTTTTTTAAACTTTGCTTCAGGCTGGGTAGTGGTAGAGGTTTGAATTAAAAT
GTTTTCCTGTCAGTAGTTGTATGAGGTGTGGCTTCTTAATCTAGAAGTAATGGAAATTTCTTGCTT
AAATGAAGTGCACTTTTCTTCCTGCAGAAGTTAGAACTCTTTTAGACAAGCTTAACACTTCAAAA
CTGTATATCTTTAACAGAAACTTTTGGAAATTTTTAAATTCCATTTGTGTAGATTAAAAATATCAAT
GCTAATACTTTCTATTAAATAAGGATTTATCTATTTTCATAAAACCTCTAATTATATTCCTTGAATT
GCTATGAGTAGAATTTTGTTGTAAACACAATATTTTTAAAAAGTCAAATATATTTTAAAAATCTTA

ACATTATATTACTATAACTTAGAGAACAGGTGTTTACATGTTTAAAATTTTCTAGGCCTTAAATTT
GTGAAATCAGTCTTTCAGAAGTTATTTTTAATATAGTTATCTGTGTTACAGGTTTATAAGTTGACA
TTTCAATTTAAAATTTTTTCAATTTATATTTCAATGACCAGGAAAAAATAGTTAAACTTATTCCTAA
ATTTTAATAGAAAAATAAAAAACCACATAGTTGTTAATTTCTGGTAATTTATCTTGAGACTCCAGG
CATGTTTTTAGAACATTTTTACCTTAAAAGCAATTTTGAAATAGGGAAATTAAAGTTTAAAGCAG
AGTTTTCACTTTAATGAGGTAATTTTATACTTCCCAGATAGGTAGGCCCATATTCAAGAACACTCT
GATCTCTGTTTATAGTAAGTTTTGATGCCATTATATCATTTACTTATTCACGCATTATAAGCATAGT
AGATAGCCCCATTGTCACGAAGCTGGCATTATTTTCTTGTCTGTCATTCAATATTGTCCCCTTTTTT
TTTTCTGTTTTCATCATAGCCGTCCTTTTCATTTTATTGTAGCCCTGACTCGAAATGAAAGTAAGTT
TGAACTCTTAAAAAAGAGTTCCTCAAACTCTTTTTTAAGAATATACAGTGAAATGTCTTAAATAAT
ATTATCTAAATAATACCTAAAGAATGCAAATGACTTGCAATGCTGAATAGTCAGACACAGGATG
TTCCATGCTAAGGGTAGACTCATATTTCCACTTTATAAAATGTGGTTTCAGAAAACAAATAGATG
TGTTTCTTCCTTAATAATTAGTGTTTCAGGACCAAAGTTGACTAAATATGGAAGTTTAAGATAAAA
CCCGGTTTCTTAGGGAATCTTTCTCAGTCCTTAGTAATTCTGTAGAATTGATGATAGAGTGGCAA
CTTCAATACACTCAGTATTCTTTTCTGTTTGAAGTCTGTTTTGCCATTGGAAAAAACTTATTGTTGT
TTGAATTTGGGTATATGTATTCACAGATGGTTTACTTTATTTTTATTTTGTTTAAACAAATACTTTCA
TGTCCCAAACACTGAGACATGTTTCATGTCCTAAACACTTTATAAATATTAGAATATTTAATCCCC
TTAACTCTGTAGTTATAATTATCTTCATTTTACAATTGAGAAAACTAAGATACGACGTTTTATTTAA
TGGTAACGCCTTAAGTAAGGGGAGAGTGATAAGTGTAAGCAGAGTGGAAAACTTAGTAGTGCT
AAGGAGCAACAGAAGTATGTGCAAGAGTTTGGGAAGATGTGTCTGCTTGGAAAGGATCAGGGA
CTCAGCTGGGATCAGTCTTAAAGAGCAACATGTCCTCATTCCTCAATTTTAAGCCTTTGGGCTCT
AGAAGTAAAGGGCAAACTTAGAGGAGGCACTCCTCAGGTTTCAGCTGCCTGTTCCGGCCTGGT
GAGGTGGCTGTACTGGCTCATGTGTTTCTCATAGGGCTACATGAGCCTTTATGTCTTACCTCCTC
CTCATTTCTTGGCTGACTCCTGCTTTTTTGGAGGGTTTAACTTCTCTCTCAGCTAGAAGGTTAGGC
ATTGCAAATACCAGTGGATAATTTTTTTCTTAGCTTTAACCCCAGCCCATTTCAACCCCCTCTTTG
CCCTTTGTATATTCTTTTGAAAATATGATCCAGTAGTGTTTATGAATGTGTGTTGTGTAAAATTTAG
AGATTGATGTTAAACAACAGAATTAAAGGACAAAGCTGTCTTTTTTGTTGGAATTGGGGATGGG
AGAGCAGCTCAAAGTGGGAAATATGGAGAAAGGAGAGACATTGTGGAAAAGAAGAGATAAAT
TGAGGGGAAAAAAAGAGGATGAAAAAATGAAGAGAGAGAATTACTGATGTGCTTCACACCCAC
ACCTCCTCTCCAACTTCACAGTAACAGAGGCAATGTCACTATCTTTTACCTGACAAGTGCTTTAC
CAGGGAGTTGTGCCTGGCATCGTGGGAGTGGAAAATTTTTCCCTTTTGCATTTTGAGACTTGGGT
GTGCTGCTTTAAGATGCCACTGGTATCTAAGTAATGTTTGTTTATACTGGATATTTTTCTCTGTAG
CCTTTGTGGATTGGACTTATAATATAAACAATATTGTTAGCAGAACTGCAGGTACTCTATTGCTAT
GTTTATTTTATTAGTTAAAAAACTACCAGAGCCCTAGGACTTCTGAGCACATTTAGAAAATACCA
GAGGCAATTTTAAGACTAGCATTAGAAAGAAAGAAAAGAATAAGAATAAAACTAAGACTTGCA
TACCTTAGAATCCTAACCAATGTGGTGATTTTTAAAAATTAATGTTTCAGAGACTCTAAATTAGG
GTAGTTGGTTTCATCAATTACATTTTTTAAGTTAATATAAAGTGCTTTTGTATGTAAATTCTCTTAA
TAGATAATTTAGGTAAAATGAAAGTGTGGCATTTTTTGTTCCATAGAATGTAAAACTAACGTCAG
AATAAGAATTCTTTTGGGAATTTAGGATTTTTTTGATGCTTCTCAAATCATCTGTAATGACTTTTAT
TTATAGAAAAGGTAGATTATTCATTAGTTTTTATGAAAAACACTCAGTTTAAATAGCCGGGTATG
GTGGCGCATGGCTGTAGTCCCAGCTACTCAGGAGGCTGGGCACGAGAATTGCTTGAACCTGTG
AGGCAGAGGTTGCAGTGAGCCAAGATCGCACCACTGCACTCCAGCCTGGTCCAGAGTGAGATT
CCGTCTCGAACAAAACAAAACAAAAATATTGAGCTAATCTAAATGGTAAAAATCTAGATTTGAA
GAAATTTATTTTAAAATAGTTGTTCATTTGTCAAATAGAAAAGTATGATCTTAGCAGGTTTGGTTA

GATCCCTATCTGAAACAAAGTAAACTAAAGATGTTTTTCTTGATCAATGCATAGCAATAACTGCT
GTTCAAACATGTGAGGAATCACCAGTAAATTGGGGGATAGAAAGCAGGAAAAGGAGCTTTTTTC
TCTTATTTTTTTTCATTTATCAGTTTTTCCCATTTGTCCATCCTTTCCTATAATCATGGAAATTTTTAT
TTTTTACACTCCTCTCCCCATTTTTTTATCCTAGATTGCCACCACACCAAGGTTTTCTAGAGTTAG
GCAGAAAGAGAACCTGGGCCTAGATGCTAATGGAAACTATCCCATGCTTTCTCAGACTCTCTTT
CCTCCCCAGAGACAAGAGGAGCAAAACAGGAGTTGATGGCTCATTGCTGTTGCTTCTGTGGCTG
CCCTGTTTGGTTGACCTGAGCTTTTTTTAAAAACCTCCACTCTTGACGCTTAACTCTATGAATAAA
ATTAGGGCCAGTCACACTGTATTTTCATCATCAGTTATCCCTAGTGATTCAAATTTAGCACAAAC
CTGCTGTGAGTACATATTAATTAGGTGAATACAGTAGAATGCAATCATAACATGAGTCTTTTAAT
AACTATTCTTTGTTTGATTTTTATTTTAGAAGTTTGAAGTTATTTTGTTGAAAATCTAGAAAATCTTT
ATGTGAGCAACTGAGGTTCGGGTAGCCCACAACAGTTTAAATCAAACATTAACTTGCAGTAAAA
CACTACTGAAAGTGTCTTTGATTTTGAGGTGCAGAGAGCTGTTTCAAGTTAAGTTGCTATATTAGT
CTGTTCTCATGCTGCTAATAAAGACATACCTGCAACCGGGCAATTTATTAAAAAAAAAAAAGAGG
TTTAATTGACTGACAGTTCCACATGGCTGGGGAGGCCTCACCGTCATGGTGAAAGAGCAAGGG
ATGTCTTACATGGTGGCAGGCAAGAAGTTTGTGCAGAGGAACTCCTGTTTATAAAACCACCAGA
TCTCATGAGACTTACTCACTACCAGGAGAACAGGATGCGGGAAACCACCTCGGTGATTCAGTTA
TCTGCACCTGGCCCCACCCTTGACACATGTTGATTATTACAATTCAAGGTGAGATTTGGGTAGGT
ACACAGCCAAACCATATCAGCTGCCCATTCTCCTGTGCAGTGACATAAAGGTCTGATAACCTGA
TTTAAAGCTTGGACTCTAAGATTTTTTTTTTTCTTTTTTTGAGTCAGGTTATCTCTGTCGCTCAAGCT
GGAGTTCAGTGGCATGAACAGGGCTCATTGCAGCCTCAACCTTCCAGGTTCAAGTGATTCTCTT
GCCTCAACCTCCTGAGTATCTGGGGCTACAGGCATGTGCCACCGTGCCAGGCTAATATTTTTATT
TTTATTTTTGTAAAGATGGGGTCTCACTTCTGGGCTCAAGCAATCTTTCCTCAGCCTCCCAAAGTG
CTGGGATTATAGGTGCGAGCCACTGCACCCTGCTGTAAAGAATTTCATATCCCTGTTTTCGGTGT
GCTGTGTCACTGTTTGCCATGGGTAAAGGTATATGAATCTCTTTTATTTTGCTGCTTGCAACCCAA
TTCCCATGCCAAACCCAATTTGTATCCAAACAAACAATATTGGAGACAAGTTCTTCTATAGAAAG
CTTCATAAGCCAGTTACTGACTACTGTGTCTGTAAATATACTCAAACTATTTTGTAGTCTTGCTAT
TTTAAAAAATGTGTGGGTGTGGGAAGTGATGTTGAATGAATTCAGGGTAATTGGTGGTTCTTTGT
ATTAGGAAAACTCACTAGTTAAGGGAAGTCTGTTTTCATGATTCATGTATGTTTAACTTAAAAAA
AAATTCTTCCATCCTTCTCTCCAGTTTCCACTCTACCTCATAAATTCAAGTAATGAAGGAATTTAA
ATAATAGAATTTTATAACTGAAAGGATTTTGGAGAGAATCTATTAACCCTCCTACTACAGTTGAG
GAAATAGATGTCCAGTGAGGTCAAATGAGATCATGTAGCTAGTTTAAAAACCCAAATAACATTT
TAGTAGTTTGTTTCCATTAGTAAACACTGTTCTGTTTGATTTGCCCTAACATGTTCATAATCAGAG
TCATAATAAGGAAAAATCACTAATATGTGTGTGGTACTTTCTTGTGCATACCTCAGTCACTTCTCA
AAAGAGGTCTGTAAGGTGTCGGTATCCTGGTTTCCATTCAGGGAGAGAACAGTCTATGAGCCTA
AGAGACCTGTCTGTGCCAGTCACTATCAGAACAGTCTAATGCTATGTGTCTTCCTACCTTGGCAA
AAGCATTGGCTGGTCTCTGGACAGTTCTCATAGACCACAGGATTGGGCAGGGGGGAGCCACAT
CTTCATGTTAGGGCTCTAACCTGTACCAATTATTAACTGTTGTCTCTATTTCATCATTTCTTAAAGG
GAAAAAAAAGCACTTTTTAAAGAGCATGAGATTTTAAATTTTTCAAAAGAAGGATGCTGGATCAT
TATAAGTAAGGCATGTTTTAAAGATCTTCTTATCAACAGCTTAAAAATTAGCCAGTATATACTGA
ACATCCACTGTATAAAAAGCACATTATGGGGTGCTCCAGTAGGTACAGCAGTGATCGAGACAG
AATCATAGCCTTCAGGGAAGAGGTATATGCAATGTTCTCTCCTTCCAAAGATTACCTAGATAGAG
AACTGTTCATAAGAAATATCTTGGTACTGTCTACATTACTCTTGAGATATAAAATATATATTATTT
CTTACGTCTGAAATGAGTCAGTTGTTTCTAAAACAATTTGTATTAAAGTATTCACATTAAAAATTT
GTAAAATACAGAAAAGTAGAGAGAAAAAATCATGAATTTTTAACATGGTAATGATTATATCAGTT

TCGTTCTTGGCGTTTCTTTAACTTGATGGGATAGCTAATGTTTTTTGTCATTGCATAGCTTTGTAAA
CTTGTCCTAAGGATTATATAATTTCAGCATGAGTTAGTTTCTAAGTATGTCAGAAATCTAGCTTTT
TTGTGGTAATTGTTGTTTTACACAAAATGATTAATAAAAGACTCTAAACTAACCTTGGCTTTAGAA
ATGCTTTTTAAATTTTAAAATAGTGAATGAGTAGCAGATTACTTTCTAGTATTTCTTTAAGGTCAC
CAAAATCACTTAACTTGCTTAAAATATTGATGGAAATATCAACTACGTACAGTTGACCTAAATTA
GAAATGGGTGTTTTTTAAATAGTGAAATTTATAAATATAATATGTATATTTTAAAATACAGTTTATA
TTATAGAGTTATGTTCCTCTGTAATAATTTCTCTTTACACTGAATCATGTTCAGTTTTTATTATGCA
GATAATTTAATCTTACAGCTTTGATCTTTTTTAAAAAATAAAACAGCCATTTGGAGTCTAAA
ID|25797|gene_id|79940|transcript_id|NR_121614 Homo sapiens lncRNA
ATGCGAGGCTGGGGCCGGTTGCCTACCGGCCGCTTCTCGCCGAGGCAGTCCAGACTTTTC
CCCCGGCGGTGCCCGCTCCAAGACAGCATCTGTCAACGCTCCTCTTCTCCCCTCCTCCTCCTGC
CGGGCCGGGCTCCGCCGGCTGCGGCCGAGAGGACGCGGGACCCGGCGCGGTGAGCCCATCA
GCTGTCAGGCGAGCGGCGAAGCGGCTGGAGGGCGGCGAGAGACACACAAAGAACGCGGTGG
GCGGCGGCGGCGAAAGGGGACGGCAACTCCTCCCCGCGCCCGCCGGTGCCACCGCCGGCCGT
GCTTGTTCCGAGGCCGCGCAGACAATGCGGCCGGGCTCGTCCCCGCGTGCCCCAGAGTGCGG
AGCGCCCGCGCTCCCCCGACCCCAACTTGACCGTCTCCCGGCTCGCCCAGCCCCCTCCCGGGG
TAGGGGCGCCCCCTCCCTCCGGTGGCCGGCGAAGGAAGTCGGTCCGCGGCCGCAGATCCCGG
CAACTTGCGAACCGGGAAAAGTTTGCGGCGCCTCCGCGGGGCGGCGCGACGCGGCCCGCCCC
TCGCGTCCGCGGTCATCGCGGGTGACTTTCTCGACTCGTCGTCAGCCGGGGCCGCAGCGCGGC
CGGTGGGGACTGCGGGGCGGGCCGGAGTCCGTCCGAGGGCTCCCGCACCTCGGGCTGCGGCA
CCAGACATGTTGGCATCATCTCTTAGTTGGGCCATGTGGCCAGGGAACCCTTCATGAAACCACC
TCTGCAGTCCTGGAGAGTTGCTGTAGAAGAAGGAAAAGAGAGGAAGACAACACAACACAGGA
GGGGATAAGTACCTCTGGACACAATGGAAGTAGTAGTTGTTGAGCAGAGGGAAAGGGATAGGA
GAAGGATGTGGACTGCAGTGCTGCAGTGCCAGGTAATAACTTTGATCATGTACCTCCTTGGCTC
AGGTGCTGTCTCTTCTACTCTCCTCCTTACTTGTACTACCTTATTTAGCACTTAATTATATACTTTCT
TGACTCACTTTGAGACACACTGATTATGTGGTCTGCCTCAAAGACAGAATCCTAGTTTTGGATTT
TAGCATTACTGATCTATAGCCTTGCAGGGGCTTAAGGATGGCAGCTGTCTCTCTCCCTTGGGATC
TAGCTTTTGCAAATATTGAAGGGAAGAGATTTCTTTGATCTTATAGGAATAGATCCACAGTTCCT
CTCCAGATTTGCTTTTATAGAGAAGTGTGTGTCACCAAAACAATGGAGATACGGTCAGAGAGGC
CCCTACCCCCAGAATTTTAATATAATTTTACTTTCTGGGGCATTTATTCTTGACAAATACTTTCTCT
CCTATCAGCTCACTTGTTCCTCACAAAAACCCTATAAGATATTAATATTATTAAACAGACCCCTAT
GAAGCACCTCAAGTCTTTCTCTCTTCCCACTTCCATTCTTGCACACCTTTTGCTAGACTAGTCTCT
GCGTTCATCACATCAGGTTTCTAAAATAAAAGTCCCCTAATAACTTTCTATTTGTTCTCTCATCAT
ATTGAACCTTTTTTGCCTGGCTTATAAGGTGGCCTTAAAACCCCATTTTCCTCTAAACATTGTCTC
TGTAGTTCAGGAATATGGCAGGCTCAACACTATGCTTGAGCCCATATTGCTTCCCTCATTAGTGA
TGCCTCCTCTCAGACCACCCAGATTCTACTCATTCTTCAATTTCCAGCTTCTCCTTGGAGCCTTCC
CAGATCATTAGCTTCTTAAAAATCCCAGCTTTCTCTGAATTCTGATGGTAGCCCCCGTTATTTGGA
CAATTATGTCCTGGTCTTAAAGTCCTAATGGGAAAAAAAAGTACATGATGGACTTCATGTAGAG
AAGAAAGCACTGGACTGAGTTTTAAGACAGCTGCTAAGGAGCTGTGTGAGCCAAACCAGGTTA
CTTTACCTCTCTGGATCTATATTTGCTGATCTGTTAAGTGGAGACAATTGGGTCAGATAATCTTGG
TGGTCTTTGCTTATTCTGAGGTTCTTGGACAACTTGGCCTAGTTCACGTGGGATGGGATTGAAGA
TCTTACTGAGTTCTTGGATGGAAATGTCAGTAATGGGCACTTAGTGCCTTCAGGACTTGTTCTAT
ACTATCCCAGAAATTTCTGATGCTAAACTTGGCAATTTTTAAATGTCATATTCCTTTTCTCCAAACT
CTTTAACCACAAGAATTCAGGGAAGGTTATGGGGGGTGACTTAGTTATCTGGATAATACATATTG

TGGACAATATAGCATTATCCTTGGGAATGATTTCATATTGTTGAATAAATAGGATCAACTTCTTGA
TAAACCTTAGTCCCAAGCATTTCATCTTCATTGGATAGTGTTACATAGTAATATATTTATGTTTTCT
TTTAATCATTTCATAACTTGGAAAATACTAACATAGTCAAAACTCTAGGGTAGGTGATACATGAG
TTTCTGTAGTAATCTGGTTGGAGACATGTTGTAATTCTGTATATATATGTACATTTATCCCATGCAT
GTTATGCCTAAACTAAGACGGATACCCCTGAATTAAGAGGTGCTGTTATACATTGACCAGGCTT
AAGAATATCTCTTTAAAGTGTGTCGACATTTAATTGACCTTTGGAAGTTCATTCTGTTAATCATAC
TCAAAGTGCTAAAGCTATGGTTGACTGCTCTGGTGTTTTTATATTCATTCGTGCTTTAGCATATAA
ATTCTTCAGCATAATTGCTACTTATTTAGCAAGAGTTTCCTTTATTTGAAAATGTGAGTTGTGCTTG
TATTTTTGTGTCTTTCTTTCTTTCTTTCTTTTTTTAAACTTTGCTTCAGGCTGGGTAGTGGTAGAGGT
TTGAATTAAAATGTTTTCCTGTCAGTAGTTGTATGAGGTGTGGCTTCTTAATCTAGAAGTAATGGA
AATTTCTTGCTTAAATGAAGTGCACTTTTCTTCCTGCAGAAGTTAGAACTCTTTTAGACAAGCTTA
ACACTTCAAAACTGTATATCTTTAACAGAAACTTTTGGAAATTTTTAAATTCCATTTGTGTAGATT
AAAAATATCAATGCTAATACTTTCTATTAAATAAGGATTTATCTATTTTCATAAAACCTCTAATTAT
ATTCCTTGAATTGCTATGAGTAGAATTTTGTTGTAAACACAATATTTTTAAAAAGTCAAATATATTT
TAAAAATCTTAACATTATATTACTATAACTTAGAGAACAGGTGTTTACATGTTTAAAATTTTCTAG
GCCTTAAATTTGTGAAATCAGTCTTTCAGAAGTTATTTTTAATATAGTTATCTGTGTTACAGGTTTA
TAAGTTGACATTTCAATTTAAAATTTTTTCAATTTATATTTCAATGACCAGGAAAAAATAGTTAAA
CTTATTCCTAAATTTTAATAGAAAAATAAAAAACCACATAGTTGTTAATTTCTGGTAATTTATCTTG
AGACTCCAGGCATGTTTTTAGAACATTTTTACCTTAAAAGCAATTTTGAAATAGGGAAATTAAAG
TTTAAAGCAGAGTTTTCACTTTAATGAGGTAATTTTATACTTCCCAGATAGGTAGGCCCATATTCA
AGAACACTCTGATCTCTGTTTATAGTAAGTTTTGATGCCATTATATCATTTACTTATTCACGCATTA
TAAGCATAGTAGATAGCCCCATTGTCACGAAGCTGGCATTATTTTCTTGTCTGTCATTCAATATTG
TCCCCTTTTTTTTTTCTGTTTTCATCATAGCCGTCCTTTTCATTTTATTGTAGCCCTGACTCGAAATG
AAAGTAAGTTTGAACTCTTAAAAAAGAGTTCCTCAAACTCTTTTTTAAGAATATACAGTGAAATG
TCTTAAATAATATTATCTAAATAATACCTAAAGAATGCAAATGACTTGCAATGCTGAATAGTCAG
ACACAGGATGTTCCATGCTAAGGGTAGACTCATATTTCCACTTTATAAAATGTGGTTTCAGAAAA
CAAATAGATGTGTTTCTTCCTTAATAATTAGTGTTTCAGGACCAAAGTTGACTAAATATGGAAGTT
TAAGATAAAACCCGGTTTCTTAGGGAATCTTTCTCAGTCCTTAGTAATTCTGTAGAATTGATGATA
GAGTGGCAACTTCAATACACTCAGTATTCTTTTCTGTTTGAAGTCTGTTTTGCCATTGGAAAAAAC
TTATTGTTGTTTGAATTTGGGTATATGTATTCACAGATGGTTTACTTTATTTTTATTTTGTTTAAACA
AATACTTTCATGTCCCAAACACTGAGACATGTTTCATGTCCTAAACACTTTATAAATATTAGAATA
TTTAATCCCCTTAACTCTGTAGTTATAATTATCTTCATTTTACAATTGAGAAAACTAAGATACGAC
GTTTTATTTAATGGTAACGCCTTAAGTAAGGGGAGAGTGATAAGTGTAAGCAGAGTGGAAAACT
TAGTAGTGCTAAGGAGCAACAGAAGTATGTGCAAGAGTTTGGGAAGATGTGTCTGCTTGGAAA
GGATCAGGGACTCAGCTGGGATCAGTCTTAAAGAGCAACATGTCCTCATTCCTCAATTTTAAGC
CTTTGGGCTCTAGAAGTAAAGGGCAAACTTAGAGGAGGCACTCCTCAGGTTTCAGCTGCCTGTT
CCGGCCTGGTGAGGTGGCTGTACTGGCTCATGTGTTTCTCATAGGGCTACATGAGCCTTTATGTC
TTACCTCCTCCTCATTTCTTGGCTGACTCCTGCTTTTTTGGAGGGTTTAACTTCTCTCTCAGCTAGA
AGGTTAGGCATTGCAAATACCAGTGGATAATTTTTTTCTTAGCTTTAACCCCAGCCCATTTCAACC
CCCTCTTTGCCCTTTGTATATTCTTTTGAAAATATGATCCAGTAGTGTTTATGAATGTGTGTTGTGT
AAAATTTAGAGATTGATGTTAAACAACAGAATTAAAGGACAAAGCTGTCTTTTTTGTTGGAATTG
GGGATGGGAGAGCAGCTCAAAGTGGGAAATATGGAGAAAGGAGAGACATTGTGGAAAAGAAG
AGATAAATTGAGGGGAAAAAAAGAGGATGAAAAAATGAAGAGAGAGAATTACTGATGTGCTTC
ACACCCACACCTCCTCTCCAACTTCACAGTAACAGAGGCAATGTCACTATCTTTTACCTGACAAG

TGCTTTACCAGGGAGTTGTGCCTGGCATCGTGGGAGTGGAAAATTTTTCCCTTTTGCATTTTGAG
ACTTGGGTGTGCTGCTTTAAGATGCCACTGGTATCTAAGTAATGTTTGTTTATACTGGATATTTTT
CTCTGTAGCCTTTGTGGATTGGACTTATAATATAAACAATATTGTTAGCAGAACTGCAGGTACTC
TATTGCTATGTTTATTTTATTAGTTAAAAAACTACCAGAGCCCTAGGACTTCTGAGCACATTTAGA
AAATACCAGAGGCAATTTTAAGACTAGCATTAGAAAGAAAGAAAAGAATAAGAATAAAACTAA
GACTTGCATACCTTAGAATCCTAACCAATGTGGTGATTTTTAAAAATTAATGTTTCAGAGACTCTA
AATTAGGGTAGTTGGTTTCATCAATTACATTTTTTAAGTTAATATAAAGTGCTTTTGTATGTAAATT
CTCTTAATAGATAATTTAGGTAAAATGAAAGTGTGGCATTTTTTGTTCCATAGAATGTAAAACTAA
CGTCAGAATAAGAATTCTTTTGGGAATTTAGGATTTTTTTGATGCTTCTCAAATCATCTGTAATGA
CTTTTATTTATAGAAAAGGTAGATTATTCATTAGTTTTTATGAAAAACACTCAGTTTAAATAGCCG
GGTATGGTGGCGCATGGCTGTAGTCCCAGCTACTCAGGAGGCTGGGCACGAGAATTGCTTGAA
CCTGTGAGGCAGAGGTTGCAGTGAGCCAAGATCGCACCACTGCACTCCAGCCTGGTCCAGAGT
GAGATTCCGTCTCGAACAAAACAAAACAAAAATATTGAGCTAATCTAAATGGTAAAAATCTAGA
TTTGAAGAAATTTATTTTAAAATAGTTGTTCATTTGTCAAATAGAAAAGTATGATCTTAGCAGGTT
TGGTTAGATCCCTATCTGAAACAAAGTAAACTAAAGATGTTTTTCTTGATCAATGCATAGCAATA
ACTGCTGTTCAAACATGTGAGGAATCACCAGTAAATTGGGGGATAGAAAGCAGGAAAAGGAGC
TTTTTTCTCTTATTTTTTTTCATTTATCAGTTTTTCCCATTTGTCCATCCTTTCCTATAATCATGGAAA
TTTTTATTTTTTACACTCCTCTCCCCATTTTTTTATCCTAGATTGCCACCACACCAAGGTTTTCTAG
AGTTAGGCAGAAAGAGAACCTGGGCCTAGATGCTAATGGAAACTATCCCATGCTTTCTCAGACT
CTCTTTCCTCCCCAGAGACAAGAGGAGCAAAACAGGAGTTGATGGCTCATTGCTGTTGCTTCTG
TGGCTGCCCTGTTTGGTTGACCTGAGCTTTTTTTAAAAACCTCCACTCTTGACGCTTAACTCTATG
AATAAAATTAGGGCCAGTCACACTGTATTTTCATCATCAGTTATCCCTAGTGATTCAAATTTAGCA
CAAACCTGCTGTGAGTACATATTAATTAGGTGAATACAGTAGAATGCAATCATAACATGAGTCTT
TTAATAACTATTCTTTGTTTGATTTTTATTTTAGAAGTTTGAAGTTATTTTGTTGAAAATCTAGAAAA
TCTTTATGTGAGCAACTGAGGTTCGGGTAGCCCACAACAGTTTAAATCAAACATTAACTTGCAGT
AAAACACTACTGAAAGTGTCTTTGATTTTGAGGTGCAGAGAGCTGTTTCAAGTTAAGTTGCTATA
TTAGTCTGTTCTCATGCTGCTAATAAAGACATACCTGCAACCGGGCAATTTATTAAAAAAAAAAA
GAGGTTTAATTGACTGACAGTTCCACATGGCTGGGGAGGCCTCACCGTCATGGTGAAAGAGCA
AGGGATGTCTTACATGGTGGCAGGCAAGAAGTTTGTGCAGAGGAACTCCTGTTTATAAAACCAC
CAGATCTCATGAGACTTACTCACTACCAGGAGAACAGGATGCGGGAAACCACCTCGGTGATTC
AGTTATCTGCACCTGGCCCCACCCTTGACACATGTTGATTATTACAATTCAAGGTGAGATTTGGG
TAGGTACACAGCCAAACCATATCAGCTGCCCATTCTCCTGTGCAGTGACATAAAGGTCTGATAA
CCTGATTTAAAGCTTGGACTCTAAGATTTTTTTTTTTCTTTTTTTGAGTCAGGTTATCTCTGTCGCTC
AAGCTGGAGTTCAGTGGCATGAACAGGGCTCATTGCAGCCTCAACCTTCCAGGTTCAAGTGATT
CTCTTGCCTCAACCTCCTGAGTATCTGGGGCTACAGGCATGTGCCACCGTGCCAGGCTAATATTT
TTATTTTTATTTTTGTAAAGATGGGGTCTCACTTCTGGGCTCAAGCAATCTTTCCTCAGCCTCCCA
AAGTGCTGGGATTATAGGTGCGAGCCACTGCACCCTGCTGTAAAGAATTTCATATCCCTGTTTTC
GGTGTGCTGTGTCACTGTTTGCCATGGGTAAAGGTATATGAATCTCTTTTATTTTGCTGCTTGCAA
CCCAATTCCCATGCCAAACCCAATTTGTATCCAAACAAACAATATTGGAGACAAGTTCTTCTATA
GAAAGCTTCATAAGCCAGTTACTGACTACTGTGTCTGTAAATATACTCAAACTATTTTGTAGTCTT
GCTATTTTAAAAAATGTGTGGGTGTGGGAAGTGATGTTGAATGAATTCAGGGTAATTGGTGGTTC
TTTGTATTAGGAAAACTCACTAGTTAAGGGAAGTCTGTTTTCATGATTCATGTATGTTTAACTTAA
AAAAAAATTCTTCCATCCTTCTCTCCAGTTTCCACTCTACCTCATAAATTCAAGTAATGAAGGAAT
TTAAATAATAGAATTTTATAACTGAAAGGATTTTGGAGAGAATCTATTAACCCTCCTACTACAGTT

GAGGAAATAGATGTCCAGTGAGGTCAAATGAGATCATGTAGCTAGTTTAAAAACCCAAATAACA
TTTTAGTAGTTTGTTTCCATTAGTAAACACTGTTCTGTTTGATTTGCCCTAACATGTTCATAATCAG
AGTCATAATAAGGAAAAATCACTAATATGTGTGTGGTACTTTCTTGTGCATACCTCAGTCACTTCT
CAAAAGAGGTCTGTAAGGTGTCGGTATCCTGGTTTCCATTCAGGGAGAGAACAGTCTATGAGCC
TAAGAGACCTGTCTGTGCCAGTCACTATCAGAACAGTCTAATGCTATGTGTCTTCCTACCTTGGC
AAAAGCATTGGCTGGTCTCTGGACAGTTCTCATAGACCACAGGATTGGGCAGGGGGGAGCCAC
ATCTTCATGTTAGGGCTCTAACCTGTACCAATTATTAACTGTTGTCTCTATTTCATCATTTCTTAAA
GGGAAAAAAAAGCACTTTTTAAAGAGCATGAGATTTTAAATTTTTCAAAAGAAGGATGCTGGAT
CATTATAAGTAAGGCATGTTTTAAAGATCTTCTTATCAACAGCTTAAAAATTAGCCAGTATATACT
GAACATCCACTGTATAAAAAGCACATTATGGGGTGCTCCAGTAGGTACAGCAGTGATCGAGAC
AGAATCATAGCCTTCAGGGAAGAGGTATATGCAATGTTCTCTCCTTCCAAAGATTACCTAGATAG
AGAACTGTTCATAAGAAATATCTTGGTACTGTCTACATTACTCTTGAGATATAAAATATATATTAT
TTCTTACGTCTGAAATGAGTCAGTTGTTTCTAAAACAATTTGTATTAAAGTATTCACATTAAAAAT
TTGTAAAATACAGAAAAGTAGAGAGAAAAAATCATGAATTTTTAACATGGTAATGATTATATCAG
TTTCGTTCTTGGCGTTTCTTTAACTTGATGGGATAGCTAATGTTTTTTGTCATTGCATAGCTTTGTA
AACTTGTCCTAAGGATTATATAATTTCAGCATGAGTTAGTTTCTAAGTATGTCAGAAATCTAGCTT
TTTTGTGGTAATTGTTGTTTTACACAAAATGATTAATAAAAGACTCTAAACTAACCTTGGCTTTAG
AAATGCTTTTTAAATTTTAAAATAGTGAATGAGTAGCAGATTACTTTCTAGTATTTCTTTAAGGTC
ACCAAAATCACTTAACTTGCTTAAAATATTGATGGAAATATCAACTACGTACAGTTGACCTAAAT
TAGAAATGGGTGTTTTTTAAATAGTGAAATTTATAAATATAATATGTATATTTTAAAATACAGTTTA
TATTATAGAGTTATGTTCCTCTGTAATAATTTCTCTTTACACTGAATCATGTTCAGTTTTTATTATGC
AGATAATTTAATCTTACAGCTTTGATCTTTTTTAAAAAATAAAACAGCCATTTGGAGTCTAAA
ID|27172|gene_id|84973|transcript_id|NR_003672 Homo sapiens lncRNA
CTCTGCGTGCGCCGGAGGCTGCCGTGGCGGGTGGGCCGCCTGACTTCTCCTCCCGGCCAG
TTCTCGAGCGCCTCACCGGGCCTCGCCCTGCAGCCTCGCTCTCGCTGGCGCTGCGCGGCCTAG
GGGACTGGGCTGCTGGCCTCCGGGTGCGGGGTGGGGGCAGGCTCCGACCTGGGGCGTCCTGG
CCGCGCGAGCCGCGGGATGGGGGCCCGGGCCGCGGAGGAGGCGCCGCTGGTGTGTCCCTTGG
TGGAGAGGGCGCTGCCGGCCCTGCGCGGTTTCCAGCCAGGAAGCTTCGGGAAGCCTGGACGT
CTGCTCACTGGAGATGACACGTGCGTGGGGTGTTGGGTAAGTTGGTGCATTGGAGCTGGAACA
GGGTCAATCCTCCAATGTAACTAAGCCCTCCTGTGCAACATCTTAAGTGACTGGAGGGACCAGT
CGGTCTTGTGTTCACATTGAGGCTAGAAGCTGTCTCCAGGGGAGTAAAAACGGATTATTTAGTCT
TCAACAGGACACTGCCCCTAGAGGCGTTGCAGCTGTGGCTGCCGTGTCACATCTGTGTCATTAG
GTGGCAGAGATTAGAGAGGCTATGTCTACGCTCAGCGTTCTGCCCCGTGAACGTTTGAATGTTT
GATAGTCTCACACTCCTGTTTTGTGGACAGGCATCTGTTTTCCTTCGCTGGTATCTGGGAGAGAG
GGTGGGCCCTGTGAGTGGGCTTTCTAACTCTCGCGCTGGGCTAGGCTGGCCTCTAGGGGTCTGA
AGGGATAGTGGGAAGGTGGAGGGTGCCTTCACCAGGAGCAGCCTTCATGAGTGAGTTTTAAGA
ACCTGTGAGGATCGGTCAGGTCAGGTGAGGTACGCGCTGGATCATGCCGTACACCGTGTCAGG
ATCACGCAGGACAGGACTGGGGAAGGGTTTGCTGGCGTCTCGGTTAATAGCAGTAACGGCAGC
CGCTTGTGTTCTTGATTCTTTGCCAAGCATTCTTGTTATTTAACACGGGAAGGAGGTGACTTCGC
CTGTGATGGACTTCCAGTGTGAGCACTGGCCAGAGTGACCAGGCTGACCAGCACCAGCCCTGA
TCCAGGTAAGAGGTGAGGCGGAGGCTTGCCCTCCTTCCCTGCCTGCCGGGTGCGTGTGAGCGG
CTGCCCTGCGGATGCTGTCCTGGACCTGTTGGCACCACAGACAGTTGCTCTGCTGTGCCTGTGG
CCTCGGGGCAAAGAGAAAGTGGCGATTTCTACACTCAGTGCTCGGGAACCAGTGGGCACTGAG
AATGGTTTATGGCCTGACATTACTTGCAACCAAAGCCAGGCAGCATTTGGATTTTCGCTTTTGGA

AACCACCTTACCTGGTTTGCTCCATGAGGGAGGGGCAGCAACATCGCTGGGAACCCTGAGGTG
TAGATATGTATAAATGAGCTGTGTGTGTATCTGAAGCTCGGGCACTTGTGGGGTTACCAGGAGT
GTGCGTCTCACTCCCAGGCCTCGTTCTGGAAACGATTCTGTAGGTCTGTCTGGGAGGCCTTAGG
CAAGTTTGATTAAGAACAGTCGAGCTGGATAAACAGGCTGTTCCTTGTCCAGCACTAGTCATCC
CAGGGGGGCGGGGGCGGGGCTGCAACTCCTGTGCACAAAGGAAGGGCCCTGGGTAGGGGTC
AGGAGCTGCCTTGCCCAGAGATGTGTCTGGGTCACTTTGGAAACAGGAATCCTCTCCCCAAAAT
GGGTAGCAATGGGGGTACAGAGGCCAAAGACGCAGCTGCATGTCCTGCAGTGCCTGGGACGC
CCCGTAGTGAAGAGTGATGTGGCCCAAATGTCAGCAGTGCCAGTGTCAGACTCCTGCTAAGAG
ACAGTATCAAGAAAGATAACTTCAAAATACACTTTCTGGAGGCCTGGCGCGGTGGCTCAGGCCT
GTAATCCCAGCACTCTGGGAGGCCAATGCATGTGGATCACCTGAGGTCAGGAGTTCGAGACCA
GCCTGGCCAACTGCCTGAAACCCCATCTCTACTAAAAATACAAAAATTAGCCAGGTGTGGTGGC
GCAAGCCTGTAATCCCAGCTATTTGGGAGGCTGAGGCAGGAGAATCGCTTGAACCCGGGAGGC
AGAGGTTGCAGTGAGCCGAGATCGCACCACTGCACTCCAGCCTGGACAACAGAGTGGGACTCT
GTCTCAAAAAAAAAAAAAAAAAAA

ID|27173|gene_id|84973|transcript_id|NR_024542 Homo sapiens lncRNA

CTCTGCGTGCGCCGGAGGCTGCCGTGGCGGGTGGGCCGCCTGACTTCTCCTCCCGGCCAG
TTCTCGAGCGCCTCACCGGGCCTCGCCCTGCAGCCTCGCTCTCGCTGGCGCTGCGCGGCCTAG
GGGACTGGGCTGCTGGCCTCCGGGTGCGGGGTGGGGGCAGGCTCCGACCTGGGGCGTCCTGG
CCGCGCGAGCCGCGGGATGGGGGCCCGGGCCGCGGAGGAGGCGCCGCTGGTGTGTCCCTTGG
TGGAGAGGGCGCTGCCGGCCCTGCGCGGTTTCCAGCCAGGAAGCTTCGGGAAGCCTGGACGT
CTGCTCACTGGAGATGACACGTGCGTGGGGTGTTGGCATTCTTGTTATTTAACACGGGAAGGAG
GTGACTTCGCCTGTGATGGACTTCCAGTGTGAGCACTGGCCAGAGTGACCAGGCTGACCAGCA
CCAGCCCTGATCCAGATGCAGAGGCCAGGATGTGGGCCCAGCCCTGTGCCAGGAGGCTGGCT
GGAATAAAGGTACAGATAGAGGCCTCACCCCCTCTGGGACCACTGGCACTCAGGGTGTTTGCA
GCCTCAGAGCCCACCTGCCCCCAGGGCCACAGCTGCATCTCCTGCCCTGCTGTCATTACAGGG
ATGGGCAGGCTGGCATGGGGGCAGCCGCTGCCCCTGCCTGGGTGTTGCTGTGTATTCCTGCCG
GCCAGGGGCCACTGCCAGGACCACGCCTCCCTTTTCATATCCCGATTCTTAAGTTCTGCTATTGT
GGTATTCTGGTGGAGAAAAAAGAACCGCGTGGCTGTTTTTGAACTGCCTGGAACCTAAGACCCT
GAATTCTTTTCCCCCCCAAGGGGAAAATCTATATGGAAAACATTTATTTTAAAATACAGGATGAA
GTGAATTAAAAGATTTAAATGCACATTTCTTTAAGGATAATATTTCTGTGTTGGCAAAATTTGAGA
GTAAATTGGTCTTGAATGGAAAAAAAAAAAAAAAAAAA

ID|27174|gene_id|84973|transcript_id|NR_024543 Homo sapiens lncRNA

CTCTGCGTGCGCCGGAGGCTGCCGTGGCGGGTGGGCCGCCTGACTTCTCCTCCCGGCCAG
TTCTCGAGCGCCTCACCGGGCCTCGCCCTGCAGCCTCGCTCTCGCTGGCGCTGCGCGGCCTAG
GGGACTGGGCTGCTGGCCTCCGGGTGCGGGGTGGGGGCAGGCTCCGACCTGGGGCGTCCTGG
CCGCGCGAGCCGCGGGATGGGGGCCCGGGCCGCGGAGGAGGCGCCGCTGGTGTGTCCCTTGG
TGGAGAGGGCGCTGCCGGCCCTGCGCGGTTTCCAGCCAGGAAGCTTCGGGAAGCCTGGACGT
CTGCTCACTGGAGATGACACGTGCGTGGGGTGTTGGCATTCTTGTTATTTAACACGGGAAGGAG
GTGACTTCGCCTGTGATGGACTTCCAGTGTGAGCACTGGCCAGAGTGACCAGGCTGACCAGCA
CCAGCCCTGATCCAGATGCAGAGGCCAGGATGTGGGCCCAGCCCTGTGCCAGGAGGCTGGCT
GGAATAAAGGGATGGGCAGGCTGGCATGGGGGCAGCCGCTGCCCCTGCCTGGGTGTTGCTGT
GTATTCCTGCCGGCCAGGGGCCACTGCCAGGACCACGCCTCCCTTTTCATATCCCGATTCTTAA
GTTCTGCTATTGTGGTATTCTGGTGGAGAAAAAAGAACCGCGTGGCTGTTTTTGAACTGCCTGGA
ACCTAAGACCCTGAATTCTTTTCCCCCCCAAGGGGAAAATCTATATGGAAAACATTTATTTTAAA

ATACAGGATGAAGTGAATTAAAAGATTTAAATGCACATTTCTTTAAGGATAATATTTCTGTGTTG
GCAAAATTTGAGAGTAAATTGGTCTTGAATGGAAAAAAAAAAAAAAAAAA
ID|30208|gene_id|EF565083|transcript_id|EF565083 Homo sapiens lncRNA

GGAAGAAGTGAAATCACTATATTTAGAAACGAATGCTACCATCCCACCCCAACTACATGTT
CAGGTTAAATGCGTGCACATCCTGGTAGCTGGAACATCTGTTTCTAGCTTGTTGTTCCTCTTGGCT
GCTTTTCAATTGGATTTCCTCGAGCAGGTCCCAGGCTTGTCACTGCAGCCAGGGTCTGTTAACCA
TCACCACTGGTTCTACATTAAGTAGGAGGTTCAGGGCTAAGCTAATAACTCGCCTGCCTTTCTGA
CTGATCACAGTGATGTTCGCCGTGGCCACTTATCATGCGGGCTCCTTTGGGAGGGTGCTCTTCTT
TGTCTGTACCTCTGGGTGGAAAGGGCGAATTC
ID|30582|gene_id|RLGI00550|transcript_id|RLGI00550 Homo sapiens lncRNA

TGGATCGATGATGACTTCCTTATATACATTCCTTGGAAAGCTGAACAAAATGAGTGAAAAC
TCTATACCGTCATCCTCGTCGAACTGAGGTCCAGCACATTGCTCTTACAGGGGCTAGAGAGAGA
GGGACAAATTTCATTTGATGATGCCCATTGCACCAAGGGGTTCTGTCCAGGCTTAGGATGGGGT
CTCGTTTGGGCAAAGGAGAATGGCAGGGGAGTGGAGGCTATGTACACAGGAGATTCCTTGTTT
GAAGGACTCTATTTGTGAGGCCAGGGTACCACACATGCTGTCCGCAGGAGTAGGTGAATGTGC
AGTTGCCCAGGAAGAGTTAGCCTGTAGCCTGCCTCTGCATGGCAGTTTGTCCTTGGGTCCTGGC
TCTGGATTTCCATGTTCCTTGGAGGAGGATAGGTGATTTTGCTTGAGAAGACAGCACAGTACCAT
ACTTTTGTTGTTTTTCTGCTCATTTCATCGTCCATTGAGGACAATGAAGTTGTGGTCAGCAGGCAT
AGCTTTCAGGCCAGCGTGCCCATGTTGTGTCCCATGCATTGTGAGCACATGCATGTGGCATGAA
CACATAGGCTGCCACTCCAAGCTGAGTCTGATAGGCAATGAGACTCTGGCTTATCCTGATCCCG
GTGTAGATCAAAGTCTTCCCAGTAGGATTGCATGGCCCCGAGGCTATTGTGAGCTGCATTGCAG
GTGTGGAAGCAAGGGTGTTGAGAGGGATGCTCAACATTAGTGCTCTTTAGCGAGATGATGCACT
ATAAGGGCACCCTGAACCCAGACGTGCATCCCTATGTACGTGCATTTCTGTGTCCATAAATAGTT
GAAGCCAGACAGCCAGATTCCAGATGTATCGCAGGGGGCTGGATGACATGGCCCTTGTCACCT
GTGTACCTGTCTGCCTTTCTGAAGCACGCTTGTGTTTCCTCTACACCTCCCAGGTAGCATTGGCA
TGGAAGGCAGGCCCATGTTGGTGAGGGACAATTGTTATCTTGTGTGAGCCGCAGGGATACCAG
GAAACCCCTGGACACAAATGGCAAAGGCTTCTTTGGAAGTTGTTGGATCCCTTCTGCATGTAAG
CAGTTCTTTCCCAGAGCGCTCTGATTTTCCTCATTTGCAGGGACAAACACTGTGCGTGGATCGAT
GATGACTTCCATATATACATTCCTTGGAAAGCTGAACAAAATGAGTGAAAACTCTATACCGTCAT
CCTCGTCGAACTGAGTGGATCGATGATGACTTCCATATATACATTCCTTGGAAAGCTGAACAAA
ATGAGTGAAAACTCTATACTGTCATCCTCGTCGAACTGAGGTCCAGCACATTACTCCAACAGGG
GCTAGACAGAGAAGGCCAACATCCGTTTGTTGACATGGGTTATATCAAGGCGTCTGTTCAGGCT
TAGAATGTGGTCTCTTATGGGTGATGGGGGTCACAGGAGAGTGGTGGCTCCCATGTATAGGAAA
TTTCTTGTTTGAAGGACTGTCAGTGAGGGTGGGTAACACATGCATTGTCTGCAGGACTAGGTGA
ATGTCCATGTGGCCTAGCAAGAGTTAGCTGGTAGCCCGCCTCTGGTTGCCAATTTGTTCTTGAGT
CCTTGTTCTGGGTTCTCAGGTCCCACGGAGGAAAACAGATCTGTGTGGTTGAGAGGTGGGTACA
AGGCCGCATCTTTGTCATTTGTTGGCTAACTTTGTCCTTGGTTGAGGACATTAGAGTTTTGGTCAC
CAGGCATAGCCTATGTGCCTTTGTGCCCGTGTTGTATCCCACGTGTTTTGAGGACATGTATTTTG
CACGTAAAGGTGAGCTCCTGCTCCAAGCTGGTTCTGATACCAAAGGAGTCCCTGGCTTATCCTA
AACTCATGGTAGGTTAAAGCCTTCCTCCTTAGGGGTTCAGGGCCGCAAGGCTTTTGTGAGTGGC
ATTGCAGGCGTTGAAGCAGTGATGTTGAGAGGGATGGTCAATGTCAGTGCTCTTTAGCAGGATG
GTGTACTGCAGGGGCCCCCAGCCCCGAGACGAGCATCCCTGCATCCATGCATTTCTGCCTCCAT
GAACAGGGGAGGCCAGAGACAGGCAGATAGTAGATAAATTGCAGGGGACTGGATGACATGGC

CCTCGTGACCTGTGCACCTGTCTGTCTTTCTGAAGCACGCCTGTGTTAACTCTGCACCTCCCAGG
TAGCACTGGCATGGAGGGCAGGCACATGTTGGTGAGGGACAATTGTTACCTTGTGTGAGCTGC
GGAGATACCAGGAAGCCCCTGGACACAAATGGCAAAGGCTCCTTCGGAAGTTGTTGGATCCCT
TCTGAATGTAAGCACTTCTTTCCCAGAGCACTCTGAGTTTCCTCATTTGCAGGGACAAATACTGT
GCGTGGATCGATGATGACTTCCACATATACATTCCTTGGAAAGCTGAACAAAATGAGTGAAAAC
TCTATACCGTCATCCTCGTCGAACTGAGGTCCATGGATCGATGATGACTATAAAAAAAATGGAT
CTCATCGGAATCTGAACAAAATGAGTGACCAAATCATTTCTGTGCCACTTCTGTGAGCTGAGGTC
CAGCACTGGGACCATGCCAGGCAAACATGAGGAGGGAGTTTACAGGGACTAGGGTATTGGAA
AACTAACCTACCCATATATATTAGAGCAAATGAATTGTTCTCAGTTCTGTAAATATCCAGCTGTG
GTTTGACATAGCAACTCTCTTTGTATTTGCTTGGCAGCATTTATAGAAACAGCTGGTAGACCCTG
GGGAACCAGGGCATAGTGAGATTCATGGGACGTTATTTCACCTACTCTTGAGCCATTCACACAT
TTGAGCTCGCAGAATTATCTGGTCAATGAGCAGTCTCTTGAGGGTAATGAGATGGTCAACATCC
AAATCCAAAGATACTAATGAGATTTTACTTTTTTTTTTGAGATGGAGTCTCATTCTGTTGCCCAGG
CTGGAGTGCAGTGGCATAATCTCGGCTCACTGCAACCTCCACCTCCTGGGTTCAAGAGATTCTC
CCGCCCCAGCATCCTGAGTAGCTGGGATTACAGGCGCCCGCCACCACCCCTGGCTAATTTTTGT
GTTTGTAGTAGAGATGGAGTTTCACCATATTGGCCAAGCTGGTCTCGAACTCCCAACCTCAGGT
GATCCAGCCACTGCAGCCTCTCAAAGTGTTGGGATTACAGGCATGAGCCGCCGTGCCCGGCCT
TACTTTTTATTTTCAATTAAGTGAAACAATTACATGGAAATTGAAAAAAAAAAAAAGGTACTCTAA
GTAGACTACTCTAGTGTATTTTAATTTTCTTGCCAATTCAGCCACTAGGATTGTGTCACTTGTTTTT
TTGATTTTAGTTTTTCGTATCAAAAAGCAAACCACTGTTTATATTTGAGTGCTGGTTTCAACTGTAT
GCTGCCTCAAATAAACTTAGATTATATTACAAATTCTTTTTTACATAGATTTCATTGGCTATATTCA
TATATCCTCAAAGGTTTCCATTGTGTTTGAAGAGGTATCCTAATTGTAATAAATGCTTTTGATTGG
GGATGCCAGGGAAGCTTTGAGACTAATGTCTCAGATTTTTTTTAAAAAAGTTTATATTCTATAGTG
ATCAAAGATGTAAGAATAATTTTTCTTGTCATTCATTGGATAGTCCAGTGAGTGTCTACTGTGAAT
TCTTATATTCACTTGTAACTAAACATTCCATTTGGCAAATTTCGTTTCATAACTTCCCTAATTTCCT
CTACACTTTGTAGATTGGGTGAGATACAATTCCATGGAATTTTACCACTGAACCAGTGGAGGGC
TAATTTTGTCATTTGTACCAGTTAACTAACTTGGGAGGAAACAGAACAACACACTCCTGAGGTA
GTATCCAATTTTTAGTAAATTTATTTTTATTATTTTTCAAACTGTCAGTGATTTGGTTTGGTGAACA
ATAGGTTAATACAGACAATGTTAGGAATTATTAATCTGATGCGCTATCTGAAGCATCAGTTTCCT
TTATTTTTAAGATTTTTTTCTTCTCTGTAGTTTGGATAATATTTAAAACATTTCCAAGAGTTATAAAT
TAGTATATTTTCTATGCTGATTAATAATGAAACCTTTTGTCTTATACAGATTTTTAAAAAATGGAC
GTGTGCGTGCATGTGTATATATATAAATATATATATATATAAAATCTCTTCTCACAAGATGATGCA
GACTAAAACTGCATTTTACATTTTATATGGCTAAGTTTATTGTTTCCTTGTGTTGTAATATGATGGA
AAGTAATGGGAATAATTCTGCTTCCTGTCCTTAGGGACCAGTAGGAATTTTCCCATGATAAGCAT
TTTGTCCTTAAAATACTGACATCCTGTTAAGAGATCATGACACTCCAATGACCATGAGACCCTGG
CTATGCTAGTCAGCCAGGGGGCTGACCATTGACAGTGATGCTGGTCTCCATGATTAAATAGTGT
GGGTCAGCATGTCCCAGGGTGACAGAAAGCTGTGTGATCAGTGTCTGTCACATATCTCAGACTC
AGGGTTCCTTTTTAAAATGTGCCTGGATCGATGATGACTTAAAGATTTATCTAATTTAAATCTGAA
CAAAATGAGTGACCAAAACACTTCTGTACCACTTCTGTGAGCTGAGGTCCAGCACAGAG
ID|30583|gene_id|RLGI00551|transcript_id|RLGI00551 Homo sapiens lncRNA

TGGATCGATGATGACTTCCTTATATACATTCCTTGGAAAGCTGAACAAAATGAGTGAAAAC
TCTATACCGTCATCCTCGTCGAACTGAGGTCCAGCACATTGCTCTTACAGGGGCTAGAGAGAGA
GGGACAAATTTCATTTGATGATGCCCATTGCACCAAGGGGTTCTGTCCAGGCTTAGGATGGGGT

CTCGTTTGGGCAAAGGAGAATGGCAGGGGAGTGGAGGCTATGTACACAGGAGATTCCTTGTTT
GAAGGACTCTATTTGTGAGGCCAGGGTACCACACATGCTGTCCGCAGGAGTAGGTGAATGTGC
AGTTGCCCAGGAAGAGTTAGCCTGTAGCCTGCCTCTGCATGGCAGTTTGTCCTTGGGTCCTGGC
TCTGGATTTCCATGTTCCTTGGAGGAGGATAGGTGATTTTGCTTGAGAAGACAGCACAGTACCAT
ACTTTTGTTGTTTTTCTGCTCATTTCATCGTCCATTGAGGACAATGAAGTTGTGGTCAGCAGGCAT
AGCTTTCAGGCCAGCGTGCCCATGTTGTGTCCCATGCATTGTGAGCACATGCATGTGGCATGAA
CACATAGGCTGCCACTCCAAGCTGAGTCTGATAGGCAATGAGACTCTGGCTTATCCTGATCCCG
GTGTAGATCAAAGTCTTCCCAGTAGGATTGCATGGCCCCGAGGCTATTGTGAGCTGCATTGCAG
GTGTGGAAGCAAGGGTGTTGAGAGGGATGCTCAACATTAGTGCTCTTTAGCGAGATGATGCACT
ATAAGGGCACCCTGAACCCAGACGTGCATCCCTATGTACGTGCATTTCTGTGTCCATAAATAGTT
GAAGCCAGACAGCCAGATTCCAGATGTATCGCAGGGGGCTGGATGACATGGCCCTTGTCACCT
GTGTACCTGTCTGCCTTTCTGAAGCACGCTTGTGTTTCCTCTACACCTCCCAGGTAGCATTGGCA
TGGAAGGCAGGCCCATGTTGGTGAGGGACAATTGTTATCTTGTGTGAGCCGCAGGGATACCAG
GAAACCCCTGGACACAAATGGCAAAGGCTTCTTTGGAAGTTGTTGGATCCCTTCTGCATGTAAG
CAGTTCTTTCCCAGAGCGCTCTGATTTTCCTCATTTGCAGGGACAAACACTGTGCGTGGATCGAT
GATGACTTCCATATATACATTCCTTGGAAAGCTGAACAAAATGAGTGAAAACTCTATACCGTCAT
CCTCGTCGAACTGAGTGGATCGATGATGACTTCCATATATACATTCCTTGGAAAGCTGAACAAA
ATGAGTGAAAACTCTATACTGTCATCCTCGTCGAACTGAGGTCCAGCACATTACTCCAACAGGG
GCTAGACAGAGAAGGCCAACATCCGTTTGTTGACATGGGTTATATCAAGGCGTCTGTTCAGGCT
TAGAATGTGGTCTCTTATGGGTGATGGGGGTCACAGGAGAGTGGTGGCTCCCATGTATAGGAAA
TTTCTTGTTTGAAGGACTGTCAGTGAGGGTGGGTAACACATGCATTGTCTGCAGGACTAGGTGA
ATGTCCATGTGGCCTAGCAAGAGTTAGCTGGTAGCCCGCCTCTGGTTGCCAATTTGTTCTTGAGT
CCTTGTTCTGGGTTCTCAGGTCCCACGGAGGAAAACAGATCTGTGTGGTTGAGAGGTGGGTACA
AGGCCGCATCTTTGTCATTTGTTGGCTAACTTTGTCCTTGGTTGAGGACATTAGAGTTTTGGTCAC
CAGGCATAGCCTATGTGCCTTTGTGCCCGTGTTGTATCCCACGTGTTTTGAGGACATGTATTTTG
CACGTAAAGGTGAGCTCCTGCTCCAAGCTGGTTCTGATACCAAAGGAGTCCCTGGCTTATCCTA
AACTCATGGTAGGTTAAAGCCTTCCTCCTTAGGGGTTCAGGGCCGCAAGGCTTTTGTGAGTGGC
ATTGCAGGCGTTGAAGCAGTGATGTTGAGAGGGATGGTCAATGTCAGTGCTCTTTAGCAGGATG
GTGTACTGCAGGGGCCCCCAGCCCCGAGACGAGCATCCCTGCATCCATGCATTTCTGCCTCCAT
GAACAGGGGAGGCCAGAGACAGGCAGATAGTAGATAAATTGCAGGGGACTGGATGACATGGC
CCTCGTGACCTGTGCACCTGTCTGTCTTTCTGAAGCACGCCTGTGTTAACTCTGCACCTCCCAGG
TAGCACTGGCATGGAGGGCAGGCACATGTTGGTGAGGGACAATTGTTACCTTGTGTGAGCTGC
GGAGATACCAGGAAGCCCCTGGACACAAATGGCAAAGGCTCCTTCGGAAGTTGTTGGATCCCT
TCTGAATGTAAGCACTTCTTTCCCAGAGCACTCTGAGTTTCCTCATTTGCAGGGACAAATACTGT
GCGTGGATCGATGATGACTTCCACATATACATTCCTTGGAAAGCTGAACAAAATGAGTGAAAAC
TCTATACCGTCATCCTCGTCGAACTGAGGTCCA
ID|30584|gene_id|RLGI00552|transcript_id|RLGI00552 Homo sapiens lncRNA

TGGATCGATGATGACTTCCTTATATACATTCCTTGGAAAGCTGAACAAAATGAGTGAAAAC
TCTATACCGTCATCCTCGTCGAACTGAGGTCCAGCACATTGCTCTTACAGGGGCTAGAGAGAGA
GGGACAAATTTCATTTGATGATGCCCATTGCACCAAGGGGTTCTGTCCAGGCTTAGGATGGGGT
CTCGTTTGGGCAAAGGAGAATGGCAGGGGAGTGGAGGCTATGTACACAGGAGATTCCTTGTTT
GAAGGACTCTATTTGTGAGGCCAGGGTACCACACATGCTGTCCGCAGGAGTAGGTGAATGTGC
AGTTGCCCAGGAAGAGTTAGCCTGTAGCCTGCCTCTGCATGGCAGTTTGTCCTTGGGTCCTGGC

TCTGGATTTCCATGTTCCTTGGAGGAGGATAGGTGATTTTGCTTGAGAAGACAGCACAGTACCAT
ACTTTTGTTGTTTTTCTGCTCATTTCATCGTCCATTGAGGACAATGAAGTTGTGGTCAGCAGGCAT
AGCTTTCAGGCCAGCGTGCCCATGTTGTGTCCCATGCATTGTGAGCACATGCATGTGGCATGAA
CACATAGGCTGCCACTCCAAGCTGAGTCTGATAGGCAATGAGACTCTGGCTTATCCTGATCCCG
GTGTAGATCAAAGTCTTCCCAGTAGGATTGCATGGCCCCGAGGCTATTGTGAGCTGCATTGCAG
GTGTGGAAGCAAGGGTGTTGAGAGGGATGCTCAACATTAGTGCTCTTTAGCGAGATGATGCACT
ATAAGGGCACCCTGAACCCAGACGTGCATCCCTATGTACGTGCATTTCTGTGTCCATAAATAGTT
GAAGCCAGACAGCCAGATTCCAGATGTATCGCAGGGGGCTGGATGACATGGCCCTTGTCACCT
GTGTACCTGTCTGCCTTTCTGAAGCACGCTTGTGTTTCCTCTACACCTCCCAGGTAGCATTGGCA
TGGAAGGCAGGCCCATGTTGGTGAGGGACAATTGTTATCTTGTGTGAGCCGCAGGGATACCAG
GAAACCCCTGGACACAAATGGCAAAGGCTTCTTTGGAAGTTGTTGGATCCCTTCTGCATGTAAG
CAGTTCTTTCCCAGAGCGCTCTGATTTTCCTCATTTGCAGGGACAAACACTGTGCGTGGATCGAT
GATGACTTCCATATATACATTCCTTGGAAAGCTGAACAAAATGAGTGAAAACTCTATACCGTCAT
CCTCGTCGAACTGAG
ID|30585|gene_id|RLGI00553|transcript_id|RLGI00553 Homo sapiens lncRNA

TGGATCGATGATGAGTCCTCCAAAAAAAACATTCCTTGGAAAAGCTGAACAAAATGAGTG
AAAACTCATACCGTCATTCTCATCGGAACTGAGGTCCAGCACATTGCTTCCTCTGGGGCCTTTAC
TAGTGAGGGACAACTTCCACTAGTCCCTATAGGTTCTAACACCCATGGCGTTCACAGGTGAAGG
TCTCCTGTCATATTACAAACATTCCCCCCACTCCCCTCCCAAAAAAAGGAACATTTCCTCTGTGT
CAGGTACACAGGTCAGAGAGTCATAGGGTGACTGGCTGCTCTGTGATGGCATAGTGGCCGTGG
AAGTGGATGGAAATGTCGCAGGCTTAGGGGAGTGTGGGGCTGGCCTCGGCTTGGTGAGCTCCC
AGGAAGGGGGACCTGGAAATCGCTGCGGGCATCCTTGCATAGAGGACCAGGTACGTGTTCCAG
TTCTGCTTTCAAGGAGAGCATGAGGCCCAGGCCTCAGACAGTCCTTGGTCTTGATCTTTGGTTAG
CTGAGGACAGGGGAGTGCCAAGGGTGATAGGGGAGAGCGCACAGCAATGGATGTTGTGGCAG
ACAGCTAAAGCCTTTCTGTCCCTCCTTTCACGGTCAGTCCCAGGGGGCCCCCAGCACAGACTTT
TTTCACCTCTCGAGCTGTGAGATTCCTGTGGTGTTGTGGCCAAGCCTGGGCAGGAAGGTACCCC
AGAGAAAAGGTTGTAGAGGGCTCACGTGGACATGCATGCCATCAGTGGTCTTGGGCTGTCATTT
GGTGGTGATGGGCTTTCCAGGGATTTCTCTGGTGGATGACAGGTTGGCCATTGGGGGTCTTCAA
GGGCAGGTCCTAGGGAGCATGAGGGTCGGGCTGATGCCTGTGGTCCTGGGTCACTCCATTCTTC
CTGGCAGAGTAGTAGAAGTGTCCATCTTCCTGGAAATGGTCTCTTGGCCCGCCATTGCCAGGCG
GCCCCACCTGCTGCAGCTCGTGTGAGCAAATACTGCTCTGGATCCCATTTCCCGCTTTGAAAGC
AGCTTCCATCCCAAAGGCGTCCAGAGGGAATGTGCCCTGGCCCTGGCCCTGGCCTGGGACTCT
CCGATGGGGTCCAGTCACACTTGGCGTATTCATGGAGGTCCTTGTGCATTCCAGGCAACACATG
TGCAATACCAGGGGCACTGCCGTGGAGAGTCTCTTGATTTGGGATGCCTCCTCGCCTTTGTGTGT
TGGCGATGTTCCAGTATTGGATTCTGCTACATCCTCAGGGCAATGAAGCGTTTCAGCTCACTTAG
CCGGTTTGACATCATAGTGCAGCCAGGACAGCCGTGCCACATTGGCATGGCACATAGTTGACTT
AGGACAGTCGGAAAGGGTTGGTGGTTCTATGTCTCGCAGCTCACTTCATAGCTGTTTGCATGTA
AACCTGGTGATGGTGTTGAGATGGTGTTGACTTGCTGACGTTGGGGTGGTTGGTAGTGCAGTTA
AGTTGGCTGCATGATGGGATGTTGCCTCAGATGTCGGTGCAGCGAAGCCCTGTAGGTGTGTCCA
ATGCAAGGAAAATGAAAGGTGGTGTGTCCTGTCCACCTTGTCTTTAGGGGTTCAGGCAGGAATG
TCCTGTTGGAAAACTAGCCATTCCCTTTGTGTGTCCTGAAGGCATGGTGCGGGCATGTGAACTG
ATTGGAGGAAGGAAGTGTGTGTCTTGAGGTCCTTTGGCTGGGTTCAGAAGACTGTCTCTGGCCT
TGATGCCGTGTGCCGTGTTTAAGACCAACGTGGTGGCCATTGCAAAGGTGCCTTCCAGTGCAAG

TTTTGGTTGGTTTGGTGTCAGGTGTGCCTTACCTGGGAGAGCGGCGAGAGACAGTGTCTGGTCA
CGACATTATATGCCCCGTAAGGAGGACTGTGTGCTTGCACCGCCAGGGGTGCTGGCTGTTAGTC
GAGGGACATCAGGCCAATGTCTGTCTGACCCCTAGGTCAAAATTCGCATCCTACTGATTGAAGG
CCTTGGGTCTCGAGCCTTTTGGTTGCAGGTGTGGGGATGGTTTCCAGGTCTGGAGGCTGCCAAC
TATGACAACCCGTTGTGCTGTTGGGAAACATCTGTGTGGGGGAGACACATACACATAGACACAC
TCACTCTGACATACACACCTACACATGCACAGGCGTGCACAGCCACAGGCAGATAGATGCAGC
AATGGGTGTGTGCTATTGTTGTGGTCACATGTGCTTCCTAGGTGTCCACAGAGTGACATGGGTCA
GGTATATTCATGAAGATGGAGGTCTGTCATGGAGCCCAAGGAGAAGCCGGTGTGCAAGTTTCC
CTGTGGGAAAGAATGGGATTGAAGTGGTGACCAGTGGACTTGGGCCCGTTTCTCAAGCATGCAT
TTGATTTCTTTACACTTCTCAGCAGCAGTTGTCATGAGAGGTCTGCCGCTTGCTGGCAAGGAAGA
TGGTCAATTCTTCTGACCAACAGAGGCTGCCTTGAGTCCTGATTTGGTGTGGCCTGGCATCTATG
GTGCACCCAATGCTGGTGAGTGGCTCAGGGTGGTAAGCATTCCTCTGCCCTGTGTGCAGAGGTG
TCTTTTGAATTTTGAGACCTGCAAAGAGCTGTTTCTCAGCAGGCCACTAATGGCATGGCGAGTTC
CACTCCCAGAGCTGATTGTCTGTGTTCATTGCTCAGTGGTGTGGCTCTGGGTTCCCCCCAGGATG
CTGAAGGTGTTTAAGGTCATCAGCTCTCCCACTGCCCAGCCTGTGGTGTCAAATGTTCTGCCTCT
TCGAACGTGCTTGGATCGATGATGAGTCCCCCATAAAAACATTCCTTGGAAAAGCTGAACAAAA
TGAGTGAGAACTCATACCGTCGTTCTCATCAGAACTGAGGTCCTGGACCAATGATGACTTCCAT
ACATGCATTCCTTGGAAAGCTGAACAAAATGAGTGGGAACTCTGTACTATCATCTTAGTTGAACT
GAGGTCCACCGGGGGCTATAGAGGGACAACTGTCATTTTTTTTGATGCCCGTTGCATGAAGCAT
TCTTCCAGGCTTAGGAAGGGATTCCGTTTGGGTGAAGGAGAGTCACAGGGGAGTTGAGGCTAT
GTGCACAGGACTTCTGTCTACAAGGCTGGGGTAGCACACATGCAGTCCGTGGGACTAGGTGAA
TGTGCAGGTTGCTCAGGGAGAGTTAACCTGTAGCCTGCCTCTGGGTGGCCAAGATTGTCCTTGA
CTCCTGGCTCTGGGTTCCCACAACCCCTGGAGGAAGACAAGTAATTGTGCTTGGGAAGAGAGA
ACAAGGCCATACTTTTGCCTTTCTTTTTTTCATTCTTAGTTTGAGAACAGTGGAGTTCCAATCACC
TGGCATAGCTTTCATGTTGTGTTCCATGCATTGTGAGTACATGCATATGGCAGGAACACACAGGT
TCCCACTGCAAGCTGAGCCTGATAGGCAAGGAGTCTCTAGCTTAACCCCAGCCCAGTGTACGTC
AATGGCTTCCTCATTAAAATTGCATGGCCCTGTAGCTTTTGTGAGTGACATTATAGGTATCGATG
TAAGGGTGTTGAGAGGGATGCTCAATGTCAGTGCTCTTTAGTGTGCTGATGCACTACTACAAGG
GCACCTGAGCCCTTGTCCATGCATTTCTGTGTCCAAAATCAGGTAAAGCCAGAGTTGGGTATCA
ACCAGATACACTGCAGGGGATCAGAGGACATGGCCTTTGTGTACTGCACATCTGTCAGATTTTC
TCAAGCACACCCGTGTTTCCTCTTCACCTCCCACGTGGAAGTGATGAATTGGCCAGGACCATGT
TGGTGAGGGACAATCATCATCTCATGTTAACCTCAGAGAAGCCAGGAAGCTCCTGGACACACAT
GGTGTAGGCTTCCTTGGAGGCTGTTGGATCTCTCCTGAATGTAAGCAATTCCTTCCCAGTGCACC
CTGATTTTCCTCATTTGCAGAAACAAAGATTGTGTGTGGATCGATGATGACTTCCAT
ID|30586|gene_id|RLGI00554|transcript_id|RLGI00554 Homo sapiens lncRNA

TGGATCGATGATGAGTCCTCCAAAAAAAACATTCCTTGGAAAAGCTGAACAAAATGAGTG
AAAACTCATACCGTCATTCTCATCGGAACTGAGGTCCAGCACATTGCTTCCTCTGGGGCCTTTAC
TAGTGAGGGACAACTTCCACTAGTCCCTATAGGTTCTAACACCCATGGCGTTCACAGGTGAAGG
TCTCCTGTCATATTACAAACATTCCCCCCACTCCCCTCCCAAAAAAAGGAACATTTCCTCTGTGT
CAGGTACACAGGTCAGAGAGTCATAGGGTGACTGGCTGCTCTGTGATGGCATAGTGGCCGTGG
AAGTGGATGGAAATGTCGCAGGCTTAGGGGAGTGTGGGGCTGGCCTCGGCTTGGTGAGCTCCC
AGGAAGGGGGACCTGGAAATCGCTGCGGGCATCCTTGCATAGAGGACCAGGTACGTGTTCCAG
TTCTGCTTTCAAGGAGAGCATGAGGCCCAGGCCTCAGACAGTCCTTGGTCTTGATCTTTGGTTAG

CTGAGGACAGGGGAGTGCCAAGGGTGATAGGGGAGAGCGCACAGCAATGGATGTTGTGGCAG ACAGCTAAAGCCTTTCTGTCCCTCCTTTCACGGTCAGTCCCAGGGGGCCCCCAGCACAGACTTT TTTCACCTCTCGAGCTGTGAGATTCCTGTGGTGTTGTGGCCAAGCCTGGGCAGGAAGGTACCCC AGAGAAAAGGTTGTAGAGGGCTCACGTGGACATGCATGCCATCAGTGGTCTTGGGCTGTCATTT GGTGGTGATGGGCTTTCCAGGGATTTCTCTGGTGGATGACAGGTTGGCCATTGGGGGTCTTCAA GGGCAGGTCCTAGGGAGCATGAGGGTCGGGCTGATGCCTGTGGTCCTGGGTCACTCCATTCTTC CTGGCAGAGTAGTAGAAGTGTCCATCTTCCTGGAAATGGTCTCTTGGCCCGCCATTGCCAGGCG GCCCCACCTGCTGCAGCTCGTGTGAGCAAATACTGCTCTGGATCCCATTTCCCGCTTTGAAAGC AGCTTCCATCCCAAAGGCGTCCAGAGGGAATGTGCCCTGGCCCTGGCCCTGGCCTGGGACTCT CCGATGGGGTCCAGTCACACTTGGCGTATTCATGGAGGTCCTTGTGCATTCCAGGCAACACATG TGCAATACCAGGGGCACTGCCGTGGAGAGTCTCTTGATTTGGGATGCCTCCTCGCCTTTGTGTGT TGGCGATGTTCCAGTATTGGATTCTGCTACATCCTCAGGGCAATGAAGCGTTTCAGCTCACTTAG CCGGTTTGACATCATAGTGCAGCCAGGACAGCCGTGCCACATTGGCATGGCACATAGTTGACTT AGGACAGTCGGAAAGGGTTGGTGGTTCTATGTCTCGCAGCTCACTTCATAGCTGTTTGCATGTA AACCTGGTGATGGTGTTGAGATGGTGTTGACTTGCTGACGTTGGGGTGGTTGGTAGTGCAGTTA AGTTGGCTGCATGATGGGATGTTGCCTCAGATGTCGGTGCAGCGAAGCCCTGTAGGTGTGTCCA ATGCAAGGAAAATGAAAGGTGGTGTGTCCTGTCCACCTTGTCTTTAGGGGTTCAGGCAGGAATG TCCTGTTGGAAAACTAGCCATTCCCTTTGTGTGTCCTGAAGGCATGGTGCGGGCATGTGAACTG ATTGGAGGAAGGAAGTGTGTGTCTTGAGGTCCTTTGGCTGGGTTCAGAAGACTGTCTCTGGCCT TGATGCCGTGTGCCGTGTTTAAGACCAACGTGGTGGCCATTGCAAAGGTGCCTTCCAGTGCAAG TTTTGGTTGGTTTGGTGTCAGGTGTGCCTTACCTGGGAGAGCGGCGAGAGACAGTGTCTGGTCA CGACATTATATGCCCCGTAAGGAGGACTGTGTGCTTGCACCGCCAGGGGTGCTGGCTGTTAGTC GAGGGACATCAGGCCAATGTCTGTCTGACCCCTAGGTCAAAATTCGCATCCTACTGATTGAAGG CCTTGGGTCTCGAGCCTTTTGGTTGCAGGTGTGGGGATGGTTTCCAGGTCTGGAGGCTGCCAAC TATGACAACCCGTTGTGCTGTTGGGAAACATCTGTGTGGGGGAGACACATACACATAGACACAC TCACTCTGACATACACACCTACACATGCACAGGCGTGCACAGCCACAGGCAGATAGATGCAGC AATGGGTGTGTGCTATTGTTGTGGTCACATGTGCTTCCTAGGTGTCCACAGAGTGACATGGGTCA GGTATATTCATGAAGATGGAGGTCTGTCATGGAGCCCAAGGAGAAGCCGGTGTGCAAGTTTCC CTGTGGGAAAGAATGGGATTGAAGTGGTGACCAGTGGACTTGGGCCCGTTTCTCAAGCATGCAT TTGATTTCTTTACACTTCTCAGCAGCAGTTGTCATGAGAGGTCTGCCGCTTGCTGGCAAGGAAGA TGGTCAATTCTTCTGACCAACAGAGGCTGCCTTGAGTCCTGATTTGGTGTGGCCTGGCATCTATG GTGCACCCAATGCTGGTGAGTGGCTCAGGGTGGTAAGCATTCCTCTGCCCTGTGTGCAGAGGTG TCTTTTGAATTTTGAGACCTGCAAAGAGCTGTTTCTCAGCAGGCCACTAATGGCATGGCGAGTTC CACTCCCAGAGCTGATTGTCTGTGTTCATTGCTCAGTGGTGTGGCTCTGGGTTCCCCCCAGGATG CTGAAGGTGTTTAAGGTCATCAGCTCTCCCACTGCCCAGCCTGTGGTGTCAAATGTTCTGCCTCT TCGAACGTGCTTGGATCGATGATGAGTCCCCCATAAAAACATTCCTTGGAAAAGCTGAACAAAA TGAGTGAGAACTCATACCGTCGTTCTCATCAGAACTGAGGTCC

ID|30598|gene_id|RLGI01027|transcript_id|RLGI01027 Homo sapiens lncRNA

ATTCGACGCCGTCCACCGGTCCTGTCCATCCTACTGACCCGATCGTCCAAGACTCGGAGAA GAGGGGGTCCCGACCACGACCAGTGGCTACGGTAGGGACGGAACCCGGACCCGTTGCCGGCC GTGTGCGACCCTGTCGTCCTTCACCTTCACCTGCCAGACTGCCGGATGCACCGTCCACTCGCGG GACTGGGTGACCCAGGGTCCAGGGTCGGGCGTACGGTCCACCGGGTGCTGGGGGGGTCTCGG ACGGGAGAGACGAGAGTTCCGTGGTTCTGCGACTCACCGTCGTATCGGGGTTACCTGCAGACA

CAGGCCGTGAAGGACGTCCGGTGTCCAGTCACTCGTCGTGCCCACGCCCAAATCCACCAGACA
CTCACCACGCAGGGGTTCCCCGACCTGGCCCACGGGCCTGACATCGGGTTCGAGACGGGTGTC
CTCGAGGACCCGTAACCCATGAACCACTGAGTCCGGGTCCCACTGTCCGTCTGGTCGTCCCAG
GAACAATCGGACGACGTCTCAGGGACTCTGTCCCTCACGACCACACCAACCCCCGACAACCGT
TTCGGTACACCCGAACCCCTGACAGTGGATCGACACAGGGGGTTCGTCCGACGTCGTACCTCA
GCCGGGACCTCCGACGTAGGGACGTGGGGCGGGTCAACGACCCCGACCTCTTCTCATTCCCCT
GGGACCTGAAACCACGACTGCGTCCACTCCCGGACAGCGTCCCAGTGGCCCGTCGGACCGGG
ACGGACATCTGAGACAACAAGGACCCCGCCGGACCTGTCCCGGTCCCTCCCACCCAGGAGGG
ATCGAGGGAGGAGAGTCCACCCGACGGCCGACGGTCGGAGTCAACATCGGGGCACAACGGTA
GTCCGGCCCGAAAGACCACAACGTCTGTTCCGGTCCGTTCCCCAACGTCCCTCGTAACAGGTCA
TGGAGACAGGTAGACACTACCCAGTCCCACAGAACGTGATCAGCACAGGGACCCGGAGTCAA
AGGGGTGGTCGCAGCCCAACAACCCCTCGTCGAGACCCCATCCACCCGCCGGGGTGAGGACG
GGGGATGAGTAACGGGCCGAGACAGGGTCTGAAGCACCACGAGCTGCTGA
ID|55925|gene_id|ENSG00000189229|transcript_id|ENST00000342990 Homo sapiens lncRNA

GGGACTACAGGCGCCCGCCATCACGCCCGGCTGCTTTTTTTGTTTTAGTAGAGAGGAGGTT
TCACCGTGTTAGCCAGGATGGTCTCAATCTCCTGACCTCGTGATCCGCCCGCCTCGGCCTCTCA
AAGTGCTGGGATTACACGCGTGAGCCACCGCGCCTGGCCTGAAGAAAGTTAAGTTCAATTTTGA
GTTTTTTTCATACGTATAAAGCTGTGATATTTAGATGGAAAAAGACAAAATACAAACAACAGAAA
CTCCAGACTTTATTTGGTAAAGGGGTCGTAAGCGATTGTTGTCTAGAATTAATTATTTAACTATTT
TTGGAATGGTGTTTTGCATTATTTAGAAAGAGAAAAAAGTATAAAAAATAAAATACATATAATCT
AACAATTCCATTATAAACACCGACCGCACTGCTTATTTCCTTCCCATTGGTCAGTTGTTTGCTTCC
TTCTTTCCTTCCTTCTCAGTTCCGCTCCTTTTTCCCCTTCTTTTCCTTTCCTTTCTTTTCTCCCCGTTT
GTGTTCATTTCCTTTTCTCTTCTCCTTCTTTTCTTCATCTATCTACTTACTTTTGTAATATTATATGGG
TATAATGGAAACATTTTAATATAATGTTTAATATCTTTTAAAATAGGATTCTGTGTTGCCACTATAT
TCCCATTATATGGAGACACCCTAAGTTGTTAACCTTCTTTGGTTTGAACATTGGCTTCTATCATTT
GGTAGTGAATTTCTGTCGTTTTGCATATGACCATGATTATCCTCAAGACAGAGTGACAGTCTAAT
TTATCTTTGGGTTTTGTCTTTGTCCTTCCAAAATAGTAACAGCACACGCTAAGTACTCAGTGTTAG
TTTTTCTGACTAAAGAAATAAGTTTGCTTGTTTCCTGCTTTGAATGTTAACTGTATCTGACATGAA
GATCAGTTATGGAATTCACCAGTTAGCATTGTGTTTACATGTTTACATGTATTTACATCTATTAAG
AACAGTGCTTTTCGGAAGAAAGTAATATTCCTCTGCTTTAATTAAACATATGCATGTTTTCAGTAA
AGAATAATGTTTCTGTTTCTTTAAGTATACCTTAATATTATAGGCCTTATATTTTACCAGAAGGAC
CCGATTTAAAGCAAAAGTTGAATGCTCTGTAACCTATAATCAGCCCACACACAGTCTATCAAAAT
AGCAAAATATCATTTCTTTCCTATAGAAATATATAAGCTTCTAATTTACAAATCTGTATGTTTATGT
TATCAACATGAACTTGTATCTCATCTTTCTGTTTATGGGTGCGTGTTTCTTACATTCACCATTTTTA
CTGAAACGCAAGTGGCAACTCTTATTTGCCAAGAAAAGATGACAAAAAATAAAGAAATGCTTTC
ACAATAAAGGGAGAATTTGCAGTAGCAGAAAATTATTAGCTGAGAAAAAATGTTAAAATAAGCT
TATTACTTGAGCCATGCTTTTTACTATGAAAGTGAAAAGTTAGAAATCATTATTTTAAAACTTTCA
CAAGGAATAAAACGTTTATGCATATAGTCAGGGATTTGAGGTCTGAATTATTCAGAGTATTTATC
TGTATAGTTCATATTCCATGGATATTGGCTCAAATCTCTATTTTGCCTGATAAAGATTTCATTTTCA
GTTTGACCACATTGAACTGGGTACTAAGCATTCAAAAGGTTGTTACATCTCATTTTTTGCAGAAA
AAGAATGAAATAATGGAAAACTTATTGACATACATAATAAGTGCAAACCAAAGACTGTGTAGGC
ATTATTATGTAAAAAGTTCATGCCCAGAGAGAAATTAGAGCAGGATACAGTTCCTCAGGGCAAG
GAACTGCTAACTATACACCCAGCTCTGTGACATTTAGCATTCTGTGGCCAGCCAAAAATAATAAT

GTTAGTAAGTGTATTGCCAGTTTTAATTAAGTCGCAAGTGAAATATTTGTCATTCCAGTTTCTCAG
AACTTTTGTGCTGTGTATATGTTTCCATTGTTTATTGTTTTATGATTCTTCTGTAGATTGGTGAATG
GAAATACCTGAAATTGCAAGATTAGTCCGGCTGCAATGATATAATGCATACGTCTATACTTTATA
AAATGCAGAGTGTTGTAAACATTTAGACATTGTCACTTCTTATCATTGCGTGTATCATGCAAGTAT
ATAATCCAGATATTGTGGAAAGTCCCAAGCCAACATCTGTAGTCATTTTAAATGTATGGAGACTA
TTAACACTTCCAAATGTCATTCCCTCTGGATGAGACACAAATCACTTAAAAAGAACCAGTGACTA
CTTAATACCCCAGCGAAAAACAAAGACAATGAGTACAACTTTTCTGTGTGCATCAGTTTACCATT
GTTTCCAAAGCTAATAGAAAGATCCTCAAATGCATACTTTTACATACTAATATGTTGCATTTCACA
CTATAAACCAGACCCAGCATATCTGTTTAAGCATCAGGTGCTTTATCTAATCCAAGTATACTTAA
TTACTTAAATTGTAATTAATTCAAAAAATGATGTGAATAAGCAGGAAAATATTCAAGAATTTACA
TATTCTCCATTTCAGCGACCGTTAGCCAAACTCAGATTTAGCTGACAAGCTTATCCCAGTTTTTGT
TCTCTCCCTCTTTCTTAGTATCTTCCATTAAGTTTCTTGAAACTTATTAATCTCATCAGTAGGAGAC
CATATCAGTCTTCTTACTTATTAACTAACATGGGTCAACTTCAAAGTTAACTTTTGCTAGATTCTAT
AATCTAAACTTGATGTAAAGAACAAAAATAAGGCTTTCTATATTTATAGCCCACTTATTTTCAGTT
TGGAGAAACATGGAGAACCGTTCAGAAACCAATGGTCAGCTAATTGTGCAAACACTAAACTTTT
CAAAAGAAAACTTACTTAGGAAAGAAAATAAATGTTTAAGAAACTAAATTGGATAAATATATAA
CATTTTTCCAGGTATTTAACTATAATAGTTTCTCTACATATCTTTTCCTGTGAATTTTCTTTGGAAA
GCTGACTTTAAGTACACAAAATTAAGATGAATTGTTCATCTTCAGTTAACACGATAGTATTTTGTC
TCAGACTCATGAACTGACAAAGAAAGCAGATAATCCTAGGTTGATTTACAGAGCAACAGCTATC
TTTGCAACCAGAGGGTTAGAAGTGAAAAGGAAAAAAAATCTTATCTTAAATAATAAGACTCCCT
ACATATTTGCAGAATAAATAAAAATGAAATATCTACAGAGAGGACCTAAATTCCCAGCCACAAT
ATCCATATTTTTGGATATCATTCTGCTGTTGATACTATCTGTAGTCTTTCCACACTTGCTTAGCTTG
TCATCAGTTTCCTTCAATAGTTTGAAACCTGCTTCAGAGACAGATGTGTACCAATGGAGGCATGA
TTACATGTGAGAGGCTGGACTGTGAATAGCAGAAAAAATACAGGGGCAGAGACAAATAAATAT
TTTTTGTTATAGTCGCAAACCAAGTGGATGGTGCCCAATTCGTAATGCTGTTGGTCATGATTTTGA
TTTTATTAATATAGGACCAGATTTCTAGGTGTATGTTGTATTCTTTGTCCAGGCTAGAGAGTAAAG
AAAAGGAAGTAGGGTAATTCCCTATTTCTTTTTCTTTATTTTTTAAGTTGTATTGATATATAACAGT
TGTACGTATTTTGAGGGTACATGTGACATTTTGATACCTGTATACGTTGTGTAATGATCAAATTAG
GGTGATTTGGATATCTATCACCTCAAACATTTGTCTTTTCTTTGTCTTGGGAACATTACAATTCTTC
TGGCTGTTTTGAAATATAAAATAAGCTATTGTTAACTATAACCTCACTAATATACTATTGAACACT
AGAACTTATTTTTTCTATCTAATTGTGTTTTTGTACTGATGAACTAATTTCTTTTCATCCAACTTCCC
TTCTTCTTTGTCTTCTACCGATCCTAAGTCTAATTTGACCACTTTTCTGCCGGTCCTGTATTTAAAA
AAGAAGGAACTCAAGAAGTATCAAAAGAAGGAGTGTTCAAGTGGAAAACACTGAAGTAGATTT
TACTTGCTCAGCATCCCACGGATTTGGGGGAGATTTTTGTGTCCATTGCTTACCTTCAGGTGGCC
AGCAAAGCCAACCAGTGGAATGAATTTAACATGTCCACAACTCTGGAAGAAAAGCTGTTGTACA
ATGTGCAGTGGGTACAGGTGCACACAGTCTACCGCCATGGAGTATAAATATAAAACAGAATTTA
TTGTGAGGAAAAACCTAGAAGCAGAAAAGCACTACAGAGGTTCCCCCGAGGTCTCATTATTCCA
CACAAAAATGTGCTTAGGCTGGGTGTGGTTGAGCTGGATATAGCACGGAAGTTTCCAACACAG
GGCTCCTATTCCTTCAAGCCCCTCAGATTTAAAGGGAACAGGGCGGTAGGACACATCTCTTTGA
TGCTGTAACAATGGTAATTTATAGCTAAGGAAGTGGCTTCCTTGGTAAAAAGCCTCTGGATCTCT
ATGACAAGTCCTAGTCTGATCTGCTGATGTGGCTCAGACATGCAGAGTCCACCCACTGCTTGCC
CAACAAAATGGGGAGGACTGAAGCTTCCCATGCCATCTGGATGTGTCTGTCTTCACAGGTCCAA
CTCTTTGGCACCTTCTATTGTACTGAGGAATAAAGCTTTAATTTAGAGCATGTCTCAGTCAACAA
GGCACCTGTGTAAACAGCCCCCTGCTCAAAAGGCTGCTGTCTGGACTCTCTCCACAAACACTCA

GAGGCTCCAGCCAGCAAGGGAAAGCCACCCTGTACAGTTTGGAAGACAGCTCTTTGAAAACCC
ATTGTGGATGTCACAGTGATGCATTGCCTTATCTTAGGGATGGAGCTCTGCCGAGGACAACCAG
TTGCTGAGAGGCAAAGCTCATCATTTTGAAGGGCTAGGTGAAGGCCCATTTAATTTCCTCCAGG
AGAAAATGTCACCACCACCATTGGTAAGCACAGGGGATGCAGAGTGAAATTTGGCTACATCTTC
ATACAGAGTTGCTCCTAGAAGAGAAAGACATGATTTATGACTGAAGAGCCAGTATTTTCCCCTA
GGAAATTTATTTCATTTATGAATAAAAAAAGGGAAAGATATTTAAACAAGAAATTTCCTAGTTTT
GTTGATGATCTGAAAACCTCCCCTGCCACTTCGGCTGTAATAAAAAAAAAAGTAAACTGAATTC
ACAACAACTGGTGATTTCTATGCCAGTGACTGAGAAGCAGAGAGATTAATATCTCCCCAAACAA
AATTCACTAATGATGCTTCTCTCCCCAAAAGAGTGAGTATTACTTGGTTTCTAGATAACACATTTT
ATTTCACATTTCTTAATGCCCAGTGAGTTTCAGGTAAACACACACATAAACACACACACACACAC
ACACACATGCACACATCTTACATAAAATTATATTTATTAAGTGCCCTCAAGTCAACTTCTTTTTAC
TTTGGAACATCACAAAACTTATTTTATGAGGCACATCTTTTGGTACACCAGATGTTTTTGAACTAA
ACAGGTTTATTTATTTATTTTATTATTATTTTTATTTATTATTATTATTTTTTTGAGATGGAATCTTGC
TCTGTTGCCAGGCTGGAGTGCAGTAGCACTATCTTGGCTCACTGCAGCCTCCGCCTCCCAGGTT
CAAACGATTCTCCTGCCTCAGCCTCCCAAGCAGCTCTGACTACAGGCGTGCACCACACCCAGCT
AAGTTTTGTATTTTTAGTAGAGATGGGGTTTCACCATGTTGGCCAGGATGGTCTCAATCTCTTGA
CCTCATGATCCACTCACCTCGGCCTCCCAAAGTGCCAGGATTACAGATGTGAGCCACTGTGCCT
GGCCTATTTATTTATATTTTTTGAGTCAGGGTCTCACTCTTTCACCCAGGCTGGAGTTAAGTGGTG
CGATCACGGCTCACTGCAGCCCTCACCTCCCTGGGCTTAGGTGATCTTCCGACCTTAGACTCCT
GAGTAACTGGGCCTACAAGCAAGCCTGGTTAATTTTTCTGTTTTTTGTAGAGACAGGGTTTTGCC
ATGTTGCCCAGGCTAGTCTCTAACTTCTAAGCTCACGTGATCTACCCTTCTCAGCTCCCAAAGGC
CTGGCATTACAGGCGTGAGCCACCACACCTGACCCTAAACAAGTTTATTAAAATAAGTGGAAAA
TAAACAACTGCCTTTTTTTTTTGGTTAAGTAATGATTTGATAGGCAATTACAATGTTTTATAAAGAT
GCTCTGGATAAATATGTTTTTAATTTGAAACACAATCACATAAACTCTACATCTGTCAAGAAAAT
ATTTTTCTTCGTGTCATTTTCTACAGCATTGCAGCCAGCACAGGACACATAAAATGTGGAAATAT
AAACCCTATACCAACCTCATGGTGGTGTTACAGGAACTCATTAATACCTTTGTCTTACAATTGCC
TTTGCATTGCTTAGTTGATGCTAGCTACTATGATTATTATTACTCTTTTCCAAAATTTTCATGTATTG
CACTTTTTATCTTTTCTTAATTAAATGTCGTGTTATTGTATTTGTTTTATTCATCCCAGCTTACACGA
TGTATTTTGAATTACTGCACCCAATCTCTTTATATCTCTCCCAATCCATGCTATATTCCCATTTGAT
GAGCTTTGCATAAAGGTGATGTTGAACAAGACAGGCCTTATGATAGAGCTCTGGAAGTTACCTA
GAAGCCTCCTTTATCCCTACCTGAGTATTTAGCATTACTTTTGAAGGGTGGCAGAAGATGCCACC
CTACACACTATGCCCCTTTGACATGAGAATTAATTTGAGCTAAACGCACTTGAAAAACAGCCGA
TGCAATAAAGGCATTCTCATCCACCCTCTTATTTCCCCCTGAAAACAGGAGATAAAAACTTGCAT
GTGAAAGATGCCCTCCCTCTACCAGGAGAAAAGAAATATTCTTCATTAAAGAGTCATAGATGAG
AGAATTCTGTACAAACAGAACTTGCCCAAATAATTCTTATCTTTCTTTAGCCTCTTCACATAATTT
ACTTTCTTTTCCACAATTACCTCTCTTTGTTCAACCTAATACTAAAGAATTCAGATTGTACAACTTC
TTTGAGTCTTCATTTCCTTATGAGGGCTCCTGTGTCACATAAAGCTTATGTTATATTTACAAGCTTT
TTTTCCTGTTAATCTGTGTTATGTCAGTTTCATTCTCAGGCCCACCCCAAAATCCTAAGAGGGTAG
AAGTAAAATTTTGCTTCTCCTACACTTCAACAAATTCATTCAACATGTCATTAATTCATCTACCCA
TCTGCATTCTTCTCCATGCTGTAAGATCTCACCATGTCAATAAGGAAATCATGAAAACACTATCA
AATATACATGCACTGAGGCTGTGGCATTCTTCTGATCTACATTTTTTTTTTTTTAGACGGAGTCTC
GCTCTGTCGTCCAGGCTAGAGTGCAGTGGCGCGATCTTGGCTCACTGCAAGCTCCGCCTCCCAG
GTTCACGCCATTCTCCTGCCTCAGCCTCCCTAGTAGCTGAGACTCCAGGTGCCCGCCACCATGC
CCGGCTAATGTTTTGTATTTTTTTTTTTTTTTTAGTAGAGACAGGGTTTCACCGTGTTAGCCAGGAT

GGTCTCAATCCCCTGACCTCGTGATCCACCCGCCTCGGCCTCCCAAAGTGCTGGGATTATAGGT
GTGAGCCACTGCGCCGGCCCTGATCTACATTTTAATAACTCTACTCGCAAAAACAAAACTAAATT
AAAATCAAGCAAATAAAAGGTAGGGAGACATATGCTTGAAGCAAGATATATAAAAACCAAAGA
TAAGTCAGTGCAGGAATTTTAATTCAGTTCAACAGACATTCATTGCATTCTAGAAGGTGGCACAC
CTCAGTATGAGCAGCCCACTCAAGTCTTAATCCTTCTGAGGCCCCCTTTGTGGGCCTAGGTCCAT
CTGTTACACTTTCTATGAAATATCTGGCGTCAAGTTGGATTTGATGATGTTACTTTGTAATAACAT
TCTGTCTACATCACTTCCTCTTGTTATCCAGGAGCACTTGGCAACTCCCACTATATTTTTTCAGCA
CCCCCACTCTCCTTTCCTGTGTATTCATGCCCTTAACAGACATAGGAGGTTAACTATTTCAGAAG
GAATTGTACCAAGCATCAGGTGGGCAGAGTCTCTATGAATTCCTGCTTTGAATCCTTGCTGAGCC
AGTTTTCAATAGGAAGATAATATTTCGTATTGTTCAGATTAGTACAATCAAGCCATGGCAATAAT
GGAGACGCTTAGAAAATGGGGGCTAGGTAAATTTAGCAATAGGCTCAGTTATATTTTGTCCAGG
TAATAAGTGGCTCTTTTGAAATAAGACAAAGCTGATCACTTCATTATTTGATTAGATTCTTCTAAT
TGTGCCCAGGTGATCAGTTAATTCTCTGCTAAGTCTCAAAACAAATTTAGAAAATCAAAAGAGG
ATGCTGAGAATAGAAACCAAACAACACTTTGCTATCTTGTACAGATAGTGCTAATTGACCTTTCT
CCTGTTACTTTCAAGGCAATTAGGGAGGATAATTGTAGCTTAAGAAACTTAGCACAAGTTAGAG
GGAAGAGTAACAACAGATGGAAACAGGGATATATTTTGCCTTGAGAAAGTTAAGTGATACCATT
TGTGACAAGGTCTCTGATGCTGGGAGTGGGAGGTAATTTCTACAGATTCACTGGCATATAAGGC
CTGCATGTATCTATATATGTTGTAGGAAGTTCCAATTTCTAACTTTCTCTTAGATCTTTTGTCCTAA
AAGTCAGGGGAACATCCATGACCCTGATTCATTTATTTCAAAGAGAGGTATGCTGGCATCTGTT
GTGGAATATTTCTAAGGGTCAAACTGCACATTATTCTGAACTGACGCCAGAGCAGGCAAACTCT
TCATTTGTAAGAAAAAAATGATAGATGCTGCCTTTAGGAGCTATCATATTTACTTCATGCACCTC
AGCAGGCTTTGTTCTTGAACATTAGGAAAGTGAGCAGGACTTGGTTTCAGAATGAGGTTGGATG
AATCACATTGACTAGAAACTGTATTATCATATTCCGTTGTGTCTAATCTTGAAACAGGCAGAATG
GTTTTGGTACTGCTGGGTAGAGGCTGTTTGTCTCTCTTCTGGCCTGGGATGATGCAGTAACTCTG
TCTCCTTGGCTATAGAGAAAAATATGATGCCCCTGTCCTGATTCTGTCCTAGCCAAAGAAGATTC
ATTTGGGGAGCTCAGCACATTTTTGGTATGTATTTGTTTAGTGAATGGAGATGACGAATTCAGGC
CTTTTTTGGGTTTAAGGATGGGGGTTGGTAGGCACATTAAAAGGTGGGGAGGTAAAGAAGAAA
GTGTTGTTGGGGGATGCAAGTTGTGCTGAGAGACAGGCAAAGAGAAGTGTGCAATTGGAAATT
TCAGTAACAAGCCTCCAGCAATGACAGTTTGCTTGGATCTCTTTCTCGTTTGCATGTCGCTAGAG
GAATCTTCAGACTGAGCAATATGACCCTAGGATGCCAGGGATGACAGAGAAAGGATCTTTTCAA
AAAACCTATTAAAATTGCTTGGAAATCAAATTTGCAAAGAGTGCAATTTTAAAAGGAGGCATCC
CATGTTTTTCCGGTTATTACAAGTGGGGTTTTACTAAATTACCAAATCTCTCTGTTCCTCCTTCATC
TGACAAAGTGGCTAATAAAAGCCCCCTCACTGGGTGATTGATGTTAGTAACACTAAACCCATAG
GAAATACATATTTTAAATATTCTTTGTTACAGTTAGTTGTTCTTACTTACATAACAATATTAAAATA
TCCTATATAAATTTTGAGTAAAAAATAGAAAAACACTTAGTATTTAAAATAATATTTTACCAATAT
TTGGCCATCTAGTATTTATTTGCCAAGTAGAGCTGCCTGCTGATAATGTAAAGAAAAACAAGTCA
TGGCCTCTATCCTTATGAAAGGTACAACCTATTAACGAATACTATTAGTGTTTATTTTCTTTTTTTT
TCCCCTTTTCTCATAGGTTATTTTAATATAGTAGCAAAGATTCTGCACATGTATGTGGCAGATACA
CATTCTTTTCCTTGTTTTTGAAGAGTTTCATTTGTCTTTTACTCCTACCTATTCTTTAAACAAAGTCA
GTTCCTTCCGCCCTTGACCAACAAAACCCTTGACCAGCTTAAAATATCAGTAGATGCCAGAATAT
TAACAAGGATGAAACTAAATGGTTATGTTTACAGAGATTTAAAAATTCCATTTCCCCAGGAATAT
GCCATTTCAAGATTTGTGTAAAAACAGGAAGAATGAAGAAAAGGTTCTTGTTTCACCTTGTCTTT
TTACGGATGAAGGTAGTCAACCAATAGTCCCAAACGTGAGAATTTCTACTTTTAAAAAACATTTT
GGAATAATTCCAGATTCATACAAAAGTAAACATGGGATTTTTTTAATAAAAAATAAATAAATGTT

TGTCTTGGAATGCCAGATTCCAAGTATTGGACAATTTGACCAACGGCTTAGAATTTAAAGTCATT
TCAAAAATTAGTACTTGAAAACACTTTCTTAAATAGCCTGATTTCTTTCCAGAGTGAGCATGTGG
ATGAGCCTTAGTCTACCATTATTAGCAAATTATTTATATTCTTACTTTATTCCCCTTCTAATTAACC
TATCATCACTTCTTAATCTATGTCAATTTTTTTTCCCCTAGTCAAACACAAACAGAATCAGGAACT
TCATTAAAATATTCCAACTGTCATTATTAGTGGCTGCTATGATAATAATATTTATTTTGTATCATTC
TTGACAATTCTATTTGGAAAATATTGCAATGATTTTGTCACTACTTATATAATTACAATAGGAAAT
GTTAATCATGAAAATTTTCTCCAGCTCATTTTCTAGTCCCCAGTCATCACACATTCTTGGGTTCTA
ATAAGCTATGTGTCAGATATAAGAAGAAGTTCCTCTTCAAAGGTTTAGCTTGTTTAGTTTCCTTGT
TCTTTGTTCTCTACTTTCAAGGCCAAACTTCCTTACTCTCTATGCCTCCCTGTCTCGGTTTCAGTAA
ACAACTCTCCCTCCAGTTGTTATCTACAGAGCTCACATCTGCTACCCATTCTGTAAATTACCCCTC
CCATCACAATGGCCCTTCCCGCCAAAATTGTCCTTCCTGCCAGTGTAACCGCATTCCTGCACTTT
TCAAGTTAGCCAACCAGGTTCAGCTTAAGTTGTGTGGTCCAACTCCAGCCAGTGGAGGCAGGAC
ACAGTAGCAGGGACAAGTTGCGTTAAGGATAAAAACCCCTTCCCTCCTTTGTTCAGTGTGCTCTT
GTGGCATCCAGACCTGCGAGAAGCACCCTTCTGCAGAAGTAAATTTTCTTTGCTGTCTGGTGTCT
GGTGAGGGTCTGCTTCCTGGTTCATATACAGCTGTCTTTATGCTGTGTCTTCATCTGGTAGAAGG
GACAAGGCCACTCTGTGTCTTCTTTAAAGGACATGAATCTTATTCATTATGGCTCTGCCCTCATG
ACTTAATACCCTCCCGAAGCTCTCACCCCCTACTACCATCATCTTGGAGGCTAGAATTTCAACAT
ATGAATTTTGGGGAGACACAATCATTTAGACCATAGCATTTGGGCCTGCTTCTTCTAAATTTATGT
TATTCTCACATGAAAAAATATATCCATTTTATTTCAGTTAAGCCCAATAATCCCCAAAGCCTTAAT
TCATTCCAACATCAACTCAAAAGTCTAAAGCACAATGTTTCATCTAAATATCGTCTAAGTCAGAT
ATGGATGAGACCCAAGTTACTATTCATTCTAAGGCAAATTACTCTCCAACTGTGAGCCTATAAAA
CCGAGAAAAGTTATGTGCTTTCAACATACAATGATGGAACAGGCATAGGACAGACATTTCCATT
CCAAAAGAGTCAAATGAGAAAGAAGAAAGGAGTGGCAGATCCCAAACAAGTCCAAAACCTAA
CGGGATAAAATACATTAAACTTCAAGGCTAGAGAATAATTCTCTTTGGCTTGATGCTCCGCTCTC
TAGGCCCACTTGGTATGCACTGCCACTTTCTGGATACAATGACGTGGAGGGGCACACTGGGAAT
GTGGCCCCACAGATTTGCCAAGACCCTCCAGGGGTCCCAGTGGCCCTGCTCCCATGGCTTTGAA
TGACCCACCCCTGTGGCAACTCTGCCTGGGCCCTCCCCTTAAAGTGGTTCTCTGTGATGGCCCC
ACCTCCCCAAGACTGTGTGCATTGGTCATGCCCCAAGATCACTTTAGGCTCTAATTGTAGGCCA
GATATTGTGCTACCCACATTATAACAAGGATGACCTTTCCTCCATTGCCCACTGGGTAGCATAGT
ATCGGGTGTACAATTAGAGCCTGGAGTGGGGGTCTGGGTTTCCGGTGGTGGTGGTCACACCCA
CATGTCCTTGCTGGTCTGGGGTTTTGGGGGTGGCCACACCCACATGTTCTTGCTGGGCTTTTTCC
TAATGGTAGTTCTCTGCAGTGGCCCTTCACCCACTGGGACTCTTGCACTTTGGACCTGTGATGGG
AGGGGCAGCTCTAATGAATTTTGAATTCCTTAATGGGTTAATCTTCTATTGTCTTGGACAATAGGT
ACTTACTTCTGTTTAGATGGCCTATAATCTCCTTCTCAGATGGTTGTTTGGCCACAACCTTTATGTT
CTCTCTTGAACATGTGCTCTCATTCTTTTCAATATGGATAAAATGAAAATGTTCCAAGTCTTTAAG
TTCTACTTCCCTTCTGATTACCAATTCCATCTTTAAGTCATTTGTCTCTTCTTACATTTTACTATGAG
CAGTCCAAAGGAGCCAAGCCAAACTTTCAAAGCTTTTCTTAGATATTTCCCCAGTCAAATATTCA
TCTTCACCTCTGAAAAGCTCTACCTTCCCCAAAACACTAAAACAAAAACACAATCCAGCCAAGT
TTTTTGCCACTTTATAACAGGGATGGCCTTTCCTCCATTGCCTAATAACATGTTCTTCATTTCCAC
CTGAGGCCTCATCAGAATGGTCTTTACTGTCTGTGTTTCTACCAACATTCTGTCTATGTTTACGTA
GGTAATCTCTAAAAGGAAGTTTTCTGTACAACTCTCCTCCTCTCCTTCTGAGCTTTCACCAGAATA
GCCCTTAATAGTCTGCTCTTGGTAATGCAGGGTTTTTCTAGCATGCACCTCAAAACTCTTTCAGC
ATCTACTCATTACACAGTTCCAAAACCACTTTTACATTTTAAGGTATTTGCTGCAACAGCACTCCA
TTCTCAGTACCAATTTTCTGTCTTAGCCTTTCCAGGATTTTATAACAAAATATTATAAATGGGTGG

CTTATAAACAGCAGAAATTTATTTCTCACAGTTTTGGAGGCTAGAAGTCCAAGACAAGGCACCA
GCAAATTCAGTGCCTGGTGAGTGGAAAGACCCCTCTTGGGCCTCTCTTTTAGGGCTAATCCCATT
TATGAGGGCTCTATCCTCACGACCCATACCCTCCCAAAGGTACCACGTCCCAATACCATTATCTT
GGAGGCTAGGTTTTCAACGTATACATTTTGAAGAGACACAAACATTTAGACCATAGCAGTACCC
TCGGCACTACATTCTGTGGTCCTGCTTGTTATTGTATTGGATAGCTAGAAGAACCCTTAGTTTCTA
TTATGTGTACATTCTCATTGAGTAAAACTAGCTGACAGTTCAGGCAAAGCCCAGTTCTGCCAGCC
AGAGCCTATCTCATTGCATCAGAAATGATACATAACCTCTTTGAGTCTAATTGTCTCATCTGTGAT
ATTATTTAGCTCATGATATTATTGGAAGGATTACATGAGCTAACACCATGATTATTAGCCCGTAAT
AATGTTTCTAGCATACCTTGTAAAACTTGTAAGAACATGCACAGAAAAATGGAATTTAGAAAAA
GAGGTAAATTTGAAGAAGAGAGAAACCCAGAATTAATTTTATGTTTGTGTCTAATAATAGTGTTG
CTTAATGGAAAAATAAAAATTCATGATGTCAGCTGGCTTCATGTATTGTATAATGCAGGGTGAAT
CATGCAGCCTCTCTGAGTTAGTGTTTCCTGATTAACTAATGACATATACCTCATGGAGTCATTATA
GTGATTAAATGAGGTGATGTATGTAAAGTAGGTAGAACAGTATCTGGTATATGACATACACACA
ATTTAGTATTTTGAAGGGTTGTAGCATAGTCATATGAATGGGTCCAGCACACTCTTGGAAATGAA
GGTTGCAACTCAGATGAGCAACCAGGCTTGAAGACATGCATTTGGGAATCTAAAATAAGAAAGT
CATTTGTAAGAAAACTGTGATAACAAGTAAGATTACAAGAAAAAAAAAAAAAAGCAGTGGTTCCC
AATTTGGCTGTGTTTCTCTGTCTCATGTAGAACCTATATCAGTTAAGTTTCCAAAGTTCTTACTCA
AACCCACTGATTTACAATTTCCAGGTGAGAGCCACAGAGGATGCTTATGAAGTCAGTTGTGCAT
TTGGGCATTAAGGGACAAGAATTTGAAGGATGACTTAATTTAGAGGGCAGAAGGTTTCCTAAGA
GAAATTCCCAAAAGGCCTGAAGAGTCCAGATGTTTGGGGAGTGAGGCCAAGAAAGAGGAAAG
TTAGGATAAAGAAATGGACTACATATAGATAATGTTTTAGAAAAAGGGTTAAAGAATGTTGAAA
TAAAGCAAAAACCTTAAATAGTTTTTATGTGGCACACCATGTTAATAAGCTTAATGTAAATAAGC
CAAACATGTGAATAAATTTCTTTTGCCAGGGCAAGAAAAAAAGTCAATAGAAGAAAATGTTTTT
GACAAAAAAAAAAAAAAAAGCTTTTAAAAAATCTCCTCTTTTCAGCCTTGGTCCATGAAAACCATT
TGATAAAGGAGATGGGGTATGCAGTGACCCTAAATGCATCTGAATCATGAATTCATACATCAAA
AAAGAGAAACTATCAGGAAGTCCATATAACCGTACAGCATAACAGCTAAATAGTAGCCATTGAT
AAAGTGCGAAAGAAACAGAAGGATATTTTCTGTACTGTTCATAGTGCTATGCAAGATATTTATTT
TCCTGGCCACTTGAACGCATCCAGGGTCAGAATGCAAATACCTCCTGATTTTGTTATTTGCACAC
GTTCAAACAAGTGGTGAGTGAAATGGTCCGCTCAAAATGCAAGAATCAGAAGCCTACTCTAAG
GTTTTACTTTAGGAAAATAATTTAAAGGAATGAGACTTAAAACATAAAACCTCCTGTAAGAGAGA
GATTAAAAACGATTAGCTTCCTTCACTGTTCCTCTGCTTTTCTAGGGGGATGCATATGCACTCAC
ATTCTATCATTGATTTATCCTTCTAGTGGAAACACAATGCTGACTATAGCATGGGCCTCTCTGTGA
CCAGAATGCTCAACAAGCAACTTAACAGTTTCCATGTTTCAGAATATGTCATTCACACACCCCTG
GGGAGAATGTTTAACATTTCCTTAAATTGGTGTGAGTTTTCACTAAGGCTACTGTCATACCAACC
AAGCAACAACTATCATAGATAGCTCCAGTGTGCATAACATGCTAACTCCTTTTAACCCGTCAGTG
TCCTGATATGCTTATTCAGTGTACAAGCTGCTATAAAATCATGTATTATCAATTCTAGAAAATATT
AGGAGTGCACCGTGGAACTTAAGTTCTAAATTATTTTGCAGTAGATGGCTTTTTAAAGCTAAATA
GGGTCTGGATAGAGCTTATAAACATTTGATTGATTAAGTTGTGGATTAGCTGATTTATGAACTAT
ACTTCAGGTGTTATGAATCACTTTGAAGTAAGTACCCCATGGGGCATTCCTGGGGTGTGGGTTTC
CTGGTGGTTTGGTAGACAAGCTACACATTTCTCTTTTCAAAGGGGATGATGACTTAGTACACACT
GCCCCAGATGAAACCTCTTCTTTCTGAACAACCTTCACAGAAATCTTGCCAGAAACTGTGTAACC
AAAGTCGAAACATGTCGCTCCTTAATAGATTGGGGGCTTATGTTGGCAGGTTCAGTGACAGACA
CTGGGACAGAGTGCCAAAACAAAAGTTGAGGCTGAGTTTCAAAGCAATGTTATGAGAAAAGTA
GTATTAATTAAACAAAACAGATTTATTGCAATTTTATCTTTTGTGAACTCCTTACATATGTCTCTAT

TAGAACATACCAAATTATGCTTTAACTTTTGGCACATGTCCTCTGCCTAGGGCTGCTGCATCAGC
CCATCTTTGTAATCCAAAAATGCTTTGTATGAAGTAGATGCTTAATCATGCTGGCATCATTTATCA
ACTTGCCATTCAATTTCTTCACATTTAAAAATATTTGGTGCATACCTGCCATAACCCTATTGATAA
CTGGCAATATGATACAGAGCCATAAACTAAGCTGATAATATCCCTGTCTTCTGGGACTTCCCATT
CAGTGGAGGTAGGGATGGAAGTAGGAGAACACAGAATGAAAAATAAACAAAATGAATACCAA
AGAAAATTTCAGAAAGTTGTGTTAGAACACATAGAGTTAATGTGAATAAATTAATGAATATATTC
ATGAATGAGCTGTCTAAAGTTGACTAATGGACATTTTTAATATATGCTGTCTACTTGAGTGAGGG
TACAAAGCAACAAATAAAACAGAGCTTAAGTTAGAACAATTTGTTTCGTTTACTTAATTTTATTAT
TCTAATTATTATTATTTTTTTTTAGATTTTGTTAACTCTAGATATAAGGACAGCTTGGTAAACACTA
AACTGGGTTACCAAAGTAAAGTTACATAATGCTGTTTCTTGGAGCTGTTTAAAAATAGAAGGTAC
TTTAGTGAGTTGCAAGGTCTAGTTGCTAATCTGCCTGGAGGCAGGGAGACAGGCAGGGTGACC
TCAGATGGACTTCTCCAGCCAAGGCTTGTTTCTGCATTCAGCATTTAGACCAACTGAAAACTTAG
ATGGGCTAGAAACATCAGCTAACCAAAAGTGCAAAGACTTTATAACCAGGTAGGCTGAAGTTCT
TTGATTCTGGTGTATGAAAAAGGGAAACCCATTCCCTTTACACAACCTACAACATCAATTCAGAT
GGAGACTGTTTCCCTCTTCTCTCTAAAACATCCTTCATTTAGAAGATTTTGCAGCCTGGAGTATTA
ATGCTGTGAGGTTGGCAGGAAAGGGTCATGGGAAAACAAGGCTCCAAAGTGCAGCTTTCTCGA
TGAACCATCACTGGCAATAAAGTCCAAAGTCAATGGAAACATGAAGGAGGTTTCTGTGTACAAG
GGTTATTAACTTTATCCTTCCAAGAGCCTTAATTATTCATTCTGATCATCTATAAATGGAAAGGCA
TAACCAGCTCAGAACATAGTCTGCAAAGTCAGATGTTCAAGAAAGTCTATTTATTCATTGTTTTA
AAATCAAAATCATTCTGACTCAAGGGTTCCATACTATCTATCAGCTGGCAGAAAAAGTGATATAA
ACCATTATTGGGGTTGTTTTGTCCACAGTTGTGCAACATTCCTAGAGATTCTTCAGGCTTATGGA
ATTCAGCCCATCCATAAGTCATTGGTCATTGATGATCCATCCACAGCGTTGATAGAGATGCCCTT
TGTACCAGTCTCCATGATACCTTTGGACATACCTTTGCTGCTTTGATGCTCTGTTTGGATAAAAGG
CAATTACTGGGAAAATAAAATCTTTGTATCTAGACTTCCACATCCTTAGAAACACCAAAAAACCA
TCTCCTTTCAGTCAGCTCTTACCTACTGCCTGAGATATTGATACCAAAGTCTAAAGGATCCCATT
CTCTCTGGCTTATTTTCTTACTTCCACAACCTCTAATGTCTTTTTAGCCTTTGGAAAATTTCTTTTCT
CTTCTTGCCCTTGAAGGCACTCACTAACATAATTGTTTTAAAGTTTTTTATAAAAGACTGGAAAAA
GATTTTAGCTGTAAGTGGCATCTGTTTTCTACTGTATAAACATCTGTGATTAAGACATGTCTTCTA
GCTTGCAGTGAGGGAAAGGAAAAAGAAGAAGAACTAATACAAGTTTATCTCTCAGTAAATTATT
CTTGCACAAAATATCCTAAGTGTCAGGAAAAAAGAAAGAGAAAGAAGGAACATTTGGTTCAGT
CAACTTTAAAAGGACAAAGAAAATATATTAAGAGCATTTTACTTAATAGTTCATATTTCCCTTTGT
GACTCTTTTCAATTACATTATCCAAATCTAAATTGCCCACATCTCTCCTTTGCTTTTCTATCTATAC
TTTAAAAAGTACTAGGATAGAAAAAAAAAAATTAATGCAGGTTTTTATTGAAGTAAATAATTTAAA
AAATCTATTTGCACTGAAGTGAAAGTCTATTCTCTGATGAAAAATAGGCATGTTTCCAGATAGTC
TTTTTTAGCTGCAATGTTGTGGGCCAATTTTTCTAATATTCTACACCAAAATGTTGGGGCAGACCA
AGATGGTTTGTCTGCAGAGACAGAACAACCCATAGCTGAGAACCCATCTGTTAGTGACAAAAG
CAGCTGCGGAAGGTTTGGTGGTGGCCAGGATATGGAGTTTGATGAGTTTCTTCTGCACCTGGGA
AAAAAAACTTGCCACTAAGCAGGTAAGTGCAAAAAGGAACTGCAGCTGCCAGCGTGGAAGTTT
GTACTGTATTCATCTAGGCTGACTCAGAATCTAGGGTGTGGGCCATGGAATTCCACTGATTACGT
TTCTCAAAGGCTCGCAGTGTGGTGAAGACTCACAGCCTATAGCAAAAGCTCTACAGAAGTGGTG
AGTGGGTCGGGTGCAGTAGCTCACACCTGTAATCCCAGCACTTTGAGAGGCCGAGGCGGGTGG
ATCACCTGAGGTCAGGAGCTGGAGATCAGCCTCGCCAACATGGTGAAACCCCATCTCTACTAA
AAATACAAAAATTAGCCAGGCATGGTGGTGCGCACCTGTAATCCCAGCTACATGGGAGGCTGA
GGCAGGAGGATCACTTGAACCTAGGAGGTGGAGATTGCAGTGAGCCAAGATCACGCCGCTGC

ACTGCAGCCTGGCTGACAGAACAAGGCTCTGTCTCAAAAAAAAAAAAAAAAAGAAAAAAAAAG
AGAAGTGGGTGATGAGAAAATGTGCTTATTTTTGGTCGTCATGAATAGACTGCCTGTGGAGAGA
AAGAGGAAGAAGGGAGAAGACAGGACTAAGTCAGAGGGTTCTACCAAGTCTTTTGTTTACAGT
TGGCTCCAAGTGAGACTAACACTCGACCAGAGTCTGCCAAGGTTGAATCAAGGTCACAGAGGA
GTGCAAGTTTGAATTGAATCTTGCTCTGTGGTGAGGTTAATGTGGTTTGTGTCCTCTAAAACCAA
AGCAACCAACCTGTCAGAGGTCTTAAGGCCTACCATGTCCTGTTAATGGTGAGTCTAGCTCTTAA
AAATTTAGAGAATGCTACCTTTAAGGTCCCCCTAATAACTATTCTTGGTCAGAAAAAGACTCTGA
AGAAATATGACATGTCTGAAAATGATTGAGTTTACTTAGATCCTGGTCGAAGATATAAGCTGATT
CTGCTTATAAAAGTCAAGATAGTAAATATTTTAGGATTTGCAGGCCACACATATTAGCAGCATTC
TCTCTGTTGCATATTCTTTTTTTTACTGGTTAATGTTGTTTTGTTTACAACACTTTAAAGATATCAGC
ACTATTCTTACCTTGAGGACTATACAAGAACAGGTCCTGGACTGGATCTCACCCTTGAACCATAG
TTTACTAGCCACTAATCTAGAGCATTTCAATTCCAAACTATATGTGACCCAAACAAACAGATGAA
TGGAAGAATGCAATGAGGAATGACAGTGGGGGGACAGCCTGGAATACTCTGGAAGGCCAGAT
TTCAAGGATCATGTGTTGCGCATGGTCCTCACAAATACACAGAGTATCTTGGTTTCAACAGTGTC
AGAAACAACAGAGAGAGTTTCCCTAAACTGAGGACACAGCCCCTGAGCCTGTGAATCAAAGGA
GGGTTGGATGTTAACAATAGGTATATGGCATGTGGTTGGCTATAGGTCTCTTATCGTGGTGCGAC
CGACTTAGTTCCAATTCGTGCTTTTTCTTTTAAAATTTCCATGAGAGATTTTTGTCAGGATTTTGTC
AGGTACAGCTAGACCTGACTGTGGATGGACTCTAGTTCCCTACTATAAAGTTAGTACCCTTACAG
AGACAAAGCATAGGCTTGTCTACCTGGAATTAGTCTGTTCCCCTCCCCTTTGTCACCCAGATTAC
ATCTATTTCCACAGTGTTATTCAAGACCCAGTAAAATACCTGTCTTTTTTTTTCTTCTCTAGACAAT
TCAATTGTTTCCATGAGTTTGAATATGATCCTCTTCTGGTCCTTTGAATAAAGGGCCCTATAGTTT
CATTTTGCATTGAACTCTGAAAATTAGGTAGCTGATTCTGCTTGCATTTGACAGCAGCTACCTAAT
TTGCAAGGTTCAGTGCAAAATGAAAATATGGGGCCCTTTATTCAAACAGTATTACAAAGTCCAA
GATATCAACATCAGAACGTTAATGCAAGCCTGGGGCCCTTCTGATCTTGGACTCTGAGCCACTG
CATAGATAGCTCTGAGCCACTGCTTAGGTAGCTCTGAGCCACTGCATAGGTAGCTCTGAGCCAC
TGCATAGGTAACTTGCCCAGGAAGCTGGCCGTGAATGGAAGTAATGACACACACATTTTCTTGA
GATTCAAATGAGAGTATGTACATACAAATACTAAGTAAAGCACAATGTGGAAAAATAATTTACC
AGGATTTGTTAAATATCTTAATAAAGAATTTATTGTTACAAGCCTTGTAAATGTTTTCATGAATTTT
TTGACCATTTCCATGGCCCTGATTTTGTGAATAAACTGAAAATCAGAAAACTCGCATGCATTTCA
TATCTTCTACAGTCCTTCCTGATCTGGACTTATGCCTAATATGTTGTTTTTCATCCAACAGGTTCTC
AACAGGGCTAGCAAATATCTAAGTGGTCAGAGAAAAAAATTATTGCCTGTGATGTCTTGAGACT
CAGGCAACATGGAAATGTGCTTAACTTTTTGAATTTGCTTTGGCCATAAAACTTTTATTACCTTCT
CTAGAATTAAATTAATATGCACCCCCCTATTTATCTATATTGGATTTATTTAATTTTTGACAGCTTG
TGCTAATACTAAACACAAATTTCTTCTCAGCACTAAGAAGAAATTATTGTAACTCAAGTATAGTA
CTAGTACTGTCTGACTGAAATGAAATAAAACCCATGACTGATTGAATTCCTGGCCACTTCAAAGC
AGGTATGAAAGTGATTCATACCCAAACATCACAGGTTCATTTGAGAGTTAAGAGATTGAATAGA
TCATGAGTAACTGCAGTTAATTTAAACCATGTATGTACAAGTAAGAATGTAGTGTCAAAGGTCTG
TGTTTGTCAGGAGAAAATTGGTTTTTTCTCTTTCAGTGTTTGTTGGCAGAATTGCTGAAACATACA
AGAACCATTCAAAGTCTAATATCTGCTACTAAGCTGAAATGAGCTAGAAAGATGGTCTCCCACG
ATCATCTTTTCAATAATCCTATGGGCTTTGCCGATTCTGTTACCACTATTTAAATTTTATTTTATTAT
TACTTTTTTTTAGTAGCATGCCTTGAAGATAATTTAAAGTGAGTGTCCTCCAAACACAGGATTTTT
AGTGCAATGCAGAATATTTGGAGGTGATGATCAAAGCGAGCATTGATAATAATATTCATAATGTT
AATTCATAATGTTAATGCTTTGAGGAAATAAGACAGCAGTAACCCTGTCTTCTTTAGAGGACCTC
TGGATTTATATCCATTTAGAACATTCATATTGTTAAATTAATAAAACTTCTTTAGGAGATTGATTAT

TTTACCCAAGTATGCTTTCCATATTAATGTAAACATTAATATCAATAACTTTATATGTTCCTCAGA
GTGAGGGAAAAGTCAACCTTAACATAGAGTCAAAAATAAACACAAGAACCTGAAGACTTTAAC
CAAAAGTTTTTACTAACCTGTTCCCTAAAACTTGAGAAATATAAAGATAGTGAAAGTGGTTTCCT
CCCAATACATTTATTGAGCATTTTAAGTTTATGGAACTGTGTGGTGGATTTTCCAGCATATCAGG
GAAGCAAAAGCGATGTCTCTTGTCTTCACAAGCTTAAAATGTCATTGGGGAGACAAAGCAGGCT
GAATAAATGTTAAATAATTTTTTTAAAGAATGGAAAAATTAAGAACCAAATATATGGGGTAATTT
GCATTGGTAAGAATTCTGAAGAAGTAAACACTTTCTAGTTTATCTTAAACAGGCAGGAAAGTGG
ATATTTGCCATTACTTTATTTGATACAAATTCATCTTTTAAACTAATATTTTTTGTCCTGAACTCATG
GATGTTCATATATTACTTCTTGGACATATGAGTAATTTACTTTGCTTCCTCATTTTTAAAAATTATT
ATGTGTTCATTAGCATGTACATAATTTAAAATTTAAATTATTTTAAAATAATAACTAAAAATACAA
ATAAACATGTGGCCCGGTGCAGTGGCTCACACCTGTAATCCTAGCACTTTGGGAGGCCGAGGT
GGGTGGATCACCAGGAGTACAAGACCAGCCTGGCCATTATGGTGAAGCCCCGTCTCTATTAAA
AATACAAAAAGTTAGTTGGGCGTGGTGGCAGACGTCTGTAATTTCAGCTACTCAGGAGGCTGAG
CCAGGAGAATCGCTTGAACCCAGGAAGTGGAGATTGCAGTAAGCTGAGATCGCGACATTGCAC
TCCAGCCTGGACAACAAGAATGAAACTCCATCTCAAAAAAAAAAAAAATTAAAAAAAATAAATA
AACGTAATTATTTTTACCTGTCCATTAGTATGTAATAATTTAAAAATTATTACATGTTCTTCCTTTG
CATATTGCCTCTCTTTGTCATTATTATTCATGTTAGATATTAAGTTTCTAGGAGGTATAAACAGTAT
TTATTGAATTTCTTCTTTGTAAAGTCTAGCCTAGAGAATTTGGAGTTCCTGATACGTTATTTTTGGT
TCTTTTTAAAATAATTATCACGATAGACACCATGTGGAAATGTTAAAGTAACTCAACCTAATTCTC
AAAATGACCTATGGTTTGTAAGTGGGGTTTTTAAATCATGCTTATTTTGTAATTATATCCAGCATA
TATACAGAATAGAATCAGAGAATATCTGGAAGAAACTTTTCAGTTTACCACCCTAAAAATTTTAT
TAACATCTTCAAGGGACATTTGTGGAAGACTTGCTGGTCAGGTTGGAGCACATGATCGGAGGTG
TTCCTGAAGCCAGATACATAGCTTCAGCGCCCTCACGATCCCATCACGAAGAGCAGACTTAAAT
AATTAACTGTTAACGCTGGGGCAAGTCCATAGGGTTATGGAGAATGCACTTTGGAAGTACAGGA
GCAGGATTGATTAATTCCTCATGAGAGAGGTCAGGAAAATCATTACAGAGGAGGTACTATTTGA
AATGGATTTGGAAGAAGGGCTGGAATTTCACCCAGTGAAGATTAATGGTGTTCCAGTCAGAGGG
AGTCACATGAGCAAAGGCAGGGAGACCTGAAACTCTGCAGTGTTTTTCTTTATTTCAAGTGTAAA
AATTTCTTTTATTGCAAATTTGAGCTTTTAATGGCTATGAAAAGAAGTTTTATGTCGAGGGGGAG
CAACAACTATTTGGGCCGGTGGCACAGGGATAAAATAATTTATCAAGACAATTGTAGGTAGAGA
AAGGCATATTTATTAAAGAAAGTAGAAAAATACATTGCCTTCTCTCAACGGGCAGGATCAGCAG
AAGAGAGGCTGACTGCAAGGAAACAAAGGCTTGCTGGAGATTTTATAGAATAATGTGTATCTGT
CTGTTGAAGAGGGCTTTGTGCCGTATCGATAATGCCAAGGTTGCAGTGACGGGGAAGGGGCAT
ATGACAACAAACTAGACCACTTAAGTACTCTTTCTCATAAATCCAACTCTTGAGCATCCGTGACA
CCAAGGCAAAAAAAAAAAGGGATGGAGTCATTTGTCAAATCAGTCTCATCCTGGAAAAATCATCT
ATTCTGCCTCCTAGAAAGCCAGAGTGCTTGGAAGCTAGTTCCACCTTCTCCTAACAGTAAATTGC
TTTGTTTGTTGGAGTTAGCTAGAGTTGCTTTTTGTTTTTGTTTTTGTTTTTTTTTTTTTGCAATTCTTAT
TTAGTAATGAAGGAGATAACTCCCTCAGGCAAGTTAAAAGGATGGTGAAGATAAGCATAGAAA
GAACGAGGTTTGGCTAAGTAAAGGTTAAAGTTAAGGCTTGAAGTAAAATCATAGCTAAGTAAGG
TGGTAGGAGCTACTGGCTGGAGTTTCATCTTTTCAGTTCTCCATTCATCTTTATAAATTCTATTTAG
CGACTCATGATTAGAGCCAAATCACTACATTCTATTTAGTGACTCACCGGGTATACCTGAGAGTC
AGGTATACAACAGATACCTCTTTAGAGTTGCTCAATATGGAGGCTTGATGAATATTGACTCAGTG
TTTATACTAGAAAAATTGTCTATAAGTTTTGGTGCACTTTAACATAATTCTCTGAAATGATTTTGA
AAATAAAGCTAGAGATTATGAGTTAAATATGTAATATCTTTCAAAATTTCAGCACTTACTGGCTTT
CATTCTTCAACTTTTCTAGACAGACTAAGCGAGTCTAAATCTGACTTTCATCTTTGTTAATTTGCCT

TAAATCATTTTTTGATTGAGTCATTAGTGATTAATGTCACTGCATTTTCCCATTCTGGTGGGATTCA
AATTTGAAAGAAAAATCACTATTCTATGTTTGTCTGAAATTGTTTGAGCATTTTATTTTTCAATAAA
TGCATGTATACAGGAGGATGAACTTTTAAACAAATGCAAATAAGAACCCAAGCAAGCTAGCAAT
TTGAAAATAACCTGCTAATCTGTATGTATTGCAAGGTTTTGAAAATAATTTGCCTTAGTTTATACA
ATCAGTGCTCTTTTTTTTTTTGACAAAATTCACTAAAAGGGTAGCTGCAGAAAATAAATTCCCTGTG
CAGAGAAGGTCTTTATAAAAGCAATGCCTTTGTTGATCTGACATTTTTTCTTCTGTAGTTAGTTGT
TTTTCACTTAGAGACTGAATTTTTTTTAACACATCGATAGAAGTAGATTTGAACAGAGAGGCTTTT
TGTTTCTTGTATGATTTGATGAATTAACATTTTGTTGAATGTCTACTCCGTATAAAATGCTTTTGCA
GATGTAAAGATGAACAACACACAAACTTGTCCTCACAGAGCACACACTCTAGTAGGAGATCAA
ATATAACTCCGTGCATTATAACACAGAATACATTAGAAGACAGGTAGAGTATACAGTAGTGATT
CAGAGAAAGTCTAAATTGAGGTCATTGGAAATGGCTTCTAGAAGGAAGTTGCATTTAAAACGGT
CCTTAAAGATTTTCTACCATCAGAGGAAGGGAAACAGACAATCTAGAAGGAGAGTACAAAACC
TACCCTCTTTAGATATTGATAGCCTCCCTGGTGTCACTGGAGTAGGAAAAAAAATCGAGGCATTTA
AAGGTATTTCAGGTCAGCTTTACTTGCCACTTATCCTAGCATGTGTAAGGAAATACTTTGTAGTAT
CACATCATCATCACCATAATCACCTCCTCATCCTCACCCTCAAAGTCTGTGCACTTTACATTTGGC
ATATAAGAAAGAACAGATTTTTTTCTATTCTTGCCGCAAACCAATTTCTAACAACTTGGAGTCAG
AAAACTGAAGGTACTATTCAAGGAAAAAGAAAATACACCAGTTAAATTGGAACAGACAGAATG
TTTTAAAAAAATTAGAATGAAAGAAAAGCTCAATTTTTTTTGTTGTGCAAGAGCACTGTGGAGCTG
TGATGTCAAATGAATCTAACATGTCCCTGGAATTTTTTAGACAGATTCATATTACGATTTGTGATT
CAGGAAAACTCATTACCAAGATGTAGGTTGTATTGAAGAGGAATAATAAAGAATGTAGTTAATA
TTTGATGTGGTTCCATTTAGAGAATATCAAAGTATTAAACATCCAGGCTCAATAATACAGACTTA
ATTTTGTATAGCATATCCCAGTTTATAATGAATTTTCCCATCTACTTCCTTAATTCATCCTCACAGT
ATTGTAAGGTAGTAGAGAGGAGGGAGAGTTTGCTCTCCTCTAAAATAAAAGTATTACTGACACT
TGAAAATAGCACGAAAACAAGATTCAACTAAAGTCACTCAACTAATTCACTAAATGGACACAAA
TTGTTAAATTGCTTGTTTTTACTATTCTCTGCTTTGATGTCCTGTGAAGGTTAACATTTGTCACCAT
ACATGATAATTAAATTAGTCTCCCTCATTTTCCTTATCCTGAATAAAAATGATTTCTAACCTGGGC
ATGATGTTTTGCACCTGTAGTCTCAGCCACAGGACTGGGGACTGGAGGCTGAGGCAGGAGGAT
CACTTGAGCCCAGGAGTCTGAGCCTGTAGTAAGCTGTGATCATTCCTGTGAATCCAGTGGTGAC
TGCATTCCAGTTCAGGAAACATAGCAAAACTTTGTCTCTGAAAATAAAAAAAAATTAAAAATATT
TCTGTAGGTGTTTATTTGATATTATAGAACAGTAATATATGCACATTATACAGTGTTTGAAATATA
TAAAAAACATGTACAGTGAAATGTAGGTCTCCTCCCCCTGTCCACAGTCATTCTGTTCCCTTCCA
ACAAAAATAAATTGCTAGTATCTTGTGTATGCTTCAAGAAATATTCCATATCTTTATGGTCATATT
TATGTCTTTAAATACATTTTCACACACACACACACAAAGCATCTTGCTTTTCTTCACTTAGTAACA
CCAAGGAGGGTGTTCCATAATATTACATCCTTTTAAATGACTGCATTATTTGCCACTATACAAATA
TCCCATGTTTTGTGTGACCAGATATAGAGTTTTTTTATATTTCATACCTCCTTTTTATTTATTTATTT
ATTTATTTATTTATTTTATTTTATGTTTTATTATACATTAAGTTGTGGGGTACCTGTGCAGAACGTG
CAGGTTTGTTACATAGGTATACATGTGCCATGGTGGTTTGCTGTACCCATTAACCTGTCATCTAC
ATTAGGCATTTCTCCTAATGCTATCCTTCCCCTAGTCCCCATCCCGCTCCCAACAGGCCCCAGTG
TGTGATATTCCGCTCCCTGTGTCCATGTGTTCTCATTGTTCAACTCCCACTTATGAGTGAGAACAT
GCAGTGTTTGGTTTTCTGTTCTTGTGTTAGTTTGCTGAGAATGATGGTTTCCAGCTTCATCCATGT
CCCTACAAAGGACATGAACTCATCCTTTTTATGGCTGCATAGTATTCCATGGTGTATATGTGCCA
CATTTTCTAAATCCAGTCTATCATTGATGGATATTTGGGTTGGTTTCAAGTCTTTGCAATTGTGAA
CAGTGCCACAATAAACATACGTGTGCATGTGTCTTTATAGTAGAATGATTTATAATCCTTTGGGT
ACATACCCTGTAATGGGATTGCTGGGTCAAATGGTATTTCTAGTTCTAATCCTTGAGGAATCGCC

ACACTGACTTCCACAATGGTTGAACTAATTTACGCTCCCACCAACAGTGTAAAAGCATTCCTATT
TCTCCATATCCTCTCCAGCATCTGTTGTTTCCTGACGTTTTAATGATTGCCATTTTAACTGTCATGA
GACGGTATCTCATTGTGGTTTTGATGTGCATTTCTCTAATGACCAGTGATGATGAGCTTTTTTTCA
TGTGTCTGTTGGCTGCATAAATGCCTTCTTTTGAGAAATGCCTGTTCATATCCTTTGCCCACTTTTT
GATGGGTTTGTTAGTTTCTTTCTTGTAAATTTGTTTAAGTTCTTTGTAGATTCTGGATATTAGCCCT
TTGTCAGATGGATAGATTGCAAAAATTTTCTCCCATTCTGTAGGTTGCCTGTTCACGCTGATGATA
GTTTCTTTGACTGTGCAGAAGCTCTTTAGTTTAATTAGATCCCGTTTGTCTTTTGTTGCCATTGCTT
TTGGTGTTTTAGTCATGAAGTCTTTGCCCATCCCTGTGTCCCGAATGGCATTGCCTAGGTTTTCTT
CTAGGGTTTTTATGGCTTTAGGTCTTACGTTTAAGTCTTTAATCCATCTTGAGTTAATTTTTGTAAG
GAAGGGATCCAGTTTCAGCTTTCTACATATAGCTAGCCAGTTTTCCCAACACCATTTATTAAATAT
GCAATCCTTTCACCATTGCTTGTTTCTATCAGGTTTGTCAAAGATCGGATGGTTGTAGATGTGTGG
CATTATTTCTGAGGTCTCTGTTCTGTTCCATTGGTCTATATATCTGTTTTAGTACAAGTATCATGCT
GTTTTTGTTACTGTAGCCTTGTAGTATAGTTTGAAGTCAGGTAGTGTGATGCCTCCAGCTTTGTTC
TTTTTGCTTACTATTGTCTTGGCTATGTGGGCTCCTTTTGGTTCCATATGAAATTTAAAGTAGTTTT
TTCCAGTTCTGTGAAGAAAGTCATTGGTAGCTTGATGGGGCTAGCATTGAATCTATAAATTACTT
TGGGCAGTATGGCCATTTTCATGATATTCATTCTTCCTGTCCATGAGCATAGAATGTTTTTCCATT
TGTTTGTGTCCTCTCTTATTTCTTTGAGCAGAGATTTCTAGTTCTACTTGAAGAGTTCCTTCACATC
CCTCGTAAGCTGTATTCCTAGGTATTTTGTTCTCTTTGTAGCAATCGTGAACGGGAGTTCACTCAT
TTTCTATAAGGTGTAAGGAAGGGATCCAGTTTCAGCTTTCTACATATGACTAGCCAGTTTTCCCA
GCACCATTTATTAAATAGGGAATCCTTTCCCCATTGCTTGTTTTTCTCAGGTTTGTCAAAGATCAG
ACAGTTGTAGATATGCGGCGTTATTTCTGAGGGCTCTGTTCTGTTCCGTTGGTCTATATCTCTGTT
TTGGTACCAGTACCATGCTGTTTTGGTTACTGTAGCCTTGTAGTATAGTTTGAAGTCAGGTAGCA
TGATGCCTCCAGCTTTGTTCTTTTGGTTTAGGATTGTCTTGGCGATGCGGGCTCTTTTTTGGTTCC
ATATGAACTTTAAAGTAGTTTTTTCCAATTCTGTGAAGAAAGTCATTGGTAGCTTGATGGGGATG
GCATTGAATCTATAAATTACCTTGGGCAGTATGGCCATTTTCAAGATATTGATTCTTCCTACCCAT
GAGCATGGAATGTTCTTCCATTTGTTTGTGTCCTCTTTTATTTCATTGAGCAGTGGTTTGAAGTTCT
CCTTGAAGAGGTCCTTCACATCCCTTGTAAGTTGGATTCCTAGGTATTTTATTCTCTTTGAAGCAA
TTGTGAATGGGACTTCACTCATGATTTGGCTCTCTGTTAGTCTGTTATTGGTGTATAGGAATGTTT
GTCATTTTTGCACATTGATGTTGTATCCTGAGACTTTGCTGAAGTTGCTTATCAGCTTAAGGAGAT
TTTGGGCTGAGATGATGGGGTTTTCTAAATACACAGTCCTGTCATCTGCAAACAGAGACAATTTG
ACTTCCTCTTTTCCTAATTAAATACCCTTTATTTCTTTTTCCTGCCTGATTGTCCTGGCCAGAAGTT
CCAATACTATGTTGAATAGGAGTAGTGAGAGAGGGCATCCTTATCTTGTGCTGGTTTTCAAAGG
GAATGCTTCCAGTTTTTGCCTATTCAGTATGATATTTACTGTGGGTTTGTCATAAACTGCTCTTATT
ATTTATGAGCTCTTATTATTTATATTTGAGGCATTCCATCAATGCCTAGTTTATTGAGAGTTTTTAG
CATGAGGGGTTGTTGAATTTTGTTGAAGGCTTTTTCTGTGTCTTTTGAGATAATCAGGTGGCTTTT
GTCATTGGTTCTGTTTATGTGATGGATTACGTTTATTGATTTGTATATGTTGAGCCAGCCTTGCAT
CCCAGGGATGAAGCCGATTTGATCATGGTGGATAAGCTTTTTGATGTGCTGCTGAATTTGGTTTG
CCAGTATTTTATTGAGGGTTTTTGCATCGATGTTCATCAGGGATATTGGCCTGAAATTTTATTTTTC
TGTTGTGTCTCTGCCAGGTTTTGGTATGAGGATGATGCTGGCCTCATAAAATGAGTTAGGACGG
ATTCCCTGATTTTCTATTGTTTGGAATAGTTTCAGAAGGAATGGTACTAGCTCCTCTTTGTACCTC
TGGTAGAATTCGGCTGTGAATCTGTCCAGTGTAGGGCTTTTTTTGTTTGGCAGGCTATTTATTGCT
GCCTCCATTTCAGAACTTGCTATTGGTCTATTCTGGAATTCAGCTTCCTGGTTGAGTCTTGGGAG
GGTGTATGTGTCCAGGAATTTATCTATTTCTTCCAGATTTTCTAGTTTATTTGCATAAAGGTGTTTA
TAGTATTCTCTGATGGTAGTTTGTATTTCTGTGGAATCAGTGGTGATATCCCCTTTATCATTTTTTA

TTGCATCTATTTTGTTCTTCTCTCTTTTCTTCTTTATTTGTCTGGCTAGTGGTCTATCTATTTTGTTTA
TCTCTTCAAAAAACCAGCTCCTGGATTCATTGATTTTTTTGAAGGGTTTTTGTGTCTCTCTCTCCTT
CAGTTCTGCTCTGACCTTAGTTATTTCTTGTCTTCTGCTAGCTTTTGAATTTGTTTGCTCTTGCTTCT
CTAGTTCTTTTAATTGTGATGTTAGGGTGTTGATTTTAGATCTGTCTTGCTGTCTCTTGTGGGCATT
TAGTGCTCTAAATTTCCCTCTACACACTGCTTTACATATGTCCCAGAGATTCTGGAATGTTGTGTC
TTTGTTCTCATTGGTTTGAAAGAACATCTTTATTTCTGCCTTCATTTCGTTGTTCACCCAGTAGTCA
TTCAGGAGCAAGTTGTTCAGTTTTCATGTAGTTGTGTGGTTTTGAGTGAGTTTGTTAATCCTGAGT
TCTAATTTGATTGCACTATGGTCTGAGAGACTGTTATTATTTCCATTCTTTTGAATTTGCTGAGGA
GTGTTTTACTTCCAATTATGTGGTCAATTTTAGAATAAGTGTGATGTGGTGCTGAGAAGAATGTAT
ATTCTGTTGATTTAGGGTGAAGAGTTCTTTAGATGTCAATTAGTTCCACTTGATCCAGAGCTGAGT
TCAAGTCCTGGATATCCTTGTCAATTTTCTATCTCGTTGATCTGTCTAATATTGACAATGGGGTGT
TAAAGTCTCCGGCTATTATTGTGTGGGAGTCTGTCTGTAGGTCTCTAAGAACCTCCTTTATGAATC
TGTGTGCTCCTGTATCGGGTGGATATATATTTAGGATAGTTAGCTCTTCTTGTTGCATTGATCCCT
TTACCATTATGTAATGGCCTTCTTTGTCTCTTTTGATCTCTGTTGGTTTGAAGTCTGTTTTATCAGA
GACTAGGACTGCAACCCCTGCTATTTTCTGCTTTCCATTTGCTTGGTAAATAGTCCTCCATCCCTT
TATTTTGAGCCTTTGTGTGTCTTTGCACGTGAGATGGGTCTCCTGAATACAGCACACTGATGTGT
CTGGACTCTTTATCCAATTTGCCAGTCTGTGTCTTTGGATTGGGGCACTTTGCCTGTTTACATTTA
AGATTAATATTGATATGTGTTAGTTTGATCCTGCCATTATGATGCTAGCTGGTAATTTTGCCCGTT
AGTTTATGCAGTTTCTTCATAACGTTGACAGTCTTTACAATTTGGTATGTTTTTGCAGTGGCTGGT
ACAAGTTGTTCCTTTCAATGTTTAGTGCTTCCTTCAGGAGCTCTTGTAAGGTAGGCCTGGTGGTG
ACAAAATCTCTCAGCATTTGCTTGTCTGTAAAGGATTTTATTTCCCCTTCACTTATGAACCTTAGTT
TCGCTGGATATGAAATTCTGGGTTGAAAATTCTTTTCTTTAAGAATGTTGAATATTGGCCTCCACT
CTCTTCTGGATTGTAGGGTTTCTGCAGAGAGATCTGCTATTAGACTTACGGGCTTCCCTTTGTGG
GTAACCTGACCTTTCTCTCTGGCTGCCCTTAACATTTTTCCCTTCATTTCAACTTTGGTTAATCTGA
TGATTATGTGTATTGGGGTTGCTCTTCTCAAGGAGTAACTTTGTGGTGTTCTCTGTATTTCCTGAA
TTTGAATGTTAGCCTGCCTTGCTAAATTGGGGAAGTTCTCCTGGATAATATCCTGAAGAGTGTTTT
CCAACTTGGATCCATTCTCCCTGTCACTTTCAGGTATACCAATCAAATGTAGATTTGGTCTTTTCA
CATAGTCCCATATTTCTTGGAGGCTTTGTTCATTTCTTTTTCACTCTTTTTTTTTCTCTGATCTTGTC
TTTTATCTTTATTTCATTGAGTTGATCTTCAATCTCTGATATCTTTTCTTCCGCTTGATTGATTTGGC
TATTGATACTTGTGTATGCTTCACACAGTTCTCGTGCTCTGTTTTTCGGCTCCATCAGGTCATTTAT
GTTCTTCTCCAAACTGGTTATTCTAGTTAGCAATTCATCTAACTTTTTTTCAAGGTTCTTAGCTTCC
TTTCATTGGGTTAAAACATGCTCCTTTAGCTTGGAGGAGTTTGTTATTACCCACCTTCTGAAGCCT
ACCTCTGTCAATTCATCACAGTCATTCTCTGTCCAGTTTTGCTCCCTTGCTGGCGAGG
ID|55939|gene_id|ENSG00000228043|transcript_id|ENST00000414911 Homo sapiens lncRNA

AAGCCGCCACCGCCGCCGCTGCTGCATGGCACTAGGCTCCCGGCGGGGCAGGTTCCGCA
GCCGGCACCGCTCTGGCTAGGCGCACGGAGGCGCCTTGCTTAGTCTGACCCTTATTTGGCCAG
GCGTAAAACTTGTCCTGCCTAAAAGCCTACTGAGACCTCCTAACTGGTCTAATCCGCGGGAGAC
GCCAAAGGCGGCGGCCGTTTGTGCGCACAGGATTTTTCTTGGAGCTGTAGCAGTCTAGGAAGC
CAGGGTGGAACTTTTGCTAAGCTCCCGTTGTAAGCCCAGAGCTGTAATAACTGCTGAGACACAA
CCAGCAGTATACAGGCTCCAGTTGGAAATCGAGAACTTTCCAGGTAGATGAAGTGCCCCATGG
AGCAGTTTTCAGGTGGAGGGGATGAGGCTCAGCTGGAAGTCTCAGGCCAGAAGGAGAGGCTG
CCGAGGGGCGGCCAGGGATGCCTAGGAAGTGAGCGCAGAAGACCAGGCGGCCTCCTGGGAC
CGCACGGTGGGAAATAAAGCTGCACCTGCACAAATGTGTGAAGAGAGGATGGGATTGCCTTGT

CTCTTCGGACATTTAGTTTAAAAAGGGGGTGACAACTGAACTTTTTATTCCCCACGGCCATGGGA
CAAAGATACTTAATCGAGCTACCAGGAGTAAACTTCTTTAAAGAAGTTTGGGGGACCAACAATC
CCGGCTTTGCCAGAACAGACATGAACGTCATTCAAGGATCCCAACTCTTTTTGCTTTAACTGTGC
ATACGTCAGCAATTTTTGTTTTGTTTTGTTTTTGACAGGGCTGTCACCCAGGCTGGAGTGCAGTG
GTGCCATCACAGCTCACTGCAGCCTCGACCTCCTAGGCTAAAGTGATCCTCCCAACTCAGCCTC
CCGTAGCTGGGACTACAGGAATGTGCCACGCCCAGGTAATCTTAATTTTTTGTGGAGACGAGGT
CTTGCTATGTGGCCCAAGCAGATCTCGAACTCCTGAGCTCAAGGGATCCTCCCACCTTGGTCTC
CCAAAGTGCTGGGATTTGCAGGCGTGAACCACCACACCTGGCCATAAACCAACTTCTCATTGCT
TACCTGGCTTTCCAAGAAAAGAGAGAGGGCAGAGGGAAAATATTTATTATTTTGCTATGTACTG
AGTATTCTAAGCATGGACTTGTATCGTCTCTGTTCTCTCAACAACCTCTCTAAAACTTCAACGTAG
AAATTTCCATCTTAAAATTAAGCGACAAGAACTAGAAATGGCAAGTAATTCATCCAGAGGTTAC
TGGGATTTGAATACTGGACAGGCAGATCCCCCTCATTTCAAATTAATACTACATTAGCTGTCTTTT
TTATTTCTTTTTTCTTTTTGTTTGTTTGAGACAGGATCTCGCTTTGTACGCCAGGCTGGAGTGCAGT
GGCGCGATCCTGGGTCACCTCCCAGATTCAAGCAATTCTGTGCCTCAGCCACCAAGTATCTGGG
ACTACAGGCGTGAGCCCGGCTAATTTTTGTATTTTTAGTAGAAACAGAGTCTCACCATATTGCCC
AGGCTGGGCTCAAACTCCTGGCCTCAAGTGATCCTCCCACTTCTGCCTCCCAAAATGCTGGGAT
TAGCACCTGACCTTACGTTAGCAATCCTGTTGACTTATTTAAGACAAAACGTGCTTTAAGACAAA
ATCTGTGCCAAAGGTTTTCAGATAAAGATAAAATTAGCAATGTAACCATAAAATTCAAAGTTGCC
TTGGGCTGAGTTGGTAGAATGTTTATGCAGATCAAGGAACTCCAAAAGTTAAAAAACAGAAGTG
GAAGTGAGCATTAGGGAATGGGGAAAGAGTTGGTGGAGAGAGGAAATATTTGTGTAAACATTT
AACCATCTCCAAACACCAGCAAAGAACAGTTGAAACGATTTGCTATACTCACTGTCCACCCGTG
TTAACTTTATGGTATGTGACATACAGCAAAAATTACATGTAATTTTTGACTAAGCCTTTTAGCTCT
TTCAATCCCTTCGTAGTATGTCACCCCCTCTCTTCCCATAATTAAAAATTATAAGACACCTAGTAA
ATATAATACACTCATAGAATTAAGTTATAAGTCCATCAAGTACATTTCCAATTGGAAGTTAAGAG
TGATGCTGACCCTTCACAGTTGACTGGGCTGAAGTTGGGAACTGATTCAATAAACTTCCACCAC
AAGGATGTCAAATTGCTCCTCAGGGTATTCTCAGAGAGTCTTTTCCCTAGGCTTGATCTCCTTAC
CCTAAGTGTTAGGACACACCATTCCTCTCTTGCTTGCAGTATTCAATGACTTTACCCACTAATAAA
ATGATAGTAAATAGTAAGAGTGAATATGGCTTGTAAGTAATGCTAACAAAACTGTGATGGCTTA
GCAAAGTAAAAGATGGTTTTATGGTCAGTAAGTTTGGATTAAAGCTTACTAAAGCTTGATGCTTT
GTTACCAGTCTGCTAAAGAACTACCTTCTTAATTTGATTTCCTACTTACTGGATACCTATTGGAAA
ACTAGAGAGATATGATAGGCTTTCATTTATTGCTGCCGTAAATAAATTTTTAAAATAGAGAAATA
ACAGCTTTGTCATTAGTGTAGAGCACAGTTTGCCTCTGCTAGGTCAGTATTGGGCTTGCTTCGTTT
AAGTCATTAACCATTAGGATCACCCAACTTGTCCTGCCTAAAAGCCTACTGAAAAAACATGCAT
ATAACTTTTGCATATTTATTTTGCTACTTATTGTTGATTTTCTATAGGAAACTATTTATACTCCTCAG
TGTATATGGCCAACTTGGAAGTTCTACTTCCTATAATTATGTCAGCAGTTAGTGAAGATGATCAA
AACAGCCATTCTGAATTTAATAAACAGATGAGCCTCAGACTCTGGATCTCCATGACCCAACCCT
ATGAGGCTCTGCCTGAAGAACCAATCAAGCTTCCCACTTTGATATTTTAGTGACTGTAGCATCTT
GTTCCTCTTCTAAATTAAACACCACAGATGCTCCCATTTCATGGCTGTGTAGGCACGATGGACTC
ACCACTGCCTTCCATACCACCACCTGGTTTATACAATAATTTTCCAATTCTAGTTGTCCAAAACGA
AGCTATACAAAACTCACAAATCCATTATTGTCTTCCATGTAGAGATAAAAAAGCAAGGAATATG
CTTGTTAAAAAGCATGCAATACTTTATGAATCCTCGGTGATGTACTTTTTGGGCTCGCTGCTGCTG
GAGATTTTGCTAATGCCCCTACACGTTTTAATGCAGTCCACCAAGCTCTTTGCTAGTAAGCTGGT
TTCCACTTTTTTTTTTTCCTTTTTCTTTTTCTTTTCTTTTCTTTTTTTTTTTTAAACAGACAGGATCTCC
CTTTGTTGCCCAGGCTGGAGTGCAGTGGTTATTCATAGGCATGATGATGGCTCACTACAGTTTCA

AACTCCTGGACTTAAACAATCCTGTCTCAGCCTCCTGAGTAGCTGGGACTTACAGATGCGCACC
ACCATACCTGGCATCCCACATATTTTTCTAATCATTATCCCCTTCTTTCCTAAATTTTCTTCAAGGA
AACCAAACTCAGTCCCAACTTCCATAAGCTAACACTTATACAATTAAAATGACATTTAGCTTCTG
GAATCAATGTGGAAAGAAATGACAACTTATTTGCTGATTATTTTTCACTGTAATAAATATTAGGC
CTTTTATCTGGAAGGAAATACCAAACCCTTGTTTAATATTAATAACTTGTAAATTATTGTACACGT
AATTTCATTCTCTCTTCCCATGTCTTCATTTTTCTGCCTCTGGCTTTTCACCTTGTTGTGAATTCCTA
GTTTTATAGTACTTTTATACTGTATGATACTGACTTCTTAAGCATACAAAAAACTTCAGAACTCAA
GCAATTCCACCTGTAAGCCTTGTCTAGTGATGCCTATGGCAAAAAAGGACCCATACATTTGATGT
ACTTATCACTCAAAAATCATTCCCAGAGAATTCTCAGGAAACTCAGAACAGGGCCTACCCTGAA
TTCTGAGAACAGGACCATTTTTAAGTCCTTTAAGTCCTTCATTGACACCCTTCCTTTCTCTTACTTT
CTTTTCTTTCTTATTTTTCTCTTTCTTTCTTTTCTTTTCTTTTTTTCTTTTTCTTTTCTTTCCTTTCTTCCT
TCTTTCCTTTCCTTTCCTTTTTTCCTTTCTTTTACTTTTCTCTTTCTTTCTTTCTCTCCCTTTCCTTTCCT
TTTCCTTTTCCTTCCCCGTCCCTTCCCCGTCCCGTTCCATTCCGTTCCATTCTGTCTTGCTCTGTTA
CCCAGGCTGGAGGGCAGTGGTGCAATCATAACTCAGTGCAGTCTTGACCTCTTGGGCCCAAGG
GATCCTCCCATCTCAGCCTCCTGCAGGCATGCACTATCAAGTCCAGCTGATTTTTTTTTTTTTTTC
ATTTTATTTTTTCTAAACACCGGGTTTCACCATGTTGCCCAGGCTGGTCTGGAACTCCTGGCTTCA
AGTGATCTTCCTGCCTTGGCCTCCCAAAGTGCTGAGATTACAGGCATGAGCCACAGTGCCCGGC
CAACACCCTTACCTTTATGTTCCCAGTCACATTATGTCAAATATTTCAGAATGCTTCTTTGTTTCAA
ATTAAATACACATTCATAAATTTACTGCTTTGGTTCTTTGGATAATCAATCAGTCAGGTCTATAGG
AGAGATGATTTTTGTTGTTTTTCTTGTGAAAAATGTGAAGAATAAGTTGAATGGTTGGCATACTGC
CACATCTTATAGCCATTGAGTTAAATATTCATTTGAATTAGGTTTAAAACGTGGTTGAGGCTCTTG
AAATGCTAGTAATCCATAGATTCTTTATCATTAGTGCCTCACGCATTAGAAATAGCAGTACTTCT
GCTGGGGAATGTATCATCTGGGAAAAGTCTTGGCTACCCTACCTGCAGGTCCTCTATTCATAGTG
TAATAAAAGAAAAACAATAAAAATGATCTTTTCTACAAGATTATCCAAAACTCCTACAGCATCCC
TCCAGGCATCAGCTGAGGGCTCTTATTCCAAAGTCACTTACTGTTGCTGGAATTCTTTTATTCTGC
CAGGATCCAGGCAGTCGCTGCTGCCATGATCTAGTCCCTAATGACCTCTCTCTTCAGCTTGGCCA
GCTAAGCTCTCTCCTGCTGGCGCCCTGGCCCTGCCCCTTCTCTGCTCTCAGGTAACAGAGAAAG
AGGAGTTGTGGTTGGCCCTGGAACCTGGATACAGACCCAGGGGAAGTCTAAAGCTCTCCACTC
TGTTCAGCCAGAGGCCACCAGCCTCTGCACCAGCCCTAATTGGCTCTAGAGGTGCAACTGATCG
GTCTCCTCCTCTTTTATCTCTGCTCAGTCATCACCATGTCTCCAGGTCAGGGTCTGTGGTCTTCTC
AGAGAGACACACCCTCCTCCCTATCAGGGCTGTACCTGGCTAGAAGAGTTTGTTTAAACATCTG
CCCCTGCAGTGCGCACTGGGTGGTTTCCTGTCTTTGTGTCCTTCTCCTAAATTGGAGCCACTTCAT
CCCAGTCACCTTGGAACCTGACTCAAGAAATGGCAGTTCCATGCCTATTTCTCTGGACACACAA
AGGGGTCCTGTCTCCAGGGTAATCTCAGATTGGATCACCCCTTGTTTCACTTAGACTCAGGTGCC
CCTTCTCCCAACGAGGATGCATATAGAATAGAATTAGGCCCTGCTCCCAGACAAGCAATCTAGA
AGTCCATTCTCATTGGTTGACCTTCTGTGGTGTTCCTGAAATTGCCTTGTAGCCAATCAGACACTA
AGTAAACCCAGGAGCCTTGGAACCTCTGGACCTCAGTTTTCCTTGACTCACTGATTTTCCACTGG
TCTGGTGCCTCAGAAGAACCAGATTCTCCTGTTCAGTCGTCACTTCCCTTCTGCTGCTTCCCTGCT
TTAGAAACTAAGCCCTGTCTTGCTTTTGTCAATTCTGTATTTATACCCCTACACAAAACTGAATGG
CCACTGGCCTCCAGATGGTTCTCCTACACTTCAGTCTTTCACTGCTAGCCTGCCTGCCGAATTGG
AAACCCTGGTATGACCATTTCCAAAATTAGAAACCAGACTGCACCATCTCCCAGGGGCACTATG
CCTGCATCTCTCAGCACTCAGTCGAGCCAACCTCTGCAAGTGTCCCAGACACCCTTCCTTCCAGT
CTCTTCTACTCCCCTCCCCCATATTTCTGGCTATGGTGATTTGGGCTTTTGCCTTCCACTCTTTGCC
CAACCTTGCTGAAGTCTTAACACATTGGACCTCCCCTGCCTGAACTATGCCAAGCAGAAGAAAG

GCGAATTCAAGGGTTACCTAAACCCTGGATATTCTTTCAGAGGCCTCATCACACATCCTATCAAA
CACTGGAAAATGTCCTATCCCACATGAAAAGCACGACGTTTTGTTATGAAAATTAAAGGAAATC
ATATAATTTTCCTCTTGAAACATTTATTAATTGCATTTTATAATTTGCTTTGTGTTTTAGAGCCAGG
CCATTTTCTTGTCCAGCTCTCAAACAATTTGATAAGACTGGGAAATGCTTATGAGCTCTGGGCAC
TGCACCTATAGTGAGAACTTGAGCCTTAAAGGATTAGAAAAATGCCTCTGGGAGGAAAGCAAA
CACACACGTCTCTATGATGCAGTTCAAGCCCCTCACCCCCATCATCACTTAAATCTCTCTCTTGC
CCAGTTTCTATATCCTAATGGGCTTCTTTGCCTGATCCCCTGTCTTGGCTACTCACCTTCTGCCAG
CAGCTCTCAGACAAACACTCCCCTTATTCCCAAAGCAAGCTGGCCTGCCTGGATTTCAAGAGTT
TCACCAGCCCCTAGATTCCCTTTCAAGCCCTTGTTCCACCTCTGAGGACCCTGCCCCCAGGCGA
CTGCCACTTTGGACTATGGCATTTGTTTGCTTGACCCTGTAGAAATGATAACTGAACTCTCTTAA
GGCTGCAGCTTCATCTGGCTCTCTGGAGCCCCAGCCCCCTTCAAAGCCCTGATCTGGGATTGCA
TATTGGGTGCTTCAGTCTTTCCCTGCCTGCTGGGACTGACCAGCAAAGGCCATTTGTGTCCCAAG
TAGGACAGCTACCAGAAATCACACTTCACATTTTCCTACTTAAGGTGCCTGCTTACATTTCCCTG
TGCTTTGAGTGTCTCCTTCTCTAAAGGAGCCTTTTATGAGTCAGTTACATGTGGCATCCCTGCTAC
ACTTCTCTGTGGCTTTTCTGATCAGTTATACAGATTAAGAACATGTACAAGAAAGCCAAAATATC
CCCAGCAATTACCCCAGGTTACGTCTCTTGTAAATGTTAACCATTACTCAGTTGAAATAGCCATT
AATAGTTGGCTTTTGTGTTCCAGGATTTCTGAGGTCACTGAATTACTAGTGATTCTGCAATTTCAA
TATATTTCTTAAAATTAAATTACACATATTTTGGGTACACAGTGATCCAGTTAAGAGGGAGAGTC
TGCAATTATATATTTTTGTCTCTTTAGTTTCCTTTCTCATTTTTTTCCCCCAGAAAGGAAATTAGACT
AGCCTTTTAAAAATGGTGTTCTGCTACAGTCTGCAATTTTGAGAGGAAAGGTGATGAATGCCTTT
ATTTTCATGTCGTCAAAAGCTCAGCAAGAATTTGCGTCTATTTTGACTGTGGAGCCTACTGTGTG
CAAAACAGGTGTGACAAAATCCCCCCTATTAGAGCTGTTTTCTTTCCATTTTCTATTTTAAAAAAA
CTTTTCTCTCTGTGGTAATCACTTAACCCTAAAAGGCCTGGATATTTATACAAAAAGGTACCATG
CTTCATTTGTTAAATACTCTTTCTCTTCTGAGTTTGATTTTAATTTTAGTTATTTAATTGTTTGGTCC
AAGATAGACTTAAAACATCTTATTTTGAAAGAAAACATGATTAAGTTCCTGGAATATTTACTTGAT
CACTTGAACCAATCCATGGTGTTCCTCCATTGGCATTCCTCCGCGGGACGTGTGATGACTAGCCT
GCTAGTCTTGGAAAATACTCCTCCCCTGCCCTTCTCTGTCCTTATCTAAGCTGGCTTCTACAGGCT
GTTTTCCAGACCCATGTGTCAGCTGGCTTCCTACTGGGCTAGGCTAACAGGGAGCACTGTCAGG
TGATTGGAGGGTAGGAGAAACCAATATATCTCCCCATCTCTTTCTGCCTTAGGCACCATCCCCTG
CAGCTGTCTCCCCAGCGGCTCCCTCTCTTGCTGGACAGGCCTACCATGAAATCAGCCCTGGGAT
CTGACAATACTACCTCCTCCTGCCACCTACTTAAAAGTGTAATAGTTTCCTGTGGTTGCTTCTCTG
GGGAAGAATTTCCCCTGGATTCTTCTCAGTTTTTCCATCACCTGTGCAACAGATTCCTTGTATCAC
ATTCTTTGTTTGAAGAAATAAGTGTGGTTTCTGTTTTTCTAGTTGAATTCAGCAGATTTATCCATTT
TGTGTTTTCGTAAGGGTCTTACCCTGCTTAGGGGAGAAGGAATGATTTTCACAGGAAAGCAGTC
ACATTCAGTTTAGGAATCTGCCTGTGCTTCCCCCGATGGCTTTAGTAATGTCATTATTTTTTCCAC
CTCTTACTCCCCATGCTTGCTCCTCACCTCCCATACCCAACCTGTGTCTCTCCTGCTTCCTCCATT
CTTAGAGCATATGACACCTATCTTCTGGGGTCACATCCCCAGAGGTACACAATGATTTGTTTTTG
TTTATTTTTAAATTTCTTTTCATATAGTGTCCTTAACAAGAATGTCACTGCTGTAGGATAGGATAG
CAAAAGAGCACTGGGTTTTTCATTCAGAATGTGAATCAGTGTGGCTTGTTTGAATCCTATAGCCT
TGTCCTGCACAACAGTGCCCTTGATAGCAGAACAGGAAAAAAGATAATTTCACCACAAATCTAT
GAGGAGAAAATGAGATGAAACATATGAAAGGATTGTGCAAAAGATAAAAGGCTAAAAGAAAAT
AAAAAGAAAAGGCCAGGTATGGTGTTTCATGCCTATAATCCCAGCATTGTGGGAGCCCAAGGC
AGGAGGATCACTTGAGCCCAGGAGTTCAATATCAGCCTGGGCAGCATAGAGAGGTCCTGTCTG
TATTTAAAAAAAATTTTTTTAACGATAAAATGCTAAGCAAATAAGAAGTTATTATTTTGAAATAG

TAAATAAGCCTAAATTAGTTGTTTTCCTTGAAACAATTGTAATTTTAAAAGTTACCACAAAGGTAT
TTCTCAACTAGAATTTATACAGAAGCTTAACAACATCACAGTCTTGATTTAACACATGATTTCTCA
ATCTCAGTTCTATTGACCTTTGAGGTTGGATAATTCTTTGTTGTAGGGAGCTGTCTTGTGCATTGT
AAGATTTTTGGTGGCATCTCTACCAGTAGCACCCACCTGTCTAGTTATAACAAAACATTTCTCAA
CATTGCCAAATGTCCCCTAGGAAAAAAAAAAAAAAGAGACAAAACCGCCCCAAATTGAAAACCA
CTGATTTAACAAAACCTACTTGGAAAGATGTGCCTTCTTTTTGATATAAGTTGTTAAAACAAACA
ATAATTAAAAGAAGTCTGGCTTTTCATAGATTATTTGGGAAAAAATCTTTTAAAAATGCAGTTCAGT
CATAAAATATAACCTTAAAGATCCAAAAACCCTACAGTGAAAGTCAGTGGTGATTGTCACAGCA
GTAGTGTGTGTTACTAAGTATTTACGTTGGAGAAGCTAATCTTCCTGTTAAGTATTGTGGTCGGA
CTACCTAACCAAGATGTCCTTTCTTCTTCATACAATTAATAACATTGTTATTTTTACTAGGTGTTAA
AAAAAAACACACTAACACTTTATATCTGACTACTGTATGTTGAAATCCTTTGATGTCCTTTAAAAG
GCAAGGTAACTCAGATTGAATATTAGTAGGAACATATATTTCACTCTTGACTCATATATTCTGGG
TTTGTACTTGTATGGACTACCTTGAGTAGCTGCAGGACTTAGAATTTTATGAGCATCACAGCTAA
GTCATGGTCTGTAGAATCATTCATATTTCAATTCCTAATATGGTAGTCTTAGCTACCATTTAATTTC
TAGAGAACTGGAAACCAAAGTATAACTATGTAAAGTCTAGAAATTATTGTATTTAAAATATTGAT
TCCAGGTCTCCAAATATGTCTCTTCTAGCTTATATCAATAGGTTTCTAAAACCAGTTTTTCATCTCC
ACTGTGTCAGTTAGCTTTCTTCCCAGCTAAAGAAAATATTGACCAAAGCAAGTTGCAAAACAGG
TGAAACTGGTTGTAGTAAAAGAGTTACTTTTTGCTGGGAAGATAAAAGAGATGTAACTACTGTCC
TTGACATGAGTATATTGCTAAGCTTTGTGGACAAACATTCTGAGATTGGTAAAATACTTACTGCTT
CAGGAAAATTACACTTATTTCCTCATCTATGATTTTGCATTCTCCTAGTTAATCTTCTTTTGGGGGA
CAGTGAGCCTCCTTTGAAGGACTTACGGGCATTAATAATAATATCACTGTACAAAAAATTAATAAT
ATGTATTGCTCTTTTAAAGATACTTTTGTACAAGATATTTTAATAAAACAATCTTATTCAGTGCTGC
TTATTGTGGTTGGTGAAAAATAAATAAATAAAAACTATCTTATAACTGAGGCGGGTGGTCAGAGT
TTTGGATGCAGACAGTGGGATTAAGTTCAGTCAGAAAGTTAGGGCACTTTTATTTGCCGTCATGC
TTTTGAATTTGAAGGACTTTGGACCAAGCCTGCCTCTCCAGTTCACTGTCATGTTCTCAGTTCATC
ACATATTACATCTGGCCTACGCTTCACATCTGTCAAGCCTCTGTGCAGACATTTGGATCTGTAAA
CTCTGCCAGGACAAAGCTTGGGCTAGCATCCCATACTATAAATGACTATTCCTCAGTGGAATATT
TTGTTTCCAAAAAAGAAATCTATTTGAGGTATCAGTGCTTGCCCCATCTGTTTTTTAAGATCTGAG
AAACAACTATGTAAATATACATAGAAATGATTTGAGTCAAAGCTCTCATTCTCATCATAAATAAG
GAAGGAAAGGCACTGACGATGGGCCATTTCAGAGTCCTCCCTGCTATCTCTGATTTAGAAAAAA
TATATGATCAAGGTGGAGTAATAGAACTAGATTTACCCTCCTACCTGTAACAACTAAACAACAA
CAAAAACAGACAAAAGACATGAAACAATGGTTTTTATGACACTAGATATCAAGCAATGGACATG
GTGATTCTTGAAATATAGGAAATAAAATGGATGAGACCTACTATTGCCCCAGTTTACTAATTTGA
GAGAGTTTTCAGGTCATGACATGTGGTAGGAGAACAGAGGTGAAGCCCAGTTATGATTCTGAAT
TGAGGCAACATTGCTGAGAGTCCAGGAAGATCAACGCAGTGAGATTTCTTAGGAAAGAGTGAT
AGAGGAGAGAGAGAGCTACACAGAGTAAAGCCTGGGGGTCTGCAGAGGGATCTCTGTTAAGT
ATTCAGAAGAGTTTTGATCATGACTATGAAGAAACTACATGAGGCCTAGAAAAGGACCATCTGG
AGGAGGTAGAGAGAACATGTGGCCATAACATACATAGGACCAGAAGTAGTGTCTGTTCCCACT
ACCGGCCAGATTGGAAAAACTCATAATTCATAGACACTGGATAAAGTACTCAAGAAGGTCTTGC
CTCAGTGGTGGGAAATAATTAGCCCTAGCCTGAGAACTGCTCTCAACCTGCCAAACAAATCATA
AAAGCAACATCTGAAAAGATCAAACTGTTTCCAAGTAACTTAACATCATACCTGAACAAAACTC
AAGAAGATTTATGAGAGTTCAAAAGTATCCAGCACCCAACAAATTCACAAAGTATGCATGCAAT
CAAAGATTACCGGGCATGCAAAGAAAAAGAAAAACATATCTCATAATGGGGAGAGCAACCAGT
CAATCAAAACAGACCCAAAATTGGCACAGATGATATAATTAGCAGAGAGAGACACTAAAACAA

TTATTGTGACTATGTTCCAAATGTTCAGAAAGCTAAGTAGAGATGGAATATATTTTTAAAAGACC
CAAATCTGGTTGGAATTTCTGGTGGGAATATAAAATGGTGCAGCCACTTTAGAAAAGTCTGGCA
GTTCCTCAAATTGCTAAACATATAGTTACTACATGACATAGCAATTCTACTCCTAGGACGTACCC
AAGAGAAATGAAAATATTTTCGCACAAAAACTCATACATGAATGCTTATAGCAGCCTTATCTTTA
ACAGCCGAAAGGTAGAAACAACCCAAATGTCTATCATCTGATAAACAGATAAAATATGGTATAT
CCATAAAATAATTGAATATATTTAGTCATAGAAAGGAATAAAGCATGGACAGACACTGCAACCT
TGCAAACTGCTAAGCGAAAGAACCAGACACAAAGGCCCATGTATTGTATGACTTCACTTGCATG
AAATGTCCAGAGTAGGCAAATTCATAGAGACAGAAAGAAGATAGTGATTGGCAGGGATTGGGA
GATTGGAAAGTGACTGCTAATAAAGGTGAGGTTTCTTTGGGAAGTATTGAAAATGTTCTGGAATT
AGATGGTAGTGATGGTTGTACAATCTTTTGAATACACTGAATACCACTGAATTGTATACTTTACAT
GGGTAAATTTATGGTATGTTAATTATATCACAACAATGATGGTTAAAAATAGATGCAATCAGAAT
CAATTTATCTTTCTACTACCATGTTAGTTGTATAAACTATCTTCCATTTCACTCCTTCTATTCTGTCC
CTAACCCATCCCATCTTTCCACTAATAAGGTTACACAAGGTTACCAATGACCTGAATGTATCTAA
TTCATTGTCAGCCTCTGTTCAGTTTTTCCTAAATCTCTCAGCAGCATCAAAATCTCTCAACATTGC
ATCCTTTTAGTAACAGCAGCATCTTCTATAGATTTCATGAAATACACTCACCTAGTTTTCTCTTAC
CAGACTTTATAGCTGCATACTCATAATCTTCACTTAAATGTCCAGTAGGTACCTAAAGCCTAACA
TGTATATGACAGAATTCGGATCTACTTGCTGTAACCACATCCTACAAATACGCATTAGGTAGGCC
CATACCTACTCCTGCCCTAGTCTTTCCCATGAGAGTTAACACACAATCATATATCCAGGTGCTCA
GGCCAAAAACTAGGGAGTTGTTTACCATGTTTTATTGCTCTCTTTCCCTAATCCTACCACATCAAA
TCCACTGGCAGGTCTTAATAGTTCTGCCTCCAAAAATCTATGTGGAACCTATCTCCATTCTCCAG
TTATTTATTCAGAGGTTAAAGAGGAGACTGGAATGGTGCCTGAGGGATTAGATTCTATCTGGTCT
TGTGAACTGTTACATGAACATTCACCTTTATTCCGAATAAGATACAGAGTTTTGACCAAAGTAAT
AACATGATCTGATTTAAGTCTTAAAAGGATCACTCTGACTTCTGTAGTGAAATTAGACTGTGGGG
GAAAATGAAAATAGGGAACCCAATCAGGAGGCTATTGAAATTTTAAAGAAGGATTATGGTTCGG
ACTAGAAAGTTGGGGGCAATGGAGGTGCAAGTGGTGACTGAGTTCTATTTATACTTTTAATATAA
ATAGAGCCAAAAAGATTTGCTGATAAAGTAAGGATGTAGAGTGTGAAAGAGAAATTAAATGAT
GCCAGGACTTTGGTCTGAGCAAATGGAATGATGAGTATGCCATTTCCTAAGTAGAAGATTATTG
AGTGAGCATGTTTGGATAGGAGGATATAAATTTGGTTTTTAAAATTTCCAGTTGGAACTATTTATC
AGGTACCAGATAGAGCTATCAAAAAGGATAGTGGATATATAAGTCTGGAATTTAGAGGTGAGAT
CAAACTGGATAAATAAATTCTGGAATTTTCATTTTATATGTGATATTGAAAGCCACAGAAATTAA
CAAAATCATAATGTAAGCAGCATAGATAGAGAAAAGTTCATGGGCTTAGCCCTGGGGGCCCTC
CAATAGTTAGAATCAAAGAGACAAGGCAAAACTAGCAAAGTAGGTTGTGGTAAAGTAGCCAGT
GAAGTAGAATAAAAACCAGTTGAGCATATTATCATTTTCAGATAGGCAGAATTGCAGCATATTTG
ACTGCTATGAAGAGGGAAAAATTTGGGACGCAGGTAGAGCAAGATGAATTGCTAGAATTATAT
CCTTCAGTGGGGAGGAAAGTATAGGATTAGAACTAGAAGACATGTTGGTCTGGATAGGCACAG
GGTGTTCATTTATAGTAACAGAAAAGAAGGCAGTAGGTGGGCACAGATGCAGAGATACAGGTA
GGTATTTGGTAGGAGATTCTGGAGGTTCTCTTATGAAGGACAGATAAGATCATTGAAGAAGAGA
AGAGAGAAAGTCAAGGAACTGAGAGGCCATGGAGTTGAAAGGATCATCCACGTGAATATTACA
ATCACCAAGAACTATTATAGGAATAGTGTTTGATAAAATGTCAATCAGCTAAAGGCTAAATCTTT
AAGGAATAAGGAGGAGTGGTCAAGGGAAATACAGTGAATCATAGCAATATGGTGGGAAAATAT
GATATTAAAAGCTGGGGTTTTAGGGGAAGAAGAGAAAATGATCTGGAAGCAGTAAGGAGCCCC
CCACTGGCCTAGTGGTATGTGGCATCTGAGAGAAGATATTGGCTCTACTTGAGAGGGCTACAGG
AAAAGCTGTGTTTCCAGGGGAGAGAGAAAGACACGTGGAAATATGGAAATGCATGTATTAAGT
TTTTGGCTGTCACAGTAACTAGTGGAATTCTGTGTCCAAAGAAGAGACATGCTAAACATCCTGA

AAGGTAATGGCAAGATTATATGACATGGACATATGCTACCCAAATGCCAGTAGCACCCCTGTTA
GGAATCTAAAAGTAGAAAGAACATTCAGAGAAAAGGCTTGGATTGATTGTCTTAGTTGAATTTCT
CCAGAAACAAATCTTGATTGTAAGTAATTTATTCAGAAGTACTGGGAAAACCAGTAAAGGAAGA
GGGAAGTGATCCAGGGAAAAGAAGGCAGTCATCCAATGCAGTATCGAGCCAAGGTACTGCCAT
GGGCAACTGGAGTTTAACCTTATGGGAAGCTCAAATGAGGAAATCTGAGAAAGGAGAAAAACA
CACTCCTCACAGTTATTTAACCTCAAGAGTGAGGTAGCTGAAGTGAATACACACCAGCTTCTGA
GAGTCATTGGTAGAAGGCTGCAGATCTGATGAACATGAGCAGAGCACTGACAGCCTCTGTTATA
GGATGTAGAATAAAGGTTTATCTCTAGGATAAATTACAATGAGGAGAATCTTCTGGGTCTAAGA
ATAAGTGCTTGTGTGATTTTGATAGATACCTGCAAATTGTCCATCACAGACAGTATACTAATTTAC
ATTCACCCCAGAAATGTATGAGAATATTTCTTTCTACCCTCCCCAAGTTTATATTCTATTAAGCTC
TCTTCTTTTTGCTAATCTTTAGTAAATATACATTTCAGTGTAATTTTCATTTTCATTTATCTTATTAA
GAGTGAAATCGAACAGCTTAACATATATTTAGCATTTTTTATTTCCTTTTCTGCAAACTGCCTGTT
AACATTCCTTGTTCATTTTACTTTCAGTTACCAGTCTTTTTCTTACTGATCTGTAGAAAAGCAATAT
GAGGTTCAAATATTTTCCCAGTCCGTTATGTCTTTGGAATTTCTTTTTCTTTTTTCTTTCTTTCTTTCT
TTCTTTTTTTTTTTTTTTTTTGAGATAGAGTCTTGCTCTGTCGCCCAGGCTGGAGTGCAGTGGCATG
TTCTTGGCTCACCACAACCTCTGCCTCCTGGGTTCAAGCGATTTTCCTGCCTCAGCCTCCTGAGT
AGCTGGGAGTACAGGTGTGCACCACCATGCTCAGCTAATTTTTGTATTTTAGTAGAGATGGGGTT
TCACCATGTTAGCCAGGCTGGTCTCCAACTCTTGACCTCAGGTGATCTGCCCGCCTCAGCCTCC
CAAAGTGCTGGGATTACAGGTGTGAGCCACTGCGCCCAGCCTGTCTTTAGAATTTCTTTATGGA
ATTCTGTCCATTTCTCTTCATCTCCTCTACTGATCCAAGCCTCCTTCTCTCAGCTCCCACTTACACT
AAACCCTCGCCTCCTATTAAATCTCTCCTTCCACTCTGCCACCAAGACACTATTATTTTCTATGTA
CAAGATAGGGAGCAATGTGAAAAATATGAATCAGTTCATACTACATCCCTACAAAAAATACCCC
AAGGACTGCTTCCTAGTACACATAAATCTAGACCCCATGTTATGGCCATTTTGGCCTGGTATGAT
TCACACCTTGCCTCCTCATGTCCTACCCCTAAAATTTCACATTGTTTACTTTCTTCTTCATTTAGCA
ATCAGCATGATATCACCCTCTGAGAAAAGCCAACCACTGCCTTCTGAGTCAAAATGGCATGCAC
ACCACAGTGTGTGAGATACCAGTGAGGTACCCACATCTCCAGCTTTCTGATCTCTTTCTGCCTCT
TTGTATTATATTTCTCTTATTATTTAGTATGATGCCAAGCACATAATAGACTCTTAATACTTTGTCC
AAAAGTATTCCTCAAAATTAAAGGATATTGTCTTACATTCACATTCTTGTAAGTCTTTCACATTTTA
GAGCATTTTCTAAATTACTTTATTTTGAACAAATATTATTTGGAGAACACAAACTGAAATAACCCA
AATCAATACTAGATTTATTTATTTATTTATTTTTCCTTCAACTTTTAAGTTCCACGGTACTTGTGCA
GGATGTGCAGGTTTGTTACACAGGTAAACATGTGCCATGATGGTTTGCTGCACACATCAACCCA
TCACCTAGGTAGTTTTTATATATATATATATTTTTATTACACTTTAAGTTCTAGGGTACATGTGCACAA
CCTGCAGTTTTGTTAACGTATGTATACATGTGCCATGTTGGTGTGCTGCACCCATTAACTCATCAT
TTACATTAGGTATATCTCCTAATGCTATCCCTCCCCACTCCCCCCATGCCATGACAGGCCCTGGT
GTGTGATGTTCCCCTTTCTGTGTCCAAGTGTTCTCATTGTTCAATTCCCACCTATGAGTGAGAACA
TGCGGTGTTTTTTTGTCCTTGCGATAGTTTGCTGAGAATGATGGTTTCCAGCTTCATCCATGTCCC
TACAAAGGACATGAACTCATCCTTTTTTATCACTGCATAGTATTCCATGGTGTATATGTGCCACAT
TTTCTTAATCCAGTCTATCATTGTTAGACATTTGGGTTGGTTCCAAGTTTTTGCTATTGTGAATAGT
GCCATAATAAACATACGTGTGCATGTGTCTTTATAGCAGCATGATTTATAATCCTTTGGGTATATA
CCCAGTAATGGGATGGCTGGGTCAAATGGTATTTCTAGTTCTAGATCCCTGAGGAATCGCCACA
CTGTCTTGCACAATGGTTGAACTAGTTTACAGTCCCACCAACAGTGTAAAAGTGTTCCTATTTCT
CCACGTCCTCTCCAGCACCTGTTGTTTCCTGACTTTTTAATGATCGCCATTCTAACTGGTGTGAGA
TGGTATCTCATTGTGGTTTTGATTTGCATTTCTCTGATGGCCAGTGATGATGAGCATTTTTTCATGT
GTCTGTTGGCTGCATAAATGTCTTCTTTTGAGAAGTGTTGGTTCATATCCTTCAGCCACTTTTTGA

TGGGGTTGTTTGTTTTTTTCTTGTAAATTTGTTTTAGTTCATTGTAGATTCTGGATATTAGCCCTTTG
TCAGATGAGTAGATTGCAAACATTTTCTCCCATTCTGTAGGTTGCCTGTTCACTCTGATGGTAGTT
TCTTTTGCTGTGCAGAAGCTCTTTAGTTTAATTAGATCCCATTTGTCAATTTTGGCTTTTGTTGCCA
TTGCTTTTGGTGTTTTAGACATAAAGTCCTTGCCCATGCCTATGTCCTGAATGGTATTGCCTAGGT
TTTCTTATGGGGTTTTTATGGTTTTAGGTTTAACATTTAAGTCTTTAATCCATCTTGAATTAATTTTT
GTATAAGGTGTAAGGAAGGGATCTAGTTTCAGCTTTCTACATATGGCTAGCCAGTTTTCCCAGCA
CCATTTGTTGAATAGGGAATCCTTTCCCCATTTCTTGTTTTTGTCAGGTTTGTCAAAGATCAGATA
GTTGTAGATGTGTGGTATTATTTCTGAGGGCTCTGTTCTGTTCCATTGGTCTATATCTGTGTTTTGT
TACCAGTACCATGCTGTTTTGGTTACTGTAGCCTTGTAATATAGTTTGAAGTCAGGTAGCGTGAT
GCCTCCAGCTTTGTTCTTTTGGCTTAGGATTGACTTGGCAATGCAGGCTCTTTTTTGGTTCCAAAT
GAACTTTAAAGTAGTTTTTTCCAATTCTGCATCACCTAGGTATTAAGCTCAGTGTTCATTAGCTGG
TCTTCCTGATGGCCTCACTCCCCCTACCTCCCCACATGAAAGGCCCCAGTTTTTGTTGTTCCCCTC
CCTGTGTCCATGTGTTCTCATTGTTTAGCTCCCACTTATAAGTGAGAGCATCTAGTGTTTGGTTTT
CTGTTTCTGTGTTAGTTTGCTGAAGATAATGGATTCCAGCTCCCTCCATATCCTTGCAAAGGAAA
TGATCTCATTCCTTTGTATGGCTGCATACTATTCCATGGTATATATTTTCTTTATCCAGTCTATCAC
TGATGGGCATTTGGGTTGATTCCATGTCTTTGCTATTGTGAATAGTGCTGCAATGAACATGCAGG
TGCATATATCTTTATAATAGAGTGGTTTATTTATTTATTTATTTTTTGAGACTGAGCCTTGCTCTGTC
ACCCCAGGCTGGAGTGCAGTGGTGCGATCTCGACTCACTGCAGCCCCTGCCATCCAGGTTCAA
GTGATTCTTATGCCTAAGCCTCCTGAGTAGCTGGGACTAAAGGCACATGACATCACACCTGTCT
AATTTTTGTATTTTTAGTAGAGATAGGGTTTCACTCGTTGGTCAGGCTGGTCTCAAACTCCTGACC
TCTAGTGATCCGCCCACCTCAGCCTCCCAAAGTGCTGGGATTACAGGCATGAGTCGCTGCACCC
GGACTGTAATAGAATGATTTATATTCCTTTGGGTATATACCCAGTAATGAGATTGTTGGATTAAAT
GGTATTTCTGCCTCTAGATCTTTGAGAAATCACAACACTATCTTCCACAATTGAACTAATTTCAAT
TGAACTAATTTACTAATTTGTTGAACTAATTTACACTCCCACTAACAGTGTAAAAGTGTTCCTTTTT
CTCTGCATCCTCACCAGCATCTGTTGTTTCTGGACTTTTTAATAATCGCCATTCTGACTAGTGTGA
GATGGTATCACTTTAAATCCCTATCAAGGTCACTGGTTTTAAATGCTTATAGAACTGGTTTAAAG
GGAGGGAAAATAAGGCATGATAATGTAAAAGATTCAGTTTTTCTGGGAGGATTTTACAATTGCA
AGAATACCATGTAAACACGCTTTTCAAATATCACTTAGAAAGCAATGTGACAAGTATAAATATTG
TGAAGATACAATGAATGAAAACAAATGAAGTGAGAGACAAACCTACCCTGCAAAATACAAAAT
AGGAGAAAATATTTGCAAACCATATGTCTGTATAAAGAATTAGTATCCAAAATATATAAAGAAC
CCTTACAGCTCCATACCAAAAAATCCTCCAAAAGCCCGACAACAAATAATCTGGTTAAAAATTG
GCAAAGGATCACAATAGATATTTCTCCGAAGAAGACATGCAAATGCCCAACACTTATATCAAAA
GTGCTCAACATCACTAATCATCAGGGAAAAACAAATCAAAACCACAGTGAGATATTTCCTCATA
CATATTAGGAGGAGTATTAATAATAAAAAAAGTTAAGAAGTATTGGCCAGGATGTGGAAAAAAA
AGGAGCACTTGTATACTGTTGGTGAGAATGTAAACTGGTGCAGCTGCTATGGAAAACAATAAGA
AGATTCCTCAAAAAATTATAAATAACCCCACCATATGATCCAATAAACCTACTTCTTGGTATTTAT
ACAAAGGAATTAATGTTAGGATCTTGAAGAGATATCTGTATTACTAGGTTCACGACAATAGCCA
AGAAATGGAAACAACCTAAATGTCTGTTGATGGATGTATAGATAAAGAAAATGTGGTATATACA
TACAATGAAATATTATTCAGCCTTAAAAATGTAAGAAATCCTGCCATTTGCAACACCATGGATGA
ACCCAGAAGATATTATTCTAAGTGAAATAAACCAGACACAGAAAGACAAATACTGCATTATCTC
GATTATATGATGAATCTAAAATAGTCAAATTCATAGAAGCAGAGAGTGGAATGATGGTTGGCAG
GGGCTGGGGGGAGGTATTAATGGGGAAGTATTAGTCAGAGTGTACAAAGTATCAATTATACAA
GATGAATAAATTCTAGAGATCTACTGTACAGTATAATGCGTATAGTTAACAATACTGTAATGTAT
AAGGTAGACTTTTTGTTACATATTCTTATCATACACACATACAATAATAATAAGGGCAGGAGAAA

ACTTTGGAGGTGATGTGTAGATTTATGGCATAGATTATGGTGATGGTTTCATGGGTGTATGCCTA
CTTCCAAACCCATCATGTTGTGTACATTTAATATTTATGACCTTTAATATATCAATCATACTTCAAT
AAAGTGGTTTAACAAAAAAATTACACACAAAAAAAATTTAGTAAGCATTGACATGAACTTATTTG
TTATTCTAGAAACAATTTCAATGTGCAAATATATATAGGCATCATCCTCCCTAAAACACAAAAAC
AGAAAACAAAAAAATATATATCCTAACATTATATCAATAGATGCTTGTGGCTTAGAACAAAATTT
AGAATTGATAGCATCATATACCATTTAAATGATTCTTTAAAAAAAATTTTGACAAAACTAAAAAT
GATATGCTCTGAAACCCACTGGAAGATGATAAATGCAGCATCAATTTTTTGAGTCTTTGGATGAA
GGACTTTAGCCTGAAGAGTCACCTTGTCCTACAACAGACTTATAGTAAACAAGTTACAAAATTTT
GTTTTATTAATCTACTAATATATGTTTTTCAACCAAAGTATAACCTACCCTATACTGACAAATACA
ACTGTCTACAGTATTTCCTTGAATTAACCTAGCCATCTGAACATAAATTCATACGGTTTTTACCTT
TGTAATGTCCCTTACTTTCTTTTAGGAACAAACCAGTATATAAAATAACACTTATATGAATAAATC
AGTCTAGACATGCTCTCCAGAAATATTCTTACCTACAAGTGTAAAATTTAAAAGACTACCACTTT
TGAAAAATAAAAATATTTCTAAGTATCATCAATCATATCATGTTTATATTAATTTGCCTTTAAATAT
TGTTACCACAGTGTTTGAAAAATCAATTTGTAGATCTTGTTATAAAATATCTGCATTTTTACAAGC
AATATAAAAACCAAAAAAATTTTATTCTAAAATAAGATAGGAATATGTATGGTCACATGCACTTG
GAATTAGAAATGATAATTTACCAGATAGAGGTCTTTGTCTACACTGTTAATGTAAAAAATCAAAC
CTCTAGTTTCATAAAATGTATGTTAAAAATATTTCACAGAAATATATGCTTCAATAACTTTAATAA
CATTTTATAACAAGATGTATTTTGTTTGTTTGTTTGAAATGGAGTCTCGCTGCGATGCCCAGACTA
GGGTGCGGTGATGTGATCTTGGCTCACTGCAACCTCCGCTTCCCAGGTTCAAGCAATTCTTGTGC
CTCAGCCTCCCGAGTAGCTGGGACTACAGGTCCCCGCCACCATGCCCAGCTACTTTTTGTATTTT
TAGTAGAGACGGAGTTTCACCATATTGGCCGGGCTGGTCTTGAACTCCTGACCTCAAGTGATCC
ACCTGCCTTGGCTTCCCAAAGTATTGGGATTACAGGCGTGAGCCACTGGGCCCAGCCTATAACA
AGATGTATTTTTGATAGTAAACTGTATTTTCTATATCCCCACTTTTTCCATTGGAAATAAGGATTTC
TCAAAGGCTGGCTTGAGTCACCACCCAACCATCTTCAGACTATCTGCAGAAACACCACTTCTCA
CTTCTTAGTGCATACCATGTGGTAATCTGCTACCTCGGAAAACCAGGGTTTTGGCTTAGCTATGT
TTGGTGGAGAATGATGGCTTTCTCTGGAATAAGGGTAGCATAGTGTAAAGCCCATTATTCCAATT
TGGAAATATCCCATTTTAGGTTTCCTATATCCTTCTTTATCAGGTCTTCTAGTCTTATCCACCAAGG
GGCACAGGGAACACATGCAAATTTTTTTACCACACTTCACCAGCATGTGCTACCATATCTTTCTG
TATAGTGATTTCATTTTTTTTTTTGTTTCTTTGATTACAGTCTTGACTTGAATTTTATCCCCAACCAGA
ATATATTCACTGTGATATGAAGGAATTGTATAATTATAAGCATTGGTATGAGAATATTACAAATTA
TAAACAATGACTCTCCTATTATTTACTGCTTTACTAAGAGGAAAAATGTGCATTATTACCCACACT
TACTCTACAAGACCTATTTAAATTTTAATATTAAAGTTGTTTTTGAGAAGACACTCTTTAATCACCT
TCATCTAGCTTGGAAATTTCACAAAACTCAATTACTGAGTTAATGTCTATGGCCTGGTTTATAATA
ACACAGCTTAAATCAGCCACAGATCTAGAAGAGAAGCAGCATGGTGTGGTAGATCAAGGCTCT
CCCCATGAGGTAAACATGTCCTGGATTCTCTACCCTCTTATTTGCTGTGAGACCTGAGCGTAGCA
TTGAACTCCATAAAATGAAGGCATGTGTCTACTTATCTTTTATTGGTTAGAATATAGGGGATAGG
GCAAGATAATCTTTTCAGACCTCTTATAGCACTAAAACCATTGGCTGTTATATTAGGGTTAAATTA
AAAGTTAAAAGTTACAGAAATTACTTGACTCTTTGTAGGAGATCTATTTATCTAGAAATGATGTG
CCTGTCTATCCCCAAACCATTAACTAGATAGAAAAAGTTACCTATCCTCTTAAAAAAAAAGTATC
CCAACTACTAAAGGATATTTTAATTTTAATTTTTGTTGTTAAATAGTAGAACTCTGAGAAATGAAA
TAAAGAAAAGCCACTGAATGAATATTCTGTGAATCAGGACAGCCTTTATAAGGGGTGACTTCAT
AATTTTTCCCTTGAAAGTATATCAGAGGGACAGATGAAGATGTATTCCAAGTTTTCCAGGTCCAG
GTTTGCTGTATCTGTGGACTGAGGGGAAAGTAATTGGATTGGGTTGCTGAATAAAGCTCTTTTGG
TAGGCCAAGGCATTTTTCCATCCACTTTAAGTCTTCTCTTACCAAAGAAAGTAGAATGACAGCCC

ATTCAGACTTGGAAAAGTGCTGATTGATACCCTGCCATTCAACATCCTACCACCAACACTCTGTG
GGATCAGTTGGGATGAAGCAGGAAAAAACAAAACAACAAAAACACCTTCAGCCACTTCATTAA
AGATGTATCTGTTTAAAATCTCTTTTTGACTGGTTGCTTTTCTTGATGACTTCTTCAGACATCTGTT
TTTGTTTGTTTGTTTTTTTGAGACAGAGTCTCGCTCTGTCACCCAGGCTGGAGTGCAGTGGTGCG
ATCTCGGTTCACTGCAACCCCAGCCTCTTGGGTTCAAGTGATTCTCCTGCATCAGCCTCCACAGT
AGCTGGGATTACAGGCTTGCGCCACCATGCCCGGCTAAATTCTGTATTTTTAATAGAGACGGGG
TTTCGCCATGTTGGCCCGGCTGGTCTTGAACTCTTGATCTCAGGTGATCCACCTGCCTTGGCCTC
CCAAAGTGCTGGGATTACAGGCGTGAGCCACCGCACCCGGCCTAAGACATCTGCTTTTATTTCT
CAAGTTTTTTCAAAGGCTAATGAGATTTTTTTTTCACACTCTACTGGAGAAATAAAGTATAGATGT
ATAGATTATTTATATCCTTGCTTTGATCATCATTTCCATGATTGTCCAGTAGGCTTCATGAACTATT
ACTGAGATCAAAACATAACACTTCCTGTTACCACACTGAACAAATATCTGTCTGAGTAAATGTGC
AGGAGTTTATTTTCATTCACCTGCTTAGAAATGTATAGCATAGGACTCCCATAACCCTATTCATTA
AAAACTCTGATTCCTTACTGACAAGGGTTCCTTGGACACAGCATATATAATAGCTTTGAAATAGG
ACTCGCATCGCTTCTTTTTATGTAGTTAGAGATATGTGCCTGTTGTCCTTAATGAACTTATTTTCTT
TACTTTTGTATTCTCCAGGTCTATCCAGCTGAAGTCAAAAATGCCCCTCTCAGGCTGCCCCCAGT
GACTCCAGTTTCCTAGCAGAGCCTAACCAGGTCAGGTAGTAAAAGCAGTTTTCTTAGCACCACC
TATAGACAGTCTAGAAGTATTATTCCTGATTTGTAATTGGTATGGTCCTGCCAAGCTCCTGGAGC
ATGTAAACCTTCTGTTGCAGGGTTTTTGTTTCTCCCCTGTTACCCACCATATTAATTCAGATCATG
AATCTGCTACGCTTTCCTTGCTGGGCTTGAGATACTCCTCAAGCTGCAATTCGCTTGGCTGTGAC
TCCAACTCCCTATGTGGCTTGTATCAAGCCTCTCTCCTGTGGATCCTGGCTCCTGGCAAGAAGCT
TGCTGTGACAAGAGTTATAATCAACATCAGATGGATTTCTGATGCTTTCCCTACCTGACTGATGG
GGACCCTTAGTTACTCAGCATTGGGCTTCCATATCTTTTGATTTTTGGGCATTGGAATTCCTGGCT
AGTCCCCCAGAAGCTAGCTGTTCAAGATTGGCAGGTCTTATGTTCTTCAAAGTCTCAGCTTCTGC
TCCTTTCTTATTGATACAAATTGCGAAGGTACCAGGATGCCTCATGTCTCAGTCTGCCTAATTTGT
AATACTATAGTGCTTATCCATATATACACATATAAATGCATTTGTTTTTATTACTGTAAGACAAAA
TACACTAAAGAATAAGGCATTGTATCTCCAATTCTTTCATTAGAATCCTCAGATCATGATGTACA
AGAAATATCTTTAAAGCCCTGAAGCCACTGCTTGTTTGATCCTTGAGAGCCATAACTACTATCTA
TAACTTGTTCATGCACTTCTGCATCTATGACTCACCTAAATGAAATTTCATAAACCTACCTGAGGT
CTAATAGAATGCACTGCTCTTAATTCCTTGCTTAATTTCATTACTTGATGGTTTCTGGACTACCCA
GAACTTCAGAAGGCATTCCTTTCCCTAACAATCTAATTTACATCTAAAGTTGCATTAAATTTAGTC
TTTGACTAAGTTGAGCCAAAAACATGATGCTGATATCTAAATAACATGATTTTACTTTATAGACTC
TGATAGTTGAGAGATATCAAAGTTGTCACTCTGGTACGAAAGTTTTATTCTCCACAGCACAACCT
GAATAGTTCTGAATTTCTTTCTATCAACTAAACACATTTTTCTATCAACACATGTTTTAATTCCCCT
TTGCCTCCTGGAAACCTTTTCCTTCCTGGCATATTTCCATGATCATTTTTTAAATGCTATTCAGAAA
CCTTCATTTTCTCTAATACAAGAAACCATGGTGAAGCGTTCATTGCTCAGACTTTCTTTACCTTGG
GCTTTTGTAATGGAGAGTAGATTCTTCTTTGGAATGTGAAGTTTATATTAATAGTAATCTCAGGCA
AAAGCATAATTATGGCACATGCAAGGCTCCAGAACAGTCTCATTGATGTCCCTGCTTCCTGGGC
TTACAGTGAACTCCAGACAGACTTCTGTGGTTCCACCAATGAACTGCTGGGCAACTTTTGTCTTA
GTCACTAAGAATTACTGTCAATAATGTTTTAAATTCTTTCTGACTTTTGTCTTCCTCTCAAGCTACT
AACTTCCACTAATCTGACACGTGTGAGTAAACTTCACTTTGCAAGTATCTGTTCCTGTTTACCTTG
TGGACGTTAGACAGCCTATTGAATACGGTTCACAAGTGTTGTCCCAGGGATTTTTAGTCAGTTAC
CATCATCGGGGGATTCCCTTTATCACTCCTTTAAATTTAATCCTCTGTTTGCTTGACACTAGTCTTC
CTCTTTCTTTATTTACTTCTTTTTGATGAAGACTTTGCCTTTGTCAGGCAACTAATGGAGAAGTTCA
GTCAGAGTTCAACCAGAGAAACAGAACTAATAGGCAATATACACTAAGGATTTTTTTTTTTTTTT

TGAGTCAGGGTCTCACTCTGTCACCCAGGCTGCAGTTGTGGGAGCTGGTTAGACAAGTCCAAAA
TGTATCAAGCTGAAGCTGATAGGTAGAATTTCTTCTTCCAGGAAACCATTCTGCTGTAAAGCTTT
TCAACTGATTGAATTAGGGCCACTCAGATGATATAGGATTATCTCTTTTACTTAAAGTCAACTGA
CTGTAGATGTCTATTTTATCTATTCTTTCTTTCTTGAAATCTGAAAGATTGTCTCCTGTCTGTAAGA
AAGCTTGAGATCTGGAATATTGTCTTAGCAGGCTGTAAGCTTGGTGAACCACCCTTATTTATTGA
TAGAATTTACCTCAGGTGACCTAGATATAGTCTCTCTTCTTCTTGGATAGTACAAATTTTTATAAA
TCTTTAGTTTTGATACCACCTTTTCTAGCTTCGGTTTGAATTACTGGGCTTCTATAAACACATCCCA
TCCTTTCTACAGATGGATTTGATTGATTGTTATATTTTTTATTTACATTTGATTATTTGACTGGATTT
TGATCTCCTCAGGAAAAAGAAAATGTCTCCTCAGGAAAAAGAAAATATCTCTTGAATATGAAAC
TTCTACAGTTGGTGACTTTCAACTTCTGTATTTTAATTCTCAGTGGTTTCATTCTCATATTTTGGCCT
TTTTATGATCTGGCCTAAGCTTCTGAGAGTTTATAATTGTAGCCATCCAGATAACATAAGCTACAT
GTAAATTTGAACTTCTAGTATGGAGATTCTAAGAATCTGGACATTCTGTTCTTTAATCACTGGAAA
GCAACTTAAAAGTTTAGCATCCAATTTCCTGCTACTATGACAAATTCCACACTAAACATCATGGG
AAAAATACATTCCAGCTTCATCCACATTAGCAATGAATTTCAATTCATAACCTTACACAGTTGTCT
TTAGAAGTACTAGCATTCCCTTCCCACGTTCTTATCTATAGCTACAGCCTCCAACCCATTTCCTGA
GACATATGGTTCTGTATTTCTTGGAAGGTTCGCAGCAATTCATTACTACATTCAAAATCCCCTGA
GTCCAGCTACAATGCAGGGTAATTACTCCAACGTGGAGAAGAGTCAAACTGCATGTGAACGTG
CTTCTCCAATAATTATAACCCATATCTTCAAAAGAGTGACAAGCATTTTTGCTTAGCTTTTAGAAT
CATAAGGTGGAAGCTCAGTTAAGTACTTTGCATAATGCTATTTTGGACAGTAATCATTGGGAGTA
CATAGGCTTTATTCAGTATTGAACCATTGGTAAGCATTTAAAAATATTTGTACACACACACACAC
ACACACACACACACACACACACACAAACAGGACTTGACAATATCACTGCCCCTGCTCAGTAGT
AGCTTTGTCAACAAACAAGTAGAGTCACTGAAGTGAAATATTCACTGTCAATTAGCAGCTGGGT
CAATGTTACTAATTGTCAAACTATTTTTTTTGCTATAAAACACATTTACACACAATCTTGTGTAGA
AACCCACTATATAAAACAGACAAGGGCCAGGTGCAGTGTCTCTTGCCCATAATCCCAGCACTTT
GGGAGGCTGAGGCAGGAGGATTGCTTGAAACTAGATGTTTGAGACTAGCCTGGGCAACATAGG
GAGACCCCACGTCTAAAATCATACAAAAATTAGCTGAGTGTGGTGGCACATGCCTGTAGTCCCA
GCTACTCAGGAGGCTGAGGCAGGACGATTACTTGATCCCAGGAGGTCGAGGCTGCAGTGAGCT
GTGATGGAGCCACTGCACTCCAGTCTAGATGACAGAGTGAGACCCTGTCTCAATAAATAAATAA
AAAGAAAAAGGATTAGGACAGAGTTGCTCAGCTTGAAGGATATGGGATGCGCAGACTGACATG
AGTTTAGCTGCCTCCTCATATCACAGTGGTCAGTGAAGTACATCTAGAGAATCCCAGAGCTCTG
CAGAGTATACTAGGAAACCACAAATTTATGTTATTTACAAGAACATTTTGGTTATTCACTTAGCA
CAATGGCTATTTTCGCCATATAAAATTGTCAAAAGACAAAATTACAACAAATTTAGTTATAGATA
GAATTGGTTTTTATCTGTGGCTCATGAATTGGGGCAGCTTCTATTCTTCAAGATAGAGTAAAGCT
CCCACTGGGCAATGGCAGAACAGTGGGTTTTGTAAAGGGGGGACAAGGAAAAGAGCAATGTA
AAAATAACTGAGGCTGGGCACGGTGGTTCACACCTGTAATCCTAACACTTTGGGAGGCTGAGGT
GGGCGGATCAGGAGGCCAGGAGTTCGAGAGCAGCCTGGCCAACATGGTGAAATCCCATCTCTA
CTAATAATACAAAAATTAGCCAGGTGTGGTGGTGTGCGCGTATAGTCCCAGCTACTCAGGAGGC
TGAGGCAAGAGAATCGCTTGAACCTGGGAGGCGGAGGTTGCAGTGAGCCAAGATCACACCACT
GCACTCCAGCCTGGGTGACAGTGCAAGACTCCATCTGAAAAATAAAAAAAAATTAGAATAAAAT
AAAATAAAATAAAATAAATAAAATAAAAATCTGAGTGGGGCTGGGCCCAGTGGCTCATGCCTAT
AATTTCAGCACTTTGGGAGGATGAGGCAAGAGAATTGCTTGATGTCCAGAGTTTGAGAGCAGCC
TGGGTAACAAAGTGAGACCTCATCTCTACAAAAAACAAAATAAAAAATTGGCTGGGCGTGGTA
GTGTGTGCCTGTAGTTAGTCCCAGCTACTCAGGAGGGTGAGGGGAGAGGATCTCTTGAACCTA
GTATTTCTATGCTGCAATGACCCATTATTGCACCTCTGCACCCCAGCTTGGGTGACAGAGTGAG

ACCCCACATAAAAAAAAGAATAAAAATAACTGATTATTTAATATCAGGTTACTTTTTTGTGCAGG
TTCAAAGAGAGAGGACTTCCTTATTACTCTGACTCAGGTAGACTGGAGTCTCCTATTTTCAGGAA
AAGTTGGTCCATTTGGGATCTATTTGATTTCTTAAAGCTTTAGTTTGATTATGTGCCATTTAGCATG
AGTGACCCTGTTTTGGTTTGGTCCAATTTGTTAGGGACTAGTGCAGGAATTCCATCCTAAAACAA
TGGCCTCCTGTAATTTTTGTTTCACAAAACATAGCCAGTAAGTACCAGTTAGTGGCTAGTTTTTGA
GGAATTTATTGTGCATAGCATTAAAATAAGCATCGAGGGGAAGAAATTGCTATGAAAAAATAAA
ATCTTGGAGCCTGGAGACAATCTGAAATGAAAACAAAAGAAGGAGTCTATTGTGAAAAAAAAC
CTAAAACATAAATGAATGTAATCACAGGAAGACGTTAAACTGCACTAAATAGAGCACAACTGAT
CTTGTTTGATCTGGGAAACTAAGTAGAGCTGATCTTGTTATGTTGACAGCAAATGCCTAGGGTTA
CCATGAGCTATCTAGATACCAGATAGTTTCAGGGCAAAGATGTTCTCGGGTTTAGCAGGAATCT
ATTCAATATAATGCTTCACTCACCAGCACTTTGGCATAGTTGTTTTGCTTTTGTGAGCATCAATAA
AACAGACAAACCATGATCCTAAAATACTTGATGAGTCACCAGGAGAAGATTATACTACATTCAG
CACAAGTACCCATATATTATTATAATATGATTTAATATAATATGATGTAATAGAATATCATGTATT
ATTATTGTTCGAAACCTGTTGAAAATGTTATATGGTATGTCAAAAGCATATCTTTCCAGAAGCCA
GAATATTGTAGAGCTGGGATATTGCATTCCCTGACTGTATCATATAAGAGAAGTTTCTCTGTATA
AGTACTAAAGAGTAGCTAAAGTCATTGAAGAGACCAGAATCAAGGACATGAAACAAAAAGGTT
TTGAGAACTTTAAATGTAGTAAGAAGATTTGGCAGAGAAACTTAGAAGAGGGCATTTCTGTTAA
AGAATCTCCTCATACTTACGTAGCTTAAATTCACTTTGCTTGTTTTTCAATTGGTGTATTTATAAGG
CACTTGTCTTCCAAAAGTAGATTTGTATTTCGATGATAATGGCAATAGTGCTTTCTTTGTAGAACC
TATCTGTGTGATCTTGGCCAAATCAGACTGGGTGAAACATAGAGCACACATTTGCTTCTTCTGAA
TGGTGCTTCATCTCAGTGGCTGAGCCACGTGGATGGCATTCAGAAATAACAGTTGTGCTTCCAC
ACAGCGGTGAGCCAATCCCCAGTGATTCTAAAGGCTGAATGTTCTGGTTCTGCAACCAAATGAA
TACAATTATAGATACTTAGCCGACCCTGACTAGGTTTCATTCTTTCATTAATTAACACATGTATTA
TAGGCACTTATTTGGGCACTGCAGAGCTGCTGTAGATTATCTAAAGTTTAATTCAATATGGCTTTC
CCCCTTAGAATCTTGTTATGATTATTCTTATATCTTCCCTTCTGCCCATCTCTGAAAATTTCTCACT
TCCCCTCTTGGAACACCATACTATGATTAAATTCACTTCCAGGGACTGGCCATGGCTTAGAAAAA
TGATTCTCTTACTTGCCAGGATCCCTGTACAGACGCTGCCGAGTCAAATTTAGTGCCAAAGGGA
ATGTGTGCTTTATTTGATCAGCGCTGATTTCTGAGCTGGGCCCTGGAAAAAGAAAACATGGATTT
TGTCAGTATCTGTGGACCCAGAAAAAGGTATCCTTATTGCAATCTGTCCTGATTACAGCTGTGCT
GGTACCAAGTGTGGCACTCAGAATATCCAGTATGCTCTCATGTCAACCAAACTTAATTTCTGTAT
GCAGCAAATGCTAGGGCCTGTTAAATGGATGAAATGTACAGCTTGTAAAATATCTGCTTCTTAAG
TTGGAAGTGACTTTGTCTTTGATAACTTTACCTCTCCAAAGAAAATCTTCTACCACCCACTCAAAA
TGTCAGGTTCCTCCTGGTCTCCATAATGATCCATAAATATCTACCCTGAACTCATGATGATGATT
GGATTCAACATTATAATTGCATATGCCTGACTTTTCCACTCTGTCAACAGCCTCTTTTCTGGAGTA
TCTCCCAGTGATCATGTCATATCCTGTTAGGTAAATTGTGAACCTGTAGGATTGTATCTCCTAGTT
TTATGAGTGAAGATCACATTTAATTCTCTGTCATTTTCAGAACATAACACATCGTTAGTGCTTTAA
AAGGATGAAACATGCAGGGCATTTGTAAACTTTAAAAGTACTTTTTTATCTGTTATTTTATGCAAT
CCCTACAATTATCCTTTGAGGGCTGGCAAATCCCGGTTTGACAAATGAGAAAACTGAGGCATAA
AGGCTAAGTGACTTCTCTGAAGTCACACAGTGACAGAACCAGATTTAGGATCCAGTTCCCTTGA
CTCCTAGACTCAGACTTTTTTTTTTGTTAAATATTGTCTAGTCATATTACTACTCTCTGAAAGCTAT
TCCCACCAGAAGTCTCATTTTAATTAGATATTTCATACCTTTAAACTCTGTAGTGTGGCTAGCATT
TACAAAATCATGCTGAGTAGAGGCAGTGAACAAGGCTTGGGTTGCCCAAAGCAGAAAAAAGAT
ATAAAGATGAAACCACCAGACAGATTTTATTTATGACTATTTTAGAGAGGGTAGTGCAACCTCTG
ATTCTCCTTAACACCAAGTTCTGTTGAAACTCAGTCTTTATCACAGGAACCACTGGGTGCCTTTCT

CATGGCCACAGCAGCTTGGCTTACAACCGTCTTCAAACAGCCAGGCTGTGCCCCAGAACTTCAC
TGGGCTTCCTTCCATAACTATGGATCTGTGAGCATCACTTTAATTTCAGAGTGTGGAAGACACCT
TAATAAGAATCATGAATCACATTTTACAAATCAGGATACACAGGATGTAAGGTTAAGTGACCTG
TCCTATCAGGGCCACAAAGCCAGTTAAACTTCTTTATTAACTGAGTCACTTAAAAATCATTTATTT
AAAAACCTATTTTGTTCCAGGAACTATTCTGGGTACTGGAAATAAAACAGTGGAGACAGAGAGA
GGGGAAATAAAAAACAAGACAAAAATGATTGCTTTGGTGGAGTTTATATATTCCACTGGAAGAA
GGTAGATAATAAATAAAAGTGAAAAAGTACATTATAAGGTGGGAGGAAGGCTTGAGCCTAGGA
GGTTGAGGTTGCAGTGGGCCGAGATTGTGCCACTGCACTCTAGACTGGGCAACAGGGCAAGAC
GTTGTCTCAAAAAAAAAAAAAAAAAAAAAGAAAAGTAAATTATAGAGTATCTTAAAAGGTGACATT
ACATGAAAGAAAAACAAAGCAGAAAAAAGGGTAAGTAGTTCTGGGCACTAAGTACATTTTAAA
CAGGGTAGTCGGGGTTATTGACTGAGAAAGT

ID|55951|gene_id|ENSG00000234142|transcript_id|ENST00000426519 Homo sapiens lncRNA

ATTCCTGGAAGCCCGTGAGACCAGGAACCCTTCGATCGAGAAAAGACCTTCCATTGGGAG
AAGACTTCTCGTCTTATTTCCACCTATAATCTCTTCAAAACATAGCAGTCAGAGTTACCTGCTGAA
CTCATAAGTCACTCATCTGCTCAAAGCCTCCCAGTGGCTTCTCATCTCACAGAGTAAGTCAGGCT
GCTTACAGTGGCCCACGAGGAGCTTCACAGTTTAACTGGCTCTCTAGCTCTCTCTTGCTCTCTCT
CTCTCTCTCTCTCAATGACCTCACCCTTCACTTCTTCCCCTTGCACACACTGAACATGCCACACCT
CAGACATGCCAGGCACACTCCTGACTCAGGGCTTTTGCCCTTGCTTTTCCCTGGACCTAGGCATC
CTTCCCCTAAATAGTGCCAATGCACCCTCTCTACCTTCAAGTCTTTGCTCTTTCTCAAATGCCACT
TTCCAAGTCCCTTTCTGGCCCCCTGCTCTTCCTCTTTCTTATTACGCCCTTTTCTTTCCCATGTCAC
AATCTCTTCTAACTATATAATTGTCTATTTTACTTTATATAAACCTCTTTCGCCAGAATCTGTAAGC
TCCAGAAACATAAGGGCTTTTGTTGGTTTTGTGTATTTCCCTATTCCTAGATCCTAGAACAGGGC
CTGGCACATGATATAGGCTCAATAAATATTAGTGGTATAGATGAAATTATTAATTCTATGGACCT
TCATCCTTCATTGAGAAACAGTGGTTACAGGGAGGGAAGGGGGAAAGAATTGGCTTAAGACCT
AAATGATTTACATTTACTCCCTTCAAATGTAAACAACTGCTGACCATATGAATATTCTTGGACAA
ATCTCTTTATCTCTCTGGGCTTCAGTTCTCTTATGTGTATAAATAATGGGTTAAACTAAGTAGTCT
CCATAAACATTCTTACTTTAGTATTCTTTAATTCTCTAAACCTCTTGTCCCTGCACTGTCTCTGGGA
TTTACTCTCTATCTCTCTCCCTTTTAAATGTTCTTTATCCATTCATCAATTCTATAAGCAATAATTGA
GCATATTCAATGGGCCAATCCCTGGTCTGAGCCCTGAGGATATACGGTTGAATAAAATAAGAAT
TTCCACGGGGGAGAAGGTAAGTGAAGAAAGGCAGCTCATATACTTCAACAAGGTGGTCACTGT
TGTCCTATGGAGGCCAGAGCCTTGAGTGTATGTTGGGTGCCGGTGGGAGATGGTGGGGAAGTT
GGAAGAGGATGGGGCTGGGCTTCACTCATGGCTGCATAATGCACAGGAAATCCAGAAGCTGAC
CCTCTATAATAAAGAAGGCTATTGATTTTAGCAGTTTTCTGGTATCAGAGTTCTGGTTTTGTGGCT
GTAGTATTTGTAGTAGTGGCAAAACGCTAGCCCATCTCTGCTACAAAACATACAGAGAAGATGA
GGCTTCTACAAAAGGCTTAGCTCATTACAGTTGGGGTCAACCTGTCTTTCTGGCTTGATGCCTAC
CTGAGTGAGCAAGCAAAAAAGATTGCTGGCTGAAGCACTCTCTACCCTGCTCACCCAAGGGCC
ACCTTAGGACAGCTTCTGACTGATGTTTGGAGAATTTCCCAGGAAGCTAACTTGAGGCAAGGGG
AGGACATGAGAAATGGAAGACAACAAATTAAACATTGCCCATCCCTTCTGAAACATACAAAGC
CTTCGGAACACCCTCCATTTGCAGAAGATTTATTTCTCGAATTATATATTTAAGGGCCTGAGTCA
GTCAATTATCTACCTTCACAGACCTATCTTGCTGCATAAACAGATTTCCATTTCCTGATGCTGTCC
TTAAATGGCAAATAGTAAAACACTGGGAGAAGGTGGCTGACATTGCTCTGTAGGTGTCTCTAAA
GAGTCTAGTGTGATCTGATGCATCTCCTGGGGCAAACACAGATGCATGTGAGAAGAGAAGGTG
CAGAAGGGCTCAGCATATATGGGAGTGGACCATCACCTCTCACTCCAAATCCTGGAGCTGCTTT

CTGCTGTCCTTTCTTTTTATGCCAATGATGTAGCCAAAACTGCCTGCATTGTTGTCACTTCAGATT
TTGGACTCTTGTAGGGATGTTCTAACATACTCCAGTCCTTGCTTTTGTGGCTTTTCCCTCAGTCAT
CCAGTCAACAAACCTTTACTGTGAGCCTAGTATAGGCTGCTCTCCAGGCATTGCTAAAGGAACA
AATATTAACCTGGCACCGTCCAAACCCAACAAACTCAATACTCTAGCTGGGAGACAAGCATTAA
GCAATAATTACAATGCAGTATGTTATAGGCAGTAACATAAATACGTTTAAGAAATGTAAGTGTCA
CAAGAAATAAAAATGTATAACTTCAAGAGAGGCAGGAACAGAGAAGATTCTCAAAAGAGATGT
TATCTGAGCTAGGTTTCTAAGAATCAATAGAAATTTATCACATGGATAAGAGTCAGAACAACTG
AGGAATCTGCACATGCACAGGCACAGAAGTGAGCCGCAGTAGACCCATGTGCAGGGAACCAC
AGGTCCTTAAAGCATTCTCAGAGTGCAGCACAAAAGCCAAGGTACTGAGAGAAGACAGGTTCC
CAGGGGTCTGCTGGCGAAGGTGGTATTTGTCATAAGGAAGACTTTTCTTACTGACATGAAGCAT
CACTGGAGGCTTTCAAACAAGACAGTGCCATGGTCAGGTTGGGGTTTTAGAAATATCACCCTAT
AAATAGTGGAGAAAAGATCAAGCAGTAAACGTGATCAGAATGAAAGGGGGTAAGTGACAAGT
GAAGGTAAGGGCGTGCAAAGATGGAGGAGGCAGACAACACTGGCATTTAGGAGGCAGAGATA
CAGAGCTCAGGGACTGCTGGATTGCTTGGTTGTGGAAGAAAGGGAGAATGAAGCAACAAGCTG
GGTGGCAGGCTCTGATTTGGTGGCTGGCTCAGTGGTGACCCCTCCAACCTCCAGGCCTCTGTTT
GGACACATGTGACAACAATGGCCACCCTGTTGGAGTGTATGAACTGCCTTTCTTCACTCACATTC
CTCTCTCCAGTTCTGAGGTCAGGGGTCTTATCTTATTCAACAAGTGTCCTGCCCTGTGTTTGGCTT
ACAGAATATGCTCAATTATTGCTTTTAGAATTGGTGAATGAATAAAGGAAGTCTAAAAGGGAGA
GAGAGAGTAAAACCTGGAGACAGTGCAGGGACATGAAGTTTAGAGCATTAAAGAATGTTAAAG
TAAAAACATTCATGGAGATTATTTAGTTCAACCTGGTAATTATACAGATGAAGAAACTGAGGCCC
AGAGCACTAAGATATTTGTCCAACGTCACACACCTGAGATAATAAGACAGAGTAATGGGCAAGT
TGGGGATGGAGAGATGATGAATTCAGTTTGGATGCCATTACATTTGTGATGCTTGTGGTGGTAA
ATGCAGATTATCATATCCAATGTGCTGTTGGAATGTGATGTTCAAGAGAAGGGTCAAGTCTAGA
CATACAAATTTGGGATACAGATCATACTAAGACCATACCTCATGAGAACAGGCTGAGTCAGGAG
CCAGGAGGGCCACAAGGGGAGCTCTAGTAATCTCAGAGATGGCATAACATGGTAGGAAGCAG
CACACCAAATTGATCGGCCTTCCTGTGCCTCAGTTTCCCCAATCTATTTTCTAAGGTTGTCATGTG
TTATATAAAATGAATGTATATTTATAAGACAGCAGTGCCTGGCATGAACTAAGGTCTAAGTAAGT
GTGAAAATGAATATATAAACACTAATATTTGATGGTGGACAGAGGAAAAAGACTCAGAAAATCA
GTAAACTAGGTATGACAAGAATGGTAGGAGGAAATCAGGCAACTATGGGAACATCAATGACCA
AGGAAGAGACAGTGTAAAGAAGGCAGGAGCAATTATGTGGCAGATGCTAAAGAAAGACTAAA
ATCAGAAAGTGCCTATTGGCTTTGGCAGTTGAAAGTTCACTGGTGATTTTAGAGCCTCGTTGGTG
GGTGCTCAGGGGGAAGAGGGGTGGGCTGAAAAGTGAGTTAGGGGCAAGAAGTGGGAGGGCT
GATGTGGGCAGTTGGCTCAGGGTGAGGCTGGCAAAGTGGAGGAACCCTGAGCTGTTGAACTCC
TTTCTAGTGAGGCTCTGCAACAAGTCTCACCTACAACACAGGCCGAGTCTCTTCCCCATCCCAA
AGTCATCATCTGTAAAATGAAGGCAAACTTGACCTCCACATTCCTATCAGCTATGATGGTTGAAT
ACTTTTCTTGTTACTAACATATGATGGTGATCAAATTCTTTTTCACTTAGAAATGTACCTGAGTCA
GCTAGTGGCATTCTTGTTAAAATCTTGTTTTGAGTTTATGGAAATAGATGCTATGAGGGCTGGTTT
ATTTTTAAATTTATGTAGTGTTTTCAATGTACAATTTTGCACTTGATCCTTAGGCATGCACGTTGTT
GGTATTGTCACTTTATCTGTGAAGCCGACTTCAAACAATAGGGTAATTTTTCTCAAAGTCACAAG
AGAAGTAACCCAAGAATTGAAGCCAGGACTTCTGGCAGGCAAATCCTGTGCCTGAGAGCTGCT
GGGAGTACCTGGGGCAGGTGGCACAAAACTACTTAAGAACTGGGCCTCTAAAGAACAATTGTT
TTTGTTTATGAAAAATGCCTTTCAGCACAAAATATCTTTGATATATTTATCAAAATAATAGGTTTTT
CTGCCACATCCTCTTCTCCTGCAGTAGGATATAAGATTTCTCATTTGGAGAAATAGGCTTTTAAA
ATAAATATTTTGGTTTCTCCTGCAGTGATAGGGTGTTTAACAATATGATCATTTATATTTTATTAGA

CTGATGTGGCTTTGTATTCACCTAGACAATTTCAAAGCACTTTTGCATACATTATTCATCTGATTC
TCCTCGCCACTCTTTGAGAATGGTAAAACAGGAATGACTAGCTTCATTTTAGACATAAGAATCTG
GTGTTAGAAAGATTACGTGACCAATGCAGGCTCGCTCAGAATTGCTCAGGAGCACCATTTACAC
ACTGAGTTTCCTCATCTCGAAGCTGGAGATAATTTCAAGCTTGGGGAATCATTGTGCAGCTGCC
GATCAGATAGGGTCAGTAGGTCTCTAGTCTATAATTGGCACTCGTAAATTAGTGAGCATTAGGA
GAAAAAAGTGCCCCACCTCTCTATCCAGTCCTCATCTTTATTGTCAGGATGTTGGTGGTGGTGTC
ACTGAAACTTAAGGGAAAACTGAAAATAATTCATAAGATGGGAGAGAAAGAGAAAGGAAAGA
ACAGGAGAAGGAAAAGAGAAGAAACCTTAGCAGTAGTTAAACATTTCACAAATGGTGGCTTTG
GCTGTTTCCAATGTTTGTGTAAAGTGCTGACTTCATCATTCTATAAGTGTTTTATGGAATAATATTT
AAAAAGCCCTTTGAGTGCCTTTGAAAACAGTGTTAGATAAATATAAAATACTGGGTGCATAAAA
GTGGAAGACAACCTCAGATATATAGTCTATGTGTGAAAAGAAATAAAATTATTCATCAAAAAAT
AATCACTGTAAAGTGAAGAAAGCAGAGTTTTTTTTTTTCCCCTTGGCAGAGTAACCAGCCTGGGT
TTCATAAGGATTAATGTGGTATAAACATATTTATCACTAATAAAAGAGTCTATAGAAATACTGCG
GCTCTTCTTCAGCATGAGGATGAAACACTGCTAATTTAATCTCAGCAAGAACAATTCATCTGTCT
TTGGGGCACAAACTTGTATAGTAAGTAAAAAAATTCTATGATCAAGTAATTTATTTTATCAGGGT
GTCAGGTTTAGAGTGTGACCTGATTTAATCTCAGAAAGATATCTTTTTGGATGTGGTTCCAGAAT
ATTCTGGGTATTATCTTTTAAACTAATTAGCACCATAATTTCAAAGGACGACAAAATAAAGAGAA
AAGCATGAATATCTTTTCTTAATTTCATTAGTACTAAAATTCTTGCACCAATTTCAAGAAAAGGGT
AAGTTTGACTGTAAATGCCTTACTGCAAAACTGAAAGAAAAACATTTATTTATTTTCTTTAGCCCA
AGAAAGTACTTTTGAAAAAGAAAAAACGCTATATGTATAAAAAGATCAAATAATGAACAATTAA
TAAGTAGTTGAATGTTAATATGGTAGGCTGTTTTATAACTATTAAAATTATAAATATGCTGTCCAT
GAAAAAGCTGGGGAAATGTTTATAATATAATATTTTGAAAAGTGTACAACAAAGAAAAGTATATT
ATATACTATTATTATGTCCACGTAAAAACTCTATGTCTATATGGACAAAGTGTGGATGGGAACAT
GAAGAAACAGAAACCATTGGTTGTGTCGGTATATCAGTCAGGATAAGCTAGGTTATGCTATGGT
AACAAACAATACCTATATATCAGTGACTCACAACAAAAGCTCATGCTACCCGATCAGACTCTGC
TCATTGTAATCATTTTGGTACTCCGGCTGAAGAAGGTTCCATCTTTACACATCCTTCCTTAATTGT
CTCAATGTTGGCAGAGGAAAGATATGGTTGGTACTATATCTAAAAGATTGTACATGGAAGTGAA
ATATGTCACTTCTACTTACATTTATTTGGCCAAAGCAAGTCACATTGGCATGTCAACTCCAAAGG
GGACAGGGAAGCTAAATCCTACTAGGTGATCAGAAACAGAACCAGATATATTTGAATGACACT
AACACGCTCAATAAGATTGATAAAATTACAGATGCTTTTTCAAATCTTTTATAATGTTAAAATATA
TTTTTATTATTATTACCTCAGATTCCACTTGTGAGCCAGAAATTGGGACTTGATGATGTATATGAT
ACTAATCTTTCAAAGAAAGGCTTTTAACTGAAAATTCTTTAACATGCATATTCTTTTATTCAAATA
GTTTAAATGGATCAAGATGATAAACATTTTCACATTAGGAAATACAGTTTGATTGAAAAATATTT
CTGGCTTCACTCATTGTAATATGCAATTTGTAAATTACATTAAGGAAACAACTTGCAAGAAGGCA
TTCTGTTATAAATATTTATAAATGTAGTTAGAAAAATACGTCTTTTGCCTATGTACAATAGAACAA
TTTGCTTGCTTCAGCAAGCAATCAGCAATAAAGAAAAACATGTCACATTGGTAGGGTTTTAAATA
TTTCAGTTTATAAACTCCTGAAAAACATCAGTTGAGCACGAATGGAAGACAGTATGATGTACAG
CAAAGGACACTGGCTTTGGCATCTGGTAGGTTCAAATGCTGAACCACATTTATTACACTACCTGG
GGCAATCAGGCTAACTTCCTCCACACAGTTCCCAGCACACAGTAAGCACTCATATTTGTTTCATA
AGTGAATTTCTTCTTTATTTCTATCATTTCTGCATTATGAAAAAAGTTTTCTCTTGGCTTGTAAGAT
AGTCAAATTATACCTGAATATAAAAACCAAGGGCAGAGTAAGTGTCTGCAATAAGGAGTTTTGT
ATTCTTTCTTTTTTTTTTTTTTTTTTTTTTTAGCATTCCTGAAAACTGCCTTCCTGACATTAGAAGTTC
TATCTAAAATCTGACTTATGGATTTGACCACACACCCCTAAGCCCATAAATTCAGTAATTGCCTG
TGGTGAAGGACCCTACTTTTCCCTGTGGCCTCTATTCTCTGGACCATGTCAGCCATCCTCTCCAT

GGAGGACCGGACTGTGTTCAGTCCTATGAAGGCAAACACAGGATAAGGAAAGGTTTAGGAAAG
GCCAAGGAGTATAGTTCCAGGAAAGTAAAAGCGTGGGAGTGCGAGTTCTGAGCTTCTCCAAAG
CTAATTGAAAGGACCTAAAAGAAGAGTTCTAGAAAGAAAATGAAGACCTGACTTTTTTCTTTCCC
TGTTAGCAATATAAATGGGAGTTTTTATTCCTATATTTTTAAAGAGAATTGCTGAGAATGGCATGT
TTTTCCTTCTCTGGCTCCTCAATTACTGTCTGTTCCACAAAATAGATAAAAAGAAAAAGAATGAA
GGAGAAATCTCATCCCCAAAATAATTTTAGTACAAGTTCTAAATGGAACCCAGGGCCCTGGAGT
GATGCTGATGCTGGCGGCTGATTCTGGACTATCCTGGTTTTGTGCTGTGGGAGAGCCCAGAAAG
TGTGTACCAGGATGGGGGATTCTCTCAGTTTTGTAAAAATTGTTATGAATGAGTTTCCCACCCTT
GATAACAATTTTTTAGGCTTTTCGGGCCTTTCTAAATTCTCCAAAAATAATTTTAAAAAAGGAAGA
TGTGTCCCTGTTATTGCCTTTAAGCTAAATAAAATTTATCTGGATAAAAAAATTATTTTAAACAAA
GTTAGTGAACAGTAATTTCGGTTGTAATTGACCCATTCTTTCATTTAAACTGACTCAATTTTAATG
CTCCTTTGGTTCAAGGTGGCTGGCTGTAGCAAAAAGGAAGCCACAAGAATTCATTCCTTTTAACT
GTGTTACCTTTTAACACTCAGGTGGCCCAGAGAGTTTGGAATTTCCTTGTTGGCATTACAGCTCC
CGATGGCTGCATTAACAACAACAACCAAAGTCACTTTTTCCACATGAAGTGGGAATACCTAATT
GTATAGGAATATCTGGTTATCAGTGATATTCACAGAATCTTCAAAGAAAGGAAATGAGAAGGTG
TGTTATTTGGCAAAGATGACATCAACATTCTCCACAGTGCTACGGTGTTAAAAAGTGGCAGCTAT
TTCCGTGAATTCTTTCACATTGGGTTTGATCATTATTCTGACAGAAATGGATGTTTTGGGAAGGTT
GTCGCTAAGCTCTCACTGTCATTGAGAGTTGAGAAATCTTCAAAACTGGGGAAGATGGCTCATA
TTAGTATTTGTCTCACTTCATTTACTTATCCATTTATTCATTCTGGAAAATGTTATTGAATACTCTTT
TTTCCCCTTCTGTTTCTACAGATGTATTAAAAAATGGTCTCTAGGAAAGATTTGGACTCCAGGGTT
CTCCTTTTCCTCCTCCTTTCCTCTTCCTTCACTCTCCCTATATGAGGTTGATCTCATACTAAGAAAA
GTAAAAAGCCAGGAAGGCAAAGCTCTGGTCTTCCACTGGAAACATCTTCACCTGTCTTTCCACG
GTAGGGCCTTAAGCACAGCCTGTCTACACTAGCACAAGACAGAGGCACCTATAAAAGCTGGCT
CTGGGGACTCATTCTCAGCTCTGATCTAGCATTTATCACAACCCCTGGTTCTCTTTGTTCTGAGAT
ATTTAGCTGAATTTCACATAGTGGTGATGATTGGCACACAATCATATAGCATATAGCAAAGTCTC
ATTTGTCTGGCACTGTCATATTATCAGGATGTTGAGGAAACTTACATTTATACAGTTAAGAAAAT
ACTTCTTTTACAATTTCTTTACTTTTTCACTTTGTTTTGATTAAAATATTTCTAAAATTGATTAAGCT
CTTGTCTCATTTCTTAAACAGTGTGCAACGAACATAACTTAACCTTTTTTATTGCTTATTCTGATCA
TTAAGCACCTTAATTACTGCTCAAATGCTATAATGCAGTTTTAATGACTCCTCCCTGTGTTGTTCA
TTATTATAGCTCTTGTCACTGAATCTGCAGCCAGCAGTATTTCCCTTCTCACTTGTGGTTCTGGCC
TGTCAAACCAGACAACCCATGGTTAGAATTATCTGTAAAGTAAGGGCCAATAAATACTGAGTGG
TGGCGGGTGCCTGTAGTCCCAGCTACTCGGGAGGCTGAGGCAGGAGAATGGCGTGAACCCGG
GAGGCGGAGCTTGCAGTGAGCGGAGCTGAGATCACGCCACTGCACTCCAGCCTGGGCAACAG
AGAGACTCCGTCTCAAAAAAAAAAAGACTTGGAAAACTAACACTTTACAATCTTTGGTGGTGTGA
CTATGTACTTTTCCTGCTAATCTATAGGCTTCACAAGATCAAGTACTTTGTCCTTTACCTATCTAA
ACCCCAGCAGTGCCTGGTGCTCCTGGGCTCTCTCACCTTCTTGATCTTCTAAACTCTGCCAGTCT
TCAGTCCTTAATTAGCATAACCATTTCCAAGCTGGGGATCCTAAATCTTAAATTTGCAAAGGGCT
GGGATCAGCTTCCCTCACTTTTACTTCTCAATTCACTCTCTAGTATCCTCATCCAATTTCATGCTTA
AGCAATTTGCTTCTGTGTGAATGAACCCTAAATAAATATCTCTACACAAACCATTTAAATTAATCC
CTATGCATTTCCTTTGTCAAGTCAGTGATCATTGCTTACATCTGTAAAAAGAAAAAAAAAAGTTGA
TTATTTCTTTTATTCTTAGGTTGAAATGTAAGCAAATCTTCTAGGAAGGAGATGTCGATTTTAGTA
CGAGCCTTTTAAGTAATTTCCTGCCTTCCTTTACACTAACTATTAATGTCTTGGACCCTCAGTTTTC
TTGGCTGTAAAATATGGGGGTTGAACTAAATAATTTCTTCAGCAATGCCAAGCTTTAAGATGCTA
TAATATTTTATTGATTACAGGAATTTAACCTCATTTAATTGGCTCAAAGAACTACTATCAGAATTT

CTTTAAAATAATATTGTTTTATTTGTCGTATTTTTCAGACTTTTTTTGCTCTTTACATTGGATATTTTT
AGTGATAAAATATTTTACACATGCAAAAAAGTAAAGAGAATCATACAACAGACACTTGTATCAT
GTATGATAATGTACCTGGCACACAGTAGGTGAATAATAAGTACTCTTGAAAGAATGATCTGGAC
CATCAGAGATAACTACAGTTGCTATAAAATAGCTGTGATTTTCCCAGAATGGCCAGCAGAGGGC
GGACTTGCACATTGACAAAACTGACCCTTGGTTTAGGTAAATTACCTTGGTGTCTTAGCTCCCCT
GGTACAAATTAATTATTGTGAAATTGTTTAGCACATAGCCAACAGCTGATCTATCTACCCCTTGTT
AACCACATTAAATGAACACAACTACTGTTCACAGATTGTTCAGAGACTTCAAAGTCAGTGCATTG
TCAAATAACAGTTTGGAAAATTTGCAGTATTTAAACCGCCTATCTAGATCACTCTAAATCTCCCC
AAATCTTCTTTGCCAGTTGTAGCTATATTTCTAAACCTTGTCATTAGGAGTAGCAGAGTGATGGTC
TGAATTAATTATCAAAGTTGCTGAATATTTTGTTTCACTAGTTAAGTATTGTAATCTCACTCTCGAT
CTGTTAACAAAATATATATAACCCCCTCACCCCCCCGCCACACACATGCATATCCATTCTTTATG
CCCAGAGGAAGCCACTGTTAACGGTTACTTGAATCTTTTTTGAAATTGCATATGCACATATAACC
ATAGATATATAGGTTTTTTTTTTTTTAACCATAAATGGGATCACAAATGCCTGGAGTCCTATAATG
TAATTTTTTTTAATAACCATGTAATCATCTACAATATATCTGTGTTAACTAGTAGAGATCTTGGCA
GAGAGCAAACGACCTGAATATTTCTGATCTGATTACTGGGAATCCATTTGGCCCAGCCAACACA
CTTAATCCTTTGTTGTCTCTTGTGTGTAGATAAGGTGTTTTCAGTGGGAAGCCATAAAAATAACCC
CTGAATCTTGGTTATCCTATACTTTGTGCCTCTCCATGCAGGTGGAGGATTACCACAACAACAAG
ATGCAGCTGTAAGAAAATAGGCTTGCTTTGCAGTGTGGCTGAGCACCACAGCCAGATCAGAGC
CAGAATTTCCAATGCCAGTCATGAGGATTCTGTTTCCCATAAATCCCTCTAAATTTTATATTTCTC
ACTGTGGAAATATTTGCCTTTGAATTTCTCAATCTCTATTTAAGGAAAATAAATGGAAAATAATTG
TTTAGATTCAAGAAATGCAAAGGTTACTTTAGCTTTTGCATTGCATTTGTGCAGTCTAACAGTAAA
CCCATTTTATAACTAGAATTTATATATAGTACCATGTTCAACTTCTTTTACTACTGGTCCTTCTGTG
TCAGAGATCCATAATCCGTGGGCCATGGACTGGTACTGGTCTGTGGCCTGTTAGGAACTGGGCT
GCACAGCAGGAGGTGAGCGGTGGACTAGCAAGCAAAGCTTCATCTGTATTTGCAGCCACTGCC
CATTGCTTGCATTACTGCCTGAGTTTTGCCTCCTGTCAGATCAGTGGTGGCATTGGATTCTCACA
GGAGTGCAAACGCTATTGTGGACTGCACATGTGAAGGATCTAGGTTGCATGCTCCTTATGAGAA
TCTAATATCTAATGATCTGTTACTGTCTCCTATCCCTTGAGATGGGACCATCTAGTTTCAGGAAAA
CAAGCTCAGGGCTCCCACTGATTCTACATGATGGTGAATTGGATAATCATTTCATTATATATTAC
AATGTAATAATAATAGAAATAGAGTGCACAATAAGTGTAATGCATTTGAATCTTCCCCAAATCAT
CCCCACCCCACCCCACTCTGTGGAAAAATTGTCTTCCACAAAACTCTTCCCTGGTGCCAAAAAG
ATTGGAGACCACTGCTCTATGTTACACCAACATTAACTGATACTAACAGAAATCTTATTTACCAT
AGTAAACACATGAAGTTATTTTATATACATTTTCACAGAGCAAGTTCATTTTGGTAATACCCAAAT
CCATACAGCATACTATGTTTTTTCTATCATTATCATCATGGACTCTCAAAATTGGAAAATGAGTCC
ATCTGGTCCAATTTTACATCATATATTAGCAGAGACTTGCTATGCAAAATTAACCCACAGACCGG
CAGTAGCAGCAGCAGCAGCACCAGAGAACTTAGGCAAAGGCAAATTCAATTCAGAGGACCCA
CTCCACACATGTTGAATTGGAATTTTGGGGAGTTGGAACTAATAATCTGATTTAACAAGGACTTT
GGGTGATTCTTGCCATACTAAAGTTTGAGAACCATTGATCTATAAACTCCTCATTAATGATCATTA
GTTTCTGCTTAAATATTTCCTTAATTAGGAAATCTACTACTTCACAAGGCAGACCGTTTCATTTTA
GATCATTACTTCTTGTTGGAAATCCCTTCTTATTTAGATCCAAGTCGACCTTCCTGGAGCCTCTAT
CCACTACTTGTTTTGCTCAACAAGAACACACATCGTGTTGACTCCATCTTTTATATGATGGTGAGT
TGTGGTCAAACACAGCTATCCTGCTCCTCTTCCTCCCTCCGTGAGTGTCCTCCTCCCCCAGTGAA
TGTCCCTCTCAGGTTAAATAACTTTAGTGGTTTTTAAATGTTCACCAAGCTGCATTTATCTCTTTAA
TATTCTTATTGTTCTCCTTTAATGAAGTTCCCTTTTGTCACCATTCCTTCAAAGTGAAACACTTCAA
AGAGTACAATAAAAAATAAATAAATAAATTTTTAAAAATAAATTTAAAATGAGAACAGAACATA
[truncated: 577,182 more chars]
